# Sulfur alleviates arsenic toxicity by reducing its accumulation and modulating proteome, amino acids and thiol metabolism in rice leaves

**DOI:** 10.1038/srep16205

**Published:** 2015-11-10

**Authors:** Garima Dixit, Amit Pal Singh, Amit Kumar, Sanjay Dwivedi, Farah Deeba, Smita Kumar, Shankar Suman, Bijan Adhikari, Yogeshwar Shukla, Prabodh Kumar Trivedi, Vivek Pandey, Rudra Deo Tripathi

**Affiliations:** 1 CSIR-National Botanical Research Institute, Rana Pratap Marg, Lucknow – 226001, Uttar Pradesh, India; 2 CSIR-Indian Institute of Toxicology Research, Rana Pratap Marg, Lucknow – 226001, Uttar Pradesh, India; 3 Rice Research Station, Government of West Bengal, Chinsurah, Hooghly – 712102, West Bengal, India

## Abstract

Arsenic (As) contamination of water is a global concern and rice consumption is the biggest dietary exposure to human posing carcinogenic risks, predominantly in Asia. Sulfur (S) is involved in di-sulfide linkage in many proteins and plays crucial role in As detoxification. Present study explores role of variable S supply on rice leaf proteome, its inclination towards amino acids (AA) profile and non protein thiols under arsenite exposure. Analysis of 282 detected proteins on 2-DE gel revealed 113 differentially expressed proteins, out of which 80 were identified by MALDI-TOF-TOF. The identified proteins were mostly involved in glycolysis, TCA cycle, AA biosynthesis, photosynthesis, protein metabolism, stress and energy metabolism. Among these, glycolytic enzymes play a major role in AA biosynthesis that leads to change in AAs profiling. Proteins of glycolytic pathway, photosynthesis and energy metabolism were also validated by western blot analysis. Conclusively S supplementation reduced the As accumulation in shoot positively skewed thiol metabolism and glycolysis towards AA accumulation under AsIII stress.

Arsenic (As) exposure is a serious threat to human beings and its key source of exposure is the As tainted water. Unfortunately, in many parts of the world rice is grown in highly As contaminated areas which leads to contamination of food chain^1^. Rice is the staple crop of 3 billion people worldwide and Asian countries account for 90% of its production as well as consumption^2^. Unlike other cereal crops, rice is particularly efficient AsIII accumulator that leads to impaired cellular functions through strapping of sulfhydryl groups of enzymes and proteins^3^. In addition, inhibition of SH containing enzymes by As, alters cellular redox state and finally leads to cytotoxicity^4^.

Sulfur, an essential element, plays vital role in the plant growth and defense response by regulating the expression of genes involved in the uptake and assimilation of sulfate^5^,^6^. Plant deficient in sulfur status have lower non protein thiol content, metal(loid) complexing ligands such as PCs^7^, thus they have higher As translocation from root to shoot^8^. In plants, inorganic sulfate is reduced to sulfide and further assimilated to form Cys^9^. Cysteine serves as a precursor for a range of S-containing defense compounds, such as methionine, S-adenosylmethionine (SAM), glucosinolates, and GSH^10^. Sulfur moiety of Cys is responsible for di-sulfide bonding in proteins for their proper function and also important for the formation of the Fe–S cluster in the photosynthetic apparatus and electron transport chain^11^. A set of Cys containing redox-sensitive proteins e.g., glyceraldehyde-3-P-dehydrogenase (GAPDH), malate dehydrogenase (MDH) and elongation factor-thermo unstable (EF-TU) are well known for their roles in oxidative thiol modifications^12^.

Rice grain contains 3–7% protein, comprising of different essential and non essential amino acids (EAAs and NEAAs), in which glutamic acid (Glu), proline (Pro) and lysine (Lys) are the major components. Various studies^13^,^14^ indicate variable responses of AAs metabolism including EAAs and NEAAs during heavy metal stresses. Dwivedi *et al.* (2010a)^15^ demonstrated a positive correlation between total AAs content and As accumulation. Further, the stress responsive AAs like Pro, glycine (Gly), Cys, Glu and methionine (Met) showed higher accumulation in high As accumulating rice genotype in comparison to low As accumulating rice genotype^16^.

Genome-wide gene expression profiling experiments have revealed that a wide array of genes (stress-responsive gene, heat-shock proteins, metallothioniens, sulfate-metabolizing proteins and assimilation pathway) are differentially expressed during As stress in rice plant^17^. Over the years, the proteomic approach has been used extensively to analyze the proteins involved in various stress responses in plants^18^,^19^. Arsenate (AsV) induced NADP-dependent malic enzyme (NADP-ME), aspartate aminotransferase, NAD-dependent formate dehydrogenase (FDH), GAPDH and ATP-dependent protease, proteolytic subunit ClpP-like protein have been reported in rice leaves^19^. In another study, AsV stress altered the chloroplast 23 kDa polypeptide of photosystem II, Chloroplastic aldolase and Photosystem II stability/assembly factor HCF136 that leads to reduced photosynthetic rate and distorted chloroplast structure in rice leaves^20^. Studies on nutritional aspects vis-à-vis As have been targeted only to nitrogen^21^ and phosphorus supplementations^22^. As AsIII is more toxic than AsV in fields and also limits uptake of macro and micro nutrients^23^, it is important to study AsIII and S interaction in rice. In order to gain an insight into AsIII induced stress under different S regimes in rice leaves, hydroponic studies were conducted in rice (IR-36) with respect to combined proteome, AA, thiol metabolism and western blot analysis. Besides deprivation, higher S dose was also used during experiments to observe role of S amelioration of As toxicity as S has potential to mitigate As toxicity through chelation. The study emphasizes role of SH under AsIII toxicity as S containing enzymes are prime target during AsIII exposure. It is hypothesized that S supplementation would alleviate the AsIII toxicity in rice plants.

## Results

### Impact of Sulfur and Arsenite on Rice Leaf Morphology, Photosynthetic Pigments and Arsenic Accumulation

**Figure 1:**
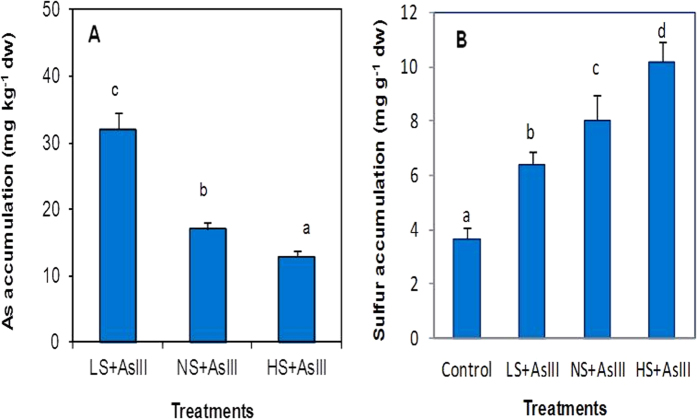
Effect of different sulfur doses on arsenic (A) sulfur (B) accumulation under arsenite stress in leaves of rice (*Oryza sativa* L.) plant. All the values are means of triplicate ± S.D. ANOVA significant at *p* ≤ 0.01. Different letters indicate significantly different values at a particular treatment (DMRT, *p* ≤ 0.05).

The S conditions were abbreviated as follows: LS for the low sulfur conditions, NS for standard sulfur conditions, and HS for the high sulfur concentration. All the treatments were compared to NS control unless otherwise mentioned in the text. AsIII hampered plant growth through reduction of shoot length and weight. LS + AsIII showed considerable reduction (20%) in shoot length; however, HS + AsIII ameliorated this effect by increasing shoot length by 17%. Similarly LS + AsIII reduced shoot weight by 47% and HS + AsIII ameliorated it by 68% (Supplemental Information 3 Fig. S1 and S2 A, B). Chl a, b and total Chl contents were substantially reduced (31, 43 and 31%) in LS + AsIII while S supplementation elevated Chl levels (19, 44 and 21%) in NS + AsIII and (41, 91 and 32%) in HS + AsIII, respectively. Carotenoid contents were enhanced in LS + AsIII (108%) and decreased in NS + AsIII (19%) and (53%) in HS + AsIII, respectively as compared to LS + AsIII (Supplemental Information 3 Fig. S2 C, D, E, F). Arsenic accumulation was analyzed in twenty days old hydroponically grown rice seedling leaves (Fig. 1A). Results indicated that LS + AsIII increased As accumulation (32.12 mg kg^−1^ dw) and reduced in HS + AsIII (12.92 mg kg^−1^ dw) treatments in comparison to NS + AsIII (17.03 mg kg^−1^ dw). Arsenic exposure enhanced the S accumulation in shoot than the plants not exposed to As. Sulfur accumulation in shoot was allied to S concentration in the nutrient solution (Fig. 1B).

### Rice leaf proteome modulation during S and AsIII interaction

**Figure 2:**
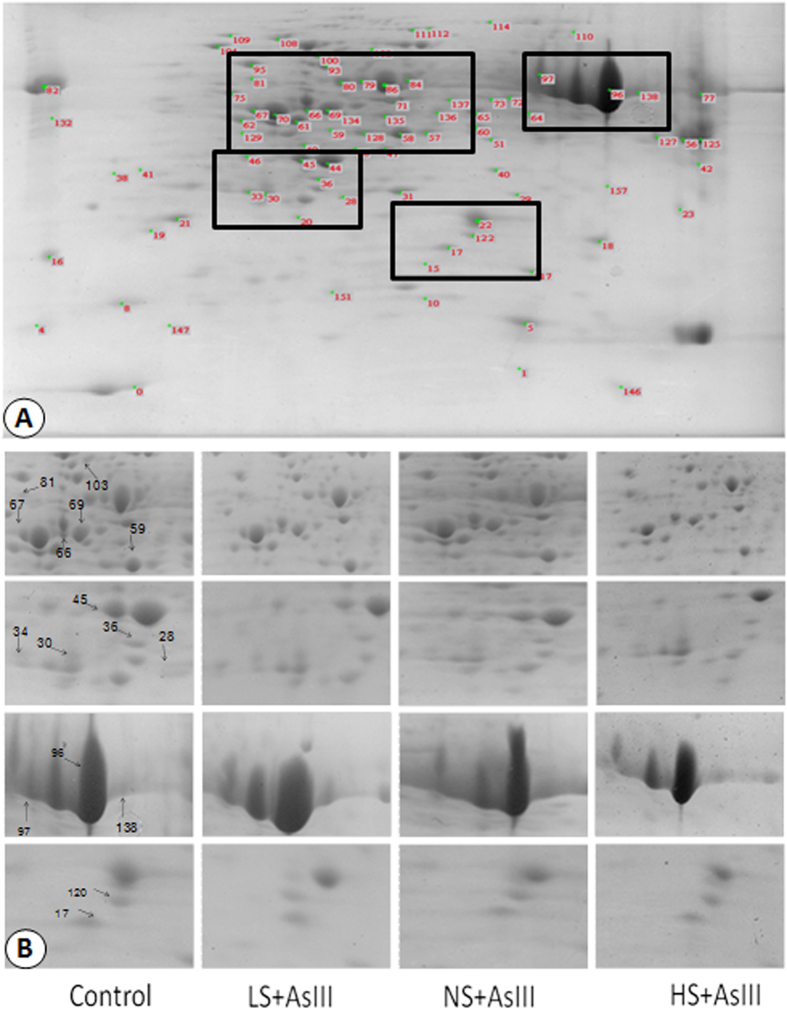
(**A**) Reference 2-DE image of rice leaves proteome. Proteins were stained with CBB G-250. (**B**) Close-up of some up and down regulated proteins detected by 2-DE (gel image with identified proteins) and their expression profile patterns.

**Figure 3:**
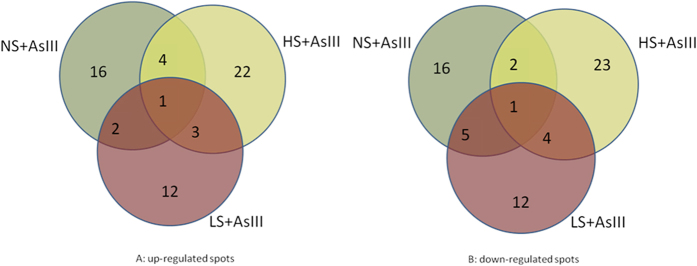
Venn diagram analysis of the differentially expressed protein spots in rice (*Oryza sativa* L.) leaves. The numbers of differentially expressed protein spots with up- or down-regulation under different concentration of sulfur and equimolar concentration of arsenite are shown in the different segments. (**A**) The up regulated protein spots. (**B**) The down regulated protein spots.

A total 282 spots were detected using CBB staining; reference gel depicting all spots and four typical enlarged regions are shown in Fig. 2A,B. Out of 282 spots, 113 spots were differentially expressed in comparison to control. 60 spots were up regulated while 53 spots were down regulated. All the spots showed at least 1.5 fold changes in abundance. Out of these 113 (60 + 53), 80 spots showed more than 2 fold changes in abundance (Table 1). Venn diagrams of differentially expressed proteins indicated that out of 60 up regulated spots, one common spot was present in all the treatments, 9 spots showed the enhanced abundance in any of two different treatments (Fig. 3A). Out of 53 down regulated spots, one spot was down regulated under all three treatments and 11 spots diminished in abundance in any of two different treatments (Fig. 3B).

### Identification and functional categorization of the differentially expressed proteins

**Figure 4:**
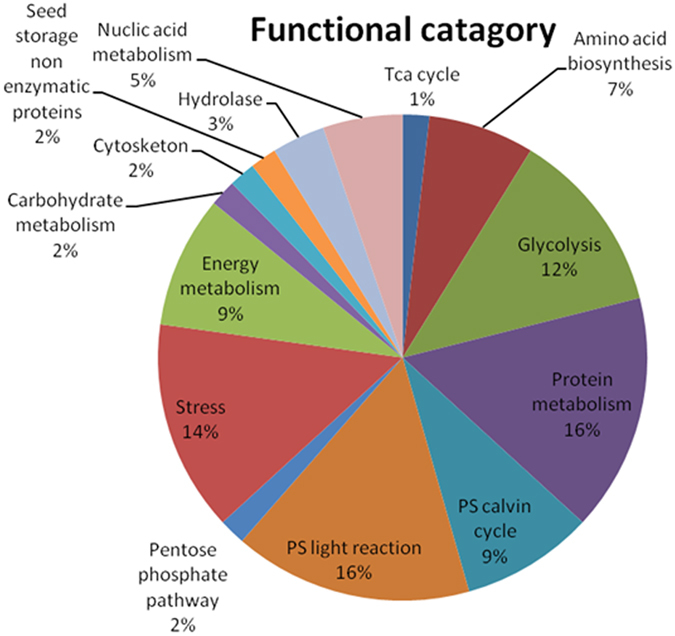
Functional classification and distribution of all identified proteins of rice (*Oryza sativa* L.) leaves proteome as classified by Bevan *et al.* (1998) on non-redundant basis.

A total 113 differentially expressed protein spots were analyzed by MALDI-TOF-TOF. Of these, 80 spots were successfully identified by MS/MS as listed in Table 1. Proteins were identified, and their functions were determined using the classifications by Bevan *et al.* (1998)^24^. On the basis of functionality, proteins were classified into 14 major categories (Fig. 4).

Functional categorization of proteins was done on non-redundant basis. Taken together, the 80 identified proteins represented 57 unique proteins; rest may be their corresponding isoforms. The proteins involved in photosynthesis and protein metabolism were represented more (16% each) of total identified proteins. Notably LS + AsIII had a positive influence on photosynthetic light reaction proteins (Spots n^o^0, 20, 24, 30, 34 and 50). Proteins influencing AA metabolism included glycolysis (12%), AA biosynthesis (7%) and TCA cycle (1%) proteins, comprising 20% of whole proteome. Energy metabolism related proteins (9%) primarily involved divergent subunits of ATP synthase. ATP synthase (Spots n^o^79, 85) and iron-sulfur protein (Spot n^o^16) levels were enhanced considerably by higher S supply while Ferredoxin NADP reductase (Spot n^o^50) level was increased at all the S supplies + AsIII. Many proteins with a potential role in protection against As induced oxidative damage changed in abundance in rice leaves during As stress e.g. Cu, Zn SOD, 2-cys peroxiredoxin, glyoxalase, heat shock proteins though their expression levels varied at different S supplies (Table 1). Among stress proteins (14%), iron-sulfur protein (Spot n^o^16) was up regulated under HS + AsIII, while LS + AsIII conditions had negative effect on its abundance. Though nucleic acid metabolic proteins (5%) were not detected in LS + AsIII, however, HS + AsIII treatment up regulated these proteins (Spots n^o^129 and 134). Cytoskeleton (2%) and carbohydrate metabolism (2%) proteins were enhanced during AsIII stress throughout all the S regimes except HS + AsIII where NAD dependent epimerase was not detectable. Hydrolase and seed storage non enzymatic proteins were down regulated in all the NS + AsIII exposure conditions.

### Biochemical changes during AsIII Stress under different S conditions

**Figure 5:**
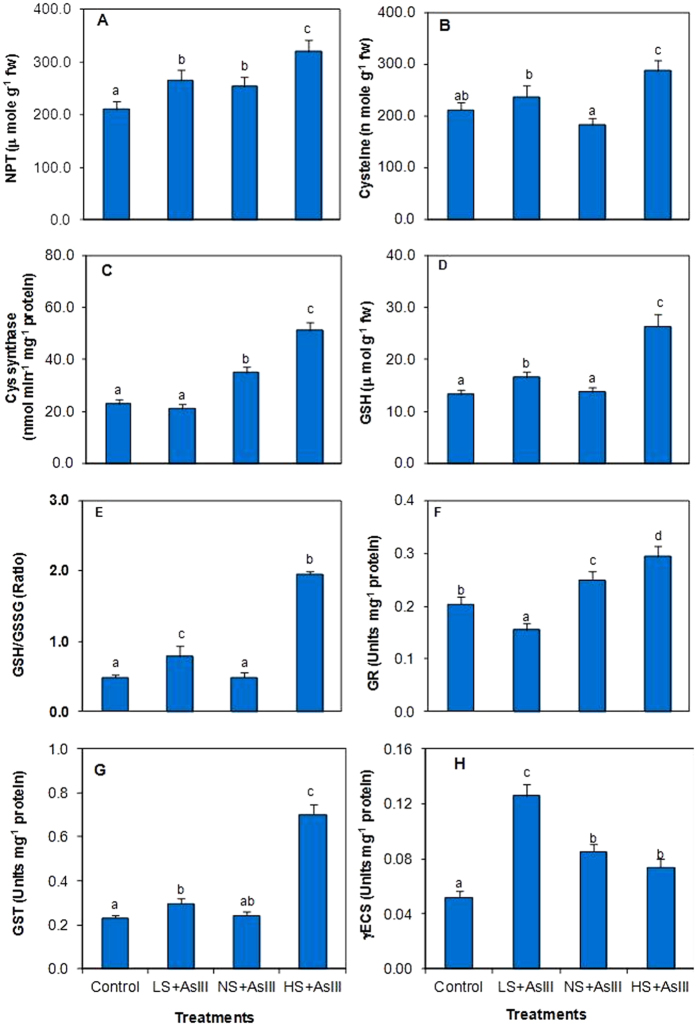
Effect of different S doses on the level of (A) non protein thiols (NPTs), (B) cysteine, (C) reduced glutathione (GSH), (D) ratio of reduced to oxidized glutathione (E), cysteine synthase (CS), (F) glutathione reeducates (GR), (G) glutathione-S-transferase (GST), (H) γ-glutamylcysteine synthetase (γ-ECS) under arsenite stress in rice (*Oryza sativa* L.) leaves. All the values are means of triplicate ± S.D. ANOVA significant at *p* ≤ 0.01. Different letters indicate significantly different values at a particular treatment (DMRT, *p* ≤ 0.05).

Different S treatments illustrated statistically significant variation in thiolic ligands. Plants exposed to HS + AsIII had elevated levels of thiolic compounds. Total NPTs, Cys and GSH levels were enhanced up to 52%, 46% and 99%, respectively in HS + AsIII plants. LS + AsIII exposed plants also had enhanced thiolic compounds as NPTs (26%) and GSH (24%), while Cys level declined by 9%. Activities of enzymes related to thiol metabolism (CS, GR, GST and γ-ECS) modulated considerably in various S treatments. Cysteine synthase was enhanced (139%) in NS + AsIII treatment, whereas 9% reduction was observed in LS + AsIII plants. GR activity was substantially different under different S treatments, while LS + AsIII exposure resulted in 24% decline, HS + AsIII plants demonstrated higher increase (204%). Similarly activity of GST was higher (189%) in HS + AsIII than NS + AsIII and activity of γ-ECS was increased by 142% LS + AsIII exposure than NS + AsIII (Fig. 5A–H).

### Gene expression analysis and western blot analysis

To investigate the changes in gene expression at the mRNA level, qPCR analysis was performed (Supplemental Information 3, Fig. S3). The genes encoding 15 proteins (PKR, 2Fe-2S, TPI, GAPDH, OEE, FNR, PsbR, Fru-bisP aldolase, GLN1, GLN2, AtpB, RuP3-E, aminotransferase) in *O. sativa* (Table 1) were analyzed. Transcript level analysis revealed induction at mRNA level in most of the proteins under LS + AsIII. However, in seedlings exposed to HS + AsIII and NS + AsIII decrease in expression level was observed.

**Figure 6:**
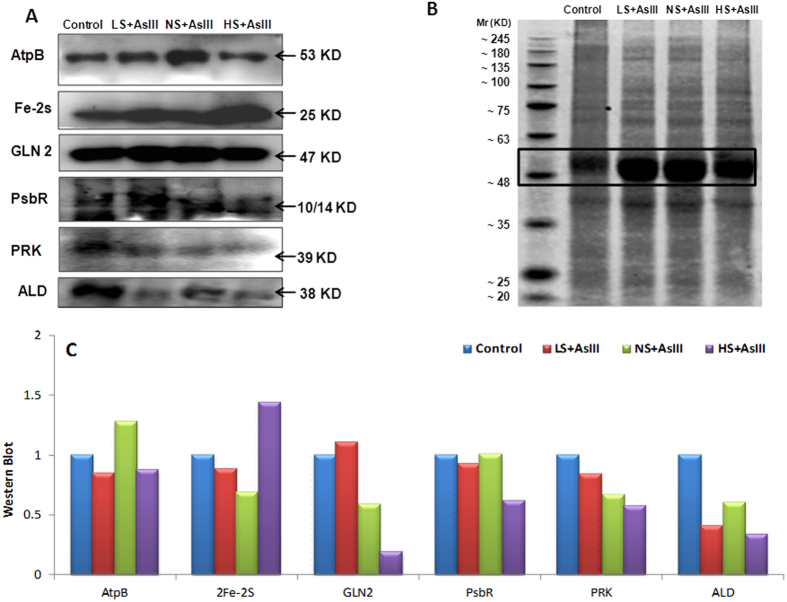
Western blot analysis of selected candidate proteins (AtpB, 2Fe-2S, GLN2, Psb R, PRK, ALD) with their corresponding molecular weight in rice leaves during As stress under various S regimes (A); RuBisCo large subunit a band in coomassie blue staining (CBB) SDS gel served as a loading control (B), which used for normalization of detected proteins in densitometry studies (C) fold change indicated with respect of control samples.

To validate the results of 2DE gel analysis, western blot of selected candidate proteins were performed (Fig. 6A–C). Based on our matching rationales from the literatures, supporting their roles on the cellular physiology of sulfur and arsenic interactions, we selected AtpB, 2Fe-2S, GLN2, PsbR, PRK, and ALD proteins to verify their expression in treated conditions by western blotting. The densitometry analysis of these proteins on blot showed a similar trends expression by the effect of treatment as it observed by 2DE analysis.

### Amino acid profiling

**Figure 7:**
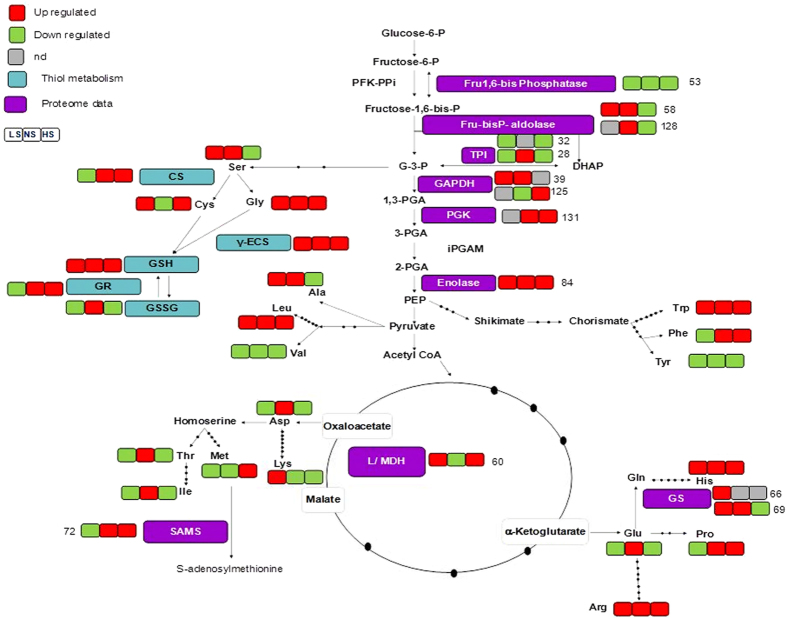
Pathways involved in the biosynthesis of amino acids. All data were extracted from Tables 1; Purple boxes represent protein change from proteome analysis. Metabolite abbreviations are as follows: nd, not detected; LS, low sulfur; S, optimum sulfur; HS, high sulfur; PFK-PPi, PPi-Fru-6-P 1-phosphotransferase; fructose 1,6-bisphosphatase, FBPase-1; fructose bisphosphate aldolase, Fru-bisP aldolase; DHAP, dihydroxyacetone-phosphate; TPI, triosephosphate isomerase; GAPDH, glyceraldehyde-3-phosphate dehydrogenase; PGK, phosphoglycerate kinase; G-3P, glyceraldehyde-3-phosphate; 1,3PGA, 1,3-bisphosphoglycerate; 3PGA, 3-phosphoglycerate; 2PGA, 2-phosphoglycerate; PEP, phosphoenolpyruvate; L/MDH, lactate/malate dehydrogenase; SAMS, S-adenosine methionine synthetase; GS, glutamine synthetase; GAPDH, glyceraldehyde 3-phosphate dehydrogenase; PGK, phosphoglycerate kinase; CS, cysteine synthase; γECS, glutamylcysteine synthetase; GSH, reduced glutathione; GSSG, oxidized glutathione; GR, glutathione reductase; Aspartic Acid(Asp), Threonine (Thr), Serine (Ser), Glutamic Acid (Glu), Proline (Pro), Glycine (Gly), Alanine (Ala), Valine (Val), Methionine (Met), Leucine (Leu), Tyrosine (Tyr), Phenylalanine (Phe), Lysine (Lys), Histidine (His), Arginine (Arg), Isoleucine (Ile), Cysteine (Cys).

The fueling reactions of central metabolism provide precursor metabolites for synthesis of the 20 AAs which are incorporated into proteins. The diversions of AA biosynthetic pathways from the glycolytic pathway and the TCA of central metabolism are shown in (Fig. 7). Free AAs were examined *viz.*, aspartic acid (Asp), threonine (Thr), serine (Ser), glutamic acid (Glu), proline (Pro), glycine (Gly), alanine (Ala), valine (Val), methionine (Met), leucine (Leu), tyrosine (Tyr), phenylalanine (Phe), lysine (Lys), histidine (His), arginine (Arg), isoleucine (Ile) and cysteine (Cys) in rice shoot during As stress with different S doses. Histidine was induced maximally (333%) among all the AAs followed by gly (274%) during HS + AsIII. Valine showed highest reduction of 97% under LS + AsIII followed by Lys (94%) HS + AsIII. In all the treatments, levels of His, Trp, Gly, Arg and Leu enhanced, however, content of Val and Tyr were declined (Table 2).

## Discussion

The aim of the present study was to validate the hypothesis that high and adequate S may mitigate As stress through various metabolic pathways. In this study results obtained provide insight on the influence of different S conditions on proteomic changes in rice leaves during As stress. Results indicated that As accumulation is considerably affected by different S regimes. Limiting S enhanced As accumulation in aerial part of the plants, perhaps due to lesser chelation of As by thiols in root^25^. Contrastingly high S supplementation results in lesser As accumulation in leaves, as high As-phytochelatin complexation in root restricts entry of As in shoot. High PC-As chelation has been found to immobilize As in the root and minimizing its level in shoot and grain^26^ in transgenic rice. In HS + AsIII leaves, the excess thiolic metabolites such as glutathione may serve as antioxidant leading to reduction in toxicity. Limiting S reduced shoot length as well as weight, while HS + AsIII reverted this effect. Similar study in *Arabidopsis* plants supported the finding^27^. Decline in growth under stress has been regarded as an effect of reduced photosynthetic content^28^ (Supplemental Information 3 Fig. S1, S2 C–F).

In the present proteomic study, a number of proteins were differentially expressed among different S treatments (Table 1). Functional classification of differently expressed proteins reveled that major proteins were dedicated to central metabolic pathways (glycolysis, TCA cycle and AA biosynthesis) followed by photosynthesis (electron transport chain and Calvin cycle), energy, stress, nucleic acid and protein metabolism (Fig. 4).

### Effect of sulfur and arsenic interaction on glycolysis and TCA cycle

In the glycolysis, preparatory phase is energy consuming, starting from glucose and ending with splitting of hexose to triose. During the present study different enzymes of preparatory and payoff phase of glycolysis behaved differentially during low and high S regimes in combination with As. Results indicated that enzymes of preparatory phase specifically fructose 1,6-bisphosphatase (FBPase-1), fructose bisphosphate aldolase (Fru-bisP aldolase) and triose phosphate isomerase (TPI) were down regulated in LS + AsIII and HS + AsIII combinations except upregulatory response of Fru-bisP aldolase under LS + AsIII. To abate the As toxicity, plant required more reducing equivalents so that FBPase-1 might be inhibited to compel glycolysis to move in forward direction^29^. Though LS + AsIII enhanced the Fru-bisP aldolase while decreased during HS + AsIII exposure, this may be due to the excess GSH formation that inhibits enzyme activity^30^. Higher level of GSSG and GSH may reduce TPI level in LS + AsIII and HS + AsIII treatments respectively and it is also evident by study of Ito *et al.* (2003)^30^.

In contrast to preparatory phase, enzymes of payoff phase were up regulated under low and high S regimes along with As. However, similar to preparatory phase, enzymes of payoff phase (PGK and enolase) also showed up-regulation under As exposure alone except GAPDH, which showed differential response, might be due to the different isoforms (Table 1, spots n^o^39 and 125) of this enzyme. Differential expression of GAPDH has also been observed in several other proteomic studies in response to metal and metalloid stress^19^,^31^. In the present study enzymes of payoff phase of glycolysis *viz.*, GAPDH, phosphoglycerate kinase (PGK) and enolase played active role to fulfill the energy requirement during stress conditions probably by using photo assimilates directly from chloroplasts for mitochondrial respiration^19^. The enzymes of preparatory phase were down regulated in high sulfur combined with AsIII, and enzymes of pay off phase were up regulated under same circumstances, then there must be any alternative source of substrate. Pentose phosphate pathway may provide substrate to pay off phase, as glyceraldehyde 3-phosphate and fructose 6-phosphate are the byproduct of pentose phosphate pathway^32^.

Lactate/malate dehydrogenase (L/MDH) is chloroplast localized enzyme of TCA cycle and catalyses malate to oxaloacetate or *vice-versa* was positively regulated in HS + AsIII treatment. Higher requirement of oxaloacetate occurs during stress conditions in cells to synthesize various stress related AAs (Met, Lys, Thr) to maintain the homeostasis. While malate counterbalance the uptake of anions and cations in plant cells in order to maintain the anion-cation charge balance and the cytoplasmic pH, oxaloacetate is required for synthesis of Met involving various steps. S-adenosylmethionine synthetase (SAMS) catalyzes the biosynthesis of S-adenosyl-L-methionine (SAM) from Met and ATP, which is a universal methyl group donor in several transmethylation reactions^33^. SAMS were elevated at HS + AsIII in the present study, similarly^34^ concluded that different isogenes of SAMS play important role during hexavalent Cr stress.

Sulfur deficiency is considered as S shortage relative to nitrogen (N) nutrition, depicting symptoms of excess nitrogen^35^. Enhanced levels of glutamine synthetase (GS) as observed during LS + AsIII, suggest that low S nutrition mimics excess N nutrition. Glutamine synthetase functions as the major assimilatory enzyme for ammonia produced from N fixation or nitrate reduction pathway^36^. Additionally GS is involved in the synthesis of GSH through Glu biosynthesis pathway^29^. The enhanced expression of GS leads to more GSH formation which would enhance cellular defense against oxidative stress by participating in the ascorbate/GSH cycle^37^. At HS + AsIII, glutamine synthetase activity was down regulated imitating deficiency of N in comparison to S nutrition. This result is further corroborated by the downregulation of aminotrasferases at almost all the S supply. Aminotransferases are involved in number of cellular processes including glycolysis, AA metabolism, photorespiration and N use efficiency^38^. Therefore down regulation of this enzyme would have resulted in lesser nitrogen in rice leaves.

### Effect of arsenic sulfur interaction on amino acid level and glutathione metabolism

Protein and AA composition are the vital component of rice nutrient quality, however, literature indicated As induced changes in various AAs due their role for chelation of metal(iod)s and cellular homeostasis^16^. During the present study As and S interaction differentially affected the AAs content, e.g. NS + AsIII enhanced the Thr, Met, Ile, Asp except Lys. HS + AsIII exposure resulted in high Met level and also the downstream enzyme S-adenosyl methionine synthatase (SAMS). Similarly HS + AsIII treatment resulted in higher Cys and Met levels and associated enzymes in submerged macrophyte *Hydrilla verticillata*^39^. Induced Met level also indicates its role in AdoMet-dependent reactions, the nitrogen–carbon skeleton and its S derivatives recycling^40^. These cycles could be of importance in situations of low S availability for Met synthesis as a consequence of a high demand of Cys for GSH synthesis under stress conditions^39^. Glutamic acid, Cys and Gly are the main components of GSH, induction of these AAs in all the treatments except Glu and Cys at LS + AsIII may be due to the limiting S in the medium which is essential for the formation of GSH^39^. In addition, higher level of GSH is maintained in HS + AsIII due to higher activity of GR^41^,^42^,^43^. Glutathione is involved in detoxification processes, and plays a role as an enzyme cofactor, and a storage and transport form of Cys^44^. GSTs are involved in conjugation of GSH to metal or metalloid and metabolites induced due to oxidative stress^39^,^45^ and its enhanced activity would be helpful in preventing oxidative stress^16^. At LS + AsIII higher activity of γ-ECS that catalyses the ATP-dependent ligation of Cys and Glu to form γ-EC may occur to fulfill demand of GSH. The low levels of Glu and Cys were obtained in the present study suggesting rationale for higher rate of enzymatic activity of γ-EC. In LS + AsIII, As accumulation enhanced NPTs to chelate endogenous AsIII using most of the available GSH pool. However, HS + AsIII induced NPTs because of higher exogenous supply of S resulting into higher GSH pool^37^. Which may have played crucial role in detoxification of As during high S supply. Ratio of GSH:GSSG is crucial to maintain cellular homeostasis, HS + AsIII treatment has been found to achieve this aim through maintaining this homeostasis when plant was suffering from AsIII toxicity. This is in accordance with study demonstrating protection of *Arabidopsis* plants from heavy metal toxicity by maintaining GSH homeostasis through recycling Glu^46^.

### Effect of S and As interaction on proteins of photosynthesis, energy and stress

Various proteins of photosynthesis machinery have been shown to be differentially affected by metal stress^19^.Proteomic analysis revealed oxygen evolving enhancer (OEE) protein was down regulated under AsIII stress with various S regimes. Leaf proteome of *Agrostis tenuis* has shown drastic reduction of OEE protein in response to 134 μM As(V)^47^,^48^. Considerable reduction in photo system I reaction center subunit IV (Spot n^o^17) was observed under HS + AsIII condition, which is in agreement with earlier study showing similar trend^45^ irrespective of As stress. This contrast may be due to the varietal difference among the rice genotypes, behaving differentially under As stress^15^,^23^.

Recently, D’Hooghe *et al.* (2013)^49^ concluded that S limitation results in reduction of plastocyanin and Ferredoxin–NADP reductase (FNR) in *Brassica napus*. However, current study on rice demonstrating up regulation of plastocyanin may be due to additional As stress. Sulfate restriction reduces S assimilation into cysteine^27^, also reduces carbon assimilation and photosynthetic activity, resulting into distortion of glycolytic flux that leads to reduction of AA accumulation.

In the present study, NS + AsIII and HS + AsIII up regulated FNR level, that catalyses the production of NADPH + H^+^ required for CO_2_ assimilation and energy production. Srivastava *et al.* (2011)^4^ reported that plant requires more energy during As stress to maintain cellular homeostasis. Recycling of NADPH serves to strengthen antioxidant system under salt stress in olive plants^50^.Thus it can be inferred that to full-fill energy requirement, S enhanced energy production through enhancing NADPH + H^+^ availability to cope up As stress, as lower S limits the availability of NADPH + H^+^ 50.

Many proteins which showed different pIs and/or Mws were identified as ribulose-1,5-biphosphate carboxylase/oxygenase (RuBisCo) subunits (Table 1). Interestingly ribulose bisphosphate carboxylase (RuBisCo) small chain precursor (Spots n^o^4 and 8) was down regulated during all the AsIII treatments irrespective of S condition, whereas RuBisCo large chain precursor was both up regulated (Spots n^o^96, 132 and 138) and down regulated (Spot n^o^42 and 82). Small subunit being a limiting factor, may down regulate overall RuBisCo mediated reaction leading to reduced carbon fixation (Calvin cycle) that lead to reduced photosynthesis rate^19^,^49^.

Reduction in ATP synthase along with its subunits during LS + AsIII exposure could be drawn in, for S remobilization processes through the maintenance of an efficient sulfate efflux from the vacuole. Similar results were reported in leaves of *Brassica* involving tonoplast sulfate transporters (BnSult4;1 and BnSult4;2)^51^. At higher S, increased abundance of ATP synthase (alpha and beta subunits) and Ferredoxin-NADP reductase suggest their likely role in As tolerance mechanism. In general, plants exposed to abiotic stresses show downregulation of linear electron flow (LEF) and activation of cyclic electron transport (CET) when LEF becomes saturated^52^. It could be possible that under high S the LEF is partially replaced by up regulated CET which provides energy to Calvin cycle^53^, thereby meeting energy demand.

Iron-sulfur clusters is essential and versatile cofactors of proteins involved in catalysis, electron transport, vitamin B1 synthesis and sensing of ambient conditions^54^,^55^. During current study this protein was found up regulated with HS + AsIII while LS + AsIII reduced this protein, suggesting an imbalance of this protein under deprived S condition, and this modulation by S concentration is first time reported during current study.

IAP100 is a member of ‘inhibitor of apoptosis’ (IAP) gene family which was discovered less than a decade ago, serves as anti-apoptotic protein^56^. This protein, reported for the first time in rice, was found up regulated in HS + ASIII condition tentatively suggesting role of S in apoptosis inhibition, though this requires further investigation.

## Materials and Methods

### Plant Material and Stress Treatment

Rice seeds (*Oryza sativa* L.), cultivars IR-36, collected from RRS, Chinsurah India, were surface sterilized using 10% H_2_O_2_ for 30 s, followed by thorough washing with de-ionized water, and then were soaked in distilled water for 24 h. Seeds were germinated in the dark for 4 d at 37 ± 1 °C. Uniform germinated seedlings were selected and transplanted to trays containing fixed PVC cups (4 cm diameter and 5 cm height, 10 plants per cup) and grown in modified Hewitt’s media^57^,^58^ supplemented with low sulfur (0.5 mM), normal sulfur (3.5 mM) as used in standard Hewitt media, or high sulfur (5.0 mM)^39^ for 10 d. Then As was added as AsIII (NaAsO_2_; 25 μM)^56^ for 7 d in a controlled growth environment at 28/21 °C at light intensity of 210 μ mol cm^−2^s^−1^ (16-h light/8-h dark) with relative humidity of 70%. The S conditions were abbreviated as follows: LS for the low sulfur conditions (0.5 mM), NS for standard sulfur conditions (3.5 mM), and HS for the high sulfur concentration (5.0 mM). All the experiments were conducted with three replicates (biological replicates) for each treatment combination. Plants were harvested, washed three times with milli-Q water, and the plant material was divided up into different aliquots for various analyses. In all the analyses only plant leaves were used, except for the determination of total S and As in which both roots and leaves were used.

### Protein Extraction, 2-DE, Gel Staining, and Image Analysis

Leaf proteins from three biological replicates were extracted using a trichloroacetic acid/acetone precipitation method followed by phenol extraction as per method described by Deeba *et al.* (2012)^59^. Protein were collected from three biological replicates and pooled to make technical replicates to normalize the effect of variation in the biological replicates. Total protein was estimated by Bradford reagent (Bradford 1976)^60^ using BSA as standard. For First-dimensional electrophoresis an equal amount (100 μg) of protein was loaded on each immobilized pH gradient (IPG) strips (7 cm, pH 4–7 linear) diluted with an iso electric focusing (IEF) rehydration solution (7 M urea, 2 M Thiourea, 2% CHAPS, 20 mM DTT, 0.5% v/v IPG buffers) in a re-swelling tray (GE Healthcare, USA) at room temperature for overnight. Then focusing was performed on the IPGphore-3 (GE Healthcare, USA) under the following conditions: ramping to 200 V for 1 h, ramping to 500 V for 1 h, 3000 V for 2 h, and 8000 V for 2 h for a total of 12 kVh.

Before the SDS-PAGE, the strips were in 10 ml of reducing equilibration buffer [6 M urea, 50 mM Tris-HCl (pH 8.8), 30% (v/v) glycerol, 2% (w/v) SDS, a trace of bromophenol blue, and 1% (w/v) DTT] for 15 min, and another 15 min in alkylating equilibration buffer that contained 2.5% (w/v) iodoacetamide instead of 1% DTT. The strips were placed on the top of 10% SDS-polyacrylamide gels and sealed with 0.5% agarose solution. The electrophoresis was carried out at a constant voltage of 200 V at 25 °C (Bio-Rad mini-gel apparatus, BioRad) with a standard Tris Glycine running buffer. Protein spots in 2-DE gels were detected by CBB G-250 staining. The 2-DE gels were scanned using scanner (HP precision scan pro 3.02, USA). 2-DE gels were analyzed using the Image Master^TM^ 2-D platinum software (version 7.0; GE Healthcare, USA). Spot detection parameters were as follows: smoothness 2; saliency 1; minimum area 5. Quantification was carried out using the percent volume criterion. The match analysis was performed in an automatic mode, and further manual editing was performed to correct the mismatched and unmatched spots (Supplemental Information 1, Table S1 A–B). The relative volume of each spot was represented as expression level. Total spot intensity per gel was used to normalize spot intensities (% of individual spot intensity/% spot intensity of each gel) to compensate for variations between gel replicates. A criterion of *p* < 0.001 was used to define the significant difference when analyzing the parallel spots between groups with analysis of one-way variance (ANOVA). Protein spots with an abundance ratio of at least 1.5 folds were selected as differentially expressed.

### In-Gel Digestion, MS Analysis, and Database Searching

Protein spots showing significant changes in abundance between different treatments were selected and excised manually with scalpel. Excised gel pieces were destained with 50% MetOH and 50 mM ammonium bicarbonate (ABC) followed by a washing with 25 mM ABC. Selected gel pieces were dehydrated with 50% acetonitrile (ACN) and 50 mM ABC mixed in 2:1 ratio. The cycle of dehydration followed by rehydration by 25 mM ABC was repeated 3 times. Destained gel pieces were dried in speed vac (Labconco, USA) and rehydrated in trypsin solution (20 μg/ml) at 1:20 ratio of protein. Gel particles were immersed in 25 mM ammonium bicarbonate (ABC) and samples were digested overnight at 37 ^o^C (about 16–18 h digestion). Peptides were extracted twice with 50% ACN/1% tri flouro acetic acid (TFA). Extracted peptides were mixed with matrix solution (5 mg/ml a-Cyano-4-hydroxycinnamic acid in 50% ACN containing 0.1% TFA) in 1:1 ratio. Samples were spotted and air dried at room temperature on stainless steel 384 wells target plate. External calibration of mass spectrometer was performed with a mixture of angiotensin I, Glufibrino-peptide B, ACTH (1–17), and ACTH (18–39) while for MS/MS with fragment of Glufibrino-peptide B.

All samples were analyzed using a MALDI-TOF-TOF (Model 4800, Applied Biosystems, USA). The monoisotopic peptide masses obtained from MALDI-TOF-TOF were analyzed by the 4000 Series Explorer software (version 3.5, Applied Biosystems, USA). On the basis of mass signals, protein identification was performed with the Mascot software (http://www.matrixscience.com) to search proteins against SwissProt, NCBInr and MSDB databases (Supplemental Information 1, Table S1 C). The following parameters were used for database searches: monoisotopic mass accuracy, <100 ppm; missed cleavages, 1; carbamidomethylation of Cys as fixed modification and oxidation of Met; keratin, known contaminant was excluded. Additionally the theoretical values of molecular weight (*M*r) and pI of identified proteins were calculated by using the Peptide Mass program (ExPASy). The score threshold to achieve *p* < 0.05 was set by the Mascot algorithm and was based on the size of the database used in the search. Peptides were again searched against Rice Genome Database Project to identify the locus ID and encoded proteins names are assigned (Supplemental Information 1, Table S1 D). Peptide view of Mascot search results is mentioned in Supplemental Information 2, Table S2. Hierarchical clustering of S responsive proteins was conducted using MeV software (Supplemental Information 3, Figure S4).

### Expression Analysis using Quantitative RT-PCR

Approximately, 5 μg RNase free DNase-treated total RNA (5 μg) isolated from leaves of rice plants exposed to various treatments and control was reverse-transcribed using SuperScriptII (Fermentas, USA), following the manufacturer’s recommendation. The synthesized cDNA was diluted 1:5 in DEPC water and subjected to quantitative RT-PCR (qRT-PCR) analysis. The qRT-PCR was performed using an ABI 7500 instrument (ABI Biosystems, USA) using primers (Supplemental Information 3, Table S1). Each qPCR reaction contained 5 μl of SYBR Green Supermix (ABI Biosystems, USA), 1 μl of the diluted cDNA reaction mixture (corresponding to 5 ng of starting amount of RNA) and 10 pM of each primer in a total reaction volume of 10 μl. qPCR reactions were performed under the following conditions: 10 min at 95 °C and 40 cycles of the one step thermal cycling of 3 s at 95 °C and 30 s at 60 °C in a 96-well reaction plate. Actin gene was used as an internal control to estimate the relative transcript levels of the target gene. Specificity of amplicons generated in qPCR reactions was verified by melt curve analysis. Each qPCR reaction was performed in triplicate (technical replicates) for each biological replicate (three for each treatment). Relative gene expression was calculated using ^∆∆^CT method^61^.

### Crude extracts of plant tissues and western blot analysis

Protein extraction was carried out by Tanou *et al.* (2009)^62^ with slight modifications. Leaf tissues were ground in liquid N_2_ using a mortar and pestle. Soluble proteins were extracted from the powdered tissue at 4 °C in 1 ml of buffer (pH = 7.5) containing 50 mM HEPES-KOH, 1 mM EDTA, 5 mM DTT, 10% (v/v) glycerol, 2 mM benzamidine, 2 mM aminocuprioc acid, protease inhibitor cocktail mini from Roche Diagnostic. The extract was stirred for 20 min at 4 °C then centrifuged (14000 g for 10 min at 4 °C). Supernatant was used for further analysis. Protein estimation in supernatant was carried out by Bradford method (1976)^60^.

Western blotting is carried out by method of Mitani *et al.* (2009)^63^. Briefly, 50 μg of proteins of control and treated samples were resolved on 12% 1D-PAGE and blotted onto PVDF membrane. Further, the blocking of blotting membrane was done for 1 h using 1x blocking buffer (Sigma-Aldrich). Further, these membranes were probed by polyclonal primary antibodies against AtpB (AS05 085), GLN2 (AS08 296), 2Fe-2S (AS12 1852), PsbR (AS 05–059), PRK (AS09 464), ALD (AS08 294) proteins at recommended dilution for overnight. All antibodies were procured from Agrisera, Sweden. In the next step, HRP conjugated cross-reactive secondary antibodies was incubated and their bands were visualized by chemiluminescence system. For the analysis of their expression equal amount of proteins was loaded on another 12% 1D-PAGE for normalization of protein expression of the corresponding western blot^64^. The intensities of each blot were quantified by using UN-SCAN IT software (Orem, UT, USA). Data were analyzed by comparing relative pixel density of the protein bands normalized to with bands of single lane RuBisCo large subunit of commassie stained gel.

### Arsenic and Sulfur Estimation

For analysis of total As the analytical procedure was performed according to Dwivedi *et al.* (2010b)^23^ using Inductively Coupled Plasma-Mass Spectrometry (ICP-MS) (7500 cx; Agilent, Tokyo, Japan). Total S concentration was estimated by Chesnin and Yien (1951)^65^. Detailed methodology is provided in Supplemental Information 3 Text S1 A.

### Plant growth parameters

Shoot length was measured on a metric scale and samples were oven dried for biomass measurements. Photosynthetic pigments measurement was carried out after extraction in 80% chilled acetone^66^ and carotenoids by Duxbury and Yenstch (1956)^67^ method.

### Amino Acid Profiling

Amino acid analysis was performed by HPLC (Waters model 2475, column-C18) using the pico tag method^14^,^68^. Detailed methodology is provided in Supplemental information (Detailed methodology is provided in Supplemental Information 3 Text S1 B).

### Estimation of Thiol Compounds and Enzymes

The level of nonprotein thiols (NPTs) was measured using Ellman’s *et al.* (1959)^69^ reagent. Estimation of Cys was performed using acid ninhydrin reagent^70^. The levels of reduced (GSH) and oxidized (GSSG) glutathione were determined fluorometrically using o-phthaldialdehyde (OPT) as fluorophore on fluorescence spectrophotometer (Hitachi F 7000, Japan)^71^. For the assay of cysteine synthase (CS; EC 2.5.1.47) and γ-glutamylcysteine synthetase (γECS; EC 6.3.2.2) activities, homogenization and assay were performed following Saito *et al.* (1994)^72^ and Seelig and Meister (1984)^73^ respectively, with slight modifications. Glutathione S-transferase (GST; EC 2.5.1.18) activity was assayed following Habig and Jacoby (1981)^74^. The GR activity was assayed by following Smith *et al.* (1988)^75^. Detailed methodology is provided in Supplemental Information 3 Text S1 C. The concentration of total phytochelatins (PCs) was calculated as PCs = NPTs − total GSH (Hartley‐Whitaker *et al.* 2001)^76^.

## Additional Information

**How to cite this article**: Dixit, G. *et al.* Sulfur alleviates arsenic toxicity by reducing its accumulation and modulating proteome, amino acids and thiol metabolism in rice leaves. *Sci. Rep.* **5**, 16205; doi: 10.1038/srep16205 (2015).

## Supplementary Material

Supplemental Information 1

Supplemental Information 2

Supplemental Information 3

## Figures and Tables

**Table 1:** Differentially expressed proteins identified by MALDI-TOF-TOF (negative sign represents down regulation of protein).

Functional category & Match ID	NCBI accession no.	Protein name	Average fold change			Score	Mass	% coverage
			LS + AsIII	NS + AsIII	HS + AsIII			
Amino acid biosynthesis								
64	gi|29468084	aminotransferase, classes I and II, domain containing protein	−2.599	−2.269	nd	51	46016	13%
66	gi|19387272	glutamine synthetase, catalytic domain containing protein	8.316	nd	nd	336	49770	17%
69	gi|19387272	glutamine synthetase, catalytic domain containing protein	1.015	1.105	−2.004	626	49770	30%
72	gi|127046	S-adenosylmethionine synthetase, putative	−1.082	1.248	2.178	46	43618	3%
77	gi|29569153	aminotransferase, classes I and II, domain containing protein	−1.09	−1.648	−3.419	467	53947	27%
136	gi|255571784	aminotransferase, putative	nd	1.049	−2.63	57	50836	5%
Carbohydrate metabolism								
57	gi|115471157	NAD dependent epimerase/dehydratase family protein, putative	1.501	1.065	1.202	267	41268	14%
73	gi|110289082	NAD dependent epimerase/dehydratase family protein, putative	1.462	−2.479	nd	64	41235	11%
Cell wall polysaccharide metabolism								
40	gi|297604125	glycosyl hydrolase, putative	1.674	1.319	1.436	277	32757	28%
Cytosketon								
81	gi|493725	tubulin/FtsZ domain containing protein, putative	1.903	1.376	2.575	199	50703	18%
Energy metabolism								
23	gi|3345477	carbonic anhydrase, chloroplast precursor, putative	−2.651	2.695	−1.202	220	29498	22%
67	gi|8918361	AAA-type ATPase family protein, putative	−1.141	−1.053	−1.575	335	48128	19%
74	gi|8918361	AAA-type ATPase family protein, putative	−0.684	nd	−2.52341	276	48128	22%
75	gi|8918361	AAA-type ATPase family protein, putative	−1.685	−2.492	−2.556	586	48128	30%
79	gi|11466794	ATP synthase subunit beta, putative	−1.265	1.712	1.174	846	54037	45%
85	gi|3676294	ATP synthase, putative	−1.183	−4.003	2.670	708	60045	24%
87	gi|11466794	ATP synthase subunit beta, putative	−1.577	−1.173	−1.127	1680	54037	40%
97	gi|11466784	ATP synthase subunit alpha, mitochondrial, putative	1.965	1.937	2.704	1323	55687	37%
114	gi|11583	ATP synthase subunit beta, putative	1.077	−3.633	−1.042	285	53899	18%
127	gi|285014508	ATP synthase gamma chain, putative	nd	nd	2.359	144	40012	12%
Glycolysis								
28	gi|553107	triosephosphate isomerase, cytosolic, putative	−1.268	1.574	−2.254	413	27816	26%
32	gi|553107	triosephosphate isomerase, cytosolic, putative	−2.040	nd	−1.197	474	27816	31%
58	gi|108864048	fructose-bisphospate aldolase isozyme, putative	1.132	1.043	−1.657	41808	527	27%
53	gi|118175929	fructose-1,6-bisphosphatase, putative	−3.535	−0.949	−2.043	79	42825	12%
39	gi|115450493	glyceraldehyde-3-phosphate dehydrogenase, putative	2.612	2.568	nd	259	47537	16%
84	gi|780372	enolase, putative	1.15	1.594	1.318	222	48299	10%
125	gi|968996	glyceraldehyde-3-phosphate dehydrogenase, putative	nd	−1.659	1.244	545	36641	32%
128	gi|108864048	fructose-bisphospate aldolase isozyme, putative	nd	1.688	−2.57	685	41808	45%
131	gi|114386664	phosphoglycerate kinase protein, putative	nd	1.568	0.934	71	42224	12%
Hydrolase								
36	gi|115453797	cbbY, putative	−5.075	nd	−1.487	225	34138	23%
Pentose phosphate pathway								
56	gi|125580	phosphoribulokinase/Uridine kinase family protein	−2.784	nd	nd	400	45512	21%
Protein metabolism								
10	gi|18103931	RNA recognition motif containing protein	1.15	−1.525	nd	207	19579	30%
38	gi|125559266	RNA recognition motif containing protein, putative	1.128	−1.597	nd	59	27820	9%
41	gi|56784713	nascent polypeptide-associated complex subunit alpha, putative	−1.201	1.599	nd	47	57717	3%
48	gi|115444057	peptidase, T1 family, putative	2.012	nd	nd	284	29897	22%
71	gi|6525065	chloroplast translational elongation factor Tu [Oryza sativa Japonica Group]	1.185	1.035	−1.451	390	50555	12%
92	gi|15231255	T-complex protein, putative	−3.235	nd	−1.721	347	63702	14%
90	gi|115488160	T-complex protein, putative	1.129	1.514	1.115	323	61150	17%
101	gi|75114857	OsFtsH2 FtsH protease, homologue of AtFtsH2/8	1.2003	2.495	2.507	120	72607	6%
110	gi|18423214	ATP-dependent Clp protease ATP-binding subunit clpA homolog CD4B,chloroplast precursor, putative	1.442	nd	2.636	455	102241	18%
118	gi|108711192	eukaryotic translation initiation factor 5A, putative	nd	0.679	1.553	188	17930	25%
154	gi|108706511	peptidase, T1 family, putative	nd	2.2917	nd	99	32472	12%
PS calvin cycle								
4	gi|671740	ribulose bisphosphate carboxylase small chain, chloroplast precursor, putative	−1.275	−1.303	−14.731	168	15111	35%
8	gi|56966763	ribulose bisphosphate carboxylase small chain, chloroplast precursor, putative	−1.164	1.199	−1.521	352	15091	47%
29	gi|4105561	ribulose-phosphate 3-epimerase, chloroplast precursor, putative	−1.371	−1.575	2.835	416	29234	34%
42	gi|2961307	ribulose bisphosphate carboxylase large chain precursor, putative	−2.962	−1.52	nd	165	53618	6%
82	gi|11466795	ribulose bisphosphate carboxylase large chain precursor, putative	−2.429	−1.812	nd	1020	53418	27%
96	gi|11466795	ribulose bisphosphate carboxylase large chain precursor, putative	1.395	1.107	1.564	1025	53418	30%
113	gi|28190676	transketolase, chloroplast precursor, putative	1.064	nd	4.902	104	80549	8%
112	gi|28190676	transketolase, chloroplast precursor, putative	1.255	−2.032	−2.085	388	80549	23%
132	gi|328682245	ribulose bisphosphate carboxylase large chain precursor, putative	nd	−6.512	11.681	77	50092	7%
148	gi|146741370	dehydrogenase, putative	nd	2.399	nd	169	48012	6%
138	gi|11466795	ribulose bisphosphate carboxylase large chain precursor, putative	nd	1.717	1.657	1018	53418	40%
PS light reaction								
0	gi|115465862	proteinplastocyanin, chloroplast precursor, putative	3.045	−2.879	nd	72	15624	15%
2	gi|1835731	photosystem II 10 kDa polypeptide, chloroplast precursor, putative	−1.663	nd	nd	329	12885	24%
17	gi|34394725	photosystem I reaction center subunit IV A, chloroplast precursor, putative	−1.132	1.107	−1.601	182	15537	31%
20	gi|115467828	chlorophyll A-B binding protein, putative	1.76	1.226	1.242	70	26397	4%
24	gi|115470529	PsbP, putative	2.184	nd	4.424	694	27094	42%
30	gi|62733870	chlorophyll A-B binding protein, putative	1.634	nd	−1.505	79	24317	17%
37	gi|19184	chlorophyll A-B binding protein, putative	−3.581	−1.069	nd	58	30575	6%
34	gi|108864186	chlorophyll A-B binding protein, putative	2.859	2.712	1.374	240	24038	27%
45	gi|739292	oxygen-evolving enhancer protein 1, chloroplast precursor, putative	−1.355	−1.031	−1.937	92	26603	19%
49	gi|27261025	FAD dependent oxidoreductase domain containing protein	−3.744	−1.163	nd	239	37156	19%
50	gi|41052915	ferredoxin–NADP reductase, chloroplast precursor, putative	1.031	1.943	1.956	199	41095	19%
144	gi|11466848	photosystem I iron-S center, putative	nd	nd	−1.55	252	9406	54%
Nuclic acid metaboism								
129	gi|186463816	phosphoribosylformylglycinamidine synthase, putative	nd	nd	3.345	53	11324	27%
134	gi|78708842	IAP100, putative	nd	nd	1.621	125	47305	14%
147	gi|115466468	profilin domain containing protein	nd	2.63	nd	77	14352	24%
Stress								
13	gi|42408425	copper/zinc superoxide dismutase, putative	1.216	2.087	nd	341	20633	46%
16	gi|18698985	2Fe-2S iron-S cluster binding domain containing protein	−1.534	−1.679	5.052	123	15082	33%
18	gi|15667623	abscisic stress-ripening, putative	−1.717	1.429	nd	398	15923	24%
19	gi|6002472	2-Cys peroxiredoxin BAS1, chloroplast precursor, putative	−1.351	1.357	2.051	223	29490	37%
21	gi|115446541	peroxiredoxin, putative	1.008	1.318	−1.595	233	28307	37%
47	gi|162461576	glyoxalase family protein, putative	−1.925	1.407	1.626	80	32450	10%
102	gi|115448989	DnaK family protein, putative	1.995	nd	nd	130	73081	10%
103	gi|6746592	DnaK family protein, putative	−1.601	nd	1.637	151	77230	6%
108	gi|39104468	heat shock protein, putative	−1.384	1.287	1.877	315	80449	11%
116	gi|115477014	heat shock protein, putative	nd	1.5903	0.088	286	88749	14%
151	gi|42408425	copper/zinc superoxide dismutase, putative	nd	−1.52	nd	116	20633	18%
Seed storage non enzymatic proteins								
120	gi|4239821	Cupin domain containing protein	nd	−1.532	−1.075	155	22017	10%
TCA Cycle								
60	gi|15982948	lactate/malate dehydrogenase, putative	1.079	−1.323	1.643	285	35817	27%

nd: not detected.

**Table 2:** Amino acid content was expressed in mg kg^−1^ fw.

Amino acid	Control	LS + AsIII	NS + AsIII	HS + AsIII
SER	4.72^b^ ± 0.31	16.13^d^ ± 1.06	12.70^c^ ± 0.84	1.73^a^ ± 0.11
THR	5.75^c^ ± 0.38	1.49^a^ ± 0.10	13.56^d^ ± 0.91	3.73^b^ ± 0.25
HIS	2.23^a^ ± 0.17	3.94^b^ ± 0.30	5.23^c^ ± 0.39	9.64^d^ ± 0.72
ALA	4.95^b^ ± 0.39	5.73^b^ ± 0.45	8.71^c^ ± 0.69	3.12^a^ ± 0.25
MET	4.83^b^ ± 0.35	3.37^a^ ± 0.24	3.65^a^ ± 0.26	5.65^c^ ± 0.41
TRP	2.36^a^ ± 0.16	3.80^b^ ± 0.25	2.65^a^ ± 0.18	5.08^c^ ± 0.34
GLY	7.22^a^ ± 0.64	18.86^c^ ± 1.68	10.41^b^ ± 0.93	26.98^d^ ± 2.41
ARG	3.20^a^ ± 0.19	8.63^c^ ± 0.51	5.68^b^ ± 0.33	10.77^d^ ± 0.63
ILE	8.05^b^ ± 0.61	4.18^a^ ± 0.32	12.50^c^ ± 0.95	3.75^a^ ± 0.29
PRO	11.26^b^ ± 0.89	1.85^a^ ± 0.15	15.32^c^ ± 1.21	15.08^c^ ± 1.19
LYS	3.78^c^ ± 0.27	6.94^d^ ± 0.49	1.40^b^ ± 0.10	0.21^a^ ± 0.01
TYR	1.34^d^ ± 0.08	0.69^b^ ± 0.05	0.39^a^ ± 0.03	1.13^c^ ± 0.08
LEU	5.58^a^ ± 0.49	11.96^b^ ± 1.06	15.51^c^ ± 1.37	10.78^b^ ± 0.95
CYS	3.10^b^ ± 0.25	1.13^a^ ± 0.09	7.99^d^ ± 0.63	5.83^c^ ± 0.46
ASP	8.96^b^ ± 0.55	5.25^a^ ± 0.32	14.01^c^ ± 0.87	4.64^a^ ± 0.29
VAL	8.23^a^ ± 0.69	0.27^b^ ± 0.02	3.78^b^ ± 0.32	3.11^c^ ± 0.26
PHE	3.92^b^ ± 0.32	2.00^a^ ± 0.16	4.58^b^ ± 0.37	6.08^c^ ± 0.49
GLU	26.97^b^ ± 2.31	3.89^a^ ± 0.33	60.75^c^ ± 5.21	3.19^a^ ± 0.27

Chromatograms were kintegrated using Em power 2 HPLC software v 6.0. All values are mean of triplicates ± S.D. ANOVA significant at p ≤ 0.01.
