# Supplementary material for: Sulfur alleviates arsenic toxicity by reducing its accumulation and modulating proteome, amino acids and thiol metabolism in rice leaves
Source: Sci Rep. 2015 Nov 10;5:16205. doi: 10.1038/srep16205 (PMC4639781; doi:10.1038/srep16205)
Supplement: Supplemental Information 2 [file srep16205-s2.pdf]

# MATRIX SCIENCE Mascot Search Results

## Peptide View Spot no 0

MS/MS Fragmentation of **NNAGFPHNVVFDEDAVPSGVDVSK**
Found in **gi|115465862** in **NCBInr**, Os06g0101600 [Oryza sativa Japonica Group]

Match to Query 31: 2513.339624 from(2514.346900,1+) intensity(0.0000) index(12)
Title: Label: H6, Spot_Id: 228929, Peak_List_Id: 257551, MSMS Job_Run_Id: 24925, Comment:
Data file ppw_H6_138985133400.txt

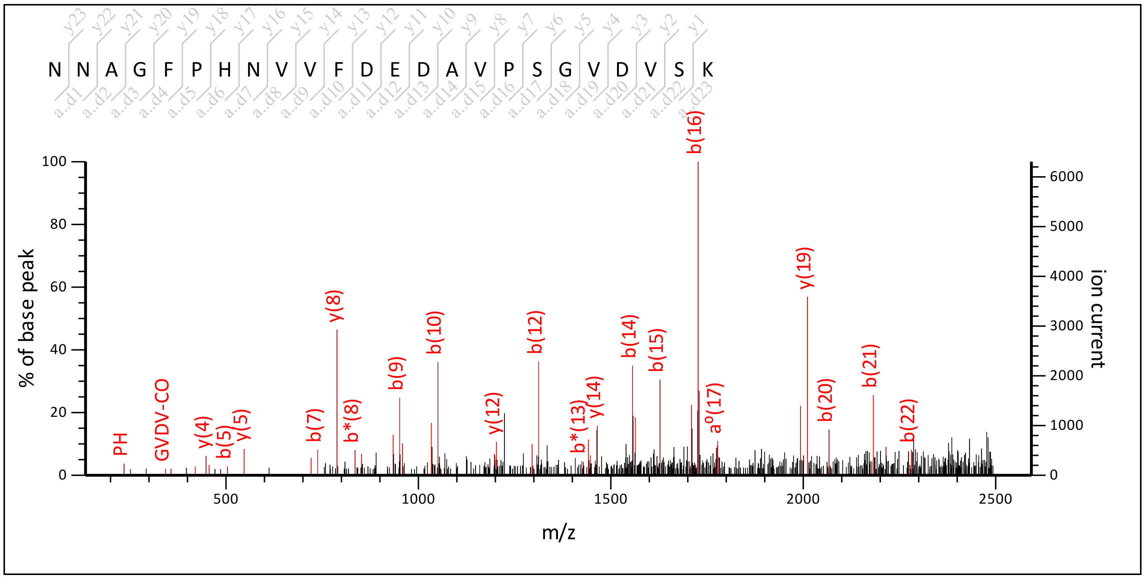


135.09 to 2592.79

Label all possible matches ○ Label matches used for scoring ◉

**Monoisotopic mass of neutral peptide Mr(calc):** 2513.1823
**Fixed modifications:** Carbamidomethyl (C) (apply to specified residues or termini only)
**Ions Score:** 72 **Expect:** 7.9e-05
**Matches :** 45/461 fragment ions using 86 most intense peaks (help)

| # | Immon. | a | a* | $a^0$ | b | b* | $b^0$ | d | Seq. | v | w | y | y* | $y^0$ | # |
|---|---|---|---|---|---|---|---|---|---|---|---|---|---|---|---|
| 1 | 87.0553 | 87.0553 | 70.0287 | | 115.0502 | 98.0237 | | 44.0495 | N | | | | | | 24 |
| 2 | 87.0553 | 201.0982 | 184.0717 | | 229.0931 | 212.0666 | | 158.0924 | N | 2341.1095 | 2340.1143 | 2400.1466 | 2383.1201 | 2382.1361 | 23 |
| 3 | 44.0495 | 272.1353 | 255.1088 | | 300.1302 | 283.1037 | | | A | 2270.0724 | | 2286.1037 | 2269.0772 | 2268.0931 | 22 |
| 4 | 30.0338 | 329.1568 | 312.1302 | | ***357.1517*** | 340.1252 | | | G | | | **2215.0666** | 2198.0400 | 2197.0560 | 21 |
| 5 | 120.0808 | 476.2252 | 459.1987 | | ***504.2201*** | 487.1936 | | | F | 2065.9825 | | 2158.0451 | 2141.0186 | 2140.0346 | 20 |
| 6 | 70.0651 | 573.2780 | 556.2514 | | 601.2729 | 584.2463 | | 547.2623 | P | 1968.9298 | 1967.9345 | **2010.9767** | 1993.9502 | 1992.9661 | 19 |
| 7 | 110.0713 | 710.3369 | 693.3103 | | ***738.3318*** | 721.3052 | | | H | 1831.8708 | | 1913.9239 | 1896.8974 | 1895.9134 | 18 |
| 8 | 87.0553 | 824.3798 | 807.3533 | | ***852.3747*** | 835.3482 | | 781.3740 | N | 1717.8279 | 1716.8327 | **1776.8650** | 1759.8385 | 1758.8545 | 17 |
| 9 | 72.0808 | 923.4482 | 906.4217 | | ***951.4431*** | 934.4166 | | 909.4326 | V | 1618.7595 | 1631.7799 | 1662.8221 | 1645.7956 | 1644.8115 | 16 |
| 10 | 72.0808 | 1022.5166 | 1005.4901 | | ***1050.5116*** | 1033.4850 | | 1008.5010 | V | 1519.6911 | 1532.7115 | **1563.7537** | 1546.7271 | 1545.7431 | 15 |
| 11 | 120.0808 | 1169.5851 | 1152.5585 | | ***1197.5800*** | 1180.5534 | | | F | 1372.6227 | | **1464.6853** | 1447.6587 | 1446.6747 | 14 |
| 12 | 88.0393 | 1284.6120 | 1267.5854 | 1266.6014 | ***1312.6069*** | 1295.5804 | 1294.5963 | 1240.6222 | D | 1257.5957 | 1256.6005 | 1317.6169 | 1300.5903 | 1299.6063 | 13 |
| 13 | 102.0550 | 1413.6546 | 1396.6280 | 1395.6440 | ***1441.6495*** | 1424.6230 | 1423.6389 | 1355.6491 | E | 1128.5531 | 1127.5579 | **1202.5899** | 1185.5634 | 1184.5794 | 12 |
| 14 | 88.0393 | 1528.6815 | 1511.6550 | 1510.6710 | ***1556.6764*** | 1539.6499 | 1538.6659 | 1484.6917 | D | 1013.5262 | 1012.5310 | 1073.5473 | 1056.5208 | 1055.5368 | 11 |
| 15 | 44.0495 | 1599.7186 | 1582.6921 | 1581.7081 | ***1627.7136*** | 1610.6870 | 1609.7030 | | A | 942.4891 | | **958.5204** | 941.4938 | 940.5098 | 10 |
| 16 | 72.0808 | 1698.7871 | 1681.7605 | 1680.7765 | ***1726.7820*** | 1709.7554 | 1708.7714 | 1684.7714 | V | 843.4207 | 856.4411 | 887.4833 | 870.4567 | 869.4727 | 9 |
| 17 | 70.0651 | 1795.8398 | 1778.8133 | 1777.8293 | 1823.8347 | 1806.8082 | 1805.8242 | 1769.8242 | P | 746.3679 | 745.3727 | **788.4149** | 771.3883 | 770.4043 | 8 |
| 18 | 60.0444 | 1882.8719 | 1865.8453 | 1864.8613 | 1910.8668 | 1893.8402 | 1892.8562 | 1866.8769 | S | 659.3359 | 658.3406 | 691.3621 | 674.3355 | 673.3515 | 7 |
| 19 | 30.0338 | 1939.8933 | 1922.8668 | 1921.8828 | 1967.8882 | 1950.8617 | 1949.8777 | | G | | | 604.3301 | 587.3035 | 586.3195 | 6 |

| | | | | | | | | | | | | | | | |
|---|---|---|---|---|---|---|---|---|---|---|---|---|---|---|---|
| **20** | 72.0808 | 2038.9617 | 2021.9352 | 2020.9512 | ***2066.9566*** | 2049.9301 | 2048.9461 | 2024.9461 | **V** | 503.2460 | 516.2664 | **547.3086** | 530.2821 | 529.2980 | **5** |
| **21** | 88.0393 | 2153.9887 | 2136.9621 | 2135.9781 | ***2181.9836*** | 2164.9570 | 2163.9730 | 2109.9988 | **D** | 388.2191 | 387.2238 | **448.2402** | 431.2136 | 430.2296 | **4** |
| **22** | 72.0808 | 2253.0571 | 2236.0305 | 2235.0465 | ***2281.0520*** | 2264.0255 | 2263.0414 | 2239.0414 | **V** | 289.1506 | 302.1710 | 333.2132 | 316.1867 | 315.2027 | **3** |
| **23** | 60.0444 | 2340.0891 | 2323.0626 | 2322.0785 | 2368.0840 | 2351.0575 | 2350.0735 | 2324.0942 | **S** | 202.1186 | 201.1234 | 234.1448 | 217.1183 | 216.1343 | **2** |
| **24** | 101.1073 | | | | | | | | **K** | 74.0237 | 73.0284 | 147.1128 | 130.0863 | | **1** |

| Seq | ya | yb | Seq | ya | yb | Seq | ya | yb |
|---|---|---|---|---|---|---|---|---|
| **NA** | 158.0924 | 186.0873 | **NAG** | 215.1139 | 243.1088 | **NAGF** | 362.1823 | 390.1772 |
| **NAGFP** | 459.2350 | 487.2300 | **NAGFPH** | 596.2940 | 624.2889 | **AG** | 101.0709 | 129.0659 |
| **AGF** | 248.1394 | 276.1343 | **AGFP** | 345.1921 | 373.1870 | **AGFPH** | 482.2510 | 510.2459 |
| **AGFPHN** | 596.2940 | 624.2889 | **AGFPHNV** | 695.3624 | 723.3573 | **GF** | 177.1022 | 205.0972 |
| **GFP** | 274.1550 | 302.1499 | **GFPH** | 411.2139 | 439.2088 | **GFPHN** | 525.2568 | 553.2518 |
| **GFPHNV** | 624.3253 | 652.3202 | **FP** | 217.1335 | 245.1285 | **FPH** | 354.1925 | 382.1874 |
| **FPHN** | 468.2354 | 496.2303 | **FPHNV** | 567.3038 | 595.2987 | **FPHNVV** | 666.3722 | 694.3671 |
| **PH** | 207.1240 | 235.1190 | **PHN** | 321.1670 | 349.1619 | **PHNV** | 420.2354 | 448.2303 |
| **PHNVV** | 519.3038 | 547.2987 | **PHNVVF** | 666.3722 | 694.3671 | **HN** | 224.1142 | 252.1091 |
| **HNV** | 323.1826 | 351.1775 | **HNVV** | 422.2510 | 450.2459 | **HNVVF** | 569.3194 | 597.3144 |
| **HNVVFD** | 684.3464 | 712.3413 | **NV** | 186.1237 | 214.1186 | **NVV** | 285.1921 | 313.1870 |
| **NVVF** | 432.2605 | 460.2554 | **NVVFD** | 547.2875 | 575.2824 | **NVVFDE** | 676.3301 | 704.3250 |
| **VV** | 171.1492 | 199.1441 | **VVF** | 318.2176 | 346.2125 | **VVFD** | 433.2445 | 461.2395 |
| **VVFDE** | 562.2871 | 590.2821 | **VVFDED** | 677.3141 | 705.3090 | **VF** | 219.1492 | 247.1441 |
| **VFD** | 334.1761 | 362.1710 | **VFDE** | 463.2187 | 491.2136 | **VFDED** | 578.2457 | 606.2406 |
| **VFDEDA** | 649.2828 | 677.2777 | **FD** | 235.1077 | 263.1026 | **FDE** | 364.1503 | 392.1452 |
| **FDED** | 479.1773 | 507.1722 | **FDEDA** | 550.2144 | 578.2093 | **FDEDAV** | 649.2828 | 677.2777 |
| **DE** | 217.0819 | 245.0768 | **DED** | 332.1088 | 360.1038 | **DEDA** | 403.1460 | 431.1409 |
| **DEDAV** | 502.2144 | 530.2093 | **DEDAVP** | 599.2671 | 627.2620 | **DEDAVPS** | 686.2992 | 714.2941 |
| **ED** | 217.0819 | 245.0768 | **EDA** | 288.1190 | 316.1139 | **EDAV** | 387.1874 | 415.1823 |
| **EDAVP** | 484.2402 | 512.2351 | **EDAVPS** | 571.2722 | 599.2671 | **EDAVPSG** | 628.2937 | 656.2886 |
| **DA** | 159.0764 | 187.0713 | **DAV** | 258.1448 | 286.1397 | **DAVP** | 355.1976 | 383.1925 |
| **DAVPS** | 442.2296 | 470.2245 | **DAVPSG** | 499.2511 | 527.2460 | **DAVPSGV** | 598.3195 | 626.3144 |
| **AV** | 143.1179 | 171.1128 | **AVP** | 240.1707 | 268.1656 | **AVPS** | 327.2027 | 355.1976 |
| **AVPSG** | 384.2241 | 412.2191 | **AVPSGV** | 483.2926 | 511.2875 | **AVPSGVD** | 598.3195 | 626.3144 |
| **AVPSGVDV** | 697.3879 | 725.3828 | **VP** | 169.1335 | 197.1285 | **VPS** | 256.1656 | 284.1605 |
| **VPSG** | 313.1870 | 341.1819 | **VPSGV** | 412.2554 | 440.2504 | **VPSGVD** | 527.2824 | 555.2773 |
| **VPSGVDV** | 626.3508 | 654.3457 | **PS** | 157.0972 | 185.0921 | **PSG** | 214.1186 | 242.1135 |
| **PSGV** | 313.1870 | 341.1819 | **PSGVD** | 428.2140 | 456.2089 | **PSGVDV** | 527.2824 | 555.2773 |
| **PSGVDVS** | 614.3144 | 642.3093 | **SG** | 117.0659 | 145.0608 | **SGV** | 216.1343 | 244.1292 |
| **SGVD** | 331.1612 | 359.1561 | **SGVDV** | 430.2296 | 458.2245 | **SGVDVS** | 517.2617 | 545.2566 |
| **GV** | 129.1022 | 157.0972 | **GVD** | 244.1292 | 272.1241 | **GVDV** | 343.1976 | 371.1925 |
| **GVDVS** | 430.2296 | 458.2245 | **VD** | 187.1077 | 215.1026 | **VDV** | 286.1761 | 314.1710 |
| **VDVS** | 373.2082 | 401.2031 | **DV** | 187.1077 | 215.1026 | **DVS** | 274.1397 | 302.1347 |
| **VS** | 159.1128 | 187.1077 | | | | | | |

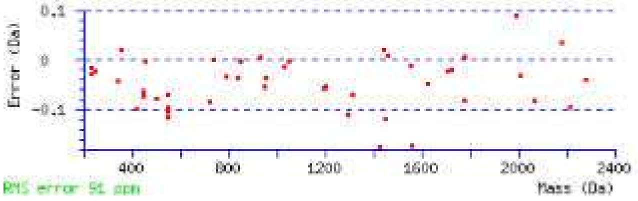


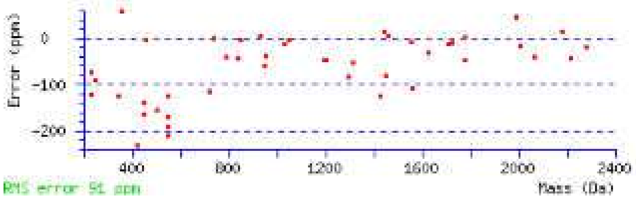


NCBI **BLAST** search of NNAGFPHNVVFDEDAVPSGVDVSK
(Parameters: blastp, nr protein database, expect=20000, no filter, PAM30)
Other BLAST web gateways

**All matches to this query**

| Score | Mr(calc) | Delta | Sequence |
|---|---|---|---|
| 72.4 | 2513.1823 | 0.1573 | NNAGFPHNVVFDEDAVPSGVDVSK |
| 34.0 | 2513.1459 | 0.1937 | NNAGFPHNVVFDEDEVPGGVDASK |
| 6.8 | 2513.3278 | 0.0118 | DARIFPQEIIEFPAVLVDGATGR |
| 3.3 | 2513.2142 | 0.1255 | KTMDVQLYTEVMESVQTIDAGR |
| 2.6 | 2513.2299 | 0.1097 | GLEQSYDTPNPQLIAVSNQHFR |
| 1.9 | 2513.2591 | 0.0805 | GNQFEVLVDSINGDFFLTKGWK |
| 0.9 | 2513.1203 | 0.2194 | AGDPYEMFTHDELIVMGRDASK |
| 0.8 | 2513.2121 | 0.1275 | YVQLANVYAAEGNWAEMGRISR |
| 0.2 | 2513.0958 | 0.2438 | KDFGYSFLCHGLGQGNTYDFST |

**Mascot:** http://www.matrixscience.com/

# MATRIX SCIENCE Mascot Search Results

## Peptide View Spot no 2

MS/MS Fragmentation of **GVYQFVDK**
Found in **gi|1835731** in **NCBInr**, photosystem II 10 kDa polypeptide [Oryza sativa Indica Group]

Match to Query 51: 954.464404 from(955.471680,1+) intensity(0.0000) index(6)
Title: Label: K5, Spot_Id: 219748, Peak_List_Id: 225860, MSMS Job_Run_Id: 21792, Comment:
Data file C:\Documents and Settings\Administrator\Desktop\farah BHU\spot set plate\Farah-Garima 11May 2013\ppw_K5_136842223800.txt

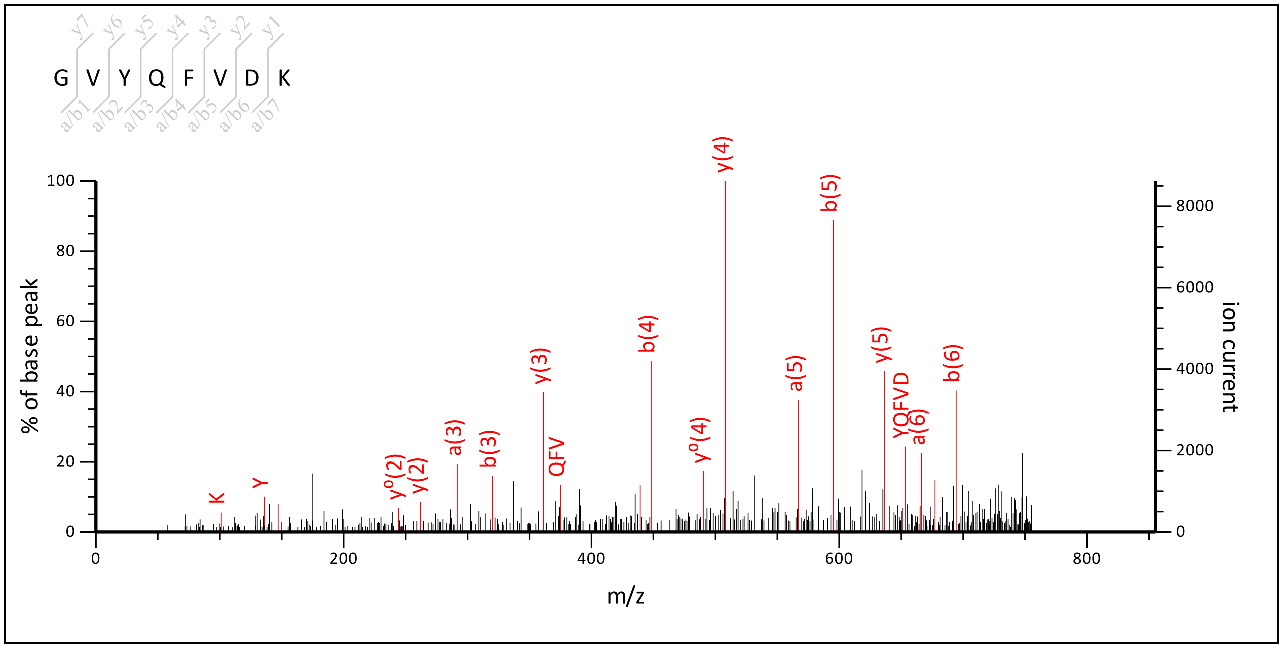


0 to 855.27

Label all possible matches ○ Label matches used for scoring ◉

**Monoisotopic mass of neutral peptide Mr(calc):** 954.4811
**Fixed modifications:** Carbamidomethyl (C) (apply to specified residues or termini only)
**Ions Score:** 34 **Expect:** 0.67
**Matches :** 23/97 fragment ions using 28 most intense peaks (help)

| # | Immon. | a | a* | $a^0$ | b | b* | $b^0$ | d | Seq. | v | w | y | y* | $y^0$ | # |
|---|---|---|---|---|---|---|---|---|---|---|---|---|---|---|---|
| 1 | 30.0338 | 30.0338 | | | 58.0287 | | | 44.0495 | G | | | | | | 8 |
| 2 | 72.0808 | 129.1022 | | | 157.0972 | | | 115.0866 | V | 854.4043 | 867.4247 | 898.4669 | 881.4403 | 880.4563 | 7 |
| 3 | 136.0757 | **292.1656** | | | **320.1605** | | | | Y | 691.3410 | | 799.3985 | 782.3719 | 781.3879 | 6 |
| 4 | 101.0709 | 420.2241 | 403.1976 | | **448.2191** | 431.1925 | | 363.2027 | Q | 563.2824 | 562.2871 | ***636.3352*** | 619.3086 | 618.3246 | 5 |
| 5 | 120.0808 | **567.2926** | 550.2660 | | **595.2875** | 578.2609 | | | F | 416.2140 | | ***508.2766*** | 491.2500 | 490.2660 | 4 |
| 6 | 72.0808 | **666.3610** | 649.3344 | | **694.3559** | 677.3293 | | 652.3453 | V | 317.1456 | 330.1660 | ***361.2082*** | 344.1816 | 343.1976 | 3 |
| 7 | 88.0393 | 781.3879 | 764.3614 | 763.3774 | 809.3828 | 792.3563 | 791.3723 | 737.3981 | D | 202.1186 | 201.1234 | ***262.1397*** | 245.1132 | 244.1292 | 2 |
| 8 | 101.1073 | | | | | | | | K | 74.0237 | 73.0284 | ***147.1128*** | 130.0863 | | 1 |

| Seq | ya | yb | Seq | ya | yb | Seq | ya | yb |
|---|---|---|---|---|---|---|---|---|
| VY | 235.1441 | 263.1390 | VYQ | 363.2027 | 391.1976 | VYQF | 510.2711 | 538.2660 |
| VYQFV | 609.3395 | 637.3344 | YQ | 264.1343 | 292.1292 | YQF | 411.2027 | 439.1976 |
| YQFV | 510.2711 | 538.2660 | YQFVD | 625.2980 | 653.2930 | QF | 248.1394 | 276.1343 |
| QFV | 347.2078 | 375.2027 | QFVD | 462.2347 | 490.2296 | FV | 219.1492 | 247.1441 |

| | | | | | | | | |
|---|---|---|---|---|---|---|---|---|
| **FVD** | 334.1761 | 362.1710 | **VD** | 187.1077 | 215.1026 | | | |

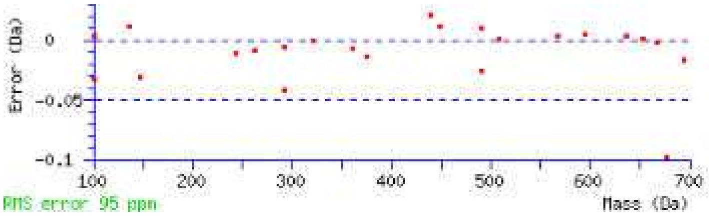


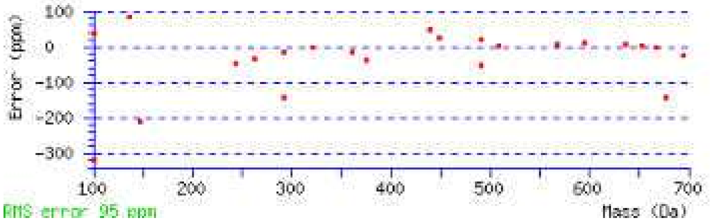


NCBI **BLAST** search of GVYQFVDK
(Parameters: blastp, nr protein database, expect=20000, no filter, PAM30)
Other BLAST web gateways

**All matches to this query**

| Score | Mr(calc) | Delta | Sequence |
|---|---|---|---|
| 34.3 | 954.4811 | -0.0167 | GVYQFVDK |
| 28.3 | 954.3964 | 0.0680 | SSDMASVDK |
| 26.9 | 954.3964 | 0.0680 | TDGSSMVDK |
| 25.6 | 954.4117 | 0.0527 | FNDAMVDK |
| 24.4 | 954.4077 | 0.0568 | MTSSTSDAR |
| 24.4 | 954.3865 | 0.0779 | NFADMGER |
| 24.3 | 954.5076 | -0.0432 | VYGKFWR |
| 24.2 | 954.4447 | 0.0197 | NFGEFVDK |
| 24.2 | 954.4845 | -0.0200 | TMKSFVDK |
| 23.8 | 954.4593 | 0.0051 | VYGRMADK |

**Mascot:** http://www.matrixscience.com/

# MATRIX SCIENCE Mascot Search Results

## Peptide View Spot no 2

MS/MS Fragmentation of **TDKPYGIGGGMSVDIDASGR**
Found in **gi|1835731** in **NCBInr**, photosystem II 10 kDa polypeptide [Oryza sativa Indica Group]

Match to Query 175: 1994.933424 from(1995.940700,1+) intensity(0.0000) index(27)
Title: Label: K5, Spot_Id: 219748, Peak_List_Id: 225865, MSMS Job_Run_Id: 21792, Comment:
Data file C:\Documents and Settings\Administrator\Desktop\farah BHU\spot set plate\Farah-Garima 11May 2013\ppw_K5_136842223800.txt

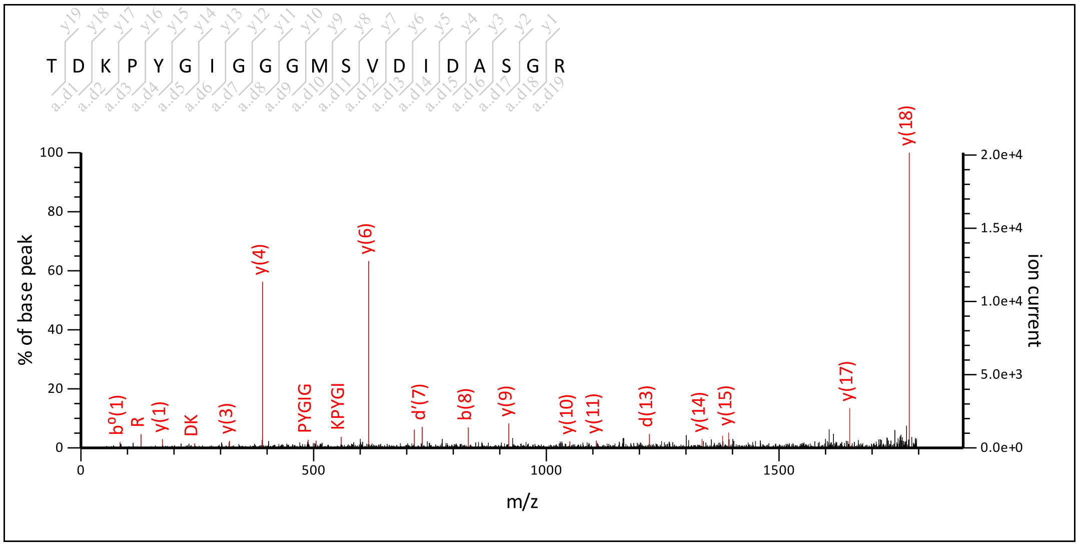


0 to 1895.7

Label all possible matches ○ Label matches used for scoring ◉

```
Monoisotopic mass of neutral peptide Mr(calc): 1994.9368
Fixed modifications: Carbamidomethyl (C) (apply to specified residues or termini only)
Ions Score: 124  Expect: 6.1e-10
Matches : 33/401 fragment ions using 36 most intense peaks   (help)
```

| # | Immon. | a | a* | $a^0$ | b | b* | $b^0$ | d | d' | Seq. | v | w | w' | y | y* | |
|---|---|---|---|---|---|---|---|---|---|---|---|---|---|---|---|---|
| 1 | 74.0600 | 74.0600 | | 56.0495 | 102.0550 | | 84.0444 | 44.0495 | | T | | | | | | |
| 2 | 88.0393 | 189.0870 | | 171.0764 | 217.0819 | | 199.0713 | 145.0972 | | D | 1834.8752 | 1833.8800 | | 1894.8964 | 1877.8698 | [illegible] |
| 3 | 101.1073 | 317.1819 | 300.1554 | 299.1714 | 345.1769 | 328.1503 | 327.1663 | 260.1241 | | K | 1706.7803 | 1705.7850 | | **1779.8694** | 1762.8429 | [illegible] |
| 4 | 70.0651 | 414.2347 | 397.2082 | 396.2241 | 442.2296 | 425.2031 | 424.2191 | 388.2191 | | P | 1609.7275 | 1608.7322 | | **1651.7744** | 1634.7479 | [illegible] |
| 5 | 136.0757 | 577.2980 | 560.2715 | 559.2875 | 605.2930 | 588.2664 | 587.2824 | | | Y | 1446.6642 | | | 1554.7217 | 1537.6951 | [illegible] |
| 6 | 30.0338 | 634.3195 | 617.2930 | 616.3089 | 662.3144 | 645.2879 | 644.3039 | | | G | | | | **1391.6584** | 1374.6318 | [illegible] |
| 7 | 86.0964 | 747.4036 | 730.3770 | 729.3930 | 775.3985 | 758.3719 | 757.3879 | 719.3723 | 733.3879 | I | 1276.5586 | 1289.5790 | 1303.5947 | **1334.6369** | 1317.6103 | [illegible] |
| 8 | 30.0338 | 804.4250 | 787.3985 | 786.4145 | **832.4199** | 815.3934 | 814.4094 | | | G | | | | **1221.5528** | 1204.5263 | [illegible] |
| 9 | 30.0338 | 861.4465 | 844.4199 | 843.4359 | 889.4414 | 872.4149 | 871.4308 | | | G | | | | 1164.5314 | 1147.5048 | [illegible] |
| 10 | 30.0338 | 918.4680 | 901.4414 | 900.4574 | 946.4629 | 929.4363 | 928.4523 | | | G | | | | **1107.5099** | 1090.4834 | [illegible] |
| 11 | 104.0528 | 1049.5084 | 1032.4819 | 1031.4979 | 1077.5034 | 1060.4768 | 1059.4928 | 989.5051 | | M | 974.4538 | 973.4585 | | **1050.4884** | 1033.4619 | [illegible] |
| 12 | 60.0444 | 1136.5405 | 1119.5139 | 1118.5299 | 1164.5354 | 1147.5088 | 1146.5248 | 1120.5456 | | S | 887.4217 | 886.4265 | | **919.4480** | 902.4214 | [illegible] |
| 13 | 72.0808 | 1235.6089 | 1218.5823 | 1217.5983 | 1263.6038 | 1246.5773 | 1245.5932 | 1221.5932 | | V | 788.3533 | 801.3737 | | **832.4159** | 815.3894 | [illegible] |
| 14 | 88.0393 | 1350.6358 | 1333.6093 | 1332.6253 | **1378.6307** | 1361.6042 | 1360.6202 | 1306.6460 | | D | 673.3264 | 672.3311 | | **733.3475** | 716.3210 | [illegible] |
| 15 | 86.0964 | 1463.7199 | 1446.6933 | 1445.7093 | 1491.7148 | 1474.6883 | 1473.7042 | 1435.6886 | 1449.7042 | I | 560.2423 | 573.2627 | 587.2784 | **618.3206** | 601.2940 | [illegible] |
| 16 | 88.0393 | 1578.7468 | 1561.7203 | 1560.7363 | 1606.7418 | 1589.7152 | 1588.7312 | 1534.7570 | | D | 445.2154 | 444.2201 | | **505.2365** | 488.2100 | [illegible] |
| 17 | 44.0495 | 1649.7840 | 1632.7574 | 1631.7734 | 1677.7789 | 1660.7523 | 1659.7683 | | | A | 374.1783 | | | **390.2096** | 373.1830 | [illegible] |
| 18 | 60.0444 | 1736.8160 | 1719.7894 | 1718.8054 | 1764.8109 | 1747.7843 | 1746.8003 | 1720.8211 | | S | 287.1462 | 286.1510 | | **319.1724** | 302.1459 | [illegible] |
| 19 | 30.0338 | 1793.8374 | 1776.8109 | 1775.8269 | 1821.8324 | 1804.8058 | 1803.8218 | | | G | | | | 232.1404 | 215.1139 | |
| 20 | 129.1135 | | | | | | | | | R | 74.0237 | 73.0284 | | **175.1190** | 158.0924 | |

| Seq | ya | yb | Seq | ya | yb | Seq | ya | yb |
|---|---|---|---|---|---|---|---|---|
| DK | 216.1343 | 244.1292 | DKP | 313.1870 | 341.1819 | DKPY | 476.2504 | 504.2453 |

| | | | | | | | | |
|---|---|---|---|---|---|---|---|---|
| **DKPYG** | 533.2718 | 561.2667 | **DKPYGI** | 646.3559 | 674.3508 | **KP** | 198.1601 | 226.1550 |
| **KPY** | 361.2234 | 389.2183 | **KPYG** | 418.2449 | 446.2398 | **KPYGI** | 531.3289 | 559.3239 |
| **KPYGIG** | 588.3504 | 616.3453 | **KPYGIGG** | 645.3719 | 673.3668 | **PY** | 233.1285 | 261.1234 |
| **PYG** | 290.1499 | 318.1448 | **PYGI** | 403.2340 | 431.2289 | **PYGIG** | 460.2554 | 488.2504 |
| **PYGIGG** | 517.2769 | 545.2718 | **PYGIGGG** | 574.2984 | 602.2933 | **YG** | 193.0972 | 221.0921 |
| **YGI** | 306.1812 | 334.1761 | **YGIG** | 363.2027 | 391.1976 | **YGIGG** | 420.2241 | 448.2191 |
| **YGIGGG** | 477.2456 | 505.2405 | **YGIGGGM** | 608.2861 | 636.2810 | **YGIGGGMS** | 695.3181 | 723.3130 |
| **GI** | 143.1179 | 171.1128 | **GIG** | 200.1394 | 228.1343 | **GIGG** | 257.1608 | 285.1557 |
| **GIGGG** | 314.1823 | 342.1772 | **GIGGGM** | 445.2228 | 473.2177 | **GIGGGMS** | 532.2548 | 560.2497 |
| **GIGGGMSV** | 631.3232 | 659.3181 | **IG** | 143.1179 | 171.1128 | **IGG** | 200.1394 | 228.1343 |
| **IGGG** | 257.1608 | 285.1557 | **IGGGM** | 388.2013 | 416.1962 | **IGGGMS** | 475.2333 | 503.2282 |
| **IGGGMSV** | 574.3017 | 602.2967 | **IGGGMSVD** | 689.3287 | 717.3236 | **GG** | 87.0553 | 115.0502 |
| **GGG** | 144.0768 | 172.0717 | **GGGM** | 275.1172 | 303.1122 | **GGGMS** | 362.1493 | 390.1442 |
| **GGGMSV** | 461.2177 | 489.2126 | **GGGMSVD** | 576.2446 | 604.2395 | **GGGMSVDI** | 689.3287 | 717.3236 |
| **GG** | 87.0553 | 115.0502 | **GGM** | 218.0958 | 246.0907 | **GGMS** | 305.1278 | 333.1227 |
| **GGMSV** | 404.1962 | 432.1911 | **GGMSVD** | 519.2232 | 547.2181 | **GGMSVDI** | 632.3072 | 660.3021 |
| **GM** | 161.0743 | 189.0692 | **GMS** | 248.1063 | 276.1013 | **GMSV** | 347.1748 | 375.1697 |
| **GMSVD** | 462.2017 | 490.1966 | **GMSVDI** | 575.2858 | 603.2807 | **GMSVDID** | 690.3127 | 718.3076 |
| **MS** | 191.0849 | 219.0798 | **MSV** | 290.1533 | 318.1482 | **MSVD** | 405.1802 | 433.1751 |
| **MSVDI** | 518.2643 | 546.2592 | **MSVDID** | 633.2912 | 661.2862 | **SV** | 159.1128 | 187.1077 |
| **SVD** | 274.1397 | 302.1347 | **SVDI** | 387.2238 | 415.2187 | **SVDID** | 502.2508 | 530.2457 |
| **SVDIDA** | 573.2879 | 601.2828 | **SVDIDAS** | 660.3199 | 688.3148 | **VD** | 187.1077 | 215.1026 |
| **VDI** | 300.1918 | 328.1867 | **VDID** | 415.2187 | 443.2136 | **VDIDA** | 486.2558 | 514.2508 |
| **VDIDAS** | 573.2879 | 601.2828 | **VDIDASG** | 630.3093 | 658.3042 | **DI** | 201.1234 | 229.1183 |
| **DID** | 316.1503 | 344.1452 | **DIDA** | 387.1874 | 415.1823 | **DIDAS** | 474.2195 | 502.2144 |
| **DIDASG** | 531.2409 | 559.2358 | **ID** | 201.1234 | 229.1183 | **IDA** | 272.1605 | 300.1554 |
| **IDAS** | 359.1925 | 387.1874 | **IDASG** | 416.2140 | 444.2089 | **DA** | 159.0764 | 187.0713 |
| **DAS** | 246.1084 | 274.1034 | **DASG** | 303.1299 | 331.1248 | **AS** | 131.0815 | 159.0764 |
| **ASG** | 188.1030 | 216.0979 | **SG** | 117.0659 | 145.0608 | | | |

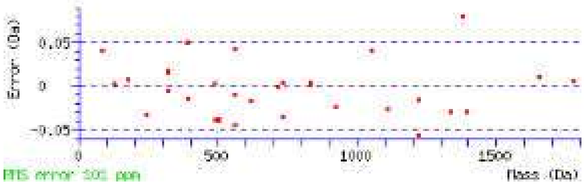


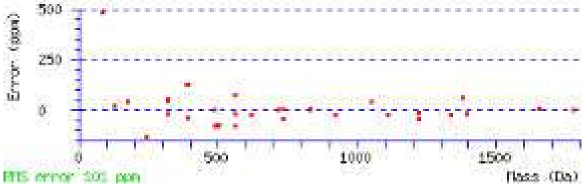


NCBI **BLAST** search of TDKPYGIGGGMSVDIDASGR
(Parameters: blastp, nr protein database, expect=20000, no filter, PAM30)
Other BLAST web gateways

**All matches to this query**

| Score | Mr(calc) | Delta | Sequence |
|---|---|---|---|
| 123.8 | 1994.9368 | -0.0033 | TDKPYGIGGGMSVDIDASGR |
| 14.8 | 1994.8826 | 0.0508 | MGCASASWIEAVVDEASGR |
| 14.6 | 1994.8795 | 0.0539 | GHGMCGMNTMVSAVVTSTR |
| 13.2 | 1995.0115 | -0.0781 | RNTWVFVFGNIGWSQGK |
| 12.9 | 1994.9983 | -0.0649 | DTMLANVAATSELFLLDR |
| 12.1 | 1994.9553 | -0.0219 | TNMAAEDVEFLEAMLRR |
| 11.4 | 1994.9078 | 0.0257 | VASCDIPIPYMDEGSSVR |
| 11.1 | 1994.9302 | 0.0032 | CPPHLNPSSSSCISSPIR |
| 10.9 | 1995.0637 | -0.1302 | EILSGIAVPGLNVVDENTR |
| 10.7 | 1995.0762 | -0.1428 | ISITAPLHASPHRPAGAGSR |

**Mascot:** http://www.matrixscience.com/

# MATRIX SCIENCE Mascot Search Results

## Peptide View Spot no 2

MS/MS Fragmentation of **TDKPYGIGGGMSVDIDASGRK**
Found in **gi|1835731** in **NCBInr**, photosystem II 10 kDa polypeptide [Oryza sativa Indica Group]

Match to Query 179: 2123.028324 from(2124.035600,1+) intensity(0.0000) index(29)
Title: Label: K5, Spot_Id: 219748, Peak_List_Id: 225867, MSMS Job_Run_Id: 21792, Comment:
Data file C:\Documents and Settings\Administrator\Desktop\farah BHU\spot set plate\Farah-Garima 11May 2013\ppw_K5_136842223800.txt

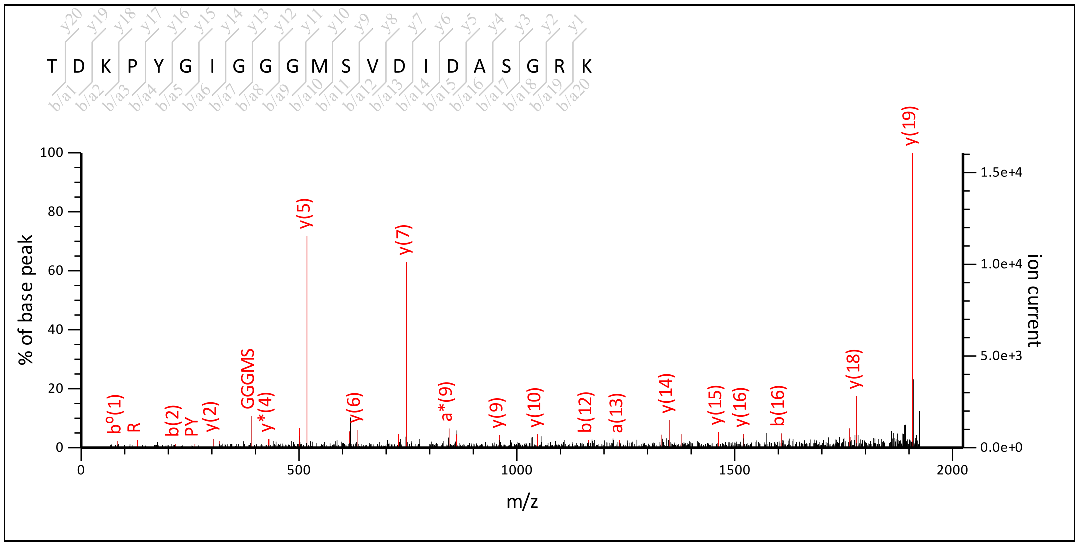


0 to 2023.87

Label all possible matches ○ Label matches used for scoring ◉

**Monoisotopic mass of neutral peptide Mr(calc):** 2123.0317
**Fixed modifications:** Carbamidomethyl (C) (apply to specified residues or termini only)
**Ions Score:** 79 **Expect:** 1.9e-05
**Matches :** 43/424 fragment ions using 57 most intense peaks (help)

| # | Immon. | a | a* | $a^0$ | b | b* | $b^0$ | d | d' | Seq. | v | w | w' | y | y* |
|---|---|---|---|---|---|---|---|---|---|---|---|---|---|---|---|
| 1 | 74.0600 | 74.0600 | | 56.0495 | 102.0550 | | 84.0444 | 44.0495 | | T | | | | | |
| 2 | 88.0393 | 189.0870 | | 171.0764 | **217.0819** | | 199.0713 | 145.0972 | | D | 1962.9702 | 1961.9749 | | 2022.9913 | 2005.9648 |
| 3 | 101.1073 | 317.1819 | 300.1554 | 299.1714 | 345.1769 | 328.1503 | 327.1663 | 260.1241 | | K | 1834.8752 | 1833.8800 | | ***1907.9644*** | 1890.9378 |
| 4 | 70.0651 | 414.2347 | 397.2082 | 396.2241 | 442.2296 | 425.2031 | 424.2191 | 388.2191 | | P | 1737.8225 | 1736.8272 | | ***1779.8694*** | 1762.8429 |
| 5 | 136.0757 | 577.2980 | 560.2715 | 559.2875 | 605.2930 | 588.2664 | 587.2824 | | | Y | 1574.7591 | | | 1682.8166 | 1665.7901 |
| 6 | 30.0338 | 634.3195 | 617.2930 | 616.3089 | 662.3144 | 645.2879 | 644.3039 | | | G | | | | ***1519.7533*** | 1502.7268 |
| 7 | 86.0964 | 747.4036 | 730.3770 | 729.3930 | 775.3985 | 758.3719 | 757.3879 | 719.3723 | 733.3879 | I | 1404.6536 | 1417.6740 | 1431.6897 | ***1462.7319*** | 1445.7053 |
| 8 | 30.0338 | 804.4250 | 787.3985 | 786.4145 | 832.4199 | 815.3934 | 814.4094 | | | G | | | | ***1349.6478*** | 1332.6212 |
| 9 | 30.0338 | **861.4465** | 844.4199 | 843.4359 | 889.4414 | 872.4149 | 871.4308 | | | G | | | | 1292.6263 | 1275.5998 |
| 10 | 30.0338 | 918.4680 | 901.4414 | 900.4574 | 946.4629 | 929.4363 | 928.4523 | | | G | | | | ***1235.6049*** | 1218.5783 |
| 11 | 104.0528 | 1049.5084 | 1032.4819 | 1031.4979 | 1077.5034 | 1060.4768 | 1059.4928 | 989.5051 | | M | 1102.5487 | 1101.5535 | | 1178.5834 | 1161.5568 |
| 12 | 60.0444 | 1136.5405 | 1119.5139 | 1118.5299 | **1164.5354** | 1147.5088 | 1146.5248 | 1120.5456 | | S | 1015.5167 | 1014.5215 | | ***1047.5429*** | 1030.5164 |
| 13 | 72.0808 | **1235.6089** | 1218.5823 | 1217.5983 | 1263.6038 | 1246.5773 | 1245.5932 | 1221.5932 | | V | 916.4483 | 929.4687 | | ***960.5109*** | 943.4843 |
| 14 | 88.0393 | 1350.6358 | 1333.6093 | 1332.6253 | **1378.6307** | 1361.6042 | 1360.6202 | 1306.6460 | | D | 801.4213 | 800.4261 | | ***861.4425*** | 844.4159 |
| 15 | 86.0964 | 1463.7199 | 1446.6933 | 1445.7093 | 1491.7148 | 1474.6883 | 1473.7042 | 1435.6886 | 1449.7042 | I | 688.3373 | 701.3577 | 715.3733 | ***746.4155*** | 729.3890 |
| 16 | 88.0393 | 1578.7468 | 1561.7203 | 1560.7363 | **1606.7418** | 1589.7152 | 1588.7312 | 1534.7570 | | D | 573.3103 | 572.3151 | | ***633.3315*** | 616.3049 |
| 17 | 44.0495 | 1649.7840 | 1632.7574 | 1631.7734 | 1677.7789 | 1660.7523 | 1659.7683 | | | A | 502.2732 | | | ***518.3045*** | 501.2780 |
| 18 | 60.0444 | 1736.8160 | 1719.7894 | 1718.8054 | **1764.8109** | 1747.7843 | 1746.8003 | 1720.8211 | | S | 415.2412 | 414.2459 | | 447.2674 | 430.2409 |
| 19 | 30.0338 | 1793.8374 | 1776.8109 | 1775.8269 | 1821.8324 | 1804.8058 | 1803.8218 | | | G | | | | 360.2354 | 343.2088 |
| 20 | 129.1135 | 1949.9386 | 1932.9120 | 1931.9280 | 1977.9335 | 1960.9069 | 1959.9229 | 1864.8746 | | R | 202.1186 | 201.1234 | | ***303.2139*** | 286.1874 |
| 21 | 101.1073 | | | | | | | | | K | 74.0237 | 73.0284 | | 147.1128 | 130.0863 |

| Seq | ya | yb | Seq | ya | yb | Seq | ya | yb |
|---|---|---|---|---|---|---|---|---|

| | | | | | | | | |
|---|---|---|---|---|---|---|---|---|
| **DK** | 216.1343 | 244.1292 | **DKP** | 313.1870 | 341.1819 | **DKPY** | 476.2504 | 504.2453 |
| **DKPYG** | 533.2718 | 561.2667 | **DKPYGI** | 646.3559 | 674.3508 | **KP** | 198.1601 | 226.1550 |
| **KPY** | 361.2234 | 389.2183 | **KPYG** | 418.2449 | 446.2398 | **KPYGI** | 531.3289 | 559.3239 |
| **KPYGIG** | 588.3504 | 616.3453 | **KPYGIGG** | 645.3719 | 673.3668 | **PY** | 233.1285 | 261.1234 |
| **PYG** | 290.1499 | 318.1448 | **PYGI** | 403.2340 | 431.2289 | **PYGIG** | 460.2554 | 488.2504 |
| **PYGIGG** | 517.2769 | 545.2718 | **PYGIGGG** | 574.2984 | 602.2933 | **YG** | 193.0972 | 221.0921 |
| **YGI** | 306.1812 | 334.1761 | **YGIG** | 363.2027 | 391.1976 | **YGIGG** | 420.2241 | 448.2191 |
| **YGIGGG** | 477.2456 | 505.2405 | **YGIGGGM** | 608.2861 | 636.2810 | **YGIGGGMS** | 695.3181 | 723.3130 |
| **GI** | 143.1179 | 171.1128 | **GIG** | 200.1394 | 228.1343 | **GIGG** | 257.1608 | 285.1557 |
| **GIGGG** | 314.1823 | 342.1772 | **GIGGGM** | 445.2228 | 473.2177 | **GIGGGMS** | 532.2548 | 560.2497 |
| **GIGGGMSV** | 631.3232 | 659.3181 | **IG** | 143.1179 | 171.1128 | **IGG** | 200.1394 | 228.1343 |
| **IGGG** | 257.1608 | 285.1557 | **IGGGM** | 388.2013 | 416.1962 | **IGGGMS** | 475.2333 | 503.2282 |
| **IGGGMSV** | 574.3017 | 602.2967 | **IGGGMSVD** | 689.3287 | 717.3236 | **GG** | 87.0553 | 115.0502 |
| **GGG** | 144.0768 | 172.0717 | **GGGM** | 275.1172 | 303.1122 | **GGGMS** | 362.1493 | 390.1442 |
| **GGGMSV** | 461.2177 | 489.2126 | **GGGMSVD** | 576.2446 | 604.2395 | **GGGMSVDI** | 689.3287 | 717.3236 |
| **GG** | 87.0553 | 115.0502 | **GGM** | 218.0958 | 246.0907 | **GGMS** | 305.1278 | 333.1227 |
| **GGMSV** | 404.1962 | 432.1911 | **GGMSVD** | 519.2232 | 547.2181 | **GGMSVDI** | 632.3072 | 660.3021 |
| **GM** | 161.0743 | 189.0692 | **GMS** | 248.1063 | 276.1013 | **GMSV** | 347.1748 | 375.1697 |
| **GMSVD** | 462.2017 | 490.1966 | **GMSVDI** | 575.2858 | 603.2807 | **GMSVDID** | 690.3127 | 718.3076 |
| **MS** | 191.0849 | 219.0798 | **MSV** | 290.1533 | 318.1482 | **MSVD** | 405.1802 | 433.1751 |
| **MSVDI** | 518.2643 | 546.2592 | **MSVDID** | 633.2912 | 661.2862 | **SV** | 159.1128 | 187.1077 |
| **SVD** | 274.1397 | 302.1347 | **SVDI** | 387.2238 | 415.2187 | **SVDID** | 502.2508 | 530.2457 |
| **SVDIDA** | 573.2879 | 601.2828 | **SVDIDAS** | 660.3199 | 688.3148 | **VD** | 187.1077 | 215.1026 |
| **VDI** | 300.1918 | 328.1867 | **VDID** | 415.2187 | 443.2136 | **VDIDA** | 486.2558 | 514.2508 |
| **VDIDAS** | 573.2879 | 601.2828 | **VDIDASG** | 630.3093 | 658.3042 | **DI** | 201.1234 | 229.1183 |
| **DID** | 316.1503 | 344.1452 | **DIDA** | 387.1874 | 415.1823 | **DIDAS** | 474.2195 | 502.2144 |
| **DIDASG** | 531.2409 | 559.2358 | **DIDASGR** | 687.3420 | 715.3369 | **ID** | 201.1234 | 229.1183 |
| **IDA** | 272.1605 | 300.1554 | **IDAS** | 359.1925 | 387.1874 | **IDASG** | 416.2140 | 444.2089 |
| **IDASGR** | 572.3151 | 600.3100 | **DA** | 159.0764 | 187.0713 | **DAS** | 246.1084 | 274.1034 |
| **DASG** | 303.1299 | 331.1248 | **DASGR** | 459.2310 | 487.2259 | **AS** | 131.0815 | 159.0764 |
| **ASG** | 188.1030 | 216.0979 | **ASGR** | 344.2041 | 372.1990 | **SG** | 117.0659 | 145.0608 |
| **SGR** | 273.1670 | 301.1619 | **GR** | 186.1349 | 214.1299 | | | |

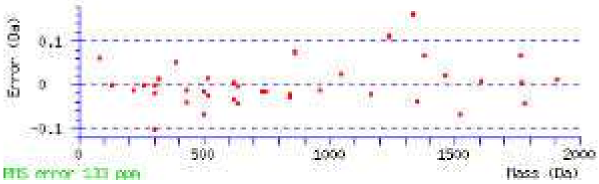


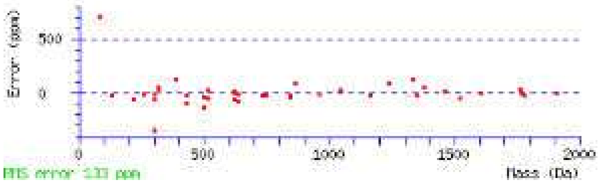


NCBI **BLAST** search of TDKPYGIGGGMSVDIDASGRK
(Parameters: blastp, nr protein database, expect=20000, no filter, PAM30)
Other BLAST web gateways

**All matches to this query**

| Score | Mr(calc) | Delta | Sequence |
|---|---|---|---|
| 78.7 | 2123.0317 | -0.0034 | TDKPYGIGGGMSVDIDASGRK |
| 17.4 | 2123.0504 | -0.0220 | TFNMGVGMVLVVNQEVSQR |
| 14.7 | 2122.9953 | 0.0330 | MVEVNADGKIYAAEGAADAGR |
| 13.3 | 2122.9841 | 0.0442 | TMEISINDYPLTNPDTSGR |
| 12.7 | 2122.9511 | 0.0773 | LNEMAEVQSAMELGDDTVR |
| 12.7 | 2122.9511 | 0.0773 | LNEMAEVQSAMELGDDTVR |
| 12.6 | 2123.0205 | 0.0078 | GLSSPSMDEGALFATPSTSIR |
| 12.0 | 2123.0019 | 0.0265 | SVEDDSIPSFSGGIDDSLRK |
| 11.7 | 2123.0429 | -0.0146 | ISYNLAMANVDEIRQSQR |
| 11.2 | 2123.1627 | -0.1343 | SLTGFVGLIASLVFLDQSTR |

**Mascot:** http://www.matrixscience.com/

# MATRIX SCIENCE Mascot Search Results

## Peptide View Spot no 2

MS/MS Fragmentation of **TDKPYGIGGGMSVDIDASGR**
Found in **gi|1835731** in **NCBInr**, photosystem II 10 kDa polypeptide [Oryza sativa Indica Group]

Match to Query 177: 2010.909624 from(2011.916900,1+) intensity(0.0000) index(28)
Title: Label: K5, Spot_Id: 219748, Peak_List_Id: 225866, MSMS Job_Run_Id: 21792, Comment:
Data file C:\Documents and Settings\Administrator\Desktop\farah BHU\spot set plate\Farah-Garima 11May 2013\ppw_K5_136842223800.txt

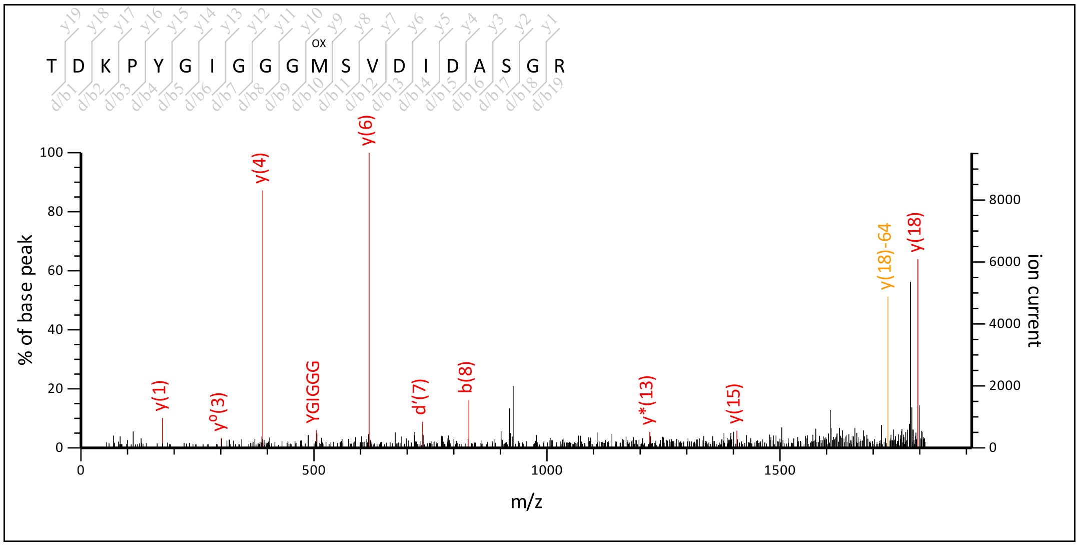


0 to 1911.46

Label all possible matches ○ Label matches used for scoring ◉

**Monoisotopic mass of neutral peptide Mr(calc):** 2010.9317
**Fixed modifications:** Carbamidomethyl (C) (apply to specified residues or termini only)
**Variable modifications:**
**M11** : Oxidation (M), with neutral losses 0.0000(shown in table), 63.9983
**Ions Score:** 49 **Expect:** 0.017
**Matches** : 16/551 fragment ions using 18 most intense peaks (help)

| # | Immon. | a | a* | $a^0$ | b | b* | $b^0$ | d | d' | Seq. | v | w | w' | y | y* | |
|---|---|---|---|---|---|---|---|---|---|---|---|---|---|---|---|---|
| 1 | 74.0600 | 74.0600 | | 56.0495 | 102.0550 | | 84.0444 | 44.0495 | | T | | | | | | |
| 2 | 88.0393 | 189.0870 | | 171.0764 | 217.0819 | | 199.0713 | 145.0972 | | D | 1850.8701 | 1849.8749 | | 1910.8913 | 1893.8647 | [illegible] |
| 3 | 101.1073 | 317.1819 | 300.1554 | 299.1714 | 345.1769 | 328.1503 | 327.1663 | 260.1241 | | K | 1722.7752 | 1721.7799 | | ***1795.8643*** | 1778.8378 | [illegible] |
| 4 | 70.0651 | 414.2347 | 397.2082 | 396.2241 | 442.2296 | 425.2031 | 424.2191 | 388.2191 | | P | 1625.7224 | 1624.7272 | | 1667.7694 | 1650.7428 | [illegible] |
| 5 | 136.0757 | 577.2980 | 560.2715 | 559.2875 | 605.2930 | 588.2664 | 587.2824 | | | Y | 1462.6591 | | | 1570.7166 | 1553.6901 | [illegible] |
| 6 | 30.0338 | 634.3195 | 617.2930 | 616.3089 | 662.3144 | 645.2879 | 644.3039 | | | G | | | | ***1407.6533*** | 1390.6267 | [illegible] |
| 7 | 86.0964 | 747.4036 | 730.3770 | 729.3930 | 775.3985 | 758.3719 | 757.3879 | 719.3723 | 733.3879 | I | 1292.5536 | 1305.5740 | 1319.5896 | 1350.6318 | 1333.6053 | [illegible] |
| 8 | 30.0338 | 804.4250 | 787.3985 | 786.4145 | 832.4199 | 815.3934 | 814.4094 | | | G | | | | 1237.5477 | 1220.5212 | [illegible] |
| 9 | 30.0338 | 861.4465 | 844.4199 | 843.4359 | 889.4414 | 872.4149 | 871.4308 | | | G | | | | 1180.5263 | 1163.4997 | [illegible] |
| 10 | 30.0338 | 918.4680 | 901.4414 | 900.4574 | 946.4629 | 929.4363 | 928.4523 | | | G | | | | 1123.5048 | 1106.4783 | [illegible] |
| 11 | 120.0478 | 1065.5034 | 1048.4768 | 1047.4928 | 1093.4983 | 1076.4717 | 1075.4877 | 989.5051 | | M | 974.4538 | 973.4585 | | 1066.4834 | 1049.4568 | [illegible] |
| 12 | 60.0444 | 1152.5354 | 1135.5088 | 1134.5248 | 1180.5303 | 1163.5038 | 1162.5197 | 1136.5405 | | S | 887.4217 | 886.4265 | | 919.4480 | 902.4214 | [illegible] |
| 13 | 72.0808 | 1251.6038 | 1234.5773 | 1233.5932 | 1279.5987 | 1262.5722 | 1261.5882 | 1237.5882 | | V | 788.3533 | 801.3737 | | ***832.4159*** | 815.3894 | [illegible] |
| 14 | 88.0393 | 1366.6307 | 1349.6042 | 1348.6202 | 1394.6257 | 1377.5991 | 1376.6151 | 1322.6409 | | D | 673.3264 | 672.3311 | | ***733.3475*** | 716.3210 | [illegible] |
| 15 | 86.0964 | 1479.7148 | 1462.6883 | 1461.7042 | 1507.7097 | 1490.6832 | 1489.6992 | 1451.6835 | 1465.6992 | I | 560.2423 | 573.2627 | 587.2784 | ***618.3206*** | 601.2940 | [illegible] |
| 16 | 88.0393 | 1594.7418 | 1577.7152 | 1576.7312 | 1622.7367 | 1605.7101 | 1604.7261 | 1550.7519 | | D | 445.2154 | 444.2201 | | ***505.2365*** | 488.2100 | [illegible] |
| 17 | 44.0495 | 1665.7789 | 1648.7523 | 1647.7683 | 1693.7738 | 1676.7472 | 1675.7632 | | | A | 374.1783 | | | ***390.2096*** | 373.1830 | [illegible] |
| 18 | 60.0444 | 1752.8109 | 1735.7843 | 1734.8003 | 1780.8058 | 1763.7793 | 1762.7952 | 1736.8160 | | S | 287.1462 | 286.1510 | | 319.1724 | 302.1459 | [illegible] |
| 19 | 30.0338 | 1809.8324 | 1792.8058 | 1791.8218 | 1837.8273 | 1820.8007 | 1819.8167 | | | G | | | | 232.1404 | 215.1139 | |
| 20 | 129.1135 | | | | | | | | | R | 74.0237 | 73.0284 | | ***175.1190*** | 158.0924 | |

| Seq | ya | yb | Seq | ya | yb | Seq | ya | yb |
|---|---|---|---|---|---|---|---|---|
| DK | 216.1343 | 244.1292 | DKP | 313.1870 | 341.1819 | DKPY | 476.2504 | 504.2453 |
| DKPYG | 533.2718 | 561.2667 | DKPYGI | 646.3559 | 674.3508 | KP | 198.1601 | 226.1550 |
| KPY | 361.2234 | 389.2183 | KPYG | 418.2449 | 446.2398 | KPYGI | 531.3289 | 559.3239 |
| KPYGIG | 588.3504 | 616.3453 | KPYGIGG | 645.3719 | 673.3668 | PY | 233.1285 | 261.1234 |
| PYG | 290.1499 | 318.1448 | PYGI | 403.2340 | 431.2289 | PYGIG | 460.2554 | 488.2504 |
| PYGIGG | 517.2769 | 545.2718 | PYGIGGG | 574.2984 | 602.2933 | YG | 193.0972 | 221.0921 |
| YGI | 306.1812 | 334.1761 | YGIG | 363.2027 | 391.1976 | YGIGG | 420.2241 | 448.2191 |
| YGIGGG | 477.2456 | 505.2405 | YGIGGGM | 624.2810 | 652.2759 | GI | 143.1179 | 171.1128 |
| GIG | 200.1394 | 228.1343 | GIGG | 257.1608 | 285.1557 | GIGGG | 314.1823 | 342.1772 |
| GIGGGM | 461.2177 | 489.2126 | GIGGGMS | 548.2497 | 576.2446 | GIGGGMSV | 647.3181 | 675.3130 |
| IG | 143.1179 | 171.1128 | IGG | 200.1394 | 228.1343 | IGGG | 257.1608 | 285.1557 |
| IGGGM | 404.1962 | 432.1911 | IGGGMS | 491.2282 | 519.2232 | IGGGMSV | 590.2967 | 618.2916 |
| GG | 87.0553 | 115.0502 | GGG | 144.0768 | 172.0717 | GGGM | 291.1122 | 319.1071 |
| GGGMS | 378.1442 | 406.1391 | GGGMSV | 477.2126 | 505.2075 | GGGMSVD | 592.2395 | 620.2345 |
| GG | 87.0553 | 115.0502 | GGM | 234.0907 | 262.0856 | GGMS | 321.1227 | 349.1176 |
| GGMSV | 420.1911 | 448.1860 | GGMSVD | 535.2181 | 563.2130 | GGMSVDI | 648.3021 | 676.2971 |
| GM | 177.0692 | 205.0641 | GMS | 264.1013 | 292.0962 | GMSV | 363.1697 | 391.1646 |
| GMSVD | 478.1966 | 506.1915 | GMSVDI | 591.2807 | 619.2756 | MS | 207.0798 | 235.0747 |
| MSV | 306.1482 | 334.1431 | MSVD | 421.1751 | 449.1701 | MSVDI | 534.2592 | 562.2541 |
| MSVDID | 649.2862 | 677.2811 | SV | 159.1128 | 187.1077 | SVD | 274.1397 | 302.1347 |
| SVDI | 387.2238 | 415.2187 | SVDID | 502.2508 | 530.2457 | SVDIDA | 573.2879 | 601.2828 |
| SVDIDAS | 660.3199 | 688.3148 | VD | 187.1077 | 215.1026 | VDI | 300.1918 | 328.1867 |
| VDID | 415.2187 | 443.2136 | VDIDA | 486.2558 | 514.2508 | VDIDAS | 573.2879 | 601.2828 |
| VDIDASG | 630.3093 | 658.3042 | DI | 201.1234 | 229.1183 | DID | 316.1503 | 344.1452 |
| DIDA | 387.1874 | 415.1823 | DIDAS | 474.2195 | 502.2144 | DIDASG | 531.2409 | 559.2358 |
| ID | 201.1234 | 229.1183 | IDA | 272.1605 | 300.1554 | IDAS | 359.1925 | 387.1874 |
| IDASG | 416.2140 | 444.2089 | DA | 159.0764 | 187.0713 | DAS | 246.1084 | 274.1034 |
| DASG | 303.1299 | 331.1248 | AS | 131.0815 | 159.0764 | ASG | 188.1030 | 216.0979 |
| SG | 117.0659 | 145.0608 | | | | | | |

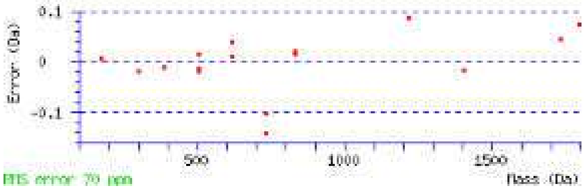


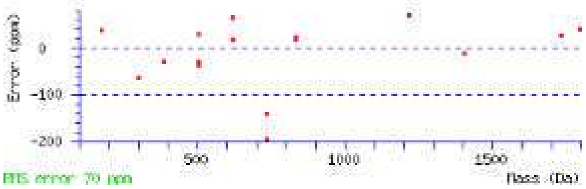


NCBI **BLAST** search of TDKPYGIGGGMSVDIDASGR
(Parameters: blastp, nr protein database, expect=20000, no filter, PAM30)
Other BLAST web gateways

**All matches to this query**

| Score | Mr(calc) | Delta | Sequence |
|---|---|---|---|
| 49.2 | 2010.9317 | -0.0221 | TDKPYGIGGGMSVDIDASGR |
| 11.0 | 2010.8614 | 0.0482 | SEATSSIGAESDDGSSAPTSR |
| 10.1 | 2010.9342 | -0.0245 | ESSSTIDVSDSDNVVSSKR |
| 9.9 | 2010.9027 | 0.0069 | VASCDIPIPYMDEGSSVR |
| 8.1 | 2010.8371 | 0.0725 | SDSSNNECVLQCVEGASR |
| 8.1 | 2010.9763 | -0.0667 | MILMPCYLQLDLGLCR |
| 8.1 | 2010.9763 | -0.0667 | MILMPCYLQLDLGLCR |
| 8.0 | 2010.8991 | 0.0105 | TDNNSVRSGYNADNVTER |
| 7.6 | 2010.8614 | 0.0482 | ESSATTVSPDSENDTTNTR |
| 6.9 | 2011.0011 | -0.0915 | SQPYGISTNGVVGSLFETR |

**Mascot:** http://www.matrixscience.com/

# MATRIX SCIENCE Mascot Search Results

## Peptide View Spot no 2

MS/MS Fragmentation of **TDKPYGIGGGMSVDIDASGRK**
Found in **gi|1835731** in **NCBInr**, photosystem II 10 kDa polypeptide [Oryza sativa Indica Group]

Match to Query 181: 2139.007324 from(2140.014600,1+) intensity(0.0000) index(30)
Title: Label: K5, Spot_Id: 219748, Peak_List_Id: 225869, MSMS Job_Run_Id: 21792, Comment:
Data file C:\Documents and Settings\Administrator\Desktop\farah BHU\spot set plate\Farah-Garima 11May 2013\ppw_K5_136842223800.txt

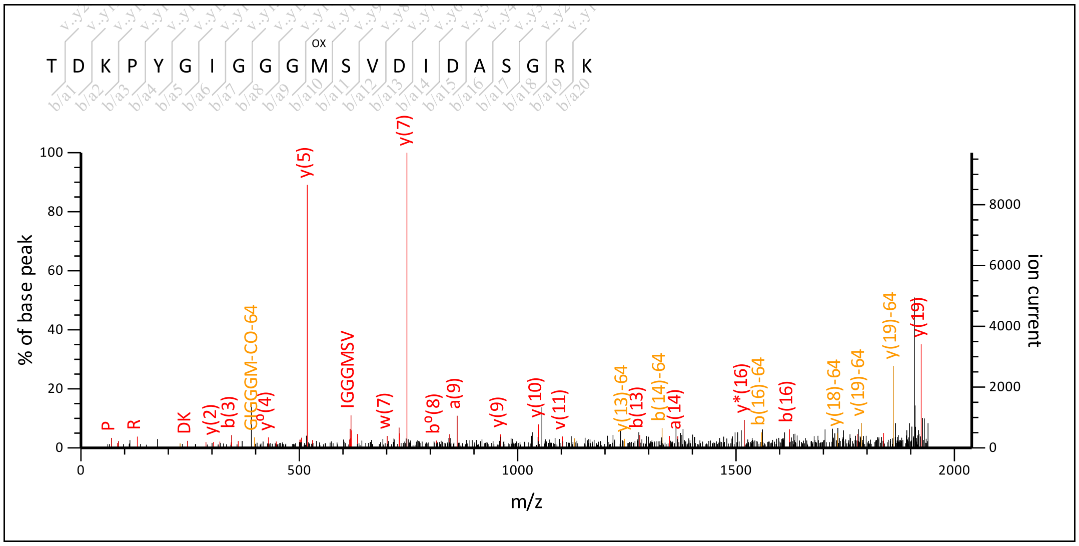


0 to 2039.73

Label all possible matches ○ Label matches used for scoring ◉

**Monoisotopic mass of neutral peptide Mr(calc):** 2139.0266
**Fixed modifications:** Carbamidomethyl (C) (apply to specified residues or termini only)
**Variable modifications:**
**M11** : Oxidation (M), with neutral losses 63.9983(shown in table), 0.0000
**Ions Score:** 44 **Expect:** 0.05
**Matches** : 64/609 fragment ions using 112 most intense peaks (help)

| # | Immon. | a | a* | $a^0$ | b | b* | $b^0$ | d | d' | Seq. | v | w | w' | y | y* |
|---|---|---|---|---|---|---|---|---|---|---|---|---|---|---|---|
| 1 | 74.0600 | 74.0600 | | 56.0495 | 102.0550 | | 84.0444 | 44.0495 | | T | | | | | |
| 2 | 88.0393 | 189.0870 | | 171.0764 | 217.0819 | | 199.0713 | 145.0972 | | D | 1914.9668 | 1913.9716 | | 1974.9879 | 1957.9614 |
| 3 | 101.1073 | 317.1819 | 300.1554 | 299.1714 | **345.1769** | 328.1503 | 327.1663 | 260.1241 | | K | 1786.8719 | 1785.8766 | | ***1859.9610*** | 1842.9345 |
| 4 | 70.0651 | 414.2347 | 397.2082 | 396.2241 | 442.2296 | 425.2031 | 424.2191 | 388.2191 | | P | 1689.8191 | 1688.8238 | | ***1731.8660*** | 1714.8395 |
| 5 | 136.0757 | 577.2980 | 560.2715 | 559.2875 | 605.2930 | 588.2664 | 587.2824 | | | Y | 1526.7558 | | | 1634.8133 | 1617.7867 |
| 6 | 30.0338 | 634.3195 | 617.2930 | 616.3089 | 662.3144 | 645.2879 | 644.3039 | | | G | | | | 1471.7499 | 1454.7234 |
| 7 | 86.0964 | 747.4036 | 730.3770 | 729.3930 | 775.3985 | 758.3719 | 757.3879 | 719.3723 | 733.3879 | I | 1356.6502 | 1369.6706 | 1383.6863 | 1414.7285 | 1397.7019 |
| 8 | 30.0338 | 804.4250 | 787.3985 | 786.4145 | 832.4199 | 815.3934 | 814.4094 | | | G | | | | 1301.6444 | 1284.6179 |
| 9 | 30.0338 | 861.4465 | 844.4199 | 843.4359 | 889.4414 | 872.4149 | 871.4308 | | | G | | | | ***1244.6230*** | 1227.5964 |
| 10 | 30.0338 | 918.4680 | 901.4414 | 900.4574 | 946.4629 | 929.4363 | 928.4523 | | | G | | | | 1187.6015 | 1170.5749 |
| 11 | 56.0495 | 1001.5051 | 984.4785 | 983.4945 | 1029.5000 | 1012.4734 | 1011.4894 | 989.5051 | | M | 1102.5487 | 1101.5535 | | ***1130.5800*** | 1113.5535 |
| 12 | 60.0444 | 1088.5371 | 1071.5106 | 1070.5265 | 1116.5320 | 1099.5055 | 1098.5215 | 1072.5422 | | S | 1015.5167 | 1014.5215 | | ***1047.5429*** | 1030.5164 |
| 13 | 72.0808 | 1187.6055 | 1170.5790 | 1169.5950 | 1215.6004 | 1198.5739 | 1197.5899 | 1173.5899 | | V | 916.4483 | 929.4687 | | ***960.5109*** | 943.4843 |
| 14 | 88.0393 | 1302.6325 | 1285.6059 | 1284.6219 | **1330.6274** | 1313.6008 | 1312.6168 | 1258.6426 | | D | 801.4213 | 800.4261 | | ***861.4425*** | 844.4159 |
| 15 | 86.0964 | 1415.7165 | 1398.6900 | 1397.7060 | 1443.7114 | 1426.6849 | 1425.7009 | 1387.6852 | 1401.7009 | I | 688.3373 | 701.3577 | 715.3733 | ***746.4155*** | 729.3890 |
| 16 | 88.0393 | 1530.7435 | 1513.7169 | 1512.7329 | **1558.7384** | 1541.7118 | 1540.7278 | 1486.7536 | | D | 573.3103 | 572.3151 | | ***633.3315*** | 616.3049 |
| 17 | 44.0495 | 1601.7806 | 1584.7540 | 1583.7700 | 1629.7755 | 1612.7489 | 1611.7649 | | | A | 502.2732 | | | ***518.3045*** | 501.2780 |
| 18 | 60.0444 | 1688.8126 | 1671.7861 | 1670.8020 | 1716.8075 | 1699.7810 | 1698.7970 | 1672.8177 | | S | 415.2412 | 414.2459 | | ***447.2674*** | 430.2409 |
| 19 | 30.0338 | 1745.8341 | 1728.8075 | 1727.8235 | 1773.8290 | 1756.8024 | 1755.8184 | | | G | | | | ***360.2354*** | 343.2088 |
| 20 | 129.1135 | 1901.9352 | 1884.9086 | 1883.9246 | 1929.9301 | 1912.9035 | 1911.9195 | 1816.8712 | | R | 202.1186 | 201.1234 | | ***303.2139*** | 286.1874 |

| | | | | | | | | | | | | | | | |
|---|---|---|---|---|---|---|---|---|---|---|---|---|---|---|---|
| 21 | 101.1073 | | | | | | | | | K | 74.0237 | 73.0284 | | 147.1128 | 130.0863 |

| Seq | ya | yb | Seq | ya | yb | Seq | ya | yb |
|---|---|---|---|---|---|---|---|---|
| DK | 216.1343 | 244.1292 | DKP | 313.1870 | 341.1819 | DKPY | 476.2504 | 504.2453 |
| DKPYG | 533.2718 | 561.2667 | DKPYGI | 646.3559 | 674.3508 | KP | 198.1601 | 226.1550 |
| KPY | 361.2234 | 389.2183 | KPYG | 418.2449 | 446.2398 | KPYGI | 531.3289 | 559.3239 |
| KPYGIG | 588.3504 | 616.3453 | KPYGIGG | 645.3719 | 673.3668 | PY | 233.1285 | 261.1234 |
| PYG | 290.1499 | 318.1448 | PYGI | 403.2340 | 431.2289 | PYGIG | 460.2554 | 488.2504 |
| PYGIGG | 517.2769 | 545.2718 | PYGIGGG | 574.2984 | 602.2933 | PYGIGGGM | 657.3355 | 685.3304 |
| YG | 193.0972 | 221.0921 | YGI | 306.1812 | 334.1761 | YGIG | 363.2027 | 391.1976 |
| YGIGG | 420.2241 | 448.2191 | YGIGGG | 477.2456 | 505.2405 | YGIGGGM | 560.2827 | 588.2776 |
| YGIGGGMS | 647.3148 | 675.3097 | GI | 143.1179 | 171.1128 | GIG | 200.1394 | 228.1343 |
| GIGG | 257.1608 | 285.1557 | GIGGG | 314.1823 | 342.1772 | GIGGGM | 397.2194 | 425.2143 |
| GIGGGMS | 484.2514 | 512.2463 | GIGGGMSV | 583.3198 | 611.3148 | GIGGGMSVD | 698.3468 | 726.3417 |
| IG | 143.1179 | 171.1128 | IGG | 200.1394 | 228.1343 | IGGG | 257.1608 | 285.1557 |
| IGGGM | 340.1979 | 368.1928 | IGGGMS | 427.2300 | 455.2249 | IGGGMSV | 526.2984 | 554.2933 |
| IGGGMSVD | 641.3253 | 669.3202 | GG | 87.0553 | 115.0502 | GGG | 144.0768 | 172.0717 |
| GGGM | 227.1139 | 255.1088 | GGGMS | 314.1459 | 342.1408 | GGGMSV | 413.2143 | 441.2092 |
| GGGMSVD | 528.2413 | 556.2362 | GGGMSVDI | 641.3253 | 669.3202 | GG | 87.0553 | 115.0502 |
| GGM | 170.0924 | 198.0873 | GGMS | 257.1244 | 285.1193 | GGMSV | 356.1928 | 384.1878 |
| GGMSVD | 471.2198 | 499.2147 | GGMSVDI | 584.3039 | 612.2988 | GGMSVDID | 699.3308 | 727.3257 |
| GM | 113.0709 | 141.0659 | GMS | 200.1030 | 228.0979 | GMSV | 299.1714 | 327.1663 |
| GMSVD | 414.1983 | 442.1932 | GMSVDI | 527.2824 | 555.2773 | GMSVDID | 642.3093 | 670.3042 |
| MS | 143.0815 | 171.0764 | MSV | 242.1499 | 270.1448 | MSVD | 357.1769 | 385.1718 |
| MSVDI | 470.2609 | 498.2558 | MSVDID | 585.2879 | 613.2828 | MSVDIDA | 656.3250 | 684.3199 |
| SV | 159.1128 | 187.1077 | SVD | 274.1397 | 302.1347 | SVDI | 387.2238 | 415.2187 |
| SVDID | 502.2508 | 530.2457 | SVDIDA | 573.2879 | 601.2828 | SVDIDAS | 660.3199 | 688.3148 |
| VD | 187.1077 | 215.1026 | VDI | 300.1918 | 328.1867 | VDID | 415.2187 | 443.2136 |
| VDIDA | 486.2558 | 514.2508 | VDIDAS | 573.2879 | 601.2828 | VDIDASG | 630.3093 | 658.3042 |
| DI | 201.1234 | 229.1183 | DID | 316.1503 | 344.1452 | DIDA | 387.1874 | 415.1823 |
| DIDAS | 474.2195 | 502.2144 | DIDASG | 531.2409 | 559.2358 | DIDASGR | 687.3420 | 715.3369 |
| ID | 201.1234 | 229.1183 | IDA | 272.1605 | 300.1554 | IDAS | 359.1925 | 387.1874 |
| IDASG | 416.2140 | 444.2089 | IDASGR | 572.3151 | 600.3100 | DA | 159.0764 | 187.0713 |
| DAS | 246.1084 | 274.1034 | DASG | 303.1299 | 331.1248 | DASGR | 459.2310 | 487.2259 |
| AS | 131.0815 | 159.0764 | ASG | 188.1030 | 216.0979 | ASGR | 344.2041 | 372.1990 |
| SG | 117.0659 | 145.0608 | SGR | 273.1670 | 301.1619 | GR | 186.1349 | 214.1299 |

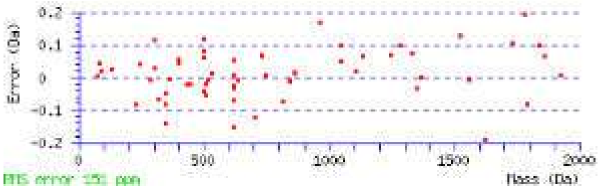


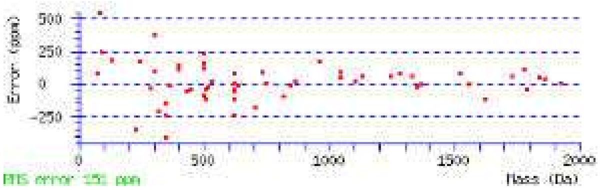


NCBI **BLAST** search of TDKPYGIGGGMSVDIDASGRK
(Parameters: blastp, nr protein database, expect=20000, no filter, PAM30)
Other BLAST web gateways

**All matches to this query**

| Score | Mr(calc) | Delta | Sequence |
|---|---|---|---|
| 44.3 | 2139.0266 | -0.0193 | TDKPYGIGGGMSVDIDASGRK |
| 11.5 | 2138.9976 | 0.0097 | AFGDALDSIPMMLAENSGLR |
| 9.4 | 2139.0354 | -0.0280 | DVNAWSTMITGFARNGMLR |
| 7.7 | 2138.9725 | 0.0348 | TTDPDDIMLNFARGECIR |
| 7.5 | 2139.0444 | -0.0370 | GVLEVNPDALAEADKADQER |
| 7.2 | 2138.9392 | 0.0681 | SSEVDVEYPSWLSENDQR |
| 6.9 | 2139.0300 | -0.0227 | NAMSTTVNDVMVGITSAGLSR |

| | | | |
|---|---|---|---|
| 6.8 | 2139.0241 | -0.0168 | SRPESMAALMEGFWIGIGR |
| 6.7 | 2138.9539 | 0.0534 | TDTPYGTGGGMALPDGKDASGR |
| 6.6 | 2139.0630 | -0.0557 | MASKAATPSSPSFILTSGASGR |

**Mascot:** http://www.matrixscience.com/

# MATRIX SCIENCE Mascot Search Results

## Peptide View Spot no 2

MS/MS Fragmentation of **IKTDKPYGIGGGMSVDIDASGR**
Found in **gi|1835731** in **NCBInr**, photosystem II 10 kDa polypeptide [Oryza sativa Indica Group]

Match to Query 185: 2236.110624 from(2237.117900,1+) intensity(0.0000) index(31)
Title: Label: K5, Spot_Id: 219748, Peak_List_Id: 225862, MSMS Job_Run_Id: 21792, Comment:
Data file C:\Documents and Settings\Administrator\Desktop\farah BHU\spot set plate\Farah-Garima 11May 2013\ppw_K5_136842223800.txt

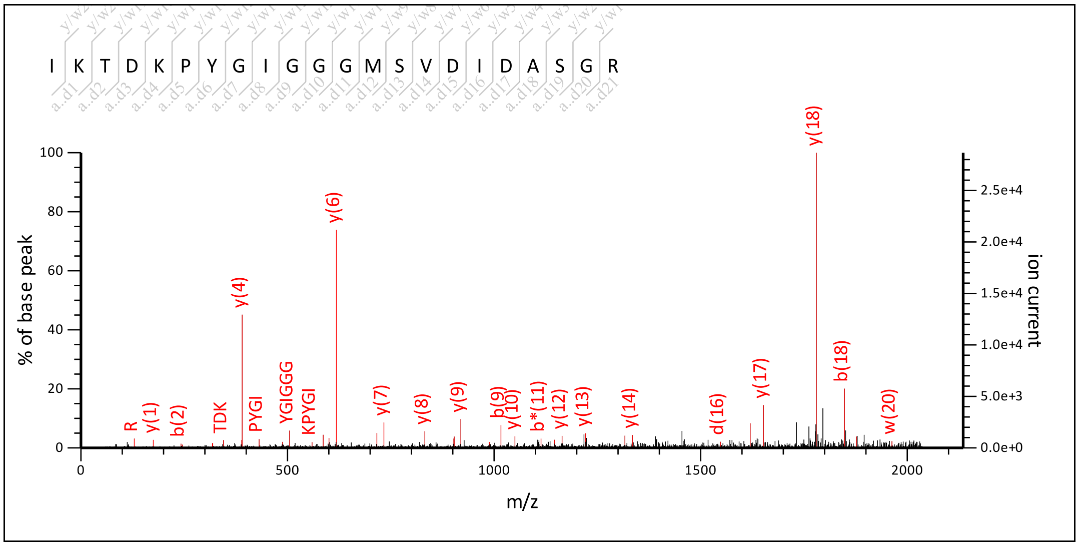


0 to 2135.37

Label all possible matches ○ Label matches used for scoring ◉

**Monoisotopic mass of neutral peptide Mr(calc):** 2236.1158
**Fixed modifications:** Carbamidomethyl (C) (apply to specified residues or termini only)
**Ions Score:** 92 **Expect:** 7.7e-07
**Matches :** 45/445 fragment ions using 60 most intense peaks (help)

| # | Immon. | a | a* | $a^0$ | b | b* | $b^0$ | d | d' | Seq. | v | w | w' | y | y* | |
|---|---|---|---|---|---|---|---|---|---|---|---|---|---|---|---|---|
| 1 | 86.0964 | 86.0964 | | | 114.0913 | | | 44.0495 | | I | | | | | | |
| 2 | 101.1073 | 214.1914 | 197.1648 | | **242.1863** | 225.1598 | | 157.1335 | | K | 2050.9498 | 2049.9546 | | 2124.0390 | 2107.0124 | [illegible] |
| 3 | 74.0600 | 315.2391 | 298.2125 | 297.2285 | 343.2340 | 326.2074 | 325.2234 | 299.2442 | 301.2234 | T | 1949.9022 | 1962.9226 | 1964.9018 | 1995.9440 | 1978.9175 | [illegible] |
| 4 | 88.0393 | 430.2660 | 413.2395 | 412.2554 | 458.2609 | 441.2344 | 440.2504 | 386.2762 | | D | 1834.8752 | 1833.8800 | | 1894.8964 | 1877.8698 | [illegible] |
| 5 | 101.1073 | 558.3610 | 541.3344 | 540.3504 | **586.3559** | 569.3293 | 568.3453 | 501.3031 | | K | 1706.7803 | 1705.7850 | | ***1779.8694*** | 1762.8429 | [illegible] |
| 6 | 70.0651 | 655.4137 | 638.3872 | 637.4032 | 683.4087 | 666.3821 | 665.3981 | 629.3981 | | P | 1609.7275 | 1608.7322 | | ***1651.7744*** | 1634.7479 | [illegible] |
| 7 | 136.0757 | 818.4771 | 801.4505 | 800.4665 | 846.4720 | 829.4454 | 828.4614 | | | Y | 1446.6642 | | | 1554.7217 | 1537.6951 | [illegible] |
| 8 | 30.0338 | 875.4985 | 858.4720 | 857.4880 | **903.4934** | 886.4669 | 885.4829 | | | G | | | | 1391.6584 | 1374.6318 | [illegible] |
| 9 | 86.0964 | 988.5826 | 971.5560 | 970.5720 | **1016.5775** | 999.5510 | 998.5669 | 960.5513 | 974.5669 | I | 1276.5586 | 1289.5790 | 1303.5947 | ***1334.6369*** | 1317.6103 | [illegible] |
| 10 | 30.0338 | 1045.6041 | 1028.5775 | 1027.5935 | 1073.5990 | 1056.5724 | 1055.5884 | | | G | | | | ***1221.5528*** | 1204.5263 | [illegible] |
| 11 | 30.0338 | 1102.6255 | 1085.5990 | 1084.6150 | 1130.6204 | 1113.5939 | 1112.6099 | | | G | | | | ***1164.5314*** | 1147.5048 | [illegible] |
| 12 | 30.0338 | 1159.6470 | 1142.6204 | 1141.6364 | 1187.6419 | 1170.6154 | 1169.6313 | | | G | | | | 1107.5099 | 1090.4834 | [illegible] |
| 13 | 104.0528 | 1290.6875 | 1273.6609 | 1272.6769 | 1318.6824 | 1301.6558 | 1300.6718 | 1230.6841 | | M | 974.4538 | 973.4585 | | ***1050.4884*** | 1033.4619 | [illegible] |
| 14 | 60.0444 | 1377.7195 | 1360.6930 | 1359.7089 | 1405.7144 | 1388.6879 | 1387.7038 | 1361.7246 | | S | 887.4217 | 886.4265 | | ***919.4480*** | 902.4214 | [illegible] |
| 15 | 72.0808 | 1476.7879 | 1459.7614 | 1458.7773 | 1504.7828 | 1487.7563 | 1486.7723 | 1462.7723 | | V | 788.3533 | 801.3737 | | ***832.4159*** | 815.3894 | [illegible] |
| 16 | 88.0393 | 1591.8149 | 1574.7883 | 1573.8043 | **1619.8098** | 1602.7832 | 1601.7992 | 1547.8250 | | D | 673.3264 | 672.3311 | | ***733.3475*** | 716.3210 | [illegible] |
| 17 | 86.0964 | 1704.8989 | 1687.8724 | 1686.8884 | 1732.8938 | 1715.8673 | 1714.8833 | 1676.8676 | 1690.8833 | I | 560.2423 | 573.2627 | 587.2784 | ***618.3206*** | 601.2940 | [illegible] |
| 18 | 88.0393 | 1819.9259 | 1802.8993 | 1801.9153 | **1847.9208** | 1830.8942 | 1829.9102 | 1775.9360 | | D | 445.2154 | 444.2201 | | ***505.2365*** | 488.2100 | [illegible] |
| 19 | 44.0495 | 1890.9630 | 1873.9364 | 1872.9524 | 1918.9579 | 1901.9313 | 1900.9473 | | | A | 374.1783 | | | ***390.2096*** | 373.1830 | [illegible] |
| 20 | 60.0444 | 1977.9950 | 1960.9685 | 1959.9844 | 2005.9899 | 1988.9634 | 1987.9794 | 1962.0001 | | S | 287.1462 | 286.1510 | | ***319.1724*** | 302.1459 | [illegible] |
| 21 | 30.0338 | 2035.0165 | 2017.9899 | 2017.0059 | 2063.0114 | 2045.9848 | 2045.0008 | | | G | | | | 232.1404 | 215.1139 | |
| 22 | 129.1135 | | | | | | | | | R | 74.0237 | 73.0284 | | ***175.1190*** | 158.0924 | |

| Seq | ya | yb | Seq | ya | yb | Seq | ya | yb |
|---|---|---|---|---|---|---|---|---|
| KT | 202.1550 | 230.1499 | KTD | 317.1819 | 345.1769 | KTDK | 445.2769 | 473.2718 |
| KTDKP | 542.3297 | 570.3246 | TD | 189.0870 | 217.0819 | TDK | 317.1819 | 345.1769 |
| TDKP | 414.2347 | 442.2296 | TDKPY | 577.2980 | 605.2930 | TDKPYG | 634.3195 | 662.3144 |
| DK | 216.1343 | 244.1292 | DKP | 313.1870 | 341.1819 | DKPY | 476.2504 | 504.2453 |
| DKPYG | 533.2718 | 561.2667 | DKPYGI | 646.3559 | 674.3508 | KP | 198.1601 | 226.1550 |
| KPY | 361.2234 | 389.2183 | KPYG | 418.2449 | 446.2398 | KPYGI | 531.3289 | 559.3239 |
| KPYGIG | 588.3504 | 616.3453 | KPYGIGG | 645.3719 | 673.3668 | PY | 233.1285 | 261.1234 |
| PYG | 290.1499 | 318.1448 | PYGI | 403.2340 | 431.2289 | PYGIG | 460.2554 | 488.2504 |
| PYGIGG | 517.2769 | 545.2718 | PYGIGGG | 574.2984 | 602.2933 | YG | 193.0972 | 221.0921 |
| YGI | 306.1812 | 334.1761 | YGIG | 363.2027 | 391.1976 | YGIGG | 420.2241 | 448.2191 |
| YGIGGG | 477.2456 | 505.2405 | YGIGGGM | 608.2861 | 636.2810 | YGIGGGMS | 695.3181 | 723.3130 |
| GI | 143.1179 | 171.1128 | GIG | 200.1394 | 228.1343 | GIGG | 257.1608 | 285.1557 |
| GIGGG | 314.1823 | 342.1772 | GIGGGM | 445.2228 | 473.2177 | GIGGGMS | 532.2548 | 560.2497 |
| GIGGGMSV | 631.3232 | 659.3181 | IG | 143.1179 | 171.1128 | IGG | 200.1394 | 228.1343 |
| IGGG | 257.1608 | 285.1557 | IGGGM | 388.2013 | 416.1962 | IGGGMS | 475.2333 | 503.2282 |
| IGGGMSV | 574.3017 | 602.2967 | IGGGMSVD | 689.3287 | 717.3236 | GG | 87.0553 | 115.0502 |
| GGG | 144.0768 | 172.0717 | GGGM | 275.1172 | 303.1122 | GGGMS | 362.1493 | 390.1442 |
| GGGMSV | 461.2177 | 489.2126 | GGGMSVD | 576.2446 | 604.2395 | GGGMSVDI | 689.3287 | 717.3236 |
| GG | 87.0553 | 115.0502 | GGM | 218.0958 | 246.0907 | GGMS | 305.1278 | 333.1227 |
| GGMSV | 404.1962 | 432.1911 | GGMSVD | 519.2232 | 547.2181 | GGMSVDI | 632.3072 | 660.3021 |
| GM | 161.0743 | 189.0692 | GMS | 248.1063 | 276.1013 | GMSV | 347.1748 | 375.1697 |
| GMSVD | 462.2017 | 490.1966 | GMSVDI | 575.2858 | 603.2807 | GMSVDID | 690.3127 | 718.3076 |
| MS | 191.0849 | 219.0798 | MSV | 290.1533 | 318.1482 | MSVD | 405.1802 | 433.1751 |
| MSVDI | 518.2643 | 546.2592 | MSVDID | 633.2912 | 661.2862 | SV | 159.1128 | 187.1077 |
| SVD | 274.1397 | 302.1347 | SVDI | 387.2238 | 415.2187 | SVDID | 502.2508 | 530.2457 |
| SVDIDA | 573.2879 | 601.2828 | SVDIDAS | 660.3199 | 688.3148 | VD | 187.1077 | 215.1026 |
| VDI | 300.1918 | 328.1867 | VDID | 415.2187 | 443.2136 | VDIDA | 486.2558 | 514.2508 |
| VDIDAS | 573.2879 | 601.2828 | VDIDASG | 630.3093 | 658.3042 | DI | 201.1234 | 229.1183 |
| DID | 316.1503 | 344.1452 | DIDA | 387.1874 | 415.1823 | DIDAS | 474.2195 | 502.2144 |
| DIDASG | 531.2409 | 559.2358 | ID | 201.1234 | 229.1183 | IDA | 272.1605 | 300.1554 |
| IDAS | 359.1925 | 387.1874 | IDASG | 416.2140 | 444.2089 | DA | 159.0764 | 187.0713 |
| DAS | 246.1084 | 274.1034 | DASG | 303.1299 | 331.1248 | AS | 131.0815 | 159.0764 |
| ASG | 188.1030 | 216.0979 | SG | 117.0659 | 145.0608 | | | |

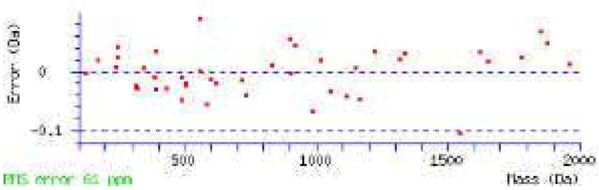


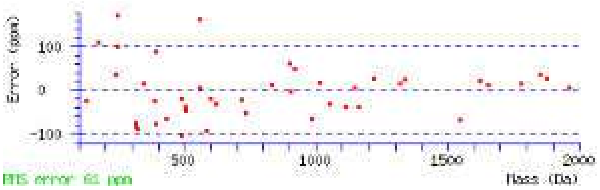


NCBI **BLAST** search of IKTDKPYGIGGGMSVDIDASGR
(Parameters: blastp, nr protein database, expect=20000, no filter, PAM30)
Other BLAST web gateways

**All matches to this query**

| Score | Mr(calc) | Delta | Sequence |
|---|---|---|---|
| 92.2 | 2236.1158 | -0.0052 | IKTDKPYGIGGGMSVDIDASGR |
| 13.5 | 2236.0212 | 0.0894 | DDLLESGMADAMSVSANQRAR |
| 13.3 | 2236.0179 | 0.0927 | SASPRFSGGGGSGSGSGSSMPPTPK |
| 11.9 | 2235.9855 | 0.1251 | FFGKGSSTQSGSMSPTSDWAR |
| 11.8 | 2236.0819 | 0.0287 | VTEEGSSSQSEDVKAISTNLR |
| 11.4 | 2236.0946 | 0.0160 | KEDPWNSIMAGAATGGFLSLR |
| 11.2 | 2236.0286 | 0.0820 | EMHMKGSASVEDGQMVSLLR |
| 10.9 | 2236.0980 | 0.0126 | VNFATITPRETNMDGCVALK |
| 10.7 | 2236.0356 | 0.0750 | IGGFRSAVDAAATTGEEQADDR |

| | | | |
|---|---|---|---|
| 9.8 | 2236.0286 | 0.0820 | EMHMKGSASVEDGQMVSLLR |

**Mascot:** http://www.matrixscience.com/

# MATRIX SCIENCE Mascot Search Results

## Peptide View Spot no 2

MS/MS Fragmentation of **IKTDKPYGIGGGMSVDIDASGR**
Found in **gi|1835731** in **NCBInr**, photosystem II 10 kDa polypeptide [Oryza sativa Indica Group]

Match to Query 187: 2252.092624 from(2253.099900,1+) intensity(0.0000) index(32)
Title: Label: K5, Spot_Id: 219748, Peak_List_Id: 225863, MSMS Job_Run_Id: 21792, Comment:
Data file C:\Documents and Settings\Administrator\Desktop\farah BHU\spot set plate\Farah-Garima 11May 2013\ppw_K5_136842223800.txt

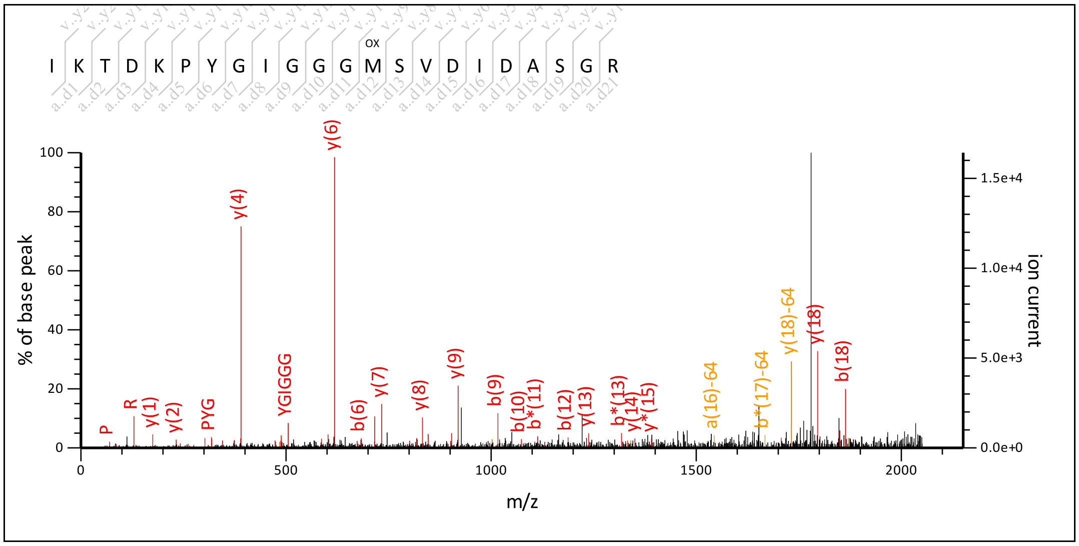


0 to 2150.76

Label all possible matches ○ Label matches used for scoring ◉

**Monoisotopic mass of neutral peptide Mr(calc):** 2252.1107
**Fixed modifications:** Carbamidomethyl (C) (apply to specified residues or termini only)
**Variable modifications:**
**M13** : Oxidation (M), with neutral losses 0.0000(shown in table), 63.9983
**Ions Score:** 68 **Expect:** 0.0002
**Matches** : 75/606 fragment ions using 140 most intense peaks (help)

| # | Immon. | a | a* | a⁰ | b | b* | b⁰ | d | d' | Seq. | v | w | w' | y | y* | |
|---|---|---|---|---|---|---|---|---|---|---|---|---|---|---|---|---|
| 1 | 86.0964 | 86.0964 | | | ***114.0913*** | | | 44.0495 | | I | | | | | | |
| 2 | 101.1073 | 214.1914 | 197.1648 | | ***242.1863*** | 225.1598 | | 157.1335 | | K | 2066.9448 | 2065.9495 | | 2140.0339 | 2123.0074 | [illegible] |
| 3 | 74.0600 | 315.2391 | 298.2125 | 297.2285 | 343.2340 | 326.2074 | 325.2234 | 299.2442 | 301.2234 | T | 1965.8971 | 1978.9175 | 1980.8967 | 2011.9389 | 1994.9124 | [illegible] |
| 4 | 88.0393 | 430.2660 | 413.2395 | 412.2554 | 458.2609 | 441.2344 | 440.2504 | 386.2762 | | D | 1850.8701 | 1849.8749 | | 1910.8913 | 1893.8647 | [illegible] |
| 5 | 101.1073 | 558.3610 | 541.3344 | 540.3504 | ***586.3559*** | 569.3293 | 568.3453 | 501.3031 | | K | 1722.7752 | 1721.7799 | | ***1795.8643*** | 1778.8378 | [illegible] |
| 6 | 70.0651 | 655.4137 | 638.3872 | 637.4032 | ***683.4087*** | 666.3821 | 665.3981 | 629.3981 | | P | 1625.7224 | 1624.7272 | | ***1667.7694*** | 1650.7428 | [illegible] |
| 7 | 136.0757 | 818.4771 | 801.4505 | 800.4665 | ***846.4720*** | 829.4454 | 828.4614 | | | Y | 1462.6591 | | | 1570.7166 | 1553.6901 | [illegible] |
| 8 | 30.0338 | 875.4985 | 858.4720 | 857.4880 | ***903.4934*** | 886.4669 | 885.4829 | | | G | | | | 1407.6533 | 1390.6267 | [illegible] |
| 9 | 86.0964 | 988.5826 | 971.5560 | 970.5720 | ***1016.5775*** | 999.5510 | 998.5669 | 960.5513 | 974.5669 | I | 1292.5536 | 1305.5740 | 1319.5896 | ***1350.6318*** | 1333.6053 | [illegible] |
| 10 | 30.0338 | 1045.6041 | 1028.5775 | 1027.5935 | ***1073.5990*** | 1056.5724 | 1055.5884 | | | G | | | | ***1237.5477*** | 1220.5212 | [illegible] |
| 11 | 30.0338 | 1102.6255 | 1085.5990 | 1084.6150 | ***1130.6204*** | 1113.5939 | 1112.6099 | | | G | | | | 1180.5263 | 1163.4997 | [illegible] |
| 12 | 30.0338 | 1159.6470 | 1142.6204 | 1141.6364 | ***1187.6419*** | 1170.6154 | 1169.6313 | | | G | | | | 1123.5048 | 1106.4783 | [illegible] |
| 13 | 120.0478 | 1306.6824 | 1289.6558 | 1288.6718 | 1334.6773 | 1317.6508 | 1316.6667 | 1230.6841 | | M | 974.4538 | 973.4585 | | 1066.4834 | 1049.4568 | [illegible] |
| 14 | 60.0444 | 1393.7144 | 1376.6879 | 1375.7038 | 1421.7093 | 1404.6828 | 1403.6988 | 1377.7195 | | S | 887.4217 | 886.4265 | | ***919.4480*** | 902.4214 | [illegible] |
| 15 | 72.0808 | 1492.7828 | 1475.7563 | 1474.7723 | 1520.7777 | 1503.7512 | 1502.7672 | 1478.7672 | | V | 788.3533 | 801.3737 | | ***832.4159*** | 815.3894 | [illegible] |
| 16 | 88.0393 | 1607.8098 | 1590.7832 | 1589.7992 | 1635.8047 | 1618.7781 | 1617.7941 | 1563.8199 | | D | 673.3264 | 672.3311 | | ***733.3475*** | 716.3210 | [illegible] |
| 17 | 86.0964 | 1720.8938 | 1703.8673 | 1702.8833 | 1748.8888 | 1731.8622 | 1730.8782 | 1692.8625 | 1706.8782 | I | 560.2423 | 573.2627 | 587.2784 | ***618.3206*** | 601.2940 | [illegible] |
| 18 | 88.0393 | 1835.9208 | 1818.8942 | 1817.9102 | ***1863.9157*** | 1846.8891 | 1845.9051 | 1791.9309 | | D | 445.2154 | 444.2201 | | ***505.2365*** | 488.2100 | [illegible] |
| 19 | 44.0495 | 1906.9579 | 1889.9313 | 1888.9473 | 1934.9528 | 1917.9263 | 1916.9422 | | | A | 374.1783 | | | ***390.2096*** | 373.1830 | [illegible] |
| 20 | 60.0444 | 1993.9899 | 1976.9634 | 1975.9794 | 2021.9848 | 2004.9583 | 2003.9743 | 1977.9950 | | S | 287.1462 | 286.1510 | | ***319.1724*** | 302.1459 | [illegible] |

| | | | | | | | | | | | | | | | | |
|---|---|---|---|---|---|---|---|---|---|---|---|---|---|---|---|---|
| 21 | 30.0338 | 2051.0114 | 2033.9848 | 2033.0008 | 2079.0063 | 2061.9797 | 2060.9957 | | | G | | | | ***232.1404*** | 215.1139 | |
| 22 | 129.1135 | | | | | | | | | R | 74.0237 | 73.0284 | | ***175.1190*** | 158.0924 | |

| Seq | ya | yb | Seq | ya | yb | Seq | ya | yb |
|---|---|---|---|---|---|---|---|---|
| **KT** | 202.1550 | 230.1499 | **KTD** | 317.1819 | 345.1769 | **KTDK** | 445.2769 | 473.2718 |
| **KTDKP** | 542.3297 | 570.3246 | **TD** | 189.0870 | 217.0819 | **TDK** | 317.1819 | 345.1769 |
| **TDKP** | 414.2347 | 442.2296 | **TDKPY** | 577.2980 | 605.2930 | **TDKPYG** | 634.3195 | 662.3144 |
| **DK** | 216.1343 | 244.1292 | **DKP** | 313.1870 | 341.1819 | **DKPY** | 476.2504 | 504.2453 |
| **DKPYG** | 533.2718 | 561.2667 | **DKPYGI** | 646.3559 | 674.3508 | **KP** | 198.1601 | 226.1550 |
| **KPY** | 361.2234 | 389.2183 | **KPYG** | 418.2449 | 446.2398 | **KPYGI** | 531.3289 | 559.3239 |
| **KPYGIG** | 588.3504 | 616.3453 | **KPYGIGG** | 645.3719 | 673.3668 | **PY** | 233.1285 | 261.1234 |
| **PYG** | 290.1499 | 318.1448 | **PYGI** | 403.2340 | 431.2289 | **PYGIG** | 460.2554 | 488.2504 |
| **PYGIGG** | 517.2769 | 545.2718 | **PYGIGGG** | 574.2984 | 602.2933 | **YG** | 193.0972 | 221.0921 |
| **YGI** | 306.1812 | 334.1761 | **YGIG** | 363.2027 | 391.1976 | **YGIGG** | 420.2241 | 448.2191 |
| **YGIGGG** | 477.2456 | 505.2405 | **YGIGGGM** | 624.2810 | 652.2759 | **GI** | 143.1179 | 171.1128 |
| **GIG** | 200.1394 | 228.1343 | **GIGG** | 257.1608 | 285.1557 | **GIGGG** | 314.1823 | 342.1772 |
| **GIGGGM** | 461.2177 | 489.2126 | **GIGGGMS** | 548.2497 | 576.2446 | **GIGGGMSV** | 647.3181 | 675.3130 |
| **IG** | 143.1179 | 171.1128 | **IGG** | 200.1394 | 228.1343 | **IGGG** | 257.1608 | 285.1557 |
| **IGGGM** | 404.1962 | 432.1911 | **IGGGMS** | 491.2282 | 519.2232 | **IGGGMSV** | 590.2967 | 618.2916 |
| **GG** | 87.0553 | 115.0502 | **GGG** | 144.0768 | 172.0717 | **GGGM** | 291.1122 | 319.1071 |
| **GGGMS** | 378.1442 | 406.1391 | **GGGMSV** | 477.2126 | 505.2075 | **GGGMSVD** | 592.2395 | 620.2345 |
| **GG** | 87.0553 | 115.0502 | **GGM** | 234.0907 | 262.0856 | **GGMS** | 321.1227 | 349.1176 |
| **GGMSV** | 420.1911 | 448.1860 | **GGMSVD** | 535.2181 | 563.2130 | **GGMSVDI** | 648.3021 | 676.2971 |
| **GM** | 177.0692 | 205.0641 | **GMS** | 264.1013 | 292.0962 | **GMSV** | 363.1697 | 391.1646 |
| **GMSVD** | 478.1966 | 506.1915 | **GMSVDI** | 591.2807 | 619.2756 | **MS** | 207.0798 | 235.0747 |
| **MSV** | 306.1482 | 334.1431 | **MSVD** | 421.1751 | 449.1701 | **MSVDI** | 534.2592 | 562.2541 |
| **MSVDID** | 649.2862 | 677.2811 | **SV** | 159.1128 | 187.1077 | **SVD** | 274.1397 | 302.1347 |
| **SVDI** | 387.2238 | 415.2187 | **SVDID** | 502.2508 | 530.2457 | **SVDIDA** | 573.2879 | 601.2828 |
| **SVDIDAS** | 660.3199 | 688.3148 | **VD** | 187.1077 | 215.1026 | **VDI** | 300.1918 | 328.1867 |
| **VDID** | 415.2187 | 443.2136 | **VDIDA** | 486.2558 | 514.2508 | **VDIDAS** | 573.2879 | 601.2828 |
| **VDIDASG** | 630.3093 | 658.3042 | **DI** | 201.1234 | 229.1183 | **DID** | 316.1503 | 344.1452 |
| **DIDA** | 387.1874 | 415.1823 | **DIDAS** | 474.2195 | 502.2144 | **DIDASG** | 531.2409 | 559.2358 |
| **ID** | 201.1234 | 229.1183 | **IDA** | 272.1605 | 300.1554 | **IDAS** | 359.1925 | 387.1874 |
| **IDASG** | 416.2140 | 444.2089 | **DA** | 159.0764 | 187.0713 | **DAS** | 246.1084 | 274.1034 |
| **DASG** | 303.1299 | 331.1248 | **AS** | 131.0815 | 159.0764 | **ASG** | 188.1030 | 216.0979 |
| **SG** | 117.0659 | 145.0608 | | | | | | |

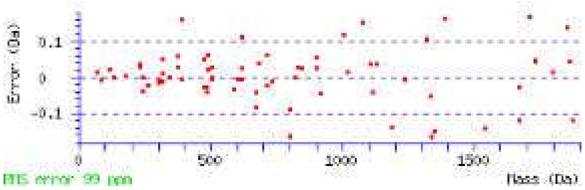


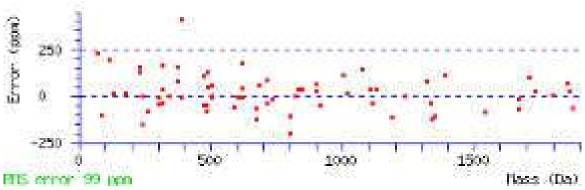


NCBI **BLAST** search of IKTDKPYGIGGGMSVDIDASGR
(Parameters: blastp, nr protein database, expect=20000, no filter, PAM30)
Other BLAST web gateways

**All matches to this query**

| Score | Mr(calc) | Delta | Sequence |
|---|---|---|---|
| 68.0 | 2252.1107 | -0.0181 | IKTDKPYGIGGGMSVDIDASGR |
| 14.4 | 2252.1140 | -0.0214 | QSSSLVETAIMSTMNNISIAR |
| 11.4 | 2251.8958 | 0.1969 | MGEADDARDAVEEMDVDGGDR |
| 10.8 | 2252.1073 | -0.0147 | TVVAGQNEFEVFKADLENSR |
| 10.6 | 2252.1037 | -0.0110 | KCSIIMELMDGDLLALMQR |
| 9.8 | 2252.1284 | -0.0358 | LEAHANDVLETLDTLLSENR |
| 9.2 | 2252.1702 | -0.0776 | NSFRPNFTAEGLYGGALLGLR |

| | | | |
|---|---|---|---|
| 8.9 | 2252.0610 | 0.0316 | TVSDRPFNDHRYFIDDQK |
| 8.7 | 2251.9751 | 0.1176 | MATQTLDSHQTSTTDEETEK |
| 8.5 | 2251.9677 | 0.1249 | DTQSTQDVADGTTTDVDNVDR |

**Mascot:** http://www.matrixscience.com/

# MATRIX SCIENCE Mascot Search Results

## Peptide View Spot no 4

MS/MS Fragmentation of **SPGYYDGR**
Found in **gi|671740** in **NCBInr**, ribulose-bisphosphate carboxylase [synthetic construct]

Match to Query 7: 913.384024 from(914.391300,1+) intensity(0.0000) index(1)
Title: Label: J5, Spot_Id: 219747, Peak_List_Id: 225845, MSMS Job_Run_Id: 21791, Comment:
Data file C:\Documents and Settings\Administrator\Desktop\farah BHU\spot set plate\Farah-Garima 11May 2013\ppw_J5_136842222100.txt

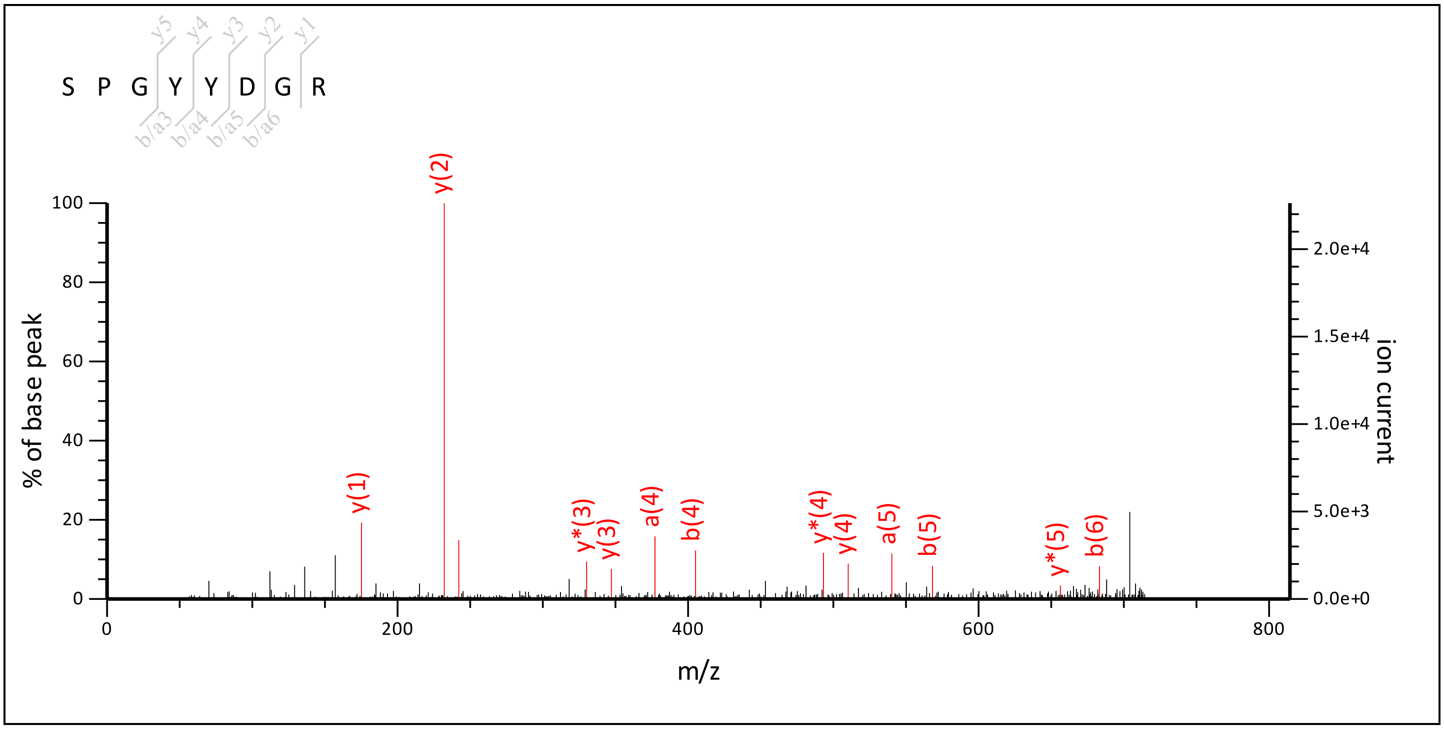


0 to 814.34

Label all possible matches ○ Label matches used for scoring ◉

**Monoisotopic mass of neutral peptide Mr(calc):** 913.3930
**Fixed modifications:** Carbamidomethyl (C) (apply to specified residues or termini only)
**Ions Score:** 27 **Expect:** 3.9
**Matches :** 13/28 fragment ions using 21 most intense peaks (help)

| # | a | b | Seq. | y | y* | # |
|---|---|---|---|---|---|---|
| 1 | 60.0444 | 88.0393 | S | | | 8 |
| 2 | 157.0972 | 185.0921 | P | 827.3682 | 810.3417 | 7 |
| 3 | 214.1186 | ***242.1135*** | G | 730.3155 | 713.2889 | 6 |
| 4 | **377.1819** | ***405.1769*** | Y | 673.2940 | 656.2675 | 5 |
| 5 | **540.2453** | ***568.2402*** | Y | ***510.2307*** | 493.2041 | 4 |
| 6 | 655.2722 | ***683.2671*** | D | ***347.1674*** | 330.1408 | 3 |
| 7 | 712.2937 | 740.2886 | G | ***232.1404*** | 215.1139 | 2 |
| 8 | | | R | ***175.1190*** | 158.0924 | 1 |

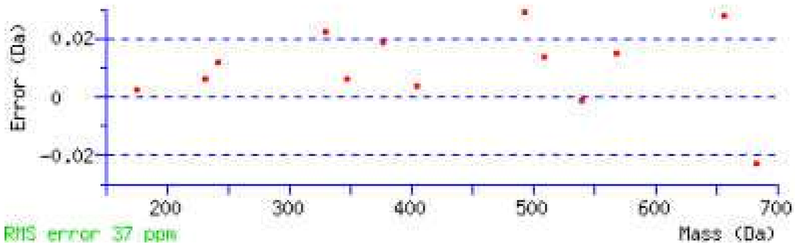


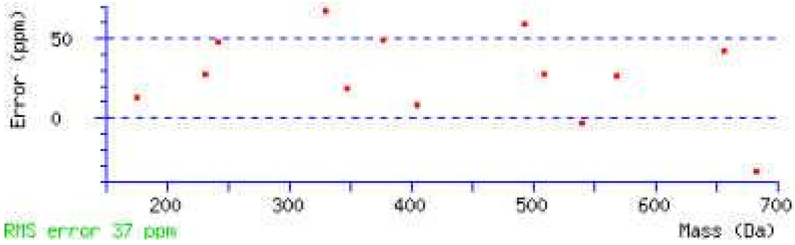


NCBI **BLAST** search of SPGYYDGR
(Parameters: blastp, nr protein database, expect=20000, no filter, PAM30)
Other BLAST web gateways

**All matches to this query**

| Score | Mr(calc) | Delta | Sequence |
|---|---|---|---|
| 27.0 | 913.3930 | -0.0090 | SPGYYDGR |
| 27.0 | 913.3930 | -0.0090 | SPGYYDGR |
| 24.2 | 913.4076 | -0.0236 | RMTYDGR |
| 22.1 | 913.3964 | -0.0123 | ASVMYDGR |
| 22.1 | 913.4294 | -0.0453 | FEKYDGR |
| 22.1 | 913.3786 | 0.0054 | MCLYDGR |
| 16.3 | 913.4042 | -0.0202 | GGSGYFNGR |
| 15.7 | 913.4657 | -0.0817 | QLYYATR |
| 15.2 | 913.3964 | -0.0123 | YMSLGDGR |
| 14.1 | 913.4254 | -0.0413 | QVPENDGR |

**Mascot:** http://www.matrixscience.com/

# Mascot Search Results

## Peptide View Spot no 4

MS/MS Fragmentation of **IIGFDNVR**
Found in **gi|671740** in **NCBInr**, ribulose-bisphosphate carboxylase [synthetic construct]

Match to Query 11: 932.502614 from(933.509890,1+) intensity(0.0000) index(2)
Title: Label: J5, Spot_Id: 219747, Peak_List_Id: 225844, MSMS Job_Run_Id: 21791, Comment:
Data file C:\Documents and Settings\Administrator\Desktop\farah BHU\spot set plate\Farah-Garima 11May 2013\ppw_J5_136842222100.txt

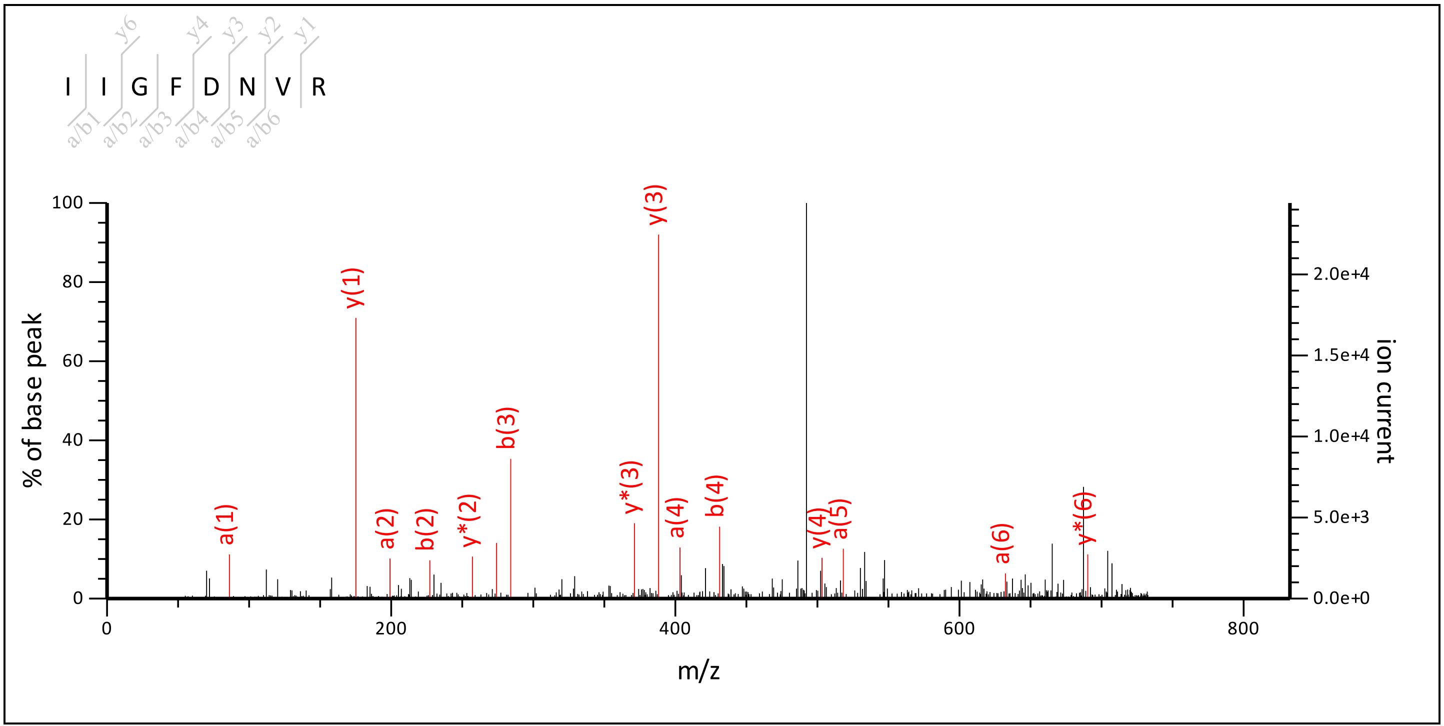


0 to 832.59

Label all possible matches ○ Label matches used for scoring ◉

**Monoisotopic mass of neutral peptide Mr(calc):** 932.5080
**Fixed modifications:** Carbamidomethyl (C) (apply to specified residues or termini only)
**Ions Score:** 28 **Expect:** 4.8
**Matches :** 15/32 fragment ions using 28 most intense peaks (help)

| # | a | a* | b | b* | Seq. | y | y* | # |
|---|---|---|---|---|---|---|---|---|
| 1 | ***86.0964*** | | 114.0913 | | I | | | 8 |
| 2 | ***199.1805*** | | **227.1754** | | I | 820.4312 | 803.4046 | 7 |
| 3 | 256.2020 | | **284.1969** | | G | 707.3471 | 690.3206 | 6 |
| 4 | ***403.2704*** | | **431.2653** | | F | 650.3256 | 633.2991 | 5 |
| 5 | ***518.2973*** | | 546.2922 | | D | ***503.2572*** | 486.2307 | 4 |
| 6 | ***632.3402*** | 615.3137 | 660.3352 | 643.3086 | N | ***388.2303*** | 371.2037 | 3 |
| 7 | 731.4087 | 714.3821 | 759.4036 | 742.3770 | V | ***274.1874*** | 257.1608 | 2 |
| 8 | | | | | R | ***175.1190*** | 158.0924 | 1 |

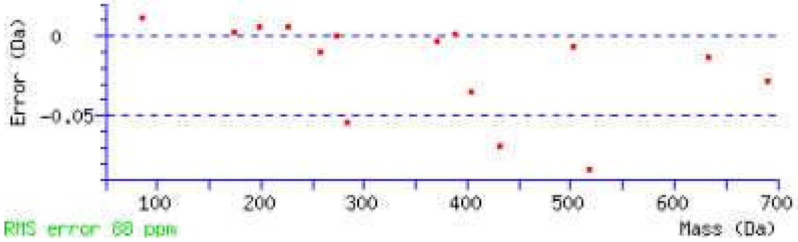


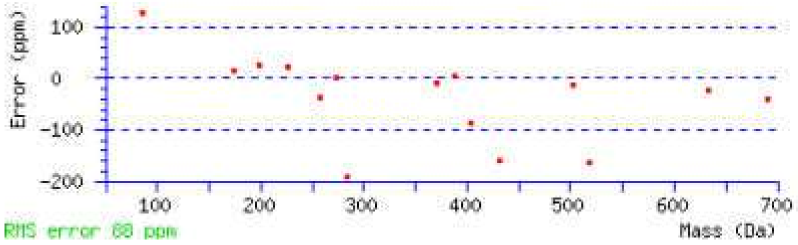


NCBI **BLAST** search of IIGFDNVR
(Parameters: blastp, nr protein database, expect=20000, no filter, PAM30)
Other BLAST web gateways

**All matches to this query**

| Score | Mr(calc) | Delta | Sequence |
|---|---|---|---|
| 27.8 | 932.5080 | -0.0054 | IIGFDNVR |
| 24.8 | 932.5728 | -0.0702 | LITAMKLK |
| 24.5 | 932.4352 | 0.0674 | DNADPFVR |
| 23.5 | 932.5113 | -0.0087 | KTAMVEVR |
| 22.3 | 932.5080 | -0.0054 | VFLADNVR |
| 21.9 | 932.4749 | 0.0277 | VMSIDNVR |
| 21.8 | 932.5331 | -0.0305 | LLGFDQIK |
| 21.1 | 932.4862 | 0.0165 | LERMNVR |
| 21.1 | 932.5080 | -0.0054 | LVAFDNVR |
| 20.9 | 932.4134 | 0.0892 | GDGAMNNVR |

**Mascot:** http://www.matrixscience.com/

# Mascot Search Results

## Peptide View **Spot no 4**

MS/MS Fragmentation of **AYPDAFVR**
Found in **gi|671740** in **NCBInr**, ribulose-bisphosphate carboxylase [synthetic construct]

Match to Query 13: 937.455374 from(938.462650,1+) intensity(0.0000) index(3)
Title: Label: J5, Spot_Id: 219747, Peak_List_Id: 225843, MSMS Job_Run_Id: 21791, Comment:
Data file C:\Documents and Settings\Administrator\Desktop\farah BHU\spot set plate\Farah-Garima 11May 2013\ppw_J5_136842222100.txt

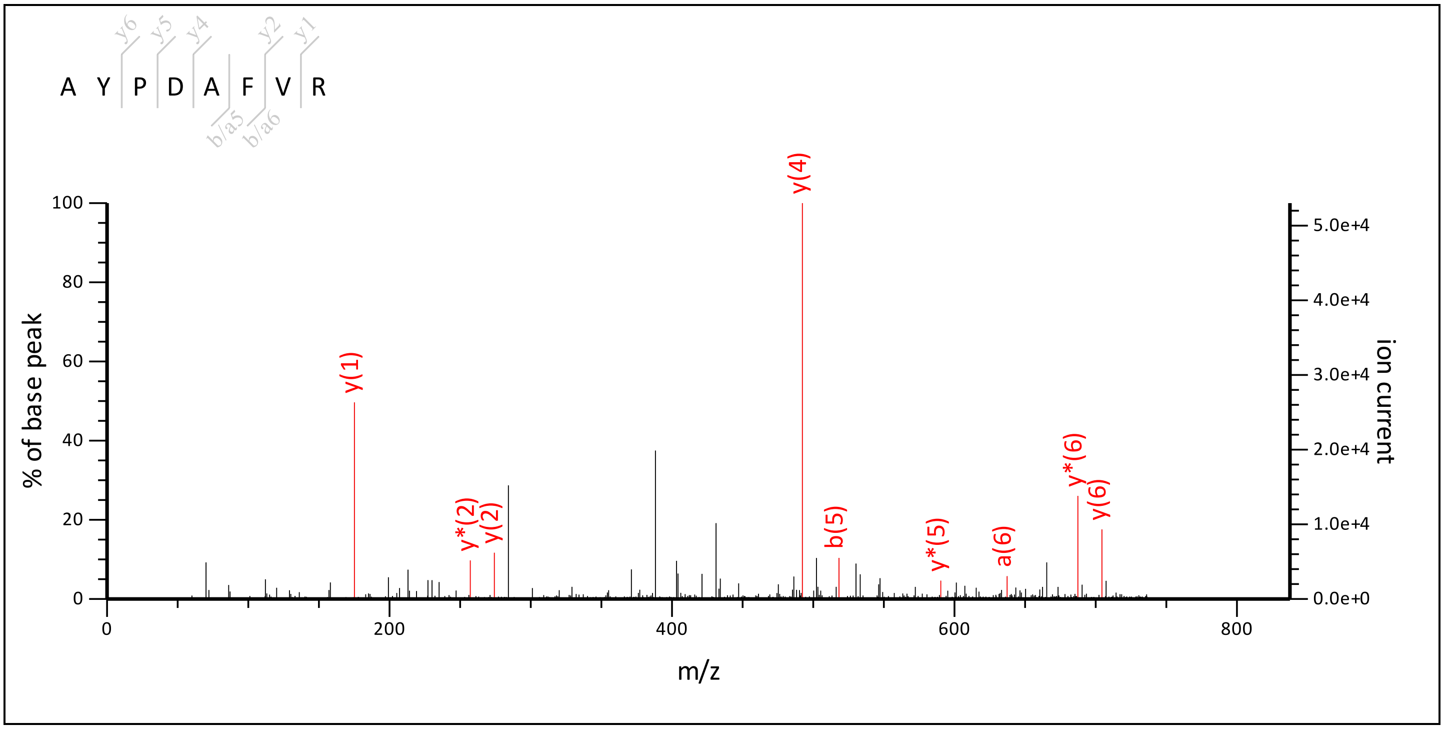


0 to 837.51

Label all possible matches ○ Label matches used for scoring ◉

**Monoisotopic mass of neutral peptide Mr(calc):** 937.4658
**Fixed modifications:** Carbamidomethyl (C) (apply to specified residues or termini only)
**Ions Score:** 25 **Expect:** 7
**Matches :** 9/28 fragment ions using 14 most intense peaks (help)

| # | a | b | Seq. | y | y* | # |
|---|---|---|---|---|---|---|
| 1 | 44.0495 | 72.0444 | A | | | 8 |
| 2 | 207.1128 | 235.1077 | Y | 867.4359 | 850.4094 | 7 |
| 3 | 304.1656 | 332.1605 | P | ***704.3726*** | 687.3461 | 6 |
| 4 | 419.1925 | 447.1874 | D | 607.3198 | 590.2933 | 5 |
| 5 | 490.2296 | **518.2245** | A | ***492.2929*** | 475.2663 | 4 |
| 6 | **637.2980** | 665.2930 | F | 421.2558 | 404.2292 | 3 |
| 7 | 736.3665 | 764.3614 | V | ***274.1874*** | 257.1608 | 2 |
| 8 | | | R | ***175.1190*** | 158.0924 | 1 |

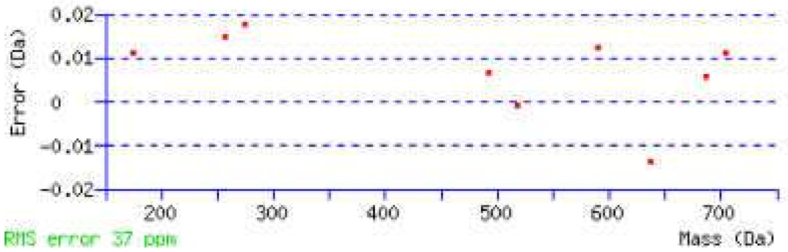


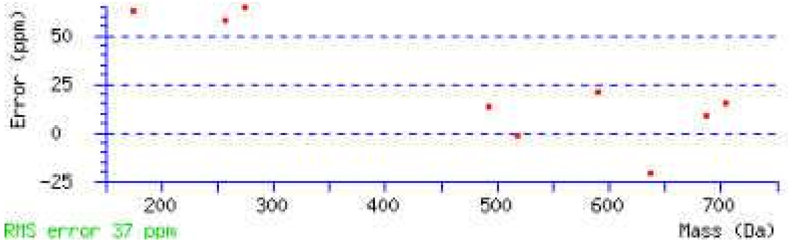


NCBI **BLAST** search of AYPDAFVR
(Parameters: blastp, nr protein database, expect=20000, no filter, PAM30)
Other BLAST web gateways

**All matches to this query**

| Score | Mr(calc) | Delta | Sequence |
|---|---|---|---|
| 24.9 | 937.4658 | -0.0104 | AYPDAFVR |
| 21.6 | 937.5055 | -0.0502 | FSSVLMVR |
| 18.6 | 937.4254 | 0.0300 | HPGEEDVR |
| 18.4 | 937.4552 | 0.0002 | FSNRCVR |
| 18.3 | 937.4658 | -0.0104 | SWYVEVR |
| 17.1 | 937.4691 | -0.0138 | AVPYSMVR |
| 16.9 | 937.4658 | -0.0104 | SFPADFVR |
| 16.7 | 937.5021 | -0.0468 | AYVPAYVR |
| 16.2 | 937.4691 | -0.0138 | EMAVFAVR |
| 15.4 | 937.5022 | -0.0468 | IFADAFVR |

**Mascot:** http://www.matrixscience.com/

# MATRIX SCIENCE Mascot Search Results

## Peptide View Spot no 4

MS/MS Fragmentation of **KAYPDAFVR**
Found in **gi|671740** in **NCBInr**, ribulose-bisphosphate carboxylase [synthetic construct]

Match to Query 27: 1065.567024 from(1066.574300,1+) intensity(0.0000) index(8)
Title: Label: J5, Spot_Id: 219747, Peak_List_Id: 225847, MSMS Job_Run_Id: 21791, Comment:
Data file C:\Documents and Settings\Administrator\Desktop\farah BHU\spot set plate\Farah-Garima 11May 2013\ppw_J5_136842222100.txt

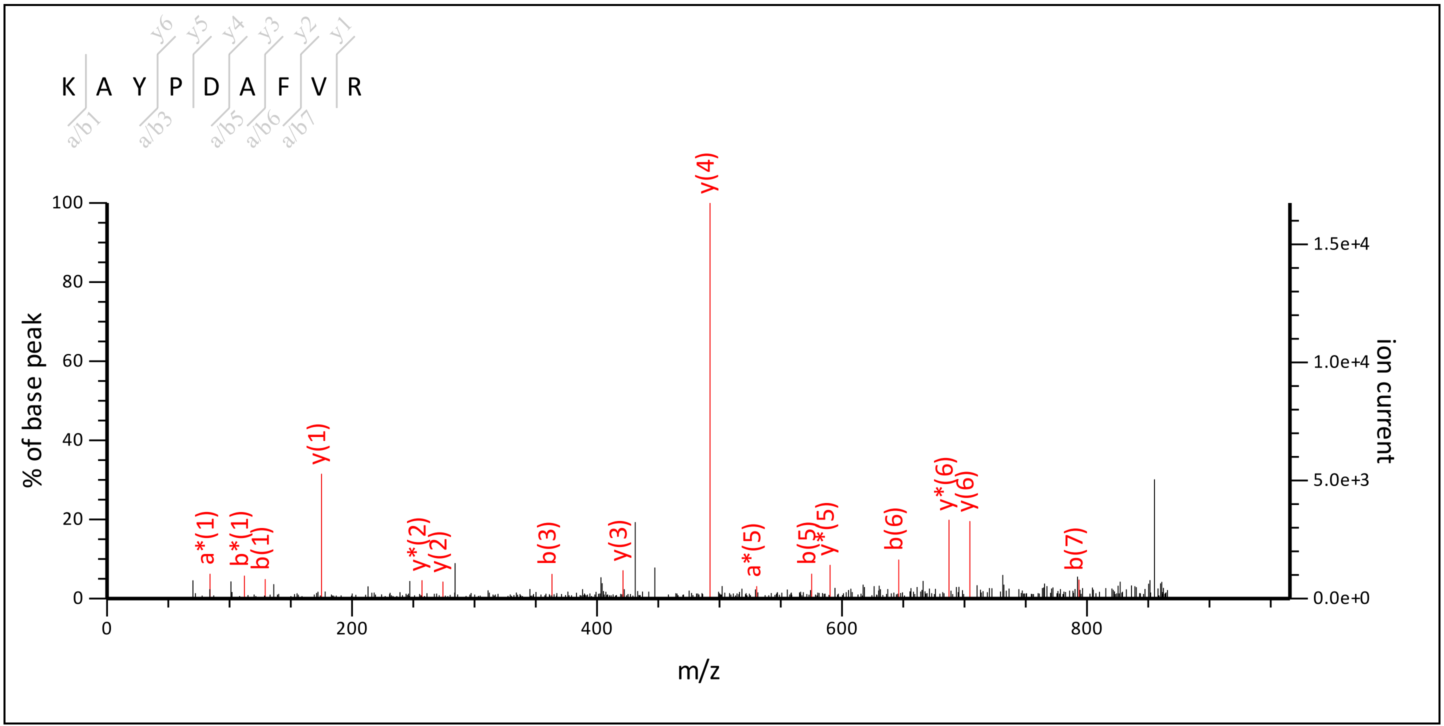


0 to 965.68

Label all possible matches ○ Label matches used for scoring ◉

**Monoisotopic mass of neutral peptide Mr(calc):** 1065.5607
**Fixed modifications:** Carbamidomethyl (C) (apply to specified residues or termini only)
**Ions Score:** 33 **Expect:** 1.2
**Matches :** 16/48 fragment ions using 24 most intense peaks (help)

| # | a | a* | b | b* | Seq. | y | y* | # |
|---|---|---|---|---|---|---|---|---|
| 1 | 101.1073 | 84.0808 | ***129.1022*** | 112.0757 | K | | | 9 |
| 2 | 172.1444 | 155.1179 | 200.1394 | 183.1128 | A | 938.4730 | 921.4465 | 8 |
| 3 | 335.2078 | 318.1812 | ***363.2027*** | 346.1761 | Y | 867.4359 | 850.4094 | 7 |
| 4 | 432.2605 | 415.2340 | 460.2554 | 443.2289 | P | ***704.3726*** | 687.3461 | 6 |
| 5 | 547.2875 | 530.2609 | ***575.2824*** | 558.2558 | D | 607.3198 | 590.2933 | 5 |
| 6 | 618.3246 | 601.2980 | ***646.3195*** | 629.2930 | A | ***492.2929*** | 475.2663 | 4 |
| 7 | 765.3930 | 748.3665 | ***793.3879*** | 776.3614 | F | ***421.2558*** | 404.2292 | 3 |
| 8 | 864.4614 | 847.4349 | 892.4563 | 875.4298 | V | ***274.1874*** | 257.1608 | 2 |

| | | | | | | | | |
|---|---|---|---|---|---|---|---|---|
| **9** | | | | | **R** | ***175.1190*** | 158.0924 | **1** |

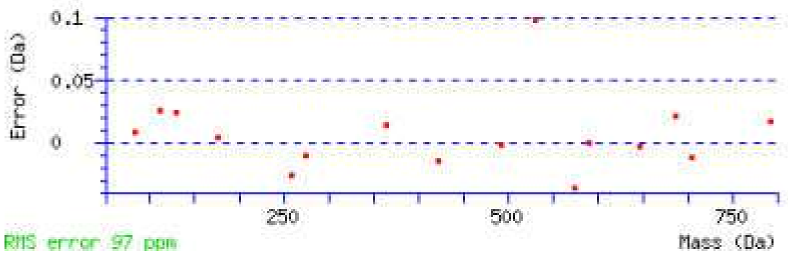


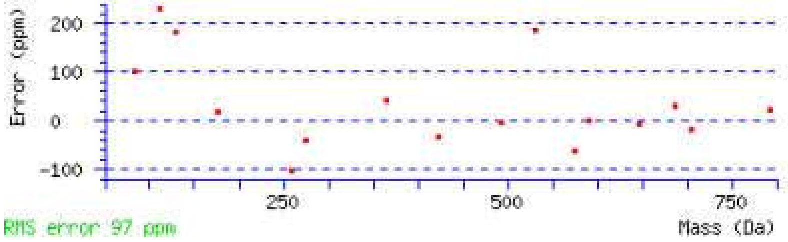


NCBI **BLAST** search of KAYPDAFVR
(Parameters: blastp, nr protein database, expect=20000, no filter, PAM30)
Other BLAST web gateways

**All matches to this query**

| Score | Mr(calc) | Delta | Sequence |
|---|---|---|---|
| 32.8 | 1065.5607 | 0.0063 | KAYPDAFVR |
| 20.8 | 1065.5091 | 0.0579 | KTDDDAFVR |
| 18.1 | 1065.5390 | 0.0281 | RVFDGMAVR |
| 16.4 | 1065.4695 | 0.0975 | MVRDGEMGR |
| 14.5 | 1065.5311 | 0.0360 | KMMTATEVR |
| 14.2 | 1065.5125 | 0.0546 | VSSMAKDGQK |
| 13.1 | 1065.5100 | 0.0571 | MICAFGPVR |
| 13.0 | 1065.5641 | 0.0029 | LKDAMAVFR |
| 12.8 | 1065.4938 | 0.0732 | TLSTDGESTR |
| 12.5 | 1065.5567 | 0.0103 | SQFNKTSVR |

**Mascot:** http://www.matrixscience.com/

# Mascot Search Results

## Peptide View Spot no 4

MS/MS Fragmentation of **MQVWPIEGIK**
Found in **gi|671740** in **NCBInr**, ribulose-bisphosphate carboxylase [synthetic construct]

Match to Query 42: 1215.620924 from(1216.628200,1+) intensity(0.0000) index(13)
Title: Label: J5, Spot_Id: 219747, Peak_List_Id: 225851, MSMS Job_Run_Id: 21791, Comment:
Data file C:\Documents and Settings\Administrator\Desktop\farah BHU\spot set plate\Farah-Garima 11May 2013\ppw_J5_136842222100.txt

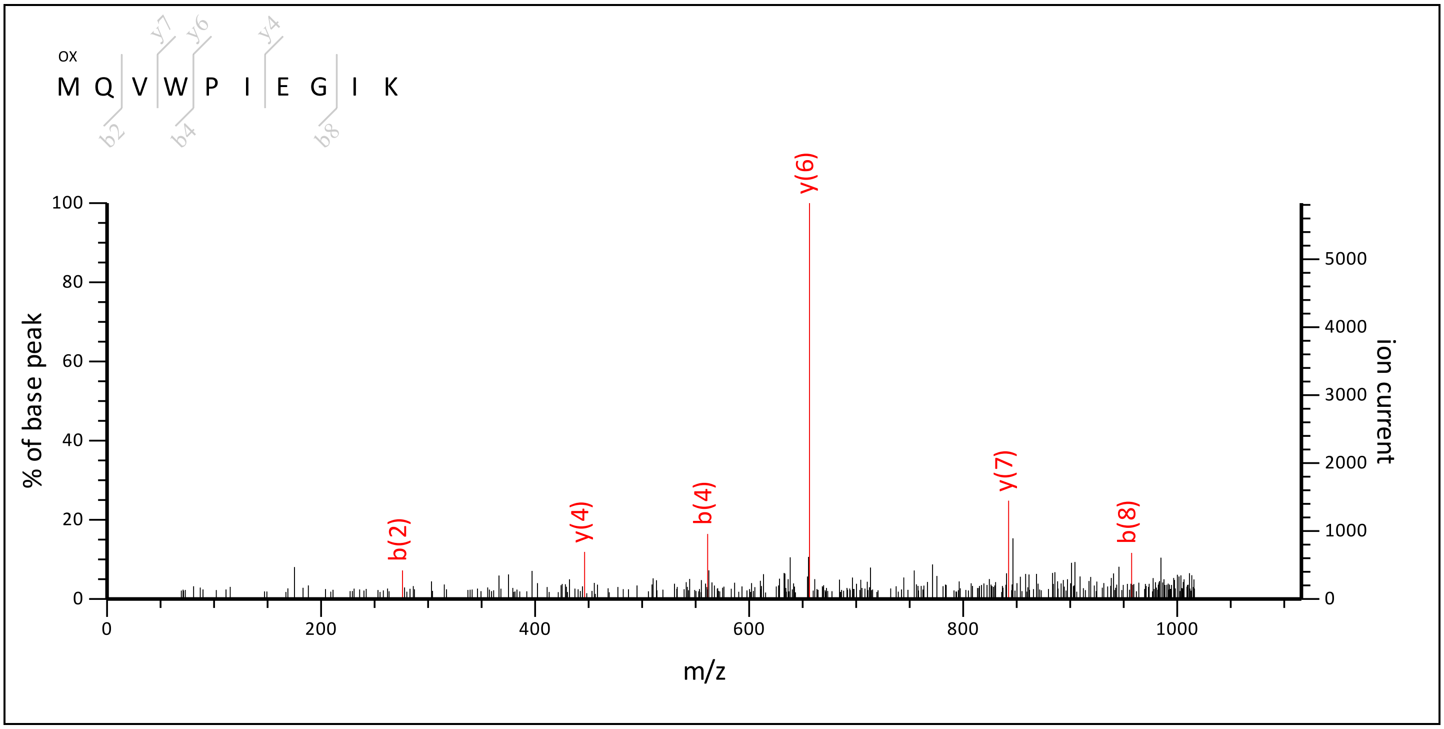


0 to 1116.05

Label all possible matches ○ Label matches used for scoring ◉

**Monoisotopic mass of neutral peptide Mr(calc):** 1215.6322
**Fixed modifications:** Carbamidomethyl (C) (apply to specified residues or termini only)
**Variable modifications:**
**M1** : Oxidation (M), with neutral losses 0.0000(shown in table), 63.9983
**Ions Score:** 20 **Expect:** 25
**Matches :** 6/86 fragment ions using 10 most intense peaks (help)

| # | a | a* | b | b* | Seq. | y | y* | # |
|---|---|---|---|---|---|---|---|---|
| 1 | 120.0478 | | 148.0427 | | M | | | 10 |
| 2 | 248.1063 | 231.0798 | *276.1013* | 259.0747 | Q | 1069.6041 | 1052.5775 | 9 |
| 3 | 347.1748 | 330.1482 | 375.1697 | 358.1431 | V | 941.5455 | 924.5189 | 8 |
| 4 | 533.2541 | 516.2275 | *561.2490* | 544.2224 | W | *842.4771* | 825.4505 | 7 |
| 5 | 630.3068 | 613.2803 | 658.3017 | 641.2752 | P | *656.3978* | 639.3712 | 6 |
| 6 | 743.3909 | 726.3643 | 771.3858 | 754.3593 | I | 559.3450 | 542.3184 | 5 |

| | | | | | | | | |
|---|---|---|---|---|---|---|---|---|
| 7 | 872.4335 | 855.4069 | 900.4284 | 883.4019 | E | 446.2609 | 429.2344 | 4 |
| 8 | 929.4550 | 912.4284 | 957.4499 | 940.4233 | G | 317.2183 | 300.1918 | 3 |
| 9 | 1042.5390 | 1025.5125 | 1070.5339 | 1053.5074 | I | 260.1969 | 243.1703 | 2 |
| 10 | | | | | K | 147.1128 | 130.0863 | 1 |

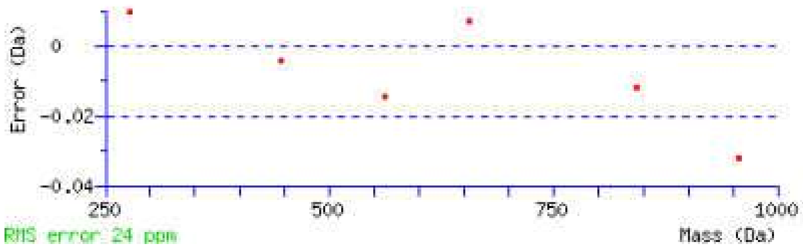


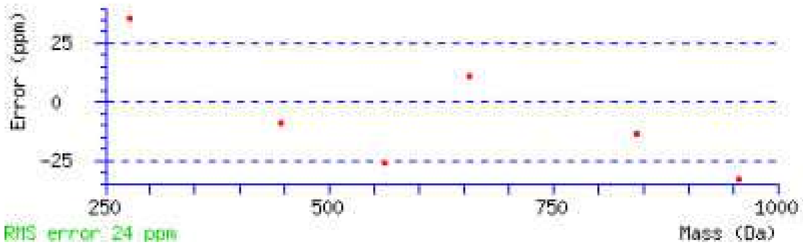


NCBI **BLAST** search of MQVWPIEGIK
(Parameters: blastp, nr protein database, expect=20000, no filter, PAM30)
Other BLAST web gateways

**All matches to this query**

| Score | Mr(calc) | Delta | Sequence |
|---|---|---|---|
| 31.5 | 1215.6169 | 0.0040 | MNSILPGEDIK |
| 26.8 | 1215.6360 | -0.0151 | GVFVRPDVDGR |
| 20.4 | 1215.6652 | -0.0443 | FQVWPIEGIK |
| 20.4 | 1215.6322 | -0.0112 | MQVWPIEGIK |
| 20.4 | 1215.6322 | -0.0112 | MQVWPIEGIK |
| 20.3 | 1215.5965 | 0.0245 | MATASVRHMGR |
| 19.0 | 1215.6533 | -0.0324 | QVALMIGEDIK |
| 19.0 | 1215.6459 | -0.0250 | RLSDLAGEDLK |
| 18.8 | 1215.5732 | 0.0478 | DLDLVQGEDGR |
| 17.2 | 1215.6976 | -0.0766 | KVIYGLVPDGR |

**Mascot:** http://www.matrixscience.com/

# MATRIX SCIENCE Mascot Search Results

## Peptide View Spot no 4

MS/MS Fragmentation of **VGFVYRENHR**
Found in **gi|671740** in **NCBInr**, ribulose-bisphosphate carboxylase [synthetic construct]

Match to Query 54: 1275.644624 from(1276.651900,1+) intensity(0.0000) index(16)
Title: Label: J5, Spot_Id: 219747, Peak_List_Id: 225842, MSMS Job_Run_Id: 21791, Comment:
Data file C:\Documents and Settings\Administrator\Desktop\farah BHU\spot set plate\Farah-Garima 11May 2013\ppw_J5_136842222100.txt

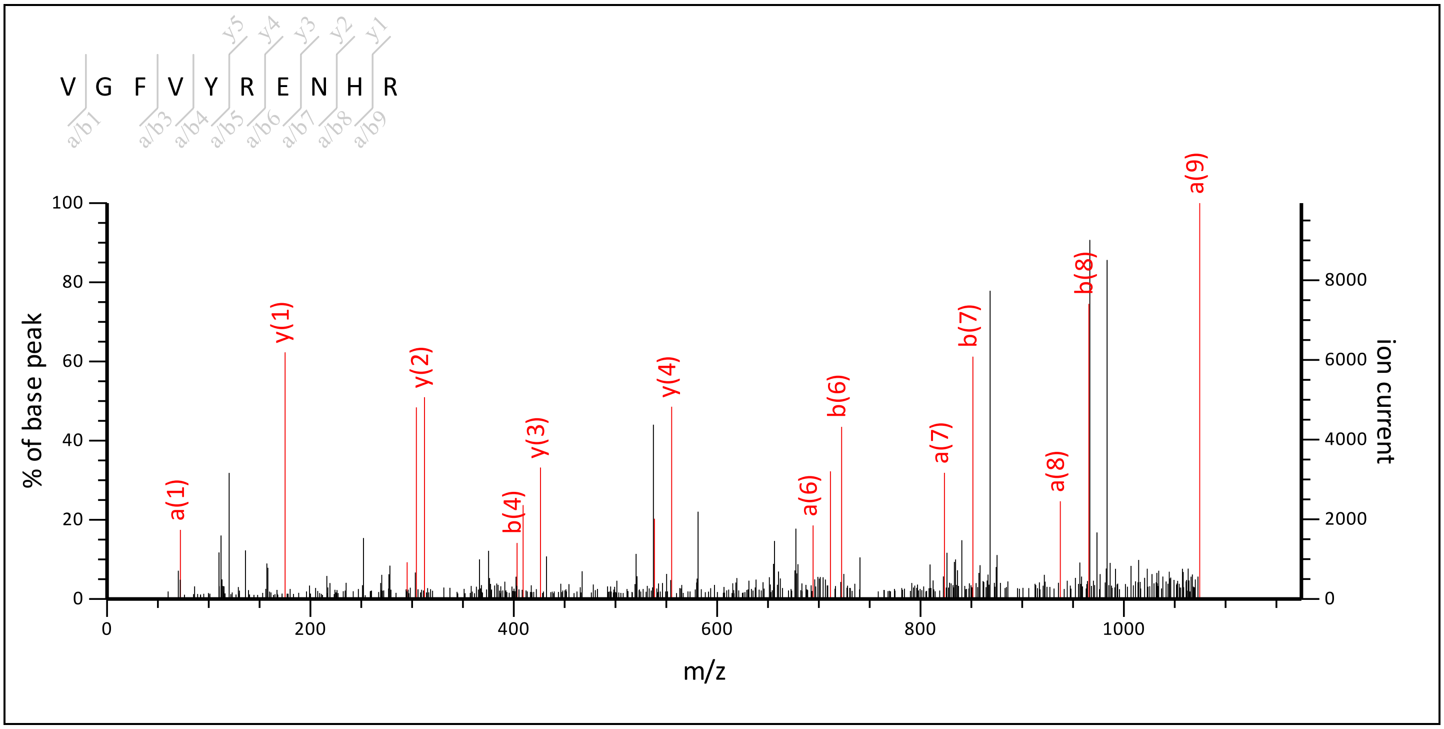


0 to 1174.58

Label all possible matches ○ Label matches used for scoring ◉

**Monoisotopic mass of neutral peptide Mr(calc):** 1275.6472
**Fixed modifications:** Carbamidomethyl (C) (apply to specified residues or termini only)
**Ions Score:** 36 **Expect:** 0.66
**Matches :** 20/44 fragment ions using 33 most intense peaks (help)

| # | a | a* | b | b* | Seq. | y | y* | # |
|---|---|---|---|---|---|---|---|---|
| 1 | *72.0808* | | 100.0757 | | V | | | 10 |
| 2 | 129.1022 | | 157.0972 | | G | 1177.5861 | 1160.5596 | 9 |
| 3 | 276.1707 | | *304.1656* | | F | 1120.5647 | 1103.5381 | 8 |
| 4 | 375.2391 | | *403.2340* | | V | 973.4962 | 956.4697 | 7 |
| 5 | *538.3024* | | 566.2973 | | Y | 874.4278 | 857.4013 | 6 |
| 6 | *694.4035* | 677.3770 | *722.3984* | 705.3719 | R | **711.3645** | 694.3379 | 5 |
| 7 | *823.4461* | 806.4196 | *851.4410* | 834.4145 | E | **555.2634** | 538.2368 | 4 |
| 8 | *937.4890* | 920.4625 | *965.4839* | 948.4574 | N | **426.2208** | 409.1942 | 3 |

| | | | | | | | | |
|---|---|---|---|---|---|---|---|---|
| 9 | *1074.5479* | 1057.5214 | 1102.5429 | 1085.5163 | H | 312.1779 | 295.1513 | 2 |
| 10 | | | | | R | 175.1190 | 158.0924 | 1 |

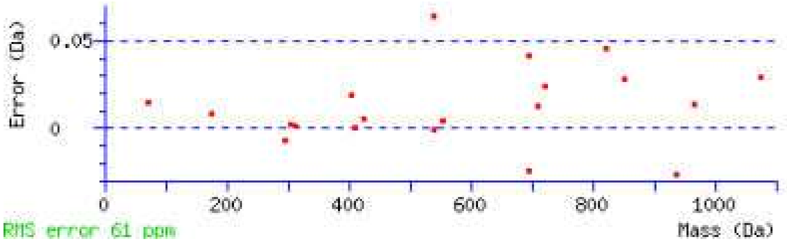


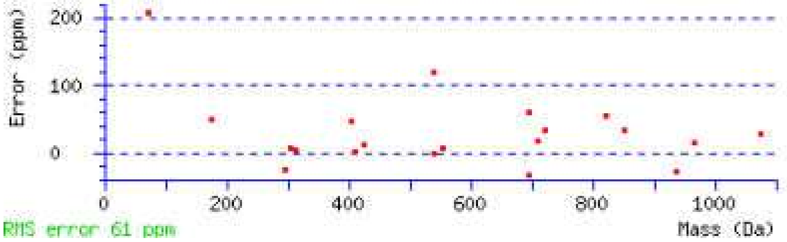


NCBI **BLAST** search of VGFVYRENHR
(Parameters: blastp, nr protein database, expect=20000, no filter, PAM30)
Other BLAST web gateways

**All matches to this query**

| Score | Mr(calc) | Delta | Sequence |
|---|---|---|---|
| 35.6 | 1275.6472 | -0.0026 | VGFVYRENHR |
| 23.6 | 1275.6221 | 0.0226 | HWRELENHR |
| 21.3 | 1275.6798 | -0.0352 | FYMITVPIHR |
| 21.0 | 1275.6472 | -0.0026 | GPSPSPAKGWHR |
| 21.0 | 1275.5381 | 0.1066 | WYSGNNENHR |
| 18.7 | 1275.6545 | -0.0098 | GRPTRGPQDHR |
| 16.7 | 1275.5732 | 0.0715 | YDDDGLAPPGTR |
| 16.7 | 1275.6823 | -0.0377 | LPTNEIFSSLR |
| 14.7 | 1275.6432 | 0.0014 | NKPPRENHER |
| 14.7 | 1275.6255 | 0.0192 | CRPGGAPATPHR |

**Mascot:** http://www.matrixscience.com/

# MATRIX SCIENCE Mascot Search Results

## Peptide View Spot no 8

MS/MS Fragmentation of **SPGYYDGR**
Found in **gi|56966763** in **NCBInr**, Chain S, Crystal Structure Of Activated Rice Rubisco Complexed With 2- Carboxyarabinitol-1,5-Bisphosphate

Match to Query 23: 913.473924 from(914.481200,1+) intensity(0.0000) index(1)
Title: Label: N9, Spot_Id: 219815, Peak_List_Id: 227090, MSMS Job_Run_Id: 21951, Comment:
Data file C:\Documents and Settings\Administrator\Desktop\Farah-Garima 11May 2013\ppw_N9_136868212700.txt

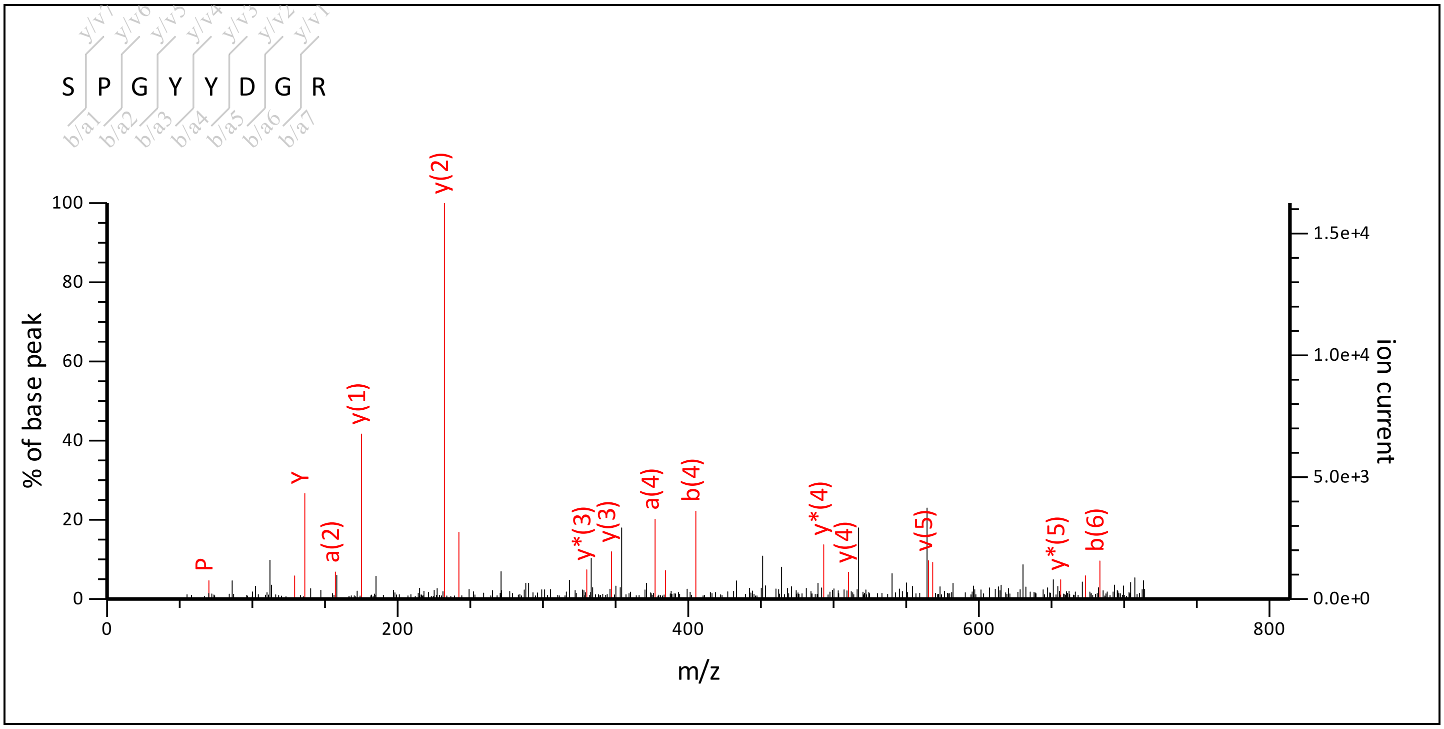


0 to 814.08

Label all possible matches ○ Label matches used for scoring ◉

**Monoisotopic mass of neutral peptide Mr(calc):** 913.3930
**Fixed modifications:** Carbamidomethyl (C) (apply to specified residues or termini only)
**Ions Score:** 33 **Expect:** 22
**Matches :** 22/96 fragment ions using 28 most intense peaks (help)

| # | Immon. | a | $a^0$ | b | $b^0$ | d | Seq. | v | w | y | y* | $y^0$ | # |
|---|---|---|---|---|---|---|---|---|---|---|---|---|---|
| 1 | 60.0444 | 60.0444 | 42.0338 | 88.0393 | 70.0287 | 44.0495 | S | | | | | | 8 |
| 2 | 70.0651 | **157.0972** | 139.0866 | 185.0921 | 167.0815 | 131.0815 | P | 785.3213 | 784.3260 | 827.3682 | 810.3417 | 809.3577 | 7 |
| 3 | 30.0338 | 214.1186 | 196.1081 | **242.1135** | 224.1030 | | G | | | 730.3155 | 713.2889 | 712.3049 | 6 |
| 4 | 136.0757 | **377.1819** | 359.1714 | **405.1769** | 387.1663 | | Y | 565.2365 | | ***673.2940*** | 656.2675 | 655.2835 | 5 |
| 5 | 136.0757 | 540.2453 | 522.2347 | **568.2402** | 550.2296 | | Y | 402.1732 | | ***510.2307*** | 493.2041 | 492.2201 | 4 |
| 6 | 88.0393 | 655.2722 | 637.2617 | **683.2671** | 665.2566 | 611.2824 | D | 287.1462 | 286.1510 | ***347.1674*** | 330.1408 | 329.1568 | 3 |
| 7 | 30.0338 | 712.2937 | 694.2831 | 740.2886 | 722.2780 | | G | | | ***232.1404*** | 215.1139 | | 2 |

| | | | | | | | | | | | | | |
|---|---|---|---|---|---|---|---|---|---|---|---|---|---|
| 8 | 129.1135 | | | | | | R | 74.0237 | 73.0284 | *175.1190* | 158.0924 | | 1 |

| Seq | ya | yb | Seq | ya | yb | Seq | ya | yb |
|---|---|---|---|---|---|---|---|---|
| **PG** | 127.0866 | 155.0815 | **PGY** | 290.1499 | 318.1448 | **PGYY** | 453.2132 | 481.2082 |
| **PGYYD** | 568.2402 | 596.2351 | **PGYYDG** | 625.2617 | 653.2566 | **GY** | 193.0972 | 221.0921 |
| **GYY** | 356.1605 | 384.1554 | **GYYD** | 471.1874 | 499.1823 | **GYYDG** | 528.2089 | 556.2038 |
| **YY** | 299.1390 | 327.1339 | **YYD** | 414.1660 | 442.1609 | **YYDG** | 471.1874 | 499.1823 |
| **YD** | 251.1026 | 279.0975 | **YDG** | 308.1241 | 336.1190 | **DG** | 145.0608 | 173.0557 |

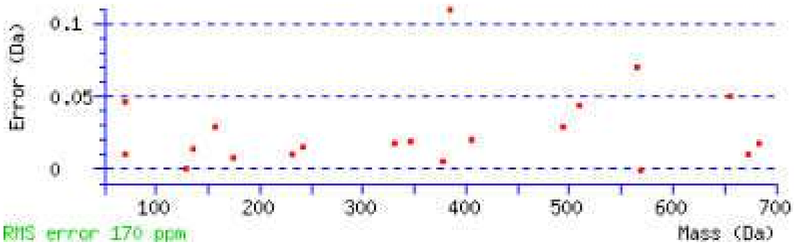


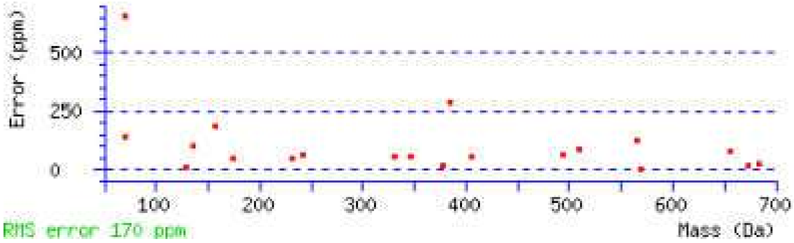


NCBI **BLAST** search of SPGYYDGR
(Parameters: blastp, nr protein database, expect=20000, no filter, PAM30)
Other BLAST web gateways

**All matches to this query**

| Score | Mr(calc) | Delta | Sequence |
|---|---|---|---|
| 33.7 | 913.4294 | 0.0446 | QLYYDGR |
| 32.9 | 913.3930 | 0.0809 | SPGYYDGR |
| 32.9 | 913.3930 | 0.0809 | SPGYYDGR |
| 32.3 | 913.3964 | 0.0776 | DYATMTGR |
| 32.2 | 913.4294 | 0.0446 | GAIYYDGR |
| 30.1 | 913.5094 | -0.0354 | SPGRLATGR |
| 29.3 | 913.5094 | -0.0354 | QIRAVDGR |
| 28.3 | 913.4981 | -0.0242 | QLGVSPTGR |
| 27.3 | 913.3964 | 0.0776 | YMSLGDGR |
| 26.7 | 913.5094 | -0.0354 | AVARAVDGR |

**Mascot:** http://www.matrixscience.com/

# Mascot Search Results

## Peptide View Spot no 8

MS/MS Fragmentation of **IIGFDNVR**
Found in **gi|56966763** in **NCBInr**, Chain S, Crystal Structure Of Activated Rice Rubisco Complexed With 2- Carboxyarabinitol-1,5-Bisphosphate

Match to Query 33: 932.585134 from(933.592410,1+) intensity(0.0000) index(2)
Title: Label: N9, Spot_Id: 219815, Peak_List_Id: 227087, MSMS Job_Run_Id: 21951, Comment:
Data file C:\Documents and Settings\Administrator\Desktop\Farah-Garima 11May 2013\ppw_N9_136868212700.txt

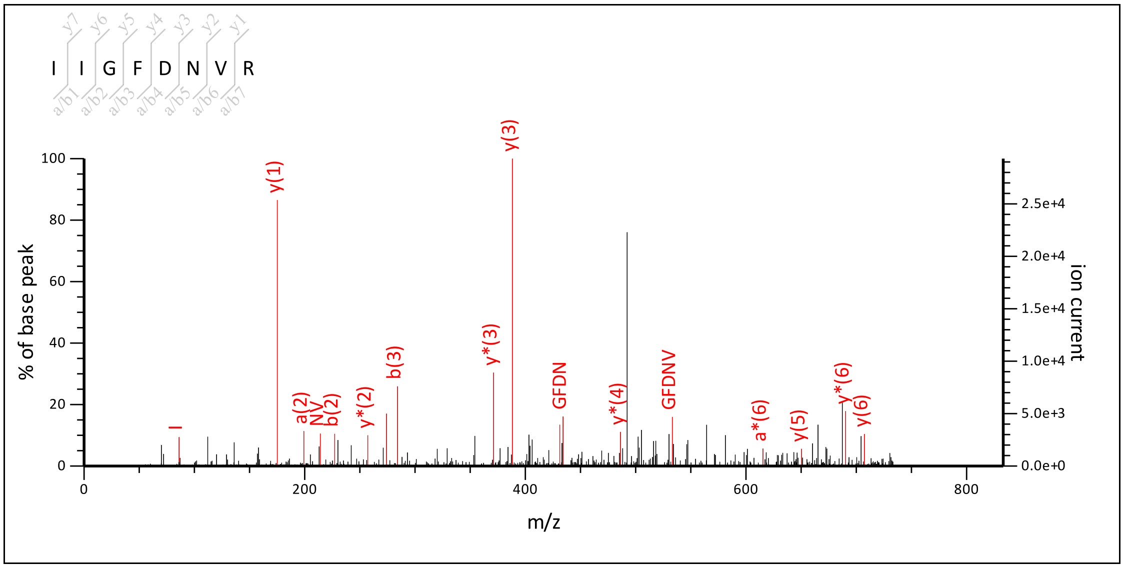


0 to 833.2

Label all possible matches ○ Label matches used for scoring ◉

**Monoisotopic mass of neutral peptide Mr(calc):** 932.5080
**Fixed modifications:** Carbamidomethyl (C) (apply to specified residues or termini only)
**Ions Score:** 32 **Expect:** 13
**Matches :** 21/98 fragment ions using 28 most intense peaks (help)

| # | Immon. | a | a* | $a^0$ | b | b* | $b^0$ | d | d' | Seq. | v | w | w' | y | y* | $y^0$ | # |
|---|---|---|---|---|---|---|---|---|---|---|---|---|---|---|---|---|---|
| 1 | 86.0964 | **86.0964** | | | 114.0913 | | | 44.0495 | | **I** | | | | | | | 8 |
| 2 | 86.0964 | **199.1805** | | | **227.1754** | | | 171.1492 | 185.1648 | **I** | 762.3529 | 775.3733 | 789.3890 | 820.4312 | 803.4046 | 802.4206 | 7 |
| 3 | 30.0338 | 256.2020 | | | **284.1969** | | | | | **G** | | | | ***707.3471*** | 690.3206 | 689.3365 | 6 |
| 4 | 120.0808 | 403.2704 | | | **431.2653** | | | | | **F** | 558.2630 | | | ***650.3256*** | 633.2991 | 632.3151 | 5 |
| 5 | 88.0393 | 518.2973 | | 500.2867 | 546.2922 | | 528.2817 | 474.3075 | | **D** | 443.2361 | 442.2409 | | 503.2572 | 486.2307 | 485.2467 | 4 |
| 6 | 87.0553 | 632.3402 | 615.3137 | 614.3297 | 660.3352 | 643.3086 | 642.3246 | 589.3344 | | **N** | 329.1932 | 328.1979 | | ***388.2303*** | 371.2037 | | 3 |
| 7 | 72.0808 | 731.4087 | 714.3821 | 713.3981 | 759.4036 | 742.3770 | 741.3930 | 717.3930 | | **V** | 230.1248 | 243.1452 | | ***274.1874*** | 257.1608 | | 2 |
| 8 | 129.1135 | | | | | | | | | **R** | 74.0237 | 73.0284 | | ***175.1190*** | 158.0924 | | 1 |

| Seq | ya | yb | Seq | ya | yb | Seq | ya | yb |
|---|---|---|---|---|---|---|---|---|
| **IG** | 143.1179 | 171.1128 | **IGF** | 290.1863 | 318.1812 | **IGFD** | 405.2132 | 433.2082 |
| **IGFDN** | 519.2562 | 547.2511 | **IGFDNV** | 618.3246 | 646.3195 | **GF** | 177.1022 | 205.0972 |
| **GFD** | 292.1292 | 320.1241 | **GFDN** | 406.1721 | 434.1670 | **GFDNV** | 505.2405 | 533.2354 |
| **FD** | 235.1077 | 263.1026 | **FDN** | 349.1506 | 377.1456 | **FDNV** | 448.2191 | 476.2140 |
| **DN** | 202.0822 | 230.0771 | **DNV** | 301.1506 | 329.1456 | **NV** | 186.1237 | 214.1186 |

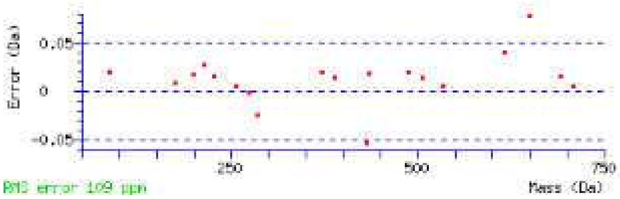


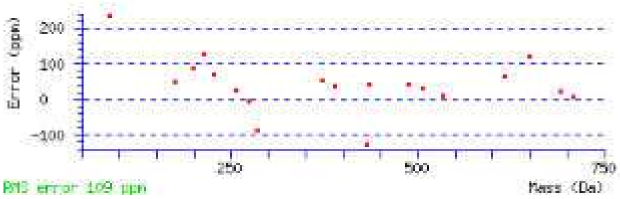


NCBI **BLAST** search of IIGFDNVR
(Parameters: blastp, nr protein database, expect=20000, no filter, PAM30)
Other BLAST web gateways

**All matches to this query**

| Score | Mr(calc) | Delta | Sequence |
|---|---|---|---|
| 32.5 | 932.5080 | 0.0772 | IIGFDNVR |
| 30.2 | 932.5444 | 0.0408 | VGTFGAILR |
| 26.7 | 932.5807 | 0.0044 | KTFGVLLR |
| 26.6 | 932.5113 | 0.0738 | LLGSMVASR |
| 26.0 | 932.5807 | 0.0044 | TKFGVLLR |
| 25.0 | 932.4927 | 0.0924 | ILGDTTSAR |
| 24.7 | 932.5080 | 0.0772 | LPTSFNVR |
| 24.4 | 932.5556 | 0.0296 | RTFGALLR |
| 24.0 | 932.5444 | 0.0408 | AGFTGVLLR |
| 23.9 | 932.5113 | 0.0738 | LIGMSGALR |

**Mascot:** http://www.matrixscience.com/

# MATRIX SCIENCE Mascot Search Results

## Peptide View Spot no 8

MS/MS Fragmentation of **KAYPDAFVR**
Found in **gi|56966763** in **NCBInr**, Chain S, Crystal Structure Of Activated Rice Rubisco Complexed With 2- Carboxyarabinitol-1,5-Bisphosphate

Match to Query 91: 1065.665124 from(1066.672400,1+) intensity(0.0000) index(9)
Title: Label: N9, Spot_Id: 219815, Peak_List_Id: 227091, MSMS Job_Run_Id: 21951, Comment:
Data file C:\Documents and Settings\Administrator\Desktop\Farah-Garima 11May 2013\ppw_N9_136868212700.txt

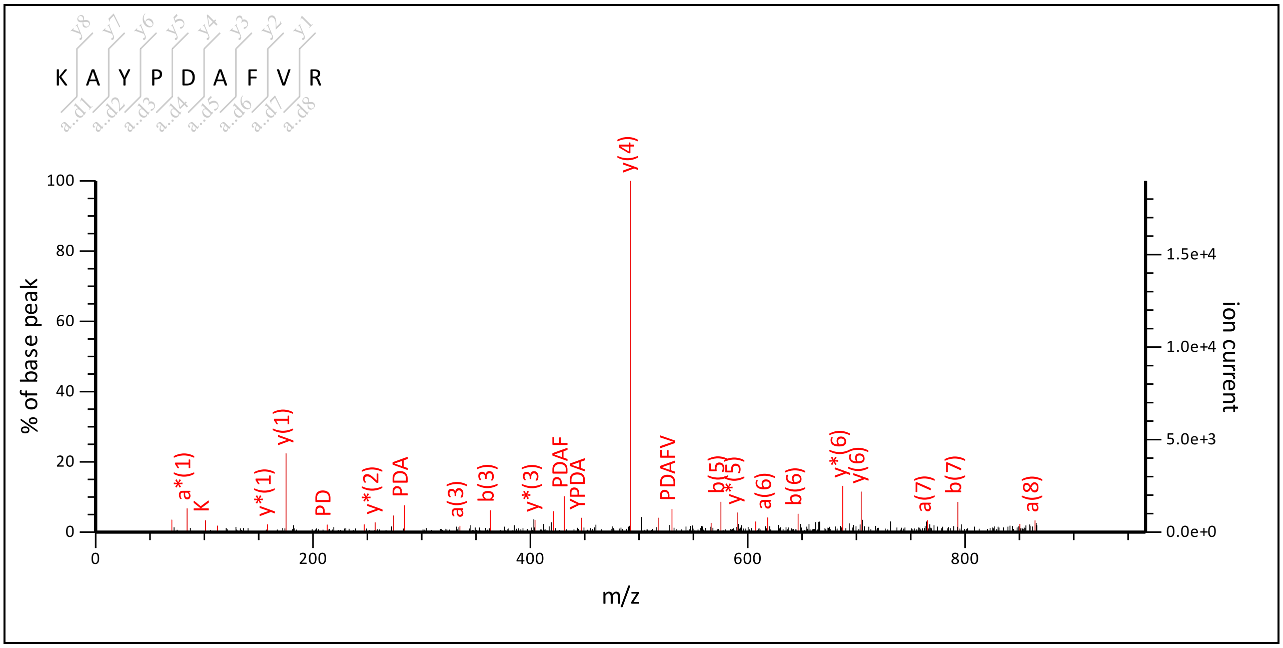


0 to 966.02

Label all possible matches ○ Label matches used for scoring ◉

**Monoisotopic mass of neutral peptide Mr(calc):** 1065.5607
**Fixed modifications:** Carbamidomethyl (C) (apply to specified residues or termini only)
**Ions Score:** 51 **Expect:** 0.13
**Matches :** 38/125 fragment ions using 40 most intense peaks (help)

| # | Immon. | a | a* | $a^0$ | b | b* | $b^0$ | d | Seq. | v | w | y | y* | $y^0$ | # |
|---|---|---|---|---|---|---|---|---|---|---|---|---|---|---|---|
| 1 | 101.1073 | ***101.1073*** | 84.0808 | | 129.1022 | 112.0757 | | 44.0495 | K | | | | | | 9 |
| 2 | 44.0495 | 172.1444 | 155.1179 | | 200.1394 | 183.1128 | | | A | 922.4417 | | 938.4730 | 921.4465 | 920.4625 | 8 |
| 3 | 136.0757 | ***335.2078*** | 318.1812 | | **363.2027** | 346.1761 | | | Y | 759.3784 | | 867.4359 | 850.4094 | 849.4254 | 7 |
| 4 | 70.0651 | 432.2605 | 415.2340 | | 460.2554 | 443.2289 | | 406.2449 | P | 662.3257 | 661.3304 | ***704.3726*** | 687.3461 | 686.3620 | 6 |
| 5 | 88.0393 | 547.2875 | 530.2609 | 529.2769 | **575.2824** | 558.2558 | 557.2718 | 503.2976 | D | 547.2987 | 546.3035 | ***607.3198*** | 590.2933 | 589.3093 | 5 |
| 6 | 44.0495 | ***618.3246*** | 601.2980 | 600.3140 | **646.3195** | 629.2930 | 628.3089 | | A | 476.2616 | | ***492.2929*** | 475.2663 | | 4 |
| 7 | 120.0808 | ***765.3930*** | 748.3665 | 747.3824 | **793.3879** | 776.3614 | 775.3774 | | F | 329.1932 | | ***421.2558*** | 404.2292 | | 3 |
| 8 | 72.0808 | ***864.4614*** | 847.4349 | 846.4509 | 892.4563 | 875.4298 | 874.4458 | 850.4458 | V | 230.1248 | 243.1452 | ***274.1874*** | 257.1608 | | 2 |
| 9 | 129.1135 | | | | | | | | R | 74.0237 | 73.0284 | ***175.1190*** | 158.0924 | | 1 |

| Seq | ya | yb | Seq | ya | yb | Seq | ya | yb |
|---|---|---|---|---|---|---|---|---|
| AY | 207.1128 | 235.1077 | AYP | 304.1656 | 332.1605 | AYPD | 419.1925 | 447.1874 |
| AYPDA | 490.2296 | 518.2245 | AYPDAF | 637.2980 | 665.2930 | YP | 233.1285 | 261.1234 |
| YPD | 348.1554 | 376.1503 | YPDA | 419.1925 | 447.1874 | YPDAF | 566.2609 | 594.2558 |

| | | | | | | | | |
|---|---|---|---|---|---|---|---|---|
| **YPDAFV** | 665.3293 | 693.3243 | **PD** | 185.0921 | 213.0870 | **PDA** | 256.1292 | 284.1241 |
| **PDAF** | 403.1976 | 431.1925 | **PDAFV** | 502.2660 | 530.2609 | **DA** | 159.0764 | 187.0713 |
| **DAF** | 306.1448 | 334.1397 | **DAFV** | 405.2132 | 433.2082 | **AF** | 191.1179 | 219.1128 |
| **AFV** | 290.1863 | 318.1812 | **FV** | 219.1492 | 247.1441 | | | |

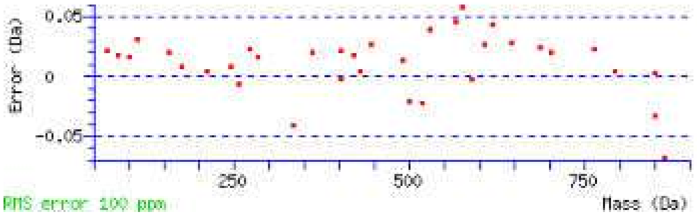


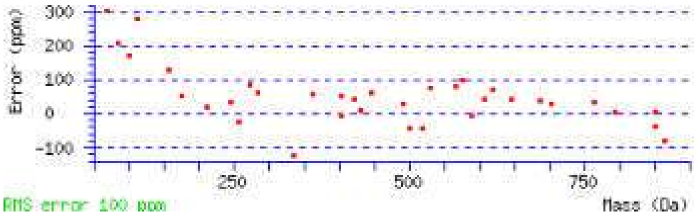


NCBI **BLAST** search of KAYPDAFVR
(Parameters: blastp, nr protein database, expect=20000, no filter, PAM30)
Other BLAST web gateways

**All matches to this query**

| Score | Mr(calc) | Delta | Sequence |
|---|---|---|---|
| 50.6 | 1065.5607 | 0.1044 | KAYPDAFVR |
| 34.9 | 1065.5641 | 0.1010 | KYVSPAMVR |
| 34.3 | 1065.5607 | 0.1044 | QSFPVSFVR |
| 32.6 | 1065.5641 | 0.1010 | MVISQVFAR |
| 31.7 | 1065.5641 | 0.1010 | MVLQSVFAR |
| 30.9 | 1065.5641 | 0.1010 | MEAFVKAVR |
| 30.6 | 1065.5753 | 0.0898 | AFLSRAMVR |
| 29.4 | 1065.5641 | 0.1010 | KEFAVMAVR |
| 29.2 | 1065.5971 | 0.0680 | KAIFDAFVR |
| 28.1 | 1065.5607 | 0.1044 | FKSFDPAVR |

**Mascot:** http://www.matrixscience.com/

# Mascot Search Results

## Peptide View Spot no 8

MS/MS Fragmentation of **AYPDAFVR**
Found in **gi|56966763** in **NCBInr**, Chain S, Crystal Structure Of Activated Rice Rubisco Complexed With 2- Carboxyarabinitol-1,5-Bisphosphate

Match to Query 35: 937.542044 from(938.549320,1+) intensity(0.0000) index(3)
Title: Label: N9, Spot_Id: 219815, Peak_List_Id: 227089, MSMS Job_Run_Id: 21951, Comment:
Data file C:\Documents and Settings\Administrator\Desktop\Farah-Garima 11May 2013\ppw_N9_136868212700.txt

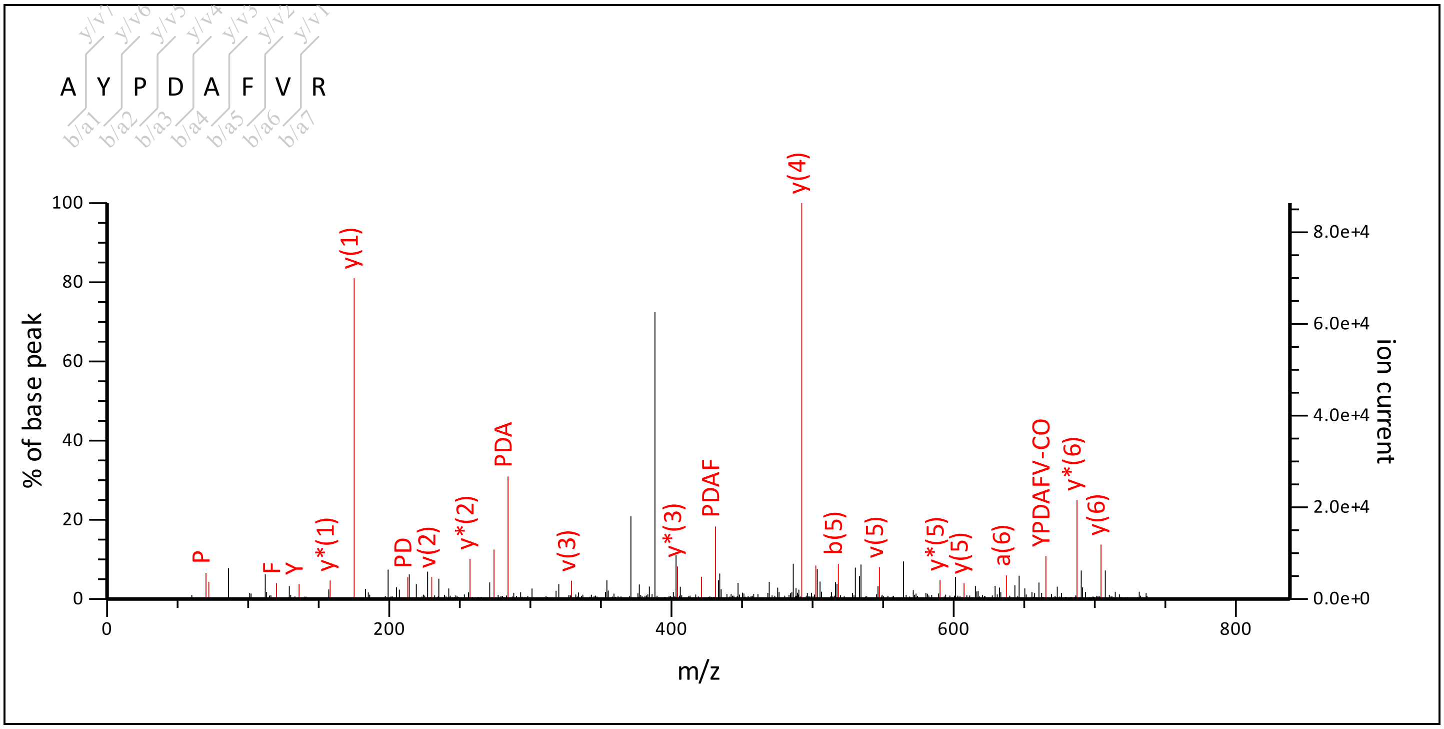


0 to 838.29

Label all possible matches ○ Label matches used for scoring ◉

**Monoisotopic mass of neutral peptide Mr(calc):** 937.4658
**Fixed modifications:** Carbamidomethyl (C) (apply to specified residues or termini only)
**Ions Score:** 33 **Expect:** 14
**Matches :** 29/92 fragment ions using 49 most intense peaks (help)

| # | Immon. | a | $a^0$ | b | $b^0$ | d | Seq. | v | w | y | y* | $y^0$ | # |
|---|---|---|---|---|---|---|---|---|---|---|---|---|---|
| 1 | 44.0495 | 44.0495 | | **72.0444** | | 44.0495 | A | | | | | | 8 |
| 2 | 136.0757 | 207.1128 | | 235.1077 | | | Y | 759.3784 | | 867.4359 | 850.4094 | 849.4254 | 7 |
| 3 | 70.0651 | 304.1656 | | 332.1605 | | 278.1499 | P | 662.3257 | 661.3304 | ***704.3726*** | 687.3461 | 686.3620 | 6 |
| 4 | 88.0393 | 419.1925 | 401.1819 | 447.1874 | 429.1769 | 375.2027 | D | 547.2987 | 546.3035 | ***607.3198*** | 590.2933 | 589.3093 | 5 |
| 5 | 44.0495 | 490.2296 | 472.2191 | **518.2245** | 500.2140 | | A | 476.2616 | | ***492.2929*** | 475.2663 | | 4 |
| 6 | 120.0808 | 637.2980 | 619.2875 | **665.2930** | 647.2824 | | F | 329.1932 | | ***421.2558*** | 404.2292 | | 3 |
| 7 | 72.0808 | 736.3665 | 718.3559 | 764.3614 | 746.3508 | 722.3508 | V | 230.1248 | 243.1452 | ***274.1874*** | 257.1608 | | 2 |

| | | | | | | | | | | | | | |
|---|---|---|---|---|---|---|---|---|---|---|---|---|---|
| 8 | 129.1135 | | | | | | R | 74.0237 | 73.0284 | *175.1190* | 158.0924 | | 1 |

| Seq | ya | yb | Seq | ya | yb | Seq | ya | yb |
|---|---|---|---|---|---|---|---|---|
| YP | 233.1285 | 261.1234 | YPD | 348.1554 | 376.1503 | YPDA | 419.1925 | 447.1874 |
| YPDAF | 566.2609 | 594.2558 | YPDAFV | 665.3293 | 693.3243 | PD | 185.0921 | 213.0870 |
| PDA | 256.1292 | 284.1241 | PDAF | 403.1976 | 431.1925 | PDAFV | 502.2660 | 530.2609 |
| DA | 159.0764 | 187.0713 | DAF | 306.1448 | 334.1397 | DAFV | 405.2132 | 433.2082 |
| AF | 191.1179 | 219.1128 | AFV | 290.1863 | 318.1812 | FV | 219.1492 | 247.1441 |

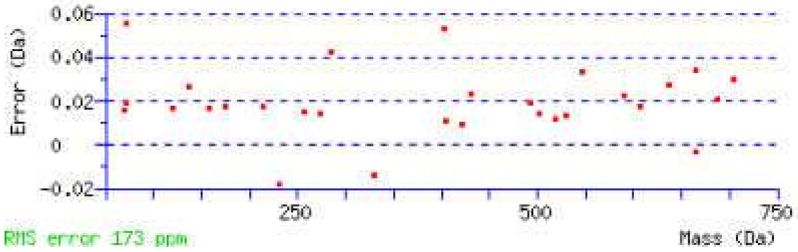


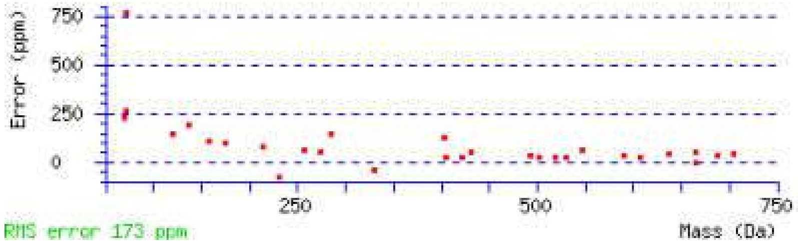


NCBI **BLAST** search of AYPDAFVR
(Parameters: blastp, nr protein database, expect=20000, no filter, PAM30)
Other BLAST web gateways

**All matches to this query**

| Score | Mr(calc) | Delta | Sequence |
|---|---|---|---|
| 32.7 | 937.4658 | 0.0763 | AYPDAFVR |
| 25.9 | 937.5134 | 0.0287 | HPIGYPVR |
| 25.5 | 937.5385 | 0.0035 | YALLGFVR |
| 25.4 | 937.4658 | 0.0763 | YAEGFPVR |
| 25.2 | 937.5168 | 0.0253 | HPAMIVVR |
| 25.2 | 937.5168 | 0.0253 | HPAMVIVR |
| 25.2 | 937.5168 | 0.0253 | HPSMVIVR |
| 25.0 | 937.4617 | 0.0803 | LSDHDPVR |
| 25.0 | 937.4617 | 0.0803 | SLDHDPVR |
| 24.8 | 937.5055 | 0.0365 | SFVLAMVR |

**Mascot:** http://www.matrixscience.com/

# MATRIX SCIENCE Mascot Search Results

## Peptide View Spot no 8

MS/MS Fragmentation of **MQVWPIEGIK**
Found in **gi|56966763** in **NCBInr**, Chain S, Crystal Structure Of Activated Rice Rubisco Complexed With 2- Carboxyarabinitol-1,5-Bisphosphate

Match to Query 125: 1199.741024 from(1200.748300,1+) intensity(0.0000) index(13)
Title: Label: N9, Spot_Id: 219815, Peak_List_Id: 227093, MSMS Job_Run_Id: 21951, Comment:
Data file C:\Documents and Settings\Administrator\Desktop\Farah-Garima 11May 2013\ppw_N9_136868212700.txt

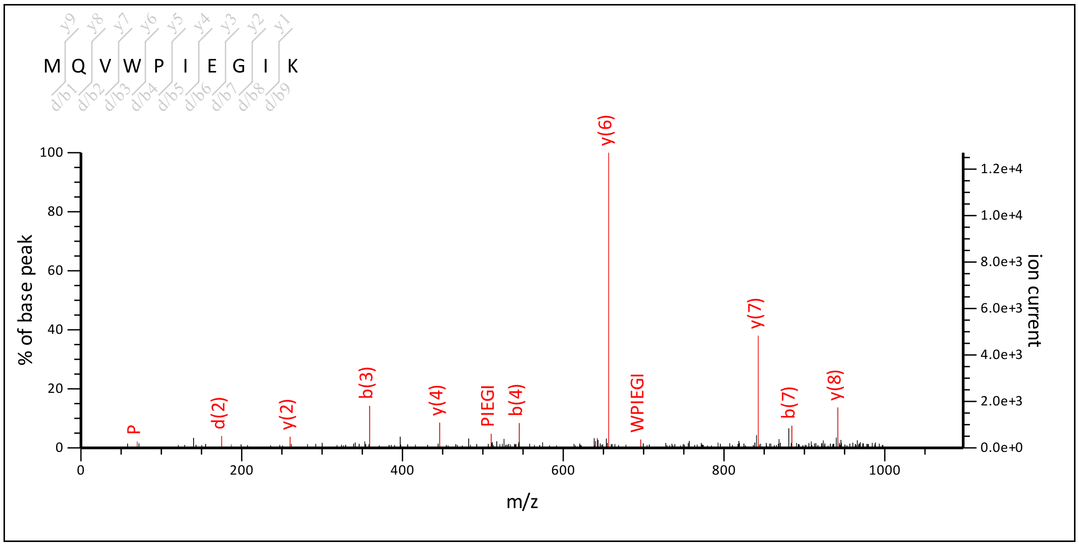


0 to 1097.53

Label all possible matches ○ Label matches used for scoring ◉

**Monoisotopic mass of neutral peptide Mr(calc):** 1199.6373
**Fixed modifications:** Carbamidomethyl (C) (apply to specified residues or termini only)
**Ions Score:** 46 **Expect:** 0.54
**Matches :** 16/148 fragment ions using 20 most intense peaks (help)

| # | Immon. | a | a* | $a^0$ | b | b* | $b^0$ | d | d' | Seq. | v | w | w' | y | y* | $y^0$ |
|---|---|---|---|---|---|---|---|---|---|---|---|---|---|---|---|---|
| 1 | 104.0528 | 104.0528 | | | 132.0478 | | | 44.0495 | | M | | | | | | |
| 2 | 101.0709 | 232.1114 | 215.0849 | | ***260.1063*** | 243.0798 | | 175.0900 | | Q | 996.5513 | 995.5560 | | 1069.6041 | 1052.5775 | 1051.5 |
| 3 | 72.0808 | 331.1798 | 314.1533 | | ***359.1748*** | 342.1482 | | 317.1642 | | V | 897.4829 | 910.5033 | | ***941.5455*** | 924.5189 | 923.5 |
| 4 | 159.0917 | 517.2592 | 500.2326 | | ***545.2541*** | 528.2275 | | | | W | 711.4036 | | | ***842.4771*** | 825.4505 | 824.4 |
| 5 | 70.0651 | 614.3119 | 597.2854 | | ***642.3068*** | 625.2803 | | 588.2963 | | P | 614.3508 | 613.3556 | | ***656.3978*** | 639.3712 | 638.3 |
| 6 | 86.0964 | 727.3960 | 710.3694 | | 755.3909 | 738.3643 | | 699.3647 | 713.3803 | I | 501.2667 | 514.2871 | 528.3028 | 559.3450 | 542.3184 | 541.3 |
| 7 | 102.0550 | 856.4386 | 839.4120 | 838.4280 | ***884.4335*** | 867.4069 | 866.4229 | 798.4331 | | E | 372.2241 | 371.2289 | | ***446.2609*** | 429.2344 | 428.2 |
| 8 | 30.0338 | 913.4600 | 896.4335 | 895.4495 | ***941.4550*** | 924.4284 | 923.4444 | | | G | | | | 317.2183 | 300.1918 | |
| 9 | 86.0964 | 1026.5441 | 1009.5176 | 1008.5335 | 1054.5390 | 1037.5125 | 1036.5285 | 998.5128 | 1012.5285 | I | 202.1186 | 215.1390 | 229.1547 | ***260.1969*** | 243.1703 | |
| 10 | 101.1073 | | | | | | | | | K | 74.0237 | 73.0284 | | 147.1128 | 130.0863 | |

| Seq | ya | yb | Seq | ya | yb | Seq | ya | yb |
|---|---|---|---|---|---|---|---|---|
| QV | 200.1394 | 228.1343 | QVW | 386.2187 | 414.2136 | QVWP | 483.2714 | 511.2663 |
| QVWPI | 596.3555 | 624.3504 | VW | 258.1601 | 286.1550 | VWP | 355.2129 | 383.2078 |
| VWPI | 468.2969 | 496.2918 | VWPIE | 597.3395 | 625.3344 | VWPIEG | 654.3610 | 682.3559 |
| WP | 256.1444 | 284.1394 | WPI | 369.2285 | 397.2234 | WPIE | 498.2711 | 526.2660 |
| WPIEG | 555.2926 | 583.2875 | WPIEGI | 668.3766 | 696.3715 | PI | 183.1492 | 211.1441 |
| PIE | 312.1918 | 340.1867 | PIEG | 369.2132 | 397.2082 | PIEGI | 482.2973 | 510.2922 |
| IE | 215.1390 | 243.1339 | IEG | 272.1605 | 300.1554 | IEGI | 385.2445 | 413.2395 |
| EG | 159.0764 | 187.0713 | EGI | 272.1605 | 300.1554 | GI | 143.1179 | 171.1128 |

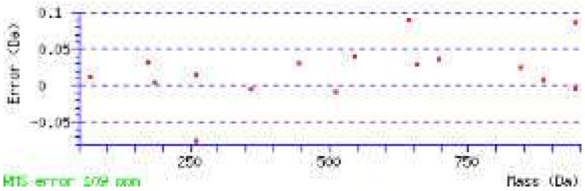


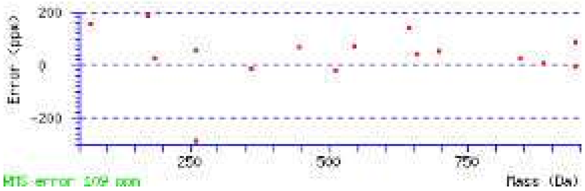


NCBI **BLAST** search of [MQVWPIEGIK]
(Parameters: blastp, nr protein database, expect=20000, no filter, PAM30)
Other BLAST [web gateways]

**All matches to this query**

| Score | Mr(calc) | Delta | Sequence |
|---|---|---|---|
| 46.2 | 1199.6373 | 0.1038 | MQVWPIEGIK |
| 46.2 | 1199.6373 | 0.1038 | MQVWPIEGIK |
| 43.4 | 1199.6761 | 0.0649 | ATSVAPEALTIK |
| 40.8 | 1199.6696 | 0.0714 | MQVVSSPILAR |
| 40.7 | 1199.6398 | 0.1013 | VSDGSVPDIAIK |
| 40.0 | 1199.6914 | 0.0496 | ASTVLSPWVLK |
| 37.2 | 1199.6874 | 0.0536 | TVTGSVPLLASR |
| 32.7 | 1199.6220 | 0.1190 | AMGVPVEAELGK |
| 31.7 | 1199.6696 | 0.0714 | LSVVCPVSALR |
| 30.7 | 1199.6332 | 0.1078 | EGVCVPLLASR |

**Mascot:** http://www.matrixscience.com/

# MATRIX SCIENCE Mascot Search Results

## Peptide View Spot no 8

MS/MS Fragmentation of **FQVWPIEGIK**
Found in **gi|56966763** in **NCBInr**, Chain S, Crystal Structure Of Activated Rice Rubisco Complexed With 2- Carboxyarabinitol-1,5-Bisphosphate

Match to Query 130: 1215.734924 from(1216.742200,1+) intensity(0.0000) index(14)
Title: Label: N9, Spot_Id: 219815, Peak_List_Id: 227094, MSMS Job_Run_Id: 21951, Comment:
Data file C:\Documents and Settings\Administrator\Desktop\Farah-Garima 11May 2013\ppw_N9_136868212700.txt

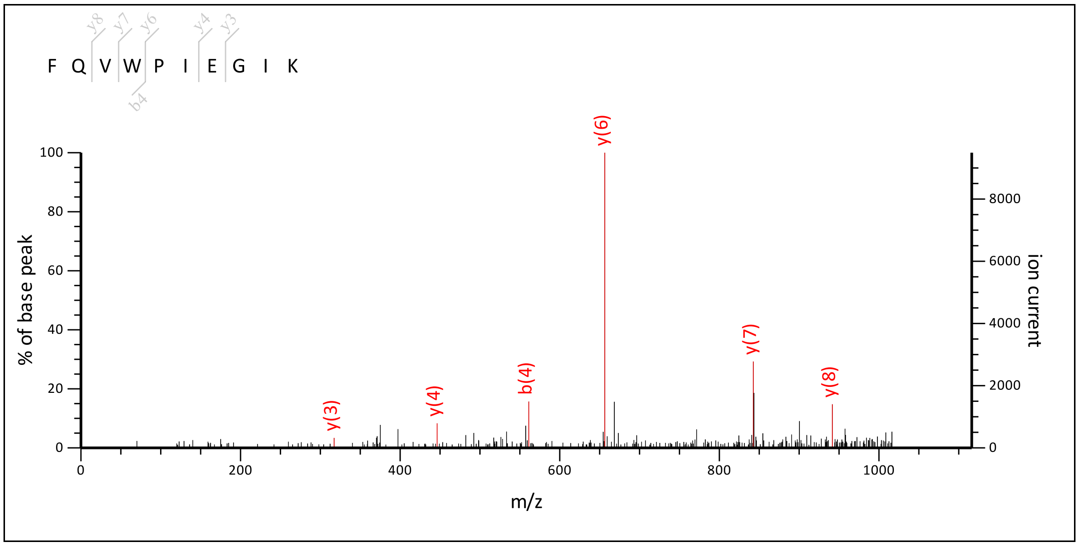


0 to 1116.27

Label all possible matches ○ Label matches used for scoring ◉

**Monoisotopic mass of neutral peptide Mr(calc):** 1215.6652
**Fixed modifications:** Carbamidomethyl (C) (apply to specified residues or termini only)
**Ions Score:** 33 **Expect:** 14
**Matches :** 6/148 fragment ions using 10 most intense peaks (help)

| # | Immon. | a | a* | $a^0$ | b | b* | $b^0$ | d | d' | Seq. | v | w | w' | y | y* | y |
|---|---|---|---|---|---|---|---|---|---|---|---|---|---|---|---|---|
| 1 | 120.0808 | 120.0808 | | | 148.0757 | | | 44.0495 | | F | | | | | | |
| 2 | 101.0709 | 248.1394 | 231.1128 | | 276.1343 | 259.1077 | | 191.1179 | | Q | 996.5513 | 995.5560 | | 1069.6041 | 1052.5775 | 1051. |
| 3 | 72.0808 | 347.2078 | 330.1812 | | 375.2027 | 358.1761 | | 333.1921 | | V | 897.4829 | 910.5033 | | ***941.5455*** | 924.5189 | 923. |
| 4 | 159.0917 | 533.2871 | 516.2605 | | **561.2820** | 544.2554 | | | | W | 711.4036 | | | ***842.4771*** | 825.4505 | 824. |
| 5 | 70.0651 | 630.3398 | 613.3133 | | 658.3348 | 641.3082 | | 604.3242 | | P | 614.3508 | 613.3556 | | ***656.3978*** | 639.3712 | 638. |
| 6 | 86.0964 | 743.4239 | 726.3974 | | 771.4188 | 754.3923 | | 715.3926 | 729.4083 | I | 501.2667 | 514.2871 | 528.3028 | 559.3450 | 542.3184 | 541. |
| 7 | 102.0550 | 872.4665 | 855.4400 | 854.4559 | 900.4614 | 883.4349 | 882.4509 | 814.4610 | | E | 372.2241 | 371.2289 | | ***446.2609*** | 429.2344 | 428. |
| 8 | 30.0338 | 929.4880 | 912.4614 | 911.4774 | 957.4829 | 940.4563 | 939.4723 | | | G | | | | ***317.2183*** | 300.1918 | |
| 9 | 86.0964 | 1042.5720 | 1025.5455 | 1024.5615 | 1070.5669 | 1053.5404 | 1052.5564 | 1014.5407 | 1028.5564 | I | 202.1186 | 215.1390 | 229.1547 | 260.1969 | 243.1703 | |
| 10 | 101.1073 | | | | | | | | | K | 74.0237 | 73.0284 | | 147.1128 | 130.0863 | |

| Seq | ya | yb | Seq | ya | yb | Seq | ya | yb |
|---|---|---|---|---|---|---|---|---|
| QV | 200.1394 | 228.1343 | QVW | 386.2187 | 414.2136 | QVWP | 483.2714 | 511.2663 |
| QVWPI | 596.3555 | 624.3504 | VW | 258.1601 | 286.1550 | VWP | 355.2129 | 383.2078 |
| VWPI | 468.2969 | 496.2918 | VWPIE | 597.3395 | 625.3344 | VWPIEG | 654.3610 | 682.3559 |
| WP | 256.1444 | 284.1394 | WPI | 369.2285 | 397.2234 | WPIE | 498.2711 | 526.2660 |
| WPIEG | 555.2926 | 583.2875 | WPIEGI | 668.3766 | 696.3715 | PI | 183.1492 | 211.1441 |
| PIE | 312.1918 | 340.1867 | PIEG | 369.2132 | 397.2082 | PIEGI | 482.2973 | 510.2922 |
| IE | 215.1390 | 243.1339 | IEG | 272.1605 | 300.1554 | IEGI | 385.2445 | 413.2395 |
| EG | 159.0764 | 187.0713 | EGI | 272.1605 | 300.1554 | GI | 143.1179 | 171.1128 |

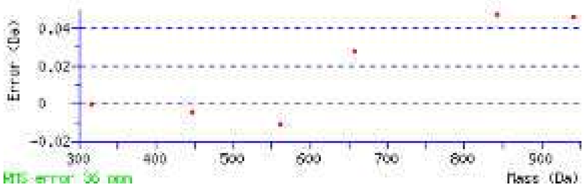


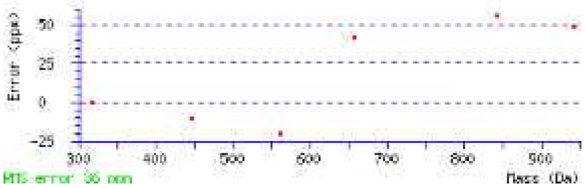


NCBI **BLAST** search of FQVWPIEGIK
(Parameters: blastp, nr protein database, expect=20000, no filter, PAM30)
Other BLAST web gateways

**All matches to this query**

| Score | Mr(calc) | Delta | Sequence |
|---|---|---|---|
| 32.7 | 1215.6652 | 0.0697 | FQVWPIEGIK |
| 32.7 | 1215.6322 | 0.1028 | MQVWPIEGIK |
| 32.7 | 1215.6322 | 0.1028 | MQVWPIEGIK |
| 29.6 | 1215.6281 | 0.1068 | MAALPTLTEGGR |
| 29.6 | 1215.6506 | 0.0843 | KMGGRPGSLASR |
| 28.5 | 1215.6459 | 0.0890 | TASDADLLALAR |
| 26.8 | 1215.6281 | 0.1068 | TASGLMLPEAAR |
| 26.5 | 1215.6281 | 0.1068 | GMAVTPREVEK |
| 25.5 | 1215.6645 | 0.0704 | MQVVSSPILAR |
| 25.5 | 1215.6758 | 0.0591 | AMGVVVKLGNGR |

**Mascot:** http://www.matrixscience.com/

# MATRIX SCIENCE Mascot Search Results

## Peptide View Spot no 8

MS/MS Fragmentation of **MQVWPIEGIKK**
Found in **gi|56966763** in **NCBInr**, Chain S, Crystal Structure Of Activated Rice Rubisco Complexed With 2- Carboxyarabinitol-1,5-Bisphosphate

Match to Query 174: 1327.848024 from(1328.855300,1+) intensity(0.0000) index(19)
Title: Label: N9, Spot_Id: 219815, Peak_List_Id: 227107, MSMS Job_Run_Id: 21951, Comment:
Data file C:\Documents and Settings\Administrator\Desktop\Farah-Garima 11May 2013\ppw_N9_136868212700.txt

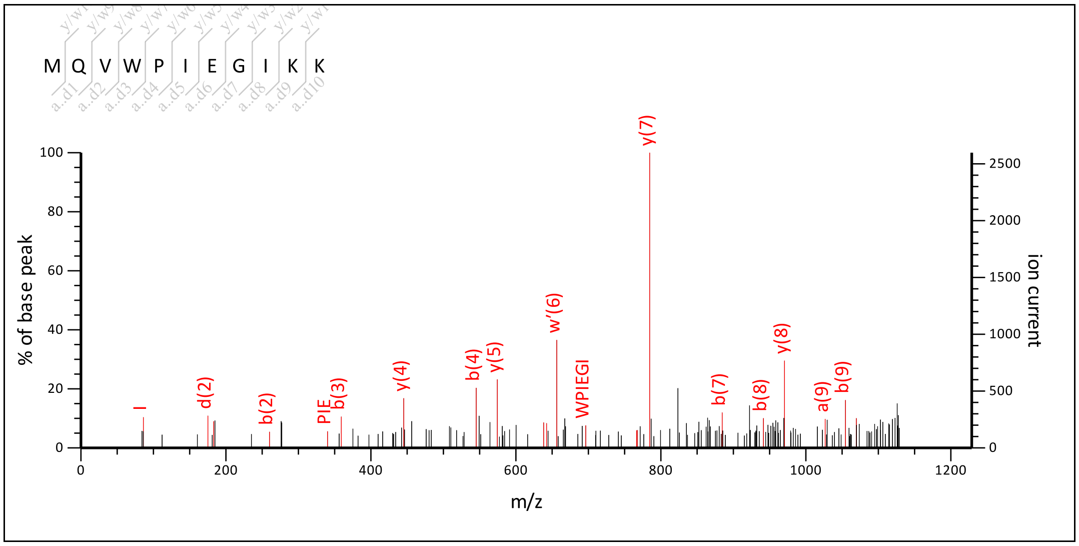


0 to 1228.83

Label all possible matches ○ Label matches used for scoring ◉

**Monoisotopic mass of neutral peptide Mr(calc):** 1327.7322
**Fixed modifications:** Carbamidomethyl (C) (apply to specified residues or termini only)
**Ions Score:** 27 **Expect:** 22
**Matches :** 24/170 fragment ions using 55 most intense peaks (help)

| # | Immon. | a | a* | $a^0$ | b | b* | $b^0$ | d | d' | Seq. | v | w | w' | y | y* | [illegible] |
|---|---|---|---|---|---|---|---|---|---|---|---|---|---|---|---|---|
| 1 | 104.0528 | 104.0528 | | | 132.0478 | | | 44.0495 | | M | | | | | | |
| 2 | 101.0709 | 232.1114 | 215.0849 | | ***260.1063*** | 243.0798 | | 175.0900 | | Q | 1124.6463 | 1123.6510 | | 1197.6990 | 1180.6725 | [illegible] |
| 3 | 72.0808 | 331.1798 | 314.1533 | | ***359.1748*** | 342.1482 | | 317.1642 | | V | 1025.5778 | 1038.5982 | | **1069.6404** | 1052.6139 | [illegible] |
| 4 | 159.0917 | 517.2592 | 500.2326 | | ***545.2541*** | 528.2275 | | | | W | 839.4985 | | | **970.5720** | 953.5455 | [illegible] |
| 5 | 70.0651 | 614.3119 | 597.2854 | | ***642.3068*** | 625.2803 | | 588.2963 | | P | 742.4458 | 741.4505 | | **784.4927** | 767.4662 | [illegible] |
| 6 | 86.0964 | 727.3960 | 710.3694 | | 755.3909 | 738.3643 | | 699.3647 | 713.3803 | I | 629.3617 | 642.3821 | 656.3978 | 687.4400 | 670.4134 | [illegible] |
| 7 | 102.0550 | 856.4386 | 839.4120 | 838.4280 | ***884.4335*** | 867.4069 | 866.4229 | 798.4331 | | E | 500.3191 | 499.3239 | | **574.3559** | 557.3293 | [illegible] |
| 8 | 30.0338 | 913.4600 | 896.4335 | 895.4495 | ***941.4550*** | 924.4284 | 923.4444 | | | G | | | | **445.3133** | 428.2867 | |
| 9 | 86.0964 | 1026.5441 | 1009.5176 | 1008.5335 | ***1054.5390*** | 1037.5125 | 1036.5285 | 998.5128 | 1012.5285 | I | 330.2136 | 343.2340 | 357.2496 | 388.2918 | 371.2653 | |
| 10 | 101.1073 | 1154.6391 | 1137.6125 | 1136.6285 | 1182.6340 | 1165.6074 | 1164.6234 | 1097.5812 | | K | 202.1186 | 201.1234 | | 275.2078 | 258.1812 | |
| 11 | 101.1073 | | | | | | | | | K | 74.0237 | 73.0284 | | 147.1128 | 130.0863 | |

| Seq | ya | yb | Seq | ya | yb | Seq | ya | yb |
|---|---|---|---|---|---|---|---|---|
| QV | 200.1394 | 228.1343 | QVW | 386.2187 | 414.2136 | QVWP | 483.2714 | 511.2663 |
| QVWPI | 596.3555 | 624.3504 | VW | 258.1601 | 286.1550 | VWP | 355.2129 | 383.2078 |
| VWPI | 468.2969 | 496.2918 | VWPIE | 597.3395 | 625.3344 | VWPIEG | 654.3610 | 682.3559 |
| WP | 256.1444 | 284.1394 | WPI | 369.2285 | 397.2234 | WPIE | 498.2711 | 526.2660 |
| WPIEG | 555.2926 | 583.2875 | WPIEGI | 668.3766 | 696.3715 | PI | 183.1492 | 211.1441 |
| PIE | 312.1918 | 340.1867 | PIEG | 369.2132 | 397.2082 | PIEGI | 482.2973 | 510.2922 |
| PIEGIK | 610.3923 | 638.3872 | IE | 215.1390 | 243.1339 | IEG | 272.1605 | 300.1554 |
| IEGI | 385.2445 | 413.2395 | IEGIK | 513.3395 | 541.3344 | EG | 159.0764 | 187.0713 |
| EGI | 272.1605 | 300.1554 | EGIK | 400.2554 | 428.2504 | GI | 143.1179 | 171.1128 |
| GIK | 271.2129 | 299.2078 | IK | 214.1914 | 242.1863 | | | |

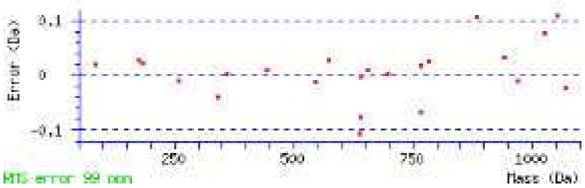


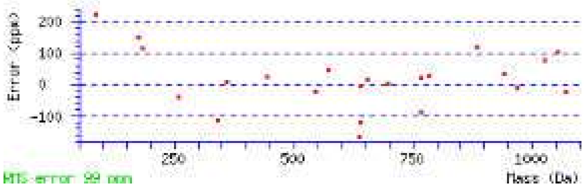


NCBI **BLAST** search of MQVWPIEGIKK
(Parameters: blastp, nr protein database, expect=20000, no filter, PAM30)
Other BLAST web gateways

**All matches to this query**

| Score | Mr(calc) | Delta | Sequence |
|---|---|---|---|
| 27.4 | 1327.7322 | 0.1158 | MQVWPIEGIKK |
| 27.4 | 1327.7322 | 0.1158 | MQVWPIEGIKK |
| 26.9 | 1327.7459 | 0.1021 | EKTVSPLQSAIR |
| 21.6 | 1327.7646 | 0.0835 | NCLTIALLALAR |
| 20.2 | 1327.7646 | 0.0835 | ADMKVGALLAALR |
| 19.5 | 1327.7646 | 0.0835 | QKMGLLLIGAER |
| 18.4 | 1327.8340 | 0.0141 | VAVKFKPSLAIR |
| 18.1 | 1327.7459 | 0.1021 | LTSENKAIISPR |
| 17.9 | 1327.7572 | 0.0909 | IIGISSIAGERGR |
| 17.9 | 1327.7572 | 0.0908 | RSDGIILGTGLAR |

**Mascot:** http://www.matrixscience.com/

# MATRIX SCIENCE Mascot Search Results

## Peptide View Spot no 8

MS/MS Fragmentation of **SKWVPCLEFSK**
Found in **gi|56966763** in **NCBInr**, Chain S, Crystal Structure Of Activated Rice Rubisco Complexed With 2- Carboxyarabinitol-1,5-Bisphosphate

Match to Query 183: 1379.818024 from(1380.825300,1+) intensity(0.0000) index(22)
Title: Label: N9, Spot_Id: 219815, Peak_List_Id: 227096, MSMS Job_Run_Id: 21951, Comment:
Data file C:\Documents and Settings\Administrator\Desktop\Farah-Garima 11May 2013\ppw_N9_136868212700.txt

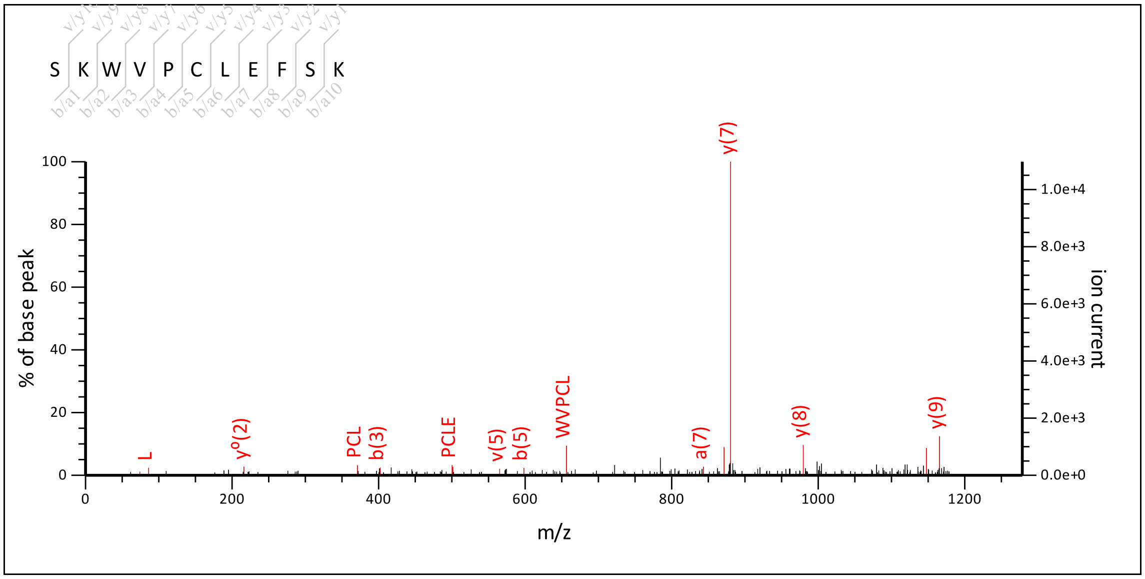


0 to 1278.55

Label all possible matches Label matches used for scoring

**Monoisotopic mass of neutral peptide Mr(calc):** 1379.6907
**Fixed modifications:** Carbamidomethyl (C) (apply to specified residues or termini only)
**Ions Score:** 24 **Expect:** 77
**Matches :** 18/176 fragment ions using 36 most intense peaks (help)

| # | Immon. | a | a* | $a^0$ | b | b* | $b^0$ | d | Seq. | v | w | y | y* | $y^0$ | # |
|---|---|---|---|---|---|---|---|---|---|---|---|---|---|---|---|
| 1 | 60.0444 | 60.0444 | | 42.0338 | 88.0393 | | 70.0287 | 44.0495 | S | | | | | | 11 |
| 2 | 101.1073 | 188.1394 | 171.1128 | 170.1288 | ***216.1343*** | 199.1077 | 198.1237 | 131.0815 | K | 1220.5769 | 1219.5816 | 1293.6660 | 1276.6395 | 1275.6554 | 10 |
| 3 | 159.0917 | 374.2187 | 357.1921 | 356.2081 | ***402.2136*** | 385.1870 | 384.2030 | | W | 1034.4975 | | **1165.5710** | 1148.5445 | 1147.5605 | 9 |
| 4 | 72.0808 | 473.2871 | 456.2605 | 455.2765 | ***501.2820*** | 484.2554 | 483.2714 | 459.2714 | V | 935.4291 | 948.4495 | **979.4917** | 962.4652 | 961.4812 | 8 |
| 5 | 70.0651 | 570.3398 | 553.3133 | 552.3293 | ***598.3348*** | 581.3082 | 580.3242 | 544.3242 | P | 838.3764 | 837.3811 | **880.4233** | 863.3968 | 862.4128 | 7 |
| 6 | 133.0430 | 730.3705 | 713.3439 | 712.3599 | 758.3654 | 741.3389 | 740.3548 | 641.3770 | C | 678.3457 | 677.3505 | 783.3706 | 766.3440 | 765.3600 | 6 |
| 7 | 86.0964 | 843.4546 | 826.4280 | 825.4440 | ***871.4495*** | 854.4229 | 853.4389 | 801.4076 | L | 565.2617 | 564.2664 | 623.3399 | 606.3134 | 605.3293 | 5 |
| 8 | 102.0550 | 972.4971 | 955.4706 | 954.4866 | 1000.4921 | 983.4655 | 982.4815 | 914.4917 | E | 436.2191 | 435.2238 | 510.2558 | 493.2293 | 492.2453 | 4 |
| 9 | 120.0808 | 1119.5656 | 1102.5390 | 1101.5550 | ***1147.5605*** | 1130.5339 | 1129.5499 | | F | 289.1506 | | 381.2132 | 364.1867 | 363.2027 | 3 |
| 10 | 60.0444 | 1206.5976 | 1189.5710 | 1188.5870 | 1234.5925 | 1217.5660 | 1216.5819 | 1190.6027 | S | 202.1186 | 201.1234 | 234.1448 | 217.1183 | 216.1343 | 2 |
| 11 | 101.1073 | | | | | | | | K | 74.0237 | 73.0284 | 147.1128 | 130.0863 | | 1 |

| Seq | ya | yb | Seq | ya | yb | Seq | ya | yb |
|---|---|---|---|---|---|---|---|---|
| KW | 287.1866 | 315.1816 | KWV | 386.2551 | 414.2500 | KWVP | 483.3078 | 511.3027 |
| KWVPC | 643.3385 | 671.3334 | WV | 258.1601 | 286.1550 | WVP | 355.2129 | 383.2078 |
| WVPC | 515.2435 | 543.2384 | WVPCL | 628.3276 | 656.3225 | VP | 169.1335 | 197.1285 |
| VPC | 329.1642 | 357.1591 | VPCL | 442.2483 | 470.2432 | VPCLE | 571.2908 | 599.2858 |
| PC | 230.0958 | 258.0907 | PCL | 343.1798 | 371.1748 | PCLE | 472.2224 | 500.2173 |
| PCLEF | 619.2908 | 647.2858 | CL | 246.1271 | 274.1220 | CLE | 375.1697 | 403.1646 |
| | | | | | | | | |

| | | | | | | | | |
|---|---|---|---|---|---|---|---|---|
| **CLEF** | 522.2381 | 550.2330 | **CLEFS** | 609.2701 | 637.2650 | **LE** | 215.1390 | 243.1339 |
| **LEF** | 362.2074 | 390.2023 | **LEFS** | 449.2395 | 477.2344 | **EF** | 249.1234 | 277.1183 |
| **EFS** | 336.1554 | 364.1503 | **FS** | 207.1128 | 235.1077 | | | |

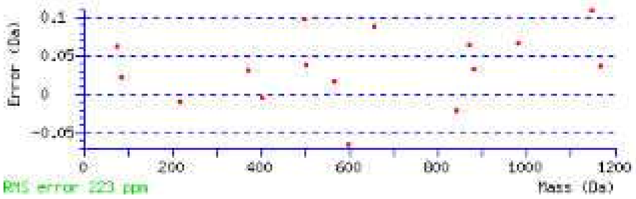


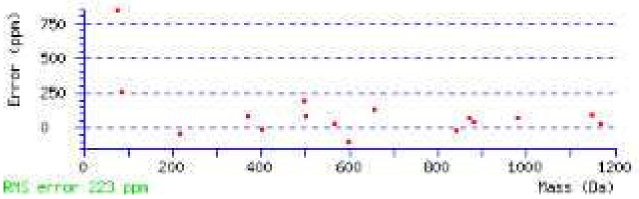


NCBI **BLAST** search of SKWVPCLEFSK
(Parameters: blastp, nr protein database, expect=20000, no filter, PAM30)
Other BLAST web gateways

**All matches to this query**

| Score | Mr(calc) | Delta | Sequence |
|---|---|---|---|
| 24.4 | 1379.6907 | 0.1273 | SKWVPCLEFSK |
| 20.9 | 1379.6867 | 0.1313 | IREEHMIPDPK |
| 20.4 | 1379.7231 | 0.0949 | LMGFLDGTGAVRK |
| 20.3 | 1379.7885 | 0.0295 | LTPSERPVVLNR |
| 18.7 | 1379.6867 | 0.1313 | LREYCDAVQVK |
| 18.7 | 1379.7343 | 0.0837 | LGIDMQPHVARK |
| 18.6 | 1379.8249 | -0.0068 | IVSSLRQIHISK |
| 18.4 | 1379.7521 | 0.0659 | ADVIERQLAHTK |
| 16.5 | 1379.8249 | -0.0068 | AGSVSLLIRISHK |
| 16.4 | 1379.7674 | 0.0507 | VWIATIHSVAQR |

**Mascot:** http://www.matrixscience.com/

# MATRIX SCIENCE Mascot Search Results

## Peptide View Spot no 8

MS/MS Fragmentation of **LPMFGCTDATQVLK**
Found in **gi|56966763** in **NCBInr**, Chain S, Crystal Structure Of Activated Rice Rubisco Complexed With 2- Carboxyarabinitol-1,5-Bisphosphate

Match to Query 205: 1579.924724 from(1580.932000,1+) intensity(0.0000) index(26)
Title: Label: N9, Spot_Id: 219815, Peak_List_Id: 227100, MSMS Job_Run_Id: 21951, Comment:
Data file C:\Documents and Settings\Administrator\Desktop\Farah-Garima 11May 2013\ppw_N9_136868212700.txt

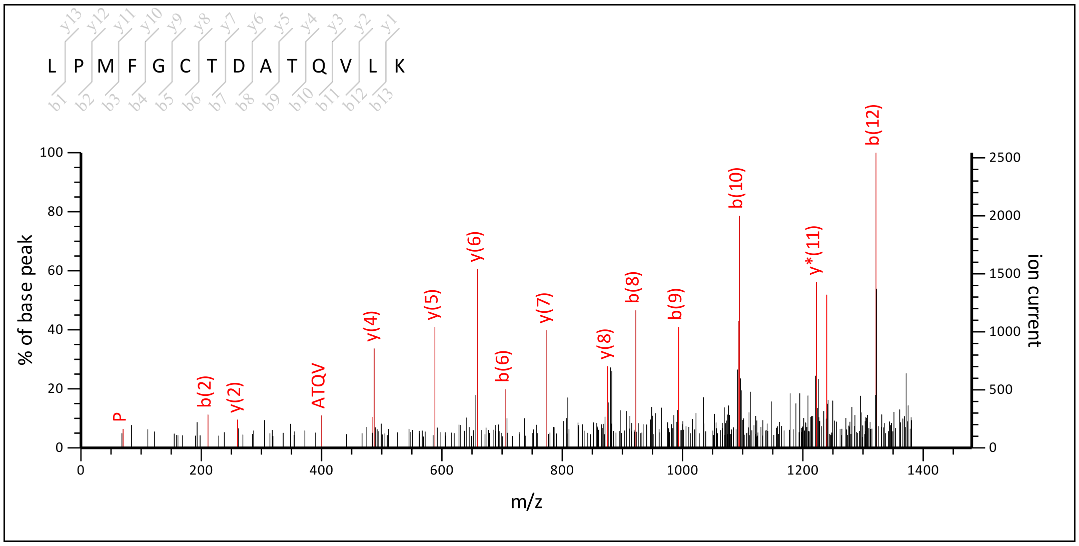


0 to 1480.69

Label all possible matches Label matches used for scoring

**Monoisotopic mass of neutral peptide Mr(calc):** 1579.7738
**Fixed modifications:** Carbamidomethyl (C) (apply to specified residues or termini only)
**Ions Score:** 60 **Expect:** 0.019
**Matches :** 24/220 fragment ions using 28 most intense peaks (help)

| # | Immon. | a | a* | a⁰ | b | b* | b⁰ | d | d' | Seq. | v | w | w' | y | y* | |
|---|---|---|---|---|---|---|---|---|---|---|---|---|---|---|---|---|
| 1 | 86.0964 | 86.0964 | | | 114.0913 | | | 44.0495 | | L | | | | | | |
| 2 | 70.0651 | 183.1492 | | | ***211.1441*** | | | 157.1335 | | P | 1425.6501 | 1424.6549 | | 1467.6971 | 1450.6705 | [illegible] |
| 3 | 104.0528 | 314.1897 | | | 342.1846 | | | 254.1863 | | M | 1294.6096 | 1293.6144 | | 1370.6443 | 1353.6177 | [illegible] |
| 4 | 120.0808 | 461.2581 | | | 489.2530 | | | | | F | 1147.5412 | | | ***1239.6038*** | 1222.5773 | [illegible] |
| 5 | 30.0338 | 518.2796 | | | 546.2745 | | | | | G | | | | ***1092.5354*** | 1075.5088 | [illegible] |
| 6 | 133.0430 | 678.3102 | | | ***706.3051*** | | | 589.3167 | | C | 930.4891 | 929.4938 | | 1035.5139 | 1018.4874 | [illegible] |
| 7 | 74.0600 | 779.3579 | | 761.3473 | 807.3528 | | 789.3422 | 763.3630 | 765.3422 | T | 829.4414 | 842.4618 | 844.4411 | ***875.4833*** | 858.4567 | [illegible] |
| 8 | 88.0393 | 894.3848 | | 876.3743 | ***922.3797*** | | 904.3692 | 850.3950 | | D | 714.4145 | 713.4192 | | ***774.4356*** | 757.4090 | [illegible] |
| 9 | 44.0495 | 965.4219 | | 947.4114 | ***993.4169*** | | 975.4063 | | | A | 643.3774 | | | ***659.4087*** | 642.3821 | [illegible] |
| 10 | 74.0600 | 1066.4696 | | 1048.4591 | ***1094.4645*** | | 1076.4540 | 1050.4747 | 1052.4540 | T | 542.3297 | 555.3501 | 557.3293 | ***588.3715*** | 571.3450 | [illegible] |
| 11 | 101.0709 | 1194.5282 | 1177.5016 | 1176.5176 | ***1222.5231*** | 1205.4966 | 1204.5125 | 1137.5067 | | Q | 414.2711 | 413.2758 | | ***487.3239*** | 470.2973 | |
| 12 | 72.0808 | 1293.5966 | 1276.5701 | 1275.5860 | ***1321.5915*** | 1304.5650 | 1303.5810 | 1279.5810 | | V | 315.2027 | 328.2231 | | 359.2653 | 342.2387 | |
| 13 | 86.0964 | 1406.6807 | 1389.6541 | 1388.6701 | 1434.6756 | 1417.6490 | 1416.6650 | 1364.6337 | | L | 202.1186 | 201.1234 | | ***260.1969*** | 243.1703 | |
| 14 | 101.1073 | | | | | | | | | K | 74.0237 | 73.0284 | | 147.1128 | 130.0863 | |

| Seq | ya | yb | Seq | ya | yb | Seq | ya | yb |
|---|---|---|---|---|---|---|---|---|
| PM | 201.1056 | 229.1005 | PMF | 348.1740 | 376.1689 | PMFG | 405.1955 | 433.1904 |
| PMFGC | 565.2261 | 593.2211 | PMFGCT | 666.2738 | 694.2687 | MF | 251.1213 | 279.1162 |
| MFG | 308.1427 | 336.1376 | MFGC | 468.1734 | 496.1683 | MFGCT | 569.2211 | 597.2160 |
| MFGCTD | 684.2480 | 712.2429 | FG | 177.1022 | 205.0972 | FGC | 337.1329 | 365.1278 |
| FGCT | 438.1806 | 466.1755 | FGCTD | 553.2075 | 581.2024 | FGCTDA | 624.2446 | 652.2395 |
| GC | 190.0645 | 218.0594 | GCT | 291.1122 | 319.1071 | GCTD | 406.1391 | 434.1340 |
| GCTDA | 477.1762 | 505.1711 | GCTDAT | 578.2239 | 606.2188 | CT | 234.0907 | 262.0856 |

| | | | | | | | | |
|---|---|---|---|---|---|---|---|---|
| **CTD** | 349.1176 | 377.1125 | **CTDA** | 420.1547 | 448.1497 | **CTDAT** | 521.2024 | 549.1973 |
| **CTDATQ** | 649.2610 | 677.2559 | **TD** | 189.0870 | 217.0819 | **TDA** | 260.1241 | 288.1190 |
| **TDAT** | 361.1718 | 389.1667 | **TDATQ** | 489.2304 | 517.2253 | **TDATQV** | 588.2988 | 616.2937 |
| **DA** | 159.0764 | 187.0713 | **DAT** | 260.1241 | 288.1190 | **DATQ** | 388.1827 | 416.1776 |
| **DATQV** | 487.2511 | 515.2460 | **DATQVL** | 600.3352 | 628.3301 | **AT** | 145.0972 | 173.0921 |
| **ATQ** | 273.1557 | 301.1506 | **ATQV** | 372.2241 | 400.2191 | **ATQVL** | 485.3082 | 513.3031 |
| **TQ** | 202.1186 | 230.1135 | **TQV** | 301.1870 | 329.1819 | **TQVL** | 414.2711 | 442.2660 |
| **QV** | 200.1394 | 228.1343 | **QVL** | 313.2234 | 341.2183 | **VL** | 185.1648 | 213.1598 |

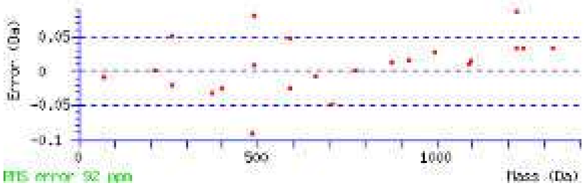


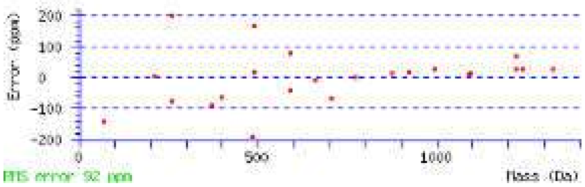


NCBI **BLAST** search of LPMFGCTDATQVLK
(Parameters: blastp, nr protein database, expect=20000, no filter, PAM30)
Other BLAST web gateways

**All matches to this query**

| Score | Mr(calc) | Delta | Sequence |
|---|---|---|---|
| 60.5 | 1579.7738 | 0.1509 | LPMFGCTDATQVLK |
| 24.4 | 1579.8756 | 0.0491 | FLLSRMSSATQVLK |
| 23.0 | 1579.8570 | 0.0678 | LPLPGDLTDAQTALR |
| 21.0 | 1579.7916 | 0.1331 | QMGFEESLATQLVK |
| 18.3 | 1579.8029 | 0.1219 | MFDTVAIVGATGAVGR |
| 17.9 | 1579.8868 | 0.0379 | RMIAEHALATNLIK |
| 17.6 | 1579.8206 | 0.1042 | ENKLSSDTSFILAR |
| 17.5 | 1579.7850 | 0.1397 | KQNGMMLFATQSPK |
| 16.3 | 1579.8643 | 0.0604 | VYTLIMQGATLNLK |
| 16.2 | 1579.8933 | 0.0314 | ELVQELLPATGAAIR |

**Mascot:** http://www.matrixscience.com/

# MATRIX SCIENCE Mascot Search Results

## Peptide View **Spot no 8**

MS/MS Fragmentation of **LPMFGCTDATQVLK**
Found in **gi|56966763** in **NCBInr**, Chain S, Crystal Structure Of Activated Rice Rubisco Complexed With 2- Carboxyarabinitol-1,5-Bisphosphate

Match to Query 207: 1595.917624 from(1596.924900,1+) intensity(0.0000) index(27)
Title: Label: N9, Spot_Id: 219815, Peak_List_Id: 227095, MSMS Job_Run_Id: 21951, Comment:
Data file C:\Documents and Settings\Administrator\Desktop\Farah-Garima 11May 2013\ppw_N9_136868212700.txt

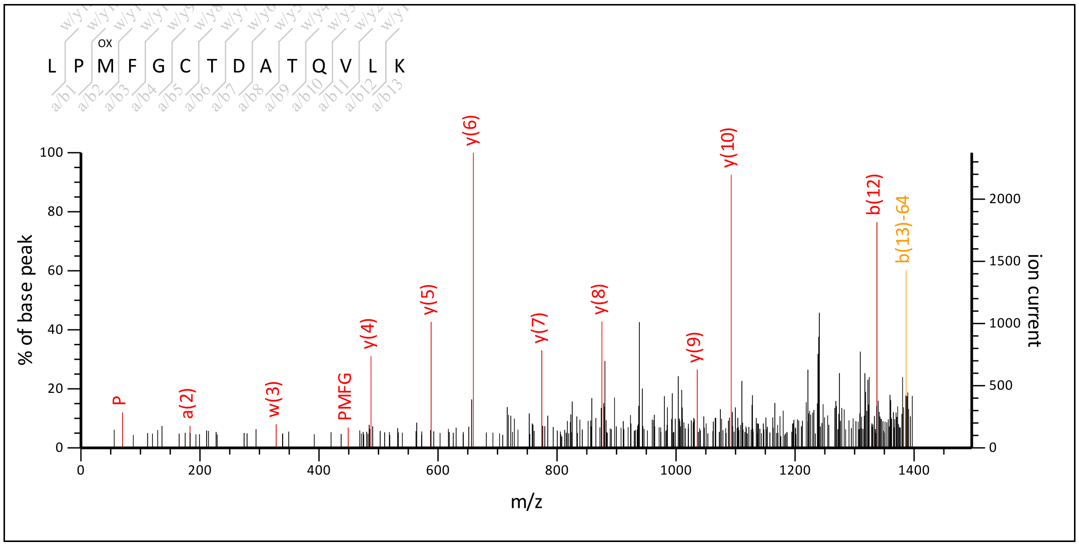


0 to 1496.86

Label all possible matches ○ Label matches used for scoring ◉

**Monoisotopic mass of neutral peptide Mr(calc):** 1595.7688
**Fixed modifications:** Carbamidomethyl (C) (apply to specified residues or termini only)
**Variable modifications:**
**M3** : Oxidation (M), with neutral losses 0.0000(shown in table), 63.9983
**Ions Score:** 74 **Expect:** 0.00091
**Matches :** 18/295 fragment ions using 14 most intense peaks (help)

| # | Immon. | a | a* | $a^0$ | b | b* | $b^0$ | d | d' | Seq. | v | w | w' | y | y* | [illegible] |
|---|---|---|---|---|---|---|---|---|---|---|---|---|---|---|---|---|
| 1 | 86.0964 | 86.0964 | | | 114.0913 | | | 44.0495 | | L | | | | | | |
| 2 | 70.0651 | **183.1492** | | | 211.1441 | | | 157.1335 | | P | 1441.6450 | 1440.6498 | | 1483.6920 | 1466.6654 | [illegible] |
| 3 | 120.0478 | 330.1846 | | | 358.1795 | | | 254.1863 | | M | 1294.6096 | 1293.6144 | | ***1386.6392*** | 1369.6127 | [illegible] |
| 4 | 120.0808 | 477.2530 | | | 505.2479 | | | | | F | 1147.5412 | | | 1239.6038 | 1222.5773 | [illegible] |
| 5 | 30.0338 | 534.2745 | | | 562.2694 | | | | | G | | | | ***1092.5354*** | 1075.5088 | [illegible] |
| 6 | 133.0430 | 694.3051 | | | 722.3000 | | | 605.3116 | | C | 930.4891 | 929.4938 | | ***1035.5139*** | 1018.4874 | [illegible] |
| 7 | 74.0600 | 795.3528 | | 777.3422 | 823.3477 | | 805.3371 | 779.3579 | 781.3371 | T | 829.4414 | 842.4618 | 844.4411 | ***875.4833*** | 858.4567 | [illegible] |
| 8 | 88.0393 | 910.3797 | | 892.3692 | 938.3747 | | 920.3641 | 866.3899 | | D | 714.4145 | 713.4192 | | ***774.4356*** | 757.4090 | [illegible] |
| 9 | 44.0495 | 981.4169 | | 963.4063 | 1009.4118 | | 991.4012 | | | A | 643.3774 | | | ***659.4087*** | 642.3821 | [illegible] |
| 10 | 74.0600 | 1082.4645 | | 1064.4540 | 1110.4594 | | 1092.4489 | 1066.4696 | 1068.4489 | T | 542.3297 | 555.3501 | 557.3293 | ***588.3715*** | 571.3450 | [illegible] |
| 11 | 101.0709 | 1210.5231 | 1193.4966 | 1192.5125 | 1238.5180 | 1221.4915 | 1220.5075 | 1153.5016 | | Q | 414.2711 | 413.2758 | | ***487.3239*** | 470.2973 | |
| 12 | 72.0808 | 1309.5915 | 1292.5650 | 1291.5810 | **1337.5864** | 1320.5599 | 1319.5759 | 1295.5759 | | V | 315.2027 | 328.2231 | | 359.2653 | 342.2387 | |
| 13 | 86.0964 | 1422.6756 | 1405.6490 | 1404.6650 | 1450.6705 | 1433.6440 | 1432.6599 | 1380.6286 | | L | 202.1186 | 201.1234 | | 260.1969 | 243.1703 | |
| 14 | 101.1073 | | | | | | | | | K | 74.0237 | 73.0284 | | 147.1128 | 130.0863 | |

| Seq | ya | yb | Seq | ya | yb | Seq | ya | yb |
|---|---|---|---|---|---|---|---|---|
| **PM** | 217.1005 | 245.0954 | **PMF** | 364.1689 | 392.1639 | **PMFG** | 421.1904 | 449.1853 |
| **PMFGC** | 581.2211 | 609.2160 | **PMFGCT** | 682.2687 | 710.2636 | **MF** | 267.1162 | 295.1111 |
| **MFG** | 324.1376 | 352.1326 | **MFGC** | 484.1683 | 512.1632 | **MFGCT** | 585.2160 | 613.2109 |
| **FG** | 177.1022 | 205.0972 | **FGC** | 337.1329 | 365.1278 | **FGCT** | 438.1806 | 466.1755 |
| **FGCTD** | 553.2075 | 581.2024 | **FGCTDA** | 624.2446 | 652.2395 | **GC** | 190.0645 | 218.0594 |
| **GCT** | 291.1122 | 319.1071 | **GCTD** | 406.1391 | 434.1340 | **GCTDA** | 477.1762 | 505.1711 |

| | | | | | | | | |
|---|---|---|---|---|---|---|---|---|
| **GCTDAT** | 578.2239 | 606.2188 | **CT** | 234.0907 | 262.0856 | **CTD** | 349.1176 | 377.1125 |
| **CTDA** | 420.1547 | 448.1497 | **CTDAT** | 521.2024 | 549.1973 | **CTDATQ** | 649.2610 | 677.2559 |
| **TD** | 189.0870 | 217.0819 | **TDA** | 260.1241 | 288.1190 | **TDAT** | 361.1718 | 389.1667 |
| **TDATQ** | 489.2304 | 517.2253 | **TDATQV** | 588.2988 | 616.2937 | **DA** | 159.0764 | 187.0713 |
| **DAT** | 260.1241 | 288.1190 | **DATQ** | 388.1827 | 416.1776 | **DATQV** | 487.2511 | 515.2460 |
| **DATQVL** | 600.3352 | 628.3301 | **AT** | 145.0972 | 173.0921 | **ATQ** | 273.1557 | 301.1506 |
| **ATQV** | 372.2241 | 400.2191 | **ATQVL** | 485.3082 | 513.3031 | **TQ** | 202.1186 | 230.1135 |
| **TQV** | 301.1870 | 329.1819 | **TQVL** | 414.2711 | 442.2660 | **QV** | 200.1394 | 228.1343 |
| **QVL** | 313.2234 | 341.2183 | **VL** | 185.1648 | 213.1598 | | | |

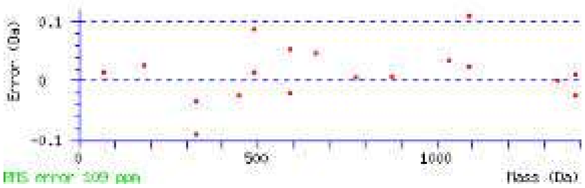


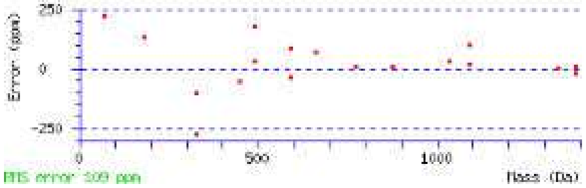


NCBI **BLAST** search of LPMFGCTDATQVLK
(Parameters: blastp, nr protein database, expect=20000, no filter, PAM30)
Other BLAST web gateways

**All matches to this query**

| Score | Mr(calc) | Delta | Sequence |
|---|---|---|---|
| 74.2 | 1595.7688 | 0.1489 | LPMFGCTDATQVLK |
| 74.2 | 1595.7688 | 0.1489 | LPMYGCTDATQVLK |
| 26.0 | 1595.8276 | 0.0900 | TLLLMVSTCLHHR |
| 24.1 | 1595.8882 | 0.0294 | VGSSRTAEEPILPLK |
| 16.1 | 1595.7692 | 0.1484 | HDHPTADALYTSIR |
| 15.8 | 1595.8379 | 0.0797 | EQRAQLPDATIAQR |
| 15.1 | 1595.8269 | 0.0907 | VFLDALDVMGYPIK |
| 14.7 | 1595.7614 | 0.1562 | VCVAGGDVVSSEYVR |
| 14.1 | 1595.7687 | 0.1489 | EIILMFDMDKPGR |
| 13.9 | 1595.8415 | 0.0761 | LMMLFGEKSGLNIK |

**Mascot:** http://www.matrixscience.com/

# MATRIX SCIENCE Mascot Search Results

## Peptide View **Spot no 10**

MS/MS Fragmentation of **SGGGGGGGYGQR**
Found in **gi|18103931** in **NCBInr**, glycine rich RNA binding protein [Oryza sativa Indica Group]

Match to Query 12: 1008.498624 from(1009.505900,1+) intensity(0.0000) index(5)
Title: Label: E6, Spot_Id: 228926, Peak_List_Id: 257517, MSMS Job_Run_Id: 24923, Comment:
Data file ppw_E6_138985130300.txt

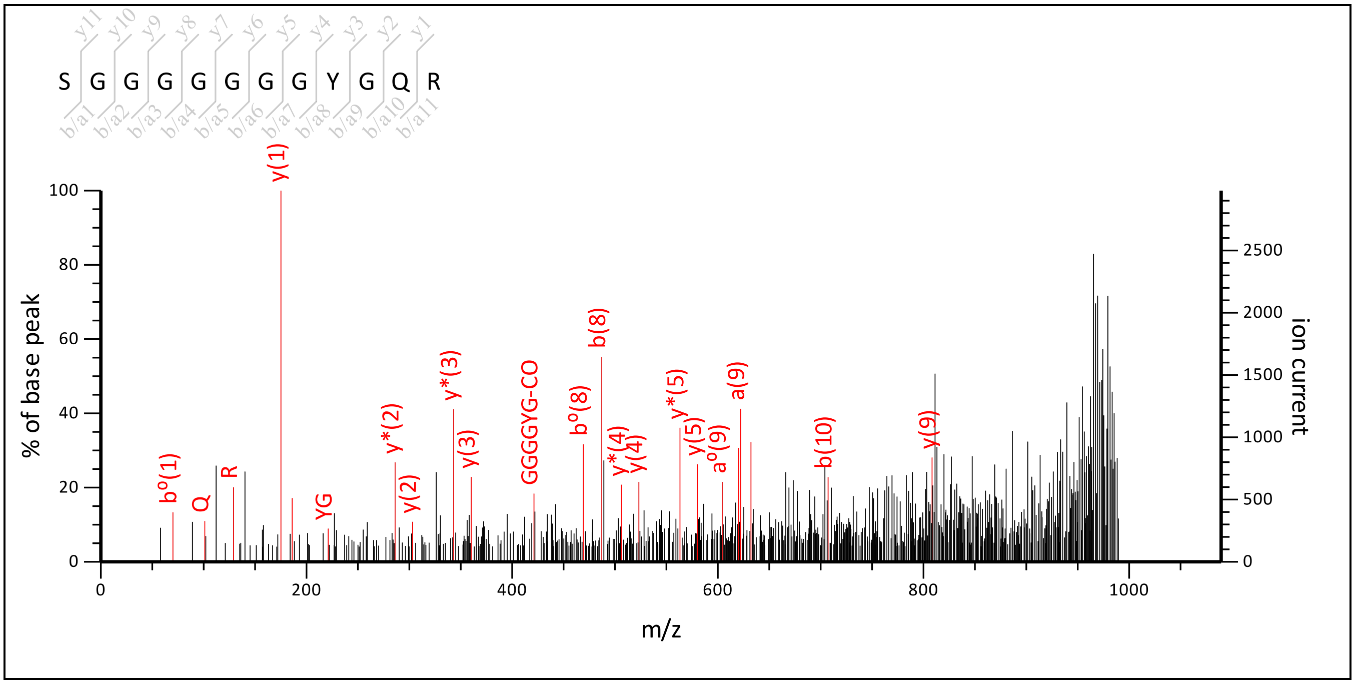


0 to 1089.46

Label all possible matches ○ Label matches used for scoring ◉

**Monoisotopic mass of neutral peptide Mr(calc):** 1008.4373
**Fixed modifications:** Carbamidomethyl (C) (apply to specified residues or termini only)
**Ions Score:** 17 **Expect:** 50
**Matches :** 37/175 fragment ions using 60 most intense peaks (help)

| # | Immon. | a | a* | $a^0$ | b | b* | $b^0$ | d | Seq. | v | w | y | y* | # |
|---|---|---|---|---|---|---|---|---|---|---|---|---|---|---|
| 1 | 60.0444 | 60.0444 | | 42.0338 | 88.0393 | | 70.0287 | 44.0495 | S | | | | | 12 |
| 2 | 30.0338 | 117.0659 | | 99.0553 | 145.0608 | | 127.0502 | | G | | | 922.4126 | 905.3860 | 11 |
| 3 | 30.0338 | 174.0873 | | 156.0768 | 202.0822 | | 184.0717 | | G | | | 865.3911 | 848.3646 | 10 |
| 4 | 30.0338 | 231.1088 | | 213.0982 | 259.1037 | | 241.0931 | | G | | | ***808.3696*** | 791.3431 | 9 |
| 5 | 30.0338 | 288.1302 | | 270.1197 | 316.1252 | | 298.1146 | | G | | | 751.3482 | 734.3216 | 8 |
| 6 | 30.0338 | 345.1517 | | 327.1411 | 373.1466 | | 355.1361 | | G | | | 694.3267 | 677.3002 | 7 |
| 7 | 30.0338 | 402.1732 | | 384.1626 | 430.1681 | | 412.1575 | | G | | | 637.3053 | 620.2787 | 6 |
| 8 | 30.0338 | 459.1946 | | 441.1841 | **487.1896** | | 469.1790 | | G | | | ***580.2838*** | 563.2572 | 5 |
| 9 | 136.0757 | 622.2580 | | 604.2474 | 650.2529 | | 632.2423 | | Y | 415.2048 | | ***523.2623*** | 506.2358 | 4 |
| 10 | 30.0338 | 679.2794 | | 661.2689 | **707.2743** | | 689.2638 | | G | | | ***360.1990*** | 343.1724 | 3 |

| | | | | | | | | | | | | | | |
|---|---|---|---|---|---|---|---|---|---|---|---|---|---|---|
| 11 | 101.0709 | 807.3380 | 790.3115 | 789.3274 | 835.3329 | 818.3064 | 817.3224 | 750.3165 | Q | 230.1248 | 229.1295 | ***303.1775*** | 286.1510 | 2 |
| 12 | 129.1135 | | | | | | | | R | 74.0237 | 73.0284 | ***175.1190*** | 158.0924 | 1 |

| Seq | ya | yb | Seq | ya | yb | Seq | ya | yb |
|---|---|---|---|---|---|---|---|---|
| GG | 87.0553 | 115.0502 | GGG | 144.0768 | 172.0717 | GGGG | 201.0982 | 229.0931 |
| GGGGG | 258.1197 | 286.1146 | GGGGGG | 315.1411 | 343.1361 | GGGGGGG | 372.1626 | 400.1575 |
| GGGGGGGY | 535.2259 | 563.2209 | GGGGGGGYG | 592.2474 | 620.2423 | GG | 87.0553 | 115.0502 |
| GGG | 144.0768 | 172.0717 | GGGG | 201.0982 | 229.0931 | GGGGG | 258.1197 | 286.1146 |
| GGGGGG | 315.1411 | 343.1361 | GGGGGGY | 478.2045 | 506.1994 | GGGGGGYG | 535.2259 | 563.2209 |
| GGGGGGYGQ | 663.2845 | 691.2794 | GG | 87.0553 | 115.0502 | GGG | 144.0768 | 172.0717 |
| GGGG | 201.0982 | 229.0931 | GGGGG | 258.1197 | 286.1146 | GGGGGY | 421.1830 | 449.1779 |
| GGGGGYG | 478.2045 | 506.1994 | GGGGGYGQ | 606.2631 | 634.2580 | GG | 87.0553 | 115.0502 |
| GGG | 144.0768 | 172.0717 | GGGG | 201.0982 | 229.0931 | GGGGY | 364.1615 | 392.1565 |
| GGGGYG | 421.1830 | 449.1779 | GGGGYGQ | 549.2416 | 577.2365 | GG | 87.0553 | 115.0502 |
| GGG | 144.0768 | 172.0717 | GGGY | 307.1401 | 335.1350 | GGGYG | 364.1615 | 392.1565 |
| GGGYGQ | 492.2201 | 520.2150 | GG | 87.0553 | 115.0502 | GGY | 250.1186 | 278.1135 |
| GGYG | 307.1401 | 335.1350 | GGYGQ | 435.1987 | 463.1936 | GY | 193.0972 | 221.0921 |
| GYG | 250.1186 | 278.1135 | GYGQ | 378.1772 | 406.1721 | YG | 193.0972 | 221.0921 |
| YGQ | 321.1557 | 349.1506 | GQ | 158.0924 | 186.0873 | | | |

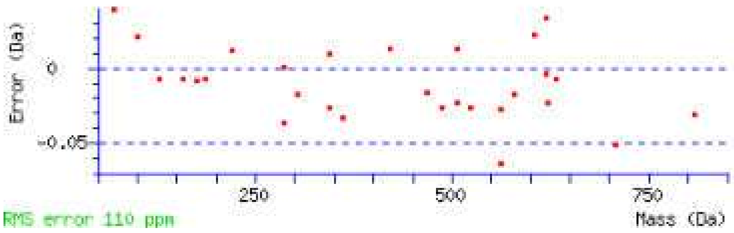


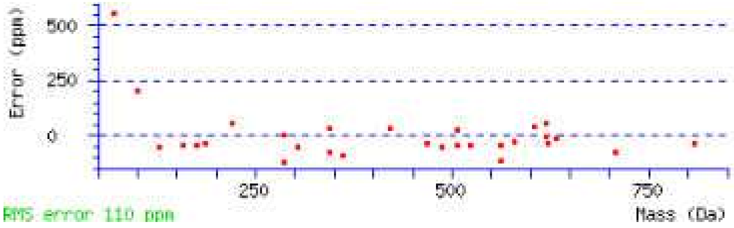


NCBI **BLAST** search of SGGGGGGGYGQR
(Parameters: blastp, nr protein database, expect=20000, no filter, PAM30)
Other BLAST web gateways

**All matches to this query**

| Score | Mr(calc) | Delta | Sequence |
|---|---|---|---|
| 17.2 | 1008.4373 | 0.0613 | SGGGGGGGYGQR |
| 9.2 | 1008.5352 | -0.0366 | YGVVSGGSKR |
| 8.6 | 1008.4413 | 0.0573 | QDWGYANR |
| 8.1 | 1008.5101 | -0.0115 | RVGSSYGQR |
| 8.0 | 1008.4373 | 0.0613 | SGGGGGGGAGYGR |
| 7.4 | 1008.5716 | -0.0730 | VVSANPVPAR |
| 7.2 | 1008.5287 | -0.0301 | QGAMPPRPR |
| | | | |

| | | | |
|---|---|---|---|
| 7.1 | 1008.4295 | 0.0692 | MASDGDGKGR |
| 6.6 | 1008.4889 | 0.0097 | RWYNSQR |
| 6.3 | 1008.5604 | -0.0618 | KSSLFGGVSK |

**Mascot:** http://www.matrixscience.com/

# Mascot Search Results

## Peptide View **Spot no 10**

MS/MS Fragmentation of **RSGGGGGGGYGQR**
Found in **gi|18103931** in **NCBInr**, glycine rich RNA binding protein [Oryza sativa Indica Group]

Match to Query 22: 1164.605024 from(1165.612300,1+) intensity(0.0000) index(9)
Title: Label: E6, Spot_Id: 228926, Peak_List_Id: 257520, MSMS Job_Run_Id: 24923, Comment:
Data file ppw_E6_138985130300.txt

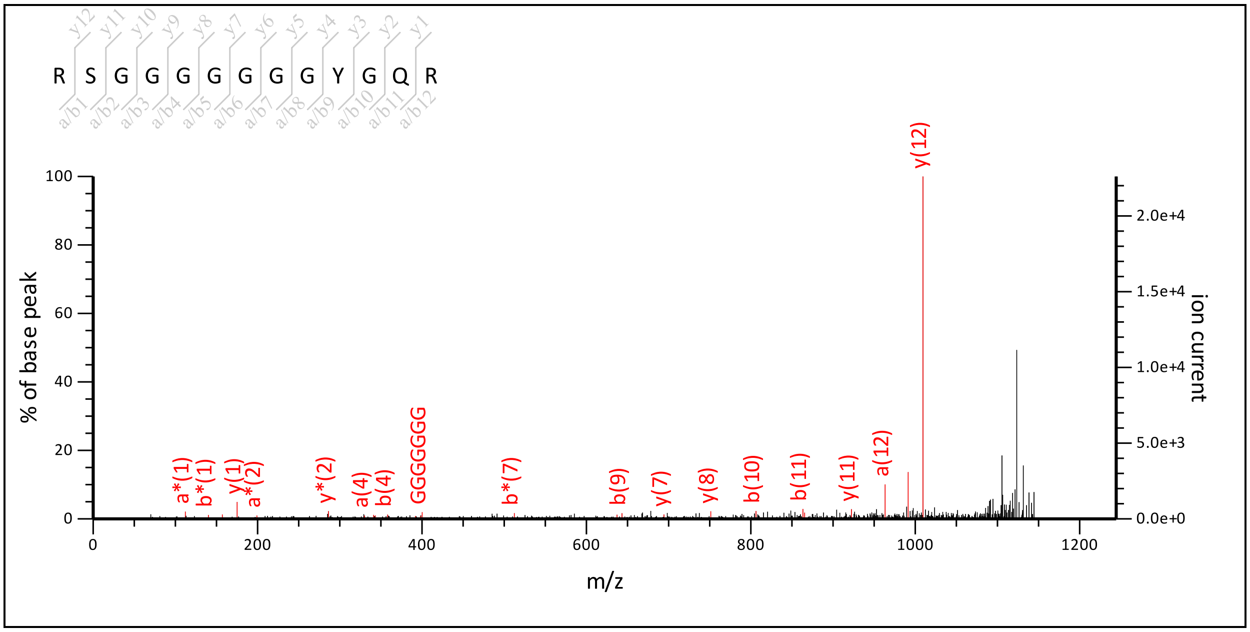


0 to 1244.44

Label all possible matches ○ Label matches used for scoring ◉

**Monoisotopic mass of neutral peptide Mr(calc):** 1164.5385
**Fixed modifications:** Carbamidomethyl (C) (apply to specified residues or termini only)
**Ions Score:** 30 **Expect:** 2.6
**Matches :** 32/223 fragment ions using 77 most intense peaks (help)

| # | Immon. | a | a* | $a^0$ | b | b* | $b^0$ | d | Seq. | v | w | y | y* | $y^0$ | # |
|---|---|---|---|---|---|---|---|---|---|---|---|---|---|---|---|
| 1 | 129.1135 | 129.1135 | 112.0869 | | ***157.1084*** | 140.0818 | | 44.0495 | R | | | | | | 13 |
| 2 | 60.0444 | 216.1455 | 199.1190 | 198.1349 | 244.1404 | 227.1139 | 226.1298 | 200.1506 | S | 977.4184 | 976.4231 | ***1009.4446*** | 992.4181 | 991.4340 | 12 |
| 3 | 30.0338 | 273.1670 | 256.1404 | 255.1564 | 301.1619 | 284.1353 | 283.1513 | | G | | | ***922.4126*** | 905.3860 | | 11 |
| 4 | 30.0338 | **330.1884** | 313.1619 | 312.1779 | ***358.1833*** | 341.1568 | 340.1728 | | G | | | ***865.3911*** | 848.3646 | | 10 |
| 5 | 30.0338 | 387.2099 | 370.1833 | 369.1993 | 415.2048 | 398.1783 | 397.1942 | | G | | | 808.3696 | 791.3431 | | 9 |
| 6 | 30.0338 | 444.2314 | 427.2048 | 426.2208 | 472.2263 | 455.1997 | 454.2157 | | G | | | ***751.3482*** | 734.3216 | | 8 |
| 7 | 30.0338 | 501.2528 | 484.2263 | 483.2423 | 529.2477 | 512.2212 | 511.2372 | | G | | | ***694.3267*** | 677.3002 | | 7 |
| 8 | 30.0338 | 558.2743 | 541.2477 | 540.2637 | 586.2692 | 569.2427 | 568.2586 | | G | | | ***637.3053*** | 620.2787 | | 6 |
| 9 | 30.0338 | 615.2957 | 598.2692 | 597.2852 | ***643.2907*** | 626.2641 | 625.2801 | | G | | | 580.2838 | 563.2572 | | 5 |
| 10 | 136.0757 | 778.3591 | 761.3325 | 760.3485 | ***806.3540*** | 789.3274 | 788.3434 | | Y | 415.2048 | | 523.2623 | 506.2358 | | 4 |
| 11 | 30.0338 | 835.3805 | 818.3540 | 817.3700 | ***863.3755*** | 846.3489 | 845.3649 | | G | | | 360.1990 | 343.1724 | | 3 |
| 12 | 101.0709 | **963.4391** | 946.4126 | 945.4286 | ***991.4340*** | 974.4075 | 973.4235 | 906.4177 | Q | 230.1248 | 229.1295 | 303.1775 | 286.1510 | | 2 |
| 13 | 129.1135 | | | | | | | | R | 74.0237 | 73.0284 | ***175.1190*** | 158.0924 | | 1 |

| Seq | ya | yb | Seq | ya | yb | Seq | ya | yb |
|---|---|---|---|---|---|---|---|---|
| SG | 117.0659 | 145.0608 | SGG | 174.0873 | 202.0822 | SGGG | 231.1088 | 259.1037 |
| SGGGG | 288.1302 | 316.1252 | SGGGGG | 345.1517 | 373.1466 | SGGGGGG | 402.1732 | 430.1681 |
| SGGGGGGG | 459.1946 | 487.1896 | SGGGGGGGY | 622.2580 | 650.2529 | SGGGGGGGYG | 679.2794 | 707.2743 |
| GG | 87.0553 | 115.0502 | GGG | 144.0768 | 172.0717 | GGGG | 201.0982 | 229.0931 |
| GGGGG | 258.1197 | 286.1146 | GGGGGG | 315.1411 | 343.1361 | GGGGGGG | 372.1626 | 400.1575 |
| GGGGGGGY | 535.2259 | 563.2209 | GGGGGGGYG | 592.2474 | 620.2423 | GG | 87.0553 | 115.0502 |
| GGG | 144.0768 | 172.0717 | GGGG | 201.0982 | 229.0931 | GGGGG | 258.1197 | 286.1146 |
| GGGGGG | 315.1411 | 343.1361 | GGGGGGY | 478.2045 | 506.1994 | GGGGGGYG | 535.2259 | 563.2209 |
| GGGGGGYGQ | 663.2845 | 691.2794 | GG | 87.0553 | 115.0502 | GGG | 144.0768 | 172.0717 |
| GGGG | 201.0982 | 229.0931 | GGGGG | 258.1197 | 286.1146 | GGGGGY | 421.1830 | 449.1779 |
| GGGGGYG | 478.2045 | 506.1994 | GGGGGYGQ | 606.2631 | 634.2580 | GG | 87.0553 | 115.0502 |
| GGG | 144.0768 | 172.0717 | GGGG | 201.0982 | 229.0931 | GGGGY | 364.1615 | 392.1565 |
| GGGGYG | 421.1830 | 449.1779 | GGGGYGQ | 549.2416 | 577.2365 | GG | 87.0553 | 115.0502 |
| GGG | 144.0768 | 172.0717 | GGGY | 307.1401 | 335.1350 | GGGYG | 364.1615 | 392.1565 |
| GGGYGQ | 492.2201 | 520.2150 | GG | 87.0553 | 115.0502 | GGY | 250.1186 | 278.1135 |
| GGYG | 307.1401 | 335.1350 | GGYGQ | 435.1987 | 463.1936 | GY | 193.0972 | 221.0921 |
| GYG | 250.1186 | 278.1135 | GYGQ | 378.1772 | 406.1721 | YG | 193.0972 | 221.0921 |
| YGQ | 321.1557 | 349.1506 | GQ | 158.0924 | 186.0873 | | | |

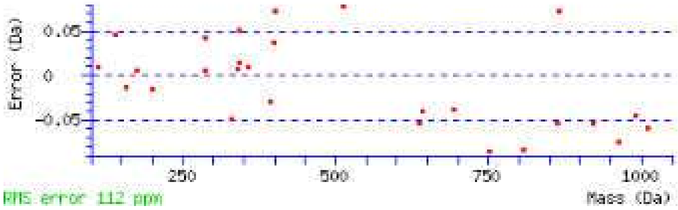


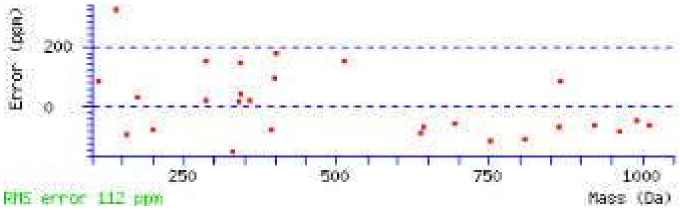


NCBI **BLAST** search of RSGGGGGGGYGQR
(Parameters: blastp, nr protein database, expect=20000, no filter, PAM30)
Other BLAST web gateways

**All matches to this query**

| Score | Mr(calc) | Delta | Sequence |
|---|---|---|---|
| 30.3 | 1164.5385 | 0.0666 | RSGGGGGGGYGQR |
| 19.2 | 1164.5636 | 0.0414 | VGQDSYRGAGR |
| 19.0 | 1164.5385 | 0.0666 | RSGGGGGGGAGYGR |
| 15.9 | 1164.6251 | -0.0201 | RELVYTAASR |
| 15.9 | 1164.5974 | 0.0076 | RTLXVSVIXR |
| 14.0 | 1164.5676 | 0.0374 | RPEDAFFQR |
| 14.0 | 1164.5709 | 0.0341 | RFIAENEMR |
| 13.9 | 1164.5128 | 0.0922 | REMMGGNSQR |
| 13.9 | 1164.6040 | 0.0011 | RENAYFPLR |
| 12.3 | 1164.6476 | -0.0425 | RPRVDAAQPR |

**Mascot:** http://www.matrixscience.com/

# MATRIX SCIENCE Mascot Search Results

## Peptide View **Spot no 10**

MS/MS Fragmentation of **NITVNEAQSRR**
Found in **gi|18103931** in **NCBInr**, glycine rich RNA binding protein [Oryza sativa Indica Group]

Match to Query 26: 1286.750124 from(1287.757400,1+) intensity(0.0000) index(11)
Title: Label: E6, Spot_Id: 228926, Peak_List_Id: 257529, MSMS Job_Run_Id: 24923, Comment:
Data file ppw_E6_138985130300.txt

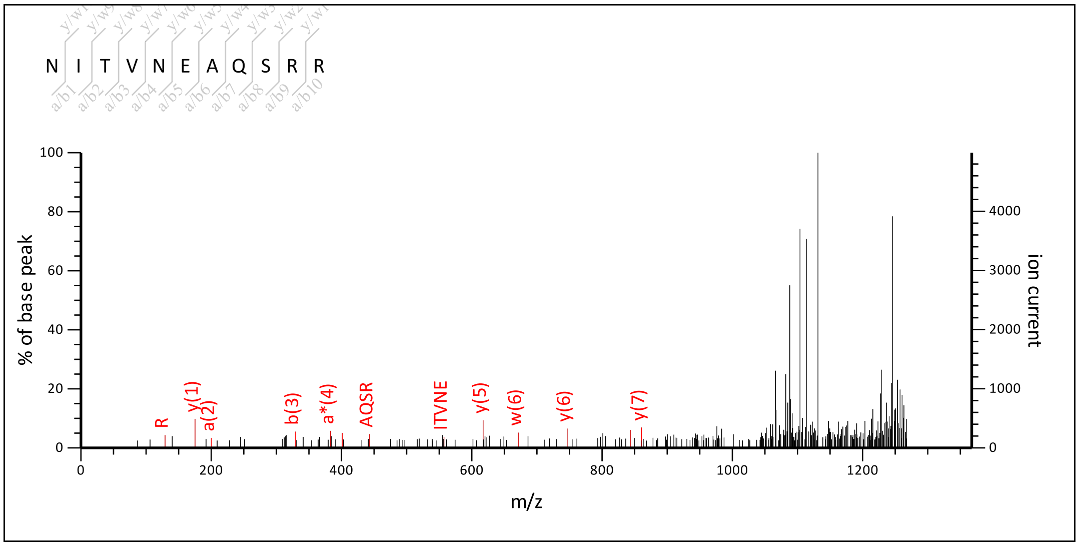


0 to 1367.5

Label all possible matches ○ Label matches used for scoring ◉

**Monoisotopic mass of neutral peptide Mr(calc):** 1286.6691
**Fixed modifications:** Carbamidomethyl (C) (apply to specified residues or termini only)
**Ions Score:** 10 **Expect:** 1.9e+02
**Matches :** 18/187 fragment ions using 24 most intense peaks (help)

| # | Immon. | a | a* | a⁰ | b | b* | b⁰ | d | d' | Seq. | v | w | w' | y | y* | |
|---|---|---|---|---|---|---|---|---|---|---|---|---|---|---|---|---|
| 1 | 87.0553 | 87.0553 | 70.0287 | | 115.0502 | 98.0237 | | 44.0495 | | N | | | | | | |
| 2 | 86.0964 | 200.1394 | 183.1128 | | 228.1343 | 211.1077 | | 172.1081 | 186.1237 | I | 1115.5552 | 1128.5756 | 1142.5913 | 1173.6335 | 1156.6069 | [illegible] |
| 3 | 74.0600 | 301.1870 | 284.1605 | 283.1765 | **329.1819** | 312.1554 | 311.1714 | 285.1921 | 287.1714 | T | 1014.5075 | 1027.5279 | 1029.5072 | 1060.5494 | 1043.5228 | [illegible] |
| 4 | 72.0808 | 400.2554 | 383.2289 | 382.2449 | 428.2504 | 411.2238 | 410.2398 | 386.2398 | | V | 915.4391 | 928.4595 | | 959.5017 | 942.4752 | [illegible] |
| 5 | 87.0553 | 514.2984 | 497.2718 | 496.2878 | 542.2933 | 525.2667 | 524.2827 | 471.2926 | | N | 801.3962 | 800.4009 | | ***860.4333*** | 843.4068 | [illegible] |
| 6 | 102.0550 | 643.3410 | 626.3144 | 625.3304 | **671.3359** | 654.3093 | 653.3253 | 585.3355 | | E | 672.3536 | 671.3583 | | ***746.3904*** | 729.3638 | [illegible] |
| 7 | 44.0495 | 714.3781 | 697.3515 | 696.3675 | 742.3730 | 725.3464 | 724.3624 | | | A | 601.3165 | | | ***617.3478*** | 600.3212 | [illegible] |
| 8 | 101.0709 | 842.4367 | 825.4101 | 824.4261 | 870.4316 | 853.4050 | 852.4210 | 785.4152 | | Q | 473.2579 | 472.2627 | | 546.3107 | 529.2841 | [illegible] |
| 9 | 60.0444 | 929.4687 | 912.4421 | 911.4581 | 957.4636 | 940.4371 | 939.4530 | 913.4738 | | S | 386.2259 | 385.2306 | | 418.2521 | 401.2255 | [illegible] |
| 10 | 129.1135 | 1085.5698 | 1068.5432 | 1067.5592 | 1113.5647 | 1096.5382 | 1095.5541 | 1000.5058 | | R | 230.1248 | 229.1295 | | 331.2201 | 314.1935 | |
| 11 | 129.1135 | | | | | | | | | R | 74.0237 | 73.0284 | | ***175.1190*** | 158.0924 | |

| Seq | ya | yb | Seq | ya | yb | Seq | ya | yb |
|---|---|---|---|---|---|---|---|---|
| IT | 187.1441 | 215.1390 | ITV | 286.2125 | 314.2074 | ITVN | 400.2554 | 428.2504 |
| ITVNE | 529.2980 | 557.2930 | ITVNEA | 600.3352 | 628.3301 | TV | 173.1285 | 201.1234 |
| TVN | 287.1714 | 315.1663 | TVNE | 416.2140 | 444.2089 | TVNEA | 487.2511 | 515.2460 |
| TVNEAQ | 615.3097 | 643.3046 | VN | 186.1237 | 214.1186 | VNE | 315.1663 | 343.1612 |
| VNEA | 386.2034 | 414.1983 | VNEAQ | 514.2620 | 542.2569 | VNEAQS | 601.2940 | 629.2889 |
| NE | 216.0979 | 244.0928 | NEA | 287.1350 | 315.1299 | NEAQ | 415.1936 | 443.1885 |
| NEAQS | 502.2256 | 530.2205 | NEAQSR | 658.3267 | 686.3216 | EA | 173.0921 | 201.0870 |
| EAQ | 301.1506 | 329.1456 | EAQS | 388.1827 | 416.1776 | EAQSR | 544.2838 | 572.2787 |
| AQ | 172.1081 | 200.1030 | AQS | 259.1401 | 287.1350 | AQSR | 415.2412 | 443.2361 |

| | | | | | | | | |
|---|---|---|---|---|---|---|---|---|
| **QS** | 188.1030 | 216.0979 | **QSR** | 344.2041 | 372.1990 | **SR** | 216.1455 | 244.1404 |

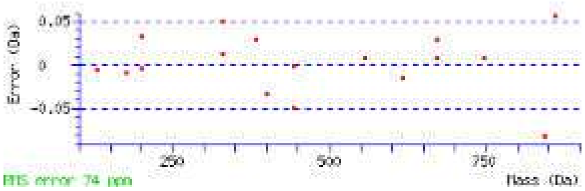


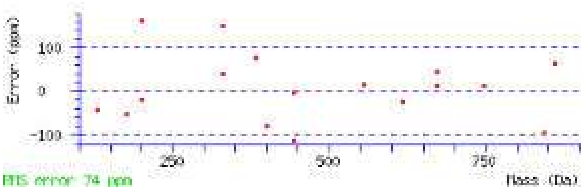


NCBI **BLAST** search of NITVNEAQSRR
(Parameters: blastp, nr protein database, expect=20000, no filter, PAM30)
Other BLAST web gateways

**All matches to this query**

| Score | Mr(calc) | Delta | Sequence |
|---|---|---|---|
| 16.8 | 1286.6579 | 0.0923 | RGQSIEVVDER |
| 15.1 | 1286.6983 | 0.0519 | GVATALNFPLER |
| 14.7 | 1286.6830 | 0.0671 | RATVNEDEILK |
| 14.5 | 1286.6731 | 0.0770 | RSPSPAPPPEPR |
| 11.2 | 1286.6466 | 0.1035 | AIASQLANQDEK |
| 11.0 | 1286.6830 | 0.0671 | LNEVQSSLLER |
| 10.9 | 1286.7194 | 0.0307 | KVLGEGSLSLER |
| 10.8 | 1286.6691 | 0.0810 | QVTVNEAQSRR |
| 10.4 | 1286.6691 | 0.0810 | NITVNEAQSRR |
| 10.1 | 1286.6327 | 0.1174 | RQEQESAAAAAR |

**Mascot:** http://www.matrixscience.com/

# MATRIX SCIENCE Mascot Search Results

## Peptide View Spot no 10

MS/MS Fragmentation of **GGGGGYGGGGYGGGGGGGGYGQR**
Found in **gi|18103931** in **NCBInr**, glycine rich RNA binding protein [Oryza sativa Indica Group]

Match to Query 46: 1817.858424 from(1818.865700,1+) intensity(0.0000) index(21)
Title: Label: E6, Spot_Id: 228926, Peak_List_Id: 257514, MSMS Job_Run_Id: 24923, Comment:
Data file ppw_E6_138985130300.txt

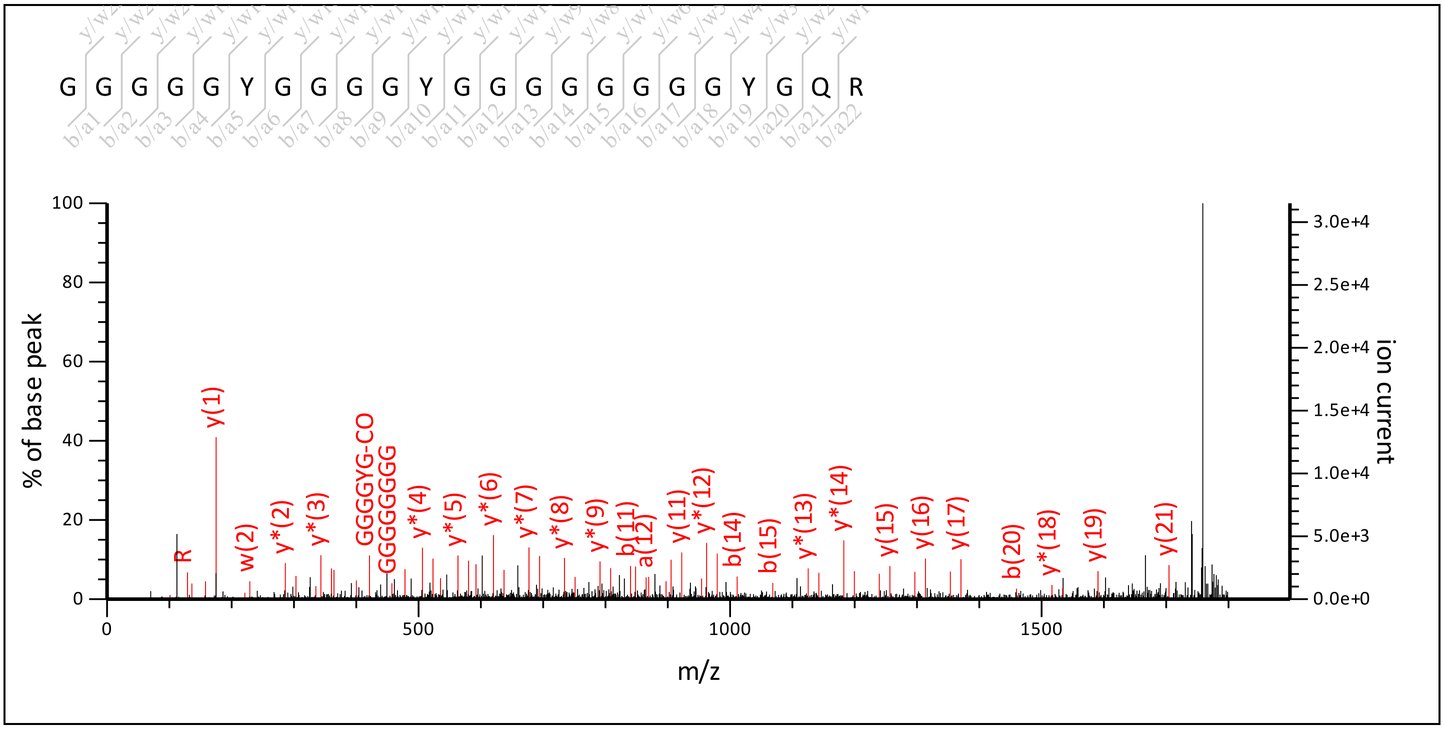


0 to 1898.59

Label all possible matches ○ Label matches used for scoring ◉

**Monoisotopic mass of neutral peptide Mr(calc):** 1817.7467
**Fixed modifications:** Carbamidomethyl (C) (apply to specified residues or termini only)
**Ions Score:** 124 **Expect:** 8.8e-10
**Matches :** 207/392 fragment ions using 126 most intense peaks (help)

| # | Immon. | a | a* | b | b* | d | Seq. | v | w | y | y* | # |
|---|---|---|---|---|---|---|---|---|---|---|---|---|
| 1 | 30.0338 | 30.0338 | | 58.0287 | | 44.0495 | G | | | | | 23 |
| 2 | 30.0338 | 87.0553 | | 115.0502 | | | G | | | 1761.7324 | 1744.7059 | 22 |
| 3 | 30.0338 | 144.0768 | | 172.0717 | | | G | | | ***1704.7109*** | 1687.6844 | 21 |
| 4 | 30.0338 | 201.0982 | | **229.0931** | | | G | | | 1647.6895 | 1630.6629 | 20 |
| 5 | 30.0338 | 258.1197 | | **286.1146** | | | G | | | ***1590.6680*** | 1573.6415 | 19 |
| 6 | 136.0757 | **421.1830** | | **449.1779** | | | Y | 1425.5890 | | ***1533.6465*** | 1516.6200 | 18 |
| 7 | 30.0338 | **478.2045** | | **506.1994** | | | G | | | ***1370.5832*** | 1353.5567 | 17 |
| 8 | 30.0338 | **535.2259** | | **563.2209** | | | G | | | ***1313.5618*** | 1296.5352 | 16 |

| | | | | | | | | | | | | |
|---|---|---|---|---|---|---|---|---|---|---|---|---|
| **9** | 30.0338 | **592.2474** | | **620.2423** | | | **G** | | | ***1256.5403*** | 1239.5137 | **15** |
| **10** | 30.0338 | 649.2689 | | **677.2638** | | | **G** | | | ***1199.5188*** | 1182.4923 | **14** |
| **11** | 136.0757 | 812.3322 | | **840.3271** | | | **Y** | 1034.4398 | | ***1142.4974*** | 1125.4708 | **13** |
| **12** | 30.0338 | **869.3537** | | **897.3486** | | | **G** | | | ***979.4340*** | 962.4075 | **12** |
| **13** | 30.0338 | 926.3751 | | **954.3700** | | | **G** | | | ***922.4126*** | 905.3860 | **11** |
| **14** | 30.0338 | 983.3966 | | **1011.3915** | | | **G** | | | ***865.3911*** | 848.3646 | **10** |
| **15** | 30.0338 | 1040.4181 | | **1068.4130** | | | **G** | | | ***808.3696*** | 791.3431 | **9** |
| **16** | 30.0338 | 1097.4395 | | **1125.4344** | | | **G** | | | ***751.3482*** | 734.3216 | **8** |
| **17** | 30.0338 | 1154.4610 | | **1182.4559** | | | **G** | | | ***694.3267*** | 677.3002 | **7** |
| **18** | 30.0338 | 1211.4824 | | **1239.4774** | | | **G** | | | ***637.3053*** | 620.2787 | **6** |
| **19** | 30.0338 | 1268.5039 | | **1296.4988** | | | **G** | | | ***580.2838*** | 563.2572 | **5** |
| **20** | 136.0757 | 1431.5672 | | **1459.5622** | | | **Y** | 415.2048 | | ***523.2623*** | 506.2358 | **4** |
| **21** | 30.0338 | 1488.5887 | | **1516.5836** | | | **G** | | | ***360.1990*** | 343.1724 | **3** |
| **22** | 101.0709 | 1616.6473 | 1599.6207 | 1644.6422 | 1627.6156 | 1559.6258 | **Q** | 230.1248 | 229.1295 | ***303.1775*** | 286.1510 | **2** |
| **23** | 129.1135 | | | | | | **R** | 74.0237 | 73.0284 | ***175.1190*** | 158.0924 | **1** |

| Seq | ya | yb | Seq | ya | yb | Seq | ya | yb |
|---|---|---|---|---|---|---|---|---|
| **GG** | 87.0553 | 115.0502 | **GGG** | 144.0768 | 172.0717 | **GGGG** | 201.0982 | 229.0931 |
| **GGGGY** | 364.1615 | 392.1565 | **GGGGYG** | 421.1830 | 449.1779 | **GGGGYGG** | 478.2045 | 506.1994 |
| **GGGGYGGG** | 535.2259 | 563.2209 | **GGGGYGGGG** | 592.2474 | 620.2423 | **GG** | 87.0553 | 115.0502 |
| **GGG** | 144.0768 | 172.0717 | **GGGY** | 307.1401 | 335.1350 | **GGGYG** | 364.1615 | 392.1565 |
| **GGGYGG** | 421.1830 | 449.1779 | **GGGYGGG** | 478.2045 | 506.1994 | **GGGYGGGG** | 535.2259 | 563.2209 |
| **GGGYGGGGY** | 698.2893 | 726.2842 | **GG** | 87.0553 | 115.0502 | **GGY** | 250.1186 | 278.1135 |
| **GGYG** | 307.1401 | 335.1350 | **GGYGG** | 364.1615 | 392.1565 | **GGYGGG** | 421.1830 | 449.1779 |
| **GGYGGGG** | 478.2045 | 506.1994 | **GGYGGGGY** | 641.2678 | 669.2627 | **GGYGGGGYG** | 698.2893 | 726.2842 |
| **GY** | 193.0972 | 221.0921 | **GYG** | 250.1186 | 278.1135 | **GYGG** | 307.1401 | 335.1350 |
| **GYGGG** | 364.1615 | 392.1565 | **GYGGGG** | 421.1830 | 449.1779 | **GYGGGGY** | 584.2463 | 612.2413 |
| **GYGGGGYG** | 641.2678 | 669.2627 | **GYGGGGYGG** | 698.2893 | 726.2842 | **YG** | 193.0972 | 221.0921 |
| **YGG** | 250.1186 | 278.1135 | **YGGG** | 307.1401 | 335.1350 | **YGGGG** | 364.1615 | 392.1565 |
| **YGGGGY** | 527.2249 | 555.2198 | **YGGGGYG** | 584.2463 | 612.2413 | **YGGGGYGG** | 641.2678 | 669.2627 |
| **YGGGGYGGG** | 698.2893 | 726.2842 | **GG** | 87.0553 | 115.0502 | **GGG** | 144.0768 | 172.0717 |
| **GGGG** | 201.0982 | 229.0931 | **GGGGY** | 364.1615 | 392.1565 | **GGGGYG** | 421.1830 | 449.1779 |
| **GGGGYGG** | 478.2045 | 506.1994 | **GGGGYGGG** | 535.2259 | 563.2209 | **GGGGYGGGG** | 592.2474 | 620.2423 |
| **GGGGYGGGGG** | 649.2689 | 677.2638 | **GG** | 87.0553 | 115.0502 | **GGG** | 144.0768 | 172.0717 |
| **GGGY** | 307.1401 | 335.1350 | **GGGYG** | 364.1615 | 392.1565 | **GGGYGG** | 421.1830 | 449.1779 |
| **GGGYGGG** | 478.2045 | 506.1994 | **GGGYGGGG** | 535.2259 | 563.2209 | **GGGYGGGGG** | 592.2474 | 620.2423 |
| **GGGYGGGGGG** | 649.2689 | 677.2638 | **GG** | 87.0553 | 115.0502 | **GGY** | 250.1186 | 278.1135 |
| **GGYG** | 307.1401 | 335.1350 | **GGYGG** | 364.1615 | 392.1565 | **GGYGGG** | 421.1830 | 449.1779 |
| **GGYGGGG** | 478.2045 | 506.1994 | **GGYGGGGG** | 535.2259 | 563.2209 | **GGYGGGGGG** | 592.2474 | 620.2423 |
| **GGYGGGGGGG** | 649.2689 | 677.2638 | **GY** | 193.0972 | 221.0921 | **GYG** | 250.1186 | 278.1135 |
| **GYGG** | 307.1401 | 335.1350 | **GYGGG** | 364.1615 | 392.1565 | **GYGGGG** | 421.1830 | 449.1779 |
| | | | | | | | | |

| | | | | | | | | |
|---|---|---|---|---|---|---|---|---|
| GYGGGGG | 478.2045 | 506.1994 | GYGGGGGG | 535.2259 | 563.2209 | GYGGGGGGG | 592.2474 | 620.2423 |
| GYGGGGGGGG | 649.2689 | 677.2638 | YG | 193.0972 | 221.0921 | YGG | 250.1186 | 278.1135 |
| YGGG | 307.1401 | 335.1350 | YGGGG | 364.1615 | 392.1565 | YGGGGG | 421.1830 | 449.1779 |
| YGGGGGG | 478.2045 | 506.1994 | YGGGGGGG | 535.2259 | 563.2209 | YGGGGGGGG | 592.2474 | 620.2423 |
| GG | 87.0553 | 115.0502 | GGG | 144.0768 | 172.0717 | GGGG | 201.0982 | 229.0931 |
| GGGGG | 258.1197 | 286.1146 | GGGGGG | 315.1411 | 343.1361 | GGGGGGG | 372.1626 | 400.1575 |
| GGGGGGGG | 429.1841 | 457.1790 | GGGGGGGGY | 592.2474 | 620.2423 | GGGGGGGGYG | 649.2689 | 677.2638 |
| GG | 87.0553 | 115.0502 | GGG | 144.0768 | 172.0717 | GGGG | 201.0982 | 229.0931 |
| GGGGG | 258.1197 | 286.1146 | GGGGGG | 315.1411 | 343.1361 | GGGGGGG | 372.1626 | 400.1575 |
| GGGGGGGY | 535.2259 | 563.2209 | GGGGGGGYG | 592.2474 | 620.2423 | GG | 87.0553 | 115.0502 |
| GGG | 144.0768 | 172.0717 | GGGG | 201.0982 | 229.0931 | GGGGG | 258.1197 | 286.1146 |
| GGGGGG | 315.1411 | 343.1361 | GGGGGGY | 478.2045 | 506.1994 | GGGGGGYG | 535.2259 | 563.2209 |
| GGGGGGYGQ | 663.2845 | 691.2794 | GG | 87.0553 | 115.0502 | GGG | 144.0768 | 172.0717 |
| GGGG | 201.0982 | 229.0931 | GGGGG | 258.1197 | 286.1146 | GGGGGY | 421.1830 | 449.1779 |
| GGGGGYG | 478.2045 | 506.1994 | GGGGGYGQ | 606.2631 | 634.2580 | GG | 87.0553 | 115.0502 |
| GGG | 144.0768 | 172.0717 | GGGG | 201.0982 | 229.0931 | GGGGY | 364.1615 | 392.1565 |
| GGGGYG | 421.1830 | 449.1779 | GGGGYGQ | 549.2416 | 577.2365 | GG | 87.0553 | 115.0502 |
| GGG | 144.0768 | 172.0717 | GGGY | 307.1401 | 335.1350 | GGGYG | 364.1615 | 392.1565 |
| GGGYGQ | 492.2201 | 520.2150 | GG | 87.0553 | 115.0502 | GGY | 250.1186 | 278.1135 |
| GGYG | 307.1401 | 335.1350 | GGYGQ | 435.1987 | 463.1936 | GY | 193.0972 | 221.0921 |
| GYG | 250.1186 | 278.1135 | GYGQ | 378.1772 | 406.1721 | YG | 193.0972 | 221.0921 |
| YGQ | 321.1557 | 349.1506 | GQ | 158.0924 | 186.0873 | | | |

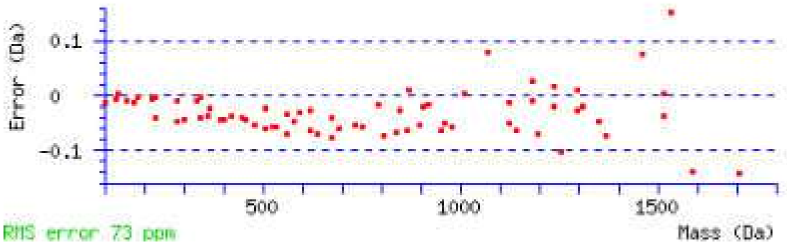


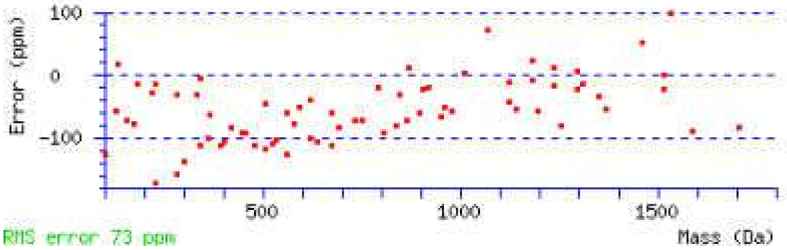


NCBI **BLAST** search of GGGGGYGGGGYGGGGGGGGYGQR
(Parameters: blastp, nr protein database, expect=20000, no filter, PAM30)
Other BLAST web gateways

**All matches to this query**

| Score | Mr(calc) | Delta | Sequence |
|---|---|---|---|
| 123.7 | 1817.7467 | 0.1118 | GGGGGYGGGGYGGGGGGGGYGQR |
| | | | |

| | | | |
|---|---|---|---|
| 115.9 | 1817.7467 | 0.1118 | GGGGGYGGGGGYGGGGGGGYGQR |
| 17.8 | 1817.7360 | 0.1224 | GDHGNRGNDEGMNHHR |
| 14.6 | 1817.7825 | 0.0759 | WVCEGAGPPGGMGESTAR |
| 14.5 | 1817.9577 | -0.0993 | QGVIFLTDHGFFPGKR |
| 13.0 | 1817.8472 | 0.0112 | NFPIYSDGLYTESGQK |
| 12.8 | 1817.8584 | -0.0000 | DFIFGVSSSAYQVEGGR |
| 12.6 | 1817.8730 | -0.0146 | ASMKEEHSLIGGPGFSR |
| 12.3 | 1817.7937 | 0.0647 | NNNFTVSAMHGDMPQR |
| 11.1 | 1817.9247 | -0.0663 | VLRMHGYEVTPDVFR |

**Mascot:** http://www.matrixscience.com/

# MATRIX SCIENCE Mascot Search Results

## Peptide View Spot no 10

MS/MS Fragmentation of **CFVGGLAWATDDR**
Found in **gi|18103931** in **NCBInr**, glycine rich RNA binding protein [Oryza sativa Indica Group]

Match to Query 34: 1466.759724 from(1467.767000,1+) intensity(0.0000) index(15)
Title: Label: E6, Spot_Id: 228926, Peak_List_Id: 257530, MSMS Job_Run_Id: 24923, Comment:
Data file ppw_E6_138985130300.txt

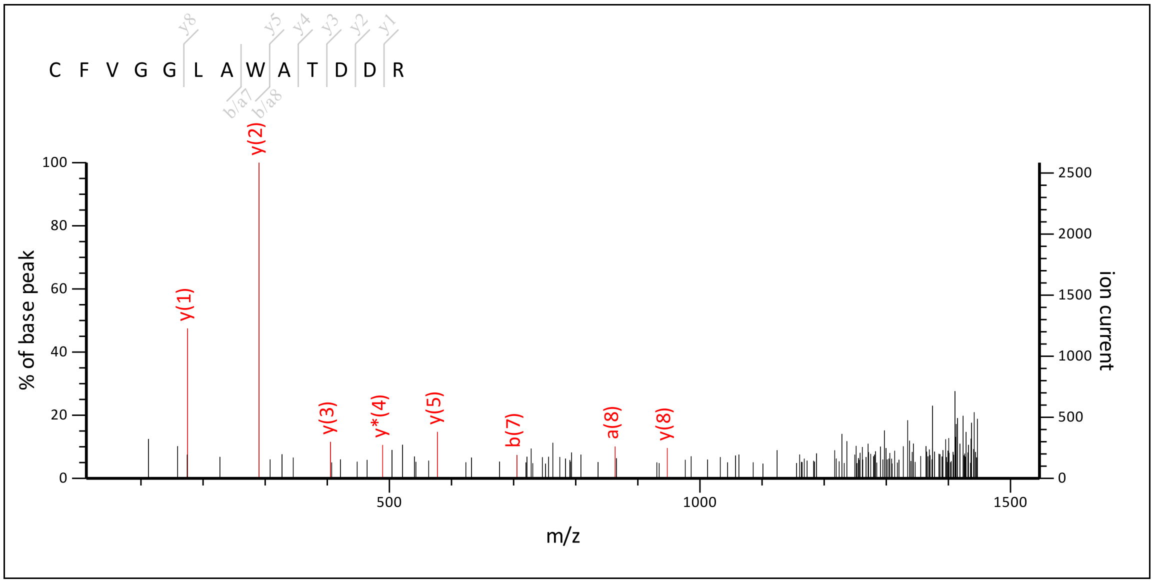


12.09 to 1546.66

Label all possible matches ○ Label matches used for scoring ◉

**Monoisotopic mass of neutral peptide Mr(calc):** 1466.6613
**Fixed modifications:** Carbamidomethyl (C) (apply to specified residues or termini only)
**Ions Score:** 25 **Expect:** 7.9
**Matches :** 8/187 fragment ions using 14 most intense peaks (help)

| # | Immon. | a | $a^0$ | b | $b^0$ | d | d' | Seq. | v | w | w' | y | y* | $y^0$ | # |
|---|---|---|---|---|---|---|---|---|---|---|---|---|---|---|---|
| 1 | 133.0430 | 133.0430 | | 161.0379 | | 44.0495 | | C | | | | | | | 13 |
| 2 | 120.0808 | 280.1114 | | 308.1063 | | | | F | 1215.5753 | | | 1307.6379 | 1290.6113 | 1289.6273 | 12 |
| 3 | 72.0808 | 379.1798 | | 407.1748 | | 365.1642 | | V | 1116.5069 | 1129.5273 | | 1160.5695 | 1143.5429 | 1142.5589 | 11 |
| 4 | 30.0338 | 436.2013 | | 464.1962 | | | | G | | | | 1061.5011 | 1044.4745 | 1043.4905 | 10 |
| 5 | 30.0338 | 493.2228 | | 521.2177 | | | | G | | | | 1004.4796 | 987.4530 | 986.4690 | 9 |
| 6 | 86.0964 | 606.3068 | | 634.3017 | | 564.2599 | | L | 889.3799 | 888.3846 | | ***947.4581*** | 930.4316 | 929.4476 | 8 |
| 7 | 44.0495 | 677.3439 | | **705.3389** | | | | A | 818.3428 | | | 834.3741 | 817.3475 | 816.3635 | 7 |
| 8 | 159.0917 | **863.4233** | | 891.4182 | | | | W | 632.2634 | | | 763.3369 | 746.3104 | 745.3264 | 6 |
| 9 | 44.0495 | 934.4604 | | 962.4553 | | | | A | 561.2263 | | | ***577.2576*** | 560.2311 | 559.2471 | 5 |
| 10 | 74.0600 | 1035.5081 | 1017.4975 | 1063.5030 | 1045.4924 | 1019.5131 | 1021.4924 | T | 460.1787 | 473.1991 | 475.1783 | 506.2205 | 489.1940 | 488.2100 | 4 |
| 11 | 88.0393 | 1150.5350 | 1132.5244 | 1178.5299 | 1160.5193 | 1106.5452 | | D | 345.1517 | 344.1565 | | ***405.1728*** | 388.1463 | 387.1623 | 3 |
| 12 | 88.0393 | 1265.5619 | 1247.5514 | 1293.5569 | 1275.5463 | 1221.5721 | | D | 230.1248 | 229.1295 | | ***290.1459*** | 273.1193 | 272.1353 | 2 |
| 13 | 129.1135 | | | | | | | R | 74.0237 | 73.0284 | | ***175.1190*** | 158.0924 | | 1 |

| Seq | ya | yb | Seq | ya | yb | Seq | ya | yb |
|---|---|---|---|---|---|---|---|---|
| FV | 219.1492 | 247.1441 | FVG | 276.1707 | 304.1656 | FVGG | 333.1921 | 361.1870 |
| FVGGL | 446.2762 | 474.2711 | FVGGLA | 517.3133 | 545.3082 | VG | 129.1022 | 157.0972 |
| VGG | 186.1237 | 214.1186 | VGGL | 299.2078 | 327.2027 | VGGLA | 370.2449 | 398.2398 |
| | | | | | | | | |

| | | | | | | | | |
|---|---|---|---|---|---|---|---|---|
| **VGGLAW** | 556.3242 | 584.3191 | **VGGLAWA** | 627.3613 | 655.3562 | **GG** | 87.0553 | 115.0502 |
| **GGL** | 200.1394 | 228.1343 | **GGLA** | 271.1765 | 299.1714 | **GGLAW** | 457.2558 | 485.2507 |
| **GGLAWA** | 528.2929 | 556.2878 | **GGLAWAT** | 629.3406 | 657.3355 | **GL** | 143.1179 | 171.1128 |
| **GLA** | 214.1550 | 242.1499 | **GLAW** | 400.2343 | 428.2292 | **GLAWA** | 471.2714 | 499.2663 |
| **GLAWAT** | 572.3191 | 600.3140 | **GLAWATD** | 687.3461 | 715.3410 | **LA** | 157.1335 | 185.1285 |
| **LAW** | 343.2129 | 371.2078 | **LAWA** | 414.2500 | 442.2449 | **LAWAT** | 515.2976 | 543.2926 |
| **LAWATD** | 630.3246 | 658.3195 | **AW** | 230.1288 | 258.1237 | **AWA** | 301.1659 | 329.1608 |
| **AWAT** | 402.2136 | 430.2085 | **AWATD** | 517.2405 | 545.2354 | **AWATDD** | 632.2675 | 660.2624 |
| **WA** | 230.1288 | 258.1237 | **WAT** | 331.1765 | 359.1714 | **WATD** | 446.2034 | 474.1983 |
| **WATDD** | 561.2304 | 589.2253 | **AT** | 145.0972 | 173.0921 | **ATD** | 260.1241 | 288.1190 |
| **ATDD** | 375.1510 | 403.1460 | **TD** | 189.0870 | 217.0819 | **TDD** | 304.1139 | 332.1088 |
| **DD** | 203.0662 | 231.0612 | | | | | | |

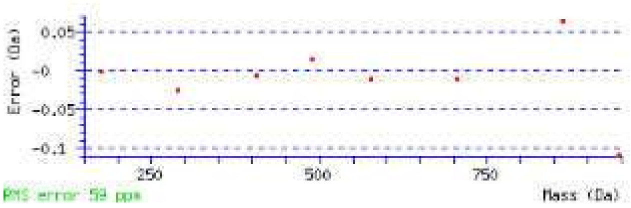


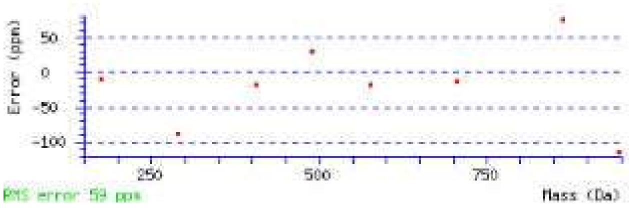


NCBI **BLAST** search of CFVGGLAWATDDR
(Parameters: blastp, nr protein database, expect=20000, no filter, PAM30)
Other BLAST web gateways

**All matches to this query**

| Score | Mr(calc) | Delta | Sequence |
|---|---|---|---|
| 24.9 | 1466.6613 | 0.0985 | CFVGGLAWATDDR |
| 14.9 | 1466.6274 | 0.1323 | GNVFTADADADTDR |
| 11.0 | 1466.6638 | 0.0960 | GLVSYGDASPDSGSR |
| 9.2 | 1466.6903 | 0.0695 | GGQFSRPFSVDDR |
| 9.1 | 1466.6691 | 0.0906 | GYNAEGWRFPDR |
| 8.1 | 1466.7551 | 0.0046 | GKQIMFIGDSISR |
| 8.1 | 1466.6282 | 0.1315 | GLMTSDAEMWNGR |
| 8.1 | 1466.6686 | 0.0911 | GTPYWMAPEMIR |
| 7.7 | 1466.6613 | 0.0985 | HGFDVSQDIFMR |
| 7.7 | 1466.6671 | 0.0926 | KLSMDASTQTDDR |

**Mascot:** http://www.matrixscience.com/

# MATRIX SCIENCE Mascot Search Results

## Peptide View Spot no 13

MS/MS Fragmentation of **QIPLSGPNSVVGR**
Found in **gi|42408425** in **NCBInr**, putative superoxide dismutase [Cu-Zn], chloroplast precursor [Oryza sativa Japonica Group]

Match to Query 216: 1322.758024 from(1323.765300,1+) intensity(0.0000) index(23)
Title: Label: G6, Spot_Id: 219760, Peak_List_Id: 225740, MSMS Job_Run_Id: 21788, Comment:
Data file C:\Documents and Settings\Administrator\Desktop\farah BHU\spot set plate\Farah-Garima 11May 2013\ppw_G6_136842238300.txt

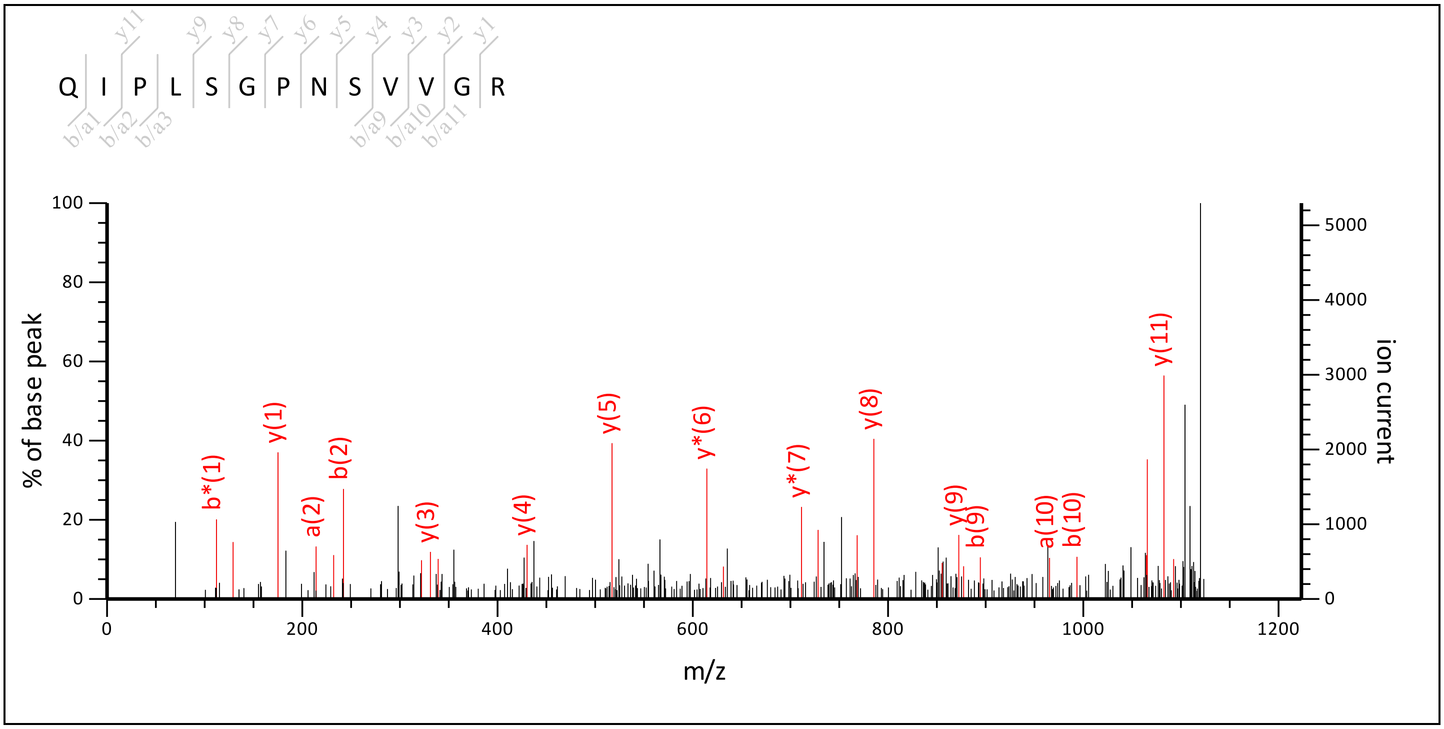


0 to 1223.41

Label all possible matches ○ Label matches used for scoring ◉

**Monoisotopic mass of neutral peptide Mr(calc):** 1322.7307
**Fixed modifications:** Carbamidomethyl (C) (apply to specified residues or termini only)
**Ions Score:** 59 **Expect:** 0.0022
**Matches :** 27/72 fragment ions using 55 most intense peaks (help)

| # | a | a* | b | b* | Seq. | y | y* | # |
|---|---|---|---|---|---|---|---|---|
| 1 | 101.0709 | 84.0444 | **129.0659** | 112.0393 | Q | | | 13 |
| 2 | **214.1550** | 197.1285 | **242.1499** | 225.1234 | I | 1195.6793 | 1178.6528 | 12 |
| 3 | 311.2078 | 294.1812 | **339.2027** | 322.1761 | P | ***1082.5953*** | 1065.5687 | 11 |
| 4 | 424.2918 | 407.2653 | 452.2867 | 435.2602 | L | 985.5425 | 968.5160 | 10 |
| 5 | 511.3239 | 494.2973 | 539.3188 | 522.2922 | S | ***872.4585*** | 855.4319 | 9 |
| 6 | 568.3453 | 551.3188 | 596.3402 | 579.3137 | G | ***785.4264*** | 768.3999 | 8 |
| 7 | 665.3981 | 648.3715 | 693.3930 | 676.3665 | P | ***728.4050*** | 711.3784 | 7 |

| | | | | | | | | |
|---|---|---|---|---|---|---|---|---|
| 8 | 779.4410 | 762.4145 | 807.4359 | 790.4094 | N | 631.3522 | 614.3256 | 6 |
| 9 | 866.4730 | 849.4465 | 894.4680 | 877.4414 | S | 517.3093 | 500.2827 | 5 |
| 10 | 965.5415 | 948.5149 | 993.5364 | 976.5098 | V | 430.2772 | 413.2507 | 4 |
| 11 | 1064.6099 | 1047.5833 | 1092.6048 | 1075.5782 | V | 331.2088 | 314.1823 | 3 |
| 12 | 1121.6313 | 1104.6048 | 1149.6262 | 1132.5997 | G | 232.1404 | 215.1139 | 2 |
| 13 | | | | | R | 175.1190 | 158.0924 | 1 |

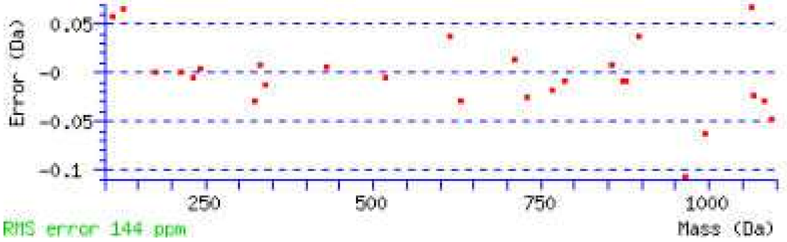


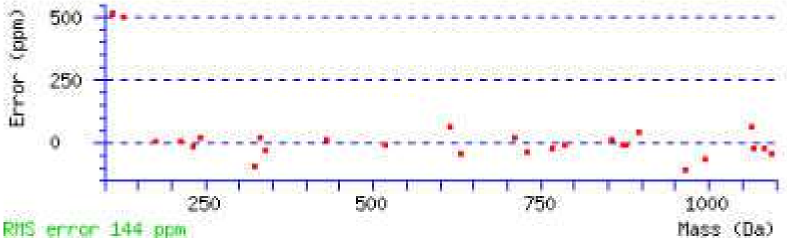


NCBI **BLAST** search of QIPLSGPNSVVGR
(Parameters: blastp, nr protein database, expect=20000, no filter, PAM30)
Other BLAST web gateways

**All matches to this query**

| Score | Mr(calc) | Delta | Sequence |
|---|---|---|---|
| 58.8 | 1322.7307 | 0.0274 | QIPLSGPNSVVGR |
| 12.1 | 1322.8034 | -0.0454 | QLPTNVKLALAR |
| 11.9 | 1322.7207 | 0.0373 | QLPAFLADHRR |
| 10.6 | 1322.6488 | 0.1092 | MNMHVPPWRR |
| 10.4 | 1322.6691 | 0.0889 | QLPQERPADGGR |
| 10.2 | 1322.7921 | -0.0341 | KLLEELAPIAAR |
| 9.8 | 1322.8034 | -0.0454 | KLIALSQPALNR |
| 9.2 | 1322.7493 | 0.0088 | KLLPSHGTLMAR |
| 8.9 | 1322.6653 | 0.0928 | QLGEKGLEMFR |
| 8.7 | 1322.6322 | 0.1258 | KINAMGSMSQQL |

**Mascot:** http://www.matrixscience.com/

# Mascot Search Results

## Peptide View Spot no 13

MS/MS Fragmentation of **HAGDLGNIVANAEGVAEATIVDK**
Found in **gi|42408425** in **NCBInr**, putative superoxide dismutase [Cu-Zn], chloroplast precursor [Oryza sativa Japonica Group]

Match to Query 255: 2263.154124 from(2264.161400,1+) intensity(0.0000) index(30)
Title: Label: G6, Spot_Id: 219760, Peak_List_Id: 225757, MSMS Job_Run_Id: 21788, Comment:
Data file C:\Documents and Settings\Administrator\Desktop\farah BHU\spot set plate\Farah-Garima 11May 2013\ppw_G6_136842238300.txt

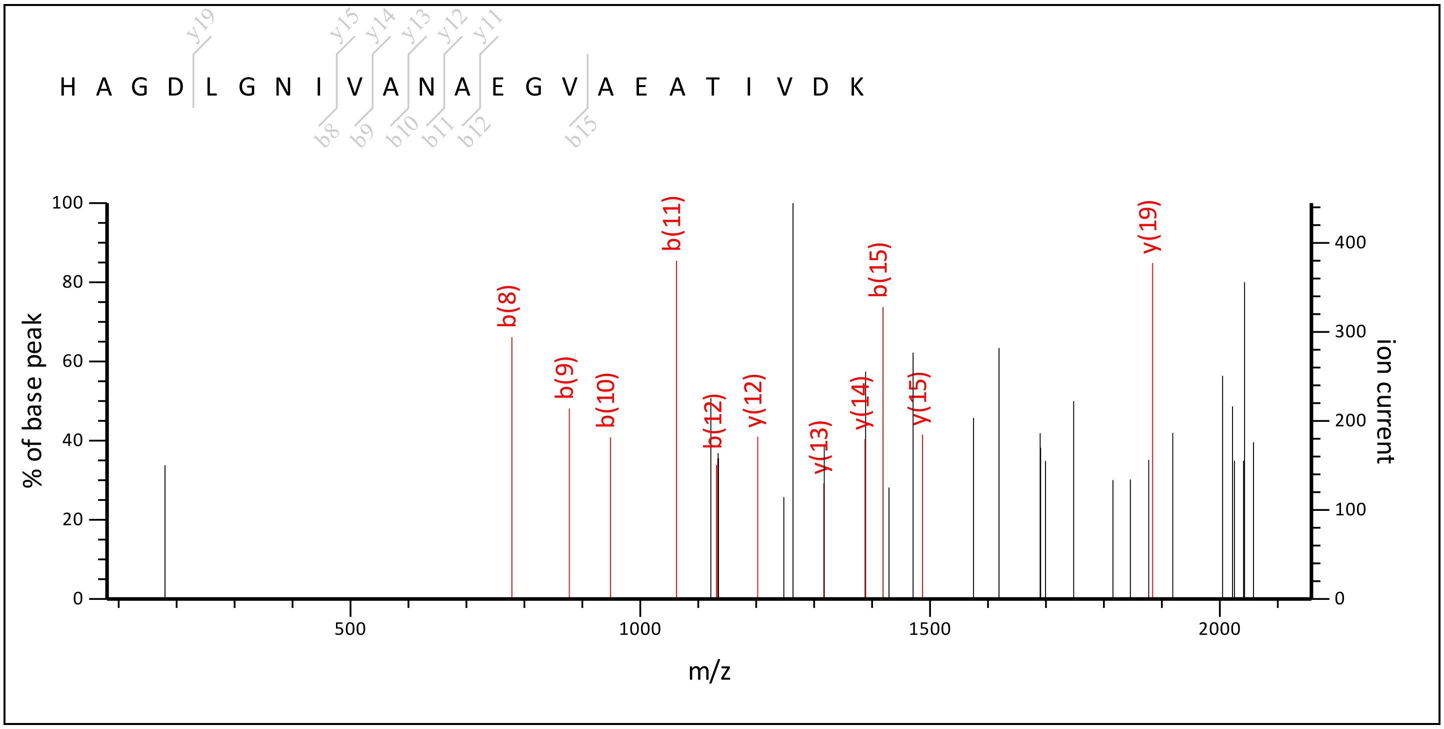


79.92 to 2158.07

Label all possible matches ○ Label matches used for scoring ◉

**Monoisotopic mass of neutral peptide Mr(calc):** 2263.1444
**Fixed modifications:** Carbamidomethyl (C) (apply to specified residues or termini only)
**Ions Score:** 17 **Expect:** 28
**Matches :** 12/120 fragment ions using 37 most intense peaks (help)

| # | a | a* | b | b* | Seq. | y | y* | # |
|---|---|---|---|---|---|---|---|---|
| 1 | 110.0713 | | 138.0662 | | H | | | 23 |
| 2 | 181.1084 | | 209.1033 | | A | 2127.0928 | 2110.0663 | 22 |
| 3 | 238.1299 | | 266.1248 | | G | 2056.0557 | 2039.0291 | 21 |
| 4 | 353.1568 | | 381.1517 | | D | 1999.0342 | 1982.0077 | 20 |
| 5 | 466.2409 | | 494.2358 | | L | ***1884.0073*** | 1866.9807 | 19 |
| 6 | 523.2623 | | 551.2572 | | G | 1770.9232 | 1753.8967 | 18 |
| 7 | 637.3052 | 620.2787 | 665.3002 | 648.2736 | N | 1713.9018 | 1696.8752 | 17 |

| | | | | | | | | |
|---|---|---|---|---|---|---|---|---|
| 8 | 750.3893 | 733.3628 | 778.3842 | 761.3577 | I | 1599.8588 | 1582.8323 | 16 |
| 9 | 849.4577 | 832.4312 | 877.4526 | 860.4261 | V | 1486.7748 | 1469.7482 | 15 |
| 10 | 920.4948 | 903.4683 | 948.4898 | 931.4632 | A | 1387.7064 | 1370.6798 | 14 |
| 11 | 1034.5378 | 1017.5112 | 1062.5327 | 1045.5061 | N | 1316.6692 | 1299.6427 | 13 |
| 12 | 1105.5749 | 1088.5483 | 1133.5698 | 1116.5432 | A | 1202.6263 | 1185.5998 | 12 |
| 13 | 1234.6175 | 1217.5909 | 1262.6124 | 1245.5858 | E | 1131.5892 | 1114.5626 | 11 |
| 14 | 1291.6389 | 1274.6124 | 1319.6339 | 1302.6073 | G | 1002.5466 | 985.5201 | 10 |
| 15 | 1390.7074 | 1373.6808 | 1418.7023 | 1401.6757 | V | 945.5251 | 928.4986 | 9 |
| 16 | 1461.7445 | 1444.7179 | 1489.7394 | 1472.7128 | A | 846.4567 | 829.4302 | 8 |
| 17 | 1590.7871 | 1573.7605 | 1618.7820 | 1601.7554 | E | 775.4196 | 758.3931 | 7 |
| 18 | 1661.8242 | 1644.7976 | 1689.8191 | 1672.7925 | A | 646.3770 | 629.3505 | 6 |
| 19 | 1762.8719 | 1745.8453 | 1790.8668 | 1773.8402 | T | 575.3399 | 558.3134 | 5 |
| 20 | 1875.9559 | 1858.9294 | 1903.9508 | 1886.9243 | I | 474.2922 | 457.2657 | 4 |
| 21 | 1975.0243 | 1957.9978 | 2003.0192 | 1985.9927 | V | 361.2082 | 344.1816 | 3 |
| 22 | 2090.0513 | 2073.0247 | 2118.0462 | 2101.0196 | D | 262.1397 | 245.1132 | 2 |
| 23 | | | | | K | 147.1128 | 130.0863 | 1 |

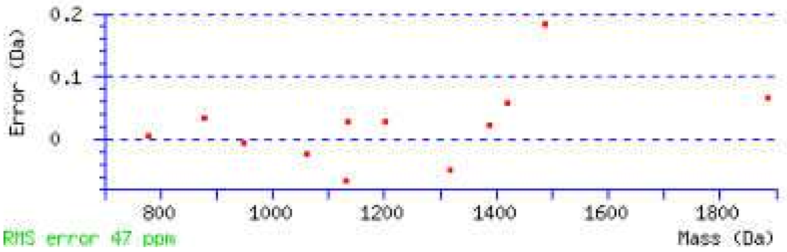


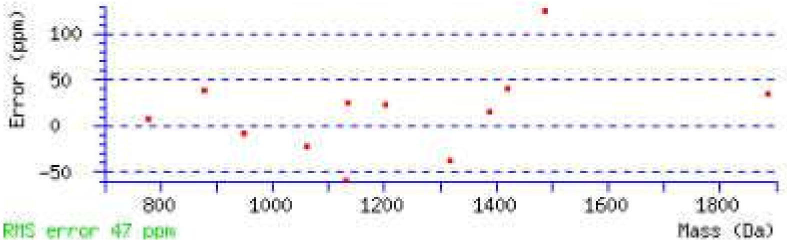


NCBI **BLAST** search of HAGDLGNIVANAEGVAEATIVDK
(Parameters: blastp, nr protein database, expect=20000, no filter, PAM30)
Other BLAST web gateways

**All matches to this query**

| Score | Mr(calc) | Delta | Sequence |
|---|---|---|---|
| 16.8 | 2263.1444 | 0.0097 | HAGDLGNIVANAEGVAEATIVDK |
| 3.1 | 2263.1228 | 0.0313 | QMGLAENQLSIDMLELWLK |
| 1.4 | 2263.0869 | 0.0672 | FEQSGGWIRTLLEEAENER |
| 0.7 | 2263.1444 | 0.0097 | NQAEGSFITNNDSKQSALVLK |
| 0.1 | 2263.1696 | -0.0155 | TFKSGVEGAGSGGAVLPSSVESIK |

**Mascot:** http://www.matrixscience.com/

# MATRIX SCIENCE Mascot Search Results

## Peptide View Spot no 13

MS/MS Fragmentation of **GTSQVEGVVTLTQDDQGPTTVNVR**
Found in **gi|42408425** in **NCBInr**, putative superoxide dismutase [Cu-Zn], chloroplast precursor [Oryza sativa Japonica Group]

Match to Query 257: 2500.258824 from(2501.266100,1+) intensity(0.0000) index(31)
Title: Label: G6, Spot_Id: 219760, Peak_List_Id: 225741, MSMS Job_Run_Id: 21788, Comment:
Data file C:\Documents and Settings\Administrator\Desktop\farah BHU\spot set plate\Farah-Garima 11May 2013\ppw_G6_136842238300.txt

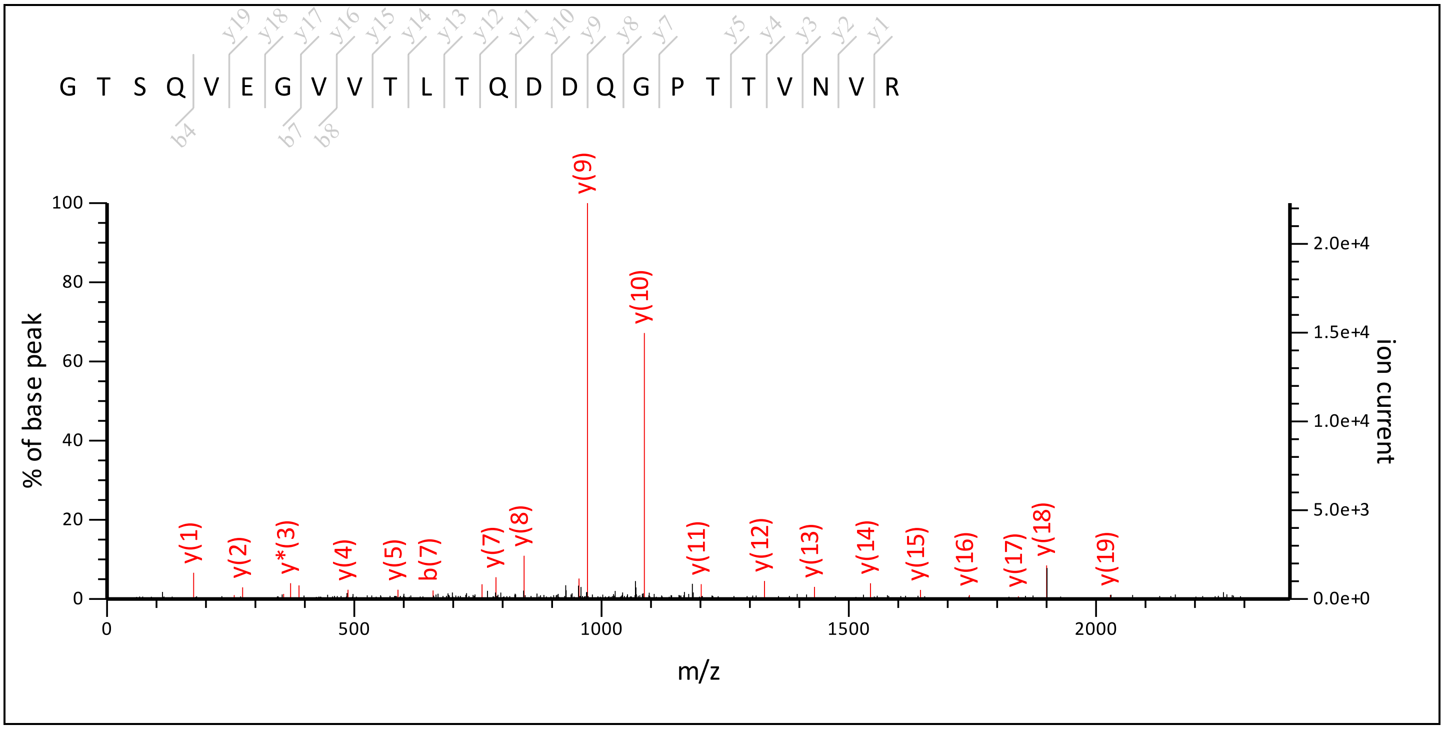


0 to 2392.08

Label all possible matches ○ Label matches used for scoring ◉

**Monoisotopic mass of neutral peptide Mr(calc):** 2500.2406
**Fixed modifications:** Carbamidomethyl (C) (apply to specified residues or termini only)
**Ions Score:** 129 **Expect:** 1.8e-10
**Matches :** 24/132 fragment ions using 46 most intense peaks (help)

| # | a | a* | b | b* | Seq. | y | y* | # |
|---|---|---|---|---|---|---|---|---|
| 1 | 30.0338 | | 58.0287 | | G | | | 24 |
| 2 | 131.0815 | | 159.0764 | | T | 2444.2263 | 2427.1998 | 23 |
| 3 | 218.1135 | | 246.1084 | | S | 2343.1787 | 2326.1521 | 22 |
| 4 | 346.1721 | 329.1456 | 374.1670 | 357.1405 | Q | 2256.1466 | 2239.1201 | 21 |
| 5 | 445.2405 | 428.2140 | 473.2354 | 456.2089 | V | 2128.0881 | 2111.0615 | 20 |
| 6 | 574.2831 | 557.2566 | 602.2780 | 585.2515 | E | ***2029.0196*** | 2011.9931 | 19 |
| 7 | 631.3046 | 614.2780 | **659.2995** | 642.2729 | G | ***1899.9770*** | 1882.9505 | 18 |

| | | | | | | | | |
|---|---|---|---|---|---|---|---|---|
| 8 | 730.3730 | 713.3464 | 758.3679 | 741.3414 | V | 1842.9556 | 1825.9290 | 17 |
| 9 | 829.4414 | 812.4149 | 857.4363 | 840.4098 | V | 1743.8872 | 1726.8606 | 16 |
| 10 | 930.4891 | 913.4625 | 958.4840 | 941.4575 | T | 1644.8188 | 1627.7922 | 15 |
| 11 | 1043.5732 | 1026.5466 | 1071.5681 | 1054.5415 | L | 1543.7711 | 1526.7445 | 14 |
| 12 | 1144.6208 | 1127.5943 | 1172.6157 | 1155.5892 | T | 1430.6870 | 1413.6605 | 13 |
| 13 | 1272.6794 | 1255.6529 | 1300.6743 | 1283.6478 | Q | 1329.6393 | 1312.6128 | 12 |
| 14 | 1387.7064 | 1370.6798 | 1415.7013 | 1398.6747 | D | 1201.5808 | 1184.5542 | 11 |
| 15 | 1502.7333 | 1485.7067 | 1530.7282 | 1513.7017 | D | 1086.5538 | 1069.5273 | 10 |
| 16 | 1630.7919 | 1613.7653 | 1658.7868 | 1641.7602 | Q | 971.5269 | 954.5003 | 9 |
| 17 | 1687.8133 | 1670.7868 | 1715.8083 | 1698.7817 | G | 843.4683 | 826.4417 | 8 |
| 18 | 1784.8661 | 1767.8396 | 1812.8610 | 1795.8345 | P | 786.4468 | 769.4203 | 7 |
| 19 | 1885.9138 | 1868.8872 | 1913.9087 | 1896.8821 | T | 689.3941 | 672.3675 | 6 |
| 20 | 1986.9615 | 1969.9349 | 2014.9564 | 1997.9298 | T | 588.3464 | 571.3198 | 5 |
| 21 | 2086.0299 | 2069.0033 | 2114.0248 | 2096.9982 | V | 487.2987 | 470.2722 | 4 |
| 22 | 2200.0728 | 2183.0463 | 2228.0677 | 2211.0412 | N | 388.2303 | 371.2037 | 3 |
| 23 | 2299.1412 | 2282.1147 | 2327.1361 | 2310.1096 | V | 274.1874 | 257.1608 | 2 |
| 24 | | | | | R | 175.1190 | 158.0924 | 1 |

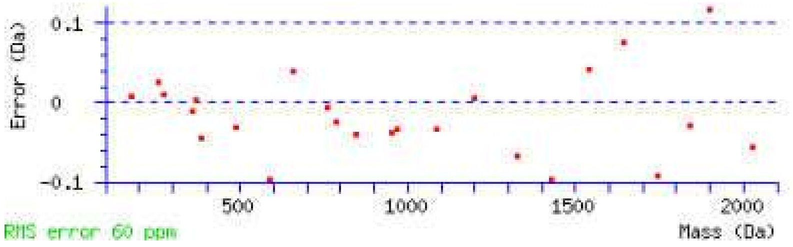


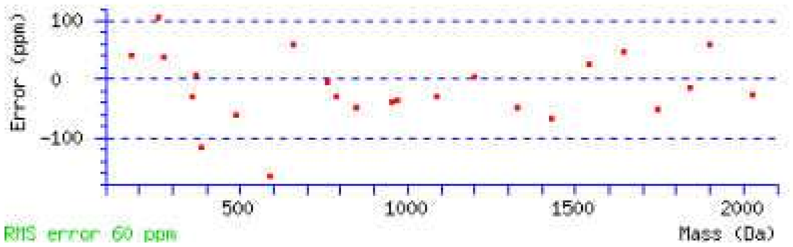


NCBI **BLAST** search of GTSQVEGVVTLTQDDQGPTTVNVR
(Parameters: blastp, nr protein database, expect=20000, no filter, PAM30)
Other BLAST web gateways

**All matches to this query**

| Score | Mr(calc) | Delta | Sequence |
|---|---|---|---|
| 129.3 | 2500.2406 | 0.0182 | GTSQVEGVVTLTQDDQGPTTVNVR |
| 16.6 | 2500.2406 | 0.0183 | GNSTVEGVVTLTQENEGPTTVNVR |
| 7.6 | 2500.2881 | -0.0293 | SVAVASSRLTSLEQQGAASTSTPPR |
| 6.0 | 2500.3537 | -0.0948 | EKTLEIQLLELYGLQEQGAAVR |
| 5.4 | 2500.1758 | 0.0830 | EDETEFSPFYGIDKGAVLQEAR |
| 5.2 | 2500.1978 | 0.0610 | YLCVAADLDNESDVVMLLAAFR |
| | | | |

| | | | |
|---|---|---|---|
| 5.1 | 2500.3108 | -0.0519 | EWVIGVGETLAEAASMLRTQALR |
| 4.2 | 2500.2647 | -0.0058 | CLPGIISAYLKLNSYFPCAWK |
| 3.6 | 2500.3286 | -0.0697 | AVDENPPLQPVQIEKQVAGVDVR |
| 3.4 | 2500.1039 | 0.1549 | VVPEFEGLQDFGGSVMHACDYK |

**Mascot:** http://www.matrixscience.com/

# MATRIX SCIENCE Mascot Search Results

## Peptide View Spot no 13

MS/MS Fragmentation of **AFVVHELEDDLGKGGHELSLSTGNAGGR**
Found in **gi|42408425** in **NCBInr**, putative superoxide dismutase [Cu-Zn], chloroplast precursor [Oryza sativa Japonica Group]

Match to Query 259: 2864.417024 from(2865.424300,1+) intensity(0.0000) index(32)
Title: Label: G6, Spot_Id: 219760, Peak_List_Id: 225744, MSMS Job_Run_Id: 21788, Comment:
Data file C:\Documents and Settings\Administrator\Desktop\farah BHU\spot set plate\Farah-Garima 11May 2013\ppw_G6_136842238300.txt

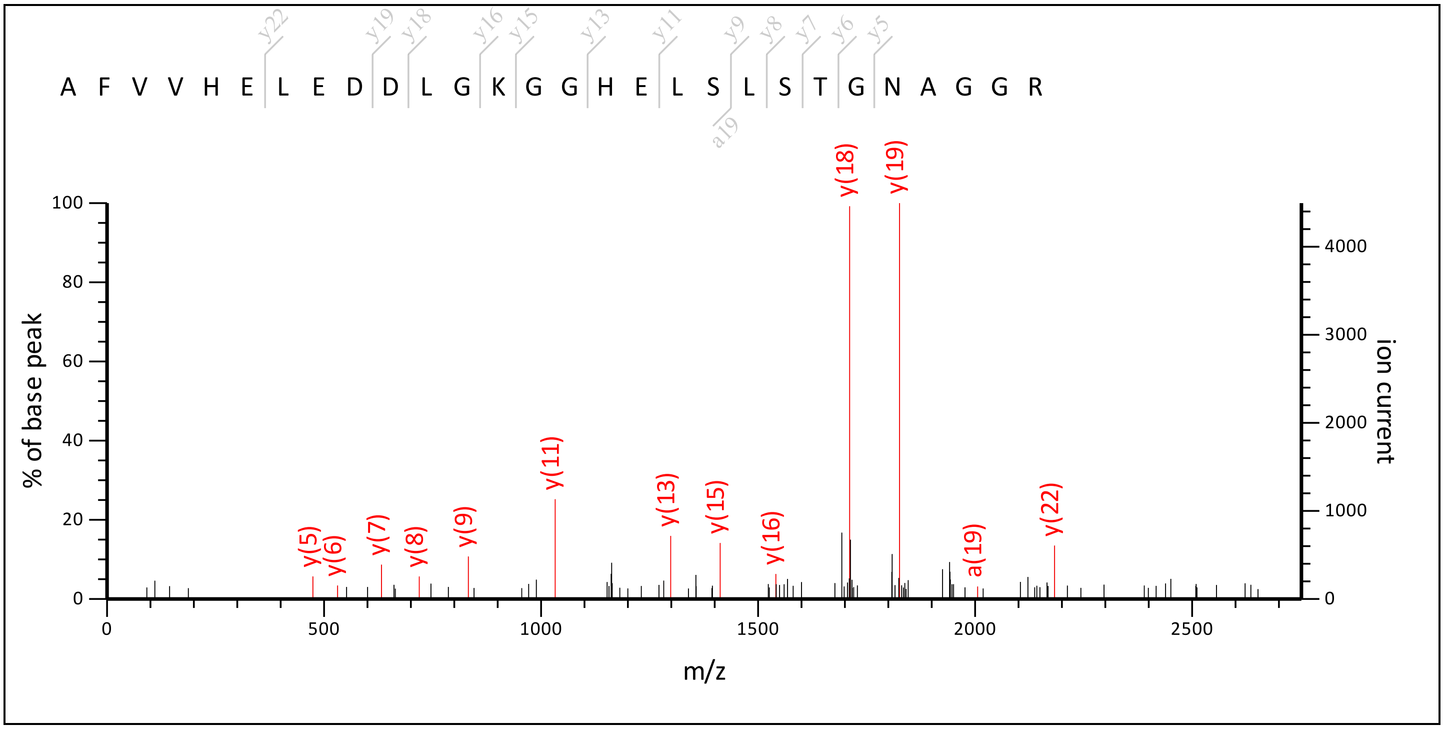


0 to 2751.78

Label all possible matches ○ Label matches used for scoring ◉

**Monoisotopic mass of neutral peptide Mr(calc):** 2864.4053
**Fixed modifications:** Carbamidomethyl (C) (apply to specified residues or termini only)
**Ions Score:** 70 **Expect:** 9.8e-05
**Matches :** 13/138 fragment ions using 24 most intense peaks (help)

| # | a | a* | b | b* | Seq. | y | y* | # |
|---|---|---|---|---|---|---|---|---|
| 1 | 44.0495 | | 72.0444 | | A | | | 28 |
| 2 | 191.1179 | | 219.1128 | | F | 2794.3755 | 2777.3489 | 27 |
| 3 | 290.1863 | | 318.1812 | | V | 2647.3070 | 2630.2805 | 26 |
| 4 | 389.2547 | | 417.2496 | | V | 2548.2386 | 2531.2121 | 25 |
| 5 | 526.3136 | | 554.3085 | | H | 2449.1702 | 2432.1437 | 24 |
| 6 | 655.3562 | | 683.3511 | | E | 2312.1113 | 2295.0848 | 23 |
| 7 | 768.4403 | | 796.4352 | | L | *2183.0687* | 2166.0422 | 22 |

| | | | | | | | | |
|---|---|---|---|---|---|---|---|---|
| 8 | 897.4829 | | 925.4778 | | E | 2069.9847 | 2052.9581 | 21 |
| 9 | 1012.5098 | | 1040.5047 | | D | 1940.9421 | 1923.9155 | 20 |
| 10 | 1127.5368 | | 1155.5317 | | D | 1825.9151 | 1808.8886 | 19 |
| 11 | 1240.6208 | | 1268.6157 | | L | 1710.8882 | 1693.8616 | 18 |
| 12 | 1297.6423 | | 1325.6372 | | G | 1597.8041 | 1580.7776 | 17 |
| 13 | 1425.7373 | 1408.7107 | 1453.7322 | 1436.7056 | K | 1540.7826 | 1523.7561 | 16 |
| 14 | 1482.7587 | 1465.7322 | 1510.7536 | 1493.7271 | G | 1412.6877 | 1395.6611 | 15 |
| 15 | 1539.7802 | 1522.7536 | 1567.7751 | 1550.7486 | G | 1355.6662 | 1338.6397 | 14 |
| 16 | 1676.8391 | 1659.8125 | 1704.8340 | 1687.8075 | H | 1298.6448 | 1281.6182 | 13 |
| 17 | 1805.8817 | 1788.8551 | 1833.8766 | 1816.8501 | E | 1161.5858 | 1144.5593 | 12 |
| 18 | 1918.9658 | 1901.9392 | 1946.9607 | 1929.9341 | L | 1032.5432 | 1015.5167 | 11 |
| 19 | 2005.9978 | 1988.9712 | 2033.9927 | 2016.9661 | S | 919.4592 | 902.4326 | 10 |
| 20 | 2119.0818 | 2102.0553 | 2147.0768 | 2130.0502 | L | 832.4272 | 815.4006 | 9 |
| 21 | 2206.1139 | 2189.0873 | 2234.1088 | 2217.0822 | S | 719.3431 | 702.3165 | 8 |
| 22 | 2307.1616 | 2290.1350 | 2335.1565 | 2318.1299 | T | 632.3111 | 615.2845 | 7 |
| 23 | 2364.1830 | 2347.1565 | 2392.1779 | 2375.1514 | G | 531.2634 | 514.2368 | 6 |
| 24 | 2478.2259 | 2461.1994 | 2506.2209 | 2489.1943 | N | 474.2419 | 457.2154 | 5 |
| 25 | 2549.2631 | 2532.2365 | 2577.2580 | 2560.2314 | A | 360.1990 | 343.1724 | 4 |
| 26 | 2606.2845 | 2589.2580 | 2634.2794 | 2617.2529 | G | 289.1619 | 272.1353 | 3 |
| 27 | 2663.3060 | 2646.2794 | 2691.3009 | 2674.2744 | G | 232.1404 | 215.1139 | 2 |
| 28 | | | | | R | 175.1190 | 158.0924 | 1 |

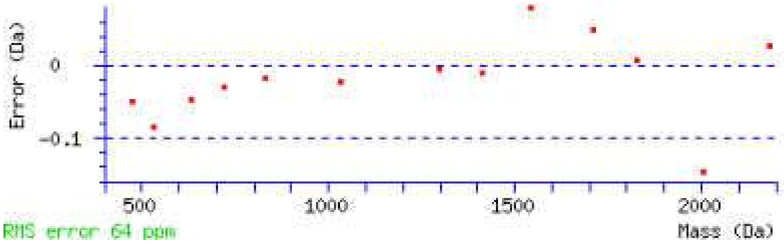


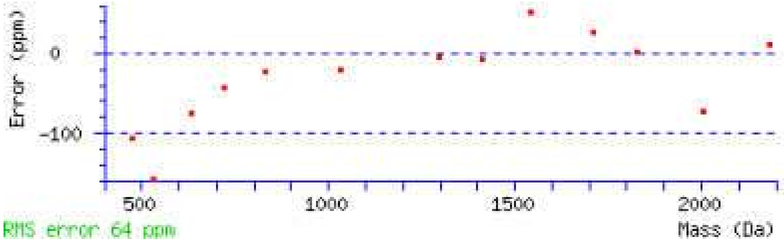


NCBI **BLAST** search of AFVVHELEDDLGKGGHELSLSTGNAGGR
(Parameters: blastp, nr protein database, expect=20000, no filter, PAM30)
Other BLAST web gateways

**All matches to this query**

| Score | Mr(calc) | Delta | Sequence |
|---|---|---|---|
| 70.0 | 2864.4053 | 0.0117 | AFVVHELEDDLGKGGHELSLSTGNAGGR |
| 9.1 | 2864.4879 | -0.0709 | ALESPAPIANQGVITQLSDQISTLNER |
| | | | |

| | | | |
|---|---|---|---|
| 0.9 | 2864.3022 | 0.1148 | DDSDGGISLAPAGARTHMGHAHHHHHK |
| 0.8 | 2864.4451 | -0.0281 | HTQGDGSAVAVLESMGSKGSVLVNGTHVK |
| 0.3 | 2864.5398 | -0.1227 | KEKPPLADPDAVYLIPMEWVLTPVK |
| 0.2 | 2864.1973 | 0.2198 | EVESGMMEDDPINFQQAMKSSNSMK |

**Mascot:** http://www.matrixscience.com/

# Mascot Search Results

## Peptide View Spot no 13

MS/MS Fragmentation of **AVAVLKGTSQVEGVVTLTQDDQGPTTVNVR**
Found in **gi|42408425** in **NCBInr**, putative superoxide dismutase [Cu-Zn], chloroplast precursor [Oryza sativa Japonica Group]

Match to Query 261: 3081.629924 from(3082.637200,1+) intensity(0.0000) index(33)
Title: Label: G6, Spot_Id: 219760, Peak_List_Id: 225743, MSMS Job_Run_Id: 21788, Comment:
Data file C:\Documents and Settings\Administrator\Desktop\farah BHU\spot set plate\Farah-Garima 11May 2013\ppw_G6_136842238300.txt

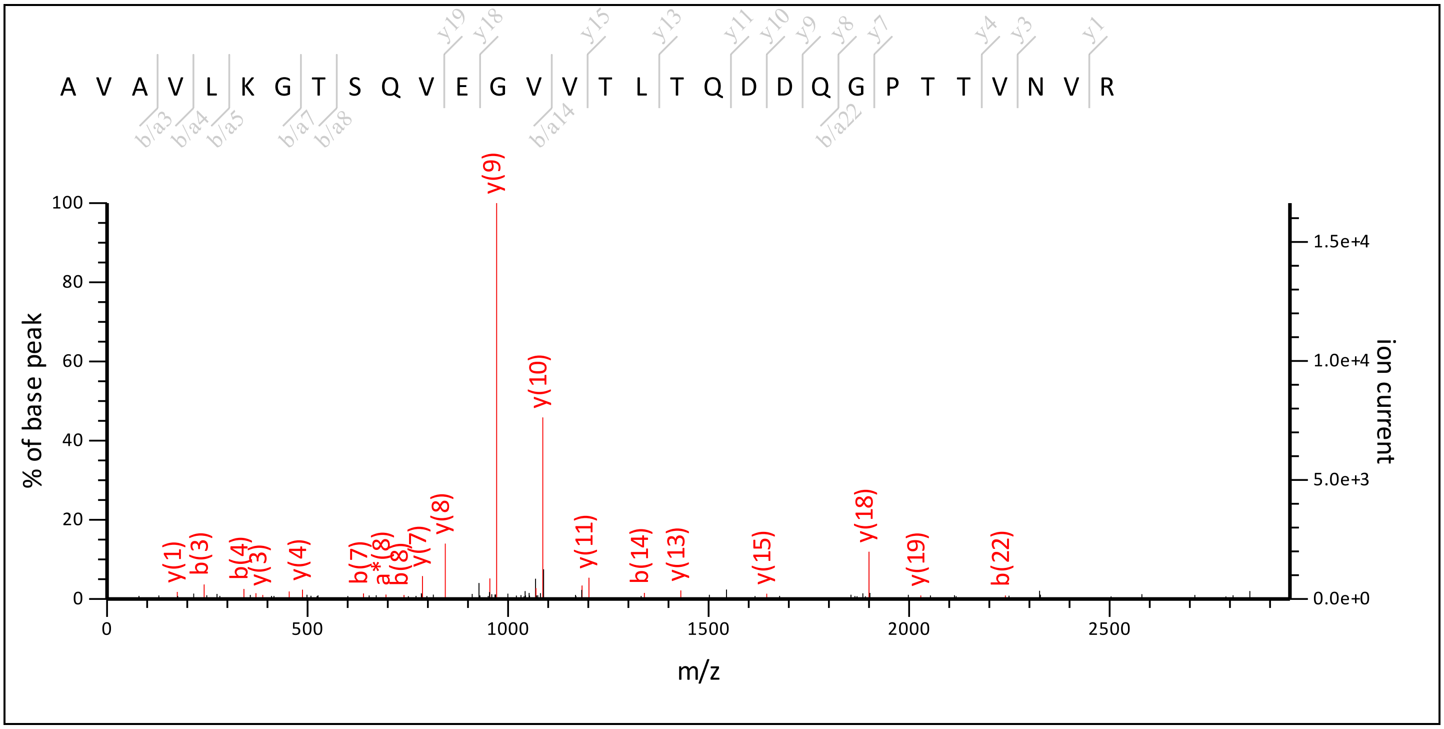


0 to 2949.57

Label all possible matches Label matches used for scoring

**Monoisotopic mass of neutral peptide Mr(calc):** 3081.6307
**Fixed modifications:** Carbamidomethyl (C) (apply to specified residues or termini only)
**Ions Score:** 66 **Expect:** 0.0002
**Matches :** 24/164 fragment ions using 48 most intense peaks (help)

| # | a | a* | b | b* | Seq. | y | y* | # |
|---|---|---|---|---|---|---|---|---|
| 1 | 44.0495 | | 72.0444 | | A | | | 30 |
| 2 | 143.1179 | | 171.1128 | | V | 3011.6008 | 2994.5742 | 29 |
| 3 | 214.1550 | | *242.1499* | | A | 2912.5324 | 2895.5058 | 28 |
| 4 | 313.2234 | | *341.2183* | | V | 2841.4952 | 2824.4687 | 27 |
| 5 | 426.3075 | | *454.3024* | | L | 2742.4268 | 2725.4003 | 26 |
| 6 | 554.4024 | 537.3759 | 582.3974 | 565.3708 | K | 2629.3428 | 2612.3162 | 25 |
| 7 | 611.4239 | 594.3974 | *639.4188* | 622.3923 | G | 2501.2478 | 2484.2213 | 24 |

| | | | | | | | | |
|---|---|---|---|---|---|---|---|---|
| 8 | 712.4716 | 695.4450 | 740.4665 | 723.4400 | T | 2444.2263 | 2427.1998 | 23 |
| 9 | 799.5036 | 782.4771 | 827.4985 | 810.4720 | S | 2343.1787 | 2326.1521 | 22 |
| 10 | 927.5622 | 910.5356 | 955.5571 | 938.5306 | Q | 2256.1466 | 2239.1201 | 21 |
| 11 | 1026.6306 | 1009.6041 | 1054.6255 | 1037.5990 | V | 2128.0881 | 2111.0615 | 20 |
| 12 | 1155.6732 | 1138.6467 | 1183.6681 | 1166.6416 | E | 2029.0196 | 2011.9931 | 19 |
| 13 | 1212.6947 | 1195.6681 | 1240.6896 | 1223.6630 | G | 1899.9770 | 1882.9505 | 18 |
| 14 | 1311.7631 | 1294.7365 | 1339.7580 | 1322.7314 | V | 1842.9556 | 1825.9290 | 17 |
| 15 | 1410.8315 | 1393.8049 | 1438.8264 | 1421.7999 | V | 1743.8872 | 1726.8606 | 16 |
| 16 | 1511.8792 | 1494.8526 | 1539.8741 | 1522.8475 | T | 1644.8188 | 1627.7922 | 15 |
| 17 | 1624.9632 | 1607.9367 | 1652.9582 | 1635.9316 | L | 1543.7711 | 1526.7445 | 14 |
| 18 | 1726.0109 | 1708.9844 | 1754.0058 | 1736.9793 | T | 1430.6870 | 1413.6605 | 13 |
| 19 | 1854.0695 | 1837.0429 | 1882.0644 | 1865.0379 | Q | 1329.6393 | 1312.6128 | 12 |
| 20 | 1969.0964 | 1952.0699 | 1997.0914 | 1980.0648 | D | 1201.5808 | 1184.5542 | 11 |
| 21 | 2084.1234 | 2067.0968 | 2112.1183 | 2095.0917 | D | 1086.5538 | 1069.5273 | 10 |
| 22 | 2212.1820 | 2195.1554 | 2240.1769 | 2223.1503 | Q | 971.5269 | 954.5003 | 9 |
| 23 | 2269.2034 | 2252.1769 | 2297.1983 | 2280.1718 | G | 843.4683 | 826.4417 | 8 |
| 24 | 2366.2562 | 2349.2296 | 2394.2511 | 2377.2245 | P | 786.4468 | 769.4203 | 7 |
| 25 | 2467.3039 | 2450.2773 | 2495.2988 | 2478.2722 | T | 689.3941 | 672.3675 | 6 |
| 26 | 2568.3515 | 2551.3250 | 2596.3465 | 2579.3199 | T | 588.3464 | 571.3198 | 5 |
| 27 | 2667.4200 | 2650.3934 | 2695.4149 | 2678.3883 | V | 487.2987 | 470.2722 | 4 |
| 28 | 2781.4629 | 2764.4363 | 2809.4578 | 2792.4312 | N | 388.2303 | 371.2037 | 3 |
| 29 | 2880.5313 | 2863.5047 | 2908.5262 | 2891.4997 | V | 274.1874 | 257.1608 | 2 |
| 30 | | | | | R | 175.1190 | 158.0924 | 1 |

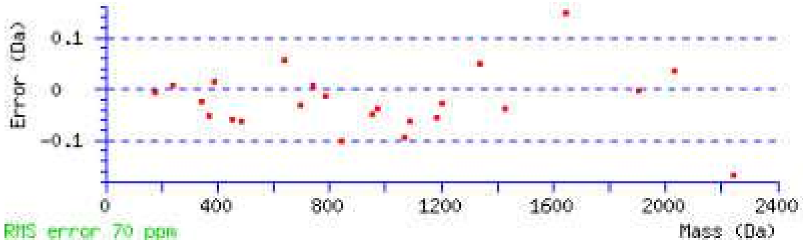


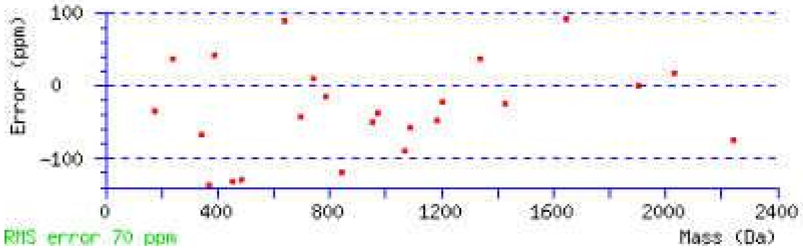


NCBI **BLAST** search of AVAVLKGTSQVEGVVTLTQDDQGPTTVNVR
(Parameters: blastp, nr protein database, expect=20000, no filter, PAM30)
Other BLAST web gateways

**All matches to this query**

| Score | Mr(calc) | Delta | Sequence |
|---|---|---|---|
| | | | |

| | | | |
|---|---|---|---|
| 66.2 | 3081.6307 | -0.0007 | AVAVLKGTSQVEGVVTLTQDDQGPTTVNVR |
| 12.9 | 3081.6306 | -0.0007 | AVAVLKGNSTVEGVVTLTQENEGPTTVNVR |
| 10.7 | 3081.4834 | 0.1465 | QAAADALMHYSQFAMVCISQGVSPSKLR |
| 6.7 | 3081.5403 | 0.0897 | LVKLGSPMETVYIAEQATPMVVEWSGSK |
| 5.4 | 3081.4834 | 0.1465 | QAAADALMHYSQFAMVCISQGVSPSKLR |
| 4.6 | 3081.3855 | 0.2444 | MEFSMVDHPPCARHLGDDVAIAEAGQGR |
| 4.6 | 3081.3855 | 0.2444 | MEFSMVDHPPCARHLGDDVAIAEAGQGR |
| 4.5 | 3081.4615 | 0.1685 | GSFETIHVQDSTGHEFATRMGNVFTIGK |
| 4.3 | 3081.3891 | 0.2409 | RMPFGLCNAPATFQECMLSIFSDMVK |
| 3.5 | 3081.4727 | 0.1572 | IRVSDGDGNGETTAGQGGVVGVFFAGCWLR |

**Mascot:** http://www.matrixscience.com/

# MATRIX SCIENCE Mascot Search Results

## Peptide View Spot no 41

MS/MS Fragmentation of **IEDLSSQLQAQAAQQFR**
Found in **gi|56784713** in **NCBInr**, alpha NAC-like protein [Oryza sativa Japonica Group]

Match to Query 41: 1932.133724 from(1933.141000,1+) intensity(0.0000) index(14)
Title: Label: L6, Spot_Id: 228933, Peak_List_Id: 257650, MSMS Job_Run_Id: 24929, Comment:
Data file ppw_L6_138985137000.txt

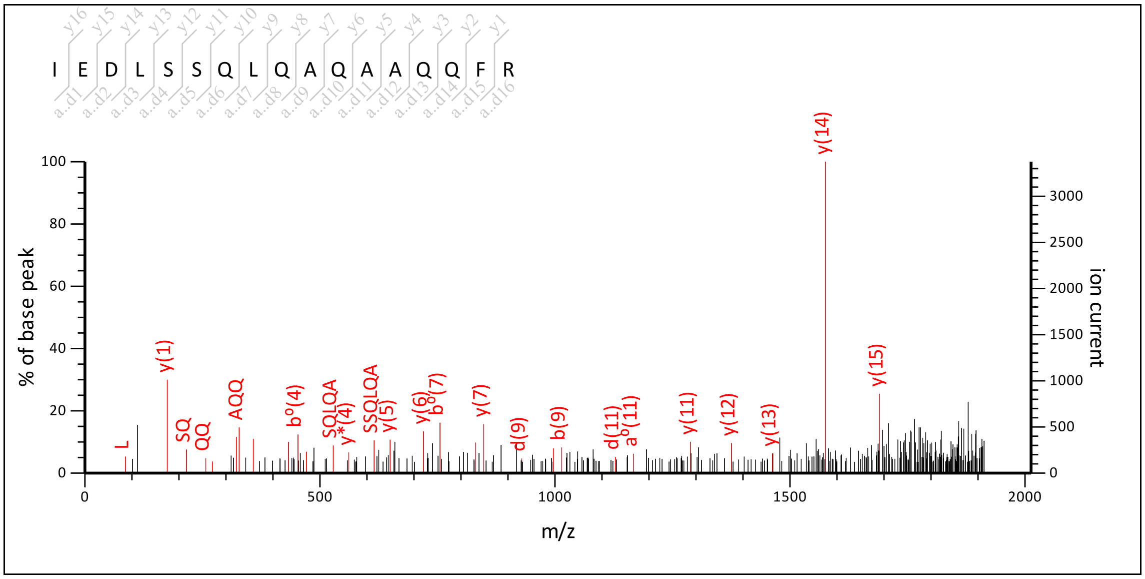


0 to 2012.89

Label all possible matches ○ Label matches used for scoring ◉

**Monoisotopic mass of neutral peptide Mr(calc):** 1931.9701
**Fixed modifications:** Carbamidomethyl (C) (apply to specified residues or termini only)
**Ions Score:** 47 **Expect:** 0.025
**Matches :** 37/300 fragment ions using 57 most intense peaks (help)

| # | Immon. | a | a* | $a^0$ | b | b* | $b^0$ | d | Seq. | v | w | y | y* | $y^0$ | # |
|---|---|---|---|---|---|---|---|---|---|---|---|---|---|---|---|
| 1 | 86.0964 | 86.0964 | | | 114.0913 | | | 44.0495 | I | | | | | | 17 |
| 2 | 102.0550 | 215.1390 | | 197.1285 | 243.1339 | | 225.1234 | 157.1335 | E | 1745.8565 | 1744.8613 | 1819.8933 | 1802.8668 | 1801.8828 | 16 |
| 3 | 88.0393 | 330.1660 | | 312.1554 | **358.1609** | | 340.1503 | 286.1761 | D | 1630.8296 | 1629.8343 | ***1690.8507*** | 1673.8242 | 1672.8402 | 15 |
| 4 | 86.0964 | 443.2500 | | 425.2395 | **471.2449** | | 453.2344 | 401.2031 | L | 1517.7455 | 1516.7503 | ***1575.8238*** | 1558.7972 | 1557.8132 | 14 |
| 5 | 60.0444 | 530.2821 | | 512.2715 | 558.2770 | | 540.2664 | 514.2871 | S | 1430.7135 | 1429.7183 | ***1462.7397*** | 1445.7132 | 1444.7292 | 13 |
| 6 | 60.0444 | 617.3141 | | 599.3035 | 645.3090 | | 627.2984 | 601.3192 | S | 1343.6815 | 1342.6862 | ***1375.7077*** | 1358.6811 | 1357.6971 | 12 |
| 7 | 101.0709 | 745.3727 | 728.3461 | 727.3621 | 773.3676 | 756.3410 | 755.3570 | 688.3512 | Q | 1215.6229 | 1214.6276 | ***1288.6757*** | 1271.6491 | | 11 |
| 8 | 86.0964 | 858.4567 | 841.4302 | 840.4462 | 886.4516 | 869.4251 | 868.4411 | 816.4098 | L | 1102.5388 | 1101.5436 | 1160.6171 | 1143.5905 | | 10 |
| 9 | 101.0709 | 986.5153 | 969.4888 | 968.5047 | **1014.5102** | 997.4837 | 996.4997 | 929.4938 | Q | 974.4803 | 973.4850 | 1047.5330 | 1030.5065 | | 9 |
| 10 | 44.0495 | 1057.5524 | 1040.5259 | 1039.5419 | 1085.5473 | 1068.5208 | 1067.5368 | | A | 903.4431 | | 919.4744 | 902.4479 | | 8 |
| 11 | 101.0709 | 1185.6110 | 1168.5844 | 1167.6004 | 1213.6059 | 1196.5794 | 1195.5953 | 1128.5895 | Q | 775.3846 | 774.3893 | ***848.4373*** | 831.4108 | | 7 |
| 12 | 44.0495 | 1256.6481 | 1239.6216 | 1238.6375 | 1284.6430 | 1267.6165 | 1266.6325 | | A | 704.3475 | | ***720.3788*** | 703.3522 | | 6 |
| 13 | 44.0495 | 1327.6852 | 1310.6587 | 1309.6747 | 1355.6801 | 1338.6536 | 1337.6696 | | A | 633.3103 | | ***649.3416*** | 632.3151 | | 5 |
| 14 | 101.0709 | 1455.7438 | 1438.7173 | 1437.7332 | 1483.7387 | 1466.7122 | 1465.7281 | 1398.7223 | Q | 505.2518 | 504.2565 | 578.3045 | 561.2780 | | 4 |
| 15 | 101.0709 | 1583.8024 | 1566.7758 | 1565.7918 | 1611.7973 | 1594.7707 | 1593.7867 | 1526.7809 | Q | 377.1932 | 376.1979 | 450.2459 | 433.2194 | | 3 |
| 16 | 120.0808 | 1730.8708 | 1713.8442 | 1712.8602 | 1758.8657 | 1741.8392 | 1740.8551 | | F | 230.1248 | | ***322.1874*** | 305.1608 | | 2 |
| 17 | 129.1135 | | | | | | | | R | 74.0237 | 73.0284 | ***175.1190*** | 158.0924 | | 1 |

| Seq | ya | yb | Seq | ya | yb | Seq | ya | yb |
|---|---|---|---|---|---|---|---|---|

| | | | | | | | | |
|---|---|---|---|---|---|---|---|---|
| **ED** | 217.0819 | 245.0768 | **EDL** | 330.1660 | 358.1609 | **EDLS** | 417.1980 | 445.1929 |
| **EDLSS** | 504.2300 | 532.2249 | **EDLSSQ** | 632.2886 | 660.2835 | **DL** | 201.1234 | 229.1183 |
| **DLS** | 288.1554 | 316.1503 | **DLSS** | 375.1874 | 403.1823 | **DLSSQ** | 503.2460 | 531.2409 |
| **DLSSQL** | 616.3301 | 644.3250 | **LS** | 173.1285 | 201.1234 | **LSS** | 260.1605 | 288.1554 |
| **LSSQ** | 388.2191 | 416.2140 | **LSSQL** | 501.3031 | 529.2980 | **LSSQLQ** | 629.3617 | 657.3566 |
| **SS** | 147.0764 | 175.0713 | **SSQ** | 275.1350 | 303.1299 | **SSQL** | 388.2191 | 416.2140 |
| **SSQLQ** | 516.2776 | 544.2726 | **SSQLQA** | 587.3148 | 615.3097 | **SQ** | 188.1030 | 216.0979 |
| **SQL** | 301.1870 | 329.1819 | **SQLQ** | 429.2456 | 457.2405 | **SQLQA** | 500.2827 | 528.2776 |
| **SQLQAQ** | 628.3413 | 656.3362 | **SQLQAQA** | 699.3784 | 727.3733 | **QL** | 214.1550 | 242.1499 |
| **QLQ** | 342.2136 | 370.2085 | **QLQA** | 413.2507 | 441.2456 | **QLQAQ** | 541.3093 | 569.3042 |
| **QLQAQA** | 612.3464 | 640.3413 | **QLQAQAA** | 683.3835 | 711.3784 | **LQ** | 214.1550 | 242.1499 |
| **LQA** | 285.1921 | 313.1870 | **LQAQ** | 413.2507 | 441.2456 | **LQAQA** | 484.2878 | 512.2827 |
| **LQAQAA** | 555.3249 | 583.3198 | **LQAQAAQ** | 683.3835 | 711.3784 | **QA** | 172.1081 | 200.1030 |
| **QAQ** | 300.1666 | 328.1615 | **QAQA** | 371.2037 | 399.1987 | **QAQAA** | 442.2409 | 470.2358 |
| **QAQAAQ** | 570.2994 | 598.2944 | **QAQAAQQ** | 698.3580 | 726.3529 | **AQ** | 172.1081 | 200.1030 |
| **AQA** | 243.1452 | 271.1401 | **AQAA** | 314.1823 | 342.1772 | **AQAAQ** | 442.2409 | 470.2358 |
| **AQAAQQ** | 570.2994 | 598.2944 | **QA** | 172.1081 | 200.1030 | **QAA** | 243.1452 | 271.1401 |
| **QAAQ** | 371.2037 | 399.1987 | **QAAQQ** | 499.2623 | 527.2572 | **QAAQQF** | 646.3307 | 674.3257 |
| **AA** | 115.0866 | 143.0815 | **AAQ** | 243.1452 | 271.1401 | **AAQQ** | 371.2037 | 399.1987 |
| **AAQQF** | 518.2722 | 546.2671 | **AQ** | 172.1081 | 200.1030 | **AQQ** | 300.1666 | 328.1615 |
| **AQQF** | 447.2350 | 475.2300 | **QQ** | 229.1295 | 257.1244 | **QQF** | 376.1979 | 404.1928 |
| **QF** | 248.1394 | 276.1343 | | | | | | |

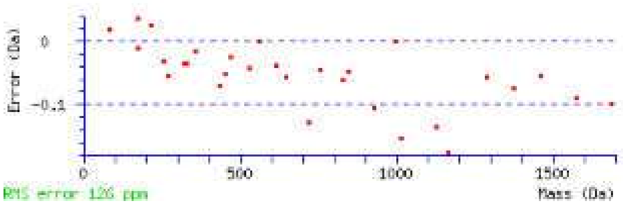


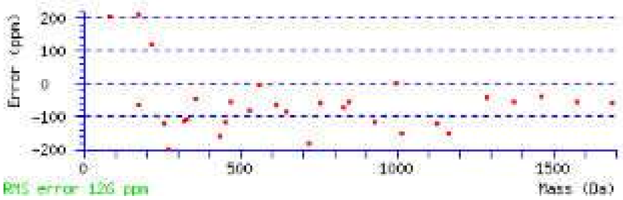


NCBI **BLAST** search of IEDLSSQLQAQAAQQFR
(Parameters: blastp, nr protein database, expect=20000, no filter, PAM30)
Other BLAST web gateways

**All matches to this query**

| Score | Mr(calc) | Delta | Sequence |
|---|---|---|---|
| 46.8 | 1931.9701 | 0.1636 | IEDLSSQLQAQAAQQFR |
| 7.1 | 1932.0065 | 0.1273 | GQGDASQEELLFLLRTR |
| 3.8 | 1932.0330 | 0.1008 | QSALAHFTQTKPLHTPR |
| 3.3 | 1931.9775 | 0.1562 | LFPLGNLDGASITMGSSPR |
| 2.6 | 1931.9424 | 0.1913 | HRLSIYNHMSDFIQR |
| 2.2 | 1932.0204 | 0.1133 | GDNGSVFVLIKEGSSLLTP |
| 1.6 | 1932.0350 | 0.0988 | QKTLVSLNSAESNALVMK |
| 1.5 | 1931.9734 | 0.1603 | LSMLQRNAEEQEIISR |
| 1.1 | 1931.9775 | 0.1562 | EILTQVVTMDTAAQAWR |
| 1.0 | 1931.9926 | 0.1412 | EVKNGHPRPSQDGGLSVR |

**Mascot:** http://www.matrixscience.com/

# MATRIX SCIENCE Mascot Search Results

## Peptide View Spot no 42

MS/MS Fragmentation of **TFQGPPHGIQVER**
Found in **gi|2961307** in **NCBInr**, ribulose-1,5-bisphosphate carboxylase [Rondeletia odorata]

Match to Query 83: 1464.871524 from(1465.878800,1+) intensity(0.0000) index(16)
Title: Label: N6, Spot_Id: 228935, Peak_List_Id: 257665, MSMS Job_Run_Id: 24931, Comment:
Data file ppw_N6_138985138100.txt

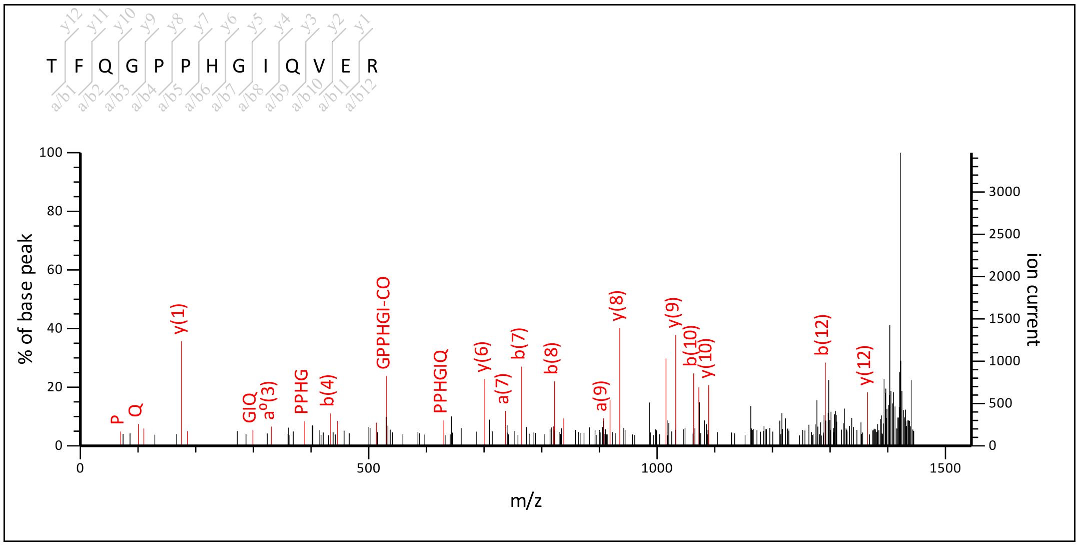


0 to 1545.05

Label all possible matches Label matches used for scoring

**Monoisotopic mass of neutral peptide Mr(calc):** 1464.7474
**Fixed modifications:** Carbamidomethyl (C) (apply to specified residues or termini only)
**Ions Score:** 60 **Expect:** 0.0031
**Matches :** 40/229 fragment ions using 41 most intense peaks (help)

| # | Immon. | a | a* | $a^0$ | b | b* | $b^0$ | d | d' | Seq. | v | w | w' | y | y* | |
|---|---|---|---|---|---|---|---|---|---|---|---|---|---|---|---|---|
| 1 | 74.0600 | 74.0600 | | 56.0495 | 102.0550 | | 84.0444 | 44.0495 | | T | | | | | | |
| 2 | 120.0808 | 221.1285 | | 203.1179 | 249.1234 | | 231.1128 | | | F | 1272.6444 | | | ***1364.7070*** | 1347.6804 | 134 |
| 3 | 101.0709 | 349.1870 | 332.1605 | 331.1765 | 377.1819 | 360.1554 | 359.1714 | 292.1656 | | Q | 1144.5858 | 1143.5905 | | 1217.6385 | 1200.6120 | 119 |
| 4 | 30.0338 | 406.2085 | 389.1819 | 388.1979 | ***434.2034*** | 417.1769 | 416.1928 | | | G | | | | ***1089.5800*** | 1072.5534 | 107 |
| 5 | 70.0651 | 503.2613 | 486.2347 | 485.2507 | ***531.2562*** | 514.2296 | 513.2456 | 477.2456 | | P | 990.5116 | 989.5163 | | ***1032.5585*** | 1015.5320 | 101 |
| 6 | 70.0651 | 600.3140 | 583.2875 | 582.3035 | 628.3089 | 611.2824 | 610.2984 | 574.2984 | | P | 893.4588 | 892.4635 | | ***935.5057*** | 918.4792 | 91 |
| 7 | 110.0713 | ***737.3729*** | 720.3464 | 719.3624 | ***765.3679*** | 748.3413 | 747.3573 | | | H | 756.3999 | | | ***838.4530*** | 821.4264 | 82 |
| 8 | 30.0338 | 794.3944 | 777.3679 | 776.3838 | ***822.3893*** | 805.3628 | 804.3787 | | | G | | | | ***701.3941*** | 684.3675 | 68 |
| 9 | 86.0964 | ***907.4785*** | 890.4519 | 889.4679 | ***935.4734*** | 918.4468 | 917.4628 | 879.4472 | 893.4628 | I | 586.2944 | 599.3148 | 613.3304 | 644.3726 | 627.3461 | 62 |
| 10 | 101.0709 | 1035.5370 | 1018.5105 | 1017.5265 | ***1063.5320*** | 1046.5054 | 1045.5214 | 978.5156 | | Q | 458.2358 | 457.2405 | | ***531.2885*** | 514.2620 | 51 |
| 11 | 72.0808 | 1134.6055 | 1117.5789 | 1116.5949 | 1162.6004 | 1145.5738 | 1144.5898 | 1120.5898 | | V | 359.1674 | 372.1878 | | 403.2300 | 386.2034 | 38 |
| 12 | 102.0550 | 1263.6480 | 1246.6215 | 1245.6375 | ***1291.6430*** | 1274.6164 | 1273.6324 | 1205.6426 | | E | 230.1248 | 229.1295 | | 304.1615 | 287.1350 | 28 |
| 13 | 129.1135 | | | | | | | | | R | 74.0237 | 73.0284 | | ***175.1190*** | 158.0924 | |

| Seq | ya | yb | Seq | ya | yb | Seq | ya | yb |
|---|---|---|---|---|---|---|---|---|
| **FQ** | 248.1394 | 276.1343 | **FQG** | 305.1608 | 333.1557 | **FQGP** | 402.2136 | 430.2085 |
| **FQGPP** | 499.2663 | 527.2613 | **FQGPPH** | 636.3253 | 664.3202 | **FQGPPHG** | 693.3467 | 721.3416 |
| **QG** | 158.0924 | 186.0873 | **QGP** | 255.1452 | 283.1401 | **QGPP** | 352.1979 | 380.1928 |
| **QGPPH** | 489.2568 | 517.2518 | **QGPPHG** | 546.2783 | 574.2732 | **QGPPHGI** | 659.3624 | 687.3573 |
| **GP** | 127.0866 | 155.0815 | **GPP** | 224.1394 | 252.1343 | **GPPH** | 361.1983 | 389.1932 |
| **GPPHG** | 418.2197 | 446.2146 | **GPPHGI** | 531.3038 | 559.2987 | **GPPHGIQ** | 659.3624 | 687.3573 |
| **PP** | 167.1179 | 195.1128 | **PPH** | 304.1768 | 332.1717 | **PPHG** | 361.1983 | 389.1932 |

| | | | | | | | | |
|---|---|---|---|---|---|---|---|---|
| **PPHGI** | 474.2823 | 502.2772 | **PPHGIQ** | 602.3409 | 630.3358 | **PH** | 207.1240 | 235.1190 |
| **PHG** | 264.1455 | 292.1404 | **PHGI** | 377.2296 | 405.2245 | **PHGIQ** | 505.2881 | 533.2831 |
| **PHGIQV** | 604.3566 | 632.3515 | **HG** | 167.0927 | 195.0877 | **HGI** | 280.1768 | 308.1717 |
| **HGIQ** | 408.2354 | 436.2303 | **HGIQV** | 507.3038 | 535.2987 | **HGIQVE** | 636.3464 | 664.3413 |
| **GI** | 143.1179 | 171.1128 | **GIQ** | 271.1765 | 299.1714 | **GIQV** | 370.2449 | 398.2398 |
| **GIQVE** | 499.2875 | 527.2824 | **IQ** | 214.1550 | 242.1499 | **IQV** | 313.2234 | 341.2183 |
| **IQVE** | 442.2660 | 470.2609 | **QV** | 200.1394 | 228.1343 | **QVE** | 329.1819 | 357.1769 |
| **VE** | 201.1234 | 229.1183 | | | | | | |

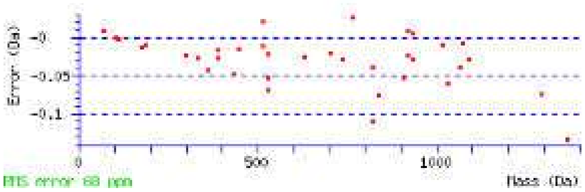


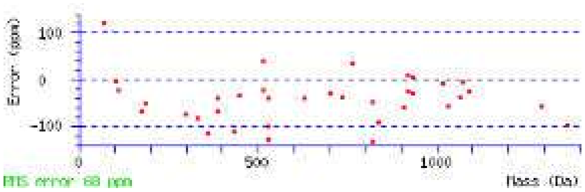


NCBI **BLAST** search of TFQGPPHGIQVER
(Parameters: blastp, nr protein database, expect=20000, no filter, PAM30)
Other BLAST web gateways

**All matches to this query**

| Score | Mr(calc) | Delta | Sequence |
|---|---|---|---|
| 60.9 | 1464.7474 | 0.1241 | TFQGPPHGLQVER |
| 60.9 | 1464.7474 | 0.1241 | TFQGPPHGLQVER |
| 60.0 | 1464.7838 | 0.0878 | TFKGPPHGIQVER |
| 60.0 | 1464.7838 | 0.0878 | TFKGPPHGIQVER |
| 60.0 | 1464.7838 | 0.0878 | TFQGPPHGIKVER |
| 60.0 | 1464.7838 | 0.0878 | TFQGPPHGIKVER |
| 60.0 | 1464.7474 | 0.1241 | TFQGPPHGIQVER |
| 60.0 | 1464.7474 | 0.1241 | TFQGPPHGIQVER |
| 60.0 | 1464.7474 | 0.1241 | TFQGPPHGIQVER |
| 60.0 | 1464.7474 | 0.1241 | TFQGPPHGIQVER |

**Mascot:** http://www.matrixscience.com/

# MATRIX SCIENCE Mascot Search Results

## Peptide View Spot no 42

MS/MS Fragmentation of **GGLDFTKDDENVNSQPFMR**
Found in **gi|2961307** in **NCBInr**, ribulose-1,5-bisphosphate carboxylase [Rondeletia odorata]

Match to Query 113: 2169.179224 from(2170.186500,1+) intensity(0.0000) index(24)
Title: Label: N6, Spot_Id: 228935, Peak_List_Id: 257671, MSMS Job_Run_Id: 24931, Comment:
Data file ppw_N6_138985138100.txt

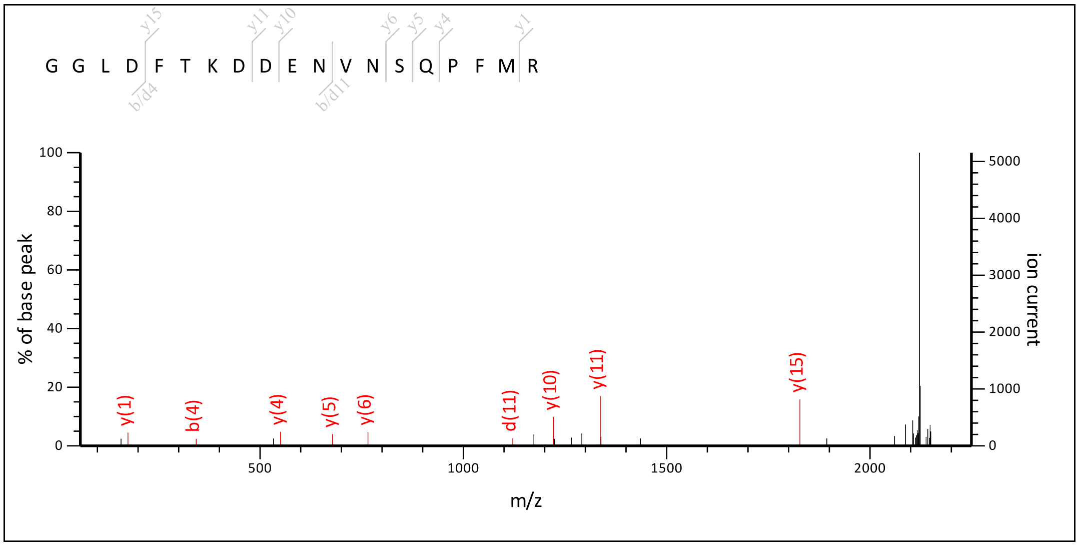


58.11 to 2249.42

Label all possible matches ○ Label matches used for scoring ◉

**Monoisotopic mass of neutral peptide Mr(calc):** 2168.9797
**Fixed modifications:** Carbamidomethyl (C) (apply to specified residues or termini only)
**Ions Score:** 48 **Expect:** 0.053
**Matches :** 10/340 fragment ions using 12 most intense peaks (help)

| # | Immon. | a | a* | $a^0$ | b | b* | $b^0$ | d | d' | Seq. | v | w | w' | y | y* | |
|---|---|---|---|---|---|---|---|---|---|---|---|---|---|---|---|---|
| 1 | 30.0338 | 30.0338 | | | 58.0287 | | | 44.0495 | | G | | | | | | |
| 2 | 30.0338 | 87.0553 | | | 115.0502 | | | | | G | | | | 2112.9655 | 2095.9389 | 20 |
| 3 | 86.0964 | 200.1394 | | | 228.1343 | | | 158.0924 | | L | 1997.8658 | 1996.8705 | | 2055.9440 | 2038.9175 | 20 |
| 4 | 88.0393 | 315.1663 | | 297.1557 | **343.1612** | | 325.1506 | 271.1765 | | D | 1882.8388 | 1881.8436 | | 1942.8600 | 1925.8334 | 19 |
| 5 | 120.0808 | 462.2347 | | 444.2241 | 490.2296 | | 472.2191 | | | F | 1735.7704 | | | ***1827.8330*** | 1810.8065 | 18 |
| 6 | 74.0600 | 563.2824 | | 545.2718 | 591.2773 | | 573.2667 | 547.2875 | 549.2667 | T | 1634.7227 | 1647.7431 | 1649.7224 | 1680.7646 | 1663.7381 | 16 |
| 7 | 101.1073 | 691.3774 | 674.3508 | 673.3668 | 719.3723 | 702.3457 | 701.3617 | 634.3195 | | K | 1506.6278 | 1505.6325 | | 1579.7169 | 1562.6904 | 15 |
| 8 | 88.0393 | 806.4043 | 789.3777 | 788.3937 | 834.3992 | 817.3727 | 816.3886 | 762.4145 | | D | 1391.6008 | 1390.6056 | | 1451.6220 | 1434.5954 | 14 |
| 9 | 88.0393 | 921.4312 | 904.4047 | 903.4207 | 949.4262 | 932.3996 | 931.4156 | 877.4414 | | D | 1276.5739 | 1275.5786 | | ***1336.5950*** | 1319.5685 | 13 |
| 10 | 102.0550 | 1050.4738 | 1033.4473 | 1032.4633 | 1078.4687 | 1061.4422 | 1060.4582 | 992.4684 | | E | 1147.5313 | 1146.5361 | | ***1221.5681*** | 1204.5415 | 12 |
| 11 | 87.0553 | 1164.5168 | 1147.4902 | 1146.5062 | 1192.5117 | 1175.4851 | 1174.5011 | 1121.5109 | | N | 1033.4884 | 1032.4931 | | 1092.5255 | 1075.4989 | 10 |
| 12 | 72.0808 | 1263.5852 | 1246.5586 | 1245.5746 | 1291.5801 | 1274.5535 | 1273.5695 | 1249.5695 | | V | 934.4200 | 947.4404 | | 978.4826 | 961.4560 | 9 |
| 13 | 87.0553 | 1377.6281 | 1360.6016 | 1359.6175 | 1405.6230 | 1388.5965 | 1387.6124 | 1334.6223 | | N | 820.3770 | 819.3818 | | 879.4141 | 862.3876 | 8 |
| 14 | 60.0444 | 1464.6601 | 1447.6336 | 1446.6496 | 1492.6550 | 1475.6285 | 1474.6445 | 1448.6652 | | S | 733.3450 | 732.3498 | | ***765.3712*** | 748.3447 | 7 |
| 15 | 101.0709 | 1592.7187 | 1575.6922 | 1574.7081 | 1620.7136 | 1603.6871 | 1602.7031 | 1535.6972 | | Q | 605.2864 | 604.2912 | | ***678.3392*** | 661.3126 | |
| 16 | 70.0651 | 1689.7715 | 1672.7449 | 1671.7609 | 1717.7664 | 1700.7398 | 1699.7558 | 1663.7558 | | P | 508.2337 | 507.2384 | | ***550.2806*** | 533.2541 | |
| 17 | 120.0808 | 1836.8399 | 1819.8133 | 1818.8293 | 1864.8348 | 1847.8082 | 1846.8242 | | | F | 361.1653 | | | 453.2279 | 436.2013 | |
| 18 | 104.0528 | 1967.8804 | 1950.8538 | 1949.8698 | 1995.8753 | 1978.8487 | 1977.8647 | 1907.8770 | | M | 230.1248 | 229.1295 | | 306.1594 | 289.1329 | |
| 19 | 129.1135 | | | | | | | | | R | 74.0237 | 73.0284 | | ***175.1190*** | 158.0924 | |

| Seq | ya | yb | Seq | ya | yb | Seq | ya | yb |
|---|---|---|---|---|---|---|---|---|
| GL | 143.1179 | 171.1128 | GLD | 258.1448 | 286.1397 | GLDF | 405.2132 | 433.2082 |
| | | | | | | | | |

| | | | | | | | | |
|---|---|---|---|---|---|---|---|---|
| **GLDFT** | 506.2609 | 534.2558 | **GLDFTK** | 634.3559 | 662.3508 | **LD** | 201.1234 | 229.1183 |
| **LDF** | 348.1918 | 376.1867 | **LDFT** | 449.2395 | 477.2344 | **LDFTK** | 577.3344 | 605.3293 |
| **LDFTKD** | 692.3614 | 720.3563 | **DF** | 235.1077 | 263.1026 | **DFT** | 336.1554 | 364.1503 |
| **DFTK** | 464.2504 | 492.2453 | **DFTKD** | 579.2773 | 607.2722 | **DFTKDD** | 694.3042 | 722.2992 |
| **FT** | 221.1285 | 249.1234 | **FTK** | 349.2234 | 377.2183 | **FTKD** | 464.2504 | 492.2453 |
| **FTKDD** | 579.2773 | 607.2722 | **TK** | 202.1550 | 230.1499 | **TKD** | 317.1819 | 345.1769 |
| **TKDD** | 432.2089 | 460.2038 | **TKDDE** | 561.2515 | 589.2464 | **TKDDEN** | 675.2944 | 703.2893 |
| **KD** | 216.1343 | 244.1292 | **KDD** | 331.1612 | 359.1561 | **KDDE** | 460.2038 | 488.1987 |
| **KDDEN** | 574.2467 | 602.2416 | **KDDENV** | 673.3151 | 701.3101 | **DD** | 203.0662 | 231.0612 |
| **DDE** | 332.1088 | 360.1038 | **DDEN** | 446.1518 | 474.1467 | **DDENV** | 545.2202 | 573.2151 |
| **DDENVN** | 659.2631 | 687.2580 | **DE** | 217.0819 | 245.0768 | **DEN** | 331.1248 | 359.1197 |
| **DENV** | 430.1932 | 458.1882 | **DENVN** | 544.2362 | 572.2311 | **DENVNS** | 631.2682 | 659.2631 |
| **EN** | 216.0979 | 244.0928 | **ENV** | 315.1663 | 343.1612 | **ENVN** | 429.2092 | 457.2041 |
| **ENVNS** | 516.2413 | 544.2362 | **ENVNSQ** | 644.2998 | 672.2947 | **NV** | 186.1237 | 214.1186 |
| **NVN** | 300.1666 | 328.1615 | **NVNS** | 387.1987 | 415.1936 | **NVNSQ** | 515.2572 | 543.2522 |
| **NVNSQP** | 612.3100 | 640.3049 | **VN** | 186.1237 | 214.1186 | **VNS** | 273.1557 | 301.1506 |
| **VNSQ** | 401.2143 | 429.2092 | **VNSQP** | 498.2671 | 526.2620 | **VNSQPF** | 645.3355 | 673.3304 |
| **NS** | 174.0873 | 202.0822 | **NSQ** | 302.1459 | 330.1408 | **NSQP** | 399.1987 | 427.1936 |
| **NSQPF** | 546.2671 | 574.2620 | **NSQPFM** | 677.3076 | 705.3025 | **SQ** | 188.1030 | 216.0979 |
| **SQP** | 285.1557 | 313.1506 | **SQPF** | 432.2241 | 460.2191 | **SQPFM** | 563.2646 | 591.2595 |
| **QP** | 198.1237 | 226.1186 | **QPF** | 345.1921 | 373.1870 | **QPFM** | 476.2326 | 504.2275 |
| **PF** | 217.1335 | 245.1285 | **PFM** | 348.1740 | 376.1689 | **FM** | 251.1213 | 279.1162 |

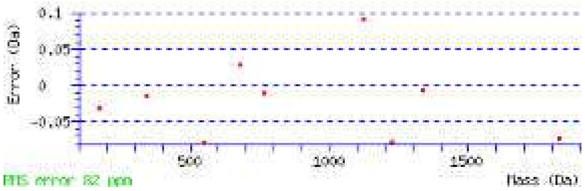


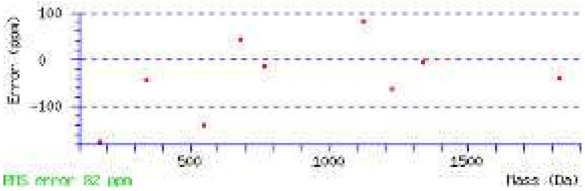


NCBI **BLAST** search of GGLDFTKDDENVNSQPFMR
(Parameters: blastp, nr protein database, expect=20000, no filter, PAM30)
Other BLAST web gateways

**All matches to this query**

| Score | Mr(calc) | Delta | Sequence |
|---|---|---|---|
| 48.0 | 2168.9797 | 0.1995 | GGIDFTKDDENVNSQPFMR |
| 48.0 | 2168.9797 | 0.1995 | GGIDFTKDDENVNSQPFMR |
| 48.0 | 2169.0161 | 0.1632 | GGLDFTKDDENVNSKPFMR |
| 48.0 | 2168.9797 | 0.1995 | GGLDFTKDDENVNSQPFMR |
| 48.0 | 2168.9797 | 0.1995 | GGLDFTKDDENVNSQPFMR |
| 48.0 | 2168.9797 | 0.1995 | GGLDFTKDDENVNSQPFMR |
| 48.0 | 2168.9797 | 0.1995 | GGLDFTKDDENVNSQPFMR |
| 48.0 | 2168.9797 | 0.1995 | GGLDFTKDDENVNSQPFMR |
| 48.0 | 2168.9797 | 0.1995 | GGLDFTKDDENVNSQPFMR |
| 48.0 | 2168.9797 | 0.1995 | GGLDFTKDDENVNSQPFMR |

**Mascot:** http://www.matrixscience.com/

# MATRIX SCIENCE Mascot Search Results

## Peptide View Spot no 42

MS/MS Fragmentation of **GGLMFTKDDENVNSQPFMR**
Found in **gi|2961307** in **NCBInr**, ribulose-1,5-bisphosphate carboxylase [Rondeletia odorata]

Match to Query 115: 2185.160924 from(2186.168200,1+) intensity(0.0000) index(25)
Title: Label: N6, Spot_Id: 228935, Peak_List_Id: 257674, MSMS Job_Run_Id: 24931, Comment:
Data file ppw_N6_138985138100.txt

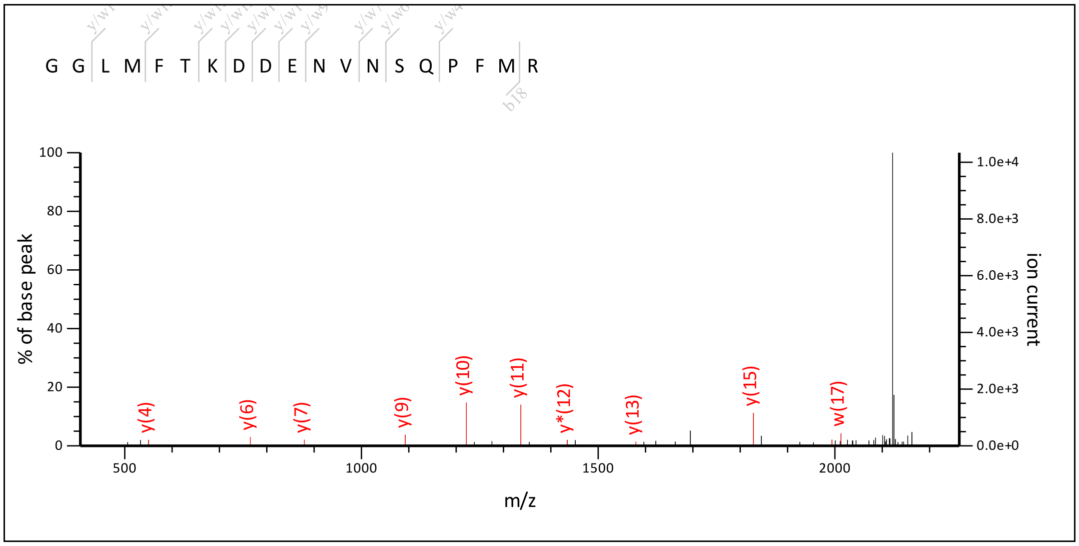


406.24 to 2262.72

Label all possible matches ○ Label matches used for scoring ◉

**Monoisotopic mass of neutral peptide Mr(calc):** 2184.9932
**Fixed modifications:** Carbamidomethyl (C) (apply to specified residues or termini only)
**Ions Score:** 59 **Expect:** 0.0046
**Matches :** 12/334 fragment ions using 13 most intense peaks (help)

| # | Immon. | a | a* | $a^0$ | b | b* | $b^0$ | d | d' | Seq. | v | w | w' | y | y* | |
|---|---|---|---|---|---|---|---|---|---|---|---|---|---|---|---|---|
| 1 | 30.0338 | 30.0338 | | | 58.0287 | | | 44.0495 | | G | | | | | | |
| 2 | 30.0338 | 87.0553 | | | 115.0502 | | | | | G | | | | 2128.9790 | 2111.9525 | 21 |
| 3 | 86.0964 | 200.1394 | | | 228.1343 | | | 158.0924 | | L | 2013.8793 | 2012.8841 | | 2071.9576 | 2054.9310 | 20 |
| 4 | 104.0528 | 331.1798 | | | 359.1748 | | | 271.1765 | | M | 1882.8388 | 1881.8436 | | 1958.8735 | 1941.8470 | 19 |
| 5 | 120.0808 | 478.2483 | | | 506.2432 | | | | | F | 1735.7704 | | | ***1827.8330*** | 1810.8065 | 18 |
| 6 | 74.0600 | 579.2959 | | 561.2854 | 607.2908 | | 589.2803 | 563.3010 | 565.2803 | T | 1634.7227 | 1647.7431 | 1649.7224 | 1680.7646 | 1663.7381 | 16 |
| 7 | 101.1073 | 707.3909 | 690.3643 | 689.3803 | 735.3858 | 718.3593 | 717.3752 | 650.3330 | | K | 1506.6278 | 1505.6325 | | ***1579.7169*** | 1562.6904 | 15 |
| 8 | 88.0393 | 822.4178 | 805.3913 | 804.4073 | 850.4128 | 833.3862 | 832.4022 | 778.4280 | | D | 1391.6008 | 1390.6056 | | 1451.6220 | 1434.5954 | 14 |
| 9 | 88.0393 | 937.4448 | 920.4182 | 919.4342 | 965.4397 | 948.4131 | 947.4291 | 893.4550 | | D | 1276.5739 | 1275.5786 | | ***1336.5950*** | 1319.5685 | 13 |
| 10 | 102.0550 | 1066.4874 | 1049.4608 | 1048.4768 | 1094.4823 | 1077.4557 | 1076.4717 | 1008.4819 | | E | 1147.5313 | 1146.5361 | | ***1221.5681*** | 1204.5415 | 12 |
| 11 | 87.0553 | 1180.5303 | 1163.5038 | 1162.5197 | 1208.5252 | 1191.4987 | 1190.5146 | 1137.5245 | | N | 1033.4884 | 1032.4931 | | ***1092.5255*** | 1075.4989 | 10 |
| 12 | 72.0808 | 1279.5987 | 1262.5722 | 1261.5881 | 1307.5936 | 1290.5671 | 1289.5831 | 1265.5831 | | V | 934.4200 | 947.4404 | | 978.4826 | 961.4560 | 9 |
| 13 | 87.0553 | 1393.6416 | 1376.6151 | 1375.6311 | 1421.6366 | 1404.6100 | 1403.6260 | 1350.6358 | | N | 820.3770 | 819.3818 | | ***879.4141*** | 862.3876 | 8 |
| 14 | 60.0444 | 1480.6737 | 1463.6471 | 1462.6631 | 1508.6686 | 1491.6420 | 1490.6580 | 1464.6788 | | S | 733.3450 | 732.3498 | | ***765.3712*** | 748.3447 | 7 |
| 15 | 101.0709 | 1608.7322 | 1591.7057 | 1590.7217 | 1636.7272 | 1619.7006 | 1618.7166 | 1551.7108 | | Q | 605.2864 | 604.2912 | | 678.3392 | 661.3126 | |
| 16 | 70.0651 | 1705.7850 | 1688.7585 | 1687.7744 | 1733.7799 | 1716.7534 | 1715.7694 | 1679.7694 | | P | 508.2337 | 507.2384 | | ***550.2806*** | 533.2541 | |
| 17 | 120.0808 | 1852.8534 | 1835.8269 | 1834.8429 | 1880.8483 | 1863.8218 | 1862.8378 | | | F | 361.1653 | | | 453.2279 | 436.2013 | |
| 18 | 104.0528 | 1983.8939 | 1966.8674 | 1965.8833 | 2011.8888 | 1994.8623 | 1993.8783 | 1923.8905 | | M | 230.1248 | 229.1295 | | 306.1594 | 289.1329 | |
| 19 | 129.1135 | | | | | | | | | R | 74.0237 | 73.0284 | | 175.1190 | 158.0924 | |

| Seq | ya | yb | Seq | ya | yb | Seq | ya | yb |
|---|---|---|---|---|---|---|---|---|
| GL | 143.1179 | 171.1128 | GLM | 274.1584 | 302.1533 | GLMF | 421.2268 | 449.2217 |
| | | | | | | | | |

| | | | | | | | | |
|---|---|---|---|---|---|---|---|---|
| **GLMFT** | 522.2745 | 550.2694 | **GLMFTK** | 650.3694 | 678.3643 | **LM** | 217.1369 | 245.1318 |
| **LMF** | 364.2053 | 392.2002 | **LMFT** | 465.2530 | 493.2479 | **LMFTK** | 593.3480 | 621.3429 |
| **MF** | 251.1213 | 279.1162 | **MFT** | 352.1689 | 380.1639 | **MFTK** | 480.2639 | 508.2588 |
| **MFTKD** | 595.2908 | 623.2858 | **FT** | 221.1285 | 249.1234 | **FTK** | 349.2234 | 377.2183 |
| **FTKD** | 464.2504 | 492.2453 | **FTKDD** | 579.2773 | 607.2722 | **TK** | 202.1550 | 230.1499 |
| **TKD** | 317.1819 | 345.1769 | **TKDD** | 432.2089 | 460.2038 | **TKDDE** | 561.2515 | 589.2464 |
| **TKDDEN** | 675.2944 | 703.2893 | **KD** | 216.1343 | 244.1292 | **KDD** | 331.1612 | 359.1561 |
| **KDDE** | 460.2038 | 488.1987 | **KDDEN** | 574.2467 | 602.2416 | **KDDENV** | 673.3151 | 701.3101 |
| **DD** | 203.0662 | 231.0612 | **DDE** | 332.1088 | 360.1038 | **DDEN** | 446.1518 | 474.1467 |
| **DDENV** | 545.2202 | 573.2151 | **DDENVN** | 659.2631 | 687.2580 | **DE** | 217.0819 | 245.0768 |
| **DEN** | 331.1248 | 359.1197 | **DENV** | 430.1932 | 458.1882 | **DENVN** | 544.2362 | 572.2311 |
| **DENVNS** | 631.2682 | 659.2631 | **EN** | 216.0979 | 244.0928 | **ENV** | 315.1663 | 343.1612 |
| **ENVN** | 429.2092 | 457.2041 | **ENVNS** | 516.2413 | 544.2362 | **ENVNSQ** | 644.2998 | 672.2947 |
| **NV** | 186.1237 | 214.1186 | **NVN** | 300.1666 | 328.1615 | **NVNS** | 387.1987 | 415.1936 |
| **NVNSQ** | 515.2572 | 543.2522 | **NVNSQP** | 612.3100 | 640.3049 | **VN** | 186.1237 | 214.1186 |
| **VNS** | 273.1557 | 301.1506 | **VNSQ** | 401.2143 | 429.2092 | **VNSQP** | 498.2671 | 526.2620 |
| **VNSQPF** | 645.3355 | 673.3304 | **NS** | 174.0873 | 202.0822 | **NSQ** | 302.1459 | 330.1408 |
| **NSQP** | 399.1987 | 427.1936 | **NSQPF** | 546.2671 | 574.2620 | **NSQPFM** | 677.3076 | 705.3025 |
| **SQ** | 188.1030 | 216.0979 | **SQP** | 285.1557 | 313.1506 | **SQPF** | 432.2241 | 460.2191 |
| **SQPFM** | 563.2646 | 591.2595 | **QP** | 198.1237 | 226.1186 | **QPF** | 345.1921 | 373.1870 |
| **QPFM** | 476.2326 | 504.2275 | **PF** | 217.1335 | 245.1285 | **PFM** | 348.1740 | 376.1689 |
| **FM** | 251.1213 | 279.1162 | | | | | | |

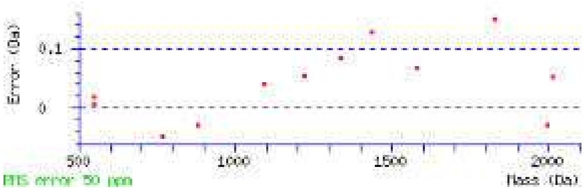


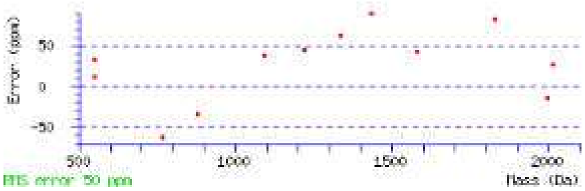


NCBI **BLAST** search of GGLMFTKDDENVNSQPFMR
(Parameters: blastp, nr protein database, expect=20000, no filter, PAM30)
Other BLAST web gateways

**All matches to this query**

| Score | Mr(calc) | Delta | Sequence |
|---|---|---|---|
| 58.7 | 2184.9932 | 0.1677 | GGLMFTKDDENVNSQPFMR |
| 43.8 | 2184.9746 | 0.1863 | GGLDYTKDDENVNSQPFMR |
| 28.3 | 2184.9932 | 0.1677 | GGLDFTKMDENVNSQPFMR |
| 16.8 | 2185.1201 | 0.0408 | NMLHALLTILEDEGAGGLYR |
| 16.5 | 2184.9932 | 0.1677 | GGLDFTKDMENVNSQPFMR |
| 11.8 | 2185.0586 | 0.1023 | LISGYMAQLVEDGGNHGGVPR |
| 11.5 | 2185.0361 | 0.1248 | HLQIIESGLEEGGDEVWMK |
| 11.5 | 2185.0262 | 0.1347 | DWLYASGNIRQLYCLQGE |
| 11.4 | 2185.0725 | 0.0884 | CVATSFVNLNDQLTDIFTK |
| 10.1 | 2185.0646 | 0.0963 | IYAELCKLETDEMSTAAIK |

**Mascot:** http://www.matrixscience.com/

# MATRIX SCIENCE Mascot Search Results

## Peptide View Spot no 45

MS/MS Fragmentation of **RLTFDEIQSK**
Found in **gi|739292** in **NCBInr**, oxygen-evolving complex protein 1

Match to Query 22: 1235.688724 from(1236.696000,1+) intensity(0.0000) index(21)
Title: Label: N3, Spot_Id: 219719, Peak_List_Id: 225482, MSMS Job_Run_Id: 21780, Comment:
Data file C:\Documents and Settings\Administrator\Desktop\farah BHU\spot set plate\Farah-Garima 11May 2013\ppw_N3_136842149300.txt

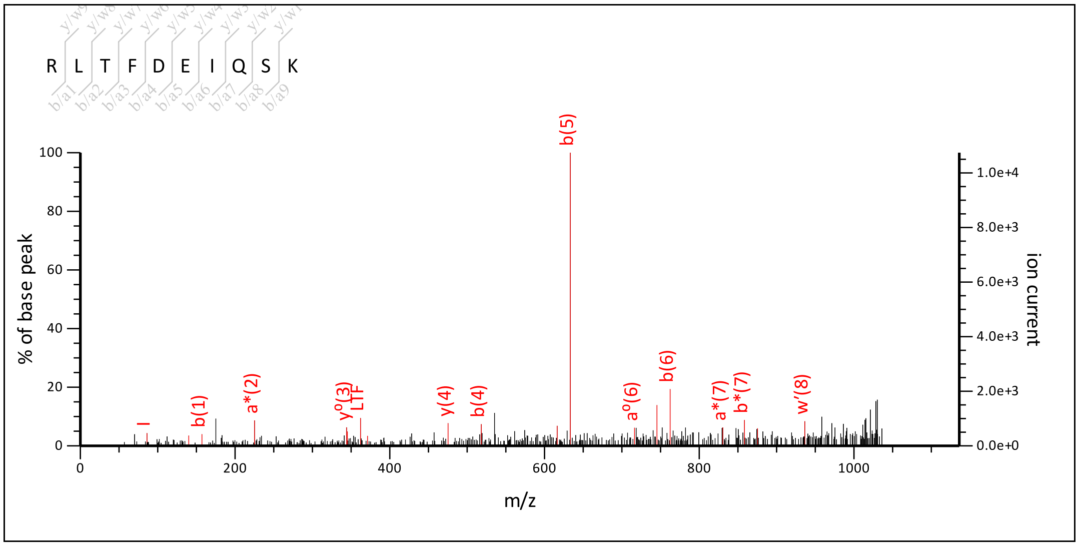


0 to 1136.14

Label all possible matches ○ Label matches used for scoring ◉

**Monoisotopic mass of neutral peptide Mr(calc):** 1235.6510
**Fixed modifications:** Carbamidomethyl (C) (apply to specified residues or termini only)
**Ions Score:** 31 **Expect:** 1.7
**Matches :** 23/161 fragment ions using 30 most intense peaks (help)

| # | Immon. | a | a* | $a^0$ | b | b* | $b^0$ | d | d' | Seq. | v | w | w' | y | y* | |
|---|---|---|---|---|---|---|---|---|---|---|---|---|---|---|---|---|
| 1 | 129.1135 | 129.1135 | 112.0869 | | ***157.1084*** | 140.0818 | | 44.0495 | | **R** | | | | | | |
| 2 | 86.0964 | 242.1975 | 225.1710 | | 270.1925 | 253.1659 | | 200.1506 | | **L** | 1022.4789 | 1021.4837 | | 1080.5572 | 1063.5306 | 106[illegible] |
| 3 | 74.0600 | 343.2452 | 326.2187 | 325.2347 | ***371.2401*** | 354.2136 | 353.2296 | 327.2503 | 329.2296 | **T** | 921.4312 | 934.4516 | 936.4309 | 967.4731 | 950.4466 | 94[illegible] |
| 4 | 120.0808 | 490.3136 | 473.2871 | 472.3031 | ***518.3085*** | 501.2820 | 500.2980 | | | **F** | 774.3628 | | | 866.4254 | 849.3989 | 84[illegible] |
| 5 | 88.0393 | 605.3406 | 588.3140 | 587.3300 | ***633.3355*** | 616.3089 | 615.3249 | 561.3507 | | **D** | 659.3359 | 658.3406 | | 719.3570 | 702.3305 | 70[illegible] |
| 6 | 102.0550 | 734.3832 | 717.3566 | 716.3726 | ***762.3781*** | 745.3515 | 744.3675 | 676.3777 | | **E** | 530.2933 | 529.2980 | | 604.3301 | 587.3035 | 58[illegible] |
| 7 | 86.0964 | 847.4672 | 830.4407 | 829.4567 | ***875.4621*** | 858.4356 | 857.4516 | 819.4359 | 833.4516 | **I** | 417.2092 | 430.2296 | 444.2453 | **475.2875** | 458.2609 | 45[illegible] |
| 8 | 101.0709 | 975.5258 | 958.4993 | 957.5152 | 1003.5207 | 986.4942 | 985.5102 | 918.5043 | | **Q** | 289.1506 | 288.1554 | | **362.2034** | 345.1769 | 34[illegible] |
| 9 | 60.0444 | 1062.5578 | 1045.5313 | 1044.5473 | 1090.5528 | 1073.5262 | 1072.5422 | 1046.5629 | | **S** | 202.1186 | 201.1234 | | 234.1448 | 217.1183 | 21[illegible] |
| 10 | 101.1073 | | | | | | | | | **K** | 74.0237 | 73.0284 | | 147.1128 | 130.0863 | |

| Seq | ya | yb | Seq | ya | yb | Seq | ya | yb |
|---|---|---|---|---|---|---|---|---|
| **LT** | 187.1441 | 215.1390 | **LTF** | 334.2125 | 362.2074 | **LTFD** | 449.2395 | 477.2344 |
| **LTFDE** | 578.2821 | 606.2770 | **LTFDEI** | 691.3661 | 719.3610 | **TF** | 221.1285 | 249.1234 |
| **TFD** | 336.1554 | 364.1503 | **TFDE** | 465.1980 | 493.1929 | **TFDEI** | 578.2821 | 606.2770 |
| **FD** | 235.1077 | 263.1026 | **FDE** | 364.1503 | 392.1452 | **FDEI** | 477.2344 | 505.2293 |
| **FDEIQ** | 605.2930 | 633.2879 | **FDEIQS** | 692.3250 | 720.3199 | **DE** | 217.0819 | 245.0768 |
| **DEI** | 330.1660 | 358.1609 | **DEIQ** | 458.2245 | 486.2195 | **DEIQS** | 545.2566 | 573.2515 |
| **EI** | 215.1390 | 243.1339 | **EIQ** | 343.1976 | 371.1925 | **EIQS** | 430.2296 | 458.2245 |
| **IQ** | 214.1550 | 242.1499 | **IQS** | 301.1870 | 329.1819 | **QS** | 188.1030 | 216.0979 |

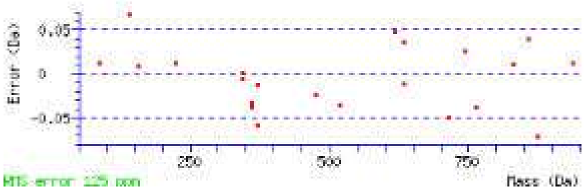


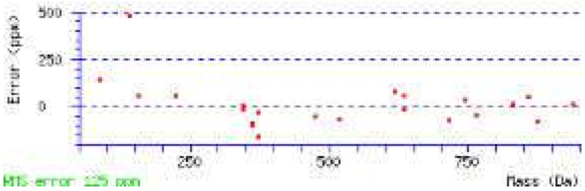


NCBI **BLAST** search of RLTFDEIQSK
(Parameters: blastp, nr protein database, expect=20000, no filter, PAM30)
Other BLAST web gateways

**All matches to this query**

| Score | Mr(calc) | Delta | Sequence |
|---|---|---|---|
| 30.7 | 1235.6510 | 0.0377 | RLTFDEIQSK |
| 16.8 | 1235.6007 | 0.0880 | HHSLETDNKR |
| 14.7 | 1235.5895 | 0.0993 | LQNFTDAQGSR |
| 14.4 | 1235.6033 | 0.0854 | IFLTEAEEER |
| 14.4 | 1235.5935 | 0.0953 | LQFNSAENWK |
| 14.1 | 1235.6106 | 0.0781 | VGSSSGNLSVSSR |
| 13.6 | 1235.6663 | 0.0225 | YPTLQVVNFR |
| 12.4 | 1235.5969 | 0.0919 | CVNTEPTFLR |
| 12.2 | 1235.5968 | 0.0919 | SAYGLAMPSSPR |
| 12.1 | 1235.6007 | 0.0880 | RFGGGEGGSAVSR |

**Mascot:** http://www.matrixscience.com/

# MATRIX SCIENCE Mascot Search Results

## Peptide View Spot no 45

MS/MS Fragmentation of **DGIDYAAVTVQLPGGER**
Found in **gi|739292** in **NCBInr**, oxygen-evolving complex protein 1

Match to Query 35: 1759.930824 from(1760.938100,1+) intensity(0.0000) index(32)
Title: Label: N3, Spot_Id: 219719, Peak_List_Id: 225497, MSMS Job_Run_Id: 21780, Comment:
Data file C:\Documents and Settings\Administrator\Desktop\farah BHU\spot set plate\Farah-Garima 11May 2013\ppw_N3_136842149300.txt

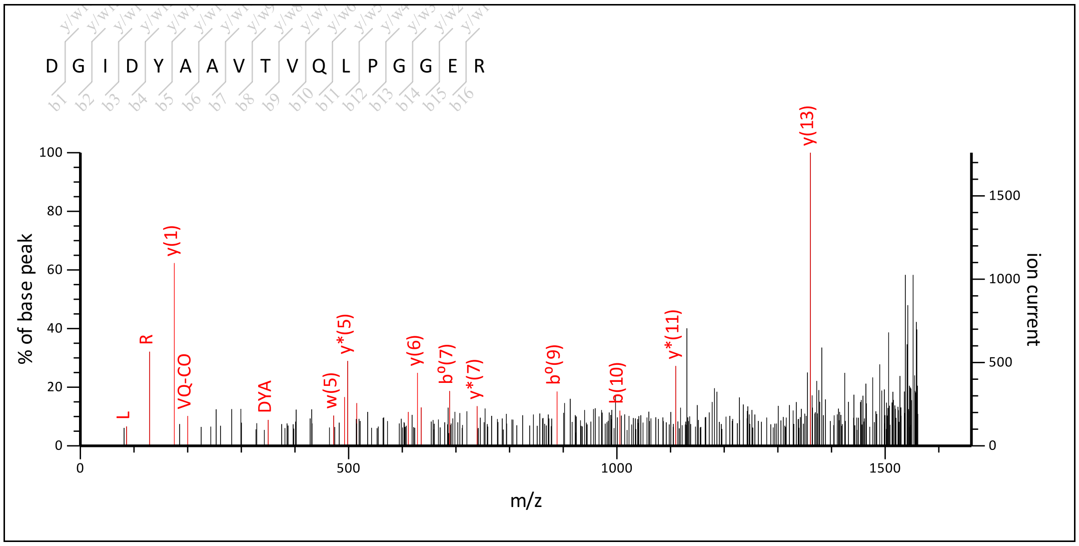


0 to 1660.79

Label all possible matches ○ Label matches used for scoring ◉

**Monoisotopic mass of neutral peptide Mr(calc):** 1759.8741
**Fixed modifications:** Carbamidomethyl (C) (apply to specified residues or termini only)
**Ions Score:** 4 **Expect:** 6.1e+02
**Matches :** 20/309 fragment ions using 45 most intense peaks (help)

| # | Immon. | a | a* | a⁰ | b | b* | b⁰ | d | d' | Seq. | v | w | w' | y | y* | [illegible] |
|---|---|---|---|---|---|---|---|---|---|---|---|---|---|---|---|---|
| 1 | 88.0393 | 88.0393 | | 70.0287 | 116.0342 | | 98.0237 | 44.0495 | | D | | | | | | |
| 2 | 30.0338 | 145.0608 | | 127.0502 | 173.0557 | | 155.0451 | | | G | | | | 1645.8544 | 1628.8279 | [illegible] |
| 3 | 86.0964 | 258.1448 | | 240.1343 | 286.1397 | | 268.1292 | 230.1135 | 244.1292 | I | 1530.7547 | 1543.7751 | 1557.7908 | 1588.8329 | 1571.8064 | [illegible] |
| 4 | 88.0393 | 373.1718 | | 355.1612 | 401.1667 | | 383.1561 | 329.1819 | | D | 1415.7278 | 1414.7325 | | 1475.7489 | 1458.7223 | [illegible] |
| 5 | 136.0757 | 536.2351 | | 518.2245 | 564.2300 | | 546.2195 | | | Y | 1252.6644 | | | ***1360.7219*** | 1343.6954 | [illegible] |
| 6 | 44.0495 | 607.2722 | | 589.2617 | **635.2671** | | 617.2566 | | | A | 1181.6273 | | | 1197.6586 | 1180.6321 | [illegible] |
| 7 | 44.0495 | 678.3093 | | 660.2988 | 706.3042 | | 688.2937 | | | A | 1110.5902 | | | 1126.6215 | 1109.5950 | [illegible] |
| 8 | 72.0808 | 777.3777 | | 759.3672 | 805.3727 | | 787.3621 | 763.3621 | | V | 1011.5218 | 1024.5422 | | 1055.5844 | 1038.5578 | [illegible] |
| 9 | 74.0600 | 878.4254 | | 860.4149 | 906.4203 | | 888.4098 | 862.4305 | 864.4098 | T | 910.4741 | 923.4945 | 925.4738 | 956.5160 | 939.4894 | [illegible] |
| 10 | 72.0808 | 977.4938 | | 959.4833 | **1005.4888** | | 987.4782 | 963.4782 | | V | 811.4057 | 824.4261 | | 855.4683 | 838.4417 | [illegible] |
| 11 | 101.0709 | 1105.5524 | 1088.5259 | 1087.5419 | 1133.5473 | 1116.5208 | 1115.5368 | 1048.5310 | | Q | 683.3471 | 682.3519 | | 756.3999 | 739.3733 | [illegible] |
| 12 | 86.0964 | 1218.6365 | 1201.6099 | 1200.6259 | 1246.6314 | 1229.6048 | 1228.6208 | 1176.5895 | | L | 570.2631 | 569.2678 | | ***628.3413*** | 611.3148 | [illegible] |
| 13 | 70.0651 | 1315.6892 | 1298.6627 | 1297.6787 | 1343.6842 | 1326.6576 | 1325.6736 | 1289.6736 | | P | 473.2103 | 472.2150 | | ***515.2572*** | 498.2307 | [illegible] |
| 14 | 30.0338 | 1372.7107 | 1355.6842 | 1354.7001 | 1400.7056 | 1383.6791 | 1382.6951 | | | G | | | | 418.2045 | 401.1779 | [illegible] |
| 15 | 30.0338 | 1429.7322 | 1412.7056 | 1411.7216 | 1457.7271 | 1440.7005 | 1439.7165 | | | G | | | | 361.1830 | 344.1565 | [illegible] |
| 16 | 102.0550 | 1558.7748 | 1541.7482 | 1540.7642 | 1586.7697 | 1569.7431 | 1568.7591 | 1500.7693 | | E | 230.1248 | 229.1295 | | 304.1615 | 287.1350 | [illegible] |
| 17 | 129.1135 | | | | | | | | | R | 74.0237 | 73.0284 | | ***175.1190*** | 158.0924 | |

| Seq | ya | yb | Seq | ya | yb | Seq | ya | yb |
|---|---|---|---|---|---|---|---|---|
| GI | 143.1179 | 171.1128 | GID | 258.1448 | 286.1397 | GIDY | 421.2082 | 449.2031 |
| GIDYA | 492.2453 | 520.2402 | GIDYAA | 563.2824 | 591.2773 | GIDYAAV | 662.3508 | 690.3457 |
| ID | 201.1234 | 229.1183 | IDY | 364.1867 | 392.1816 | IDYA | 435.2238 | 463.2187 |
| IDYAA | 506.2609 | 534.2558 | IDYAAV | 605.3293 | 633.3243 | DY | 251.1026 | 279.0975 |

| | | | | | | | | |
|---|---|---|---|---|---|---|---|---|
| **DYA** | 322.1397 | 350.1347 | **DYAA** | 393.1769 | 421.1718 | **DYAAV** | 492.2453 | 520.2402 |
| **DYAAVT** | 593.2930 | 621.2879 | **DYAAVTV** | 692.3614 | 720.3563 | **YA** | 207.1128 | 235.1077 |
| **YAA** | 278.1499 | 306.1448 | **YAAV** | 377.2183 | 405.2132 | **YAAVT** | 478.2660 | 506.2609 |
| **YAAVTV** | 577.3344 | 605.3293 | **AA** | 115.0866 | 143.0815 | **AAV** | 214.1550 | 242.1499 |
| **AAVT** | 315.2027 | 343.1976 | **AAVTV** | 414.2711 | 442.2660 | **AAVTVQ** | 542.3297 | 570.3246 |
| **AAVTVQL** | 655.4137 | 683.4087 | **AV** | 143.1179 | 171.1128 | **AVT** | 244.1656 | 272.1605 |
| **AVTV** | 343.2340 | 371.2289 | **AVTVQ** | 471.2926 | 499.2875 | **AVTVQL** | 584.3766 | 612.3715 |
| **AVTVQLP** | 681.4294 | 709.4243 | **VT** | 173.1285 | 201.1234 | **VTV** | 272.1969 | 300.1918 |
| **VTVQ** | 400.2554 | 428.2504 | **VTVQL** | 513.3395 | 541.3344 | **VTVQLP** | 610.3923 | 638.3872 |
| **VTVQLPG** | 667.4137 | 695.4087 | **TV** | 173.1285 | 201.1234 | **TVQ** | 301.1870 | 329.1819 |
| **TVQL** | 414.2711 | 442.2660 | **TVQLP** | 511.3239 | 539.3188 | **TVQLPG** | 568.3453 | 596.3402 |
| **TVQLPGG** | 625.3668 | 653.3617 | **VQ** | 200.1394 | 228.1343 | **VQL** | 313.2234 | 341.2183 |
| **VQLP** | 410.2762 | 438.2711 | **VQLPG** | 467.2976 | 495.2926 | **VQLPGG** | 524.3191 | 552.3140 |
| **VQLPGGE** | 653.3617 | 681.3566 | **QL** | 214.1550 | 242.1499 | **QLP** | 311.2078 | 339.2027 |
| **QLPG** | 368.2292 | 396.2241 | **QLPGG** | 425.2507 | 453.2456 | **QLPGGE** | 554.2933 | 582.2882 |
| **LP** | 183.1492 | 211.1441 | **LPG** | 240.1707 | 268.1656 | **LPGG** | 297.1921 | 325.1870 |
| **LPGGE** | 426.2347 | 454.2296 | **PG** | 127.0866 | 155.0815 | **PGG** | 184.1081 | 212.1030 |
| **PGGE** | 313.1506 | 341.1456 | **GG** | 87.0553 | 115.0502 | **GGE** | 216.0979 | 244.0928 |
| **GE** | 159.0764 | 187.0713 | | | | | | |

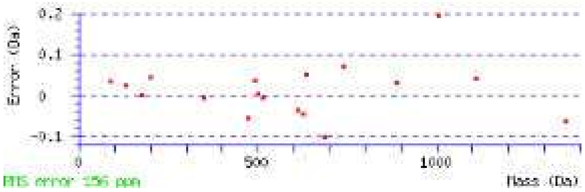


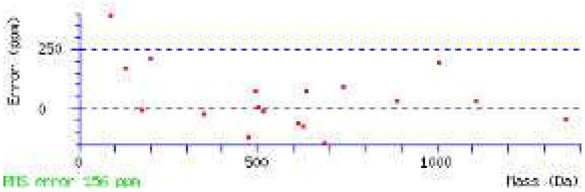


NCBI **BLAST** search of DGIDYAAVTVQLPGGER
(Parameters: blastp, nr protein database, expect=20000, no filter, PAM30)
Other BLAST web gateways

**All matches to this query**

| Score | Mr(calc) | Delta | Sequence |
|---|---|---|---|
| 9.4 | 1759.8840 | 0.0469 | IVAESTDDVIDGPSSKK |
| 8.9 | 1759.9026 | 0.0282 | SDLKVINDCLDDLIK |
| 6.8 | 1759.9316 | -0.0007 | ISTVETELQKLSQER |
| 6.5 | 1759.9832 | -0.0524 | LPASPPVILAAAGTTPER |
| 4.8 | 1759.8159 | 0.1149 | EEMPQQVASGEQAGKR |
| 4.8 | 1759.8597 | 0.0711 | GCALLLDLDCNDLIR |
| 4.7 | 1759.8523 | 0.0786 | AESNPDAASMVDRLLR |
| 4.4 | 1759.8741 | 0.0567 | DGIDYAAVTVQLPGGER |
| 4.3 | 1759.9502 | -0.0194 | LSSIVTGLQMQEVSLR |
| 3.9 | 1759.9356 | -0.0048 | AVGVLYAVVDDIIEER |

**Mascot:** http://www.matrixscience.com/

# MATRIX SCIENCE Mascot Search Results

## Peptide View Spot no 45

MS/MS Fragmentation of **NLVATGKPESFGGPFLVPSYR**
Found in **gi|739292** in **NCBInr**, oxygen-evolving complex protein 1

Match to Query 159: 2235.221024 from(2236.228300,1+) intensity(0.0000) index(34)
Title: Label: N3, Spot_Id: 219719, Peak_List_Id: 225485, MSMS Job_Run_Id: 21780, Comment:
Data file C:\Documents and Settings\Administrator\Desktop\farah BHU\spot set plate\Farah-Garima 11May 2013\ppw_N3_136842149300.txt

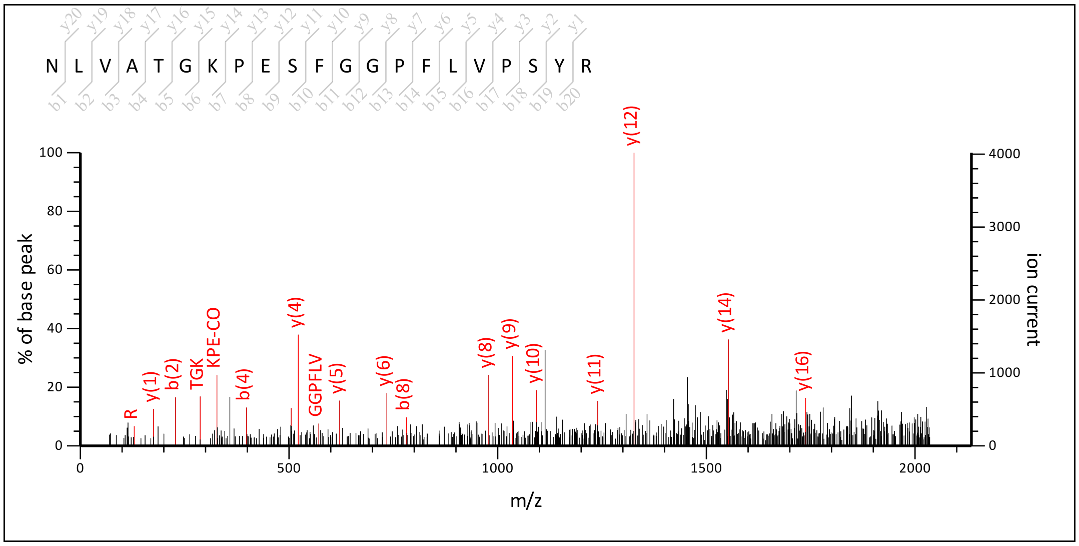


0 to 2135.14

Label all possible matches ○ Label matches used for scoring ◉

**Monoisotopic mass of neutral peptide Mr(calc):** 2235.1688
**Fixed modifications:** Carbamidomethyl (C) (apply to specified residues or termini only)
**Ions Score:** 57 **Expect:** 0.0027
**Matches :** 20/412 fragment ions using 40 most intense peaks (help)

| # | Immon. | a | a* | a0 | b | b* | b0 | d | d' | Seq. | v | w | w' | y | y* | |
|---|---|---|---|---|---|---|---|---|---|---|---|---|---|---|---|---|
| 1 | 87.0553 | 87.0553 | 70.0287 | | 115.0502 | 98.0237 | | 44.0495 | | N | | | | | | |
| 2 | 86.0964 | 200.1394 | 183.1128 | | **228.1343** | 211.1077 | | 158.0924 | | L | 2064.0549 | 2063.0597 | | 2122.1332 | 2105.1066 | 21[illegible] |
| 3 | 72.0808 | 299.2078 | 282.1812 | | **327.2027** | 310.1761 | | 285.1921 | | V | 1964.9865 | 1978.0069 | | 2009.0491 | 1992.0225 | 19[illegible] |
| 4 | 44.0495 | 370.2449 | 353.2183 | | **398.2398** | 381.2132 | | | | A | 1893.9494 | | | 1909.9807 | 1892.9541 | 18[illegible] |
| 5 | 74.0600 | 471.2926 | 454.2660 | 453.2820 | 499.2875 | 482.2609 | 481.2769 | 455.2976 | 457.2769 | T | 1792.9017 | 1805.9221 | 1807.9014 | 1838.9436 | 1821.9170 | 18[illegible] |
| 6 | 30.0338 | 528.3140 | 511.2875 | 510.3035 | 556.3089 | 539.2824 | 538.2984 | | | G | | | | ***1737.8959*** | 1720.8693 | 17[illegible] |
| 7 | 101.1073 | 656.4090 | 639.3824 | 638.3984 | 684.4039 | 667.3774 | 666.3933 | 599.3511 | | K | 1607.7853 | 1606.7900 | | 1680.8744 | 1663.8479 | 16[illegible] |
| 8 | 70.0651 | 753.4618 | 736.4352 | 735.4512 | **781.4567** | 764.4301 | 763.4461 | 727.4461 | | P | 1510.7325 | 1509.7373 | | ***1552.7795*** | 1535.7529 | 15[illegible] |
| 9 | 102.0550 | 882.5043 | 865.4778 | 864.4938 | 910.4993 | 893.4727 | 892.4887 | 824.4989 | | E | 1381.6899 | 1380.6947 | | 1455.7267 | 1438.7001 | 14[illegible] |
| 10 | 60.0444 | 969.5364 | 952.5098 | 951.5258 | 997.5313 | 980.5047 | 979.5207 | 953.5415 | | S | 1294.6579 | 1293.6626 | | ***1326.6841*** | 1309.6576 | 13[illegible] |
| 11 | 120.0808 | 1116.6048 | 1099.5782 | 1098.5942 | 1144.5997 | 1127.5732 | 1126.5891 | | | F | 1147.5895 | | | ***1239.6521*** | 1222.6255 | 12[illegible] |
| 12 | 30.0338 | 1173.6262 | 1156.5997 | 1155.6157 | 1201.6212 | 1184.5946 | 1183.6106 | | | G | | | | ***1092.5837*** | 1075.5571 | 10[illegible] |
| 13 | 30.0338 | 1230.6477 | 1213.6212 | 1212.6371 | 1258.6426 | 1241.6161 | 1240.6321 | | | G | | | | ***1035.5622*** | 1018.5356 | 10[illegible] |
| 14 | 70.0651 | 1327.7005 | 1310.6739 | 1309.6899 | 1355.6954 | 1338.6688 | 1337.6848 | 1301.6848 | | P | 936.4938 | 935.4985 | | ***978.5407*** | 961.5142 | 9[illegible] |
| 15 | 120.0808 | 1474.7689 | 1457.7423 | 1456.7583 | 1502.7638 | 1485.7373 | 1484.7532 | | | F | 789.4254 | | | 881.4880 | 864.4614 | 8[illegible] |
| 16 | 86.0964 | 1587.8530 | 1570.8264 | 1569.8424 | 1615.8479 | 1598.8213 | 1597.8373 | 1545.8060 | | L | 676.3413 | 675.3461 | | ***734.4196*** | 717.3930 | 7[illegible] |
| 17 | 72.0808 | 1686.9214 | 1669.8948 | 1668.9108 | 1714.9163 | 1697.8897 | 1696.9057 | 1672.9057 | | V | 577.2729 | 590.2933 | | ***621.3355*** | 604.3089 | 6[illegible] |
| 18 | 70.0651 | 1783.9741 | 1766.9476 | 1765.9636 | 1811.9690 | 1794.9425 | 1793.9585 | 1757.9585 | | P | 480.2201 | 479.2249 | | ***522.2671*** | 505.2405 | 5[illegible] |
| 19 | 60.0444 | 1871.0062 | 1853.9796 | 1852.9956 | 1899.0011 | 1881.9745 | 1880.9905 | 1855.0112 | | S | 393.1881 | 392.1928 | | 425.2143 | 408.1878 | 4[illegible] |
| 20 | 136.0757 | 2034.0695 | 2017.0429 | 2016.0589 | 2062.0644 | 2045.0379 | 2044.0538 | | | Y | 230.1248 | | | 338.1823 | 321.1557 | |
| 21 | 129.1135 | | | | | | | | | R | 74.0237 | 73.0284 | | ***175.1190*** | 158.0924 | |

| Seq | ya | yb | Seq | ya | yb | Seq | ya | yb |
|---|---|---|---|---|---|---|---|---|

| | | | | | | | | |
|---|---|---|---|---|---|---|---|---|
| LV | 185.1648 | 213.1598 | LVA | 256.2020 | 284.1969 | LVAT | 357.2496 | 385.2445 |
| LVATG | 414.2711 | 442.2660 | LVATGK | 542.3661 | 570.3610 | LVATGKP | 639.4188 | 667.4137 |
| VA | 143.1179 | 171.1128 | VAT | 244.1656 | 272.1605 | VATG | 301.1870 | 329.1819 |
| VATGK | 429.2820 | 457.2769 | VATGKP | 526.3348 | 554.3297 | VATGKPE | 655.3774 | 683.3723 |
| AT | 145.0972 | 173.0921 | ATG | 202.1186 | 230.1135 | ATGK | 330.2136 | 358.2085 |
| ATGKP | 427.2663 | 455.2613 | ATGKPE | 556.3089 | 584.3039 | ATGKPES | 643.3410 | 671.3359 |
| TG | 131.0815 | 159.0764 | TGK | 259.1765 | 287.1714 | TGKP | 356.2292 | 384.2241 |
| TGKPE | 485.2718 | 513.2667 | TGKPES | 572.3039 | 600.2988 | GK | 158.1288 | 186.1237 |
| GKP | 255.1816 | 283.1765 | GKPE | 384.2241 | 412.2191 | GKPES | 471.2562 | 499.2511 |
| GKPESF | 618.3246 | 646.3195 | GKPESFG | 675.3461 | 703.3410 | KP | 198.1601 | 226.1550 |
| KPE | 327.2027 | 355.1976 | KPES | 414.2347 | 442.2296 | KPESF | 561.3031 | 589.2980 |
| KPESFG | 618.3246 | 646.3195 | KPESFGG | 675.3461 | 703.3410 | PE | 199.1077 | 227.1026 |
| PES | 286.1397 | 314.1347 | PESF | 433.2082 | 461.2031 | PESFG | 490.2296 | 518.2245 |
| PESFGG | 547.2511 | 575.2460 | PESFGGP | 644.3039 | 672.2988 | ES | 189.0870 | 217.0819 |
| ESF | 336.1554 | 364.1503 | ESFG | 393.1769 | 421.1718 | ESFGG | 450.1983 | 478.1932 |
| ESFGGP | 547.2511 | 575.2460 | ESFGGPF | 694.3195 | 722.3144 | SF | 207.1128 | 235.1077 |
| SFG | 264.1343 | 292.1292 | SFGG | 321.1557 | 349.1506 | SFGGP | 418.2085 | 446.2034 |
| SFGGPF | 565.2769 | 593.2718 | SFGGPFL | 678.3610 | 706.3559 | FG | 177.1022 | 205.0972 |
| FGG | 234.1237 | 262.1186 | FGGP | 331.1765 | 359.1714 | FGGPF | 478.2449 | 506.2398 |
| FGGPFL | 591.3289 | 619.3239 | FGGPFLV | 690.3974 | 718.3923 | GG | 87.0553 | 115.0502 |
| GGP | 184.1081 | 212.1030 | GGPF | 331.1765 | 359.1714 | GGPFL | 444.2605 | 472.2554 |
| GGPFLV | 543.3289 | 571.3239 | GGPFLVP | 640.3817 | 668.3766 | GP | 127.0866 | 155.0815 |
| GPF | 274.1550 | 302.1499 | GPFL | 387.2391 | 415.2340 | GPFLV | 486.3075 | 514.3024 |
| GPFLVP | 583.3602 | 611.3552 | GPFLVPS | 670.3923 | 698.3872 | PF | 217.1335 | 245.1285 |
| PFL | 330.2176 | 358.2125 | PFLV | 429.2860 | 457.2809 | PFLVP | 526.3388 | 554.3337 |
| PFLVPS | 613.3708 | 641.3657 | FL | 233.1648 | 261.1598 | FLV | 332.2333 | 360.2282 |
| FLVP | 429.2860 | 457.2809 | FLVPS | 516.3180 | 544.3130 | FLVPSY | 679.3814 | 707.3763 |
| LV | 185.1648 | 213.1598 | LVP | 282.2176 | 310.2125 | LVPS | 369.2496 | 397.2445 |
| LVPSY | 532.3130 | 560.3079 | VP | 169.1335 | 197.1285 | VPS | 256.1656 | 284.1605 |
| VPSY | 419.2289 | 447.2238 | PS | 157.0972 | 185.0921 | PSY | 320.1605 | 348.1554 |
| SY | 223.1077 | 251.1026 | | | | | | |

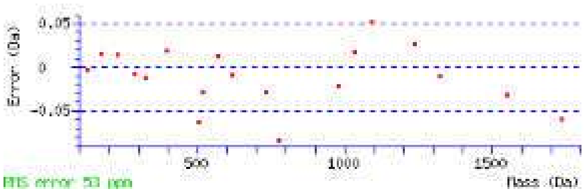


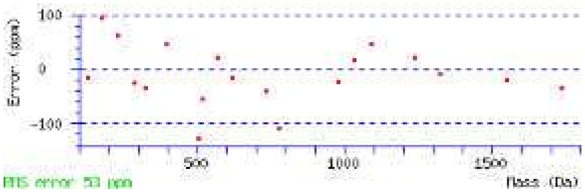


NCBI **BLAST** search of NLVATGKPESFGGPFLVPSYR
(Parameters: blastp, nr protein database, expect=20000, no filter, PAM30)
Other BLAST web gateways

**All matches to this query**

| Score | Mr(calc) | Delta | Sequence |
|---|---|---|---|
| 57.4 | 2235.1688 | 0.0522 | NLVATGKPESFGGPFLVPSYR |
| 32.7 | 2235.1688 | 0.0522 | QLVATGKPDSFGGPFLVPSYR |
| 7.9 | 2235.0916 | 0.1295 | LAVLVGDYMGGPVVDPDSMLR |
| 5.5 | 2235.1933 | 0.0277 | KVVISGSAEGVQMANYLLTQK |
| 4.7 | 2235.1259 | 0.0951 | QPTASAQMFTRPWLALFDR |
| 4.4 | 2235.0452 | 0.1758 | WKEIPFQSPASDLPNGMMR |
| 4.4 | 2235.1102 | 0.1108 | MCELETVVGGFVIVATHVMK |
| 4.0 | 2235.2331 | -0.0120 | VRVALMMSAGVTTAAVLVLSSK |
| 3.4 | 2235.1899 | 0.0311 | IIETSFVSVPANAAPQPEPIR |
| 3.1 | 2235.1859 | 0.0352 | LALGDNALPEAGIGAIASQLADR |

**Mascot:** http://www.matrixscience.com/

# MATRIX SCIENCE Mascot Search Results

## Peptide View Spot no 47

MS/MS Fragmentation of **ITREPGPVK**
Found in **gi|162461576** in **NCBInr**, glyoxylase1 [Zea mays]

Match to Query 27: 995.564684 from(996.571960,1+) intensity(0.0000) index(7)
Title: Label: M3, Spot_Id: 219718, Peak_List_Id: 225458, MSMS Job_Run_Id: 21779, Comment:
Data file C:\Documents and Settings\Administrator\Desktop\farah BHU\spot set plate\Farah-Garima 11May 2013\ppw_M3_136842147700.txt

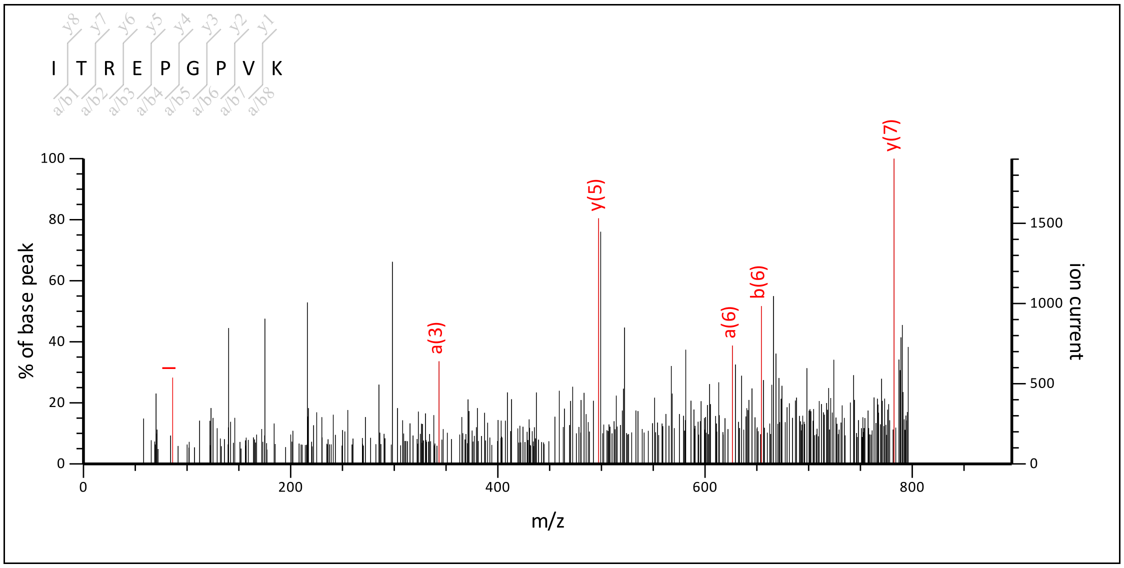


0 to 896.13

Label all possible matches ○ Label matches used for scoring ◉

**Monoisotopic mass of neutral peptide Mr(calc):** 995.5764
**Fixed modifications:** Carbamidomethyl (C) (apply to specified residues or termini only)
**Ions Score:** 12 **Expect:** 1.1e+02
**Matches :** 9/133 fragment ions using 16 most intense peaks (help)

| # | Immon. | a | a* | a⁰ | b | b* | b⁰ | d | d' | Seq. | v | w | w' | y | y* | y⁰ | # |
|---|---|---|---|---|---|---|---|---|---|---|---|---|---|---|---|---|---|
| 1 | 86.0964 | ***86.0964*** | | | 114.0913 | | | 44.0495 | | I | | | | | | | 9 |
| 2 | 74.0600 | 187.1441 | | 169.1335 | 215.1390 | | 197.1285 | 171.1492 | 173.1285 | T | 837.4577 | 850.4781 | 852.4574 | 883.4996 | 866.4730 | 865.4890 | 8 |
| 3 | 129.1135 | ***343.2452*** | 326.2187 | 325.2346 | 371.2401 | 354.2136 | 353.2296 | 258.1812 | | R | 681.3566 | 680.3614 | | ***782.4519*** | 765.4254 | 764.4413 | 7 |
| 4 | 102.0550 | 472.2878 | 455.2613 | 454.2772 | 500.2827 | 483.2562 | 482.2722 | 414.2823 | | E | 552.3140 | 551.3188 | | ***626.3508*** | 609.3243 | 608.3402 | 6 |
| 5 | 70.0651 | 569.3406 | 552.3140 | 551.3300 | 597.3355 | 580.3089 | 579.3249 | 543.3249 | | P | 455.2613 | 454.2660 | | ***497.3082*** | 480.2817 | | 5 |
| 6 | 30.0338 | ***626.3620*** | 609.3355 | 608.3515 | 654.3570 | 637.3304 | 636.3464 | | | G | | | | 400.2554 | 383.2289 | | 4 |
| 7 | 70.0651 | 723.4148 | 706.3883 | 705.4042 | 751.4097 | 734.3832 | 733.3992 | 697.3992 | | P | 301.1870 | 300.1918 | | ***343.2340*** | 326.2074 | | 3 |
| 8 | 72.0808 | 822.4832 | 805.4567 | 804.4726 | 850.4781 | 833.4516 | 832.4676 | 808.4676 | | V | 202.1186 | 215.1390 | | 246.1812 | 229.1547 | | 2 |
| 9 | 101.1073 | | | | | | | | | K | 74.0237 | 73.0284 | | 147.1128 | 130.0863 | | 1 |

| Seq | ya | yb | Seq | ya | yb | Seq | ya | yb |
|---|---|---|---|---|---|---|---|---|
| TR | 230.1612 | 258.1561 | TRE | 359.2037 | 387.1987 | TREP | 456.2565 | 484.2514 |
| TREPG | 513.2780 | 541.2729 | TREPGP | 610.3307 | 638.3257 | RE | 258.1561 | 286.1510 |
| REP | 355.2088 | 383.2037 | REPG | 412.2303 | 440.2252 | REPGP | 509.2831 | 537.2780 |
| REPGPV | 608.3515 | 636.3464 | EP | 199.1077 | 227.1026 | EPG | 256.1292 | 284.1241 |
| EPGP | 353.1819 | 381.1769 | EPGPV | 452.2504 | 480.2453 | PG | 127.0866 | 155.0815 |
| PGP | 224.1394 | 252.1343 | PGPV | 323.2078 | 351.2027 | GP | 127.0866 | 155.0815 |
| GPV | 226.1550 | 254.1499 | PV | 169.1335 | 197.1285 | | | |

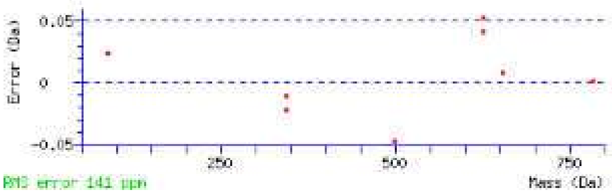


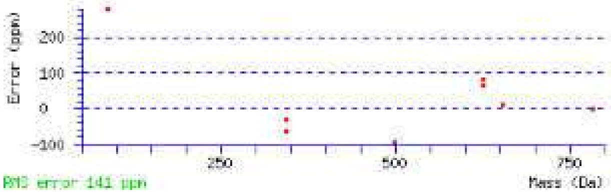


NCBI **BLAST** search of ITREPGPVK
(Parameters: blastp, nr protein database, expect=20000, no filter, PAM30)
Other BLAST web gateways

**All matches to this query**

| Score | Mr(calc) | Delta | Sequence |
|---|---|---|---|
| 12.0 | 995.5764 | -0.0117 | ITREPGPVK |
| 10.2 | 995.6128 | -0.0481 | VDKGPLLVR |
| 8.6 | 995.6240 | -0.0593 | LSAILRAPR |
| 8.3 | 995.6127 | -0.0481 | TINLLIPGR |
| 8.2 | 995.6128 | -0.0481 | LQTLVVPAR |
| 8.1 | 995.5764 | -0.0117 | LLVAAGAEPR |
| 8.1 | 995.6128 | -0.0481 | AVQILTVPR |
| 8.1 | 995.6128 | -0.0481 | LLVATPVAGR |
| 8.0 | 995.6240 | -0.0593 | ILASRLAPR |
| 8.0 | 995.6240 | -0.0593 | LLASRLAPR |

**Mascot:** http://www.matrixscience.com/

# MATRIX SCIENCE Mascot Search Results

## Peptide View Spot no 47

MS/MS Fragmentation of **SIKFYEK**
Found in **gi|162461576** in **NCBInr**, glyoxylase1 [Zea mays]

Match to Query 11: 913.476614 from(914.483890,1+) intensity(0.0000) index(3)
Title: Label: M3, Spot_Id: 219718, Peak_List_Id: 225455, MSMS Job_Run_Id: 21779, Comment:
Data file C:\Documents and Settings\Administrator\Desktop\farah BHU\spot set plate\Farah-Garima 11May 2013\ppw_M3_136842147700.txt

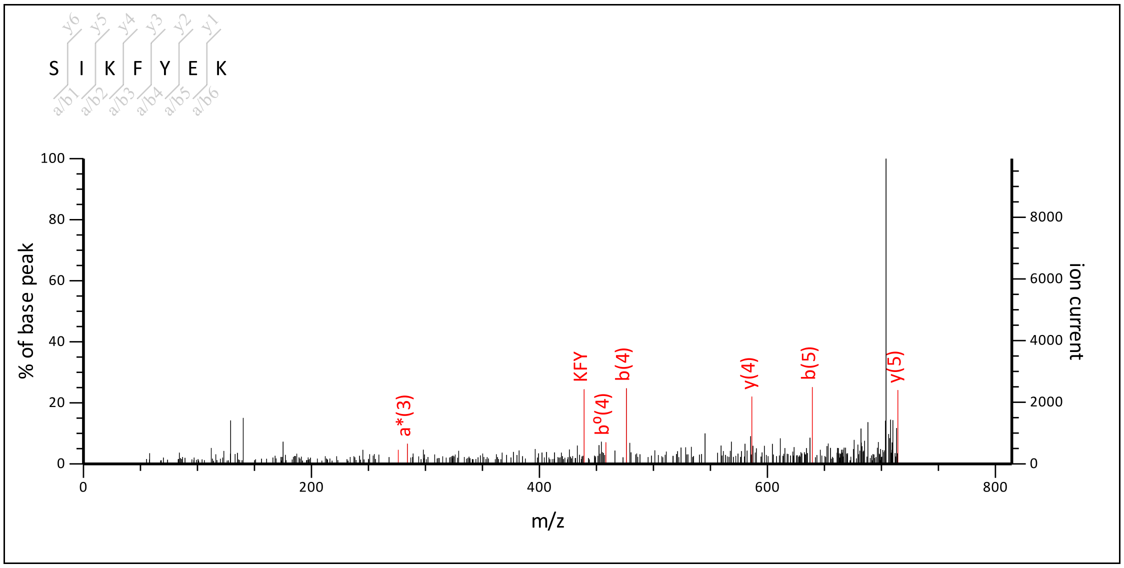


0 to 814.38

Label all possible matches ○ Label matches used for scoring ◉

**Monoisotopic mass of neutral peptide Mr(calc):** 913.4909
**Fixed modifications:** Carbamidomethyl (C) (apply to specified residues or termini only)
**Ions Score:** 13 **Expect:** 1.6e+02
**Matches :** 10/92 fragment ions using 21 most intense peaks (help)

| # | Immon. | a | a* | $a^0$ | b | b* | $b^0$ | d | d' | Seq. | v | w | w' | y | y* | $y^0$ | # |
|---|---|---|---|---|---|---|---|---|---|---|---|---|---|---|---|---|---|
| 1 | 60.0444 | 60.0444 | | 42.0338 | 88.0393 | | 70.0287 | 44.0495 | | S | | | | | | | 7 |
| 2 | 86.0964 | 173.1285 | | 155.1179 | 201.1234 | | 183.1128 | 145.0972 | 159.1128 | I | 769.3879 | 782.4083 | 796.4240 | 827.4662 | 810.4396 | 809.4556 | 6 |
| 3 | 101.1073 | 301.2234 | 284.1969 | 283.2129 | 329.2183 | 312.1918 | 311.2078 | 244.1656 | | K | 641.2930 | 640.2977 | | ***714.3821*** | 697.3556 | 696.3715 | 5 |
| 4 | 120.0808 | 448.2918 | 431.2653 | 430.2813 | **476.2867** | 459.2602 | 458.2762 | | | F | 494.2245 | | | ***586.2871*** | 569.2606 | 568.2766 | 4 |
| 5 | 136.0757 | 611.3552 | 594.3286 | 593.3446 | **639.3501** | 622.3235 | 621.3395 | | | Y | 331.1612 | | | ***439.2187*** | 422.1922 | 421.2082 | 3 |
| 6 | 102.0550 | 740.3978 | 723.3712 | 722.3872 | 768.3927 | 751.3661 | 750.3821 | 682.3923 | | E | 202.1186 | 201.1234 | | ***276.1554*** | 259.1288 | 258.1448 | 2 |
| 7 | 101.1073 | | | | | | | | | K | 74.0237 | 73.0284 | | 147.1128 | 130.0863 | | 1 |

| Seq | ya | yb | Seq | ya | yb | Seq | ya | yb |
|---|---|---|---|---|---|---|---|---|
| IK | 214.1914 | 242.1863 | IKF | 361.2598 | 389.2547 | IKFY | 524.3231 | 552.3180 |
| IKFYE | 653.3657 | 681.3606 | KF | 248.1757 | 276.1707 | KFY | 411.2391 | 439.2340 |
| KFYE | 540.2817 | 568.2766 | FY | 283.1441 | 311.1390 | FYE | 412.1867 | 440.1816 |
| YE | 265.1183 | 293.1132 | | | | | | |

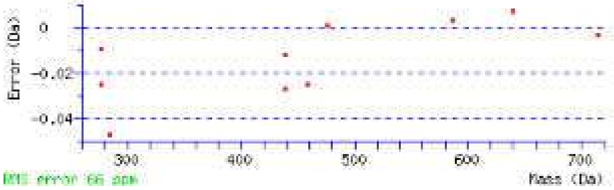


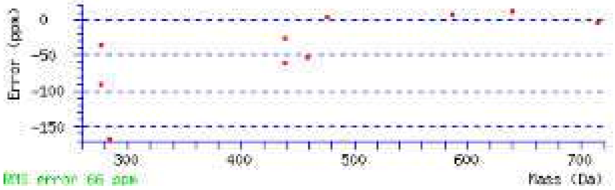


NCBI **BLAST** search of SIKFYEK

(Parameters: blastp, nr protein database, expect=20000, no filter, PAM30)
Other BLAST web gateways

**All matches to this query**

| Score | Mr(calc) | Delta | Sequence |
|---|---|---|---|
| 16.8 | 913.5021 | -0.0255 | KFYISTR |
| 15.5 | 913.4691 | 0.0075 | LSMKYTR |
| 14.0 | 913.4658 | 0.0109 | SIFGAYTR |
| 14.0 | 913.4505 | 0.0261 | SSTLSYTR |
| 13.9 | 913.4658 | 0.0109 | ALGSFYTR |
| 13.9 | 913.4658 | 0.0109 | TNIFYTR |
| 13.0 | 913.4909 | -0.0143 | SIKFYEK |
| 12.4 | 913.4658 | 0.0109 | SLGAYFTR |
| 12.0 | 913.4658 | 0.0109 | FQYTLSR |
| 12.0 | 913.4294 | 0.0472 | KFYDDAR |

**Mascot:** http://www.matrixscience.com/

# MATRIX SCIENCE Mascot Search Results

## Peptide View Spot no 47

MS/MS Fragmentation of **ILRQPGPLPGINTK**
Found in **gi|162461576** in **NCBInr**, glyoxylase1 [Zea mays]

Match to Query 63: 1502.895824 from(1503.903100,1+) intensity(0.0000) index(19)
Title: Label: M3, Spot_Id: 219718, Peak_List_Id: 225450, MSMS Job_Run_Id: 21779, Comment:
Data file C:\Documents and Settings\Administrator\Desktop\farah BHU\spot set plate\Farah-Garima 11May 2013\ppw_M3_136842147700.txt

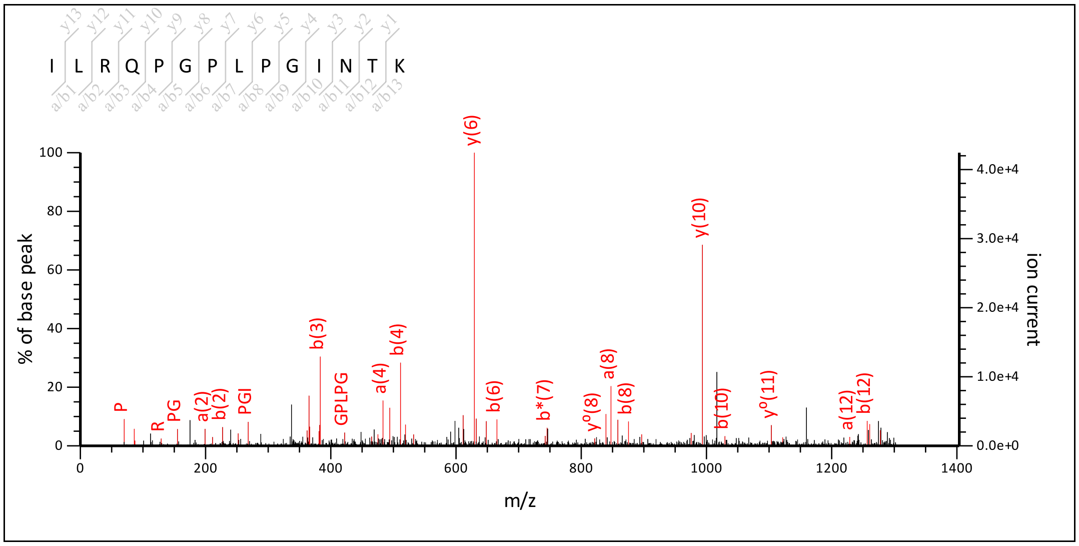


0 to 1403.8

Label all possible matches ○ Label matches used for scoring ◉

**Monoisotopic mass of neutral peptide Mr(calc):** 1502.8933
**Fixed modifications:** Carbamidomethyl (C) (apply to specified residues or termini only)
**Ions Score:** 55 **Expect:** 0.0038
**Matches :** 69/237 fragment ions using 91 most intense peaks (help)

| # | Immon. | a | a* | a0 | b | b* | b0 | d | d' | Seq. | v | w | w' | y | y* | [illegible] |
|---|---|---|---|---|---|---|---|---|---|---|---|---|---|---|---|---|
| 1 | 86.0964 | **86.0964** | | | 114.0913 | | | 44.0495 | | I | | | | | | |
| 2 | 86.0964 | **199.1805** | | | **227.1754** | | | 157.1335 | | L | 1332.7383 | 1331.7430 | | 1390.8165 | 1373.7900 | [illegible] |
| 3 | 129.1135 | 355.2816 | 338.2551 | | **383.2765** | 366.2500 | | 270.2176 | | R | 1176.6372 | 1175.6419 | | ***1277.7324*** | 1260.7059 | [illegible] |
| 4 | 101.0709 | **483.3402** | 466.3136 | | **511.3351** | 494.3085 | | 426.3187 | | Q | 1048.5786 | 1047.5833 | | ***1121.6313*** | 1104.6048 | [illegible] |
| 5 | 70.0651 | 580.3929 | 563.3664 | | 608.3879 | 591.3613 | | 554.3773 | | P | 951.5258 | 950.5306 | | ***993.5728*** | 976.5462 | [illegible] |
| 6 | 30.0338 | 637.4144 | 620.3879 | | **665.4093** | 648.3828 | | | | G | | | | ***896.5200*** | 879.4934 | [illegible] |
| 7 | 70.0651 | 734.4672 | 717.4406 | | 762.4621 | 745.4355 | | 708.4515 | | P | 797.4516 | 796.4563 | | ***839.4985*** | 822.4720 | [illegible] |
| 8 | 86.0964 | **847.5512** | 830.5247 | | **875.5461** | 858.5196 | | 805.5043 | | L | 684.3675 | 683.3723 | | ***742.4458*** | 725.4192 | [illegible] |
| 9 | 70.0651 | 944.6040 | 927.5774 | | 972.5989 | 955.5724 | | 918.5883 | | P | 587.3148 | 586.3195 | | ***629.3617*** | 612.3352 | [illegible] |
| 10 | 30.0338 | 1001.6255 | 984.5989 | | **1029.6204** | 1012.5938 | | | | G | | | | ***532.3089*** | 515.2824 | [illegible] |
| 11 | 86.0964 | 1114.7095 | 1097.6830 | | 1142.7044 | 1125.6779 | | 1086.6782 | 1100.6939 | I | 417.2092 | 430.2296 | 444.2453 | ***475.2875*** | 458.2609 | [illegible] |
| 12 | 87.0553 | **1228.7525** | 1211.7259 | | **1256.7474** | 1239.7208 | | 1185.7466 | | N | 303.1663 | 302.1710 | | ***362.2034*** | 345.1769 | [illegible] |
| 13 | 74.0600 | 1329.8001 | 1312.7736 | 1311.7896 | 1357.7950 | 1340.7685 | 1339.7845 | 1313.8052 | 1315.7845 | T | 202.1186 | 215.1390 | 217.1183 | 248.1605 | 231.1339 | [illegible] |
| 14 | 101.1073 | | | | | | | | | K | 74.0237 | 73.0284 | | 147.1128 | 130.0863 | |

| Seq | ya | yb | Seq | ya | yb | Seq | ya | yb |
|---|---|---|---|---|---|---|---|---|
| LR | 242.1975 | 270.1925 | LRQ | 370.2561 | 398.2510 | LRQP | 467.3089 | 495.3038 |
| LRQPG | 524.3303 | 552.3253 | LRQPGP | 621.3831 | 649.3780 | RQ | 257.1720 | 285.1670 |
| RQP | 354.2248 | 382.2197 | RQPG | 411.2463 | 439.2412 | RQPGP | 508.2990 | 536.2940 |
| RQPGPL | 621.3831 | 649.3780 | QP | 198.1237 | 226.1186 | QPG | 255.1452 | 283.1401 |
| QPGP | 352.1979 | 380.1928 | QPGPL | 465.2820 | 493.2769 | QPGPLP | 562.3348 | 590.3297 |
| QPGPLPG | 619.3562 | 647.3511 | PG | 127.0866 | 155.0815 | PGP | 224.1394 | 252.1343 |
| PGPL | 337.2234 | 365.2183 | PGPLP | 434.2762 | 462.2711 | PGPLPG | 491.2976 | 519.2926 |

| | | | | | | | | |
|---|---|---|---|---|---|---|---|---|
| **PGPLPGI** | 604.3817 | 632.3766 | **GP** | 127.0866 | 155.0815 | **GPL** | 240.1707 | 268.1656 |
| **GPLP** | 337.2234 | 365.2183 | **GPLPG** | 394.2449 | 422.2398 | **GPLPGI** | 507.3289 | 535.3239 |
| **GPLPGIN** | 621.3719 | 649.3668 | **PL** | 183.1492 | 211.1441 | **PLP** | 280.2020 | 308.1969 |
| **PLPG** | 337.2234 | 365.2183 | **PLPGI** | 450.3075 | 478.3024 | **PLPGIN** | 564.3504 | 592.3453 |
| **PLPGINT** | 665.3981 | 693.3930 | **LP** | 183.1492 | 211.1441 | **LPG** | 240.1707 | 268.1656 |
| **LPGI** | 353.2547 | 381.2496 | **LPGIN** | 467.2976 | 495.2926 | **LPGINT** | 568.3453 | 596.3402 |
| **PG** | 127.0866 | 155.0815 | **PGI** | 240.1707 | 268.1656 | **PGIN** | 354.2136 | 382.2085 |
| **PGINT** | 455.2613 | 483.2562 | **GI** | 143.1179 | 171.1128 | **GIN** | 257.1608 | 285.1557 |
| **GINT** | 358.2085 | 386.2034 | **IN** | 200.1394 | 228.1343 | **INT** | 301.1870 | 329.1819 |
| **NT** | 188.1030 | 216.0979 | | | | | | |

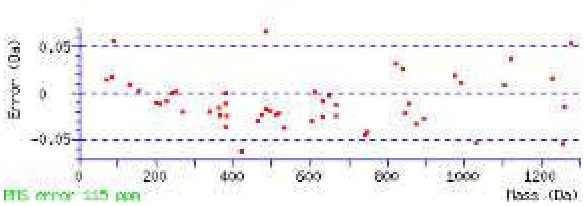


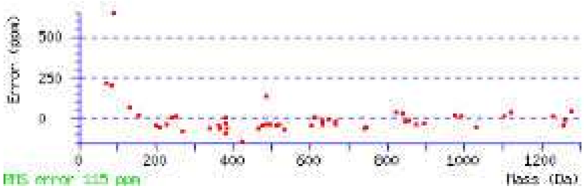


NCBI **BLAST** search of ILRQPGPLPGINTK
(Parameters: blastp, nr protein database, expect=20000, no filter, PAM30)
Other BLAST web gateways

**All matches to this query**

| Score | Mr(calc) | Delta | Sequence |
|---|---|---|---|
| 55.1 | 1502.8933 | 0.0025 | ILRQPGPLPGINTK |
| 55.1 | 1502.8933 | 0.0025 | ILRQPGPLPGLNTK |
| 15.9 | 1502.9548 | -0.0590 | LPSILLHTKQILK |
| 15.1 | 1502.8245 | 0.0713 | GPPLGWIPTPEIAR |
| 13.4 | 1502.8167 | 0.0791 | LIIDAMGNFSPVVK |
| 13.4 | 1502.8167 | 0.0791 | LLIDAMGNFSPVVK |
| 13.3 | 1502.8820 | 0.0138 | LLRQAFVESIVTK |
| 13.1 | 1502.8708 | 0.0250 | ILLPEVIPPDEIR |
| 12.8 | 1502.7702 | 0.1256 | RRPTPEPSSSHPR |
| 11.4 | 1502.8430 | 0.0528 | APGRAVLQVHGTAAR |

**Mascot:** http://www.matrixscience.com/

# Mascot Search Results

## Peptide View Spot no 48

MS/MS Fragmentation of **LLHAVYR**
Found in **gi|115475151** in **NCBInr**, Os08g0191700 [Oryza sativa Japonica Group]

Match to Query 3: 870.583724 from(871.591000,1+) intensity(0.0000) index(0)
Title: Label: E19, Spot_Id: 219966, Peak_List_Id: 229275, MSMS Job_Run_Id: 22016, Comment:
Data file C:\Documents and Settings\Administrator\Desktop\Farah-Garima 11May 2013\ppw_E19_136868347200.txt

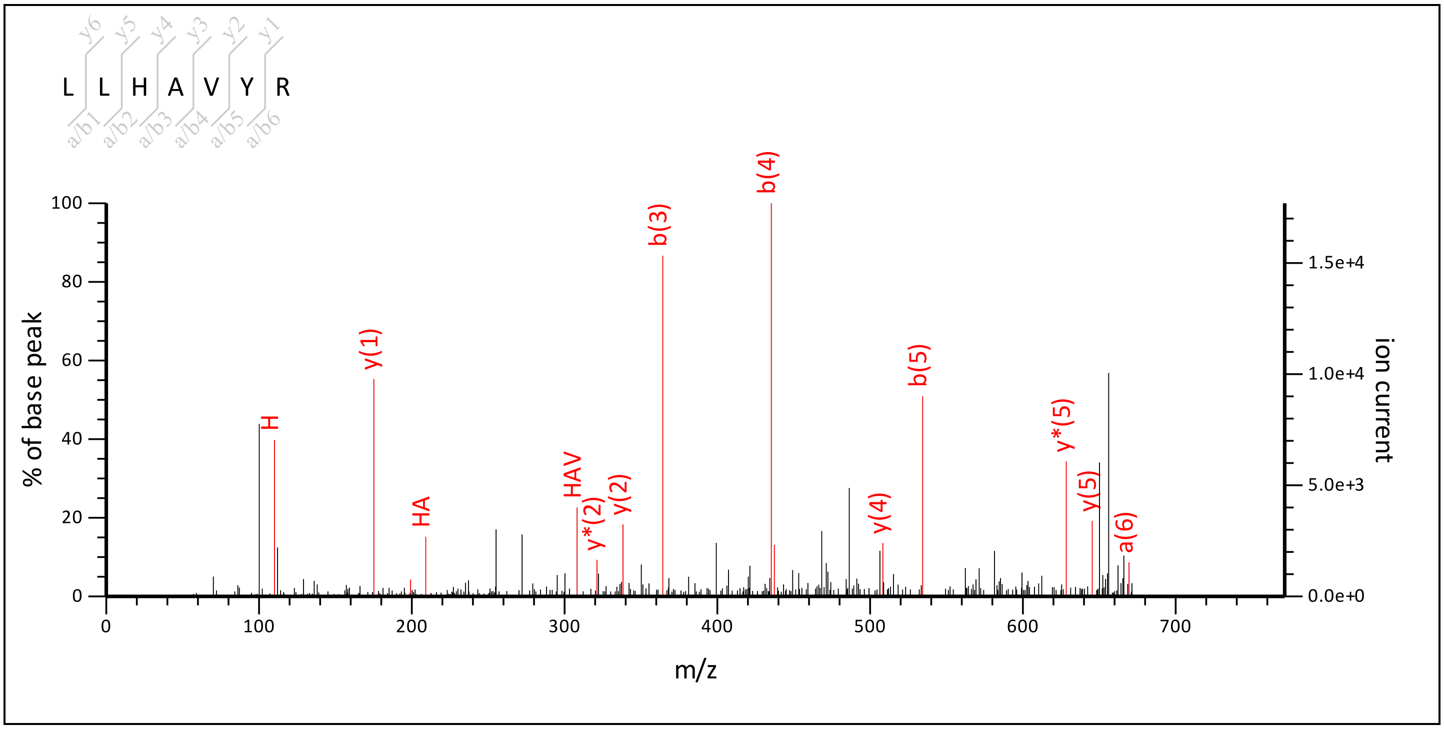


0 to 771.37

Label all possible matches ○ Label matches used for scoring ◉

**Monoisotopic mass of neutral peptide Mr(calc):** 870.5076
**Fixed modifications:** Carbamidomethyl (C) (apply to specified residues or termini only)
**Ions Score:** 31 **Expect:** 29
**Matches** : 15/63 fragment ions using 28 most intense peaks (help)

| # | Immon. | a | b | d | Seq. | v | w | y | y* | # |
|---|---|---|---|---|---|---|---|---|---|---|
| 1 | 86.0964 | 86.0964 | 114.0913 | 44.0495 | L | | | | | 7 |
| 2 | 86.0964 | **199.1805** | 227.1754 | 157.1335 | L | 700.3525 | 699.3573 | 758.4308 | 741.4042 | 6 |
| 3 | 110.0713 | 336.2394 | **364.2343** | | H | 563.2936 | | ***645.3467*** | 628.3202 | 5 |
| 4 | 44.0495 | 407.2765 | **435.2714** | | A | 492.2565 | | ***508.2878*** | 491.2613 | 4 |
| 5 | 72.0808 | 506.3449 | **534.3398** | 492.3293 | V | 393.1881 | 406.2085 | ***437.2507*** | 420.2241 | 3 |
| 6 | 136.0757 | **669.4083** | 697.4032 | | Y | 230.1248 | | ***338.1823*** | 321.1557 | 2 |
| 7 | 129.1135 | | | | R | 74.0237 | 73.0284 | ***175.1190*** | 158.0924 | 1 |

| Seq | ya | yb | Seq | ya | yb | Seq | ya | yb |
|---|---|---|---|---|---|---|---|---|

| | | | | | | | | | |
|---|---|---|---|---|---|---|---|---|---|
| **LH** | 223.1553 | 251.1503 | **LHA** | 294.1925 | 322.1874 | **LHAV** | 393.2609 | 421.2558 |
| **LHAVY** | 556.3242 | 584.3191 | **HA** | 181.1084 | 209.1033 | **HAV** | 280.1768 | 308.1717 |
| **HAVY** | 443.2401 | 471.2350 | **AV** | 143.1179 | 171.1128 | **AVY** | 306.1812 | 334.1761 |
| **VY** | 235.1441 | 263.1390 | | | | | | |

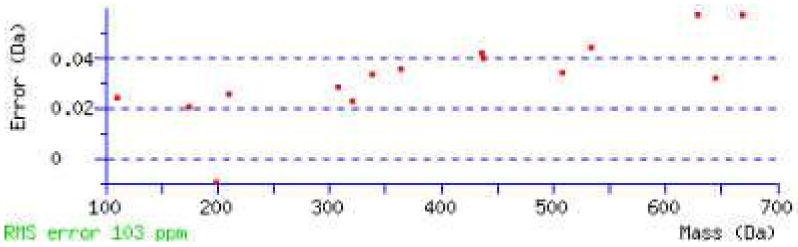


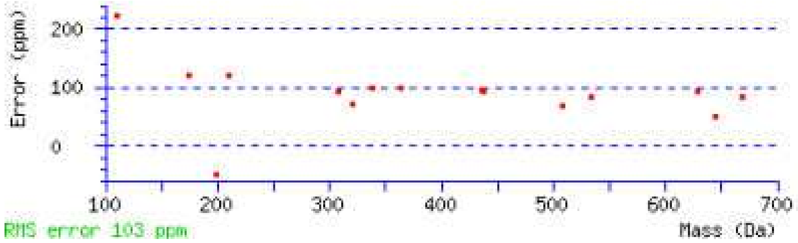


NCBI **BLAST** search of LLHAVYR
(Parameters: blastp, nr protein database, expect=20000, no filter, PAM30)
Other BLAST web gateways

**All matches to this query**

| Score | Mr(calc) | Delta | Sequence |
|---|---|---|---|
| 30.6 | 870.5076 | 0.0762 | ILHAVYR |
| 30.6 | 870.5076 | 0.0762 | LLHAVYR |
| 27.0 | 870.5076 | 0.0762 | HVLLAYR |
| 22.9 | 870.5076 | 0.0762 | LLHVAYR |
| 22.8 | 870.5076 | 0.0762 | VHLIAYR |
| 22.7 | 870.5076 | 0.0762 | VHIAIYR |
| 22.6 | 870.5076 | 0.0762 | VHAILYR |
| 22.6 | 870.5076 | 0.0762 | VHALLYR |
| 22.4 | 870.5076 | 0.0761 | VVHVVYR |
| 21.8 | 870.5076 | 0.0762 | VHLLAYR |

**Mascot:** http://www.matrixscience.com/

# MATRIX SCIENCE Mascot Search Results

## Peptide View **Spot no 48**

MS/MS Fragmentation of **VADHAGVALAGLTADGR**
Found in **gi|115444057** in **NCBInr**, Os02g0133800 [Oryza sativa Japonica Group]

Match to Query 159: 1592.901024 from(1593.908300,1+) intensity(0.0000) index(26)
Title: Label: E19, Spot_Id: 219966, Peak_List_Id: 229271, MSMS Job_Run_Id: 22016, Comment:
Data file C:\Documents and Settings\Administrator\Desktop\Farah-Garima 11May 2013\ppw_E19_136868347200.txt

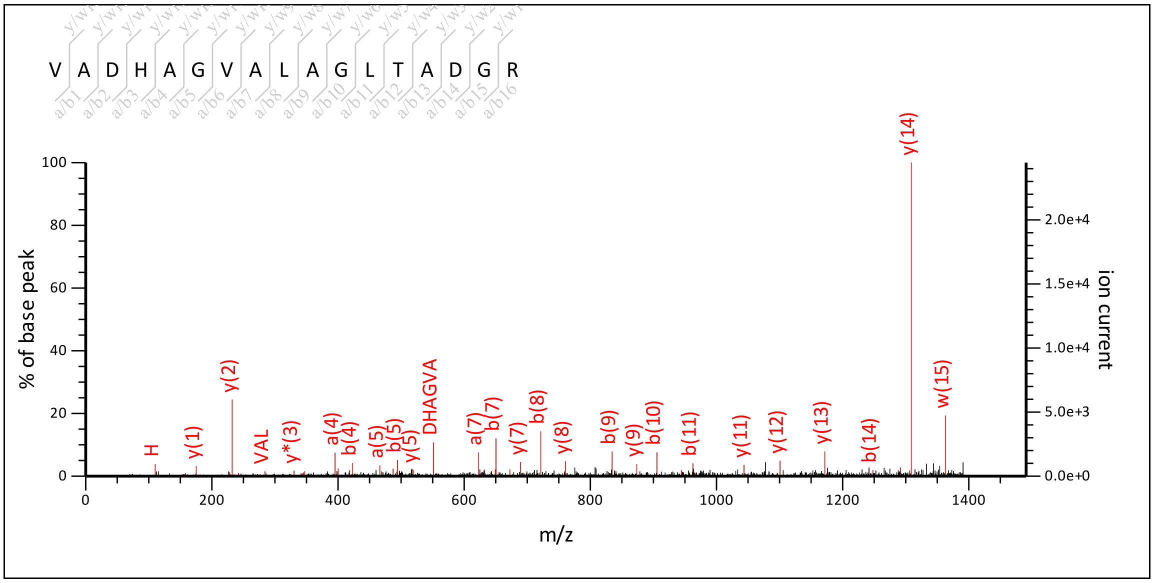


0 to 1490.9

Label all possible matches ○ Label matches used for scoring ◉

**Monoisotopic mass of neutral peptide Mr(calc):** 1592.8271
**Fixed modifications:** Carbamidomethyl (C) (apply to specified residues or termini only)
**Ions Score:** 84 **Expect:** 0.00021
**Matches :** 50/301 fragment ions using 84 most intense peaks (help)

| # | Immon. | a | $a^0$ | b | $b^0$ | d | d' | Seq. | v | w | w' | y | y* | $y^0$ | # |
|---|---|---|---|---|---|---|---|---|---|---|---|---|---|---|---|
| 1 | 72.0808 | 72.0808 | | 100.0757 | | 44.0495 | | V | | | | | | | 17 |
| 2 | 44.0495 | 143.1179 | | 171.1128 | | | | A | 1478.7346 | | | 1494.7659 | 1477.7394 | 1476.7554 | 16 |
| 3 | 88.0393 | 258.1448 | 240.1343 | 286.1397 | 268.1292 | 214.1550 | | D | 1363.7077 | 1362.7124 | | 1423.7288 | 1406.7023 | 1405.7183 | 15 |
| 4 | 110.0713 | **395.2037** | 377.1932 | **423.1987** | 405.1881 | | | H | 1226.6488 | | | ***1308.7019*** | 1291.6753 | 1290.6913 | 14 |
| 5 | 44.0495 | **466.2409** | 448.2303 | **494.2358** | 476.2252 | | | A | 1155.6117 | | | ***1171.6430*** | 1154.6164 | 1153.6324 | 13 |
| 6 | 30.0338 | 523.2623 | 505.2518 | **551.2572** | 533.2467 | | | G | | | | ***1100.6059*** | 1083.5793 | 1082.5953 | 12 |
| 7 | 72.0808 | **622.3307** | 604.3202 | **650.3257** | 632.3151 | 608.3151 | | V | 999.5218 | 1012.5422 | | ***1043.5844*** | 1026.5578 | 1025.5738 | 11 |
| 8 | 44.0495 | 693.3678 | 675.3573 | **721.3628** | 703.3522 | | | A | 928.4847 | | | ***944.5160*** | 927.4894 | 926.5054 | 10 |
| 9 | 86.0964 | 806.4519 | 788.4413 | **834.4468** | 816.4363 | 764.4050 | | L | 815.4006 | 814.4054 | | ***873.4789*** | 856.4523 | 855.4683 | 9 |
| 10 | 44.0495 | 877.4890 | 859.4785 | **905.4839** | 887.4734 | | | A | 744.3635 | | | ***760.3948*** | 743.3682 | 742.3842 | 8 |
| 11 | 30.0338 | 934.5105 | 916.4999 | **962.5054** | 944.4948 | | | G | | | | ***689.3577*** | 672.3311 | 671.3471 | 7 |
| 12 | 86.0964 | 1047.5946 | 1029.5840 | 1075.5895 | 1057.5789 | 1005.5476 | | L | 574.2580 | 573.2627 | | ***632.3362*** | 615.3097 | 614.3257 | 6 |
| 13 | 74.0600 | 1148.6422 | 1130.6317 | 1176.6372 | 1158.6266 | 1132.6473 | 1134.6266 | T | 473.2103 | 486.2307 | 488.2100 | ***519.2522*** | 502.2256 | 501.2416 | 5 |
| 14 | 44.0495 | 1219.6793 | 1201.6688 | **1247.6743** | 1229.6637 | | | A | 402.1732 | | | ***418.2045*** | 401.1779 | 400.1939 | 4 |
| 15 | 88.0393 | 1334.7063 | 1316.6957 | **1362.7012** | 1344.6906 | 1290.7165 | | D | 287.1462 | 286.1510 | | ***347.1674*** | 330.1408 | 329.1568 | 3 |
| 16 | 30.0338 | 1391.7278 | 1373.7172 | 1419.7227 | 1401.7121 | | | G | | | | ***232.1404*** | 215.1139 | | 2 |
| 17 | 129.1135 | | | | | | | R | 74.0237 | 73.0284 | | ***175.1190*** | 158.0924 | | 1 |

| Seq | ya | yb | Seq | ya | yb | Seq | ya | yb |
|---|---|---|---|---|---|---|---|---|
| | | | | | | | | |

| | | | | | | | | |
|---|---|---|---|---|---|---|---|---|
| **AD** | 159.0764 | 187.0713 | **ADH** | 296.1353 | 324.1302 | **ADHA** | 367.1724 | 395.1674 |
| **ADHAG** | 424.1939 | 452.1888 | **ADHAGV** | 523.2623 | 551.2572 | **ADHAGVA** | 594.2994 | 622.2944 |
| **DH** | 225.0982 | 253.0931 | **DHA** | 296.1353 | 324.1302 | **DHAG** | 353.1568 | 381.1517 |
| **DHAGV** | 452.2252 | 480.2201 | **DHAGVA** | 523.2623 | 551.2572 | **DHAGVAL** | 636.3464 | 664.3413 |
| **HA** | 181.1084 | 209.1033 | **HAG** | 238.1299 | 266.1248 | **HAGV** | 337.1983 | 365.1932 |
| **HAGVA** | 408.2354 | 436.2303 | **HAGVAL** | 521.3194 | 549.3144 | **HAGVALA** | 592.3566 | 620.3515 |
| **HAGVALAG** | 649.3780 | 677.3729 | **AG** | 101.0709 | 129.0659 | **AGV** | 200.1394 | 228.1343 |
| **AGVA** | 271.1765 | 299.1714 | **AGVAL** | 384.2605 | 412.2554 | **AGVALA** | 455.2976 | 483.2926 |
| **AGVALAG** | 512.3191 | 540.3140 | **AGVALAGL** | 625.4032 | 653.3981 | **GV** | 129.1022 | 157.0972 |
| **GVA** | 200.1394 | 228.1343 | **GVAL** | 313.2234 | 341.2183 | **GVALA** | 384.2605 | 412.2554 |
| **GVALAG** | 441.2820 | 469.2769 | **GVALAGL** | 554.3661 | 582.3610 | **GVALAGLT** | 655.4137 | 683.4087 |
| **VA** | 143.1179 | 171.1128 | **VAL** | 256.2020 | 284.1969 | **VALA** | 327.2391 | 355.2340 |
| **VALAG** | 384.2605 | 412.2554 | **VALAGL** | 497.3446 | 525.3395 | **VALAGLT** | 598.3923 | 626.3872 |
| **VALAGLTA** | 669.4294 | 697.4243 | **AL** | 157.1335 | 185.1285 | **ALA** | 228.1707 | 256.1656 |
| **ALAG** | 285.1921 | 313.1870 | **ALAGL** | 398.2762 | 426.2711 | **ALAGLT** | 499.3239 | 527.3188 |
| **ALAGLTA** | 570.3610 | 598.3559 | **ALAGLTAD** | 685.3879 | 713.3828 | **LA** | 157.1335 | 185.1285 |
| **LAG** | 214.1550 | 242.1499 | **LAGL** | 327.2391 | 355.2340 | **LAGLT** | 428.2867 | 456.2817 |
| **LAGLTA** | 499.3239 | 527.3188 | **LAGLTAD** | 614.3508 | 642.3457 | **LAGLTADG** | 671.3723 | 699.3672 |
| **AG** | 101.0709 | 129.0659 | **AGL** | 214.1550 | 242.1499 | **AGLT** | 315.2027 | 343.1976 |
| **AGLTA** | 386.2398 | 414.2347 | **AGLTAD** | 501.2667 | 529.2617 | **AGLTADG** | 558.2882 | 586.2831 |
| **GL** | 143.1179 | 171.1128 | **GLT** | 244.1656 | 272.1605 | **GLTA** | 315.2027 | 343.1976 |
| **GLTAD** | 430.2296 | 458.2245 | **GLTADG** | 487.2511 | 515.2460 | **LT** | 187.1441 | 215.1390 |
| **LTA** | 258.1812 | 286.1761 | **LTAD** | 373.2082 | 401.2031 | **LTADG** | 430.2296 | 458.2245 |
| **TA** | 145.0972 | 173.0921 | **TAD** | 260.1241 | 288.1190 | **TADG** | 317.1456 | 345.1405 |
| **AD** | 159.0764 | 187.0713 | **ADG** | 216.0979 | 244.0928 | **DG** | 145.0608 | 173.0557 |

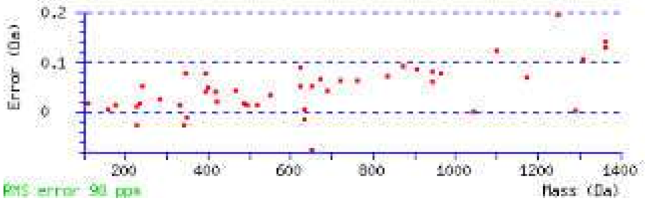


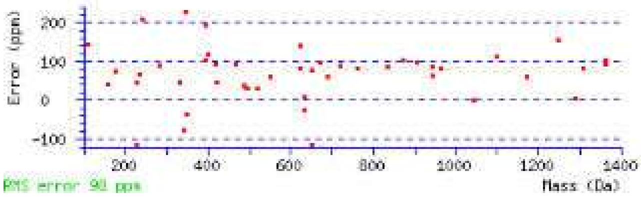


NCBI **BLAST** search of VADHAGVALAGLTADGR
(Parameters: blastp, nr protein database, expect=20000, no filter, PAM30)
Other BLAST web gateways

**All matches to this query**

| Score | Mr(calc) | Delta | Sequence |
|---|---|---|---|
| 83.9 | 1592.8271 | 0.0740 | VADHAGVALAGLTADGR |
| 33.4 | 1592.8131 | 0.0879 | ARHEGIGIEQRDGR |
| 27.3 | 1592.7794 | 0.1216 | GYDSAGVATVAAGELGR |
| 26.5 | 1592.8046 | 0.0964 | GYDSAGVALLDGNTLK |
| 25.9 | 1592.8423 | 0.0587 | FAFGAGVTAIVGRDGR |
| 25.8 | 1592.9038 | -0.0028 | GFKFAGIIAGDKNKK |
| 24.0 | 1592.8383 | 0.0628 | REHQAALVKQEER |
| 24.0 | 1592.8385 | 0.0626 | ACVYGVAAILWATAK |
| 23.8 | 1592.8674 | 0.0336 | AFLTGAAGALAAAAYVR |
| 22.9 | 1592.8133 | 0.0877 | MKFALNIVDWQGR |

**Mascot:** http://www.matrixscience.com/

# MATRIX SCIENCE Mascot Search Results

## Peptide View Spot no 48

MS/MS Fragmentation of **NQYDTDVTTWSPAGR**
Found in **gi|115444057** in **NCBInr**, Os02g0133800 [Oryza sativa Japonica Group]

Match to Query 172: 1709.843224 from(1710.850500,1+) intensity(0.0000) index(30)
Title: Label: E19, Spot_Id: 219966, Peak_List_Id: 229270, MSMS Job_Run_Id: 22016, Comment:
Data file C:\Documents and Settings\Administrator\Desktop\Farah-Garima 11May 2013\ppw_E19_136868347200.txt

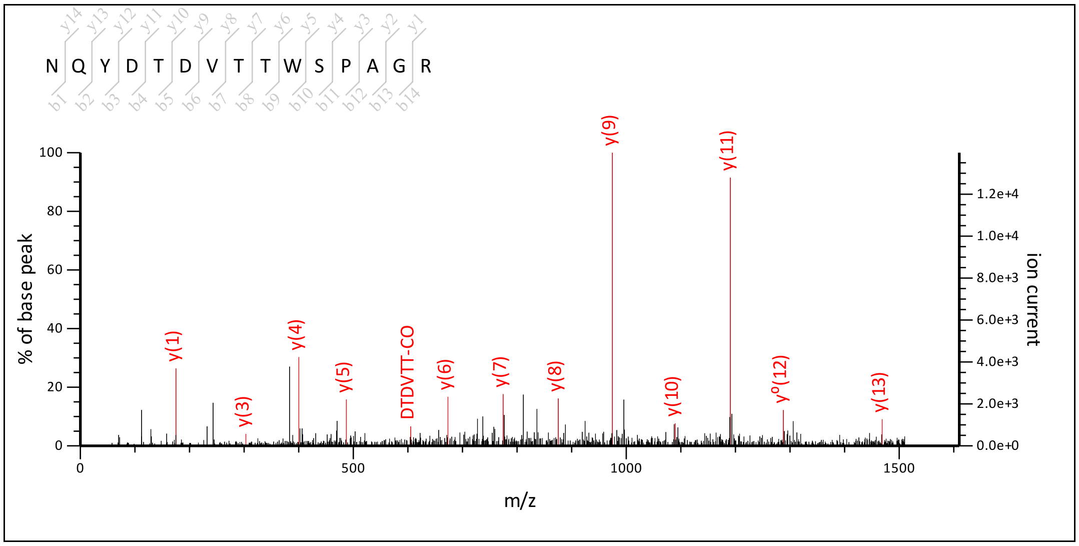


0 to 1610.06

Label all possible matches ○ Label matches used for scoring ◉

**Monoisotopic mass of neutral peptide Mr(calc):** 1709.7645
**Fixed modifications:** Carbamidomethyl (C) (apply to specified residues or termini only)
**Ions Score:** 108 **Expect:** 7.6e-07
**Matches :** 16/269 fragment ions using 15 most intense peaks (help)

| # | Immon. | a | a* | a0 | b | b* | b0 | d | d' | Seq. | v | w | w' | y | y* | |
|---|---|---|---|---|---|---|---|---|---|---|---|---|---|---|---|---|
| 1 | 87.0553 | 87.0553 | 70.0287 | | 115.0502 | 98.0237 | | 44.0495 | | N | | | | | | |
| 2 | 101.0709 | 215.1139 | 198.0873 | | 243.1088 | 226.0822 | | 158.0924 | | Q | 1523.6761 | 1522.6809 | | 1596.7289 | 1579.7023 | [illegible] |
| 3 | 136.0757 | 378.1772 | 361.1506 | | 406.1721 | 389.1456 | | | | Y | 1360.6128 | | | ***1468.6703*** | 1451.6438 | [illegible] |
| 4 | 88.0393 | 493.2041 | 476.1776 | 475.1936 | 521.1991 | 504.1725 | 503.1885 | 449.2143 | | D | 1245.5858 | 1244.5906 | | 1305.6070 | 1288.5804 | [illegible] |
| 5 | 74.0600 | 594.2518 | 577.2253 | 576.2413 | 622.2467 | 605.2202 | 604.2362 | 578.2569 | 580.2362 | T | 1144.5382 | 1157.5586 | 1159.5378 | ***1190.5800*** | 1173.5535 | [illegible] |
| 6 | 88.0393 | 709.2788 | 692.2522 | 691.2682 | 737.2737 | 720.2471 | 719.2631 | 665.2889 | | D | 1029.5112 | 1028.5160 | | ***1089.5323*** | 1072.5058 | [illegible] |
| 7 | 72.0808 | 808.3472 | 791.3206 | 790.3366 | 836.3421 | 819.3155 | 818.3315 | 794.3315 | | V | 930.4428 | 943.4632 | | ***974.5054*** | 957.4789 | [illegible] |
| 8 | 74.0600 | 909.3949 | 892.3683 | 891.3843 | 937.3898 | 920.3632 | 919.3792 | 893.3999 | 895.3792 | T | 829.3951 | 842.4155 | 844.3948 | ***875.4370*** | 858.4104 | [illegible] |
| 9 | 74.0600 | 1010.4425 | 993.4160 | 992.4320 | 1038.4374 | 1021.4109 | 1020.4269 | 994.4476 | 996.4269 | T | 728.3474 | 741.3678 | 743.3471 | ***774.3893*** | 757.3628 | [illegible] |
| 10 | 159.0917 | 1196.5218 | 1179.4953 | 1178.5113 | 1224.5168 | 1207.4902 | 1206.5062 | | | W | 542.2681 | | | ***673.3416*** | 656.3151 | [illegible] |
| 11 | 60.0444 | 1283.5539 | 1266.5273 | 1265.5433 | 1311.5488 | 1294.5222 | 1293.5382 | 1267.5590 | | S | 455.2361 | 454.2409 | | ***487.2623*** | 470.2358 | [illegible] |
| 12 | 70.0651 | 1380.6066 | 1363.5801 | 1362.5961 | 1408.6016 | 1391.5750 | 1390.5910 | 1354.5910 | | P | 358.1833 | 357.1881 | | ***400.2303*** | 383.2037 | |
| 13 | 44.0495 | 1451.6438 | 1434.6172 | 1433.6332 | 1479.6387 | 1462.6121 | 1461.6281 | | | A | 287.1462 | | | ***303.1775*** | 286.1510 | |
| 14 | 30.0338 | 1508.6652 | 1491.6387 | 1490.6546 | 1536.6601 | 1519.6336 | 1518.6496 | | | G | | | | 232.1404 | 215.1139 | |
| 15 | 129.1135 | | | | | | | | | R | 74.0237 | 73.0284 | | ***175.1190*** | 158.0924 | |

| Seq | ya | yb | Seq | ya | yb | Seq | ya | yb |
|---|---|---|---|---|---|---|---|---|
| QY | 264.1343 | 292.1292 | QYD | 379.1612 | 407.1561 | QYDT | 480.2089 | 508.2038 |
| QYDTD | 595.2358 | 623.2307 | QYDTDV | 694.3042 | 722.2992 | YD | 251.1026 | 279.0975 |
| YDT | 352.1503 | 380.1452 | YDTD | 467.1773 | 495.1722 | YDTDV | 566.2457 | 594.2406 |
| YDTDVT | 667.2933 | 695.2883 | DT | 189.0870 | 217.0819 | DTD | 304.1139 | 332.1088 |
| DTDV | 403.1823 | 431.1773 | DTDVT | 504.2300 | 532.2249 | DTDVTT | 605.2777 | 633.2726 |
| TD | 189.0870 | 217.0819 | TDV | 288.1554 | 316.1503 | TDVT | 389.2031 | 417.1980 |

| | | | | | | | | |
|---|---|---|---|---|---|---|---|---|
| **TDVTT** | 490.2508 | 518.2457 | **TDVTTW** | 676.3301 | 704.3250 | **DV** | 187.1077 | 215.1026 |
| **DVT** | 288.1554 | 316.1503 | **DVTT** | 389.2031 | 417.1980 | **DVTTW** | 575.2824 | 603.2773 |
| **DVTTWS** | 662.3144 | 690.3093 | **VT** | 173.1285 | 201.1234 | **VTT** | 274.1761 | 302.1710 |
| **VTTW** | 460.2554 | 488.2504 | **VTTWS** | 547.2875 | 575.2824 | **VTTWSP** | 644.3402 | 672.3352 |
| **TT** | 175.1077 | 203.1026 | **TTW** | 361.1870 | 389.1819 | **TTWS** | 448.2191 | 476.2140 |
| **TTWSP** | 545.2718 | 573.2667 | **TTWSPA** | 616.3089 | 644.3039 | **TTWSPAG** | 673.3304 | 701.3253 |
| **TW** | 260.1394 | 288.1343 | **TWS** | 347.1714 | 375.1663 | **TWSP** | 444.2241 | 472.2191 |
| **TWSPA** | 515.2613 | 543.2562 | **TWSPAG** | 572.2827 | 600.2776 | **WS** | 246.1237 | 274.1186 |
| **WSP** | 343.1765 | 371.1714 | **WSPA** | 414.2136 | 442.2085 | **WSPAG** | 471.2350 | 499.2300 |
| **SP** | 157.0972 | 185.0921 | **SPA** | 228.1343 | 256.1292 | **SPAG** | 285.1557 | 313.1506 |
| **PA** | 141.1022 | 169.0972 | **PAG** | 198.1237 | 226.1186 | **AG** | 101.0709 | 129.0659 |

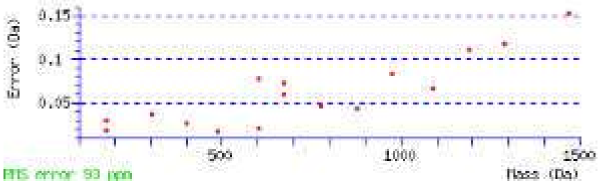


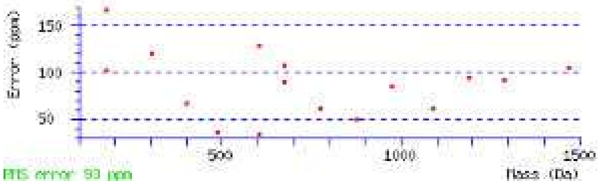


NCBI **BLAST** search of NQYDTDVTTWSPAGR
(Parameters: blastp, nr protein database, expect=20000, no filter, PAM30)
Other BLAST web gateways

**All matches to this query**

| Score | Mr(calc) | Delta | Sequence |
|---|---|---|---|
| 108.4 | 1709.7645 | 0.0787 | NQYDTDVTTWSPAGR |
| 36.2 | 1709.8883 | -0.0450 | HKDKLDMVQESPKR |
| 34.9 | 1709.8809 | -0.0377 | IHNGVTDVTRNETVR |
| 25.2 | 1709.8948 | -0.0516 | KPVVPSEEEIEGNKR |
| 22.1 | 1709.7719 | 0.0713 | EFMPTFPALNEQDR |
| 22.0 | 1709.8948 | -0.0516 | LNVPSEPVTVIGDTNR |
| 21.7 | 1709.8559 | -0.0127 | KATVSIGSMVDWFNR |
| 21.7 | 1709.9536 | -0.1104 | LAKNLQPARATASVDR |
| 21.6 | 1709.8566 | -0.0134 | HKMGAPMNRGKSPGAR |
| 21.4 | 1709.8696 | -0.0264 | LEPLAGRAEAAEAEQR |

**Mascot:** http://www.matrixscience.com/

# MATRIX SCIENCE Mascot Search Results

## Peptide View Spot no 48

MS/MS Fragmentation of **SECINHAFVYDAPLPVSR**
Found in **gi|115444057** in **NCBInr**, Os02g0133800 [Oryza sativa Japonica Group]

Match to Query 186: 2074.099124 from(2075.106400,1+) intensity(0.0000) index(32)
Title: Label: E19, Spot_Id: 219966, Peak_List_Id: 229273, MSMS Job_Run_Id: 22016, Comment:
Data file C:\Documents and Settings\Administrator\Desktop\Farah-Garima 11May 2013\ppw_E19_136868347200.txt

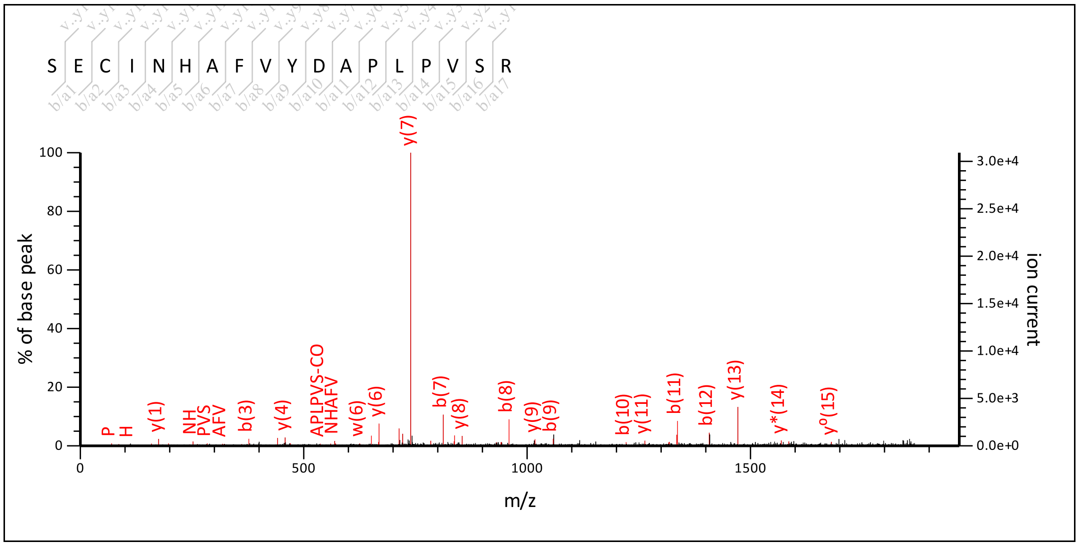


0 to 1967.39

Label all possible matches ○ Label matches used for scoring ◉

**Monoisotopic mass of neutral peptide Mr(calc):** 2073.9942
**Fixed modifications:** Carbamidomethyl (C) (apply to specified residues or termini only)
**Ions Score:** 74 **Expect:** 0.0022
**Matches :** 57/330 fragment ions using 90 most intense peaks (help)

| # | Immon. | a | a* | $a^0$ | b | b* | $b^0$ | d | d' | Seq. | v | w | w' | y | y* | |
|---|---|---|---|---|---|---|---|---|---|---|---|---|---|---|---|---|
| 1 | 60.0444 | 60.0444 | | 42.0338 | 88.0393 | | 70.0287 | 44.0495 | | S | | | | | | |
| 2 | 102.0550 | 189.0870 | | 171.0764 | 217.0819 | | 199.0713 | 131.0815 | | E | 1913.9327 | 1912.9374 | | 1987.9695 | 1970.9429 | 19 |
| 3 | 133.0430 | 349.1176 | | 331.1071 | **377.1125** | | 359.1020 | 260.1241 | | C | 1753.9020 | 1752.9068 | | 1858.9269 | 1841.9003 | 18 |
| 4 | 86.0964 | 462.2017 | | 444.1911 | 490.1966 | | 472.1860 | 434.1704 | 448.1860 | I | 1640.8180 | 1653.8384 | 1667.8540 | 1698.8962 | 1681.8697 | 16 |
| 5 | 87.0553 | 576.2446 | 559.2181 | 558.2341 | 604.2395 | 587.2130 | 586.2290 | 533.2388 | | N | 1526.7750 | 1525.7798 | | ***1585.8122*** | 1568.7856 | 15 |
| 6 | 110.0713 | **713.3035** | 696.2770 | 695.2930 | 741.2984 | 724.2719 | 723.2879 | | | H | 1389.7161 | | | ***1471.7692*** | 1454.7427 | 14 |
| 7 | 44.0495 | **784.3406** | 767.3141 | 766.3301 | **812.3356** | 795.3090 | 794.3250 | | | A | 1318.6790 | | | ***1334.7103*** | 1317.6838 | 13 |
| 8 | 120.0808 | **931.4091** | 914.3825 | 913.3985 | **959.4040** | 942.3774 | 941.3934 | | | F | 1171.6106 | | | ***1263.6732*** | 1246.6467 | 12 |
| 9 | 72.0808 | 1030.4775 | 1013.4509 | 1012.4669 | **1058.4724** | 1041.4458 | 1040.4618 | 1016.4618 | | V | 1072.5422 | 1085.5626 | | 1116.6048 | 1099.5782 | 10 |
| 10 | 136.0757 | 1193.5408 | 1176.5143 | 1175.5302 | **1221.5357** | 1204.5092 | 1203.5252 | | | Y | 909.4789 | | | ***1017.5364*** | 1000.5098 | 9 |
| 11 | 88.0393 | 1308.5677 | 1291.5412 | 1290.5572 | **1336.5627** | 1319.5361 | 1318.5521 | 1264.5779 | | D | 794.4519 | 793.4567 | | ***854.4730*** | 837.4465 | 8 |
| 12 | 44.0495 | 1379.6049 | 1362.5783 | 1361.5943 | **1407.5998** | 1390.5732 | 1389.5892 | | | A | 723.4148 | | | ***739.4461*** | 722.4196 | 7 |
| 13 | 70.0651 | 1476.6576 | 1459.6311 | 1458.6471 | 1504.6525 | 1487.6260 | 1486.6420 | 1450.6420 | | P | 626.3620 | 625.3668 | | ***668.4090*** | 651.3824 | 6 |
| 14 | 86.0964 | 1589.7417 | 1572.7151 | 1571.7311 | 1617.7366 | 1600.7101 | 1599.7260 | 1547.6947 | | L | 513.2780 | 512.2827 | | ***571.3562*** | 554.3297 | 5 |
| 15 | 70.0651 | 1686.7945 | 1669.7679 | 1668.7839 | 1714.7894 | 1697.7628 | 1696.7788 | 1660.7788 | | P | 416.2252 | 415.2300 | | ***458.2722*** | 441.2456 | 4 |
| 16 | 72.0808 | 1785.8629 | 1768.8363 | 1767.8523 | 1813.8578 | 1796.8312 | 1795.8472 | 1771.8472 | | V | 317.1568 | 330.1772 | | ***361.2194*** | 344.1928 | 3 |
| 17 | 60.0444 | 1872.8949 | 1855.8683 | 1854.8843 | 1900.8898 | 1883.8633 | 1882.8792 | 1856.9000 | | S | 230.1248 | 229.1295 | | ***262.1510*** | 245.1244 | 2 |
| 18 | 129.1135 | | | | | | | | | R | 74.0237 | 73.0284 | | ***175.1190*** | 158.0924 | |

| Seq | ya | yb | Seq | ya | yb | Seq | ya | yb |
|---|---|---|---|---|---|---|---|---|
| EC | 262.0856 | 290.0805 | ECI | 375.1697 | 403.1646 | ECIN | 489.2126 | 517.2075 |
| ECINH | 626.2715 | 654.2664 | ECINHA | 697.3086 | 725.3035 | CI | 246.1271 | 274.1220 |
| CIN | 360.1700 | 388.1649 | CINH | 497.2289 | 525.2238 | CINHA | 568.2660 | 596.2609 |

| | | | | | | | | |
|---|---|---|---|---|---|---|---|---|
| **IN** | 200.1394 | 228.1343 | **INH** | 337.1983 | 365.1932 | **INHA** | 408.2354 | 436.2303 |
| **INHAF** | 555.3038 | 583.2987 | **INHAFV** | 654.3722 | 682.3671 | **NH** | 224.1142 | 252.1091 |
| **NHA** | 295.1513 | 323.1462 | **NHAF** | 442.2197 | 470.2146 | **NHAFV** | 541.2881 | 569.2831 |
| **HA** | 181.1084 | 209.1033 | **HAF** | 328.1768 | 356.1717 | **HAFV** | 427.2452 | 455.2401 |
| **HAFVY** | 590.3085 | 618.3035 | **AF** | 191.1179 | 219.1128 | **AFV** | 290.1863 | 318.1812 |
| **AFVY** | 453.2496 | 481.2445 | **AFVYD** | 568.2766 | 596.2715 | **AFVYDA** | 639.3137 | 667.3086 |
| **FV** | 219.1492 | 247.1441 | **FVY** | 382.2125 | 410.2074 | **FVYD** | 497.2395 | 525.2344 |
| **FVYDA** | 568.2766 | 596.2715 | **FVYDAP** | 665.3293 | 693.3243 | **VY** | 235.1441 | 263.1390 |
| **VYD** | 350.1710 | 378.1660 | **VYDA** | 421.2082 | 449.2031 | **VYDAP** | 518.2609 | 546.2558 |
| **VYDAPL** | 631.3450 | 659.3399 | **YD** | 251.1026 | 279.0975 | **YDA** | 322.1397 | 350.1347 |
| **YDAP** | 419.1925 | 447.1874 | **YDAPL** | 532.2766 | 560.2715 | **YDAPLP** | 629.3293 | 657.3243 |
| **DA** | 159.0764 | 187.0713 | **DAP** | 256.1292 | 284.1241 | **DAPL** | 369.2132 | 397.2082 |
| **DAPLP** | 466.2660 | 494.2609 | **DAPLPV** | 565.3344 | 593.3293 | **DAPLPVS** | 652.3665 | 680.3614 |
| **AP** | 141.1022 | 169.0972 | **APL** | 254.1863 | 282.1812 | **APLP** | 351.2391 | 379.2340 |
| **APLPV** | 450.3075 | 478.3024 | **APLPVS** | 537.3395 | 565.3344 | **PL** | 183.1492 | 211.1441 |
| **PLP** | 280.2020 | 308.1969 | **PLPV** | 379.2704 | 407.2653 | **PLPVS** | 466.3024 | 494.2973 |
| **LP** | 183.1492 | 211.1441 | **LPV** | 282.2176 | 310.2125 | **LPVS** | 369.2496 | 397.2445 |
| **PV** | 169.1335 | 197.1285 | **PVS** | 256.1656 | 284.1605 | **VS** | 159.1128 | 187.1077 |

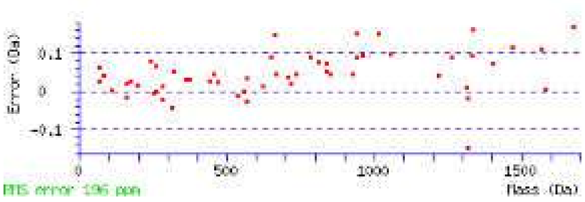


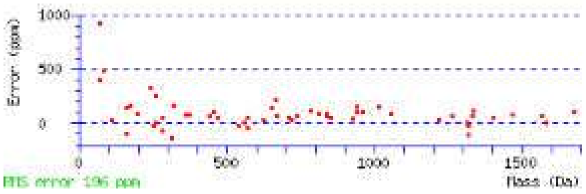


NCBI **BLAST** search of SECINHAFVYDAPLPVSR
(Parameters: blastp, nr protein database, expect=20000, no filter, PAM30)
Other BLAST web gateways

**All matches to this query**

| Score | Mr(calc) | Delta | Sequence |
|---|---|---|---|
| 74.3 | 2073.9942 | 0.1049 | SECINHAFVYDAPLPVSR |
| 28.2 | 2074.1106 | -0.0114 | METTYGLVLRRAGVSIHR |
| 20.0 | 2074.1833 | -0.0842 | KVGGGRIAAHEIMIGIPAIR |
| 20.0 | 2074.1833 | -0.0842 | KVGGGRIAAHEIMLGIPAIR |
| 17.5 | 2074.1833 | -0.0842 | KIGGGRVAAHEIMIAVPAIR |
| 17.5 | 2074.1833 | -0.0842 | KIGGGRVAAHEIMLAVPAIR |
| 16.7 | 2074.1826 | -0.0835 | LSIVAALAPVEKAEFVYVR |
| 15.7 | 2074.1946 | -0.0954 | MGHDKVADKLAPLLRRVR |
| 15.3 | 2074.1833 | -0.0842 | KGIRVNALCPGPVNTPLLR |
| 15.3 | 2074.1535 | -0.0543 | ENLLRLVNPTLSGPEIPGR |

**Mascot:** http://www.matrixscience.com/

# MATRIX SCIENCE Mascot Search Results

## Peptide View Spot no 49

MS/MS Fragmentation of **FSPIKESIVSR**
Found in **gi|27261025** in **NCBInr**, putative thiamine biosynthesis protein [Oryza sativa Japonica Group]

Match to Query 25: 1261.788524 from(1262.795800,1+) intensity(0.0000) index(11)
Title: Label: H9, Spot_Id: 228977, Peak_List_Id: 257861, MSMS Job_Run_Id: 24940, Comment:
Data file ppw_H9_138985153400.txt

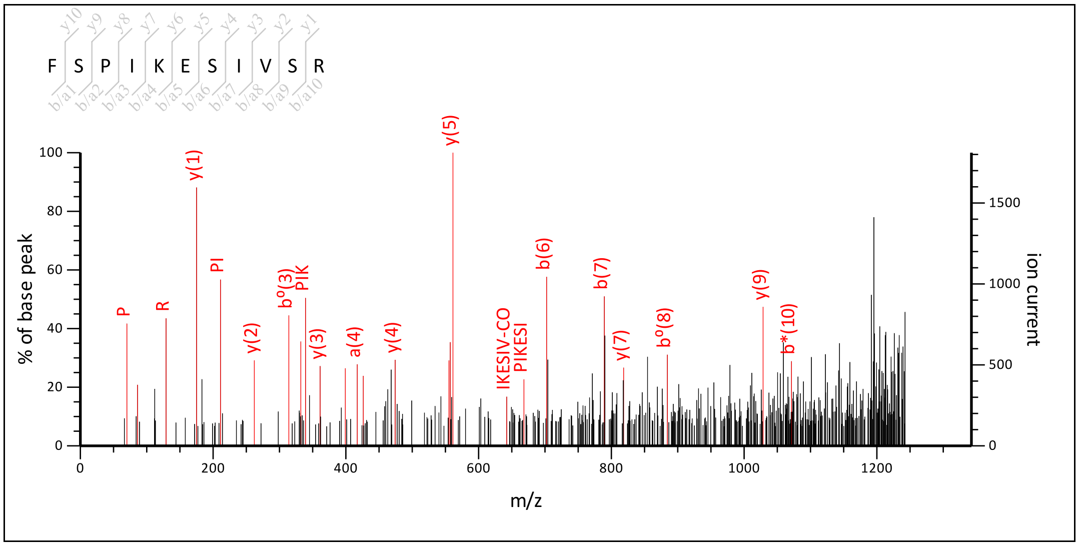


0 to 1342.47

Label all possible matches ○ Label matches used for scoring ◉

**Monoisotopic mass of neutral peptide Mr(calc):** 1261.7030
**Fixed modifications:** Carbamidomethyl (C) (apply to specified residues or termini only)
**Ions Score:** 33 **Expect:** 0.52
**Matches :** 30/184 fragment ions using 48 most intense peaks (help)

| # | Immon. | a | a* | $a^0$ | b | b* | $b^0$ | d | d' | Seq. | v | w | w' | y | y* | |
|---|---|---|---|---|---|---|---|---|---|---|---|---|---|---|---|---|
| 1 | 120.0808 | 120.0808 | | | 148.0757 | | | 44.0495 | | F | | | | | | |
| 2 | 60.0444 | 207.1128 | | 189.1022 | 235.1077 | | 217.0972 | 191.1179 | | S | 1083.6157 | 1082.6204 | | 1115.6419 | 1098.6153 | [illegible] |
| 3 | 70.0651 | 304.1656 | | 286.1550 | **332.1605** | | 314.1499 | 278.1499 | | P | 986.5629 | 985.5677 | | ***1028.6099*** | 1011.5833 | [illegible] |
| 4 | 86.0964 | 417.2496 | | 399.2391 | 445.2445 | | 427.2340 | 389.2183 | 403.2340 | I | 873.4789 | 886.4993 | 900.5149 | 931.5571 | 914.5306 | [illegible] |
| 5 | 101.1073 | 545.3446 | 528.3180 | 527.3340 | 573.3395 | 556.3130 | 555.3289 | 488.2867 | | K | 745.3839 | 744.3886 | | ***818.4730*** | 801.4465 | [illegible] |
| 6 | 102.0550 | 674.3872 | 657.3606 | 656.3766 | **702.3821** | 685.3556 | 684.3715 | 616.3817 | | E | 616.3413 | 615.3461 | | 690.3781 | 673.3515 | [illegible] |
| 7 | 60.0444 | 761.4192 | 744.3927 | 743.4086 | **789.4141** | 772.3876 | 771.4036 | 745.4243 | | S | 529.3093 | 528.3140 | | ***561.3355*** | 544.3089 | [illegible] |
| 8 | 86.0964 | 874.5033 | 857.4767 | 856.4927 | 902.4982 | 885.4716 | 884.4876 | 846.4720 | 860.4876 | I | 416.2252 | 429.2456 | 443.2613 | ***474.3035*** | 457.2769 | [illegible] |
| 9 | 72.0808 | 973.5717 | 956.5451 | 955.5611 | 1001.5666 | 984.5401 | 983.5560 | 959.5560 | | V | 317.1568 | 330.1772 | | ***361.2194*** | 344.1928 | [illegible] |
| 10 | 60.0444 | 1060.6037 | 1043.5772 | 1042.5932 | 1088.5986 | 1071.5721 | 1070.5881 | 1044.6088 | | S | 230.1248 | 229.1295 | | ***262.1510*** | 245.1244 | [illegible] |
| 11 | 129.1135 | | | | | | | | | R | 74.0237 | 73.0284 | | ***175.1190*** | 158.0924 | |

| Seq | ya | yb | Seq | ya | yb | Seq | ya | yb |
|---|---|---|---|---|---|---|---|---|
| SP | 157.0972 | 185.0921 | SPI | 270.1812 | 298.1761 | SPIK | 398.2762 | 426.2711 |
| SPIKE | 527.3188 | 555.3137 | SPIKES | 614.3508 | 642.3457 | PI | 183.1492 | 211.1441 |
| PIK | 311.2442 | 339.2391 | PIKE | 440.2867 | 468.2817 | PIKES | 527.3188 | 555.3137 |
| PIKESI | 640.4028 | 668.3978 | IK | 214.1914 | 242.1863 | IKE | 343.2340 | 371.2289 |
| IKES | 430.2660 | 458.2609 | IKESI | 543.3501 | 571.3450 | IKESIV | 642.4185 | 670.4134 |
| KE | 230.1499 | 258.1448 | KES | 317.1819 | 345.1769 | KESI | 430.2660 | 458.2609 |
| KESIV | 529.3344 | 557.3293 | KESIVS | 616.3665 | 644.3614 | ES | 189.0870 | 217.0819 |
| ESI | 302.1710 | 330.1660 | ESIV | 401.2395 | 429.2344 | ESIVS | 488.2715 | 516.2664 |
| SI | 173.1285 | 201.1234 | SIV | 272.1969 | 300.1918 | SIVS | 359.2289 | 387.2238 |

| | | | | | | | | |
|---|---|---|---|---|---|---|---|---|
| **IV** | 185.1648 | 213.1598 | **IVS** | 272.1969 | 300.1918 | **VS** | 159.1128 | 187.1077 |

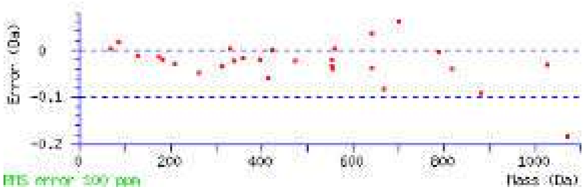


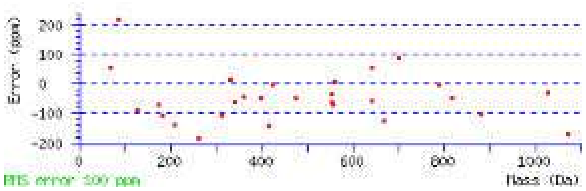


NCBI **BLAST** search of FSPIKESIVSR
(Parameters: blastp, nr protein database, expect=20000, no filter, PAM30)
Other BLAST web gateways

**All matches to this query**

| Score | Mr(calc) | Delta | Sequence |
|---|---|---|---|
| 33.1 | 1261.7030 | 0.0855 | FSPIKESIVSR |
| 14.9 | 1261.6666 | 0.1219 | ALAFASIPETSR |
| 10.1 | 1261.7030 | 0.0855 | IYNPTLSLVSR |
| 8.7 | 1261.6779 | 0.1107 | GRLFVESLEGR |
| 8.6 | 1261.6813 | 0.1073 | MSVIVRTEVGR |
| 8.3 | 1261.6853 | 0.1033 | YRPGSPMLLTK |
| 8.2 | 1261.7030 | 0.0855 | AKELFLSLEGR |
| 8.2 | 1261.6626 | 0.1259 | QNSGSSVTITLR |
| 8.0 | 1261.6666 | 0.1219 | AFSSSPIVGEIR |
| 7.7 | 1261.6779 | 0.1107 | AALHDLPQLER |

**Mascot:** http://www.matrixscience.com/

# MATRIX SCIENCE Mascot Search Results

## Peptide View Spot no 49

MS/MS Fragmentation of **LFNAVAVEDLIVKEGR**
Found in **gi|27261025** in **NCBInr**, putative thiamine biosynthesis protein [Oryza sativa Japonica Group]

Match to Query 28: 1772.097624 from(1773.104900,1+) intensity(0.0000) index(12)
Title: Label: H9, Spot_Id: 228977, Peak_List_Id: 257859, MSMS Job_Run_Id: 24940, Comment:
Data file ppw_H9_138985153400.txt

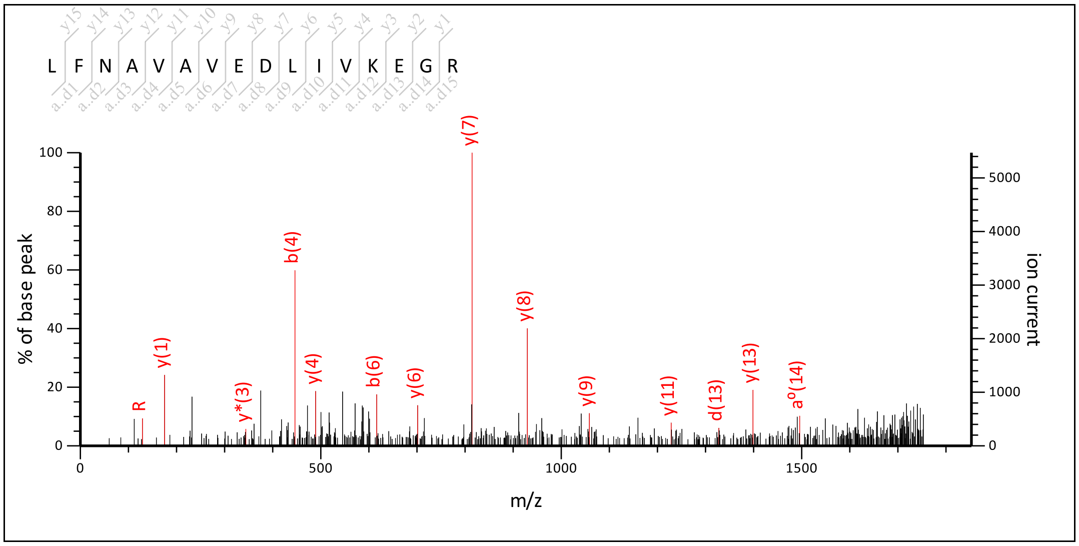


0 to 1852.94

Label all possible matches ○ Label matches used for scoring ◉

**Monoisotopic mass of neutral peptide Mr(calc):** 1771.9832
**Fixed modifications:** Carbamidomethyl (C) (apply to specified residues or termini only)
**Ions Score:** 77 **Expect:** 1.5e-05
**Matches :** 17/283 fragment ions using 17 most intense peaks (help)

| # | Immon. | a | a* | $a^0$ | b | b* | $b^0$ | d | d' | Seq. | v | w | w' | y | y* | [illegible] |
|---|---|---|---|---|---|---|---|---|---|---|---|---|---|---|---|---|
| 1 | 86.0964 | 86.0964 | | | 114.0913 | | | 44.0495 | | L | | | | | | |
| 2 | 120.0808 | 233.1648 | | | 261.1598 | | | | | F | 1567.8438 | | | 1659.9064 | 1642.8799 | [illegible] |
| 3 | 87.0553 | 347.2078 | 330.1812 | | 375.2027 | 358.1761 | | 304.2020 | | N | 1453.8009 | 1452.8057 | | 1512.8380 | 1495.8115 | [illegible] |
| 4 | 44.0495 | 418.2449 | 401.2183 | | **446.2398** | 429.2132 | | | | A | 1382.7638 | | | ***1398.7951*** | 1381.7686 | [illegible] |
| 5 | 72.0808 | 517.3133 | 500.2867 | | 545.3082 | 528.2817 | | 503.2976 | | V | 1283.6954 | 1296.7158 | | ***1327.7580*** | 1310.7314 | [illegible] |
| 6 | 44.0495 | 588.3504 | 571.3239 | | **616.3453** | 599.3188 | | | | A | 1212.6583 | | | ***1228.6896*** | 1211.6630 | [illegible] |
| 7 | 72.0808 | 687.4188 | 670.3923 | | 715.4137 | 698.3872 | | 673.4032 | | V | 1113.5899 | 1126.6103 | | 1157.6525 | 1140.6259 | [illegible] |
| 8 | 102.0550 | 816.4614 | 799.4349 | 798.4508 | 844.4563 | 827.4298 | 826.4458 | 758.4559 | | E | 984.5473 | 983.5520 | | ***1058.5840*** | 1041.5575 | [illegible] |
| 9 | 88.0393 | 931.4884 | 914.4618 | 913.4778 | 959.4833 | 942.4567 | 941.4727 | 887.4985 | | D | 869.5203 | 868.5251 | | ***929.5415*** | 912.5149 | [illegible] |
| 10 | 86.0964 | 1044.5724 | 1027.5459 | 1026.5619 | 1072.5673 | 1055.5408 | 1054.5568 | 1002.5255 | | L | 756.4363 | 755.4410 | | ***814.5145*** | 797.4880 | [illegible] |
| 11 | 86.0964 | 1157.6565 | 1140.6299 | 1139.6459 | 1185.6514 | 1168.6249 | 1167.6408 | 1129.6252 | 1143.6408 | I | 643.3522 | 656.3726 | 670.3883 | ***701.4304*** | 684.4039 | [illegible] |
| 12 | 72.0808 | 1256.7249 | 1239.6984 | 1238.7143 | 1284.7198 | 1267.6933 | 1266.7092 | 1242.7092 | | V | 544.2838 | 557.3042 | | 588.3464 | 571.3198 | [illegible] |
| 13 | 101.1073 | 1384.8199 | 1367.7933 | 1366.8093 | 1412.8148 | 1395.7882 | 1394.8042 | 1327.7620 | | K | 416.1888 | 415.1936 | | ***489.2780*** | 472.2514 | [illegible] |
| 14 | 102.0550 | 1513.8625 | 1496.8359 | 1495.8519 | 1541.8574 | 1524.8308 | 1523.8468 | 1455.8570 | | E | 287.1462 | 286.1510 | | 361.1830 | 344.1565 | [illegible] |
| 15 | 30.0338 | 1570.8839 | 1553.8574 | 1552.8734 | 1598.8788 | 1581.8523 | 1580.8683 | | | G | | | | 232.1404 | 215.1139 | |
| 16 | 129.1135 | | | | | | | | | R | 74.0237 | 73.0284 | | ***175.1190*** | 158.0924 | |

| Seq | ya | yb | Seq | ya | yb | Seq | ya | yb |
|---|---|---|---|---|---|---|---|---|
| FN | 234.1237 | 262.1186 | FNA | 305.1608 | 333.1557 | FNAV | 404.2292 | 432.2241 |
| FNAVA | 475.2663 | 503.2613 | FNAVAV | 574.3348 | 602.3297 | NA | 158.0924 | 186.0873 |
| NAV | 257.1608 | 285.1557 | NAVA | 328.1979 | 356.1928 | NAVAV | 427.2663 | 455.2613 |
| NAVAVE | 556.3089 | 584.3039 | NAVAVED | 671.3359 | 699.3308 | AV | 143.1179 | 171.1128 |

| | | | | | | | | |
|---|---|---|---|---|---|---|---|---|
| **AVA** | 214.1550 | 242.1499 | **AVAV** | 313.2234 | 341.2183 | **AVAVE** | 442.2660 | 470.2609 |
| **AVAVED** | 557.2930 | 585.2879 | **AVAVEDL** | 670.3770 | 698.3719 | **VA** | 143.1179 | 171.1128 |
| **VAV** | 242.1863 | 270.1812 | **VAVE** | 371.2289 | 399.2238 | **VAVED** | 486.2558 | 514.2508 |
| **VAVEDL** | 599.3399 | 627.3348 | **AV** | 143.1179 | 171.1128 | **AVE** | 272.1605 | 300.1554 |
| **AVED** | 387.1874 | 415.1823 | **AVEDL** | 500.2715 | 528.2664 | **AVEDLI** | 613.3556 | 641.3505 |
| **VE** | 201.1234 | 229.1183 | **VED** | 316.1503 | 344.1452 | **VEDL** | 429.2344 | 457.2293 |
| **VEDLI** | 542.3184 | 570.3134 | **VEDLIV** | 641.3869 | 669.3818 | **ED** | 217.0819 | 245.0768 |
| **EDL** | 330.1660 | 358.1609 | **EDLI** | 443.2500 | 471.2449 | **EDLIV** | 542.3184 | 570.3134 |
| **EDLIVK** | 670.4134 | 698.4083 | **DL** | 201.1234 | 229.1183 | **DLI** | 314.2074 | 342.2023 |
| **DLIV** | 413.2758 | 441.2708 | **DLIVK** | 541.3708 | 569.3657 | **DLIVKE** | 670.4134 | 698.4083 |
| **LI** | 199.1805 | 227.1754 | **LIV** | 298.2489 | 326.2438 | **LIVK** | 426.3439 | 454.3388 |
| **LIVKE** | 555.3865 | 583.3814 | **LIVKEG** | 612.4079 | 640.4028 | **IV** | 185.1648 | 213.1598 |
| **IVK** | 313.2598 | 341.2547 | **IVKE** | 442.3024 | 470.2973 | **IVKEG** | 499.3239 | 527.3188 |
| **VK** | 200.1757 | 228.1707 | **VKE** | 329.2183 | 357.2132 | **VKEG** | 386.2398 | 414.2347 |
| **KE** | 230.1499 | 258.1448 | **KEG** | 287.1714 | 315.1663 | **EG** | 159.0764 | 187.0713 |

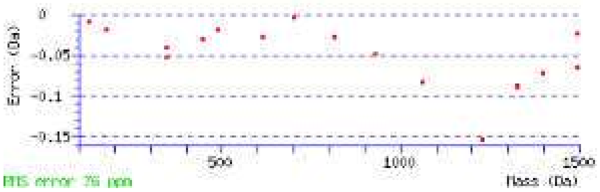


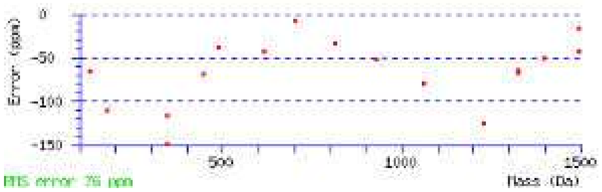


NCBI **BLAST** search of LFNAVAVEDLIVKEGR
(Parameters: blastp, nr protein database, expect=20000, no filter, PAM30)
Other BLAST web gateways

**All matches to this query**

| Score | Mr(calc) | Delta | Sequence |
|---|---|---|---|
| 76.5 | 1771.9832 | 0.1144 | LFNAVAVEDLIVKEGR |
| 19.6 | 1771.9502 | 0.1474 | RSAMLILGSDSPAVVEK |
| 18.4 | 1771.9250 | 0.1726 | EMNLRAELISQLEAR |
| 16.4 | 1771.9581 | 0.1396 | LFNAVAAEDLIIRGDR |
| 16.0 | 1771.9325 | 0.1652 | QVDEVLLVGGMTRMPK |
| 15.9 | 1771.9291 | 0.1685 | MARIGSPEEVVVGVWK |
| 14.0 | 1771.9316 | 0.1660 | DDSKVLLTSPTANGINK |
| 12.7 | 1771.9693 | 0.1283 | NIFIVRAINEGGNTVR |
| 10.8 | 1771.9614 | 0.1362 | SCIIADALITAIREAR |
| 10.8 | 1772.0084 | 0.0892 | LNKILFLDDDIVVQK |

**Mascot:** http://www.matrixscience.com/

# Mascot Search Results

## Peptide View Spot no 49

MS/MS Fragmentation of **VVVSSCGHDGPFGATGVKR**
Found in **gi|27261025** in **NCBInr**, putative thiamine biosynthesis protein [Oryza sativa Japonica Group]

Match to Query 32: 1929.079224 from(1930.086500,1+) intensity(0.0000) index(14)
Title: Label: H9, Spot_Id: 228977, Peak_List_Id: 257872, MSMS Job_Run_Id: 24940, Comment:
Data file ppw_H9_138985153400.txt

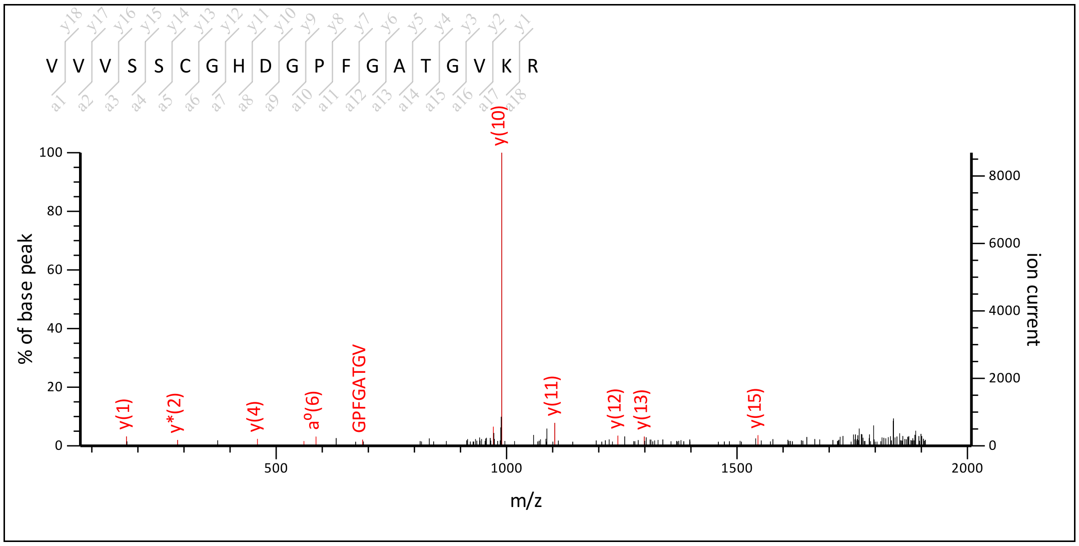


75.12 to 2008.65

Label all possible matches ○ Label matches used for scoring ◉

**Monoisotopic mass of neutral peptide Mr(calc):** 1928.9527
**Fixed modifications:** Carbamidomethyl (C) (apply to specified residues or termini only)
**Ions Score:** 53 **Expect:** 0.0082
**Matches :** 16/333 fragment ions using 18 most intense peaks (help)

| # | Immon. | a | a* | a⁰ | b | b* | b⁰ | d | d' | Seq. | v | w | w' | y | y* | |
|---|---|---|---|---|---|---|---|---|---|---|---|---|---|---|---|---|
| 1 | 72.0808 | 72.0808 | | | 100.0757 | | | 44.0495 | | V | | | | | | |
| 2 | 72.0808 | 171.1492 | | | 199.1441 | | | 157.1335 | | V | 1786.8289 | 1799.8493 | | 1830.8915 | 1813.8650 | [illegible] |
| 3 | 72.0808 | 270.2176 | | | 298.2125 | | | 256.2020 | | V | 1687.7605 | 1700.7809 | | 1731.8231 | 1714.7966 | [illegible] |
| 4 | 60.0444 | 357.2496 | | 339.2391 | 385.2445 | | 367.2340 | 341.2547 | | S | 1600.7285 | 1599.7333 | | 1632.7547 | 1615.7282 | [illegible] |
| 5 | 60.0444 | 444.2817 | | 426.2711 | 472.2766 | | 454.2660 | 428.2867 | | S | 1513.6965 | 1512.7012 | | ***1545.7227*** | 1528.6961 | [illegible] |
| 6 | 133.0430 | 604.3123 | | 586.3017 | 632.3072 | | 614.2967 | 515.3188 | | C | 1353.6658 | 1352.6706 | | 1458.6907 | 1441.6641 | [illegible] |
| 7 | 30.0338 | 661.3338 | | 643.3232 | 689.3287 | | 671.3181 | | | G | | | | ***1298.6600*** | 1281.6335 | [illegible] |
| 8 | 110.0713 | 798.3927 | | 780.3821 | 826.3876 | | 808.3770 | | | H | 1159.5854 | | | ***1241.6385*** | 1224.6120 | [illegible] |
| 9 | 88.0393 | 913.4196 | | 895.4091 | 941.4145 | | 923.4040 | 869.4298 | | D | 1044.5585 | 1043.5633 | | ***1104.5796*** | 1087.5531 | [illegible] |
| 10 | 30.0338 | 970.4411 | | 952.4305 | 998.4360 | | 980.4254 | | | G | | | | ***989.5527*** | 972.5261 | [illegible] |
| 11 | 70.0651 | 1067.4939 | | 1049.4833 | 1095.4888 | | 1077.4782 | 1041.4782 | | P | 890.4843 | 889.4890 | | 932.5312 | 915.5047 | [illegible] |
| 12 | 120.0808 | 1214.5623 | | 1196.5517 | 1242.5572 | | 1224.5466 | | | F | 743.4159 | | | 835.4785 | 818.4519 | [illegible] |
| 13 | 30.0338 | 1271.5837 | | 1253.5732 | 1299.5786 | | 1281.5681 | | | G | | | | 688.4100 | 671.3835 | [illegible] |
| 14 | 44.0495 | 1342.6208 | | 1324.6103 | 1370.6158 | | 1352.6052 | | | A | 615.3573 | | | 631.3886 | 614.3620 | [illegible] |
| 15 | 74.0600 | 1443.6685 | | 1425.6580 | 1471.6634 | | 1453.6529 | 1427.6736 | 1429.6529 | T | 514.3096 | 527.3300 | 529.3093 | ***560.3515*** | 543.3249 | [illegible] |
| 16 | 30.0338 | 1500.6900 | | 1482.6794 | 1528.6849 | | 1510.6743 | | | G | | | | ***459.3038*** | 442.2772 | |
| 17 | 72.0808 | 1599.7584 | | 1581.7478 | 1627.7533 | | 1609.7428 | 1585.7428 | | V | 358.2197 | 371.2401 | | 402.2823 | 385.2558 | |
| 18 | 101.1073 | 1727.8534 | 1710.8268 | 1709.8428 | 1755.8483 | 1738.8217 | 1737.8377 | 1670.7955 | | K | 230.1248 | 229.1295 | | 303.2139 | 286.1874 | |
| 19 | 129.1135 | | | | | | | | | R | 74.0237 | 73.0284 | | ***175.1190*** | 158.0924 | |

| Seq | ya | yb | Seq | ya | yb | Seq | ya | yb |
|---|---|---|---|---|---|---|---|---|
| VV | 171.1492 | 199.1441 | VVS | 258.1812 | 286.1761 | VVSS | 345.2132 | 373.2082 |
| | | | | | | | | |

| | | | | | | | | |
|---|---|---|---|---|---|---|---|---|
| **VVSSC** | 505.2439 | 533.2388 | **VVSSCG** | 562.2654 | 590.2603 | **VVSSCGH** | 699.3243 | 727.3192 |
| **VS** | 159.1128 | 187.1077 | **VSS** | 246.1448 | 274.1397 | **VSSC** | 406.1755 | 434.1704 |
| **VSSCG** | 463.1969 | 491.1919 | **VSSCGH** | 600.2559 | 628.2508 | **SS** | 147.0764 | 175.0713 |
| **SSC** | 307.1071 | 335.1020 | **SSCG** | 364.1285 | 392.1234 | **SSCGH** | 501.1874 | 529.1824 |
| **SSCGHD** | 616.2144 | 644.2093 | **SSCGHDG** | 673.2358 | 701.2308 | **SC** | 220.0750 | 248.0700 |
| **SCG** | 277.0965 | 305.0914 | **SCGH** | 414.1554 | 442.1503 | **SCGHD** | 529.1824 | 557.1773 |
| **SCGHDG** | 586.2038 | 614.1987 | **SCGHDGP** | 683.2566 | 711.2515 | **CG** | 190.0645 | 218.0594 |
| **CGH** | 327.1234 | 355.1183 | **CGHD** | 442.1503 | 470.1452 | **CGHDG** | 499.1718 | 527.1667 |
| **CGHDGP** | 596.2246 | 624.2195 | **GH** | 167.0927 | 195.0877 | **GHD** | 282.1197 | 310.1146 |
| **GHDG** | 339.1411 | 367.1361 | **GHDGP** | 436.1939 | 464.1888 | **GHDGPF** | 583.2623 | 611.2572 |
| **GHDGPFG** | 640.2838 | 668.2787 | **HD** | 225.0982 | 253.0931 | **HDG** | 282.1197 | 310.1146 |
| **HDGP** | 379.1724 | 407.1674 | **HDGPF** | 526.2409 | 554.2358 | **HDGPFG** | 583.2623 | 611.2572 |
| **HDGPFGA** | 654.2994 | 682.2944 | **DG** | 145.0608 | 173.0557 | **DGP** | 242.1135 | 270.1084 |
| **DGPF** | 389.1819 | 417.1769 | **DGPFG** | 446.2034 | 474.1983 | **DGPFGA** | 517.2405 | 545.2354 |
| **DGPFGAT** | 618.2882 | 646.2831 | **DGPFGATG** | 675.3097 | 703.3046 | **GP** | 127.0866 | 155.0815 |
| **GPF** | 274.1550 | 302.1499 | **GPFG** | 331.1765 | 359.1714 | **GPFGA** | 402.2136 | 430.2085 |
| **GPFGAT** | 503.2613 | 531.2562 | **GPFGATG** | 560.2827 | 588.2776 | **GPFGATGV** | 659.3511 | 687.3461 |
| **PF** | 217.1335 | 245.1285 | **PFG** | 274.1550 | 302.1499 | **PFGA** | 345.1921 | 373.1870 |
| **PFGAT** | 446.2398 | 474.2347 | **PFGATG** | 503.2613 | 531.2562 | **PFGATGV** | 602.3297 | 630.3246 |
| **FG** | 177.1022 | 205.0972 | **FGA** | 248.1394 | 276.1343 | **FGAT** | 349.1870 | 377.1819 |
| **FGATG** | 406.2085 | 434.2034 | **FGATGV** | 505.2769 | 533.2718 | **FGATGVK** | 633.3719 | 661.3668 |
| **GA** | 101.0709 | 129.0659 | **GAT** | 202.1186 | 230.1135 | **GATG** | 259.1401 | 287.1350 |
| **GATGV** | 358.2085 | 386.2034 | **GATGVK** | 486.3035 | 514.2984 | **AT** | 145.0972 | 173.0921 |
| **ATG** | 202.1186 | 230.1135 | **ATGV** | 301.1870 | 329.1819 | **ATGVK** | 429.2820 | 457.2769 |
| **TG** | 131.0815 | 159.0764 | **TGV** | 230.1499 | 258.1448 | **TGVK** | 358.2449 | 386.2398 |
| **GV** | 129.1022 | 157.0972 | **GVK** | 257.1972 | 285.1921 | **VK** | 200.1757 | 228.1707 |

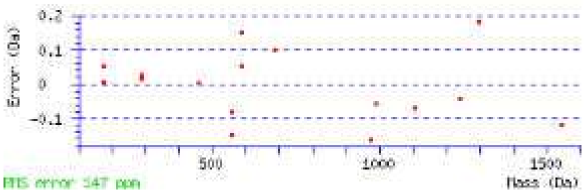


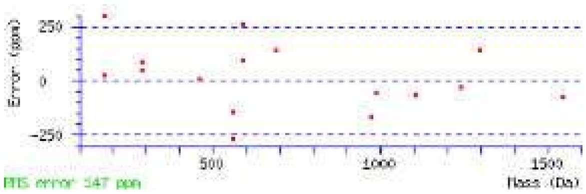


NCBI **BLAST** search of VVVSSCGHDGPFGATGVKR
(Parameters: blastp, nr protein database, expect=20000, no filter, PAM30)
Other BLAST web gateways

**All matches to this query**

| Score | Mr(calc) | Delta | Sequence |
|---|---|---|---|
| 53.0 | 1928.9527 | 0.1265 | VVVSSCGHDGPFGATGVKR |
| 40.5 | 1928.9197 | 0.1595 | VVVSSCGHDGPMGATGVKR |
| 9.3 | 1928.8878 | 0.1915 | FGGNGQQHYTDAPEPRR |
| 6.7 | 1929.0935 | -0.0143 | IPILVYELVSNGSLEKR |
| 6.5 | 1928.9495 | 0.1297 | WVVELSQFDIYFMPR |
| 5.6 | 1929.0068 | 0.0724 | NAGSKHFTVSLLDTGNLR |
| 4.3 | 1928.9884 | 0.0908 | SKETAPVIDVTQFGYFK |
| 3.3 | 1928.9998 | 0.0794 | LCMQRAADALLGICLPK |
| 3.1 | 1928.9052 | 0.1740 | FVEMTSASIAMSFFAFK |
| 3.0 | 1928.9076 | 0.1717 | LAESEQAGDLVVGGEGGEGR |

**Mascot:** http://www.matrixscience.com/

# MATRIX SCIENCE Mascot Search Results

## Peptide View Spot no 49

MS/MS Fragmentation of **EVVPGMIVTGMEVAEIDGAPR**
Found in **gi|27261025** in **NCBInr**, putative thiamine biosynthesis protein [Oryza sativa Japonica Group]

Match to Query 36: 2169.228524 from(2170.235800,1+) intensity(0.0000) index(16)
Title: Label: H9, Spot_Id: 228977, Peak_List_Id: 257865, MSMS Job_Run_Id: 24940, Comment:
Data file ppw_H9_138985153400.txt

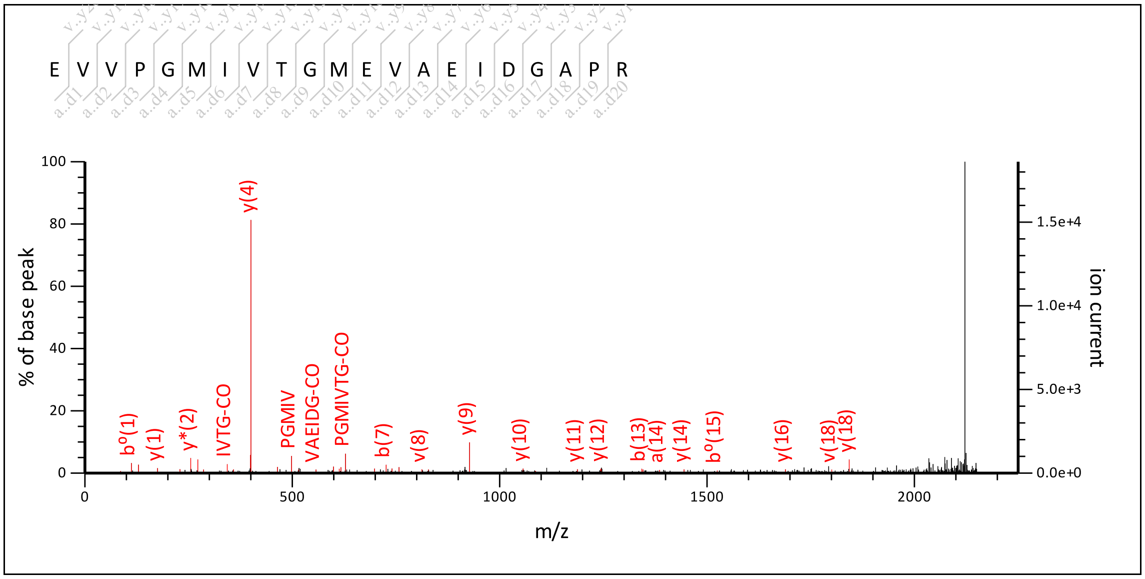


0 to 2250.16

Label all possible matches ○ Label matches used for scoring ◉

**Monoisotopic mass of neutral peptide Mr(calc):** 2169.0810
**Fixed modifications:** Carbamidomethyl (C) (apply to specified residues or termini only)
**Ions Score:** 76 **Expect:** 3.5e-05
**Matches :** 61/386 fragment ions using 105 most intense peaks (help)

| # | Immon. | a | $a^0$ | b | $b^0$ | d | d' | Seq. | v | w | w' | y | y* | $y^0$ | # |
|---|---|---|---|---|---|---|---|---|---|---|---|---|---|---|---|
| 1 | 102.0550 | 102.0550 | 84.0444 | 130.0499 | 112.0393 | 44.0495 | | E | | | | | | | 21 |
| 2 | 72.0808 | 201.1234 | 183.1128 | **229.1183** | 211.1077 | 187.1077 | | V | 1996.9831 | 2010.0035 | | 2041.0457 | 2024.0191 | 2023.0351 | 20 |
| 3 | 72.0808 | 300.1918 | 282.1812 | 328.1867 | 310.1761 | 286.1761 | | V | 1897.9146 | 1910.9350 | | 1941.9772 | 1924.9507 | 1923.9667 | 19 |
| 4 | 70.0651 | 397.2445 | 379.2340 | 425.2395 | 407.2289 | 371.2289 | | P | 1800.8619 | 1799.8666 | | ***1842.9088*** | 1825.8823 | 1824.8983 | 18 |
| 5 | 30.0338 | 454.2660 | 436.2554 | 482.2609 | 464.2504 | | | G | | | | 1745.8561 | 1728.8295 | 1727.8455 | 17 |
| 6 | 104.0528 | 585.3065 | 567.2959 | **613.3014** | 595.2908 | 525.3031 | | M | 1612.7999 | 1611.8047 | | ***1688.8346*** | 1671.8081 | 1670.8240 | 16 |
| 7 | 86.0964 | **698.3906** | 680.3800 | **726.3855** | 708.3749 | 670.3593 | 684.3749 | I | 1499.7159 | 1512.7363 | 1526.7519 | 1557.7941 | 1540.7676 | 1539.7836 | 15 |
| 8 | 72.0808 | 797.4590 | 779.4484 | 825.4539 | 807.4433 | 783.4433 | | V | 1400.6475 | 1413.6679 | | ***1444.7101*** | 1427.6835 | 1426.6995 | 14 |
| 9 | 74.0600 | 898.5067 | 880.4961 | 926.5016 | 908.4910 | 882.5117 | 884.4910 | T | 1299.5998 | 1312.6202 | 1314.5994 | ***1345.6416*** | 1328.6151 | 1327.6311 | 13 |
| 10 | 30.0338 | 955.5281 | 937.5176 | 983.5230 | 965.5125 | | | G | | | | ***1244.5940*** | 1227.5674 | 1226.5834 | 12 |
| 11 | 104.0528 | **1086.5686** | 1068.5580 | 1114.5635 | 1096.5530 | 1026.5652 | | M | 1111.5378 | 1110.5426 | | ***1187.5725*** | 1170.5460 | 1169.5619 | 11 |
| 12 | 102.0550 | 1215.6112 | 1197.6006 | **1243.6061** | 1225.5955 | 1157.6057 | | E | 982.4952 | 981.5000 | | ***1056.5320*** | 1039.5055 | 1038.5215 | 10 |
| 13 | 72.0808 | 1314.6796 | 1296.6690 | **1342.6745** | 1324.6640 | 1300.6640 | | V | 883.4268 | 896.4472 | | ***927.4894*** | 910.4629 | 909.4789 | 9 |
| 14 | 44.0495 | **1385.7167** | 1367.7062 | 1413.7116 | 1395.7011 | | | A | 812.3897 | | | ***828.4210*** | 811.3945 | 810.4104 | 8 |
| 15 | 102.0550 | 1514.7593 | 1496.7488 | 1542.7542 | 1524.7437 | 1456.7538 | | E | 683.3471 | 682.3519 | | ***757.3839*** | 740.3573 | 739.3733 | 7 |
| 16 | 86.0964 | 1627.8434 | 1609.8328 | 1655.8383 | 1637.8277 | 1599.8121 | 1613.8277 | I | 570.2631 | 583.2835 | 597.2991 | ***628.3413*** | 611.3148 | 610.3307 | 6 |
| 17 | 88.0393 | 1742.8703 | 1724.8598 | 1770.8652 | 1752.8547 | 1698.8805 | | D | 455.2361 | 454.2409 | | ***515.2572*** | 498.2307 | 497.2467 | 5 |
| 18 | 30.0338 | 1799.8918 | 1781.8812 | 1827.8867 | 1809.8761 | | | G | | | | ***400.2303*** | 383.2037 | | 4 |
| 19 | 44.0495 | 1870.9289 | 1852.9183 | 1898.9238 | 1880.9133 | | | A | 327.1775 | | | ***343.2088*** | 326.1823 | | 3 |

| | | | | | | | | | | | | | | | |
|---|---|---|---|---|---|---|---|---|---|---|---|---|---|---|---|
| 20 | 70.0651 | 1967.9817 | 1949.9711 | 1995.9766 | 1977.9660 | 1941.9660 | | P | 230.1248 | 229.1295 | | 272.1717 | 255.1452 | | 2 |
| 21 | 129.1135 | | | | | | | R | 74.0237 | 73.0284 | | 175.1190 | 158.0924 | | 1 |

| Seq | ya | yb | Seq | ya | yb | Seq | ya | yb |
|---|---|---|---|---|---|---|---|---|
| VV | 171.1492 | 199.1441 | VVP | 268.2020 | 296.1969 | VVPG | 325.2234 | 353.2183 |
| VVPGM | 456.2639 | 484.2588 | VVPGMI | 569.3480 | 597.3429 | VVPGMIV | 668.4164 | 696.4113 |
| VP | 169.1335 | 197.1285 | VPG | 226.1550 | 254.1499 | VPGM | 357.1955 | 385.1904 |
| VPGMI | 470.2796 | 498.2745 | VPGMIV | 569.3480 | 597.3429 | VPGMIVT | 670.3956 | 698.3906 |
| PG | 127.0866 | 155.0815 | PGM | 258.1271 | 286.1220 | PGMI | 371.2111 | 399.2061 |
| PGMIV | 470.2796 | 498.2745 | PGMIVT | 571.3272 | 599.3221 | PGMIVTG | 628.3487 | 656.3436 |
| GM | 161.0743 | 189.0692 | GMI | 274.1584 | 302.1533 | GMIV | 373.2268 | 401.2217 |
| GMIVT | 474.2745 | 502.2694 | GMIVTG | 531.2959 | 559.2908 | GMIVTGM | 662.3364 | 690.3313 |
| MI | 217.1369 | 245.1318 | MIV | 316.2053 | 344.2002 | MIVT | 417.2530 | 445.2479 |
| MIVTG | 474.2745 | 502.2694 | MIVTGM | 605.3150 | 633.3099 | IV | 185.1648 | 213.1598 |
| IVT | 286.2125 | 314.2074 | IVTG | 343.2340 | 371.2289 | IVTGM | 474.2745 | 502.2694 |
| IVTGME | 603.3171 | 631.3120 | VT | 173.1285 | 201.1234 | VTG | 230.1499 | 258.1448 |
| VTGM | 361.1904 | 389.1853 | VTGME | 490.2330 | 518.2279 | VTGMEV | 589.3014 | 617.2963 |
| VTGMEVA | 660.3385 | 688.3334 | TG | 131.0815 | 159.0764 | TGM | 262.1220 | 290.1169 |
| TGME | 391.1646 | 419.1595 | TGMEV | 490.2330 | 518.2279 | TGMEVA | 561.2701 | 589.2650 |
| TGMEVAE | 690.3127 | 718.3076 | GM | 161.0743 | 189.0692 | GME | 290.1169 | 318.1118 |
| GMEV | 389.1853 | 417.1802 | GMEVA | 460.2224 | 488.2173 | GMEVAE | 589.2650 | 617.2599 |
| ME | 233.0954 | 261.0904 | MEV | 332.1639 | 360.1588 | MEVA | 403.2010 | 431.1959 |
| MEVAE | 532.2436 | 560.2385 | MEVAEI | 645.3276 | 673.3225 | EV | 201.1234 | 229.1183 |
| EVA | 272.1605 | 300.1554 | EVAE | 401.2031 | 429.1980 | EVAEI | 514.2871 | 542.2821 |
| EVAEID | 629.3141 | 657.3090 | EVAEIDG | 686.3355 | 714.3305 | VA | 143.1179 | 171.1128 |
| VAE | 272.1605 | 300.1554 | VAEI | 385.2445 | 413.2395 | VAEID | 500.2715 | 528.2664 |
| VAEIDG | 557.2930 | 585.2879 | VAEIDGA | 628.3301 | 656.3250 | AE | 173.0921 | 201.0870 |
| AEI | 286.1761 | 314.1710 | AEID | 401.2031 | 429.1980 | AEIDG | 458.2245 | 486.2195 |
| AEIDGA | 529.2617 | 557.2566 | AEIDGAP | 626.3144 | 654.3093 | EI | 215.1390 | 243.1339 |
| EID | 330.1660 | 358.1609 | EIDG | 387.1874 | 415.1823 | EIDGA | 458.2245 | 486.2195 |
| EIDGAP | 555.2773 | 583.2722 | ID | 201.1234 | 229.1183 | IDG | 258.1448 | 286.1397 |
| IDGA | 329.1819 | 357.1769 | IDGAP | 426.2347 | 454.2296 | DG | 145.0608 | 173.0557 |
| DGA | 216.0979 | 244.0928 | DGAP | 313.1506 | 341.1456 | GA | 101.0709 | 129.0659 |
| GAP | 198.1237 | 226.1186 | AP | 141.1022 | 169.0972 | | | |

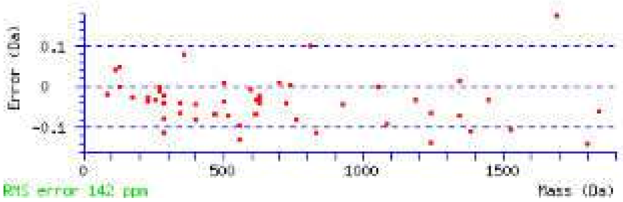


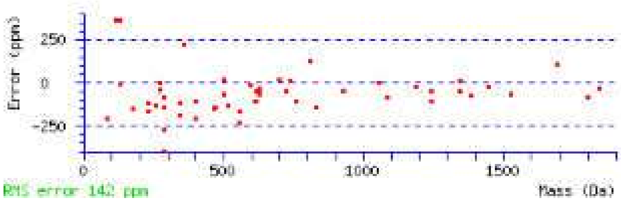


NCBI **BLAST** search of EVVPGMIVTGMEVAEIDGAPR
(Parameters: blastp, nr protein database, expect=20000, no filter, PAM30)
Other BLAST web gateways

**All matches to this query**

| Score | Mr(calc) | Delta | Sequence |
|---|---|---|---|
| 75.9 | 2169.0810 | 0.1475 | EVVPGMIVTGMEVAEIDGAPR |
| 7.0 | 2169.1218 | 0.1067 | GVLEVEIFHGEYNATVKHK |
| 6.1 | 2169.1389 | 0.0896 | NQIVLVAEDTDGSAIAELRR |
| 6.0 | 2169.2330 | -0.0045 | VAEKDLVTVIELLMNELIK |
| 5.1 | 2169.1001 | 0.1284 | FTVTGRLSVFAVMGDLNNGR |

| | | | |
|---|---|---|---|
| 4.6 | 2169.1688 | 0.0597 | SLMSTARASLRPAAPSSTLPR |
| 4.6 | 2169.0161 | 0.2125 | ELPNPTNPMASSSNPTPPFR |
| 4.5 | 2169.1827 | 0.0458 | RLDMLPSLLSENLASLLER |
| 4.4 | 2169.1389 | 0.0896 | SSQAENIEINTGQILQGVIR |
| 4.4 | 2169.1165 | 0.1121 | DADQLPKVLEDGVTAAAIESK |

**Mascot:** http://www.matrixscience.com/

# MATRIX SCIENCE Mascot Search Results

## Peptide View Spot no 49

MS/MS Fragmentation of **EVVPGMIVTGMEVAEIDGAPR**
Found in **gi|27261025** in **NCBInr**, putative thiamine biosynthesis protein [Oryza sativa Japonica Group]

Match to Query 38: 2185.194624 from(2186.201900,1+) intensity(0.0000) index(17)
Title: Label: H9, Spot_Id: 228977, Peak_List_Id: 257876, MSMS Job_Run_Id: 24940, Comment:
Data file ppw_H9_138985153400.txt

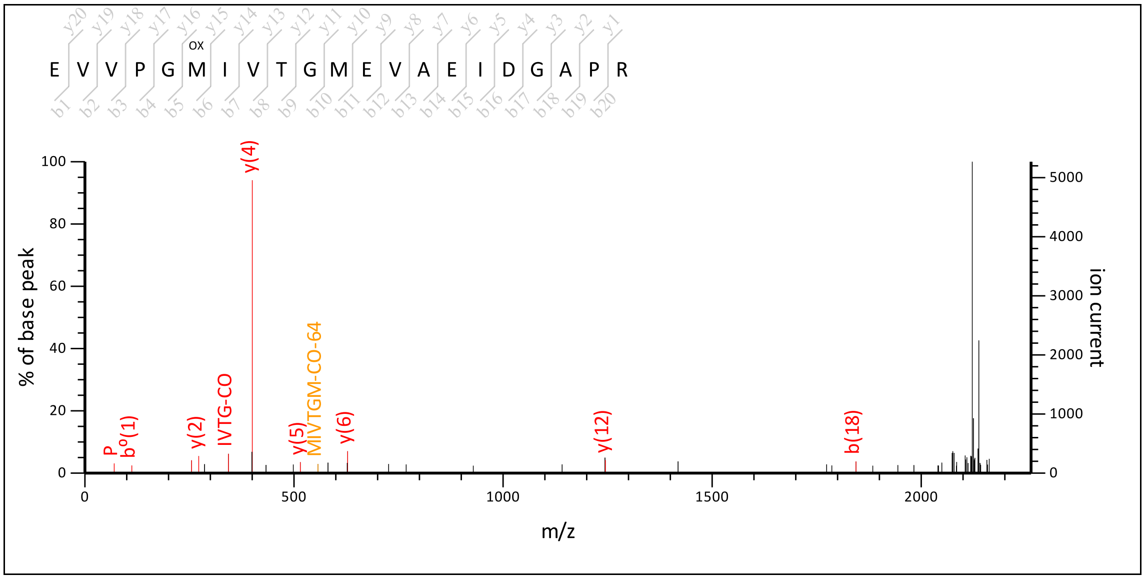


0 to 2262.68

Label all possible matches ○ Label matches used for scoring ◉

**Monoisotopic mass of neutral peptide Mr(calc):** 2185.0759
**Fixed modifications:** Carbamidomethyl (C) (apply to specified residues or termini only)
**Variable modifications:**
**M6** : Oxidation (M), with neutral losses 0.0000(shown in table), 63.9983
**Ions Score:** 21 **Expect:** 11
**Matches :** 18/521 fragment ions using 26 most intense peaks (help)

| # | Immon. | a | $a^0$ | b | $b^0$ | d | d' | Seq. | v | w | w' | y | y* | $y^0$ | # |
|---|---|---|---|---|---|---|---|---|---|---|---|---|---|---|---|
| 1 | 102.0550 | 102.0550 | 84.0444 | 130.0499 | 112.0393 | 44.0495 | | E | | | | | | | 21 |
| 2 | 72.0808 | 201.1234 | 183.1128 | 229.1183 | 211.1077 | 187.1077 | | V | 2012.9780 | 2025.9984 | | 2057.0406 | 2040.0140 | 2039.0300 | 20 |
| 3 | 72.0808 | 300.1918 | 282.1812 | 328.1867 | 310.1761 | 286.1761 | | V | 1913.9096 | 1926.9300 | | 1957.9722 | 1940.9456 | 1939.9616 | 19 |
| 4 | 70.0651 | 397.2445 | 379.2340 | 425.2395 | 407.2289 | 371.2289 | | P | 1816.8568 | 1815.8616 | | 1858.9037 | 1841.8772 | 1840.8932 | 18 |
| 5 | 30.0338 | 454.2660 | 436.2554 | 482.2609 | 464.2504 | | | G | | | | 1761.8510 | 1744.8244 | 1743.8404 | 17 |
| 6 | 120.0478 | 601.3014 | 583.2908 | 629.2963 | 611.2858 | 525.3031 | | M | 1612.7999 | 1611.8047 | | 1704.8295 | 1687.8030 | 1686.8190 | 16 |
| 7 | 86.0964 | 714.3855 | 696.3749 | 742.3804 | 724.3698 | 686.3542 | 700.3698 | I | 1499.7159 | 1512.7363 | 1526.7519 | 1557.7941 | 1540.7676 | 1539.7836 | 15 |
| 8 | 72.0808 | 813.4539 | 795.4433 | 841.4488 | 823.4382 | 799.4382 | | V | 1400.6475 | 1413.6679 | | 1444.7101 | 1427.6835 | 1426.6995 | 14 |
| 9 | 74.0600 | 914.5016 | 896.4910 | 942.4965 | 924.4859 | 898.5067 | 900.4859 | T | 1299.5998 | 1312.6202 | 1314.5994 | 1345.6416 | 1328.6151 | 1327.6311 | 13 |
| 10 | 30.0338 | 971.5230 | 953.5125 | 999.5179 | 981.5074 | | | G | | | | ***1244.5940*** | 1227.5674 | 1226.5834 | 12 |
| 11 | 104.0528 | 1102.5635 | 1084.5530 | 1130.5584 | 1112.5479 | 1042.5601 | | M | 1111.5378 | 1110.5426 | | 1187.5725 | 1170.5460 | 1169.5619 | 11 |
| 12 | 102.0550 | 1231.6061 | 1213.5955 | 1259.6010 | 1241.5905 | 1173.6006 | | E | 982.4952 | 981.5000 | | 1056.5320 | 1039.5055 | 1038.5215 | 10 |
| 13 | 72.0808 | 1330.6745 | 1312.6640 | 1358.6694 | 1340.6589 | 1316.6589 | | V | 883.4268 | 896.4472 | | 927.4894 | 910.4629 | 909.4789 | 9 |
| 14 | 44.0495 | 1401.7116 | 1383.7011 | 1429.7066 | 1411.6960 | | | A | 812.3897 | | | 828.4210 | 811.3945 | 810.4104 | 8 |
| 15 | 102.0550 | 1530.7542 | 1512.7437 | 1558.7491 | 1540.7386 | 1472.7488 | | E | 683.3471 | 682.3519 | | 757.3839 | 740.3573 | 739.3733 | 7 |
| 16 | 86.0964 | 1643.8383 | 1625.8277 | 1671.8332 | 1653.8226 | 1615.8070 | 1629.8226 | I | 570.2631 | 583.2835 | 597.2991 | ***628.3413*** | 611.3148 | 610.3307 | 6 |
| 17 | 88.0393 | 1758.8652 | 1740.8547 | 1786.8602 | 1768.8496 | 1714.8754 | | D | 455.2361 | 454.2409 | | ***515.2572*** | 498.2307 | 497.2467 | 5 |
| 18 | 30.0338 | 1815.8867 | 1797.8761 | 1843.8816 | 1825.8711 | | | G | | | | ***400.2303*** | 383.2037 | | 4 |

| | | | | | | | | | | | | | | | |
|---|---|---|---|---|---|---|---|---|---|---|---|---|---|---|---|
| 19 | 44.0495 | 1886.9238 | 1868.9133 | 1914.9187 | 1896.9082 | | | A | 327.1775 | | | ***343.2088*** | 326.1823 | | 3 |
| 20 | 70.0651 | 1983.9766 | 1965.9660 | 2011.9715 | 1993.9609 | 1957.9609 | | P | 230.1248 | 229.1295 | | ***272.1717*** | 255.1452 | | 2 |
| 21 | 129.1135 | | | | | | | R | 74.0237 | 73.0284 | | 175.1190 | 158.0924 | | 1 |

| Seq | ya | yb | Seq | ya | yb | Seq | ya | yb |
|---|---|---|---|---|---|---|---|---|
| VV | 171.1492 | 199.1441 | VVP | 268.2020 | 296.1969 | VVPG | 325.2234 | 353.2183 |
| VVPGM | 472.2588 | 500.2537 | VVPGMI | 585.3429 | 613.3378 | VVPGMIV | 684.4113 | 712.4062 |
| VP | 169.1335 | 197.1285 | VPG | 226.1550 | 254.1499 | VPGM | 373.1904 | 401.1853 |
| VPGMI | 486.2745 | 514.2694 | VPGMIV | 585.3429 | 613.3378 | VPGMIVT | 686.3906 | 714.3855 |
| PG | 127.0866 | 155.0815 | PGM | 274.1220 | 302.1169 | PGMI | 387.2061 | 415.2010 |
| PGMIV | 486.2745 | 514.2694 | PGMIVT | 587.3221 | 615.3171 | PGMIVTG | 644.3436 | 672.3385 |
| GM | 177.0692 | 205.0641 | GMI | 290.1533 | 318.1482 | GMIV | 389.2217 | 417.2166 |
| GMIVT | 490.2694 | 518.2643 | GMIVTG | 547.2908 | 575.2858 | GMIVTGM | 678.3313 | 706.3262 |
| MI | 233.1318 | 261.1267 | MIV | 332.2002 | 360.1952 | MIVT | 433.2479 | 461.2428 |
| MIVTG | 490.2694 | 518.2643 | MIVTGM | 621.3099 | 649.3048 | IV | 185.1648 | 213.1598 |
| IVT | 286.2125 | 314.2074 | IVTG | 343.2340 | 371.2289 | IVTGM | 474.2745 | 502.2694 |
| IVTGME | 603.3171 | 631.3120 | VT | 173.1285 | 201.1234 | VTG | 230.1499 | 258.1448 |
| VTGM | 361.1904 | 389.1853 | VTGME | 490.2330 | 518.2279 | VTGMEV | 589.3014 | 617.2963 |
| VTGMEVA | 660.3385 | 688.3334 | TG | 131.0815 | 159.0764 | TGM | 262.1220 | 290.1169 |
| TGME | 391.1646 | 419.1595 | TGMEV | 490.2330 | 518.2279 | TGMEVA | 561.2701 | 589.2650 |
| TGMEVAE | 690.3127 | 718.3076 | GM | 161.0743 | 189.0692 | GME | 290.1169 | 318.1118 |
| GMEV | 389.1853 | 417.1802 | GMEVA | 460.2224 | 488.2173 | GMEVAE | 589.2650 | 617.2599 |
| ME | 233.0954 | 261.0904 | MEV | 332.1639 | 360.1588 | MEVA | 403.2010 | 431.1959 |
| MEVAE | 532.2436 | 560.2385 | MEVAEI | 645.3276 | 673.3225 | EV | 201.1234 | 229.1183 |
| EVA | 272.1605 | 300.1554 | EVAE | 401.2031 | 429.1980 | EVAEI | 514.2871 | 542.2821 |
| EVAEID | 629.3141 | 657.3090 | EVAEIDG | 686.3355 | 714.3305 | VA | 143.1179 | 171.1128 |
| VAE | 272.1605 | 300.1554 | VAEI | 385.2445 | 413.2395 | VAEID | 500.2715 | 528.2664 |
| VAEIDG | 557.2930 | 585.2879 | VAEIDGA | 628.3301 | 656.3250 | AE | 173.0921 | 201.0870 |
| AEI | 286.1761 | 314.1710 | AEID | 401.2031 | 429.1980 | AEIDG | 458.2245 | 486.2195 |
| AEIDGA | 529.2617 | 557.2566 | AEIDGAP | 626.3144 | 654.3093 | EI | 215.1390 | 243.1339 |
| EID | 330.1660 | 358.1609 | EIDG | 387.1874 | 415.1823 | EIDGA | 458.2245 | 486.2195 |
| EIDGAP | 555.2773 | 583.2722 | ID | 201.1234 | 229.1183 | IDG | 258.1448 | 286.1397 |
| IDGA | 329.1819 | 357.1769 | IDGAP | 426.2347 | 454.2296 | DG | 145.0608 | 173.0557 |
| DGA | 216.0979 | 244.0928 | DGAP | 313.1506 | 341.1456 | GA | 101.0709 | 129.0659 |
| GAP | 198.1237 | 226.1186 | AP | 141.1022 | 169.0972 | | | |

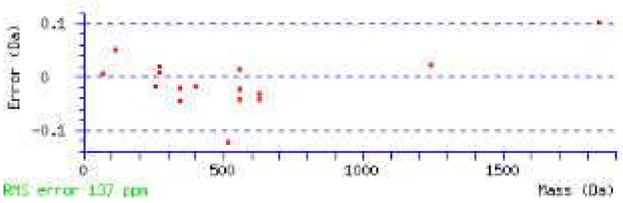


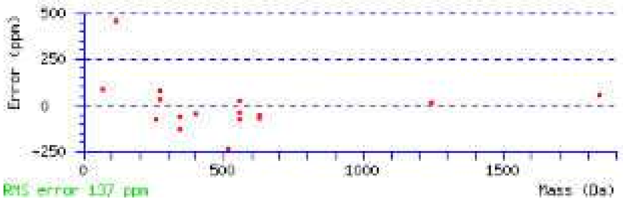


NCBI **BLAST** search of EVVPGMIVTGMEVAEIDGAPR
(Parameters: blastp, nr protein database, expect=20000, no filter, PAM30)
Other BLAST web gateways

**All matches to this query**

| Score | Mr(calc) | Delta | Sequence |
|---|---|---|---|
| 21.5 | 2185.0759 | 0.1187 | EVVPGMIVTGMEVAEIDGAPR |
| 20.4 | 2185.0759 | 0.1187 | EVVPGMIVTGMEVAEIDGAPR |
| 12.8 | 2185.0345 | 0.1601 | FMAVPHYTYIMMKMPGPR |
| 11.8 | 2185.0345 | 0.1601 | FMAVPHYTYMIMKMPGPR |
| 11.6 | 2185.0797 | 0.1149 | GDLNNNQVGEAMERVLALDK |

| | | | |
|---|---|---|---|
| 10.1 | 2185.1703 | 0.0243 | VSVGVGGVLTNNNGGISDGITKK |
| 10.1 | 2185.1703 | 0.0243 | VSVGVGGVLTNNNGGLSDGITKK |
| 9.9 | 2185.1242 | 0.0705 | VWLNPTGTIEMLLWQGPSK |
| 9.4 | 2185.1024 | 0.0923 | SLLNLMGVKDNSPMFAPAHK |
| 9.3 | 2185.0838 | 0.1109 | DGILGTAGAPIHMVAASEGGSFK |

**Mascot:** http://www.matrixscience.com/

# MATRIX SCIENCE Mascot Search Results

## Peptide View **Spot no 16**

MS/MS Fragmentation of **SDVVIETHKEDDLI**
Found in **gi|18698985** in **NCBInr**, anti-disease protein 1 [Oryza sativa]

Match to Query 11: 1611.876224 from(1612.883500,1+) intensity(0.0000) index(5)
Title: Label: L7, Spot_Id: 219781, Peak_List_Id: 226463, MSMS Job_Run_Id: 21845, Comment:
Data file C:\Documents and Settings\Administrator\Desktop\Farah-Garima 11May 2013\ppw_L7_136859918200.txt

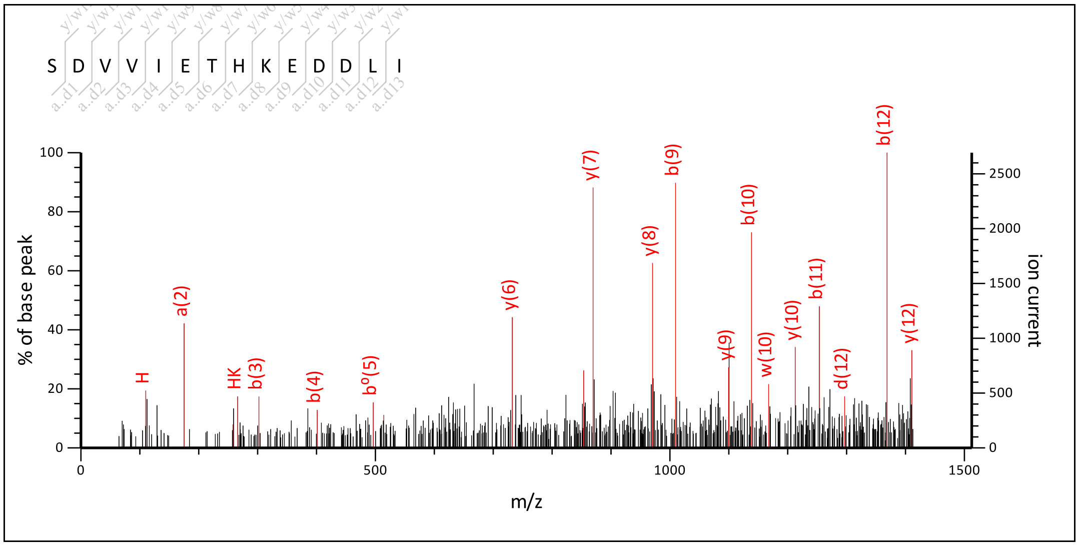


0 to 1512.48

Label all possible matches Label matches used for scoring

**Monoisotopic mass of neutral peptide Mr(calc):** 1611.7992
**Fixed modifications:** Carbamidomethyl (C) (apply to specified residues or termini only)
**Ions Score:** 45 **Expect:** 0.99
**Matches :** 24/231 fragment ions using 42 most intense peaks (help)

| # | Immon. | a | a* | $a^0$ | b | b* | $b^0$ | d | d' | Seq. | v | w | w' | y | y* | |
|---|---|---|---|---|---|---|---|---|---|---|---|---|---|---|---|---|
| 1 | 60.0444 | 60.0444 | | 42.0338 | 88.0393 | | 70.0287 | 44.0495 | | S | | | | | | |
| 2 | 88.0393 | 175.0713 | | 157.0608 | 203.0662 | | 185.0557 | 131.0815 | | D | 1465.7533 | 1464.7581 | | 1525.7744 | 1508.7479 | 15[illegible] |
| 3 | 72.0808 | 274.1397 | | 256.1292 | *302.1347* | | 284.1241 | 260.1241 | | V | 1366.6849 | 1379.7053 | | *1410.7475* | 1393.7209 | 13[illegible] |
| 4 | 72.0808 | 373.2082 | | 355.1976 | *401.2031* | | 383.1925 | 359.1925 | | V | 1267.6165 | 1280.6369 | | 1311.6791 | 1294.6525 | 12[illegible] |
| 5 | 86.0964 | 486.2922 | | 468.2817 | *514.2871* | | 496.2766 | 458.2609 | 472.2766 | I | 1154.5324 | 1167.5528 | 1181.5685 | *1212.6107* | 1195.5841 | 11[illegible] |
| 6 | 102.0550 | 615.3348 | | 597.3243 | 643.3297 | | 625.3192 | 557.3293 | | E | 1025.4898 | 1024.4946 | | *1099.5266* | 1082.5000 | 10[illegible] |
| 7 | 74.0600 | 716.3825 | | 698.3719 | 744.3774 | | 726.3668 | 700.3876 | 702.3668 | T | 924.4421 | 937.4625 | 939.4418 | *970.4840* | 953.4575 | 9[illegible] |
| 8 | 110.0713 | 853.4414 | | 835.4308 | 881.4363 | | 863.4258 | | | H | 787.3832 | | | *869.4363* | 852.4098 | 8[illegible] |
| 9 | 101.1073 | 981.5364 | 964.5098 | 963.5258 | *1009.5313* | 992.5047 | 991.5207 | 924.4785 | | K | 659.2883 | 658.2930 | | *732.3774* | 715.3509 | 7[illegible] |
| 10 | 102.0550 | 1110.5790 | 1093.5524 | 1092.5684 | *1138.5739* | 1121.5473 | 1120.5633 | 1052.5735 | | E | 530.2457 | 529.2504 | | 604.2824 | | 5[illegible] |
| 11 | 88.0393 | 1225.6059 | 1208.5794 | 1207.5953 | *1253.6008* | 1236.5743 | 1235.5903 | 1181.6161 | | D | 415.2187 | 414.2235 | | 475.2399 | | 4[illegible] |
| 12 | 88.0393 | 1340.6329 | 1323.6063 | 1322.6223 | *1368.6278* | 1351.6012 | 1350.6172 | 1296.6430 | | D | 300.1918 | 299.1965 | | 360.2129 | | 3[illegible] |
| 13 | 86.0964 | 1453.7169 | 1436.6904 | 1435.7063 | 1481.7118 | 1464.6853 | 1463.7013 | 1411.6700 | | L | 187.1077 | 186.1125 | | 245.1860 | | |
| 14 | 86.0964 | | | | | | | | | I | 74.0237 | 73.0284 | | 132.1019 | | |

| Seq | ya | yb | Seq | ya | yb | Seq | ya | yb |
|---|---|---|---|---|---|---|---|---|
| DV | 187.1077 | 215.1026 | DVV | 286.1761 | 314.1710 | DVVI | 399.2602 | 427.2551 |
| DVVIE | 528.3028 | 556.2977 | DVVIET | 629.3505 | 657.3454 | VV | 171.1492 | 199.1441 |
| VVI | 284.2333 | 312.2282 | VVIE | 413.2758 | 441.2708 | VVIET | 514.3235 | 542.3184 |
| VVIETH | 651.3824 | 679.3774 | VI | 185.1648 | 213.1598 | VIE | 314.2074 | 342.2023 |
| VIET | 415.2551 | 443.2500 | VIETH | 552.3140 | 580.3089 | VIETHK | 680.4090 | 708.4039 |
| IE | 215.1390 | 243.1339 | IET | 316.1867 | 344.1816 | IETH | 453.2456 | 481.2405 |
| IETHK | 581.3406 | 609.3355 | ET | 203.1026 | 231.0975 | ETH | 340.1615 | 368.1565 |

| | | | | | | | | |
|---|---|---|---|---|---|---|---|---|
| **ETHK** | 468.2565 | 496.2514 | **ETHKE** | 597.2991 | 625.2940 | **TH** | 211.1190 | 239.1139 |
| **THK** | 339.2139 | 367.2088 | **THKE** | 468.2565 | 496.2514 | **THKED** | 583.2835 | 611.2784 |
| **THKEDD** | 698.3104 | 726.3053 | **HK** | 238.1662 | 266.1612 | **HKE** | 367.2088 | 395.2037 |
| **HKED** | 482.2358 | 510.2307 | **HKEDD** | 597.2627 | 625.2576 | **KE** | 230.1499 | 258.1448 |
| **KED** | 345.1769 | 373.1718 | **KEDD** | 460.2038 | 488.1987 | **KEDDL** | 573.2879 | 601.2828 |
| **ED** | 217.0819 | 245.0768 | **EDD** | 332.1088 | 360.1038 | **EDDL** | 445.1929 | 473.1878 |
| **DD** | 203.0662 | 231.0612 | **DDL** | 316.1503 | 344.1452 | **DL** | 201.1234 | 229.1183 |

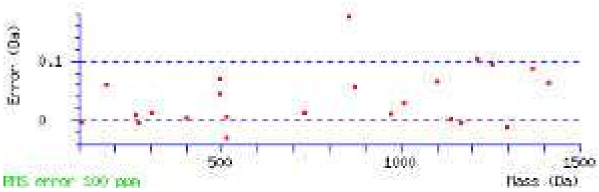


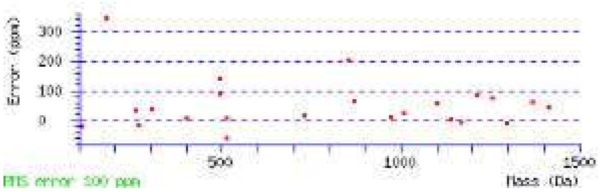


NCBI **BLAST** search of SDVVIETHKEDDLI
(Parameters: blastp, nr protein database, expect=20000, no filter, PAM30)
Other BLAST web gateways

**All matches to this query**

| Score | Mr(calc) | Delta | Sequence |
|---|---|---|---|
| 44.6 | 1611.7992 | 0.0770 | SDVVIETHKEDDLI |
| 27.9 | 1611.8216 | 0.0546 | AEVTIEGTTSAGALHR |
| 20.9 | 1611.8580 | 0.0182 | NNATLLDGSTGALLPR |
| 20.6 | 1611.8733 | 0.0029 | ERVLAAFPGLGETPR |
| 18.6 | 1611.8481 | 0.0281 | RQSDTGVHYALPLR |
| 18.4 | 1611.7781 | 0.0982 | DEDDVLGSFFSLIR |
| 18.3 | 1611.8766 | -0.0004 | AMPLTAGQEVKALQR |
| 17.6 | 1611.7311 | 0.1451 | GEILEHNPMASENR |
| 17.5 | 1611.8832 | -0.0069 | LDATIETLPDLQKR |
| 17.4 | 1611.9195 | -0.0433 | ELGITEDKVNIILR |

**Mascot:** http://www.matrixscience.com/

# MATRIX SCIENCE Mascot Search Results

## Peptide View Spot no 16

MS/MS Fragmentation of **VVSGEIDQSDQSFLDDDQVAAGWVLTCHAYPK**
Found in **gi|18698985** in **NCBInr**, anti-disease protein 1 [Oryza sativa]

Match to Query 19: 3549.797124 from(3550.804400,1+) intensity(0.0000) index(9)
Title: Label: L7, Spot_Id: 219781, Peak_List_Id: 226457, MSMS Job_Run_Id: 21845, Comment:
Data file C:\Documents and Settings\Administrator\Desktop\Farah-Garima 11May 2013\ppw_L7_136859918200.txt

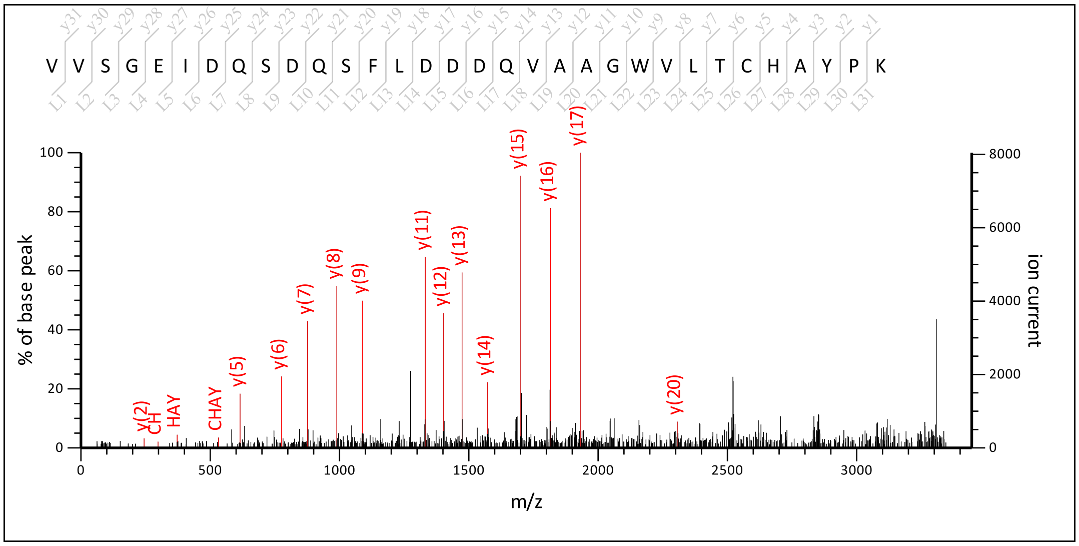


0 to 3444.93

Label all possible matches ○ Label matches used for scoring ◉

**Monoisotopic mass of neutral peptide Mr(calc):** 3549.6359
**Fixed modifications:** Carbamidomethyl (C) (apply to specified residues or termini only)
**Ions Score:** 78 **Expect:** 0.00017
**Matches :** 22/632 fragment ions using 33 most intense peaks (help)

| # | Immon. | a | a* | a⁰ | b | b* | b⁰ | d | d' | Seq. | v | w | w' | y | y* | |
|---|---|---|---|---|---|---|---|---|---|---|---|---|---|---|---|---|
| 1 | 72.0808 | 72.0808 | | | 100.0757 | | | 44.0495 | | V | | | | | | |
| 2 | 72.0808 | 171.1492 | | | 199.1441 | | | 157.1335 | | V | 3407.5121 | 3420.5325 | | 3451.5747 | 3434.5481 | 34 |
| 3 | 60.0444 | 258.1812 | | 240.1707 | 286.1761 | | 268.1656 | 242.1863 | | S | 3320.4801 | 3319.4848 | | 3352.5063 | 3335.4797 | 33 |
| 4 | 30.0338 | 315.2027 | | 297.1921 | 343.1976 | | 325.1870 | | | G | | | | 3265.4743 | 3248.4477 | 32 |
| 5 | 102.0550 | 444.2453 | | 426.2347 | 472.2402 | | 454.2296 | 386.2398 | | E | 3134.4160 | 3133.4208 | | 3208.4528 | 3191.4262 | 31 |
| 6 | 86.0964 | 557.3293 | | 539.3188 | 585.3243 | | 567.3137 | 529.2980 | 543.3137 | I | 3021.3319 | 3034.3523 | 3048.3680 | 3079.4102 | 3062.3836 | 30 |
| 7 | 88.0393 | 672.3563 | | 654.3457 | 700.3512 | | 682.3406 | 628.3665 | | D | 2906.3050 | 2905.3098 | | 2966.3261 | 2949.2996 | 29 |
| 8 | 101.0709 | 800.4149 | 783.3883 | 782.4043 | 828.4098 | 811.3832 | 810.3992 | 743.3934 | | Q | 2778.2464 | 2777.2512 | | 2851.2992 | 2834.2726 | 28 |
| 9 | 60.0444 | 887.4469 | 870.4203 | 869.4363 | 915.4418 | 898.4153 | 897.4312 | 871.4520 | | S | 2691.2144 | 2690.2191 | | 2723.2406 | 2706.2141 | 27 |
| 10 | 88.0393 | 1002.4738 | 985.4473 | 984.4633 | 1030.4687 | 1013.4422 | 1012.4582 | 958.4840 | | D | 2576.1875 | 2575.1922 | | 2636.2086 | 2619.1820 | 26 |
| 11 | 101.0709 | 1130.5324 | 1113.5059 | 1112.5218 | 1158.5273 | 1141.5008 | 1140.5168 | 1073.5109 | | Q | 2448.1289 | 2447.1336 | | 2521.1816 | 2504.1551 | 25 |
| 12 | 60.0444 | 1217.5644 | 1200.5379 | 1199.5539 | 1245.5594 | 1228.5328 | 1227.5488 | 1201.5695 | | S | 2361.0968 | 2360.1016 | | 2393.1231 | 2376.0965 | 23 |
| 13 | 120.0808 | 1364.6329 | 1347.6063 | 1346.6223 | 1392.6278 | 1375.6012 | 1374.6172 | | | F | 2214.0284 | | | ***2306.0910*** | 2289.0645 | 22 |
| 14 | 86.0964 | 1477.7169 | 1460.6904 | 1459.7063 | 1505.7118 | 1488.6853 | 1487.7013 | 1435.6700 | | L | 2100.9444 | 2099.9491 | | 2159.0226 | 2141.9961 | 21 |
| 15 | 88.0393 | 1592.7439 | 1575.7173 | 1574.7333 | 1620.7388 | 1603.7122 | 1602.7282 | 1548.7540 | | D | 1985.9174 | 1984.9222 | | 2045.9386 | 2028.9120 | 20 |
| 16 | 88.0393 | 1707.7708 | 1690.7443 | 1689.7602 | 1735.7657 | 1718.7392 | 1717.7552 | 1663.7810 | | D | 1870.8905 | 1869.8952 | | ***1930.9116*** | 1913.8851 | 19 |
| 17 | 88.0393 | 1822.7977 | 1805.7712 | 1804.7872 | 1850.7927 | 1833.7661 | 1832.7821 | 1778.8079 | | D | 1755.8635 | 1754.8683 | | ***1815.8847*** | 1798.8581 | 17 |
| 18 | 101.0709 | 1950.8563 | 1933.8298 | 1932.8458 | 1978.8512 | 1961.8247 | 1960.8407 | 1893.8349 | | Q | 1627.8050 | 1626.8097 | | ***1700.8577*** | 1683.8312 | 16 |
| 19 | 72.0808 | 2049.9247 | 2032.8982 | 2031.9142 | 2077.9196 | 2060.8931 | 2059.9091 | 2035.9091 | | V | 1528.7365 | 1541.7569 | | ***1572.7991*** | 1555.7726 | 15 |
| 20 | 44.0495 | 2120.9618 | 2103.9353 | 2102.9513 | 2148.9568 | 2131.9302 | 2130.9462 | | | A | 1457.6994 | | | ***1473.7307*** | 1456.7042 | 14 |
| 21 | 44.0495 | 2191.9990 | 2174.9724 | 2173.9884 | 2219.9939 | 2202.9673 | 2201.9833 | | | A | 1386.6623 | | | ***1402.6936*** | 1385.6671 | 13 |
| 22 | 30.0338 | 2249.0204 | 2231.9939 | 2231.0099 | 2277.0153 | 2259.9888 | 2259.0048 | | | G | | | | ***1331.6565*** | 1314.6300 | 13 |
| 23 | 159.0917 | 2435.0997 | 2418.0732 | 2417.0892 | 2463.0947 | 2446.0681 | 2445.0841 | | | W | 1143.5615 | | | 1274.6350 | 1257.6085 | 12 |

| | | | | | | | | | | | | | | | | |
|---|---|---|---|---|---|---|---|---|---|---|---|---|---|---|---|---|
| 24 | 72.0808 | 2534.1682 | 2517.1416 | 2516.1576 | 2562.1631 | 2545.1365 | 2544.1525 | 2520.1525 | | V | 1044.4931 | 1057.5135 | | ***1088.5557*** | 1071.5292 | [illegible] |
| 25 | 86.0964 | 2647.2522 | 2630.2257 | 2629.2417 | 2675.2471 | 2658.2206 | 2657.2366 | 2605.2053 | | L | 931.4091 | 930.4138 | | ***989.4873*** | 972.4608 | [illegible] |
| 26 | 74.0600 | 2748.2999 | 2731.2733 | 2730.2893 | 2776.2948 | 2759.2683 | 2758.2842 | 2732.3050 | 2734.2842 | T | 830.3614 | 843.3818 | 845.3611 | ***876.4033*** | 859.3767 | [illegible] |
| 27 | 133.0430 | 2908.3305 | 2891.3040 | 2890.3200 | 2936.3255 | 2919.2989 | 2918.3149 | 2819.3370 | | C | 670.3307 | 669.3355 | | ***775.3556*** | 758.3290 | |
| 28 | 110.0713 | 3045.3895 | 3028.3629 | 3027.3789 | 3073.3844 | 3056.3578 | 3055.3738 | | | H | 533.2718 | | | ***615.3249*** | 598.2984 | |
| 29 | 44.0495 | 3116.4266 | 3099.4000 | 3098.4160 | 3144.4215 | 3127.3949 | 3126.4109 | | | A | 462.2347 | | | 478.2660 | 461.2395 | |
| 30 | 136.0757 | 3279.4899 | 3262.4634 | 3261.4793 | 3307.4848 | 3290.4583 | 3289.4743 | | | Y | 299.1714 | | | 407.2289 | 390.2023 | |
| 31 | 70.0651 | 3376.5427 | 3359.5161 | 3358.5321 | 3404.5376 | 3387.5110 | 3386.5270 | 3350.5270 | | P | 202.1186 | 201.1234 | | ***244.1656*** | 227.1390 | |
| 32 | 101.1073 | | | | | | | | | K | 74.0237 | 73.0284 | | 147.1128 | 130.0863 | |

| Seq | ya | yb | Seq | ya | yb | Seq | ya | yb |
|---|---|---|---|---|---|---|---|---|
| VS | 159.1128 | 187.1077 | VSG | 216.1343 | 244.1292 | VSGE | 345.1769 | 373.1718 |
| VSGEI | 458.2609 | 486.2558 | VSGEID | 573.2879 | 601.2828 | SG | 117.0659 | 145.0608 |
| SGE | 246.1084 | 274.1034 | SGEI | 359.1925 | 387.1874 | SGEID | 474.2195 | 502.2144 |
| SGEIDQ | 602.2780 | 630.2729 | SGEIDQS | 689.3101 | 717.3050 | GE | 159.0764 | 187.0713 |
| GEI | 272.1605 | 300.1554 | GEID | 387.1874 | 415.1823 | GEIDQ | 515.2460 | 543.2409 |
| GEIDQS | 602.2780 | 630.2729 | EI | 215.1390 | 243.1339 | EID | 330.1660 | 358.1609 |
| EIDQ | 458.2245 | 486.2195 | EIDQS | 545.2566 | 573.2515 | EIDQSD | 660.2835 | 688.2784 |
| ID | 201.1234 | 229.1183 | IDQ | 329.1819 | 357.1769 | IDQS | 416.2140 | 444.2089 |
| IDQSD | 531.2409 | 559.2358 | IDQSDQ | 659.2995 | 687.2944 | DQ | 216.0979 | 244.0928 |
| DQS | 303.1299 | 331.1248 | DQSD | 418.1569 | 446.1518 | DQSDQ | 546.2154 | 574.2103 |
| DQSDQS | 633.2475 | 661.2424 | QS | 188.1030 | 216.0979 | QSD | 303.1299 | 331.1248 |
| QSDQ | 431.1885 | 459.1834 | QSDQS | 518.2205 | 546.2154 | QSDQSF | 665.2889 | 693.2838 |
| SD | 175.0713 | 203.0662 | SDQ | 303.1299 | 331.1248 | SDQS | 390.1619 | 418.1569 |
| SDQSF | 537.2304 | 565.2253 | SDQSFL | 650.3144 | 678.3093 | DQ | 216.0979 | 244.0928 |
| DQS | 303.1299 | 331.1248 | DQSF | 450.1983 | 478.1932 | DQSFL | 563.2824 | 591.2773 |
| DQSFLD | 678.3093 | 706.3042 | QS | 188.1030 | 216.0979 | QSF | 335.1714 | 363.1663 |
| QSFL | 448.2554 | 476.2504 | QSFLD | 563.2824 | 591.2773 | QSFLDD | 678.3093 | 706.3042 |
| SF | 207.1128 | 235.1077 | SFL | 320.1969 | 348.1918 | SFLD | 435.2238 | 463.2187 |
| SFLDD | 550.2508 | 578.2457 | SFLDDD | 665.2777 | 693.2726 | FL | 233.1648 | 261.1598 |
| FLD | 348.1918 | 376.1867 | FLDD | 463.2187 | 491.2136 | FLDDD | 578.2457 | 606.2406 |
| LD | 201.1234 | 229.1183 | LDD | 316.1503 | 344.1452 | LDDD | 431.1773 | 459.1722 |
| LDDDQ | 559.2358 | 587.2307 | LDDDQV | 658.3042 | 686.2992 | DD | 203.0662 | 231.0612 |
| DDD | 318.0932 | 346.0881 | DDDQ | 446.1518 | 474.1467 | DDDQV | 545.2202 | 573.2151 |
| DDDQVA | 616.2573 | 644.2522 | DDDQVAA | 687.2944 | 715.2893 | DD | 203.0662 | 231.0612 |
| DDQ | 331.1248 | 359.1197 | DDQV | 430.1932 | 458.1882 | DDQVA | 501.2304 | 529.2253 |
| DDQVAA | 572.2675 | 600.2624 | DDQVAAG | 629.2889 | 657.2838 | DQ | 216.0979 | 244.0928 |
| DQV | 315.1663 | 343.1612 | DQVA | 386.2034 | 414.1983 | DQVAA | 457.2405 | 485.2354 |
| DQVAAG | 514.2620 | 542.2569 | QV | 200.1394 | 228.1343 | QVA | 271.1765 | 299.1714 |
| QVAA | 342.2136 | 370.2085 | QVAAG | 399.2350 | 427.2300 | QVAAGW | 585.3144 | 613.3093 |
| QVAAGWV | 684.3828 | 712.3777 | VA | 143.1179 | 171.1128 | VAA | 214.1550 | 242.1499 |
| VAAG | 271.1765 | 299.1714 | VAAGW | 457.2558 | 485.2507 | VAAGWV | 556.3242 | 584.3191 |
| VAAGWVL | 669.4083 | 697.4032 | AA | 115.0866 | 143.0815 | AAG | 172.1081 | 200.1030 |
| AAGW | 358.1874 | 386.1823 | AAGWV | 457.2558 | 485.2507 | AAGWVL | 570.3398 | 598.3348 |
| AAGWVLT | 671.3875 | 699.3824 | AG | 101.0709 | 129.0659 | AGW | 287.1503 | 315.1452 |
| AGWV | 386.2187 | 414.2136 | AGWVL | 499.3027 | 527.2976 | AGWVLT | 600.3504 | 628.3453 |
| GW | 216.1131 | 244.1081 | GWV | 315.1816 | 343.1765 | GWVL | 428.2656 | 456.2605 |
| GWVLT | 529.3133 | 557.3082 | GWVLTC | 689.3439 | 717.3389 | WV | 258.1601 | 286.1550 |
| WVL | 371.2442 | 399.2391 | WVLT | 472.2918 | 500.2867 | WVLTC | 632.3225 | 660.3174 |
| VL | 185.1648 | 213.1598 | VLT | 286.2125 | 314.2074 | VLTC | 446.2432 | 474.2381 |
| VLTCH | 583.3021 | 611.2970 | VLTCHA | 654.3392 | 682.3341 | LT | 187.1441 | 215.1390 |
| LTC | 347.1748 | 375.1697 | LTCH | 484.2337 | 512.2286 | LTCHA | 555.2708 | 583.2657 |
| TC | 234.0907 | 262.0856 | TCH | 371.1496 | 399.1445 | TCHA | 442.1867 | 470.1816 |
| TCHAY | 605.2500 | 633.2450 | CH | 270.1019 | 298.0968 | CHA | 341.1390 | 369.1340 |
| CHAY | 504.2024 | 532.1973 | CHAYP | 601.2551 | 629.2500 | HA | 181.1084 | 209.1033 |

| | | | | | | | | |
|---|---|---|---|---|---|---|---|---|
| **HAY** | 344.1717 | 372.1666 | **HAYP** | 441.2245 | 469.2194 | **AY** | 207.1128 | 235.1077 |
| **AYP** | 304.1656 | 332.1605 | **YP** | 233.1285 | 261.1234 | | | |

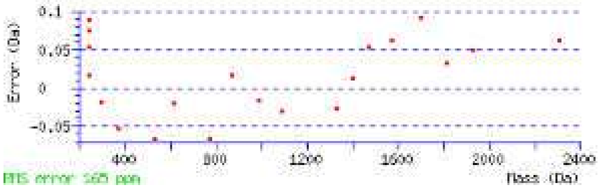


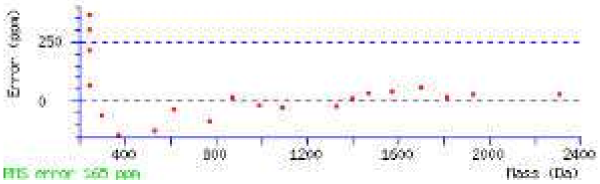


NCBI **BLAST** search of VVSGEIDQSDQSFLDDDQVAAGWVLTCHAYPK
(Parameters: blastp, nr protein database, expect=20000, no filter, PAM30)
Other BLAST web gateways

**All matches to this query**

| Score | Mr(calc) | Delta | Sequence |
|---|---|---|---|
| 78.3 | 3549.6359 | 0.1613 | VVSGEIDQSDQSFLDDDQVAAGWVLTCHAYPK |
| 14.5 | 3549.7774 | 0.0197 | SWNRTTVTAVTAAAAVVVACGVTGFLLLGDDGDDK |
| 6.1 | 3549.9177 | -0.1206 | GWRWLFIIEGAATVGFAIISLFILPDFPGTSR |
| 5.9 | 3549.7912 | 0.0059 | EFIKPEDVSAEVVEKEYQVQLDIAMQSGKPK |
| 5.7 | 3549.7005 | 0.0966 | AATTTAAAVGATTTAAAAGPESTAPDMHQATGPPLSER |
| 5.4 | 3549.9489 | -0.1518 | MGFLLLLGGVVGGTLGVQLIMVLRAMGNADFVIK |
| 4.5 | 3549.8103 | -0.0132 | KDFIDALHNATSEEHVVELVNTVIHPAPETPK |
| 4.0 | 3549.8081 | -0.0109 | SVLIMAHRPAAIAECDMLLVLDHGVRMAFGPK |
| 3.8 | 3549.8640 | -0.0669 | MLLSALSAVLSLAGSAAAALTYKGVDWSSVPVEEK |
| 3.5 | 3549.5525 | 0.2446 | GGNGGGIEACSSAAAAAAVRDACETFAEAFETFER |

**Mascot:** http://www.matrixscience.com/

# MATRIX SCIENCE Mascot Search Results

## Peptide View **Spot no 17**

MS/MS Fragmentation of **VNYAGVSTNNYALDEIQEVK**
Found in **gi|34394725** in **NCBInr**, putative Photosystem I reaction center subunit IV [Oryza sativa Japonica Group]

Match to Query 23: 2226.235424 from(2227.242700,1+) intensity(0.0000) index(9)
Title: Label: O9, Spot_Id: 228984, Peak_List_Id: 257934, MSMS Job_Run_Id: 24944, Comment:
Data file ppw_O9_138985154400.txt

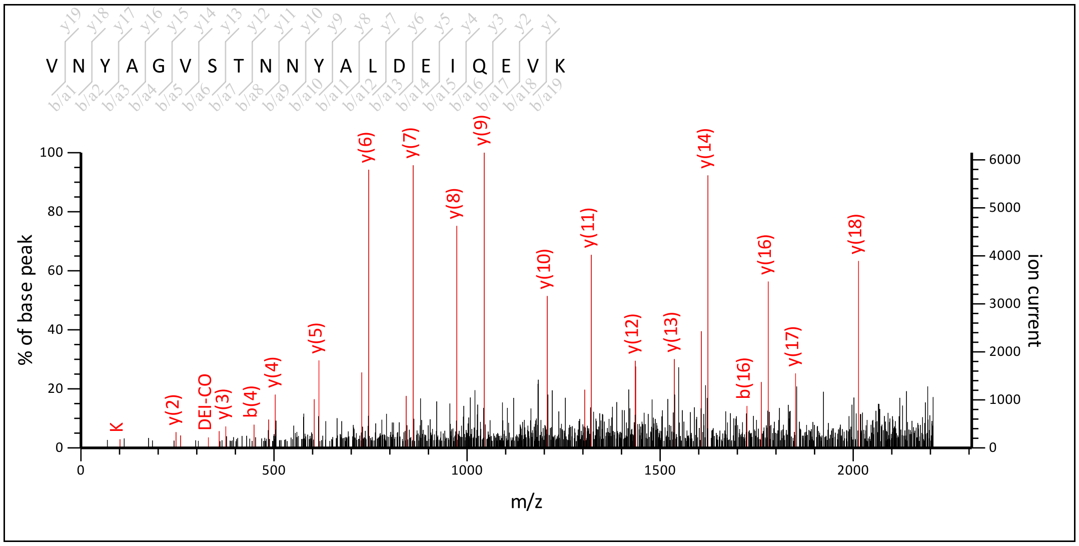


0 to 2307.02

Label all possible matches ○ Label matches used for scoring ◉

**Monoisotopic mass of neutral peptide Mr(calc):** 2226.0804
**Fixed modifications:** Carbamidomethyl (C) (apply to specified residues or termini only)
**Ions Score:** 128 **Expect:** 2.3e-10
**Matches :** 40/376 fragment ions using 44 most intense peaks (help)

| # | Immon. | a | a* | $a^0$ | b | b* | $b^0$ | d | d' | Seq. | v | w | w' | y | y* | |
|---|---|---|---|---|---|---|---|---|---|---|---|---|---|---|---|---|
| 1 | 72.0808 | 72.0808 | | | 100.0757 | | | 44.0495 | | V | | | | | | |
| 2 | 87.0553 | 186.1237 | 169.0972 | | 214.1186 | 197.0921 | | 143.1179 | | N | 2068.9822 | 2067.9869 | | 2128.0193 | 2110.9928 | [illegible] |
| 3 | 136.0757 | 349.1870 | 332.1605 | | 377.1819 | 360.1554 | | | | Y | 1905.9189 | | | ***2013.9764*** | 1996.9498 | [illegible] |
| 4 | 44.0495 | 420.2241 | 403.1976 | | **448.2191** | 431.1925 | | | | A | 1834.8817 | | | ***1850.9130*** | 1833.8865 | [illegible] |
| 5 | 30.0338 | 477.2456 | 460.2191 | | 505.2405 | 488.2140 | | | | G | | | | ***1779.8759*** | 1762.8494 | [illegible] |
| 6 | 72.0808 | 576.3140 | 559.2875 | | **604.3089** | 587.2824 | | 562.2984 | | V | 1678.7919 | 1691.8123 | | 1722.8545 | 1705.8279 | [illegible] |
| 7 | 60.0444 | 663.3461 | 646.3195 | 645.3355 | 691.3410 | 674.3144 | 673.3304 | 647.3511 | | S | 1591.7598 | 1590.7646 | | ***1623.7861*** | 1606.7595 | [illegible] |
| 8 | 74.0600 | 764.3937 | 747.3672 | 746.3832 | 792.3886 | 775.3621 | 774.3781 | 748.3988 | 750.3781 | T | 1490.7122 | 1503.7326 | 1505.7118 | ***1536.7540*** | 1519.7275 | [illegible] |
| 9 | 87.0553 | 878.4367 | 861.4101 | 860.4261 | 906.4316 | 889.4050 | 888.4210 | 835.4308 | | N | 1376.6692 | 1375.6740 | | ***1435.7063*** | 1418.6798 | [illegible] |
| 10 | 87.0553 | 992.4796 | 975.4530 | 974.4690 | 1020.4745 | 1003.4479 | 1002.4639 | 949.4738 | | N | 1262.6263 | 1261.6311 | | ***1321.6634*** | 1304.6369 | [illegible] |
| 11 | 136.0757 | 1155.5429 | 1138.5164 | 1137.5323 | 1183.5378 | 1166.5113 | 1165.5273 | | | Y | 1099.5630 | | | ***1207.6205*** | 1190.5939 | [illegible] |
| 12 | 44.0495 | 1226.5800 | 1209.5535 | 1208.5695 | 1254.5749 | 1237.5484 | 1236.5644 | | | A | 1028.5259 | | | ***1044.5572*** | 1027.5306 | [illegible] |
| 13 | 86.0964 | 1339.6641 | 1322.6375 | 1321.6535 | 1367.6590 | 1350.6325 | 1349.6484 | 1297.6171 | | L | 915.4418 | 914.4466 | | ***973.5201*** | 956.4935 | [illegible] |
| 14 | 88.0393 | 1454.6910 | 1437.6645 | 1436.6805 | 1482.6859 | 1465.6594 | 1464.6754 | 1410.7012 | | D | 800.4149 | 799.4196 | | ***860.4360*** | 843.4094 | [illegible] |
| 15 | 102.0550 | 1583.7336 | 1566.7071 | 1565.7231 | 1611.7285 | 1594.7020 | 1593.7180 | 1525.7281 | | E | 671.3723 | 670.3770 | | ***745.4090*** | 728.3825 | [illegible] |
| 16 | 86.0964 | 1696.8177 | 1679.7911 | 1678.8071 | **1724.8126** | 1707.7861 | 1706.8020 | 1668.7864 | 1682.8020 | I | 558.2882 | 571.3086 | 585.3243 | ***616.3665*** | 599.3399 | [illegible] |
| 17 | 101.0709 | 1824.8763 | 1807.8497 | 1806.8657 | 1852.8712 | 1835.8446 | 1834.8606 | 1767.8548 | | Q | 430.2296 | 429.2344 | | ***503.2824*** | 486.2558 | [illegible] |
| 18 | 102.0550 | 1953.9189 | 1936.8923 | 1935.9083 | 1981.9138 | 1964.8872 | 1963.9032 | 1895.9134 | | E | 301.1870 | 300.1918 | | ***375.2238*** | 358.1973 | [illegible] |
| 19 | 72.0808 | 2052.9873 | 2035.9607 | 2034.9767 | 2080.9822 | 2063.9556 | 2062.9716 | 2038.9716 | | V | 202.1186 | 215.1390 | | ***246.1812*** | 229.1547 | |
| 20 | 101.1073 | | | | | | | | | K | 74.0237 | 73.0284 | | 147.1128 | 130.0863 | |

| Seq | ya | yb | Seq | ya | yb | Seq | ya | yb |
|---|---|---|---|---|---|---|---|---|
| NY | 250.1186 | 278.1135 | NYA | 321.1557 | 349.1506 | NYAG | 378.1772 | 406.1721 |

| | | | | | | | | |
|---|---|---|---|---|---|---|---|---|
| **NYAGV** | 477.2456 | 505.2405 | **NYAGVS** | 564.2776 | 592.2726 | **NYAGVST** | 665.3253 | 693.3202 |
| **YA** | 207.1128 | 235.1077 | **YAG** | 264.1343 | 292.1292 | **YAGV** | 363.2027 | 391.1976 |
| **YAGVS** | 450.2347 | 478.2296 | **YAGVST** | 551.2824 | 579.2773 | **YAGVSTN** | 665.3253 | 693.3202 |
| **AG** | 101.0709 | 129.0659 | **AGV** | 200.1394 | 228.1343 | **AGVS** | 287.1714 | 315.1663 |
| **AGVST** | 388.2191 | 416.2140 | **AGVSTN** | 502.2620 | 530.2569 | **AGVSTNN** | 616.3049 | 644.2998 |
| **GV** | 129.1022 | 157.0972 | **GVS** | 216.1343 | 244.1292 | **GVST** | 317.1819 | 345.1769 |
| **GVSTN** | 431.2249 | 459.2198 | **GVSTNN** | 545.2678 | 573.2627 | **VS** | 159.1128 | 187.1077 |
| **VST** | 260.1605 | 288.1554 | **VSTN** | 374.2034 | 402.1983 | **VSTNN** | 488.2463 | 516.2413 |
| **VSTNNY** | 651.3097 | 679.3046 | **ST** | 161.0921 | 189.0870 | **STN** | 275.1350 | 303.1299 |
| **STNN** | 389.1779 | 417.1728 | **STNNY** | 552.2413 | 580.2362 | **STNNYA** | 623.2784 | 651.2733 |
| **TN** | 188.1030 | 216.0979 | **TNN** | 302.1459 | 330.1408 | **TNNY** | 465.2092 | 493.2041 |
| **TNNYA** | 536.2463 | 564.2413 | **TNNYAL** | 649.3304 | 677.3253 | **NN** | 201.0982 | 229.0931 |
| **NNY** | 364.1615 | 392.1565 | **NNYA** | 435.1987 | 463.1936 | **NNYAL** | 548.2827 | 576.2776 |
| **NNYALD** | 663.3097 | 691.3046 | **NY** | 250.1186 | 278.1135 | **NYA** | 321.1557 | 349.1506 |
| **NYAL** | 434.2398 | 462.2347 | **NYALD** | 549.2667 | 577.2617 | **NYALDE** | 678.3093 | 706.3042 |
| **YA** | 207.1128 | 235.1077 | **YAL** | 320.1969 | 348.1918 | **YALD** | 435.2238 | 463.2187 |
| **YALDE** | 564.2664 | 592.2613 | **YALDEI** | 677.3505 | 705.3454 | **AL** | 157.1335 | 185.1285 |
| **ALD** | 272.1605 | 300.1554 | **ALDE** | 401.2031 | 429.1980 | **ALDEI** | 514.2871 | 542.2821 |
| **ALDEIQ** | 642.3457 | 670.3406 | **LD** | 201.1234 | 229.1183 | **LDE** | 330.1660 | 358.1609 |
| **LDEI** | 443.2500 | 471.2449 | **LDEIQ** | 571.3086 | 599.3035 | **DE** | 217.0819 | 245.0768 |
| **DEI** | 330.1660 | 358.1609 | **DEIQ** | 458.2245 | 486.2195 | **DEIQE** | 587.2671 | 615.2620 |
| **DEIQEV** | 686.3355 | 714.3305 | **EI** | 215.1390 | 243.1339 | **EIQ** | 343.1976 | 371.1925 |
| **EIQE** | 472.2402 | 500.2351 | **EIQEV** | 571.3086 | 599.3035 | **IQ** | 214.1550 | 242.1499 |
| **IQE** | 343.1976 | 371.1925 | **IQEV** | 442.2660 | 470.2609 | **QE** | 230.1135 | 258.1084 |
| **QEV** | 329.1819 | 357.1769 | **EV** | 201.1234 | 229.1183 | | | |

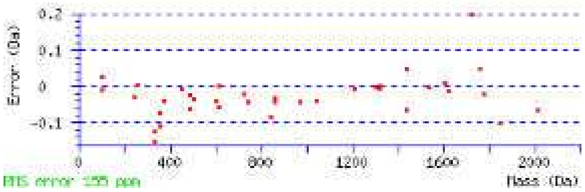


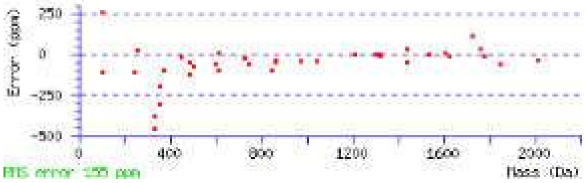


NCBI **BLAST** search of VNYAGVSTNNYALDEIQEVK
(Parameters: blastp, nr protein database, expect=20000, no filter, PAM30)
Other BLAST web gateways

**All matches to this query**

| Score | Mr(calc) | Delta | Sequence |
|---|---|---|---|
| 127.7 | 2226.0804 | 0.1550 | VNYAGVSTNNYALDEIQEVK |
| 8.8 | 2226.0990 | 0.1364 | QMDALGLPFSFLSNKETSNK |
| 8.2 | 2226.1492 | 0.0863 | ASVSGTLEQIIDKHGDIASASK |
| 7.0 | 2226.1063 | 0.1291 | LGQCPSNVELHIDTSKSSVR |
| 4.5 | 2226.1141 | 0.1213 | GAHQQDAAAPPPLVLGQQDSAR |
| 4.4 | 2226.0382 | 0.1972 | FFYETGIDFSNANSPSFKR |
| 4.2 | 2226.1128 | 0.1226 | DSEKGSTSNNLVIASTHIPEK |
| 4.1 | 2226.0725 | 0.1629 | EEEEELVEYILSTSRCLK |
| 4.0 | 2226.0625 | 0.1729 | VDSSSHSPHANSASLSSRVSSK |
| 3.9 | 2226.0725 | 0.1629 | YEKILEMISDEDIQSELR |

**Mascot:** http://www.matrixscience.com/

# MATRIX SCIENCE Mascot Search Results

## Peptide View **Spot no 17**

MS/MS Fragmentation of **ESYWYNGTGSVVTVDQDPNTRYPVVVR**
Found in **gi|34394725** in **NCBInr**, putative Photosystem I reaction center subunit IV [Oryza sativa Japonica Group]

Match to Query 26: 3100.773724 from(3101.781000,1+) intensity(0.0000) index(10)
Title: Label: O9, Spot_Id: 228984, Peak_List_Id: 257937, MSMS Job_Run_Id: 24944, Comment:
Data file ppw_O9_138985154400.txt

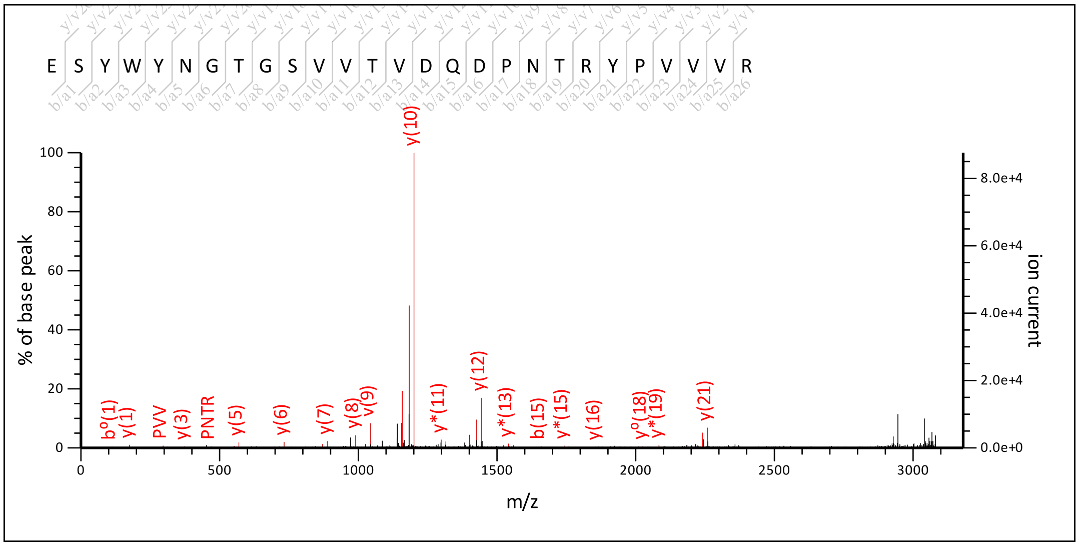


0 to 3180.62

Label all possible matches ○ Label matches used for scoring ◉

**Monoisotopic mass of neutral peptide Mr(calc):** 3100.4891
**Fixed modifications:** Carbamidomethyl (C) (apply to specified residues or termini only)
**Ions Score:** 54 **Expect:** 0.0033
**Matches :** 44/532 fragment ions using 62 most intense peaks (help)

| # | Immon. | a | a* | a0 | b | b* | b0 | d | d' | Seq. | v | w | w' | y | y* | |
|---|---|---|---|---|---|---|---|---|---|---|---|---|---|---|---|---|
| 1 | 102.0550 | 102.0550 | | 84.0444 | 130.0499 | | 112.0393 | 44.0495 | | E | | | | | | |
| 2 | 60.0444 | 189.0870 | | 171.0764 | 217.0819 | | 199.0713 | 173.0921 | | S | 2940.4275 | 2939.4323 | | 2972.4537 | 2955.4272 | 29 |
| 3 | 136.0757 | 352.1503 | | 334.1397 | 380.1452 | | 362.1347 | | | Y | 2777.3642 | | | 2885.4217 | 2868.3951 | 28 |
| 4 | 159.0917 | 538.2296 | | 520.2191 | 566.2245 | | 548.2140 | | | W | 2591.2849 | | | 2722.3584 | 2705.3318 | 27 |
| 5 | 136.0757 | 701.2930 | | 683.2824 | 729.2879 | | 711.2773 | | | Y | 2428.2215 | | | 2536.2790 | 2519.2525 | 25 |
| 6 | 87.0553 | 815.3359 | 798.3093 | 797.3253 | 843.3308 | 826.3042 | 825.3202 | 772.3301 | | N | 2314.1786 | 2313.1834 | | 2373.2157 | 2356.1892 | 23 |
| 7 | 30.0338 | 872.3573 | 855.3308 | 854.3468 | 900.3523 | 883.3257 | 882.3417 | | | G | | | | ***2259.1728*** | 2242.1462 | 22 |
| 8 | 74.0600 | 973.4050 | 956.3785 | 955.3945 | 1001.3999 | 984.3734 | 983.3894 | 957.4101 | 959.3894 | T | 2156.1095 | 2169.1299 | 2171.1091 | 2202.1513 | 2185.1248 | 21 |
| 9 | 30.0338 | 1030.4265 | 1013.3999 | 1012.4159 | 1058.4214 | 1041.3949 | 1040.4108 | | | G | | | | 2101.1036 | 2084.0771 | 20 |
| 10 | 60.0444 | 1117.4585 | 1100.4320 | 1099.4480 | 1145.4534 | 1128.4269 | 1127.4429 | 1101.4636 | | S | 2012.0560 | 2011.0607 | | 2044.0822 | 2027.0556 | 20 |
| 11 | 72.0808 | 1216.5269 | 1199.5004 | 1198.5164 | 1244.5218 | 1227.4953 | 1226.5113 | 1202.5113 | | V | 1912.9876 | 1926.0080 | | 1957.0502 | 1940.0236 | 19 |
| 12 | 72.0808 | **1315.5953** | 1298.5688 | 1297.5848 | 1343.5903 | 1326.5637 | 1325.5797 | 1301.5797 | | V | 1813.9191 | 1826.9395 | | ***1857.9817*** | 1840.9552 | 18 |
| 13 | 74.0600 | 1416.6430 | 1399.6165 | 1398.6325 | 1444.6379 | 1427.6114 | 1426.6274 | 1400.6481 | 1402.6274 | T | 1712.8715 | 1725.8919 | 1727.8711 | ***1758.9133*** | 1741.8868 | 17 |
| 14 | 72.0808 | 1515.7114 | 1498.6849 | 1497.7009 | 1543.7064 | 1526.6798 | 1525.6958 | 1501.6958 | | V | 1613.8030 | 1626.8234 | | 1657.8656 | 1640.8391 | 16 |
| 15 | 88.0393 | 1630.7384 | 1613.7118 | 1612.7278 | 1658.7333 | 1641.7067 | 1640.7227 | 1586.7486 | | D | 1498.7761 | 1497.7809 | | 1558.7972 | 1541.7707 | 15 |
| 16 | 101.0709 | **1758.7970** | 1741.7704 | 1740.7864 | 1786.7919 | 1769.7653 | 1768.7813 | 1701.7755 | | Q | 1370.7175 | 1369.7223 | | ***1443.7703*** | 1426.7437 | 14 |
| 17 | 88.0393 | 1873.8239 | 1856.7974 | 1855.8133 | 1901.8188 | 1884.7923 | 1883.8083 | 1829.8341 | | D | 1255.6906 | 1254.6953 | | ***1315.7117*** | 1298.6852 | 12 |
| 18 | 70.0651 | 1970.8767 | 1953.8501 | 1952.8661 | 1998.8716 | 1981.8450 | 1980.8610 | 1944.8610 | | P | 1158.6378 | 1157.6426 | | ***1200.6848*** | 1183.6582 | 11 |
| 19 | 87.0553 | 2084.9196 | 2067.8930 | 2066.9090 | 2112.9145 | 2095.8880 | 2094.9039 | 2041.9138 | | N | 1044.5949 | 1043.5996 | | 1103.6320 | 1086.6055 | 10 |
| 20 | 74.0600 | 2185.9673 | 2168.9407 | 2167.9567 | 2213.9622 | 2196.9356 | 2195.9516 | 2169.9724 | 2171.9516 | T | 943.5472 | 956.5676 | 958.5469 | ***989.5891*** | 972.5625 | 9 |
| 21 | 129.1135 | 2342.0684 | 2325.0418 | 2324.0578 | 2370.0633 | 2353.0367 | 2352.0527 | 2257.0044 | | R | 787.4461 | 786.4509 | | ***888.5414*** | 871.5148 | |
| 22 | 136.0757 | 2505.1317 | 2488.1052 | 2487.1211 | 2533.1266 | 2516.1001 | 2515.1161 | | | Y | 624.3828 | | | ***732.4403*** | 715.4137 | |
| 23 | 70.0651 | 2602.1845 | 2585.1579 | 2584.1739 | 2630.1794 | 2613.1528 | 2612.1688 | 2576.1688 | | P | 527.3300 | 526.3348 | | ***569.3770*** | 552.3504 | |

| | | | | | | | | | | | | | | | |
|---|---|---|---|---|---|---|---|---|---|---|---|---|---|---|---|
| 24 | 72.0808 | 2701.2529 | 2684.2263 | 2683.2423 | 2729.2478 | 2712.2213 | 2711.2372 | 2687.2372 | | V | 428.2616 | 441.2820 | | 472.3242 | 455.2976 |
| 25 | 72.0808 | 2800.3213 | 2783.2948 | 2782.3107 | 2828.3162 | 2811.2897 | 2810.3057 | 2786.3057 | | V | 329.1932 | 342.2136 | | ***373.2558*** | 356.2292 |
| 26 | 72.0808 | 2899.3897 | 2882.3632 | 2881.3792 | 2927.3846 | 2910.3581 | 2909.3741 | 2885.3741 | | V | 230.1248 | 243.1452 | | 274.1874 | 257.1608 |
| 27 | 129.1135 | | | | | | | | | R | 74.0237 | 73.0284 | | ***175.1190*** | 158.0924 |

| Seq | ya | yb | Seq | ya | yb | Seq | ya | yb |
|---|---|---|---|---|---|---|---|---|
| SY | 223.1077 | 251.1026 | SYW | 409.1870 | 437.1819 | SYWY | 572.2504 | 600.2453 |
| SYWYN | 686.2933 | 714.2882 | YW | 322.1550 | 350.1499 | YWY | 485.2183 | 513.2132 |
| YWYN | 599.2613 | 627.2562 | YWYNG | 656.2827 | 684.2776 | WY | 322.1550 | 350.1499 |
| WYN | 436.1979 | 464.1928 | WYNG | 493.2194 | 521.2143 | WYNGT | 594.2671 | 622.2620 |
| WYNGTG | 651.2885 | 679.2835 | YN | 250.1186 | 278.1135 | YNG | 307.1401 | 335.1350 |
| YNGT | 408.1878 | 436.1827 | YNGTG | 465.2092 | 493.2041 | YNGTGS | 552.2413 | 580.2362 |
| YNGTGSV | 651.3097 | 679.3046 | NG | 144.0768 | 172.0717 | NGT | 245.1244 | 273.1193 |
| NGTG | 302.1459 | 330.1408 | NGTGS | 389.1779 | 417.1728 | NGTGSV | 488.2463 | 516.2413 |
| NGTGSVV | 587.3148 | 615.3097 | NGTGSVVT | 688.3624 | 716.3573 | GT | 131.0815 | 159.0764 |
| GTG | 188.1030 | 216.0979 | GTGS | 275.1350 | 303.1299 | GTGSV | 374.2034 | 402.1983 |
| GTGSVV | 473.2718 | 501.2667 | GTGSVVT | 574.3195 | 602.3144 | GTGSVVTV | 673.3879 | 701.3828 |
| TG | 131.0815 | 159.0764 | TGS | 218.1135 | 246.1084 | TGSV | 317.1819 | 345.1769 |
| TGSVV | 416.2504 | 444.2453 | TGSVVT | 517.2980 | 545.2930 | TGSVVTV | 616.3665 | 644.3614 |
| GS | 117.0659 | 145.0608 | GSV | 216.1343 | 244.1292 | GSVV | 315.2027 | 343.1976 |
| GSVVT | 416.2504 | 444.2453 | GSVVTV | 515.3188 | 543.3137 | GSVVTVD | 630.3457 | 658.3406 |
| SV | 159.1128 | 187.1077 | SVV | 258.1812 | 286.1761 | SVVT | 359.2289 | 387.2238 |
| SVVTV | 458.2973 | 486.2922 | SVVTVD | 573.3243 | 601.3192 | VV | 171.1492 | 199.1441 |
| VVT | 272.1969 | 300.1918 | VVTV | 371.2653 | 399.2602 | VVTVD | 486.2922 | 514.2871 |
| VVTVDQ | 614.3508 | 642.3457 | VT | 173.1285 | 201.1234 | VTV | 272.1969 | 300.1918 |
| VTVD | 387.2238 | 415.2187 | VTVDQ | 515.2824 | 543.2773 | VTVDQD | 630.3093 | 658.3042 |
| TV | 173.1285 | 201.1234 | TVD | 288.1554 | 316.1503 | TVDQ | 416.2140 | 444.2089 |
| TVDQD | 531.2409 | 559.2358 | TVDQDP | 628.2937 | 656.2886 | VD | 187.1077 | 215.1026 |
| VDQ | 315.1663 | 343.1612 | VDQD | 430.1932 | 458.1882 | VDQDP | 527.2460 | 555.2409 |
| VDQDPN | 641.2889 | 669.2838 | DQ | 216.0979 | 244.0928 | DQD | 331.1248 | 359.1197 |
| DQDP | 428.1776 | 456.1725 | DQDPN | 542.2205 | 570.2154 | DQDPNT | 643.2682 | 671.2631 |
| QD | 216.0979 | 244.0928 | QDP | 313.1506 | 341.1456 | QDPN | 427.1936 | 455.1885 |
| QDPNT | 528.2413 | 556.2362 | QDPNTR | 684.3424 | 712.3373 | DP | 185.0921 | 213.0870 |
| DPN | 299.1350 | 327.1299 | DPNT | 400.1827 | 428.1776 | DPNTR | 556.2838 | 584.2787 |
| PN | 184.1081 | 212.1030 | PNT | 285.1557 | 313.1506 | PNTR | 441.2568 | 469.2518 |
| PNTRY | 604.3202 | 632.3151 | NT | 188.1030 | 216.0979 | NTR | 344.2041 | 372.1990 |
| NTRY | 507.2674 | 535.2623 | NTRYP | 604.3202 | 632.3151 | TR | 230.1612 | 258.1561 |
| TRY | 393.2245 | 421.2194 | TRYP | 490.2772 | 518.2722 | TRYPV | 589.3457 | 617.3406 |
| TRYPVV | 688.4141 | 716.4090 | RY | 292.1768 | 320.1717 | RYP | 389.2296 | 417.2245 |
| RYPV | 488.2980 | 516.2929 | RYPVV | 587.3664 | 615.3613 | RYPVVV | 686.4348 | 714.4297 |
| YP | 233.1285 | 261.1234 | YPV | 332.1969 | 360.1918 | YPVV | 431.2653 | 459.2602 |
| YPVVV | 530.3337 | 558.3286 | PV | 169.1335 | 197.1285 | PVV | 268.2020 | 296.1969 |
| PVVV | 367.2704 | 395.2653 | VV | 171.1492 | 199.1441 | VVV | 270.2176 | 298.2125 |
| VV | 171.1492 | 199.1441 | | | | | | |

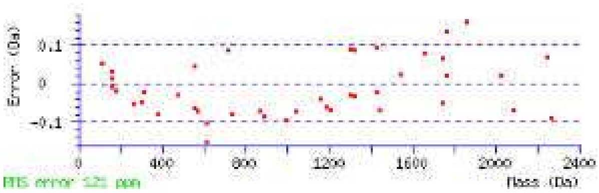


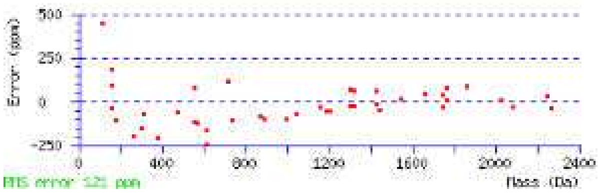


NCBI **BLAST** search of ESYWYNGTGSVVTVDQDPNTRYPVVVR
(Parameters: blastp, nr protein database, expect=20000, no filter, PAM30)
Other BLAST web gateways

**All matches to this query**

| Score | Mr(calc) | Delta | Sequence |
|---|---|---|---|
| 53.8 | 3100.4891 | 0.2847 | ESYWYNGTGSVVTVDQDPNTRYPVVVR |
| 2.4 | 3100.4648 | 0.3089 | GNPGFGVSEMSGGVGFGWMGRTLVVASAWR |
| 0.9 | 3100.4949 | 0.2788 | NTSSTSSEFLYLGTIVNSRGGVDELSDPR |
| 0.8 | 3100.5646 | 0.2091 | VGGTVVMAMNGTVDGGSVVGPVTVNQNVTAVR |
| 0.5 | 3100.5011 | 0.2726 | FANWRDSLGCSMAPNPPKPENLPTVFR |

**Mascot:** http://www.matrixscience.com/

# MATRIX SCIENCE Mascot Search Results

## Peptide View **Spot no 18**

MS/MS Fragmentation of **HHHHLFHHK**
Found in **gi|15667623** in **NCBInr**, drought inducible 22 kD protein [Saccharum officinarum]

Match to Query 7: 1228.673524 from(1229.680800,1+) intensity(0.0000) index(2)
Title: Label: K8, Spot_Id: 219796, Peak_List_Id: 226437, MSMS Job_Run_Id: 21844, Comment:
Data file C:\Documents and Settings\Administrator\Desktop\Farah-Garima 11May 2013\ppw_K8_136859930300.txt

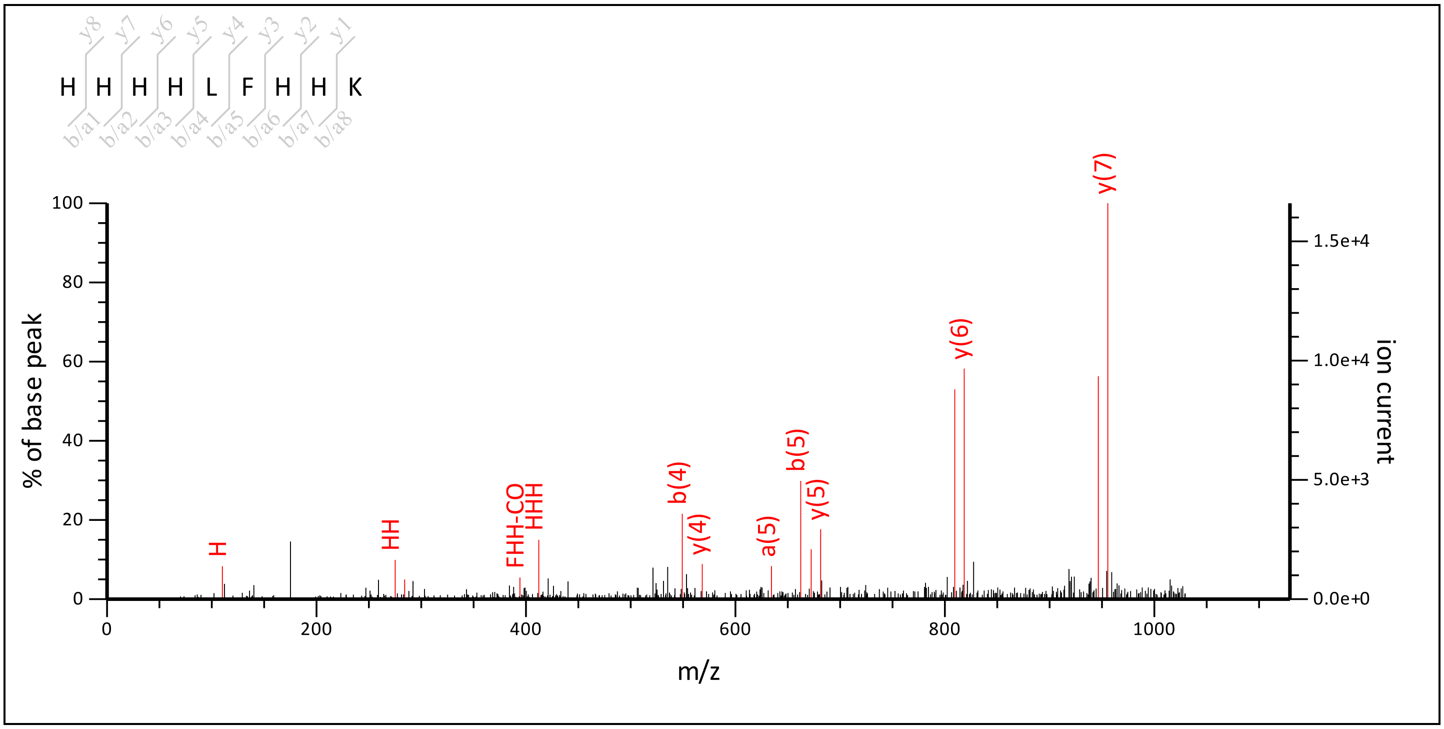


0 to 1129.46

Label all possible matches ○ Label matches used for scoring ◉

**Monoisotopic mass of neutral peptide Mr(calc):** 1228.6115
**Fixed modifications:** Carbamidomethyl (C) (apply to specified residues or termini only)
**Ions Score:** 45 **Expect:** 1.1
**Matches :** 27/89 fragment ions using 20 most intense peaks (help)

| # | Immon. | a | b | d | Seq. | v | w | y | y* | # |
|---|---|---|---|---|---|---|---|---|---|---|
| 1 | 110.0713 | **110.0713** | 138.0662 | 44.0495 | H | | | | | 9 |
| 2 | 110.0713 | 247.1302 | ***275.1251*** | | H | 1010.5067 | | 1092.5598 | 1075.5333 | 8 |
| 3 | 110.0713 | 384.1891 | ***412.1840*** | | H | 873.4478 | | **955.5009** | 938.4744 | 7 |
| 4 | 110.0713 | 521.2480 | ***549.2429*** | | H | 736.3889 | | **818.4420** | 801.4155 | 6 |
| 5 | 86.0964 | **634.3321** | ***662.3270*** | 592.2851 | L | 623.3049 | 622.3096 | **681.3831** | 664.3566 | 5 |
| 6 | 120.0808 | 781.4005 | ***809.3954*** | | F | 476.2364 | | **568.2990** | 551.2725 | 4 |
| 7 | 110.0713 | 918.4594 | ***946.4543*** | | H | 339.1775 | | 421.2306 | 404.2041 | 3 |
| 8 | 110.0713 | 1055.5183 | 1083.5132 | | H | 202.1186 | | **284.1717** | 267.1452 | 2 |

| | | | | | | | | | | |
|---|---|---|---|---|---|---|---|---|---|---|
| 9 | 101.1073 | | | | K | 74.0237 | 73.0284 | 147.1128 | 130.0863 | 1 |

| Seq | ya | yb | Seq | ya | yb | Seq | ya | yb |
|---|---|---|---|---|---|---|---|---|
| HH | 247.1302 | 275.1251 | HHH | 384.1891 | 412.1840 | HHHL | 497.2732 | 525.2681 |
| HHHLF | 644.3416 | 672.3365 | HH | 247.1302 | 275.1251 | HHL | 360.2142 | 388.2092 |
| HHLF | 507.2827 | 535.2776 | HHLFH | 644.3416 | 672.3365 | HL | 223.1553 | 251.1503 |
| HLF | 370.2238 | 398.2187 | HLFH | 507.2827 | 535.2776 | HLFHH | 644.3416 | 672.3365 |
| LF | 233.1648 | 261.1598 | LFH | 370.2238 | 398.2187 | LFHH | 507.2827 | 535.2776 |
| FH | 257.1397 | 285.1346 | FHH | 394.1986 | 422.1935 | HH | 247.1302 | 275.1251 |

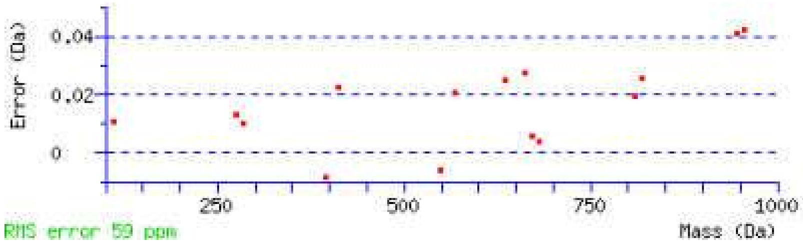


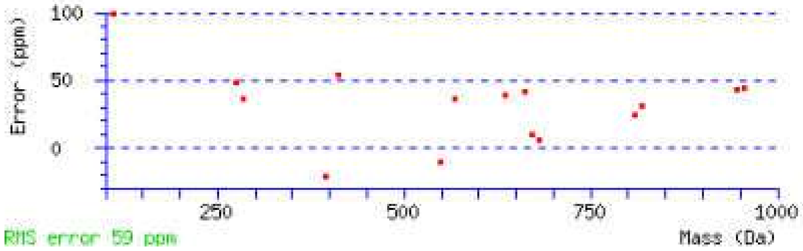


NCBI **BLAST** search of HHHHLFHHK
(Parameters: blastp, nr protein database, expect=20000, no filter, PAM30)
Other BLAST web gateways

**All matches to this query**

| Score | Mr(calc) | Delta | Sequence |
|---|---|---|---|
| 45.2 | 1228.6115 | 0.0620 | HHHHLFHHK |
| 30.6 | 1228.7153 | -0.0417 | RAALVAVHHQK |
| 28.6 | 1228.7292 | -0.0557 | GLIAGVGLQVFR |
| 27.7 | 1228.6313 | 0.0423 | GSQEFIRHGAK |
| 27.7 | 1228.6941 | -0.0206 | RPWQLHHKK |
| 27.7 | 1228.6651 | 0.0084 | WMHLLHHKK |
| 27.5 | 1228.5533 | 0.1202 | HHHHHHMTR |
| 27.5 | 1228.6075 | 0.0661 | HHHHHQLQR |
| 27.5 | 1228.6075 | 0.0661 | HHHHQQHLR |
| 24.9 | 1228.5983 | 0.0753 | ADAGPRLVDCR |

**Mascot:** http://www.matrixscience.com/

# MATRIX SCIENCE Mascot Search Results

## Peptide View Spot no 18

MS/MS Fragmentation of **HHHHLFHHKK**
Found in **gi|15667623** in **NCBInr**, drought inducible 22 kD protein [Saccharum officinarum]

Match to Query 10: 1356.769724 from(1357.777000,1+) intensity(0.0000) index(4)
Title: Label: K8, Spot_Id: 219796, Peak_List_Id: 226451, MSMS Job_Run_Id: 21844, Comment:
Data file C:\Documents and Settings\Administrator\Desktop\Farah-Garima 11May 2013\ppw_K8_136859930300.txt

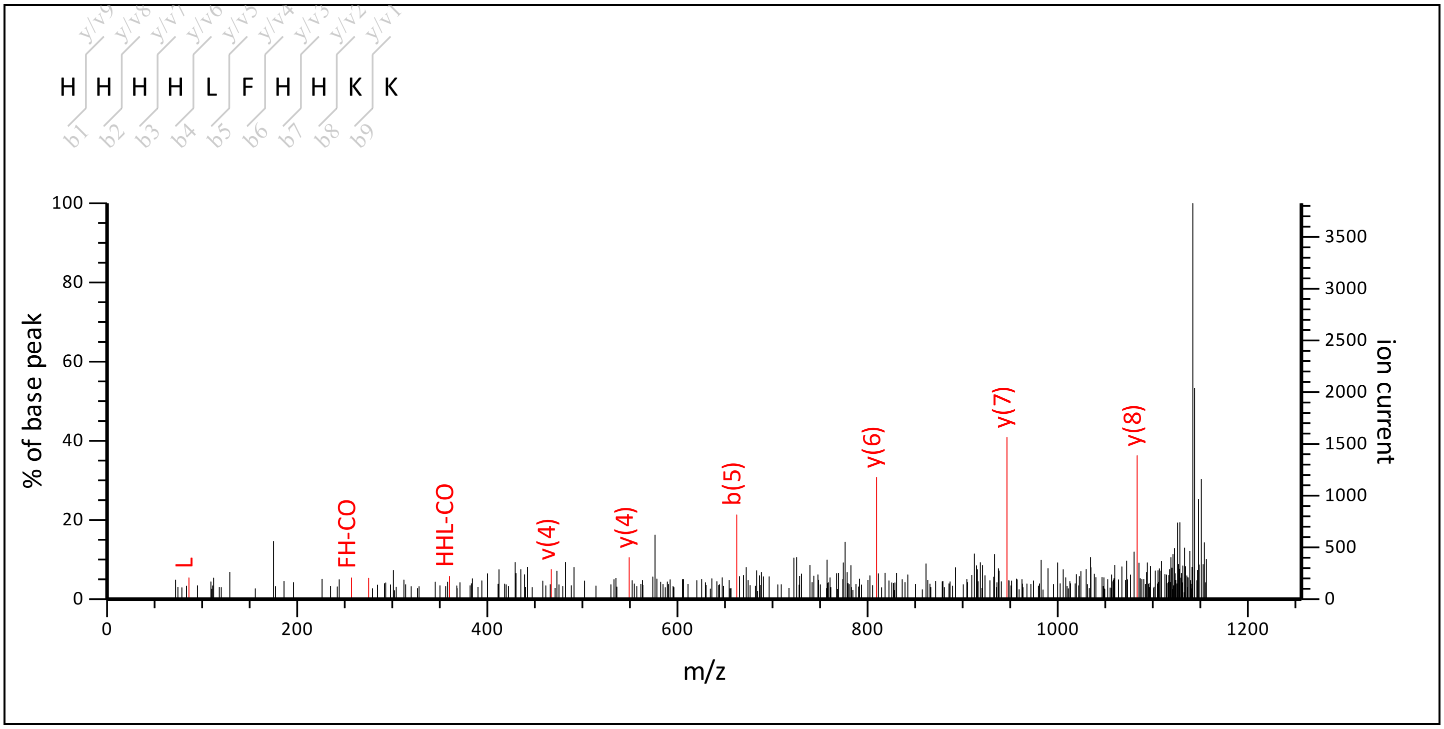


0 to 1256.32

Label all possible matches ○ Label matches used for scoring ◉

**Monoisotopic mass of neutral peptide Mr(calc):** 1356.7064
**Fixed modifications:** Carbamidomethyl (C) (apply to specified residues or termini only)
**Ions Score:** 17 **Expect:** 6.7e+02
**Matches :** 18/107 fragment ions using 33 most intense peaks (help)

| # | Immon. | a | a* | b | b* | d | Seq. | v | w | y | y* | # |
|---|---|---|---|---|---|---|---|---|---|---|---|---|
| 1 | 110.0713 | 110.0713 | | 138.0662 | | 44.0495 | H | | | | | 10 |
| 2 | 110.0713 | 247.1302 | | ***275.1251*** | | | H | 1138.6017 | | 1220.6548 | 1203.6283 | 9 |
| 3 | 110.0713 | 384.1891 | | 412.1840 | | | H | 1001.5428 | | **1083.5959** | 1066.5693 | 8 |
| 4 | 110.0713 | 521.2480 | | ***549.2429*** | | | H | 864.4839 | | **946.5370** | 929.5104 | 7 |
| 5 | 86.0964 | 634.3321 | | ***662.3270*** | | 592.2851 | L | 751.3998 | 750.4046 | **809.4781** | 792.4515 | 6 |
| 6 | 120.0808 | 781.4005 | | ***809.3954*** | | | F | 604.3314 | | 696.3940 | 679.3675 | 5 |
| 7 | 110.0713 | 918.4594 | | ***946.4543*** | | | H | 467.2725 | | **549.3256** | 532.2990 | 4 |
| 8 | 110.0713 | 1055.5183 | | ***1083.5132*** | | | H | 330.2136 | | 412.2667 | 395.2401 | 3 |

| | | | | | | | | | | | | |
|---|---|---|---|---|---|---|---|---|---|---|---|---|
| 9 | 101.1073 | 1183.6133 | 1166.5867 | 1211.6082 | 1194.5816 | 1126.5554 | K | 202.1186 | 201.1234 | **275.2078** | 258.1812 | 2 |
| 10 | 101.1073 | | | | | | K | 74.0237 | 73.0284 | 147.1128 | 130.0863 | 1 |

| Seq | ya | yb | Seq | ya | yb | Seq | ya | yb |
|---|---|---|---|---|---|---|---|---|
| HH | 247.1302 | 275.1251 | HHH | 384.1891 | 412.1840 | HHHL | 497.2732 | 525.2681 |
| HHHLF | 644.3416 | 672.3365 | HH | 247.1302 | 275.1251 | HHL | 360.2142 | 388.2092 |
| HHLF | 507.2827 | 535.2776 | HHLFH | 644.3416 | 672.3365 | HL | 223.1553 | 251.1503 |
| HLF | 370.2238 | 398.2187 | HLFH | 507.2827 | 535.2776 | HLFHH | 644.3416 | 672.3365 |
| LF | 233.1648 | 261.1598 | LFH | 370.2238 | 398.2187 | LFHH | 507.2827 | 535.2776 |
| LFHHK | 635.3776 | 663.3725 | FH | 257.1397 | 285.1346 | FHH | 394.1986 | 422.1935 |
| FHHK | 522.2936 | 550.2885 | HH | 247.1302 | 275.1251 | HHK | 375.2251 | 403.2201 |
| HK | 238.1662 | 266.1612 | | | | | | |

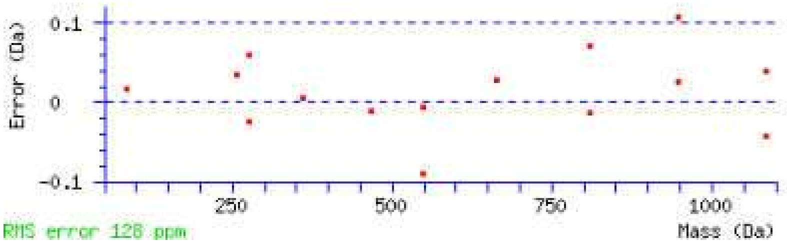


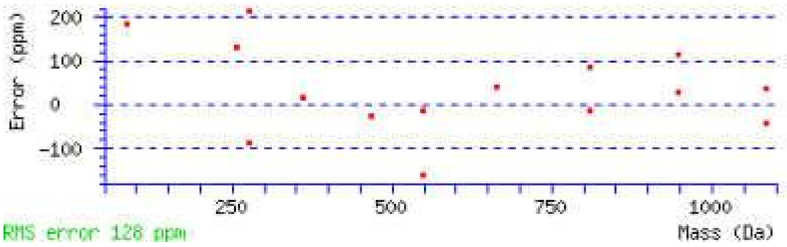


NCBI **BLAST** search of HHHHLFHHKK
(Parameters: blastp, nr protein database, expect=20000, no filter, PAM30)
Other BLAST web gateways

**All matches to this query**

| Score | Mr(calc) | Delta | Sequence |
|---|---|---|---|
| 23.8 | 1356.8030 | -0.0333 | ILKGHFLQVFR |
| 20.2 | 1356.6422 | 0.1275 | AGDAPHFSSLEAR |
| 17.7 | 1356.6568 | 0.1129 | DPGAAGRALENMR |
| 17.7 | 1356.8493 | -0.0796 | IPLATSLLKVFR |
| 17.6 | 1356.7361 | 0.0336 | IGLQIGNLSGETR |
| 17.0 | 1356.7302 | 0.0395 | ADPKHLFNFLR |
| 16.7 | 1356.7064 | 0.0633 | HHHHLFHHKK |
| 16.5 | 1356.6820 | 0.0878 | SSMRSLFSSSLR |
| 15.6 | 1356.7184 | 0.0514 | KGAPLPASSGGLMR |
| 15.3 | 1356.7262 | 0.0435 | AGTQHGLIPAHAGK |

**Mascot:** http://www.matrixscience.com/

# MATRIX SCIENCE Mascot Search Results

## Peptide View **Spot no 18**

MS/MS Fragmentation of **QHLGEAGAIAAGAFALYEK**
Found in **gi|15667623** in **NCBInr**, drought inducible 22 kD protein [Saccharum officinarum]

Match to Query 24: 1916.054524 from(1917.061800,1+) intensity(0.0000) index(10)
Title: Label: K8, Spot_Id: 219796, Peak_List_Id: 226444, MSMS Job_Run_Id: 21844, Comment:
Data file C:\Documents and Settings\Administrator\Desktop\Farah-Garima 11May 2013\ppw_K8_136859930300.txt

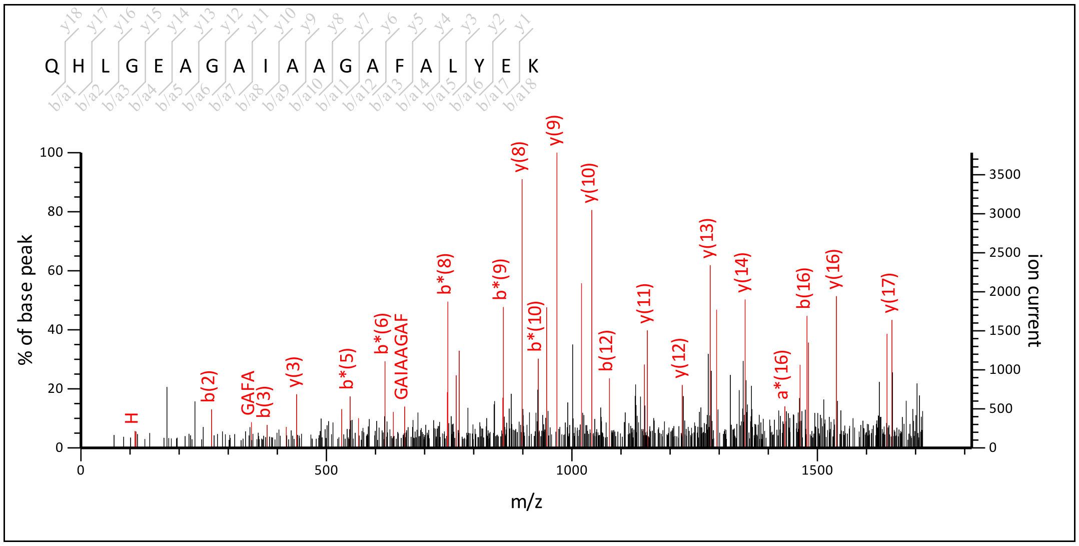


0 to 1814.36

Label all possible matches Label matches used for scoring

**Monoisotopic mass of neutral peptide Mr(calc):** 1915.9792
**Fixed modifications:** Carbamidomethyl (C) (apply to specified residues or termini only)
**Ions Score:** 105 **Expect:** 8.1e-07
**Matches :** 44/377 fragment ions using 51 most intense peaks (help)

| # | Immon. | a | a* | a0 | b | b* | b0 | d | d' | Seq. | v | w | w' | y | y* | |
|---|---|---|---|---|---|---|---|---|---|---|---|---|---|---|---|---|
| 1 | 101.0709 | 101.0709 | 84.0444 | | 129.0659 | 112.0393 | | 44.0495 | | Q | | | | | | |
| 2 | 110.0713 | 238.1299 | 221.1033 | | ***266.1248*** | 249.0982 | | | | H | 1706.8748 | | | 1788.9279 | 1771.9014 | 17 |
| 3 | 86.0964 | 351.2139 | 334.1874 | | ***379.2088*** | 362.1823 | | 309.1670 | | L | 1593.7908 | 1592.7955 | | ***1651.8690*** | 1634.8425 | 16 |
| 4 | 30.0338 | 408.2354 | 391.2088 | | 436.2303 | 419.2037 | | | | G | | | | ***1538.7849*** | 1521.7584 | 15 |
| 5 | 102.0550 | 537.2780 | 520.2514 | 519.2674 | ***565.2729*** | 548.2463 | 547.2623 | 479.2725 | | E | 1407.7267 | 1406.7314 | | ***1481.7635*** | 1464.7369 | 14 |
| 6 | 44.0495 | 608.3151 | 591.2885 | 590.3045 | ***636.3100*** | 619.2835 | 618.2994 | | | A | 1336.6896 | | | ***1352.7209*** | 1335.6943 | 13 |
| 7 | 30.0338 | 665.3366 | 648.3100 | 647.3260 | 693.3315 | 676.3049 | 675.3209 | | | G | | | | ***1281.6838*** | 1264.6572 | 12 |
| 8 | 44.0495 | 736.3737 | 719.3471 | 718.3631 | ***764.3686*** | 747.3420 | 746.3580 | | | A | 1208.6310 | | | ***1224.6623*** | 1207.6358 | 12 |
| 9 | 86.0964 | 849.4577 | 832.4312 | 831.4472 | 877.4526 | 860.4261 | 859.4421 | 821.4264 | 835.4421 | I | 1095.5469 | 1108.5673 | 1122.5830 | ***1153.6252*** | 1136.5986 | 11 |
| 10 | 44.0495 | 920.4948 | 903.4683 | 902.4843 | ***948.4898*** | 931.4632 | 930.4792 | | | A | 1024.5098 | | | ***1040.5411*** | 1023.5146 | 10 |
| 11 | 44.0495 | 991.5320 | 974.5054 | 973.5214 | ***1019.5269*** | 1002.5003 | 1001.5163 | | | A | 953.4727 | | | ***969.5040*** | 952.4775 | 9 |
| 12 | 30.0338 | 1048.5534 | 1031.5269 | 1030.5429 | ***1076.5483*** | 1059.5218 | 1058.5378 | | | G | | | | ***898.4669*** | 881.4403 | 8 |
| 13 | 44.0495 | 1119.5905 | 1102.5640 | 1101.5800 | ***1147.5854*** | 1130.5589 | 1129.5749 | | | A | 825.4141 | | | 841.4454 | 824.4189 | 8 |
| 14 | 120.0808 | 1266.6589 | 1249.6324 | 1248.6484 | ***1294.6539*** | 1277.6273 | 1276.6433 | | | F | 678.3457 | | | ***770.4083*** | 753.3818 | 7 |
| 15 | 44.0495 | 1337.6961 | 1320.6695 | 1319.6855 | 1365.6910 | 1348.6644 | 1347.6804 | | | A | 607.3086 | | | 623.3399 | 606.3134 | 6 |
| 16 | 86.0964 | 1450.7801 | 1433.7536 | 1432.7696 | ***1478.7750*** | 1461.7485 | 1460.7645 | 1408.7332 | | L | 494.2245 | 493.2293 | | 552.3028 | 535.2762 | 5 |
| 17 | 136.0757 | 1613.8435 | 1596.8169 | 1595.8329 | ***1641.8384*** | 1624.8118 | 1623.8278 | | | Y | 331.1612 | | | ***439.2187*** | 422.1922 | 4 |
| 18 | 102.0550 | 1742.8860 | 1725.8595 | 1724.8755 | 1770.8810 | 1753.8544 | 1752.8704 | 1684.8806 | | E | 202.1186 | 201.1234 | | 276.1554 | 259.1288 | 2 |
| 19 | 101.1073 | | | | | | | | | K | 74.0237 | 73.0284 | | 147.1128 | 130.0863 | |

| Seq | ya | yb | Seq | ya | yb | Seq | ya | yb |
|---|---|---|---|---|---|---|---|---|
| HL | 223.1553 | 251.1503 | HLG | 280.1768 | 308.1717 | HLGE | 409.2194 | 437.2143 |
| HLGEA | 480.2565 | 508.2514 | HLGEAG | 537.2780 | 565.2729 | HLGEAGA | 608.3151 | 636.3100 |

| | | | | | | | | |
|---|---|---|---|---|---|---|---|---|
| LG | 143.1179 | 171.1128 | LGE | 272.1605 | 300.1554 | LGEA | 343.1976 | 371.1925 |
| LGEAG | 400.2191 | 428.2140 | LGEAGA | 471.2562 | 499.2511 | LGEAGAI | 584.3402 | 612.3352 |
| LGEAGAIA | 655.3774 | 683.3723 | GE | 159.0764 | 187.0713 | GEA | 230.1135 | 258.1084 |
| GEAG | 287.1350 | 315.1299 | GEAGA | 358.1721 | 386.1670 | GEAGAI | 471.2562 | 499.2511 |
| GEAGAIA | 542.2933 | 570.2882 | GEAGAIAA | 613.3304 | 641.3253 | GEAGAIAAG | 670.3519 | 698.3468 |
| EA | 173.0921 | 201.0870 | EAG | 230.1135 | 258.1084 | EAGA | 301.1506 | 329.1456 |
| EAGAI | 414.2347 | 442.2296 | EAGAIA | 485.2718 | 513.2667 | EAGAIAA | 556.3089 | 584.3039 |
| EAGAIAAG | 613.3304 | 641.3253 | EAGAIAAGA | 684.3675 | 712.3624 | AG | 101.0709 | 129.0659 |
| AGA | 172.1081 | 200.1030 | AGAI | 285.1921 | 313.1870 | AGAIA | 356.2292 | 384.2241 |
| AGAIAA | 427.2663 | 455.2613 | AGAIAAG | 484.2878 | 512.2827 | AGAIAAGA | 555.3249 | 583.3198 |
| GA | 101.0709 | 129.0659 | GAI | 214.1550 | 242.1499 | GAIA | 285.1921 | 313.1870 |
| GAIAA | 356.2292 | 384.2241 | GAIAAG | 413.2507 | 441.2456 | GAIAAGA | 484.2878 | 512.2827 |
| GAIAAGAF | 631.3562 | 659.3511 | AI | 157.1335 | 185.1285 | AIA | 228.1707 | 256.1656 |
| AIAA | 299.2078 | 327.2027 | AIAAG | 356.2292 | 384.2241 | AIAAGA | 427.2663 | 455.2613 |
| AIAAGAF | 574.3348 | 602.3297 | AIAAGAFA | 645.3719 | 673.3668 | IA | 157.1335 | 185.1285 |
| IAA | 228.1707 | 256.1656 | IAAG | 285.1921 | 313.1870 | IAAGA | 356.2292 | 384.2241 |
| IAAGAF | 503.2976 | 531.2926 | IAAGAFA | 574.3348 | 602.3297 | IAAGAFAL | 687.4188 | 715.4137 |
| AA | 115.0866 | 143.0815 | AAG | 172.1081 | 200.1030 | AAGA | 243.1452 | 271.1401 |
| AAGAF | 390.2136 | 418.2085 | AAGAFA | 461.2507 | 489.2456 | AAGAFAL | 574.3348 | 602.3297 |
| AG | 101.0709 | 129.0659 | AGA | 172.1081 | 200.1030 | AGAF | 319.1765 | 347.1714 |
| AGAFA | 390.2136 | 418.2085 | AGAFAL | 503.2976 | 531.2926 | AGAFALY | 666.3610 | 694.3559 |
| GA | 101.0709 | 129.0659 | GAF | 248.1394 | 276.1343 | GAFA | 319.1765 | 347.1714 |
| GAFAL | 432.2605 | 460.2554 | GAFALY | 595.3239 | 623.3188 | AF | 191.1179 | 219.1128 |
| AFA | 262.1550 | 290.1499 | AFAL | 375.2391 | 403.2340 | AFALY | 538.3024 | 566.2973 |
| AFALYE | 667.3450 | 695.3399 | FA | 191.1179 | 219.1128 | FAL | 304.2020 | 332.1969 |
| FALY | 467.2653 | 495.2602 | FALYE | 596.3079 | 624.3028 | AL | 157.1335 | 185.1285 |
| ALY | 320.1969 | 348.1918 | ALYE | 449.2395 | 477.2344 | LY | 249.1598 | 277.1547 |
| LYE | 378.2023 | 406.1973 | YE | 265.1183 | 293.1132 | | | |

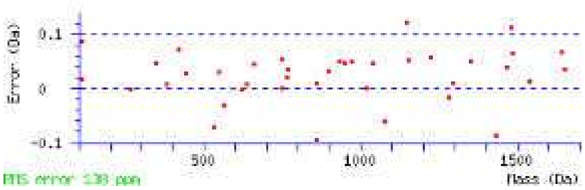


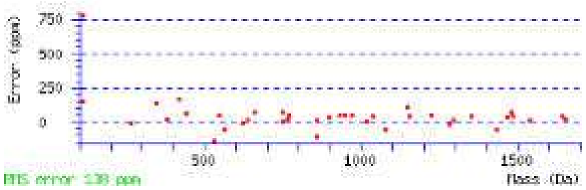


NCBI **BLAST** search of QHLGEAGAIAAGAFALYEK
(Parameters: blastp, nr protein database, expect=20000, no filter, PAM30)
Other BLAST web gateways

**All matches to this query**

| Score | Mr(calc) | Delta | Sequence |
|---|---|---|---|
| 104.9 | 1915.9792 | 0.0754 | QHLGEAGAIAAGAFALYEK |
| 104.9 | 1915.9792 | 0.0754 | QHLGEAGALAAGAFALYEK |
| 21.1 | 1916.0592 | -0.0047 | LLLTHDAHRITVTVGDR |
| 20.6 | 1915.8768 | 0.1778 | MDSASMTAAAKALADAGFR |
| 16.1 | 1916.0150 | 0.0396 | IGDVVTKGQAIALMGNTGR |
| 15.4 | 1916.0731 | -0.0186 | TVAVIGGGVSGLAAAKAFAEK |
| 14.7 | 1915.9534 | 0.1011 | GSGKGGQLQAAMAQIAEAGR |
| 14.6 | 1915.9561 | 0.0984 | DVMGTDDPLILSLISGDR |
| 14.4 | 1915.9866 | 0.0679 | DFLLMKPSAYFINVSR |
| 14.2 | 1915.9064 | 0.1481 | AGDYFSEAGATPATKAFGR |

**Mascot:** http://www.matrixscience.com/

# MATRIX SCIENCE Mascot Search Results

## Peptide View Spot no 18

MS/MS Fragmentation of **HKQHLGEAGAIAAGAFALYEK**
Found in **gi|15667623** in **NCBInr**, drought inducible 22 kD protein [Saccharum officinarum]

Match to Query 26: 2181.221024 from(2182.228300,1+) intensity(0.0000) index(11)
Title: Label: K8, Spot_Id: 219796, Peak_List_Id: 226439, MSMS Job_Run_Id: 21844, Comment:
Data file C:\Documents and Settings\Administrator\Desktop\Farah-Garima 11May 2013\ppw_K8_136859930300.txt

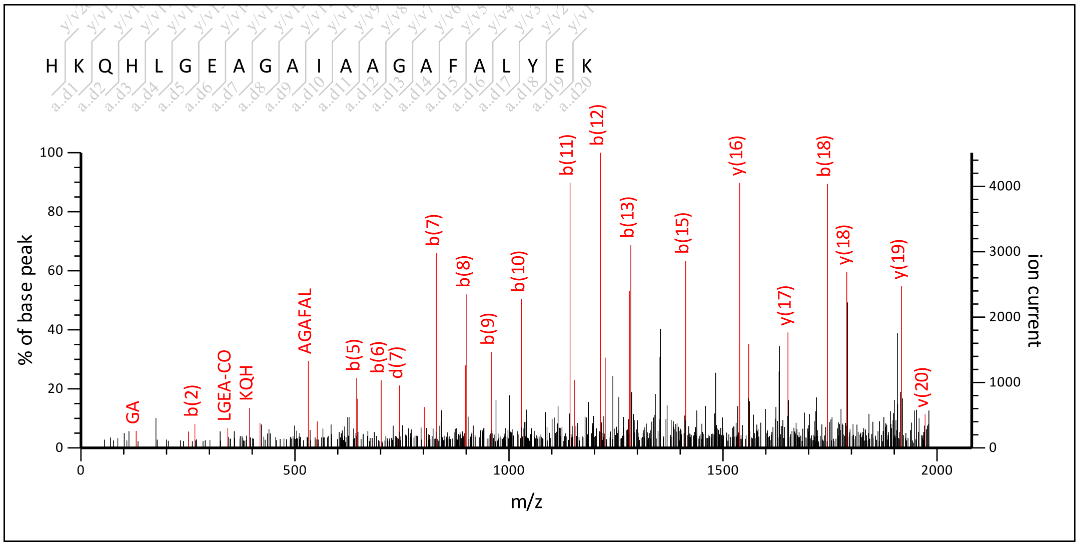


0 to 2081.08

Label all possible matches ○ Label matches used for scoring ◉

**Monoisotopic mass of neutral peptide Mr(calc):** 2181.1330
**Fixed modifications:** Carbamidomethyl (C) (apply to specified residues or termini only)
**Ions Score:** 131 **Expect:** 1.7e-09
**Matches :** 40/419 fragment ions using 40 most intense peaks (help)

| # | Immon. | a | a* | $a^0$ | b | b* | $b^0$ | d | d' | Seq. | v | w | w' | y | y* |
|---|---|---|---|---|---|---|---|---|---|---|---|---|---|---|---|
| 1 | 110.0713 | 110.0713 | | | 138.0662 | | | 44.0495 | | H | | | | | |
| 2 | 101.1073 | 238.1662 | 221.1397 | | ***266.1612*** | 249.1346 | | 181.1084 | | K | 1971.9923 | 1970.9971 | | 2045.0815 | 2028.0549 |
| 3 | 101.0709 | 366.2248 | 349.1983 | | ***394.2197*** | 377.1932 | | 309.2034 | | Q | 1843.9337 | 1842.9385 | | **1916.9865** | 1899.9599 |
| 4 | 110.0713 | 503.2837 | 486.2572 | | ***531.2786*** | 514.2521 | | | | H | 1706.8748 | | | **1788.9279** | 1771.9014 |
| 5 | 86.0964 | 616.3678 | 599.3412 | | ***644.3627*** | 627.3362 | | 574.3208 | | L | 1593.7908 | 1592.7955 | | **1651.8690** | 1634.8425 |
| 6 | 30.0338 | 673.3893 | 656.3627 | | ***701.3842*** | 684.3576 | | | | G | | | | **1538.7849** | 1521.7584 |
| 7 | 102.0550 | 802.4318 | 785.4053 | 784.4213 | ***830.4268*** | 813.4002 | 812.4162 | 744.4264 | | E | 1407.7267 | 1406.7314 | | 1481.7635 | 1464.7369 |
| 8 | 44.0495 | 873.4690 | 856.4424 | 855.4584 | ***901.4639*** | 884.4373 | 883.4533 | | | A | 1336.6896 | | | 1352.7209 | 1335.6943 |
| 9 | 30.0338 | 930.4904 | 913.4639 | 912.4799 | ***958.4853*** | 941.4588 | 940.4748 | | | G | | | | **1281.6838** | 1264.6572 |
| 10 | 44.0495 | 1001.5275 | 984.5010 | 983.5170 | ***1029.5225*** | 1012.4959 | 1011.5119 | | | A | 1208.6310 | | | **1224.6623** | 1207.6358 |
| 11 | 86.0964 | 1114.6116 | 1097.5851 | 1096.6010 | ***1142.6065*** | 1125.5800 | 1124.5960 | 1086.5803 | 1100.5960 | I | 1095.5469 | 1108.5673 | 1122.5830 | **1153.6252** | 1136.5986 |
| 12 | 44.0495 | 1185.6487 | 1168.6222 | 1167.6382 | ***1213.6436*** | 1196.6171 | 1195.6331 | | | A | 1024.5098 | | | 1040.5411 | 1023.5146 |
| 13 | 44.0495 | 1256.6858 | 1239.6593 | 1238.6753 | ***1284.6807*** | 1267.6542 | 1266.6702 | | | A | 953.4727 | | | 969.5040 | 952.4775 |
| 14 | 30.0338 | 1313.7073 | 1296.6807 | 1295.6967 | 1341.7022 | 1324.6757 | 1323.6916 | | | G | | | | **898.4669** | 881.4403 |
| 15 | 44.0495 | 1384.7444 | 1367.7179 | 1366.7338 | ***1412.7393*** | 1395.7128 | 1394.7288 | | | A | 825.4141 | | | 841.4454 | 824.4189 |
| 16 | 120.0808 | 1531.8128 | 1514.7863 | 1513.8023 | ***1559.8077*** | 1542.7812 | 1541.7972 | | | F | 678.3457 | | | 770.4083 | 753.3818 |
| 17 | 44.0495 | 1602.8499 | 1585.8234 | 1584.8394 | 1630.8449 | 1613.8183 | 1612.8343 | | | A | 607.3086 | | | 623.3399 | 606.3134 |
| 18 | 86.0964 | 1715.9340 | 1698.9075 | 1697.9234 | ***1743.9289*** | 1726.9024 | 1725.9184 | 1673.8871 | | L | 494.2245 | 493.2293 | | **552.3028** | 535.2762 |
| 19 | 136.0757 | 1878.9973 | 1861.9708 | 1860.9868 | 1906.9922 | 1889.9657 | 1888.9817 | | | Y | 331.1612 | | | 439.2187 | 422.1922 |
| 20 | 102.0550 | 2008.0399 | 1991.0134 | 1990.0294 | 2036.0348 | 2019.0083 | 2018.0243 | 1950.0344 | | E | 202.1186 | 201.1234 | | 276.1554 | 259.1288 |
| 21 | 101.1073 | | | | | | | | | K | 74.0237 | 73.0284 | | 147.1128 | 130.0863 |

| Seq | ya | yb | Seq | ya | yb | Seq | ya | yb |
|---|---|---|---|---|---|---|---|---|

| | | | | | | | | |
|---|---|---|---|---|---|---|---|---|
| **KQ** | 229.1659 | 257.1608 | **KQH** | 366.2248 | 394.2197 | **KQHL** | 479.3089 | 507.3038 |
| **KQHLG** | 536.3303 | 564.3253 | **KQHLGE** | 665.3729 | 693.3679 | **QH** | 238.1299 | 266.1248 |
| **QHL** | 351.2139 | 379.2088 | **QHLG** | 408.2354 | 436.2303 | **QHLGE** | 537.2780 | 565.2729 |
| **QHLGEA** | 608.3151 | 636.3100 | **QHLGEAG** | 665.3366 | 693.3315 | **HL** | 223.1553 | 251.1503 |
| **HLG** | 280.1768 | 308.1717 | **HLGE** | 409.2194 | 437.2143 | **HLGEA** | 480.2565 | 508.2514 |
| **HLGEAG** | 537.2780 | 565.2729 | **HLGEAGA** | 608.3151 | 636.3100 | **LG** | 143.1179 | 171.1128 |
| **LGE** | 272.1605 | 300.1554 | **LGEA** | 343.1976 | 371.1925 | **LGEAG** | 400.2191 | 428.2140 |
| **LGEAGA** | 471.2562 | 499.2511 | **LGEAGAI** | 584.3402 | 612.3352 | **LGEAGAIA** | 655.3774 | 683.3723 |
| **GE** | 159.0764 | 187.0713 | **GEA** | 230.1135 | 258.1084 | **GEAG** | 287.1350 | 315.1299 |
| **GEAGA** | 358.1721 | 386.1670 | **GEAGAI** | 471.2562 | 499.2511 | **GEAGAIA** | 542.2933 | 570.2882 |
| **GEAGAIAA** | 613.3304 | 641.3253 | **GEAGAIAAG** | 670.3519 | 698.3468 | **EA** | 173.0921 | 201.0870 |
| **EAG** | 230.1135 | 258.1084 | **EAGA** | 301.1506 | 329.1456 | **EAGAI** | 414.2347 | 442.2296 |
| **EAGAIA** | 485.2718 | 513.2667 | **EAGAIAA** | 556.3089 | 584.3039 | **EAGAIAAG** | 613.3304 | 641.3253 |
| **EAGAIAAGA** | 684.3675 | 712.3624 | **AG** | 101.0709 | 129.0659 | **AGA** | 172.1081 | 200.1030 |
| **AGAI** | 285.1921 | 313.1870 | **AGAIA** | 356.2292 | 384.2241 | **AGAIAA** | 427.2663 | 455.2613 |
| **AGAIAAG** | 484.2878 | 512.2827 | **AGAIAAGA** | 555.3249 | 583.3198 | **GA** | 101.0709 | 129.0659 |
| **GAI** | 214.1550 | 242.1499 | **GAIA** | 285.1921 | 313.1870 | **GAIAA** | 356.2292 | 384.2241 |
| **GAIAAG** | 413.2507 | 441.2456 | **GAIAAGA** | 484.2878 | 512.2827 | **GAIAAGAF** | 631.3562 | 659.3511 |
| **AI** | 157.1335 | 185.1285 | **AIA** | 228.1707 | 256.1656 | **AIAA** | 299.2078 | 327.2027 |
| **AIAAG** | 356.2292 | 384.2241 | **AIAAGA** | 427.2663 | 455.2613 | **AIAAGAF** | 574.3348 | 602.3297 |
| **AIAAGAFA** | 645.3719 | 673.3668 | **IA** | 157.1335 | 185.1285 | **IAA** | 228.1707 | 256.1656 |
| **IAAG** | 285.1921 | 313.1870 | **IAAGA** | 356.2292 | 384.2241 | **IAAGAF** | 503.2976 | 531.2926 |
| **IAAGAFA** | 574.3348 | 602.3297 | **IAAGAFAL** | 687.4188 | 715.4137 | **AA** | 115.0866 | 143.0815 |
| **AAG** | 172.1081 | 200.1030 | **AAGA** | 243.1452 | 271.1401 | **AAGAF** | 390.2136 | 418.2085 |
| **AAGAFA** | 461.2507 | 489.2456 | **AAGAFAL** | 574.3348 | 602.3297 | **AG** | 101.0709 | 129.0659 |
| **AGA** | 172.1081 | 200.1030 | **AGAF** | 319.1765 | 347.1714 | **AGAFA** | 390.2136 | 418.2085 |
| **AGAFAL** | 503.2976 | 531.2926 | **AGAFALY** | 666.3610 | 694.3559 | **GA** | 101.0709 | 129.0659 |
| **GAF** | 248.1394 | 276.1343 | **GAFA** | 319.1765 | 347.1714 | **GAFAL** | 432.2605 | 460.2554 |
| **GAFALY** | 595.3239 | 623.3188 | **AF** | 191.1179 | 219.1128 | **AFA** | 262.1550 | 290.1499 |
| **AFAL** | 375.2391 | 403.2340 | **AFALY** | 538.3024 | 566.2973 | **AFALYE** | 667.3450 | 695.3399 |
| **FA** | 191.1179 | 219.1128 | **FAL** | 304.2020 | 332.1969 | **FALY** | 467.2653 | 495.2602 |
| **FALYE** | 596.3079 | 624.3028 | **AL** | 157.1335 | 185.1285 | **ALY** | 320.1969 | 348.1918 |
| **ALYE** | 449.2395 | 477.2344 | **LY** | 249.1598 | 277.1547 | **LYE** | 378.2023 | 406.1973 |
| **YE** | 265.1183 | 293.1132 | | | | | | |

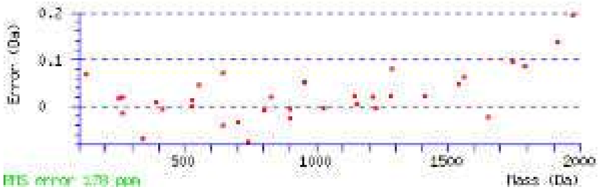


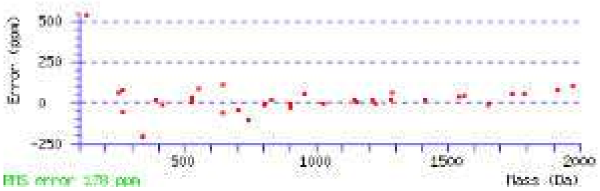


NCBI **BLAST** search of HKQHLGEAGAIAAGAFALYEK
(Parameters: blastp, nr protein database, expect=20000, no filter, PAM30)
Other BLAST web gateways

**All matches to this query**

| Score | Mr(calc) | Delta | Sequence |
|---|---|---|---|
| 130.7 | 2181.1330 | 0.0880 | HKQHLGEAGAIAAGAFALYEK |
| 130.7 | 2181.1330 | 0.0880 | HKQHLGEAGALAAGAFALYEK |
| 29.5 | 2181.2344 | -0.0133 | MAIGAALGAAVSLALLVVWSPR |
| 25.2 | 2181.2382 | -0.0172 | GHTGHLLGGAGALAAVVVLEALR |
| 25.1 | 2181.1688 | 0.0522 | HIAGMDNTLSLTQVGIRSLR |
| 21.9 | 2181.2018 | 0.0192 | QHQIASYLGITPTQLSRIR |
| 21.1 | 2181.0675 | 0.1535 | HQPAPLGPPAQEGGQEGAERR |
| 20.8 | 2181.2020 | 0.0190 | LFIPYTAVALAGAVNVFLMR |
| 20.6 | 2181.1100 | 0.1111 | IGDNMLGISQLNDTVHVLDK |

| | | | |
|---|---|---|---|
| 20.0 | 2181.2633 | -0.0423 | GLGFRLLVLALAAAAAAEVEAR |

**Mascot:** http://www.matrixscience.com/

# MATRIX SCIENCE Mascot Search Results

## Peptide View Spot no 18

MS/MS Fragmentation of **QHLGEAGAIAAGAFALYEKHEAK**
Found in **gi|15667623** in **NCBInr**, drought inducible 22 kD protein [Saccharum officinarum]

Match to Query 35: 2381.304724 from(2382.312000,1+) intensity(0.0000) index(15)
Title: Label: K8, Spot_Id: 219796, Peak_List_Id: 226441, MSMS Job_Run_Id: 21844, Comment:
Data file C:\Documents and Settings\Administrator\Desktop\Farah-Garima 11May 2013\ppw_K8_136859930300.txt

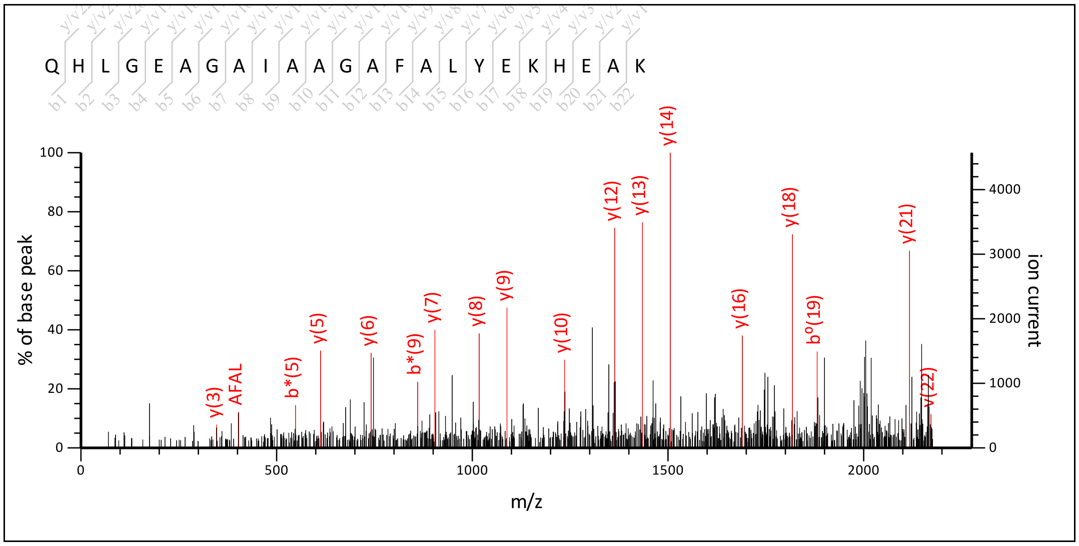


0 to 2276.14

Label all possible matches ○ Label matches used for scoring ◉

**Monoisotopic mass of neutral peptide Mr(calc):** 2381.2127
**Fixed modifications:** Carbamidomethyl (C) (apply to specified residues or termini only)
**Ions Score:** 100 **Expect:** 1.9e-06
**Matches :** 21/456 fragment ions using 22 most intense peaks (help)

| # | Immon. | a | a* | a⁰ | b | b* | b⁰ | d | d' | Seq. | v | w | w' | y | y* | [illegible] |
|---|---|---|---|---|---|---|---|---|---|---|---|---|---|---|---|---|
| 1 | 101.0709 | 101.0709 | 84.0444 | | 129.0659 | 112.0393 | | 44.0495 | | Q | | | | | | |
| 2 | 110.0713 | 238.1299 | 221.1033 | | 266.1248 | 249.0982 | | | | H | 2172.1084 | | | 2254.1615 | 2237.1349 | [illegible] |
| 3 | 86.0964 | 351.2139 | 334.1874 | | 379.2088 | 362.1823 | | 309.1670 | | L | 2059.0243 | 2058.0291 | | ***2117.1026*** | 2100.0760 | [illegible] |
| 4 | 30.0338 | 408.2354 | 391.2088 | | 436.2303 | 419.2037 | | | | G | | | | 2004.0185 | 1986.9920 | [illegible] |
| 5 | 102.0550 | 537.2780 | 520.2514 | 519.2674 | 565.2729 | 548.2463 | 547.2623 | 479.2725 | | E | 1872.9603 | 1871.9650 | | 1946.9971 | 1929.9705 | [illegible] |
| 6 | 44.0495 | 608.3151 | 591.2885 | 590.3045 | 636.3100 | 619.2835 | 618.2994 | | | A | 1801.9232 | | | ***1817.9545*** | 1800.9279 | [illegible] |
| 7 | 30.0338 | 665.3366 | 648.3100 | 647.3260 | 693.3315 | 676.3049 | 675.3209 | | | G | | | | 1746.9173 | 1729.8908 | [illegible] |
| 8 | 44.0495 | 736.3737 | 719.3471 | 718.3631 | 764.3686 | 747.3420 | 746.3580 | | | A | 1673.8646 | | | ***1689.8959*** | 1672.8693 | [illegible] |
| 9 | 86.0964 | 849.4577 | 832.4312 | 831.4472 | 877.4526 | 860.4261 | 859.4421 | 821.4264 | 835.4421 | I | 1560.7805 | 1573.8009 | 1587.8166 | 1618.8588 | 1601.8322 | [illegible] |
| 10 | 44.0495 | 920.4948 | 903.4683 | 902.4843 | 948.4898 | 931.4632 | 930.4792 | | | A | 1489.7434 | | | ***1505.7747*** | 1488.7482 | [illegible] |
| 11 | 44.0495 | 991.5320 | 974.5054 | 973.5214 | 1019.5269 | 1002.5003 | 1001.5163 | | | A | 1418.7063 | | | ***1434.7376*** | 1417.7110 | [illegible] |
| 12 | 30.0338 | 1048.5534 | 1031.5269 | 1030.5429 | 1076.5483 | 1059.5218 | 1058.5378 | | | G | | | | ***1363.7005*** | 1346.6739 | [illegible] |
| 13 | 44.0495 | 1119.5905 | 1102.5640 | 1101.5800 | 1147.5854 | 1130.5589 | 1129.5749 | | | A | 1290.6477 | | | 1306.6790 | 1289.6525 | [illegible] |
| 14 | 120.0808 | 1266.6589 | 1249.6324 | 1248.6484 | 1294.6539 | 1277.6273 | 1276.6433 | | | F | 1143.5793 | | | ***1235.6419*** | 1218.6154 | [illegible] |
| 15 | 44.0495 | 1337.6961 | 1320.6695 | 1319.6855 | 1365.6910 | 1348.6644 | 1347.6804 | | | A | 1072.5422 | | | ***1088.5735*** | 1071.5469 | [illegible] |
| 16 | 86.0964 | 1450.7801 | 1433.7536 | 1432.7696 | 1478.7750 | 1461.7485 | 1460.7645 | 1408.7332 | | L | 959.4581 | 958.4629 | | ***1017.5364*** | 1000.5098 | [illegible] |
| 17 | 136.0757 | 1613.8435 | 1596.8169 | 1595.8329 | 1641.8384 | 1624.8118 | 1623.8278 | | | Y | 796.3948 | | | ***904.4523*** | 887.4258 | [illegible] |
| 18 | 102.0550 | 1742.8860 | 1725.8595 | 1724.8755 | 1770.8810 | 1753.8544 | 1752.8704 | 1684.8806 | | E | 667.3522 | 666.3570 | | ***741.3890*** | 724.3624 | [illegible] |
| 19 | 101.1073 | 1870.9810 | 1853.9545 | 1852.9704 | 1898.9759 | 1881.9494 | 1880.9654 | 1813.9232 | | K | 539.2572 | 538.2620 | | ***612.3464*** | 595.3198 | [illegible] |
| 20 | 110.0713 | 2008.0399 | 1991.0134 | 1990.0294 | 2036.0348 | 2019.0083 | 2018.0243 | | | H | 402.1983 | | | 484.2514 | 467.2249 | [illegible] |
| 21 | 102.0550 | 2137.0825 | 2120.0560 | 2119.0720 | 2165.0774 | 2148.0509 | 2147.0669 | 2079.0770 | | E | 273.1557 | 272.1605 | | ***347.1925*** | 330.1660 | [illegible] |
| 22 | 44.0495 | 2208.1196 | 2191.0931 | 2190.1091 | 2236.1145 | 2219.0880 | 2218.1040 | | | A | 202.1186 | | | 218.1499 | 201.1234 | |
| 23 | 101.1073 | | | | | | | | | K | 74.0237 | 73.0284 | | 147.1128 | 130.0863 | |

| Seq | ya | yb | Seq | ya | yb | Seq | ya | yb |
|---|---|---|---|---|---|---|---|---|
| HL | 223.1553 | 251.1503 | HLG | 280.1768 | 308.1717 | HLGE | 409.2194 | 437.2143 |
| HLGEA | 480.2565 | 508.2514 | HLGEAG | 537.2780 | 565.2729 | HLGEAGA | 608.3151 | 636.3100 |
| LG | 143.1179 | 171.1128 | LGE | 272.1605 | 300.1554 | LGEA | 343.1976 | 371.1925 |
| LGEAG | 400.2191 | 428.2140 | LGEAGA | 471.2562 | 499.2511 | LGEAGAI | 584.3402 | 612.3352 |
| LGEAGAIA | 655.3774 | 683.3723 | GE | 159.0764 | 187.0713 | GEA | 230.1135 | 258.1084 |
| GEAG | 287.1350 | 315.1299 | GEAGA | 358.1721 | 386.1670 | GEAGAI | 471.2562 | 499.2511 |
| GEAGAIA | 542.2933 | 570.2882 | GEAGAIAA | 613.3304 | 641.3253 | GEAGAIAAG | 670.3519 | 698.3468 |
| EA | 173.0921 | 201.0870 | EAG | 230.1135 | 258.1084 | EAGA | 301.1506 | 329.1456 |
| EAGAI | 414.2347 | 442.2296 | EAGAIA | 485.2718 | 513.2667 | EAGAIAA | 556.3089 | 584.3039 |
| EAGAIAAG | 613.3304 | 641.3253 | EAGAIAAGA | 684.3675 | 712.3624 | AG | 101.0709 | 129.0659 |
| AGA | 172.1081 | 200.1030 | AGAI | 285.1921 | 313.1870 | AGAIA | 356.2292 | 384.2241 |
| AGAIAA | 427.2663 | 455.2613 | AGAIAAG | 484.2878 | 512.2827 | AGAIAAGA | 555.3249 | 583.3198 |
| GA | 101.0709 | 129.0659 | GAI | 214.1550 | 242.1499 | GAIA | 285.1921 | 313.1870 |
| GAIAA | 356.2292 | 384.2241 | GAIAAG | 413.2507 | 441.2456 | GAIAAGA | 484.2878 | 512.2827 |
| GAIAAGAF | 631.3562 | 659.3511 | AI | 157.1335 | 185.1285 | AIA | 228.1707 | 256.1656 |
| AIAA | 299.2078 | 327.2027 | AIAAG | 356.2292 | 384.2241 | AIAAGA | 427.2663 | 455.2613 |
| AIAAGAF | 574.3348 | 602.3297 | AIAAGAFA | 645.3719 | 673.3668 | IA | 157.1335 | 185.1285 |
| IAA | 228.1707 | 256.1656 | IAAG | 285.1921 | 313.1870 | IAAGA | 356.2292 | 384.2241 |
| IAAGAF | 503.2976 | 531.2926 | IAAGAFA | 574.3348 | 602.3297 | IAAGAFAL | 687.4188 | 715.4137 |
| AA | 115.0866 | 143.0815 | AAG | 172.1081 | 200.1030 | AAGA | 243.1452 | 271.1401 |
| AAGAF | 390.2136 | 418.2085 | AAGAFA | 461.2507 | 489.2456 | AAGAFAL | 574.3348 | 602.3297 |
| AG | 101.0709 | 129.0659 | AGA | 172.1081 | 200.1030 | AGAF | 319.1765 | 347.1714 |
| AGAFA | 390.2136 | 418.2085 | AGAFAL | 503.2976 | 531.2926 | AGAFALY | 666.3610 | 694.3559 |
| GA | 101.0709 | 129.0659 | GAF | 248.1394 | 276.1343 | GAFA | 319.1765 | 347.1714 |
| GAFAL | 432.2605 | 460.2554 | GAFALY | 595.3239 | 623.3188 | AF | 191.1179 | 219.1128 |
| AFA | 262.1550 | 290.1499 | AFAL | 375.2391 | 403.2340 | AFALY | 538.3024 | 566.2973 |
| AFALYE | 667.3450 | 695.3399 | FA | 191.1179 | 219.1128 | FAL | 304.2020 | 332.1969 |
| FALY | 467.2653 | 495.2602 | FALYE | 596.3079 | 624.3028 | AL | 157.1335 | 185.1285 |
| ALY | 320.1969 | 348.1918 | ALYE | 449.2395 | 477.2344 | ALYEK | 577.3344 | 605.3293 |
| LY | 249.1598 | 277.1547 | LYE | 378.2023 | 406.1973 | LYEK | 506.2973 | 534.2922 |
| LYEKH | 643.3562 | 671.3511 | YE | 265.1183 | 293.1132 | YEK | 393.2132 | 421.2082 |
| YEKH | 530.2722 | 558.2671 | YEKHE | 659.3148 | 687.3097 | EK | 230.1499 | 258.1448 |
| EKH | 367.2088 | 395.2037 | EKHE | 496.2514 | 524.2463 | EKHEA | 567.2885 | 595.2835 |
| KH | 238.1662 | 266.1612 | KHE | 367.2088 | 395.2037 | KHEA | 438.2459 | 466.2409 |
| HE | 239.1139 | 267.1088 | HEA | 310.1510 | 338.1459 | EA | 173.0921 | 201.0870 |

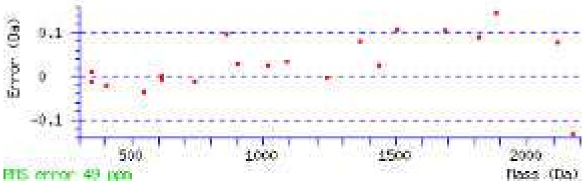


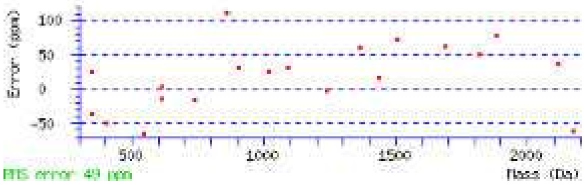


NCBI **BLAST** search of QHLGEAGAIAAGAFALYEKHEAK
(Parameters: blastp, nr protein database, expect=20000, no filter, PAM30)
Other BLAST web gateways

**All matches to this query**

| Score | Mr(calc) | Delta | Sequence |
|---|---|---|---|
| 100.1 | 2381.2127 | 0.0920 | QHLGEAGAIAAGAFALYEKHEAK |
| 100.1 | 2381.2127 | 0.0920 | QHLGEAGALAAGAFALYEKHEAK |
| 17.7 | 2381.1971 | 0.1077 | MSVADLPTTALFQALLSGIMDR |
| 17.1 | 2381.1436 | 0.1611 | SDIVVMVATLAFFGFACGEFGK |
| 16.4 | 2381.2664 | 0.0383 | LLEQDYSVQFALALMLKDLR |
| 14.5 | 2381.1144 | 0.1903 | DVSHPNMGYAFKAVGNGLEMTK |
| | | | |

| | | | |
|---|---|---|---|
| 12.9 | 2381.1719 | 0.1328 | HMNALLSKTATSDEGYVMLIR |
| 11.7 | 2381.1177 | 0.1870 | HSMAMVGMAAKQLYVGEEAQAK |
| 11.5 | 2381.1256 | 0.1791 | AQCPDGYVGQFAADVDRMLLR |
| 11.5 | 2381.1733 | 0.1314 | GGSQTWRVFGAMAGGGCLLATLR |

**Mascot:** http://www.matrixscience.com/

# MATRIX SCIENCE Mascot Search Results

## Peptide View **Spot no 19**

MS/MS Fragmentation of **SFGVLIPDQGIALR**
Found in **gi|115446541** in **NCBInr**, Os02g0537700 [Oryza sativa Japonica Group]

Match to Query 81: 1484.901924 from(1485.909200,1+) intensity(0.0000) index(13)
Title: Label: H17, Spot_Id: 219937, Peak_List_Id: 228919, MSMS Job_Run_Id: 22005, Comment:
Data file C:\Documents and Settings\Administrator\Desktop\Farah-Garima 11May 2013\ppw_H17_136868316000.txt

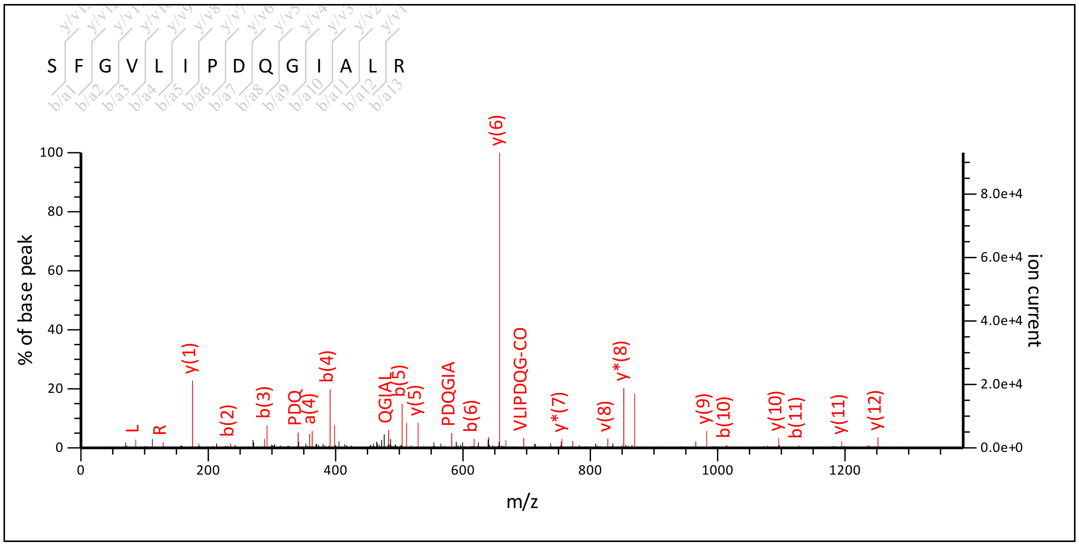


0 to 1385.58

Label all possible matches ○ Label matches used for scoring ◉

**Monoisotopic mass of neutral peptide Mr(calc):** 1484.8351
**Fixed modifications:** Carbamidomethyl (C) (apply to specified residues or termini only)
**Ions Score:** 90 **Expect:** 1.7e-05
**Matches :** 56/238 fragment ions using 52 most intense peaks (help)

| # | Immon. | a | a* | a⁰ | b | b* | b⁰ | d | d' | Seq. | v | w | w' | y | y* | |
|---|---|---|---|---|---|---|---|---|---|---|---|---|---|---|---|---|
| 1 | 60.0444 | 60.0444 | | 42.0338 | 88.0393 | | 70.0287 | 44.0495 | | S | | | | | | |
| 2 | 120.0808 | 207.1128 | | 189.1022 | **235.1077** | | 217.0972 | | | F | 1306.7478 | | | 1398.8104 | 1381.7838 | 138[illegible] |
| 3 | 30.0338 | 264.1343 | | 246.1237 | **292.1292** | | 274.1186 | | | G | | | | ***1251.7419*** | 1234.7154 | 123[illegible] |
| 4 | 72.0808 | 363.2027 | | 345.1921 | **391.1976** | | 373.1870 | 349.1870 | | V | 1150.6579 | 1163.6783 | | ***1194.7205*** | 1177.6939 | 117[illegible] |
| 5 | 86.0964 | 476.2867 | | 458.2762 | **504.2817** | | 486.2711 | 434.2398 | | L | 1037.5738 | 1036.5786 | | ***1095.6521*** | 1078.6255 | 107[illegible] |
| 6 | 86.0964 | 589.3708 | | 571.3602 | **617.3657** | | 599.3552 | 561.3395 | 575.3552 | I | 924.4898 | 937.5102 | 951.5258 | ***982.5680*** | 965.5415 | 96[illegible] |
| 7 | 70.0651 | 686.4236 | | 668.4130 | 714.4185 | | 696.4079 | 660.4079 | | P | 827.4370 | 826.4417 | | ***869.4839*** | 852.4574 | 85[illegible] |
| 8 | 88.0393 | 801.4505 | | 783.4400 | 829.4454 | | 811.4349 | 757.4607 | | D | 712.4100 | 711.4148 | | ***772.4312*** | 755.4046 | 75[illegible] |
| 9 | 101.0709 | 929.5091 | 912.4825 | 911.4985 | 957.5040 | 940.4775 | 939.4934 | 872.4876 | | Q | 584.3515 | 583.3562 | | ***657.4042*** | 640.3777 | |
| 10 | 30.0338 | 986.5306 | 969.5040 | 968.5200 | **1014.5255** | 997.4989 | 996.5149 | | | G | | | | ***529.3457*** | 512.3191 | |
| 11 | 86.0964 | 1099.6146 | 1082.5881 | 1081.6041 | **1127.6095** | 1110.5830 | 1109.5990 | 1071.5833 | 1085.5990 | I | 414.2459 | 427.2663 | 441.2820 | 472.3242 | 455.2976 | |
| 12 | 44.0495 | 1170.6517 | 1153.6252 | 1152.6412 | 1198.6467 | 1181.6201 | 1180.6361 | | | A | 343.2088 | | | ***359.2401*** | 342.2136 | |
| 13 | 86.0964 | 1283.7358 | 1266.7093 | 1265.7252 | 1311.7307 | 1294.7042 | 1293.7201 | 1241.6889 | | L | 230.1248 | 229.1295 | | ***288.2030*** | 271.1765 | |
| 14 | 129.1135 | | | | | | | | | R | 74.0237 | 73.0284 | | ***175.1190*** | 158.0924 | |

| Seq | ya | yb | Seq | ya | yb | Seq | ya | yb |
|---|---|---|---|---|---|---|---|---|
| FG | 177.1022 | 205.0972 | FGV | 276.1707 | 304.1656 | FGVL | 389.2547 | 417.2496 |
| FGVLI | 502.3388 | 530.3337 | FGVLIP | 599.3915 | 627.3865 | GV | 129.1022 | 157.0972 |
| GVL | 242.1863 | 270.1812 | GVLI | 355.2704 | 383.2653 | GVLIP | 452.3231 | 480.3180 |
| GVLIPD | 567.3501 | 595.3450 | GVLIPDQ | 695.4087 | 723.4036 | VL | 185.1648 | 213.1598 |
| VLI | 298.2489 | 326.2438 | VLIP | 395.3017 | 423.2966 | VLIPD | 510.3286 | 538.3235 |
| VLIPDQ | 638.3872 | 666.3821 | VLIPDQG | 695.4087 | 723.4036 | LI | 199.1805 | 227.1754 |
| LIP | 296.2333 | 324.2282 | LIPD | 411.2602 | 439.2551 | LIPDQ | 539.3188 | 567.3137 |

| | | | | | | | | |
|---|---|---|---|---|---|---|---|---|
| **LIPDQG** | 596.3402 | 624.3352 | **IP** | 183.1492 | 211.1441 | **IPD** | 298.1761 | 326.1710 |
| **IPDQ** | 426.2347 | 454.2296 | **IPDQG** | 483.2562 | 511.2511 | **IPDQGI** | 596.3402 | 624.3352 |
| **IPDQGIA** | 667.3774 | 695.3723 | **PD** | 185.0921 | 213.0870 | **PDQ** | 313.1506 | 341.1456 |
| **PDQG** | 370.1721 | 398.1670 | **PDQGI** | 483.2562 | 511.2511 | **PDQGIA** | 554.2933 | 582.2882 |
| **PDQGIAL** | 667.3774 | 695.3723 | **DQ** | 216.0979 | 244.0928 | **DQG** | 273.1193 | 301.1143 |
| **DQGI** | 386.2034 | 414.1983 | **DQGIA** | 457.2405 | 485.2354 | **DQGIAL** | 570.3246 | 598.3195 |
| **QG** | 158.0924 | 186.0873 | **QGI** | 271.1765 | 299.1714 | **QGIA** | 342.2136 | 370.2085 |
| **QGIAL** | 455.2976 | 483.2926 | **GI** | 143.1179 | 171.1128 | **GIA** | 214.1550 | 242.1499 |
| **GIAL** | 327.2391 | 355.2340 | **IA** | 157.1335 | 185.1285 | **IAL** | 270.2176 | 298.2125 |
| **AL** | 157.1335 | 185.1285 | | | | | | |

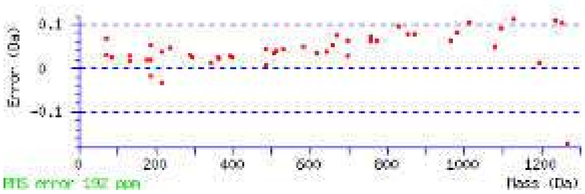


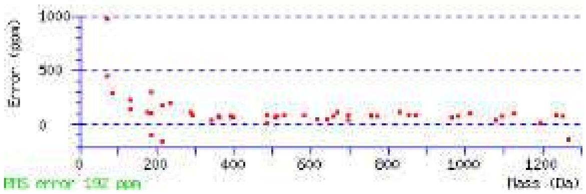


NCBI **BLAST** search of SFGVLIPDQGIALR
(Parameters: blastp, nr protein database, expect=20000, no filter, PAM30)
Other BLAST web gateways

**All matches to this query**

| Score | Mr(calc) | Delta | Sequence |
|---|---|---|---|
| 90.4 | 1484.8351 | 0.0668 | SFGVLIPDQGIALR |
| 32.3 | 1484.8674 | 0.0345 | SSLALAVARLIESR |
| 32.0 | 1484.7835 | 0.1185 | VTSAIANVLPDETR |
| 30.4 | 1484.8212 | 0.0807 | FSRIIVSASHVNR |
| 30.4 | 1484.8576 | 0.0444 | HPRLLTITEHIR |
| 30.4 | 1484.8212 | 0.0807 | SFRLLSQLTQHR |
| 30.0 | 1484.8861 | 0.0158 | LSSIAAVRIVAMVR |
| 29.9 | 1484.7697 | 0.1322 | MSRLFAFLIDEK |
| 29.9 | 1484.8562 | 0.0457 | LVQIITSNILDTR |
| 27.7 | 1484.7875 | 0.1144 | FDKILTITYESR |

**Mascot:** http://www.matrixscience.com/

# MATRIX SCIENCE Mascot Search Results

## Peptide View Spot no 19

MS/MS Fragmentation of **APDFDAEAVFDQEFINVK**
Found in **gi|115446541** in **NCBInr**, Os02g0537700 [Oryza sativa Japonica Group]

Match to Query 92: 2054.045724 from(2055.053000,1+) intensity(0.0000) index(18)
Title: Label: H17, Spot_Id: 219937, Peak_List_Id: 228922, MSMS Job_Run_Id: 22005, Comment:
Data file C:\Documents and Settings\Administrator\Desktop\Farah-Garima 11May 2013\ppw_H17_136868316000.txt

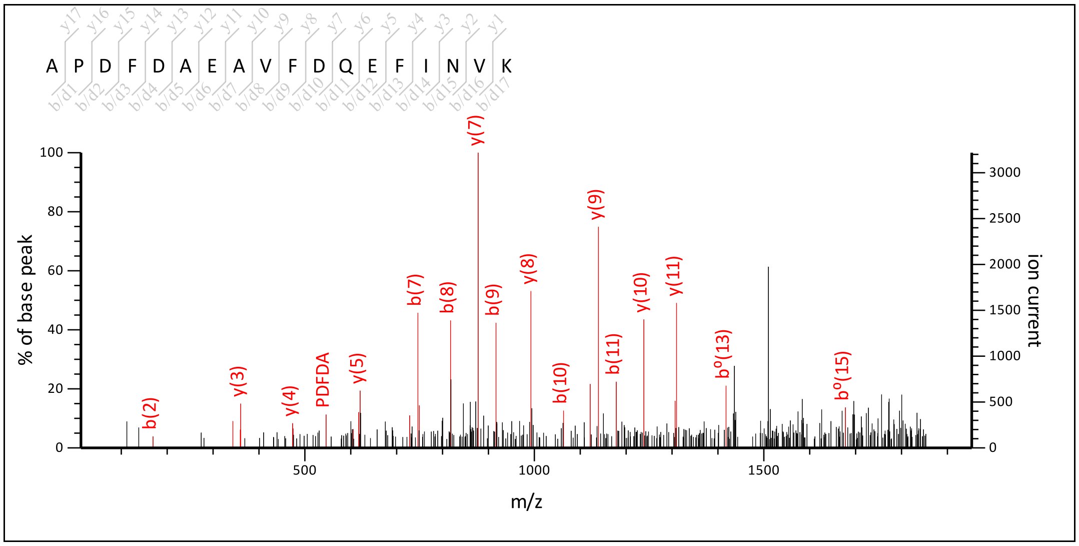


12.11 to 1953.01

Label all possible matches ○ Label matches used for scoring ◉

**Monoisotopic mass of neutral peptide Mr(calc):** 2053.9633
**Fixed modifications:** Carbamidomethyl (C) (apply to specified residues or termini only)
**Ions Score:** 50 **Expect:** 0.28
**Matches :** 33/305 fragment ions using 53 most intense peaks (help)

| # | Immon. | a | a* | $a^0$ | b | b* | $b^0$ | d | d' | Seq. | v | w | w' | y | y* | |
|---|---|---|---|---|---|---|---|---|---|---|---|---|---|---|---|---|
| 1 | 44.0495 | 44.0495 | | | 72.0444 | | | 44.0495 | | A | | | | | | |
| 2 | 70.0651 | 141.1022 | | | ***169.0972*** | | | 115.0866 | | P | 1941.8865 | 1940.8913 | | 1983.9335 | 1966.9069 | 196 |
| 3 | 88.0393 | 256.1292 | | 238.1186 | 284.1241 | | 266.1135 | 212.1394 | | D | 1826.8596 | 1825.8643 | | 1886.8807 | 1869.8541 | 186 |
| 4 | 120.0808 | 403.1976 | | 385.1870 | 431.1925 | | 413.1819 | | | F | 1679.7911 | | | 1771.8537 | 1754.8272 | 17 |
| 5 | 88.0393 | 518.2245 | | 500.2140 | ***546.2195*** | | 528.2089 | 474.2347 | | D | 1564.7642 | 1563.7690 | | 1624.7853 | 1607.7588 | 160 |
| 6 | 44.0495 | 589.2617 | | 571.2511 | ***617.2566*** | | 599.2460 | | | A | 1493.7271 | | | 1509.7584 | 1492.7318 | 149 |
| 7 | 102.0550 | 718.3042 | | 700.2937 | ***746.2992*** | | 728.2886 | 660.2988 | | E | 1364.6845 | 1363.6892 | | 1438.7213 | 1421.6947 | 142 |
| 8 | 44.0495 | 789.3414 | | 771.3308 | ***817.3363*** | | 799.3257 | | | A | 1293.6474 | | | ***1309.6787*** | 1292.6521 | 129 |
| 9 | 72.0808 | 888.4098 | | 870.3992 | ***916.4047*** | | 898.3941 | 874.3941 | | V | 1194.5790 | 1207.5994 | | ***1238.6416*** | 1221.6150 | 122 |
| 10 | 120.0808 | 1035.4782 | | 1017.4676 | ***1063.4731*** | | 1045.4625 | | | F | 1047.5106 | | | ***1139.5732*** | 1122.5466 | 112 |
| 11 | 88.0393 | 1150.5051 | | 1132.4946 | ***1178.5000*** | | 1160.4895 | 1106.5153 | | D | 932.4836 | 931.4884 | | ***992.5047*** | 975.4782 | 97 |
| 12 | 101.0709 | 1278.5637 | 1261.5372 | 1260.5531 | ***1306.5586*** | 1289.5321 | 1288.5481 | 1221.5422 | | Q | 804.4250 | 803.4298 | | ***877.4778*** | 860.4512 | 85 |
| 13 | 102.0550 | 1407.6063 | 1390.5798 | 1389.5957 | 1435.6012 | 1418.5747 | 1417.5907 | 1349.6008 | | E | 675.3824 | 674.3872 | | ***749.4192*** | 732.3927 | 73 |
| 14 | 120.0808 | 1554.6747 | 1537.6482 | 1536.6642 | 1582.6696 | 1565.6431 | 1564.6591 | | | F | 528.3140 | | | ***620.3766*** | 603.3501 | |
| 15 | 86.0964 | 1667.7588 | 1650.7322 | 1649.7482 | 1695.7537 | 1678.7271 | 1677.7431 | 1639.7275 | 1653.7431 | I | 415.2300 | 428.2504 | 442.2660 | ***473.3082*** | 456.2817 | |
| 16 | 87.0553 | 1781.8017 | 1764.7752 | 1763.7911 | 1809.7966 | 1792.7701 | 1791.7861 | 1738.7959 | | N | 301.1870 | 300.1918 | | ***360.2241*** | 343.1976 | |
| 17 | 72.0808 | 1880.8701 | 1863.8436 | 1862.8596 | 1908.8650 | 1891.8385 | 1890.8545 | 1866.8545 | | V | 202.1186 | 215.1390 | | 246.1812 | 229.1547 | |
| 18 | 101.1073 | | | | | | | | | K | 74.0237 | 73.0284 | | 147.1128 | 130.0863 | |

| Seq | ya | yb | Seq | ya | yb | Seq | ya | yb |
|---|---|---|---|---|---|---|---|---|
| PD | 185.0921 | 213.0870 | PDF | 332.1605 | 360.1554 | PDFD | 447.1874 | 475.1823 |
| PDFDA | 518.2245 | 546.2195 | PDFDAE | 647.2671 | 675.2620 | DF | 235.1077 | 263.1026 |
| DFD | 350.1347 | 378.1296 | DFDA | 421.1718 | 449.1667 | DFDAE | 550.2144 | 578.2093 |

| | | | | | | | | |
|---|---|---|---|---|---|---|---|---|
| **DFDAEA** | 621.2515 | 649.2464 | **FD** | 235.1077 | 263.1026 | **FDA** | 306.1448 | 334.1397 |
| **FDAE** | 435.1874 | 463.1823 | **FDAEA** | 506.2245 | 534.2195 | **FDAEAV** | 605.2930 | 633.2879 |
| **DA** | 159.0764 | 187.0713 | **DAE** | 288.1190 | 316.1139 | **DAEA** | 359.1561 | 387.1510 |
| **DAEAV** | 458.2245 | 486.2195 | **DAEAVF** | 605.2930 | 633.2879 | **AE** | 173.0921 | 201.0870 |
| **AEA** | 244.1292 | 272.1241 | **AEAV** | 343.1976 | 371.1925 | **AEAVF** | 490.2660 | 518.2609 |
| **AEAVFD** | 605.2930 | 633.2879 | **EA** | 173.0921 | 201.0870 | **EAV** | 272.1605 | 300.1554 |
| **EAVF** | 419.2289 | 447.2238 | **EAVFD** | 534.2558 | 562.2508 | **EAVFDQ** | 662.3144 | 690.3093 |
| **AV** | 143.1179 | 171.1128 | **AVF** | 290.1863 | 318.1812 | **AVFD** | 405.2132 | 433.2082 |
| **AVFDQ** | 533.2718 | 561.2667 | **AVFDQE** | 662.3144 | 690.3093 | **VF** | 219.1492 | 247.1441 |
| **VFD** | 334.1761 | 362.1710 | **VFDQ** | 462.2347 | 490.2296 | **VFDQE** | 591.2773 | 619.2722 |
| **FD** | 235.1077 | 263.1026 | **FDQ** | 363.1663 | 391.1612 | **FDQE** | 492.2089 | 520.2038 |
| **FDQEF** | 639.2773 | 667.2722 | **DQ** | 216.0979 | 244.0928 | **DQE** | 345.1405 | 373.1354 |
| **DQEF** | 492.2089 | 520.2038 | **DQEFI** | 605.2930 | 633.2879 | **QE** | 230.1135 | 258.1084 |
| **QEF** | 377.1819 | 405.1769 | **QEFI** | 490.2660 | 518.2609 | **QEFIN** | 604.3089 | 632.3039 |
| **EF** | 249.1234 | 277.1183 | **EFI** | 362.2074 | 390.2023 | **EFIN** | 476.2504 | 504.2453 |
| **EFINV** | 575.3188 | 603.3137 | **FI** | 233.1648 | 261.1598 | **FIN** | 347.2078 | 375.2027 |
| **FINV** | 446.2762 | 474.2711 | **IN** | 200.1394 | 228.1343 | **INV** | 299.2078 | 327.2027 |
| **NV** | 186.1237 | 214.1186 | | | | | | |

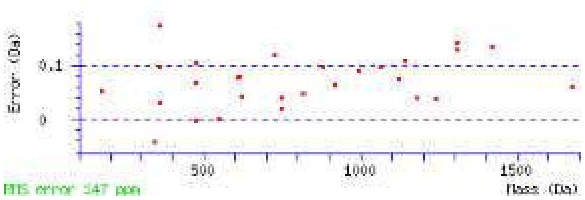


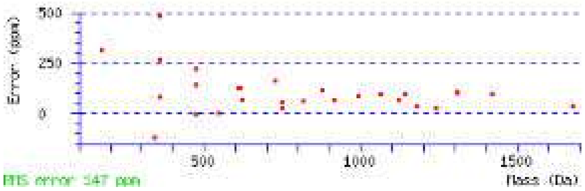


NCBI **BLAST** search of APDFDAEAVFDQEFINVK
(Parameters: blastp, nr protein database, expect=20000, no filter, PAM30)
Other BLAST web gateways

**All matches to this query**

| Score | Mr(calc) | Delta | Sequence |
|---|---|---|---|
| 49.5 | 2053.9633 | 0.0824 | APDFDAEAVFDQEFINVK |
| 14.1 | 2054.0507 | -0.0049 | ENHLPIIVFDMDTVGNLK |
| 13.6 | 2054.0029 | 0.0429 | HALTAVAVQTADETTADNAR |
| 12.4 | 2053.9592 | 0.0865 | VVEYEAATEADFLAQADGR |
| 12.4 | 2054.0428 | 0.0030 | MDKILEAVMLSSYPTNVK |
| 12.2 | 2054.0684 | -0.0227 | SVLDGELSVYENLYLRGK |
| 11.7 | 2053.9819 | 0.0638 | QNQFDIAVMSALTYFYK |
| 10.8 | 2054.0652 | -0.0195 | VPPGLLVCRAMVAELEER |
| 10.3 | 2053.8964 | 0.1493 | EGSQSGEAFVELESEDDVK |
| 9.7 | 2053.9482 | 0.0975 | MVEMVTGEKTSLEEMGGAR |

**Mascot:** http://www.matrixscience.com/

# MATRIX SCIENCE Mascot Search Results

## Peptide View Spot no 19

MS/MS Fragmentation of **SGGLGDLKYPLISDVTK**
Found in **gi|115446541** in **NCBInr**, Os02g0537700 [Oryza sativa Japonica Group]

Match to Query 88: 1762.020824 from(1763.028100,1+) intensity(0.0000) index(16)
Title: Label: H17, Spot_Id: 219937, Peak_List_Id: 228921, MSMS Job_Run_Id: 22005, Comment:
Data file C:\Documents and Settings\Administrator\Desktop\Farah-Garima 11May 2013\ppw_H17_136868316000.txt

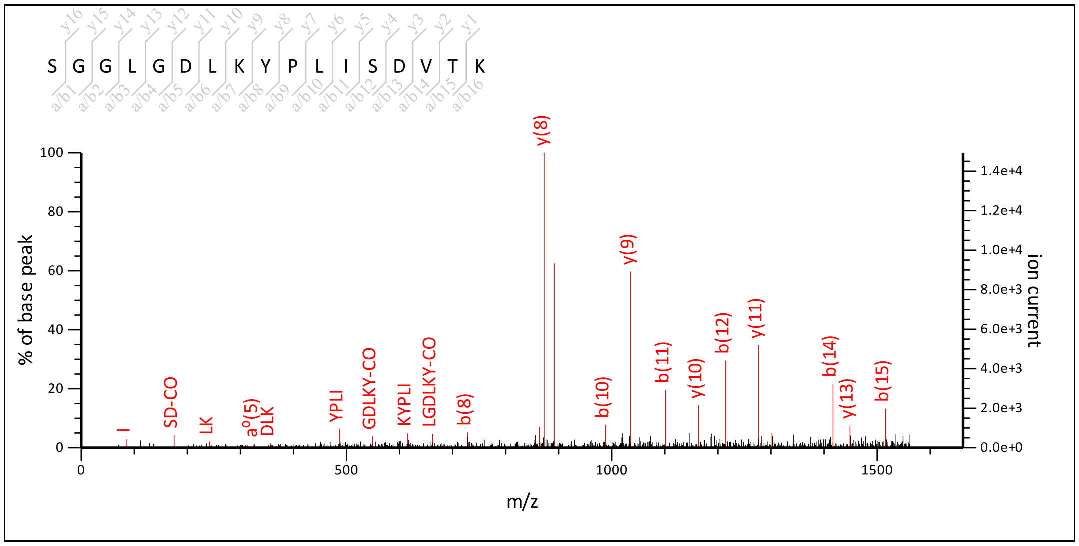


0 to 1661.88

Label all possible matches ○ Label matches used for scoring ◉

**Monoisotopic mass of neutral peptide Mr(calc):** 1761.9513
**Fixed modifications:** Carbamidomethyl (C) (apply to specified residues or termini only)
**Ions Score:** 71 **Expect:** 0.0018
**Matches :** 31/305 fragment ions using 30 most intense peaks (help)

| # | Immon. | a | a* | a0 | b | b* | b0 | d | d' | Seq. | v | w | w' | y | y* | |
|---|---|---|---|---|---|---|---|---|---|---|---|---|---|---|---|---|
| 1 | 60.0444 | 60.0444 | | 42.0338 | 88.0393 | | 70.0287 | 44.0495 | | S | | | | | | |
| 2 | 30.0338 | 117.0659 | | 99.0553 | 145.0608 | | 127.0502 | | | G | | | | 1675.9265 | 1658.9000 | 16[illegible] |
| 3 | 30.0338 | 174.0873 | | 156.0768 | 202.0822 | | 184.0717 | | | G | | | | 1618.9051 | 1601.8785 | 16[illegible] |
| 4 | 86.0964 | 287.1714 | | 269.1608 | 315.1663 | | 297.1557 | 245.1244 | | L | 1503.8053 | 1502.8101 | | 1561.8836 | 1544.8570 | 15[illegible] |
| 5 | 30.0338 | 344.1928 | | 326.1823 | 372.1878 | | 354.1772 | | | G | | | | ***1448.7995*** | 1431.7730 | 14[illegible] |
| 6 | 88.0393 | 459.2198 | | 441.2092 | ***487.2147*** | | 469.2041 | 415.2300 | | D | 1331.7569 | 1330.7617 | | 1391.7781 | 1374.7515 | 13[illegible] |
| 7 | 86.0964 | 572.3039 | | 554.2933 | 600.2988 | | 582.2882 | 530.2569 | | L | 1218.6729 | 1217.6776 | | ***1276.7511*** | 1259.7246 | 12[illegible] |
| 8 | 101.1073 | 700.3988 | 683.3723 | 682.3882 | ***728.3937*** | 711.3672 | 710.3832 | 643.3410 | | K | 1090.5779 | 1089.5827 | | ***1163.6671*** | 1146.6405 | 11[illegible] |
| 9 | 136.0757 | 863.4621 | 846.4356 | 845.4516 | ***891.4571*** | 874.4305 | 873.4465 | | | Y | 927.5146 | | | ***1035.5721*** | 1018.5455 | 10[illegible] |
| 10 | 70.0651 | 960.5149 | 943.4884 | 942.5043 | ***988.5098*** | 971.4833 | 970.4993 | 934.4993 | | P | 830.4618 | 829.4666 | | ***872.5088*** | 855.4822 | 8[illegible] |
| 11 | 86.0964 | 1073.5990 | 1056.5724 | 1055.5884 | ***1101.5939*** | 1084.5673 | 1083.5833 | 1031.5520 | | L | 717.3777 | 716.3825 | | 775.4560 | 758.4294 | 7[illegible] |
| 12 | 86.0964 | 1186.6830 | 1169.6565 | 1168.6725 | ***1214.6780*** | 1197.6514 | 1196.6674 | 1158.6517 | 1172.6674 | I | 604.2937 | 617.3141 | 631.3297 | ***662.3719*** | 645.3454 | 6[illegible] |
| 13 | 60.0444 | 1273.7151 | 1256.6885 | 1255.7045 | ***1301.7100*** | 1284.6834 | 1283.6994 | 1257.7201 | | S | 517.2617 | 516.2664 | | ***549.2879*** | 532.2613 | 5[illegible] |
| 14 | 88.0393 | 1388.7420 | 1371.7155 | 1370.7314 | ***1416.7369*** | 1399.7104 | 1398.7264 | 1344.7522 | | D | 402.2347 | 401.2395 | | 462.2558 | 445.2293 | 4[illegible] |
| 15 | 72.0808 | 1487.8104 | 1470.7839 | 1469.7999 | ***1515.8053*** | 1498.7788 | 1497.7948 | 1473.7948 | | V | 303.1663 | 316.1867 | | 347.2289 | 330.2023 | 3[illegible] |
| 16 | 74.0600 | 1588.8581 | 1571.8316 | 1570.8475 | 1616.8530 | 1599.8265 | 1598.8425 | 1572.8632 | 1574.8425 | T | 202.1186 | 215.1390 | 217.1183 | 248.1605 | 231.1339 | 2[illegible] |
| 17 | 101.1073 | | | | | | | | | K | 74.0237 | 73.0284 | | 147.1128 | 130.0863 | |

| Seq | ya | yb | Seq | ya | yb | Seq | ya | yb |
|---|---|---|---|---|---|---|---|---|
| GG | 87.0553 | 115.0502 | GGL | 200.1394 | 228.1343 | GGLG | 257.1608 | 285.1557 |
| GGLGD | 372.1878 | 400.1827 | GGLGDL | 485.2718 | 513.2667 | GGLGDLK | 613.3668 | 641.3617 |
| GL | 143.1179 | 171.1128 | GLG | 200.1394 | 228.1343 | GLGD | 315.1663 | 343.1612 |
| GLGDL | 428.2504 | 456.2453 | GLGDLK | 556.3453 | 584.3402 | LG | 143.1179 | 171.1128 |

| | | | | | | | | |
|---|---|---|---|---|---|---|---|---|
| **LGD** | 258.1448 | 286.1397 | **LGDL** | 371.2289 | 399.2238 | **LGDLK** | 499.3239 | 527.3188 |
| **LGDLKY** | 662.3872 | 690.3821 | **GD** | 145.0608 | 173.0557 | **GDL** | 258.1448 | 286.1397 |
| **GDLK** | 386.2398 | 414.2347 | **GDLKY** | 549.3031 | 577.2980 | **GDLKYP** | 646.3559 | 674.3508 |
| **DL** | 201.1234 | 229.1183 | **DLK** | 329.2183 | 357.2132 | **DLKY** | 492.2817 | 520.2766 |
| **DLKYP** | 589.3344 | 617.3293 | **LK** | 214.1914 | 242.1863 | **LKY** | 377.2547 | 405.2496 |
| **LKYP** | 474.3075 | 502.3024 | **LKYPL** | 587.3915 | 615.3865 | **KY** | 264.1707 | 292.1656 |
| **KYP** | 361.2234 | 389.2183 | **KYPL** | 474.3075 | 502.3024 | **KYPLI** | 587.3915 | 615.3865 |
| **KYPLIS** | 674.4236 | 702.4185 | **YP** | 233.1285 | 261.1234 | **YPL** | 346.2125 | 374.2074 |
| **YPLI** | 459.2966 | 487.2915 | **YPLIS** | 546.3286 | 574.3235 | **YPLISD** | 661.3556 | 689.3505 |
| **PL** | 183.1492 | 211.1441 | **PLI** | 296.2333 | 324.2282 | **PLIS** | 383.2653 | 411.2602 |
| **PLISD** | 498.2922 | 526.2871 | **PLISDV** | 597.3606 | 625.3556 | **PLISDVT** | 698.4083 | 726.4032 |
| **LI** | 199.1805 | 227.1754 | **LIS** | 286.2125 | 314.2074 | **LISD** | 401.2395 | 429.2344 |
| **LISDV** | 500.3079 | 528.3028 | **LISDVT** | 601.3556 | 629.3505 | **IS** | 173.1285 | 201.1234 |
| **ISD** | 288.1554 | 316.1503 | **ISDV** | 387.2238 | 415.2187 | **ISDVT** | 488.2715 | 516.2664 |
| **SD** | 175.0713 | 203.0662 | **SDV** | 274.1397 | 302.1347 | **SDVT** | 375.1874 | 403.1823 |
| **DV** | 187.1077 | 215.1026 | **DVT** | 288.1554 | 316.1503 | **VT** | 173.1285 | 201.1234 |

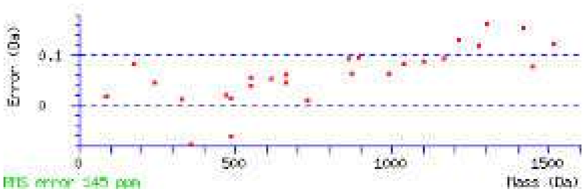


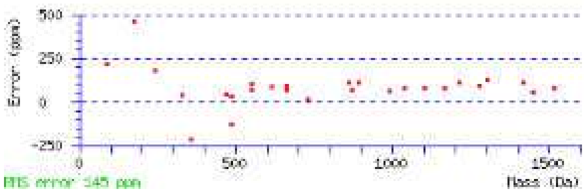


NCBI **BLAST** search of SGGLGDLKYPLISDVTK
(Parameters: blastp, nr protein database, expect=20000, no filter, PAM30)
Other BLAST web gateways

**All matches to this query**

| Score | Mr(calc) | Delta | Sequence |
|---|---|---|---|
| 70.7 | 1761.9513 | 0.0696 | SGGLGDLKYPLISDVTK |
| 42.3 | 1761.9149 | 0.1059 | TGGLGDLNYPLISDVTK |
| 39.4 | 1761.9513 | 0.0696 | SGGLGDLKYPLVSDLTK |
| 24.4 | 1761.8897 | 0.1311 | IATTQKAWNSLSDAEK |
| 23.4 | 1761.9447 | 0.0761 | KPLYCDGGSGLTLLLR |
| 22.5 | 1761.8679 | 0.1529 | AAGMSDTQIQQELAKR |
| 22.0 | 1761.8614 | 0.1594 | AVGEQLCAAAARGSMVR |
| 19.6 | 1761.8872 | 0.1336 | GFGQPQLAMIFPLDGR |
| 19.5 | 1761.9586 | 0.0622 | MLPEEFKIPLTTSIK |
| 19.4 | 1761.9989 | 0.0219 | TIGSKQLTATLLDFVR |

**Mascot:** http://www.matrixscience.com/

# MATRIX SCIENCE Mascot Search Results

## Peptide View Spot no 19

MS/MS Fragmentation of **EGVIQHSTINNLAIGR**
Found in **gi|115446541** in **NCBInr**, Os02g0537700 [Oryza sativa Japonica Group]

Match to Query 86: 1721.003924 from(1722.011200,1+) intensity(0.0000) index(15)
Title: Label: H17, Spot_Id: 219937, Peak_List_Id: 228932, MSMS Job_Run_Id: 22005, Comment:
Data file C:\Documents and Settings\Administrator\Desktop\Farah-Garima 11May 2013\ppw_H17_136868316000.txt

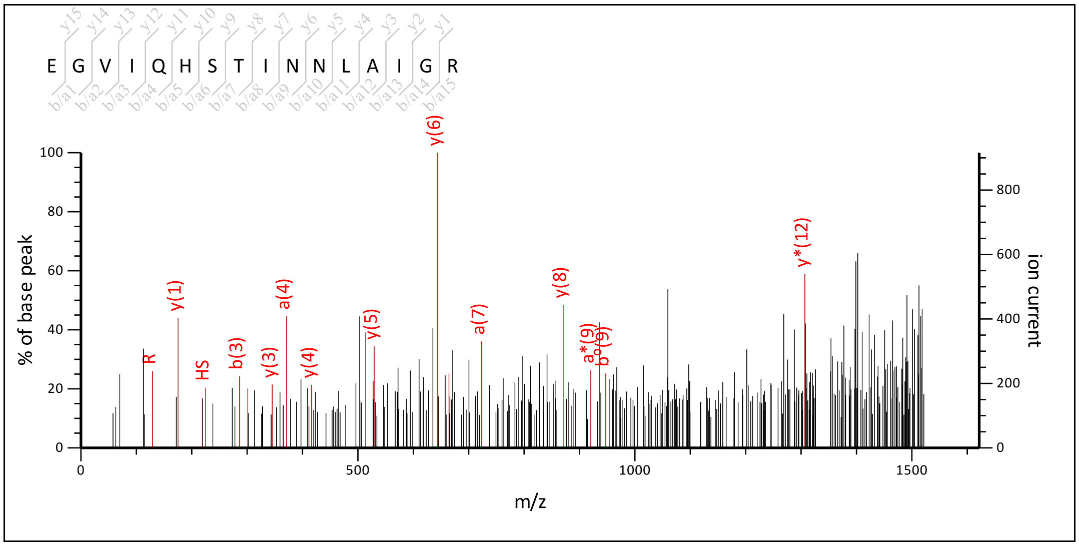


0 to 1621.59

Label all possible matches ○ Label matches used for scoring ◉

**Monoisotopic mass of neutral peptide Mr(calc):** 1720.9220
**Fixed modifications:** Carbamidomethyl (C) (apply to specified residues or termini only)
**Ions Score:** 11 **Expect:** 1.5e+03
**Matches :** 21/292 fragment ions using 60 most intense peaks (help)

| # | Immon. | a | a* | a$^0$ | b | b* | b$^0$ | d | d' | Seq. | v | w | w' | y | y* | |
|---|---|---|---|---|---|---|---|---|---|---|---|---|---|---|---|---|
| 1 | 102.0550 | 102.0550 | | 84.0444 | 130.0499 | | 112.0393 | 44.0495 | | E | | | | | | |
| 2 | 30.0338 | 159.0764 | | 141.0659 | 187.0713 | | 169.0608 | | | G | | | | 1592.8867 | 1575.8602 | [illegible] |
| 3 | 72.0808 | 258.1448 | | 240.1343 | ***286.1397*** | | 268.1292 | 244.1292 | | V | 1491.8027 | 1504.8231 | | 1535.8653 | 1518.8387 | [illegible] |
| 4 | 86.0964 | **371.2289** | | 353.2183 | 399.2238 | | 381.2132 | 343.1976 | 357.2132 | I | 1378.7186 | 1391.7390 | 1405.7546 | 1436.7968 | 1419.7703 | [illegible] |
| 5 | 101.0709 | 499.2875 | 482.2609 | 481.2769 | ***527.2824*** | 510.2558 | 509.2718 | 442.2660 | | Q | 1250.6600 | 1249.6648 | | 1323.7128 | 1306.6862 | [illegible] |
| 6 | 110.0713 | 636.3464 | 619.3198 | 618.3358 | ***664.3413*** | 647.3148 | 646.3307 | | | H | 1113.6011 | | | 1195.6542 | 1178.6276 | [illegible] |
| 7 | 60.0444 | **723.3784** | 706.3519 | 705.3678 | 751.3733 | 734.3468 | 733.3628 | 707.3835 | | S | 1026.5691 | 1025.5738 | | 1058.5953 | 1041.5687 | [illegible] |
| 8 | 74.0600 | 824.4261 | 807.3995 | 806.4155 | 852.4210 | 835.3945 | 834.4104 | 808.4312 | 810.4104 | T | 925.5214 | 938.5418 | 940.5211 | 971.5633 | 954.5367 | [illegible] |
| 9 | 86.0964 | 937.5102 | 920.4836 | 919.4996 | 965.5051 | 948.4785 | 947.4945 | 909.4789 | 923.4945 | I | 812.4373 | 825.4577 | 839.4734 | ***870.5156*** | 853.4890 | |
| 10 | 87.0553 | 1051.5531 | 1034.5265 | 1033.5425 | 1079.5480 | 1062.5215 | 1061.5374 | 1008.5473 | | N | 698.3944 | 697.3991 | | 757.4315 | 740.4050 | |
| 11 | 87.0553 | 1165.5960 | 1148.5695 | 1147.5854 | 1193.5909 | 1176.5644 | 1175.5804 | 1122.5902 | | N | 584.3515 | 583.3562 | | ***643.3886*** | 626.3620 | |
| 12 | 86.0964 | 1278.6801 | 1261.6535 | 1260.6695 | ***1306.6750*** | 1289.6484 | 1288.6644 | 1236.6331 | | L | 471.2674 | 470.2722 | | ***529.3457*** | 512.3191 | |
| 13 | 44.0495 | 1349.7172 | 1332.6906 | 1331.7066 | 1377.7121 | 1360.6856 | 1359.7015 | | | A | 400.2303 | | | ***416.2616*** | 399.2350 | |
| 14 | 86.0964 | 1462.8013 | 1445.7747 | 1444.7907 | 1490.7962 | 1473.7696 | 1472.7856 | 1434.7700 | 1448.7856 | I | 287.1462 | 300.1666 | 314.1823 | ***345.2245*** | 328.1979 | |
| 15 | 30.0338 | 1519.8227 | 1502.7962 | 1501.8122 | 1547.8176 | 1530.7911 | 1529.8071 | | | G | | | | 232.1404 | 215.1139 | |
| 16 | 129.1135 | | | | | | | | | R | 74.0237 | 73.0284 | | ***175.1190*** | 158.0924 | |

| Seq | ya | yb | Seq | ya | yb | Seq | ya | yb |
|---|---|---|---|---|---|---|---|---|
| GV | 129.1022 | 157.0972 | GVI | 242.1863 | 270.1812 | GVIQ | 370.2449 | 398.2398 |
| GVIQH | 507.3038 | 535.2987 | GVIQHS | 594.3358 | 622.3307 | GVIQHST | 695.3835 | 723.3784 |
| VI | 185.1648 | 213.1598 | VIQ | 313.2234 | 341.2183 | VIQH | 450.2823 | 478.2772 |
| VIQHS | 537.3144 | 565.3093 | VIQHST | 638.3620 | 666.3570 | IQ | 214.1550 | 242.1499 |
| IQH | 351.2139 | 379.2088 | IQHS | 438.2459 | 466.2409 | IQHST | 539.2936 | 567.2885 |

| | | | | | | | | |
|---|---|---|---|---|---|---|---|---|
| **IQHSTI** | 652.3777 | 680.3726 | **QH** | 238.1299 | 266.1248 | **QHS** | 325.1619 | 353.1568 |
| **QHST** | 426.2096 | 454.2045 | **QHSTI** | 539.2936 | 567.2885 | **QHSTIN** | 653.3365 | 681.3315 |
| **HS** | 197.1033 | 225.0982 | **HST** | 298.1510 | 326.1459 | **HSTI** | 411.2350 | 439.2300 |
| **HSTIN** | 525.2780 | 553.2729 | **HSTINN** | 639.3209 | 667.3158 | **ST** | 161.0921 | 189.0870 |
| **STI** | 274.1761 | 302.1710 | **STIN** | 388.2191 | 416.2140 | **STINN** | 502.2620 | 530.2569 |
| **STINNL** | 615.3460 | 643.3410 | **STINNLA** | 686.3832 | 714.3781 | **TI** | 187.1441 | 215.1390 |
| **TIN** | 301.1870 | 329.1819 | **TINN** | 415.2300 | 443.2249 | **TINNL** | 528.3140 | 556.3089 |
| **TINNLA** | 599.3511 | 627.3461 | **IN** | 200.1394 | 228.1343 | **INN** | 314.1823 | 342.1772 |
| **INNL** | 427.2663 | 455.2613 | **INNLA** | 498.3035 | 526.2984 | **INNLAI** | 611.3875 | 639.3824 |
| **INNLAIG** | 668.4090 | 696.4039 | **NN** | 201.0982 | 229.0931 | **NNL** | 314.1823 | 342.1772 |
| **NNLA** | 385.2194 | 413.2143 | **NNLAI** | 498.3035 | 526.2984 | **NNLAIG** | 555.3249 | 583.3198 |
| **NL** | 200.1394 | 228.1343 | **NLA** | 271.1765 | 299.1714 | **NLAI** | 384.2605 | 412.2554 |
| **NLAIG** | 441.2820 | 469.2769 | **LA** | 157.1335 | 185.1285 | **LAI** | 270.2176 | 298.2125 |
| **LAIG** | 327.2391 | 355.2340 | **AI** | 157.1335 | 185.1285 | **AIG** | 214.1550 | 242.1499 |
| **IG** | 143.1179 | 171.1128 | | | | | | |

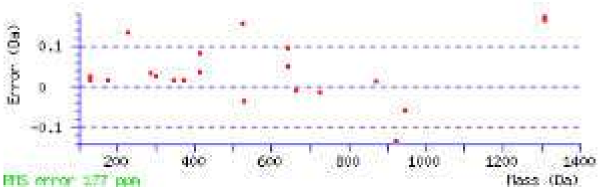


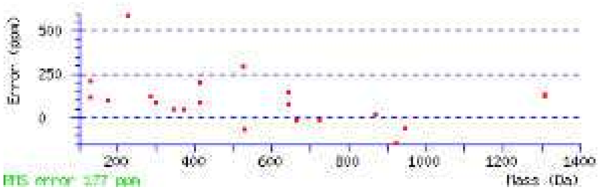


NCBI **BLAST** search of EGVIQHSTINNLAIGR
(Parameters: blastp, nr protein database, expect=20000, no filter, PAM30)
Other BLAST web gateways

**All matches to this query**

| Score | Mr(calc) | Delta | Sequence |
|---|---|---|---|
| 12.7 | 1720.9108 | 0.0932 | NANDQLPQKLPSAPTK |
| 11.8 | 1720.8605 | 0.1434 | RDVSGGVGLAHSAQPDR |
| 11.3 | 1720.9220 | 0.0819 | EGVIQHSTINNLAIGR |
| 9.2 | 1720.8566 | 0.1473 | QTFHEMAGLSITKSR |
| 9.1 | 1720.8488 | 0.1551 | QMVVASVGPTTSEMLR |
| 8.9 | 1721.0386 | -0.0347 | VTSIVVRPMLPPLKR |
| 8.5 | 1720.9624 | 0.0415 | ATGWVLVGPTPANIIGR |
| 8.5 | 1720.9624 | 0.0415 | AWGTVLVGPTPANIIGR |
| 8.5 | 1720.9625 | 0.0414 | AWGTVLVGPTPVNVVGR |
| 8.5 | 1720.9948 | 0.0091 | RTGTVLVGPTPANIIGR |

**Mascot:** http://www.matrixscience.com/

# MATRIX SCIENCE Mascot Search Results

## Peptide View Spot no 19

MS/MS Fragmentation of **GLFIIDKEGVIQHSTINNLAIGR**
Found in **gi|115446541** in **NCBInr**, Os02g0537700 [Oryza sativa Japonica Group]

Match to Query 99: 2507.505424 from(2508.512700,1+) intensity(0.0000) index(20)
Title: Label: H17, Spot_Id: 219937, Peak_List_Id: 228929, MSMS Job_Run_Id: 22005, Comment:
Data file C:\Documents and Settings\Administrator\Desktop\Farah-Garima 11May 2013\ppw_H17_136868316000.txt

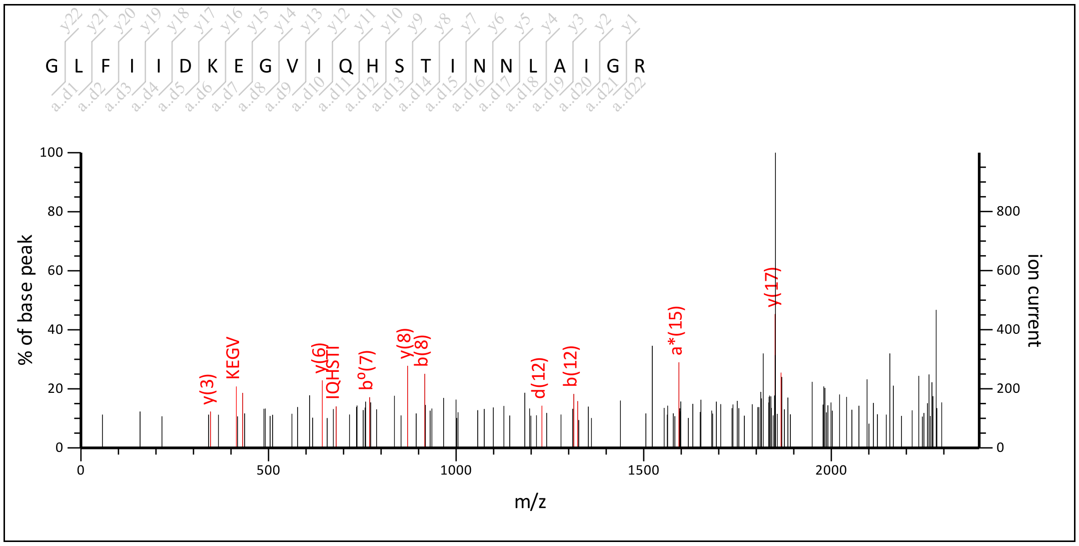


0 to 2394.22

Label all possible matches Label matches used for scoring

**Monoisotopic mass of neutral peptide Mr(calc):** 2507.3860
**Fixed modifications:** Carbamidomethyl (C) (apply to specified residues or termini only)
**Ions Score:** 6 **Expect:** 2.6e+03
**Matches :** 17/437 fragment ions using 45 most intense peaks (help)

| # | Immon. | a | a* | a⁰ | b | b* | b⁰ | d | d' | Seq. | v | w | w' | y | y* | [illegible] |
|---|---|---|---|---|---|---|---|---|---|---|---|---|---|---|---|---|
| 1 | 30.0338 | 30.0338 | | | 58.0287 | | | 44.0495 | | G | | | | | | |
| 2 | 86.0964 | 143.1179 | | | 171.1128 | | | 101.0709 | | L | 2393.2936 | 2392.2983 | | 2451.3718 | 2434.3453 | 24 |
| 3 | 120.0808 | 290.1863 | | | 318.1812 | | | | | F | 2246.2252 | | | 2338.2878 | 2321.2612 | 23 |
| 4 | 86.0964 | 403.2704 | | | 431.2653 | | | 375.2391 | 389.2547 | I | 2133.1411 | 2146.1615 | 2160.1771 | 2191.2193 | 2174.1928 | [illegible] |
| 5 | 86.0964 | 516.3544 | | | 544.3493 | | | 488.3231 | 502.3388 | I | 2020.0570 | 2033.0774 | 2047.0931 | 2078.1353 | 2061.1087 | [illegible] |
| 6 | 88.0393 | 631.3814 | | 613.3708 | 659.3763 | | 641.3657 | 587.3915 | | D | 1905.0301 | 1904.0348 | | 1965.0512 | 1948.0247 | [illegible] |
| 7 | 101.1073 | 759.4763 | 742.4498 | 741.4658 | 787.4713 | 770.4447 | 769.4607 | 702.4185 | | K | 1776.9351 | 1775.9399 | | 1850.0243 | 1832.9977 | [illegible] |
| 8 | 102.0550 | 888.5189 | 871.4924 | 870.5084 | 916.5138 | 899.4873 | 898.5033 | 830.5135 | | E | 1647.8925 | 1646.8973 | | 1721.9293 | 1704.9028 | [illegible] |
| 9 | 30.0338 | 945.5404 | 928.5138 | 927.5298 | 973.5353 | 956.5088 | 955.5247 | | | G | | | | 1592.8867 | 1575.8602 | [illegible] |
| 10 | 72.0808 | 1044.6088 | 1027.5823 | 1026.5982 | 1072.6037 | 1055.5772 | 1054.5932 | 1030.5932 | | V | 1491.8027 | 1504.8231 | | 1535.8653 | 1518.8387 | [illegible] |
| 11 | 86.0964 | 1157.6929 | 1140.6663 | 1139.6823 | 1185.6878 | 1168.6612 | 1167.6772 | 1129.6616 | 1143.6772 | I | 1378.7186 | 1391.7390 | 1405.7546 | 1436.7968 | 1419.7703 | [illegible] |
| 12 | 101.0709 | 1285.7515 | 1268.7249 | 1267.7409 | 1313.7464 | 1296.7198 | 1295.7358 | 1228.7300 | | Q | 1250.6600 | 1249.6648 | | 1323.7128 | 1306.6862 | [illegible] |
| 13 | 110.0713 | 1422.8104 | 1405.7838 | 1404.7998 | 1450.8053 | 1433.7787 | 1432.7947 | | | H | 1113.6011 | | | 1195.6542 | 1178.6276 | [illegible] |
| 14 | 60.0444 | 1509.8424 | 1492.8158 | 1491.8318 | 1537.8373 | 1520.8108 | 1519.8267 | 1493.8475 | | S | 1026.5691 | 1025.5738 | | 1058.5953 | 1041.5687 | [illegible] |
| 15 | 74.0600 | 1610.8901 | 1593.8635 | 1592.8795 | 1638.8850 | 1621.8584 | 1620.8744 | 1594.8952 | 1596.8744 | T | 925.5214 | 938.5418 | 940.5211 | 971.5633 | 954.5367 | [illegible] |
| 16 | 86.0964 | 1723.9741 | 1706.9476 | 1705.9636 | 1751.9690 | 1734.9425 | 1733.9585 | 1695.9428 | 1709.9585 | I | 812.4373 | 825.4577 | 839.4734 | 870.5156 | 853.4890 | |
| 17 | 87.0553 | 1838.0171 | 1820.9905 | 1820.0065 | 1866.0120 | 1848.9854 | 1848.0014 | 1795.0112 | | N | 698.3944 | 697.3992 | | 757.4315 | 740.4050 | |
| 18 | 87.0553 | 1952.0600 | 1935.0334 | 1934.0494 | 1980.0549 | 1963.0284 | 1962.0443 | 1909.0542 | | N | 584.3515 | 583.3562 | | 643.3886 | 626.3620 | |
| 19 | 86.0964 | 2065.1441 | 2048.1175 | 2047.1335 | 2093.1390 | 2076.1124 | 2075.1284 | 2023.0971 | | L | 471.2674 | 470.2722 | | 529.3457 | 512.3191 | |
| 20 | 44.0495 | 2136.1812 | 2119.1546 | 2118.1706 | 2164.1761 | 2147.1495 | 2146.1655 | | | A | 400.2303 | | | 416.2616 | 399.2350 | |
| 21 | 86.0964 | 2249.2652 | 2232.2387 | 2231.2547 | 2277.2601 | 2260.2336 | 2259.2496 | 2221.2339 | 2235.2496 | I | 287.1462 | 300.1666 | 314.1823 | 345.2245 | 328.1979 | |
| 22 | 30.0338 | 2306.2867 | 2289.2601 | 2288.2761 | 2334.2816 | 2317.2551 | 2316.2710 | | | G | | | | 232.1404 | 215.1139 | |
| 23 | 129.1135 | | | | | | | | | R | 74.0237 | 73.0284 | | 175.1190 | 158.0924 | |

| Seq | ya | yb | Seq | ya | yb | Seq | ya | yb |
|---|---|---|---|---|---|---|---|---|
| LF | 233.1648 | 261.1598 | LFI | 346.2489 | 374.2438 | LFII | 459.3330 | 487.3279 |
| LFIID | 574.3599 | 602.3548 | FI | 233.1648 | 261.1598 | FII | 346.2489 | 374.2438 |
| FIID | 461.2758 | 489.2708 | FIIDK | 589.3708 | 617.3657 | II | 199.1805 | 227.1754 |
| IID | 314.2074 | 342.2023 | IIDK | 442.3024 | 470.2973 | IIDKE | 571.3450 | 599.3399 |
| IIDKEG | 628.3665 | 656.3614 | ID | 201.1234 | 229.1183 | IDK | 329.2183 | 357.2132 |
| IDKE | 458.2609 | 486.2558 | IDKEG | 515.2824 | 543.2773 | IDKEGV | 614.3508 | 642.3457 |
| DK | 216.1343 | 244.1292 | DKE | 345.1769 | 373.1718 | DKEG | 402.1983 | 430.1932 |
| DKEGV | 501.2667 | 529.2617 | DKEGVI | 614.3508 | 642.3457 | KE | 230.1499 | 258.1448 |
| KEG | 287.1714 | 315.1663 | KEGV | 386.2398 | 414.2347 | KEGVI | 499.3239 | 527.3188 |
| KEGVIQ | 627.3824 | 655.3774 | EG | 159.0764 | 187.0713 | EGV | 258.1448 | 286.1397 |
| EGVI | 371.2289 | 399.2238 | EGVIQ | 499.2875 | 527.2824 | EGVIQH | 636.3464 | 664.3413 |
| GV | 129.1022 | 157.0972 | GVI | 242.1863 | 270.1812 | GVIQ | 370.2449 | 398.2398 |
| GVIQH | 507.3038 | 535.2987 | GVIQHS | 594.3358 | 622.3307 | GVIQHST | 695.3835 | 723.3784 |
| VI | 185.1648 | 213.1598 | VIQ | 313.2234 | 341.2183 | VIQH | 450.2823 | 478.2772 |
| VIQHS | 537.3144 | 565.3093 | VIQHST | 638.3620 | 666.3570 | IQ | 214.1550 | 242.1499 |
| IQH | 351.2139 | 379.2088 | IQHS | 438.2459 | 466.2409 | IQHST | 539.2936 | 567.2885 |
| IQHSTI | 652.3777 | 680.3726 | QH | 238.1299 | 266.1248 | QHS | 325.1619 | 353.1568 |
| QHST | 426.2096 | 454.2045 | QHSTI | 539.2936 | 567.2885 | QHSTIN | 653.3365 | 681.3315 |
| HS | 197.1033 | 225.0982 | HST | 298.1510 | 326.1459 | HSTI | 411.2350 | 439.2300 |
| HSTIN | 525.2780 | 553.2729 | HSTINN | 639.3209 | 667.3158 | ST | 161.0921 | 189.0870 |
| STI | 274.1761 | 302.1710 | STIN | 388.2191 | 416.2140 | STINN | 502.2620 | 530.2569 |
| STINNL | 615.3461 | 643.3410 | STINNLA | 686.3832 | 714.3781 | TI | 187.1441 | 215.1390 |
| TIN | 301.1870 | 329.1819 | TINN | 415.2300 | 443.2249 | TINNL | 528.3140 | 556.3089 |
| TINNLA | 599.3511 | 627.3461 | IN | 200.1394 | 228.1343 | INN | 314.1823 | 342.1772 |
| INNL | 427.2663 | 455.2613 | INNLA | 498.3035 | 526.2984 | INNLAI | 611.3875 | 639.3824 |
| INNLAIG | 668.4090 | 696.4039 | NN | 201.0982 | 229.0931 | NNL | 314.1823 | 342.1772 |
| NNLA | 385.2194 | 413.2143 | NNLAI | 498.3035 | 526.2984 | NNLAIG | 555.3249 | 583.3198 |
| NL | 200.1394 | 228.1343 | NLA | 271.1765 | 299.1714 | NLAI | 384.2605 | 412.2554 |
| NLAIG | 441.2820 | 469.2769 | LA | 157.1335 | 185.1285 | LAI | 270.2176 | 298.2125 |
| LAIG | 327.2391 | 355.2340 | AI | 157.1335 | 185.1285 | AIG | 214.1550 | 242.1499 |
| IG | 143.1179 | 171.1128 | | | | | | |

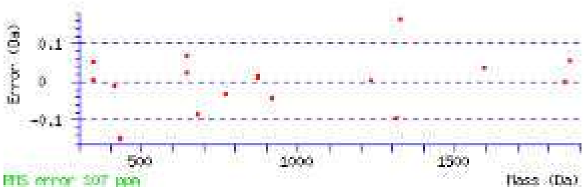


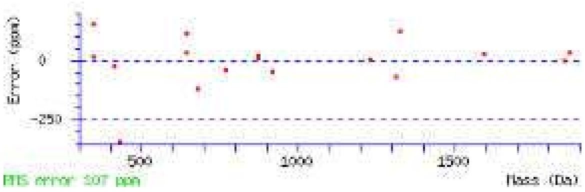


NCBI **BLAST** search of GLFIIDKEGVIQHSTINNLAIGR
(Parameters: blastp, nr protein database, expect=20000, no filter, PAM30)
Other BLAST web gateways

**All matches to this query**

| Score | Mr(calc) | Delta | Sequence |
|---|---|---|---|
| 5.6 | 2507.3860 | 0.1194 | GLFIIDKEGVIQHSTINNLAIGR |

**Mascot:** http://www.matrixscience.com/

# MATRIX SCIENCE Mascot Search Results

## Peptide View Spot no 19

MS/MS Fragmentation of **TLQALQYVQDNPDEVCPAGWKPGDK**
Found in **gi|115446541** in **NCBInr**, Os02g0537700 [Oryza sativa Japonica Group]

Match to Query 101: 2828.457824 from(2829.465100,1+) intensity(0.0000) index(21)
Title: Label: H17, Spot_Id: 219937, Peak_List_Id: 228940, MSMS Job_Run_Id: 22005, Comment:
Data file C:\Documents and Settings\Administrator\Desktop\Farah-Garima 11May 2013\ppw_H17_136868316000.txt

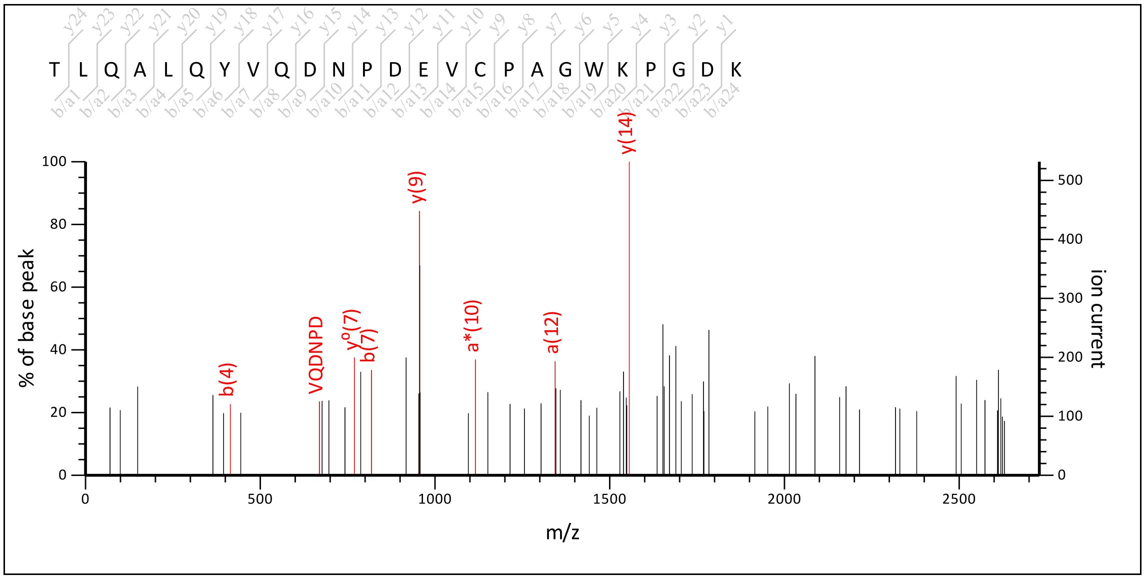


0 to 2729.35

Label all possible matches Label matches used for scoring

**Monoisotopic mass of neutral peptide Mr(calc):** 2828.3439
**Fixed modifications:** Carbamidomethyl (C) (apply to specified residues or termini only)
**Ions Score:** 5 **Expect:** 4.9e+03
**Matches :** 11/487 fragment ions using 23 most intense peaks (help)

| # | Immon. | a | a* | $a^0$ | b | b* | $b^0$ | d | Seq. | v | w | y | y* | $y^0$ | # |
|---|---|---|---|---|---|---|---|---|---|---|---|---|---|---|---|
| 1 | 74.0600 | 74.0600 | | 56.0495 | 102.0550 | | 84.0444 | 44.0495 | T | | | | | | 25 |
| 2 | 86.0964 | 187.1441 | | 169.1335 | 215.1390 | | 197.1285 | 145.0972 | L | 2670.2253 | 2669.2300 | 2728.3035 | 2711.2770 | 2710.2930 | 24 |
| 3 | 101.0709 | 315.2027 | 298.1761 | 297.1921 | 343.1976 | 326.1710 | 325.1870 | 258.1812 | Q | 2542.1667 | 2541.1715 | 2615.2195 | 2598.1929 | 2597.2089 | 23 |
| 4 | 44.0495 | 386.2398 | 369.2132 | 368.2292 | ***414.2347*** | 397.2082 | 396.2241 | | A | 2471.1296 | | 2487.1609 | 2470.1344 | 2469.1503 | 22 |
| 5 | 86.0964 | 499.3239 | 482.2973 | 481.3133 | 527.3188 | 510.2922 | 509.3082 | 457.2769 | L | 2358.0455 | 2357.0503 | 2416.1238 | 2399.0972 | 2398.1132 | 21 |
| 6 | 101.0709 | 627.3824 | 610.3559 | 609.3719 | 655.3774 | 638.3508 | 637.3668 | 570.3610 | Q | 2229.9870 | 2228.9917 | 2303.0397 | 2286.0132 | 2285.0292 | 20 |
| 7 | 136.0757 | 790.4458 | 773.4192 | 772.4352 | ***818.4407*** | 801.4141 | 800.4301 | | Y | 2066.9236 | | 2174.9811 | 2157.9546 | 2156.9706 | 19 |
| 8 | 72.0808 | 889.5142 | 872.4876 | 871.5036 | 917.5091 | 900.4825 | 899.4985 | 875.4985 | V | 1967.8552 | 1980.8756 | 2011.9178 | 1994.8913 | 1993.9073 | 18 |
| 9 | 101.0709 | 1017.5728 | 1000.5462 | 999.5622 | 1045.5677 | 1028.5411 | 1027.5571 | 960.5513 | Q | 1839.7966 | 1838.8014 | 1912.8494 | 1895.8229 | 1894.8388 | 17 |
| 10 | 88.0393 | 1132.5997 | 1115.5732 | 1114.5891 | 1160.5946 | 1143.5681 | 1142.5841 | 1088.6099 | D | 1724.7697 | 1723.7744 | 1784.7908 | 1767.7643 | 1766.7803 | 16 |
| 11 | 87.0553 | 1246.6426 | 1229.6161 | 1228.6321 | 1274.6375 | 1257.6110 | 1256.6270 | 1203.6368 | N | 1610.7268 | 1609.7315 | 1669.7639 | 1652.7373 | 1651.7533 | 15 |
| 12 | 70.0651 | 1343.6954 | 1326.6688 | 1325.6848 | 1371.6903 | 1354.6638 | 1353.6797 | 1317.6797 | P | 1513.6740 | 1512.6788 | ***1555.7210*** | 1538.6944 | 1537.7104 | 14 |
| 13 | 88.0393 | 1458.7223 | 1441.6958 | 1440.7118 | 1486.7173 | 1469.6907 | 1468.7067 | 1414.7325 | D | 1398.6471 | 1397.6518 | 1458.6682 | 1441.6416 | 1440.6576 | 13 |
| 14 | 102.0550 | 1587.7649 | 1570.7384 | 1569.7544 | 1615.7598 | 1598.7333 | 1597.7493 | 1529.7595 | E | 1269.6045 | 1268.6092 | ***1343.6412*** | 1326.6147 | 1325.6307 | 12 |
| 15 | 72.0808 | 1686.8333 | 1669.8068 | 1668.8228 | 1714.8283 | 1697.8017 | 1696.8177 | 1672.8177 | V | 1170.5361 | 1183.5565 | 1214.5987 | 1197.5721 | 1196.5881 | 11 |
| 16 | 133.0430 | 1846.8640 | 1829.8374 | 1828.8534 | 1874.8589 | 1857.8324 | 1856.8483 | 1757.8705 | C | 1010.5054 | 1009.5102 | ***1115.5302*** | 1098.5037 | 1097.5197 | 10 |
| 17 | 70.0651 | 1943.9168 | 1926.8902 | 1925.9062 | 1971.9117 | 1954.8851 | 1953.9011 | 1917.9011 | P | 913.4526 | 912.4574 | ***955.4996*** | 938.4730 | 937.4890 | 9 |
| 18 | 44.0495 | 2014.9539 | 1997.9273 | 1996.9433 | 2042.9488 | 2025.9222 | 2024.9382 | | A | 842.4155 | | 858.4468 | 841.4203 | 840.4363 | 8 |
| 19 | 30.0338 | 2071.9753 | 2054.9488 | 2053.9648 | 2099.9702 | 2082.9437 | 2081.9597 | | G | | | 787.4097 | 770.3832 | 769.3992 | 7 |

| | | | | | | | | | | | | | | | |
|---|---|---|---|---|---|---|---|---|---|---|---|---|---|---|---|
| 20 | 159.0917 | 2258.0546 | 2241.0281 | 2240.0441 | 2286.0496 | 2269.0230 | 2268.0390 | | W | 599.3148 | | 730.3883 | 713.3617 | 712.3777 | 6 |
| 21 | 101.1073 | 2386.1496 | 2369.1231 | 2368.1390 | 2414.1445 | 2397.1180 | 2396.1340 | 2329.0918 | K | 471.2198 | 470.2245 | 544.3089 | 527.2824 | 526.2984 | 5 |
| 22 | 70.0651 | 2483.2024 | 2466.1758 | 2465.1918 | 2511.1973 | 2494.1707 | 2493.1867 | 2457.1867 | P | 374.1670 | 373.1718 | 416.2140 | 399.1874 | 398.2034 | 4 |
| 23 | 30.0338 | 2540.2238 | 2523.1973 | 2522.2133 | 2568.2188 | 2551.1922 | 2550.2082 | | G | | | 319.1612 | 302.1347 | 301.1506 | 3 |
| 24 | 88.0393 | 2655.2508 | 2638.2242 | 2637.2402 | 2683.2457 | 2666.2191 | 2665.2351 | 2611.2610 | D | 202.1186 | 201.1234 | 262.1397 | 245.1132 | 244.1292 | 2 |
| 25 | 101.1073 | | | | | | | | K | 74.0237 | 73.0284 | 147.1128 | 130.0863 | | 1 |

| Seq | ya | yb | Seq | ya | yb | Seq | ya | yb |
|---|---|---|---|---|---|---|---|---|
| LQ | 214.1550 | 242.1499 | LQA | 285.1921 | 313.1870 | LQAL | 398.2762 | 426.2711 |
| LQALQ | 526.3348 | 554.3297 | LQALQY | 689.3981 | 717.3930 | QA | 172.1081 | 200.1030 |
| QAL | 285.1921 | 313.1870 | QALQ | 413.2507 | 441.2456 | QALQY | 576.3140 | 604.3089 |
| QALQYV | 675.3824 | 703.3774 | AL | 157.1335 | 185.1285 | ALQ | 285.1921 | 313.1870 |
| ALQY | 448.2554 | 476.2504 | ALQYV | 547.3239 | 575.3188 | ALQYVQ | 675.3824 | 703.3774 |
| LQ | 214.1550 | 242.1499 | LQY | 377.2183 | 405.2132 | LQYV | 476.2867 | 504.2817 |
| LQYVQ | 604.3453 | 632.3402 | QY | 264.1343 | 292.1292 | QYV | 363.2027 | 391.1976 |
| QYVQ | 491.2613 | 519.2562 | QYVQD | 606.2882 | 634.2831 | YV | 235.1441 | 263.1390 |
| YVQ | 363.2027 | 391.1976 | YVQD | 478.2296 | 506.2245 | YVQDN | 592.2726 | 620.2675 |
| YVQDNP | 689.3253 | 717.3202 | VQ | 200.1394 | 228.1343 | VQD | 315.1663 | 343.1612 |
| VQDN | 429.2092 | 457.2041 | VQDNP | 526.2620 | 554.2569 | VQDNPD | 641.2889 | 669.2838 |
| QD | 216.0979 | 244.0928 | QDN | 330.1408 | 358.1357 | QDNP | 427.1936 | 455.1885 |
| QDNPD | 542.2205 | 570.2154 | QDNPDE | 671.2631 | 699.2580 | DN | 202.0822 | 230.0771 |
| DNP | 299.1350 | 327.1299 | DNPD | 414.1619 | 442.1569 | DNPDE | 543.2045 | 571.1994 |
| DNPDEV | 642.2729 | 670.2679 | NP | 184.1081 | 212.1030 | NPD | 299.1350 | 327.1299 |
| NPDE | 428.1776 | 456.1725 | NPDEV | 527.2460 | 555.2409 | NPDEVC | 687.2767 | 715.2716 |
| PD | 185.0921 | 213.0870 | PDE | 314.1347 | 342.1296 | PDEV | 413.2031 | 441.1980 |
| PDEVC | 573.2337 | 601.2286 | PDEVCP | 670.2865 | 698.2814 | DE | 217.0819 | 245.0768 |
| DEV | 316.1503 | 344.1452 | DEVC | 476.1810 | 504.1759 | DEVCP | 573.2337 | 601.2286 |
| DEVCPA | 644.2708 | 672.2658 | EV | 201.1234 | 229.1183 | EVC | 361.1540 | 389.1489 |
| EVCP | 458.2068 | 486.2017 | EVCPA | 529.2439 | 557.2388 | EVCPAG | 586.2654 | 614.2603 |
| VC | 232.1114 | 260.1063 | VCP | 329.1642 | 357.1591 | VCPA | 400.2013 | 428.1962 |
| VCPAG | 457.2228 | 485.2177 | VCPAGW | 643.3021 | 671.2970 | CP | 230.0958 | 258.0907 |
| CPA | 301.1329 | 329.1278 | CPAG | 358.1544 | 386.1493 | CPAGW | 544.2337 | 572.2286 |
| CPAGWK | 672.3286 | 700.3235 | PA | 141.1022 | 169.0972 | PAG | 198.1237 | 226.1186 |
| PAGW | 384.2030 | 412.1979 | PAGWK | 512.2980 | 540.2929 | PAGWKP | 609.3507 | 637.3457 |
| PAGWKPG | 666.3722 | 694.3671 | AG | 101.0709 | 129.0659 | AGW | 287.1503 | 315.1452 |
| AGWK | 415.2452 | 443.2401 | AGWKP | 512.2980 | 540.2929 | AGWKPG | 569.3194 | 597.3144 |
| AGWKPGD | 684.3464 | 712.3413 | GW | 216.1131 | 244.1081 | GWK | 344.2081 | 372.2030 |
| GWKP | 441.2609 | 469.2558 | GWKPG | 498.2823 | 526.2772 | GWKPGD | 613.3093 | 641.3042 |
| WK | 287.1866 | 315.1816 | WKP | 384.2394 | 412.2343 | WKPG | 441.2609 | 469.2558 |
| WKPGD | 556.2878 | 584.2827 | KP | 198.1601 | 226.1550 | KPG | 255.1816 | 283.1765 |
| KPGD | 370.2085 | 398.2034 | PG | 127.0866 | 155.0815 | PGD | 242.1135 | 270.1084 |
| GD | 145.0608 | 173.0557 | | | | | | |

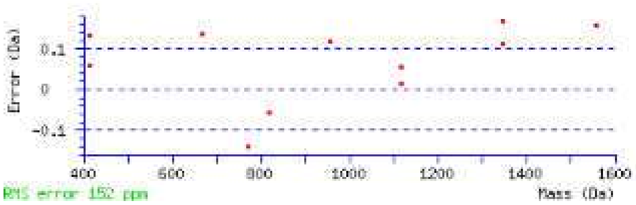


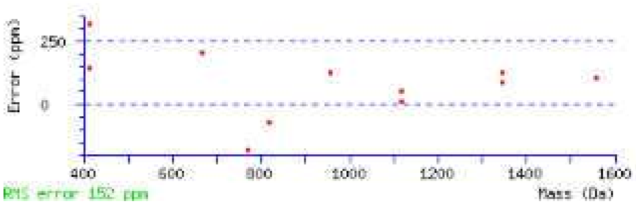


NCBI **BLAST** search of TLQALQYVQDNPDEVCPAGWKPGDK
(Parameters: blastp, nr protein database, expect=20000, no filter, PAM30)
Other BLAST web gateways

**All matches to this query**

| Score | Mr(calc) | Delta | Sequence |
|---|---|---|---|
| 5.5 | 2828.3439 | 0.1139 | TLQALQYVQDNPDEVCPAGWKPGDK |
| 4.4 | 2828.4598 | -0.0020 | LLVMQMAQQQKLDQQQMDDLLLAK |
| 2.8 | 2828.4929 | -0.0351 | DGAVAYPSGHTTLMVVVLGMVILVAGAR |
| 2.5 | 2828.4531 | 0.0047 | LSWNTVDVVTDAGGVPVMSKTGHAFIK |
| 0.9 | 2828.3551 | 0.1027 | GSPLNEIFPGESNLSQLMRAHDWSK |
| 0.3 | 2828.3837 | 0.0741 | ATQIEFLDGPTHIVPVMIGDAATCTR |
| 0.1 | 2828.3433 | 0.1145 | SQGLSQEPESGTLIQMQGQSKDMPPR |

**Mascot:** http://www.matrixscience.com/

# MATRIX SCIENCE Mascot Search Results

## Peptide View Spot no 20

MS/MS Fragmentation of **FKESEVYHCR**
Found in **gi|115467828** in **NCBInr**, Os06g0320500 [Oryza sativa Japonica Group]

Match to Query 255: 1353.666024 from(1354.673300,1+) intensity(0.0000) index(28)
Title: Label: H13, Spot_Id: 219873, Peak_List_Id: 227935, MSMS Job_Run_Id: 21976, Comment:
Data file C:\Documents and Settings\Administrator\Desktop\Farah-Garima 11May 2013\ppw_H13_136868249100.txt

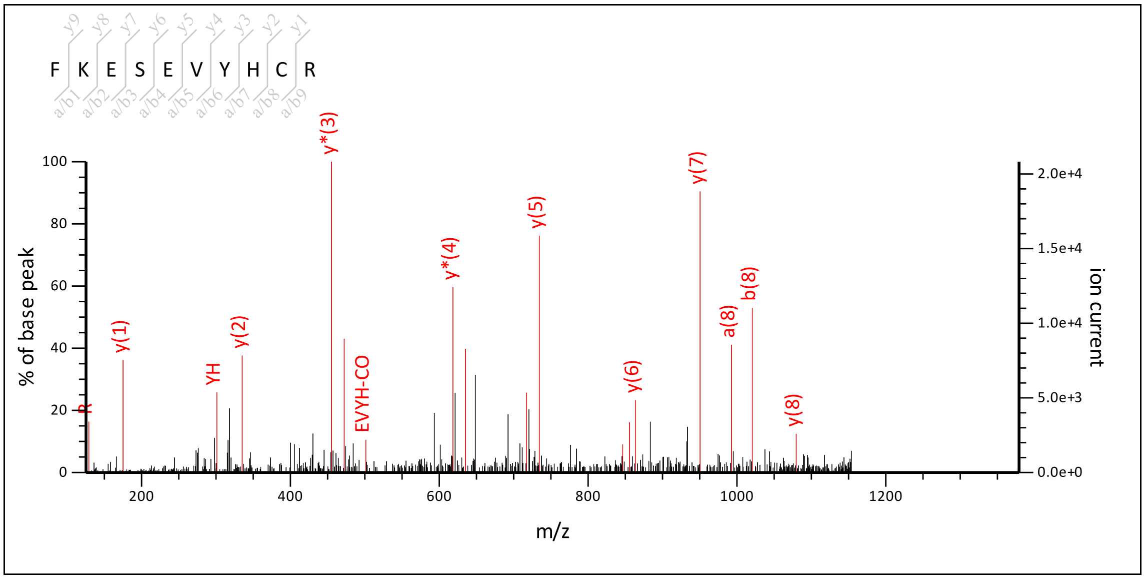


125.37 to 1379.05

Label all possible matches ○ Label matches used for scoring ◉

**Monoisotopic mass of neutral peptide Mr(calc):** 1353.6136
**Fixed modifications:** Carbamidomethyl (C) (apply to specified residues or termini only)
**Ions Score:** 70 **Expect:** 0.0034
**Matches :** 19/147 fragment ions using 22 most intense peaks (help)

| # | Immon. | a | a* | $a^0$ | b | b* | $b^0$ | d | Seq. | v | w | y | y* | $y^0$ | # |
|---|---|---|---|---|---|---|---|---|---|---|---|---|---|---|---|
| 1 | 120.0808 | 120.0808 | | | 148.0757 | | | 44.0495 | F | | | | | | 10 |
| 2 | 101.1073 | 248.1757 | 231.1492 | | 276.1707 | 259.1441 | | 191.1179 | K | 1134.4633 | 1133.4680 | 1207.5524 | 1190.5259 | 1189.5419 | 9 |
| 3 | 102.0550 | 377.2183 | 360.1918 | 359.2078 | 405.2132 | 388.1867 | 387.2027 | 319.2129 | E | 1005.4207 | 1004.4254 | ***1079.4575*** | 1062.4309 | 1061.4469 | 8 |
| 4 | 60.0444 | 464.2504 | 447.2238 | 446.2398 | 492.2453 | 475.2187 | 474.2347 | 448.2554 | S | 918.3887 | 917.3934 | ***950.4149*** | 933.3883 | 932.4043 | 7 |
| 5 | 102.0550 | 593.2930 | 576.2664 | 575.2824 | 621.2879 | 604.2613 | 603.2773 | 535.2875 | E | 789.3461 | 788.3508 | ***863.3828*** | 846.3563 | 845.3723 | 6 |
| 6 | 72.0808 | 692.3614 | 675.3348 | 674.3508 | 720.3563 | 703.3297 | 702.3457 | 678.3457 | V | 690.2777 | 703.2981 | ***734.3403*** | 717.3137 | | 5 |
| 7 | 136.0757 | **855.4247** | 838.3981 | 837.4141 | 883.4196 | 866.3931 | 865.4090 | | Y | 527.2143 | | ***635.2718*** | 618.2453 | | 4 |
| 8 | 110.0713 | **992.4836** | 975.4571 | 974.4730 | 1020.4785 | 1003.4520 | 1002.4680 | | H | 390.1554 | | ***472.2085*** | 455.1820 | | 3 |
| 9 | 133.0430 | 1152.5143 | 1135.4877 | 1134.5037 | 1180.5092 | 1163.4826 | 1162.4986 | 1063.5207 | C | 230.1248 | 229.1295 | ***335.1496*** | 318.1231 | | 2 |
| 10 | 129.1135 | | | | | | | | R | 74.0237 | 73.0284 | ***175.1190*** | 158.0924 | | 1 |

| Seq | ya | yb | Seq | ya | yb | Seq | ya | yb |
|---|---|---|---|---|---|---|---|---|
| **KE** | 230.1499 | 258.1448 | **KES** | 317.1819 | 345.1769 | **KESE** | 446.2245 | 474.2195 |
| **KESEV** | 545.2930 | 573.2879 | **ES** | 189.0870 | 217.0819 | **ESE** | 318.1296 | 346.1245 |
| **ESEV** | 417.1980 | 445.1929 | **ESEVY** | 580.2613 | 608.2562 | **SE** | 189.0870 | 217.0819 |
| **SEV** | 288.1554 | 316.1503 | **SEVY** | 451.2187 | 479.2136 | **SEVYH** | 588.2776 | 616.2726 |
| **EV** | 201.1234 | 229.1183 | **EVY** | 364.1867 | 392.1816 | **EVYH** | 501.2456 | 529.2405 |
| **EVYHC** | 661.2763 | 689.2712 | **VY** | 235.1441 | 263.1390 | **VYH** | 372.2030 | 400.1979 |
| **VYHC** | 532.2337 | 560.2286 | **YH** | 273.1346 | 301.1295 | **YHC** | 433.1653 | 461.1602 |

| | | | | | | | | |
|---|---|---|---|---|---|---|---|---|
| **HC** | 270.1019 | 298.0968 | | | | | | |

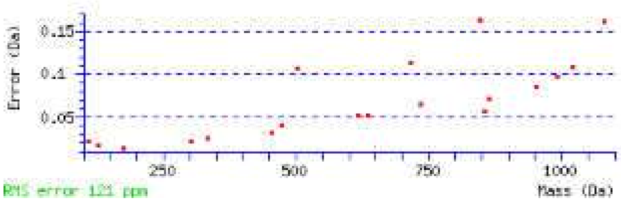


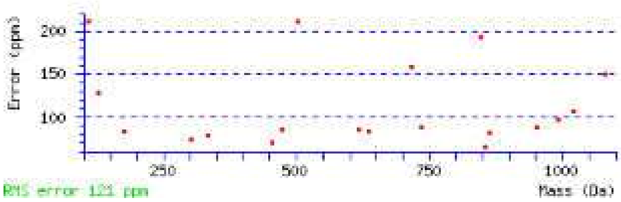


NCBI **BLAST** search of FKESEVYHCR
(Parameters: blastp, nr protein database, expect=20000, no filter, PAM30)
Other BLAST web gateways

**All matches to this query**

| Score | Mr(calc) | Delta | Sequence |
|---|---|---|---|
| 69.9 | 1353.6136 | 0.0525 | FKESEVYHCR |
| 32.8 | 1353.6048 | 0.0612 | QISEAFQSSDDK |
| 27.5 | 1353.7306 | -0.0646 | HPKHYVTAVFR |
| 25.9 | 1353.6161 | 0.0499 | TDTSAPGSQFSTR |
| 25.4 | 1353.6564 | 0.0096 | KHFSYLSSEEK |
| 24.3 | 1353.5401 | 0.1259 | RMEMGDANGSDR |
| 23.7 | 1353.7656 | -0.0996 | YISKEVYALLR |
| 23.6 | 1353.6976 | -0.0315 | IKYIHLDHCR |
| 23.4 | 1353.6048 | 0.0612 | AYKEELSEDDR |
| 23.3 | 1353.6381 | 0.0279 | STSMVVVGGGTMGR |

**Mascot:** http://www.matrixscience.com/

# MATRIX SCIENCE Mascot Search Results

## Peptide View Spot no 21

MS/MS Fragmentation of **SFGVLIPDQGIALR**
Found in **gi|115446541** in **NCBInr**, Os02g0537700 [Oryza sativa Japonica Group]

Match to Query 13: 1484.907024 from(1485.914300,1+) intensity(0.0000) index(4)
Title: Label: J7, Spot_Id: 219779, Peak_List_Id: 226419, MSMS Job_Run_Id: 21843, Comment:
Data file C:\Documents and Settings\Administrator\Desktop\Farah-Garima 11May 2013\ppw_J7_136859916700.txt

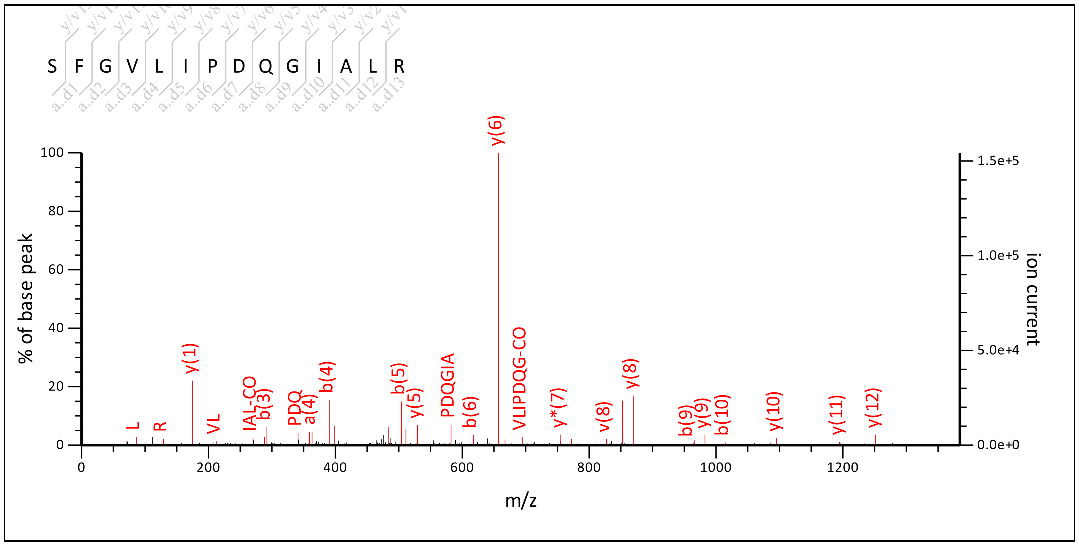


0 to 1384.47

Label all possible matches ○ Label matches used for scoring ◉

**Monoisotopic mass of neutral peptide Mr(calc):** 1484.8351
**Fixed modifications:** Carbamidomethyl (C) (apply to specified residues or termini only)
**Ions Score:** 94 **Expect:** 6.6e-06
**Matches :** 57/238 fragment ions using 52 most intense peaks (help)

| # | Immon. | a | a* | $a^0$ | b | b* | $b^0$ | d | d' | Seq. | v | w | w' | y | y* | |
|---|---|---|---|---|---|---|---|---|---|---|---|---|---|---|---|---|
| 1 | 60.0444 | 60.0444 | | 42.0338 | 88.0393 | | 70.0287 | 44.0495 | | S | | | | | | |
| 2 | 120.0808 | 207.1128 | | 189.1022 | 235.1077 | | 217.0972 | | | F | 1306.7478 | | | 1398.8104 | 1381.7838 | 138 |
| 3 | 30.0338 | 264.1343 | | 246.1237 | 292.1292 | | 274.1186 | | | G | | | | 1251.7419 | 1234.7154 | 123 |
| 4 | 72.0808 | 363.2027 | | 345.1921 | 391.1976 | | 373.1870 | 349.1870 | | V | 1150.6579 | 1163.6783 | | 1194.7205 | 1177.6939 | 11' |
| 5 | 86.0964 | 476.2867 | | 458.2762 | 504.2817 | | 486.2711 | 434.2398 | | L | 1037.5738 | 1036.5786 | | 1095.6521 | 1078.6255 | 10' |
| 6 | 86.0964 | 589.3708 | | 571.3602 | 617.3657 | | 599.3552 | 561.3395 | 575.3552 | I | 924.4898 | 937.5102 | 951.5258 | 982.5680 | 965.5415 | 96 |
| 7 | 70.0651 | 686.4236 | | 668.4130 | 714.4185 | | 696.4079 | 660.4079 | | P | 827.4370 | 826.4417 | | 869.4839 | 852.4574 | 8: |
| 8 | 88.0393 | 801.4505 | | 783.4400 | 829.4454 | | 811.4349 | 757.4607 | | D | 712.4100 | 711.4148 | | 772.4312 | 755.4046 | 7: |
| 9 | 101.0709 | 929.5091 | 912.4825 | 911.4985 | 957.5040 | 940.4775 | 939.4934 | 872.4876 | | Q | 584.3515 | 583.3562 | | 657.4042 | 640.3777 | |
| 10 | 30.0338 | 986.5306 | 969.5040 | 968.5200 | 1014.5255 | 997.4989 | 996.5149 | | | G | | | | 529.3457 | 512.3191 | |
| 11 | 86.0964 | 1099.6146 | 1082.5881 | 1081.6041 | 1127.6095 | 1110.5830 | 1109.5990 | 1071.5833 | 1085.5990 | I | 414.2459 | 427.2663 | 441.2820 | 472.3242 | 455.2976 | |
| 12 | 44.0495 | 1170.6517 | 1153.6252 | 1152.6412 | 1198.6467 | 1181.6201 | 1180.6361 | | | A | 343.2088 | | | 359.2401 | 342.2136 | |
| 13 | 86.0964 | 1283.7358 | 1266.7093 | 1265.7252 | 1311.7307 | 1294.7042 | 1293.7201 | 1241.6889 | | L | 230.1248 | 229.1295 | | 288.2030 | 271.1765 | |
| 14 | 129.1135 | | | | | | | | | R | 74.0237 | 73.0284 | | 175.1190 | 158.0924 | |

| Seq | ya | yb | Seq | ya | yb | Seq | ya | yb |
|---|---|---|---|---|---|---|---|---|
| FG | 177.1022 | 205.0972 | FGV | 276.1707 | 304.1656 | FGVL | 389.2547 | 417.2496 |
| FGVLI | 502.3388 | 530.3337 | FGVLIP | 599.3915 | 627.3865 | GV | 129.1022 | 157.0972 |
| GVL | 242.1863 | 270.1812 | GVLI | 355.2704 | 383.2653 | GVLIP | 452.3231 | 480.3180 |
| GVLIPD | 567.3501 | 595.3450 | GVLIPDQ | 695.4087 | 723.4036 | VL | 185.1648 | 213.1598 |
| VLI | 298.2489 | 326.2438 | VLIP | 395.3017 | 423.2966 | VLIPD | 510.3286 | 538.3235 |
| VLIPDQ | 638.3872 | 666.3821 | VLIPDQG | 695.4087 | 723.4036 | LI | 199.1805 | 227.1754 |
| LIP | 296.2333 | 324.2282 | LIPD | 411.2602 | 439.2551 | LIPDQ | 539.3188 | 567.3137 |

| | | | | | | | | |
|---|---|---|---|---|---|---|---|---|
| **LIPDQG** | 596.3402 | 624.3352 | **IP** | 183.1492 | 211.1441 | **IPD** | 298.1761 | 326.1710 |
| **IPDQ** | 426.2347 | 454.2296 | **IPDQG** | 483.2562 | 511.2511 | **IPDQGI** | 596.3402 | 624.3352 |
| **IPDQGIA** | 667.3774 | 695.3723 | **PD** | 185.0921 | 213.0870 | **PDQ** | 313.1506 | 341.1456 |
| **PDQG** | 370.1721 | 398.1670 | **PDQGI** | 483.2562 | 511.2511 | **PDQGIA** | 554.2933 | 582.2882 |
| **PDQGIAL** | 667.3774 | 695.3723 | **DQ** | 216.0979 | 244.0928 | **DQG** | 273.1193 | 301.1143 |
| **DQGI** | 386.2034 | 414.1983 | **DQGIA** | 457.2405 | 485.2354 | **DQGIAL** | 570.3246 | 598.3195 |
| **QG** | 158.0924 | 186.0873 | **QGI** | 271.1765 | 299.1714 | **QGIA** | 342.2136 | 370.2085 |
| **QGIAL** | 455.2976 | 483.2926 | **GI** | 143.1179 | 171.1128 | **GIA** | 214.1550 | 242.1499 |
| **GIAL** | 327.2391 | 355.2340 | **IA** | 157.1335 | 185.1285 | **IAL** | 270.2176 | 298.2125 |
| **AL** | 157.1335 | 185.1285 | | | | | | |

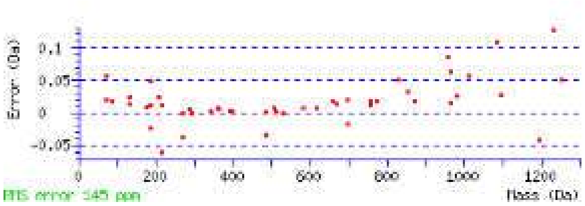


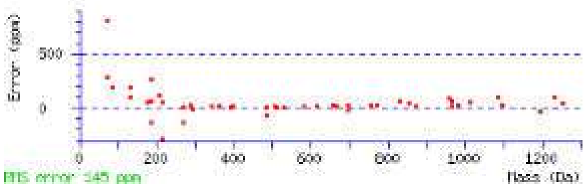


NCBI **BLAST** search of SFGVLIPDQGIALR
(Parameters: blastp, nr protein database, expect=20000, no filter, PAM30)
Other BLAST web gateways

**All matches to this query**

| Score | Mr(calc) | Delta | Sequence |
|---|---|---|---|
| 94.1 | 1484.8351 | 0.0719 | SFGVLIPDQGIALR |
| 33.7 | 1484.8562 | 0.0508 | LVQIITSNILDTR |
| 30.3 | 1484.8674 | 0.0396 | SSLALAVARLIESR |
| 29.7 | 1484.7697 | 0.1373 | MSRLFAFLIDEK |
| 29.7 | 1484.8198 | 0.0872 | ALTSLLVGLNAEER |
| 29.2 | 1484.8311 | 0.0759 | IVSTTDPSIGARIR |
| 29.2 | 1484.8311 | 0.0760 | LSEARAGIVIAETR |
| 28.6 | 1484.8450 | 0.0620 | VLVVKQDDLSIEK |
| 28.6 | 1484.7947 | 0.1123 | REDVGKPIDITSR |
| 28.2 | 1484.8311 | 0.0760 | RETVAQALLLSER |

**Mascot:** http://www.matrixscience.com/

# MATRIX SCIENCE Mascot Search Results

## Peptide View Spot no 21

MS/MS Fragmentation of **EGVIQHSTINNLAIGR**
Found in **gi|115446541** in **NCBInr**, Os02g0537700 [Oryza sativa Japonica Group]

Match to Query 21: 1721.009124 from(1722.016400,1+) intensity(0.0000) index(7)
Title: Label: J7, Spot_Id: 219779, Peak_List_Id: 226428, MSMS Job_Run_Id: 21843, Comment:
Data file C:\Documents and Settings\Administrator\Desktop\Farah-Garima 11May 2013\ppw_J7_136859916700.txt

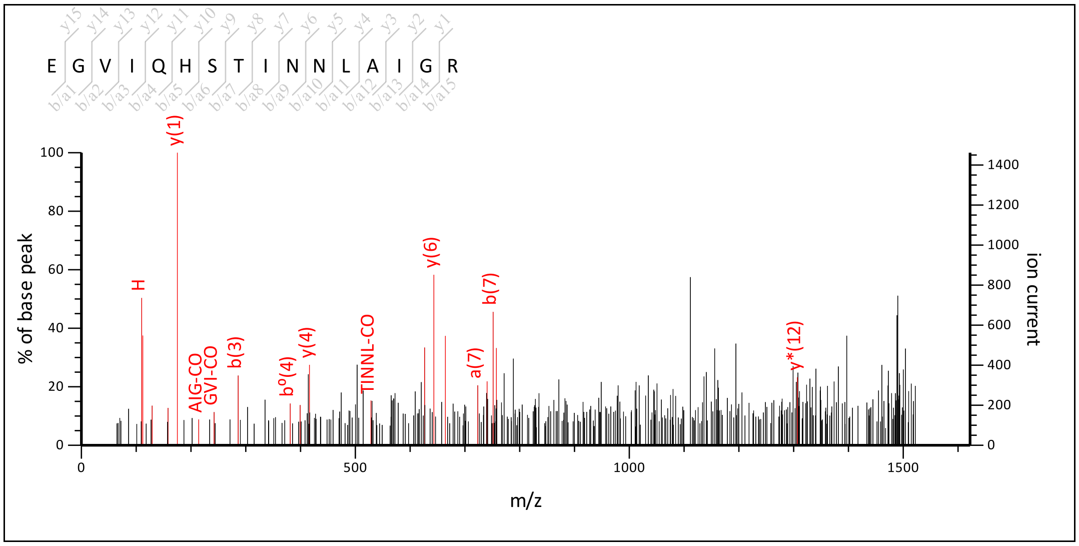


0 to 1621.94

Label all possible matches ○ Label matches used for scoring ◉

**Monoisotopic mass of neutral peptide Mr(calc):** 1720.9220
**Fixed modifications:** Carbamidomethyl (C) (apply to specified residues or termini only)
**Ions Score:** 10 **Expect:** 1.9e+03
**Matches :** 28/292 fragment ions using 60 most intense peaks (help)

| # | Immon. | a | a* | $a^0$ | b | b* | $b^0$ | d | d' | Seq. | v | w | w' | y | y* |
|---|---|---|---|---|---|---|---|---|---|---|---|---|---|---|---|
| 1 | 102.0550 | 102.0550 | | 84.0444 | 130.0499 | | 112.0393 | 44.0495 | | E | | | | | |
| 2 | 30.0338 | 159.0764 | | 141.0659 | 187.0713 | | 169.0608 | | | G | | | | 1592.8867 | 1575.8602 |
| 3 | 72.0808 | 258.1448 | | 240.1343 | ***286.1397*** | | 268.1292 | 244.1292 | | V | 1491.8027 | 1504.8231 | | 1535.8653 | 1518.8387 |
| 4 | 86.0964 | 371.2289 | | 353.2183 | ***399.2238*** | | 381.2132 | 343.1976 | 357.2132 | I | 1378.7186 | 1391.7390 | 1405.7546 | 1436.7968 | 1419.7703 |
| 5 | 101.0709 | 499.2875 | 482.2609 | 481.2769 | 527.2824 | 510.2558 | 509.2718 | 442.2660 | | Q | 1250.6600 | 1249.6648 | | 1323.7128 | 1306.6862 |
| 6 | 110.0713 | 636.3464 | 619.3198 | 618.3358 | ***664.3413*** | 647.3148 | 646.3307 | | | H | 1113.6011 | | | 1195.6542 | 1178.6276 |
| 7 | 60.0444 | 723.3784 | 706.3519 | 705.3678 | ***751.3733*** | 734.3468 | 733.3628 | 707.3835 | | S | 1026.5691 | 1025.5738 | | 1058.5953 | 1041.5687 |
| 8 | 74.0600 | 824.4261 | 807.3995 | 806.4155 | 852.4210 | 835.3945 | 834.4104 | 808.4312 | 810.4104 | T | 925.5214 | 938.5418 | 940.5211 | 971.5633 | 954.5367 |
| 9 | 86.0964 | 937.5102 | 920.4836 | 919.4996 | 965.5051 | 948.4785 | 947.4945 | 909.4789 | 923.4945 | I | 812.4373 | 825.4577 | 839.4734 | 870.5156 | 853.4890 |
| 10 | 87.0553 | 1051.5531 | 1034.5265 | 1033.5425 | 1079.5480 | 1062.5215 | 1061.5374 | 1008.5473 | | N | 698.3944 | 697.3991 | | ***757.4315*** | 740.4050 |
| 11 | 87.0553 | 1165.5960 | 1148.5695 | 1147.5854 | 1193.5909 | 1176.5644 | 1175.5804 | 1122.5902 | | N | 584.3515 | 583.3562 | | ***643.3886*** | 626.3620 |
| 12 | 86.0964 | 1278.6801 | 1261.6535 | 1260.6695 | ***1306.6750*** | 1289.6484 | 1288.6644 | 1236.6331 | | L | 471.2674 | 470.2722 | | 529.3457 | 512.3191 |
| 13 | 44.0495 | 1349.7172 | 1332.6906 | 1331.7066 | 1377.7121 | 1360.6856 | 1359.7015 | | | A | 400.2303 | | | ***416.2616*** | 399.2350 |
| 14 | 86.0964 | 1462.8013 | 1445.7747 | 1444.7907 | 1490.7962 | 1473.7696 | 1472.7856 | 1434.7700 | 1448.7856 | I | 287.1462 | 300.1666 | 314.1823 | 345.2245 | 328.1979 |
| 15 | 30.0338 | 1519.8227 | 1502.7962 | 1501.8122 | 1547.8176 | 1530.7911 | 1529.8071 | | | G | | | | 232.1404 | 215.1139 |
| 16 | 129.1135 | | | | | | | | | R | 74.0237 | 73.0284 | | ***175.1190*** | 158.0924 |

| Seq | ya | yb | Seq | ya | yb | Seq | ya | yb |
|---|---|---|---|---|---|---|---|---|
| GV | 129.1022 | 157.0972 | GVI | 242.1863 | 270.1812 | GVIQ | 370.2449 | 398.2398 |
| GVIQH | 507.3038 | 535.2987 | GVIQHS | 594.3358 | 622.3307 | GVIQHST | 695.3835 | 723.3784 |
| VI | 185.1648 | 213.1598 | VIQ | 313.2234 | 341.2183 | VIQH | 450.2823 | 478.2772 |
| VIQHS | 537.3144 | 565.3093 | VIQHST | 638.3620 | 666.3570 | IQ | 214.1550 | 242.1499 |
| IQH | 351.2139 | 379.2088 | IQHS | 438.2459 | 466.2409 | IQHST | 539.2936 | 567.2885 |

| | | | | | | | | |
|---|---|---|---|---|---|---|---|---|
| **IQHSTI** | 652.3777 | 680.3726 | **QH** | 238.1299 | 266.1248 | **QHS** | 325.1619 | 353.1568 |
| **QHST** | 426.2096 | 454.2045 | **QHSTI** | 539.2936 | 567.2885 | **QHSTIN** | 653.3365 | 681.3315 |
| **HS** | 197.1033 | 225.0982 | **HST** | 298.1510 | 326.1459 | **HSTI** | 411.2350 | 439.2300 |
| **HSTIN** | 525.2780 | 553.2729 | **HSTINN** | 639.3209 | 667.3158 | **ST** | 161.0921 | 189.0870 |
| **STI** | 274.1761 | 302.1710 | **STIN** | 388.2191 | 416.2140 | **STINN** | 502.2620 | 530.2569 |
| **STINNL** | 615.3460 | 643.3410 | **STINNLA** | 686.3832 | 714.3781 | **TI** | 187.1441 | 215.1390 |
| **TIN** | 301.1870 | 329.1819 | **TINN** | 415.2300 | 443.2249 | **TINNL** | 528.3140 | 556.3089 |
| **TINNLA** | 599.3511 | 627.3461 | **IN** | 200.1394 | 228.1343 | **INN** | 314.1823 | 342.1772 |
| **INNL** | 427.2663 | 455.2613 | **INNLA** | 498.3035 | 526.2984 | **INNLAI** | 611.3875 | 639.3824 |
| **INNLAIG** | 668.4090 | 696.4039 | **NN** | 201.0982 | 229.0931 | **NNL** | 314.1823 | 342.1772 |
| **NNLA** | 385.2194 | 413.2143 | **NNLAI** | 498.3035 | 526.2984 | **NNLAIG** | 555.3249 | 583.3198 |
| **NL** | 200.1394 | 228.1343 | **NLA** | 271.1765 | 299.1714 | **NLAI** | 384.2605 | 412.2554 |
| **NLAIG** | 441.2820 | 469.2769 | **LA** | 157.1335 | 185.1285 | **LAI** | 270.2176 | 298.2125 |
| **LAIG** | 327.2391 | 355.2340 | **AI** | 157.1335 | 185.1285 | **AIG** | 214.1550 | 242.1499 |
| **IG** | 143.1179 | 171.1128 | | | | | | |

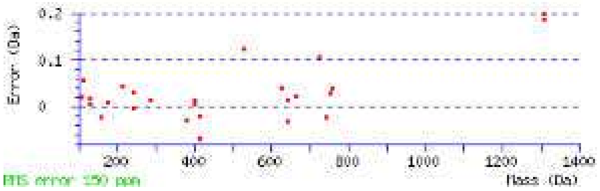


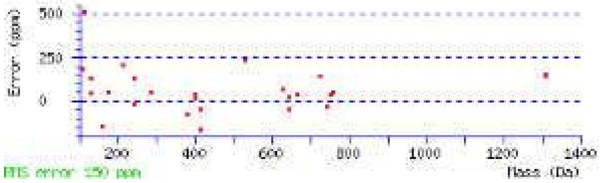


NCBI **BLAST** search of EGVIQHSTINNLAIGR
(Parameters: blastp, nr protein database, expect=20000, no filter, PAM30)
Other BLAST web gateways

**All matches to this query**

| Score | Mr(calc) | Delta | Sequence |
|---|---|---|---|
| 13.9 | 1720.9472 | 0.0619 | QLVTNSVNTVYSIKR |
| 13.0 | 1720.8744 | 0.1348 | NAETTGNYLLDNLKR |
| 12.9 | 1720.9162 | 0.0930 | FPWPSGPPRGLNLAGR |
| 12.8 | 1720.9359 | 0.0732 | RLEELATGTTFVAVSK |
| 12.0 | 1720.8937 | 0.1155 | NYHIFYQLLAGAPSK |
| 11.3 | 1720.9332 | 0.0759 | RGEPAAALQGQVGALGAR |
| 11.1 | 1720.9334 | 0.0757 | SLLTPCYFGGPILKR |
| 10.3 | 1720.9049 | 0.1042 | NYHIFYQLLAGLNR |
| 10.3 | 1720.9220 | 0.0871 | EGVIQHSTINNLAIGR |
| 10.1 | 1720.9108 | 0.0983 | VDSLISHSADALPIQR |

**Mascot:** http://www.matrixscience.com/

# MATRIX SCIENCE Mascot Search Results

## Peptide View Spot no 21

MS/MS Fragmentation of **SGGLGDLKYPLISDVTK**
Found in **gi|115446541** in **NCBInr**, Os02g0537700 [Oryza sativa Japonica Group]

Match to Query 24: 1762.019224 from(1763.026500,1+) intensity(0.0000) index(8)
Title: Label: J7, Spot_Id: 219779, Peak_List_Id: 226422, MSMS Job_Run_Id: 21843, Comment:
Data file C:\Documents and Settings\Administrator\Desktop\Farah-Garima 11May 2013\ppw_J7_136859916700.txt

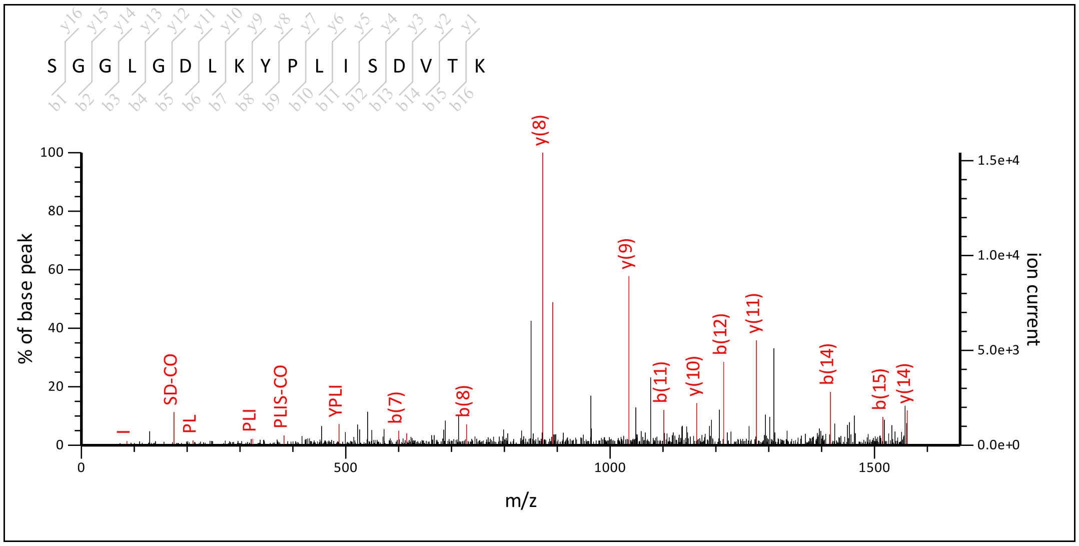


0 to 1661.93

Label all possible matches ○ Label matches used for scoring ◉

**Monoisotopic mass of neutral peptide Mr(calc):** 1761.9513
**Fixed modifications:** Carbamidomethyl (C) (apply to specified residues or termini only)
**Ions Score:** 33 **Expect:** 10
**Matches :** 24/305 fragment ions using 45 most intense peaks (help)

| # | Immon. | a | a* | a⁰ | b | b* | b⁰ | d | d' | Seq. | v | w | w' | y | y* | |
|---|---|---|---|---|---|---|---|---|---|---|---|---|---|---|---|---|
| 1 | 60.0444 | 60.0444 | | 42.0338 | 88.0393 | | 70.0287 | 44.0495 | | S | | | | | | |
| 2 | 30.0338 | 117.0659 | | 99.0553 | 145.0608 | | 127.0502 | | | G | | | | 1675.9265 | 1658.9000 | 16[illegible] |
| 3 | 30.0338 | 174.0873 | | 156.0768 | 202.0822 | | 184.0717 | | | G | | | | 1618.9051 | 1601.8785 | 16[illegible] |
| 4 | **86.0964** | 287.1714 | | 269.1608 | 315.1663 | | 297.1557 | 245.1244 | | L | 1503.8053 | 1502.8101 | | **1561.8836** | 1544.8570 | 15[illegible] |
| 5 | 30.0338 | 344.1928 | | 326.1823 | 372.1878 | | 354.1772 | | | G | | | | 1448.7995 | 1431.7730 | 14[illegible] |
| 6 | 88.0393 | 459.2198 | | 441.2092 | ***487.2147*** | | 469.2041 | 415.2300 | | D | 1331.7569 | 1330.7617 | | 1391.7781 | 1374.7515 | 13[illegible] |
| 7 | **86.0964** | 572.3039 | | 554.2933 | ***600.2988*** | | 582.2882 | 530.2569 | | L | 1218.6729 | 1217.6776 | | **1276.7511** | 1259.7246 | 12[illegible] |
| 8 | 101.1073 | 700.3988 | 683.3723 | 682.3882 | ***728.3937*** | 711.3672 | 710.3832 | 643.3410 | | K | 1090.5779 | 1089.5827 | | **1163.6671** | 1146.6405 | 11[illegible] |
| 9 | 136.0757 | 863.4621 | 846.4356 | 845.4516 | ***891.4571*** | 874.4305 | 873.4465 | | | Y | 927.5146 | | | **1035.5721** | 1018.5455 | 10[illegible] |
| 10 | 70.0651 | 960.5149 | 943.4884 | 942.5043 | 988.5098 | 971.4833 | 970.4993 | 934.4993 | | P | 830.4618 | 829.4666 | | **872.5088** | 855.4822 | 8[illegible] |
| 11 | **86.0964** | 1073.5990 | 1056.5724 | 1055.5884 | ***1101.5939*** | 1084.5673 | 1083.5833 | 1031.5520 | | L | 717.3777 | 716.3825 | | 775.4560 | 758.4294 | 7[illegible] |
| 12 | **86.0964** | 1186.6830 | 1169.6565 | 1168.6725 | ***1214.6780*** | 1197.6514 | 1196.6674 | 1158.6517 | 1172.6674 | I | 604.2937 | 617.3141 | 631.3297 | 662.3719 | 645.3454 | 6[illegible] |
| 13 | 60.0444 | 1273.7151 | 1256.6885 | 1255.7045 | 1301.7100 | 1284.6834 | 1283.6994 | 1257.7201 | | S | 517.2617 | 516.2664 | | 549.2879 | 532.2613 | 5[illegible] |
| 14 | 88.0393 | 1388.7420 | 1371.7155 | 1370.7314 | ***1416.7369*** | 1399.7104 | 1398.7264 | 1344.7522 | | D | 402.2347 | 401.2395 | | 462.2558 | 445.2293 | 4[illegible] |
| 15 | 72.0808 | 1487.8104 | 1470.7839 | 1469.7999 | ***1515.8053*** | 1498.7788 | 1497.7948 | 1473.7948 | | V | 303.1663 | 316.1867 | | 347.2289 | 330.2023 | 3[illegible] |
| 16 | 74.0600 | 1588.8581 | 1571.8316 | 1570.8475 | 1616.8530 | 1599.8265 | 1598.8425 | 1572.8632 | 1574.8425 | T | 202.1186 | 215.1390 | 217.1183 | 248.1605 | 231.1339 | 2[illegible] |
| 17 | 101.1073 | | | | | | | | | K | 74.0237 | 73.0284 | | 147.1128 | 130.0863 | |

| Seq | ya | yb | Seq | ya | yb | Seq | ya | yb |
|---|---|---|---|---|---|---|---|---|
| GG | 87.0553 | 115.0502 | GGL | 200.1394 | 228.1343 | GGLG | 257.1608 | 285.1557 |
| GGLGD | 372.1878 | 400.1827 | GGLGDL | 485.2718 | 513.2667 | GGLGDLK | 613.3668 | 641.3617 |
| GL | 143.1179 | 171.1128 | GLG | 200.1394 | 228.1343 | GLGD | 315.1663 | 343.1612 |
| GLGDL | 428.2504 | 456.2453 | GLGDLK | 556.3453 | 584.3402 | LG | 143.1179 | 171.1128 |

| | | | | | | | | |
|---|---|---|---|---|---|---|---|---|
| **LGD** | 258.1448 | 286.1397 | **LGDL** | 371.2289 | 399.2238 | **LGDLK** | 499.3239 | 527.3188 |
| **LGDLKY** | 662.3872 | 690.3821 | **GD** | 145.0608 | 173.0557 | **GDL** | 258.1448 | 286.1397 |
| **GDLK** | 386.2398 | 414.2347 | **GDLKY** | 549.3031 | 577.2980 | **GDLKYP** | 646.3559 | 674.3508 |
| **DL** | 201.1234 | 229.1183 | **DLK** | 329.2183 | 357.2132 | **DLKY** | 492.2817 | 520.2766 |
| **DLKYP** | 589.3344 | 617.3293 | **LK** | 214.1914 | 242.1863 | **LKY** | 377.2547 | 405.2496 |
| **LKYP** | 474.3075 | 502.3024 | **LKYPL** | 587.3915 | 615.3865 | **KY** | 264.1707 | 292.1656 |
| **KYP** | 361.2234 | 389.2183 | **KYPL** | 474.3075 | 502.3024 | **KYPLI** | 587.3915 | 615.3865 |
| **KYPLIS** | 674.4236 | 702.4185 | **YP** | 233.1285 | 261.1234 | **YPL** | 346.2125 | 374.2074 |
| **YPLI** | 459.2966 | 487.2915 | **YPLIS** | 546.3286 | 574.3235 | **YPLISD** | 661.3556 | 689.3505 |
| **PL** | 183.1492 | 211.1441 | **PLI** | 296.2333 | 324.2282 | **PLIS** | 383.2653 | 411.2602 |
| **PLISD** | 498.2922 | 526.2871 | **PLISDV** | 597.3606 | 625.3556 | **PLISDVT** | 698.4083 | 726.4032 |
| **LI** | 199.1805 | 227.1754 | **LIS** | 286.2125 | 314.2074 | **LISD** | 401.2395 | 429.2344 |
| **LISDV** | 500.3079 | 528.3028 | **LISDVT** | 601.3556 | 629.3505 | **IS** | 173.1285 | 201.1234 |
| **ISD** | 288.1554 | 316.1503 | **ISDV** | 387.2238 | 415.2187 | **ISDVT** | 488.2715 | 516.2664 |
| **SD** | 175.0713 | 203.0662 | **SDV** | 274.1397 | 302.1347 | **SDVT** | 375.1874 | 403.1823 |
| **DV** | 187.1077 | 215.1026 | **DVT** | 288.1554 | 316.1503 | **VT** | 173.1285 | 201.1234 |

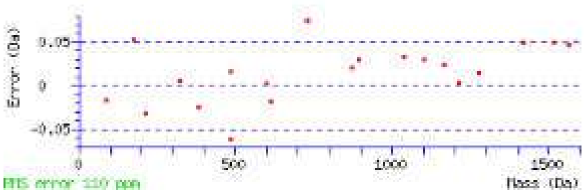


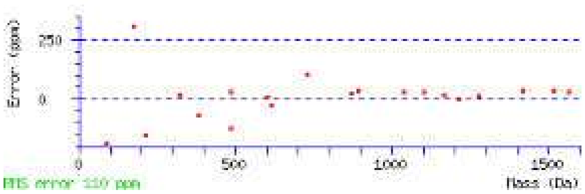


NCBI **BLAST** search of SGGLGDLKYPLISDVTK
(Parameters: blastp, nr protein database, expect=20000, no filter, PAM30)
Other BLAST web gateways

**All matches to this query**

| Score | Mr(calc) | Delta | Sequence |
|---|---|---|---|
| 33.2 | 1761.9513 | 0.0680 | SGGLGDLKYPLISDVTK |
| 24.9 | 1762.0213 | -0.0021 | TTRQISLLPAASHLVR |
| 23.9 | 1762.0352 | -0.0160 | IPEAADPLRLSLQVLK |
| 22.0 | 1761.8719 | 0.1473 | MQSLPEYNSLLTPNR |
| 21.4 | 1761.9598 | 0.0594 | ALEVGSRNHALAGSKPR |
| 21.3 | 1761.9447 | 0.0745 | SSLLNLLMGFIAPSSGR |
| 21.2 | 1761.9737 | 0.0455 | KPDISTSPLHSLPSRK |
| 20.9 | 1761.9261 | 0.0931 | QVAATVETLYSLSPQR |
| 20.4 | 1761.9665 | 0.0527 | QFLNPLESFSLGVLAK |
| 19.1 | 1761.9513 | 0.0680 | SGGLGDLKYPLVSDLTK |

**Mascot:** http://www.matrixscience.com/

# MATRIX SCIENCE Mascot Search Results

## Peptide View Spot no 21

MS/MS Fragmentation of **APDFDAEAVFDQEFINVK**
Found in **gi|115446541** in **NCBInr**, Os02g0537700 [Oryza sativa Japonica Group]

Match to Query 30: 2054.050824 from(2055.058100,1+) intensity(0.0000) index(10)
Title: Label: J7, Spot_Id: 219779, Peak_List_Id: 226423, MSMS Job_Run_Id: 21843, Comment:
Data file C:\Documents and Settings\Administrator\Desktop\Farah-Garima 11May 2013\ppw_J7_136859916700.txt

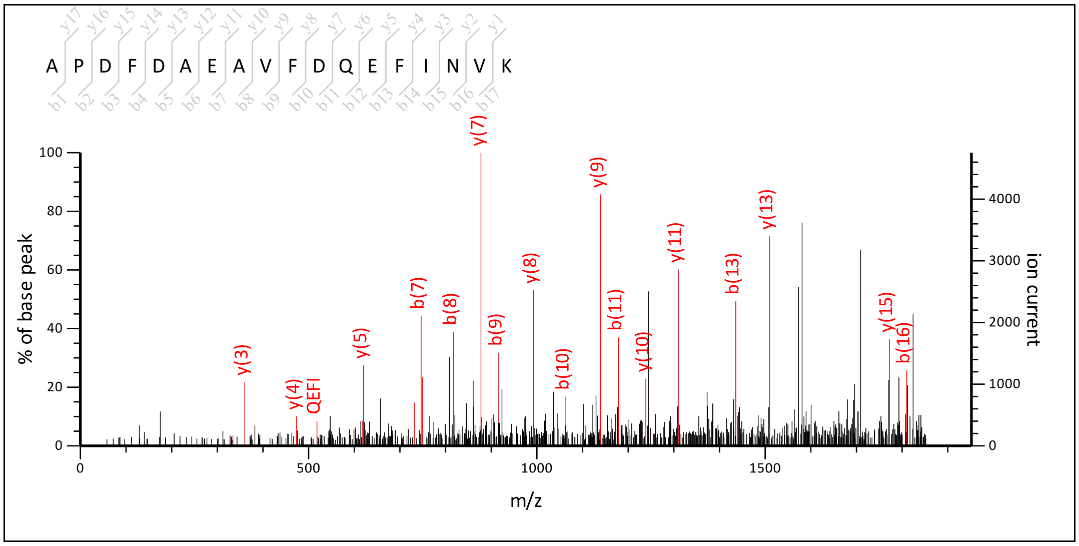


0 to 1951.84

Label all possible matches ○ Label matches used for scoring ◉

**Monoisotopic mass of neutral peptide Mr(calc):** 2053.9633
**Fixed modifications:** Carbamidomethyl (C) (apply to specified residues or termini only)
**Ions Score:** 54 **Expect:** 0.098
**Matches :** 29/305 fragment ions using 54 most intense peaks (help)

| # | Immon. | a | a* | a0 | b | b* | b0 | d | d' | Seq. | v | w | w' | y | y* | |
|---|---|---|---|---|---|---|---|---|---|---|---|---|---|---|---|---|
| 1 | 44.0495 | 44.0495 | | | 72.0444 | | | 44.0495 | | A | | | | | | |
| 2 | 70.0651 | 141.1022 | | | 169.0972 | | | 115.0866 | | P | 1941.8865 | 1940.8913 | | 1983.9335 | 1966.9069 | 196[illegible] |
| 3 | 88.0393 | 256.1292 | | 238.1186 | 284.1241 | | 266.1135 | 212.1394 | | D | 1826.8596 | 1825.8643 | | 1886.8807 | 1869.8541 | 186[illegible] |
| 4 | 120.0808 | 403.1976 | | 385.1870 | 431.1925 | | 413.1819 | | | F | 1679.7911 | | | ***1771.8537*** | 1754.8272 | 17[illegible] |
| 5 | 88.0393 | 518.2245 | | 500.2140 | 546.2195 | | 528.2089 | 474.2347 | | D | 1564.7642 | 1563.7690 | | 1624.7853 | 1607.7588 | 16[illegible] |
| 6 | 44.0495 | 589.2617 | | 571.2511 | **617.2566** | | 599.2460 | | | A | 1493.7271 | | | ***1509.7584*** | 1492.7318 | 14[illegible] |
| 7 | 102.0550 | 718.3042 | | 700.2937 | **746.2992** | | 728.2886 | 660.2988 | | E | 1364.6845 | 1363.6892 | | 1438.7213 | 1421.6947 | 14[illegible] |
| 8 | 44.0495 | 789.3414 | | 771.3308 | **817.3363** | | 799.3257 | | | A | 1293.6474 | | | ***1309.6787*** | 1292.6521 | 12[illegible] |
| 9 | 72.0808 | 888.4098 | | 870.3992 | **916.4047** | | 898.3941 | 874.3941 | | V | 1194.5790 | 1207.5994 | | ***1238.6416*** | 1221.6150 | 12[illegible] |
| 10 | 120.0808 | 1035.4782 | | 1017.4676 | **1063.4731** | | 1045.4625 | | | F | 1047.5106 | | | ***1139.5732*** | 1122.5466 | 11[illegible] |
| 11 | 88.0393 | 1150.5051 | | 1132.4946 | **1178.5000** | | 1160.4895 | 1106.5153 | | D | 932.4836 | 931.4884 | | ***992.5047*** | 975.4782 | 9[illegible] |
| 12 | 101.0709 | 1278.5637 | 1261.5372 | 1260.5531 | 1306.5586 | 1289.5321 | 1288.5481 | 1221.5422 | | Q | 804.4250 | 803.4298 | | ***877.4778*** | 860.4512 | 8[illegible] |
| 13 | 102.0550 | 1407.6063 | 1390.5798 | 1389.5957 | **1435.6012** | 1418.5747 | 1417.5907 | 1349.6008 | | E | 675.3824 | 674.3872 | | ***749.4192*** | 732.3927 | 7[illegible] |
| 14 | 120.0808 | 1554.6747 | 1537.6482 | 1536.6642 | 1582.6696 | 1565.6431 | 1564.6591 | | | F | 528.3140 | | | ***620.3766*** | 603.3501 | |
| 15 | 86.0964 | 1667.7588 | 1650.7322 | 1649.7482 | 1695.7537 | 1678.7271 | 1677.7431 | 1639.7275 | 1653.7431 | I | 415.2300 | 428.2504 | 442.2660 | ***473.3082*** | 456.2817 | |
| 16 | 87.0553 | 1781.8017 | 1764.7752 | 1763.7911 | **1809.7966** | 1792.7701 | 1791.7861 | 1738.7959 | | N | 301.1870 | 300.1918 | | ***360.2241*** | 343.1976 | |
| 17 | 72.0808 | 1880.8701 | 1863.8436 | 1862.8596 | 1908.8650 | 1891.8385 | 1890.8545 | 1866.8545 | | V | 202.1186 | 215.1390 | | 246.1812 | 229.1547 | |
| 18 | 101.1073 | | | | | | | | | K | 74.0237 | 73.0284 | | 147.1128 | 130.0863 | |

| Seq | ya | yb | Seq | ya | yb | Seq | ya | yb |
|---|---|---|---|---|---|---|---|---|
| PD | 185.0921 | 213.0870 | PDF | 332.1605 | 360.1554 | PDFD | 447.1874 | 475.1823 |
| PDFDA | 518.2245 | 546.2195 | PDFDAE | 647.2671 | 675.2620 | DF | 235.1077 | 263.1026 |
| DFD | 350.1347 | 378.1296 | DFDA | 421.1718 | 449.1667 | DFDAE | 550.2144 | 578.2093 |

| | | | | | | | | |
|---|---|---|---|---|---|---|---|---|
| **DFDAEA** | 621.2515 | 649.2464 | **FD** | 235.1077 | 263.1026 | **FDA** | 306.1448 | 334.1397 |
| **FDAE** | 435.1874 | 463.1823 | **FDAEA** | 506.2245 | 534.2195 | **FDAEAV** | 605.2930 | 633.2879 |
| **DA** | 159.0764 | 187.0713 | **DAE** | 288.1190 | 316.1139 | **DAEA** | 359.1561 | 387.1510 |
| **DAEAV** | 458.2245 | 486.2195 | **DAEAVF** | 605.2930 | 633.2879 | **AE** | 173.0921 | 201.0870 |
| **AEA** | 244.1292 | 272.1241 | **AEAV** | 343.1976 | 371.1925 | **AEAVF** | 490.2660 | 518.2609 |
| **AEAVFD** | 605.2930 | 633.2879 | **EA** | 173.0921 | 201.0870 | **EAV** | 272.1605 | 300.1554 |
| **EAVF** | 419.2289 | 447.2238 | **EAVFD** | 534.2558 | 562.2508 | **EAVFDQ** | 662.3144 | 690.3093 |
| **AV** | 143.1179 | 171.1128 | **AVF** | 290.1863 | 318.1812 | **AVFD** | 405.2132 | 433.2082 |
| **AVFDQ** | 533.2718 | 561.2667 | **AVFDQE** | 662.3144 | 690.3093 | **VF** | 219.1492 | 247.1441 |
| **VFD** | 334.1761 | 362.1710 | **VFDQ** | 462.2347 | 490.2296 | **VFDQE** | 591.2773 | 619.2722 |
| **FD** | 235.1077 | 263.1026 | **FDQ** | 363.1663 | 391.1612 | **FDQE** | 492.2089 | 520.2038 |
| **FDQEF** | 639.2773 | 667.2722 | **DQ** | 216.0979 | 244.0928 | **DQE** | 345.1405 | 373.1354 |
| **DQEF** | 492.2089 | 520.2038 | **DQEFI** | 605.2930 | 633.2879 | **QE** | 230.1135 | 258.1084 |
| **QEF** | 377.1819 | 405.1769 | **QEFI** | 490.2660 | 518.2609 | **QEFIN** | 604.3089 | 632.3039 |
| **EF** | 249.1234 | 277.1183 | **EFI** | 362.2074 | 390.2023 | **EFIN** | 476.2504 | 504.2453 |
| **EFINV** | 575.3188 | 603.3137 | **FI** | 233.1648 | 261.1598 | **FIN** | 347.2078 | 375.2027 |
| **FINV** | 446.2762 | 474.2711 | **IN** | 200.1394 | 228.1343 | **INV** | 299.2078 | 327.2027 |
| **NV** | 186.1237 | 214.1186 | | | | | | |

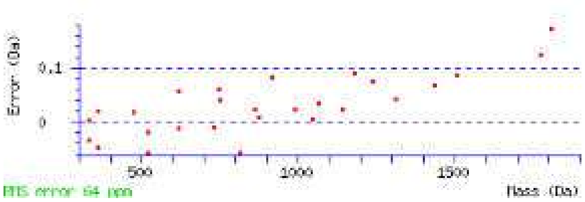


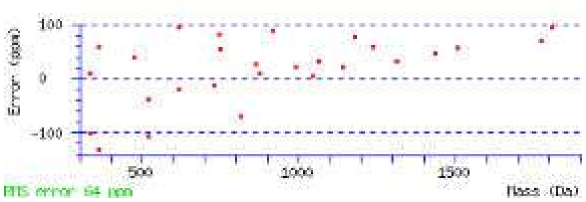


NCBI **BLAST** search of APDFDAEAVFDQEFINVK
(Parameters: blastp, nr protein database, expect=20000, no filter, PAM30)
Other BLAST web gateways

**All matches to this query**

| Score | Mr(calc) | Delta | Sequence |
|---|---|---|---|
| 54.0 | 2053.9633 | 0.0875 | APDFDAEAVFDQEFINVK |
| 20.8 | 2054.0029 | 0.0480 | HALTAVAVQTADETTADNAR |
| 19.2 | 2053.9858 | 0.0650 | TQDQFKPFADPFTGAQTR |
| 18.7 | 2053.9482 | 0.1026 | MVEMVTGEKTSLEEMGGAR |
| 18.5 | 2053.9819 | 0.0689 | QNQFDIAVMSALTYFYK |
| 15.5 | 2054.0255 | 0.0253 | GVQAAAYLAAFVEDMKASGR |
| 15.0 | 2054.1097 | -0.0589 | MLVLYLCVFLGDVPLFR |
| 14.0 | 2054.0657 | -0.0149 | HAPAAVSDAFTATRLAGVSGR |
| 13.9 | 2054.1637 | -0.1128 | RLFTGGNVALTALNGLPGVGK |
| 13.7 | 2054.0003 | 0.0505 | MNNEIKVFSNNQFGQIR |

**Mascot:** http://www.matrixscience.com/

# MATRIX SCIENCE Mascot Search Results

## Peptide View Spot no 21

MS/MS Fragmentation of **GLFIIDKEGVIQHSTINNLAIGR**
Found in **gi|115446541** in **NCBInr**, Os02g0537700 [Oryza sativa Japonica Group]

Match to Query 35: 2507.492924 from(2508.500200,1+) intensity(0.0000) index(12)
Title: Label: J7, Spot_Id: 219779, Peak_List_Id: 226420, MSMS Job_Run_Id: 21843, Comment:
Data file C:\Documents and Settings\Administrator\Desktop\Farah-Garima 11May 2013\ppw_J7_136859916700.txt

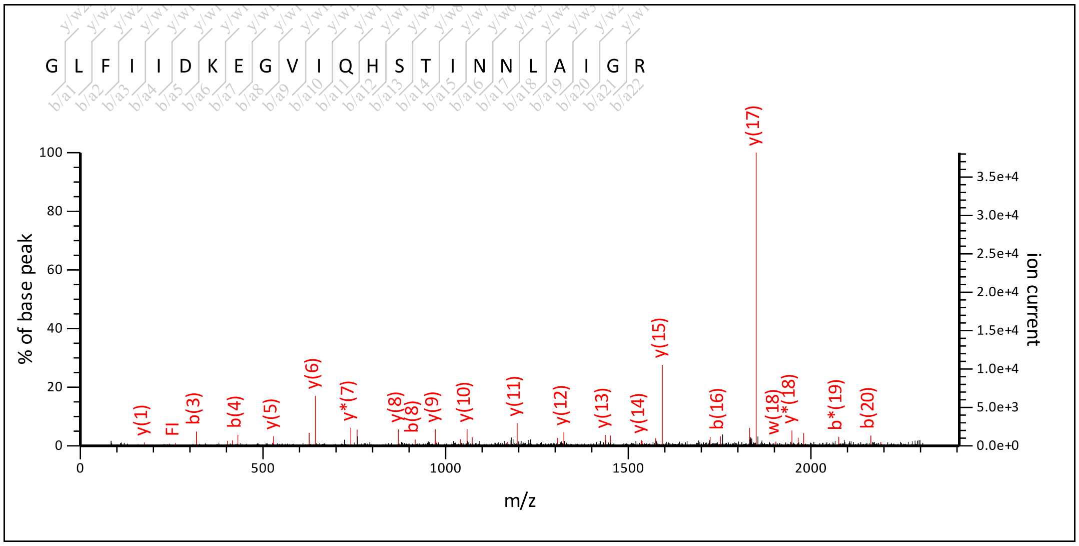


0 to 2406.12

Label all possible matches Label matches used for scoring

**Monoisotopic mass of neutral peptide Mr(calc):** 2507.3860
**Fixed modifications:** Carbamidomethyl (C) (apply to specified residues or termini only)
**Ions Score:** 110 **Expect:** 1e-07
**Matches :** 53/437 fragment ions using 69 most intense peaks (help)

| # | Immon. | a | a* | $a^0$ | b | b* | $b^0$ | d | d' | Seq. | v | w | w' | y | y* | |
|---|---|---|---|---|---|---|---|---|---|---|---|---|---|---|---|---|
| 1 | 30.0338 | 30.0338 | | | 58.0287 | | | 44.0495 | | G | | | | | | |
| 2 | 86.0964 | 143.1179 | | | 171.1128 | | | 101.0709 | | L | 2393.2936 | 2392.2983 | | 2451.3718 | 2434.3453 | [illegible] |
| 3 | 120.0808 | 290.1863 | | | **318.1812** | | | | | F | 2246.2252 | | | 2338.2878 | 2321.2612 | [illegible] |
| 4 | 86.0964 | **403.2704** | | | **431.2653** | | | 375.2391 | 389.2547 | I | 2133.1411 | 2146.1615 | 2160.1771 | ***2191.2193*** | 2174.1928 | [illegible] |
| 5 | 86.0964 | 516.3544 | | | 544.3493 | | | 488.3231 | 502.3388 | I | 2020.0570 | 2033.0774 | 2047.0931 | 2078.1353 | 2061.1087 | [illegible] |
| 6 | 88.0393 | 631.3814 | | 613.3708 | 659.3763 | | 641.3657 | 587.3915 | | D | 1905.0301 | 1904.0348 | | ***1965.0512*** | 1948.0247 | [illegible] |
| 7 | 101.1073 | 759.4763 | 742.4498 | 741.4658 | 787.4713 | 770.4447 | 769.4607 | 702.4185 | | K | 1776.9351 | 1775.9399 | | ***1850.0243*** | 1832.9977 | [illegible] |
| 8 | 102.0550 | 888.5189 | 871.4924 | 870.5084 | **916.5138** | 899.4873 | 898.5033 | 830.5135 | | E | 1647.8925 | 1646.8973 | | 1721.9293 | 1704.9028 | [illegible] |
| 9 | 30.0338 | 945.5404 | 928.5138 | 927.5298 | **973.5353** | 956.5088 | 955.5247 | | | G | | | | ***1592.8867*** | 1575.8602 | [illegible] |
| 10 | 72.0808 | 1044.6088 | 1027.5823 | 1026.5982 | **1072.6037** | 1055.5772 | 1054.5932 | 1030.5932 | | V | 1491.8027 | 1504.8231 | | ***1535.8653*** | 1518.8387 | [illegible] |
| 11 | 86.0964 | 1157.6929 | 1140.6663 | 1139.6823 | 1185.6878 | 1168.6612 | 1167.6772 | 1129.6616 | 1143.6772 | I | 1378.7186 | 1391.7390 | 1405.7546 | ***1436.7968*** | 1419.7703 | [illegible] |
| 12 | 101.0709 | 1285.7515 | 1268.7249 | 1267.7409 | 1313.7464 | 1296.7198 | 1295.7358 | 1228.7300 | | Q | 1250.6600 | 1249.6648 | | ***1323.7128*** | 1306.6862 | [illegible] |
| 13 | 110.0713 | 1422.8104 | 1405.7838 | 1404.7998 | **1450.8053** | 1433.7787 | 1432.7947 | | | H | 1113.6011 | | | ***1195.6542*** | 1178.6276 | [illegible] |
| 14 | 60.0444 | 1509.8424 | 1492.8158 | 1491.8318 | **1537.8373** | 1520.8108 | 1519.8267 | 1493.8475 | | S | 1026.5691 | 1025.5738 | | ***1058.5953*** | 1041.5687 | [illegible] |
| 15 | 74.0600 | 1610.8901 | 1593.8635 | 1592.8795 | 1638.8850 | 1621.8584 | 1620.8744 | 1594.8952 | 1596.8744 | T | 925.5214 | 938.5418 | 940.5211 | ***971.5633*** | 954.5367 | [illegible] |
| 16 | 86.0964 | **1723.9741** | 1706.9476 | 1705.9636 | **1751.9690** | 1734.9425 | 1733.9585 | 1695.9428 | 1709.9585 | I | 812.4373 | 825.4577 | 839.4734 | ***870.5156*** | 853.4890 | |
| 17 | 87.0553 | 1838.0171 | 1820.9905 | 1820.0065 | 1866.0120 | 1848.9854 | 1848.0014 | 1795.0112 | | N | 698.3944 | 697.3992 | | ***757.4315*** | 740.4050 | |
| 18 | 87.0553 | 1952.0600 | 1935.0334 | 1934.0494 | **1980.0549** | 1963.0284 | 1962.0443 | 1909.0542 | | N | 584.3515 | 583.3562 | | ***643.3886*** | 626.3620 | |
| 19 | 86.0964 | 2065.1441 | 2048.1175 | 2047.1335 | 2093.1390 | 2076.1124 | 2075.1284 | 2023.0971 | | L | 471.2674 | 470.2722 | | ***529.3457*** | 512.3191 | |
| 20 | 44.0495 | 2136.1812 | 2119.1546 | 2118.1706 | **2164.1761** | 2147.1495 | 2146.1655 | | | A | 400.2303 | | | ***416.2616*** | 399.2350 | |
| 21 | 86.0964 | 2249.2652 | 2232.2387 | 2231.2547 | 2277.2601 | 2260.2336 | 2259.2496 | 2221.2339 | 2235.2496 | I | 287.1462 | 300.1666 | 314.1823 | 345.2245 | 328.1979 | |
| 22 | 30.0338 | 2306.2867 | 2289.2601 | 2288.2761 | 2334.2816 | 2317.2551 | 2316.2710 | | | G | | | | 232.1404 | 215.1139 | |
| 23 | 129.1135 | | | | | | | | | R | 74.0237 | 73.0284 | | ***175.1190*** | 158.0924 | |

| Seq | ya | yb | Seq | ya | yb | Seq | ya | yb |
|---|---|---|---|---|---|---|---|---|
| LF | 233.1648 | 261.1598 | LFI | 346.2489 | 374.2438 | LFII | 459.3330 | 487.3279 |
| LFIID | 574.3599 | 602.3548 | FI | 233.1648 | 261.1598 | FII | 346.2489 | 374.2438 |
| FIID | 461.2758 | 489.2708 | FIIDK | 589.3708 | 617.3657 | II | 199.1805 | 227.1754 |
| IID | 314.2074 | 342.2023 | IIDK | 442.3024 | 470.2973 | IIDKE | 571.3450 | 599.3399 |
| IIDKEG | 628.3665 | 656.3614 | ID | 201.1234 | 229.1183 | IDK | 329.2183 | 357.2132 |
| IDKE | 458.2609 | 486.2558 | IDKEG | 515.2824 | 543.2773 | IDKEGV | 614.3508 | 642.3457 |
| DK | 216.1343 | 244.1292 | DKE | 345.1769 | 373.1718 | DKEG | 402.1983 | 430.1932 |
| DKEGV | 501.2667 | 529.2617 | DKEGVI | 614.3508 | 642.3457 | KE | 230.1499 | 258.1448 |
| KEG | 287.1714 | 315.1663 | KEGV | 386.2398 | 414.2347 | KEGVI | 499.3239 | 527.3188 |
| KEGVIQ | 627.3824 | 655.3774 | EG | 159.0764 | 187.0713 | EGV | 258.1448 | 286.1397 |
| EGVI | 371.2289 | 399.2238 | EGVIQ | 499.2875 | 527.2824 | EGVIQH | 636.3464 | 664.3413 |
| GV | 129.1022 | 157.0972 | GVI | 242.1863 | 270.1812 | GVIQ | 370.2449 | 398.2398 |
| GVIQH | 507.3038 | 535.2987 | GVIQHS | 594.3358 | 622.3307 | GVIQHST | 695.3835 | 723.3784 |
| VI | 185.1648 | 213.1598 | VIQ | 313.2234 | 341.2183 | VIQH | 450.2823 | 478.2772 |
| VIQHS | 537.3144 | 565.3093 | VIQHST | 638.3620 | 666.3570 | IQ | 214.1550 | 242.1499 |
| IQH | 351.2139 | 379.2088 | IQHS | 438.2459 | 466.2409 | IQHST | 539.2936 | 567.2885 |
| IQHSTI | 652.3777 | 680.3726 | QH | 238.1299 | 266.1248 | QHS | 325.1619 | 353.1568 |
| QHST | 426.2096 | 454.2045 | QHSTI | 539.2936 | 567.2885 | QHSTIN | 653.3365 | 681.3315 |
| HS | 197.1033 | 225.0982 | HST | 298.1510 | 326.1459 | HSTI | 411.2350 | 439.2300 |
| HSTIN | 525.2780 | 553.2729 | HSTINN | 639.3209 | 667.3158 | ST | 161.0921 | 189.0870 |
| STI | 274.1761 | 302.1710 | STIN | 388.2191 | 416.2140 | STINN | 502.2620 | 530.2569 |
| STINNL | 615.3461 | 643.3410 | STINNLA | 686.3832 | 714.3781 | TI | 187.1441 | 215.1390 |
| TIN | 301.1870 | 329.1819 | TINN | 415.2300 | 443.2249 | TINNL | 528.3140 | 556.3089 |
| TINNLA | 599.3511 | 627.3461 | IN | 200.1394 | 228.1343 | INN | 314.1823 | 342.1772 |
| INNL | 427.2663 | 455.2613 | INNLA | 498.3035 | 526.2984 | INNLAI | 611.3875 | 639.3824 |
| INNLAIG | 668.4090 | 696.4039 | NN | 201.0982 | 229.0931 | NNL | 314.1823 | 342.1772 |
| NNLA | 385.2194 | 413.2143 | NNLAI | 498.3035 | 526.2984 | NNLAIG | 555.3249 | 583.3198 |
| NL | 200.1394 | 228.1343 | NLA | 271.1765 | 299.1714 | NLAI | 384.2605 | 412.2554 |
| NLAIG | 441.2820 | 469.2769 | LA | 157.1335 | 185.1285 | LAI | 270.2176 | 298.2125 |
| LAIG | 327.2391 | 355.2340 | AI | 157.1335 | 185.1285 | AIG | 214.1550 | 242.1499 |
| IG | 143.1179 | 171.1128 | | | | | | |

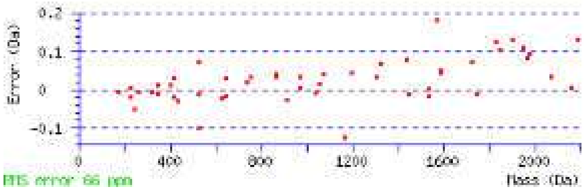


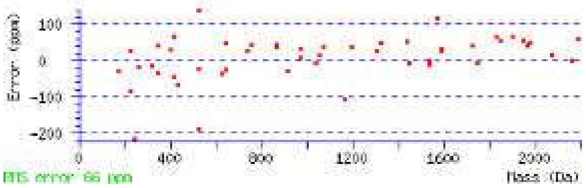


NCBI **BLAST** search of GLFIIDKEGVIQHSTINNLAIGR
(Parameters: blastp, nr protein database, expect=20000, no filter, PAM30)
Other BLAST web gateways

**All matches to this query**

| Score | Mr(calc) | Delta | Sequence |
|---|---|---|---|
| 110.2 | 2507.3860 | 0.1069 | GLFIIDKEGVIQHSTINNLAIGR |
| 72.1 | 2507.3860 | 0.1069 | GLFIIDKEGVIQHSTIQNLGIGR |
| 13.8 | 2507.3860 | 0.1069 | GLFIIDKEGIIQHSTINNLGIGR |
| 12.6 | 2507.2703 | 0.2226 | MPHIRPDGDPVRLTSNFQNGLK |
| 11.9 | 2507.2512 | 0.2417 | GNQTIQMANFGAGDIVAIMIGNLK |
| 11.1 | 2507.4111 | 0.0818 | EATAPSRLFLVGAVLPELVEAAVR |
| 10.5 | 2507.2631 | 0.2298 | WGERPMAFVILTPAAEKEYAGR |
| 9.8 | 2507.3384 | 0.1545 | TGNYLGQNFGLITGVSEVEIKLR |
| 9.8 | 2507.2626 | 0.2303 | LPAYMIPSAFVMLSTMPLNPNGK |
| 9.7 | 2507.2763 | 0.2166 | LGLMEIENRLQSDLAELFLCK |

**Mascot:** http://www.matrixscience.com/

# Mascot Search Results

## Peptide View Spot no 21

MS/MS Fragmentation of **TLQALQYVQDNPDEVCPAGWKPGDK**
Found in **gi|115446541** in **NCBInr**, Os02g0537700 [Oryza sativa Japonica Group]

Match to Query 37: 2828.469024 from(2829.476300,1+) intensity(0.0000) index(13)
Title: Label: J7, Spot_Id: 219779, Peak_List_Id: 226427, MSMS Job_Run_Id: 21843, Comment:
Data file C:\Documents and Settings\Administrator\Desktop\Farah-Garima 11May 2013\ppw_J7_136859916700.txt

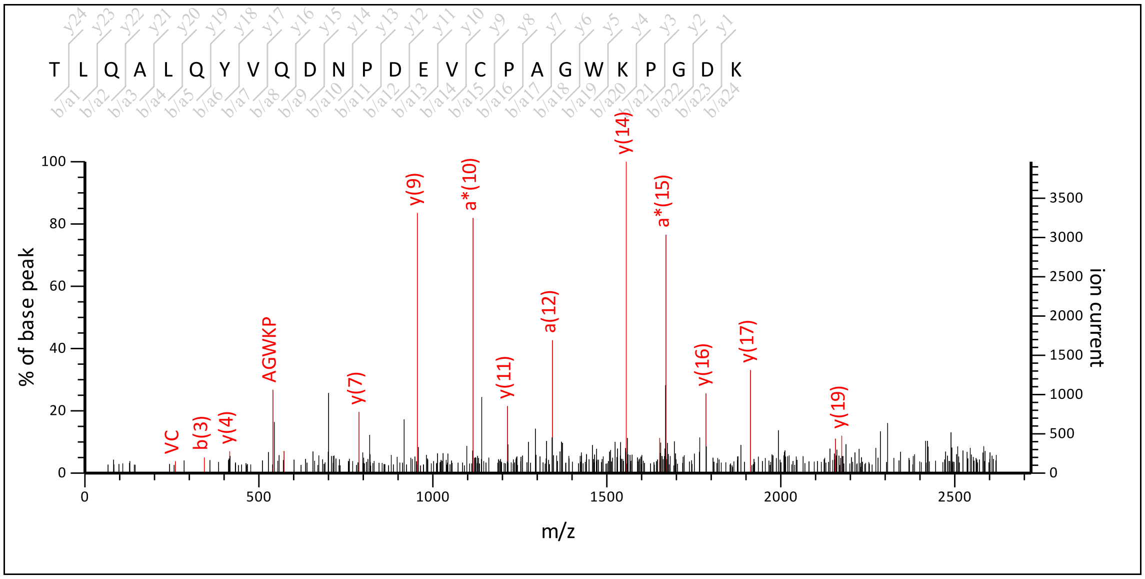


0 to 2719.26

Label all possible matches ○ Label matches used for scoring ◉

**Monoisotopic mass of neutral peptide Mr(calc):** 2828.3439
**Fixed modifications:** Carbamidomethyl (C) (apply to specified residues or termini only)
**Ions Score:** 71 **Expect:** 0.0015
**Matches :** 22/487 fragment ions using 26 most intense peaks (help)

| # | Immon. | a | a* | a$^0$ | b | b* | b$^0$ | d | Seq. | v | w | y | y* | y$^0$ | # |
|---|---|---|---|---|---|---|---|---|---|---|---|---|---|---|---|
| 1 | 74.0600 | 74.0600 | | 56.0495 | 102.0550 | | 84.0444 | 44.0495 | T | | | | | | 25 |
| 2 | 86.0964 | 187.1441 | | 169.1335 | 215.1390 | | 197.1285 | 145.0972 | L | 2670.2253 | 2669.2300 | 2728.3035 | 2711.2770 | 2710.2930 | 24 |
| 3 | 101.0709 | 315.2027 | 298.1761 | 297.1921 | 343.1976 | 326.1710 | 325.1870 | 258.1812 | Q | 2542.1667 | 2541.1715 | 2615.2195 | 2598.1929 | 2597.2089 | 23 |
| 4 | 44.0495 | 386.2398 | 369.2132 | 368.2292 | 414.2347 | 397.2082 | 396.2241 | | A | 2471.1296 | | 2487.1609 | 2470.1344 | 2469.1503 | 22 |
| 5 | 86.0964 | 499.3239 | 482.2973 | 481.3133 | 527.3188 | 510.2922 | 509.3082 | 457.2769 | L | 2358.0455 | 2357.0503 | 2416.1238 | 2399.0972 | 2398.1132 | 21 |
| 6 | 101.0709 | 627.3824 | 610.3559 | 609.3719 | 655.3774 | 638.3508 | 637.3668 | 570.3610 | Q | 2229.9870 | 2228.9917 | 2303.0397 | 2286.0132 | 2285.0292 | 20 |
| 7 | 136.0757 | 790.4458 | 773.4192 | 772.4352 | 818.4407 | 801.4141 | 800.4301 | | Y | 2066.9236 | | ***2174.9811*** | 2157.9546 | 2156.9706 | 19 |
| 8 | 72.0808 | 889.5142 | 872.4876 | 871.5036 | 917.5091 | 900.4825 | 899.4985 | 875.4985 | V | 1967.8552 | 1980.8756 | 2011.9178 | 1994.8913 | 1993.9073 | 18 |
| 9 | 101.0709 | 1017.5728 | 1000.5462 | 999.5622 | 1045.5677 | 1028.5411 | 1027.5571 | 960.5513 | Q | 1839.7966 | 1838.8014 | ***1912.8494*** | 1895.8229 | 1894.8388 | 17 |
| 10 | 88.0393 | 1132.5997 | 1115.5732 | 1114.5891 | 1160.5946 | 1143.5681 | 1142.5841 | 1088.6099 | D | 1724.7697 | 1723.7744 | ***1784.7908*** | 1767.7643 | 1766.7803 | 16 |
| 11 | 87.0553 | 1246.6426 | 1229.6161 | 1228.6321 | 1274.6375 | 1257.6110 | 1256.6270 | 1203.6368 | N | 1610.7268 | 1609.7315 | ***1669.7639*** | 1652.7373 | 1651.7533 | 15 |
| 12 | 70.0651 | 1343.6954 | 1326.6688 | 1325.6848 | 1371.6903 | 1354.6638 | 1353.6797 | 1317.6797 | P | 1513.6740 | 1512.6788 | ***1555.7210*** | 1538.6944 | 1537.7104 | 14 |
| 13 | 88.0393 | 1458.7223 | 1441.6958 | 1440.7118 | 1486.7173 | 1469.6907 | 1468.7067 | 1414.7325 | D | 1398.6471 | 1397.6518 | 1458.6682 | 1441.6416 | 1440.6576 | 13 |
| 14 | 102.0550 | 1587.7649 | 1570.7384 | 1569.7544 | 1615.7598 | 1598.7333 | 1597.7493 | 1529.7595 | E | 1269.6045 | 1268.6092 | ***1343.6412*** | 1326.6147 | 1325.6307 | 12 |
| 15 | 72.0808 | 1686.8333 | 1669.8068 | 1668.8228 | 1714.8283 | 1697.8017 | 1696.8177 | 1672.8177 | V | 1170.5361 | 1183.5565 | ***1214.5987*** | 1197.5721 | 1196.5881 | 11 |
| 16 | 133.0430 | 1846.8640 | 1829.8374 | 1828.8534 | 1874.8589 | 1857.8324 | 1856.8483 | 1757.8705 | C | 1010.5054 | 1009.5102 | ***1115.5302*** | 1098.5037 | 1097.5197 | 10 |
| 17 | 70.0651 | 1943.9168 | 1926.8902 | 1925.9062 | 1971.9117 | 1954.8851 | 1953.9011 | 1917.9011 | P | 913.4526 | 912.4574 | ***955.4996*** | 938.4730 | 937.4890 | 9 |
| 18 | 44.0495 | 2014.9539 | 1997.9273 | 1996.9433 | 2042.9488 | 2025.9222 | 2024.9382 | | A | 842.4155 | | 858.4468 | 841.4203 | 840.4363 | 8 |
| 19 | 30.0338 | 2071.9753 | 2054.9488 | 2053.9648 | 2099.9702 | 2082.9437 | 2081.9597 | | G | | | ***787.4097*** | 770.3832 | 769.3992 | 7 |

| | | | | | | | | | | | | | | | |
|---|---|---|---|---|---|---|---|---|---|---|---|---|---|---|---|
| 20 | 159.0917 | 2258.0546 | 2241.0281 | 2240.0441 | 2286.0496 | 2269.0230 | 2268.0390 | | W | 599.3148 | | 730.3883 | 713.3617 | 712.3777 | 6 |
| 21 | 101.1073 | 2386.1496 | 2369.1231 | 2368.1390 | 2414.1445 | 2397.1180 | 2396.1340 | 2329.0918 | K | 471.2198 | 470.2245 | 544.3089 | 527.2824 | 526.2984 | 5 |
| 22 | 70.0651 | 2483.2024 | 2466.1758 | 2465.1918 | 2511.1973 | 2494.1707 | 2493.1867 | 2457.1867 | P | 374.1670 | 373.1718 | ***416.2140*** | 399.1874 | 398.2034 | 4 |
| 23 | 30.0338 | 2540.2238 | 2523.1973 | 2522.2133 | 2568.2188 | 2551.1922 | 2550.2082 | | G | | | 319.1612 | 302.1347 | 301.1506 | 3 |
| 24 | 88.0393 | 2655.2508 | 2638.2242 | 2637.2402 | 2683.2457 | 2666.2191 | 2665.2351 | 2611.2610 | D | 202.1186 | 201.1234 | 262.1397 | 245.1132 | 244.1292 | 2 |
| 25 | 101.1073 | | | | | | | | K | 74.0237 | 73.0284 | 147.1128 | 130.0863 | | 1 |

| Seq | ya | yb | Seq | ya | yb | Seq | ya | yb |
|---|---|---|---|---|---|---|---|---|
| LQ | 214.1550 | 242.1499 | LQA | 285.1921 | 313.1870 | LQAL | 398.2762 | 426.2711 |
| LQALQ | 526.3348 | 554.3297 | LQALQY | 689.3981 | 717.3930 | QA | 172.1081 | 200.1030 |
| QAL | 285.1921 | 313.1870 | QALQ | 413.2507 | 441.2456 | QALQY | 576.3140 | 604.3089 |
| QALQYV | 675.3824 | 703.3774 | AL | 157.1335 | 185.1285 | ALQ | 285.1921 | 313.1870 |
| ALQY | 448.2554 | 476.2504 | ALQYV | 547.3239 | 575.3188 | ALQYVQ | 675.3824 | 703.3774 |
| LQ | 214.1550 | 242.1499 | LQY | 377.2183 | 405.2132 | LQYV | 476.2867 | 504.2817 |
| LQYVQ | 604.3453 | 632.3402 | QY | 264.1343 | 292.1292 | QYV | 363.2027 | 391.1976 |
| QYVQ | 491.2613 | 519.2562 | QYVQD | 606.2882 | 634.2831 | YV | 235.1441 | 263.1390 |
| YVQ | 363.2027 | 391.1976 | YVQD | 478.2296 | 506.2245 | YVQDN | 592.2726 | 620.2675 |
| YVQDNP | 689.3253 | 717.3202 | VQ | 200.1394 | 228.1343 | VQD | 315.1663 | 343.1612 |
| VQDN | 429.2092 | 457.2041 | VQDNP | 526.2620 | 554.2569 | VQDNPD | 641.2889 | 669.2838 |
| QD | 216.0979 | 244.0928 | QDN | 330.1408 | 358.1357 | QDNP | 427.1936 | 455.1885 |
| QDNPD | 542.2205 | 570.2154 | QDNPDE | 671.2631 | 699.2580 | DN | 202.0822 | 230.0771 |
| DNP | 299.1350 | 327.1299 | DNPD | 414.1619 | 442.1569 | DNPDE | 543.2045 | 571.1994 |
| DNPDEV | 642.2729 | 670.2679 | NP | 184.1081 | 212.1030 | NPD | 299.1350 | 327.1299 |
| NPDE | 428.1776 | 456.1725 | NPDEV | 527.2460 | 555.2409 | NPDEVC | 687.2767 | 715.2716 |
| PD | 185.0921 | 213.0870 | PDE | 314.1347 | 342.1296 | PDEV | 413.2031 | 441.1980 |
| PDEVC | 573.2337 | 601.2286 | PDEVCP | 670.2865 | 698.2814 | DE | 217.0819 | 245.0768 |
| DEV | 316.1503 | 344.1452 | DEVC | 476.1810 | 504.1759 | DEVCP | 573.2337 | 601.2286 |
| DEVCPA | 644.2708 | 672.2658 | EV | 201.1234 | 229.1183 | EVC | 361.1540 | 389.1489 |
| EVCP | 458.2068 | 486.2017 | EVCPA | 529.2439 | 557.2388 | EVCPAG | 586.2654 | 614.2603 |
| VC | 232.1114 | 260.1063 | VCP | 329.1642 | 357.1591 | VCPA | 400.2013 | 428.1962 |
| VCPAG | 457.2228 | 485.2177 | VCPAGW | 643.3021 | 671.2970 | CP | 230.0958 | 258.0907 |
| CPA | 301.1329 | 329.1278 | CPAG | 358.1544 | 386.1493 | CPAGW | 544.2337 | 572.2286 |
| CPAGWK | 672.3286 | 700.3235 | PA | 141.1022 | 169.0972 | PAG | 198.1237 | 226.1186 |
| PAGW | 384.2030 | 412.1979 | PAGWK | 512.2980 | 540.2929 | PAGWKP | 609.3507 | 637.3457 |
| PAGWKPG | 666.3722 | 694.3671 | AG | 101.0709 | 129.0659 | AGW | 287.1503 | 315.1452 |
| AGWK | 415.2452 | 443.2401 | AGWKP | 512.2980 | 540.2929 | AGWKPG | 569.3194 | 597.3144 |
| AGWKPGD | 684.3464 | 712.3413 | GW | 216.1131 | 244.1081 | GWK | 344.2081 | 372.2030 |
| GWKP | 441.2609 | 469.2558 | GWKPG | 498.2823 | 526.2772 | GWKPGD | 613.3093 | 641.3042 |
| WK | 287.1866 | 315.1816 | WKP | 384.2394 | 412.2343 | WKPG | 441.2609 | 469.2558 |
| WKPGD | 556.2878 | 584.2827 | KP | 198.1601 | 226.1550 | KPG | 255.1816 | 283.1765 |
| KPGD | 370.2085 | 398.2034 | PG | 127.0866 | 155.0815 | PGD | 242.1135 | 270.1084 |
| GD | 145.0608 | 173.0557 | | | | | | |

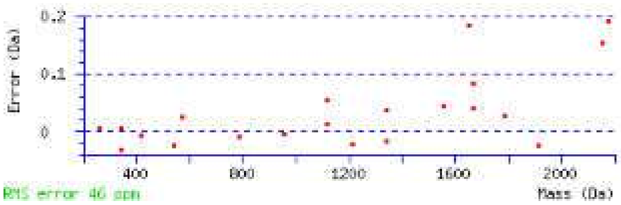


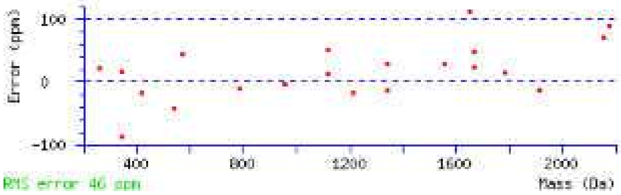


NCBI **BLAST** search of TLQALQYVQDNPDEVCPAGWKPGDK
(Parameters: blastp, nr protein database, expect=20000, no filter, PAM30)
Other BLAST web gateways

**All matches to this query**

| Score | Mr(calc) | Delta | Sequence |
|---|---|---|---|
| 70.5 | 2828.3439 | 0.1251 | TLQALQYVQDNPDEVCPAGWKPGDK |
| 11.4 | 2828.4630 | 0.0061 | KMVAEEGGVSPEEVILVELYPSGLQR |
| 9.6 | 2828.4888 | -0.0198 | MQVVAMASALLSQGPALSQAGSDLLRSK |
| 9.1 | 2828.3941 | 0.0750 | QGGSDVRANTTTAIPIDDGTYELLGHK |
| 8.3 | 2828.4234 | 0.0456 | GIPVAAMEIMDEVQMKVVNIGGATAPR |
| 8.3 | 2828.4234 | 0.0456 | GIPVAAMEIMDEVQMKVVNLGGATAPR |
| 8.2 | 2828.3076 | 0.1615 | DLFSDHKWNILCAPDGAVSSDPQEK |
| 7.7 | 2828.3763 | 0.0927 | VLDEVVACAGSGNPADTAGGALAAFTAPAR |
| 7.6 | 2828.2877 | 0.1813 | AMAQQTEGMDIESEAAALDSKASAMLK |
| 7.6 | 2828.2877 | 0.1813 | AMAQQTEGMDIESEAAALDSKASAMLK |

**Mascot:** http://www.matrixscience.com/

# Mascot Search Results

## Peptide View Spot no 23

MS/MS Fragmentation of YMVFSCADSR
Found in gi|3345477 in NCBInr, carbonic anhydrase [Oryza sativa]

Match to Query 21: 1234.592824 from(1235.600100,1+) intensity(0.0000) index(9)
Title: Label: G9, Spot_Id: 228976, Peak_List_Id: 257846, MSMS Job_Run_Id: 24939, Comment:
Data file ppw_G9_138985152400.txt

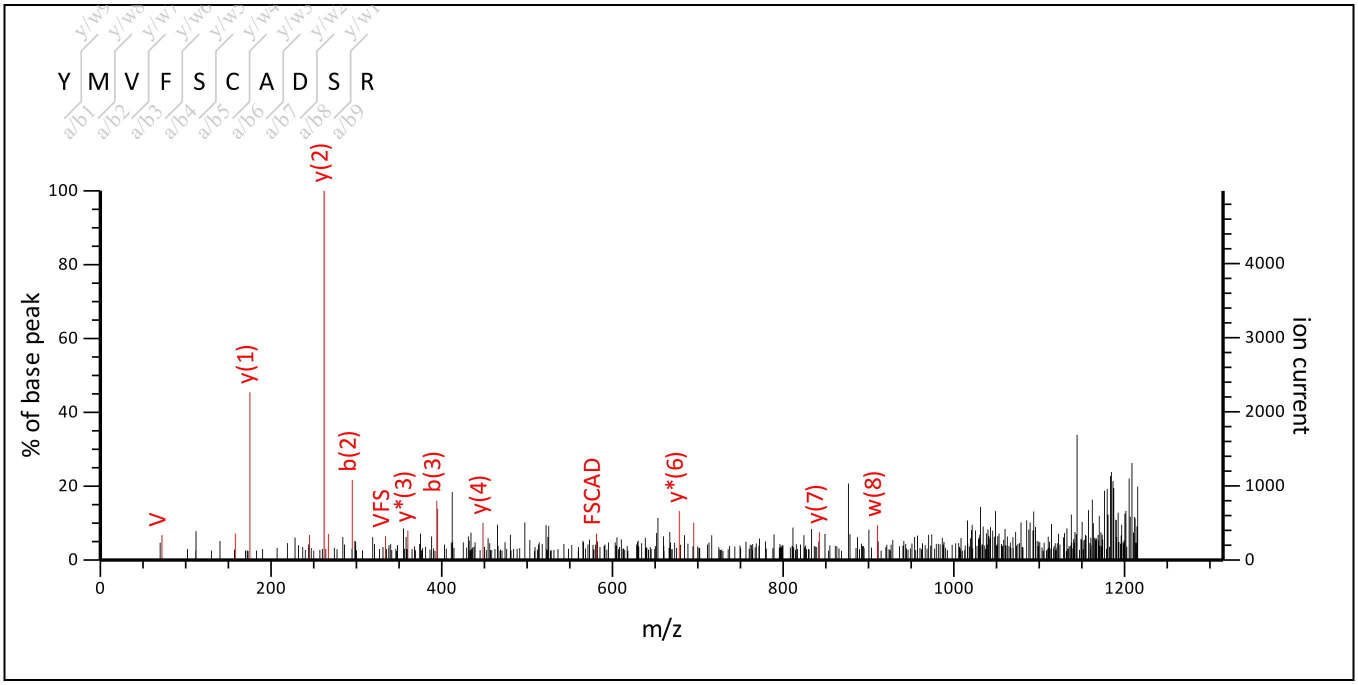


0 to 1315.41

Label all possible matches ○ Label matches used for scoring ◉

**Monoisotopic mass of neutral peptide Mr(calc):** 1234.5111
**Fixed modifications:** Carbamidomethyl (C) (apply to specified residues or termini only)
**Ions Score:** 10 **Expect:** 2.6e+02
**Matches :** 18/137 fragment ions using 48 most intense peaks (help)

| # | Immon. | a | $a^0$ | b | $b^0$ | d | Seq. | v | w | y | y* | $y^0$ | # |
|---|---|---|---|---|---|---|---|---|---|---|---|---|---|
| 1 | 136.0757 | 136.0757 | | 164.0706 | | 44.0495 | Y | | | | | | 10 |
| 2 | 104.0528 | 267.1162 | | **295.1111** | | 207.1128 | M | 996.4204 | 995.4251 | 1072.4550 | 1055.4285 | 1054.4445 | 9 |
| 3 | 72.0808 | 366.1846 | | **394.1795** | | 352.1689 | V | 897.3519 | 910.3723 | 941.4145 | 924.3880 | 923.4040 | 8 |
| 4 | 120.0808 | 513.2530 | | 541.2479 | | | F | 750.2835 | | ***842.3461*** | 825.3196 | 824.3356 | 7 |
| 5 | 60.0444 | 600.2850 | 582.2745 | 628.2799 | 610.2694 | 584.2901 | S | 663.2515 | 662.2563 | ***695.2777*** | 678.2512 | 677.2671 | 6 |
| 6 | 133.0430 | 760.3157 | 742.3051 | 788.3106 | 770.3000 | 671.3221 | C | 503.2209 | 502.2256 | 608.2457 | 591.2191 | 590.2351 | 5 |
| 7 | 44.0495 | 831.3528 | 813.3422 | 859.3477 | 841.3371 | | A | 432.1837 | | ***448.2150*** | 431.1885 | 430.2045 | 4 |
| 8 | 88.0393 | 946.3797 | 928.3692 | 974.3747 | 956.3641 | 902.3899 | D | 317.1568 | 316.1615 | 377.1779 | 360.1514 | 359.1674 | 3 |
| 9 | 60.0444 | 1033.4118 | 1015.4012 | 1061.4067 | 1043.3961 | 1017.4169 | S | 230.1248 | 229.1295 | ***262.1510*** | 245.1244 | 244.1404 | 2 |
| 10 | 129.1135 | | | | | | R | 74.0237 | 73.0284 | ***175.1190*** | 158.0924 | | 1 |

| Seq | ya | yb | Seq | ya | yb | Seq | ya | yb |
|---|---|---|---|---|---|---|---|---|
| **MV** | 203.1213 | 231.1162 | **MVF** | 350.1897 | 378.1846 | **MVFS** | 437.2217 | 465.2166 |
| **MVFSC** | 597.2524 | 625.2473 | **MVFSCA** | 668.2895 | 696.2844 | **VF** | 219.1492 | 247.1441 |
| **VFS** | 306.1812 | 334.1761 | **VFSC** | 466.2119 | 494.2068 | **VFSCA** | 537.2490 | 565.2439 |
| **VFSCAD** | 652.2759 | 680.2708 | **FS** | 207.1128 | 235.1077 | **FSC** | 367.1435 | 395.1384 |
| **FSCA** | 438.1806 | 466.1755 | **FSCAD** | 553.2075 | 581.2024 | **FSCADS** | 640.2395 | 668.2345 |
| **SC** | 220.0750 | 248.0700 | **SCA** | 291.1122 | 319.1071 | **SCAD** | 406.1391 | 434.1340 |
| **SCADS** | 493.1711 | 521.1660 | **CA** | 204.0801 | 232.0750 | **CAD** | 319.1071 | 347.1020 |
| **CADS** | 406.1391 | 434.1340 | **AD** | 159.0764 | 187.0713 | **ADS** | 246.1084 | 274.1034 |
| **DS** | 175.0713 | 203.0662 | | | | | | |

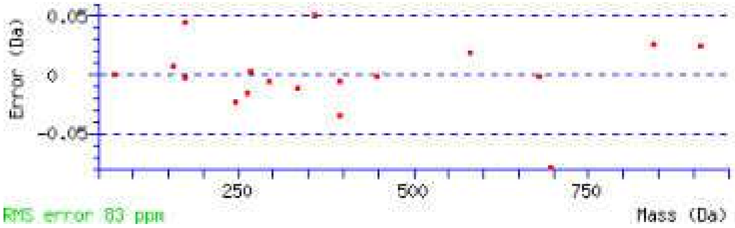


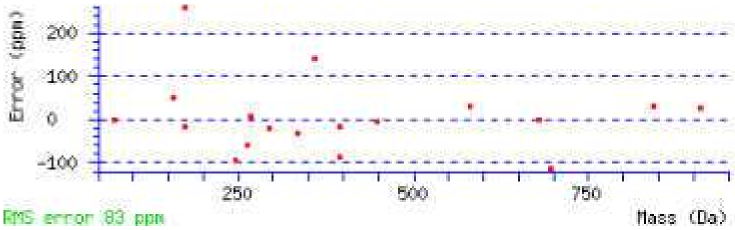


NCBI **BLAST** search of YMVFSCADSR
(Parameters: blastp, nr protein database, expect=20000, no filter, PAM30)
Other BLAST web gateways

**All matches to this query**

| Score | Mr(calc) | Delta | Sequence |
|---|---|---|---|
| 10.4 | 1234.5111 | 0.0817 | YMVFSCADSR |
| 6.6 | 1234.6670 | -0.0742 | VAIQYGTGAISR |
| 6.3 | 1234.5441 | 0.0487 | FFVFACSDSR |
| 6.3 | 1234.5111 | 0.0817 | FMVFACSDSR |
| 5.8 | 1234.6459 | -0.0530 | VPWGLAGGEPPR |
| 5.5 | 1234.5111 | 0.0817 | YMVFACSDSR |
| 5.3 | 1234.6050 | -0.0121 | IMVGLPSMESR |
| 5.2 | 1234.6054 | -0.0126 | ASNEGVRPYSR |
| 5.1 | 1234.6016 | -0.0088 | FPDQLIMESR |
| 5.0 | 1234.6306 | -0.0377 | IEDAERIFSR |

**Mascot:** http://www.matrixscience.com/

# Mascot Search Results

## Peptide View Spot no 23

MS/MS Fragmentation of **DGAPDSFHFVEDWVR**
Found in **gi|3345477** in **NCBInr**, carbonic anhydrase [Oryza sativa]

Match to Query 27: 1775.892724 from(1776.900000,1+) intensity(0.0000) index(12)
Title: Label: G9, Spot_Id: 228976, Peak_List_Id: 257844, MSMS Job_Run_Id: 24939, Comment:
Data file ppw_G9_138985152400.txt

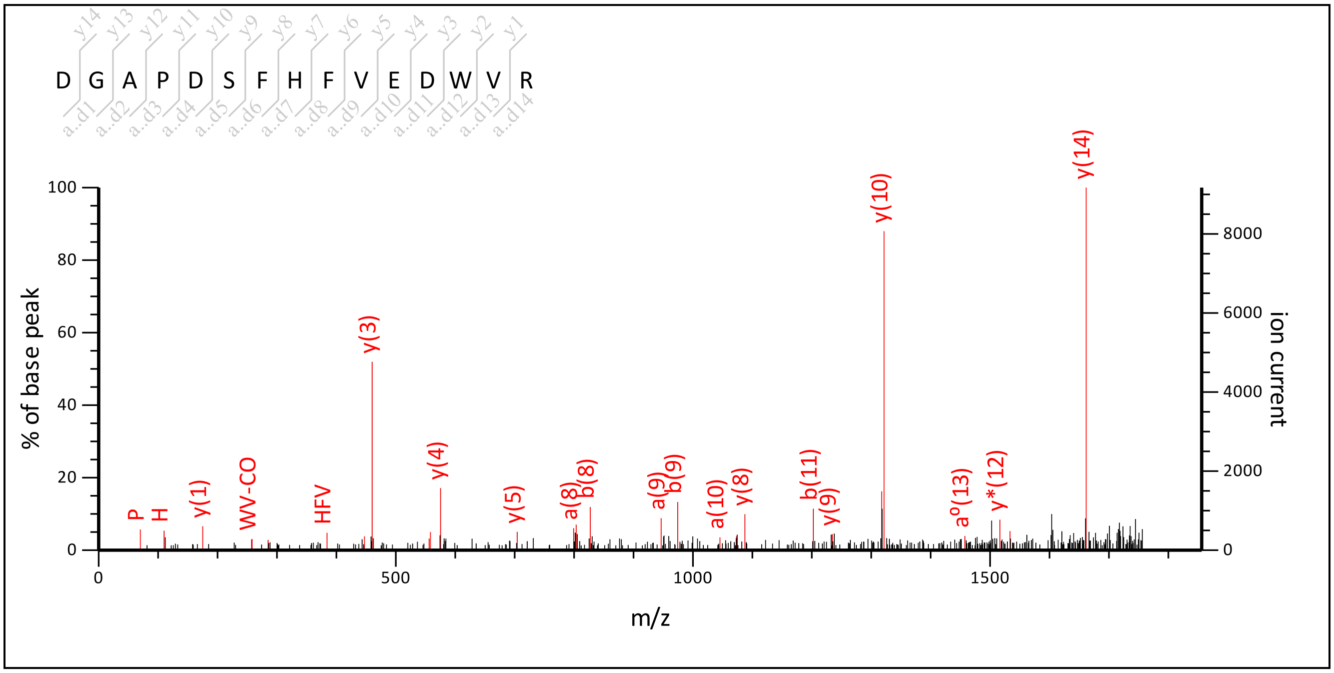


0 to 1856.1

Label all possible matches ○ Label matches used for scoring ◉

**Monoisotopic mass of neutral peptide Mr(calc):** 1775.7904
**Fixed modifications:** Carbamidomethyl (C) (apply to specified residues or termini only)
**Ions Score:** 60 **Expect:** 0.0023
**Matches :** 36/228 fragment ions using 51 most intense peaks (help)

| # | Immon. | a | $a^0$ | b | $b^0$ | d | Seq. | v | w | y | y* | $y^0$ | # |
|---|---|---|---|---|---|---|---|---|---|---|---|---|---|
| 1 | 88.0393 | 88.0393 | 70.0287 | 116.0342 | 98.0237 | 44.0495 | D | | | | | | 15 |
| 2 | 30.0338 | 145.0608 | 127.0502 | 173.0557 | 155.0451 | | G | | | ***1661.7707*** | 1644.7441 | 1643.7601 | 14 |
| 3 | 44.0495 | 216.0979 | 198.0873 | 244.0928 | 226.0822 | | A | 1588.7179 | | 1604.7492 | 1587.7227 | 1586.7387 | 13 |
| 4 | 70.0651 | 313.1506 | 295.1401 | 341.1456 | 323.1350 | 287.1350 | P | 1491.6652 | 1490.6699 | ***1533.7121*** | 1516.6856 | 1515.7015 | 12 |
| 5 | 88.0393 | 428.1776 | 410.1670 | 456.1725 | 438.1619 | 384.1878 | D | 1376.6382 | 1375.6430 | 1436.6593 | 1419.6328 | 1418.6488 | 11 |
| 6 | 60.0444 | 515.2096 | 497.1991 | 543.2045 | 525.1940 | 499.2147 | S | 1289.6062 | 1288.6109 | ***1321.6324*** | 1304.6058 | 1303.6218 | 10 |
| 7 | 120.0808 | 662.2780 | 644.2675 | 690.2729 | 672.2624 | | F | 1142.5378 | | ***1234.6004*** | 1217.5738 | 1216.5898 | 9 |
| 8 | 110.0713 | **799.3369** | 781.3264 | **827.3319** | 809.3213 | | H | 1005.4789 | | ***1087.5320*** | 1070.5054 | 1069.5214 | 8 |
| 9 | 120.0808 | **946.4054** | 928.3948 | **974.4003** | 956.3897 | | F | 858.4104 | | 950.4730 | 933.4465 | 932.4625 | 7 |
| 10 | 72.0808 | **1045.4738** | 1027.4632 | **1073.4687** | 1055.4581 | 1031.4581 | V | 759.3420 | 772.3624 | ***803.4046*** | 786.3781 | 785.3941 | 6 |
| 11 | 102.0550 | 1174.5164 | 1156.5058 | **1202.5113** | 1184.5007 | 1116.5109 | E | 630.2994 | 629.3042 | ***704.3362*** | 687.3097 | 686.3257 | 5 |

| | | | | | | | | | | | | |
|---|---|---|---|---|---|---|---|---|---|---|---|---|
| 12 | 88.0393 | 1289.5433 | 1271.5327 | **1317.5382** | 1299.5277 | 1245.5535 | D | 515.2725 | 514.2772 | ***575.2936*** | 558.2671 | 557.2831 | 4 |
| 13 | 159.0917 | 1475.6226 | 1457.6121 | 1503.6175 | 1485.6070 | | W | 329.1932 | | ***460.2667*** | 443.2401 | | 3 |
| 14 | 72.0808 | 1574.6910 | 1556.6805 | 1602.6859 | 1584.6754 | 1560.6754 | V | 230.1248 | 243.1452 | 274.1874 | 257.1608 | | 2 |
| 15 | 129.1135 | | | | | | R | 74.0237 | 73.0284 | ***175.1190*** | 158.0924 | | 1 |

| Seq | ya | yb | Seq | ya | yb | Seq | ya | yb |
|---|---|---|---|---|---|---|---|---|
| GA | 101.0709 | 129.0659 | GAP | 198.1237 | 226.1186 | GAPD | 313.1506 | 341.1456 |
| GAPDS | 400.1827 | 428.1776 | GAPDSF | 547.2511 | 575.2460 | GAPDSFH | 684.3100 | 712.3049 |
| AP | 141.1022 | 169.0972 | APD | 256.1292 | 284.1241 | APDS | 343.1612 | 371.1561 |
| APDSF | 490.2296 | 518.2245 | APDSFH | 627.2885 | 655.2835 | PD | 185.0921 | 213.0870 |
| PDS | 272.1241 | 300.1190 | PDSF | 419.1925 | 447.1874 | PDSFH | 556.2514 | 584.2463 |
| DS | 175.0713 | 203.0662 | DSF | 322.1397 | 350.1347 | DSFH | 459.1987 | 487.1936 |
| DSFHF | 606.2671 | 634.2620 | SF | 207.1128 | 235.1077 | SFH | 344.1717 | 372.1666 |
| SFHF | 491.2401 | 519.2350 | SFHFV | 590.3085 | 618.3035 | FH | 257.1397 | 285.1346 |
| FHF | 404.2081 | 432.2030 | FHFV | 503.2765 | 531.2714 | FHFVE | 632.3191 | 660.3140 |
| HF | 257.1397 | 285.1346 | HFV | 356.2081 | 384.2030 | HFVE | 485.2507 | 513.2456 |
| HFVED | 600.2776 | 628.2726 | FV | 219.1492 | 247.1441 | FVE | 348.1918 | 376.1867 |
| FVED | 463.2187 | 491.2136 | FVEDW | 649.2980 | 677.2930 | VE | 201.1234 | 229.1183 |
| VED | 316.1503 | 344.1452 | VEDW | 502.2296 | 530.2245 | VEDWV | 601.2980 | 629.2930 |
| ED | 217.0819 | 245.0768 | EDW | 403.1612 | 431.1561 | EDWV | 502.2296 | 530.2245 |
| DW | 274.1186 | 302.1135 | DWV | 373.1870 | 401.1819 | WV | 258.1601 | 286.1550 |

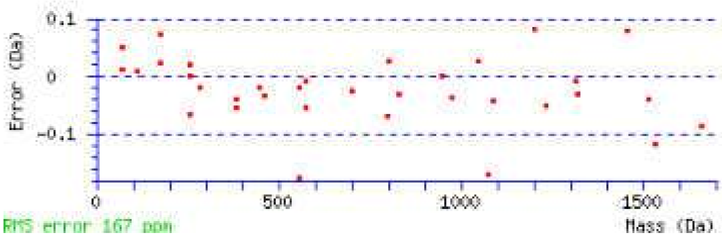


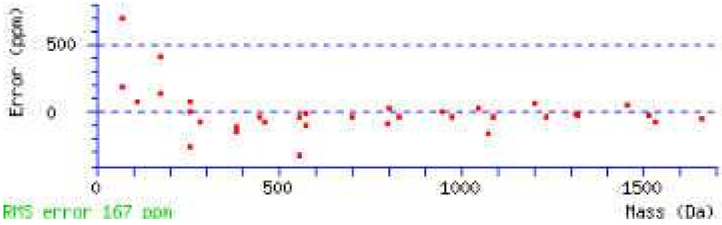


NCBI **BLAST** search of DGAPDSFHFVEDWVR
(Parameters: blastp, nr protein database, expect=20000, no filter, PAM30)
Other BLAST web gateways

**All matches to this query**

| Score | Mr(calc) | Delta | Sequence |
|---|---|---|---|
| 59.7 | 1775.7904 | 0.1023 | DGAPDSFHFVEDWVR |
| 15.4 | 1775.8916 | 0.0011 | EYYSFLKILCGVER |
| 14.8 | 1775.8074 | 0.0853 | DGFPDAPSSQNNITASR |
| 13.8 | 1775.8818 | 0.0110 | DPKDMLVSFWHFVR |
| 12.5 | 1775.8115 | 0.0812 | DDPAGSTVPQADYVWR |
| 11.7 | 1775.7598 | 0.1329 | DRPSFEESPESDPER |

| | | | |
|---|---|---|---|
| 11.7 | 1775.8672 | 0.0256 | MLSARDLGHMHHISR |
| 10.3 | 1775.8479 | 0.0449 | DHLNYSVYIPDADVR |
| 9.7 | 1775.8876 | 0.0051 | DAVKLTGGPAYEVPMGR |
| 9.7 | 1775.8108 | 0.0819 | DQDQMNVQSLSVEQR |

**Mascot:** http://www.matrixscience.com/

# MATRIX SCIENCE Mascot Search Results

## Peptide View Spot no 23

MS/MS Fragmentation of **VCPSVTMGLEPGEAFTVR**
Found in **gi|3345477** in **NCBInr**, carbonic anhydrase [Oryza sativa]

Match to Query 31: 1965.031224 from(1966.038500,1+) intensity(0.0000) index(14)
Title: Label: G9, Spot_Id: 228976, Peak_List_Id: 257845, MSMS Job_Run_Id: 24939, Comment:
Data file ppw_G9_138985152400.txt

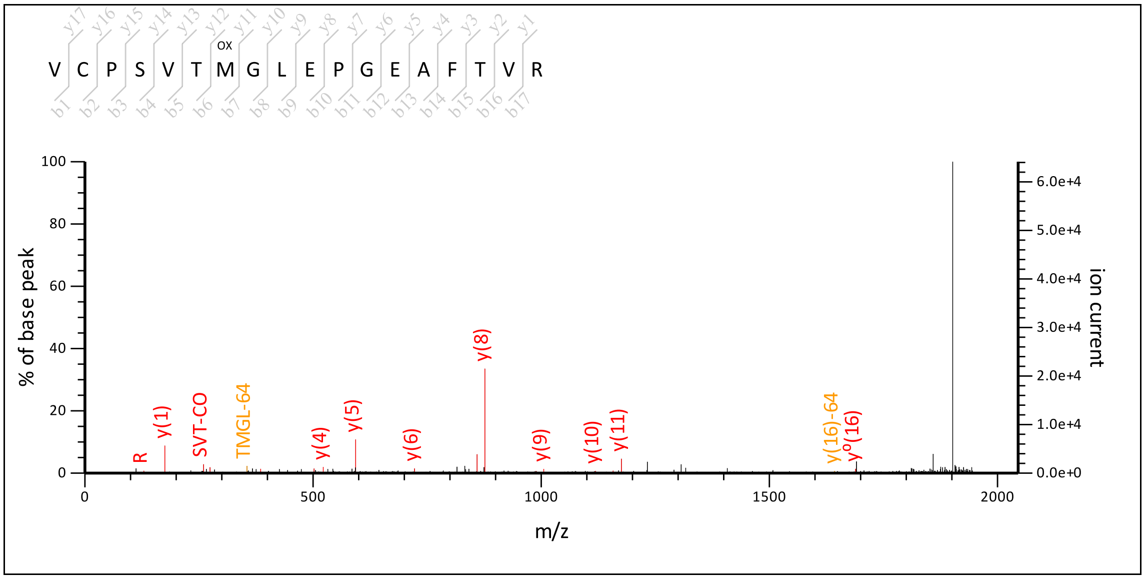


0 to 2045.23

Label all possible matches Label matches used for scoring

**Monoisotopic mass of neutral peptide Mr(calc):** 1964.9336
**Fixed modifications:** Carbamidomethyl (C) (apply to specified residues or termini only)
**Variable modifications:**
**M7** : Oxidation (M), with neutral losses 63.9983(shown in table), 0.0000
**Ions Score:** 57 **Expect:** 0.0041
**Matches :** 23/452 fragment ions using 38 most intense peaks (help)

| # | Immon. | a | $a^0$ | b | $b^0$ | d | d' | Seq. | v | w | w' | y | y* | $y^0$ | # |
|---|---|---|---|---|---|---|---|---|---|---|---|---|---|---|---|
| 1 | 72.0808 | 72.0808 | | 100.0757 | | 44.0495 | | V | | | | | | | 18 |
| 2 | 133.0430 | 232.1114 | | 260.1063 | | 143.1179 | | C | 1697.8493 | 1696.8541 | | 1802.8742 | 1785.8476 | 1784.8636 | 17 |
| 3 | 70.0651 | 329.1642 | | 357.1591 | | 303.1485 | | P | 1600.7966 | 1599.8013 | | ***1642.8435*** | 1625.8170 | 1624.8329 | 16 |
| 4 | 60.0444 | 416.1962 | 398.1857 | 444.1911 | 426.1806 | 400.2013 | | S | 1513.7645 | 1512.7693 | | 1545.7908 | 1528.7642 | 1527.7802 | 15 |
| 5 | 72.0808 | 515.2646 | 497.2541 | 543.2595 | 525.2490 | 501.2490 | | V | 1414.6961 | 1427.7165 | | 1458.7587 | 1441.7322 | 1440.7482 | 14 |
| 6 | 74.0600 | 616.3123 | 598.3017 | 644.3072 | 626.2967 | 600.3174 | 602.2967 | T | 1313.6484 | 1326.6688 | 1328.6481 | 1359.6903 | 1342.6638 | 1341.6797 | 13 |
| 7 | 56.0495 | 699.3494 | 681.3389 | 727.3443 | 709.3338 | 687.3494 | | M | 1230.6113 | 1229.6161 | | 1258.6426 | 1241.6161 | 1240.6321 | 12 |
| 8 | 30.0338 | 756.3709 | 738.3603 | 784.3658 | 766.3552 | | | G | | | | ***1175.6055*** | 1158.5790 | 1157.5949 | 11 |
| 9 | 86.0964 | 869.4550 | 851.4444 | 897.4499 | 879.4393 | 827.4080 | | L | 1060.5058 | 1059.5106 | | ***1118.5841*** | 1101.5575 | 1100.5735 | 10 |
| 10 | 102.0550 | 998.4975 | 980.4870 | 1026.4925 | 1008.4819 | 940.4921 | | E | 931.4632 | 930.4680 | | ***1005.5000*** | 988.4734 | 987.4894 | 9 |
| 11 | 70.0651 | 1095.5503 | 1077.5397 | 1123.5452 | 1105.5347 | 1069.5347 | | P | 834.4104 | 833.4152 | | ***876.4574*** | 859.4308 | 858.4468 | 8 |
| 12 | 30.0338 | 1152.5718 | 1134.5612 | 1180.5667 | 1162.5561 | | | G | | | | 779.4046 | 762.3781 | 761.3941 | 7 |
| 13 | 102.0550 | 1281.6144 | 1263.6038 | 1309.6093 | 1291.5987 | 1223.6089 | | E | 648.3464 | 647.3511 | | ***722.3832*** | 705.3566 | 704.3726 | 6 |
| 14 | 44.0495 | 1352.6515 | 1334.6409 | 1380.6464 | 1362.6358 | | | A | 577.3093 | | | ***593.3406*** | 576.3140 | 575.3300 | 5 |
| 15 | 120.0808 | 1499.7199 | 1481.7093 | 1527.7148 | 1509.7042 | | | F | 430.2409 | | | ***522.3035*** | 505.2769 | 504.2929 | 4 |
| 16 | 74.0600 | 1600.7676 | 1582.7570 | 1628.7625 | 1610.7519 | 1584.7727 | 1586.7519 | T | 329.1932 | 342.2136 | 344.1928 | 375.2350 | 358.2085 | 357.2245 | 3 |
| 17 | 72.0808 | 1699.8360 | 1681.8254 | 1727.8309 | 1709.8203 | 1685.8203 | | V | 230.1248 | 243.1452 | | ***274.1874*** | 257.1608 | | 2 |
| 18 | 129.1135 | | | | | | | R | 74.0237 | 73.0284 | | ***175.1190*** | 158.0924 | | 1 |

| Seq | ya | yb | Seq | ya | yb | Seq | ya | yb |
|---|---|---|---|---|---|---|---|---|
| **CP** | 230.0958 | 258.0907 | **CPS** | 317.1278 | 345.1227 | **CPSV** | 416.1962 | 444.1911 |
| **CPSVT** | 517.2439 | 545.2388 | **CPSVTM** | 600.2810 | 628.2759 | **CPSVTMG** | 657.3025 | 685.2974 |
| **PS** | 157.0972 | 185.0921 | **PSV** | 256.1656 | 284.1605 | **PSVT** | 357.2132 | 385.2082 |
| **PSVTM** | 440.2504 | 468.2453 | **PSVTMG** | 497.2718 | 525.2667 | **PSVTMGL** | 610.3559 | 638.3508 |
| **SV** | 159.1128 | 187.1077 | **SVT** | 260.1605 | 288.1554 | **SVTM** | 343.1976 | 371.1925 |
| **SVTMG** | 400.2191 | 428.2140 | **SVTMGL** | 513.3031 | 541.2980 | **SVTMGLE** | 642.3457 | 670.3406 |
| **VT** | 173.1285 | 201.1234 | **VTM** | 256.1656 | 284.1605 | **VTMG** | 313.1870 | 341.1819 |
| **VTMGL** | 426.2711 | 454.2660 | **VTMGLE** | 555.3137 | 583.3086 | **VTMGLEP** | 652.3665 | 680.3614 |
| **TM** | 157.0972 | 185.0921 | **TMG** | 214.1186 | 242.1135 | **TMGL** | 327.2027 | 355.1976 |
| **TMGLE** | 456.2453 | 484.2402 | **TMGLEP** | 553.2980 | 581.2930 | **TMGLEPG** | 610.3195 | 638.3144 |
| **MG** | 113.0709 | 141.0659 | **MGL** | 226.1550 | 254.1499 | **MGLE** | 355.1976 | 383.1925 |
| **MGLEP** | 452.2504 | 480.2453 | **MGLEPG** | 509.2718 | 537.2667 | **MGLEPGE** | 638.3144 | 666.3093 |
| **GL** | 143.1179 | 171.1128 | **GLE** | 272.1605 | 300.1554 | **GLEP** | 369.2132 | 397.2082 |
| **GLEPG** | 426.2347 | 454.2296 | **GLEPGE** | 555.2773 | 583.2722 | **GLEPGEA** | 626.3144 | 654.3093 |
| **LE** | 215.1390 | 243.1339 | **LEP** | 312.1918 | 340.1867 | **LEPG** | 369.2132 | 397.2082 |
| **LEPGE** | 498.2558 | 526.2508 | **LEPGEA** | 569.2930 | 597.2879 | **EP** | 199.1077 | 227.1026 |
| **EPG** | 256.1292 | 284.1241 | **EPGE** | 385.1718 | 413.1667 | **EPGEA** | 456.2089 | 484.2038 |
| **EPGEAF** | 603.2773 | 631.2722 | **PG** | 127.0866 | 155.0815 | **PGE** | 256.1292 | 284.1241 |
| **PGEA** | 327.1663 | 355.1612 | **PGEAF** | 474.2347 | 502.2296 | **PGEAFT** | 575.2824 | 603.2773 |
| **PGEAFTV** | 674.3508 | 702.3457 | **GE** | 159.0764 | 187.0713 | **GEA** | 230.1135 | 258.1084 |
| **GEAF** | 377.1819 | 405.1769 | **GEAFT** | 478.2296 | 506.2245 | **GEAFTV** | 577.2980 | 605.2930 |
| **EA** | 173.0921 | 201.0870 | **EAF** | 320.1605 | 348.1554 | **EAFT** | 421.2082 | 449.2031 |
| **EAFTV** | 520.2766 | 548.2715 | **AF** | 191.1179 | 219.1128 | **AFT** | 292.1656 | 320.1605 |
| **AFTV** | 391.2340 | 419.2289 | **FT** | 221.1285 | 249.1234 | **FTV** | 320.1969 | 348.1918 |
| **TV** | 173.1285 | 201.1234 | | | | | | |

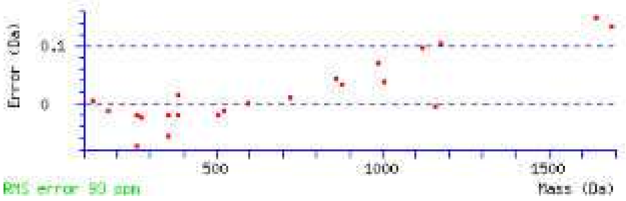


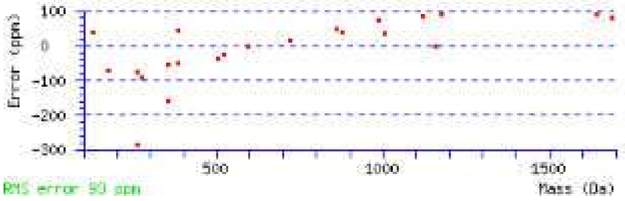


NCBI **BLAST** search of VCPSVTMGLEPGEAFTVR
(Parameters: blastp, nr protein database, expect=20000, no filter, PAM30)
Other BLAST web gateways

**All matches to this query**

| Score | Mr(calc) | Delta | Sequence |
|---|---|---|---|
| 56.7 | 1964.9336 | 0.0976 | VCPSVTMGLEPGEAFTVR |
| 17.1 | 1964.9778 | 0.0534 | LKGMGFEQSLHEAAIYR |
| 16.5 | 1965.0419 | -0.0106 | VSDLGSTSTLRYLDPTLK |
| 13.4 | 1964.9183 | 0.1129 | MQEPCEVSIDISVSLSR |
| 12.2 | 1964.8997 | 0.1316 | VERSSMAVEIADSPSDEK |
| 11.8 | 1964.9448 | 0.0864 | VASSALGFGPQMAMQVAER |
| 11.8 | 1965.0030 | 0.0282 | MVGLEGFGLIDQIYGPTR |
| 11.6 | 1964.9473 | 0.0839 | EVRGSLTDMLNEEVTTR |
| 11.1 | 1964.9990 | 0.0322 | ILGEGGQGTVYKGMLVDGR |
| 10.4 | 1964.9051 | 0.1262 | TYVSQYDLHHAEMTVR |

**Mascot:** http://www.matrixscience.com/

# MATRIX SCIENCE Mascot Search Results

## Peptide View Spot no 23

MS/MS Fragmentation of **VCPSVTMGLEPGEAFTVR**
Found in **gi|3345477** in **NCBInr**, carbonic anhydrase [Oryza sativa]

Match to Query 29: 1949.058124 from(1950.065400,1+) intensity(0.0000) index(13)
Title: Label: G9, Spot_Id: 228976, Peak_List_Id: 257843, MSMS Job_Run_Id: 24939, Comment:
Data file ppw_G9_138985152400.txt

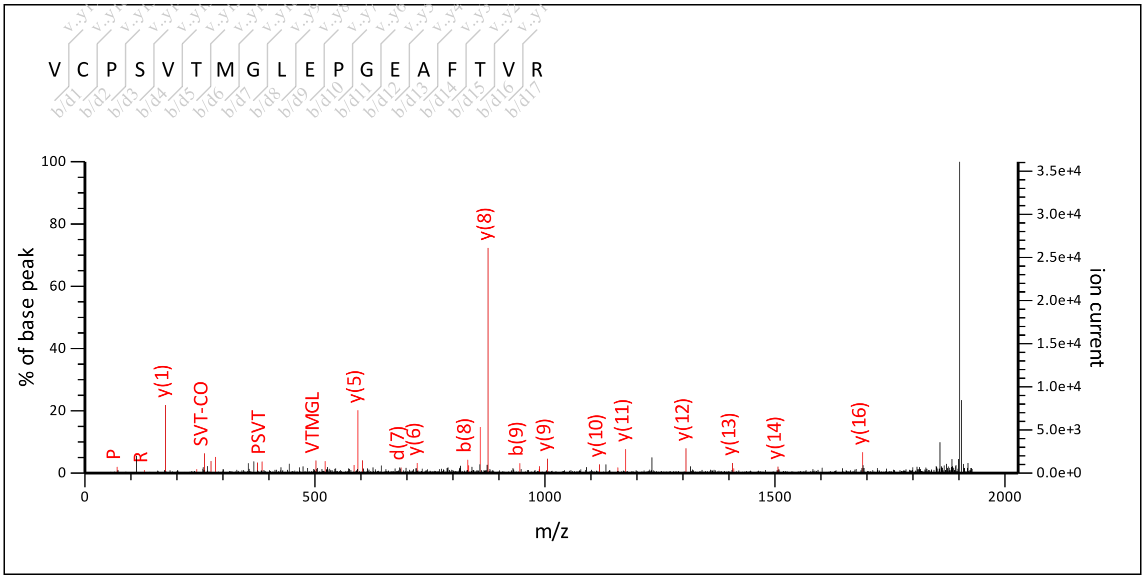


0 to 2028.71

Label all possible matches ○ Label matches used for scoring ◉

**Monoisotopic mass of neutral peptide Mr(calc):** 1948.9387
**Fixed modifications:** Carbamidomethyl (C) (apply to specified residues or termini only)
**Ions Score:** 101 **Expect:** 1.4e-07
**Matches :** 49/313 fragment ions using 57 most intense peaks (help)

| # | Immon. | a | $a^0$ | b | $b^0$ | d | d' | Seq. | v | w | w' | y | y* | $y^0$ | # |
|---|---|---|---|---|---|---|---|---|---|---|---|---|---|---|---|
| 1 | 72.0808 | 72.0808 | | 100.0757 | | 44.0495 | | V | | | | | | | 18 |
| 2 | 133.0430 | 232.1114 | | **260.1063** | | 143.1179 | | C | 1745.8527 | 1744.8575 | | 1850.8775 | 1833.8510 | 1832.8670 | 17 |
| 3 | 70.0651 | 329.1642 | | 357.1591 | | 303.1485 | | P | 1648.7999 | 1647.8047 | | ***1690.8469*** | 1673.8203 | 1672.8363 | 16 |
| 4 | 60.0444 | 416.1962 | 398.1857 | 444.1911 | 426.1806 | 400.2013 | | S | 1561.7679 | 1560.7727 | | 1593.7941 | 1576.7676 | 1575.7836 | 15 |
| 5 | 72.0808 | 515.2646 | 497.2541 | 543.2595 | 525.2490 | 501.2490 | | V | 1462.6995 | 1475.7199 | | ***1506.7621*** | 1489.7355 | 1488.7515 | 14 |
| 6 | 74.0600 | 616.3123 | 598.3017 | 644.3072 | 626.2967 | 600.3174 | 602.2967 | T | 1361.6518 | 1374.6722 | 1376.6515 | ***1407.6937*** | 1390.6671 | 1389.6831 | 13 |
| 7 | 104.0528 | 747.3528 | 729.3422 | 775.3477 | 757.3371 | 687.3494 | | M | 1230.6113 | 1229.6161 | | ***1306.6460*** | 1289.6195 | 1288.6354 | 12 |
| 8 | 30.0338 | 804.3743 | 786.3637 | **832.3692** | 814.3586 | | | G | | | | ***1175.6055*** | 1158.5790 | 1157.5950 | 11 |
| 9 | 86.0964 | 917.4583 | 899.4478 | **945.4532** | 927.4427 | 875.4114 | | L | 1060.5058 | 1059.5106 | | ***1118.5841*** | 1101.5575 | 1100.5735 | 10 |
| 10 | 102.0550 | 1046.5009 | 1028.4904 | 1074.4958 | 1056.4853 | 988.4954 | | E | 931.4632 | 930.4680 | | ***1005.5000*** | 988.4734 | 987.4894 | 9 |
| 11 | 70.0651 | 1143.5537 | 1125.5431 | 1171.5486 | 1153.5380 | 1117.5380 | | P | 834.4104 | 833.4152 | | ***876.4574*** | 859.4308 | 858.4468 | 8 |
| 12 | 30.0338 | 1200.5751 | 1182.5646 | 1228.5701 | 1210.5595 | | | G | | | | 779.4046 | 762.3781 | 761.3941 | 7 |
| 13 | 102.0550 | 1329.6177 | 1311.6072 | 1357.6127 | 1339.6021 | 1271.6123 | | E | 648.3464 | 647.3511 | | ***722.3832*** | 705.3566 | 704.3726 | 6 |
| 14 | 44.0495 | 1400.6549 | 1382.6443 | 1428.6498 | 1410.6392 | | | A | 577.3093 | | | ***593.3406*** | 576.3140 | 575.3300 | 5 |
| 15 | 120.0808 | 1547.7233 | 1529.7127 | 1575.7182 | 1557.7076 | | | F | 430.2409 | | | ***522.3035*** | 505.2769 | 504.2929 | 4 |
| 16 | 74.0600 | 1648.7709 | 1630.7604 | 1676.7659 | 1658.7553 | 1632.7760 | 1634.7553 | T | 329.1932 | 342.2136 | 344.1928 | ***375.2350*** | 358.2085 | 357.2245 | 3 |
| 17 | 72.0808 | 1747.8394 | 1729.8288 | 1775.8343 | 1757.8237 | 1733.8237 | | V | 230.1248 | 243.1452 | | ***274.1874*** | 257.1608 | | 2 |
| 18 | 129.1135 | | | | | | | R | 74.0237 | 73.0284 | | ***175.1190*** | 158.0924 | | 1 |

| Seq | ya | yb | Seq | ya | yb | Seq | ya | yb |
|---|---|---|---|---|---|---|---|---|
| CP | 230.0958 | 258.0907 | CPS | 317.1278 | 345.1227 | CPSV | 416.1962 | 444.1911 |
| CPSVT | 517.2439 | 545.2388 | CPSVTM | 648.2844 | 676.2793 | PS | 157.0972 | 185.0921 |
| PSV | 256.1656 | 284.1605 | PSVT | 357.2132 | 385.2082 | PSVTM | 488.2537 | 516.2486 |
| PSVTMG | 545.2752 | 573.2701 | PSVTMGL | 658.3593 | 686.3542 | SV | 159.1128 | 187.1077 |
| SVT | 260.1605 | 288.1554 | SVTM | 391.2010 | 419.1959 | SVTMG | 448.2224 | 476.2173 |
| SVTMGL | 561.3065 | 589.3014 | SVTMGLE | 690.3491 | 718.3440 | VT | 173.1285 | 201.1234 |
| VTM | 304.1689 | 332.1639 | VTMG | 361.1904 | 389.1853 | VTMGL | 474.2745 | 502.2694 |
| VTMGLE | 603.3171 | 631.3120 | TM | 205.1005 | 233.0954 | TMG | 262.1220 | 290.1169 |
| TMGL | 375.2061 | 403.2010 | TMGLE | 504.2486 | 532.2436 | TMGLEP | 601.3014 | 629.2963 |
| TMGLEPG | 658.3229 | 686.3178 | MG | 161.0743 | 189.0692 | MGL | 274.1584 | 302.1533 |
| MGLE | 403.2010 | 431.1959 | MGLEP | 500.2537 | 528.2486 | MGLEPG | 557.2752 | 585.2701 |
| MGLEPGE | 686.3178 | 714.3127 | GL | 143.1179 | 171.1128 | GLE | 272.1605 | 300.1554 |
| GLEP | 369.2132 | 397.2082 | GLEPG | 426.2347 | 454.2296 | GLEPGE | 555.2773 | 583.2722 |
| GLEPGEA | 626.3144 | 654.3093 | LE | 215.1390 | 243.1339 | LEP | 312.1918 | 340.1867 |
| LEPG | 369.2132 | 397.2082 | LEPGE | 498.2558 | 526.2508 | LEPGEA | 569.2930 | 597.2879 |
| EP | 199.1077 | 227.1026 | EPG | 256.1292 | 284.1241 | EPGE | 385.1718 | 413.1667 |
| EPGEA | 456.2089 | 484.2038 | EPGEAF | 603.2773 | 631.2722 | PG | 127.0866 | 155.0815 |
| PGE | 256.1292 | 284.1241 | PGEA | 327.1663 | 355.1612 | PGEAF | 474.2347 | 502.2296 |
| PGEAFT | 575.2824 | 603.2773 | PGEAFTV | 674.3508 | 702.3457 | GE | 159.0764 | 187.0713 |
| GEA | 230.1135 | 258.1084 | GEAF | 377.1819 | 405.1769 | GEAFT | 478.2296 | 506.2245 |
| GEAFTV | 577.2980 | 605.2930 | EA | 173.0921 | 201.0870 | EAF | 320.1605 | 348.1554 |
| EAFT | 421.2082 | 449.2031 | EAFTV | 520.2766 | 548.2715 | AF | 191.1179 | 219.1128 |
| AFT | 292.1656 | 320.1605 | AFTV | 391.2340 | 419.2289 | FT | 221.1285 | 249.1234 |
| FTV | 320.1969 | 348.1918 | TV | 173.1285 | 201.1234 | | | |

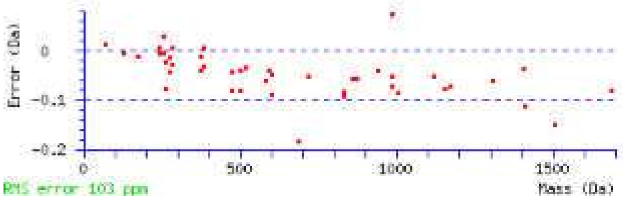


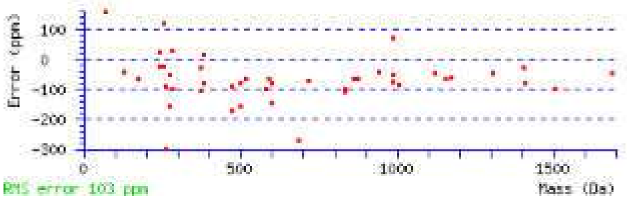


NCBI **BLAST** search of VCPSVTMGLEPGEAFTVR
(Parameters: blastp, nr protein database, expect=20000, no filter, PAM30)
Other BLAST web gateways

**All matches to this query**

| Score | Mr(calc) | Delta | Sequence |
|---|---|---|---|
| 101.5 | 1948.9387 | 0.1194 | VCPSVTMGLEPGEAFTVR |
| 13.9 | 1949.0155 | 0.0427 | AMAVAVCEIVWLISFLQ |
| 12.3 | 1948.8684 | 0.1897 | DGSDADESVEVQVTMPVR |
| 11.8 | 1948.9928 | 0.0653 | CVGSAEDLIDFVISVLGR |
| 11.2 | 1949.0027 | 0.0554 | ITKTSIMLGLGESDDELK |
| 10.2 | 1949.0081 | 0.0501 | LLGEFAELCGPFAGVLTR |
| 9.1 | 1948.9564 | 0.1017 | PTLTKLYSMEEAATHNK |
| 9.0 | 1948.9061 | 0.1520 | SRSDSHCDEIIGFVTAR |
| 8.7 | 1948.9829 | 0.0752 | LKGMGFEQSLHEAAIYR |
| 8.7 | 1948.9234 | 0.1347 | MQEPCEVSIDISVSLSR |

**Mascot:** http://www.matrixscience.com/

# MATRIX SCIENCE Mascot Search Results

## Peptide View Spot no 24

MS/MS Fragmentation of **EFPGQVLR**
Found in **gi|115470529** in **NCBInr**, Os07g0141400 [Oryza sativa Japonica Group]

Match to Query 9: 944.618394 from(945.625670,1+) intensity(0.0000) index(2)
Title: Label: B12, Spot_Id: 216654, Peak_List_Id: 221696, MSMS Job_Run_Id: 21388, Comment:
Data file C:\Documents and Settings\Administrator\Desktop\farah BHU\Cotton leaves\RAJESH\12-1-13 raj\ppw_B12_136117695600.txt

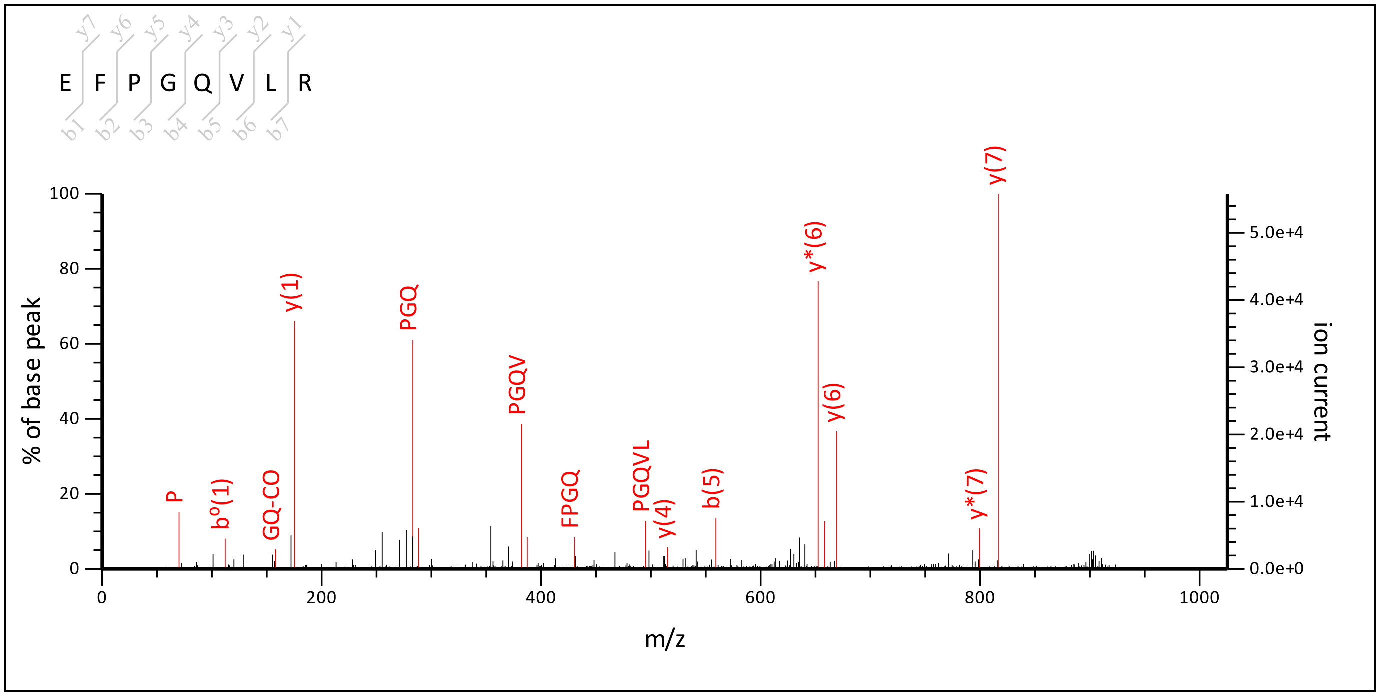


0 to 1025.33

Label all possible matches ○ Label matches used for scoring ◉

**Monoisotopic mass of neutral peptide Mr(calc):** 944.5080
**Fixed modifications:** Carbamidomethyl (C) (apply to specified residues or termini only)
**Ions Score:** 46 **Expect:** 0.22
**Matches :** 20/102 fragment ions using 27 most intense peaks (help)

| # | Immon. | a | a* | $a^0$ | b | b* | $b^0$ | d | Seq. | v | w | y | y* | # |
|---|---|---|---|---|---|---|---|---|---|---|---|---|---|---|
| 1 | 102.0550 | 102.0550 | | 84.0444 | 130.0499 | | 112.0393 | 44.0495 | E | | | | | 8 |
| 2 | 120.0808 | 249.1234 | | 231.1128 | 277.1183 | | 259.1077 | | F | 724.4101 | | ***816.4727*** | 799.4461 | 7 |
| 3 | 70.0651 | 346.1761 | | 328.1656 | 374.1710 | | 356.1605 | 320.1605 | P | 627.3573 | 626.3620 | ***669.4042*** | 652.3777 | 6 |
| 4 | 30.0338 | 403.1976 | | 385.1870 | 431.1925 | | 413.1819 | | G | | | 572.3515 | 555.3249 | 5 |
| 5 | 101.0709 | 531.2562 | 514.2296 | 513.2456 | **559.2511** | 542.2245 | 541.2405 | 474.2347 | Q | 442.2772 | 441.2820 | ***515.3300*** | 498.3035 | 4 |
| 6 | 72.0808 | 630.3246 | 613.2980 | 612.3140 | **658.3195** | 641.2930 | 640.3089 | 616.3089 | V | 343.2088 | 356.2292 | ***387.2714*** | 370.2449 | 3 |
| 7 | 86.0964 | 743.4087 | 726.3821 | 725.3981 | 771.4036 | 754.3770 | 753.3930 | 701.3617 | L | 230.1248 | 229.1295 | ***288.2030*** | 271.1765 | 2 |
| 8 | 129.1135 | | | | | | | | R | 74.0237 | 73.0284 | ***175.1190*** | 158.0924 | 1 |

| Seq | ya | yb | Seq | ya | yb | Seq | ya | yb |
|---|---|---|---|---|---|---|---|---|
| FP | 217.1335 | 245.1285 | FPG | 274.1550 | 302.1499 | FPGQ | 402.2136 | 430.2085 |

| | | | | | | | | |
|---|---|---|---|---|---|---|---|---|
| **FPGQV** | 501.2820 | 529.2769 | **FPGQVL** | 614.3661 | 642.3610 | **PG** | 127.0866 | 155.0815 |
| **PGQ** | 255.1452 | 283.1401 | **PGQV** | 354.2136 | 382.2085 | **PGQVL** | 467.2976 | 495.2926 |
| **GQ** | 158.0924 | 186.0873 | **GQV** | 257.1608 | 285.1557 | **GQVL** | 370.2449 | 398.2398 |
| **QV** | 200.1394 | 228.1343 | **QVL** | 313.2234 | 341.2183 | **VL** | 185.1648 | 213.1598 |

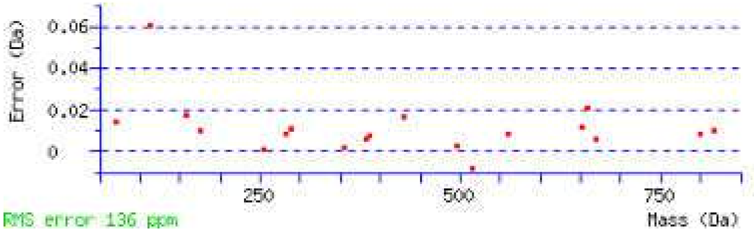


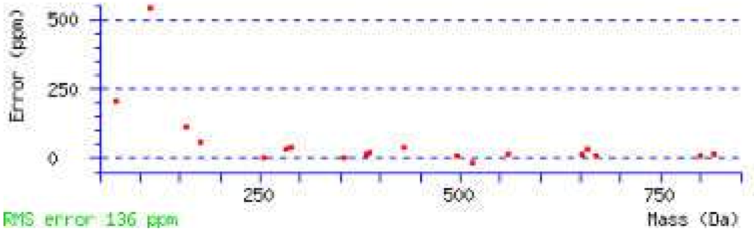


NCBI **BLAST** search of EFPGQVLR
(Parameters: blastp, nr protein database, expect=20000, no filter, PAM30)
Other BLAST web gateways

**All matches to this query**

| Score | Mr(calc) | Delta | Sequence |
|---|---|---|---|
| 45.8 | 944.5080 | 0.1104 | EFPGQVLR |
| 26.7 | 944.5114 | 0.1070 | MTGVGPVLR |
| 25.8 | 944.4862 | 0.1322 | EMQRPLR |
| 25.2 | 943.4611 | 1.1573 | QGPLADSEK |
| 25.2 | 945.5430 | -0.9246 | NVMAKVLR |
| 23.8 | 944.4749 | 0.1435 | MEAAPAALR |
| 22.6 | 944.4927 | 0.1257 | GEEDKVLR |
| 21.7 | 944.4563 | 0.1621 | EEQEAAIR |
| 21.5 | 943.4723 | 1.1461 | AGEQEAALR |
| 20.9 | 943.5239 | 1.0945 | AGFAVNPIR |

**Mascot:** http://www.matrixscience.com/

# MATRIX SCIENCE Mascot Search Results

## Peptide View Spot no 24

MS/MS Fragmentation of **QYYSVTVLTR**
Found in **gi|115470529** in **NCBInr**, Os07g0141400 [Oryza sativa Japonica Group]

Match to Query 13: 1228.790724 from(1229.798000,1+) intensity(0.0000) index(4)
Title: Label: B12, Spot_Id: 216654, Peak_List_Id: 221697, MSMS Job_Run_Id: 21388, Comment:
Data file C:\Documents and Settings\Administrator\Desktop\farah BHU\Cotton leaves\RAJESH\12-1-13 raj\ppw_B12_136117695600.txt

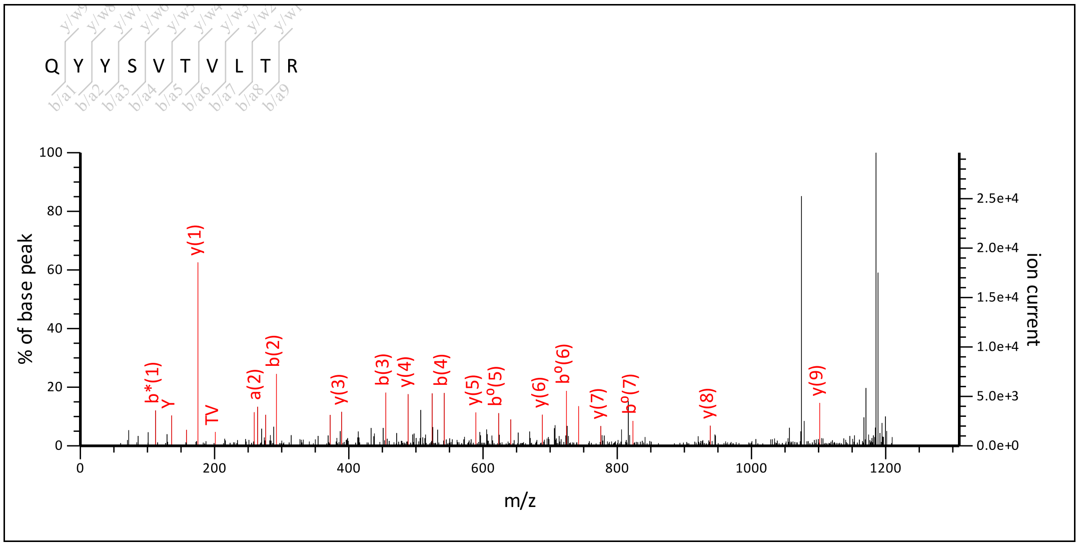


0 to 1309.63

Label all possible matches ○ Label matches used for scoring ◉

**Monoisotopic mass of neutral peptide Mr(calc):** 1228.6452
**Fixed modifications:** Carbamidomethyl (C) (apply to specified residues or termini only)
**Ions Score:** 67 **Expect:** 0.0012
**Matches :** 28/160 fragment ions using 36 most intense peaks (help)

| # | Immon. | a | a* | $a^0$ | b | b* | $b^0$ | d | d' | Seq. | v | w | w' | y | y* | y |
|---|---|---|---|---|---|---|---|---|---|---|---|---|---|---|---|---|
| 1 | 101.0709 | 101.0709 | 84.0444 | | 129.0659 | 112.0393 | | 44.0495 | | Q | | | | | | |
| 2 | 136.0757 | 264.1343 | 247.1077 | | **292.1292** | 275.1026 | | | | Y | 993.5364 | | | ***1101.5939*** | 1084.5673 | 1083. |
| 3 | 136.0757 | 427.1976 | 410.1710 | | **455.1925** | 438.1660 | | | | Y | 830.4730 | | | ***938.5306*** | 921.5040 | 920. |
| 4 | 60.0444 | 514.2296 | 497.2031 | 496.2191 | **542.2245** | 525.1980 | 524.2140 | 498.2347 | | S | 743.4410 | 742.4458 | | ***775.4672*** | 758.4407 | 757. |
| 5 | 72.0808 | 613.2980 | 596.2715 | 595.2875 | **641.2930** | 624.2664 | 623.2824 | 599.2824 | | V | 644.3726 | 657.3930 | | ***688.4352*** | 671.4087 | 670. |
| 6 | 74.0600 | 714.3457 | 697.3192 | 696.3352 | **742.3406** | 725.3141 | 724.3301 | 698.3508 | 700.3301 | T | 543.3249 | 556.3453 | 558.3246 | ***589.3668*** | 572.3402 | 571. |
| 7 | 72.0808 | 813.4141 | 796.3876 | 795.4036 | 841.4090 | 824.3825 | 823.3985 | 799.3985 | | V | 444.2565 | 457.2769 | | ***488.3191*** | 471.2926 | 470. |
| 8 | 86.0964 | 926.4982 | 909.4716 | 908.4876 | 954.4931 | 937.4666 | 936.4825 | 884.4512 | | L | 331.1724 | 330.1772 | | ***389.2507*** | 372.2241 | 371. |
| 9 | 74.0600 | 1027.5459 | 1010.5193 | 1009.5353 | 1055.5408 | 1038.5142 | 1037.5302 | 1011.5510 | 1013.5302 | T | 230.1248 | 243.1452 | 245.1244 | ***276.1666*** | 259.1401 | 258. |
| 10 | 129.1135 | | | | | | | | | R | 74.0237 | 73.0284 | | ***175.1190*** | 158.0924 | |

| Seq | ya | yb | Seq | ya | yb | Seq | ya | yb |
|---|---|---|---|---|---|---|---|---|
| YY | 299.1390 | 327.1339 | YYS | 386.1710 | 414.1660 | YYSV | 485.2395 | 513.2344 |
| YYSVT | 586.2871 | 614.2821 | YYSVTV | 685.3556 | 713.3505 | YS | 223.1077 | 251.1026 |
| YSV | 322.1761 | 350.1710 | YSVT | 423.2238 | 451.2187 | YSVTV | 522.2922 | 550.2871 |
| YSVTVL | 635.3763 | 663.3712 | SV | 159.1128 | 187.1077 | SVT | 260.1605 | 288.1554 |
| SVTV | 359.2289 | 387.2238 | SVTVL | 472.3130 | 500.3079 | SVTVLT | 573.3606 | 601.3556 |
| VT | 173.1285 | 201.1234 | VTV | 272.1969 | 300.1918 | VTVL | 385.2809 | 413.2758 |
| VTVLT | 486.3286 | 514.3235 | TV | 173.1285 | 201.1234 | TVL | 286.2125 | 314.2074 |
| TVLT | 387.2602 | 415.2551 | VL | 185.1648 | 213.1598 | VLT | 286.2125 | 314.2074 |
| LT | 187.1441 | 215.1390 | | | | | | |

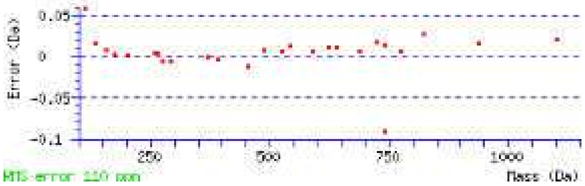


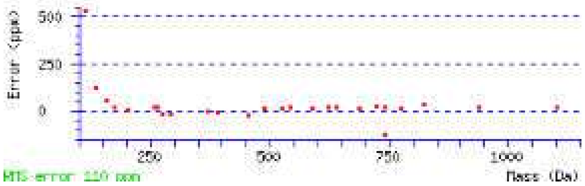


NCBI **BLAST** search of QYYSVTVLTR
(Parameters: blastp, nr protein database, expect=20000, no filter, PAM30)
Other BLAST web gateways

**All matches to this query**

| Score | Mr(calc) | Delta | Sequence |
|---|---|---|---|
| 66.7 | 1228.6452 | 0.1455 | QYYSVTVLTR |
| 21.7 | 1227.6572 | 1.1336 | ANPGSNVTVTLR |
| 19.4 | 1229.6802 | -0.8895 | ISVPQVIMSTR |
| 17.5 | 1227.6823 | 1.1085 | LNNEAVEALKK |
| 17.1 | 1227.6823 | 1.1084 | DIPQSISVITR |
| 16.9 | 1229.7092 | -0.9184 | RSVLDALSLTR |
| 16.8 | 1228.6564 | 0.1343 | SGNWPVSIITR |
| 15.3 | 1227.7187 | 1.0721 | VALEKLDAITR |
| 14.3 | 1228.7139 | 0.0768 | KGTIQITEALR |
| 14.0 | 1228.6160 | 0.1747 | SLVNQAEAQNR |

**Mascot:** http://www.matrixscience.com/

# MATRIX SCIENCE Mascot Search Results

## Peptide View **Spot no 24**

MS/MS Fragmentation of **KFVESAASSFSVA**
Found in **gi|115470529** in **NCBInr**, Os07g0141400 [Oryza sativa Japonica Group]

Match to Query 16: 1328.787024 from(1329.794300,1+) intensity(0.0000) index(5)
Title: Label: B12, Spot_Id: 216654, Peak_List_Id: 221715, MSMS Job_Run_Id: 21388, Comment:
Data file C:\Documents and Settings\Administrator\Desktop\farah BHU\Cotton leaves\RAJESH\12-1-13 raj\ppw_B12_136117695600.txt

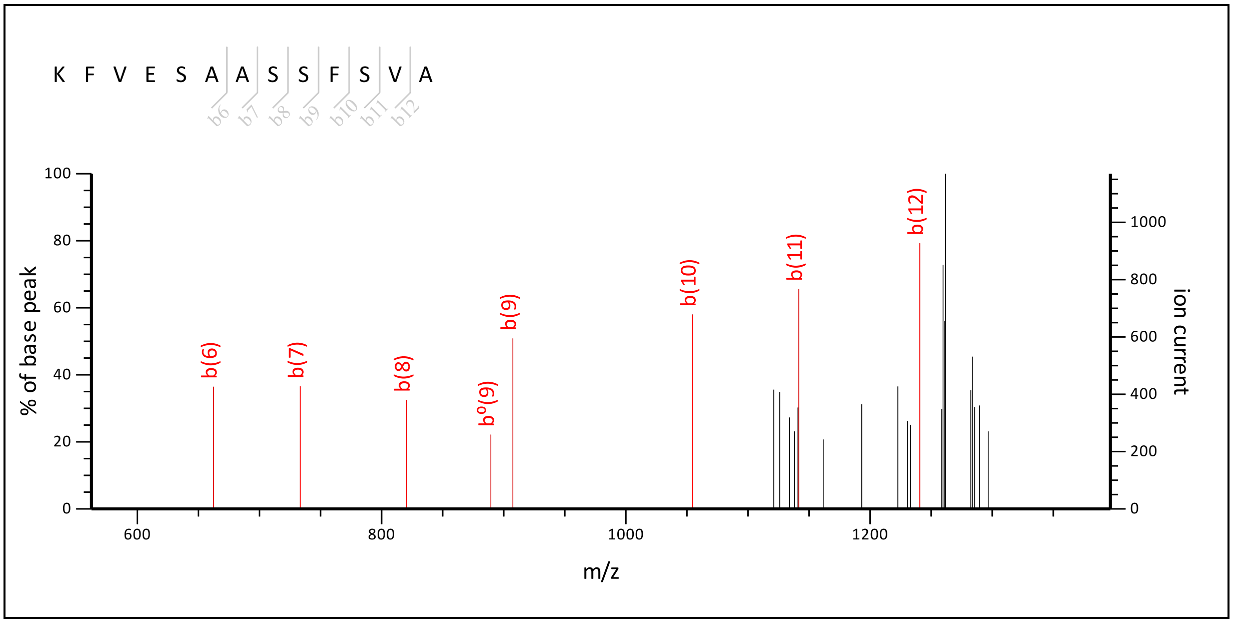


562.38 to 1396.72

Label all possible matches ○ Label matches used for scoring ●

**Monoisotopic mass of neutral peptide Mr(calc):** 1328.6612
**Fixed modifications:** Carbamidomethyl (C) (apply to specified residues or termini only)
**Ions Score:** 53 **Expect:** 0.029
**Matches :** 9/219 fragment ions using 12 most intense peaks (help)

| # | Immon. | a | a* | $a^0$ | b | b* | $b^0$ | d | Seq. | v | w | y | $y^0$ | # |
|---|---|---|---|---|---|---|---|---|---|---|---|---|---|---|
| 1 | 101.1073 | 101.1073 | 84.0808 | | 129.1022 | 112.0757 | | 44.0495 | K | | | | | 13 |
| 2 | 120.0808 | 248.1757 | 231.1492 | | 276.1707 | 259.1441 | | | F | 1109.5109 | | 1201.5735 | 1183.5630 | 12 |
| 3 | 72.0808 | 347.2442 | 330.2176 | | 375.2391 | 358.2125 | | 333.2285 | V | 1010.4425 | 1023.4629 | 1054.5051 | 1036.4946 | 11 |
| 4 | 102.0550 | 476.2867 | 459.2602 | 458.2762 | 504.2817 | 487.2551 | 486.2711 | 418.2813 | E | 881.3999 | 880.4047 | 955.4367 | 937.4262 | 10 |
| 5 | 60.0444 | 563.3188 | 546.2922 | 545.3082 | 591.3137 | 574.2871 | 573.3031 | 547.3239 | S | 794.3679 | 793.3727 | 826.3941 | 808.3836 | 9 |
| 6 | 44.0495 | 634.3559 | 617.3293 | 616.3453 | ***662.3508*** | 645.3243 | 644.3402 | | A | 723.3308 | | 739.3621 | 721.3515 | 8 |
| 7 | 44.0495 | 705.3930 | 688.3665 | 687.3824 | ***733.3879*** | 716.3614 | 715.3774 | | A | 652.2937 | | 668.3250 | 650.3144 | 7 |
| 8 | 60.0444 | 792.4250 | 775.3985 | 774.4145 | ***820.4199*** | 803.3934 | 802.4094 | 776.4301 | S | 565.2617 | 564.2664 | 597.2879 | 579.2773 | 6 |
| 9 | 60.0444 | 879.4571 | 862.4305 | 861.4465 | ***907.4520*** | 890.4254 | 889.4414 | 863.4621 | S | 478.2296 | 477.2344 | 510.2558 | 492.2453 | 5 |
| 10 | 120.0808 | 1026.5255 | 1009.4989 | 1008.5149 | ***1054.5204*** | 1037.4938 | 1036.5098 | | F | 331.1612 | | 423.2238 | 405.2132 | 4 |
| 11 | 60.0444 | 1113.5575 | 1096.5310 | 1095.5469 | ***1141.5524*** | 1124.5259 | 1123.5418 | 1097.5626 | S | 244.1292 | 243.1339 | 276.1554 | 258.1448 | 3 |
| 12 | 72.0808 | 1212.6259 | 1195.5994 | 1194.6153 | ***1240.6208*** | 1223.5943 | 1222.6103 | 1198.6103 | V | 145.0608 | 158.0812 | 189.1234 | | 2 |
| 13 | 44.0495 | | | | | | | | A | 74.0237 | 73.0284 | 90.0550 | | 1 |

| Seq | ya | yb | Seq | ya | yb | Seq | ya | yb |
|---|---|---|---|---|---|---|---|---|
| FV | 219.1492 | 247.1441 | FVE | 348.1918 | 376.1867 | FVES | 435.2238 | 463.2187 |

| | | | | | | | | |
|---|---|---|---|---|---|---|---|---|
| **FVESA** | 506.2609 | 534.2558 | **FVESAA** | 577.2980 | 605.2930 | **FVESAAS** | 664.3301 | 692.3250 |
| **VE** | 201.1234 | 229.1183 | **VES** | 288.1554 | 316.1503 | **VESA** | 359.1925 | 387.1874 |
| **VESAA** | 430.2296 | 458.2245 | **VESAAS** | 517.2617 | 545.2566 | **VESAASS** | 604.2937 | 632.2886 |
| **ES** | 189.0870 | 217.0819 | **ESA** | 260.1241 | 288.1190 | **ESAA** | 331.1612 | 359.1561 |
| **ESAAS** | 418.1932 | 446.1882 | **ESAASS** | 505.2253 | 533.2202 | **ESAASSF** | 652.2937 | 680.2886 |
| **SA** | 131.0815 | 159.0764 | **SAA** | 202.1186 | 230.1135 | **SAAS** | 289.1506 | 317.1456 |
| **SAASS** | 376.1827 | 404.1776 | **SAASSF** | 523.2511 | 551.2460 | **SAASSFS** | 610.2831 | 638.2780 |
| **AA** | 115.0866 | 143.0815 | **AAS** | 202.1186 | 230.1135 | **AASS** | 289.1506 | 317.1456 |
| **AASSF** | 436.2191 | 464.2140 | **AASSFS** | 523.2511 | 551.2460 | **AASSFSV** | 622.3195 | 650.3144 |
| **AS** | 131.0815 | 159.0764 | **ASS** | 218.1135 | 246.1084 | **ASSF** | 365.1819 | 393.1769 |
| **ASSFS** | 452.2140 | 480.2089 | **ASSFSV** | 551.2824 | 579.2773 | **SS** | 147.0764 | 175.0713 |
| **SSF** | 294.1448 | 322.1397 | **SSFS** | 381.1769 | 409.1718 | **SSFSV** | 480.2453 | 508.2402 |
| **SF** | 207.1128 | 235.1077 | **SFS** | 294.1448 | 322.1397 | **SFSV** | 393.2132 | 421.2082 |
| **FS** | 207.1128 | 235.1077 | **FSV** | 306.1812 | 334.1761 | **SV** | 159.1128 | 187.1077 |

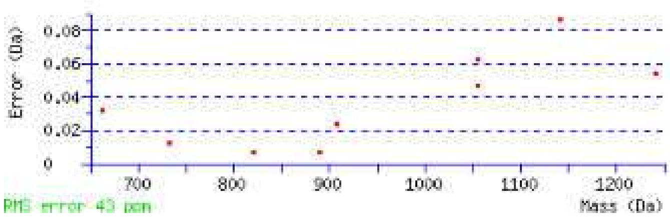


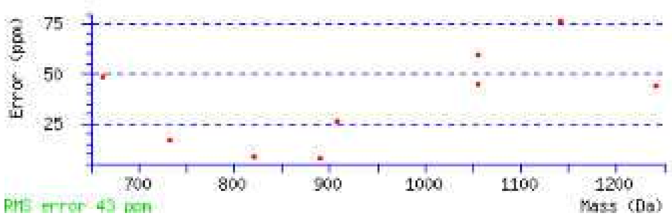


NCBI **BLAST** search of KFVESAASSFSVA
(Parameters: blastp, nr protein database, expect=20000, no filter, PAM30)
Other BLAST web gateways

**All matches to this query**

| Score | Mr(calc) | Delta | Sequence |
|---|---|---|---|
| 52.9 | 1328.6612 | 0.1258 | KFVESAASSFSVA |
| 28.7 | 1328.6183 | 0.1687 | TARMDWSSFTK |
| 28.7 | 1328.5997 | 0.1873 | TNASSWNSSFTK |
| 25.5 | 1327.6806 | 1.1065 | NSISIMASSFKK |
| 24.5 | 1328.6096 | 0.1774 | TSSFSLQESTDK |
| 20.6 | 1328.6282 | 0.1588 | ASALNYISSMEK |
| 19.7 | 1327.6289 | 1.1581 | STLSSEQSSMKK |
| 19.6 | 1329.7517 | -0.9647 | AAAIPSSAFARIR |
| 16.8 | 1327.6554 | 1.1316 | AHNVSTGEAMAIK |
| 16.4 | 1328.7048 | 0.0822 | TSSESRLSPLPR |

**Mascot:** http://www.matrixscience.com/

# MATRIX SCIENCE Mascot Search Results

## Peptide View **Spot no 24**

MS/MS Fragmentation of **YEDNFDANSNVSVIINPTTK**
Found in **gi|115470529** in **NCBInr**, Os07g0141400 [Oryza sativa Japonica Group]

Match to Query 28: 2240.296424 from(2241.303700,1+) intensity(0.0000) index(10)
Title: Label: B12, Spot_Id: 216654, Peak_List_Id: 221710, MSMS Job_Run_Id: 21388, Comment:
Data file C:\Documents and Settings\Administrator\Desktop\farah BHU\Cotton leaves\RAJESH\12-1-13 raj\ppw_B12_136117695600.txt

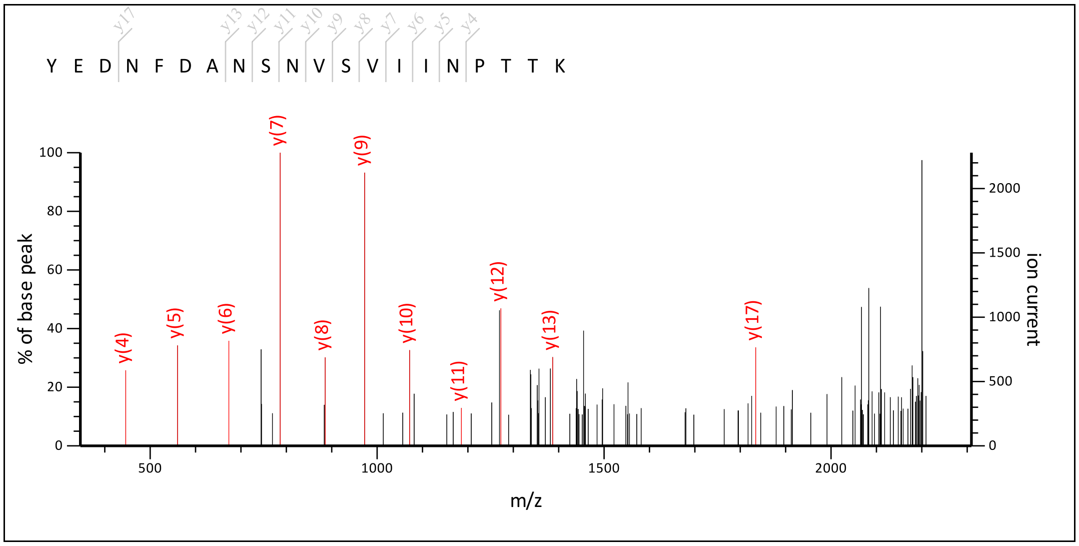


346.21 to 2309.31

Label all possible matches ○ Label matches used for scoring ◉

**Monoisotopic mass of neutral peptide Mr(calc):** 2240.0597
**Fixed modifications:** Carbamidomethyl (C) (apply to specified residues or termini only)
**Ions Score:** 77 **Expect:** 6.3e-05
**Matches :** 11/401 fragment ions using 18 most intense peaks (help)

| # | Immon. | a | a* | a0 | b | b* | b0 | d | d' | Seq. | v | w | w' | y | y* | |
|---|---|---|---|---|---|---|---|---|---|---|---|---|---|---|---|---|
| 1 | 136.0757 | 136.0757 | | | 164.0706 | | | 44.0495 | | Y | | | | | | |
| 2 | 102.0550 | 265.1183 | | 247.1077 | 293.1132 | | 275.1026 | 207.1128 | | E | 2003.9669 | 2002.9716 | | 2078.0037 | 2060.9771 | [illegible] |
| 3 | 88.0393 | 380.1452 | | 362.1347 | 408.1401 | | 390.1296 | 336.1554 | | D | 1888.9399 | 1887.9447 | | 1948.9611 | 1931.9345 | [illegible] |
| 4 | 87.0553 | 494.1882 | 477.1616 | 476.1776 | 522.1831 | 505.1565 | 504.1725 | 451.1823 | | N | 1774.8970 | 1773.9018 | | ***1833.9341*** | 1816.9076 | [illegible] |
| 5 | 120.0808 | 641.2566 | 624.2300 | 623.2460 | 669.2515 | 652.2249 | 651.2409 | | | F | 1627.8286 | | | 1719.8912 | 1702.8646 | [illegible] |
| 6 | 88.0393 | 756.2835 | 739.2570 | 738.2729 | 784.2784 | 767.2519 | 766.2679 | 712.2937 | | D | 1512.8016 | 1511.8064 | | 1572.8228 | 1555.7962 | [illegible] |
| 7 | 44.0495 | 827.3206 | 810.2941 | 809.3101 | 855.3155 | 838.2890 | 837.3050 | | | A | 1441.7645 | | | 1457.7958 | 1440.7693 | [illegible] |
| 8 | 87.0553 | 941.3636 | 924.3370 | 923.3530 | 969.3585 | 952.3319 | 951.3479 | 898.3577 | | N | 1327.7216 | 1326.7264 | | ***1386.7587*** | 1369.7322 | [illegible] |
| 9 | 60.0444 | 1028.3956 | 1011.3690 | 1010.3850 | 1056.3905 | 1039.3639 | 1038.3799 | 1012.4007 | | S | 1240.6896 | 1239.6943 | | ***1272.7158*** | 1255.6892 | [illegible] |
| 10 | 87.0553 | 1142.4385 | 1125.4120 | 1124.4279 | 1170.4334 | 1153.4069 | 1152.4229 | 1099.4327 | | N | 1126.6467 | 1125.6514 | | ***1185.6838*** | 1168.6572 | [illegible] |
| 11 | 72.0808 | 1241.5069 | 1224.4804 | 1223.4964 | 1269.5018 | 1252.4753 | 1251.4913 | 1227.4913 | | V | 1027.5782 | 1040.5986 | | ***1071.6408*** | 1054.6143 | [illegible] |
| 12 | 60.0444 | 1328.5389 | 1311.5124 | 1310.5284 | 1356.5339 | 1339.5073 | 1338.5233 | 1312.5440 | | S | 940.5462 | 939.5510 | | ***972.5724*** | 955.5459 | [illegible] |
| 13 | 72.0808 | 1427.6074 | 1410.5808 | 1409.5968 | 1455.6023 | 1438.5757 | 1437.5917 | 1413.5917 | | V | 841.4778 | 854.4982 | | ***885.5404*** | 868.5138 | [illegible] |
| 14 | 86.0964 | 1540.6914 | 1523.6649 | 1522.6809 | 1568.6863 | 1551.6598 | 1550.6758 | 1512.6601 | 1526.6758 | I | 728.3937 | 741.4141 | 755.4298 | ***786.4720*** | 769.4454 | [illegible] |
| 15 | 86.0964 | 1653.7755 | 1636.7489 | 1635.7649 | 1681.7704 | 1664.7439 | 1663.7598 | 1625.7442 | 1639.7598 | I | 615.3097 | 628.3301 | 642.3457 | ***673.3879*** | 656.3614 | [illegible] |
| 16 | 87.0553 | 1767.8184 | 1750.7919 | 1749.8079 | 1795.8133 | 1778.7868 | 1777.8028 | 1724.8126 | | N | 501.2667 | 500.2715 | | ***560.3039*** | 543.2773 | [illegible] |
| 17 | 70.0651 | 1864.8712 | 1847.8446 | 1846.8606 | 1892.8661 | 1875.8395 | 1874.8555 | 1838.8555 | | P | 404.2140 | 403.2187 | | ***446.2609*** | 429.2344 | [illegible] |
| 18 | 74.0600 | 1965.9189 | 1948.8923 | 1947.9083 | 1993.9138 | 1976.8872 | 1975.9032 | 1949.9239 | 1951.9032 | T | 303.1663 | 316.1867 | 318.1660 | 349.2082 | 332.1816 | [illegible] |
| 19 | 74.0600 | 2066.9665 | 2049.9400 | 2048.9560 | 2094.9615 | 2077.9349 | 2076.9509 | 2050.9716 | 2052.9509 | T | 202.1186 | 215.1390 | 217.1183 | 248.1605 | 231.1339 | [illegible] |
| 20 | 101.1073 | | | | | | | | | K | 74.0237 | 73.0284 | | 147.1128 | 130.0863 | |

| Seq | ya | yb | Seq | ya | yb | Seq | ya | yb |
|---|---|---|---|---|---|---|---|---|
| ED | 217.0819 | 245.0768 | EDN | 331.1248 | 359.1197 | EDNF | 478.1932 | 506.1882 |

| | | | | | | | | |
|---|---|---|---|---|---|---|---|---|
| **EDNFD** | 593.2202 | 621.2151 | **EDNFDA** | 664.2573 | 692.2522 | **DN** | 202.0822 | 230.0771 |
| **DNF** | 349.1506 | 377.1456 | **DNFD** | 464.1776 | 492.1725 | **DNFDA** | 535.2147 | 563.2096 |
| **DNFDAN** | 649.2576 | 677.2525 | **NF** | 234.1237 | 262.1186 | **NFD** | 349.1506 | 377.1456 |
| **NFDA** | 420.1878 | 448.1827 | **NFDAN** | 534.2307 | 562.2256 | **NFDANS** | 621.2627 | 649.2576 |
| **FD** | 235.1077 | 263.1026 | **FDA** | 306.1448 | 334.1397 | **FDAN** | 420.1878 | 448.1827 |
| **FDANS** | 507.2198 | 535.2147 | **FDANSN** | 621.2627 | 649.2576 | **DA** | 159.0764 | 187.0713 |
| **DAN** | 273.1193 | 301.1143 | **DANS** | 360.1514 | 388.1463 | **DANSN** | 474.1943 | 502.1892 |
| **DANSNV** | 573.2627 | 601.2576 | **DANSNVS** | 660.2947 | 688.2897 | **AN** | 158.0924 | 186.0873 |
| **ANS** | 245.1244 | 273.1193 | **ANSN** | 359.1674 | 387.1623 | **ANSNV** | 458.2358 | 486.2307 |
| **ANSNVS** | 545.2678 | 573.2627 | **ANSNVSV** | 644.3362 | 672.3311 | **NS** | 174.0873 | 202.0822 |
| **NSN** | 288.1302 | 316.1252 | **NSNV** | 387.1987 | 415.1936 | **NSNVS** | 474.2307 | 502.2256 |
| **NSNVSV** | 573.2991 | 601.2940 | **NSNVSVI** | 686.3832 | 714.3781 | **SN** | 174.0873 | 202.0822 |
| **SNV** | 273.1557 | 301.1506 | **SNVS** | 360.1878 | 388.1827 | **SNVSV** | 459.2562 | 487.2511 |
| **SNVSVI** | 572.3402 | 600.3352 | **SNVSVII** | 685.4243 | 713.4192 | **NV** | 186.1237 | 214.1186 |
| **NVS** | 273.1557 | 301.1506 | **NVSV** | 372.2241 | 400.2191 | **NVSVI** | 485.3082 | 513.3031 |
| **NVSVII** | 598.3923 | 626.3872 | **VS** | 159.1128 | 187.1077 | **VSV** | 258.1812 | 286.1761 |
| **VSVI** | 371.2653 | 399.2602 | **VSVII** | 484.3493 | 512.3443 | **VSVIIN** | 598.3923 | 626.3872 |
| **VSVIINP** | 695.4450 | 723.4400 | **SV** | 159.1128 | 187.1077 | **SVI** | 272.1969 | 300.1918 |
| **SVII** | 385.2809 | 413.2758 | **SVIIN** | 499.3239 | 527.3188 | **SVIINP** | 596.3766 | 624.3715 |
| **SVIINPT** | 697.4243 | 725.4192 | **VI** | 185.1648 | 213.1598 | **VII** | 298.2489 | 326.2438 |
| **VIIN** | 412.2918 | 440.2867 | **VIINP** | 509.3446 | 537.3395 | **VIINPT** | 610.3923 | 638.3872 |
| **II** | 199.1805 | 227.1754 | **IIN** | 313.2234 | 341.2183 | **IINP** | 410.2762 | 438.2711 |
| **IINPT** | 511.3239 | 539.3188 | **IINPTT** | 612.3715 | 640.3665 | **IN** | 200.1394 | 228.1343 |
| **INP** | 297.1921 | 325.1870 | **INPT** | 398.2398 | 426.2347 | **INPTT** | 499.2875 | 527.2824 |
| **NP** | 184.1081 | 212.1030 | **NPT** | 285.1557 | 313.1506 | **NPTT** | 386.2034 | 414.1983 |
| **PT** | 171.1128 | 199.1077 | **PTT** | 272.1605 | 300.1554 | **TT** | 175.1077 | 203.1026 |

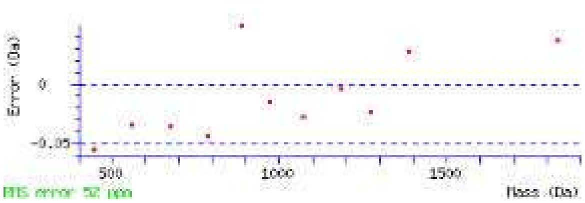


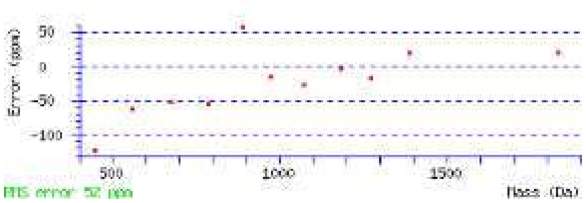


NCBI **BLAST** search of YEDNFDANSNVSVIINPTTK
(Parameters: blastp, nr protein database, expect=20000, no filter, PAM30)
Other BLAST web gateways

**All matches to this query**

| Score | Mr(calc) | Delta | Sequence |
|---|---|---|---|
| 76.5 | 2240.0597 | 0.2367 | YEDNFDANSNVSVIINPTTK |
| 62.2 | 2240.0597 | 0.2367 | YEDNFDANSNVSVIIQPTSK |
| 17.0 | 2241.0801 | -0.7837 | YEDNFDATSNLSVIINPTTK |
| 10.7 | 2241.0437 | -0.7473 | AEDDELIVEGSISDAYFNVR |
| 8.7 | 2240.0359 | 0.2605 | NNTSGSLPNSWGGNSKNGFFR |
| 8.6 | 2241.2077 | -0.9113 | LTLNIDGSVKASTNNAGIGGALR |
| 5.3 | 2240.1107 | 0.1857 | GMQVIDTGAPLSEPVGENTLGR |
| 3.3 | 2240.1722 | 0.1242 | DLLDILMDAAADPAAEVKLTR |
| 3.0 | 2239.1531 | 1.1433 | AQAAEAMLVDATRHLPATAFR |
| 2.9 | 2241.2369 | -0.9405 | FAVEIIQAVVDEIGADKVGIR |

**Mascot:** http://www.matrixscience.com/

# MATRIX SCIENCE Mascot Search Results

## Peptide View Spot no 24

MS/MS Fragmentation of **TITEFGSPEEFLAQVDFLLGK**
Found in **gi|115470529** in **NCBInr**, Os07g0141400 [Oryza sativa Japonica Group]

Match to Query 30: 2340.437524 from(2341.444800,1+) intensity(0.0000) index(11)
Title: Label: B12, Spot_Id: 216654, Peak_List_Id: 221706, MSMS Job_Run_Id: 21388, Comment:
Data file C:\Documents and Settings\Administrator\Desktop\farah BHU\Cotton leaves\RAJESH\12-1-13 raj\ppw_B12_136117695600.txt

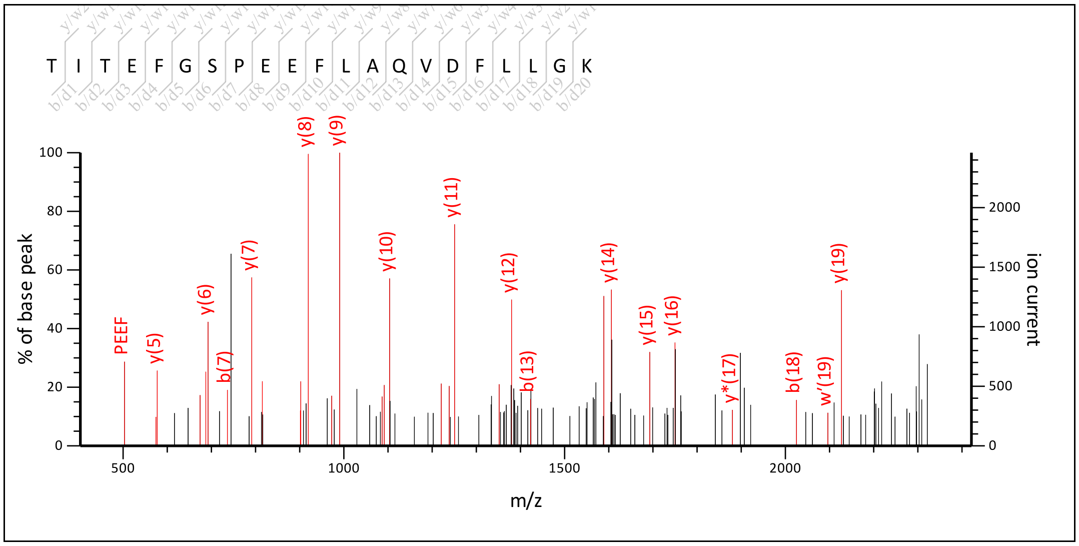


403.2 to 2421.37

Label all possible matches ○ Label matches used for scoring ◉

**Monoisotopic mass of neutral peptide Mr(calc):** 2340.1889
**Fixed modifications:** Carbamidomethyl (C) (apply to specified residues or termini only)
**Ions Score:** 67 **Expect:** 0.00048
**Matches :** 36/377 fragment ions using 55 most intense peaks (help)

| # | Immon. | a | a* | $a^0$ | b | b* | $b^0$ | d | d' | Seq. | v | w | w' | y | y* | |
|---|---|---|---|---|---|---|---|---|---|---|---|---|---|---|---|---|
| 1 | 74.0600 | 74.0600 | | 56.0495 | 102.0550 | | 84.0444 | 44.0495 | | T | | | | | | |
| 2 | 86.0964 | 187.1441 | | 169.1335 | 215.1390 | | 197.1285 | 159.1128 | 173.1285 | I | 2182.0703 | 2195.0907 | 2209.1063 | 2240.1485 | 2223.1220 | 222 |
| 3 | 74.0600 | 288.1918 | | 270.1812 | 316.1867 | | 298.1761 | 272.1969 | 274.1761 | T | 2081.0226 | 2094.0430 | 2096.0223 | ***2127.0645*** | 2110.0379 | 21 |
| 4 | 102.0550 | 417.2344 | | 399.2238 | 445.2293 | | 427.2187 | 359.2289 | | E | 1951.9800 | 1950.9848 | | 2026.0168 | 2008.9902 | 20 |
| 5 | 120.0808 | 564.3028 | | 546.2922 | 592.2977 | | 574.2871 | | | F | 1804.9116 | | | 1896.9742 | 1879.9476 | 18 |
| 6 | 30.0338 | 621.3243 | | 603.3137 | 649.3192 | | 631.3086 | | | G | | | | ***1749.9058*** | 1732.8792 | 17 |
| 7 | 60.0444 | 708.3563 | | 690.3457 | **736.3512** | | 718.3406 | 692.3614 | | S | 1660.8581 | 1659.8629 | | ***1692.8843*** | 1675.8578 | 16 |
| 8 | 70.0651 | 805.4090 | | 787.3985 | 833.4040 | | 815.3934 | 779.3934 | | P | 1563.8053 | 1562.8101 | | ***1605.8523*** | 1588.8257 | 15 |
| 9 | 102.0550 | 934.4516 | | 916.4411 | 962.4466 | | 944.4360 | 876.4462 | | E | 1434.7627 | 1433.7675 | | 1508.7995 | 1491.7730 | 14 |
| 10 | 102.0550 | 1063.4942 | | 1045.4837 | **1091.4891** | | 1073.4786 | 1005.4888 | | E | 1305.7202 | 1304.7249 | | ***1379.7569*** | 1362.7304 | 13 |
| 11 | 120.0808 | 1210.5626 | | 1192.5521 | **1238.5576** | | 1220.5470 | | | F | 1158.6517 | | | ***1250.7143*** | 1233.6878 | 12 |
| 12 | 86.0964 | 1323.6467 | | 1305.6361 | **1351.6416** | | 1333.6311 | 1281.5998 | | L | 1045.5677 | 1044.5724 | | ***1103.6459*** | 1086.6194 | 10 |
| 13 | 44.0495 | 1394.6838 | | 1376.6733 | **1422.6787** | | 1404.6682 | | | A | 974.5306 | | | ***990.5619*** | 973.5353 | 9 |
| 14 | 101.0709 | 1522.7424 | 1505.7159 | 1504.7318 | 1550.7373 | 1533.7108 | 1532.7268 | 1465.7209 | | Q | 846.4720 | 845.4767 | | ***919.5247*** | 902.4982 | 9 |
| 15 | 72.0808 | 1621.8108 | 1604.7843 | 1603.8003 | 1649.8057 | 1632.7792 | 1631.7952 | 1607.7952 | | V | 747.4036 | 760.4240 | | ***791.4662*** | 774.4396 | 7 |
| 16 | 88.0393 | 1736.8378 | 1719.8112 | 1718.8272 | 1764.8327 | 1747.8061 | 1746.8221 | 1692.8479 | | D | 632.3766 | 631.3814 | | ***692.3978*** | 675.3712 | 6 |
| 17 | 120.0808 | 1883.9062 | 1866.8796 | 1865.8956 | 1911.9011 | 1894.8745 | 1893.8905 | | | F | 485.3082 | | | ***577.3708*** | 560.3443 | |
| 18 | 86.0964 | 1996.9902 | 1979.9637 | 1978.9797 | **2024.9852** | 2007.9586 | 2006.9746 | 1954.9433 | | L | 372.2241 | 371.2289 | | 430.3024 | 413.2758 | |
| 19 | 86.0964 | 2110.0743 | 2093.0478 | 2092.0637 | 2138.0692 | 2121.0427 | 2120.0587 | 2068.0274 | | L | 259.1401 | 258.1448 | | 317.2183 | 300.1918 | |
| 20 | 30.0338 | 2167.0958 | 2150.0692 | 2149.0852 | 2195.0907 | 2178.0641 | 2177.0801 | | | G | | | | 204.1343 | 187.1077 | |
| 21 | 101.1073 | | | | | | | | | K | 74.0237 | 73.0284 | | 147.1128 | 130.0863 | |

| Seq | ya | yb | Seq | ya | yb | Seq | ya | yb |
|---|---|---|---|---|---|---|---|---|

| | | | | | | | | |
|---|---|---|---|---|---|---|---|---|
| **IT** | 187.1441 | 215.1390 | **ITE** | 316.1867 | 344.1816 | **ITEF** | 463.2551 | 491.2500 |
| **ITEFG** | 520.2766 | 548.2715 | **ITEFGS** | 607.3086 | 635.3035 | **TE** | 203.1026 | 231.0975 |
| **TEF** | 350.1710 | 378.1660 | **TEFG** | 407.1925 | 435.1874 | **TEFGS** | 494.2245 | 522.2195 |
| **TEFGSP** | 591.2773 | 619.2722 | **EF** | 249.1234 | 277.1183 | **EFG** | 306.1448 | 334.1397 |
| **EFGS** | 393.1769 | 421.1718 | **EFGSP** | 490.2296 | 518.2245 | **EFGSPE** | 619.2722 | 647.2671 |
| **FG** | 177.1022 | 205.0972 | **FGS** | 264.1343 | 292.1292 | **FGSP** | 361.1870 | 389.1819 |
| **FGSPE** | 490.2296 | 518.2245 | **FGSPEE** | 619.2722 | 647.2671 | **GS** | 117.0659 | 145.0608 |
| **GSP** | 214.1186 | 242.1135 | **GSPE** | 343.1612 | 371.1561 | **GSPEE** | 472.2038 | 500.1987 |
| **GSPEEF** | 619.2722 | 647.2671 | **SP** | 157.0972 | 185.0921 | **SPE** | 286.1397 | 314.1347 |
| **SPEE** | 415.1823 | 443.1773 | **SPEEF** | 562.2508 | 590.2457 | **SPEEFL** | 675.3348 | 703.3297 |
| **PE** | 199.1077 | 227.1026 | **PEE** | 328.1503 | 356.1452 | **PEEF** | 475.2187 | 503.2136 |
| **PEEFL** | 588.3028 | 616.2977 | **PEEFLA** | 659.3399 | 687.3348 | **EE** | 231.0975 | 259.0925 |
| **EEF** | 378.1660 | 406.1609 | **EEFL** | 491.2500 | 519.2449 | **EEFLA** | 562.2871 | 590.2821 |
| **EEFLAQ** | 690.3457 | 718.3406 | **EF** | 249.1234 | 277.1183 | **EFL** | 362.2074 | 390.2023 |
| **EFLA** | 433.2445 | 461.2395 | **EFLAQ** | 561.3031 | 589.2980 | **EFLAQV** | 660.3715 | 688.3665 |
| **FL** | 233.1648 | 261.1598 | **FLA** | 304.2020 | 332.1969 | **FLAQ** | 432.2605 | 460.2554 |
| **FLAQV** | 531.3289 | 559.3239 | **FLAQVD** | 646.3559 | 674.3508 | **LA** | 157.1335 | 185.1285 |
| **LAQ** | 285.1921 | 313.1870 | **LAQV** | 384.2605 | 412.2554 | **LAQVD** | 499.2875 | 527.2824 |
| **LAQVDF** | 646.3559 | 674.3508 | **AQ** | 172.1081 | 200.1030 | **AQV** | 271.1765 | 299.1714 |
| **AQVD** | 386.2034 | 414.1983 | **AQVDF** | 533.2718 | 561.2667 | **AQVDFL** | 646.3559 | 674.3508 |
| **QV** | 200.1394 | 228.1343 | **QVD** | 315.1663 | 343.1612 | **QVDF** | 462.2347 | 490.2296 |
| **QVDFL** | 575.3188 | 603.3137 | **QVDFLL** | 688.4028 | 716.3978 | **VD** | 187.1077 | 215.1026 |
| **VDF** | 334.1761 | 362.1710 | **VDFL** | 447.2602 | 475.2551 | **VDFLL** | 560.3443 | 588.3392 |
| **VDFLLG** | 617.3657 | 645.3606 | **DF** | 235.1077 | 263.1026 | **DFL** | 348.1918 | 376.1867 |
| **DFLL** | 461.2758 | 489.2708 | **DFLLG** | 518.2973 | 546.2922 | **FL** | 233.1648 | 261.1598 |
| **FLL** | 346.2489 | 374.2438 | **FLLG** | 403.2704 | 431.2653 | **LL** | 199.1805 | 227.1754 |
| **LLG** | 256.2020 | 284.1969 | **LG** | 143.1179 | 171.1128 | | | |

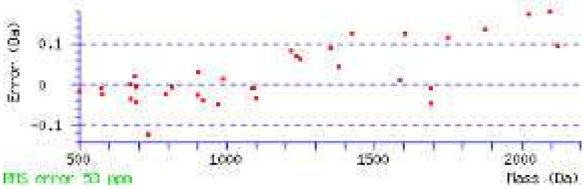


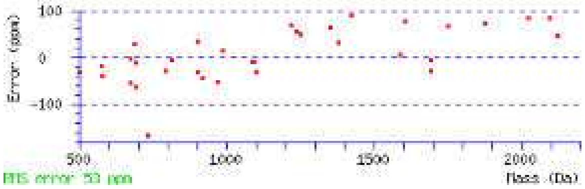


NCBI **BLAST** search of TITEFGSPEEFLAQVDFLLGK
(Parameters: blastp, nr protein database, expect=20000, no filter, PAM30)
Other BLAST web gateways

**All matches to this query**

| Score | Mr(calc) | Delta | Sequence |
|---|---|---|---|
| 66.8 | 2340.1889 | 0.2486 | TITEFGSPEEFLAQVDFLLGK |
| 10.1 | 2341.1413 | -0.7037 | SEFVEAQEALVQMKDWFLR |
| 8.4 | 2340.1541 | 0.2834 | LCAVLGGTFAVTGMLDRWMSR |
| 7.8 | 2340.0991 | 0.3384 | LDGQWSTMALMGGFARIDNNK |
| 6.4 | 2341.1882 | -0.7507 | MATSTMPLGGASPSGRVLGPALDR |
| 5.9 | 2340.1566 | 0.2809 | TVFSTVIKNMGTTLGACGDVNR |
| 5.5 | 2340.2325 | 0.2050 | LTFPSSSGGLAITRLPFSSTSSK |
| 4.8 | 2339.9780 | 0.4595 | AVGCNDAACALGSETQCLSDNK |
| 2.9 | 2341.1606 | -0.7231 | GHPPNRPVYTMLITMCGRGGK |
| 2.4 | 2339.2705 | 1.1670 | MLTSRVLNSTTMAIVILYAAR |

**Mascot:** http://www.matrixscience.com/

# MATRIX SCIENCE Mascot Search Results

## Peptide View Spot no 24

MS/MS Fragmentation of **YEDNFDANSNVSVIINPTTKK**
Found in **gi|115470529** in **NCBInr**, Os07g0141400 [Oryza sativa Japonica Group]

Match to Query 32: 2368.419724 from(2369.427000,1+) intensity(0.0000) index(12)
Title: Label: B12, Spot_Id: 216654, Peak_List_Id: 221707, MSMS Job_Run_Id: 21388, Comment:
Data file C:\Documents and Settings\Administrator\Desktop\farah BHU\Cotton leaves\RAJESH\12-1-13 raj\ppw_B12_136117695600.txt

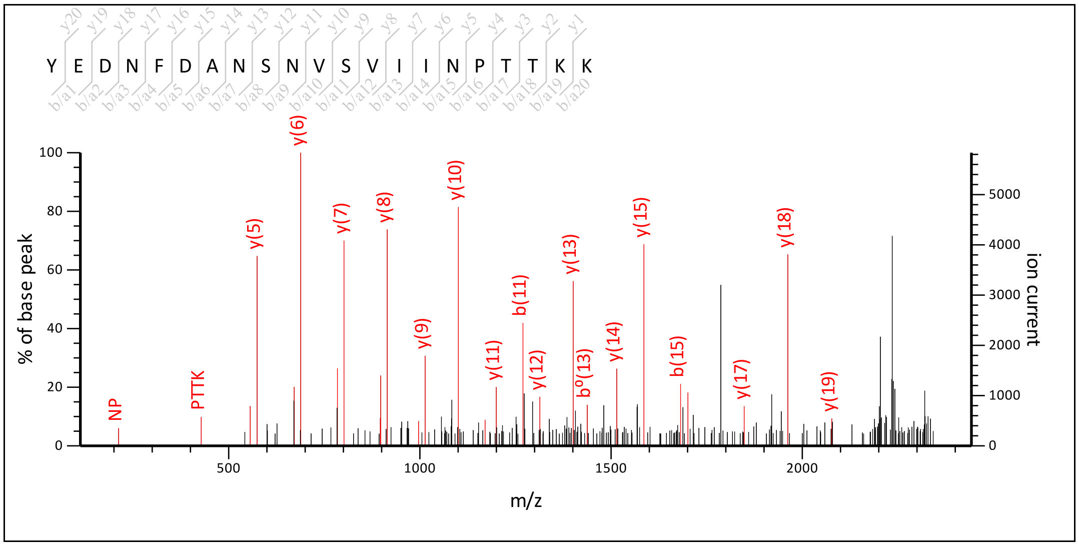


112.11 to 2442.32

Label all possible matches ○ Label matches used for scoring ◉

**Monoisotopic mass of neutral peptide Mr(calc):** 2368.1546
**Fixed modifications:** Carbamidomethyl (C) (apply to specified residues or termini only)
**Ions Score:** 116 **Expect:** 6.1e-09
**Matches :** 31/423 fragment ions using 40 most intense peaks (help)

| # | Immon. | a | a* | a⁰ | b | b* | b⁰ | d | d' | Seq. | v | w | w' | y | y* | |
|---|---|---|---|---|---|---|---|---|---|---|---|---|---|---|---|---|
| 1 | 136.0757 | 136.0757 | | | 164.0706 | | | 44.0495 | | Y | | | | | | |
| 2 | 102.0550 | 265.1183 | | 247.1077 | 293.1132 | | 275.1026 | 207.1128 | | E | 2132.0618 | 2131.0666 | | 2206.0986 | 2189.0721 | 21 |
| 3 | 88.0393 | 380.1452 | | 362.1347 | 408.1401 | | 390.1296 | 336.1554 | | D | 2017.0349 | 2016.0396 | | ***2077.0560*** | 2060.0295 | 20 |
| 4 | 87.0553 | 494.1882 | 477.1616 | 476.1776 | 522.1831 | 505.1565 | 504.1725 | 451.1823 | | N | 1902.9920 | 1901.9967 | | ***1962.0291*** | 1945.0025 | 19 |
| 5 | 120.0808 | 641.2566 | 624.2300 | 623.2460 | 669.2515 | 652.2249 | 651.2409 | | | F | 1755.9236 | | | ***1847.9862*** | 1830.9596 | 18 |
| 6 | 88.0393 | 756.2835 | 739.2570 | 738.2729 | **784.2784** | 767.2519 | 766.2679 | 712.2937 | | D | 1640.8966 | 1639.9014 | | ***1700.9177*** | 1683.8912 | 16 |
| 7 | 44.0495 | 827.3206 | 810.2941 | 809.3101 | 855.3155 | 838.2890 | 837.3050 | | | A | 1569.8595 | | | ***1585.8908*** | 1568.8642 | 15 |
| 8 | 87.0553 | 941.3636 | 924.3370 | 923.3530 | 969.3585 | 952.3319 | 951.3479 | 898.3577 | | N | 1455.8166 | 1454.8213 | | ***1514.8537*** | 1497.8271 | 14 |
| 9 | 60.0444 | 1028.3956 | 1011.3690 | 1010.3850 | 1056.3905 | 1039.3639 | 1038.3799 | 1012.4007 | | S | 1368.7845 | 1367.7893 | | ***1400.8108*** | 1383.7842 | 13 |
| 10 | 87.0553 | 1142.4385 | 1125.4120 | 1124.4279 | **1170.4334** | 1153.4069 | 1152.4229 | 1099.4327 | | N | 1254.7416 | 1253.7464 | | ***1313.7787*** | 1296.7522 | 12 |
| 11 | 72.0808 | 1241.5069 | 1224.4804 | 1223.4964 | **1269.5018** | 1252.4753 | 1251.4913 | 1227.4913 | | V | 1155.6732 | 1168.6936 | | ***1199.7358*** | 1182.7093 | 11 |
| 12 | 60.0444 | 1328.5389 | 1311.5124 | 1310.5284 | 1356.5339 | 1339.5073 | 1338.5233 | 1312.5440 | | S | 1068.6412 | 1067.6459 | | ***1100.6674*** | 1083.6408 | 10 |
| 13 | 72.0808 | 1427.6074 | 1410.5808 | 1409.5968 | 1455.6023 | 1438.5757 | 1437.5917 | 1413.5917 | | V | 969.5728 | 982.5932 | | ***1013.6354*** | 996.6088 | 9 |
| 14 | 86.0964 | 1540.6914 | 1523.6649 | 1522.6809 | 1568.6863 | 1551.6598 | 1550.6758 | 1512.6601 | 1526.6758 | I | 856.4887 | 869.5091 | 883.5247 | ***914.5669*** | 897.5404 | 8 |
| 15 | 86.0964 | 1653.7755 | 1636.7489 | 1635.7649 | **1681.7704** | 1664.7439 | 1663.7598 | 1625.7442 | 1639.7598 | I | 743.4046 | 756.4250 | 770.4407 | ***801.4829*** | 784.4563 | 7 |
| 16 | 87.0553 | 1767.8184 | 1750.7919 | 1749.8079 | 1795.8133 | 1778.7868 | 1777.8028 | 1724.8126 | | N | 629.3617 | 628.3665 | | ***688.3988*** | 671.3723 | 6 |
| 17 | 70.0651 | 1864.8712 | 1847.8446 | 1846.8606 | 1892.8661 | 1875.8395 | 1874.8555 | 1838.8555 | | P | 532.3089 | 531.3137 | | ***574.3559*** | 557.3293 | 5 |
| 18 | 74.0600 | 1965.9189 | 1948.8923 | 1947.9083 | 1993.9138 | 1976.8872 | 1975.9032 | 1949.9239 | 1951.9032 | T | 431.2613 | 444.2817 | 446.2609 | 477.3031 | 460.2766 | 4 |
| 19 | 74.0600 | 2066.9665 | 2049.9400 | 2048.9560 | 2094.9615 | 2077.9349 | 2076.9509 | 2050.9716 | 2052.9509 | T | 330.2136 | 343.2340 | 345.2132 | 376.2554 | 359.2289 | 3 |
| 20 | 101.1073 | 2195.0615 | 2178.0350 | 2177.0509 | 2223.0564 | 2206.0299 | 2205.0459 | 2138.0037 | | K | 202.1186 | 201.1234 | | 275.2078 | 258.1812 | |
| 21 | 101.1073 | | | | | | | | | K | 74.0237 | 73.0284 | | 147.1128 | 130.0863 | |

| Seq | ya | yb | Seq | ya | yb | Seq | ya | yb |
|---|---|---|---|---|---|---|---|---|

| | | | | | | | | |
|---|---|---|---|---|---|---|---|---|
| ED | 217.0819 | 245.0768 | EDN | 331.1248 | 359.1197 | EDNF | 478.1932 | 506.1882 |
| EDNFD | 593.2202 | 621.2151 | EDNFDA | 664.2573 | 692.2522 | DN | 202.0822 | 230.0771 |
| DNF | 349.1506 | 377.1456 | DNFD | 464.1776 | 492.1725 | DNFDA | 535.2147 | 563.2096 |
| DNFDAN | 649.2576 | 677.2525 | NF | 234.1237 | 262.1186 | NFD | 349.1506 | 377.1456 |
| NFDA | 420.1878 | 448.1827 | NFDAN | 534.2307 | 562.2256 | NFDANS | 621.2627 | 649.2576 |
| FD | 235.1077 | 263.1026 | FDA | 306.1448 | 334.1397 | FDAN | 420.1878 | 448.1827 |
| FDANS | 507.2198 | 535.2147 | FDANSN | 621.2627 | 649.2576 | DA | 159.0764 | 187.0713 |
| DAN | 273.1193 | 301.1143 | DANS | 360.1514 | 388.1463 | DANSN | 474.1943 | 502.1892 |
| DANSNV | 573.2627 | 601.2576 | DANSNVS | 660.2947 | 688.2897 | AN | 158.0924 | 186.0873 |
| ANS | 245.1244 | 273.1193 | ANSN | 359.1674 | 387.1623 | ANSNV | 458.2358 | 486.2307 |
| ANSNVS | 545.2678 | 573.2627 | ANSNVSV | 644.3362 | 672.3311 | NS | 174.0873 | 202.0822 |
| NSN | 288.1302 | 316.1252 | NSNV | 387.1987 | 415.1936 | NSNVS | 474.2307 | 502.2256 |
| NSNVSV | 573.2991 | 601.2940 | NSNVSVI | 686.3832 | 714.3781 | SN | 174.0873 | 202.0822 |
| SNV | 273.1557 | 301.1506 | SNVS | 360.1878 | 388.1827 | SNVSV | 459.2562 | 487.2511 |
| SNVSVI | 572.3402 | 600.3352 | SNVSVII | 685.4243 | 713.4192 | NV | 186.1237 | 214.1186 |
| NVS | 273.1557 | 301.1506 | NVSV | 372.2241 | 400.2191 | NVSVI | 485.3082 | 513.3031 |
| NVSVII | 598.3923 | 626.3872 | VS | 159.1128 | 187.1077 | VSV | 258.1812 | 286.1761 |
| VSVI | 371.2653 | 399.2602 | VSVII | 484.3493 | 512.3443 | VSVIIN | 598.3923 | 626.3872 |
| VSVIINP | 695.4450 | 723.4400 | SV | 159.1128 | 187.1077 | SVI | 272.1969 | 300.1918 |
| SVII | 385.2809 | 413.2758 | SVIIN | 499.3239 | 527.3188 | SVIINP | 596.3766 | 624.3715 |
| SVIINPT | 697.4243 | 725.4192 | VI | 185.1648 | 213.1598 | VII | 298.2489 | 326.2438 |
| VIIN | 412.2918 | 440.2867 | VIINP | 509.3446 | 537.3395 | VIINPT | 610.3923 | 638.3872 |
| II | 199.1805 | 227.1754 | IIN | 313.2234 | 341.2183 | IINP | 410.2762 | 438.2711 |
| IINPT | 511.3239 | 539.3188 | IINPTT | 612.3715 | 640.3665 | IN | 200.1394 | 228.1343 |
| INP | 297.1921 | 325.1870 | INPT | 398.2398 | 426.2347 | INPTT | 499.2875 | 527.2824 |
| INPTTK | 627.3824 | 655.3774 | NP | 184.1081 | 212.1030 | NPT | 285.1557 | 313.1506 |
| NPTT | 386.2034 | 414.1983 | NPTTK | 514.2984 | 542.2933 | PT | 171.1128 | 199.1077 |
| PTT | 272.1605 | 300.1554 | PTTK | 400.2554 | 428.2504 | TT | 175.1077 | 203.1026 |
| TTK | 303.2027 | 331.1976 | TK | 202.1550 | 230.1499 | | | |

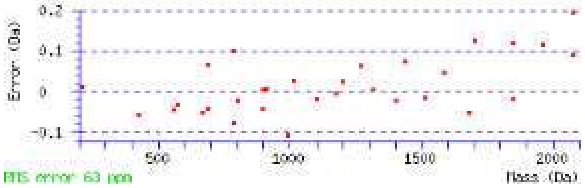


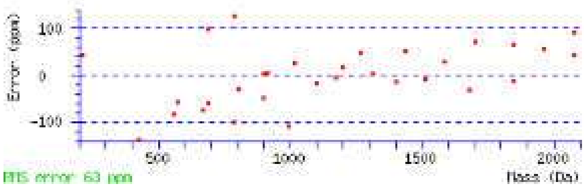


NCBI **BLAST** search of YEDNFDANSNVSVIINPTTKK
(Parameters: blastp, nr protein database, expect=20000, no filter, PAM30)
Other BLAST web gateways

**All matches to this query**

| Score | Mr(calc) | Delta | Sequence |
|---|---|---|---|
| 115.8 | 2368.1546 | 0.2651 | YEDNFDANSNVSVIINPTTKK |
| 93.7 | 2368.1546 | 0.2651 | YEDNFDANSNVSVIIQPTSKK |
| 14.3 | 2369.1750 | -0.7553 | YEDNFDATSNLSVIINPTTKK |
| 7.5 | 2369.2168 | -0.7971 | GYHRTIEGVGFGFISILNDFK |
| 6.9 | 2368.1771 | 0.2426 | TTNPWANEDAVVNVSLETPRR |
| 6.8 | 2368.2461 | 0.1736 | LTDRSDVFSFGVMLLELITGR |
| 6.8 | 2368.2461 | 0.1736 | LTDRSDVFSFGVMLLELLTGR |
| 6.0 | 2369.1362 | -0.7165 | FITHTVPFSEINKAFDNMDK |
| 6.0 | 2369.1548 | -0.7351 | FITHTVPFSEINKAFDNMMK |
| 6.0 | 2369.2955 | -0.8757 | TPVLRFLTLEGNDLIDLDGLR |

**Mascot:** http://www.matrixscience.com/

# MATRIX SCIENCE Mascot Search Results

## Peptide View **Spot no 24**

MS/MS Fragmentation of **KTITEFGSPEEFLAQVDFLLGK**
Found in **gi|115470529** in **NCBInr**, Os07g0141400 [Oryza sativa Japonica Group]

Match to Query 34: 2468.557924 from(2469.565200,1+) intensity(0.0000) index(13)
Title: Label: B12, Spot_Id: 216654, Peak_List_Id: 221708, MSMS Job_Run_Id: 21388, Comment:
Data file C:\Documents and Settings\Administrator\Desktop\farah BHU\Cotton leaves\RAJESH\12-1-13 raj\ppw_B12_136117695600.txt

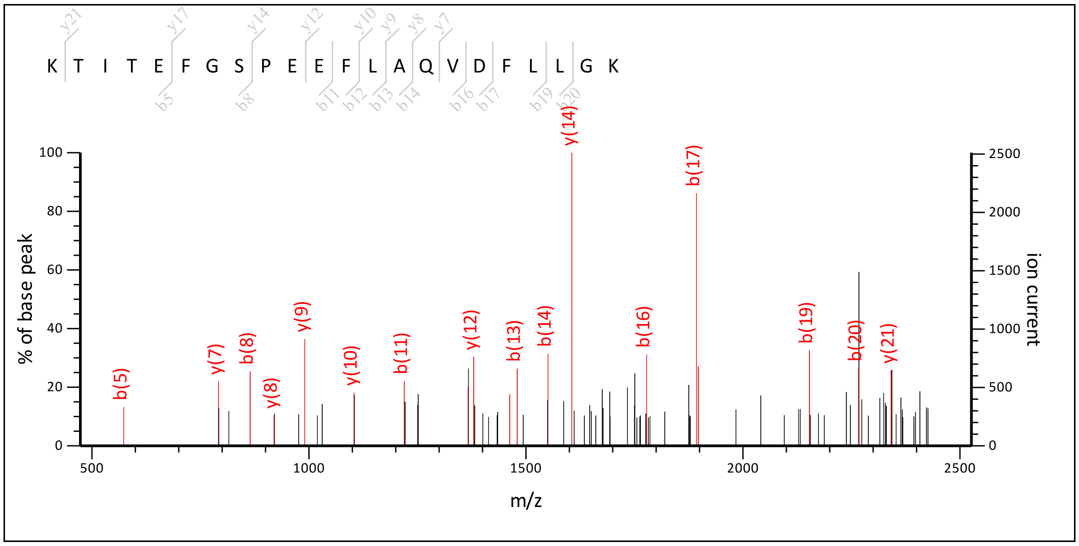


473.3 to 2526.45

Label all possible matches ○ Label matches used for scoring ◉

**Monoisotopic mass of neutral peptide Mr(calc):** 2468.2839
**Fixed modifications:** Carbamidomethyl (C) (apply to specified residues or termini only)
**Ions Score:** 60 **Expect:** 0.0022
**Matches :** 19/426 fragment ions using 35 most intense peaks (help)

| # | Immon. | a | a* | $a^0$ | b | b* | $b^0$ | d | d' | Seq. | v | w | w' | y | y* | |
|---|---|---|---|---|---|---|---|---|---|---|---|---|---|---|---|---|
| 1 | 101.1073 | 101.1073 | 84.0808 | | 129.1022 | 112.0757 | | 44.0495 | | K | | | | | | |
| 2 | 74.0600 | 202.1550 | 185.1285 | 184.1444 | 230.1499 | 213.1234 | 212.1394 | 186.1601 | 188.1394 | T | 2295.1543 | 2308.1747 | 2310.1540 | ***2341.1962*** | 2324.1697 | [illegible] |
| 3 | 86.0964 | 315.2391 | 298.2125 | 297.2285 | 343.2340 | 326.2074 | 325.2234 | 287.2078 | 301.2234 | I | 2182.0703 | 2195.0907 | 2209.1063 | 2240.1485 | 2223.1220 | [illegible] |
| 4 | 74.0600 | 416.2867 | 399.2602 | 398.2762 | 444.2817 | 427.2551 | 426.2711 | 400.2918 | 402.2711 | T | 2081.0226 | 2094.0430 | 2096.0223 | 2127.0645 | 2110.0379 | [illegible] |
| 5 | 102.0550 | 545.3293 | 528.3028 | 527.3188 | ***573.3243*** | 556.2977 | 555.3137 | 487.3239 | | E | 1951.9800 | 1950.9848 | | 2026.0168 | 2008.9902 | [illegible] |
| 6 | 120.0808 | 692.3978 | 675.3712 | 674.3872 | 720.3927 | 703.3661 | 702.3821 | | | F | 1804.9116 | | | ***1896.9742*** | 1879.9476 | [illegible] |
| 7 | 30.0338 | 749.4192 | 732.3927 | 731.4087 | 777.4141 | 760.3876 | 759.4036 | | | G | | | | 1749.9058 | 1732.8792 | [illegible] |
| 8 | 60.0444 | 836.4512 | 819.4247 | 818.4407 | ***864.4462*** | 847.4196 | 846.4356 | 820.4563 | | S | 1660.8581 | 1659.8629 | | 1692.8843 | 1675.8578 | [illegible] |
| 9 | 70.0651 | 933.5040 | 916.4775 | 915.4934 | 961.4989 | 944.4724 | 943.4884 | 907.4884 | | P | 1563.8053 | 1562.8101 | | ***1605.8523*** | 1588.8257 | [illegible] |
| 10 | 102.0550 | 1062.5466 | 1045.5201 | 1044.5360 | 1090.5415 | 1073.5150 | 1072.5310 | 1004.5411 | | E | 1434.7627 | 1433.7675 | | 1508.7995 | 1491.7730 | [illegible] |
| 11 | 102.0550 | 1191.5892 | 1174.5626 | 1173.5786 | ***1219.5841*** | 1202.5576 | 1201.5735 | 1133.5837 | | E | 1305.7202 | 1304.7249 | | ***1379.7569*** | 1362.7304 | [illegible] |
| 12 | 120.0808 | 1338.6576 | 1321.6311 | 1320.6470 | ***1366.6525*** | 1349.6260 | 1348.6420 | | | F | 1158.6517 | | | 1250.7143 | 1233.6878 | [illegible] |
| 13 | 86.0964 | 1451.7417 | 1434.7151 | 1433.7311 | ***1479.7366*** | 1462.7100 | 1461.7260 | 1409.6947 | | L | 1045.5677 | 1044.5724 | | ***1103.6459*** | 1086.6194 | [illegible] |
| 14 | 44.0495 | 1522.7788 | 1505.7522 | 1504.7682 | ***1550.7737*** | 1533.7472 | 1532.7631 | | | A | 974.5306 | | | ***990.5619*** | 973.5353 | [illegible] |
| 15 | 101.0709 | 1650.8374 | 1633.8108 | 1632.8268 | 1678.8323 | 1661.8057 | 1660.8217 | 1593.8159 | | Q | 846.4720 | 845.4767 | | ***919.5247*** | 902.4982 | [illegible] |
| 16 | 72.0808 | 1749.9058 | 1732.8792 | 1731.8952 | ***1777.9007*** | 1760.8741 | 1759.8901 | 1735.8901 | | V | 747.4036 | 760.4240 | | ***791.4662*** | 774.4396 | [illegible] |
| 17 | 88.0393 | 1864.9327 | 1847.9062 | 1846.9222 | ***1892.9276*** | 1875.9011 | 1874.9171 | 1820.9429 | | D | 632.3766 | 631.3814 | | 692.3978 | 675.3712 | [illegible] |
| 18 | 120.0808 | 2012.0011 | 1994.9746 | 1993.9906 | 2039.9961 | 2022.9695 | 2021.9855 | | | F | 485.3082 | | | 577.3708 | 560.3443 | |
| 19 | 86.0964 | 2125.0852 | 2108.0587 | 2107.0746 | ***2153.0801*** | 2136.0536 | 2135.0695 | 2083.0383 | | L | 372.2241 | 371.2289 | | 430.3024 | 413.2758 | |
| 20 | 86.0964 | 2238.1693 | 2221.1427 | 2220.1587 | ***2266.1642*** | 2249.1376 | 2248.1536 | 2196.1223 | | L | 259.1401 | 258.1448 | | 317.2183 | 300.1918 | |
| 21 | 30.0338 | 2295.1907 | 2278.1642 | 2277.1802 | 2323.1856 | 2306.1591 | 2305.1751 | | | G | | | | 204.1343 | 187.1077 | |
| 22 | 101.1073 | | | | | | | | | K | 74.0237 | 73.0284 | | 147.1128 | 130.0863 | |

| Seq | ya | yb | Seq | ya | yb | Seq | ya | yb |
|---|---|---|---|---|---|---|---|---|
| TI | 187.1441 | 215.1390 | TIT | 288.1918 | 316.1867 | TITE | 417.2344 | 445.2293 |
| TITEF | 564.3028 | 592.2977 | TITEFG | 621.3243 | 649.3192 | IT | 187.1441 | 215.1390 |
| ITE | 316.1867 | 344.1816 | ITEF | 463.2551 | 491.2500 | ITEFG | 520.2766 | 548.2715 |
| ITEFGS | 607.3086 | 635.3035 | TE | 203.1026 | 231.0975 | TEF | 350.1710 | 378.1660 |
| TEFG | 407.1925 | 435.1874 | TEFGS | 494.2245 | 522.2195 | TEFGSP | 591.2773 | 619.2722 |
| EF | 249.1234 | 277.1183 | EFG | 306.1448 | 334.1397 | EFGS | 393.1769 | 421.1718 |
| EFGSP | 490.2296 | 518.2245 | EFGSPE | 619.2722 | 647.2671 | FG | 177.1022 | 205.0972 |
| FGS | 264.1343 | 292.1292 | FGSP | 361.1870 | 389.1819 | FGSPE | 490.2296 | 518.2245 |
| FGSPEE | 619.2722 | 647.2671 | GS | 117.0659 | 145.0608 | GSP | 214.1186 | 242.1135 |
| GSPE | 343.1612 | 371.1561 | GSPEE | 472.2038 | 500.1987 | GSPEEF | 619.2722 | 647.2671 |
| SP | 157.0972 | 185.0921 | SPE | 286.1397 | 314.1347 | SPEE | 415.1823 | 443.1773 |
| SPEEF | 562.2508 | 590.2457 | SPEEFL | 675.3348 | 703.3297 | PE | 199.1077 | 227.1026 |
| PEE | 328.1503 | 356.1452 | PEEF | 475.2187 | 503.2136 | PEEFL | 588.3028 | 616.2977 |
| PEEFLA | 659.3399 | 687.3348 | EE | 231.0975 | 259.0925 | EEF | 378.1660 | 406.1609 |
| EEFL | 491.2500 | 519.2449 | EEFLA | 562.2871 | 590.2821 | EEFLAQ | 690.3457 | 718.3406 |
| EF | 249.1234 | 277.1183 | EFL | 362.2074 | 390.2023 | EFLA | 433.2445 | 461.2395 |
| EFLAQ | 561.3031 | 589.2980 | EFLAQV | 660.3715 | 688.3665 | FL | 233.1648 | 261.1598 |
| FLA | 304.2020 | 332.1969 | FLAQ | 432.2605 | 460.2554 | FLAQV | 531.3289 | 559.3239 |
| FLAQVD | 646.3559 | 674.3508 | LA | 157.1335 | 185.1285 | LAQ | 285.1921 | 313.1870 |
| LAQV | 384.2605 | 412.2554 | LAQVD | 499.2875 | 527.2824 | LAQVDF | 646.3559 | 674.3508 |
| AQ | 172.1081 | 200.1030 | AQV | 271.1765 | 299.1714 | AQVD | 386.2034 | 414.1983 |
| AQVDF | 533.2718 | 561.2667 | AQVDFL | 646.3559 | 674.3508 | QV | 200.1394 | 228.1343 |
| QVD | 315.1663 | 343.1612 | QVDF | 462.2347 | 490.2296 | QVDFL | 575.3188 | 603.3137 |
| QVDFLL | 688.4028 | 716.3978 | VD | 187.1077 | 215.1026 | VDF | 334.1761 | 362.1710 |
| VDFL | 447.2602 | 475.2551 | VDFLL | 560.3443 | 588.3392 | VDFLLG | 617.3657 | 645.3606 |
| DF | 235.1077 | 263.1026 | DFL | 348.1918 | 376.1867 | DFLL | 461.2758 | 489.2708 |
| DFLLG | 518.2973 | 546.2922 | FL | 233.1648 | 261.1598 | FLL | 346.2489 | 374.2438 |
| FLLG | 403.2704 | 431.2653 | LL | 199.1805 | 227.1754 | LLG | 256.2020 | 284.1969 |
| LG | 143.1179 | 171.1128 | | | | | | |

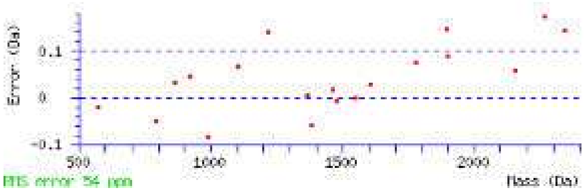


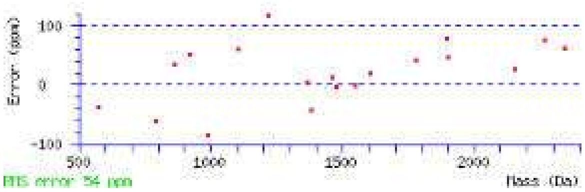


NCBI **BLAST** search of [KTITEFGSPEEFLAQVDFLLGK]
(Parameters: blastp, nr protein database, expect=20000, no filter, PAM30)
Other BLAST [web gateways]

**All matches to this query**

| Score | Mr(calc) | Delta | Sequence |
|---|---|---|---|
| 59.6 | 2468.2839 | 0.2740 | KTITEFGSPEEFLAQVDFLLGK |
| 10.4 | 2468.2369 | 0.3210 | QLTSCESLALGDYPVEVRLYR |
| 8.6 | 2468.2482 | 0.3097 | NDEVLTFELMSQKGQGPHLLGR |
| 7.1 | 2468.2071 | 0.3508 | NQTDNSSIFPFDVVADAVSTLTK |
| 6.7 | 2468.1749 | 0.3830 | LADIFLDMALGCMQKQLDDSGK |
| 6.7 | 2468.1386 | 0.4194 | LADIFLDMALGCMQQQLDDSGK |
| 4.2 | 2468.1399 | 0.4180 | KIHDMNPGPEHYACMVDLLGR |
| 4.1 | 2468.1449 | 0.4130 | MATVDGTTAPSSGGKTATVASESGGGR |
| 3.9 | 2468.1716 | 0.3864 | AKVFMADLEDALSPTWENLMR |
| 3.8 | 2469.1733 | -0.6154 | EAYELAVAEGADFIECDVVLTR |

**Mascot:** http://www.matrixscience.com/

# MATRIX SCIENCE Mascot Search Results

## Peptide View **Spot no 24**

MS/MS Fragmentation of **TDSEGGFESDAVATANILESSAPVVGGK**
Found in **gi|115470529** in **NCBInr**, Os07g0141400 [Oryza sativa Japonica Group]

Match to Query 38: 2707.608424 from(2708.615700,1+) intensity(0.0000) index(15)
Title: Label: B12, Spot_Id: 216654, Peak_List_Id: 221717, MSMS Job_Run_Id: 21388, Comment:
Data file C:\Documents and Settings\Administrator\Desktop\farah BHU\Cotton leaves\RAJESH\12-1-13 raj\ppw_B12_136117695600.txt

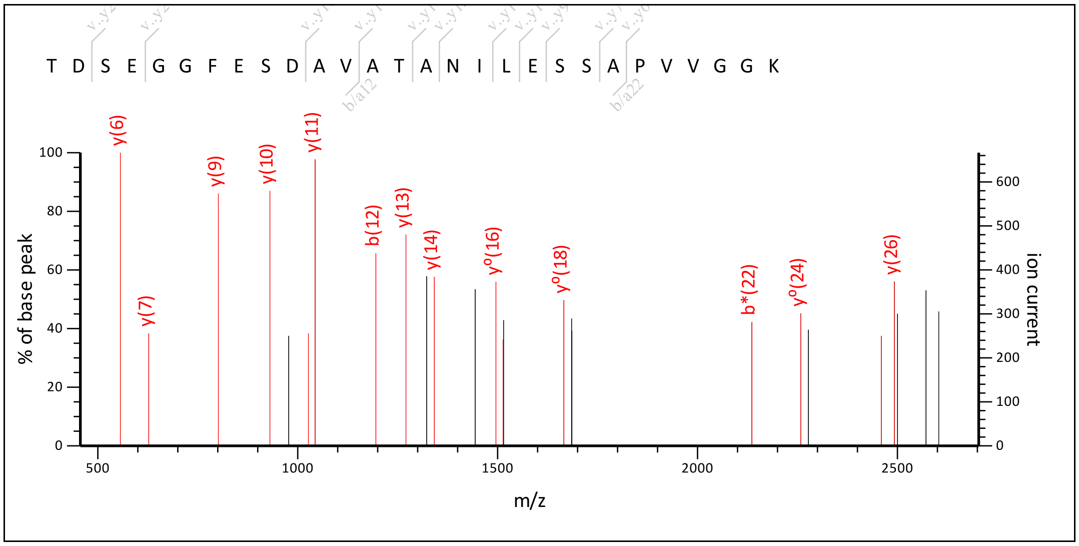


456.3 to 2703.34

Label all possible matches ○ Label matches used for scoring ◉

**Monoisotopic mass of neutral peptide Mr(calc):** 2707.2825
**Fixed modifications:** Carbamidomethyl (C) (apply to specified residues or termini only)
**Ions Score:** 51 **Expect:** 0.017
**Matches :** 22/561 fragment ions using 26 most intense peaks (help)

| # | Immon. | a | a* | a0 | b | b* | b0 | d | d' | Seq. | v | w | w' | y | y* | |
|---|---|---|---|---|---|---|---|---|---|---|---|---|---|---|---|---|
| 1 | 74.0600 | 74.0600 | | 56.0495 | 102.0550 | | 84.0444 | 44.0495 | | T | | | | | | |
| 2 | 88.0393 | 189.0870 | | 171.0764 | 217.0819 | | 199.0713 | 145.0972 | | D | 2547.2209 | 2546.2257 | | 2607.2420 | 2590.2155 | [illegible] |
| 3 | 60.0444 | 276.1190 | | 258.1084 | 304.1139 | | 286.1034 | 260.1241 | | S | 2460.1889 | 2459.1936 | | **_2492.2151_** | 2475.1886 | [illegible] |
| 4 | 102.0550 | 405.1616 | | 387.1510 | 433.1565 | | 415.1460 | 347.1561 | | E | 2331.1463 | 2330.1510 | | 2405.1831 | 2388.1565 | [illegible] |
| 5 | 30.0338 | 462.1831 | | 444.1725 | 490.1780 | | 472.1674 | | | G | | | | 2276.1405 | 2259.1139 | [illegible] |
| 6 | 30.0338 | 519.2045 | | 501.1940 | 547.1994 | | 529.1889 | | | G | | | | 2219.1190 | 2202.0925 | [illegible] |
| 7 | 120.0808 | 666.2729 | | 648.2624 | 694.2679 | | 676.2573 | | | F | 2070.0350 | | | 2162.0976 | 2145.0710 | [illegible] |
| 8 | 102.0550 | 795.3155 | | 777.3050 | 823.3105 | | 805.2999 | 737.3101 | | E | 1940.9924 | 1939.9971 | | 2015.0291 | 1998.0026 | [illegible] |
| 9 | 60.0444 | 882.3476 | | 864.3370 | 910.3425 | | 892.3319 | 866.3527 | | S | 1853.9603 | 1852.9651 | | 1885.9865 | 1868.9600 | [illegible] |
| 10 | 88.0393 | 997.3745 | | 979.3639 | 1025.3694 | | 1007.3589 | 953.3847 | | D | 1738.9334 | 1737.9381 | | 1798.9545 | 1781.9280 | [illegible] |
| 11 | 44.0495 | 1068.4116 | | 1050.4011 | 1096.4065 | | 1078.3960 | | | A | 1667.8963 | | | 1683.9276 | 1666.9010 | [illegible] |
| 12 | 72.0808 | 1167.4800 | | 1149.4695 | **1195.4750** | | 1177.4644 | 1153.4644 | | V | 1568.8279 | 1581.8483 | | 1612.8905 | 1595.8639 | [illegible] |
| 13 | 44.0495 | 1238.5172 | | 1220.5066 | 1266.5121 | | 1248.5015 | | | A | 1497.7907 | | | **_1513.8220_** | 1496.7955 | [illegible] |
| 14 | 74.0600 | 1339.5648 | | 1321.5543 | 1367.5597 | | 1349.5492 | 1323.5699 | 1325.5492 | T | 1396.7431 | 1409.7635 | 1411.7427 | 1442.7849 | 1425.7584 | [illegible] |
| 15 | 44.0495 | 1410.6019 | | 1392.5914 | 1438.5969 | | 1420.5863 | | | A | 1325.7060 | | | **_1341.7373_** | 1324.7107 | [illegible] |
| 16 | 87.0553 | 1524.6449 | 1507.6183 | 1506.6343 | 1552.6398 | 1535.6132 | 1534.6292 | 1481.6391 | | N | 1211.6630 | 1210.6678 | | **_1270.7001_** | 1253.6736 | [illegible] |
| 17 | 86.0964 | 1637.7289 | 1620.7024 | 1619.7184 | **1665.7239** | 1648.6973 | 1647.7133 | 1609.6976 | 1623.7133 | I | 1098.5790 | 1111.5994 | 1125.6150 | 1156.6572 | 1139.6307 | [illegible] |
| 18 | 86.0964 | 1750.8130 | 1733.7865 | 1732.8024 | 1778.8079 | 1761.7814 | 1760.7973 | 1708.7660 | | L | 985.4949 | 984.4997 | | **_1043.5732_** | 1026.5466 | [illegible] |
| 19 | 102.0550 | 1879.8556 | 1862.8290 | 1861.8450 | 1907.8505 | 1890.8240 | 1889.8399 | 1821.8501 | | E | 856.4523 | 855.4571 | | **_930.4891_** | 913.4625 | [illegible] |
| 20 | 60.0444 | 1966.8876 | 1949.8611 | 1948.8771 | 1994.8825 | 1977.8560 | 1976.8720 | 1950.8927 | | S | 769.4203 | 768.4250 | | **_801.4465_** | 784.4199 | [illegible] |
| 21 | 60.0444 | 2053.9196 | 2036.8931 | 2035.9091 | 2081.9146 | 2064.8880 | 2063.9040 | 2037.9247 | | S | 682.3883 | 681.3930 | | 714.4145 | 697.3879 | [illegible] |
| 22 | 44.0495 | 2124.9568 | 2107.9302 | 2106.9462 | 2152.9517 | 2135.9251 | 2134.9411 | | | A | 611.3511 | | | **_627.3824_** | 610.3559 | |
| 23 | 70.0651 | 2222.0095 | 2204.9830 | 2203.9990 | 2250.0044 | 2232.9779 | 2231.9939 | 2195.9939 | | P | 514.2984 | 513.3031 | | **_556.3453_** | 539.3188 | |

| | | | | | | | | | | | | | | | |
|---|---|---|---|---|---|---|---|---|---|---|---|---|---|---|---|
| 24 | 72.0808 | 2321.0779 | 2304.0514 | 2303.0674 | 2349.0729 | 2332.0463 | 2331.0623 | 2307.0623 | | V | 415.2300 | 428.2504 | | 459.2926 | 442.2660 |
| 25 | 72.0808 | 2420.1464 | 2403.1198 | 2402.1358 | 2448.1413 | 2431.1147 | 2430.1307 | 2406.1307 | | V | 316.1615 | 329.1819 | | 360.2241 | 343.1976 |
| 26 | 30.0338 | 2477.1678 | 2460.1413 | 2459.1573 | 2505.1627 | 2488.1362 | 2487.1522 | | | G | | | | 261.1557 | 244.1292 |
| 27 | 30.0338 | 2534.1893 | 2517.1627 | 2516.1787 | 2562.1842 | 2545.1576 | 2544.1736 | | | G | | | | 204.1343 | 187.1077 |
| 28 | 101.1073 | | | | | | | | | K | 74.0237 | 73.0284 | | 147.1128 | 130.0863 |

| Seq | ya | yb | Seq | ya | yb | Seq | ya | yb |
|---|---|---|---|---|---|---|---|---|
| DS | 175.0713 | 203.0662 | DSE | 304.1139 | 332.1088 | DSEG | 361.1354 | 389.1303 |
| DSEGG | 418.1569 | 446.1518 | DSEGGF | 565.2253 | 593.2202 | DSEGGFE | 694.2679 | 722.2628 |
| SE | 189.0870 | 217.0819 | SEG | 246.1084 | 274.1034 | SEGG | 303.1299 | 331.1248 |
| SEGGF | 450.1983 | 478.1932 | SEGGFE | 579.2409 | 607.2358 | SEGGFES | 666.2729 | 694.2679 |
| EG | 159.0764 | 187.0713 | EGG | 216.0979 | 244.0928 | EGGF | 363.1663 | 391.1612 |
| EGGFE | 492.2089 | 520.2038 | EGGFES | 579.2409 | 607.2358 | EGGFESD | 694.2679 | 722.2628 |
| GG | 87.0553 | 115.0502 | GGF | 234.1237 | 262.1186 | GGFE | 363.1663 | 391.1612 |
| GGFES | 450.1983 | 478.1932 | GGFESD | 565.2253 | 593.2202 | GGFESDA | 636.2624 | 664.2573 |
| GF | 177.1022 | 205.0972 | GFE | 306.1448 | 334.1397 | GFES | 393.1769 | 421.1718 |
| GFESD | 508.2038 | 536.1987 | GFESDA | 579.2409 | 607.2358 | GFESDAV | 678.3093 | 706.3042 |
| FE | 249.1234 | 277.1183 | FES | 336.1554 | 364.1503 | FESD | 451.1823 | 479.1773 |
| FESDA | 522.2195 | 550.2144 | FESDAV | 621.2879 | 649.2828 | FESDAVA | 692.3250 | 720.3199 |
| ES | 189.0870 | 217.0819 | ESD | 304.1139 | 332.1088 | ESDA | 375.1510 | 403.1460 |
| ESDAV | 474.2195 | 502.2144 | ESDAVA | 545.2566 | 573.2515 | ESDAVAT | 646.3042 | 674.2992 |
| SD | 175.0713 | 203.0662 | SDA | 246.1084 | 274.1034 | SDAV | 345.1769 | 373.1718 |
| SDAVA | 416.2140 | 444.2089 | SDAVAT | 517.2617 | 545.2566 | SDAVATA | 588.2988 | 616.2937 |
| DA | 159.0764 | 187.0713 | DAV | 258.1448 | 286.1397 | DAVA | 329.1819 | 357.1769 |
| DAVAT | 430.2296 | 458.2245 | DAVATA | 501.2667 | 529.2617 | DAVATAN | 615.3097 | 643.3046 |
| AV | 143.1179 | 171.1128 | AVA | 214.1550 | 242.1499 | AVAT | 315.2027 | 343.1976 |
| AVATA | 386.2398 | 414.2347 | AVATAN | 500.2827 | 528.2776 | AVATANI | 613.3668 | 641.3617 |
| VA | 143.1179 | 171.1128 | VAT | 244.1656 | 272.1605 | VATA | 315.2027 | 343.1976 |
| VATAN | 429.2456 | 457.2405 | VATANI | 542.3297 | 570.3246 | VATANIL | 655.4137 | 683.4087 |
| AT | 145.0972 | 173.0921 | ATA | 216.1343 | 244.1292 | ATAN | 330.1772 | 358.1721 |
| ATANI | 443.2613 | 471.2562 | ATANIL | 556.3453 | 584.3402 | ATANILE | 685.3879 | 713.3828 |
| TA | 145.0972 | 173.0921 | TAN | 259.1401 | 287.1350 | TANI | 372.2241 | 400.2191 |
| TANIL | 485.3082 | 513.3031 | TANILE | 614.3508 | 642.3457 | AN | 158.0924 | 186.0873 |
| ANI | 271.1765 | 299.1714 | ANIL | 384.2605 | 412.2554 | ANILE | 513.3031 | 541.2980 |
| ANILES | 600.3352 | 628.3301 | ANILESS | 687.3672 | 715.3621 | NI | 200.1394 | 228.1343 |
| NIL | 313.2234 | 341.2183 | NILE | 442.2660 | 470.2609 | NILES | 529.2980 | 557.2930 |
| NILESS | 616.3301 | 644.3250 | NILESSA | 687.3672 | 715.3621 | IL | 199.1805 | 227.1754 |
| ILE | 328.2231 | 356.2180 | ILES | 415.2551 | 443.2500 | ILESS | 502.2871 | 530.2821 |
| ILESSA | 573.3243 | 601.3192 | ILESSAP | 670.3770 | 698.3719 | LE | 215.1390 | 243.1339 |
| LES | 302.1710 | 330.1660 | LESS | 389.2031 | 417.1980 | LESSA | 460.2402 | 488.2351 |
| LESSAP | 557.2930 | 585.2879 | LESSAPV | 656.3614 | 684.3563 | ES | 189.0870 | 217.0819 |
| ESS | 276.1190 | 304.1139 | ESSA | 347.1561 | 375.1510 | ESSAP | 444.2089 | 472.2038 |
| ESSAPV | 543.2773 | 571.2722 | ESSAPVV | 642.3457 | 670.3406 | ESSAPVVG | 699.3672 | 727.3621 |
| SS | 147.0764 | 175.0713 | SSA | 218.1135 | 246.1084 | SSAP | 315.1663 | 343.1612 |
| SSAPV | 414.2347 | 442.2296 | SSAPVV | 513.3031 | 541.2980 | SSAPVVG | 570.3246 | 598.3195 |
| SSAPVVGG | 627.3461 | 655.3410 | SA | 131.0815 | 159.0764 | SAP | 228.1343 | 256.1292 |
| SAPV | 327.2027 | 355.1976 | SAPVV | 426.2711 | 454.2660 | SAPVVG | 483.2926 | 511.2875 |
| SAPVVGG | 540.3140 | 568.3089 | AP | 141.1022 | 169.0972 | APV | 240.1707 | 268.1656 |
| APVV | 339.2391 | 367.2340 | APVVG | 396.2605 | 424.2554 | APVVGG | 453.2820 | 481.2769 |
| PV | 169.1335 | 197.1285 | PVV | 268.2020 | 296.1969 | PVVG | 325.2234 | 353.2183 |
| PVVGG | 382.2449 | 410.2398 | VV | 171.1492 | 199.1441 | VVG | 228.1707 | 256.1656 |
| VVGG | 285.1921 | 313.1870 | VG | 129.1022 | 157.0972 | VGG | 186.1237 | 214.1186 |
| GG | 87.0553 | 115.0502 | | | | | | |

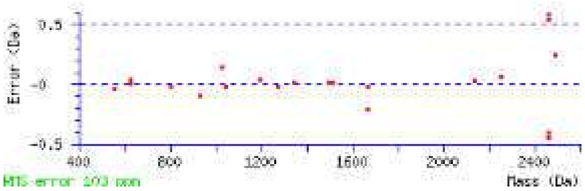

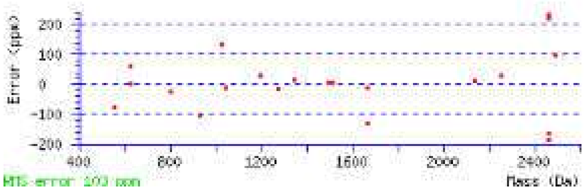

NCBI **BLAST** search of TDSEGGFESDAVATANILESSAPVVGGK
(Parameters: blastp, nr protein database, expect=20000, no filter, PAM30)
Other BLAST web gateways

**All matches to this query**

| Score | Mr(calc) | Delta | Sequence |
|---|---|---|---|
| 50.7 | 2707.2825 | 0.3260 | TDSEGGFESDAVATANILESSAPVVGGK |
| 8.6 | 2708.0980 | -0.4896 | WGSDGGTMGERWGNDGGTMTTFSGGR |
| 8.0 | 2707.3925 | 0.2160 | LMTEAELAAMFRDVAIVDEVTVIR |
| 7.9 | 2707.3541 | 0.2544 | VAVNEGRGIFAVGEGGGVYALAQCWK |
| 5.2 | 2707.3970 | 0.2115 | AVLGWEPTPFRVVAAALGEAGADADPK |
| 5.1 | 2707.3925 | 0.2160 | LMTEAELAAMFRDVAIVDEVTVIR |
| 4.8 | 2708.4095 | -0.8011 | VIMTLTRVILEGEFQESFPIDGAK |
| 4.6 | 2707.4466 | 0.1618 | QAMLEPLVDTVEPNQIVTQTQLIK |
| 4.6 | 2707.4116 | 0.1969 | FNRRPSDGLNLLQVYGDGSMLVIK |
| 4.2 | 2707.6186 | -0.0102 | WELRVMVLLSLSLQILLILLGNR |

**Mascot:** http://www.matrixscience.com/

# MATRIX SCIENCE Mascot Search Results

## Peptide View Spot no 24

MS/MS Fragmentation of **EFPGQVLRYEDNFDANSNVSVIINPTTK**
Found in **gi|115470529** in **NCBInr**, Os07g0141400 [Oryza sativa Japonica Group]

Match to Query 46: 3166.926324 from(3167.933600,1+) intensity(0.0000) index(19)
Title: Label: B12, Spot_Id: 216654, Peak_List_Id: 221705, MSMS Job_Run_Id: 21388, Comment:
Data file C:\Documents and Settings\Administrator\Desktop\farah BHU\Cotton leaves\RAJESH\12-1-13 raj\ppw_B12_136117695600.txt

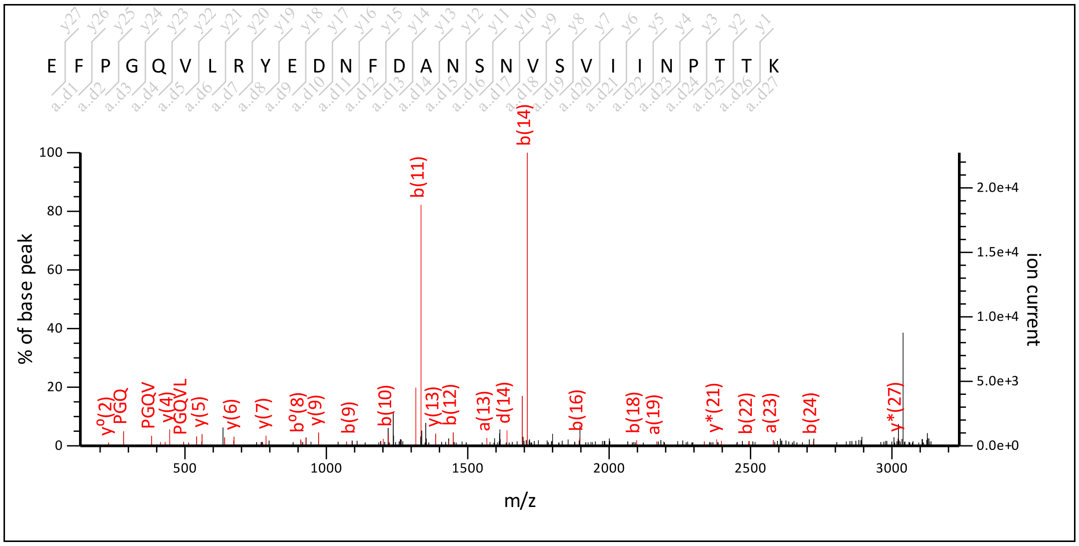


130.1 to 3238.17

Label all possible matches ○ Label matches used for scoring ◉

**Monoisotopic mass of neutral peptide Mr(calc):** 3166.5571
**Fixed modifications:** Carbamidomethyl (C) (apply to specified residues or termini only)
**Ions Score:** 45 **Expect:** 0.039
**Matches :** 51/567 fragment ions using 109 most intense peaks (help)

| # | Immon. | a | a* | $a^0$ | b | b* | $b^0$ | d | d' | Seq. | v | w | w' | y | y* | |
|---|---|---|---|---|---|---|---|---|---|---|---|---|---|---|---|---|
| 1 | 102.0550 | 102.0550 | | 84.0444 | 130.0499 | | 112.0393 | 44.0495 | | E | | | | | | |
| 2 | 120.0808 | 249.1234 | | 231.1128 | 277.1183 | | 259.1077 | | | F | 2946.4592 | | | 3038.5218 | 3021.4952 | 302 |
| 3 | 70.0651 | 346.1761 | | 328.1656 | 374.1710 | | 356.1605 | 320.1605 | | P | 2849.4064 | 2848.4112 | | 2891.4534 | 2874.4268 | 287 |
| 4 | 30.0338 | 403.1976 | | 385.1870 | 431.1925 | | 413.1819 | | | G | | | | 2794.4006 | 2777.3741 | 277 |
| 5 | 101.0709 | 531.2562 | 514.2296 | 513.2456 | ***559.2511*** | 542.2245 | 541.2405 | 474.2347 | | Q | 2664.3264 | 2663.3311 | | 2737.3791 | 2720.3526 | 271 |
| 6 | 72.0808 | 630.3246 | 613.2980 | 612.3140 | 658.3195 | 641.2930 | 640.3089 | 616.3089 | | V | 2565.2580 | 2578.2784 | | 2609.3206 | 2592.2940 | 259 |
| 7 | 86.0964 | 743.4087 | 726.3821 | 725.3981 | ***771.4036*** | 754.3770 | 753.3930 | 701.3617 | | L | 2452.1739 | 2451.1787 | | 2510.2522 | 2493.2256 | 249 |
| 8 | 129.1135 | 899.5098 | 882.4832 | 881.4992 | ***927.5047*** | 910.4781 | 909.4941 | 814.4458 | | R | 2296.0728 | 2295.0775 | | ***2397.1681*** | 2380.1415 | 237 |
| 9 | 136.0757 | 1062.5731 | 1045.5465 | 1044.5625 | ***1090.5680*** | 1073.5415 | 1072.5574 | | | Y | 2133.0095 | | | 2241.0670 | 2224.0404 | 222 |
| 10 | 102.0550 | **1191.6157** | 1174.5891 | 1173.6051 | ***1219.6106*** | 1202.5841 | 1201.6000 | 1133.6102 | | E | 2003.9669 | 2002.9716 | | 2078.0037 | 2060.9771 | 205 |
| 11 | 88.0393 | 1306.6426 | 1289.6161 | 1288.6321 | ***1334.6375*** | 1317.6110 | 1316.6270 | 1262.6528 | | D | 1888.9399 | 1887.9447 | | 1948.9611 | 1931.9345 | 193 |
| 12 | 87.0553 | 1420.6856 | 1403.6590 | 1402.6750 | ***1448.6805*** | 1431.6539 | 1430.6699 | 1377.6797 | | N | 1774.8970 | 1773.9018 | | 1833.9341 | 1816.9076 | 181 |
| 13 | 120.0808 | **1567.7540** | 1550.7274 | 1549.7434 | 1595.7489 | 1578.7223 | 1577.7383 | | | F | 1627.8286 | | | 1719.8912 | 1702.8646 | 170 |
| 14 | 88.0393 | 1682.7809 | 1665.7544 | 1664.7703 | ***1710.7758*** | 1693.7493 | 1692.7653 | 1638.7911 | | D | 1512.8016 | 1511.8064 | | 1572.8228 | 1555.7962 | 155 |
| 15 | 44.0495 | 1753.8180 | 1736.7915 | 1735.8075 | 1781.8129 | 1764.7864 | 1763.8024 | | | A | 1441.7645 | | | 1457.7958 | 1440.7693 | 143 |
| 16 | 87.0553 | 1867.8610 | 1850.8344 | 1849.8504 | ***1895.8559*** | 1878.8293 | 1877.8453 | 1824.8551 | | N | 1327.7216 | 1326.7264 | | ***1386.7587*** | 1369.7322 | 136 |
| 17 | 60.0444 | 1954.8930 | 1937.8664 | 1936.8824 | 1982.8879 | 1965.8613 | 1964.8773 | 1938.8981 | | S | 1240.6896 | 1239.6943 | | 1272.7158 | 1255.6892 | 125 |
| 18 | 87.0553 | 2068.9359 | 2051.9094 | 2050.9253 | ***2096.9308*** | 2079.9043 | 2078.9203 | 2025.9301 | | N | 1126.6467 | 1125.6514 | | 1185.6838 | 1168.6572 | 116 |
| 19 | 72.0808 | **2168.0043** | 2150.9778 | 2149.9938 | 2195.9992 | 2178.9727 | 2177.9887 | 2153.9887 | | V | 1027.5782 | 1040.5986 | | ***1071.6408*** | 1054.6143 | 105 |
| 20 | 60.0444 | 2255.0363 | 2238.0098 | 2237.0258 | 2283.0313 | 2266.0047 | 2265.0207 | 2239.0414 | | S | 940.5462 | 939.5510 | | ***972.5724*** | 955.5459 | 95 |
| 21 | 72.0808 | 2354.1048 | 2337.0782 | 2336.0942 | 2382.0997 | 2365.0731 | 2364.0891 | 2340.0891 | | V | 841.4778 | 854.4982 | | 885.5404 | 868.5138 | 86 |
| 22 | 86.0964 | 2467.1888 | 2450.1623 | 2449.1783 | ***2495.1837*** | 2478.1572 | 2477.1732 | 2439.1575 | 2453.1732 | I | 728.3937 | 741.4141 | 755.4298 | ***786.4720*** | 769.4454 | 76 |
| 23 | 86.0964 | **2580.2729** | 2563.2463 | 2562.2623 | 2608.2678 | 2591.2413 | 2590.2572 | 2552.2416 | 2566.2572 | I | 615.3097 | 628.3301 | 642.3457 | ***673.3879*** | 656.3614 | 65 |

| | | | | | | | | | | | | | | | | |
|---|---|---|---|---|---|---|---|---|---|---|---|---|---|---|---|---|
| 24 | 87.0553 | 2694.3158 | 2677.2893 | 2676.3053 | 2722.3107 | 2705.2842 | 2704.3002 | 2651.3100 | | N | 501.2667 | 500.2715 | | 560.3039 | 543.2773 | [illegible] |
| 25 | 70.0651 | 2791.3686 | 2774.3420 | 2773.3580 | 2819.3635 | 2802.3369 | 2801.3529 | 2765.3529 | | P | 404.2140 | 403.2187 | | 446.2609 | 429.2344 | [illegible] |
| 26 | 74.0600 | 2892.4163 | 2875.3897 | 2874.4057 | 2920.4112 | 2903.3846 | 2902.4006 | 2876.4213 | 2878.4006 | T | 303.1663 | 316.1867 | 318.1660 | 349.2082 | 332.1816 | [illegible] |
| 27 | 74.0600 | 2993.4639 | 2976.4374 | 2975.4534 | 3021.4589 | 3004.4323 | 3003.4483 | 2977.4690 | 2979.4483 | T | 202.1186 | 215.1390 | 217.1183 | 248.1605 | 231.1339 | [illegible] |
| 28 | 101.1073 | | | | | | | | | K | 74.0237 | 73.0284 | | 147.1128 | 130.0863 | |

| Seq | ya | yb | Seq | ya | yb | Seq | ya | yb |
|---|---|---|---|---|---|---|---|---|
| FP | 217.1335 | 245.1285 | FPG | 274.1550 | 302.1499 | FPGQ | 402.2136 | 430.2085 |
| FPGQV | 501.2820 | 529.2769 | FPGQVL | 614.3661 | 642.3610 | PG | 127.0866 | 155.0815 |
| PGQ | 255.1452 | 283.1401 | PGQV | 354.2136 | 382.2085 | PGQVL | 467.2976 | 495.2926 |
| PGQVLR | 623.3988 | 651.3937 | GQ | 158.0924 | 186.0873 | GQV | 257.1608 | 285.1557 |
| GQVL | 370.2449 | 398.2398 | GQVLR | 526.3460 | 554.3409 | GQVLRY | 689.4093 | 717.4042 |
| QV | 200.1394 | 228.1343 | QVL | 313.2234 | 341.2183 | QVLR | 469.3245 | 497.3194 |
| QVLRY | 632.3879 | 660.3828 | VL | 185.1648 | 213.1598 | VLR | 341.2659 | 369.2609 |
| VLRY | 504.3293 | 532.3242 | VLRYE | 633.3719 | 661.3668 | LR | 242.1975 | 270.1925 |
| LRY | 405.2609 | 433.2558 | LRYE | 534.3035 | 562.2984 | LRYED | 649.3304 | 677.3253 |
| RY | 292.1768 | 320.1717 | RYE | 421.2194 | 449.2143 | RYED | 536.2463 | 564.2413 |
| RYEDN | 650.2893 | 678.2842 | YE | 265.1183 | 293.1132 | YED | 380.1452 | 408.1401 |
| YEDN | 494.1882 | 522.1831 | YEDNF | 641.2566 | 669.2515 | ED | 217.0819 | 245.0768 |
| EDN | 331.1248 | 359.1197 | EDNF | 478.1932 | 506.1882 | EDNFD | 593.2202 | 621.2151 |
| EDNFDA | 664.2573 | 692.2522 | DN | 202.0822 | 230.0771 | DNF | 349.1506 | 377.1456 |
| DNFD | 464.1776 | 492.1725 | DNFDA | 535.2147 | 563.2096 | DNFDAN | 649.2576 | 677.2525 |
| NF | 234.1237 | 262.1186 | NFD | 349.1506 | 377.1456 | NFDA | 420.1878 | 448.1827 |
| NFDAN | 534.2307 | 562.2256 | NFDANS | 621.2627 | 649.2576 | FD | 235.1077 | 263.1026 |
| FDA | 306.1448 | 334.1397 | FDAN | 420.1878 | 448.1827 | FDANS | 507.2198 | 535.2147 |
| FDANSN | 621.2627 | 649.2576 | DA | 159.0764 | 187.0713 | DAN | 273.1193 | 301.1143 |
| DANS | 360.1514 | 388.1463 | DANSN | 474.1943 | 502.1892 | DANSNV | 573.2627 | 601.2576 |
| DANSNVS | 660.2947 | 688.2897 | AN | 158.0924 | 186.0873 | ANS | 245.1244 | 273.1193 |
| ANSN | 359.1674 | 387.1623 | ANSNV | 458.2358 | 486.2307 | ANSNVS | 545.2678 | 573.2627 |
| ANSNVSV | 644.3362 | 672.3311 | NS | 174.0873 | 202.0822 | NSN | 288.1302 | 316.1252 |
| NSNV | 387.1987 | 415.1936 | NSNVS | 474.2307 | 502.2256 | NSNVSV | 573.2991 | 601.2940 |
| NSNVSVI | 686.3832 | 714.3781 | SN | 174.0873 | 202.0822 | SNV | 273.1557 | 301.1506 |
| SNVS | 360.1878 | 388.1827 | SNVSV | 459.2562 | 487.2511 | SNVSVI | 572.3402 | 600.3352 |
| SNVSVII | 685.4243 | 713.4192 | NV | 186.1237 | 214.1186 | NVS | 273.1557 | 301.1506 |
| NVSV | 372.2241 | 400.2191 | NVSVI | 485.3082 | 513.3031 | NVSVII | 598.3923 | 626.3872 |
| VS | 159.1128 | 187.1077 | VSV | 258.1812 | 286.1761 | VSVI | 371.2653 | 399.2602 |
| VSVII | 484.3493 | 512.3443 | VSVIIN | 598.3923 | 626.3872 | VSVIINP | 695.4450 | 723.4400 |
| SV | 159.1128 | 187.1077 | SVI | 272.1969 | 300.1918 | SVII | 385.2809 | 413.2758 |
| SVIIN | 499.3239 | 527.3188 | SVIINP | 596.3766 | 624.3715 | SVIINPT | 697.4243 | 725.4192 |
| VI | 185.1648 | 213.1598 | VII | 298.2489 | 326.2438 | VIIN | 412.2918 | 440.2867 |
| VIINP | 509.3446 | 537.3395 | VIINPT | 610.3923 | 638.3872 | II | 199.1805 | 227.1754 |
| IIN | 313.2234 | 341.2183 | IINP | 410.2762 | 438.2711 | IINPT | 511.3239 | 539.3188 |
| IINPTT | 612.3715 | 640.3665 | IN | 200.1394 | 228.1343 | INP | 297.1921 | 325.1870 |
| INPT | 398.2398 | 426.2347 | INPTT | 499.2875 | 527.2824 | NP | 184.1081 | 212.1030 |
| NPT | 285.1557 | 313.1506 | NPTT | 386.2034 | 414.1983 | PT | 171.1128 | 199.1077 |
| PTT | 272.1605 | 300.1554 | TT | 175.1077 | 203.1026 | | | |

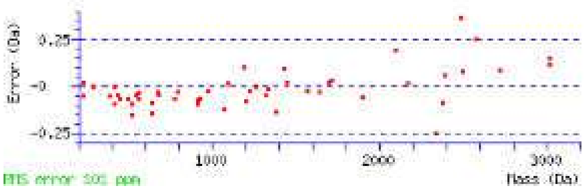


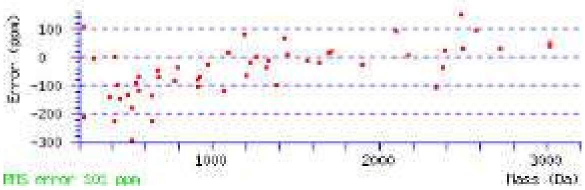


NCBI **BLAST** search of EFPGQVLRYEDNFDANSNVSVIINPTTK
(Parameters: blastp, nr protein database, expect=20000, no filter, PAM30)
Other BLAST web gateways

**All matches to this query**

| Score | Mr(calc) | Delta | Sequence |
|---|---|---|---|
| 45.5 | 3166.5571 | 0.3692 | EFPGQVLRYEDNFDANSNVSVIINPTTK |
| 39.2 | 3166.5571 | 0.3692 | EFPGQVLRYEDNFDANSNVSVIIQPTSK |
| 32.5 | 3167.5775 | -0.6512 | EFPGQVLRYEDNFDATSNLSVIINPTTK |
| 5.4 | 3167.4005 | -0.4742 | VSSHLFSQGFMPDYYVSYMHGEEQLGK |
| 5.4 | 3166.6849 | 0.2414 | WIQDEGFIAVGNMSAVDKPNILPLLATVR |
| 3.9 | 3167.5333 | -0.6069 | SVVAPPPTDDTRDPEGTPGYMAPELLLGEK |
| 3.8 | 3167.4630 | -0.5366 | EIASNEKPSDPEEKPSDPTEKPSDPKDTT |
| 1.5 | 3166.4165 | 0.5099 | SDQNAAARAVDFMFGWFMGPLTEGEYPK |
| 1.1 | 3166.6056 | 0.3207 | WRTAVTVAGDNASPMMLWAVEVVPPKPGR |
| 0.7 | 3166.4403 | 0.4860 | GASEIIVQMCDMMIDGDGNSVPLSEAQRK |

**Mascot:** http://www.matrixscience.com/

# MATRIX SCIENCE Mascot Search Results

## Peptide View Spot no 24

MS/MS Fragmentation of **QAYSGKTDSEGGFESDAVATANILESSAPVVGGK**
Found in **gi|115470529** in **NCBInr**, Os07g0141400 [Oryza sativa Japonica Group]

Match to Query 50: 3342.033224 from(3343.040500,1+) intensity(0.0000) index(21)
Title: Label: B12, Spot_Id: 216654, Peak_List_Id: 221714, MSMS Job_Run_Id: 21388, Comment:
Data file C:\Documents and Settings\Administrator\Desktop\farah BHU\Cotton leaves\RAJESH\12-1-13 raj\ppw_B12_136117695600.txt

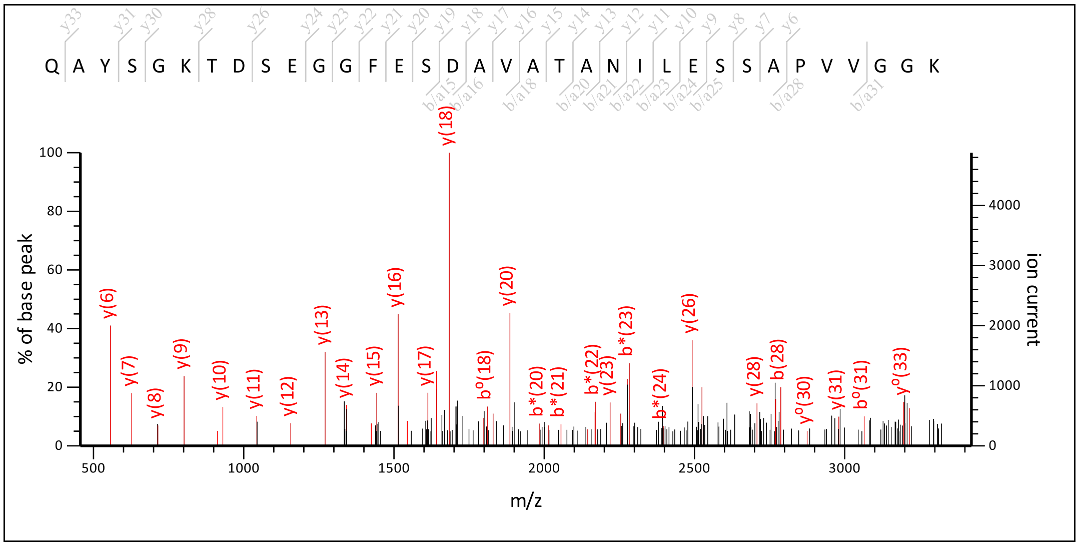


456.31 to 3421.71

Label all possible matches ○ Label matches used for scoring ◉

**Monoisotopic mass of neutral peptide Mr(calc):** 3341.5899
**Fixed modifications:** Carbamidomethyl (C) (apply to specified residues or termini only)
**Ions Score:** 115 **Expect:** 3.5e-09
**Matches :** 46/726 fragment ions using 93 most intense peaks (help)

| # | Immon. | a | a* | a$^0$ | b | b* | b$^0$ | d | d' | Seq. | v | w | w' | y | y* | |
|---|---|---|---|---|---|---|---|---|---|---|---|---|---|---|---|---|
| 1 | 101.0709 | 101.0709 | 84.0444 | | 129.0659 | 112.0393 | | 44.0495 | | Q | | | | | | |
| 2 | 44.0495 | 172.1081 | 155.0815 | | 200.1030 | 183.0764 | | | | A | 3198.5073 | | | ***3214.5386*** | 3197.5121 | [illegible] |
| 3 | 136.0757 | 335.1714 | 318.1448 | | 363.1663 | 346.1397 | | | | Y | 3035.4440 | | | 3143.5015 | 3126.4750 | [illegible] |
| 4 | 60.0444 | 422.2034 | 405.1769 | 404.1928 | 450.1983 | 433.1718 | 432.1878 | 406.2085 | | S | 2948.4120 | 2947.4167 | | ***2980.4382*** | 2963.4116 | [illegible] |
| 5 | 30.0338 | 479.2249 | 462.1983 | 461.2143 | 507.2198 | 490.1932 | 489.2092 | | | G | | | | 2893.4062 | 2876.3796 | [illegible] |
| 6 | 101.1073 | 607.3198 | 590.2933 | 589.3093 | 635.3148 | 618.2882 | 617.3042 | 550.2620 | | K | 2763.2955 | 2762.3003 | | 2836.3847 | 2819.3581 | [illegible] |
| 7 | 74.0600 | 708.3675 | 691.3410 | 690.3570 | 736.3624 | 719.3359 | 718.3519 | 692.3726 | 694.3519 | T | 2662.2479 | 2675.2683 | 2677.2475 | ***2708.2897*** | 2691.2632 | [illegible] |
| 8 | 88.0393 | 823.3945 | 806.3679 | 805.3839 | 851.3894 | 834.3628 | 833.3788 | 779.4046 | | D | 2547.2209 | 2546.2257 | | 2607.2420 | 2590.2155 | [illegible] |
| 9 | 60.0444 | 910.4265 | 893.3999 | 892.4159 | 938.4214 | 921.3949 | 920.4108 | 894.4316 | | S | 2460.1889 | 2459.1936 | | ***2492.2151*** | 2475.1886 | [illegible] |
| 10 | 102.0550 | 1039.4691 | 1022.4425 | 1021.4585 | 1067.4640 | 1050.4374 | 1049.4534 | 981.4636 | | E | 2331.1463 | 2330.1510 | | 2405.1831 | 2388.1565 | [illegible] |
| 11 | 30.0338 | 1096.4905 | 1079.4640 | 1078.4800 | 1124.4855 | 1107.4589 | 1106.4749 | | | G | | | | ***2276.1405*** | 2259.1139 | [illegible] |
| 12 | 30.0338 | 1153.5120 | 1136.4855 | 1135.5014 | 1181.5069 | 1164.4804 | 1163.4964 | | | G | | | | ***2219.1190*** | 2202.0925 | [illegible] |
| 13 | 120.0808 | 1300.5804 | 1283.5539 | 1282.5699 | 1328.5753 | 1311.5488 | 1310.5648 | | | F | 2070.0350 | | | 2162.0976 | 2145.0710 | [illegible] |
| 14 | 102.0550 | 1429.6230 | 1412.5965 | 1411.6124 | 1457.6179 | 1440.5914 | 1439.6074 | 1371.6175 | | E | 1940.9924 | 1939.9971 | | ***2015.0291*** | 1998.0026 | [illegible] |
| 15 | 60.0444 | 1516.6550 | 1499.6285 | 1498.6445 | **1544.6500** | 1527.6234 | 1526.6394 | 1500.6601 | | S | 1853.9603 | 1852.9651 | | ***1885.9865*** | 1868.9600 | [illegible] |
| 16 | 88.0393 | 1631.6820 | 1614.6554 | 1613.6714 | 1659.6769 | 1642.6504 | 1641.6663 | 1587.6922 | | D | 1738.9334 | 1737.9381 | | ***1798.9545*** | 1781.9280 | [illegible] |
| 17 | 44.0495 | 1702.7191 | 1685.6926 | 1684.7085 | 1730.7140 | 1713.6875 | 1712.7034 | | | A | 1667.8963 | | | ***1683.9276*** | 1666.9010 | [illegible] |
| 18 | 72.0808 | 1801.7875 | 1784.7610 | 1783.7769 | **1829.7824** | 1812.7559 | 1811.7719 | 1787.7719 | | V | 1568.8279 | 1581.8483 | | ***1612.8905*** | 1595.8639 | [illegible] |
| 19 | 44.0495 | 1872.8246 | 1855.7981 | 1854.8141 | 1900.8195 | 1883.7930 | 1882.8090 | | | A | 1497.7907 | | | ***1513.8220*** | 1496.7955 | [illegible] |
| 20 | 74.0600 | 1973.8723 | 1956.8458 | 1955.8617 | 2001.8672 | 1984.8407 | 1983.8567 | 1957.8774 | 1959.8567 | T | 1396.7431 | 1409.7635 | 1411.7427 | ***1442.7849*** | 1425.7584 | [illegible] |
| 21 | 44.0495 | 2044.9094 | 2027.8829 | 2026.8989 | 2072.9043 | 2055.8778 | 2054.8938 | | | A | 1325.7060 | | | ***1341.7373*** | 1324.7107 | [illegible] |
| 22 | 87.0553 | 2158.9523 | 2141.9258 | 2140.9418 | 2186.9473 | 2169.9207 | 2168.9367 | 2115.9465 | | N | 1211.6630 | 1210.6678 | | ***1270.7001*** | 1253.6736 | [illegible] |
| 23 | 86.0964 | 2272.0364 | 2255.0099 | 2254.0258 | 2300.0313 | 2283.0048 | 2282.0208 | 2244.0051 | 2258.0208 | I | 1098.5790 | 1111.5994 | 1125.6150 | ***1156.6572*** | 1139.6307 | [illegible] |

| | | | | | | | | | | | | | | | | |
|---|---|---|---|---|---|---|---|---|---|---|---|---|---|---|---|---|
| 24 | 86.0964 | 2385.1205 | 2368.0939 | 2367.1099 | 2413.1154 | 2396.0888 | 2395.1048 | 2343.0735 | | L | 985.4949 | 984.4997 | | ***1043.5732*** | 1026.5466 | [illegible] |
| 25 | 102.0550 | 2514.1631 | 2497.1365 | 2496.1525 | 2542.1580 | 2525.1314 | 2524.1474 | 2456.1576 | | E | 856.4523 | 855.4571 | | ***930.4891*** | 913.4625 | [illegible] |
| 26 | 60.0444 | 2601.1951 | 2584.1685 | 2583.1845 | 2629.1900 | 2612.1635 | 2611.1794 | 2585.2002 | | S | 769.4203 | 768.4250 | | ***801.4465*** | 784.4199 | [illegible] |
| 27 | 60.0444 | 2688.2271 | 2671.2006 | 2670.2166 | 2716.2220 | 2699.1955 | 2698.2115 | 2672.2322 | | S | 682.3883 | 681.3930 | | ***714.4145*** | 697.3879 | [illegible] |
| 28 | 44.0495 | 2759.2642 | 2742.2377 | 2741.2537 | **2787.2592** | 2770.2326 | 2769.2486 | | | A | 611.3511 | | | ***627.3824*** | 610.3559 | |
| 29 | 70.0651 | 2856.3170 | 2839.2905 | 2838.3064 | 2884.3119 | 2867.2854 | 2866.3014 | 2830.3014 | | P | 514.2984 | 513.3031 | | ***556.3453*** | 539.3188 | |
| 30 | 72.0808 | 2955.3854 | 2938.3589 | 2937.3749 | 2983.3803 | 2966.3538 | 2965.3698 | 2941.3698 | | V | 415.2300 | 428.2504 | | 459.2926 | 442.2660 | |
| 31 | 72.0808 | 3054.4538 | 3037.4273 | 3036.4433 | 3082.4487 | 3065.4222 | 3064.4382 | 3040.4382 | | V | 316.1615 | 329.1819 | | 360.2241 | 343.1976 | |
| 32 | 30.0338 | 3111.4753 | 3094.4487 | 3093.4647 | 3139.4702 | 3122.4437 | 3121.4596 | | | G | | | | 261.1557 | 244.1292 | |
| 33 | 30.0338 | 3168.4968 | 3151.4702 | 3150.4862 | **3196.4917** | 3179.4651 | 3178.4811 | | | G | | | | 204.1343 | 187.1077 | |
| 34 | 101.1073 | | | | | | | | | K | 74.0237 | 73.0284 | | 147.1128 | 130.0863 | |

| Seq | ya | yb | Seq | ya | yb | Seq | ya | yb |
|---|---|---|---|---|---|---|---|---|
| AY | 207.1128 | 235.1077 | AYS | 294.1448 | 322.1397 | AYSG | 351.1663 | 379.1612 |
| AYSGK | 479.2613 | 507.2562 | AYSGKT | 580.3089 | 608.3039 | AYSGKTD | 695.3359 | 723.3308 |
| YS | 223.1077 | 251.1026 | YSG | 280.1292 | 308.1241 | YSGK | 408.2241 | 436.2191 |
| YSGKT | 509.2718 | 537.2667 | YSGKTD | 624.2988 | 652.2937 | SG | 117.0659 | 145.0608 |
| SGK | 245.1608 | 273.1557 | SGKT | 346.2085 | 374.2034 | SGKTD | 461.2354 | 489.2304 |
| SGKTDS | 548.2675 | 576.2624 | SGKTDSE | 677.3101 | 705.3050 | GK | 158.1288 | 186.1237 |
| GKT | 259.1765 | 287.1714 | GKTD | 374.2034 | 402.1983 | GKTDS | 461.2354 | 489.2304 |
| GKTDSE | 590.2780 | 618.2729 | GKTDSEG | 647.2995 | 675.2944 | KT | 202.1550 | 230.1499 |
| KTD | 317.1819 | 345.1769 | KTDS | 404.2140 | 432.2089 | KTDSE | 533.2566 | 561.2515 |
| KTDSEG | 590.2780 | 618.2729 | KTDSEGG | 647.2995 | 675.2944 | TD | 189.0870 | 217.0819 |
| TDS | 276.1190 | 304.1139 | TDSE | 405.1616 | 433.1565 | TDSEG | 462.1831 | 490.1780 |
| TDSEGG | 519.2045 | 547.1994 | TDSEGGF | 666.2729 | 694.2679 | DS | 175.0713 | 203.0662 |
| DSE | 304.1139 | 332.1088 | DSEG | 361.1354 | 389.1303 | DSEGG | 418.1569 | 446.1518 |
| DSEGGF | 565.2253 | 593.2202 | DSEGGFE | 694.2679 | 722.2628 | SE | 189.0870 | 217.0819 |
| SEG | 246.1084 | 274.1034 | SEGG | 303.1299 | 331.1248 | SEGGF | 450.1983 | 478.1932 |
| SEGGFE | 579.2409 | 607.2358 | SEGGFES | 666.2729 | 694.2679 | EG | 159.0764 | 187.0713 |
| EGG | 216.0979 | 244.0928 | EGGF | 363.1663 | 391.1612 | EGGFE | 492.2089 | 520.2038 |
| EGGFES | 579.2409 | 607.2358 | EGGFESD | 694.2679 | 722.2628 | GG | 87.0553 | 115.0502 |
| GGF | 234.1237 | 262.1186 | GGFE | 363.1663 | 391.1612 | GGFES | 450.1983 | 478.1932 |
| GGFESD | 565.2253 | 593.2202 | GGFESDA | 636.2624 | 664.2573 | GF | 177.1022 | 205.0972 |
| GFE | 306.1448 | 334.1397 | GFES | 393.1769 | 421.1718 | GFESD | 508.2038 | 536.1987 |
| GFESDA | 579.2409 | 607.2358 | GFESDAV | 678.3093 | 706.3042 | FE | 249.1234 | 277.1183 |
| FES | 336.1554 | 364.1503 | FESD | 451.1823 | 479.1773 | FESDA | 522.2195 | 550.2144 |
| FESDAV | 621.2879 | 649.2828 | FESDAVA | 692.3250 | 720.3199 | ES | 189.0870 | 217.0819 |
| ESD | 304.1139 | 332.1088 | ESDA | 375.1510 | 403.1460 | ESDAV | 474.2195 | 502.2144 |
| ESDAVA | 545.2566 | 573.2515 | ESDAVAT | 646.3042 | 674.2992 | SD | 175.0713 | 203.0662 |
| SDA | 246.1084 | 274.1034 | SDAV | 345.1769 | 373.1718 | SDAVA | 416.2140 | 444.2089 |
| SDAVAT | 517.2617 | 545.2566 | SDAVATA | 588.2988 | 616.2937 | DA | 159.0764 | 187.0713 |
| DAV | 258.1448 | 286.1397 | DAVA | 329.1819 | 357.1769 | DAVAT | 430.2296 | 458.2245 |
| DAVATA | 501.2667 | 529.2617 | DAVATAN | 615.3097 | 643.3046 | AV | 143.1179 | 171.1128 |
| AVA | 214.1550 | 242.1499 | AVAT | 315.2027 | 343.1976 | AVATA | 386.2398 | 414.2347 |
| AVATAN | 500.2827 | 528.2776 | AVATANI | 613.3668 | 641.3617 | VA | 143.1179 | 171.1128 |
| VAT | 244.1656 | 272.1605 | VATA | 315.2027 | 343.1976 | VATAN | 429.2456 | 457.2405 |
| VATANI | 542.3297 | 570.3246 | VATANIL | 655.4137 | 683.4087 | AT | 145.0972 | 173.0921 |
| ATA | 216.1343 | 244.1292 | ATAN | 330.1772 | 358.1721 | ATANI | 443.2613 | 471.2562 |
| ATANIL | 556.3453 | 584.3402 | ATANILE | 685.3879 | 713.3828 | TA | 145.0972 | 173.0921 |
| TAN | 259.1401 | 287.1350 | TANI | 372.2241 | 400.2191 | TANIL | 485.3082 | 513.3031 |
| TANILE | 614.3508 | 642.3457 | AN | 158.0924 | 186.0873 | ANI | 271.1765 | 299.1714 |
| ANIL | 384.2605 | 412.2554 | ANILE | 513.3031 | 541.2980 | ANILES | 600.3352 | 628.3301 |
| ANILESS | 687.3672 | 715.3621 | NI | 200.1394 | 228.1343 | NIL | 313.2234 | 341.2183 |
| NILE | 442.2660 | 470.2609 | NILES | 529.2980 | 557.2930 | NILESS | 616.3301 | 644.3250 |
| NILESSA | 687.3672 | 715.3621 | IL | 199.1805 | 227.1754 | ILE | 328.2231 | 356.2180 |
| | | | | | | | | |

| | | | | | | | | |
|---|---|---|---|---|---|---|---|---|
| **ILES** | 415.2551 | 443.2500 | **ILESS** | 502.2871 | 530.2821 | **ILESSA** | 573.3243 | 601.3192 |
| **ILESSAP** | 670.3770 | 698.3719 | **LE** | 215.1390 | 243.1339 | **LES** | 302.1710 | 330.1660 |
| **LESS** | 389.2031 | 417.1980 | **LESSA** | 460.2402 | 488.2351 | **LESSAP** | 557.2930 | 585.2879 |
| **LESSAPV** | 656.3614 | 684.3563 | **ES** | 189.0870 | 217.0819 | **ESS** | 276.1190 | 304.1139 |
| **ESSA** | 347.1561 | 375.1510 | **ESSAP** | 444.2089 | 472.2038 | **ESSAPV** | 543.2773 | 571.2722 |
| **ESSAPVV** | 642.3457 | 670.3406 | **ESSAPVVG** | 699.3672 | 727.3621 | **SS** | 147.0764 | 175.0713 |
| **SSA** | 218.1135 | 246.1084 | **SSAP** | 315.1663 | 343.1612 | **SSAPV** | 414.2347 | 442.2296 |
| **SSAPVV** | 513.3031 | 541.2980 | **SSAPVVG** | 570.3246 | 598.3195 | **SSAPVVGG** | 627.3461 | 655.3410 |
| **SA** | 131.0815 | 159.0764 | **SAP** | 228.1343 | 256.1292 | **SAPV** | 327.2027 | 355.1976 |
| **SAPVV** | 426.2711 | 454.2660 | **SAPVVG** | 483.2926 | 511.2875 | **SAPVVGG** | 540.3140 | 568.3089 |
| **AP** | 141.1022 | 169.0972 | **APV** | 240.1707 | 268.1656 | **APVV** | 339.2391 | 367.2340 |
| **APVVG** | 396.2605 | 424.2554 | **APVVGG** | 453.2820 | 481.2769 | **PV** | 169.1335 | 197.1285 |
| **PVV** | 268.2020 | 296.1969 | **PVVG** | 325.2234 | 353.2183 | **PVVGG** | 382.2449 | 410.2398 |
| **VV** | 171.1492 | 199.1441 | **VVG** | 228.1707 | 256.1656 | **VVGG** | 285.1921 | 313.1870 |
| **VG** | 129.1022 | 157.0972 | **VGG** | 186.1237 | 214.1186 | **GG** | 87.0553 | 115.0502 |

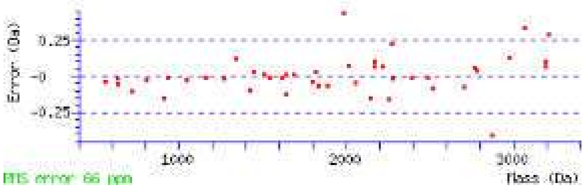


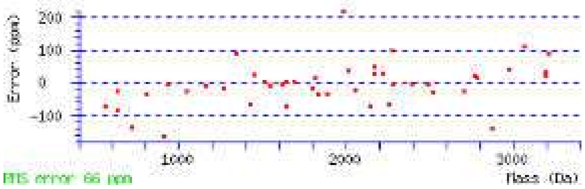


NCBI **BLAST** search of QAYSGKTDSEGGFESDAVATANILESSAPVVGGK
(Parameters: blastp, nr protein database, expect=20000, no filter, PAM30)
Other BLAST web gateways

**All matches to this query**

| Score | Mr(calc) | Delta | Sequence |
|---|---|---|---|
| 115.4 | 3341.5899 | 0.4433 | QAYSGKTDSEGGFESDAVATANILESSAPVVGGK |
| 1.7 | 3342.6112 | -0.5780 | KACSEPISVPTNDANVGAMPTYLSSFSLSGVK |
| 1.2 | 3342.4301 | -0.3969 | INMETEVLELFNHAEDNMEKGMDMWER |
| 1.2 | 3341.7383 | 0.2949 | DYLVKPAQMWWPQNLVQVSLFRTLHEK |
| 0.0 | 3341.4704 | 0.5628 | DEIQARTDNAVGTYGYMSPEYAMDGVISEK |

**Mascot:** http://www.matrixscience.com/

# Mascot Search Results

## Peptide View Spot no 28

MS/MS Fragmentation of **FFVGGNWK**
Found in **gi|553107** in **NCBInr**, triosephosphate isomerase, partial [Oryza sativa Japonica Group]

Match to Query 7: 953.494984 from(954.502260,1+) intensity(0.0000) index(1)
Title: Label: J9, Spot_Id: 219811, Peak_List_Id: 226895, MSMS Job_Run_Id: 21859, Comment:
Data file C:\Documents and Settings\Administrator\Desktop\Farah-Garima 11May 2013\ppw_J9_136859946600.txt

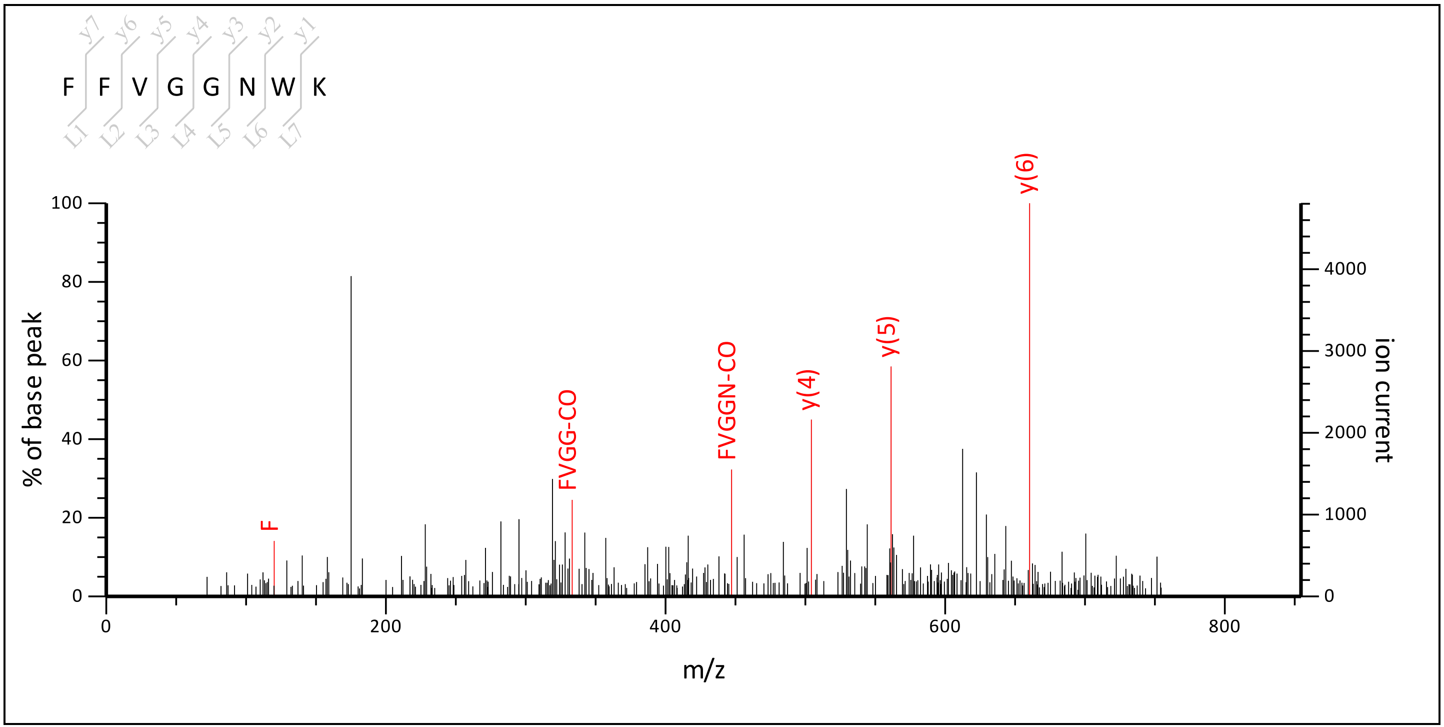


0 to 854.48

Label all possible matches Label matches used for scoring

**Monoisotopic mass of neutral peptide Mr(calc):** 953.4760
**Fixed modifications:** Carbamidomethyl (C) (apply to specified residues or termini only)
**Ions Score:** 26 **Expect:** 83
**Matches :** 10/81 fragment ions using 14 most intense peaks (help)

| # | Immon. | a | a* | b | b* | d | Seq. | v | w | y | y* | # |
|---|---|---|---|---|---|---|---|---|---|---|---|---|
| 1 | 120.0808 | **120.0808** | | 148.0757 | | 44.0495 | F | | | | | 8 |
| 2 | 120.0808 | 267.1492 | | 295.1441 | | | F | 715.3522 | | 807.4148 | 790.3883 | 7 |
| 3 | 72.0808 | 366.2176 | | 394.2125 | | 352.2020 | V | 616.2838 | 629.3042 | ***660.3464*** | 643.3198 | 6 |
| 4 | 30.0338 | 423.2391 | | 451.2340 | | | G | | | ***561.2780*** | 544.2514 | 5 |
| 5 | 30.0338 | 480.2605 | | 508.2554 | | | G | | | ***504.2565*** | 487.2300 | 4 |
| 6 | 87.0553 | 594.3035 | 577.2769 | 622.2984 | 605.2718 | 551.2976 | N | 388.1979 | 387.2027 | ***447.2350*** | 430.2085 | 3 |
| 7 | 159.0917 | 780.3828 | 763.3562 | 808.3777 | 791.3511 | | W | 202.1186 | | ***333.1921*** | 316.1656 | 2 |
| 8 | 101.1073 | | | | | | K | 74.0237 | 73.0284 | 147.1128 | 130.0863 | 1 |

| Seq | ya | yb | Seq | ya | yb | Seq | ya | yb |
|---|---|---|---|---|---|---|---|---|
| **FV** | 219.1492 | 247.1441 | **FVG** | 276.1707 | 304.1656 | **FVGG** | 333.1921 | 361.1870 |
| **FVGGN** | 447.2350 | 475.2300 | **FVGGNW** | 633.3144 | 661.3093 | **VG** | 129.1022 | 157.0972 |
| **VGG** | 186.1237 | 214.1186 | **VGGN** | 300.1666 | 328.1615 | **VGGNW** | 486.2459 | 514.2409 |
| **GG** | 87.0553 | 115.0502 | **GGN** | 201.0982 | 229.0931 | **GGNW** | 387.1775 | 415.1724 |
| **GN** | 144.0768 | 172.0717 | **GNW** | 330.1561 | 358.1510 | **NW** | 273.1346 | 301.1295 |

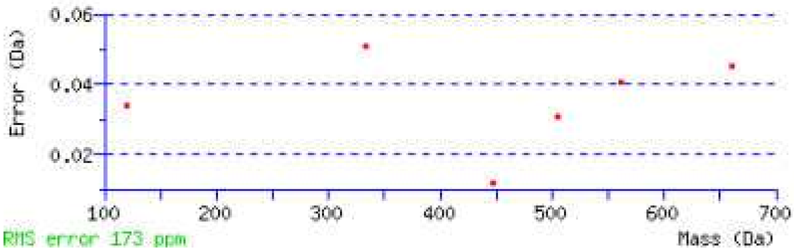


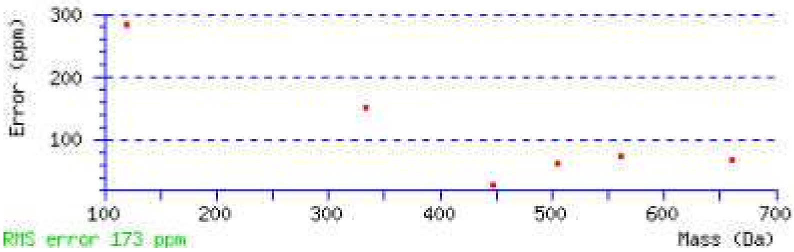


NCBI **BLAST** search of FFVGGNWK
(Parameters: blastp, nr protein database, expect=20000, no filter, PAM30)
Other BLAST web gateways

**All matches to this query**

| Score | Mr(calc) | Delta | Sequence |
|---|---|---|---|
| 28.8 | 953.4832 | 0.0118 | FFRGQGSR |
| 28.8 | 953.4832 | 0.0118 | FFRGQSGR |
| 27.8 | 953.5083 | -0.0133 | FFVNGSKR |
| 27.8 | 953.4389 | 0.0561 | MFVNSANR |
| 27.7 | 953.4641 | 0.0309 | MFVEIGSR |
| 26.9 | 953.4641 | 0.0309 | MFVNKDGK |
| 26.5 | 953.4059 | 0.0891 | MDMNKSGR |
| 26.5 | 953.4059 | 0.0891 | MDMNKSGR |
| 25.6 | 953.4760 | 0.0190 | FFVGGNWK |
| 25.6 | 953.4760 | 0.0190 | FFVGGNWK |

**Mascot:** http://www.matrixscience.com/

# MATRIX SCIENCE Mascot Search Results

## Peptide View **Spot no 28**

MS/MS Fragmentation of **VIACVGETLEQR**
Found in **gi|553107** in **NCBInr**, triosephosphate isomerase, partial [Oryza sativa Japonica Group]

Match to Query 55: 1373.738124 from(1374.745400,1+) intensity(0.0000) index(14)
Title: Label: J9, Spot_Id: 219811, Peak_List_Id: 226893, MSMS Job_Run_Id: 21859, Comment:
Data file C:\Documents and Settings\Administrator\Desktop\Farah-Garima 11May 2013\ppw_J9_136859946600.txt

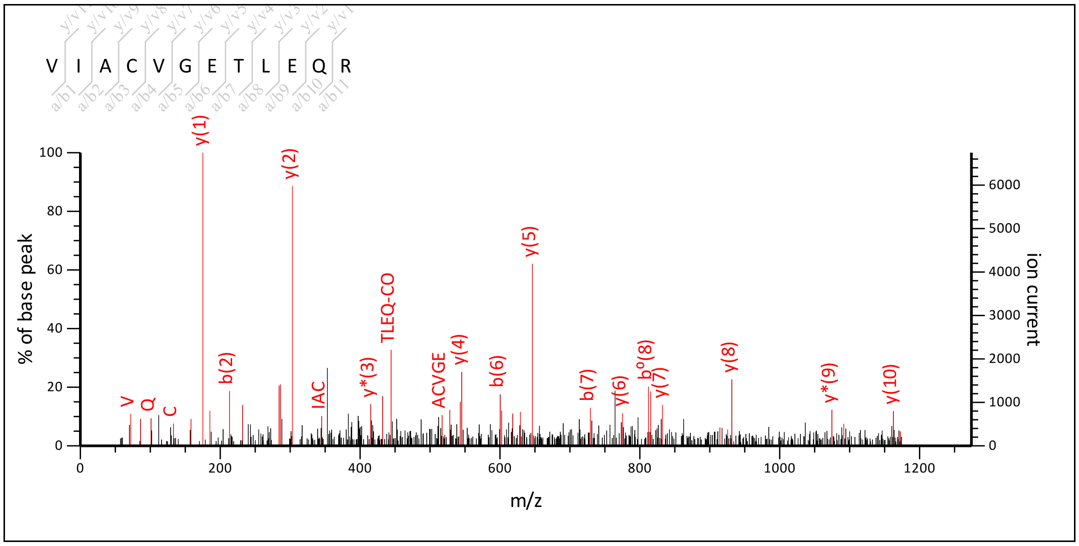


0 to 1274.12

Label all possible matches ○ Label matches used for scoring ◉

**Monoisotopic mass of neutral peptide Mr(calc):** 1373.6973
**Fixed modifications:** Carbamidomethyl (C) (apply to specified residues or termini only)
**Ions Score:** 68 **Expect:** 0.0055
**Matches :** 48/179 fragment ions using 60 most intense peaks (help)

| # | Immon. | a | a* | $a^0$ | b | b* | $b^0$ | d | d' | Seq. | v | w | w' | y | y* | |
|---|---|---|---|---|---|---|---|---|---|---|---|---|---|---|---|---|
| 1 | 72.0808 | **72.0808** | | | 100.0757 | | | 44.0495 | | V | | | | | | |
| 2 | 86.0964 | **185.1648** | | | **213.1598** | | | 157.1335 | 171.1492 | I | 1217.5579 | 1230.5783 | 1244.5940 | 1275.6362 | 1258.6096 | 125[illegible] |
| 3 | 44.0495 | 256.2020 | | | **284.1969** | | | | | A | 1146.5208 | | | ***1162.5521*** | 1145.5256 | 114[illegible] |
| 4 | 133.0430 | 416.2326 | | | **444.2275** | | | 327.2391 | | C | 986.4902 | 985.4949 | | ***1091.5150*** | 1074.4884 | 107[illegible] |
| 5 | 72.0808 | 515.3010 | | | **543.2959** | | | 501.2854 | | V | 887.4217 | 900.4421 | | ***931.4843*** | 914.4578 | 91[illegible] |
| 6 | 30.0338 | 572.3225 | | | **600.3174** | | | | | G | | | | ***832.4159*** | 815.3894 | 81[illegible] |
| 7 | 102.0550 | 701.3651 | | 683.3545 | **729.3600** | | 711.3494 | 643.3596 | | E | 701.3577 | 700.3624 | | ***775.3945*** | 758.3679 | 75[illegible] |
| 8 | 74.0600 | 802.4128 | | 784.4022 | 830.4077 | | 812.3971 | 786.4178 | 788.3971 | T | 600.3100 | 613.3304 | 615.3097 | ***646.3519*** | 629.3253 | 62[illegible] |
| 9 | 86.0964 | 915.4968 | | 897.4863 | 943.4917 | | 925.4812 | 873.4499 | | L | 487.2259 | 486.2307 | | ***545.3042*** | 528.2776 | 52[illegible] |
| 10 | 102.0550 | 1044.5394 | | 1026.5288 | 1072.5343 | | 1054.5238 | 986.5339 | | E | 358.1833 | 357.1881 | | ***432.2201*** | 415.1936 | 41[illegible] |
| 11 | 101.0709 | **1172.5980** | 1155.5714 | 1154.5874 | 1200.5929 | 1183.5664 | 1182.5823 | 1115.5765 | | Q | 230.1248 | 229.1295 | | ***303.1775*** | 286.1510 | |
| 12 | 129.1135 | | | | | | | | | R | 74.0237 | 73.0284 | | ***175.1190*** | 158.0924 | |

| Seq | ya | yb | Seq | ya | yb | Seq | ya | yb |
|---|---|---|---|---|---|---|---|---|
| IA | 157.1335 | 185.1285 | IAC | 317.1642 | 345.1591 | IACV | 416.2326 | 444.2275 |
| IACVG | 473.2541 | 501.2490 | IACVGE | 602.2967 | 630.2916 | AC | 204.0801 | 232.0750 |
| ACV | 303.1485 | 331.1435 | ACVG | 360.1700 | 388.1649 | ACVGE | 489.2126 | 517.2075 |
| ACVGET | 590.2603 | 618.2552 | CV | 232.1114 | 260.1063 | CVG | 289.1329 | 317.1278 |
| CVGE | 418.1755 | 446.1704 | CVGET | 519.2232 | 547.2181 | CVGETL | 632.3072 | 660.3021 |
| VG | 129.1022 | 157.0972 | VGE | 258.1448 | 286.1397 | VGET | 359.1925 | 387.1874 |
| VGETL | 472.2766 | 500.2715 | VGETLE | 601.3192 | 629.3141 | GE | 159.0764 | 187.0713 |
| GET | 260.1241 | 288.1190 | GETL | 373.2082 | 401.2031 | GETLE | 502.2508 | 530.2457 |
| GETLEQ | 630.3093 | 658.3042 | ET | 203.1026 | 231.0975 | ETL | 316.1867 | 344.1816 |

| | | | | | | | | |
|---|---|---|---|---|---|---|---|---|
| **ETLE** | 445.2293 | 473.2242 | **ETLEQ** | 573.2879 | 601.2828 | **TL** | 187.1441 | 215.1390 |
| **TLE** | 316.1867 | 344.1816 | **TLEQ** | 444.2453 | 472.2402 | **LE** | 215.1390 | 243.1339 |
| **LEQ** | 343.1976 | 371.1925 | **EQ** | 230.1135 | 258.1084 | | | |

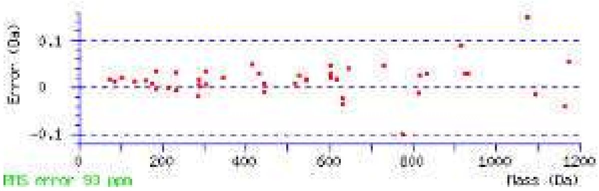


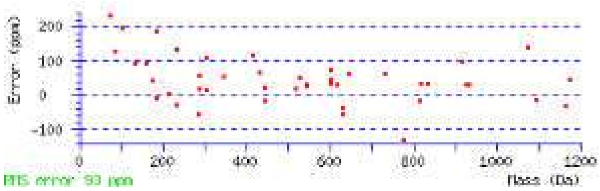


NCBI **BLAST** search of VIACVGETLEQR
(Parameters: blastp, nr protein database, expect=20000, no filter, PAM30)
Other BLAST web gateways

**All matches to this query**

| Score | Mr(calc) | Delta | Sequence |
|---|---|---|---|
| 68.0 | 1373.6973 | 0.0408 | VIACVGETLEQR |
| 58.0 | 1373.6973 | 0.0408 | VIACVGETLQER |
| 39.6 | 1373.6972 | 0.0409 | QMLAADEAIEKR |
| 37.0 | 1373.6575 | 0.0806 | NWDIAETELQR |
| 37.0 | 1373.7701 | -0.0319 | LVCSGLVTELKR |
| 36.4 | 1373.7337 | 0.0044 | IVSCADITVLAGR |
| 36.1 | 1373.6609 | 0.0772 | CVALEGEPGVSTR |
| 35.3 | 1373.7150 | 0.0231 | AITEQTLSDALGR |
| 33.8 | 1373.7263 | 0.0119 | ATGITRETLEGAR |
| 33.8 | 1373.7336 | 0.0045 | DSQIAMIAELKR |

**Mascot:** http://www.matrixscience.com/

# MATRIX SCIENCE Mascot Search Results

## Peptide View **Spot no 28**

MS/MS Fragmentation of **WLAANVSAEVAESTR**
Found in **gi|553107** in **NCBInr**, triosephosphate isomerase, partial [Oryza sativa Japonica Group]

Match to Query 71: 1602.848324 from(1603.855600,1+) intensity(0.0000) index(20)
Title: Label: J9, Spot_Id: 219811, Peak_List_Id: 226889, MSMS Job_Run_Id: 21859, Comment:
Data file C:\Documents and Settings\Administrator\Desktop\Farah-Garima 11May 2013\ppw_J9_136859946600.txt

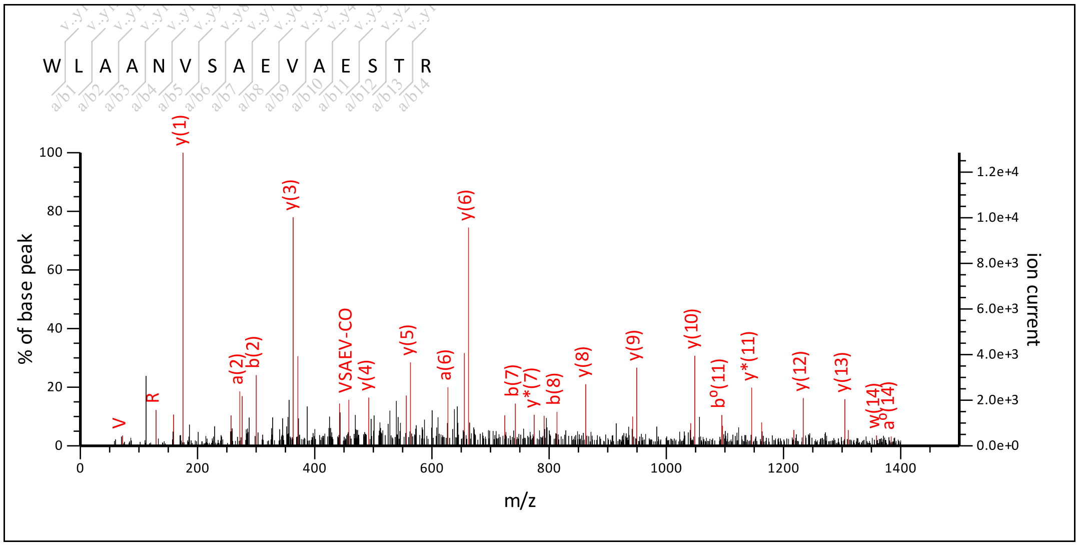


0 to 1499.97

Label all possible matches ○ Label matches used for scoring ◉

**Monoisotopic mass of neutral peptide Mr(calc):** 1602.8002
**Fixed modifications:** Carbamidomethyl (C) (apply to specified residues or termini only)
**Ions Score:** 137 **Expect:** 6e-10
**Matches :** 53/270 fragment ions using 42 most intense peaks (help)

| # | Immon. | a | a* | a0 | b | b* | b0 | d | d' | Seq. | v | w | w' | y | y* | |
|---|---|---|---|---|---|---|---|---|---|---|---|---|---|---|---|---|
| 1 | 159.0917 | **159.0917** | | | 187.0866 | | | 44.0495 | | W | | | | | | |
| 2 | 86.0964 | **272.1757** | | | **300.1707** | | | 230.1288 | | L | 1359.6499 | 1358.6546 | | 1417.7281 | 1400.7016 | 13[illegible] |
| 3 | 44.0495 | 343.2129 | | | **371.2078** | | | | | A | 1288.6128 | | | ***1304.6441*** | 1287.6175 | 12[illegible] |
| 4 | 44.0495 | 414.2500 | | | **442.2449** | | | | | A | 1217.5757 | | | ***1233.6070*** | 1216.5804 | 12[illegible] |
| 5 | 87.0553 | 528.2929 | 511.2663 | | **556.2878** | 539.2613 | | 485.2871 | | N | 1103.5327 | 1102.5375 | | ***1162.5699*** | 1145.5433 | 11[illegible] |
| 6 | 72.0808 | **627.3613** | 610.3348 | | **655.3562** | 638.3297 | | 613.3457 | | V | 1004.4643 | 1017.4847 | | ***1048.5269*** | 1031.5004 | 10[illegible] |
| 7 | 60.0444 | 714.3933 | 697.3668 | 696.3828 | **742.3882** | 725.3617 | 724.3777 | 698.3984 | | S | 917.4323 | 916.4371 | | ***949.4585*** | 932.4320 | 9[illegible] |
| 8 | 44.0495 | 785.4304 | 768.4039 | 767.4199 | **813.4254** | 796.3988 | 795.4148 | | | A | 846.3952 | | | ***862.4265*** | 845.3999 | 8[illegible] |
| 9 | 102.0550 | 914.4730 | 897.4465 | 896.4625 | **942.4680** | 925.4414 | 924.4574 | 856.4676 | | E | 717.3526 | 716.3573 | | ***791.3894*** | 774.3628 | 7[illegible] |
| 10 | 72.0808 | 1013.5415 | 996.5149 | 995.5309 | **1041.5364** | 1024.5098 | 1023.5258 | 999.5258 | | V | 618.2842 | 631.3046 | | ***662.3468*** | 645.3202 | 6[illegible] |
| 11 | 44.0495 | 1084.5786 | 1067.5520 | 1066.5680 | 1112.5735 | 1095.5469 | 1094.5629 | | | A | 547.2471 | | | ***563.2784*** | 546.2518 | 5[illegible] |
| 12 | 102.0550 | 1213.6212 | 1196.5946 | 1195.6106 | 1241.6161 | 1224.5895 | 1223.6055 | 1155.6157 | | E | 418.2045 | 417.2092 | | ***492.2413*** | 475.2147 | 4[illegible] |
| 13 | 60.0444 | 1300.6532 | 1283.6266 | 1282.6426 | 1328.6481 | 1311.6216 | 1310.6375 | 1284.6583 | | S | 331.1724 | 330.1772 | | ***363.1987*** | 346.1721 | 3[illegible] |
| 14 | 74.0600 | 1401.7009 | 1384.6743 | 1383.6903 | 1429.6958 | 1412.6692 | 1411.6852 | 1385.7060 | 1387.6852 | T | 230.1248 | 243.1452 | 245.1244 | ***276.1666*** | 259.1401 | 2[illegible] |
| 15 | 129.1135 | | | | | | | | | R | 74.0237 | 73.0284 | | ***175.1190*** | 158.0924 | |

| Seq | ya | yb | Seq | ya | yb | Seq | ya | yb |
|---|---|---|---|---|---|---|---|---|
| LA | 157.1335 | 185.1285 | LAA | 228.1707 | 256.1656 | LAAN | 342.2136 | 370.2085 |
| LAANV | 441.2820 | 469.2769 | LAANVS | 528.3140 | 556.3089 | LAANVSA | 599.3511 | 627.3461 |
| AA | 115.0866 | 143.0815 | AAN | 229.1295 | 257.1244 | AANV | 328.1979 | 356.1928 |
| AANVS | 415.2300 | 443.2249 | AANVSA | 486.2671 | 514.2620 | AANVSAE | 615.3097 | 643.3046 |
| AN | 158.0924 | 186.0873 | ANV | 257.1608 | 285.1557 | ANVS | 344.1928 | 372.1878 |
| ANVSA | 415.2300 | 443.2249 | ANVSAE | 544.2726 | 572.2675 | ANVSAEV | 643.3410 | 671.3359 |

| | | | | | | | | |
|---|---|---|---|---|---|---|---|---|
| **NV** | 186.1237 | 214.1186 | **NVS** | 273.1557 | 301.1506 | **NVSA** | 344.1928 | 372.1878 |
| **NVSAE** | 473.2354 | 501.2304 | **NVSAEV** | 572.3039 | 600.2988 | **NVSAEVA** | 643.3410 | 671.3359 |
| **VS** | 159.1128 | 187.1077 | **VSA** | 230.1499 | 258.1448 | **VSAE** | 359.1925 | 387.1874 |
| **VSAEV** | 458.2609 | 486.2558 | **VSAEVA** | 529.2980 | 557.2930 | **VSAEVAE** | 658.3406 | 686.3355 |
| **SA** | 131.0815 | 159.0764 | **SAE** | 260.1241 | 288.1190 | **SAEV** | 359.1925 | 387.1874 |
| **SAEVA** | 430.2296 | 458.2245 | **SAEVAE** | 559.2722 | 587.2671 | **SAEVAES** | 646.3042 | 674.2992 |
| **AE** | 173.0921 | 201.0870 | **AEV** | 272.1605 | 300.1554 | **AEVA** | 343.1976 | 371.1925 |
| **AEVAE** | 472.2402 | 500.2351 | **AEVAES** | 559.2722 | 587.2671 | **AEVAEST** | 660.3199 | 688.3148 |
| **EV** | 201.1234 | 229.1183 | **EVA** | 272.1605 | 300.1554 | **EVAE** | 401.2031 | 429.1980 |
| **EVAES** | 488.2351 | 516.2300 | **EVAEST** | 589.2828 | 617.2777 | **VA** | 143.1179 | 171.1128 |
| **VAE** | 272.1605 | 300.1554 | **VAES** | 359.1925 | 387.1874 | **VAEST** | 460.2402 | 488.2351 |
| **AE** | 173.0921 | 201.0870 | **AES** | 260.1241 | 288.1190 | **AEST** | 361.1718 | 389.1667 |
| **ES** | 189.0870 | 217.0819 | **EST** | 290.1347 | 318.1296 | **ST** | 161.0921 | 189.0870 |

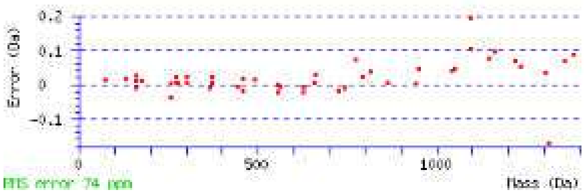


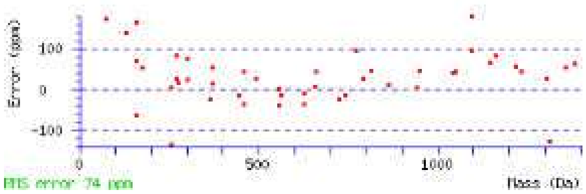


NCBI **BLAST** search of WLAANVSAEVAESTR
(Parameters: blastp, nr protein database, expect=20000, no filter, PAM30)
Other BLAST web gateways

**All matches to this query**

| Score | Mr(calc) | Delta | Sequence |
|---|---|---|---|
| 137.3 | 1602.8002 | 0.0482 | WLAANVSAEVAESTR |
| 34.3 | 1602.7896 | 0.0587 | GTLDAQGVANRMQAR |
| 34.3 | 1602.7494 | 0.0989 | MQSEADIAMVHLAR |
| 33.3 | 1602.8148 | 0.0336 | SVDLAAGRATVEAGMR |
| 33.0 | 1602.7784 | 0.0700 | TRSPNDAAEMAALTR |
| 33.0 | 1602.7889 | 0.0594 | DYYVNSSLVLSTSR |
| 32.8 | 1602.7121 | 0.1362 | LDAAAAADAAEEDSQR |
| 32.7 | 1602.7889 | 0.0594 | DKPSQVSWLAESEK |
| 30.4 | 1602.8399 | 0.0084 | KSNILVSAEDMQLR |
| 29.4 | 1602.7671 | 0.0812 | IAGASEGPSAEVASAMR |

**Mascot:** http://www.matrixscience.com/

# MATRIX SCIENCE Mascot Search Results

## Peptide View **Spot no 28**

MS/MS Fragmentation of **VATPDQAQEVHDGLR**
Found in **gi|553107** in **NCBInr**, triosephosphate isomerase, partial [Oryza sativa Japonica Group]

Match to Query 76: 1634.847224 from(1635.854500,1+) intensity(0.0000) index(21)
Title: Label: J9, Spot_Id: 219811, Peak_List_Id: 226913, MSMS Job_Run_Id: 21859, Comment:
Data file C:\Documents and Settings\Administrator\Desktop\Farah-Garima 11May 2013\ppw_J9_136859946600.txt

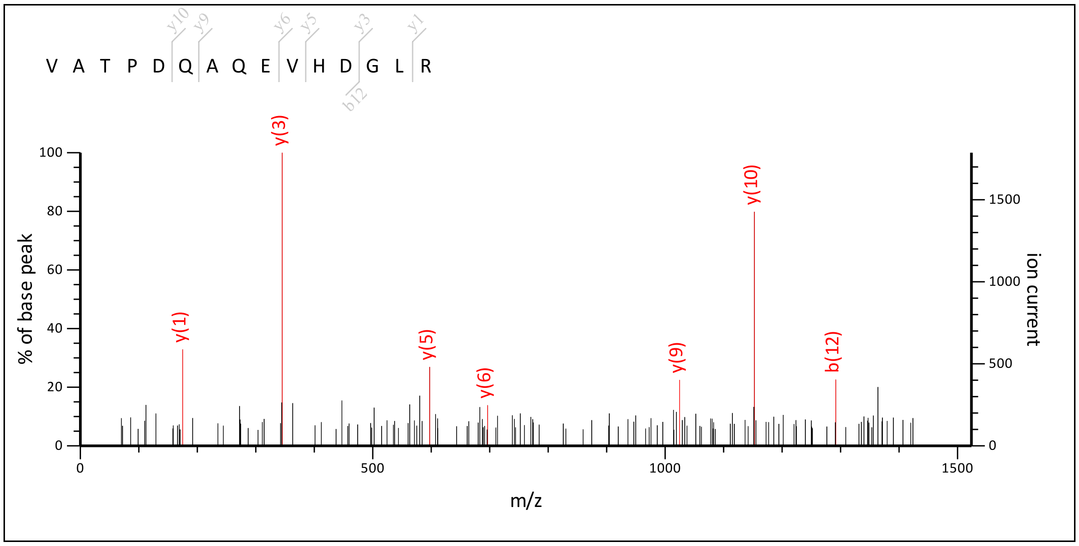


0 to 1523.75

Label all possible matches ○ Label matches used for scoring ◉

**Monoisotopic mass of neutral peptide Mr(calc):** 1634.8013
**Fixed modifications:** Carbamidomethyl (C) (apply to specified residues or termini only)
**Ions Score:** 31 **Expect:** 27
**Matches :** 7/260 fragment ions using 14 most intense peaks (help)

| # | Immon. | a | a* | a⁰ | b | b* | b⁰ | d | d' | Seq. | v | w | w' | y | y* | |
|---|---|---|---|---|---|---|---|---|---|---|---|---|---|---|---|---|
| 1 | 72.0808 | 72.0808 | | | 100.0757 | | | 44.0495 | | V | | | | | | |
| 2 | 44.0495 | 143.1179 | | | 171.1128 | | | | | A | 1520.7088 | | | 1536.7401 | 1519.7136 | 15[illegible] |
| 3 | 74.0600 | 244.1656 | | 226.1550 | 272.1605 | | 254.1499 | 228.1707 | 230.1499 | T | 1419.6611 | 1432.6815 | 1434.6608 | 1465.7030 | 1448.6764 | 14[illegible] |
| 4 | 70.0651 | 341.2183 | | 323.2078 | 369.2132 | | 351.2027 | 315.2027 | | P | 1322.6084 | 1321.6131 | | 1364.6553 | 1347.6288 | 13[illegible] |
| 5 | 88.0393 | 456.2453 | | 438.2347 | 484.2402 | | 466.2296 | 412.2554 | | D | 1207.5814 | 1206.5862 | | 1267.6026 | 1250.5760 | 12[illegible] |
| 6 | 101.0709 | 584.3039 | 567.2773 | 566.2933 | 612.2988 | 595.2722 | 594.2882 | 527.2824 | | Q | 1079.5228 | 1078.5276 | | ***1152.5756*** | 1135.5491 | 11[illegible] |
| 7 | 44.0495 | 655.3410 | 638.3144 | 637.3304 | 683.3359 | 666.3093 | 665.3253 | | | A | 1008.4857 | | | ***1024.5170*** | 1007.4905 | 10[illegible] |
| 8 | 101.0709 | 783.3995 | 766.3730 | 765.3890 | 811.3945 | 794.3679 | 793.3839 | 726.3781 | | Q | 880.4272 | 879.4319 | | 953.4799 | 936.4534 | 9[illegible] |
| 9 | 102.0550 | 912.4421 | 895.4156 | 894.4316 | 940.4371 | 923.4105 | 922.4265 | 854.4367 | | E | 751.3846 | 750.3893 | | 825.4213 | 808.3948 | 8[illegible] |
| 10 | 72.0808 | 1011.5106 | 994.4840 | 993.5000 | 1039.5055 | 1022.4789 | 1021.4949 | 997.4949 | | V | 652.3161 | 665.3365 | | ***696.3787*** | 679.3522 | 6[illegible] |
| 11 | 110.0713 | 1148.5695 | 1131.5429 | 1130.5589 | 1176.5644 | 1159.5378 | 1158.5538 | | | H | 515.2572 | | | ***597.3103*** | 580.2838 | 5[illegible] |
| 12 | 88.0393 | 1263.5964 | 1246.5699 | 1245.5858 | **1291.5913** | 1274.5648 | 1273.5808 | 1219.6066 | | D | 400.2303 | 399.2350 | | 460.2514 | 443.2249 | 4[illegible] |
| 13 | 30.0338 | 1320.6179 | 1303.5913 | 1302.6073 | 1348.6128 | 1331.5862 | 1330.6022 | | | G | | | | ***345.2245*** | 328.1979 | |
| 14 | 86.0964 | 1433.7019 | 1416.6754 | 1415.6914 | 1461.6969 | 1444.6703 | 1443.6863 | 1391.6550 | | L | 230.1248 | 229.1295 | | 288.2030 | 271.1765 | |
| 15 | 129.1135 | | | | | | | | | R | 74.0237 | 73.0284 | | ***175.1190*** | 158.0924 | |

| Seq | ya | yb | Seq | ya | yb | Seq | ya | yb |
|---|---|---|---|---|---|---|---|---|
| AT | 145.0972 | 173.0921 | ATP | 242.1499 | 270.1448 | ATPD | 357.1769 | 385.1718 |
| ATPDQ | 485.2354 | 513.2304 | ATPDQA | 556.2726 | 584.2675 | ATPDQAQ | 684.3311 | 712.3260 |
| TP | 171.1128 | 199.1077 | TPD | 286.1397 | 314.1347 | TPDQ | 414.1983 | 442.1932 |
| TPDQA | 485.2354 | 513.2304 | TPDQAQ | 613.2940 | 641.2889 | PD | 185.0921 | 213.0870 |
| PDQ | 313.1506 | 341.1456 | PDQA | 384.1878 | 412.1827 | PDQAQ | 512.2463 | 540.2413 |
| PDQAQE | 641.2889 | 669.2838 | DQ | 216.0979 | 244.0928 | DQA | 287.1350 | 315.1299 |

| | | | | | | | | |
|---|---|---|---|---|---|---|---|---|
| **DQAQ** | 415.1936 | 443.1885 | **DQAQE** | 544.2362 | 572.2311 | **DQAQEV** | 643.3046 | 671.2995 |
| **QA** | 172.1081 | 200.1030 | **QAQ** | 300.1666 | 328.1615 | **QAQE** | 429.2092 | 457.2041 |
| **QAQEV** | 528.2776 | 556.2726 | **QAQEVH** | 665.3366 | 693.3315 | **AQ** | 172.1081 | 200.1030 |
| **AQE** | 301.1506 | 329.1456 | **AQEV** | 400.2191 | 428.2140 | **AQEVH** | 537.2780 | 565.2729 |
| **AQEVHD** | 652.3049 | 680.2998 | **QE** | 230.1135 | 258.1084 | **QEV** | 329.1819 | 357.1769 |
| **QEVH** | 466.2409 | 494.2358 | **QEVHD** | 581.2678 | 609.2627 | **QEVHDG** | 638.2893 | 666.2842 |
| **EV** | 201.1234 | 229.1183 | **EVH** | 338.1823 | 366.1772 | **EVHD** | 453.2092 | 481.2041 |
| **EVHDG** | 510.2307 | 538.2256 | **EVHDGL** | 623.3148 | 651.3097 | **VH** | 209.1397 | 237.1346 |
| **VHD** | 324.1666 | 352.1615 | **VHDG** | 381.1881 | 409.1830 | **VHDGL** | 494.2722 | 522.2671 |
| **HD** | 225.0982 | 253.0931 | **HDG** | 282.1197 | 310.1146 | **HDGL** | 395.2037 | 423.1987 |
| **DG** | 145.0608 | 173.0557 | **DGL** | 258.1448 | 286.1397 | **GL** | 143.1179 | 171.1128 |

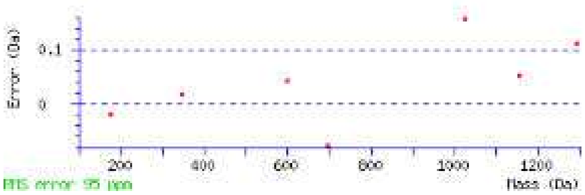


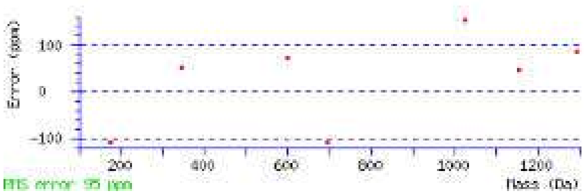


NCBI **BLAST** search of VATPDQAQEVHDGLR
(Parameters: blastp, nr protein database, expect=20000, no filter, PAM30)
Other BLAST web gateways

**All matches to this query**

| Score | Mr(calc) | Delta | Sequence |
|---|---|---|---|
| 30.6 | 1634.8013 | 0.0460 | VATPDQAQEVHDGLR |
| 22.2 | 1634.8601 | -0.0129 | QLNQQGLTRHDLGR |
| 14.4 | 1634.9355 | -0.0883 | KIQLEQLSVLPPDR |
| 13.9 | 1634.9104 | -0.0632 | IDVRPAVDTLPGGIGR |
| 13.7 | 1635.0083 | -0.1611 | SLLVAGATALAAPLVLR |
| 13.2 | 1634.8628 | -0.0155 | KESQGALILVDGYSR |
| 12.9 | 1634.8297 | 0.0175 | AALDLMTSLQEKSGR |
| 12.9 | 1634.9290 | -0.0818 | KGINAILGPCIGPLGR |
| 12.4 | 1634.8239 | 0.0233 | LLEQGAVCFSWLGR |
| 12.4 | 1634.8273 | 0.0200 | ALLWGGATLGVGMAMR |

**Mascot:** http://www.matrixscience.com/

# MATRIX SCIENCE Mascot Search Results

## Peptide View Spot no 28

MS/MS Fragmentation of **SQLRPEIQVAAQNCWVK**
Found in **gi|553107** in **NCBInr**, triosephosphate isomerase, partial [Oryza sativa Japonica Group]

Match to Query 105: 2026.095624 from(2027.102900,1+) intensity(0.0000) index(30)
Title: Label: J9, Spot_Id: 219811, Peak_List_Id: 226888, MSMS Job_Run_Id: 21859, Comment:
Data file C:\Documents and Settings\Administrator\Desktop\Farah-Garima 11May 2013\ppw_J9_136859946600.txt

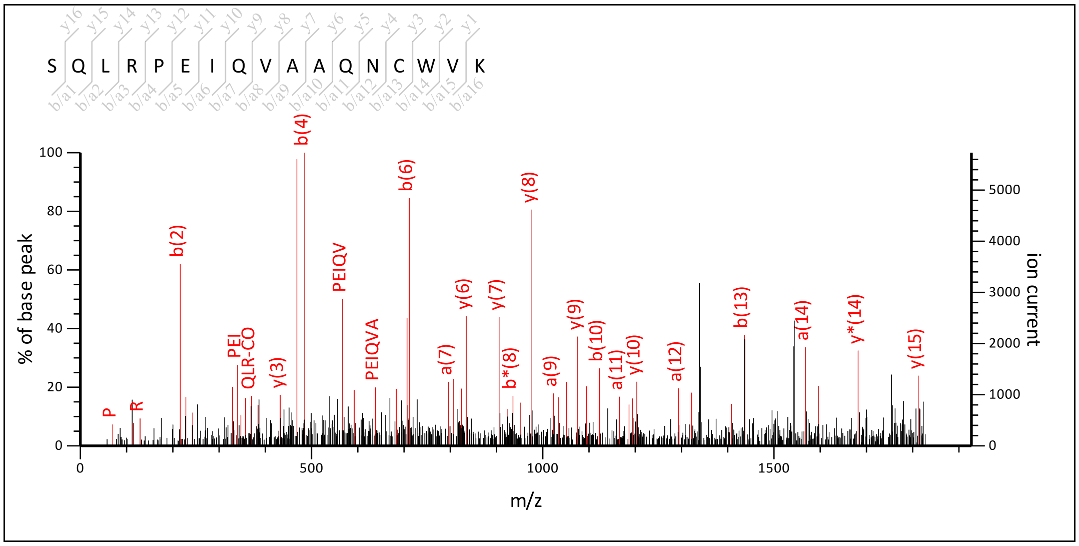


0 to 1927.01

Label all possible matches ○ Label matches used for scoring ◉

**Monoisotopic mass of neutral peptide Mr(calc):** 2026.0418
**Fixed modifications:** Carbamidomethyl (C) (apply to specified residues or termini only)
**Ions Score:** 79 **Expect:** 0.00034
**Matches :** 57/305 fragment ions using 72 most intense peaks (help)

| # | Immon. | a | a* | a⁰ | b | b* | b⁰ | d | d' | Seq. | v | w | w' | y | y* | |
|---|---|---|---|---|---|---|---|---|---|---|---|---|---|---|---|---|
| 1 | 60.0444 | 60.0444 | | 42.0338 | 88.0393 | | 70.0287 | 44.0495 | | S | | | | | | |
| 2 | 101.0709 | 188.1030 | 171.0764 | 170.0924 | **216.0979** | 199.0713 | 198.0873 | 131.0815 | | Q | 1866.9643 | 1865.9691 | | 1940.0171 | 1922.9905 | 192 |
| 3 | 86.0964 | 301.1870 | 284.1605 | 283.1765 | **329.1819** | 312.1554 | 311.1714 | 259.1401 | | L | 1753.8803 | 1752.8850 | | **1811.9585** | 1794.9320 | 179 |
| 4 | 129.1135 | 457.2881 | 440.2616 | 439.2776 | **485.2831** | 468.2565 | 467.2725 | 372.2241 | | R | 1597.7791 | 1596.7839 | | 1698.8744 | 1681.8479 | 168 |
| 5 | 70.0651 | 554.3409 | 537.3144 | 536.3303 | 582.3358 | 565.3093 | 564.3253 | 528.3253 | | P | 1500.7264 | 1499.7311 | | 1542.7733 | 1525.7468 | 152 |
| 6 | 102.0550 | **683.3835** | 666.3569 | 665.3729 | **711.3784** | 694.3519 | 693.3678 | 625.3780 | | E | 1371.6838 | 1370.6885 | | 1445.7206 | 1428.6940 | 142 |
| 7 | 86.0964 | **796.4676** | 779.4410 | 778.4570 | **824.4625** | 807.4359 | 806.4519 | 768.4363 | 782.4519 | I | 1258.5997 | 1271.6201 | 1285.6358 | 1316.6780 | 1299.6514 | |
| 8 | 101.0709 | **924.5261** | 907.4996 | 906.5156 | **952.5211** | 935.4945 | 934.5105 | 867.5047 | | Q | 1130.5411 | 1129.5459 | | **1203.5939** | 1186.5674 | |
| 9 | 72.0808 | **1023.5946** | 1006.5680 | 1005.5840 | **1051.5895** | 1034.5629 | 1033.5789 | 1009.5789 | | V | 1031.4727 | 1044.4931 | | **1075.5353** | 1058.5088 | |
| 10 | 44.0495 | **1094.6317** | 1077.6051 | 1076.6211 | **1122.6266** | 1105.6000 | 1104.6160 | | | A | 960.4356 | | | **976.4669** | 959.4404 | |
| 11 | 44.0495 | **1165.6688** | 1148.6422 | 1147.6582 | **1193.6637** | 1176.6371 | 1175.6531 | | | A | 889.3985 | | | **905.4298** | 888.4033 | |
| 12 | 101.0709 | **1293.7274** | 1276.7008 | 1275.7168 | **1321.7223** | 1304.6957 | 1303.7117 | 1236.7059 | | Q | 761.3399 | 760.3447 | | **834.3927** | 817.3661 | |
| 13 | 87.0553 | **1407.7703** | 1390.7437 | 1389.7597 | **1435.7652** | 1418.7387 | 1417.7546 | 1364.7645 | | N | 647.2970 | 646.3017 | | **706.3341** | 689.3076 | |
| 14 | 133.0430 | **1567.8009** | 1550.7744 | 1549.7904 | **1595.7959** | 1578.7693 | 1577.7853 | 1478.8074 | | C | 487.2663 | 486.2711 | | **592.2912** | 575.2646 | |
| 15 | 159.0917 | 1753.8803 | 1736.8537 | 1735.8697 | 1781.8752 | 1764.8486 | 1763.8646 | | | W | 301.1870 | | | **432.2605** | 415.2340 | |
| 16 | 72.0808 | 1852.9487 | 1835.9221 | 1834.9381 | 1880.9436 | 1863.9170 | 1862.9330 | 1838.9330 | | V | 202.1186 | 215.1390 | | 246.1812 | 229.1547 | |
| 17 | 101.1073 | | | | | | | | | K | 74.0237 | 73.0284 | | 147.1128 | 130.0863 | |

| Seq | ya | yb | Seq | ya | yb | Seq | ya | yb |
|---|---|---|---|---|---|---|---|---|
| QL | 214.1550 | 242.1499 | QLR | 370.2561 | 398.2510 | QLRP | 467.3089 | 495.3038 |
| QLRPE | 596.3515 | 624.3464 | LR | 242.1975 | 270.1925 | LRP | 339.2503 | 367.2452 |
| LRPE | 468.2929 | 496.2878 | LRPEI | 581.3770 | 609.3719 | RP | 226.1662 | 254.1612 |
| RPE | 355.2088 | 383.2037 | RPEI | 468.2929 | 496.2878 | RPEIQ | 596.3515 | 624.3464 |

| | | | | | | | | |
|---|---|---|---|---|---|---|---|---|
| **RPEIQV** | 695.4199 | 723.4148 | **PE** | 199.1077 | 227.1026 | **PEI** | 312.1918 | 340.1867 |
| **PEIQ** | 440.2504 | 468.2453 | **PEIQV** | 539.3188 | 567.3137 | **PEIQVA** | 610.3559 | 638.3508 |
| **PEIQVAA** | 681.3930 | 709.3879 | **EI** | 215.1390 | 243.1339 | **EIQ** | 343.1976 | 371.1925 |
| **EIQV** | 442.2660 | 470.2609 | **EIQVA** | 513.3031 | 541.2980 | **EIQVAA** | 584.3402 | 612.3352 |
| **IQ** | 214.1550 | 242.1499 | **IQV** | 313.2234 | 341.2183 | **IQVA** | 384.2605 | 412.2554 |
| **IQVAA** | 455.2976 | 483.2926 | **IQVAAQ** | 583.3562 | 611.3511 | **IQVAAQN** | 697.3992 | 725.3941 |
| **QV** | 200.1394 | 228.1343 | **QVA** | 271.1765 | 299.1714 | **QVAA** | 342.2136 | 370.2085 |
| **QVAAQ** | 470.2722 | 498.2671 | **QVAAQN** | 584.3151 | 612.3100 | **VA** | 143.1179 | 171.1128 |
| **VAA** | 214.1550 | 242.1499 | **VAAQ** | 342.2136 | 370.2085 | **VAAQN** | 456.2565 | 484.2514 |
| **VAAQNC** | 616.2872 | 644.2821 | **AA** | 115.0866 | 143.0815 | **AAQ** | 243.1452 | 271.1401 |
| **AAQN** | 357.1881 | 385.1830 | **AAQNC** | 517.2187 | 545.2137 | **AQ** | 172.1081 | 200.1030 |
| **AQN** | 286.1510 | 314.1459 | **AQNC** | 446.1816 | 474.1765 | **AQNCW** | 632.2609 | 660.2559 |
| **QN** | 215.1139 | 243.1088 | **QNC** | 375.1445 | 403.1394 | **QNCW** | 561.2238 | 589.2187 |
| **QNCWV** | 660.2922 | 688.2872 | **NC** | 247.0859 | 275.0809 | **NCW** | 433.1652 | 461.1602 |
| **NCWV** | 532.2337 | 560.2286 | **CW** | 319.1223 | 347.1172 | **CWV** | 418.1907 | 446.1857 |
| **WV** | 258.1601 | 286.1550 | | | | | | |

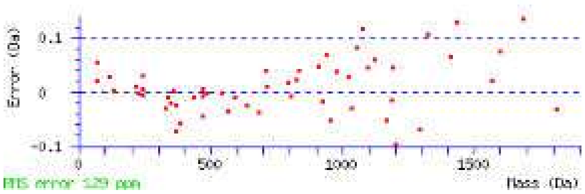


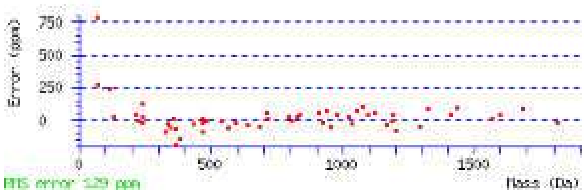


NCBI **BLAST** search of SQLRPEIQVAAQNCWVK
(Parameters: blastp, nr protein database, expect=20000, no filter, PAM30)
Other BLAST web gateways

**All matches to this query**

| Score | Mr(calc) | Delta | Sequence |
|---|---|---|---|
| 78.5 | 2026.0418 | 0.0538 | SQLRPEIQVAAQNCWVK |
| 29.1 | 2026.1324 | -0.0368 | DVPAGAVTLAGVAHVPAIGGVR |
| 24.0 | 2026.1211 | -0.0255 | VKEINIAPSVNLPPPDAPR |
| 22.3 | 2026.1146 | -0.0190 | VVRVLPGMQAAFVDIGQAR |
| 21.9 | 2026.1575 | -0.0619 | LILQVAVGSAASSGVFLAAPR |
| 21.4 | 2025.9136 | 0.1821 | CSPSYGGENVAAQLAEVMK |
| 19.4 | 2026.0378 | 0.0578 | QSLSLLAVQAMAQSDRHR |
| 19.1 | 2026.0847 | 0.0109 | RLDPSIPSNAAVFELIER |
| 18.7 | 2026.1397 | -0.0441 | LLLRIPQTAPAMAALFER |
| 18.2 | 2026.1609 | -0.0652 | LTGGGIALIVGAGMALSAIGRL |

**Mascot:** http://www.matrixscience.com/

# MATRIX SCIENCE Mascot Search Results

## Peptide View Spot no 29

MS/MS Fragmentation of **GVNPWIEVDGGVGPK**
Found in **gi|4105561** in **NCBInr**, ribulose-5-phosphate-3-epimerase [Oryza sativa]

Match to Query 77: 1522.849324 from(1523.856600,1+) intensity(0.0000) index(21)
Title: Label: J20, Spot_Id: 219987, Peak_List_Id: 229446, MSMS Job_Run_Id: 22021, Comment:
Data file C:\Documents and Settings\Administrator\Desktop\Farah-Garima 11May 2013\ppw_J20_136868359600.txt

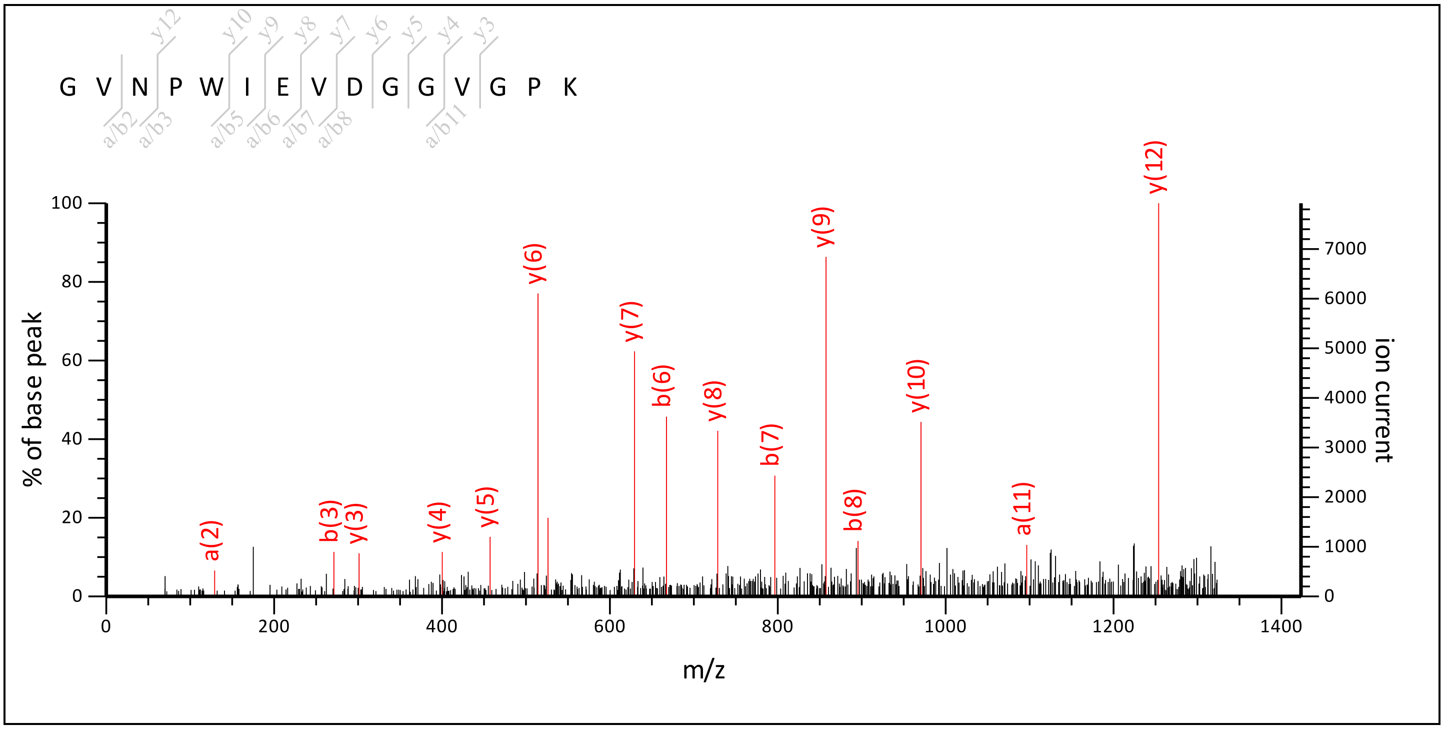


0 to 1423.3

Label all possible matches ○ Label matches used for scoring ●

**Monoisotopic mass of neutral peptide Mr(calc):** 1522.7780
**Fixed modifications:** Carbamidomethyl (C) (apply to specified residues or termini only)
**Ions Score:** 63 **Expect:** 0.00085
**Matches :** 16/80 fragment ions using 26 most intense peaks (help)

| # | a | a* | b | b* | Seq. | y | y* | # |
|---|---|---|---|---|---|---|---|---|
| 1 | 30.0338 | | 58.0287 | | G | | | 15 |
| 2 | **129.1022** | | 157.0972 | | V | 1466.7638 | 1449.7373 | 14 |
| 3 | 243.1452 | 226.1186 | **271.1401** | 254.1135 | N | 1367.6954 | 1350.6688 | 13 |
| 4 | 340.1979 | 323.1714 | 368.1928 | 351.1663 | P | ***1253.6525*** | 1236.6259 | 12 |
| 5 | **526.2772** | 509.2507 | 554.2722 | 537.2456 | W | 1156.5997 | 1139.5732 | 11 |
| 6 | 639.3613 | 622.3348 | **667.3562** | 650.3297 | I | ***970.5204*** | 953.4938 | 10 |
| 7 | 768.4039 | 751.3774 | **796.3988** | 779.3723 | E | ***857.4363*** | 840.4098 | 9 |
| 8 | 867.4723 | 850.4458 | **895.4672** | 878.4407 | V | ***728.3937*** | 711.3672 | 8 |

| | | | | | | | | |
|---|---|---|---|---|---|---|---|---|
| 9 | 982.4993 | 965.4727 | 1010.4942 | 993.4676 | D | 629.3253 | 612.2988 | 7 |
| 10 | 1039.5207 | 1022.4942 | 1067.5156 | 1050.4891 | G | 514.2984 | 497.2718 | 6 |
| 11 | 1096.5422 | 1079.5156 | 1124.5371 | 1107.5106 | G | 457.2769 | 440.2504 | 5 |
| 12 | 1195.6106 | 1178.5840 | 1223.6055 | 1206.5790 | V | 400.2554 | 383.2289 | 4 |
| 13 | 1252.6321 | 1235.6055 | 1280.6270 | 1263.6004 | G | 301.1870 | 284.1605 | 3 |
| 14 | 1349.6848 | 1332.6583 | 1377.6797 | 1360.6532 | P | 244.1656 | 227.1390 | 2 |
| 15 | | | | | K | 147.1128 | 130.0863 | 1 |

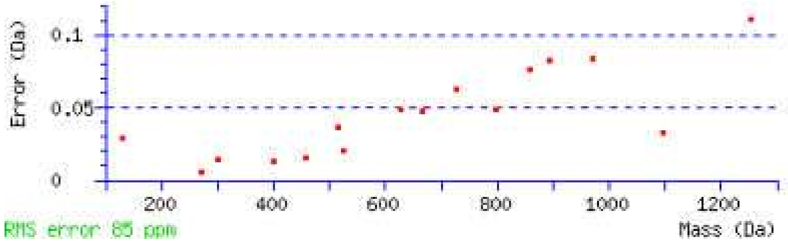


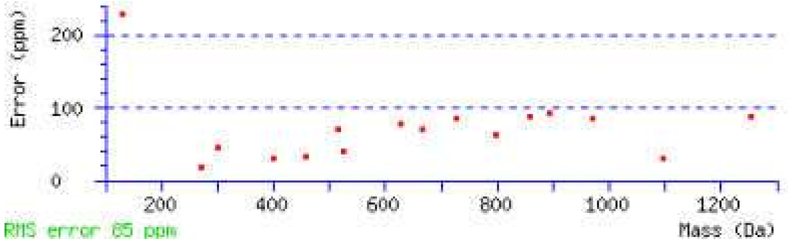


NCBI **BLAST** search of GVNPWIEVDGGVGPK
(Parameters: blastp, nr protein database, expect=20000, no filter, PAM30)
Other BLAST web gateways

**All matches to this query**

| Score | Mr(calc) | Delta | Sequence |
|---|---|---|---|
| 63.0 | 1522.7780 | 0.0713 | GVNPWIEVDGGVGPK |
| 22.3 | 1522.8943 | -0.0450 | NRGLLLEQQIALR |
| 13.6 | 1522.9096 | -0.0603 | RGGILGLLLAGWAAR |
| 11.8 | 1522.7739 | 0.0754 | NTQIGKSQLEYSR |
| 11.7 | 1522.8944 | -0.0450 | RIGVGAAGGGVLVLER |
| 9.2 | 1522.8103 | 0.0390 | QAQLDSITIPQGPR |
| 8.9 | 1522.7164 | 0.1329 | NLAENSATFGGTWR |
| 8.8 | 1522.8580 | -0.0086 | LNILVNNAGTGKPGR |
| 8.5 | 1522.7052 | 0.1441 | AKVWGDNQEDAYK |
| 7.9 | 1522.8216 | 0.0277 | RGQDPIVNVIEQR |

**Mascot:** http://www.matrixscience.com/

# MATRIX SCIENCE Mascot Search Results

## Peptide View Spot no 29

MS/MS Fragmentation of **NDIIVSPSILSANFSK**
Found in **gi|4105561** in **NCBInr**, ribulose-5-phosphate-3-epimerase [Oryza sativa]

Match to Query 89: 1703.984524 from(1704.991800,1+) intensity(0.0000) index(25)
Title: Label: J20, Spot_Id: 219987, Peak_List_Id: 229444, MSMS Job_Run_Id: 22021, Comment:
Data file C:\Documents and Settings\Administrator\Desktop\Farah-Garima 11May 2013\ppw_J20_136868359600.txt

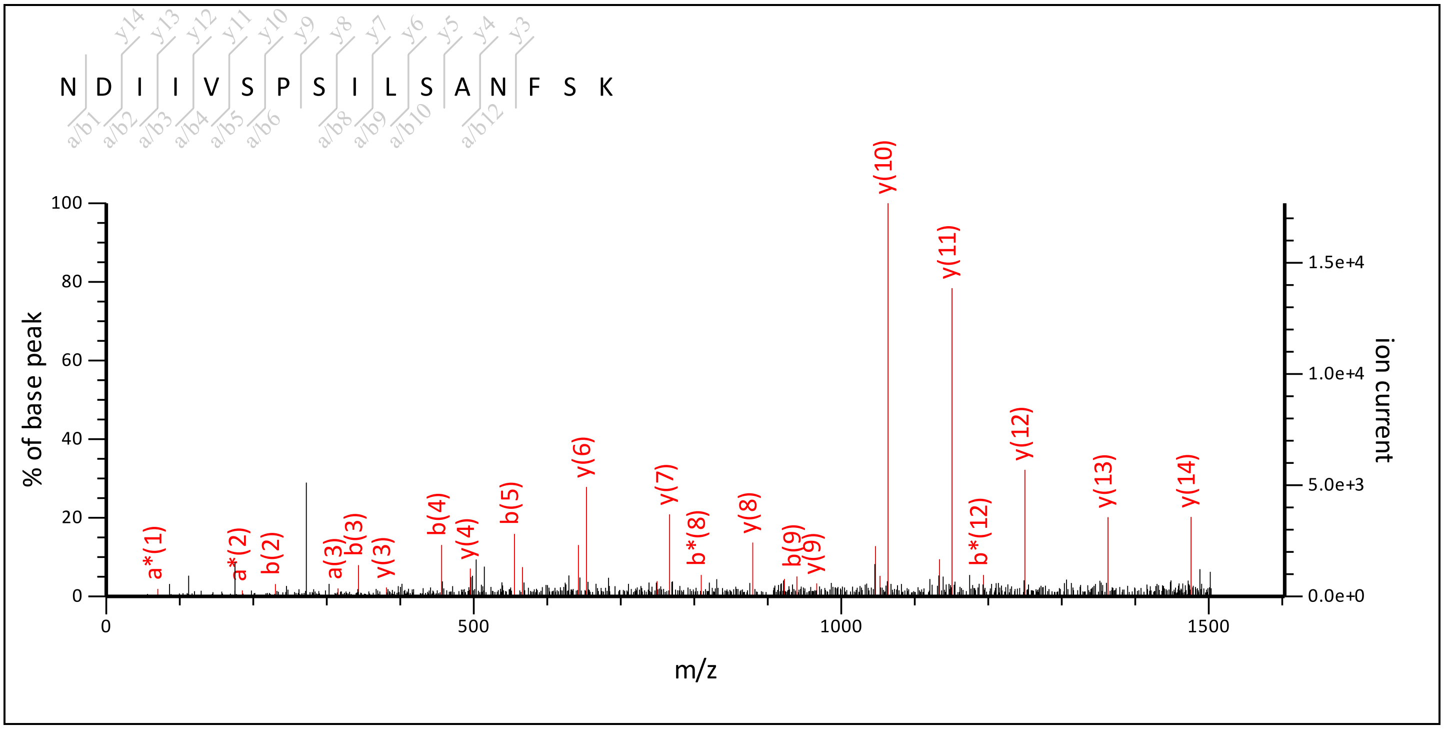


0 to 1603.33

Label all possible matches ○ Label matches used for scoring ◉

**Monoisotopic mass of neutral peptide Mr(calc):** 1703.9094
**Fixed modifications:** Carbamidomethyl (C) (apply to specified residues or termini only)
**Ions Score:** 53 **Expect:** 0.006
**Matches** : 28/90 fragment ions using 75 most intense peaks (help)

| # | a | a* | b | b* | Seq. | y | y* | # |
|---|---|---|---|---|---|---|---|---|
| 1 | 87.0553 | 70.0287 | 115.0502 | 98.0237 | N | | | 16 |
| 2 | 202.0822 | 185.0557 | **230.0771** | 213.0506 | D | 1590.8737 | 1573.8472 | 15 |
| 3 | 315.1663 | 298.1397 | **343.1612** | 326.1347 | I | ***1475.8468*** | 1458.8203 | 14 |
| 4 | 428.2504 | 411.2238 | **456.2453** | 439.2187 | I | ***1362.7627*** | 1345.7362 | 13 |
| 5 | 527.3188 | 510.2922 | **555.3137** | 538.2871 | V | ***1249.6787*** | 1232.6521 | 12 |
| 6 | 614.3508 | 597.3243 | **642.3457** | 625.3192 | S | ***1150.6103*** | 1133.5837 | 11 |
| 7 | 711.4036 | 694.3770 | 739.3985 | 722.3719 | P | ***1063.5782*** | 1046.5517 | 10 |
| 8 | 798.4356 | 781.4090 | 826.4305 | 809.4040 | S | ***966.5255*** | 949.4989 | 9 |

| | | | | | | | | |
|---|---|---|---|---|---|---|---|---|
| 9 | 911.5197 | 894.4931 | **939.5146** | 922.4880 | I | ***879.4934*** | 862.4669 | 8 |
| 10 | 1024.6037 | 1007.5772 | **1052.5986** | 1035.5721 | L | ***766.4094*** | 749.3828 | 7 |
| 11 | 1111.6357 | 1094.6092 | 1139.6307 | 1122.6041 | S | ***653.3253*** | 636.2988 | 6 |
| 12 | 1182.6729 | 1165.6463 | 1210.6678 | 1193.6412 | A | ***566.2933*** | 549.2667 | 5 |
| 13 | 1296.7158 | 1279.6892 | 1324.7107 | 1307.6842 | N | ***495.2562*** | 478.2296 | 4 |
| 14 | 1443.7842 | 1426.7577 | 1471.7791 | 1454.7526 | F | ***381.2132*** | 364.1867 | 3 |
| 15 | 1530.8162 | 1513.7897 | 1558.8111 | 1541.7846 | S | 234.1448 | 217.1183 | 2 |
| 16 | | | | | K | 147.1128 | 130.0863 | 1 |

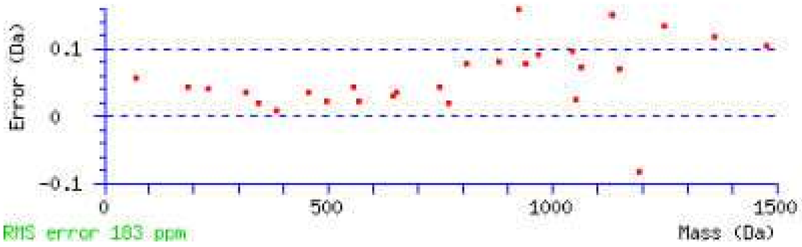


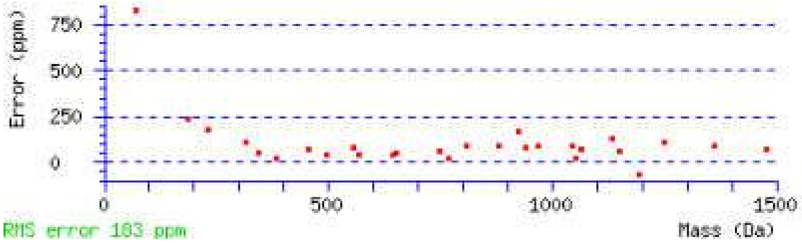


NCBI **BLAST** search of NDIIVSPSILSANFSK
(Parameters: blastp, nr protein database, expect=20000, no filter, PAM30)
Other BLAST web gateways

**All matches to this query**

| Score | Mr(calc) | Delta | Sequence |
|---|---|---|---|
| 53.5 | 1703.9094 | 0.0751 | NDIIVSPSILSANFSK |
| 45.9 | 1703.9458 | 0.0388 | KDVIVSPSILSANFSK |
| 17.5 | 1703.9682 | 0.0163 | SAALADLIPIRPSGAPR |
| 13.1 | 1703.9028 | 0.0817 | EEVFRTLIAMNLPR |
| 12.1 | 1703.9359 | 0.0486 | HLTTSLLSPLSPPWR |
| 11.7 | 1703.8665 | 0.1180 | GDGGGMYLSAIQAVLPR |
| 11.1 | 1703.8955 | 0.0890 | GEGGGNAVTVPLGLPPNR |
| 9.1 | 1703.8168 | 0.1677 | LWVWGQNQGSNYPR |
| 8.6 | 1703.9795 | 0.0051 | LVQGAPISASQPRPKR |
| 8.6 | 1703.8512 | 0.1333 | ERLQDALESIVEMR |

**Mascot:** http://www.matrixscience.com/

# MATRIX SCIENCE Mascot Search Results

## Peptide View Spot no 29

MS/MS Fragmentation of **AVEVAGCDWIHVDVMDGR**
Found in **gi|4105561** in **NCBInr**, ribulose-5-phosphate-3-epimerase [Oryza sativa]

Match to Query 95: 2028.020924 from(2029.028200,1+) intensity(0.0000) index(27)
Title: Label: J20, Spot_Id: 219987, Peak_List_Id: 229448, MSMS Job_Run_Id: 22021, Comment:
Data file C:\Documents and Settings\Administrator\Desktop\Farah-Garima 11May 2013\ppw_J20_136868359600.txt

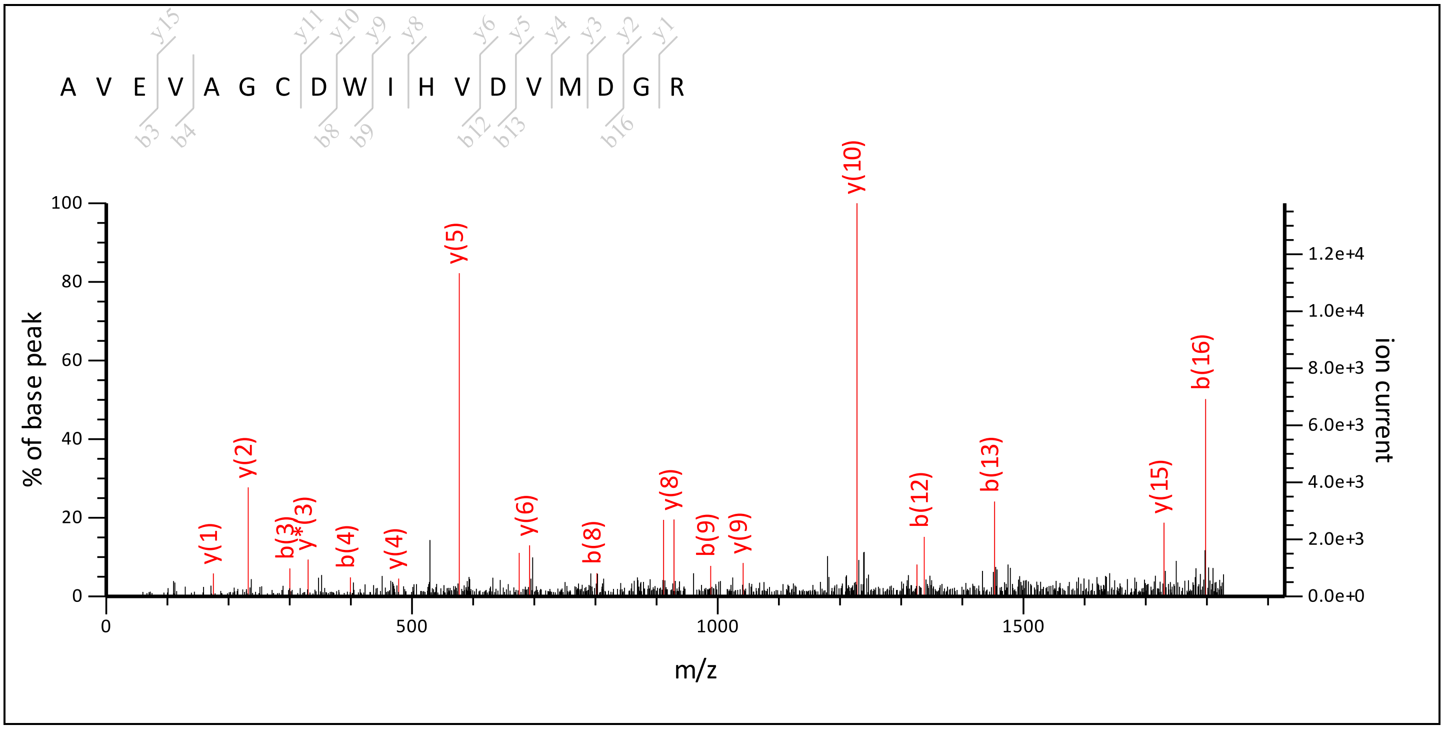


0 to 1927.21

Label all possible matches ○ Label matches used for scoring ◉

**Monoisotopic mass of neutral peptide Mr(calc):** 2027.9194
**Fixed modifications:** Carbamidomethyl (C) (apply to specified residues or termini only)
**Ions Score:** 49 **Expect:** 0.018
**Matches :** 20/68 fragment ions using 36 most intense peaks (help)

| # | a | b | Seq. | y | y* | # |
|---|---|---|---|---|---|---|
| 1 | 44.0495 | 72.0444 | A | | | 18 |
| 2 | 143.1179 | 171.1128 | V | 1957.8895 | 1940.8629 | 17 |
| 3 | 272.1605 | **300.1554** | E | 1858.8211 | 1841.7945 | 16 |
| 4 | 371.2289 | **399.2238** | V | ***1729.7785*** | 1712.7519 | 15 |
| 5 | 442.2660 | 470.2609 | A | 1630.7101 | 1613.6835 | 14 |
| 6 | 499.2875 | 527.2824 | G | 1559.6730 | 1542.6464 | 13 |
| 7 | 659.3181 | 687.3130 | C | 1502.6515 | 1485.6249 | 12 |
| 8 | 774.3451 | **802.3400** | D | 1342.6208 | 1325.5943 | 11 |

| | | | | | | |
|---|---|---|---|---|---|---|
| 9 | 960.4244 | 988.4193 | W | 1227.5939 | 1210.5674 | 10 |
| 10 | 1073.5084 | 1101.5034 | I | 1041.5146 | 1024.4880 | 9 |
| 11 | 1210.5674 | 1238.5623 | H | 928.4305 | 911.4040 | 8 |
| 12 | 1309.6358 | 1337.6307 | V | 791.3716 | 774.3451 | 7 |
| 13 | 1424.6627 | 1452.6576 | D | 692.3032 | 675.2767 | 6 |
| 14 | 1523.7311 | 1551.7260 | V | 577.2763 | 560.2497 | 5 |
| 15 | 1654.7716 | 1682.7665 | M | 478.2078 | 461.1813 | 4 |
| 16 | 1769.7986 | 1797.7935 | D | 347.1674 | 330.1408 | 3 |
| 17 | 1826.8200 | 1854.8149 | G | 232.1404 | 215.1139 | 2 |
| 18 | | | R | 175.1190 | 158.0924 | 1 |

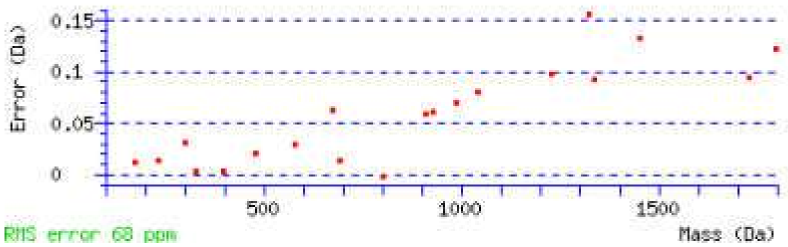


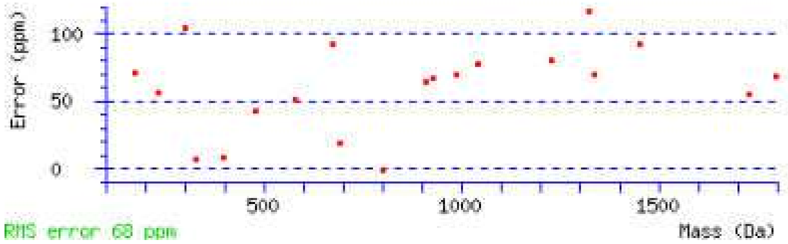


NCBI **BLAST** search of AVEVAGCDWIHVDVMDGR
(Parameters: blastp, nr protein database, expect=20000, no filter, PAM30)
Other BLAST web gateways

**All matches to this query**

| Score | Mr(calc) | Delta | Sequence |
|---|---|---|---|
| 49.0 | 2027.9194 | 0.1016 | AVEVAGCDWIHVDVMDGR |
| 22.7 | 2027.9194 | 0.1016 | AVELAGCDWVHVDVMDGR |
| 7.3 | 2027.9701 | 0.0508 | KLYGDNGGGVNYVHSGYTK |
| 4.4 | 2027.9775 | 0.0434 | FVNLMEGHIWIDSEGPGK |
| 2.9 | 2027.8855 | 0.1355 | ADSFVGDMERGTLAEDSGR |
| 0.9 | 2027.9185 | 0.1025 | RLLLGDDGSGGGEYEYDGR |
| 0.5 | 2028.1480 | -0.1271 | LGDHLRAGALPGVAGVPTSLK |
| 0.4 | 2027.9377 | 0.0832 | VKDYADVHWEYYQQGK |
| 0.3 | 2028.0245 | -0.0036 | VSLLCTQGDPQLRPTMGR |
| 0.1 | 2027.9872 | 0.0338 | LALAAAATETGADAAAAGENGGR |

**Mascot:** http://www.matrixscience.com/

# Mascot Search Results

## Peptide View **Spot no 29**

MS/MS Fragmentation of **AVEVAGCDWIHVDVMDGR**
Found in **gi|4105561** in **NCBInr**, ribulose-5-phosphate-3-epimerase [Oryza sativa]

Match to Query 97: 2044.010324 from(2045.017600,1+) intensity(0.0000) index(28)
Title: Label: J20, Spot_Id: 219987, Peak_List_Id: 229443, MSMS Job_Run_Id: 22021, Comment:
Data file C:\Documents and Settings\Administrator\Desktop\Farah-Garima 11May 2013\ppw_J20_136868359600.txt

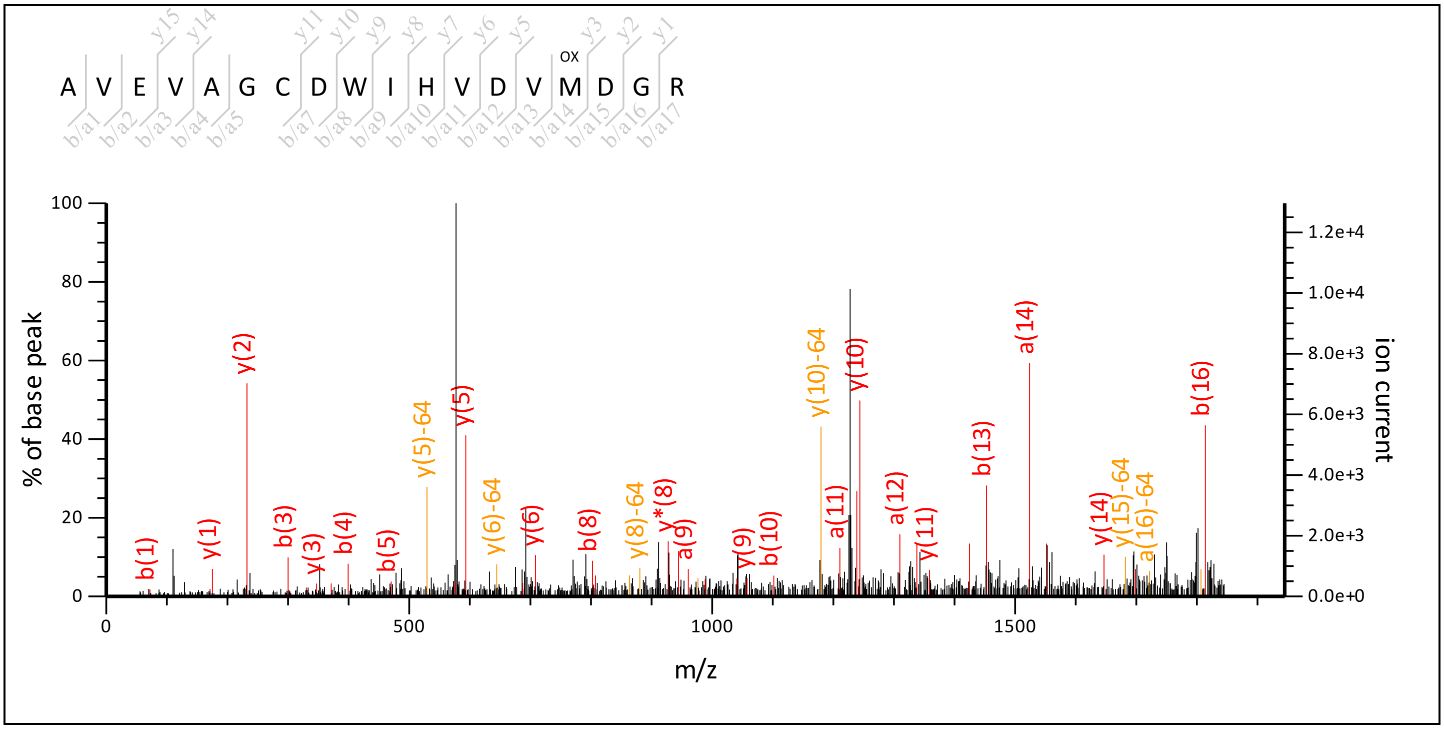


0 to 1944.91

Label all possible matches ○ Label matches used for scoring ◉

**Monoisotopic mass of neutral peptide Mr(calc):** 2043.9143
**Fixed modifications:** Carbamidomethyl (C) (apply to specified residues or termini only)
**Variable modifications:**
**M15** : Oxidation (M), with neutral losses 0.0000(shown in table), 63.9983
**Ions Score:** 42 **Expect:** 0.088
**Matches** : 45/102 fragment ions using 180 most intense peaks (help)

| # | a | b | Seq. | y | y* | # |
|---|---|---|---|---|---|---|
| 1 | 44.0495 | *72.0444* | A | | | 18 |
| 2 | 143.1179 | *171.1128* | V | 1973.8844 | 1956.8579 | 17 |
| 3 | 272.1605 | *300.1554* | E | 1874.8160 | 1857.7894 | 16 |
| 4 | **371.2289** | *399.2238* | V | 1745.7734 | 1728.7469 | 15 |
| 5 | 442.2660 | *470.2609* | A | **1646.7050** | 1629.6784 | 14 |
| 6 | 499.2875 | 527.2824 | G | 1575.6679 | 1558.6413 | 13 |
| 7 | 659.3181 | *687.3130* | C | 1518.6464 | 1501.6199 | 12 |

| | | | | | | |
|---|---|---|---|---|---|---|
| 8 | 774.3451 | 802.3400 | D | 1358.6158 | 1341.5892 | 11 |
| 9 | 960.4244 | 988.4193 | W | 1243.5888 | 1226.5623 | 10 |
| 10 | 1073.5084 | 1101.5034 | I | 1057.5095 | 1040.4830 | 9 |
| 11 | 1210.5674 | 1238.5623 | H | 944.4254 | 927.3989 | 8 |
| 12 | 1309.6358 | 1337.6307 | V | 807.3665 | 790.3400 | 7 |
| 13 | 1424.6627 | 1452.6576 | D | 708.2981 | 691.2716 | 6 |
| 14 | 1523.7311 | 1551.7260 | V | 593.2712 | 576.2446 | 5 |
| 15 | 1670.7665 | 1698.7614 | M | 494.2028 | 477.1762 | 4 |
| 16 | 1785.7935 | 1813.7884 | D | 347.1674 | 330.1408 | 3 |
| 17 | 1842.8149 | 1870.8098 | G | 232.1404 | 215.1139 | 2 |
| 18 | | | R | 175.1190 | 158.0924 | 1 |

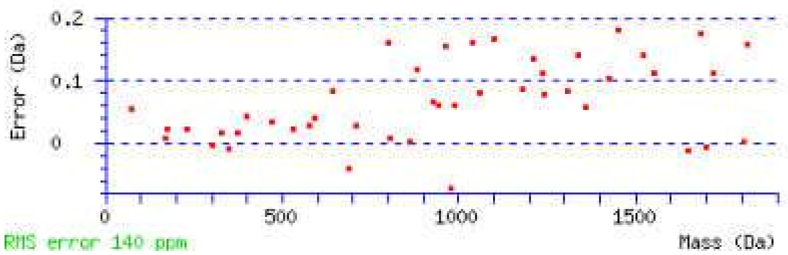


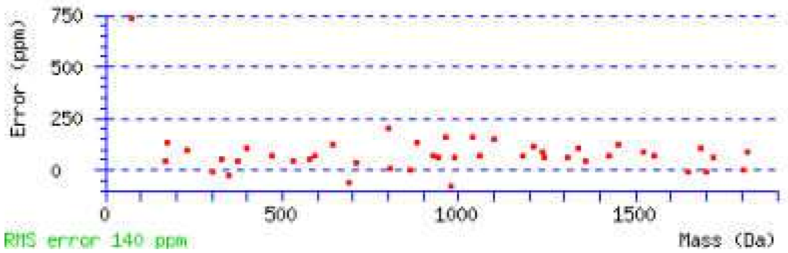


NCBI **BLAST** search of AVEVAGCDWIHVDVMDGR
(Parameters: blastp, nr protein database, expect=20000, no filter, PAM30)
Other BLAST web gateways

**All matches to this query**

| Score | Mr(calc) | Delta | Sequence |
|---|---|---|---|
| 42.0 | 2043.9143 | 0.0960 | AVEVAGCDWIHVDVMDGR |
| 16.5 | 2043.9143 | 0.0960 | AVELAGCDWVHVDVMDGR |
| 7.3 | 2043.9498 | 0.0606 | GATATSEATRDLYDDVFGR |
| 4.2 | 2043.9585 | 0.0518 | MYGAAYTGEGAAIVPSHHGR |
| 3.5 | 2044.0517 | -0.0414 | HTPEQLELLLYDLFEGK |
| 3.2 | 2044.0953 | -0.0850 | DDKLVAGALNLIGGDTLFGR |
| 1.6 | 2044.0751 | -0.0647 | HVGMAPFVMAALQVVFATR |

**Mascot:** http://www.matrixscience.com/

# MATRIX SCIENCE Mascot Search Results

## Peptide View Spot no 29

MS/MS Fragmentation of **AGADIVSVHCEQSSTIHLHR**
Found in **gi|4105561** in **NCBInr**, ribulose-5-phosphate-3-epimerase [Oryza sativa]

Match to Query 106: 2216.185824 from(2217.193100,1+) intensity(0.0000) index(31)
Title: Label: J20, Spot_Id: 219987, Peak_List_Id: 229442, MSMS Job_Run_Id: 22021, Comment:
Data file C:\Documents and Settings\Administrator\Desktop\Farah-Garima 11May 2013\ppw_J20_136868359600.txt

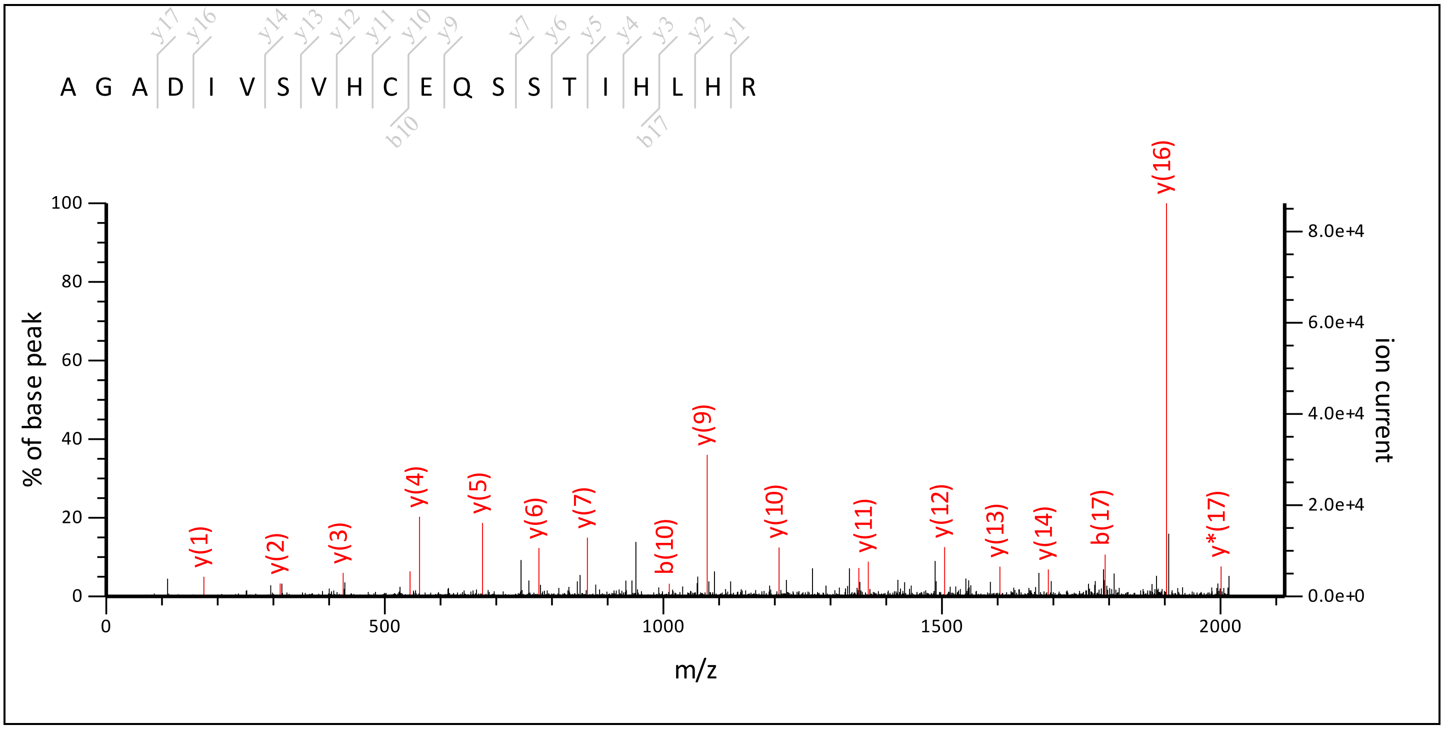


0 to 2115.19

Label all possible matches ○ Label matches used for scoring ●

**Monoisotopic mass of neutral peptide Mr(calc):** 2216.0757
**Fixed modifications:** Carbamidomethyl (C) (apply to specified residues or termini only)
**Ions Score:** 149 **Expect:** 1.7e-12
**Matches :** 19/92 fragment ions using 20 most intense peaks (help)

| # | a | a* | b | b* | Seq. | y | y* | # |
|---|---|---|---|---|---|---|---|---|
| 1 | 44.0495 | | 72.0444 | | A | | | 20 |
| 2 | 101.0709 | | 129.0659 | | G | 2146.0458 | 2129.0193 | 19 |
| 3 | 172.1081 | | 200.1030 | | A | 2089.0243 | 2071.9978 | 18 |
| 4 | 287.1350 | | 315.1299 | | D | 2017.9872 | 2000.9607 | 17 |
| 5 | 400.2191 | | 428.2140 | | I | ***1902.9603*** | 1885.9337 | 16 |
| 6 | 499.2875 | | 527.2824 | | V | 1789.8762 | 1772.8497 | 15 |
| 7 | 586.3195 | | 614.3144 | | S | ***1690.8078*** | 1673.7813 | 14 |
| 8 | 685.3879 | | 713.3828 | | V | ***1603.7758*** | 1586.7492 | 13 |

| | | | | | | | | |
|---|---|---|---|---|---|---|---|---|
| **9** | 822.4468 | | 850.4417 | | **H** | 1504.7074 | 1487.6808 | **12** |
| **10** | 982.4775 | | **1010.4724** | | **C** | 1367.6485 | 1350.6219 | **11** |
| **11** | 1111.5201 | | 1139.5150 | | **E** | 1207.6178 | 1190.5913 | **10** |
| **12** | 1239.5786 | 1222.5521 | 1267.5736 | 1250.5470 | **Q** | 1078.5752 | 1061.5487 | **9** |
| **13** | 1326.6107 | 1309.5841 | 1354.6056 | 1337.5790 | **S** | 950.5166 | 933.4901 | **8** |
| **14** | 1413.6427 | 1396.6162 | 1441.6376 | 1424.6111 | **S** | 863.4846 | 846.4581 | **7** |
| **15** | 1514.6904 | 1497.6638 | 1542.6853 | 1525.6587 | **T** | 776.4526 | 759.4260 | **6** |
| **16** | 1627.7744 | 1610.7479 | 1655.7694 | 1638.7428 | **I** | 675.4049 | 658.3784 | **5** |
| **17** | 1764.8334 | 1747.8068 | **1792.8283** | 1775.8017 | **H** | 562.3208 | 545.2943 | **4** |
| **18** | 1877.9174 | 1860.8909 | 1905.9123 | 1888.8858 | **L** | 425.2619 | 408.2354 | **3** |
| **19** | 2014.9763 | 1997.9498 | 2042.9713 | 2025.9447 | **H** | 312.1779 | 295.1513 | **2** |
| **20** | | | | | **R** | 175.1190 | 158.0924 | **1** |

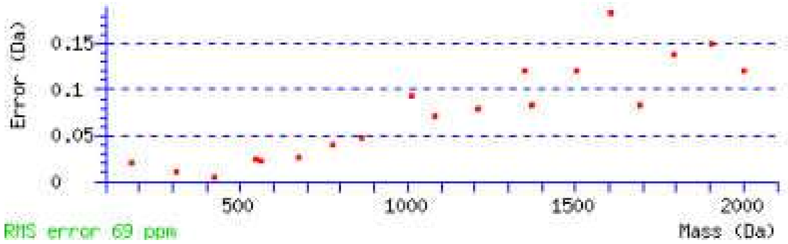


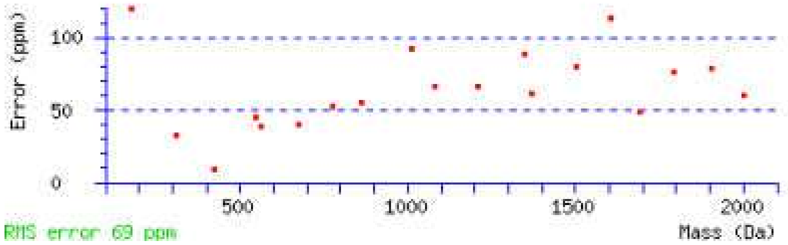


NCBI **BLAST** search of AGADIVSVHCEQSSTIHLHR
(Parameters: blastp, nr protein database, expect=20000, no filter, PAM30)
Other BLAST web gateways

**All matches to this query**

| Score | Mr(calc) | Delta | Sequence |
|---|---|---|---|
| 148.6 | 2216.0757 | 0.1102 | AGADIVSVHCEQSSTIHLHR |
| 12.5 | 2216.1524 | 0.0334 | IHGNKLAEMPFIATRPSYR |
| 12.3 | 2216.0875 | 0.0983 | GTRSTGNPHPIYNFLSYHR |
| 10.5 | 2216.0929 | 0.0929 | ELIGILNNNPEMSAECKLR |
| 7.5 | 2216.0202 | 0.1657 | MNYSNIQKVLGCSSTEVDR |
| 4.0 | 2216.0731 | 0.1127 | VFDIGEMARLCPHEHLHR |
| 3.9 | 2216.1082 | 0.0776 | GRTFQLTMASLLEMGYQVR |
| 3.9 | 2216.1082 | 0.0776 | GRTFQLTMASLLEMGYQVR |
| 2.6 | 2216.1915 | -0.0057 | YSLVSSKQMVGIFLTIWTK |
| 2.4 | 2216.0822 | 0.1037 | DAQVALDHLYQRTDIDTSR |

**Mascot:** http://www.matrixscience.com/

# Mascot Search Results

## Peptide View Spot no 29

MS/MS Fragmentation of **VIEAGANAIVAGSAVFGAPDYAEAIK**
Found in **gi|4105561** in **NCBInr**, ribulose-5-phosphate-3-epimerase [Oryza sativa]

Match to Query 112: 2503.411624 from(2504.418900,1+) intensity(0.0000) index(34)
Title: Label: J20, Spot_Id: 219987, Peak_List_Id: 229447, MSMS Job_Run_Id: 22021, Comment:
Data file C:\Documents and Settings\Administrator\Desktop\Farah-Garima 11May 2013\ppw_J20_136868359600.txt

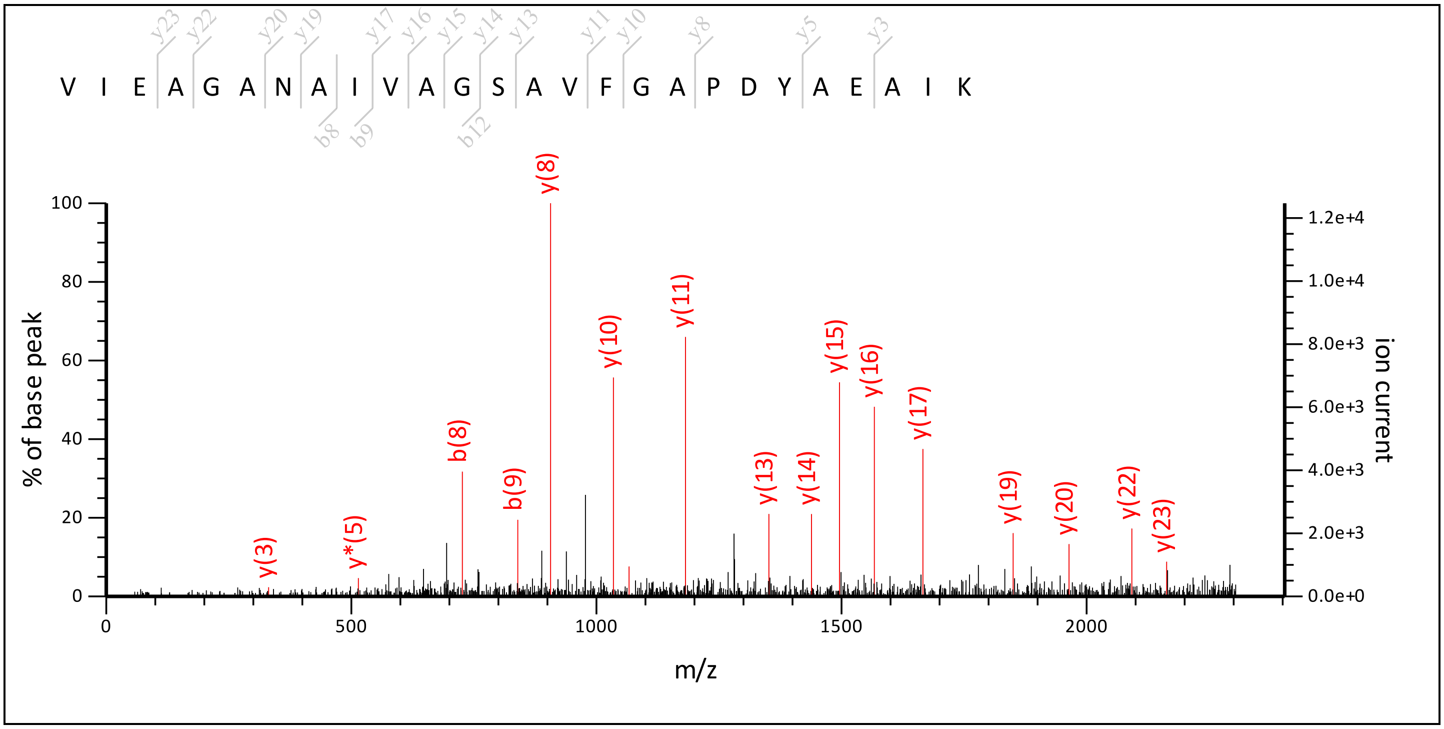


0 to 2404.11

Label all possible matches ○ Label matches used for scoring ◉

**Monoisotopic mass of neutral peptide Mr(calc):** 2503.2958
**Fixed modifications:** Carbamidomethyl (C) (apply to specified residues or termini only)
**Ions Score:** 102 **Expect:** 5.9e-08
**Matches :** 17/138 fragment ions using 23 most intense peaks (help)

| # | a | a* | b | b* | Seq. | y | y* | # |
|---|---|---|---|---|---|---|---|---|
| 1 | 72.0808 | | 100.0757 | | V | | | 26 |
| 2 | 185.1648 | | 213.1598 | | I | 2405.2347 | 2388.2082 | 25 |
| 3 | 314.2074 | | 342.2023 | | E | 2292.1507 | 2275.1241 | 24 |
| 4 | 385.2445 | | 413.2395 | | A | ***2163.1081*** | 2146.0815 | 23 |
| 5 | 442.2660 | | 470.2609 | | G | ***2092.0709*** | 2075.0444 | 22 |
| 6 | 513.3031 | | 541.2980 | | A | 2035.0495 | 2018.0229 | 21 |
| 7 | 627.3461 | 610.3195 | 655.3410 | 638.3144 | N | ***1964.0124*** | 1946.9858 | 20 |
| 8 | 698.3832 | 681.3566 | **726.3781** | 709.3515 | A | ***1849.9694*** | 1832.9429 | 19 |

| | | | | | | | | |
|---|---|---|---|---|---|---|---|---|
| 9 | 811.4672 | 794.4407 | **839.4621** | 822.4356 | I | 1778.9323 | 1761.9058 | 18 |
| 10 | 910.5356 | 893.5091 | 938.5306 | 921.5040 | V | ***1665.8483*** | 1648.8217 | 17 |
| 11 | 981.5728 | 964.5462 | 1009.5677 | 992.5411 | A | ***1566.7799*** | 1549.7533 | 16 |
| 12 | 1038.5942 | 1021.5677 | **1066.5891** | 1049.5626 | G | ***1495.7427*** | 1478.7162 | 15 |
| 13 | 1125.6262 | 1108.5997 | 1153.6212 | 1136.5946 | S | ***1438.7213*** | 1421.6947 | 14 |
| 14 | 1196.6634 | 1179.6368 | 1224.6583 | 1207.6317 | A | ***1351.6892*** | 1334.6627 | 13 |
| 15 | 1295.7318 | 1278.7052 | 1323.7267 | 1306.7001 | V | 1280.6521 | 1263.6256 | 12 |
| 16 | 1442.8002 | 1425.7736 | 1470.7951 | 1453.7686 | F | ***1181.5837*** | 1164.5572 | 11 |
| 17 | 1499.8217 | 1482.7951 | 1527.8166 | 1510.7900 | G | ***1034.5153*** | 1017.4888 | 10 |
| 18 | 1570.8588 | 1553.8322 | 1598.8537 | 1581.8271 | A | 977.4938 | 960.4673 | 9 |
| 19 | 1667.9115 | 1650.8850 | 1695.9064 | 1678.8799 | P | ***906.4567*** | 889.4302 | 8 |
| 20 | 1782.9385 | 1765.9119 | 1810.9334 | 1793.9068 | D | 809.4040 | 792.3774 | 7 |
| 21 | 1946.0018 | 1928.9753 | 1973.9967 | 1956.9702 | Y | 694.3770 | 677.3505 | 6 |
| 22 | 2017.0389 | 2000.0124 | 2045.0338 | 2028.0073 | A | 531.3137 | 514.2871 | 5 |
| 23 | 2146.0815 | 2129.0550 | 2174.0764 | 2157.0499 | E | 460.2766 | 443.2500 | 4 |
| 24 | 2217.1186 | 2200.0921 | 2245.1135 | 2228.0870 | A | ***331.2340*** | 314.2074 | 3 |
| 25 | 2330.2027 | 2313.1761 | 2358.1976 | 2341.1711 | I | 260.1969 | 243.1703 | 2 |
| 26 | | | | | K | 147.1128 | 130.0863 | 1 |

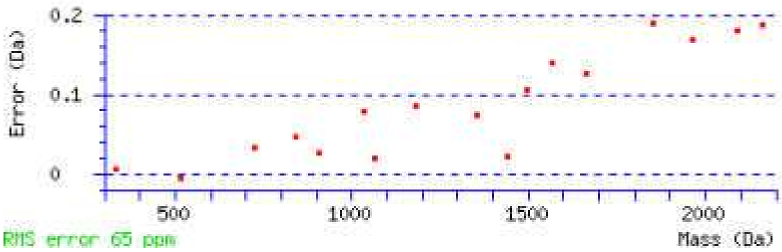


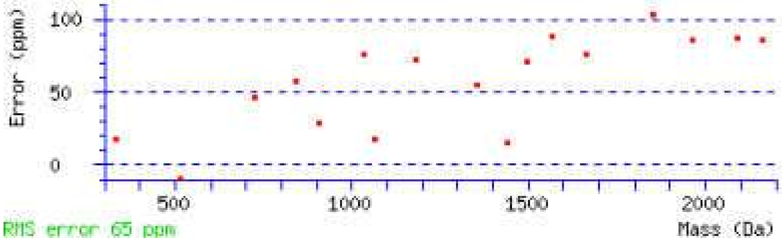


NCBI **BLAST** search of VIEAGANAIVAGSAVFGAPDYAEAIK
(Parameters: blastp, nr protein database, expect=20000, no filter, PAM30)
Other BLAST web gateways

**All matches to this query**

| Score | Mr(calc) | Delta | Sequence |
|---|---|---|---|
| 101.9 | 2503.2958 | 0.1158 | VIEAGANAIVAGSAVFGAPDYAEAIK |
| 101.9 | 2503.2958 | 0.1158 | VIEAGANALVAGSAVFGAPDYAEAIK |
| 11.9 | 2503.3192 | 0.0924 | LHVPVGGDVAPPPPHMLMLQINR |
| 6.3 | 2503.3608 | 0.0508 | SPDTLVALIVGVTGMVGFSLAEALK |
| 6.0 | 2503.2390 | 0.1726 | VSISQENHLGGSNVTAALPWMHR |
| | | | |

| | | | |
|---|---|---|---|
| 1.3 | 2503.3369 | 0.0747 | MQSLQHQLPFTLRPSPLEPLR |
| 1.2 | 2503.3223 | 0.0893 | LGYVYQDVDIPWLTSKPTPRR |
| 0.2 | 2503.2410 | 0.1706 | QGITPEIIKELTDCNAMLSSQR |
| 0.0 | 2503.4373 | -0.0257 | ITLEVIVLQIDNQIEELLPRR |

**Mascot:** http://www.matrixscience.com/

# MATRIX SCIENCE Mascot Search Results

## Peptide View **Spot no 30**

MS/MS Fragmentation of **IFLPEGLLDR**
Found in **gi|62733870** in **NCBInr**, chlorophyll a/b-binding protein CP26 precursor - maize [Oryza sativa Japonica Group]

Match to Query 120: 1171.732724 from(1172.740000,1+) intensity(0.0000) index(10)
Title: Label: G8, Spot_Id: 219792, Peak_List_Id: 226318, MSMS Job_Run_Id: 21840, Comment:
Data file C:\Documents and Settings\Administrator\Desktop\Farah-Garima 11May 2013\ppw_G8_136859928300.txt

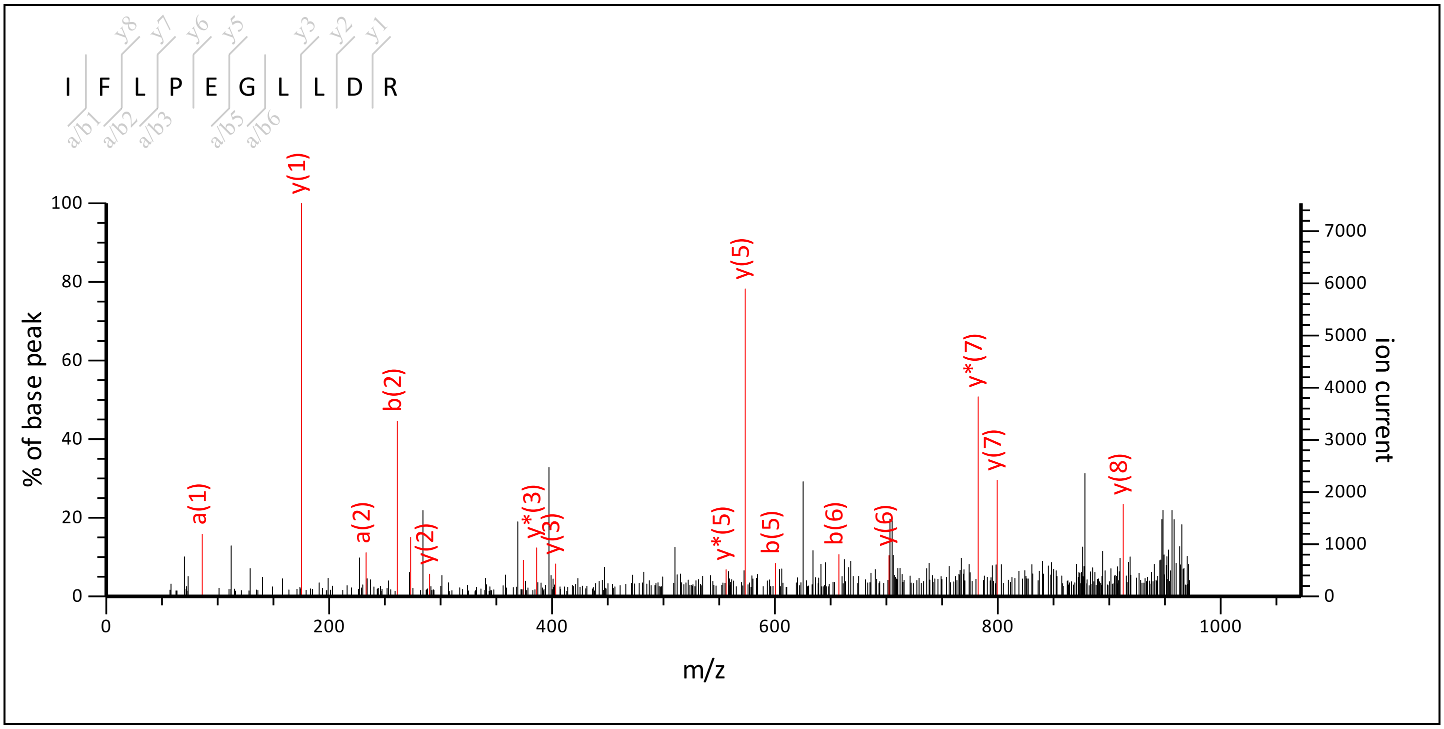


0 to 1072.04

Label all possible matches ○ Label matches used for scoring ◉

**Monoisotopic mass of neutral peptide Mr(calc):** 1171.6601
**Fixed modifications:** Carbamidomethyl (C) (apply to specified residues or termini only)
**Ions Score:** 26 **Expect:** 50
**Matches :** 17/36 fragment ions using 50 most intense peaks (help)

| # | a | b | Seq. | y | y* | # |
|---|---|---|---|---|---|---|
| 1 | **86.0964** | 114.0913 | I | | | 10 |
| 2 | **233.1648** | **261.1598** | F | 1059.5833 | 1042.5568 | 9 |
| 3 | 346.2489 | **374.2438** | L | ***912.5149*** | 895.4884 | 8 |
| 4 | 443.3017 | 471.2966 | P | ***799.4308*** | 782.4043 | 7 |
| 5 | 572.3443 | **600.3392** | E | ***702.3781*** | 685.3515 | 6 |
| 6 | 629.3657 | **657.3606** | G | ***573.3355*** | 556.3089 | 5 |
| 7 | 742.4498 | 770.4447 | L | 516.3140 | 499.2875 | 4 |
| 8 | 855.5339 | 883.5288 | L | ***403.2300*** | 386.2034 | 3 |

| | | | | | | |
|---|---|---|---|---|---|---|
| **9** | 970.5608 | 998.5557 | **D** | ***290.1459*** | 273.1193 | **2** |
| **10** | | | **R** | ***175.1190*** | 158.0924 | **1** |

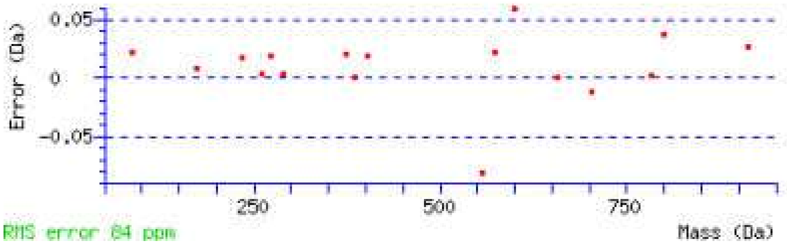


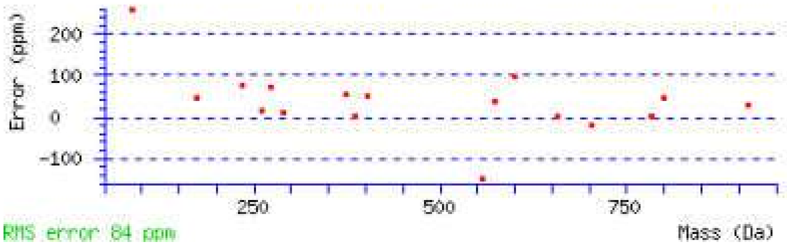


NCBI **BLAST** search of IFLPEGLLDR
(Parameters: blastp, nr protein database, expect=20000, no filter, PAM30)
Other BLAST web gateways

**All matches to this query**

| Score | Mr(calc) | Delta | Sequence |
|---|---|---|---|
| 26.3 | 1171.6601 | 0.0726 | IFLPEGLLDR |
| 17.2 | 1171.7329 | -0.0001 | LFLLLPLSTR |
| 14.6 | 1171.7441 | -0.0114 | LFLLIAGAAKR |
| 13.6 | 1171.6561 | 0.0767 | IIAATNTNLNK |
| 13.3 | 1171.6635 | 0.0693 | ILMSLDVLPR |
| 13.2 | 1171.6423 | 0.0904 | IFLPEPMGLR |
| 13.2 | 1171.7441 | -0.0114 | LFILLNGKVR |
| 13.2 | 1171.7441 | -0.0114 | LFILLREIR |
| 13.2 | 1171.6601 | 0.0726 | LFLLIPGDER |
| 13.2 | 1171.7362 | -0.0035 | LMLLLLATLR |

**Mascot:** http://www.matrixscience.com/

# MATRIX SCIENCE Mascot Search Results

## Peptide View Spot no 30

MS/MS Fragmentation of **YQAYELIHAR**
Found in **gi|62733870** in **NCBInr**, chlorophyll a/b-binding protein CP26 precursor - maize [Oryza sativa Japonica Group]

Match to Query 131: 1262.721024 from(1263.728300,1+) intensity(0.0000) index(13)
Title: Label: G8, Spot_Id: 219792, Peak_List_Id: 226319, MSMS Job_Run_Id: 21840, Comment:
Data file C:\Documents and Settings\Administrator\Desktop\Farah-Garima 11May 2013\ppw_G8_136859928300.txt

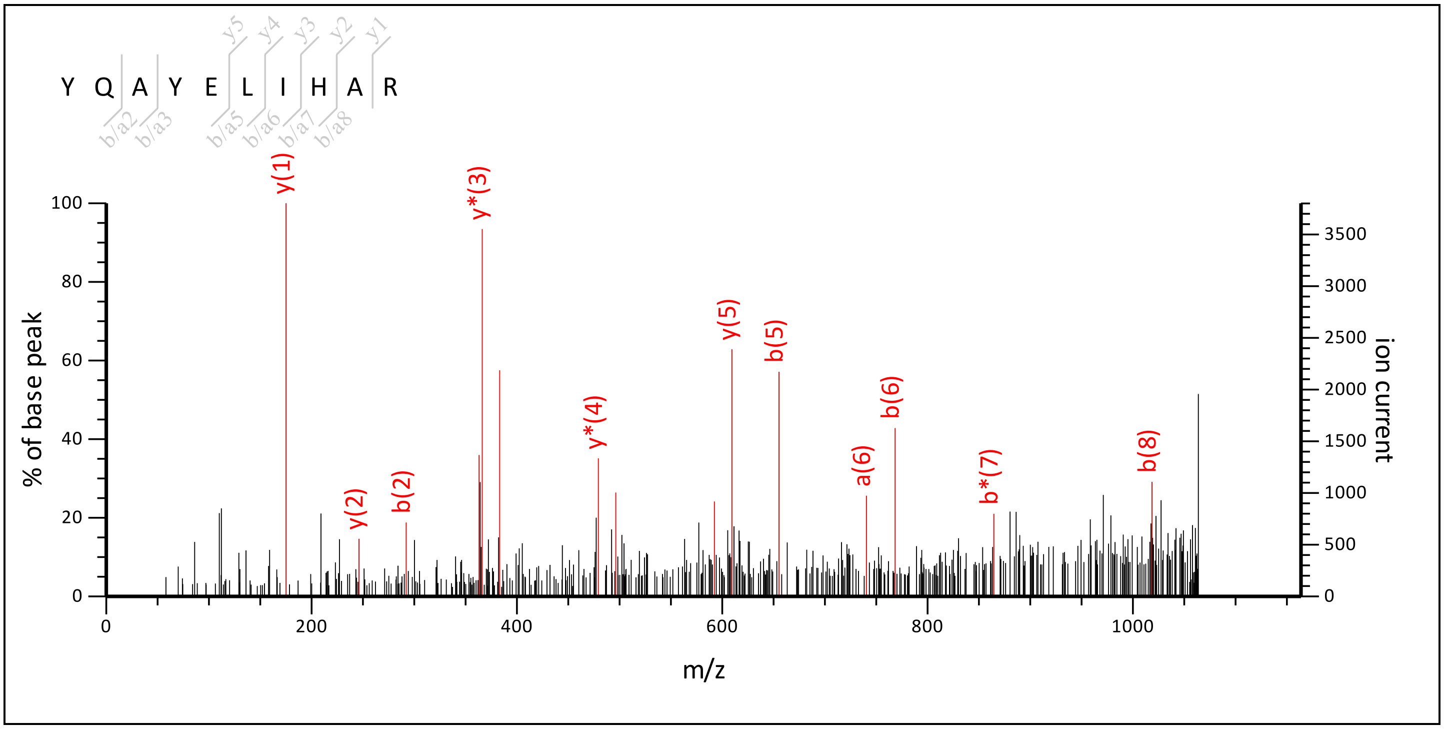


0 to 1163.72

Label all possible matches ○ Label matches used for scoring ◉

**Monoisotopic mass of neutral peptide Mr(calc):** 1262.6407
**Fixed modifications:** Carbamidomethyl (C) (apply to specified residues or termini only)
**Ions Score:** 23 **Expect:** 1.6e+02
**Matches :** 15/52 fragment ions using 33 most intense peaks (help)

| # | a | a* | b | b* | Seq. | y | y* | # |
|---|---|---|---|---|---|---|---|---|
| 1 | 136.0757 | | 164.0706 | | Y | | | 10 |
| 2 | 264.1343 | 247.1077 | **292.1292** | 275.1026 | Q | 1100.5847 | 1083.5582 | 9 |
| 3 | 335.1714 | 318.1448 | **363.1663** | 346.1397 | A | 972.5261 | 955.4996 | 8 |
| 4 | 498.2347 | 481.2082 | 526.2296 | 509.2031 | Y | 901.4890 | 884.4625 | 7 |
| 5 | 627.2773 | 610.2508 | **655.2722** | 638.2457 | E | 738.4257 | 721.3992 | 6 |
| 6 | 740.3614 | 723.3348 | **768.3563** | 751.3297 | L | **609.3831** | 592.3566 | 5 |
| 7 | 853.4454 | 836.4189 | 881.4403 | 864.4138 | I | **496.2990** | 479.2725 | 4 |
| 8 | 990.5043 | 973.4778 | **1018.4993** | 1001.4727 | H | **383.2150** | 366.1884 | 3 |

| | | | | | | | | |
|---|---|---|---|---|---|---|---|---|
| **9** | 1061.5415 | 1044.5149 | 1089.5364 | 1072.5098 | **A** | ***246.1561*** | 229.1295 | **2** |
| **10** | | | | | **R** | ***175.1190*** | 158.0924 | **1** |

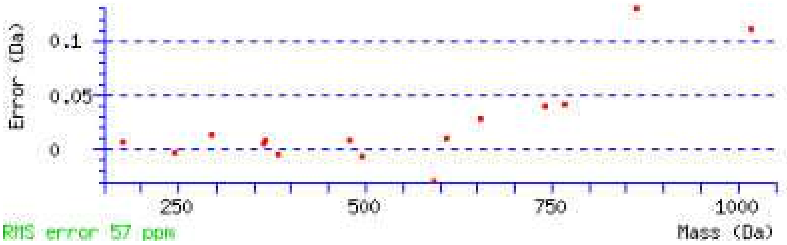


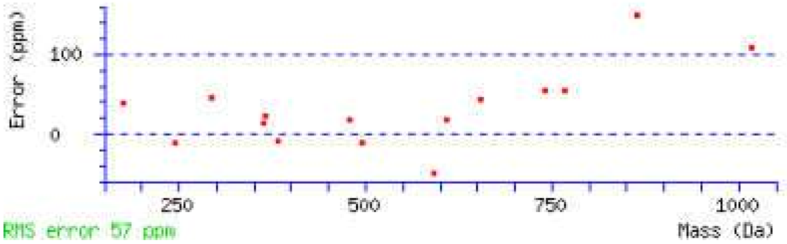


NCBI **BLAST** search of YQAYELIHAR
(Parameters: blastp, nr protein database, expect=20000, no filter, PAM30)
Other BLAST web gateways

**All matches to this query**

| Score | Mr(calc) | Delta | Sequence |
|---|---|---|---|
| 23.5 | 1262.6407 | 0.0803 | YQAYELIHAR |
| 17.1 | 1262.7247 | -0.0037 | HKEFIIIHAR |
| 17.1 | 1262.6884 | 0.0327 | HQIFELIHAR |
| 17.1 | 1262.7207 | 0.0003 | NNVQAKLLHAR |
| 16.1 | 1262.7247 | -0.0037 | QHLLYILHAR |
| 15.4 | 1262.6441 | 0.0769 | LSGSMYLHLAR |
| 15.0 | 1262.7571 | -0.0361 | ARVGTAVLHLAR |
| 15.0 | 1262.7571 | -0.0361 | GIGRSIALHLAR |
| 14.9 | 1262.7207 | 0.0003 | AAGVEARLIHAR |
| 14.3 | 1262.7095 | 0.0115 | LLIEGGANIHAR |

**Mascot:** http://www.matrixscience.com/

# MATRIX SCIENCE Mascot Search Results

## Peptide View Spot no 30

MS/MS Fragmentation of **HLSDPFGNNLLTVISGAAER**
Found in **gi|62733870** in **NCBInr**, chlorophyll a/b-binding protein CP26 precursor - maize [Oryza sativa Japonica Group]

Match to Query 251: 2110.183424 from(2111.190700,1+) intensity(0.0000) index(33)
Title: Label: G8, Spot_Id: 219792, Peak_List_Id: 226327, MSMS Job_Run_Id: 21840, Comment:
Data file C:\Documents and Settings\Administrator\Desktop\Farah-Garima 11May 2013\ppw_G8_136859928300.txt

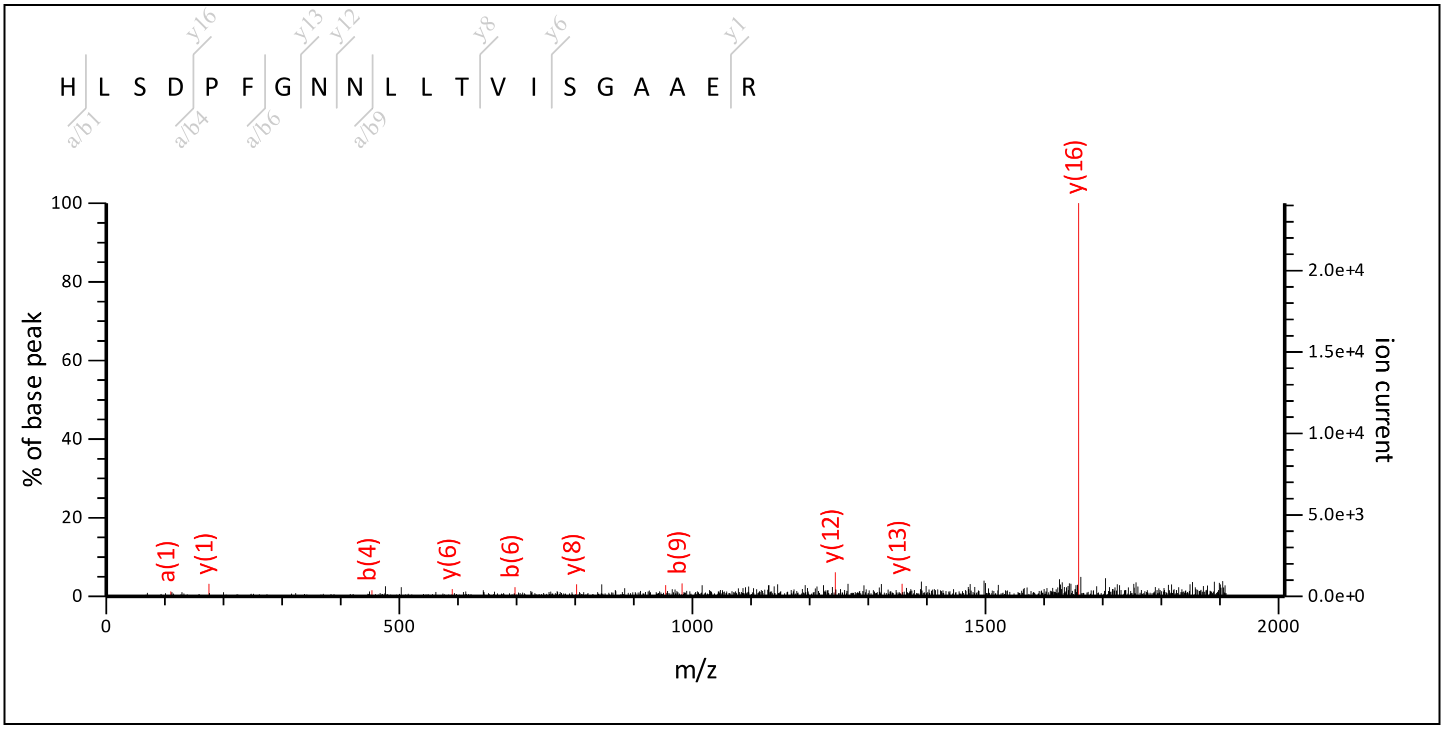


0 to 2010.46

Label all possible matches ○ Label matches used for scoring ◉

**Monoisotopic mass of neutral peptide Mr(calc):** 2110.0807
**Fixed modifications:** Carbamidomethyl (C) (apply to specified residues or termini only)
**Ions Score:** 29 **Expect:** 26
**Matches :** 11/100 fragment ions using 19 most intense peaks (help)

| # | a | a* | b | b* | Seq. | y | y* | # |
|---|---|---|---|---|---|---|---|---|
| 1 | **110.0713** | | 138.0662 | | H | | | 20 |
| 2 | 223.1553 | | 251.1503 | | L | 1974.0291 | 1957.0025 | 19 |
| 3 | 310.1874 | | 338.1823 | | S | 1860.9450 | 1843.9185 | 18 |
| 4 | 425.2143 | | **453.2092** | | D | 1773.9130 | 1756.8864 | 17 |
| 5 | 522.2671 | | 550.2620 | | P | ***1658.8860*** | 1641.8595 | 16 |
| 6 | 669.3355 | | **697.3304** | | F | 1561.8333 | 1544.8067 | 15 |
| 7 | 726.3569 | | 754.3519 | | G | 1414.7649 | 1397.7383 | 14 |
| 8 | 840.3999 | 823.3733 | 868.3948 | 851.3682 | N | ***1357.7434*** | 1340.7169 | 13 |

| | | | | | | | | |
|---|---|---|---|---|---|---|---|---|
| 9 | 954.4428 | 937.4163 | 982.4377 | 965.4112 | N | 1243.7005 | 1226.6739 | 12 |
| 10 | 1067.5269 | 1050.5003 | 1095.5218 | 1078.4952 | L | 1129.6576 | 1112.6310 | 11 |
| 11 | 1180.6109 | 1163.5844 | 1208.6058 | 1191.5793 | L | 1016.5735 | 999.5469 | 10 |
| 12 | 1281.6586 | 1264.6321 | 1309.6535 | 1292.6270 | T | 903.4894 | 886.4629 | 9 |
| 13 | 1380.7270 | 1363.7005 | 1408.7219 | 1391.6954 | V | 802.4417 | 785.4152 | 8 |
| 14 | 1493.8111 | 1476.7845 | 1521.8060 | 1504.7795 | I | 703.3733 | 686.3468 | 7 |
| 15 | 1580.8431 | 1563.8166 | 1608.8380 | 1591.8115 | S | 590.2893 | 573.2627 | 6 |
| 16 | 1637.8646 | 1620.8380 | 1665.8595 | 1648.8329 | G | 503.2572 | 486.2307 | 5 |
| 17 | 1708.9017 | 1691.8751 | 1736.8966 | 1719.8701 | A | 446.2358 | 429.2092 | 4 |
| 18 | 1779.9388 | 1762.9123 | 1807.9337 | 1790.9072 | A | 375.1987 | 358.1721 | 3 |
| 19 | 1908.9814 | 1891.9549 | 1936.9763 | 1919.9498 | E | 304.1615 | 287.1350 | 2 |
| 20 | | | | | R | 175.1190 | 158.0924 | 1 |

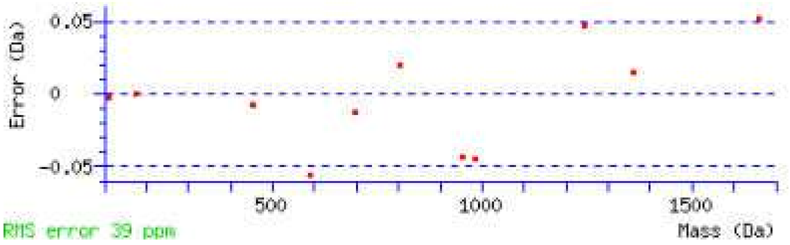


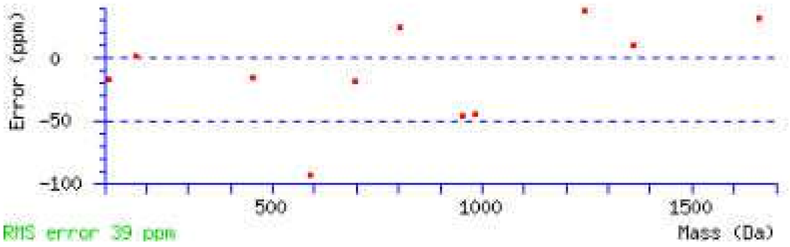


NCBI **BLAST** search of HLSDPFGNNLLTVISGAAER
(Parameters: blastp, nr protein database, expect=20000, no filter, PAM30)
Other BLAST web gateways

**All matches to this query**

| Score | Mr(calc) | Delta | Sequence |
|---|---|---|---|
| 28.8 | 2110.0807 | 0.1027 | HLSDPFGNNLLTVIAGSAER |
| 28.8 | 2110.0807 | 0.1027 | HLSDPFGNNLLTVISAGAER |
| 28.8 | 2110.0807 | 0.1027 | HLSDPFGNNLLTVISGAAER |
| 28.8 | 2110.0807 | 0.1027 | HLSDPFGNNLLTVLAGSAER |
| 9.2 | 2110.0192 | 0.1642 | ARQPDWVTASHSLVSEDGR |
| 8.9 | 2110.0742 | 0.1092 | QHGGNIIVATPGRLEDMFR |
| 6.9 | 2110.0847 | 0.0987 | ASALELPAASAYRLPFYDR |
| 5.2 | 2110.0153 | 0.1681 | YSDSMLSEPFSTKAAAHIR |
| 5.0 | 2110.0551 | 0.1283 | MLQVPGFVASAGAASMLVSSR |
| 5.0 | 2110.0881 | 0.0953 | LEPLQMTWGTLSPPLGNTR |

**Mascot:** http://www.matrixscience.com/

# Mascot Search Results

## Peptide View Spot no 32

MS/MS Fragmentation of **FFVGGNWK**
Found in **gi|553107** in **NCBInr**, triosephosphate isomerase, partial [Oryza sativa Japonica Group]

Match to Query 18: 953.513784 from(954.521060,1+) intensity(0.0000) index(2)
Title: Label: H19, Spot_Id: 219969, Peak_List_Id: 229374, MSMS Job_Run_Id: 22019, Comment:
Data file C:\Documents and Settings\Administrator\Desktop\Farah-Garima 11May 2013\ppw_H19_136868348800.txt

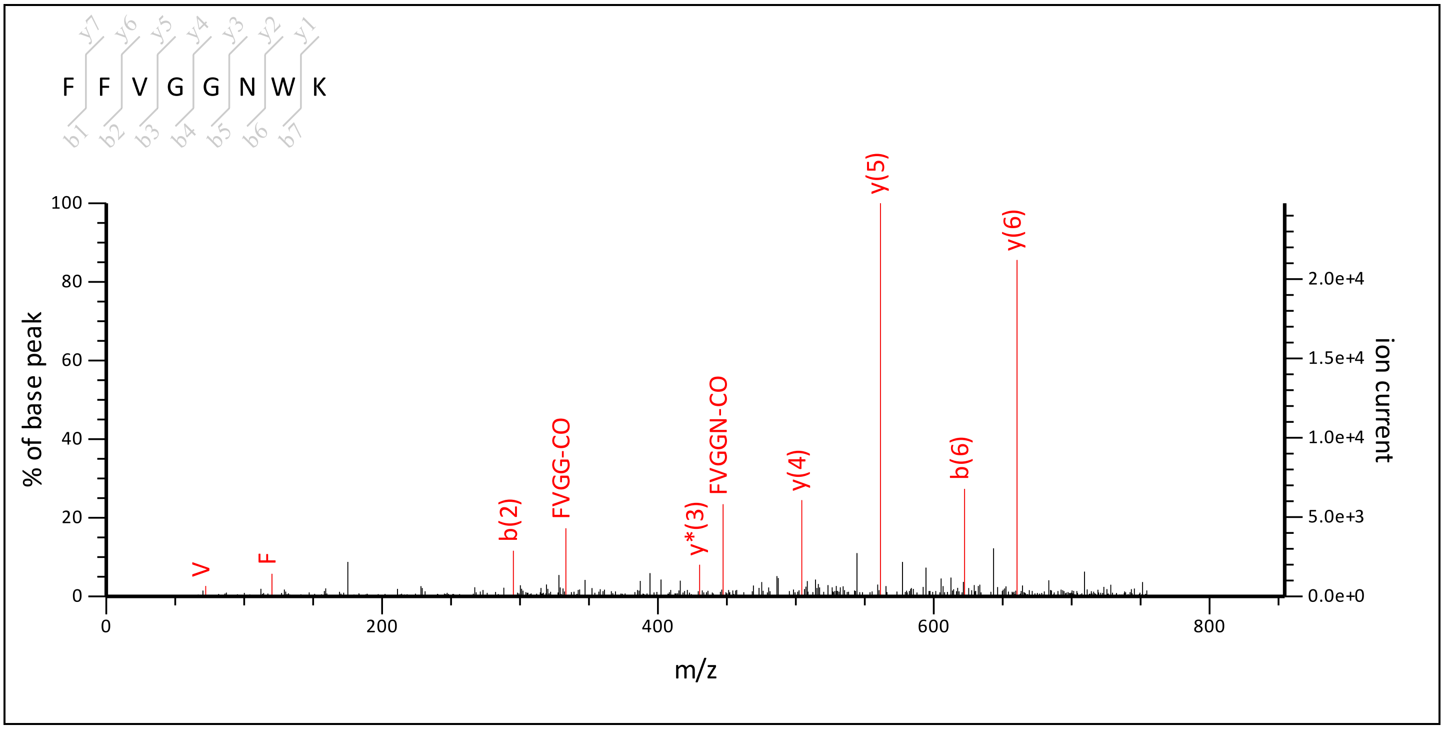


0 to 854.47

Label all possible matches Label matches used for scoring

**Monoisotopic mass of neutral peptide Mr(calc):** 953.4760
**Fixed modifications:** Carbamidomethyl (C) (apply to specified residues or termini only)
**Ions Score:** 39 **Expect:** 3.6
**Matches :** 14/81 fragment ions using 14 most intense peaks (help)

| # | Immon. | a | a* | b | b* | d | Seq. | v | w | y | y* | # |
|---|---|---|---|---|---|---|---|---|---|---|---|---|
| 1 | 120.0808 | **120.0808** | | 148.0757 | | 44.0495 | F | | | | | 8 |
| 2 | 120.0808 | 267.1492 | | **295.1441** | | | F | 715.3522 | | 807.4148 | 790.3883 | 7 |
| 3 | 72.0808 | 366.2176 | | 394.2125 | | 352.2020 | V | 616.2838 | 629.3042 | ***660.3464*** | 643.3198 | 6 |
| 4 | 30.0338 | 423.2391 | | 451.2340 | | | G | | | ***561.2780*** | 544.2514 | 5 |
| 5 | 30.0338 | 480.2605 | | 508.2554 | | | G | | | ***504.2565*** | 487.2300 | 4 |
| 6 | 87.0553 | 594.3035 | 577.2769 | **622.2984** | 605.2718 | 551.2976 | N | 388.1979 | 387.2027 | ***447.2350*** | 430.2085 | 3 |
| 7 | 159.0917 | 780.3828 | 763.3562 | 808.3777 | 791.3511 | | W | 202.1186 | | ***333.1921*** | 316.1656 | 2 |
| 8 | 101.1073 | | | | | | K | 74.0237 | 73.0284 | 147.1128 | 130.0863 | 1 |

| Seq | ya | yb | Seq | ya | yb | Seq | ya | yb |
|---|---|---|---|---|---|---|---|---|
| **FV** | 219.1492 | 247.1441 | **FVG** | 276.1707 | 304.1656 | **FVGG** | 333.1921 | 361.1870 |
| **FVGGN** | 447.2350 | 475.2300 | **FVGGNW** | 633.3144 | 661.3093 | **VG** | 129.1022 | 157.0972 |
| **VGG** | 186.1237 | 214.1186 | **VGGN** | 300.1666 | 328.1615 | **VGGNW** | 486.2459 | 514.2409 |
| **GG** | 87.0553 | 115.0502 | **GGN** | 201.0982 | 229.0931 | **GGNW** | 387.1775 | 415.1724 |
| **GN** | 144.0768 | 172.0717 | **GNW** | 330.1561 | 358.1510 | **NW** | 273.1346 | 301.1295 |

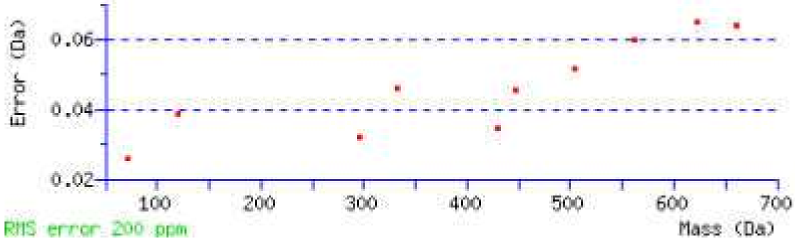


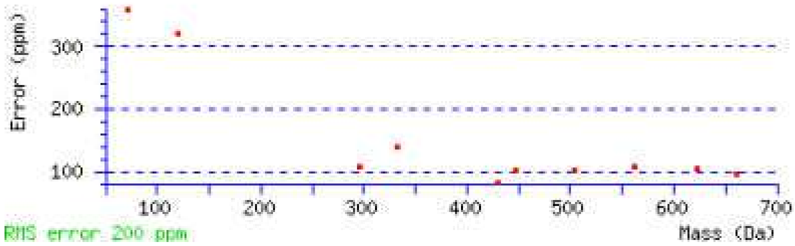


NCBI **BLAST** search of FFVGGNWK
(Parameters: blastp, nr protein database, expect=20000, no filter, PAM30)
Other BLAST web gateways

**All matches to this query**

| Score | Mr(calc) | Delta | Sequence |
|---|---|---|---|
| 39.3 | 953.4760 | 0.0378 | FFVGGNWK |
| 39.3 | 953.4760 | 0.0378 | FFVGGNWK |
| 39.0 | 953.4389 | 0.0749 | MFVNSANR |
| 35.4 | 953.4389 | 0.0749 | VMYGGNASR |
| 34.2 | 953.5083 | 0.0055 | FFVNGSKR |
| 34.2 | 953.4641 | 0.0497 | MFVEVASR |
| 30.1 | 953.4640 | 0.0497 | MYVVESAR |
| 28.9 | 953.4858 | 0.0279 | FFVDIDAK |
| 28.6 | 953.4202 | 0.0935 | TYENNSAR |
| 26.2 | 953.4277 | 0.0861 | FMVGAEER |

**Mascot:** http://www.matrixscience.com/

# Mascot Search Results

## Peptide View Spot no 32

MS/MS Fragmentation of **IIYGGSVTGANCK**
Found in **gi|553107** in **NCBInr**, triosephosphate isomerase, partial [Oryza sativa Japonica Group]

Match to Query 81: 1338.724624 from(1339.731900,1+) intensity(0.0000) index(15)
Title: Label: H19, Spot_Id: 219969, Peak_List_Id: 229382, MSMS Job_Run_Id: 22019, Comment:
Data file C:\Documents and Settings\Administrator\Desktop\Farah-Garima 11May 2013\ppw_H19_136868348800.txt

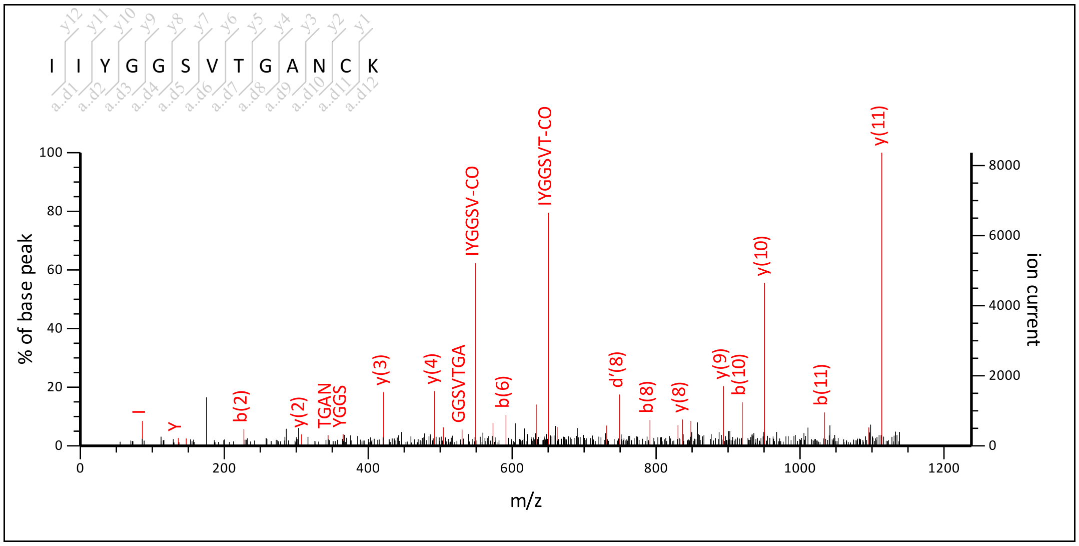


0 to 1238.14

Label all possible matches ○ Label matches used for scoring ◉

**Monoisotopic mass of neutral peptide Mr(calc):** 1338.6602
**Fixed modifications:** Carbamidomethyl (C) (apply to specified residues or termini only)
**Ions Score:** 89 **Expect:** 4e-05
**Matches :** 37/207 fragment ions using 44 most intense peaks (help)

| # | Immon. | a | a* | a0 | b | b* | b0 | d | d' | Seq. | v | w | w' | y | y* | |
|---|---|---|---|---|---|---|---|---|---|---|---|---|---|---|---|---|
| 1 | 86.0964 | **86.0964** | | | 114.0913 | | | 44.0495 | | I | | | | | | |
| 2 | 86.0964 | 199.1805 | | | **227.1754** | | | 171.1492 | 185.1648 | I | 1168.5051 | 1181.5256 | 1195.5412 | 1226.5834 | 1209.5569 | 12[illegible] |
| 3 | 136.0757 | 362.2438 | | | 390.2387 | | | | | Y | 1005.4418 | | | ***1113.4993*** | 1096.4728 | 10[illegible] |
| 4 | 30.0338 | 419.2653 | | | 447.2602 | | | | | G | | | | ***950.4360*** | 933.4095 | 9[illegible] |
| 5 | 30.0338 | 476.2867 | | | **504.2817** | | | | | G | | | | ***893.4145*** | 876.3880 | 8[illegible] |
| 6 | 60.0444 | 563.3188 | | 545.3082 | **591.3137** | | 573.3031 | 547.3239 | | S | 804.3669 | 803.3716 | | ***836.3931*** | 819.3665 | 8[illegible] |
| 7 | 72.0808 | **662.3872** | | 644.3766 | 690.3821 | | 672.3715 | 648.3715 | | V | 705.2985 | 718.3189 | | ***749.3611*** | 732.3345 | 7[illegible] |
| 8 | 74.0600 | 763.4349 | | 745.4243 | **791.4298** | | 773.4192 | 747.4400 | 749.4192 | T | 604.2508 | 617.2712 | 619.2504 | ***650.2926*** | 633.2661 | 6[illegible] |
| 9 | 30.0338 | 820.4563 | | 802.4458 | **848.4512** | | 830.4407 | | | G | | | | ***549.2450*** | 532.2184 | |
| 10 | 44.0495 | 891.4934 | | 873.4829 | **919.4884** | | 901.4778 | | | A | 476.1922 | | | ***492.2235*** | 475.1969 | |
| 11 | 87.0553 | 1005.5364 | 988.5098 | 987.5258 | **1033.5313** | 1016.5047 | 1015.5207 | 962.5306 | | N | 362.1493 | 361.1540 | | ***421.1864*** | 404.1598 | |
| 12 | 133.0430 | 1165.5670 | 1148.5405 | 1147.5565 | 1193.5619 | 1176.5354 | 1175.5514 | 1076.5735 | | C | 202.1186 | 201.1234 | | ***307.1435*** | 290.1169 | |
| 13 | 101.1073 | | | | | | | | | K | 74.0237 | 73.0284 | | ***147.1128*** | 130.0863 | |

| Seq | ya | yb | Seq | ya | yb | Seq | ya | yb |
|---|---|---|---|---|---|---|---|---|
| IY | 249.1598 | 277.1547 | IYG | 306.1812 | 334.1761 | IYGG | 363.2027 | 391.1976 |
| IYGGS | 450.2347 | 478.2296 | IYGGSV | 549.3031 | 577.2980 | IYGGSVT | 650.3508 | 678.3457 |
| YG | 193.0972 | 221.0921 | YGG | 250.1186 | 278.1135 | YGGS | 337.1506 | 365.1456 |
| YGGSV | 436.2191 | 464.2140 | YGGSVT | 537.2667 | 565.2617 | YGGSVTG | 594.2882 | 622.2831 |
| YGGSVTGA | 665.3253 | 693.3202 | GG | 87.0553 | 115.0502 | GGS | 174.0873 | 202.0822 |
| GGSV | 273.1557 | 301.1506 | GGSVT | 374.2034 | 402.1983 | GGSVTG | 431.2249 | 459.2198 |
| GGSVTGA | 502.2620 | 530.2569 | GGSVTGAN | 616.3049 | 644.2998 | GS | 117.0659 | 145.0608 |
| GSV | 216.1343 | 244.1292 | GSVT | 317.1819 | 345.1769 | GSVTG | 374.2034 | 402.1983 |

| | | | | | | | | |
|---|---|---|---|---|---|---|---|---|
| **GSVTGA** | 445.2405 | 473.2354 | **GSVTGAN** | 559.2835 | 587.2784 | **SV** | 159.1128 | 187.1077 |
| **SVT** | 260.1605 | 288.1554 | **SVTG** | 317.1819 | 345.1769 | **SVTGA** | 388.2191 | 416.2140 |
| **SVTGAN** | 502.2620 | 530.2569 | **SVTGANC** | 662.2926 | 690.2876 | **VT** | 173.1285 | 201.1234 |
| **VTG** | 230.1499 | 258.1448 | **VTGA** | 301.1870 | 329.1819 | **VTGAN** | 415.2300 | 443.2249 |
| **VTGANC** | 575.2606 | 603.2555 | **TG** | 131.0815 | 159.0764 | **TGA** | 202.1186 | 230.1135 |
| **TGAN** | 316.1615 | 344.1565 | **TGANC** | 476.1922 | 504.1871 | **GA** | 101.0709 | 129.0659 |
| **GAN** | 215.1139 | 243.1088 | **GANC** | 375.1445 | 403.1394 | **AN** | 158.0924 | 186.0873 |
| **ANC** | 318.1231 | 346.1180 | **NC** | 247.0859 | 275.0809 | | | |

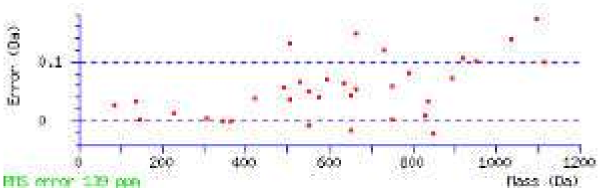


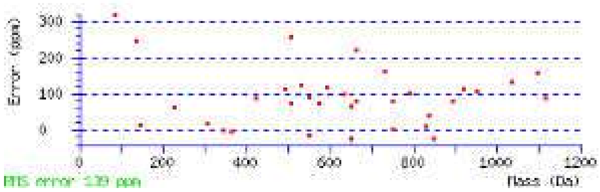


NCBI **BLAST** search of IIYGGSVTGANCK
(Parameters: blastp, nr protein database, expect=20000, no filter, PAM30)
Other BLAST web gateways

**All matches to this query**

| Score | Mr(calc) | Delta | Sequence |
|---|---|---|---|
| 89.1 | 1338.6602 | 0.0644 | IIYGGSVTGANCK |
| 89.1 | 1338.6602 | 0.0644 | ILYGGSVTGANCK |
| 77.7 | 1338.6602 | 0.0644 | IIYGGSVTAGNCK |
| 77.7 | 1338.6602 | 0.0644 | ILYGGSVTAGNCK |
| 76.2 | 1338.6602 | 0.0645 | IIYGGSVSAANCK |
| 76.2 | 1338.6602 | 0.0645 | ILYGGSVSAANCK |
| 32.8 | 1338.6714 | 0.0532 | MLENSPHVIQR |
| 30.5 | 1338.6932 | 0.0314 | IYHYTVNSSKK |
| 29.9 | 1338.7296 | -0.0049 | LNYLGETKFVR |
| 29.5 | 1338.6833 | 0.0413 | NQFNWSIFKR |

**Mascot:** http://www.matrixscience.com/

# MATRIX SCIENCE Mascot Search Results

## Peptide View Spot no 32

MS/MS Fragmentation of **VIACVGETLEQR**
Found in **gi|553107** in **NCBInr**, triosephosphate isomerase, partial [Oryza sativa Japonica Group]

Match to Query 87: 1373.778124 from(1374.785400,1+) intensity(0.0000) index(16)
Title: Label: H19, Spot_Id: 219969, Peak_List_Id: 229376, MSMS Job_Run_Id: 22019, Comment:
Data file C:\Documents and Settings\Administrator\Desktop\Farah-Garima 11May 2013\ppw_H19_136868348800.txt

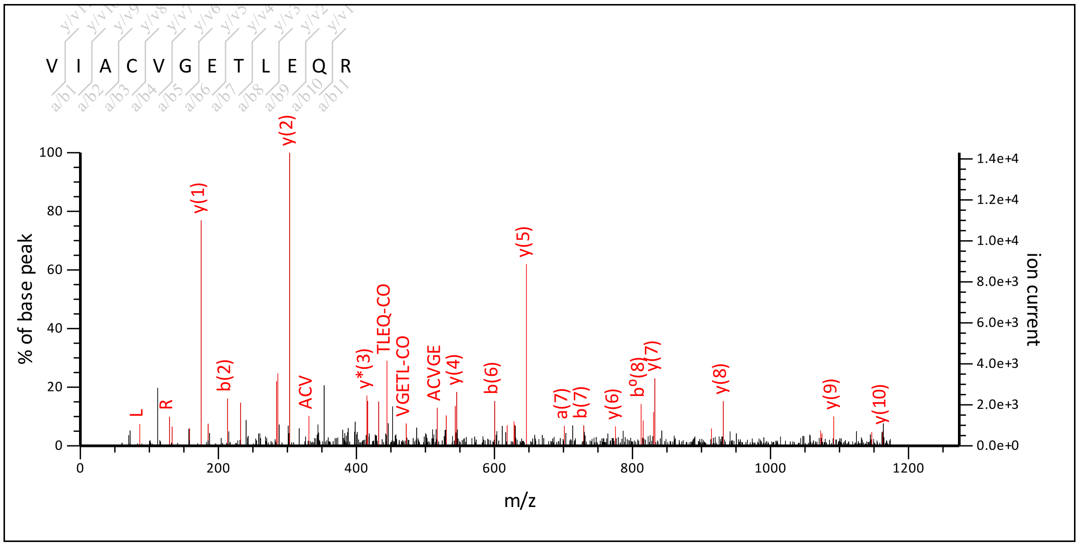


0 to 1273.56

Label all possible matches ○ Label matches used for scoring ◉

**Monoisotopic mass of neutral peptide Mr(calc):** 1373.6973
**Fixed modifications:** Carbamidomethyl (C) (apply to specified residues or termini only)
**Ions Score:** 72 **Expect:** 0.0018
**Matches :** 53/179 fragment ions using 60 most intense peaks (help)

| # | Immon. | a | a* | a0 | b | b* | b0 | d | d' | Seq. | v | w | w' | y | y* | |
|---|---|---|---|---|---|---|---|---|---|---|---|---|---|---|---|---|
| 1 | 72.0808 | 72.0808 | | | 100.0757 | | | 44.0495 | | V | | | | | | |
| 2 | 86.0964 | **185.1648** | | | **213.1598** | | | 157.1335 | 171.1492 | I | 1217.5579 | 1230.5783 | 1244.5940 | 1275.6362 | 1258.6096 | [illegible] |
| 3 | 44.0495 | 256.2020 | | | **284.1969** | | | | | A | 1146.5208 | | | ***1162.5521*** | 1145.5256 | [illegible] |
| 4 | 133.0430 | **416.2326** | | | **444.2275** | | | 327.2391 | | C | 986.4902 | 985.4949 | | ***1091.5150*** | 1074.4884 | [illegible] |
| 5 | 72.0808 | 515.3010 | | | **543.2959** | | | 501.2854 | | V | 887.4217 | 900.4421 | | ***931.4843*** | 914.4578 | [illegible] |
| 6 | 30.0338 | 572.3225 | | | **600.3174** | | | | | G | | | | ***832.4159*** | 815.3894 | [illegible] |
| 7 | 102.0550 | **701.3651** | | 683.3545 | **729.3600** | | 711.3494 | 643.3596 | | E | 701.3577 | 700.3624 | | ***775.3945*** | 758.3679 | [illegible] |
| 8 | 74.0600 | 802.4128 | | 784.4022 | **830.4077** | | 812.3971 | 786.4178 | 788.3971 | T | 600.3100 | 613.3304 | 615.3097 | ***646.3519*** | 629.3253 | [illegible] |
| 9 | 86.0964 | 915.4968 | | 897.4863 | 943.4917 | | 925.4812 | 873.4499 | | L | 487.2259 | 486.2307 | | ***545.3042*** | 528.2776 | [illegible] |
| 10 | 102.0550 | 1044.5394 | | 1026.5288 | **1072.5343** | | 1054.5238 | 986.5339 | | E | 358.1833 | 357.1881 | | ***432.2201*** | 415.1936 | [illegible] |
| 11 | 101.0709 | 1172.5980 | 1155.5714 | 1154.5874 | 1200.5929 | 1183.5664 | 1182.5823 | 1115.5765 | | Q | 230.1248 | 229.1295 | | ***303.1775*** | 286.1510 | |
| 12 | 129.1135 | | | | | | | | | R | 74.0237 | 73.0284 | | ***175.1190*** | 158.0924 | |

| Seq | ya | yb | Seq | ya | yb | Seq | ya | yb |
|---|---|---|---|---|---|---|---|---|
| IA | 157.1335 | 185.1285 | IAC | 317.1642 | 345.1591 | IACV | 416.2326 | 444.2275 |
| IACVG | 473.2541 | 501.2490 | IACVGE | 602.2967 | 630.2916 | AC | 204.0801 | 232.0750 |
| ACV | 303.1485 | 331.1435 | ACVG | 360.1700 | 388.1649 | ACVGE | 489.2126 | 517.2075 |
| ACVGET | 590.2603 | 618.2552 | CV | 232.1114 | 260.1063 | CVG | 289.1329 | 317.1278 |
| CVGE | 418.1755 | 446.1704 | CVGET | 519.2232 | 547.2181 | CVGETL | 632.3072 | 660.3021 |
| VG | 129.1022 | 157.0972 | VGE | 258.1448 | 286.1397 | VGET | 359.1925 | 387.1874 |
| VGETL | 472.2766 | 500.2715 | VGETLE | 601.3192 | 629.3141 | GE | 159.0764 | 187.0713 |
| GET | 260.1241 | 288.1190 | GETL | 373.2082 | 401.2031 | GETLE | 502.2508 | 530.2457 |
| GETLEQ | 630.3093 | 658.3042 | ET | 203.1026 | 231.0975 | ETL | 316.1867 | 344.1816 |

| | | | | | | | | |
|---|---|---|---|---|---|---|---|---|
| **ETLE** | 445.2293 | 473.2242 | **ETLEQ** | 573.2879 | 601.2828 | **TL** | 187.1441 | 215.1390 |
| **TLE** | 316.1867 | 344.1816 | **TLEQ** | 444.2453 | 472.2402 | **LE** | 215.1390 | 243.1339 |
| **LEQ** | 343.1976 | 371.1925 | **EQ** | 230.1135 | 258.1084 | | | |

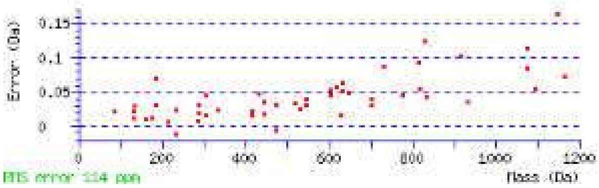


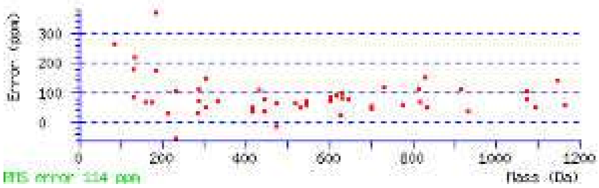


NCBI **BLAST** search of VIACVGETLEQR
(Parameters: blastp, nr protein database, expect=20000, no filter, PAM30)
Other BLAST web gateways

**All matches to this query**

| Score | Mr(calc) | Delta | Sequence |
|---|---|---|---|
| 72.3 | 1373.6973 | 0.0808 | VIACVGETLEQR |
| 58.4 | 1373.6973 | 0.0808 | VIACVGETLQER |
| 48.9 | 1373.6722 | 0.1060 | VGGDDVMREQLR |
| 48.0 | 1373.6973 | 0.0808 | GPGSMVTIAEIAGR |
| 46.5 | 1373.7085 | 0.0696 | LTNDVMERLAGR |
| 42.5 | 1373.7489 | 0.0292 | IETAGAWMVLKR |
| 42.5 | 1373.7085 | 0.0696 | ENGLEGMLARIR |
| 42.2 | 1373.6787 | 0.0995 | ETTNVDDLAQLR |
| 42.0 | 1373.7262 | 0.0519 | LTASAAERSALER |
| 40.2 | 1373.7514 | 0.0267 | LTASAVLSAGITDR |

**Mascot:** http://www.matrixscience.com/

# MATRIX SCIENCE Mascot Search Results

## Peptide View Spot no 32

MS/MS Fragmentation of **WLAANVSAEVAESTR**
Found in **gi|553107** in **NCBInr**, triosephosphate isomerase, partial [Oryza sativa Japonica Group]

Match to Query 121: 1602.898224 from(1603.905500,1+) intensity(0.0000) index(25)
Title: Label: H19, Spot_Id: 219969, Peak_List_Id: 229381, MSMS Job_Run_Id: 22019, Comment:
Data file C:\Documents and Settings\Administrator\Desktop\Farah-Garima 11May 2013\ppw_H19_136868348800.txt

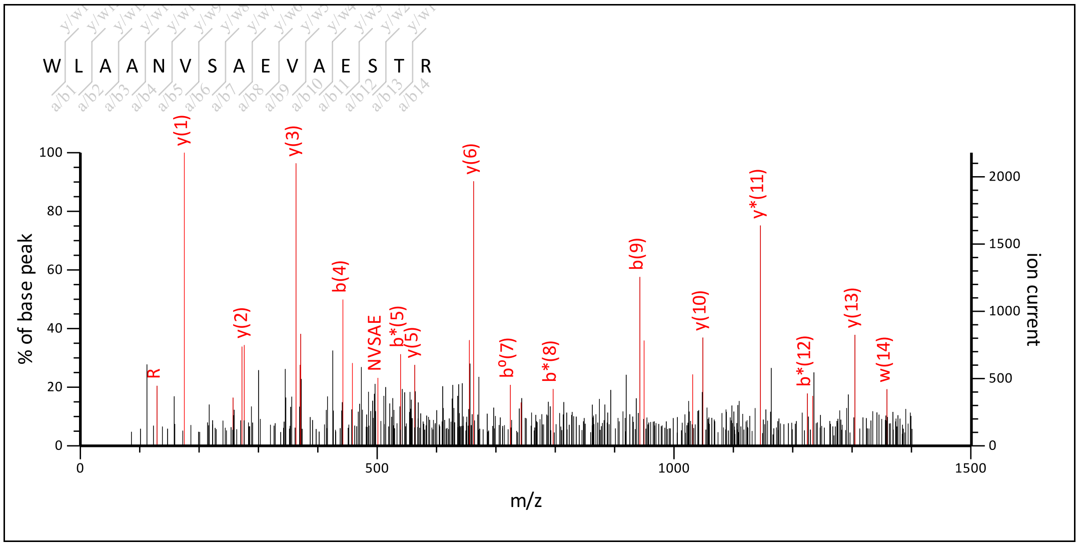


0 to 1501.03

Label all possible matches ○ Label matches used for scoring ◉

**Monoisotopic mass of neutral peptide Mr(calc):** 1602.8002
**Fixed modifications:** Carbamidomethyl (C) (apply to specified residues or termini only)
**Ions Score:** 58 **Expect:** 0.045
**Matches :** 33/270 fragment ions using 42 most intense peaks (help)

| # | Immon. | a | a* | $a^0$ | b | b* | $b^0$ | d | d' | Seq. | v | w | w' | y | y* | |
|---|---|---|---|---|---|---|---|---|---|---|---|---|---|---|---|---|
| 1 | 159.0917 | 159.0917 | | | 187.0866 | | | 44.0495 | | W | | | | | | |
| 2 | 86.0964 | 272.1757 | | | 300.1707 | | | 230.1288 | | L | 1359.6499 | 1358.6546 | | 1417.7281 | 1400.7016 | 139 |
| 3 | 44.0495 | 343.2129 | | | **371.2078** | | | | | A | 1288.6128 | | | ***1304.6441*** | 1287.6175 | 128 |
| 4 | 44.0495 | 414.2500 | | | **442.2449** | | | | | A | 1217.5757 | | | ***1233.6070*** | 1216.5804 | 12 |
| 5 | 87.0553 | 528.2929 | 511.2663 | | 556.2878 | 539.2613 | | 485.2871 | | N | 1103.5327 | 1102.5375 | | 1162.5699 | 1145.5433 | 114 |
| 6 | 72.0808 | 627.3613 | 610.3348 | | **655.3562** | 638.3297 | | 613.3457 | | V | 1004.4643 | 1017.4847 | | ***1048.5269*** | 1031.5004 | 103 |
| 7 | 60.0444 | 714.3933 | 697.3668 | 696.3828 | **742.3882** | 725.3617 | 724.3777 | 698.3984 | | S | 917.4323 | 916.4371 | | ***949.4585*** | 932.4320 | 93 |
| 8 | 44.0495 | 785.4304 | 768.4039 | 767.4199 | 813.4254 | 796.3988 | 795.4148 | | | A | 846.3952 | | | 862.4265 | 845.3999 | 84 |
| 9 | 102.0550 | 914.4730 | 897.4465 | 896.4625 | **942.4680** | 925.4414 | 924.4574 | 856.4676 | | E | 717.3526 | 716.3573 | | 791.3894 | 774.3628 | 77 |
| 10 | 72.0808 | 1013.5415 | 996.5149 | 995.5309 | 1041.5364 | 1024.5098 | 1023.5258 | 999.5258 | | V | 618.2842 | 631.3046 | | ***662.3468*** | 645.3202 | 64 |
| 11 | 44.0495 | 1084.5786 | 1067.5520 | 1066.5680 | 1112.5735 | 1095.5469 | 1094.5629 | | | A | 547.2471 | | | ***563.2784*** | 546.2518 | 54 |
| 12 | 102.0550 | 1213.6212 | 1196.5946 | 1195.6106 | 1241.6161 | 1224.5895 | 1223.6055 | 1155.6157 | | E | 418.2045 | 417.2092 | | 492.2413 | 475.2147 | 47 |
| 13 | 60.0444 | 1300.6532 | 1283.6266 | 1282.6426 | 1328.6481 | 1311.6216 | 1310.6375 | 1284.6583 | | S | 331.1724 | 330.1772 | | ***363.1987*** | 346.1721 | 34 |
| 14 | 74.0600 | 1401.7009 | 1384.6743 | 1383.6903 | 1429.6958 | 1412.6692 | 1411.6852 | 1385.7060 | 1387.6852 | T | 230.1248 | 243.1452 | 245.1244 | ***276.1666*** | 259.1401 | 25 |
| 15 | 129.1135 | | | | | | | | | R | 74.0237 | 73.0284 | | ***175.1190*** | 158.0924 | |

| Seq | ya | yb | Seq | ya | yb | Seq | ya | yb |
|---|---|---|---|---|---|---|---|---|
| LA | 157.1335 | 185.1285 | LAA | 228.1707 | 256.1656 | LAAN | 342.2136 | 370.2085 |
| LAANV | 441.2820 | 469.2769 | LAANVS | 528.3140 | 556.3089 | LAANVSA | 599.3511 | 627.3461 |
| AA | 115.0866 | 143.0815 | AAN | 229.1295 | 257.1244 | AANV | 328.1979 | 356.1928 |
| AANVS | 415.2300 | 443.2249 | AANVSA | 486.2671 | 514.2620 | AANVSAE | 615.3097 | 643.3046 |
| AN | 158.0924 | 186.0873 | ANV | 257.1608 | 285.1557 | ANVS | 344.1928 | 372.1878 |
| ANVSA | 415.2300 | 443.2249 | ANVSAE | 544.2726 | 572.2675 | ANVSAEV | 643.3410 | 671.3359 |

| | | | | | | | | |
|---|---|---|---|---|---|---|---|---|
| **NV** | 186.1237 | 214.1186 | **NVS** | 273.1557 | 301.1506 | **NVSA** | 344.1928 | 372.1878 |
| **NVSAE** | 473.2354 | 501.2304 | **NVSAEV** | 572.3039 | 600.2988 | **NVSAEVA** | 643.3410 | 671.3359 |
| **VS** | 159.1128 | 187.1077 | **VSA** | 230.1499 | 258.1448 | **VSAE** | 359.1925 | 387.1874 |
| **VSAEV** | 458.2609 | 486.2558 | **VSAEVA** | 529.2980 | 557.2930 | **VSAEVAE** | 658.3406 | 686.3355 |
| **SA** | 131.0815 | 159.0764 | **SAE** | 260.1241 | 288.1190 | **SAEV** | 359.1925 | 387.1874 |
| **SAEVA** | 430.2296 | 458.2245 | **SAEVAE** | 559.2722 | 587.2671 | **SAEVAES** | 646.3042 | 674.2992 |
| **AE** | 173.0921 | 201.0870 | **AEV** | 272.1605 | 300.1554 | **AEVA** | 343.1976 | 371.1925 |
| **AEVAE** | 472.2402 | 500.2351 | **AEVAES** | 559.2722 | 587.2671 | **AEVAEST** | 660.3199 | 688.3148 |
| **EV** | 201.1234 | 229.1183 | **EVA** | 272.1605 | 300.1554 | **EVAE** | 401.2031 | 429.1980 |
| **EVAES** | 488.2351 | 516.2300 | **EVAEST** | 589.2828 | 617.2777 | **VA** | 143.1179 | 171.1128 |
| **VAE** | 272.1605 | 300.1554 | **VAES** | 359.1925 | 387.1874 | **VAEST** | 460.2402 | 488.2351 |
| **AE** | 173.0921 | 201.0870 | **AES** | 260.1241 | 288.1190 | **AEST** | 361.1718 | 389.1667 |
| **ES** | 189.0870 | 217.0819 | **EST** | 290.1347 | 318.1296 | **ST** | 161.0921 | 189.0870 |

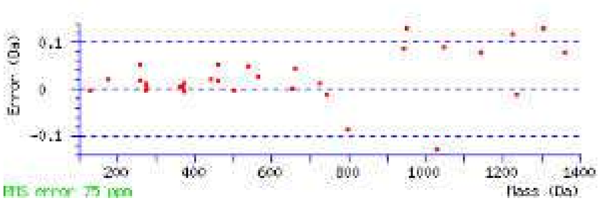


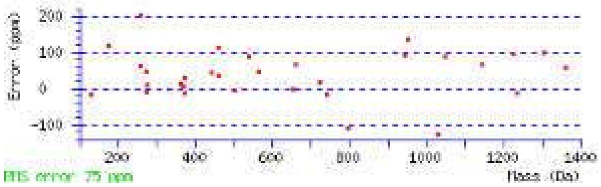


NCBI **BLAST** search of WLAANVSAEVAESTR
(Parameters: blastp, nr protein database, expect=20000, no filter, PAM30)
Other BLAST web gateways

**All matches to this query**

| Score | Mr(calc) | Delta | Sequence |
|---|---|---|---|
| 58.0 | 1602.8002 | 0.0981 | WLAANVSAEVAESTR |
| 24.7 | 1602.8399 | 0.0583 | KSNILVSAEDMQLR |
| 20.6 | 1602.8213 | 0.0769 | TVVVGGDISNPTTSTR |
| 20.6 | 1602.8994 | -0.0012 | VATPAAPRPGFPAVPR |
| 20.4 | 1602.7962 | 0.1021 | ARADAATSPTTPTSTR |
| 20.4 | 1602.7784 | 0.1199 | TRSPNDAAEMAALTR |
| 18.9 | 1602.8253 | 0.0729 | VVPYVTEPGAEVSTR |
| 18.3 | 1602.8626 | 0.0356 | MVVLPSWICLLTR |
| 17.8 | 1602.8114 | 0.0868 | AQYEVADSAIPARGR |
| 17.3 | 1602.7534 | 0.1448 | MSSAVDGFMKYVPR |

**Mascot:** http://www.matrixscience.com/

# MATRIX SCIENCE Mascot Search Results

## Peptide View Spot no 32

MS/MS Fragmentation of **VATPDQAQEVHDGLR**
Found in **gi|553107** in **NCBInr**, triosephosphate isomerase, partial [Oryza sativa Japonica Group]

Match to Query 125: 1634.902224 from(1635.909500,1+) intensity(0.0000) index(26)
Title: Label: H19, Spot_Id: 219969, Peak_List_Id: 229380, MSMS Job_Run_Id: 22019, Comment:
Data file C:\Documents and Settings\Administrator\Desktop\Farah-Garima 11May 2013\ppw_H19_136868348800.txt

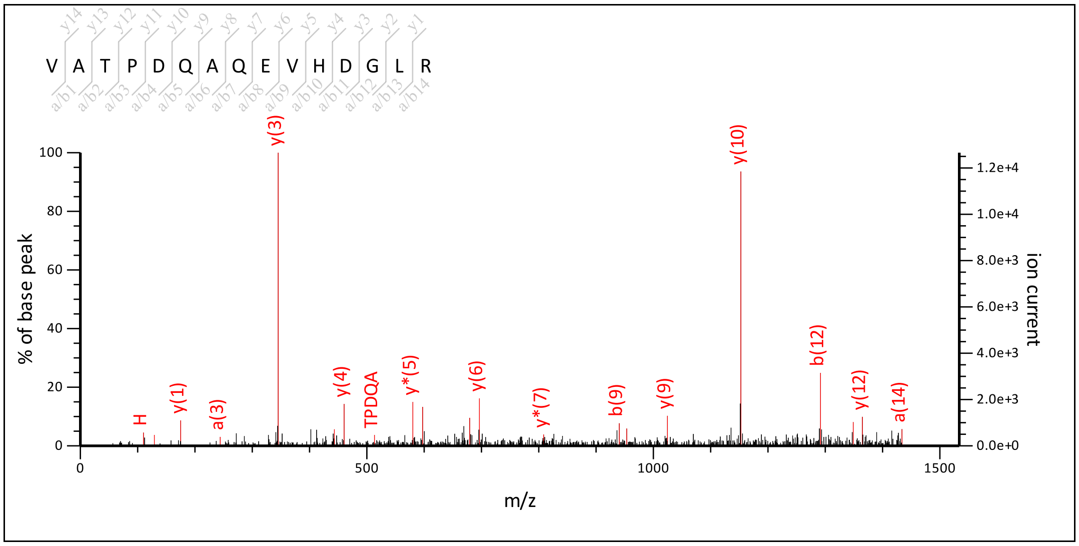


0 to 1533.87

Label all possible matches ○ Label matches used for scoring ◉

**Monoisotopic mass of neutral peptide Mr(calc):** 1634.8013
**Fixed modifications:** Carbamidomethyl (C) (apply to specified residues or termini only)
**Ions Score:** 71 **Expect:** 0.0022
**Matches :** 24/260 fragment ions using 28 most intense peaks (help)

| # | Immon. | a | a* | a$^0$ | b | b* | b$^0$ | d | d' | Seq. | v | w | w' | y | y* | |
|---|---|---|---|---|---|---|---|---|---|---|---|---|---|---|---|---|
| 1 | 72.0808 | 72.0808 | | | 100.0757 | | | 44.0495 | | V | | | | | | |
| 2 | 44.0495 | 143.1179 | | | 171.1128 | | | | | A | 1520.7088 | | | 1536.7401 | 1519.7136 | 15 |
| 3 | 74.0600 | **244.1656** | | 226.1550 | 272.1605 | | 254.1499 | 228.1707 | 230.1499 | T | 1419.6611 | 1432.6815 | 1434.6608 | 1465.7030 | 1448.6764 | 14 |
| 4 | 70.0651 | 341.2183 | | 323.2078 | 369.2132 | | 351.2027 | 315.2027 | | P | 1322.6084 | 1321.6131 | | ***1364.6553*** | 1347.6288 | 13 |
| 5 | 88.0393 | 456.2453 | | 438.2347 | 484.2402 | | 466.2296 | 412.2554 | | D | 1207.5814 | 1206.5862 | | 1267.6026 | 1250.5760 | 12 |
| 6 | 101.0709 | 584.3039 | 567.2773 | 566.2933 | 612.2988 | 595.2722 | 594.2882 | 527.2824 | | Q | 1079.5228 | 1078.5276 | | ***1152.5756*** | 1135.5491 | 11 |
| 7 | 44.0495 | 655.3410 | 638.3144 | 637.3304 | 683.3359 | 666.3093 | 665.3253 | | | A | 1008.4857 | | | ***1024.5170*** | 1007.4905 | 10 |
| 8 | 101.0709 | 783.3995 | 766.3730 | 765.3890 | 811.3945 | 794.3679 | 793.3839 | 726.3781 | | Q | 880.4272 | 879.4319 | | ***953.4799*** | 936.4534 | 9 |
| 9 | 102.0550 | 912.4421 | 895.4156 | 894.4316 | **940.4371** | 923.4105 | 922.4265 | 854.4367 | | E | 751.3846 | 750.3893 | | 825.4213 | 808.3948 | 8 |
| 10 | 72.0808 | 1011.5106 | 994.4840 | 993.5000 | 1039.5055 | 1022.4789 | 1021.4949 | 997.4949 | | V | 652.3161 | 665.3365 | | ***696.3787*** | 679.3522 | 6 |
| 11 | 110.0713 | 1148.5695 | 1131.5429 | 1130.5589 | 1176.5644 | 1159.5378 | 1158.5538 | | | H | 515.2572 | | | ***597.3103*** | 580.2838 | 5 |
| 12 | 88.0393 | 1263.5964 | 1246.5699 | 1245.5858 | **1291.5913** | 1274.5648 | 1273.5808 | 1219.6066 | | D | 400.2303 | 399.2350 | | ***460.2514*** | 443.2249 | 4 |
| 13 | 30.0338 | 1320.6179 | 1303.5913 | 1302.6073 | **1348.6128** | 1331.5862 | 1330.6022 | | | G | | | | ***345.2245*** | 328.1979 | |
| 14 | 86.0964 | **1433.7019** | 1416.6754 | 1415.6914 | 1461.6969 | 1444.6703 | 1443.6863 | 1391.6550 | | L | 230.1248 | 229.1295 | | 288.2030 | 271.1765 | |
| 15 | 129.1135 | | | | | | | | | R | 74.0237 | 73.0284 | | ***175.1190*** | 158.0924 | |

| Seq | ya | yb | Seq | ya | yb | Seq | ya | yb |
|---|---|---|---|---|---|---|---|---|
| AT | 145.0972 | 173.0921 | ATP | 242.1499 | 270.1448 | ATPD | 357.1769 | 385.1718 |
| ATPDQ | 485.2354 | 513.2304 | ATPDQA | 556.2726 | 584.2675 | ATPDQAQ | 684.3311 | 712.3260 |
| TP | 171.1128 | 199.1077 | TPD | 286.1397 | 314.1347 | TPDQ | 414.1983 | 442.1932 |
| TPDQA | 485.2354 | 513.2304 | TPDQAQ | 613.2940 | 641.2889 | PD | 185.0921 | 213.0870 |
| PDQ | 313.1506 | 341.1456 | PDQA | 384.1878 | 412.1827 | PDQAQ | 512.2463 | 540.2413 |
| PDQAQE | 641.2889 | 669.2838 | DQ | 216.0979 | 244.0928 | DQA | 287.1350 | 315.1299 |

| | | | | | | | | |
|---|---|---|---|---|---|---|---|---|
| **DQAQ** | 415.1936 | 443.1885 | **DQAQE** | 544.2362 | 572.2311 | **DQAQEV** | 643.3046 | 671.2995 |
| **QA** | 172.1081 | 200.1030 | **QAQ** | 300.1666 | 328.1615 | **QAQE** | 429.2092 | 457.2041 |
| **QAQEV** | 528.2776 | 556.2726 | **QAQEVH** | 665.3366 | 693.3315 | **AQ** | 172.1081 | 200.1030 |
| **AQE** | 301.1506 | 329.1456 | **AQEV** | 400.2191 | 428.2140 | **AQEVH** | 537.2780 | 565.2729 |
| **AQEVHD** | 652.3049 | 680.2998 | **QE** | 230.1135 | 258.1084 | **QEV** | 329.1819 | 357.1769 |
| **QEVH** | 466.2409 | 494.2358 | **QEVHD** | 581.2678 | 609.2627 | **QEVHDG** | 638.2893 | 666.2842 |
| **EV** | 201.1234 | 229.1183 | **EVH** | 338.1823 | 366.1772 | **EVHD** | 453.2092 | 481.2041 |
| **EVHDG** | 510.2307 | 538.2256 | **EVHDGL** | 623.3148 | 651.3097 | **VH** | 209.1397 | 237.1346 |
| **VHD** | 324.1666 | 352.1615 | **VHDG** | 381.1881 | 409.1830 | **VHDGL** | 494.2722 | 522.2671 |
| **HD** | 225.0982 | 253.0931 | **HDG** | 282.1197 | 310.1146 | **HDGL** | 395.2037 | 423.1987 |
| **DG** | 145.0608 | 173.0557 | **DGL** | 258.1448 | 286.1397 | **GL** | 143.1179 | 171.1128 |

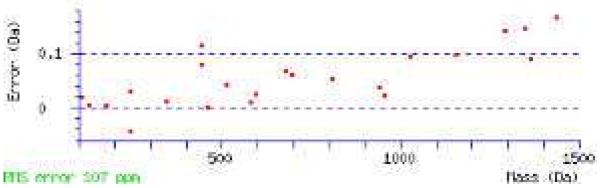


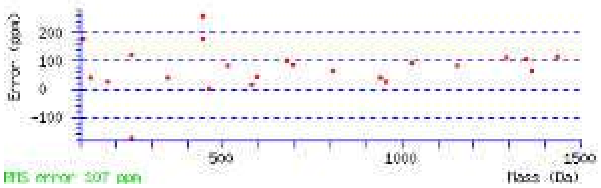


NCBI **BLAST** search of VATPDQAQEVHDGLR
(Parameters: blastp, nr protein database, expect=20000, no filter, PAM30)
Other BLAST web gateways

**All matches to this query**

| Score | Mr(calc) | Delta | Sequence |
|---|---|---|---|
| 71.2 | 1634.8013 | 0.1010 | VATPDQAQEVHDGLR |
| 27.3 | 1634.8601 | 0.0421 | QLNQQGLTRHDLGR |
| 26.5 | 1634.8264 | 0.0758 | RDIVDDLTYEAGIR |
| 26.3 | 1634.8740 | 0.0282 | RDIVDHVIDDLVAR |
| 26.3 | 1634.8814 | 0.0208 | DRLVVEMLYATGIR |
| 26.1 | 1634.8297 | 0.0725 | IASLVISKDENGMSR |
| 25.6 | 1634.9832 | -0.0810 | RDVAIVGTGVVLAPIR |
| 25.5 | 1634.9104 | -0.0082 | AVSSPDLRPSALIGPR |
| 25.5 | 1634.8125 | 0.0897 | RDTGFDSTATVGRPR |
| 25.4 | 1634.9355 | -0.0333 | ISAPPKDNLILEGIR |

**Mascot:** http://www.matrixscience.com/

# MATRIX SCIENCE Mascot Search Results

## Peptide View Spot no 32

MS/MS Fragmentation of **VATPDQAQEVHDGLRK**
Found in **gi|553107** in **NCBInr**, triosephosphate isomerase, partial [Oryza sativa Japonica Group]

Match to Query 137: 1763.004524 from(1764.011800,1+) intensity(0.0000) index(29)
Title: Label: H19, Spot_Id: 219969, Peak_List_Id: 229373, MSMS Job_Run_Id: 22019, Comment:
Data file C:\Documents and Settings\Administrator\Desktop\Farah-Garima 11May 2013\ppw_H19_136868348800.txt

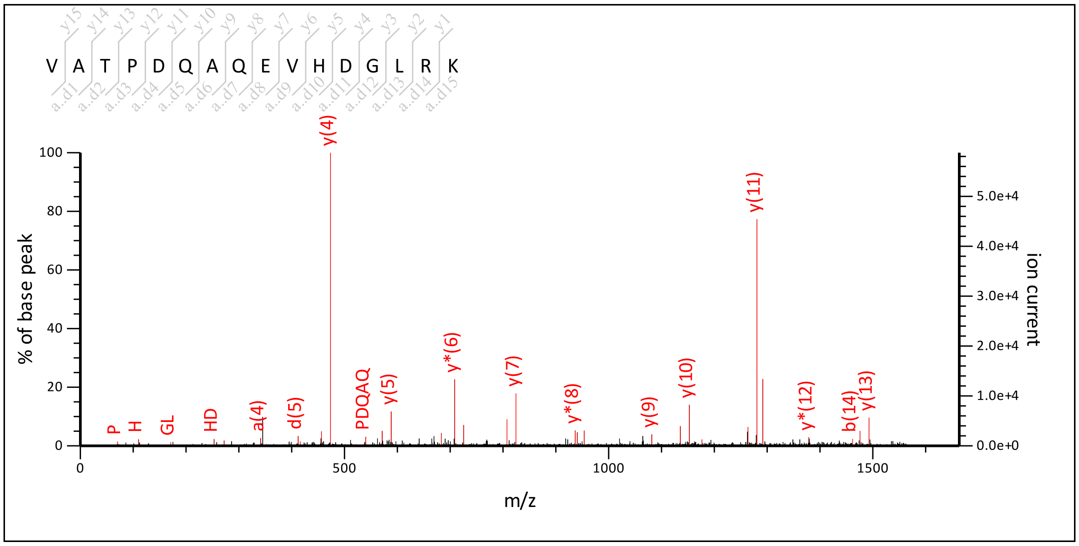


0 to 1663.64

Label all possible matches ○ Label matches used for scoring ●

**Monoisotopic mass of neutral peptide Mr(calc):** 1762.8962
**Fixed modifications:** Carbamidomethyl (C) (apply to specified residues or termini only)
**Ions Score:** 69 **Expect:** 0.0029
**Matches :** 37/282 fragment ions using 48 most intense peaks (help)

| # | Immon. | a | a* | a0 | b | b* | b0 | d | d' | Seq. | v | w | w' | y | y* | |
|---|---|---|---|---|---|---|---|---|---|---|---|---|---|---|---|---|
| 1 | 72.0808 | 72.0808 | | | 100.0757 | | | 44.0495 | | V | | | | | | |
| 2 | 44.0495 | 143.1179 | | | **171.1128** | | | | | A | 1648.8038 | | | 1664.8351 | 1647.8085 | 164 |
| 3 | 74.0600 | 244.1656 | | 226.1550 | **272.1605** | | 254.1499 | 228.1707 | 230.1499 | T | 1547.7561 | 1560.7765 | 1562.7558 | 1593.7980 | 1576.7714 | 15 |
| 4 | 70.0651 | **341.2183** | | 323.2078 | 369.2132 | | 351.2027 | 315.2027 | | P | 1450.7033 | 1449.7081 | | ***1492.7503*** | 1475.7237 | 14 |
| 5 | 88.0393 | **456.2453** | | 438.2347 | 484.2402 | | 466.2296 | 412.2554 | | D | 1335.6764 | 1334.6811 | | 1395.6975 | 1378.6710 | 13 |
| 6 | 101.0709 | 584.3039 | 567.2773 | 566.2933 | 612.2988 | 595.2722 | 594.2882 | 527.2824 | | Q | 1207.6178 | 1206.6226 | | ***1280.6706*** | 1263.6440 | 12 |
| 7 | 44.0495 | 655.3410 | 638.3144 | 637.3304 | **683.3359** | 666.3093 | 665.3253 | | | A | 1136.5807 | | | ***1152.6120*** | 1135.5854 | 11 |
| 8 | 101.0709 | 783.3995 | 766.3730 | 765.3890 | 811.3945 | 794.3679 | 793.3839 | 726.3781 | | Q | 1008.5221 | 1007.5269 | | ***1081.5749*** | 1064.5483 | 10 |
| 9 | 102.0550 | 912.4421 | 895.4156 | 894.4316 | **940.4371** | 923.4105 | 922.4265 | 854.4367 | | E | 879.4795 | 878.4843 | | ***953.5163*** | 936.4898 | 9 |
| 10 | 72.0808 | 1011.5106 | 994.4840 | 993.5000 | 1039.5055 | 1022.4789 | 1021.4949 | 997.4949 | | V | 780.4111 | 793.4315 | | ***824.4737*** | 807.4472 | 8 |
| 11 | 110.0713 | 1148.5695 | 1131.5429 | 1130.5589 | **1176.5644** | 1159.5378 | 1158.5538 | | | H | 643.3522 | | | ***725.4053*** | 708.3787 | 7 |
| 12 | 88.0393 | 1263.5964 | 1246.5699 | 1245.5858 | **1291.5913** | 1274.5648 | 1273.5808 | 1219.6066 | | D | 528.3253 | 527.3300 | | ***588.3464*** | 571.3198 | 5 |
| 13 | 30.0338 | 1320.6179 | 1303.5913 | 1302.6073 | 1348.6128 | 1331.5862 | 1330.6022 | | | G | | | | ***473.3194*** | 456.2929 | |
| 14 | 86.0964 | 1433.7019 | 1416.6754 | 1415.6914 | **1461.6969** | 1444.6703 | 1443.6863 | 1391.6550 | | L | 358.2197 | 357.2245 | | ***416.2980*** | 399.2714 | |
| 15 | 129.1135 | 1589.8030 | 1572.7765 | 1571.7925 | 1617.7980 | 1600.7714 | 1599.7874 | 1504.7390 | | R | 202.1186 | 201.1234 | | 303.2139 | 286.1874 | |
| 16 | 101.1073 | | | | | | | | | K | 74.0237 | 73.0284 | | 147.1128 | 130.0863 | |

| Seq | ya | yb | Seq | ya | yb | Seq | ya | yb |
|---|---|---|---|---|---|---|---|---|
| AT | 145.0972 | 173.0921 | ATP | 242.1499 | 270.1448 | ATPD | 357.1769 | 385.1718 |
| ATPDQ | 485.2354 | 513.2304 | ATPDQA | 556.2726 | 584.2675 | ATPDQAQ | 684.3311 | 712.3260 |
| TP | 171.1128 | 199.1077 | TPD | 286.1397 | 314.1347 | TPDQ | 414.1983 | 442.1932 |
| TPDQA | 485.2354 | 513.2304 | TPDQAQ | 613.2940 | 641.2889 | PD | 185.0921 | 213.0870 |
| PDQ | 313.1506 | 341.1456 | PDQA | 384.1878 | 412.1827 | PDQAQ | 512.2463 | 540.2413 |

| | | | | | | | | |
|---|---|---|---|---|---|---|---|---|
| **PDQAQE** | 641.2889 | 669.2838 | **DQ** | 216.0979 | 244.0928 | **DQA** | 287.1350 | 315.1299 |
| **DQAQ** | 415.1936 | 443.1885 | **DQAQE** | 544.2362 | 572.2311 | **DQAQEV** | 643.3046 | 671.2995 |
| **QA** | 172.1081 | 200.1030 | **QAQ** | 300.1666 | 328.1615 | **QAQE** | 429.2092 | 457.2041 |
| **QAQEV** | 528.2776 | 556.2726 | **QAQEVH** | 665.3366 | 693.3315 | **AQ** | 172.1081 | 200.1030 |
| **AQE** | 301.1506 | 329.1456 | **AQEV** | 400.2191 | 428.2140 | **AQEVH** | 537.2780 | 565.2729 |
| **AQEVHD** | 652.3049 | 680.2998 | **QE** | 230.1135 | 258.1084 | **QEV** | 329.1819 | 357.1769 |
| **QEVH** | 466.2409 | 494.2358 | **QEVHD** | 581.2678 | 609.2627 | **QEVHDG** | 638.2893 | 666.2842 |
| **EV** | 201.1234 | 229.1183 | **EVH** | 338.1823 | 366.1772 | **EVHD** | 453.2092 | 481.2041 |
| **EVHDG** | 510.2307 | 538.2256 | **EVHDGL** | 623.3148 | 651.3097 | **VH** | 209.1397 | 237.1346 |
| **VHD** | 324.1666 | 352.1615 | **VHDG** | 381.1881 | 409.1830 | **VHDGL** | 494.2722 | 522.2671 |
| **VHDGLR** | 650.3733 | 678.3682 | **HD** | 225.0982 | 253.0931 | **HDG** | 282.1197 | 310.1146 |
| **HDGL** | 395.2037 | 423.1987 | **HDGLR** | 551.3049 | 579.2998 | **DG** | 145.0608 | 173.0557 |
| **DGL** | 258.1448 | 286.1397 | **DGLR** | 414.2459 | 442.2409 | **GL** | 143.1179 | 171.1128 |
| **GLR** | 299.2190 | 327.2139 | **LR** | 242.1975 | 270.1925 | | | |

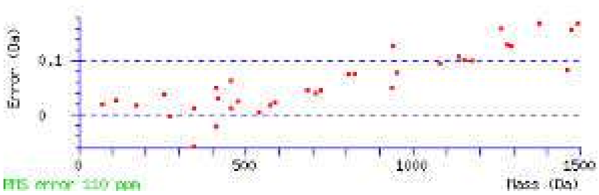


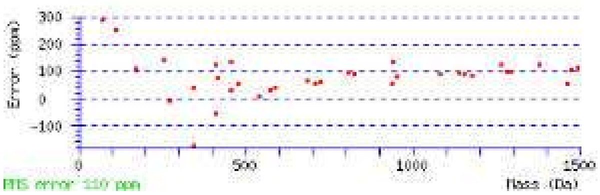


NCBI **BLAST** search of VATPDQAQEVHDGLRK
(Parameters: blastp, nr protein database, expect=20000, no filter, PAM30)
Other BLAST web gateways

**All matches to this query**

| Score | Mr(calc) | Delta | Sequence |
|---|---|---|---|
| 69.1 | 1762.8962 | 0.1083 | VATPDQAQEVHDGLRK |
| 32.3 | 1762.9074 | 0.0971 | RPTEAQQEDRPAIPR |
| 30.7 | 1762.9400 | 0.0645 | LVCLNQTLPPGDLAPR |
| 29.0 | 1762.9479 | 0.0567 | KAAGIFHVTNPGSVTHK |
| 27.6 | 1762.9829 | 0.0217 | LIEQPVEAPNLDGLKK |
| 26.3 | 1763.0417 | -0.0372 | VGDLRAVLQLDKPALR |
| 26.1 | 1762.8594 | 0.1451 | IVVTDIQMPGSMDGLR |
| 25.8 | 1762.9326 | 0.0719 | ERVAAVGAGLDLPPDQR |
| 25.6 | 1762.9942 | 0.0104 | AVTQTVDGAPVIIVAGPR |
| 24.7 | 1762.8850 | 0.1195 | VSVSQTYTDNVNLAPR |

**Mascot:** http://www.matrixscience.com/

# MATRIX SCIENCE Mascot Search Results

## Peptide View Spot no 32

MS/MS Fragmentation of **SQLRPEIQVAAQNCWVK**
Found in **gi|553107** in **NCBInr**, triosephosphate isomerase, partial [Oryza sativa Japonica Group]

Match to Query 151: 2026.161324 from(2027.168600,1+) intensity(0.0000) index(33)
Title: Label: H19, Spot_Id: 219969, Peak_List_Id: 229378, MSMS Job_Run_Id: 22019, Comment:
Data file C:\Documents and Settings\Administrator\Desktop\Farah-Garima 11May 2013\ppw_H19_136868348800.txt

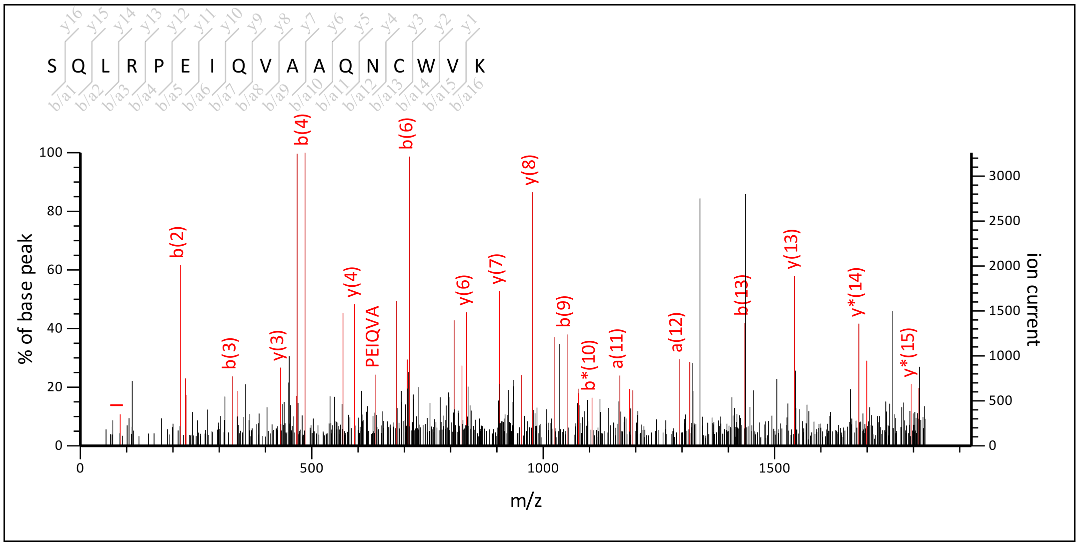


0 to 1925.25

Label all possible matches ○ Label matches used for scoring ◉

**Monoisotopic mass of neutral peptide Mr(calc):** 2026.0418
**Fixed modifications:** Carbamidomethyl (C) (apply to specified residues or termini only)
**Ions Score:** 76 **Expect:** 0.00055
**Matches :** 45/305 fragment ions using 54 most intense peaks (help)

| # | Immon. | a | a* | $a^0$ | b | b* | $b^0$ | d | d' | Seq. | v | w | w' | y | y* | |
|---|---|---|---|---|---|---|---|---|---|---|---|---|---|---|---|---|
| 1 | 60.0444 | 60.0444 | | 42.0338 | 88.0393 | | 70.0287 | 44.0495 | | S | | | | | | |
| 2 | 101.0709 | 188.1030 | 171.0764 | 170.0924 | **216.0979** | 199.0713 | 198.0873 | 131.0815 | | Q | 1866.9643 | 1865.9691 | | 1940.0171 | 1922.9905 | 19[illegible] |
| 3 | 86.0964 | 301.1870 | 284.1605 | 283.1765 | **329.1819** | 312.1554 | 311.1714 | 259.1401 | | L | 1753.8803 | 1752.8850 | | ***1811.9585*** | 1794.9320 | 17[illegible] |
| 4 | 129.1135 | 457.2881 | 440.2616 | 439.2776 | **485.2831** | 468.2565 | 467.2725 | 372.2241 | | R | 1597.7791 | 1596.7839 | | ***1698.8744*** | 1681.8479 | 16[illegible] |
| 5 | 70.0651 | 554.3409 | 537.3144 | 536.3303 | 582.3358 | 565.3093 | 564.3253 | 528.3253 | | P | 1500.7264 | 1499.7311 | | ***1542.7733*** | 1525.7468 | 15[illegible] |
| 6 | 102.0550 | **683.3835** | 666.3569 | 665.3729 | **711.3784** | 694.3519 | 693.3678 | 625.3780 | | E | 1371.6838 | 1370.6885 | | 1445.7206 | 1428.6940 | 14[illegible] |
| 7 | 86.0964 | 796.4676 | 779.4410 | 778.4570 | **824.4625** | 807.4359 | 806.4519 | 768.4363 | 782.4519 | I | 1258.5997 | 1271.6201 | 1285.6358 | ***1316.6780*** | 1299.6514 | |
| 8 | 101.0709 | 924.5261 | 907.4996 | 906.5156 | **952.5211** | 935.4945 | 934.5105 | 867.5047 | | Q | 1130.5411 | 1129.5459 | | 1203.5939 | 1186.5674 | |
| 9 | 72.0808 | **1023.5946** | 1006.5680 | 1005.5840 | **1051.5895** | 1034.5629 | 1033.5789 | 1009.5789 | | V | 1031.4727 | 1044.4931 | | ***1075.5353*** | 1058.5088 | |
| 10 | 44.0495 | 1094.6317 | 1077.6051 | 1076.6211 | 1122.6266 | 1105.6000 | 1104.6160 | | | A | 960.4356 | | | ***976.4669*** | 959.4404 | |
| 11 | 44.0495 | **1165.6688** | 1148.6422 | 1147.6582 | **1193.6637** | 1176.6371 | 1175.6531 | | | A | 889.3985 | | | ***905.4298*** | 888.4033 | |
| 12 | 101.0709 | **1293.7274** | 1276.7008 | 1275.7168 | 1321.7223 | 1304.6957 | 1303.7117 | 1236.7059 | | Q | 761.3399 | 760.3447 | | ***834.3927*** | 817.3661 | |
| 13 | 87.0553 | 1407.7703 | 1390.7437 | 1389.7597 | **1435.7652** | 1418.7387 | 1417.7546 | 1364.7645 | | N | 647.2970 | 646.3017 | | ***706.3341*** | 689.3076 | |
| 14 | 133.0430 | 1567.8009 | 1550.7744 | 1549.7904 | 1595.7959 | 1578.7693 | 1577.7853 | 1478.8074 | | C | 487.2663 | 486.2711 | | ***592.2912*** | 575.2646 | |
| 15 | 159.0917 | 1753.8803 | 1736.8537 | 1735.8697 | 1781.8752 | 1764.8486 | 1763.8646 | | | W | 301.1870 | | | ***432.2605*** | 415.2340 | |
| 16 | 72.0808 | 1852.9487 | 1835.9221 | 1834.9381 | 1880.9436 | 1863.9170 | 1862.9330 | 1838.9330 | | V | 202.1186 | 215.1390 | | 246.1812 | 229.1547 | |
| 17 | 101.1073 | | | | | | | | | K | 74.0237 | 73.0284 | | 147.1128 | 130.0863 | |

| Seq | ya | yb | Seq | ya | yb | Seq | ya | yb |
|---|---|---|---|---|---|---|---|---|
| QL | 214.1550 | 242.1499 | QLR | 370.2561 | 398.2510 | QLRP | 467.3089 | 495.3038 |
| QLRPE | 596.3515 | 624.3464 | LR | 242.1975 | 270.1925 | LRP | 339.2503 | 367.2452 |
| LRPE | 468.2929 | 496.2878 | LRPEI | 581.3770 | 609.3719 | RP | 226.1662 | 254.1612 |
| RPE | 355.2088 | 383.2037 | RPEI | 468.2929 | 496.2878 | RPEIQ | 596.3515 | 624.3464 |

| | | | | | | | | |
|---|---|---|---|---|---|---|---|---|
| **RPEIQV** | 695.4199 | 723.4148 | **PE** | 199.1077 | 227.1026 | **PEI** | 312.1918 | 340.1867 |
| **PEIQ** | 440.2504 | 468.2453 | **PEIQV** | 539.3188 | 567.3137 | **PEIQVA** | 610.3559 | 638.3508 |
| **PEIQVAA** | 681.3930 | 709.3879 | **EI** | 215.1390 | 243.1339 | **EIQ** | 343.1976 | 371.1925 |
| **EIQV** | 442.2660 | 470.2609 | **EIQVA** | 513.3031 | 541.2980 | **EIQVAA** | 584.3402 | 612.3352 |
| **IQ** | 214.1550 | 242.1499 | **IQV** | 313.2234 | 341.2183 | **IQVA** | 384.2605 | 412.2554 |
| **IQVAA** | 455.2976 | 483.2926 | **IQVAAQ** | 583.3562 | 611.3511 | **IQVAAQN** | 697.3992 | 725.3941 |
| **QV** | 200.1394 | 228.1343 | **QVA** | 271.1765 | 299.1714 | **QVAA** | 342.2136 | 370.2085 |
| **QVAAQ** | 470.2722 | 498.2671 | **QVAAQN** | 584.3151 | 612.3100 | **VA** | 143.1179 | 171.1128 |
| **VAA** | 214.1550 | 242.1499 | **VAAQ** | 342.2136 | 370.2085 | **VAAQN** | 456.2565 | 484.2514 |
| **VAAQNC** | 616.2872 | 644.2821 | **AA** | 115.0866 | 143.0815 | **AAQ** | 243.1452 | 271.1401 |
| **AAQN** | 357.1881 | 385.1830 | **AAQNC** | 517.2187 | 545.2137 | **AQ** | 172.1081 | 200.1030 |
| **AQN** | 286.1510 | 314.1459 | **AQNC** | 446.1816 | 474.1765 | **AQNCW** | 632.2609 | 660.2559 |
| **QN** | 215.1139 | 243.1088 | **QNC** | 375.1445 | 403.1394 | **QNCW** | 561.2238 | 589.2187 |
| **QNCWV** | 660.2922 | 688.2872 | **NC** | 247.0859 | 275.0809 | **NCW** | 433.1652 | 461.1602 |
| **NCWV** | 532.2337 | 560.2286 | **CW** | 319.1223 | 347.1172 | **CWV** | 418.1907 | 446.1857 |
| **WV** | 258.1601 | 286.1550 | | | | | | |

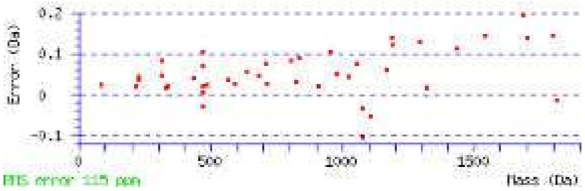


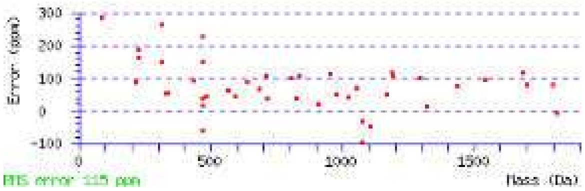


NCBI **BLAST** search of SQLRPEIQVAAQNCWVK
(Parameters: blastp, nr protein database, expect=20000, no filter, PAM30)
Other BLAST web gateways

**All matches to this query**

| Score | Mr(calc) | Delta | Sequence |
|---|---|---|---|
| 75.5 | 2026.0418 | 0.1195 | SQLRPEIQVAAQNCWVK |
| 20.3 | 2026.1324 | 0.0289 | DVPAGAVTLAGVAHVPAIGGVR |
| 18.0 | 2026.1323 | 0.0290 | LGSAADIRPIGAAATLVFQR |
| 16.3 | 2026.0636 | 0.0977 | LRPTAVVSQYSNIFEFR |
| 14.5 | 2026.0517 | 0.1096 | VAEVLPDLDVAAIENRMR |
| 14.2 | 2026.0259 | 0.1355 | FAGTTLLFTVAAEAAEEASK |
| 13.4 | 2025.9868 | 0.1746 | QSYAEAIAAAEEDLPRHR |
| 13.0 | 2026.1171 | 0.0442 | LRPQLVSLGSAALTSQETR |
| 13.0 | 2026.1184 | 0.0429 | LRQLAENLLLNATHHGAR |
| 12.8 | 2026.1575 | 0.0038 | LILQVAVGSAASSGVFLAAPR |

**Mascot:** http://www.matrixscience.com/

# MATRIX SCIENCE Mascot Search Results

## Peptide View Spot no 34

MS/MS Fragmentation of **IFLPEGLLDR**
Found in **gi|108864186** in **NCBInr**, Chlorophyll A-B binding protein, expressed [Oryza sativa Japonica Group]

Match to Query 115: 1171.705624 from(1172.712900,1+) intensity(0.0000) index(6)
Title: Label: F7, Spot_Id: 219775, Peak_List_Id: 226288, MSMS Job_Run_Id: 21839, Comment:
Data file C:\Documents and Settings\Administrator\Desktop\Farah-Garima 11May 2013\ppw_F7_136859913100.txt

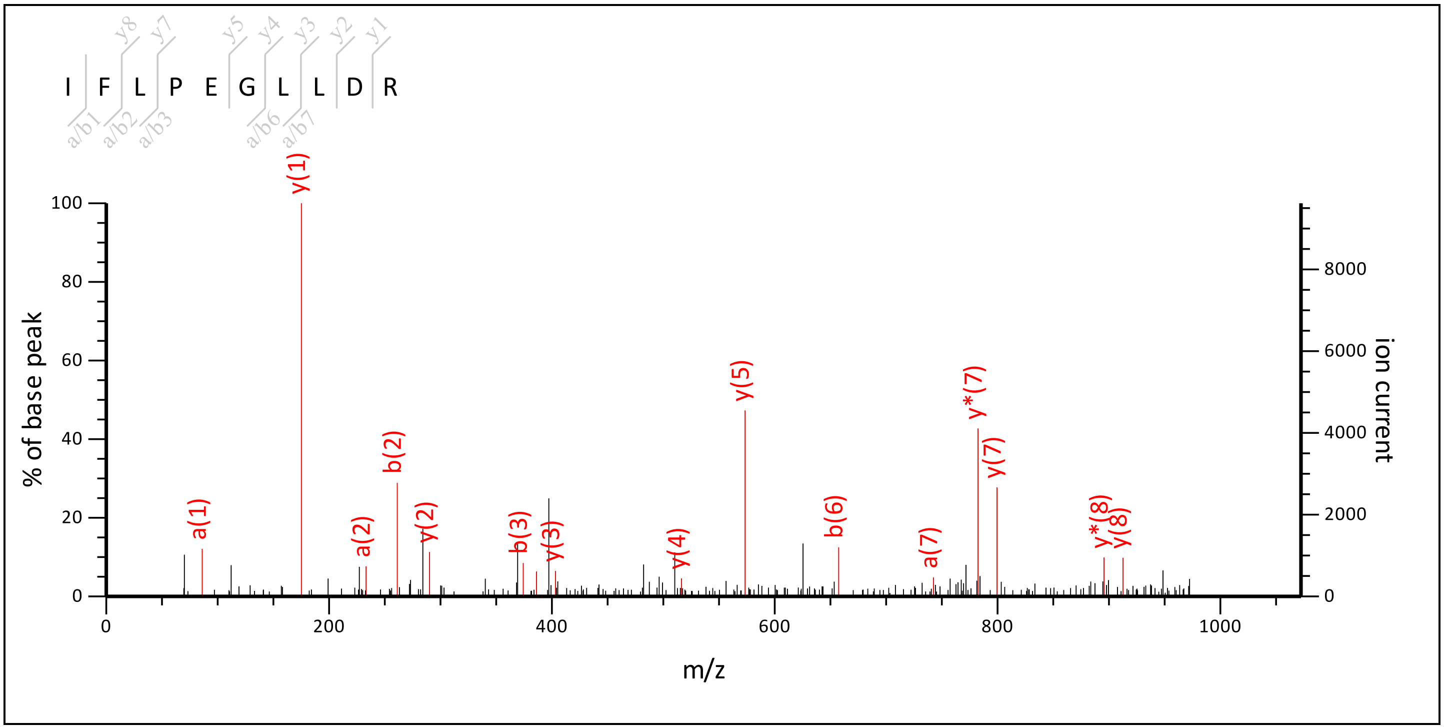


0 to 1072.37

Label all possible matches ○ Label matches used for scoring ◉

**Monoisotopic mass of neutral peptide Mr(calc):** 1171.6601
**Fixed modifications:** Carbamidomethyl (C) (apply to specified residues or termini only)
**Ions Score:** 35 **Expect:** 9.1
**Matches :** 16/36 fragment ions using 38 most intense peaks (help)

| # | a | b | Seq. | y | y* | # |
|---|---|---|---|---|---|---|
| 1 | **86.0964** | 114.0913 | I | | | 10 |
| 2 | **233.1648** | **261.1598** | F | 1059.5833 | 1042.5568 | 9 |
| 3 | 346.2489 | **374.2438** | L | ***912.5149*** | 895.4884 | 8 |
| 4 | 443.3017 | 471.2966 | P | ***799.4308*** | 782.4043 | 7 |
| 5 | 572.3443 | 600.3392 | E | 702.3781 | 685.3515 | 6 |
| 6 | 629.3657 | **657.3606** | G | ***573.3355*** | 556.3089 | 5 |
| 7 | **742.4498** | 770.4447 | L | ***516.3140*** | 499.2875 | 4 |
| 8 | 855.5339 | 883.5288 | L | ***403.2300*** | 386.2034 | 3 |

| | | | | | | |
|---|---|---|---|---|---|---|
| **9** | 970.5608 | 998.5557 | **D** | ***290.1459*** | 273.1193 | **2** |
| **10** | | | **R** | ***175.1190*** | 158.0924 | **1** |

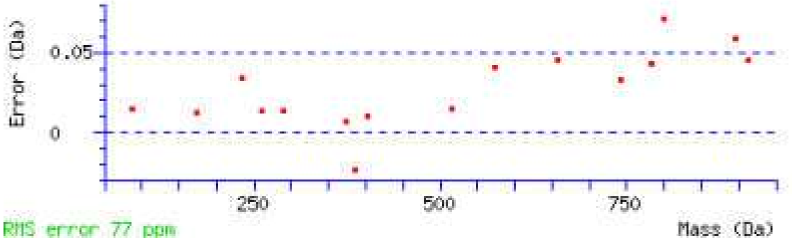


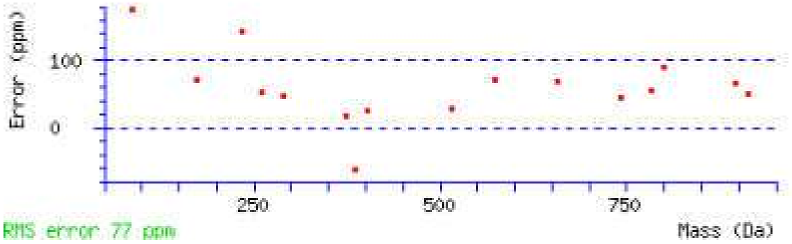


NCBI **BLAST** search of IFLPEGLLDR
(Parameters: blastp, nr protein database, expect=20000, no filter, PAM30)
Other BLAST web gateways

**All matches to this query**

| Score | Mr(calc) | Delta | Sequence |
|---|---|---|---|
| 34.9 | 1171.6601 | 0.0455 | IFLPEGLLDR |
| 23.5 | 1171.6866 | 0.0190 | IFIFGILGHR |
| 23.4 | 1171.7077 | -0.0021 | FLLGAAALGLAR |
| 23.3 | 1171.6965 | 0.0091 | FLLPVSLIDR |
| 23.3 | 1171.6998 | 0.0058 | MIILGIILDR |
| 22.4 | 1171.6561 | 0.0496 | LVAILGTNSER |
| 22.2 | 1171.7441 | -0.0385 | LFLLIAGAAKR |
| 21.7 | 1171.6998 | 0.0058 | LMDLLLIVAR |
| 21.3 | 1171.6495 | 0.0561 | IMIGRSLNPR |
| 21.1 | 1171.6635 | 0.0422 | VPSLLSMALNK |

**Mascot:** http://www.matrixscience.com/

# MATRIX SCIENCE Mascot Search Results

## Peptide View Spot no 34

MS/MS Fragmentation of **YQAYELIHAR**
Found in **gi|108864186** in **NCBInr**, Chlorophyll A-B binding protein, expressed [Oryza sativa Japonica Group]

Match to Query 137: 1262.691324 from(1263.698600,1+) intensity(0.0000) index(8)
Title: Label: F7, Spot_Id: 219775, Peak_List_Id: 226283, MSMS Job_Run_Id: 21839, Comment:
Data file C:\Documents and Settings\Administrator\Desktop\Farah-Garima 11May 2013\ppw_F7_136859913100.txt

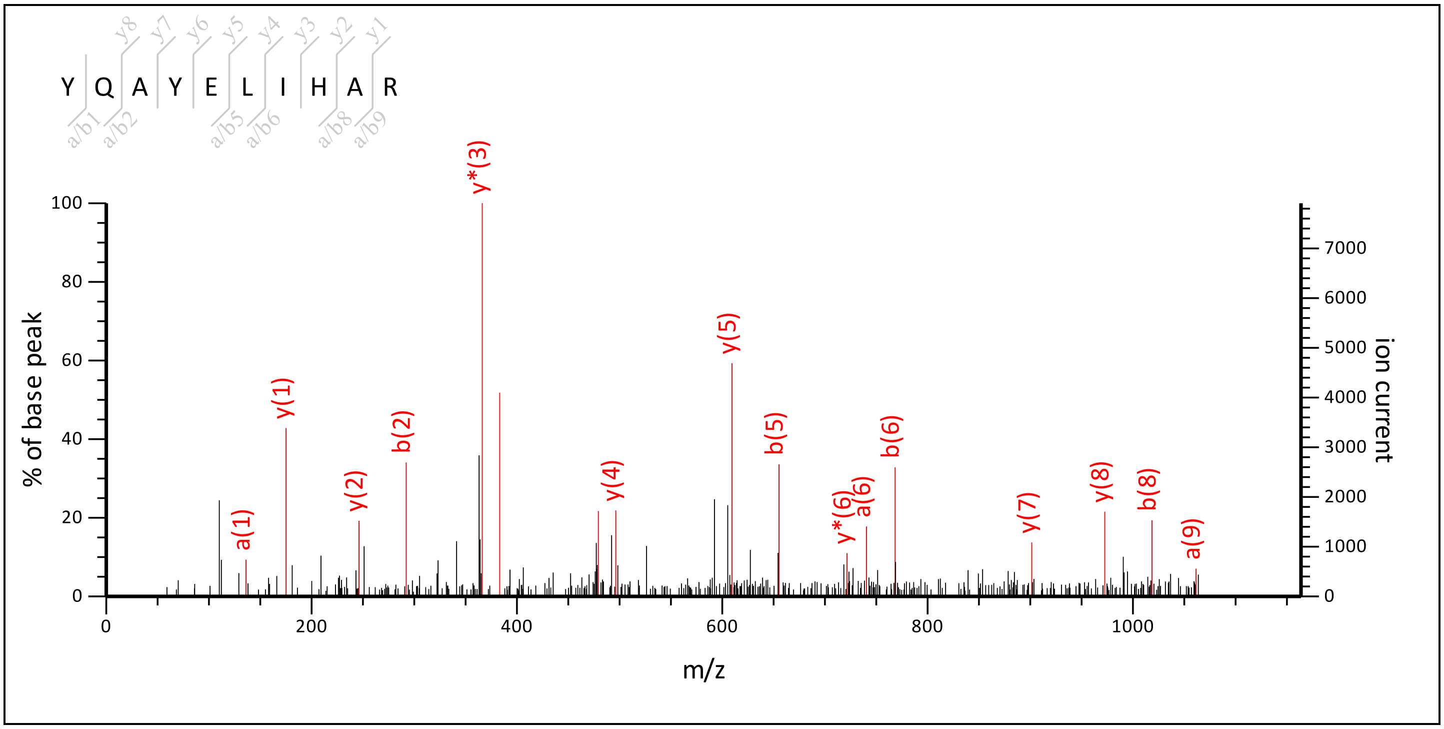


0 to 1163.68

Label all possible matches ○ Label matches used for scoring ◉

**Monoisotopic mass of neutral peptide Mr(calc):** 1262.6407
**Fixed modifications:** Carbamidomethyl (C) (apply to specified residues or termini only)
**Ions Score:** 56 **Expect:** 0.1
**Matches :** 17/52 fragment ions using 22 most intense peaks (help)

| # | a | a* | b | b* | Seq. | y | y* | # |
|---|---|---|---|---|---|---|---|---|
| 1 | **136.0757** | | 164.0706 | | Y | | | 10 |
| 2 | 264.1343 | 247.1077 | **292.1292** | 275.1026 | Q | 1100.5847 | 1083.5582 | 9 |
| 3 | 335.1714 | 318.1448 | 363.1663 | 346.1397 | A | ***972.5261*** | 955.4996 | 8 |
| 4 | 498.2347 | 481.2082 | 526.2296 | 509.2031 | Y | ***901.4890*** | 884.4625 | 7 |
| 5 | 627.2773 | 610.2508 | **655.2722** | 638.2457 | E | 738.4257 | 721.3992 | 6 |
| 6 | **740.3614** | 723.3348 | **768.3563** | 751.3297 | L | ***609.3831*** | 592.3566 | 5 |
| 7 | 853.4454 | 836.4189 | 881.4403 | 864.4138 | I | ***496.2990*** | 479.2725 | 4 |
| 8 | 990.5043 | 973.4778 | **1018.4993** | 1001.4727 | H | ***383.2150*** | 366.1884 | 3 |

| | | | | | | | | |
|---|---|---|---|---|---|---|---|---|
| 9 | 1061.5415 | 1044.5149 | 1089.5364 | 1072.5098 | A | *246.1561* | 229.1295 | 2 |
| 10 | | | | | R | *175.1190* | 158.0924 | 1 |

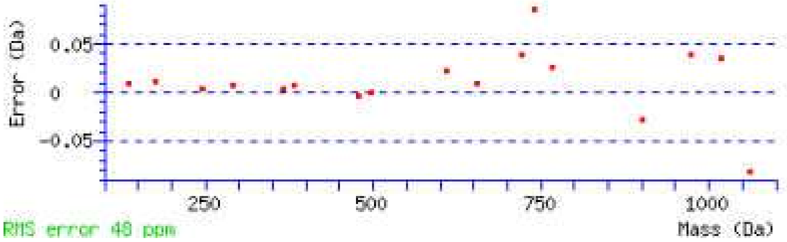


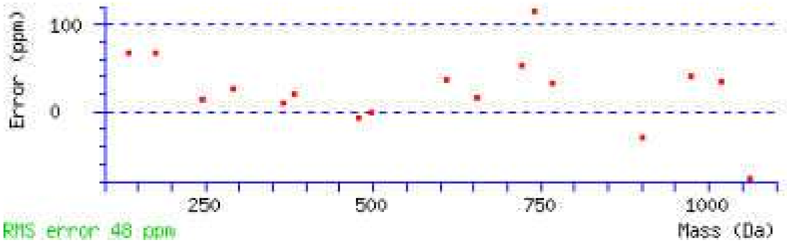


NCBI **BLAST** search of [YQAYELIHAR]
(Parameters: blastp, nr protein database, expect=20000, no filter, PAM30)
Other BLAST [web gateways]

**All matches to this query**

| Score | Mr(calc) | Delta | Sequence |
|---|---|---|---|
| 56.0 | 1262.6407 | 0.0506 | YQAYELIHAR |
| 28.7 | 1262.6077 | 0.0836 | YAGLDMTLHAR |
| 28.3 | 1262.6884 | 0.0030 | HQIFELIHAR |
| 27.4 | 1262.6077 | 0.0836 | YESVGTMIHAR |
| 26.6 | 1262.7247 | -0.0334 | HKEFIIIHAR |
| 26.4 | 1262.6632 | 0.0281 | FGSGKHPGLHAR |
| 24.9 | 1262.7207 | -0.0294 | AAGVEARLIHAR |
| 24.9 | 1262.6190 | 0.0724 | EVCEHILHAR |
| 24.9 | 1262.7207 | -0.0294 | NNVQAKLLHAR |
| 24.9 | 1262.7247 | -0.0334 | QHLLYILHAR |

**Mascot:** http://www.matrixscience.com/

# MATRIX SCIENCE Mascot Search Results

## Peptide View **Spot no 34**

MS/MS Fragmentation of **FGANCGPEAVWFK**
Found in **gi|108864186** in **NCBInr**, Chlorophyll A-B binding protein, expressed [Oryza sativa Japonica Group]

Match to Query 204: 1481.717824 from(1482.725100,1+) intensity(0.0000) index(17)
Title: Label: F7, Spot_Id: 219775, Peak_List_Id: 226297, MSMS Job_Run_Id: 21839, Comment:
Data file C:\Documents and Settings\Administrator\Desktop\Farah-Garima 11May 2013\ppw_F7_136859913100.txt

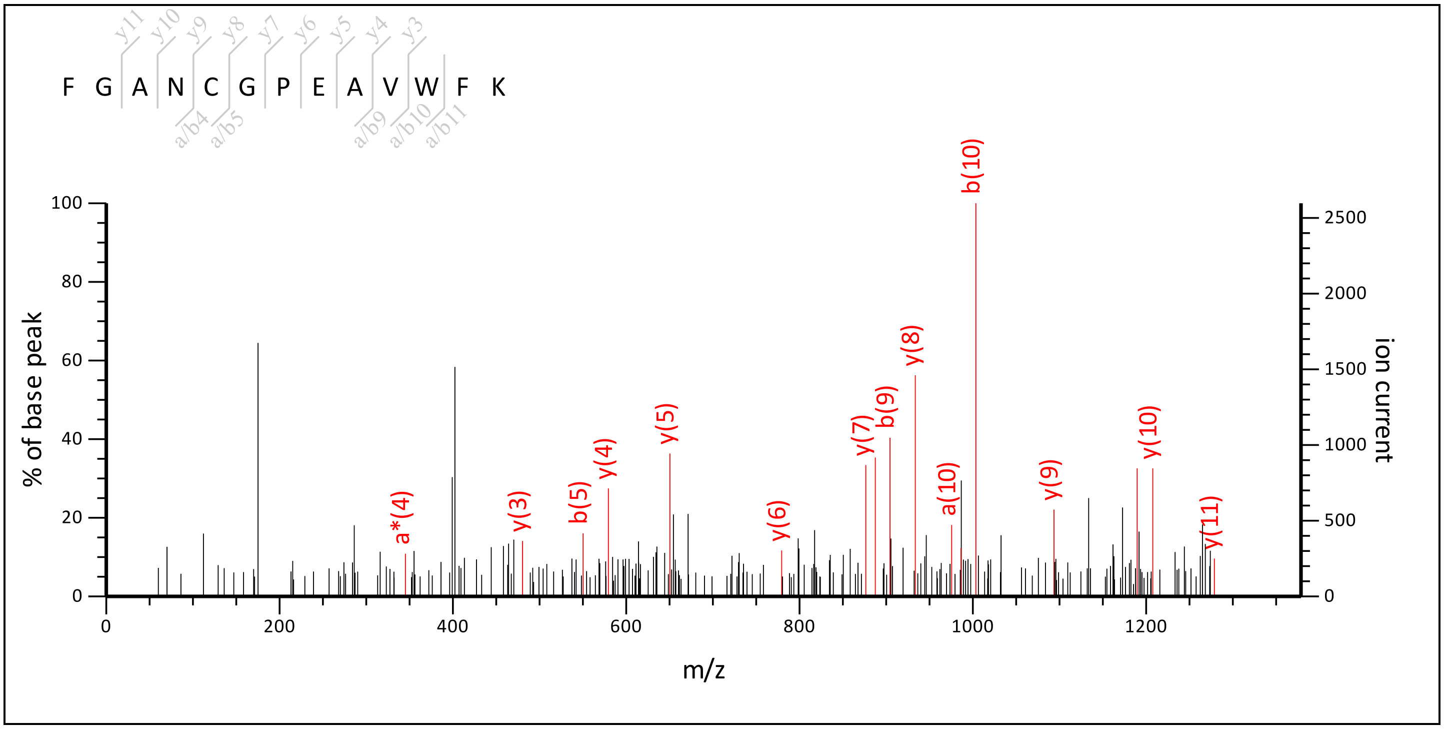


0 to 1378.66

Label all possible matches Label matches used for scoring

**Monoisotopic mass of neutral peptide Mr(calc):** 1481.6762
**Fixed modifications:** Carbamidomethyl (C) (apply to specified residues or termini only)
**Ions Score:** 27 **Expect:** 63
**Matches :** 19/66 fragment ions using 65 most intense peaks (help)

| # | a | a* | b | b* | Seq. | y | y* | # |
|---|---|---|---|---|---|---|---|---|
| 1 | 120.0808 | | 148.0757 | | F | | | 13 |
| 2 | 177.1022 | | 205.0972 | | G | 1335.6150 | 1318.5885 | 12 |
| 3 | 248.1394 | | 276.1343 | | A | *1278.5936* | 1261.5670 | 11 |
| 4 | 362.1823 | 345.1557 | 390.1772 | 373.1506 | N | *1207.5565* | 1190.5299 | 10 |
| 5 | 522.2129 | 505.1864 | **550.2078** | 533.1813 | C | *1093.5135* | 1076.4870 | 9 |
| 6 | **579.2344** | 562.2078 | 607.2293 | 590.2028 | G | *933.4829* | 916.4563 | 8 |
| 7 | 676.2872 | 659.2606 | 704.2821 | 687.2555 | P | *876.4614* | 859.4349 | 7 |
| 8 | 805.3298 | 788.3032 | 833.3247 | 816.2981 | E | *779.4087* | 762.3821 | 6 |

| | | | | | | | | |
|---|---|---|---|---|---|---|---|---|
| 9 | 876.3669 | 859.3403 | 904.3618 | 887.3352 | A | 650.3661 | 633.3395 | 5 |
| 10 | 975.4353 | 958.4087 | 1003.4302 | 986.4036 | V | 579.3289 | 562.3024 | 4 |
| 11 | 1161.5146 | 1144.4880 | 1189.5095 | 1172.4830 | W | 480.2605 | 463.2340 | 3 |
| 12 | 1308.5830 | 1291.5565 | 1336.5779 | 1319.5514 | F | 294.1812 | 277.1547 | 2 |
| 13 | | | | | K | 147.1128 | 130.0863 | 1 |

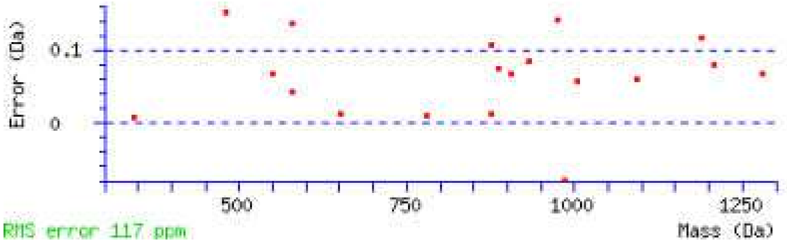


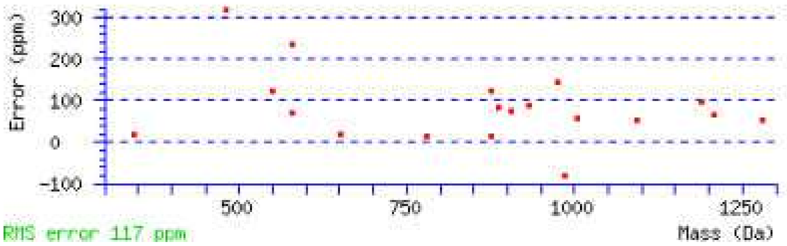


NCBI **BLAST** search of FGANCGPEAVWFK
(Parameters: blastp, nr protein database, expect=20000, no filter, PAM30)
Other BLAST web gateways

**All matches to this query**

| Score | Mr(calc) | Delta | Sequence |
|---|---|---|---|
| 26.9 | 1481.6762 | 0.0416 | FGANCGPEAVWFK |
| 16.5 | 1481.6789 | 0.0389 | KMNCVPSNITMR |
| 15.7 | 1481.6966 | 0.0212 | SMDLSMTELGRAR |
| 15.2 | 1481.7548 | -0.0370 | TMTTVFDGTPKLR |
| 14.4 | 1481.7303 | -0.0125 | RPESIAYDAWFK |
| 13.7 | 1481.6892 | 0.0286 | QTEVGSSASRAMSR |
| 13.6 | 1481.7514 | -0.0336 | DYAISPISAYINR |
| 13.6 | 1481.7667 | -0.0489 | VSYISPFTWINR |
| 13.3 | 1481.7548 | -0.0370 | LTAISMSLEFAQR |
| 13.2 | 1481.7184 | -0.0006 | RTDLMAAEGVEFK |

**Mascot:** http://www.matrixscience.com/

# MATRIX SCIENCE Mascot Search Results

## Peptide View Spot no 34

MS/MS Fragmentation of **KPEDFSKYQAYELIHAR**
Found in **gi|108864186** in **NCBInr**, Chlorophyll A-B binding protein, expressed [Oryza sativa Japonica Group]

Match to Query 302: 2094.141924 from(2095.149200,1+) intensity(0.0000) index(32)
Title: Label: F7, Spot_Id: 219775, Peak_List_Id: 226300, MSMS Job_Run_Id: 21839, Comment:
Data file C:\Documents and Settings\Administrator\Desktop\Farah-Garima 11May 2013\ppw_F7_136859913100.txt

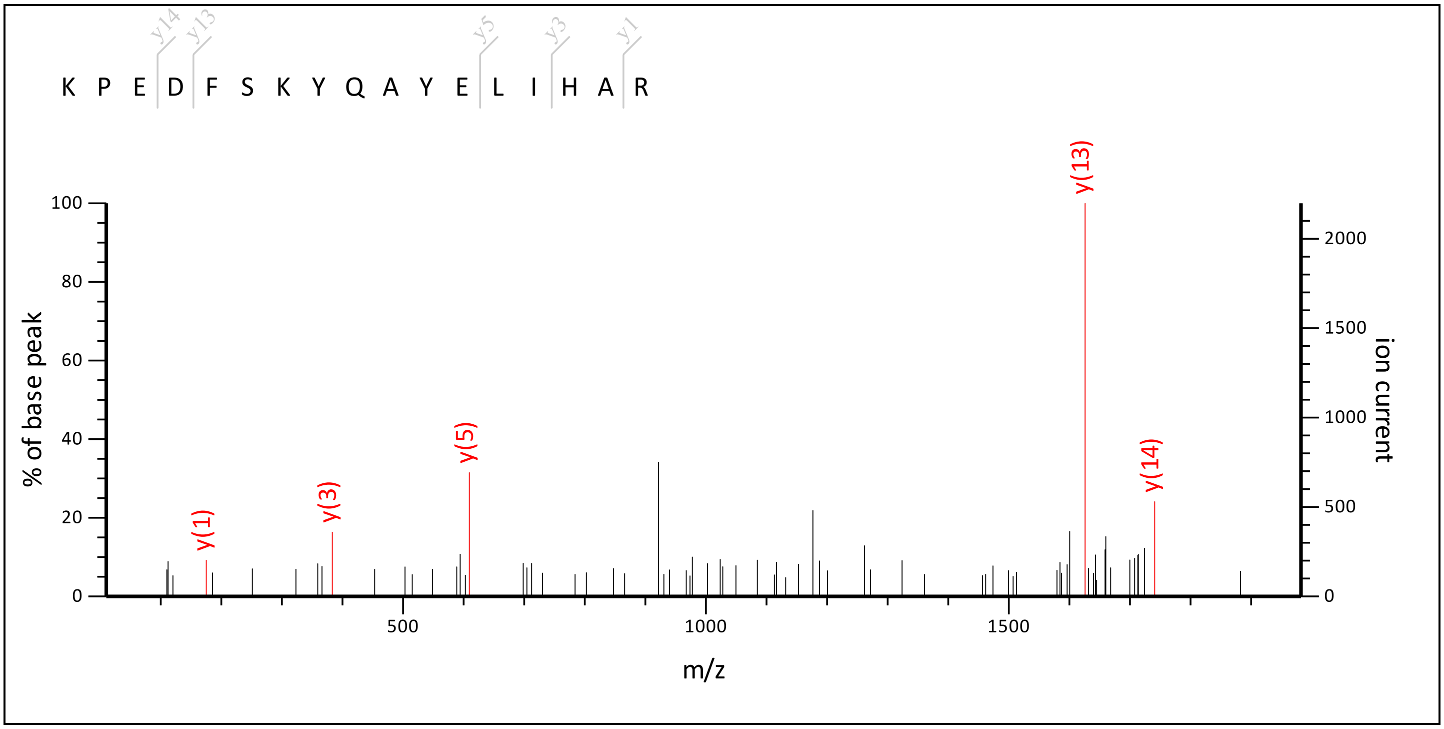


10.07 to 1982.31

Label all possible matches ○ Label matches used for scoring ◉

**Monoisotopic mass of neutral peptide Mr(calc):** 2094.0534
**Fixed modifications:** Carbamidomethyl (C) (apply to specified residues or termini only)
**Ions Score:** 12 **Expect:** 1.3e+03
**Matches :** 5/96 fragment ions using 18 most intense peaks (help)

| # | a | a* | b | b* | Seq. | y | y* | # |
|---|---|---|---|---|---|---|---|---|
| 1 | 101.1073 | 84.0808 | 129.1022 | 112.0757 | K | | | 17 |
| 2 | 198.1601 | 181.1335 | 226.1550 | 209.1285 | P | 1966.9658 | 1949.9392 | 16 |
| 3 | 327.2027 | 310.1761 | 355.1976 | 338.1710 | E | 1869.9130 | 1852.8864 | 15 |
| 4 | 442.2296 | 425.2031 | 470.2245 | 453.1980 | D | *1740.8704* | 1723.8438 | 14 |
| 5 | 589.2980 | 572.2715 | 617.2930 | 600.2664 | F | *1625.8435* | 1608.8169 | 13 |
| 6 | 676.3301 | 659.3035 | 704.3250 | 687.2984 | S | 1478.7750 | 1461.7485 | 12 |
| 7 | 804.4250 | 787.3985 | 832.4199 | 815.3934 | K | 1391.7430 | 1374.7165 | 11 |
| 8 | 967.4884 | 950.4618 | 995.4833 | 978.4567 | Y | 1263.6480 | 1246.6215 | 10 |

| | | | | | | | | |
|---|---|---|---|---|---|---|---|---|
| 9 | 1095.5469 | 1078.5204 | 1123.5419 | 1106.5153 | Q | 1100.5847 | 1083.5582 | 9 |
| 10 | 1166.5841 | 1149.5575 | 1194.5790 | 1177.5524 | A | 972.5261 | 955.4996 | 8 |
| 11 | 1329.6474 | 1312.6208 | 1357.6423 | 1340.6157 | Y | 901.4890 | 884.4625 | 7 |
| 12 | 1458.6900 | 1441.6634 | 1486.6849 | 1469.6583 | E | 738.4257 | 721.3991 | 6 |
| 13 | 1571.7740 | 1554.7475 | 1599.7690 | 1582.7424 | L | ***609.3831*** | 592.3566 | 5 |
| 14 | 1684.8581 | 1667.8316 | 1712.8530 | 1695.8265 | I | 496.2990 | 479.2725 | 4 |
| 15 | 1821.9170 | 1804.8905 | 1849.9119 | 1832.8854 | H | ***383.2150*** | 366.1884 | 3 |
| 16 | 1892.9541 | 1875.9276 | 1920.9490 | 1903.9225 | A | 246.1561 | 229.1295 | 2 |
| 17 | | | | | R | ***175.1190*** | 158.0924 | 1 |

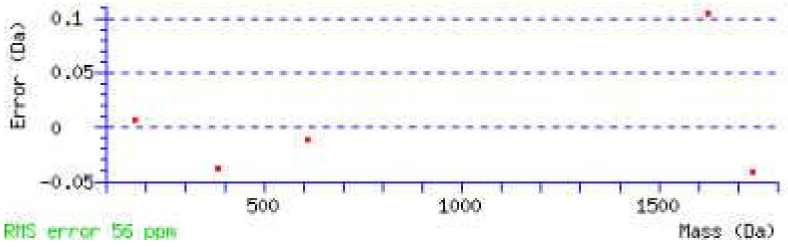


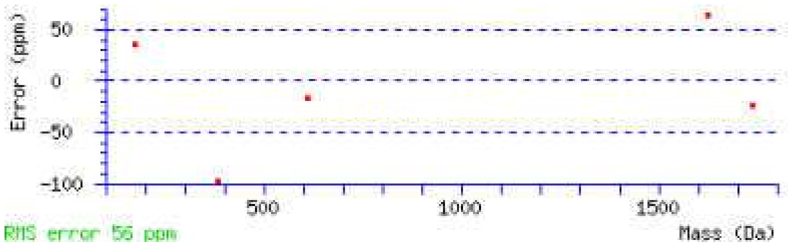


NCBI **BLAST** search of KPEDFSKYQAYELIHAR
(Parameters: blastp, nr protein database, expect=20000, no filter, PAM30)
Other BLAST web gateways

**All matches to this query**

| Score | Mr(calc) | Delta | Sequence |
|---|---|---|---|
| 12.4 | 2094.0534 | 0.0885 | KPEDFSKYQAYELIHAR |
| 11.8 | 2094.0528 | 0.0892 | KILDHISDVNMTLSEHAR |
| 9.5 | 2094.0337 | 0.1082 | LLAVPMTILAASTSDSEGMR |
| 9.0 | 2094.1249 | 0.0170 | KLLDIGDFIGVEGELFTTK |
| 8.3 | 2094.0382 | 0.1038 | SINGFDVTDPQKALEAYAR |
| 3.9 | 2094.0390 | 0.1029 | LVPCNYQWLDTMISKAR |
| 3.3 | 2093.9726 | 0.1693 | KPDEDAVGSNRVDDVDAHR |
| 3.2 | 2094.0164 | 0.1256 | IIENNQHDPAEMLAQLSR |
| 3.1 | 2094.1222 | 0.0197 | TVVVASGASYRKPAVTGFER |
| 3.1 | 2094.0858 | 0.0561 | KIIDHTSDVNFVLTEHAR |

**Mascot:** http://www.matrixscience.com/

# Mascot Search Results

## Peptide View Spot no 34

MS/MS Fragmentation of **HLSDPFGNNLLTVISGAAER**
Found in **gi|108864186** in **NCBInr**, Chlorophyll A-B binding protein, expressed [Oryza sativa Japonica Group]

Match to Query 305: 2110.167524 from(2111.174800,1+) intensity(0.0000) index(33)
Title: Label: F7, Spot_Id: 219775, Peak_List_Id: 226287, MSMS Job_Run_Id: 21839, Comment:
Data file C:\Documents and Settings\Administrator\Desktop\Farah-Garima 11May 2013\ppw_F7_136859913100.txt

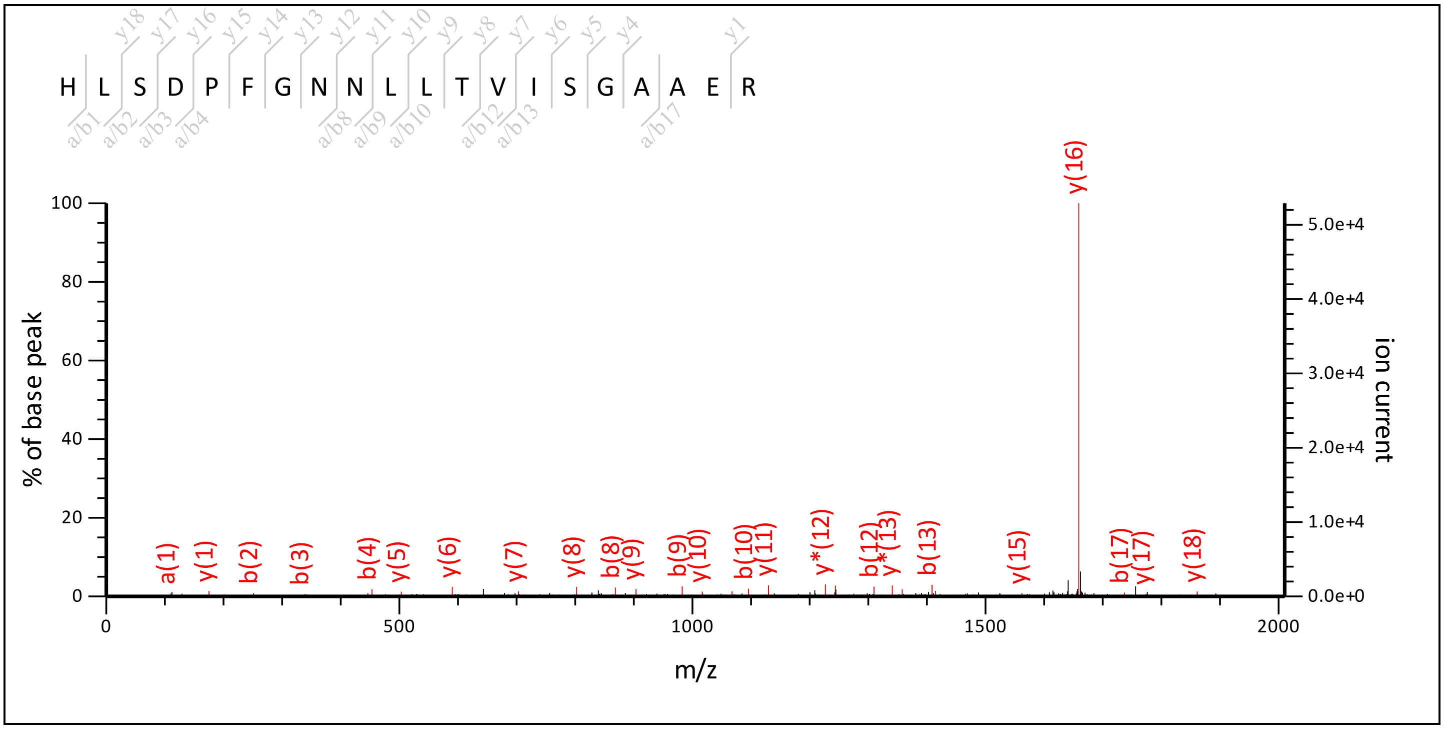


0 to 2010.39

Label all possible matches Label matches used for scoring

**Monoisotopic mass of neutral peptide Mr(calc):** 2110.0807
**Fixed modifications:** Carbamidomethyl (C) (apply to specified residues or termini only)
**Ions Score:** 110 **Expect:** 2.2e-07
**Matches :** 29/100 fragment ions using 57 most intense peaks (help)

| # | a | a* | b | b* | Seq. | y | y* | # |
|---|---|---|---|---|---|---|---|---|
| 1 | 110.0713 | | 138.0662 | | H | | | 20 |
| 2 | 223.1553 | | 251.1503 | | L | 1974.0291 | 1957.0025 | 19 |
| 3 | 310.1874 | | 338.1823 | | S | 1860.9450 | 1843.9185 | 18 |
| 4 | 425.2143 | | 453.2092 | | D | 1773.9130 | 1756.8864 | 17 |
| 5 | 522.2671 | | 550.2620 | | P | 1658.8860 | 1641.8595 | 16 |
| 6 | 669.3355 | | 697.3304 | | F | 1561.8333 | 1544.8067 | 15 |
| 7 | 726.3569 | | 754.3519 | | G | 1414.7649 | 1397.7383 | 14 |
| 8 | 840.3999 | 823.3733 | 868.3948 | 851.3682 | N | 1357.7434 | 1340.7169 | 13 |

| | | | | | | | | |
|---|---|---|---|---|---|---|---|---|
| 9 | 954.4428 | 937.4163 | 982.4377 | 965.4112 | N | 1243.7005 | 1226.6739 | 12 |
| 10 | 1067.5269 | 1050.5003 | 1095.5218 | 1078.4952 | L | 1129.6576 | 1112.6310 | 11 |
| 11 | 1180.6109 | 1163.5844 | 1208.6058 | 1191.5793 | L | 1016.5735 | 999.5469 | 10 |
| 12 | 1281.6586 | 1264.6321 | 1309.6535 | 1292.6270 | T | 903.4894 | 886.4629 | 9 |
| 13 | 1380.7270 | 1363.7005 | 1408.7219 | 1391.6954 | V | 802.4417 | 785.4152 | 8 |
| 14 | 1493.8111 | 1476.7845 | 1521.8060 | 1504.7795 | I | 703.3733 | 686.3468 | 7 |
| 15 | 1580.8431 | 1563.8166 | 1608.8380 | 1591.8115 | S | 590.2893 | 573.2627 | 6 |
| 16 | 1637.8646 | 1620.8380 | 1665.8595 | 1648.8329 | G | 503.2572 | 486.2307 | 5 |
| 17 | 1708.9017 | 1691.8751 | 1736.8966 | 1719.8701 | A | 446.2358 | 429.2092 | 4 |
| 18 | 1779.9388 | 1762.9123 | 1807.9337 | 1790.9072 | A | 375.1987 | 358.1721 | 3 |
| 19 | 1908.9814 | 1891.9549 | 1936.9763 | 1919.9498 | E | 304.1615 | 287.1350 | 2 |
| 20 | | | | | R | 175.1190 | 158.0924 | 1 |

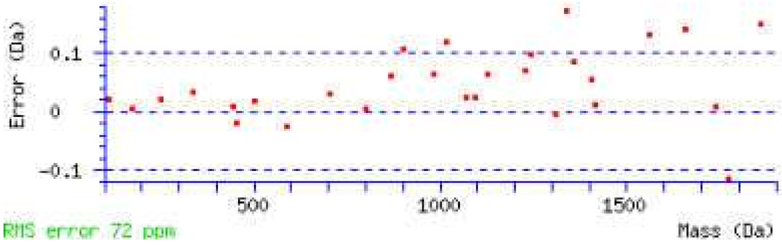


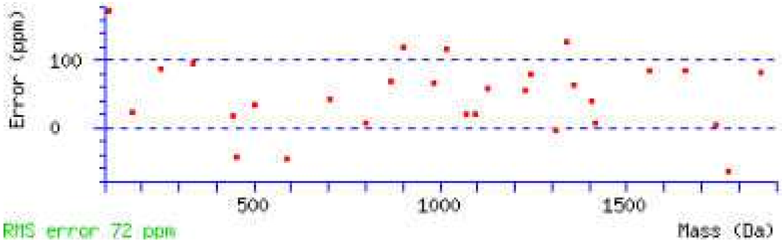


NCBI **BLAST** search of HLSDPFGNNLLTVISGAAER
(Parameters: blastp, nr protein database, expect=20000, no filter, PAM30)
Other BLAST web gateways

**All matches to this query**

| Score | Mr(calc) | Delta | Sequence |
|---|---|---|---|
| 109.8 | 2110.0807 | 0.0868 | HLSDPFGNNLLTVISGAAER |
| 96.2 | 2110.0807 | 0.0868 | HLSDPFGNNLLTVISAGAER |
| 83.5 | 2110.0807 | 0.0868 | HLSDPFGNNLLTVIAGSAER |
| 83.5 | 2110.0807 | 0.0868 | HLSDPFGNNLLTVLAGSAER |
| 27.2 | 2110.2626 | -0.0951 | RFNQIIIPLLSLISINTR |
| 21.0 | 2110.0980 | 0.0695 | KYPEVVSIIDTVSSMSALR |
| 16.9 | 2110.1521 | 0.0154 | LEIPASLDIQAISGLSNELK |
| 16.8 | 2110.0232 | 0.1443 | VGWDSITVDIQHGLHDYR |
| 14.1 | 2110.2051 | -0.0376 | LLEPGYLVRPRSPVPVYR |
| 14.1 | 2110.0775 | 0.0900 | MAHPQALATLLATEGQRMR |

**Mascot:** http://www.matrixscience.com/

# Mascot Search Results

## Peptide View Spot no 36

MS/MS Fragmentation of **LLPLRPGVQR**
Found in **gi|115453797** in **NCBInr**, Os03g0565200 [Oryza sativa Japonica Group]

Match to Query 34: 1147.747824 from(1148.755100,1+) intensity(0.0000) index(8)
Title: Label: I10, Spot_Id: 219826, Peak_List_Id: 226858, MSMS Job_Run_Id: 21858, Comment:
Data file C:\Documents and Settings\Administrator\Desktop\Farah-Garima 11May 2013\ppw_I10_136859951300.txt

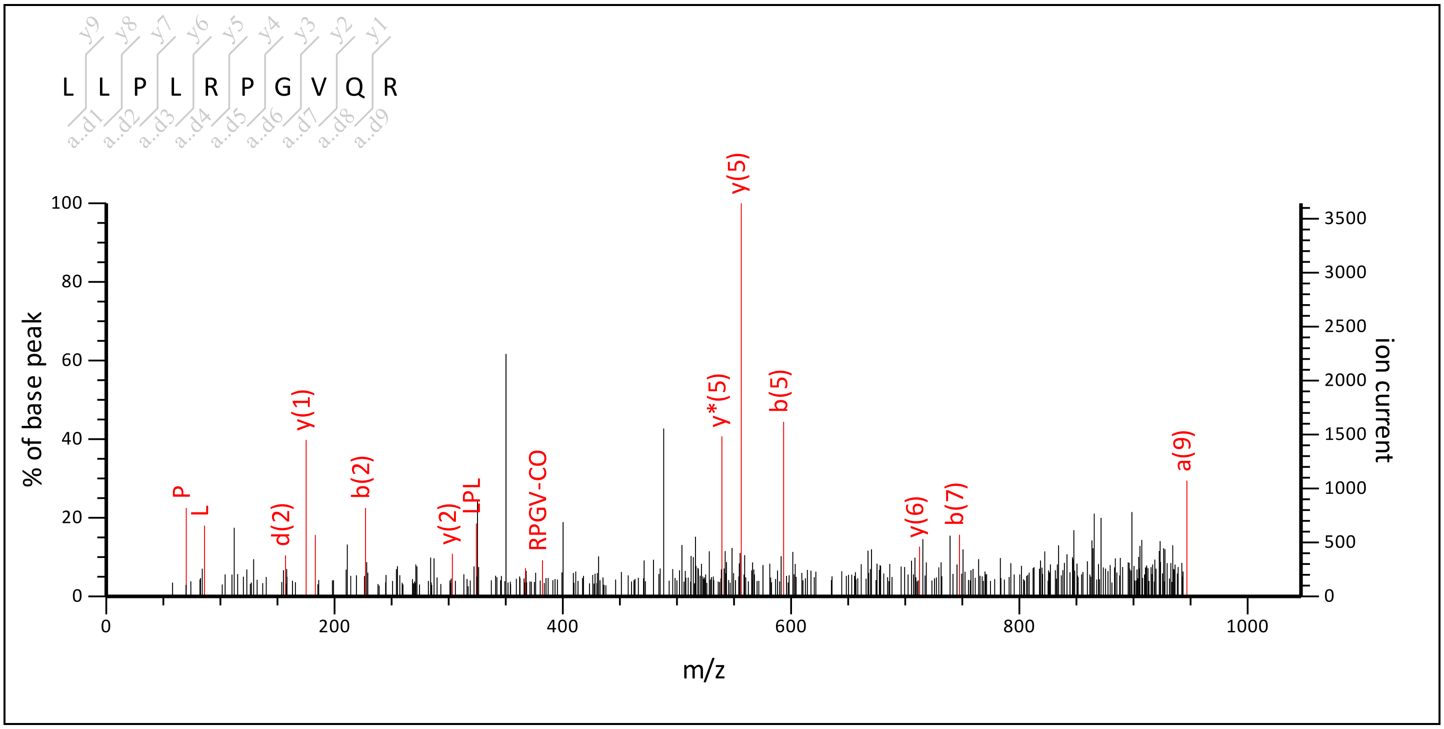


0 to 1046.73

Label all possible matches ○ Label matches used for scoring ◉

**Monoisotopic mass of neutral peptide Mr(calc):** 1147.7189
**Fixed modifications:** Carbamidomethyl (C) (apply to specified residues or termini only)
**Ions Score:** 21 **Expect:** 60
**Matches :** 28/130 fragment ions using 36 most intense peaks (help)

| # | Immon. | a | a* | b | b* | d | Seq. | v | w | y | y* | # |
|---|---|---|---|---|---|---|---|---|---|---|---|---|
| 1 | 86.0964 | **86.0964** | | 114.0913 | | 44.0495 | L | | | | | 10 |
| 2 | 86.0964 | 199.1805 | | ***227.1754*** | | 157.1335 | L | 977.5639 | 976.5687 | 1035.6422 | 1018.6156 | 9 |
| 3 | 70.0651 | 296.2333 | | ***324.2282*** | | 270.2176 | P | 880.5112 | 879.5159 | 922.5581 | 905.5316 | 8 |
| 4 | 86.0964 | 409.3173 | | 437.3122 | | 367.2704 | L | 767.4271 | 766.4318 | 825.5053 | 808.4788 | 7 |
| 5 | 129.1135 | 565.4184 | 548.3919 | ***593.4133*** | 576.3868 | 480.3544 | R | 611.3260 | 610.3307 | ***712.4213*** | 695.3947 | 6 |
| 6 | 70.0651 | 662.4712 | 645.4446 | 690.4661 | 673.4396 | 636.4555 | P | 514.2732 | 513.2780 | ***556.3202*** | 539.2936 | 5 |
| 7 | 30.0338 | 719.4927 | 702.4661 | ***747.4876*** | 730.4610 | | G | | | 459.2674 | 442.2409 | 4 |
| 8 | 72.0808 | 818.5611 | 801.5345 | 846.5560 | 829.5294 | 804.5454 | V | 358.1833 | 371.2037 | 402.2459 | 385.2194 | 3 |

| | | | | | | | | | | | | |
|---|---|---|---|---|---|---|---|---|---|---|---|---|
| 9 | 101.0709 | **946.6196** | 929.5931 | 974.6146 | 957.5880 | 889.5982 | Q | 230.1248 | 229.1295 | ***303.1775*** | 286.1510 | 2 |
| 10 | 129.1135 | | | | | | R | 74.0237 | 73.0284 | ***175.1190*** | 158.0924 | 1 |

| Seq | ya | yb | Seq | ya | yb | Seq | ya | yb |
|---|---|---|---|---|---|---|---|---|
| LP | 183.1492 | 211.1441 | LPL | 296.2333 | 324.2282 | LPLR | 452.3344 | 480.3293 |
| LPLRP | 549.3871 | 577.3820 | LPLRPG | 606.4086 | 634.4035 | PL | 183.1492 | 211.1441 |
| PLR | 339.2503 | 367.2452 | PLRP | 436.3031 | 464.2980 | PLRPG | 493.3245 | 521.3194 |
| PLRPGV | 592.3929 | 620.3879 | LR | 242.1975 | 270.1925 | LRP | 339.2503 | 367.2452 |
| LRPG | 396.2718 | 424.2667 | LRPGV | 495.3402 | 523.3351 | LRPGVQ | 623.3988 | 651.3937 |
| RP | 226.1662 | 254.1612 | RPG | 283.1877 | 311.1826 | RPGV | 382.2561 | 410.2510 |
| RPGVQ | 510.3147 | 538.3096 | PG | 127.0866 | 155.0815 | PGV | 226.1550 | 254.1499 |
| PGVQ | 354.2136 | 382.2085 | GV | 129.1022 | 157.0972 | GVQ | 257.1608 | 285.1557 |
| VQ | 200.1394 | 228.1343 | | | | | | |

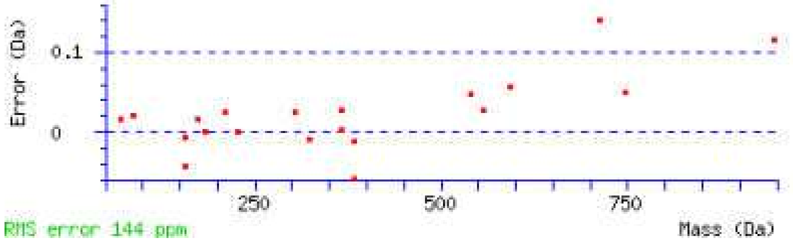


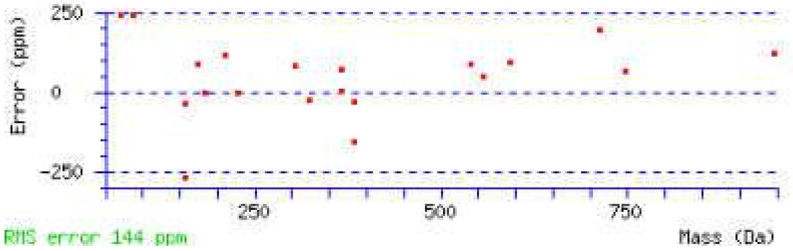


NCBI **BLAST** search of LLPLRPGVQR
(Parameters: blastp, nr protein database, expect=20000, no filter, PAM30)
Other BLAST web gateways

**All matches to this query**

| Score | Mr(calc) | Delta | Sequence |
|---|---|---|---|
| 25.5 | 1147.7189 | 0.0289 | RVPGIAPAIVR |
| 23.4 | 1147.6601 | 0.0877 | SDVVFILLSR |
| 22.6 | 1147.6350 | 0.1129 | QVSIGVYVAGR |
| 21.3 | 1147.7189 | 0.0289 | LLPLRPGVQR |
| 20.9 | 1147.6502 | 0.0976 | RVPYPVYVR |
| 19.9 | 1147.6462 | 0.1016 | IIGTAHGPGAVR |
| 19.8 | 1147.6462 | 0.1017 | PSPIATAHAKR |
| 19.4 | 1147.6614 | 0.0864 | LPPVWRPAGR |
| 19.3 | 1147.6687 | 0.0792 | RTVARPGHVR |
| 19.1 | 1147.7077 | 0.0401 | ILGPVLGPQVR |

**Mascot:** http://www.matrixscience.com/

# MATRIX SCIENCE Mascot Search Results

## Peptide View Spot no 36

MS/MS Fragmentation of **ISFNETFAER**
Found in **gi|115453797** in **NCBInr**, Os03g0565200 [Oryza sativa Japonica Group]

Match to Query 41: 1212.614924 from(1213.622200,1+) intensity(0.0000) index(11)
Title: Label: I10, Spot_Id: 219826, Peak_List_Id: 226860, MSMS Job_Run_Id: 21858, Comment:
Data file C:\Documents and Settings\Administrator\Desktop\Farah-Garima 11May 2013\ppw_I10_136859951300.txt

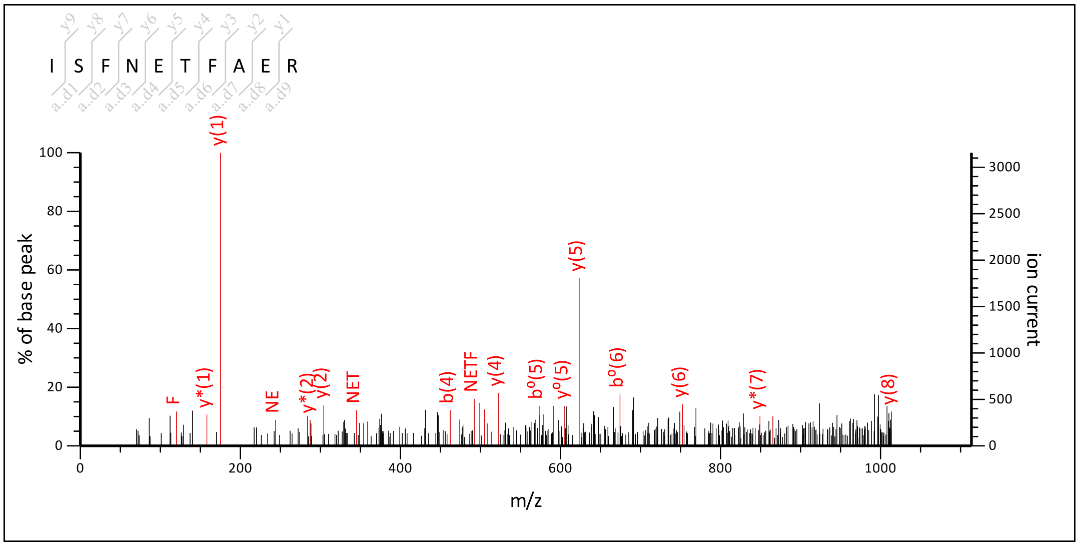


0 to 1113.59

Label all possible matches  Label matches used for scoring

**Monoisotopic mass of neutral peptide Mr(calc):** 1212.5775
**Fixed modifications:** Carbamidomethyl (C) (apply to specified residues or termini only)
**Ions Score:** 26 **Expect:** 88
**Matches :** 23/153 fragment ions using 40 most intense peaks (help)

| # | Immon. | a | a* | a⁰ | b | b* | b⁰ | d | d' | Seq. | v | w | w' | y | y* | y⁰ |
|---|---|---|---|---|---|---|---|---|---|---|---|---|---|---|---|---|
| 1 | 86.0964 | 86.0964 | | | 114.0913 | | | 44.0495 | | I | | | | | | |
| 2 | 60.0444 | 173.1285 | | 155.1179 | 201.1234 | | 183.1128 | 157.1335 | | S | 1068.4745 | 1067.4793 | | 1100.5007 | 1083.4742 | 1082.49 |
| 3 | 120.0808 | 320.1969 | | 302.1863 | 348.1918 | | 330.1812 | | | F | 921.4061 | | | ***1013.4687*** | 996.4421 | 995.45 |
| 4 | 87.0553 | 434.2398 | 417.2132 | 416.2292 | **462.2347** | 445.2082 | 444.2241 | 391.2340 | | N | 807.3632 | 806.3679 | | 866.4003 | 849.3737 | 848.38 |
| 5 | 102.0550 | 563.2824 | 546.2558 | 545.2718 | **591.2773** | 574.2508 | 573.2667 | 505.2769 | | E | 678.3206 | 677.3253 | | ***752.3573*** | 735.3308 | 734.34 |
| 6 | 74.0600 | 664.3301 | 647.3035 | 646.3195 | 692.3250 | 675.2984 | 674.3144 | 648.3352 | 650.3144 | T | 577.2729 | 590.2933 | 592.2726 | ***623.3148*** | 606.2882 | 605.30 |
| 7 | 120.0808 | 811.3985 | 794.3719 | 793.3879 | 839.3934 | 822.3668 | 821.3828 | | | F | 430.2045 | | | ***522.2671*** | 505.2405 | 504.25 |
| 8 | 44.0495 | 882.4356 | 865.4090 | 864.4250 | 910.4305 | 893.4040 | 892.4199 | | | A | 359.1674 | | | 375.1987 | 358.1721 | 357.18 |
| 9 | 102.0550 | 1011.4782 | 994.4516 | 993.4676 | 1039.4731 | 1022.4466 | 1021.4625 | 953.4727 | | E | 230.1248 | 229.1295 | | ***304.1615*** | 287.1350 | 286.15 |
| 10 | 129.1135 | | | | | | | | | R | 74.0237 | 73.0284 | | ***175.1190*** | 158.0924 | |

| Seq | ya | yb | Seq | ya | yb | Seq | ya | yb |
|---|---|---|---|---|---|---|---|---|
| SF | 207.1128 | 235.1077 | SFN | 321.1557 | 349.1506 | SFNE | 450.1983 | 478.1932 |
| SFNET | 551.2460 | 579.2409 | SFNETF | 698.3144 | 726.3093 | FN | 234.1237 | 262.1186 |
| FNE | 363.1663 | 391.1612 | FNET | 464.2140 | 492.2089 | FNETF | 611.2824 | 639.2773 |
| FNETFA | 682.3195 | 710.3144 | NE | 216.0979 | 244.0928 | NET | 317.1456 | 345.1405 |
| NETF | 464.2140 | 492.2089 | NETFA | 535.2511 | 563.2460 | NETFAE | 664.2937 | 692.2886 |
| ET | 203.1026 | 231.0975 | ETF | 350.1710 | 378.1660 | ETFA | 421.2082 | 449.2031 |
| ETFAE | 550.2508 | 578.2457 | TF | 221.1285 | 249.1234 | TFA | 292.1656 | 320.1605 |
| TFAE | 421.2082 | 449.2031 | FA | 191.1179 | 219.1128 | FAE | 320.1605 | 348.1554 |
| AE | 173.0921 | 201.0870 | | | | | | |

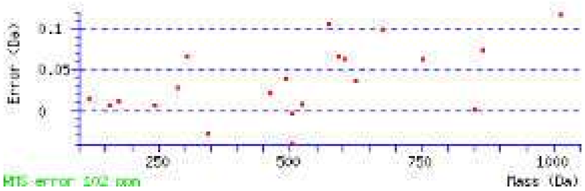


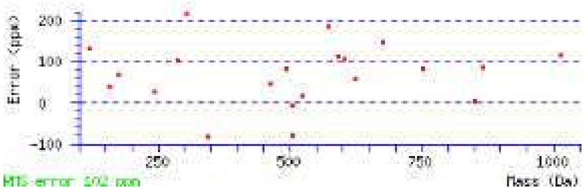


NCBI **BLAST** search of ISFNETFAER
(Parameters: blastp, nr protein database, expect=20000, no filter, PAM30)
Other BLAST web gateways

**All matches to this query**

| Score | Mr(calc) | Delta | Sequence |
|---|---|---|---|
| 26.3 | 1212.5775 | 0.0374 | ISFNETFAER |
| 24.8 | 1212.5696 | 0.0453 | SEASSTMIFPK |
| 19.4 | 1212.5921 | 0.0228 | YGSGKMKPSSR |
| 18.9 | 1212.6172 | -0.0023 | TAAFSLTMLSR |
| 18.6 | 1212.6099 | 0.0051 | QPQADTPTISR |
| 18.6 | 1212.5258 | 0.0891 | SESAFETASER |
| 18.2 | 1212.5775 | 0.0374 | QDYFPTISSR |
| 17.1 | 1212.5631 | 0.0518 | MFVEAGAMAVR |
| 17.0 | 1212.5445 | 0.0704 | LSDSTFADMAR |
| 16.9 | 1212.5775 | 0.0374 | QDYFPTLSSR |

**Mascot:** http://www.matrixscience.com/

# MATRIX SCIENCE Mascot Search Results

## Peptide View Spot no 36

MS/MS Fragmentation of **AVSAIVSCLLGPDR**
Found in **gi|115453797** in **NCBInr**, Os03g0565200 [Oryza sativa Japonica Group]

Match to Query 54: 1456.804124 from(1457.811400,1+) intensity(0.0000) index(17)
Title: Label: I10, Spot_Id: 219826, Peak_List_Id: 226864, MSMS Job_Run_Id: 21858, Comment:
Data file C:\Documents and Settings\Administrator\Desktop\Farah-Garima 11May 2013\ppw_I10_136859951300.txt

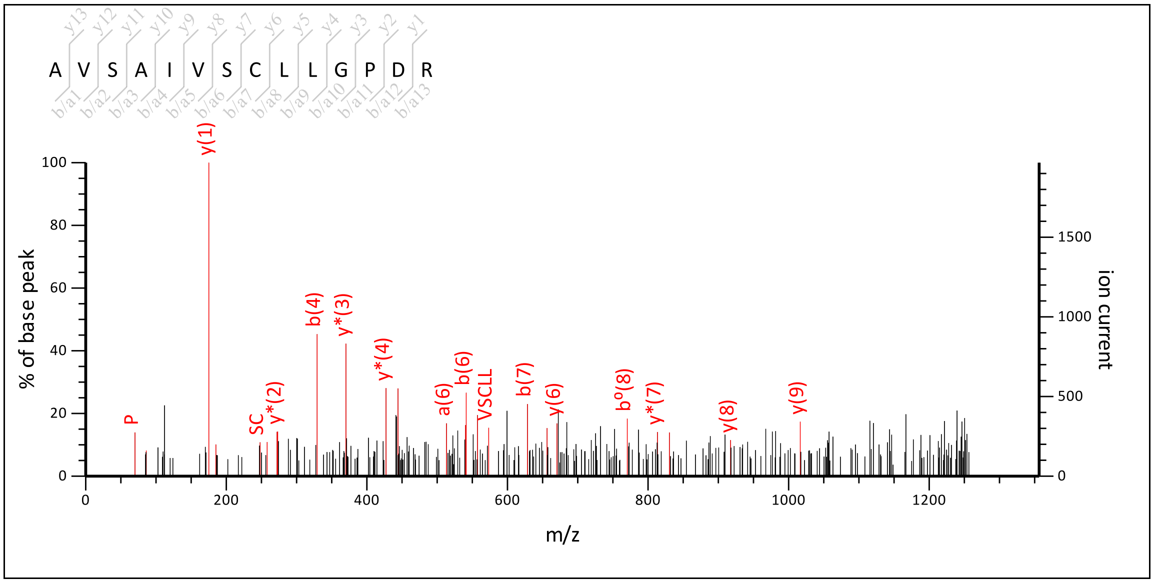


0 to 1356.41

Label all possible matches ○ Label matches used for scoring ◉

**Monoisotopic mass of neutral peptide Mr(calc):** 1456.7708
**Fixed modifications:** Carbamidomethyl (C) (apply to specified residues or termini only)
**Ions Score:** 32 **Expect:** 19
**Matches :** 40/228 fragment ions using 48 most intense peaks (help)

| # | Immon. | a | $a^0$ | b | $b^0$ | d | d' | Seq. | v | w | w' | y | y* | $y^0$ | # |
|---|---|---|---|---|---|---|---|---|---|---|---|---|---|---|---|
| 1 | 44.0495 | 44.0495 | | 72.0444 | | 44.0495 | | A | | | | | | | 14 |
| 2 | 72.0808 | 143.1179 | | 171.1128 | | 129.1022 | | V | 1342.6784 | 1355.6988 | | 1386.7410 | 1369.7144 | 1368.7304 | 13 |
| 3 | 60.0444 | 230.1499 | 212.1394 | ***258.1448*** | 240.1343 | 214.1550 | | S | 1255.6463 | 1254.6511 | | 1287.6725 | 1270.6460 | 1269.6620 | 12 |
| 4 | 44.0495 | 301.1870 | 283.1765 | ***329.1819*** | 311.1714 | | | A | 1184.6092 | | | 1200.6405 | 1183.6140 | 1182.6300 | 11 |
| 5 | 86.0964 | 414.2711 | 396.2605 | 442.2660 | 424.2554 | 386.2398 | 400.2554 | I | 1071.5252 | 1084.5456 | 1098.5612 | 1129.6034 | 1112.5769 | 1111.5928 | 10 |
| 6 | 72.0808 | 513.3395 | 495.3289 | ***541.3344*** | 523.3239 | 499.3239 | | V | 972.4567 | 985.4771 | | ***1016.5193*** | 999.4928 | 998.5088 | 9 |
| 7 | 60.0444 | 600.3715 | 582.3610 | ***628.3665*** | 610.3559 | 584.3766 | | S | 885.4247 | 884.4295 | | ***917.4509*** | 900.4244 | 899.4404 | 8 |
| 8 | 133.0430 | 760.4022 | 742.3916 | 788.3971 | 770.3865 | 671.4087 | | C | 725.3941 | 724.3988 | | ***830.4189*** | 813.3924 | 812.4083 | 7 |
| 9 | 86.0964 | 873.4863 | 855.4757 | 901.4812 | 883.4706 | 831.4393 | | L | 612.3100 | 611.3148 | | ***670.3883*** | 653.3617 | 652.3777 | 6 |
| 10 | 86.0964 | 986.5703 | 968.5597 | 1014.5652 | 996.5547 | 944.5234 | | L | 499.2259 | 498.2307 | | ***557.3042*** | 540.2776 | 539.2936 | 5 |
| 11 | 30.0338 | 1043.5918 | 1025.5812 | 1071.5867 | 1053.5761 | | | G | | | | ***444.2201*** | 427.1936 | 426.2096 | 4 |
| 12 | 70.0651 | 1140.6445 | 1122.6340 | 1168.6395 | 1150.6289 | 1114.6289 | | P | 345.1517 | 344.1565 | | 387.1987 | 370.1721 | 369.1881 | 3 |
| 13 | 88.0393 | 1255.6715 | 1237.6609 | 1283.6664 | 1265.6558 | 1211.6817 | | D | 230.1248 | 229.1295 | | 290.1459 | 273.1193 | 272.1353 | 2 |
| 14 | 129.1135 | | | | | | | R | 74.0237 | 73.0284 | | ***175.1190*** | 158.0924 | | 1 |

| Seq | ya | yb | Seq | ya | yb | Seq | ya | yb |
|---|---|---|---|---|---|---|---|---|
| VS | 159.1128 | 187.1077 | VSA | 230.1499 | 258.1448 | VSAI | 343.2340 | 371.2289 |
| VSAIV | 442.3024 | 470.2973 | VSAIVS | 529.3344 | 557.3293 | VSAIVSC | 689.3651 | 717.3600 |
| SA | 131.0815 | 159.0764 | SAI | 244.1656 | 272.1605 | SAIV | 343.2340 | 371.2289 |
| | | | | | | | | |

| | | | | | | | | |
|---|---|---|---|---|---|---|---|---|
| **SAIVS** | 430.2660 | 458.2609 | **SAIVSC** | 590.2967 | 618.2916 | **AI** | 157.1335 | 185.1285 |
| **AIV** | 256.2020 | 284.1969 | **AIVS** | 343.2340 | 371.2289 | **AIVSC** | 503.2646 | 531.2595 |
| **AIVSCL** | 616.3487 | 644.3436 | **IV** | 185.1648 | 213.1598 | **IVS** | 272.1969 | 300.1918 |
| **IVSC** | 432.2275 | 460.2224 | **IVSCL** | 545.3116 | 573.3065 | **IVSCLL** | 658.3956 | 686.3906 |
| **VS** | 159.1128 | 187.1077 | **VSC** | 319.1435 | 347.1384 | **VSCL** | 432.2275 | 460.2224 |
| **VSCLL** | 545.3116 | 573.3065 | **VSCLLG** | 602.3330 | 630.3280 | **VSCLLGP** | 699.3858 | 727.3807 |
| **SC** | 220.0750 | 248.0700 | **SCL** | 333.1591 | 361.1540 | **SCLL** | 446.2432 | 474.2381 |
| **SCLLG** | 503.2646 | 531.2595 | **SCLLGP** | 600.3174 | 628.3123 | **CL** | 246.1271 | 274.1220 |
| **CLL** | 359.2111 | 387.2061 | **CLLG** | 416.2326 | 444.2275 | **CLLGP** | 513.2854 | 541.2803 |
| **CLLGPD** | 628.3123 | 656.3072 | **LL** | 199.1805 | 227.1754 | **LLG** | 256.2020 | 284.1969 |
| **LLGP** | 353.2547 | 381.2496 | **LLGPD** | 468.2817 | 496.2766 | **LG** | 143.1179 | 171.1128 |
| **LGP** | 240.1707 | 268.1656 | **LGPD** | 355.1976 | 383.1925 | **GP** | 127.0866 | 155.0815 |
| **GPD** | 242.1135 | 270.1084 | **PD** | 185.0921 | 213.0870 | | | |

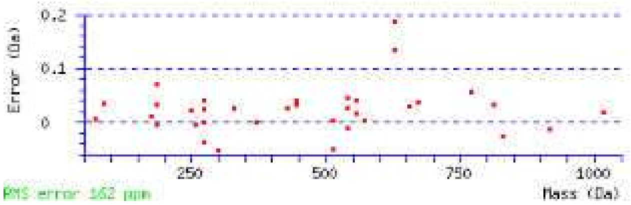


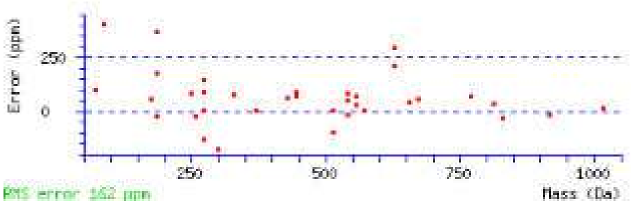


NCBI **BLAST** search of AVSAIVSCLLGPDR
(Parameters: blastp, nr protein database, expect=20000, no filter, PAM30)
Other BLAST web gateways

**All matches to this query**

| Score | Mr(calc) | Delta | Sequence |
|---|---|---|---|
| 32.1 | 1456.7708 | 0.0333 | AVSAIVSCLLGPDR |
| 28.9 | 1456.8110 | -0.0069 | ALSGIVSGQNSRLR |
| 24.9 | 1456.7787 | 0.0255 | FAVAAGPDGVERIR |
| 23.6 | 1456.7998 | 0.0044 | AAELGDAILSRSVR |
| 23.3 | 1456.7820 | 0.0221 | TRIVAMASAGVEPR |
| 22.8 | 1456.7860 | 0.0181 | SLGALMGISVWAPR |
| 21.9 | 1456.7708 | 0.0334 | QEALADLLMAQVR |
| 21.6 | 1456.7932 | 0.0109 | RANANAVGVGLMLR |
| 21.4 | 1456.7708 | 0.0333 | LVDCPLAISDRAK |
| 20.6 | 1456.7886 | 0.0155 | SSVEVAVTGSGLPVR |

**Mascot:** http://www.matrixscience.com/

# MATRIX SCIENCE Mascot Search Results

## Peptide View Spot no 36

MS/MS Fragmentation of **AVSAIVSCLLGPDRAEK**
Found in **gi|115453797** in **NCBInr**, Os03g0565200 [Oryza sativa Japonica Group]

Match to Query 72: 1784.986024 from(1785.993300,1+) intensity(0.0000) index(25)
Title: Label: I10, Spot_Id: 219826, Peak_List_Id: 226859, MSMS Job_Run_Id: 21858, Comment:
Data file C:\Documents and Settings\Administrator\Desktop\Farah-Garima 11May 2013\ppw_I10_136859951300.txt

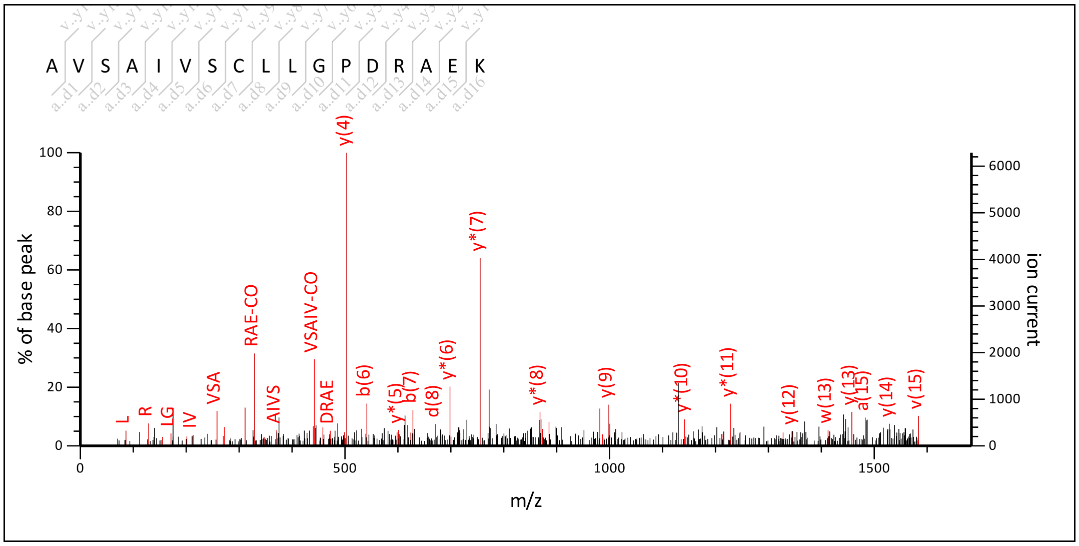


0 to 1683.66

Label all possible matches ○ Label matches used for scoring ◉

**Monoisotopic mass of neutral peptide Mr(calc):** 1784.9454
**Fixed modifications:** Carbamidomethyl (C) (apply to specified residues or termini only)
**Ions Score:** 43 **Expect:** 1.3
**Matches :** 89/296 fragment ions using 158 most intense peaks (help)

| # | Immon. | a | a* | a$^0$ | b | b* | b$^0$ | d | d' | Seq. | v | w | w' | y | y* | |
|---|---|---|---|---|---|---|---|---|---|---|---|---|---|---|---|---|
| 1 | 44.0495 | 44.0495 | | | 72.0444 | | | 44.0495 | | A | | | | | | |
| 2 | 72.0808 | 143.1179 | | | **171.1128** | | | 129.1022 | | V | 1670.8530 | 1683.8734 | | 1714.9156 | 1697.8891 | 169 |
| 3 | 60.0444 | 230.1499 | | 212.1394 | **258.1448** | | 240.1343 | 214.1550 | | S | 1583.8210 | 1582.8258 | | 1615.8472 | 1598.8207 | 159 |
| 4 | 44.0495 | 301.1870 | | 283.1765 | **329.1819** | | 311.1714 | | | A | 1512.7839 | | | ***1528.8152*** | 1511.7886 | 151 |
| 5 | 86.0964 | 414.2711 | | 396.2605 | **442.2660** | | 424.2554 | 386.2398 | 400.2554 | I | 1399.6998 | 1412.7202 | 1426.7359 | ***1457.7781*** | 1440.7515 | 143 |
| 6 | 72.0808 | 513.3395 | | 495.3289 | **541.3344** | | 523.3239 | 499.3239 | | V | 1300.6314 | 1313.6518 | | ***1344.6940*** | 1327.6675 | 132 |
| 7 | 60.0444 | 600.3715 | | 582.3610 | **628.3665** | | 610.3559 | 584.3766 | | S | 1213.5994 | 1212.6041 | | ***1245.6256*** | 1228.5990 | 122 |
| 8 | 133.0430 | 760.4022 | | 742.3916 | 788.3971 | | 770.3865 | 671.4087 | | C | 1053.5687 | 1052.5735 | | ***1158.5936*** | 1141.5670 | 114 |
| 9 | 86.0964 | 873.4863 | | 855.4757 | 901.4812 | | 883.4706 | 831.4393 | | L | 940.4847 | 939.4894 | | ***998.5629*** | 981.5364 | 98 |
| 10 | 86.0964 | 986.5703 | | 968.5597 | 1014.5652 | | 996.5547 | 944.5234 | | L | 827.4006 | 826.4054 | | ***885.4789*** | 868.4523 | 86 |
| 11 | 30.0338 | 1043.5918 | | 1025.5812 | 1071.5867 | | 1053.5761 | | | G | | | | ***772.3948*** | 755.3682 | 75 |
| 12 | 70.0651 | 1140.6445 | | 1122.6340 | 1168.6395 | | 1150.6289 | 1114.6289 | | P | 673.3264 | 672.3311 | | ***715.3733*** | 698.3468 | 69 |
| 13 | 88.0393 | 1255.6715 | | 1237.6609 | 1283.6664 | | 1265.6558 | 1211.6817 | | D | 558.2994 | 557.3042 | | ***618.3206*** | 601.2940 | 60 |
| 14 | 129.1135 | 1411.7726 | 1394.7460 | 1393.7620 | 1439.7675 | 1422.7410 | 1421.7569 | 1326.7086 | | R | 402.1983 | 401.2031 | | ***503.2936*** | 486.2671 | 48 |
| 15 | 44.0495 | 1482.8097 | 1465.7832 | 1464.7991 | 1510.8046 | 1493.7781 | 1492.7941 | | | A | 331.1612 | | | ***347.1925*** | 330.1660 | 32 |
| 16 | 102.0550 | 1611.8523 | 1594.8258 | 1593.8417 | 1639.8472 | 1622.8207 | 1621.8367 | 1553.8468 | | E | 202.1186 | 201.1234 | | 276.1554 | 259.1288 | 25 |
| 17 | 101.1073 | | | | | | | | | K | 74.0237 | 73.0284 | | 147.1128 | 130.0863 | |

| Seq | ya | yb | Seq | ya | yb | Seq | ya | yb |
|---|---|---|---|---|---|---|---|---|
| VS | 159.1128 | 187.1077 | VSA | 230.1499 | 258.1448 | VSAI | 343.2340 | 371.2289 |
| VSAIV | 442.3024 | 470.2973 | VSAIVS | 529.3344 | 557.3293 | VSAIVSC | 689.3651 | 717.3600 |
| SA | 131.0815 | 159.0764 | SAI | 244.1656 | 272.1605 | SAIV | 343.2340 | 371.2289 |
| SAIVS | 430.2660 | 458.2609 | SAIVSC | 590.2967 | 618.2916 | AI | 157.1335 | 185.1285 |

| | | | | | | | | |
|---|---|---|---|---|---|---|---|---|
| **AIV** | 256.2020 | 284.1969 | **AIVS** | 343.2340 | 371.2289 | **AIVSC** | 503.2646 | 531.2595 |
| **AIVSCL** | 616.3487 | 644.3436 | **IV** | 185.1648 | 213.1598 | **IVS** | 272.1969 | 300.1918 |
| **IVSC** | 432.2275 | 460.2224 | **IVSCL** | 545.3116 | 573.3065 | **IVSCLL** | 658.3956 | 686.3906 |
| **VS** | 159.1128 | 187.1077 | **VSC** | 319.1435 | 347.1384 | **VSCL** | 432.2275 | 460.2224 |
| **VSCLL** | 545.3116 | 573.3065 | **VSCLLG** | 602.3330 | 630.3280 | **VSCLLGP** | 699.3858 | 727.3807 |
| **SC** | 220.0750 | 248.0700 | **SCL** | 333.1591 | 361.1540 | **SCLL** | 446.2432 | 474.2381 |
| **SCLLG** | 503.2646 | 531.2595 | **SCLLGP** | 600.3174 | 628.3123 | **CL** | 246.1271 | 274.1220 |
| **CLL** | 359.2111 | 387.2061 | **CLLG** | 416.2326 | 444.2275 | **CLLGP** | 513.2854 | 541.2803 |
| **CLLGPD** | 628.3123 | 656.3072 | **LL** | 199.1805 | 227.1754 | **LLG** | 256.2020 | 284.1969 |
| **LLGP** | 353.2547 | 381.2496 | **LLGPD** | 468.2817 | 496.2766 | **LLGPDR** | 624.3828 | 652.3777 |
| **LLGPDRA** | 695.4199 | 723.4148 | **LG** | 143.1179 | 171.1128 | **LGP** | 240.1707 | 268.1656 |
| **LGPD** | 355.1976 | 383.1925 | **LGPDR** | 511.2987 | 539.2936 | **LGPDRA** | 582.3358 | 610.3307 |
| **GP** | 127.0866 | 155.0815 | **GPD** | 242.1135 | 270.1084 | **GPDR** | 398.2146 | 426.2096 |
| **GPDRA** | 469.2518 | 497.2467 | **GPDRAE** | 598.2944 | 626.2893 | **PD** | 185.0921 | 213.0870 |
| **PDR** | 341.1932 | 369.1881 | **PDRA** | 412.2303 | 440.2252 | **PDRAE** | 541.2729 | 569.2678 |
| **DR** | 244.1404 | 272.1353 | **DRA** | 315.1775 | 343.1724 | **DRAE** | 444.2201 | 472.2150 |
| **RA** | 200.1506 | 228.1455 | **RAE** | 329.1932 | 357.1881 | **AE** | 173.0921 | 201.0870 |

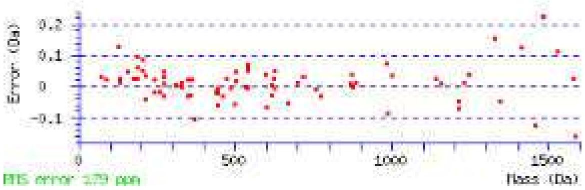


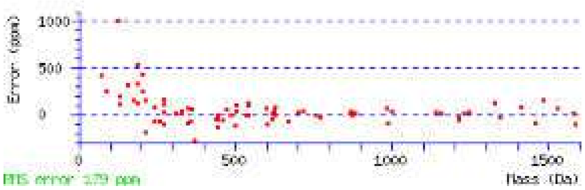


NCBI **BLAST** search of AVSAIVSCLLGPDRAEK
(Parameters: blastp, nr protein database, expect=20000, no filter, PAM30)
Other BLAST web gateways

**All matches to this query**

| Score | Mr(calc) | Delta | Sequence |
|---|---|---|---|
| 45.2 | 1784.9454 | 0.0406 | LNEIVACLLSAKSDPR |
| 43.0 | 1784.9454 | 0.0406 | AVSAIVSCLLGPDRAEK |
| 33.0 | 1784.9632 | 0.0228 | TIAASDPELGLSLKNTR |
| 32.8 | 1784.9156 | 0.0704 | QELAPSEDQASVLTAVK |
| 30.6 | 1784.9380 | 0.0480 | AGLSLEGEIRDSQAALR |
| 29.2 | 1784.8839 | 0.1021 | QLDLMAQQNDPQKTR |
| 28.5 | 1784.9533 | 0.0327 | LDAAAVSFILRHSETR |
| 27.6 | 1784.9897 | -0.0037 | FPGTQLRLLTATPSQR |
| 26.6 | 1784.9632 | 0.0228 | GISAKLSDLLQDLQER |
| 26.6 | 1784.9057 | 0.0803 | EAGALQQGLPVFSSPER |

**Mascot:** http://www.matrixscience.com/

# MATRIX SCIENCE Mascot Search Results

## Peptide View Spot no 36

MS/MS Fragmentation of **SGYTAEEDFATADAVFDCIGDPPEVR**
Found in **gi|115453797** in **NCBInr**, Os03g0565200 [Oryza sativa Japonica Group]

Match to Query 79: 2831.283024 from(2832.290300,1+) intensity(0.0000) index(27)
Title: Label: I10, Spot_Id: 219826, Peak_List_Id: 226876, MSMS Job_Run_Id: 21858, Comment:
Data file C:\Documents and Settings\Administrator\Desktop\Farah-Garima 11May 2013\ppw_I10_136859951300.txt

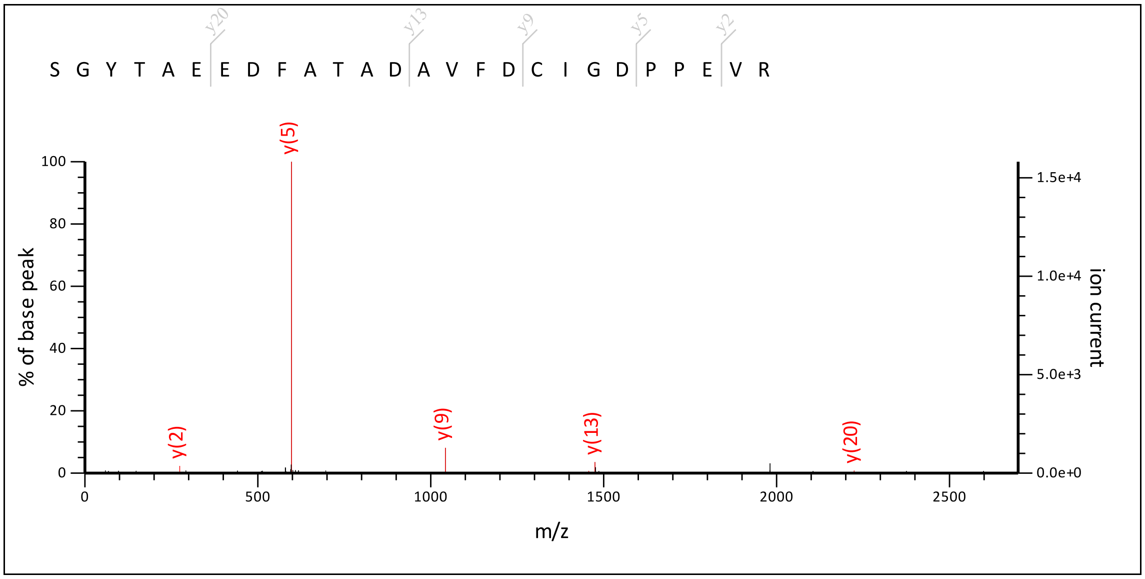


0 to 2698.47

Label all possible matches ○ Label matches used for scoring ◉

**Monoisotopic mass of neutral peptide Mr(calc):** 2831.2232
**Fixed modifications:** Carbamidomethyl (C) (apply to specified residues or termini only)
**Ions Score:** 22 **Expect:** 1.1e+02
**Matches :** 8/474 fragment ions using 14 most intense peaks (help)

| # | Immon. | a | $a^0$ | b | $b^0$ | d | d' | Seq. | v | w | w' | y | y* | $y^0$ | # |
|---|---|---|---|---|---|---|---|---|---|---|---|---|---|---|---|
| 1 | 60.0444 | 60.0444 | 42.0338 | 88.0393 | 70.0287 | 44.0495 | | S | | | | | | | 26 |
| 2 | 30.0338 | 117.0659 | 99.0553 | 145.0608 | 127.0502 | | | G | | | | 2745.1985 | 2728.1719 | 2727.1879 | 25 |
| 3 | 136.0757 | 280.1292 | 262.1186 | 308.1241 | 290.1135 | | | Y | 2580.1195 | | | 2688.1770 | 2671.1505 | 2670.1664 | 24 |
| 4 | 74.0600 | 381.1769 | 363.1663 | 409.1718 | 391.1612 | 365.1819 | 367.1612 | T | 2479.0718 | 2492.0922 | 2494.0715 | 2525.1137 | 2508.0871 | 2507.1031 | 23 |
| 5 | 44.0495 | 452.2140 | 434.2034 | 480.2089 | 462.1983 | | | A | 2408.0347 | | | 2424.0660 | 2407.0394 | 2406.0554 | 22 |
| 6 | 102.0550 | 581.2566 | 563.2460 | 609.2515 | 591.2409 | 523.2511 | | E | 2278.9921 | 2277.9969 | | 2353.0289 | 2336.0023 | 2335.0183 | 21 |
| 7 | 102.0550 | 710.2992 | 692.2886 | 738.2941 | 720.2835 | 652.2937 | | E | 2149.9495 | 2148.9543 | | ***2223.9863*** | 2206.9597 | 2205.9757 | 20 |
| 8 | 88.0393 | 825.3261 | 807.3155 | 853.3210 | 835.3105 | 781.3363 | | D | 2034.9226 | 2033.9273 | | 2094.9437 | 2077.9171 | 2076.9331 | 19 |
| 9 | 120.0808 | 972.3945 | 954.3840 | 1000.3894 | 982.3789 | | | F | 1887.8542 | | | 1979.9168 | 1962.8902 | 1961.9062 | 18 |
| 10 | 44.0495 | 1043.4316 | 1025.4211 | 1071.4265 | 1053.4160 | | | A | 1816.8170 | | | 1832.8483 | 1815.8218 | 1814.8378 | 17 |
| 11 | 74.0600 | 1144.4793 | 1126.4687 | 1172.4742 | 1154.4637 | 1128.4844 | 1130.4637 | T | 1715.7694 | 1728.7898 | 1730.7690 | 1761.8112 | 1744.7847 | 1743.8007 | 16 |
| 12 | 44.0495 | 1215.5164 | 1197.5059 | 1243.5113 | 1225.5008 | | | A | 1644.7322 | | | 1660.7635 | 1643.7370 | 1642.7530 | 15 |
| 13 | 88.0393 | 1330.5434 | 1312.5328 | 1358.5383 | 1340.5277 | 1286.5535 | | D | 1529.7053 | 1528.7101 | | 1589.7264 | 1572.6999 | 1571.7159 | 14 |
| 14 | 44.0495 | 1401.5805 | 1383.5699 | 1429.5754 | 1411.5648 | | | A | 1458.6682 | | | ***1474.6995*** | 1457.6729 | 1456.6889 | 13 |
| 15 | 72.0808 | 1500.6489 | 1482.6383 | 1528.6438 | 1510.6332 | 1486.6332 | | V | 1359.5998 | 1372.6202 | | 1403.6624 | 1386.6358 | 1385.6518 | 12 |
| 16 | 120.0808 | 1647.7173 | 1629.7067 | 1675.7122 | 1657.7017 | | | F | 1212.5314 | | | 1304.5940 | 1287.5674 | 1286.5834 | 11 |
| 17 | 88.0393 | 1762.7443 | 1744.7337 | 1790.7392 | 1772.7286 | 1718.7544 | | D | 1097.5044 | 1096.5092 | | 1157.5255 | 1140.4990 | 1139.5150 | 10 |
| 18 | 133.0430 | 1922.7749 | 1904.7643 | 1950.7698 | 1932.7593 | 1833.7814 | | C | 937.4738 | 936.4785 | | ***1042.4986*** | 1025.4721 | 1024.4880 | 9 |
| 19 | 86.0964 | 2035.8590 | 2017.8484 | 2063.8539 | 2045.8433 | 2007.8277 | 2021.8433 | I | 824.3897 | 837.4101 | 851.4258 | 882.4680 | 865.4414 | 864.4574 | 8 |

| | | | | | | | | | | | | | | | |
|---|---|---|---|---|---|---|---|---|---|---|---|---|---|---|---|
| 20 | 30.0338 | 2092.8804 | 2074.8699 | 2120.8753 | 2102.8648 | | | G | | | | 769.3839 | 752.3573 | 751.3733 | 7 |
| 21 | 88.0393 | 2207.9074 | 2189.8968 | 2235.9023 | 2217.8917 | 2163.9175 | | D | 652.3413 | 651.3461 | | 712.3624 | 695.3359 | 694.3519 | 6 |
| 22 | 70.0651 | 2304.9601 | 2286.9496 | 2332.9551 | 2314.9445 | 2278.9445 | | P | 555.2885 | 554.2933 | | ***597.3355*** | 580.3089 | 579.3249 | 5 |
| 23 | 70.0651 | 2402.0129 | 2384.0023 | 2430.0078 | 2411.9973 | 2375.9973 | | P | 458.2358 | 457.2405 | | 500.2827 | 483.2562 | 482.2722 | 4 |
| 24 | 102.0550 | 2531.0555 | 2513.0449 | 2559.0504 | 2541.0398 | 2473.0500 | | E | 329.1932 | 328.1979 | | 403.2300 | 386.2034 | 385.2194 | 3 |
| 25 | 72.0808 | 2630.1239 | 2612.1133 | 2658.1188 | 2640.1083 | 2616.1083 | | V | 230.1248 | 243.1452 | | ***274.1874*** | 257.1608 | | 2 |
| 26 | 129.1135 | | | | | | | R | 74.0237 | 73.0284 | | 175.1190 | 158.0924 | | 1 |

| Seq | ya | yb | Seq | ya | yb | Seq | ya | yb |
|---|---|---|---|---|---|---|---|---|
| GY | 193.0972 | 221.0921 | GYT | 294.1448 | 322.1397 | GYTA | 365.1819 | 393.1769 |
| GYTAE | 494.2245 | 522.2195 | GYTAEE | 623.2671 | 651.2620 | YT | 237.1234 | 265.1183 |
| YTA | 308.1605 | 336.1554 | YTAE | 437.2031 | 465.1980 | YTAEE | 566.2457 | 594.2406 |
| YTAEED | 681.2726 | 709.2675 | TA | 145.0972 | 173.0921 | TAE | 274.1397 | 302.1347 |
| TAEE | 403.1823 | 431.1773 | TAEED | 518.2093 | 546.2042 | TAEEDF | 665.2777 | 693.2726 |
| AE | 173.0921 | 201.0870 | AEE | 302.1347 | 330.1296 | AEED | 417.1616 | 445.1565 |
| AEEDF | 564.2300 | 592.2249 | AEEDFA | 635.2671 | 663.2620 | EE | 231.0975 | 259.0925 |
| EED | 346.1245 | 374.1194 | EEDF | 493.1929 | 521.1878 | EEDFA | 564.2300 | 592.2249 |
| EEDFAT | 665.2777 | 693.2726 | ED | 217.0819 | 245.0768 | EDF | 364.1503 | 392.1452 |
| EDFA | 435.1874 | 463.1823 | EDFAT | 536.2351 | 564.2300 | EDFATA | 607.2722 | 635.2671 |
| DF | 235.1077 | 263.1026 | DFA | 306.1448 | 334.1397 | DFAT | 407.1925 | 435.1874 |
| DFATA | 478.2296 | 506.2245 | DFATAD | 593.2566 | 621.2515 | DFATADA | 664.2937 | 692.2886 |
| FA | 191.1179 | 219.1128 | FAT | 292.1656 | 320.1605 | FATA | 363.2027 | 391.1976 |
| FATAD | 478.2296 | 506.2245 | FATADA | 549.2667 | 577.2617 | FATADAV | 648.3352 | 676.3301 |
| AT | 145.0972 | 173.0921 | ATA | 216.1343 | 244.1292 | ATAD | 331.1612 | 359.1561 |
| ATADA | 402.1983 | 430.1932 | ATADAV | 501.2667 | 529.2617 | ATADAVF | 648.3352 | 676.3301 |
| TA | 145.0972 | 173.0921 | TAD | 260.1241 | 288.1190 | TADA | 331.1612 | 359.1561 |
| TADAV | 430.2296 | 458.2245 | TADAVF | 577.2980 | 605.2930 | TADAVFD | 692.3250 | 720.3199 |
| AD | 159.0764 | 187.0713 | ADA | 230.1135 | 258.1084 | ADAV | 329.1819 | 357.1769 |
| ADAVF | 476.2504 | 504.2453 | ADAVFD | 591.2773 | 619.2722 | DA | 159.0764 | 187.0713 |
| DAV | 258.1448 | 286.1397 | DAVF | 405.2132 | 433.2082 | DAVFD | 520.2402 | 548.2351 |
| DAVFDC | 680.2708 | 708.2658 | AV | 143.1179 | 171.1128 | AVF | 290.1863 | 318.1812 |
| AVFD | 405.2132 | 433.2082 | AVFDC | 565.2439 | 593.2388 | AVFDCI | 678.3280 | 706.3229 |
| VF | 219.1492 | 247.1441 | VFD | 334.1761 | 362.1710 | VFDC | 494.2068 | 522.2017 |
| VFDCI | 607.2908 | 635.2858 | VFDCIG | 664.3123 | 692.3072 | FD | 235.1077 | 263.1026 |
| FDC | 395.1384 | 423.1333 | FDCI | 508.2224 | 536.2173 | FDCIG | 565.2439 | 593.2388 |
| FDCIGD | 680.2708 | 708.2658 | DC | 248.0700 | 276.0649 | DCI | 361.1540 | 389.1489 |
| DCIG | 418.1755 | 446.1704 | DCIGD | 533.2024 | 561.1973 | DCIGDP | 630.2552 | 658.2501 |
| CI | 246.1271 | 274.1220 | CIG | 303.1485 | 331.1435 | CIGD | 418.1755 | 446.1704 |
| CIGDP | 515.2282 | 543.2232 | CIGDPP | 612.2810 | 640.2759 | IG | 143.1179 | 171.1128 |
| IGD | 258.1448 | 286.1397 | IGDP | 355.1976 | 383.1925 | IGDPP | 452.2504 | 480.2453 |
| IGDPPE | 581.2930 | 609.2879 | IGDPPEV | 680.3614 | 708.3563 | GD | 145.0608 | 173.0557 |
| GDP | 242.1135 | 270.1084 | GDPP | 339.1663 | 367.1612 | GDPPE | 468.2089 | 496.2038 |
| GDPPEV | 567.2773 | 595.2722 | DP | 185.0921 | 213.0870 | DPP | 282.1448 | 310.1397 |
| DPPE | 411.1874 | 439.1823 | DPPEV | 510.2558 | 538.2508 | PP | 167.1179 | 195.1128 |
| PPE | 296.1605 | 324.1554 | PPEV | 395.2289 | 423.2238 | PE | 199.1077 | 227.1026 |
| PEV | 298.1761 | 326.1710 | EV | 201.1234 | 229.1183 | | | |

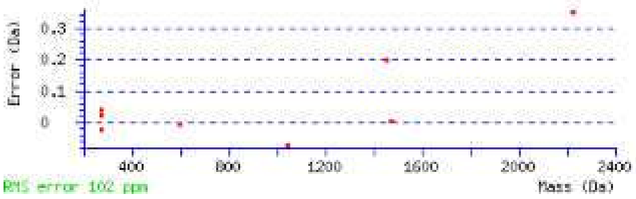


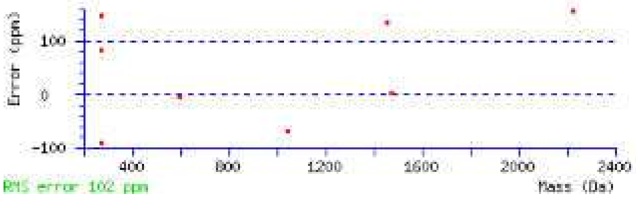

NCBI **BLAST** search of SGYTAEEDFATADAVFDCIGDPPEVR
(Parameters: blastp, nr protein database, expect=20000, no filter, PAM30)
Other BLAST web gateways

**All matches to this query**

| Score | Mr(calc) | Delta | Sequence |
|---|---|---|---|
| 21.7 | 2831.2232 | 0.0598 | SGYTAEEDFATADAVFDCIGDPPEVR |
| 12.5 | 2831.4388 | -0.1558 | MSFQPLPPGAAADDAVLVQPETRLHR |
| 12.4 | 2831.4488 | -0.1658 | NGAVVGAVQVFDGQEIMLISDQGTLVR |
| 12.3 | 2831.1181 | 0.1649 | DDYGSSYNYYAMDYWGQGTSVTVSS |
| 11.6 | 2831.4084 | -0.1253 | MSTSTTASVSTHILDTSVGRPAGDVAVR |
| 11.5 | 2831.2894 | -0.0064 | SMSSAYLEWSSLRASDTAIYYCVR |
| 10.9 | 2831.2717 | 0.0113 | SFGCMPNAATLGGITPAQWLMEEYR |
| 10.9 | 2831.2162 | 0.0668 | YAMQEFMILPTGAANFTEAMKMGSE |
| 10.7 | 2831.4501 | -0.1671 | MSGQLFDVVQGNSPLIAVALHNGHGVR |
| 10.7 | 2831.3990 | -0.1160 | AQANLVVRENYAQVNFYNEISFSR |

**Mascot:** http://www.matrixscience.com/

# MATRIX SCIENCE Mascot Search Results

## Peptide View **Spot no 37**

MS/MS Fragmentation of **IFLPEGLLDR**
Found in **gi|19184** in **NCBInr**, Type I (26 kD) CP29 polypeptide [Solanum lycopersicum]

Match to Query 175: 1171.674124 from(1172.681400,1+) intensity(0.0000) index(12)
Title: Label: E5, Spot_Id: 219742, Peak_List_Id: 225695, MSMS Job_Run_Id: 21786, Comment:
Data file C:\Documents and Settings\Administrator\Desktop\farah BHU\spot set plate\Farah-Garima 11May 2013\ppw_E5_136842184900.txt

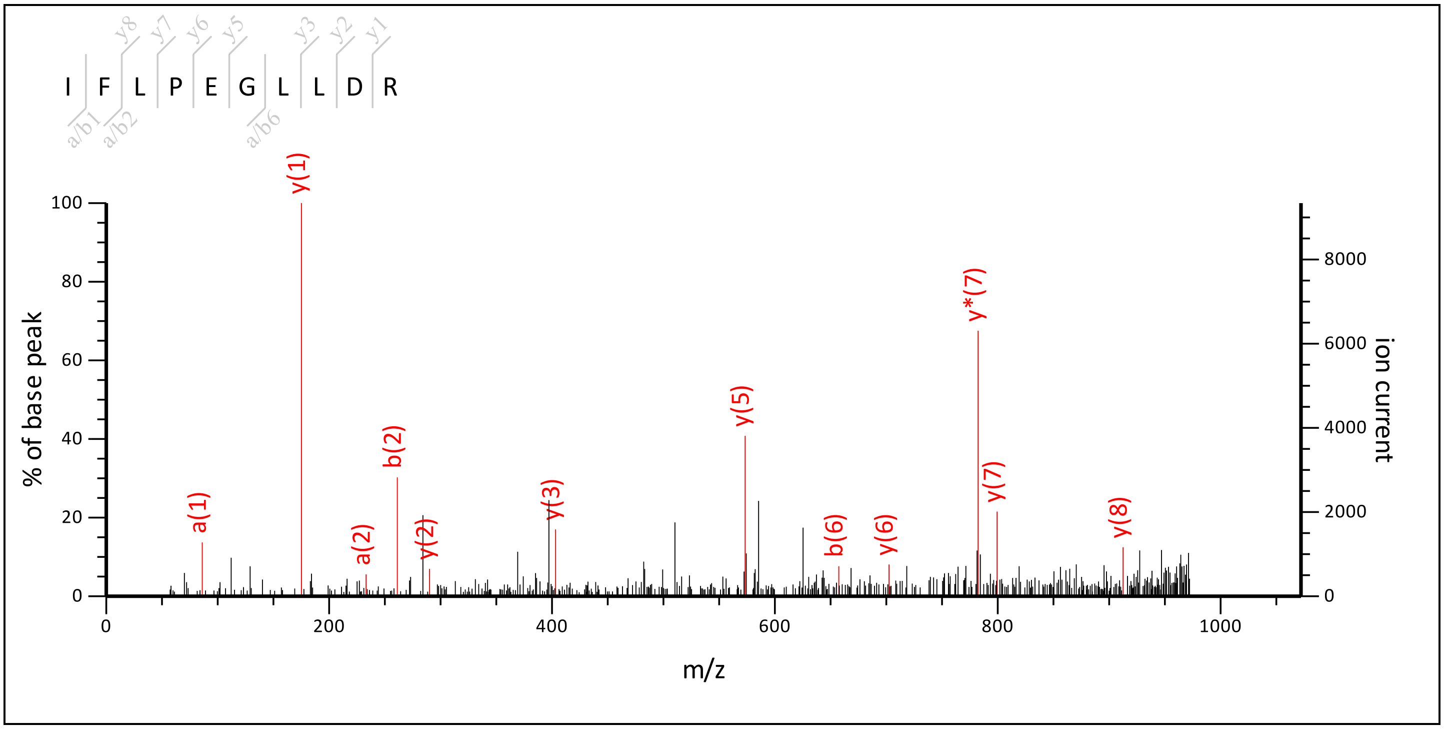


0 to 1072.13

Label all possible matches ○ Label matches used for scoring ◉

**Monoisotopic mass of neutral peptide Mr(calc):** 1171.6601
**Fixed modifications:** Carbamidomethyl (C) (apply to specified residues or termini only)
**Ions Score:** 35 **Expect:** 0.64
**Matches :** 12/36 fragment ions using 30 most intense peaks (help)

| # | a | b | Seq. | y | y* | # |
|---|---|---|---|---|---|---|
| 1 | **86.0964** | 114.0913 | I | | | 10 |
| 2 | **233.1648** | **261.1598** | F | 1059.5833 | 1042.5568 | 9 |
| 3 | 346.2489 | 374.2438 | L | ***912.5149*** | 895.4884 | 8 |
| 4 | 443.3017 | 471.2966 | P | ***799.4308*** | 782.4043 | 7 |
| 5 | 572.3443 | 600.3392 | E | ***702.3781*** | 685.3515 | 6 |
| 6 | 629.3657 | **657.3606** | G | ***573.3355*** | 556.3089 | 5 |
| 7 | 742.4498 | 770.4447 | L | 516.3140 | 499.2875 | 4 |
| 8 | 855.5339 | 883.5288 | L | ***403.2300*** | 386.2034 | 3 |

| | | | | | | |
|---|---|---|---|---|---|---|
| **9** | 970.5608 | 998.5557 | **D** | ***290.1459*** | 273.1193 | **2** |
| **10** | | | **R** | ***175.1190*** | 158.0924 | **1** |

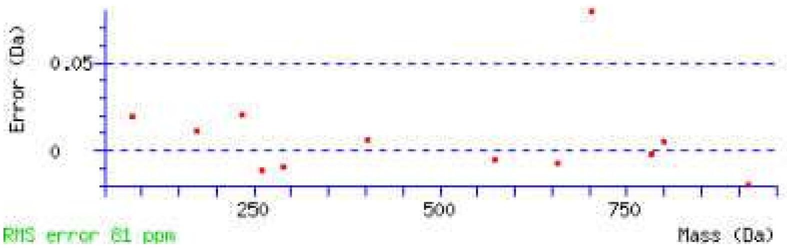


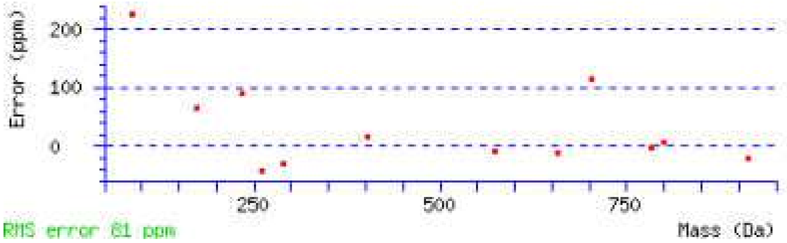


NCBI **BLAST** search of IFLPEGLLDR
(Parameters: blastp, nr protein database, expect=20000, no filter, PAM30)
Other BLAST web gateways

**All matches to this query**

| Score | Mr(calc) | Delta | Sequence |
|---|---|---|---|
| 34.7 | 1171.6601 | 0.0140 | IFLPEGLLDR |
| 15.3 | 1171.6462 | 0.0279 | RSGSKPWAVGK |
| 12.8 | 1171.6999 | -0.0257 | LMIPTTVILR |
| 12.7 | 1171.6495 | 0.0246 | IMIGRSLNPR |
| 11.5 | 1171.5768 | 0.0973 | AATGLQCPAGAR |
| 11.5 | 1171.7216 | -0.0475 | IFLPETVLLK |
| 11.5 | 1171.6713 | 0.0028 | LHPKPEPLNK |
| 11.5 | 1171.6859 | -0.0118 | LMLINRAGIR |
| 11.3 | 1171.6383 | 0.0358 | MEIILNVANR |
| 11.2 | 1171.5833 | 0.0908 | QNEVEGLIDR |

**Mascot:** http://www.matrixscience.com/

# MATRIX SCIENCE Mascot Search Results

## Peptide View Spot no 37

MS/MS Fragmentation of **YQAYELIHAR**
Found in **gi|19184** in **NCBInr**, Type I (26 kD) CP29 polypeptide [Solanum lycopersicum]

Match to Query 218: 1262.659024 from(1263.666300,1+) intensity(0.0000) index(15)
Title: Label: E5, Spot_Id: 219742, Peak_List_Id: 225693, MSMS Job_Run_Id: 21786, Comment:
Data file C:\Documents and Settings\Administrator\Desktop\farah BHU\spot set plate\Farah-Garima 11May 2013\ppw_E5_136842184900.txt

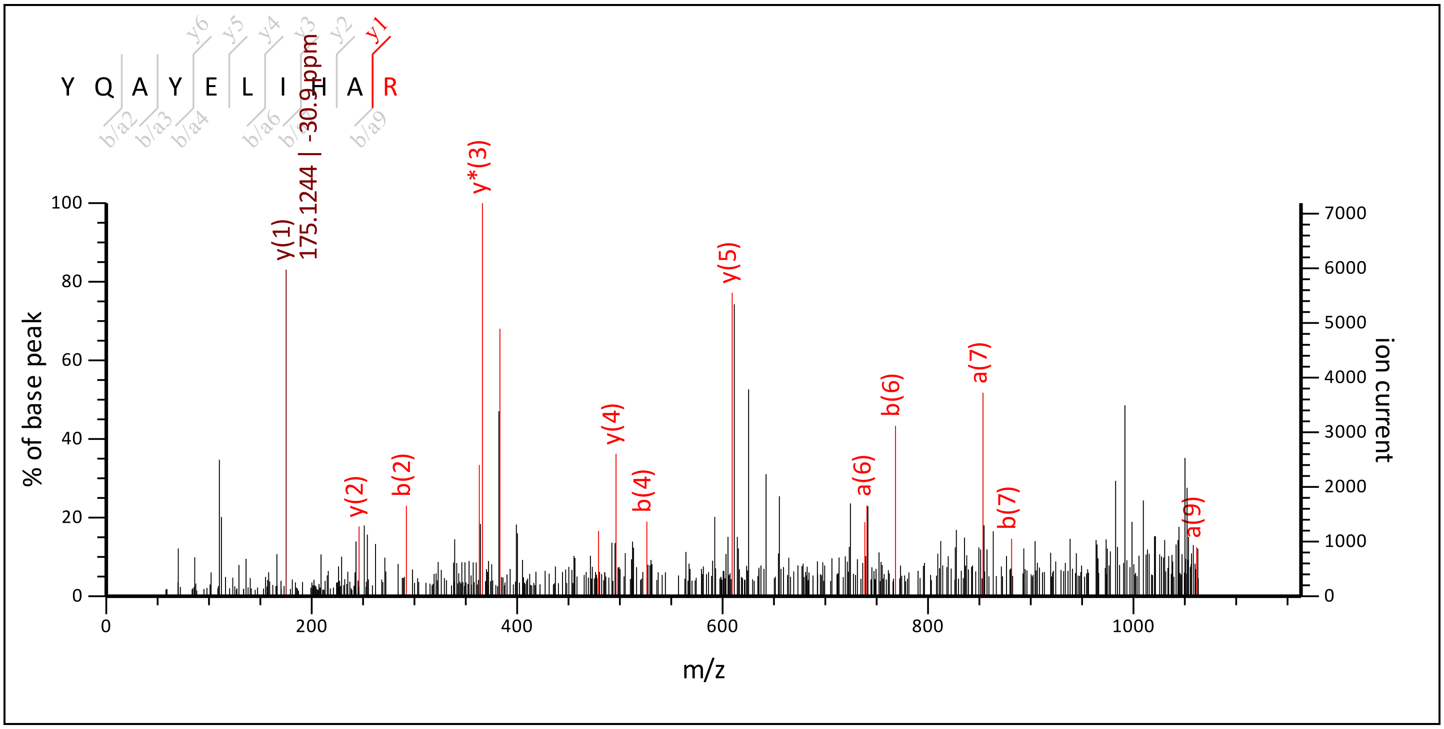


0 to 1163.09

Label all possible matches ○ Label matches used for scoring ◉

**Monoisotopic mass of neutral peptide Mr(calc):** 1262.6407
**Fixed modifications:** Carbamidomethyl (C) (apply to specified residues or termini only)
**Ions Score:** 23 **Expect:** 11
**Matches :** 16/52 fragment ions using 44 most intense peaks (help)

| # | a | a* | b | b* | Seq. | y | y* | # |
|---|---|---|---|---|---|---|---|---|
| 1 | 136.0757 | | 164.0706 | | Y | | | 10 |
| 2 | 264.1343 | 247.1077 | 292.1292 | 275.1026 | Q | 1100.5847 | 1083.5582 | 9 |
| 3 | 335.1714 | 318.1448 | 363.1663 | 346.1397 | A | 972.5261 | 955.4996 | 8 |
| 4 | 498.2347 | 481.2082 | 526.2296 | 509.2031 | Y | 901.4890 | 884.4625 | 7 |
| 5 | 627.2773 | 610.2508 | 655.2722 | 638.2457 | E | 738.4257 | 721.3992 | 6 |
| 6 | 740.3614 | 723.3348 | 768.3563 | 751.3297 | L | 609.3831 | 592.3566 | 5 |
| 7 | 853.4454 | 836.4189 | 881.4403 | 864.4138 | I | 496.2990 | 479.2725 | 4 |
| 8 | 990.5043 | 973.4778 | 1018.4993 | 1001.4727 | H | 383.2150 | 366.1884 | 3 |

| | | | | | | | | |
|---|---|---|---|---|---|---|---|---|
| 9 | **1061.5415** | 1044.5149 | 1089.5364 | 1072.5098 | **A** | ***246.1561*** | 229.1295 | **2** |
| 10 | | | | | **R** | ***175.1190*** | 158.0924 | **1** |

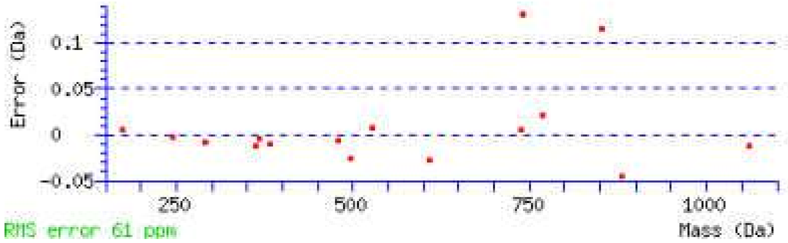


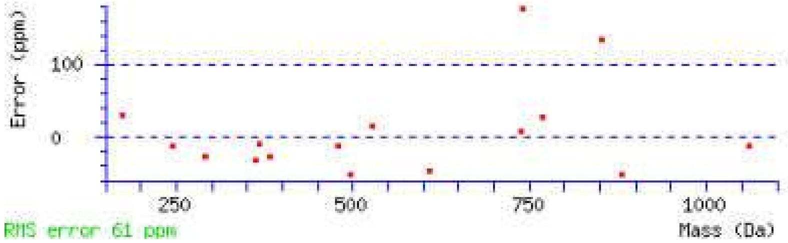


NCBI **BLAST** search of YQAYELIHAR
(Parameters: blastp, nr protein database, expect=20000, no filter, PAM30)
Other BLAST web gateways

**All matches to this query**

| Score | Mr(calc) | Delta | Sequence |
|---|---|---|---|
| 23.3 | 1262.6407 | 0.0183 | YQAYELIHAR |
| 19.5 | 1262.6884 | -0.0293 | HQIFELIHAR |
| 11.1 | 1262.7057 | -0.0466 | VLLTIPMSFSR |
| 11.1 | 1262.6619 | -0.0029 | LNGLAADISFSR |
| 10.7 | 1262.6480 | 0.0110 | TGGAPVGVGANHAR |
| 10.0 | 1262.5527 | 0.1063 | HEDGSELFSSR |
| 9.6 | 1262.7095 | -0.0505 | VEALLNQIHAR |
| 8.9 | 1262.6731 | -0.0141 | AALRQLDGTYR |
| 8.4 | 1262.6506 | 0.0084 | APELELYTTAR |
| 8.2 | 1262.6996 | -0.0406 | RYHPVVTHVR |

**Mascot:** http://www.matrixscience.com/

# Mascot Search Results

## Peptide View **Spot no 38**

MS/MS Fragmentation of **VIYDRESGR**
Found in **gi|115473531** in **NCBInr**, Os07g0631900 [Oryza sativa Japonica Group]

Match to Query 149: 1093.530824 from(1094.538100,1+) intensity(0.0000) index(22)
Title: Label: C5, Spot_Id: 219740, Peak_List_Id: 225626, MSMS Job_Run_Id: 21784, Comment:
Data file C:\Documents and Settings\Administrator\Desktop\farah BHU\spot set plate\Farah-Garima 11May 2013\ppw_C5_136842183200.txt

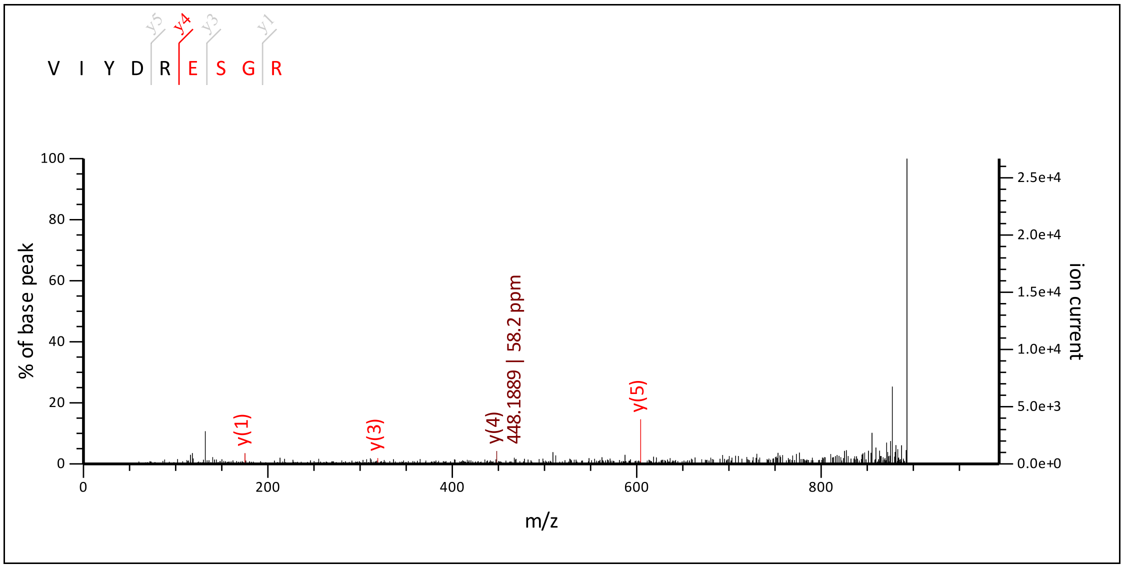


0 to 993.07

Label all possible matches ○ Label matches used for scoring ◉

**Monoisotopic mass of neutral peptide Mr(calc):** 1093.5516
**Fixed modifications:** Carbamidomethyl (C) (apply to specified residues or termini only)
**Ions Score:** 13 **Expect:** 1.4e+02
**Matches :** 4/123 fragment ions using 9 most intense peaks (help)

| # | Immon. | a | a* | $a^0$ | b | b* | $b^0$ | d | d' | Seq. | v | w | w' | y | y* | $y^0$ | # |
|---|---|---|---|---|---|---|---|---|---|---|---|---|---|---|---|---|---|
| 1 | 72.0808 | 72.0808 | | | 100.0757 | | | 44.0495 | | V | | | | | | | 9 |
| 2 | 86.0964 | 185.1648 | | | 213.1598 | | | 157.1335 | 171.1492 | I | 937.4122 | 950.4326 | 964.4483 | 995.4905 | 978.4639 | 977.4799 | 8 |
| 3 | 136.0757 | 348.2282 | | | 376.2231 | | | | | Y | 774.3489 | | | 882.4064 | 865.3799 | 864.3959 | 7 |
| 4 | 88.0393 | 463.2551 | | 445.2445 | 491.2500 | | 473.2395 | 419.2653 | | D | 659.3220 | 658.3267 | | 719.3431 | 702.3165 | 701.3325 | 6 |
| 5 | 129.1135 | 619.3562 | 602.3297 | 601.3457 | 647.3511 | 630.3246 | 629.3406 | 534.2922 | | R | 503.2209 | 502.2256 | | ***604.3161*** | 587.2896 | 586.3056 | 5 |
| 6 | 102.0550 | 748.3988 | 731.3723 | 730.3883 | 776.3937 | 759.3672 | 758.3832 | 690.3933 | | E | 374.1783 | 373.1830 | | ***448.2150*** | 431.1885 | 430.2045 | 4 |
| 7 | 60.0444 | 835.4308 | 818.4043 | 817.4203 | 863.4258 | 846.3992 | 845.4152 | 819.4359 | | S | 287.1462 | 286.1510 | | ***319.1724*** | 302.1459 | 301.1619 | 3 |
| 8 | 30.0338 | 892.4523 | 875.4258 | 874.4417 | 920.4472 | 903.4207 | 902.4367 | | | G | | | | 232.1404 | 215.1139 | | 2 |
| 9 | 129.1135 | | | | | | | | | R | 74.0237 | 73.0284 | | ***175.1190*** | 158.0924 | | 1 |

| Seq | ya | yb | Seq | ya | yb | Seq | ya | yb |
|---|---|---|---|---|---|---|---|---|
| IY | 249.1598 | 277.1547 | IYD | 364.1867 | 392.1816 | IYDR | 520.2878 | 548.2827 |
| IYDRE | 649.3304 | 677.3253 | YD | 251.1026 | 279.0975 | YDR | 407.2037 | 435.1987 |
| YDRE | 536.2463 | 564.2413 | YDRES | 623.2784 | 651.2733 | YDRESG | 680.2998 | 708.2947 |
| DR | 244.1404 | 272.1353 | DRE | 373.1830 | 401.1779 | DRES | 460.2150 | 488.2100 |
| DRESG | 517.2365 | 545.2314 | RE | 258.1561 | 286.1510 | RES | 345.1881 | 373.1830 |
| RESG | 402.2096 | 430.2045 | ES | 189.0870 | 217.0819 | ESG | 246.1084 | 274.1034 |
| SG | 117.0659 | 145.0608 | | | | | | |

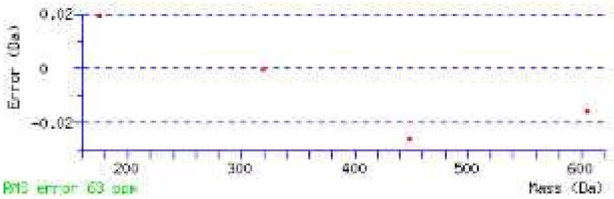


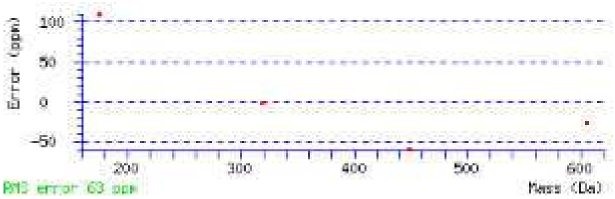


NCBI **BLAST** search of VIYDRESGR
(Parameters: blastp, nr protein database, expect=20000, no filter, PAM30)
Other BLAST web gateways

**All matches to this query**

| Score | Mr(calc) | Delta | Sequence |
|---|---|---|---|
| 13.1 | 1093.5013 | 0.0295 | EHHSRESGR |
| 13.1 | 1093.5265 | 0.0044 | FQTNRESGR |
| 13.1 | 1093.5516 | -0.0208 | IVYDRESGR |
| 13.1 | 1093.5516 | -0.0208 | VIYDRESGR |
| 7.1 | 1093.4896 | 0.0412 | ISPMMAEGSR |
| 6.5 | 1093.4975 | 0.0334 | DPYDRAMAR |
| 6.5 | 1093.5008 | 0.0300 | VMMERDTGR |
| 5.3 | 1093.5121 | 0.0188 | RSGTGMAMAGR |
| 5.1 | 1093.4822 | 0.0486 | CASKAEESGR |
| 5.1 | 1093.5074 | 0.0235 | DGTLMLEGSR |

**Mascot:** http://www.matrixscience.com/

# MATRIX SCIENCE Mascot Search Results

## Peptide View **Spot no 38**

MS/MS Fragmentation of **VNSGPPPPRDDFAPR**
Found in **gi|115473531** in **NCBInr**, Os07g0631900 [Oryza sativa Japonica Group]

Match to Query 298: 1620.784424 from(1621.791700,1+) intensity(0.0000) index(34)
Title: Label: C5, Spot_Id: 219740, Peak_List_Id: 225628, MSMS Job_Run_Id: 21784, Comment:
Data file C:\Documents and Settings\Administrator\Desktop\farah BHU\spot set plate\Farah-Garima 11May 2013\ppw_C5_136842183200.txt

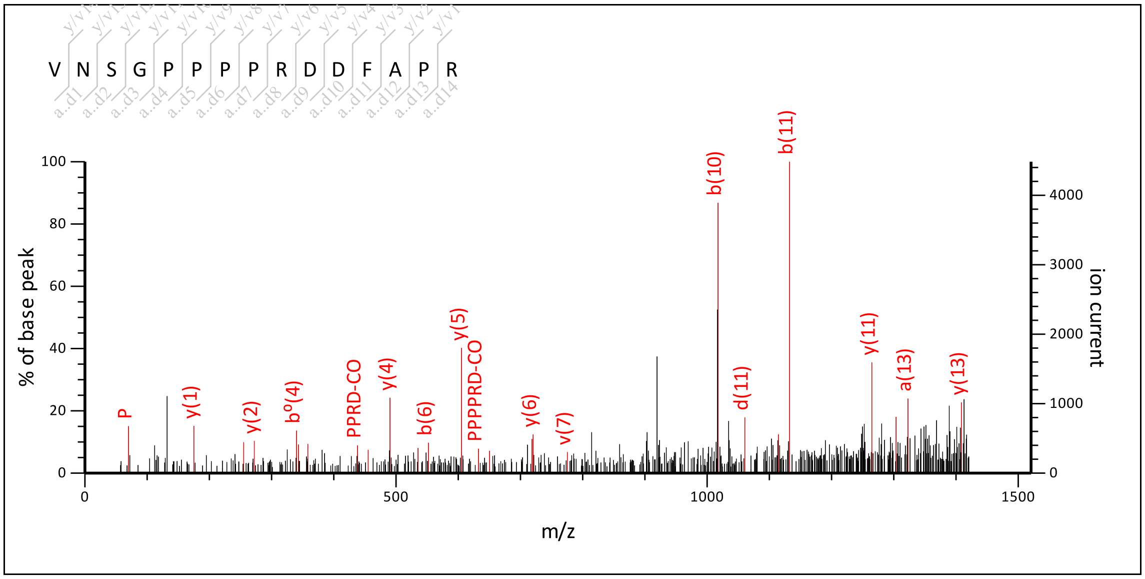


0 to 1520.61

Label all possible matches ○ Label matches used for scoring ◉

**Monoisotopic mass of neutral peptide Mr(calc):** 1620.8009
**Fixed modifications:** Carbamidomethyl (C) (apply to specified residues or termini only)
**Ions Score:** 46 **Expect:** 0.05
**Matches :** 36/267 fragment ions using 42 most intense peaks (help)

| # | Immon. | a | a* | $a^0$ | b | b* | $b^0$ | d | Seq. | v | w | y | y* | $y^0$ | # |
|---|---|---|---|---|---|---|---|---|---|---|---|---|---|---|---|
| 1 | 72.0808 | 72.0808 | | | 100.0757 | | | 44.0495 | V | | | | | | 15 |
| 2 | 87.0553 | 186.1237 | 169.0972 | | 214.1186 | 197.0921 | | 143.1179 | N | 1463.7026 | 1462.7074 | 1522.7397 | 1505.7132 | 1504.7292 | 14 |
| 3 | 60.0444 | 273.1557 | 256.1292 | 255.1452 | 301.1506 | 284.1241 | 283.1401 | 257.1608 | S | 1376.6706 | 1375.6753 | ***1408.6968*** | 1391.6702 | 1390.6862 | 13 |
| 4 | 30.0338 | 330.1772 | 313.1506 | 312.1666 | **358.1721** | 341.1456 | 340.1615 | | G | | | 1321.6648 | 1304.6382 | 1303.6542 | 12 |
| 5 | 70.0651 | 427.2300 | 410.2034 | 409.2194 | **455.2249** | 438.1983 | 437.2143 | 401.2143 | P | 1222.5963 | 1221.6011 | ***1264.6433*** | 1247.6167 | 1246.6327 | 11 |
| 6 | 70.0651 | 524.2827 | 507.2562 | 506.2722 | **552.2776** | 535.2511 | 534.2671 | 498.2671 | P | 1125.5436 | 1124.5483 | 1167.5905 | 1150.5640 | 1149.5800 | 10 |
| 7 | 70.0651 | 621.3355 | 604.3089 | 603.3249 | 649.3304 | 632.3039 | 631.3198 | 595.3198 | P | 1028.4908 | 1027.4956 | 1070.5378 | 1053.5112 | 1052.5272 | 9 |
| 8 | 70.0651 | **718.3883** | 701.3617 | 700.3777 | 746.3832 | 729.3566 | 728.3726 | 692.3726 | P | 931.4381 | 930.4428 | 973.4850 | 956.4585 | 955.4744 | 8 |
| 9 | 129.1135 | 874.4894 | 857.4628 | 856.4788 | 902.4843 | 885.4577 | 884.4737 | 789.4254 | R | 775.3369 | 774.3417 | 876.4322 | 859.4057 | 858.4217 | 7 |
| 10 | 88.0393 | 989.5163 | 972.4898 | 971.5057 | **1017.5112** | 1000.4847 | 999.5007 | 945.5265 | D | 660.3100 | 659.3148 | ***720.3311*** | 703.3046 | 702.3206 | 6 |
| 11 | 88.0393 | 1104.5432 | 1087.5167 | 1086.5327 | **1132.5382** | 1115.5116 | 1114.5276 | 1060.5534 | D | 545.2831 | 544.2878 | ***605.3042*** | 588.2776 | 587.2936 | 5 |
| 12 | 120.0808 | 1251.6117 | 1234.5851 | 1233.6011 | 1279.6066 | 1262.5800 | 1261.5960 | | F | 398.2146 | | ***490.2772*** | 473.2507 | | 4 |
| 13 | 44.0495 | **1322.6488** | 1305.6222 | 1304.6382 | 1350.6437 | 1333.6171 | 1332.6331 | | A | 327.1775 | | ***343.2088*** | 326.1823 | | 3 |
| 14 | 70.0651 | 1419.7015 | 1402.6750 | 1401.6910 | 1447.6965 | 1430.6699 | 1429.6859 | 1393.6859 | P | 230.1248 | 229.1295 | ***272.1717*** | 255.1452 | | 2 |
| 15 | 129.1135 | | | | | | | | R | 74.0237 | 73.0284 | ***175.1190*** | 158.0924 | | 1 |

| Seq | ya | yb | Seq | ya | yb | Seq | ya | yb |
|---|---|---|---|---|---|---|---|---|
| NS | 174.0873 | 202.0822 | NSG | 231.1088 | 259.1037 | NSGP | 328.1615 | 356.1565 |
| NSGPP | 425.2143 | 453.2092 | NSGPPP | 522.2671 | 550.2620 | NSGPPPP | 619.3198 | 647.3148 |
| SG | 117.0659 | 145.0608 | SGP | 214.1186 | 242.1135 | SGPP | 311.1714 | 339.1663 |

| | | | | | | | | |
|---|---|---|---|---|---|---|---|---|
| **SGPPP** | 408.2241 | 436.2191 | **SGPPPP** | 505.2769 | 533.2718 | **SGPPPPR** | 661.3780 | 689.3729 |
| **GP** | 127.0866 | 155.0815 | **GPP** | 224.1394 | 252.1343 | **GPPP** | 321.1921 | 349.1870 |
| **GPPPP** | 418.2449 | 446.2398 | **GPPPPR** | 574.3460 | 602.3409 | **GPPPPRD** | 689.3729 | 717.3679 |
| **PP** | 167.1179 | 195.1128 | **PPP** | 264.1707 | 292.1656 | **PPPP** | 361.2234 | 389.2183 |
| **PPPPR** | 517.3245 | 545.3194 | **PPPPRD** | 632.3515 | 660.3464 | **PP** | 167.1179 | 195.1128 |
| **PPP** | 264.1707 | 292.1656 | **PPPR** | 420.2718 | 448.2667 | **PPPRD** | 535.2987 | 563.2936 |
| **PPPRDD** | 650.3257 | 678.3206 | **PP** | 167.1179 | 195.1128 | **PPR** | 323.2190 | 351.2139 |
| **PPRD** | 438.2459 | 466.2409 | **PPRDD** | 553.2729 | 581.2678 | **PR** | 226.1662 | 254.1612 |
| **PRD** | 341.1932 | 369.1881 | **PRDD** | 456.2201 | 484.2150 | **PRDDF** | 603.2885 | 631.2835 |
| **PRDDFA** | 674.3257 | 702.3206 | **RD** | 244.1404 | 272.1353 | **RDD** | 359.1674 | 387.1623 |
| **RDDF** | 506.2358 | 534.2307 | **RDDFA** | 577.2729 | 605.2678 | **RDDFAP** | 674.3257 | 702.3206 |
| **DD** | 203.0662 | 231.0612 | **DDF** | 350.1347 | 378.1296 | **DDFA** | 421.1718 | 449.1667 |
| **DDFAP** | 518.2245 | 546.2195 | **DF** | 235.1077 | 263.1026 | **DFA** | 306.1448 | 334.1397 |
| **DFAP** | 403.1976 | 431.1925 | **FA** | 191.1179 | 219.1128 | **FAP** | 288.1707 | 316.1656 |
| **AP** | 141.1022 | 169.0972 | | | | | | |

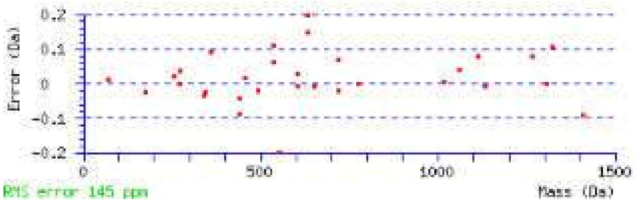


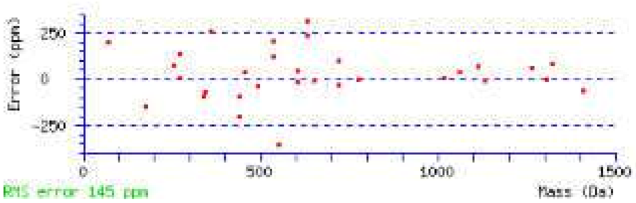


NCBI **BLAST** search of VNSGPPPPRDDFAPR
(Parameters: blastp, nr protein database, expect=20000, no filter, PAM30)
Other BLAST web gateways

**All matches to this query**

| Score | Mr(calc) | Delta | Sequence |
|---|---|---|---|
| 45.7 | 1620.8009 | -0.0164 | VNSGPPPPRDDFAPR |
| 16.0 | 1620.7777 | 0.0067 | SKDMIQNSLDESVR |
| 13.9 | 1620.6912 | 0.0932 | MPSSSDLYNRTFGM |
| 13.7 | 1620.7858 | -0.0013 | MLLFQPEWSDDLK |
| 13.6 | 1620.6329 | 0.1516 | SSFDFGFNDDDAER |
| 12.9 | 1620.8141 | -0.0297 | SAIMTTANNLDNTKK |
| 12.9 | 1620.8909 | -0.1065 | SSSIVVMKAAAAPVFK |
| 10.9 | 1620.7665 | 0.0179 | MEVVVLSDSGDIDSR |
| 10.7 | 1620.8042 | -0.0198 | FPCSLLSNGRDSIR |
| 10.4 | 1620.7678 | 0.0166 | AGDAASARAVFDAMPR |

**Mascot:** http://www.matrixscience.com/

# MATRIX SCIENCE Mascot Search Results

## Peptide View Spot no 39

MS/MS Fragmentation of **VVDLAHLVASK**
Found in **gi|115450493** in **NCBInr**, Os03g0129300 [Oryza sativa Japonica Group]

Match to Query 168: 1150.728824 from(1151.736100,1+) intensity(0.0000) index(15)
Title: Label: H12, Spot_Id: 219857, Peak_List_Id: 227353, MSMS Job_Run_Id: 21959, Comment:
Data file C:\Documents and Settings\Administrator\Desktop\Farah-Garima 11May 2013\ppw_H12_136868235200.txt

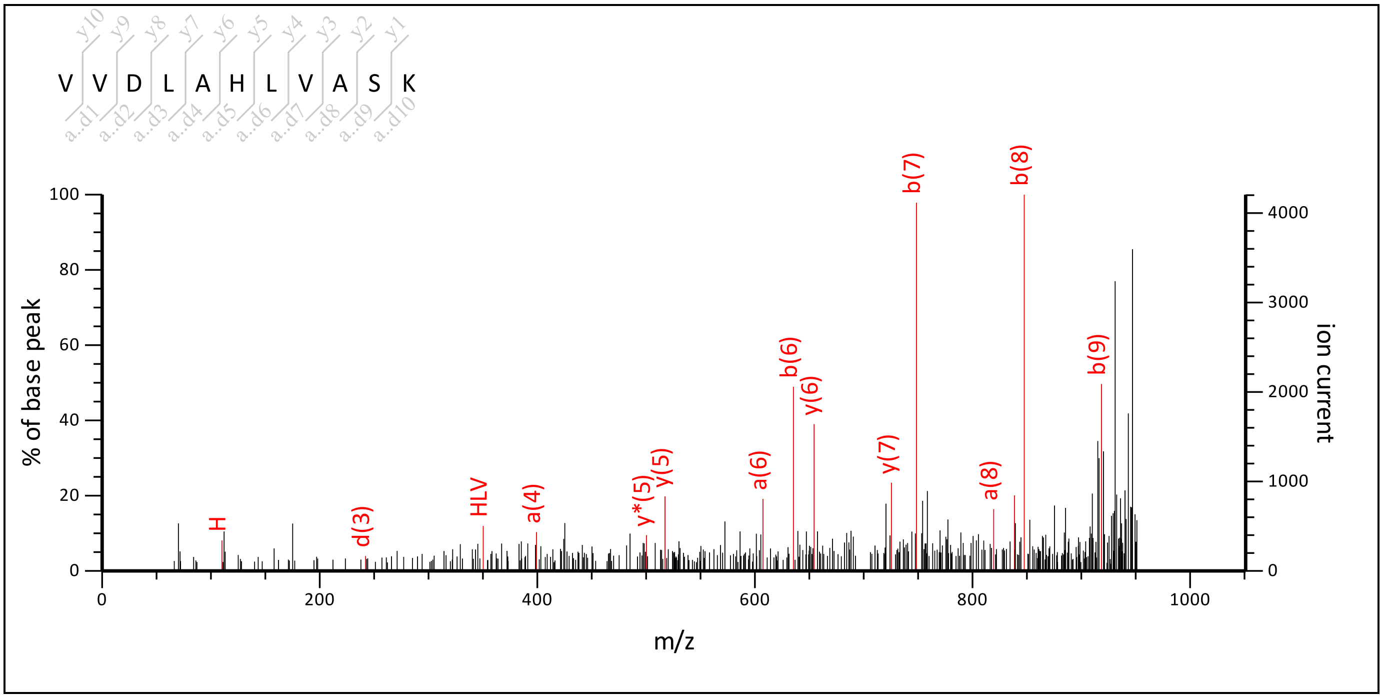


0 to 1051.06

Label all possible matches ○ Label matches used for scoring ◉

**Monoisotopic mass of neutral peptide Mr(calc):** 1150.6710
**Fixed modifications:** Carbamidomethyl (C) (apply to specified residues or termini only)
**Ions Score:** 27 **Expect:** 25
**Matches :** 16/163 fragment ions using 27 most intense peaks (help)

| # | Immon. | a | $a^0$ | b | $b^0$ | d | Seq. | v | w | y | y* | $y^0$ | # |
|---|---|---|---|---|---|---|---|---|---|---|---|---|---|
| 1 | 72.0808 | 72.0808 | | 100.0757 | | 44.0495 | V | | | | | | 11 |
| 2 | 72.0808 | 171.1492 | | 199.1441 | | 157.1335 | V | 1008.5473 | 1021.5677 | 1052.6099 | 1035.5833 | 1034.5993 | 10 |
| 3 | 88.0393 | 286.1761 | 268.1656 | 314.1710 | 296.1605 | 242.1863 | D | 893.5203 | 892.5251 | 953.5415 | 936.5149 | 935.5309 | 9 |
| 4 | 86.0964 | **399.2602** | 381.2496 | 427.2551 | 409.2445 | 357.2132 | L | 780.4363 | 779.4410 | ***838.5145*** | 821.4880 | 820.5039 | 8 |
| 5 | 44.0495 | 470.2973 | 452.2867 | 498.2922 | 480.2817 | | A | 709.3991 | | ***725.4304*** | 708.4039 | 707.4199 | 7 |
| 6 | 110.0713 | **607.3562** | 589.3457 | ***635.3511*** | 617.3406 | | H | 572.3402 | | ***654.3933*** | 637.3668 | 636.3828 | 6 |
| 7 | 86.0964 | 720.4403 | 702.4297 | ***748.4352*** | 730.4246 | 678.3933 | L | 459.2562 | 458.2609 | ***517.3344*** | 500.3079 | 499.3239 | 5 |
| 8 | 72.0808 | **819.5087** | 801.4981 | ***847.5036*** | 829.4930 | 805.4930 | V | 360.1878 | 373.2082 | 404.2504 | 387.2238 | 386.2398 | 4 |
| 9 | 44.0495 | 890.5458 | 872.5352 | ***918.5407*** | 900.5302 | | A | 289.1506 | | 305.1819 | 288.1554 | 287.1714 | 3 |
| 10 | 60.0444 | 977.5778 | 959.5673 | 1005.5728 | 987.5622 | 961.5829 | S | 202.1186 | 201.1234 | 234.1448 | 217.1183 | 216.1343 | 2 |

| | | | | | | | | | | | | |
|---|---|---|---|---|---|---|---|---|---|---|---|---|
| 11 | 101.1073 | | | | | K | 74.0237 | 73.0284 | 147.1128 | 130.0863 | | 1 |

| Seq | ya | yb | Seq | ya | yb | Seq | ya | yb |
|---|---|---|---|---|---|---|---|---|
| VD | 187.1077 | 215.1026 | VDL | 300.1918 | 328.1867 | VDLA | 371.2289 | 399.2238 |
| VDLAH | 508.2878 | 536.2827 | VDLAHL | 621.3719 | 649.3668 | DL | 201.1234 | 229.1183 |
| DLA | 272.1605 | 300.1554 | DLAH | 409.2194 | 437.2143 | DLAHL | 522.3035 | 550.2984 |
| DLAHLV | 621.3719 | 649.3668 | DLAHLVA | 692.4090 | 720.4039 | LA | 157.1335 | 185.1285 |
| LAH | 294.1925 | 322.1874 | LAHL | 407.2765 | 435.2714 | LAHLV | 506.3449 | 534.3398 |
| LAHLVA | 577.3820 | 605.3770 | LAHLVAS | 664.4141 | 692.4090 | AH | 181.1084 | 209.1033 |
| AHL | 294.1925 | 322.1874 | AHLV | 393.2609 | 421.2558 | AHLVA | 464.2980 | 492.2929 |
| AHLVAS | 551.3300 | 579.3249 | HL | 223.1553 | 251.1503 | HLV | 322.2238 | 350.2187 |
| HLVA | 393.2609 | 421.2558 | HLVAS | 480.2929 | 508.2878 | LV | 185.1648 | 213.1598 |
| LVA | 256.2020 | 284.1969 | LVAS | 343.2340 | 371.2289 | VA | 143.1179 | 171.1128 |
| VAS | 230.1499 | 258.1448 | AS | 131.0815 | 159.0764 | | | |

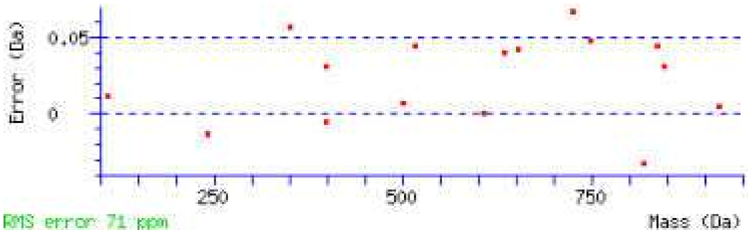


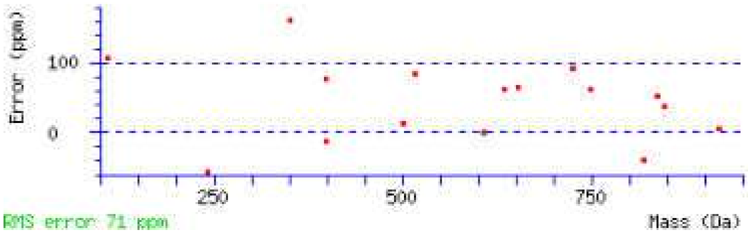


NCBI **BLAST** search of VVDLAHLVASK
(Parameters: blastp, nr protein database, expect=20000, no filter, PAM30)
Other BLAST web gateways

**All matches to this query**

| Score | Mr(calc) | Delta | Sequence |
|---|---|---|---|
| 26.6 | 1150.6710 | 0.0578 | VVDLAHLVASK |
| 22.1 | 1150.6194 | 0.1095 | IKSTSSSGAVSK |
| 21.4 | 1150.6359 | 0.0929 | HEHLLPLHR |
| 21.4 | 1150.6458 | 0.0830 | SNTTIPILHR |
| 21.4 | 1150.6458 | 0.0830 | STQSLPLLHR |
| 20.0 | 1150.6169 | 0.1120 | GCSVLFSILR |
| 20.0 | 1150.6169 | 0.1120 | GCSVLFSLLR |
| 18.6 | 1150.6710 | 0.0578 | ELAAIHVLASK |
| 15.3 | 1150.6207 | 0.1082 | AEIIAHRDAR |
| 14.6 | 1150.6532 | 0.0756 | GVHMLPLAVSK |

**Mascot:** http://www.matrixscience.com/

# MATRIX SCIENCE Mascot Search Results

## Peptide View Spot no 39

MS/MS Fragmentation of **TGITADDVNAAFR**
Found in **gi|115450493** in **NCBInr**, Os03g0129300 [Oryza sativa Japonica Group]

Match to Query 273: 1349.752224 from(1350.759500,1+) intensity(0.0000) index(24)
Title: Label: H12, Spot_Id: 219857, Peak_List_Id: 227347, MSMS Job_Run_Id: 21959, Comment:
Data file C:\Documents and Settings\Administrator\Desktop\Farah-Garima 11May 2013\ppw_H12_136868235200.txt

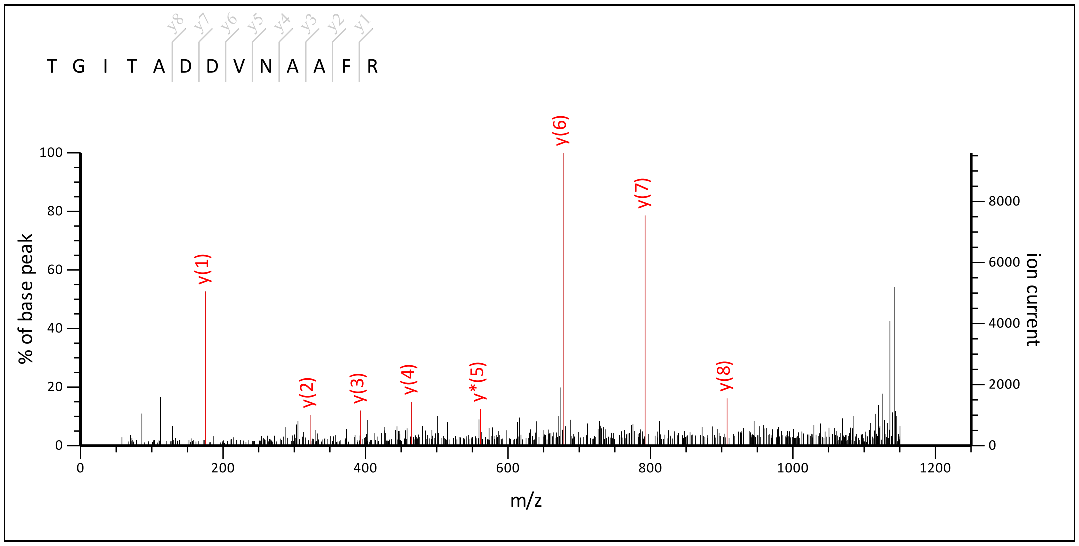


0 to 1250.3

Label all possible matches ○ Label matches used for scoring ◉

**Monoisotopic mass of neutral peptide Mr(calc):** 1349.6575
**Fixed modifications:** Carbamidomethyl (C) (apply to specified residues or termini only)
**Ions Score:** 55 **Expect:** 0.092
**Matches :** 8/214 fragment ions using 11 most intense peaks (help)

| # | Immon. | a | a* | $a^0$ | b | b* | $b^0$ | d | d' | Seq. | v | w | w' | y | y* | |
|---|---|---|---|---|---|---|---|---|---|---|---|---|---|---|---|---|
| 1 | 74.0600 | 74.0600 | | 56.0495 | 102.0550 | | 84.0444 | 44.0495 | | T | | | | | | |
| 2 | 30.0338 | 131.0815 | | 113.0709 | 159.0764 | | 141.0659 | | | G | | | | 1249.6171 | 1232.5906 | 123[illegible] |
| 3 | 86.0964 | 244.1656 | | 226.1550 | 272.1605 | | 254.1499 | 216.1343 | 230.1499 | I | 1134.5174 | 1147.5378 | 1161.5535 | 1192.5957 | 1175.5691 | 117[illegible] |
| 4 | 74.0600 | 345.2132 | | 327.2027 | 373.2082 | | 355.1976 | 329.2183 | 331.1976 | T | 1033.4697 | 1046.4902 | 1048.4694 | 1079.5116 | 1062.4851 | 106[illegible] |
| 5 | 44.0495 | 416.2504 | | 398.2398 | 444.2453 | | 426.2347 | | | A | 962.4326 | | | 978.4639 | 961.4374 | 96[illegible] |
| 6 | 88.0393 | 531.2773 | | 513.2667 | 559.2722 | | 541.2617 | 487.2875 | | D | 847.4057 | 846.4104 | | ***907.4268*** | 890.4003 | 88[illegible] |
| 7 | 88.0393 | 646.3042 | | 628.2937 | 674.2992 | | 656.2886 | 602.3144 | | D | 732.3787 | 731.3835 | | ***792.3999*** | 775.3733 | 77[illegible] |
| 8 | 72.0808 | 745.3727 | | 727.3621 | 773.3676 | | 755.3570 | 731.3570 | | V | 633.3103 | 646.3307 | | ***677.3729*** | 660.3464 | |
| 9 | 87.0553 | 859.4156 | 842.3890 | 841.4050 | 887.4105 | 870.3840 | 869.3999 | 816.4098 | | N | 519.2674 | 518.2722 | | 578.3045 | 561.2780 | |
| 10 | 44.0495 | 930.4527 | 913.4262 | 912.4421 | 958.4476 | 941.4211 | 940.4371 | | | A | 448.2303 | | | ***464.2616*** | 447.2350 | |
| 11 | 44.0495 | 1001.4898 | 984.4633 | 983.4793 | 1029.4847 | 1012.4582 | 1011.4742 | | | A | 377.1932 | | | ***393.2245*** | 376.1979 | |
| 12 | 120.0808 | 1148.5582 | 1131.5317 | 1130.5477 | 1176.5531 | 1159.5266 | 1158.5426 | | | F | 230.1248 | | | ***322.1874*** | 305.1608 | |
| 13 | 129.1135 | | | | | | | | | R | 74.0237 | 73.0284 | | ***175.1190*** | 158.0924 | |

| Seq | ya | yb | Seq | ya | yb | Seq | ya | yb |
|---|---|---|---|---|---|---|---|---|
| GI | 143.1179 | 171.1128 | GIT | 244.1656 | 272.1605 | GITA | 315.2027 | 343.1976 |
| GITAD | 430.2296 | 458.2245 | GITADD | 545.2566 | 573.2515 | GITADDV | 644.3250 | 672.3199 |
| IT | 187.1441 | 215.1390 | ITA | 258.1812 | 286.1761 | ITAD | 373.2082 | 401.2031 |
| ITADD | 488.2351 | 516.2300 | ITADDV | 587.3035 | 615.2984 | TA | 145.0972 | 173.0921 |
| TAD | 260.1241 | 288.1190 | TADD | 375.1510 | 403.1460 | TADDV | 474.2195 | 502.2144 |
| TADDVN | 588.2624 | 616.2573 | TADDVNA | 659.2995 | 687.2944 | AD | 159.0764 | 187.0713 |
| ADD | 274.1034 | 302.0983 | ADDV | 373.1718 | 401.1667 | ADDVN | 487.2147 | 515.2096 |
| ADDVNA | 558.2518 | 586.2467 | ADDVNAA | 629.2889 | 657.2838 | DD | 203.0662 | 231.0612 |

| | | | | | | | | |
|---|---|---|---|---|---|---|---|---|
| **DDV** | 302.1347 | 330.1296 | **DDVN** | 416.1776 | 444.1725 | **DDVNA** | 487.2147 | 515.2096 |
| **DDVNAA** | 558.2518 | 586.2467 | **DV** | 187.1077 | 215.1026 | **DVN** | 301.1506 | 329.1456 |
| **DVNA** | 372.1878 | 400.1827 | **DVNAA** | 443.2249 | 471.2198 | **DVNAAF** | 590.2933 | 618.2882 |
| **VN** | 186.1237 | 214.1186 | **VNA** | 257.1608 | 285.1557 | **VNAA** | 328.1979 | 356.1928 |
| **VNAAF** | 475.2663 | 503.2613 | **NA** | 158.0924 | 186.0873 | **NAA** | 229.1295 | 257.1244 |
| **NAAF** | 376.1979 | 404.1928 | **AA** | 115.0866 | 143.0815 | **AAF** | 262.1550 | 290.1499 |
| **AF** | 191.1179 | 219.1128 | | | | | | |

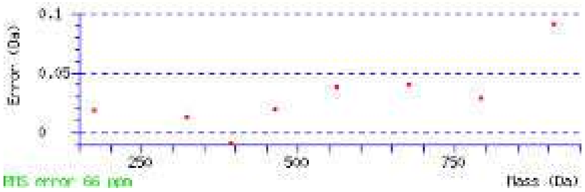


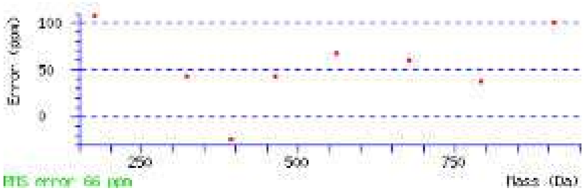


NCBI **BLAST** search of TGITADDVNAAFR
(Parameters: blastp, nr protein database, expect=20000, no filter, PAM30)
Other BLAST web gateways

**All matches to this query**

| Score | Mr(calc) | Delta | Sequence |
|---|---|---|---|
| 55.4 | 1349.6575 | 0.0947 | TGITADDVNAAFR |
| 33.6 | 1349.6907 | 0.0615 | RVGMLAATSAAMR |
| 30.5 | 1349.6874 | 0.0648 | QAIMDGLSRGFR |
| 30.3 | 1349.6575 | 0.0947 | SDFAQAVTDAGIR |
| 28.1 | 1349.6687 | 0.0835 | DYNSPSATARLR |
| 27.2 | 1349.6323 | 0.1199 | DAYAAAAADARER |
| 27.2 | 1349.6324 | 0.1199 | SEADRDGLNAFR |
| 26.7 | 1349.6688 | 0.0835 | IENDGSVRSAFR |
| 26.4 | 1349.6952 | 0.0570 | EFSRNRPAAFR |
| 26.4 | 1349.7204 | 0.0318 | ERGALFVNAAFR |

**Mascot:** http://www.matrixscience.com/

# MATRIX SCIENCE Mascot Search Results

## Peptide View Spot no 39

MS/MS Fragmentation of **TGITADDVNAAFRK**
Found in **gi|115450493** in **NCBInr**, Os03g0129300 [Oryza sativa Japonica Group]

Match to Query 329: 1477.854824 from(1478.862100,1+) intensity(0.0000) index(27)
Title: Label: H12, Spot_Id: 219857, Peak_List_Id: 227345, MSMS Job_Run_Id: 21959, Comment:
Data file C:\Documents and Settings\Administrator\Desktop\Farah-Garima 11May 2013\ppw_H12_136868235200.txt

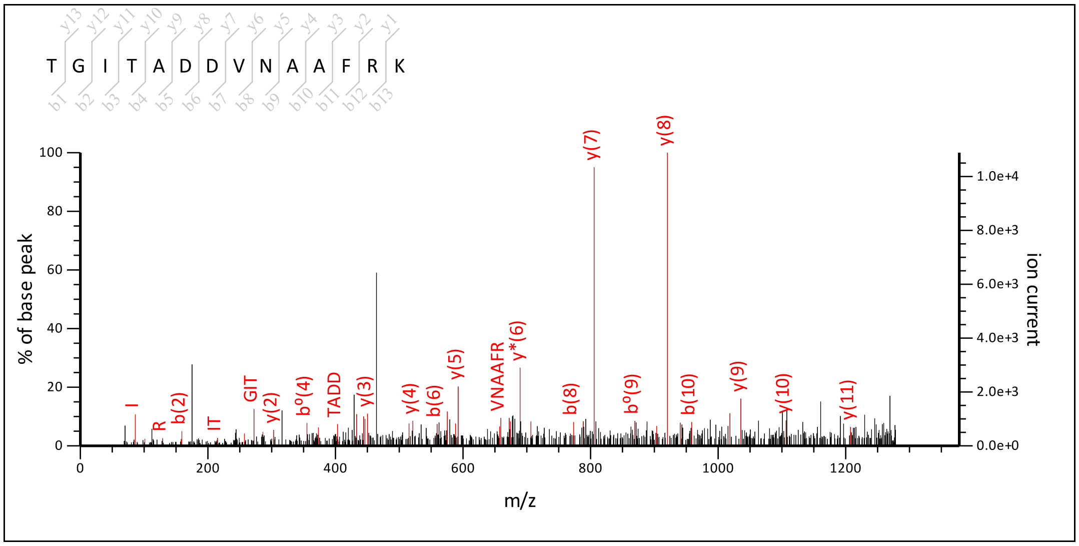


0 to 1378.08

Label all possible matches ○ Label matches used for scoring ◉

**Monoisotopic mass of neutral peptide Mr(calc):** 1477.7525
**Fixed modifications:** Carbamidomethyl (C) (apply to specified residues or termini only)
**Ions Score:** 42 **Expect:** 1.5
**Matches :** 53/236 fragment ions using 104 most intense peaks (help)

| # | Immon. | a | a* | $a^0$ | b | b* | $b^0$ | d | d' | Seq. | v | w | w' | y | y* | |
|---|---|---|---|---|---|---|---|---|---|---|---|---|---|---|---|---|
| 1 | 74.0600 | 74.0600 | | 56.0495 | 102.0550 | | 84.0444 | 44.0495 | | T | | | | | | |
| 2 | 30.0338 | 131.0815 | | 113.0709 | **159.0764** | | 141.0659 | | | G | | | | 1377.7121 | 1360.6856 | [illegible] |
| 3 | 86.0964 | 244.1656 | | 226.1550 | **272.1605** | | 254.1499 | 216.1343 | 230.1499 | I | 1262.6124 | 1275.6328 | 1289.6484 | 1320.6906 | 1303.6641 | [illegible] |
| 4 | 74.0600 | 345.2132 | | 327.2027 | **373.2082** | | 355.1976 | 329.2183 | 331.1976 | T | 1161.5647 | 1174.5851 | 1176.5644 | ***1207.6066*** | 1190.5800 | [illegible] |
| 5 | 44.0495 | 416.2504 | | 398.2398 | **444.2453** | | 426.2347 | | | A | 1090.5276 | | | ***1106.5589*** | 1089.5323 | [illegible] |
| 6 | 88.0393 | 531.2773 | | 513.2667 | **559.2722** | | 541.2617 | 487.2875 | | D | 975.5007 | 974.5054 | | ***1035.5218*** | 1018.4952 | [illegible] |
| 7 | 88.0393 | 646.3042 | | 628.2937 | **674.2992** | | 656.2886 | 602.3144 | | D | 860.4737 | 859.4785 | | ***920.4948*** | 903.4683 | [illegible] |
| 8 | 72.0808 | 745.3727 | | 727.3621 | **773.3676** | | 755.3570 | 731.3570 | | V | 761.4053 | 774.4257 | | ***805.4679*** | 788.4413 | |
| 9 | 87.0553 | 859.4156 | 842.3890 | 841.4050 | 887.4105 | 870.3840 | 869.3999 | 816.4098 | | N | 647.3624 | 646.3671 | | ***706.3995*** | 689.3729 | |
| 10 | 44.0495 | 930.4527 | 913.4262 | 912.4421 | **958.4476** | 941.4211 | 940.4371 | | | A | 576.3253 | | | ***592.3566*** | 575.3300 | |
| 11 | 44.0495 | 1001.4898 | 984.4633 | 983.4793 | 1029.4847 | 1012.4582 | 1011.4742 | | | A | 505.2881 | | | ***521.3194*** | 504.2929 | |
| 12 | 120.0808 | 1148.5582 | 1131.5317 | 1130.5477 | 1176.5531 | 1159.5266 | 1158.5426 | | | F | 358.2197 | | | ***450.2823*** | 433.2558 | |
| 13 | 129.1135 | 1304.6593 | 1287.6328 | 1286.6488 | 1332.6543 | 1315.6277 | 1314.6437 | 1219.5953 | | R | 202.1186 | 201.1234 | | ***303.2139*** | 286.1874 | |
| 14 | 101.1073 | | | | | | | | | K | 74.0237 | 73.0284 | | 147.1128 | 130.0863 | |

| Seq | ya | yb | Seq | ya | yb | Seq | ya | yb |
|---|---|---|---|---|---|---|---|---|
| GI | 143.1179 | 171.1128 | GIT | 244.1656 | 272.1605 | GITA | 315.2027 | 343.1976 |
| GITAD | 430.2296 | 458.2245 | GITADD | 545.2566 | 573.2515 | GITADDV | 644.3250 | 672.3199 |
| IT | 187.1441 | 215.1390 | ITA | 258.1812 | 286.1761 | ITAD | 373.2082 | 401.2031 |
| ITADD | 488.2351 | 516.2300 | ITADDV | 587.3035 | 615.2984 | TA | 145.0972 | 173.0921 |
| TAD | 260.1241 | 288.1190 | TADD | 375.1510 | 403.1460 | TADDV | 474.2195 | 502.2144 |
| TADDVN | 588.2624 | 616.2573 | TADDVNA | 659.2995 | 687.2944 | AD | 159.0764 | 187.0713 |
| ADD | 274.1034 | 302.0983 | ADDV | 373.1718 | 401.1667 | ADDVN | 487.2147 | 515.2096 |

| | | | | | | | | |
|---|---|---|---|---|---|---|---|---|
| **ADDVNA** | 558.2518 | 586.2467 | **ADDVNAA** | 629.2889 | 657.2838 | **DD** | 203.0662 | 231.0612 |
| **DDV** | 302.1347 | 330.1296 | **DDVN** | 416.1776 | 444.1725 | **DDVNA** | 487.2147 | 515.2096 |
| **DDVNAA** | 558.2518 | 586.2467 | **DV** | 187.1077 | 215.1026 | **DVN** | 301.1506 | 329.1456 |
| **DVNA** | 372.1878 | 400.1827 | **DVNAA** | 443.2249 | 471.2198 | **DVNAAF** | 590.2933 | 618.2882 |
| **VN** | 186.1237 | 214.1186 | **VNA** | 257.1608 | 285.1557 | **VNAA** | 328.1979 | 356.1928 |
| **VNAAF** | 475.2663 | 503.2613 | **VNAAFR** | 631.3675 | 659.3624 | **NA** | 158.0924 | 186.0873 |
| **NAA** | 229.1295 | 257.1244 | **NAAF** | 376.1979 | 404.1928 | **NAAFR** | 532.2990 | 560.2940 |
| **AA** | 115.0866 | 143.0815 | **AAF** | 262.1550 | 290.1499 | **AAFR** | 418.2561 | 446.2510 |
| **AF** | 191.1179 | 219.1128 | **AFR** | 347.2190 | 375.2139 | **FR** | 276.1819 | 304.1768 |

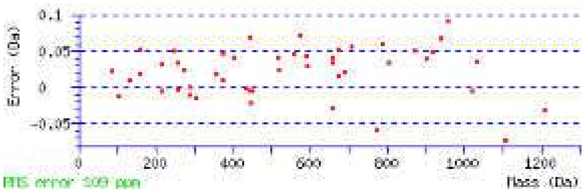


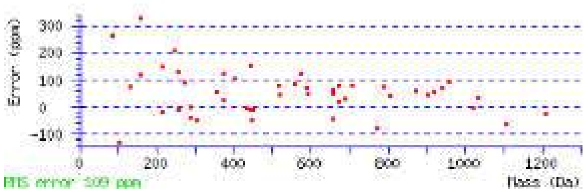


NCBI **BLAST** search of TGITADDVNAAFRK
(Parameters: blastp, nr protein database, expect=20000, no filter, PAM30)
Other BLAST web gateways

**All matches to this query**

| Score | Mr(calc) | Delta | Sequence |
|---|---|---|---|
| 42.0 | 1477.7525 | 0.1023 | TGITADDVNAAFRK |
| 26.9 | 1477.7381 | 0.1167 | QAIMDGKLTGGMTR |
| 26.6 | 1477.7599 | 0.0950 | MHSGYIKSDAIIK |
| 26.0 | 1477.7273 | 0.1275 | YAASVGRDAGQEVR |
| 24.8 | 1477.7776 | 0.0772 | SDAFAKAAADLIASK |
| 23.6 | 1477.7307 | 0.1241 | RMSTQGDAGATVIR |
| 23.1 | 1477.7776 | 0.0772 | LTEGYIQDLTGIR |
| 23.0 | 1477.7082 | 0.1466 | SGEVTEADMISALR |
| 22.3 | 1477.7082 | 0.1466 | NKTILMVDDEER |
| 21.7 | 1477.7373 | 0.1176 | PSTSAASSVVQSTTR |

**Mascot:** http://www.matrixscience.com/

# MATRIX SCIENCE Mascot Search Results

## Peptide View **Spot no 39**

MS/MS Fragmentation of **GTMTTTHSYTGDQR**
Found in **gi|115450493** in **NCBInr**, Os03g0129300 [Oryza sativa Japonica Group]

Match to Query 354: 1554.784824 from(1555.792100,1+) intensity(0.0000) index(29)
Title: Label: H12, Spot_Id: 219857, Peak_List_Id: 227352, MSMS Job_Run_Id: 21959, Comment:
Data file C:\Documents and Settings\Administrator\Desktop\Farah-Garima 11May 2013\ppw_H12_136868235200.txt

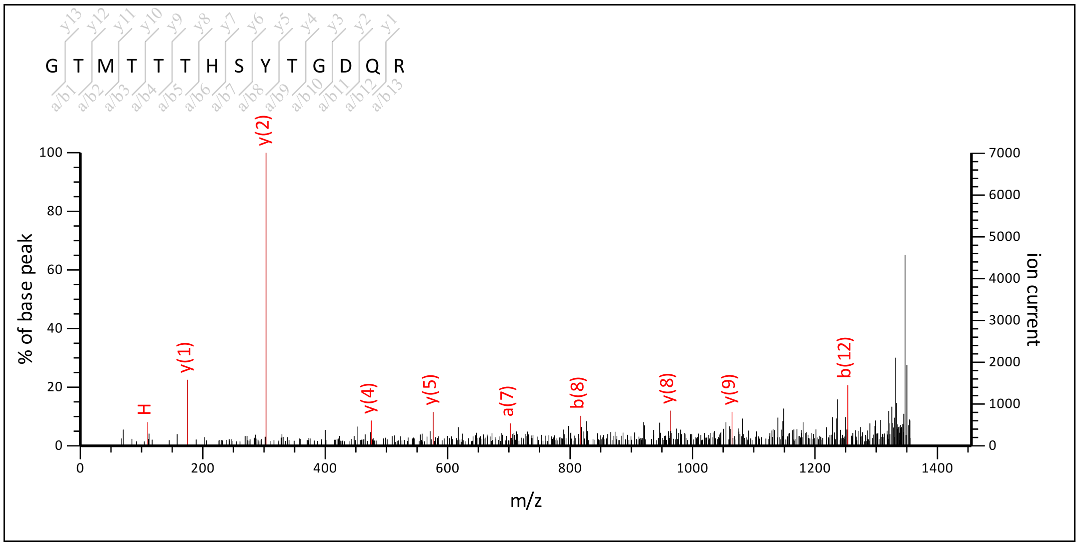


0 to 1455.54

Label all possible matches ○ Label matches used for scoring ◉

**Monoisotopic mass of neutral peptide Mr(calc):** 1554.6733
**Fixed modifications:** Carbamidomethyl (C) (apply to specified residues or termini only)
**Ions Score:** 42 **Expect:** 2.2
**Matches :** 12/235 fragment ions using 13 most intense peaks (help)

| # | Immon. | a | a* | $a^0$ | b | b* | $b^0$ | d | d' | Seq. | v | w | w' | y | y* | [illegible] |
|---|---|---|---|---|---|---|---|---|---|---|---|---|---|---|---|---|
| 1 | 30.0338 | 30.0338 | | | 58.0287 | | | 44.0495 | | G | | | | | | |
| 2 | 74.0600 | 131.0815 | | 113.0709 | 159.0764 | | 141.0659 | 115.0866 | 117.0659 | T | 1452.6172 | 1465.6376 | 1467.6169 | 1498.6591 | 1481.6325 | [illegible] |
| 3 | 104.0528 | 262.1220 | | 244.1114 | 290.1169 | | 272.1063 | 202.1186 | | M | 1321.5767 | 1320.5815 | | 1397.6114 | 1380.5849 | [illegible] |
| 4 | 74.0600 | 363.1697 | | 345.1591 | 391.1646 | | 373.1540 | 347.1748 | 349.1540 | T | 1220.5291 | 1233.5495 | 1235.5287 | 1266.5709 | 1249.5444 | [illegible] |
| 5 | 74.0600 | 464.2173 | | 446.2068 | 492.2123 | | 474.2017 | 448.2224 | 450.2017 | T | 1119.4814 | 1132.5018 | 1134.4810 | 1165.5232 | 1148.4967 | [illegible] |
| 6 | 74.0600 | 565.2650 | | 547.2545 | 593.2599 | | 575.2494 | 549.2701 | 551.2494 | T | 1018.4337 | 1031.4541 | 1033.4334 | ***1064.4756*** | 1047.4490 | [illegible] |
| 7 | 110.0713 | **702.3239** | | 684.3134 | 730.3189 | | 712.3083 | | | H | 881.3748 | | | ***963.4279*** | 946.4013 | [illegible] |
| 8 | 60.0444 | 789.3560 | | 771.3454 | **817.3509** | | 799.3403 | 773.3611 | | S | 794.3428 | 793.3475 | | 826.3690 | 809.3424 | [illegible] |
| 9 | 136.0757 | 952.4193 | | 934.4087 | 980.4142 | | 962.4036 | | | Y | 631.2794 | | | 739.3369 | 722.3104 | [illegible] |
| 10 | 74.0600 | 1053.4670 | | 1035.4564 | 1081.4619 | | 1063.4513 | 1037.4721 | 1039.4513 | T | 530.2318 | 543.2522 | 545.2314 | ***576.2736*** | 559.2471 | [illegible] |
| 11 | 30.0338 | 1110.4884 | | 1092.4779 | 1138.4834 | | 1120.4728 | | | G | | | | ***475.2259*** | 458.1994 | [illegible] |
| 12 | 88.0393 | 1225.5154 | | 1207.5048 | **1253.5103** | | 1235.4997 | 1181.5256 | | D | 358.1833 | 357.1881 | | 418.2045 | 401.1779 | [illegible] |
| 13 | 101.0709 | 1353.5740 | 1336.5474 | 1335.5634 | 1381.5689 | 1364.5423 | 1363.5583 | 1296.5525 | | Q | 230.1248 | 229.1295 | | ***303.1775*** | 286.1510 | |
| 14 | 129.1135 | | | | | | | | | R | 74.0237 | 73.0284 | | ***175.1190*** | 158.0924 | |

| Seq | ya | yb | Seq | ya | yb | Seq | ya | yb |
|---|---|---|---|---|---|---|---|---|
| TM | 205.1005 | 233.0954 | TMT | 306.1482 | 334.1431 | TMTT | 407.1959 | 435.1908 |
| TMTTT | 508.2436 | 536.2385 | TMTTTH | 645.3025 | 673.2974 | MT | 205.1005 | 233.0954 |
| MTT | 306.1482 | 334.1431 | MTTT | 407.1959 | 435.1908 | MTTTH | 544.2548 | 572.2497 |
| MTTTHS | 631.2868 | 659.2817 | TT | 175.1077 | 203.1026 | TTT | 276.1554 | 304.1503 |
| TTTH | 413.2143 | 441.2092 | TTTHS | 500.2463 | 528.2413 | TTTHSY | 663.3097 | 691.3046 |
| TT | 175.1077 | 203.1026 | TTH | 312.1666 | 340.1615 | TTHS | 399.1987 | 427.1936 |
| TTHSY | 562.2620 | 590.2569 | TTHSYT | 663.3097 | 691.3046 | TH | 211.1190 | 239.1139 |

| | | | | | | | | |
|---|---|---|---|---|---|---|---|---|
| **THS** | 298.1510 | 326.1459 | **THSY** | 461.2143 | 489.2092 | **THSYT** | 562.2620 | 590.2569 |
| **THSYTG** | 619.2835 | 647.2784 | **HS** | 197.1033 | 225.0982 | **HSY** | 360.1666 | 388.1615 |
| **HSYT** | 461.2143 | 489.2092 | **HSYTG** | 518.2358 | 546.2307 | **HSYTGD** | 633.2627 | 661.2576 |
| **SY** | 223.1077 | 251.1026 | **SYT** | 324.1554 | 352.1503 | **SYTG** | 381.1769 | 409.1718 |
| **SYTGD** | 496.2038 | 524.1987 | **SYTGDQ** | 624.2624 | 652.2573 | **YT** | 237.1234 | 265.1183 |
| **YTG** | 294.1448 | 322.1397 | **YTGD** | 409.1718 | 437.1667 | **YTGDQ** | 537.2304 | 565.2253 |
| **TG** | 131.0815 | 159.0764 | **TGD** | 246.1084 | 274.1034 | **TGDQ** | 374.1670 | 402.1619 |
| **GD** | 145.0608 | 173.0557 | **GDQ** | 273.1193 | 301.1143 | **DQ** | 216.0979 | 244.0928 |

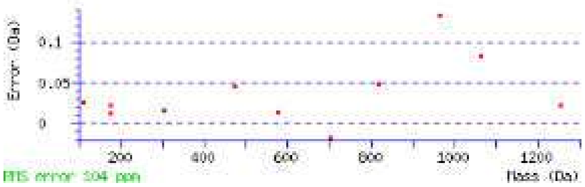


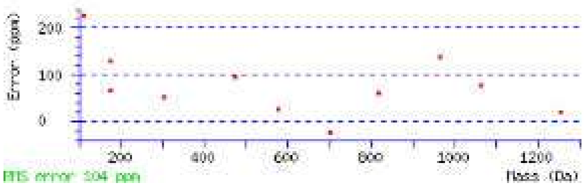


NCBI **BLAST** search of GTMTTTHSYTGDQR
(Parameters: blastp, nr protein database, expect=20000, no filter, PAM30)
Other BLAST web gateways

**All matches to this query**

| Score | Mr(calc) | Delta | Sequence |
|---|---|---|---|
| 41.7 | 1554.6733 | 0.1115 | GTMTTTHSYTGDQR |
| 32.6 | 1554.8266 | -0.0418 | YLQSTHAAIPNGKR |
| 28.5 | 1554.6733 | 0.1115 | GSMTTVHSYTGDQR |
| 23.4 | 1554.6953 | 0.0896 | MEQSTQMQCIKR |
| 19.6 | 1554.8188 | -0.0340 | SFRTTLSTVLMGAR |
| 19.6 | 1554.6998 | 0.0851 | NDNMFWRTTAQR |
| 18.3 | 1554.7130 | 0.0718 | TTKATTACGTQCQK |
| 17.4 | 1554.8267 | -0.0418 | QGIATPNGKLWSQR |
| 17.4 | 1554.8631 | -0.0782 | GVLHKFNALTATQR |
| 17.4 | 1554.7977 | -0.0128 | HKLLEMIDSWQR |

**Mascot:** http://www.matrixscience.com/

# MATRIX SCIENCE Mascot Search Results

## Peptide View Spot no 39

MS/MS Fragmentation of **ENSPLEVVVVNDSGGVR**
Found in **gi|115450493** in **NCBInr**, Os03g0129300 [Oryza sativa Japonica Group]

Match to Query 377: 1769.017024 from(1770.024300,1+) intensity(0.0000) index(33)
Title: Label: H12, Spot_Id: 219857, Peak_List_Id: 227348, MSMS Job_Run_Id: 21959, Comment:
Data file C:\Documents and Settings\Administrator\Desktop\Farah-Garima 11May 2013\ppw_H12_136868235200.txt

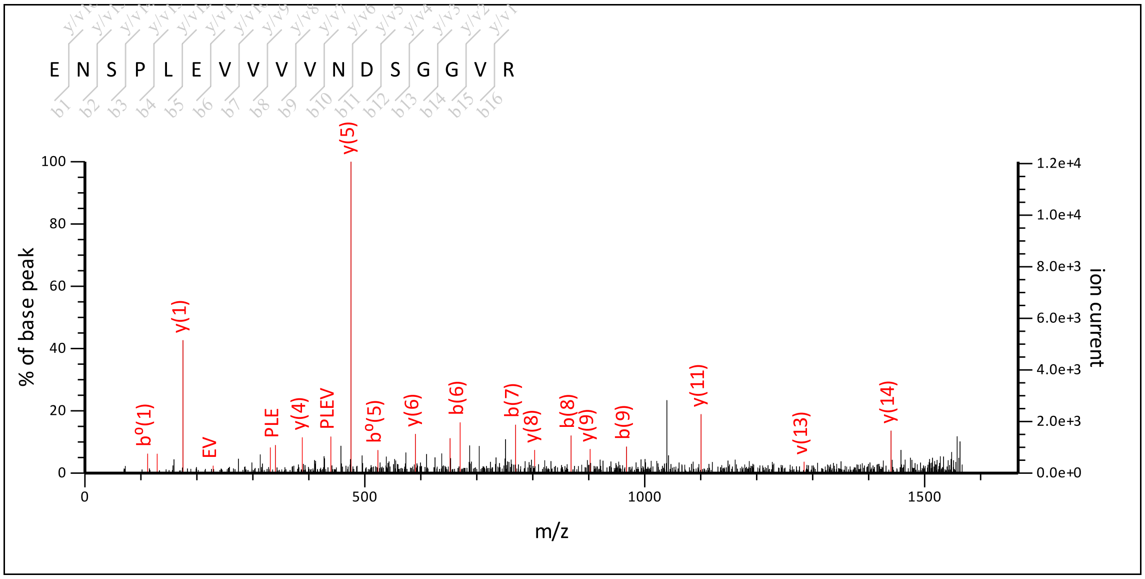


0 to 1667.05

Label all possible matches ○ Label matches used for scoring ◉

**Monoisotopic mass of neutral peptide Mr(calc):** 1768.8956
**Fixed modifications:** Carbamidomethyl (C) (apply to specified residues or termini only)
**Ions Score:** 63 **Expect:** 0.012
**Matches :** 26/325 fragment ions using 30 most intense peaks (help)

| # | Immon. | a | a* | $a^0$ | b | b* | $b^0$ | d | Seq. | v | w | y | y* | $y^0$ | # |
|---|---|---|---|---|---|---|---|---|---|---|---|---|---|---|---|
| 1 | 102.0550 | 102.0550 | | 84.0444 | 130.0499 | | 112.0393 | 44.0495 | E | | | | | | 17 |
| 2 | 87.0553 | 216.0979 | 199.0713 | 198.0873 | 244.0928 | 227.0662 | 226.0822 | 173.0921 | N | 1581.8231 | 1580.8279 | 1640.8602 | 1623.8337 | 1622.8497 | 16 |
| 3 | 60.0444 | 303.1299 | 286.1034 | 285.1193 | **331.1248** | 314.0983 | 313.1143 | 287.1350 | S | 1494.7911 | 1493.7958 | 1526.8173 | 1509.7907 | 1508.8067 | 15 |
| 4 | 70.0651 | 400.1827 | 383.1561 | 382.1721 | 428.1776 | 411.1510 | 410.1670 | 374.1670 | P | 1397.7383 | 1396.7431 | ***1439.7853*** | 1422.7587 | 1421.7747 | 14 |
| 5 | 86.0964 | 513.2667 | 496.2402 | 495.2562 | 541.2617 | 524.2351 | 523.2511 | 471.2198 | L | 1284.6543 | 1283.6590 | 1342.7325 | 1325.7060 | 1324.7219 | 13 |
| 6 | 102.0550 | 642.3093 | 625.2828 | 624.2988 | **670.3042** | 653.2777 | 652.2937 | 584.3039 | E | 1155.6117 | 1154.6164 | 1229.6484 | 1212.6219 | 1211.6379 | 12 |
| 7 | 72.0808 | 741.3777 | 724.3512 | 723.3672 | **769.3727** | 752.3461 | 751.3621 | 727.3621 | V | 1056.5432 | 1069.5636 | ***1100.6058*** | 1083.5793 | 1082.5953 | 11 |
| 8 | 72.0808 | 840.4462 | 823.4196 | 822.4356 | **868.4411** | 851.4145 | 850.4305 | 826.4305 | V | 957.4748 | 970.4952 | 1001.5374 | 984.5109 | 983.5269 | 10 |
| 9 | 72.0808 | 939.5146 | 922.4880 | 921.5040 | **967.5095** | 950.4829 | 949.4989 | 925.4989 | V | 858.4064 | 871.4268 | ***902.4690*** | 885.4425 | 884.4585 | 9 |
| 10 | 72.0808 | 1038.5830 | 1021.5564 | 1020.5724 | 1066.5779 | 1049.5514 | 1048.5673 | 1024.5673 | V | 759.3380 | 772.3584 | ***803.4006*** | 786.3741 | 785.3900 | 8 |
| 11 | 87.0553 | 1152.6259 | 1135.5994 | 1134.6153 | 1180.6208 | 1163.5943 | 1162.6103 | 1109.6201 | N | 645.2951 | 644.2998 | 704.3322 | 687.3056 | 686.3216 | 7 |
| 12 | 88.0393 | 1267.6529 | 1250.6263 | 1249.6423 | 1295.6478 | 1278.6212 | 1277.6372 | 1223.6630 | D | 530.2681 | 529.2729 | ***590.2893*** | 573.2627 | 572.2787 | 6 |
| 13 | 60.0444 | 1354.6849 | 1337.6583 | 1336.6743 | 1382.6798 | 1365.6533 | 1364.6692 | 1338.6900 | S | 443.2361 | 442.2409 | ***475.2623*** | 458.2358 | 457.2518 | 5 |
| 14 | 30.0338 | 1411.7063 | 1394.6798 | 1393.6958 | 1439.7013 | 1422.6747 | 1421.6907 | | G | | | ***388.2303*** | 371.2037 | | 4 |
| 15 | 30.0338 | 1468.7278 | 1451.7013 | 1450.7172 | 1496.7227 | 1479.6962 | 1478.7122 | | G | | | ***331.2088*** | 314.1823 | | 3 |
| 16 | 72.0808 | 1567.7962 | 1550.7697 | 1549.7857 | 1595.7911 | 1578.7646 | 1577.7806 | 1553.7806 | V | 230.1248 | 243.1452 | 274.1874 | 257.1608 | | 2 |
| 17 | 129.1135 | | | | | | | | R | 74.0237 | 73.0284 | ***175.1190*** | 158.0924 | | 1 |

| Seq | ya | yb | Seq | ya | yb | Seq | ya | yb |
|---|---|---|---|---|---|---|---|---|
| NS | 174.0873 | 202.0822 | NSP | 271.1401 | 299.1350 | NSPL | 384.2241 | 412.2191 |

| | | | | | | | | |
|---|---|---|---|---|---|---|---|---|
| **NSPLE** | 513.2667 | 541.2617 | **NSPLEV** | 612.3352 | 640.3301 | **SP** | 157.0972 | 185.0921 |
| **SPL** | 270.1812 | 298.1761 | **SPLE** | 399.2238 | 427.2187 | **SPLEV** | 498.2922 | 526.2871 |
| **SPLEVV** | 597.3606 | 625.3556 | **SPLEVVV** | 696.4291 | 724.4240 | **PL** | 183.1492 | 211.1441 |
| **PLE** | 312.1918 | 340.1867 | **PLEV** | 411.2602 | 439.2551 | **PLEVV** | 510.3286 | 538.3235 |
| **PLEVVV** | 609.3970 | 637.3919 | **LE** | 215.1390 | 243.1339 | **LEV** | 314.2074 | 342.2023 |
| **LEVV** | 413.2758 | 441.2708 | **LEVVV** | 512.3443 | 540.3392 | **LEVVVV** | 611.4127 | 639.4076 |
| **EV** | 201.1234 | 229.1183 | **EVV** | 300.1918 | 328.1867 | **EVVV** | 399.2602 | 427.2551 |
| **EVVVV** | 498.3286 | 526.3235 | **EVVVVN** | 612.3715 | 640.3665 | **VV** | 171.1492 | 199.1441 |
| **VVV** | 270.2176 | 298.2125 | **VVVV** | 369.2860 | 397.2809 | **VVVVN** | 483.3289 | 511.3239 |
| **VVVVND** | 598.3559 | 626.3508 | **VVVVNDS** | 685.3879 | 713.3828 | **VV** | 171.1492 | 199.1441 |
| **VVV** | 270.2176 | 298.2125 | **VVVN** | 384.2605 | 412.2554 | **VVVND** | 499.2875 | 527.2824 |
| **VVVNDS** | 586.3195 | 614.3144 | **VVVNDSG** | 643.3410 | 671.3359 | **VV** | 171.1492 | 199.1441 |
| **VVN** | 285.1921 | 313.1870 | **VVND** | 400.2191 | 428.2140 | **VVNDS** | 487.2511 | 515.2460 |
| **VVNDSG** | 544.2726 | 572.2675 | **VVNDSGG** | 601.2940 | 629.2889 | **VN** | 186.1237 | 214.1186 |
| **VND** | 301.1506 | 329.1456 | **VNDS** | 388.1827 | 416.1776 | **VNDSG** | 445.2041 | 473.1991 |
| **VNDSGG** | 502.2256 | 530.2205 | **VNDSGGV** | 601.2940 | 629.2889 | **ND** | 202.0822 | 230.0771 |
| **NDS** | 289.1143 | 317.1092 | **NDSG** | 346.1357 | 374.1306 | **NDSGG** | 403.1572 | 431.1521 |
| **NDSGGV** | 502.2256 | 530.2205 | **DS** | 175.0713 | 203.0662 | **DSG** | 232.0928 | 260.0877 |
| **DSGG** | 289.1143 | 317.1092 | **DSGGV** | 388.1827 | 416.1776 | **SG** | 117.0659 | 145.0608 |
| **SGG** | 174.0873 | 202.0822 | **SGGV** | 273.1557 | 301.1506 | **GG** | 87.0553 | 115.0502 |
| **GGV** | 186.1237 | 214.1186 | **GV** | 129.1022 | 157.0972 | | | |

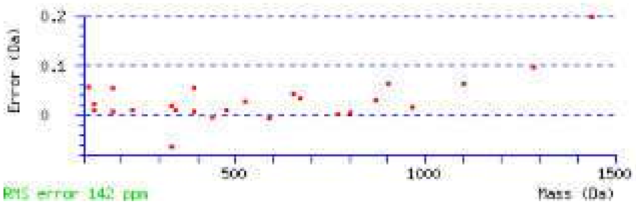


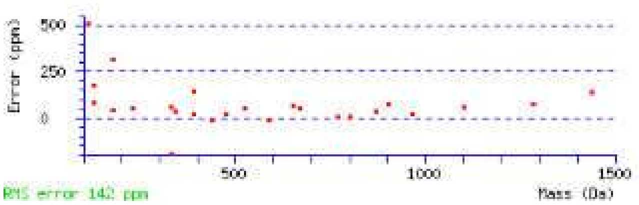


NCBI **BLAST** search of ENSPLEVVVVNDSGGVR
(Parameters: blastp, nr protein database, expect=20000, no filter, PAM30)
Other BLAST web gateways

**All matches to this query**

| Score | Mr(calc) | Delta | Sequence |
|---|---|---|---|
| 62.9 | 1768.8956 | 0.1215 | ENSPLEVVVVNDSGGVR |
| 28.5 | 1768.9571 | 0.0599 | KDSPLEVVVINDTGGVK |
| 21.0 | 1768.9068 | 0.1103 | GSEGTELEVPVNAVGRR |
| 18.4 | 1768.9207 | 0.0963 | ENSPLEVVVINDTGGVK |
| 18.1 | 1768.8492 | 0.1678 | EGDPSQYHSVPEIRR |
| 17.6 | 1768.8778 | 0.1392 | QCVSDAKIHQDELVK |
| 16.9 | 1768.9544 | 0.0627 | KGGAAGLNAGAGASLVSAAAR |
| 16.1 | 1768.9571 | 0.0599 | KDSPLDIVVINDTGGVK |
| 16.1 | 1768.8956 | 0.1215 | AADISDPVVVTTPNASGR |
| 15.9 | 1768.9757 | 0.0413 | ELPAGSTIVVVLMDGIR |

**Mascot:** http://www.matrixscience.com/

# Mascot Search Results

## Peptide View Spot no 39

MS/MS Fragmentation of **VPTPNVSVVDLVINTVK**
Found in **gi|115450493** in **NCBInr**, Os03g0129300 [Oryza sativa Japonica Group]

Match to Query 383: 1793.118824 from(1794.126100,1+) intensity(0.0000) index(34)
Title: Label: H12, Spot_Id: 219857, Peak_List_Id: 227356, MSMS Job_Run_Id: 21959, Comment:
Data file C:\Documents and Settings\Administrator\Desktop\Farah-Garima 11May 2013\ppw_H12_136868235200.txt

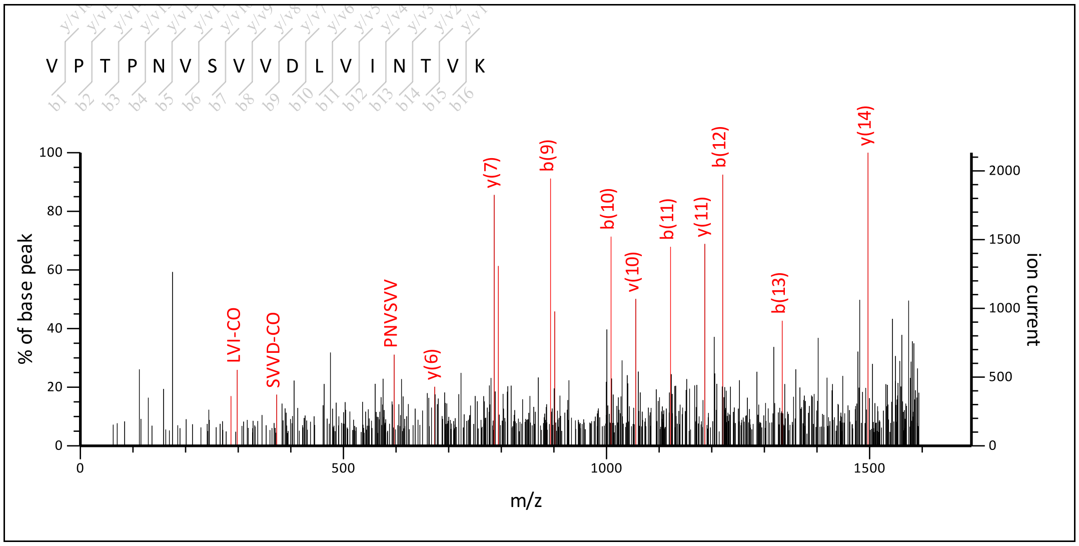


0 to 1693.52

Label all possible matches ○ Label matches used for scoring ◉

**Monoisotopic mass of neutral peptide Mr(calc):** 1793.0299
**Fixed modifications:** Carbamidomethyl (C) (apply to specified residues or termini only)
**Ions Score:** 30 **Expect:** 7.6
**Matches :** 20/329 fragment ions using 32 most intense peaks (help)

| # | Immon. | a | a* | a0 | b | b* | b0 | d | d' | Seq. | v | w | w' | y | y* | |
|---|---|---|---|---|---|---|---|---|---|---|---|---|---|---|---|---|
| 1 | 72.0808 | 72.0808 | | | 100.0757 | | | 44.0495 | | V | | | | | | |
| 2 | 70.0651 | 169.1335 | | | 197.1285 | | | 143.1179 | | P | 1652.9218 | 1651.9265 | | 1694.9687 | 1677.9422 | [illegible] |
| 3 | 74.0600 | 270.1812 | | 252.1707 | ***298.1761*** | | 280.1656 | 254.1863 | 256.1656 | T | 1551.8741 | 1564.8945 | 1566.8738 | 1597.9159 | 1580.8894 | [illegible] |
| 4 | 70.0651 | 367.2340 | | 349.2234 | 395.2289 | | 377.2183 | 341.2183 | | P | 1454.8213 | 1453.8261 | | **1496.8683** | 1479.8417 | [illegible] |
| 5 | 87.0553 | 481.2769 | 464.2504 | 463.2663 | 509.2718 | 492.2453 | 491.2613 | 438.2711 | | N | 1340.7784 | 1339.7831 | | 1399.8155 | 1382.7890 | [illegible] |
| 6 | 72.0808 | 580.3453 | 563.3188 | 562.3348 | 608.3402 | 591.3137 | 590.3297 | 566.3297 | | V | 1241.7100 | 1254.7304 | | 1285.7726 | 1268.7460 | [illegible] |
| 7 | 60.0444 | 667.3774 | 650.3508 | 649.3668 | 695.3723 | 678.3457 | 677.3617 | 651.3824 | | S | 1154.6780 | 1153.6827 | | **1186.7042** | 1169.6776 | [illegible] |
| 8 | 72.0808 | 766.4458 | 749.4192 | 748.4352 | ***794.4407*** | 777.4141 | 776.4301 | 752.4301 | | V | 1055.6095 | 1068.6299 | | 1099.6721 | 1082.6456 | [illegible] |
| 9 | 72.0808 | 865.5142 | 848.4876 | 847.5036 | ***893.5091*** | 876.4825 | 875.4985 | 851.4985 | | V | 956.5411 | 969.5615 | | 1000.6037 | 983.5772 | [illegible] |
| 10 | 88.0393 | 980.5411 | 963.5146 | 962.5306 | ***1008.5360*** | 991.5095 | 990.5255 | 936.5513 | | D | 841.5142 | 840.5189 | | **901.5353** | 884.5088 | [illegible] |
| 11 | 86.0964 | 1093.6252 | 1076.5986 | 1075.6146 | ***1121.6201*** | 1104.5936 | 1103.6095 | 1051.5782 | | L | 728.4301 | 727.4349 | | **786.5084** | 769.4818 | [illegible] |
| 12 | 72.0808 | 1192.6936 | 1175.6671 | 1174.6830 | ***1220.6885*** | 1203.6620 | 1202.6780 | 1178.6780 | | V | 629.3617 | 642.3821 | | **673.4243** | 656.3978 | [illegible] |
| 13 | 86.0964 | 1305.7777 | 1288.7511 | 1287.7671 | ***1333.7726*** | 1316.7460 | 1315.7620 | 1277.7464 | 1291.7620 | I | 516.2776 | 529.2980 | 543.3137 | 574.3559 | 557.3293 | [illegible] |
| 14 | 87.0553 | 1419.8206 | 1402.7940 | 1401.8100 | 1447.8155 | 1430.7890 | 1429.8049 | 1376.8148 | | N | 402.2347 | 401.2395 | | 461.2718 | 444.2453 | [illegible] |
| 15 | 74.0600 | 1520.8683 | 1503.8417 | 1502.8577 | 1548.8632 | 1531.8366 | 1530.8526 | 1504.8734 | 1506.8526 | T | 301.1870 | 314.2074 | 316.1867 | 347.2289 | 330.2023 | [illegible] |
| 16 | 72.0808 | 1619.9367 | 1602.9101 | 1601.9261 | 1647.9316 | 1630.9051 | 1629.9210 | 1605.9210 | | V | 202.1186 | 215.1390 | | 246.1812 | 229.1547 | |
| 17 | 101.1073 | | | | | | | | | K | 74.0237 | 73.0284 | | 147.1128 | 130.0863 | |

| Seq | ya | yb | Seq | ya | yb | Seq | ya | yb |
|---|---|---|---|---|---|---|---|---|
| PT | 171.1128 | 199.1077 | PTP | 268.1656 | 296.1605 | PTPN | 382.2085 | 410.2034 |
| PTPNV | 481.2769 | 509.2718 | PTPNVS | 568.3089 | 596.3039 | PTPNVSV | 667.3774 | 695.3723 |
| TP | 171.1128 | 199.1077 | TPN | 285.1557 | 313.1506 | TPNV | 384.2241 | 412.2191 |
| TPNVS | 471.2562 | 499.2511 | TPNVSV | 570.3246 | 598.3195 | TPNVSVV | 669.3930 | 697.3879 |

| | | | | | | | | |
|---|---|---|---|---|---|---|---|---|
| PN | 184.1081 | 212.1030 | PNV | 283.1765 | 311.1714 | PNVS | 370.2085 | 398.2034 |
| PNVSV | 469.2769 | 497.2718 | PNVSVV | 568.3453 | 596.3402 | PNVSVVD | 683.3723 | 711.3672 |
| NV | 186.1237 | 214.1186 | NVS | 273.1557 | 301.1506 | NVSV | 372.2241 | 400.2191 |
| NVSVV | 471.2926 | 499.2875 | NVSVVD | 586.3195 | 614.3144 | NVSVVDL | 699.4036 | 727.3985 |
| VS | 159.1128 | 187.1077 | VSV | 258.1812 | 286.1761 | VSVV | 357.2496 | 385.2445 |
| VSVVD | 472.2766 | 500.2715 | VSVVDL | 585.3606 | 613.3556 | VSVVDLV | 684.4291 | 712.4240 |
| SV | 159.1128 | 187.1077 | SVV | 258.1812 | 286.1761 | SVVD | 373.2082 | 401.2031 |
| SVVDL | 486.2922 | 514.2871 | SVVDLV | 585.3606 | 613.3556 | SVVDLVI | 698.4447 | 726.4396 |
| VV | 171.1492 | 199.1441 | VVD | 286.1761 | 314.1710 | VVDL | 399.2602 | 427.2551 |
| VVDLV | 498.3286 | 526.3235 | VVDLVI | 611.4127 | 639.4076 | VD | 187.1077 | 215.1026 |
| VDL | 300.1918 | 328.1867 | VDLV | 399.2602 | 427.2551 | VDLVI | 512.3443 | 540.3392 |
| VDLVIN | 626.3872 | 654.3821 | DL | 201.1234 | 229.1183 | DLV | 300.1918 | 328.1867 |
| DLVI | 413.2758 | 441.2708 | DLVIN | 527.3188 | 555.3137 | DLVINT | 628.3665 | 656.3614 |
| LV | 185.1648 | 213.1598 | LVI | 298.2489 | 326.2438 | LVIN | 412.2918 | 440.2867 |
| LVINT | 513.3395 | 541.3344 | LVINTV | 612.4079 | 640.4028 | VI | 185.1648 | 213.1598 |
| VIN | 299.2078 | 327.2027 | VINT | 400.2554 | 428.2504 | VINTV | 499.3239 | 527.3188 |
| IN | 200.1394 | 228.1343 | INT | 301.1870 | 329.1819 | INTV | 400.2554 | 428.2504 |
| NT | 188.1030 | 216.0979 | NTV | 287.1714 | 315.1663 | TV | 173.1285 | 201.1234 |

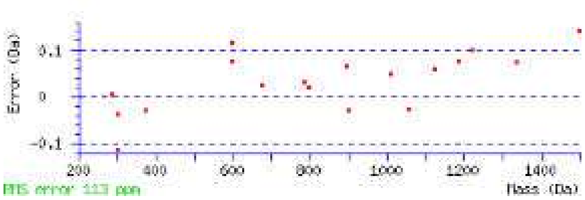


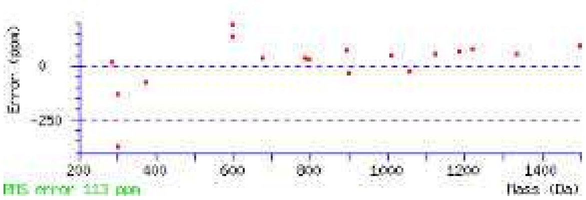


NCBI **BLAST** search of VPTPNVSVVDLVINTVK
(Parameters: blastp, nr protein database, expect=20000, no filter, PAM30)
Other BLAST web gateways

**All matches to this query**

| Score | Mr(calc) | Delta | Sequence |
|---|---|---|---|
| 30.6 | 1792.9935 | 0.1253 | VPTPNVSVVDLVVNVDK |
| 30.1 | 1793.0299 | 0.0889 | VPTPNVSVVDLVINTVK |
| 29.1 | 1793.0299 | 0.0889 | VPTPNVSVVDLVVQISK |
| 29.1 | 1793.0299 | 0.0889 | VPTPNVSVVDLVVQVTK |
| 24.9 | 1793.0411 | 0.0777 | VPTPNVSVVDLKAVLSR |
| 23.3 | 1792.9505 | 0.1683 | MANKLVLIDGNSIAYR |
| 22.7 | 1793.0411 | 0.0777 | VPTPNVSVVDLTVRLGK |
| 22.5 | 1792.9935 | 0.1253 | VPTPNVSVVDLVAQVEK |
| 17.2 | 1793.0451 | 0.0737 | LIAFEIYGTILAKVSR |
| 16.8 | 1792.9472 | 0.1716 | HPELPVEVVSGGLFTGR |

**Mascot:** http://www.matrixscience.com/

# MATRIX SCIENCE Mascot Search Results

## Peptide View Spot no 40

MS/MS Fragmentation of **CVFPDQR**
Found in **gi|297604125** in **NCBInr**, Os05g0247100 [Oryza sativa Japonica Group]

Match to Query 3: 920.402754 from(921.410030,1+) intensity(0.0000) index(2)
Title: Label: B5, Spot_Id: 219739, Peak_List_Id: 225587, MSMS Job_Run_Id: 21783, Comment:
Data file C:\Documents and Settings\Administrator\Desktop\farah BHU\spot set plate\Farah-Garima 11May 2013\ppw_B5_136842181400.txt

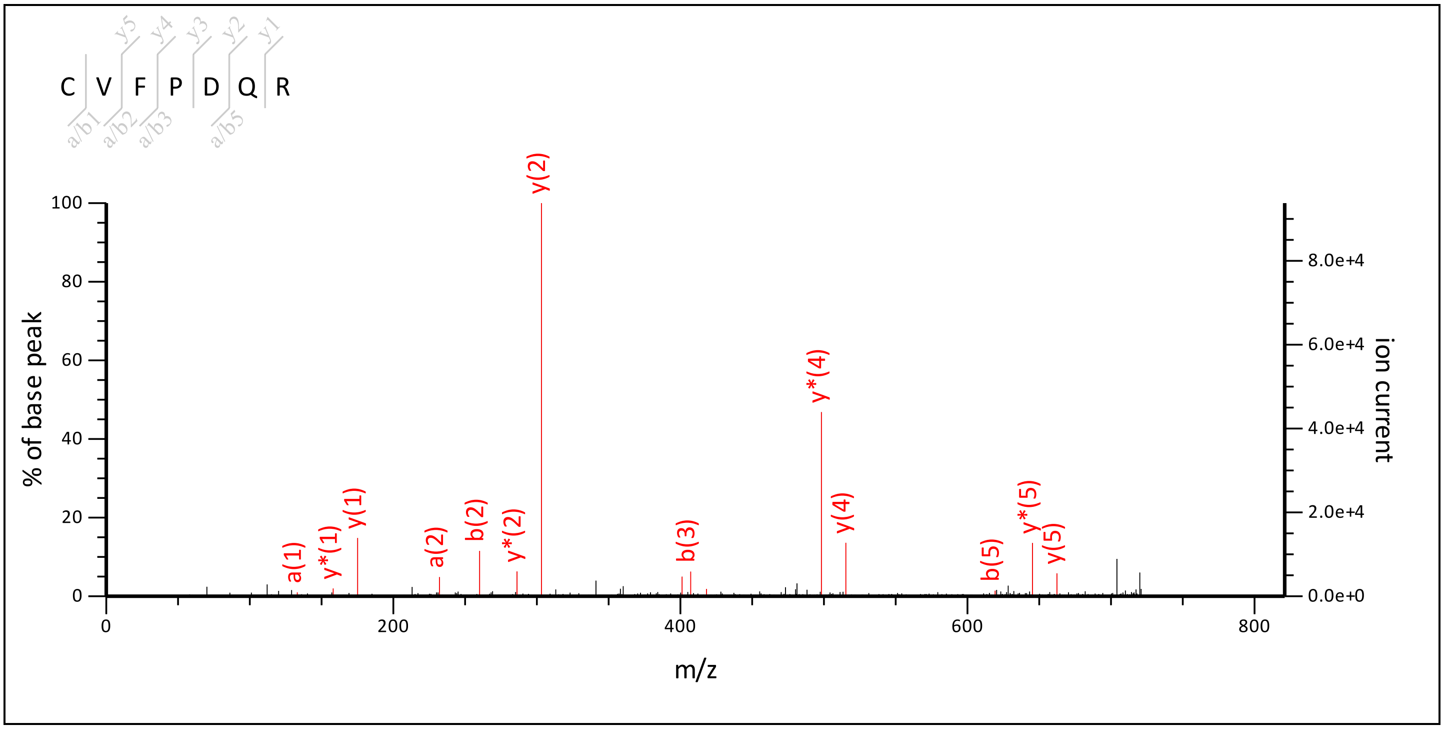


0 to 821.02

Label all possible matches ○ Label matches used for scoring ◉

**Monoisotopic mass of neutral peptide Mr(calc):** 920.4175
**Fixed modifications:** Carbamidomethyl (C) (apply to specified residues or termini only)
**Ions Score:** 24 **Expect:** 9.1
**Matches :** 15/26 fragment ions using 35 most intense peaks (help)

| # | a | a* | b | b* | Seq. | y | y* | # |
|---|---|---|---|---|---|---|---|---|
| 1 | **133.0430** | | 161.0379 | | C | | | 7 |
| 2 | **232.1114** | | **260.1063** | | V | 761.3941 | 744.3675 | 6 |
| 3 | 379.1798 | | **407.1748** | | F | ***662.3257*** | 645.2991 | 5 |
| 4 | 476.2326 | | 504.2275 | | P | ***515.2572*** | 498.2307 | 4 |
| 5 | 591.2595 | | **619.2545** | | D | ***418.2045*** | 401.1779 | 3 |
| 6 | 719.3181 | 702.2916 | 747.3130 | 730.2865 | Q | ***303.1775*** | 286.1510 | 2 |
| 7 | | | | | R | ***175.1190*** | 158.0924 | 1 |

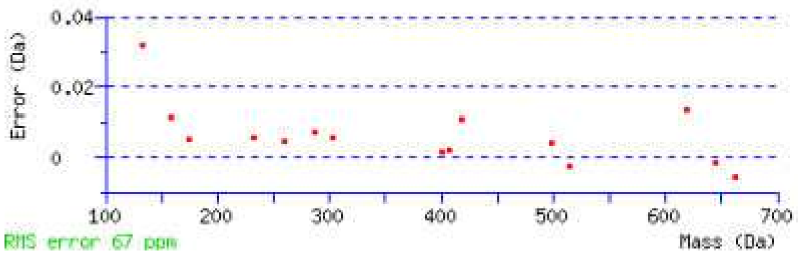


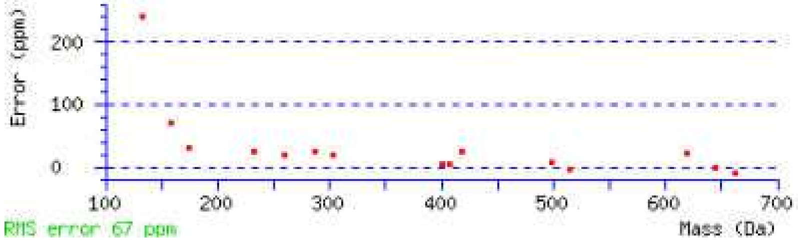


NCBI **BLAST** search of CVFPDQR
(Parameters: blastp, nr protein database, expect=20000, no filter, PAM30)
Other BLAST web gateways

**All matches to this query**

| Score | Mr(calc) | Delta | Sequence |
|---|---|---|---|
| 29.3 | 920.4749 | -0.0722 | KTIEMAGR |
| 23.6 | 920.4175 | -0.0147 | CVFPDQR |
| 20.5 | 920.4749 | -0.0722 | MTSAVLAGR |
| 19.0 | 920.4174 | -0.0147 | QMFPADGR |
| 18.9 | 920.4936 | -0.0908 | MKMLVQR |
| 18.9 | 920.4902 | -0.0874 | MQFIVQR |
| 18.6 | 920.4803 | -0.0775 | XXLALVKR |
| 18.3 | 920.4385 | -0.0358 | NDLEMKR |
| 18.1 | 920.4749 | -0.0722 | VLMSDGKR |
| 17.9 | 920.3472 | 0.0556 | SDGEDDAGR |

**Mascot:** http://www.matrixscience.com/

# Mascot Search Results

## Peptide View Spot no 40

MS/MS Fragmentation of **LGVMLTATAR**
Found in **gi|297604125** in **NCBInr**, Os05g0247100 [Oryza sativa Japonica Group]

Match to Query 9: 1031.571624 from(1032.578900,1+) intensity(0.0000) index(8)
Title: Label: B5, Spot_Id: 219739, Peak_List_Id: 225593, MSMS Job_Run_Id: 21783, Comment:
Data file C:\Documents and Settings\Administrator\Desktop\farah BHU\spot set plate\Farah-Garima 11May 2013\ppw_B5_136842181400.txt

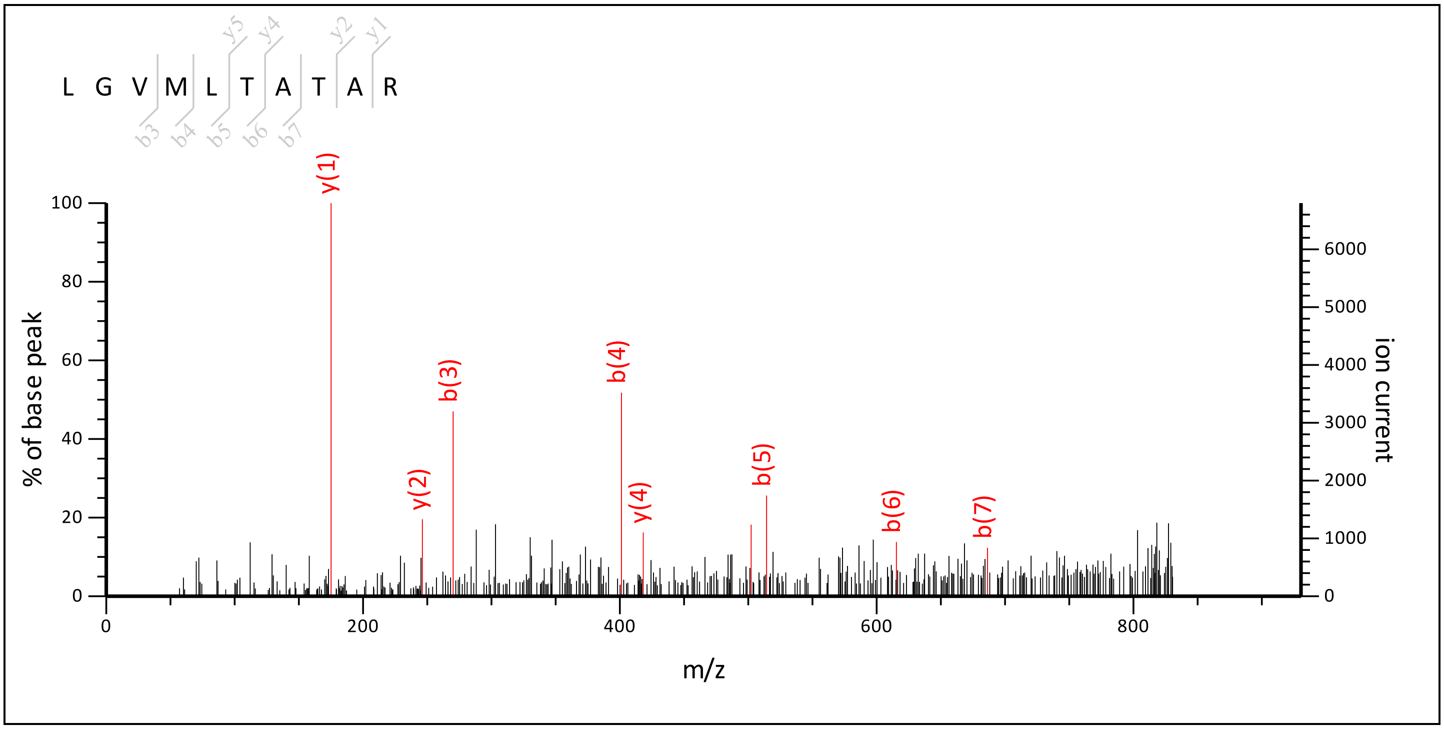


0 to 930.49

Label all possible matches ○ Label matches used for scoring ◉

**Monoisotopic mass of neutral peptide Mr(calc):** 1031.5797
**Fixed modifications:** Carbamidomethyl (C) (apply to specified residues or termini only)
**Ions Score:** 32 **Expect:** 1.7
**Matches :** 11/36 fragment ions using 16 most intense peaks (help)

| # | a | b | Seq. | y | y* | # |
|---|---|---|---|---|---|---|
| 1 | 86.0964 | 114.0913 | L | | | 10 |
| 2 | 143.1179 | 171.1128 | G | 919.5030 | 902.4764 | 9 |
| 3 | 242.1863 | ***270.1812*** | V | 862.4815 | 845.4550 | 8 |
| 4 | 373.2268 | ***401.2217*** | M | 763.4131 | 746.3865 | 7 |
| 5 | 486.3109 | ***514.3058*** | L | 632.3726 | 615.3461 | 6 |
| 6 | 587.3585 | ***615.3534*** | T | 519.2885 | 502.2620 | 5 |
| 7 | 658.3956 | ***686.3906*** | A | ***418.2409*** | 401.2143 | 4 |
| 8 | 759.4433 | 787.4382 | T | 347.2037 | 330.1772 | 3 |

| | | | | | | |
|---|---|---|---|---|---|---|
| 9 | 830.4804 | 858.4754 | A | *246.1561* | 229.1295 | 2 |
| 10 | | | R | *175.1190* | 158.0924 | 1 |

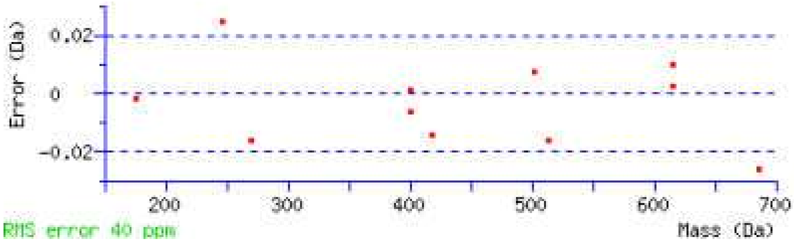


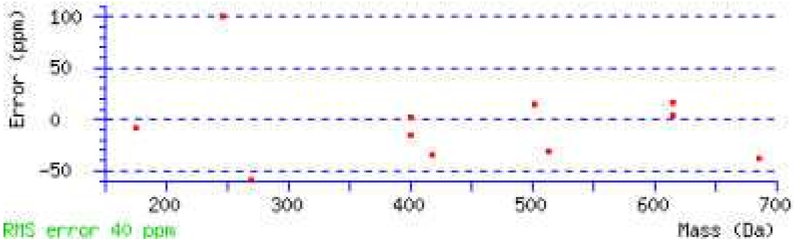


NCBI **BLAST** search of LGVMLTATAR
(Parameters: blastp, nr protein database, expect=20000, no filter, PAM30)
Other BLAST web gateways

**All matches to this query**

| Score | Mr(calc) | Delta | Sequence |
|---|---|---|---|
| 31.8 | 1031.5797 | -0.0081 | LGVMLTATAR |
| 23.6 | 1031.5400 | 0.0316 | KFPELADGR |
| 19.6 | 1031.5433 | 0.0283 | AANDMLIIR |
| 19.6 | 1031.5797 | -0.0081 | DQKMLLLR |
| 18.7 | 1031.5433 | 0.0283 | CEQTLILR |
| 18.7 | 1031.5910 | -0.0193 | MNRTIILR |
| 18.2 | 1031.5508 | 0.0209 | VIGMIPGMAK |
| 18.2 | 1031.5508 | 0.0209 | VLGMIPGMAK |
| 18.1 | 1031.5182 | 0.0534 | MTNAGAALGAR |
| 17.5 | 1031.4996 | 0.0720 | GASGAAVTADGR |

**Mascot:** http://www.matrixscience.com/

# Mascot Search Results

## Peptide View Spot no 40

MS/MS Fragmentation of **IHVKVFGDGR**
Found in **gi|297604125** in **NCBInr**, Os05g0247100 [Oryza sativa Japonica Group]

Match to Query 13: 1126.616524 from(1127.623800,1+) intensity(0.0000) index(12)
Title: Label: B5, Spot_Id: 219739, Peak_List_Id: 225590, MSMS Job_Run_Id: 21783, Comment:
Data file C:\Documents and Settings\Administrator\Desktop\farah BHU\spot set plate\Farah-Garima 11May 2013\ppw_B5_136842181400.txt

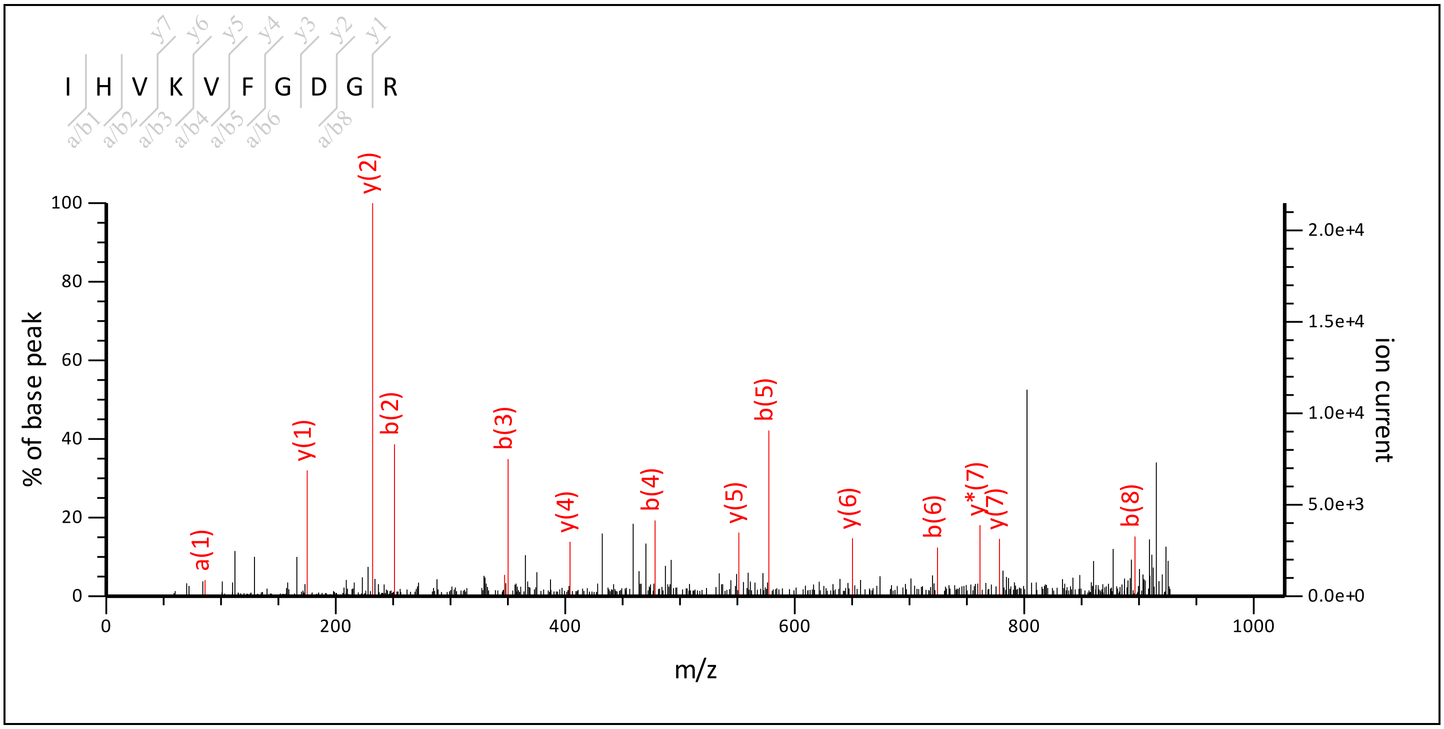


0 to 1026.97

Label all possible matches ○ Label matches used for scoring ◉

**Monoisotopic mass of neutral peptide Mr(calc):** 1126.6247
**Fixed modifications:** Carbamidomethyl (C) (apply to specified residues or termini only)
**Ions Score:** 44 **Expect:** 0.084
**Matches :** 15/48 fragment ions using 27 most intense peaks (help)

| # | a | a* | b | b* | Seq. | y | y* | # |
|---|---|---|---|---|---|---|---|---|
| 1 | 86.0964 | | 114.0913 | | I | | | 10 |
| 2 | 223.1553 | | ***251.1503*** | | H | 1014.5479 | 997.5214 | 9 |
| 3 | 322.2238 | | ***350.2187*** | | V | 877.4890 | 860.4625 | 8 |
| 4 | 450.3187 | 433.2922 | ***478.3136*** | 461.2871 | K | ***778.4206*** | 761.3941 | 7 |
| 5 | 549.3871 | 532.3606 | ***577.3820*** | 560.3555 | V | ***650.3257*** | 633.2991 | 6 |
| 6 | 696.4555 | 679.4290 | ***724.4505*** | 707.4239 | F | ***551.2572*** | 534.2307 | 5 |
| 7 | 753.4770 | 736.4505 | 781.4719 | 764.4454 | G | ***404.1888*** | 387.1623 | 4 |
| 8 | 868.5039 | 851.4774 | ***896.4989*** | 879.4723 | D | ***347.1674*** | 330.1408 | 3 |

| | | | | | | | | |
|---|---|---|---|---|---|---|---|---|
| 9 | 925.5254 | 908.4989 | 953.5203 | 936.4938 | G | 232.1404 | 215.1139 | 2 |
| 10 | | | | | R | 175.1190 | 158.0924 | 1 |

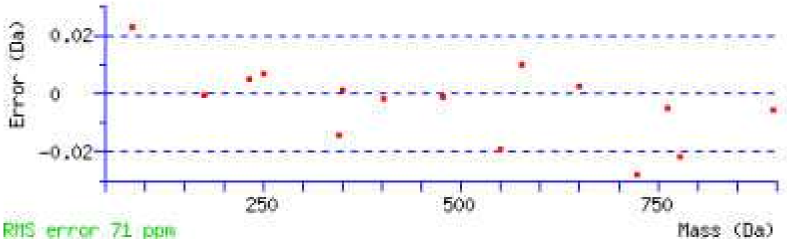


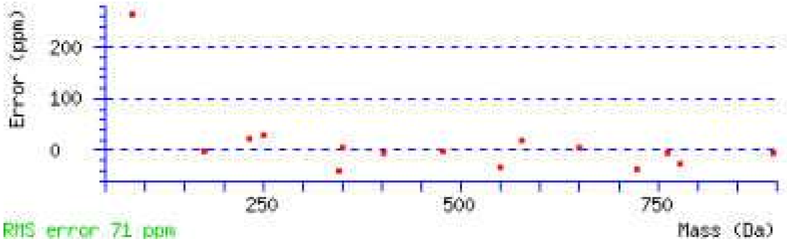


NCBI **BLAST** search of IHVKVFGDGR
(Parameters: blastp, nr protein database, expect=20000, no filter, PAM30)
Other BLAST web gateways

**All matches to this query**

| Score | Mr(calc) | Delta | Sequence |
|---|---|---|---|
| 43.6 | 1126.6247 | -0.0082 | IHVKVFGDGR |
| 14.0 | 1126.5553 | 0.0612 | HTKMLPGDGR |
| 12.6 | 1126.5619 | 0.0547 | DLPPSTIGDGR |
| 12.5 | 1126.5441 | 0.0724 | TPAPSPPMTGR |
| 11.4 | 1126.5376 | 0.0789 | VCSGFVMRR |
| 11.0 | 1126.5376 | 0.0790 | ARGMMAVGFR |
| 10.6 | 1126.5515 | 0.0651 | MFNLAMGLSK |
| 10.4 | 1126.5255 | 0.0911 | IDPTGPAEDGR |
| 9.8 | 1126.5454 | 0.0711 | RLHGWMDGR |
| 8.2 | 1126.5441 | 0.0725 | SYVKAMEQR |

**Mascot:** http://www.matrixscience.com/

# MATRIX SCIENCE Mascot Search Results

## Peptide View **Spot no 40**

MS/MS Fragmentation of **LYAHNKDYK**
Found in **gi|297604125** in **NCBInr**, Os05g0247100 [Oryza sativa Japonica Group]

Match to Query 14: 1150.566224 from(1151.573500,1+) intensity(0.0000) index(13)
Title: Label: B5, Spot_Id: 219739, Peak_List_Id: 225597, MSMS Job_Run_Id: 21783, Comment:
Data file C:\Documents and Settings\Administrator\Desktop\farah BHU\spot set plate\Farah-Garima 11May 2013\ppw_B5_136842181400.txt

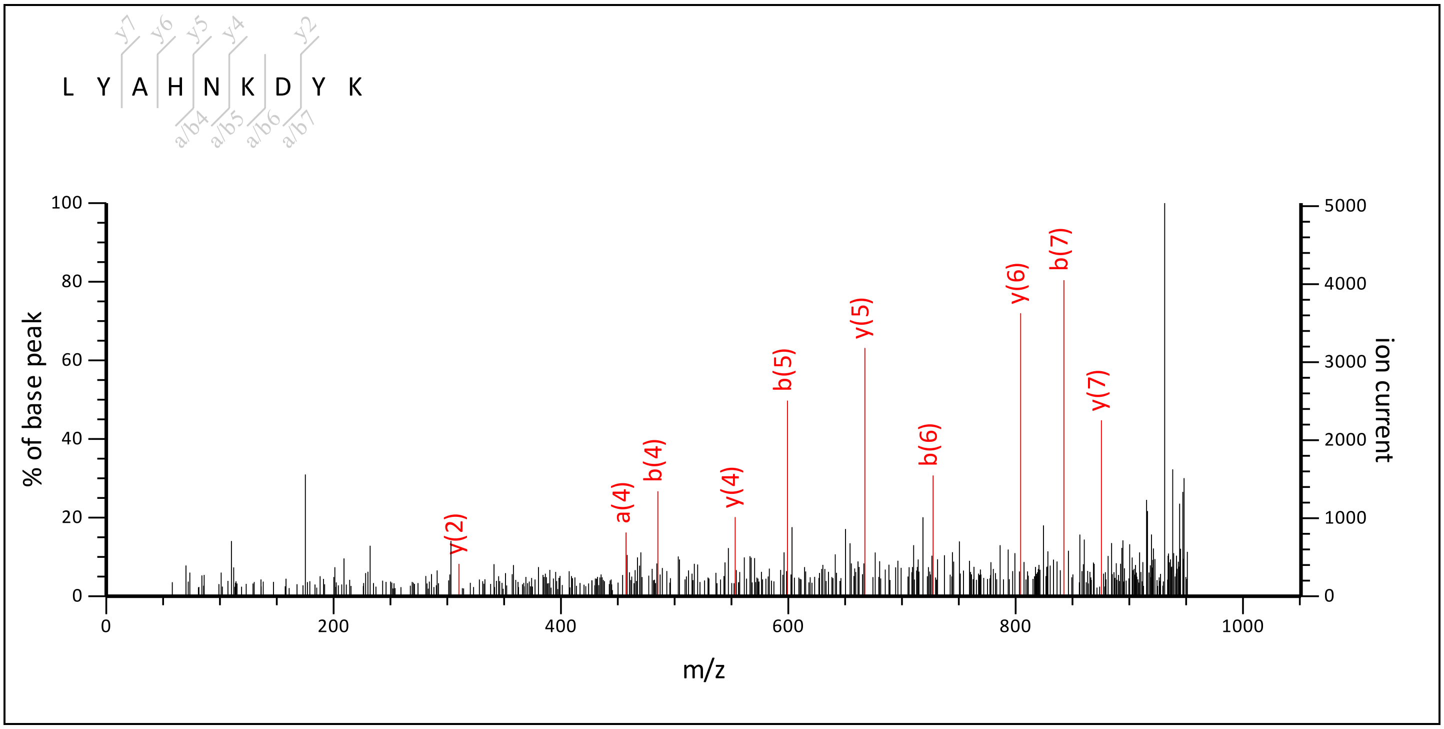


0 to 1051.03

Label all possible matches ○ Label matches used for scoring ◉

**Monoisotopic mass of neutral peptide Mr(calc):** 1150.5771
**Fixed modifications:** Carbamidomethyl (C) (apply to specified residues or termini only)
**Ions Score:** 26 **Expect:** 6.3
**Matches :** 10/40 fragment ions using 18 most intense peaks (help)

| # | a | a* | b | b* | Seq. | y | y* | # |
|---|---|---|---|---|---|---|---|---|
| 1 | 86.0964 | | 114.0913 | | L | | | 9 |
| 2 | 249.1598 | | 277.1547 | | Y | 1038.5003 | 1021.4738 | 8 |
| 3 | 320.1969 | | 348.1918 | | A | ***875.4370*** | 858.4104 | 7 |
| 4 | 457.2558 | | ***485.2507*** | | H | ***804.3999*** | 787.3733 | 6 |
| 5 | 571.2987 | 554.2722 | ***599.2936*** | 582.2671 | N | ***667.3410*** | 650.3144 | 5 |
| 6 | 699.3937 | 682.3671 | ***727.3886*** | 710.3620 | K | ***553.2980*** | 536.2715 | 4 |
| 7 | 814.4206 | 797.3941 | ***842.4155*** | 825.3890 | D | 425.2031 | 408.1765 | 3 |
| 8 | 977.4839 | 960.4574 | 1005.4789 | 988.4523 | Y | ***310.1761*** | 293.1496 | 2 |

| | | | | | | | | |
|---|---|---|---|---|---|---|---|---|
| 9 | | | | | K | 147.1128 | 130.0863 | 1 |

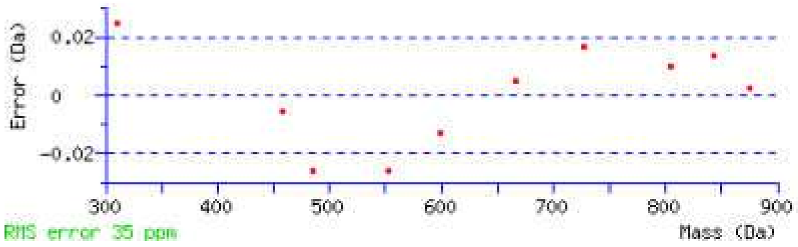


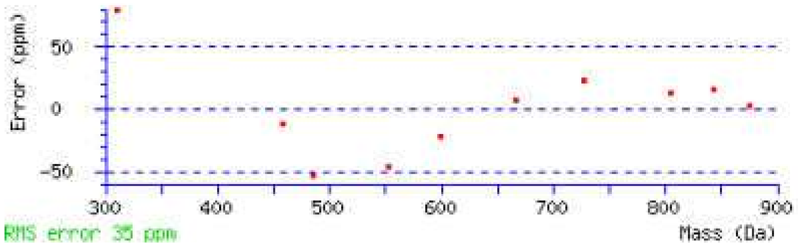


NCBI **BLAST** search of LYAHNKDYK
(Parameters: blastp, nr protein database, expect=20000, no filter, PAM30)
Other BLAST web gateways

**All matches to this query**

| Score | Mr(calc) | Delta | Sequence |
|---|---|---|---|
| 26.5 | 1150.5771 | -0.0108 | LYAHNKDYK |
| 13.7 | 1150.5587 | 0.0075 | TQMQNMVKR |
| 13.5 | 1150.5618 | 0.0044 | EELLNYSQR |
| 13.5 | 1150.5982 | -0.0320 | IIEENYSKR |
| 13.2 | 1150.6359 | -0.0697 | HHPLLPAPNR |
| 8.9 | 1150.4859 | 0.0803 | NDALACGMQR |
| 8.9 | 1150.5843 | -0.0181 | QADAVVGHAQR |
| 8.9 | 1150.6207 | -0.0545 | TAQALINHQR |
| 8.9 | 1150.6571 | -0.0909 | VSGKIIHGGQR |
| 8.9 | 1150.5995 | -0.0333 | YHPSLNHKR |

**Mascot:** http://www.matrixscience.com/

# MATRIX SCIENCE Mascot Search Results

## Peptide View Spot no 40

MS/MS Fragmentation of **LQAALSTGLFSR**
Found in **gi|297604125** in **NCBInr**, Os05g0247100 [Oryza sativa Japonica Group]

Match to Query 20: 1262.689824 from(1263.697100,1+) intensity(0.0000) index(19)
Title: Label: B5, Spot_Id: 219739, Peak_List_Id: 225586, MSMS Job_Run_Id: 21783, Comment:
Data file C:\Documents and Settings\Administrator\Desktop\farah BHU\spot set plate\Farah-Garima 11May 2013\ppw_B5_136842181400.txt

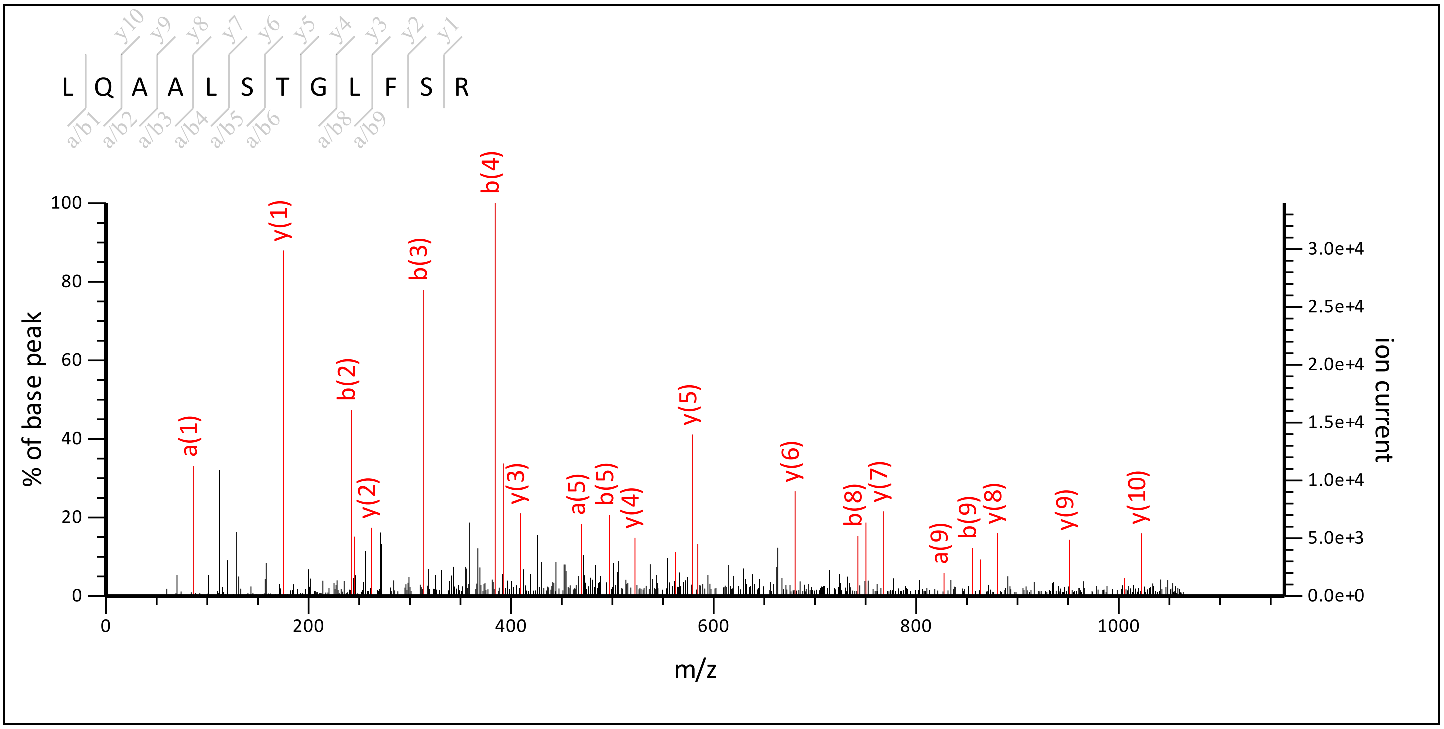


0 to 1163.59

Label all possible matches ○ Label matches used for scoring ◉

**Monoisotopic mass of neutral peptide Mr(calc):** 1262.6983
**Fixed modifications:** Carbamidomethyl (C) (apply to specified residues or termini only)
**Ions Score:** 93 **Expect:** 1e-06
**Matches :** 26/64 fragment ions using 33 most intense peaks (help)

| # | a | a* | b | b* | Seq. | y | y* | # |
|---|---|---|---|---|---|---|---|---|
| 1 | **86.0964** | | 114.0913 | | L | | | 12 |
| 2 | 214.1550 | 197.1285 | **242.1499** | 225.1234 | Q | 1150.6215 | 1133.5950 | 11 |
| 3 | 285.1921 | 268.1656 | **313.1870** | 296.1605 | A | ***1022.5629*** | 1005.5364 | 10 |
| 4 | 356.2292 | 339.2027 | **384.2241** | 367.1976 | A | ***951.5258*** | 934.4993 | 9 |
| 5 | **469.3133** | 452.2867 | **497.3082** | 480.2817 | L | ***880.4887*** | 863.4621 | 8 |
| 6 | 556.3453 | 539.3188 | **584.3402** | 567.3137 | S | ***767.4046*** | 750.3781 | 7 |
| 7 | 657.3930 | 640.3665 | 685.3879 | 668.3614 | T | ***680.3726*** | 663.3461 | 6 |
| 8 | 714.4145 | 697.3879 | **742.4094** | 725.3828 | G | ***579.3249*** | 562.2984 | 5 |

| | | | | | | | | |
|---|---|---|---|---|---|---|---|---|
| 9 | 827.4985 | 810.4720 | 855.4934 | 838.4669 | L | 522.3035 | 505.2769 | 4 |
| 10 | 974.5669 | 957.5404 | 1002.5619 | 985.5353 | F | 409.2194 | 392.1928 | 3 |
| 11 | 1061.5990 | 1044.5724 | 1089.5939 | 1072.5673 | S | 262.1510 | 245.1244 | 2 |
| 12 | | | | | R | 175.1190 | 158.0924 | 1 |

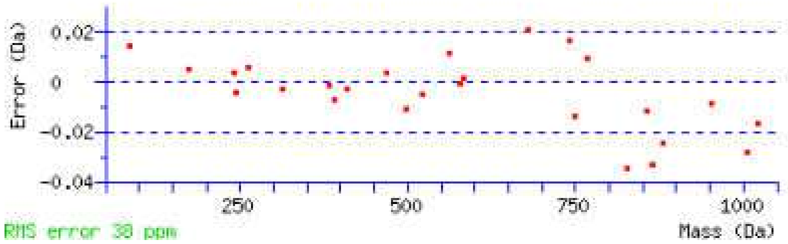


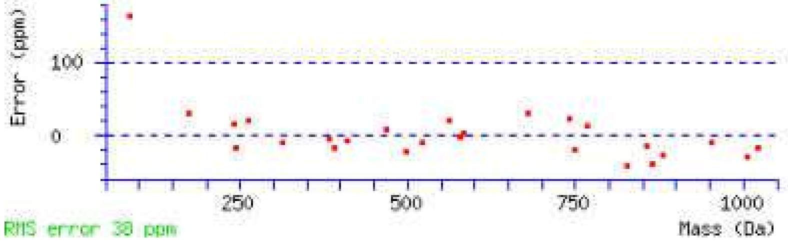


NCBI **BLAST** search of LQAALSTGLFSR
(Parameters: blastp, nr protein database, expect=20000, no filter, PAM30)
Other BLAST web gateways

**All matches to this query**

| Score | Mr(calc) | Delta | Sequence |
|---|---|---|---|
| 93.0 | 1262.6983 | -0.0084 | LQAALSTGLFSR |
| 26.6 | 1262.7360 | -0.0462 | LGAAALWHRLR |
| 22.5 | 1262.6223 | 0.0675 | LRNLDMGLCR |
| 20.4 | 1262.6553 | 0.0345 | IKAANPEMPHR |
| 20.3 | 1262.6255 | 0.0644 | LAQAEAAEAAYR |
| 19.9 | 1262.7247 | -0.0349 | IQAAFRAFLAR |
| 18.8 | 1262.6983 | -0.0084 | AVAAAASYTVAIR |
| 18.4 | 1262.6982 | -0.0084 | KGLISIENYAR |
| 17.3 | 1262.6289 | 0.0609 | LQSGIGEAVMSR |
| 16.4 | 1262.6553 | 0.0345 | LRHSLSTYMR |

**Mascot:** http://www.matrixscience.com/

# Mascot Search Results

## Peptide View Spot no 40

MS/MS Fragmentation of **LDISGHTVSAVGPDIK**
Found in **gi|297604125** in **NCBInr**, Os05g0247100 [Oryza sativa Japonica Group]

Match to Query 31: 1607.820524 from(1608.827800,1+) intensity(0.0000) index(30)
Title: Label: B5, Spot_Id: 219739, Peak_List_Id: 225604, MSMS Job_Run_Id: 21783, Comment:
Data file C:\Documents and Settings\Administrator\Desktop\farah BHU\spot set plate\Farah-Garima 11May 2013\ppw_B5_136842181400.txt

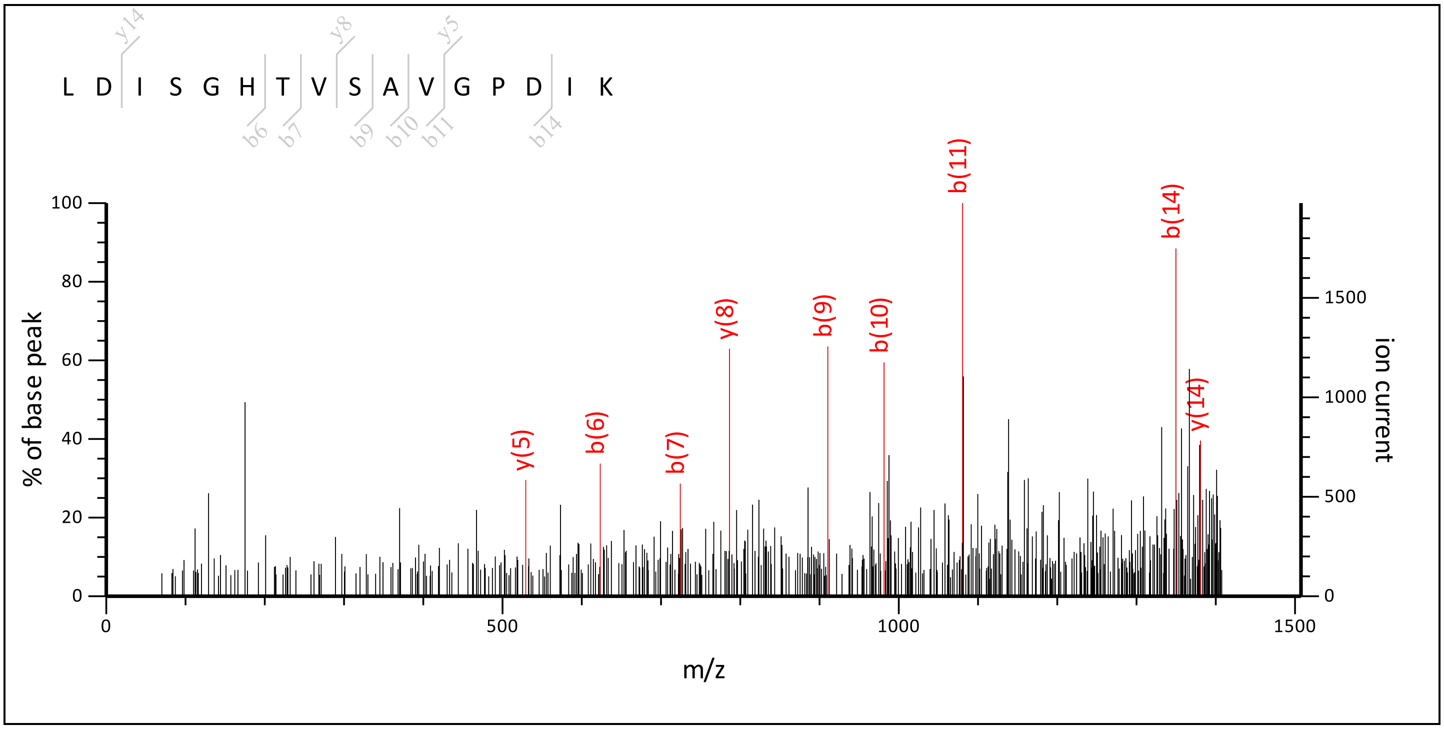


0 to 1507.52

Label all possible matches ○ Label matches used for scoring ◉

**Monoisotopic mass of neutral peptide Mr(calc):** 1607.8519
**Fixed modifications:** Carbamidomethyl (C) (apply to specified residues or termini only)
**Ions Score:** 34 **Expect:** 0.67
**Matches :** 9/60 fragment ions using 14 most intense peaks (help)

| # | a | b | Seq. | y | y* | # |
|---|---|---|---|---|---|---|
| 1 | 86.0964 | 114.0913 | L | | | 16 |
| 2 | 201.1234 | 229.1183 | D | 1495.7751 | 1478.7486 | 15 |
| 3 | 314.2074 | 342.2023 | I | *1380.7482* | 1363.7216 | 14 |
| 4 | 401.2395 | 429.2344 | S | 1267.6641 | 1250.6375 | 13 |
| 5 | 458.2609 | 486.2558 | G | 1180.6321 | 1163.6055 | 12 |
| 6 | 595.3198 | *623.3148* | H | 1123.6106 | 1106.5841 | 11 |
| 7 | 696.3675 | *724.3624* | T | 986.5517 | 969.5251 | 10 |
| 8 | 795.4359 | 823.4308 | V | 885.5040 | 868.4775 | 9 |

| | | | | | | |
|---|---|---|---|---|---|---|
| **9** | 882.4680 | ***910.4629*** | **S** | ***786.4356*** | 769.4090 | **8** |
| **10** | 953.5051 | ***981.5000*** | **A** | 699.4036 | 682.3770 | **7** |
| **11** | 1052.5735 | ***1080.5684*** | **V** | 628.3665 | 611.3399 | **6** |
| **12** | 1109.5949 | 1137.5899 | **G** | ***529.2980*** | 512.2715 | **5** |
| **13** | 1206.6477 | 1234.6426 | **P** | 472.2766 | 455.2500 | **4** |
| **14** | 1321.6747 | ***1349.6696*** | **D** | 375.2238 | 358.1973 | **3** |
| **15** | 1434.7587 | 1462.7536 | **I** | 260.1969 | 243.1703 | **2** |
| **16** | | | **K** | 147.1128 | 130.0863 | **1** |

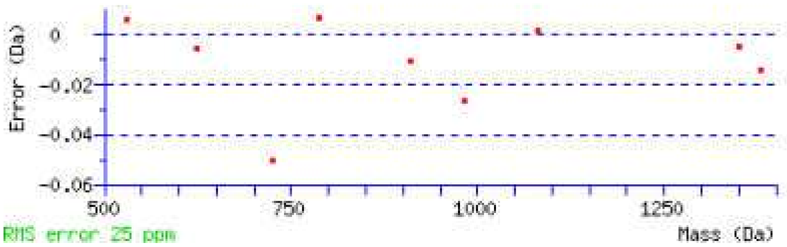


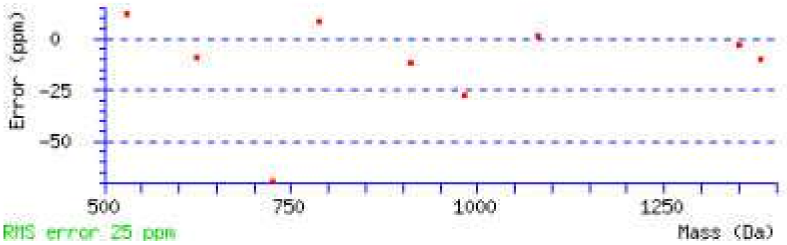


NCBI **BLAST** search of LDISGHTVSAVGPDIK
(Parameters: blastp, nr protein database, expect=20000, no filter, PAM30)
Other BLAST web gateways

**All matches to this query**

| Score | Mr(calc) | Delta | Sequence |
|---|---|---|---|
| 34.2 | 1607.8519 | -0.0314 | LDISGHTVSAVGPDIK |
| 13.5 | 1607.7210 | 0.0996 | RPSTSVVDESDMGGR |
| 12.8 | 1607.8128 | 0.0077 | RPGRGVEADGDGLPGR |
| 11.1 | 1607.8155 | 0.0050 | LDLKSAAYLDEGTGR |
| 10.7 | 1607.7501 | 0.0704 | YIPDLPSVTDEMGR |
| 8.7 | 1607.7180 | 0.1026 | KYMMLSMMISGPR |
| 8.7 | 1607.7097 | 0.1108 | QEASLLGSDEMEQR |
| 7.3 | 1607.8631 | -0.0426 | QVAVAAGVDAVVGEAPR |
| 7.1 | 1607.8784 | -0.0579 | EVSVIGGSGVFRFVR |
| 6.4 | 1607.8519 | -0.0313 | EVPPVDDADARLALK |

**Mascot:** http://www.matrixscience.com/

# Mascot Search Results

## Peptide View Spot no 40

MS/MS Fragmentation of **VLVGVVASPEADRDGYVSHK**
Found in **gi|297604125** in **NCBInr**, Os05g0247100 [Oryza sativa Japonica Group]

Match to Query 127: 2097.057224 from(2098.064500,1+) intensity(0.0000) index(34)
Title: Label: B5, Spot_Id: 219739, Peak_List_Id: 225596, MSMS Job_Run_Id: 21783, Comment:
Data file C:\Documents and Settings\Administrator\Desktop\farah BHU\spot set plate\Farah-Garima 11May 2013\ppw_B5_136842181400.txt

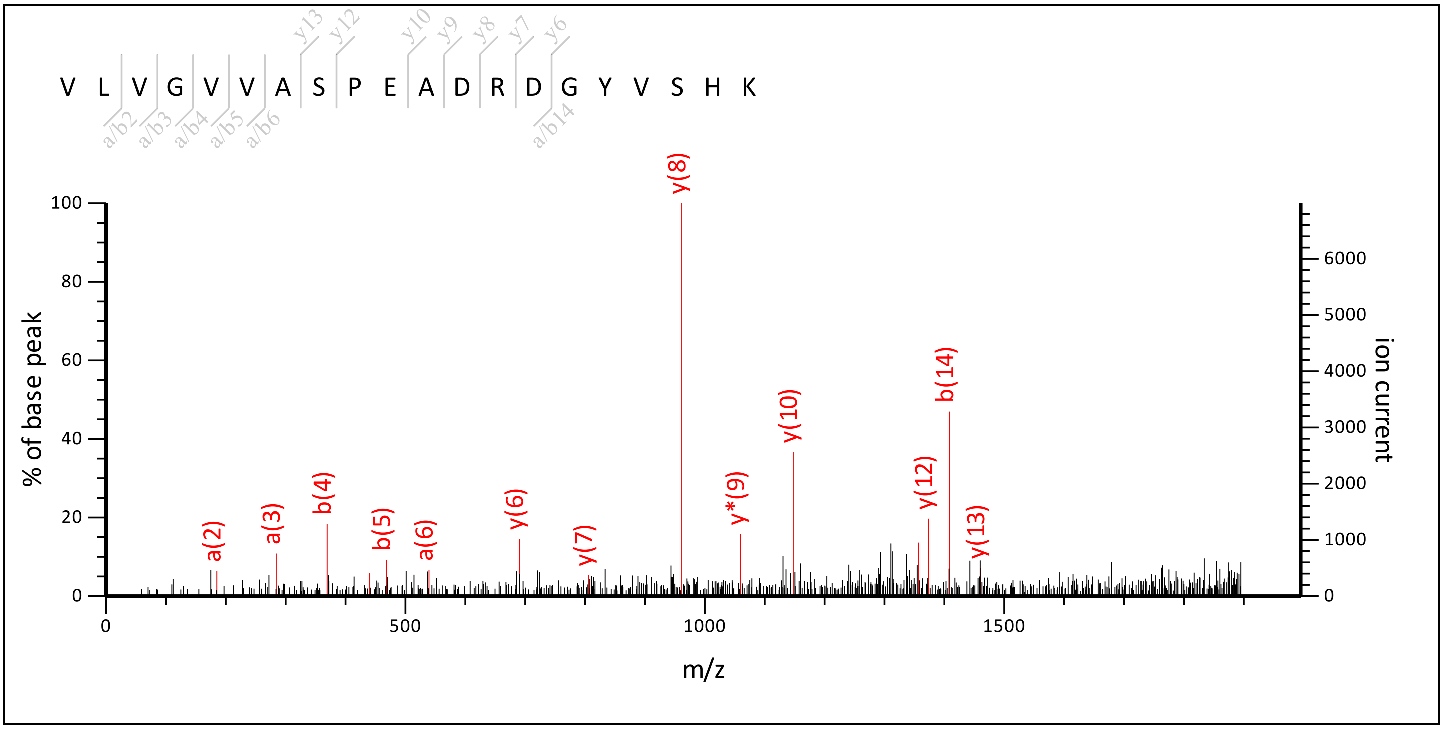


0 to 1995.17

Label all possible matches ○ Label matches used for scoring ◉

**Monoisotopic mass of neutral peptide Mr(calc):** 2097.0855
**Fixed modifications:** Carbamidomethyl (C) (apply to specified residues or termini only)
**Ions Score:** 25 **Expect:** 5
**Matches :** 16/90 fragment ions using 38 most intense peaks (help)

| # | a | a* | b | b* | Seq. | y | y* | # |
|---|---|---|---|---|---|---|---|---|
| 1 | 72.0808 | | 100.0757 | | V | | | 20 |
| 2 | *185.1648* | | 213.1598 | | L | 1999.0243 | 1981.9978 | 19 |
| 3 | *284.2333* | | 312.2282 | | V | 1885.9403 | 1868.9137 | 18 |
| 4 | 341.2547 | | **369.2496** | | G | 1786.8719 | 1769.8453 | 17 |
| 5 | *440.3231* | | **468.3180** | | V | 1729.8504 | 1712.8238 | 16 |
| 6 | *539.3915* | | 567.3865 | | V | 1630.7820 | 1613.7554 | 15 |
| 7 | 610.4287 | | 638.4236 | | A | 1531.7136 | 1514.6870 | 14 |
| 8 | 697.4607 | | 725.4556 | | S | *1460.6764* | 1443.6499 | 13 |

| | | | | | | | | |
|---|---|---|---|---|---|---|---|---|
| 9 | 794.5135 | | 822.5084 | | P | *1373.6444* | 1356.6179 | 12 |
| 10 | 923.5560 | | 951.5510 | | E | 1276.5917 | 1259.5651 | 11 |
| 11 | 994.5932 | | 1022.5881 | | A | *1147.5491* | 1130.5225 | 10 |
| 12 | 1109.6201 | | 1137.6150 | | D | 1076.5119 | 1059.4854 | 9 |
| 13 | 1265.7212 | 1248.6947 | 1293.7161 | 1276.6896 | R | *961.4850* | 944.4585 | 8 |
| 14 | 1380.7482 | 1363.7216 | **1408.7431** | 1391.7165 | D | *805.3839* | 788.3573 | 7 |
| 15 | 1437.7696 | 1420.7431 | 1465.7645 | 1448.7380 | G | *690.3570* | 673.3304 | 6 |
| 16 | 1600.8329 | 1583.8064 | 1628.8279 | 1611.8013 | Y | 633.3355 | 616.3089 | 5 |
| 17 | 1699.9014 | 1682.8748 | 1727.8963 | 1710.8697 | V | 470.2722 | 453.2456 | 4 |
| 18 | 1786.9334 | 1769.9068 | 1814.9283 | 1797.9018 | S | 371.2037 | 354.1772 | 3 |
| 19 | 1923.9923 | 1906.9658 | 1951.9872 | 1934.9607 | H | *284.1717* | 267.1452 | 2 |
| 20 | | | | | K | 147.1128 | 130.0863 | 1 |

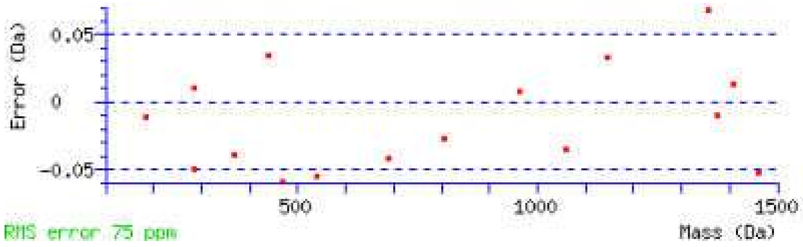


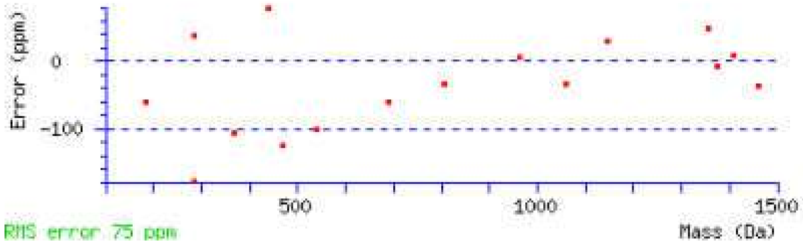


NCBI **BLAST** search of VLVGVVASPEADRDGYVSHK
(Parameters: blastp, nr protein database, expect=20000, no filter, PAM30)
Other BLAST web gateways

**All matches to this query**

| Score | Mr(calc) | Delta | Sequence |
|---|---|---|---|
| 24.5 | 2097.0855 | -0.0283 | VLVGVVASPEADRDGYVSHK |
| 5.3 | 2097.0789 | -0.0217 | SLMLVHRLLVDGDSSFHR |
| 2.1 | 2097.0288 | 0.0284 | DPALAHCIPMQLHFLYR |
| 0.8 | 2096.9440 | 0.1133 | WGIPPSTPDWGADDEELGR |

**Mascot:** http://www.matrixscience.com/

# MATRIX SCIENCE Mascot Search Results

## Peptide View Spot no 50

MS/MS Fragmentation of **YRPKEPYTGR**
Found in **gi|41052915** in **NCBInr**, putative ferredoxin-NADP(H) oxidoreductase [Oryza sativa Japonica Group]

Match to Query 20: 1265.645424 from(1266.652700,1+) intensity(0.0000) index(8)
Title: Label: L3, Spot_Id: 219717, Peak_List_Id: 225433, MSMS Job_Run_Id: 21778, Comment:
Data file C:\Documents and Settings\Administrator\Desktop\farah BHU\spot set plate\Farah-Garima 11May 2013\ppw_L3_136842146000.txt

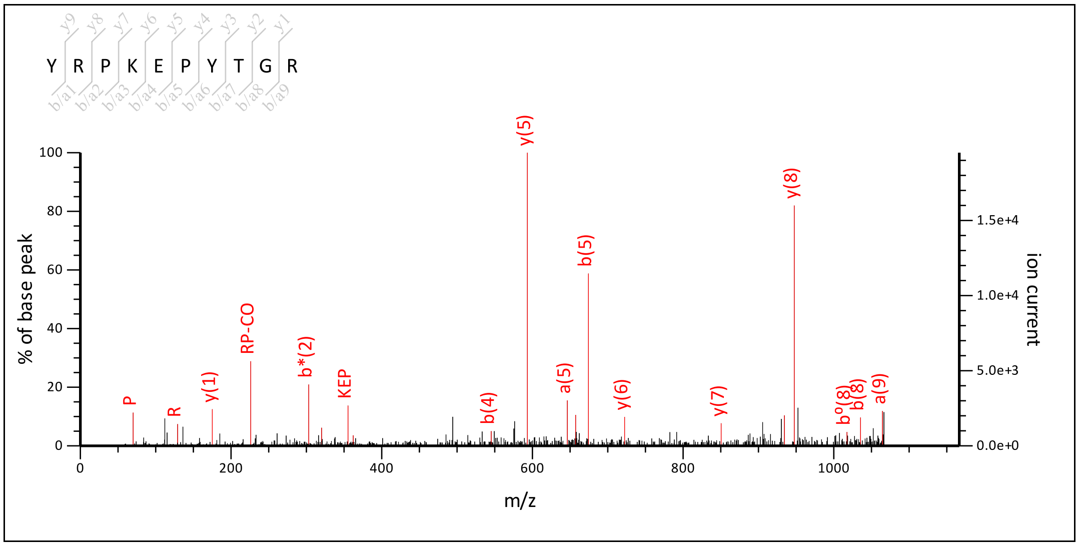


0 to 1166.55

Label all possible matches ○ Label matches used for scoring ◉

**Monoisotopic mass of neutral peptide Mr(calc):** 1265.6516
**Fixed modifications:** Carbamidomethyl (C) (apply to specified residues or termini only)
**Ions Score:** 33 **Expect:** 1.1
**Matches :** 25/150 fragment ions using 33 most intense peaks (help)

| # | Immon. | a | a* | $a^0$ | b | b* | $b^0$ | d | d' | Seq. | v | w | w' | y | y* | y |
|---|---|---|---|---|---|---|---|---|---|---|---|---|---|---|---|---|
| 1 | 136.0757 | 136.0757 | | | 164.0706 | | | 44.0495 | | Y | | | | | | |
| 2 | 129.1135 | 292.1768 | 275.1503 | | ***320.1717*** | 303.1452 | | 207.1128 | | R | 1002.5003 | 1001.5051 | | 1103.5956 | 1086.5691 | 1085. |
| 3 | 70.0651 | 389.2296 | 372.2030 | | 417.2245 | 400.1979 | | 363.2139 | | P | 905.4476 | 904.4523 | | ***947.4945*** | 930.4680 | 929. |
| 4 | 101.1073 | 517.3245 | 500.2980 | | ***545.3194*** | 528.2929 | | 460.2667 | | K | 777.3526 | 776.3573 | | ***850.4417*** | 833.4152 | 832. |
| 5 | 102.0550 | ***646.3671*** | 629.3406 | 628.3566 | ***674.3620*** | 657.3355 | 656.3515 | 588.3616 | | E | 648.3100 | 647.3148 | | ***722.3468*** | 705.3202 | 704. |
| 6 | 70.0651 | 743.4199 | 726.3933 | 725.4093 | 771.4148 | 754.3883 | 753.4042 | 717.4042 | | P | 551.2572 | 550.2620 | | ***593.3042*** | 576.2776 | 575. |
| 7 | 136.0757 | 906.4832 | 889.4567 | 888.4726 | ***934.4781*** | 917.4516 | 916.4676 | | | Y | 388.1939 | | | 496.2514 | 479.2249 | 478. |
| 8 | 74.0600 | 1007.5309 | 990.5043 | 989.5203 | ***1035.5258*** | 1018.4993 | 1017.5152 | 991.5360 | 993.5152 | T | 287.1462 | 300.1666 | 302.1459 | 333.1881 | 316.1615 | 315. |
| 9 | 30.0338 | **1064.5524** | 1047.5258 | 1046.5418 | 1092.5473 | 1075.5207 | 1074.5367 | | | G | | | | 232.1404 | 215.1139 | |
| 10 | 129.1135 | | | | | | | | | R | 74.0237 | 73.0284 | | ***175.1190*** | 158.0924 | |

| Seq | ya | yb | Seq | ya | yb | Seq | ya | yb |
|---|---|---|---|---|---|---|---|---|
| **RP** | 226.1662 | 254.1612 | **RPK** | 354.2612 | 382.2561 | **RPKE** | 483.3038 | 511.2987 |
| **RPKEP** | 580.3566 | 608.3515 | **PK** | 198.1601 | 226.1550 | **PKE** | 327.2027 | 355.1976 |
| **PKEP** | 424.2554 | 452.2504 | **PKEPY** | 587.3188 | 615.3137 | **PKEPYT** | 688.3665 | 716.3614 |
| **KE** | 230.1499 | 258.1448 | **KEP** | 327.2027 | 355.1976 | **KEPY** | 490.2660 | 518.2609 |
| **KEPYT** | 591.3137 | 619.3086 | **KEPYTG** | 648.3352 | 676.3301 | **EP** | 199.1077 | 227.1026 |
| **EPY** | 362.1710 | 390.1660 | **EPYT** | 463.2187 | 491.2136 | **EPYTG** | 520.2402 | 548.2351 |
| **PY** | 233.1285 | 261.1234 | **PYT** | 334.1761 | 362.1710 | **PYTG** | 391.1976 | 419.1925 |
| **YT** | 237.1234 | 265.1183 | **YTG** | 294.1448 | 322.1397 | **TG** | 131.0815 | 159.0764 |

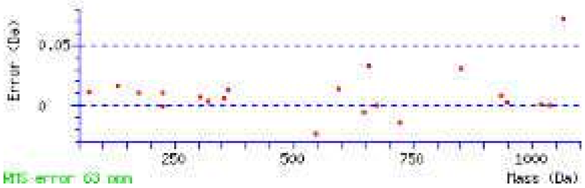


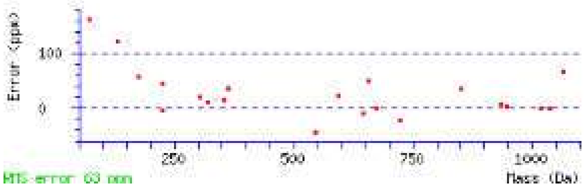


NCBI **BLAST** search of YRPKEPYTGR
(Parameters: blastp, nr protein database, expect=20000, no filter, PAM30)
Other BLAST web gateways

**All matches to this query**

| Score | Mr(calc) | Delta | Sequence |
|---|---|---|---|
| 32.9 | 1265.6516 | -0.0062 | YRPKEPYTGR |
| 19.7 | 1265.6000 | 0.0454 | EANSFATSVGAGR |
| 16.3 | 1265.5888 | 0.0566 | SDNFEEKNGVK |
| 16.1 | 1265.5968 | 0.0486 | TMAGRAAASMAGR |
| 15.5 | 1265.5960 | 0.0494 | SNSTGSSSKQQR |
| 15.4 | 1265.6113 | 0.0342 | SGSGAGAPGPPQAGR |
| 15.2 | 1265.5524 | 0.0930 | DAADTAEVFGDR |
| 15.1 | 1265.6364 | 0.0090 | RTYTAEEIQR |
| 15.1 | 1265.6802 | -0.0348 | SGGIMVLTFIGR |
| 15.0 | 1265.6108 | 0.0346 | DAVMKEMATVR |

**Mascot:** http://www.matrixscience.com/

# MATRIX SCIENCE Mascot Search Results

## Peptide View Spot no 50

MS/MS Fragmentation of **LVYTNDQGEIVK**
Found in **gi|41052915** in **NCBInr**, putative ferredoxin-NADP(H) oxidoreductase [Oryza sativa Japonica Group]

Match to Query 28: 1377.684424 from(1378.691700,1+) intensity(0.0000) index(11)
Title: Label: L3, Spot_Id: 219717, Peak_List_Id: 225437, MSMS Job_Run_Id: 21778, Comment:
Data file C:\Documents and Settings\Administrator\Desktop\farah BHU\spot set plate\Farah-Garima 11May 2013\ppw_L3_136842146000.txt

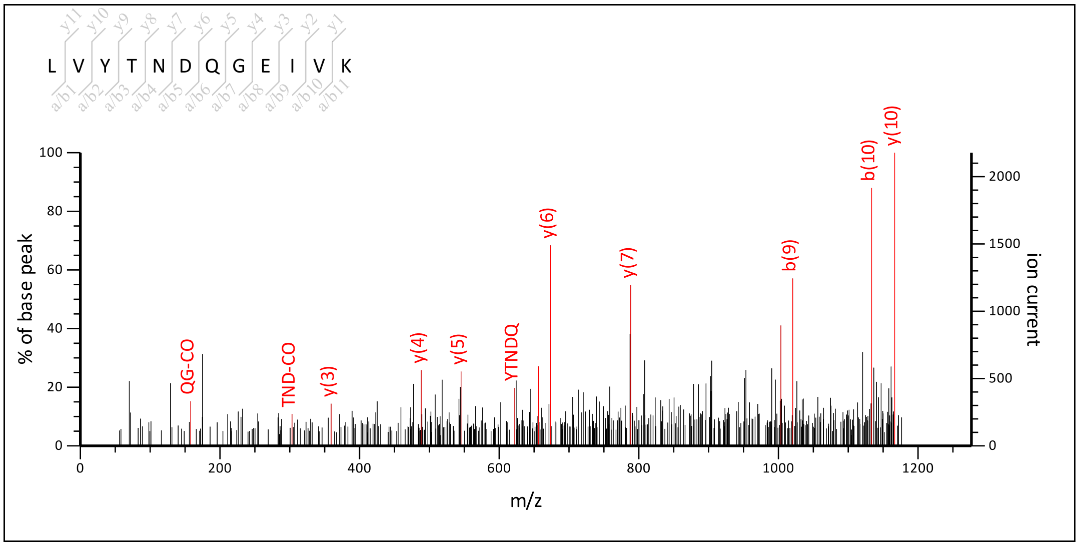


0 to 1276.49

Label all possible matches ○ Label matches used for scoring ◉

**Monoisotopic mass of neutral peptide Mr(calc):** 1377.7140
**Fixed modifications:** Carbamidomethyl (C) (apply to specified residues or termini only)
**Ions Score:** 42 **Expect:** 0.15
**Matches :** 17/195 fragment ions using 24 most intense peaks (help)

| # | Immon. | a | a* | $a^0$ | b | b* | $b^0$ | d | d' | Seq. | v | w | w' | y | y* | [illegible] |
|---|---|---|---|---|---|---|---|---|---|---|---|---|---|---|---|---|
| 1 | 86.0964 | 86.0964 | | | 114.0913 | | | 44.0495 | | L | | | | | | |
| 2 | 72.0808 | 185.1648 | | | 213.1598 | | | 171.1492 | | V | 1221.5746 | 1234.5950 | | 1265.6372 | 1248.6107 | [illegible] |
| 3 | 136.0757 | 348.2282 | | | 376.2231 | | | | | Y | 1058.5113 | | | ***1166.5688*** | 1149.5422 | [illegible] |
| 4 | 74.0600 | 449.2758 | | 431.2653 | 477.2708 | | 459.2602 | 433.2809 | 435.2602 | T | 957.4636 | 970.4840 | 972.4633 | ***1003.5055*** | 986.4789 | [illegible] |
| 5 | 87.0553 | 563.3188 | 546.2922 | 545.3082 | 591.3137 | 574.2871 | 573.3031 | 520.3130 | | N | 843.4207 | 842.4254 | | 902.4578 | 885.4312 | [illegible] |
| 6 | 88.0393 | 678.3457 | 661.3192 | 660.3352 | 706.3406 | 689.3141 | 688.3301 | 634.3559 | | D | 728.3937 | 727.3985 | | ***788.4149*** | 771.3883 | [illegible] |
| 7 | 101.0709 | 806.4043 | 789.3777 | 788.3937 | 834.3992 | 817.3727 | 816.3886 | 749.3828 | | Q | 600.3352 | 599.3399 | | ***673.3879*** | 656.3614 | [illegible] |
| 8 | 30.0338 | 863.4258 | 846.3992 | 845.4152 | 891.4207 | 874.3941 | 873.4101 | | | G | | | | ***545.3293*** | 528.3028 | [illegible] |
| 9 | 102.0550 | 992.4684 | 975.4418 | 974.4578 | **1020.4633** | 1003.4367 | 1002.4527 | 934.4629 | | E | 414.2711 | 413.2758 | | ***488.3079*** | 471.2813 | [illegible] |
| 10 | 86.0964 | 1105.5524 | 1088.5259 | 1087.5419 | **1133.5473** | 1116.5208 | 1115.5368 | 1077.5211 | 1091.5368 | I | 301.1870 | 314.2074 | 328.2231 | ***359.2653*** | 342.2387 | |
| 11 | 72.0808 | 1204.6208 | 1187.5943 | 1186.6103 | 1232.6157 | 1215.5892 | 1214.6052 | 1190.6052 | | V | 202.1186 | 215.1390 | | 246.1812 | 229.1547 | |
| 12 | 101.1073 | | | | | | | | | K | 74.0237 | 73.0284 | | 147.1128 | 130.0863 | |

| Seq | ya | yb | Seq | ya | yb | Seq | ya | yb |
|---|---|---|---|---|---|---|---|---|
| VY | 235.1441 | 263.1390 | VYT | 336.1918 | 364.1867 | VYTN | 450.2347 | 478.2296 |
| VYTND | 565.2617 | 593.2566 | VYTNDQ | 693.3202 | 721.3151 | YT | 237.1234 | 265.1183 |
| YTN | 351.1663 | 379.1612 | YTND | 466.1932 | 494.1882 | YTNDQ | 594.2518 | 622.2467 |
| YTNDQG | 651.2733 | 679.2682 | TN | 188.1030 | 216.0979 | TND | 303.1299 | 331.1248 |
| TNDQ | 431.1885 | 459.1834 | TNDQG | 488.2100 | 516.2049 | TNDQGE | 617.2525 | 645.2475 |
| ND | 202.0822 | 230.0771 | NDQ | 330.1408 | 358.1357 | NDQG | 387.1623 | 415.1572 |
| NDQGE | 516.2049 | 544.1998 | NDQGEI | 629.2889 | 657.2838 | DQ | 216.0979 | 244.0928 |
| DQG | 273.1193 | 301.1143 | DQGE | 402.1619 | 430.1569 | DQGEI | 515.2460 | 543.2409 |
| DQGEIV | 614.3144 | 642.3093 | QG | 158.0924 | 186.0873 | QGE | 287.1350 | 315.1299 |

| | | | | | | | | |
|---|---|---|---|---|---|---|---|---|
| **QGEI** | 400.2191 | 428.2140 | **QGEIV** | 499.2875 | 527.2824 | **GE** | 159.0764 | 187.0713 |
| **GEI** | 272.1605 | 300.1554 | **GEIV** | 371.2289 | 399.2238 | **EI** | 215.1390 | 243.1339 |
| **EIV** | 314.2074 | 342.2023 | **IV** | 185.1648 | 213.1598 | | | |

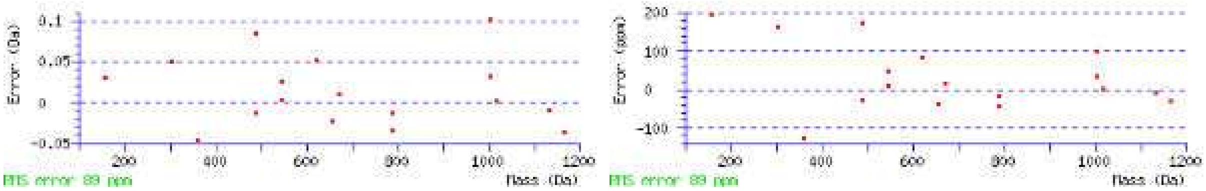


NCBI **BLAST** search of LVYTNDQGEIVK
(Parameters: blastp, nr protein database, expect=20000, no filter, PAM30)
Other BLAST web gateways

**All matches to this query**

| Score | Mr(calc) | Delta | Sequence |
|---|---|---|---|
| 41.6 | 1377.7504 | -0.0659 | LVYTNDKGEIVK |
| 41.6 | 1377.7504 | -0.0659 | LVYTNDKGELVK |
| 41.6 | 1377.7140 | -0.0296 | LVYTNDQGEIVK |
| 25.9 | 1377.7140 | -0.0295 | LVYTNENGEIVK |
| 25.9 | 1377.7140 | -0.0295 | LVYTNENGELVK |
| 19.9 | 1377.6345 | 0.0499 | AGGGSGSAGRSGGSSAR |
| 18.8 | 1377.6922 | -0.0078 | TTLAEGKAVDAMR |
| 17.3 | 1377.7252 | -0.0408 | QTEFSLQLEKR |
| 16.5 | 1377.6380 | 0.0464 | MADAADMKADLAR |
| 15.2 | 1377.6711 | 0.0133 | VLDFCAEAGIGAR |

**Mascot:** http://www.matrixscience.com/

# MATRIX SCIENCE Mascot Search Results

## Peptide View **Spot no 50**

MS/MS Fragmentation of **RLVYTNDQGEIVK**
Found in **gi|41052915** in **NCBInr**, putative ferredoxin-NADP(H) oxidoreductase [Oryza sativa Japonica Group]

Match to Query 30: 1533.810024 from(1534.817300,1+) intensity(0.0000) index(12)
Title: Label: L3, Spot_Id: 219717, Peak_List_Id: 225436, MSMS Job_Run_Id: 21778, Comment:
Data file C:\Documents and Settings\Administrator\Desktop\farah BHU\spot set plate\Farah-Garima 11May 2013\ppw_L3_136842146000.txt

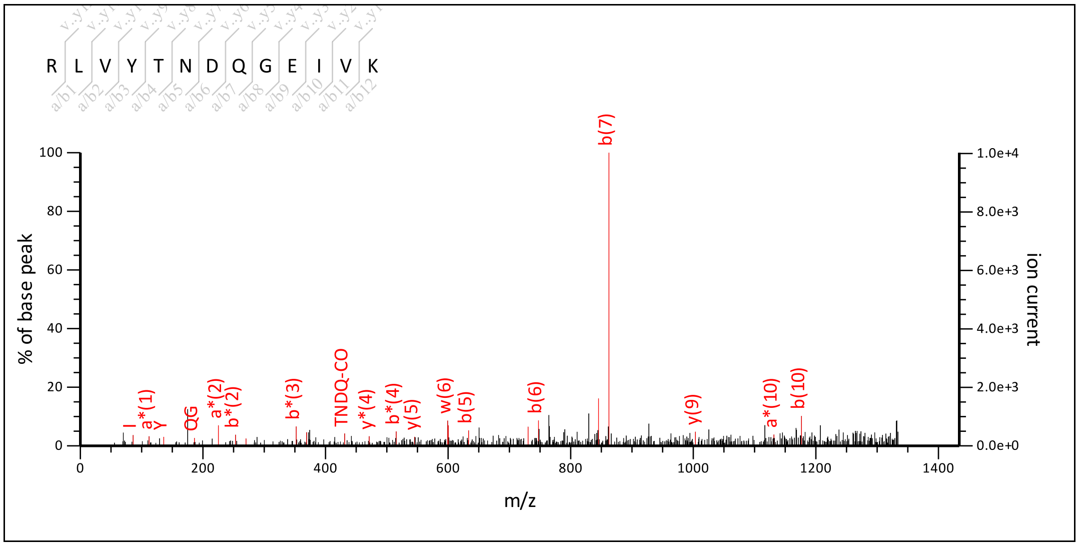


0 to 1433.98

Label all possible matches Label matches used for scoring

**Monoisotopic mass of neutral peptide Mr(calc):** 1533.8151
**Fixed modifications:** Carbamidomethyl (C) (apply to specified residues or termini only)
**Ions Score:** 17 **Expect:** 35
**Matches :** 25/223 fragment ions using 52 most intense peaks (help)

| # | Immon. | a | a* | a0 | b | b* | b0 | d | d' | Seq. | v | w | w' | y | y* | |
|---|---|---|---|---|---|---|---|---|---|---|---|---|---|---|---|---|
| 1 | 129.1135 | 129.1135 | 112.0869 | | 157.1084 | 140.0818 | | 44.0495 | | R | | | | | | |
| 2 | 86.0964 | 242.1975 | 225.1710 | | ***270.1925*** | 253.1659 | | 200.1506 | | L | 1320.6430 | 1319.6478 | | 1378.7213 | 1361.6947 | 13[illegible] |
| 3 | 72.0808 | 341.2660 | 324.2394 | | ***369.2609*** | 352.2343 | | 327.2503 | | V | 1221.5746 | 1234.5950 | | 1265.6372 | 1248.6107 | 12[illegible] |
| 4 | 136.0757 | 504.3293 | 487.3027 | | 532.3242 | 515.2976 | | | | Y | 1058.5113 | | | 1166.5688 | 1149.5422 | 11[illegible] |
| 5 | 74.0600 | 605.3770 | 588.3504 | 587.3664 | ***633.3719*** | 616.3453 | 615.3613 | 589.3820 | 591.3613 | T | 957.4636 | 970.4840 | 972.4633 | **1003.5055** | 986.4789 | 9[illegible] |
| 6 | 87.0553 | 719.4199 | 702.3933 | 701.4093 | ***747.4148*** | 730.3883 | 729.4042 | 676.4141 | | N | 843.4207 | 842.4254 | | 902.4578 | 885.4312 | 8[illegible] |
| 7 | 88.0393 | 834.4468 | 817.4203 | 816.4363 | ***862.4417*** | 845.4152 | 844.4312 | 790.4570 | | D | 728.3937 | 727.3985 | | 788.4149 | 771.3883 | 7[illegible] |
| 8 | 101.0709 | 962.5054 | 945.4789 | 944.4948 | 990.5003 | 973.4738 | 972.4898 | 905.4839 | | Q | 600.3352 | 599.3399 | | 673.3879 | 656.3614 | 6[illegible] |
| 9 | 30.0338 | 1019.5269 | 1002.5003 | 1001.5163 | 1047.5218 | 1030.4952 | 1029.5112 | | | G | | | | **545.3293** | 528.3028 | 5[illegible] |
| 10 | 102.0550 | 1148.5695 | 1131.5429 | 1130.5589 | ***1176.5644*** | 1159.5378 | 1158.5538 | 1090.5640 | | E | 414.2711 | 413.2758 | | 488.3079 | 471.2813 | 4[illegible] |
| 11 | 86.0964 | 1261.6535 | 1244.6270 | 1243.6430 | 1289.6484 | 1272.6219 | 1271.6379 | 1233.6222 | 1247.6379 | I | 301.1870 | 314.2074 | 328.2231 | 359.2653 | 342.2387 | |
| 12 | 72.0808 | 1360.7219 | 1343.6954 | 1342.7114 | 1388.7169 | 1371.6903 | 1370.7063 | 1346.7063 | | V | 202.1186 | 215.1390 | | 246.1812 | 229.1547 | |
| 13 | 101.1073 | | | | | | | | | K | 74.0237 | 73.0284 | | 147.1128 | 130.0863 | |

| Seq | ya | yb | Seq | ya | yb | Seq | ya | yb |
|---|---|---|---|---|---|---|---|---|
| LV | 185.1648 | 213.1598 | LVY | 348.2282 | 376.2231 | LVYT | 449.2758 | 477.2708 |
| LVYTN | 563.3188 | 591.3137 | LVYTND | 678.3457 | 706.3406 | VY | 235.1441 | 263.1390 |
| VYT | 336.1918 | 364.1867 | VYTN | 450.2347 | 478.2296 | VYTND | 565.2617 | 593.2566 |
| VYTNDQ | 693.3202 | 721.3151 | YT | 237.1234 | 265.1183 | YTN | 351.1663 | 379.1612 |
| YTND | 466.1932 | 494.1882 | YTNDQ | 594.2518 | 622.2467 | YTNDQG | 651.2733 | 679.2682 |
| TN | 188.1030 | 216.0979 | TND | 303.1299 | 331.1248 | TNDQ | 431.1885 | 459.1834 |
| TNDQG | 488.2100 | 516.2049 | TNDQGE | 617.2525 | 645.2475 | ND | 202.0822 | 230.0771 |
| NDQ | 330.1408 | 358.1357 | NDQG | 387.1623 | 415.1572 | NDQGE | 516.2049 | 544.1998 |

| | | | | | | | | |
|---|---|---|---|---|---|---|---|---|
| **NDQGEI** | 629.2889 | 657.2838 | **DQ** | 216.0979 | 244.0928 | **DQG** | 273.1193 | 301.1143 |
| **DQGE** | 402.1619 | 430.1569 | **DQGEI** | 515.2460 | 543.2409 | **DQGEIV** | 614.3144 | 642.3093 |
| **QG** | 158.0924 | 186.0873 | **QGE** | 287.1350 | 315.1299 | **QGEI** | 400.2191 | 428.2140 |
| **QGEIV** | 499.2875 | 527.2824 | **GE** | 159.0764 | 187.0713 | **GEI** | 272.1605 | 300.1554 |
| **GEIV** | 371.2289 | 399.2238 | **EI** | 215.1390 | 243.1339 | **EIV** | 314.2074 | 342.2023 |
| **IV** | 185.1648 | 213.1598 | | | | | | |

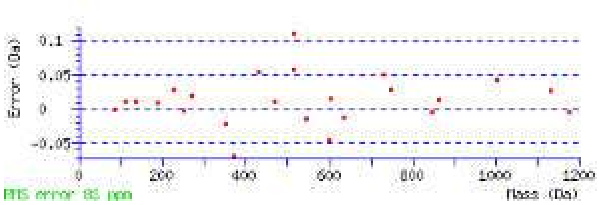


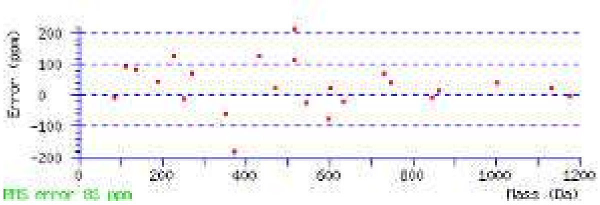


NCBI **BLAST** search of RLVYTNDQGEIVK
(Parameters: blastp, nr protein database, expect=20000, no filter, PAM30)
Other BLAST web gateways

**All matches to this query**

| Score | Mr(calc) | Delta | Sequence |
|---|---|---|---|
| 17.4 | 1533.8151 | -0.0051 | RLVYTNDQGEIVK |
| 10.0 | 1533.8627 | -0.0527 | ILQHTPDKSINLR |
| 9.9 | 1533.8159 | -0.0059 | WTNITGAMKILMR |
| 9.8 | 1533.7722 | 0.0379 | LARAFGGDAMDAALR |
| 9.7 | 1533.8047 | 0.0053 | VLELAFPNMTMIR |
| 9.3 | 1533.7205 | 0.0895 | VANGTLAPNSMSSNR |
| 9.1 | 1533.8012 | 0.0089 | EFQRTISSARPSR |
| 9.0 | 1533.7271 | 0.0829 | SSSVSQPDSSPTVTR |
| 8.8 | 1533.7254 | 0.0846 | YMFTMACGRLIR |
| 8.7 | 1533.7874 | 0.0226 | ALAWWMSASRALR |

**Mascot:** http://www.matrixscience.com/

# MATRIX SCIENCE Mascot Search Results

## Peptide View Spot no 50

MS/MS Fragmentation of **LYSIASSAIGDFADSK**
Found in **gi|41052915** in **NCBInr**, putative ferredoxin-NADP(H) oxidoreductase [Oryza sativa Japonica Group]

Match to Query 32: 1643.778324 from(1644.785600,1+) intensity(0.0000) index(13)
Title: Label: L3, Spot_Id: 219717, Peak_List_Id: 225444, MSMS Job_Run_Id: 21778, Comment:
Data file C:\Documents and Settings\Administrator\Desktop\farah BHU\spot set plate\Farah-Garima 11May 2013\ppw_L3_136842146000.txt

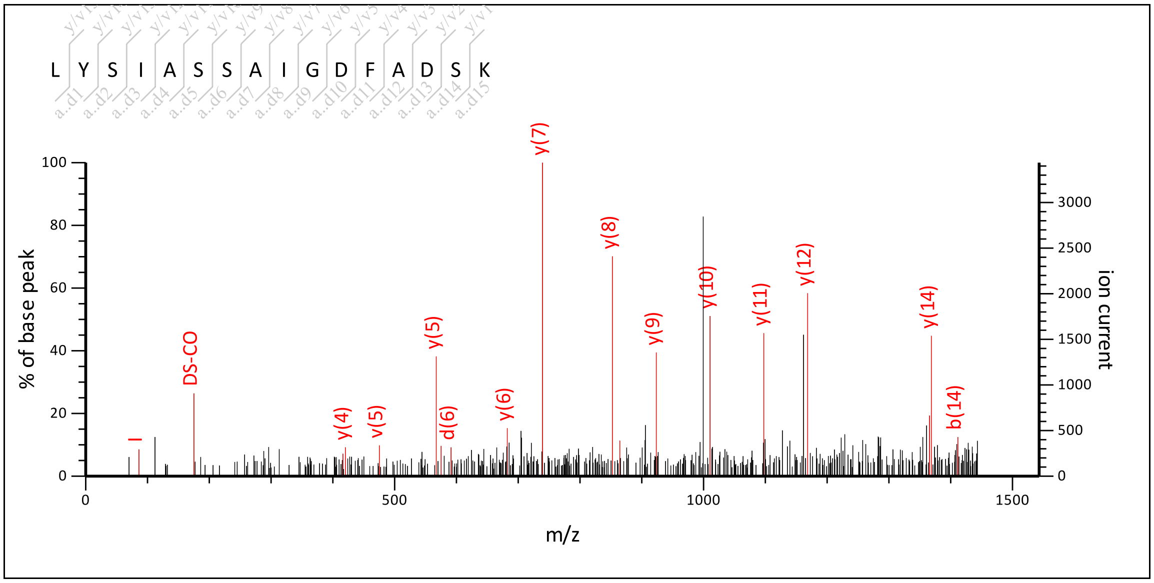


0 to 1543.16

Label all possible matches ○ Label matches used for scoring ◉

**Monoisotopic mass of neutral peptide Mr(calc):** 1643.8042
**Fixed modifications:** Carbamidomethyl (C) (apply to specified residues or termini only)
**Ions Score:** 70 **Expect:** 0.0002
**Matches :** 26/280 fragment ions using 28 most intense peaks (help)

| # | Immon. | a | $a^0$ | b | $b^0$ | d | d' | Seq. | v | w | w' | y | y* | $y^0$ | # |
|---|---|---|---|---|---|---|---|---|---|---|---|---|---|---|---|
| 1 | 86.0964 | 86.0964 | | 114.0913 | | 44.0495 | | L | | | | | | | 16 |
| 2 | 136.0757 | 249.1598 | | 277.1547 | | | | Y | 1423.6700 | | | 1531.7275 | 1514.7009 | 1513.7169 | 15 |
| 3 | 60.0444 | 336.1918 | 318.1812 | 364.1867 | 346.1761 | 320.1969 | | S | 1336.6379 | 1335.6427 | | 1368.6642 | 1351.6376 | 1350.6536 | 14 |
| 4 | 86.0964 | 449.2758 | 431.2653 | 477.2708 | 459.2602 | 421.2445 | 435.2602 | I | 1223.5539 | 1236.5743 | 1250.5899 | 1281.6321 | 1264.6056 | 1263.6216 | 13 |
| 5 | 44.0495 | 520.3130 | 502.3024 | 548.3079 | 530.2973 | | | A | 1152.5168 | | | 1168.5481 | 1151.5215 | 1150.5375 | 12 |
| 6 | 60.0444 | 607.3450 | 589.3344 | 635.3399 | 617.3293 | 591.3501 | | S | 1065.4847 | 1064.4895 | | 1097.5109 | 1080.4844 | 1079.5004 | 11 |
| 7 | 60.0444 | 694.3770 | 676.3665 | 722.3719 | 704.3614 | 678.3821 | | S | 978.4527 | 977.4575 | | 1010.4789 | 993.4524 | 992.4684 | 10 |
| 8 | 44.0495 | 765.4141 | 747.4036 | 793.4090 | 775.3985 | | | A | 907.4156 | | | 923.4469 | 906.4203 | 905.4363 | 9 |
| 9 | 86.0964 | 878.4982 | 860.4876 | 906.4931 | 888.4825 | 850.4669 | 864.4825 | I | 794.3315 | 807.3519 | 821.3676 | 852.4098 | 835.3832 | 834.3992 | 8 |
| 10 | 30.0338 | 935.5197 | 917.5091 | 963.5146 | 945.5040 | | | G | | | | 739.3257 | 722.2992 | 721.3151 | 7 |
| 11 | 88.0393 | 1050.5466 | 1032.5360 | 1078.5415 | 1060.5310 | 1006.5568 | | D | 622.2831 | 621.2879 | | 682.3042 | 665.2777 | 664.2937 | 6 |
| 12 | 120.0808 | 1197.6150 | 1179.6045 | 1225.6099 | 1207.5994 | | | F | 475.2147 | | | 567.2773 | 550.2508 | 549.2667 | 5 |
| 13 | 44.0495 | 1268.6521 | 1250.6416 | 1296.6470 | 1278.6365 | | | A | 404.1776 | | | 420.2089 | 403.1823 | 402.1983 | 4 |
| 14 | 88.0393 | 1383.6791 | 1365.6685 | 1411.6740 | 1393.6634 | 1339.6892 | | D | 289.1506 | 288.1554 | | 349.1718 | 332.1452 | 331.1612 | 3 |
| 15 | 60.0444 | 1470.7111 | 1452.7005 | 1498.7060 | 1480.6955 | 1454.7162 | | S | 202.1186 | 201.1234 | | 234.1448 | 217.1183 | 216.1343 | 2 |
| 16 | 101.1073 | | | | | | | K | 74.0237 | 73.0284 | | 147.1128 | 130.0863 | | 1 |

| Seq | ya | yb | Seq | ya | yb | Seq | ya | yb |
|---|---|---|---|---|---|---|---|---|
| YS | 223.1077 | 251.1026 | YSI | 336.1918 | 364.1867 | YSIA | 407.2289 | 435.2238 |

| | | | | | | | | |
|---|---|---|---|---|---|---|---|---|
| **YSIAS** | 494.2609 | 522.2558 | **YSIASS** | 581.2930 | 609.2879 | **YSIASSA** | 652.3301 | 680.3250 |
| **SI** | 173.1285 | 201.1234 | **SIA** | 244.1656 | 272.1605 | **SIAS** | 331.1976 | 359.1925 |
| **SIASS** | 418.2296 | 446.2245 | **SIASSA** | 489.2667 | 517.2617 | **SIASSAI** | 602.3508 | 630.3457 |
| **SIASSAIG** | 659.3723 | 687.3672 | **IA** | 157.1335 | 185.1285 | **IAS** | 244.1656 | 272.1605 |
| **IASS** | 331.1976 | 359.1925 | **IASSA** | 402.2347 | 430.2296 | **IASSAI** | 515.3188 | 543.3137 |
| **IASSAIG** | 572.3402 | 600.3352 | **IASSAIGD** | 687.3672 | 715.3621 | **AS** | 131.0815 | 159.0764 |
| **ASS** | 218.1135 | 246.1084 | **ASSA** | 289.1506 | 317.1456 | **ASSAI** | 402.2347 | 430.2296 |
| **ASSAIG** | 459.2562 | 487.2511 | **ASSAIGD** | 574.2831 | 602.2780 | **SS** | 147.0764 | 175.0713 |
| **SSA** | 218.1135 | 246.1084 | **SSAI** | 331.1976 | 359.1925 | **SSAIG** | 388.2191 | 416.2140 |
| **SSAIGD** | 503.2460 | 531.2409 | **SSAIGDF** | 650.3144 | 678.3093 | **SA** | 131.0815 | 159.0764 |
| **SAI** | 244.1656 | 272.1605 | **SAIG** | 301.1870 | 329.1819 | **SAIGD** | 416.2140 | 444.2089 |
| **SAIGDF** | 563.2824 | 591.2773 | **SAIGDFA** | 634.3195 | 662.3144 | **AI** | 157.1335 | 185.1285 |
| **AIG** | 214.1550 | 242.1499 | **AIGD** | 329.1819 | 357.1769 | **AIGDF** | 476.2504 | 504.2453 |
| **AIGDFA** | 547.2875 | 575.2824 | **AIGDFAD** | 662.3144 | 690.3093 | **IG** | 143.1179 | 171.1128 |
| **IGD** | 258.1448 | 286.1397 | **IGDF** | 405.2132 | 433.2082 | **IGDFA** | 476.2504 | 504.2453 |
| **IGDFAD** | 591.2773 | 619.2722 | **IGDFADS** | 678.3093 | 706.3042 | **GD** | 145.0608 | 173.0557 |
| **GDF** | 292.1292 | 320.1241 | **GDFA** | 363.1663 | 391.1612 | **GDFAD** | 478.1932 | 506.1882 |
| **GDFADS** | 565.2253 | 593.2202 | **DF** | 235.1077 | 263.1026 | **DFA** | 306.1448 | 334.1397 |
| **DFAD** | 421.1718 | 449.1667 | **DFADS** | 508.2038 | 536.1987 | **FA** | 191.1179 | 219.1128 |
| **FAD** | 306.1448 | 334.1397 | **FADS** | 393.1769 | 421.1718 | **AD** | 159.0764 | 187.0713 |
| **ADS** | 246.1084 | 274.1034 | **DS** | 175.0713 | 203.0662 | | | |

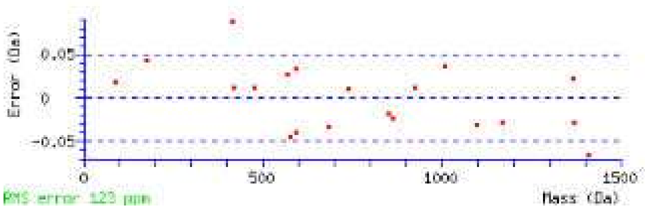


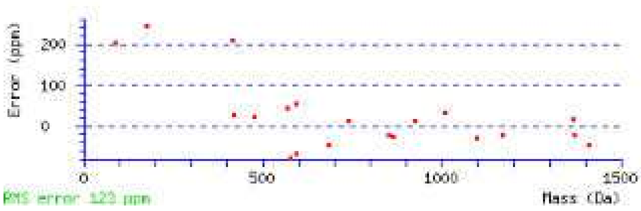


NCBI **BLAST** search of LYSIASSAIGDFADSK
(Parameters: blastp, nr protein database, expect=20000, no filter, PAM30)
Other BLAST web gateways

**All matches to this query**

| Score | Mr(calc) | Delta | Sequence |
|---|---|---|---|
| 69.6 | 1643.8042 | -0.0259 | LYSIASSAIGDFADSK |
| 64.5 | 1643.8043 | -0.0259 | LYSIASSALGDFGDTK |
| 20.5 | 1643.6742 | 0.1041 | CGELDMAGEVFGGMR |
| 15.6 | 1643.7032 | 0.0751 | RFSGEQDMSSTMPR |
| 14.0 | 1643.8705 | -0.0922 | LYTVVYIMDLRSR |
| 10.8 | 1643.7825 | -0.0041 | ESIFNASSSSSKCIK |
| 10.4 | 1643.8242 | -0.0459 | MRAIFYWSLGTGSR |
| 10.2 | 1643.8705 | -0.0922 | YLAVMQAEPVVGVPR |
| 9.7 | 1643.7766 | 0.0017 | GCTTFEALAAYVWR |
| 9.7 | 1643.7568 | 0.0215 | LKGMEMALTASAMDK |

**Mascot:** http://www.matrixscience.com/

# Mascot Search Results

## Peptide View **Spot no 50**

MS/MS Fragmentation of **EQTNAAGEKMYIQTR**
Found in **gi|41052915** in **NCBInr**, putative ferredoxin-NADP(H) oxidoreductase [Oryza sativa Japonica Group]

Match to Query 34: 1754.808624 from(1755.815900,1+) intensity(0.0000) index(14)
Title: Label: L3, Spot_Id: 219717, Peak_List_Id: 225434, MSMS Job_Run_Id: 21778, Comment:
Data file C:\Documents and Settings\Administrator\Desktop\farah BHU\spot set plate\Farah-Garima 11May 2013\ppw_L3_136842146000.txt

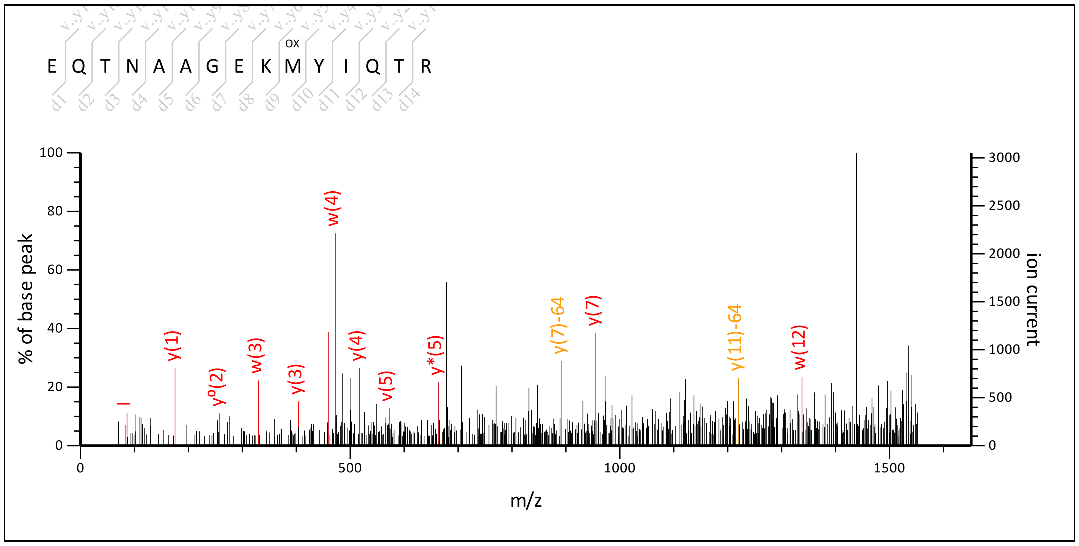


0 to 1651.57

Label all possible matches ○ Label matches used for scoring ◉

**Monoisotopic mass of neutral peptide Mr(calc):** 1754.8257
**Fixed modifications:** Carbamidomethyl (C) (apply to specified residues or termini only)
**Variable modifications:**
**M10** : Oxidation (M), with neutral losses 63.9983(shown in table), 0.0000
**Ions Score:** 20 **Expect:** 16
**Matches** : 25/395 fragment ions using 30 most intense peaks (help)

| # | Immon. | a | a* | $a^0$ | b | b* | $b^0$ | d | d' | Seq. | v | w | w' | y | y* | [illegible] |
|---|---|---|---|---|---|---|---|---|---|---|---|---|---|---|---|---|
| 1 | 102.0550 | 102.0550 | | 84.0444 | 130.0499 | | 112.0393 | 44.0495 | | E | | | | | | |
| 2 | 101.0709 | 230.1135 | 213.0870 | 212.1030 | 258.1084 | 241.0819 | 240.0979 | 173.0921 | | Q | 1489.7394 | 1488.7441 | | 1562.7921 | 1545.7656 | [illegible] |
| 3 | 74.0600 | 331.1612 | 314.1347 | 313.1506 | 359.1561 | 342.1296 | 341.1456 | 315.1663 | 317.1456 | T | 1388.6917 | 1401.7121 | 1403.6914 | 1434.7336 | 1417.7070 | [illegible] |
| 4 | 87.0553 | 445.2041 | 428.1776 | 427.1936 | 473.1991 | 456.1725 | 455.1885 | 402.1983 | | N | 1274.6488 | 1273.6535 | | 1333.6859 | 1316.6593 | [illegible] |
| 5 | 44.0495 | 516.2413 | 499.2147 | 498.2307 | 544.2362 | 527.2096 | 526.2256 | | | A | 1203.6117 | | | ***1219.6430*** | 1202.6164 | [illegible] |
| 6 | 44.0495 | 587.2784 | 570.2518 | 569.2678 | 615.2733 | 598.2467 | 597.2627 | | | A | 1132.5746 | | | 1148.6059 | 1131.5793 | [illegible] |
| 7 | 30.0338 | 644.2998 | 627.2733 | 626.2893 | 672.2947 | 655.2682 | 654.2842 | | | G | | | | 1077.5687 | 1060.5422 | [illegible] |
| 8 | 102.0550 | 773.3424 | 756.3159 | 755.3319 | 801.3373 | 784.3108 | 783.3268 | 715.3369 | | E | 946.5105 | 945.5152 | | 1020.5473 | 1003.5207 | [illegible] |
| 9 | 101.1073 | 901.4374 | 884.4108 | 883.4268 | 929.4323 | 912.4058 | 911.4217 | 844.3795 | | K | 818.4155 | 817.4203 | | ***891.5047*** | 874.4781 | [illegible] |
| 10 | 56.0495 | 984.4745 | 967.4480 | 966.4639 | 1012.4694 | 995.4429 | 994.4589 | 972.4745 | | M | 735.3784 | 734.3832 | | 763.4097 | 746.3832 | [illegible] |
| 11 | 136.0757 | 1147.5378 | 1130.5113 | 1129.5273 | 1175.5327 | 1158.5062 | 1157.5222 | | | Y | 572.3151 | | | 680.3726 | 663.3461 | [illegible] |
| 12 | 86.0964 | 1260.6219 | 1243.5953 | 1242.6113 | 1288.6168 | 1271.5903 | 1270.6062 | 1232.5906 | 1246.6062 | I | 459.2310 | 472.2514 | 486.2671 | ***517.3093*** | 500.2827 | [illegible] |
| 13 | 101.0709 | 1388.6805 | 1371.6539 | 1370.6699 | 1416.6754 | 1399.6488 | 1398.6648 | 1331.6590 | | Q | 331.1724 | 330.1772 | | ***404.2252*** | 387.1987 | [illegible] |
| 14 | 74.0600 | 1489.7282 | 1472.7016 | 1471.7176 | 1517.7231 | 1500.6965 | 1499.7125 | 1473.7332 | 1475.7125 | T | 230.1248 | 243.1452 | 245.1244 | ***276.1666*** | 259.1401 | [illegible] |
| 15 | 129.1135 | | | | | | | | | R | 74.0237 | 73.0284 | | ***175.1190*** | 158.0924 | |

| Seq | ya | yb | Seq | ya | yb | Seq | ya | yb |
|---|---|---|---|---|---|---|---|---|
| QT | 202.1186 | 230.1135 | QTN | 316.1615 | 344.1565 | QTNA | 387.1987 | 415.1936 |
| QTNAA | 458.2358 | 486.2307 | QTNAAG | 515.2572 | 543.2522 | QTNAAGE | 644.2998 | 672.2947 |
| TN | 188.1030 | 216.0979 | TNA | 259.1401 | 287.1350 | TNAA | 330.1772 | 358.1721 |
| TNAAG | 387.1987 | 415.1936 | TNAAGE | 516.2413 | 544.2362 | TNAAGEK | 644.3362 | 672.3311 |
| NA | 158.0924 | 186.0873 | NAA | 229.1295 | 257.1244 | NAAG | 286.1510 | 314.1459 |

| | | | | | | | | |
|---|---|---|---|---|---|---|---|---|
| **NAAGE** | 415.1936 | 443.1885 | **NAAGEK** | 543.2885 | 571.2835 | **NAAGEKM** | 626.3257 | 654.3206 |
| **AA** | 115.0866 | 143.0815 | **AAG** | 172.1081 | 200.1030 | **AAGE** | 301.1506 | 329.1456 |
| **AAGEK** | 429.2456 | 457.2405 | **AAGEKM** | 512.2827 | 540.2776 | **AAGEKMY** | 675.3461 | 703.3410 |
| **AG** | 101.0709 | 129.0659 | **AGE** | 230.1135 | 258.1084 | **AGEK** | 358.2085 | 386.2034 |
| **AGEKM** | 441.2456 | 469.2405 | **AGEKMY** | 604.3089 | 632.3039 | **GE** | 159.0764 | 187.0713 |
| **GEK** | 287.1714 | 315.1663 | **GEKM** | 370.2085 | 398.2034 | **GEKMY** | 533.2718 | 561.2667 |
| **GEKMYI** | 646.3559 | 674.3508 | **EK** | 230.1499 | 258.1448 | **EKM** | 313.1870 | 341.1819 |
| **EKMY** | 476.2504 | 504.2453 | **EKMYI** | 589.3344 | 617.3293 | **KM** | 184.1444 | 212.1394 |
| **KMY** | 347.2078 | 375.2027 | **KMYI** | 460.2918 | 488.2867 | **KMYIQ** | 588.3504 | 616.3453 |
| **KMYIQT** | 689.3981 | 717.3930 | **MY** | 219.1128 | 247.1077 | **MYI** | 332.1969 | 360.1918 |
| **MYIQ** | 460.2554 | 488.2504 | **MYIQT** | 561.3031 | 589.2980 | **YI** | 249.1598 | 277.1547 |
| **YIQ** | 377.2183 | 405.2132 | **YIQT** | 478.2660 | 506.2609 | **IQ** | 214.1550 | 242.1499 |
| **IQT** | 315.2027 | 343.1976 | **QT** | 202.1186 | 230.1135 | | | |

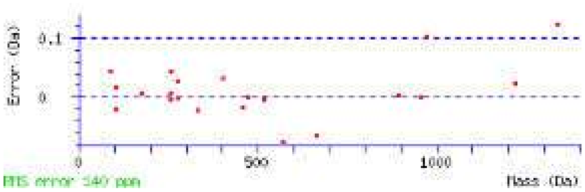


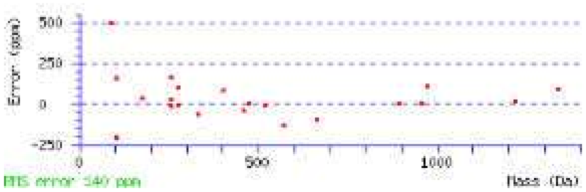


NCBI **BLAST** search of EQTNAAGEKMYIQTR
(Parameters: blastp, nr protein database, expect=20000, no filter, PAM30)
Other BLAST web gateways

**All matches to this query**

| Score | Mr(calc) | Delta | Sequence |
|---|---|---|---|
| 20.2 | 1754.8257 | -0.0171 | EQTNAAGEKMYIQTR |
| 12.8 | 1754.9389 | -0.1303 | FLGTCVLTSVYLINR |
| 12.3 | 1754.8509 | -0.0423 | AGMVEDDILHLIEER |
| 9.0 | 1754.7460 | 0.0626 | MGILDAVTEMCACPR |
| 8.9 | 1754.9567 | -0.1481 | FAVTGTVDGPLPEIAIR |
| 8.9 | 1754.8911 | -0.0825 | LEGTTASTASHAAVNLGR |
| 8.8 | 1754.8542 | -0.0456 | SVISSNSGKVTMAAMEK |
| 8.6 | 1754.8153 | -0.0067 | IWSAAMEELMSKGMR |
| 8.6 | 1754.7669 | 0.0418 | EEAEIYEGEAVCTQK |
| 8.3 | 1754.8509 | -0.0423 | SSPSICKAFGIESTGSK |

**Mascot:** http://www.matrixscience.com/

# MATRIX SCIENCE Mascot Search Results

## Peptide View **Spot no 50**

MS/MS Fragmentation of **DPNATIIMLGTGTGIAPFR**
Found in **gi|41052915** in **NCBInr**, putative ferredoxin-NADP(H) oxidoreductase [Oryza sativa Japonica Group]

Match to Query 37: 1959.991124 from(1960.998400,1+) intensity(0.0000) index(15)
Title: Label: L3, Spot_Id: 219717, Peak_List_Id: 225438, MSMS Job_Run_Id: 21778, Comment:
Data file C:\Documents and Settings\Administrator\Desktop\farah BHU\spot set plate\Farah-Garima 11May 2013\ppw_L3_136842146000.txt

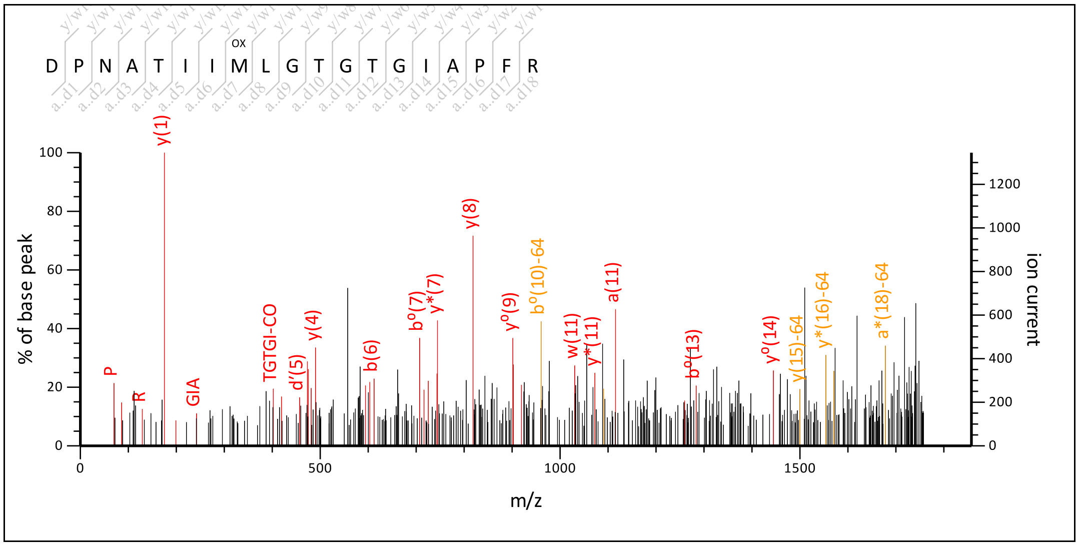


0 to 1857.47

Label all possible matches ○ Label matches used for scoring ◉

**Monoisotopic mass of neutral peptide Mr(calc):** 1960.0088
**Fixed modifications:** Carbamidomethyl (C) (apply to specified residues or termini only)
**Variable modifications:**
**M8** : Oxidation (M), with neutral losses 63.9983(shown in table), 0.0000
**Ions Score:** 17 **Expect:** 31
**Matches :** 46/558 fragment ions using 85 most intense peaks (help)

| # | Immon. | a | a* | a⁰ | b | b* | b⁰ | d | d' | Seq. | v | w | w' | y | y* | |
|---|---|---|---|---|---|---|---|---|---|---|---|---|---|---|---|---|
| 1 | 88.0393 | 88.0393 | | 70.0287 | 116.0342 | | 98.0237 | 44.0495 | | D | | | | | | |
| 2 | 70.0651 | 185.0921 | | 167.0815 | 213.0870 | | 195.0764 | 159.0764 | | P | 1739.9439 | 1738.9487 | | 1781.9908 | 1764.9643 | [illegible] |
| 3 | 87.0553 | 299.1350 | 282.1084 | 281.1244 | 327.1299 | 310.1034 | 309.1193 | 256.1292 | | N | 1625.9010 | 1624.9057 | | 1684.9381 | 1667.9115 | [illegible] |
| 4 | 44.0495 | 370.1721 | 353.1456 | 352.1615 | 398.1670 | 381.1405 | 380.1565 | | | A | 1554.8639 | | | ***1570.8952*** | 1553.8686 | [illegible] |
| 5 | 74.0600 | 471.2198 | 454.1932 | 453.2092 | 499.2147 | 482.1882 | 481.2041 | 455.2249 | 457.2041 | T | 1453.8162 | 1466.8366 | 1468.8158 | ***1499.8580*** | 1482.8315 | [illegible] |
| 6 | 86.0964 | 584.3039 | 567.2773 | 566.2933 | **612.2988** | 595.2722 | 594.2882 | 556.2726 | 570.2882 | I | 1340.7321 | 1353.7525 | 1367.7682 | 1398.8104 | 1381.7838 | [illegible] |
| 7 | 86.0964 | 697.3879 | 680.3614 | 679.3774 | **725.3828** | 708.3563 | 707.3723 | 669.3566 | 683.3723 | I | 1227.6481 | 1240.6685 | 1254.6841 | 1285.7263 | 1268.6998 | [illegible] |
| 8 | 56.0495 | 780.4250 | 763.3985 | 762.4145 | 808.4199 | 791.3934 | 790.4094 | 768.4250 | | M | 1144.6109 | 1143.6157 | | 1172.6422 | 1155.6157 | [illegible] |
| 9 | 86.0964 | 893.5091 | 876.4825 | 875.4985 | 921.5040 | 904.4775 | 903.4934 | 851.4621 | | L | 1031.5269 | 1030.5316 | | 1089.6051 | 1072.5786 | [illegible] |
| 10 | 30.0338 | 950.5306 | 933.5040 | 932.5200 | 978.5255 | 961.4989 | 960.5149 | | | G | | | | 976.5211 | 959.4945 | [illegible] |
| 11 | 74.0600 | 1051.5782 | 1034.5517 | 1033.5677 | 1079.5732 | 1062.5466 | 1061.5626 | 1035.5833 | 1037.5626 | T | 873.4577 | 886.4781 | 888.4574 | ***919.4996*** | 902.4730 | [illegible] |
| 12 | 30.0338 | 1108.5997 | 1091.5732 | 1090.5891 | 1136.5946 | 1119.5681 | 1118.5841 | | | G | | | | ***818.4519*** | 801.4254 | [illegible] |
| 13 | 74.0600 | 1209.6474 | 1192.6208 | 1191.6368 | 1237.6423 | 1220.6157 | 1219.6317 | 1193.6525 | 1195.6317 | T | 715.3886 | 728.4090 | 730.3883 | 761.4305 | 744.4039 | [illegible] |
| 14 | 30.0338 | 1266.6688 | 1249.6423 | 1248.6583 | 1294.6638 | 1277.6372 | 1276.6532 | | | G | | | | 660.3828 | 643.3562 | |
| 15 | 86.0964 | 1379.7529 | 1362.7264 | 1361.7423 | 1407.7478 | 1390.7213 | 1389.7373 | 1351.7216 | 1365.7373 | I | 545.2831 | 558.3035 | 572.3191 | ***603.3613*** | 586.3348 | |
| 16 | 44.0495 | 1450.7900 | 1433.7635 | 1432.7795 | 1478.7849 | 1461.7584 | 1460.7744 | | | A | 474.2459 | | | ***490.2772*** | 473.2507 | |
| 17 | 70.0651 | 1547.8428 | 1530.8162 | 1529.8322 | 1575.8377 | 1558.8112 | 1557.8271 | 1521.8271 | | P | 377.1932 | 376.1979 | | ***419.2401*** | 402.2136 | |
| 18 | 120.0808 | 1694.9112 | 1677.8847 | 1676.9006 | 1722.9061 | 1705.8796 | 1704.8956 | | | F | 230.1248 | | | 322.1874 | 305.1608 | |
| 19 | 129.1135 | | | | | | | | | R | 74.0237 | 73.0284 | | ***175.1190*** | 158.0924 | |

| Seq | ya | yb | Seq | ya | yb | Seq | ya | yb |
|---|---|---|---|---|---|---|---|---|
| PN | 184.1081 | 212.1030 | PNA | 255.1452 | 283.1401 | PNAT | 356.1928 | 384.1878 |

| | | | | | | | | |
|---|---|---|---|---|---|---|---|---|
| **PNATI** | 469.2769 | 497.2718 | **PNATII** | 582.3610 | 610.3559 | **PNATIIM** | 665.3981 | 693.3930 |
| **NA** | 158.0924 | 186.0873 | **NAT** | 259.1401 | 287.1350 | **NATI** | 372.2241 | 400.2191 |
| **NATII** | 485.3082 | 513.3031 | **NATIIM** | 568.3453 | 596.3402 | **NATIIML** | 681.4294 | 709.4243 |
| **AT** | 145.0972 | 173.0921 | **ATI** | 258.1812 | 286.1761 | **ATII** | 371.2653 | 399.2602 |
| **ATIIM** | 454.3024 | 482.2973 | **ATIIML** | 567.3865 | 595.3814 | **ATIIMLG** | 624.4079 | 652.4028 |
| **TI** | 187.1441 | 215.1390 | **TII** | 300.2282 | 328.2231 | **TIIM** | 383.2653 | 411.2602 |
| **TIIML** | 496.3493 | 524.3443 | **TIIMLG** | 553.3708 | 581.3657 | **TIIMLGT** | 654.4185 | 682.4134 |
| **II** | 199.1805 | 227.1754 | **IIM** | 282.2176 | 310.2125 | **IIML** | 395.3017 | 423.2966 |
| **IIMLG** | 452.3231 | 480.3180 | **IIMLGT** | 553.3708 | 581.3657 | **IIMLGTG** | 610.3923 | 638.3872 |
| **IM** | 169.1335 | 197.1285 | **IML** | 282.2176 | 310.2125 | **IMLG** | 339.2391 | 367.2340 |
| **IMLGT** | 440.2867 | 468.2817 | **IMLGTG** | 497.3082 | 525.3031 | **IMLGTGT** | 598.3559 | 626.3508 |
| **IMLGTGTG** | 655.3774 | 683.3723 | **ML** | 169.1335 | 197.1285 | **MLG** | 226.1550 | 254.1499 |
| **MLGT** | 327.2027 | 355.1976 | **MLGTG** | 384.2241 | 412.2191 | **MLGTGT** | 485.2718 | 513.2667 |
| **MLGTGTG** | 542.2933 | 570.2882 | **MLGTGTGI** | 655.3774 | 683.3723 | **LG** | 143.1179 | 171.1128 |
| **LGT** | 244.1656 | 272.1605 | **LGTG** | 301.1870 | 329.1819 | **LGTGT** | 402.2347 | 430.2296 |
| **LGTGTG** | 459.2562 | 487.2511 | **LGTGTGI** | 572.3402 | 600.3352 | **LGTGTGIA** | 643.3774 | 671.3723 |
| **GT** | 131.0815 | 159.0764 | **GTG** | 188.1030 | 216.0979 | **GTGT** | 289.1506 | 317.1456 |
| **GTGTG** | 346.1721 | 374.1670 | **GTGTGI** | 459.2562 | 487.2511 | **GTGTGIA** | 530.2933 | 558.2882 |
| **GTGTGIAP** | 627.3461 | 655.3410 | **TG** | 131.0815 | 159.0764 | **TGT** | 232.1292 | 260.1241 |
| **TGTG** | 289.1506 | 317.1456 | **TGTGI** | 402.2347 | 430.2296 | **TGTGIA** | 473.2718 | 501.2667 |
| **TGTGIAP** | 570.3246 | 598.3195 | **GT** | 131.0815 | 159.0764 | **GTG** | 188.1030 | 216.0979 |
| **GTGI** | 301.1870 | 329.1819 | **GTGIA** | 372.2241 | 400.2191 | **GTGIAP** | 469.2769 | 497.2718 |
| **GTGIAPF** | 616.3453 | 644.3402 | **TG** | 131.0815 | 159.0764 | **TGI** | 244.1656 | 272.1605 |
| **TGIA** | 315.2027 | 343.1976 | **TGIAP** | 412.2554 | 440.2504 | **TGIAPF** | 559.3239 | 587.3188 |
| **GI** | 143.1179 | 171.1128 | **GIA** | 214.1550 | 242.1499 | **GIAP** | 311.2078 | 339.2027 |
| **GIAPF** | 458.2762 | 486.2711 | **IA** | 157.1335 | 185.1285 | **IAP** | 254.1863 | 282.1812 |
| **IAPF** | 401.2547 | 429.2496 | **AP** | 141.1022 | 169.0972 | **APF** | 288.1707 | 316.1656 |
| **PF** | 217.1335 | 245.1285 | | | | | | |

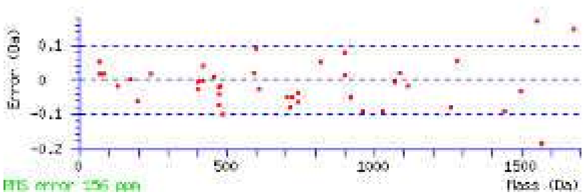


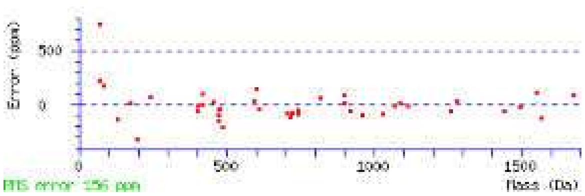


NCBI **BLAST** search of DPNATIIMLGTGTGIAPFR
(Parameters: blastp, nr protein database, expect=20000, no filter, PAM30)
Other BLAST web gateways

**All matches to this query**

| Score | Mr(calc) | Delta | Sequence |
|---|---|---|---|
| 17.2 | 1960.0088 | -0.0177 | DPNATIIMLGTGTGIAPFR |
| 11.5 | 1960.0088 | -0.0177 | DPNATVIMLATGTGIAPFR |
| 10.8 | 1960.0088 | -0.0177 | DPNATIVMLATGTGIAPFR |
| 7.5 | 1959.9133 | 0.0778 | DSEKNDAEVQAEGVLNSR |
| 4.5 | 1959.9610 | 0.0301 | GGDNNLATLDATTARVDTR |
| 4.5 | 1960.0234 | -0.0323 | VVKPTSVMVELCEARAR |
| 3.9 | 1960.0485 | -0.0574 | RLTAMSTVVALPNMLDTK |
| 3.7 | 1960.0234 | -0.0323 | AAEILKGMVTLGCKPNSR |
| 3.7 | 1959.8592 | 0.1319 | QDVCDAVAAAVADGGEGEAR |
| 3.6 | 1959.9974 | -0.0063 | VTATTINDGKRPATSSEGR |

**Mascot:** http://www.matrixscience.com/

# MATRIX SCIENCE Mascot Search Results

## Peptide View Spot no 53

MS/MS Fragmentation of **LLFEVAPLGFLIEK**
Found in **gi|118175929** in **NCBInr**, chloroplast sedoheptulose-1,7-bisphosphatase [Morus alba var. multicaulis]

Match to Query 75: 1587.902624 from(1588.909900,1+) intensity(0.0000) index(11)
Title: Label: H4, Spot_Id: 219729, Peak_List_Id: 225332, MSMS Job_Run_Id: 21774, Comment:
Data file C:\Documents and Settings\Administrator\Desktop\farah BHU\spot set plate\Farah-Garima 11May 2013\ppw_H4_136842165600.txt

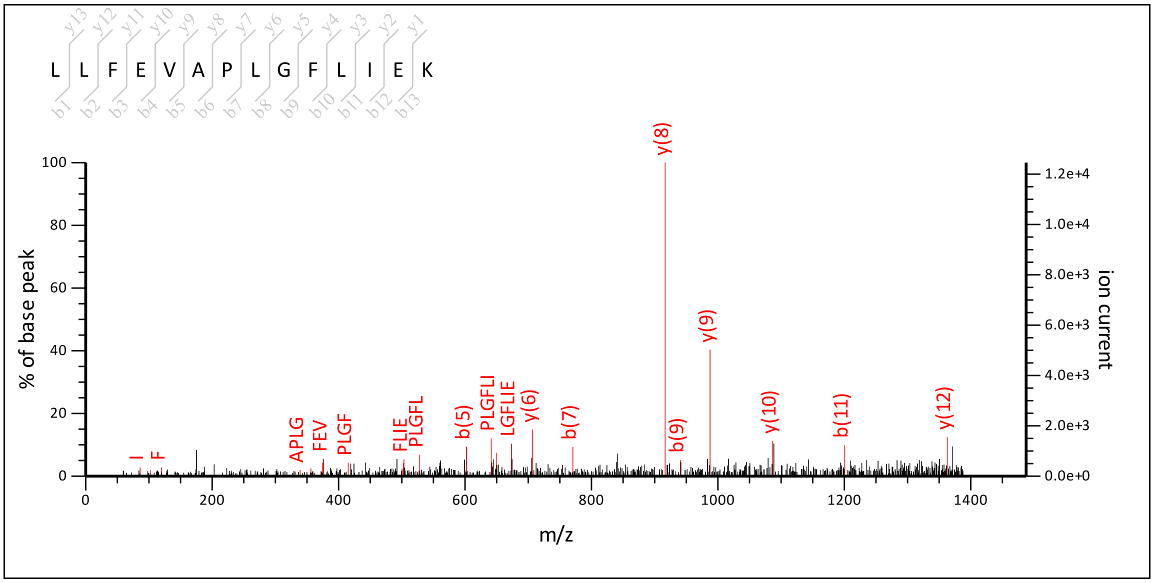


0 to 1487.58

Label all possible matches ○ Label matches used for scoring ◉

**Monoisotopic mass of neutral peptide Mr(calc):** 1587.9276
**Fixed modifications:** Carbamidomethyl (C) (apply to specified residues or termini only)
**Ions Score:** 34 **Expect:** 0.61
**Matches :** 35/225 fragment ions using 56 most intense peaks (help)

| # | Immon. | a | $a^0$ | b | $b^0$ | d | d' | Seq. | v | w | w' | y | y* | $y^0$ | # |
|---|---|---|---|---|---|---|---|---|---|---|---|---|---|---|---|
| 1 | 86.0964 | **86.0964** | | 114.0913 | | 44.0495 | | L | | | | | | | 14 |
| 2 | 86.0964 | 199.1805 | | 227.1754 | | 157.1335 | | L | 1417.7726 | 1416.7773 | | 1475.8508 | 1458.8243 | 1457.8403 | 13 |
| 3 | 120.0808 | 346.2489 | | ***374.2438*** | | | | F | 1270.7042 | | | ***1362.7668*** | 1345.7402 | 1344.7562 | 12 |
| 4 | 102.0550 | 475.2915 | 457.2809 | ***503.2864*** | 485.2758 | 417.2860 | | E | 1141.6616 | 1140.6663 | | 1215.6984 | 1198.6718 | 1197.6878 | 11 |
| 5 | 72.0808 | 574.3599 | 556.3493 | ***602.3548*** | 584.3443 | 560.3443 | | V | 1042.5932 | 1055.6136 | | ***1086.6558*** | 1069.6292 | 1068.6452 | 10 |
| 6 | 44.0495 | **645.3970** | 627.3865 | ***673.3919*** | 655.3814 | | | A | 971.5560 | | | ***987.5873*** | 970.5608 | 969.5768 | 9 |
| 7 | 70.0651 | 742.4498 | 724.4392 | ***770.4447*** | 752.4341 | 716.4341 | | P | 874.5033 | 873.5080 | | ***916.5502*** | 899.5237 | 898.5397 | 8 |
| 8 | 86.0964 | 855.5339 | 837.5233 | 883.5288 | 865.5182 | 813.4869 | | L | 761.4192 | 760.4240 | | 819.4975 | 802.4709 | 801.4869 | 7 |
| 9 | 30.0338 | 912.5553 | 894.5448 | ***940.5502*** | 922.5397 | | | G | | | | ***706.4134*** | 689.3869 | 688.4028 | 6 |
| 10 | 120.0808 | 1059.6237 | 1041.6132 | 1087.6186 | 1069.6081 | | | F | 557.3293 | | | ***649.3919*** | 632.3654 | 631.3814 | 5 |
| 11 | 86.0964 | 1172.7078 | 1154.6972 | ***1200.7027*** | 1182.6921 | 1130.6608 | | L | 444.2453 | 443.2500 | | ***502.3235*** | 485.2970 | 484.3130 | 4 |
| 12 | 86.0964 | 1285.7919 | 1267.7813 | 1313.7868 | 1295.7762 | 1257.7606 | 1271.7762 | I | 331.1612 | 344.1816 | 358.1973 | 389.2395 | 372.2129 | 371.2289 | 3 |
| 13 | 102.0550 | 1414.8345 | 1396.8239 | 1442.8294 | 1424.8188 | 1356.8290 | | E | 202.1186 | 201.1234 | | 276.1554 | 259.1288 | 258.1448 | 2 |
| 14 | 101.1073 | | | | | | | K | 74.0237 | 73.0284 | | 147.1128 | 130.0863 | | 1 |

| Seq | ya | yb | Seq | ya | yb | Seq | ya | yb |
|---|---|---|---|---|---|---|---|---|
| LF | 233.1648 | 261.1598 | LFE | 362.2074 | 390.2023 | LFEV | 461.2758 | 489.2708 |
| LFEVA | 532.3130 | 560.3079 | LFEVAP | 629.3657 | 657.3606 | FE | 249.1234 | 277.1183 |
| FEV | 348.1918 | 376.1867 | FEVA | 419.2289 | 447.2238 | FEVAP | 516.2817 | 544.2766 |

| | | | | | | | | |
|---|---|---|---|---|---|---|---|---|
| **FEVAPL** | 629.3657 | 657.3606 | **FEVAPLG** | 686.3872 | 714.3821 | **EV** | 201.1234 | 229.1183 |
| **EVA** | 272.1605 | 300.1554 | **EVAP** | 369.2132 | 397.2082 | **EVAPL** | 482.2973 | 510.2922 |
| **EVAPLG** | 539.3188 | 567.3137 | **EVAPLGF** | 686.3872 | 714.3821 | **VA** | 143.1179 | 171.1128 |
| **VAP** | 240.1707 | 268.1656 | **VAPL** | 353.2547 | 381.2496 | **VAPLG** | 410.2762 | 438.2711 |
| **VAPLGF** | 557.3446 | 585.3395 | **VAPLGFL** | 670.4287 | 698.4236 | **AP** | 141.1022 | 169.0972 |
| **APL** | 254.1863 | 282.1812 | **APLG** | 311.2078 | 339.2027 | **APLGF** | 458.2762 | 486.2711 |
| **APLGFL** | 571.3602 | 599.3552 | **APLGFLI** | 684.4443 | 712.4392 | **PL** | 183.1492 | 211.1441 |
| **PLG** | 240.1707 | 268.1656 | **PLGF** | 387.2391 | 415.2340 | **PLGFL** | 500.3231 | 528.3180 |
| **PLGFLI** | 613.4072 | 641.4021 | **LG** | 143.1179 | 171.1128 | **LGF** | 290.1863 | 318.1812 |
| **LGFL** | 403.2704 | 431.2653 | **LGFLI** | 516.3544 | 544.3493 | **LGFLIE** | 645.3970 | 673.3919 |
| **GF** | 177.1022 | 205.0972 | **GFL** | 290.1863 | 318.1812 | **GFLI** | 403.2704 | 431.2653 |
| **GFLIE** | 532.3130 | 560.3079 | **FL** | 233.1648 | 261.1598 | **FLI** | 346.2489 | 374.2438 |
| **FLIE** | 475.2915 | 503.2864 | **LI** | 199.1805 | 227.1754 | **LIE** | 328.2231 | 356.2180 |
| **IE** | 215.1390 | 243.1339 | | | | | | |

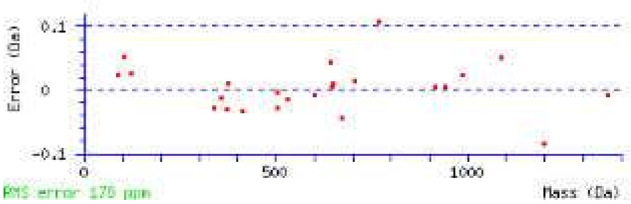


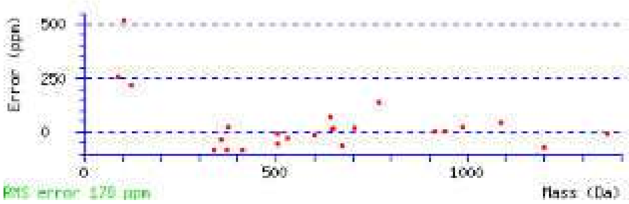


NCBI **BLAST** search of LLFEVAPLGFLIEK
(Parameters: blastp, nr protein database, expect=20000, no filter, PAM30)
Other BLAST web gateways

**All matches to this query**

| Score | Mr(calc) | Delta | Sequence |
|---|---|---|---|
| 34.0 | 1587.9276 | -0.0250 | LLFEVAPLGFLIEK |
| 15.5 | 1587.9276 | -0.0250 | IVLEAFPAVFLLEK |
| 14.9 | 1587.8304 | 0.0723 | ATQAIAPMHAAVVHR |
| 14.7 | 1587.7881 | 0.1145 | WMAGRGWAAGLAWR |
| 14.1 | 1587.7650 | 0.1377 | INWMKAGMLESHR |
| 13.5 | 1587.8151 | 0.0875 | MGGAAILLSNRQADR |
| 13.2 | 1587.8005 | 0.1021 | GFLSINSQPAVNGER |
| 13.1 | 1587.8654 | 0.0372 | MDLILAQLTTINAR |
| 12.8 | 1587.7675 | 0.1352 | NMKPDAAAASLNDVR |
| 12.7 | 1587.7536 | 0.1490 | MGQDAVAANGTAGGGRR |

**Mascot:** http://www.matrixscience.com/

# MATRIX SCIENCE Mascot Search Results

## Peptide View Spot no 53

MS/MS Fragmentation of **NEIIRFEETLYGSSR**
Found in **gi|118175929** in **NCBInr**, chloroplast sedoheptulose-1,7-bisphosphatase [Morus alba var. multicaulis]

Match to Query 82: 1812.887224 from(1813.894500,1+) intensity(0.0000) index(14)
Title: Label: H4, Spot_Id: 219729, Peak_List_Id: 225328, MSMS Job_Run_Id: 21774, Comment:
Data file C:\Documents and Settings\Administrator\Desktop\farah BHU\spot set plate\Farah-Garima 11May 2013\ppw_H4_136842165600.txt

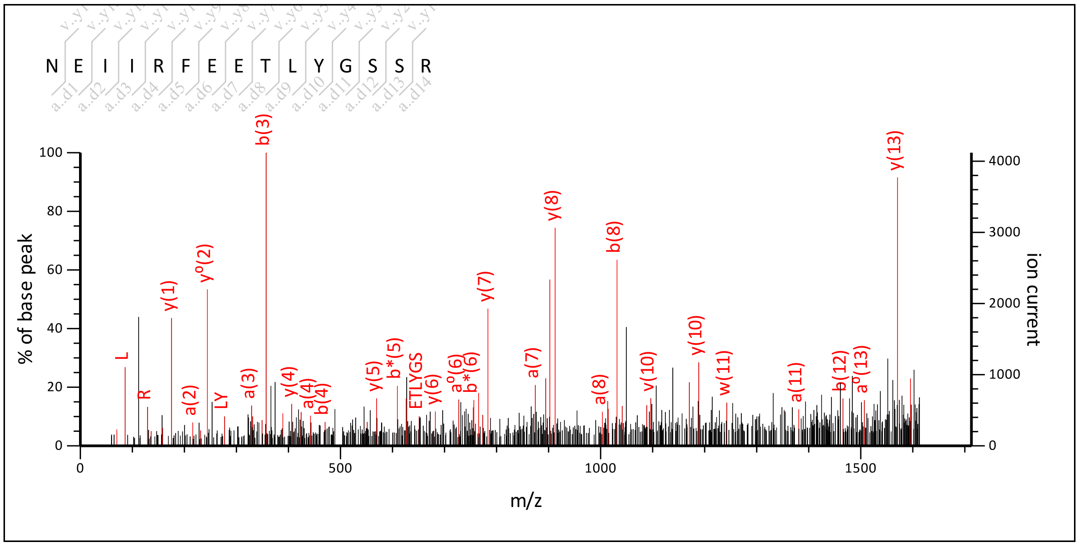


0 to 1712.7

Label all possible matches ○ Label matches used for scoring ◉

**Monoisotopic mass of neutral peptide Mr(calc):** 1812.9006
**Fixed modifications:** Carbamidomethyl (C) (apply to specified residues or termini only)
**Ions Score:** 33 **Expect:** 1.1
**Matches :** 61/269 fragment ions using 128 most intense peaks (help)

| # | Immon. | a | a* | a⁰ | b | b* | b⁰ | d | d' | Seq. | v | w | w' | y | y* | |
|---|---|---|---|---|---|---|---|---|---|---|---|---|---|---|---|---|
| 1 | 87.0553 | 87.0553 | 70.0287 | | 115.0502 | 98.0237 | | 44.0495 | | N | | | | | | |
| 2 | 102.0550 | 216.0979 | 199.0713 | 198.0873 | 244.0928 | 227.0662 | 226.0822 | 158.0924 | | E | 1625.8282 | 1624.8329 | | 1699.8650 | 1682.8384 | 16 |
| 3 | 86.0964 | 329.1819 | 312.1554 | 311.1714 | 357.1769 | 340.1503 | 339.1663 | 301.1506 | 315.1663 | I | 1512.7441 | 1525.7645 | 1539.7802 | 1570.8224 | 1553.7958 | 1 |
| 4 | 86.0964 | 442.2660 | 425.2395 | 424.2554 | 470.2609 | 453.2344 | 452.2504 | 414.2347 | 428.2504 | I | 1399.6601 | 1412.6805 | 1426.6961 | 1457.7383 | 1440.7118 | 14 |
| 5 | 129.1135 | 598.3671 | 581.3406 | 580.3566 | 626.3620 | 609.3355 | 608.3515 | 513.3031 | | R | 1243.5590 | 1242.5637 | | 1344.6543 | 1327.6277 | 1 |
| 6 | 120.0808 | 745.4355 | 728.4090 | 727.4250 | 773.4304 | 756.4039 | 755.4199 | | | F | 1096.4905 | | | 1188.5531 | 1171.5266 | 1 |
| 7 | 102.0550 | 874.4781 | 857.4516 | 856.4676 | 902.4730 | 885.4465 | 884.4625 | 816.4726 | | E | 967.4480 | 966.4527 | | 1041.4847 | 1024.4582 | 1 |
| 8 | 102.0550 | 1003.5207 | 986.4942 | 985.5102 | 1031.5156 | 1014.4891 | 1013.5051 | 945.5152 | | E | 838.4054 | 837.4101 | | 912.4421 | 895.4156 | [illegible] |
| 9 | 74.0600 | 1104.5684 | 1087.5419 | 1086.5578 | 1132.5633 | 1115.5368 | 1114.5527 | 1088.5735 | 1090.5527 | T | 737.3577 | 750.3781 | 752.3573 | 783.3995 | 766.3730 | [illegible] |
| 10 | 86.0964 | 1217.6525 | 1200.6259 | 1199.6419 | 1245.6474 | 1228.6208 | 1227.6368 | 1175.6055 | | L | 624.2736 | 623.2784 | | 682.3519 | 665.3253 | [illegible] |
| 11 | 136.0757 | 1380.7158 | 1363.6892 | 1362.7052 | 1408.7107 | 1391.6842 | 1390.7001 | | | Y | 461.2103 | | | 569.2678 | 552.2413 | [illegible] |
| 12 | 30.0338 | 1437.7373 | 1420.7107 | 1419.7267 | 1465.7322 | 1448.7056 | 1447.7216 | | | G | | | | 406.2045 | 389.1779 | [illegible] |
| 13 | 60.0444 | 1524.7693 | 1507.7427 | 1506.7587 | 1552.7642 | 1535.7377 | 1534.7536 | 1508.7744 | | S | 317.1568 | 316.1615 | | 349.1830 | 332.1565 | [illegible] |
| 14 | 60.0444 | 1611.8013 | 1594.7748 | 1593.7907 | 1639.7962 | 1622.7697 | 1621.7857 | 1595.8064 | | S | 230.1248 | 229.1295 | | 262.1510 | 245.1244 | [illegible] |
| 15 | 129.1135 | | | | | | | | | R | 74.0237 | 73.0284 | | 175.1190 | 158.0924 | |

| Seq | ya | yb | Seq | ya | yb | Seq | ya | yb |
|---|---|---|---|---|---|---|---|---|
| EI | 215.1390 | 243.1339 | EII | 328.2231 | 356.2180 | EIIR | 484.3242 | 512.3191 |
| EIIRF | 631.3926 | 659.3875 | II | 199.1805 | 227.1754 | IIR | 355.2816 | 383.2765 |
| IIRF | 502.3500 | 530.3449 | IIRFE | 631.3926 | 659.3875 | IR | 242.1975 | 270.1925 |
| IRF | 389.2659 | 417.2609 | IRFE | 518.3085 | 546.3035 | IRFEE | 647.3511 | 675.3461 |
| RF | 276.1819 | 304.1768 | RFE | 405.2245 | 433.2194 | RFEE | 534.2671 | 562.2620 |
| RFEET | 635.3148 | 663.3097 | FE | 249.1234 | 277.1183 | FEE | 378.1660 | 406.1609 |

| | | | | | | | | |
|---|---|---|---|---|---|---|---|---|
| **FEET** | 479.2136 | 507.2086 | **FEETL** | 592.2977 | 620.2926 | **EE** | 231.0975 | 259.0925 |
| **EET** | 332.1452 | 360.1401 | **EETL** | 445.2293 | 473.2242 | **EETLY** | 608.2926 | 636.2875 |
| **EETLYG** | 665.3141 | 693.3090 | **ET** | 203.1026 | 231.0975 | **ETL** | 316.1867 | 344.1816 |
| **ETLY** | 479.2500 | 507.2449 | **ETLYG** | 536.2715 | 564.2664 | **ETLYGS** | 623.3035 | 651.2984 |
| **TL** | 187.1441 | 215.1390 | **TLY** | 350.2074 | 378.2023 | **TLYG** | 407.2289 | 435.2238 |
| **TLYGS** | 494.2609 | 522.2558 | **TLYGSS** | 581.2930 | 609.2879 | **LY** | 249.1598 | 277.1547 |
| **LYG** | 306.1812 | 334.1761 | **LYGS** | 393.2132 | 421.2082 | **LYGSS** | 480.2453 | 508.2402 |
| **YG** | 193.0972 | 221.0921 | **YGS** | 280.1292 | 308.1241 | **YGSS** | 367.1612 | 395.1561 |
| **GS** | 117.0659 | 145.0608 | **GSS** | 204.0979 | 232.0928 | **SS** | 147.0764 | 175.0713 |

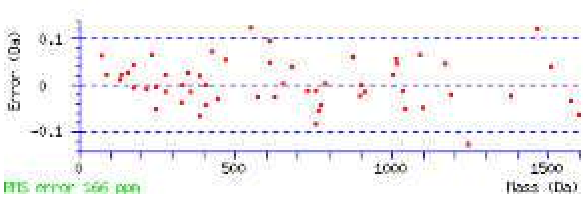


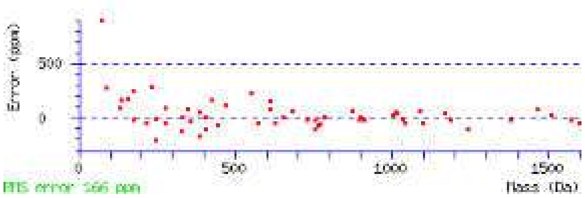


NCBI **BLAST** search of NEIIRFEETLYGSSR
(Parameters: blastp, nr protein database, expect=20000, no filter, PAM30)
Other BLAST web gateways

**All matches to this query**

| Score | Mr(calc) | Delta | Sequence |
|---|---|---|---|
| 32.6 | 1812.9006 | -0.0134 | NEIIRFEETLYGSSR |
| 14.8 | 1812.9330 | -0.0457 | QDSELAQLRVQEIER |
| 14.5 | 1812.8755 | 0.0118 | QSLAFILEDGNHEQGR |
| 13.4 | 1812.9330 | -0.0457 | QDLSTNEGSKQPAIVAR |
| 10.1 | 1812.9621 | -0.0749 | GGETIFPEAEGKLLQPK |
| 9.6 | 1812.8604 | 0.0268 | RLGSVFLEEFVMEEE |
| 9.4 | 1812.9523 | -0.0651 | GVPLNAGRFEWTPVATV |
| 9.1 | 1812.8014 | 0.0858 | QDITTNSDVGSTEDFGK |
| 8.9 | 1813.0058 | -0.1185 | SALLATTDLAGLTRGTPR |
| 8.9 | 1812.9330 | -0.0457 | RVVDLEAELSSPTNQR |

**Mascot:** http://www.matrixscience.com/

# MATRIX SCIENCE Mascot Search Results

## Peptide View Spot no 53

MS/MS Fragmentation of **LRLLFEVAPLGFLIEK**
Found in **gi|118175929** in **NCBInr**, chloroplast sedoheptulose-1,7-bisphosphatase [Morus alba var. multicaulis]

Match to Query 86: 1857.107224 from(1858.114500,1+) intensity(0.0000) index(16)
Title: Label: H4, Spot_Id: 219729, Peak_List_Id: 225334, MSMS Job_Run_Id: 21774, Comment:
Data file C:\Documents and Settings\Administrator\Desktop\farah BHU\spot set plate\Farah-Garima 11May 2013\ppw_H4_136842165600.txt

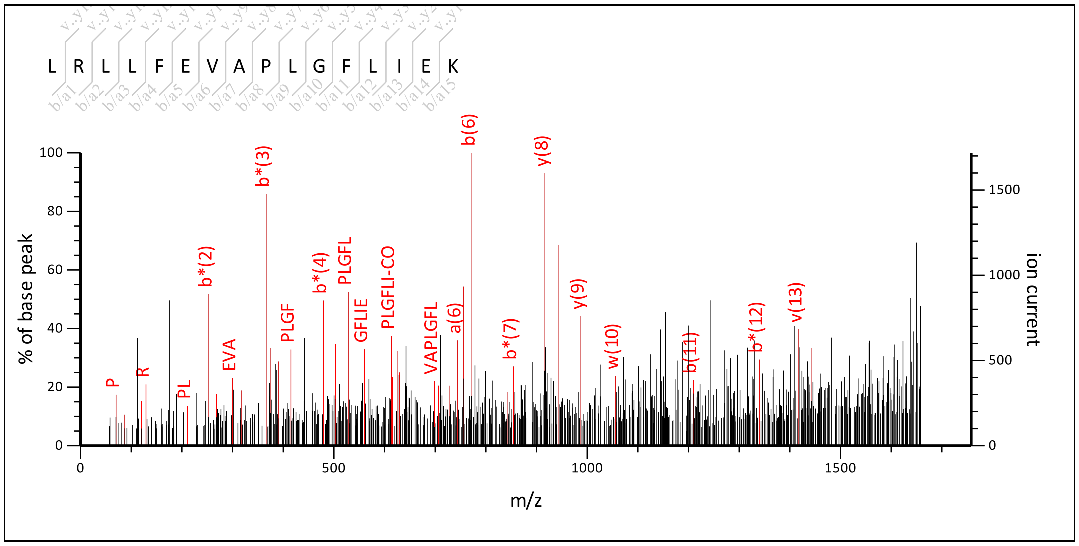


0 to 1758.02

Label all possible matches ○ Label matches used for scoring ◉

**Monoisotopic mass of neutral peptide Mr(calc):** 1857.1128
**Fixed modifications:** Carbamidomethyl (C) (apply to specified residues or termini only)
**Ions Score:** 5 **Expect:** 8.2e+02
**Matches :** 52/289 fragment ions using 114 most intense peaks (help)

| # | Immon. | a | a* | a⁰ | b | b* | b⁰ | d | d' | Seq. | v | w | w' | y | y* | |
|---|---|---|---|---|---|---|---|---|---|---|---|---|---|---|---|---|
| 1 | 86.0964 | ***86.0964*** | | | 114.0913 | | | 44.0495 | | L | | | | | | |
| 2 | 129.1135 | 242.1975 | 225.1710 | | 270.1925 | 253.1659 | | 157.1335 | | R | 1643.9407 | 1642.9455 | | 1745.0360 | 1728.0095 | [illegible] |
| 3 | 86.0964 | 355.2816 | 338.2551 | | 383.2765 | 366.2500 | | 313.2347 | | L | 1530.8566 | 1529.8614 | | 1588.9349 | 1571.9083 | [illegible] |
| 4 | 86.0964 | 468.3657 | 451.3391 | | 496.3606 | 479.3340 | | 426.3187 | | L | 1417.7726 | 1416.7773 | | 1475.8508 | 1458.8243 | [illegible] |
| 5 | 120.0808 | ***615.4341*** | 598.4075 | | 643.4290 | 626.4024 | | | | F | 1270.7042 | | | 1362.7668 | 1345.7402 | [illegible] |
| 6 | 102.0550 | ***744.4767*** | 727.4501 | 726.4661 | **772.4716** | 755.4450 | 754.4610 | 686.4712 | | E | 1141.6616 | 1140.6663 | | 1215.6984 | 1198.6718 | [illegible] |
| 7 | 72.0808 | ***843.5451*** | 826.5185 | 825.5345 | 871.5400 | 854.5135 | 853.5294 | 829.5294 | | V | 1042.5932 | 1055.6136 | | 1086.6558 | 1069.6292 | [illegible] |
| 8 | 44.0495 | 914.5822 | 897.5557 | 896.5716 | **942.5771** | 925.5506 | 924.5665 | | | A | 971.5560 | | | **987.5873** | 970.5608 | [illegible] |
| 9 | 70.0651 | 1011.6350 | 994.6084 | 993.6244 | 1039.6299 | 1022.6033 | 1021.6193 | 985.6193 | | P | 874.5033 | 873.5080 | | **916.5502** | 899.5237 | [illegible] |
| 10 | 86.0964 | 1124.7190 | 1107.6925 | 1106.7085 | 1152.7139 | 1135.6874 | 1134.7034 | 1082.6721 | | L | 761.4192 | 760.4240 | | 819.4975 | 802.4709 | [illegible] |
| 11 | 30.0338 | 1181.7405 | 1164.7139 | 1163.7299 | **1209.7354** | 1192.7089 | 1191.7248 | | | G | | | | **706.4134** | 689.3869 | [illegible] |
| 12 | 120.0808 | 1328.8089 | 1311.7824 | 1310.7983 | 1356.8038 | 1339.7773 | 1338.7933 | | | F | 557.3293 | | | 649.3919 | 632.3654 | [illegible] |
| 13 | 86.0964 | ***1441.8930*** | 1424.8664 | 1423.8824 | 1469.8879 | 1452.8613 | 1451.8773 | 1399.8460 | | L | 444.2453 | 443.2500 | | 502.3235 | 485.2970 | [illegible] |
| 14 | 86.0964 | 1554.9770 | 1537.9505 | 1536.9665 | 1582.9719 | 1565.9454 | 1564.9614 | 1526.9457 | 1540.9614 | I | 331.1612 | 344.1816 | 358.1973 | 389.2395 | 372.2129 | [illegible] |
| 15 | 102.0550 | 1684.0196 | 1666.9931 | 1666.0091 | 1712.0145 | 1694.9880 | 1694.0040 | 1626.0141 | | E | 202.1186 | 201.1234 | | 276.1554 | 259.1288 | [illegible] |
| 16 | 101.1073 | | | | | | | | | K | 74.0237 | 73.0284 | | 147.1128 | 130.0863 | |

| Seq | ya | yb | Seq | ya | yb | Seq | ya | yb |
|---|---|---|---|---|---|---|---|---|
| RL | 242.1975 | 270.1925 | RLL | 355.2816 | 383.2765 | RLLF | 502.3500 | 530.3449 |
| RLLFE | 631.3926 | 659.3875 | LL | 199.1805 | 227.1754 | LLF | 346.2489 | 374.2438 |
| LLFE | 475.2915 | 503.2864 | LLFEV | 574.3599 | 602.3548 | LLFEVA | 645.3970 | 673.3919 |
| LF | 233.1648 | 261.1598 | LFE | 362.2074 | 390.2023 | LFEV | 461.2758 | 489.2708 |
| LFEVA | 532.3130 | 560.3079 | LFEVAP | 629.3657 | 657.3606 | FE | 249.1234 | 277.1183 |

| | | | | | | | | |
|---|---|---|---|---|---|---|---|---|
| **FEV** | 348.1918 | 376.1867 | **FEVA** | 419.2289 | 447.2238 | **FEVAP** | 516.2817 | 544.2766 |
| **FEVAPL** | 629.3657 | 657.3606 | **FEVAPLG** | 686.3872 | 714.3821 | **EV** | 201.1234 | 229.1183 |
| **EVA** | 272.1605 | 300.1554 | **EVAP** | 369.2132 | 397.2082 | **EVAPL** | 482.2973 | 510.2922 |
| **EVAPLG** | 539.3188 | 567.3137 | **EVAPLGF** | 686.3872 | 714.3821 | **VA** | 143.1179 | 171.1128 |
| **VAP** | 240.1707 | 268.1656 | **VAPL** | 353.2547 | 381.2496 | **VAPLG** | 410.2762 | 438.2711 |
| **VAPLGF** | 557.3446 | 585.3395 | **VAPLGFL** | 670.4287 | 698.4236 | **AP** | 141.1022 | 169.0972 |
| **APL** | 254.1863 | 282.1812 | **APLG** | 311.2078 | 339.2027 | **APLGF** | 458.2762 | 486.2711 |
| **APLGFL** | 571.3602 | 599.3552 | **APLGFLI** | 684.4443 | 712.4392 | **PL** | 183.1492 | 211.1441 |
| **PLG** | 240.1707 | 268.1656 | **PLGF** | 387.2391 | 415.2340 | **PLGFL** | 500.3231 | 528.3180 |
| **PLGFLI** | 613.4072 | 641.4021 | **LG** | 143.1179 | 171.1128 | **LGF** | 290.1863 | 318.1812 |
| **LGFL** | 403.2704 | 431.2653 | **LGFLI** | 516.3544 | 544.3493 | **LGFLIE** | 645.3970 | 673.3919 |
| **GF** | 177.1022 | 205.0972 | **GFL** | 290.1863 | 318.1812 | **GFLI** | 403.2704 | 431.2653 |
| **GFLIE** | 532.3130 | 560.3079 | **FL** | 233.1648 | 261.1598 | **FLI** | 346.2489 | 374.2438 |
| **FLIE** | 475.2915 | 503.2864 | **LI** | 199.1805 | 227.1754 | **LIE** | 328.2231 | 356.2180 |
| **IE** | 215.1390 | 243.1339 | | | | | | |

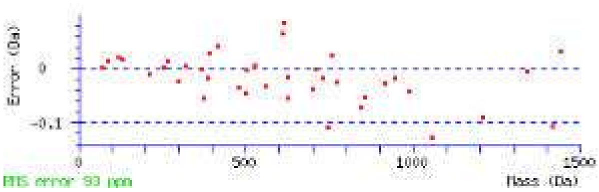


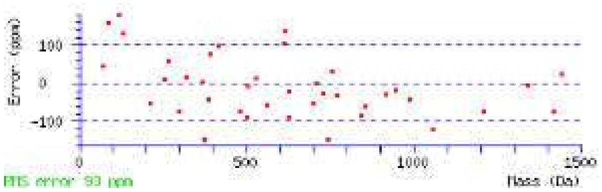


NCBI **BLAST** search of LRLLFEVAPLGFLIEK
(Parameters: blastp, nr protein database, expect=20000, no filter, PAM30)
Other BLAST web gateways

**All matches to this query**

| Score | Mr(calc) | Delta | Sequence |
|---|---|---|---|
| 6.6 | 1857.0823 | 0.0249 | SLTLLSLTSVNLTGTIPK |
| 6.0 | 1857.0281 | 0.0791 | MGLLVKGGDVLESLGEIK |
| 5.1 | 1857.1128 | -0.0055 | LRLLFEVAPLGFLIEK |
| 4.2 | 1856.9605 | 0.1467 | RSQIVGNAVFTGHASASR |
| 4.2 | 1856.9778 | 0.1294 | LADNMVQLGKEGTLAAAR |
| 4.0 | 1856.9592 | 0.1480 | ISEDVVIDATERGNIAR |
| 4.0 | 1857.0030 | 0.1043 | LKMEINGGVATPTASAVAK |
| 4.0 | 1857.0207 | 0.0865 | RALQETSGVLVISDLEK |
| 4.0 | 1857.0935 | 0.0137 | VTEVIITTTKLNAVSLR |
| 3.9 | 1856.9553 | 0.1519 | SLEVPAEGVELMLSDLR |

**Mascot:** http://www.matrixscience.com/

# MATRIX SCIENCE Mascot Search Results

## Peptide View Spot no 53

MS/MS Fragmentation of **ATFDNPEYNKLIDYYVK**
Found in **gi|118175929** in **NCBInr**, chloroplast sedoheptulose-1,7-bisphosphatase [Morus alba var. multicaulis]

Match to Query 90: 2091.987124 from(2092.994400,1+) intensity(0.0000) index(18)
Title: Label: H4, Spot_Id: 219729, Peak_List_Id: 225335, MSMS Job_Run_Id: 21774, Comment:
Data file C:\Documents and Settings\Administrator\Desktop\farah BHU\spot set plate\Farah-Garima 11May 2013\ppw_H4_136842165600.txt

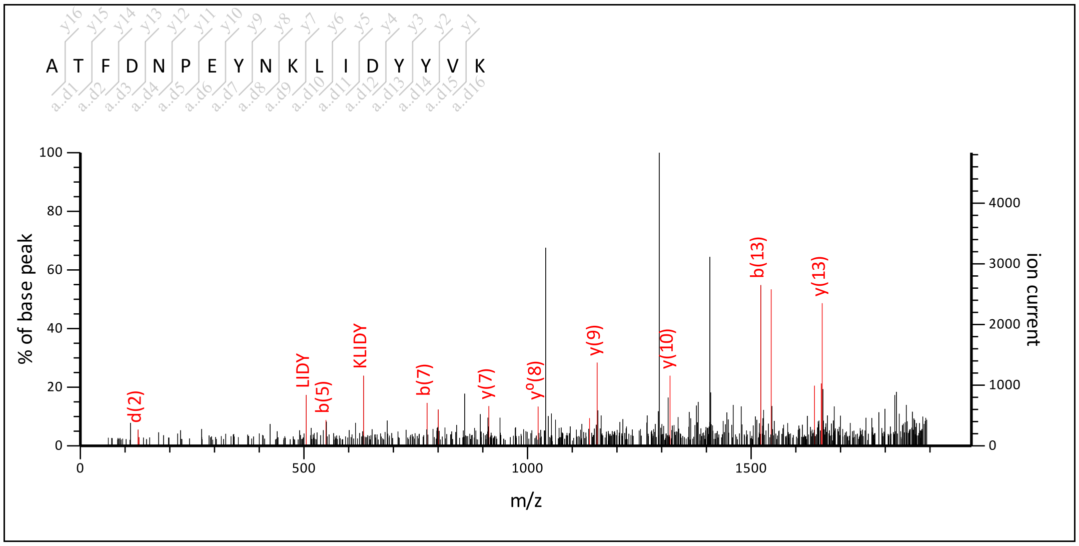


0 to 1992.68

Label all possible matches ○ Label matches used for scoring ◉

**Monoisotopic mass of neutral peptide Mr(calc):** 2092.0153
**Fixed modifications:** Carbamidomethyl (C) (apply to specified residues or termini only)
**Ions Score:** 7 **Expect:** 2.4e+02
**Matches :** 17/292 fragment ions using 57 most intense peaks (help)

| # | Immon. | a | a* | $a^0$ | b | b* | $b^0$ | d | d' | Seq. | v | w | w' | y | y* | |
|---|---|---|---|---|---|---|---|---|---|---|---|---|---|---|---|---|
| 1 | 44.0495 | 44.0495 | | | 72.0444 | | | 44.0495 | | A | | | | | | |
| 2 | 74.0600 | 145.0972 | | 127.0866 | 173.0921 | | 155.0815 | 129.1022 | 131.0815 | T | 1975.9436 | 1988.9640 | 1990.9433 | 2021.9855 | 2004.9589 | [illegible] |
| 3 | 120.0808 | 292.1656 | | 274.1550 | 320.1605 | | 302.1499 | | | F | 1828.8752 | | | 1920.9378 | 1903.9113 | [illegible] |
| 4 | 88.0393 | 407.1925 | | 389.1819 | 435.1874 | | 417.1769 | 363.2027 | | D | 1713.8483 | 1712.8530 | | 1773.8694 | 1756.8428 | [illegible] |
| 5 | 87.0553 | 521.2354 | 504.2089 | 503.2249 | **549.2304** | 532.2038 | 531.2198 | 478.2296 | | N | 1599.8053 | 1598.8101 | | ***1658.8425*** | 1641.8159 | [illegible] |
| 6 | 70.0651 | 618.2882 | 601.2617 | 600.2776 | 646.2831 | 629.2566 | 628.2726 | 592.2726 | | P | 1502.7526 | 1501.7573 | | ***1544.7995*** | 1527.7730 | [illegible] |
| 7 | 102.0550 | 747.3308 | 730.3042 | 729.3202 | **775.3257** | 758.2992 | 757.3151 | 689.3253 | | E | 1373.7100 | 1372.7147 | | 1447.7468 | 1430.7202 | [illegible] |
| 8 | 136.0757 | 910.3941 | 893.3676 | 892.3836 | 938.3890 | 921.3625 | 920.3785 | | | Y | 1210.6467 | | | ***1318.7042*** | 1301.6776 | [illegible] |
| 9 | 87.0553 | 1024.4371 | 1007.4105 | 1006.4265 | 1052.4320 | 1035.4054 | 1034.4214 | 981.4312 | | N | 1096.6037 | 1095.6085 | | ***1155.6408*** | 1138.6143 | [illegible] |
| 10 | 101.1073 | 1152.5320 | 1135.5055 | 1134.5214 | 1180.5269 | 1163.5004 | 1162.5164 | 1095.4742 | | K | 968.5088 | 967.5135 | | 1041.5979 | 1024.5714 | [illegible] |
| 11 | 86.0964 | 1265.6161 | 1248.5895 | 1247.6055 | 1293.6110 | 1276.5844 | 1275.6004 | 1223.5691 | | L | 855.4247 | 854.4294 | | ***913.5029*** | 896.4764 | [illegible] |
| 12 | 86.0964 | 1378.7001 | 1361.6736 | 1360.6896 | 1406.6951 | 1389.6685 | 1388.6845 | 1350.6688 | 1364.6845 | I | 742.3406 | 755.3610 | 769.3767 | ***800.4189*** | 783.3923 | [illegible] |
| 13 | 88.0393 | 1493.7271 | 1476.7005 | 1475.7165 | **1521.7220** | 1504.6955 | 1503.7114 | 1449.7373 | | D | 627.3137 | 626.3184 | | 687.3348 | 670.3083 | [illegible] |
| 14 | 136.0757 | 1656.7904 | 1639.7639 | 1638.7798 | 1684.7853 | 1667.7588 | 1666.7748 | | | Y | 464.2504 | | | 572.3079 | 555.2813 | |
| 15 | 136.0757 | 1819.8537 | 1802.8272 | 1801.8432 | 1847.8487 | 1830.8221 | 1829.8381 | | | Y | 301.1870 | | | 409.2445 | 392.2180 | |
| 16 | 72.0808 | 1918.9222 | 1901.8956 | 1900.9116 | 1946.9171 | 1929.8905 | 1928.9065 | 1904.9065 | | V | 202.1186 | 215.1390 | | 246.1812 | 229.1547 | |
| 17 | 101.1073 | | | | | | | | | K | 74.0237 | 73.0284 | | 147.1128 | 130.0863 | |

| Seq | ya | yb | Seq | ya | yb | Seq | ya | yb |
|---|---|---|---|---|---|---|---|---|
| TF | 221.1285 | 249.1234 | TFD | 336.1554 | 364.1503 | TFDN | 450.1983 | 478.1932 |
| TFDNP | 547.2511 | 575.2460 | TFDNPE | 676.2937 | 704.2886 | FD | 235.1077 | 263.1026 |
| FDN | 349.1506 | 377.1456 | FDNP | 446.2034 | 474.1983 | FDNPE | 575.2460 | 603.2409 |
| DN | 202.0822 | 230.0771 | DNP | 299.1350 | 327.1299 | DNPE | 428.1776 | 456.1725 |

| | | | | | | | | |
|---|---|---|---|---|---|---|---|---|
| **DNPEY** | 591.2409 | 619.2358 | **NP** | 184.1081 | 212.1030 | **NPE** | 313.1506 | 341.1456 |
| **NPEY** | 476.2140 | 504.2089 | **NPEYN** | 590.2569 | 618.2518 | **PE** | 199.1077 | 227.1026 |
| **PEY** | 362.1710 | 390.1660 | **PEYN** | 476.2140 | 504.2089 | **PEYNK** | 604.3089 | 632.3039 |
| **EY** | 265.1183 | 293.1132 | **EYN** | 379.1612 | 407.1561 | **EYNK** | 507.2562 | 535.2511 |
| **EYNKL** | 620.3402 | 648.3352 | **YN** | 250.1186 | 278.1135 | **YNK** | 378.2136 | 406.2085 |
| **YNKL** | 491.2976 | 519.2926 | **YNKLI** | 604.3817 | 632.3766 | **NK** | 215.1503 | 243.1452 |
| **NKL** | 328.2343 | 356.2292 | **NKLI** | 441.3184 | 469.3133 | **NKLID** | 556.3453 | 584.3402 |
| **KL** | 214.1914 | 242.1863 | **KLI** | 327.2755 | 355.2704 | **KLID** | 442.3024 | 470.2973 |
| **KLIDY** | 605.3657 | 633.3606 | **LI** | 199.1805 | 227.1754 | **LID** | 314.2074 | 342.2023 |
| **LIDY** | 477.2708 | 505.2657 | **LIDYY** | 640.3341 | 668.3290 | **ID** | 201.1234 | 229.1183 |
| **IDY** | 364.1867 | 392.1816 | **IDYY** | 527.2500 | 555.2449 | **IDYYV** | 626.3184 | 654.3134 |
| **DY** | 251.1026 | 279.0975 | **DYY** | 414.1660 | 442.1609 | **DYYV** | 513.2344 | 541.2293 |
| **YY** | 299.1390 | 327.1339 | **YYV** | 398.2074 | 426.2023 | **YV** | 235.1441 | 263.1390 |

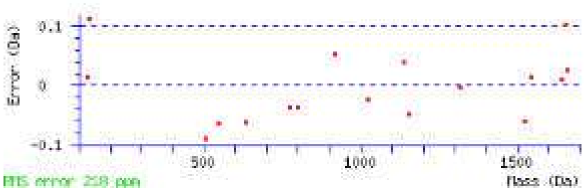


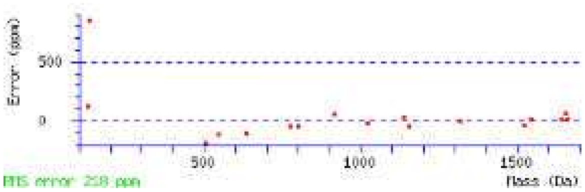


NCBI **BLAST** search of ATFDNPEYNKLIDYYVK
(Parameters: blastp, nr protein database, expect=20000, no filter, PAM30)
Other BLAST web gateways

**All matches to this query**

| Score | Mr(calc) | Delta | Sequence |
|---|---|---|---|
| 21.3 | 2092.0153 | -0.0282 | ATFDNPEYDKLINYYVK |
| 9.5 | 2092.0153 | -0.0282 | ATFDNPDYEKLINYYVK |
| 8.8 | 2092.0598 | -0.0726 | MPRVWLDYLQLLMDQR |
| 7.4 | 2092.0153 | -0.0282 | ATFDNPEYNKLIDYYVK |
| 4.4 | 2091.9305 | 0.0566 | QQPSGPGSDGAATSNSTAASSSK |
| 4.1 | 2092.0623 | -0.0752 | VIAEDTGIHSTGPSISHLMK |
| 3.0 | 2091.9618 | 0.0253 | MRFNYNNIGSSIGEFMGR |
| 2.7 | 2092.1099 | -0.1228 | RNVSVAMVLSSTPLNVSFR |
| 2.7 | 2092.0701 | -0.0830 | DALTPPRLDADELNHFLR |
| 2.5 | 2092.0636 | -0.0765 | LNQLKCHTADLLHPYNR |

**Mascot:** http://www.matrixscience.com/

# MATRIX SCIENCE Mascot Search Results

## Peptide View Spot no 56

MS/MS Fragmentation of **KLTCSYPGIK**
Found in **gi|125580** in **NCBInr**, RecName: Full=Phosphoribulokinase, chloroplastic; Short=PRK; Short=PRKase; AltName: Full=Phosphopentokinase; Flags: Precursor

Match to Query 2: 1165.594024 from(1166.601300,1+) intensity(0.0000) index(1)
Title: Label: I4, Spot_Id: 219730, Peak_List_Id: 225358, MSMS Job_Run_Id: 21775, Comment:
Data file C:\Documents and Settings\Administrator\Desktop\farah BHU\spot set plate\Farah-Garima 11May 2013\ppw_I4_136842168700.txt

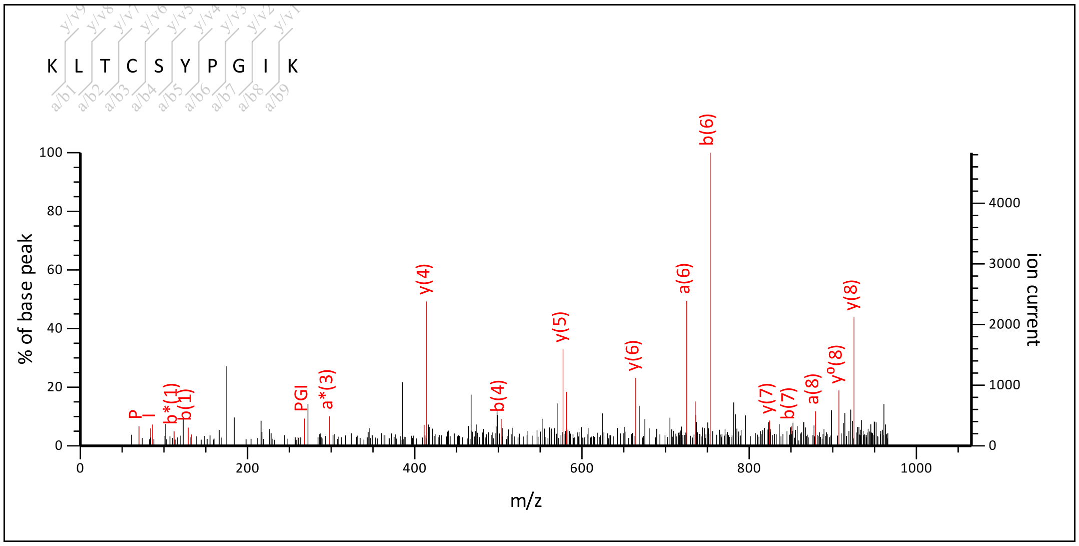


0 to 1065.85

Label all possible matches ○ Label matches used for scoring ◉

**Monoisotopic mass of neutral peptide Mr(calc):** 1165.6165
**Fixed modifications:** Carbamidomethyl (C) (apply to specified residues or termini only)
**Ions Score:** 17 **Expect:** 41
**Matches :** 27/157 fragment ions using 86 most intense peaks (help)

| # | Immon. | a | a* | $a^0$ | b | b* | $b^0$ | d | d' | Seq. | v | w | w' | y | y* | $y^0$ |
|---|---|---|---|---|---|---|---|---|---|---|---|---|---|---|---|---|
| 1 | 101.1073 | 101.1073 | 84.0808 | | ***129.1022*** | 112.0757 | | 44.0495 | | K | | | | | | |
| 2 | 86.0964 | 214.1914 | 197.1648 | | 242.1863 | 225.1598 | | 172.1444 | | L | 980.4506 | 979.4553 | | 1038.5288 | 1021.5023 | 1020.5183 |
| 3 | 74.0600 | 315.2391 | 298.2125 | 297.2285 | 343.2340 | 326.2074 | 325.2234 | 299.2442 | 301.2234 | T | 879.4029 | 892.4233 | 894.4026 | ***925.4448*** | 908.4182 | 907.4342 |
| 4 | 133.0430 | 475.2697 | 458.2432 | 457.2592 | ***503.2646*** | 486.2381 | 485.2541 | 386.2762 | | C | 719.3723 | 718.3770 | | ***824.3971*** | 807.3706 | 806.3865 |
| 5 | 60.0444 | 562.3017 | 545.2752 | 544.2912 | 590.2967 | 573.2701 | 572.2861 | 546.3068 | | S | 632.3402 | 631.3450 | | ***664.3665*** | 647.3399 | 646.3559 |
| 6 | 136.0757 | **725.3651** | 708.3385 | 707.3545 | ***753.3600*** | 736.3334 | 735.3494 | | | Y | 469.2769 | | | ***577.3344*** | 560.3079 | |
| 7 | 70.0651 | 822.4178 | 805.3913 | 804.4073 | ***850.4128*** | 833.3862 | 832.4022 | 796.4022 | | P | 372.2241 | 371.2289 | | ***414.2711*** | 397.2445 | |
| 8 | 30.0338 | **879.4393** | 862.4128 | 861.4287 | ***907.4342*** | 890.4077 | 889.4237 | | | G | | | | 317.2183 | 300.1918 | |
| 9 | 86.0964 | 992.5234 | 975.4968 | 974.5128 | 1020.5183 | 1003.4917 | 1002.5077 | 964.4921 | 978.5077 | I | 202.1186 | 215.1390 | 229.1547 | ***260.1969*** | 243.1703 | |
| 10 | 101.1073 | | | | | | | | | K | 74.0237 | 73.0284 | | 147.1128 | 130.0863 | |

| Seq | ya | yb | Seq | ya | yb | Seq | ya | yb |
|---|---|---|---|---|---|---|---|---|
| **LT** | 187.1441 | 215.1390 | **LTC** | 347.1748 | 375.1697 | **LTCS** | 434.2068 | 462.2017 |
| **LTCSY** | 597.2701 | 625.2650 | **LTCSYP** | 694.3229 | 722.3178 | **TC** | 234.0907 | 262.0856 |
| **TCS** | 321.1227 | 349.1176 | **TCSY** | 484.1860 | 512.1810 | **TCSYP** | 581.2388 | 609.2337 |
| **TCSYPG** | 638.2603 | 666.2552 | **CS** | 220.0750 | 248.0700 | **CSY** | 383.1384 | 411.1333 |
| **CSYP** | 480.1911 | 508.1860 | **CSYPG** | 537.2126 | 565.2075 | **CSYPGI** | 650.2967 | 678.2916 |
| **SY** | 223.1077 | 251.1026 | **SYP** | 320.1605 | 348.1554 | **SYPG** | 377.1819 | 405.1769 |
| **SYPGI** | 490.2660 | 518.2609 | **YP** | 233.1285 | 261.1234 | **YPG** | 290.1499 | 318.1448 |
| **YPGI** | 403.2340 | 431.2289 | **PG** | 127.0866 | 155.0815 | **PGI** | 240.1707 | 268.1656 |
| **GI** | 143.1179 | 171.1128 | | | | | | |

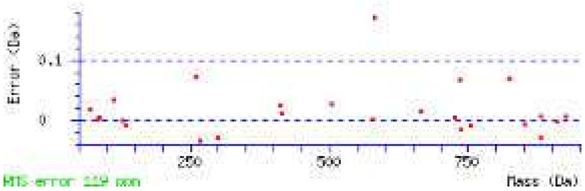


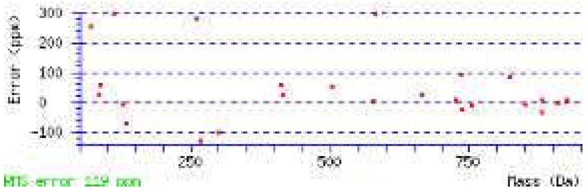


NCBI **BLAST** search of KLTCSYPGIK
(Parameters: blastp, nr protein database, expect=20000, no filter, PAM30)
Other BLAST web gateways

**All matches to this query**

| Score | Mr(calc) | Delta | Sequence |
|---|---|---|---|
| 18.6 | 1165.6819 | -0.0878 | KILPTEAAAPR |
| 17.3 | 1165.6165 | -0.0225 | KLTCSYPGIK |
| 16.3 | 1165.6026 | -0.0086 | QLPKCPGNPR |
| 15.9 | 1165.6819 | -0.0879 | NDGVGLLLLPR |
| 15.4 | 1165.6316 | -0.0376 | LAGSGQRQPPR |
| 15.4 | 1165.7070 | -0.1130 | TNLPLITAPVK |
| 14.5 | 1165.6819 | -0.0879 | AIGQALEVIPR |
| 14.5 | 1165.6819 | -0.0879 | AVAQALEVIPR |
| 14.3 | 1165.5550 | 0.0390 | MAAAAAGDAVFR |
| 14.2 | 1165.6819 | -0.0879 | GAQEAVILLPR |

**Mascot:** http://www.matrixscience.com/

# MATRIX SCIENCE Mascot Search Results

## Peptide View **Spot no 56**

MS/MS Fragmentation of **DLYEQIIAER**
Found in **gi|125580** in **NCBInr**, RecName: Full=Phosphoribulokinase, chloroplastic; Short=PRK; Short=PRKase; AltName: Full=Phosphopentokinase; Flags: Precursor

Match to Query 4: 1248.615624 from(1249.622900,1+) intensity(0.0000) index(3)
Title: Label: I4, Spot_Id: 219730, Peak_List_Id: 225357, MSMS Job_Run_Id: 21775, Comment:
Data file C:\Documents and Settings\Administrator\Desktop\farah BHU\spot set plate\Farah-Garima 11May 2013\ppw_I4_136842168700.txt

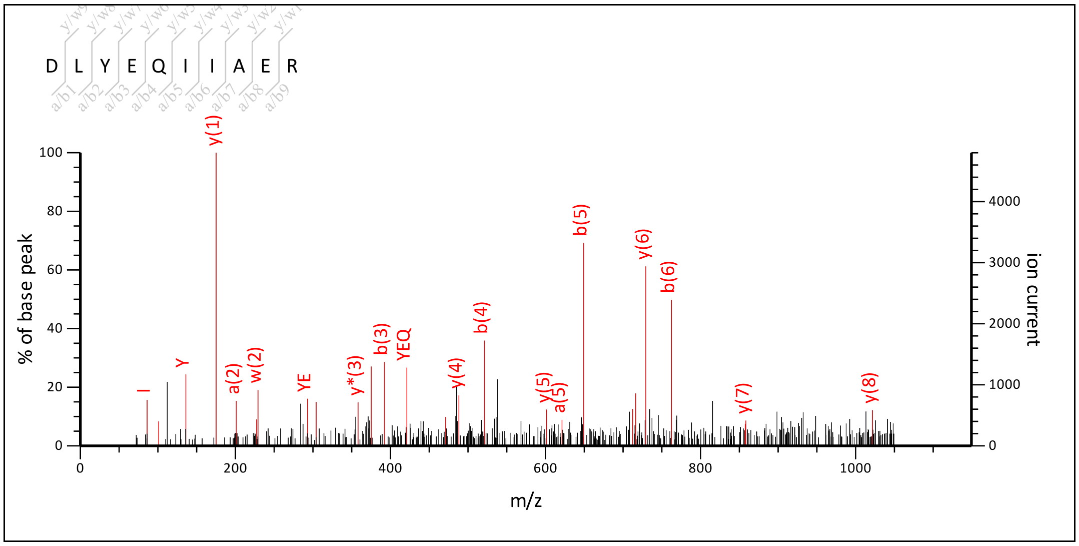


0 to 1149.34

Label all possible matches ○ Label matches used for scoring ◉

**Monoisotopic mass of neutral peptide Mr(calc):** 1248.6350
**Fixed modifications:** Carbamidomethyl (C) (apply to specified residues or termini only)
**Ions Score:** 55 **Expect:** 0.0077
**Matches :** 31/156 fragment ions using 40 most intense peaks (help)

| # | Immon. | a | a* | $a^0$ | b | b* | $b^0$ | d | d' | Seq. | v | w | w' | y | y* | y |
|---|---|---|---|---|---|---|---|---|---|---|---|---|---|---|---|---|
| 1 | 88.0393 | 88.0393 | | 70.0287 | 116.0342 | | 98.0237 | 44.0495 | | D | | | | | | |
| 2 | 86.0964 | 201.1234 | | 183.1128 | 229.1183 | | 211.1077 | 159.0764 | | L | 1076.5371 | 1075.5419 | | 1134.6154 | 1117.5888 | 1116. |
| 3 | 136.0757 | 364.1867 | | 346.1761 | 392.1816 | | 374.1710 | | | Y | 913.4738 | | | 1021.5313 | 1004.5047 | 1003. |
| 4 | 102.0550 | 493.2293 | | 475.2187 | 521.2242 | | 503.2136 | 435.2238 | | E | 784.4312 | 783.4359 | | 858.4680 | 841.4414 | 840. |
| 5 | 101.0709 | 621.2879 | 604.2613 | 603.2773 | 649.2828 | 632.2562 | 631.2722 | 564.2664 | | Q | 656.3726 | 655.3774 | | 729.4254 | 712.3988 | 711. |
| 6 | 86.0964 | 734.3719 | 717.3454 | 716.3614 | 762.3668 | 745.3403 | 744.3563 | 706.3406 | 720.3563 | I | 543.2885 | 556.3089 | 570.3246 | 601.3668 | 584.3402 | 583. |
| 7 | 86.0964 | 847.4560 | 830.4294 | 829.4454 | 875.4509 | 858.4244 | 857.4403 | 819.4247 | 833.4403 | I | 430.2045 | 443.2249 | 457.2405 | 488.2827 | 471.2562 | 470. |
| 8 | 44.0495 | 918.4931 | 901.4666 | 900.4825 | 946.4880 | 929.4615 | 928.4775 | | | A | 359.1674 | | | 375.1987 | 358.1721 | 357. |
| 9 | 102.0550 | 1047.5357 | 1030.5092 | 1029.5251 | 1075.5306 | 1058.5041 | 1057.5201 | 989.5302 | | E | 230.1248 | 229.1295 | | 304.1615 | 287.1350 | 286. |
| 10 | 129.1135 | | | | | | | | | R | 74.0237 | 73.0284 | | 175.1190 | 158.0924 | |

| Seq | ya | yb | Seq | ya | yb | Seq | ya | yb |
|---|---|---|---|---|---|---|---|---|
| LY | 249.1598 | 277.1547 | LYE | 378.2023 | 406.1973 | LYEQ | 506.2609 | 534.2558 |
| LYEQI | 619.3450 | 647.3399 | YE | 265.1183 | 293.1132 | YEQ | 393.1769 | 421.1718 |
| YEQI | 506.2609 | 534.2558 | YEQII | 619.3450 | 647.3399 | YEQIIA | 690.3821 | 718.3770 |
| EQ | 230.1135 | 258.1084 | EQI | 343.1976 | 371.1925 | EQII | 456.2817 | 484.2766 |
| EQIIA | 527.3188 | 555.3137 | EQIIAE | 656.3614 | 684.3563 | QI | 214.1550 | 242.1499 |
| QII | 327.2391 | 355.2340 | QIIA | 398.2762 | 426.2711 | QIIAE | 527.3188 | 555.3137 |
| II | 199.1805 | 227.1754 | IIA | 270.2176 | 298.2125 | IIAE | 399.2602 | 427.2551 |
| IA | 157.1335 | 185.1285 | IAE | 286.1761 | 314.1710 | AE | 173.0921 | 201.0870 |

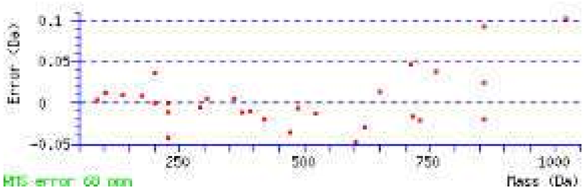


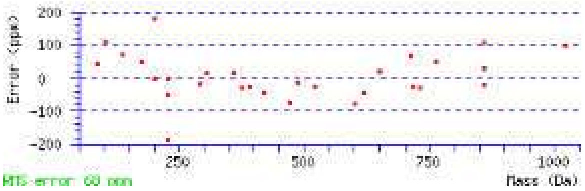


NCBI **BLAST** search of DLYEQIIAER
(Parameters: blastp, nr protein database, expect=20000, no filter, PAM30)
Other BLAST web gateways

**All matches to this query**

| Score | Mr(calc) | Delta | Sequence |
|---|---|---|---|
| 54.8 | 1248.6350 | -0.0194 | DLYEQIIAER |
| 25.2 | 1248.6285 | -0.0129 | LFVQLQEGCR |
| 20.3 | 1248.6714 | -0.0557 | LDYLLEKAER |
| 19.8 | 1248.6714 | -0.0557 | EYEVKLAELR |
| 18.6 | 1248.5267 | 0.0889 | IDYCCYTVR |
| 17.2 | 1248.6649 | -0.0492 | WTSLLQKMAR |
| 16.1 | 1248.6132 | 0.0024 | DDMRDLILSR |
| 15.9 | 1248.6462 | -0.0306 | DIEYVRGELR |
| 15.9 | 1248.5834 | 0.0323 | DSSDEDLLISR |
| 15.3 | 1248.6318 | -0.0162 | MDMVRLLAER |

**Mascot:** http://www.matrixscience.com/

# MATRIX SCIENCE Mascot Search Results

## Peptide View Spot no 56

MS/MS Fragmentation of **FYGEVTQQMLK**
Found in **gi|125580** in **NCBInr**, RecName: Full=Phosphoribulokinase, chloroplastic; Short=PRK; Short=PRKase; AltName: Full=Phosphopentokinase; Flags: Precursor

Match to Query 7: 1358.616524 from(1359.623800,1+) intensity(0.0000) index(6)
Title: Label: I4, Spot_Id: 219730, Peak_List_Id: 225348, MSMS Job_Run_Id: 21775, Comment:
Data file C:\Documents and Settings\Administrator\Desktop\farah BHU\spot set plate\Farah-Garima 11May 2013\ppw_I4_136842168700.txt

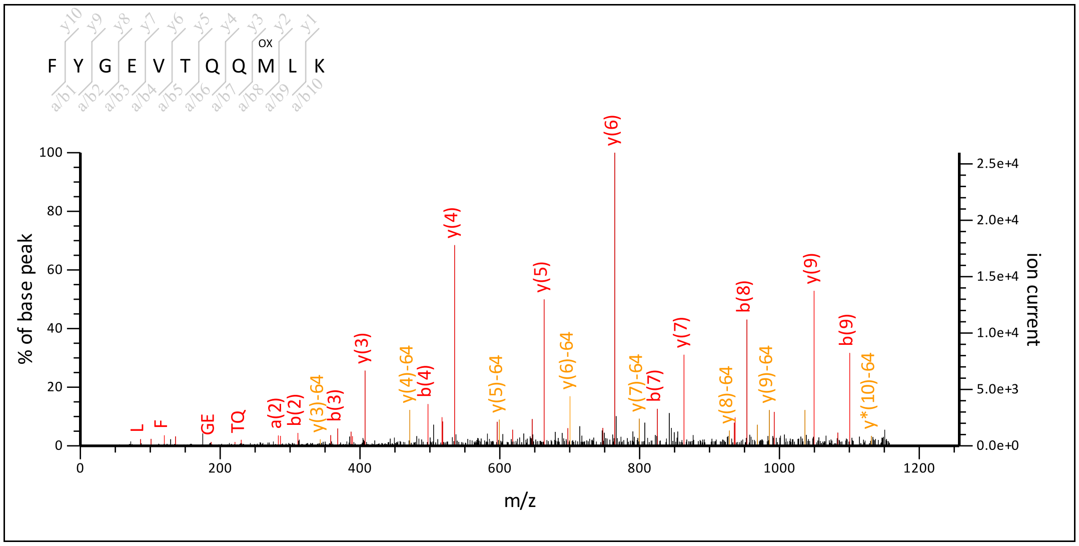


0 to 1257.23

Label all possible matches ○ Label matches used for scoring ◉

```
Monoisotopic mass of neutral peptide Mr(calc): 1358.6540
Fixed modifications: Carbamidomethyl (C) (apply to specified residues or termini only)
Variable modifications:
M9      : Oxidation (M), with neutral losses 0.0000(shown in table), 63.9983
Ions Score: 58  Expect: 0.0027
Matches : 50/226 fragment ions using 57 most intense peaks   (help)
```

| # | Immon. | a | a* | $a^0$ | b | b* | $b^0$ | d | d' | Seq. | v | w | w' | y | y* | y |
|---|---|---|---|---|---|---|---|---|---|---|---|---|---|---|---|---|
| 1 | 120.0808 | 120.0808 | | | 148.0757 | | | 44.0495 | | F | | | | | | |
| 2 | 136.0757 | 283.1441 | | | 311.1390 | | | | | Y | 1104.5354 | | | 1212.5929 | 1195.5664 | 1194. |
| 3 | 30.0338 | 340.1656 | | | 368.1605 | | | | | G | | | | 1049.5296 | 1032.5030 | 1031. |
| 4 | 102.0550 | 469.2082 | | 451.1976 | 497.2031 | | 479.1925 | 411.2027 | | E | 918.4713 | 917.4761 | | 992.5081 | 975.4816 | 974. |
| 5 | 72.0808 | 568.2766 | | 550.2660 | 596.2715 | | 578.2609 | 554.2609 | | V | 819.4029 | 832.4233 | | 863.4655 | 846.4390 | 845. |
| 6 | 74.0600 | 669.3243 | | 651.3137 | 697.3192 | | 679.3086 | 653.3293 | 655.3086 | T | 718.3552 | 731.3756 | 733.3549 | 764.3971 | 747.3706 | 746. |
| 7 | 101.0709 | 797.3828 | 780.3563 | 779.3723 | 825.3777 | 808.3512 | 807.3672 | 740.3614 | | Q | 590.2967 | 589.3014 | | 663.3494 | 646.3229 | |
| 8 | 101.0709 | 925.4414 | 908.4149 | 907.4308 | 953.4363 | 936.4098 | 935.4258 | 868.4199 | | Q | 462.2381 | 461.2428 | | 535.2908 | 518.2643 | |
| 9 | 120.0478 | 1072.4768 | 1055.4503 | 1054.4662 | 1100.4717 | 1083.4452 | 1082.4612 | 996.4785 | | M | 315.2027 | 314.2074 | | 407.2323 | 390.2057 | |
| 10 | 86.0964 | 1185.5609 | 1168.5343 | 1167.5503 | 1213.5558 | 1196.5292 | 1195.5452 | 1143.5139 | | L | 202.1186 | 201.1234 | | 260.1969 | 243.1703 | |
| 11 | 101.1073 | | | | | | | | | K | 74.0237 | 73.0284 | | 147.1128 | 130.0863 | |

| Seq | ya | yb | Seq | ya | yb | Seq | ya | yb |
|---|---|---|---|---|---|---|---|---|
| YG | 193.0972 | 221.0921 | YGE | 322.1397 | 350.1347 | YGEV | 421.2082 | 449.2031 |
| YGEVT | 522.2558 | 550.2508 | YGEVTQ | 650.3144 | 678.3093 | GE | 159.0764 | 187.0713 |
| GEV | 258.1448 | 286.1397 | GEVT | 359.1925 | 387.1874 | GEVTQ | 487.2511 | 515.2460 |
| GEVTQQ | 615.3097 | 643.3046 | EV | 201.1234 | 229.1183 | EVT | 302.1710 | 330.1660 |
| EVTQ | 430.2296 | 458.2245 | EVTQQ | 558.2882 | 586.2831 | VT | 173.1285 | 201.1234 |
| VTQ | 301.1870 | 329.1819 | VTQQ | 429.2456 | 457.2405 | VTQQM | 576.2810 | 604.2759 |
| VTQQML | 689.3651 | 717.3600 | TQ | 202.1186 | 230.1135 | TQQ | 330.1772 | 358.1721 |
| TQQM | 477.2126 | 505.2075 | TQQML | 590.2967 | 618.2916 | QQ | 229.1295 | 257.1244 |
| QQM | 376.1649 | 404.1598 | QQML | 489.2490 | 517.2439 | QM | 248.1063 | 276.1013 |

| | | | | | | | | |
|---|---|---|---|---|---|---|---|---|
| QML | 361.1904 | 389.1853 | ML | 233.1318 | 261.1267 | | | |

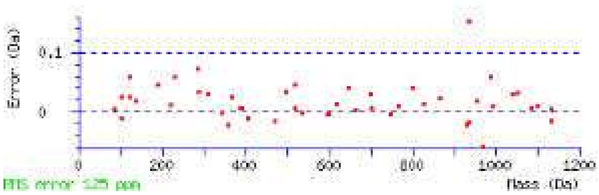


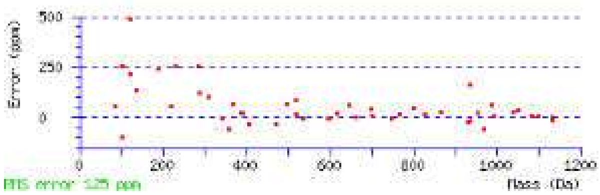


NCBI **BLAST** search of FYGEVTQQMLK
(Parameters: blastp, nr protein database, expect=20000, no filter, PAM30)
Other BLAST web gateways

**All matches to this query**

| Score | Mr(calc) | Delta | Sequence |
|---|---|---|---|
| 58.3 | 1358.6540 | -0.0375 | FYGEVTQQMLK |
| 17.7 | 1358.5674 | 0.0492 | MDGDFEGGRFGR |
| 15.9 | 1358.6302 | -0.0137 | CVFSHHGFVNR |
| 15.1 | 1358.7089 | -0.0923 | DKPVRTQISCR |
| 14.4 | 1358.6844 | -0.0678 | YIHDAPQRFGR |
| 13.8 | 1358.7041 | -0.0876 | GQSLEVVEELTR |
| 13.7 | 1358.6976 | -0.0811 | LAQIAVEKDCGR |
| 13.4 | 1358.7095 | -0.0930 | YTIGYVARGFGR |
| 13.2 | 1358.6904 | -0.0739 | WLGIEEPQLMK |
| 13.1 | 1358.6765 | -0.0600 | AGTLPHFMERGK |

**Mascot:** http://www.matrixscience.com/

# MATRIX SCIENCE Mascot Search Results

## Peptide View Spot no 56

MS/MS Fragmentation of **KPDFDAFIDPQK**
Found in **gi|125580** in **NCBInr**, RecName: Full=Phosphoribulokinase, chloroplastic; Short=PRK; Short=PRKase; AltName: Full=Phosphopentokinase; Flags: Precursor

Match to Query 8: 1419.661524 from(1420.668800,1+) intensity(0.0000) index(7)
Title: Label: I4, Spot_Id: 219730, Peak_List_Id: 225353, MSMS Job_Run_Id: 21775, Comment:
Data file C:\Documents and Settings\Administrator\Desktop\farah BHU\spot set plate\Farah-Garima 11May 2013\ppw_I4_136842168700.txt

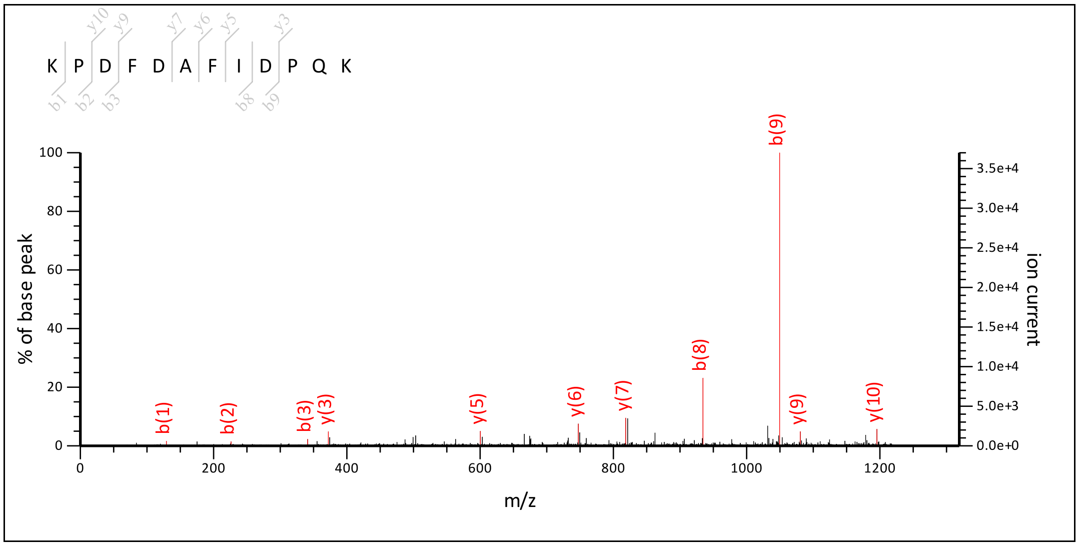


0 to 1319.14

Label all possible matches ○ Label matches used for scoring ◉

**Monoisotopic mass of neutral peptide Mr(calc):** 1419.7034
**Fixed modifications:** Carbamidomethyl (C) (apply to specified residues or termini only)
**Ions Score:** 63 **Expect:** 0.001
**Matches :** 13/201 fragment ions using 12 most intense peaks (help)

| # | Immon. | a | a* | $a^0$ | b | b* | $b^0$ | d | d' | Seq. | v | w | w' | y | y* | |
|---|---|---|---|---|---|---|---|---|---|---|---|---|---|---|---|---|
| 1 | 101.1073 | 101.1073 | 84.0808 | | ***129.1022*** | 112.0757 | | 44.0495 | | K | | | | | | |
| 2 | 70.0651 | 198.1601 | 181.1335 | | ***226.1550*** | 209.1285 | | 172.1444 | | P | 1250.5688 | 1249.5735 | | 1292.6157 | 1275.5892 | 1274 |
| 3 | 88.0393 | 313.1870 | 296.1605 | 295.1765 | ***341.1819*** | 324.1554 | 323.1714 | 269.1972 | | D | 1135.5419 | 1134.5466 | | ***1195.5630*** | 1178.5364 | 1177 |
| 4 | 120.0808 | 460.2554 | 443.2289 | 442.2449 | 488.2504 | 471.2238 | 470.2398 | | | F | 988.4734 | | | ***1080.5360*** | 1063.5095 | 1062 |
| 5 | 88.0393 | 575.2824 | 558.2558 | 557.2718 | 603.2773 | 586.2508 | 585.2667 | 531.2926 | | D | 873.4465 | 872.4512 | | 933.4676 | 916.4411 | 915 |
| 6 | 44.0495 | 646.3195 | 629.2930 | 628.3089 | 674.3144 | 657.2879 | 656.3039 | | | A | 802.4094 | | | ***818.4407*** | 801.4141 | 800 |
| 7 | 120.0808 | 793.3879 | 776.3614 | 775.3774 | 821.3828 | 804.3563 | 803.3723 | | | F | 655.3410 | | | ***747.4036*** | 730.3770 | 729 |
| 8 | 86.0964 | 906.4720 | 889.4454 | 888.4614 | ***934.4669*** | 917.4403 | 916.4563 | 878.4407 | 892.4563 | I | 542.2569 | 555.2773 | 569.2930 | ***600.3352*** | 583.3086 | 582 |
| 9 | 88.0393 | 1021.4989 | 1004.4724 | 1003.4884 | ***1049.4938*** | 1032.4673 | 1031.4833 | 977.5091 | | D | 427.2300 | 426.2347 | | 487.2511 | 470.2245 | 469 |
| 10 | 70.0651 | 1118.5517 | 1101.5251 | 1100.5411 | 1146.5466 | 1129.5201 | 1128.5360 | 1092.5360 | | P | 330.1772 | 329.1819 | | ***372.2241*** | 355.1976 | |
| 11 | 101.0709 | 1246.6103 | 1229.5837 | 1228.5997 | 1274.6052 | 1257.5786 | 1256.5946 | 1189.5888 | | Q | 202.1186 | 201.1234 | | 275.1714 | 258.1448 | |
| 12 | 101.1073 | | | | | | | | | K | 74.0237 | 73.0284 | | 147.1128 | 130.0863 | |

| Seq | ya | yb | Seq | ya | yb | Seq | ya | yb |
|---|---|---|---|---|---|---|---|---|
| PD | 185.0921 | 213.0870 | PDF | 332.1605 | 360.1554 | PDFD | 447.1874 | 475.1823 |
| PDFDA | 518.2245 | 546.2195 | PDFDAF | 665.2930 | 693.2879 | DF | 235.1077 | 263.1026 |
| DFD | 350.1347 | 378.1296 | DFDA | 421.1718 | 449.1667 | DFDAF | 568.2402 | 596.2351 |
| DFDAFI | 681.3243 | 709.3192 | FD | 235.1077 | 263.1026 | FDA | 306.1448 | 334.1397 |
| FDAF | 453.2132 | 481.2082 | FDAFI | 566.2973 | 594.2922 | FDAFID | 681.3243 | 709.3192 |
| DA | 159.0764 | 187.0713 | DAF | 306.1448 | 334.1397 | DAFI | 419.2289 | 447.2238 |
| DAFID | 534.2558 | 562.2508 | DAFIDP | 631.3086 | 659.3035 | AF | 191.1179 | 219.1128 |
| AFI | 304.2020 | 332.1969 | AFID | 419.2289 | 447.2238 | AFIDP | 516.2817 | 544.2766 |
| AFIDPQ | 644.3402 | 672.3352 | FI | 233.1648 | 261.1598 | FID | 348.1918 | 376.1867 |

| | | | | | | | | |
|---|---|---|---|---|---|---|---|---|
| **FIDP** | 445.2445 | 473.2395 | **FIDPQ** | 573.3031 | 601.2980 | **ID** | 201.1234 | 229.1183 |
| **IDP** | 298.1761 | 326.1710 | **IDPQ** | 426.2347 | 454.2296 | **DP** | 185.0921 | 213.0870 |
| **DPQ** | 313.1506 | 341.1456 | **PQ** | 198.1237 | 226.1186 | | | |

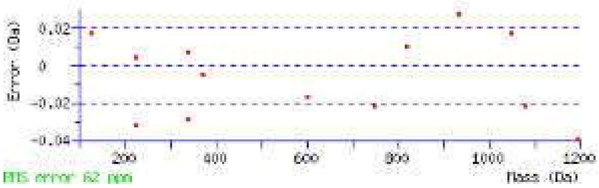


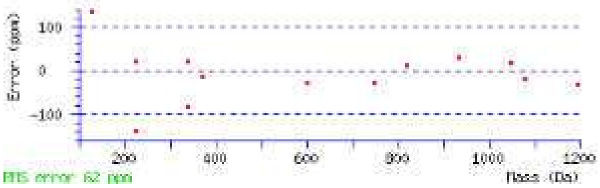


NCBI **BLAST** search of KPDFDAFIDPQK
(Parameters: blastp, nr protein database, expect=20000, no filter, PAM30)
Other BLAST web gateways

**All matches to this query**

| Score | Mr(calc) | Delta | Sequence |
|---|---|---|---|
| 63.0 | 1419.7034 | -0.0419 | KPDFDAFIDPQK |
| 22.0 | 1419.6001 | 0.0614 | ELEDLEESDEGR |
| 17.7 | 1419.7214 | -0.0599 | AGTAAMNSVVMPKK |
| 15.4 | 1419.6452 | 0.0163 | ELEDNMWAEKR |
| 15.2 | 1419.7180 | -0.0565 | KGGMGTAAGDLAFPK |
| 14.8 | 1419.7066 | -0.0451 | SGLNSTSGGESRLR |
| 14.3 | 1419.6776 | -0.0161 | RNSSASISDMQPK |
| 14.3 | 1419.7214 | -0.0599 | AGTAAMNSVVMPKK |
| 13.5 | 1419.7027 | -0.0412 | SVANDLEMELKR |
| 12.8 | 1419.5862 | 0.0753 | KGGEGGEGGGDEESR |

**Mascot:** http://www.matrixscience.com/

# MATRIX SCIENCE Mascot Search Results

## Peptide View Spot no 56

MS/MS Fragmentation of **ANDFDLMYEQVK**
Found in **gi|125580** in **NCBInr**, RecName: Full=Phosphoribulokinase, chloroplastic; Short=PRK; Short=PRKase; AltName: Full=Phosphopentokinase; Flags: Precursor

Match to Query 10: 1487.624324 from(1488.631600,1+) intensity(0.0000) index(9)
Title: Label: I4, Spot_Id: 219730, Peak_List_Id: 225349, MSMS Job_Run_Id: 21775, Comment:
Data file C:\Documents and Settings\Administrator\Desktop\farah BHU\spot set plate\Farah-Garima 11May 2013\ppw_I4_136842168700.txt

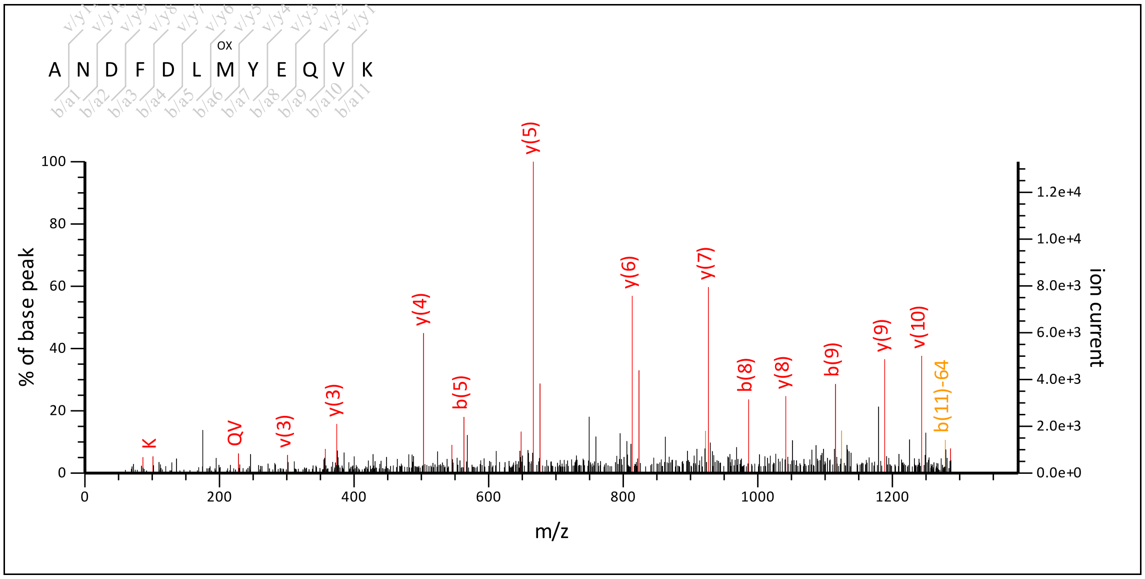


0 to 1386.91

Label all possible matches ○ Label matches used for scoring ◉

**Monoisotopic mass of neutral peptide Mr(calc):** 1487.6602
**Fixed modifications:** Carbamidomethyl (C) (apply to specified residues or termini only)
**Variable modifications:**
**M7** : Oxidation (M), with neutral losses 0.0000(shown in table), 63.9983
**Ions Score:** 70 **Expect:** 0.00013
**Matches :** 29/280 fragment ions using 26 most intense peaks (help)

| # | Immon. | a | a* | $a^0$ | b | b* | $b^0$ | d | Seq. | v | w | y | y* | $y^0$ | # |
|---|---|---|---|---|---|---|---|---|---|---|---|---|---|---|---|
| 1 | 44.0495 | 44.0495 | | | 72.0444 | | | 44.0495 | A | | | | | | 12 |
| 2 | 87.0553 | 158.0924 | 141.0659 | | 186.0873 | 169.0608 | | 115.0866 | N | 1358.5933 | 1357.5980 | 1417.6304 | 1400.6039 | 1399.6198 | 11 |
| 3 | 88.0393 | 273.1193 | 256.0928 | 255.1088 | 301.1143 | 284.0877 | 283.1037 | 229.1295 | D | 1243.5664 | 1242.5711 | 1303.5875 | 1286.5609 | 1285.5769 | 10 |
| 4 | 120.0808 | 420.1878 | 403.1612 | 402.1772 | 448.1827 | 431.1561 | 430.1721 | | F | 1096.4979 | | 1188.5605 | 1171.5340 | 1170.5500 | 9 |
| 5 | 88.0393 | 535.2147 | 518.1882 | 517.2041 | 563.2096 | 546.1831 | 545.1991 | 491.2249 | D | 981.4710 | 980.4757 | 1041.4921 | 1024.4656 | 1023.4816 | 8 |
| 6 | 86.0964 | 648.2988 | 631.2722 | 630.2882 | 676.2937 | 659.2671 | 658.2831 | 606.2518 | L | 868.3869 | 867.3917 | 926.4652 | 909.4386 | 908.4546 | 7 |
| 7 | 120.0478 | 795.3342 | 778.3076 | 777.3236 | 823.3291 | 806.3025 | 805.3185 | 719.3359 | M | 721.3515 | 720.3563 | 813.3811 | 796.3546 | 795.3706 | 6 |
| 8 | 136.0757 | 958.3975 | 941.3709 | 940.3869 | 986.3924 | 969.3659 | 968.3818 | | Y | 558.2882 | | 666.3457 | 649.3192 | 648.3352 | 5 |
| 9 | 102.0550 | 1087.4401 | 1070.4135 | 1069.4295 | 1115.4350 | 1098.4085 | 1097.4244 | 1029.4346 | E | 429.2456 | 428.2504 | 503.2824 | 486.2558 | 485.2718 | 4 |
| 10 | 101.0709 | 1215.4987 | 1198.4721 | 1197.4881 | 1243.4936 | 1226.4670 | 1225.4830 | 1158.4772 | Q | 301.1870 | 300.1918 | 374.2398 | 357.2132 | | 3 |
| 11 | 72.0808 | 1314.5671 | 1297.5405 | 1296.5565 | 1342.5620 | 1325.5354 | 1324.5514 | 1300.5514 | V | 202.1186 | 215.1390 | 246.1812 | 229.1547 | | 2 |
| 12 | 101.1073 | | | | | | | | K | 74.0237 | 73.0284 | 147.1128 | 130.0863 | | 1 |

| Seq | ya | yb | Seq | ya | yb | Seq | ya | yb |
|---|---|---|---|---|---|---|---|---|
| ND | 202.0822 | 230.0771 | NDF | 349.1506 | 377.1456 | NDFD | 464.1776 | 492.1725 |
| NDFDL | 577.2617 | 605.2566 | DF | 235.1077 | 263.1026 | DFD | 350.1347 | 378.1296 |
| DFDL | 463.2187 | 491.2136 | DFDLM | 610.2541 | 638.2490 | FD | 235.1077 | 263.1026 |
| FDL | 348.1918 | 376.1867 | FDLM | 495.2272 | 523.2221 | FDLMY | 658.2905 | 686.2854 |
| | | | | | | | | |

| | | | | | | | | |
|---|---|---|---|---|---|---|---|---|
| **DL** | 201.1234 | 229.1183 | **DLM** | 348.1588 | 376.1537 | **DLMY** | 511.2221 | 539.2170 |
| **DLMYE** | 640.2647 | 668.2596 | **LM** | 233.1318 | 261.1267 | **LMY** | 396.1952 | 424.1901 |
| **LMYE** | 525.2377 | 553.2327 | **LMYEQ** | 653.2963 | 681.2912 | **MY** | 283.1111 | 311.1060 |
| **MYE** | 412.1537 | 440.1486 | **MYEQ** | 540.2123 | 568.2072 | **MYEQV** | 639.2807 | 667.2756 |
| **YE** | 265.1183 | 293.1132 | **YEQ** | 393.1769 | 421.1718 | **YEQV** | 492.2453 | 520.2402 |
| **EQ** | 230.1135 | 258.1084 | **EQV** | 329.1819 | 357.1769 | **QV** | 200.1394 | 228.1343 |

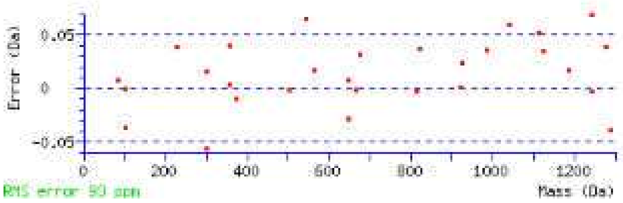


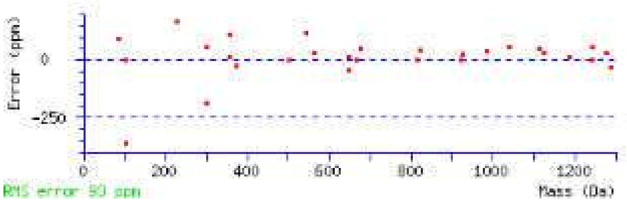


NCBI **BLAST** search of ANDFDLMYEQVK
(Parameters: blastp, nr protein database, expect=20000, no filter, PAM30)
Other BLAST web gateways

**All matches to this query**

| Score | Mr(calc) | Delta | Sequence |
|---|---|---|---|
| 70.1 | 1487.6602 | -0.0359 | ANDFDLMYEQVK |
| 22.8 | 1487.7402 | -0.1159 | DSMVNVPKNLEAR |
| 21.8 | 1487.6602 | -0.0359 | AQDFELMYEQAK |
| 19.9 | 1487.6966 | -0.0723 | LLEMYFDLGDTR |
| 19.2 | 1487.7270 | -0.1026 | SDRFGLFAAHPDR |
| 18.2 | 1487.7514 | -0.1271 | NQMEGRIPTSLSR |
| 17.9 | 1487.7270 | -0.1026 | LDHHYHSQPQVK |
| 17.9 | 1487.6899 | -0.0656 | AREGGAMGAGAGDAAAR |
| 17.1 | 1487.7402 | -0.1159 | AGEAQVLVASDAMAR |
| 16.9 | 1487.7402 | -0.1159 | AKDTMIPLNDAQR |

**Mascot:** http://www.matrixscience.com/

# MATRIX SCIENCE Mascot Search Results

## Peptide View Spot no 56

MS/MS Fragmentation of **GHSLESIKASIEAR**
Found in **gi|125580** in **NCBInr**, RecName: Full=Phosphoribulokinase, chloroplastic; Short=PRK; Short=PRKase; AltName: Full=Phosphopentokinase; Flags: Precursor

Match to Query 11: 1496.772024 from(1497.779300,1+) intensity(0.0000) index(10)
Title: Label: I4, Spot_Id: 219730, Peak_List_Id: 225352, MSMS Job_Run_Id: 21775, Comment:
Data file C:\Documents and Settings\Administrator\Desktop\farah BHU\spot set plate\Farah-Garima 11May 2013\ppw_I4_136842168700.txt

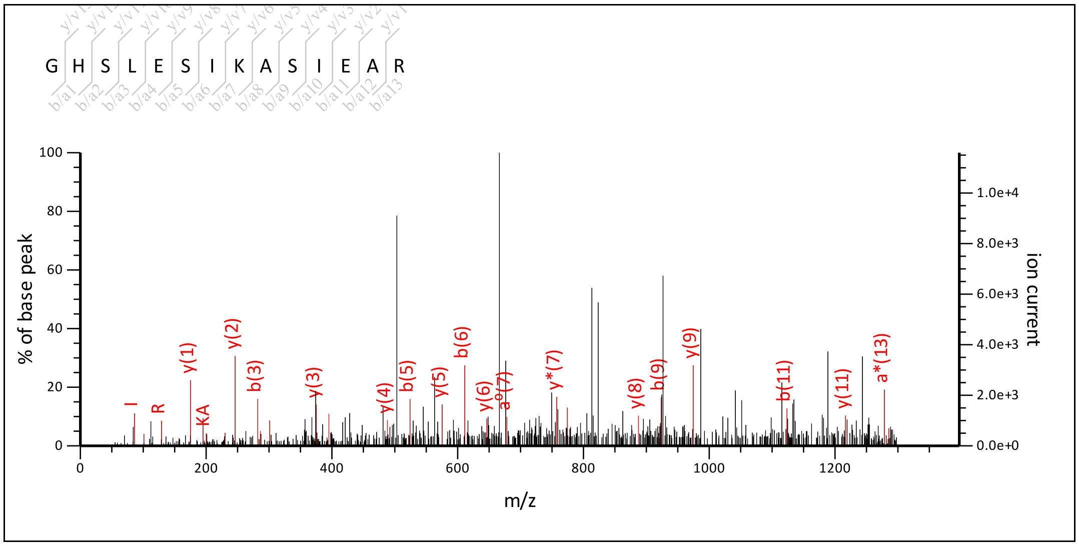


0 to 1397.61

Label all possible matches ○ Label matches used for scoring ◉

**Monoisotopic mass of neutral peptide Mr(calc):** 1496.7947
**Fixed modifications:** Carbamidomethyl (C) (apply to specified residues or termini only)
**Ions Score:** 45 **Expect:** 0.083
**Matches :** 31/239 fragment ions using 65 most intense peaks (help)

| # | Immon. | a | a* | a0 | b | b* | b0 | d | d' | Seq. | v | w | w' | y | y* | [illegible] |
|---|---|---|---|---|---|---|---|---|---|---|---|---|---|---|---|---|
| 1 | 30.0338 | 30.0338 | | | 58.0287 | | | 44.0495 | | G | | | | | | |
| 2 | 110.0713 | 167.0927 | | | **195.0877** | | | | | H | 1358.7274 | | | 1440.7805 | 1423.7540 | 142[illegible] |
| 3 | 60.0444 | 254.1248 | | 236.1142 | **282.1197** | | 264.1091 | 238.1299 | | S | 1271.6954 | 1270.7001 | | 1303.7216 | 1286.6951 | 128[illegible] |
| 4 | 86.0964 | 367.2088 | | 349.1983 | **395.2037** | | 377.1932 | 325.1619 | | L | 1158.6113 | 1157.6161 | | ***1216.6896*** | 1199.6630 | 119[illegible] |
| 5 | 102.0550 | 496.2514 | | 478.2409 | **524.2463** | | 506.2358 | 438.2459 | | E | 1029.5687 | 1028.5735 | | 1103.6055 | 1086.5790 | 108[illegible] |
| 6 | 60.0444 | 583.2835 | | 565.2729 | **611.2784** | | 593.2678 | 567.2885 | | S | 942.5367 | 941.5415 | | ***974.5629*** | 957.5364 | 9[illegible] |
| 7 | 86.0964 | 696.3675 | | 678.3569 | 724.3624 | | 706.3519 | 668.3362 | 682.3519 | I | 829.4526 | 842.4730 | 856.4887 | ***887.5309*** | 870.5043 | 8[illegible] |
| 8 | 101.1073 | 824.4625 | 807.4359 | 806.4519 | 852.4574 | 835.4308 | 834.4468 | 767.4046 | | K | 701.3577 | 700.3624 | | ***774.4468*** | 757.4203 | 7[illegible] |
| 9 | 44.0495 | 895.4996 | 878.4730 | 877.4890 | **923.4945** | 906.4680 | 905.4839 | | | A | 630.3206 | | | ***646.3519*** | 629.3253 | 6[illegible] |
| 10 | 60.0444 | 982.5316 | 965.5051 | 964.5211 | 1010.5265 | 993.5000 | 992.5160 | 966.5367 | | S | 543.2885 | 542.2933 | | ***575.3148*** | 558.2882 | 5[illegible] |
| 11 | 86.0964 | 1095.6157 | 1078.5891 | 1077.6051 | **1123.6106** | 1106.5840 | 1105.6000 | 1067.5844 | 1081.6000 | I | 430.2045 | 443.2249 | 457.2405 | ***488.2827*** | 471.2562 | 4[illegible] |
| 12 | 102.0550 | 1224.6583 | 1207.6317 | 1206.6477 | 1252.6532 | 1235.6266 | 1234.6426 | 1166.6528 | | E | 301.1619 | 300.1666 | | ***375.1987*** | 358.1721 | 3[illegible] |
| 13 | 44.0495 | 1295.6954 | 1278.6688 | 1277.6848 | 1323.6903 | 1306.6638 | 1305.6797 | | | A | 230.1248 | | | ***246.1561*** | 229.1295 | |
| 14 | 129.1135 | | | | | | | | | R | 74.0237 | 73.0284 | | ***175.1190*** | 158.0924 | |

| Seq | ya | yb | Seq | ya | yb | Seq | ya | yb |
|---|---|---|---|---|---|---|---|---|
| HS | 197.1033 | 225.0982 | HSL | 310.1874 | 338.1823 | HSLE | 439.2300 | 467.2249 |
| HSLES | 526.2620 | 554.2569 | HSLESI | 639.3460 | 667.3410 | SL | 173.1285 | 201.1234 |
| SLE | 302.1710 | 330.1660 | SLES | 389.2031 | 417.1980 | SLESI | 502.2871 | 530.2821 |
| SLESIK | 630.3821 | 658.3770 | LE | 215.1390 | 243.1339 | LES | 302.1710 | 330.1660 |
| LESI | 415.2551 | 443.2500 | LESIK | 543.3501 | 571.3450 | LESIKA | 614.3872 | 642.3821 |
| ES | 189.0870 | 217.0819 | ESI | 302.1710 | 330.1660 | ESIK | 430.2660 | 458.2609 |
| ESIKA | 501.3031 | 529.2980 | ESIKAS | 588.3352 | 616.3301 | SI | 173.1285 | 201.1234 |

| | | | | | | | | |
|---|---|---|---|---|---|---|---|---|
| **SIK** | 301.2234 | 329.2183 | **SIKA** | 372.2605 | 400.2554 | **SIKAS** | 459.2926 | 487.2875 |
| **SIKASI** | 572.3766 | 600.3715 | **IK** | 214.1914 | 242.1863 | **IKA** | 285.2285 | 313.2234 |
| **IKAS** | 372.2605 | 400.2554 | **IKASI** | 485.3446 | 513.3395 | **IKASIE** | 614.3872 | 642.3821 |
| **IKASIEA** | 685.4243 | 713.4192 | **KA** | 172.1444 | 200.1394 | **KAS** | 259.1765 | 287.1714 |
| **KASI** | 372.2605 | 400.2554 | **KASIE** | 501.3031 | 529.2980 | **KASIEA** | 572.3402 | 600.3352 |
| **AS** | 131.0815 | 159.0764 | **ASI** | 244.1656 | 272.1605 | **ASIE** | 373.2082 | 401.2031 |
| **ASIEA** | 444.2453 | 472.2402 | **SI** | 173.1285 | 201.1234 | **SIE** | 302.1710 | 330.1660 |
| **SIEA** | 373.2082 | 401.2031 | **IE** | 215.1390 | 243.1339 | **IEA** | 286.1761 | 314.1710 |
| **EA** | 173.0921 | 201.0870 | | | | | | |

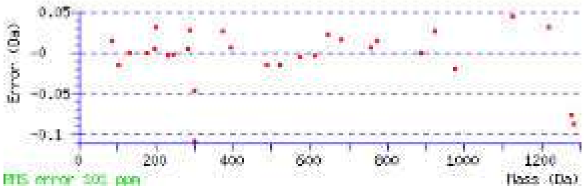


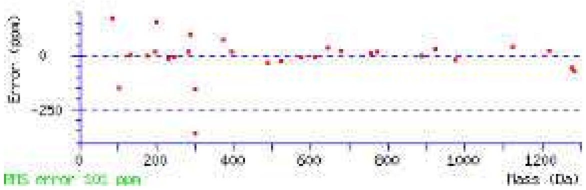


NCBI **BLAST** search of GHSLESIKASIEAR
(Parameters: blastp, nr protein database, expect=20000, no filter, PAM30)
Other BLAST web gateways

**All matches to this query**

| Score | Mr(calc) | Delta | Sequence |
|---|---|---|---|
| 44.6 | 1496.7947 | -0.0226 | GHSLESIKASIEAR |
| 34.3 | 1496.8198 | -0.0478 | GHSLESIKASIEVK |
| 22.2 | 1496.7583 | 0.0137 | SPNTDGIHITSSIR |
| 17.3 | 1496.6718 | 0.1002 | SDAFVMTHGGNFAK |
| 15.5 | 1496.8351 | -0.0631 | DPWVLKLAEVTAR |
| 13.4 | 1496.7583 | 0.0137 | DPVVLASGQQQEAR |
| 12.8 | 1496.7471 | 0.0249 | VLPQDDPSATVAER |
| 12.7 | 1496.8497 | -0.0777 | ICGALGPVVSLRQK |
| 12.6 | 1496.7471 | 0.0250 | KADADSSFITSSLR |
| 11.9 | 1496.8861 | -0.1140 | ICLIARNLLNGLK |

**Mascot:** http://www.matrixscience.com/

# MATRIX SCIENCE Mascot Search Results

## Peptide View Spot no 56

MS/MS Fragmentation of **IRDLYEQIIAER**
Found in **gi|125580** in **NCBInr**, RecName: Full=Phosphoribulokinase, chloroplastic; Short=PRK; Short=PRKase; AltName: Full=Phosphopentokinase; Flags: Precursor

Match to Query 12: 1517.790924 from(1518.798200,1+) intensity(0.0000) index(11)
Title: Label: I4, Spot_Id: 219730, Peak_List_Id: 225347, MSMS Job_Run_Id: 21775, Comment:
Data file C:\Documents and Settings\Administrator\Desktop\farah BHU\spot set plate\Farah-Garima 11May 2013\ppw_I4_136842168700.txt

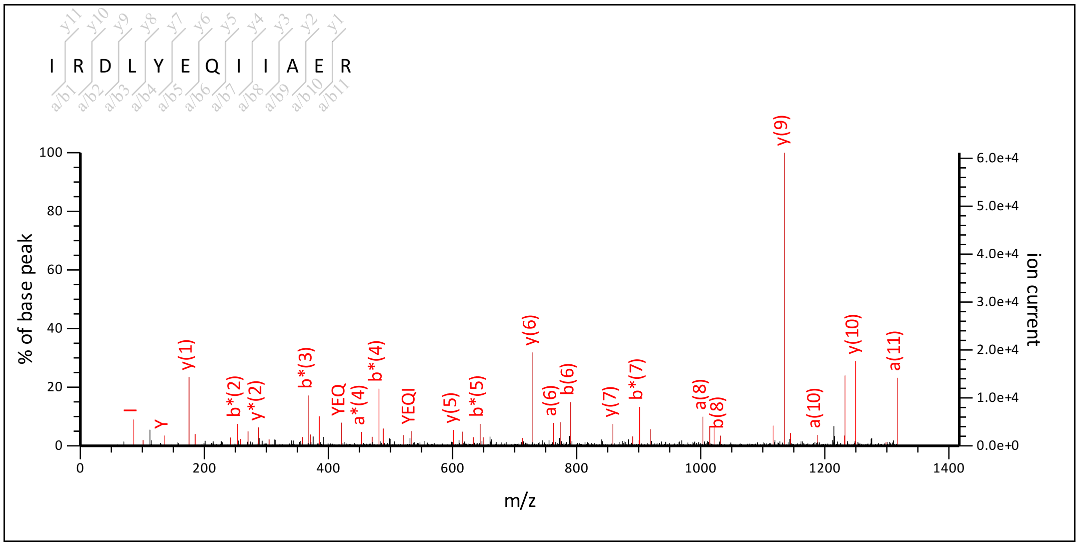


0 to 1416.73

Label all possible matches ○ Label matches used for scoring ◉

**Monoisotopic mass of neutral peptide Mr(calc):** 1517.8201
**Fixed modifications:** Carbamidomethyl (C) (apply to specified residues or termini only)
**Ions Score:** 78 **Expect:** 3.9e-05
**Matches :** 62/200 fragment ions using 65 most intense peaks (help)

| # | Immon. | a | a* | $a^0$ | b | b* | $b^0$ | d | d' | Seq. | v | w | w' | y | y* | |
|---|---|---|---|---|---|---|---|---|---|---|---|---|---|---|---|---|
| 1 | 86.0964 | ***86.0964*** | | | 114.0913 | | | 44.0495 | | I | | | | | | |
| 2 | 129.1135 | ***242.1975*** | 225.1710 | | **270.1925** | 253.1659 | | 157.1335 | | R | 1304.6481 | 1303.6529 | | 1405.7434 | 1388.7169 | [illegible] |
| 3 | 88.0393 | 357.2245 | 340.1979 | 339.2139 | **385.2194** | 368.1928 | 367.2088 | 313.2347 | | D | 1189.6212 | 1188.6259 | | **1249.6423** | 1232.6157 | [illegible] |
| 4 | 86.0964 | ***470.3085*** | 453.2820 | 452.2980 | 498.3035 | 481.2769 | 480.2929 | 428.2616 | | L | 1076.5371 | 1075.5419 | | **1134.6154** | 1117.5888 | [illegible] |
| 5 | 136.0757 | ***633.3719*** | 616.3453 | 615.3613 | 661.3668 | 644.3402 | 643.3562 | | | Y | 913.4738 | | | **1021.5313** | 1004.5047 | [illegible] |
| 6 | 102.0550 | ***762.4145*** | 745.3879 | 744.4039 | **790.4094** | 773.3828 | 772.3988 | 704.4090 | | E | 784.4312 | 783.4359 | | **858.4680** | 841.4414 | [illegible] |
| 7 | 101.0709 | ***890.4730*** | 873.4465 | 872.4625 | **918.4680** | 901.4414 | 900.4574 | 833.4516 | | Q | 656.3726 | 655.3774 | | **729.4254** | 712.3988 | [illegible] |
| 8 | 86.0964 | ***1003.5571*** | 986.5306 | 985.5465 | **1031.5520** | 1014.5255 | 1013.5415 | 975.5258 | 989.5415 | I | 543.2885 | 556.3089 | 570.3246 | **601.3668** | 584.3402 | [illegible] |
| 9 | 86.0964 | ***1116.6412*** | 1099.6146 | 1098.6306 | **1144.6361** | 1127.6095 | 1126.6255 | 1088.6099 | 1102.6255 | I | 430.2045 | 443.2249 | 457.2405 | **488.2827** | 471.2562 | [illegible] |
| 10 | 44.0495 | ***1187.6783*** | 1170.6517 | 1169.6677 | 1215.6732 | 1198.6467 | 1197.6626 | | | A | 359.1674 | | | 375.1987 | 358.1721 | [illegible] |
| 11 | 102.0550 | ***1316.7209*** | 1299.6943 | 1298.7103 | 1344.7158 | 1327.6892 | 1326.7052 | 1258.7154 | | E | 230.1248 | 229.1295 | | **304.1615** | 287.1350 | [illegible] |
| 12 | 129.1135 | | | | | | | | | R | 74.0237 | 73.0284 | | **175.1190** | 158.0924 | |

| Seq | ya | yb | Seq | ya | yb | Seq | ya | yb |
|---|---|---|---|---|---|---|---|---|
| RD | 244.1404 | 272.1353 | RDL | 357.2245 | 385.2194 | RDLY | 520.2878 | 548.2827 |
| RDLYE | 649.3304 | 677.3253 | DL | 201.1234 | 229.1183 | DLY | 364.1867 | 392.1816 |
| DLYE | 493.2293 | 521.2242 | DLYEQ | 621.2879 | 649.2828 | LY | 249.1598 | 277.1547 |
| LYE | 378.2023 | 406.1973 | LYEQ | 506.2609 | 534.2558 | LYEQI | 619.3450 | 647.3399 |
| YE | 265.1183 | 293.1132 | YEQ | 393.1769 | 421.1718 | YEQI | 506.2609 | 534.2558 |
| YEQII | 619.3450 | 647.3399 | YEQIIA | 690.3821 | 718.3770 | EQ | 230.1135 | 258.1084 |
| EQI | 343.1976 | 371.1925 | EQII | 456.2817 | 484.2766 | EQIIA | 527.3188 | 555.3137 |
| EQIIAE | 656.3614 | 684.3563 | QI | 214.1550 | 242.1499 | QII | 327.2391 | 355.2340 |
| QIIA | 398.2762 | 426.2711 | QIIAE | 527.3188 | 555.3137 | II | 199.1805 | 227.1754 |

| | | | | | | | | |
|---|---|---|---|---|---|---|---|---|
| **IIA** | 270.2176 | 298.2125 | **IIAE** | 399.2602 | 427.2551 | **IA** | 157.1335 | 185.1285 |
| **IAE** | 286.1761 | 314.1710 | **AE** | 173.0921 | 201.0870 | | | |

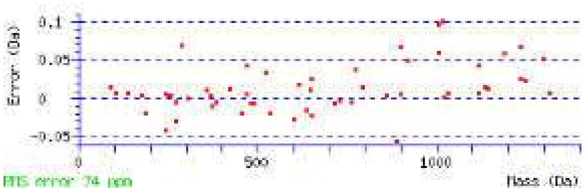


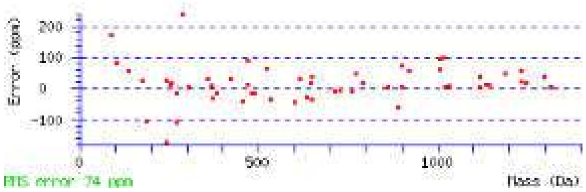


NCBI **BLAST** search of IRDLYEQIIAER
(Parameters: blastp, nr protein database, expect=20000, no filter, PAM30)
Other BLAST web gateways

**All matches to this query**

| Score | Mr(calc) | Delta | Sequence |
|---|---|---|---|
| 77.8 | 1517.8201 | -0.0292 | IRDLYEQIIAER |
| 30.6 | 1517.7395 | 0.0514 | AMAEEEEKLLEAR |
| 27.4 | 1517.7950 | -0.0041 | QYNQQQQLLISR |
| 26.8 | 1517.8024 | -0.0115 | IPSSSFKHMISLR |
| 26.7 | 1517.7474 | 0.0435 | EWSLGALDGSQISR |
| 24.9 | 1517.8929 | -0.1020 | ILSAVYSREVIIR |
| 24.4 | 1517.8202 | -0.0292 | EVLRQNQYDLLK |
| 23.6 | 1517.8049 | -0.0140 | DVSKSETLGNLSLR |
| 23.5 | 1517.8202 | -0.0293 | LYVDADLRLDGIR |
| 23.4 | 1517.6780 | 0.1129 | MAISGPSLDGNEDGR |

**Mascot:** http://www.matrixscience.com/

# MATRIX SCIENCE Mascot Search Results

## Peptide View Spot no 56

MS/MS Fragmentation of **IFVIEGLHPMYDER**
Found in **gi|125580** in **NCBInr**, RecName: Full=Phosphoribulokinase, chloroplastic; Short=PRK; Short=PRKase; AltName: Full=Phosphopentokinase; Flags: Precursor

Match to Query 14: 1717.816624 from(1718.823900,1+) intensity(0.0000) index(13)
Title: Label: I4, Spot_Id: 219730, Peak_List_Id: 225350, MSMS Job_Run_Id: 21775, Comment:
Data file C:\Documents and Settings\Administrator\Desktop\farah BHU\spot set plate\Farah-Garima 11May 2013\ppw_I4_136842168700.txt

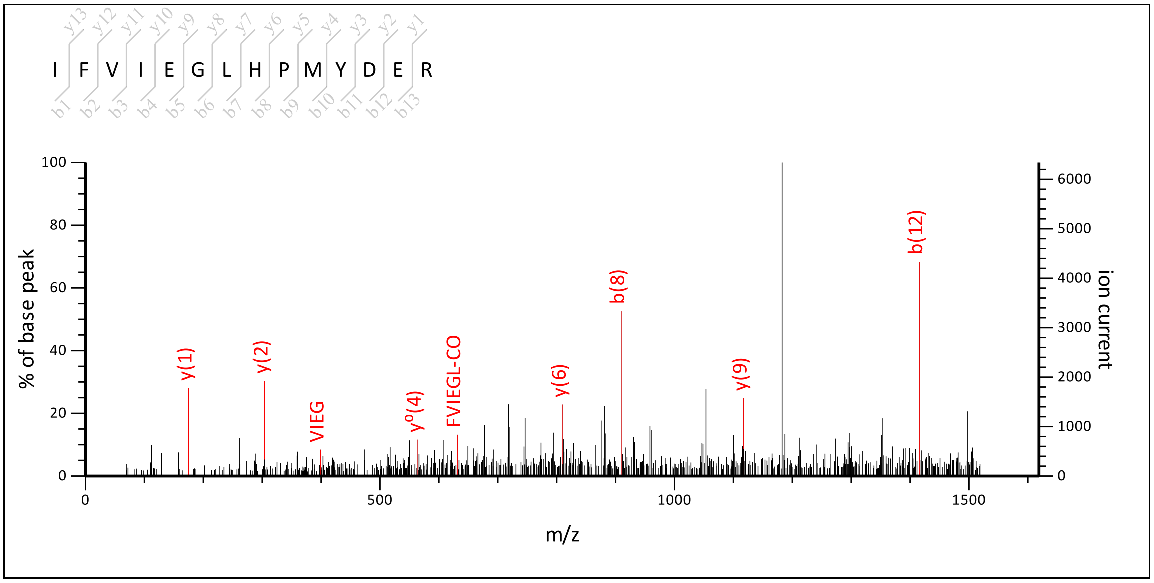


0 to 1618.76

Label all possible matches ○ Label matches used for scoring ●

**Monoisotopic mass of neutral peptide Mr(calc):** 1717.8498
**Fixed modifications:** Carbamidomethyl (C) (apply to specified residues or termini only)
**Ions Score:** 15 **Expect:** 55
**Matches :** 10/214 fragment ions using 15 most intense peaks (help)

| # | Immon. | a | $a^0$ | b | $b^0$ | d | d' | Seq. | v | w | w' | y | y* | $y^0$ | # |
|---|---|---|---|---|---|---|---|---|---|---|---|---|---|---|---|
| 1 | 86.0964 | 86.0964 | | 114.0913 | | 44.0495 | | I | | | | | | | 14 |
| 2 | 120.0808 | 233.1648 | | 261.1598 | | | | F | 1513.7104 | | | 1605.7730 | 1588.7464 | 1587.7624 | 13 |
| 3 | 72.0808 | 332.2333 | | 360.2282 | | 318.2176 | | V | 1414.6420 | 1427.6624 | | 1458.7046 | 1441.6780 | 1440.6940 | 12 |
| 4 | 86.0964 | 445.3173 | | 473.3122 | | 417.2860 | 431.3017 | I | 1301.5579 | 1314.5783 | 1328.5940 | 1359.6362 | 1342.6096 | 1341.6256 | 11 |
| 5 | 102.0550 | 574.3599 | 556.3493 | 602.3548 | 584.3443 | 516.3544 | | E | 1172.5153 | 1171.5201 | | 1246.5521 | 1229.5256 | 1228.5415 | 10 |
| 6 | 30.0338 | 631.3814 | 613.3708 | 659.3763 | 641.3657 | | | G | | | | ***1117.5095*** | 1100.4830 | 1099.4989 | 9 |
| 7 | 86.0964 | 744.4654 | 726.4549 | 772.4604 | 754.4498 | 702.4185 | | L | 1002.4098 | 1001.4145 | | 1060.4880 | 1043.4615 | 1042.4775 | 8 |
| 8 | 110.0713 | 881.5244 | 863.5138 | **909.5193** | 891.5087 | | | H | 865.3509 | | | 947.4040 | 930.3774 | 929.3934 | 7 |
| 9 | 70.0651 | 978.5771 | 960.5665 | 1006.5720 | 988.5615 | 952.5615 | | P | 768.2981 | 767.3029 | | ***810.3451*** | 793.3185 | 792.3345 | 6 |
| 10 | 104.0528 | 1109.6176 | 1091.6070 | 1137.6125 | 1119.6019 | 1049.6142 | | M | 637.2576 | 636.2624 | | 713.2923 | 696.2658 | 695.2817 | 5 |
| 11 | 136.0757 | 1272.6809 | 1254.6704 | 1300.6758 | 1282.6653 | | | Y | 474.1943 | | | 582.2518 | 565.2253 | 564.2413 | 4 |
| 12 | 88.0393 | 1387.7079 | 1369.6973 | **1415.7028** | 1397.6922 | 1343.7180 | | D | 359.1674 | 358.1721 | | 419.1885 | 402.1619 | 401.1779 | 3 |
| 13 | 102.0550 | 1516.7505 | 1498.7399 | 1544.7454 | 1526.7348 | 1458.7450 | | E | 230.1248 | 229.1295 | | ***304.1615*** | 287.1350 | 286.1510 | 2 |
| 14 | 129.1135 | | | | | | | R | 74.0237 | 73.0284 | | ***175.1190*** | 158.0924 | | 1 |

| Seq | ya | yb | Seq | ya | yb | Seq | ya | yb |
|---|---|---|---|---|---|---|---|---|
| FV | 219.1492 | 247.1441 | FVI | 332.2333 | 360.2282 | FVIE | 461.2758 | 489.2708 |
| FVIEG | 518.2973 | 546.2922 | FVIEGL | 631.3814 | 659.3763 | VI | 185.1648 | 213.1598 |

| | | | | | | | | |
|---|---|---|---|---|---|---|---|---|
| **VIE** | 314.2074 | 342.2023 | **VIEG** | 371.2289 | 399.2238 | **VIEGL** | 484.3130 | 512.3079 |
| **VIEGLH** | 621.3719 | 649.3668 | **IE** | 215.1390 | 243.1339 | **IEG** | 272.1605 | 300.1554 |
| **IEGL** | 385.2445 | 413.2395 | **IEGLH** | 522.3035 | 550.2984 | **IEGLHP** | 619.3562 | 647.3511 |
| **EG** | 159.0764 | 187.0713 | **EGL** | 272.1605 | 300.1554 | **EGLH** | 409.2194 | 437.2143 |
| **EGLHP** | 506.2722 | 534.2671 | **EGLHPM** | 637.3126 | 665.3076 | **GL** | 143.1179 | 171.1128 |
| **GLH** | 280.1768 | 308.1717 | **GLHP** | 377.2296 | 405.2245 | **GLHPM** | 508.2701 | 536.2650 |
| **GLHPMY** | 671.3334 | 699.3283 | **LH** | 223.1553 | 251.1503 | **LHP** | 320.2081 | 348.2030 |
| **LHPM** | 451.2486 | 479.2435 | **LHPMY** | 614.3119 | 642.3068 | **HP** | 207.1240 | 235.1190 |
| **HPM** | 338.1645 | 366.1594 | **HPMY** | 501.2279 | 529.2228 | **HPMYD** | 616.2548 | 644.2497 |
| **PM** | 201.1056 | 229.1005 | **PMY** | 364.1689 | 392.1639 | **PMYD** | 479.1959 | 507.1908 |
| **PMYDE** | 608.2385 | 636.2334 | **MY** | 267.1162 | 295.1111 | **MYD** | 382.1431 | 410.1380 |
| **MYDE** | 511.1857 | 539.1806 | **YD** | 251.1026 | 279.0975 | **YDE** | 380.1452 | 408.1401 |
| **DE** | 217.0819 | 245.0768 | | | | | | |

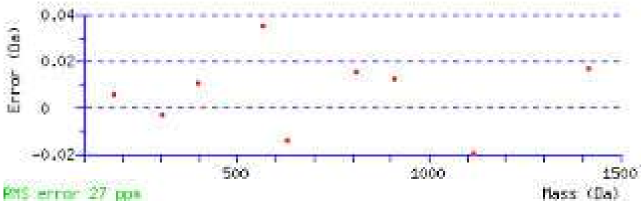


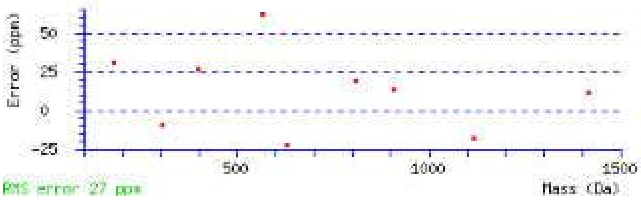


NCBI **BLAST** search of IFVIEGLHPMYDER
(Parameters: blastp, nr protein database, expect=20000, no filter, PAM30)
Other BLAST web gateways

**All matches to this query**

| Score | Mr(calc) | Delta | Sequence |
|---|---|---|---|
| 32.7 | 1717.8498 | -0.0332 | IFVIEGLHPMFDER |
| 19.5 | 1717.8419 | -0.0252 | MLEYEVKYVMIER |
| 14.9 | 1717.8498 | -0.0331 | IFVIEGLHPMYDER |
| 13.1 | 1717.8167 | -0.0001 | MLDYETRFVMIER |
| 12.8 | 1717.8457 | -0.0291 | MAETLIHYSVNPSTR |
| 12.5 | 1717.8536 | -0.0370 | WPEPRGDAAGAPPLER |
| 11.9 | 1717.8206 | -0.0040 | IASCLNSSHFQVAER |
| 10.9 | 1717.8648 | -0.0482 | GKYLHAAPGASAGPHER |
| 9.7 | 1717.7036 | 0.1130 | MSGGNSNCTVYIDER |
| 9.4 | 1717.8496 | -0.0330 | DHNLGSDVPLRNPER |

**Mascot:** http://www.matrixscience.com/

# Mascot Search Results

## Peptide View **Spot no 57**

MS/MS Fragmentation of **FAEYASSGR**
Found in **gi|115471157** in **NCBInr**, Os07g0212200 [Oryza sativa Japonica Group]

Match to Query 58: 986.477164 from(987.484440,1+) intensity(0.0000) index(3)
Title: Label: N11, Spot_Id: 219847, Peak_List_Id: 227553, MSMS Job_Run_Id: 21965, Comment:
Data file C:\Documents and Settings\Administrator\Desktop\Farah-Garima 11May 2013\ppw_N11_136868230800.txt

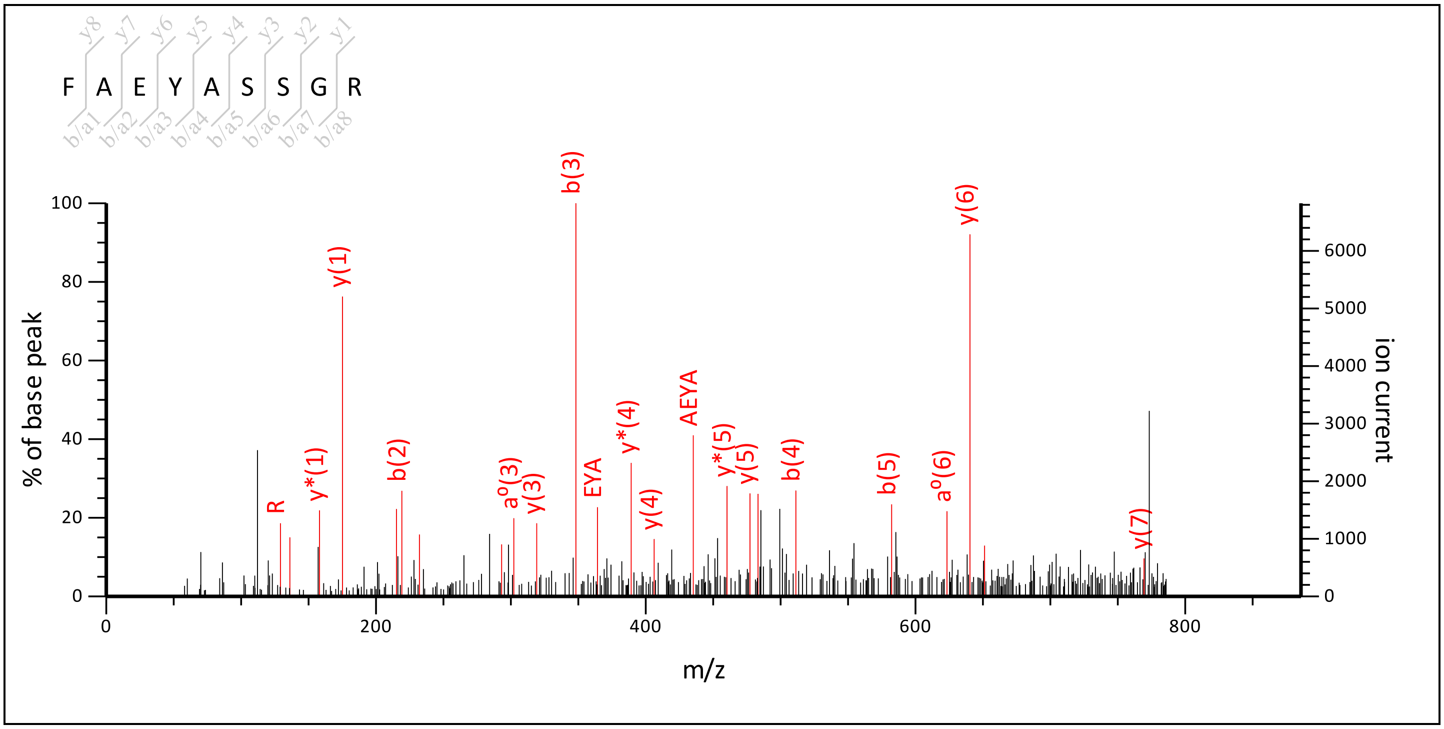


0 to 885.83

Label all possible matches ○ Label matches used for scoring ◉

**Monoisotopic mass of neutral peptide Mr(calc):** 986.4457
**Fixed modifications:** Carbamidomethyl (C) (apply to specified residues or termini only)
**Ions Score:** 47 **Expect:** 0.86
**Matches :** 29/116 fragment ions using 40 most intense peaks (help)

| # | Immon. | a | $a^0$ | b | $b^0$ | d | Seq. | v | w | y | y* | $y^0$ | # |
|---|---|---|---|---|---|---|---|---|---|---|---|---|---|
| 1 | 120.0808 | 120.0808 | | 148.0757 | | 44.0495 | F | | | | | | 9 |
| 2 | 44.0495 | 191.1179 | | **219.1128** | | | A | 824.3533 | | 840.3846 | 823.3581 | 822.3741 | 8 |
| 3 | 102.0550 | 320.1605 | 302.1499 | **348.1554** | 330.1448 | 262.1550 | E | 695.3107 | 694.3155 | ***769.3475*** | 752.3210 | 751.3369 | 7 |
| 4 | 136.0757 | 483.2238 | 465.2132 | **511.2187** | 493.2082 | | Y | 532.2474 | | ***640.3049*** | 623.2784 | 622.2944 | 6 |
| 5 | 44.0495 | 554.2609 | 536.2504 | **582.2558** | 564.2453 | | A | 461.2103 | | ***477.2416*** | 460.2150 | 459.2310 | 5 |
| 6 | 60.0444 | 641.2930 | 623.2824 | 669.2879 | 651.2773 | 625.2980 | S | 374.1783 | 373.1830 | ***406.2045*** | 389.1779 | 388.1939 | 4 |
| 7 | 60.0444 | 728.3250 | 710.3144 | 756.3199 | 738.3093 | 712.3301 | S | 287.1462 | 286.1510 | ***319.1724*** | 302.1459 | 301.1619 | 3 |
| 8 | 30.0338 | 785.3464 | 767.3359 | 813.3414 | 795.3308 | | G | | | ***232.1404*** | 215.1139 | | 2 |

| | | | | | | | | | | | | | |
|---|---|---|---|---|---|---|---|---|---|---|---|---|---|
| 9 | 129.1135 | | | | | | R | 74.0237 | 73.0284 | *175.1190* | 158.0924 | | 1 |

| Seq | ya | yb | Seq | ya | yb | Seq | ya | yb |
|---|---|---|---|---|---|---|---|---|
| AE | 173.0921 | 201.0870 | AEY | 336.1554 | 364.1503 | AEYA | 407.1925 | 435.1874 |
| AEYAS | 494.2245 | 522.2195 | AEYASS | 581.2566 | 609.2515 | AEYASSG | 638.2780 | 666.2729 |
| EY | 265.1183 | 293.1132 | EYA | 336.1554 | 364.1503 | EYAS | 423.1874 | 451.1823 |
| EYASS | 510.2195 | 538.2144 | EYASSG | 567.2409 | 595.2358 | YA | 207.1128 | 235.1077 |
| YAS | 294.1448 | 322.1397 | YASS | 381.1769 | 409.1718 | YASSG | 438.1983 | 466.1932 |
| AS | 131.0815 | 159.0764 | ASS | 218.1135 | 246.1084 | ASSG | 275.1350 | 303.1299 |
| SS | 147.0764 | 175.0713 | SSG | 204.0979 | 232.0928 | SG | 117.0659 | 145.0608 |

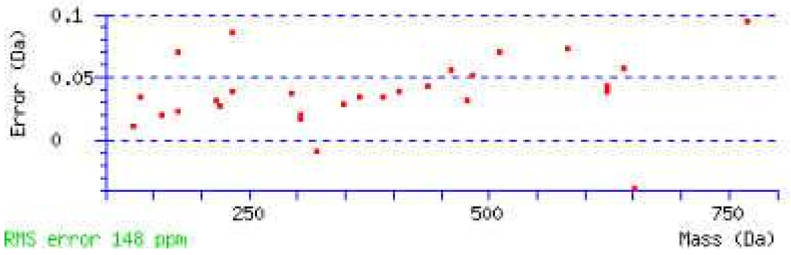


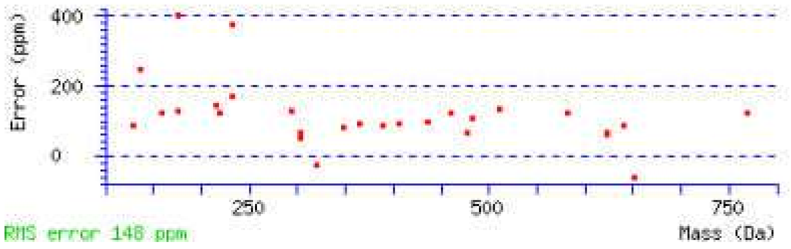


NCBI **BLAST** search of FAEYASSGR
(Parameters: blastp, nr protein database, expect=20000, no filter, PAM30)
Other BLAST web gateways

**All matches to this query**

| Score | Mr(calc) | Delta | Sequence |
|---|---|---|---|
| 46.7 | 986.4457 | 0.0314 | FAEYASSGR |
| 33.6 | 986.4127 | 0.0644 | ESMYASSGR |
| 25.2 | 986.3950 | 0.0822 | MAEYMNGR |
| 25.1 | 986.3950 | 0.0822 | MSEYAACR |
| 24.1 | 986.4127 | 0.0644 | AEYMASSGR |
| 23.2 | 986.5073 | -0.0301 | SSTLAYFAK |
| 22.2 | 986.4716 | 0.0056 | RPPCASSGR |
| 21.6 | 986.3983 | 0.0788 | SMMMTSSGR |
| 21.2 | 986.4821 | -0.0050 | ALYFSSSGR |
| 20.9 | 986.5509 | -0.0737 | ILGAGVTGSGR |

**Mascot:** http://www.matrixscience.com/

# MATRIX SCIENCE Mascot Search Results

## Peptide View Spot no 57

MS/MS Fragmentation of **IFNCVSDR**
Found in **gi|115471157** in **NCBInr**, Os07g0212200 [Oryza sativa Japonica Group]

Match to Query 73: 1009.493824 from(1010.501100,1+) intensity(0.0000) index(5)
Title: Label: N11, Spot_Id: 219847, Peak_List_Id: 227550, MSMS Job_Run_Id: 21965, Comment:
Data file C:\Documents and Settings\Administrator\Desktop\Farah-Garima 11May 2013\ppw_N11_136868230800.txt

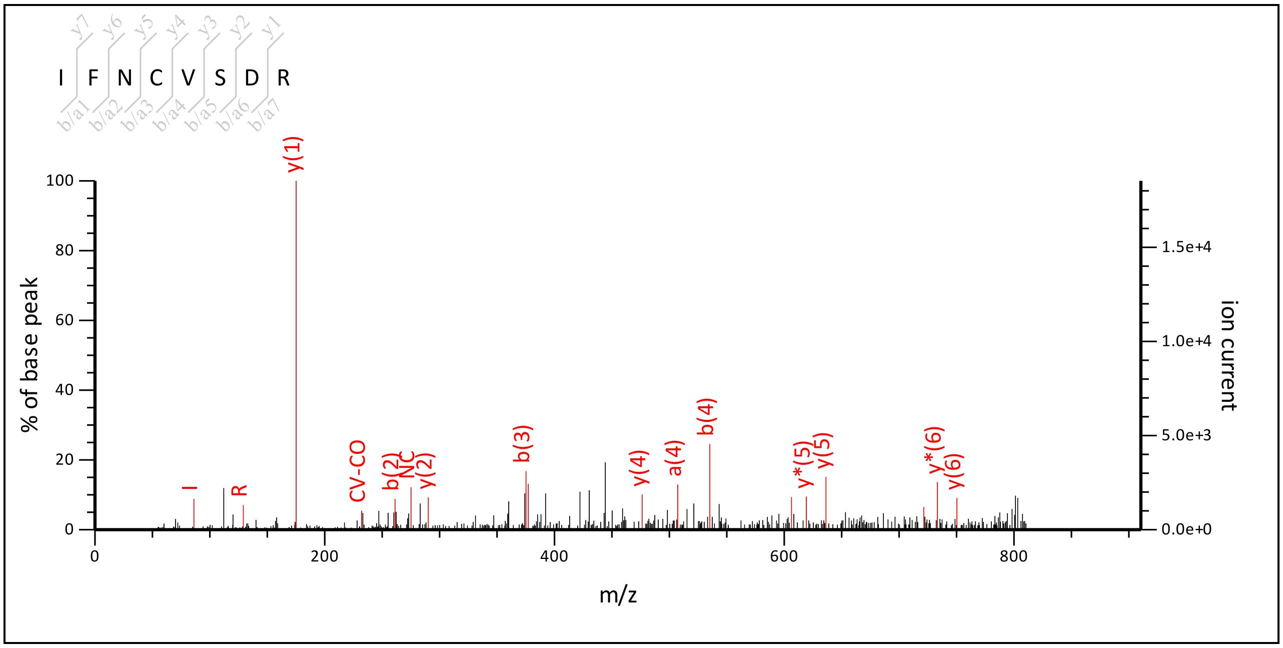


0 to 910.45

Label all possible matches ○ Label matches used for scoring ◉

**Monoisotopic mass of neutral peptide Mr(calc):** 1009.4651
**Fixed modifications:** Carbamidomethyl (C) (apply to specified residues or termini only)
**Ions Score:** 48 **Expect:** 0.51
**Matches :** 21/104 fragment ions using 24 most intense peaks (help)

| # | Immon. | a | a* | $a^0$ | b | b* | $b^0$ | d | Seq. | v | w | y | y* | $y^0$ | # |
|---|---|---|---|---|---|---|---|---|---|---|---|---|---|---|---|
| 1 | 86.0964 | **86.0964** | | | 114.0913 | | | 44.0495 | I | | | | | | 8 |
| 2 | 120.0808 | 233.1648 | | | **261.1598** | | | | F | 805.3257 | | 897.3883 | 880.3618 | 879.3778 | 7 |
| 3 | 87.0553 | 347.2078 | 330.1812 | | **375.2027** | 358.1761 | | 304.2020 | N | 691.2828 | 690.2876 | ***750.3199*** | 733.2934 | 732.3093 | 6 |
| 4 | 133.0430 | **507.2384** | 490.2119 | | **535.2333** | 518.2068 | | 418.2449 | C | 531.2522 | 530.2569 | ***636.2770*** | 619.2504 | 618.2664 | 5 |
| 5 | 72.0808 | **606.3068** | 589.2803 | | 634.3017 | 617.2752 | | 592.2912 | V | 432.1837 | 445.2041 | ***476.2463*** | 459.2198 | 458.2358 | 4 |
| 6 | 60.0444 | 693.3389 | 676.3123 | 675.3283 | **721.3338** | 704.3072 | 703.3232 | 677.3439 | S | 345.1517 | 344.1565 | ***377.1779*** | 360.1514 | 359.1674 | 3 |
| 7 | 88.0393 | 808.3658 | 791.3393 | 790.3552 | 836.3607 | 819.3342 | 818.3501 | 764.3760 | D | 230.1248 | 229.1295 | ***290.1459*** | 273.1193 | 272.1353 | 2 |
| 8 | 129.1135 | | | | | | | | R | 74.0237 | 73.0284 | ***175.1190*** | 158.0924 | | 1 |

| Seq | ya | yb | Seq | ya | yb | Seq | ya | yb |
|---|---|---|---|---|---|---|---|---|
| FN | 234.1237 | 262.1186 | FNC | 394.1544 | 422.1493 | FNCV | 493.2228 | 521.2177 |
| FNCVS | 580.2548 | 608.2497 | FNCVSD | 695.2817 | 723.2767 | NC | 247.0859 | 275.0809 |
| NCV | 346.1544 | 374.1493 | NCVS | 433.1864 | 461.1813 | NCVSD | 548.2133 | 576.2082 |
| CV | 232.1114 | 260.1063 | CVS | 319.1435 | 347.1384 | CVSD | 434.1704 | 462.1653 |

| | | | | | | | | |
|---|---|---|---|---|---|---|---|---|
| **VS** | 159.1128 | 187.1077 | **VSD** | 274.1397 | 302.1347 | **SD** | 175.0713 | 203.0662 |

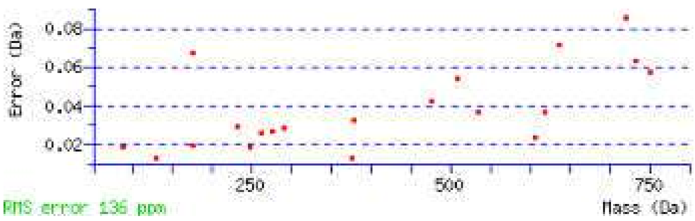


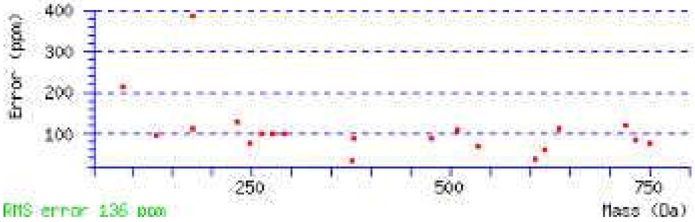


NCBI **BLAST** search of IFNCVSDR
(Parameters: blastp, nr protein database, expect=20000, no filter, PAM30)
Other BLAST web gateways

**All matches to this query**

| Score | Mr(calc) | Delta | Sequence |
|---|---|---|---|
| 48.0 | 1009.4651 | 0.0287 | IFNCVSDR |
| 34.7 | 1009.4829 | 0.0110 | LFGSSGAESR |
| 33.1 | 1009.5015 | -0.0077 | IFGGTLCSR |
| 31.6 | 1009.4685 | 0.0253 | MLNCTLSR |
| 30.4 | 1009.5015 | -0.0077 | FLNCTLSR |
| 29.4 | 1009.5094 | -0.0155 | FIHHVSDR |
| 29.2 | 1009.4651 | 0.0287 | FLNTCAER |
| 29.1 | 1009.4498 | 0.0440 | MLGSGSSEAR |
| 28.9 | 1009.3957 | 0.0981 | MVAGCADDR |
| 28.0 | 1009.5920 | -0.0982 | NIPPLTISR |

**Mascot:** http://www.matrixscience.com/

# MATRIX SCIENCE Mascot Search Results

## Peptide View Spot no 57

MS/MS Fragmentation of **NMHFYAEPR**
Found in **gi|115471157** in **NCBInr**, Os07g0212200 [Oryza sativa Japonica Group]

Match to Query 143: 1163.567524 from(1164.574800,1+) intensity(0.0000) index(13)
Title: Label: N11, Spot_Id: 219847, Peak_List_Id: 227555, MSMS Job_Run_Id: 21965, Comment:
Data file C:\Documents and Settings\Administrator\Desktop\Farah-Garima 11May 2013\ppw_N11_136868230800.txt

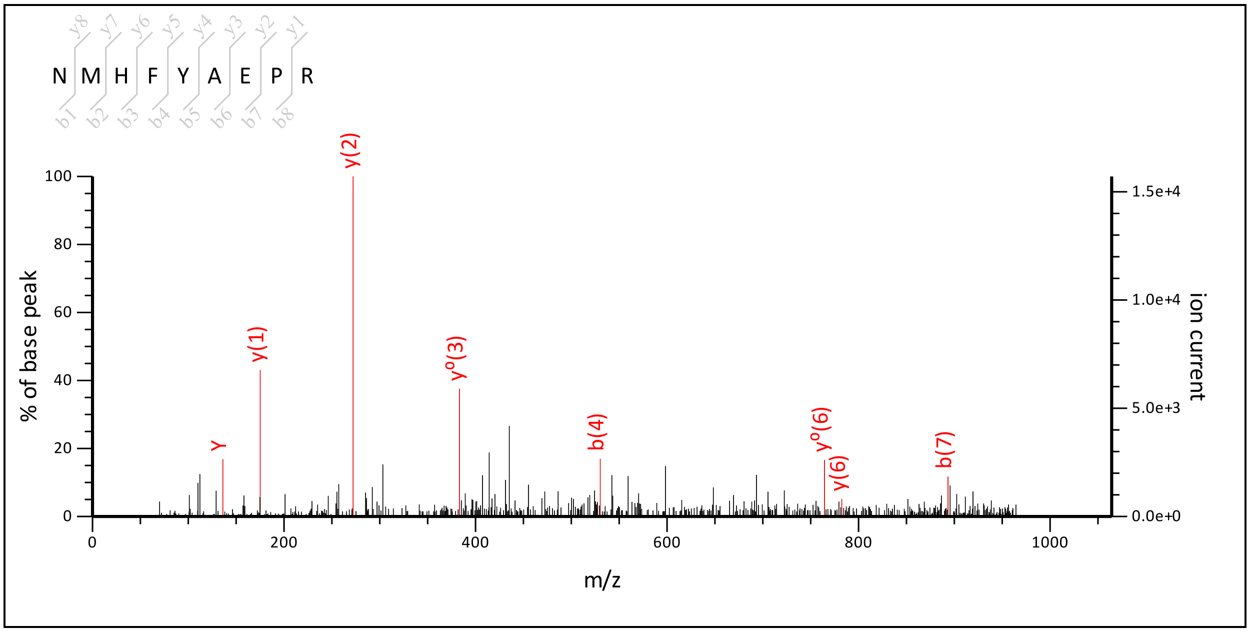


0 to 1064.32

Label all possible matches ○ Label matches used for scoring ◉

**Monoisotopic mass of neutral peptide Mr(calc):** 1163.5182
**Fixed modifications:** Carbamidomethyl (C) (apply to specified residues or termini only)
**Ions Score:** 34 **Expect:** 16
**Matches :** 10/119 fragment ions using 9 most intense peaks (help)

| # | Immon. | a | a* | $a^0$ | b | b* | $b^0$ | d | Seq. | v | w | y | y* | $y^0$ | # |
|---|---|---|---|---|---|---|---|---|---|---|---|---|---|---|---|
| 1 | 87.0553 | 87.0553 | 70.0287 | | 115.0502 | 98.0237 | | 44.0495 | N | | | | | | 9 |
| 2 | 104.0528 | 218.0958 | 201.0692 | | 246.0907 | 229.0641 | | 158.0924 | M | 974.4479 | 973.4526 | 1050.4826 | 1033.4560 | 1032.4720 | 8 |
| 3 | 110.0713 | 355.1547 | 338.1281 | | ***383.1496*** | 366.1231 | | | H | 837.3890 | | 919.4421 | 902.4155 | 901.4315 | 7 |
| 4 | 120.0808 | 502.2231 | 485.1966 | | ***530.2180*** | 513.1915 | | | F | 690.3206 | | ***782.3832*** | 765.3566 | 764.3726 | 6 |
| 5 | 136.0757 | 665.2864 | 648.2599 | | 693.2813 | 676.2548 | | | Y | 527.2572 | | 635.3148 | 618.2882 | 617.3042 | 5 |
| 6 | 44.0495 | 736.3235 | 719.2970 | | ***764.3185*** | 747.2919 | | | A | 456.2201 | | 472.2514 | 455.2249 | 454.2409 | 4 |
| 7 | 102.0550 | 865.3661 | 848.3396 | 847.3556 | ***893.3611*** | 876.3345 | 875.3505 | 807.3607 | E | 327.1775 | 326.1823 | 401.2143 | 384.1878 | 383.2037 | 3 |
| 8 | 70.0651 | 962.4189 | 945.3924 | 944.4083 | 990.4138 | 973.3873 | 972.4032 | 936.4032 | P | 230.1248 | 229.1295 | ***272.1717*** | 255.1452 | | 2 |
| 9 | 129.1135 | | | | | | | | R | 74.0237 | 73.0284 | ***175.1190*** | 158.0924 | | 1 |

| Seq | ya | yb | Seq | ya | yb | Seq | ya | yb |
|---|---|---|---|---|---|---|---|---|
| MH | 241.1118 | 269.1067 | MHF | 388.1802 | 416.1751 | MHFY | 551.2435 | 579.2384 |
| MHFYA | 622.2806 | 650.2755 | HF | 257.1397 | 285.1346 | HFY | 420.2030 | 448.1979 |
| HFYA | 491.2401 | 519.2350 | HFYAE | 620.2827 | 648.2776 | FY | 283.1441 | 311.1390 |
| FYA | 354.1812 | 382.1761 | FYAE | 483.2238 | 511.2187 | FYAEP | 580.2766 | 608.2715 |

| | | | | | | | | |
|---|---|---|---|---|---|---|---|---|
| **YA** | 207.1128 | 235.1077 | **YAE** | 336.1554 | 364.1503 | **YAEP** | 433.2082 | 461.2031 |
| **AE** | 173.0921 | 201.0870 | **AEP** | 270.1448 | 298.1397 | **EP** | 199.1077 | 227.1026 |

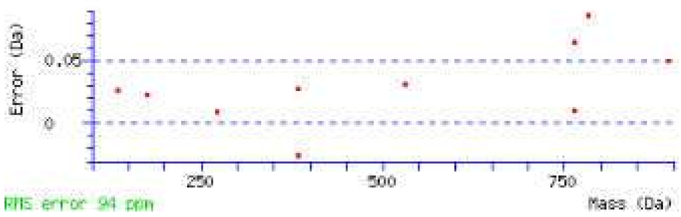


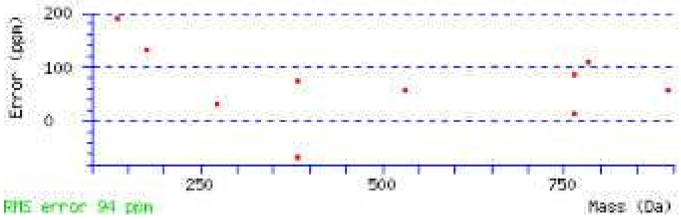


NCBI **BLAST** search of NMHFYAEPR
(Parameters: blastp, nr protein database, expect=20000, no filter, PAM30)
Other BLAST web gateways

**All matches to this query**

| Score | Mr(calc) | Delta | Sequence |
|---|---|---|---|
| 33.6 | 1163.5182 | 0.0493 | NMHFYAEPR |
| 32.5 | 1163.5935 | -0.0259 | ITGEAYLNQR |
| 27.5 | 1163.5683 | -0.0008 | AAEEAYRQAR |
| 27.5 | 1163.5934 | -0.0259 | DREYAEIIR |
| 27.5 | 1163.5822 | -0.0147 | GVDEYAELLR |
| 27.5 | 1163.6186 | -0.0511 | IASEYAELIR |
| 27.5 | 1163.5142 | 0.0534 | RDEAYHSMR |
| 27.5 | 1163.5934 | -0.0259 | RDEAYIEIR |
| 27.5 | 1163.5934 | -0.0259 | SALEYAGNLAR |
| 27.3 | 1163.5294 | 0.0381 | ASLXTALXNPR |

**Mascot:** http://www.matrixscience.com/

# MATRIX SCIENCE Mascot Search Results

## Peptide View Spot no 57

MS/MS Fragmentation of **SSTNLPEDLKER**
Found in **gi|115471157** in **NCBInr**, Os07g0212200 [Oryza sativa Japonica Group]

Match to Query 203: 1387.755224 from(1388.762500,1+) intensity(0.0000) index(23)
Title: Label: N11, Spot_Id: 219847, Peak_List_Id: 227554, MSMS Job_Run_Id: 21965, Comment:
Data file C:\Documents and Settings\Administrator\Desktop\Farah-Garima 11May 2013\ppw_N11_136868230800.txt

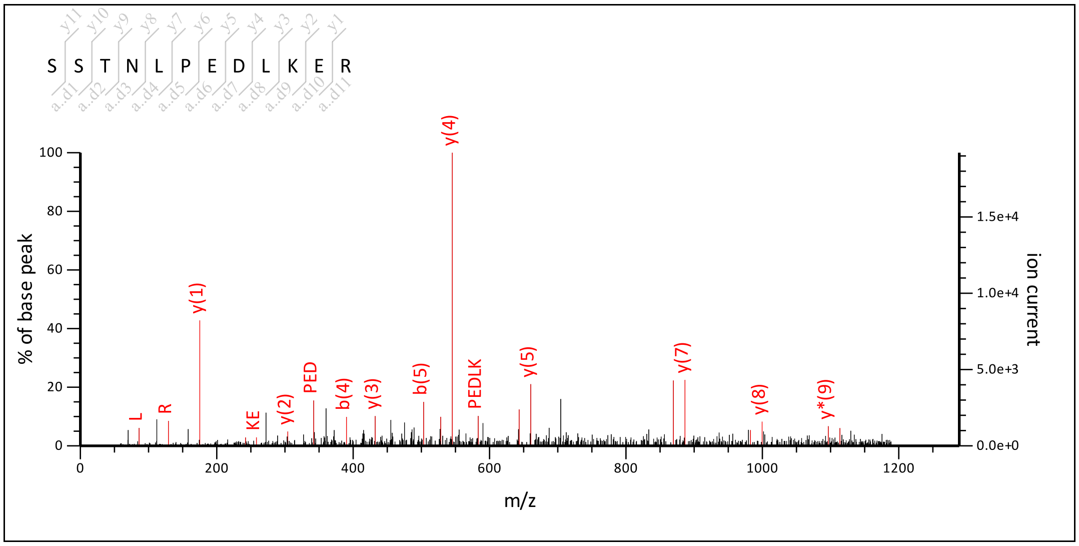


0 to 1288.72

Label all possible matches ○ Label matches used for scoring ◉

**Monoisotopic mass of neutral peptide Mr(calc):** 1387.6943
**Fixed modifications:** Carbamidomethyl (C) (apply to specified residues or termini only)
**Ions Score:** 50 **Expect:** 0.34
**Matches :** 27/208 fragment ions using 36 most intense peaks (help)

| # | Immon. | a | a* | $a^0$ | b | b* | $b^0$ | d | d' | Seq. | v | w | w' | y | y* | |
|---|---|---|---|---|---|---|---|---|---|---|---|---|---|---|---|---|
| 1 | 60.0444 | 60.0444 | | 42.0338 | 88.0393 | | 70.0287 | 44.0495 | | S | | | | | | |
| 2 | 60.0444 | 147.0764 | | 129.0659 | **175.0713** | | 157.0608 | 131.0815 | | S | 1269.6434 | 1268.6481 | | 1301.6696 | 1284.6430 | [illegible] |
| 3 | 74.0600 | 248.1241 | | 230.1135 | 276.1190 | | 258.1084 | 232.1292 | 234.1084 | T | 1168.5957 | 1181.6161 | 1183.5953 | 1214.6375 | 1197.6110 | [illegible] |
| 4 | 87.0553 | 362.1670 | 345.1405 | 344.1565 | **390.1619** | 373.1354 | 372.1514 | 319.1612 | | N | 1054.5528 | 1053.5575 | | ***1113.5899*** | 1096.5633 | [illegible] |
| 5 | 86.0964 | 475.2511 | 458.2245 | 457.2405 | **503.2460** | 486.2195 | 485.2354 | 433.2041 | | L | 941.4687 | 940.4734 | | ***999.5469*** | 982.5204 | [illegible] |
| 6 | 70.0651 | 572.3039 | 555.2773 | 554.2933 | 600.2988 | 583.2722 | 582.2882 | 546.2882 | | P | 844.4159 | 843.4207 | | ***886.4629*** | 869.4363 | [illegible] |
| 7 | 102.0550 | 701.3464 | 684.3199 | 683.3359 | 729.3414 | 712.3148 | 711.3308 | 643.3410 | | E | 715.3733 | 714.3781 | | 789.4101 | 772.3836 | [illegible] |
| 8 | 88.0393 | 816.3734 | 799.3468 | 798.3628 | 844.3683 | 827.3418 | 826.3577 | 772.3836 | | D | 600.3464 | 599.3511 | | ***660.3675*** | 643.3410 | [illegible] |
| 9 | 86.0964 | 929.4575 | 912.4309 | 911.4469 | 957.4524 | 940.4258 | 939.4418 | 887.4105 | | L | 487.2623 | 486.2671 | | ***545.3406*** | 528.3140 | [illegible] |
| 10 | 101.1073 | 1057.5524 | 1040.5259 | 1039.5418 | 1085.5473 | 1068.5208 | 1067.5368 | 1000.4946 | | K | 359.1674 | 358.1721 | | ***432.2565*** | 415.2300 | [illegible] |
| 11 | 102.0550 | 1186.5950 | 1169.5685 | 1168.5844 | 1214.5899 | 1197.5634 | 1196.5794 | 1128.5895 | | E | 230.1248 | 229.1295 | | ***304.1615*** | 287.1350 | [illegible] |
| 12 | 129.1135 | | | | | | | | | R | 74.0237 | 73.0284 | | ***175.1190*** | 158.0924 | |

| Seq | ya | yb | Seq | ya | yb | Seq | ya | yb |
|---|---|---|---|---|---|---|---|---|
| ST | 161.0921 | 189.0870 | STN | 275.1350 | 303.1299 | STNL | 388.2191 | 416.2140 |
| STNLP | 485.2718 | 513.2667 | STNLPE | 614.3144 | 642.3093 | TN | 188.1030 | 216.0979 |
| TNL | 301.1870 | 329.1819 | TNLP | 398.2398 | 426.2347 | TNLPE | 527.2824 | 555.2773 |
| TNLPED | 642.3093 | 670.3042 | NL | 200.1394 | 228.1343 | NLP | 297.1921 | 325.1870 |
| NLPE | 426.2347 | 454.2296 | NLPED | 541.2617 | 569.2566 | NLPEDL | 654.3457 | 682.3406 |
| LP | 183.1492 | 211.1441 | LPE | 312.1918 | 340.1867 | LPED | 427.2187 | 455.2136 |
| LPEDL | 540.3028 | 568.2977 | LPEDLK | 668.3978 | 696.3927 | PE | 199.1077 | 227.1026 |
| PED | 314.1347 | 342.1296 | PEDL | 427.2187 | 455.2136 | PEDLK | 555.3137 | 583.3086 |
| PEDLKE | 684.3563 | 712.3512 | ED | 217.0819 | 245.0768 | EDL | 330.1660 | 358.1609 |

| | | | | | | | | |
|---|---|---|---|---|---|---|---|---|
| **EDLK** | 458.2609 | 486.2558 | **EDLKE** | 587.3035 | 615.2984 | **DL** | 201.1234 | 229.1183 |
| **DLK** | 329.2183 | 357.2132 | **DLKE** | 458.2609 | 486.2558 | **LK** | 214.1914 | 242.1863 |
| **LKE** | 343.2340 | 371.2289 | **KE** | 230.1499 | 258.1448 | | | |

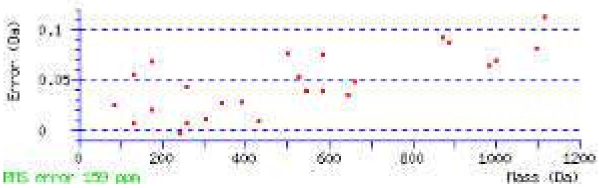


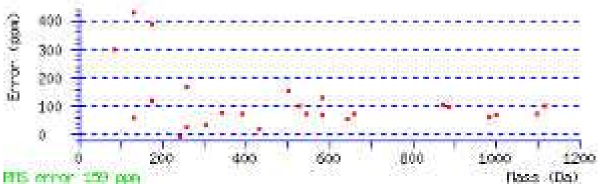


NCBI **BLAST** search of SSTNLPEDLKER
(Parameters: blastp, nr protein database, expect=20000, no filter, PAM30)
Other BLAST web gateways

**All matches to this query**

| Score | Mr(calc) | Delta | Sequence |
|---|---|---|---|
| 50.0 | 1387.6943 | 0.0609 | SSTNLPEDLKER |
| 37.1 | 1387.6765 | 0.0787 | MQNIEPDKLER |
| 36.1 | 1387.6943 | 0.0609 | SDDGKLIDLGAER |
| 34.3 | 1387.6765 | 0.0787 | MKNLPEDDAIAR |
| 33.1 | 1387.7783 | -0.0231 | KSGLVINLSSSAGR |
| 29.9 | 1387.7605 | -0.0053 | GRAMLDLLLTNR |
| 29.7 | 1387.7459 | 0.0093 | LFELINALDANR |
| 27.6 | 1387.6514 | 0.1038 | IDCNINNLQER |
| 27.5 | 1387.7572 | -0.0019 | IQFNINNIKER |
| 27.4 | 1387.6803 | 0.0749 | EAAAAAASSAREGAR |

**Mascot:** http://www.matrixscience.com/

# Mascot Search Results

## Peptide View Spot no 57

MS/MS Fragmentation of **DLASMVALAVESPGAAAGR**
Found in **gi|115471157** in **NCBInr**, Os07g0212200 [Oryza sativa Japonica Group]

Match to Query 245: 1784.997524 from(1786.004800,1+) intensity(0.0000) index(30)
Title: Label: N11, Spot_Id: 219847, Peak_List_Id: 227560, MSMS Job_Run_Id: 21965, Comment:
Data file C:\Documents and Settings\Administrator\Desktop\Farah-Garima 11May 2013\ppw_N11_136868230800.txt

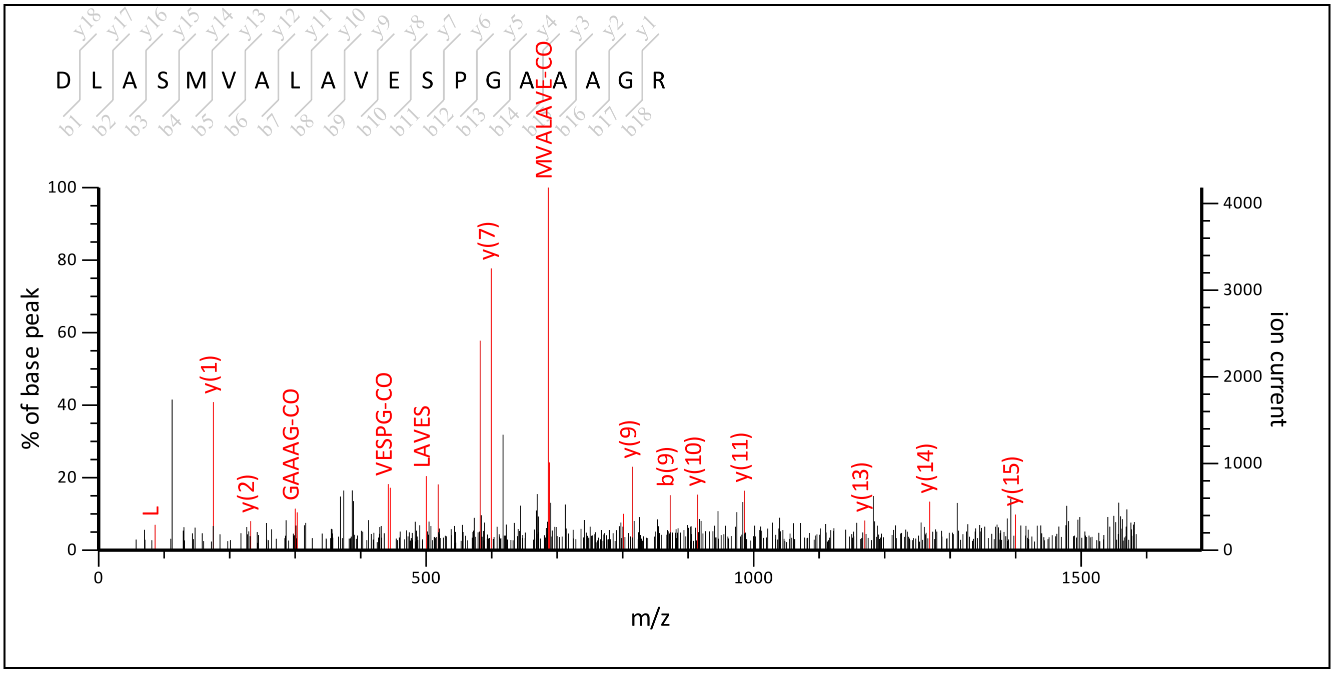


0 to 1684.04

Label all possible matches ○ Label matches used for scoring ◉

**Monoisotopic mass of neutral peptide Mr(calc):** 1784.9091
**Fixed modifications:** Carbamidomethyl (C) (apply to specified residues or termini only)
**Ions Score:** 88 **Expect:** 3.8e-05
**Matches :** 30/343 fragment ions using 32 most intense peaks (help)

| # | Immon. | a | a0 | b | b0 | d | Seq. | v | w | y | y* | y0 | # |
|---|---|---|---|---|---|---|---|---|---|---|---|---|---|
| 1 | 88.0393 | 88.0393 | 70.0287 | 116.0342 | 98.0237 | 44.0495 | D | | | | | | 19 |
| 2 | 86.0964 | 201.1234 | 183.1128 | 229.1183 | 211.1077 | 159.0764 | L | 1612.8112 | 1611.8159 | 1670.8894 | 1653.8629 | 1652.8789 | 18 |
| 3 | 44.0495 | 272.1605 | 254.1499 | **300.1554** | 282.1448 | | A | 1541.7741 | | 1557.8054 | 1540.7788 | 1539.7948 | 17 |
| 4 | 60.0444 | 359.1925 | 341.1819 | 387.1874 | 369.1769 | 343.1976 | S | 1454.7420 | 1453.7468 | 1486.7682 | 1469.7417 | 1468.7577 | 16 |
| 5 | 104.0528 | 490.2330 | 472.2224 | **518.2279** | 500.2173 | 430.2296 | M | 1323.7015 | 1322.7063 | ***1399.7362*** | 1382.7097 | 1381.7256 | 15 |
| 6 | 72.0808 | 589.3014 | 571.2908 | 617.2963 | 599.2858 | 575.2858 | V | 1224.6331 | 1237.6535 | ***1268.6957*** | 1251.6692 | 1250.6852 | 14 |
| 7 | 44.0495 | 660.3385 | 642.3280 | **688.3334** | 670.3229 | | A | 1153.5960 | | ***1169.6273*** | 1152.6008 | 1151.6167 | 13 |
| 8 | 86.0964 | 773.4226 | 755.4120 | **801.4175** | 783.4069 | 731.3756 | L | 1040.5119 | 1039.5167 | 1098.5902 | 1081.5637 | 1080.5796 | 12 |
| 9 | 44.0495 | 844.4597 | 826.4491 | **872.4546** | 854.4441 | | A | 969.4748 | | ***985.5061*** | 968.4796 | 967.4956 | 11 |
| 10 | 72.0808 | 943.5281 | 925.5175 | 971.5230 | 953.5125 | 929.5125 | V | 870.4064 | 883.4268 | ***914.4690*** | 897.4425 | 896.4585 | 10 |
| 11 | 102.0550 | 1072.5707 | 1054.5601 | 1100.5656 | 1082.5551 | 1014.5652 | E | 741.3638 | 740.3686 | ***815.4006*** | 798.3741 | 797.3900 | 9 |
| 12 | 60.0444 | 1159.6027 | 1141.5922 | 1187.5977 | 1169.5871 | 1143.6078 | S | 654.3318 | 653.3366 | ***686.3580*** | 669.3315 | 668.3474 | 8 |

| | | | | | | | | | | | | | |
|---|---|---|---|---|---|---|---|---|---|---|---|---|---|
| 13 | 70.0651 | 1256.6555 | 1238.6449 | 1284.6504 | 1266.6399 | 1230.6399 | P | 557.2790 | 556.2838 | ***599.3260*** | 582.2994 | | 7 |
| 14 | 30.0338 | 1313.6770 | 1295.6664 | 1341.6719 | 1323.6613 | | G | | | 502.2732 | 485.2467 | | 6 |
| 15 | 44.0495 | 1384.7141 | 1366.7035 | 1412.7090 | 1394.6984 | | A | 429.2205 | | ***445.2518*** | 428.2252 | | 5 |
| 16 | 44.0495 | 1455.7512 | 1437.7406 | 1483.7461 | 1465.7355 | | A | 358.1833 | | 374.2146 | 357.1881 | | 4 |
| 17 | 44.0495 | 1526.7883 | 1508.7777 | 1554.7832 | 1536.7727 | | A | 287.1462 | | ***303.1775*** | 286.1510 | | 3 |
| 18 | 30.0338 | 1583.8098 | 1565.7992 | 1611.8047 | 1593.7941 | | G | | | ***232.1404*** | 215.1139 | | 2 |
| 19 | 129.1135 | | | | | | R | 74.0237 | 73.0284 | ***175.1190*** | 158.0924 | | 1 |

| Seq | ya | yb | Seq | ya | yb | Seq | ya | yb |
|---|---|---|---|---|---|---|---|---|
| LA | 157.1335 | 185.1285 | LAS | 244.1656 | 272.1605 | LASM | 375.2061 | 403.2010 |
| LASMV | 474.2745 | 502.2694 | LASMVA | 545.3116 | 573.3065 | LASMVAL | 658.3956 | 686.3906 |
| AS | 131.0815 | 159.0764 | ASM | 262.1220 | 290.1169 | ASMV | 361.1904 | 389.1853 |
| ASMVA | 432.2275 | 460.2224 | ASMVAL | 545.3116 | 573.3065 | ASMVALA | 616.3487 | 644.3436 |
| SM | 191.0849 | 219.0798 | SMV | 290.1533 | 318.1482 | SMVA | 361.1904 | 389.1853 |
| SMVAL | 474.2745 | 502.2694 | SMVALA | 545.3116 | 573.3065 | SMVALAV | 644.3800 | 672.3749 |
| MV | 203.1213 | 231.1162 | MVA | 274.1584 | 302.1533 | MVAL | 387.2424 | 415.2374 |
| MVALA | 458.2796 | 486.2745 | MVALAV | 557.3480 | 585.3429 | MVALAVE | 686.3906 | 714.3855 |
| VA | 143.1179 | 171.1128 | VAL | 256.2020 | 284.1969 | VALA | 327.2391 | 355.2340 |
| VALAV | 426.3075 | 454.3024 | VALAVE | 555.3501 | 583.3450 | VALAVES | 642.3821 | 670.3770 |
| AL | 157.1335 | 185.1285 | ALA | 228.1707 | 256.1656 | ALAV | 327.2391 | 355.2340 |
| ALAVE | 456.2817 | 484.2766 | ALAVES | 543.3137 | 571.3086 | ALAVESP | 640.3665 | 668.3614 |
| ALAVESPG | 697.3879 | 725.3828 | LA | 157.1335 | 185.1285 | LAV | 256.2020 | 284.1969 |
| LAVE | 385.2445 | 413.2395 | LAVES | 472.2766 | 500.2715 | LAVESP | 569.3293 | 597.3243 |
| LAVESPG | 626.3508 | 654.3457 | LAVESPGA | 697.3879 | 725.3828 | AV | 143.1179 | 171.1128 |
| AVE | 272.1605 | 300.1554 | AVES | 359.1925 | 387.1874 | AVESP | 456.2453 | 484.2402 |
| AVESPG | 513.2667 | 541.2617 | AVESPGA | 584.3039 | 612.2988 | AVESPGAA | 655.3410 | 683.3359 |
| VE | 201.1234 | 229.1183 | VES | 288.1554 | 316.1503 | VESP | 385.2082 | 413.2031 |
| VESPG | 442.2296 | 470.2245 | VESPGA | 513.2667 | 541.2617 | VESPGAA | 584.3039 | 612.2988 |
| VESPGAAA | 655.3410 | 683.3359 | ES | 189.0870 | 217.0819 | ESP | 286.1397 | 314.1347 |
| ESPG | 343.1612 | 371.1561 | ESPGA | 414.1983 | 442.1932 | ESPGAA | 485.2354 | 513.2304 |
| ESPGAAA | 556.2726 | 584.2675 | ESPGAAAG | 613.2940 | 641.2889 | SP | 157.0972 | 185.0921 |
| SPG | 214.1186 | 242.1135 | SPGA | 285.1557 | 313.1506 | SPGAA | 356.1928 | 384.1878 |
| SPGAAA | 427.2300 | 455.2249 | SPGAAAG | 484.2514 | 512.2463 | PG | 127.0866 | 155.0815 |
| PGA | 198.1237 | 226.1186 | PGAA | 269.1608 | 297.1557 | PGAAA | 340.1979 | 368.1928 |
| PGAAAG | 397.2194 | 425.2143 | GA | 101.0709 | 129.0659 | GAA | 172.1081 | 200.1030 |
| GAAA | 243.1452 | 271.1401 | GAAAG | 300.1666 | 328.1615 | AA | 115.0866 | 143.0815 |
| AAA | 186.1237 | 214.1186 | AAAG | 243.1452 | 271.1401 | AA | 115.0866 | 143.0815 |
| AAG | 172.1081 | 200.1030 | AG | 101.0709 | 129.0659 | | | |

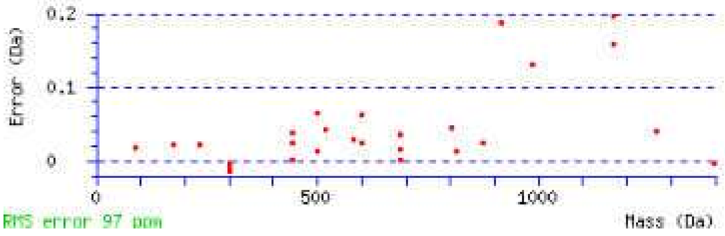

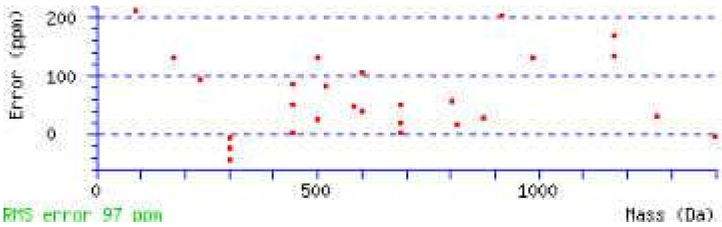


NCBI **BLAST** search of DLASMVALAVESPGAAAGR
(Parameters: blastp, nr protein database, expect=20000, no filter, PAM30)
Other BLAST web gateways

**All matches to this query**

| Score | Mr(calc) | Delta | Sequence |
|---|---|---|---|
| 88.3 | 1784.9091 | 0.0885 | DLASMVALAVESPGAAAGR |
| 30.8 | 1784.9454 | 0.0521 | EREIAAMLNLNPSTVK |
| 29.9 | 1785.0148 | -0.0173 | ESRAPFIIALAGSVAVGK |
| 29.9 | 1785.0620 | -0.0645 | LAMLMGALALLTSLLVR |
| 28.1 | 1784.8978 | 0.0997 | AGVALPVEAVEVEAMER |
| 26.9 | 1784.9892 | 0.0083 | IEASLMALAVNKPMVAK |
| 25.4 | 1784.8978 | 0.0997 | EMIAKLDAIGPEVDER |
| 25.0 | 1784.9342 | 0.0633 | LELATVSDQMPNGAIVK |
| 24.8 | 1784.9594 | 0.0381 | ESATLVDLPITVGMPVK |
| 24.8 | 1784.8918 | 0.1058 | WLSDGAGRAALADAQQR |

**Mascot:** http://www.matrixscience.com/

# MATRIX SCIENCE Mascot Search Results

## Peptide View Spot no 57

MS/MS Fragmentation of **DLASMVALAVESPGAAAGR**
Found in **gi|115471157** in **NCBInr**, Os07g0212200 [Oryza sativa Japonica Group]

Match to Query 247: 1800.980924 from(1801.988200,1+) intensity(0.0000) index(31)
Title: Label: N11, Spot_Id: 219847, Peak_List_Id: 227578, MSMS Job_Run_Id: 21965, Comment:
Data file C:\Documents and Settings\Administrator\Desktop\Farah-Garima 11May 2013\ppw_N11_136868230800.txt

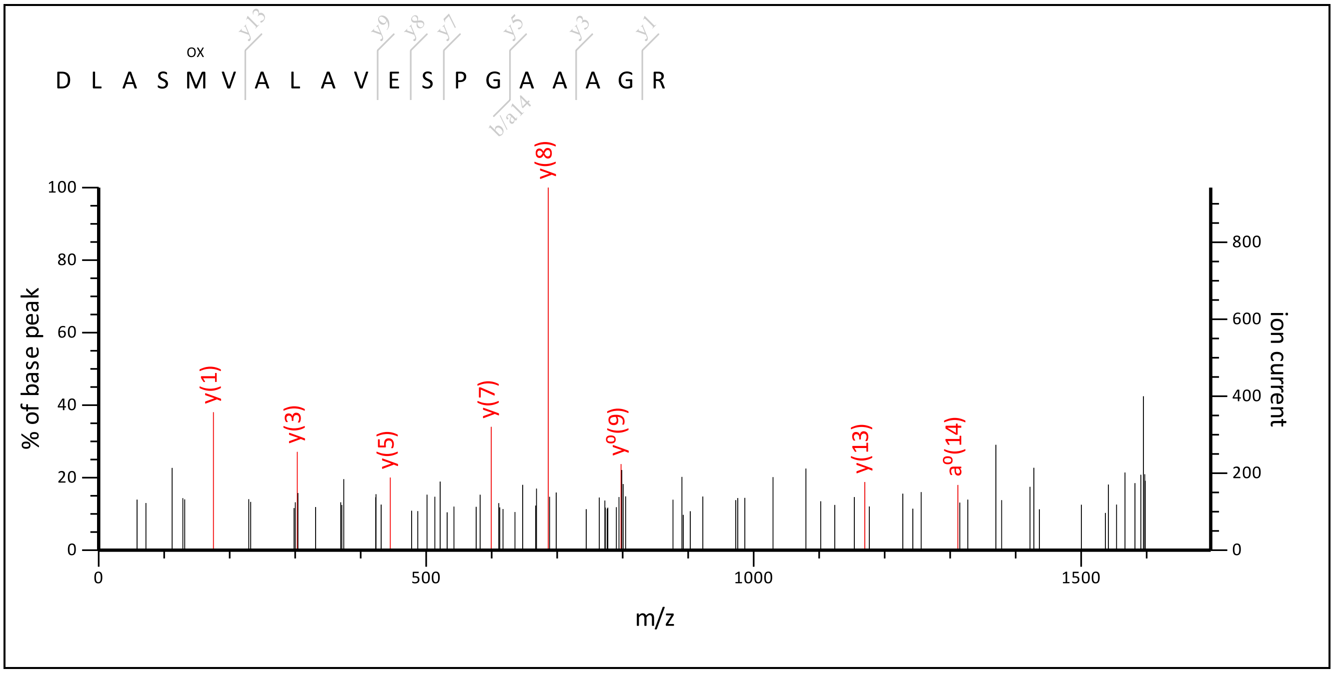


0 to 1697.79

Label all possible matches ○ Label matches used for scoring ◉

**Monoisotopic mass of neutral peptide Mr(calc):** 1800.9040
**Fixed modifications:** Carbamidomethyl (C) (apply to specified residues or termini only)
**Variable modifications:**
**M5** : Oxidation (M), with neutral losses 0.0000(shown in table), 63.9983
**Ions Score:** 23 **Expect:** 1.3e+02
**Matches :** 9/460 fragment ions using 16 most intense peaks (help)

| # | Immon. | a | $a^0$ | b | $b^0$ | d | Seq. | v | w | y | y* | $y^0$ | # |
|---|---|---|---|---|---|---|---|---|---|---|---|---|---|
| 1 | 88.0393 | 88.0393 | 70.0287 | 116.0342 | 98.0237 | 44.0495 | D | | | | | | 19 |
| 2 | 86.0964 | 201.1234 | 183.1128 | 229.1183 | 211.1077 | 159.0764 | L | 1628.8061 | 1627.8108 | 1686.8843 | 1669.8578 | 1668.8738 | 18 |
| 3 | 44.0495 | 272.1605 | 254.1499 | 300.1554 | 282.1448 | | A | 1557.7690 | | 1573.8003 | 1556.7737 | 1555.7897 | 17 |
| 4 | 60.0444 | 359.1925 | 341.1819 | 387.1874 | 369.1769 | 343.1976 | S | 1470.7369 | 1469.7417 | 1502.7632 | 1485.7366 | 1484.7526 | 16 |
| 5 | 120.0478 | 506.2279 | 488.2173 | 534.2228 | 516.2123 | 430.2296 | M | 1323.7015 | 1322.7063 | 1415.7311 | 1398.7046 | 1397.7206 | 15 |
| 6 | 72.0808 | 605.2963 | 587.2858 | 633.2912 | 615.2807 | 591.2807 | V | 1224.6331 | 1237.6535 | 1268.6957 | 1251.6692 | 1250.6852 | 14 |
| 7 | 44.0495 | 676.3334 | 658.3229 | 704.3284 | 686.3178 | | A | 1153.5960 | | ***1169.6273*** | 1152.6008 | 1151.6167 | 13 |
| 8 | 86.0964 | 789.4175 | 771.4069 | 817.4124 | 799.4019 | 747.3706 | L | 1040.5119 | 1039.5167 | 1098.5902 | 1081.5637 | 1080.5796 | 12 |
| 9 | 44.0495 | 860.4546 | 842.4441 | 888.4495 | 870.4390 | | A | 969.4748 | | 985.5061 | 968.4796 | 967.4956 | 11 |

| | | | | | | | | | | | | | |
|---|---|---|---|---|---|---|---|---|---|---|---|---|---|
| 10 | 72.0808 | 959.5230 | 941.5125 | 987.5179 | 969.5074 | 945.5074 | V | 870.4064 | 883.4268 | 914.4690 | 897.4425 | 896.4585 | 10 |
| 11 | 102.0550 | 1088.5656 | 1070.5551 | 1116.5605 | 1098.5500 | 1030.5601 | E | 741.3638 | 740.3686 | 815.4006 | 798.3741 | 797.3900 | 9 |
| 12 | 60.0444 | 1175.5977 | 1157.5871 | 1203.5926 | 1185.5820 | 1159.6027 | S | 654.3318 | 653.3366 | 686.3580 | 669.3315 | 668.3474 | 8 |
| 13 | 70.0651 | 1272.6504 | 1254.6399 | 1300.6453 | 1282.6348 | 1246.6348 | P | 557.2790 | 556.2838 | 599.3260 | 582.2994 | | 7 |
| 14 | 30.0338 | 1329.6719 | 1311.6613 | 1357.6668 | 1339.6562 | | G | | | 502.2732 | 485.2467 | | 6 |
| 15 | 44.0495 | 1400.7090 | 1382.6984 | 1428.7039 | 1410.6933 | | A | 429.2205 | | 445.2518 | 428.2252 | | 5 |
| 16 | 44.0495 | 1471.7461 | 1453.7355 | 1499.7410 | 1481.7305 | | A | 358.1833 | | 374.2146 | 357.1881 | | 4 |
| 17 | 44.0495 | 1542.7832 | 1524.7727 | 1570.7781 | 1552.7676 | | A | 287.1462 | | 303.1775 | 286.1510 | | 3 |
| 18 | 30.0338 | 1599.8047 | 1581.7941 | 1627.7996 | 1609.7890 | | G | | | 232.1404 | 215.1139 | | 2 |
| 19 | 129.1135 | | | | | | R | 74.0237 | 73.0284 | 175.1190 | 158.0924 | | 1 |

| Seq | ya | yb | Seq | ya | yb | Seq | ya | yb |
|---|---|---|---|---|---|---|---|---|
| LA | 157.1335 | 185.1285 | LAS | 244.1656 | 272.1605 | LASM | 391.2010 | 419.1959 |
| LASMV | 490.2694 | 518.2643 | LASMVA | 561.3065 | 589.3014 | LASMVAL | 674.3906 | 702.3855 |
| AS | 131.0815 | 159.0764 | ASM | 278.1169 | 306.1118 | ASMV | 377.1853 | 405.1802 |
| ASMVA | 448.2224 | 476.2173 | ASMVAL | 561.3065 | 589.3014 | ASMVALA | 632.3436 | 660.3385 |
| SM | 207.0798 | 235.0747 | SMV | 306.1482 | 334.1431 | SMVA | 377.1853 | 405.1802 |
| SMVAL | 490.2694 | 518.2643 | SMVALA | 561.3065 | 589.3014 | SMVALAV | 660.3749 | 688.3698 |
| MV | 219.1162 | 247.1111 | MVA | 290.1533 | 318.1482 | MVAL | 403.2374 | 431.2323 |
| MVALA | 474.2745 | 502.2694 | MVALAV | 573.3429 | 601.3378 | VA | 143.1179 | 171.1128 |
| VAL | 256.2020 | 284.1969 | VALA | 327.2391 | 355.2340 | VALAV | 426.3075 | 454.3024 |
| VALAVE | 555.3501 | 583.3450 | VALAVES | 642.3821 | 670.3770 | AL | 157.1335 | 185.1285 |
| ALA | 228.1707 | 256.1656 | ALAV | 327.2391 | 355.2340 | ALAVE | 456.2817 | 484.2766 |
| ALAVES | 543.3137 | 571.3086 | ALAVESP | 640.3665 | 668.3614 | ALAVESPG | 697.3879 | 725.3828 |
| LA | 157.1335 | 185.1285 | LAV | 256.2020 | 284.1969 | LAVE | 385.2445 | 413.2395 |
| LAVES | 472.2766 | 500.2715 | LAVESP | 569.3293 | 597.3243 | LAVESPG | 626.3508 | 654.3457 |
| LAVESPGA | 697.3879 | 725.3828 | AV | 143.1179 | 171.1128 | AVE | 272.1605 | 300.1554 |
| AVES | 359.1925 | 387.1874 | AVESP | 456.2453 | 484.2402 | AVESPG | 513.2667 | 541.2617 |
| AVESPGA | 584.3039 | 612.2988 | AVESPGAA | 655.3410 | 683.3359 | VE | 201.1234 | 229.1183 |
| VES | 288.1554 | 316.1503 | VESP | 385.2082 | 413.2031 | VESPG | 442.2296 | 470.2245 |
| VESPGA | 513.2667 | 541.2617 | VESPGAA | 584.3039 | 612.2988 | VESPGAAA | 655.3410 | 683.3359 |
| ES | 189.0870 | 217.0819 | ESP | 286.1397 | 314.1347 | ESPG | 343.1612 | 371.1561 |
| ESPGA | 414.1983 | 442.1932 | ESPGAA | 485.2354 | 513.2304 | ESPGAAA | 556.2726 | 584.2675 |
| ESPGAAAG | 613.2940 | 641.2889 | SP | 157.0972 | 185.0921 | SPG | 214.1186 | 242.1135 |
| SPGA | 285.1557 | 313.1506 | SPGAA | 356.1928 | 384.1878 | SPGAAA | 427.2300 | 455.2249 |
| SPGAAAG | 484.2514 | 512.2463 | PG | 127.0866 | 155.0815 | PGA | 198.1237 | 226.1186 |
| PGAA | 269.1608 | 297.1557 | PGAAA | 340.1979 | 368.1928 | PGAAAG | 397.2194 | 425.2143 |
| GA | 101.0709 | 129.0659 | GAA | 172.1081 | 200.1030 | GAAA | 243.1452 | 271.1401 |
| GAAAG | 300.1666 | 328.1615 | AA | 115.0866 | 143.0815 | AAA | 186.1237 | 214.1186 |
| AAAG | 243.1452 | 271.1401 | AA | 115.0866 | 143.0815 | AAG | 172.1081 | 200.1030 |
| AG | 101.0709 | 129.0659 | | | | | | |

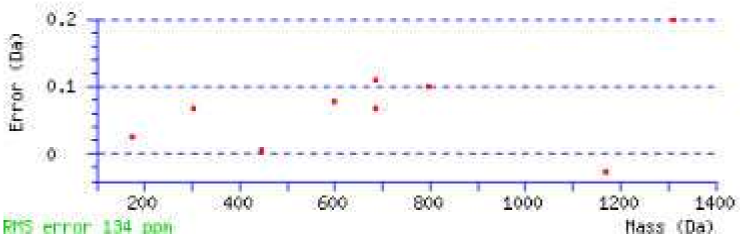


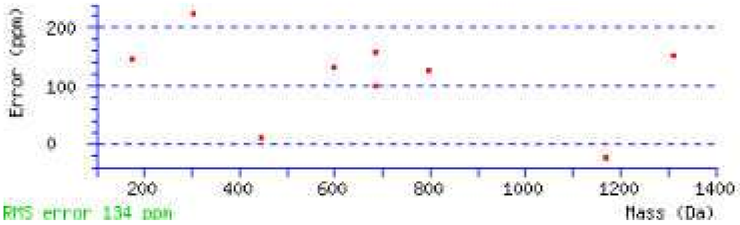


NCBI **BLAST** search of DLASMVALAVESPGAAAGR
(Parameters: blastp, nr protein database, expect=20000, no filter, PAM30)
Other BLAST web gateways

**All matches to this query**

| Score | Mr(calc) | Delta | Sequence |
|---|---|---|---|
| 23.5 | 1800.9040 | 0.0769 | DLASMVALAVESPGAAAGR |
| 15.9 | 1801.0686 | -0.0877 | KPRLPGPGSLLSKPAQR |
| 14.5 | 1800.9078 | 0.0731 | SQQLGSATGNTASAKPQR |
| 14.4 | 1800.8828 | 0.0981 | QLTWGETPELMAAAQR |
| 12.0 | 1800.9516 | 0.0294 | IMNIQARLETEIAAGR |
| 10.3 | 1800.8684 | 0.1125 | IESMRALGMYLSMQR |
| 10.2 | 1800.8101 | 0.1709 | MEPEIDDFHKDAAAGR |
| 10.1 | 1800.8900 | 0.0909 | MTTAAAAARGAAPESAAQR |
| 9.3 | 1800.9330 | 0.0480 | ALDASADEILGVRTSAGR |
| 9.2 | 1800.8966 | 0.0843 | TTALEEQNTQQAAIAGR |

**Mascot:** http://www.matrixscience.com/

# MATRIX SCIENCE Mascot Search Results

## Peptide View Spot no 58

MS/MS Fragmentation of **EAAYYQQGAR**
Found in **gi|108864048** in **NCBInr**, Fructose-bisphosphate aldolase, chloroplast precursor, putative, expressed [Oryza sativa Japonica Group]

Match to Query 82: 1155.571324 from(1156.578600,1+) intensity(0.0000) index(9)
Title: Label: C7, Spot_Id: 219772, Peak_List_Id: 226176, MSMS Job_Run_Id: 21836, Comment:
Data file C:\Documents and Settings\Administrator\Desktop\Farah-Garima 11May 2013\ppw_C7_136859909200.txt

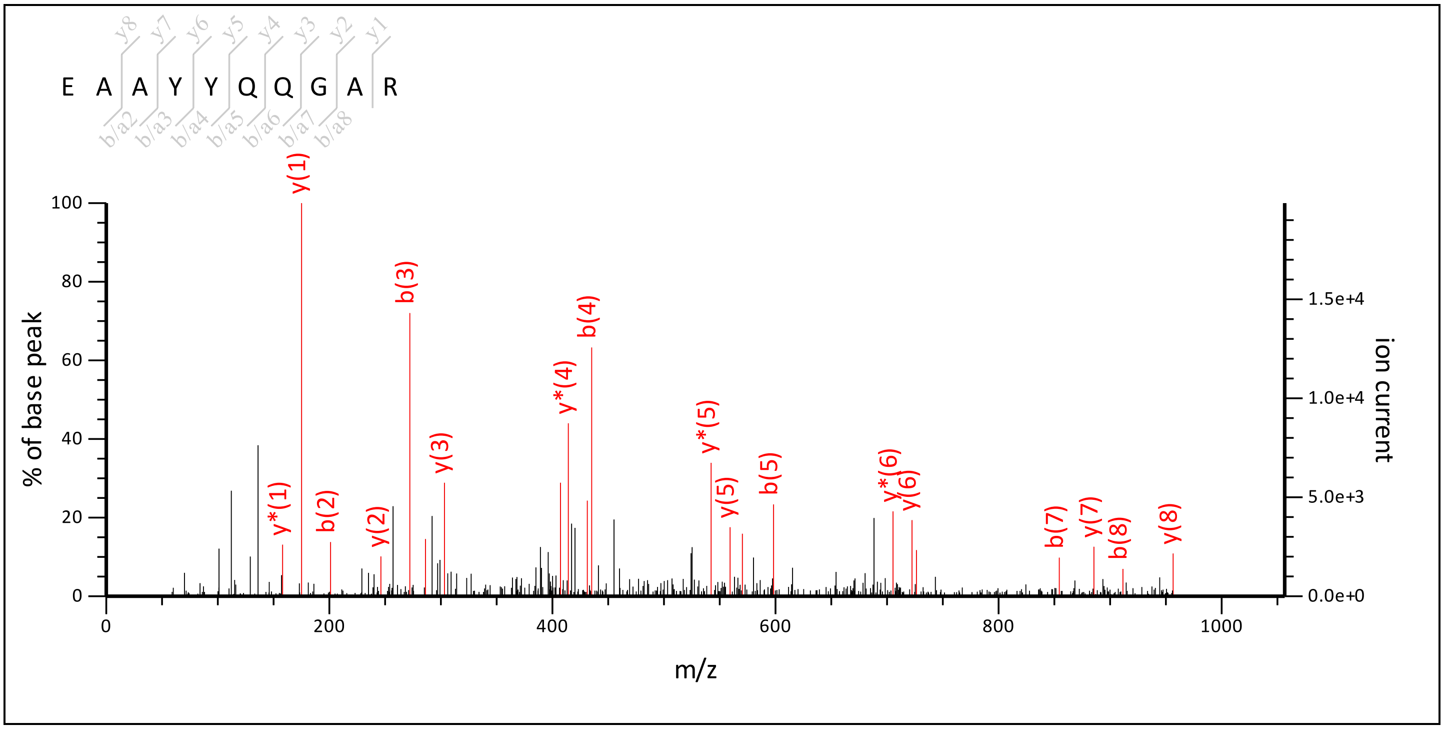


0 to 1056.51

Label all possible matches ○ Label matches used for scoring ●

**Monoisotopic mass of neutral peptide Mr(calc):** 1155.5309
**Fixed modifications:** Carbamidomethyl (C) (apply to specified residues or termini only)
**Ions Score:** 56 **Expect:** 0.086
**Matches :** 22/44 fragment ions using 36 most intense peaks (help)

| # | a | a* | b | b* | Seq. | y | y* | # |
|---|---|---|---|---|---|---|---|---|
| 1 | 102.0550 | | 130.0499 | | E | | | 10 |
| 2 | 173.0921 | | **201.0870** | | A | 1027.4956 | 1010.4690 | 9 |
| 3 | 244.1292 | | **272.1241** | | A | ***956.4585*** | 939.4319 | 8 |
| 4 | **407.1925** | | **435.1874** | | Y | ***885.4213*** | 868.3948 | 7 |
| 5 | **570.2558** | | **598.2508** | | Y | ***722.3580*** | 705.3315 | 6 |
| 6 | 698.3144 | 681.2879 | **726.3093** | 709.2828 | Q | ***559.2947*** | 542.2681 | 5 |
| 7 | 826.3730 | 809.3464 | **854.3679** | 837.3414 | Q | ***431.2361*** | 414.2096 | 4 |

| | | | | | | | | |
|---|---|---|---|---|---|---|---|---|
| **8** | 883.3945 | 866.3679 | **911.3894** | 894.3628 | **G** | ***303.1775*** | 286.1510 | **3** |
| **9** | 954.4316 | 937.4050 | 982.4265 | 965.3999 | **A** | ***246.1561*** | 229.1295 | **2** |
| **10** | | | | | **R** | ***175.1190*** | 158.0924 | **1** |

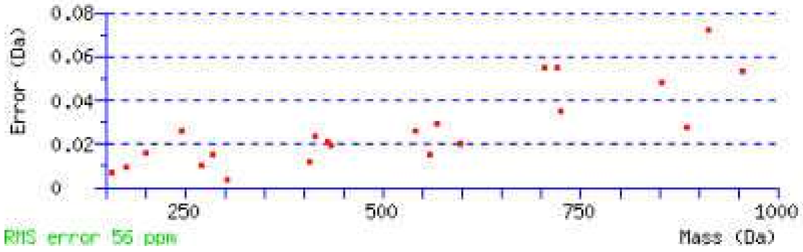


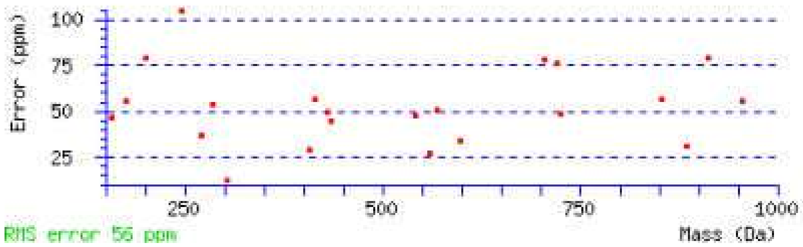


NCBI **BLAST** search of EAAYYQQGAR
(Parameters: blastp, nr protein database, expect=20000, no filter, PAM30)
Other BLAST web gateways

**All matches to this query**

| Score | Mr(calc) | Delta | Sequence |
|---|---|---|---|
| 56.3 | 1155.5309 | 0.0405 | EAAYYQQGAR |
| 42.3 | 1155.5672 | 0.0041 | AAEYYKQGAR |
| 35.6 | 1155.6360 | -0.0647 | TVAIAAAQQGAR |
| 29.7 | 1155.5461 | 0.0252 | RDYYTWPR |
| 27.3 | 1155.5713 | 0.0001 | AYPNLYYPR |
| 27.3 | 1155.5672 | 0.0041 | DREASYFIR |
| 27.3 | 1155.5924 | -0.0211 | ESTLKYYPR |
| 27.3 | 1155.6110 | -0.0397 | MKIVSYYPR |
| 27.3 | 1155.5746 | -0.0033 | NAILMYYPR |
| 24.7 | 1155.6360 | -0.0647 | SIAAAQQALAGR |

**Mascot:** http://www.matrixscience.com/

# MATRIX SCIENCE Mascot Search Results

## Peptide View Spot no 58

MS/MS Fragmentation of **LASIGLENTEANR**
Found in **gi|108864048** in **NCBInr**, Fructose-bisphosphate aldolase, chloroplast precursor, putative, expressed [Oryza sativa Japonica Group]

Match to Query 134: 1386.757224 from(1387.764500,1+) intensity(0.0000) index(15)
Title: Label: C7, Spot_Id: 219772, Peak_List_Id: 226182, MSMS Job_Run_Id: 21836, Comment:
Data file C:\Documents and Settings\Administrator\Desktop\Farah-Garima 11May 2013\ppw_C7_136859909200.txt

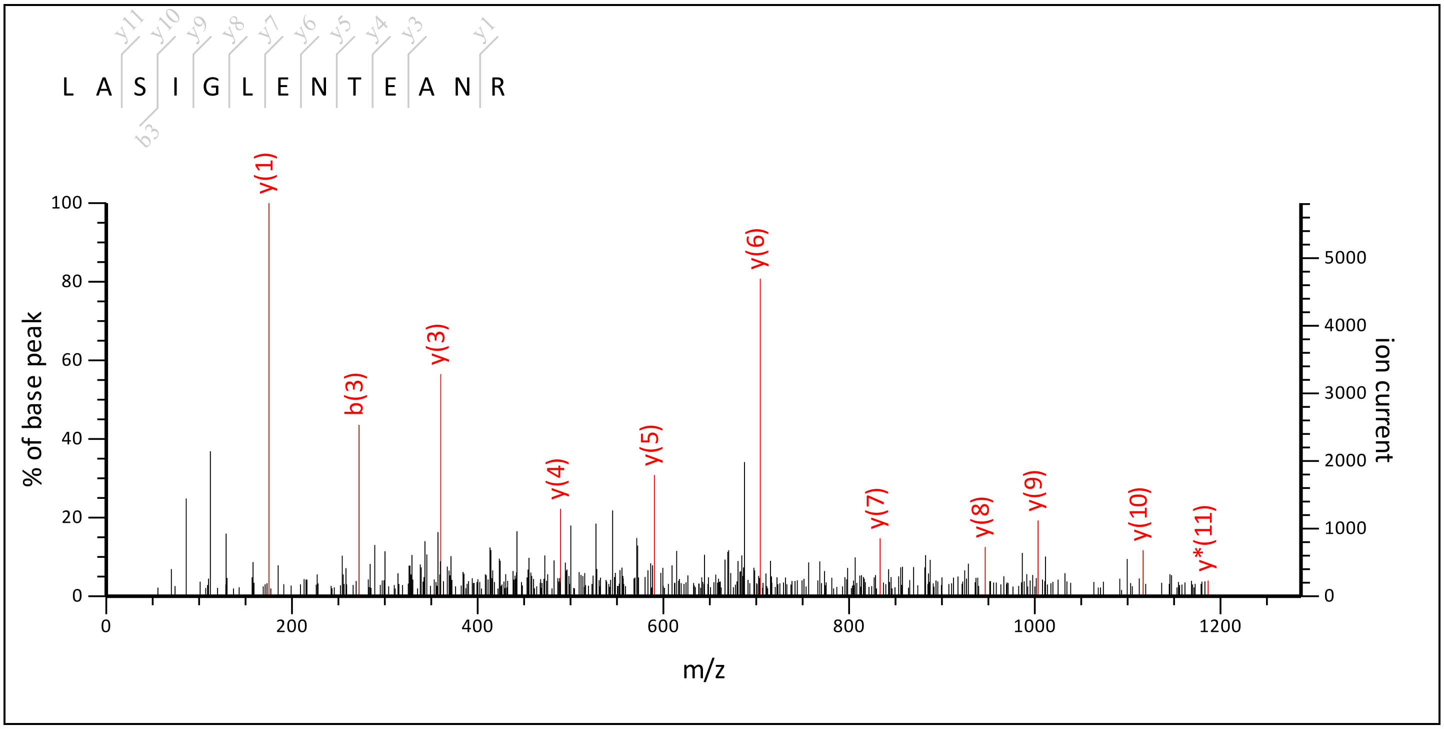


0 to 1286.68

Label all possible matches ○ Label matches used for scoring ◉

**Monoisotopic mass of neutral peptide Mr(calc):** 1386.7103
**Fixed modifications:** Carbamidomethyl (C) (apply to specified residues or termini only)
**Ions Score:** 91 **Expect:** 2.4e-05
**Matches :** 12/58 fragment ions using 12 most intense peaks (help)

| # | a | a* | b | b* | Seq. | y | y* | # |
|---|---|---|---|---|---|---|---|---|
| 1 | 86.0964 | | 114.0913 | | L | | | 13 |
| 2 | 157.1335 | | 185.1285 | | A | 1274.6335 | 1257.6070 | 12 |
| 3 | 244.1656 | | **272.1605** | | S | 1203.5964 | 1186.5699 | 11 |
| 4 | 357.2496 | | 385.2445 | | I | ***1116.5644*** | 1099.5378 | 10 |
| 5 | 414.2711 | | 442.2660 | | G | ***1003.4803*** | 986.4538 | 9 |
| 6 | 527.3552 | | 555.3501 | | L | ***946.4588*** | 929.4323 | 8 |
| 7 | 656.3978 | | 684.3927 | | E | ***833.3748*** | 816.3482 | 7 |

| | | | | | | | | |
|---|---|---|---|---|---|---|---|---|
| 8 | 770.4407 | 753.4141 | 798.4356 | 781.4090 | N | *704.3322* | 687.3056 | 6 |
| 9 | 871.4884 | 854.4618 | 899.4833 | 882.4567 | T | *590.2893* | 573.2627 | 5 |
| 10 | 1000.5310 | 983.5044 | 1028.5259 | 1011.4993 | E | *489.2416* | 472.2150 | 4 |
| 11 | 1071.5681 | 1054.5415 | 1099.5630 | 1082.5364 | A | *360.1990* | 343.1724 | 3 |
| 12 | 1185.6110 | 1168.5844 | 1213.6059 | 1196.5794 | N | 289.1619 | 272.1353 | 2 |
| 13 | | | | | R | *175.1190* | 158.0924 | 1 |

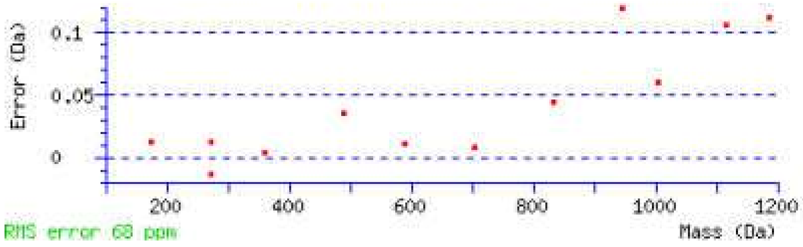


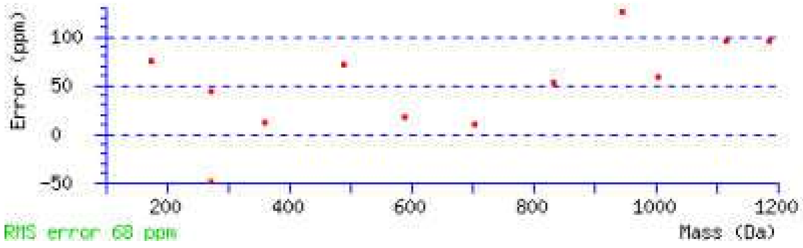


NCBI **BLAST** search of LASIGLENTEANR
(Parameters: blastp, nr protein database, expect=20000, no filter, PAM30)
Other BLAST web gateways

**All matches to this query**

| Score | Mr(calc) | Delta | Sequence |
|---|---|---|---|
| 91.4 | 1386.7103 | 0.0470 | LASIGLENTEANR |
| 47.2 | 1386.7619 | -0.0047 | AAELGLEIRYPR |
| 37.4 | 1386.7467 | 0.0106 | AVTLLADSKEQGR |
| 36.7 | 1386.7507 | 0.0065 | VTALFDGELGLPR |
| 34.6 | 1386.6739 | 0.0833 | SLAAVLGGEDDGAGR |
| 34.2 | 1386.7177 | 0.0396 | LEARMDEIGIPK |
| 33.9 | 1386.6375 | 0.1197 | TELPNENTEQGR |
| 33.2 | 1386.7289 | 0.0283 | TVAAVLEEMRPR |
| 32.1 | 1386.6449 | 0.1123 | AAELGLAEMDDPR |
| 28.1 | 1386.7871 | -0.0299 | LASIFSDVLGLPR |

**Mascot:** http://www.matrixscience.com/

# MATRIX SCIENCE Mascot Search Results

## Peptide View Spot no 58

MS/MS Fragmentation of **TVVSIPNGPSELAVK**
Found in **gi|108864048** in **NCBInr**, Fructose-bisphosphate aldolase, chloroplast precursor, putative, expressed [Oryza sativa Japonica Group]

Match to Query 159: 1509.866724 from(1510.874000,1+) intensity(0.0000) index(18)
Title: Label: C7, Spot_Id: 219772, Peak_List_Id: 226201, MSMS Job_Run_Id: 21836, Comment:
Data file C:\Documents and Settings\Administrator\Desktop\Farah-Garima 11May 2013\ppw_C7_136859909200.txt

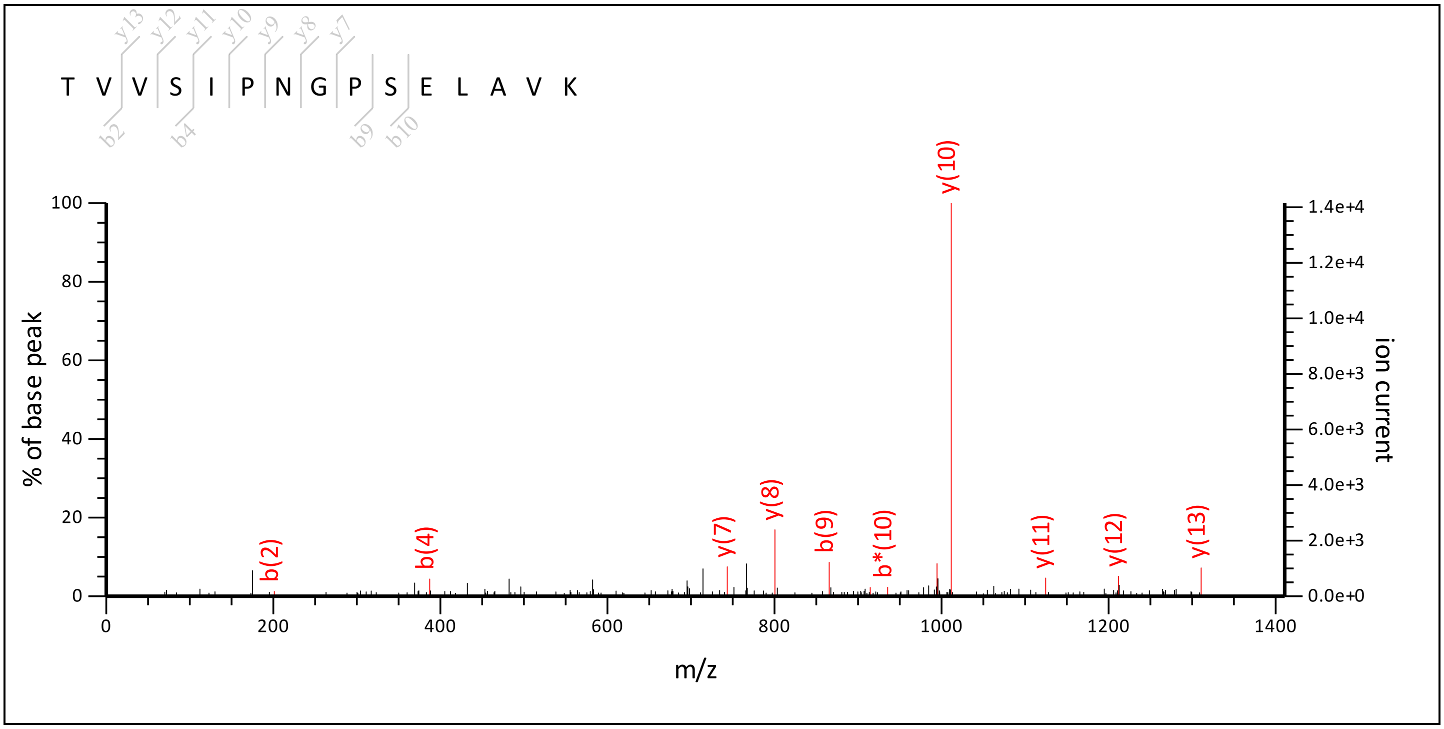


0 to 1410.8

Label all possible matches ○ Label matches used for scoring ◉

**Monoisotopic mass of neutral peptide Mr(calc):** 1509.8403
**Fixed modifications:** Carbamidomethyl (C) (apply to specified residues or termini only)
**Ions Score:** 35 **Expect:** 7.3
**Matches :** 12/72 fragment ions using 26 most intense peaks (help)

| # | a | a* | b | b* | Seq. | y | y* | # |
|---|---|---|---|---|---|---|---|---|
| 1 | 74.0600 | | 102.0550 | | T | | | 15 |
| 2 | 173.1285 | | **201.1234** | | V | 1409.7999 | 1392.7733 | 14 |
| 3 | 272.1969 | | 300.1918 | | V | ***1310.7314*** | 1293.7049 | 13 |
| 4 | 359.2289 | | **387.2238** | | S | ***1211.6630*** | 1194.6365 | 12 |
| 5 | 472.3130 | | 500.3079 | | I | ***1124.6310*** | 1107.6045 | 11 |
| 6 | 569.3657 | | 597.3606 | | P | ***1011.5469*** | 994.5204 | 10 |
| 7 | 683.4087 | 666.3821 | 711.4036 | 694.3770 | N | ***914.4942*** | 897.4676 | 9 |

| | | | | | | | | |
|---|---|---|---|---|---|---|---|---|
| **8** | 740.4301 | 723.4036 | 768.4250 | 751.3985 | **G** | ***800.4512*** | 783.4247 | **8** |
| **9** | 837.4829 | 820.4563 | **865.4778** | 848.4512 | **P** | ***743.4298*** | 726.4032 | **7** |
| **10** | 924.5149 | 907.4884 | 952.5098 | 935.4833 | **S** | 646.3770 | 629.3505 | **6** |
| **11** | 1053.5575 | 1036.5310 | 1081.5524 | 1064.5259 | **E** | 559.3450 | 542.3184 | **5** |
| **12** | 1166.6416 | 1149.6150 | 1194.6365 | 1177.6099 | **L** | 430.3024 | 413.2758 | **4** |
| **13** | 1237.6787 | 1220.6521 | 1265.6736 | 1248.6470 | **A** | 317.2183 | 300.1918 | **3** |
| **14** | 1336.7471 | 1319.7205 | 1364.7420 | 1347.7155 | **V** | 246.1812 | 229.1547 | **2** |
| **15** | | | | | **K** | 147.1128 | 130.0863 | **1** |

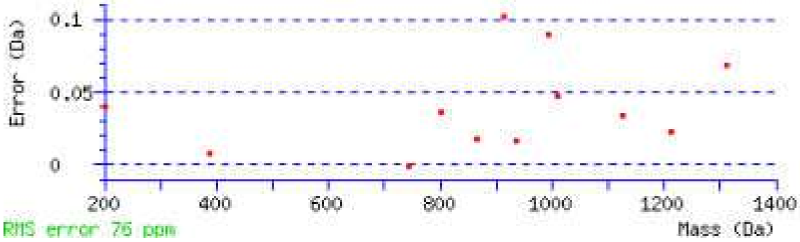


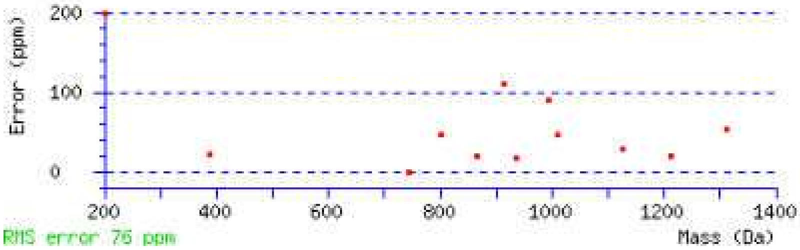


NCBI **BLAST** search of TVVSIPNGPSELAVK
(Parameters: blastp, nr protein database, expect=20000, no filter, PAM30)
Other BLAST web gateways

**All matches to this query**

| Score | Mr(calc) | Delta | Sequence |
|---|---|---|---|
| 35.2 | 1509.8403 | 0.0265 | TVVSIPNGPSELAVK |
| 30.7 | 1509.7180 | 0.1487 | AEVSMGAVQRCFR |
| 26.9 | 1509.8589 | 0.0079 | LSVSLPMPLPSTLR |
| 24.6 | 1509.8402 | 0.0265 | EAVSLPNPISLKDK |
| 24.2 | 1509.9395 | -0.0728 | TVVSLFPLLRLPR |
| 24.2 | 1509.7675 | 0.0992 | VTVSLDSLSDATFR |
| 24.2 | 1509.9243 | -0.0575 | VTVTVSSPKKPVIR |
| 24.1 | 1509.7787 | 0.0880 | DALSLESAIEAHVR |
| 23.6 | 1509.7787 | 0.0880 | LSVSLTFSGTGTNAR |
| 23.0 | 1509.8151 | 0.0516 | EAVSIPIAAAGGLDAR |

**Mascot:** http://www.matrixscience.com/

# Mascot Search Results

## Peptide View Spot no 58

MS/MS Fragmentation of **GILAMDESNATCGKR**
Found in **gi|108864048** in **NCBInr**, Fructose-bisphosphate aldolase, chloroplast precursor, putative, expressed [Oryza sativa Japonica Group]

Match to Query 171: 1621.811924 from(1622.819200,1+) intensity(0.0000) index(20)
Title: Label: C7, Spot_Id: 219772, Peak_List_Id: 226181, MSMS Job_Run_Id: 21836, Comment:
Data file C:\Documents and Settings\Administrator\Desktop\Farah-Garima 11May 2013\ppw_C7_136859909200.txt

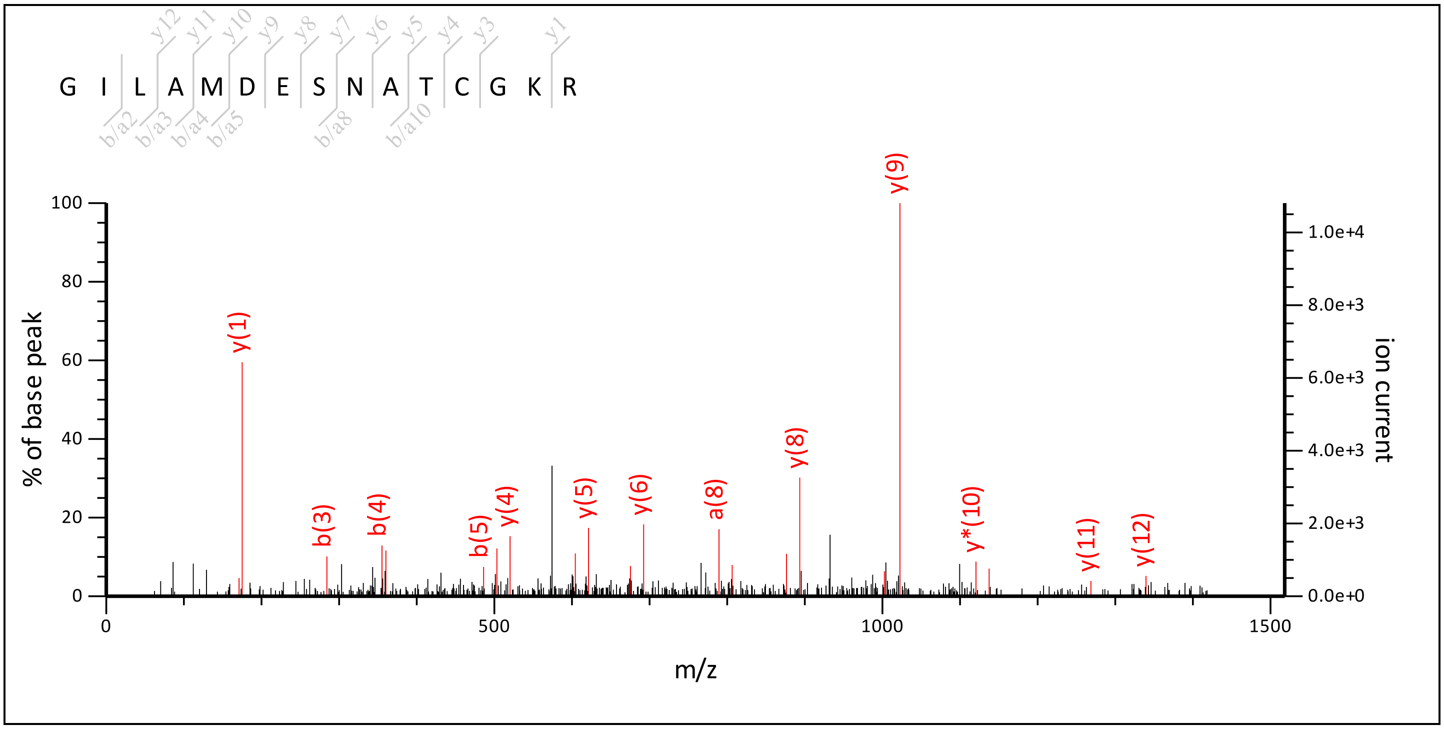


0 to 1518.28

Label all possible matches ○ Label matches used for scoring ◉

**Monoisotopic mass of neutral peptide Mr(calc):** 1621.7552
**Fixed modifications:** Carbamidomethyl (C) (apply to specified residues or termini only)
**Ions Score:** 72 **Expect:** 0.002
**Matches :** 23/68 fragment ions using 42 most intense peaks (help)

| # | a | a* | b | b* | Seq. | y | y* | # |
|---|---|---|---|---|---|---|---|---|
| 1 | 30.0338 | | 58.0287 | | G | | | 15 |
| 2 | 143.1179 | | **171.1128** | | I | 1565.7410 | 1548.7145 | 14 |
| 3 | 256.2020 | | **284.1969** | | L | 1452.6570 | 1435.6304 | 13 |
| 4 | 327.2391 | | **355.2340** | | A | ***1339.5729*** | 1322.5464 | 12 |
| 5 | 458.2796 | | **486.2745** | | M | ***1268.5358*** | 1251.5092 | 11 |
| 6 | 573.3065 | | 601.3014 | | D | ***1137.4953*** | 1120.4688 | 10 |
| 7 | 702.3491 | | 730.3440 | | E | ***1022.4684*** | 1005.4418 | 9 |

| | | | | | | | | |
|---|---|---|---|---|---|---|---|---|
| 8 | 789.3811 | | 817.3760 | | S | 893.4258 | 876.3992 | 8 |
| 9 | 903.4240 | 886.3975 | 931.4190 | 914.3924 | N | 806.3937 | 789.3672 | 7 |
| 10 | 974.4612 | 957.4346 | 1002.4561 | 985.4295 | A | 692.3508 | 675.3243 | 6 |
| 11 | 1075.5088 | 1058.4823 | 1103.5038 | 1086.4772 | T | 621.3137 | 604.2872 | 5 |
| 12 | 1235.5395 | 1218.5129 | 1263.5344 | 1246.5079 | C | 520.2660 | 503.2395 | 4 |
| 13 | 1292.5609 | 1275.5344 | 1320.5559 | 1303.5293 | G | 360.2354 | 343.2088 | 3 |
| 14 | 1420.6559 | 1403.6294 | 1448.6508 | 1431.6243 | K | 303.2139 | 286.1874 | 2 |
| 15 | | | | | R | 175.1190 | 158.0924 | 1 |

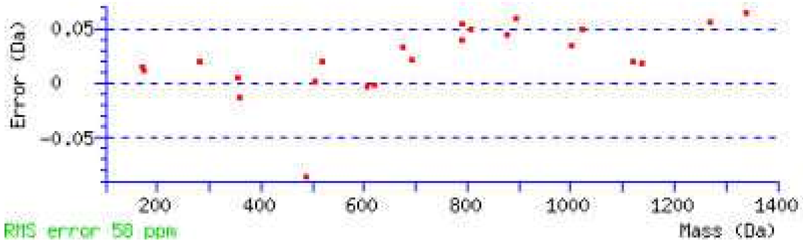


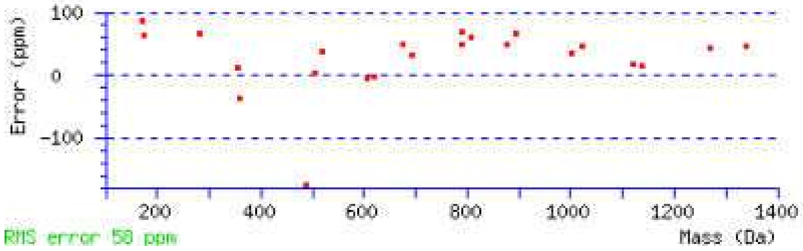


NCBI **BLAST** search of GILAMDESNATCGKR
(Parameters: blastp, nr protein database, expect=20000, no filter, PAM30)
Other BLAST web gateways

**All matches to this query**

| Score | Mr(calc) | Delta | Sequence |
|---|---|---|---|
| 71.8 | 1621.7552 | 0.0567 | GILAMDESNATCGKR |
| 32.5 | 1621.7552 | 0.0567 | GLLAMDESNGTCAKR |
| 16.3 | 1621.8464 | -0.0345 | GLIFLGTSGDAATFPR |
| 15.5 | 1621.8134 | -0.0015 | TTYGPTAPSMISAAVR |
| 14.9 | 1621.8610 | -0.0491 | LVQFLESNRLMTR |
| 13.3 | 1621.9225 | -0.1106 | LNPLVEKITVPMPR |
| 13.2 | 1621.8940 | -0.0821 | IGLFGLEKLHNPER |
| 13.0 | 1621.7738 | 0.0381 | ALAVMMSRGMSPPSR |
| 12.2 | 1621.7657 | 0.0463 | TGTVGTGGTGNTTTGGQR |
| 12.2 | 1621.8172 | -0.0053 | LGLDVQAHSAGSAPSGR |

**Mascot:** http://www.matrixscience.com/

# Mascot Search Results

## Peptide View Spot no 58

MS/MS Fragmentation of **GLVPLAGSNNESWCQGLDGLASR**
Found in **gi|108864048** in **NCBInr**, Fructose-bisphosphate aldolase, chloroplast precursor, putative, expressed [Oryza sativa Japonica Group]

Match to Query 273: 2400.240524 from(2401.247800,1+) intensity(0.0000) index(32)
Title: Label: C7, Spot_Id: 219772, Peak_List_Id: 226180, MSMS Job_Run_Id: 21836, Comment:
Data file C:\Documents and Settings\Administrator\Desktop\Farah-Garima 11May 2013\ppw_C7_136859909200.txt

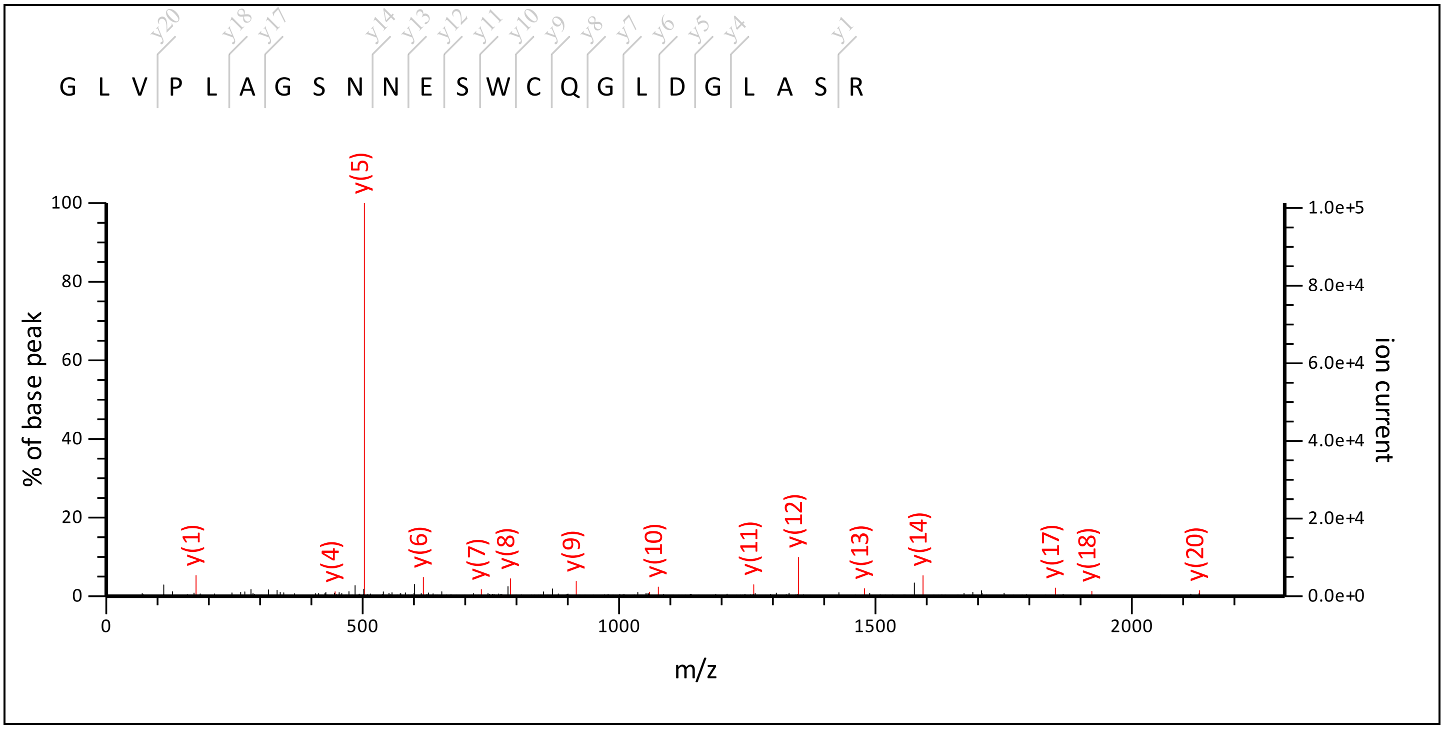


0 to 2298.04

Label all possible matches ○ Label matches used for scoring ◉

**Monoisotopic mass of neutral peptide Mr(calc):** 2400.1492
**Fixed modifications:** Carbamidomethyl (C) (apply to specified residues or termini only)
**Ions Score:** 132 **Expect:** 1.4e-09
**Matches :** 16/116 fragment ions using 22 most intense peaks (help)

| # | a | a* | b | b* | Seq. | y | y* | # |
|---|---|---|---|---|---|---|---|---|
| 1 | 30.0338 | | 58.0287 | | G | | | 23 |
| 2 | 143.1179 | | 171.1128 | | L | 2344.1350 | 2327.1085 | 22 |
| 3 | 242.1863 | | 270.1812 | | V | 2231.0510 | 2214.0244 | 21 |
| 4 | 339.2391 | | 367.2340 | | P | ***2131.9825*** | 2114.9560 | 20 |
| 5 | 452.3231 | | 480.3180 | | L | 2034.9298 | 2017.9032 | 19 |
| 6 | 523.3602 | | 551.3552 | | A | ***1921.8457*** | 1904.8192 | 18 |
| 7 | 580.3817 | | 608.3766 | | G | ***1850.8086*** | 1833.7821 | 17 |

| | | | | | | | | |
|---|---|---|---|---|---|---|---|---|
| 8 | 667.4137 | | 695.4087 | | S | 1793.7871 | 1776.7606 | 16 |
| 9 | 781.4567 | 764.4301 | 809.4516 | 792.4250 | N | 1706.7551 | 1689.7286 | 15 |
| 10 | 895.4996 | 878.4730 | 923.4945 | 906.4680 | N | 1592.7122 | 1575.6856 | 14 |
| 11 | 1024.5422 | 1007.5156 | 1052.5371 | 1035.5105 | E | 1478.6693 | 1461.6427 | 13 |
| 12 | 1111.5742 | 1094.5477 | 1139.5691 | 1122.5426 | S | 1349.6267 | 1332.6001 | 12 |
| 13 | 1297.6535 | 1280.6270 | 1325.6484 | 1308.6219 | W | 1262.5946 | 1245.5681 | 11 |
| 14 | 1457.6842 | 1440.6576 | 1485.6791 | 1468.6525 | C | 1076.5153 | 1059.4888 | 10 |
| 15 | 1585.7428 | 1568.7162 | 1613.7377 | 1596.7111 | Q | 916.4847 | 899.4581 | 9 |
| 16 | 1642.7642 | 1625.7377 | 1670.7591 | 1653.7326 | G | 788.4261 | 771.3995 | 8 |
| 17 | 1755.8483 | 1738.8217 | 1783.8432 | 1766.8166 | L | 731.4046 | 714.3781 | 7 |
| 18 | 1870.8752 | 1853.8487 | 1898.8701 | 1881.8436 | D | 618.3206 | 601.2940 | 6 |
| 19 | 1927.8967 | 1910.8701 | 1955.8916 | 1938.8651 | G | 503.2936 | 486.2671 | 5 |
| 20 | 2040.9808 | 2023.9542 | 2068.9757 | 2051.9491 | L | 446.2722 | 429.2456 | 4 |
| 21 | 2112.0179 | 2094.9913 | 2140.0128 | 2122.9862 | A | 333.1881 | 316.1615 | 3 |
| 22 | 2199.0499 | 2182.0233 | 2227.0448 | 2210.0183 | S | 262.1510 | 245.1244 | 2 |
| 23 | | | | | R | 175.1190 | 158.0924 | 1 |

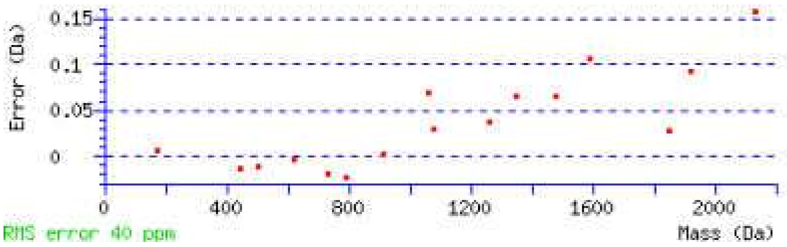


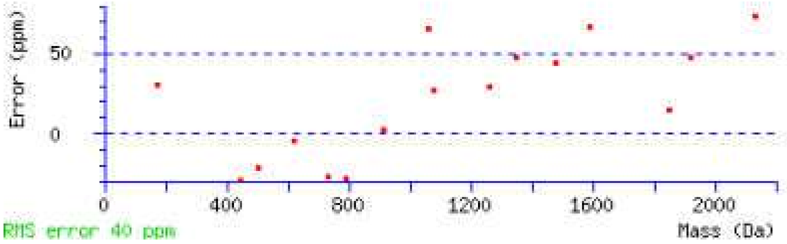


NCBI **BLAST** search of GLVPLAGSNNESWCQGLDGLASR
(Parameters: blastp, nr protein database, expect=20000, no filter, PAM30)
Other BLAST web gateways

**All matches to this query**

| Score | Mr(calc) | Delta | Sequence |
|---|---|---|---|
| 131.8 | 2400.1492 | 0.0913 | GLVPLAGSNNESWCQGLDGLASR |
| 17.6 | 2400.2649 | -0.0243 | NPRTGNIVAESFGTIILDENLK |
| 17.1 | 2400.1890 | 0.0515 | NVMVVELMVGGTGGRPGLDGVDGR |
| 17.1 | 2400.1890 | 0.0515 | NVMVVELMVGGTGGRPGLDGVDGR |
| 16.9 | 2400.1193 | 0.1212 | WASSAPATAVAEAEAEALGDGDGVR |
| 12.8 | 2400.1961 | 0.0444 | RIDLYYGQFGENLTVEGLADK |
| 11.1 | 2400.1211 | 0.1194 | DSMCGFRVYPLAPCMAILQR |
| | | | |

| | | | |
|---|---|---|---|
| 9.7 | 2400.1591 | 0.0814 | SSASVAQTMGTVSYVGALVGEQSR |
| 9.3 | 2400.0990 | 0.1415 | GIQTMEAANAWAPSFMTAYNAR |
| 9.2 | 2400.0335 | 0.2070 | CDTPGCRPYAEEIANGSPHNR |

**Mascot:** http://www.matrixscience.com/

# Mascot Search Results

## Peptide View **Spot no 58**

MS/MS Fragmentation of **VDKGLVPLAGSNNESWCQGLDGLASR**
Found in **gi|108864048** in **NCBInr**, Fructose-bisphosphate aldolase, chloroplast precursor, putative, expressed [Oryza sativa Japonica Group]

Match to Query 279: 2742.446624 from(2743.453900,1+) intensity(0.0000) index(33)
Title: Label: C7, Spot_Id: 219772, Peak_List_Id: 226210, MSMS Job_Run_Id: 21836, Comment:
Data file C:\Documents and Settings\Administrator\Desktop\Farah-Garima 11May 2013\ppw_C7_136859909200.txt

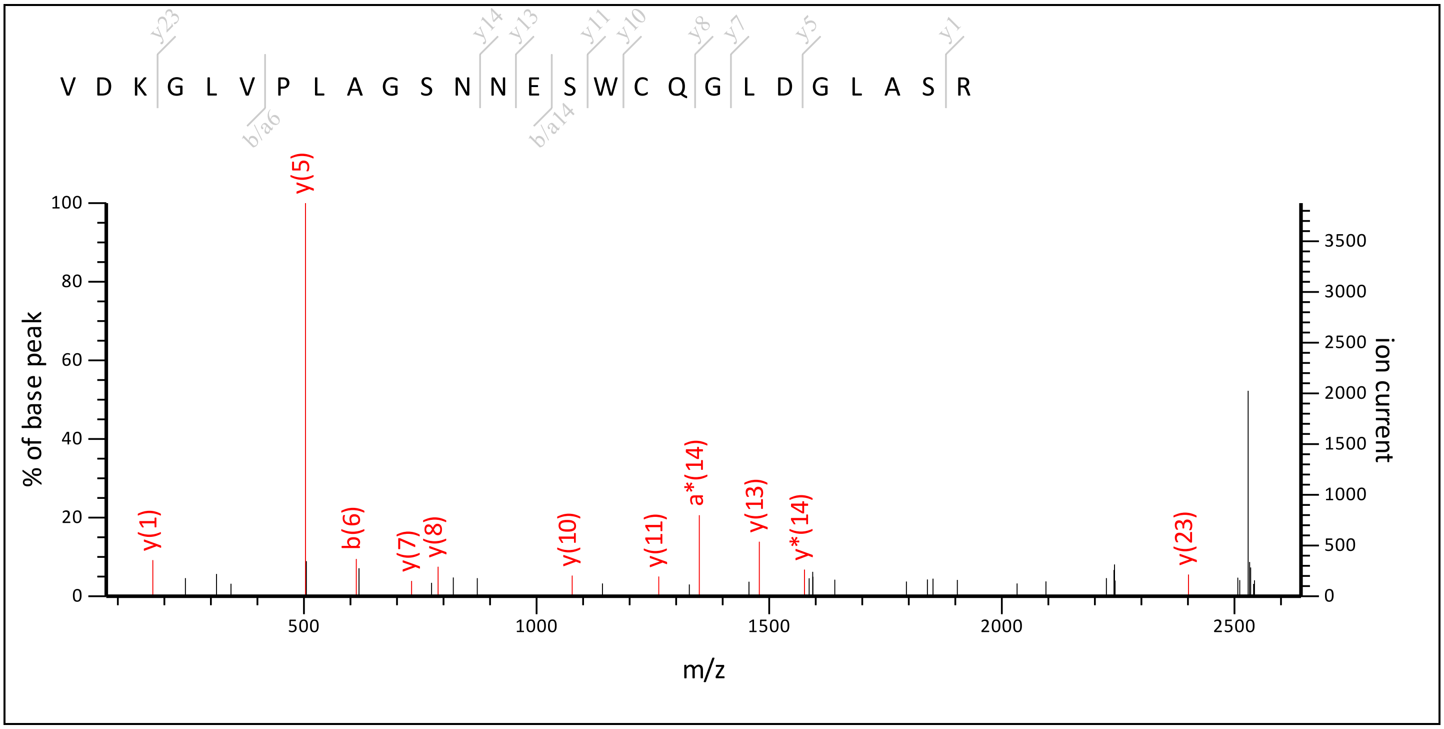


75.14 to 2642.99

Label all possible matches ○ Label matches used for scoring ◉

**Monoisotopic mass of neutral peptide Mr(calc):** 2742.3395
**Fixed modifications:** Carbamidomethyl (C) (apply to specified residues or termini only)
**Ions Score:** 50 **Expect:** 0.17
**Matches :** 12/146 fragment ions using 19 most intense peaks (help)

| # | a | a* | b | b* | Seq. | y | y* | # |
|---|---|---|---|---|---|---|---|---|
| 1 | 72.0808 | | 100.0757 | | V | | | 26 |
| 2 | 187.1077 | | 215.1026 | | D | 2644.2784 | 2627.2518 | 25 |
| 3 | 315.2027 | 298.1761 | 343.1976 | 326.1710 | K | 2529.2514 | 2512.2249 | 24 |
| 4 | 372.2241 | 355.1976 | 400.2191 | 383.1925 | G | ***2401.1565*** | 2384.1299 | 23 |
| 5 | 485.3082 | 468.2817 | 513.3031 | 496.2766 | L | 2344.1350 | 2327.1085 | 22 |
| 6 | 584.3766 | 567.3501 | 612.3715 | 595.3450 | V | 2231.0510 | 2214.0244 | 21 |
| 7 | 681.4294 | 664.4028 | 709.4243 | 692.3978 | P | 2131.9825 | 2114.9560 | 20 |

| | | | | | | | | |
|---|---|---|---|---|---|---|---|---|
| 8 | 794.5135 | 777.4869 | 822.5084 | 805.4818 | L | 2034.9298 | 2017.9032 | 19 |
| 9 | 865.5506 | 848.5240 | 893.5455 | 876.5189 | A | 1921.8457 | 1904.8192 | 18 |
| 10 | 922.5720 | 905.5455 | 950.5669 | 933.5404 | G | 1850.8086 | 1833.7821 | 17 |
| 11 | 1009.6041 | 992.5775 | 1037.5990 | 1020.5724 | S | 1793.7871 | 1776.7606 | 16 |
| 12 | 1123.6470 | 1106.6204 | 1151.6419 | 1134.6154 | N | 1706.7551 | 1689.7286 | 15 |
| 13 | 1237.6899 | 1220.6634 | 1265.6848 | 1248.6583 | N | 1592.7122 | 1575.6856 | 14 |
| 14 | 1366.7325 | 1349.7060 | 1394.7274 | 1377.7009 | E | ***1478.6693*** | 1461.6427 | 13 |
| 15 | 1453.7645 | 1436.7380 | 1481.7594 | 1464.7329 | S | ***1349.6267*** | 1332.6001 | 12 |
| 16 | 1639.8438 | 1622.8173 | 1667.8388 | 1650.8122 | W | ***1262.5946*** | 1245.5681 | 11 |
| 17 | 1799.8745 | 1782.8479 | 1827.8694 | 1810.8429 | C | ***1076.5153*** | 1059.4888 | 10 |
| 18 | 1927.9331 | 1910.9065 | 1955.9280 | 1938.9014 | Q | 916.4847 | 899.4581 | 9 |
| 19 | 1984.9545 | 1967.9280 | 2012.9495 | 1995.9229 | G | ***788.4261*** | 771.3995 | 8 |
| 20 | 2098.0386 | 2081.0121 | 2126.0335 | 2109.0070 | L | ***731.4046*** | 714.3781 | 7 |
| 21 | 2213.0655 | 2196.0390 | 2241.0605 | 2224.0339 | D | 618.3206 | 601.2940 | 6 |
| 22 | 2270.0870 | 2253.0605 | 2298.0819 | 2281.0554 | G | ***503.2936*** | 486.2671 | 5 |
| 23 | 2383.1711 | 2366.1445 | 2411.1660 | 2394.1394 | L | 446.2722 | 429.2456 | 4 |
| 24 | 2454.2082 | 2437.1816 | 2482.2031 | 2465.1766 | A | 333.1881 | 316.1615 | 3 |
| 25 | 2541.2402 | 2524.2137 | 2569.2351 | 2552.2086 | S | 262.1510 | 245.1244 | 2 |
| 26 | | | | | R | ***175.1190*** | 158.0924 | 1 |

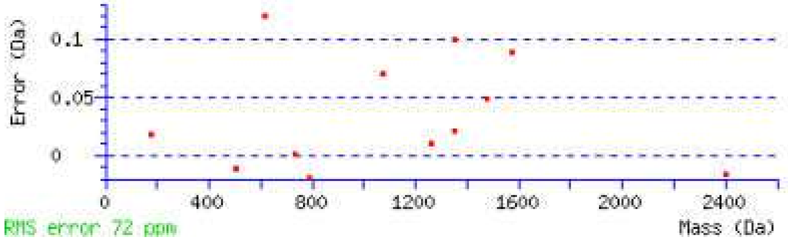


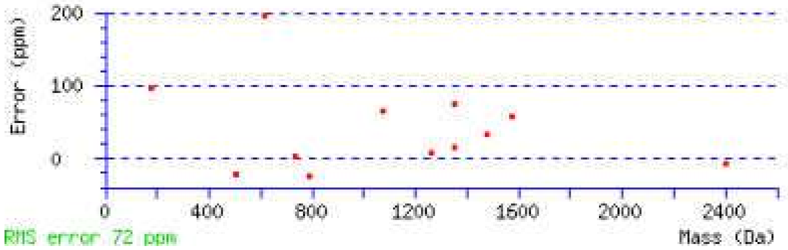


NCBI **BLAST** search of VDKGLVPLAGSNNESWCQGLDGLASR
(Parameters: blastp, nr protein database, expect=20000, no filter, PAM30)
Other BLAST web gateways

**All matches to this query**

| Score | Mr(calc) | Delta | Sequence |
|---|---|---|---|
| 50.0 | 2742.3395 | 0.1071 | VDKGLVPLAGSNNESWCQGLDGLASR |
| 11.8 | 2742.3622 | 0.0844 | AFVVNIGDTFMALTNGIYKSCLHR |
| 10.0 | 2742.4262 | 0.0204 | LVLTDHDMAPITASVTALFIDVATGR |
| 9.4 | 2742.3759 | 0.0707 | GQPVAPGLPPVDEHMAASSNSTPSLKR |
| | | | |

| | | | |
|---|---|---|---|
| 9.3 | 2742.2551 | 0.1916 | TGYPVEMLEPGMGMDSDLGIDSIKR |
| 9.0 | 2742.3422 | 0.1044 | FEPETTGTTIMAVIYDGGLIIGADSR |
| 9.0 | 2742.3422 | 0.1044 | FEPETTGTTIMAVIYDGGLLIGADSR |
| 8.8 | 2742.2290 | 0.2176 | DLNVGDPNDPETDVGPMISEEEAKR |
| 8.2 | 2742.3383 | 0.1083 | MPAPRTLHLNAFLMNAGHHDAAWR |
| 7.6 | 2742.3978 | 0.0488 | DYPFVKIMDNLEWYLALILNEK |

**Mascot:** http://www.matrixscience.com/

# MATRIX SCIENCE Mascot Search Results

## Peptide View **Spot no 58**

MS/MS Fragmentation of **YAAISQDNGLVPIVEPEILLDGEHGIDR**
Found in **gi|108864048** in **NCBInr**, Fructose-bisphosphate aldolase, chloroplast precursor, putative, expressed [Oryza sativa Japonica Group]

Match to Query 281: 3032.674824 from(3033.682100,1+) intensity(0.0000) index(34)
Title: Label: C7, Spot_Id: 219772, Peak_List_Id: 226185, MSMS Job_Run_Id: 21836, Comment:
Data file C:\Documents and Settings\Administrator\Desktop\Farah-Garima 11May 2013\ppw_C7_136859909200.txt

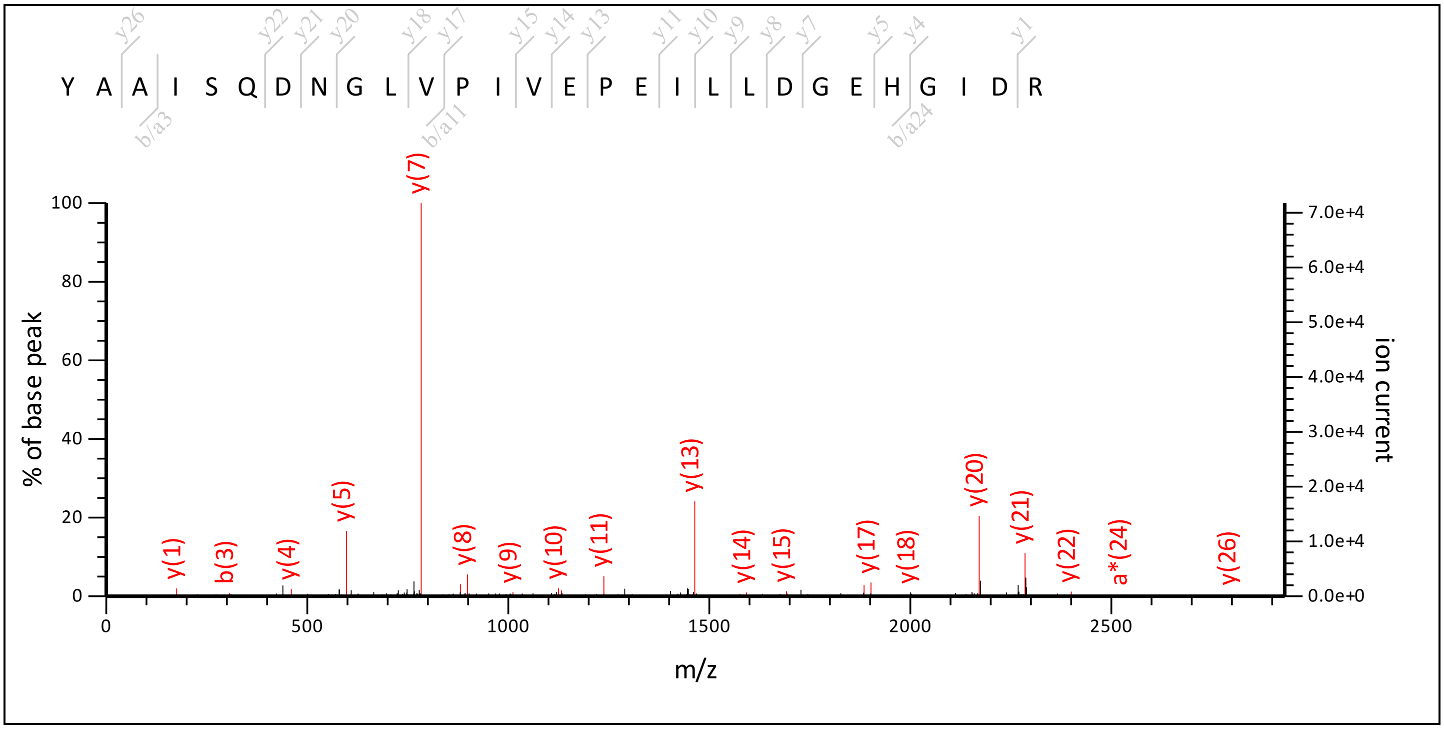


0 to 2931.19

Label all possible matches ○ Label matches used for scoring ◉

**Monoisotopic mass of neutral peptide Mr(calc):** 3032.5455
**Fixed modifications:** Carbamidomethyl (C) (apply to specified residues or termini only)
**Ions Score:** 91 **Expect:** 1e-05
**Matches :** 24/152 fragment ions using 56 most intense peaks (help)

| # | a | a* | b | b* | Seq. | y | y* | # |
|---|---|---|---|---|---|---|---|---|
| 1 | 136.0757 | | 164.0706 | | Y | | | 28 |
| 2 | 207.1128 | | 235.1077 | | A | 2870.4894 | 2853.4629 | 27 |
| 3 | 278.1499 | | **306.1448** | | A | ***2799.4523*** | 2782.4258 | 26 |
| 4 | 391.2340 | | 419.2289 | | I | 2728.4152 | 2711.3887 | 25 |
| 5 | 478.2660 | | 506.2609 | | S | 2615.3311 | 2598.3046 | 24 |
| 6 | 606.3246 | 589.2980 | 634.3195 | 617.2930 | Q | 2528.2991 | 2511.2726 | 23 |
| 7 | 721.3515 | 704.3250 | 749.3464 | 732.3199 | D | ***2400.2405*** | 2383.2140 | 22 |

| | | | | | | | | |
|---|---|---|---|---|---|---|---|---|
| **8** | 835.3945 | 818.3679 | 863.3894 | 846.3628 | **N** | ***2285.2136*** | 2268.1870 | **21** |
| **9** | 892.4159 | 875.3894 | 920.4108 | 903.3843 | **G** | ***2171.1707*** | 2154.1441 | **20** |
| **10** | 1005.5000 | 988.4734 | 1033.4949 | 1016.4684 | **L** | 2114.1492 | 2097.1226 | **19** |
| **11** | 1104.5684 | 1087.5419 | **1132.5633** | 1115.5368 | **V** | ***2001.0651*** | 1984.0386 | **18** |
| **12** | 1201.6212 | 1184.5946 | 1229.6161 | 1212.5895 | **P** | ***1901.9967*** | 1884.9702 | **17** |
| **13** | 1314.7052 | 1297.6787 | 1342.7001 | 1325.6736 | **I** | 1804.9440 | 1787.9174 | **16** |
| **14** | 1413.7736 | 1396.7471 | 1441.7686 | 1424.7420 | **V** | ***1691.8599*** | 1674.8333 | **15** |
| **15** | 1542.8162 | 1525.7897 | 1570.8112 | 1553.7846 | **E** | ***1592.7915*** | 1575.7649 | **14** |
| **16** | 1639.8690 | 1622.8425 | 1667.8639 | 1650.8374 | **P** | ***1463.7489*** | 1446.7223 | **13** |
| **17** | 1768.9116 | 1751.8850 | 1796.9065 | 1779.8800 | **E** | 1366.6961 | 1349.6696 | **12** |
| **18** | 1881.9957 | 1864.9691 | 1909.9906 | 1892.9640 | **I** | ***1237.6535*** | 1220.6270 | **11** |
| **19** | 1995.0797 | 1978.0532 | 2023.0746 | 2006.0481 | **L** | ***1124.5695*** | 1107.5429 | **10** |
| **20** | 2108.1638 | 2091.1372 | 2136.1587 | 2119.1321 | **L** | ***1011.4854*** | 994.4588 | **9** |
| **21** | 2223.1907 | 2206.1642 | 2251.1856 | 2234.1591 | **D** | ***898.4013*** | 881.3748 | **8** |
| **22** | 2280.2122 | 2263.1856 | 2308.2071 | 2291.1806 | **G** | ***783.3744*** | 766.3478 | **7** |
| **23** | 2409.2548 | 2392.2282 | 2437.2497 | 2420.2231 | **E** | 726.3529 | 709.3264 | **6** |
| **24** | 2546.3137 | 2529.2871 | 2574.3086 | 2557.2821 | **H** | ***597.3103*** | 580.2838 | **5** |
| **25** | 2603.3352 | 2586.3086 | 2631.3301 | 2614.3035 | **G** | ***460.2514*** | 443.2249 | **4** |
| **26** | 2716.4192 | 2699.3927 | 2744.4141 | 2727.3876 | **I** | 403.2300 | 386.2034 | **3** |
| **27** | 2831.4462 | 2814.4196 | 2859.4411 | 2842.4145 | **D** | 290.1459 | 273.1193 | **2** |
| **28** | | | | | **R** | ***175.1190*** | 158.0924 | **1** |

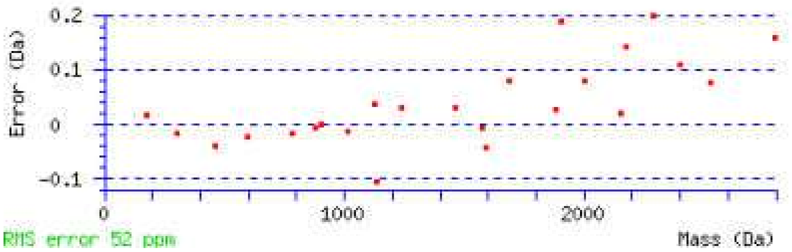


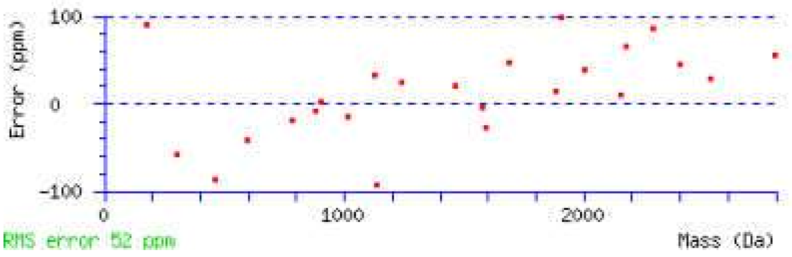


NCBI **BLAST** search of YAAISQDNGLVPIVEPEILLDGEHGIDR
(Parameters: blastp, nr protein database, expect=20000, no filter, PAM30)
Other BLAST web gateways

**All matches to this query**

| Score | Mr(calc) | Delta | Sequence |
|---|---|---|---|
| 91.1 | 3032.5455 | 0.1294 | YAAISQDNGLVPIVEPEILLDGEHGIDR |
| 74.2 | 3032.5455 | 0.1294 | YAAISQDNGLVPIVEPEILLDGDHGIER |
| | | | |

| | | | |
|---|---|---|---|
| 19.4 | 3032.6290 | 0.0458 | GACDVVEDLAKPMPSLLILDILGLPLDR |
| 6.4 | 3032.5502 | 0.1246 | LGGTARAYYASGGATGVALDGLLDLHQMVR |
| 5.9 | 3032.9531 | -0.2783 | TNPLNPLVILGILLVIPMILLAVLLLVR |
| 5.8 | 3032.4735 | 0.2013 | LQNKIVSDIIQEYAHEPLMWDTMAR |
| 5.5 | 3032.4590 | 0.2159 | FASMSGGETAQPLADYYVQQWKEIGLK |
| 5.0 | 3032.5825 | 0.0923 | GIATEVIVATISPADAQAHLRNGLAMGANR |
| 4.9 | 3032.4558 | 0.2190 | MGFLEDFQASVEALPAMLQRNYSLMR |
| 4.7 | 3032.4410 | 0.2338 | MTTPPHAAGTPPPGDAAGRASPPDSEPTPPR |

**Mascot:** http://www.matrixscience.com/

# MATRIX SCIENCE Mascot Search Results

## Peptide View Spot no 60

MS/MS Fragmentation of **MELVDAAFPLLK**
Found in **gi|15982948** in **NCBInr**, NAD-dependent malate dehydrogenase [Prunus persica]

Match to Query 159: 1345.749924 from(1346.757200,1+) intensity(0.0000) index(15)
Title: Label: E10, Spot_Id: 219822, Peak_List_Id: 226724, MSMS Job_Run_Id: 21854, Comment:
Data file C:\Documents and Settings\Administrator\Desktop\Farah-Garima 11May 2013\ppw_E10_136859948400.txt

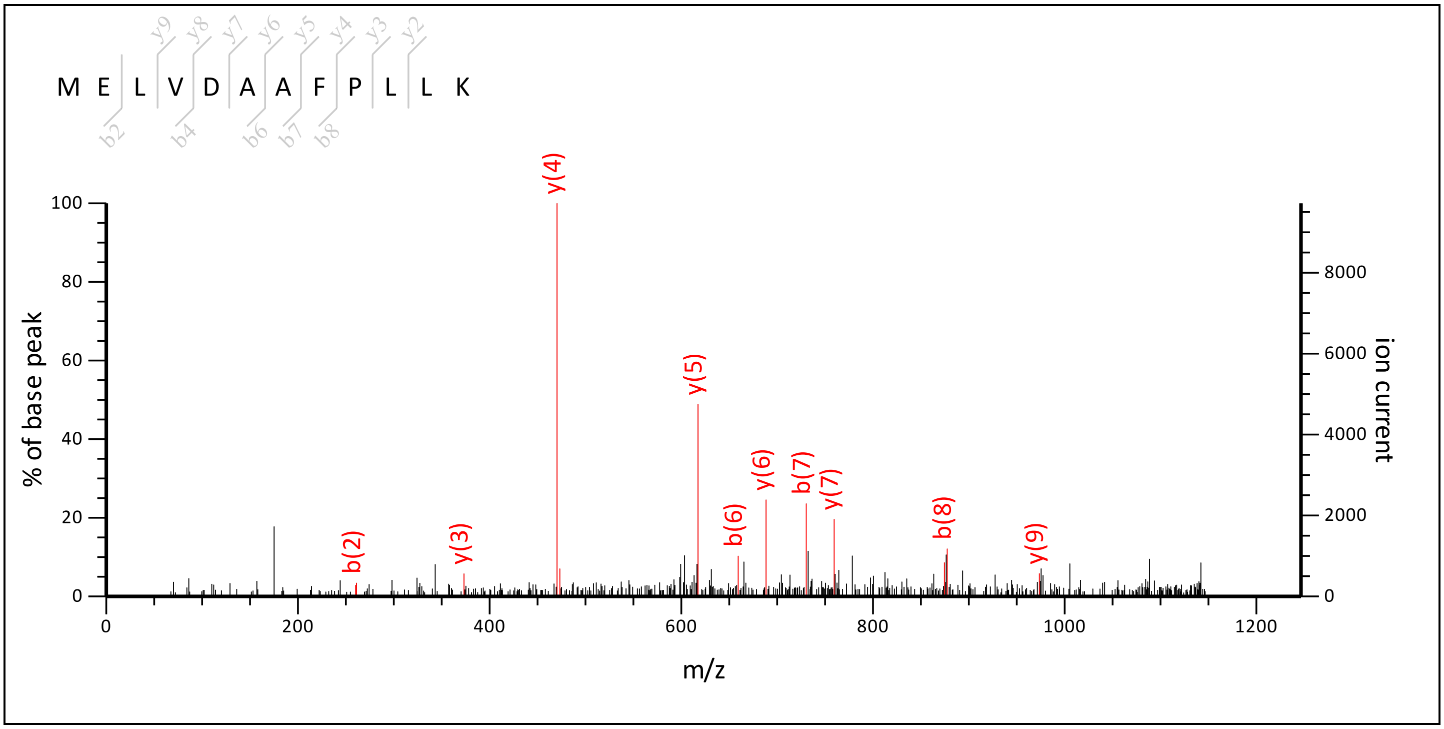


0 to 1246.57

Label all possible matches ○ Label matches used for scoring ◉

**Monoisotopic mass of neutral peptide Mr(calc):** 1345.7315
**Fixed modifications:** Carbamidomethyl (C) (apply to specified residues or termini only)
**Ions Score:** 30 **Expect:** 37
**Matches :** 13/44 fragment ions using 44 most intense peaks (help)

| # | a | b | Seq. | y | y* | # |
|---|---|---|---|---|---|---|
| 1 | 104.0528 | 132.0478 | M | | | 12 |
| 2 | 233.0954 | **261.0904** | E | 1215.6984 | 1198.6718 | 11 |
| 3 | 346.1795 | 374.1744 | L | 1086.6558 | 1069.6292 | 10 |
| 4 | 445.2479 | **473.2428** | V | ***973.5717*** | 956.5451 | 9 |
| 5 | 560.2749 | 588.2698 | D | ***874.5033*** | 857.4767 | 8 |
| 6 | 631.3120 | **659.3069** | A | ***759.4763*** | 742.4498 | 7 |
| 7 | 702.3491 | **730.3440** | A | ***688.4392*** | 671.4127 | 6 |
| 8 | 849.4175 | **877.4124** | F | ***617.4021*** | 600.3756 | 5 |

| | | | | | | |
|---|---|---|---|---|---|---|
| **9** | 946.4703 | 974.4652 | **P** | *470.3337* | 453.3071 | **4** |
| **10** | 1059.5543 | 1087.5492 | **L** | *373.2809* | 356.2544 | **3** |
| **11** | 1172.6384 | 1200.6333 | **L** | *260.1969* | 243.1703 | **2** |
| **12** | | | **K** | 147.1128 | 130.0863 | **1** |

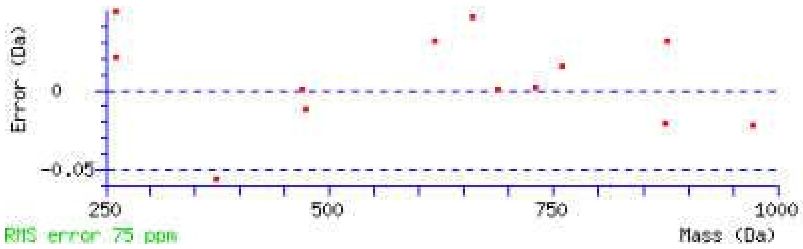


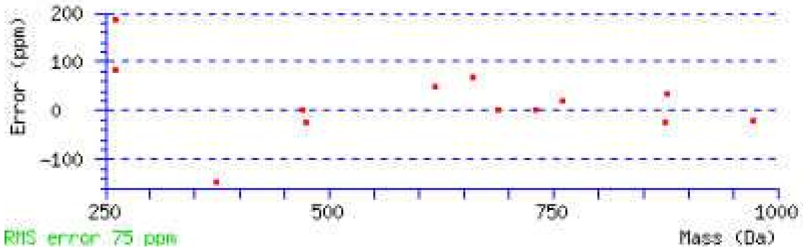


NCBI **BLAST** search of [MELVDAAFPLLK]
(Parameters: blastp, nr protein database, expect=20000, no filter, PAM30)
Other BLAST [web gateways]

**All matches to this query**

| Score | Mr(calc) | Delta | Sequence |
|---|---|---|---|
| 29.6 | 1345.7315 | 0.0184 | MELVDAAFPLLK |
| 19.9 | 1345.7354 | 0.0145 | IEGAVVFITGANR |
| 19.5 | 1345.7969 | -0.0470 | LASLIFLQLSNK |
| 17.9 | 1345.7354 | 0.0146 | LVEQFALKENR |
| 17.9 | 1345.7136 | 0.0363 | ILRSMATAGSAPR |
| 17.9 | 1345.8373 | -0.0874 | EVLKFFLPILK |
| 17.8 | 1345.6772 | 0.0727 | EIVQMAREAGAR |
| 17.8 | 1345.6626 | 0.0873 | GNSGGPVVDELFR |
| 17.2 | 1345.7103 | 0.0397 | VRDVFAADQVAR |
| 17.1 | 1345.6738 | 0.0761 | RSEPLFQQGER |

**Mascot:** http://www.matrixscience.com/

# MATRIX SCIENCE Mascot Search Results

## Peptide View Spot no 60

MS/MS Fragmentation of **VLVVANPANTNALILK**
Found in **gi|15982948** in **NCBInr**, NAD-dependent malate dehydrogenase [Prunus persica]

Match to Query 207: 1649.011524 from(1650.018800,1+) intensity(0.0000) index(19)
Title: Label: E10, Spot_Id: 219822, Peak_List_Id: 226722, MSMS Job_Run_Id: 21854, Comment:
Data file C:\Documents and Settings\Administrator\Desktop\Farah-Garima 11May 2013\ppw_E10_136859948400.txt

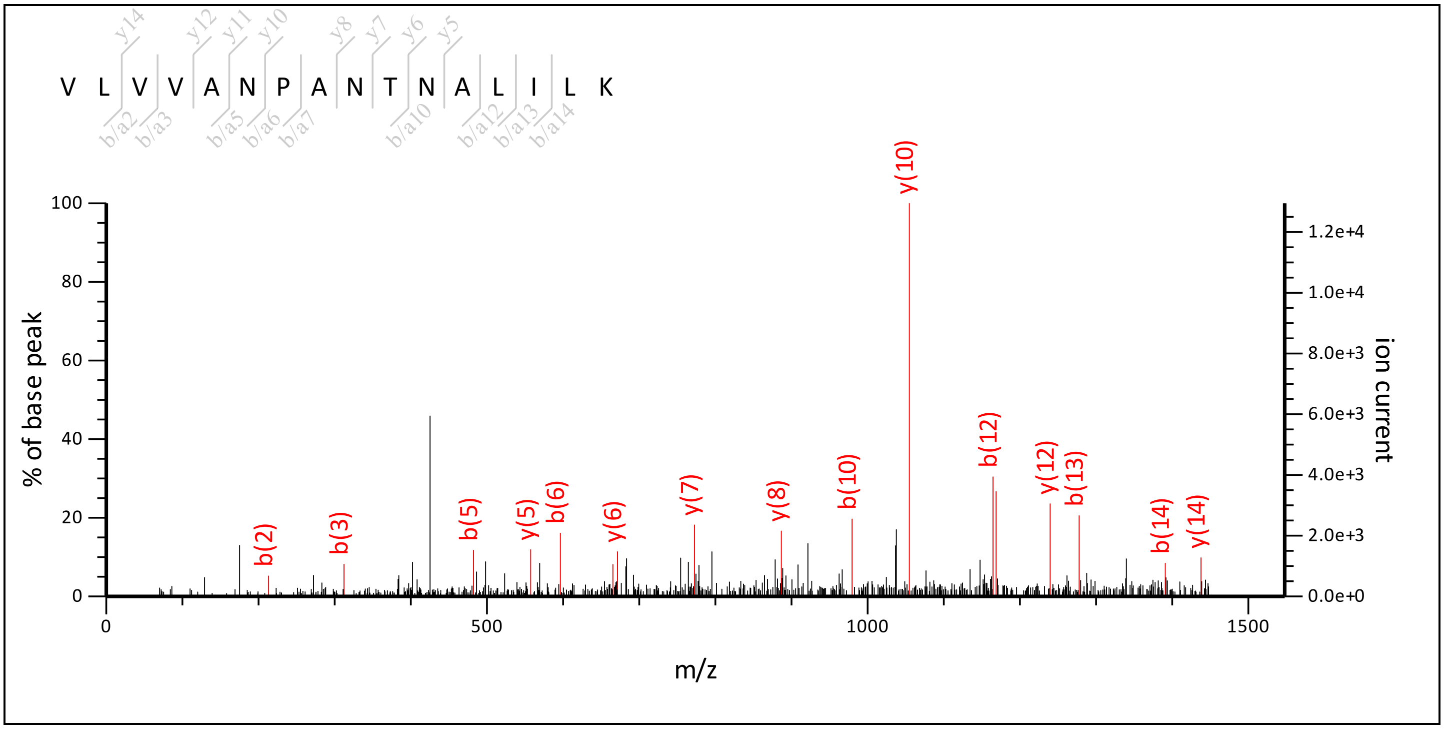


0 to 1547.75

Label all possible matches ○ Label matches used for scoring ◉

**Monoisotopic mass of neutral peptide Mr(calc):** 1648.9876
**Fixed modifications:** Carbamidomethyl (C) (apply to specified residues or termini only)
**Ions Score:** 65 **Expect:** 0.0045
**Matches :** 17/80 fragment ions using 28 most intense peaks (help)

| # | a | a* | b | b* | Seq. | y | y* | # |
|---|---|---|---|---|---|---|---|---|
| 1 | 72.0808 | | 100.0757 | | V | | | 16 |
| 2 | 185.1648 | | ***213.1598*** | | L | 1550.9265 | 1533.8999 | 15 |
| 3 | 284.2333 | | ***312.2282*** | | V | ***1437.8424*** | 1420.8158 | 14 |
| 4 | 383.3017 | | 411.2966 | | V | 1338.7740 | 1321.7474 | 13 |
| 5 | 454.3388 | | ***482.3337*** | | A | ***1239.7056*** | 1222.6790 | 12 |
| 6 | 568.3817 | 551.3552 | ***596.3766*** | 579.3501 | N | ***1168.6684*** | 1151.6419 | 11 |
| 7 | 665.4345 | 648.4079 | 693.4294 | 676.4028 | P | ***1054.6255*** | 1037.5990 | 10 |
| 8 | 736.4716 | 719.4450 | 764.4665 | 747.4400 | A | 957.5728 | 940.5462 | 9 |

| | | | | | | | | |
|---|---|---|---|---|---|---|---|---|
| **9** | 850.5145 | 833.4880 | 878.5094 | 861.4829 | **N** | ***886.5356*** | 869.5091 | **8** |
| **10** | 951.5622 | 934.5356 | ***979.5571*** | 962.5306 | **T** | ***772.4927*** | 755.4662 | **7** |
| **11** | 1065.6051 | 1048.5786 | 1093.6000 | 1076.5735 | **N** | ***671.4450*** | 654.4185 | **6** |
| **12** | 1136.6422 | 1119.6157 | ***1164.6371*** | 1147.6106 | **A** | ***557.4021*** | 540.3756 | **5** |
| **13** | 1249.7263 | 1232.6997 | ***1277.7212*** | 1260.6947 | **L** | 486.3650 | 469.3384 | **4** |
| **14** | 1362.8104 | 1345.7838 | ***1390.8053*** | 1373.7787 | **I** | 373.2809 | 356.2544 | **3** |
| **15** | 1475.8944 | 1458.8679 | 1503.8893 | 1486.8628 | **L** | 260.1969 | 243.1703 | **2** |
| **16** | | | | | **K** | 147.1128 | 130.0863 | **1** |

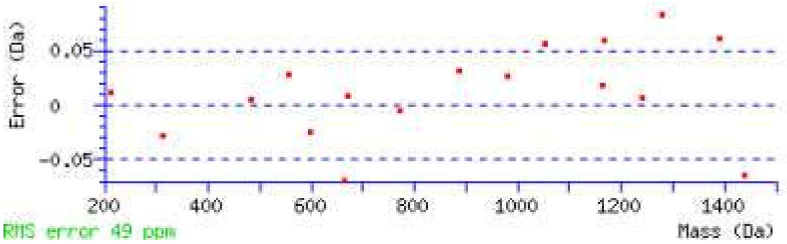


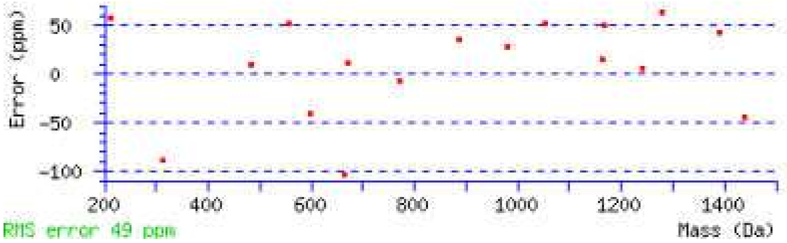


NCBI **BLAST** search of VLVVANPANTNALILK
(Parameters: blastp, nr protein database, expect=20000, no filter, PAM30)
Other BLAST web gateways

**All matches to this query**

| Score | Mr(calc) | Delta | Sequence |
|---|---|---|---|
| 64.6 | 1648.9876 | 0.0240 | VLVVANPANTNALILK |
| 17.9 | 1648.9875 | 0.0240 | EKDLQQLILLPAIR |
| 17.2 | 1648.9121 | 0.0994 | LTGAALRTAQANGLHR |
| 16.8 | 1649.0240 | -0.0124 | LTGPLNLLVLLTGIGR |
| 16.4 | 1648.9413 | 0.0702 | LVRIDLLIESWHR |
| 14.8 | 1648.9624 | 0.0491 | LVLTAAGQTLLSHAVR |
| 14.6 | 1648.9736 | 0.0379 | STLALGLALSAARLHR |
| 14.4 | 1648.9261 | 0.0855 | KDITVPVTISNHLGR |
| 14.3 | 1648.8494 | 0.1621 | VDASIMFNLIEELR |
| 13.5 | 1648.8896 | 0.1219 | VLVVGSGAAEAAEALHR |

**Mascot:** http://www.matrixscience.com/

# MATRIX SCIENCE Mascot Search Results

## Peptide View Spot no 60

MS/MS Fragmentation of **EFAPSIPEKNITCLTR**
Found in **gi|15982948** in **NCBInr**, NAD-dependent malate dehydrogenase [Prunus persica]

Match to Query 242: 1875.006224 from(1876.013500,1+) intensity(0.0000) index(23)
Title: Label: E10, Spot_Id: 219822, Peak_List_Id: 226718, MSMS Job_Run_Id: 21854, Comment:
Data file C:\Documents and Settings\Administrator\Desktop\Farah-Garima 11May 2013\ppw_E10_136859948400.txt

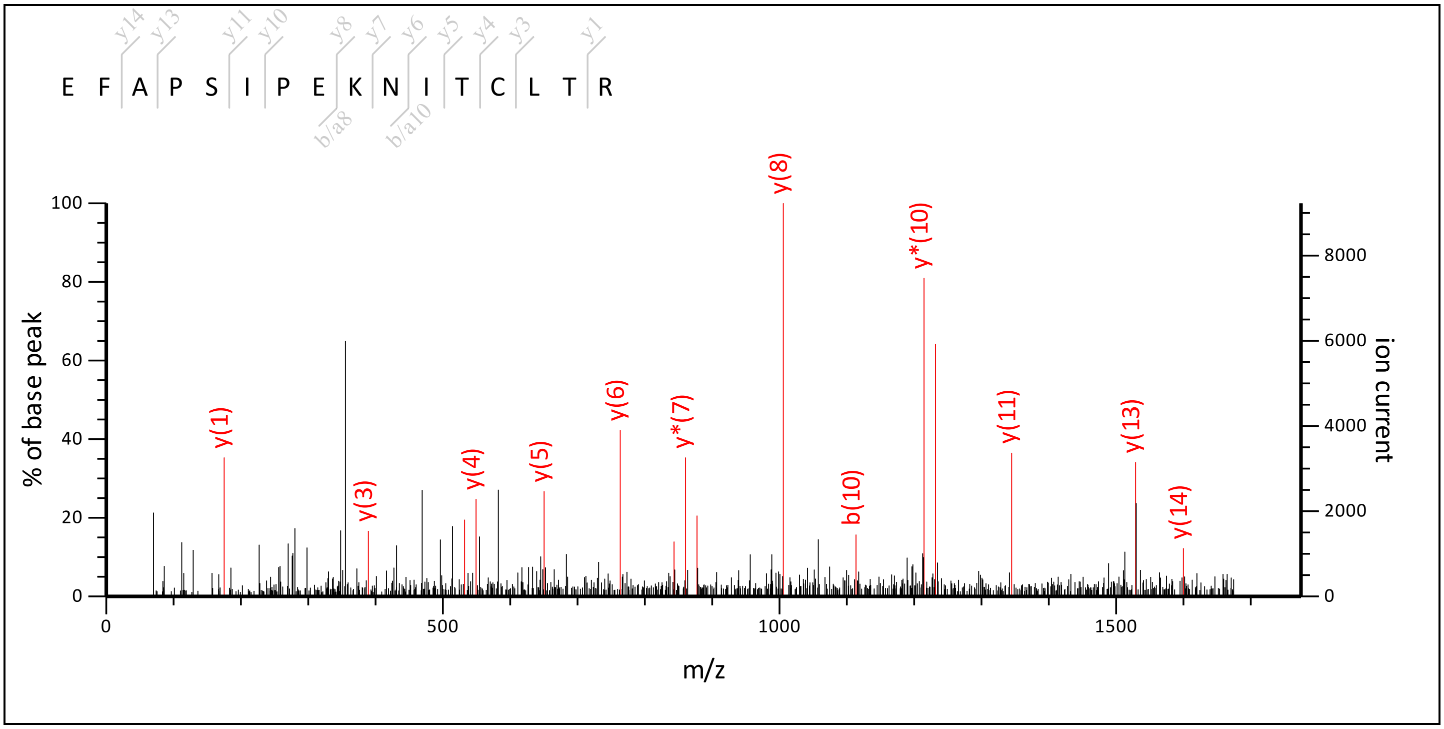


0 to 1774.59

Label all possible matches ○ Label matches used for scoring ◉

**Monoisotopic mass of neutral peptide Mr(calc):** 1874.9560
**Fixed modifications:** Carbamidomethyl (C) (apply to specified residues or termini only)
**Ions Score:** 63 **Expect:** 0.013
**Matches :** 17/74 fragment ions using 34 most intense peaks (help)

| # | a | a* | b | b* | Seq. | y | y* | # |
|---|---|---|---|---|---|---|---|---|
| 1 | 102.0550 | | 130.0499 | | E | | | 16 |
| 2 | 249.1234 | | 277.1183 | | F | 1746.9207 | 1729.8942 | 15 |
| 3 | 320.1605 | | 348.1554 | | A | ***1599.8523*** | 1582.8258 | 14 |
| 4 | 417.2132 | | 445.2082 | | P | ***1528.8152*** | 1511.7886 | 13 |
| 5 | 504.2453 | | **532.2402** | | S | 1431.7624 | 1414.7359 | 12 |
| 6 | 617.3293 | | 645.3243 | | I | ***1344.7304*** | 1327.7038 | 11 |
| 7 | 714.3821 | | 742.3770 | | P | ***1231.6463*** | 1214.6198 | 10 |
| 8 | 843.4247 | | 871.4196 | | E | 1134.5936 | 1117.5670 | 9 |
| | | | | | | | | |

| | | | | | | | | |
|---|---|---|---|---|---|---|---|---|
| 9 | 971.5197 | 954.4931 | 999.5146 | 982.4880 | K | 1005.5510 | 988.5244 | 8 |
| 10 | 1085.5626 | 1068.5360 | 1113.5575 | 1096.5310 | N | 877.4560 | 860.4295 | 7 |
| 11 | 1198.6466 | 1181.6201 | 1226.6416 | 1209.6150 | I | 763.4131 | 746.3865 | 6 |
| 12 | 1299.6943 | 1282.6678 | 1327.6892 | 1310.6627 | T | 650.3290 | 633.3025 | 5 |
| 13 | 1459.7250 | 1442.6984 | 1487.7199 | 1470.6933 | C | 549.2813 | 532.2548 | 4 |
| 14 | 1572.8090 | 1555.7825 | 1600.8040 | 1583.7774 | L | 389.2507 | 372.2241 | 3 |
| 15 | 1673.8567 | 1656.8302 | 1701.8516 | 1684.8251 | T | 276.1666 | 259.1401 | 2 |
| 16 | | | | | R | 175.1190 | 158.0924 | 1 |

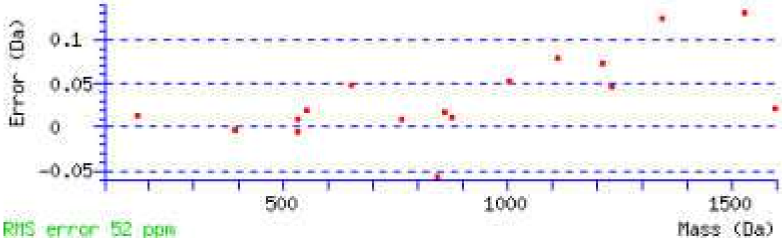


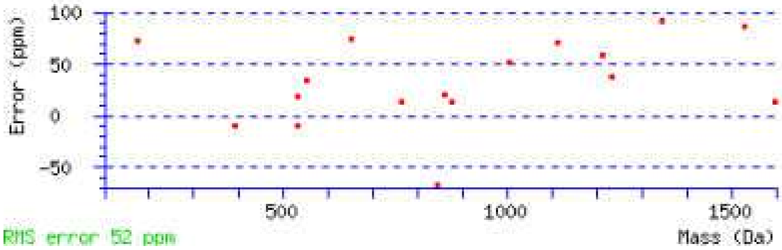


NCBI **BLAST** search of EFAPSIPEKNITCLTR
(Parameters: blastp, nr protein database, expect=20000, no filter, PAM30)
Other BLAST web gateways

**All matches to this query**

| Score | Mr(calc) | Delta | Sequence |
|---|---|---|---|
| 63.2 | 1874.9560 | 0.0502 | EFAPSIPEKNITCLTR |
| 25.1 | 1874.9309 | 0.0753 | EFAPSIPDRNVTCLTR |
| 16.5 | 1874.9117 | 0.0945 | MSESPLLILEQAMEER |
| 15.4 | 1874.9958 | 0.0105 | MSDMVNINKLILVTER |
| 13.4 | 1874.8330 | 0.1732 | VPWNDVCQQSTGGSGQR |
| 13.1 | 1874.9962 | 0.0100 | TAEAARGTLQNGLTVAFR |
| 11.2 | 1874.9407 | 0.0655 | LENAEISNIEITCTIR |
| 10.4 | 1874.9295 | 0.0767 | MSEPSAESTLLLEIEAR |
| 10.1 | 1874.8985 | 0.1077 | MSVYFNEGANTKYVPR |
| 9.9 | 1874.9519 | 0.0543 | ALMKANEILETQSSNAR |

**Mascot:** http://www.matrixscience.com/

# MATRIX SCIENCE Mascot Search Results

## Peptide View Spot no 60

MS/MS Fragmentation of **VLVTGAAGQIGYALVPMIAR**
Found in **gi|15982948** in **NCBInr**, NAD-dependent malate dehydrogenase [Prunus persica]

Match to Query 254: 1999.182624 from(2000.189900,1+) intensity(0.0000) index(25)
Title: Label: E10, Spot_Id: 219822, Peak_List_Id: 226717, MSMS Job_Run_Id: 21854, Comment:
Data file C:\Documents and Settings\Administrator\Desktop\Farah-Garima 11May 2013\ppw_E10_136859948400.txt

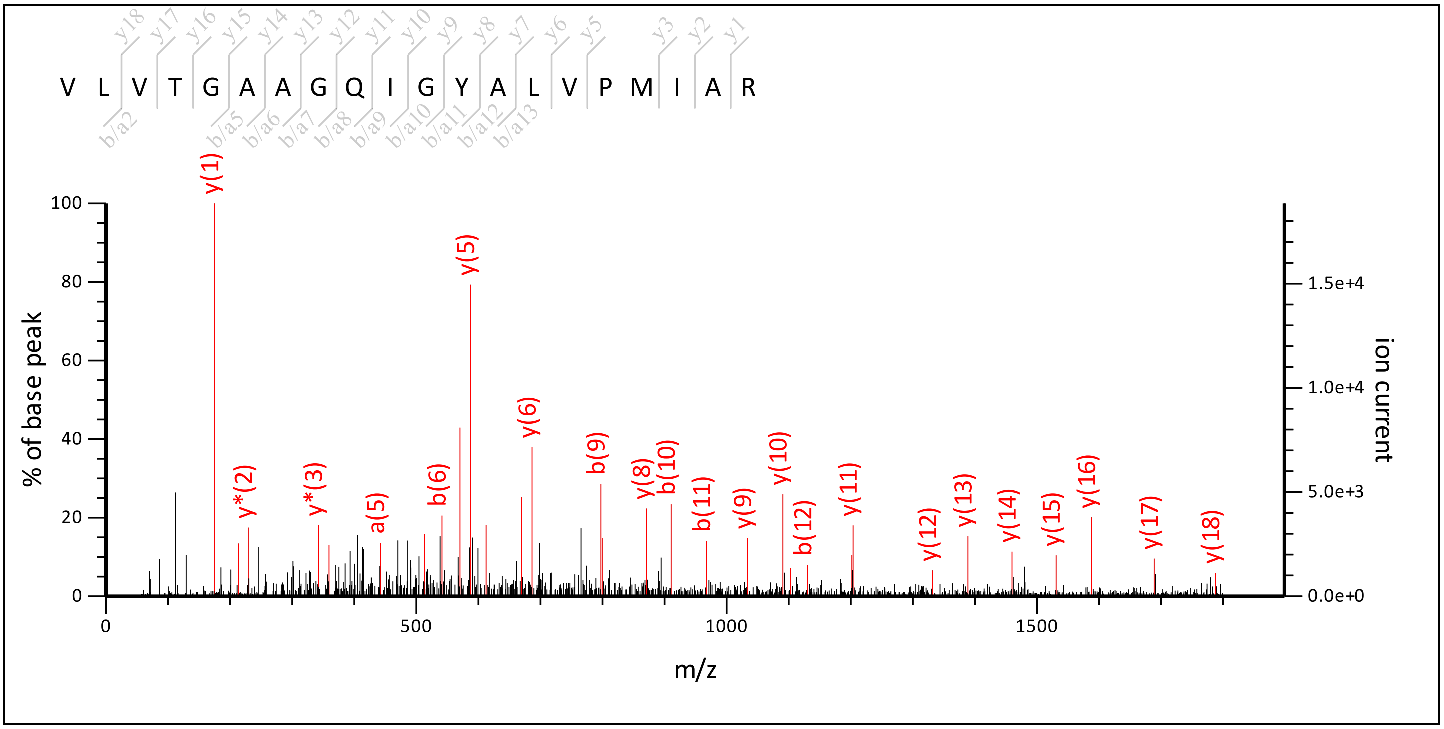


0 to 1898.85

Label all possible matches ○ Label matches used for scoring ◉

**Monoisotopic mass of neutral peptide Mr(calc):** 1999.1288
**Fixed modifications:** Carbamidomethyl (C) (apply to specified residues or termini only)
**Ions Score:** 117 **Expect:** 2.9e-08
**Matches :** 32/98 fragment ions using 54 most intense peaks (help)

| # | a | a* | b | b* | Seq. | y | y* | # |
|---|---|---|---|---|---|---|---|---|
| 1 | 72.0808 | | 100.0757 | | V | | | 20 |
| 2 | 185.1648 | | **213.1598** | | L | 1901.0677 | 1884.0412 | 19 |
| 3 | 284.2333 | | 312.2282 | | V | ***1787.9837*** | 1770.9571 | 18 |
| 4 | 385.2809 | | 413.2758 | | T | ***1688.9152*** | 1671.8887 | 17 |
| 5 | **442.3024** | | 470.2973 | | G | ***1587.8676*** | 1570.8410 | 16 |
| 6 | **513.3395** | | **541.3344** | | A | ***1530.8461*** | 1513.8195 | 15 |
| 7 | 584.3766 | | **612.3715** | | A | ***1459.8090*** | 1442.7824 | 14 |
| 8 | 641.3981 | | **669.3930** | | G | ***1388.7719*** | 1371.7453 | 13 |

| | | | | | | | | |
|---|---|---|---|---|---|---|---|---|
| 9 | 769.4567 | 752.4301 | 797.4516 | 780.4250 | Q | 1331.7504 | 1314.7239 | 12 |
| 10 | 882.5407 | 865.5142 | 910.5356 | 893.5091 | I | 1203.6918 | 1186.6653 | 11 |
| 11 | 939.5622 | 922.5356 | 967.5571 | 950.5306 | G | 1090.6078 | 1073.5812 | 10 |
| 12 | 1102.6255 | 1085.5990 | 1130.6204 | 1113.5939 | Y | 1033.5863 | 1016.5598 | 9 |
| 13 | 1173.6626 | 1156.6361 | 1201.6576 | 1184.6310 | A | 870.5230 | 853.4964 | 8 |
| 14 | 1286.7467 | 1269.7202 | 1314.7416 | 1297.7151 | L | 799.4859 | 782.4593 | 7 |
| 15 | 1385.8151 | 1368.7886 | 1413.8100 | 1396.7835 | V | 686.4018 | 669.3752 | 6 |
| 16 | 1482.8679 | 1465.8413 | 1510.8628 | 1493.8362 | P | 587.3334 | 570.3068 | 5 |
| 17 | 1613.9084 | 1596.8818 | 1641.9033 | 1624.8767 | M | 490.2806 | 473.2541 | 4 |
| 18 | 1726.9924 | 1709.9659 | 1754.9873 | 1737.9608 | I | 359.2401 | 342.2136 | 3 |
| 19 | 1798.0295 | 1781.0030 | 1826.0245 | 1808.9979 | A | 246.1561 | 229.1295 | 2 |
| 20 | | | | | R | 175.1190 | 158.0924 | 1 |

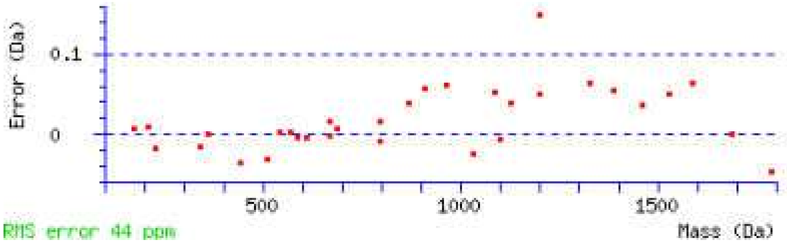


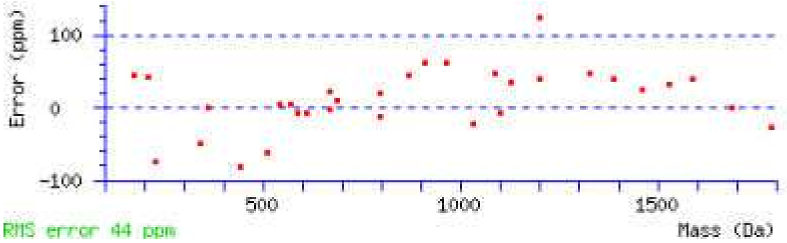


NCBI **BLAST** search of VLVTGAAGQIGYALVPMIAR
(Parameters: blastp, nr protein database, expect=20000, no filter, PAM30)
Other BLAST web gateways

**All matches to this query**

| Score | Mr(calc) | Delta | Sequence |
|---|---|---|---|
| 117.2 | 1999.1288 | 0.0538 | VLVTGAAGQIGYAIVPMIAR |
| 117.2 | 1999.1288 | 0.0538 | VLVTGAAGQIGYALVPMIAR |
| 35.3 | 1999.0521 | 0.1306 | LVSLQQQIASQGGIVMDGR |
| 24.8 | 1999.1327 | 0.0499 | VRVDAAQGLLHADLGVPLR |
| 24.0 | 1999.1037 | 0.0790 | NLLAEAGKLAKPGMLGFGGR |
| 22.1 | 1999.1024 | 0.0803 | IVVDSVLAIGILDQLMER |
| 22.0 | 1999.1288 | 0.0538 | EKVIASIVHLLQVTFMR |
| 20.9 | 1999.1215 | 0.0612 | VSVAALAGQGLVAVAKDAFGR |
| 20.9 | 1999.1003 | 0.0823 | VVAITGAGQGIGLAAAWAFAR |
| 19.2 | 1999.0963 | 0.0863 | DAVAALKQHGVDSVLIAHR |

**Mascot:** http://www.matrixscience.com/

# Mascot Search Results

## Peptide View Spot no 60

MS/MS Fragmentation of **VLVTGAAGQIGYALVPMIAR**
Found in **gi|15982948** in **NCBInr**, NAD-dependent malate dehydrogenase [Prunus persica]

Match to Query 256: 2015.162524 from(2016.169800,1+) intensity(0.0000) index(26)
Title: Label: E10, Spot_Id: 219822, Peak_List_Id: 226720, MSMS Job_Run_Id: 21854, Comment:
Data file C:\Documents and Settings\Administrator\Desktop\Farah-Garima 11May 2013\ppw_E10_136859948400.txt

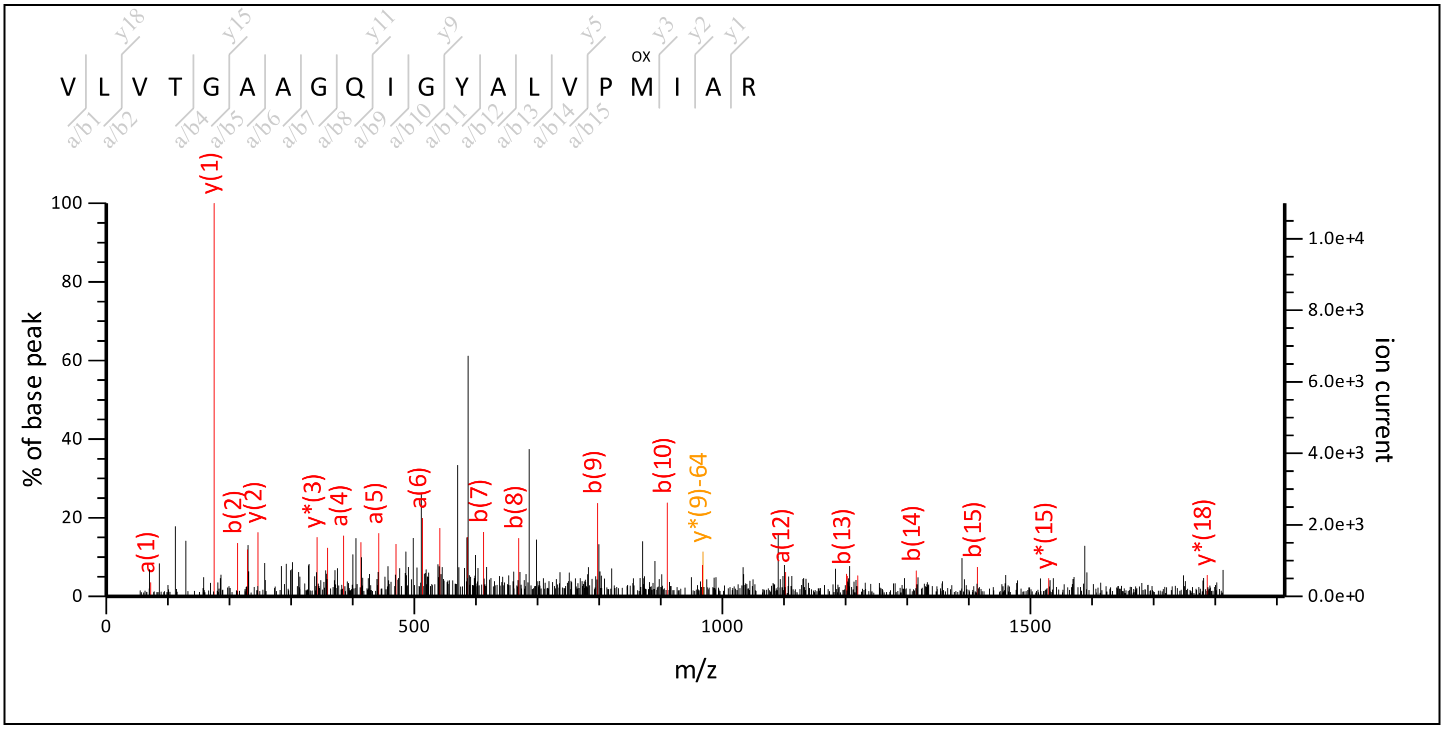


0 to 1912.69

Label all possible matches ○ Label matches used for scoring ◉

**Monoisotopic mass of neutral peptide Mr(calc):** 2015.1238
**Fixed modifications:** Carbamidomethyl (C) (apply to specified residues or termini only)
**Variable modifications:**
**M17** : Oxidation (M), with neutral losses 0.0000(shown in table), 63.9983
**Ions Score:** 37 **Expect:** 4.1
**Matches** : 29/142 fragment ions using 90 most intense peaks (help)

| # | a | a* | b | b* | Seq. | y | y* | # |
|---|---|---|---|---|---|---|---|---|
| 1 | 72.0808 | | 100.0757 | | V | | | 20 |
| 2 | 185.1648 | | 213.1598 | | L | 1917.0626 | 1900.0361 | 19 |
| 3 | 284.2333 | | 312.2282 | | V | 1803.9786 | 1786.9520 | 18 |
| 4 | 385.2809 | | 413.2758 | | T | 1704.9102 | 1687.8836 | 17 |
| 5 | 442.3024 | | 470.2973 | | G | 1603.8625 | 1586.8359 | 16 |
| 6 | 513.3395 | | 541.3344 | | A | 1546.8410 | 1529.8145 | 15 |
| 7 | 584.3766 | | 612.3715 | | A | 1475.8039 | 1458.7774 | 14 |

| | | | | | | | | |
|---|---|---|---|---|---|---|---|---|
| 8 | 641.3981 | | *669.3930* | | G | 1404.7668 | 1387.7402 | 13 |
| 9 | 769.4567 | 752.4301 | *797.4516* | 780.4250 | Q | 1347.7453 | 1330.7188 | 12 |
| 10 | 882.5407 | 865.5142 | *910.5356* | 893.5091 | I | **1219.6867** | 1202.6602 | 11 |
| 11 | 939.5622 | 922.5356 | *967.5571* | 950.5306 | G | 1106.6027 | 1089.5761 | 10 |
| 12 | **1102.6255** | 1085.5990 | 1130.6204 | 1113.5939 | Y | 1049.5812 | 1032.5547 | 9 |
| 13 | 1173.6626 | 1156.6361 | *1201.6576* | 1184.6310 | A | 886.5179 | 869.4913 | 8 |
| 14 | 1286.7467 | 1269.7202 | *1314.7416* | 1297.7151 | L | 815.4808 | 798.4542 | 7 |
| 15 | 1385.8151 | 1368.7886 | *1413.8100* | 1396.7835 | V | 702.3967 | 685.3702 | 6 |
| 16 | 1482.8679 | 1465.8413 | 1510.8628 | 1493.8362 | P | 603.3283 | 586.3017 | 5 |
| 17 | 1629.9033 | 1612.8767 | 1657.8982 | 1640.8716 | M | 506.2755 | 489.2490 | 4 |
| 18 | 1742.9873 | 1725.9608 | 1770.9823 | 1753.9557 | I | **359.2401** | 342.2136 | 3 |
| 19 | 1814.0245 | 1796.9979 | 1842.0194 | 1824.9928 | A | **246.1561** | 229.1295 | 2 |
| 20 | | | | | R | **175.1190** | 158.0924 | 1 |

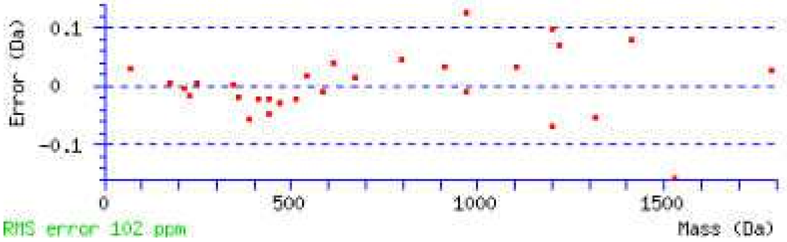


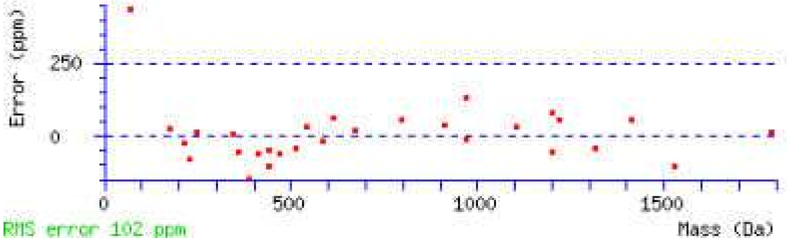


NCBI **BLAST** search of VLVTGAAGQIGYALVPMIAR
(Parameters: blastp, nr protein database, expect=20000, no filter, PAM30)
Other BLAST web gateways

**All matches to this query**

| Score | Mr(calc) | Delta | Sequence |
|---|---|---|---|
| 36.8 | 2015.1238 | 0.0388 | VLVTGAAGQIGYAIVPMIAR |
| 36.8 | 2015.1238 | 0.0388 | VLVTGAAGQIGYALVPMIAR |
| 14.8 | 2015.0795 | 0.0831 | LMLLDAAKLPAIVEEMAR |
| 12.5 | 2015.1415 | 0.0210 | ILVSGKVLSPSELQLNYR |
| 12.1 | 2015.1098 | 0.0527 | AFTCQLAVLAALAVGAGRAR |
| 10.4 | 2015.0575 | 0.1050 | QAALDELSDYIPIIEVAR |
| 9.1 | 2015.1891 | -0.0266 | LSDAGLAKVAAHPVLSVVLR |
| 8.9 | 2015.0470 | 0.1156 | VSSAVLAGGRPDTAMVIAER |
| 8.8 | 2015.0800 | 0.0826 | GTKFSSNVNNGLIPVEIAR |
| 8.6 | 2015.0874 | 0.0752 | IDAFADLMRPLGLIDIAR |

**Mascot:** http://www.matrixscience.com/

# MATRIX SCIENCE Mascot Search Results

## Peptide View Spot no 60

MS/MS Fragmentation of **GVVATTDVVEACTGVNIAVMVGGFPR**
Found in **gi|15982948** in **NCBInr**, NAD-dependent malate dehydrogenase [Prunus persica]

Match to Query 291: 2618.380924 from(2619.388200,1+) intensity(0.0000) index(33)
Title: Label: E10, Spot_Id: 219822, Peak_List_Id: 226731, MSMS Job_Run_Id: 21854, Comment:
Data file C:\Documents and Settings\Administrator\Desktop\Farah-Garima 11May 2013\ppw_E10_136859948400.txt

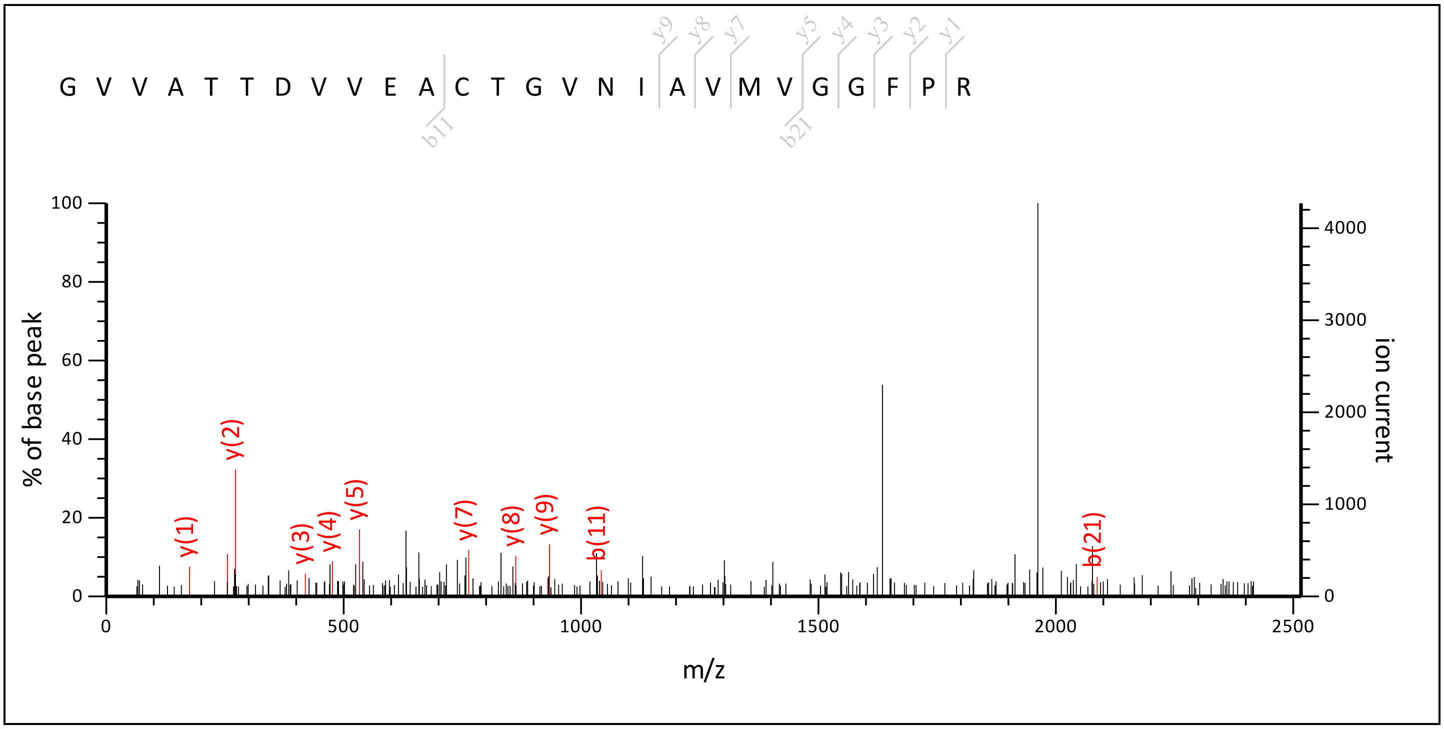


0 to 2516.29

Label all possible matches ○ Label matches used for scoring ◉

**Monoisotopic mass of neutral peptide Mr(calc):** 2618.3197
**Fixed modifications:** Carbamidomethyl (C) (apply to specified residues or termini only)
**Ions Score:** 10 **Expect:** 1.8e+03
**Matches :** 11/120 fragment ions using 48 most intense peaks (help)

| # | a | a* | b | b* | Seq. | y | y* | # |
|---|---|---|---|---|---|---|---|---|
| 1 | 30.0338 | | 58.0287 | | G | | | 26 |
| 2 | 129.1022 | | 157.0972 | | V | 2562.3055 | 2545.2789 | 25 |
| 3 | 228.1707 | | 256.1656 | | V | 2463.2370 | 2446.2105 | 24 |
| 4 | 299.2078 | | 327.2027 | | A | 2364.1686 | 2347.1421 | 23 |
| 5 | 400.2554 | | 428.2504 | | T | 2293.1315 | 2276.1050 | 22 |
| 6 | 501.3031 | | 529.2980 | | T | 2192.0838 | 2175.0573 | 21 |
| 7 | 616.3301 | | 644.3250 | | D | 2091.0362 | 2074.0096 | 20 |
| 8 | 715.3985 | | 743.3934 | | V | 1976.0092 | 1958.9827 | 19 |

| | | | | | | | | |
|---|---|---|---|---|---|---|---|---|
| **9** | 814.4669 | | 842.4618 | | **V** | 1876.9408 | 1859.9143 | **18** |
| **10** | 943.5095 | | 971.5044 | | **E** | 1777.8724 | 1760.8458 | **17** |
| **11** | 1014.5466 | | **1042.5415** | | **A** | 1648.8298 | 1631.8032 | **16** |
| **12** | 1174.5773 | | 1202.5722 | | **C** | 1577.7927 | 1560.7661 | **15** |
| **13** | 1275.6249 | | 1303.6198 | | **T** | 1417.7620 | 1400.7355 | **14** |
| **14** | 1332.6464 | | 1360.6413 | | **G** | 1316.7144 | 1299.6878 | **13** |
| **15** | 1431.7148 | | 1459.7097 | | **V** | 1259.6929 | 1242.6663 | **12** |
| **16** | 1545.7577 | 1528.7312 | 1573.7527 | 1556.7261 | **N** | 1160.6245 | 1143.5979 | **11** |
| **17** | 1658.8418 | 1641.8153 | 1686.8367 | 1669.8102 | **I** | 1046.5815 | 1029.5550 | **10** |
| **18** | 1729.8789 | 1712.8524 | 1757.8738 | 1740.8473 | **A** | ***933.4975*** | 916.4709 | **9** |
| **19** | 1828.9473 | 1811.9208 | 1856.9422 | 1839.9157 | **V** | ***862.4604*** | 845.4338 | **8** |
| **20** | 1959.9878 | 1942.9613 | 1987.9827 | 1970.9562 | **M** | ***763.3920*** | 746.3654 | **7** |
| **21** | 2059.0562 | 2042.0297 | **2087.0511** | 2070.0246 | **V** | 632.3515 | 615.3249 | **6** |
| **22** | 2116.0777 | 2099.0511 | 2144.0726 | 2127.0461 | **G** | ***533.2831*** | 516.2565 | **5** |
| **23** | 2173.0992 | 2156.0726 | 2201.0941 | 2184.0675 | **G** | ***476.2616*** | 459.2350 | **4** |
| **24** | 2320.1676 | 2303.1410 | 2348.1625 | 2331.1359 | **F** | ***419.2401*** | 402.2136 | **3** |
| **25** | 2417.2203 | 2400.1938 | 2445.2152 | 2428.1887 | **P** | ***272.1717*** | 255.1452 | **2** |
| **26** | | | | | **R** | ***175.1190*** | 158.0924 | **1** |

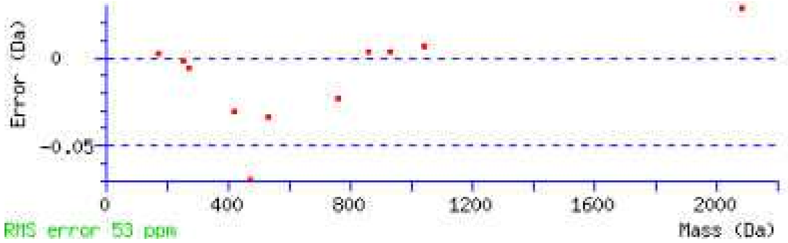


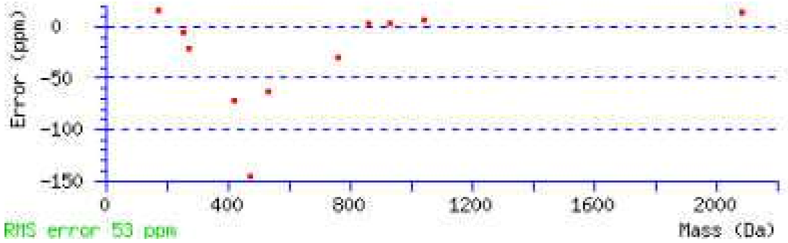


NCBI **BLAST** search of GVVATTDVVEACTGVNIAVMVGGFPR
(Parameters: blastp, nr protein database, expect=20000, no filter, PAM30)
Other BLAST web gateways

**All matches to this query**

| Score | Mr(calc) | Delta | Sequence |
|---|---|---|---|
| 70.2 | 2618.3197 | 0.0612 | GIVATTDVVEACTGVNVAVMVGGFPR |
| 45.0 | 2618.3197 | 0.0612 | GIVATTDVAEACKGVDVAVMVGGFPR |
| 10.1 | 2618.3197 | 0.0612 | GVVATTDVVEACTGVNIAVMVGGFPR |
| 7.1 | 2618.3275 | 0.0535 | WKETLTTALQEHAAANPLAPGMPR |
| 3.5 | 2618.2040 | 0.1769 | QVGVSCASGLQVWGDVVLCCDGPR |
| | | | |

| | | | |
|---|---|---|---|
| 2.7 | 2618.3309 | 0.0500 | NIGCDIVMIDVNQDVAGFITRLR |
| 2.4 | 2618.2395 | 0.1414 | MLASGGDDDTVILWDLSDRDRPR |
| 1.2 | 2618.2283 | 0.1527 | TLCDAGYHVSLETSGALDVSGVDPR |
| 1.2 | 2618.2039 | 0.1770 | TMLALQSHNMTAWLGSESQNMPR |
| 1.1 | 2618.2567 | 0.1242 | IASALECSTAVLMSEAESQLAAAAQP |

**Mascot:** http://www.matrixscience.com/

# Mascot Search Results

## Peptide View Spot no 64

MS/MS Fragmentation of **VPWSEYR**
Found in **gi|29468084** in **NCBInr**, aspartate aminotransferase [Oryza sativa]

Match to Query 13: 935.521044 from(936.528320,1+) intensity(0.0000) index(2)
Title: Label: K7, Spot_Id: 228948, Peak_List_Id: 257615, MSMS Job_Run_Id: 24928, Comment:
Data file ppw_K7_138985140200.txt

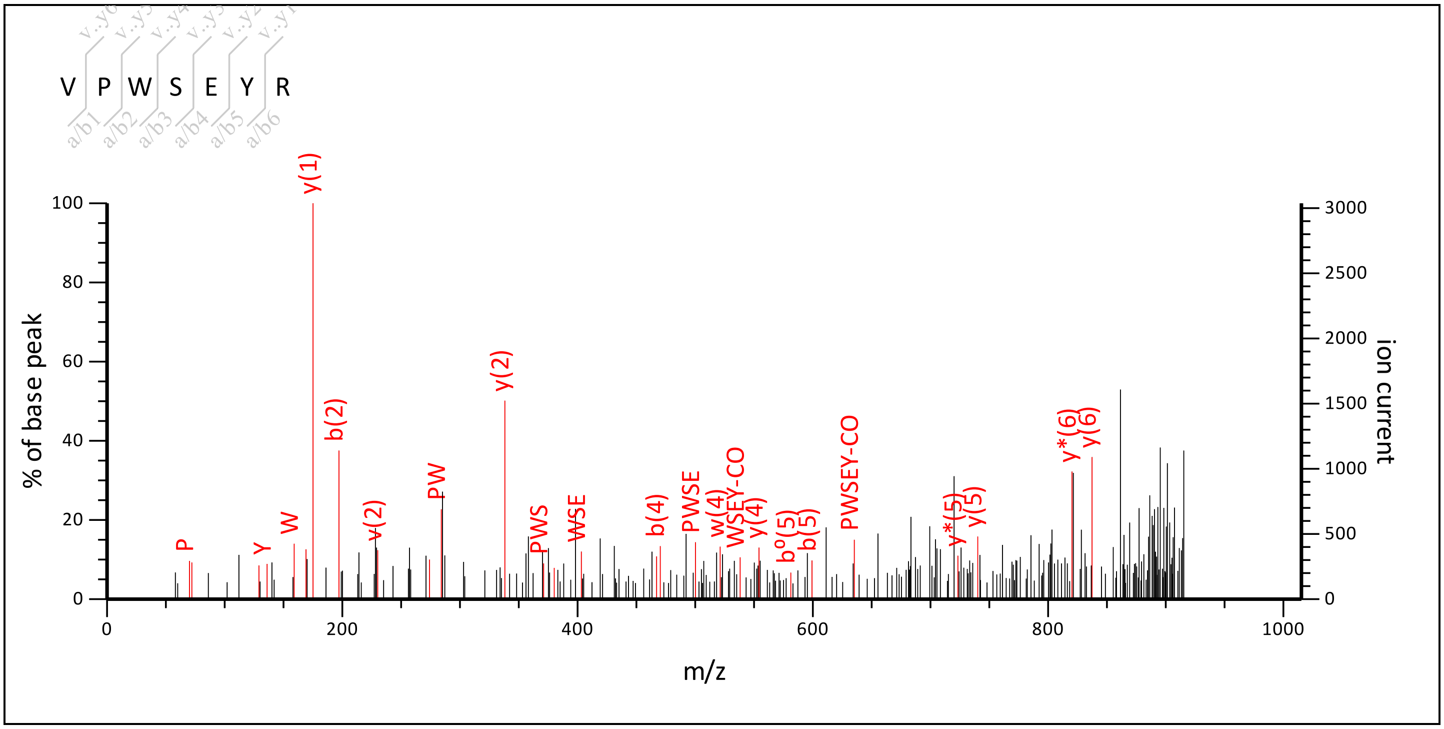


0 to 1015.32

Label all possible matches ○ Label matches used for scoring ◉

**Monoisotopic mass of neutral peptide Mr(calc):** 935.4501
**Fixed modifications:** Carbamidomethyl (C) (apply to specified residues or termini only)
**Ions Score:** 20 **Expect:** 29
**Matches :** 31/75 fragment ions using 90 most intense peaks (help)

| # | Immon. | a | $a^0$ | b | $b^0$ | d | Seq. | v | w | y | y* | $y^0$ | # |
|---|---|---|---|---|---|---|---|---|---|---|---|---|---|
| 1 | 72.0808 | **72.0808** | | 100.0757 | | 44.0495 | V | | | | | | 7 |
| 2 | 70.0651 | **169.1335** | | **197.1285** | | 143.1179 | P | 795.3420 | 794.3468 | ***837.3890*** | 820.3624 | 819.3784 | 6 |
| 3 | 159.0917 | 355.2129 | | 383.2078 | | | W | 609.2627 | | ***740.3362*** | 723.3097 | 722.3257 | 5 |
| 4 | 60.0444 | 442.2449 | 424.2343 | **470.2398** | 452.2292 | 426.2500 | S | 522.2307 | 521.2354 | ***554.2569*** | 537.2304 | 536.2463 | 4 |
| 5 | 102.0550 | 571.2875 | 553.2769 | **599.2824** | 581.2718 | 513.2820 | E | 393.1881 | 392.1928 | ***467.2249*** | 450.1983 | 449.2143 | 3 |
| 6 | 136.0757 | 734.3508 | 716.3402 | 762.3457 | 744.3352 | | Y | 230.1248 | | ***338.1823*** | 321.1557 | | 2 |
| 7 | 129.1135 | | | | | | R | 74.0237 | 73.0284 | ***175.1190*** | 158.0924 | | 1 |

| Seq | ya | yb | Seq | ya | yb | Seq | ya | yb |
|---|---|---|---|---|---|---|---|---|
| PW | 256.1444 | 284.1394 | PWS | 343.1765 | 371.1714 | PWSE | 472.2191 | 500.2140 |
| PWSEY | 635.2824 | 663.2773 | WS | 246.1237 | 274.1186 | WSE | 375.1663 | 403.1612 |
| WSEY | 538.2296 | 566.2245 | SE | 189.0870 | 217.0819 | SEY | 352.1503 | 380.1452 |
| EY | 265.1183 | 293.1132 | | | | | | |

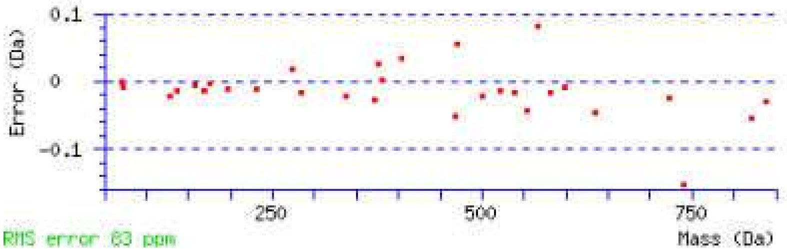


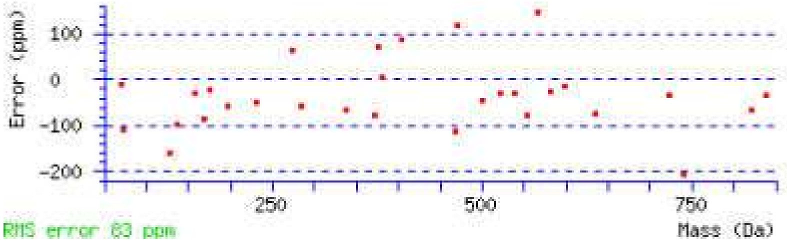


NCBI **BLAST** search of VPWSEYR
(Parameters: blastp, nr protein database, expect=20000, no filter, PAM30)
Other BLAST web gateways

**All matches to this query**

| Score | Mr(calc) | Delta | Sequence |
|---|---|---|---|
| 20.0 | 935.4501 | 0.0709 | VPWSEYR |
| 17.1 | 935.5262 | -0.0052 | VILCIYR |
| 17.1 | 935.5076 | 0.0134 | VLIDTGYR |
| 13.0 | 935.4712 | 0.0498 | DLFSAEVR |
| 12.3 | 935.5076 | 0.0134 | DLIKEYR |
| 12.3 | 935.5076 | 0.0134 | DLLKEYR |
| 10.7 | 935.5188 | 0.0022 | VKNASVYR |
| 10.6 | 935.4825 | 0.0386 | AANTYIAGR |
| 10.3 | 935.4825 | 0.0386 | EVHAEPVR |
| 10.2 | 935.4865 | 0.0345 | VPPASFYR |

**Mascot:** http://www.matrixscience.com/

# MATRIX SCIENCE Mascot Search Results

## Peptide View Spot no 64

MS/MS Fragmentation of **GLEVFVAQSYSK**
Found in **gi|29468084** in **NCBInr**, aspartate aminotransferase [Oryza sativa]

Match to Query 45: 1326.763924 from(1327.771200,1+) intensity(0.0000) index(10)
Title: Label: K7, Spot_Id: 228948, Peak_List_Id: 257625, MSMS Job_Run_Id: 24928, Comment:
Data file ppw_K7_138985140200.txt

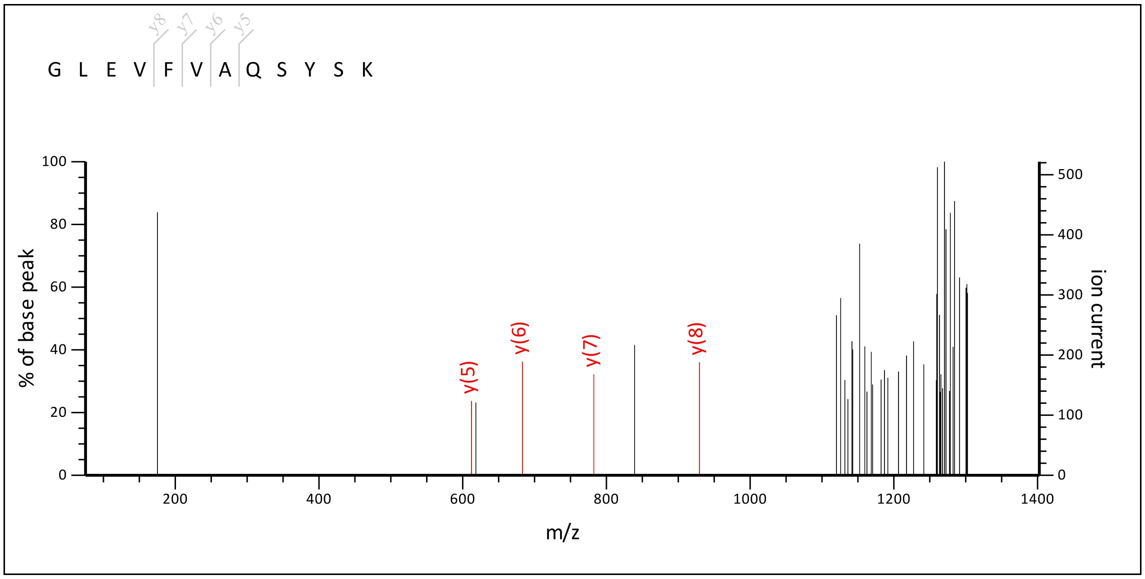


75.1 to 1402.47

Label all possible matches Label matches used for scoring

**Monoisotopic mass of neutral peptide Mr(calc):** 1326.6820
**Fixed modifications:** Carbamidomethyl (C) (apply to specified residues or termini only)
**Ions Score:** 7 **Expect:** 3.9e+02
**Matches :** 4/189 fragment ions using 13 most intense peaks (help)

| # | Immon. | a | a* | $a^0$ | b | b* | $b^0$ | d | Seq. | v | w | y | y* | $y^0$ | # |
|---|---|---|---|---|---|---|---|---|---|---|---|---|---|---|---|
| 1 | 30.0338 | 30.0338 | | | 58.0287 | | | 44.0495 | G | | | | | | 12 |
| 2 | 86.0964 | 143.1179 | | | 171.1128 | | | 101.0709 | L | 1212.5895 | 1211.5943 | 1270.6678 | 1253.6412 | 1252.6572 | 11 |
| 3 | 102.0550 | 272.1605 | | 254.1499 | 300.1554 | | 282.1448 | 214.1550 | E | 1083.5469 | 1082.5517 | 1157.5837 | 1140.5572 | 1139.5732 | 10 |
| 4 | 72.0808 | 371.2289 | | 353.2183 | 399.2238 | | 381.2132 | 357.2132 | V | 984.4785 | 997.4989 | 1028.5411 | 1011.5146 | 1010.5306 | 9 |
| 5 | 120.0808 | 518.2973 | | 500.2867 | 546.2922 | | 528.2817 | | F | 837.4101 | | ***929.4727*** | 912.4462 | 911.4621 | 8 |
| 6 | 72.0808 | 617.3657 | | 599.3552 | 645.3606 | | 627.3501 | 603.3501 | V | 738.3417 | 751.3621 | ***782.4043*** | 765.3777 | 764.3937 | 7 |
| 7 | 44.0495 | 688.4028 | | 670.3923 | 716.3978 | | 698.3872 | | A | 667.3046 | | ***683.3359*** | 666.3093 | 665.3253 | 6 |
| 8 | 101.0709 | 816.4614 | 799.4349 | 798.4509 | 844.4563 | 827.4298 | 826.4458 | 759.4400 | Q | 539.2460 | 538.2508 | ***612.2988*** | 595.2722 | 594.2882 | 5 |
| 9 | 60.0444 | 903.4934 | 886.4669 | 885.4829 | 931.4884 | 914.4618 | 913.4778 | 887.4985 | S | 452.2140 | 451.2187 | 484.2402 | 467.2136 | 466.2296 | 4 |
| 10 | 136.0757 | 1066.5568 | 1049.5302 | 1048.5462 | 1094.5517 | 1077.5251 | 1076.5411 | | Y | 289.1506 | | 397.2082 | 380.1816 | 379.1976 | 3 |
| 11 | 60.0444 | 1153.5888 | 1136.5623 | 1135.5782 | 1181.5837 | 1164.5572 | 1163.5732 | 1137.5939 | S | 202.1186 | 201.1234 | 234.1448 | 217.1183 | 216.1343 | 2 |
| 12 | 101.1073 | | | | | | | | K | 74.0237 | 73.0284 | 147.1128 | 130.0863 | | 1 |

| Seq | ya | yb | Seq | ya | yb | Seq | ya | yb |
|---|---|---|---|---|---|---|---|---|
| LE | 215.1390 | 243.1339 | LEV | 314.2074 | 342.2023 | LEVF | 461.2758 | 489.2708 |
| LEVFV | 560.3443 | 588.3392 | LEVFVA | 631.3814 | 659.3763 | EV | 201.1234 | 229.1183 |
| EVF | 348.1918 | 376.1867 | EVFV | 447.2602 | 475.2551 | EVFVA | 518.2973 | 546.2922 |
| EVFVAQ | 646.3559 | 674.3508 | VF | 219.1492 | 247.1441 | VFV | 318.2176 | 346.2125 |
| VFVA | 389.2547 | 417.2496 | VFVAQ | 517.3133 | 545.3082 | VFVAQS | 604.3453 | 632.3402 |

| | | | | | | | | |
|---|---|---|---|---|---|---|---|---|
| **FV** | 219.1492 | 247.1441 | **FVA** | 290.1863 | 318.1812 | **FVAQ** | 418.2449 | 446.2398 |
| **FVAQS** | 505.2769 | 533.2718 | **FVAQSY** | 668.3402 | 696.3352 | **VA** | 143.1179 | 171.1128 |
| **VAQ** | 271.1765 | 299.1714 | **VAQS** | 358.2085 | 386.2034 | **VAQSY** | 521.2718 | 549.2667 |
| **VAQSYS** | 608.3039 | 636.2988 | **AQ** | 172.1081 | 200.1030 | **AQS** | 259.1401 | 287.1350 |
| **AQSY** | 422.2034 | 450.1983 | **AQSYS** | 509.2354 | 537.2304 | **QS** | 188.1030 | 216.0979 |
| **QSY** | 351.1663 | 379.1612 | **QSYS** | 438.1983 | 466.1932 | **SY** | 223.1077 | 251.1026 |
| **SYS** | 310.1397 | 338.1347 | **YS** | 223.1077 | 251.1026 | | | |

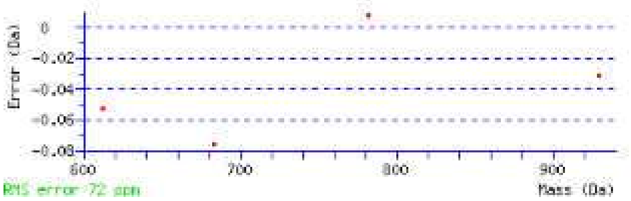


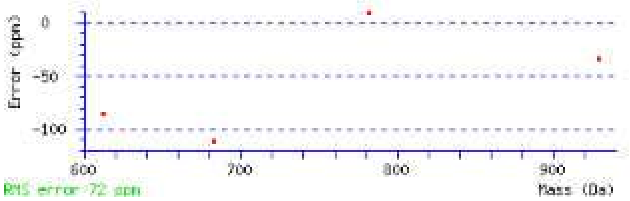


NCBI **BLAST** search of GLEVFVAQSYSK
(Parameters: blastp, nr protein database, expect=20000, no filter, PAM30)
Other BLAST web gateways

**All matches to this query**

| Score | Mr(calc) | Delta | Sequence |
|---|---|---|---|
| 7.8 | 1326.6602 | 0.1038 | DSVRVMNLYSK |
| 7.8 | 1326.6979 | 0.0660 | KVFNRPPPGCR |
| 7.5 | 1326.6503 | 0.1136 | VMGNLPARFGEH |
| 7.2 | 1326.6826 | 0.0813 | MATAAPPAASGARR |
| 7.2 | 1326.6640 | 0.0999 | VGASSSVGHDGAKR |
| 6.7 | 1326.6820 | 0.0820 | GLEVFVAQSYSK |
| 6.3 | 1326.6317 | 0.1323 | GGLGFGSNVYNSR |
| 5.8 | 1326.7408 | 0.0231 | IADVLKASFAHR |
| 5.8 | 1326.7520 | 0.0119 | VLWRGAGLAQTR |
| 5.6 | 1326.6891 | 0.0748 | VALEAQVQARNE |

**Mascot:** http://www.matrixscience.com/

# Mascot Search Results

## Peptide View Spot no 64

MS/MS Fragmentation of **VATLQSLSGTGSLR**
Found in **gi|29468084** in **NCBInr**, aspartate aminotransferase [Oryza sativa]

Match to Query 54: 1388.883824 from(1389.891100,1+) intensity(0.0000) index(13)
Title: Label: K7, Spot_Id: 228948, Peak_List_Id: 257621, MSMS Job_Run_Id: 24928, Comment:
Data file ppw_K7_138985140200.txt

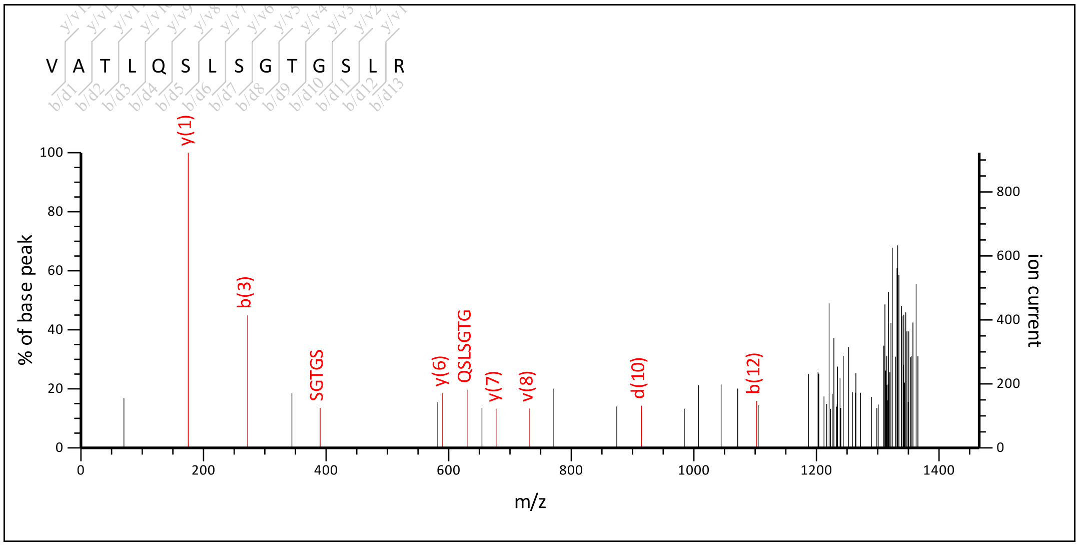


0 to 1465.57

Label all possible matches ○ Label matches used for scoring ◉

**Monoisotopic mass of neutral peptide Mr(calc):** 1388.7623
**Fixed modifications:** Carbamidomethyl (C) (apply to specified residues or termini only)
**Ions Score:** 8 **Expect:** 1.1e+02
**Matches :** 10/255 fragment ions using 20 most intense peaks (help)

| # | Immon. | a | a* | $a^0$ | b | b* | $b^0$ | d | d' | Seq. | v | w | w' | y | y* | |
|---|---|---|---|---|---|---|---|---|---|---|---|---|---|---|---|---|
| 1 | 72.0808 | 72.0808 | | | 100.0757 | | | 44.0495 | | V | | | | | | |
| 2 | 44.0495 | 143.1179 | | | 171.1128 | | | | | A | 1274.6699 | | | 1290.7012 | 1273.6747 | [illegible] |
| 3 | 74.0600 | 244.1656 | | 226.1550 | ***272.1605*** | | 254.1499 | 228.1707 | 230.1499 | T | 1173.6222 | 1186.6426 | 1188.6219 | 1219.6641 | 1202.6375 | [illegible] |
| 4 | 86.0964 | 357.2496 | | 339.2391 | 385.2445 | | 367.2340 | 315.2027 | | L | 1060.5382 | 1059.5429 | | 1118.6164 | 1101.5899 | [illegible] |
| 5 | 101.0709 | 485.3082 | 468.2817 | 467.2976 | 513.3031 | 496.2766 | 495.2926 | 428.2867 | | Q | 932.4796 | 931.4843 | | 1005.5323 | 988.5058 | [illegible] |
| 6 | 60.0444 | 572.3402 | 555.3137 | 554.3297 | 600.3352 | 583.3086 | 582.3246 | 556.3453 | | S | 845.4476 | 844.4523 | | 877.4738 | 860.4472 | [illegible] |
| 7 | 86.0964 | 685.4243 | 668.3978 | 667.4137 | 713.4192 | 696.3927 | 695.4087 | 643.3774 | | L | 732.3635 | 731.3682 | | 790.4417 | 773.4152 | [illegible] |
| 8 | 60.0444 | 772.4563 | 755.4298 | 754.4458 | 800.4512 | 783.4247 | 782.4407 | 756.4614 | | S | 645.3315 | 644.3362 | | ***677.3577*** | 660.3311 | [illegible] |
| 9 | 30.0338 | 829.4778 | 812.4512 | 811.4672 | 857.4727 | 840.4462 | 839.4621 | | | G | | | | ***590.3257*** | 573.2991 | [illegible] |
| 10 | 74.0600 | 930.5255 | 913.4989 | 912.5149 | 958.5204 | 941.4938 | 940.5098 | 914.5306 | 916.5098 | T | 487.2623 | 500.2827 | 502.2620 | 533.3042 | 516.2776 | [illegible] |
| 11 | 30.0338 | 987.5469 | 970.5204 | 969.5364 | 1015.5419 | 998.5153 | 997.5313 | | | G | | | | 432.2565 | 415.2300 | [illegible] |
| 12 | 60.0444 | 1074.5790 | 1057.5524 | 1056.5684 | ***1102.5739*** | 1085.5473 | 1084.5633 | 1058.5841 | | S | 343.2088 | 342.2136 | | 375.2350 | 358.2085 | [illegible] |
| 13 | 86.0964 | 1187.6630 | 1170.6365 | 1169.6525 | 1215.6579 | 1198.6314 | 1197.6474 | 1145.6161 | | L | 230.1248 | 229.1295 | | 288.2030 | 271.1765 | |
| 14 | 129.1135 | | | | | | | | | R | 74.0237 | 73.0284 | | ***175.1190*** | 158.0924 | |

| Seq | ya | yb | Seq | ya | yb | Seq | ya | yb |
|---|---|---|---|---|---|---|---|---|
| AT | 145.0972 | 173.0921 | ATL | 258.1812 | 286.1761 | ATLQ | 386.2398 | 414.2347 |
| ATLQS | 473.2718 | 501.2667 | ATLQSL | 586.3559 | 614.3508 | ATLQSLS | 673.3879 | 701.3828 |
| TL | 187.1441 | 215.1390 | TLQ | 315.2027 | 343.1976 | TLQS | 402.2347 | 430.2296 |
| TLQSL | 515.3188 | 543.3137 | TLQSLS | 602.3508 | 630.3457 | TLQSLSG | 659.3723 | 687.3672 |
| LQ | 214.1550 | 242.1499 | LQS | 301.1870 | 329.1819 | LQSL | 414.2711 | 442.2660 |
| LQSLS | 501.3031 | 529.2980 | LQSLSG | 558.3246 | 586.3195 | LQSLSGT | 659.3723 | 687.3672 |

| | | | | | | | | |
|---|---|---|---|---|---|---|---|---|
| **QS** | 188.1030 | 216.0979 | **QSL** | 301.1870 | 329.1819 | **QSLS** | 388.2191 | 416.2140 |
| **QSLSG** | 445.2405 | 473.2354 | **QSLSGT** | 546.2882 | 574.2831 | **QSLSGTG** | 603.3097 | 631.3046 |
| **QSLSGTGS** | 690.3417 | 718.3366 | **SL** | 173.1285 | 201.1234 | **SLS** | 260.1605 | 288.1554 |
| **SLSG** | 317.1819 | 345.1769 | **SLSGT** | 418.2296 | 446.2245 | **SLSGTG** | 475.2511 | 503.2460 |
| **SLSGTGS** | 562.2831 | 590.2780 | **SLSGTGSL** | 675.3672 | 703.3621 | **LS** | 173.1285 | 201.1234 |
| **LSG** | 230.1499 | 258.1448 | **LSGT** | 331.1976 | 359.1925 | **LSGTG** | 388.2191 | 416.2140 |
| **LSGTGS** | 475.2511 | 503.2460 | **LSGTGSL** | 588.3352 | 616.3301 | **SG** | 117.0659 | 145.0608 |
| **SGT** | 218.1135 | 246.1084 | **SGTG** | 275.1350 | 303.1299 | **SGTGS** | 362.1670 | 390.1619 |
| **SGTGSL** | 475.2511 | 503.2460 | **GT** | 131.0815 | 159.0764 | **GTG** | 188.1030 | 216.0979 |
| **GTGS** | 275.1350 | 303.1299 | **GTGSL** | 388.2191 | 416.2140 | **TG** | 131.0815 | 159.0764 |
| **TGS** | 218.1135 | 246.1084 | **TGSL** | 331.1976 | 359.1925 | **GS** | 117.0659 | 145.0608 |
| **GSL** | 230.1499 | 258.1448 | **SL** | 173.1285 | 201.1234 | | | |

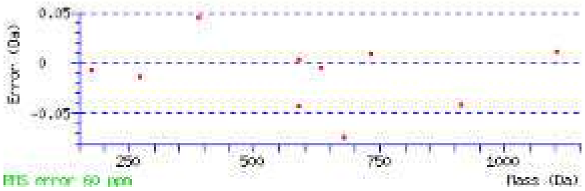


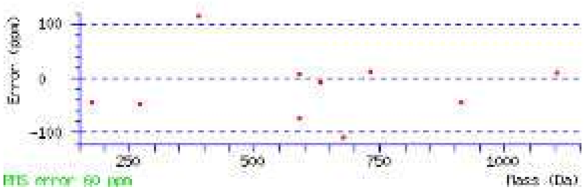


NCBI **BLAST** search of VATLQSLSGTGSLR
(Parameters: blastp, nr protein database, expect=20000, no filter, PAM30)
Other BLAST web gateways

**All matches to this query**

| Score | Mr(calc) | Delta | Sequence |
|---|---|---|---|
| 7.8 | 1388.7623 | 0.1215 | VATIQSLSGTGSLR |
| 7.8 | 1388.7623 | 0.1215 | VATLQSLSGTGSLR |
| 7.2 | 1388.7623 | 0.1215 | SSITIETLRNQK |
| 7.0 | 1388.7452 | 0.1386 | GLIFQEYLPGPR |
| 7.0 | 1388.7875 | 0.0963 | SDKSISIIQTGLK |
| 6.9 | 1388.7565 | 0.1274 | GGRIFVYDLPPR |
| 6.9 | 1388.7663 | 0.1175 | GVLISVYEEKPR |
| 6.5 | 1388.7452 | 0.1386 | GSSFPLIPPFATR |
| 6.0 | 1388.7598 | 0.1240 | LKSFQIQCIPR |
| 6.0 | 1388.7452 | 0.1386 | VTQFLPAPYTPR |

**Mascot:** http://www.matrixscience.com/

# MATRIX SCIENCE Mascot Search Results

## Peptide View Spot no 64

MS/MS Fragmentation of **LARPMYSNPPIHGAR**
Found in **gi|29468084** in **NCBInr**, aspartate aminotransferase [Oryza sativa]

Match to Query 73: 1678.996124 from(1680.003400,1+) intensity(0.0000) index(20)
Title: Label: K7, Spot_Id: 228948, Peak_List_Id: 257619, MSMS Job_Run_Id: 24928, Comment:
Data file ppw_K7_138985140200.txt

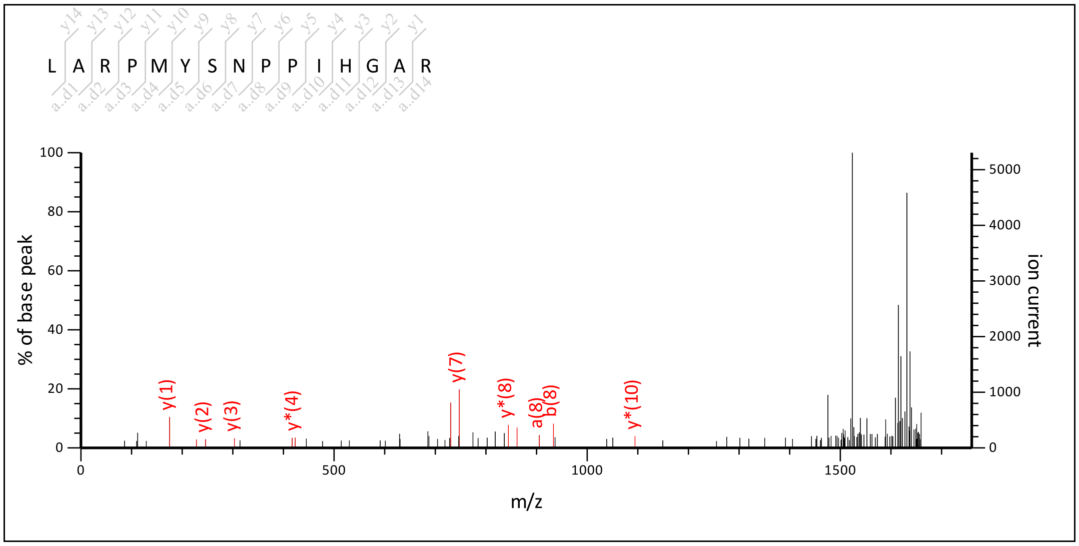


0 to 1759.61

Label all possible matches ○ Label matches used for scoring ◉

**Monoisotopic mass of neutral peptide Mr(calc):** 1678.8725
**Fixed modifications:** Carbamidomethyl (C) (apply to specified residues or termini only)
**Ions Score:** 8 **Expect:** 1.7e+02
**Matches :** 14/250 fragment ions using 32 most intense peaks (help)

| # | Immon. | a | a* | $a^0$ | b | b* | $b^0$ | d | d' | Seq. | v | w | w' | y | y* | |
|---|---|---|---|---|---|---|---|---|---|---|---|---|---|---|---|---|
| 1 | 86.0964 | 86.0964 | | | 114.0913 | | | 44.0495 | | L | | | | | | |
| 2 | 44.0495 | 157.1335 | | | 185.1285 | | | | | A | 1550.7645 | | | 1566.7958 | 1549.7692 | 154 |
| 3 | 129.1135 | 313.2346 | 296.2081 | | 341.2296 | 324.2030 | | 228.1707 | | R | 1394.6634 | 1393.6681 | | 1495.7587 | 1478.7321 | 147 |
| 4 | 70.0651 | 410.2874 | 393.2609 | | 438.2823 | 421.2558 | | 384.2718 | | P | 1297.6106 | 1296.6154 | | 1339.6576 | 1322.6310 | 132 |
| 5 | 104.0528 | 541.3279 | 524.3013 | | 569.3228 | 552.2963 | | 481.3245 | | M | 1166.5701 | 1165.5749 | | 1242.6048 | 1225.5783 | 122 |
| 6 | 136.0757 | 704.3912 | 687.3647 | | 732.3861 | 715.3596 | | | | Y | 1003.5068 | | | 1111.5643 | 1094.5378 | 109 |
| 7 | 60.0444 | 791.4233 | 774.3967 | 773.4127 | 819.4182 | 802.3916 | 801.4076 | 775.4283 | | S | 916.4748 | 915.4795 | | 948.5010 | 931.4744 | 93 |
| 8 | 87.0553 | 905.4662 | 888.4396 | 887.4556 | 933.4611 | 916.4345 | 915.4505 | 862.4604 | | N | 802.4318 | 801.4366 | | ***861.4690*** | 844.4424 | |
| 9 | 70.0651 | 1002.5189 | 985.4924 | 984.5084 | 1030.5139 | 1013.4873 | 1012.5033 | 976.5033 | | P | 705.3791 | 704.3838 | | ***747.4260*** | 730.3995 | |
| 10 | 70.0651 | 1099.5717 | 1082.5452 | 1081.5611 | 1127.5666 | 1110.5401 | 1109.5561 | 1073.5561 | | P | 608.3263 | 607.3311 | | 650.3733 | 633.3467 | |
| 11 | 86.0964 | 1212.6558 | 1195.6292 | 1194.6452 | 1240.6507 | 1223.6241 | 1222.6401 | 1184.6245 | 1198.6401 | I | 495.2423 | 508.2627 | 522.2783 | 553.3205 | 536.2940 | |
| 12 | 110.0713 | 1349.7147 | 1332.6881 | 1331.7041 | 1377.7096 | 1360.6831 | 1359.6990 | | | H | 358.1833 | | | 440.2364 | 423.2099 | |
| 13 | 30.0338 | 1406.7362 | 1389.7096 | 1388.7256 | 1434.7311 | 1417.7045 | 1416.7205 | | | G | | | | ***303.1775*** | 286.1510 | |
| 14 | 44.0495 | 1477.7733 | 1460.7467 | 1459.7627 | 1505.7682 | 1488.7416 | 1487.7576 | | | A | 230.1248 | | | ***246.1561*** | 229.1295 | |
| 15 | 129.1135 | | | | | | | | | R | 74.0237 | 73.0284 | | ***175.1190*** | 158.0924 | |

| Seq | ya | yb | Seq | ya | yb | Seq | ya | yb |
|---|---|---|---|---|---|---|---|---|
| AR | 200.1506 | 228.1455 | ARP | 297.2033 | 325.1983 | ARPM | 428.2438 | 456.2387 |
| ARPMY | 591.3072 | 619.3021 | ARPMYS | 678.3392 | 706.3341 | RP | 226.1662 | 254.1612 |
| RPM | 357.2067 | 385.2016 | RPMY | 520.2700 | 548.2650 | RPMYS | 607.3021 | 635.2970 |
| PM | 201.1056 | 229.1005 | PMY | 364.1689 | 392.1639 | PMYS | 451.2010 | 479.1959 |
| PMYSN | 565.2439 | 593.2388 | PMYSNP | 662.2967 | 690.2916 | MY | 267.1162 | 295.1111 |
| | | | | | | | | |

| | | | | | | | | |
|---|---|---|---|---|---|---|---|---|
| **MYS** | 354.1482 | 382.1431 | **MYSN** | 468.1911 | 496.1860 | **MYSNP** | 565.2439 | 593.2388 |
| **MYSNPP** | 662.2967 | 690.2916 | **YS** | 223.1077 | 251.1026 | **YSN** | 337.1506 | 365.1456 |
| **YSNP** | 434.2034 | 462.1983 | **YSNPP** | 531.2562 | 559.2511 | **YSNPPI** | 644.3402 | 672.3352 |
| **SN** | 174.0873 | 202.0822 | **SNP** | 271.1401 | 299.1350 | **SNPP** | 368.1928 | 396.1878 |
| **SNPPI** | 481.2769 | 509.2718 | **SNPPIH** | 618.3358 | 646.3307 | **SNPPIHG** | 675.3573 | 703.3522 |
| **NP** | 184.1081 | 212.1030 | **NPP** | 281.1608 | 309.1557 | **NPPI** | 394.2449 | 422.2398 |
| **NPPIH** | 531.3038 | 559.2987 | **NPPIHG** | 588.3253 | 616.3202 | **NPPIHGA** | 659.3624 | 687.3573 |
| **PP** | 167.1179 | 195.1128 | **PPI** | 280.2020 | 308.1969 | **PPIH** | 417.2609 | 445.2558 |
| **PPIHG** | 474.2823 | 502.2772 | **PPIHGA** | 545.3194 | 573.3144 | **PI** | 183.1492 | 211.1441 |
| **PIH** | 320.2081 | 348.2030 | **PIHG** | 377.2296 | 405.2245 | **PIHGA** | 448.2667 | 476.2616 |
| **IH** | 223.1553 | 251.1503 | **IHG** | 280.1768 | 308.1717 | **IHGA** | 351.2139 | 379.2088 |
| **HG** | 167.0927 | 195.0877 | **HGA** | 238.1299 | 266.1248 | **GA** | 101.0709 | 129.0659 |

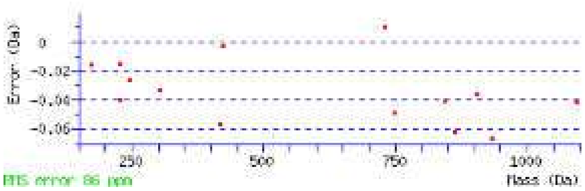


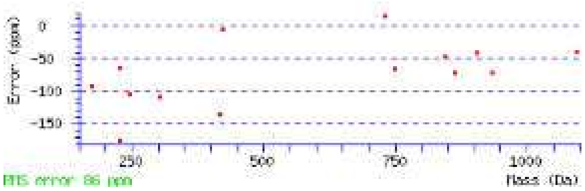


NCBI **BLAST** search of LARPMYSNPPIHGAR
(Parameters: blastp, nr protein database, expect=20000, no filter, PAM30)
Other BLAST web gateways

**All matches to this query**

| Score | Mr(calc) | Delta | Sequence |
|---|---|---|---|
| 13.7 | 1678.8362 | 0.1599 | RSNCSFWLVGGLGAR |
| 9.8 | 1678.9114 | 0.0847 | RLLLENPSVGSNGPAR |
| 9.7 | 1678.8573 | 0.1388 | MVTGDNIHTAKHIAR |
| 9.6 | 1678.8499 | 0.1462 | RSSSSNHNNIPKPNK |
| 9.2 | 1678.8322 | 0.1640 | RHVAGAGTPSSAPMGGAR |
| 9.1 | 1678.8317 | 0.1644 | RIGMSMKPCLELGR |
| 8.9 | 1678.9036 | 0.0925 | RSTLSTTTTIAIMQR |
| 8.5 | 1678.8725 | 0.1236 | IARPMYSNPPIHGAR |
| 8.5 | 1678.8725 | 0.1236 | LARPMYSNPPIHGAR |
| 8.2 | 1678.8751 | 0.1210 | RGVAVSQSTYTVQQR |

**Mascot:** http://www.matrixscience.com/

# MATRIX SCIENCE Mascot Search Results

## Peptide View Spot no 64

MS/MS Fragmentation of **LARPMYSNPPIHGAR**
Found in **gi|29468084** in **NCBInr**, aspartate aminotransferase [Oryza sativa]

Match to Query 75: 1694.980024 from(1695.987300,1+) intensity(0.0000) index(21)
Title: Label: K7, Spot_Id: 228948, Peak_List_Id: 257620, MSMS Job_Run_Id: 24928, Comment:
Data file ppw_K7_138985140200.txt

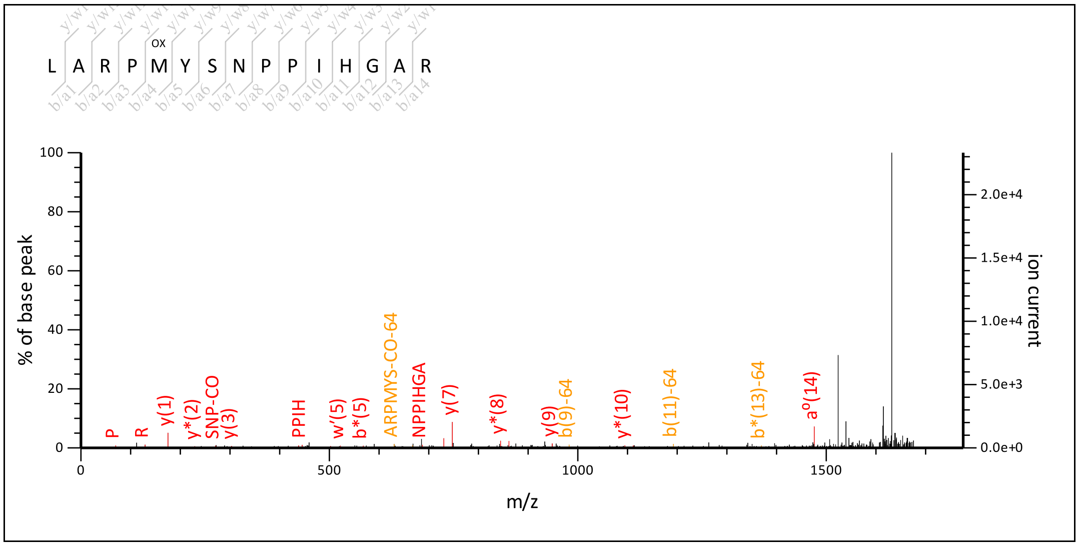


0 to 1775.59

Label all possible matches ○ Label matches used for scoring ◉

**Monoisotopic mass of neutral peptide Mr(calc):** 1694.8675
**Fixed modifications:** Carbamidomethyl (C) (apply to specified residues or termini only)
**Variable modifications:**
**M5** : Oxidation (M), with neutral losses 63.9983(shown in table), 0.0000
**Ions Score:** 13 **Expect:** 82
**Matches :** 29/365 fragment ions using 77 most intense peaks (help)

| # | Immon. | a | a* | $a^0$ | b | b* | $b^0$ | d | d' | Seq. | v | w | w' | y | y* | [illegible] |
|---|---|---|---|---|---|---|---|---|---|---|---|---|---|---|---|---|
| 1 | 86.0964 | 86.0964 | | | 114.0913 | | | 44.0495 | | L | | | | | | |
| 2 | 44.0495 | 157.1335 | | | 185.1285 | | | | | A | 1502.7611 | | | 1518.7924 | 1501.7659 | [illegible] |
| 3 | 129.1135 | 313.2346 | 296.2081 | | 341.2296 | 324.2030 | | 228.1707 | | R | 1346.6600 | 1345.6648 | | 1447.7553 | 1430.7288 | [illegible] |
| 4 | 70.0651 | 410.2874 | 393.2609 | | **438.2823** | 421.2558 | | 384.2718 | | P | 1249.6072 | 1248.6120 | | 1291.6542 | 1274.6276 | [illegible] |
| 5 | 56.0495 | 493.3245 | 476.2980 | | 521.3194 | 504.2929 | | 481.3245 | | M | 1166.5701 | 1165.5749 | | 1194.6014 | 1177.5749 | [illegible] |
| 6 | 136.0757 | 656.3879 | 639.3613 | | 684.3828 | 667.3562 | | | | Y | 1003.5068 | | | ***1111.5643*** | 1094.5378 | [illegible] |
| 7 | 60.0444 | 743.4199 | 726.3933 | 725.4093 | 771.4148 | 754.3883 | 753.4042 | 727.4250 | | S | 916.4748 | 915.4795 | | ***948.5010*** | 931.4744 | [illegible] |
| 8 | 87.0553 | 857.4628 | 840.4363 | 839.4522 | 885.4577 | 868.4312 | 867.4472 | 814.4570 | | N | 802.4318 | 801.4366 | | ***861.4690*** | 844.4424 | |
| 9 | 70.0651 | 954.5156 | 937.4890 | 936.5050 | **982.5105** | 965.4839 | 964.4999 | 928.4999 | | P | 705.3791 | 704.3838 | | ***747.4260*** | 730.3995 | |
| 10 | 70.0651 | 1051.5683 | 1034.5418 | 1033.5578 | 1079.5633 | 1062.5367 | 1061.5527 | 1025.5527 | | P | 608.3263 | 607.3311 | | 650.3733 | 633.3467 | |
| 11 | 86.0964 | 1164.6524 | 1147.6259 | 1146.6418 | **1192.6473** | 1175.6208 | 1174.6368 | 1136.6211 | 1150.6368 | I | 495.2423 | 508.2627 | 522.2783 | 553.3205 | 536.2940 | |
| 12 | 110.0713 | 1301.7113 | 1284.6848 | 1283.7008 | 1329.7062 | 1312.6797 | 1311.6957 | | | H | 358.1833 | | | 440.2364 | 423.2099 | |
| 13 | 30.0338 | 1358.7328 | 1341.7062 | 1340.7222 | 1386.7277 | 1369.7011 | 1368.7171 | | | G | | | | ***303.1775*** | 286.1510 | |
| 14 | 44.0495 | 1429.7699 | 1412.7433 | 1411.7593 | 1457.7648 | 1440.7383 | 1439.7542 | | | A | 230.1248 | | | 246.1561 | 229.1295 | |
| 15 | 129.1135 | | | | | | | | | R | 74.0237 | 73.0284 | | ***175.1190*** | 158.0924 | |

| Seq | ya | yb | Seq | ya | yb | Seq | ya | yb |
|---|---|---|---|---|---|---|---|---|
| AR | 200.1506 | 228.1455 | ARP | 297.2033 | 325.1983 | ARPM | 380.2405 | 408.2354 |
| ARPMY | 543.3038 | 571.2987 | ARPMYS | 630.3358 | 658.3307 | RP | 226.1662 | 254.1612 |
| RPM | 309.2034 | 337.1983 | RPMY | 472.2667 | 500.2616 | RPMYS | 559.2987 | 587.2936 |
| RPMYSN | 673.3416 | 701.3365 | PM | 153.1022 | 181.0972 | PMY | 316.1656 | 344.1605 |

| | | | | | | | | |
|---|---|---|---|---|---|---|---|---|
| **PMYS** | 403.1976 | 431.1925 | **PMYSN** | 517.2405 | 545.2354 | **PMYSNP** | 614.2933 | 642.2882 |
| **MY** | 219.1128 | 247.1077 | **MYS** | 306.1448 | 334.1397 | **MYSN** | 420.1878 | 448.1827 |
| **MYSNP** | 517.2405 | 545.2354 | **MYSNPP** | 614.2933 | 642.2882 | **YS** | 223.1077 | 251.1026 |
| **YSN** | 337.1506 | 365.1456 | **YSNP** | 434.2034 | 462.1983 | **YSNPP** | 531.2562 | 559.2511 |
| **YSNPPI** | 644.3402 | 672.3352 | **SN** | 174.0873 | 202.0822 | **SNP** | 271.1401 | 299.1350 |
| **SNPP** | 368.1928 | 396.1878 | **SNPPI** | 481.2769 | 509.2718 | **SNPPIH** | 618.3358 | 646.3307 |
| **SNPPIHG** | 675.3573 | 703.3522 | **NP** | 184.1081 | 212.1030 | **NPP** | 281.1608 | 309.1557 |
| **NPPI** | 394.2449 | 422.2398 | **NPPIH** | 531.3038 | 559.2987 | **NPPIHG** | 588.3253 | 616.3202 |
| **NPPIHGA** | 659.3624 | 687.3573 | **PP** | 167.1179 | 195.1128 | **PPI** | 280.2020 | 308.1969 |
| **PPIH** | 417.2609 | 445.2558 | **PPIHG** | 474.2823 | 502.2772 | **PPIHGA** | 545.3194 | 573.3144 |
| **PI** | 183.1492 | 211.1441 | **PIH** | 320.2081 | 348.2030 | **PIHG** | 377.2296 | 405.2245 |
| **PIHGA** | 448.2667 | 476.2616 | **IH** | 223.1553 | 251.1503 | **IHG** | 280.1768 | 308.1717 |
| **IHGA** | 351.2139 | 379.2088 | **HG** | 167.0927 | 195.0877 | **HGA** | 238.1299 | 266.1248 |
| **GA** | 101.0709 | 129.0659 | | | | | | |

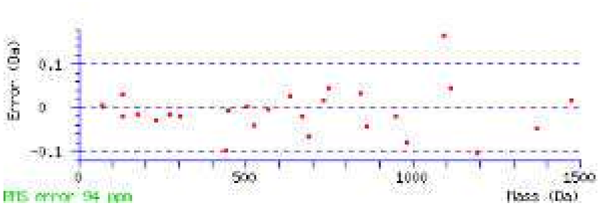


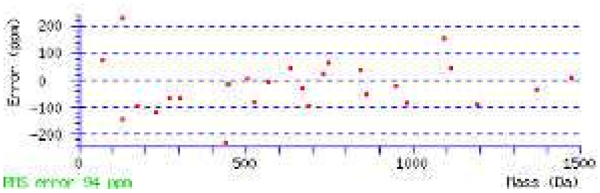


NCBI **BLAST** search of LARPMYSNPPIHGAR
(Parameters: blastp, nr protein database, expect=20000, no filter, PAM30)
Other BLAST web gateways

**All matches to this query**

| Score | Mr(calc) | Delta | Sequence |
|---|---|---|---|
| 21.0 | 1694.8926 | 0.0874 | RALPHSLNTMVASIW |
| 12.5 | 1694.8675 | 0.1126 | IARPMYSNPPIHGAR |
| 12.5 | 1694.8675 | 0.1126 | LARPMYSNPPIHGAR |
| 11.5 | 1694.9540 | 0.0260 | RTAASGRPLTLPVSNR |
| 11.0 | 1694.8199 | 0.1601 | GSHGMVYKGVLFNDR |
| 10.0 | 1694.8886 | 0.0914 | VGESLPLSATHLRCR |
| 10.0 | 1695.0043 | -0.0242 | VLDIAAIITRTEKPR |
| 9.7 | 1694.9540 | 0.0261 | VGAAAILLSNRPSDRR |
| 9.6 | 1695.0043 | -0.0242 | RVTEALSSLALPLGLR |
| 9.6 | 1694.9250 | 0.0551 | RAQLLIHEMAGLTSR |

**Mascot:** http://www.matrixscience.com/

# MATRIX SCIENCE Mascot Search Results

## Peptide View Spot no 64

MS/MS Fragmentation of **NIFNDAKVPWSEYR**
Found in **gi|29468084** in **NCBInr**, aspartate aminotransferase [Oryza sativa]

Match to Query 81: 1737.993624 from(1739.000900,1+) intensity(0.0000) index(23)
Title: Label: K7, Spot_Id: 228948, Peak_List_Id: 257624, MSMS Job_Run_Id: 24928, Comment:
Data file ppw_K7_138985140200.txt

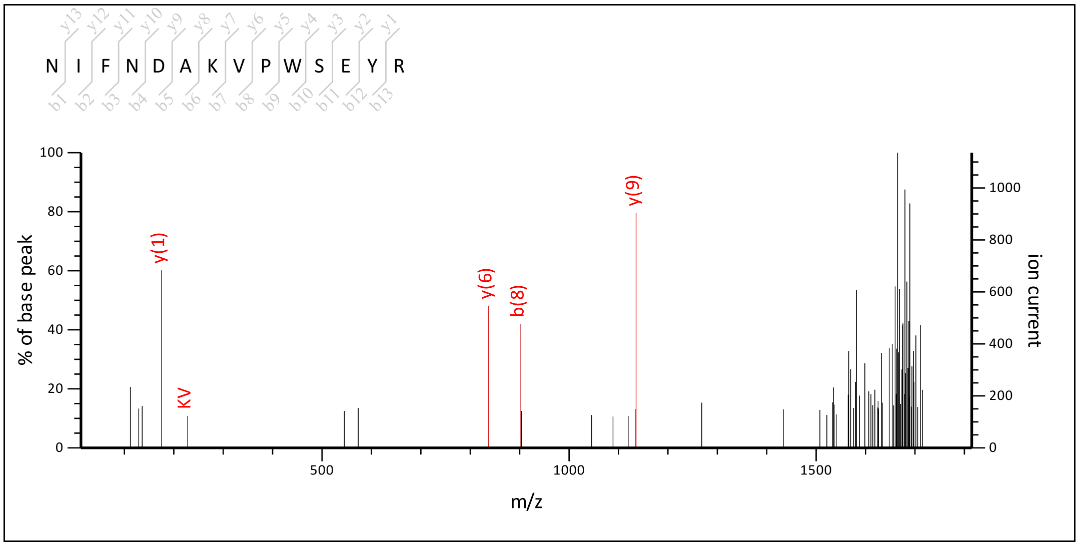


12.09 to 1814.9

Label all possible matches ○ Label matches used for scoring ◉

**Monoisotopic mass of neutral peptide Mr(calc):** 1737.8474
**Fixed modifications:** Carbamidomethyl (C) (apply to specified residues or termini only)
**Ions Score:** 5 **Expect:** 5.9e+02
**Matches :** 6/241 fragment ions using 19 most intense peaks (help)

| # | Immon. | a | a* | $a^0$ | b | b* | $b^0$ | d | d' | Seq. | v | w | w' | y | y* | [illegible] |
|---|---|---|---|---|---|---|---|---|---|---|---|---|---|---|---|---|
| 1 | 87.0553 | 87.0553 | 70.0287 | | 115.0502 | 98.0237 | | 44.0495 | | N | | | | | | |
| 2 | 86.0964 | 200.1394 | 183.1128 | | ***228.1343*** | 211.1077 | | 172.1081 | 186.1237 | I | 1566.7336 | 1579.7540 | 1593.7696 | 1624.8118 | 1607.7853 | [illegible] |
| 3 | 120.0808 | 347.2078 | 330.1812 | | 375.2027 | 358.1761 | | | | F | 1419.6652 | | | 1511.7278 | 1494.7012 | [illegible] |
| 4 | 87.0553 | 461.2507 | 444.2241 | | 489.2456 | 472.2191 | | 418.2449 | | N | 1305.6222 | 1304.6270 | | 1364.6593 | 1347.6328 | [illegible] |
| 5 | 88.0393 | 576.2776 | 559.2511 | 558.2671 | 604.2726 | 587.2460 | 586.2620 | 532.2878 | | D | 1190.5953 | 1189.6000 | | 1250.6164 | 1233.5899 | [illegible] |
| 6 | 44.0495 | 647.3148 | 630.2882 | 629.3042 | 675.3097 | 658.2831 | 657.2991 | | | A | 1119.5582 | | | ***1135.5895*** | 1118.5629 | [illegible] |
| 7 | 101.1073 | 775.4097 | 758.3832 | 757.3991 | 803.4046 | 786.3781 | 785.3941 | 718.3519 | | K | 991.4632 | 990.4680 | | 1064.5524 | 1047.5258 | [illegible] |
| 8 | 72.0808 | 874.4781 | 857.4516 | 856.4676 | ***902.4730*** | 885.4465 | 884.4625 | 860.4625 | | V | 892.3948 | 905.4152 | | 936.4574 | 919.4308 | [illegible] |
| 9 | 70.0651 | 971.5309 | 954.5043 | 953.5203 | 999.5258 | 982.4993 | 981.5152 | 945.5152 | | P | 795.3420 | 794.3468 | | ***837.3890*** | 820.3624 | [illegible] |
| 10 | 159.0917 | 1157.6102 | 1140.5837 | 1139.5996 | 1185.6051 | 1168.5786 | 1167.5946 | | | W | 609.2627 | | | 740.3362 | 723.3097 | [illegible] |
| 11 | 60.0444 | 1244.6422 | 1227.6157 | 1226.6317 | 1272.6371 | 1255.6106 | 1254.6266 | 1228.6473 | | S | 522.2307 | 521.2354 | | 554.2569 | 537.2304 | [illegible] |
| 12 | 102.0550 | 1373.6848 | 1356.6583 | 1355.6743 | 1401.6797 | 1384.6532 | 1383.6692 | 1315.6793 | | E | 393.1881 | 392.1928 | | 467.2249 | 450.1983 | [illegible] |
| 13 | 136.0757 | 1536.7482 | 1519.7216 | 1518.7376 | 1564.7431 | 1547.7165 | 1546.7325 | | | Y | 230.1248 | | | 338.1823 | 321.1557 | |
| 14 | 129.1135 | | | | | | | | | R | 74.0237 | 73.0284 | | ***175.1190*** | 158.0924 | |

| Seq | ya | yb | Seq | ya | yb | Seq | ya | yb |
|---|---|---|---|---|---|---|---|---|
| IF | 233.1648 | 261.1598 | IFN | 347.2078 | 375.2027 | IFND | 462.2347 | 490.2296 |
| IFNDA | 533.2718 | 561.2667 | IFNDAK | 661.3668 | 689.3617 | FN | 234.1237 | 262.1186 |
| FND | 349.1506 | 377.1456 | FNDA | 420.1878 | 448.1827 | FNDAK | 548.2827 | 576.2776 |
| FNDAKV | 647.3511 | 675.3461 | ND | 202.0822 | 230.0771 | NDA | 273.1193 | 301.1143 |
| NDAK | 401.2143 | 429.2092 | NDAKV | 500.2827 | 528.2776 | NDAKVP | 597.3355 | 625.3304 |
| DA | 159.0764 | 187.0713 | DAK | 287.1714 | 315.1663 | DAKV | 386.2398 | 414.2347 |

| | | | | | | | | |
|---|---|---|---|---|---|---|---|---|
| **DAKVP** | 483.2926 | 511.2875 | **DAKVPW** | 669.3719 | 697.3668 | **AK** | 172.1444 | 200.1394 |
| **AKV** | 271.2129 | 299.2078 | **AKVP** | 368.2656 | 396.2605 | **AKVPW** | 554.3449 | 582.3398 |
| **AKVPWS** | 641.3770 | 669.3719 | **KV** | 200.1757 | 228.1707 | **KVP** | 297.2285 | 325.2234 |
| **KVPW** | 483.3078 | 511.3027 | **KVPWS** | 570.3398 | 598.3348 | **KVPWSE** | 699.3824 | 727.3774 |
| **VP** | 169.1335 | 197.1285 | **VPW** | 355.2129 | 383.2078 | **VPWS** | 442.2449 | 470.2398 |
| **VPWSE** | 571.2875 | 599.2824 | **PW** | 256.1444 | 284.1394 | **PWS** | 343.1765 | 371.1714 |
| **PWSE** | 472.2191 | 500.2140 | **PWSEY** | 635.2824 | 663.2773 | **WS** | 246.1237 | 274.1186 |
| **WSE** | 375.1663 | 403.1612 | **WSEY** | 538.2296 | 566.2245 | **SE** | 189.0870 | 217.0819 |
| **SEY** | 352.1503 | 380.1452 | **EY** | 265.1183 | 293.1132 | | | |

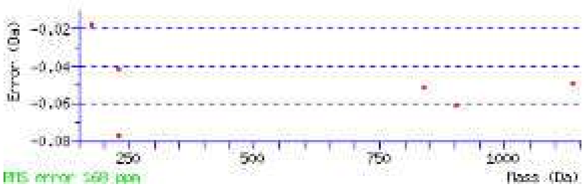


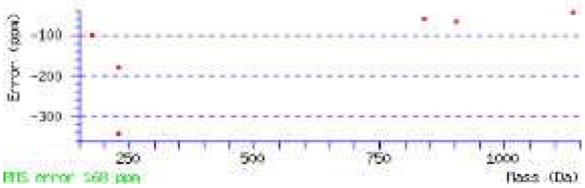


NCBI **BLAST** search of NIFNDAKVPWSEYR
(Parameters: blastp, nr protein database, expect=20000, no filter, PAM30)
Other BLAST web gateways

**All matches to this query**

| Score | Mr(calc) | Delta | Sequence |
|---|---|---|---|
| 8.9 | 1737.8435 | 0.1502 | HADFIGPVQVADAGEGR |
| 5.5 | 1737.8257 | 0.1680 | QMVVARSNWEAEFR |
| 5.5 | 1737.8533 | 0.1403 | QSETTGIQVYSQIER |
| 4.9 | 1737.8463 | 0.1473 | QVTMDLITCLLEMR |
| 4.5 | 1737.8474 | 0.1462 | NIFNDAKVPWSEYR |
| 3.9 | 1737.9058 | 0.0878 | QMKISAMTLLHFFR |
| 3.5 | 1737.8798 | 0.1138 | GWLDTTYLSRSGNIR |
| 3.5 | 1737.8798 | 0.1138 | GWLDTTYLSRSGNLR |
| 3.3 | 1737.8291 | 0.1646 | KVNYVICTMSGSHSR |
| 3.3 | 1737.8621 | 0.1316 | QLGGVMAYPSPYARGR |

**Mascot:** http://www.matrixscience.com/

# MATRIX SCIENCE Mascot Search Results

## Peptide View Spot no 66

MS/MS Fragmentation of **HDLHISAYGEGNER**
Found in **gi|19387272** in **NCBInr**, putative precursor chloroplastic glutamine synthetase [Oryza sativa Japonica Group]

Match to Query 69: 1596.843724 from(1597.851000,1+) intensity(0.0000) index(20)
Title: Label: C8, Spot_Id: 228956, Peak_List_Id: 257807, MSMS Job_Run_Id: 24936, Comment:
Data file ppw_C8_138985146000.txt

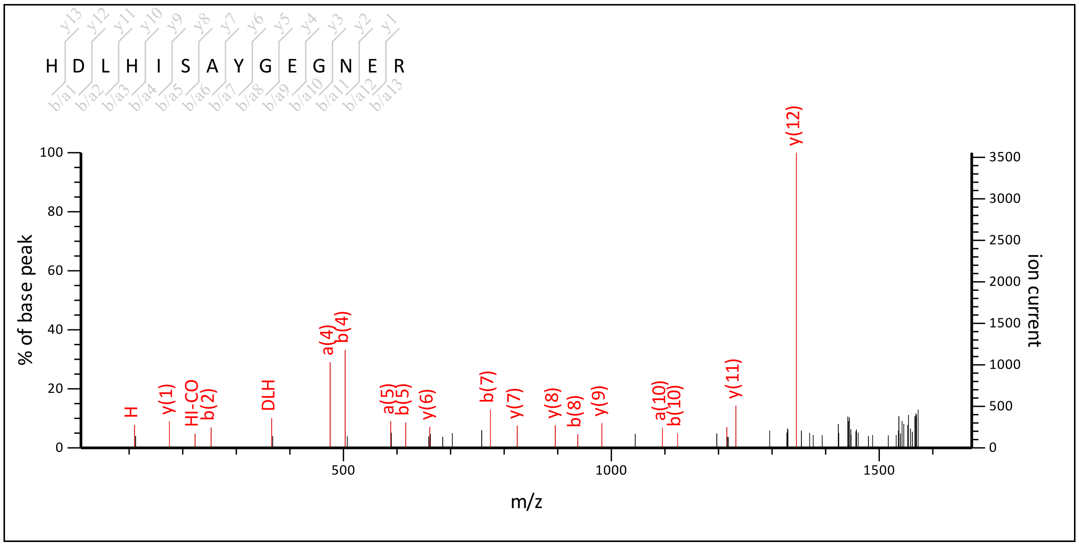


10.07 to 1672.66

Label all possible matches ○ Label matches used for scoring ◉

**Monoisotopic mass of neutral peptide Mr(calc):** 1596.7281
**Fixed modifications:** Carbamidomethyl (C) (apply to specified residues or termini only)
**Ions Score:** 65 **Expect:** 0.00069
**Matches :** 26/230 fragment ions using 30 most intense peaks (help)

| # | Immon. | a | a* | $a^0$ | b | b* | $b^0$ | d | d' | Seq. | v | w | w' | y | y* | |
|---|---|---|---|---|---|---|---|---|---|---|---|---|---|---|---|---|
| 1 | 110.0713 | **110.0713** | | | 138.0662 | | | 44.0495 | | H | | | | | | |
| 2 | 88.0393 | 225.0982 | | 207.0877 | **253.0931** | | 235.0826 | 181.1084 | | D | 1400.6553 | 1399.6601 | | 1460.6764 | 1443.6499 | [illegible] |
| 3 | 86.0964 | 338.1823 | | 320.1717 | **366.1772** | | 348.1666 | 296.1353 | | L | 1287.5713 | 1286.5760 | | ***1345.6495*** | 1328.6230 | [illegible] |
| 4 | 110.0713 | **475.2412** | | 457.2306 | **503.2361** | | 485.2255 | | | H | 1150.5123 | | | ***1232.5654*** | 1215.5389 | [illegible] |
| 5 | 86.0964 | **588.3253** | | 570.3147 | **616.3202** | | 598.3096 | 560.2940 | 574.3096 | I | 1037.4283 | 1050.4487 | 1064.4643 | ***1095.5065*** | 1078.4800 | [illegible] |
| 6 | 60.0444 | 675.3573 | | 657.3467 | 703.3522 | | 685.3416 | 659.3624 | | S | 950.3963 | 949.4010 | | ***982.4225*** | 965.3959 | [illegible] |
| 7 | 44.0495 | 746.3944 | | 728.3838 | **774.3893** | | 756.3787 | | | A | 879.3591 | | | ***895.3904*** | 878.3639 | [illegible] |
| 8 | 136.0757 | 909.4577 | | 891.4472 | **937.4526** | | 919.4421 | | | Y | 716.2958 | | | ***824.3533*** | 807.3268 | [illegible] |
| 9 | 30.0338 | 966.4792 | | 948.4686 | 994.4741 | | 976.4635 | | | G | | | | ***661.2900*** | 644.2634 | [illegible] |
| 10 | 102.0550 | **1095.5218** | | 1077.5112 | **1123.5167** | | 1105.5061 | 1037.5163 | | E | 530.2317 | 529.2365 | | 604.2685 | 587.2420 | [illegible] |
| 11 | 30.0338 | 1152.5432 | | 1134.5327 | 1180.5382 | | 1162.5276 | | | G | | | | ***475.2259*** | 458.1994 | [illegible] |
| 12 | 87.0553 | 1266.5862 | 1249.5596 | 1248.5756 | 1294.5811 | 1277.5545 | 1276.5705 | 1223.5804 | | N | 359.1674 | 358.1721 | | 418.2045 | 401.1779 | [illegible] |
| 13 | 102.0550 | 1395.6288 | 1378.6022 | 1377.6182 | 1423.6237 | 1406.5971 | 1405.6131 | 1337.6233 | | E | 230.1248 | 229.1295 | | 304.1615 | 287.1350 | [illegible] |
| 14 | 129.1135 | | | | | | | | | R | 74.0237 | 73.0284 | | ***175.1190*** | 158.0924 | |

| Seq | ya | yb | Seq | ya | yb | Seq | ya | yb |
|---|---|---|---|---|---|---|---|---|
| DL | 201.1234 | 229.1183 | DLH | 338.1823 | 366.1772 | DLHI | 451.2663 | 479.2613 |
| DLHIS | 538.2984 | 566.2933 | DLHISA | 609.3355 | 637.3304 | LH | 223.1553 | 251.1503 |
| LHI | 336.2394 | 364.2343 | LHIS | 423.2714 | 451.2663 | LHISA | 494.3085 | 522.3035 |
| LHISAY | 657.3719 | 685.3668 | HI | 223.1553 | 251.1503 | HIS | 310.1874 | 338.1823 |
| HISA | 381.2245 | 409.2194 | HISAY | 544.2878 | 572.2827 | HISAYG | 601.3093 | 629.3042 |
| IS | 173.1285 | 201.1234 | ISA | 244.1656 | 272.1605 | ISAY | 407.2289 | 435.2238 |
| ISAYG | 464.2504 | 492.2453 | ISAYGE | 593.2930 | 621.2879 | ISAYGEG | 650.3144 | 678.3093 |

| | | | | | | | | |
|---|---|---|---|---|---|---|---|---|
| **SA** | 131.0815 | 159.0764 | **SAY** | 294.1448 | 322.1397 | **SAYG** | 351.1663 | 379.1612 |
| **SAYGE** | 480.2089 | 508.2038 | **SAYGEG** | 537.2304 | 565.2253 | **SAYGEGN** | 651.2733 | 679.2682 |
| **AY** | 207.1128 | 235.1077 | **AYG** | 264.1343 | 292.1292 | **AYGE** | 393.1769 | 421.1718 |
| **AYGEG** | 450.1983 | 478.1932 | **AYGEGN** | 564.2413 | 592.2362 | **AYGEGNE** | 693.2838 | 721.2788 |
| **YG** | 193.0972 | 221.0921 | **YGE** | 322.1397 | 350.1347 | **YGEG** | 379.1612 | 407.1561 |
| **YGEGN** | 493.2041 | 521.1991 | **YGEGNE** | 622.2467 | 650.2416 | **GE** | 159.0764 | 187.0713 |
| **GEG** | 216.0979 | 244.0928 | **GEGN** | 330.1408 | 358.1357 | **GEGNE** | 459.1834 | 487.1783 |
| **EG** | 159.0764 | 187.0713 | **EGN** | 273.1193 | 301.1143 | **EGNE** | 402.1619 | 430.1569 |
| **GN** | 144.0768 | 172.0717 | **GNE** | 273.1193 | 301.1143 | **NE** | 216.0979 | 244.0928 |

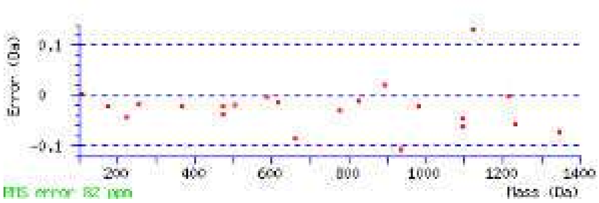


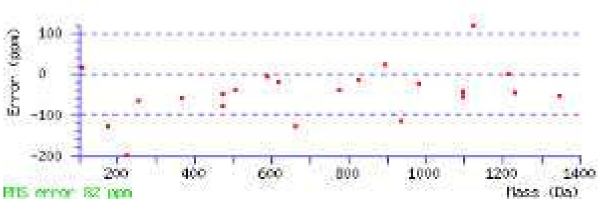


NCBI **BLAST** search of HDLHISAYGEGNER
(Parameters: blastp, nr protein database, expect=20000, no filter, PAM30)
Other BLAST web gateways

**All matches to this query**

| Score | Mr(calc) | Delta | Sequence |
|---|---|---|---|
| 65.1 | 1596.7281 | 0.1157 | HDLHISAYGEGNER |
| 48.5 | 1596.7281 | 0.1157 | HEVHISAYGEGNER |
| 48.5 | 1596.7281 | 0.1157 | HVEHISAYGEGNER |
| 35.9 | 1596.7644 | 0.0793 | HVEHISSYGLGNER |
| 16.6 | 1596.8004 | 0.0434 | SFMVECEVLKSIR |
| 11.5 | 1596.7566 | 0.0871 | VGFTQTTCDLATQR |
| 10.1 | 1596.7678 | 0.0759 | RMAAGLNGFSSVSER |
| 9.9 | 1596.7049 | 0.1388 | MENQLSSSSEAASTR |
| 9.5 | 1596.8493 | -0.0056 | RCWLLPIARPCR |
| 8.6 | 1596.7177 | 0.1260 | AKQMWSDMLNWR |

**Mascot:** http://www.matrixscience.com/

# MATRIX SCIENCE Mascot Search Results

## Peptide View Spot no 66

MS/MS Fragmentation of **LTGLHETASIDNFSWGVANR**
Found in **gi|19387272** in **NCBInr**, putative precursor chloroplastic glutamine synthetase [Oryza sativa Japonica Group]

Match to Query 83: 2187.221024 from(2188.228300,1+) intensity(0.0000) index(24)
Title: Label: C8, Spot_Id: 228956, Peak_List_Id: 257806, MSMS Job_Run_Id: 24936, Comment:
Data file ppw_C8_138985146000.txt

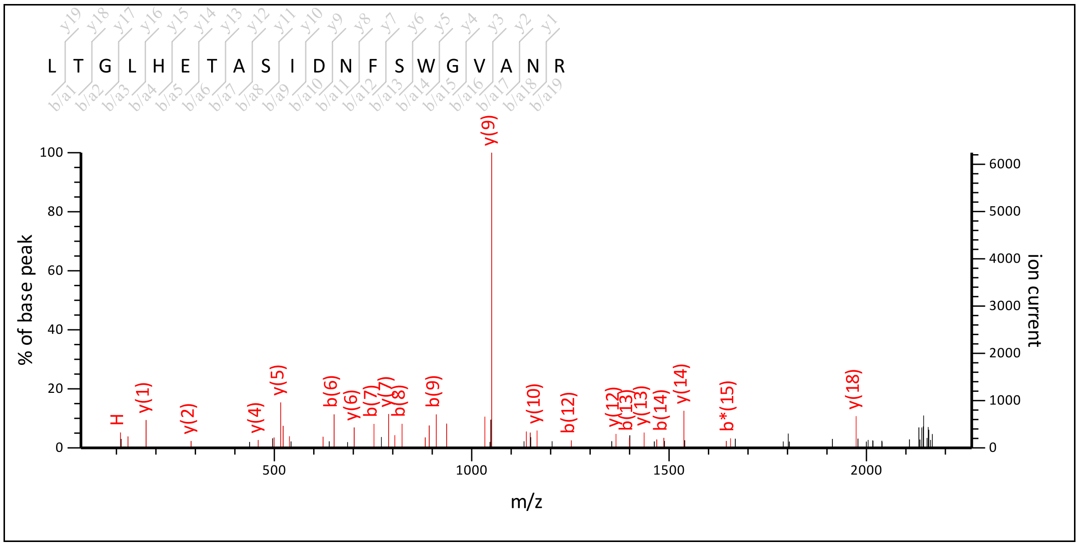


10.07 to 2266.76

Label all possible matches ○ Label matches used for scoring ◉

**Monoisotopic mass of neutral peptide Mr(calc):** 2187.0709
**Fixed modifications:** Carbamidomethyl (C) (apply to specified residues or termini only)
**Ions Score:** 96 **Expect:** 3.5e-07
**Matches :** 37/358 fragment ions using 51 most intense peaks (help)

| # | Immon. | a | a* | a0 | b | b* | b0 | d | d' | Seq. | v | w | w' | y | y* | |
|---|---|---|---|---|---|---|---|---|---|---|---|---|---|---|---|---|
| 1 | 86.0964 | 86.0964 | | | 114.0913 | | | 44.0495 | | L | | | | | | |
| 2 | 74.0600 | 187.1441 | | 169.1335 | 215.1390 | | 197.1285 | 171.1492 | 173.1285 | T | 2028.9522 | 2041.9726 | 2043.9519 | 2074.9941 | 2057.9675 | [illegible] |
| 3 | 30.0338 | 244.1656 | | 226.1550 | 272.1605 | | 254.1499 | | | G | | | | 1973.9464 | 1956.9199 | [illegible] |
| 4 | 86.0964 | 357.2496 | | 339.2391 | 385.2445 | | 367.2340 | 315.2027 | | L | 1858.8467 | 1857.8515 | | 1916.9250 | 1899.8984 | [illegible] |
| 5 | 110.0713 | 494.3085 | | 476.2980 | 522.3035 | | 504.2929 | | | H | 1721.7878 | | | 1803.8409 | 1786.8143 | [illegible] |
| 6 | 102.0550 | 623.3511 | | 605.3406 | 651.3461 | | 633.3355 | 565.3457 | | E | 1592.7452 | 1591.7499 | | 1666.7820 | 1649.7554 | [illegible] |
| 7 | 74.0600 | 724.3988 | | 706.3883 | 752.3937 | | 734.3832 | 708.4039 | 710.3832 | T | 1491.6975 | 1504.7179 | 1506.6972 | 1537.7394 | 1520.7128 | [illegible] |
| 8 | 44.0495 | 795.4359 | | 777.4254 | 823.4308 | | 805.4203 | | | A | 1420.6604 | | | 1436.6917 | 1419.6652 | [illegible] |
| 9 | 60.0444 | 882.4680 | | 864.4574 | 910.4629 | | 892.4523 | 866.4730 | | S | 1333.6284 | 1332.6331 | | 1365.6546 | 1348.6280 | [illegible] |
| 10 | 86.0964 | 995.5520 | | 977.5415 | 1023.5469 | | 1005.5364 | 967.5207 | 981.5364 | I | 1220.5443 | 1233.5647 | 1247.5804 | 1278.6226 | 1261.5960 | [illegible] |
| 11 | 88.0393 | 1110.5790 | | 1092.5684 | 1138.5739 | | 1120.5633 | 1066.5891 | | D | 1105.5174 | 1104.5221 | | 1165.5385 | 1148.5119 | [illegible] |
| 12 | 87.0553 | 1224.6219 | 1207.5953 | 1206.6113 | 1252.6168 | 1235.5903 | 1234.6062 | 1181.6161 | | N | 991.4744 | 990.4792 | | 1050.5116 | 1033.4850 | [illegible] |
| 13 | 120.0808 | 1371.6903 | 1354.6638 | 1353.6797 | 1399.6852 | 1382.6587 | 1381.6747 | | | F | 844.4060 | | | 936.4686 | 919.4421 | [illegible] |
| 14 | 60.0444 | 1458.7223 | 1441.6958 | 1440.7118 | 1486.7172 | 1469.6907 | 1468.7067 | 1442.7274 | | S | 757.3740 | 756.3787 | | 789.4002 | 772.3737 | [illegible] |
| 15 | 159.0917 | 1644.8016 | 1627.7751 | 1626.7911 | 1672.7966 | 1655.7700 | 1654.7860 | | | W | 571.2947 | | | 702.3682 | 685.3416 | |
| 16 | 30.0338 | 1701.8231 | 1684.7966 | 1683.8125 | 1729.8180 | 1712.7915 | 1711.8075 | | | G | | | | 516.2889 | 499.2623 | |
| 17 | 72.0808 | 1800.8915 | 1783.8650 | 1782.8810 | 1828.8864 | 1811.8599 | 1810.8759 | 1786.8759 | | V | 415.2048 | 428.2252 | | 459.2674 | 442.2409 | |
| 18 | 44.0495 | 1871.9286 | 1854.9021 | 1853.9181 | 1899.9236 | 1882.8970 | 1881.9130 | | | A | 344.1677 | | | 360.1990 | 343.1724 | |
| 19 | 87.0553 | 1985.9716 | 1968.9450 | 1967.9610 | 2013.9665 | 1996.9399 | 1995.9559 | 1942.9658 | | N | 230.1248 | 229.1295 | | 289.1619 | 272.1353 | |
| 20 | 129.1135 | | | | | | | | | R | 74.0237 | 73.0284 | | 175.1190 | 158.0924 | |

| Seq | ya | yb | Seq | ya | yb | Seq | ya | yb |
|---|---|---|---|---|---|---|---|---|
| TG | 131.0815 | 159.0764 | TGL | 244.1656 | 272.1605 | TGLH | 381.2245 | 409.2194 |

| | | | | | | | | |
|---|---|---|---|---|---|---|---|---|
| **TGLHE** | 510.2671 | 538.2620 | **TGLHET** | 611.3148 | 639.3097 | **TGLHETA** | 682.3519 | 710.3468 |
| **GL** | 143.1179 | 171.1128 | **GLH** | 280.1768 | 308.1717 | **GLHE** | 409.2194 | 437.2143 |
| **GLHET** | 510.2671 | 538.2620 | **GLHETA** | 581.3042 | 609.2991 | **GLHETAS** | 668.3362 | 696.3311 |
| **LH** | 223.1553 | 251.1503 | **LHE** | 352.1979 | 380.1928 | **LHET** | 453.2456 | 481.2405 |
| **LHETA** | 524.2827 | 552.2776 | **LHETAS** | 611.3148 | 639.3097 | **HE** | 239.1139 | 267.1088 |
| **HET** | 340.1615 | 368.1565 | **HETA** | 411.1987 | 439.1936 | **HETAS** | 498.2307 | 526.2256 |
| **HETASI** | 611.3148 | 639.3097 | **ET** | 203.1026 | 231.0975 | **ETA** | 274.1397 | 302.1347 |
| **ETAS** | 361.1718 | 389.1667 | **ETASI** | 474.2558 | 502.2508 | **ETASID** | 589.2828 | 617.2777 |
| **TA** | 145.0972 | 173.0921 | **TAS** | 232.1292 | 260.1241 | **TASI** | 345.2132 | 373.2082 |
| **TASID** | 460.2402 | 488.2351 | **TASIDN** | 574.2831 | 602.2780 | **AS** | 131.0815 | 159.0764 |
| **ASI** | 244.1656 | 272.1605 | **ASID** | 359.1925 | 387.1874 | **ASIDN** | 473.2354 | 501.2304 |
| **ASIDNF** | 620.3039 | 648.2988 | **SI** | 173.1285 | 201.1234 | **SID** | 288.1554 | 316.1503 |
| **SIDN** | 402.1983 | 430.1932 | **SIDNF** | 549.2667 | 577.2617 | **SIDNFS** | 636.2988 | 664.2937 |
| **ID** | 201.1234 | 229.1183 | **IDN** | 315.1663 | 343.1612 | **IDNF** | 462.2347 | 490.2296 |
| **IDNFS** | 549.2667 | 577.2617 | **DN** | 202.0822 | 230.0771 | **DNF** | 349.1506 | 377.1456 |
| **DNFS** | 436.1827 | 464.1776 | **DNFSW** | 622.2620 | 650.2569 | **DNFSWG** | 679.2835 | 707.2784 |
| **NF** | 234.1237 | 262.1186 | **NFS** | 321.1557 | 349.1506 | **NFSW** | 507.2350 | 535.2300 |
| **NFSWG** | 564.2565 | 592.2514 | **NFSWGV** | 663.3249 | 691.3198 | **FS** | 207.1128 | 235.1077 |
| **FSW** | 393.1921 | 421.1870 | **FSWG** | 450.2136 | 478.2085 | **FSWGV** | 549.2820 | 577.2769 |
| **FSWGVA** | 620.3191 | 648.3140 | **SW** | 246.1237 | 274.1186 | **SWG** | 303.1452 | 331.1401 |
| **SWGV** | 402.2136 | 430.2085 | **SWGVA** | 473.2507 | 501.2456 | **SWGVAN** | 587.2936 | 615.2885 |
| **WG** | 216.1131 | 244.1081 | **WGV** | 315.1816 | 343.1765 | **WGVA** | 386.2187 | 414.2136 |
| **WGVAN** | 500.2616 | 528.2565 | **GV** | 129.1022 | 157.0972 | **GVA** | 200.1394 | 228.1343 |
| **GVAN** | 314.1823 | 342.1772 | **VA** | 143.1179 | 171.1128 | **VAN** | 257.1608 | 285.1557 |
| **AN** | 158.0924 | 186.0873 | | | | | | |

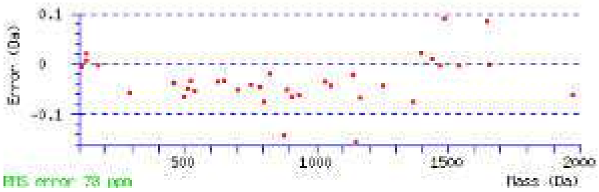


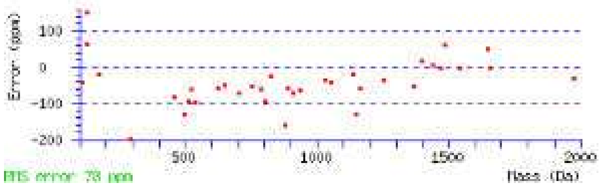


NCBI **BLAST** search of LTGLHETASIDNFSWGVANR
(Parameters: blastp, nr protein database, expect=20000, no filter, PAM30)
Other BLAST web gateways

**All matches to this query**

| Score | Mr(calc) | Delta | Sequence |
|---|---|---|---|
| 96.0 | 2187.0709 | 0.1501 | LTGLHETASIDNFSWGVANR |
| 19.1 | 2187.0789 | 0.1421 | LRHNCAAVQMPIGLEGEHR |
| 10.7 | 2187.1834 | 0.0376 | IGVVAAVATATLAAFGCVGAVGGR |
| 10.4 | 2187.0637 | 0.1573 | FFLENPENFDTLSVVFNR |
| 9.4 | 2187.0266 | 0.1944 | QMSSPTFLDDATGIGEPLHR |
| 8.9 | 2187.0392 | 0.1818 | CGAHLGFGYDDTAAARRPPR |
| 8.9 | 2187.0565 | 0.1646 | KVHCALAANLDSMYVTNHK |
| 7.2 | 2187.0169 | 0.2041 | MLEGTRALSHLLCSCHMR |
| 7.2 | 2187.0533 | 0.1677 | MLIGTRALSHLLCSCHMR |
| 7.2 | 2187.0533 | 0.1677 | MLLGTRALSHLLCSCHMR |

**Mascot:** http://www.matrixscience.com/

# MATRIX SCIENCE Mascot Search Results

## Peptide View Spot no 66

MS/MS Fragmentation of **VVSQVPWFGIEQEYTLLQR**
Found in **gi|19387272** in **NCBInr**, putative precursor chloroplastic glutamine synthetase [Oryza sativa Japonica Group]

Match to Query 86: 2291.353824 from(2292.361100,1+) intensity(0.0000) index(25)
Title: Label: C8, Spot_Id: 228956, Peak_List_Id: 257805, MSMS Job_Run_Id: 24936, Comment:
Data file ppw_C8_138985146000.txt

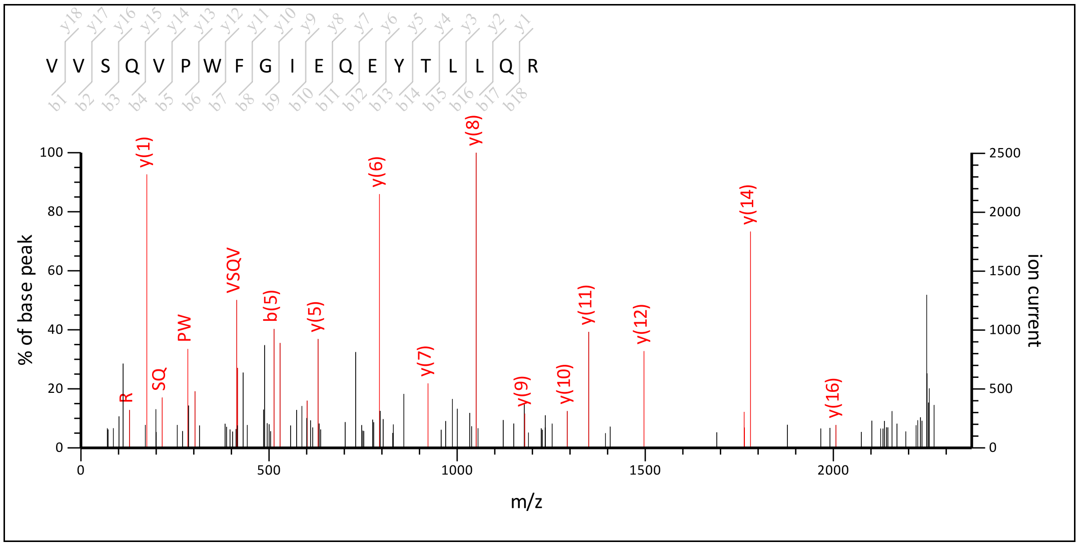


0 to 2367.85

Label all possible matches ○ Label matches used for scoring ◉

**Monoisotopic mass of neutral peptide Mr(calc):** 2291.1950
**Fixed modifications:** Carbamidomethyl (C) (apply to specified residues or termini only)
**Ions Score:** 98 **Expect:** 1.3e-07
**Matches :** 25/338 fragment ions using 40 most intense peaks (help)

| # | Immon. | a | a* | a$^0$ | b | b* | b$^0$ | d | d' | Seq. | v | w | w' | y | y* | |
|---|---|---|---|---|---|---|---|---|---|---|---|---|---|---|---|---|
| 1 | 72.0808 | 72.0808 | | | 100.0757 | | | 44.0495 | | V | | | | | | |
| 2 | 72.0808 | 171.1492 | | | 199.1441 | | | 157.1335 | | V | 2149.0713 | 2162.0917 | | 2193.1339 | 2176.1073 | [illegible] |
| 3 | 60.0444 | 258.1812 | | 240.1707 | 286.1761 | | 268.1656 | 242.1863 | | S | 2062.0393 | 2061.0440 | | 2094.0655 | 2077.0389 | [illegible] |
| 4 | 101.0709 | 386.2398 | 369.2132 | 368.2292 | 414.2347 | 397.2082 | 396.2241 | 329.2183 | | Q | 1933.9807 | 1932.9854 | | 2007.0334 | 1990.0069 | [illegible] |
| 5 | 72.0808 | 485.3082 | 468.2817 | 467.2976 | 513.3031 | 496.2766 | 495.2926 | 471.2926 | | V | 1834.9123 | 1847.9327 | | 1878.9749 | 1861.9483 | [illegible] |
| 6 | 70.0651 | 582.3610 | 565.3344 | 564.3504 | 610.3559 | 593.3293 | 592.3453 | 556.3453 | | P | 1737.8595 | 1736.8643 | | 1779.9064 | 1762.8799 | [illegible] |
| 7 | 159.0917 | 768.4403 | 751.4137 | 750.4297 | 796.4352 | 779.4087 | 778.4246 | | | W | 1551.7802 | | | 1682.8537 | 1665.8271 | [illegible] |
| 8 | 120.0808 | 915.5087 | 898.4822 | 897.4981 | 943.5036 | 926.4771 | 925.4930 | | | F | 1404.7118 | | | 1496.7744 | 1479.7478 | [illegible] |
| 9 | 30.0338 | 972.5302 | 955.5036 | 954.5196 | 1000.5251 | 983.4985 | 982.5145 | | | G | | | | 1349.7060 | 1332.6794 | [illegible] |
| 10 | 86.0964 | 1085.6142 | 1068.5877 | 1067.6037 | 1113.6091 | 1096.5826 | 1095.5986 | 1057.5829 | 1071.5986 | I | 1234.6062 | 1247.6266 | 1261.6423 | 1292.6845 | 1275.6579 | [illegible] |
| 11 | 102.0550 | 1214.6568 | 1197.6303 | 1196.6463 | 1242.6517 | 1225.6252 | 1224.6412 | 1156.6513 | | E | 1105.5637 | 1104.5684 | | 1179.6004 | 1162.5739 | [illegible] |
| 12 | 101.0709 | 1342.7154 | 1325.6889 | 1324.7048 | 1370.7103 | 1353.6838 | 1352.6997 | 1285.6939 | | Q | 977.5051 | 976.5098 | | 1050.5578 | 1033.5313 | [illegible] |
| 13 | 102.0550 | 1471.7580 | 1454.7314 | 1453.7474 | 1499.7529 | 1482.7264 | 1481.7423 | 1413.7525 | | E | 848.4625 | 847.4672 | | 922.4993 | 905.4727 | [illegible] |
| 14 | 136.0757 | 1634.8213 | 1617.7948 | 1616.8108 | 1662.8162 | 1645.7897 | 1644.8057 | | | Y | 685.3992 | | | 793.4567 | 776.4301 | [illegible] |
| 15 | 74.0600 | 1735.8690 | 1718.8425 | 1717.8584 | 1763.8639 | 1746.8374 | 1745.8534 | 1719.8741 | 1721.8534 | T | 584.3515 | 597.3719 | 599.3511 | 630.3933 | 613.3668 | [illegible] |
| 16 | 86.0964 | 1848.9531 | 1831.9265 | 1830.9425 | 1876.9480 | 1859.9214 | 1858.9374 | 1806.9061 | | L | 471.2674 | 470.2722 | | 529.3457 | 512.3191 | |
| 17 | 86.0964 | 1962.0371 | 1945.0106 | 1944.0266 | 1990.0320 | 1973.0055 | 1972.0215 | 1919.9902 | | L | 358.1833 | 357.1881 | | 416.2616 | 399.2350 | |
| 18 | 101.0709 | 2090.0957 | 2073.0692 | 2072.0851 | 2118.0906 | 2101.0641 | 2100.0801 | 2033.0742 | | Q | 230.1248 | 229.1295 | | 303.1775 | 286.1510 | |
| 19 | 129.1135 | | | | | | | | | R | 74.0237 | 73.0284 | | 175.1190 | 158.0924 | |

| Seq | ya | yb | Seq | ya | yb | Seq | ya | yb |
|---|---|---|---|---|---|---|---|---|
| VS | 159.1128 | 187.1077 | VSQ | 287.1714 | 315.1663 | VSQV | 386.2398 | 414.2347 |
| VSQVP | 483.2926 | 511.2875 | VSQVPW | 669.3719 | 697.3668 | SQ | 188.1030 | 216.0979 |

| | | | | | | | | |
|---|---|---|---|---|---|---|---|---|
| **SQV** | 287.1714 | 315.1663 | **SQVP** | 384.2241 | 412.2191 | **SQVPW** | 570.3035 | 598.2984 |
| **QV** | 200.1394 | 228.1343 | **QVP** | 297.1921 | 325.1870 | **QVPW** | 483.2714 | 511.2663 |
| **QVPWF** | 630.3398 | 658.3348 | **QVPWFG** | 687.3613 | 715.3562 | **VP** | 169.1335 | 197.1285 |
| **VPW** | 355.2129 | 383.2078 | **VPWF** | 502.2813 | 530.2762 | **VPWFG** | 559.3027 | 587.2976 |
| **VPWFGI** | 672.3868 | 700.3817 | **PW** | 256.1444 | 284.1394 | **PWF** | 403.2129 | 431.2078 |
| **PWFG** | 460.2343 | 488.2292 | **PWFGI** | 573.3184 | 601.3133 | **WF** | 306.1601 | 334.1550 |
| **WFG** | 363.1816 | 391.1765 | **WFGI** | 476.2656 | 504.2605 | **WFGIE** | 605.3082 | 633.3031 |
| **FG** | 177.1022 | 205.0972 | **FGI** | 290.1863 | 318.1812 | **FGIE** | 419.2289 | 447.2238 |
| **FGIEQ** | 547.2875 | 575.2824 | **FGIEQE** | 676.3301 | 704.3250 | **GI** | 143.1179 | 171.1128 |
| **GIE** | 272.1605 | 300.1554 | **GIEQ** | 400.2191 | 428.2140 | **GIEQE** | 529.2617 | 557.2566 |
| **GIEQEY** | 692.3250 | 720.3199 | **IE** | 215.1390 | 243.1339 | **IEQ** | 343.1976 | 371.1925 |
| **IEQE** | 472.2402 | 500.2351 | **IEQEY** | 635.3035 | 663.2984 | **EQ** | 230.1135 | 258.1084 |
| **EQE** | 359.1561 | 387.1510 | **EQEY** | 522.2195 | 550.2144 | **EQEYT** | 623.2671 | 651.2620 |
| **QE** | 230.1135 | 258.1084 | **QEY** | 393.1769 | 421.1718 | **QEYT** | 494.2245 | 522.2195 |
| **QEYTL** | 607.3086 | 635.3035 | **EY** | 265.1183 | 293.1132 | **EYT** | 366.1660 | 394.1609 |
| **EYTL** | 479.2500 | 507.2449 | **EYTLL** | 592.3341 | 620.3290 | **YT** | 237.1234 | 265.1183 |
| **YTL** | 350.2074 | 378.2023 | **YTLL** | 463.2915 | 491.2864 | **YTLLQ** | 591.3501 | 619.3450 |
| **TL** | 187.1441 | 215.1390 | **TLL** | 300.2282 | 328.2231 | **TLLQ** | 428.2867 | 456.2817 |
| **LL** | 199.1805 | 227.1754 | **LLQ** | 327.2391 | 355.2340 | **LQ** | 214.1550 | 242.1499 |

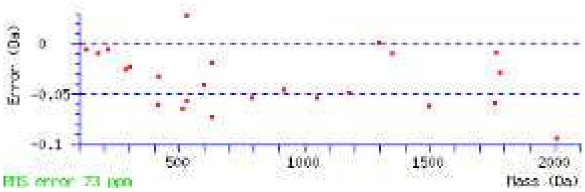


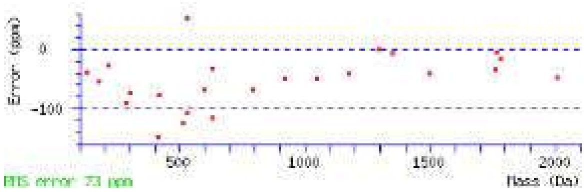


NCBI **BLAST** search of VVSQVPWFGIEQEYTLLQR
(Parameters: blastp, nr protein database, expect=20000, no filter, PAM30)
Other BLAST web gateways

**All matches to this query**

| Score | Mr(calc) | Delta | Sequence |
|---|---|---|---|
| 98.0 | 2291.1950 | 0.1588 | VVSQVPWFGIEQEYTLLQR |
| 12.3 | 2291.1645 | 0.1894 | EGDNLVEEAPADIPSLPELKR |
| 9.9 | 2291.1952 | 0.1586 | RAMPLNMLLTGGLDVQMFLR |
| 9.3 | 2291.1757 | 0.1781 | EDQHTIETLTHEITDLIKR |
| 8.8 | 2291.1249 | 0.2289 | MRPEAETLALMSELVTSNQR |
| 8.6 | 2291.1883 | 0.1655 | QVRPPYLPGGGGGLGGGAAGQAAQR |
| 8.2 | 2291.2307 | 0.1231 | AAATTICYGPSSIVLGSNLLKR |
| 8.2 | 2291.2784 | 0.0755 | VEVPAGLQPAMGGITAGLRSIVR |
| 8.1 | 2291.1952 | 0.1586 | RAMPLNMLLTGGLDVQMFLR |
| 8.0 | 2291.3287 | 0.0251 | RSSLLVFTILSGVCSILSVLK |

**Mascot:** http://www.matrixscience.com/

# Mascot Search Results

## Peptide View Spot no 66

MS/MS Fragmentation of **RLTGLHETASIDNFSWGVANR**
Found in **gi|19387272** in **NCBInr**, putative precursor chloroplastic glutamine synthetase [Oryza sativa Japonica Group]

Match to Query 89: 2343.326224 from(2344.333500,1+) intensity(0.0000) index(26)
Title: Label: C8, Spot_Id: 228956, Peak_List_Id: 257809, MSMS Job_Run_Id: 24936, Comment:
Data file ppw_C8_138985146000.txt

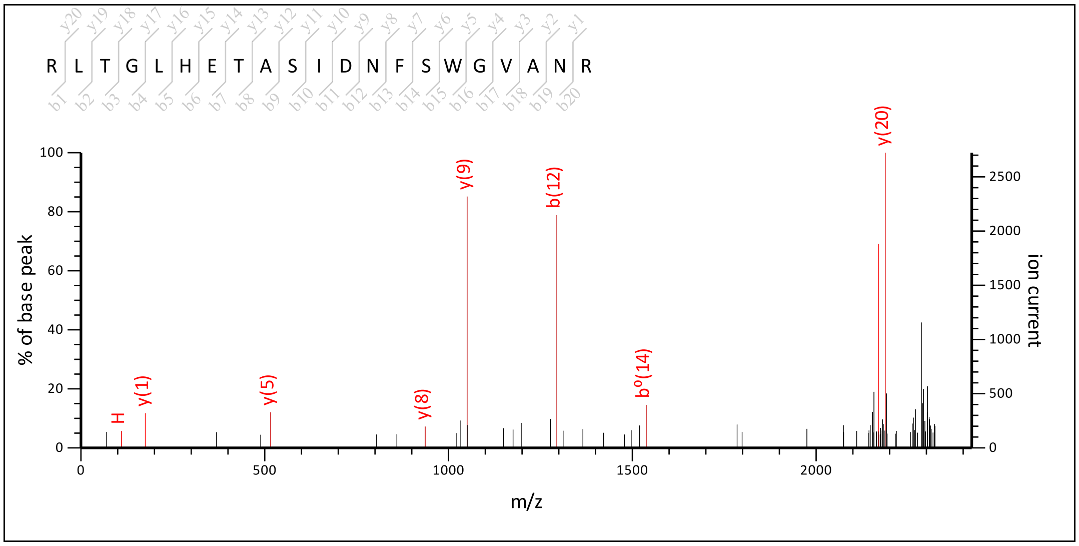


0 to 2423.35

Label all possible matches ○ Label matches used for scoring ◉

**Monoisotopic mass of neutral peptide Mr(calc):** 2343.1720
**Fixed modifications:** Carbamidomethyl (C) (apply to specified residues or termini only)
**Ions Score:** 29 **Expect:** 1.5
**Matches :** 11/401 fragment ions using 17 most intense peaks (help)

| # | Immon. | a | a* | $a^0$ | b | b* | $b^0$ | d | d' | Seq. | v | w | w' | y | y* | |
|---|---|---|---|---|---|---|---|---|---|---|---|---|---|---|---|---|
| 1 | 129.1135 | 129.1135 | 112.0869 | | 157.1084 | 140.0818 | | 44.0495 | | R | | | | | | |
| 2 | 86.0964 | 242.1975 | 225.1710 | | 270.1925 | 253.1659 | | 200.1506 | | L | 2129.9999 | 2129.0047 | | ***2188.0782*** | 2171.0516 | [illegible] |
| 3 | 74.0600 | 343.2452 | 326.2187 | 325.2347 | 371.2401 | 354.2136 | 353.2296 | 327.2503 | 329.2296 | T | 2028.9522 | 2041.9726 | 2043.9519 | 2074.9941 | 2057.9675 | [illegible] |
| 4 | 30.0338 | 400.2667 | 383.2401 | 382.2561 | 428.2616 | 411.2350 | 410.2510 | | | G | | | | 1973.9464 | 1956.9199 | [illegible] |
| 5 | 86.0964 | 513.3507 | 496.3242 | 495.3402 | 541.3457 | 524.3191 | 523.3351 | 471.3038 | | L | 1858.8467 | 1857.8515 | | 1916.9250 | 1899.8984 | [illegible] |
| 6 | 110.0713 | 650.4097 | 633.3831 | 632.3991 | 678.4046 | 661.3780 | 660.3940 | | | H | 1721.7878 | | | 1803.8409 | 1786.8143 | [illegible] |
| 7 | 102.0550 | 779.4522 | 762.4257 | 761.4417 | 807.4472 | 790.4206 | 789.4366 | 721.4468 | | E | 1592.7452 | 1591.7499 | | 1666.7820 | 1649.7554 | [illegible] |
| 8 | 74.0600 | 880.4999 | 863.4734 | 862.4894 | 908.4948 | 891.4683 | 890.4843 | 864.5050 | 866.4843 | T | 1491.6975 | 1504.7179 | 1506.6972 | ***1537.7394*** | 1520.7128 | [illegible] |
| 9 | 44.0495 | 951.5370 | 934.5105 | 933.5265 | 979.5320 | 962.5054 | 961.5214 | | | A | 1420.6604 | | | 1436.6917 | 1419.6652 | [illegible] |
| 10 | 60.0444 | 1038.5691 | 1021.5425 | 1020.5585 | 1066.5640 | 1049.5374 | 1048.5534 | 1022.5742 | | S | 1333.6284 | 1332.6331 | | 1365.6546 | 1348.6280 | [illegible] |
| 11 | 86.0964 | 1151.6531 | 1134.6266 | 1133.6426 | 1179.6480 | 1162.6215 | 1161.6375 | 1123.6218 | 1137.6375 | I | 1220.5443 | 1233.5647 | 1247.5804 | 1278.6226 | 1261.5960 | [illegible] |
| 12 | 88.0393 | 1266.6801 | 1249.6535 | 1248.6695 | **1294.6750** | 1277.6484 | 1276.6644 | 1222.6902 | | D | 1105.5174 | 1104.5221 | | 1165.5385 | 1148.5119 | [illegible] |
| 13 | 87.0553 | 1380.7230 | 1363.6965 | 1362.7124 | 1408.7179 | 1391.6914 | 1390.7074 | 1337.7172 | | N | 991.4744 | 990.4792 | | ***1050.5116*** | 1033.4850 | [illegible] |
| 14 | 120.0808 | 1527.7914 | 1510.7649 | 1509.7809 | 1555.7863 | 1538.7598 | 1537.7758 | | | F | 844.4060 | | | ***936.4686*** | 919.4421 | [illegible] |
| 15 | 60.0444 | 1614.8234 | 1597.7969 | 1596.8129 | 1642.8184 | 1625.7918 | 1624.8078 | 1598.8285 | | S | 757.3740 | 756.3787 | | 789.4002 | 772.3737 | [illegible] |
| 16 | 159.0917 | 1800.9028 | 1783.8762 | 1782.8922 | 1828.8977 | 1811.8711 | 1810.8871 | | | W | 571.2947 | | | 702.3682 | 685.3416 | |
| 17 | 30.0338 | 1857.9242 | 1840.8977 | 1839.9137 | 1885.9191 | 1868.8926 | 1867.9086 | | | G | | | | ***516.2889*** | 499.2623 | |
| 18 | 72.0808 | 1956.9926 | 1939.9661 | 1938.9821 | 1984.9876 | 1967.9610 | 1966.9770 | 1942.9770 | | V | 415.2048 | 428.2252 | | 459.2674 | 442.2409 | |
| 19 | 44.0495 | 2028.0298 | 2011.0032 | 2010.0192 | 2056.0247 | 2038.9981 | 2038.0141 | | | A | 344.1677 | | | 360.1990 | 343.1724 | |
| 20 | 87.0553 | 2142.0727 | 2125.0461 | 2124.0621 | **2170.0676** | 2153.0410 | 2152.0570 | 2099.0669 | | N | 230.1248 | 229.1295 | | 289.1619 | 272.1353 | |
| 21 | 129.1135 | | | | | | | | | R | 74.0237 | 73.0284 | | ***175.1190*** | 158.0924 | |

| Seq | ya | yb | Seq | ya | yb | Seq | ya | yb |
|---|---|---|---|---|---|---|---|---|

| | | | | | | | | |
|---|---|---|---|---|---|---|---|---|
| LT | 187.1441 | 215.1390 | LTG | 244.1656 | 272.1605 | LTGL | 357.2496 | 385.2445 |
| LTGLH | 494.3085 | 522.3035 | LTGLHE | 623.3511 | 651.3461 | TG | 131.0815 | 159.0764 |
| TGL | 244.1656 | 272.1605 | TGLH | 381.2245 | 409.2194 | TGLHE | 510.2671 | 538.2620 |
| TGLHET | 611.3148 | 639.3097 | TGLHETA | 682.3519 | 710.3468 | GL | 143.1179 | 171.1128 |
| GLH | 280.1768 | 308.1717 | GLHE | 409.2194 | 437.2143 | GLHET | 510.2671 | 538.2620 |
| GLHETA | 581.3042 | 609.2991 | GLHETAS | 668.3362 | 696.3311 | LH | 223.1553 | 251.1503 |
| LHE | 352.1979 | 380.1928 | LHET | 453.2456 | 481.2405 | LHETA | 524.2827 | 552.2776 |
| LHETAS | 611.3148 | 639.3097 | HE | 239.1139 | 267.1088 | HET | 340.1615 | 368.1565 |
| HETA | 411.1987 | 439.1936 | HETAS | 498.2307 | 526.2256 | HETASI | 611.3148 | 639.3097 |
| ET | 203.1026 | 231.0975 | ETA | 274.1397 | 302.1347 | ETAS | 361.1718 | 389.1667 |
| ETASI | 474.2558 | 502.2508 | ETASID | 589.2828 | 617.2777 | TA | 145.0972 | 173.0921 |
| TAS | 232.1292 | 260.1241 | TASI | 345.2132 | 373.2082 | TASID | 460.2402 | 488.2351 |
| TASIDN | 574.2831 | 602.2780 | AS | 131.0815 | 159.0764 | ASI | 244.1656 | 272.1605 |
| ASID | 359.1925 | 387.1874 | ASIDN | 473.2354 | 501.2304 | ASIDNF | 620.3039 | 648.2988 |
| SI | 173.1285 | 201.1234 | SID | 288.1554 | 316.1503 | SIDN | 402.1983 | 430.1932 |
| SIDNF | 549.2667 | 577.2617 | SIDNFS | 636.2988 | 664.2937 | ID | 201.1234 | 229.1183 |
| IDN | 315.1663 | 343.1612 | IDNF | 462.2347 | 490.2296 | IDNFS | 549.2667 | 577.2617 |
| DN | 202.0822 | 230.0771 | DNF | 349.1506 | 377.1456 | DNFS | 436.1827 | 464.1776 |
| DNFSW | 622.2620 | 650.2569 | DNFSWG | 679.2835 | 707.2784 | NF | 234.1237 | 262.1186 |
| NFS | 321.1557 | 349.1506 | NFSW | 507.2350 | 535.2300 | NFSWG | 564.2565 | 592.2514 |
| NFSWGV | 663.3249 | 691.3198 | FS | 207.1128 | 235.1077 | FSW | 393.1921 | 421.1870 |
| FSWG | 450.2136 | 478.2085 | FSWGV | 549.2820 | 577.2769 | FSWGVA | 620.3191 | 648.3140 |
| SW | 246.1237 | 274.1186 | SWG | 303.1452 | 331.1401 | SWGV | 402.2136 | 430.2085 |
| SWGVA | 473.2507 | 501.2456 | SWGVAN | 587.2936 | 615.2885 | WG | 216.1131 | 244.1081 |
| WGV | 315.1816 | 343.1765 | WGVA | 386.2187 | 414.2136 | WGVAN | 500.2616 | 528.2565 |
| GV | 129.1022 | 157.0972 | GVA | 200.1394 | 228.1343 | GVAN | 314.1823 | 342.1772 |
| VA | 143.1179 | 171.1128 | VAN | 257.1608 | 285.1557 | AN | 158.0924 | 186.0873 |

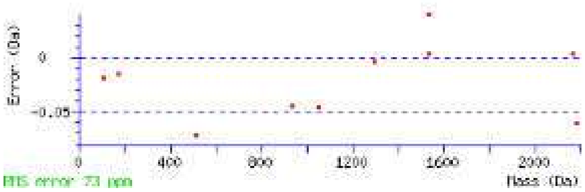


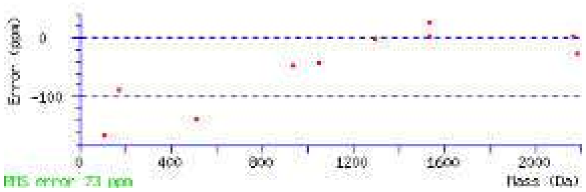


NCBI **BLAST** search of RLTGLHETASIDNFSWGVANR
(Parameters: blastp, nr protein database, expect=20000, no filter, PAM30)
Other BLAST web gateways

**All matches to this query**

| Score | Mr(calc) | Delta | Sequence |
|---|---|---|---|
| 28.9 | 2343.1720 | 0.1542 | RLTGLHETASIDNFSWGVANR |
| 17.1 | 2343.1892 | 0.1370 | LEELNLSHNMLSGLIPASFSR |
| 13.5 | 2343.1416 | 0.1846 | SSTIYEVGVQMVEIYNEQVR |
| 11.9 | 2343.3314 | -0.0052 | IVLKPLPETVGEDRPALFPPR |
| 11.3 | 2343.1722 | 0.1541 | WAAEHPDMLKVWYVIDQVK |
| 11.2 | 2343.1940 | 0.1323 | RTPLSELRPMNTMYNIHVR |
| 11.1 | 2343.1980 | 0.1282 | QMNLSHVISHFSFGMKVLPR |
| 10.6 | 2343.2086 | 0.1177 | FLSPALFHHFKDPIMITEGK |
| 10.0 | 2343.1792 | 0.1471 | RAAAAASAHGSGGLGQENAIEHLR |
| 9.7 | 2343.2838 | 0.0424 | LVNALVSAGKPYELLIFPDER |

**Mascot:** http://www.matrixscience.com/

# MATRIX SCIENCE Mascot Search Results

## Peptide View Spot no 66

MS/MS Fragmentation of **GGNNILVMCDTYTPAGEPIPTNKR**
Found in **gi|19387272** in **NCBInr**, putative precursor chloroplastic glutamine synthetase [Oryza sativa Japonica Group]

Match to Query 93: 2617.441224 from(2618.448500,1+) intensity(0.0000) index(28)
Title: Label: C8, Spot_Id: 228956, Peak_List_Id: 257811, MSMS Job_Run_Id: 24936, Comment:
Data file ppw_C8_138985146000.txt

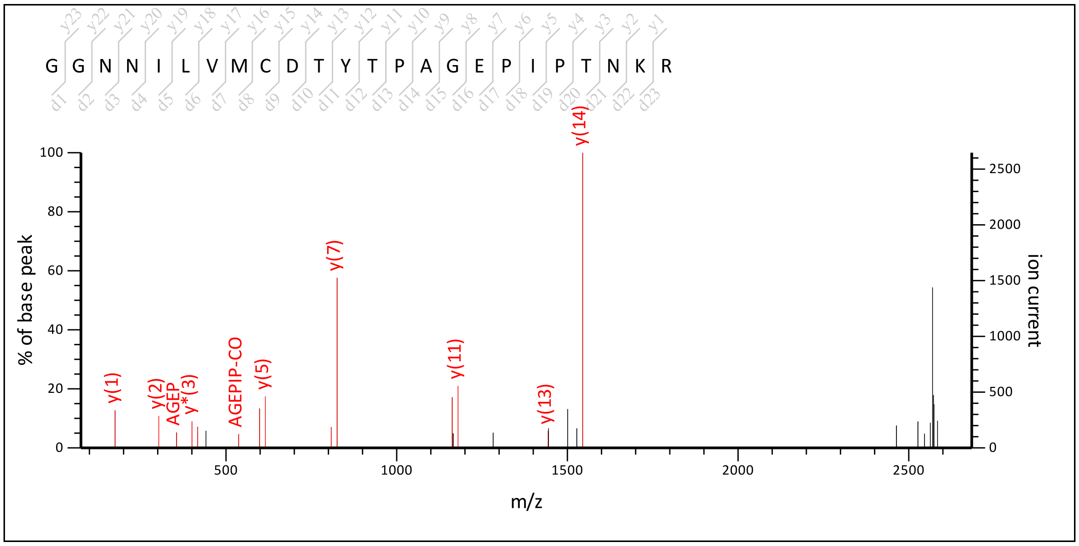


75.11 to 2684.47

Label all possible matches ○ Label matches used for scoring ◉

**Monoisotopic mass of neutral peptide Mr(calc):** 2617.2628
**Fixed modifications:** Carbamidomethyl (C) (apply to specified residues or termini only)
**Ions Score:** 48 **Expect:** 0.019
**Matches :** 17/464 fragment ions using 22 most intense peaks (help)

| # | Immon. | a | a* | $a^0$ | b | b* | $b^0$ | d | d' | Seq. | v | w | w' | y | y* | |
|---|---|---|---|---|---|---|---|---|---|---|---|---|---|---|---|---|
| 1 | 30.0338 | 30.0338 | | | 58.0287 | | | 44.0495 | | G | | | | | | |
| 2 | 30.0338 | 87.0553 | | | 115.0502 | | | | | G | | | | 2561.2487 | 2544.2221 | [illegible] |
| 3 | 87.0553 | 201.0982 | 184.0717 | | 229.0931 | 212.0666 | | 158.0924 | | N | 2445.1901 | 2444.1948 | | 2504.2272 | 2487.2007 | [illegible] |
| 4 | 87.0553 | 315.1411 | 298.1146 | | 343.1361 | 326.1095 | | 272.1353 | | N | 2331.1472 | 2330.1519 | | 2390.1843 | 2373.1577 | [illegible] |
| 5 | 86.0964 | 428.2252 | 411.1987 | | 456.2201 | 439.1936 | | 400.1939 | 414.2096 | I | 2218.0631 | 2231.0835 | 2245.0992 | 2276.1414 | 2259.1148 | [illegible] |
| 6 | 86.0964 | 541.3093 | 524.2827 | | 569.3042 | 552.2776 | | 499.2623 | | L | 2104.9790 | 2103.9838 | | 2163.0573 | 2146.0307 | [illegible] |
| 7 | 72.0808 | 640.3777 | 623.3511 | | 668.3726 | 651.3461 | | 626.3620 | | V | 2005.9106 | 2018.9310 | | 2049.9732 | 2032.9467 | [illegible] |
| 8 | 104.0528 | 771.4182 | 754.3916 | | 799.4131 | 782.3865 | | 711.4148 | | M | 1874.8701 | 1873.8749 | | 1950.9048 | 1933.8783 | [illegible] |
| 9 | 133.0430 | 931.4488 | 914.4223 | | 959.4437 | 942.4172 | | 842.4553 | | C | 1714.8395 | 1713.8442 | | 1819.8643 | 1802.8378 | [illegible] |
| 10 | 88.0393 | 1046.4758 | 1029.4492 | 1028.4652 | 1074.4707 | 1057.4441 | 1056.4601 | 1002.4859 | | D | 1599.8125 | 1598.8173 | | 1659.8337 | 1642.8071 | [illegible] |
| 11 | 74.0600 | 1147.5234 | 1130.4969 | 1129.5129 | 1175.5184 | 1158.4918 | 1157.5078 | 1131.5285 | 1133.5078 | T | 1498.7649 | 1511.7853 | 1513.7645 | ***1544.8067*** | 1527.7802 | [illegible] |
| 12 | 136.0757 | 1310.5868 | 1293.5602 | 1292.5762 | 1338.5817 | 1321.5551 | 1320.5711 | | | Y | 1335.7015 | | | ***1443.7591*** | 1426.7325 | [illegible] |
| 13 | 74.0600 | 1411.6344 | 1394.6079 | 1393.6239 | 1439.6294 | 1422.6028 | 1421.6188 | 1395.6395 | 1397.6188 | T | 1234.6539 | 1247.6743 | 1249.6535 | 1280.6957 | 1263.6692 | [illegible] |
| 14 | 70.0651 | 1508.6872 | 1491.6607 | 1490.6766 | 1536.6821 | 1519.6556 | 1518.6716 | 1482.6716 | | P | 1137.6011 | 1136.6058 | | ***1179.6480*** | 1162.6215 | [illegible] |
| 15 | 44.0495 | 1579.7243 | 1562.6978 | 1561.7138 | 1607.7192 | 1590.6927 | 1589.7087 | | | A | 1066.5640 | | | 1082.5953 | 1065.5687 | [illegible] |
| 16 | 30.0338 | 1636.7458 | 1619.7192 | 1618.7352 | 1664.7407 | 1647.7142 | 1646.7301 | | | G | | | | 1011.5582 | 994.5316 | [illegible] |
| 17 | 102.0550 | 1765.7884 | 1748.7618 | 1747.7778 | 1793.7833 | 1776.7568 | 1775.7727 | 1707.7829 | | E | 880.4999 | 879.5047 | | 954.5367 | 937.5102 | [illegible] |
| 18 | 70.0651 | 1862.8411 | 1845.8146 | 1844.8306 | 1890.8361 | 1873.8095 | 1872.8255 | 1836.8255 | | P | 783.4472 | 782.4519 | | ***825.4941*** | 808.4676 | [illegible] |
| 19 | 86.0964 | 1975.9252 | 1958.8987 | 1957.9146 | 2003.9201 | 1986.8936 | 1985.9096 | 1947.8939 | 1961.9096 | I | 670.3631 | 683.3835 | 697.3991 | 728.4413 | 711.4148 | [illegible] |
| 20 | 70.0651 | 2072.9780 | 2055.9514 | 2054.9674 | 2100.9729 | 2083.9463 | 2082.9623 | 2046.9623 | | P | 573.3103 | 572.3151 | | ***615.3573*** | 598.3307 | [illegible] |
| 21 | 74.0600 | 2174.0257 | 2156.9991 | 2156.0151 | 2202.0206 | 2184.9940 | 2184.0100 | 2158.0307 | 2160.0100 | T | 472.2627 | 485.2831 | 487.2623 | 518.3045 | 501.2780 | [illegible] |
| 22 | 87.0553 | 2288.0686 | 2271.0420 | 2270.0580 | 2316.0635 | 2299.0369 | 2298.0529 | 2245.0628 | | N | 358.2197 | 357.2245 | | ***417.2568*** | 400.2303 | |
| 23 | 101.1073 | 2416.1635 | 2399.1370 | 2398.1530 | 2444.1585 | 2427.1319 | 2426.1479 | 2359.1057 | | K | 230.1248 | 229.1295 | | ***303.2139*** | 286.1874 | |

| | | | | | | | | | | | | | | | | |
|---|---|---|---|---|---|---|---|---|---|---|---|---|---|---|---|---|
| 24 | 129.1135 | | | | | | | | | R | 74.0237 | 73.0284 | | *175.1190* | 158.0924 | |

| Seq | ya | yb | Seq | ya | yb | Seq | ya | yb |
|---|---|---|---|---|---|---|---|---|
| GN | 144.0768 | 172.0717 | GNN | 258.1197 | 286.1146 | GNNI | 371.2037 | 399.1987 |
| GNNIL | 484.2878 | 512.2827 | GNNILV | 583.3562 | 611.3511 | NN | 201.0982 | 229.0931 |
| NNI | 314.1823 | 342.1772 | NNIL | 427.2663 | 455.2613 | NNILV | 526.3348 | 554.3297 |
| NNILVM | 657.3752 | 685.3702 | NI | 200.1394 | 228.1343 | NIL | 313.2234 | 341.2183 |
| NILV | 412.2918 | 440.2867 | NILVM | 543.3323 | 571.3272 | IL | 199.1805 | 227.1754 |
| ILV | 298.2489 | 326.2438 | ILVM | 429.2894 | 457.2843 | ILVMC | 589.3200 | 617.3150 |
| LV | 185.1648 | 213.1598 | LVM | 316.2053 | 344.2002 | LVMC | 476.2360 | 504.2309 |
| LVMCD | 591.2629 | 619.2578 | LVMCDT | 692.3106 | 720.3055 | VM | 203.1213 | 231.1162 |
| VMC | 363.1519 | 391.1468 | VMCD | 478.1789 | 506.1738 | VMCDT | 579.2265 | 607.2214 |
| MC | 264.0835 | 292.0784 | MCD | 379.1104 | 407.1054 | MCDT | 480.1581 | 508.1530 |
| MCDTY | 643.2214 | 671.2164 | CD | 248.0700 | 276.0649 | CDT | 349.1176 | 377.1125 |
| CDTY | 512.1810 | 540.1759 | CDTYT | 613.2286 | 641.2236 | DT | 189.0870 | 217.0819 |
| DTY | 352.1503 | 380.1452 | DTYT | 453.1980 | 481.1929 | DTYTP | 550.2508 | 578.2457 |
| DTYTPA | 621.2879 | 649.2828 | DTYTPAG | 678.3093 | 706.3042 | TY | 237.1234 | 265.1183 |
| TYT | 338.1710 | 366.1660 | TYTP | 435.2238 | 463.2187 | TYTPA | 506.2609 | 534.2558 |
| TYTPAG | 563.2824 | 591.2773 | TYTPAGE | 692.3250 | 720.3199 | YT | 237.1234 | 265.1183 |
| YTP | 334.1761 | 362.1710 | YTPA | 405.2132 | 433.2082 | YTPAG | 462.2347 | 490.2296 |
| YTPAGE | 591.2773 | 619.2722 | YTPAGEP | 688.3301 | 716.3250 | TP | 171.1128 | 199.1077 |
| TPA | 242.1499 | 270.1448 | TPAG | 299.1714 | 327.1663 | TPAGE | 428.2140 | 456.2089 |
| TPAGEP | 525.2667 | 553.2617 | TPAGEPI | 638.3508 | 666.3457 | PA | 141.1022 | 169.0972 |
| PAG | 198.1237 | 226.1186 | PAGE | 327.1663 | 355.1612 | PAGEP | 424.2191 | 452.2140 |
| PAGEPI | 537.3031 | 565.2980 | PAGEPIP | 634.3559 | 662.3508 | AG | 101.0709 | 129.0659 |
| AGE | 230.1135 | 258.1084 | AGEP | 327.1663 | 355.1612 | AGEPI | 440.2504 | 468.2453 |
| AGEPIP | 537.3031 | 565.2980 | AGEPIPT | 638.3508 | 666.3457 | GE | 159.0764 | 187.0713 |
| GEP | 256.1292 | 284.1241 | GEPI | 369.2132 | 397.2082 | GEPIP | 466.2660 | 494.2609 |
| GEPIPT | 567.3137 | 595.3086 | GEPIPTN | 681.3566 | 709.3515 | EP | 199.1077 | 227.1026 |
| EPI | 312.1918 | 340.1867 | EPIP | 409.2445 | 437.2395 | EPIPT | 510.2922 | 538.2871 |
| EPIPTN | 624.3352 | 652.3301 | PI | 183.1492 | 211.1441 | PIP | 280.2020 | 308.1969 |
| PIPT | 381.2496 | 409.2445 | PIPTN | 495.2926 | 523.2875 | PIPTNK | 623.3875 | 651.3824 |
| IP | 183.1492 | 211.1441 | IPT | 284.1969 | 312.1918 | IPTN | 398.2398 | 426.2347 |
| IPTNK | 526.3348 | 554.3297 | PT | 171.1128 | 199.1077 | PTN | 285.1557 | 313.1506 |
| PTNK | 413.2507 | 441.2456 | TN | 188.1030 | 216.0979 | TNK | 316.1979 | 344.1928 |
| NK | 215.1503 | 243.1452 | | | | | | |

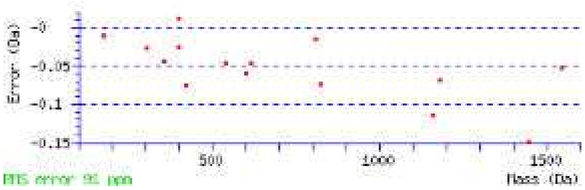


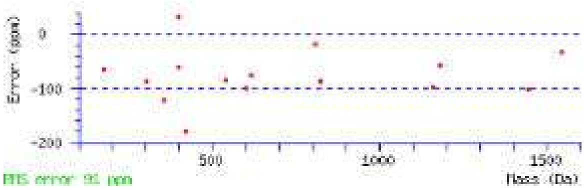


NCBI **BLAST** search of GGNNILVMCDTYTPAGEPIPTNKR
(Parameters: blastp, nr protein database, expect=20000, no filter, PAM30)
Other BLAST web gateways

**All matches to this query**

| Score | Mr(calc) | Delta | Sequence |
|---|---|---|---|
| 48.0 | 2617.2628 | 0.1784 | GGNNILVMCDTYTPAGEPIPTNKR |
| 11.3 | 2617.4228 | 0.0185 | QQSSPPLPLPELAPQPLQPSPLQR |
| 7.2 | 2617.2094 | 0.2319 | DMFGPDIPNATDILVPMWWNNR |
| 5.7 | 2617.2831 | 0.1582 | KLSEDLEQNATLNTSLSAAAEEQR |
| 5.3 | 2617.3951 | 0.0461 | SYGPVPMGLLQGRVTHIIWPPQR |
| 5.2 | 2617.2224 | 0.2188 | SGENLSATLHPSGRDDMSILSMQR |
| 5.1 | 2617.3026 | 0.1386 | MLPAVSMSGLAPMDVSRTQDVKPR |
| | | | |

| | | | |
|---|---|---|---|
| 5.1 | 2617.3026 | 0.1386 | MLPAVSMSGLAPMDVSRTQDVKPR |
| 4.9 | 2617.2953 | 0.1460 | VLGLDVCSETVVGNDMLRGVSGGQR |
| 4.7 | 2617.3751 | 0.0661 | LGVLLEARGESYYNDLIPPTLER |

**Mascot:** http://www.matrixscience.com/

# Mascot Search Results

## Peptide View Spot no 67

MS/MS Fragmentation of **VYDDEVR**
Found in **gi|8918361** in **NCBInr**, RuBisCO activase small isoform precursor [Oryza sativa]

Match to Query 4: 894.424424 from(895.431700,1+) intensity(0.0000) index(1)
Title: Label: D4, Spot_Id: 219725, Peak_List_Id: 225233, MSMS Job_Run_Id: 21770, Comment:
Data file C:\Documents and Settings\Administrator\Desktop\farah BHU\spot set plate\Farah-Garima 11May 2013\ppw_D4_136842162200.txt

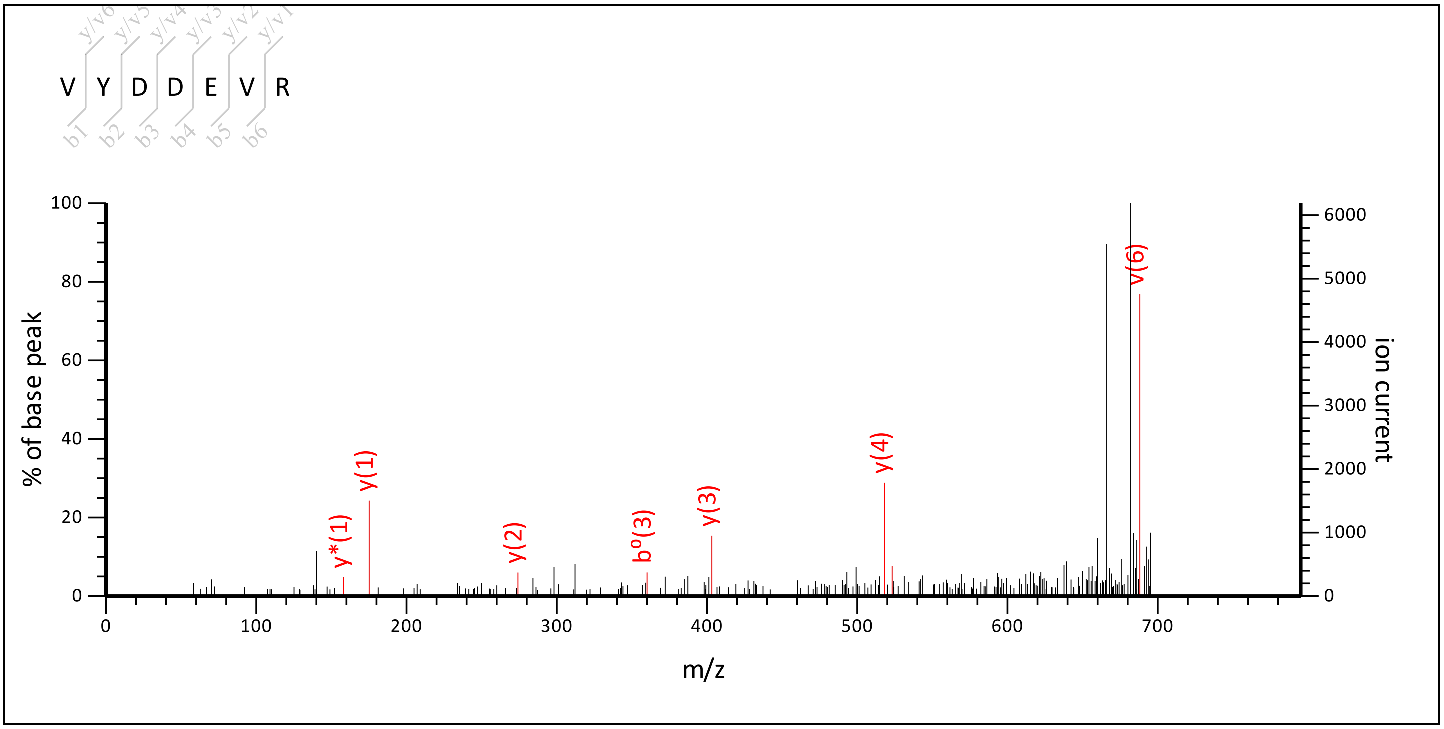


0 to 795.21

Label all possible matches ○ Label matches used for scoring ◉

**Monoisotopic mass of neutral peptide Mr(calc):** 894.4083
**Fixed modifications:** Carbamidomethyl (C) (apply to specified residues or termini only)
**Ions Score:** 15 **Expect:** 71
**Matches :** 9/79 fragment ions using 21 most intense peaks (help)

| # | Immon. | a | a$^0$ | b | b$^0$ | d | Seq. | v | w | y | y* | y$^0$ | # |
|---|---|---|---|---|---|---|---|---|---|---|---|---|---|
| 1 | 72.0808 | 72.0808 | | 100.0757 | | 44.0495 | V | | | | | | 7 |
| 2 | 136.0757 | 235.1441 | | 263.1390 | | | Y | 688.2897 | | 796.3472 | 779.3206 | 778.3366 | 6 |
| 3 | 88.0393 | 350.1710 | 332.1605 | 378.1660 | 360.1554 | 306.1812 | D | 573.2627 | 572.2675 | 633.2838 | 616.2573 | 615.2733 | 5 |
| 4 | 88.0393 | 465.1980 | 447.1874 | 493.1929 | 475.1823 | 421.2082 | D | 458.2358 | 457.2405 | ***518.2569*** | 501.2304 | 500.2463 | 4 |
| 5 | 102.0550 | 594.2406 | 576.2300 | 622.2355 | 604.2249 | 536.2351 | E | 329.1932 | 328.1979 | ***403.2300*** | 386.2034 | 385.2194 | 3 |
| 6 | 72.0808 | 693.3090 | 675.2984 | 721.3039 | 703.2933 | 679.2933 | V | 230.1248 | 243.1452 | ***274.1874*** | 257.1608 | | 2 |
| 7 | 129.1135 | | | | | | R | 74.0237 | 73.0284 | ***175.1190*** | 158.0924 | | 1 |

| Seq | ya | yb | Seq | ya | yb | Seq | ya | yb |
|---|---|---|---|---|---|---|---|---|
| YD | 251.1026 | 279.0975 | YDD | 366.1296 | 394.1245 | YDDE | 495.1722 | 523.1671 |
| YDDEV | 594.2406 | 622.2355 | DD | 203.0662 | 231.0612 | DDE | 332.1088 | 360.1038 |
| DDEV | 431.1773 | 459.1722 | DE | 217.0819 | 245.0768 | DEV | 316.1503 | 344.1452 |
| EV | 201.1234 | 229.1183 | | | | | | |

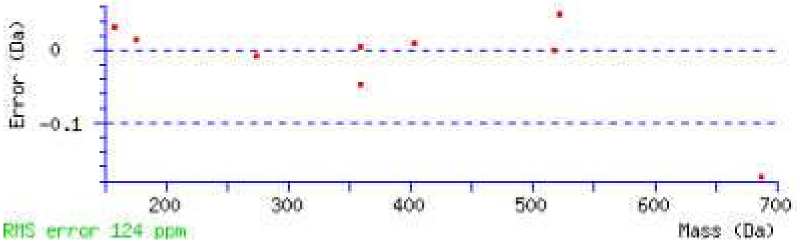


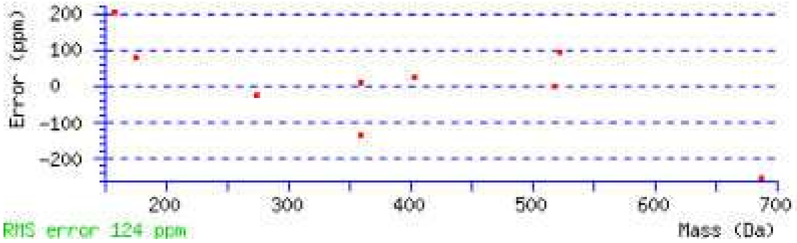


NCBI **BLAST** search of VYDDEVR
(Parameters: blastp, nr protein database, expect=20000, no filter, PAM30)
Other BLAST web gateways

**All matches to this query**

| Score | Mr(calc) | Delta | Sequence |
|---|---|---|---|
| 14.5 | 894.4083 | 0.0161 | VYDDEVR |
| 8.5 | 894.4633 | -0.0389 | VDYMIVR |
| 8.2 | 894.3719 | 0.0525 | DDFDLDR |
| 8.2 | 894.4083 | 0.0161 | EFTDEVR |
| 8.2 | 894.4083 | 0.0161 | ETFDDLR |
| 8.2 | 894.3542 | 0.0703 | FDDDMPR |
| 8.2 | 894.4447 | -0.0203 | LYTDVER |
| 8.2 | 894.3389 | 0.0855 | MDDDDLR |
| 8.2 | 894.4447 | -0.0203 | TYLDVER |
| 8.2 | 894.3905 | 0.0339 | YVDDMPR |

**Mascot:** http://www.matrixscience.com/

# MATRIX SCIENCE Mascot Search Results

## Peptide View Spot no 67

MS/MS Fragmentation of **FYWAPTR**
Found in **gi|8918361** in **NCBInr**, RuBisCO activase small isoform precursor [Oryza sativa]

Match to Query 7: 939.446334 from(940.453610,1+) intensity(0.0000) index(2)
Title: Label: D4, Spot_Id: 219725, Peak_List_Id: 225224, MSMS Job_Run_Id: 21770, Comment:
Data file C:\Documents and Settings\Administrator\Desktop\farah BHU\spot set plate\Farah-Garima 11May 2013\ppw_D4_136842162200.txt

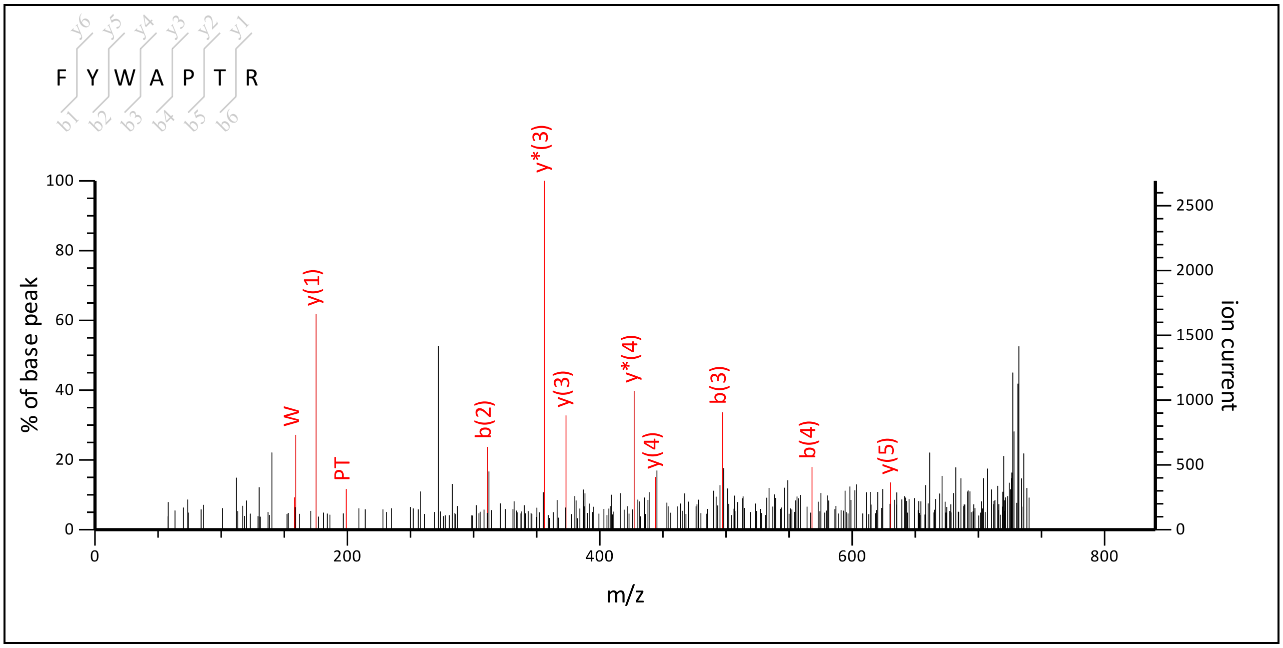


0 to 840.19

Label all possible matches ○ Label matches used for scoring ◉

**Monoisotopic mass of neutral peptide Mr(calc):** 939.4603
**Fixed modifications:** Carbamidomethyl (C) (apply to specified residues or termini only)
**Ions Score:** 14 **Expect:** 70
**Matches :** 12/72 fragment ions using 28 most intense peaks (help)

| # | Immon. | a | $a^0$ | b | $b^0$ | d | d' | Seq. | v | w | w' | y | y* | $y^0$ | # |
|---|---|---|---|---|---|---|---|---|---|---|---|---|---|---|---|
| 1 | 120.0808 | 120.0808 | | 148.0757 | | 44.0495 | | F | | | | | | | 7 |
| 2 | 136.0757 | 283.1441 | | **311.1390** | | | | Y | 685.3416 | | | 793.3992 | 776.3726 | 775.3886 | 6 |
| 3 | 159.0917 | 469.2234 | | **497.2183** | | | | W | 499.2623 | | | ***630.3358*** | 613.3093 | 612.3253 | 5 |
| 4 | 44.0495 | 540.2605 | | **568.2554** | | | | A | 428.2252 | | | ***444.2565*** | 427.2300 | 426.2459 | 4 |
| 5 | 70.0651 | 637.3133 | | 665.3082 | | 611.2976 | | P | 331.1724 | 330.1772 | | ***373.2194*** | 356.1928 | 355.2088 | 3 |
| 6 | 74.0600 | 738.3610 | 720.3504 | 766.3559 | 748.3453 | 722.3661 | 724.3453 | T | 230.1248 | 243.1452 | 245.1244 | 276.1666 | 259.1401 | 258.1561 | 2 |
| 7 | 129.1135 | | | | | | | R | 74.0237 | 73.0284 | | ***175.1190*** | 158.0924 | | 1 |

| Seq | ya | yb | Seq | ya | yb | Seq | ya | yb |
|---|---|---|---|---|---|---|---|---|
| YW | 322.1550 | 350.1499 | YWA | 393.1921 | 421.1870 | YWAP | 490.2449 | 518.2398 |
| YWAPT | 591.2926 | 619.2875 | WA | 230.1288 | 258.1237 | WAP | 327.1816 | 355.1765 |
| WAPT | 428.2292 | 456.2241 | AP | 141.1022 | 169.0972 | APT | 242.1499 | 270.1448 |
| PT | 171.1128 | 199.1077 | | | | | | |

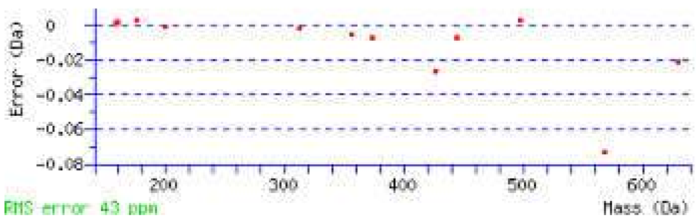


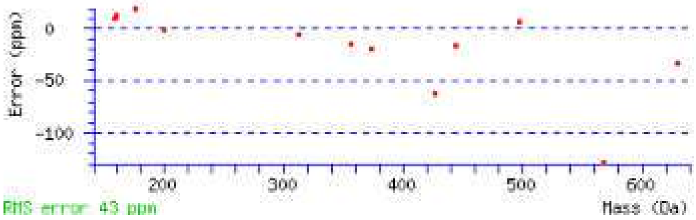


NCBI **BLAST** search of FYWAPTR
(Parameters: blastp, nr protein database, expect=20000, no filter, PAM30)
Other BLAST web gateways

**All matches to this query**

| Score | Mr(calc) | Delta | Sequence |
|---|---|---|---|
| 23.8 | 939.5362 | -0.0899 | RGTRPTPR |
| 21.7 | 939.5250 | -0.0787 | AAATPRTPR |
| 18.2 | 939.5138 | -0.0674 | LTSAAPQPR |
| 16.0 | 939.4998 | -0.0535 | RGPDSRPR |
| 14.9 | 939.5138 | -0.0674 | LGASAPATPR |
| 14.6 | 939.5250 | -0.0786 | AAAPGEIRR |
| 14.6 | 939.5362 | -0.0899 | GRPGEIRR |
| 13.9 | 939.4603 | -0.0139 | FYWAPTR |
| 13.7 | 939.4709 | -0.0245 | HCAKATPR |
| 13.2 | 939.5250 | -0.0787 | GRPTSGLPR |

**Mascot:** http://www.matrixscience.com/

# MATRIX SCIENCE Mascot Search Results

## Peptide View Spot no 67

MS/MS Fragmentation of **VYDDEVRK**
Found in **gi|8918361** in **NCBInr**, RuBisCO activase small isoform precursor [Oryza sativa]

Match to Query 19: 1022.497624 from(1023.504900,1+) intensity(0.0000) index(7)
Title: Label: D4, Spot_Id: 219725, Peak_List_Id: 225220, MSMS Job_Run_Id: 21770, Comment:
Data file C:\Documents and Settings\Administrator\Desktop\farah BHU\spot set plate\Farah-Garima 11May 2013\ppw_D4_136842162200.txt

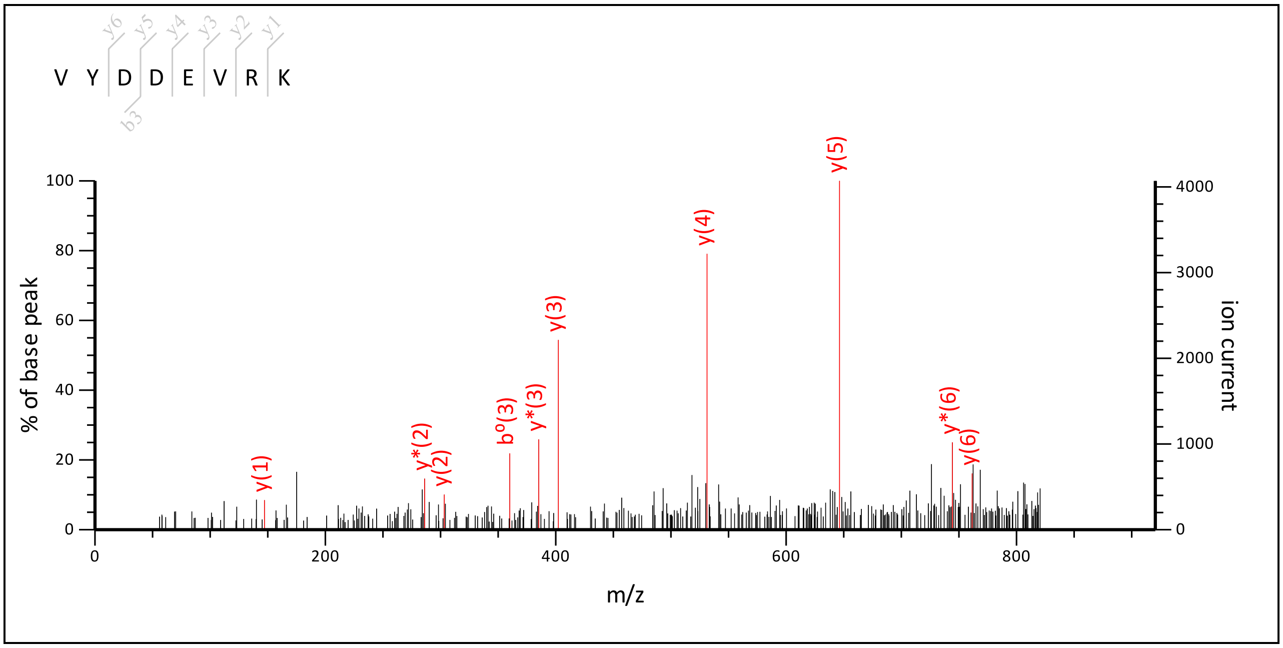


0 to 920.5

Label all possible matches ○ Label matches used for scoring ◉

**Monoisotopic mass of neutral peptide Mr(calc):** 1022.5033
**Fixed modifications:** Carbamidomethyl (C) (apply to specified residues or termini only)
**Ions Score:** 30 **Expect:** 2.2
**Matches :** 12/99 fragment ions using 24 most intense peaks (help)

| # | Immon. | a | a* | $a^0$ | b | b* | $b^0$ | d | Seq. | v | w | y | y* | $y^0$ | # |
|---|---|---|---|---|---|---|---|---|---|---|---|---|---|---|---|
| 1 | 72.0808 | 72.0808 | | | 100.0757 | | | 44.0495 | V | | | | | | 8 |
| 2 | 136.0757 | 235.1441 | | | 263.1390 | | | | Y | 816.3846 | | 924.4421 | 907.4156 | 906.4316 | 7 |
| 3 | 88.0393 | 350.1710 | | 332.1605 | 378.1660 | | 360.1554 | 306.1812 | D | 701.3577 | 700.3624 | ***761.3788*** | 744.3523 | 743.3682 | 6 |
| 4 | 88.0393 | 465.1980 | | 447.1874 | 493.1929 | | 475.1823 | 421.2082 | D | 586.3307 | 585.3355 | ***646.3519*** | 629.3253 | 628.3413 | 5 |
| 5 | 102.0550 | 594.2406 | | 576.2300 | 622.2355 | | 604.2249 | 536.2351 | E | 457.2881 | 456.2929 | ***531.3249*** | 514.2984 | 513.3144 | 4 |
| 6 | 72.0808 | 693.3090 | | 675.2984 | 721.3039 | | 703.2933 | 679.2933 | V | 358.2197 | 371.2401 | ***402.2823*** | 385.2558 | | 3 |
| 7 | 129.1135 | 849.4101 | 832.3836 | 831.3995 | 877.4050 | 860.3785 | 859.3945 | 764.3461 | R | 202.1186 | 201.1234 | ***303.2139*** | 286.1874 | | 2 |
| 8 | 101.1073 | | | | | | | | K | 74.0237 | 73.0284 | ***147.1128*** | 130.0863 | | 1 |

| Seq | ya | yb | Seq | ya | yb | Seq | ya | yb |
|---|---|---|---|---|---|---|---|---|
| **YD** | 251.1026 | 279.0975 | **YDD** | 366.1296 | 394.1245 | **YDDE** | 495.1722 | 523.1671 |
| **YDDEV** | 594.2406 | 622.2355 | **DD** | 203.0662 | 231.0612 | **DDE** | 332.1088 | 360.1038 |
| **DDEV** | 431.1773 | 459.1722 | **DDEVR** | 587.2784 | 615.2733 | **DE** | 217.0819 | 245.0768 |
| **DEV** | 316.1503 | 344.1452 | **DEVR** | 472.2514 | 500.2463 | **EV** | 201.1234 | 229.1183 |

| | | | | | | | | |
|---|---|---|---|---|---|---|---|---|
| **EVR** | 357.2245 | 385.2194 | **VR** | 228.1819 | 256.1768 | | | |

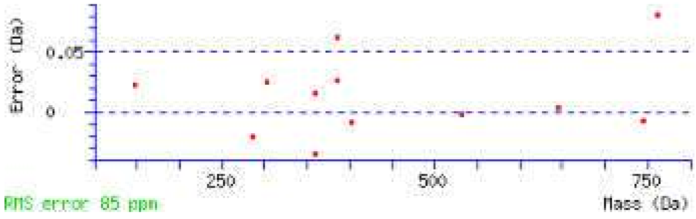


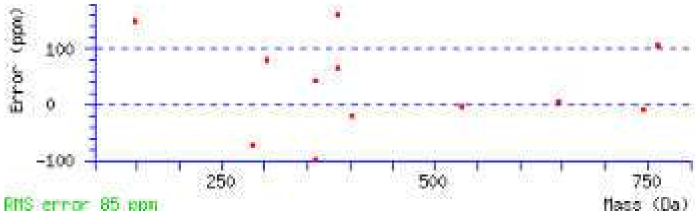


NCBI **BLAST** search of VYDDEVRK
(Parameters: blastp, nr protein database, expect=20000, no filter, PAM30)
Other BLAST web gateways

**All matches to this query**

| Score | Mr(calc) | Delta | Sequence |
|---|---|---|---|
| 34.2 | 1022.3975 | 0.1001 | DMDDEVQR |
| 30.2 | 1022.5033 | -0.0056 | VYDDEVRK |
| 20.9 | 1022.4702 | 0.0274 | DMTEEVRK |
| 18.4 | 1022.5145 | -0.0169 | KDAGEGPPPR |
| 17.4 | 1022.5734 | -0.0757 | RVQTHVQR |
| 16.2 | 1022.4274 | 0.0703 | CTACPTSAR |
| 15.7 | 1022.5066 | -0.0090 | KGMITDSGSK |
| 14.4 | 1022.5145 | -0.0169 | DYVDRLSR |
| 14.4 | 1022.5145 | -0.0169 | EFTDRLSR |
| 14.4 | 1022.5033 | -0.0057 | YVDDIVGSR |

**Mascot:** http://www.matrixscience.com/

# MATRIX SCIENCE Mascot Search Results

## Peptide View **Spot no 67**

MS/MS Fragmentation of **EGPPEFEQPK**
Found in **gi|8918361** in **NCBInr**, RuBisCO activase small isoform precursor [Oryza sativa]

Match to Query 37: 1156.524424 from(1157.531700,1+) intensity(0.0000) index(14)
Title: Label: D4, Spot_Id: 219725, Peak_List_Id: 225228, MSMS Job_Run_Id: 21770, Comment:
Data file C:\Documents and Settings\Administrator\Desktop\farah BHU\spot set plate\Farah-Garima 11May 2013\ppw_D4_136842162200.txt

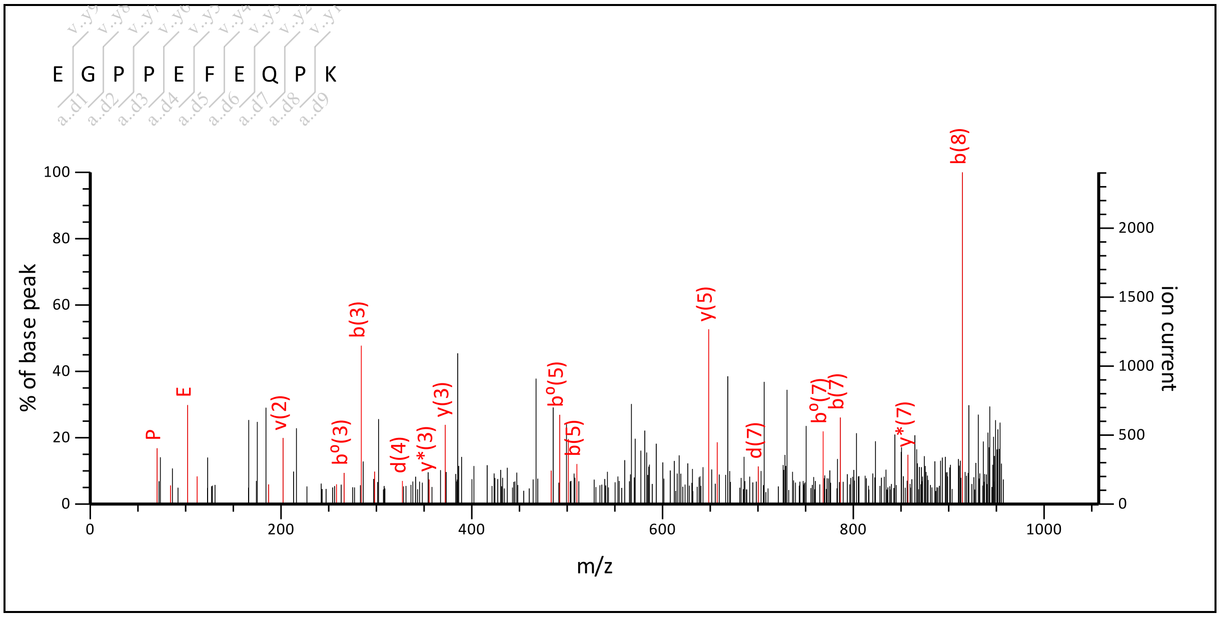


0 to 1057.26

Label all possible matches ○ Label matches used for scoring ◉

**Monoisotopic mass of neutral peptide Mr(calc):** 1156.5400
**Fixed modifications:** Carbamidomethyl (C) (apply to specified residues or termini only)
**Ions Score:** 10 **Expect:** 1.6e+02
**Matches :** 32/142 fragment ions using 90 most intense peaks (help)

| # | Immon. | a | a* | $a^0$ | b | b* | $b^0$ | d | Seq. | v | w | y | y* | $y^0$ | # |
|---|---|---|---|---|---|---|---|---|---|---|---|---|---|---|---|
| 1 | 102.0550 | 102.0550 | | 84.0444 | 130.0499 | | 112.0393 | 44.0495 | E | | | | | | 10 |
| 2 | 30.0338 | 159.0764 | | 141.0659 | ***187.0713*** | | 169.0608 | | G | | | 1028.5047 | 1011.4782 | 1010.4942 | 9 |
| 3 | 70.0651 | 256.1292 | | 238.1186 | ***284.1241*** | | 266.1135 | 230.1135 | P | 929.4363 | 928.4411 | 971.4833 | 954.4567 | 953.4727 | 8 |
| 4 | 70.0651 | 353.1819 | | 335.1714 | 381.1769 | | 363.1663 | 327.1663 | P | 832.3836 | 831.3883 | 874.4305 | 857.4040 | 856.4199 | 7 |
| 5 | 102.0550 | 482.2245 | | 464.2140 | ***510.2195*** | | 492.2089 | 424.2191 | E | 703.3410 | 702.3457 | 777.3777 | 760.3512 | 759.3672 | 6 |
| 6 | 120.0808 | 629.2930 | | 611.2824 | ***657.2879*** | | 639.2773 | | F | 556.2726 | | **648.3352** | 631.3086 | 630.3246 | 5 |
| 7 | 102.0550 | 758.3355 | | 740.3250 | ***786.3305*** | | 768.3199 | 700.3301 | E | 427.2300 | 426.2347 | **501.2667** | 484.2402 | 483.2562 | 4 |
| 8 | 101.0709 | 886.3941 | 869.3676 | 868.3836 | ***914.3890*** | 897.3625 | 896.3785 | 829.3727 | Q | 299.1714 | 298.1761 | **372.2241** | 355.1976 | | 3 |
| 9 | 70.0651 | 983.4469 | 966.4203 | 965.4363 | 1011.4418 | 994.4153 | 993.4312 | 957.4312 | P | 202.1186 | 201.1234 | 244.1656 | 227.1390 | | 2 |
| 10 | 101.1073 | | | | | | | | K | 74.0237 | 73.0284 | 147.1128 | 130.0863 | | 1 |

| Seq | ya | yb | Seq | ya | yb | Seq | ya | yb |
|---|---|---|---|---|---|---|---|---|
| **GP** | 127.0866 | 155.0815 | **GPP** | 224.1394 | 252.1343 | **GPPE** | 353.1819 | 381.1769 |
| **GPPEF** | 500.2504 | 528.2453 | **GPPEFE** | 629.2930 | 657.2879 | **PP** | 167.1179 | 195.1128 |
| **PPE** | 296.1605 | 324.1554 | **PPEF** | 443.2289 | 471.2238 | **PPEFE** | 572.2715 | 600.2664 |
| **PE** | 199.1077 | 227.1026 | **PEF** | 346.1761 | 374.1710 | **PEFE** | 475.2187 | 503.2136 |

| | | | | | | | | |
|---|---|---|---|---|---|---|---|---|
| **PEFEQ** | 603.2773 | 631.2722 | **EF** | 249.1234 | 277.1183 | **EFE** | 378.1660 | 406.1609 |
| **EFEQ** | 506.2245 | 534.2195 | **EFEQP** | 603.2773 | 631.2722 | **FE** | 249.1234 | 277.1183 |
| **FEQ** | 377.1819 | 405.1769 | **FEQP** | 474.2347 | 502.2296 | **EQ** | 230.1135 | 258.1084 |
| **EQP** | 327.1663 | 355.1612 | **QP** | 198.1237 | 226.1186 | | | |

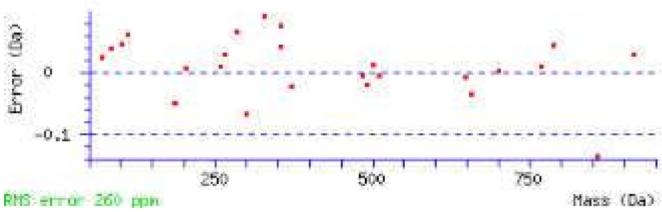


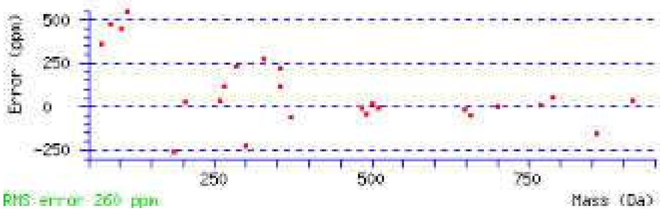


NCBI **BLAST** search of EGPPEFEQPK
(Parameters: blastp, nr protein database, expect=20000, no filter, PAM30)
Other BLAST web gateways

**All matches to this query**

| Score | Mr(calc) | Delta | Sequence |
|---|---|---|---|
| 14.8 | 1156.6088 | -0.0844 | EKDGAVQVSPK |
| 14.8 | 1156.5585 | -0.0340 | EQGADSPAARR |
| 13.3 | 1156.5989 | -0.0745 | QSVGLYTHPR |
| 12.4 | 1156.5811 | -0.0567 | GWAGLQCIPR |
| 12.2 | 1156.5877 | -0.0632 | AWDQSPTPKK |
| 10.9 | 1156.6200 | -0.0956 | TALSKGNNQPK |
| 10.4 | 1156.4852 | 0.0392 | MNKSGGGEMSK |
| 10.3 | 1156.5400 | -0.0156 | EGPPEFEQPK |
| 10.2 | 1156.5546 | -0.0302 | LMDPEGPNLR |
| 9.7 | 1156.6200 | -0.0956 | AVSGGLAEGLQR |

**Mascot:** http://www.matrixscience.com/

# MATRIX SCIENCE Mascot Search Results

## Peptide View Spot no 67

MS/MS Fragmentation of **SFQCELVFAK**
Found in **gi|8918361** in **NCBInr**, RuBisCO activase small isoform precursor [Oryza sativa]

Match to Query 45: 1227.577724 from(1228.585000,1+) intensity(0.0000) index(17)
Title: Label: D4, Spot_Id: 219725, Peak_List_Id: 225215, MSMS Job_Run_Id: 21770, Comment:
Data file C:\Documents and Settings\Administrator\Desktop\farah BHU\spot set plate\Farah-Garima 11May 2013\ppw_D4_136842162200.txt

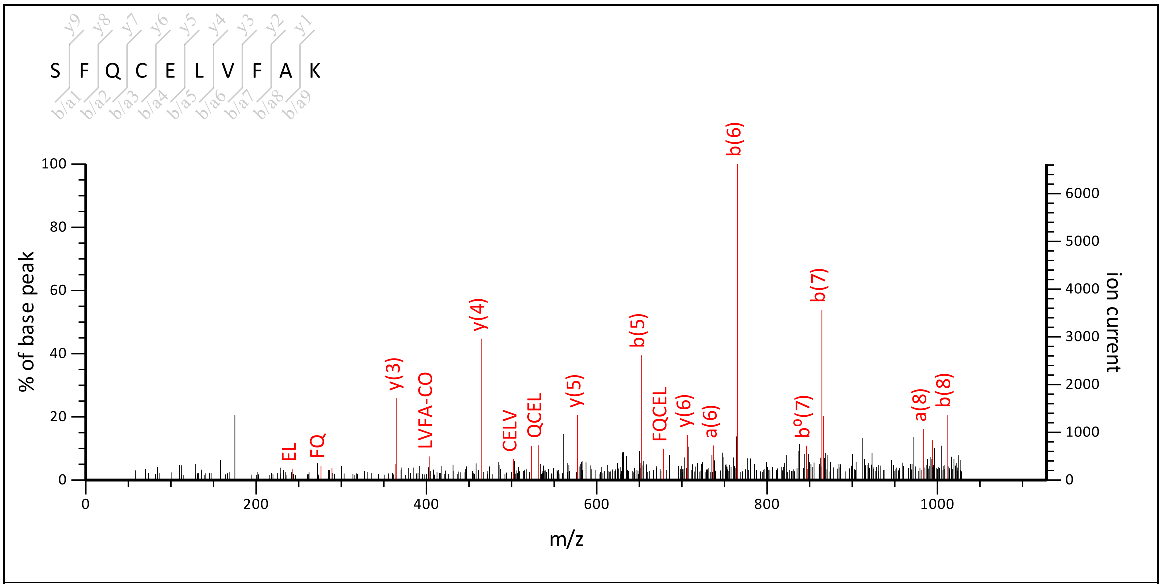


0 to 1128.36

Label all possible matches ○ Label matches used for scoring ◉

**Monoisotopic mass of neutral peptide Mr(calc):** 1227.5958
**Fixed modifications:** Carbamidomethyl (C) (apply to specified residues or termini only)
**Ions Score:** 36 **Expect:** 0.48
**Matches :** 26/148 fragment ions using 40 most intense peaks (help)

| # | Immon. | a | a* | $a^0$ | b | b* | $b^0$ | d | Seq. | v | w | y | y* | $y^0$ | # |
|---|---|---|---|---|---|---|---|---|---|---|---|---|---|---|---|
| 1 | 60.0444 | 60.0444 | | 42.0338 | 88.0393 | | 70.0287 | 44.0495 | S | | | | | | 10 |
| 2 | 120.0808 | 207.1128 | | 189.1022 | 235.1077 | | 217.0972 | | F | 1049.5084 | | 1141.5710 | 1124.5445 | 1123.5605 | 9 |
| 3 | 101.0709 | 335.1714 | 318.1448 | 317.1608 | ***363.1663*** | 346.1397 | 345.1557 | 278.1499 | Q | 921.4499 | 920.4546 | ***994.5026*** | 977.4761 | 976.4921 | 8 |
| 4 | 133.0430 | 495.2020 | 478.1755 | 477.1915 | ***523.1969*** | 506.1704 | 505.1864 | 406.2085 | C | 761.4192 | 760.4240 | ***866.4441*** | 849.4175 | 848.4335 | 7 |
| 5 | 102.0550 | 624.2446 | 607.2181 | 606.2341 | ***652.2395*** | 635.2130 | 634.2290 | 566.2391 | E | 632.3766 | 631.3814 | ***706.4134*** | 689.3869 | 688.4028 | 6 |
| 6 | 86.0964 | **737.3287** | 720.3021 | 719.3181 | ***765.3236*** | 748.2971 | 747.3130 | 695.2817 | L | 519.2926 | 518.2973 | ***577.3708*** | 560.3443 | | 5 |
| 7 | 72.0808 | 836.3971 | 819.3706 | 818.3865 | ***864.3920*** | 847.3655 | 846.3815 | 822.3815 | V | 420.2241 | 433.2445 | ***464.2867*** | 447.2602 | | 4 |
| 8 | 120.0808 | **983.4655** | 966.4390 | 965.4549 | ***1011.4604*** | 994.4339 | 993.4499 | | F | 273.1557 | | ***365.2183*** | 348.1918 | | 3 |
| 9 | 44.0495 | 1054.5026 | 1037.4761 | 1036.4921 | 1082.4975 | 1065.4710 | 1064.4870 | | A | 202.1186 | | 218.1499 | 201.1234 | | 2 |
| 10 | 101.1073 | | | | | | | | K | 74.0237 | 73.0284 | 147.1128 | 130.0863 | | 1 |

| Seq | ya | yb | Seq | ya | yb | Seq | ya | yb |
|---|---|---|---|---|---|---|---|---|
| FQ | 248.1394 | 276.1343 | FQC | 408.1700 | 436.1649 | FQCE | 537.2126 | 565.2075 |
| FQCEL | 650.2967 | 678.2916 | QC | 261.1016 | 289.0965 | QCE | 390.1442 | 418.1391 |
| QCEL | 503.2282 | 531.2232 | QCELV | 602.2967 | 630.2916 | CE | 262.0856 | 290.0805 |
| CEL | 375.1697 | 403.1646 | CELV | 474.2381 | 502.2330 | CELVF | 621.3065 | 649.3014 |
| CELVFA | 692.3436 | 720.3385 | EL | 215.1390 | 243.1339 | ELV | 314.2074 | 342.2023 |
| ELVF | 461.2758 | 489.2708 | ELVFA | 532.3130 | 560.3079 | LV | 185.1648 | 213.1598 |
| LVF | 332.2333 | 360.2282 | LVFA | 403.2704 | 431.2653 | VF | 219.1492 | 247.1441 |

| | | | | | | | | |
|---|---|---|---|---|---|---|---|---|
| **VFA** | 290.1863 | 318.1812 | **FA** | 191.1179 | 219.1128 | | | |

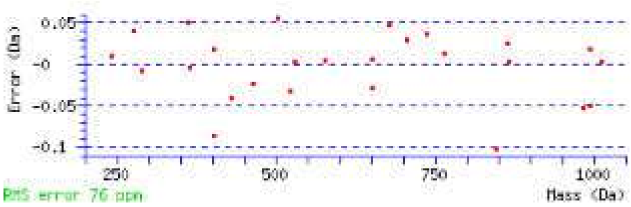


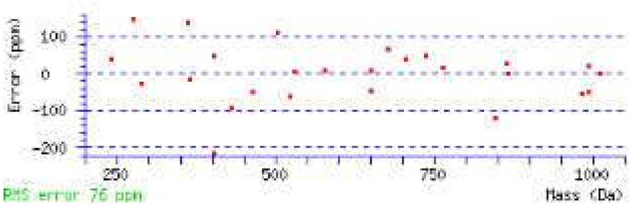


NCBI **BLAST** search of SFQCELVFAK
(Parameters: blastp, nr protein database, expect=20000, no filter, PAM30)
Other BLAST web gateways

**All matches to this query**

| Score | Mr(calc) | Delta | Sequence |
|---|---|---|---|
| 37.4 | 1227.5628 | 0.0149 | SFQCELVMAK |
| 36.1 | 1227.5958 | -0.0181 | SFQCELVFAK |
| 36.0 | 1227.6208 | -0.0430 | KVHPESSTSTR |
| 29.6 | 1227.5628 | 0.0149 | SFQCELVMSK |
| 29.4 | 1227.5376 | 0.0401 | FGCVSTMASPR |
| 24.2 | 1227.5992 | -0.0214 | GCFVIKTEMK |
| 23.3 | 1227.6248 | -0.0470 | HKPTSESPFAK |
| 23.2 | 1227.4860 | 0.0917 | ECSSLTSECR |
| 20.8 | 1227.5488 | 0.0289 | RDCYGLAMAR |
| 20.1 | 1227.6499 | -0.0722 | KFSVDISYAAK |

**Mascot:** http://www.matrixscience.com/

# Mascot Search Results

## Peptide View **Spot no 67**

MS/MS Fragmentation of **FYWAPTRDDR**
Found in **gi|8918361** in **NCBInr**, RuBisCO activase small isoform precursor [Oryza sativa]

Match to Query 58: 1325.603924 from(1326.611200,1+) intensity(0.0000) index(20)
Title: Label: D4, Spot_Id: 219725, Peak_List_Id: 225217, MSMS Job_Run_Id: 21770, Comment:
Data file C:\Documents and Settings\Administrator\Desktop\farah BHU\spot set plate\Farah-Garima 11May 2013\ppw_D4_136842162200.txt

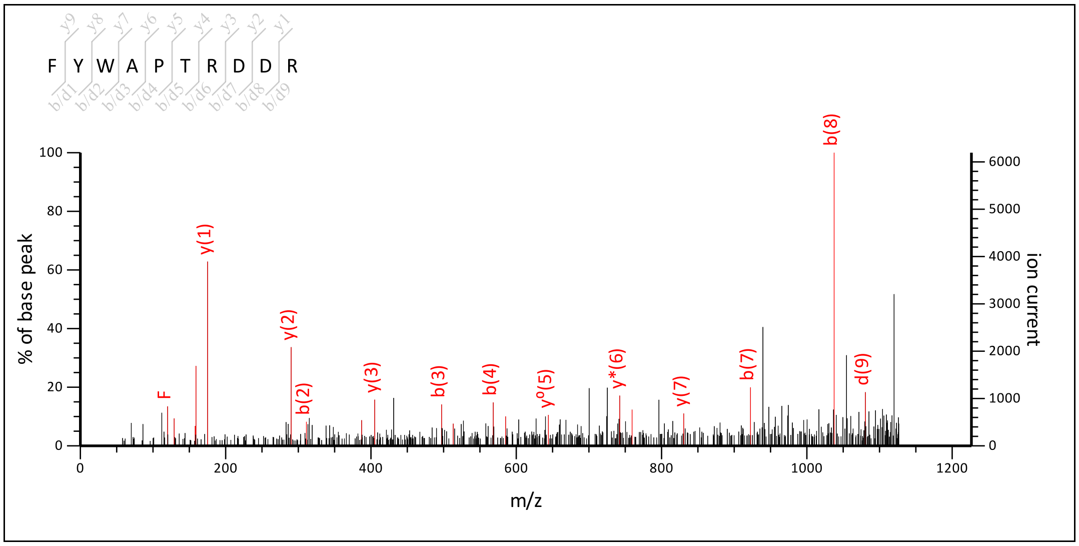


0 to 1226.51

Label all possible matches ○ Label matches used for scoring ◉

**Monoisotopic mass of neutral peptide Mr(calc):** 1325.6153
**Fixed modifications:** Carbamidomethyl (C) (apply to specified residues or termini only)
**Ions Score:** 27 **Expect:** 3.7
**Matches :** 23/138 fragment ions using 33 most intense peaks (help)

| # | Immon. | a | a* | $a^0$ | b | b* | $b^0$ | d | d' | Seq. | v | w | w' | y | y* | y |
|---|---|---|---|---|---|---|---|---|---|---|---|---|---|---|---|---|
| 1 | 120.0808 | 120.0808 | | | 148.0757 | | | 44.0495 | | F | | | | | | |
| 2 | 136.0757 | 283.1441 | | | ***311.1390*** | | | | | Y | 1071.4966 | | | 1179.5541 | 1162.5276 | 1161. |
| 3 | 159.0917 | 469.2234 | | | ***497.2183*** | | | | | W | 885.4173 | | | 1016.4908 | 999.4643 | 998. |
| 4 | 44.0495 | 540.2605 | | | ***568.2554*** | | | | | A | 814.3802 | | | ***830.4115*** | 813.3850 | 812. |
| 5 | 70.0651 | 637.3133 | | | 665.3082 | | | 611.2976 | | P | 717.3274 | 716.3322 | | ***759.3744*** | 742.3478 | 741. |
| 6 | 74.0600 | 738.3610 | | 720.3504 | 766.3559 | | 748.3453 | 722.3661 | 724.3453 | T | 616.2798 | 629.3002 | 631.2794 | 662.3216 | 645.2951 | 644. |
| 7 | 129.1135 | 894.4621 | 877.4355 | 876.4515 | ***922.4570*** | 905.4305 | 904.4464 | 809.3981 | | R | 460.1787 | 459.1834 | | 561.2739 | 544.2474 | 543. |
| 8 | 88.0393 | 1009.4890 | 992.4625 | 991.4785 | ***1037.4839*** | 1020.4574 | 1019.4734 | 965.4992 | | D | 345.1517 | 344.1565 | | ***405.1728*** | 388.1463 | 387. |
| 9 | 88.0393 | 1124.5160 | 1107.4894 | 1106.5054 | 1152.5109 | 1135.4843 | 1134.5003 | 1080.5261 | | D | 230.1248 | 229.1295 | | ***290.1459*** | 273.1193 | 272. |
| 10 | 129.1135 | | | | | | | | | R | 74.0237 | 73.0284 | | ***175.1190*** | 158.0924 | |

| Seq | ya | yb | Seq | ya | yb | Seq | ya | yb |
|---|---|---|---|---|---|---|---|---|
| YW | 322.1550 | 350.1499 | YWA | 393.1921 | 421.1870 | YWAP | 490.2449 | 518.2398 |
| YWAPT | 591.2926 | 619.2875 | WA | 230.1288 | 258.1237 | WAP | 327.1816 | 355.1765 |
| WAPT | 428.2292 | 456.2241 | WAPTR | 584.3303 | 612.3253 | WAPTRD | 699.3573 | 727.3522 |
| AP | 141.1022 | 169.0972 | APT | 242.1499 | 270.1448 | APTR | 398.2510 | 426.2459 |
| APTRD | 513.2780 | 541.2729 | APTRDD | 628.3049 | 656.2998 | PT | 171.1128 | 199.1077 |
| PTR | 327.2139 | 355.2088 | PTRD | 442.2409 | 470.2358 | PTRDD | 557.2678 | 585.2627 |
| TR | 230.1612 | 258.1561 | TRD | 345.1881 | 373.1830 | TRDD | 460.2150 | 488.2100 |
| RD | 244.1404 | 272.1353 | RDD | 359.1674 | 387.1623 | DD | 203.0662 | 231.0612 |

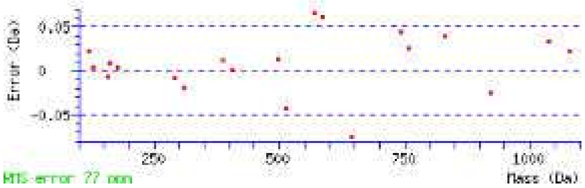


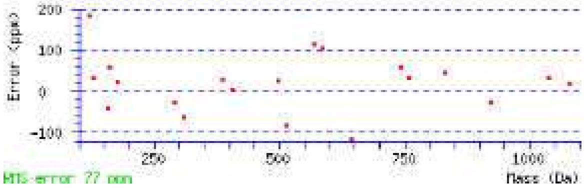


NCBI **BLAST** search of FYWAPTRDDR
(Parameters: blastp, nr protein database, expect=20000, no filter, PAM30)
Other BLAST web gateways

**All matches to this query**

| Score | Mr(calc) | Delta | Sequence |
|---|---|---|---|
| 26.9 | 1325.6153 | -0.0114 | FYWAPTRDDR |
| 16.2 | 1325.5710 | 0.0329 | MQVWVDYDDR |
| 15.2 | 1325.5921 | 0.0118 | KMETPPSSYDR |
| 15.1 | 1325.6186 | -0.0147 | MWAYNDVAKGR |
| 14.7 | 1325.6252 | -0.0212 | GIPYLNTYDDR |
| 14.0 | 1325.6649 | -0.0610 | METIKVTSFDR |
| 13.8 | 1325.7052 | -0.1012 | TPDAAVLGRSPSR |
| 13.7 | 1325.6616 | -0.0576 | TLFLVNAYDDR |
| 13.1 | 1325.6954 | -0.0915 | FFMWLALKDR |
| 12.5 | 1325.7092 | -0.1053 | VFALHADAVLDR |

**Mascot:** http://www.matrixscience.com/

# MATRIX SCIENCE Mascot Search Results

## Peptide View Spot no 67

MS/MS Fragmentation of **WVSDTGVENIGKR**
Found in **gi|8918361** in **NCBInr**, RuBisCO activase small isoform precursor [Oryza sativa]

Match to Query 69: 1459.736224 from(1460.743500,1+) intensity(0.0000) index(24)
Title: Label: D4, Spot_Id: 219725, Peak_List_Id: 225216, MSMS Job_Run_Id: 21770, Comment:
Data file C:\Documents and Settings\Administrator\Desktop\farah BHU\spot set plate\Farah-Garima 11May 2013\ppw_D4_136842162200.txt

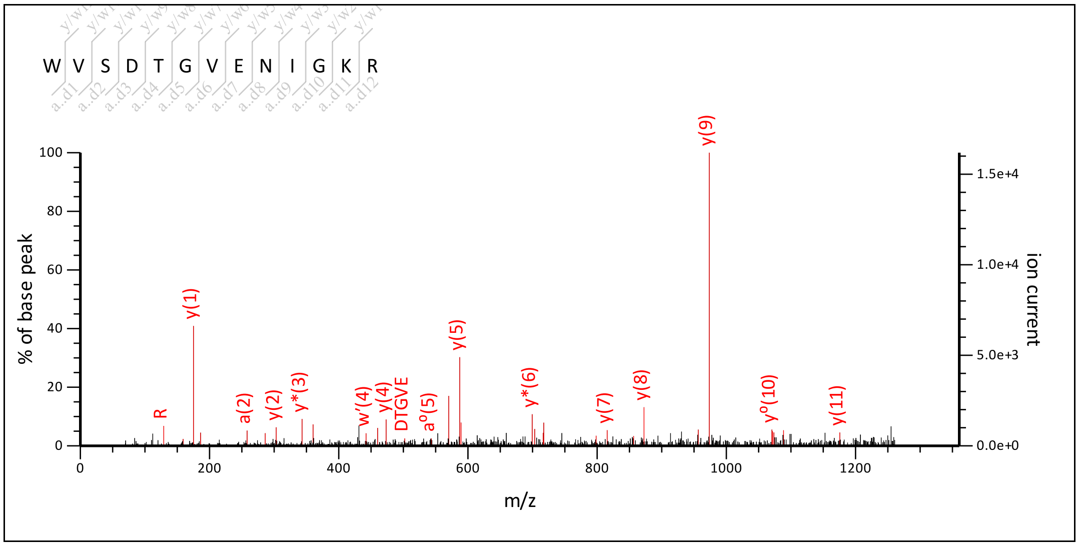


0 to 1360.59

Label all possible matches ○ Label matches used for scoring ◉

**Monoisotopic mass of neutral peptide Mr(calc):** 1459.7419
**Fixed modifications:** Carbamidomethyl (C) (apply to specified residues or termini only)
**Ions Score:** 85 **Expect:** 6.9e-06
**Matches :** 49/217 fragment ions using 48 most intense peaks (help)

#0000FF>13

| # | Immon. | a | a* | a0 | b | b* | b0 | d | d' | Seq. | v | w | w' | y | y* | |
|---|---|---|---|---|---|---|---|---|---|---|---|---|---|---|---|---|
| 1 | 159.0917 | **159.0917** | | | 187.0866 | | | 44.0495 | | **W** | | | | | | |
| 2 | 72.0808 | **258.1601** | | | **286.1550** | | | 244.1444 | | **V** | 1230.6073 | 1243.6277 | | 1274.6699 | 1257.6434 | [illegible] |
| 3 | 60.0444 | 345.1921 | | 327.1816 | 373.1870 | | 355.1765 | 329.1972 | | **S** | 1143.5753 | 1142.5800 | | ***1175.6015*** | 1158.5749 | [illegible] |
| 4 | 88.0393 | **460.2191** | | 442.2085 | 488.2140 | | 470.2034 | 416.2292 | | **D** | 1028.5483 | 1027.5531 | | ***1088.5695*** | 1071.5429 | [illegible] |
| 5 | 74.0600 | 561.2667 | | 543.2562 | **589.2617** | | 571.2511 | 545.2718 | 547.2511 | **T** | 927.5007 | 940.5211 | 942.5003 | ***973.5425*** | 956.5160 | [illegible] |
| 6 | 30.0338 | 618.2882 | | 600.2776 | 646.2831 | | 628.2726 | | | **G** | | | | ***872.4948*** | 855.4683 | [illegible] |
| 7 | 72.0808 | **717.3566** | | 699.3461 | 745.3515 | | 727.3410 | 703.3410 | | **V** | 771.4108 | 784.4312 | | ***815.4734*** | 798.4468 | [illegible] |
| 8 | 102.0550 | 846.3992 | | 828.3886 | 874.3941 | | 856.3836 | 788.3937 | | **E** | 642.3682 | 641.3729 | | ***716.4050*** | 699.3784 | [illegible] |
| 9 | 87.0553 | 960.4421 | 943.4156 | 942.4316 | 988.4371 | 971.4105 | 970.4265 | 917.4363 | | **N** | 528.3253 | 527.3300 | | ***587.3624*** | 570.3358 | |
| 10 | 86.0964 | **1073.5262** | 1056.4997 | 1055.5156 | 1101.5211 | 1084.4946 | 1083.5105 | 1045.4949 | 1059.5105 | **I** | 415.2412 | 129.1135 | | | | |

| Seq | ya | yb | Seq | ya | yb | Seq | ya | yb | | | | | | | | | |
|---|---|---|---|---|---|---|---|---|---|---|---|---|---|---|---|---|---|
| **VS** | 159.1128 | 187.1077 | **VSD** | 274.1397 | 302.1347 | **VSDT** | 375.1874 | 403.1823 | | | | | | | | | |
| **VSDTG** | 432.2089 | 460.2038 | **VSDTGV** | 531.2773 | 559.2722 | **VSDTGVE** | 660.3199 | 688.3148 | 428.2616 | 442.2772 | ***473.3194*** | 456.2929 | | **4** | | | |
| **11** | 30.0338 | 1130.5477 | 1113.5211 | 1112.5371 | 1158.5426 | 1141.5160 | 1140.5320 | | | **G** | | | | ***360.2354*** | 343.2088 | | **3** |
| **12** | 101.1073 | 1258.6426 | 1241.6161 | 1240.6321 | 1286.6375 | 1269.6110 | 1268.6270 | 1201.5848 | | **K** | 230.1248 | 229.1295 | | ***303.2139*** | 286.1874 | | **2** |
| **SD** | 175.0713 | 203.0662 | **SDT** | 276.1190 | 304.1139 | **SDTG** | 333.1405 | 361.1354 | | | | | | | | | |
| **SDTGV** | 432.2089 | 460.2038 | **SDTGVE** | 561.2515 | 589.2464 | **SDTGVEN** | 675.2944 | 703.2893 | | | | | | | | | |
| **DT** | 189.0870 | 217.0819 | **DTG** | 246.1084 | 274.1034 | **DTGV** | 345.1769 | 373.1718 | | | | | | | | | |
| **DTGVE** | 474.2195 | 502.2144 | **DTGVEN** | 588.2624 | 616.2573 | **TG** | 131.0815 | 159.0764 | | | | | | | | | |
| **TGV** | 230.1499 | 258.1448 | **TGVE** | 359.1925 | 387.1874 | **TGVEN** | 473.2354 | 501.2304 | | | | | | | | | |
| **TGVENI** | 586.3195 | 614.3144 | **TGVENIG** | 643.3410 | 671.3359 | **GV** | 129.1022 | 157.0972 | | | | | | | | | |

| | | | | | | | | |
|---|---|---|---|---|---|---|---|---|
| **GVE** | 258.1448 | 286.1397 | **GVEN** | 372.1878 | 400.1827 | **GVENI** | 485.2718 | 513.2667 |
| **GVENIG** | 542.2933 | 570.2882 | **GVENIGK** | 670.3882 | 698.3832 | **VE** | 201.1234 | 229.1183 |
| **VEN** | 315.1663 | 343.1612 | **VENI** | 428.2504 | 456.2453 | **VENIG** | 485.2718 | 513.2667 |
| **VENIGK** | 613.3668 | 641.3617 | **EN** | 216.0979 | 244.0928 | **ENI** | 329.1819 | 357.1769 |
| **ENIG** | 386.2034 | 414.1983 | **ENIGK** | 514.2984 | 542.2933 | **NI** | 200.1394 | 228.1343 |
| **NIG** | 257.1608 | 285.1557 | **NIGK** | 385.2558 | 413.2507 | **IG** | 143.1179 | 171.1128 |
| **IGK** | 271.2129 | 299.2078 | **GK** | 158.1288 | 186.1237 | | | |

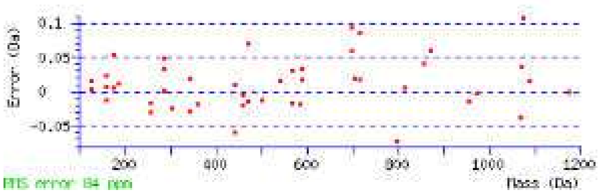


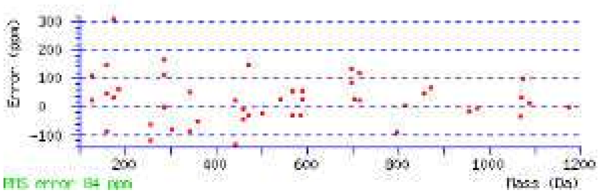


NCBI **BLAST** search of WVSDTGVENIGKR
(Parameters: blastp, nr protein database, expect=20000, no filter, PAM30)
Other BLAST web gateways

**All matches to this query**

| Score | Mr(calc) | Delta | Sequence |
|---|---|---|---|
| 84.9 | 1459.7419 | -0.0057 | WVSDTGVENIGKR |
| 30.6 | 1459.6652 | 0.0711 | TDEAGGVNQTGAGGAR |
| 30.5 | 1459.7783 | -0.0421 | WVTSTGIENIGKR |
| 25.6 | 1459.6653 | 0.0709 | YYPTTSPLSMER |
| 21.4 | 1459.7267 | 0.0095 | ATDITINGGGTIDGR |
| 21.3 | 1459.7630 | -0.0268 | TALSDVKLNESQR |
| 20.0 | 1459.6903 | 0.0459 | GNNTDSGVDLEAIR |
| 19.9 | 1459.7419 | -0.0057 | ERYVGAPVDLEGR |
| 19.1 | 1459.7089 | 0.0273 | ADVLAQQAGMDIGR |
| 18.9 | 1459.6539 | 0.0823 | EADEGANAGADSVVR |

**Mascot:** http://www.matrixscience.com/

# MATRIX SCIENCE Mascot Search Results

## Peptide View Spot no 67

MS/MS Fragmentation of **GLAYDISDDQQDITR**
Found in **gi|8918361** in **NCBInr**, RuBisCO activase small isoform precursor [Oryza sativa]

Match to Query 79: 1708.780824 from(1709.788100,1+) intensity(0.0000) index(27)
Title: Label: D4, Spot_Id: 219725, Peak_List_Id: 225214, MSMS Job_Run_Id: 21770, Comment:
Data file C:\Documents and Settings\Administrator\Desktop\farah BHU\spot set plate\Farah-Garima 11May 2013\ppw_D4_136842162200.txt

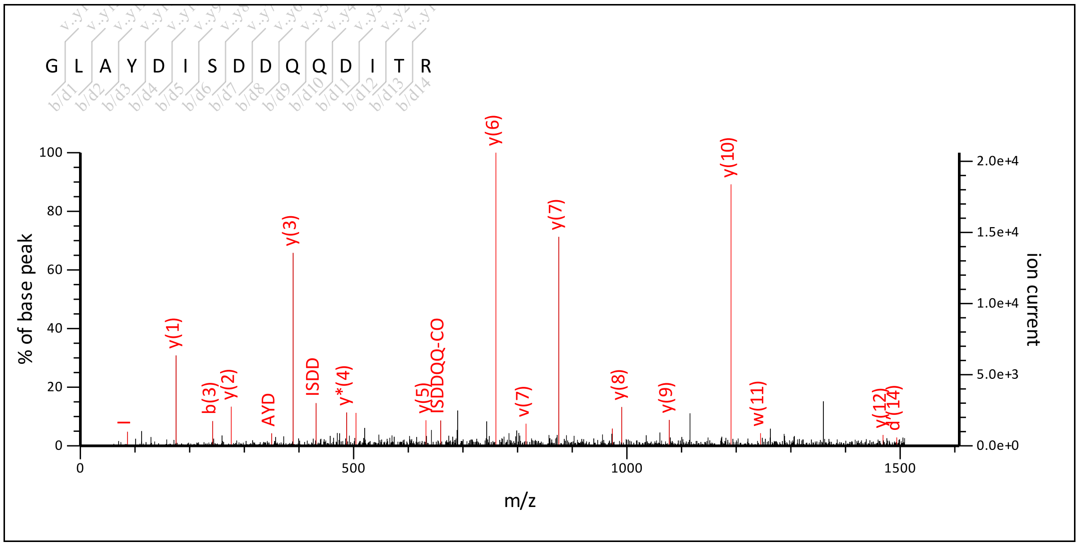


0 to 1608.15

Label all possible matches ○ Label matches used for scoring ◉

**Monoisotopic mass of neutral peptide Mr(calc):** 1708.7904
**Fixed modifications:** Carbamidomethyl (C) (apply to specified residues or termini only)
**Ions Score:** 87 **Expect:** 3.4e-06
**Matches :** 27/255 fragment ions using 30 most intense peaks (help)

| # | Immon. | a | a* | $a^0$ | b | b* | $b^0$ | d | d' | Seq. | v | w | w' | y | y* | |
|---|---|---|---|---|---|---|---|---|---|---|---|---|---|---|---|---|
| 1 | 30.0338 | 30.0338 | | | 58.0287 | | | 44.0495 | | G | | | | | | |
| 2 | 86.0964 | 143.1179 | | | 171.1128 | | | 101.0709 | | L | 1594.6980 | 1593.7027 | | 1652.7762 | 1635.7497 | [illegible] |
| 3 | 44.0495 | 214.1550 | | | 242.1499 | | | | | A | 1523.6609 | | | 1539.6922 | 1522.6656 | [illegible] |
| 4 | 136.0757 | 377.2183 | | | 405.2132 | | | | | Y | 1360.5975 | | | ***1468.6550*** | 1451.6285 | [illegible] |
| 5 | 88.0393 | 492.2453 | | 474.2347 | 520.2402 | | 502.2296 | 448.2554 | | D | 1245.5706 | 1244.5753 | | 1305.5917 | 1288.5652 | [illegible] |
| 6 | 86.0964 | 605.3293 | | 587.3188 | 633.3243 | | 615.3137 | 577.2980 | 591.3137 | I | 1132.4865 | 1145.5069 | 1159.5226 | ***1190.5648*** | 1173.5382 | [illegible] |
| 7 | 60.0444 | 692.3614 | | 674.3508 | 720.3563 | | 702.3457 | 676.3665 | | S | 1045.4545 | 1044.4592 | | ***1077.4807*** | 1060.4542 | [illegible] |
| 8 | 88.0393 | 807.3883 | | 789.3777 | 835.3832 | | 817.3727 | 763.3985 | | D | 930.4276 | 929.4323 | | ***990.4487*** | 973.4221 | [illegible] |
| 9 | 88.0393 | 922.4153 | | 904.4047 | 950.4102 | | 932.3996 | 878.4254 | | D | 815.4006 | 814.4054 | | ***875.4217*** | 858.3952 | [illegible] |
| 10 | 101.0709 | 1050.4738 | 1033.4473 | 1032.4633 | 1078.4687 | 1061.4422 | 1060.4582 | 993.4524 | | Q | 687.3420 | 686.3468 | | ***760.3948*** | 743.3682 | [illegible] |
| 11 | 101.0709 | 1178.5324 | 1161.5059 | 1160.5218 | 1206.5273 | 1189.5008 | 1188.5168 | 1121.5109 | | Q | 559.2835 | 558.2882 | | ***632.3362*** | 615.3097 | [illegible] |
| 12 | 88.0393 | 1293.5594 | 1276.5328 | 1275.5488 | 1321.5543 | 1304.5277 | 1303.5437 | 1249.5695 | | D | 444.2565 | 443.2613 | | ***504.2776*** | 487.2511 | [illegible] |
| 13 | 86.0964 | 1406.6434 | 1389.6169 | 1388.6329 | 1434.6383 | 1417.6118 | 1416.6278 | 1378.6121 | 1392.6278 | I | 331.1724 | 344.1928 | 358.2085 | ***389.2507*** | 372.2241 | [illegible] |
| 14 | 74.0600 | 1507.6911 | 1490.6645 | 1489.6805 | 1535.6860 | 1518.6595 | 1517.6754 | 1491.6962 | 1493.6754 | T | 230.1248 | 243.1452 | 245.1244 | ***276.1666*** | 259.1401 | [illegible] |
| 15 | 129.1135 | | | | | | | | | R | 74.0237 | 73.0284 | | ***175.1190*** | 158.0924 | |

| Seq | ya | yb | Seq | ya | yb | Seq | ya | yb |
|---|---|---|---|---|---|---|---|---|
| **LA** | 157.1335 | 185.1285 | **LAY** | 320.1969 | 348.1918 | **LAYD** | 435.2238 | 463.2187 |
| **LAYDI** | 548.3079 | 576.3028 | **LAYDIS** | 635.3399 | 663.3348 | **AY** | 207.1128 | 235.1077 |
| **AYD** | 322.1397 | 350.1347 | **AYDI** | 435.2238 | 463.2187 | **AYDIS** | 522.2558 | 550.2508 |
| **AYDISD** | 637.2828 | 665.2777 | **YD** | 251.1026 | 279.0975 | **YDI** | 364.1867 | 392.1816 |
| **YDIS** | 451.2187 | 479.2136 | **YDISD** | 566.2457 | 594.2406 | **YDISDD** | 681.2726 | 709.2675 |
| **DI** | 201.1234 | 229.1183 | **DIS** | 288.1554 | 316.1503 | **DISD** | 403.1823 | 431.1773 |

| | | | | | | | | |
|---|---|---|---|---|---|---|---|---|
| **DISDD** | 518.2093 | 546.2042 | **DISDDQ** | 646.2679 | 674.2628 | **IS** | 173.1285 | 201.1234 |
| **ISD** | 288.1554 | 316.1503 | **ISDD** | 403.1823 | 431.1773 | **ISDDQ** | 531.2409 | 559.2358 |
| **ISDDQQ** | 659.2995 | 687.2944 | **SD** | 175.0713 | 203.0662 | **SDD** | 290.0983 | 318.0932 |
| **SDDQ** | 418.1569 | 446.1518 | **SDDQQ** | 546.2154 | 574.2103 | **SDDQQD** | 661.2424 | 689.2373 |
| **DD** | 203.0662 | 231.0612 | **DDQ** | 331.1248 | 359.1197 | **DDQQ** | 459.1834 | 487.1783 |
| **DDQQD** | 574.2103 | 602.2053 | **DDQQDI** | 687.2944 | 715.2893 | **DQ** | 216.0979 | 244.0928 |
| **DQQ** | 344.1565 | 372.1514 | **DQQD** | 459.1834 | 487.1783 | **DQQDI** | 572.2675 | 600.2624 |
| **DQQDIT** | 673.3151 | 701.3101 | **QQ** | 229.1295 | 257.1244 | **QQD** | 344.1565 | 372.1514 |
| **QQDI** | 457.2405 | 485.2354 | **QQDIT** | 558.2882 | 586.2831 | **QD** | 216.0979 | 244.0928 |
| **QDI** | 329.1819 | 357.1769 | **QDIT** | 430.2296 | 458.2245 | **DI** | 201.1234 | 229.1183 |
| **DIT** | 302.1710 | 330.1660 | **IT** | 187.1441 | 215.1390 | | | |

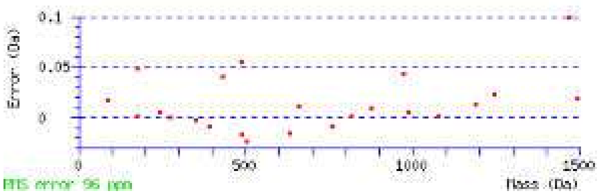


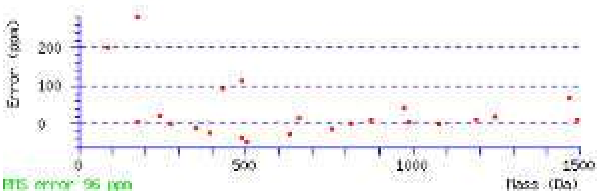


NCBI **BLAST** search of GLAYDISDDQQDITR
(Parameters: blastp, nr protein database, expect=20000, no filter, PAM30)
Other BLAST web gateways

**All matches to this query**

| Score | Mr(calc) | Delta | Sequence |
|---|---|---|---|
| 86.8 | 1708.7904 | -0.0096 | GLAYDISDDQQDITR |
| 66.9 | 1708.7540 | 0.0268 | GLAFDESDDQQDITR |
| 38.6 | 1708.7978 | -0.0170 | GLSTDSLMEAGYPGAPK |
| 18.6 | 1708.8302 | -0.0493 | MSDGITVTESISQIGR |
| 18.2 | 1708.7760 | 0.0048 | LDVMSNGATMEAAVER |
| 18.2 | 1708.9009 | -0.1201 | ANIHALSGAWGDKLTR |
| 17.3 | 1708.8679 | -0.0871 | SHIQKAGLDMSHTIR |
| 14.7 | 1708.8784 | -0.0976 | FSNGANFAIVGSSTLPK |
| 14.6 | 1708.8203 | -0.0394 | AYAMVRLEADGQVDR |
| 14.6 | 1708.8600 | -0.0792 | VASTSDLMSLMRLNR |

**Mascot:** http://www.matrixscience.com/

# MATRIX SCIENCE Mascot Search Results

## Peptide View Spot no 67

MS/MS Fragmentation of **MCCLFINDLDAGAGR**
Found in **gi|8918361** in **NCBInr**, RuBisCO activase small isoform precursor [Oryza sativa]

Match to Query 82: 1727.720424 from(1728.727700,1+) intensity(0.0000) index(28)
Title: Label: D4, Spot_Id: 219725, Peak_List_Id: 225219, MSMS Job_Run_Id: 21770, Comment:
Data file C:\Documents and Settings\Administrator\Desktop\farah BHU\spot set plate\Farah-Garima 11May 2013\ppw_D4_136842162200.txt

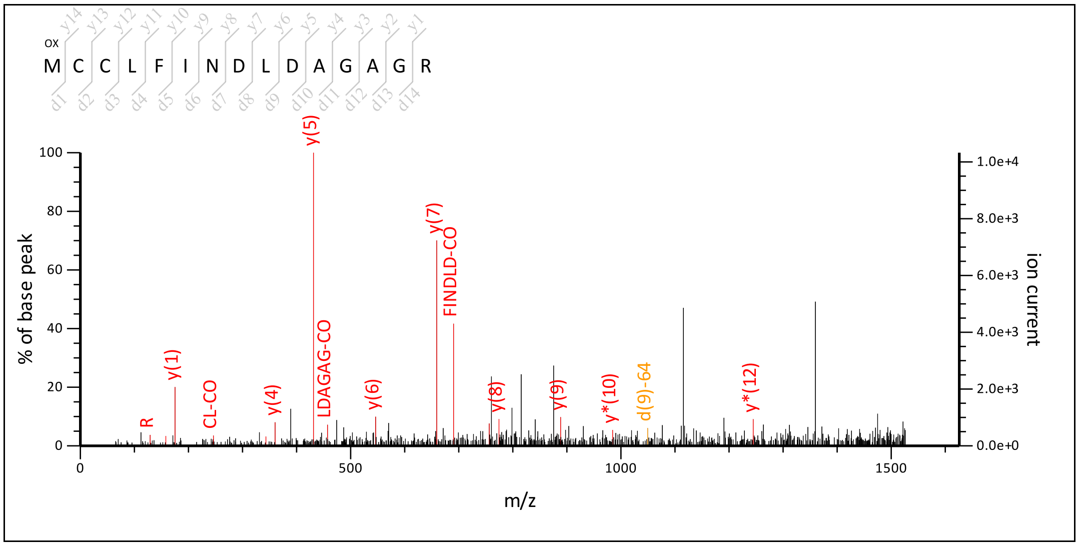


0 to 1625.91

Label all possible matches ○ Label matches used for scoring ◉

**Monoisotopic mass of neutral peptide Mr(calc):** 1727.7430
**Fixed modifications:** Carbamidomethyl (C) (apply to specified residues or termini only)
**Variable modifications:**
**M1** : Oxidation (M), with neutral losses 63.9983(shown in table), 0.0000
**Ions Score:** 22 **Expect:** 6.5
**Matches :** 23/311 fragment ions using 45 most intense peaks (help)

| # | Immon. | a | a* | $a^0$ | b | b* | $b^0$ | d | d' | Seq. | v | w | w' | y | y* | [illegible] |
|---|---|---|---|---|---|---|---|---|---|---|---|---|---|---|---|---|
| 1 | 56.0495 | 56.0495 | | | 84.0444 | | | 44.0495 | | M | | | | | | |
| 2 | 133.0430 | 216.0801 | | | 244.0750 | | | 127.0866 | | C | 1476.6900 | 1475.6947 | | 1581.7148 | 1564.6883 | 156[illegible] |
| 3 | 133.0430 | 376.1108 | | | 404.1057 | | | 287.1172 | | C | 1316.6593 | 1315.6641 | | 1421.6842 | 1404.6576 | 140[illegible] |
| 4 | 86.0964 | 489.1948 | | | 517.1898 | | | 447.1479 | | L | 1203.5753 | 1202.5800 | | 1261.6535 | 1244.6270 | 124[illegible] |
| 5 | 120.0808 | 636.2633 | | | 664.2582 | | | | | F | 1056.5069 | | | 1148.5695 | 1131.5429 | 113[illegible] |
| 6 | 86.0964 | 749.3473 | | | 777.3422 | | | 721.3160 | 735.3317 | I | 943.4228 | 956.4432 | 970.4588 | 1001.5010 | 984.4745 | 98[illegible] |
| 7 | 87.0553 | 863.3902 | 846.3637 | | 891.3852 | 874.3586 | | 820.3844 | | N | 829.3799 | 828.3846 | | ***888.4170*** | 871.3904 | 87[illegible] |
| 8 | 88.0393 | 978.4172 | 961.3906 | 960.4066 | 1006.4121 | 989.3856 | 988.4015 | 934.4274 | | D | 714.3529 | 713.3577 | | ***774.3741*** | 757.3475 | 75[illegible] |
| 9 | 86.0964 | 1091.5013 | 1074.4747 | 1073.4907 | 1119.4962 | 1102.4696 | 1101.4856 | 1049.4543 | | L | 601.2689 | 600.2736 | | ***659.3471*** | 642.3206 | 64[illegible] |
| 10 | 88.0393 | 1206.5282 | 1189.5016 | 1188.5176 | 1234.5231 | 1217.4966 | 1216.5125 | 1162.5384 | | D | 486.2419 | 485.2467 | | ***546.2631*** | 529.2365 | 52[illegible] |
| 11 | 44.0495 | 1277.5653 | 1260.5388 | 1259.5547 | 1305.5602 | 1288.5337 | 1287.5497 | | | A | 415.2048 | | | ***431.2361*** | 414.2096 | |
| 12 | 30.0338 | 1334.5868 | 1317.5602 | 1316.5762 | 1362.5817 | 1345.5551 | 1344.5711 | | | G | | | | ***360.1990*** | 343.1724 | |
| 13 | 44.0495 | 1405.6239 | 1388.5973 | 1387.6133 | 1433.6188 | 1416.5923 | 1415.6082 | | | A | 287.1462 | | | 303.1775 | 286.1510 | |
| 14 | 30.0338 | 1462.6453 | 1445.6188 | 1444.6348 | 1490.6403 | 1473.6137 | 1472.6297 | | | G | | | | 232.1404 | 215.1139 | |
| 15 | 129.1135 | | | | | | | | | R | 74.0237 | 73.0284 | | ***175.1190*** | 158.0924 | |

| Seq | ya | yb | Seq | ya | yb | Seq | ya | yb |
|---|---|---|---|---|---|---|---|---|
| CC | 293.0737 | 321.0686 | CCL | 406.1577 | 434.1526 | CCLF | 553.2261 | 581.2211 |
| CCLFI | 666.3102 | 694.3051 | CL | 246.1271 | 274.1220 | CLF | 393.1955 | 421.1904 |
| CLFI | 506.2796 | 534.2745 | CLFIN | 620.3225 | 648.3174 | LF | 233.1648 | 261.1598 |
| | | | | | | | | |

| | | | | | | | | |
|---|---|---|---|---|---|---|---|---|
| **LFI** | 346.2489 | 374.2438 | **LFIN** | 460.2918 | 488.2867 | **LFIND** | 575.3188 | 603.3137 |
| **LFINDL** | 688.4028 | 716.3978 | **FI** | 233.1648 | 261.1598 | **FIN** | 347.2078 | 375.2027 |
| **FIND** | 462.2347 | 490.2296 | **FINDL** | 575.3188 | 603.3137 | **FINDLD** | 690.3457 | 718.3406 |
| **IN** | 200.1394 | 228.1343 | **IND** | 315.1663 | 343.1612 | **INDL** | 428.2504 | 456.2453 |
| **INDLD** | 543.2773 | 571.2722 | **INDLDA** | 614.3144 | 642.3093 | **INDLDAG** | 671.3359 | 699.3308 |
| **ND** | 202.0822 | 230.0771 | **NDL** | 315.1663 | 343.1612 | **NDLD** | 430.1932 | 458.1882 |
| **NDLDA** | 501.2304 | 529.2253 | **NDLDAG** | 558.2518 | 586.2467 | **NDLDAGA** | 629.2889 | 657.2838 |
| **NDLDAGAG** | 686.3104 | 714.3053 | **DL** | 201.1234 | 229.1183 | **DLD** | 316.1503 | 344.1452 |
| **DLDA** | 387.1874 | 415.1823 | **DLDAG** | 444.2089 | 472.2038 | **DLDAGA** | 515.2460 | 543.2409 |
| **DLDAGAG** | 572.2675 | 600.2624 | **LD** | 201.1234 | 229.1183 | **LDA** | 272.1605 | 300.1554 |
| **LDAG** | 329.1819 | 357.1769 | **LDAGA** | 400.2191 | 428.2140 | **LDAGAG** | 457.2405 | 485.2354 |
| **DA** | 159.0764 | 187.0713 | **DAG** | 216.0979 | 244.0928 | **DAGA** | 287.1350 | 315.1299 |
| **DAGAG** | 344.1565 | 372.1514 | **AG** | 101.0709 | 129.0659 | **AGA** | 172.1081 | 200.1030 |
| **AGAG** | 229.1295 | 257.1244 | **GA** | 101.0709 | 129.0659 | **GAG** | 158.0924 | 186.0873 |
| **AG** | 101.0709 | 129.0659 | | | | | | |

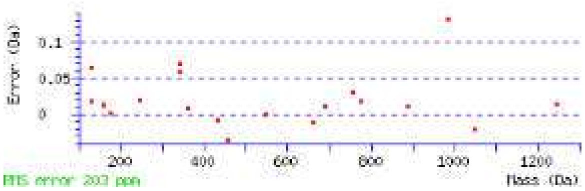


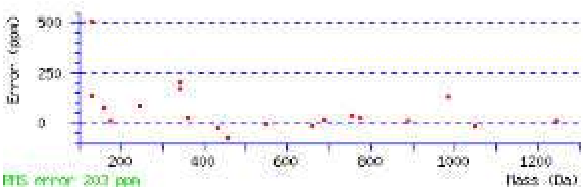


NCBI **BLAST** search of MCCLFINDLDAGAGR
(Parameters: blastp, nr protein database, expect=20000, no filter, PAM30)
Other BLAST web gateways

**All matches to this query**

| Score | Mr(calc) | Delta | Sequence |
|---|---|---|---|
| 27.1 | 1727.8559 | -0.1355 | GHVMMLNDRIQSVGR |
| 22.4 | 1727.7430 | -0.0225 | MCCLFINDLDAGAGR |
| 17.8 | 1727.8559 | -0.1355 | GHVMMLNDRIQSVGR |
| 17.2 | 1727.8559 | -0.1355 | GHVMMLNDRVQSLGR |
| 14.3 | 1727.8269 | -0.1065 | ASLCALMAPVGGHAAMR |
| 12.4 | 1727.7832 | -0.0627 | MAPAGGGDSPCASARAPR |
| 12.3 | 1727.7607 | -0.0403 | EEELDMFCRNITR |
| 11.8 | 1727.7780 | -0.0576 | IMDDESVMPTTKMSK |
| 11.6 | 1727.8764 | -0.1559 | GSNIELMLDVPNEGLK |
| 11.2 | 1727.8070 | -0.0866 | SPGTTGSVKTGMTMTEK |

**Mascot:** http://www.matrixscience.com/

# MATRIX SCIENCE Mascot Search Results

## Peptide View Spot no 67

MS/MS Fragmentation of **EGPPEFEQPKMTIEK**
Found in **gi|8918361** in **NCBInr**, RuBisCO activase small isoform precursor [Oryza sativa]

Match to Query 84: 1774.819624 from(1775.826900,1+) intensity(0.0000) index(29)
Title: Label: D4, Spot_Id: 219725, Peak_List_Id: 225223, MSMS Job_Run_Id: 21770, Comment:
Data file C:\Documents and Settings\Administrator\Desktop\farah BHU\spot set plate\Farah-Garima 11May 2013\ppw_D4_136842162200.txt

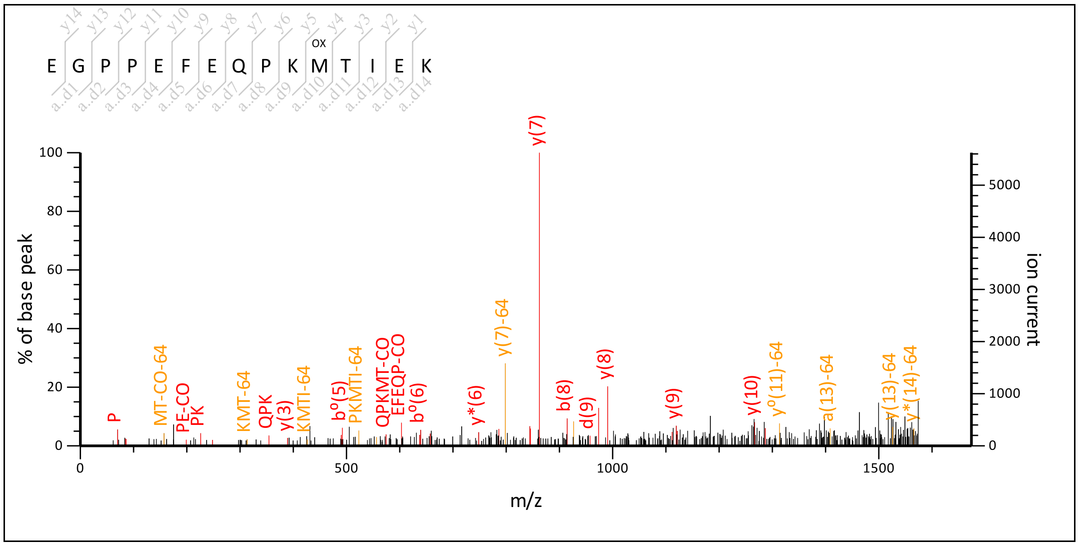


0 to 1674

Label all possible matches ○ Label matches used for scoring ◉

**Monoisotopic mass of neutral peptide Mr(calc):** 1774.8447
**Fixed modifications:** Carbamidomethyl (C) (apply to specified residues or termini only)
**Variable modifications:**
**M11** : Oxidation (M), with neutral losses 0.0000(shown in table), 63.9983
**Ions Score:** 9 **Expect:** 2e+02
**Matches :** 50/358 fragment ions using 128 most intense peaks (help)

| # | Immon. | a | a* | $a^0$ | b | b* | $b^0$ | d | d' | Seq. | v | w | w' | y | y* | [illegible] |
|---|---|---|---|---|---|---|---|---|---|---|---|---|---|---|---|---|
| 1 | 102.0550 | 102.0550 | | 84.0444 | 130.0499 | | 112.0393 | 44.0495 | | E | | | | | | |
| 2 | 30.0338 | 159.0764 | | 141.0659 | 187.0713 | | 169.0608 | | | G | | | | 1646.8094 | 1629.7829 | [illegible] |
| 3 | 70.0651 | 256.1292 | | 238.1186 | 284.1241 | | 266.1135 | 230.1135 | | P | 1547.7410 | 1546.7458 | | 1589.7880 | 1572.7614 | [illegible] |
| 4 | 70.0651 | 353.1819 | | 335.1714 | 381.1769 | | 363.1663 | 327.1663 | | P | 1450.6883 | 1449.6930 | | 1492.7352 | 1475.7087 | [illegible] |
| 5 | 102.0550 | 482.2245 | | 464.2140 | 510.2195 | | 492.2089 | 424.2191 | | E | 1321.6457 | 1320.6504 | | 1395.6824 | 1378.6559 | [illegible] |
| 6 | 120.0808 | 629.2930 | | 611.2824 | **657.2879** | | 639.2773 | | | F | 1174.5773 | | | ***1266.6399*** | 1249.6133 | [illegible] |
| 7 | 102.0550 | 758.3355 | | 740.3250 | **786.3305** | | 768.3199 | 700.3301 | | E | 1045.5347 | 1044.5394 | | ***1119.5714*** | 1102.5449 | [illegible] |
| 8 | 101.0709 | 886.3941 | 869.3676 | 868.3836 | **914.3890** | 897.3625 | 896.3785 | 829.3727 | | Q | 917.4761 | 916.4808 | | ***990.5288*** | 973.5023 | [illegible] |
| 9 | 70.0651 | 983.4469 | 966.4203 | 965.4363 | 1011.4418 | 994.4153 | 993.4312 | 957.4312 | | P | 820.4233 | 819.4281 | | ***862.4703*** | 845.4437 | [illegible] |
| 10 | 101.1073 | 1111.5419 | 1094.5153 | 1093.5313 | 1139.5368 | 1122.5102 | 1121.5262 | 1054.4840 | | K | 692.3284 | 691.3331 | | 765.4175 | 748.3910 | [illegible] |
| 11 | 120.0478 | 1258.5773 | 1241.5507 | 1240.5667 | **1286.5722** | 1269.5456 | 1268.5616 | 1182.5790 | | M | 545.2930 | 544.2977 | | ***637.3225*** | 620.2960 | [illegible] |
| 12 | 74.0600 | 1359.6249 | 1342.5984 | 1341.6144 | 1387.6198 | 1370.5933 | 1369.6093 | 1343.6300 | 1345.6093 | T | 444.2453 | 457.2657 | 459.2449 | ***490.2871*** | 473.2606 | [illegible] |
| 13 | 86.0964 | 1472.7090 | 1455.6824 | 1454.6984 | 1500.7039 | 1483.6774 | 1482.6933 | 1444.6777 | 1458.6933 | I | 331.1612 | 344.1816 | 358.1973 | ***389.2395*** | 372.2129 | [illegible] |
| 14 | 102.0550 | 1601.7516 | 1584.7250 | 1583.7410 | 1629.7465 | 1612.7200 | 1611.7359 | 1543.7461 | | E | 202.1186 | 201.1234 | | 276.1554 | 259.1288 | [illegible] |
| 15 | 101.1073 | | | | | | | | | K | 74.0237 | 73.0284 | | 147.1128 | 130.0863 | |

| Seq | ya | yb | Seq | ya | yb | Seq | ya | yb |
|---|---|---|---|---|---|---|---|---|
| GP | 127.0866 | 155.0815 | GPP | 224.1394 | 252.1343 | GPPE | 353.1819 | 381.1769 |
| GPPEF | 500.2504 | 528.2453 | GPPEFE | 629.2930 | 657.2879 | PP | 167.1179 | 195.1128 |
| PPE | 296.1605 | 324.1554 | PPEF | 443.2289 | 471.2238 | PPEFE | 572.2715 | 600.2664 |
| | | | | | | | | |

| | | | | | | | | |
|---|---|---|---|---|---|---|---|---|
| **PE** | 199.1077 | 227.1026 | **PEF** | 346.1761 | 374.1710 | **PEFE** | 475.2187 | 503.2136 |
| **PEFEQ** | 603.2773 | 631.2722 | **EF** | 249.1234 | 277.1183 | **EFE** | 378.1660 | 406.1609 |
| **EFEQ** | 506.2245 | 534.2195 | **EFEQP** | 603.2773 | 631.2722 | **FE** | 249.1234 | 277.1183 |
| **FEQ** | 377.1819 | 405.1769 | **FEQP** | 474.2347 | 502.2296 | **FEQPK** | 602.3297 | 630.3246 |
| **EQ** | 230.1135 | 258.1084 | **EQP** | 327.1663 | 355.1612 | **EQPK** | 455.2613 | 483.2562 |
| **EQPKM** | 602.2967 | 630.2916 | **QP** | 198.1237 | 226.1186 | **QPK** | 326.2187 | 354.2136 |
| **QPKM** | 473.2541 | 501.2490 | **QPKMT** | 574.3017 | 602.2967 | **QPKMTI** | 687.3858 | 715.3807 |
| **PK** | 198.1601 | 226.1550 | **PKM** | 345.1955 | 373.1904 | **PKMT** | 446.2432 | 474.2381 |
| **PKMTI** | 559.3272 | 587.3221 | **PKMTIE** | 688.3698 | 716.3647 | **KM** | 248.1427 | 276.1376 |
| **KMT** | 349.1904 | 377.1853 | **KMTI** | 462.2745 | 490.2694 | **KMTIE** | 591.3171 | 619.3120 |
| **MT** | 221.0954 | 249.0904 | **MTI** | 334.1795 | 362.1744 | **MTIE** | 463.2221 | 491.2170 |
| **TI** | 187.1441 | 215.1390 | **TIE** | 316.1867 | 344.1816 | **IE** | 215.1390 | 243.1339 |

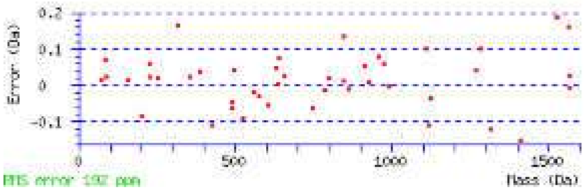


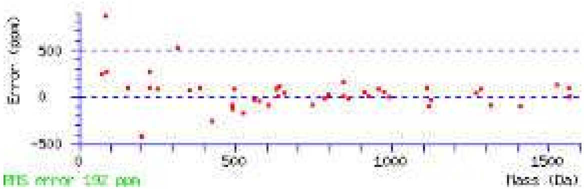


NCBI **BLAST** search of EGPPEFEQPKMTIEK
(Parameters: blastp, nr protein database, expect=20000, no filter, PAM30)
Other BLAST web gateways

**All matches to this query**

| Score | Mr(calc) | Delta | Sequence |
|---|---|---|---|
| 12.5 | 1774.7138 | 0.1058 | DSNVMYLDKDCEDR |
| 10.8 | 1774.9829 | -0.1633 | SLELDKDLSVALLGFR |
| 9.2 | 1774.9550 | -0.1354 | VQGPRAALPQAQAAQGGR |
| 9.1 | 1774.8447 | -0.0251 | EGPPEFEQPKMTIEK |
| 8.2 | 1774.9326 | -0.1130 | TTNYIRIQATGTPSPR |
| 8.2 | 1774.9260 | -0.1064 | FTAEAMVRPQLRANR |
| 7.0 | 1774.9108 | -0.0912 | VGQMNGSSTRLLTAQGR |
| 6.8 | 1774.9287 | -0.1091 | KPNELGFKELGMTPAK |
| 6.8 | 1774.9552 | -0.1356 | LWIRTFPDKPVTMR |
| 6.6 | 1774.9413 | -0.1217 | ALGTIHSHPVWMVRR |

**Mascot:** http://www.matrixscience.com/

# MATRIX SCIENCE Mascot Search Results

## Peptide View Spot no 69

MS/MS Fragmentation of **AAQVFSDPK**
Found in **gi|19387272** in **NCBInr**, putative precursor chloroplastic glutamine synthetase [Oryza sativa Japonica Group]

Match to Query 15: 961.498704 from(962.505980,1+) intensity(0.0000) index(2)
Title: Label: B19, Spot_Id: 219963, Peak_List_Id: 229185, MSMS Job_Run_Id: 22013, Comment:
Data file C:\Documents and Settings\Administrator\Desktop\Farah-Garima 11May 2013\ppw_B19_136868333300.txt

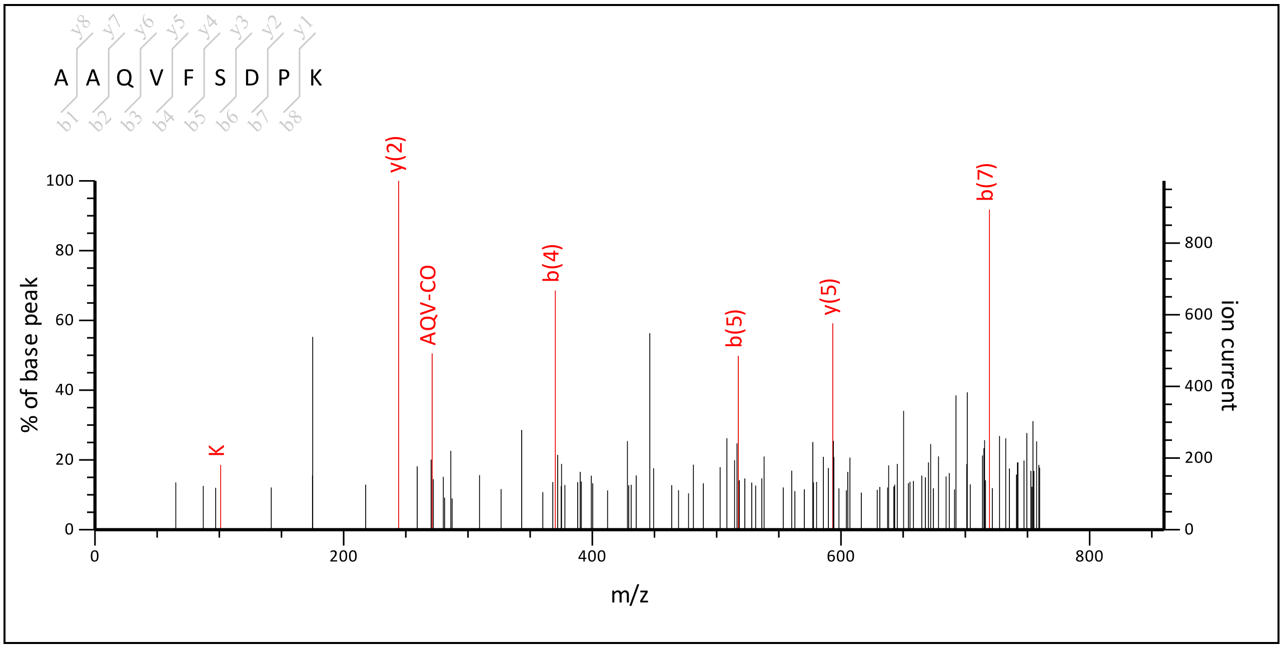


0 to 859.79

Label all possible matches ○ Label matches used for scoring ◉

**Monoisotopic mass of neutral peptide Mr(calc):** 961.4869
**Fixed modifications:** Carbamidomethyl (C) (apply to specified residues or termini only)
**Ions Score:** 36 **Expect:** 9.9
**Matches :** 9/125 fragment ions using 7 most intense peaks (help)

| # | Immon. | a | a* | a0 | b | b* | b0 | d | Seq. | v | w | y | y* | y0 | # |
|---|---|---|---|---|---|---|---|---|---|---|---|---|---|---|---|
| 1 | 44.0495 | 44.0495 | | | 72.0444 | | | 44.0495 | A | | | | | | 9 |
| 2 | 44.0495 | 115.0866 | | | 143.0815 | | | | A | 875.4258 | | 891.4571 | 874.4305 | 873.4465 | 8 |
| 3 | 101.0709 | 243.1452 | 226.1186 | | ***271.1401*** | 254.1135 | | 186.1237 | Q | 747.3672 | 746.3719 | 820.4199 | 803.3934 | 802.4094 | 7 |
| 4 | 72.0808 | 342.2136 | 325.1870 | | ***370.2085*** | 353.1819 | | 328.1979 | V | 648.2988 | 661.3192 | 692.3614 | 675.3348 | 674.3508 | 6 |
| 5 | 120.0808 | 489.2820 | 472.2554 | | ***517.2769*** | 500.2504 | | | F | 501.2304 | | **593.2930** | 576.2664 | 575.2824 | 5 |
| 6 | 60.0444 | 576.3140 | 559.2875 | 558.3035 | 604.3089 | 587.2824 | 586.2984 | 560.3191 | S | 414.1983 | 413.2031 | 446.2245 | 429.1980 | 428.2140 | 4 |
| 7 | 88.0393 | 691.3410 | 674.3144 | 673.3304 | ***719.3359*** | 702.3093 | 701.3253 | 647.3511 | D | 299.1714 | 298.1761 | 359.1925 | 342.1660 | 341.1819 | 3 |
| 8 | 70.0651 | 788.3937 | 771.3672 | 770.3832 | 816.3886 | 799.3621 | 798.3781 | 762.3781 | P | 202.1186 | 201.1234 | **244.1656** | 227.1390 | | 2 |
| 9 | 101.1073 | | | | | | | | K | 74.0237 | 73.0284 | 147.1128 | 130.0863 | | 1 |

| Seq | ya | yb | Seq | ya | yb | Seq | ya | yb |
|---|---|---|---|---|---|---|---|---|
| AQ | 172.1081 | 200.1030 | AQV | 271.1765 | 299.1714 | AQVF | 418.2449 | 446.2398 |
| AQVFS | 505.2769 | 533.2718 | AQVFSD | 620.3039 | 648.2988 | QV | 200.1394 | 228.1343 |
| QVF | 347.2078 | 375.2027 | QVFS | 434.2398 | 462.2347 | QVFSD | 549.2667 | 577.2617 |

| | | | | | | | | |
|---|---|---|---|---|---|---|---|---|
| **QVFSDP** | 646.3195 | 674.3144 | **VF** | 219.1492 | 247.1441 | **VFS** | 306.1812 | 334.1761 |
| **VFSD** | 421.2082 | 449.2031 | **VFSDP** | 518.2609 | 546.2558 | **FS** | 207.1128 | 235.1077 |
| **FSD** | 322.1397 | 350.1347 | **FSDP** | 419.1925 | 447.1874 | **SD** | 175.0713 | 203.0662 |
| **SDP** | 272.1241 | 300.1190 | **DP** | 185.0921 | 213.0870 | | | |

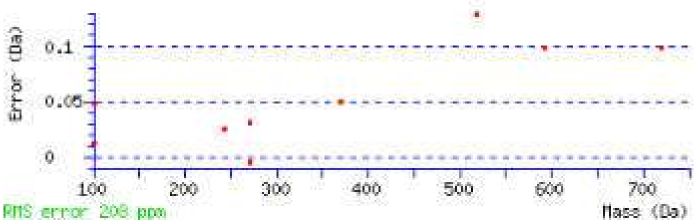


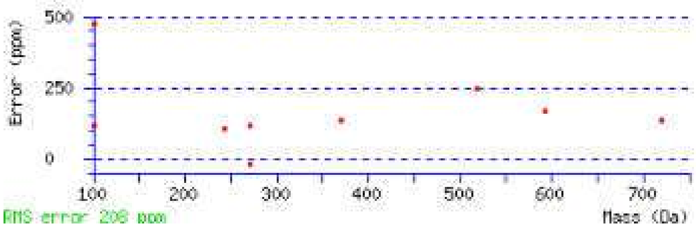


NCBI **BLAST** search of AAQVFSDPK
(Parameters: blastp, nr protein database, expect=20000, no filter, PAM30)
Other BLAST web gateways

**All matches to this query**

| Score | Mr(calc) | Delta | Sequence |
|---|---|---|---|
| 36.5 | 961.4869 | 0.0118 | AAQVFSDPK |
| 36.5 | 961.5233 | -0.0246 | AKAVFSDPK |
| 31.2 | 961.5709 | -0.0722 | KGLVFVSGR |
| 31.2 | 961.5709 | -0.0722 | KVAVFGVSR |
| 31.2 | 961.5345 | -0.0358 | QAVVFRDK |
| 31.2 | 961.5267 | -0.0280 | QAVVMLGTK |
| 31.2 | 961.5597 | -0.0610 | QGLVFAVTK |
| 31.2 | 961.4981 | 0.0006 | QVAVFQDR |
| 31.2 | 961.5345 | -0.0358 | QVAVFSGVR |
| 30.2 | 961.5345 | -0.0358 | AGAVVFGVSR |

**Mascot:** http://www.matrixscience.com/

# MATRIX SCIENCE Mascot Search Results

## Peptide View Spot no 69

MS/MS Fragmentation of **HDLHISAYGEGNER**
Found in **gi|19387272** in **NCBInr**, putative precursor chloroplastic glutamine synthetase [Oryza sativa Japonica Group]

Match to Query 112: 1596.776124 from(1597.783400,1+) intensity(0.0000) index(14)
Title: Label: B19, Spot_Id: 219963, Peak_List_Id: 229164, MSMS Job_Run_Id: 22013, Comment:
Data file C:\Documents and Settings\Administrator\Desktop\Farah-Garima 11May 2013\ppw_B19_136868333300.txt

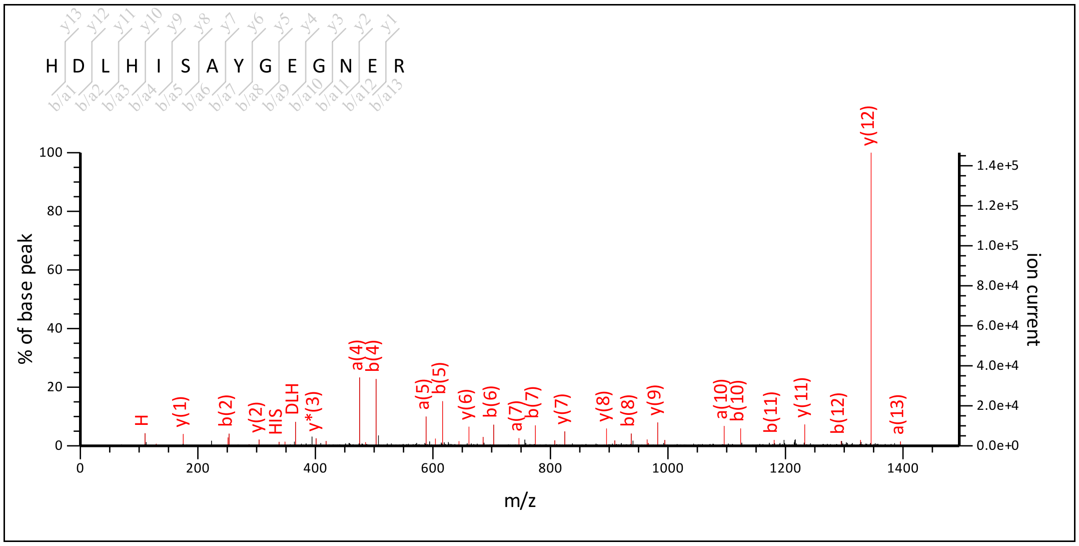


0 to 1495.79

Label all possible matches ○ Label matches used for scoring ◉

**Monoisotopic mass of neutral peptide Mr(calc):** 1596.7281
**Fixed modifications:** Carbamidomethyl (C) (apply to specified residues or termini only)
**Ions Score:** 103 **Expect:** 1.5e-06
**Matches :** 52/230 fragment ions using 56 most intense peaks (help)

| # | Immon. | a | a* | $a^0$ | b | b* | $b^0$ | d | d' | Seq. | v | w | w' | y | y* | |
|---|---|---|---|---|---|---|---|---|---|---|---|---|---|---|---|---|
| 1 | 110.0713 | **110.0713** | | | 138.0662 | | | 44.0495 | | H | | | | | | |
| 2 | 88.0393 | 225.0982 | | 207.0877 | **253.0931** | | 235.0826 | 181.1084 | | D | 1400.6553 | 1399.6601 | | 1460.6764 | 1443.6499 | 144 |
| 3 | 86.0964 | **338.1823** | | 320.1717 | **366.1772** | | 348.1666 | 296.1353 | | L | 1287.5713 | 1286.5760 | | ***1345.6495*** | 1328.6230 | 132 |
| 4 | 110.0713 | **475.2412** | | 457.2306 | **503.2361** | | 485.2255 | | | H | 1150.5123 | | | ***1232.5654*** | 1215.5389 | 12 |
| 5 | 86.0964 | **588.3253** | | 570.3147 | **616.3202** | | 598.3096 | 560.2940 | 574.3096 | I | 1037.4283 | 1050.4487 | 1064.4643 | ***1095.5065*** | 1078.4800 | 10 |
| 6 | 60.0444 | 675.3573 | | 657.3467 | **703.3522** | | 685.3416 | 659.3624 | | S | 950.3963 | 949.4010 | | ***982.4225*** | 965.3959 | 96 |
| 7 | 44.0495 | **746.3944** | | 728.3838 | **774.3893** | | 756.3787 | | | A | 879.3591 | | | ***895.3904*** | 878.3639 | 87 |
| 8 | 136.0757 | **909.4577** | | 891.4472 | **937.4526** | | 919.4421 | | | Y | 716.2958 | | | ***824.3533*** | 807.3268 | 80 |
| 9 | 30.0338 | **966.4792** | | 948.4686 | **994.4741** | | 976.4635 | | | G | | | | ***661.2900*** | 644.2634 | 64 |
| 10 | 102.0550 | **1095.5218** | | 1077.5112 | **1123.5167** | | 1105.5061 | 1037.5163 | | E | 530.2317 | 529.2365 | | ***604.2685*** | 587.2420 | 58 |
| 11 | 30.0338 | 1152.5432 | | 1134.5327 | **1180.5382** | | 1162.5276 | | | G | | | | ***475.2259*** | 458.1994 | 45 |
| 12 | 87.0553 | 1266.5862 | 1249.5596 | 1248.5756 | **1294.5811** | 1277.5545 | 1276.5705 | 1223.5804 | | N | 359.1674 | 358.1721 | | ***418.2045*** | 401.1779 | 40 |
| 13 | 102.0550 | **1395.6288** | 1378.6022 | 1377.6182 | 1423.6237 | 1406.5971 | 1405.6131 | 1337.6233 | | E | 230.1248 | 229.1295 | | ***304.1615*** | 287.1350 | 28 |
| 14 | 129.1135 | | | | | | | | | R | 74.0237 | 73.0284 | | ***175.1190*** | 158.0924 | |

| Seq | ya | yb | Seq | ya | yb | Seq | ya | yb |
|---|---|---|---|---|---|---|---|---|
| DL | 201.1234 | 229.1183 | DLH | 338.1823 | 366.1772 | DLHI | 451.2663 | 479.2613 |
| DLHIS | 538.2984 | 566.2933 | DLHISA | 609.3355 | 637.3304 | LH | 223.1553 | 251.1503 |
| LHI | 336.2394 | 364.2343 | LHIS | 423.2714 | 451.2663 | LHISA | 494.3085 | 522.3035 |
| LHISAY | 657.3719 | 685.3668 | HI | 223.1553 | 251.1503 | HIS | 310.1874 | 338.1823 |
| HISA | 381.2245 | 409.2194 | HISAY | 544.2878 | 572.2827 | HISAYG | 601.3093 | 629.3042 |
| IS | 173.1285 | 201.1234 | ISA | 244.1656 | 272.1605 | ISAY | 407.2289 | 435.2238 |
| ISAYG | 464.2504 | 492.2453 | ISAYGE | 593.2930 | 621.2879 | ISAYGEG | 650.3144 | 678.3093 |

| | | | | | | | | |
|---|---|---|---|---|---|---|---|---|
| **SA** | 131.0815 | 159.0764 | **SAY** | 294.1448 | 322.1397 | **SAYG** | 351.1663 | 379.1612 |
| **SAYGE** | 480.2089 | 508.2038 | **SAYGEG** | 537.2304 | 565.2253 | **SAYGEGN** | 651.2733 | 679.2682 |
| **AY** | 207.1128 | 235.1077 | **AYG** | 264.1343 | 292.1292 | **AYGE** | 393.1769 | 421.1718 |
| **AYGEG** | 450.1983 | 478.1932 | **AYGEGN** | 564.2413 | 592.2362 | **AYGEGNE** | 693.2838 | 721.2788 |
| **YG** | 193.0972 | 221.0921 | **YGE** | 322.1397 | 350.1347 | **YGEG** | 379.1612 | 407.1561 |
| **YGEGN** | 493.2041 | 521.1991 | **YGEGNE** | 622.2467 | 650.2416 | **GE** | 159.0764 | 187.0713 |
| **GEG** | 216.0979 | 244.0928 | **GEGN** | 330.1408 | 358.1357 | **GEGNE** | 459.1834 | 487.1783 |
| **EG** | 159.0764 | 187.0713 | **EGN** | 273.1193 | 301.1143 | **EGNE** | 402.1619 | 430.1569 |
| **GN** | 144.0768 | 172.0717 | **GNE** | 273.1193 | 301.1143 | **NE** | 216.0979 | 244.0928 |

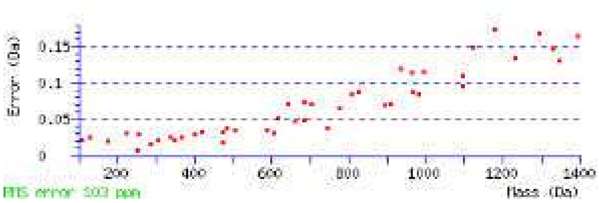


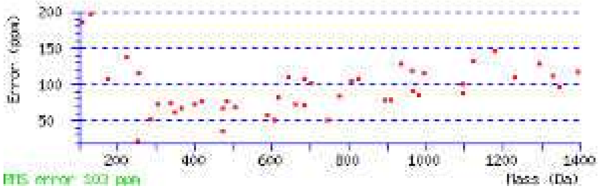


NCBI **BLAST** search of HDLHISAYGEGNER
(Parameters: blastp, nr protein database, expect=20000, no filter, PAM30)
Other BLAST web gateways

**All matches to this query**

| Score | Mr(calc) | Delta | Sequence |
|---|---|---|---|
| 103.1 | 1596.7281 | 0.0481 | HDLHISAYGEGNER |
| 85.3 | 1596.7281 | 0.0481 | HVEHISAYGEGNER |
| 84.8 | 1596.7281 | 0.0481 | HEVHISAYGEGNER |
| 70.5 | 1596.6917 | 0.0845 | HDEHIAAYGEGNER |
| 51.4 | 1596.7644 | 0.0117 | HVEHISSYGLGNER |
| 33.5 | 1596.7653 | 0.0108 | HPHMLSVSNCFIR |
| 29.6 | 1596.7427 | 0.0335 | HDCAPARDSIASAAR |
| 29.3 | 1596.6991 | 0.0771 | EKNMIDYNDHYR |
| 28.6 | 1596.7276 | 0.0485 | SMYVPSMVDLGNER |
| 27.4 | 1596.8293 | -0.0532 | EMELASLAHLSQLR |

**Mascot:** http://www.matrixscience.com/

# MATRIX SCIENCE Mascot Search Results

## Peptide View Spot no 69

MS/MS Fragmentation of **IIAEYIWVGGTGIDLR**
Found in **gi|19387272** in **NCBInr**, putative precursor chloroplastic glutamine synthetase [Oryza sativa Japonica Group]

Match to Query 143: 1775.011524 from(1776.018800,1+) intensity(0.0000) index(18)
Title: Label: B19, Spot_Id: 219963, Peak_List_Id: 229172, MSMS Job_Run_Id: 22013, Comment:
Data file C:\Documents and Settings\Administrator\Desktop\Farah-Garima 11May 2013\ppw_B19_136868333300.txt

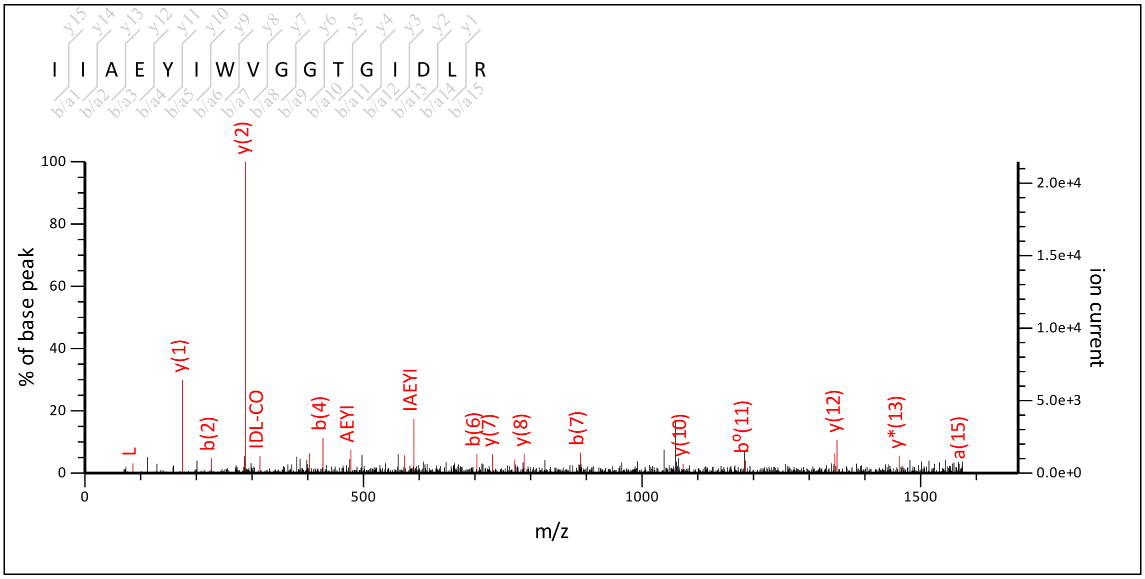


0 to 1675.04

Label all possible matches ○ Label matches used for scoring ◉

**Monoisotopic mass of neutral peptide Mr(calc):** 1774.9618
**Fixed modifications:** Carbamidomethyl (C) (apply to specified residues or termini only)
**Ions Score:** 57 **Expect:** 0.046
**Matches :** 32/264 fragment ions using 32 most intense peaks (help)

| # | Immon. | a | $a^0$ | b | $b^0$ | d | d' | Seq. | v | w | w' | y | y* | $y^0$ | # |
|---|---|---|---|---|---|---|---|---|---|---|---|---|---|---|---|
| 1 | 86.0964 | **86.0964** | | 114.0913 | | 44.0495 | | I | | | | | | | 16 |
| 2 | 86.0964 | 199.1805 | | ***227.1754*** | | 171.1492 | 185.1648 | I | 1604.8067 | 1617.8271 | 1631.8428 | 1662.8850 | 1645.8584 | 1644.8744 | 15 |
| 3 | 44.0495 | 270.2176 | | 298.2125 | | | | A | 1533.7696 | | | 1549.8009 | 1532.7744 | 1531.7904 | 14 |
| 4 | 102.0550 | 399.2602 | 381.2496 | ***427.2551*** | 409.2445 | 341.2547 | | E | 1404.7270 | 1403.7318 | | 1478.7638 | 1461.7373 | 1460.7532 | 13 |
| 5 | 136.0757 | **562.3235** | 544.3130 | ***590.3184*** | 572.3079 | | | Y | 1241.6637 | | | ***1349.7212*** | 1332.6947 | 1331.7106 | 12 |
| 6 | 86.0964 | 675.4076 | 657.3970 | ***703.4025*** | 685.3919 | 647.3763 | 661.3919 | I | 1128.5796 | 1141.6000 | 1155.6157 | 1186.6579 | 1169.6313 | 1168.6473 | 11 |
| 7 | 159.0917 | 861.4869 | 843.4763 | ***889.4818*** | 871.4713 | | | W | 942.5003 | | | ***1073.5738*** | 1056.5473 | 1055.5633 | 10 |
| 8 | 72.0808 | 960.5553 | 942.5448 | 988.5502 | 970.5397 | 946.5397 | | V | 843.4319 | 856.4523 | | 887.4945 | 870.4680 | 869.4839 | 9 |
| 9 | 30.0338 | 1017.5768 | 999.5662 | 1045.5717 | 1027.5611 | | | G | | | | ***788.4261*** | 771.3995 | 770.4155 | 8 |
| 10 | 30.0338 | 1074.5982 | 1056.5877 | 1102.5932 | 1084.5826 | | | G | | | | ***731.4046*** | 714.3781 | 713.3941 | 7 |
| 11 | 74.0600 | 1175.6459 | 1157.6354 | 1203.6408 | 1185.6303 | 1159.6510 | 1161.6303 | T | 628.3413 | 641.3617 | 643.3410 | 674.3832 | 657.3566 | 656.3726 | 6 |
| 12 | 30.0338 | 1232.6674 | 1214.6568 | 1260.6623 | 1242.6517 | | | G | | | | ***573.3355*** | 556.3089 | 555.3249 | 5 |
| 13 | 86.0964 | **1345.7515** | 1327.7409 | 1373.7464 | 1355.7358 | 1317.7202 | 1331.7358 | I | 458.2358 | 471.2562 | 485.2718 | 516.3140 | 499.2875 | 498.3035 | 4 |
| 14 | 88.0393 | 1460.7784 | 1442.7678 | 1488.7733 | 1470.7627 | 1416.7886 | | D | 343.2088 | 342.2136 | | ***403.2300*** | 386.2034 | 385.2194 | 3 |
| 15 | 86.0964 | **1573.8625** | 1555.8519 | 1601.8574 | 1583.8468 | 1531.8155 | | L | 230.1248 | 229.1295 | | ***288.2030*** | 271.1765 | | 2 |
| 16 | 129.1135 | | | | | | | R | 74.0237 | 73.0284 | | ***175.1190*** | 158.0924 | | 1 |

| Seq | ya | yb | Seq | ya | yb | Seq | ya | yb |
|---|---|---|---|---|---|---|---|---|
| IA | 157.1335 | 185.1285 | IAE | 286.1761 | 314.1710 | IAEY | 449.2395 | 477.2344 |
| | | | | | | | | |

| | | | | | | | | |
|---|---|---|---|---|---|---|---|---|
| IAEYI | 562.3235 | 590.3184 | AE | 173.0921 | 201.0870 | AEY | 336.1554 | 364.1503 |
| AEYI | 449.2395 | 477.2344 | AEYIW | 635.3188 | 663.3137 | EY | 265.1183 | 293.1132 |
| EYI | 378.2023 | 406.1973 | EYIW | 564.2817 | 592.2766 | EYIWV | 663.3501 | 691.3450 |
| YI | 249.1598 | 277.1547 | YIW | 435.2391 | 463.2340 | YIWV | 534.3075 | 562.3024 |
| YIWVG | 591.3289 | 619.3239 | YIWVGG | 648.3504 | 676.3453 | IW | 272.1757 | 300.1707 |
| IWV | 371.2442 | 399.2391 | IWVG | 428.2656 | 456.2605 | IWVGG | 485.2871 | 513.2820 |
| IWVGGT | 586.3348 | 614.3297 | IWVGGTG | 643.3562 | 671.3511 | WV | 258.1601 | 286.1550 |
| WVG | 315.1816 | 343.1765 | WVGG | 372.2030 | 400.1979 | WVGGT | 473.2507 | 501.2456 |
| WVGGTG | 530.2722 | 558.2671 | WVGGTGI | 643.3562 | 671.3511 | VG | 129.1022 | 157.0972 |
| VGG | 186.1237 | 214.1186 | VGGT | 287.1714 | 315.1663 | VGGTG | 344.1928 | 372.1878 |
| VGGTGI | 457.2769 | 485.2718 | VGGTGID | 572.3039 | 600.2988 | VGGTGIDL | 685.3879 | 713.3828 |
| GG | 87.0553 | 115.0502 | GGT | 188.1030 | 216.0979 | GGTG | 245.1244 | 273.1193 |
| GGTGI | 358.2085 | 386.2034 | GGTGID | 473.2354 | 501.2304 | GGTGIDL | 586.3195 | 614.3144 |
| GT | 131.0815 | 159.0764 | GTG | 188.1030 | 216.0979 | GTGI | 301.1870 | 329.1819 |
| GTGID | 416.2140 | 444.2089 | GTGIDL | 529.2980 | 557.2930 | TG | 131.0815 | 159.0764 |
| TGI | 244.1656 | 272.1605 | TGID | 359.1925 | 387.1874 | TGIDL | 472.2766 | 500.2715 |
| GI | 143.1179 | 171.1128 | GID | 258.1448 | 286.1397 | GIDL | 371.2289 | 399.2238 |
| ID | 201.1234 | 229.1183 | IDL | 314.2074 | 342.2023 | DL | 201.1234 | 229.1183 |

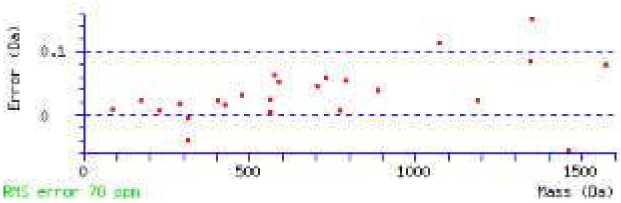


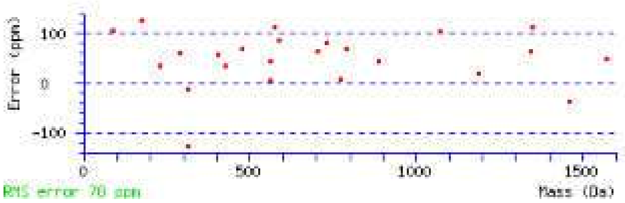


NCBI **BLAST** search of IIAEYIWVGGTGIDLR
(Parameters: blastp, nr protein database, expect=20000, no filter, PAM30)
Other BLAST web gateways

**All matches to this query**

| Score | Mr(calc) | Delta | Sequence |
|---|---|---|---|
| 57.0 | 1774.9618 | 0.0498 | IIAEYIWVGGTGIDLR |
| 45.7 | 1774.9617 | 0.0498 | IIAEYIWIGGSGIDIR |
| 45.7 | 1774.9617 | 0.0498 | IIAEYIWIGGSGIDLR |
| 45.7 | 1774.9617 | 0.0498 | IIAEYIWIGGSGLDLR |
| 45.7 | 1774.9617 | 0.0498 | IIAEYLWIGGSGLDIR |
| 37.5 | 1774.9618 | 0.0498 | IIAEYVWIGGTGIDLR |
| 26.3 | 1774.9135 | 0.0980 | ILAEMEITGITVDAQR |
| 26.1 | 1774.8638 | 0.1477 | NSLSWEAWVLPSSSGR |
| 23.7 | 1774.9618 | 0.0498 | IIAEYIWIGGTGIDVR |
| 23.3 | 1774.8962 | 0.1153 | LERVGFNSPTLSDGQR |

**Mascot:** http://www.matrixscience.com/

# MATRIX SCIENCE Mascot Search Results

## Peptide View Spot no 69

MS/MS Fragmentation of **LTGLHETASIDNFSWGVANR**
Found in **gi|19387272** in **NCBInr**, putative precursor chloroplastic glutamine synthetase [Oryza sativa Japonica Group]

Match to Query 179: 2187.135524 from(2188.142800,1+) intensity(0.0000) index(22)
Title: Label: B19, Spot_Id: 219963, Peak_List_Id: 229165, MSMS Job_Run_Id: 22013, Comment:
Data file C:\Documents and Settings\Administrator\Desktop\Farah-Garima 11May 2013\ppw_B19_136868333300.txt

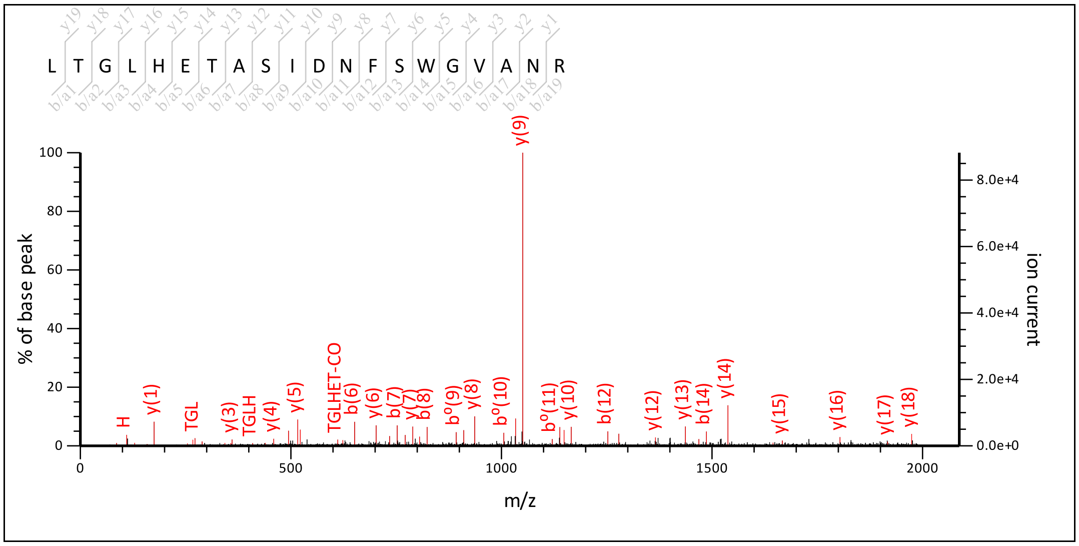


0 to 2087.21

Label all possible matches ○ Label matches used for scoring ◉

**Monoisotopic mass of neutral peptide Mr(calc):** 2187.0709
**Fixed modifications:** Carbamidomethyl (C) (apply to specified residues or termini only)
**Ions Score:** 143 **Expect:** 1.1e-10
**Matches :** 66/358 fragment ions using 80 most intense peaks (help)

| # | Immon. | a | a* | $a^0$ | b | b* | $b^0$ | d | d' | Seq. | v | w | w' | y | y* | |
|---|---|---|---|---|---|---|---|---|---|---|---|---|---|---|---|---|
| 1 | 86.0964 | **86.0964** | | | 114.0913 | | | 44.0495 | | L | | | | | | |
| 2 | 74.0600 | 187.1441 | | 169.1335 | 215.1390 | | 197.1285 | 171.1492 | 173.1285 | T | 2028.9522 | 2041.9726 | 2043.9519 | 2074.9941 | 2057.9675 | [illegible] |
| 3 | 30.0338 | 244.1656 | | 226.1550 | **272.1605** | | 254.1499 | | | G | | | | **1973.9464** | 1956.9199 | [illegible] |
| 4 | 86.0964 | **357.2496** | | 339.2391 | 385.2445 | | 367.2340 | 315.2027 | | L | 1858.8467 | 1857.8515 | | **1916.9250** | 1899.8984 | [illegible] |
| 5 | 110.0713 | **494.3085** | | 476.2980 | **522.3035** | | 504.2929 | | | H | 1721.7878 | | | **1803.8409** | 1786.8143 | [illegible] |
| 6 | 102.0550 | **623.3511** | | 605.3406 | **651.3461** | | 633.3355 | 565.3457 | | E | 1592.7452 | 1591.7499 | | **1666.7820** | 1649.7554 | [illegible] |
| 7 | 74.0600 | 724.3988 | | 706.3883 | **752.3937** | | 734.3832 | 708.4039 | 710.3832 | T | 1491.6975 | 1504.7179 | 1506.6972 | **1537.7394** | 1520.7128 | [illegible] |
| 8 | 44.0495 | 795.4359 | | 777.4254 | **823.4308** | | 805.4203 | | | A | 1420.6604 | | | **1436.6917** | 1419.6652 | [illegible] |
| 9 | 60.0444 | 882.4680 | | 864.4574 | **910.4629** | | 892.4523 | 866.4730 | | S | 1333.6284 | 1332.6331 | | **1365.6546** | 1348.6280 | [illegible] |
| 10 | 86.0964 | 995.5520 | | 977.5415 | 1023.5469 | | 1005.5364 | 967.5207 | 981.5364 | I | 1220.5443 | 1233.5647 | 1247.5804 | **1278.6226** | 1261.5960 | [illegible] |
| 11 | 88.0393 | 1110.5790 | | 1092.5684 | **1138.5739** | | 1120.5633 | 1066.5891 | | D | 1105.5174 | 1104.5221 | | **1165.5385** | 1148.5119 | [illegible] |
| 12 | 87.0553 | 1224.6219 | 1207.5953 | 1206.6113 | **1252.6168** | 1235.5903 | 1234.6062 | 1181.6161 | | N | 991.4744 | 990.4792 | | **1050.5116** | 1033.4850 | [illegible] |
| 13 | 120.0808 | 1371.6903 | 1354.6638 | 1353.6797 | 1399.6852 | 1382.6587 | 1381.6747 | | | F | 844.4060 | | | **936.4686** | 919.4421 | [illegible] |
| 14 | 60.0444 | 1458.7223 | 1441.6958 | 1440.7118 | **1486.7172** | 1469.6907 | 1468.7067 | 1442.7274 | | S | 757.3740 | 756.3787 | | **789.4002** | 772.3737 | [illegible] |
| 15 | 159.0917 | **1644.8016** | 1627.7751 | 1626.7911 | 1672.7966 | 1655.7700 | 1654.7860 | | | W | 571.2947 | | | **702.3682** | 685.3416 | |
| 16 | 30.0338 | 1701.8231 | 1684.7966 | 1683.8125 | 1729.8180 | 1712.7915 | 1711.8075 | | | G | | | | **516.2889** | 499.2623 | |
| 17 | 72.0808 | 1800.8915 | 1783.8650 | 1782.8810 | 1828.8864 | 1811.8599 | 1810.8759 | 1786.8759 | | V | 415.2048 | 428.2252 | | **459.2674** | 442.2409 | |
| 18 | 44.0495 | 1871.9286 | 1854.9021 | 1853.9181 | 1899.9236 | 1882.8970 | 1881.9130 | | | A | 344.1677 | | | **360.1990** | 343.1724 | |
| 19 | 87.0553 | 1985.9716 | 1968.9450 | 1967.9610 | 2013.9665 | 1996.9399 | 1995.9559 | 1942.9658 | | N | 230.1248 | 229.1295 | | **289.1619** | 272.1353 | |
| 20 | 129.1135 | | | | | | | | | R | 74.0237 | 73.0284 | | **175.1190** | 158.0924 | |

| Seq | ya | yb | Seq | ya | yb | Seq | ya | yb |
|---|---|---|---|---|---|---|---|---|
| TG | 131.0815 | 159.0764 | TGL | 244.1656 | 272.1605 | TGLH | 381.2245 | 409.2194 |

| | | | | | | | | |
|---|---|---|---|---|---|---|---|---|
| **TGLHE** | 510.2671 | 538.2620 | **TGLHET** | 611.3148 | 639.3097 | **TGLHETA** | 682.3519 | 710.3468 |
| **GL** | 143.1179 | 171.1128 | **GLH** | 280.1768 | 308.1717 | **GLHE** | 409.2194 | 437.2143 |
| **GLHET** | 510.2671 | 538.2620 | **GLHETA** | 581.3042 | 609.2991 | **GLHETAS** | 668.3362 | 696.3311 |
| **LH** | 223.1553 | 251.1503 | **LHE** | 352.1979 | 380.1928 | **LHET** | 453.2456 | 481.2405 |
| **LHETA** | 524.2827 | 552.2776 | **LHETAS** | 611.3148 | 639.3097 | **HE** | 239.1139 | 267.1088 |
| **HET** | 340.1615 | 368.1565 | **HETA** | 411.1987 | 439.1936 | **HETAS** | 498.2307 | 526.2256 |
| **HETASI** | 611.3148 | 639.3097 | **ET** | 203.1026 | 231.0975 | **ETA** | 274.1397 | 302.1347 |
| **ETAS** | 361.1718 | 389.1667 | **ETASI** | 474.2558 | 502.2508 | **ETASID** | 589.2828 | 617.2777 |
| **TA** | 145.0972 | 173.0921 | **TAS** | 232.1292 | 260.1241 | **TASI** | 345.2132 | 373.2082 |
| **TASID** | 460.2402 | 488.2351 | **TASIDN** | 574.2831 | 602.2780 | **AS** | 131.0815 | 159.0764 |
| **ASI** | 244.1656 | 272.1605 | **ASID** | 359.1925 | 387.1874 | **ASIDN** | 473.2354 | 501.2304 |
| **ASIDNF** | 620.3039 | 648.2988 | **SI** | 173.1285 | 201.1234 | **SID** | 288.1554 | 316.1503 |
| **SIDN** | 402.1983 | 430.1932 | **SIDNF** | 549.2667 | 577.2617 | **SIDNFS** | 636.2988 | 664.2937 |
| **ID** | 201.1234 | 229.1183 | **IDN** | 315.1663 | 343.1612 | **IDNF** | 462.2347 | 490.2296 |
| **IDNFS** | 549.2667 | 577.2617 | **DN** | 202.0822 | 230.0771 | **DNF** | 349.1506 | 377.1456 |
| **DNFS** | 436.1827 | 464.1776 | **DNFSW** | 622.2620 | 650.2569 | **DNFSWG** | 679.2835 | 707.2784 |
| **NF** | 234.1237 | 262.1186 | **NFS** | 321.1557 | 349.1506 | **NFSW** | 507.2350 | 535.2300 |
| **NFSWG** | 564.2565 | 592.2514 | **NFSWGV** | 663.3249 | 691.3198 | **FS** | 207.1128 | 235.1077 |
| **FSW** | 393.1921 | 421.1870 | **FSWG** | 450.2136 | 478.2085 | **FSWGV** | 549.2820 | 577.2769 |
| **FSWGVA** | 620.3191 | 648.3140 | **SW** | 246.1237 | 274.1186 | **SWG** | 303.1452 | 331.1401 |
| **SWGV** | 402.2136 | 430.2085 | **SWGVA** | 473.2507 | 501.2456 | **SWGVAN** | 587.2936 | 615.2885 |
| **WG** | 216.1131 | 244.1081 | **WGV** | 315.1816 | 343.1765 | **WGVA** | 386.2187 | 414.2136 |
| **WGVAN** | 500.2616 | 528.2565 | **GV** | 129.1022 | 157.0972 | **GVA** | 200.1394 | 228.1343 |
| **GVAN** | 314.1823 | 342.1772 | **VA** | 143.1179 | 171.1128 | **VAN** | 257.1608 | 285.1557 |
| **AN** | 158.0924 | 186.0873 | | | | | | |

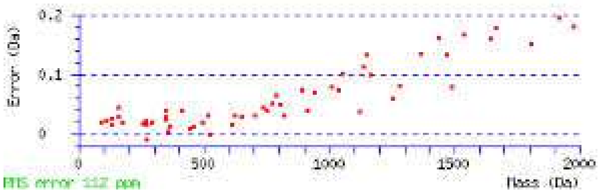


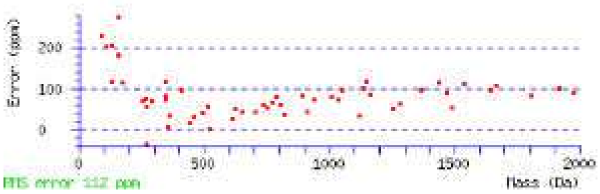


NCBI **BLAST** search of LTGLHETASIDNFSWGVANR
(Parameters: blastp, nr protein database, expect=20000, no filter, PAM30)
Other BLAST web gateways

**All matches to this query**

| Score | Mr(calc) | Delta | Sequence |
|---|---|---|---|
| 143.3 | 2187.0709 | 0.0646 | LTGLHETASIDNFSWGVANR |
| 28.0 | 2187.1775 | -0.0420 | LTHWIKVFLFNVAQMNAR |
| 20.8 | 2187.1648 | -0.0292 | ITGSKIHSIEVSFNVSNISR |
| 20.1 | 2187.1939 | -0.0584 | TIGIIIPDISNQFYPEIVR |
| 20.1 | 2187.1939 | -0.0584 | TIGILLPDISNQFYPEIVR |
| 19.9 | 2187.1067 | 0.0288 | LDPIAKNAMPLMELYESPR |
| 19.8 | 2187.1436 | -0.0081 | LVGNRPDAAAVEITFGPASFR |
| 19.7 | 2187.1647 | -0.0292 | KAQLIIDQIDLGGANNVYSR |
| 19.3 | 2187.2627 | -0.1271 | LPAVLEAESLPPRLVSVIER |
| 18.7 | 2187.2627 | -0.1271 | LPAVLEAESLPPRLVSVLER |

**Mascot:** http://www.matrixscience.com/

# MATRIX SCIENCE Mascot Search Results

## Peptide View Spot no 69

MS/MS Fragmentation of **VVSQVPWFGIEQEYTLLQR**
Found in **gi|19387272** in **NCBInr**, putative precursor chloroplastic glutamine synthetase [Oryza sativa Japonica Group]

Match to Query 199: 2291.265224 from(2292.272500,1+) intensity(0.0000) index(24)
Title: Label: B19, Spot_Id: 219963, Peak_List_Id: 229163, MSMS Job_Run_Id: 22013, Comment:
Data file C:\Documents and Settings\Administrator\Desktop\Farah-Garima 11May 2013\ppw_B19_136868333300.txt

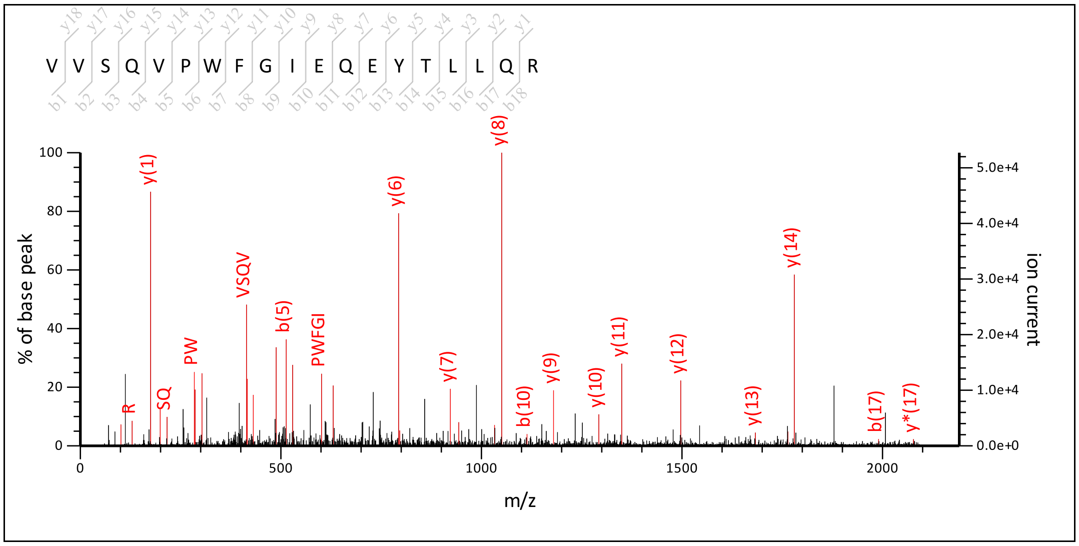


0 to 2191.39

Label all possible matches  Label matches used for scoring

**Monoisotopic mass of neutral peptide Mr(calc):** 2291.1950
**Fixed modifications:** Carbamidomethyl (C) (apply to specified residues or termini only)
**Ions Score:** 84 **Expect:** 8.7e-05
**Matches :** 40/338 fragment ions using 63 most intense peaks (help)

| # | Immon. | a | a* | $a^0$ | b | b* | $b^0$ | d | d' | Seq. | v | w | w' | y | y* |
|---|---|---|---|---|---|---|---|---|---|---|---|---|---|---|---|
| 1 | 72.0808 | 72.0808 | | | 100.0757 | | | 44.0495 | | V | | | | | |
| 2 | 72.0808 | 171.1492 | | | **199.1441** | | | 157.1335 | | V | 2149.0713 | 2162.0917 | | 2193.1339 | 2176.1073 |
| 3 | 60.0444 | 258.1812 | | 240.1707 | **286.1761** | | 268.1656 | 242.1863 | | S | 2062.0393 | 2061.0440 | | 2094.0655 | 2077.0389 |
| 4 | 101.0709 | 386.2398 | 369.2132 | 368.2292 | **414.2347** | 397.2082 | 396.2241 | 329.2183 | | Q | 1933.9807 | 1932.9854 | | 2007.0334 | 1990.0069 |
| 5 | 72.0808 | 485.3082 | 468.2817 | 467.2976 | **513.3031** | 496.2766 | 495.2926 | 471.2926 | | V | 1834.9123 | 1847.9327 | | 1878.9749 | 1861.9483 |
| 6 | 70.0651 | 582.3610 | 565.3344 | 564.3504 | 610.3559 | 593.3293 | 592.3453 | 556.3453 | | P | 1737.8595 | 1736.8643 | | **1779.9064** | 1762.8799 |
| 7 | 159.0917 | 768.4403 | 751.4137 | 750.4297 | **796.4352** | 779.4087 | 778.4246 | | | W | 1551.7802 | | | **1682.8537** | 1665.8271 |
| 8 | 120.0808 | 915.5087 | 898.4822 | 897.4981 | **943.5036** | 926.4771 | 925.4930 | | | F | 1404.7118 | | | **1496.7744** | 1479.7478 |
| 9 | 30.0338 | 972.5302 | 955.5036 | 954.5196 | 1000.5251 | 983.4985 | 982.5145 | | | G | | | | **1349.7060** | 1332.6794 |
| 10 | 86.0964 | 1085.6142 | 1068.5877 | 1067.6037 | **1113.6091** | 1096.5826 | 1095.5986 | 1057.5829 | 1071.5986 | I | 1234.6062 | 1247.6266 | 1261.6423 | **1292.6845** | 1275.6579 |
| 11 | 102.0550 | 1214.6568 | 1197.6303 | 1196.6463 | 1242.6517 | 1225.6252 | 1224.6412 | 1156.6513 | | E | 1105.5637 | 1104.5684 | | **1179.6004** | 1162.5739 |
| 12 | 101.0709 | 1342.7154 | 1325.6889 | 1324.7048 | 1370.7103 | 1353.6838 | 1352.6997 | 1285.6939 | | Q | 977.5051 | 976.5098 | | **1050.5578** | 1033.5313 |
| 13 | 102.0550 | 1471.7580 | 1454.7314 | 1453.7474 | 1499.7529 | 1482.7264 | 1481.7423 | 1413.7525 | | E | 848.4625 | 847.4672 | | **922.4993** | 905.4727 |
| 14 | 136.0757 | 1634.8213 | 1617.7948 | 1616.8108 | 1662.8162 | 1645.7897 | 1644.8057 | | | Y | 685.3992 | | | **793.4567** | 776.4301 |
| 15 | 74.0600 | 1735.8690 | 1718.8425 | 1717.8584 | **1763.8639** | 1746.8374 | 1745.8534 | 1719.8741 | 1721.8534 | T | 584.3515 | 597.3719 | 599.3511 | **630.3933** | 613.3668 |
| 16 | 86.0964 | 1848.9531 | 1831.9265 | 1830.9425 | 1876.9480 | 1859.9214 | 1858.9374 | 1806.9061 | | L | 471.2674 | 470.2722 | | **529.3457** | 512.3191 |
| 17 | 86.0964 | 1962.0371 | 1945.0106 | 1944.0266 | **1990.0320** | 1973.0055 | 1972.0215 | 1919.9902 | | L | 358.1833 | 357.1881 | | **416.2616** | 399.2350 |
| 18 | 101.0709 | 2090.0957 | 2073.0692 | 2072.0851 | 2118.0906 | 2101.0641 | 2100.0801 | 2033.0742 | | Q | 230.1248 | 229.1295 | | **303.1775** | 286.1510 |
| 19 | 129.1135 | | | | | | | | | R | 74.0237 | 73.0284 | | **175.1190** | 158.0924 |

| Seq | ya | yb | Seq | ya | yb | Seq | ya | yb |
|---|---|---|---|---|---|---|---|---|
| VS | 159.1128 | 187.1077 | VSQ | 287.1714 | 315.1663 | VSQV | 386.2398 | 414.2347 |
| VSQVP | 483.2926 | 511.2875 | VSQVPW | 669.3719 | 697.3668 | SQ | 188.1030 | 216.0979 |

| | | | | | | | | |
|---|---|---|---|---|---|---|---|---|
| **SQV** | 287.1714 | 315.1663 | **SQVP** | 384.2241 | 412.2191 | **SQVPW** | 570.3035 | 598.2984 |
| **QV** | 200.1394 | 228.1343 | **QVP** | 297.1921 | 325.1870 | **QVPW** | 483.2714 | 511.2663 |
| **QVPWF** | 630.3398 | 658.3348 | **QVPWFG** | 687.3613 | 715.3562 | **VP** | 169.1335 | 197.1285 |
| **VPW** | 355.2129 | 383.2078 | **VPWF** | 502.2813 | 530.2762 | **VPWFG** | 559.3027 | 587.2976 |
| **VPWFGI** | 672.3868 | 700.3817 | **PW** | 256.1444 | 284.1394 | **PWF** | 403.2129 | 431.2078 |
| **PWFG** | 460.2343 | 488.2292 | **PWFGI** | 573.3184 | 601.3133 | **WF** | 306.1601 | 334.1550 |
| **WFG** | 363.1816 | 391.1765 | **WFGI** | 476.2656 | 504.2605 | **WFGIE** | 605.3082 | 633.3031 |
| **FG** | 177.1022 | 205.0972 | **FGI** | 290.1863 | 318.1812 | **FGIE** | 419.2289 | 447.2238 |
| **FGIEQ** | 547.2875 | 575.2824 | **FGIEQE** | 676.3301 | 704.3250 | **GI** | 143.1179 | 171.1128 |
| **GIE** | 272.1605 | 300.1554 | **GIEQ** | 400.2191 | 428.2140 | **GIEQE** | 529.2617 | 557.2566 |
| **GIEQEY** | 692.3250 | 720.3199 | **IE** | 215.1390 | 243.1339 | **IEQ** | 343.1976 | 371.1925 |
| **IEQE** | 472.2402 | 500.2351 | **IEQEY** | 635.3035 | 663.2984 | **EQ** | 230.1135 | 258.1084 |
| **EQE** | 359.1561 | 387.1510 | **EQEY** | 522.2195 | 550.2144 | **EQEYT** | 623.2671 | 651.2620 |
| **QE** | 230.1135 | 258.1084 | **QEY** | 393.1769 | 421.1718 | **QEYT** | 494.2245 | 522.2195 |
| **QEYTL** | 607.3086 | 635.3035 | **EY** | 265.1183 | 293.1132 | **EYT** | 366.1660 | 394.1609 |
| **EYTL** | 479.2500 | 507.2449 | **EYTLL** | 592.3341 | 620.3290 | **YT** | 237.1234 | 265.1183 |
| **YTL** | 350.2074 | 378.2023 | **YTLL** | 463.2915 | 491.2864 | **YTLLQ** | 591.3501 | 619.3450 |
| **TL** | 187.1441 | 215.1390 | **TLL** | 300.2282 | 328.2231 | **TLLQ** | 428.2867 | 456.2817 |
| **LL** | 199.1805 | 227.1754 | **LLQ** | 327.2391 | 355.2340 | **LQ** | 214.1550 | 242.1499 |

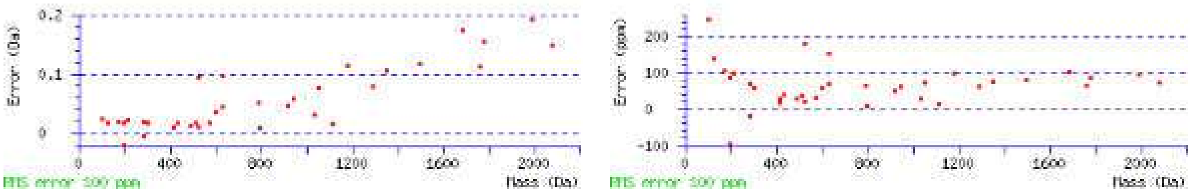


NCBI **BLAST** search of VVSQVPWFGIEQEYTLLQR
(Parameters: blastp, nr protein database, expect=20000, no filter, PAM30)
Other BLAST web gateways

**All matches to this query**

| Score | Mr(calc) | Delta | Sequence |
|---|---|---|---|
| 83.8 | 2291.1950 | 0.0702 | VVSQVPWFGIEQEYTLLQR |
| 26.9 | 2291.2750 | -0.0098 | TPLVLNGTFGAPKGGLQAGPLAGR |
| 25.3 | 2291.1402 | 0.1250 | GWVVAVLSMVMAASAVAQENSR |
| 21.5 | 2291.2386 | 0.0266 | ERLPNYGLAVEVLHLSQTPR |
| 18.4 | 2291.1508 | 0.1145 | QFMELEKGYTVLNTIFNTK |
| 17.9 | 2291.2712 | -0.0059 | VAIESPVPVVMVKPEGFVAPAR |
| 16.5 | 2291.4821 | -0.2169 | IRVATVIGALLVVAIVAFVLVR |
| 15.5 | 2291.3187 | -0.0535 | INLRLLMYGVAATIIFAIAGR |
| 15.2 | 2291.1944 | 0.0709 | IVAMDDPGRALPPGEVGEIAIR |
| 14.8 | 2291.2273 | 0.0379 | LLQSEAQLTEYIRSFPIGAR |

**Mascot:** http://www.matrixscience.com/

# MATRIX SCIENCE Mascot Search Results

## Peptide View **Spot no 69**

MS/MS Fragmentation of **RLTGLHETASIDNFSWGVANR**
Found in **gi|19387272** in **NCBInr**, putative precursor chloroplastic glutamine synthetase [Oryza sativa Japonica Group]

Match to Query 220: 2343.231724 from(2344.239000,1+) intensity(0.0000) index(27)
Title: Label: B19, Spot_Id: 219963, Peak_List_Id: 229166, MSMS Job_Run_Id: 22013, Comment:
Data file C:\Documents and Settings\Administrator\Desktop\Farah-Garima 11May 2013\ppw_B19_136868333300.txt

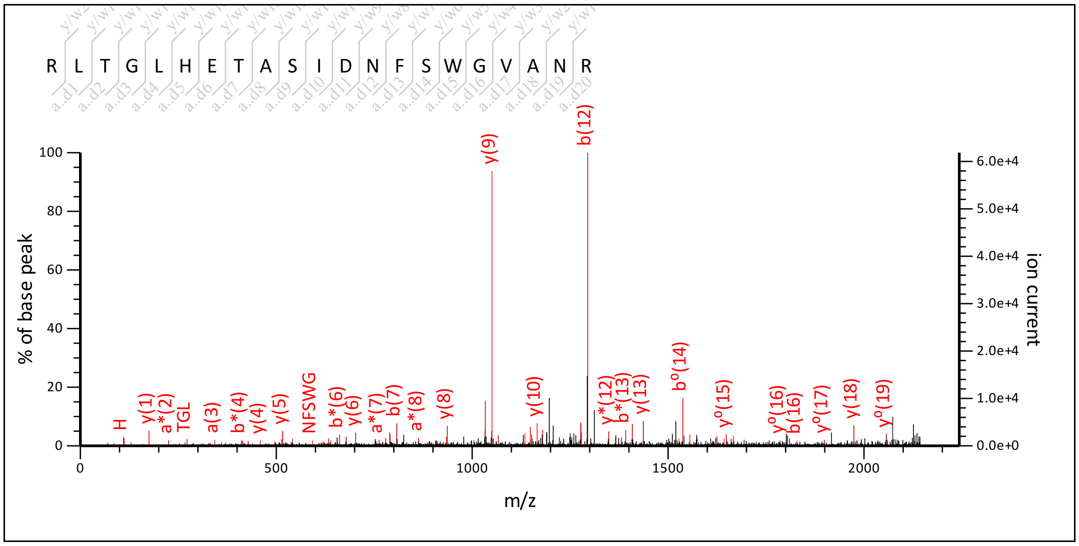


0 to 2243.19

Label all possible matches ○ Label matches used for scoring ◉

**Monoisotopic mass of neutral peptide Mr(calc):** 2343.1720
**Fixed modifications:** Carbamidomethyl (C) (apply to specified residues or termini only)
**Ions Score:** 88 **Expect:** 3.1e-05
**Matches :** 106/401 fragment ions using 147 most intense peaks (help)

| # | Immon. | a | a* | $a^0$ | b | b* | $b^0$ | d | d' | Seq. | v | w | w' | y | y* |
|---|---|---|---|---|---|---|---|---|---|---|---|---|---|---|---|
| 1 | 129.1135 | **129.1135** | 112.0869 | | 157.1084 | 140.0818 | | 44.0495 | | R | | | | | |
| 2 | 86.0964 | **242.1975** | 225.1710 | | 270.1925 | 253.1659 | | 200.1506 | | L | 2129.9999 | 2129.0047 | | 2188.0782 | 2171.0516 |
| 3 | 74.0600 | **343.2452** | 326.2187 | 325.2347 | **371.2401** | 354.2136 | 353.2296 | 327.2503 | 329.2296 | T | 2028.9522 | 2041.9726 | 2043.9519 | 2074.9941 | 2057.9675 |
| 4 | 30.0338 | 400.2667 | 383.2401 | 382.2561 | **428.2616** | 411.2350 | 410.2510 | | | G | | | | ***1973.9464*** | 1956.9199 |
| 5 | 86.0964 | **513.3507** | 496.3242 | 495.3402 | **541.3457** | 524.3191 | 523.3351 | 471.3038 | | L | 1858.8467 | 1857.8515 | | 1916.9250 | 1899.8984 |
| 6 | 110.0713 | 650.4097 | 633.3831 | 632.3991 | **678.4046** | 661.3780 | 660.3940 | | | H | 1721.7878 | | | 1803.8409 | 1786.8143 |
| 7 | 102.0550 | **779.4522** | 762.4257 | 761.4417 | **807.4472** | 790.4206 | 789.4366 | 721.4468 | | E | 1592.7452 | 1591.7499 | | ***1666.7820*** | 1649.7554 |
| 8 | 74.0600 | 880.4999 | 863.4734 | 862.4894 | **908.4948** | 891.4683 | 890.4843 | 864.5050 | 866.4843 | T | 1491.6975 | 1504.7179 | 1506.6972 | ***1537.7394*** | 1520.7128 |
| 9 | 44.0495 | 951.5370 | 934.5105 | 933.5265 | 979.5320 | 962.5054 | 961.5214 | | | A | 1420.6604 | | | ***1436.6917*** | 1419.6652 |
| 10 | 60.0444 | 1038.5691 | 1021.5425 | 1020.5585 | **1066.5640** | 1049.5374 | 1048.5534 | 1022.5742 | | S | 1333.6284 | 1332.6331 | | ***1365.6546*** | 1348.6280 |
| 11 | 86.0964 | **1151.6531** | 1134.6266 | 1133.6426 | **1179.6480** | 1162.6215 | 1161.6375 | 1123.6218 | 1137.6375 | I | 1220.5443 | 1233.5647 | 1247.5804 | ***1278.6226*** | 1261.5960 |
| 12 | 88.0393 | 1266.6801 | 1249.6535 | 1248.6695 | **1294.6750** | 1277.6484 | 1276.6644 | 1222.6902 | | D | 1105.5174 | 1104.5221 | | ***1165.5385*** | 1148.5119 |
| 13 | 87.0553 | **1380.7230** | 1363.6965 | 1362.7124 | **1408.7179** | 1391.6914 | 1390.7074 | 1337.7172 | | N | 991.4744 | 990.4792 | | ***1050.5116*** | 1033.4850 |
| 14 | 120.0808 | **1527.7914** | 1510.7649 | 1509.7809 | **1555.7863** | 1538.7598 | 1537.7758 | | | F | 844.4060 | | | ***936.4686*** | 919.4421 |
| 15 | 60.0444 | 1614.8234 | 1597.7969 | 1596.8129 | **1642.8184** | 1625.7918 | 1624.8078 | 1598.8285 | | S | 757.3740 | 756.3787 | | ***789.4002*** | 772.3737 |
| 16 | 159.0917 | 1800.9028 | 1783.8762 | 1782.8922 | **1828.8977** | 1811.8711 | 1810.8871 | | | W | 571.2947 | | | ***702.3682*** | 685.3416 |
| 17 | 30.0338 | 1857.9242 | 1840.8977 | 1839.9137 | 1885.9191 | 1868.8926 | 1867.9086 | | | G | | | | ***516.2889*** | 499.2623 |
| 18 | 72.0808 | 1956.9926 | 1939.9661 | 1938.9821 | 1984.9876 | 1967.9610 | 1966.9770 | 1942.9770 | | V | 415.2048 | 428.2252 | | ***459.2674*** | 442.2409 |
| 19 | 44.0495 | 2028.0298 | 2011.0032 | 2010.0192 | 2056.0247 | 2038.9981 | 2038.0141 | | | A | 344.1677 | | | ***360.1990*** | 343.1724 |
| 20 | 87.0553 | 2142.0727 | 2125.0461 | 2124.0621 | 2170.0676 | 2153.0410 | 2152.0570 | 2099.0669 | | N | 230.1248 | 229.1295 | | ***289.1619*** | 272.1353 |
| 21 | 129.1135 | | | | | | | | | R | 74.0237 | 73.0284 | | ***175.1190*** | 158.0924 |

| Seq | ya | yb | Seq | ya | yb | Seq | ya | yb |
|---|---|---|---|---|---|---|---|---|

| | | | | | | | | |
|---|---|---|---|---|---|---|---|---|
| **LT** | 187.1441 | 215.1390 | **LTG** | 244.1656 | 272.1605 | **LTGL** | 357.2496 | 385.2445 |
| **LTGLH** | 494.3085 | 522.3035 | **LTGLHE** | 623.3511 | 651.3461 | **TG** | 131.0815 | 159.0764 |
| **TGL** | 244.1656 | 272.1605 | **TGLH** | 381.2245 | 409.2194 | **TGLHE** | 510.2671 | 538.2620 |
| **TGLHET** | 611.3148 | 639.3097 | **TGLHETA** | 682.3519 | 710.3468 | **GL** | 143.1179 | 171.1128 |
| **GLH** | 280.1768 | 308.1717 | **GLHE** | 409.2194 | 437.2143 | **GLHET** | 510.2671 | 538.2620 |
| **GLHETA** | 581.3042 | 609.2991 | **GLHETAS** | 668.3362 | 696.3311 | **LH** | 223.1553 | 251.1503 |
| **LHE** | 352.1979 | 380.1928 | **LHET** | 453.2456 | 481.2405 | **LHETA** | 524.2827 | 552.2776 |
| **LHETAS** | 611.3148 | 639.3097 | **HE** | 239.1139 | 267.1088 | **HET** | 340.1615 | 368.1565 |
| **HETA** | 411.1987 | 439.1936 | **HETAS** | 498.2307 | 526.2256 | **HETASI** | 611.3148 | 639.3097 |
| **ET** | 203.1026 | 231.0975 | **ETA** | 274.1397 | 302.1347 | **ETAS** | 361.1718 | 389.1667 |
| **ETASI** | 474.2558 | 502.2508 | **ETASID** | 589.2828 | 617.2777 | **TA** | 145.0972 | 173.0921 |
| **TAS** | 232.1292 | 260.1241 | **TASI** | 345.2132 | 373.2082 | **TASID** | 460.2402 | 488.2351 |
| **TASIDN** | 574.2831 | 602.2780 | **AS** | 131.0815 | 159.0764 | **ASI** | 244.1656 | 272.1605 |
| **ASID** | 359.1925 | 387.1874 | **ASIDN** | 473.2354 | 501.2304 | **ASIDNF** | 620.3039 | 648.2988 |
| **SI** | 173.1285 | 201.1234 | **SID** | 288.1554 | 316.1503 | **SIDN** | 402.1983 | 430.1932 |
| **SIDNF** | 549.2667 | 577.2617 | **SIDNFS** | 636.2988 | 664.2937 | **ID** | 201.1234 | 229.1183 |
| **IDN** | 315.1663 | 343.1612 | **IDNF** | 462.2347 | 490.2296 | **IDNFS** | 549.2667 | 577.2617 |
| **DN** | 202.0822 | 230.0771 | **DNF** | 349.1506 | 377.1456 | **DNFS** | 436.1827 | 464.1776 |
| **DNFSW** | 622.2620 | 650.2569 | **DNFSWG** | 679.2835 | 707.2784 | **NF** | 234.1237 | 262.1186 |
| **NFS** | 321.1557 | 349.1506 | **NFSW** | 507.2350 | 535.2300 | **NFSWG** | 564.2565 | 592.2514 |
| **NFSWGV** | 663.3249 | 691.3198 | **FS** | 207.1128 | 235.1077 | **FSW** | 393.1921 | 421.1870 |
| **FSWG** | 450.2136 | 478.2085 | **FSWGV** | 549.2820 | 577.2769 | **FSWGVA** | 620.3191 | 648.3140 |
| **SW** | 246.1237 | 274.1186 | **SWG** | 303.1452 | 331.1401 | **SWGV** | 402.2136 | 430.2085 |
| **SWGVA** | 473.2507 | 501.2456 | **SWGVAN** | 587.2936 | 615.2885 | **WG** | 216.1131 | 244.1081 |
| **WGV** | 315.1816 | 343.1765 | **WGVA** | 386.2187 | 414.2136 | **WGVAN** | 500.2616 | 528.2565 |
| **GV** | 129.1022 | 157.0972 | **GVA** | 200.1394 | 228.1343 | **GVAN** | 314.1823 | 342.1772 |
| **VA** | 143.1179 | 171.1128 | **VAN** | 257.1608 | 285.1557 | **AN** | 158.0924 | 186.0873 |

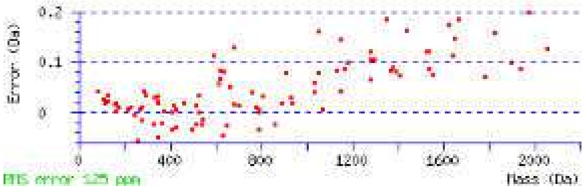


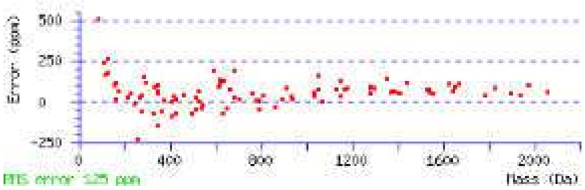


NCBI **BLAST** search of RLTGLHETASIDNFSWGVANR
(Parameters: blastp, nr protein database, expect=20000, no filter, PAM30)
Other BLAST web gateways

**All matches to this query**

| Score | Mr(calc) | Delta | Sequence |
|---|---|---|---|
| 88.5 | 2343.1720 | 0.0597 | RLTGLHETASIDNFSWGVANR |
| 21.9 | 2343.2659 | -0.0341 | SNLIASHTEPEAGIVAPLNVRR |
| 21.3 | 2343.0875 | 0.1442 | MVYDAATGQPITGSLMDYAAPR |
| 18.3 | 2343.2910 | -0.0593 | IGLSATQKPIDAVSRFLVGTDR |
| 18.3 | 2343.1648 | 0.0669 | AEAAKFDAVFFADGPALPDHVR |
| 16.9 | 2343.3275 | -0.0957 | DPGVVPEIGSVVTVQVLRINPR |
| 16.2 | 2343.3242 | -0.0925 | KFTSAFIILGSIIYYEPILR |
| 15.5 | 2343.2910 | -0.0593 | SAIDSLPPLREVIDTHQLLAR |
| 15.5 | 2343.2448 | -0.0130 | TLRAHFVRPTSPSSDFGLSIR |
| 14.8 | 2343.2508 | -0.0191 | ADIIVLAMKPQDVFAALDELR |

**Mascot:** http://www.matrixscience.com/

# MATRIX SCIENCE Mascot Search Results

## Peptide View Spot no 69

MS/MS Fragmentation of **AILNLSLRHDLHISAYGEGNER**
Found in **gi|19387272** in **NCBInr**, putative precursor chloroplastic glutamine synthetase [Oryza sativa Japonica Group]

Match to Query 229: 2477.348724 from(2478.356000,1+) intensity(0.0000) index(29)
Title: Label: B19, Spot_Id: 219963, Peak_List_Id: 229180, MSMS Job_Run_Id: 22013, Comment:
Data file C:\Documents and Settings\Administrator\Desktop\Farah-Garima 11May 2013\ppw_B19_136868333300.txt

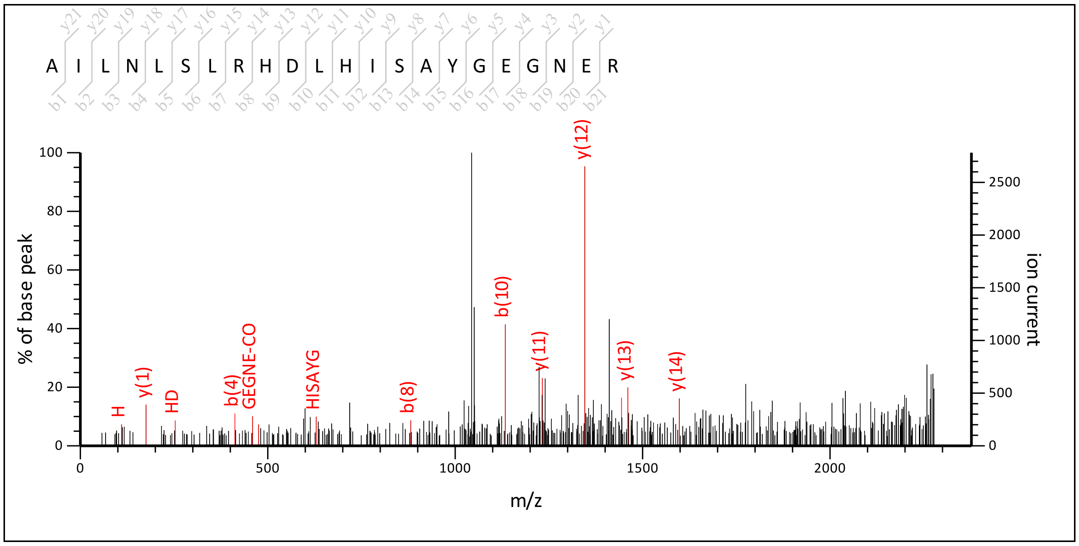


0 to 2377.19

Label all possible matches ○ Label matches used for scoring ◉

**Monoisotopic mass of neutral peptide Mr(calc):** 2477.2775
**Fixed modifications:** Carbamidomethyl (C) (apply to specified residues or termini only)
**Ions Score:** 6 **Expect:** 4.4e+03
**Matches :** 16/414 fragment ions using 46 most intense peaks (help)

| # | Immon. | a | a* | $a^0$ | b | b* | $b^0$ | d | d' | Seq. | v | w | w' | y | y* | |
|---|---|---|---|---|---|---|---|---|---|---|---|---|---|---|---|---|
| 1 | 44.0495 | 44.0495 | | | 72.0444 | | | 44.0495 | | A | | | | | | |
| 2 | 86.0964 | 157.1335 | | | 185.1285 | | | 129.1022 | 143.1179 | I | 2349.1694 | 2362.1898 | 2376.2055 | 2407.2477 | 2390.2211 | [illegible] |
| 3 | 86.0964 | 270.2176 | | | 298.2125 | | | 228.1707 | | L | 2236.0854 | 2235.0901 | | 2294.1636 | 2277.1371 | [illegible] |
| 4 | 87.0553 | 384.2605 | 367.2340 | | 412.2554 | 395.2289 | | 341.2547 | | N | 2122.0424 | 2121.0472 | | 2181.0796 | 2164.0530 | [illegible] |
| 5 | 86.0964 | 497.3446 | 480.3180 | | 525.3395 | 508.3130 | | 455.2976 | | L | 2008.9584 | 2007.9631 | | 2067.0366 | 2050.0101 | [illegible] |
| 6 | 60.0444 | 584.3766 | 567.3501 | 566.3661 | 612.3715 | 595.3450 | 594.3610 | 568.3817 | | S | 1921.9263 | 1920.9311 | | 1953.9526 | 1936.9260 | [illegible] |
| 7 | 86.0964 | 697.4607 | 680.4341 | 679.4501 | 725.4556 | 708.4291 | 707.4450 | 655.4137 | | L | 1808.8423 | 1807.8470 | | 1866.9205 | 1849.8940 | [illegible] |
| 8 | 129.1135 | 853.5618 | 836.5352 | 835.5512 | 881.5567 | 864.5302 | 863.5461 | 768.4978 | | R | 1652.7412 | 1651.7459 | | 1753.8365 | 1736.8099 | [illegible] |
| 9 | 110.0713 | 990.6207 | 973.5942 | 972.6101 | 1018.6156 | 1001.5891 | 1000.6051 | | | H | 1515.6823 | | | ***1597.7354*** | 1580.7088 | [illegible] |
| 10 | 88.0393 | 1105.6477 | 1088.6211 | 1087.6371 | 1133.6426 | 1116.6160 | 1115.6320 | 1061.6578 | | D | 1400.6553 | 1399.6601 | | ***1460.6764*** | 1443.6499 | [illegible] |
| 11 | 86.0964 | 1218.7317 | 1201.7052 | 1200.7212 | 1246.7266 | 1229.7001 | 1228.7161 | 1176.6848 | | L | 1287.5713 | 1286.5760 | | ***1345.6495*** | 1328.6230 | [illegible] |
| 12 | 110.0713 | 1355.7906 | 1338.7641 | 1337.7801 | 1383.7855 | 1366.7590 | 1365.7750 | | | H | 1150.5123 | | | ***1232.5654*** | 1215.5389 | [illegible] |
| 13 | 86.0964 | 1468.8747 | 1451.8481 | 1450.8641 | 1496.8696 | 1479.8431 | 1478.8590 | 1440.8434 | 1454.8590 | I | 1037.4283 | 1050.4487 | 1064.4643 | 1095.5065 | 1078.4800 | [illegible] |
| 14 | 60.0444 | 1555.9067 | 1538.8802 | 1537.8962 | 1583.9016 | 1566.8751 | 1565.8911 | 1539.9118 | | S | 950.3962 | 949.4010 | | 982.4225 | 965.3959 | [illegible] |
| 15 | 44.0495 | 1626.9438 | 1609.9173 | 1608.9333 | 1654.9387 | 1637.9122 | 1636.9282 | | | A | 879.3591 | | | 895.3904 | 878.3639 | [illegible] |
| 16 | 136.0757 | 1790.0072 | 1772.9806 | 1771.9966 | 1818.0021 | 1800.9755 | 1799.9915 | | | Y | 716.2958 | | | 824.3533 | 807.3268 | [illegible] |
| 17 | 30.0338 | 1847.0286 | 1830.0021 | 1829.0181 | 1875.0235 | 1857.9970 | 1857.0130 | | | G | | | | 661.2900 | 644.2634 | [illegible] |
| 18 | 102.0550 | 1976.0712 | 1959.0447 | 1958.0607 | 2004.0661 | 1987.0396 | 1986.0556 | 1918.0657 | | E | 530.2317 | 529.2365 | | 604.2685 | 587.2420 | [illegible] |
| 19 | 30.0338 | 2033.0927 | 2016.0661 | 2015.0821 | 2061.0876 | 2044.0610 | 2043.0770 | | | G | | | | ***475.2259*** | 458.1994 | [illegible] |
| 20 | 87.0553 | 2147.1356 | 2130.1091 | 2129.1250 | 2175.1305 | 2158.1040 | 2157.1200 | 2104.1298 | | N | 359.1674 | 358.1721 | | 418.2045 | 401.1779 | [illegible] |
| 21 | 102.0550 | 2276.1782 | 2259.1517 | 2258.1676 | 2304.1731 | 2287.1466 | 2286.1626 | 2218.1727 | | E | 230.1248 | 229.1295 | | 304.1615 | 287.1350 | [illegible] |
| 22 | 129.1135 | | | | | | | | | R | 74.0237 | 73.0284 | | ***175.1190*** | 158.0924 | |

| Seq | ya | yb | Seq | ya | yb | Seq | ya | yb |
|---|---|---|---|---|---|---|---|---|
| IL | 199.1805 | 227.1754 | ILN | 313.2234 | 341.2183 | ILNL | 426.3075 | 454.3024 |
| ILNLS | 513.3395 | 541.3344 | ILNLSL | 626.4236 | 654.4185 | LN | 200.1394 | 228.1343 |
| LNL | 313.2234 | 341.2183 | LNLS | 400.2554 | 428.2504 | LNLSL | 513.3395 | 541.3344 |
| LNLSLR | 669.4406 | 697.4355 | NL | 200.1394 | 228.1343 | NLS | 287.1714 | 315.1663 |
| NLSL | 400.2554 | 428.2504 | NLSLR | 556.3566 | 584.3515 | NLSLRH | 693.4155 | 721.4104 |
| LS | 173.1285 | 201.1234 | LSL | 286.2125 | 314.2074 | LSLR | 442.3136 | 470.3085 |
| LSLRH | 579.3725 | 607.3675 | LSLRHD | 694.3995 | 722.3944 | SL | 173.1285 | 201.1234 |
| SLR | 329.2296 | 357.2245 | SLRH | 466.2885 | 494.2834 | SLRHD | 581.3154 | 609.3103 |
| SLRHDL | 694.3995 | 722.3944 | LR | 242.1975 | 270.1925 | LRH | 379.2564 | 407.2514 |
| LRHD | 494.2834 | 522.2783 | LRHDL | 607.3675 | 635.3624 | RH | 266.1724 | 294.1673 |
| RHD | 381.1993 | 409.1942 | RHDL | 494.2834 | 522.2783 | RHDLH | 631.3423 | 659.3372 |
| HD | 225.0982 | 253.0931 | HDL | 338.1823 | 366.1772 | HDLH | 475.2412 | 503.2361 |
| HDLHI | 588.3253 | 616.3202 | HDLHIS | 675.3573 | 703.3522 | DL | 201.1234 | 229.1183 |
| DLH | 338.1823 | 366.1772 | DLHI | 451.2663 | 479.2613 | DLHIS | 538.2984 | 566.2933 |
| DLHISA | 609.3355 | 637.3304 | LH | 223.1553 | 251.1503 | LHI | 336.2394 | 364.2343 |
| LHIS | 423.2714 | 451.2663 | LHISA | 494.3085 | 522.3035 | LHISAY | 657.3719 | 685.3668 |
| HI | 223.1553 | 251.1503 | HIS | 310.1874 | 338.1823 | HISA | 381.2245 | 409.2194 |
| HISAY | 544.2878 | 572.2827 | HISAYG | 601.3093 | 629.3042 | IS | 173.1285 | 201.1234 |
| ISA | 244.1656 | 272.1605 | ISAY | 407.2289 | 435.2238 | ISAYG | 464.2504 | 492.2453 |
| ISAYGE | 593.2930 | 621.2879 | ISAYGEG | 650.3144 | 678.3093 | SA | 131.0815 | 159.0764 |
| SAY | 294.1448 | 322.1397 | SAYG | 351.1663 | 379.1612 | SAYGE | 480.2089 | 508.2038 |
| SAYGEG | 537.2304 | 565.2253 | SAYGEGN | 651.2733 | 679.2682 | AY | 207.1128 | 235.1077 |
| AYG | 264.1343 | 292.1292 | AYGE | 393.1769 | 421.1718 | AYGEG | 450.1983 | 478.1932 |
| AYGEGN | 564.2413 | 592.2362 | AYGEGNE | 693.2838 | 721.2788 | YG | 193.0972 | 221.0921 |
| YGE | 322.1397 | 350.1347 | YGEG | 379.1612 | 407.1561 | YGEGN | 493.2041 | 521.1991 |
| YGEGNE | 622.2467 | 650.2416 | GE | 159.0764 | 187.0713 | GEG | 216.0979 | 244.0928 |
| GEGN | 330.1408 | 358.1357 | GEGNE | 459.1834 | 487.1783 | EG | 159.0764 | 187.0713 |
| EGN | 273.1193 | 301.1143 | EGNE | 402.1619 | 430.1569 | GN | 144.0768 | 172.0717 |
| GNE | 273.1193 | 301.1143 | NE | 216.0979 | 244.0928 | | | |

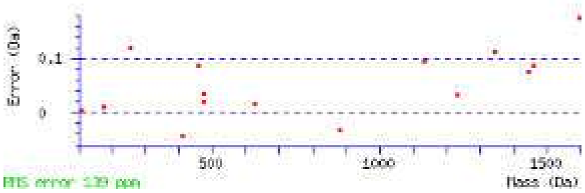


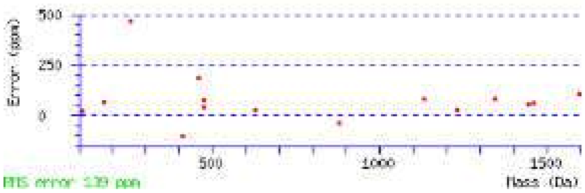


NCBI **BLAST** search of AILNLSLRHDLHISAYGEGNER
(Parameters: blastp, nr protein database, expect=20000, no filter, PAM30)
Other BLAST web gateways

**All matches to this query**

| Score | Mr(calc) | Delta | Sequence |
|---|---|---|---|
| 8.8 | 2477.1719 | 0.1768 | YTMTGVGGLFMGNDSIGSILSWR |
| 8.0 | 2477.3082 | 0.0405 | ILGVWAFALIWTIAPMFGWNR |
| 6.3 | 2477.2775 | 0.0713 | AILNLSLRHDLHISAYGEGNER |
| 5.5 | 2477.2737 | 0.0751 | LEKTYQCPLLVDHNPLGEVPR |
| 4.8 | 2477.2836 | 0.0652 | SLGNATIFNQDVLDSLLKEICK |
| 4.5 | 2477.2551 | 0.0937 | GGSGTPGLPVIKPDPDADAKHFSSK |
| 3.3 | 2477.2914 | 0.0573 | GFAHATLYKTLVEGISANETLSR |
| 3.1 | 2477.3173 | 0.0314 | HASSVGTIGSHVMILNTGSIKNVR |
| 3.0 | 2477.3087 | 0.0400 | KSSAILDEYIGMIVTDANLAIPK |
| 2.5 | 2477.1881 | 0.1606 | SNTGVGSTPAISADSGSTAEAEILSR |

**Mascot:** http://www.matrixscience.com/

# MATRIX SCIENCE Mascot Search Results

## Peptide View **Spot no 69**

MS/MS Fragmentation of **GGNNILVMCDTYTPAGEPIPTNKR**
Found in **gi|19387272** in **NCBInr**, putative precursor chloroplastic glutamine synthetase [Oryza sativa Japonica Group]

Match to Query 234: 2617.339424 from(2618.346700,1+) intensity(0.0000) index(30)
Title: Label: B19, Spot_Id: 219963, Peak_List_Id: 229168, MSMS Job_Run_Id: 22013, Comment:
Data file C:\Documents and Settings\Administrator\Desktop\Farah-Garima 11May 2013\ppw_B19_136868333300.txt

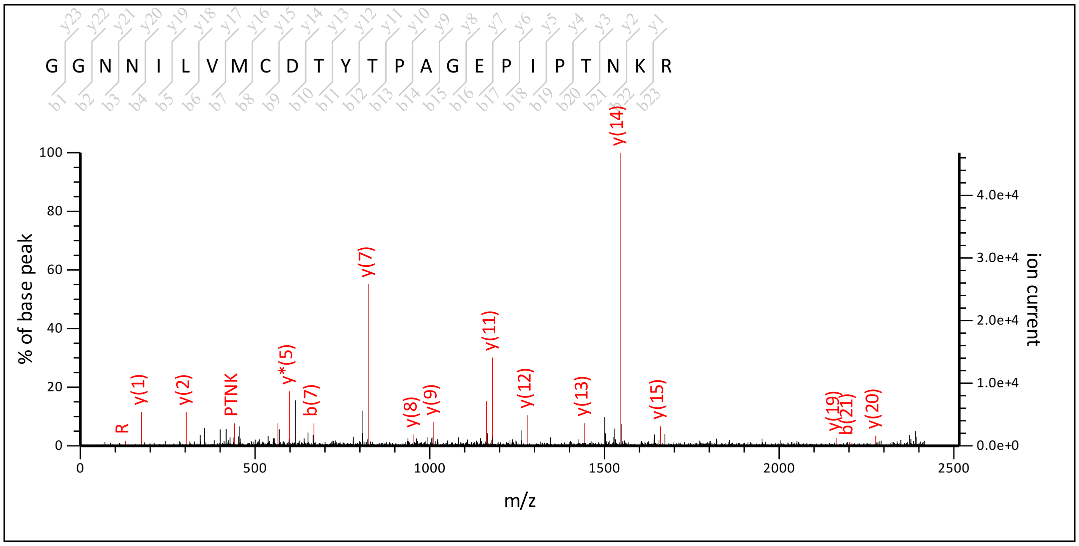


0 to 2515.52

Label all possible matches ○ Label matches used for scoring ◉

**Monoisotopic mass of neutral peptide Mr(calc):** 2617.2628
**Fixed modifications:** Carbamidomethyl (C) (apply to specified residues or termini only)
**Ions Score:** 97 **Expect:** 3.6e-06
**Matches :** 21/464 fragment ions using 24 most intense peaks (help)

| # | Immon. | a | a* | a0 | b | b* | b0 | d | d' | Seq. | v | w | w' | y | y* | [illegible] |
|---|---|---|---|---|---|---|---|---|---|---|---|---|---|---|---|---|
| 1 | 30.0338 | 30.0338 | | | 58.0287 | | | 44.0495 | | G | | | | | | |
| 2 | 30.0338 | 87.0553 | | | 115.0502 | | | | | G | | | | 2561.2487 | 2544.2221 | [illegible] |
| 3 | 87.0553 | 201.0982 | 184.0717 | | 229.0931 | 212.0666 | | 158.0924 | | N | 2445.1901 | 2444.1948 | | 2504.2272 | 2487.2007 | [illegible] |
| 4 | 87.0553 | 315.1411 | 298.1146 | | 343.1361 | 326.1095 | | 272.1353 | | N | 2331.1472 | 2330.1519 | | 2390.1843 | 2373.1577 | [illegible] |
| 5 | 86.0964 | 428.2252 | 411.1987 | | 456.2201 | 439.1936 | | 400.1939 | 414.2096 | I | 2218.0631 | 2231.0835 | 2245.0992 | ***2276.1414*** | 2259.1148 | [illegible] |
| 6 | 86.0964 | 541.3093 | 524.2827 | | 569.3042 | 552.2776 | | 499.2623 | | L | 2104.9790 | 2103.9838 | | ***2163.0573*** | 2146.0307 | [illegible] |
| 7 | 72.0808 | 640.3777 | 623.3511 | | **668.3726** | 651.3461 | | 626.3620 | | V | 2005.9106 | 2018.9310 | | 2049.9732 | 2032.9467 | [illegible] |
| 8 | 104.0528 | 771.4182 | 754.3916 | | 799.4131 | 782.3865 | | 711.4148 | | M | 1874.8701 | 1873.8749 | | 1950.9048 | 1933.8783 | [illegible] |
| 9 | 133.0430 | 931.4488 | 914.4223 | | 959.4437 | 942.4172 | | 842.4553 | | C | 1714.8395 | 1713.8442 | | 1819.8643 | 1802.8378 | [illegible] |
| 10 | 88.0393 | 1046.4758 | 1029.4492 | 1028.4652 | 1074.4707 | 1057.4441 | 1056.4601 | 1002.4859 | | D | 1599.8125 | 1598.8173 | | ***1659.8337*** | 1642.8071 | [illegible] |
| 11 | 74.0600 | 1147.5234 | 1130.4969 | 1129.5129 | 1175.5184 | 1158.4918 | 1157.5078 | 1131.5285 | 1133.5078 | T | 1498.7649 | 1511.7853 | 1513.7645 | ***1544.8067*** | 1527.7802 | [illegible] |
| 12 | 136.0757 | 1310.5868 | 1293.5602 | 1292.5762 | 1338.5817 | 1321.5551 | 1320.5711 | | | Y | 1335.7015 | | | ***1443.7591*** | 1426.7325 | [illegible] |
| 13 | 74.0600 | 1411.6344 | 1394.6079 | 1393.6239 | 1439.6294 | 1422.6028 | 1421.6188 | 1395.6395 | 1397.6188 | T | 1234.6539 | 1247.6743 | 1249.6535 | ***1280.6957*** | 1263.6692 | [illegible] |
| 14 | 70.0651 | 1508.6872 | 1491.6607 | 1490.6766 | 1536.6821 | 1519.6556 | 1518.6716 | 1482.6716 | | P | 1137.6011 | 1136.6058 | | ***1179.6480*** | 1162.6215 | [illegible] |
| 15 | 44.0495 | 1579.7243 | 1562.6978 | 1561.7138 | 1607.7192 | 1590.6927 | 1589.7087 | | | A | 1066.5640 | | | 1082.5953 | 1065.5687 | [illegible] |
| 16 | 30.0338 | 1636.7458 | 1619.7192 | 1618.7352 | 1664.7407 | 1647.7142 | 1646.7301 | | | G | | | | ***1011.5582*** | 994.5316 | [illegible] |
| 17 | 102.0550 | 1765.7884 | 1748.7618 | 1747.7778 | 1793.7833 | 1776.7568 | 1775.7727 | 1707.7829 | | E | 880.4999 | 879.5047 | | ***954.5367*** | 937.5102 | [illegible] |
| 18 | 70.0651 | 1862.8411 | 1845.8146 | 1844.8306 | 1890.8361 | 1873.8095 | 1872.8255 | 1836.8255 | | P | 783.4472 | 782.4519 | | ***825.4941*** | 808.4676 | [illegible] |
| 19 | 86.0964 | 1975.9252 | 1958.8987 | 1957.9146 | 2003.9201 | 1986.8936 | 1985.9096 | 1947.8939 | 1961.9096 | I | 670.3631 | 683.3835 | 697.3991 | 728.4413 | 711.4148 | [illegible] |
| 20 | 70.0651 | 2072.9780 | 2055.9514 | 2054.9674 | 2100.9729 | 2083.9463 | 2082.9623 | 2046.9623 | | P | 573.3103 | 572.3151 | | 615.3573 | 598.3307 | [illegible] |
| 21 | 74.0600 | 2174.0257 | 2156.9991 | 2156.0151 | **2202.0206** | 2184.9940 | 2184.0100 | 2158.0307 | 2160.0100 | T | 472.2627 | 485.2831 | 487.2623 | 518.3045 | 501.2780 | [illegible] |
| 22 | 87.0553 | 2288.0686 | 2271.0420 | 2270.0580 | 2316.0635 | 2299.0369 | 2298.0529 | 2245.0628 | | N | 358.2197 | 357.2245 | | 417.2568 | 400.2303 | |

| | | | | | | | | | | | | | | | |
|---|---|---|---|---|---|---|---|---|---|---|---|---|---|---|---|
| 23 | 101.1073 | 2416.1635 | 2399.1370 | 2398.1530 | 2444.1585 | 2427.1319 | 2426.1479 | 2359.1057 | | K | 230.1248 | 229.1295 | | ***303.2139*** | 286.1874 |
| 24 | 129.1135 | | | | | | | | | R | 74.0237 | 73.0284 | | ***175.1190*** | 158.0924 |

| Seq | ya | yb | Seq | ya | yb | Seq | ya | yb |
|---|---|---|---|---|---|---|---|---|
| GN | 144.0768 | 172.0717 | GNN | 258.1197 | 286.1146 | GNNI | 371.2037 | 399.1987 |
| GNNIL | 484.2878 | 512.2827 | GNNILV | 583.3562 | 611.3511 | NN | 201.0982 | 229.0931 |
| NNI | 314.1823 | 342.1772 | NNIL | 427.2663 | 455.2613 | NNILV | 526.3348 | 554.3297 |
| NNILVM | 657.3752 | 685.3702 | NI | 200.1394 | 228.1343 | NIL | 313.2234 | 341.2183 |
| NILV | 412.2918 | 440.2867 | NILVM | 543.3323 | 571.3272 | IL | 199.1805 | 227.1754 |
| ILV | 298.2489 | 326.2438 | ILVM | 429.2894 | 457.2843 | ILVMC | 589.3200 | 617.3150 |
| LV | 185.1648 | 213.1598 | LVM | 316.2053 | 344.2002 | LVMC | 476.2360 | 504.2309 |
| LVMCD | 591.2629 | 619.2578 | LVMCDT | 692.3106 | 720.3055 | VM | 203.1213 | 231.1162 |
| VMC | 363.1519 | 391.1468 | VMCD | 478.1789 | 506.1738 | VMCDT | 579.2265 | 607.2214 |
| MC | 264.0835 | 292.0784 | MCD | 379.1104 | 407.1054 | MCDT | 480.1581 | 508.1530 |
| MCDTY | 643.2214 | 671.2164 | CD | 248.0700 | 276.0649 | CDT | 349.1176 | 377.1125 |
| CDTY | 512.1810 | 540.1759 | CDTYT | 613.2286 | 641.2236 | DT | 189.0870 | 217.0819 |
| DTY | 352.1503 | 380.1452 | DTYT | 453.1980 | 481.1929 | DTYTP | 550.2508 | 578.2457 |
| DTYTPA | 621.2879 | 649.2828 | DTYTPAG | 678.3093 | 706.3042 | TY | 237.1234 | 265.1183 |
| TYT | 338.1710 | 366.1660 | TYTP | 435.2238 | 463.2187 | TYTPA | 506.2609 | 534.2558 |
| TYTPAG | 563.2824 | 591.2773 | TYTPAGE | 692.3250 | 720.3199 | YT | 237.1234 | 265.1183 |
| YTP | 334.1761 | 362.1710 | YTPA | 405.2132 | 433.2082 | YTPAG | 462.2347 | 490.2296 |
| YTPAGE | 591.2773 | 619.2722 | YTPAGEP | 688.3301 | 716.3250 | TP | 171.1128 | 199.1077 |
| TPA | 242.1499 | 270.1448 | TPAG | 299.1714 | 327.1663 | TPAGE | 428.2140 | 456.2089 |
| TPAGEP | 525.2667 | 553.2617 | TPAGEPI | 638.3508 | 666.3457 | PA | 141.1022 | 169.0972 |
| PAG | 198.1237 | 226.1186 | PAGE | 327.1663 | 355.1612 | PAGEP | 424.2191 | 452.2140 |
| PAGEPI | 537.3031 | 565.2980 | PAGEPIP | 634.3559 | 662.3508 | AG | 101.0709 | 129.0659 |
| AGE | 230.1135 | 258.1084 | AGEP | 327.1663 | 355.1612 | AGEPI | 440.2504 | 468.2453 |
| AGEPIP | 537.3031 | 565.2980 | AGEPIPT | 638.3508 | 666.3457 | GE | 159.0764 | 187.0713 |
| GEP | 256.1292 | 284.1241 | GEPI | 369.2132 | 397.2082 | GEPIP | 466.2660 | 494.2609 |
| GEPIPT | 567.3137 | 595.3086 | GEPIPTN | 681.3566 | 709.3515 | EP | 199.1077 | 227.1026 |
| EPI | 312.1918 | 340.1867 | EPIP | 409.2445 | 437.2395 | EPIPT | 510.2922 | 538.2871 |
| EPIPTN | 624.3352 | 652.3301 | PI | 183.1492 | 211.1441 | PIP | 280.2020 | 308.1969 |
| PIPT | 381.2496 | 409.2445 | PIPTN | 495.2926 | 523.2875 | PIPTNK | 623.3875 | 651.3824 |
| IP | 183.1492 | 211.1441 | IPT | 284.1969 | 312.1918 | IPTN | 398.2398 | 426.2347 |
| IPTNK | 526.3348 | 554.3297 | PT | 171.1128 | 199.1077 | PTN | 285.1557 | 313.1506 |
| PTNK | 413.2507 | 441.2456 | TN | 188.1030 | 216.0979 | TNK | 316.1979 | 344.1928 |
| NK | 215.1503 | 243.1452 | | | | | | |

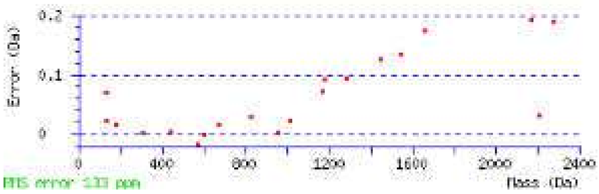


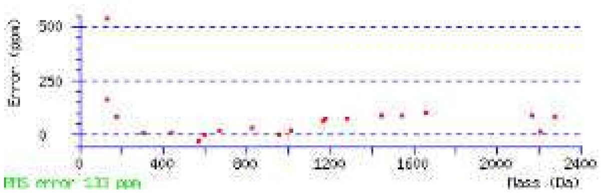


NCBI **BLAST** search of GGNNILVMCDTYTPAGEPIPTNKR
(Parameters: blastp, nr protein database, expect=20000, no filter, PAM30)
Other BLAST web gateways

**All matches to this query**

| Score | Mr(calc) | Delta | Sequence |
|---|---|---|---|
| 97.3 | 2617.2628 | 0.0766 | GGNNILVMCDTYTPAGEPIPTNKR |
| 25.6 | 2617.3284 | 0.0110 | TSFHIIFLDTYTTMDTLIAMKR |
| 15.2 | 2617.5604 | -0.2210 | AGLLPLGLGTLALLVALSMALSAPLAR |
| 14.7 | 2617.3785 | -0.0391 | QTYTQTLLAAVPKLGAMQGSDLPAK |
| 14.7 | 2617.4010 | -0.0616 | TIIVQLNESVKITCVRPYSNQR |
| 14.5 | 2617.3924 | -0.0530 | IEQLMLKILPEYTVGDPSLETTK |
| | | | |

| | | | |
|---|---|---|---|
| 13.4 | 2617.5128 | -0.1734 | LEIMKDSAIGTYGVLALILSLLIR |
| 12.2 | 2617.3612 | -0.0218 | LPREATPAQHVQYINELSDLPAR |
| 11.9 | 2617.3210 | 0.0184 | FGGDEFVVILGGSPPQEMIAAIAQR |
| 11.0 | 2617.3758 | -0.0364 | TAISVQDAAAAHMAATPGASVVPRLGR |

**Mascot:** http://www.matrixscience.com/

# Mascot Search Results

## Peptide View Spot no 69

MS/MS Fragmentation of **GGNNILVMCDTYTPAGEPIPTNKR**
Found in **gi|19387272** in **NCBInr**, putative precursor chloroplastic glutamine synthetase [Oryza sativa Japonica Group]

Match to Query 237: 2633.335024 from(2634.342300,1+) intensity(0.0000) index(31)
Title: Label: B19, Spot_Id: 219963, Peak_List_Id: 229174, MSMS Job_Run_Id: 22013, Comment:
Data file C:\Documents and Settings\Administrator\Desktop\Farah-Garima 11May 2013\ppw_B19_136868333300.txt

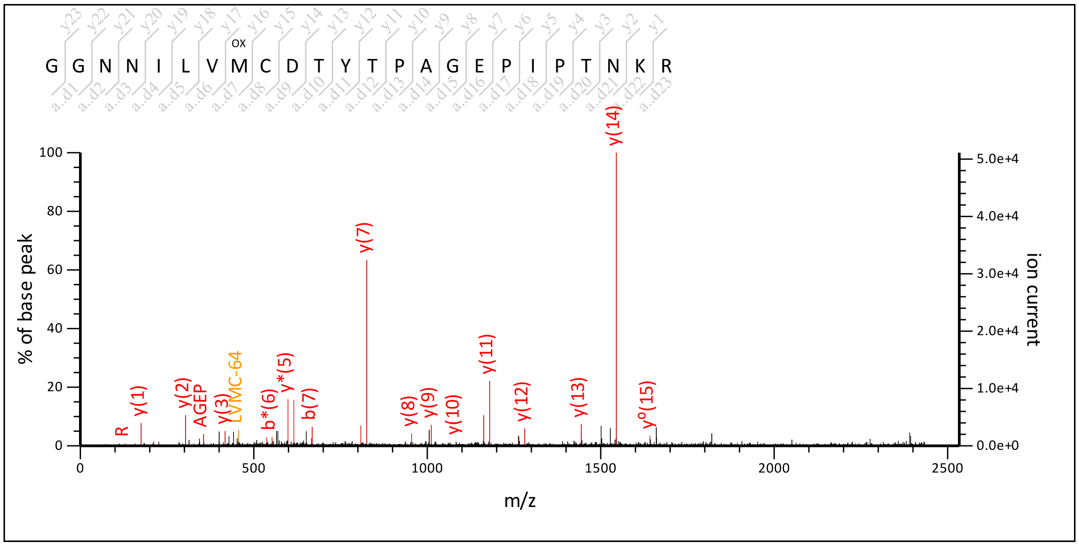


0 to 2533.53

Label all possible matches ○ Label matches used for scoring ◉

**Monoisotopic mass of neutral peptide Mr(calc):** 2633.2578
**Fixed modifications:** Carbamidomethyl (C) (apply to specified residues or termini only)
**Variable modifications:**
**M8** : Oxidation (M), with neutral losses 0.0000(shown in table), 63.9983
**Ions Score:** 66 **Expect:** 0.0043
**Matches** : 35/632 fragment ions using 48 most intense peaks (help)

| # | Immon. | a | a* | a⁰ | b | b* | b⁰ | d | d' | Seq. | v | w | w' | y | y* | |
|---|---|---|---|---|---|---|---|---|---|---|---|---|---|---|---|---|
| 1 | 30.0338 | 30.0338 | | | 58.0287 | | | 44.0495 | | G | | | | | | |
| 2 | 30.0338 | 87.0553 | | | 115.0502 | | | | | G | | | | 2577.2436 | 2560.2170 | [illegible] |
| 3 | 87.0553 | 201.0982 | 184.0717 | | 229.0931 | 212.0666 | | 158.0924 | | N | 2461.1850 | 2460.1898 | | 2520.2221 | 2503.1956 | [illegible] |
| 4 | 87.0553 | 315.1411 | 298.1146 | | 343.1361 | 326.1095 | | 272.1353 | | N | 2347.1421 | 2346.1468 | | 2406.1792 | 2389.1526 | [illegible] |
| 5 | 86.0964 | 428.2252 | 411.1987 | | **456.2201** | 439.1936 | | 400.1939 | 414.2096 | I | 2234.0580 | 2247.0784 | 2261.0941 | 2292.1363 | 2275.1097 | [illegible] |
| 6 | 86.0964 | 541.3093 | 524.2827 | | 569.3042 | 552.2776 | | 499.2623 | | L | 2120.9740 | 2119.9787 | | 2179.0522 | 2162.0257 | [illegible] |
| 7 | 72.0808 | 640.3777 | 623.3511 | | **668.3726** | 651.3461 | | 626.3620 | | V | 2021.9055 | 2034.9259 | | 2065.9681 | 2048.9416 | [illegible] |
| 8 | 120.0478 | 787.4131 | 770.3865 | | 815.4080 | 798.3815 | | 711.4148 | | M | 1874.8701 | 1873.8749 | | 1966.8997 | 1949.8732 | [illegible] |
| 9 | 133.0430 | 947.4437 | 930.4172 | | 975.4386 | 958.4121 | | 858.4502 | | C | 1714.8395 | 1713.8442 | | 1819.8643 | 1802.8378 | [illegible] |
| 10 | 88.0393 | 1062.4707 | 1045.4441 | 1044.4601 | 1090.4656 | 1073.4390 | 1072.4550 | 1018.4808 | | D | 1599.8125 | 1598.8173 | | 1659.8337 | 1642.8071 | [illegible] |
| 11 | 74.0600 | 1163.5184 | 1146.4918 | 1145.5078 | 1191.5133 | 1174.4867 | 1173.5027 | 1147.5234 | 1149.5027 | T | 1498.7649 | 1511.7853 | 1513.7645 | ***1544.8067*** | 1527.7802 | [illegible] |
| 12 | 136.0757 | 1326.5817 | 1309.5551 | 1308.5711 | 1354.5766 | 1337.5501 | 1336.5660 | | | Y | 1335.7015 | | | ***1443.7591*** | 1426.7325 | [illegible] |
| 13 | 74.0600 | 1427.6294 | 1410.6028 | 1409.6188 | 1455.6243 | 1438.5977 | 1437.6137 | 1411.6344 | 1413.6137 | T | 1234.6539 | 1247.6743 | 1249.6535 | ***1280.6957*** | 1263.6692 | [illegible] |
| 14 | 70.0651 | 1524.6821 | 1507.6556 | 1506.6716 | 1552.6770 | 1535.6505 | 1534.6665 | 1498.6665 | | P | 1137.6011 | 1136.6058 | | ***1179.6480*** | 1162.6215 | [illegible] |
| 15 | 44.0495 | 1595.7192 | 1578.6927 | 1577.7087 | 1623.7142 | 1606.6876 | 1605.7036 | | | A | 1066.5640 | | | ***1082.5953*** | 1065.5687 | [illegible] |
| 16 | 30.0338 | 1652.7407 | 1635.7142 | 1634.7301 | 1680.7356 | 1663.7091 | 1662.7251 | | | G | | | | ***1011.5582*** | 994.5316 | [illegible] |
| 17 | 102.0550 | 1781.7833 | 1764.7568 | 1763.7727 | 1809.7782 | 1792.7517 | 1791.7676 | 1723.7778 | | E | 880.4999 | 879.5047 | | ***954.5367*** | 937.5102 | [illegible] |
| 18 | 70.0651 | 1878.8361 | 1861.8095 | 1860.8255 | 1906.8310 | 1889.8044 | 1888.8204 | 1852.8204 | | P | 783.4472 | 782.4519 | | ***825.4941*** | 808.4676 | [illegible] |
| 19 | 86.0964 | 1991.9201 | 1974.8936 | 1973.9096 | 2019.9150 | 2002.8885 | 2001.9045 | 1963.8888 | 1977.9045 | I | 670.3631 | 683.3835 | 697.3991 | 728.4413 | 711.4148 | [illegible] |
| 20 | 70.0651 | 2088.9729 | 2071.9463 | 2070.9623 | 2116.9678 | 2099.9413 | 2098.9572 | 2062.9572 | | P | 573.3103 | 572.3151 | | ***615.3573*** | 598.3307 | [illegible] |
| 21 | 74.0600 | 2190.0206 | 2172.9940 | 2172.0100 | 2218.0155 | 2200.9889 | 2200.0049 | 2174.0257 | 2176.0049 | T | 472.2627 | 485.2831 | 487.2623 | 518.3045 | 501.2780 | [illegible] |

| | | | | | | | | | | | | | | | |
|---|---|---|---|---|---|---|---|---|---|---|---|---|---|---|---|
| 22 | 87.0553 | 2304.0635 | 2287.0369 | 2286.0529 | 2332.0584 | 2315.0319 | 2314.0478 | 2261.0577 | | N | 358.2197 | 357.2245 | | ***417.2568*** | 400.2303 |
| 23 | 101.1073 | 2432.1585 | 2415.1319 | 2414.1479 | 2460.1534 | 2443.1268 | 2442.1428 | 2375.1006 | | K | 230.1248 | 229.1295 | | ***303.2139*** | 286.1874 |
| 24 | 129.1135 | | | | | | | | | R | 74.0237 | 73.0284 | | ***175.1190*** | 158.0924 |

| Seq | ya | yb | Seq | ya | yb | Seq | ya | yb |
|---|---|---|---|---|---|---|---|---|
| GN | 144.0768 | 172.0717 | GNN | 258.1197 | 286.1146 | GNNI | 371.2037 | 399.1987 |
| GNNIL | 484.2878 | 512.2827 | GNNILV | 583.3562 | 611.3511 | NN | 201.0982 | 229.0931 |
| NNI | 314.1823 | 342.1772 | NNIL | 427.2663 | 455.2613 | NNILV | 526.3348 | 554.3297 |
| NNILVM | 673.3702 | 701.3651 | NI | 200.1394 | 228.1343 | NIL | 313.2234 | 341.2183 |
| NILV | 412.2918 | 440.2867 | NILVM | 559.3272 | 587.3221 | IL | 199.1805 | 227.1754 |
| ILV | 298.2489 | 326.2438 | ILVM | 445.2843 | 473.2792 | ILVMC | 605.3150 | 633.3099 |
| LV | 185.1648 | 213.1598 | LVM | 332.2002 | 360.1952 | LVMC | 492.2309 | 520.2258 |
| LVMCD | 607.2578 | 635.2527 | VM | 219.1162 | 247.1111 | VMC | 379.1468 | 407.1417 |
| VMCD | 494.1738 | 522.1687 | VMCDT | 595.2214 | 623.2164 | MC | 280.0784 | 308.0733 |
| MCD | 395.1054 | 423.1003 | MCDT | 496.1530 | 524.1479 | MCDTY | 659.2164 | 687.2113 |
| CD | 248.0700 | 276.0649 | CDT | 349.1176 | 377.1125 | CDTY | 512.1810 | 540.1759 |
| CDTYT | 613.2286 | 641.2236 | DT | 189.0870 | 217.0819 | DTY | 352.1503 | 380.1452 |
| DTYT | 453.1980 | 481.1929 | DTYTP | 550.2508 | 578.2457 | DTYTPA | 621.2879 | 649.2828 |
| DTYTPAG | 678.3093 | 706.3042 | TY | 237.1234 | 265.1183 | TYT | 338.1710 | 366.1660 |
| TYTP | 435.2238 | 463.2187 | TYTPA | 506.2609 | 534.2558 | TYTPAG | 563.2824 | 591.2773 |
| TYTPAGE | 692.3250 | 720.3199 | YT | 237.1234 | 265.1183 | YTP | 334.1761 | 362.1710 |
| YTPA | 405.2132 | 433.2082 | YTPAG | 462.2347 | 490.2296 | YTPAGE | 591.2773 | 619.2722 |
| YTPAGEP | 688.3301 | 716.3250 | TP | 171.1128 | 199.1077 | TPA | 242.1499 | 270.1448 |
| TPAG | 299.1714 | 327.1663 | TPAGE | 428.2140 | 456.2089 | TPAGEP | 525.2667 | 553.2617 |
| TPAGEPI | 638.3508 | 666.3457 | PA | 141.1022 | 169.0972 | PAG | 198.1237 | 226.1186 |
| PAGE | 327.1663 | 355.1612 | PAGEP | 424.2191 | 452.2140 | PAGEPI | 537.3031 | 565.2980 |
| PAGEPIP | 634.3559 | 662.3508 | AG | 101.0709 | 129.0659 | AGE | 230.1135 | 258.1084 |
| AGEP | 327.1663 | 355.1612 | AGEPI | 440.2504 | 468.2453 | AGEPIP | 537.3031 | 565.2980 |
| AGEPIPT | 638.3508 | 666.3457 | GE | 159.0764 | 187.0713 | GEP | 256.1292 | 284.1241 |
| GEPI | 369.2132 | 397.2082 | GEPIP | 466.2660 | 494.2609 | GEPIPT | 567.3137 | 595.3086 |
| GEPIPTN | 681.3566 | 709.3515 | EP | 199.1077 | 227.1026 | EPI | 312.1918 | 340.1867 |
| EPIP | 409.2445 | 437.2395 | EPIPT | 510.2922 | 538.2871 | EPIPTN | 624.3352 | 652.3301 |
| PI | 183.1492 | 211.1441 | PIP | 280.2020 | 308.1969 | PIPT | 381.2496 | 409.2445 |
| PIPTN | 495.2926 | 523.2875 | PIPTNK | 623.3875 | 651.3824 | IP | 183.1492 | 211.1441 |
| IPT | 284.1969 | 312.1918 | IPTN | 398.2398 | 426.2347 | IPTNK | 526.3348 | 554.3297 |
| PT | 171.1128 | 199.1077 | PTN | 285.1557 | 313.1506 | PTNK | 413.2507 | 441.2456 |
| TN | 188.1030 | 216.0979 | TNK | 316.1979 | 344.1928 | NK | 215.1503 | 243.1452 |

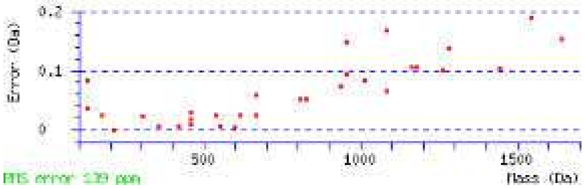

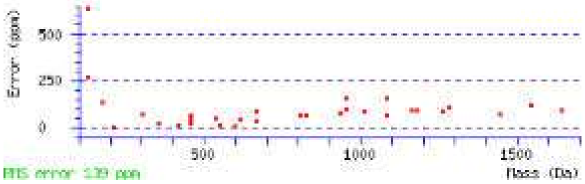


NCBI **BLAST** search of GGNNILVMCDTYTPAGEPIPTNKR
(Parameters: blastp, nr protein database, expect=20000, no filter, PAM30)
Other BLAST web gateways

**All matches to this query**

| Score | Mr(calc) | Delta | Sequence |
|---|---|---|---|
| 66.3 | 2633.2578 | 0.0773 | GGNNILVMCDTYTPAGEPIPTNKR |
| 18.6 | 2633.3912 | -0.0561 | IELDISGSKFTNLSTQLLAALDER |
| 14.8 | 2633.4323 | -0.0972 | MAQQLLPASAAPSAPRAASVNVVLGSK |
| 14.7 | 2633.4575 | -0.1224 | ALGLDVVATGLAMSVGPAISALAGVPAGR |
| 11.0 | 2633.3521 | -0.0171 | ALGLPSSVGQEARSLGQDPAGVSAPGGR |
| 9.8 | 2633.2863 | 0.0487 | GKPPKADPLLGDTAMQISTDIMMR |
| | | | |

| | | | |
|---|---|---|---|
| 9.7 | 2633.3211 | 0.0139 | HAESFRHFPDQASLADIRPLGNR |
| 9.5 | 2633.4323 | -0.0973 | LPVSSIKGAIGHSLGAASAIEAAVCVR |
| 8.4 | 2633.5852 | -0.2502 | MIVALAVAPVVLITLAIGLRGVAAMR |
| 8.2 | 2633.5052 | -0.1702 | KILPLFLTLAMIFALVGCSNIATK |

**Mascot:** http://www.matrixscience.com/

# MATRIX SCIENCE Mascot Search Results

## Peptide View Spot no 69

MS/MS Fragmentation of **DVNWPLGWPVGGYPGPQGPYYCAVGSDK**
Found in **gi|19387272** in **NCBInr**, putative precursor chloroplastic glutamine synthetase [Oryza sativa Japonica Group]

Match to Query 240: 3035.488824 from(3036.496100,1+) intensity(0.0000) index(32)
Title: Label: B19, Spot_Id: 219963, Peak_List_Id: 229184, MSMS Job_Run_Id: 22013, Comment:
Data file C:\Documents and Settings\Administrator\Desktop\Farah-Garima 11May 2013\ppw_B19_136868333300.txt

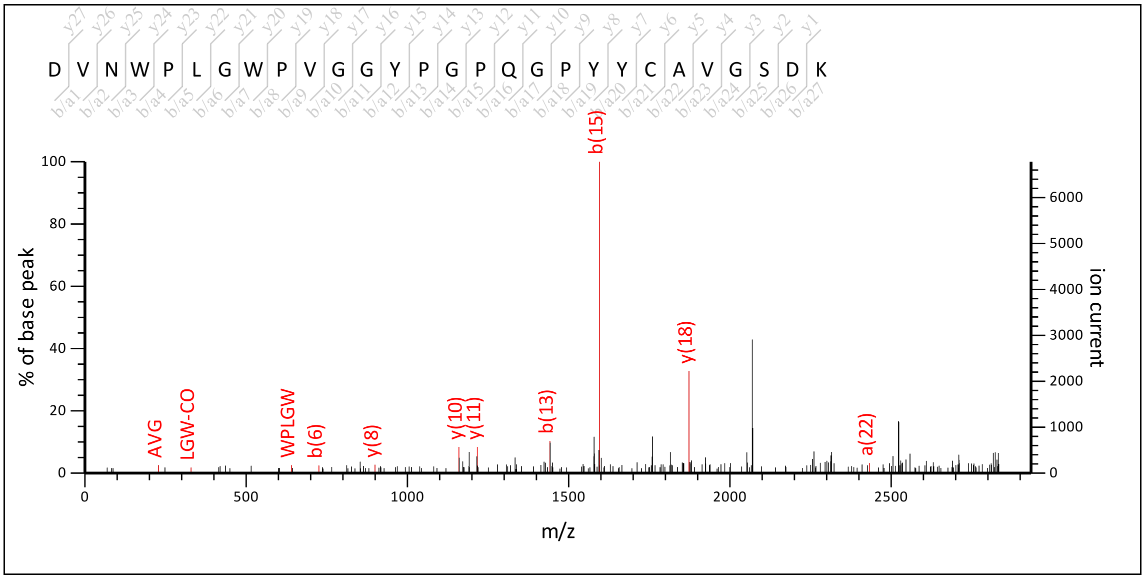


0 to 2933.75

Label all possible matches ○ Label matches used for scoring ◉

**Monoisotopic mass of neutral peptide Mr(calc):** 3035.3913
**Fixed modifications:** Carbamidomethyl (C) (apply to specified residues or termini only)
**Ions Score:** 11 **Expect:** 1.1e+03
**Matches :** 13/554 fragment ions using 28 most intense peaks (help)

| # | Immon. | a | a* | $a^0$ | b | b* | $b^0$ | d | Seq. | v | w | y | y* | $y^0$ | # |
|---|---|---|---|---|---|---|---|---|---|---|---|---|---|---|---|
| 1 | 88.0393 | 88.0393 | | 70.0287 | 116.0342 | | 98.0237 | 44.0495 | D | | | | | | 28 |
| 2 | 72.0808 | 187.1077 | | 169.0972 | 215.1026 | | 197.0921 | 173.0921 | V | 2877.3090 | 2890.3294 | 2921.3716 | 2904.3450 | 2903.3610 | 27 |
| 3 | 87.0553 | 301.1506 | 284.1241 | 283.1401 | *329.1456* | 312.1190 | 311.1350 | 258.1448 | N | 2763.2660 | 2762.2708 | 2822.3032 | 2805.2766 | 2804.2926 | 26 |
| 4 | 159.0917 | 487.2300 | 470.2034 | 469.2194 | 515.2249 | 498.1983 | 497.2143 | | W | 2577.1867 | | 2708.2602 | 2691.2337 | 2690.2497 | 25 |
| 5 | 70.0651 | 584.2827 | 567.2562 | 566.2722 | 612.2776 | 595.2511 | 594.2671 | 558.2671 | P | 2480.1340 | 2479.1387 | 2522.1809 | 2505.1544 | 2504.1703 | 24 |
| 6 | 86.0964 | 697.3668 | 680.3402 | 679.3562 | *725.3617* | 708.3352 | 707.3511 | 655.3198 | L | 2367.0499 | 2366.0546 | 2425.1281 | 2408.1016 | 2407.1176 | 23 |
| 7 | 30.0338 | 754.3883 | 737.3617 | 736.3777 | 782.3832 | 765.3566 | 764.3726 | | G | | | 2312.0441 | 2295.0175 | 2294.0335 | 22 |
| 8 | 159.0917 | 940.4676 | 923.4410 | 922.4570 | 968.4625 | 951.4359 | 950.4519 | | W | 2123.9491 | | 2255.0226 | 2237.9961 | 2237.0121 | 21 |
| 9 | 70.0651 | 1037.5203 | 1020.4938 | 1019.5098 | 1065.5152 | 1048.4887 | 1047.5047 | 1011.5047 | P | 2026.8964 | 2025.9011 | 2068.9433 | 2051.9168 | 2050.9327 | 20 |
| 10 | 72.0808 | 1136.5887 | 1119.5622 | 1118.5782 | 1164.5837 | 1147.5571 | 1146.5731 | 1122.5731 | V | 1927.8279 | 1940.8483 | 1971.8905 | 1954.8640 | 1953.8800 | 19 |
| 11 | 30.0338 | 1193.6102 | 1176.5837 | 1175.5996 | 1221.6051 | 1204.5786 | 1203.5946 | | G | | | *1872.8221* | 1855.7956 | 1854.8116 | 18 |
| 12 | 30.0338 | 1250.6317 | 1233.6051 | 1232.6211 | 1278.6266 | 1261.6000 | 1260.6160 | | G | | | 1815.8007 | 1798.7741 | 1797.7901 | 17 |
| 13 | 136.0757 | 1413.6950 | 1396.6684 | 1395.6844 | *1441.6899* | 1424.6634 | 1423.6793 | | Y | 1650.7217 | | 1758.7792 | 1741.7527 | 1740.7686 | 16 |
| 14 | 70.0651 | 1510.7478 | 1493.7212 | 1492.7372 | 1538.7427 | 1521.7161 | 1520.7321 | 1484.7321 | P | 1553.6689 | 1552.6737 | *1595.7159* | 1578.6893 | 1577.7053 | 15 |
| 15 | 30.0338 | 1567.7692 | 1550.7427 | 1549.7587 | *1595.7641* | 1578.7376 | 1577.7536 | | G | | | 1498.6631 | 1481.6366 | 1480.6525 | 14 |
| 16 | 70.0651 | 1664.8220 | 1647.7954 | 1646.8114 | 1692.8169 | 1675.7904 | 1674.8063 | 1638.8063 | P | 1399.5947 | 1398.5994 | 1441.6416 | 1424.6151 | 1423.6311 | 13 |
| 17 | 101.0709 | 1792.8806 | 1775.8540 | 1774.8700 | 1820.8755 | 1803.8489 | 1802.8649 | 1735.8591 | Q | 1271.5361 | 1270.5409 | 1344.5889 | 1327.5623 | 1326.5783 | 12 |
| 18 | 30.0338 | 1849.9020 | 1832.8755 | 1831.8915 | 1877.8969 | 1860.8704 | 1859.8864 | | G | | | *1216.5303* | 1199.5038 | 1198.5197 | 11 |
| 19 | 70.0651 | 1946.9548 | 1929.9282 | 1928.9442 | 1974.9497 | 1957.9232 | 1956.9391 | 1920.9391 | P | 1117.4619 | 1116.4666 | *1159.5088* | 1142.4823 | 1141.4983 | 10 |

| | | | | | | | | | | | | | | | |
|---|---|---|---|---|---|---|---|---|---|---|---|---|---|---|---|
| 20 | 136.0757 | 2110.0181 | 2092.9916 | 2092.0076 | 2138.0130 | 2120.9865 | 2120.0025 | | Y | 954.3986 | | 1062.4561 | 1045.4295 | 1044.4455 | 9 |
| 21 | 136.0757 | 2273.0815 | 2256.0549 | 2255.0709 | 2301.0764 | 2284.0498 | 2283.0658 | | Y | 791.3352 | | ***899.3927*** | 882.3662 | 881.3822 | 8 |
| 22 | 133.0430 | 2433.1121 | 2416.0856 | 2415.1015 | 2461.1070 | 2444.0805 | 2443.0965 | 2344.1186 | C | 631.3046 | 630.3093 | 736.3294 | 719.3029 | 718.3189 | 7 |
| 23 | 44.0495 | 2504.1492 | 2487.1227 | 2486.1387 | 2532.1441 | 2515.1176 | 2514.1336 | | A | 560.2675 | | 576.2988 | 559.2722 | 558.2882 | 6 |
| 24 | 72.0808 | 2603.2176 | 2586.1911 | 2585.2071 | 2631.2125 | 2614.1860 | 2613.2020 | 2589.2020 | V | 461.1991 | 474.2195 | 505.2617 | 488.2351 | 487.2511 | 5 |
| 25 | 30.0338 | 2660.2391 | 2643.2125 | 2642.2285 | 2688.2340 | 2671.2075 | 2670.2234 | | G | | | 406.1932 | 389.1667 | 388.1827 | 4 |
| 26 | 60.0444 | 2747.2711 | 2730.2446 | 2729.2606 | 2775.2660 | 2758.2395 | 2757.2555 | 2731.2762 | S | 317.1456 | 316.1503 | 349.1718 | 332.1452 | 331.1612 | 3 |
| 27 | 88.0393 | 2862.2981 | 2845.2715 | 2844.2875 | 2890.2930 | 2873.2664 | 2872.2824 | 2818.3082 | D | 202.1186 | 201.1234 | 262.1397 | 245.1132 | 244.1292 | 2 |
| 28 | 101.1073 | | | | | | | | K | 74.0237 | 73.0284 | 147.1128 | 130.0863 | | 1 |

| Seq | ya | yb | Seq | ya | yb | Seq | ya | yb |
|---|---|---|---|---|---|---|---|---|
| **VN** | 186.1237 | 214.1186 | **VNW** | 372.2030 | 400.1979 | **VNWP** | 469.2558 | 497.2507 |
| **VNWPL** | 582.3398 | 610.3348 | **VNWPLG** | 639.3613 | 667.3562 | **NW** | 273.1346 | 301.1295 |
| **NWP** | 370.1874 | 398.1823 | **NWPL** | 483.2714 | 511.2663 | **NWPLG** | 540.2929 | 568.2878 |
| **WP** | 256.1444 | 284.1394 | **WPL** | 369.2285 | 397.2234 | **WPLG** | 426.2500 | 454.2449 |
| **WPLGW** | 612.3293 | 640.3242 | **PL** | 183.1492 | 211.1441 | **PLG** | 240.1707 | 268.1656 |
| **PLGW** | 426.2500 | 454.2449 | **PLGWP** | 523.3027 | 551.2976 | **PLGWPV** | 622.3711 | 650.3661 |
| **PLGWPVG** | 679.3926 | 707.3875 | **LG** | 143.1179 | 171.1128 | **LGW** | 329.1972 | 357.1921 |
| **LGWP** | 426.2500 | 454.2449 | **LGWPV** | 525.3184 | 553.3133 | **LGWPVG** | 582.3398 | 610.3348 |
| **LGWPVGG** | 639.3613 | 667.3562 | **GW** | 216.1131 | 244.1081 | **GWP** | 313.1659 | 341.1608 |
| **GWPV** | 412.2343 | 440.2292 | **GWPVG** | 469.2558 | 497.2507 | **GWPVGG** | 526.2772 | 554.2722 |
| **GWPVGGY** | 689.3406 | 717.3355 | **WP** | 256.1444 | 284.1394 | **WPV** | 355.2129 | 383.2078 |
| **WPVG** | 412.2343 | 440.2292 | **WPVGG** | 469.2558 | 497.2507 | **WPVGGY** | 632.3191 | 660.3140 |
| **PV** | 169.1335 | 197.1285 | **PVG** | 226.1550 | 254.1499 | **PVGG** | 283.1765 | 311.1714 |
| **PVGGY** | 446.2398 | 474.2347 | **PVGGYP** | 543.2926 | 571.2875 | **PVGGYPG** | 600.3140 | 628.3089 |
| **PVGGYPGP** | 697.3668 | 725.3617 | **VG** | 129.1022 | 157.0972 | **VGG** | 186.1237 | 214.1186 |
| **VGGY** | 349.1870 | 377.1819 | **VGGYP** | 446.2398 | 474.2347 | **VGGYPG** | 503.2613 | 531.2562 |
| **VGGYPGP** | 600.3140 | 628.3089 | **GG** | 87.0553 | 115.0502 | **GGY** | 250.1186 | 278.1135 |
| **GGYP** | 347.1714 | 375.1663 | **GGYPG** | 404.1928 | 432.1878 | **GGYPGP** | 501.2456 | 529.2405 |
| **GGYPGPQ** | 629.3042 | 657.2991 | **GGYPGPQG** | 686.3257 | 714.3206 | **GY** | 193.0972 | 221.0921 |
| **GYP** | 290.1499 | 318.1448 | **GYPG** | 347.1714 | 375.1663 | **GYPGP** | 444.2241 | 472.2191 |
| **GYPGPQ** | 572.2827 | 600.2776 | **GYPGPQG** | 629.3042 | 657.2991 | **YP** | 233.1285 | 261.1234 |
| **YPG** | 290.1499 | 318.1448 | **YPGP** | 387.2027 | 415.1976 | **YPGPQ** | 515.2613 | 543.2562 |
| **YPGPQG** | 572.2827 | 600.2776 | **YPGPQGP** | 669.3355 | 697.3304 | **PG** | 127.0866 | 155.0815 |
| **PGP** | 224.1394 | 252.1343 | **PGPQ** | 352.1979 | 380.1928 | **PGPQG** | 409.2194 | 437.2143 |
| **PGPQGP** | 506.2722 | 534.2671 | **PGPQGPY** | 669.3355 | 697.3304 | **GP** | 127.0866 | 155.0815 |
| **GPQ** | 255.1452 | 283.1401 | **GPQG** | 312.1666 | 340.1615 | **GPQGP** | 409.2194 | 437.2143 |
| **GPQGPY** | 572.2827 | 600.2776 | **PQ** | 198.1237 | 226.1186 | **PQG** | 255.1452 | 283.1401 |
| **PQGP** | 352.1979 | 380.1928 | **PQGPY** | 515.2613 | 543.2562 | **PQGPYY** | 678.3246 | 706.3195 |
| **QG** | 158.0924 | 186.0873 | **QGP** | 255.1452 | 283.1401 | **QGPY** | 418.2085 | 446.2034 |
| **QGPYY** | 581.2718 | 609.2667 | **GP** | 127.0866 | 155.0815 | **GPY** | 290.1499 | 318.1448 |
| **GPYY** | 453.2132 | 481.2082 | **GPYYC** | 613.2439 | 641.2388 | **GPYYCA** | 684.2810 | 712.2759 |
| **PY** | 233.1285 | 261.1234 | **PYY** | 396.1918 | 424.1867 | **PYYC** | 556.2224 | 584.2173 |
| **PYYCA** | 627.2595 | 655.2545 | **YY** | 299.1390 | 327.1339 | **YYC** | 459.1697 | 487.1646 |
| **YYCA** | 530.2068 | 558.2017 | **YYCAV** | 629.2752 | 657.2701 | **YYCAVG** | 686.2967 | 714.2916 |
| **YC** | 296.1063 | 324.1013 | **YCA** | 367.1435 | 395.1384 | **YCAV** | 466.2119 | 494.2068 |
| **YCAVG** | 523.2333 | 551.2282 | **YCAVGS** | 610.2654 | 638.2603 | **CA** | 204.0801 | 232.0750 |
| **CAV** | 303.1485 | 331.1435 | **CAVG** | 360.1700 | 388.1649 | **CAVGS** | 447.2020 | 475.1969 |
| **CAVGSD** | 562.2290 | 590.2239 | **AV** | 143.1179 | 171.1128 | **AVG** | 200.1394 | 228.1343 |
| **AVGS** | 287.1714 | 315.1663 | **AVGSD** | 402.1983 | 430.1932 | **VG** | 129.1022 | 157.0972 |
| **VGS** | 216.1343 | 244.1292 | **VGSD** | 331.1612 | 359.1561 | **GS** | 117.0659 | 145.0608 |
| **GSD** | 232.0928 | 260.0877 | **SD** | 175.0713 | 203.0662 | | | |

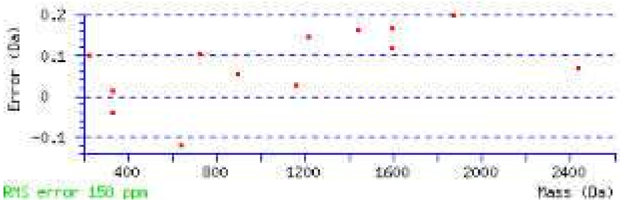


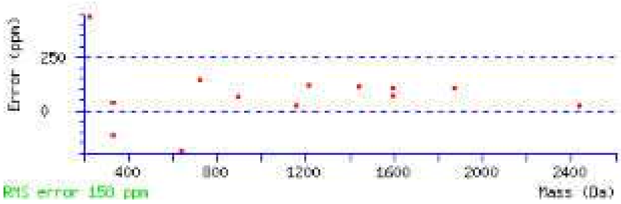


NCBI **BLAST** search of DVNWPLGWPVGGYPGPQGPYYCAVGSDK
(Parameters: blastp, nr protein database, expect=20000, no filter, PAM30)
Other BLAST web gateways

**All matches to this query**

| Score | Mr(calc) | Delta | Sequence |
|---|---|---|---|
| 11.3 | 3035.3913 | 0.0976 | DVNWPLGWPVGGYPGPQGPYYCAVGSDK |

| **Mascot:** http://www.matrixscience.com/ |
|---|

# Mascot Search Results

## Peptide View **Spot no 71**

MS/MS Fragmentation of **IVVFLNKK**
Found in **gi|6525065** in **NCBInr**, chloroplast translational elongation factor Tu [Oryza sativa Japonica Group]

Match to Query 8: 959.596854 from(960.604130,1+) intensity(0.0000) index(3)
Title: Label: B3, Spot_Id: 219707, Peak_List_Id: 225190, MSMS Job_Run_Id: 21768, Comment:
Data file C:\Documents and Settings\Administrator\Desktop\farah BHU\spot set plate\Farah-Garima 11May 2013\ppw_B3_136842116600.txt

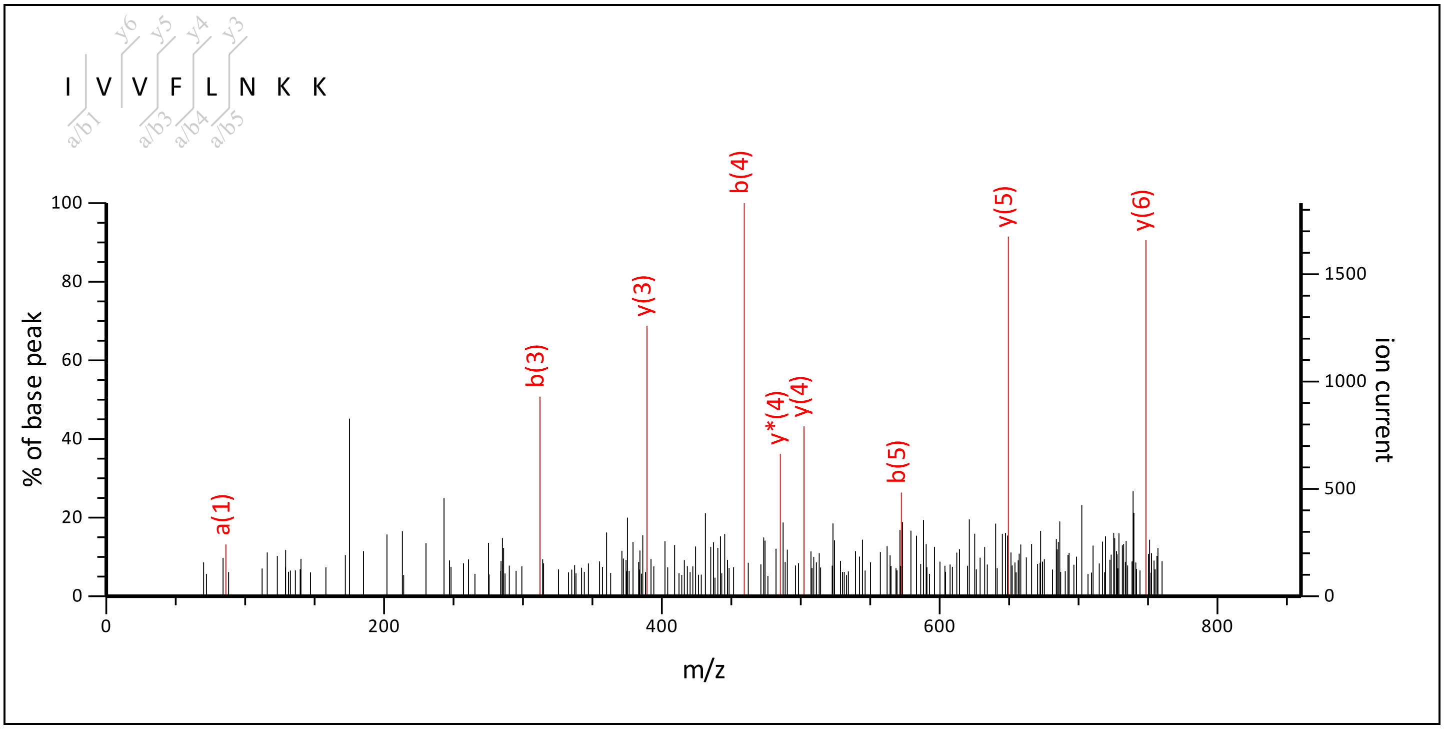


0 to 860.14

Label all possible matches ○ Label matches used for scoring ◉

**Monoisotopic mass of neutral peptide Mr(calc):** 959.6168
**Fixed modifications:** Carbamidomethyl (C) (apply to specified residues or termini only)
**Ions Score:** 24 **Expect:** 5.7
**Matches :** 9/32 fragment ions using 14 most intense peaks (help)

| # | a | a* | b | b* | Seq. | y | y* | # |
|---|---|---|---|---|---|---|---|---|
| 1 | **86.0964** | | 114.0913 | | I | | | 8 |
| 2 | 185.1648 | | 213.1598 | | V | 847.5400 | 830.5135 | 7 |
| 3 | 284.2333 | | **312.2282** | | V | ***748.4716*** | 731.4450 | 6 |
| 4 | 431.3017 | | **459.2966** | | F | ***649.4032*** | 632.3766 | 5 |
| 5 | 544.3857 | | **572.3806** | | L | ***502.3348*** | 485.3082 | 4 |
| 6 | 658.4287 | 641.4021 | 686.4236 | 669.3970 | N | ***389.2507*** | 372.2241 | 3 |
| 7 | 786.5236 | 769.4971 | 814.5185 | 797.4920 | K | 275.2078 | 258.1812 | 2 |
| 8 | | | | | K | 147.1128 | 130.0863 | 1 |

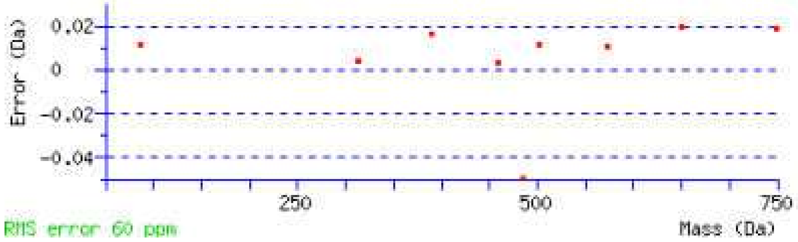


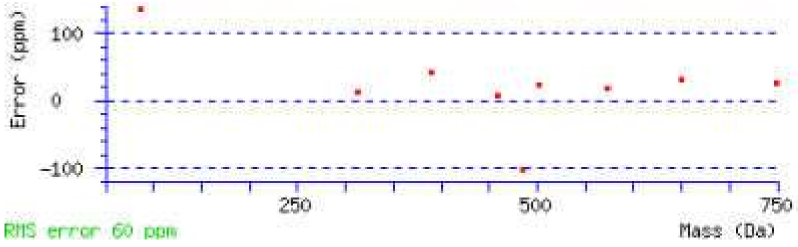


NCBI **BLAST** search of IVVFLNKK
(Parameters: blastp, nr protein database, expect=20000, no filter, PAM30)
Other BLAST web gateways

**All matches to this query**

| Score | Mr(calc) | Delta | Sequence |
|---|---|---|---|
| 36.9 | 959.5804 | 0.0164 | LVVFVLDR |
| 32.1 | 959.5804 | 0.0164 | VIVFDLVR |
| 32.1 | 959.5341 | 0.0627 | VLAFAFHR |
| 24.2 | 959.6168 | -0.0199 | IVVFLNKK |
| 23.8 | 959.5341 | 0.0627 | LQFLFHR |
| 23.8 | 959.5110 | 0.0859 | LTPMLVDR |
| 21.8 | 959.5188 | 0.0780 | SSHFIKNK |
| 21.6 | 959.5341 | 0.0627 | IGAFIFHR |
| 20.1 | 959.5705 | 0.0264 | IFLKFHR |
| 20.1 | 959.5341 | 0.0627 | IFLQFHR |

**Mascot:** http://www.matrixscience.com/

# Mascot Search Results

## Peptide View Spot no 71

MS/MS Fragmentation of **VGDTVDIVGIR**
Found in **gi|6525065** in **NCBInr**, chloroplast translational elongation factor Tu [Oryza sativa Japonica Group]

Match to Query 22: 1142.627224 from(1143.634500,1+) intensity(0.0000) index(9)
Title: Label: B3, Spot_Id: 219707, Peak_List_Id: 225183, MSMS Job_Run_Id: 21768, Comment:
Data file C:\Documents and Settings\Administrator\Desktop\farah BHU\spot set plate\Farah-Garima 11May 2013\ppw_B3_136842116600.txt

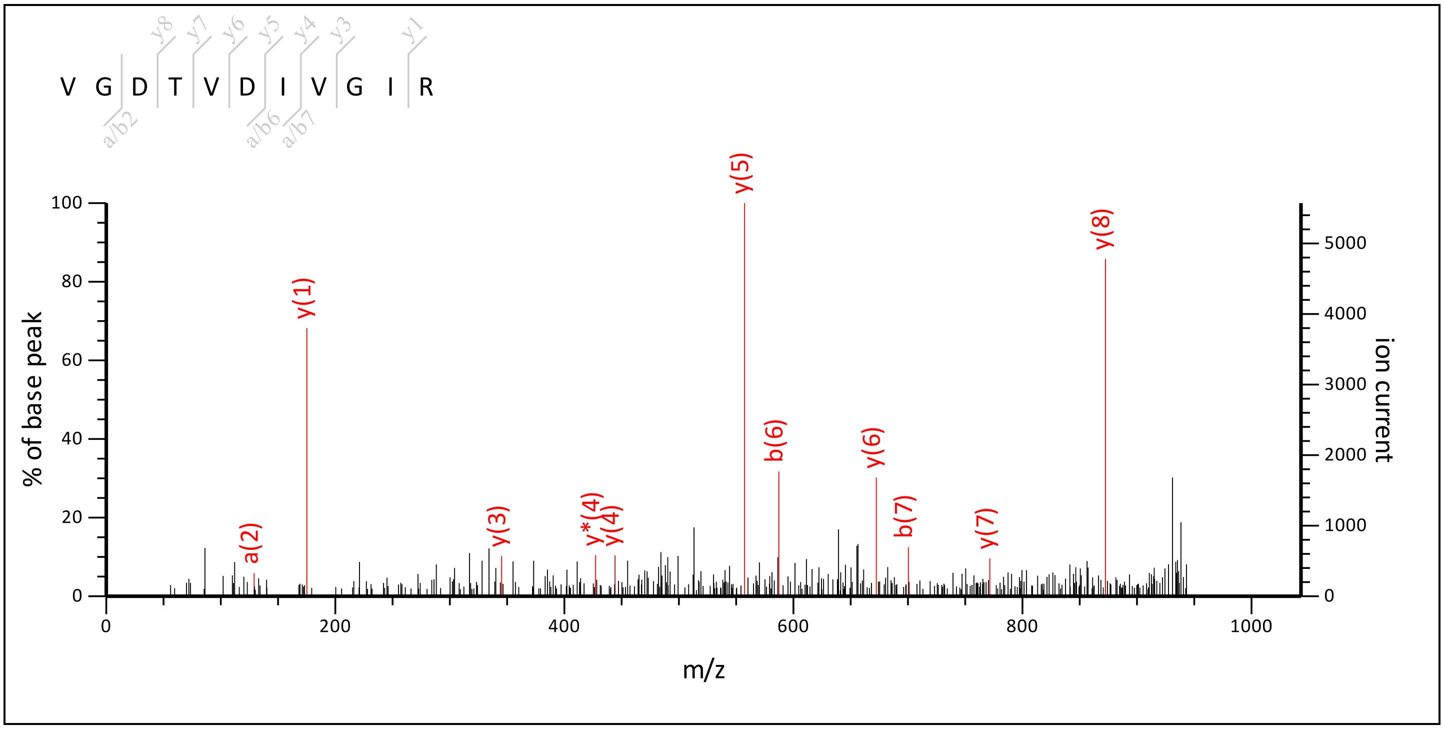


0 to 1043.2

Label all possible matches ○ Label matches used for scoring ◉

**Monoisotopic mass of neutral peptide Mr(calc):** 1142.6296
**Fixed modifications:** Carbamidomethyl (C) (apply to specified residues or termini only)
**Ions Score:** 37 **Expect:** 0.48
**Matches :** 15/40 fragment ions using 27 most intense peaks (help)

| # | a | b | Seq. | y | y* | # |
|---|---|---|---|---|---|---|
| 1 | 72.0808 | 100.0757 | V | | | 11 |
| 2 | **129.1022** | 157.0972 | G | 1044.5684 | 1027.5419 | 10 |
| 3 | 244.1292 | 272.1241 | D | 987.5469 | 970.5204 | 9 |
| 4 | **345.1769** | 373.1718 | T | ***872.5200*** | 855.4934 | 8 |
| 5 | **444.2453** | 472.2402 | V | ***771.4723*** | 754.4458 | 7 |
| 6 | 559.2722 | **587.2671** | D | ***672.4039*** | 655.3774 | 6 |
| 7 | **672.3563** | **700.3512** | I | ***557.3770*** | 540.3504 | 5 |
| 8 | **771.4247** | 799.4196 | V | ***444.2929*** | 427.2663 | 4 |

| | | | | | | |
|---|---|---|---|---|---|---|
| **9** | 828.4462 | 856.4411 | **G** | ***345.2245*** | 328.1979 | **3** |
| **10** | 941.5302 | 969.5251 | **I** | 288.2030 | 271.1765 | **2** |
| **11** | | | **R** | ***175.1190*** | 158.0924 | **1** |

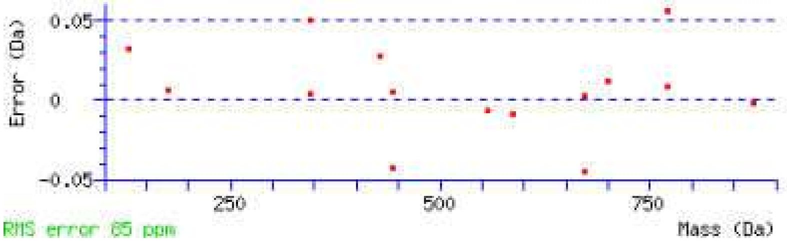


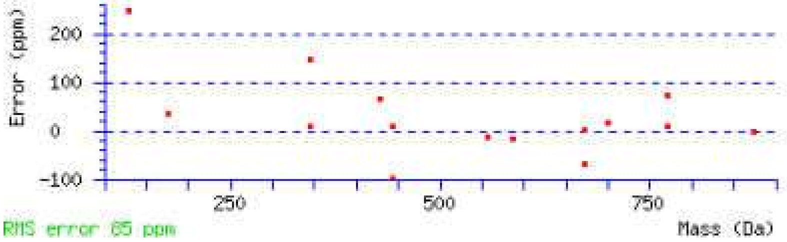


NCBI **BLAST** search of VGDTVDIVGIR
(Parameters: blastp, nr protein database, expect=20000, no filter, PAM30)
Other BLAST web gateways

**All matches to this query**

| Score | Mr(calc) | Delta | Sequence |
|---|---|---|---|
| 36.5 | 1142.6296 | -0.0023 | VGDTVDIVGIR |
| 26.8 | 1142.6084 | 0.0188 | WADDVLVGIR |
| 24.6 | 1142.6044 | 0.0228 | DRTVLDPATR |
| 20.4 | 1142.6408 | -0.0135 | SVEGVDLLRR |
| 18.6 | 1142.6118 | 0.0155 | LGAMIVDPATR |
| 18.6 | 1142.6659 | -0.0387 | LGLSESLVLGR |
| 18.6 | 1142.6746 | -0.0474 | LWMRLVGLR |
| 18.5 | 1142.6659 | -0.0387 | KISDLVDVVR |
| 18.5 | 1142.6295 | -0.0023 | SSPAITTLTPR |
| 18.4 | 1142.6156 | 0.0116 | RSADGAAAVGLR |

**Mascot:** http://www.matrixscience.com/

# MATRIX SCIENCE Mascot Search Results

## Peptide View Spot no 71

MS/MS Fragmentation of **ALENLMANPAIK**
Found in **gi|6525065** in **NCBInr**, chloroplast translational elongation factor Tu [Oryza sativa Japonica Group]

Match to Query 27: 1299.653824 from(1300.661100,1+) intensity(0.0000) index(11)
Title: Label: B3, Spot_Id: 219707, Peak_List_Id: 225185, MSMS Job_Run_Id: 21768, Comment:
Data file C:\Documents and Settings\Administrator\Desktop\farah BHU\spot set plate\Farah-Garima 11May 2013\ppw_B3_136842116600.txt

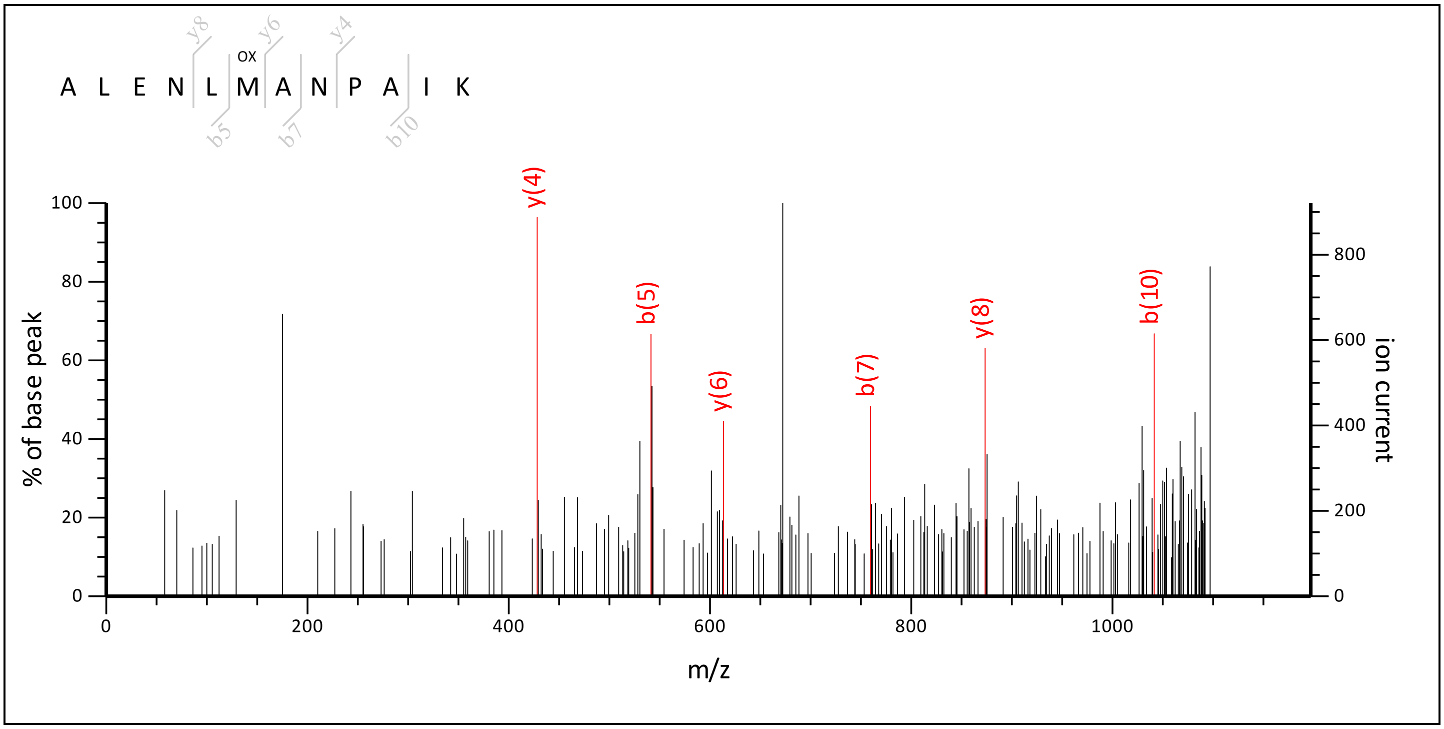


0 to 1197.05

Label all possible matches Label matches used for scoring

**Monoisotopic mass of neutral peptide Mr(calc):** 1299.6856
**Fixed modifications:** Carbamidomethyl (C) (apply to specified residues or termini only)
**Variable modifications:**
**M6** : Oxidation (M), with neutral losses 0.0000(shown in table), 63.9983
**Ions Score:** 25 **Expect:** 6.5
**Matches :** 8/94 fragment ions using 11 most intense peaks (help)

| # | a | a* | b | b* | Seq. | y | y* | # |
|---|---|---|---|---|---|---|---|---|
| 1 | 44.0495 | | 72.0444 | | A | | | 12 |
| 2 | 157.1335 | | 185.1285 | | L | 1229.6558 | 1212.6293 | 11 |
| 3 | 286.1761 | | 314.1710 | | E | 1116.5718 | 1099.5452 | 10 |
| 4 | 400.2191 | 383.1925 | ***428.2140*** | 411.1874 | N | 987.5292 | 970.5026 | 9 |
| 5 | 513.3031 | 496.2766 | ***541.2980*** | 524.2715 | L | **873.4863** | 856.4597 | 8 |
| 6 | 660.3385 | 643.3120 | 688.3334 | 671.3069 | M | 760.4022 | 743.3756 | 7 |

| | | | | | | | | |
|---|---|---|---|---|---|---|---|---|
| **7** | 731.3756 | 714.3491 | ***759.3706*** | 742.3440 | **A** | **613.3668** | 596.3402 | **6** |
| **8** | 845.4186 | 828.3920 | ***873.4135*** | 856.3869 | **N** | 542.3297 | 525.3031 | **5** |
| **9** | 942.4713 | 925.4448 | 970.4662 | 953.4397 | **P** | **428.2867** | 411.2602 | **4** |
| **10** | 1013.5084 | 996.4819 | ***1041.5034*** | 1024.4768 | **A** | 331.2340 | 314.2074 | **3** |
| **11** | 1126.5925 | 1109.5660 | 1154.5874 | 1137.5609 | **I** | 260.1969 | 243.1703 | **2** |
| **12** | | | | | **K** | 147.1128 | 130.0863 | **1** |

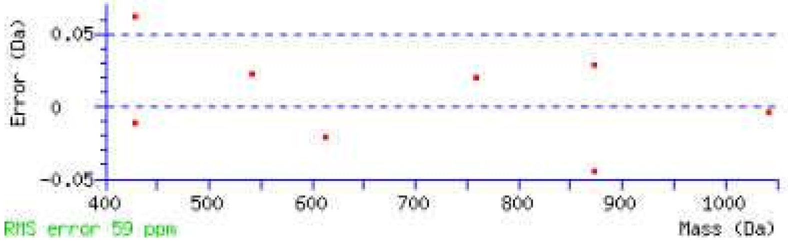


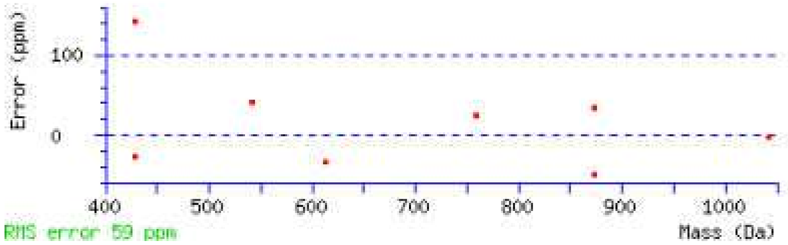


NCBI **BLAST** search of ALENLMANPAIK
(Parameters: blastp, nr protein database, expect=20000, no filter, PAM30)
Other BLAST web gateways

**All matches to this query**

| Score | Mr(calc) | Delta | Sequence |
|---|---|---|---|
| 25.4 | 1299.6856 | -0.0318 | ALENLMANPAIK |
| 21.4 | 1299.6354 | 0.0184 | GAGEKVCGNGVPR |
| 15.7 | 1299.6895 | -0.0357 | RASVAGASSPLER |
| 15.5 | 1299.6782 | -0.0244 | LQGENSAEIIAR |
| 14.9 | 1299.6870 | -0.0332 | GGPGIACFIPRR |
| 14.8 | 1299.7220 | -0.0682 | KAIDLMQPQIK |
| 14.8 | 1299.7220 | -0.0682 | KALDLMQPQIK |
| 14.8 | 1299.6857 | -0.0318 | QALDLMQPQIK |
| 14.1 | 1299.7663 | -0.1124 | KLWNSLSLLAR |
| 14.1 | 1299.7048 | -0.0509 | NLVTNSPPFRR |

**Mascot:** http://www.matrixscience.com/

# Mascot Search Results

## Peptide View Spot no 71

MS/MS Fragmentation of **KYDEIDAAPEER**
Found in **gi|6525065** in **NCBInr**, chloroplast translational elongation factor Tu [Oryza sativa Japonica Group]

Match to Query 34: 1434.649024 from(1435.656300,1+) intensity(0.0000) index(14)
Title: Label: B3, Spot_Id: 219707, Peak_List_Id: 225168, MSMS Job_Run_Id: 21768, Comment:
Data file C:\Documents and Settings\Administrator\Desktop\farah BHU\spot set plate\Farah-Garima 11May 2013\ppw_B3_136842116600.txt

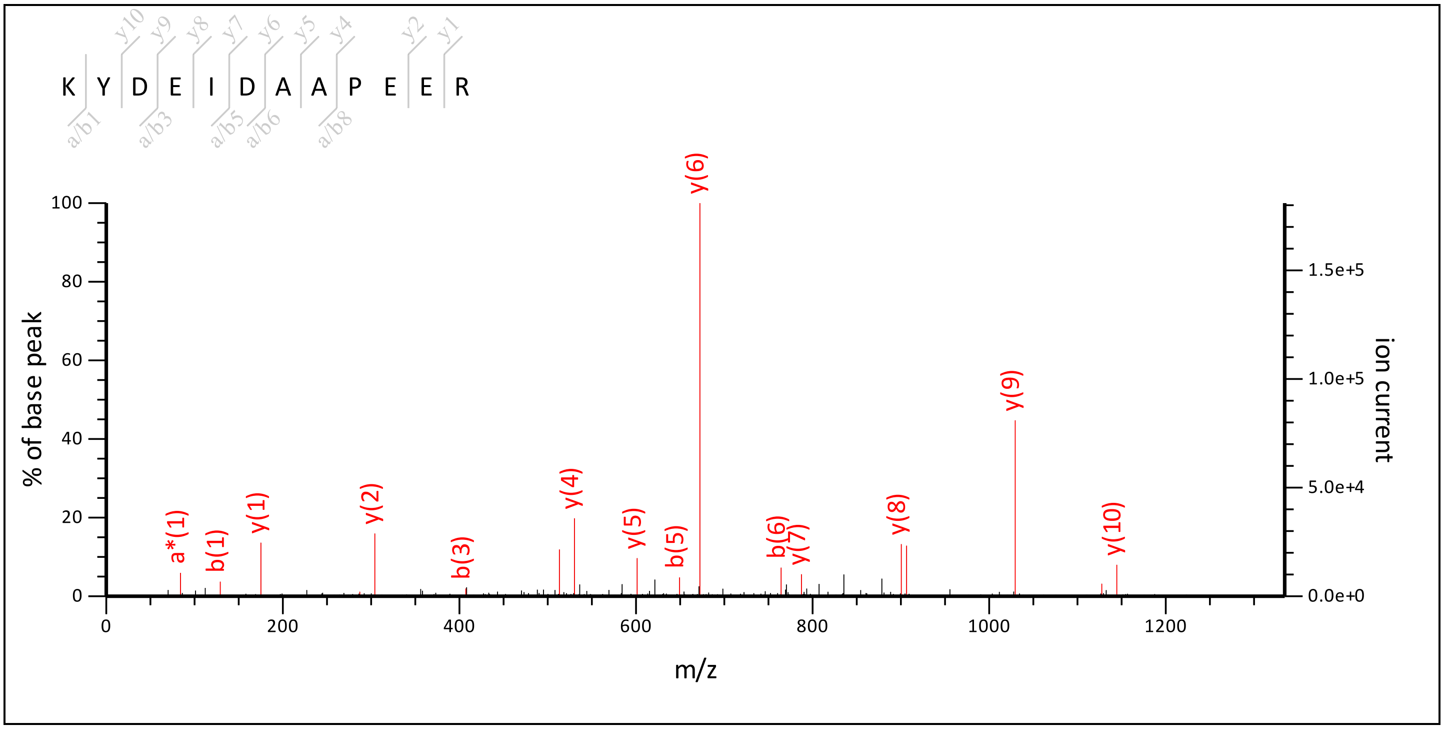


0 to 1334.71

Label all possible matches ○ Label matches used for scoring ◉

**Monoisotopic mass of neutral peptide Mr(calc):** 1434.6626
**Fixed modifications:** Carbamidomethyl (C) (apply to specified residues or termini only)
**Ions Score:** 76 **Expect:** 5e-05
**Matches :** 18/66 fragment ions using 24 most intense peaks (help)

| # | a | a* | b | b* | Seq. | y | y* | # |
|---|---|---|---|---|---|---|---|---|
| 1 | 101.1073 | 84.0808 | **129.1022** | 112.0757 | K | | | 12 |
| 2 | 264.1707 | 247.1441 | 292.1656 | 275.1390 | Y | 1307.5750 | 1290.5485 | 11 |
| 3 | 379.1976 | 362.1710 | **407.1925** | 390.1660 | D | ***1144.5117*** | 1127.4851 | 10 |
| 4 | 508.2402 | 491.2136 | 536.2351 | 519.2086 | E | ***1029.4847*** | 1012.4582 | 9 |
| 5 | 621.3243 | 604.2977 | **649.3192** | 632.2926 | I | ***900.4421*** | 883.4156 | 8 |
| 6 | 736.3512 | 719.3246 | **764.3461** | 747.3196 | D | ***787.3581*** | 770.3315 | 7 |
| 7 | 807.3883 | 790.3618 | 835.3832 | 818.3567 | A | ***672.3311*** | 655.3046 | 6 |
| 8 | 878.4254 | 861.3989 | **906.4203** | 889.3938 | A | ***601.2940*** | 584.2675 | 5 |

| | | | | | | | | |
|---|---|---|---|---|---|---|---|---|
| 9 | 975.4782 | 958.4516 | 1003.4731 | 986.4466 | P | 530.2569 | 513.2304 | 4 |
| 10 | 1104.5208 | 1087.4942 | 1132.5157 | 1115.4891 | E | 433.2041 | 416.1776 | 3 |
| 11 | 1233.5634 | 1216.5368 | 1261.5583 | 1244.5317 | E | 304.1615 | 287.1350 | 2 |
| 12 | | | | | R | 175.1190 | 158.0924 | 1 |

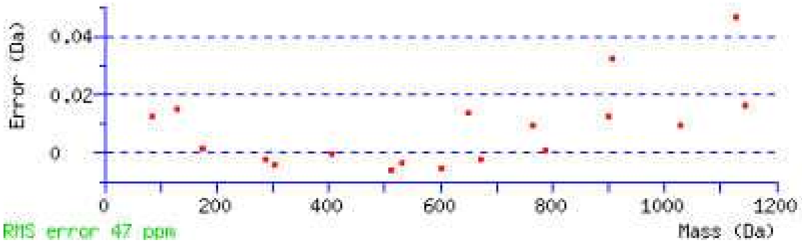


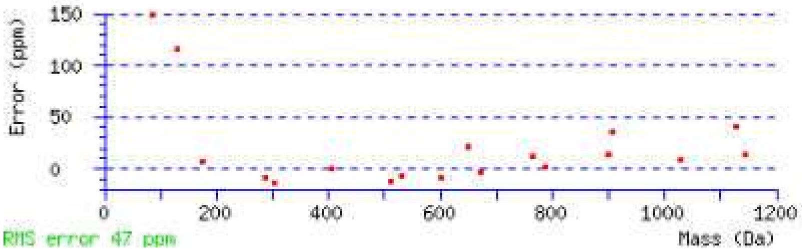


NCBI **BLAST** search of KYDEIDAAPEER
(Parameters: blastp, nr protein database, expect=20000, no filter, PAM30)
Other BLAST web gateways

**All matches to this query**

| Score | Mr(calc) | Delta | Sequence |
|---|---|---|---|
| 75.7 | 1434.6626 | -0.0136 | KYDEIDAAPEER |
| 27.4 | 1434.7541 | -0.1050 | YMTGPTLAALIER |
| 24.9 | 1434.6626 | -0.0136 | RYDEIDAAPEEK |
| 17.4 | 1434.6966 | -0.0475 | WGYEMPVDVLAR |
| 17.2 | 1434.6739 | -0.0249 | ENAKSAGFTPEER |
| 17.0 | 1434.7354 | -0.0864 | QEDSIVFSLIER |
| 16.6 | 1434.6462 | 0.0028 | HMSYHYALQNR |
| 14.3 | 1434.6991 | -0.0500 | GVSYEDPSVQLNK |
| 13.7 | 1434.6661 | -0.0170 | TMVDSSVVEEAPR |
| 11.8 | 1434.6959 | -0.0469 | VKDEVCMGNVIR |

**Mascot:** http://www.matrixscience.com/

# Mascot Search Results

## Peptide View **Spot no 71**

MS/MS Fragmentation of **VGDTVDIVGIRETR**
Found in **gi|6525065** in **NCBInr**, chloroplast translational elongation factor Tu [Oryza sativa Japonica Group]

Match to Query 40: 1528.806224 from(1529.813500,1+) intensity(0.0000) index(16)
Title: Label: B3, Spot_Id: 219707, Peak_List_Id: 225172, MSMS Job_Run_Id: 21768, Comment:
Data file C:\Documents and Settings\Administrator\Desktop\farah BHU\spot set plate\Farah-Garima 11May 2013\ppw_B3_136842116600.txt

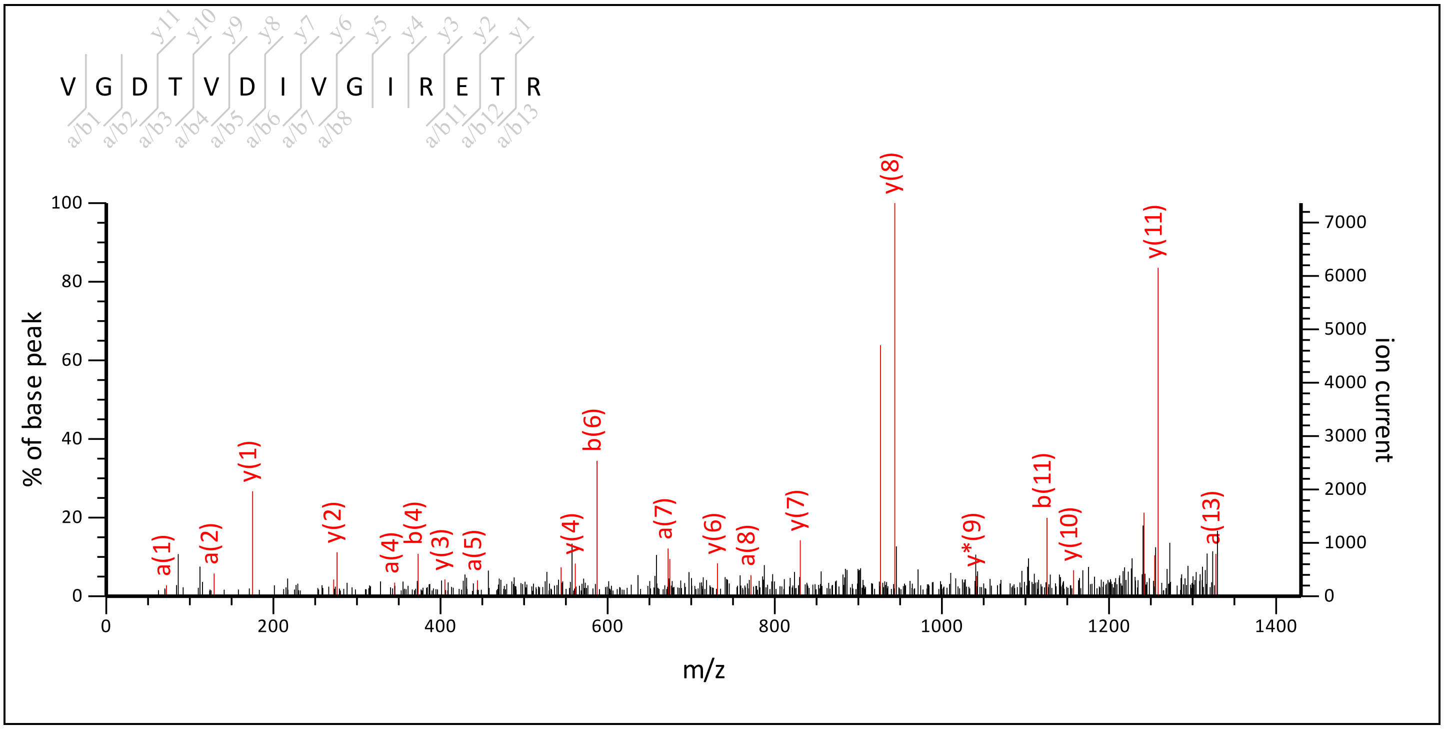


0 to 1429.7

Label all possible matches ○ Label matches used for scoring ◉

**Monoisotopic mass of neutral peptide Mr(calc):** 1528.8209
**Fixed modifications:** Carbamidomethyl (C) (apply to specified residues or termini only)
**Ions Score:** 45 **Expect:** 0.061
**Matches :** 26/58 fragment ions using 78 most intense peaks (help)

| # | a | a* | b | b* | Seq. | y | y* | # |
|---|---|---|---|---|---|---|---|---|
| 1 | *72.0808* | | 100.0757 | | V | | | 14 |
| 2 | *129.1022* | | 157.0972 | | G | 1430.7598 | 1413.7332 | 13 |
| 3 | 244.1292 | | **272.1241** | | D | 1373.7383 | 1356.7118 | 12 |
| 4 | *345.1769* | | **373.1718** | | T | *1258.7114* | 1241.6848 | 11 |
| 5 | *444.2453* | | 472.2402 | | V | *1157.6637* | 1140.6371 | 10 |
| 6 | 559.2722 | | **587.2671** | | D | 1058.5953 | 1041.5687 | 9 |
| 7 | *672.3563* | | 700.3512 | | I | *943.5683* | 926.5418 | 8 |
| 8 | *771.4247* | | 799.4196 | | V | *830.4843* | 813.4577 | 7 |

| | | | | | | | | |
|---|---|---|---|---|---|---|---|---|
| 9 | 828.4462 | | 856.4411 | | G | 731.4159 | 714.3893 | 6 |
| 10 | 941.5302 | | 969.5251 | | I | 674.3944 | 657.3679 | 5 |
| 11 | 1097.6313 | 1080.6048 | 1125.6263 | 1108.5997 | R | 561.3103 | 544.2838 | 4 |
| 12 | 1226.6739 | 1209.6474 | 1254.6688 | 1237.6423 | E | 405.2092 | 388.1827 | 3 |
| 13 | 1327.7216 | 1310.6951 | 1355.7165 | 1338.6900 | T | 276.1666 | 259.1401 | 2 |
| 14 | | | | | R | 175.1190 | 158.0924 | 1 |

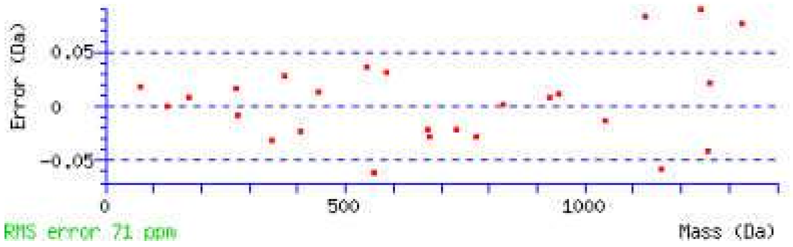


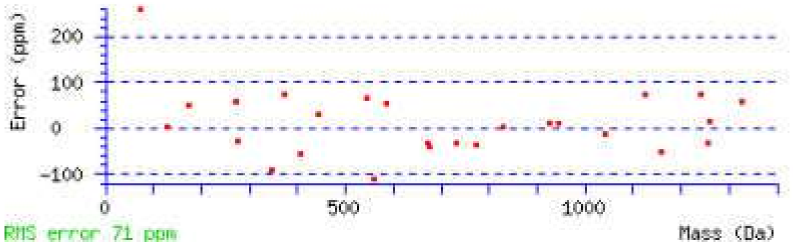


NCBI **BLAST** search of VGDTVDIVGIRETR
(Parameters: blastp, nr protein database, expect=20000, no filter, PAM30)
Other BLAST web gateways

**All matches to this query**

| Score | Mr(calc) | Delta | Sequence |
|---|---|---|---|
| 44.8 | 1528.8209 | -0.0147 | VGDTVDIVGIRETR |
| 27.6 | 1528.7555 | 0.0507 | ISAAMPVHSKDTEK |
| 23.1 | 1528.7310 | 0.0752 | EQWVEAISWEPR |
| 21.8 | 1528.8097 | -0.0034 | ISDGALVEAAILSDR |
| 21.5 | 1528.7668 | 0.0394 | MPQAQGGTAVSLTPR |
| 20.7 | 1528.7482 | 0.0581 | AVSSNGSPSPSPVTSR |
| 20.5 | 1528.8209 | -0.0147 | VGDTVEIVGVRETR |
| 18.8 | 1528.7555 | 0.0507 | EAAELAADSPMGVLR |
| 17.3 | 1528.8283 | -0.0221 | MATIPAAITTPSISR |
| 16.5 | 1528.7780 | 0.0283 | EMVNLLRNNSSPR |

**Mascot:** http://www.matrixscience.com/

# Mascot Search Results

## Peptide View Spot no 71

MS/MS Fragmentation of **TMDDAMAGDNVGLLLR**
Found in **gi|6525065** in **NCBInr**, chloroplast translational elongation factor Tu [Oryza sativa Japonica Group]

Match to Query 45: 1722.761524 from(1723.768800,1+) intensity(0.0000) index(18)
Title: Label: B3, Spot_Id: 219707, Peak_List_Id: 225171, MSMS Job_Run_Id: 21768, Comment:
Data file C:\Documents and Settings\Administrator\Desktop\farah BHU\spot set plate\Farah-Garima 11May 2013\ppw_B3_136842116600.txt

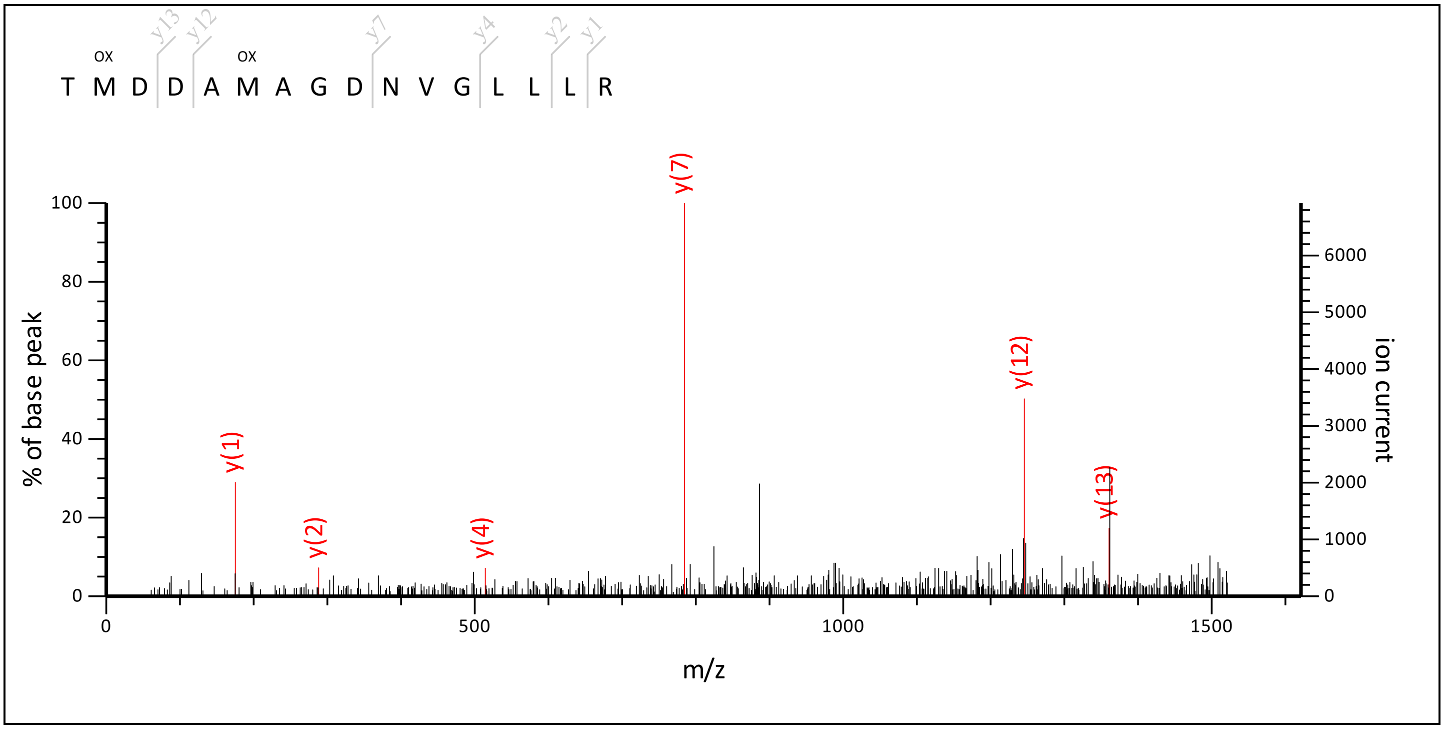


0 to 1621.14

Label all possible matches ○ Label matches used for scoring ◉

**Monoisotopic mass of neutral peptide Mr(calc):** 1722.7917
**Fixed modifications:** Carbamidomethyl (C) (apply to specified residues or termini only)
**Variable modifications:**
**M2** : Oxidation (M), with neutral losses 0.0000(shown in table), 63.9983
**M6** : Oxidation (M), with neutral losses 0.0000(shown in table), 63.9983
**Ions Score:** 25 **Expect:** 4.3
**Matches :** 7/122 fragment ions using 15 most intense peaks (help)

| # | a | a* | b | b* | Seq. | y | y* | # |
|---|---|---|---|---|---|---|---|---|
| 1 | 74.0600 | | 102.0550 | | T | | | 16 |
| 2 | 221.0954 | | 249.0904 | | M | 1622.7513 | 1605.7247 | 15 |
| 3 | 336.1224 | | 364.1173 | | D | 1475.7159 | 1458.6893 | 14 |
| 4 | 451.1493 | | 479.1442 | | D | *1360.6889* | 1343.6624 | 13 |
| 5 | 522.1864 | | 550.1814 | | A | *1245.6620* | 1228.6354 | 12 |
| 6 | 669.2218 | | 697.2168 | | M | 1174.6249 | 1157.5983 | 11 |

| | | | | | | | | |
|---|---|---|---|---|---|---|---|---|
| 7 | 740.2590 | | 768.2539 | | A | 1027.5895 | 1010.5629 | 10 |
| 8 | 797.2804 | | 825.2753 | | G | 956.5524 | 939.5258 | 9 |
| 9 | 912.3074 | | 940.3023 | | D | 899.5309 | 882.5043 | 8 |
| 10 | 1026.3503 | 1009.3237 | 1054.3452 | 1037.3187 | N | *784.5039* | 767.4774 | 7 |
| 11 | 1125.4187 | 1108.3922 | 1153.4136 | 1136.3871 | V | 670.4610 | 653.4345 | 6 |
| 12 | 1182.4402 | 1165.4136 | 1210.4351 | 1193.4085 | G | 571.3926 | 554.3661 | 5 |
| 13 | 1295.5242 | 1278.4977 | 1323.5191 | 1306.4926 | L | *514.3711* | 497.3446 | 4 |
| 14 | 1408.6083 | 1391.5817 | 1436.6032 | 1419.5767 | L | 401.2871 | 384.2605 | 3 |
| 15 | 1521.6924 | 1504.6658 | 1549.6873 | 1532.6607 | L | *288.2030* | 271.1765 | 2 |
| 16 | | | | | R | *175.1190* | 158.0924 | 1 |

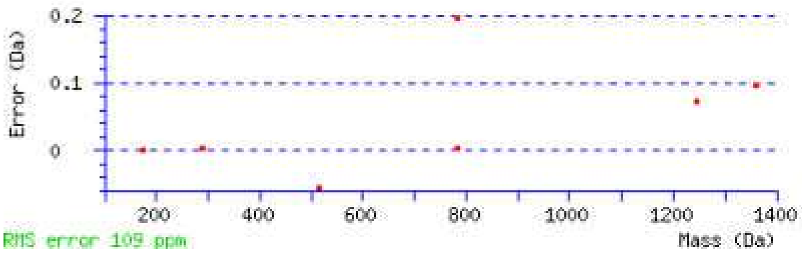


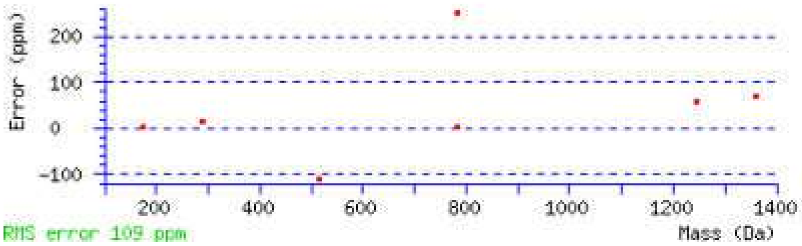


NCBI **BLAST** search of TMDDAMAGDNVGLLLR
(Parameters: blastp, nr protein database, expect=20000, no filter, PAM30)
Other BLAST web gateways

**All matches to this query**

| Score | Mr(calc) | Delta | Sequence |
|---|---|---|---|
| 25.2 | 1722.7917 | -0.0301 | TMDDAMAGDNVGLLLR |
| 10.9 | 1722.8862 | -0.1247 | ESFMELKTDELILR |
| 3.5 | 1722.7640 | -0.0025 | WIDMRELPMNCSR |
| 3.0 | 1722.8424 | -0.0809 | KSSSSEEAPASFVEIR |
| 2.0 | 1722.8901 | -0.1285 | QVITESDLTPNHTLR |
| 1.8 | 1722.7155 | 0.0460 | DSVIPADPNDSDYCR |
| 1.7 | 1722.9264 | -0.1649 | KGPSSGNLPAALDVEIR |
| 1.6 | 1722.8247 | -0.0631 | DIADNSMPNLIYVSR |
| 1.5 | 1722.7784 | -0.0169 | ASGFMGWTSHSASLER |
| 1.4 | 1722.9125 | -0.1510 | LLDHSRTVGGSQQGIR |

**Mascot:** http://www.matrixscience.com/

# MATRIX SCIENCE Mascot Search Results

## Peptide View Spot no 71

MS/MS Fragmentation of **HYAHVDCPGHADYVK**
Found in **gi|6525065** in **NCBInr**, chloroplast translational elongation factor Tu [Oryza sativa Japonica Group]

Match to Query 47: 1767.754124 from(1768.761400,1+) intensity(0.0000) index(19)
Title: Label: B3, Spot_Id: 219707, Peak_List_Id: 225169, MSMS Job_Run_Id: 21768, Comment:
Data file C:\Documents and Settings\Administrator\Desktop\farah BHU\spot set plate\Farah-Garima 11May 2013\ppw_B3_136842116600.txt

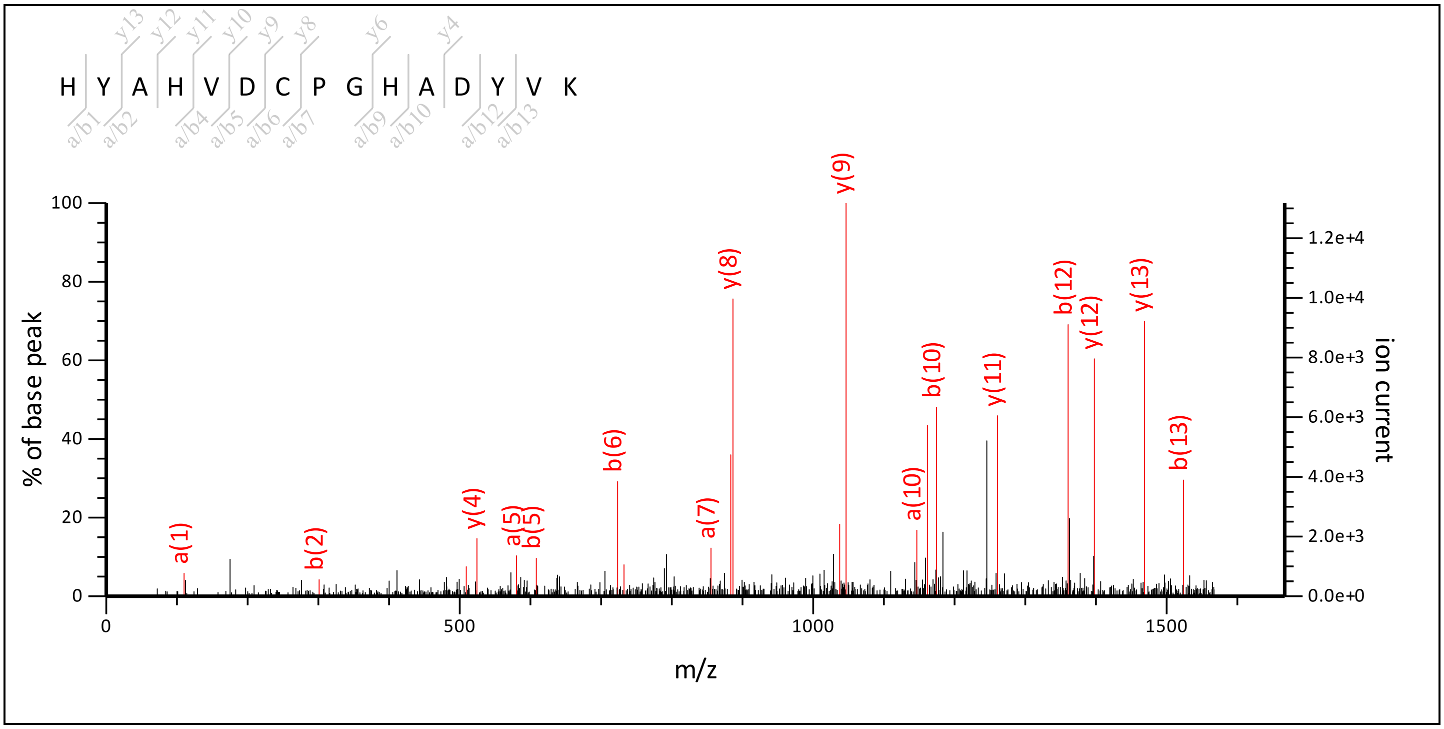


0 to 1666.98

Label all possible matches ○ Label matches used for scoring ◉

**Monoisotopic mass of neutral peptide Mr(calc):** 1767.7787
**Fixed modifications:** Carbamidomethyl (C) (apply to specified residues or termini only)
**Ions Score:** 73 **Expect:** 8e-05
**Matches :** 21/56 fragment ions using 30 most intense peaks (help)

| # | a | b | Seq. | y | y* | # |
|---|---|---|---|---|---|---|
| 1 | **110.0713** | 138.0662 | H | | | 15 |
| 2 | 273.1346 | ***301.1295*** | Y | 1631.7271 | 1614.7006 | 14 |
| 3 | 344.1717 | 372.1666 | A | ***1468.6638*** | 1451.6372 | 13 |
| 4 | 481.2306 | ***509.2255*** | H | ***1397.6267*** | 1380.6001 | 12 |
| 5 | **580.2990** | ***608.2940*** | V | ***1260.5678*** | 1243.5412 | 11 |
| 6 | 695.3260 | ***723.3209*** | D | ***1161.4993*** | 1144.4728 | 10 |
| 7 | **855.3566** | ***883.3515*** | C | ***1046.4724*** | 1029.4458 | 9 |
| 8 | 952.4094 | 980.4043 | P | ***886.4417*** | 869.4152 | 8 |

| | | | | | | |
|---|---|---|---|---|---|---|
| **9** | 1009.4309 | ***1037.4258*** | **G** | 789.3890 | 772.3624 | **7** |
| **10** | **1146.4898** | ***1174.4847*** | **H** | ***732.3675*** | 715.3410 | **6** |
| **11** | 1217.5269 | 1245.5218 | **A** | 595.3086 | 578.2821 | **5** |
| **12** | 1332.5538 | ***1360.5487*** | **D** | ***524.2715*** | 507.2449 | **4** |
| **13** | 1495.6172 | ***1523.6121*** | **Y** | 409.2445 | 392.2180 | **3** |
| **14** | 1594.6856 | 1622.6805 | **V** | 246.1812 | 229.1547 | **2** |
| **15** | | | **K** | 147.1128 | 130.0863 | **1** |

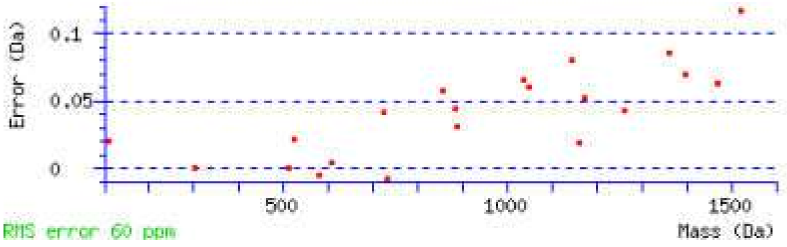


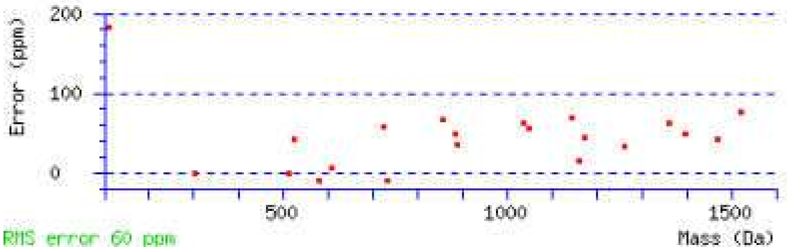


NCBI **BLAST** search of HYAHVDCPGHADYVK
(Parameters: blastp, nr protein database, expect=20000, no filter, PAM30)
Other BLAST web gateways

**All matches to this query**

| Score | Mr(calc) | Delta | Sequence |
|---|---|---|---|
| 72.6 | 1767.7787 | -0.0246 | HYAHVDCPGHADYVK |
| 9.2 | 1767.8652 | -0.1111 | HSVDVANYNHSIVSAR |
| 7.8 | 1767.8441 | -0.0900 | HINKDGPHFNEAAYR |
| 7.8 | 1767.8077 | -0.0536 | HINQDGPHFNEAAYR |
| 7.7 | 1767.8760 | -0.1219 | INTPALICHNMIEAR |
| 5.5 | 1767.8839 | -0.1297 | HVREHGVQYMLEVR |
| 5.2 | 1767.8944 | -0.1403 | NLDLVAEKYQSWFR |
| 5.2 | 1767.7291 | 0.0250 | EGEEGMVDQAQATMEK |
| 4.8 | 1767.8072 | -0.0531 | SVMEYLCEFNHLAR |
| 4.8 | 1767.8219 | -0.0678 | HVYVCATTGGKLCMR |

**Mascot:** http://www.matrixscience.com/

# MATRIX SCIENCE Mascot Search Results

## Peptide View Spot no 71

MS/MS Fragmentation of **GITINTATVEYETETR**
Found in **gi|6525065** in **NCBInr**, chloroplast translational elongation factor Tu [Oryza sativa Japonica Group]

Match to Query 51: 1796.863524 from(1797.870800,1+) intensity(0.0000) index(21)
Title: Label: B3, Spot_Id: 219707, Peak_List_Id: 225177, MSMS Job_Run_Id: 21768, Comment:
Data file C:\Documents and Settings\Administrator\Desktop\farah BHU\spot set plate\Farah-Garima 11May 2013\ppw_B3_136842116600.txt

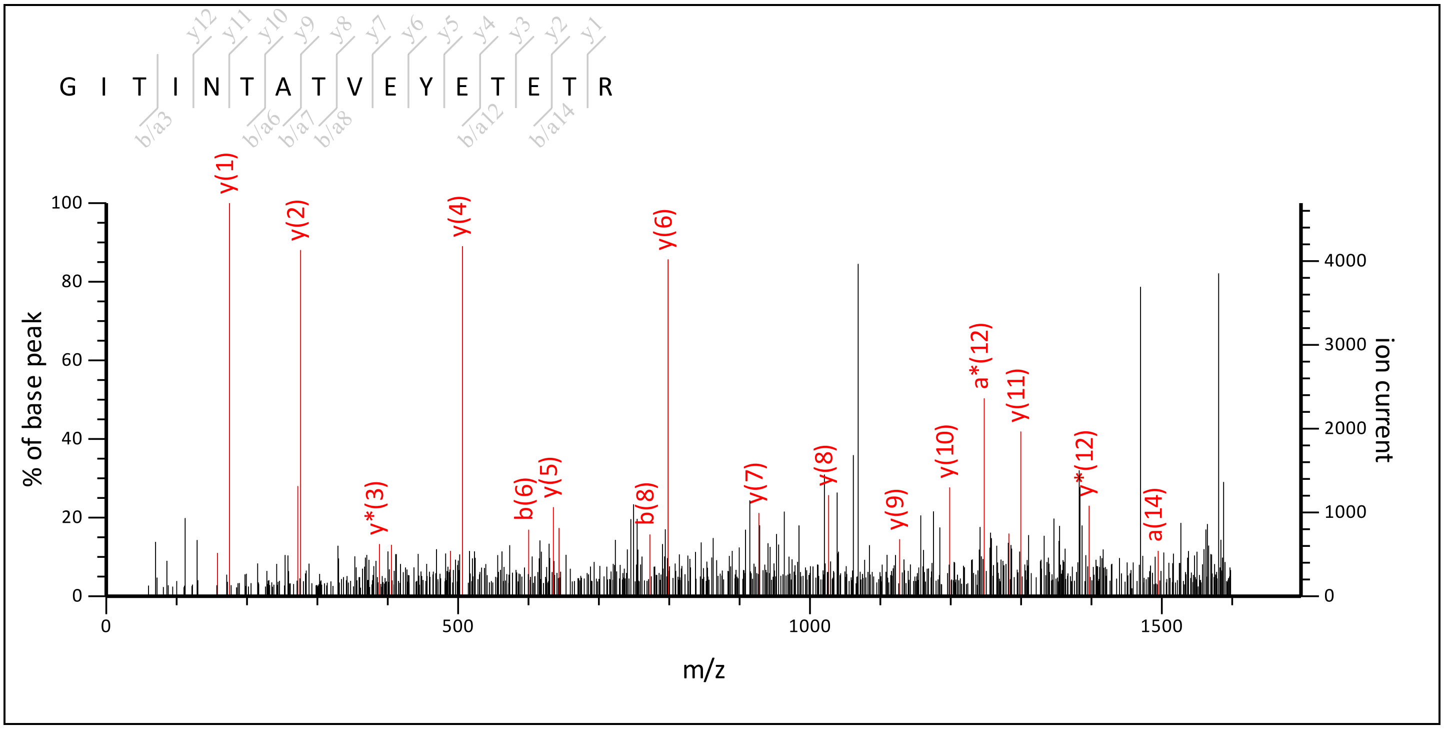


0 to 1697.75

Label all possible matches ○ Label matches used for scoring ◉

**Monoisotopic mass of neutral peptide Mr(calc):** 1796.8792
**Fixed modifications:** Carbamidomethyl (C) (apply to specified residues or termini only)
**Ions Score:** 44 **Expect:** 0.068
**Matches :** 22/82 fragment ions using 64 most intense peaks (help)

| # | a | a* | b | b* | Seq. | y | y* | # |
|---|---|---|---|---|---|---|---|---|
| 1 | 30.0338 | | 58.0287 | | G | | | 16 |
| 2 | 143.1179 | | 171.1128 | | I | 1740.8650 | 1723.8385 | 15 |
| 3 | 244.1656 | | **272.1605** | | T | 1627.7810 | 1610.7544 | 14 |
| 4 | 357.2496 | | 385.2445 | | I | 1526.7333 | 1509.7067 | 13 |
| 5 | 471.2926 | 454.2660 | 499.2875 | 482.2609 | N | 1413.6492 | 1396.6227 | 12 |
| 6 | 572.3402 | 555.3137 | **600.3352** | 583.3086 | T | ***1299.6063*** | 1282.5798 | 11 |
| 7 | **643.3774** | 626.3508 | 671.3723 | 654.3457 | A | ***1198.5586*** | 1181.5321 | 10 |
| 8 | 744.4250 | 727.3985 | **772.4199** | 755.3934 | T | ***1127.5215*** | 1110.4950 | 9 |

| | | | | | | | | |
|---|---|---|---|---|---|---|---|---|
| 9 | 843.4934 | 826.4669 | 871.4884 | 854.4618 | V | 1026.4738 | 1009.4473 | 8 |
| 10 | 972.5360 | 955.5095 | 1000.5310 | 983.5044 | E | 927.4054 | 910.3789 | 7 |
| 11 | 1135.5994 | 1118.5728 | 1163.5943 | 1146.5677 | Y | 798.3628 | 781.3363 | 6 |
| 12 | 1264.6420 | 1247.6154 | 1292.6369 | 1275.6103 | E | 635.2995 | 618.2729 | 5 |
| 13 | 1365.6896 | 1348.6631 | 1393.6846 | 1376.6580 | T | 506.2569 | 489.2304 | 4 |
| 14 | 1494.7322 | 1477.7057 | 1522.7271 | 1505.7006 | E | 405.2092 | 388.1827 | 3 |
| 15 | 1595.7799 | 1578.7534 | 1623.7748 | 1606.7483 | T | 276.1666 | 259.1401 | 2 |
| 16 | | | | | R | 175.1190 | 158.0924 | 1 |

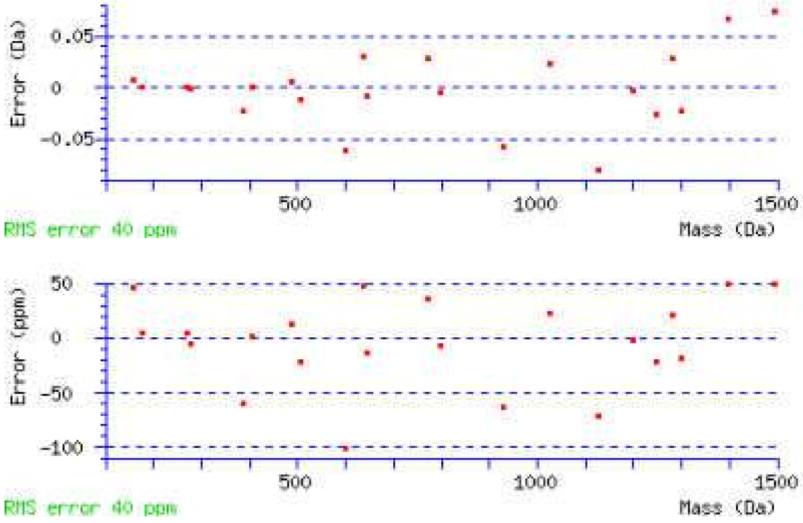


NCBI **BLAST** search of GITINTATVEYETETR
(Parameters: blastp, nr protein database, expect=20000, no filter, PAM30)
Other BLAST web gateways

**All matches to this query**

| Score | Mr(calc) | Delta | Sequence |
|---|---|---|---|
| 43.8 | 1796.8792 | -0.0157 | GITINTATVEYETETR |
| 7.7 | 1796.7119 | 0.1516 | SGSPMREDGETDDETR |
| 7.1 | 1796.9203 | -0.0568 | ISQSMASTKPYTRATR |
| 4.8 | 1796.9380 | -0.0745 | SLSNPTSSLPRISEGPR |
| 3.6 | 1796.9203 | -0.0568 | GVDGSGANHLGTLLMSIR |
| 3.5 | 1796.8776 | -0.0141 | KFEFMGLLCCHVLK |
| 3.4 | 1796.8549 | 0.0086 | RFDDALSLMENLMSR |
| 3.3 | 1796.9342 | -0.0707 | QFETSNKALASTLIMK |
| 3.2 | 1796.8358 | 0.0277 | AMVAEMESLAKDMVEK |
| 2.6 | 1796.8529 | 0.0106 | HFMNPRTYDLGPHGR |

**Mascot:** http://www.matrixscience.com/

# MATRIX SCIENCE Mascot Search Results

## Peptide View Spot no 71

MS/MS Fragmentation of **TKPHVNIGTIGHVDHGK**
Found in **gi|6525065** in **NCBInr**, chloroplast translational elongation factor Tu [Oryza sativa Japonica Group]

Match to Query 53: 1808.939124 from(1809.946400,1+) intensity(0.0000) index(22)
Title: Label: B3, Spot_Id: 219707, Peak_List_Id: 225174, MSMS Job_Run_Id: 21768, Comment:
Data file C:\Documents and Settings\Administrator\Desktop\farah BHU\spot set plate\Farah-Garima 11May 2013\ppw_B3_136842116600.txt

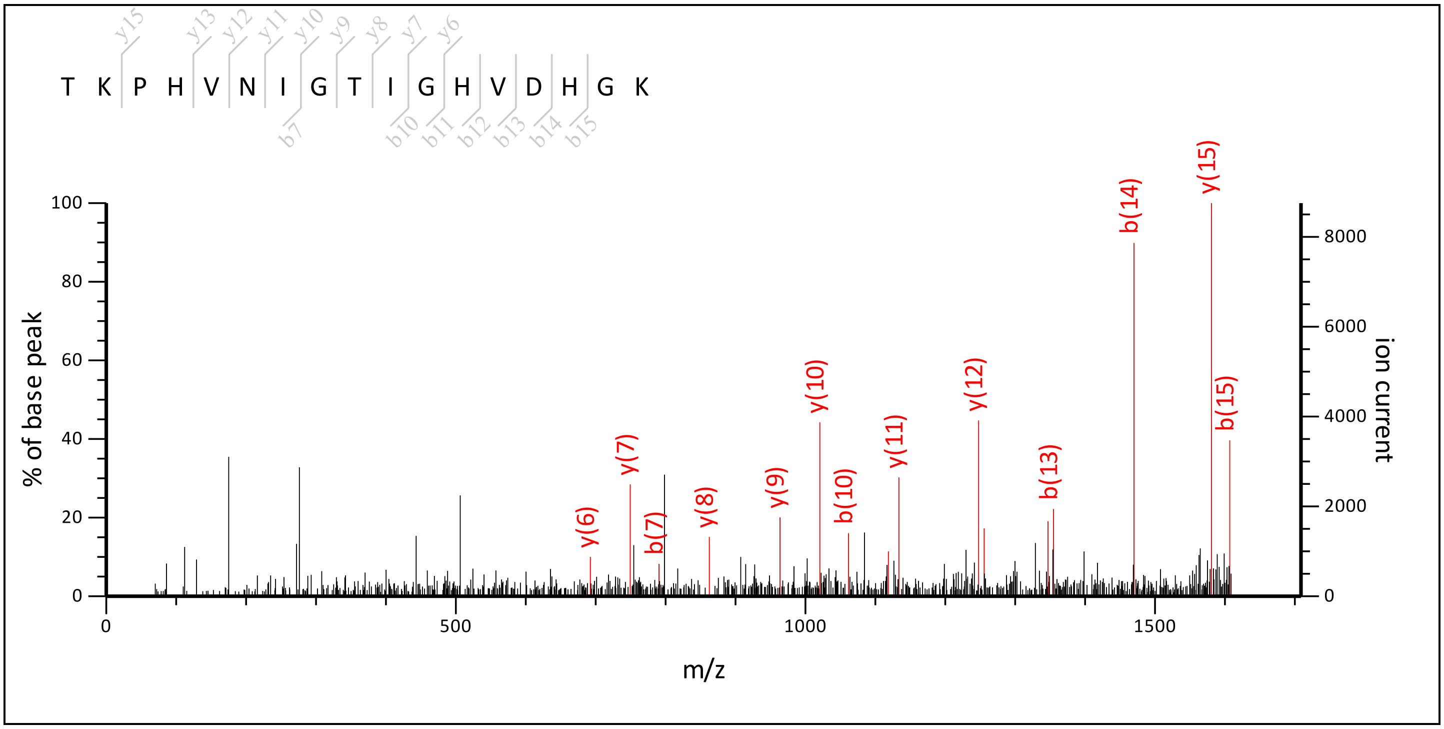


0 to 1708.78

Label all possible matches ○ Label matches used for scoring ◉

**Monoisotopic mass of neutral peptide Mr(calc):** 1808.9646
**Fixed modifications:** Carbamidomethyl (C) (apply to specified residues or termini only)
**Ions Score:** 42 **Expect:** 0.19
**Matches :** 16/94 fragment ions using 48 most intense peaks (help)

| # | a | a* | b | b* | Seq. | y | y* | # |
|---|---|---|---|---|---|---|---|---|
| 1 | 74.0600 | | 102.0550 | | T | | | 17 |
| 2 | 202.1550 | 185.1285 | 230.1499 | 213.1234 | K | 1708.9242 | 1691.8976 | 16 |
| 3 | 299.2078 | 282.1812 | 327.2027 | 310.1761 | P | *1580.8292* | 1563.8027 | 15 |
| 4 | 436.2667 | 419.2401 | 464.2616 | 447.2350 | H | 1483.7764 | 1466.7499 | 14 |
| 5 | 535.3351 | 518.3085 | 563.3300 | 546.3035 | V | *1346.7175* | 1329.6910 | 13 |
| 6 | 649.3780 | 632.3515 | 677.3729 | 660.3464 | N | *1247.6491* | 1230.6226 | 12 |
| 7 | 762.4621 | 745.4355 | *790.4570* | 773.4304 | I | *1133.6062* | 1116.5796 | 11 |
| 8 | 819.4835 | 802.4570 | 847.4785 | 830.4519 | G | *1020.5221* | 1003.4956 | 10 |

| | | | | | | | | |
|---|---|---|---|---|---|---|---|---|
| 9 | 920.5312 | 903.5047 | 948.5261 | 931.4996 | T | 963.5007 | 946.4741 | 9 |
| 10 | 1033.6153 | 1016.5887 | 1061.6102 | 1044.5837 | I | 862.4530 | 845.4264 | 8 |
| 11 | 1090.6368 | 1073.6102 | 1118.6317 | 1101.6051 | G | 749.3689 | 732.3424 | 7 |
| 12 | 1227.6957 | 1210.6691 | 1255.6906 | 1238.6640 | H | 692.3474 | 675.3209 | 6 |
| 13 | 1326.7641 | 1309.7375 | 1354.7590 | 1337.7324 | V | 555.2885 | 538.2620 | 5 |
| 14 | 1441.7910 | 1424.7645 | 1469.7859 | 1452.7594 | D | 456.2201 | 439.1936 | 4 |
| 15 | 1578.8499 | 1561.8234 | 1606.8449 | 1589.8183 | H | 341.1932 | 324.1666 | 3 |
| 16 | 1635.8714 | 1618.8449 | 1663.8663 | 1646.8398 | G | 204.1343 | 187.1077 | 2 |
| 17 | | | | | K | 147.1128 | 130.0863 | 1 |

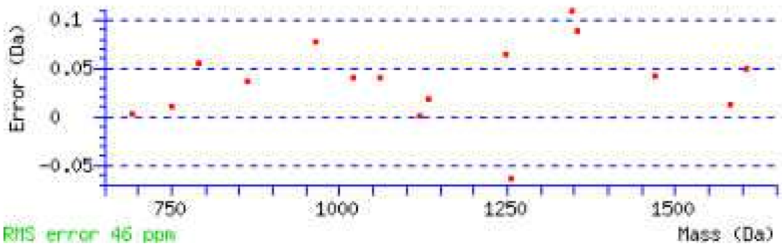


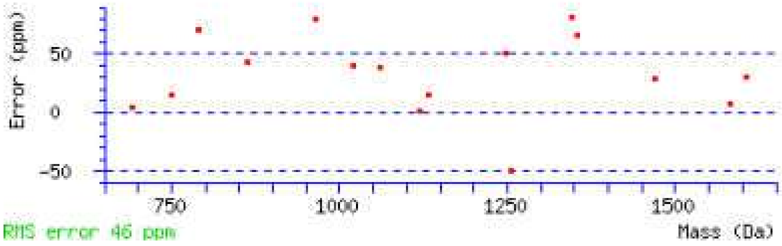


NCBI **BLAST** search of TKPHVNIGTIGHVDHGK
(Parameters: blastp, nr protein database, expect=20000, no filter, PAM30)
Other BLAST web gateways

**All matches to this query**

| Score | Mr(calc) | Delta | Sequence |
|---|---|---|---|
| 42.4 | 1808.9646 | -0.0255 | TKPHVNIGTIGHVDHGK |
| 28.3 | 1809.0010 | -0.0618 | KKPHVSIGTIGHVDHGK |
| 27.5 | 1808.9646 | -0.0255 | TKPHLNVGTIGHVDHGK |
| 11.5 | 1808.9594 | -0.0202 | MATTELKSDAIFELLK |
| 7.4 | 1808.8781 | 0.0611 | IVYPFGIGRGCYHDR |
| 7.0 | 1808.8211 | 0.1180 | SLPMQVTSNSVSGDDTR |
| 6.9 | 1808.8476 | 0.0916 | HSVSFSTTRGMLDDTR |
| 5.2 | 1808.9394 | -0.0003 | GSYREVQSIHRPQPR |
| 4.7 | 1809.0624 | -0.1233 | KHLALGNTLLNILYAR |
| 4.5 | 1808.9758 | -0.0367 | HNIRNISVIAHVDHGK |

**Mascot:** http://www.matrixscience.com/

# MATRIX SCIENCE Mascot Search Results

## Peptide View Spot no 72

MS/MS Fragmentation of **FVIGGPHGDAGLTGR**
Found in **gi|127046** in **NCBInr**, RecName: Full=S-adenosylmethionine synthase 2; Short=AdoMet synthase 2; AltName: Full=Methionine adenosyltransferase 2; Short=MAT 2

Match to Query 8: 1452.810224 from(1453.817500,1+) intensity(0.0000) index(3)
Title: Label: F9, Spot_Id: 228975, Peak_List_Id: 257839, MSMS Job_Run_Id: 24938, Comment:
Data file ppw_F9_138985151700.txt

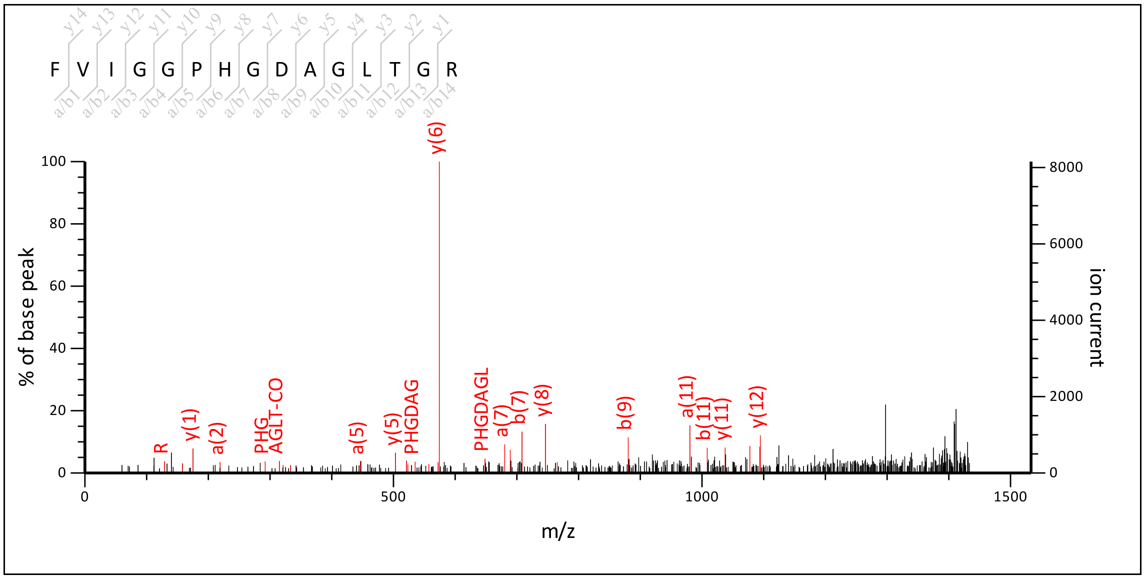


0 to 1533.27

Label all possible matches ○ Label matches used for scoring ◉

**Monoisotopic mass of neutral peptide Mr(calc):** 1452.7474
**Fixed modifications:** Carbamidomethyl (C) (apply to specified residues or termini only)
**Ions Score:** 46 **Expect:** 0.058
**Matches :** 37/241 fragment ions using 70 most intense peaks (help)

| # | Immon. | a | $a^0$ | b | $b^0$ | d | d' | Seq. | v | w | w' | y | y* | $y^0$ | # |
|---|---|---|---|---|---|---|---|---|---|---|---|---|---|---|---|
| 1 | 120.0808 | 120.0808 | | 148.0757 | | 44.0495 | | F | | | | | | | 15 |
| 2 | 72.0808 | **219.1492** | | 247.1441 | | 205.1335 | | V | 1262.6236 | 1275.6440 | | 1306.6862 | 1289.6597 | 1288.6757 | 14 |
| 3 | 86.0964 | 332.2333 | | 360.2282 | | 304.2020 | 318.2176 | I | 1149.5396 | 1162.5600 | 1176.5756 | 1207.6178 | 1190.5913 | 1189.6072 | 13 |
| 4 | 30.0338 | 389.2547 | | 417.2496 | | | | G | | | | ***1094.5337*** | 1077.5072 | 1076.5232 | 12 |
| 5 | 30.0338 | **446.2762** | | 474.2711 | | | | G | | | | ***1037.5123*** | 1020.4857 | 1019.5017 | 11 |
| 6 | 70.0651 | 543.3289 | | 571.3239 | | 517.3133 | | P | 938.4439 | 937.4486 | | ***980.4908*** | 963.4643 | 962.4803 | 10 |
| 7 | 110.0713 | **680.3879** | | **708.3828** | | | | H | 801.3850 | | | 883.4381 | 866.4115 | 865.4275 | 9 |
| 8 | 30.0338 | 737.4093 | | **765.4042** | | | | G | | | | ***746.3791*** | 729.3526 | 728.3686 | 8 |
| 9 | 88.0393 | 852.4363 | 834.4257 | **880.4312** | 862.4206 | 808.4464 | | D | 629.3366 | 628.3413 | | ***689.3577*** | 672.3311 | 671.3471 | 7 |
| 10 | 44.0495 | 923.4734 | 905.4628 | 951.4683 | 933.4577 | | | A | 558.2994 | | | ***574.3307*** | 557.3042 | 556.3202 | 6 |
| 11 | 30.0338 | **980.4948** | 962.4843 | **1008.4898** | 990.4792 | | | G | | | | ***503.2936*** | 486.2671 | 485.2831 | 5 |
| 12 | 86.0964 | **1093.5789** | 1075.5683 | 1121.5738 | 1103.5633 | 1051.5320 | | L | 388.1939 | 387.1987 | | ***446.2722*** | 429.2456 | 428.2616 | 4 |
| 13 | 74.0600 | 1194.6266 | 1176.6160 | 1222.6215 | 1204.6109 | 1178.6317 | 1180.6109 | T | 287.1462 | 300.1666 | 302.1459 | ***333.1881*** | 316.1615 | 315.1775 | 3 |
| 14 | 30.0338 | 1251.6480 | 1233.6375 | 1279.6430 | 1261.6324 | | | G | | | | 232.1404 | 215.1139 | | 2 |
| 15 | 129.1135 | | | | | | | R | 74.0237 | 73.0284 | | ***175.1190*** | 158.0924 | | 1 |

| Seq | ya | yb | Seq | ya | yb | Seq | ya | yb |
|---|---|---|---|---|---|---|---|---|
| VI | 185.1648 | 213.1598 | VIG | 242.1863 | 270.1812 | VIGG | 299.2078 | 327.2027 |
| VIGGP | 396.2605 | 424.2554 | VIGGPH | 533.3194 | 561.3144 | VIGGPHG | 590.3409 | 618.3358 |

| | | | | | | | | |
|---|---|---|---|---|---|---|---|---|
| IG | 143.1179 | 171.1128 | IGG | 200.1394 | 228.1343 | IGGP | 297.1921 | 325.1870 |
| IGGPH | 434.2510 | 462.2459 | IGGPHG | 491.2725 | 519.2674 | IGGPHGD | 606.2994 | 634.2944 |
| IGGPHGDA | 677.3365 | 705.3315 | GG | 87.0553 | 115.0502 | GGP | 184.1081 | 212.1030 |
| GGPH | 321.1670 | 349.1619 | GGPHG | 378.1884 | 406.1833 | GGPHGD | 493.2154 | 521.2103 |
| GGPHGDA | 564.2525 | 592.2474 | GGPHGDAG | 621.2740 | 649.2689 | GP | 127.0866 | 155.0815 |
| GPH | 264.1455 | 292.1404 | GPHG | 321.1670 | 349.1619 | GPHGD | 436.1939 | 464.1888 |
| GPHGDA | 507.2310 | 535.2259 | GPHGDAG | 564.2525 | 592.2474 | GPHGDAGL | 677.3365 | 705.3315 |
| PH | 207.1240 | 235.1190 | PHG | 264.1455 | 292.1404 | PHGD | 379.1724 | 407.1674 |
| PHGDA | 450.2096 | 478.2045 | PHGDAG | 507.2310 | 535.2259 | PHGDAGL | 620.3151 | 648.3100 |
| HG | 167.0927 | 195.0877 | HGD | 282.1197 | 310.1146 | HGDA | 353.1568 | 381.1517 |
| HGDAG | 410.1783 | 438.1732 | HGDAGL | 523.2623 | 551.2572 | HGDAGLT | 624.3100 | 652.3049 |
| HGDAGLTG | 681.3315 | 709.3264 | GD | 145.0608 | 173.0557 | GDA | 216.0979 | 244.0928 |
| GDAG | 273.1193 | 301.1143 | GDAGL | 386.2034 | 414.1983 | GDAGLT | 487.2511 | 515.2460 |
| GDAGLTG | 544.2726 | 572.2675 | DA | 159.0764 | 187.0713 | DAG | 216.0979 | 244.0928 |
| DAGL | 329.1819 | 357.1769 | DAGLT | 430.2296 | 458.2245 | DAGLTG | 487.2511 | 515.2460 |
| AG | 101.0709 | 129.0659 | AGL | 214.1550 | 242.1499 | AGLT | 315.2027 | 343.1976 |
| AGLTG | 372.2241 | 400.2191 | GL | 143.1179 | 171.1128 | GLT | 244.1656 | 272.1605 |
| GLTG | 301.1870 | 329.1819 | LT | 187.1441 | 215.1390 | LTG | 244.1656 | 272.1605 |
| TG | 131.0815 | 159.0764 | | | | | | |

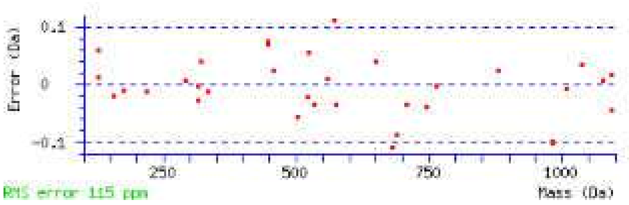


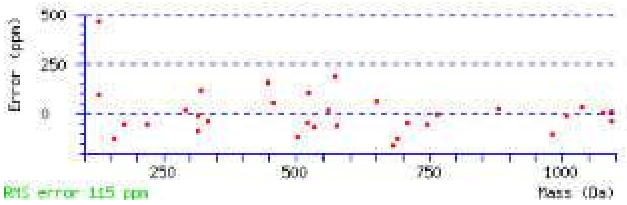


NCBI **BLAST** search of FVIGGPHGDAGLTGR
(Parameters: blastp, nr protein database, expect=20000, no filter, PAM30)
Other BLAST web gateways

**All matches to this query**

| Score | Mr(calc) | Delta | Sequence |
|---|---|---|---|
| 45.6 | 1452.7474 | 0.0628 | FVIGGPHGDAGLTGR |
| 45.6 | 1452.7474 | 0.0628 | FVIGGPHGDAGLTGR |
| 16.8 | 1452.7586 | 0.0516 | GVWLGAQPAADRGR |
| 14.0 | 1452.7209 | 0.0894 | TSDLYKGDANTLR |
| 12.7 | 1452.7143 | 0.0959 | RFSSTTLSAPACR |
| 12.7 | 1452.8161 | -0.0058 | RLLLAGQQAAAADR |
| 12.7 | 1452.7950 | 0.0153 | RPPYPAPTAATRR |
| 12.7 | 1452.7950 | 0.0153 | RPSGQQLLAAAWR |
| 12.3 | 1452.7433 | 0.0669 | SSNAPAAPSPATARR |
| 11.2 | 1452.6966 | 0.1137 | DRAMQLMTAFNR |

**Mascot:** http://www.matrixscience.com/

# MATRIX SCIENCE Mascot Search Results

## Peptide View Spot no 73

MS/MS Fragmentation of **FHNIYGPFGTWK**
Found in **gi|110289082** in **NCBInr**, NAD dependent epimerase/dehydratase family protein, expressed [Oryza sativa Japonica Group]

Match to Query 57: 1465.807224 from(1466.814500,1+) intensity(0.0000) index(18)
Title: Label: P7, Spot_Id: 228953, Peak_List_Id: 257728, MSMS Job_Run_Id: 24933, Comment:
Data file ppw_P7_138985142500.txt

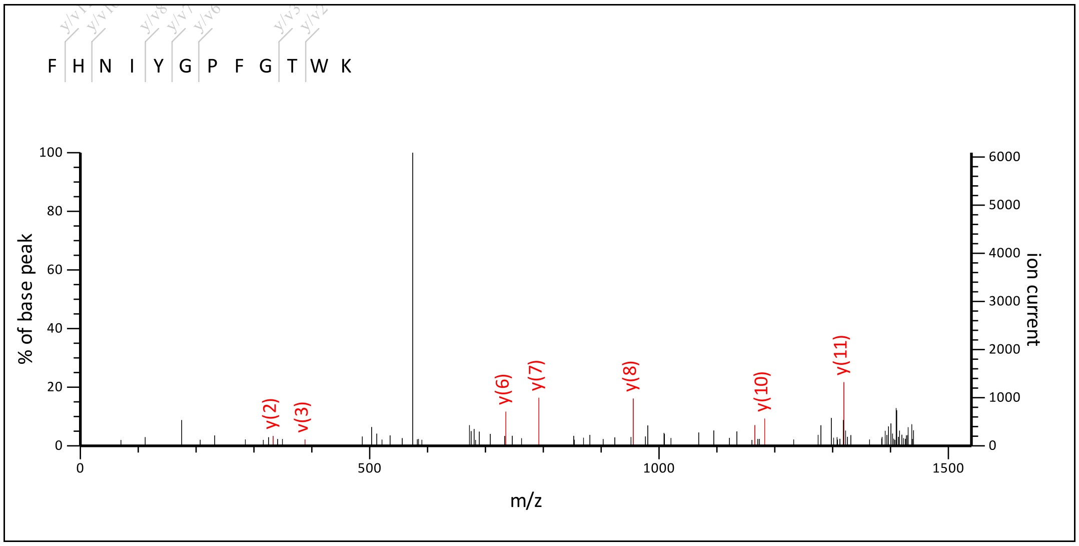


0 to 1539.84

Label all possible matches ○ Label matches used for scoring ◉

**Monoisotopic mass of neutral peptide Mr(calc):** 1465.7143
**Fixed modifications:** Carbamidomethyl (C) (apply to specified residues or termini only)
**Ions Score:** 33 **Expect:** 1.1
**Matches :** 8/180 fragment ions using 14 most intense peaks (help)

| # | Immon. | a | a* | $a^0$ | b | b* | $b^0$ | d | d' | Seq. | v | w | w' | y | y* | |
|---|---|---|---|---|---|---|---|---|---|---|---|---|---|---|---|---|
| 1 | 120.0808 | 120.0808 | | | 148.0757 | | | 44.0495 | | F | | | | | | |
| 2 | 110.0713 | 257.1397 | | | 285.1346 | | | | | H | 1237.6000 | | | ***1319.6531*** | 1302.6266 | [illegible] |
| 3 | 87.0553 | 371.1826 | 354.1561 | | 399.1775 | 382.1510 | | 328.1768 | | N | 1123.5571 | 1122.5619 | | ***1182.5942*** | 1165.5677 | [illegible] |
| 4 | 86.0964 | 484.2667 | 467.2401 | | 512.2616 | 495.2350 | | 456.2354 | 470.2510 | I | 1010.4730 | 1023.4934 | 1037.5091 | 1068.5513 | 1051.5247 | [illegible] |
| 5 | 136.0757 | 647.3300 | 630.3035 | | 675.3249 | 658.2984 | | | | Y | 847.4097 | | | ***955.4672*** | 938.4407 | [illegible] |
| 6 | 30.0338 | 704.3515 | 687.3249 | | 732.3464 | 715.3198 | | | | G | | | | ***792.4039*** | 775.3774 | [illegible] |
| 7 | 70.0651 | 801.4042 | 784.3777 | | 829.3992 | 812.3726 | | 775.3886 | | P | 693.3355 | 692.3402 | | ***735.3824*** | 718.3559 | [illegible] |
| 8 | 120.0808 | 948.4726 | 931.4461 | | 976.4676 | 959.4410 | | | | F | 546.2671 | | | 638.3297 | 621.3031 | [illegible] |
| 9 | 30.0338 | 1005.4941 | 988.4676 | | 1033.4890 | 1016.4625 | | | | G | | | | 491.2613 | 474.2347 | [illegible] |
| 10 | 74.0600 | 1106.5418 | 1089.5152 | 1088.5312 | 1134.5367 | 1117.5102 | 1116.5261 | 1090.5469 | 1092.5261 | T | 388.1979 | 401.2183 | 403.1976 | 434.2398 | 417.2132 | [illegible] |
| 11 | 159.0917 | 1292.6211 | 1275.5946 | 1274.6105 | 1320.6160 | 1303.5895 | 1302.6055 | | | W | 202.1186 | | | ***333.1921*** | 316.1656 | |
| 12 | 101.1073 | | | | | | | | | K | 74.0237 | 73.0284 | | 147.1128 | 130.0863 | |

| Seq | ya | yb | Seq | ya | yb | Seq | ya | yb |
|---|---|---|---|---|---|---|---|---|
| **HN** | 224.1142 | 252.1091 | **HNI** | 337.1983 | 365.1932 | **HNIY** | 500.2616 | 528.2565 |
| **HNIYG** | 557.2831 | 585.2780 | **HNIYGP** | 654.3358 | 682.3307 | **NI** | 200.1394 | 228.1343 |
| **NIY** | 363.2027 | 391.1976 | **NIYG** | 420.2241 | 448.2191 | **NIYGP** | 517.2769 | 545.2718 |
| **NIYGPF** | 664.3453 | 692.3402 | **IY** | 249.1598 | 277.1547 | **IYG** | 306.1812 | 334.1761 |
| **IYGP** | 403.2340 | 431.2289 | **IYGPF** | 550.3024 | 578.2973 | **IYGPFG** | 607.3239 | 635.3188 |
| **YG** | 193.0972 | 221.0921 | **YGP** | 290.1499 | 318.1448 | **YGPF** | 437.2183 | 465.2132 |
| **YGPFG** | 494.2398 | 522.2347 | **YGPFGT** | 595.2875 | 623.2824 | **GP** | 127.0866 | 155.0815 |
| **GPF** | 274.1550 | 302.1499 | **GPFG** | 331.1765 | 359.1714 | **GPFGT** | 432.2241 | 460.2191 |
| **GPFGTW** | 618.3035 | 646.2984 | **PF** | 217.1335 | 245.1285 | **PFG** | 274.1550 | 302.1499 |

| | | | | | | | | |
|---|---|---|---|---|---|---|---|---|
| **PFGT** | 375.2027 | 403.1976 | **PFGTW** | 561.2820 | 589.2769 | **FG** | 177.1022 | 205.0972 |
| **FGT** | 278.1499 | 306.1448 | **FGTW** | 464.2292 | 492.2241 | **GT** | 131.0815 | 159.0764 |
| **GTW** | 317.1608 | 345.1557 | **TW** | 260.1394 | 288.1343 | | | |

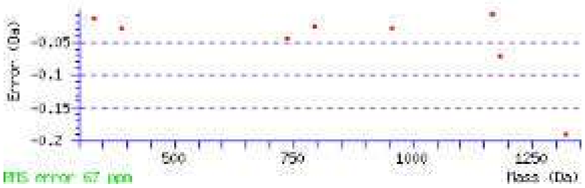


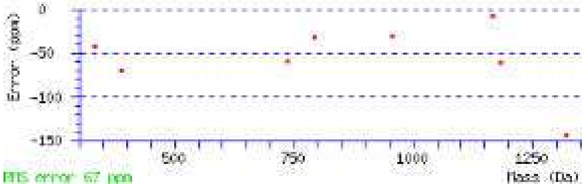


NCBI **BLAST** search of FHNIYGPFGTWK
(Parameters: blastp, nr protein database, expect=20000, no filter, PAM30)
Other BLAST web gateways

**All matches to this query**

| Score | Mr(calc) | Delta | Sequence |
|---|---|---|---|
| 33.4 | 1465.7143 | 0.0929 | FHNIYGPFGTWK |
| 21.9 | 1465.6766 | 0.1306 | MTAERACGAVAGTR |
| 16.5 | 1465.8188 | -0.0115 | AGGVCVLHVAISKR |
| 14.9 | 1465.6871 | 0.1201 | GYLPPMDESKSAR |
| 14.6 | 1465.8001 | 0.0071 | TSLPLPEGRQVNR |
| 13.5 | 1465.6797 | 0.1275 | DSLGDYNKDANVR |
| 12.9 | 1465.6983 | 0.1089 | EIKCAAFNAEGTR |
| 12.7 | 1465.7096 | 0.0976 | GINSDRAFGTCLR |
| 10.9 | 1465.8113 | -0.0041 | RPRLSGPEALGTGR |
| 10.5 | 1465.7565 | 0.0507 | GVKDSLYTELWR |

**Mascot:** http://www.matrixscience.com/

# MATRIX SCIENCE Mascot Search Results

## Peptide View Spot no 73

MS/MS Fragmentation of **ISITGAGGFIGSHIAR**
Found in **gi|110289082** in **NCBInr**, NAD dependent epimerase/dehydratase family protein, expressed [Oryza sativa Japonica Group]

Match to Query 66: 1555.982824 from(1556.990100,1+) intensity(0.0000) index(20)
Title: Label: P7, Spot_Id: 228953, Peak_List_Id: 257722, MSMS Job_Run_Id: 24933, Comment:
Data file ppw_P7_138985142500.txt

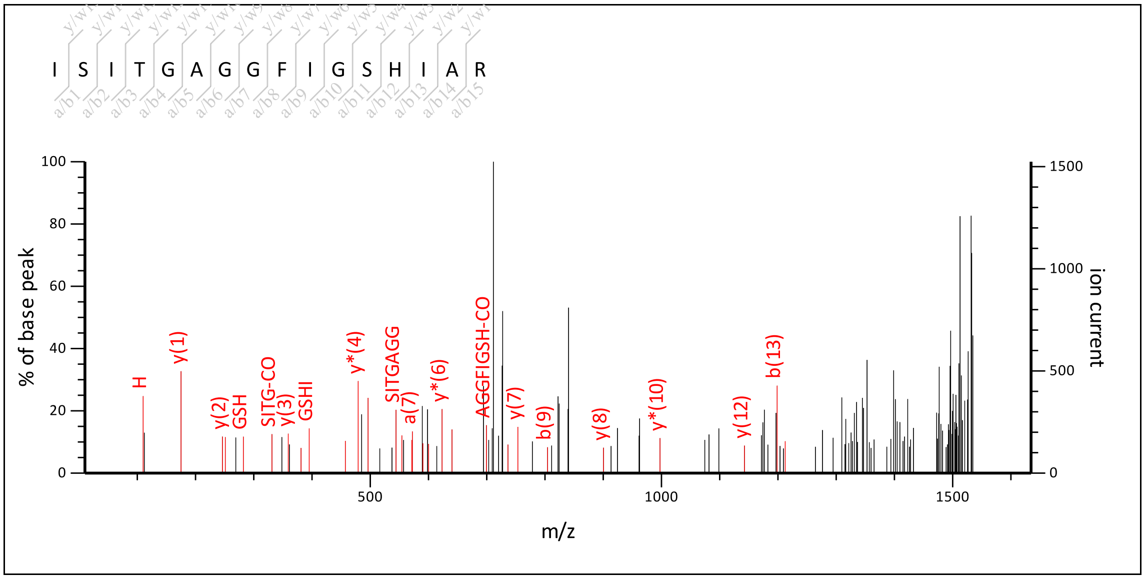


10.08 to 1634.59

Label all possible matches ○ Label matches used for scoring ◉

**Monoisotopic mass of neutral peptide Mr(calc):** 1555.8471
**Fixed modifications:** Carbamidomethyl (C) (apply to specified residues or termini only)
**Ions Score:** 15 **Expect:** 22
**Matches :** 31/280 fragment ions using 96 most intense peaks (help)

| # | Immon. | a | $a^0$ | b | $b^0$ | d | d' | Seq. | v | w | w' | y | y* | $y^0$ | # |
|---|---|---|---|---|---|---|---|---|---|---|---|---|---|---|---|
| 1 | 86.0964 | 86.0964 | | 114.0913 | | 44.0495 | | **I** | | | | | | | 16 |
| 2 | 60.0444 | 173.1285 | 155.1179 | 201.1234 | 183.1128 | 157.1335 | | **S** | 1411.7441 | 1410.7488 | | 1443.7703 | 1426.7437 | 1425.7597 | 15 |
| 3 | 86.0964 | 286.2125 | 268.2020 | 314.2074 | 296.1969 | 258.1812 | 272.1969 | **I** | 1298.6600 | 1311.6804 | 1325.6961 | 1356.7383 | 1339.7117 | 1338.7277 | 14 |
| 4 | 74.0600 | 387.2602 | 369.2496 | 415.2551 | 397.2445 | 371.2653 | 373.2445 | **T** | 1197.6123 | 1210.6327 | 1212.6120 | 1243.6542 | 1226.6276 | 1225.6436 | 13 |
| 5 | 30.0338 | 444.2817 | 426.2711 | 472.2766 | 454.2660 | | | **G** | | | | ***1142.6065*** | 1125.5800 | 1124.5960 | 12 |
| 6 | 44.0495 | 515.3188 | 497.3082 | 543.3137 | 525.3031 | | | **A** | 1069.5538 | | | 1085.5851 | 1068.5585 | 1067.5745 | 11 |
| 7 | 30.0338 | 572.3402 | 554.3297 | **600.3352** | 582.3246 | | | **G** | | | | 1014.5479 | 997.5214 | 996.5374 | 10 |
| 8 | 30.0338 | 629.3617 | 611.3511 | 657.3566 | 639.3461 | | | **G** | | | | 957.5265 | 940.4999 | 939.5159 | 9 |
| 9 | 120.0808 | 776.4301 | 758.4196 | **804.4250** | 786.4145 | | | **F** | 808.4424 | | | ***900.5050*** | 883.4785 | 882.4944 | 8 |
| 10 | 86.0964 | 889.5142 | 871.5036 | 917.5091 | 899.4985 | 861.4829 | 875.4985 | **I** | 695.3583 | 708.3787 | 722.3944 | ***753.4366*** | 736.4100 | 735.4260 | 7 |
| 11 | 30.0338 | 946.5356 | 928.5251 | 974.5306 | 956.5200 | | | **G** | | | | ***640.3525*** | 623.3260 | 622.3420 | 6 |
| 12 | 60.0444 | 1033.5677 | 1015.5571 | 1061.5626 | 1043.5520 | 1017.5728 | | **S** | 551.3049 | 550.3096 | | 583.3311 | 566.3045 | 565.3205 | 5 |
| 13 | 110.0713 | 1170.6266 | 1152.6160 | **1198.6215** | 1180.6109 | | | **H** | 414.2459 | | | ***496.2990*** | 479.2725 | | 4 |
| 14 | 86.0964 | 1283.7106 | 1265.7001 | 1311.7056 | 1293.6950 | 1255.6793 | 1269.6950 | **I** | 301.1619 | 314.1823 | 328.1979 | ***359.2401*** | 342.2136 | | 3 |
| 15 | 44.0495 | 1354.7478 | 1336.7372 | 1382.7427 | 1364.7321 | | | **A** | 230.1248 | | | ***246.1561*** | 229.1295 | | 2 |
| 16 | 129.1135 | | | | | | | **R** | 74.0237 | 73.0284 | | ***175.1190*** | 158.0924 | | 1 |

| Seq | ya | yb | Seq | ya | yb | Seq | ya | yb |
|---|---|---|---|---|---|---|---|---|
| **SI** | 173.1285 | 201.1234 | **SIT** | 274.1761 | 302.1710 | **SITG** | 331.1976 | 359.1925 |
| **SITGA** | 402.2347 | 430.2296 | **SITGAG** | 459.2562 | 487.2511 | **SITGAGG** | 516.2776 | 544.2726 |

| | | | | | | | | |
|---|---|---|---|---|---|---|---|---|
| **SITGAGGF** | 663.3461 | 691.3410 | **IT** | 187.1441 | 215.1390 | **ITG** | 244.1656 | 272.1605 |
| **ITGA** | 315.2027 | 343.1976 | **ITGAG** | 372.2241 | 400.2191 | **ITGAGG** | 429.2456 | 457.2405 |
| **ITGAGGF** | 576.3140 | 604.3089 | **ITGAGGFI** | 689.3981 | 717.3930 | **TG** | 131.0815 | 159.0764 |
| **TGA** | 202.1186 | 230.1135 | **TGAG** | 259.1401 | 287.1350 | **TGAGG** | 316.1615 | 344.1565 |
| **TGAGGF** | 463.2300 | 491.2249 | **TGAGGFI** | 576.3140 | 604.3089 | **TGAGGFIG** | 633.3355 | 661.3304 |
| **GA** | 101.0709 | 129.0659 | **GAG** | 158.0924 | 186.0873 | **GAGG** | 215.1139 | 243.1088 |
| **GAGGF** | 362.1823 | 390.1772 | **GAGGFI** | 475.2663 | 503.2613 | **GAGGFIG** | 532.2878 | 560.2827 |
| **GAGGFIGS** | 619.3198 | 647.3148 | **AG** | 101.0709 | 129.0659 | **AGG** | 158.0924 | 186.0873 |
| **AGGF** | 305.1608 | 333.1557 | **AGGFI** | 418.2449 | 446.2398 | **AGGFIG** | 475.2663 | 503.2613 |
| **AGGFIGS** | 562.2984 | 590.2933 | **AGGFIGSH** | 699.3573 | 727.3522 | **GG** | 87.0553 | 115.0502 |
| **GGF** | 234.1237 | 262.1186 | **GGFI** | 347.2078 | 375.2027 | **GGFIG** | 404.2292 | 432.2241 |
| **GGFIGS** | 491.2613 | 519.2562 | **GGFIGSH** | 628.3202 | 656.3151 | **GF** | 177.1022 | 205.0972 |
| **GFI** | 290.1863 | 318.1812 | **GFIG** | 347.2078 | 375.2027 | **GFIGS** | 434.2398 | 462.2347 |
| **GFIGSH** | 571.2987 | 599.2936 | **GFIGSHI** | 684.3828 | 712.3777 | **FI** | 233.1648 | 261.1598 |
| **FIG** | 290.1863 | 318.1812 | **FIGS** | 377.2183 | 405.2132 | **FIGSH** | 514.2772 | 542.2722 |
| **FIGSHI** | 627.3613 | 655.3562 | **FIGSHIA** | 698.3984 | 726.3933 | **IG** | 143.1179 | 171.1128 |
| **IGS** | 230.1499 | 258.1448 | **IGSH** | 367.2088 | 395.2037 | **IGSHI** | 480.2929 | 508.2878 |
| **IGSHIA** | 551.3300 | 579.3249 | **GS** | 117.0659 | 145.0608 | **GSH** | 254.1248 | 282.1197 |
| **GSHI** | 367.2088 | 395.2037 | **GSHIA** | 438.2459 | 466.2409 | **SH** | 197.1033 | 225.0982 |
| **SHI** | 310.1874 | 338.1823 | **SHIA** | 381.2245 | 409.2194 | **HI** | 223.1553 | 251.1503 |
| **HIA** | 294.1925 | 322.1874 | **IA** | 157.1335 | 185.1285 | | | |

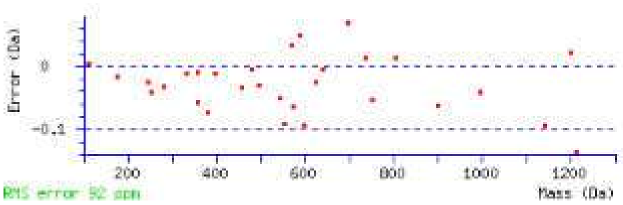


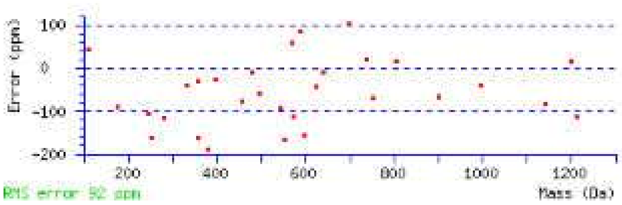


NCBI **BLAST** search of ISITGAGGFIGSHIAR
(Parameters: blastp, nr protein database, expect=20000, no filter, PAM30)
Other BLAST web gateways

**All matches to this query**

| Score | Mr(calc) | Delta | Sequence |
|---|---|---|---|
| 15.0 | 1555.8471 | 0.1357 | ISITGAGGFIGSHIAR |
| 13.6 | 1555.8504 | 0.1324 | TGSLRIMSLPPASAR |
| 6.8 | 1555.8331 | 0.1497 | HIPHIQITDRNGR |
| 5.3 | 1555.8430 | 0.1398 | SSALNAATLGVSGPRR |
| 5.1 | 1555.8457 | 0.1371 | DSSVSIQIEIVPAAK |
| 5.0 | 1555.8947 | 0.0881 | VLGHDGGVPPRLLAR |
| 4.0 | 1555.8293 | 0.1535 | VMGLAPGLEATWRR |
| 3.2 | 1555.8471 | 0.1358 | TKGSLVNYTPLHAR |
| 3.2 | 1555.8293 | 0.1536 | MHKYSPNLNLLAR |
| 3.1 | 1555.8471 | 0.1357 | VSITGAGGFIASHIAR |

**Mascot:** http://www.matrixscience.com/

# MATRIX SCIENCE Mascot Search Results

## Peptide View **Spot no 73**

MS/MS Fragmentation of **SFTFIDECVEGVLR**
Found in **gi|110289082** in **NCBInr**, NAD dependent epimerase/dehydratase family protein, expressed [Oryza sativa Japonica Group]

Match to Query 72: 1670.932624 from(1671.939900,1+) intensity(0.0000) index(23)
Title: Label: P7, Spot_Id: 228953, Peak_List_Id: 257730, MSMS Job_Run_Id: 24933, Comment:
Data file ppw_P7_138985142500.txt

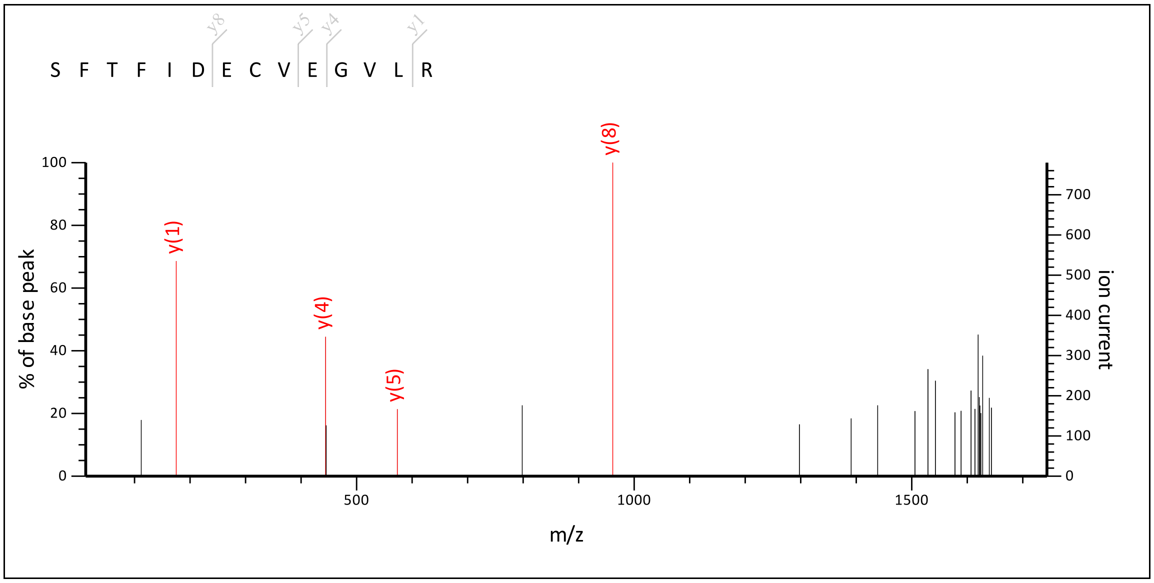


12.07 to 1743.53

Label all possible matches ○ Label matches used for scoring ◉

**Monoisotopic mass of neutral peptide Mr(calc):** 1670.7974
**Fixed modifications:** Carbamidomethyl (C) (apply to specified residues or termini only)
**Ions Score:** 15 **Expect:** 58
**Matches :** 4/219 fragment ions using 10 most intense peaks (help)

| # | Immon. | a | $a^0$ | b | $b^0$ | d | d' | Seq. | v | w | w' | y | y* | $y^0$ | # |
|---|---|---|---|---|---|---|---|---|---|---|---|---|---|---|---|
| 1 | 60.0444 | 60.0444 | 42.0338 | 88.0393 | 70.0287 | 44.0495 | | S | | | | | | | 14 |
| 2 | 120.0808 | 207.1128 | 189.1022 | 235.1077 | 217.0972 | | | F | 1492.7101 | | | 1584.7727 | 1567.7461 | 1566.7621 | 13 |
| 3 | 74.0600 | 308.1605 | 290.1499 | 336.1554 | 318.1448 | 292.1656 | 294.1448 | T | 1391.6624 | 1404.6828 | 1406.6620 | 1437.7042 | 1420.6777 | 1419.6937 | 12 |
| 4 | 120.0808 | 455.2289 | 437.2183 | 483.2238 | 465.2132 | | | F | 1244.5940 | | | 1336.6566 | 1319.6300 | 1318.6460 | 11 |
| 5 | 86.0964 | 568.3130 | 550.3024 | 596.3079 | 578.2973 | 540.2817 | 554.2973 | I | 1131.5099 | 1144.5303 | 1158.5460 | 1189.5881 | 1172.5616 | 1171.5776 | 10 |
| 6 | 88.0393 | 683.3399 | 665.3293 | 711.3348 | 693.3243 | 639.3501 | | D | 1016.4830 | 1015.4877 | | 1076.5041 | 1059.4775 | 1058.4935 | 9 |
| 7 | 102.0550 | 812.3825 | 794.3719 | 840.3774 | 822.3668 | 754.3770 | | E | 887.4404 | 886.4451 | | ***961.4771*** | 944.4506 | 943.4666 | 8 |
| 8 | 133.0430 | 972.4131 | 954.4026 | 1000.4081 | 982.3975 | 883.4196 | | C | 727.4097 | 726.4145 | | 832.4345 | 815.4080 | 814.4240 | 7 |
| 9 | 72.0808 | 1071.4816 | 1053.4710 | 1099.4765 | 1081.4659 | 1057.4659 | | V | 628.3413 | 641.3617 | | 672.4039 | 655.3774 | 654.3933 | 6 |
| 10 | 102.0550 | 1200.5242 | 1182.5136 | 1228.5191 | 1210.5085 | 1142.5187 | | E | 499.2987 | 498.3035 | | ***573.3355*** | 556.3089 | 555.3249 | 5 |
| 11 | 30.0338 | 1257.5456 | 1239.5351 | 1285.5405 | 1267.5300 | | | G | | | | ***444.2929*** | 427.2663 | | 4 |
| 12 | 72.0808 | 1356.6140 | 1338.6035 | 1384.6089 | 1366.5984 | 1342.5984 | | V | 343.2088 | 356.2292 | | 387.2714 | 370.2449 | | 3 |
| 13 | 86.0964 | 1469.6981 | 1451.6875 | 1497.6930 | 1479.6824 | 1427.6511 | | L | 230.1248 | 229.1295 | | 288.2030 | 271.1765 | | 2 |
| 14 | 129.1135 | | | | | | | R | 74.0237 | 73.0284 | | ***175.1190*** | 158.0924 | | 1 |

| Seq | ya | yb | Seq | ya | yb | Seq | ya | yb |
|---|---|---|---|---|---|---|---|---|
| FT | 221.1285 | 249.1234 | FTF | 368.1969 | 396.1918 | FTFI | 481.2809 | 509.2758 |
| FTFID | 596.3079 | 624.3028 | TF | 221.1285 | 249.1234 | TFI | 334.2125 | 362.2074 |
| TFID | 449.2395 | 477.2344 | TFIDE | 578.2821 | 606.2770 | FI | 233.1648 | 261.1598 |
| | | | | | | | | |

| | | | | | | | | |
|---|---|---|---|---|---|---|---|---|
| **FID** | 348.1918 | 376.1867 | **FIDE** | 477.2344 | 505.2293 | **FIDEC** | 637.2650 | 665.2599 |
| **ID** | 201.1234 | 229.1183 | **IDE** | 330.1660 | 358.1609 | **IDEC** | 490.1966 | 518.1915 |
| **IDECV** | 589.2650 | 617.2599 | **DE** | 217.0819 | 245.0768 | **DEC** | 377.1125 | 405.1075 |
| **DECV** | 476.1810 | 504.1759 | **DECVE** | 605.2236 | 633.2185 | **DECVEG** | 662.2450 | 690.2399 |
| **EC** | 262.0856 | 290.0805 | **ECV** | 361.1540 | 389.1489 | **ECVE** | 490.1966 | 518.1915 |
| **ECVEG** | 547.2181 | 575.2130 | **ECVEGV** | 646.2865 | 674.2814 | **CV** | 232.1114 | 260.1063 |
| **CVE** | 361.1540 | 389.1489 | **CVEG** | 418.1755 | 446.1704 | **CVEGV** | 517.2439 | 545.2388 |
| **CVEGVL** | 630.3280 | 658.3229 | **VE** | 201.1234 | 229.1183 | **VEG** | 258.1448 | 286.1397 |
| **VEGV** | 357.2132 | 385.2082 | **VEGVL** | 470.2973 | 498.2922 | **EG** | 159.0764 | 187.0713 |
| **EGV** | 258.1448 | 286.1397 | **EGVL** | 371.2289 | 399.2238 | **GV** | 129.1022 | 157.0972 |
| **GVL** | 242.1863 | 270.1812 | **VL** | 185.1648 | 213.1598 | | | |

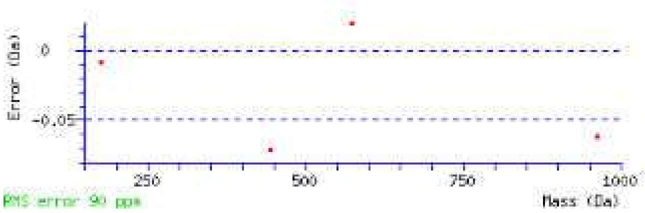


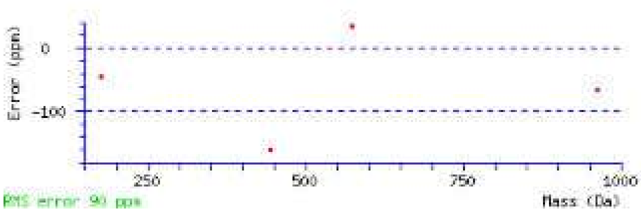


NCBI **BLAST** search of SFTFIDECVEGVLR
(Parameters: blastp, nr protein database, expect=20000, no filter, PAM30)
Other BLAST web gateways

**All matches to this query**

| Score | Mr(calc) | Delta | Sequence |
|---|---|---|---|
| 15.4 | 1670.7974 | 0.1352 | SFTFIDECVEGVLR |
| 10.9 | 1670.9315 | 0.0011 | RAVVATLSVIGDDVTR |
| 10.4 | 1670.8773 | 0.0553 | KPMSEPKNTITAQAR |
| 10.2 | 1670.9104 | 0.0223 | KFVNLPEISAAGQAAR |
| 9.5 | 1670.9468 | -0.0141 | KSANLSSWKPLVVSR |
| 9.3 | 1670.8886 | 0.0440 | MGQIGVLTGNAGEIRR |
| 8.7 | 1670.8773 | 0.0553 | KPMAEPKNTITAQAR |
| 6.7 | 1670.7900 | 0.1426 | YSYEPASRVDEISR |
| 6.1 | 1670.9032 | 0.0294 | LFPSKDSVFYILSR |
| 6.1 | 1670.7835 | 0.1492 | MPSSYEIFQQNRR |

**Mascot:** http://www.matrixscience.com/

# MATRIX SCIENCE Mascot Search Results

## Peptide View **Spot no 74**

MS/MS Fragmentation of **VYDDEVR**
Found in **gi|8918361** in **NCBInr**, RuBisCO activase small isoform precursor [Oryza sativa]

Match to Query 2: 894.452744 from(895.460020,1+) intensity(0.0000) index(0)
Title: Label: L1, Spot_Id: 219685, Peak_List_Id: 225099, MSMS Job_Run_Id: 21764, Comment:
Data file C:\Documents and Settings\Administrator\Desktop\farah BHU\spot set plate\Farah-Garima 11May 2013\ppw_L1_136842039200.txt

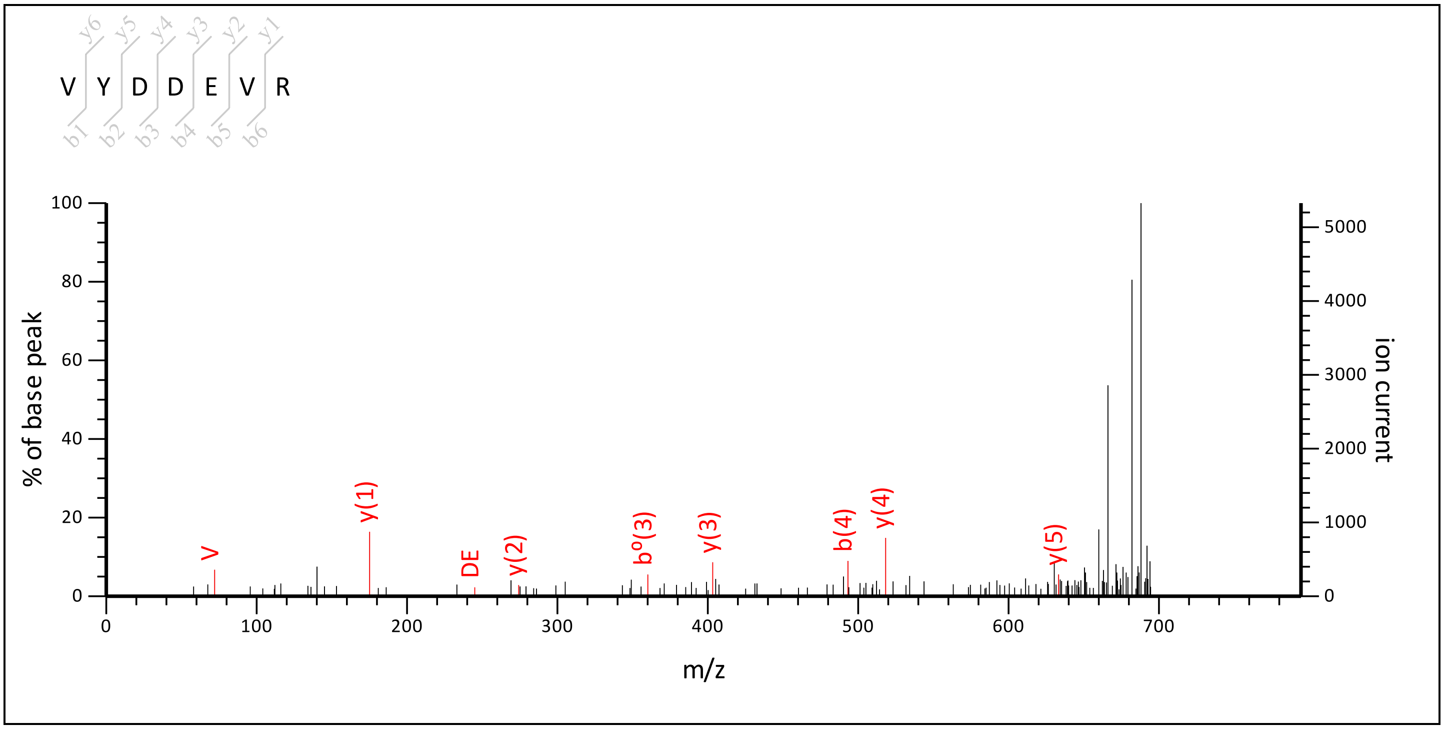


0 to 794.45

Label all possible matches ○ Label matches used for scoring ◉

**Monoisotopic mass of neutral peptide Mr(calc):** 894.4083
**Fixed modifications:** Carbamidomethyl (C) (apply to specified residues or termini only)
**Ions Score:** 14 **Expect:** 88
**Matches :** 12/79 fragment ions using 28 most intense peaks (help)

| # | Immon. | a | $a^0$ | b | $b^0$ | d | Seq. | v | w | y | y* | $y^0$ | # |
|---|---|---|---|---|---|---|---|---|---|---|---|---|---|
| 1 | 72.0808 | 72.0808 | | 100.0757 | | 44.0495 | V | | | | | | 7 |
| 2 | 136.0757 | 235.1441 | | 263.1390 | | | Y | 688.2897 | | 796.3472 | 779.3206 | 778.3366 | 6 |
| 3 | 88.0393 | 350.1710 | 332.1605 | 378.1660 | 360.1554 | 306.1812 | D | 573.2627 | 572.2675 | ***633.2838*** | 616.2573 | 615.2733 | 5 |
| 4 | 88.0393 | 465.1980 | 447.1874 | 493.1929 | 475.1823 | 421.2082 | D | 458.2358 | 457.2405 | ***518.2569*** | 501.2304 | 500.2463 | 4 |
| 5 | 102.0550 | 594.2406 | 576.2300 | 622.2355 | 604.2249 | 536.2351 | E | 329.1932 | 328.1979 | ***403.2300*** | 386.2034 | 385.2194 | 3 |
| 6 | 72.0808 | 693.3090 | 675.2984 | 721.3039 | 703.2933 | 679.2933 | V | 230.1248 | 243.1452 | ***274.1874*** | 257.1608 | | 2 |
| 7 | 129.1135 | | | | | | R | 74.0237 | 73.0284 | ***175.1190*** | 158.0924 | | 1 |

| Seq | ya | yb | Seq | ya | yb | Seq | ya | yb |
|---|---|---|---|---|---|---|---|---|
| YD | 251.1026 | 279.0975 | YDD | 366.1296 | 394.1245 | YDDE | 495.1722 | 523.1671 |
| YDDEV | 594.2406 | 622.2355 | DD | 203.0662 | 231.0612 | DDE | 332.1088 | 360.1038 |
| DDEV | 431.1773 | 459.1722 | DE | 217.0819 | 245.0768 | DEV | 316.1503 | 344.1452 |
| EV | 201.1234 | 229.1183 | | | | | | |

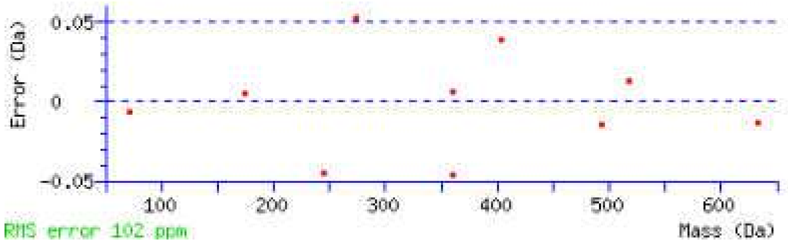


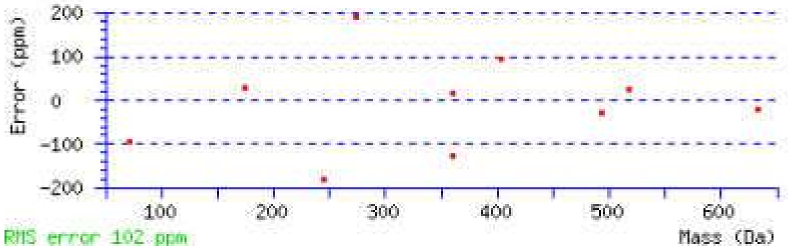


NCBI **BLAST** search of VYDDEVR
(Parameters: blastp, nr protein database, expect=20000, no filter, PAM30)
Other BLAST web gateways

**All matches to this query**

| Score | Mr(calc) | Delta | Sequence |
|---|---|---|---|
| 14.9 | 894.3905 | 0.0622 | YVDDMPR |
| 14.0 | 894.4083 | 0.0444 | VYDDEVR |
| 8.9 | 894.3719 | 0.0808 | FDDEDVR |
| 7.1 | 894.4269 | 0.0258 | FDVMDLR |
| 6.9 | 894.3719 | 0.0808 | DDFDLDR |
| 6.9 | 894.4083 | 0.0444 | EFTDEVR |
| 6.9 | 894.4083 | 0.0444 | ETFDDLR |
| 6.9 | 894.4447 | 0.0081 | LYTDVER |
| 6.9 | 894.4447 | 0.0081 | TYLDVER |
| 6.3 | 894.4083 | 0.0444 | DFETEVR |

**Mascot:** http://www.matrixscience.com/

# MATRIX SCIENCE Mascot Search Results

## Peptide View **Spot no 74**

MS/MS Fragmentation of **VYDDEVRK**
Found in **gi|8918361** in **NCBInr**, RuBisCO activase small isoform precursor [Oryza sativa]

Match to Query 14: 1022.514624 from(1023.521900,1+) intensity(0.0000) index(5)
Title: Label: L1, Spot_Id: 219685, Peak_List_Id: 225084, MSMS Job_Run_Id: 21764, Comment:
Data file C:\Documents and Settings\Administrator\Desktop\farah BHU\spot set plate\Farah-Garima 11May 2013\ppw_L1_136842039200.txt

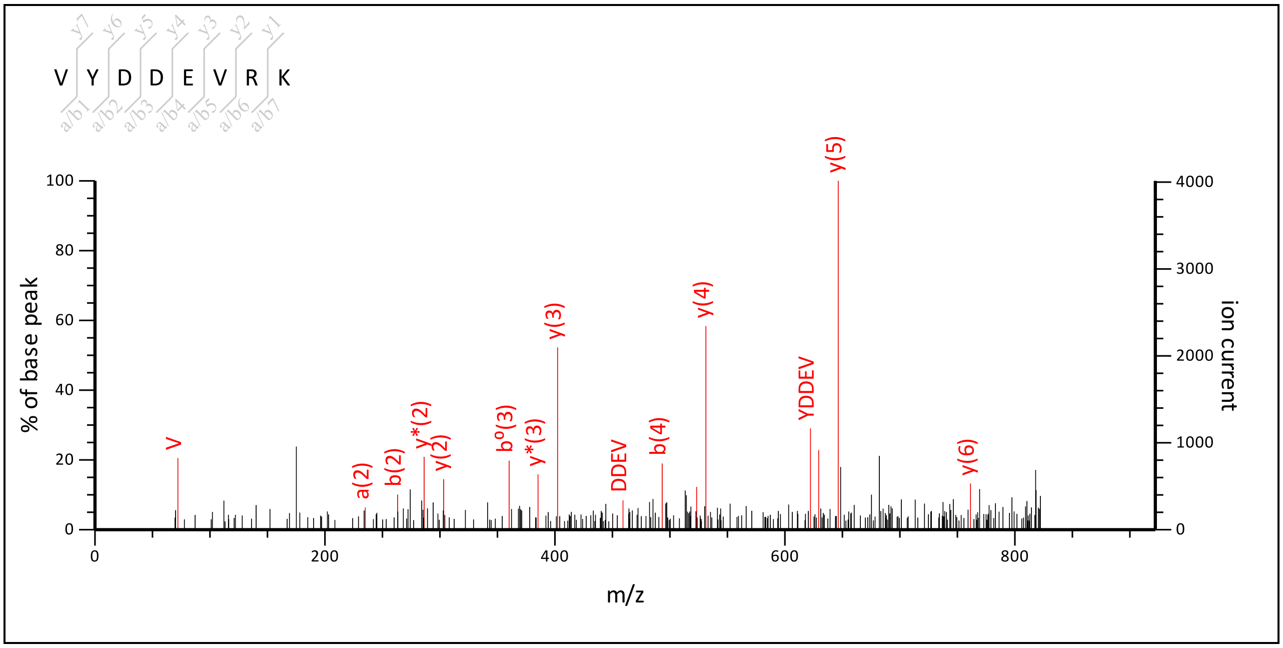


0 to 922.13

Label all possible matches ○ Label matches used for scoring ◉

**Monoisotopic mass of neutral peptide Mr(calc):** 1022.5033
**Fixed modifications:** Carbamidomethyl (C) (apply to specified residues or termini only)
**Ions Score:** 31 **Expect:** 1.7
**Matches :** 21/99 fragment ions using 24 most intense peaks (help)

| # | Immon. | a | a* | $a^0$ | b | b* | $b^0$ | d | Seq. | v | w | y | y* | $y^0$ | # |
|---|---|---|---|---|---|---|---|---|---|---|---|---|---|---|---|
| 1 | 72.0808 | **72.0808** | | | 100.0757 | | | 44.0495 | V | | | | | | 8 |
| 2 | 136.0757 | **235.1441** | | | **263.1390** | | | | Y | 816.3846 | | 924.4421 | 907.4156 | 906.4316 | 7 |
| 3 | 88.0393 | 350.1710 | | 332.1605 | 378.1660 | | 360.1554 | 306.1812 | D | 701.3577 | 700.3624 | ***761.3788*** | 744.3523 | 743.3682 | 6 |
| 4 | 88.0393 | 465.1980 | | 447.1874 | **493.1929** | | 475.1823 | 421.2082 | D | 586.3307 | 585.3355 | ***646.3519*** | 629.3253 | 628.3413 | 5 |
| 5 | 102.0550 | 594.2406 | | 576.2300 | **622.2355** | | 604.2249 | 536.2351 | E | 457.2881 | 456.2929 | ***531.3249*** | 514.2984 | 513.3144 | 4 |
| 6 | 72.0808 | 693.3090 | | 675.2984 | 721.3039 | | 703.2933 | 679.2933 | V | 358.2197 | 371.2401 | ***402.2823*** | 385.2558 | | 3 |
| 7 | 129.1135 | 849.4101 | 832.3836 | 831.3995 | 877.4050 | 860.3785 | 859.3945 | 764.3461 | R | 202.1186 | 201.1234 | ***303.2139*** | 286.1874 | | 2 |
| 8 | 101.1073 | | | | | | | | K | 74.0237 | 73.0284 | 147.1128 | 130.0863 | | 1 |

| Seq | ya | yb | Seq | ya | yb | Seq | ya | yb |
|---|---|---|---|---|---|---|---|---|
| YD | 251.1026 | 279.0975 | YDD | 366.1296 | 394.1245 | YDDE | 495.1722 | 523.1671 |
| YDDEV | 594.2406 | 622.2355 | DD | 203.0662 | 231.0612 | DDE | 332.1088 | 360.1038 |
| DDEV | 431.1773 | 459.1722 | DDEVR | 587.2784 | 615.2733 | DE | 217.0819 | 245.0768 |
| DEV | 316.1503 | 344.1452 | DEVR | 472.2514 | 500.2463 | EV | 201.1234 | 229.1183 |

| | | | | | | | | |
|---|---|---|---|---|---|---|---|---|
| **EVR** | 357.2245 | 385.2194 | **VR** | 228.1819 | 256.1768 | | | |

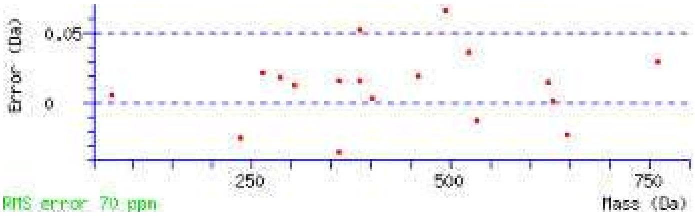


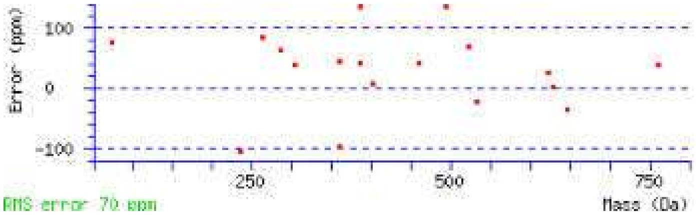


NCBI **BLAST** search of VYDDEVRK
(Parameters: blastp, nr protein database, expect=20000, no filter, PAM30)
Other BLAST web gateways

**All matches to this query**

| Score | Mr(calc) | Delta | Sequence |
|---|---|---|---|
| 31.4 | 1022.5033 | 0.0114 | VYDDEVRK |
| 19.7 | 1022.4669 | 0.0477 | YVDDAVEGR |
| 19.7 | 1022.5185 | -0.0039 | VYDPFLNR |
| 19.6 | 1022.5033 | 0.0113 | YVDDIVGSR |
| 17.9 | 1022.5873 | -0.0726 | LIHEEVKR |
| 17.8 | 1022.5145 | 0.0001 | QDTGYGVKR |
| 16.4 | 1022.5075 | 0.0071 | MMMLVVQR |
| 16.0 | 1022.4702 | 0.0444 | DMTEEVRK |
| 15.1 | 1022.5185 | -0.0039 | VYPFDNLR |
| 14.5 | 1022.4637 | 0.0509 | ANKDMICR |

**Mascot:** http://www.matrixscience.com/

# Mascot Search Results

## Peptide View Spot no 74

MS/MS Fragmentation of **EGPPEFEQPK**
Found in **gi|8918361** in **NCBInr**, RuBisCO activase small isoform precursor [Oryza sativa]

Match to Query 25: 1156.536524 from(1157.543800,1+) intensity(0.0000) index(9)
Title: Label: L1, Spot_Id: 219685, Peak_List_Id: 225091, MSMS Job_Run_Id: 21764, Comment:
Data file C:\Documents and Settings\Administrator\Desktop\farah BHU\spot set plate\Farah-Garima 11May 2013\ppw_L1_136842039200.txt

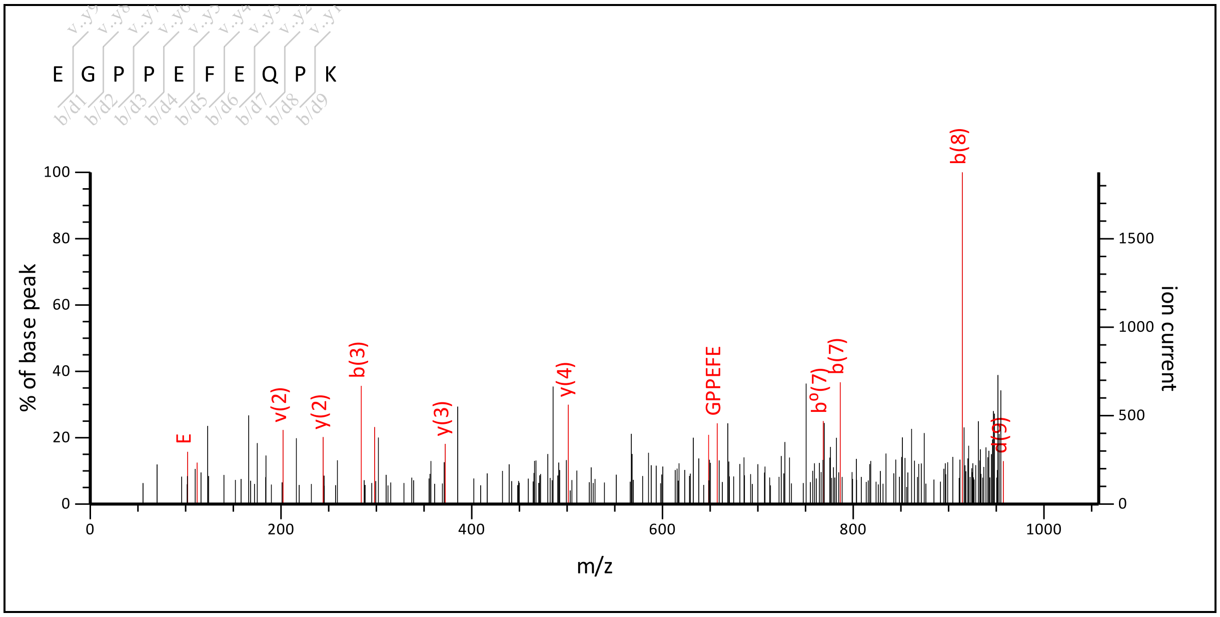


0 to 1057.39

Label all possible matches ○ Label matches used for scoring ◉

**Monoisotopic mass of neutral peptide Mr(calc):** 1156.5400
**Fixed modifications:** Carbamidomethyl (C) (apply to specified residues or termini only)
**Ions Score:** 16 **Expect:** 45
**Matches :** 18/142 fragment ions using 28 most intense peaks (help)

| # | Immon. | a | a* | $a^0$ | b | b* | $b^0$ | d | Seq. | v | w | y | y* | $y^0$ | # |
|---|---|---|---|---|---|---|---|---|---|---|---|---|---|---|---|
| 1 | 102.0550 | 102.0550 | | 84.0444 | 130.0499 | | 112.0393 | 44.0495 | E | | | | | | 10 |
| 2 | 30.0338 | 159.0764 | | 141.0659 | 187.0713 | | 169.0608 | | G | | | 1028.5047 | 1011.4782 | 1010.4942 | 9 |
| 3 | 70.0651 | 256.1292 | | 238.1186 | ***284.1241*** | | 266.1135 | 230.1135 | P | 929.4363 | 928.4411 | 971.4833 | 954.4567 | 953.4727 | 8 |
| 4 | 70.0651 | 353.1819 | | 335.1714 | 381.1769 | | 363.1663 | 327.1663 | P | 832.3836 | 831.3883 | 874.4305 | 857.4040 | 856.4199 | 7 |
| 5 | 102.0550 | 482.2245 | | 464.2140 | 510.2195 | | 492.2089 | 424.2191 | E | 703.3410 | 702.3457 | 777.3777 | 760.3512 | 759.3672 | 6 |
| 6 | 120.0808 | 629.2930 | | 611.2824 | ***657.2879*** | | 639.2773 | | F | 556.2726 | | ***648.3352*** | 631.3086 | 630.3246 | 5 |
| 7 | 102.0550 | 758.3355 | | 740.3250 | ***786.3305*** | | 768.3199 | 700.3301 | E | 427.2300 | 426.2347 | ***501.2667*** | 484.2402 | 483.2562 | 4 |
| 8 | 101.0709 | 886.3941 | 869.3676 | 868.3836 | ***914.3890*** | 897.3625 | 896.3785 | 829.3727 | Q | 299.1714 | 298.1761 | ***372.2241*** | 355.1976 | | 3 |
| 9 | 70.0651 | 983.4469 | 966.4203 | 965.4363 | 1011.4418 | 994.4153 | 993.4312 | 957.4312 | P | 202.1186 | 201.1234 | ***244.1656*** | 227.1390 | | 2 |
| 10 | 101.1073 | | | | | | | | K | 74.0237 | 73.0284 | 147.1128 | 130.0863 | | 1 |

| Seq | ya | yb | Seq | ya | yb | Seq | ya | yb |
|---|---|---|---|---|---|---|---|---|
| GP | 127.0866 | 155.0815 | GPP | 224.1394 | 252.1343 | GPPE | 353.1819 | 381.1769 |
| GPPEF | 500.2504 | 528.2453 | GPPEFE | 629.2930 | 657.2879 | PP | 167.1179 | 195.1128 |
| PPE | 296.1605 | 324.1554 | PPEF | 443.2289 | 471.2238 | PPEFE | 572.2715 | 600.2664 |
| PE | 199.1077 | 227.1026 | PEF | 346.1761 | 374.1710 | PEFE | 475.2187 | 503.2136 |

| | | | | | | | | |
|---|---|---|---|---|---|---|---|---|
| **PEFEQ** | 603.2773 | 631.2722 | **EF** | 249.1234 | 277.1183 | **EFE** | 378.1660 | 406.1609 |
| **EFEQ** | 506.2245 | 534.2195 | **EFEQP** | 603.2773 | 631.2722 | **FE** | 249.1234 | 277.1183 |
| **FEQ** | 377.1819 | 405.1769 | **FEQP** | 474.2347 | 502.2296 | **EQ** | 230.1135 | 258.1084 |
| **EQP** | 327.1663 | 355.1612 | **QP** | 198.1237 | 226.1186 | | | |

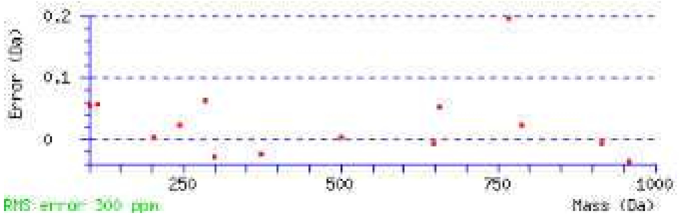


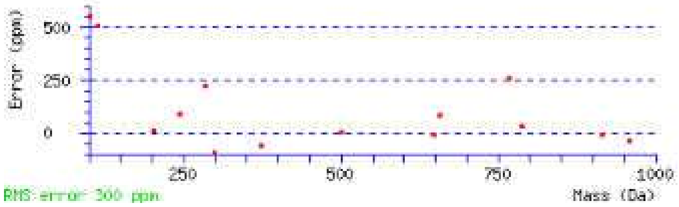


NCBI **BLAST** search of EGPPEFEQPK
(Parameters: blastp, nr protein database, expect=20000, no filter, PAM30)
Other BLAST web gateways

**All matches to this query**

| Score | Mr(calc) | Delta | Sequence |
|---|---|---|---|
| 16.2 | 1156.5400 | -0.0035 | EGPPEFEQPK |
| 15.1 | 1156.5910 | -0.0545 | SLPSCAAVQPK |
| 13.2 | 1156.6200 | -0.0835 | TALSKGNNQPK |
| 13.0 | 1156.5910 | -0.0545 | THLQSTVMPK |
| 11.9 | 1156.6063 | -0.0698 | IHQFMEKPK |
| 11.7 | 1156.5611 | -0.0246 | EEINELDAPK |
| 11.3 | 1156.5949 | -0.0583 | EATGAATARGPR |
| 11.2 | 1156.5335 | 0.0030 | HAEMKDWPK |
| 11.2 | 1156.5585 | -0.0219 | RSQNGNQEPK |
| 11.0 | 1156.6200 | -0.0835 | ENKQATNKPK |

**Mascot:** http://www.matrixscience.com/

# MATRIX SCIENCE Mascot Search Results

## Peptide View Spot no 74

MS/MS Fragmentation of **SFQCELVFAK**
Found in **gi|8918361** in **NCBInr**, RuBisCO activase small isoform precursor [Oryza sativa]

Match to Query 31: 1227.593924 from(1228.601200,1+) intensity(0.0000) index(12)
Title: Label: L1, Spot_Id: 219685, Peak_List_Id: 225081, MSMS Job_Run_Id: 21764, Comment:
Data file C:\Documents and Settings\Administrator\Desktop\farah BHU\spot set plate\Farah-Garima 11May 2013\ppw_L1_136842039200.txt

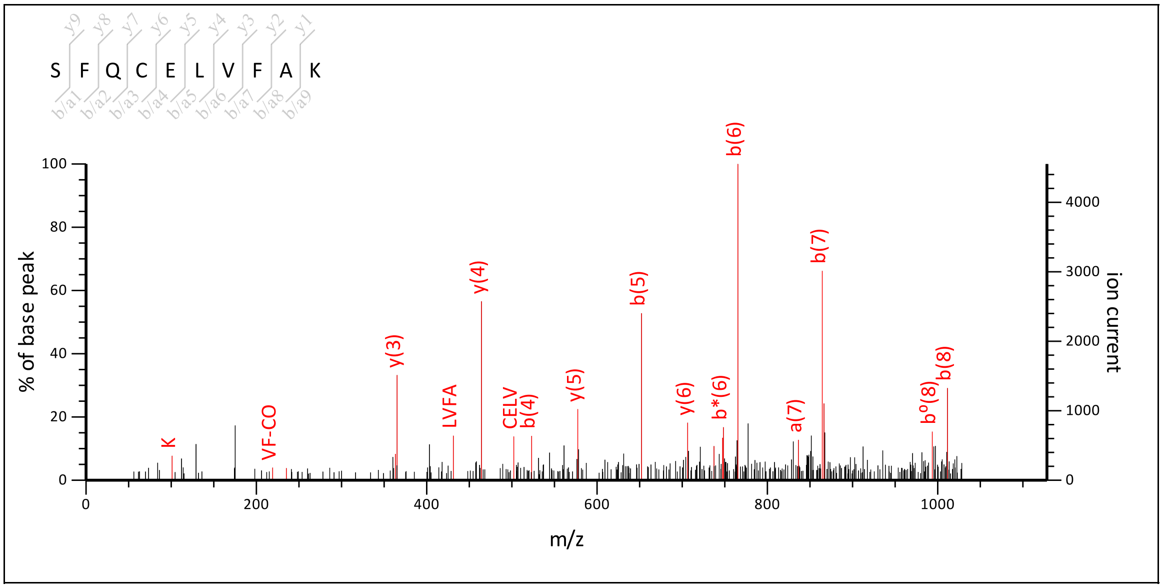


0 to 1128.23

Label all possible matches ○ Label matches used for scoring ◉

**Monoisotopic mass of neutral peptide Mr(calc):** 1227.5958
**Fixed modifications:** Carbamidomethyl (C) (apply to specified residues or termini only)
**Ions Score:** 40 **Expect:** 0.19
**Matches :** 25/148 fragment ions using 40 most intense peaks (help)

| # | Immon. | a | a* | $a^0$ | b | b* | $b^0$ | d | Seq. | v | w | y | y* | $y^0$ | # |
|---|---|---|---|---|---|---|---|---|---|---|---|---|---|---|---|
| 1 | 60.0444 | 60.0444 | | 42.0338 | 88.0393 | | 70.0287 | 44.0495 | S | | | | | | 10 |
| 2 | 120.0808 | 207.1128 | | 189.1022 | 235.1077 | | 217.0972 | | F | 1049.5084 | | 1141.5710 | 1124.5445 | 1123.5605 | 9 |
| 3 | 101.0709 | 335.1714 | 318.1448 | 317.1608 | 363.1663 | 346.1397 | 345.1557 | 278.1499 | Q | 921.4499 | 920.4546 | 994.5026 | 977.4761 | 976.4921 | 8 |
| 4 | 133.0430 | 495.2020 | 478.1755 | 477.1915 | 523.1969 | 506.1704 | 505.1864 | 406.2085 | C | 761.4192 | 760.4240 | 866.4441 | 849.4175 | 848.4335 | 7 |
| 5 | 102.0550 | 624.2446 | 607.2181 | 606.2341 | 652.2395 | 635.2130 | 634.2290 | 566.2391 | E | 632.3766 | 631.3814 | 706.4134 | 689.3869 | 688.4028 | 6 |
| 6 | 86.0964 | 737.3287 | 720.3021 | 719.3181 | 765.3236 | 748.2971 | 747.3130 | 695.2817 | L | 519.2926 | 518.2973 | 577.3708 | 560.3443 | | 5 |
| 7 | 72.0808 | 836.3971 | 819.3706 | 818.3865 | 864.3920 | 847.3655 | 846.3815 | 822.3815 | V | 420.2241 | 433.2445 | 464.2867 | 447.2602 | | 4 |
| 8 | 120.0808 | 983.4655 | 966.4390 | 965.4549 | 1011.4604 | 994.4339 | 993.4499 | | F | 273.1557 | | 365.2183 | 348.1918 | | 3 |
| 9 | 44.0495 | 1054.5026 | 1037.4761 | 1036.4921 | 1082.4975 | 1065.4710 | 1064.4870 | | A | 202.1186 | | 218.1499 | 201.1234 | | 2 |
| 10 | 101.1073 | | | | | | | | K | 74.0237 | 73.0284 | 147.1128 | 130.0863 | | 1 |

| Seq | ya | yb | Seq | ya | yb | Seq | ya | yb |
|---|---|---|---|---|---|---|---|---|
| FQ | 248.1394 | 276.1343 | FQC | 408.1700 | 436.1649 | FQCE | 537.2126 | 565.2075 |
| FQCEL | 650.2967 | 678.2916 | QC | 261.1016 | 289.0965 | QCE | 390.1442 | 418.1391 |
| QCEL | 503.2282 | 531.2232 | QCELV | 602.2967 | 630.2916 | CE | 262.0856 | 290.0805 |
| CEL | 375.1697 | 403.1646 | CELV | 474.2381 | 502.2330 | CELVF | 621.3065 | 649.3014 |
| CELVFA | 692.3436 | 720.3385 | EL | 215.1390 | 243.1339 | ELV | 314.2074 | 342.2023 |
| ELVF | 461.2758 | 489.2708 | ELVFA | 532.3130 | 560.3079 | LV | 185.1648 | 213.1598 |
| LVF | 332.2333 | 360.2282 | LVFA | 403.2704 | 431.2653 | VF | 219.1492 | 247.1441 |

| | | | | | | | | |
|---|---|---|---|---|---|---|---|---|
| **VFA** | 290.1863 | 318.1812 | **FA** | 191.1179 | 219.1128 | | | |

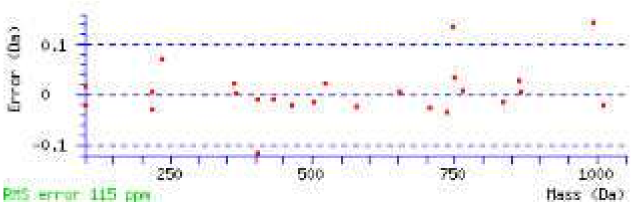


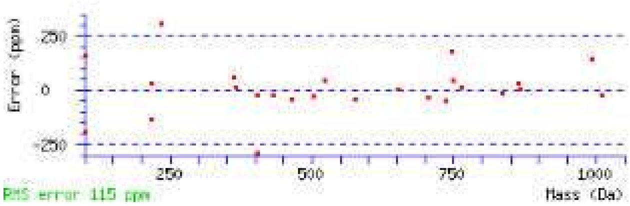


NCBI **BLAST** search of SFQCELVFAK
(Parameters: blastp, nr protein database, expect=20000, no filter, PAM30)
Other BLAST web gateways

**All matches to this query**

| Score | Mr(calc) | Delta | Sequence |
|---|---|---|---|
| 40.5 | 1227.5958 | -0.0019 | SFQCELVFAK |
| 40.5 | 1227.5628 | 0.0311 | SFQCELVMAK |
| 39.0 | 1227.5628 | 0.0311 | SFQCELVMSK |
| 24.5 | 1227.5992 | -0.0052 | GCFVIKTEMK |
| 20.6 | 1227.4860 | 0.1079 | ECSSLTSECR |
| 19.9 | 1227.5376 | 0.0563 | FGCVSTMASPR |
| 19.2 | 1227.6208 | -0.0268 | KVHPESSTSTR |
| 18.7 | 1227.6248 | -0.0308 | HKPTSESPFAK |
| 16.7 | 1227.6612 | -0.0672 | YTKALTTFQR |
| 15.9 | 1227.6499 | -0.0560 | KFSVDISYAAK |

**Mascot:** http://www.matrixscience.com/

# MATRIX SCIENCE Mascot Search Results

## Peptide View **Spot no 74**

MS/MS Fragmentation of **WVSDTGVENIGKR**
Found in **gi|8918361** in **NCBInr**, RuBisCO activase small isoform precursor [Oryza sativa]

Match to Query 43: 1459.774424 from(1460.781700,1+) intensity(0.0000) index(16)
Title: Label: L1, Spot_Id: 219685, Peak_List_Id: 225085, MSMS Job_Run_Id: 21764, Comment:
Data file C:\Documents and Settings\Administrator\Desktop\farah BHU\spot set plate\Farah-Garima 11May 2013\ppw_L1_136842039200.txt

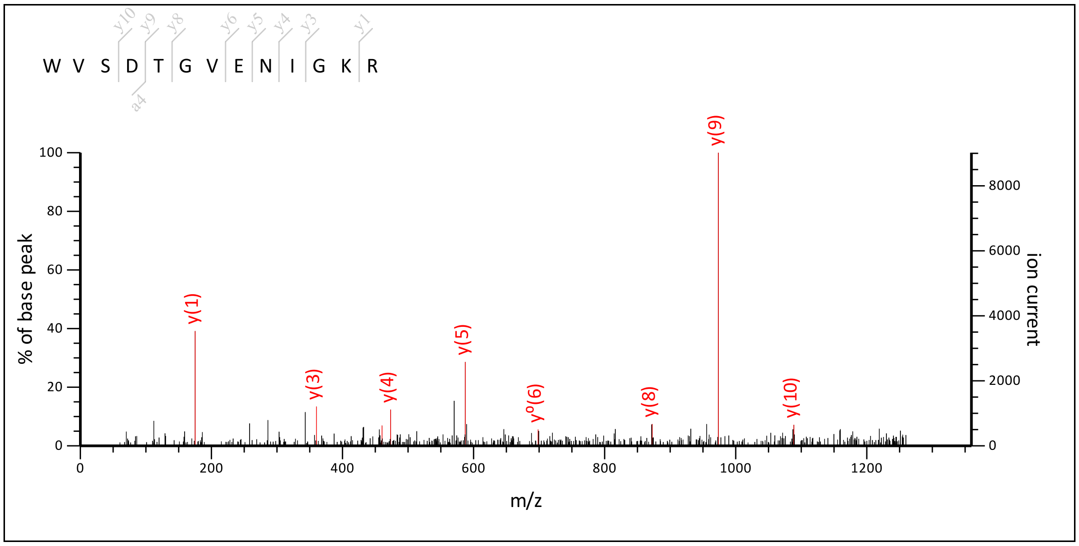


0 to 1359.64

Label all possible matches ○ Label matches used for scoring ◉

**Monoisotopic mass of neutral peptide Mr(calc):** 1459.7419
**Fixed modifications:** Carbamidomethyl (C) (apply to specified residues or termini only)
**Ions Score:** 55 **Expect:** 0.0064
**Matches :** 14/217 fragment ions using 12 most intense peaks (help)

| # | Immon. | a | a* | $a^0$ | b | b* | $b^0$ | d | d' | Seq. | v | w | w' | y | y* | [illegible] |
|---|---|---|---|---|---|---|---|---|---|---|---|---|---|---|---|---|
| 1 | 159.0917 | 159.0917 | | | 187.0866 | | | 44.0495 | | W | | | | | | |
| 2 | 72.0808 | 258.1601 | | | 286.1550 | | | 244.1444 | | V | 1230.6073 | 1243.6277 | | 1274.6699 | 1257.6434 | [illegible] |
| 3 | 60.0444 | 345.1921 | | 327.1816 | 373.1870 | | 355.1765 | 329.1972 | | S | 1143.5753 | 1142.5800 | | 1175.6015 | 1158.5749 | [illegible] |
| 4 | 88.0393 | **460.2191** | | 442.2085 | 488.2140 | | 470.2034 | 416.2292 | | D | 1028.5483 | 1027.5531 | | ***1088.5695*** | 1071.5429 | [illegible] |
| 5 | 74.0600 | 561.2667 | | 543.2562 | 589.2617 | | 571.2511 | 545.2718 | 547.2511 | T | 927.5007 | 940.5211 | 942.5003 | ***973.5425*** | 956.5160 | [illegible] |
| 6 | 30.0338 | 618.2882 | | 600.2776 | 646.2831 | | 628.2726 | | | G | | | | ***872.4948*** | 855.4683 | [illegible] |
| 7 | 72.0808 | 717.3566 | | 699.3461 | 745.3515 | | 727.3410 | 703.3410 | | V | 771.4108 | 784.4312 | | 815.4734 | 798.4468 | [illegible] |
| 8 | 102.0550 | 846.3992 | | 828.3886 | 874.3941 | | 856.3836 | 788.3937 | | E | 642.3682 | 641.3729 | | 716.4050 | 699.3784 | [illegible] |
| 9 | 87.0553 | 960.4421 | 943.4156 | 942.4316 | 988.4371 | 971.4105 | 970.4265 | 917.4363 | | N | 528.3253 | 527.3300 | | ***587.3624*** | 570.3358 | |
| 10 | 86.0964 | 1073.5262 | 1056.4997 | 1055.5156 | 1101.5211 | 1084.4946 | 1083.5105 | 1045.4949 | 1059.5105 | I | 415.2412 | 428.2616 | 442.2772 | ***473.3194*** | 456.2929 | |
| 11 | 30.0338 | 1130.5477 | 1113.5211 | 1112.5371 | 1158.5426 | 1141.5160 | 1140.5320 | | | G | | | | ***360.2354*** | 343.2088 | |
| 12 | 101.1073 | 1258.6426 | 1241.6161 | 1240.6321 | 1286.6375 | 1269.6110 | 1268.6270 | 1201.5848 | | K | 230.1248 | 229.1295 | | 303.2139 | 286.1874 | |
| 13 | 129.1135 | | | | | | | | | R | 74.0237 | 73.0284 | | ***175.1190*** | 158.0924 | |

| Seq | ya | yb | Seq | ya | yb | Seq | ya | yb |
|---|---|---|---|---|---|---|---|---|
| VS | 159.1128 | 187.1077 | VSD | 274.1397 | 302.1347 | VSDT | 375.1874 | 403.1823 |
| VSDTG | 432.2089 | 460.2038 | VSDTGV | 531.2773 | 559.2722 | VSDTGVE | 660.3199 | 688.3148 |
| SD | 175.0713 | 203.0662 | SDT | 276.1190 | 304.1139 | SDTG | 333.1405 | 361.1354 |
| SDTGV | 432.2089 | 460.2038 | SDTGVE | 561.2515 | 589.2464 | SDTGVEN | 675.2944 | 703.2893 |
| DT | 189.0870 | 217.0819 | DTG | 246.1084 | 274.1034 | DTGV | 345.1769 | 373.1718 |
| DTGVE | 474.2195 | 502.2144 | DTGVEN | 588.2624 | 616.2573 | TG | 131.0815 | 159.0764 |
| TGV | 230.1499 | 258.1448 | TGVE | 359.1925 | 387.1874 | TGVEN | 473.2354 | 501.2304 |
| TGVENI | 586.3195 | 614.3144 | TGVENIG | 643.3410 | 671.3359 | GV | 129.1022 | 157.0972 |

| | | | | | | | | |
|---|---|---|---|---|---|---|---|---|
| **GVE** | 258.1448 | 286.1397 | **GVEN** | 372.1878 | 400.1827 | **GVENI** | 485.2718 | 513.2667 |
| **GVENIG** | 542.2933 | 570.2882 | **GVENIGK** | 670.3882 | 698.3832 | **VE** | 201.1234 | 229.1183 |
| **VEN** | 315.1663 | 343.1612 | **VENI** | 428.2504 | 456.2453 | **VENIG** | 485.2718 | 513.2667 |
| **VENIGK** | 613.3668 | 641.3617 | **EN** | 216.0979 | 244.0928 | **ENI** | 329.1819 | 357.1769 |
| **ENIG** | 386.2034 | 414.1983 | **ENIGK** | 514.2984 | 542.2933 | **NI** | 200.1394 | 228.1343 |
| **NIG** | 257.1608 | 285.1557 | **NIGK** | 385.2558 | 413.2507 | **IG** | 143.1179 | 171.1128 |
| **IGK** | 271.2129 | 299.2078 | **GK** | 158.1288 | 186.1237 | | | |

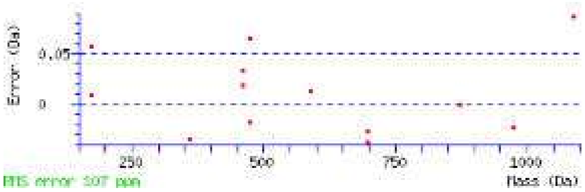


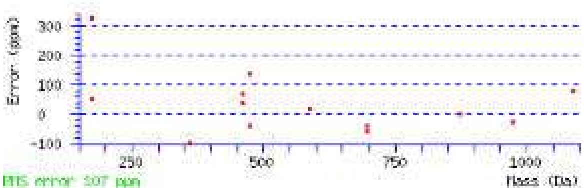


NCBI **BLAST** search of WVSDTGVENIGKR
(Parameters: blastp, nr protein database, expect=20000, no filter, PAM30)
Other BLAST web gateways

**All matches to this query**

| Score | Mr(calc) | Delta | Sequence |
|---|---|---|---|
| 55.1 | 1459.7419 | 0.0325 | WVSDTGVENIGKR |
| 31.3 | 1459.7024 | 0.0720 | CLQMGAGVGVGAGAGR |
| 24.8 | 1459.7089 | 0.0655 | ADVLAQQAGMDIGR |
| 22.0 | 1459.6950 | 0.0794 | AAARCGTSPSAAGAGR |
| 21.6 | 1459.7453 | 0.0291 | QSSVAPGASSMAVLR |
| 21.1 | 1459.6903 | 0.0841 | GNNTDSGVDLEAIR |
| 20.0 | 1459.6652 | 0.1093 | TDEAGGVNQTGAGGAR |
| 19.5 | 1459.7783 | -0.0039 | WVTSTGIENIGKR |
| 19.4 | 1459.6539 | 0.1205 | EENDVVSQQEQR |
| 19.3 | 1459.8035 | -0.0291 | LALGADGFVETLVR |

**Mascot:** http://www.matrixscience.com/

# MATRIX SCIENCE Mascot Search Results

## Peptide View Spot no 74

MS/MS Fragmentation of **GLAYDISDDQQDITR**
Found in **gi|8918361** in **NCBInr**, RuBisCO activase small isoform precursor [Oryza sativa]

Match to Query 51: 1708.823924 from(1709.831200,1+) intensity(0.0000) index(19)
Title: Label: L1, Spot_Id: 219685, Peak_List_Id: 225079, MSMS Job_Run_Id: 21764, Comment:
Data file C:\Documents and Settings\Administrator\Desktop\farah BHU\spot set plate\Farah-Garima 11May 2013\ppw_L1_136842039200.txt

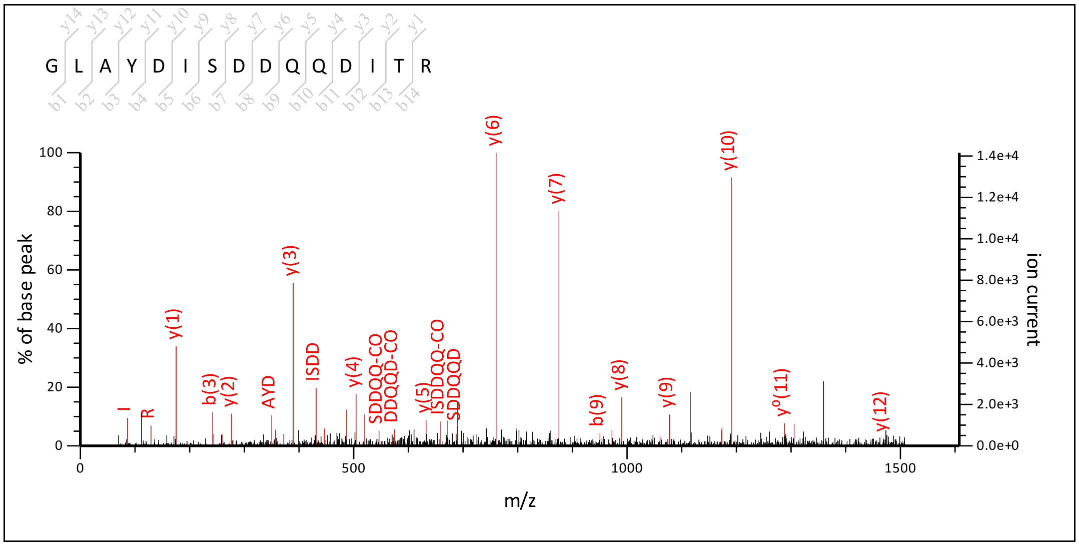


0 to 1607.7

Label all possible matches ○ Label matches used for scoring ◉

**Monoisotopic mass of neutral peptide Mr(calc):** 1708.7904
**Fixed modifications:** Carbamidomethyl (C) (apply to specified residues or termini only)
**Ions Score:** 78 **Expect:** 3.1e-05
**Matches :** 43/255 fragment ions using 60 most intense peaks (help)

| # | Immon. | a | a* | $a^0$ | b | b* | $b^0$ | d | d' | Seq. | v | w | w' | y | y* | |
|---|---|---|---|---|---|---|---|---|---|---|---|---|---|---|---|---|
| 1 | 30.0338 | 30.0338 | | | 58.0287 | | | 44.0495 | | G | | | | | | |
| 2 | 86.0964 | 143.1179 | | | 171.1128 | | | 101.0709 | | L | 1594.6980 | 1593.7027 | | 1652.7762 | 1635.7497 | [illegible] |
| 3 | 44.0495 | 214.1550 | | | 242.1499 | | | | | A | 1523.6609 | | | 1539.6922 | 1522.6656 | [illegible] |
| 4 | 136.0757 | 377.2183 | | | 405.2132 | | | | | Y | 1360.5975 | | | 1468.6550 | 1451.6285 | [illegible] |
| 5 | 88.0393 | 492.2453 | | 474.2347 | 520.2402 | | 502.2296 | 448.2554 | | D | 1245.5706 | 1244.5753 | | 1305.5917 | 1288.5652 | [illegible] |
| 6 | 86.0964 | 605.3293 | | 587.3188 | 633.3243 | | 615.3137 | 577.2980 | 591.3137 | I | 1132.4865 | 1145.5069 | 1159.5226 | 1190.5648 | 1173.5382 | [illegible] |
| 7 | 60.0444 | 692.3614 | | 674.3508 | 720.3563 | | 702.3457 | 676.3665 | | S | 1045.4545 | 1044.4592 | | 1077.4807 | 1060.4542 | [illegible] |
| 8 | 88.0393 | 807.3883 | | 789.3777 | 835.3832 | | 817.3727 | 763.3985 | | D | 930.4276 | 929.4323 | | 990.4487 | 973.4221 | [illegible] |
| 9 | 88.0393 | 922.4153 | | 904.4047 | 950.4102 | | 932.3996 | 878.4254 | | D | 815.4006 | 814.4054 | | 875.4217 | 858.3952 | [illegible] |
| 10 | 101.0709 | 1050.4738 | 1033.4473 | 1032.4633 | 1078.4687 | 1061.4422 | 1060.4582 | 993.4524 | | Q | 687.3420 | 686.3468 | | 760.3948 | 743.3682 | [illegible] |
| 11 | 101.0709 | 1178.5324 | 1161.5059 | 1160.5218 | 1206.5273 | 1189.5008 | 1188.5168 | 1121.5109 | | Q | 559.2835 | 558.2882 | | 632.3362 | 615.3097 | [illegible] |
| 12 | 88.0393 | 1293.5594 | 1276.5328 | 1275.5488 | 1321.5543 | 1304.5277 | 1303.5437 | 1249.5695 | | D | 444.2565 | 443.2613 | | 504.2776 | 487.2511 | [illegible] |
| 13 | 86.0964 | 1406.6434 | 1389.6169 | 1388.6329 | 1434.6383 | 1417.6118 | 1416.6278 | 1378.6121 | 1392.6278 | I | 331.1724 | 344.1928 | 358.2085 | 389.2507 | 372.2241 | [illegible] |
| 14 | 74.0600 | 1507.6911 | 1490.6645 | 1489.6805 | 1535.6860 | 1518.6595 | 1517.6754 | 1491.6962 | 1493.6754 | T | 230.1248 | 243.1452 | 245.1244 | 276.1666 | 259.1401 | [illegible] |
| 15 | 129.1135 | | | | | | | | | R | 74.0237 | 73.0284 | | 175.1190 | 158.0924 | |

| Seq | ya | yb | Seq | ya | yb | Seq | ya | yb |
|---|---|---|---|---|---|---|---|---|
| LA | 157.1335 | 185.1285 | LAY | 320.1969 | 348.1918 | LAYD | 435.2238 | 463.2187 |
| LAYDI | 548.3079 | 576.3028 | LAYDIS | 635.3399 | 663.3348 | AY | 207.1128 | 235.1077 |
| AYD | 322.1397 | 350.1347 | AYDI | 435.2238 | 463.2187 | AYDIS | 522.2558 | 550.2508 |
| AYDISD | 637.2828 | 665.2777 | YD | 251.1026 | 279.0975 | YDI | 364.1867 | 392.1816 |
| YDIS | 451.2187 | 479.2136 | YDISD | 566.2457 | 594.2406 | YDISDD | 681.2726 | 709.2675 |
| DI | 201.1234 | 229.1183 | DIS | 288.1554 | 316.1503 | DISD | 403.1823 | 431.1773 |

| | | | | | | | | |
|---|---|---|---|---|---|---|---|---|
| **DISDD** | 518.2093 | 546.2042 | **DISDDQ** | 646.2679 | 674.2628 | **IS** | 173.1285 | 201.1234 |
| **ISD** | 288.1554 | 316.1503 | **ISDD** | 403.1823 | 431.1773 | **ISDDQ** | 531.2409 | 559.2358 |
| **ISDDQQ** | 659.2995 | 687.2944 | **SD** | 175.0713 | 203.0662 | **SDD** | 290.0983 | 318.0932 |
| **SDDQ** | 418.1569 | 446.1518 | **SDDQQ** | 546.2154 | 574.2103 | **SDDQQD** | 661.2424 | 689.2373 |
| **DD** | 203.0662 | 231.0612 | **DDQ** | 331.1248 | 359.1197 | **DDQQ** | 459.1834 | 487.1783 |
| **DDQQD** | 574.2103 | 602.2053 | **DDQQDI** | 687.2944 | 715.2893 | **DQ** | 216.0979 | 244.0928 |
| **DQQ** | 344.1565 | 372.1514 | **DQQD** | 459.1834 | 487.1783 | **DQQDI** | 572.2675 | 600.2624 |
| **DQQDIT** | 673.3151 | 701.3101 | **QQ** | 229.1295 | 257.1244 | **QQD** | 344.1565 | 372.1514 |
| **QQDI** | 457.2405 | 485.2354 | **QQDIT** | 558.2882 | 586.2831 | **QD** | 216.0979 | 244.0928 |
| **QDI** | 329.1819 | 357.1769 | **QDIT** | 430.2296 | 458.2245 | **DI** | 201.1234 | 229.1183 |
| **DIT** | 302.1710 | 330.1660 | **IT** | 187.1441 | 215.1390 | | | |

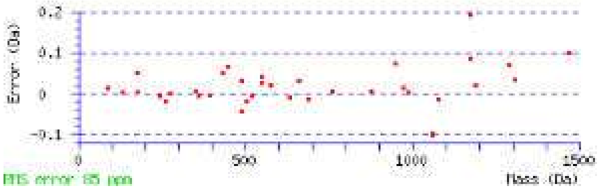


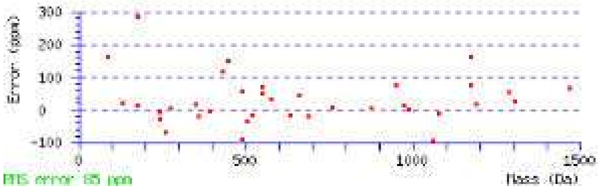


NCBI **BLAST** search of GLAYDISDDQQDITR
(Parameters: blastp, nr protein database, expect=20000, no filter, PAM30)
Other BLAST web gateways

**All matches to this query**

| Score | Mr(calc) | Delta | Sequence |
|---|---|---|---|
| 77.5 | 1708.7904 | 0.0335 | GLAYDISDDQQDITR |
| 51.6 | 1708.7540 | 0.0699 | GLAFDESDDQQDITR |
| 21.9 | 1708.8897 | -0.0658 | AGIDKFGVPVPGDPGGAR |
| 19.3 | 1708.7023 | 0.1216 | SSEQNLESDGEEETR |
| 18.9 | 1708.7978 | 0.0261 | GLSTDSLMEAGYPGAPK |
| 16.9 | 1708.8315 | -0.0076 | DMQVRSGQSIVNYGR |
| 16.4 | 1708.8931 | -0.0691 | MVRFAQVVSSLDTTR |
| 15.5 | 1708.7397 | 0.0843 | CSLADCDDGLVDITR |
| 15.3 | 1708.8753 | -0.0514 | LQGMVASMSRIPTFR |
| 15.1 | 1708.9009 | -0.0770 | ANIHALSGAWGDKLTR |

**Mascot:** http://www.matrixscience.com/

# MATRIX SCIENCE Mascot Search Results

## Peptide View Spot no 74

MS/MS Fragmentation of **MCCLFINDLDAGAGR**
Found in **gi|8918361** in **NCBInr**, RuBisCO activase small isoform precursor [Oryza sativa]

Match to Query 54: 1727.764324 from(1728.771600,1+) intensity(0.0000) index(20)
Title: Label: L1, Spot_Id: 219685, Peak_List_Id: 225086, MSMS Job_Run_Id: 21764, Comment:
Data file C:\Documents and Settings\Administrator\Desktop\farah BHU\spot set plate\Farah-Garima 11May 2013\ppw_L1_136842039200.txt

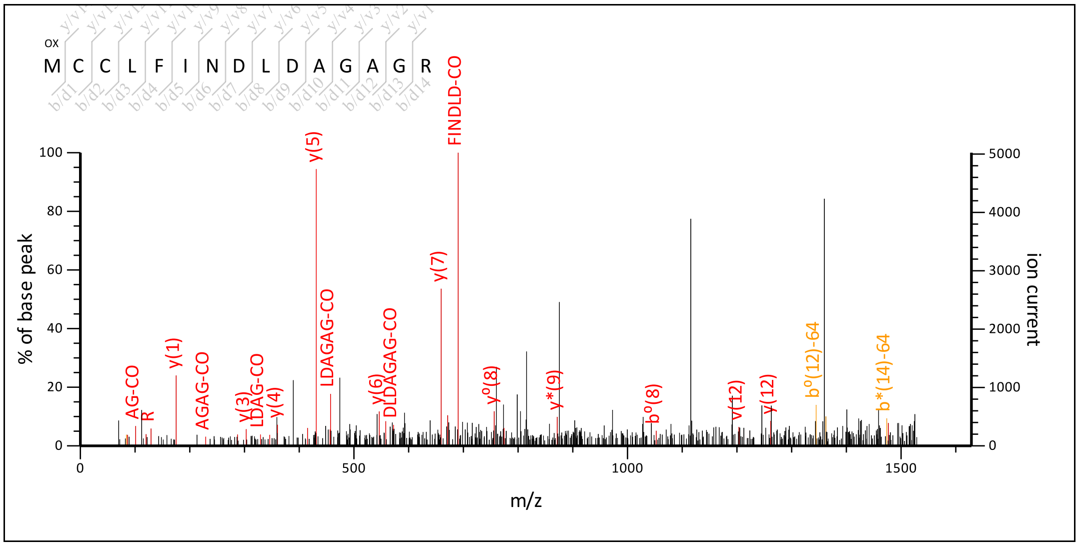


0 to 1628.57

Label all possible matches ○ Label matches used for scoring ◉

**Monoisotopic mass of neutral peptide Mr(calc):** 1727.7430
**Fixed modifications:** Carbamidomethyl (C) (apply to specified residues or termini only)
**Variable modifications:**
**M1** : Oxidation (M), with neutral losses 63.9983(shown in table), 0.0000
**Ions Score:** 16 **Expect:** 39
**Matches** : 46/311 fragment ions using 105 most intense peaks (help)

| # | Immon. | a | a* | $a^0$ | b | b* | $b^0$ | d | d' | Seq. | v | w | w' | y | y* | |
|---|---|---|---|---|---|---|---|---|---|---|---|---|---|---|---|---|
| 1 | 56.0495 | 56.0495 | | | **84.0444** | | | 44.0495 | | M | | | | | | |
| 2 | 133.0430 | 216.0801 | | | 244.0750 | | | 127.0866 | | C | 1476.6900 | 1475.6947 | | 1581.7148 | 1564.6883 | 156[illegible] |
| 3 | 133.0430 | 376.1108 | | | 404.1057 | | | 287.1172 | | C | 1316.6593 | 1315.6641 | | 1421.6842 | 1404.6576 | 140[illegible] |
| 4 | 86.0964 | 489.1948 | | | 517.1898 | | | 447.1479 | | L | 1203.5753 | 1202.5800 | | ***1261.6535*** | 1244.6270 | 124[illegible] |
| 5 | 120.0808 | 636.2633 | | | 664.2582 | | | | | F | 1056.5069 | | | 1148.5695 | 1131.5429 | 113[illegible] |
| 6 | 86.0964 | 749.3473 | | | 777.3422 | | | 721.3160 | 735.3317 | I | 943.4228 | 956.4432 | 970.4588 | 1001.5010 | 984.4745 | 98[illegible] |
| 7 | 87.0553 | 863.3902 | 846.3637 | | 891.3852 | 874.3586 | | 820.3844 | | N | 829.3799 | 828.3846 | | 888.4170 | 871.3904 | 87[illegible] |
| 8 | 88.0393 | 978.4172 | 961.3906 | 960.4066 | 1006.4121 | 989.3856 | 988.4015 | 934.4274 | | D | 714.3529 | 713.3577 | | ***774.3741*** | 757.3475 | 75[illegible] |
| 9 | 86.0964 | 1091.5013 | 1074.4747 | 1073.4907 | 1119.4962 | 1102.4696 | 1101.4856 | 1049.4543 | | L | 601.2689 | 600.2736 | | ***659.3471*** | 642.3206 | 64[illegible] |
| 10 | 88.0393 | 1206.5282 | 1189.5016 | 1188.5176 | 1234.5231 | 1217.4966 | 1216.5125 | 1162.5384 | | D | 486.2419 | 485.2467 | | ***546.2631*** | 529.2365 | 52[illegible] |
| 11 | 44.0495 | 1277.5653 | 1260.5388 | 1259.5547 | 1305.5602 | 1288.5337 | 1287.5497 | | | A | 415.2048 | | | ***431.2361*** | 414.2096 | |
| 12 | 30.0338 | 1334.5868 | 1317.5602 | 1316.5762 | **1362.5817** | 1345.5551 | 1344.5711 | | | G | | | | ***360.1990*** | 343.1724 | |
| 13 | 44.0495 | 1405.6239 | 1388.5973 | 1387.6133 | 1433.6188 | 1416.5923 | 1415.6082 | | | A | 287.1462 | | | ***303.1775*** | 286.1510 | |
| 14 | 30.0338 | 1462.6453 | 1445.6188 | 1444.6348 | 1490.6403 | 1473.6137 | 1472.6297 | | | G | | | | 232.1404 | 215.1139 | |
| 15 | 129.1135 | | | | | | | | | R | 74.0237 | 73.0284 | | ***175.1190*** | 158.0924 | |

| Seq | ya | yb | Seq | ya | yb | Seq | ya | yb |
|---|---|---|---|---|---|---|---|---|
| CC | 293.0737 | 321.0686 | CCL | 406.1577 | 434.1526 | CCLF | 553.2261 | 581.2211 |
| CCLFI | 666.3102 | 694.3051 | CL | 246.1271 | 274.1220 | CLF | 393.1955 | 421.1904 |
| CLFI | 506.2796 | 534.2745 | CLFIN | 620.3225 | 648.3174 | LF | 233.1648 | 261.1598 |
| | | | | | | | | |

| | | | | | | | | |
|---|---|---|---|---|---|---|---|---|
| **LFI** | 346.2489 | 374.2438 | **LFIN** | 460.2918 | 488.2867 | **LFIND** | 575.3188 | 603.3137 |
| **LFINDL** | 688.4028 | 716.3978 | **FI** | 233.1648 | 261.1598 | **FIN** | 347.2078 | 375.2027 |
| **FIND** | 462.2347 | 490.2296 | **FINDL** | 575.3188 | 603.3137 | **FINDLD** | 690.3457 | 718.3406 |
| **IN** | 200.1394 | 228.1343 | **IND** | 315.1663 | 343.1612 | **INDL** | 428.2504 | 456.2453 |
| **INDLD** | 543.2773 | 571.2722 | **INDLDA** | 614.3144 | 642.3093 | **INDLDAG** | 671.3359 | 699.3308 |
| **ND** | 202.0822 | 230.0771 | **NDL** | 315.1663 | 343.1612 | **NDLD** | 430.1932 | 458.1882 |
| **NDLDA** | 501.2304 | 529.2253 | **NDLDAG** | 558.2518 | 586.2467 | **NDLDAGA** | 629.2889 | 657.2838 |
| **NDLDAGAG** | 686.3104 | 714.3053 | **DL** | 201.1234 | 229.1183 | **DLD** | 316.1503 | 344.1452 |
| **DLDA** | 387.1874 | 415.1823 | **DLDAG** | 444.2089 | 472.2038 | **DLDAGA** | 515.2460 | 543.2409 |
| **DLDAGAG** | 572.2675 | 600.2624 | **LD** | 201.1234 | 229.1183 | **LDA** | 272.1605 | 300.1554 |
| **LDAG** | 329.1819 | 357.1769 | **LDAGA** | 400.2191 | 428.2140 | **LDAGAG** | 457.2405 | 485.2354 |
| **DA** | 159.0764 | 187.0713 | **DAG** | 216.0979 | 244.0928 | **DAGA** | 287.1350 | 315.1299 |
| **DAGAG** | 344.1565 | 372.1514 | **AG** | 101.0709 | 129.0659 | **AGA** | 172.1081 | 200.1030 |
| **AGAG** | 229.1295 | 257.1244 | **GA** | 101.0709 | 129.0659 | **GAG** | 158.0924 | 186.0873 |
| **AG** | 101.0709 | 129.0659 | | | | | | |

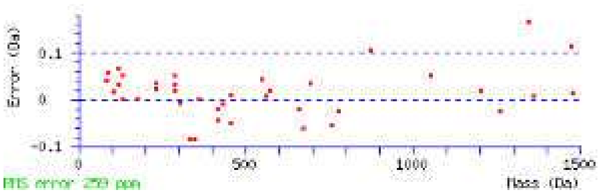


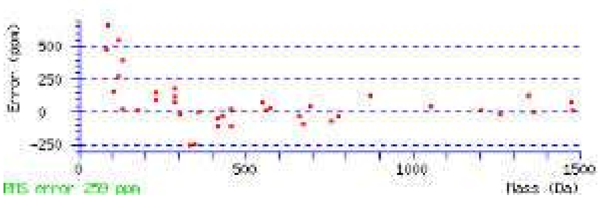


NCBI **BLAST** search of MCCLFINDLDAGAGR
(Parameters: blastp, nr protein database, expect=20000, no filter, PAM30)
Other BLAST web gateways

**All matches to this query**

| Score | Mr(calc) | Delta | Sequence |
|---|---|---|---|
| 15.8 | 1727.7430 | 0.0214 | MCCLFINDLDAGAGR |
| 15.8 | 1727.8261 | -0.0617 | QQKVNDDDAMKPPSR |
| 15.0 | 1727.9352 | -0.1709 | IPTMDLARLIASQGAR |
| 13.7 | 1727.8002 | -0.0359 | YFLVSDEESAQTPSR |
| 11.9 | 1727.8942 | -0.1298 | QQIDETQVIVTTPEK |
| 9.5 | 1727.8842 | -0.1199 | PSFVANANQQTDVLPK |
| 9.0 | 1727.9107 | -0.1464 | RNGFGGPAPSFPLGNIK |
| 8.2 | 1727.8414 | -0.0770 | EPDLGGWAVGGCVKQR |
| 8.2 | 1727.9166 | -0.1523 | VGNVSVRDVLEGLSER |
| 8.0 | 1727.7785 | -0.0141 | QEVMIGYSDSGKDAGR |

**Mascot:** http://www.matrixscience.com/

# Mascot Search Results

## Peptide View Spot no 74

MS/MS Fragmentation of **EGPPEFEQPKMTIEK**
Found in **gi|8918361** in **NCBInr**, RuBisCO activase small isoform precursor [Oryza sativa]

Match to Query 58: 1774.860424 from(1775.867700,1+) intensity(0.0000) index(22)
Title: Label: L1, Spot_Id: 219685, Peak_List_Id: 225087, MSMS Job_Run_Id: 21764, Comment:
Data file C:\Documents and Settings\Administrator\Desktop\farah BHU\spot set plate\Farah-Garima 11May 2013\ppw_L1_136842039200.txt

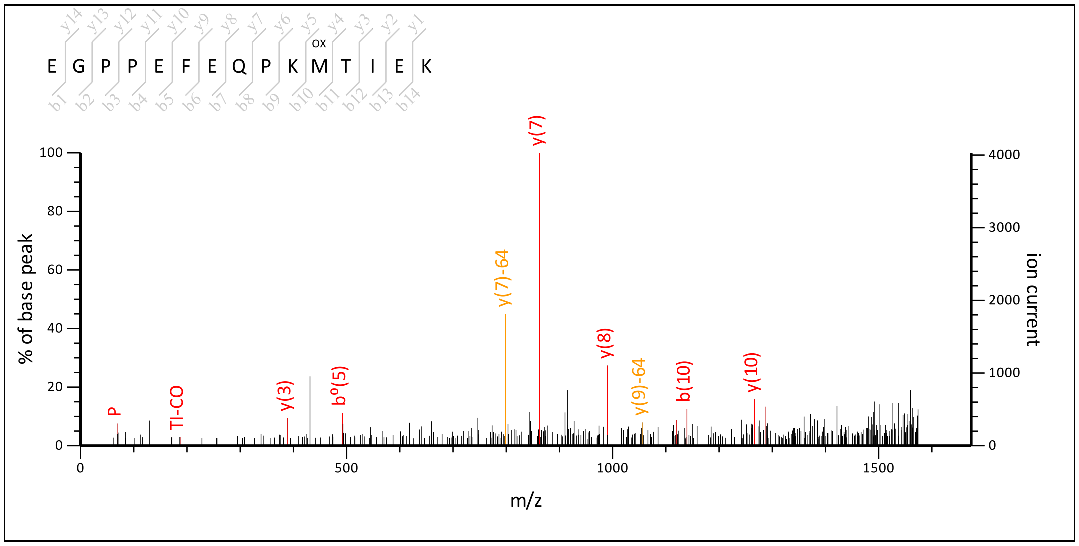


0 to 1673.97

Label all possible matches ○ Label matches used for scoring ◉

**Monoisotopic mass of neutral peptide Mr(calc):** 1774.8447
**Fixed modifications:** Carbamidomethyl (C) (apply to specified residues or termini only)
**Variable modifications:**
**M11** : Oxidation (M), with neutral losses 0.0000(shown in table), 63.9983
**Ions Score:** 17 **Expect:** 33
**Matches :** 15/358 fragment ions using 32 most intense peaks (help)

| # | Immon. | a | a* | $a^0$ | b | b* | $b^0$ | d | d' | Seq. | v | w | w' | y | y* | |
|---|---|---|---|---|---|---|---|---|---|---|---|---|---|---|---|---|
| 1 | 102.0550 | 102.0550 | | 84.0444 | 130.0499 | | 112.0393 | 44.0495 | | E | | | | | | |
| 2 | 30.0338 | 159.0764 | | 141.0659 | ***187.0713*** | | 169.0608 | | | G | | | | 1646.8094 | 1629.7829 | 162 |
| 3 | 70.0651 | 256.1292 | | 238.1186 | 284.1241 | | 266.1135 | 230.1135 | | P | 1547.7410 | 1546.7458 | | 1589.7880 | 1572.7614 | 15 |
| 4 | 70.0651 | 353.1819 | | 335.1714 | 381.1769 | | 363.1663 | 327.1663 | | P | 1450.6883 | 1449.6930 | | 1492.7352 | 1475.7087 | 14 |
| 5 | 102.0550 | 482.2245 | | 464.2140 | 510.2195 | | 492.2089 | 424.2191 | | E | 1321.6457 | 1320.6504 | | 1395.6824 | 1378.6559 | 13 |
| 6 | 120.0808 | 629.2930 | | 611.2824 | 657.2879 | | 639.2773 | | | F | 1174.5773 | | | ***1266.6399*** | 1249.6133 | 124 |
| 7 | 102.0550 | 758.3355 | | 740.3250 | 786.3305 | | 768.3199 | 700.3301 | | E | 1045.5347 | 1044.5394 | | ***1119.5714*** | 1102.5449 | 11 |
| 8 | 101.0709 | 886.3941 | 869.3676 | 868.3836 | 914.3890 | 897.3625 | 896.3785 | 829.3727 | | Q | 917.4761 | 916.4808 | | ***990.5288*** | 973.5023 | 9 |
| 9 | 70.0651 | 983.4469 | 966.4203 | 965.4363 | 1011.4418 | 994.4153 | 993.4312 | 957.4312 | | P | 820.4233 | 819.4281 | | ***862.4703*** | 845.4437 | 84 |
| 10 | 101.1073 | 1111.5419 | 1094.5153 | 1093.5313 | ***1139.5368*** | 1122.5102 | 1121.5262 | 1054.4840 | | K | 692.3284 | 691.3331 | | 765.4175 | 748.3910 | 74 |
| 11 | 120.0478 | 1258.5773 | 1241.5507 | 1240.5667 | ***1286.5722*** | 1269.5456 | 1268.5616 | 1182.5790 | | M | 545.2930 | 544.2977 | | 637.3225 | 620.2960 | 61 |
| 12 | 74.0600 | 1359.6249 | 1342.5984 | 1341.6144 | 1387.6198 | 1370.5933 | 1369.6093 | 1343.6300 | 1345.6093 | T | 444.2453 | 457.2657 | 459.2449 | 490.2871 | 473.2606 | 4 |
| 13 | 86.0964 | 1472.7090 | 1455.6824 | 1454.6984 | 1500.7039 | 1483.6774 | 1482.6933 | 1444.6777 | 1458.6933 | I | 331.1612 | 344.1816 | 358.1973 | ***389.2395*** | 372.2129 | 3 |
| 14 | 102.0550 | 1601.7516 | 1584.7250 | 1583.7410 | 1629.7465 | 1612.7200 | 1611.7359 | 1543.7461 | | E | 202.1186 | 201.1234 | | 276.1554 | 259.1288 | 25 |
| 15 | 101.1073 | | | | | | | | | K | 74.0237 | 73.0284 | | 147.1128 | 130.0863 | |

| Seq | ya | yb | Seq | ya | yb | Seq | ya | yb |
|---|---|---|---|---|---|---|---|---|
| **GP** | 127.0866 | 155.0815 | **GPP** | 224.1394 | 252.1343 | **GPPE** | 353.1819 | 381.1769 |
| **GPPEF** | 500.2504 | 528.2453 | **GPPEFE** | 629.2930 | 657.2879 | **PP** | 167.1179 | 195.1128 |
| **PPE** | 296.1605 | 324.1554 | **PPEF** | 443.2289 | 471.2238 | **PPEFE** | 572.2715 | 600.2664 |
| | | | | | | | | |

| | | | | | | | | |
|---|---|---|---|---|---|---|---|---|
| **PE** | 199.1077 | 227.1026 | **PEF** | 346.1761 | 374.1710 | **PEFE** | 475.2187 | 503.2136 |
| **PEFEQ** | 603.2773 | 631.2722 | **EF** | 249.1234 | 277.1183 | **EFE** | 378.1660 | 406.1609 |
| **EFEQ** | 506.2245 | 534.2195 | **EFEQP** | 603.2773 | 631.2722 | **FE** | 249.1234 | 277.1183 |
| **FEQ** | 377.1819 | 405.1769 | **FEQP** | 474.2347 | 502.2296 | **FEQPK** | 602.3297 | 630.3246 |
| **EQ** | 230.1135 | 258.1084 | **EQP** | 327.1663 | 355.1612 | **EQPK** | 455.2613 | 483.2562 |
| **EQPKM** | 602.2967 | 630.2916 | **QP** | 198.1237 | 226.1186 | **QPK** | 326.2187 | 354.2136 |
| **QPKM** | 473.2541 | 501.2490 | **QPKMT** | 574.3017 | 602.2967 | **QPKMTI** | 687.3858 | 715.3807 |
| **PK** | 198.1601 | 226.1550 | **PKM** | 345.1955 | 373.1904 | **PKMT** | 446.2432 | 474.2381 |
| **PKMTI** | 559.3272 | 587.3221 | **PKMTIE** | 688.3698 | 716.3647 | **KM** | 248.1427 | 276.1376 |
| **KMT** | 349.1904 | 377.1853 | **KMTI** | 462.2745 | 490.2694 | **KMTIE** | 591.3171 | 619.3120 |
| **MT** | 221.0954 | 249.0904 | **MTI** | 334.1795 | 362.1744 | **MTIE** | 463.2221 | 491.2170 |
| **TI** | 187.1441 | 215.1390 | **TIE** | 316.1867 | 344.1816 | **IE** | 215.1390 | 243.1339 |

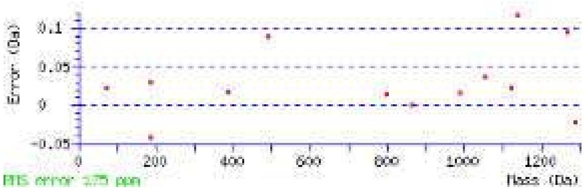


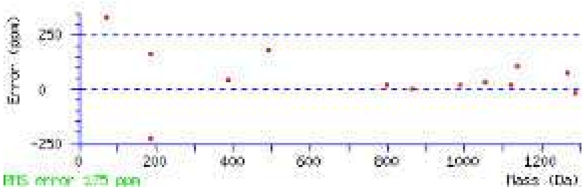


NCBI **BLAST** search of EGPPEFEQPKMTIEK
(Parameters: blastp, nr protein database, expect=20000, no filter, PAM30)
Other BLAST web gateways

**All matches to this query**

| Score | Mr(calc) | Delta | Sequence |
|---|---|---|---|
| 17.2 | 1774.8447 | 0.0157 | EGPPEFEQPKMTIEK |
| 6.2 | 1775.0067 | -0.1463 | AGIPGAAARPAGWRGVLR |
| 5.5 | 1774.8784 | -0.0180 | KNSTNMVAELFNVHR |
| 4.8 | 1774.8355 | 0.0249 | QHMTHAQTRIMSFR |
| 4.8 | 1774.8771 | -0.0167 | MATETEVVVAPVSNGGSK |
| 4.8 | 1774.8905 | -0.0301 | RPHPPVAAPPMMMRR |
| 4.8 | 1774.8957 | -0.0353 | QMDPINVETSLLLMR |
| 4.2 | 1774.9591 | -0.0987 | VVFINERPQSRQFR |
| 4.1 | 1774.9247 | -0.0643 | QMLEKVAELLASSNAR |
| 4.0 | 1774.9434 | -0.0829 | VIGMIPGMGKVSSAQIR |

**Mascot:** http://www.matrixscience.com/

# MATRIX SCIENCE Mascot Search Results

## Peptide View Spot no 74

MS/MS Fragmentation of **MGINPIMMSAGELESGNAGEPAK**
Found in **gi|8918361** in **NCBInr**, RuBisCO activase small isoform precursor [Oryza sativa]

Match to Query 62: 2351.097424 from(2352.104700,1+) intensity(0.0000) index(24)
Title: Label: L1, Spot_Id: 219685, Peak_List_Id: 225097, MSMS Job_Run_Id: 21764, Comment:
Data file C:\Documents and Settings\Administrator\Desktop\farah BHU\spot set plate\Farah-Garima 11May 2013\ppw_L1_136842039200.txt

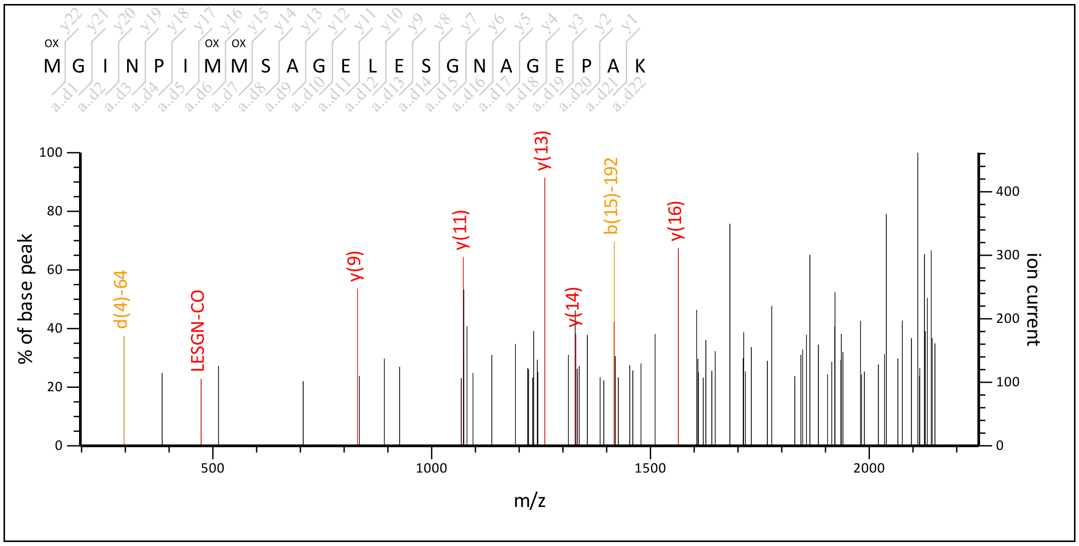


197.22 to 2250.15

Label all possible matches ○ Label matches used for scoring ◉

**Monoisotopic mass of neutral peptide Mr(calc):** 2351.0443
**Fixed modifications:** Carbamidomethyl (C) (apply to specified residues or termini only)
**Variable modifications:**
**M1** : Oxidation (M), with neutral losses 0.0000(shown in table), 63.9983
**M7** : Oxidation (M), with neutral losses 0.0000(shown in table), 63.9983
**M8** : Oxidation (M), with neutral losses 0.0000(shown in table), 63.9983
**Ions Score:** 9 **Expect:** 1.6e+02
**Matches :** 13/653 fragment ions using 32 most intense peaks (help)

| # | Immon. | a | a* | a⁰ | b | b* | b⁰ | d | d' | Seq. | v | w | w' | y | y* | |
|---|---|---|---|---|---|---|---|---|---|---|---|---|---|---|---|---|
| 1 | 120.0478 | 120.0478 | | | 148.0427 | | | 44.0495 | | M | | | | | | |
| 2 | 30.0338 | 177.0692 | | | 205.0641 | | | | | G | | | | 2205.0162 | 2187.9897 | 218 |
| 3 | 86.0964 | 290.1533 | | | 318.1482 | | | 262.1220 | 276.1376 | I | 2089.9165 | 2102.9369 | 2116.9525 | 2147.9947 | 2130.9682 | 212 |
| 4 | 87.0553 | 404.1962 | 387.1697 | | 432.1911 | 415.1646 | | 361.1904 | | N | 1975.8736 | 1974.8783 | | 2034.9107 | 2017.8841 | 201 |
| 5 | 70.0651 | 501.2490 | 484.2224 | | 529.2439 | 512.2173 | | 475.2333 | | P | 1878.8208 | 1877.8256 | | 1920.8678 | 1903.8412 | 190 |
| 6 | 86.0964 | 614.3330 | 597.3065 | | 642.3280 | 625.3014 | | 586.3017 | 600.3174 | I | 1765.7367 | 1778.7571 | 1792.7728 | 1823.8150 | 1806.7884 | 180 |
| 7 | 120.0478 | 761.3684 | 744.3419 | | 789.3634 | 772.3368 | | 685.3702 | | M | 1618.7013 | 1617.7061 | | 1710.7309 | 1693.7044 | 169 |
| 8 | 120.0478 | 908.4038 | 891.3773 | | 936.3988 | 919.3722 | | 832.4056 | | M | 1471.6659 | 1470.6707 | | ***1563.6955*** | 1546.6690 | 154 |
| 9 | 60.0444 | 995.4359 | 978.4093 | 977.4253 | 1023.4308 | 1006.4042 | 1005.4202 | 979.4410 | | S | 1384.6339 | 1383.6387 | | ***1416.6601*** | 1399.6336 | 139 |
| 10 | 44.0495 | 1066.4730 | 1049.4464 | 1048.4624 | 1094.4679 | 1077.4414 | 1076.4573 | | | A | 1313.5968 | | | ***1329.6281*** | 1312.6016 | 131 |
| 11 | 30.0338 | 1123.4944 | 1106.4679 | 1105.4839 | 1151.4894 | 1134.4628 | 1133.4788 | | | G | | | | ***1258.5910*** | 1241.5644 | 124 |
| 12 | 102.0550 | 1252.5370 | 1235.5105 | 1234.5265 | 1280.5320 | 1263.5054 | 1262.5214 | 1194.5316 | | E | 1127.5327 | 1126.5375 | | 1201.5695 | 1184.5430 | 118 |
| 13 | 86.0964 | 1365.6211 | 1348.5946 | 1347.6105 | 1393.6160 | 1376.5895 | 1375.6055 | 1323.5742 | | L | 1014.4487 | 1013.4534 | | ***1072.5269*** | 1055.5004 | 105 |
| 14 | 102.0550 | 1494.6637 | 1477.6372 | 1476.6531 | 1522.6586 | 1505.6321 | 1504.6480 | 1436.6582 | | E | 885.4061 | 884.4108 | | 959.4429 | 942.4163 | 94 |
| 15 | 60.0444 | 1581.6957 | 1564.6692 | 1563.6852 | 1609.6906 | 1592.6641 | 1591.6801 | 1565.7008 | | S | 798.3741 | 797.3788 | | ***830.4003*** | 813.3737 | 81 |
| 16 | 30.0338 | 1638.7172 | 1621.6906 | 1620.7066 | 1666.7121 | 1649.6856 | 1648.7015 | | | G | | | | 743.3682 | 726.3417 | 72 |
| 17 | 87.0553 | 1752.7601 | 1735.7336 | 1734.7496 | 1780.7550 | 1763.7285 | 1762.7445 | 1709.7543 | | N | 627.3097 | 626.3144 | | 686.3468 | 669.3202 | 66 |
| 18 | 44.0495 | 1823.7972 | 1806.7707 | 1805.7867 | 1851.7921 | 1834.7656 | 1833.7816 | | | A | 556.2726 | | | 572.3039 | 555.2773 | 55 |
| 19 | 30.0338 | 1880.8187 | 1863.7921 | 1862.8081 | 1908.8136 | 1891.7871 | 1890.8030 | | | G | | | | 501.2667 | 484.2402 | 48 |
| 20 | 102.0550 | 2009.8613 | 1992.8347 | 1991.8507 | 2037.8562 | 2020.8297 | 2019.8456 | 1951.8558 | | E | 370.2085 | 369.2132 | | 444.2453 | 427.2187 | 42 |

| | | | | | | | | | | | | | | | | |
|---|---|---|---|---|---|---|---|---|---|---|---|---|---|---|---|---|
| 21 | 70.0651 | 2106.9141 | 2089.8875 | 2088.9035 | 2134.9090 | 2117.8824 | 2116.8984 | 2080.8984 | | P | 273.1557 | 272.1605 | | 315.2027 | 298.1761 | |
| 22 | 44.0495 | 2177.9512 | 2160.9246 | 2159.9406 | 2205.9461 | 2188.9195 | 2187.9355 | | | A | 202.1186 | | | 218.1499 | 201.1234 | |
| 23 | 101.1073 | | | | | | | | | K | 74.0237 | 73.0284 | | 147.1128 | 130.0863 | |

| Seq | ya | yb | Seq | ya | yb | Seq | ya | yb |
|---|---|---|---|---|---|---|---|---|
| GI | 143.1179 | 171.1128 | GIN | 257.1608 | 285.1557 | GINP | 354.2136 | 382.2085 |
| GINPI | 467.2976 | 495.2926 | GINPIM | 614.3330 | 642.3280 | IN | 200.1394 | 228.1343 |
| INP | 297.1921 | 325.1870 | INPI | 410.2762 | 438.2711 | INPIM | 557.3116 | 585.3065 |
| NP | 184.1081 | 212.1030 | NPI | 297.1921 | 325.1870 | NPIM | 444.2275 | 472.2224 |
| NPIMM | 591.2629 | 619.2578 | NPIMMS | 678.2949 | 706.2899 | PI | 183.1492 | 211.1441 |
| PIM | 330.1846 | 358.1795 | PIMM | 477.2200 | 505.2149 | PIMMS | 564.2520 | 592.2469 |
| PIMMSA | 635.2891 | 663.2840 | PIMMSAG | 692.3106 | 720.3055 | IM | 233.1318 | 261.1267 |
| IMM | 380.1672 | 408.1621 | IMMS | 467.1993 | 495.1942 | IMMSA | 538.2364 | 566.2313 |
| IMMSAG | 595.2578 | 623.2527 | MM | 267.0832 | 295.0781 | MMS | 354.1152 | 382.1101 |
| MMSA | 425.1523 | 453.1472 | MMSAG | 482.1738 | 510.1687 | MMSAGE | 611.2164 | 639.2113 |
| MS | 207.0798 | 235.0747 | MSA | 278.1169 | 306.1118 | MSAG | 335.1384 | 363.1333 |
| MSAGE | 464.1810 | 492.1759 | MSAGEL | 577.2650 | 605.2599 | SA | 131.0815 | 159.0764 |
| SAG | 188.1030 | 216.0979 | SAGE | 317.1456 | 345.1405 | SAGEL | 430.2296 | 458.2245 |
| SAGELE | 559.2722 | 587.2671 | SAGELES | 646.3042 | 674.2992 | AG | 101.0709 | 129.0659 |
| AGE | 230.1135 | 258.1084 | AGEL | 343.1976 | 371.1925 | AGELE | 472.2402 | 500.2351 |
| AGELES | 559.2722 | 587.2671 | AGELESG | 616.2937 | 644.2886 | GE | 159.0764 | 187.0713 |
| GEL | 272.1605 | 300.1554 | GELE | 401.2031 | 429.1980 | GELES | 488.2351 | 516.2300 |
| GELESG | 545.2566 | 573.2515 | GELESGN | 659.2995 | 687.2944 | EL | 215.1390 | 243.1339 |
| ELE | 344.1816 | 372.1765 | ELES | 431.2136 | 459.2086 | ELESG | 488.2351 | 516.2300 |
| ELESGN | 602.2780 | 630.2729 | ELESGNA | 673.3151 | 701.3101 | LE | 215.1390 | 243.1339 |
| LES | 302.1710 | 330.1660 | LESG | 359.1925 | 387.1874 | LESGN | 473.2354 | 501.2304 |
| LESGNA | 544.2726 | 572.2675 | LESGNAG | 601.2940 | 629.2889 | ES | 189.0870 | 217.0819 |
| ESG | 246.1084 | 274.1034 | ESGN | 360.1514 | 388.1463 | ESGNA | 431.1885 | 459.1834 |
| ESGNAG | 488.2100 | 516.2049 | ESGNAGE | 617.2525 | 645.2475 | SG | 117.0659 | 145.0608 |
| SGN | 231.1088 | 259.1037 | SGNA | 302.1459 | 330.1408 | SGNAG | 359.1674 | 387.1623 |
| SGNAGE | 488.2100 | 516.2049 | SGNAGEP | 585.2627 | 613.2576 | SGNAGEPA | 656.2998 | 684.2947 |
| GN | 144.0768 | 172.0717 | GNA | 215.1139 | 243.1088 | GNAG | 272.1353 | 300.1302 |
| GNAGE | 401.1779 | 429.1728 | GNAGEP | 498.2307 | 526.2256 | GNAGEPA | 569.2678 | 597.2627 |
| NA | 158.0924 | 186.0873 | NAG | 215.1139 | 243.1088 | NAGE | 344.1565 | 372.1514 |
| NAGEP | 441.2092 | 469.2041 | NAGEPA | 512.2463 | 540.2413 | AG | 101.0709 | 129.0659 |
| AGE | 230.1135 | 258.1084 | AGEP | 327.1663 | 355.1612 | AGEPA | 398.2034 | 426.1983 |
| GE | 159.0764 | 187.0713 | GEP | 256.1292 | 284.1241 | GEPA | 327.1663 | 355.1612 |
| EP | 199.1077 | 227.1026 | EPA | 270.1448 | 298.1397 | PA | 141.1022 | 169.0972 |

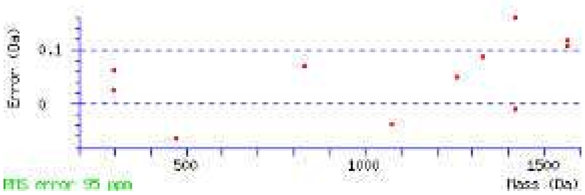


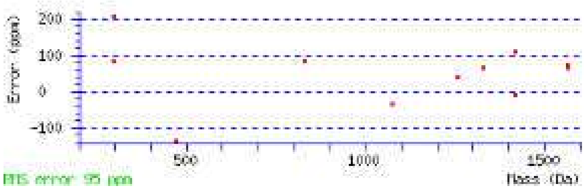


NCBI **BLAST** search of MGINPIMMSAGELESGNAGEPAK
(Parameters: blastp, nr protein database, expect=20000, no filter, PAM30)
Other BLAST web gateways

**All matches to this query**

| Score | Mr(calc) | Delta | Sequence |
|---|---|---|---|
| 10.3 | 2351.0807 | 0.0168 | MGINLIMMSAGELESGNAGEPAK |
| 8.8 | 2351.0443 | 0.0531 | MGINPIMMSAGELESGNAGEPAK |
| 3.2 | 2351.0807 | 0.0168 | MGINLIMMSAGELESGNAGEPAK |
| 1.8 | 2351.0807 | 0.0168 | MGINLIMMSAGELESGNAGEPAK |

**Mascot:** http://www.matrixscience.com/

# MATRIX SCIENCE Mascot Search Results

## Peptide View Spot no 81

MS/MS Fragmentation of **YLTASAMFR**
Found in **gi|493725** in **NCBInr**, beta tubulin [Oryza sativa Japonica Group]

Match to Query 3: 1058.621524 from(1059.628800,1+) intensity(0.0000) index(0)
Title: Label: E13, Spot_Id: 216673, Peak_List_Id: 221751, MSMS Job_Run_Id: 21390, Comment:
Data file C:\Documents and Settings\Administrator\Desktop\farah BHU\Cotton leaves\RAJESH\12-1-13 raj\ppw_E13_136117700900.txt

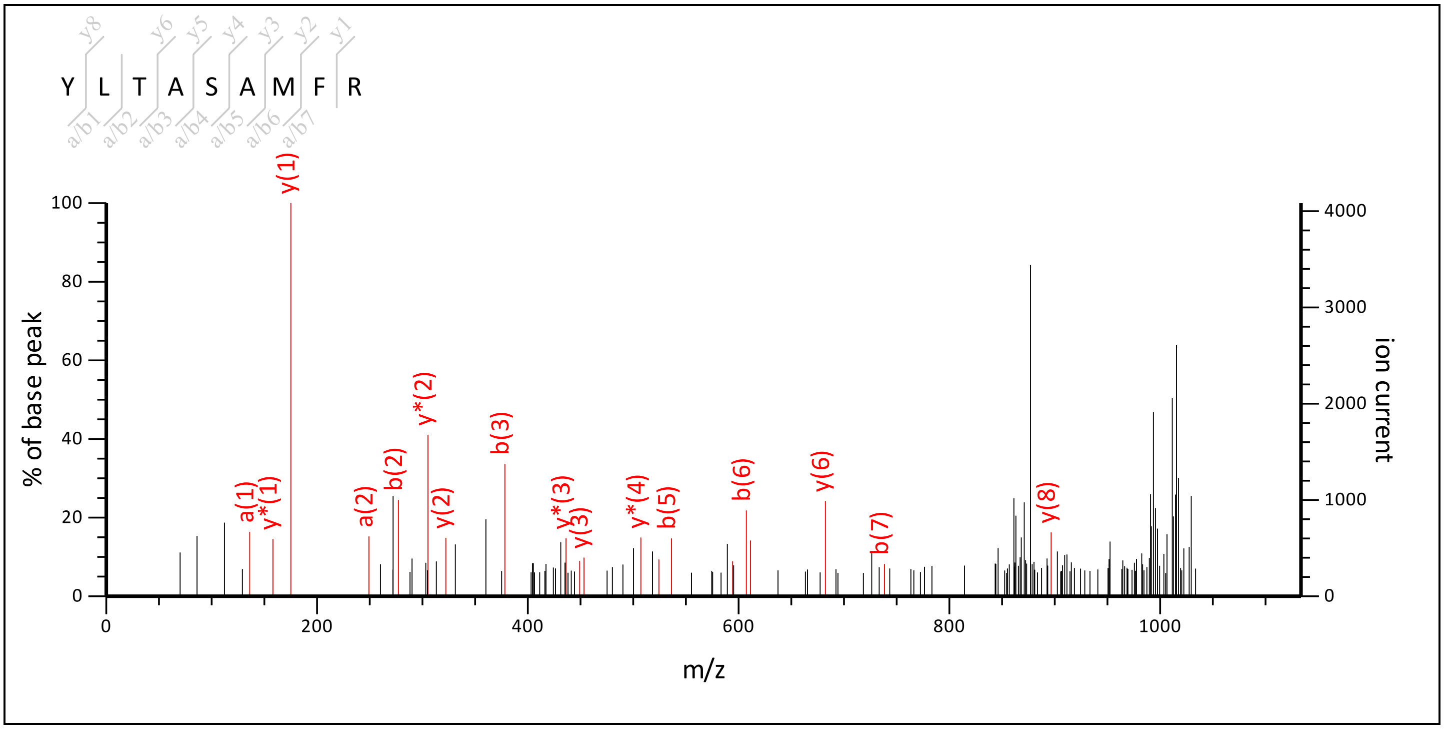


0 to 1133.64

Label all possible matches ○ Label matches used for scoring ◉

**Monoisotopic mass of neutral peptide Mr(calc):** 1058.5219
**Fixed modifications:** Carbamidomethyl (C) (apply to specified residues or termini only)
**Ions Score:** 29 **Expect:** 7.9
**Matches :** 20/32 fragment ions using 48 most intense peaks (help)

| # | a | b | Seq. | y | y* | # |
|---|---|---|---|---|---|---|
| 1 | 136.0757 | 164.0706 | Y | | | 9 |
| 2 | 249.1598 | **277.1547** | L | ***896.4659*** | 879.4393 | 8 |
| 3 | 350.2074 | **378.2023** | T | 783.3818 | 766.3552 | 7 |
| 4 | 421.2445 | **449.2395** | A | ***682.3341*** | 665.3076 | 6 |
| 5 | 508.2766 | **536.2715** | S | ***611.2970*** | 594.2704 | 5 |
| 6 | 579.3137 | **607.3086** | A | ***524.2650*** | 507.2384 | 4 |
| 7 | 710.3542 | **738.3491** | M | ***453.2279*** | 436.2013 | 3 |
| 8 | 857.4226 | 885.4175 | F | ***322.1874*** | 305.1608 | 2 |

| | | | | | | |
|---|---|---|---|---|---|---|
| **9** | | | **R** | ***175.1190*** | 158.0924 | **1** |

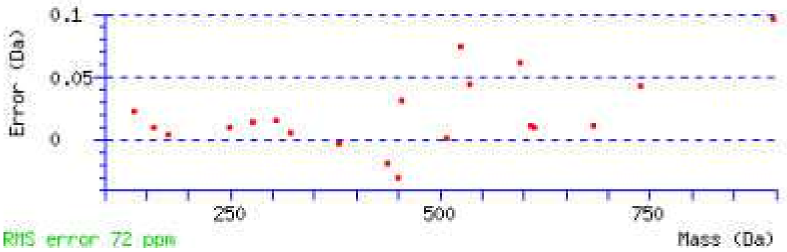


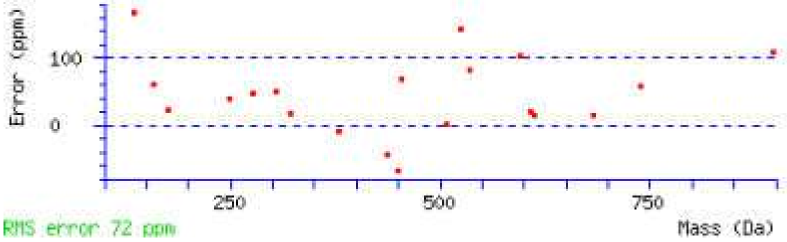


NCBI **BLAST** search of YLTASAMFR
(Parameters: blastp, nr protein database, expect=20000, no filter, PAM30)
Other BLAST web gateways

**All matches to this query**

| Score | Mr(calc) | Delta | Sequence |
|---|---|---|---|
| 29.1 | 1058.5219 | 0.0996 | YLTASAMFR |
| 9.8 | 1057.5080 | 1.1135 | YIETGAYGGK |
| 7.9 | 1059.6223 | -1.0007 | AILRVSVMR |
| 7.9 | 1059.5495 | -0.9280 | SPIRTPSMR |
| 6.1 | 1058.5873 | 0.0343 | YLTSALVHR |
| 5.7 | 1058.5185 | 0.1030 | YDVFVNFR |
| 5.7 | 1058.5549 | 0.0666 | YTLVFNFR |
| 5.7 | 1058.5219 | 0.0996 | YITMNFVR |
| 5.4 | 1059.5349 | -0.9134 | SPPPSEPPPR |
| 5.4 | 1058.5873 | 0.0343 | IYTSVAHLR |

**Mascot:** http://www.matrixscience.com/

# Mascot Search Results

## Peptide View **Spot no 81**

MS/MS Fragmentation of **LAVNLIPFPR**
Found in **gi|493725** in **NCBInr**, beta tubulin [Oryza sativa Japonica Group]

Match to Query 8: 1138.786824 from(1139.794100,1+) intensity(0.0000) index(2)
Title: Label: E13, Spot_Id: 216673, Peak_List_Id: 221744, MSMS Job_Run_Id: 21390, Comment:
Data file C:\Documents and Settings\Administrator\Desktop\farah BHU\Cotton leaves\RAJESH\12-1-13 raj\ppw_E13_136117700900.txt

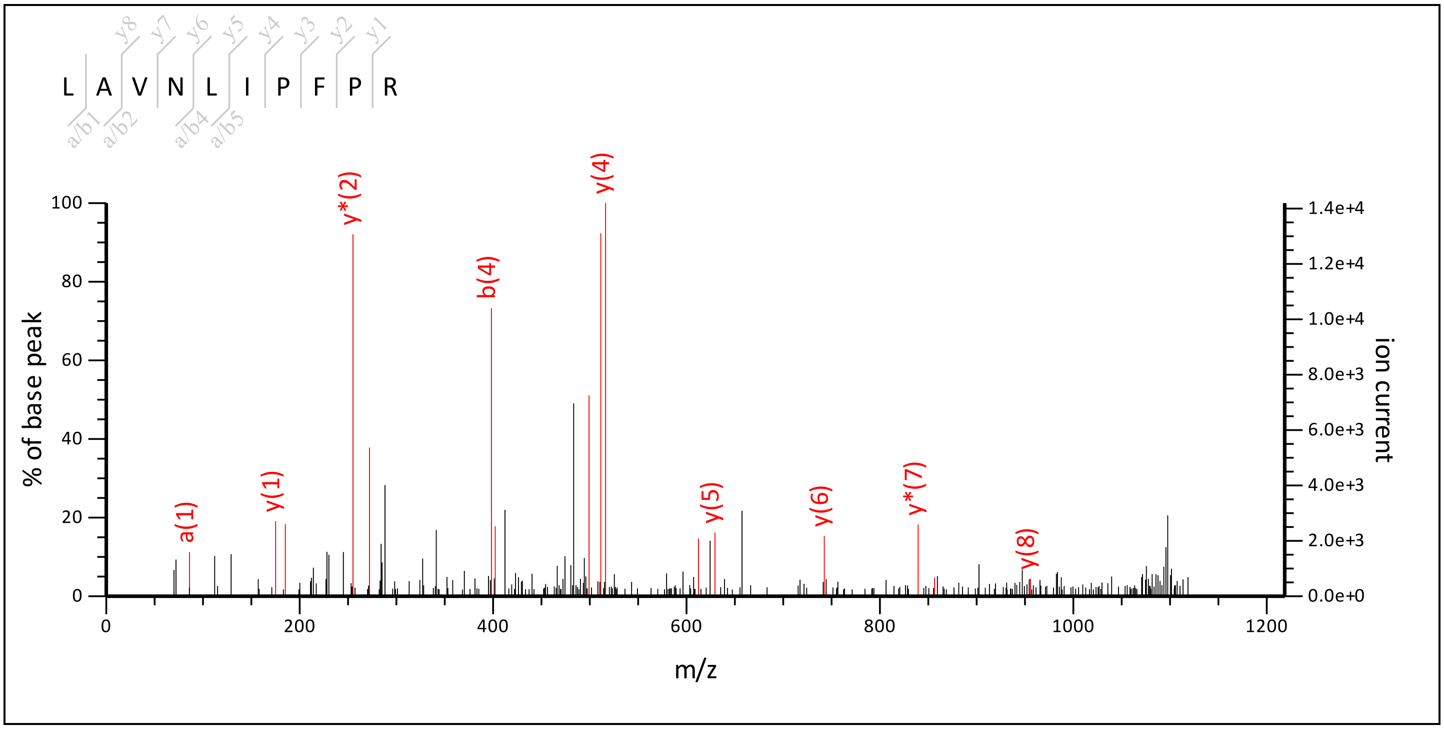


0 to 1218.55

Label all possible matches ○ Label matches used for scoring ◉

**Monoisotopic mass of neutral peptide Mr(calc):** 1138.6862
**Fixed modifications:** Carbamidomethyl (C) (apply to specified residues or termini only)
**Ions Score:** 37 **Expect:** 0.81
**Matches :** 16/48 fragment ions using 33 most intense peaks (help)

| # | a | a* | b | b* | Seq. | y | y* | # |
|---|---|---|---|---|---|---|---|---|
| 1 | 86.0964 | | 114.0913 | | L | | | 10 |
| 2 | 157.1335 | | **185.1285** | | A | 1026.6095 | 1009.5829 | 9 |
| 3 | 256.2020 | | 284.1969 | | V | ***955.5724*** | 938.5458 | 8 |
| 4 | 370.2449 | 353.2183 | **398.2398** | 381.2132 | N | ***856.5039*** | 839.4774 | 7 |
| 5 | 483.3289 | 466.3024 | **511.3239** | 494.2973 | L | ***742.4610*** | 725.4345 | 6 |
| 6 | 596.4130 | 579.3865 | 624.4079 | 607.3814 | I | ***629.3770*** | 612.3504 | 5 |
| 7 | 693.4658 | 676.4392 | 721.4607 | 704.4341 | P | ***516.2929*** | 499.2663 | 4 |
| 8 | 840.5342 | 823.5076 | 868.5291 | 851.5026 | F | 419.2401 | 402.2136 | 3 |

| | | | | | | | | | |
|---|---|---|---|---|---|---|---|---|---|
| **9** | 937.5870 | 920.5604 | 965.5819 | 948.5553 | **P** | *272.1717* | 255.1452 | **2** |
| **10** | | | | | **R** | *175.1190* | 158.0924 | **1** |

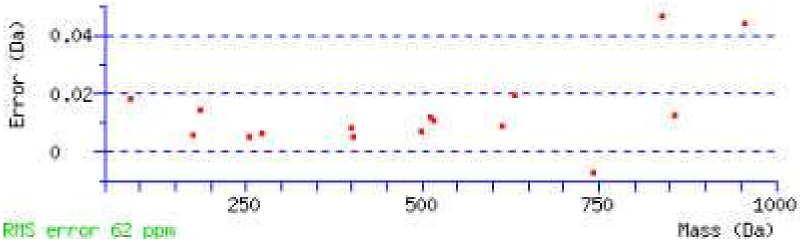


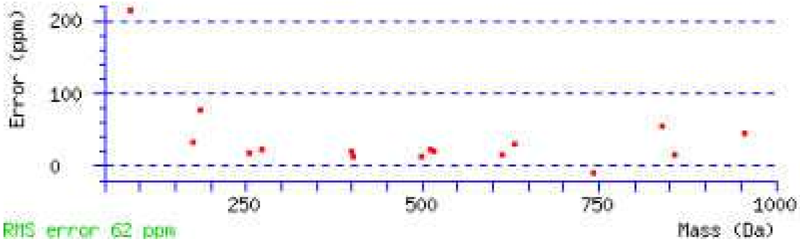


NCBI **BLAST** search of LAVNLIPFPR
(Parameters: blastp, nr protein database, expect=20000, no filter, PAM30)
Other BLAST web gateways

**All matches to this query**

| Score | Mr(calc) | Delta | Sequence |
|---|---|---|---|
| 37.3 | 1138.6862 | 0.1006 | IAVNLIPFPR |
| 37.3 | 1138.6862 | 0.1006 | LAVNLIPFPR |
| 26.0 | 1138.7074 | 0.0794 | GVLQILLDLR |
| 23.7 | 1138.6935 | 0.0934 | ARGILLNGAVR |
| 21.7 | 1137.6618 | 1.1250 | RALASSAIPPR |
| 19.5 | 1138.6281 | 0.1587 | RSPAVLMPPR |
| 18.3 | 1137.6658 | 1.1210 | LVWKTAAPPR |
| 18.1 | 1137.6870 | 1.0998 | LVSQVLLNPR |
| 17.6 | 1137.7274 | 1.0594 | LAVILIPFPR |
| 16.7 | 1138.6281 | 0.1588 | RAADPLPMLR |

**Mascot:** http://www.matrixscience.com/

# Mascot Search Results

## Peptide View Spot no 81

MS/MS Fragmentation of **FPGQLNSDLR**
Found in **gi|493725** in **NCBInr**, beta tubulin [Oryza sativa Japonica Group]

Match to Query 10: 1145.690824 from(1146.698100,1+) intensity(0.0000) index(3)
Title: Label: E13, Spot_Id: 216673, Peak_List_Id: 221745, MSMS Job_Run_Id: 21390, Comment:
Data file C:\Documents and Settings\Administrator\Desktop\farah BHU\Cotton leaves\RAJESH\12-1-13 raj\ppw_E13_136117700900.txt

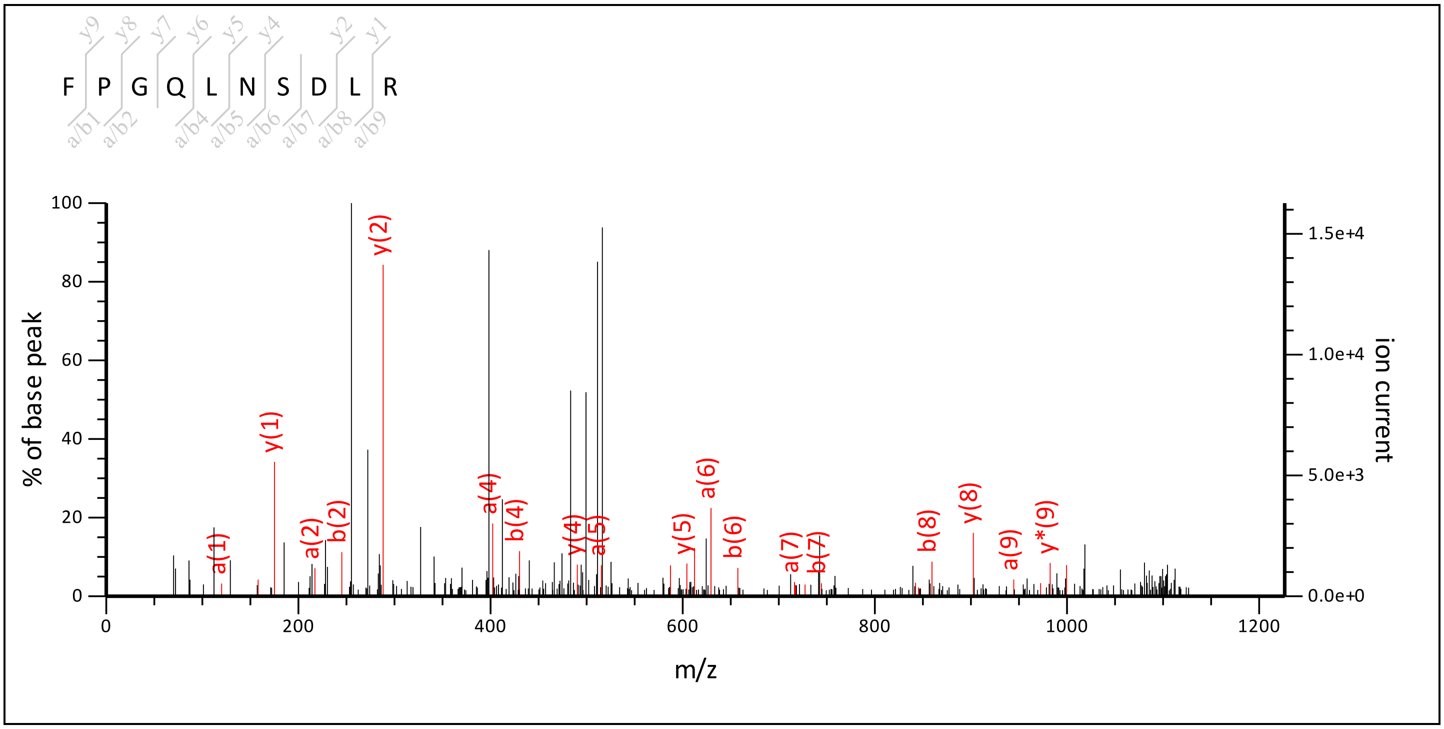


0 to 1226.58

Label all possible matches ○ Label matches used for scoring ◉

**Monoisotopic mass of neutral peptide Mr(calc):** 1145.5829
**Fixed modifications:** Carbamidomethyl (C) (apply to specified residues or termini only)
**Ions Score:** 18 **Expect:** 1e+02
**Matches :** 27/48 fragment ions using 110 most intense peaks (help)

| # | a | a* | b | b* | Seq. | y | y* | # |
|---|---|---|---|---|---|---|---|---|
| 1 | **120.0808** | | 148.0757 | | F | | | 10 |
| 2 | **217.1335** | | **245.1285** | | P | ***999.5218*** | 982.4952 | 9 |
| 3 | 274.1550 | | 302.1499 | | G | ***902.4690*** | 885.4425 | 8 |
| 4 | **402.2136** | 385.1870 | **430.2085** | 413.1819 | Q | ***845.4476*** | 828.4210 | 7 |
| 5 | **515.2976** | 498.2711 | 543.2926 | 526.2660 | L | ***717.3890*** | 700.3624 | 6 |
| 6 | **629.3406** | 612.3140 | **657.3355** | 640.3089 | N | ***604.3049*** | 587.2784 | 5 |
| 7 | **716.3726** | 699.3461 | **744.3675** | 727.3410 | S | ***490.2620*** | 473.2354 | 4 |
| 8 | 831.3995 | 814.3730 | **859.3945** | 842.3679 | D | 403.2300 | 386.2034 | 3 |

| | | | | | | | | |
|---|---|---|---|---|---|---|---|---|
| **9** | **944.4836** | 927.4571 | **972.4785** | 955.4520 | **L** | ***288.2030*** | 271.1765 | **2** |
| **10** | | | | | **R** | ***175.1190*** | 158.0924 | **1** |

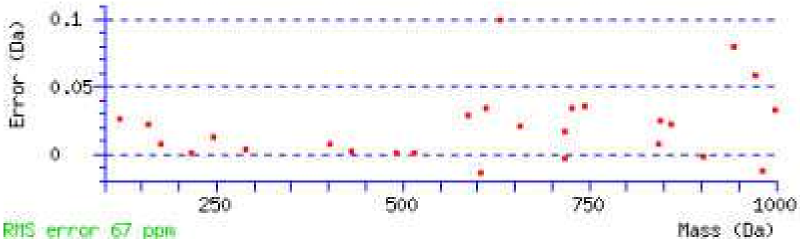


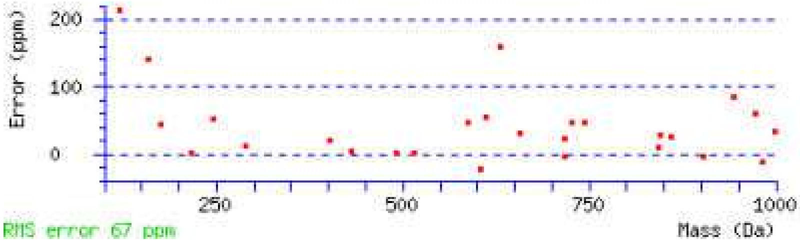


NCBI **BLAST** search of FPGQLNSDLR
(Parameters: blastp, nr protein database, expect=20000, no filter, PAM30)
Other BLAST web gateways

**All matches to this query**

| Score | Mr(calc) | Delta | Sequence |
|---|---|---|---|
| 24.1 | 1145.5750 | 0.1158 | EDCLLEVLR |
| 17.9 | 1145.6193 | 0.0715 | FPGKLNSDLR |
| 17.9 | 1145.5829 | 0.1079 | FPGQLNSDLR |
| 16.8 | 1144.5611 | 1.1297 | LAALGGEEEEK |
| 16.1 | 1144.6386 | 1.0522 | AIVRAISQCK |
| 15.9 | 1144.5975 | 1.0933 | IAGAVVEETEK |
| 15.1 | 1145.6114 | 0.0794 | MGEALLVELR |
| 14.4 | 1144.6452 | 1.0456 | GAVVLATAGGTTK |
| 13.9 | 1145.6768 | 0.0140 | KLLQSSTTIR |
| 13.4 | 1146.5161 | -0.8253 | EDCLNMLPR |

**Mascot:** http://www.matrixscience.com/

# Mascot Search Results

## Peptide View Spot no 81

MS/MS Fragmentation of **VSEQFTAMFR**
Found in **gi|493725** in **NCBInr**, beta tubulin [Oryza sativa Japonica Group]

Match to Query 15: 1214.687224 from(1215.694500,1+) intensity(0.0000) index(5)
Title: Label: E13, Spot_Id: 216673, Peak_List_Id: 221749, MSMS Job_Run_Id: 21390, Comment:
Data file C:\Documents and Settings\Administrator\Desktop\farah BHU\Cotton leaves\RAJESH\12-1-13 raj\ppw_E13_136117700900.txt

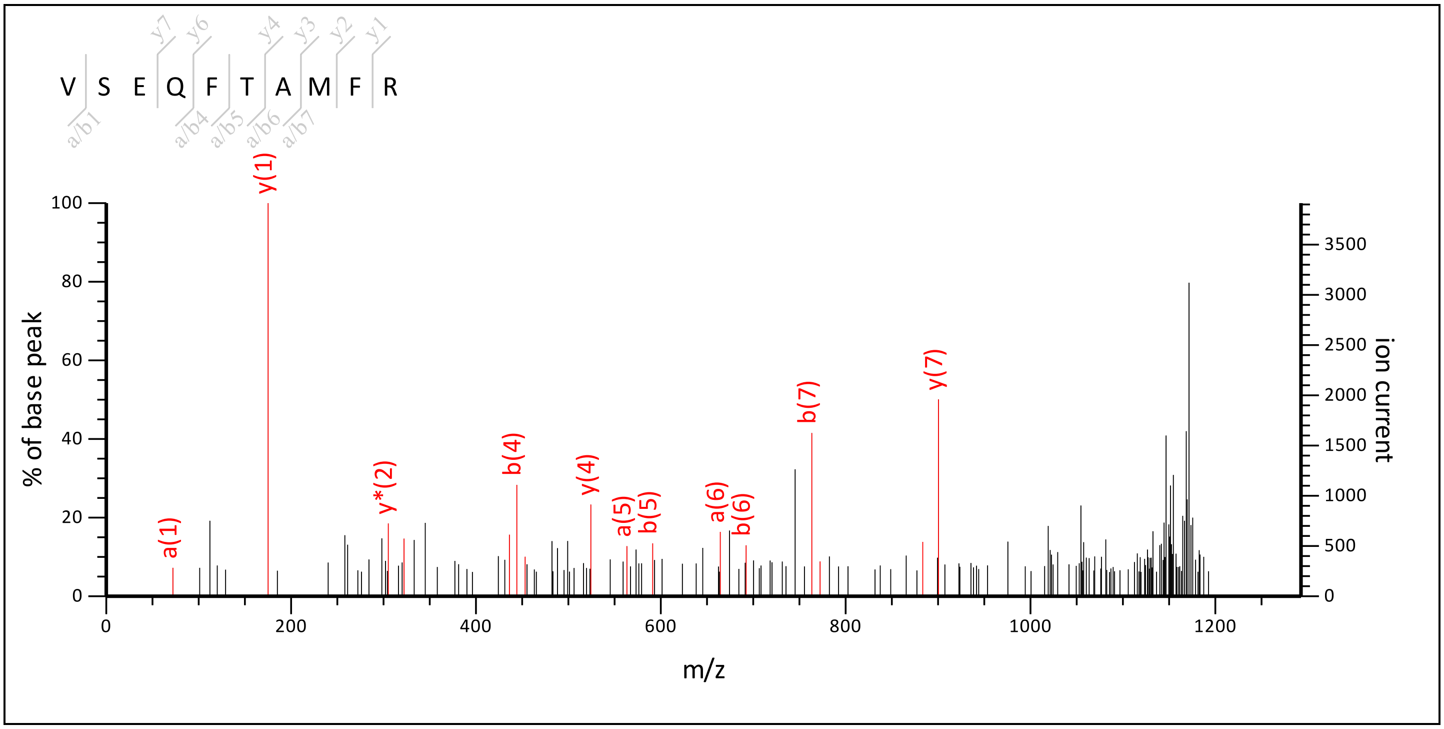


0 to 1292.69

Label all possible matches Label matches used for scoring

**Monoisotopic mass of neutral peptide Mr(calc):** 1214.5754
**Fixed modifications:** Carbamidomethyl (C) (apply to specified residues or termini only)
**Ions Score:** 18 **Expect:** 91
**Matches :** 16/48 fragment ions using 48 most intense peaks (help)

| # | a | a* | b | b* | Seq. | y | y* | # |
|---|---|---|---|---|---|---|---|---|
| 1 | *72.0808* | | 100.0757 | | V | | | 10 |
| 2 | 159.1128 | | 187.1077 | | S | 1116.5143 | 1099.4877 | 9 |
| 3 | 288.1554 | | 316.1503 | | E | 1029.4822 | 1012.4557 | 8 |
| 4 | 416.2140 | 399.1874 | **444.2089** | 427.1823 | Q | *900.4396* | 883.4131 | 7 |
| 5 | *563.2824* | 546.2558 | **591.2773** | 574.2508 | F | *772.3811* | 755.3545 | 6 |
| 6 | *664.3301* | 647.3035 | **692.3250** | 675.2984 | T | 625.3126 | 608.2861 | 5 |
| 7 | 735.3672 | 718.3406 | **763.3621** | 746.3355 | A | *524.2650* | 507.2384 | 4 |
| 8 | 866.4077 | 849.3811 | 894.4026 | 877.3760 | M | *453.2279* | 436.2013 | 3 |

| | | | | | | | | |
|---|---|---|---|---|---|---|---|---|
| 9 | 1013.4761 | 996.4495 | 1041.4710 | 1024.4444 | F | *322.1874* | 305.1608 | 2 |
| 10 | | | | | R | *175.1190* | 158.0924 | 1 |

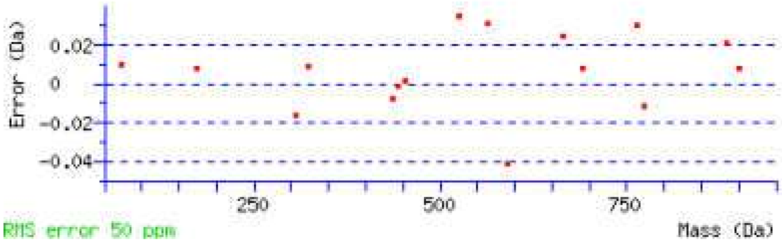


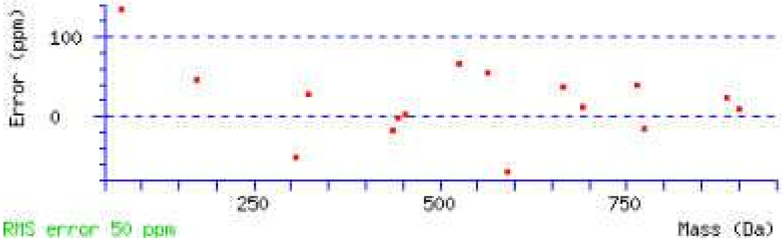


NCBI **BLAST** search of VSEQFTAMFR
(Parameters: blastp, nr protein database, expect=20000, no filter, PAM30)
Other BLAST web gateways

**All matches to this query**

| Score | Mr(calc) | Delta | Sequence |
|---|---|---|---|
| 20.7 | 1213.6568 | 1.0305 | GSIGHAFTVGIR |
| 18.4 | 1214.6118 | 0.0755 | VSEKFTAMFR |
| 18.4 | 1214.5754 | 0.1118 | VSEQFTAMFR |
| 10.3 | 1213.5696 | 1.1176 | MGSCVGYLRR |
| 8.4 | 1215.6030 | -0.9158 | RESVSMTPGPR |
| 7.3 | 1213.5509 | 1.1363 | MANSKGSSNFR |
| 7.3 | 1214.6996 | -0.0124 | NLHQAKVHLR |
| 7.3 | 1214.6924 | -0.0052 | YKLTWVHIR |
| 7.0 | 1214.6230 | 0.0642 | LYQMYGRLR |
| 6.8 | 1214.5390 | 0.1482 | GFAFEDGMGIR |

**Mascot:** http://www.matrixscience.com/

# MATRIX SCIENCE Mascot Search Results

## Peptide View Spot no 81

MS/MS Fragmentation of **INVYYNEASGGR**
Found in **gi|493725** in **NCBInr**, beta tubulin [Oryza sativa Japonica Group]

Match to Query 24: 1341.751424 from(1342.758700,1+) intensity(0.0000) index(8)
Title: Label: E13, Spot_Id: 216673, Peak_List_Id: 221750, MSMS Job_Run_Id: 21390, Comment:
Data file C:\Documents and Settings\Administrator\Desktop\farah BHU\Cotton leaves\RAJESH\12-1-13 raj\ppw_E13_136117700900.txt

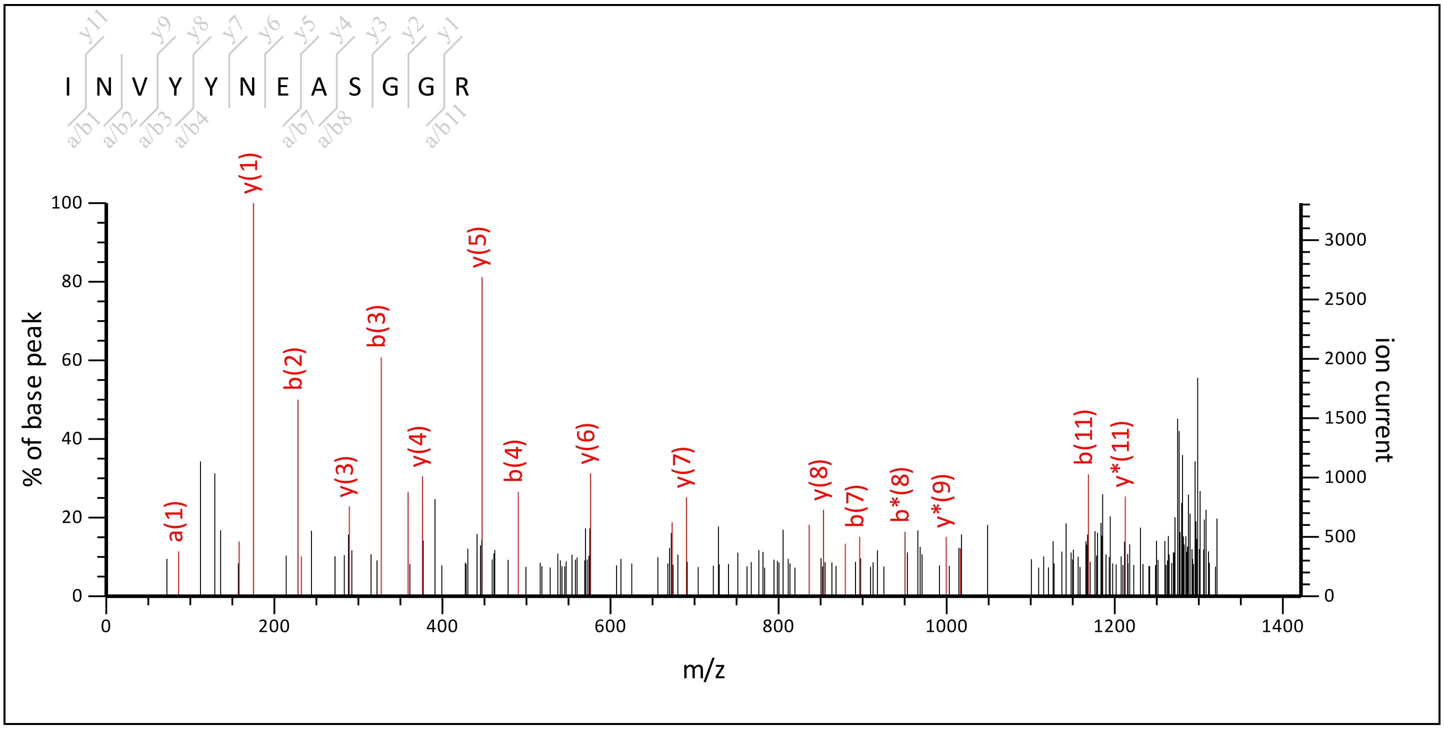


0 to 1421.59

Label all possible matches Label matches used for scoring

**Monoisotopic mass of neutral peptide Mr(calc):** 1341.6313
**Fixed modifications:** Carbamidomethyl (C) (apply to specified residues or termini only)
**Ions Score:** 35 **Expect:** 1.5
**Matches :** 23/64 fragment ions using 65 most intense peaks (help)

| # | a | a* | b | b* | Seq. | y | y* | # |
|---|---|---|---|---|---|---|---|---|
| 1 | 86.0964 | | 114.0913 | | I | | | 12 |
| 2 | 200.1394 | 183.1128 | **228.1343** | 211.1077 | N | 1229.5545 | 1212.5280 | 11 |
| 3 | 299.2078 | 282.1812 | **327.2027** | 310.1761 | V | 1115.5116 | 1098.4851 | 10 |
| 4 | 462.2711 | 445.2445 | **490.2660** | 473.2395 | Y | ***1016.4432*** | 999.4167 | 9 |
| 5 | 625.3344 | 608.3079 | 653.3293 | 636.3028 | Y | ***853.3799*** | 836.3533 | 8 |
| 6 | 739.3774 | 722.3508 | 767.3723 | 750.3457 | N | ***690.3165*** | 673.2900 | 7 |
| 7 | 868.4199 | 851.3934 | **896.4149** | 879.3883 | E | ***576.2736*** | 559.2471 | 6 |
| 8 | 939.4571 | 922.4305 | 967.4520 | 950.4254 | A | ***447.2310*** | 430.2045 | 5 |

| | | | | | | | | |
|---|---|---|---|---|---|---|---|---|
| **9** | 1026.4891 | 1009.4625 | 1054.4840 | 1037.4575 | **S** | ***376.1939*** | 359.1674 | **4** |
| **10** | 1083.5105 | 1066.4840 | 1111.5055 | 1094.4789 | **G** | ***289.1619*** | 272.1353 | **3** |
| **11** | 1140.5320 | 1123.5055 | **1168.5269** | 1151.5004 | **G** | ***232.1404*** | 215.1139 | **2** |
| **12** | | | | | **R** | ***175.1190*** | 158.0924 | **1** |

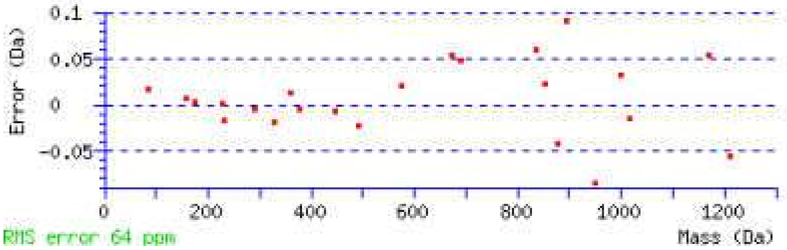


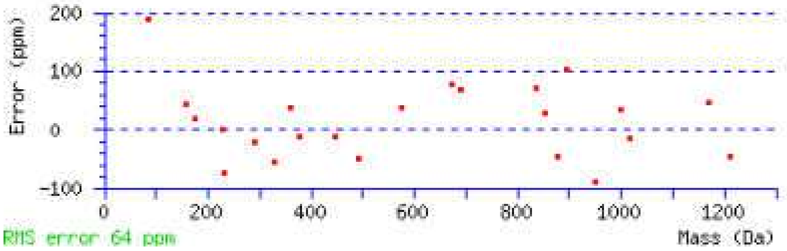


NCBI **BLAST** search of INVYYNEASGGR
(Parameters: blastp, nr protein database, expect=20000, no filter, PAM30)
Other BLAST web gateways

**All matches to this query**

| Score | Mr(calc) | Delta | Sequence |
|---|---|---|---|
| 35.5 | 1341.6313 | 0.1201 | INVYYNEASGGR |
| 35.5 | 1341.6313 | 0.1201 | LNVYYNEASGGR |
| 26.8 | 1342.6153 | -0.8639 | IDVYYNEASGGR |
| 14.0 | 1342.7027 | -0.9513 | QVVDQIAVNAMR |
| 12.6 | 1341.6499 | 0.1015 | QVVYMYPRDR |
| 9.6 | 1341.6612 | 0.0902 | KVQHCLSEWR |
| 8.9 | 1340.7776 | 0.9739 | QAATALLLALSNR |
| 8.8 | 1341.5877 | 0.1637 | YYYYLTGDER |
| 8.1 | 1341.7115 | 0.0399 | RVVDYFTALMK |
| 8.0 | 1341.5904 | 0.1610 | DSNASEMAKMVK |

**Mascot:** http://www.matrixscience.com/

# MATRIX SCIENCE Mascot Search Results

## Peptide View Spot no 81

MS/MS Fragmentation of **RVSEQFTAMFR**
Found in **gi|493725** in **NCBInr**, beta tubulin [Oryza sativa Japonica Group]

Match to Query 26: 1370.797124 from(1371.804400,1+) intensity(0.0000) index(9)
Title: Label: E13, Spot_Id: 216673, Peak_List_Id: 221754, MSMS Job_Run_Id: 21390, Comment:
Data file C:\Documents and Settings\Administrator\Desktop\farah BHU\Cotton leaves\RAJESH\12-1-13 raj\ppw_E13_136117700900.txt

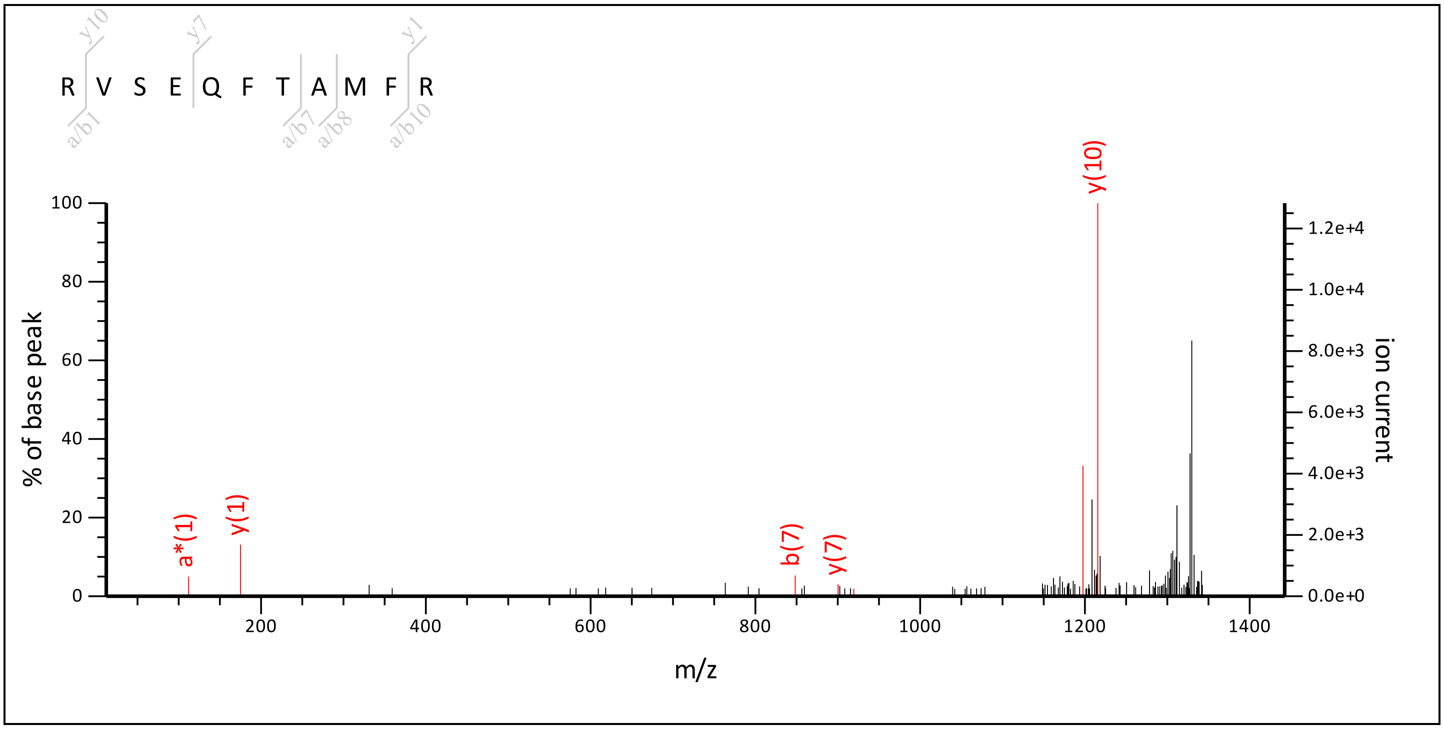


12.09 to 1442.5

Label all possible matches ○ Label matches used for scoring ◉

**Monoisotopic mass of neutral peptide Mr(calc):** 1370.6765
**Fixed modifications:** Carbamidomethyl (C) (apply to specified residues or termini only)
**Ions Score:** 7 **Expect:** 1e+03
**Matches :** 7/60 fragment ions using 22 most intense peaks (help)

| # | a | a* | b | b* | Seq. | y | y* | # |
|---|---|---|---|---|---|---|---|---|
| 1 | 129.1135 | 112.0869 | 157.1084 | 140.0818 | R | | | 11 |
| 2 | 228.1819 | 211.1553 | 256.1768 | 239.1503 | V | *1215.5827* | 1198.5561 | 10 |
| 3 | 315.2139 | 298.1874 | 343.2088 | 326.1823 | S | 1116.5143 | 1099.4877 | 9 |
| 4 | 444.2565 | 427.2300 | 472.2514 | 455.2249 | E | 1029.4822 | 1012.4557 | 8 |
| 5 | 572.3151 | 555.2885 | 600.3100 | 583.2835 | Q | *900.4396* | 883.4131 | 7 |
| 6 | 719.3835 | 702.3569 | 747.3784 | 730.3519 | F | 772.3811 | 755.3545 | 6 |
| 7 | 820.4312 | 803.4046 | *848.4261* | 831.3995 | T | 625.3126 | 608.2861 | 5 |
| 8 | 891.4683 | 874.4417 | *919.4632* | 902.4367 | A | 524.2650 | 507.2384 | 4 |

| | | | | | | | | |
|---|---|---|---|---|---|---|---|---|
| **9** | 1022.5088 | 1005.4822 | 1050.5037 | 1033.4771 | **M** | 453.2279 | 436.2013 | **3** |
| **10** | 1169.5772 | 1152.5506 | ***1197.5721*** | 1180.5456 | **F** | 322.1874 | 305.1608 | **2** |
| **11** | | | | | **R** | ***175.1190*** | 158.0924 | **1** |

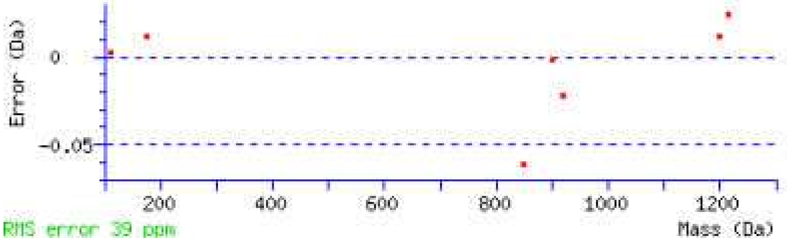


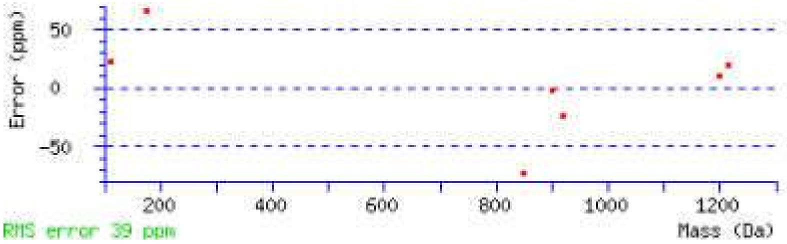


NCBI **BLAST** search of RVSEQFTAMFR
(Parameters: blastp, nr protein database, expect=20000, no filter, PAM30)
Other BLAST web gateways

**All matches to this query**

| Score | Mr(calc) | Delta | Sequence |
|---|---|---|---|
| 14.8 | 1370.6799 | 0.1173 | RMAFTMTATGIR |
| 10.6 | 1370.6361 | 0.1610 | RSSTSVGFCTNR |
| 8.3 | 1370.7168 | 0.0804 | VGPRPDTFGGGRR |
| 8.2 | 1370.7266 | 0.0705 | RGIIEQGLSDQR |
| 8.2 | 1370.7266 | 0.0705 | RLLGQSVGEEQR |
| 7.8 | 1370.7419 | 0.0553 | RHTEGTALALFR |
| 7.3 | 1370.6765 | 0.1206 | RVSEQFTAMFR |
| 6.8 | 1370.8431 | -0.0460 | SSLALVMRLIIR |
| 6.4 | 1370.7571 | 0.0400 | GVRWFQNPVLR |
| 6.4 | 1370.7419 | 0.0552 | VGVEPQFRNGLR |

**Mascot:** http://www.matrixscience.com/

# Mascot Search Results

## Peptide View Spot no 81

MS/MS Fragmentation of **LHFFMVGFAPLTSR**
Found in **gi|493725** in **NCBInr**, beta tubulin [Oryza sativa Japonica Group]

Match to Query 46: 1621.986824 from(1622.994100,1+) intensity(0.0000) index(18)
Title: Label: E13, Spot_Id: 216673, Peak_List_Id: 221756, MSMS Job_Run_Id: 21390, Comment:
Data file C:\Documents and Settings\Administrator\Desktop\farah BHU\Cotton leaves\RAJESH\12-1-13 raj\ppw_E13_136117700900.txt

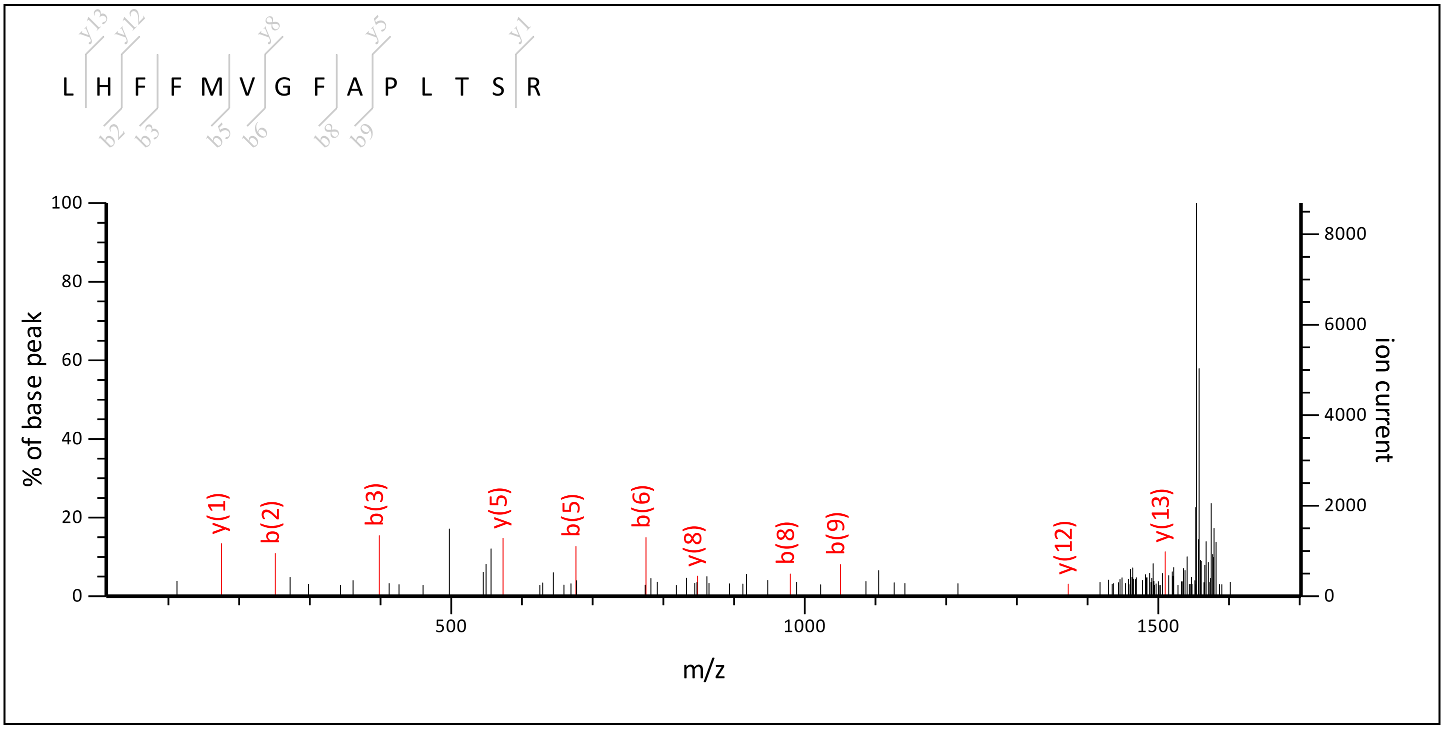


12.1 to 1701.81

Label all possible matches ○ Label matches used for scoring ◉

**Monoisotopic mass of neutral peptide Mr(calc):** 1621.8439
**Fixed modifications:** Carbamidomethyl (C) (apply to specified residues or termini only)
**Ions Score:** 45 **Expect:** 0.14
**Matches :** 11/52 fragment ions using 15 most intense peaks (help)

| # | a | b | Seq. | y | y* | # |
|---|---|---|---|---|---|---|
| 1 | 86.0964 | 114.0913 | L | | | 14 |
| 2 | 223.1553 | *251.1503* | H | *1509.7671* | 1492.7406 | 13 |
| 3 | 370.2238 | *398.2187* | F | *1372.7082* | 1355.6817 | 12 |
| 4 | 517.2922 | 545.2871 | F | 1225.6398 | 1208.6132 | 11 |
| 5 | 648.3326 | *676.3276* | M | 1078.5714 | 1061.5448 | 10 |
| 6 | 747.4011 | *775.3960* | V | 947.5309 | 930.5043 | 9 |
| 7 | 804.4225 | 832.4174 | G | *848.4625* | 831.4359 | 8 |
| 8 | 951.4909 | *979.4859* | F | 791.4410 | 774.4145 | 7 |

| | | | | | | |
|---|---|---|---|---|---|---|
| **9** | 1022.5281 | ***1050.5230*** | **A** | 644.3726 | 627.3461 | **6** |
| **10** | 1119.5808 | 1147.5757 | **P** | ***573.3355*** | 556.3089 | **5** |
| **11** | 1232.6649 | 1260.6598 | **L** | 476.2827 | 459.2562 | **4** |
| **12** | 1333.7126 | 1361.7075 | **T** | 363.1987 | 346.1721 | **3** |
| **13** | 1420.7446 | 1448.7395 | **S** | 262.1510 | 245.1244 | **2** |
| **14** | | | **R** | ***175.1190*** | 158.0924 | **1** |

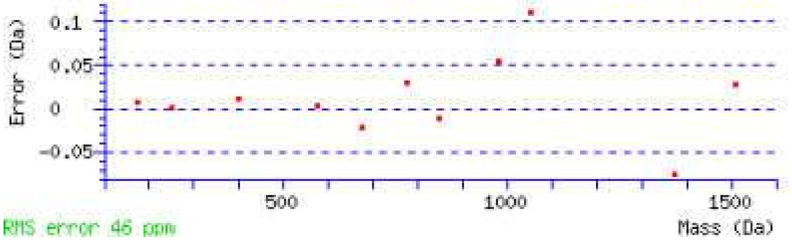


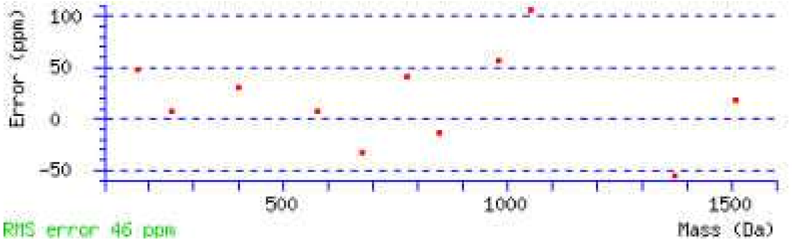


NCBI **BLAST** search of LHFFMVGFAPLTSR
(Parameters: blastp, nr protein database, expect=20000, no filter, PAM30)
Other BLAST web gateways

**All matches to this query**

| Score | Mr(calc) | Delta | Sequence |
|---|---|---|---|
| 44.8 | 1621.8439 | 0.1429 | LHFFMVGFAPLTSR |
| 11.7 | 1622.7649 | -0.7780 | NHGAPEVSGVSDEGLR |
| 11.3 | 1621.7518 | 0.2350 | LTIDGMQGNQNYPR |
| 9.9 | 1621.8075 | 0.1793 | LHPAMKPYEYAFR |
| 8.6 | 1621.9668 | 0.0200 | HLIALGFKVAVIEGR |
| 6.3 | 1621.7276 | 0.2593 | LHRCYMASMQGPR |
| 6.3 | 1621.7590 | 0.2278 | LASSNGSSAGNGGRMTR |
| 6.1 | 1621.7121 | 0.2747 | EEKWDSSTSHFNR |
| 5.7 | 1622.8013 | -0.8144 | GGHSSLPPVDGSTAVSR |
| 5.2 | 1621.7804 | 0.2065 | LLAKMMTTAGADPDR |

**Mascot:** http://www.matrixscience.com/

# MATRIX SCIENCE Mascot Search Results

## Peptide View Spot no 81

MS/MS Fragmentation of **AVLMDLEPGTMDSVR**
Found in **gi|493725** in **NCBInr**, beta tubulin [Oryza sativa Japonica Group]

Match to Query 48: 1632.939624 from(1633.946900,1+) intensity(0.0000) index(19)
Title: Label: E13, Spot_Id: 216673, Peak_List_Id: 221764, MSMS Job_Run_Id: 21390, Comment:
Data file C:\Documents and Settings\Administrator\Desktop\farah BHU\Cotton leaves\RAJESH\12-1-13 raj\ppw_E13_136117700900.txt

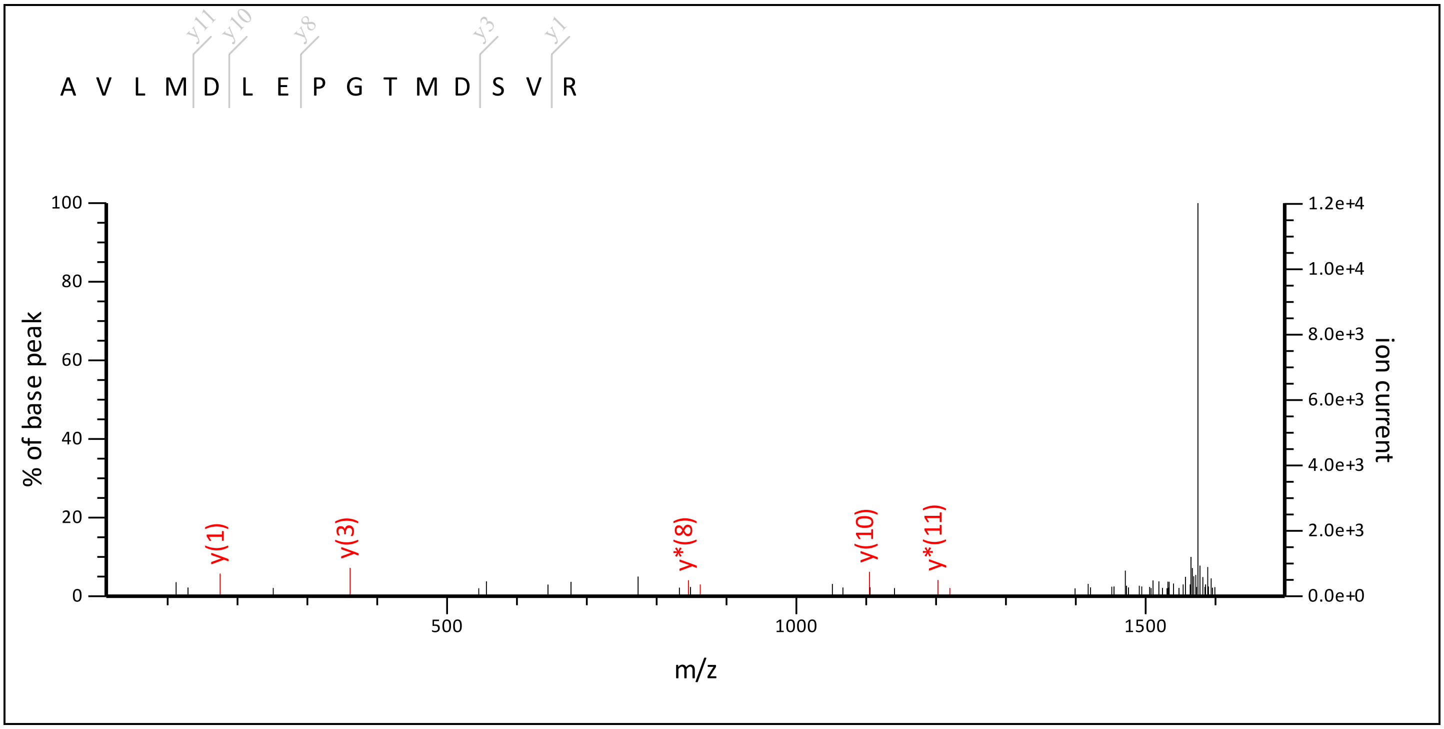


12.11 to 1699.05

Label all possible matches ○ Label matches used for scoring ◉

**Monoisotopic mass of neutral peptide Mr(calc):** 1632.7851
**Fixed modifications:** Carbamidomethyl (C) (apply to specified residues or termini only)
**Ions Score:** 11 **Expect:** 3.5e+02
**Matches :** 7/56 fragment ions using 21 most intense peaks (help)

| # | a | b | Seq. | y | y* | # |
|---|---|---|---|---|---|---|
| 1 | 44.0495 | 72.0444 | A | | | 15 |
| 2 | 143.1179 | 171.1128 | V | 1562.7553 | 1545.7287 | 14 |
| 3 | 256.2020 | 284.1969 | L | 1463.6869 | 1446.6603 | 13 |
| 4 | 387.2424 | 415.2374 | M | 1350.6028 | 1333.5763 | 12 |
| 5 | 502.2694 | 530.2643 | D | ***1219.5623*** | 1202.5358 | 11 |
| 6 | 615.3534 | 643.3484 | L | ***1104.5354*** | 1087.5088 | 10 |
| 7 | 744.3960 | 772.3910 | E | 991.4513 | 974.4248 | 9 |
| 8 | 841.4488 | 869.4437 | P | ***862.4087*** | 845.3822 | 8 |

| | | | | | | |
|---|---|---|---|---|---|---|
| **9** | 898.4703 | 926.4652 | **G** | 765.3560 | 748.3294 | **7** |
| **10** | 999.5179 | 1027.5129 | **T** | 708.3345 | 691.3080 | **6** |
| **11** | 1130.5584 | 1158.5533 | **M** | 607.2868 | 590.2603 | **5** |
| **12** | 1245.5854 | 1273.5803 | **D** | 476.2463 | 459.2198 | **4** |
| **13** | 1332.6174 | 1360.6123 | **S** | ***361.2194*** | 344.1928 | **3** |
| **14** | 1431.6858 | 1459.6807 | **V** | 274.1874 | 257.1608 | **2** |
| **15** | | | **R** | ***175.1190*** | 158.0924 | **1** |

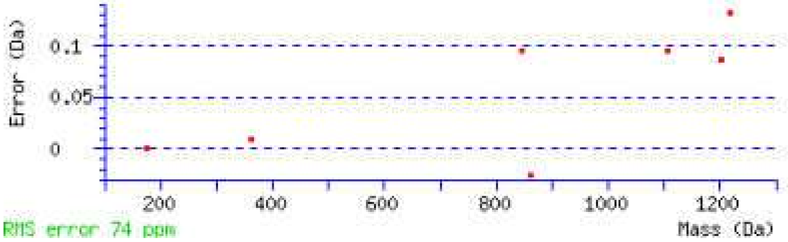


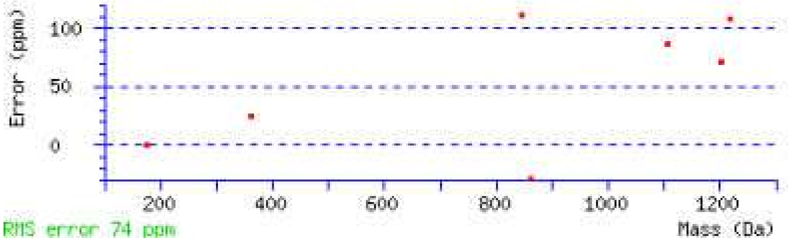


NCBI **BLAST** search of AVLMDLEPGTMDSVR
(Parameters: blastp, nr protein database, expect=20000, no filter, PAM30)
Other BLAST web gateways

**All matches to this query**

| Score | Mr(calc) | Delta | Sequence |
|---|---|---|---|
| 11.1 | 1632.7851 | 0.1545 | AVLMDLEPGTMDSVR |
| 11.1 | 1632.7851 | 0.1545 | AVLMDLEPGTMDSVR |
| 11.1 | 1632.7851 | 0.1545 | GILMDLEPGTMDSVR |
| 9.4 | 1632.6396 | 0.3000 | QTGDMMFVSEDGER |
| 8.0 | 1633.8069 | -0.8672 | VRCDMALPFGLPSR |
| 7.8 | 1633.7042 | -0.7646 | EKASDAAGEYGSYMR |
| 6.3 | 1631.8202 | 1.1194 | EAVQTCVLSQRWR |
| 6.1 | 1633.8147 | -0.8751 | RILFGWANECVNR |
| 6.1 | 1633.7817 | -0.8421 | RILMGWANECVNR |
| 5.7 | 1632.7566 | 0.1830 | SDQELVEMFDKHR |

**Mascot:** http://www.matrixscience.com/

# MATRIX SCIENCE Mascot Search Results

## Peptide View Spot no 82

MS/MS Fragmentation of **NHGMHFR**
Found in **gi|11466795** in **NCBInr**, ribulose-1,5-bisphosphate carboxylase/oxygenase large subunit [Oryza sativa Japonica Group]

Match to Query 2: 897.475204 from(898.482480,1+) intensity(0.0000) index(0)
Title: Label: C11, Spot_Id: 216639, Peak_List_Id: 221214, MSMS Job_Run_Id: 21345, Comment:
Data file C:\Documents and Settings\Administrator\Desktop\farah BHU\RAJESH\12-1-13 raj\ppw_C11_135997697400.txt

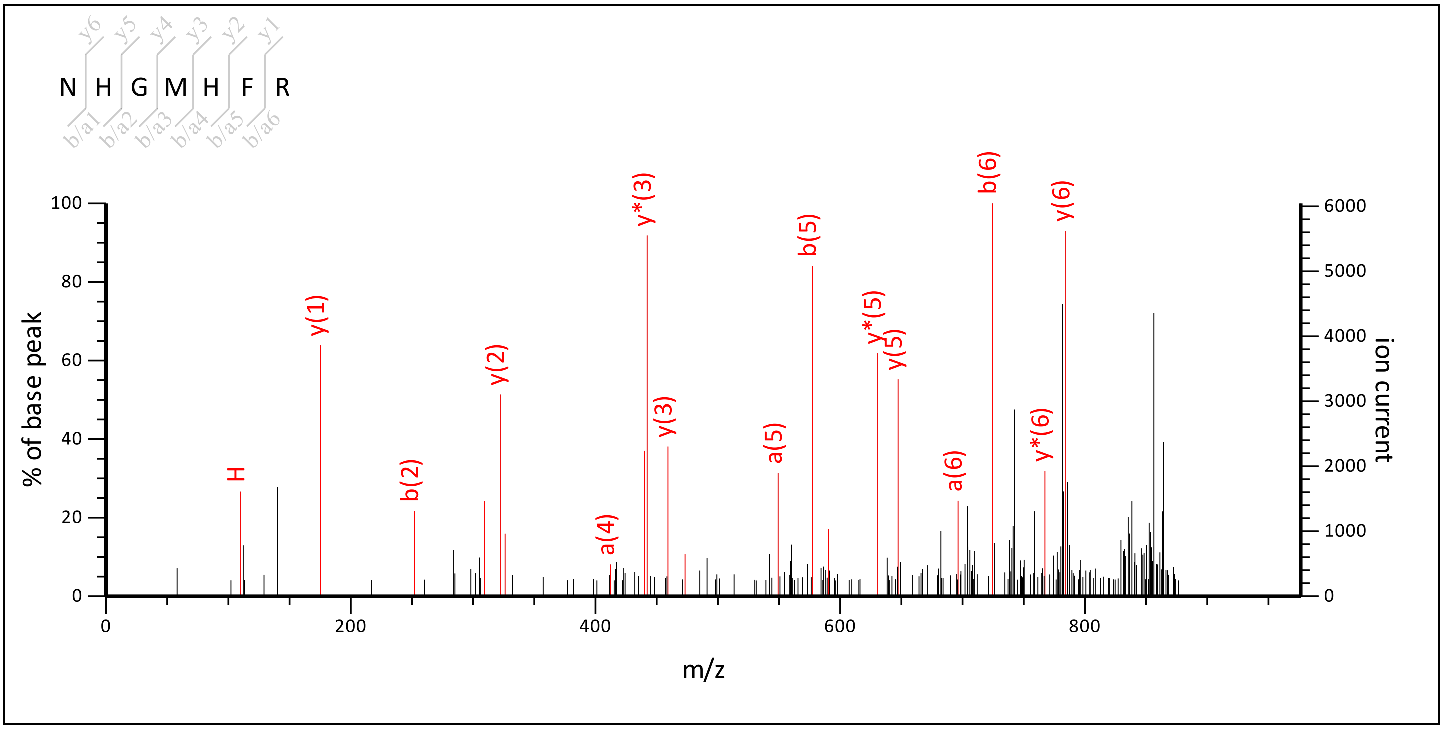


0 to 976.39

Label all possible matches ○ Label matches used for scoring ◉

**Monoisotopic mass of neutral peptide Mr(calc):** 897.4028
**Fixed modifications:** Carbamidomethyl (C) (apply to specified residues or termini only)
**Ions Score:** 39 **Expect:** 0.83
**Matches :** 22/72 fragment ions using 35 most intense peaks (help)

| # | Immon. | a | a* | b | b* | d | Seq. | v | w | y | y* | # |
|---|---|---|---|---|---|---|---|---|---|---|---|---|
| 1 | 87.0553 | 87.0553 | 70.0287 | 115.0502 | 98.0237 | 44.0495 | N | | | | | 7 |
| 2 | 110.0713 | 224.1142 | 207.0877 | **252.1091** | 235.0826 | | H | 702.3140 | | ***784.3671*** | 767.3406 | 6 |
| 3 | 30.0338 | 281.1357 | 264.1091 | **309.1306** | 292.1040 | | G | | | ***647.3082*** | 630.2817 | 5 |
| 4 | 104.0528 | **412.1761** | 395.1496 | **440.1711** | 423.1445 | 352.1728 | M | 514.2521 | 513.2568 | ***590.2868*** | 573.2602 | 4 |
| 5 | 110.0713 | **549.2351** | 532.2085 | **577.2300** | 560.2034 | | H | 377.1932 | | ***459.2463*** | 442.2197 | 3 |
| 6 | 120.0808 | **696.3035** | 679.2769 | **724.2984** | 707.2718 | | F | 230.1248 | | ***322.1874*** | 305.1608 | 2 |
| 7 | 129.1135 | | | | | | R | 74.0237 | 73.0284 | ***175.1190*** | 158.0924 | 1 |

| Seq | ya | yb | Seq | ya | yb | Seq | ya | yb |
|---|---|---|---|---|---|---|---|---|
| HG | 167.0927 | 195.0877 | HGM | 298.1332 | 326.1281 | HGMH | 435.1921 | 463.1870 |
| HGMHF | 582.2605 | 610.2555 | GM | 161.0743 | 189.0692 | GMH | 298.1332 | 326.1281 |
| GMHF | 445.2016 | 473.1966 | MH | 241.1118 | 269.1067 | MHF | 388.1802 | 416.1751 |
| HF | 257.1397 | 285.1346 | | | | | | |

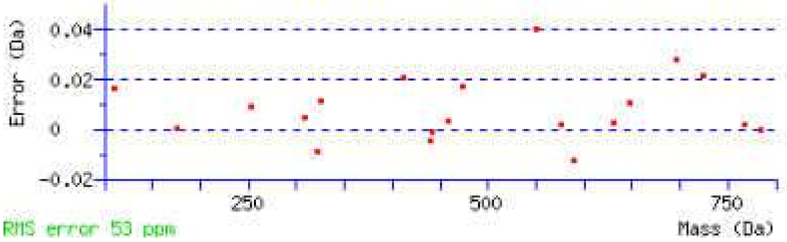


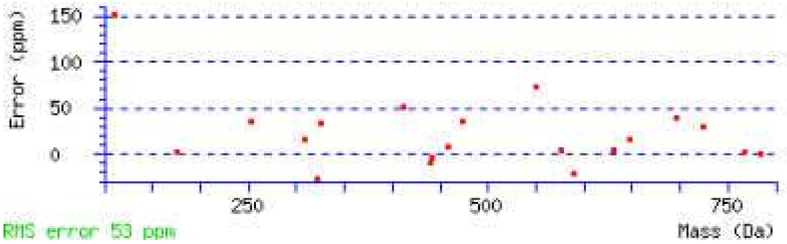


NCBI **BLAST** search of NHGMHFR
(Parameters: blastp, nr protein database, expect=20000, no filter, PAM30)
Other BLAST web gateways

**All matches to this query**

| Score | Mr(calc) | Delta | Sequence |
|---|---|---|---|
| 38.9 | 897.4028 | 0.0724 | NHGMHFR |
| 38.9 | 897.4028 | 0.0724 | NHGMHFR |
| 38.9 | 897.4028 | 0.0724 | NHGMHFR |
| 38.9 | 897.4028 | 0.0724 | NHGMHFR |
| 38.9 | 897.4028 | 0.0724 | NHGMHFR |
| 38.9 | 897.4028 | 0.0724 | NHGMHFR |
| 38.9 | 897.4028 | 0.0724 | NHGMHFR |
| 38.9 | 897.3698 | 0.1054 | NHGMHMR |
| 25.7 | 898.3868 | -0.9116 | DHGMHFR |
| 25.7 | 898.3868 | -0.9116 | DHGMHFR |

**Mascot:** http://www.matrixscience.com/

# Mascot Search Results

## Peptide View **Spot no 82**

MS/MS Fragmentation of **ACYECLR**
Found in **gi|11466795** in **NCBInr**, ribulose-1,5-bisphosphate carboxylase/oxygenase large subunit [Oryza sativa Japonica Group]

Match to Query 6: 970.475084 from(971.482360,1+) intensity(0.0000) index(3)
Title: Label: C11, Spot_Id: 216639, Peak_List_Id: 221202, MSMS Job_Run_Id: 21345, Comment:
Data file C:\Documents and Settings\Administrator\Desktop\farah BHU\RAJESH\12-1-13 raj\ppw_C11_135997697400.txt

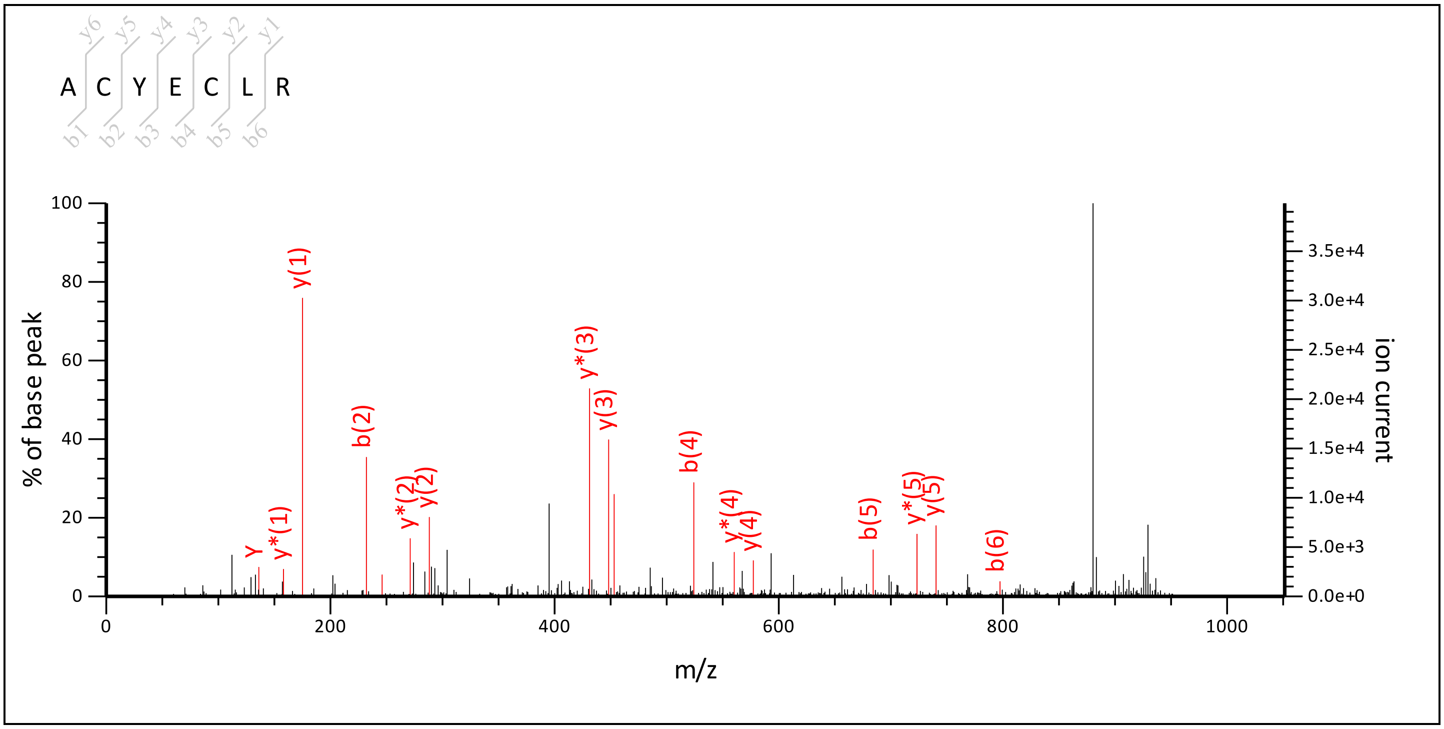


0 to 1051.37

Label all possible matches ○ Label matches used for scoring ●

**Monoisotopic mass of neutral peptide Mr(calc):** 970.4001
**Fixed modifications:** Carbamidomethyl (C) (apply to specified residues or termini only)
**Ions Score:** 30 **Expect:** 6
**Matches :** 18/75 fragment ions using 27 most intense peaks (help)

| # | Immon. | a | $a^0$ | b | $b^0$ | d | Seq. | v | w | y | y* | $y^0$ | # |
|---|---|---|---|---|---|---|---|---|---|---|---|---|---|
| 1 | 44.0495 | 44.0495 | | 72.0444 | | 44.0495 | A | | | | | | 7 |
| 2 | 133.0430 | 204.0801 | | **232.0750** | | 115.0866 | C | 795.3454 | 794.3502 | 900.3702 | 883.3437 | 882.3597 | 6 |
| 3 | 136.0757 | 367.1435 | | 395.1384 | | | Y | 632.2821 | | ***740.3396*** | 723.3130 | 722.3290 | 5 |
| 4 | 102.0550 | 496.1860 | 478.1755 | **524.1810** | 506.1704 | 438.1806 | E | 503.2395 | 502.2442 | ***577.2763*** | 560.2497 | 559.2657 | 4 |
| 5 | 133.0430 | 656.2167 | 638.2061 | **684.2116** | 666.2010 | 567.2232 | C | 343.2088 | 342.2136 | ***448.2337*** | 431.2071 | | 3 |
| 6 | 86.0964 | 769.3008 | 751.2902 | **797.2957** | 779.2851 | 727.2538 | L | 230.1248 | 229.1295 | ***288.2030*** | 271.1765 | | 2 |
| 7 | 129.1135 | | | | | | R | 74.0237 | 73.0284 | ***175.1190*** | 158.0924 | | 1 |

| Seq | ya | yb | Seq | ya | yb | Seq | ya | yb |
|---|---|---|---|---|---|---|---|---|
| CY | 296.1063 | 324.1013 | CYE | 425.1489 | 453.1438 | CYEC | 585.1796 | 613.1745 |
| CYECL | 698.2636 | 726.2586 | YE | 265.1183 | 293.1132 | YEC | 425.1489 | 453.1438 |
| YECL | 538.2330 | 566.2279 | EC | 262.0856 | 290.0805 | ECL | 375.1697 | 403.1646 |
| CL | 246.1271 | 274.1220 | | | | | | |

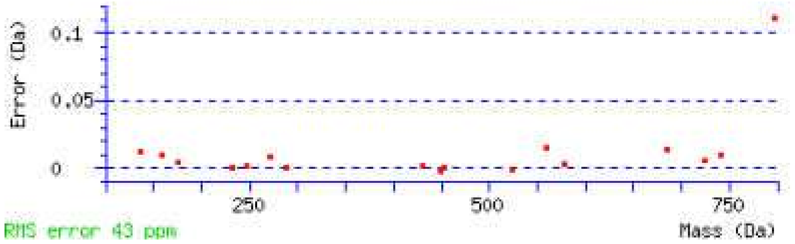


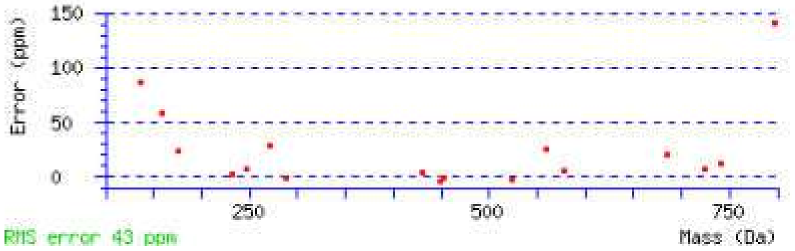


NCBI **BLAST** search of ACYECLR
(Parameters: blastp, nr protein database, expect=20000, no filter, PAM30)
Other BLAST web gateways

**All matches to this query**

| Score | Mr(calc) | Delta | Sequence |
|---|---|---|---|
| 30.4 | 970.4001 | 0.0750 | ACYECIR |
| 30.4 | 970.4001 | 0.0750 | ACYECLR |
| 30.4 | 970.4001 | 0.0750 | ACYECLR |
| 30.4 | 970.4001 | 0.0750 | ACYECLR |
| 30.4 | 970.4001 | 0.0750 | ACYECLR |
| 25.8 | 969.4412 | 1.0339 | MVYECLR |
| 25.4 | 970.4794 | -0.0043 | CVYTSTIK |
| 24.5 | 970.4113 | 0.0638 | RMCADYR |
| 22.9 | 970.5382 | -0.0631 | LANPKCLR |
| 20.9 | 969.5252 | 0.9499 | MAMLVHIR |

**Mascot:** http://www.matrixscience.com/

# MATRIX SCIENCE Mascot Search Results

## Peptide View Spot no 82

MS/MS Fragmentation of **DTDILAAFR**
Found in **gi|11466795** in **NCBInr**, ribulose-1,5-bisphosphate carboxylase/oxygenase large subunit [Oryza sativa Japonica Group]

Match to Query 9: 1020.604024 from(1021.611300,1+) intensity(0.0000) index(5)
Title: Label: C11, Spot_Id: 216639, Peak_List_Id: 221223, MSMS Job_Run_Id: 21345, Comment:
Data file C:\Documents and Settings\Administrator\Desktop\farah BHU\RAJESH\12-1-13 raj\ppw_C11_135997697400.txt

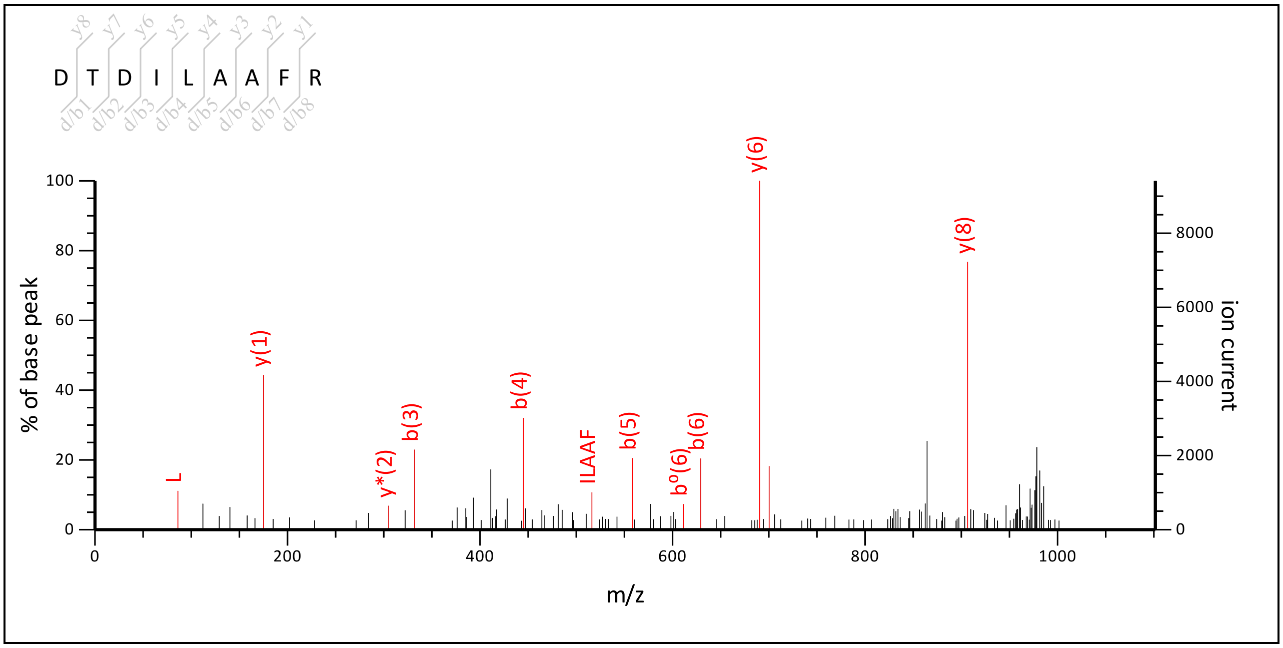


0 to 1101.53

Label all possible matches ○ Label matches used for scoring ◉

**Monoisotopic mass of neutral peptide Mr(calc):** 1020.5240
**Fixed modifications:** Carbamidomethyl (C) (apply to specified residues or termini only)
**Ions Score:** 33 **Expect:** 4.2
**Matches :** 14/121 fragment ions using 20 most intense peaks (help)

| # | Immon. | a | $a^0$ | b | $b^0$ | d | d' | Seq. | v | w | w' | y | y* | $y^0$ | # |
|---|---|---|---|---|---|---|---|---|---|---|---|---|---|---|---|
| 1 | 88.0393 | 88.0393 | 70.0287 | 116.0342 | 98.0237 | 44.0495 | | D | | | | | | | 9 |
| 2 | 74.0600 | 189.0870 | 171.0764 | 217.0819 | 199.0713 | 173.0921 | 175.0713 | T | 860.4625 | 873.4829 | 875.4621 | ***906.5043*** | 889.4778 | 888.4938 | 8 |
| 3 | 88.0393 | 304.1139 | 286.1034 | ***332.1088*** | 314.0983 | 260.1241 | | D | 745.4355 | 744.4403 | | 805.4567 | 788.4301 | 787.4461 | 7 |
| 4 | 86.0964 | 417.1980 | 399.1874 | ***445.1929*** | 427.1823 | 389.1667 | 403.1823 | I | 632.3515 | 645.3719 | 659.3875 | ***690.4297*** | 673.4032 | | 6 |
| 5 | 86.0964 | 530.2821 | 512.2715 | ***558.2770*** | 540.2664 | 488.2351 | | L | 519.2674 | 518.2722 | | 577.3457 | 560.3191 | | 5 |
| 6 | 44.0495 | 601.3192 | 583.3086 | ***629.3141*** | 611.3035 | | | A | 448.2303 | | | 464.2616 | 447.2350 | | 4 |
| 7 | 44.0495 | 672.3563 | 654.3457 | ***700.3512*** | 682.3406 | | | A | 377.1932 | | | 393.2245 | 376.1979 | | 3 |
| 8 | 120.0808 | 819.4247 | 801.4141 | 847.4196 | 829.4090 | | | F | 230.1248 | | | 322.1874 | 305.1608 | | 2 |
| 9 | 129.1135 | | | | | | | R | 74.0237 | 73.0284 | | ***175.1190*** | 158.0924 | | 1 |

| Seq | ya | yb | Seq | ya | yb | Seq | ya | yb |
|---|---|---|---|---|---|---|---|---|
| TD | 189.0870 | 217.0819 | TDI | 302.1710 | 330.1660 | TDIL | 415.2551 | 443.2500 |
| TDILA | 486.2922 | 514.2871 | TDILAA | 557.3293 | 585.3243 | DI | 201.1234 | 229.1183 |
| DIL | 314.2074 | 342.2023 | DILA | 385.2445 | 413.2395 | DILAA | 456.2817 | 484.2766 |

| | | | | | | | | |
|---|---|---|---|---|---|---|---|---|
| **DILAAF** | 603.3501 | 631.3450 | **IL** | 199.1805 | 227.1754 | **ILA** | 270.2176 | 298.2125 |
| **ILAA** | 341.2547 | 369.2496 | **ILAAF** | 488.3231 | 516.3180 | **LA** | 157.1335 | 185.1285 |
| **LAA** | 228.1707 | 256.1656 | **LAAF** | 375.2391 | 403.2340 | **AA** | 115.0866 | 143.0815 |
| **AAF** | 262.1550 | 290.1499 | **AF** | 191.1179 | 219.1128 | | | |

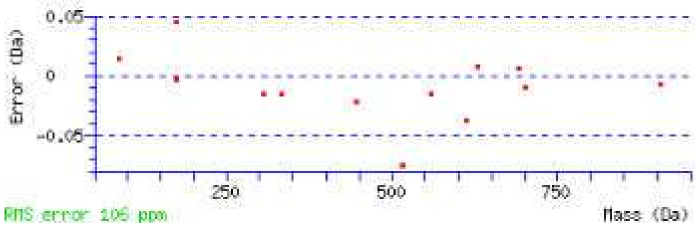


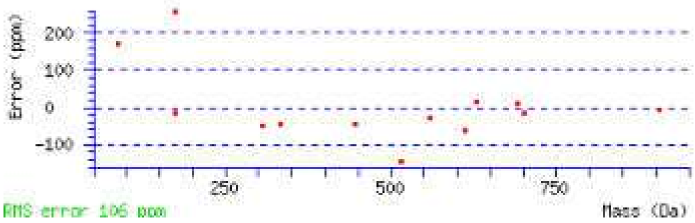


NCBI **BLAST** search of DTDILAAFR
(Parameters: blastp, nr protein database, expect=20000, no filter, PAM30)
Other BLAST web gateways

**All matches to this query**

| Score | Mr(calc) | Delta | Sequence |
|---|---|---|---|
| 33.0 | 1020.5240 | 0.0800 | DTDIIAAFR |
| 33.0 | 1020.5240 | 0.0800 | DTDIIAAFR |
| 33.0 | 1020.5240 | 0.0800 | DTDILAAFR |
| 33.0 | 1020.5240 | 0.0800 | DTDILAAFR |
| 33.0 | 1020.5240 | 0.0800 | DTDILAAFR |
| 33.0 | 1020.5240 | 0.0800 | DTDILAAFR |
| 33.0 | 1020.5240 | 0.0800 | DTDILAAFR |
| 33.0 | 1020.5240 | 0.0800 | DTDILAAFR |
| 33.0 | 1020.5240 | 0.0800 | DTDILAAFR |
| 33.0 | 1020.5240 | 0.0800 | DTDILAAFR |

**Mascot:** http://www.matrixscience.com/

# MATRIX SCIENCE Mascot Search Results

## Peptide View Spot no 82

MS/MS Fragmentation of **DNGLLLHIHR**
Found in **gi|11466795** in **NCBInr**, ribulose-1,5-bisphosphate carboxylase/oxygenase large subunit [Oryza sativa Japonica Group]

Match to Query 14: 1186.746424 from(1187.753700,1+) intensity(0.0000) index(8)
Title: Label: C11, Spot_Id: 216639, Peak_List_Id: 221215, MSMS Job_Run_Id: 21345, Comment:
Data file C:\Documents and Settings\Administrator\Desktop\farah BHU\RAJESH\12-1-13 raj\ppw_C11_135997697400.txt

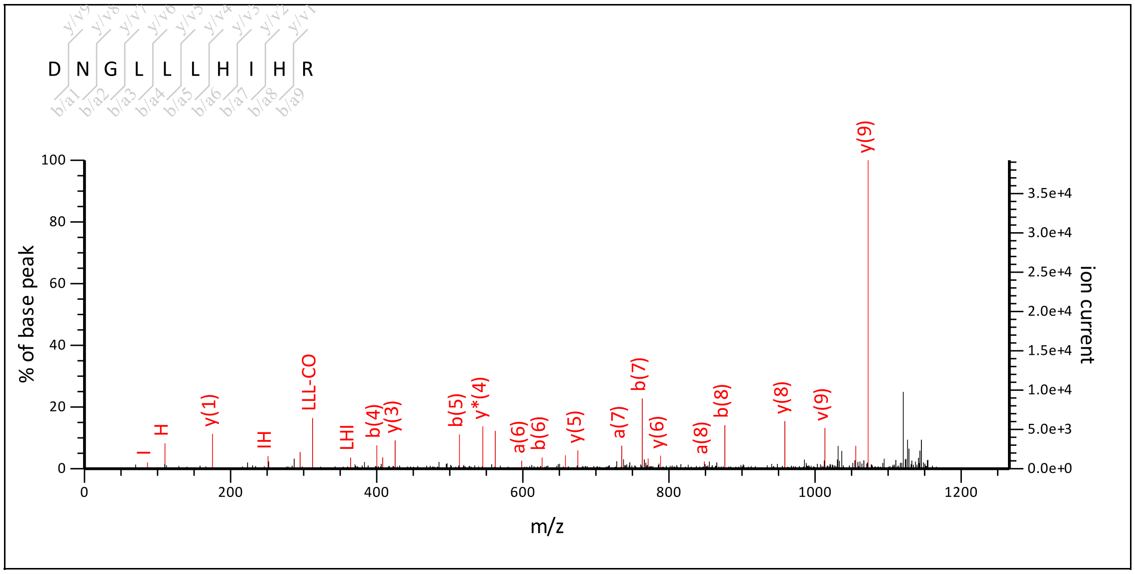


0 to 1265.95

Label all possible matches Label matches used for scoring

**Monoisotopic mass of neutral peptide Mr(calc):** 1186.6571
**Fixed modifications:** Carbamidomethyl (C) (apply to specified residues or termini only)
**Ions Score:** 75 **Expect:** 0.0002
**Matches :** 37/151 fragment ions using 33 most intense peaks (help)

| # | Immon. | a | a* | $a^0$ | b | b* | $b^0$ | d | d' | Seq. | v | w | w' | y | y* | # |
|---|---|---|---|---|---|---|---|---|---|---|---|---|---|---|---|---|
| 1 | 88.0393 | 88.0393 | | 70.0287 | 116.0342 | | 98.0237 | 44.0495 | | D | | | | | | 10 |
| 2 | 87.0553 | 202.0822 | 185.0557 | 184.0717 | 230.0771 | 213.0506 | 212.0666 | 159.0764 | | N | 1013.6003 | 1012.6051 | | ***1072.6374*** | 1055.6109 | 9 |
| 3 | 30.0338 | 259.1037 | 242.0771 | 241.0931 | 287.0986 | 270.0721 | 269.0880 | | | G | | | | ***958.5945*** | 941.5679 | 8 |
| 4 | 86.0964 | 372.1878 | 355.1612 | 354.1772 | **400.1827** | 383.1561 | 382.1721 | 330.1408 | | L | 843.4948 | 842.4995 | | 901.5730 | 884.5465 | 7 |
| 5 | 86.0964 | 485.2718 | 468.2453 | 467.2613 | **513.2667** | 496.2402 | 495.2562 | 443.2249 | | L | 730.4107 | 729.4155 | | ***788.4890*** | 771.4624 | 6 |
| 6 | 86.0964 | **598.3559** | 581.3293 | 580.3453 | **626.3508** | 609.3243 | 608.3402 | 556.3089 | | L | 617.3267 | 616.3314 | | ***675.4049*** | 658.3784 | 5 |
| 7 | 110.0713 | **735.4148** | 718.3882 | 717.4042 | **763.4097** | 746.3832 | 745.3991 | | | H | 480.2677 | | | ***562.3208*** | 545.2943 | 4 |
| 8 | 86.0964 | **848.4989** | 831.4723 | 830.4883 | **876.4938** | 859.4672 | 858.4832 | 820.4676 | 834.4832 | I | 367.1837 | 380.2041 | 394.2197 | ***425.2619*** | 408.2354 | 3 |
| 9 | 110.0713 | 985.5578 | 968.5312 | 967.5472 | **1013.5527** | 996.5261 | 995.5421 | | | H | 230.1248 | | | ***312.1779*** | 295.1513 | 2 |
| 10 | 129.1135 | | | | | | | | | R | 74.0237 | 73.0284 | | ***175.1190*** | 158.0924 | 1 |

| Seq | ya | yb | Seq | ya | yb | Seq | ya | yb |
|---|---|---|---|---|---|---|---|---|
| NG | 144.0768 | 172.0717 | NGL | 257.1608 | 285.1557 | NGLL | 370.2449 | 398.2398 |
| NGLLL | 483.3289 | 511.3239 | NGLLLH | 620.3879 | 648.3828 | GL | 143.1179 | 171.1128 |
| GLL | 256.2020 | 284.1969 | GLLL | 369.2860 | 397.2809 | GLLLH | 506.3449 | 534.3398 |
| GLLLHI | 619.4290 | 647.4239 | LL | 199.1805 | 227.1754 | LLL | 312.2646 | 340.2595 |
| LLLH | 449.3235 | 477.3184 | LLLHI | 562.4075 | 590.4024 | LLLHIH | 699.4664 | 727.4614 |
| LL | 199.1805 | 227.1754 | LLH | 336.2394 | 364.2343 | LLHI | 449.3235 | 477.3184 |
| LLHIH | 586.3824 | 614.3773 | LH | 223.1553 | 251.1503 | LHI | 336.2394 | 364.2343 |
| LHIH | 473.2983 | 501.2932 | HI | 223.1553 | 251.1503 | HIH | 360.2142 | 388.2092 |

| | | | | | | | | |
|---|---|---|---|---|---|---|---|---|
| **IH** | 223.1553 | 251.1503 | | | | | | |

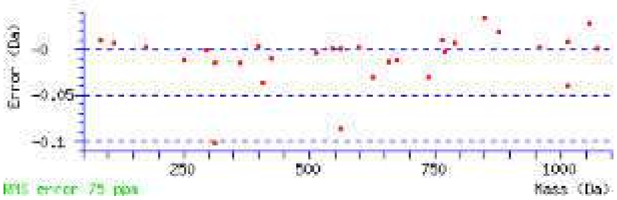


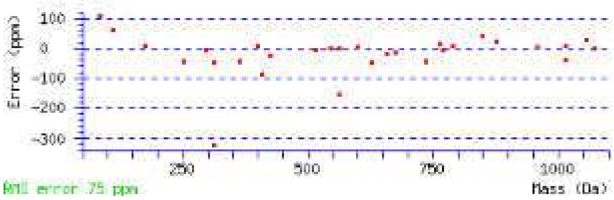


NCBI **BLAST** search of DNGLLLHIHR
(Parameters: blastp, nr protein database, expect=20000, no filter, PAM30)
Other BLAST web gateways

**All matches to this query**

| Score | Mr(calc) | Delta | Sequence |
|---|---|---|---|
| 75.5 | 1186.6571 | 0.0893 | DNGILIHIHR |
| 75.5 | 1186.6571 | 0.0893 | DNGILLHIHR |
| 75.5 | 1186.6571 | 0.0893 | DNGILLHIHR |
| 75.5 | 1186.6571 | 0.0893 | DNGILLHIHR |
| 75.5 | 1186.6571 | 0.0893 | DNGLILHIHR |
| 75.5 | 1186.6571 | 0.0893 | DNGLILHIHR |
| 75.5 | 1186.6571 | 0.0893 | DNGLLIHIHR |
| 75.5 | 1186.6571 | 0.0893 | DNGLLIHIHR |
| 75.5 | 1186.6571 | 0.0893 | DNGLLLHIHR |
| 75.5 | 1186.6571 | 0.0893 | DNGLLLHIHR |

**Mascot:** http://www.matrixscience.com/

# MATRIX SCIENCE Mascot Search Results

## Peptide View **Spot no 82**

MS/MS Fragmentation of **FVFCAEAIYK**
Found in **gi|11466795** in **NCBInr**, ribulose-1,5-bisphosphate carboxylase/oxygenase large subunit [Oryza sativa Japonica Group]

Match to Query 16: 1246.683724 from(1247.691000,1+) intensity(0.0000) index(9)
Title: Label: C11, Spot_Id: 216639, Peak_List_Id: 221211, MSMS Job_Run_Id: 21345, Comment:
Data file C:\Documents and Settings\Administrator\Desktop\farah BHU\RAJESH\12-1-13 raj\ppw_C11_135997697400.txt

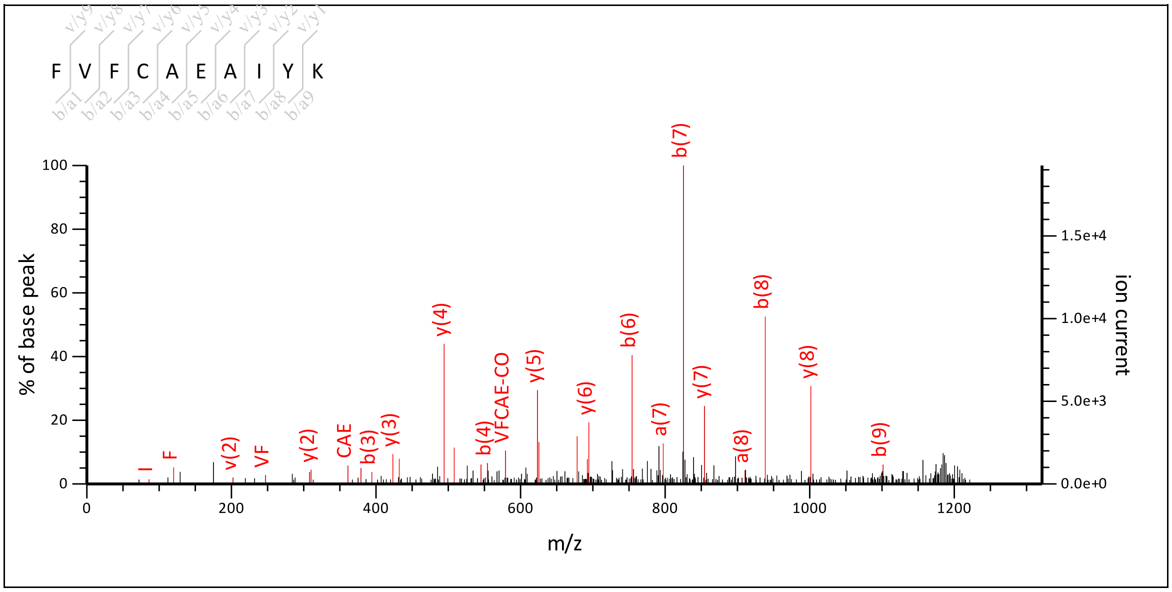


0 to 1321.38

Label all possible matches ○ Label matches used for scoring ◉

**Monoisotopic mass of neutral peptide Mr(calc):** 1246.6056
**Fixed modifications:** Carbamidomethyl (C) (apply to specified residues or termini only)
**Ions Score:** 54 **Expect:** 0.026
**Matches :** 38/129 fragment ions using 48 most intense peaks (help)

| # | Immon. | a | $a^0$ | b | $b^0$ | d | d' | Seq. | v | w | w' | y | y* | $y^0$ | # |
|---|---|---|---|---|---|---|---|---|---|---|---|---|---|---|---|
| 1 | 120.0808 | **120.0808** | | 148.0757 | | 44.0495 | | F | | | | | | | 10 |
| 2 | 72.0808 | **219.1492** | | ***247.1441*** | | 205.1335 | | V | 1056.4819 | 1069.5023 | | 1100.5445 | 1083.5179 | 1082.5339 | 9 |
| 3 | 120.0808 | 366.2176 | | ***394.2125*** | | | | F | 909.4135 | | | **1001.4761** | 984.4495 | 983.4655 | 8 |
| 4 | 133.0430 | 526.2483 | | ***554.2432*** | | 437.2547 | | C | 749.3828 | 748.3876 | | **854.4077** | 837.3811 | 836.3971 | 7 |
| 5 | 44.0495 | 597.2854 | | ***625.2803*** | | | | A | 678.3457 | | | **694.3770** | 677.3505 | 676.3665 | 6 |
| 6 | 102.0550 | 726.3280 | 708.3174 | ***754.3229*** | 736.3123 | 668.3225 | | E | 549.3031 | 548.3079 | | **623.3399** | 606.3134 | 605.3293 | 5 |
| 7 | 44.0495 | **797.3651** | 779.3545 | ***825.3600*** | 807.3494 | | | A | 478.2660 | | | **494.2973** | 477.2708 | | 4 |
| 8 | 86.0964 | **910.4491** | 892.4386 | ***938.4441*** | 920.4335 | 882.4178 | 896.4335 | I | 365.1819 | 378.2023 | 392.2180 | **423.2602** | 406.2336 | | 3 |
| 9 | 136.0757 | 1073.5125 | 1055.5019 | ***1101.5074*** | 1083.4968 | | | Y | 202.1186 | | | **310.1761** | 293.1496 | | 2 |
| 10 | 101.1073 | | | | | | | K | 74.0237 | 73.0284 | | 147.1128 | 130.0863 | | 1 |

| Seq | ya | yb | Seq | ya | yb | Seq | ya | yb |
|---|---|---|---|---|---|---|---|---|
| VF | 219.1492 | 247.1441 | VFC | 379.1798 | 407.1748 | VFCA | 450.2170 | 478.2119 |
| VFCAE | 579.2595 | 607.2545 | VFCAEA | 650.2967 | 678.2916 | FC | 280.1114 | 308.1063 |
| FCA | 351.1485 | 379.1435 | FCAE | 480.1911 | 508.1860 | FCAEA | 551.2282 | 579.2232 |
| FCAEAI | 664.3123 | 692.3072 | CA | 204.0801 | 232.0750 | CAE | 333.1227 | 361.1176 |
| CAEA | 404.1598 | 432.1547 | CAEAI | 517.2439 | 545.2388 | CAEAIY | 680.3072 | 708.3021 |
| AE | 173.0921 | 201.0870 | AEA | 244.1292 | 272.1241 | AEAI | 357.2132 | 385.2082 |

| | | | | | | | | |
|---|---|---|---|---|---|---|---|---|
| AEAIY | 520.2766 | 548.2715 | EA | 173.0921 | 201.0870 | EAI | 286.1761 | 314.1710 |
| EAIY | 449.2395 | 477.2344 | AI | 157.1335 | 185.1285 | AIY | 320.1969 | 348.1918 |
| IY | 249.1598 | 277.1547 | | | | | | |

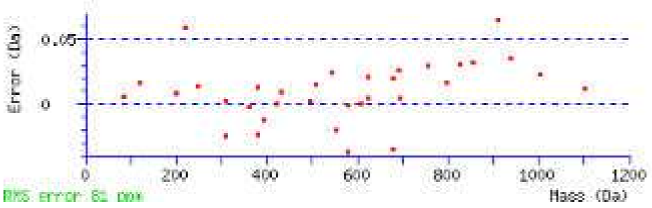


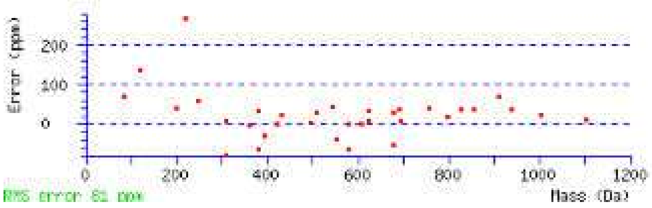


NCBI **BLAST** search of FVFCAEAIYK
(Parameters: blastp, nr protein database, expect=20000, no filter, PAM30)
Other BLAST web gateways

**All matches to this query**

| Score | Mr(calc) | Delta | Sequence |
|---|---|---|---|
| 53.9 | 1246.6056 | 0.0781 | FVFCAEAIYK |
| 53.9 | 1246.6056 | 0.0781 | FVFCAEAIYK |
| 53.9 | 1246.6056 | 0.0781 | FVFCAEAIYK |
| 53.9 | 1246.6056 | 0.0781 | FVFCAEAIYK |
| 53.9 | 1246.6056 | 0.0781 | FVFCAEAIYK |
| 53.9 | 1246.6056 | 0.0781 | FVFCAEAIYK |
| 53.9 | 1246.6056 | 0.0781 | FVFCAEAIYK |
| 53.9 | 1246.6056 | 0.0781 | FVFCAEALYK |
| 53.9 | 1246.6056 | 0.0781 | FVFCAEALYK |
| 53.9 | 1246.6056 | 0.0781 | FVFCAEALYK |

**Mascot:** http://www.matrixscience.com/

# MATRIX SCIENCE Mascot Search Results

## Peptide View Spot no 82

MS/MS Fragmentation of **LTYYTPEYETK**
Found in **gi|11466795** in **NCBInr**, ribulose-1,5-bisphosphate carboxylase/oxygenase large subunit [Oryza sativa Japonica Group]

Match to Query 18: 1406.744524 from(1407.751800,1+) intensity(0.0000) index(11)
Title: Label: C11, Spot_Id: 216639, Peak_List_Id: 221218, MSMS Job_Run_Id: 21345, Comment:
Data file C:\Documents and Settings\Administrator\Desktop\farah BHU\RAJESH\12-1-13 raj\ppw_C11_135997697400.txt

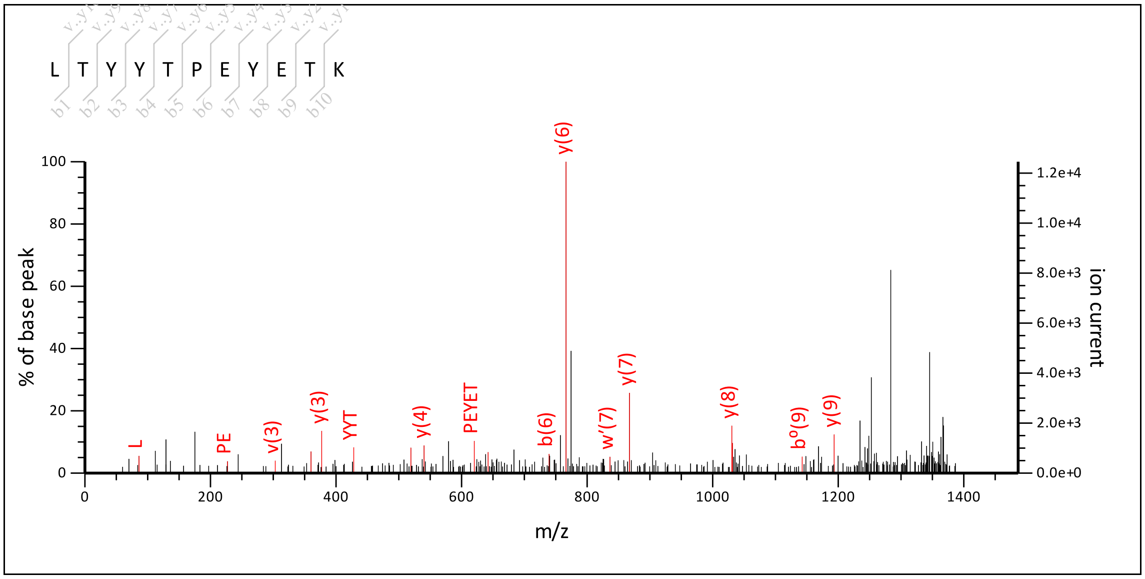


0 to 1486.6

Label all possible matches ○ Label matches used for scoring ◉

**Monoisotopic mass of neutral peptide Mr(calc):** 1406.6605
**Fixed modifications:** Carbamidomethyl (C) (apply to specified residues or termini only)
**Ions Score:** 20 **Expect:** 58
**Matches :** 22/161 fragment ions using 42 most intense peaks (help)

| # | Immon. | a | $a^0$ | b | $b^0$ | d | d' | Seq. | v | w | w' | y | y* | $y^0$ | # |
|---|---|---|---|---|---|---|---|---|---|---|---|---|---|---|---|
| 1 | 86.0964 | 86.0964 | | 114.0913 | | 44.0495 | | L | | | | | | | 11 |
| 2 | 74.0600 | 187.1441 | 169.1335 | 215.1390 | 197.1285 | 171.1492 | 173.1285 | T | 1248.5419 | 1261.5623 | 1263.5416 | 1294.5838 | 1277.5572 | 1276.5732 | 10 |
| 3 | 136.0757 | 350.2074 | 332.1969 | 378.2023 | 360.1918 | | | Y | 1085.4786 | | | ***1193.5361*** | 1176.5096 | 1175.5255 | 9 |
| 4 | 136.0757 | 513.2708 | 495.2602 | 541.2657 | 523.2551 | | | Y | 922.4153 | | | ***1030.4728*** | 1013.4462 | 1012.4622 | 8 |
| 5 | 74.0600 | 614.3184 | 596.3079 | **642.3134** | 624.3028 | 598.3235 | 600.3028 | T | 821.3676 | 834.3880 | 836.3672 | ***867.4094*** | 850.3829 | 849.3989 | 7 |
| 6 | 70.0651 | 711.3712 | 693.3606 | **739.3661** | 721.3556 | 685.3556 | | P | 724.3148 | 723.3196 | | ***766.3618*** | 749.3352 | 748.3512 | 6 |
| 7 | 102.0550 | 840.4138 | 822.4032 | 868.4087 | 850.3981 | 782.4083 | | E | 595.2722 | 594.2770 | | 669.3090 | 652.2824 | 651.2984 | 5 |
| 8 | 136.0757 | 1003.4771 | 985.4666 | **1031.4720** | 1013.4615 | | | Y | 432.2089 | | | ***540.2664*** | 523.2399 | 522.2558 | 4 |
| 9 | 102.0550 | 1132.5197 | 1114.5092 | 1160.5146 | 1142.5041 | 1074.5142 | | E | 303.1663 | 302.1710 | | ***377.2031*** | 360.1765 | 359.1925 | 3 |
| 10 | 74.0600 | 1233.5674 | 1215.5568 | 1261.5623 | 1243.5517 | 1217.5725 | 1219.5517 | T | 202.1186 | 215.1390 | 217.1183 | 248.1605 | 231.1339 | 230.1499 | 2 |
| 11 | 101.1073 | | | | | | | K | 74.0237 | 73.0284 | | 147.1128 | 130.0863 | | 1 |

| Seq | ya | yb | Seq | ya | yb | Seq | ya | yb |
|---|---|---|---|---|---|---|---|---|
| TY | 237.1234 | 265.1183 | TYY | 400.1867 | 428.1816 | TYYT | 501.2344 | 529.2293 |
| TYYTP | 598.2871 | 626.2821 | YY | 299.1390 | 327.1339 | YYT | 400.1867 | 428.1816 |
| YYTP | 497.2395 | 525.2344 | YYTPE | 626.2821 | 654.2770 | YT | 237.1234 | 265.1183 |
| YTP | 334.1761 | 362.1710 | YTPE | 463.2187 | 491.2136 | YTPEY | 626.2821 | 654.2770 |
| TP | 171.1128 | 199.1077 | TPE | 300.1554 | 328.1503 | TPEY | 463.2187 | 491.2136 |
| TPEYE | 592.2613 | 620.2562 | TPEYET | 693.3090 | 721.3039 | PE | 199.1077 | 227.1026 |
| | | | | | | | | |

| | | | | | | | | |
|---|---|---|---|---|---|---|---|---|
| **PEY** | 362.1710 | 390.1660 | **PEYE** | 491.2136 | 519.2086 | **PEYET** | 592.2613 | 620.2562 |
| **EY** | 265.1183 | 293.1132 | **EYE** | 394.1609 | 422.1558 | **EYET** | 495.2086 | 523.2035 |
| **YE** | 265.1183 | 293.1132 | **YET** | 366.1660 | 394.1609 | **ET** | 203.1026 | 231.0975 |

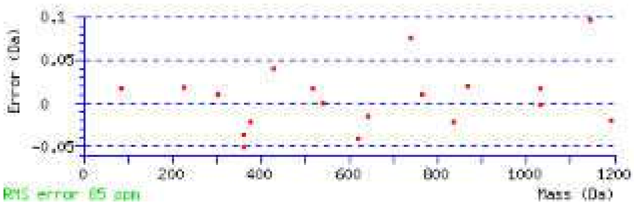


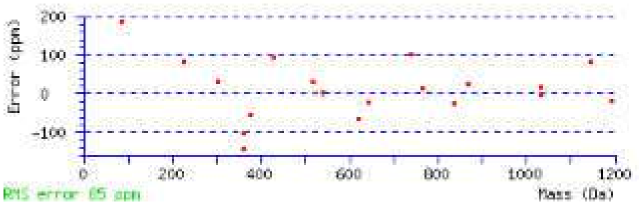


NCBI **BLAST** search of LTYYTPEYETK
(Parameters: blastp, nr protein database, expect=20000, no filter, PAM30)
Other BLAST web gateways

**All matches to this query**

| Score | Mr(calc) | Delta | Sequence |
|---|---|---|---|
| 22.3 | 1406.1685 | 0.5760 | LTYYTPEYZTK |
| 20.1 | 1406.6605 | 0.0840 | ITYYTPEYETK |
| 20.1 | 1406.6605 | 0.0840 | ITYYTPEYETK |
| 20.1 | 1406.6605 | 0.0840 | LTYYTPEYETK |
| 20.1 | 1406.6605 | 0.0840 | LTYYTPEYETK |
| 20.1 | 1406.6605 | 0.0840 | LTYYTPEYETK |
| 20.1 | 1406.6605 | 0.0840 | LTYYTPEYETK |
| 20.1 | 1406.6605 | 0.0840 | LTYYTPEYETK |
| 20.1 | 1406.6605 | 0.0840 | LTYYTPEYETK |
| 20.1 | 1406.6605 | 0.0840 | LTYYTPEYETK |

**Mascot:** http://www.matrixscience.com/

# MATRIX SCIENCE Mascot Search Results

## Peptide View Spot no 82

MS/MS Fragmentation of **LEDLRIPPTYSK**
Found in **gi|11466795** in **NCBInr**, ribulose-1,5-bisphosphate carboxylase/oxygenase large subunit [Oryza sativa Japonica Group]

Match to Query 20: 1430.873124 from(1431.880400,1+) intensity(0.0000) index(12)
Title: Label: C11, Spot_Id: 216639, Peak_List_Id: 221216, MSMS Job_Run_Id: 21345, Comment:
Data file C:\Documents and Settings\Administrator\Desktop\farah BHU\RAJESH\12-1-13 raj\ppw_C11_135997697400.txt

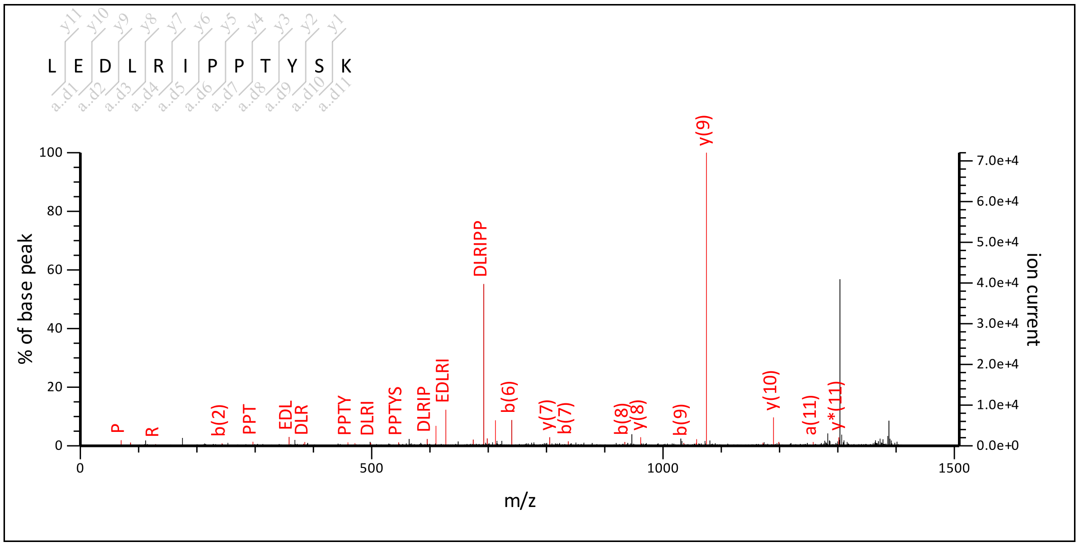


0 to 1508.44

Label all possible matches ○ Label matches used for scoring ◉

**Monoisotopic mass of neutral peptide Mr(calc):** 1430.7769
**Fixed modifications:** Carbamidomethyl (C) (apply to specified residues or termini only)
**Ions Score:** 47 **Expect:** 0.12
**Matches :** 42/202 fragment ions using 70 most intense peaks (help)

| # | Immon. | a | a* | $a^0$ | b | b* | $b^0$ | d | d' | Seq. | v | w | w' | y | y* | |
|---|---|---|---|---|---|---|---|---|---|---|---|---|---|---|---|---|
| 1 | 86.0964 | **86.0964** | | | 114.0913 | | | 44.0495 | | L | | | | | | |
| 2 | 102.0550 | 215.1390 | | 197.1285 | ***243.1339*** | | 225.1234 | 157.1335 | | E | 1244.6634 | 1243.6681 | | 1318.7001 | 1301.6736 | [illegible] |
| 3 | 88.0393 | 330.1660 | | 312.1554 | ***358.1609*** | | 340.1503 | 286.1761 | | D | 1129.6364 | 1128.6412 | | **1189.6576** | 1172.6310 | [illegible] |
| 4 | 86.0964 | 443.2500 | | 425.2395 | ***471.2449*** | | 453.2344 | 401.2031 | | L | 1016.5524 | 1015.5571 | | **1074.6306** | 1057.6041 | [illegible] |
| 5 | 129.1135 | 599.3511 | 582.3246 | 581.3406 | ***627.3461*** | 610.3195 | 609.3355 | 514.2871 | | R | 860.4512 | 859.4560 | | **961.5465** | 944.5200 | [illegible] |
| 6 | 86.0964 | **712.4352** | 695.4087 | 694.4246 | ***740.4301*** | 723.4036 | 722.4196 | 684.4039 | 698.4196 | I | 747.3672 | 760.3876 | 774.4032 | **805.4454** | 788.4189 | [illegible] |
| 7 | 70.0651 | 809.4880 | 792.4614 | 791.4774 | ***837.4829*** | 820.4563 | 819.4723 | 783.4723 | | P | 650.3144 | 649.3192 | | **692.3614** | 675.3348 | [illegible] |
| 8 | 70.0651 | 906.5407 | 889.5142 | 888.5302 | ***934.5356*** | 917.5091 | 916.5251 | 880.5251 | | P | 553.2617 | 552.2664 | | **595.3086** | 578.2821 | [illegible] |
| 9 | 74.0600 | 1007.5884 | 990.5619 | 989.5778 | ***1035.5833*** | 1018.5568 | 1017.5728 | 991.5935 | 993.5728 | T | 452.2140 | 465.2344 | 467.2136 | **498.2558** | 481.2293 | [illegible] |
| 10 | 136.0757 | 1170.6517 | 1153.6252 | 1152.6412 | ***1198.6467*** | 1181.6201 | 1180.6361 | | | Y | 289.1506 | | | 397.2082 | 380.1816 | [illegible] |
| 11 | 60.0444 | **1257.6838** | 1240.6572 | 1239.6732 | 1285.6787 | 1268.6521 | 1267.6681 | 1241.6889 | | S | 202.1186 | 201.1234 | | 234.1448 | 217.1183 | [illegible] |
| 12 | 101.1073 | | | | | | | | | K | 74.0237 | 73.0284 | | 147.1128 | 130.0863 | |

| Seq | ya | yb | Seq | ya | yb | Seq | ya | yb |
|---|---|---|---|---|---|---|---|---|
| **ED** | 217.0819 | 245.0768 | **EDL** | 330.1660 | 358.1609 | **EDLR** | 486.2671 | 514.2620 |
| **EDLRI** | 599.3511 | 627.3461 | **EDLRIP** | 696.4039 | 724.3988 | **DL** | 201.1234 | 229.1183 |
| **DLR** | 357.2245 | 385.2194 | **DLRI** | 470.3085 | 498.3035 | **DLRIP** | 567.3613 | 595.3562 |
| **DLRIPP** | 664.4141 | 692.4090 | **LR** | 242.1975 | 270.1925 | **LRI** | 355.2816 | 383.2765 |
| **LRIP** | 452.3344 | 480.3293 | **LRIPP** | 549.3871 | 577.3820 | **LRIPPT** | 650.4348 | 678.4297 |
| **RI** | 242.1975 | 270.1925 | **RIP** | 339.2503 | 367.2452 | **RIPP** | 436.3031 | 464.2980 |
| **RIPPT** | 537.3507 | 565.3457 | **IP** | 183.1492 | 211.1441 | **IPP** | 280.2020 | 308.1969 |
| **IPPT** | 381.2496 | 409.2445 | **IPPTY** | 544.3130 | 572.3079 | **IPPTYS** | 631.3450 | 659.3399 |
| **PP** | 167.1179 | 195.1128 | **PPT** | 268.1656 | 296.1605 | **PPTY** | 431.2289 | 459.2238 |

| | | | | | | | | |
|---|---|---|---|---|---|---|---|---|
| **PPTYS** | 518.2609 | 546.2558 | **PT** | 171.1128 | 199.1077 | **PTY** | 334.1761 | 362.1710 |
| **PTYS** | 421.2082 | 449.2031 | **TY** | 237.1234 | 265.1183 | **TYS** | 324.1554 | 352.1503 |
| **YS** | 223.1077 | 251.1026 | | | | | | |

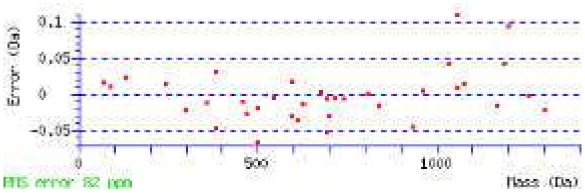


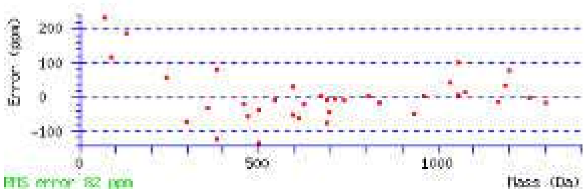


NCBI **BLAST** search of LEDLRIPPTYSK
(Parameters: blastp, nr protein database, expect=20000, no filter, PAM30)
Other BLAST web gateways

**All matches to this query**

| Score | Mr(calc) | Delta | Sequence |
|---|---|---|---|
| 46.7 | 1430.7769 | 0.0962 | LEDIRIPPTYSK |
| 46.7 | 1430.7769 | 0.0962 | LEDLRIPPTYSK |
| 46.7 | 1430.7769 | 0.0962 | LEDLRIPPTYSK |
| 46.7 | 1430.7769 | 0.0962 | LEDLRIPPTYSK |
| 46.7 | 1430.7769 | 0.0962 | LEDLRIPPTYSK |
| 37.7 | 1430.7769 | 0.0962 | LEDLRIPTPYSK |
| 30.5 | 1430.7374 | 0.1358 | EPMRLLQEMIR |
| 30.3 | 1430.7769 | 0.0962 | LEDIRIPPSYTK |
| 30.3 | 1430.7769 | 0.0962 | LEDIRIPPSYTK |
| 30.3 | 1430.7769 | 0.0962 | LEDLRIPPSYTK |

**Mascot:** http://www.matrixscience.com/

# MATRIX SCIENCE Mascot Search Results

## Peptide View Spot no 82

MS/MS Fragmentation of **TFQGPPHGIQVER**
Found in **gi|11466795** in **NCBInr**, ribulose-1,5-bisphosphate carboxylase/oxygenase large subunit [Oryza sativa Japonica Group]

Match to Query 22: 1464.842824 from(1465.850100,1+) intensity(0.0000) index(13)
Title: Label: C11, Spot_Id: 216639, Peak_List_Id: 221197, MSMS Job_Run_Id: 21345, Comment:
Data file C:\Documents and Settings\Administrator\Desktop\farah BHU\RAJESH\12-1-13 raj\ppw_C11_135997697400.txt

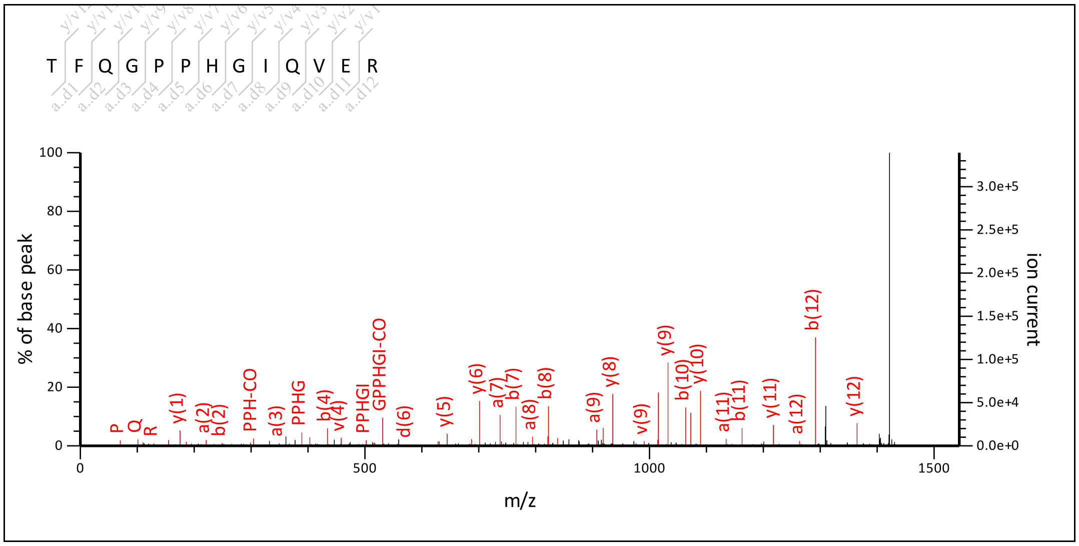


0 to 1544.27

Label all possible matches Label matches used for scoring

**Monoisotopic mass of neutral peptide Mr(calc):** 1464.7474
**Fixed modifications:** Carbamidomethyl (C) (apply to specified residues or termini only)
**Ions Score:** 104 **Expect:** 2.8e-07
**Matches :** 72/229 fragment ions using 70 most intense peaks (help)

| # | Immon. | a | a* | $a^0$ | b | b* | $b^0$ | d | d' | Seq. | v | w | w' | y | y* | |
|---|---|---|---|---|---|---|---|---|---|---|---|---|---|---|---|---|
| 1 | 74.0600 | 74.0600 | | 56.0495 | 102.0550 | | 84.0444 | 44.0495 | | T | | | | | | |
| 2 | 120.0808 | **221.1285** | | 203.1179 | **249.1234** | | 231.1128 | | | F | 1272.6444 | | | ***1364.7070*** | 1347.6804 | 134[illegible] |
| 3 | 101.0709 | **349.1870** | 332.1605 | 331.1765 | 377.1819 | 360.1554 | 359.1714 | 292.1656 | | Q | 1144.5858 | 1143.5905 | | ***1217.6385*** | 1200.6120 | 119[illegible] |
| 4 | 30.0338 | 406.2085 | 389.1819 | 388.1979 | **434.2034** | 417.1769 | 416.1928 | | | G | | | | ***1089.5800*** | 1072.5534 | 107[illegible] |
| 5 | 70.0651 | 503.2613 | 486.2347 | 485.2507 | **531.2562** | 514.2296 | 513.2456 | 477.2456 | | P | 990.5116 | 989.5163 | | ***1032.5585*** | 1015.5320 | 101[illegible] |
| 6 | 70.0651 | 600.3140 | 583.2875 | 582.3035 | **628.3089** | 611.2824 | 610.2984 | 574.2984 | | P | 893.4588 | 892.4635 | | ***935.5057*** | 918.4792 | 91[illegible] |
| 7 | 110.0713 | **737.3729** | 720.3464 | 719.3624 | **765.3679** | 748.3413 | 747.3573 | | | H | 756.3999 | | | ***838.4530*** | 821.4264 | 82[illegible] |
| 8 | 30.0338 | **794.3944** | 777.3679 | 776.3838 | **822.3893** | 805.3628 | 804.3787 | | | G | | | | ***701.3941*** | 684.3675 | 68[illegible] |
| 9 | 86.0964 | **907.4785** | 890.4519 | 889.4679 | **935.4734** | 918.4468 | 917.4628 | 879.4472 | 893.4628 | I | 586.2944 | 599.3148 | 613.3304 | ***644.3726*** | 627.3461 | 62[illegible] |
| 10 | 101.0709 | 1035.5370 | 1018.5105 | 1017.5265 | **1063.5320** | 1046.5054 | 1045.5214 | 978.5156 | | Q | 458.2358 | 457.2405 | | ***531.2885*** | 514.2620 | 51[illegible] |
| 11 | 72.0808 | **1134.6055** | 1117.5789 | 1116.5949 | **1162.6004** | 1145.5738 | 1144.5898 | 1120.5898 | | V | 359.1674 | 372.1878 | | ***403.2300*** | 386.2034 | 38[illegible] |
| 12 | 102.0550 | **1263.6480** | 1246.6215 | 1245.6375 | **1291.6430** | 1274.6164 | 1273.6324 | 1205.6426 | | E | 230.1248 | 229.1295 | | ***304.1615*** | 287.1350 | 28[illegible] |
| 13 | 129.1135 | | | | | | | | | R | 74.0237 | 73.0284 | | ***175.1190*** | 158.0924 | |

| Seq | ya | yb | Seq | ya | yb | Seq | ya | yb |
|---|---|---|---|---|---|---|---|---|
| FQ | 248.1394 | 276.1343 | FQG | 305.1608 | 333.1557 | FQGP | 402.2136 | 430.2085 |
| FQGPP | 499.2663 | 527.2613 | FQGPPH | 636.3253 | 664.3202 | FQGPPHG | 693.3467 | 721.3416 |
| QG | 158.0924 | 186.0873 | QGP | 255.1452 | 283.1401 | QGPP | 352.1979 | 380.1928 |
| QGPPH | 489.2568 | 517.2518 | QGPPHG | 546.2783 | 574.2732 | QGPPHGI | 659.3624 | 687.3573 |
| GP | 127.0866 | 155.0815 | GPP | 224.1394 | 252.1343 | GPPH | 361.1983 | 389.1932 |
| GPPHG | 418.2197 | 446.2146 | GPPHGI | 531.3038 | 559.2987 | GPPHGIQ | 659.3624 | 687.3573 |
| PP | 167.1179 | 195.1128 | PPH | 304.1768 | 332.1717 | PPHG | 361.1983 | 389.1932 |
| PPHGI | 474.2823 | 502.2772 | PPHGIQ | 602.3409 | 630.3358 | PH | 207.1240 | 235.1190 |

| | | | | | | | | |
|---|---|---|---|---|---|---|---|---|
| **PHG** | 264.1455 | 292.1404 | **PHGI** | 377.2296 | 405.2245 | **PHGIQ** | 505.2881 | 533.2831 |
| **PHGIQV** | 604.3566 | 632.3515 | **HG** | 167.0927 | 195.0877 | **HGI** | 280.1768 | 308.1717 |
| **HGIQ** | 408.2354 | 436.2303 | **HGIQV** | 507.3038 | 535.2987 | **HGIQVE** | 636.3464 | 664.3413 |
| **GI** | 143.1179 | 171.1128 | **GIQ** | 271.1765 | 299.1714 | **GIQV** | 370.2449 | 398.2398 |
| **GIQVE** | 499.2875 | 527.2824 | **IQ** | 214.1550 | 242.1499 | **IQV** | 313.2234 | 341.2183 |
| **IQVE** | 442.2660 | 470.2609 | **QV** | 200.1394 | 228.1343 | **QVE** | 329.1819 | 357.1769 |
| **VE** | 201.1234 | 229.1183 | | | | | | |

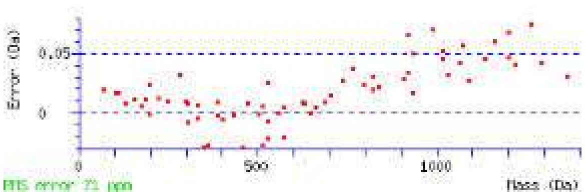


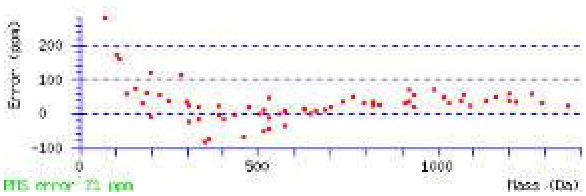


NCBI **BLAST** search of TFQGPPHGIQVER
(Parameters: blastp, nr protein database, expect=20000, no filter, PAM30)
Other BLAST web gateways

**All matches to this query**

| Score | Mr(calc) | Delta | Sequence |
|---|---|---|---|
| 104.1 | 1464.7838 | 0.0591 | TFKGPPHGIQVER |
| 104.1 | 1464.7838 | 0.0591 | TFKGPPHGIQVER |
| 104.1 | 1464.7838 | 0.0591 | TFQGPPHGIKVER |
| 104.1 | 1464.7838 | 0.0591 | TFQGPPHGIKVER |
| 104.1 | 1464.7474 | 0.0954 | TFQGPPHGIQVER |
| 104.1 | 1464.7474 | 0.0954 | TFQGPPHGIQVER |
| 104.1 | 1464.7474 | 0.0954 | TFQGPPHGIQVER |
| 104.1 | 1464.7474 | 0.0954 | TFQGPPHGIQVER |
| 104.1 | 1464.7474 | 0.0954 | TFQGPPHGIQVER |
| 104.1 | 1464.7474 | 0.0954 | TFQGPPHGIQVER |

**Mascot:** http://www.matrixscience.com/

# MATRIX SCIENCE Mascot Search Results

## Peptide View Spot no 82

MS/MS Fragmentation of **YGRPLLGCTIKPK**
Found in **gi|11466795** in **NCBInr**, ribulose-1,5-bisphosphate carboxylase/oxygenase large subunit [Oryza sativa Japonica Group]

Match to Query 24: 1501.935024 from(1502.942300,1+) intensity(0.0000) index(14)
Title: Label: C11, Spot_Id: 216639, Peak_List_Id: 221198, MSMS Job_Run_Id: 21345, Comment:
Data file C:\Documents and Settings\Administrator\Desktop\farah BHU\RAJESH\12-1-13 raj\ppw_C11_135997697400.txt

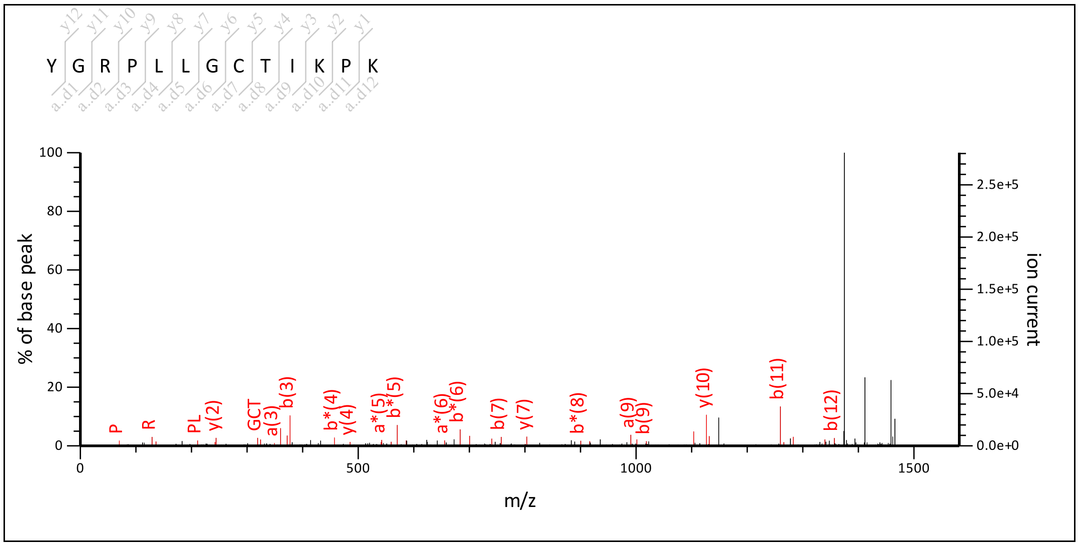


0 to 1581.57

Label all possible matches ○ Label matches used for scoring ◉

**Monoisotopic mass of neutral peptide Mr(calc):** 1501.8439
**Fixed modifications:** Carbamidomethyl (C) (apply to specified residues or termini only)
**Ions Score:** 57 **Expect:** 0.014
**Matches :** 43/211 fragment ions using 60 most intense peaks (help)

| # | Immon. | a | a* | $a^0$ | b | b* | $b^0$ | d | d' | Seq. | v | w | w' | y | y* | |
|---|---|---|---|---|---|---|---|---|---|---|---|---|---|---|---|---|
| 1 | 136.0757 | **136.0757** | | | 164.0706 | | | 44.0495 | | **Y** | | | | | | |
| 2 | 30.0338 | 193.0972 | | | 221.0921 | | | | | **G** | | | | ***1339.7879*** | 1322.7613 | [illegible] |
| 3 | 129.1135 | **349.1983** | 332.1717 | | ***377.1932*** | 360.1666 | | 264.1343 | | **R** | 1181.6711 | 1180.6758 | | ***1282.7664*** | 1265.7398 | [illegible] |
| 4 | 70.0651 | 446.2510 | 429.2245 | | 474.2459 | 457.2194 | | 420.2354 | | **P** | 1084.6183 | 1083.6231 | | ***1126.6653*** | 1109.6387 | [illegible] |
| 5 | 86.0964 | **559.3351** | 542.3085 | | 587.3300 | 570.3035 | | 517.2881 | | **L** | 971.5343 | 970.5390 | | 1029.6125 | 1012.5860 | [illegible] |
| 6 | 86.0964 | 672.4192 | 655.3926 | | ***700.4141*** | 683.3875 | | 630.3722 | | **L** | 858.4502 | 857.4550 | | ***916.5285*** | 899.5019 | [illegible] |
| 7 | 30.0338 | 729.4406 | 712.4141 | | ***757.4355*** | 740.4090 | | | | **G** | | | | ***803.4444*** | 786.4178 | [illegible] |
| 8 | 133.0430 | 889.4713 | 872.4447 | | 917.4662 | 900.4396 | | 800.4777 | | **C** | 641.3981 | 640.4028 | | 746.4229 | 729.3964 | [illegible] |
| 9 | 74.0600 | **990.5189** | 973.4924 | 972.5084 | ***1018.5139*** | 1001.4873 | 1000.5033 | 974.5240 | 976.5033 | **T** | 540.3504 | 553.3708 | 555.3501 | ***586.3923*** | 569.3657 | [illegible] |
| 10 | 86.0964 | **1103.6030** | 1086.5765 | 1085.5924 | ***1131.5979*** | 1114.5714 | 1113.5874 | 1075.5717 | 1089.5874 | **I** | 427.2663 | 440.2867 | 454.3024 | ***485.3446*** | 468.3180 | |
| 11 | 101.1073 | 1231.6980 | 1214.6714 | 1213.6874 | ***1259.6929*** | 1242.6663 | 1241.6823 | 1174.6401 | | **K** | 299.1714 | 298.1761 | | ***372.2605*** | 355.2340 | |
| 12 | 70.0651 | 1328.7507 | 1311.7242 | 1310.7402 | ***1356.7457*** | 1339.7191 | 1338.7351 | 1302.7351 | | **P** | 202.1186 | 201.1234 | | ***244.1656*** | 227.1390 | |
| 13 | 101.1073 | | | | | | | | | **K** | 74.0237 | 73.0284 | | 147.1128 | 130.0863 | |

| Seq | ya | yb | Seq | ya | yb | Seq | ya | yb |
|---|---|---|---|---|---|---|---|---|
| **GR** | 186.1349 | 214.1299 | **GRP** | 283.1877 | 311.1826 | **GRPL** | 396.2718 | 424.2667 |
| **GRPLL** | 509.3558 | 537.3507 | **GRPLLG** | 566.3773 | 594.3722 | **RP** | 226.1662 | 254.1612 |
| **RPL** | 339.2503 | 367.2452 | **RPLL** | 452.3344 | 480.3293 | **RPLLG** | 509.3558 | 537.3507 |
| **RPLLGC** | 669.3865 | 697.3814 | **PL** | 183.1492 | 211.1441 | **PLL** | 296.2333 | 324.2282 |
| **PLLG** | 353.2547 | 381.2496 | **PLLGC** | 513.2854 | 541.2803 | **PLLGCT** | 614.3330 | 642.3280 |
| **LL** | 199.1805 | 227.1754 | **LLG** | 256.2020 | 284.1969 | **LLGC** | 416.2326 | 444.2275 |
| **LLGCT** | 517.2803 | 545.2752 | **LLGCTI** | 630.3643 | 658.3593 | **LG** | 143.1179 | 171.1128 |
| **LGC** | 303.1485 | 331.1435 | **LGCT** | 404.1962 | 432.1911 | **LGCTI** | 517.2803 | 545.2752 |

| | | | | | | | | |
|---|---|---|---|---|---|---|---|---|
| **LGCTIK** | 645.3752 | 673.3702 | **GC** | 190.0645 | 218.0594 | **GCT** | 291.1122 | 319.1071 |
| **GCTI** | 404.1962 | 432.1911 | **GCTIK** | 532.2912 | 560.2861 | **GCTIKP** | 629.3439 | 657.3389 |
| **CT** | 234.0907 | 262.0856 | **CTI** | 347.1748 | 375.1697 | **CTIK** | 475.2697 | 503.2646 |
| **CTIKP** | 572.3225 | 600.3174 | **TI** | 187.1441 | 215.1390 | **TIK** | 315.2391 | 343.2340 |
| **TIKP** | 412.2918 | 440.2867 | **IK** | 214.1914 | 242.1863 | **IKP** | 311.2442 | 339.2391 |
| **KP** | 198.1601 | 226.1550 | | | | | | |

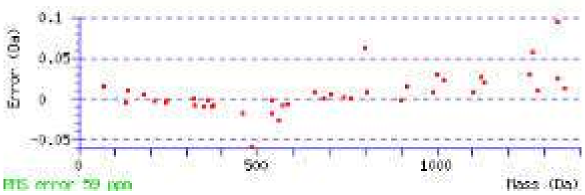


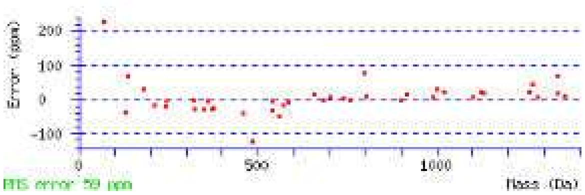


NCBI **BLAST** search of YGRPLLGCTIKPK
(Parameters: blastp, nr protein database, expect=20000, no filter, PAM30)
Other BLAST web gateways

**All matches to this query**

| Score | Mr(calc) | Delta | Sequence |
|---|---|---|---|
| 56.7 | 1501.8439 | 0.0911 | YGRPILGCTIKPK |
| 56.7 | 1501.8439 | 0.0911 | YGRPILGCTIKPK |
| 56.7 | 1501.8439 | 0.0911 | YGRPLIGCTIKPK |
| 56.7 | 1501.8439 | 0.0911 | YGRPLLGCTIKPK |
| 56.7 | 1501.8439 | 0.0911 | YGRPLLGCTIKPK |
| 56.7 | 1501.8439 | 0.0911 | YGRPLLGCTIKPK |
| 56.7 | 1501.8439 | 0.0911 | YGRPLLGCTIKPK |
| 56.7 | 1501.8439 | 0.0911 | YGRPLLGCTIKPK |
| 56.7 | 1501.8439 | 0.0911 | YGRPLLGCTIKPK |
| 56.7 | 1501.8439 | 0.0911 | YGRPLLGCTIKPK |

**Mascot:** http://www.matrixscience.com/

# MATRIX SCIENCE Mascot Search Results

## Peptide View Spot no 82

MS/MS Fragmentation of **DRFVFCAEAIYK**
Found in **gi|11466795** in **NCBInr**, ribulose-1,5-bisphosphate carboxylase/oxygenase large subunit [Oryza sativa Japonica Group]

Match to Query 25: 1517.831024 from(1518.838300,1+) intensity(0.0000) index(15)
Title: Label: C11, Spot_Id: 216639, Peak_List_Id: 221222, MSMS Job_Run_Id: 21345, Comment:
Data file C:\Documents and Settings\Administrator\Desktop\farah BHU\RAJESH\12-1-13 raj\ppw_C11_135997697400.txt

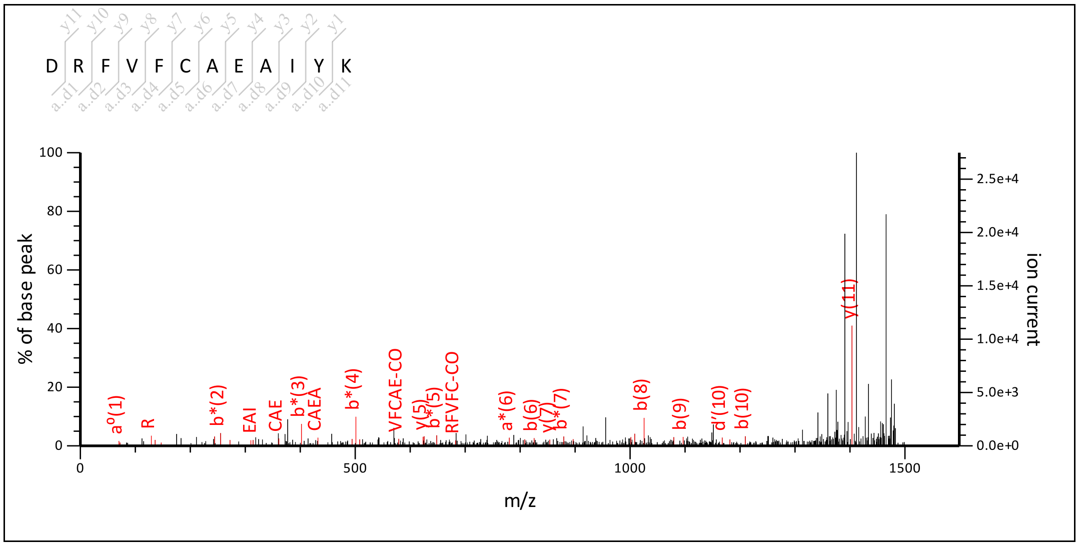


0 to 1598.83

Label all possible matches Label matches used for scoring

**Monoisotopic mass of neutral peptide Mr(calc):** 1517.7337
**Fixed modifications:** Carbamidomethyl (C) (apply to specified residues or termini only)
**Ions Score:** 15 **Expect:** 1.8e+02
**Matches :** 39/194 fragment ions using 120 most intense peaks (help)

| # | Immon. | a | a* | a0 | b | b* | b0 | d | d' | Seq. | v | w | w' | y | y* | |
|---|---|---|---|---|---|---|---|---|---|---|---|---|---|---|---|---|
| 1 | 88.0393 | 88.0393 | | 70.0287 | 116.0342 | | 98.0237 | 44.0495 | | D | | | | | | |
| 2 | 129.1135 | 244.1404 | 227.1139 | 226.1299 | 272.1353 | 255.1088 | 254.1248 | 159.0764 | | R | 1302.6187 | 1301.6235 | | 1403.7140 | 1386.6875 | [illegible] |
| 3 | 120.0808 | 391.2088 | 374.1823 | 373.1983 | 419.2037 | 402.1772 | 401.1932 | | | F | 1155.5503 | | | 1247.6129 | 1230.5864 | [illegible] |
| 4 | 72.0808 | 490.2772 | 473.2507 | 472.2667 | 518.2722 | 501.2456 | 500.2616 | 476.2616 | | V | 1056.4819 | 1069.5023 | | 1100.5445 | 1083.5179 | [illegible] |
| 5 | 120.0808 | 637.3457 | 620.3191 | 619.3351 | 665.3406 | 648.3140 | 647.3300 | | | F | 909.4135 | | | 1001.4761 | 984.4495 | [illegible] |
| 6 | 133.0430 | 797.3763 | 780.3498 | 779.3657 | 825.3712 | 808.3447 | 807.3607 | 708.3828 | | C | 749.3828 | 748.3876 | | 854.4077 | 837.3811 | [illegible] |
| 7 | 44.0495 | 868.4134 | 851.3869 | 850.4029 | 896.4083 | 879.3818 | 878.3978 | | | A | 678.3457 | | | 694.3770 | 677.3505 | [illegible] |
| 8 | 102.0550 | 997.4560 | 980.4295 | 979.4454 | 1025.4509 | 1008.4244 | 1007.4404 | 939.4505 | | E | 549.3031 | 548.3079 | | 623.3399 | 606.3134 | [illegible] |
| 9 | 44.0495 | 1068.4931 | 1051.4666 | 1050.4826 | 1096.4880 | 1079.4615 | 1078.4775 | | | A | 478.2660 | | | 494.2973 | 477.2708 | |
| 10 | 86.0964 | 1181.5772 | 1164.5506 | 1163.5666 | 1209.5721 | 1192.5456 | 1191.5615 | 1153.5459 | 1167.5615 | I | 365.1819 | 378.2023 | 392.2180 | 423.2602 | 406.2336 | |
| 11 | 136.0757 | 1344.6405 | 1327.6140 | 1326.6300 | 1372.6354 | 1355.6089 | 1354.6249 | | | Y | 202.1186 | | | 310.1761 | 293.1496 | |
| 12 | 101.1073 | | | | | | | | | K | 74.0237 | 73.0284 | | 147.1128 | 130.0863 | |

| Seq | ya | yb | Seq | ya | yb | Seq | ya | yb |
|---|---|---|---|---|---|---|---|---|
| RF | 276.1819 | 304.1768 | RFV | 375.2503 | 403.2452 | RFVF | 522.3187 | 550.3136 |
| RFVFC | 682.3494 | 710.3443 | FV | 219.1492 | 247.1441 | FVF | 366.2176 | 394.2125 |
| FVFC | 526.2483 | 554.2432 | FVFCA | 597.2854 | 625.2803 | VF | 219.1492 | 247.1441 |
| VFC | 379.1798 | 407.1748 | VFCA | 450.2170 | 478.2119 | VFCAE | 579.2595 | 607.2545 |
| VFCAEA | 650.2967 | 678.2916 | FC | 280.1114 | 308.1063 | FCA | 351.1485 | 379.1435 |
| FCAE | 480.1911 | 508.1860 | FCAEA | 551.2282 | 579.2232 | FCAEAI | 664.3123 | 692.3072 |
| CA | 204.0801 | 232.0750 | CAE | 333.1227 | 361.1176 | CAEA | 404.1598 | 432.1547 |
| CAEAI | 517.2439 | 545.2388 | CAEAIY | 680.3072 | 708.3021 | AE | 173.0921 | 201.0870 |
| AEA | 244.1292 | 272.1241 | AEAI | 357.2132 | 385.2082 | AEAIY | 520.2766 | 548.2715 |

| | | | | | | | | |
|---|---|---|---|---|---|---|---|---|
| **EA** | 173.0921 | 201.0870 | **EAI** | 286.1761 | 314.1710 | **EAIY** | 449.2395 | 477.2344 |
| **AI** | 157.1335 | 185.1285 | **AIY** | 320.1969 | 348.1918 | **IY** | 249.1598 | 277.1547 |

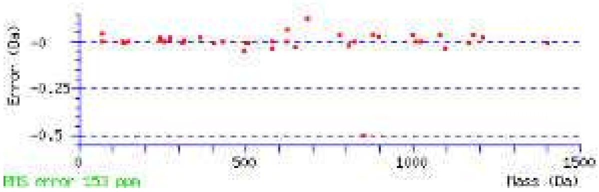


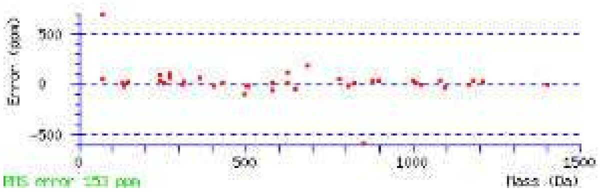


NCBI **BLAST** search of DRFVFCAEAIYK
(Parameters: blastp, nr protein database, expect=20000, no filter, PAM30)
Other BLAST web gateways

**All matches to this query**

| Score | Mr(calc) | Delta | Sequence |
|---|---|---|---|
| 15.9 | 1517.7007 | 0.1304 | DRFVMCAEAIYK |
| 14.9 | 1517.7337 | 0.0974 | DRFVFCAEAIYK |
| 14.9 | 1517.7337 | 0.0974 | DRFVFCAEAIYK |
| 14.9 | 1517.7337 | 0.0974 | DRFVFCAEAIYK |
| 14.9 | 1517.7337 | 0.0974 | DRFVFCAEAIYK |
| 14.9 | 1517.7337 | 0.0974 | DRFVFCAEAIYK |
| 14.9 | 1517.7337 | 0.0974 | DRFVFCAEAIYK |
| 14.9 | 1517.7337 | 0.0974 | DRFVFCAEAIYK |
| 14.9 | 1517.7337 | 0.0974 | DRFVFCAEAIYK |
| 14.3 | 1517.7337 | 0.0974 | DRFVFCAEALYK |

**Mascot:** http://www.matrixscience.com/

# MATRIX SCIENCE Mascot Search Results

## Peptide View Spot no 82

MS/MS Fragmentation of **WSPELAAACEIWK**
Found in **gi|11466795** in **NCBInr**, ribulose-1,5-bisphosphate carboxylase/oxygenase large subunit [Oryza sativa Japonica Group]

Match to Query 27: 1559.830624 from(1560.837900,1+) intensity(0.0000) index(16)
Title: Label: C11, Spot_Id: 216639, Peak_List_Id: 221213, MSMS Job_Run_Id: 21345, Comment:
Data file C:\Documents and Settings\Administrator\Desktop\farah BHU\RAJESH\12-1-13 raj\ppw_C11_135997697400.txt

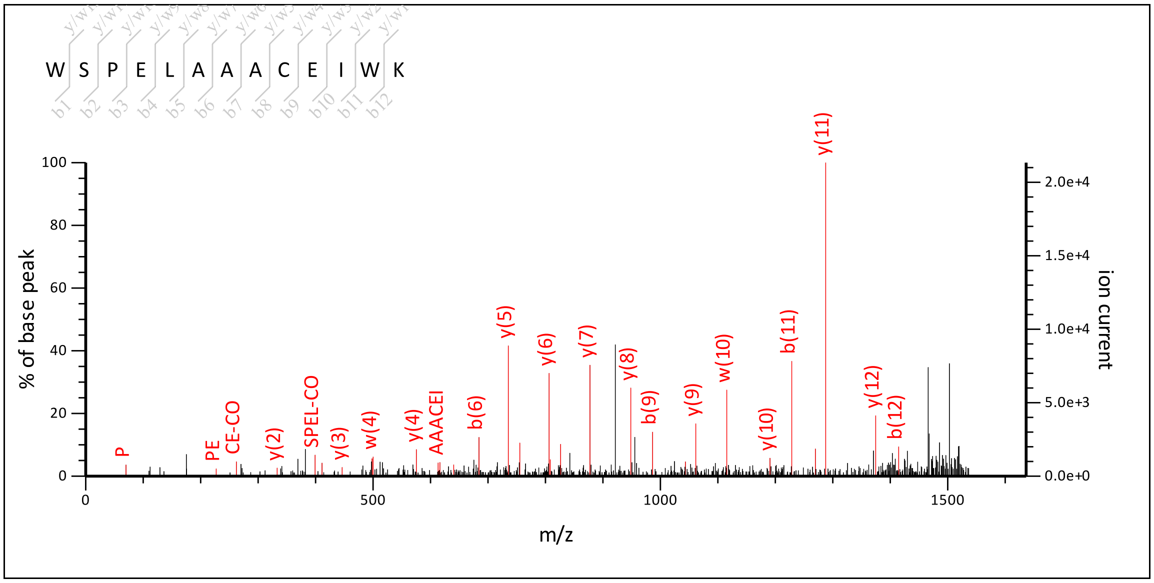


0 to 1636.45

Label all possible matches ○ Label matches used for scoring ◉

**Monoisotopic mass of neutral peptide Mr(calc):** 1559.7442
**Fixed modifications:** Carbamidomethyl (C) (apply to specified residues or termini only)
**Ions Score:** 65 **Expect:** 0.0017
**Matches :** 37/203 fragment ions using 60 most intense peaks (help)

| # | Immon. | a | $a^0$ | b | $b^0$ | d | d' | Seq. | v | w | w' | y | y* | $y^0$ | # |
|---|---|---|---|---|---|---|---|---|---|---|---|---|---|---|---|
| 1 | 159.0917 | 159.0917 | | 187.0866 | | 44.0495 | | W | | | | | | | 13 |
| 2 | 60.0444 | 246.1237 | 228.1131 | 274.1186 | 256.1081 | 230.1288 | | S | 1342.6460 | 1341.6508 | | ***1374.6722*** | 1357.6457 | 1356.6616 | 12 |
| 3 | 70.0651 | 343.1765 | 325.1659 | 371.1714 | 353.1608 | 317.1608 | | P | 1245.5932 | 1244.5980 | | ***1287.6402*** | 1270.6136 | 1269.6296 | 11 |
| 4 | 102.0550 | 472.2191 | 454.2085 | **500.2140** | 482.2034 | 414.2136 | | E | 1116.5506 | 1115.5554 | | ***1190.5874*** | 1173.5609 | 1172.5769 | 10 |
| 5 | 86.0964 | 585.3031 | 567.2926 | **613.2980** | 595.2875 | 543.2562 | | L | 1003.4666 | 1002.4713 | | ***1061.5448*** | 1044.5183 | 1043.5343 | 9 |
| 6 | 44.0495 | 656.3402 | 638.3297 | **684.3352** | 666.3246 | | | A | 932.4295 | | | ***948.4608*** | 931.4342 | 930.4502 | 8 |
| 7 | 44.0495 | 727.3774 | 709.3668 | **755.3723** | 737.3617 | | | A | 861.3924 | | | ***877.4237*** | 860.3971 | 859.4131 | 7 |
| 8 | 44.0495 | 798.4145 | 780.4039 | **826.4094** | 808.3988 | | | A | 790.3552 | | | ***806.3865*** | 789.3600 | 788.3760 | 6 |
| 9 | 133.0430 | 958.4451 | 940.4345 | **986.4400** | 968.4295 | 869.4516 | | C | 630.3246 | 629.3293 | | ***735.3494*** | 718.3229 | 717.3389 | 5 |
| 10 | 102.0550 | 1087.4877 | 1069.4771 | **1115.4826** | 1097.4721 | 1029.4822 | | E | 501.2820 | 500.2867 | | ***575.3188*** | 558.2922 | 557.3082 | 4 |
| 11 | 86.0964 | 1200.5718 | 1182.5612 | **1228.5667** | 1210.5561 | 1172.5405 | 1186.5561 | I | 388.1979 | 401.2183 | 415.2340 | ***446.2762*** | 429.2496 | | 3 |
| 12 | 159.0917 | 1386.6511 | 1368.6405 | **1414.6460** | 1396.6354 | | | W | 202.1186 | | | ***333.1921*** | 316.1656 | | 2 |
| 13 | 101.1073 | | | | | | | K | 74.0237 | 73.0284 | | 147.1128 | 130.0863 | | 1 |

| Seq | ya | yb | Seq | ya | yb | Seq | ya | yb |
|---|---|---|---|---|---|---|---|---|
| SP | 157.0972 | 185.0921 | SPE | 286.1397 | 314.1347 | SPEL | 399.2238 | 427.2187 |
| SPELA | 470.2609 | 498.2558 | SPELAA | 541.2980 | 569.2930 | SPELAAA | 612.3352 | 640.3301 |
| PE | 199.1077 | 227.1026 | PEL | 312.1918 | 340.1867 | PELA | 383.2289 | 411.2238 |
| PELAA | 454.2660 | 482.2609 | PELAAA | 525.3031 | 553.2980 | PELAAAC | 685.3338 | 713.3287 |

| | | | | | | | | |
|---|---|---|---|---|---|---|---|---|
| **EL** | 215.1390 | 243.1339 | **ELA** | 286.1761 | 314.1710 | **ELAA** | 357.2132 | 385.2082 |
| **ELAAA** | 428.2504 | 456.2453 | **ELAAAC** | 588.2810 | 616.2759 | **LA** | 157.1335 | 185.1285 |
| **LAA** | 228.1707 | 256.1656 | **LAAA** | 299.2078 | 327.2027 | **LAAAC** | 459.2384 | 487.2333 |
| **LAAACE** | 588.2810 | 616.2759 | **AA** | 115.0866 | 143.0815 | **AAA** | 186.1237 | 214.1186 |
| **AAAC** | 346.1544 | 374.1493 | **AAACE** | 475.1969 | 503.1919 | **AAACEI** | 588.2810 | 616.2759 |
| **AA** | 115.0866 | 143.0815 | **AAC** | 275.1172 | 303.1122 | **AACE** | 404.1598 | 432.1547 |
| **AACEI** | 517.2439 | 545.2388 | **AC** | 204.0801 | 232.0750 | **ACE** | 333.1227 | 361.1176 |
| **ACEI** | 446.2068 | 474.2017 | **ACEIW** | 632.2861 | 660.2810 | **CE** | 262.0856 | 290.0805 |
| **CEI** | 375.1697 | 403.1646 | **CEIW** | 561.2490 | 589.2439 | **EI** | 215.1390 | 243.1339 |
| **EIW** | 401.2183 | 429.2132 | **IW** | 272.1757 | 300.1707 | | | |

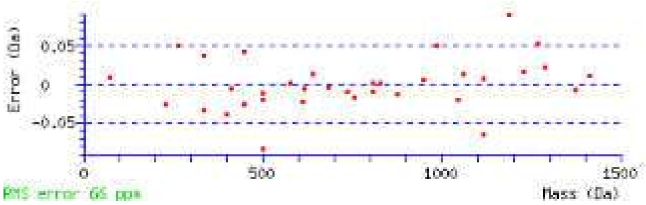


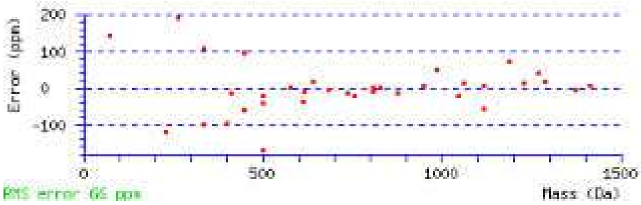


NCBI **BLAST** search of WSPELAAACEIWK
(Parameters: blastp, nr protein database, expect=20000, no filter, PAM30)
Other BLAST web gateways

**All matches to this query**

| Score | Mr(calc) | Delta | Sequence |
|---|---|---|---|
| 64.7 | 1559.7442 | 0.0864 | WSPEIAAACEIWK |
| 64.7 | 1559.7442 | 0.0864 | WSPELAAACEIWK |
| 64.7 | 1559.7442 | 0.0864 | WSPELAAACEIWK |
| 64.7 | 1559.7442 | 0.0864 | WSPELAAACEIWK |
| 64.7 | 1559.7442 | 0.0864 | WSPELAAACEIWK |
| 64.7 | 1559.7442 | 0.0864 | WSPELAAACEIWK |
| 64.7 | 1559.7442 | 0.0864 | WSPELAAACEIWK |
| 64.7 | 1559.7442 | 0.0864 | WSPELAAACEIWK |
| 64.7 | 1559.7442 | 0.0864 | WSPELAAACEIWK |
| 64.7 | 1559.7442 | 0.0864 | WSPELAAACEIWK |

**Mascot:** http://www.matrixscience.com/

# MATRIX SCIENCE Mascot Search Results

## Peptide View Spot no 82

MS/MS Fragmentation of **TFQGPPHGIQVERDK**
Found in **gi|11466795** in **NCBInr**, ribulose-1,5-bisphosphate carboxylase/oxygenase large subunit [Oryza sativa Japonica Group]

Match to Query 31: 1707.967824 from(1708.975100,1+) intensity(0.0000) index(18)
Title: Label: C11, Spot_Id: 216639, Peak_List_Id: 221209, MSMS Job_Run_Id: 21345, Comment:
Data file C:\Documents and Settings\Administrator\Desktop\farah BHU\RAJESH\12-1-13 raj\ppw_C11_135997697400.txt

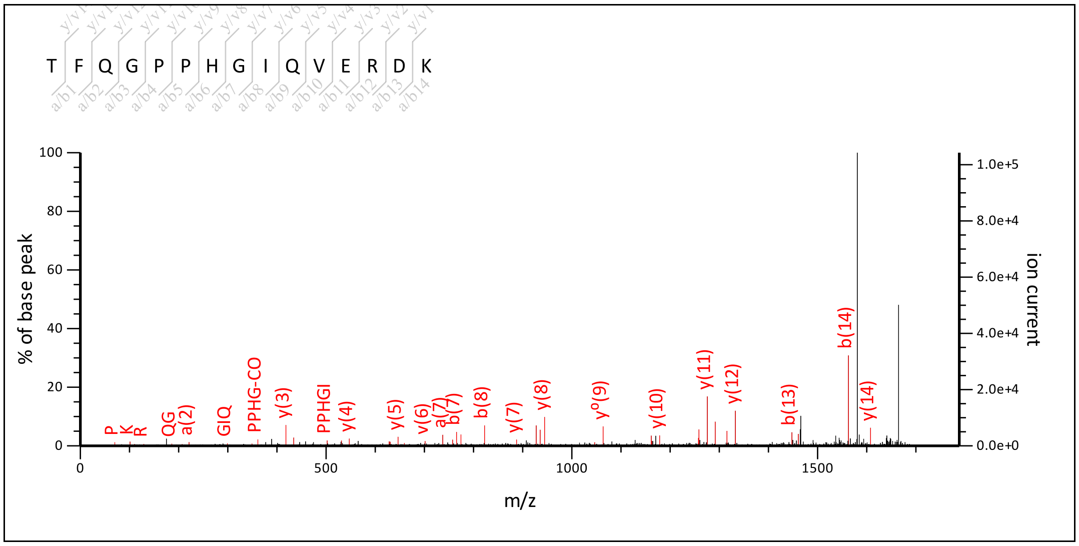


0 to 1788.28

Label all possible matches ○ Label matches used for scoring ◉

**Monoisotopic mass of neutral peptide Mr(calc):** 1707.8693
**Fixed modifications:** Carbamidomethyl (C) (apply to specified residues or termini only)
**Ions Score:** 86 **Expect:** 1.5e-05
**Matches :** 57/273 fragment ions using 68 most intense peaks (help)

| # | Immon. | a | a* | $a^0$ | b | b* | $b^0$ | d | d' | Seq. | v | w | w' | y | y* | |
|---|---|---|---|---|---|---|---|---|---|---|---|---|---|---|---|---|
| 1 | 74.0600 | 74.0600 | | 56.0495 | 102.0550 | | 84.0444 | 44.0495 | | T | | | | | | |
| 2 | 120.0808 | **221.1285** | | 203.1179 | 249.1234 | | 231.1128 | | | F | 1515.7663 | | | ***1607.8289*** | 1590.8023 | 1589 |
| 3 | 101.0709 | **349.1870** | 332.1605 | 331.1765 | 377.1819 | 360.1554 | 359.1714 | 292.1656 | | Q | 1387.7077 | 1386.7124 | | ***1460.7605*** | 1443.7339 | 1442 |
| 4 | 30.0338 | 406.2085 | 389.1819 | 388.1979 | ***434.2034*** | 417.1769 | 416.1928 | | | G | | | | ***1332.7019*** | 1315.6753 | 1314 |
| 5 | 70.0651 | 503.2613 | 486.2347 | 485.2507 | ***531.2562*** | 514.2296 | 513.2456 | 477.2456 | | P | 1233.6335 | 1232.6382 | | ***1275.6804*** | 1258.6539 | 1257 |
| 6 | 70.0651 | 600.3140 | 583.2875 | 582.3035 | ***628.3089*** | 611.2824 | 610.2984 | 574.2984 | | P | 1136.5807 | 1135.5854 | | ***1178.6276*** | 1161.6011 | 1160 |
| 7 | 110.0713 | **737.3729** | 720.3464 | 719.3624 | ***765.3679*** | 748.3413 | 747.3573 | | | H | 999.5218 | | | 1081.5749 | 1064.5483 | 1063 |
| 8 | 30.0338 | 794.3944 | 777.3679 | 776.3838 | ***822.3893*** | 805.3628 | 804.3787 | | | G | | | | ***944.5160*** | 927.4894 | 926 |
| 9 | 86.0964 | 907.4785 | 890.4519 | 889.4679 | ***935.4734*** | 918.4468 | 917.4628 | 879.4472 | 893.4628 | I | 829.4163 | 842.4367 | 856.4523 | ***887.4945*** | 870.4680 | 869 |
| 10 | 101.0709 | 1035.5370 | 1018.5105 | 1017.5265 | ***1063.5320*** | 1046.5054 | 1045.5214 | 978.5156 | | Q | 701.3577 | 700.3624 | | ***774.4104*** | 757.3839 | 756 |
| 11 | 72.0808 | 1134.6055 | 1117.5789 | 1116.5949 | ***1162.6004*** | 1145.5738 | 1144.5898 | 1120.5898 | | V | 602.2893 | 615.3097 | | ***646.3519*** | 629.3253 | 628 |
| 12 | 102.0550 | 1263.6480 | 1246.6215 | 1245.6375 | ***1291.6430*** | 1274.6164 | 1273.6324 | 1205.6426 | | E | 473.2467 | 472.2514 | | ***547.2835*** | 530.2569 | 529 |
| 13 | 129.1135 | 1419.7492 | 1402.7226 | 1401.7386 | ***1447.7441*** | 1430.7175 | 1429.7335 | 1334.6852 | | R | 317.1456 | 316.1503 | | ***418.2409*** | 401.2143 | 400 |
| 14 | 88.0393 | 1534.7761 | 1517.7496 | 1516.7655 | ***1562.7710*** | 1545.7445 | 1544.7605 | 1490.7863 | | D | 202.1186 | 201.1234 | | 262.1397 | 245.1132 | 244 |
| 15 | 101.1073 | | | | | | | | | K | 74.0237 | 73.0284 | | 147.1128 | 130.0863 | |

| Seq | ya | yb | Seq | ya | yb | Seq | ya | yb |
|---|---|---|---|---|---|---|---|---|
| FQ | 248.1394 | 276.1343 | FQG | 305.1608 | 333.1557 | FQGP | 402.2136 | 430.2085 |
| FQGPP | 499.2663 | 527.2613 | FQGPPH | 636.3253 | 664.3202 | FQGPPHG | 693.3467 | 721.3416 |
| QG | 158.0924 | 186.0873 | QGP | 255.1452 | 283.1401 | QGPP | 352.1979 | 380.1928 |
| QGPPH | 489.2568 | 517.2518 | QGPPHG | 546.2783 | 574.2732 | QGPPHGI | 659.3624 | 687.3573 |
| GP | 127.0866 | 155.0815 | GPP | 224.1394 | 252.1343 | GPPH | 361.1983 | 389.1932 |
| GPPHG | 418.2197 | 446.2146 | GPPHGI | 531.3038 | 559.2987 | GPPHGIQ | 659.3624 | 687.3573 |

| | | | | | | | | |
|---|---|---|---|---|---|---|---|---|
| **PP** | 167.1179 | 195.1128 | **PPH** | 304.1768 | 332.1717 | **PPHG** | 361.1983 | 389.1932 |
| **PPHGI** | 474.2823 | 502.2772 | **PPHGIQ** | 602.3409 | 630.3358 | **PH** | 207.1240 | 235.1190 |
| **PHG** | 264.1455 | 292.1404 | **PHGI** | 377.2296 | 405.2245 | **PHGIQ** | 505.2881 | 533.2831 |
| **PHGIQV** | 604.3566 | 632.3515 | **HG** | 167.0927 | 195.0877 | **HGI** | 280.1768 | 308.1717 |
| **HGIQ** | 408.2354 | 436.2303 | **HGIQV** | 507.3038 | 535.2987 | **HGIQVE** | 636.3464 | 664.3413 |
| **GI** | 143.1179 | 171.1128 | **GIQ** | 271.1765 | 299.1714 | **GIQV** | 370.2449 | 398.2398 |
| **GIQVE** | 499.2875 | 527.2824 | **GIQVER** | 655.3886 | 683.3835 | **IQ** | 214.1550 | 242.1499 |
| **IQV** | 313.2234 | 341.2183 | **IQVE** | 442.2660 | 470.2609 | **IQVER** | 598.3671 | 626.3620 |
| **QV** | 200.1394 | 228.1343 | **QVE** | 329.1819 | 357.1769 | **QVER** | 485.2831 | 513.2780 |
| **QVERD** | 600.3100 | 628.3049 | **VE** | 201.1234 | 229.1183 | **VER** | 357.2245 | 385.2194 |
| **VERD** | 472.2514 | 500.2463 | **ER** | 258.1561 | 286.1510 | **ERD** | 373.1830 | 401.1779 |
| **RD** | 244.1404 | 272.1353 | | | | | | |

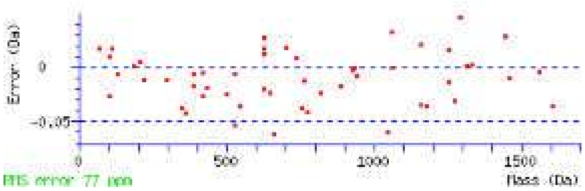


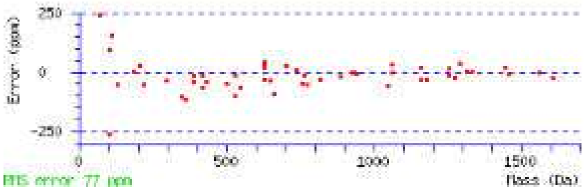


NCBI **BLAST** search of TFQGPPHGIQVERDK
(Parameters: blastp, nr protein database, expect=20000, no filter, PAM30)
Other BLAST web gateways

**All matches to this query**

| Score | Mr(calc) | Delta | Sequence |
|---|---|---|---|
| 90.9 | 1707.8805 | 0.0873 | TFQGPPHGIQVERSR |
| 87.4 | 1707.8363 | 0.1316 | TMQGPPHGIQVERDK |
| 87.4 | 1707.8693 | 0.0985 | TFQGPPHGLQVERDK |
| 87.4 | 1707.8693 | 0.0985 | TFQGPPHGLQVERDK |
| 85.9 | 1707.8693 | 0.0985 | TFQGPPHGIQVERDK |
| 85.9 | 1707.8693 | 0.0985 | TFQGPPHGIQVERDK |
| 85.9 | 1707.8693 | 0.0985 | TFQGPPHGIQVERDK |
| 85.9 | 1707.8693 | 0.0985 | TFQGPPHGIQVERDK |
| 85.9 | 1707.8693 | 0.0985 | TFQGPPHGIQVERDK |
| 85.9 | 1707.8693 | 0.0985 | TFQGPPHGIQVERDK |

**Mascot:** http://www.matrixscience.com/

# MATRIX SCIENCE Mascot Search Results

## Peptide View Spot no 82

MS/MS Fragmentation of **SACKWSPELAAACEIWK**
Found in **gi|11466795** in **NCBInr**, ribulose-1,5-bisphosphate carboxylase/oxygenase large subunit [Oryza sativa Japonica Group]

Match to Query 35: 2006.040424 from(2007.047700,1+) intensity(0.0000) index(20)
Title: Label: C11, Spot_Id: 216639, Peak_List_Id: 221203, MSMS Job_Run_Id: 21345, Comment:
Data file C:\Documents and Settings\Administrator\Desktop\farah BHU\RAJESH\12-1-13 raj\ppw_C11_135997697400.txt

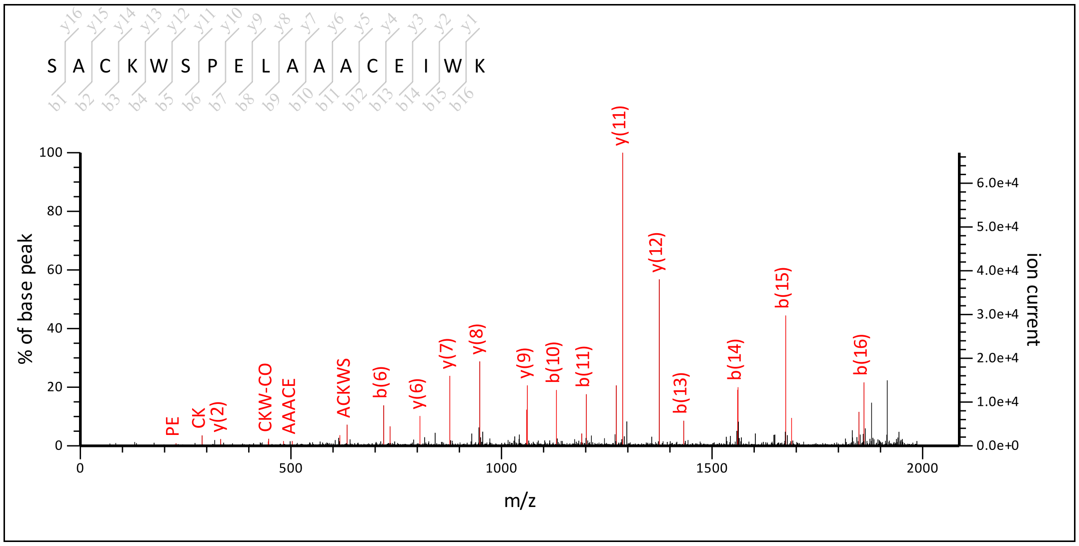


0 to 2087.04

Label all possible matches ○ Label matches used for scoring ◉

**Monoisotopic mass of neutral peptide Mr(calc):** 2005.9390
**Fixed modifications:** Carbamidomethyl (C) (apply to specified residues or termini only)
**Ions Score:** 111 **Expect:** 3.3e-08
**Matches :** 36/305 fragment ions using 40 most intense peaks (help)

| # | Immon. | a | a* | a$^0$ | b | b* | b$^0$ | d | d' | Seq. | v | w | w' | y | y* | |
|---|---|---|---|---|---|---|---|---|---|---|---|---|---|---|---|---|
| 1 | 60.0444 | 60.0444 | | 42.0338 | 88.0393 | | 70.0287 | 44.0495 | | S | | | | | | |
| 2 | 44.0495 | 131.0815 | | 113.0709 | 159.0764 | | 141.0659 | | | A | 1903.8830 | | | 1919.9143 | 1902.8877 | 19[illegible] |
| 3 | 133.0430 | 291.1122 | | 273.1016 | 319.1071 | | 301.0965 | 202.1186 | | C | 1743.8523 | 1742.8571 | | ***1848.8771*** | 1831.8506 | 18[illegible] |
| 4 | 101.1073 | 419.2071 | 402.1806 | 401.1966 | ***447.2020*** | 430.1755 | 429.1915 | 362.1493 | | K | 1615.7573 | 1614.7621 | | ***1688.8465*** | 1671.8199 | 16[illegible] |
| 5 | 159.0917 | 605.2864 | 588.2599 | 587.2759 | ***633.2813*** | 616.2548 | 615.2708 | | | W | 1429.6780 | | | ***1560.7515*** | 1543.7250 | 15[illegible] |
| 6 | 60.0444 | 692.3185 | 675.2919 | 674.3079 | ***720.3134*** | 703.2868 | 702.3028 | 676.3235 | | S | 1342.6460 | 1341.6508 | | ***1374.6722*** | 1357.6457 | 13[illegible] |
| 7 | 70.0651 | 789.3712 | 772.3447 | 771.3607 | 817.3661 | 800.3396 | 799.3556 | 763.3556 | | P | 1245.5932 | 1244.5980 | | ***1287.6402*** | 1270.6136 | 12[illegible] |
| 8 | 102.0550 | 918.4138 | 901.3873 | 900.4032 | 946.4087 | 929.3822 | 928.3982 | 860.4083 | | E | 1116.5506 | 1115.5554 | | ***1190.5874*** | 1173.5609 | 11[illegible] |
| 9 | 86.0964 | 1031.4979 | 1014.4713 | 1013.4873 | ***1059.4928*** | 1042.4662 | 1041.4822 | 989.4509 | | L | 1003.4666 | 1002.4713 | | ***1061.5448*** | 1044.5183 | 10[illegible] |
| 10 | 44.0495 | 1102.5350 | 1085.5084 | 1084.5244 | ***1130.5299*** | 1113.5034 | 1112.5193 | | | A | 932.4295 | | | ***948.4608*** | 931.4342 | 9[illegible] |
| 11 | 44.0495 | 1173.5721 | 1156.5456 | 1155.5615 | ***1201.5670*** | 1184.5405 | 1183.5565 | | | A | 861.3924 | | | ***877.4237*** | 860.3971 | 8[illegible] |
| 12 | 44.0495 | 1244.6092 | 1227.5827 | 1226.5987 | ***1272.6041*** | 1255.5776 | 1254.5936 | | | A | 790.3552 | | | ***806.3865*** | 789.3600 | 7[illegible] |
| 13 | 133.0430 | 1404.6399 | 1387.6133 | 1386.6293 | ***1432.6348*** | 1415.6082 | 1414.6242 | 1315.6463 | | C | 630.3246 | 629.3293 | | ***735.3494*** | 718.3229 | 7[illegible] |
| 14 | 102.0550 | 1533.6825 | 1516.6559 | 1515.6719 | ***1561.6774*** | 1544.6508 | 1543.6668 | 1475.6770 | | E | 501.2820 | 500.2867 | | 575.3188 | 558.2922 | 5[illegible] |
| 15 | 86.0964 | 1646.7665 | 1629.7400 | 1628.7560 | ***1674.7614*** | 1657.7349 | 1656.7509 | 1618.7352 | 1632.7509 | I | 388.1979 | 401.2183 | 415.2340 | ***446.2762*** | 429.2496 | |
| 16 | 159.0917 | 1832.8458 | 1815.8193 | 1814.8353 | ***1860.8408*** | 1843.8142 | 1842.8302 | | | W | 202.1186 | | | ***333.1921*** | 316.1656 | |
| 17 | 101.1073 | | | | | | | | | K | 74.0237 | 73.0284 | | 147.1128 | 130.0863 | |

| Seq | ya | yb | Seq | ya | yb | Seq | ya | yb |
|---|---|---|---|---|---|---|---|---|
| AC | 204.0801 | 232.0750 | ACK | 332.1751 | 360.1700 | ACKW | 518.2544 | 546.2493 |
| ACKWS | 605.2864 | 633.2813 | CK | 261.1380 | 289.1329 | CKW | 447.2173 | 475.2122 |
| CKWS | 534.2493 | 562.2442 | CKWSP | 631.3021 | 659.2970 | KW | 287.1866 | 315.1816 |
| KWS | 374.2187 | 402.2136 | KWSP | 471.2714 | 499.2663 | KWSPE | 600.3140 | 628.3089 |

| | | | | | | | | |
|---|---|---|---|---|---|---|---|---|
| **WS** | 246.1237 | 274.1186 | **WSP** | 343.1765 | 371.1714 | **WSPE** | 472.2191 | 500.2140 |
| **WSPEL** | 585.3031 | 613.2980 | **WSPELA** | 656.3402 | 684.3352 | **SP** | 157.0972 | 185.0921 |
| **SPE** | 286.1397 | 314.1347 | **SPEL** | 399.2238 | 427.2187 | **SPELA** | 470.2609 | 498.2558 |
| **SPELAA** | 541.2980 | 569.2930 | **SPELAAA** | 612.3352 | 640.3301 | **PE** | 199.1077 | 227.1026 |
| **PEL** | 312.1918 | 340.1867 | **PELA** | 383.2289 | 411.2238 | **PELAA** | 454.2660 | 482.2609 |
| **PELAAA** | 525.3031 | 553.2980 | **PELAAAC** | 685.3338 | 713.3287 | **EL** | 215.1390 | 243.1339 |
| **ELA** | 286.1761 | 314.1710 | **ELAA** | 357.2132 | 385.2082 | **ELAAA** | 428.2504 | 456.2453 |
| **ELAAAC** | 588.2810 | 616.2759 | **LA** | 157.1335 | 185.1285 | **LAA** | 228.1707 | 256.1656 |
| **LAAA** | 299.2078 | 327.2027 | **LAAAC** | 459.2384 | 487.2333 | **LAAACE** | 588.2810 | 616.2759 |
| **AA** | 115.0866 | 143.0815 | **AAA** | 186.1237 | 214.1186 | **AAAC** | 346.1544 | 374.1493 |
| **AAACE** | 475.1969 | 503.1919 | **AAACEI** | 588.2810 | 616.2759 | **AA** | 115.0866 | 143.0815 |
| **AAC** | 275.1172 | 303.1122 | **AACE** | 404.1598 | 432.1547 | **AACEI** | 517.2439 | 545.2388 |
| **AC** | 204.0801 | 232.0750 | **ACE** | 333.1227 | 361.1176 | **ACEI** | 446.2068 | 474.2017 |
| **ACEIW** | 632.2861 | 660.2810 | **CE** | 262.0856 | 290.0805 | **CEI** | 375.1697 | 403.1646 |
| **CEIW** | 561.2490 | 589.2439 | **EI** | 215.1390 | 243.1339 | **EIW** | 401.2183 | 429.2132 |
| **IW** | 272.1757 | 300.1707 | | | | | | |

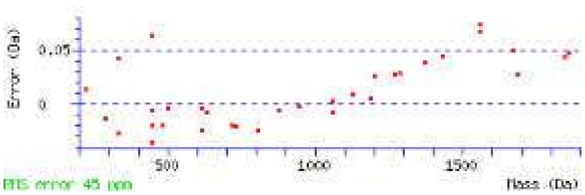


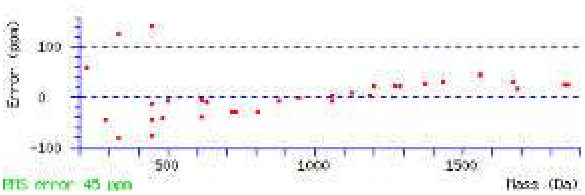


NCBI **BLAST** search of SACKWSPELAAACEIWK
(Parameters: blastp, nr protein database, expect=20000, no filter, PAM30)
Other BLAST web gateways

**All matches to this query**

| Score | Mr(calc) | Delta | Sequence |
|---|---|---|---|
| 110.6 | 2005.9390 | 0.1015 | SACKWSPELAAACEIWK |
| 79.8 | 2006.8866 | -0.8462 | SACKWSPELAAACEIWE |
| 66.8 | 2004.9615 | 1.0790 | ETSKWSPELAAACEIWK |
| 64.9 | 2006.8866 | -0.8462 | EACSWSPELAAACEIWK |
| 27.4 | 2004.9977 | 1.0428 | NSSRSYHIIAASESQSLR |
| 22.7 | 2007.0380 | -0.9976 | MVSLLLETMSVDVAITNR |
| 19.4 | 2005.0819 | 0.9585 | AAPFGFLLKIVECLQSGR |
| 18.5 | 2005.9844 | 0.0560 | KDDDDDLLISSSGLSIWK |
| 14.6 | 2006.0334 | 0.0071 | QKTGASFILPASAQHGHEK |
| 14.3 | 2005.9449 | 0.0956 | SGVGCKMEIVPETEQGSAK |

**Mascot:** http://www.matrixscience.com/

# MATRIX SCIENCE Mascot Search Results

## Peptide View Spot no 82

MS/MS Fragmentation of **GGLDFTKDDENVNSQPFMR**
Found in **gi|11466795** in **NCBInr**, ribulose-1,5-bisphosphate carboxylase/oxygenase large subunit [Oryza sativa Japonica Group]

Match to Query 39: 2169.091824 from(2170.099100,1+) intensity(0.0000) index(22)
Title: Label: C11, Spot_Id: 216639, Peak_List_Id: 221200, MSMS Job_Run_Id: 21345, Comment:
Data file C:\Documents and Settings\Administrator\Desktop\farah BHU\RAJESH\12-1-13 raj\ppw_C11_135997697400.txt

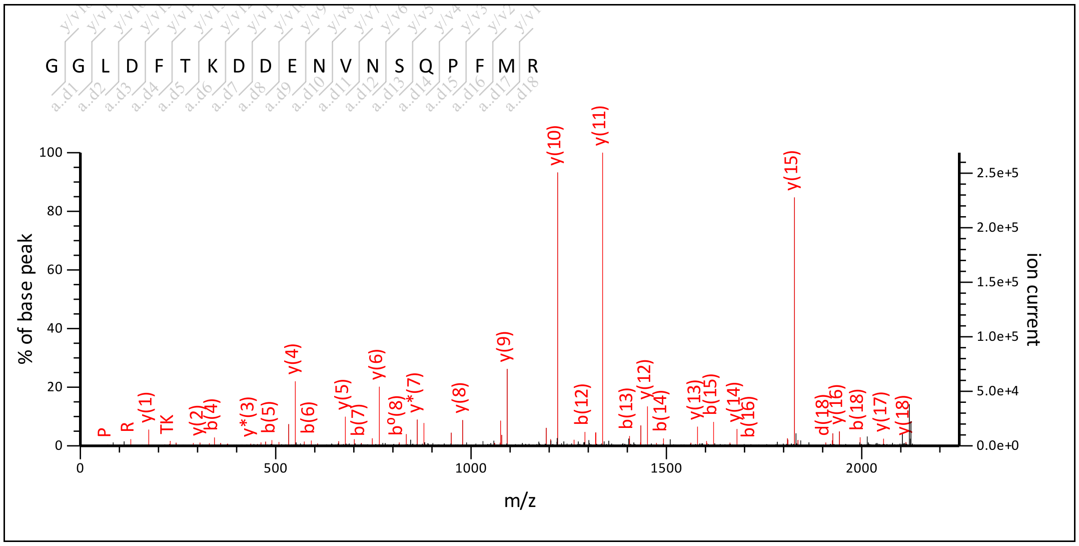


0 to 2249.63

Label all possible matches ○ Label matches used for scoring ◉

**Monoisotopic mass of neutral peptide Mr(calc):** 2168.9797
**Fixed modifications:** Carbamidomethyl (C) (apply to specified residues or termini only)
**Ions Score:** 144 **Expect:** 2.3e-11
**Matches :** 87/340 fragment ions using 105 most intense peaks (help)

| # | Immon. | a | a* | $a^0$ | b | b* | $b^0$ | d | d' | Seq. | v | w | w' | y | y* | |
|---|---|---|---|---|---|---|---|---|---|---|---|---|---|---|---|---|
| 1 | 30.0338 | 30.0338 | | | 58.0287 | | | 44.0495 | | G | | | | | | |
| 2 | 30.0338 | 87.0553 | | | 115.0502 | | | | | G | | | | ***2112.9655*** | 2095.9389 | 20 |
| 3 | 86.0964 | 200.1394 | | | **228.1343** | | | 158.0924 | | L | 1997.8658 | 1996.8705 | | ***2055.9440*** | 2038.9175 | 20 |
| 4 | 88.0393 | 315.1663 | | 297.1557 | **343.1612** | | 325.1506 | 271.1765 | | D | 1882.8388 | 1881.8436 | | ***1942.8600*** | 1925.8334 | 192 |
| 5 | 120.0808 | 462.2347 | | 444.2241 | **490.2296** | | 472.2191 | | | F | 1735.7704 | | | ***1827.8330*** | 1810.8065 | 18 |
| 6 | 74.0600 | 563.2824 | | 545.2718 | **591.2773** | | 573.2667 | 547.2875 | 549.2667 | T | 1634.7227 | 1647.7431 | 1649.7224 | ***1680.7646*** | 1663.7381 | 16 |
| 7 | 101.1073 | 691.3774 | 674.3508 | 673.3668 | **719.3723** | 702.3457 | 701.3617 | 634.3195 | | K | 1506.6278 | 1505.6325 | | ***1579.7169*** | 1562.6904 | 15 |
| 8 | 88.0393 | 806.4043 | 789.3777 | 788.3937 | **834.3992** | 817.3727 | 816.3886 | 762.4145 | | D | 1391.6008 | 1390.6056 | | ***1451.6220*** | 1434.5954 | 14 |
| 9 | 88.0393 | 921.4312 | 904.4047 | 903.4207 | **949.4262** | 932.3996 | 931.4156 | 877.4414 | | D | 1276.5739 | 1275.5786 | | ***1336.5950*** | 1319.5685 | 13 |
| 10 | 102.0550 | 1050.4738 | 1033.4473 | 1032.4633 | **1078.4687** | 1061.4422 | 1060.4582 | 992.4684 | | E | 1147.5313 | 1146.5361 | | ***1221.5681*** | 1204.5415 | 12 |
| 11 | 87.0553 | 1164.5168 | 1147.4902 | 1146.5062 | **1192.5117** | 1175.4851 | 1174.5011 | 1121.5109 | | N | 1033.4884 | 1032.4931 | | ***1092.5255*** | 1075.4989 | 10 |
| 12 | 72.0808 | 1263.5852 | 1246.5586 | 1245.5746 | **1291.5801** | 1274.5535 | 1273.5695 | 1249.5695 | | V | 934.4200 | 947.4404 | | ***978.4826*** | 961.4560 | 9 |
| 13 | 87.0553 | 1377.6281 | 1360.6016 | 1359.6175 | **1405.6230** | 1388.5965 | 1387.6124 | 1334.6223 | | N | 820.3770 | 819.3818 | | ***879.4141*** | 862.3876 | 8 |
| 14 | 60.0444 | 1464.6601 | 1447.6336 | 1446.6496 | **1492.6550** | 1475.6285 | 1474.6445 | 1448.6652 | | S | 733.3450 | 732.3498 | | ***765.3712*** | 748.3447 | 7 |
| 15 | 101.0709 | 1592.7187 | 1575.6922 | 1574.7081 | **1620.7136** | 1603.6871 | 1602.7031 | 1535.6972 | | Q | 605.2864 | 604.2912 | | ***678.3392*** | 661.3126 | |
| 16 | 70.0651 | 1689.7715 | 1672.7449 | 1671.7609 | **1717.7664** | 1700.7398 | 1699.7558 | 1663.7558 | | P | 508.2337 | 507.2384 | | ***550.2806*** | 533.2541 | |
| 17 | 120.0808 | 1836.8399 | 1819.8133 | 1818.8293 | 1864.8348 | 1847.8082 | 1846.8242 | | | F | 361.1653 | | | ***453.2279*** | 436.2013 | |
| 18 | 104.0528 | 1967.8804 | 1950.8538 | 1949.8698 | **1995.8753** | 1978.8487 | 1977.8647 | 1907.8770 | | M | 230.1248 | 229.1295 | | ***306.1594*** | 289.1329 | |
| 19 | 129.1135 | | | | | | | | | R | 74.0237 | 73.0284 | | ***175.1190*** | 158.0924 | |

| Seq | ya | yb | Seq | ya | yb | Seq | ya | yb |
|---|---|---|---|---|---|---|---|---|
| GL | 143.1179 | 171.1128 | GLD | 258.1448 | 286.1397 | GLDF | 405.2132 | 433.2082 |
| GLDFT | 506.2609 | 534.2558 | GLDFTK | 634.3559 | 662.3508 | LD | 201.1234 | 229.1183 |

| | | | | | | | | |
|---|---|---|---|---|---|---|---|---|
| **LDF** | 348.1918 | 376.1867 | **LDFT** | 449.2395 | 477.2344 | **LDFTK** | 577.3344 | 605.3293 |
| **LDFTKD** | 692.3614 | 720.3563 | **DF** | 235.1077 | 263.1026 | **DFT** | 336.1554 | 364.1503 |
| **DFTK** | 464.2504 | 492.2453 | **DFTKD** | 579.2773 | 607.2722 | **DFTKDD** | 694.3042 | 722.2992 |
| **FT** | 221.1285 | 249.1234 | **FTK** | 349.2234 | 377.2183 | **FTKD** | 464.2504 | 492.2453 |
| **FTKDD** | 579.2773 | 607.2722 | **TK** | 202.1550 | 230.1499 | **TKD** | 317.1819 | 345.1769 |
| **TKDD** | 432.2089 | 460.2038 | **TKDDE** | 561.2515 | 589.2464 | **TKDDEN** | 675.2944 | 703.2893 |
| **KD** | 216.1343 | 244.1292 | **KDD** | 331.1612 | 359.1561 | **KDDE** | 460.2038 | 488.1987 |
| **KDDEN** | 574.2467 | 602.2416 | **KDDENV** | 673.3151 | 701.3101 | **DD** | 203.0662 | 231.0612 |
| **DDE** | 332.1088 | 360.1038 | **DDEN** | 446.1518 | 474.1467 | **DDENV** | 545.2202 | 573.2151 |
| **DDENVN** | 659.2631 | 687.2580 | **DE** | 217.0819 | 245.0768 | **DEN** | 331.1248 | 359.1197 |
| **DENV** | 430.1932 | 458.1882 | **DENVN** | 544.2362 | 572.2311 | **DENVNS** | 631.2682 | 659.2631 |
| **EN** | 216.0979 | 244.0928 | **ENV** | 315.1663 | 343.1612 | **ENVN** | 429.2092 | 457.2041 |
| **ENVNS** | 516.2413 | 544.2362 | **ENVNSQ** | 644.2998 | 672.2947 | **NV** | 186.1237 | 214.1186 |
| **NVN** | 300.1666 | 328.1615 | **NVNS** | 387.1987 | 415.1936 | **NVNSQ** | 515.2572 | 543.2522 |
| **NVNSQP** | 612.3100 | 640.3049 | **VN** | 186.1237 | 214.1186 | **VNS** | 273.1557 | 301.1506 |
| **VNSQ** | 401.2143 | 429.2092 | **VNSQP** | 498.2671 | 526.2620 | **VNSQPF** | 645.3355 | 673.3304 |
| **NS** | 174.0873 | 202.0822 | **NSQ** | 302.1459 | 330.1408 | **NSQP** | 399.1987 | 427.1936 |
| **NSQPF** | 546.2671 | 574.2620 | **NSQPFM** | 677.3076 | 705.3025 | **SQ** | 188.1030 | 216.0979 |
| **SQP** | 285.1557 | 313.1506 | **SQPF** | 432.2241 | 460.2191 | **SQPFM** | 563.2646 | 591.2595 |
| **QP** | 198.1237 | 226.1186 | **QPF** | 345.1921 | 373.1870 | **QPFM** | 476.2326 | 504.2275 |
| **PF** | 217.1335 | 245.1285 | **PFM** | 348.1740 | 376.1689 | **FM** | 251.1213 | 279.1162 |

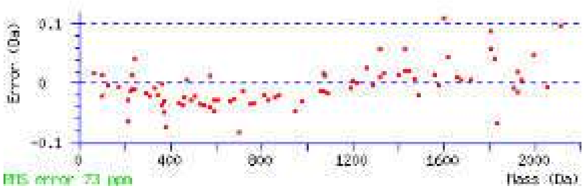


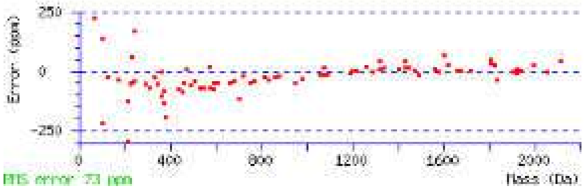


NCBI **BLAST** search of GGLDFTKDDENVNSQPFMR
(Parameters: blastp, nr protein database, expect=20000, no filter, PAM30)
Other BLAST web gateways

**All matches to this query**

| Score | Mr(calc) | Delta | Sequence |
|---|---|---|---|
| 144.9 | 2168.4877 | 0.6041 | GGLDFTKDBENVNSQPFMR |
| 143.5 | 2168.9797 | 0.1121 | GGIDFTKDDENVNSQPFMR |
| 143.5 | 2168.9797 | 0.1121 | GGIDFTKDDENVNSQPFMR |
| 143.5 | 2169.0161 | 0.0758 | GGLDFTKDDENVNSKPFMR |
| 143.5 | 2168.9797 | 0.1121 | GGLDFTKDDENVNSQPFMR |
| 143.5 | 2168.9797 | 0.1121 | GGLDFTKDDENVNSQPFMR |
| 143.5 | 2168.9797 | 0.1121 | GGLDFTKDDENVNSQPFMR |
| 143.5 | 2168.9797 | 0.1121 | GGLDFTKDDENVNSQPFMR |
| 143.5 | 2168.9797 | 0.1121 | GGLDFTKDDENVNSQPFMR |
| 143.5 | 2168.9797 | 0.1121 | GGLDFTKDDENVNSQPFMR |

**Mascot:** http://www.matrixscience.com/

# MATRIX SCIENCE Mascot Search Results

## Peptide View Spot no 82

MS/MS Fragmentation of **GGLDFTKDDENVNSQPFMR**
Found in **gi|11466795** in **NCBInr**, ribulose-1,5-bisphosphate carboxylase/oxygenase large subunit [Oryza sativa Japonica Group]

Match to Query 41: 2185.077224 from(2186.084500,1+) intensity(0.0000) index(23)
Title: Label: C11, Spot_Id: 216639, Peak_List_Id: 221210, MSMS Job_Run_Id: 21345, Comment:
Data file C:\Documents and Settings\Administrator\Desktop\farah BHU\RAJESH\12-1-13 raj\ppw_C11_135997697400.txt

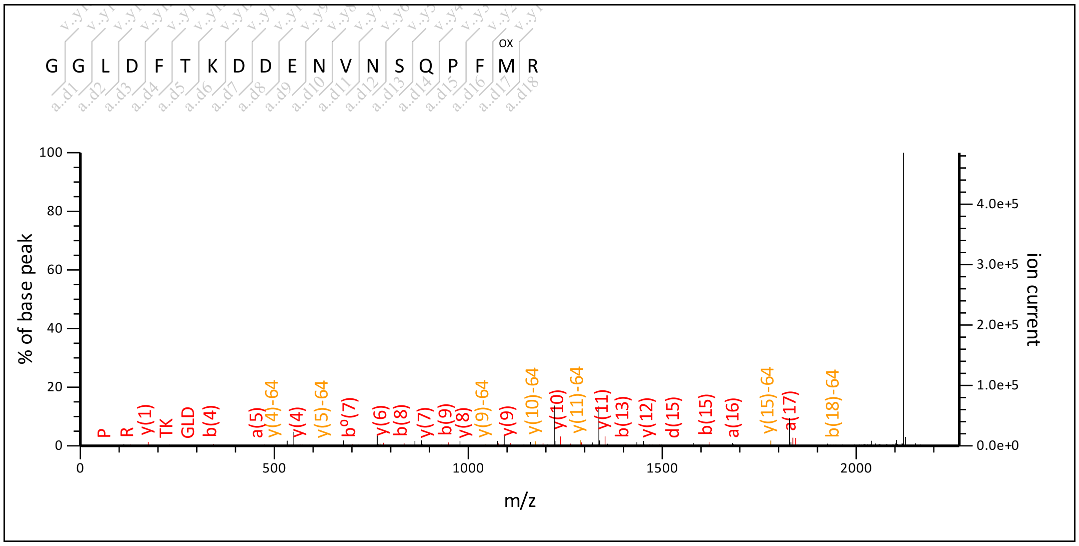


0 to 2265.68

Label all possible matches ○ Label matches used for scoring ◉

**Monoisotopic mass of neutral peptide Mr(calc):** 2184.9746
**Fixed modifications:** Carbamidomethyl (C) (apply to specified residues or termini only)
**Variable modifications:**
**M18** : Oxidation (M), with neutral losses 63.9983(shown in table), 0.0000
**Ions Score:** 42 **Expect:** 0.28
**Matches** : 94/434 fragment ions using 175 most intense peaks (help)

| # | Immon. | a | a* | $a^0$ | b | b* | $b^0$ | d | d' | Seq. | v | w | w' | y | y* | |
|---|---|---|---|---|---|---|---|---|---|---|---|---|---|---|---|---|
| 1 | 30.0338 | 30.0338 | | | 58.0287 | | | 44.0495 | | G | | | | | | |
| 2 | 30.0338 | 87.0553 | | | 115.0502 | | | | | G | | | | 2064.9621 | 2047.9356 | 204[illegible] |
| 3 | 86.0964 | 200.1394 | | | ***228.1343*** | | | 158.0924 | | L | 1949.8624 | 1948.8672 | | 2007.9407 | 1990.9141 | 19[illegible] |
| 4 | 88.0393 | 315.1663 | | 297.1557 | ***343.1612*** | | 325.1506 | 271.1765 | | D | 1834.8355 | 1833.8402 | | 1894.8566 | 1877.8300 | 18[illegible] |
| 5 | 120.0808 | **462.2347** | | 444.2241 | ***490.2296*** | | 472.2191 | | | F | 1687.7671 | | | **1779.8297** | 1762.8031 | 17[illegible] |
| 6 | 74.0600 | 563.2824 | | 545.2718 | ***591.2773*** | | 573.2667 | 547.2875 | 549.2667 | T | 1586.7194 | 1599.7398 | 1601.7190 | 1632.7612 | 1615.7347 | 16[illegible] |
| 7 | 101.1073 | 691.3774 | 674.3508 | 673.3668 | 719.3723 | 702.3457 | 701.3617 | 634.3195 | | K | 1458.6244 | 1457.6292 | | 1531.7136 | 1514.6870 | 15[illegible] |
| 8 | 88.0393 | 806.4043 | 789.3777 | 788.3937 | ***834.3992*** | 817.3727 | 816.3886 | 762.4145 | | D | 1343.5975 | 1342.6022 | | 1403.6186 | 1386.5920 | 13[illegible] |
| 9 | 88.0393 | 921.4312 | 904.4047 | 903.4207 | ***949.4262*** | 932.3996 | 931.4156 | 877.4414 | | D | 1228.5705 | 1227.5753 | | **1288.5917** | 1271.5651 | 12[illegible] |
| 10 | 102.0550 | 1050.4738 | 1033.4473 | 1032.4633 | ***1078.4687*** | 1061.4422 | 1060.4582 | 992.4684 | | E | 1099.5279 | 1098.5327 | | **1173.5647** | 1156.5382 | 11[illegible] |
| 11 | 87.0553 | 1164.5168 | 1147.4902 | 1146.5062 | ***1192.5117*** | 1175.4851 | 1174.5011 | 1121.5109 | | N | 985.4850 | 984.4898 | | **1044.5221** | 1027.4956 | 10[illegible] |
| 12 | 72.0808 | **1263.5852** | 1246.5586 | 1245.5746 | ***1291.5801*** | 1274.5535 | 1273.5695 | 1249.5695 | | V | 886.4166 | 899.4370 | | **930.4792** | 913.4526 | 9[illegible] |
| 13 | 87.0553 | 1377.6281 | 1360.6016 | 1359.6175 | ***1405.6230*** | 1388.5965 | 1387.6124 | 1334.6223 | | N | 772.3737 | 771.3784 | | 831.4108 | 814.3842 | 8[illegible] |
| 14 | 60.0444 | 1464.6601 | 1447.6336 | 1446.6496 | ***1492.6550*** | 1475.6285 | 1474.6445 | 1448.6652 | | S | 685.3416 | 684.3464 | | **717.3679** | 700.3413 | 6[illegible] |
| 15 | 101.0709 | **1592.7187** | 1575.6922 | 1574.7081 | ***1620.7136*** | 1603.6871 | 1602.7031 | 1535.6972 | | Q | 557.2831 | 556.2878 | | **630.3358** | 613.3093 | |
| 16 | 70.0651 | **1689.7715** | 1672.7449 | 1671.7609 | 1717.7664 | 1700.7398 | 1699.7558 | 1663.7558 | | P | 460.2303 | 459.2350 | | **502.2772** | 485.2507 | |
| 17 | 120.0808 | **1836.8399** | 1819.8133 | 1818.8293 | 1864.8348 | 1847.8082 | 1846.8242 | | | F | 313.1619 | | | 405.2245 | 388.1979 | |
| 18 | 56.0495 | 1919.8770 | 1902.8504 | 1901.8664 | ***1947.8719*** | 1930.8454 | 1929.8613 | 1907.8770 | | M | 230.1248 | 229.1295 | | 258.1561 | 241.1295 | |
| 19 | 129.1135 | | | | | | | | | R | 74.0237 | 73.0284 | | **175.1190** | 158.0924 | |

| Seq | ya | yb | Seq | ya | yb | Seq | ya | yb |
|---|---|---|---|---|---|---|---|---|
| GL | 143.1179 | 171.1128 | GLD | 258.1448 | 286.1397 | GLDF | 405.2132 | 433.2082 |

| | | | | | | | | |
|---|---|---|---|---|---|---|---|---|
| **GLDFT** | 506.2609 | 534.2558 | **GLDFTK** | 634.3559 | 662.3508 | **LD** | 201.1234 | 229.1183 |
| **LDF** | 348.1918 | 376.1867 | **LDFT** | 449.2395 | 477.2344 | **LDFTK** | 577.3344 | 605.3293 |
| **LDFTKD** | 692.3614 | 720.3563 | **DF** | 235.1077 | 263.1026 | **DFT** | 336.1554 | 364.1503 |
| **DFTK** | 464.2504 | 492.2453 | **DFTKD** | 579.2773 | 607.2722 | **DFTKDD** | 694.3042 | 722.2992 |
| **FT** | 221.1285 | 249.1234 | **FTK** | 349.2234 | 377.2183 | **FTKD** | 464.2504 | 492.2453 |
| **FTKDD** | 579.2773 | 607.2722 | **TK** | 202.1550 | 230.1499 | **TKD** | 317.1819 | 345.1769 |
| **TKDD** | 432.2089 | 460.2038 | **TKDDE** | 561.2515 | 589.2464 | **TKDDEN** | 675.2944 | 703.2893 |
| **KD** | 216.1343 | 244.1292 | **KDD** | 331.1612 | 359.1561 | **KDDE** | 460.2038 | 488.1987 |
| **KDDEN** | 574.2467 | 602.2416 | **KDDENV** | 673.3151 | 701.3101 | **DD** | 203.0662 | 231.0612 |
| **DDE** | 332.1088 | 360.1038 | **DDEN** | 446.1518 | 474.1467 | **DDENV** | 545.2202 | 573.2151 |
| **DDENVN** | 659.2631 | 687.2580 | **DE** | 217.0819 | 245.0768 | **DEN** | 331.1248 | 359.1197 |
| **DENV** | 430.1932 | 458.1882 | **DENVN** | 544.2362 | 572.2311 | **DENVNS** | 631.2682 | 659.2631 |
| **EN** | 216.0979 | 244.0928 | **ENV** | 315.1663 | 343.1612 | **ENVN** | 429.2092 | 457.2041 |
| **ENVNS** | 516.2413 | 544.2362 | **ENVNSQ** | 644.2998 | 672.2947 | **NV** | 186.1237 | 214.1186 |
| **NVN** | 300.1666 | 328.1615 | **NVNS** | 387.1987 | 415.1936 | **NVNSQ** | 515.2572 | 543.2522 |
| **NVNSQP** | 612.3100 | 640.3049 | **VN** | 186.1237 | 214.1186 | **VNS** | 273.1557 | 301.1506 |
| **VNSQ** | 401.2143 | 429.2092 | **VNSQP** | 498.2671 | 526.2620 | **VNSQPF** | 645.3355 | 673.3304 |
| **NS** | 174.0873 | 202.0822 | **NSQ** | 302.1459 | 330.1408 | **NSQP** | 399.1987 | 427.1936 |
| **NSQPF** | 546.2671 | 574.2620 | **NSQPFM** | 629.3042 | 657.2991 | **SQ** | 188.1030 | 216.0979 |
| **SQP** | 285.1557 | 313.1506 | **SQPF** | 432.2241 | 460.2191 | **SQPFM** | 515.2613 | 543.2562 |
| **QP** | 198.1237 | 226.1186 | **QPF** | 345.1921 | 373.1870 | **QPFM** | 428.2292 | 456.2241 |
| **PF** | 217.1335 | 245.1285 | **PFM** | 300.1707 | 328.1656 | **FM** | 203.1179 | 231.1128 |

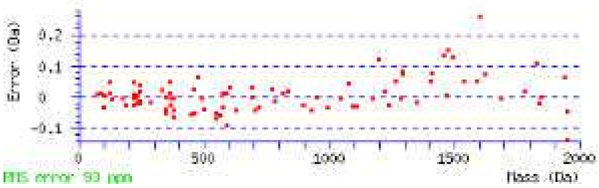


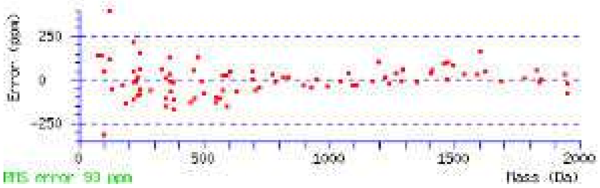


NCBI **BLAST** search of GGLDFTKDDENVNSQPFMR
(Parameters: blastp, nr protein database, expect=20000, no filter, PAM30)
Other BLAST web gateways

**All matches to this query**

| Score | Mr(calc) | Delta | Sequence |
|---|---|---|---|
| 93.7 | 2184.9382 | 0.1390 | GGEDFTKDDENVNSQPFMR |
| 93.7 | 2183.9542 | 1.1230 | GGQDFTKDDENVNSQPFMR |
| 91.4 | 2184.9932 | 0.0840 | GGLMFTKDDENVNSQPFMR |
| 75.7 | 2184.9746 | 0.1026 | GGLDYTKDDENVNSQPFMR |
| 59.2 | 2184.9932 | 0.0840 | GGLDFTKMDENVNSQPFMR |
| 49.7 | 2184.9932 | 0.0840 | GGLDFTKDMENVNSQPFMR |
| 42.5 | 2184.9746 | 0.1026 | GGIDFTKDDENVNSQPFMR |
| 42.5 | 2184.9746 | 0.1026 | GGIDFTKDDENVNSQPFMR |
| 42.5 | 2185.0110 | 0.0662 | GGLDFTKDDENVNSKPFMR |
| 42.5 | 2184.9746 | 0.1026 | GGLDFTKDDENVNSQPFMR |

**Mascot:** http://www.matrixscience.com/

# MATRIX SCIENCE Mascot Search Results

## Peptide View

MS/MS Fragmentation of **LTYYTPEYETKDTDILAAFR**
Found in **gi|11466795** in **NCBInr**, ribulose-1,5-bisphosphate carboxylase/oxygenase large subunit [Oryza sativa Japonica Group]

Match to Query 46: 2409.289324 from(2410.296600,1+) intensity(0.0000) index(26)
Title: Label: C11, Spot_Id: 216639, Peak_List_Id: 221199, MSMS Job_Run_Id: 21345, Comment:
Data file C:\Documents and Settings\Administrator\Desktop\farah BHU\RAJESH\12-1-13 raj\ppw_C11_135997697400.txt

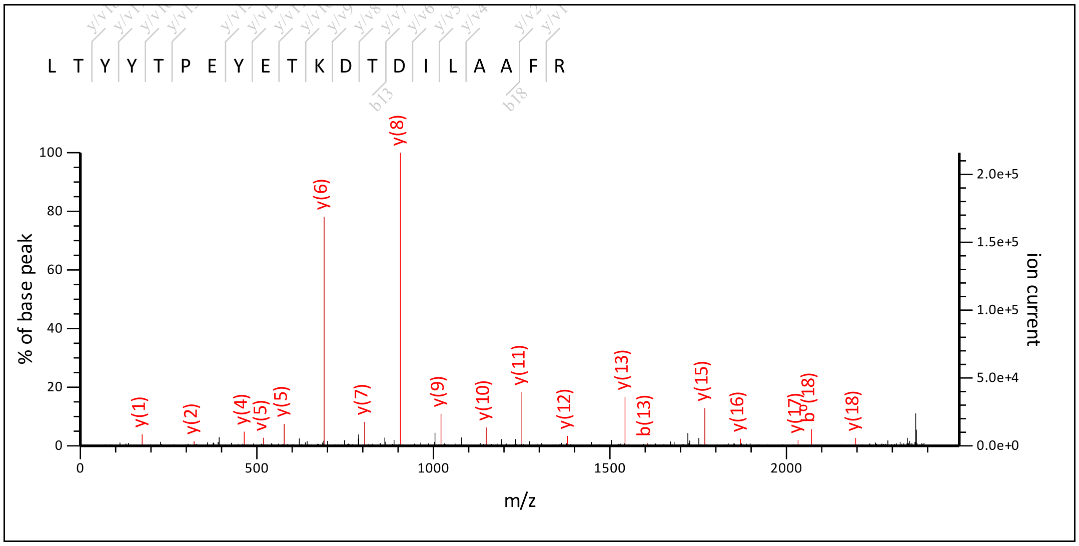


0 to 2489.58

Label all possible matches ○ Label matches used for scoring ◉

**Monoisotopic mass of neutral peptide Mr(calc):** 2409.1740
**Fixed modifications:** Carbamidomethyl (C) (apply to specified residues or termini only)
**Ions Score:** 143 **Expect:** 1.8e-11
**Matches :** 21/356 fragment ions using 24 most intense peaks (help)

| # | Immon. | a | a* | a0 | b | b* | b0 | d | d' | Seq. | v | w | w' | y | y* |
|---|---|---|---|---|---|---|---|---|---|---|---|---|---|---|---|
| 1 | 86.0964 | 86.0964 | | | 114.0913 | | | 44.0495 | | L | | | | | |
| 2 | 74.0600 | 187.1441 | | 169.1335 | 215.1390 | | 197.1285 | 171.1492 | 173.1285 | T | 2251.0554 | 2264.0758 | 2266.0550 | 2297.0972 | 2280.0707 |
| 3 | 136.0757 | 350.2074 | | 332.1969 | 378.2023 | | 360.1918 | | | Y | 2087.9920 | | | ***2196.0495*** | 2179.0230 |
| 4 | 136.0757 | 513.2708 | | 495.2602 | 541.2657 | | 523.2551 | | | Y | 1924.9287 | | | ***2032.9862*** | 2015.9597 |
| 5 | 74.0600 | 614.3184 | | 596.3079 | 642.3134 | | 624.3028 | 598.3235 | 600.3028 | T | 1823.8810 | 1836.9014 | 1838.8807 | ***1869.9229*** | 1852.8963 |
| 6 | 70.0651 | 711.3712 | | 693.3606 | 739.3661 | | 721.3556 | 685.3556 | | P | 1726.8283 | 1725.8330 | | ***1768.8752*** | 1751.8487 |
| 7 | 102.0550 | 840.4138 | | 822.4032 | 868.4087 | | 850.3981 | 782.4083 | | E | 1597.7857 | 1596.7904 | | 1671.8224 | 1654.7959 |
| 8 | 136.0757 | 1003.4771 | | 985.4666 | 1031.4720 | | 1013.4615 | | | Y | 1434.7223 | | | ***1542.7799*** | 1525.7533 |
| 9 | 102.0550 | 1132.5197 | | 1114.5092 | 1160.5146 | | 1142.5041 | 1074.5142 | | E | 1305.6797 | 1304.6845 | | ***1379.7165*** | 1362.6900 |
| 10 | 74.0600 | 1233.5674 | | 1215.5568 | 1261.5623 | | 1243.5517 | 1217.5725 | 1219.5517 | T | 1204.6321 | 1217.6525 | 1219.6317 | ***1250.6739*** | 1233.6474 |
| 11 | 101.1073 | 1361.6624 | 1344.6358 | 1343.6518 | 1389.6573 | 1372.6307 | 1371.6467 | 1304.6045 | | K | 1076.5371 | 1075.5419 | | ***1149.6262*** | 1132.5997 |
| 12 | 88.0393 | 1476.6893 | 1459.6628 | 1458.6787 | 1504.6842 | 1487.6577 | 1486.6737 | 1432.6995 | | D | 961.5102 | 960.5149 | | ***1021.5313*** | 1004.5047 |
| 13 | 74.0600 | 1577.7370 | 1560.7104 | 1559.7264 | 1605.7319 | 1588.7054 | 1587.7213 | 1561.7421 | 1563.7213 | T | 860.4625 | 873.4829 | 875.4621 | ***906.5043*** | 889.4778 |
| 14 | 88.0393 | 1692.7639 | 1675.7374 | 1674.7534 | 1720.7588 | 1703.7323 | 1702.7483 | 1648.7741 | | D | 745.4355 | 744.4403 | | ***805.4567*** | 788.4301 |
| 15 | 86.0964 | 1805.8480 | 1788.8214 | 1787.8374 | 1833.8429 | 1816.8164 | 1815.8323 | 1777.8167 | 1791.8323 | I | 632.3515 | 645.3719 | 659.3875 | ***690.4297*** | 673.4032 |
| 16 | 86.0964 | 1918.9321 | 1901.9055 | 1900.9215 | 1946.9270 | 1929.9004 | 1928.9164 | 1876.8851 | | L | 519.2674 | 518.2722 | | ***577.3457*** | 560.3191 |
| 17 | 44.0495 | 1989.9692 | 1972.9426 | 1971.9586 | 2017.9641 | 2000.9375 | 1999.9535 | | | A | 448.2303 | | | ***464.2616*** | 447.2350 |
| 18 | 44.0495 | 2061.0063 | 2043.9797 | 2042.9957 | 2089.0012 | 2071.9746 | 2070.9906 | | | A | 377.1932 | | | 393.2245 | 376.1979 |
| 19 | 120.0808 | 2208.0747 | 2191.0481 | 2190.0641 | 2236.0696 | 2219.0431 | 2218.0590 | | | F | 230.1248 | | | ***322.1874*** | 305.1608 |
| 20 | 129.1135 | | | | | | | | | R | 74.0237 | 73.0284 | | ***175.1190*** | 158.0924 |

| Seq | ya | yb | Seq | ya | yb | Seq | ya | yb |
|---|---|---|---|---|---|---|---|---|
| TY | 237.1234 | 265.1183 | TYY | 400.1867 | 428.1816 | TYYT | 501.2344 | 529.2293 |

| | | | | | | | | |
|---|---|---|---|---|---|---|---|---|
| TYYTP | 598.2871 | 626.2821 | YY | 299.1390 | 327.1339 | YYT | 400.1867 | 428.1816 |
| YYTP | 497.2395 | 525.2344 | YYTPE | 626.2821 | 654.2770 | YT | 237.1234 | 265.1183 |
| YTP | 334.1761 | 362.1710 | YTPE | 463.2187 | 491.2136 | YTPEY | 626.2821 | 654.2770 |
| TP | 171.1128 | 199.1077 | TPE | 300.1554 | 328.1503 | TPEY | 463.2187 | 491.2136 |
| TPEYE | 592.2613 | 620.2562 | TPEYET | 693.3090 | 721.3039 | PE | 199.1077 | 227.1026 |
| PEY | 362.1710 | 390.1660 | PEYE | 491.2136 | 519.2086 | PEYET | 592.2613 | 620.2562 |
| EY | 265.1183 | 293.1132 | EYE | 394.1609 | 422.1558 | EYET | 495.2086 | 523.2035 |
| EYETK | 623.3035 | 651.2984 | YE | 265.1183 | 293.1132 | YET | 366.1660 | 394.1609 |
| YETK | 494.2609 | 522.2558 | YETKD | 609.2879 | 637.2828 | ET | 203.1026 | 231.0975 |
| ETK | 331.1976 | 359.1925 | ETKD | 446.2245 | 474.2195 | ETKDT | 547.2722 | 575.2671 |
| ETKDTD | 662.2992 | 690.2941 | TK | 202.1550 | 230.1499 | TKD | 317.1819 | 345.1769 |
| TKDT | 418.2296 | 446.2245 | TKDTD | 533.2566 | 561.2515 | TKDTDI | 646.3406 | 674.3355 |
| KD | 216.1343 | 244.1292 | KDT | 317.1819 | 345.1769 | KDTD | 432.2089 | 460.2038 |
| KDTDI | 545.2930 | 573.2879 | KDTDIL | 658.3770 | 686.3719 | DT | 189.0870 | 217.0819 |
| DTD | 304.1139 | 332.1088 | DTDI | 417.1980 | 445.1929 | DTDIL | 530.2821 | 558.2770 |
| DTDILA | 601.3192 | 629.3141 | DTDILAA | 672.3563 | 700.3512 | TD | 189.0870 | 217.0819 |
| TDI | 302.1710 | 330.1660 | TDIL | 415.2551 | 443.2500 | TDILA | 486.2922 | 514.2871 |
| TDILAA | 557.3293 | 585.3243 | DI | 201.1234 | 229.1183 | DIL | 314.2074 | 342.2023 |
| DILA | 385.2445 | 413.2395 | DILAA | 456.2817 | 484.2766 | DILAAF | 603.3501 | 631.3450 |
| IL | 199.1805 | 227.1754 | ILA | 270.2176 | 298.2125 | ILAA | 341.2547 | 369.2496 |
| ILAAF | 488.3231 | 516.3180 | LA | 157.1335 | 185.1285 | LAA | 228.1707 | 256.1656 |
| LAAF | 375.2391 | 403.2340 | AA | 115.0866 | 143.0815 | AAF | 262.1550 | 290.1499 |
| AF | 191.1179 | 219.1128 | | | | | | |

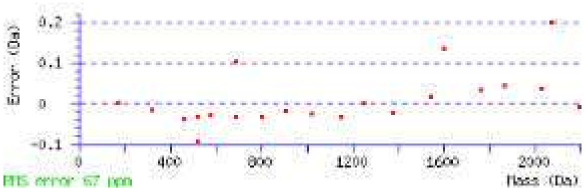


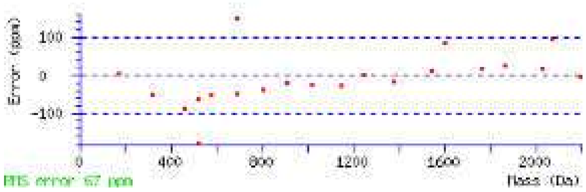


NCBI **BLAST** search of LTYYTPEYETKDTDILAAFR
(Parameters: blastp, nr protein database, expect=20000, no filter, PAM30)
Other BLAST web gateways

**All matches to this query**

| Score | Mr(calc) | Delta | Sequence |
|---|---|---|---|
| 142.9 | 2408.6820 | 0.6073 | ITYYTPEYETKDTBILAAFR |
| 142.9 | 2409.1740 | 0.1153 | ITYYTPEYETKDTDILAAFR |
| 142.9 | 2409.1740 | 0.1153 | ITYYTPEYETKDTDILAAFR |
| 142.9 | 2408.6820 | 0.6073 | LTYYTPEYETKDTBILAAFR |
| 142.9 | 2409.1740 | 0.1153 | LTYYTPEYETKDTDIIAAFR |
| 142.9 | 2409.1740 | 0.1153 | LTYYTPEYETKDTDILAAFR |
| 142.9 | 2409.1740 | 0.1153 | LTYYTPEYETKDTDILAAFR |
| 142.9 | 2409.1740 | 0.1153 | LTYYTPEYETKDTDILAAFR |
| 142.9 | 2409.1740 | 0.1153 | LTYYTPEYETKDTDILAAFR |
| 142.9 | 2409.1740 | 0.1153 | LTYYTPEYETKDTDILAAFR |

**Mascot:** http://www.matrixscience.com/

# Mascot Search Results

## Peptide View **Spot no 84**

MS/MS Fragmentation of **FRAPVEPY**
Found in **gi|780372** in **NCBInr**, enolase [Oryza sativa Japonica Group]

Match to Query 18: 977.573844 from(978.581120,1+) intensity(0.0000) index(5)
Title: Label: J6, Spot_Id: 228931, Peak_List_Id: 257585, MSMS Job_Run_Id: 24927, Comment:
Data file ppw_J6_138985135600.txt

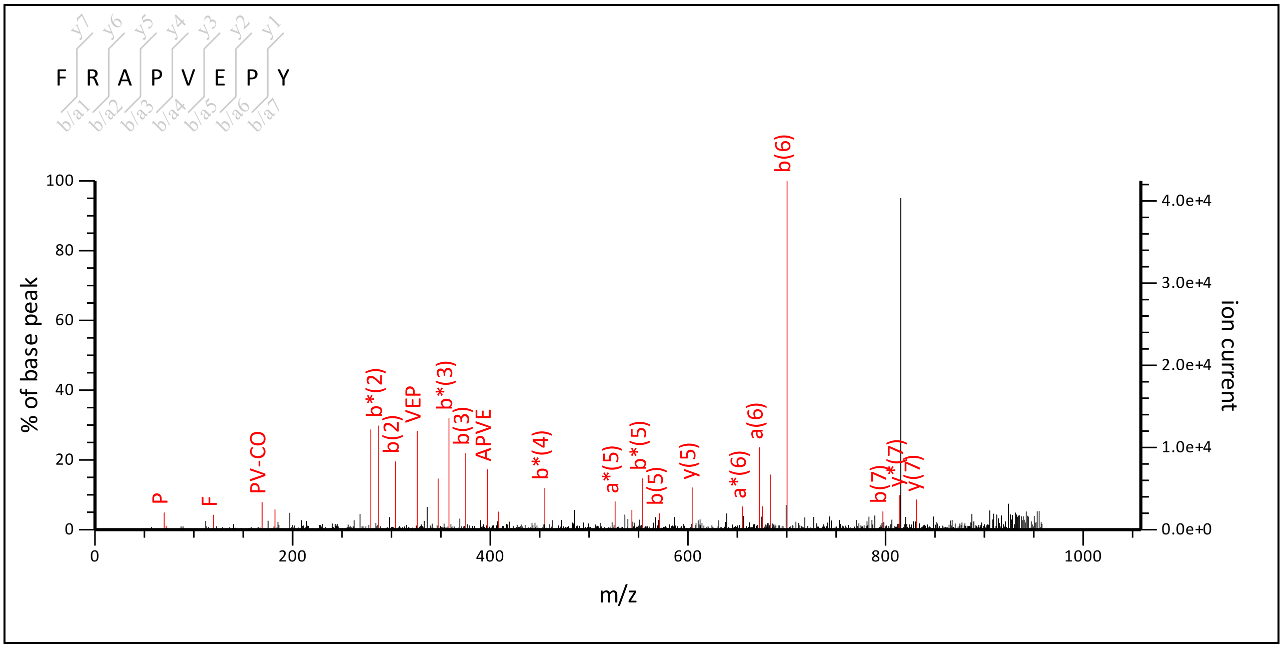


0 to 1058.34

Label all possible matches Label matches used for scoring

**Monoisotopic mass of neutral peptide Mr(calc):** 977.4971
**Fixed modifications:** Carbamidomethyl (C) (apply to specified residues or termini only)
**Ions Score:** 38 **Expect:** 0.4
**Matches :** 33/100 fragment ions using 47 most intense peaks (help)

| # | Immon. | a | a* | $a^0$ | b | b* | $b^0$ | d | Seq. | v | w | y | y* | $y^0$ | # |
|---|---|---|---|---|---|---|---|---|---|---|---|---|---|---|---|
| 1 | 120.0808 | **120.0808** | | | 148.0757 | | | 44.0495 | F | | | | | | 8 |
| 2 | 129.1135 | 276.1819 | 259.1553 | | **304.1768** | 287.1503 | | 191.1179 | R | 730.3406 | 729.3454 | ***831.4359*** | 814.4094 | 813.4254 | 7 |
| 3 | 44.0495 | **347.2190** | 330.1925 | | **375.2139** | 358.1874 | | | A | 659.3035 | | ***675.3348*** | | 657.3243 | 6 |
| 4 | 70.0651 | 444.2718 | 427.2452 | | 472.2667 | 455.2401 | | 418.2561 | P | 562.2508 | 561.2555 | ***604.2977*** | | 586.2871 | 5 |
| 5 | 72.0808 | **543.3402** | 526.3136 | | **571.3351** | 554.3085 | | 529.3245 | V | 463.1823 | 476.2027 | 507.2449 | | 489.2344 | 4 |
| 6 | 102.0550 | **672.3828** | 655.3562 | 654.3722 | **700.3777** | 683.3511 | 682.3671 | 614.3773 | E | 334.1397 | 333.1445 | ***408.1765*** | | 390.1660 | 3 |
| 7 | 70.0651 | 769.4355 | 752.4090 | 751.4250 | **797.4305** | 780.4039 | 779.4199 | 743.4199 | P | 237.0870 | 236.0917 | ***279.1339*** | | | 2 |
| 8 | 136.0757 | | | | | | | | Y | 74.0237 | 73.0284 | ***182.0812*** | | | 1 |

| Seq | ya | yb | Seq | ya | yb | Seq | ya | yb |
|---|---|---|---|---|---|---|---|---|
| RA | 200.1506 | 228.1455 | RAP | 297.2034 | 325.1983 | RAPV | 396.2718 | 424.2667 |
| RAPVE | 525.3144 | 553.3093 | RAPVEP | 622.3671 | 650.3620 | AP | 141.1022 | 169.0972 |
| APV | 240.1707 | 268.1656 | APVE | 369.2132 | 397.2082 | APVEP | 466.2660 | 494.2609 |

| | | | | | | | | |
|---|---|---|---|---|---|---|---|---|
| **PV** | 169.1335 | 197.1285 | **PVE** | 298.1761 | 326.1710 | **PVEP** | 395.2289 | 423.2238 |
| **VE** | 201.1234 | 229.1183 | **VEP** | 298.1761 | 326.1710 | **EP** | 199.1077 | 227.1026 |

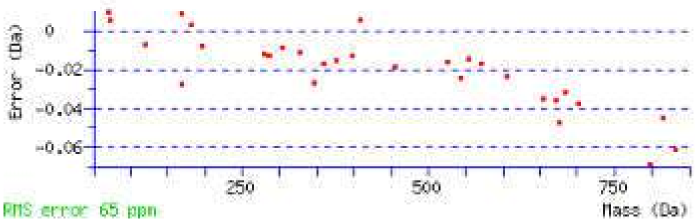


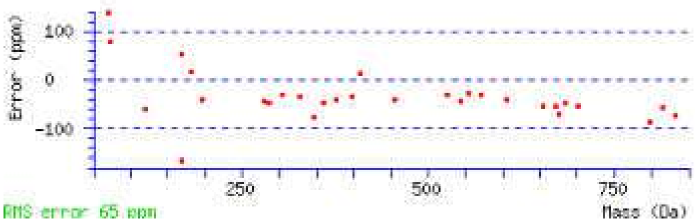


NCBI **BLAST** search of FRAPVEPY
(Parameters: blastp, nr protein database, expect=20000, no filter, PAM30)
Other BLAST web gateways

**All matches to this query**

| Score | Mr(calc) | Delta | Sequence |
|---|---|---|---|
| 37.8 | 977.4971 | 0.0768 | FRAPVEPY |
| 21.6 | 977.5004 | 0.0734 | FMEPVISR |
| 16.6 | 977.5334 | 0.0404 | YPFPVLSR |
| 15.5 | 977.5182 | 0.0557 | NATAFVLDK |
| 11.4 | 977.4818 | 0.0920 | YPEVDLSR |
| 11.3 | 977.5004 | 0.0734 | MYIVPAER |
| 10.7 | 977.5004 | 0.0734 | FMEPLVSR |
| 10.5 | 977.4865 | 0.0873 | MRAGWVSR |
| 10.4 | 977.5433 | 0.0305 | YDLVLDLK |
| 9.8 | 977.5328 | 0.0411 | RASAMVLSK |

**Mascot:** http://www.matrixscience.com/

# MATRIX SCIENCE Mascot Search Results

## Peptide View **Spot no 84**

MS/MS Fragmentation of **AAVPSGASTGVYEALELR**
Found in **gi|780372** in **NCBInr**, enolase [Oryza sativa Japonica Group]

Match to Query 65: 1790.074624 from(1791.081900,1+) intensity(0.0000) index(21)
Title: Label: J6, Spot_Id: 228931, Peak_List_Id: 257588, MSMS Job_Run_Id: 24927, Comment:
Data file ppw_J6_138985135600.txt

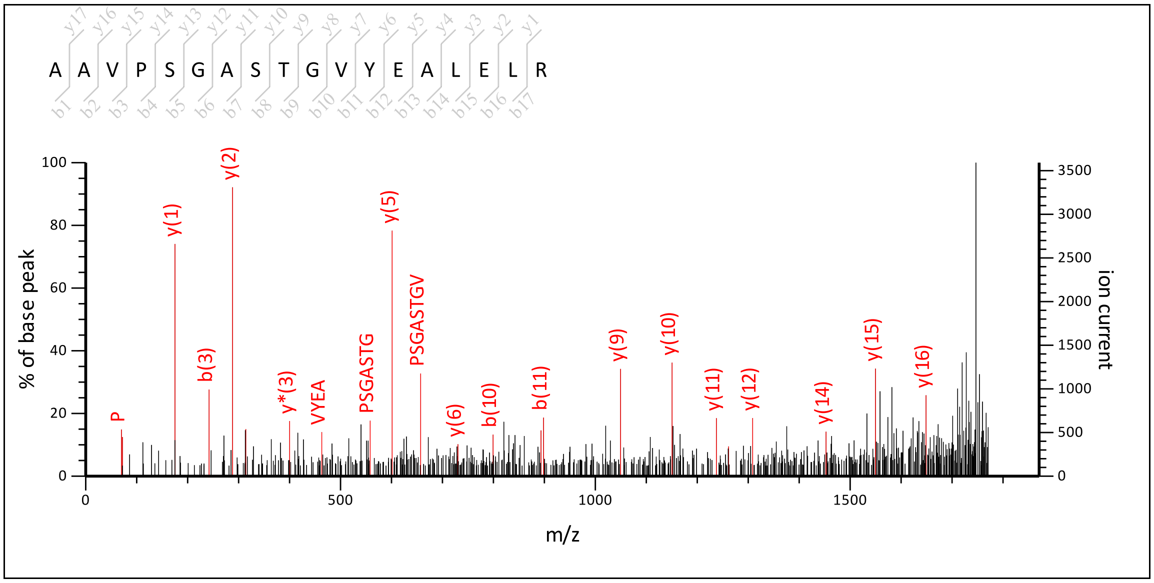


0 to 1870.88

Label all possible matches ○ Label matches used for scoring ●

**Monoisotopic mass of neutral peptide Mr(calc):** 1789.9210
**Fixed modifications:** Carbamidomethyl (C) (apply to specified residues or termini only)
**Ions Score:** 88 **Expect:** 1.7e-06
**Matches :** 29/313 fragment ions using 35 most intense peaks (help)

| # | Immon. | a | $a^0$ | b | $b^0$ | d | d' | Seq. | v | w | w' | y | y* | $y^0$ | # |
|---|---|---|---|---|---|---|---|---|---|---|---|---|---|---|---|
| 1 | 44.0495 | 44.0495 | | **72.0444** | | 44.0495 | | A | | | | | | | 18 |
| 2 | 44.0495 | 115.0866 | | 143.0815 | | | | A | 1703.8599 | | | 1719.8912 | 1702.8646 | 1701.8806 | 17 |
| 3 | 72.0808 | 214.1550 | | **242.1499** | | 200.1394 | | V | 1604.7915 | 1617.8119 | | ***1648.8541*** | 1631.8275 | 1630.8435 | 16 |
| 4 | 70.0651 | 311.2078 | | 339.2027 | | 285.1921 | | P | 1507.7387 | 1506.7435 | | ***1549.7857*** | 1532.7591 | 1531.7751 | 15 |
| 5 | 60.0444 | 398.2398 | 380.2292 | 426.2347 | 408.2241 | 382.2449 | | S | 1420.7067 | 1419.7114 | | ***1452.7329*** | 1435.7064 | 1434.7223 | 14 |
| 6 | 30.0338 | 455.2613 | 437.2507 | 483.2562 | 465.2456 | | | G | | | | 1365.7009 | 1348.6743 | 1347.6903 | 13 |
| 7 | 44.0495 | 526.2984 | 508.2878 | 554.2933 | 536.2827 | | | A | 1292.6481 | | | ***1308.6794*** | 1291.6529 | 1290.6688 | 12 |
| 8 | 60.0444 | 613.3304 | 595.3198 | 641.3253 | 623.3148 | 597.3355 | | S | 1205.6161 | 1204.6208 | | ***1237.6423*** | 1220.6157 | 1219.6317 | 11 |
| 9 | 74.0600 | 714.3781 | 696.3675 | 742.3730 | 724.3624 | 698.3832 | 700.3624 | T | 1104.5684 | 1117.5888 | 1119.5681 | ***1150.6103*** | 1133.5837 | 1132.5997 | 10 |
| 10 | 30.0338 | 771.3995 | 753.3890 | **799.3945** | 781.3839 | | | G | | | | ***1049.5626*** | 1032.5360 | 1031.5520 | 9 |
| 11 | 72.0808 | 870.4680 | 852.4574 | **898.4629** | 880.4523 | 856.4523 | | V | 948.4785 | 961.4989 | | 992.5411 | 975.5146 | 974.5306 | 8 |
| 12 | 136.0757 | 1033.5313 | 1015.5207 | 1061.5262 | 1043.5156 | | | Y | 785.4152 | | | ***893.4727*** | 876.4462 | 875.4621 | 7 |
| 13 | 102.0550 | 1162.5739 | 1144.5633 | 1190.5688 | 1172.5582 | 1104.5684 | | E | 656.3726 | 655.3774 | | ***730.4094*** | 713.3828 | 712.3988 | 6 |
| 14 | 44.0495 | 1233.6110 | 1215.6004 | **1261.6059** | 1243.5953 | | | A | 585.3355 | | | ***601.3668*** | 584.3402 | 583.3562 | 5 |
| 15 | 86.0964 | 1346.6951 | 1328.6845 | 1374.6900 | 1356.6794 | 1304.6481 | | L | 472.2514 | 471.2562 | | 530.3297 | 513.3031 | 512.3191 | 4 |
| 16 | 102.0550 | 1475.7377 | 1457.7271 | 1503.7326 | 1485.7220 | 1417.7322 | | E | 343.2088 | 342.2136 | | 417.2456 | 400.2191 | 399.2350 | 3 |
| 17 | 86.0964 | 1588.8217 | 1570.8111 | 1616.8166 | 1598.8061 | 1546.7748 | | L | 230.1248 | 229.1295 | | ***288.2030*** | 271.1765 | | 2 |
| 18 | 129.1135 | | | | | | | R | 74.0237 | 73.0284 | | ***175.1190*** | 158.0924 | | 1 |

| Seq | ya | yb | Seq | ya | yb | Seq | ya | yb |
|---|---|---|---|---|---|---|---|---|
| AV | 143.1179 | 171.1128 | AVP | 240.1707 | 268.1656 | AVPS | 327.2027 | 355.1976 |
| AVPSG | 384.2241 | 412.2191 | AVPSGA | 455.2613 | 483.2562 | AVPSGAS | 542.2933 | 570.2882 |
| AVPSGAST | 643.3410 | 671.3359 | VP | 169.1335 | 197.1285 | VPS | 256.1656 | 284.1605 |
| VPSG | 313.1870 | 341.1819 | VPSGA | 384.2241 | 412.2191 | VPSGAS | 471.2562 | 499.2511 |
| VPSGAST | 572.3039 | 600.2988 | VPSGASTG | 629.3253 | 657.3202 | PS | 157.0972 | 185.0921 |
| PSG | 214.1186 | 242.1135 | PSGA | 285.1557 | 313.1506 | PSGAS | 372.1878 | 400.1827 |
| PSGAST | 473.2354 | 501.2304 | PSGASTG | 530.2569 | 558.2518 | PSGASTGV | 629.3253 | 657.3202 |
| SG | 117.0659 | 145.0608 | SGA | 188.1030 | 216.0979 | SGAS | 275.1350 | 303.1299 |
| SGAST | 376.1827 | 404.1776 | SGASTG | 433.2041 | 461.1991 | SGASTGV | 532.2726 | 560.2675 |
| SGASTGVY | 695.3359 | 723.3308 | GA | 101.0709 | 129.0659 | GAS | 188.1030 | 216.0979 |
| GAST | 289.1506 | 317.1456 | GASTG | 346.1721 | 374.1670 | GASTGV | 445.2405 | 473.2354 |
| GASTGVY | 608.3039 | 636.2988 | AS | 131.0815 | 159.0764 | AST | 232.1292 | 260.1241 |
| ASTG | 289.1506 | 317.1456 | ASTGV | 388.2191 | 416.2140 | ASTGVY | 551.2824 | 579.2773 |
| ASTGVYE | 680.3250 | 708.3199 | ST | 161.0921 | 189.0870 | STG | 218.1135 | 246.1084 |
| STGV | 317.1819 | 345.1769 | STGVY | 480.2453 | 508.2402 | STGVYE | 609.2879 | 637.2828 |
| STGVYEA | 680.3250 | 708.3199 | TG | 131.0815 | 159.0764 | TGV | 230.1499 | 258.1448 |
| TGVY | 393.2132 | 421.2082 | TGVYE | 522.2558 | 550.2508 | TGVYEA | 593.2930 | 621.2879 |
| GV | 129.1022 | 157.0972 | GVY | 292.1656 | 320.1605 | GVYE | 421.2082 | 449.2031 |
| GVYEA | 492.2453 | 520.2402 | GVYEAL | 605.3293 | 633.3243 | VY | 235.1441 | 263.1390 |
| VYE | 364.1867 | 392.1816 | VYEA | 435.2238 | 463.2187 | VYEAL | 548.3079 | 576.3028 |
| VYEALE | 677.3505 | 705.3454 | YE | 265.1183 | 293.1132 | YEA | 336.1554 | 364.1503 |
| YEAL | 449.2395 | 477.2344 | YEALE | 578.2821 | 606.2770 | YEALEL | 691.3661 | 719.3610 |
| EA | 173.0921 | 201.0870 | EAL | 286.1761 | 314.1710 | EALE | 415.2187 | 443.2136 |
| EALEL | 528.3028 | 556.2977 | AL | 157.1335 | 185.1285 | ALE | 286.1761 | 314.1710 |
| ALEL | 399.2602 | 427.2551 | LE | 215.1390 | 243.1339 | LEL | 328.2231 | 356.2180 |
| EL | 215.1390 | 243.1339 | | | | | | |

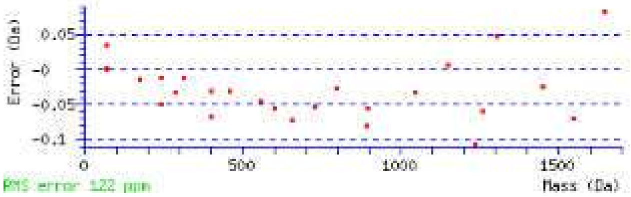


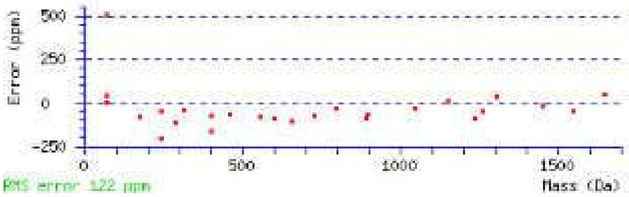


NCBI **BLAST** search of AAVPSGASTGVYEALELR
(Parameters: blastp, nr protein database, expect=20000, no filter, PAM30)
Other BLAST web gateways

**All matches to this query**

| Score | Mr(calc) | Delta | Sequence |
|---|---|---|---|
| 88.3 | 1789.9210 | 0.1536 | AAVPSGASTGVYEALELR |
| 56.6 | 1789.9210 | 0.1536 | AAVPSGASTGIYEAVELR |
| 19.9 | 1789.9071 | 0.1675 | EEGHVDEPVVARGAGLR |
| 14.7 | 1789.9210 | 0.1536 | GAVPSGASTGIYEALELR |
| 13.8 | 1789.9687 | 0.1060 | DSLVDVIPHTLQGQIR |
| 11.4 | 1789.9951 | 0.0795 | VLLHHLYSRAPSELR |
| 9.9 | 1790.0050 | 0.0696 | QQLLATTVPPGPSAAAIR |
| 8.7 | 1789.9032 | 0.1714 | AAVEDSVMPAYAALINR |
| 8.3 | 1790.0526 | 0.0220 | QLKLAGGPQQLQLLQR |
| 8.3 | 1789.9183 | 0.1563 | AAAGGGSGAGRGTFLGAAVSR |

# MATRIX SCIENCE Mascot Search Results

## Peptide View Spot no 84

MS/MS Fragmentation of **MTAEIGEQVQIVGDDLLVTNPTR**
Found in **gi|780372** in **NCBInr**, enolase [Oryza sativa Japonica Group]

Match to Query 80: 2498.474124 from(2499.481400,1+) intensity(0.0000) index(26)
Title: Label: J6, Spot_Id: 228931, Peak_List_Id: 257593, MSMS Job_Run_Id: 24927, Comment:
Data file ppw_J6_138985135600.txt

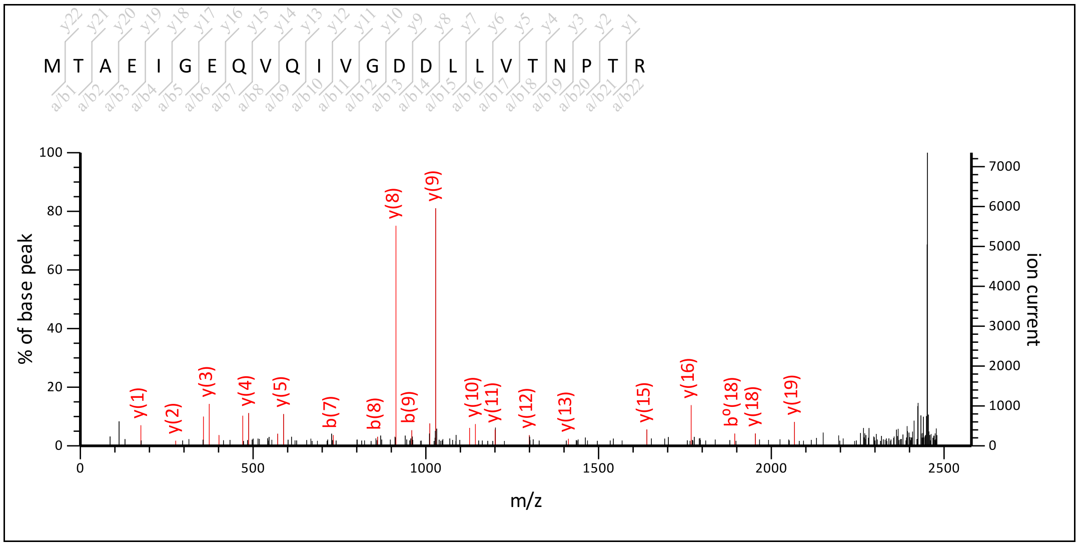


0 to 2579.21

Label all possible matches ○ Label matches used for scoring ◉

**Monoisotopic mass of neutral peptide Mr(calc):** 2498.2687
**Fixed modifications:** Carbamidomethyl (C) (apply to specified residues or termini only)
**Ions Score:** 96 **Expect:** 2.3e-07
**Matches :** 33/457 fragment ions using 47 most intense peaks (help)

| # | Immon. | a | a* | $a^0$ | b | b* | $b^0$ | d | d' | Seq. | v | w | w' | y | y* | |
|---|---|---|---|---|---|---|---|---|---|---|---|---|---|---|---|---|
| 1 | 104.0528 | 104.0528 | | | 132.0478 | | | 44.0495 | | M | | | | | | |
| 2 | 74.0600 | 205.1005 | | 187.0900 | 233.0954 | | 215.0849 | 189.1056 | 191.0849 | T | 2322.1936 | 2335.2140 | 2337.1932 | 2368.2354 | 2351.2089 | 23 |
| 3 | 44.0495 | 276.1376 | | 258.1271 | 304.1326 | | 286.1220 | | | A | 2251.1565 | | | 2267.1878 | 2250.1612 | 22 |
| 4 | 102.0550 | 405.1802 | | 387.1697 | 433.1751 | | 415.1646 | 347.1748 | | E | 2122.1139 | 2121.1186 | | 2196.1507 | 2179.1241 | 21 |
| 5 | 86.0964 | 518.2643 | | 500.2537 | 546.2592 | | 528.2486 | 490.2330 | 504.2486 | I | 2009.0298 | 2022.0502 | 2036.0659 | **2067.1081** | 2050.0815 | 20 |
| 6 | 30.0338 | 575.2858 | | 557.2752 | 603.2807 | | 585.2701 | | | G | | | | **1954.0240** | 1936.9974 | 19 |
| 7 | 102.0550 | 704.3284 | | 686.3178 | **732.3233** | | 714.3127 | 646.3229 | | E | 1822.9658 | 1821.9705 | | 1897.0025 | 1879.9760 | 18 |
| 8 | 101.0709 | 832.3869 | 815.3604 | 814.3764 | **860.3818** | 843.3553 | 842.3713 | 775.3655 | | Q | 1694.9072 | 1693.9119 | | **1767.9599** | 1750.9334 | 17 |
| 9 | 72.0808 | 931.4553 | 914.4288 | 913.4448 | **959.4503** | 942.4237 | 941.4397 | 917.4397 | | V | 1595.8388 | 1608.8592 | | **1639.9014** | 1622.8748 | 16 |
| 10 | 101.0709 | 1059.5139 | 1042.4874 | 1041.5034 | 1087.5088 | 1070.4823 | 1069.4983 | 1002.4925 | | Q | 1467.7802 | 1466.7849 | | 1540.8329 | 1523.8064 | 15 |
| 11 | 86.0964 | 1172.5980 | 1155.5714 | 1154.5874 | **1200.5929** | 1183.5664 | 1182.5823 | 1144.5667 | 1158.5823 | I | 1354.6961 | 1367.7165 | 1381.7322 | **1412.7744** | 1395.7478 | 13 |
| 12 | 72.0808 | 1271.6664 | 1254.6399 | 1253.6558 | **1299.6613** | 1282.6348 | 1281.6508 | 1257.6508 | | V | 1255.6277 | 1268.6481 | | **1299.6903** | 1282.6638 | 12 |
| 13 | 30.0338 | 1328.6879 | 1311.6613 | 1310.6773 | 1356.6828 | 1339.6562 | 1338.6722 | | | G | | | | **1200.6219** | 1183.5953 | 11 |
| 14 | 88.0393 | 1443.7148 | 1426.6883 | 1425.7042 | 1471.7097 | 1454.6832 | 1453.6992 | 1399.7250 | | D | 1083.5793 | 1082.5841 | | **1143.6004** | 1126.5739 | 11 |
| 15 | 88.0393 | 1558.7418 | 1541.7152 | 1540.7312 | 1586.7367 | 1569.7101 | 1568.7261 | 1514.7519 | | D | 968.5524 | 967.5571 | | **1028.5735** | 1011.5469 | 10 |
| 16 | 86.0964 | 1671.8258 | 1654.7993 | 1653.8152 | 1699.8207 | 1682.7942 | 1681.8102 | 1629.7789 | | L | 855.4683 | 854.4730 | | **913.5465** | 896.5200 | 8 |
| 17 | 86.0964 | 1784.9099 | 1767.8833 | 1766.8993 | 1812.9048 | 1795.8782 | 1794.8942 | 1742.8629 | | L | 742.3842 | 741.3890 | | 800.4625 | 783.4359 | 7 |
| 18 | 72.0808 | 1883.9783 | 1866.9517 | 1865.9677 | 1911.9732 | 1894.9467 | 1893.9626 | 1869.9626 | | V | 643.3158 | 656.3362 | | 687.3784 | 670.3519 | 6 |
| 19 | 74.0600 | 1985.0260 | 1967.9994 | 1967.0154 | 2013.0209 | 1995.9943 | 1995.0103 | 1969.0311 | 1971.0103 | T | 542.2681 | 555.2885 | 557.2678 | **588.3100** | 571.2835 | 5 |
| 20 | 87.0553 | 2099.0689 | 2082.0423 | 2081.0583 | 2127.0638 | 2110.0373 | 2109.0532 | 2056.0631 | | N | 428.2252 | 427.2300 | | **487.2623** | 470.2358 | 4 |
| 21 | 70.0651 | 2196.1217 | 2179.0951 | 2178.1111 | 2224.1166 | 2207.0900 | 2206.1060 | 2170.1060 | | P | 331.1724 | 330.1772 | | **373.2194** | 356.1928 | 3 |
| 22 | 74.0600 | 2297.1693 | 2280.1428 | 2279.1588 | 2325.1643 | 2308.1377 | 2307.1537 | 2281.1744 | 2283.1537 | T | 230.1248 | 243.1452 | 245.1244 | **276.1666** | 259.1401 | 2 |

| | | | | | | | | | | | | | | | |
|---|---|---|---|---|---|---|---|---|---|---|---|---|---|---|---|
| 23 | 129.1135 | | | | | | | | | R | 74.0237 | 73.0284 | | *175.1190* | 158.0924 |

| Seq | ya | yb | Seq | ya | yb | Seq | ya | yb |
|---|---|---|---|---|---|---|---|---|
| **TA** | 145.0972 | 173.0921 | **TAE** | 274.1397 | 302.1347 | **TAEI** | 387.2238 | 415.2187 |
| **TAEIG** | 444.2453 | 472.2402 | **TAEIGE** | 573.2879 | 601.2828 | **AE** | 173.0921 | 201.0870 |
| **AEI** | 286.1761 | 314.1710 | **AEIG** | 343.1976 | 371.1925 | **AEIGE** | 472.2402 | 500.2351 |
| **AEIGEQ** | 600.2988 | 628.2937 | **AEIGEQV** | 699.3672 | 727.3621 | **EI** | 215.1390 | 243.1339 |
| **EIG** | 272.1605 | 300.1554 | **EIGE** | 401.2031 | 429.1980 | **EIGEQ** | 529.2617 | 557.2566 |
| **EIGEQV** | 628.3301 | 656.3250 | **IG** | 143.1179 | 171.1128 | **IGE** | 272.1605 | 300.1554 |
| **IGEQ** | 400.2191 | 428.2140 | **IGEQV** | 499.2875 | 527.2824 | **IGEQVQ** | 627.3461 | 655.3410 |
| **GE** | 159.0764 | 187.0713 | **GEQ** | 287.1350 | 315.1299 | **GEQV** | 386.2034 | 414.1983 |
| **GEQVQ** | 514.2620 | 542.2569 | **GEQVQI** | 627.3461 | 655.3410 | **EQ** | 230.1135 | 258.1084 |
| **EQV** | 329.1819 | 357.1769 | **EQVQ** | 457.2405 | 485.2354 | **EQVQI** | 570.3246 | 598.3195 |
| **EQVQIV** | 669.3930 | 697.3879 | **QV** | 200.1394 | 228.1343 | **QVQ** | 328.1979 | 356.1928 |
| **QVQI** | 441.2820 | 469.2769 | **QVQIV** | 540.3504 | 568.3453 | **QVQIVG** | 597.3719 | 625.3668 |
| **VQ** | 200.1394 | 228.1343 | **VQI** | 313.2234 | 341.2183 | **VQIV** | 412.2918 | 440.2867 |
| **VQIVG** | 469.3133 | 497.3082 | **VQIVGD** | 584.3402 | 612.3352 | **VQIVGDD** | 699.3672 | 727.3621 |
| **QI** | 214.1550 | 242.1499 | **QIV** | 313.2234 | 341.2183 | **QIVG** | 370.2449 | 398.2398 |
| **QIVGD** | 485.2718 | 513.2667 | **QIVGDD** | 600.2988 | 628.2937 | **IV** | 185.1648 | 213.1598 |
| **IVG** | 242.1863 | 270.1812 | **IVGD** | 357.2132 | 385.2082 | **IVGDD** | 472.2402 | 500.2351 |
| **IVGDDL** | 585.3243 | 613.3192 | **IVGDDLL** | 698.4083 | 726.4032 | **VG** | 129.1022 | 157.0972 |
| **VGD** | 244.1292 | 272.1241 | **VGDD** | 359.1561 | 387.1510 | **VGDDL** | 472.2402 | 500.2351 |
| **VGDDLL** | 585.3243 | 613.3192 | **VGDDLLV** | 684.3927 | 712.3876 | **GD** | 145.0608 | 173.0557 |
| **GDD** | 260.0877 | 288.0826 | **GDDL** | 373.1718 | 401.1667 | **GDDLL** | 486.2558 | 514.2508 |
| **GDDLLV** | 585.3243 | 613.3192 | **GDDLLVT** | 686.3719 | 714.3668 | **DD** | 203.0662 | 231.0612 |
| **DDL** | 316.1503 | 344.1452 | **DDLL** | 429.2344 | 457.2293 | **DDLLV** | 528.3028 | 556.2977 |
| **DDLLVT** | 629.3505 | 657.3454 | **DL** | 201.1234 | 229.1183 | **DLL** | 314.2074 | 342.2023 |
| **DLLV** | 413.2758 | 441.2708 | **DLLVT** | 514.3235 | 542.3184 | **DLLVTN** | 628.3665 | 656.3614 |
| **LL** | 199.1805 | 227.1754 | **LLV** | 298.2489 | 326.2438 | **LLVT** | 399.2966 | 427.2915 |
| **LLVTN** | 513.3395 | 541.3344 | **LLVTNP** | 610.3923 | 638.3872 | **LV** | 185.1648 | 213.1598 |
| **LVT** | 286.2125 | 314.2074 | **LVTN** | 400.2554 | 428.2504 | **LVTNP** | 497.3082 | 525.3031 |
| **LVTNPT** | 598.3559 | 626.3508 | **VT** | 173.1285 | 201.1234 | **VTN** | 287.1714 | 315.1663 |
| **VTNP** | 384.2241 | 412.2191 | **VTNPT** | 485.2718 | 513.2667 | **TN** | 188.1030 | 216.0979 |
| **TNP** | 285.1557 | 313.1506 | **TNPT** | 386.2034 | 414.1983 | **NP** | 184.1081 | 212.1030 |
| **NPT** | 285.1557 | 313.1506 | **PT** | 171.1128 | 199.1077 | | | |

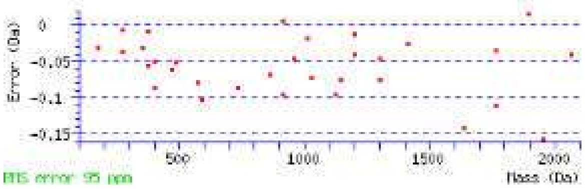


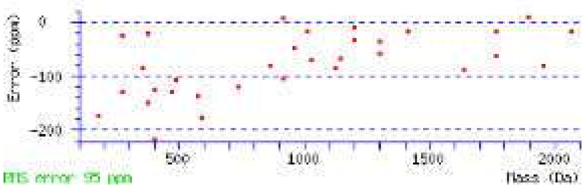


NCBI **BLAST** search of MTAEIGEQVQIVGDDLLVTNPTR
(Parameters: blastp, nr protein database, expect=20000, no filter, PAM30)
Other BLAST web gateways

**All matches to this query**

| Score | Mr(calc) | Delta | Sequence |
|---|---|---|---|
| 95.7 | 2498.2687 | 0.2055 | MTAEIGEQVQIVGDDLLVTNPTR |
| 8.3 | 2498.3063 | 0.1678 | DTAISDEKPARIMAARPPPPPPR |
| 8.0 | 2498.3493 | 0.1248 | QGLLERSGVFVVEGDINDAVLLR |
| 5.0 | 2498.3163 | 0.1579 | LSTNATASAPAATAASPGVVDVLMKR |
| 1.7 | 2498.2919 | 0.1822 | VAMFGGSILLDIYIYDYFVKR |
| 0.6 | 2498.2289 | 0.2452 | RLAEAGYVPDVSSVLHDIGDEEK |
| 0.3 | 2498.2911 | 0.1830 | MRELVSSSTSSPTAALVAAGHAALR |

**Mascot:** http://www.matrixscience.com/

# MATRIX SCIENCE Mascot Search Results

## Peptide View Spot no 85

MS/MS Fragmentation of **IGLFGGAGVGK**
Found in **gi|3676294** in **NCBInr**, mitochondrial ATPase beta subunit [Nicotiana sylvestris]

Match to Query 63: 974.560904 from(975.568180,1+) intensity(0.0000) index(3)
Title: Label: F15, Spot_Id: 219903, Peak_List_Id: 228328, MSMS Job_Run_Id: 21987, Comment:
Data file C:\Documents and Settings\Administrator\Desktop\Farah-Garima 11May 2013\ppw_F15_136868277600.txt

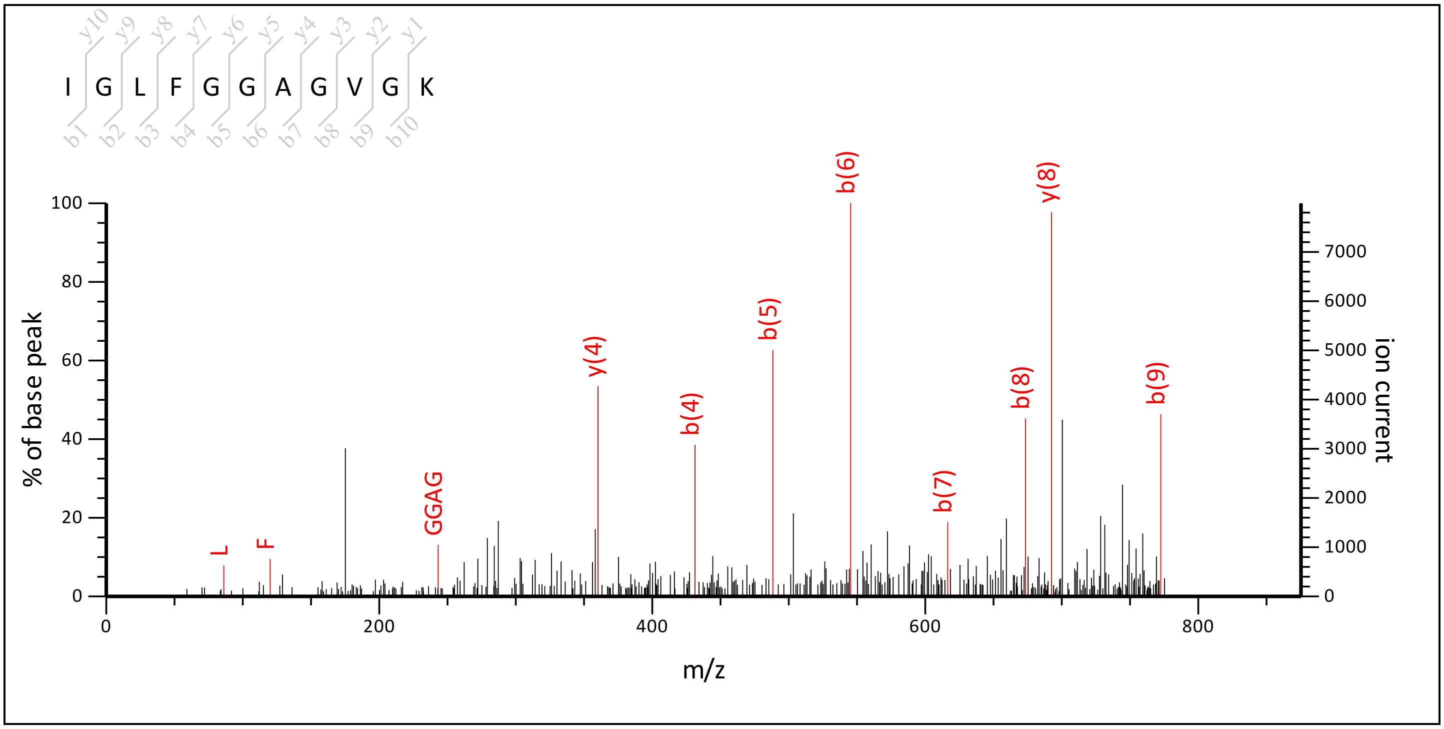


0 to 875.25

Label all possible matches ○ Label matches used for scoring ◉

**Monoisotopic mass of neutral peptide Mr(calc):** 974.5549
**Fixed modifications:** Carbamidomethyl (C) (apply to specified residues or termini only)
**Ions Score:** 46 **Expect:** 1
**Matches :** 16/133 fragment ions using 16 most intense peaks (help)

| # | Immon. | a | b | d | Seq. | v | w | y | y* | # |
|---|---|---|---|---|---|---|---|---|---|---|
| 1 | 86.0964 | 86.0964 | 114.0913 | 44.0495 | I | | | | | 11 |
| 2 | 30.0338 | 143.1179 | 171.1128 | | G | | | 862.4781 | 845.4516 | 10 |
| 3 | 86.0964 | 256.2020 | 284.1969 | 214.1550 | L | 747.3784 | 746.3832 | 805.4567 | 788.4301 | 9 |
| 4 | 120.0808 | 403.2704 | ***431.2653*** | | F | 600.3100 | | ***692.3726*** | 675.3461 | 8 |
| 5 | 30.0338 | 460.2918 | ***488.2867*** | | G | | | ***545.3042*** | 528.2776 | 7 |
| 6 | 30.0338 | 517.3133 | ***545.3082*** | | G | | | ***488.2827*** | 471.2562 | 6 |
| 7 | 44.0495 | 588.3504 | ***616.3453*** | | A | 415.2300 | | ***431.2613*** | 414.2347 | 5 |
| 8 | 30.0338 | 645.3719 | ***673.3668*** | | G | | | ***360.2241*** | 343.1976 | 4 |

| | | | | | | | | | | |
|---|---|---|---|---|---|---|---|---|---|---|
| 9 | 72.0808 | 744.4403 | *772.4352* | 730.4246 | V | 259.1401 | 272.1605 | 303.2027 | 286.1761 | 3 |
| 10 | 30.0338 | 801.4618 | 829.4567 | | G | | | 204.1343 | 187.1077 | 2 |
| 11 | 101.1073 | | | | K | 74.0237 | 73.0284 | 147.1128 | 130.0863 | 1 |

| Seq | ya | yb | Seq | ya | yb | Seq | ya | yb |
|---|---|---|---|---|---|---|---|---|
| GL | 143.1179 | 171.1128 | GLF | 290.1863 | 318.1812 | GLFG | 347.2078 | 375.2027 |
| GLFGG | 404.2292 | 432.2241 | GLFGGA | 475.2663 | 503.2613 | GLFGGAG | 532.2878 | 560.2827 |
| GLFGGAGV | 631.3562 | 659.3511 | GLFGGAGVG | 688.3777 | 716.3726 | LF | 233.1648 | 261.1598 |
| LFG | 290.1863 | 318.1812 | LFGG | 347.2078 | 375.2027 | LFGGA | 418.2449 | 446.2398 |
| LFGGAG | 475.2663 | 503.2613 | LFGGAGV | 574.3348 | 602.3297 | LFGGAGVG | 631.3562 | 659.3511 |
| FG | 177.1022 | 205.0972 | FGG | 234.1237 | 262.1186 | FGGA | 305.1608 | 333.1557 |
| FGGAG | 362.1823 | 390.1772 | FGGAGV | 461.2507 | 489.2456 | FGGAGVG | 518.2722 | 546.2671 |
| GG | 87.0553 | 115.0502 | GGA | 158.0924 | 186.0873 | GGAG | 215.1139 | 243.1088 |
| GGAGV | 314.1823 | 342.1772 | GGAGVG | 371.2037 | 399.1987 | GA | 101.0709 | 129.0659 |
| GAG | 158.0924 | 186.0873 | GAGV | 257.1608 | 285.1557 | GAGVG | 314.1823 | 342.1772 |
| AG | 101.0709 | 129.0659 | AGV | 200.1394 | 228.1343 | AGVG | 257.1608 | 285.1557 |
| GV | 129.1022 | 157.0972 | GVG | 186.1237 | 214.1186 | VG | 129.1022 | 157.0972 |

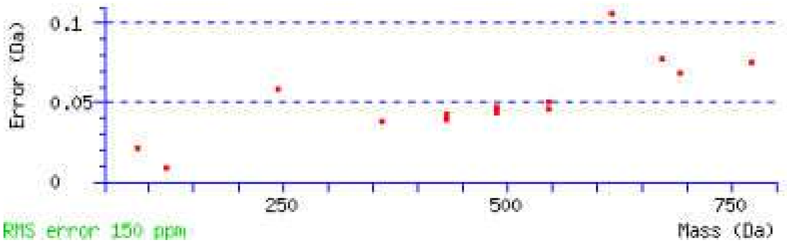


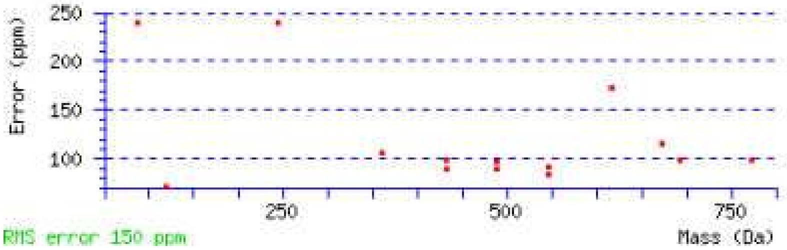


NCBI **BLAST** search of IGLFGGAGVGK
(Parameters: blastp, nr protein database, expect=20000, no filter, PAM30)
Other BLAST web gateways

**All matches to this query**

| Score | Mr(calc) | Delta | Sequence |
|---|---|---|---|
| 46.9 | 974.5549 | 0.0060 | IAVFGGAGVGK |
| 46.9 | 974.5549 | 0.0060 | IGIFGGAGVGK |
| 46.9 | 974.5549 | 0.0060 | IGIFGGAGVGK |
| 46.9 | 974.5219 | 0.0390 | IGIMGGAGVGK |
| 46.9 | 974.5549 | 0.0060 | LGIFGGAGVGK |
| 46.3 | 974.5549 | 0.0060 | IGLFGGAGVGK |
| 46.3 | 974.5549 | 0.0060 | IGLFGGAGVGK |

| | | | |
|---|---|---|---|
| 46.3 | 974.5549 | 0.0060 | IGLFGGAGVGK |
| 46.3 | 974.5549 | 0.0060 | IGLFGGAGVGK |
| 46.3 | 974.5549 | 0.0060 | IGLFGGAGVGK |

**Mascot:** http://www.matrixscience.com/

# MATRIX SCIENCE Mascot Search Results

## Peptide View Spot no 85

MS/MS Fragmentation of **TIAMDGTEGLVR**
Found in **gi|3676294** in **NCBInr**, mitochondrial ATPase beta subunit [Nicotiana sylvestris]

Match to Query 175: 1261.664924 from(1262.672200,1+) intensity(0.0000) index(13)
Title: Label: F15, Spot_Id: 219903, Peak_List_Id: 228326, MSMS Job_Run_Id: 21987, Comment:
Data file C:\Documents and Settings\Administrator\Desktop\Farah-Garima 11May 2013\ppw_F15_136868277600.txt

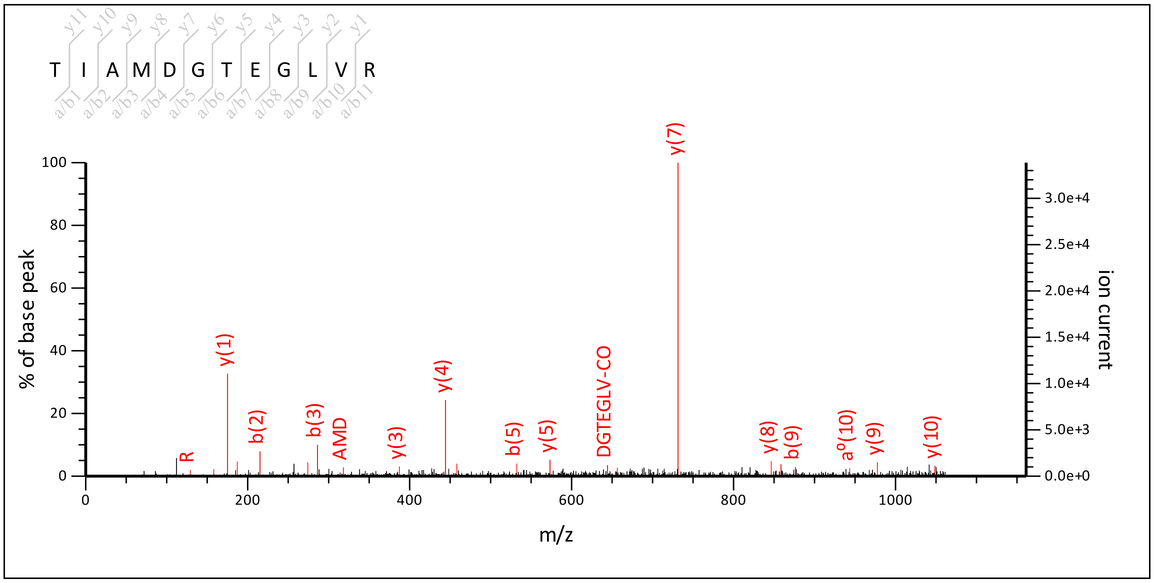


0 to 1161.31

Label all possible matches ○ Label matches used for scoring ◉

**Monoisotopic mass of neutral peptide Mr(calc):** 1261.6336
**Fixed modifications:** Carbamidomethyl (C) (apply to specified residues or termini only)
**Ions Score:** 72 **Expect:** 0.0023
**Matches :** 27/190 fragment ions using 31 most intense peaks (help)

| # | Immon. | a | $a^0$ | b | $b^0$ | d | d' | Seq. | v | w | w' | y | y* | $y^0$ | # |
|---|---|---|---|---|---|---|---|---|---|---|---|---|---|---|---|
| 1 | 74.0600 | 74.0600 | 56.0495 | 102.0550 | 84.0444 | 44.0495 | | T | | | | | | | 12 |
| 2 | 86.0964 | 187.1441 | 169.1335 | **215.1390** | 197.1285 | 159.1128 | 173.1285 | I | 1103.5150 | 1116.5354 | 1130.5510 | 1161.5932 | 1144.5667 | 1143.5827 | 11 |
| 3 | 44.0495 | 258.1812 | 240.1707 | **286.1761** | 268.1656 | | | A | 1032.4779 | | | ***1048.5092*** | 1031.4826 | 1030.4986 | 10 |
| 4 | 104.0528 | 389.2217 | 371.2111 | 417.2166 | 399.2061 | 329.2183 | | M | 901.4374 | 900.4421 | | ***977.4721*** | 960.4455 | 959.4615 | 9 |
| 5 | 88.0393 | 504.2486 | 486.2381 | **532.2436** | 514.2330 | 460.2588 | | D | 786.4104 | 785.4152 | | ***846.4316*** | 829.4050 | 828.4210 | 8 |
| 6 | 30.0338 | 561.2701 | 543.2595 | 589.2650 | 571.2545 | | | G | | | | ***731.4046*** | 714.3781 | 713.3941 | 7 |
| 7 | 74.0600 | 662.3178 | 644.3072 | 690.3127 | 672.3021 | 646.3229 | 648.3021 | T | 628.3413 | 641.3617 | 643.3410 | 674.3832 | 657.3566 | 656.3726 | 6 |
| 8 | 102.0550 | 791.3604 | 773.3498 | 819.3553 | 801.3447 | 733.3549 | | E | 499.2987 | 498.3035 | | ***573.3355*** | 556.3089 | 555.3249 | 5 |
| 9 | 30.0338 | 848.3818 | 830.3713 | **876.3768** | 858.3662 | | | G | | | | ***444.2929*** | 427.2663 | | 4 |
| 10 | 86.0964 | 961.4659 | 943.4553 | 989.4608 | 971.4503 | 919.4190 | | L | 329.1932 | 328.1979 | | ***387.2714*** | 370.2449 | | 3 |
| 11 | 72.0808 | 1060.5343 | 1042.5238 | 1088.5292 | 1070.5187 | 1046.5187 | | V | 230.1248 | 243.1452 | | ***274.1874*** | 257.1608 | | 2 |
| 12 | 129.1135 | | | | | | | R | 74.0237 | 73.0284 | | ***175.1190*** | 158.0924 | | 1 |

| Seq | ya | yb | Seq | ya | yb | Seq | ya | yb |
|---|---|---|---|---|---|---|---|---|
| IA | 157.1335 | 185.1285 | IAM | 288.1740 | 316.1689 | IAMD | 403.2010 | 431.1959 |
| IAMDG | 460.2224 | 488.2173 | IAMDGT | 561.2701 | 589.2650 | IAMDGTE | 690.3127 | 718.3076 |
| AM | 175.0900 | 203.0849 | AMD | 290.1169 | 318.1118 | AMDG | 347.1384 | 375.1333 |
| AMDGT | 448.1860 | 476.1810 | AMDGTE | 577.2286 | 605.2236 | AMDGTEG | 634.2501 | 662.2450 |
| MD | 219.0798 | 247.0747 | MDG | 276.1013 | 304.0962 | MDGT | 377.1489 | 405.1438 |
| | | | | | | | | |

| | | | | | | | | |
|---|---|---|---|---|---|---|---|---|
| **MDGTE** | 506.1915 | 534.1864 | **MDGTEG** | 563.2130 | 591.2079 | **MDGTEGL** | 676.2971 | 704.2920 |
| **DG** | 145.0608 | 173.0557 | **DGT** | 246.1084 | 274.1034 | **DGTE** | 375.1510 | 403.1460 |
| **DGTEG** | 432.1725 | 460.1674 | **DGTEGL** | 545.2566 | 573.2515 | **DGTEGLV** | 644.3250 | 672.3199 |
| **GT** | 131.0815 | 159.0764 | **GTE** | 260.1241 | 288.1190 | **GTEG** | 317.1456 | 345.1405 |
| **GTEGL** | 430.2296 | 458.2245 | **GTEGLV** | 529.2980 | 557.2930 | **TE** | 203.1026 | 231.0975 |
| **TEG** | 260.1241 | 288.1190 | **TEGL** | 373.2082 | 401.2031 | **TEGLV** | 472.2766 | 500.2715 |
| **EG** | 159.0764 | 187.0713 | **EGL** | 272.1605 | 300.1554 | **EGLV** | 371.2289 | 399.2238 |
| **GL** | 143.1179 | 171.1128 | **GLV** | 242.1863 | 270.1812 | **LV** | 185.1648 | 213.1598 |

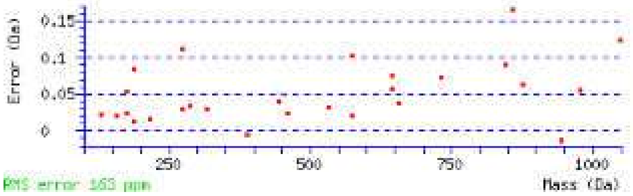


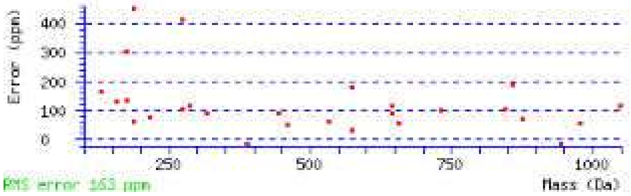


NCBI **BLAST** search of TIAMDGTEGLVR
(Parameters: blastp, nr protein database, expect=20000, no filter, PAM30)
Other BLAST web gateways

**All matches to this query**

| Score | Mr(calc) | Delta | Sequence |
|---|---|---|---|
| 72.2 | 1261.6336 | 0.0313 | TIAMDGTEGLVR |
| 72.2 | 1261.6336 | 0.0313 | TIAMDGTEGLVR |
| 60.1 | 1261.6336 | 0.0313 | TIAMDATDGLVR |
| 43.9 | 1261.6336 | 0.0313 | TIAMDGTDGLIR |
| 43.1 | 1261.6336 | 0.0313 | AIAMEGTEGLVR |
| 43.0 | 1261.6336 | 0.0313 | TIAMEGTDGLVR |
| 38.7 | 1261.6159 | 0.0491 | VDADMAAMAVLR |
| 37.5 | 1261.7143 | -0.0493 | ITAIHPGEGIVR |
| 35.6 | 1261.5721 | 0.0928 | SVVMDGAAADGNR |
| 34.5 | 1261.6667 | -0.0017 | GSFLVDAEGIVR |

**Mascot:** http://www.matrixscience.com/

# MATRIX SCIENCE Mascot Search Results

## Peptide View **Spot no 85**

MS/MS Fragmentation of **AHGGFSVFAGVGER**
Found in **gi|3676294** in **NCBInr**, mitochondrial ATPase beta subunit [Nicotiana sylvestris]

Match to Query 200: 1389.721024 from(1390.728300,1+) intensity(0.0000) index(17)
Title: Label: F15, Spot_Id: 219903, Peak_List_Id: 228324, MSMS Job_Run_Id: 21987, Comment:
Data file C:\Documents and Settings\Administrator\Desktop\Farah-Garima 11May 2013\ppw_F15_136868277600.txt

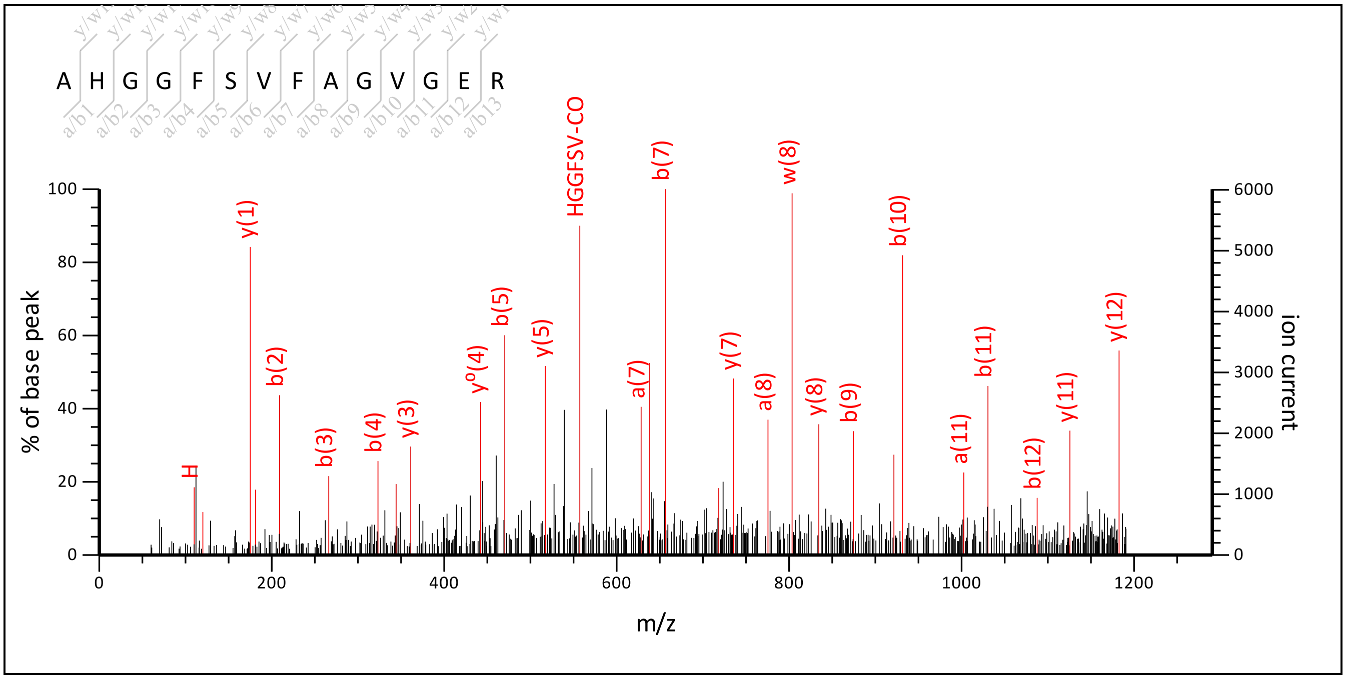


0 to 1290.69

Label all possible matches ○ Label matches used for scoring ◉

**Monoisotopic mass of neutral peptide Mr(calc):** 1389.6790
**Fixed modifications:** Carbamidomethyl (C) (apply to specified residues or termini only)
**Ions Score:** 101 **Expect:** 2.9e-06
**Matches :** 35/213 fragment ions using 36 most intense peaks (help)

| # | Immon. | a | $a^0$ | b | $b^0$ | d | Seq. | v | w | y | y* | $y^0$ | # |
|---|---|---|---|---|---|---|---|---|---|---|---|---|---|
| 1 | 44.0495 | 44.0495 | | 72.0444 | | 44.0495 | A | | | | | | 14 |
| 2 | 110.0713 | **181.1084** | | ***209.1033*** | | | H | 1237.5960 | | 1319.6491 | 1302.6226 | 1301.6385 | 13 |
| 3 | 30.0338 | 238.1299 | | ***266.1248*** | | | G | | | **1182.5902** | 1165.5636 | 1164.5796 | 12 |
| 4 | 30.0338 | 295.1513 | | ***323.1462*** | | | G | | | **1125.5687** | 1108.5422 | 1107.5582 | 11 |
| 5 | 120.0808 | **442.2197** | | ***470.2146*** | | | F | 976.4847 | | 1068.5473 | 1051.5207 | 1050.5367 | 10 |
| 6 | 60.0444 | 529.2518 | 511.2412 | ***557.2467*** | 539.2361 | 513.2568 | S | 889.4526 | 888.4574 | **921.4789** | 904.4523 | 903.4683 | 9 |
| 7 | 72.0808 | **628.3202** | 610.3096 | ***656.3151*** | 638.3045 | 614.3045 | V | 790.3842 | 803.4046 | **834.4468** | 817.4203 | 816.4363 | 8 |
| 8 | 120.0808 | **775.3886** | 757.3780 | ***803.3835*** | 785.3729 | | F | 643.3158 | | **735.3784** | 718.3519 | 717.3678 | 7 |
| 9 | 44.0495 | 846.4257 | 828.4151 | ***874.4206*** | 856.4100 | | A | 572.2787 | | 588.3100 | 571.2835 | 570.2994 | 6 |
| 10 | 30.0338 | 903.4472 | 885.4366 | ***931.4421*** | 913.4315 | | G | | | **517.2729** | 500.2463 | 499.2623 | 5 |
| 11 | 72.0808 | **1002.5156** | 984.5050 | ***1030.5105*** | 1012.4999 | 988.4999 | V | 416.1888 | 429.2092 | 460.2514 | 443.2249 | 442.2409 | 4 |

| | | | | | | | | | | | | | |
|---|---|---|---|---|---|---|---|---|---|---|---|---|---|
| 12 | 30.0338 | 1059.5370 | 1041.5265 | *1087.5320* | 1069.5214 | | G | | | **361.1830** | 344.1565 | 343.1724 | 3 |
| 13 | 102.0550 | 1188.5796 | 1170.5691 | 1216.5745 | 1198.5640 | 1130.5742 | E | 230.1248 | 229.1295 | 304.1615 | 287.1350 | 286.1510 | 2 |
| 14 | 129.1135 | | | | | | R | 74.0237 | 73.0284 | **175.1190** | 158.0924 | | 1 |

| Seq | ya | yb | Seq | ya | yb | Seq | ya | yb |
|---|---|---|---|---|---|---|---|---|
| HG | 167.0927 | 195.0877 | HGG | 224.1142 | 252.1091 | HGGF | 371.1826 | 399.1775 |
| HGGFS | 458.2146 | 486.2096 | HGGFSV | 557.2831 | 585.2780 | GG | 87.0553 | 115.0502 |
| GGF | 234.1237 | 262.1186 | GGFS | 321.1557 | 349.1506 | GGFSV | 420.2241 | 448.2191 |
| GGFSVF | 567.2926 | 595.2875 | GGFSVFA | 638.3297 | 666.3246 | GGFSVFAG | 695.3511 | 723.3461 |
| GF | 177.1022 | 205.0972 | GFS | 264.1343 | 292.1292 | GFSV | 363.2027 | 391.1976 |
| GFSVF | 510.2711 | 538.2660 | GFSVFA | 581.3082 | 609.3031 | GFSVFAG | 638.3297 | 666.3246 |
| FS | 207.1128 | 235.1077 | FSV | 306.1812 | 334.1761 | FSVF | 453.2496 | 481.2445 |
| FSVFA | 524.2867 | 552.2817 | FSVFAG | 581.3082 | 609.3031 | FSVFAGV | 680.3766 | 708.3715 |
| SV | 159.1128 | 187.1077 | SVF | 306.1812 | 334.1761 | SVFA | 377.2183 | 405.2132 |
| SVFAG | 434.2398 | 462.2347 | SVFAGV | 533.3082 | 561.3031 | SVFAGVG | 590.3297 | 618.3246 |
| VF | 219.1492 | 247.1441 | VFA | 290.1863 | 318.1812 | VFAG | 347.2078 | 375.2027 |
| VFAGV | 446.2762 | 474.2711 | VFAGVG | 503.2976 | 531.2926 | VFAGVGE | 632.3402 | 660.3352 |
| FA | 191.1179 | 219.1128 | FAG | 248.1394 | 276.1343 | FAGV | 347.2078 | 375.2027 |
| FAGVG | 404.2292 | 432.2241 | FAGVGE | 533.2718 | 561.2667 | AG | 101.0709 | 129.0659 |
| AGV | 200.1394 | 228.1343 | AGVG | 257.1608 | 285.1557 | AGVGE | 386.2034 | 414.1983 |
| GV | 129.1022 | 157.0972 | GVG | 186.1237 | 214.1186 | GVGE | 315.1663 | 343.1612 |
| VG | 129.1022 | 157.0972 | VGE | 258.1448 | 286.1397 | GE | 159.0764 | 187.0713 |

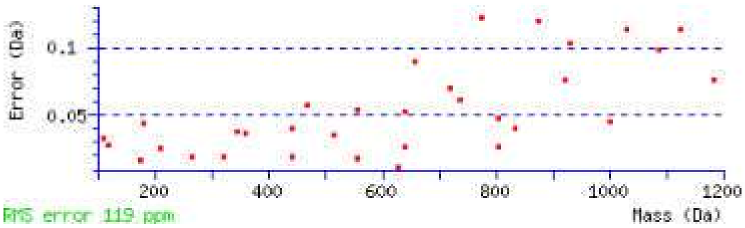


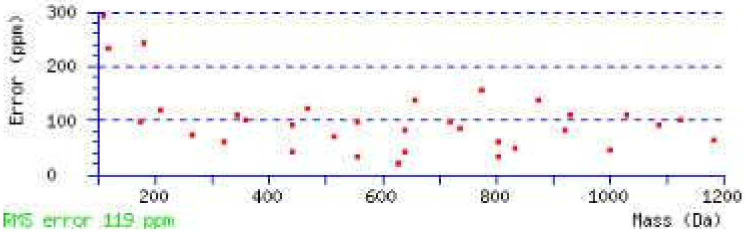


NCBI **BLAST** search of AHGGFSVFAGVGER
(Parameters: blastp, nr protein database, expect=20000, no filter, PAM30)
Other BLAST web gateways

**All matches to this query**

| Score | Mr(calc) | Delta | Sequence |
|---|---|---|---|
| 101.0 | 1389.6790 | 0.0421 | AHGGFSVFAGVGER |
| 85.1 | 1389.6790 | 0.0421 | QHGFSVFAGVGER |
| 82.5 | 1389.6790 | 0.0421 | AHGGYAVFAGVGER |
| 45.6 | 1389.6459 | 0.0751 | AHGGYSVMAGVGER |
| 40.6 | 1389.7405 | -0.0195 | AIFAGQFVDVPAR |

| | | | |
|---|---|---|---|
| 36.9 | 1389.6922 | 0.0288 | ENILVSMGAVGER |
| 31.6 | 1389.7075 | 0.0135 | MNDVPVFIVSNR |
| 31.2 | 1389.6381 | 0.0829 | CPADLAVMGVDAR |
| 31.1 | 1389.7616 | -0.0406 | VITKVDFQVDAR |
| 30.8 | 1389.6790 | 0.0421 | GFPSAQTGQGVWR |

**Mascot:** http://www.matrixscience.com/

# MATRIX SCIENCE Mascot Search Results

## Peptide View Spot no 85

MS/MS Fragmentation of VLNTGSPITVPVGR
Found in gi|3676294 in NCBInr, mitochondrial ATPase beta subunit [Nicotiana sylvestris]

Match to Query 204: 1408.845824 from(1409.853100,1+) intensity(0.0000) index(19)
Title: Label: F15, Spot_Id: 219903, Peak_List_Id: 228322, MSMS Job_Run_Id: 21987, Comment:
Data file C:\Documents and Settings\Administrator\Desktop\Farah-Garima 11May 2013\ppw_F15_136868277600.txt

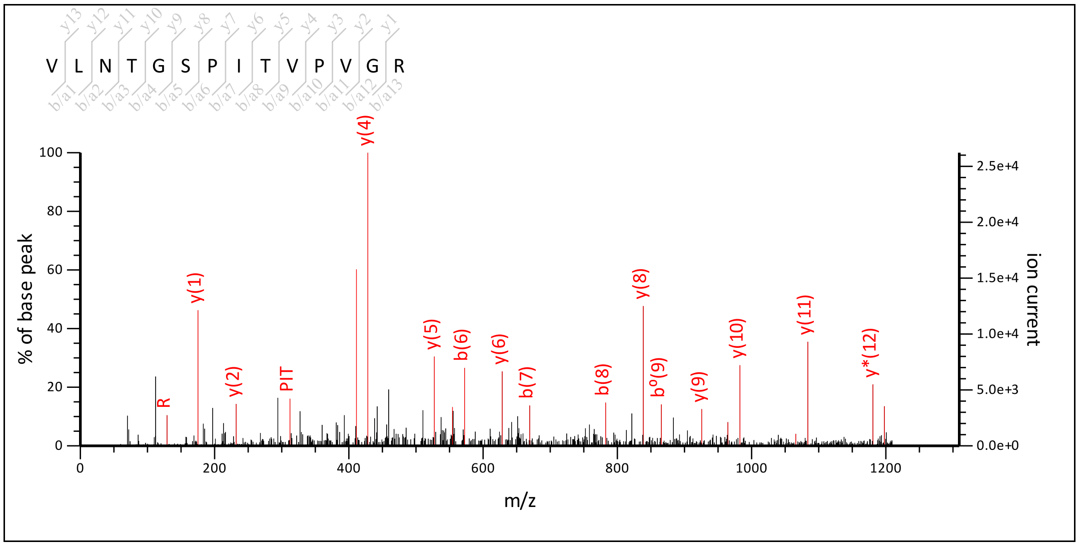


0 to 1309.38

Label all possible matches ○ Label matches used for scoring ◉

**Monoisotopic mass of neutral peptide Mr(calc):** 1408.8038
**Fixed modifications:** Carbamidomethyl (C) (apply to specified residues or termini only)
**Ions Score:** 98 **Expect:** 3.1e-06
**Matches :** 34/257 fragment ions using 24 most intense peaks (help)

| # | Immon. | a | a* | a$^0$ | b | b* | b$^0$ | d | d' | Seq. | v | w | w' | y | y* | [illegible] |
|---|---|---|---|---|---|---|---|---|---|---|---|---|---|---|---|---|
| 1 | 72.0808 | 72.0808 | | | 100.0757 | | | 44.0495 | | V | | | | | | |
| 2 | 86.0964 | 185.1648 | | | 213.1598 | | | 143.1179 | | L | 1252.6644 | 1251.6692 | | 1310.7427 | 1293.7161 | 12[illegible] |
| 3 | 87.0553 | 299.2078 | 282.1812 | | 327.2027 | 310.1761 | | 256.2020 | | N | 1138.6215 | 1137.6263 | | 1197.6586 | 1180.6321 | 11[illegible] |
| 4 | 74.0600 | 400.2554 | 383.2289 | 382.2449 | 428.2504 | 411.2238 | 410.2398 | 384.2605 | 386.2398 | T | 1037.5738 | 1050.5942 | 1052.5735 | 1083.6157 | 1066.5891 | 10[illegible] |
| 5 | 30.0338 | 457.2769 | 440.2504 | 439.2663 | 485.2718 | 468.2453 | 467.2613 | | | G | | | | 982.5680 | 965.5415 | 9[illegible] |
| 6 | 60.0444 | 544.3089 | 527.2824 | 526.2984 | 572.3039 | 555.2773 | 554.2933 | 528.3140 | | S | 893.5203 | 892.5251 | | 925.5465 | 908.5200 | 9[illegible] |
| 7 | 70.0651 | 641.3617 | 624.3352 | 623.3511 | 669.3566 | 652.3301 | 651.3461 | 615.3461 | | P | 796.4676 | 795.4723 | | 838.5145 | 821.4880 | 8[illegible] |
| 8 | 86.0964 | 754.4458 | 737.4192 | 736.4352 | 782.4407 | 765.4141 | 764.4301 | 726.4145 | 740.4301 | I | 683.3835 | 696.4039 | 710.4196 | 741.4618 | 724.4352 | 7[illegible] |
| 9 | 74.0600 | 855.4934 | 838.4669 | 837.4829 | 883.4884 | 866.4618 | 865.4778 | 839.4985 | 841.4778 | T | 582.3358 | 595.3562 | 597.3355 | 628.3777 | 611.3511 | 6[illegible] |
| 10 | 72.0808 | 954.5619 | 937.5353 | 936.5513 | 982.5568 | 965.5302 | 964.5462 | 940.5462 | | V | 483.2674 | 496.2878 | | 527.3300 | 510.3035 | |
| 11 | 70.0651 | 1051.6146 | 1034.5881 | 1033.6041 | 1079.6095 | 1062.5830 | 1061.5990 | 1025.5990 | | P | 386.2146 | 385.2194 | | 428.2616 | 411.2350 | |
| 12 | 72.0808 | 1150.6830 | 1133.6565 | 1132.6725 | 1178.6780 | 1161.6514 | 1160.6674 | 1136.6674 | | V | 287.1462 | 300.1666 | | 331.2088 | 314.1823 | |
| 13 | 30.0338 | 1207.7045 | 1190.6780 | 1189.6939 | 1235.6994 | 1218.6729 | 1217.6889 | | | G | | | | 232.1404 | 215.1139 | |
| 14 | 129.1135 | | | | | | | | | R | 74.0237 | 73.0284 | | 175.1190 | 158.0924 | |

| Seq | ya | yb | Seq | ya | yb | Seq | ya | yb |
|---|---|---|---|---|---|---|---|---|
| LN | 200.1394 | 228.1343 | LNT | 301.1870 | 329.1819 | LNTG | 358.2085 | 386.2034 |
| LNTGS | 445.2405 | 473.2354 | LNTGSP | 542.2933 | 570.2882 | LNTGSPI | 655.3774 | 683.3723 |
| NT | 188.1030 | 216.0979 | NTG | 245.1244 | 273.1193 | NTGS | 332.1565 | 360.1514 |
| NTGSP | 429.2092 | 457.2041 | NTGSPI | 542.2933 | 570.2882 | NTGSPIT | 643.3410 | 671.3359 |
| TG | 131.0815 | 159.0764 | TGS | 218.1135 | 246.1084 | TGSP | 315.1663 | 343.1612 |
| TGSPI | 428.2504 | 456.2453 | TGSPIT | 529.2980 | 557.2930 | TGSPITV | 628.3665 | 656.3614 |
| GS | 117.0659 | 145.0608 | GSP | 214.1186 | 242.1135 | GSPI | 327.2027 | 355.1976 |

| | | | | | | | | |
|---|---|---|---|---|---|---|---|---|
| **GSPIT** | 428.2504 | 456.2453 | **GSPITV** | 527.3188 | 555.3137 | **GSPITVP** | 624.3715 | 652.3665 |
| **SP** | 157.0972 | 185.0921 | **SPI** | 270.1812 | 298.1761 | **SPIT** | 371.2289 | 399.2238 |
| **SPITV** | 470.2973 | 498.2922 | **SPITVP** | 567.3501 | 595.3450 | **SPITVPV** | 666.4185 | 694.4134 |
| **PI** | 183.1492 | 211.1441 | **PIT** | 284.1969 | 312.1918 | **PITV** | 383.2653 | 411.2602 |
| **PITVP** | 480.3180 | 508.3130 | **PITVPV** | 579.3865 | 607.3814 | **PITVPVG** | 636.4079 | 664.4028 |
| **IT** | 187.1441 | 215.1390 | **ITV** | 286.2125 | 314.2074 | **ITVP** | 383.2653 | 411.2602 |
| **ITVPV** | 482.3337 | 510.3286 | **ITVPVG** | 539.3552 | 567.3501 | **TV** | 173.1285 | 201.1234 |
| **TVP** | 270.1812 | 298.1761 | **TVPV** | 369.2496 | 397.2445 | **TVPVG** | 426.2711 | 454.2660 |
| **VP** | 169.1335 | 197.1285 | **VPV** | 268.2020 | 296.1969 | **VPVG** | 325.2234 | 353.2183 |
| **PV** | 169.1335 | 197.1285 | **PVG** | 226.1550 | 254.1499 | **VG** | 129.1022 | 157.0972 |

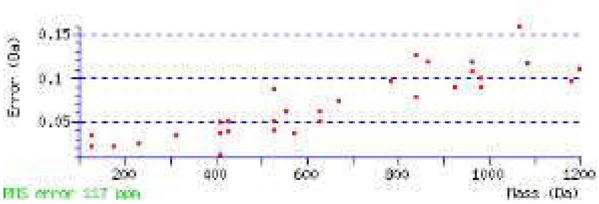


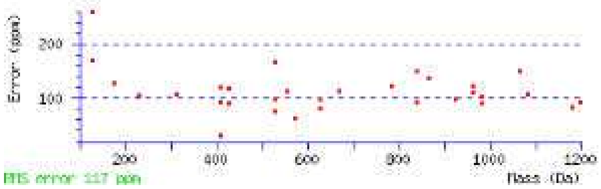


NCBI **BLAST** search of VLNTGSPITVPVGR
(Parameters: blastp, nr protein database, expect=20000, no filter, PAM30)
Other BLAST web gateways

**All matches to this query**

| Score | Mr(calc) | Delta | Sequence |
|---|---|---|---|
| 97.7 | 1408.8038 | 0.0420 | VLNTGSPITVPVGR |
| 38.9 | 1408.8038 | 0.0420 | IAEGALTAALAVPGR |
| 37.1 | 1408.7675 | 0.0784 | AVDTGQPITVPVGR |
| 36.5 | 1408.7463 | 0.0995 | GTNWLPLDALPGR |
| 34.5 | 1408.8038 | 0.0420 | LGEKGSPLISPGVR |
| 32.9 | 1408.7787 | 0.0672 | ATSAPVEARVPVGR |
| 31.8 | 1408.8402 | 0.0056 | RSIAGLALPLSVVN |
| 31.4 | 1408.8554 | -0.0096 | WLKSLLQLVPGR |
| 30.7 | 1408.8263 | 0.0196 | TQPTATRLLRPR |
| 30.6 | 1408.8402 | 0.0056 | QLDALSPLKVLGR |

**Mascot:** http://www.matrixscience.com/

# Mascot Search Results

## Peptide View Spot no 85

MS/MS Fragmentation of **FTQANSEVSALLGR**
Found in **gi|3676294** in **NCBInr**, mitochondrial ATPase beta subunit [Nicotiana sylvestris]

Match to Query 223: 1491.812224 from(1492.819500,1+) intensity(0.0000) index(22)
Title: Label: F15, Spot_Id: 219903, Peak_List_Id: 228321, MSMS Job_Run_Id: 21987, Comment:
Data file C:\Documents and Settings\Administrator\Desktop\Farah-Garima 11May 2013\ppw_F15_136868277600.txt

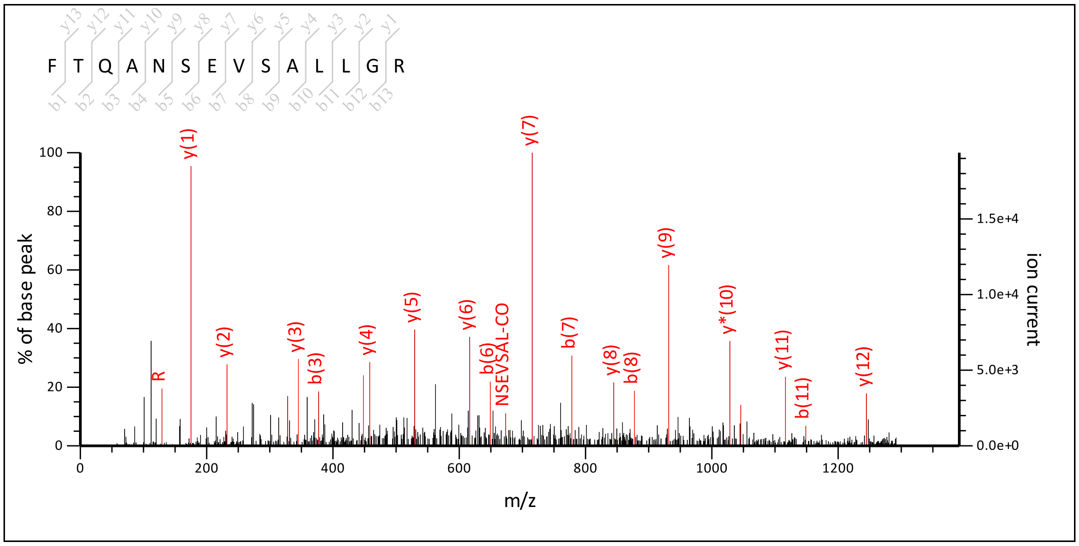


0 to 1391.82

Label all possible matches ○ Label matches used for scoring ●

**Monoisotopic mass of neutral peptide Mr(calc):** 1491.7681
**Fixed modifications:** Carbamidomethyl (C) (apply to specified residues or termini only)
**Ions Score:** 123 **Expect:** 1.5e-08
**Matches :** 23/251 fragment ions using 26 most intense peaks (help)

| # | Immon. | a | a* | a⁰ | b | b* | b⁰ | d | d' | Seq. | v | w | w' | y | y* | |
|---|---|---|---|---|---|---|---|---|---|---|---|---|---|---|---|---|
| 1 | 120.0808 | 120.0808 | | | 148.0757 | | | 44.0495 | | F | | | | | | |
| 2 | 74.0600 | 221.1285 | | 203.1179 | 249.1234 | | 231.1128 | 205.1335 | 207.1128 | T | 1299.6652 | 1312.6856 | 1314.6648 | 1345.7070 | 1328.6805 | 132[illegible] |
| 3 | 101.0709 | 349.1870 | 332.1605 | 331.1765 | **377.1819** | 360.1554 | 359.1714 | 292.1656 | | Q | 1171.6066 | 1170.6113 | | ***1244.6593*** | 1227.6328 | 122[illegible] |
| 4 | 44.0495 | 420.2241 | 403.1976 | 402.2136 | **448.2191** | 431.1925 | 430.2085 | | | A | 1100.5695 | | | ***1116.6008*** | 1099.5742 | 109[illegible] |
| 5 | 87.0553 | 534.2671 | 517.2405 | 516.2565 | 562.2620 | 545.2354 | 544.2514 | 491.2613 | | N | 986.5265 | 985.5313 | | ***1045.5636*** | 1028.5371 | 102[illegible] |
| 6 | 60.0444 | 621.2991 | 604.2726 | 603.2885 | **649.2940** | 632.2675 | 631.2835 | 605.3042 | | S | 899.4945 | 898.4993 | | ***931.5207*** | 914.4942 | 91[illegible] |
| 7 | 102.0550 | 750.3417 | 733.3151 | 732.3311 | **778.3366** | 761.3101 | 760.3260 | 692.3362 | | E | 770.4519 | 769.4567 | | ***844.4887*** | 827.4621 | 82[illegible] |
| 8 | 72.0808 | 849.4101 | 832.3836 | 831.3995 | **877.4050** | 860.3785 | 859.3945 | 835.3945 | | V | 671.3835 | 684.4039 | | ***715.4461*** | 698.4196 | 69[illegible] |
| 9 | 60.0444 | 936.4421 | 919.4156 | 918.4316 | 964.4371 | 947.4105 | 946.4265 | 920.4472 | | S | 584.3515 | 583.3562 | | ***616.3777*** | 599.3511 | 59[illegible] |
| 10 | 44.0495 | 1007.4792 | 990.4527 | 989.4687 | 1035.4742 | 1018.4476 | 1017.4636 | | | A | 513.3144 | | | ***529.3457*** | 512.3191 | |
| 11 | 86.0964 | 1120.5633 | 1103.5368 | 1102.5527 | **1148.5582** | 1131.5317 | 1130.5477 | 1078.5164 | | L | 400.2303 | 399.2350 | | ***458.3085*** | 441.2820 | |
| 12 | 86.0964 | 1233.6474 | 1216.6208 | 1215.6368 | 1261.6423 | 1244.6157 | 1243.6317 | 1191.6004 | | L | 287.1462 | 286.1510 | | ***345.2245*** | 328.1979 | |
| 13 | 30.0338 | 1290.6688 | 1273.6423 | 1272.6583 | 1318.6638 | 1301.6372 | 1300.6532 | | | G | | | | ***232.1404*** | 215.1139 | |
| 14 | 129.1135 | | | | | | | | | R | 74.0237 | 73.0284 | | ***175.1190*** | 158.0924 | |

| Seq | ya | yb | Seq | ya | yb | Seq | ya | yb |
|---|---|---|---|---|---|---|---|---|
| TQ | 202.1186 | 230.1135 | TQA | 273.1557 | 301.1506 | TQAN | 387.1987 | 415.1936 |
| TQANS | 474.2307 | 502.2256 | TQANSE | 603.2733 | 631.2682 | QA | 172.1081 | 200.1030 |
| QAN | 286.1510 | 314.1459 | QANS | 373.1830 | 401.1779 | QANSE | 502.2256 | 530.2205 |
| QANSEV | 601.2940 | 629.2889 | QANSEVS | 688.3260 | 716.3210 | AN | 158.0924 | 186.0873 |
| ANS | 245.1244 | 273.1193 | ANSE | 374.1670 | 402.1619 | ANSEV | 473.2354 | 501.2304 |
| ANSEVS | 560.2675 | 588.2624 | ANSEVSA | 631.3046 | 659.2995 | NS | 174.0873 | 202.0822 |
| NSE | 303.1299 | 331.1248 | NSEV | 402.1983 | 430.1932 | NSEVS | 489.2304 | 517.2253 |

| | | | | | | | | |
|---|---|---|---|---|---|---|---|---|
| **NSEVSA** | 560.2675 | 588.2624 | **NSEVSAL** | 673.3515 | 701.3464 | **SE** | 189.0870 | 217.0819 |
| **SEV** | 288.1554 | 316.1503 | **SEVS** | 375.1874 | 403.1823 | **SEVSA** | 446.2245 | 474.2195 |
| **SEVSAL** | 559.3086 | 587.3035 | **SEVSALL** | 672.3927 | 700.3876 | **EV** | 201.1234 | 229.1183 |
| **EVS** | 288.1554 | 316.1503 | **EVSA** | 359.1925 | 387.1874 | **EVSAL** | 472.2766 | 500.2715 |
| **EVSALL** | 585.3606 | 613.3556 | **EVSALLG** | 642.3821 | 670.3770 | **VS** | 159.1128 | 187.1077 |
| **VSA** | 230.1499 | 258.1448 | **VSAL** | 343.2340 | 371.2289 | **VSALL** | 456.3180 | 484.3130 |
| **VSALLG** | 513.3395 | 541.3344 | **SA** | 131.0815 | 159.0764 | **SAL** | 244.1656 | 272.1605 |
| **SALL** | 357.2496 | 385.2445 | **SALLG** | 414.2711 | 442.2660 | **AL** | 157.1335 | 185.1285 |
| **ALL** | 270.2176 | 298.2125 | **ALLG** | 327.2391 | 355.2340 | **LL** | 199.1805 | 227.1754 |
| **LLG** | 256.2020 | 284.1969 | **LG** | 143.1179 | 171.1128 | | | |

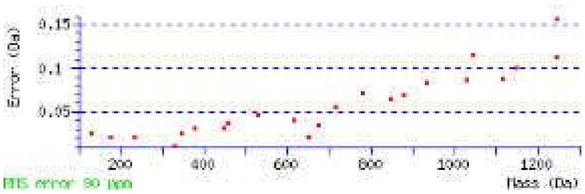


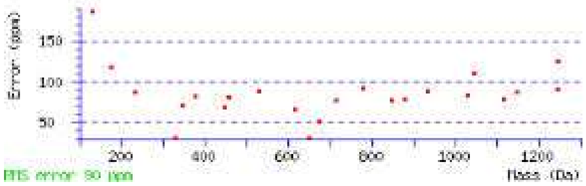


NCBI **BLAST** search of FTQANSEVSALLGR
(Parameters: blastp, nr protein database, expect=20000, no filter, PAM30)
Other BLAST web gateways

**All matches to this query**

| Score | Mr(calc) | Delta | Sequence |
|---|---|---|---|
| 123.1 | 1491.7681 | 0.0441 | FTQANSEVSALLGR |
| 84.3 | 1491.7834 | 0.0288 | FTQAGSWVSALLGR |
| 48.3 | 1491.8297 | -0.0175 | YTLAVTEVSALLGR |
| 39.4 | 1491.6987 | 0.1135 | MEATAEEVDRAVR |
| 36.1 | 1491.7794 | 0.0329 | FTQAGSEISARLGR |
| 34.8 | 1491.7318 | 0.0805 | STSTAQSDWLAAVR |
| 34.7 | 1491.7529 | 0.0593 | ITGSGSLGATTDSLGR |
| 32.0 | 1491.7967 | 0.0155 | KTTGIDTSAAVAVMK |
| 31.4 | 1491.7351 | 0.0771 | LAVSEAKGGSAMAER |
| 31.0 | 1491.7100 | 0.1022 | VGETASRGMDNLAR |

**Mascot:** http://www.matrixscience.com/

# MATRIX SCIENCE Mascot Search Results

## Peptide View **Spot no 85**

MS/MS Fragmentation of **CALVYGQMNEPPGAR**
Found in **gi|3676294** in **NCBInr**, mitochondrial ATPase beta subunit [Nicotiana sylvestris]

Match to Query 235: 1661.814424 from(1662.821700,1+) intensity(0.0000) index(24)
Title: Label: F15, Spot_Id: 219903, Peak_List_Id: 228325, MSMS Job_Run_Id: 21987, Comment:
Data file C:\Documents and Settings\Administrator\Desktop\Farah-Garima 11May 2013\ppw_F15_136868277600.txt

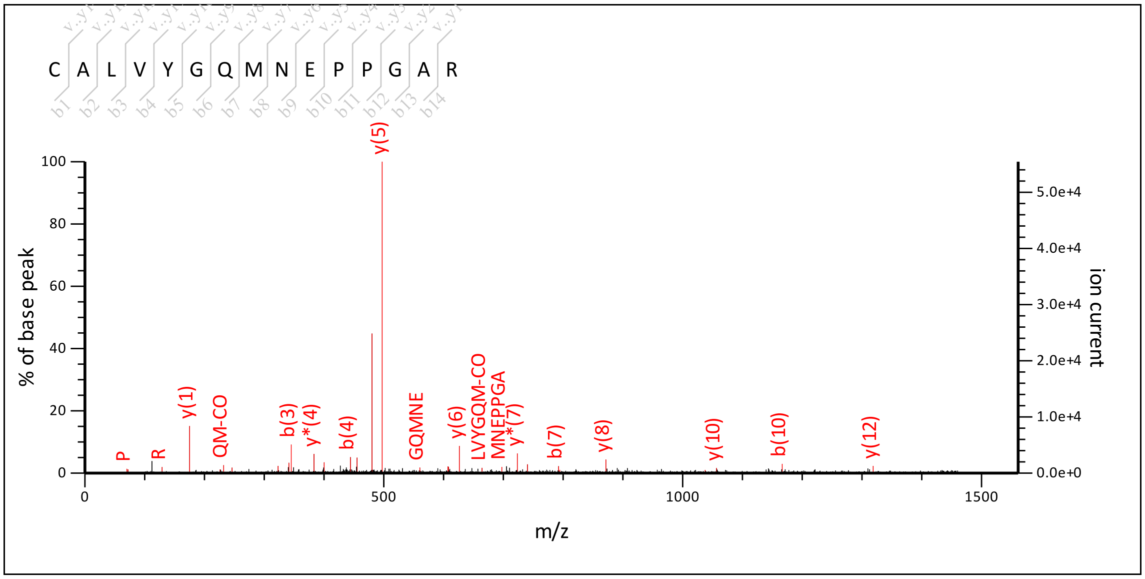


0 to 1561.27

Label all possible matches ○ Label matches used for scoring ◉

**Monoisotopic mass of neutral peptide Mr(calc):** 1661.7654
**Fixed modifications:** Carbamidomethyl (C) (apply to specified residues or termini only)
**Ions Score:** 59 **Expect:** 0.034
**Matches :** 40/237 fragment ions using 56 most intense peaks (help)

| # | Immon. | a | a* | $a^0$ | b | b* | $b^0$ | d | Seq. | v | w | y | y* | $y^0$ | # |
|---|---|---|---|---|---|---|---|---|---|---|---|---|---|---|---|
| 1 | 133.0430 | 133.0430 | | | 161.0379 | | | 44.0495 | C | | | | | | 15 |
| 2 | 44.0495 | 204.0801 | | | ***232.0750*** | | | | A | 1486.7107 | | 1502.7420 | 1485.7155 | 1484.7315 | 14 |
| 3 | 86.0964 | 317.1642 | | | ***345.1591*** | | | 275.1172 | L | 1373.6267 | 1372.6314 | 1431.7049 | 1414.6784 | 1413.6943 | 13 |
| 4 | 72.0808 | 416.2326 | | | ***444.2275*** | | | 402.2170 | V | 1274.5582 | 1287.5786 | ***1318.6208*** | 1301.5943 | 1300.6103 | 12 |
| 5 | 136.0757 | 579.2959 | | | ***607.2908*** | | | | Y | 1111.4949 | | 1219.5524 | 1202.5259 | 1201.5419 | 11 |
| 6 | 30.0338 | 636.3174 | | | ***664.3123*** | | | | G | | | ***1056.4891*** | 1039.4626 | 1038.4785 | 10 |
| 7 | 101.0709 | 764.3760 | 747.3494 | | ***792.3709*** | 775.3443 | | 707.3545 | Q | 926.4149 | 925.4196 | 999.4676 | 982.4411 | 981.4571 | 9 |
| 8 | 104.0528 | 895.4165 | 878.3899 | | 923.4114 | 906.3848 | | 835.4131 | M | 795.3744 | 794.3791 | ***871.4091*** | 854.3825 | 853.3985 | 8 |
| 9 | 87.0553 | 1009.4594 | 992.4328 | | ***1037.4543*** | 1020.4278 | | 966.4536 | N | 681.3315 | 680.3362 | ***740.3686*** | 723.3420 | 722.3580 | 7 |
| 10 | 102.0550 | 1138.5020 | 1121.4754 | 1120.4914 | ***1166.4969*** | 1149.4703 | 1148.4863 | 1080.4965 | E | 552.2889 | 551.2936 | ***626.3257*** | 609.2991 | 608.3151 | 6 |
| 11 | 70.0651 | 1235.5547 | 1218.5282 | 1217.5442 | 1263.5497 | 1246.5231 | 1245.5391 | 1209.5391 | P | 455.2361 | 454.2409 | ***497.2831*** | 480.2565 | | 5 |
| 12 | 70.0651 | 1332.6075 | 1315.5810 | 1314.5969 | 1360.6024 | 1343.5759 | 1342.5919 | 1306.5919 | P | 358.1833 | 357.1881 | ***400.2303*** | 383.2037 | | 4 |
| 13 | 30.0338 | 1389.6290 | 1372.6024 | 1371.6184 | 1417.6239 | 1400.5973 | 1399.6133 | | G | | | 303.1775 | 286.1510 | | 3 |
| 14 | 44.0495 | 1460.6661 | 1443.6395 | 1442.6555 | 1488.6610 | 1471.6345 | 1470.6504 | | A | 230.1248 | | ***246.1561*** | 229.1295 | | 2 |
| 15 | 129.1135 | | | | | | | | R | 74.0237 | 73.0284 | ***175.1190*** | 158.0924 | | 1 |

| Seq | ya | yb | Seq | ya | yb | Seq | ya | yb |
|---|---|---|---|---|---|---|---|---|
| AL | 157.1335 | 185.1285 | ALV | 256.2020 | 284.1969 | ALVY | 419.2653 | 447.2602 |
| ALVYG | 476.2867 | 504.2817 | ALVYGQ | 604.3453 | 632.3402 | LV | 185.1648 | 213.1598 |
| LVY | 348.2282 | 376.2231 | LVYG | 405.2496 | 433.2445 | LVYGQ | 533.3082 | 561.3031 |

| | | | | | | | | |
|---|---|---|---|---|---|---|---|---|
| **LVYGQM** | 664.3487 | 692.3436 | **VY** | 235.1441 | 263.1390 | **VYG** | 292.1656 | 320.1605 |
| **VYGQ** | 420.2241 | 448.2191 | **VYGQM** | 551.2646 | 579.2595 | **VYGQMN** | 665.3076 | 693.3025 |
| **YG** | 193.0972 | 221.0921 | **YGQ** | 321.1557 | 349.1506 | **YGQM** | 452.1962 | 480.1911 |
| **YGQMN** | 566.2391 | 594.2341 | **YGQMNE** | 695.2817 | 723.2767 | **GQ** | 158.0924 | 186.0873 |
| **GQM** | 289.1329 | 317.1278 | **GQMN** | 403.1758 | 431.1707 | **GQMNE** | 532.2184 | 560.2133 |
| **GQMNEP** | 629.2712 | 657.2661 | **QM** | 232.1114 | 260.1063 | **QMN** | 346.1544 | 374.1493 |
| **QMNE** | 475.1969 | 503.1919 | **QMNEP** | 572.2497 | 600.2446 | **QMNEPP** | 669.3025 | 697.2974 |
| **MN** | 218.0958 | 246.0907 | **MNE** | 347.1384 | 375.1333 | **MNEP** | 444.1911 | 472.1860 |
| **MNEPP** | 541.2439 | 569.2388 | **MNEPPG** | 598.2654 | 626.2603 | **MNEPPGA** | 669.3025 | 697.2974 |
| **NE** | 216.0979 | 244.0928 | **NEP** | 313.1506 | 341.1456 | **NEPP** | 410.2034 | 438.1983 |
| **NEPPG** | 467.2249 | 495.2198 | **NEPPGA** | 538.2620 | 566.2569 | **EP** | 199.1077 | 227.1026 |
| **EPP** | 296.1605 | 324.1554 | **EPPG** | 353.1819 | 381.1769 | **EPPGA** | 424.2191 | 452.2140 |
| **PP** | 167.1179 | 195.1128 | **PPG** | 224.1394 | 252.1343 | **PPGA** | 295.1765 | 323.1714 |
| **PG** | 127.0866 | 155.0815 | **PGA** | 198.1237 | 226.1186 | **GA** | 101.0709 | 129.0659 |

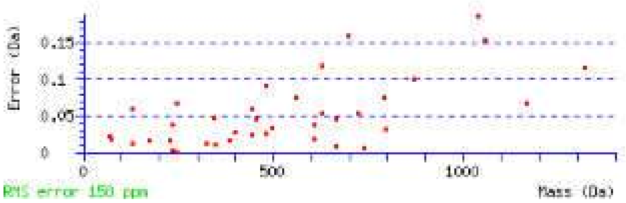


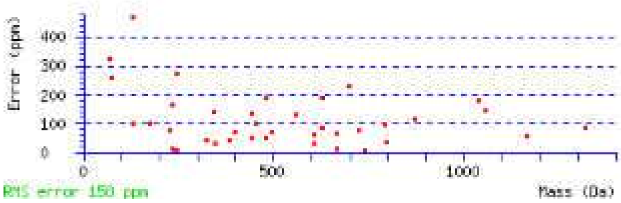


NCBI **BLAST** search of CALVYGQMNEPPGAR
(Parameters: blastp, nr protein database, expect=20000, no filter, PAM30)
Other BLAST web gateways

**All matches to this query**

| Score | Mr(calc) | Delta | Sequence |
|---|---|---|---|
| 67.3 | 1661.7654 | 0.0490 | ACLVYGQMNEPPGAR |
| 59.4 | 1661.7654 | 0.0490 | CALVYGQMNEPPGAR |
| 59.4 | 1661.7654 | 0.0490 | CALVYGQMNEPPGAR |
| 42.1 | 1661.7654 | 0.0490 | VALCYGQMNEPPGAR |
| 42.1 | 1661.7654 | 0.0490 | VALCYGQMNEPPGAR |
| 39.3 | 1661.7654 | 0.0490 | CSLVFGQMNEPPGAR |
| 29.6 | 1661.7654 | 0.0490 | CALVFGQMNEPPGAR |
| 28.9 | 1661.8737 | -0.0592 | QPGDGELKVIEPAPGR |
| 27.3 | 1661.8373 | -0.0229 | QPGDGELNIIEPAPGR |
| 26.1 | 1661.9213 | -0.1068 | QPKGSQLLAAAPSPAAR |

**Mascot:** http://www.matrixscience.com/

# MATRIX SCIENCE Mascot Search Results

## Peptide View **Spot no 85**

MS/MS Fragmentation of **CALVYGQMNEPPGAR**
Found in **gi|3676294** in **NCBInr**, mitochondrial ATPase beta subunit [Nicotiana sylvestris]

Match to Query 238: 1677.802324 from(1678.809600,1+) intensity(0.0000) index(25)
Title: Label: F15, Spot_Id: 219903, Peak_List_Id: 228336, MSMS Job_Run_Id: 21987, Comment:
Data file C:\Documents and Settings\Administrator\Desktop\Farah-Garima 11May 2013\ppw_F15_136868277600.txt

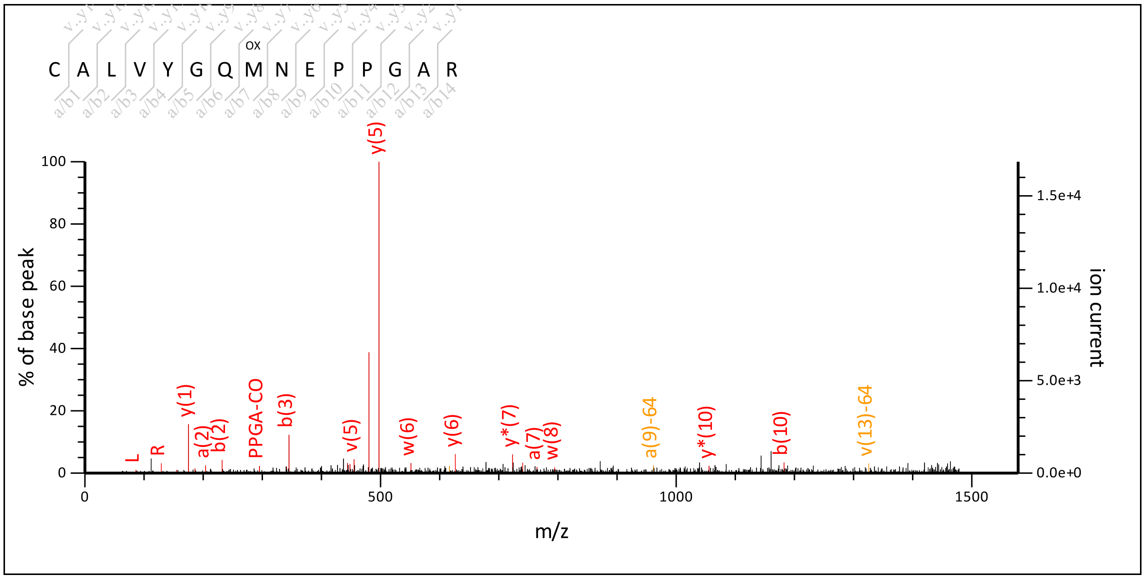


0 to 1578.39

Label all possible matches ○ Label matches used for scoring ◉

**Monoisotopic mass of neutral peptide Mr(calc):** 1677.7603
**Fixed modifications:** Carbamidomethyl (C) (apply to specified residues or termini only)
**Variable modifications:**
**M8** : Oxidation (M), with neutral losses 0.0000(shown in table), 63.9983
**Ions Score:** 13 **Expect:** 1.3e+03
**Matches :** 29/340 fragment ions using 60 most intense peaks (help)

| # | Immon. | a | a* | $a^0$ | b | b* | $b^0$ | d | Seq. | v | w | y | y* | $y^0$ | # |
|---|---|---|---|---|---|---|---|---|---|---|---|---|---|---|---|
| 1 | 133.0430 | 133.0430 | | | 161.0379 | | | 44.0495 | C | | | | | | 15 |
| 2 | 44.0495 | **204.0801** | | | ***232.0750*** | | | | A | 1502.7056 | | 1518.7369 | 1501.7104 | 1500.7264 | 14 |
| 3 | 86.0964 | 317.1642 | | | ***345.1591*** | | | 275.1172 | L | 1389.6216 | 1388.6263 | 1447.6998 | 1430.6733 | 1429.6893 | 13 |
| 4 | 72.0808 | 416.2326 | | | ***444.2275*** | | | 402.2170 | V | 1290.5532 | 1303.5736 | 1334.6158 | 1317.5892 | 1316.6052 | 12 |
| 5 | 136.0757 | 579.2959 | | | 607.2908 | | | | Y | 1127.4898 | | 1235.5473 | 1218.5208 | 1217.5368 | 11 |
| 6 | 30.0338 | 636.3174 | | | 664.3123 | | | | G | | | 1072.4840 | 1055.4575 | 1054.4735 | 10 |
| 7 | 101.0709 | **764.3760** | 747.3494 | | 792.3709 | 775.3443 | | 707.3545 | Q | 942.4098 | 941.4145 | 1015.4626 | 998.4360 | 997.4520 | 9 |
| 8 | 120.0478 | 911.4114 | 894.3848 | | 939.4063 | 922.3797 | | 835.4131 | M | 795.3744 | 794.3791 | 887.4040 | 870.3774 | 869.3934 | 8 |
| 9 | 87.0553 | 1025.4543 | 1008.4278 | | 1053.4492 | 1036.4227 | | 982.4485 | N | 681.3315 | 680.3362 | ***740.3686*** | 723.3420 | 722.3580 | 7 |
| 10 | 102.0550 | 1154.4969 | 1137.4703 | 1136.4863 | ***1182.4918*** | 1165.4653 | 1164.4812 | 1096.4914 | E | 552.2889 | 551.2936 | ***626.3257*** | 609.2991 | 608.3151 | 6 |
| 11 | 70.0651 | 1251.5497 | 1234.5231 | 1233.5391 | 1279.5446 | 1262.5180 | 1261.5340 | 1225.5340 | P | 455.2361 | 454.2409 | ***497.2831*** | 480.2565 | | 5 |
| 12 | 70.0651 | 1348.6024 | 1331.5759 | 1330.5919 | 1376.5973 | 1359.5708 | 1358.5868 | 1322.5868 | P | 358.1833 | 357.1881 | 400.2303 | 383.2037 | | 4 |
| 13 | 30.0338 | 1405.6239 | 1388.5973 | 1387.6133 | 1433.6188 | 1416.5923 | 1415.6082 | | G | | | 303.1775 | 286.1510 | | 3 |
| 14 | 44.0495 | 1476.6610 | 1459.6345 | 1458.6504 | 1504.6559 | 1487.6294 | 1486.6453 | | A | 230.1248 | | 246.1561 | 229.1295 | | 2 |
| 15 | 129.1135 | | | | | | | | R | 74.0237 | 73.0284 | ***175.1190*** | 158.0924 | | 1 |

| Seq | ya | yb | Seq | ya | yb | Seq | ya | yb |
|---|---|---|---|---|---|---|---|---|

| | | | | | | | | |
|---|---|---|---|---|---|---|---|---|
| **AL** | 157.1335 | 185.1285 | **ALV** | 256.2020 | 284.1969 | **ALVY** | 419.2653 | 447.2602 |
| **ALVYG** | 476.2867 | 504.2817 | **ALVYGQ** | 604.3453 | 632.3402 | **LV** | 185.1648 | 213.1598 |
| **LVY** | 348.2282 | 376.2231 | **LVYG** | 405.2496 | 433.2445 | **LVYGQ** | 533.3082 | 561.3031 |
| **LVYGQM** | 680.3436 | 708.3385 | **VY** | 235.1441 | 263.1390 | **VYG** | 292.1656 | 320.1605 |
| **VYGQ** | 420.2241 | 448.2191 | **VYGQM** | 567.2595 | 595.2545 | **VYGQMN** | 681.3025 | 709.2974 |
| **YG** | 193.0972 | 221.0921 | **YGQ** | 321.1557 | 349.1506 | **YGQM** | 468.1911 | 496.1860 |
| **YGQMN** | 582.2341 | 610.2290 | **GQ** | 158.0924 | 186.0873 | **GQM** | 305.1278 | 333.1227 |
| **GQMN** | 419.1707 | 447.1656 | **GQMNE** | 548.2133 | 576.2082 | **GQMNEP** | 645.2661 | 673.2610 |
| **QM** | 248.1063 | 276.1013 | **QMN** | 362.1493 | 390.1442 | **QMNE** | 491.1919 | 519.1868 |
| **QMNEP** | 588.2446 | 616.2395 | **QMNEPP** | 685.2974 | 713.2923 | **MN** | 234.0907 | 262.0856 |
| **MNE** | 363.1333 | 391.1282 | **MNEP** | 460.1860 | 488.1810 | **MNEPP** | 557.2388 | 585.2337 |
| **MNEPPG** | 614.2603 | 642.2552 | **MNEPPGA** | 685.2974 | 713.2923 | **NE** | 216.0979 | 244.0928 |
| **NEP** | 313.1506 | 341.1456 | **NEPP** | 410.2034 | 438.1983 | **NEPPG** | 467.2249 | 495.2198 |
| **NEPPGA** | 538.2620 | 566.2569 | **EP** | 199.1077 | 227.1026 | **EPP** | 296.1605 | 324.1554 |
| **EPPG** | 353.1819 | 381.1769 | **EPPGA** | 424.2191 | 452.2140 | **PP** | 167.1179 | 195.1128 |
| **PPG** | 224.1394 | 252.1343 | **PPGA** | 295.1765 | 323.1714 | **PG** | 127.0866 | 155.0815 |
| **PGA** | 198.1237 | 226.1186 | **GA** | 101.0709 | 129.0659 | | | |

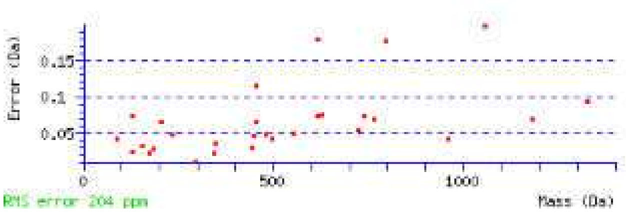


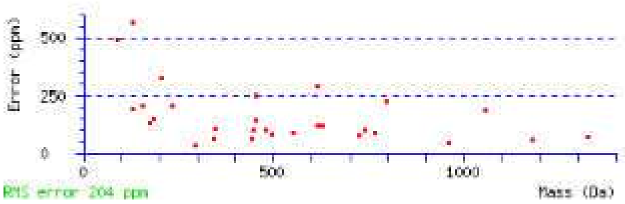


NCBI **BLAST** search of CALVYGQMNEPPGAR
(Parameters: blastp, nr protein database, expect=20000, no filter, PAM30)
Other BLAST web gateways

**All matches to this query**

| Score | Mr(calc) | Delta | Sequence |
|---|---|---|---|
| 15.3 | 1677.8951 | -0.0927 | AAERPEPGIPTAKWR |
| 14.8 | 1677.8760 | -0.0737 | TTMFSSDLPLPKIGR |
| 14.3 | 1677.8694 | -0.0671 | MAAQKAQFPIALMAR |
| 14.3 | 1677.9678 | -0.1655 | QALKLPYAALLHQGR |
| 14.2 | 1677.8911 | -0.0887 | TNALPERATVTVNHR |
| 14.1 | 1677.7087 | 0.0936 | SMMPSSSSEPTVHGGR |
| 13.7 | 1677.9050 | -0.1026 | TATAVPLPEDPAKNVR |
| 13.5 | 1677.7603 | 0.0420 | ACLVYGQMNEPPGAR |
| 13.5 | 1677.7603 | 0.0420 | CALVYGQMNEPPGAR |
| 13.5 | 1677.7603 | 0.0420 | CALVYGQMNEPPGAR |

**Mascot:** http://www.matrixscience.com/

# Mascot Search Results

## Peptide View Spot no 85

MS/MS Fragmentation of **QISELGIYPAVDPLDSTSR**
Found in **gi|3676294** in **NCBInr**, mitochondrial ATPase beta subunit [Nicotiana sylvestris]

Match to Query 260: 2060.106524 from(2061.113800,1+) intensity(0.0000) index(31)
Title: Label: F15, Spot_Id: 219903, Peak_List_Id: 228327, MSMS Job_Run_Id: 21987, Comment:
Data file C:\Documents and Settings\Administrator\Desktop\Farah-Garima 11May 2013\ppw_F15_136868277600.txt

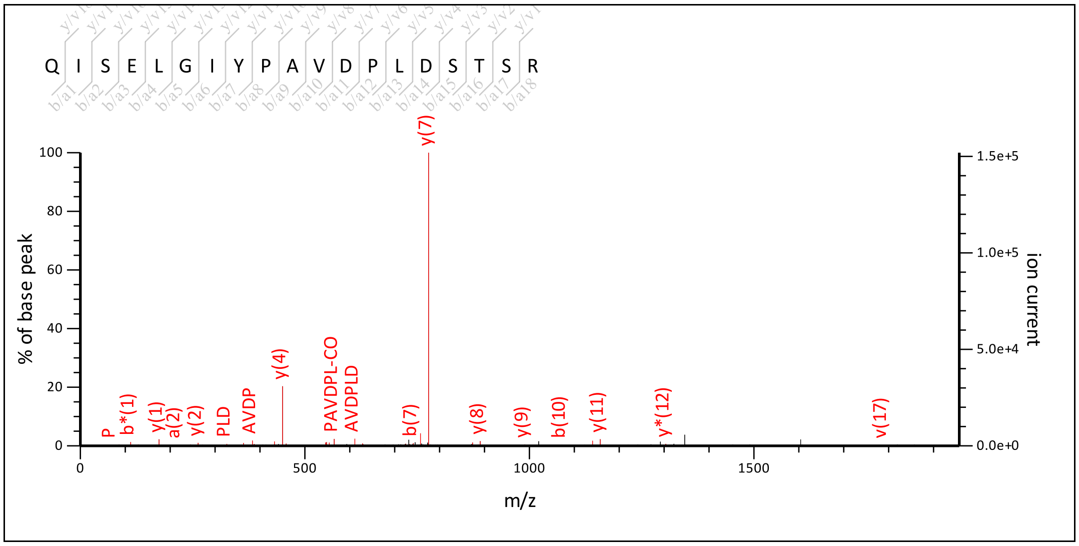


0 to 1957.3

Label all possible matches ○ Label matches used for scoring ◉

**Monoisotopic mass of neutral peptide Mr(calc):** 2060.0426
**Fixed modifications:** Carbamidomethyl (C) (apply to specified residues or termini only)
**Ions Score:** 53 **Expect:** 0.11
**Matches :** 58/375 fragment ions using 92 most intense peaks (help)

| # | Immon. | a | a* | $a^0$ | b | b* | $b^0$ | d | d' | Seq. | v | w | w' | y | y* | |
|---|---|---|---|---|---|---|---|---|---|---|---|---|---|---|---|---|
| 1 | 101.0709 | 101.0709 | 84.0444 | | ***129.0659*** | 112.0393 | | 44.0495 | | Q | | | | | | |
| 2 | 86.0964 | 214.1550 | 197.1285 | | ***242.1499*** | 225.1234 | | 186.1237 | 200.1394 | I | 1874.9130 | 1887.9335 | 1901.9491 | 1932.9913 | 1915.9648 | [illegible] |
| 3 | 60.0444 | 301.1870 | 284.1605 | 283.1765 | 329.1819 | 312.1554 | 311.1714 | 285.1921 | | S | 1787.8810 | 1786.8858 | | 1819.9072 | 1802.8807 | [illegible] |
| 4 | 102.0550 | 430.2296 | 413.2031 | 412.2191 | ***458.2245*** | 441.1980 | 440.2140 | 372.2241 | | E | 1658.8384 | 1657.8432 | | 1732.8752 | 1715.8487 | [illegible] |
| 5 | 86.0964 | 543.3137 | 526.2871 | 525.3031 | 571.3086 | 554.2821 | 553.2980 | 501.2667 | | L | 1545.7544 | 1544.7591 | | 1603.8326 | 1586.8061 | [illegible] |
| 6 | 30.0338 | 600.3352 | 583.3086 | 582.3246 | ***628.3301*** | 611.3035 | 610.3195 | | | G | | | | 1490.7486 | 1473.7220 | [illegible] |
| 7 | 86.0964 | 713.4192 | 696.3927 | 695.4087 | ***741.4141*** | 724.3876 | 723.4036 | 685.3879 | 699.4036 | I | 1375.6488 | 1388.6692 | 1402.6849 | 1433.7271 | 1416.7005 | [illegible] |
| 8 | 136.0757 | 876.4825 | 859.4560 | 858.4720 | 904.4775 | 887.4509 | 886.4669 | | | Y | 1212.5855 | | | 1320.6430 | 1303.6165 | [illegible] |
| 9 | 70.0651 | 973.5353 | 956.5088 | 955.5247 | 1001.5302 | 984.5037 | 983.5197 | 947.5197 | | P | 1115.5327 | 1114.5375 | | ***1157.5797*** | 1140.5531 | [illegible] |
| 10 | 44.0495 | 1044.5724 | 1027.5459 | 1026.5619 | ***1072.5673*** | 1055.5408 | 1054.5568 | | | A | 1044.4956 | | | ***1060.5269*** | 1043.5004 | [illegible] |
| 11 | 72.0808 | 1143.6408 | 1126.6143 | 1125.6303 | 1171.6358 | 1154.6092 | 1153.6252 | 1129.6252 | | V | 945.4272 | 958.4476 | | ***989.4898*** | 972.4633 | [illegible] |
| 12 | 88.0393 | 1258.6678 | 1241.6412 | 1240.6572 | 1286.6627 | 1269.6361 | 1268.6521 | 1214.6780 | | D | 830.4003 | 829.4050 | | ***890.4214*** | 873.3949 | [illegible] |
| 13 | 70.0651 | 1355.7205 | 1338.6940 | 1337.7100 | 1383.7155 | 1366.6889 | 1365.7049 | 1329.7049 | | P | 733.3475 | 732.3523 | | ***775.3945*** | 758.3679 | [illegible] |
| 14 | 86.0964 | 1468.8046 | 1451.7781 | 1450.7940 | 1496.7995 | 1479.7730 | 1478.7890 | 1426.7577 | | L | 620.2634 | 619.2682 | | 678.3417 | 661.3151 | [illegible] |
| 15 | 88.0393 | 1583.8316 | 1566.8050 | 1565.8210 | 1611.8265 | 1594.7999 | 1593.8159 | 1539.8417 | | D | 505.2365 | 504.2413 | | ***565.2576*** | 548.2311 | [illegible] |
| 16 | 60.0444 | 1670.8636 | 1653.8370 | 1652.8530 | 1698.8585 | 1681.8319 | 1680.8479 | 1654.8687 | | S | 418.2045 | 417.2092 | | ***450.2307*** | 433.2041 | [illegible] |
| 17 | 74.0600 | 1771.9113 | 1754.8847 | 1753.9007 | 1799.9062 | 1782.8796 | 1781.8956 | 1755.9163 | 1757.8956 | T | 317.1568 | 330.1772 | 332.1565 | ***363.1987*** | 346.1721 | [illegible] |
| 18 | 60.0444 | 1858.9433 | 1841.9167 | 1840.9327 | 1886.9382 | 1869.9117 | 1868.9276 | 1842.9484 | | S | 230.1248 | 229.1295 | | ***262.1510*** | 245.1244 | [illegible] |
| 19 | 129.1135 | | | | | | | | | R | 74.0237 | 73.0284 | | ***175.1190*** | 158.0924 | |

| Seq | ya | yb | Seq | ya | yb | Seq | ya | yb |
|---|---|---|---|---|---|---|---|---|
| IS | 173.1285 | 201.1234 | ISE | 302.1710 | 330.1660 | ISEL | 415.2551 | 443.2500 |
| ISELG | 472.2766 | 500.2715 | ISELGI | 585.3606 | 613.3556 | SE | 189.0870 | 217.0819 |

| | | | | | | | | |
|---|---|---|---|---|---|---|---|---|
| SEL | 302.1710 | 330.1660 | SELG | 359.1925 | 387.1874 | SELGI | 472.2766 | 500.2715 |
| SELGIY | 635.3399 | 663.3348 | EL | 215.1390 | 243.1339 | ELG | 272.1605 | 300.1554 |
| ELGI | 385.2445 | 413.2395 | ELGIY | 548.3079 | 576.3028 | ELGIYP | 645.3606 | 673.3556 |
| LG | 143.1179 | 171.1128 | LGI | 256.2020 | 284.1969 | LGIY | 419.2653 | 447.2602 |
| LGIYP | 516.3180 | 544.3130 | LGIYPA | 587.3552 | 615.3501 | LGIYPAV | 686.4236 | 714.4185 |
| GI | 143.1179 | 171.1128 | GIY | 306.1812 | 334.1761 | GIYP | 403.2340 | 431.2289 |
| GIYPA | 474.2711 | 502.2660 | GIYPAV | 573.3395 | 601.3344 | GIYPAVD | 688.3665 | 716.3614 |
| IY | 249.1598 | 277.1547 | IYP | 346.2125 | 374.2074 | IYPA | 417.2496 | 445.2445 |
| IYPAV | 516.3180 | 544.3130 | IYPAVD | 631.3450 | 659.3399 | YP | 233.1285 | 261.1234 |
| YPA | 304.1656 | 332.1605 | YPAV | 403.2340 | 431.2289 | YPAVD | 518.2609 | 546.2558 |
| YPAVDP | 615.3137 | 643.3086 | PA | 141.1022 | 169.0972 | PAV | 240.1707 | 268.1656 |
| PAVD | 355.1976 | 383.1925 | PAVDP | 452.2504 | 480.2453 | PAVDPL | 565.3344 | 593.3293 |
| PAVDPLD | 680.3614 | 708.3563 | AV | 143.1179 | 171.1128 | AVD | 258.1448 | 286.1397 |
| AVDP | 355.1976 | 383.1925 | AVDPL | 468.2817 | 496.2766 | AVDPLD | 583.3086 | 611.3035 |
| AVDPLDS | 670.3406 | 698.3355 | VD | 187.1077 | 215.1026 | VDP | 284.1605 | 312.1554 |
| VDPL | 397.2445 | 425.2395 | VDPLD | 512.2715 | 540.2664 | VDPLDS | 599.3035 | 627.2984 |
| DP | 185.0921 | 213.0870 | DPL | 298.1761 | 326.1710 | DPLD | 413.2031 | 441.1980 |
| DPLDS | 500.2351 | 528.2300 | DPLDST | 601.2828 | 629.2777 | DPLDSTS | 688.3148 | 716.3097 |
| PL | 183.1492 | 211.1441 | PLD | 298.1761 | 326.1710 | PLDS | 385.2082 | 413.2031 |
| PLDST | 486.2558 | 514.2508 | PLDSTS | 573.2879 | 601.2828 | LD | 201.1234 | 229.1183 |
| LDS | 288.1554 | 316.1503 | LDST | 389.2031 | 417.1980 | LDSTS | 476.2351 | 504.2300 |
| DS | 175.0713 | 203.0662 | DST | 276.1190 | 304.1139 | DSTS | 363.1510 | 391.1460 |
| ST | 161.0921 | 189.0870 | STS | 248.1241 | 276.1190 | TS | 161.0921 | 189.0870 |

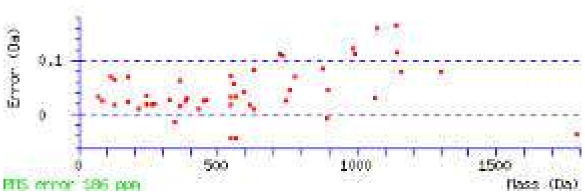


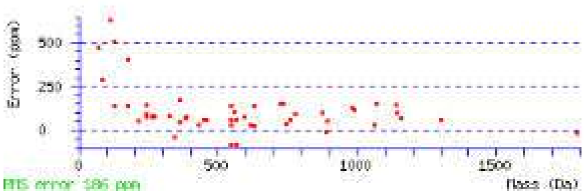


NCBI **BLAST** search of QISELGIYPAVDPLDSTSR
(Parameters: blastp, nr protein database, expect=20000, no filter, PAM30)
Other BLAST web gateways

**All matches to this query**

| Score | Mr(calc) | Delta | Sequence |
|---|---|---|---|
| 53.3 | 2060.0790 | 0.0276 | KISELGIYPAVDPLDSTSR |
| 53.3 | 2060.0426 | 0.0639 | QISELGIYPAVDPLDSTSR |
| 38.8 | 2060.0426 | 0.0640 | QISELGIYPAVDPLESSSR |
| 26.9 | 2060.0208 | 0.0857 | LINLMDGDIAVDSQAGLGSR |
| 26.1 | 2060.0426 | 0.0639 | QIAELGIYPSVDPLDSTSR |
| 16.7 | 2060.0902 | 0.0163 | ISDGVHIALEPDGTLLASPR |
| 13.0 | 2060.0724 | 0.0341 | IDAIYDRAALIALPDQMR |
| 12.9 | 2060.0071 | 0.0994 | MTNPPVFDVPALLEQMSR |
| 12.7 | 2060.0224 | 0.0842 | FLPFNKGGVMDFMSILGR |
| 12.7 | 2060.0579 | 0.0486 | DTSGPLVDGFLAISWVDLR |

**Mascot:** http://www.matrixscience.com/

# MATRIX SCIENCE Mascot Search Results

## Peptide View Spot no 85

MS/MS Fragmentation of **EAPAFVEQATEQQIIVTGIK**
Found in **gi|3676294** in **NCBInr**, mitochondrial ATPase beta subunit [Nicotiana sylvestris]

Match to Query 263: 2171.210724 from(2172.218000,1+) intensity(0.0000) index(32)
Title: Label: F15, Spot_Id: 219903, Peak_List_Id: 228335, MSMS Job_Run_Id: 21987, Comment:
Data file C:\Documents and Settings\Administrator\Desktop\Farah-Garima 11May 2013\ppw_F15_136868277600.txt

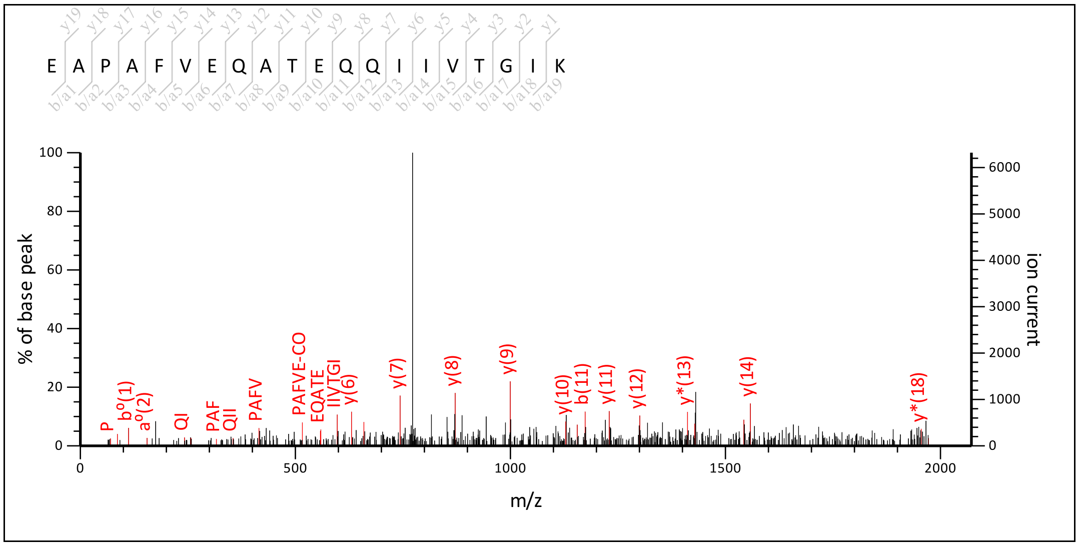


0 to 2072.01

Label all possible matches Label matches used for scoring

**Monoisotopic mass of neutral peptide Mr(calc):** 2171.1474
**Fixed modifications:** Carbamidomethyl (C) (apply to specified residues or termini only)
**Ions Score:** 30 **Expect:** 23
**Matches :** 38/379 fragment ions using 79 most intense peaks (help)

| # | Immon. | a | a* | a0 | b | b* | b0 | d | d' | Seq. | v | w | w' | y | y* | |
|---|---|---|---|---|---|---|---|---|---|---|---|---|---|---|---|---|
| 1 | 102.0550 | 102.0550 | | 84.0444 | 130.0499 | | 112.0393 | 44.0495 | | E | | | | | | |
| 2 | 44.0495 | 173.0921 | | 155.0815 | 201.0870 | | 183.0764 | | | A | 2027.0808 | | | 2043.1121 | 2026.0855 | [illegible] |
| 3 | 70.0651 | 270.1448 | | 252.1343 | 298.1397 | | 280.1292 | 244.1292 | | P | 1930.0280 | 1929.0328 | | ***1972.0750*** | 1955.0484 | [illegible] |
| 4 | 44.0495 | 341.1819 | | 323.1714 | 369.1769 | | 351.1663 | | | A | 1858.9909 | | | 1875.0222 | 1857.9957 | [illegible] |
| 5 | 120.0808 | 488.2504 | | 470.2398 | **516.2453** | | 498.2347 | | | F | 1711.9225 | | | 1803.9851 | 1786.9585 | [illegible] |
| 6 | 72.0808 | 587.3188 | | 569.3082 | 615.3137 | | 597.3031 | 573.3031 | | V | 1612.8541 | 1625.8745 | | 1656.9167 | 1639.8901 | [illegible] |
| 7 | 102.0550 | 716.3614 | | 698.3508 | 744.3563 | | 726.3457 | 658.3559 | | E | 1483.8115 | 1482.8162 | | ***1557.8483*** | 1540.8217 | [illegible] |
| 8 | 101.0709 | 844.4199 | 827.3934 | 826.4094 | 872.4149 | 855.3883 | 854.4043 | 787.3985 | | Q | 1355.7529 | 1354.7577 | | ***1428.8057*** | 1411.7791 | [illegible] |
| 9 | 44.0495 | 915.4571 | 898.4305 | 897.4465 | 943.4520 | 926.4254 | 925.4414 | | | A | 1284.7158 | | | ***1300.7471*** | 1283.7205 | [illegible] |
| 10 | 74.0600 | 1016.5047 | 999.4782 | 998.4942 | 1044.4997 | 1027.4731 | 1026.4891 | 1000.5098 | 1002.4891 | T | 1183.6681 | 1196.6885 | 1198.6678 | ***1229.7100*** | 1212.6834 | [illegible] |
| 11 | 102.0550 | 1145.5473 | 1128.5208 | 1127.5368 | **1173.5422** | 1156.5157 | 1155.5317 | 1087.5419 | | E | 1054.6255 | 1053.6303 | | ***1128.6623*** | 1111.6358 | [illegible] |
| 12 | 101.0709 | 1273.6059 | 1256.5794 | 1255.5953 | 1301.6008 | 1284.5743 | 1283.5903 | 1216.5844 | | Q | 926.5669 | 925.5717 | | ***999.6197*** | 982.5932 | [illegible] |
| 13 | 101.0709 | 1401.6645 | 1384.6379 | 1383.6539 | 1429.6594 | 1412.6329 | 1411.6488 | 1344.6430 | | Q | 798.5084 | 797.5131 | | ***871.5611*** | 854.5346 | [illegible] |
| 14 | 86.0964 | 1514.7486 | 1497.7220 | 1496.7380 | **1542.7435** | 1525.7169 | 1524.7329 | 1486.7173 | 1500.7329 | I | 685.4243 | 698.4447 | 712.4604 | ***743.5026*** | 726.4760 | [illegible] |
| 15 | 86.0964 | 1627.8326 | 1610.8061 | 1609.8220 | 1655.8275 | 1638.8010 | 1637.8170 | 1599.8013 | 1613.8170 | I | 572.3402 | 585.3606 | 599.3763 | ***630.4185*** | 613.3919 | [illegible] |
| 16 | 72.0808 | 1726.9010 | 1709.8745 | 1708.8905 | 1754.8959 | 1737.8694 | 1736.8854 | 1712.8854 | | V | 473.2718 | 486.2922 | | 517.3344 | 500.3079 | [illegible] |
| 17 | 74.0600 | 1827.9487 | 1810.9222 | 1809.9381 | 1855.9436 | 1838.9171 | 1837.9331 | 1811.9538 | 1813.9331 | T | 372.2241 | 385.2445 | 387.2238 | 418.2660 | 401.2395 | [illegible] |
| 18 | 30.0338 | 1884.9702 | 1867.9436 | 1866.9596 | 1912.9651 | 1895.9385 | 1894.9545 | | | G | | | | 317.2183 | 300.1918 | |
| 19 | 86.0964 | 1998.0542 | 1981.0277 | 1980.0437 | 2026.0492 | 2009.0226 | 2008.0386 | 1970.0229 | 1984.0386 | I | 202.1186 | 215.1390 | 229.1547 | 260.1969 | 243.1703 | |
| 20 | 101.1073 | | | | | | | | | K | 74.0237 | 73.0284 | | 147.1128 | 130.0863 | |

| Seq | ya | yb | Seq | ya | yb | Seq | ya | yb |
|---|---|---|---|---|---|---|---|---|
| AP | 141.1022 | 169.0972 | APA | 212.1394 | 240.1343 | APAF | 359.2078 | 387.2027 |

| | | | | | | | | |
|---|---|---|---|---|---|---|---|---|
| **APAFV** | 458.2762 | 486.2711 | **APAFVE** | 587.3188 | 615.3137 | **PA** | 141.1022 | 169.0972 |
| **PAF** | 288.1707 | 316.1656 | **PAFV** | 387.2391 | 415.2340 | **PAFVE** | 516.2817 | 544.2766 |
| **PAFVEQ** | 644.3402 | 672.3352 | **AF** | 191.1179 | 219.1128 | **AFV** | 290.1863 | 318.1812 |
| **AFVE** | 419.2289 | 447.2238 | **AFVEQ** | 547.2875 | 575.2824 | **AFVEQA** | 618.3246 | 646.3195 |
| **FV** | 219.1492 | 247.1441 | **FVE** | 348.1918 | 376.1867 | **FVEQ** | 476.2504 | 504.2453 |
| **FVEQA** | 547.2875 | 575.2824 | **FVEQAT** | 648.3352 | 676.3301 | **VE** | 201.1234 | 229.1183 |
| **VEQ** | 329.1819 | 357.1769 | **VEQA** | 400.2191 | 428.2140 | **VEQAT** | 501.2667 | 529.2617 |
| **VEQATE** | 630.3093 | 658.3042 | **EQ** | 230.1135 | 258.1084 | **EQA** | 301.1506 | 329.1456 |
| **EQAT** | 402.1983 | 430.1932 | **EQATE** | 531.2409 | 559.2358 | **EQATEQ** | 659.2995 | 687.2944 |
| **QA** | 172.1081 | 200.1030 | **QAT** | 273.1557 | 301.1506 | **QATE** | 402.1983 | 430.1932 |
| **QATEQ** | 530.2569 | 558.2518 | **QATEQQ** | 658.3155 | 686.3104 | **AT** | 145.0972 | 173.0921 |
| **ATE** | 274.1397 | 302.1347 | **ATEQ** | 402.1983 | 430.1932 | **ATEQQ** | 530.2569 | 558.2518 |
| **ATEQQI** | 643.3410 | 671.3359 | **TE** | 203.1026 | 231.0975 | **TEQ** | 331.1612 | 359.1561 |
| **TEQQ** | 459.2198 | 487.2147 | **TEQQI** | 572.3039 | 600.2988 | **TEQQII** | 685.3879 | 713.3828 |
| **EQ** | 230.1135 | 258.1084 | **EQQ** | 358.1721 | 386.1670 | **EQQI** | 471.2562 | 499.2511 |
| **EQQII** | 584.3402 | 612.3352 | **EQQIIV** | 683.4087 | 711.4036 | **QQ** | 229.1295 | 257.1244 |
| **QQI** | 342.2136 | 370.2085 | **QQII** | 455.2976 | 483.2926 | **QQIIV** | 554.3661 | 582.3610 |
| **QQIIVT** | 655.4137 | 683.4087 | **QI** | 214.1550 | 242.1499 | **QII** | 327.2391 | 355.2340 |
| **QIIV** | 426.3075 | 454.3024 | **QIIVT** | 527.3552 | 555.3501 | **QIIVTG** | 584.3766 | 612.3715 |
| **QIIVTGI** | 697.4607 | 725.4556 | **II** | 199.1805 | 227.1754 | **IIV** | 298.2489 | 326.2438 |
| **IIVT** | 399.2966 | 427.2915 | **IIVTG** | 456.3180 | 484.3130 | **IIVTGI** | 569.4021 | 597.3970 |
| **IV** | 185.1648 | 213.1598 | **IVT** | 286.2125 | 314.2074 | **IVTG** | 343.2340 | 371.2289 |
| **IVTGI** | 456.3180 | 484.3130 | **VT** | 173.1285 | 201.1234 | **VTG** | 230.1499 | 258.1448 |
| **VTGI** | 343.2340 | 371.2289 | **TG** | 131.0815 | 159.0764 | **TGI** | 244.1656 | 272.1605 |
| **GI** | 143.1179 | 171.1128 | | | | | | |

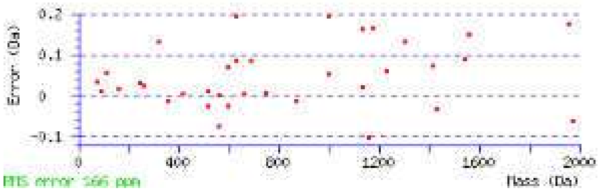


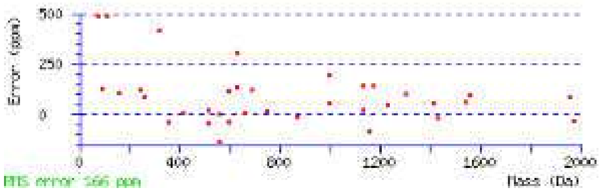


NCBI **BLAST** search of EAPAFVEQATEQQIIVTGIK
(Parameters: blastp, nr protein database, expect=20000, no filter, PAM30)
Other BLAST web gateways

**All matches to this query**

| Score | Mr(calc) | Delta | Sequence |
|---|---|---|---|
| 29.6 | 2171.1474 | 0.0633 | EAPAFVEQATEQQIIVTGIK |
| 29.6 | 2171.1474 | 0.0633 | EAPAFVEQATEQQILVTGIK |
| 18.6 | 2171.2752 | -0.0644 | LMALAAVIIFPTLSLITNVR |
| 16.2 | 2171.2413 | -0.0305 | QLKSTTLENIISTIEILQK |
| 15.7 | 2171.1004 | 0.1103 | MTAAQQDNALQVTGLAALEAR |
| 15.6 | 2171.1474 | 0.0633 | EAPAFVEQETAQQILVTGIK |
| 15.4 | 2171.2170 | -0.0063 | ICKTSIMLIIAGALGNLIDR |
| 14.9 | 2171.0970 | 0.1137 | EWLSSQAELQEQIVARER |
| 13.0 | 2171.1223 | 0.0885 | IPSAVGYQPTLGTDLGGLQER |
| 12.8 | 2171.1182 | 0.0925 | DSTGRLLTVLNDDINQLER |

**Mascot:** http://www.matrixscience.com/

# Mascot Search Results

## Peptide View **Spot no 85**

MS/MS Fragmentation of **IPSAVGYQPTLATDLGGLQER**
Found in **gi|3676294** in **NCBInr**, mitochondrial ATPase beta subunit [Nicotiana sylvestris]

Match to Query 265: 2185.212424 from(2186.219700,1+) intensity(0.0000) index(33)
Title: Label: F15, Spot_Id: 219903, Peak_List_Id: 228323, MSMS Job_Run_Id: 21987, Comment:
Data file C:\Documents and Settings\Administrator\Desktop\Farah-Garima 11May 2013\ppw_F15_136868277600.txt

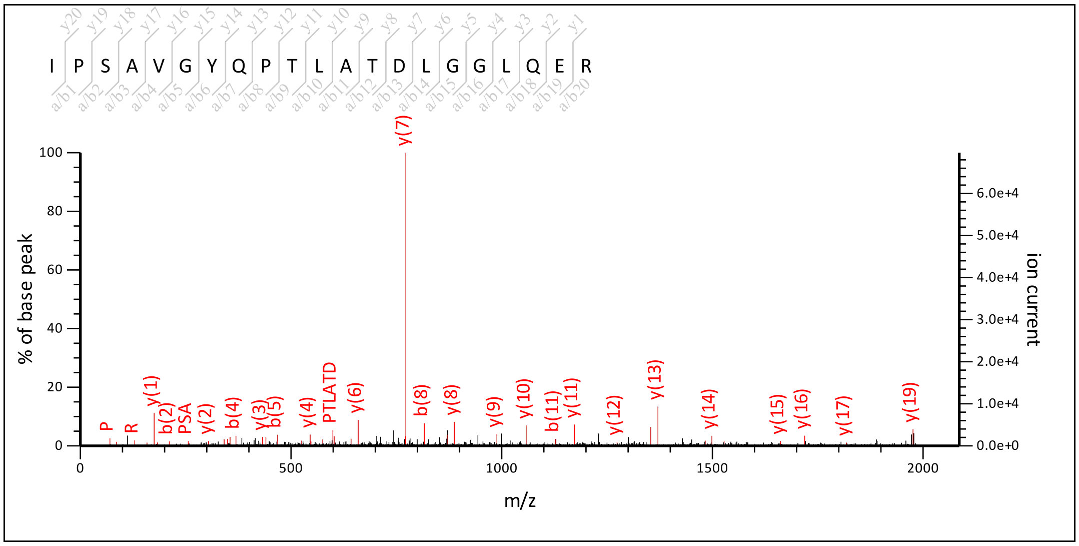


0 to 2085.93

Label all possible matches ○ Label matches used for scoring ◉

**Monoisotopic mass of neutral peptide Mr(calc):** 2185.1379
**Fixed modifications:** Carbamidomethyl (C) (apply to specified residues or termini only)
**Ions Score:** 125 **Expect:** 6.1e-09
**Matches :** 56/405 fragment ions using 80 most intense peaks (help)

| # | Immon. | a | a* | $a^0$ | b | b* | $b^0$ | d | d' | Seq. | v | w | w' | y | y* | |
|---|---|---|---|---|---|---|---|---|---|---|---|---|---|---|---|---|
| 1 | 86.0964 | **86.0964** | | | 114.0913 | | | 44.0495 | | I | | | | | | |
| 2 | 70.0651 | **183.1492** | | | **211.1441** | | | 157.1335 | | P | 2031.0142 | 2030.0189 | | 2073.0611 | 2056.0346 | [illegible] |
| 3 | 60.0444 | 270.1812 | | 252.1707 | 298.1761 | | 280.1656 | 254.1863 | | S | 1943.9821 | 1942.9869 | | ***1976.0083*** | 1958.9818 | [illegible] |
| 4 | 44.0495 | **341.2183** | | 323.2078 | **369.2132** | | 351.2027 | | | A | 1872.9450 | | | 1888.9763 | 1871.9498 | [illegible] |
| 5 | 72.0808 | **440.2867** | | 422.2762 | **468.2817** | | 450.2711 | 426.2711 | | V | 1773.8766 | 1786.8970 | | ***1817.9392*** | 1800.9127 | [illegible] |
| 6 | 30.0338 | 497.3082 | | 479.2976 | **525.3031** | | 507.2926 | | | G | | | | ***1718.8708*** | 1701.8442 | [illegible] |
| 7 | 136.0757 | 660.3715 | | 642.3610 | 688.3665 | | 670.3559 | | | Y | 1553.7918 | | | ***1661.8493*** | 1644.8228 | [illegible] |
| 8 | 101.0709 | 788.4301 | 771.4036 | 770.4196 | **816.4250** | 799.3985 | 798.4145 | 731.4087 | | Q | 1425.7332 | 1424.7380 | | ***1498.7860*** | 1481.7595 | [illegible] |
| 9 | 70.0651 | 885.4829 | 868.4563 | 867.4723 | 913.4778 | 896.4512 | 895.4672 | 859.4672 | | P | 1328.6805 | 1327.6852 | | ***1370.7274*** | 1353.7009 | [illegible] |
| 10 | 74.0600 | 986.5306 | 969.5040 | 968.5200 | 1014.5255 | 997.4989 | 996.5149 | 970.5356 | 972.5149 | T | 1227.6328 | 1240.6532 | 1242.6325 | ***1273.6747*** | 1256.6481 | [illegible] |
| 11 | 86.0964 | 1099.6146 | 1082.5881 | 1081.6041 | **1127.6095** | 1110.5830 | 1109.5990 | 1057.5677 | | L | 1114.5487 | 1113.5535 | | ***1172.6270*** | 1155.6004 | [illegible] |
| 12 | 44.0495 | 1170.6517 | 1153.6252 | 1152.6412 | 1198.6467 | 1181.6201 | 1180.6361 | | | A | 1043.5116 | | | ***1059.5429*** | 1042.5164 | [illegible] |
| 13 | 74.0600 | 1271.6994 | 1254.6729 | 1253.6889 | 1299.6943 | 1282.6678 | 1281.6838 | 1255.7045 | 1257.6838 | T | 942.4639 | 955.4843 | 957.4636 | ***988.5058*** | 971.4793 | [illegible] |
| 14 | 88.0393 | 1386.7264 | 1369.6998 | 1368.7158 | 1414.7213 | 1397.6947 | 1396.7107 | 1342.7365 | | D | 827.4370 | 826.4417 | | ***887.4581*** | 870.4316 | [illegible] |
| 15 | 86.0964 | 1499.8104 | 1482.7839 | 1481.7999 | **1527.8053** | 1510.7788 | 1509.7948 | 1457.7635 | | L | 714.3529 | 713.3577 | | ***772.4312*** | 755.4046 | [illegible] |
| 16 | 30.0338 | 1556.8319 | 1539.8053 | 1538.8213 | 1584.8268 | 1567.8003 | 1566.8162 | | | G | | | | ***659.3471*** | 642.3206 | [illegible] |
| 17 | 30.0338 | 1613.8534 | 1596.8268 | 1595.8428 | 1641.8483 | 1624.8217 | 1623.8377 | | | G | | | | ***602.3257*** | 585.2991 | [illegible] |
| 18 | 86.0964 | 1726.9374 | 1709.9109 | 1708.9268 | 1754.9323 | 1737.9058 | 1736.9218 | 1684.8905 | | L | 487.2259 | 486.2307 | | ***545.3042*** | 528.2776 | [illegible] |
| 19 | 101.0709 | 1854.9960 | 1837.9694 | 1836.9854 | 1882.9909 | 1865.9644 | 1864.9803 | 1797.9745 | | Q | 359.1674 | 358.1721 | | ***432.2201*** | 415.1936 | [illegible] |
| 20 | 102.0550 | 1984.0386 | 1967.0120 | 1966.0280 | 2012.0335 | 1995.0070 | 1994.0229 | 1926.0331 | | E | 230.1248 | 229.1295 | | ***304.1615*** | 287.1350 | [illegible] |
| 21 | 129.1135 | | | | | | | | | R | 74.0237 | 73.0284 | | ***175.1190*** | 158.0924 | |

| Seq | ya | yb | Seq | ya | yb | Seq | ya | yb |
|---|---|---|---|---|---|---|---|---|

| | | | | | | | | |
|---|---|---|---|---|---|---|---|---|
| **PS** | 157.0972 | 185.0921 | **PSA** | 228.1343 | 256.1292 | **PSAV** | 327.2027 | 355.1976 |
| **PSAVG** | 384.2241 | 412.2191 | **PSAVGY** | 547.2875 | 575.2824 | **PSAVGYQ** | 675.3461 | 703.3410 |
| **SA** | 131.0815 | 159.0764 | **SAV** | 230.1499 | 258.1448 | **SAVG** | 287.1714 | 315.1663 |
| **SAVGY** | 450.2347 | 478.2296 | **SAVGYQ** | 578.2933 | 606.2882 | **SAVGYQP** | 675.3461 | 703.3410 |
| **AV** | 143.1179 | 171.1128 | **AVG** | 200.1394 | 228.1343 | **AVGY** | 363.2027 | 391.1976 |
| **AVGYQ** | 491.2613 | 519.2562 | **AVGYQP** | 588.3140 | 616.3089 | **AVGYQPT** | 689.3617 | 717.3566 |
| **VG** | 129.1022 | 157.0972 | **VGY** | 292.1656 | 320.1605 | **VGYQ** | 420.2241 | 448.2191 |
| **VGYQP** | 517.2769 | 545.2718 | **VGYQPT** | 618.3246 | 646.3195 | **GY** | 193.0972 | 221.0921 |
| **GYQ** | 321.1557 | 349.1506 | **GYQP** | 418.2085 | 446.2034 | **GYQPT** | 519.2562 | 547.2511 |
| **GYQPTL** | 632.3402 | 660.3352 | **YQ** | 264.1343 | 292.1292 | **YQP** | 361.1870 | 389.1819 |
| **YQPT** | 462.2347 | 490.2296 | **YQPTL** | 575.3188 | 603.3137 | **YQPTLA** | 646.3559 | 674.3508 |
| **QP** | 198.1237 | 226.1186 | **QPT** | 299.1714 | 327.1663 | **QPTL** | 412.2554 | 440.2504 |
| **QPTLA** | 483.2926 | 511.2875 | **QPTLAT** | 584.3402 | 612.3352 | **QPTLATD** | 699.3672 | 727.3621 |
| **PT** | 171.1128 | 199.1077 | **PTL** | 284.1969 | 312.1918 | **PTLA** | 355.2340 | 383.2289 |
| **PTLAT** | 456.2817 | 484.2766 | **PTLATD** | 571.3086 | 599.3035 | **PTLATDL** | 684.3927 | 712.3876 |
| **TL** | 187.1441 | 215.1390 | **TLA** | 258.1812 | 286.1761 | **TLAT** | 359.2289 | 387.2238 |
| **TLATD** | 474.2558 | 502.2508 | **TLATDL** | 587.3399 | 615.3348 | **TLATDLG** | 644.3614 | 672.3563 |
| **LA** | 157.1335 | 185.1285 | **LAT** | 258.1812 | 286.1761 | **LATD** | 373.2082 | 401.2031 |
| **LATDL** | 486.2922 | 514.2871 | **LATDLG** | 543.3137 | 571.3086 | **LATDLGG** | 600.3352 | 628.3301 |
| **AT** | 145.0972 | 173.0921 | **ATD** | 260.1241 | 288.1190 | **ATDL** | 373.2082 | 401.2031 |
| **ATDLG** | 430.2296 | 458.2245 | **ATDLGG** | 487.2511 | 515.2460 | **ATDLGGL** | 600.3352 | 628.3301 |
| **TD** | 189.0870 | 217.0819 | **TDL** | 302.1710 | 330.1660 | **TDLG** | 359.1925 | 387.1874 |
| **TDLGG** | 416.2140 | 444.2089 | **TDLGGL** | 529.2980 | 557.2930 | **TDLGGLQ** | 657.3566 | 685.3515 |
| **DL** | 201.1234 | 229.1183 | **DLG** | 258.1448 | 286.1397 | **DLGG** | 315.1663 | 343.1612 |
| **DLGGL** | 428.2504 | 456.2453 | **DLGGLQ** | 556.3089 | 584.3039 | **DLGGLQE** | 685.3515 | 713.3464 |
| **LG** | 143.1179 | 171.1128 | **LGG** | 200.1394 | 228.1343 | **LGGL** | 313.2234 | 341.2183 |
| **LGGLQ** | 441.2820 | 469.2769 | **LGGLQE** | 570.3246 | 598.3195 | **GG** | 87.0553 | 115.0502 |
| **GGL** | 200.1394 | 228.1343 | **GGLQ** | 328.1979 | 356.1928 | **GGLQE** | 457.2405 | 485.2354 |
| **GL** | 143.1179 | 171.1128 | **GLQ** | 271.1765 | 299.1714 | **GLQE** | 400.2191 | 428.2140 |
| **LQ** | 214.1550 | 242.1499 | **LQE** | 343.1976 | 371.1925 | **QE** | 230.1135 | 258.1084 |

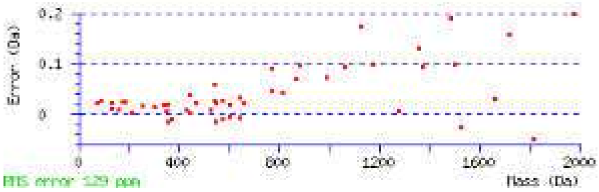


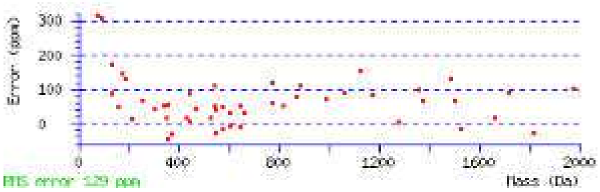


NCBI **BLAST** search of IPSAVGYQPTLATDLGGLQER
(Parameters: blastp, nr protein database, expect=20000, no filter, PAM30)
Other BLAST web gateways

**All matches to this query**

| Score | Mr(calc) | Delta | Sequence |
|---|---|---|---|
| 125.4 | 2185.1379 | 0.0745 | IPSAVGYQPTLATDLGGLQER |
| 95.5 | 2185.1379 | 0.0745 | IPSAVGYQPTLATDVGALQER |
| 68.4 | 2185.1379 | 0.0745 | IPSAVGYQPTLASDLGALQER |
| 23.3 | 2185.0909 | 0.1215 | QLAAMQGDVEQQVARLQER |
| 21.0 | 2185.2219 | -0.0095 | VLLAAQLAIGFTTAVDGRVDR |
| 15.9 | 2185.0610 | 0.1514 | ESAGRADEEEILNQVEQLR |
| 15.0 | 2185.1895 | 0.0229 | IPTAYIKTFQGPPAGIQVER |
| 14.3 | 2185.2219 | -0.0095 | VALIAFTGSVAGGLAVQKAAADR |
| 14.3 | 2185.1967 | 0.0157 | APSLPHQAIDLNAKVASIGQR |
| 14.2 | 2185.1855 | 0.0269 | KLVHIPQALLEGDDAPGAVSR |

**Mascot:** http://www.matrixscience.com/

# MATRIX SCIENCE Mascot Search Results

## Peptide View **Spot no 87**

MS/MS Fragmentation of **LSIFETGIK**
Found in **gi|11466794** in **NCBInr**, ATP synthase CF1 beta subunit [Oryza sativa Japonica Group]

Match to Query 3: 1006.647224 from(1007.654500,1+) intensity(0.0000) index(1)
Title: Label: D11, Spot_Id: 216640, Peak_List_Id: 221245, MSMS Job_Run_Id: 21346, Comment:
Data file C:\Documents and Settings\Administrator\Desktop\farah BHU\RAJESH\12-1-13 raj\ppw_D11_135997699500.txt

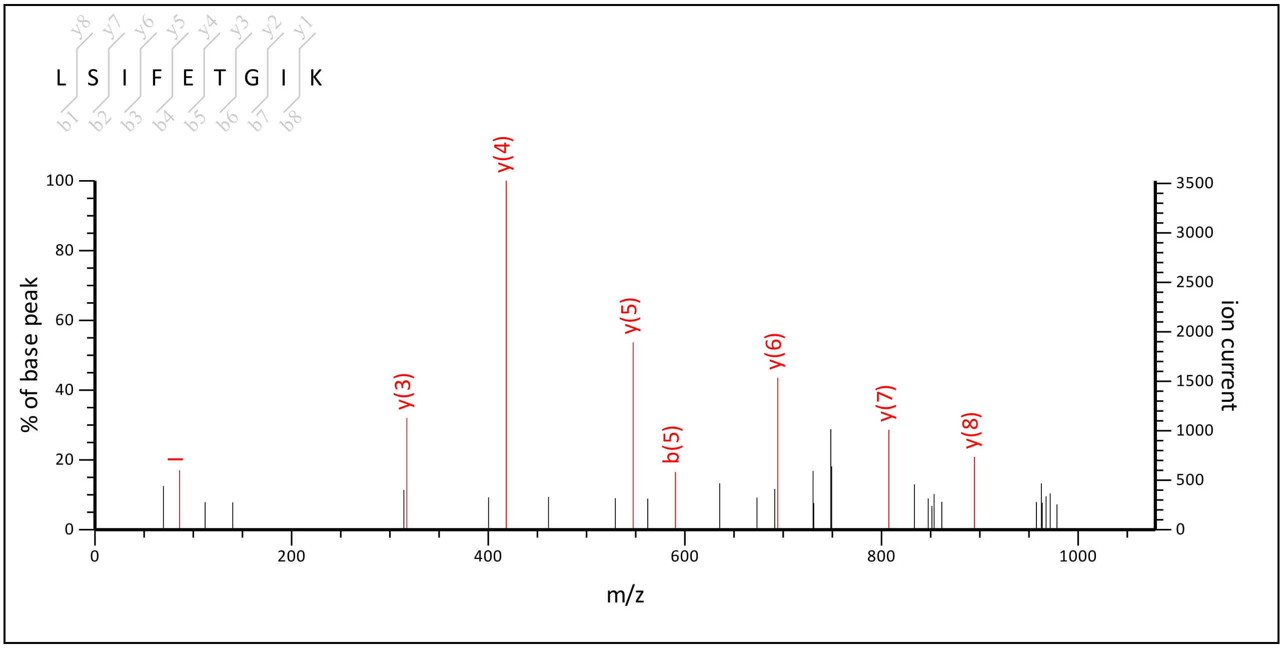


0 to 1078.49

Label all possible matches ○ Label matches used for scoring ◉

**Monoisotopic mass of neutral peptide Mr(calc):** 1006.5699
**Fixed modifications:** Carbamidomethyl (C) (apply to specified residues or termini only)
**Ions Score:** 53 **Expect:** 0.03
**Matches :** 11/125 fragment ions using 9 most intense peaks (help)

| # | Immon. | a | $a^0$ | b | $b^0$ | d | d' | Seq. | v | w | w' | y | y* | $y^0$ | # |
|---|---|---|---|---|---|---|---|---|---|---|---|---|---|---|---|
| 1 | 86.0964 | 86.0964 | | 114.0913 | | 44.0495 | | **L** | | | | | | | 9 |
| 2 | 60.0444 | 173.1285 | 155.1179 | 201.1234 | 183.1128 | 157.1335 | | **S** | 862.4669 | 861.4716 | | ***894.4931*** | 877.4666 | 876.4825 | 8 |
| 3 | 86.0964 | 286.2125 | 268.2020 | 314.2074 | 296.1969 | 258.1812 | 272.1969 | **I** | 749.3828 | 762.4032 | 776.4189 | ***807.4611*** | 790.4345 | 789.4505 | 7 |
| 4 | 120.0808 | 433.2809 | 415.2704 | 461.2758 | 443.2653 | | | **F** | 602.3144 | | | ***694.3770*** | 677.3505 | 676.3665 | 6 |
| 5 | 102.0550 | 562.3235 | 544.3130 | 590.3184 | 572.3079 | 504.3180 | | **E** | 473.2718 | 472.2766 | | ***547.3086*** | 530.2821 | 529.2980 | 5 |
| 6 | 74.0600 | 663.3712 | 645.3606 | 691.3661 | 673.3556 | 647.3763 | 649.3556 | **T** | 372.2241 | 385.2445 | 387.2238 | ***418.2660*** | 401.2395 | 400.2554 | 4 |
| 7 | 30.0338 | 720.3927 | 702.3821 | 748.3876 | 730.3770 | | | **G** | | | | ***317.2183*** | 300.1918 | | 3 |
| 8 | 86.0964 | 833.4767 | 815.4662 | 861.4716 | 843.4611 | 805.4454 | 819.4611 | **I** | 202.1186 | 215.1390 | 229.1547 | 260.1969 | 243.1703 | | 2 |
| 9 | 101.1073 | | | | | | | **K** | 74.0237 | 73.0284 | | 147.1128 | 130.0863 | | 1 |

| Seq | ya | yb | Seq | ya | yb | Seq | ya | yb |
|---|---|---|---|---|---|---|---|---|
| **SI** | 173.1285 | 201.1234 | **SIF** | 320.1969 | 348.1918 | **SIFE** | 449.2395 | 477.2344 |
| **SIFET** | 550.2871 | 578.2821 | **SIFETG** | 607.3086 | 635.3035 | **IF** | 233.1648 | 261.1598 |
| **IFE** | 362.2074 | 390.2023 | **IFET** | 463.2551 | 491.2500 | **IFETG** | 520.2766 | 548.2715 |

| | | | | | | | | |
|---|---|---|---|---|---|---|---|---|
| **IFETGI** | 633.3606 | 661.3556 | **FE** | 249.1234 | 277.1183 | **FET** | 350.1710 | 378.1660 |
| **FETG** | 407.1925 | 435.1874 | **FETGI** | 520.2766 | 548.2715 | **ET** | 203.1026 | 231.0975 |
| **ETG** | 260.1241 | 288.1190 | **ETGI** | 373.2082 | 401.2031 | **TG** | 131.0815 | 159.0764 |
| **TGI** | 244.1656 | 272.1605 | **GI** | 143.1179 | 171.1128 | | | |

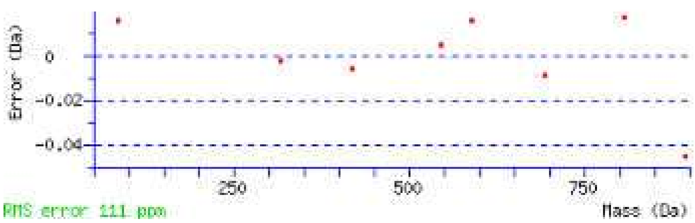


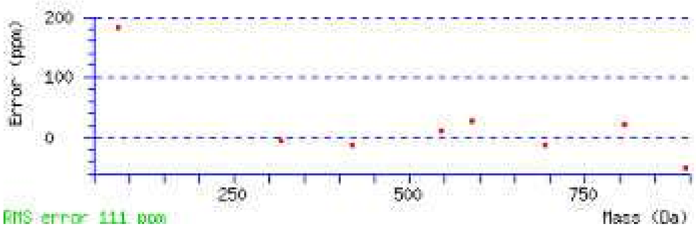


NCBI **BLAST** search of LSIFETGIK
(Parameters: blastp, nr protein database, expect=20000, no filter, PAM30)
Other BLAST web gateways

**All matches to this query**

| Score | Mr(calc) | Delta | Sequence |
|---|---|---|---|
| 53.5 | 1006.5699 | 0.0773 | LSIFETGIK |
| 53.5 | 1006.5699 | 0.0773 | LSIFETGIK |
| 53.5 | 1006.5699 | 0.0773 | LSIFETGIK |
| 53.5 | 1006.5699 | 0.0773 | LSIFETGIK |
| 53.5 | 1006.5699 | 0.0773 | LSIFETGIK |
| 53.5 | 1006.5699 | 0.0773 | LSIFETGIK |
| 53.5 | 1006.5699 | 0.0773 | LSIFETGIK |
| 53.5 | 1006.5699 | 0.0773 | LSIFETGLK |
| 53.5 | 1006.5699 | 0.0773 | LSIFETGLK |
| 53.5 | 1006.5369 | 0.1104 | LSIMETGIK |

**Mascot:** http://www.matrixscience.com/

# MATRIX SCIENCE Mascot Search Results

## Peptide View Spot no 87

MS/MS Fragmentation of **VVDLLAPYR**
Found in **gi|11466794** in **NCBInr**, ATP synthase CF1 beta subunit [Oryza sativa Japonica Group]

Match to Query 5: 1044.673224 from(1045.680500,1+) intensity(0.0000) index(2)
Title: Label: D11, Spot_Id: 216640, Peak_List_Id: 221238, MSMS Job_Run_Id: 21346, Comment:
Data file C:\Documents and Settings\Administrator\Desktop\farah BHU\RAJESH\12-1-13 raj\ppw_D11_135997699500.txt

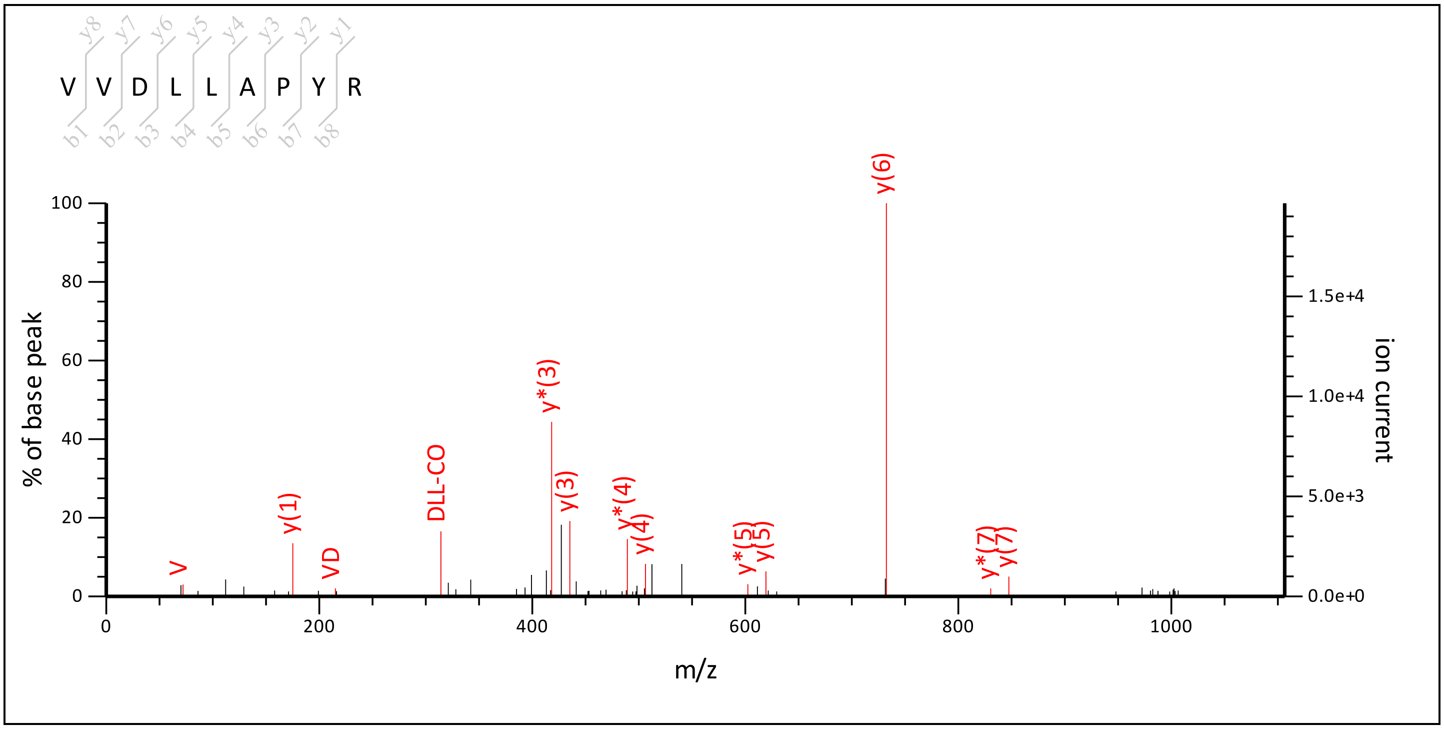


0 to 1106.43

Label all possible matches ○ Label matches used for scoring ◉

**Monoisotopic mass of neutral peptide Mr(calc):** 1044.5968
**Fixed modifications:** Carbamidomethyl (C) (apply to specified residues or termini only)
**Ions Score:** 48 **Expect:** 0.11
**Matches :** 17/115 fragment ions using 19 most intense peaks (help)

| # | Immon. | a | $a^0$ | b | $b^0$ | d | Seq. | v | w | y | y* | $y^0$ | # |
|---|---|---|---|---|---|---|---|---|---|---|---|---|---|
| 1 | 72.0808 | 72.0808 | | 100.0757 | | 44.0495 | V | | | | | | 9 |
| 2 | 72.0808 | 171.1492 | | 199.1441 | | 157.1335 | V | 902.4730 | 915.4934 | 946.5356 | 929.5091 | 928.5251 | 8 |
| 3 | 88.0393 | 286.1761 | 268.1656 | 314.1710 | 296.1605 | 242.1863 | D | 787.4461 | 786.4509 | ***847.4672*** | 830.4407 | 829.4567 | 7 |
| 4 | 86.0964 | 399.2602 | 381.2496 | 427.2551 | 409.2445 | 357.2132 | L | 674.3620 | 673.3668 | ***732.4403*** | 715.4137 | | 6 |
| 5 | 86.0964 | 512.3443 | 494.3337 | 540.3392 | 522.3286 | 470.2973 | L | 561.2780 | 560.2827 | ***619.3562*** | 602.3297 | | 5 |
| 6 | 44.0495 | 583.3814 | 565.3708 | 611.3763 | 593.3657 | | A | 490.2409 | | ***506.2722*** | 489.2456 | | 4 |
| 7 | 70.0651 | 680.4341 | 662.4236 | 708.4291 | 690.4185 | 654.4185 | P | 393.1881 | 392.1928 | ***435.2350*** | 418.2085 | | 3 |
| 8 | 136.0757 | 843.4975 | 825.4869 | 871.4924 | 853.4818 | | Y | 230.1248 | | 338.1823 | 321.1557 | | 2 |

| | | | | | | | | | | | | | |
|---|---|---|---|---|---|---|---|---|---|---|---|---|---|
| 9 | 129.1135 | | | | | | R | 74.0237 | 73.0284 | ***175.1190*** | 158.0924 | | 1 |

| Seq | ya | yb | Seq | ya | yb | Seq | ya | yb |
|---|---|---|---|---|---|---|---|---|
| **VD** | 187.1077 | 215.1026 | **VDL** | 300.1918 | 328.1867 | **VDLL** | 413.2758 | 441.2708 |
| **VDLLA** | 484.3130 | 512.3079 | **VDLLAP** | 581.3657 | 609.3606 | **DL** | 201.1234 | 229.1183 |
| **DLL** | 314.2074 | 342.2023 | **DLLA** | 385.2445 | 413.2395 | **DLLAP** | 482.2973 | 510.2922 |
| **DLLAPY** | 645.3606 | 673.3556 | **LL** | 199.1805 | 227.1754 | **LLA** | 270.2176 | 298.2125 |
| **LLAP** | 367.2704 | 395.2653 | **LLAPY** | 530.3337 | 558.3286 | **LA** | 157.1335 | 185.1285 |
| **LAP** | 254.1863 | 282.1812 | **LAPY** | 417.2496 | 445.2445 | **AP** | 141.1022 | 169.0972 |
| **APY** | 304.1656 | 332.1605 | **PY** | 233.1285 | 261.1234 | | | |

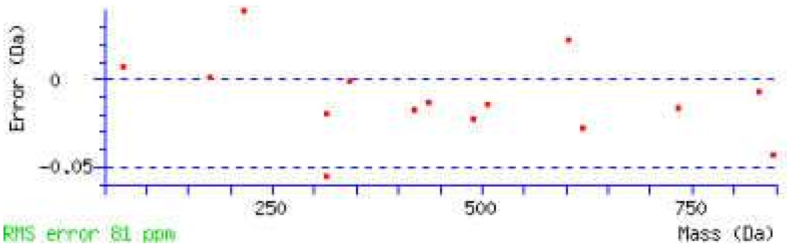


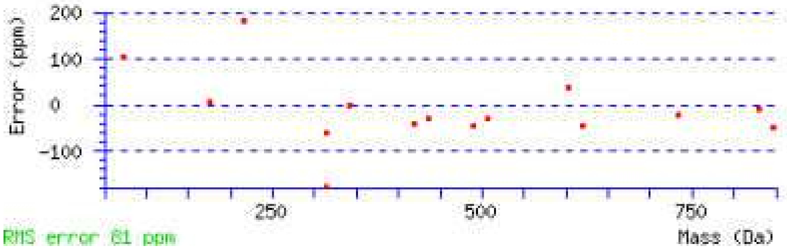


NCBI **BLAST** search of VVDLLAPYR
(Parameters: blastp, nr protein database, expect=20000, no filter, PAM30)
Other BLAST web gateways

**All matches to this query**

| Score | Mr(calc) | Delta | Sequence |
|---|---|---|---|
| 48.0 | 1044.1048 | 0.5685 | VVBLLAPYR |
| 48.0 | 1044.5968 | 0.0765 | VVDILAPYR |
| 48.0 | 1044.5968 | 0.0765 | VVDLIAPYR |
| 48.0 | 1044.5968 | 0.0765 | VVDLLAPYR |
| 48.0 | 1044.5968 | 0.0765 | VVDLLAPYR |
| 48.0 | 1044.5968 | 0.0765 | VVDLLAPYR |
| 48.0 | 1044.5968 | 0.0765 | VVDLLAPYR |
| 48.0 | 1044.5968 | 0.0765 | VVDLLAPYR |
| 48.0 | 1044.5968 | 0.0765 | VVDLLAPYR |
| 48.0 | 1044.5968 | 0.0765 | VVDLLAPYR |

**Mascot:** http://www.matrixscience.com/

# MATRIX SCIENCE Mascot Search Results

## Peptide View Spot no 87

MS/MS Fragmentation of **SAPAFIELDTK**
Found in **gi|11466794** in **NCBInr**, ATP synthase CF1 beta subunit [Oryza sativa Japonica Group]

Match to Query 6: 1190.709524 from(1191.716800,1+) intensity(0.0000) index(3)
Title: Label: D11, Spot_Id: 216640, Peak_List_Id: 221243, MSMS Job_Run_Id: 21346, Comment:
Data file C:\Documents and Settings\Administrator\Desktop\farah BHU\RAJESH\12-1-13 raj\ppw_D11_135997699500.txt

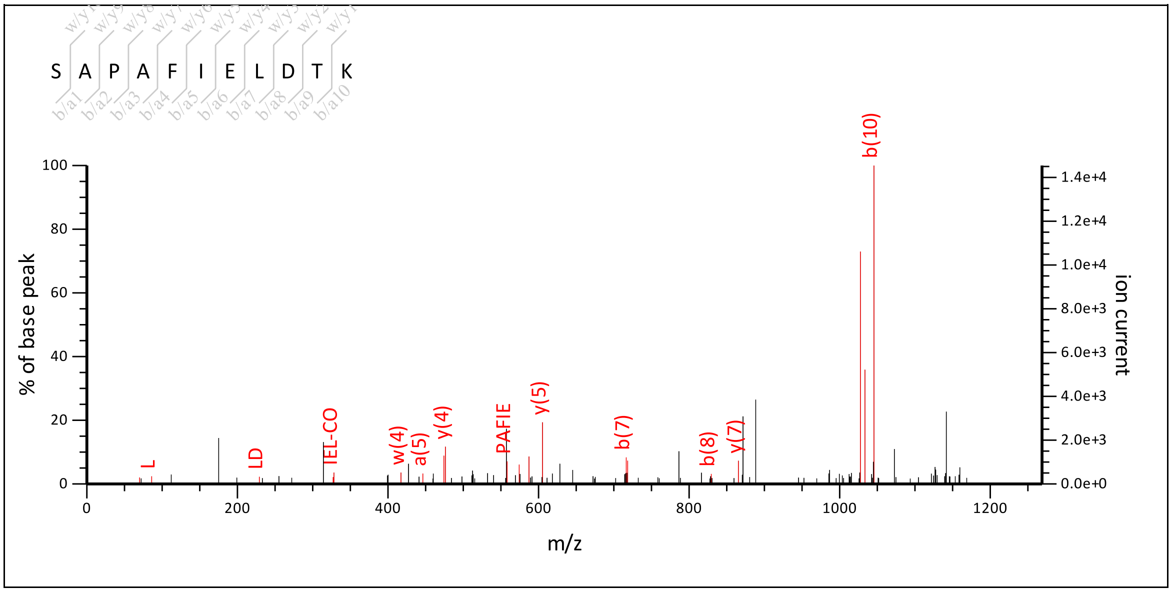


0 to 1268.77

Label all possible matches ○ Label matches used for scoring ◉

**Monoisotopic mass of neutral peptide Mr(calc):** 1190.6183
**Fixed modifications:** Carbamidomethyl (C) (apply to specified residues or termini only)
**Ions Score:** 27 **Expect:** 11
**Matches :** 23/167 fragment ions using 44 most intense peaks (help)

| # | Immon. | a | $a^0$ | b | $b^0$ | d | d' | Seq. | v | w | w' | y | y* | $y^0$ | # |
|---|---|---|---|---|---|---|---|---|---|---|---|---|---|---|---|
| 1 | 60.0444 | 60.0444 | 42.0338 | 88.0393 | 70.0287 | 44.0495 | | S | | | | | | | 11 |
| 2 | 44.0495 | 131.0815 | 113.0709 | 159.0764 | 141.0659 | | | A | 1088.5623 | | | 1104.5936 | 1087.5670 | 1086.5830 | 10 |
| 3 | 70.0651 | 228.1343 | 210.1237 | 256.1292 | 238.1186 | 202.1186 | | P | 991.5095 | 990.5142 | | ***1033.5564*** | 1016.5299 | 1015.5459 | 9 |
| 4 | 44.0495 | 299.1714 | 281.1608 | ***327.1663*** | 309.1557 | | | A | 920.4724 | | | 936.5037 | 919.4771 | 918.4931 | 8 |
| 5 | 120.0808 | 446.2398 | 428.2292 | ***474.2347*** | 456.2241 | | | F | 773.4040 | | | ***865.4666*** | 848.4400 | 847.4560 | 7 |
| 6 | 86.0964 | 559.3239 | 541.3133 | ***587.3188*** | 569.3082 | 531.2926 | 545.3082 | I | 660.3199 | 673.3403 | 687.3559 | ***718.3981*** | 701.3716 | 700.3876 | 6 |
| 7 | 102.0550 | 688.3665 | 670.3559 | ***716.3614*** | 698.3508 | 630.3610 | | E | 531.2773 | 530.2821 | | ***605.3141*** | 588.2875 | 587.3035 | 5 |
| 8 | 86.0964 | 801.4505 | 783.4400 | ***829.4454*** | 811.4349 | 759.4036 | | L | 418.1932 | 417.1980 | | ***476.2715*** | 459.2449 | 458.2609 | 4 |
| 9 | 88.0393 | 916.4775 | 898.4669 | 944.4724 | 926.4618 | 872.4876 | | D | 303.1663 | 302.1710 | | 363.1874 | 346.1609 | 345.1769 | 3 |
| 10 | 74.0600 | 1017.5251 | 999.5146 | ***1045.5201*** | 1027.5095 | 1001.5302 | 1003.5095 | T | 202.1186 | 215.1390 | 217.1183 | 248.1605 | 231.1339 | 230.1499 | 2 |
| 11 | 101.1073 | | | | | | | K | 74.0237 | 73.0284 | | 147.1128 | 130.0863 | | 1 |

| Seq | ya | yb | Seq | ya | yb | Seq | ya | yb |
|---|---|---|---|---|---|---|---|---|
| AP | 141.1022 | 169.0972 | APA | 212.1394 | 240.1343 | APAF | 359.2078 | 387.2027 |
| APAFI | 472.2918 | 500.2867 | APAFIE | 601.3344 | 629.3293 | PA | 141.1022 | 169.0972 |
| PAF | 288.1707 | 316.1656 | PAFI | 401.2547 | 429.2496 | PAFIE | 530.2973 | 558.2922 |
| PAFIEL | 643.3814 | 671.3763 | AF | 191.1179 | 219.1128 | AFI | 304.2020 | 332.1969 |
| AFIE | 433.2445 | 461.2395 | AFIEL | 546.3286 | 574.3235 | AFIELD | 661.3556 | 689.3505 |
| | | | | | | | | |

| | | | | | | | | |
|---|---|---|---|---|---|---|---|---|
| **FI** | 233.1648 | 261.1598 | **FIE** | 362.2074 | 390.2023 | **FIEL** | 475.2915 | 503.2864 |
| **FIELD** | 590.3184 | 618.3134 | **FIELDT** | 691.3661 | 719.3610 | **IE** | 215.1390 | 243.1339 |
| **IEL** | 328.2231 | 356.2180 | **IELD** | 443.2500 | 471.2449 | **IELDT** | 544.2977 | 572.2926 |
| **EL** | 215.1390 | 243.1339 | **ELD** | 330.1660 | 358.1609 | **ELDT** | 431.2136 | 459.2086 |
| **LD** | 201.1234 | 229.1183 | **LDT** | 302.1710 | 330.1660 | **DT** | 189.0870 | 217.0819 |

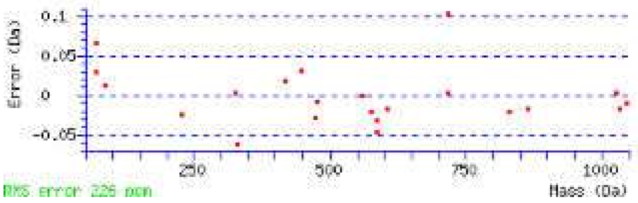


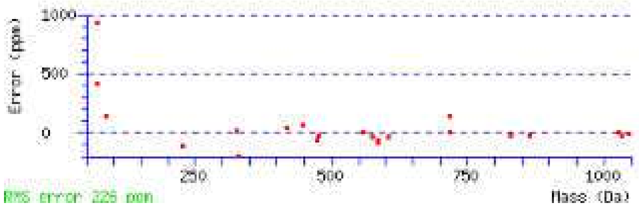


NCBI **BLAST** search of SAPAFIELDTK
(Parameters: blastp, nr protein database, expect=20000, no filter, PAM30)
Other BLAST web gateways

**All matches to this query**

| Score | Mr(calc) | Delta | Sequence |
|---|---|---|---|
| 27.3 | 1190.6183 | 0.0912 | SAPAFIELDTK |
| 27.3 | 1190.6183 | 0.0912 | SAPAFIELDTK |
| 27.3 | 1190.6183 | 0.0912 | SAPAFIELDTK |
| 27.1 | 1190.5931 | 0.1164 | SAPAFIQNDTK |
| 26.6 | 1190.5462 | 0.1633 | MVGGDAGQERR |
| 23.9 | 1189.6740 | 1.0355 | VVQKMIETVK |
| 23.3 | 1190.5779 | 0.1316 | ATAESPASTQTK |
| 22.0 | 1189.6190 | 1.0905 | LLSGGLSSEEAK |
| 20.7 | 1190.6030 | 0.1065 | GTTESEIVIDK |
| 20.4 | 1189.6343 | 1.0752 | AAPSFIQLDTK |

**Mascot:** http://www.matrixscience.com/

# MATRIX SCIENCE Mascot Search Results

## Peptide View Spot no 87

MS/MS Fragmentation of **VVDLLAPYRR**
Found in **gi|11466794** in **NCBInr**, ATP synthase CF1 beta subunit [Oryza sativa Japonica Group]

Match to Query 8: 1200.762624 from(1201.769900,1+) intensity(0.0000) index(4)
Title: Label: D11, Spot_Id: 216640, Peak_List_Id: 221234, MSMS Job_Run_Id: 21346, Comment:
Data file C:\Documents and Settings\Administrator\Desktop\farah BHU\RAJESH\12-1-13 raj\ppw_D11_135997699500.txt

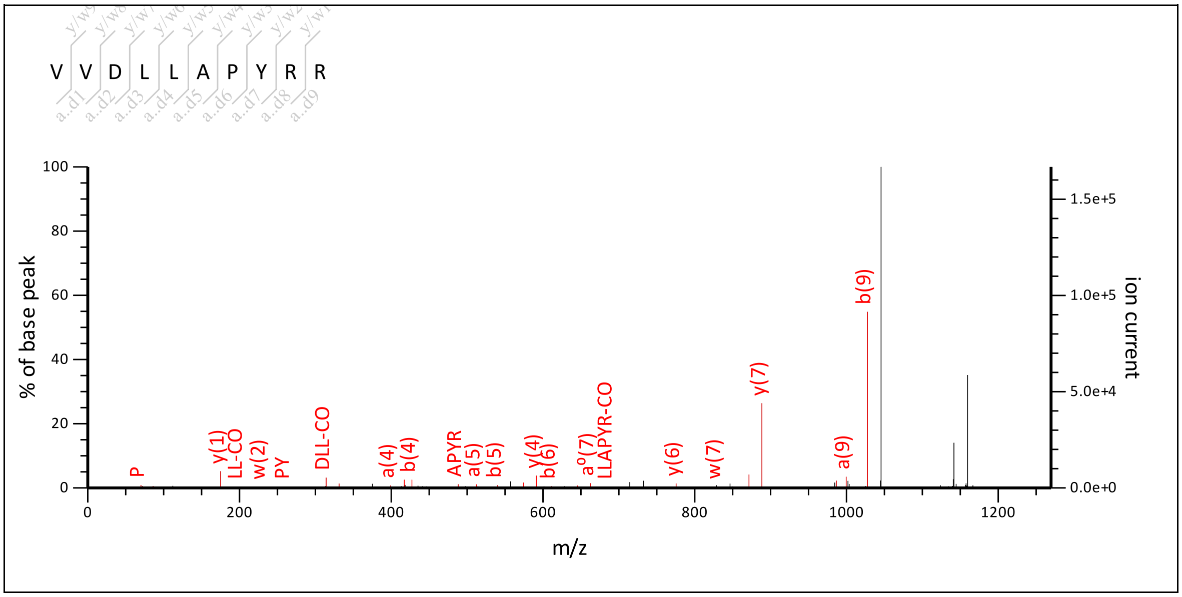


0 to 1269.64

Label all possible matches ○ Label matches used for scoring ◉

**Monoisotopic mass of neutral peptide Mr(calc):** 1200.6979
**Fixed modifications:** Carbamidomethyl (C) (apply to specified residues or termini only)
**Ions Score:** 37 **Expect:** 1.1
**Matches :** 44/136 fragment ions using 55 most intense peaks (help)

| # | Immon. | a | a* | $a^0$ | b | b* | $b^0$ | d | Seq. | v | w | y | y* | $y^0$ | # |
|---|---|---|---|---|---|---|---|---|---|---|---|---|---|---|---|
| 1 | 72.0808 | **72.0808** | | | 100.0757 | | | 44.0495 | V | | | | | | 10 |
| 2 | 72.0808 | **171.1492** | | | ***199.1441*** | | | 157.1335 | V | 1058.5742 | 1071.5946 | 1102.6368 | 1085.6102 | 1084.6262 | 9 |
| 3 | 88.0393 | 286.1761 | | 268.1656 | ***314.1710*** | | 296.1605 | 242.1863 | D | 943.5472 | 942.5520 | 1003.5683 | 986.5418 | 985.5578 | 8 |
| 4 | 86.0964 | **399.2602** | | 381.2496 | ***427.2551*** | | 409.2445 | 357.2132 | L | 830.4631 | 829.4679 | **888.5414** | 871.5148 | | 7 |
| 5 | 86.0964 | **512.3443** | | 494.3337 | ***540.3392*** | | 522.3286 | 470.2973 | L | 717.3791 | 716.3838 | **775.4573** | 758.4308 | | 6 |
| 6 | 44.0495 | 583.3814 | | 565.3708 | ***611.3763*** | | 593.3657 | | A | 646.3420 | | **662.3733** | 645.3467 | | 5 |
| 7 | 70.0651 | 680.4341 | | 662.4236 | 708.4291 | | 690.4185 | 654.4185 | P | 549.2892 | 548.2940 | **591.3362** | 574.3096 | | 4 |
| 8 | 136.0757 | 843.4975 | | 825.4869 | ***871.4924*** | | 853.4818 | | Y | 386.2259 | | 494.2834 | 477.2568 | | 3 |
| 9 | 129.1135 | **999.5986** | 982.5720 | 981.5880 | ***1027.5935*** | 1010.5669 | 1009.5829 | 914.5346 | R | 230.1248 | 229.1295 | **331.2201** | 314.1935 | | 2 |
| 10 | 129.1135 | | | | | | | | R | 74.0237 | 73.0284 | **175.1190** | 158.0924 | | 1 |

| Seq | ya | yb | Seq | ya | yb | Seq | ya | yb |
|---|---|---|---|---|---|---|---|---|
| VD | 187.1077 | 215.1026 | VDL | 300.1918 | 328.1867 | VDLL | 413.2758 | 441.2708 |
| VDLLA | 484.3130 | 512.3079 | VDLLAP | 581.3657 | 609.3606 | DL | 201.1234 | 229.1183 |
| DLL | 314.2074 | 342.2023 | DLLA | 385.2445 | 413.2395 | DLLAP | 482.2973 | 510.2922 |
| DLLAPY | 645.3606 | 673.3556 | LL | 199.1805 | 227.1754 | LLA | 270.2176 | 298.2125 |
| LLAP | 367.2704 | 395.2653 | LLAPY | 530.3337 | 558.3286 | LLAPYR | 686.4348 | 714.4297 |
| LA | 157.1335 | 185.1285 | LAP | 254.1863 | 282.1812 | LAPY | 417.2496 | 445.2445 |

| | | | | | | | | |
|---|---|---|---|---|---|---|---|---|
| **LAPYR** | 573.3507 | 601.3457 | **AP** | 141.1022 | 169.0972 | **APY** | 304.1656 | 332.1605 |
| **APYR** | 460.2667 | 488.2616 | **PY** | 233.1285 | 261.1234 | **PYR** | 389.2296 | 417.2245 |
| **YR** | 292.1768 | 320.1717 | | | | | | |

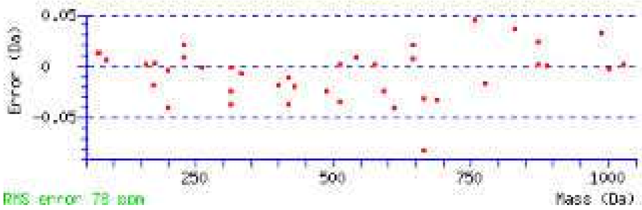


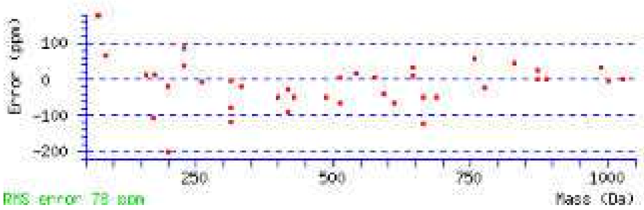


NCBI **BLAST** search of VVDLLAPYRR
(Parameters: blastp, nr protein database, expect=20000, no filter, PAM30)
Other BLAST web gateways

**All matches to this query**

| Score | Mr(calc) | Delta | Sequence |
|---|---|---|---|
| 40.7 | 1200.2059 | 0.5568 | VVBLLAPYRR |
| 38.3 | 1200.6979 | 0.0647 | VVDLIAPYRR |
| 37.4 | 1200.6979 | 0.0647 | VVDLLAPYRR |
| 37.4 | 1200.6979 | 0.0647 | VVDLLAPYRR |
| 37.4 | 1200.6979 | 0.0647 | VVDLLAPYRR |
| 37.4 | 1200.6979 | 0.0647 | VVDLLAPYRR |
| 37.4 | 1200.6979 | 0.0647 | VVDLLAPYRR |
| 37.4 | 1200.6979 | 0.0647 | VVDLLAPYRR |
| 37.4 | 1200.6979 | 0.0647 | VVDLLAPYRR |
| 37.4 | 1200.6979 | 0.0647 | VVDLLAPYRR |

**Mascot:** http://www.matrixscience.com/

# MATRIX SCIENCE Mascot Search Results

## Peptide View **Spot no 87**

MS/MS Fragmentation of **AHGGVSVFGGVGER**
Found in **gi|11466794** in **NCBInr**, ATP synthase CF1 beta subunit [Oryza sativa Japonica Group]

Match to Query 10: 1327.745924 from(1328.753200,1+) intensity(0.0000) index(5)
Title: Label: D11, Spot_Id: 216640, Peak_List_Id: 221228, MSMS Job_Run_Id: 21346, Comment:
Data file C:\Documents and Settings\Administrator\Desktop\farah BHU\RAJESH\12-1-13 raj\ppw_D11_135997699500.txt

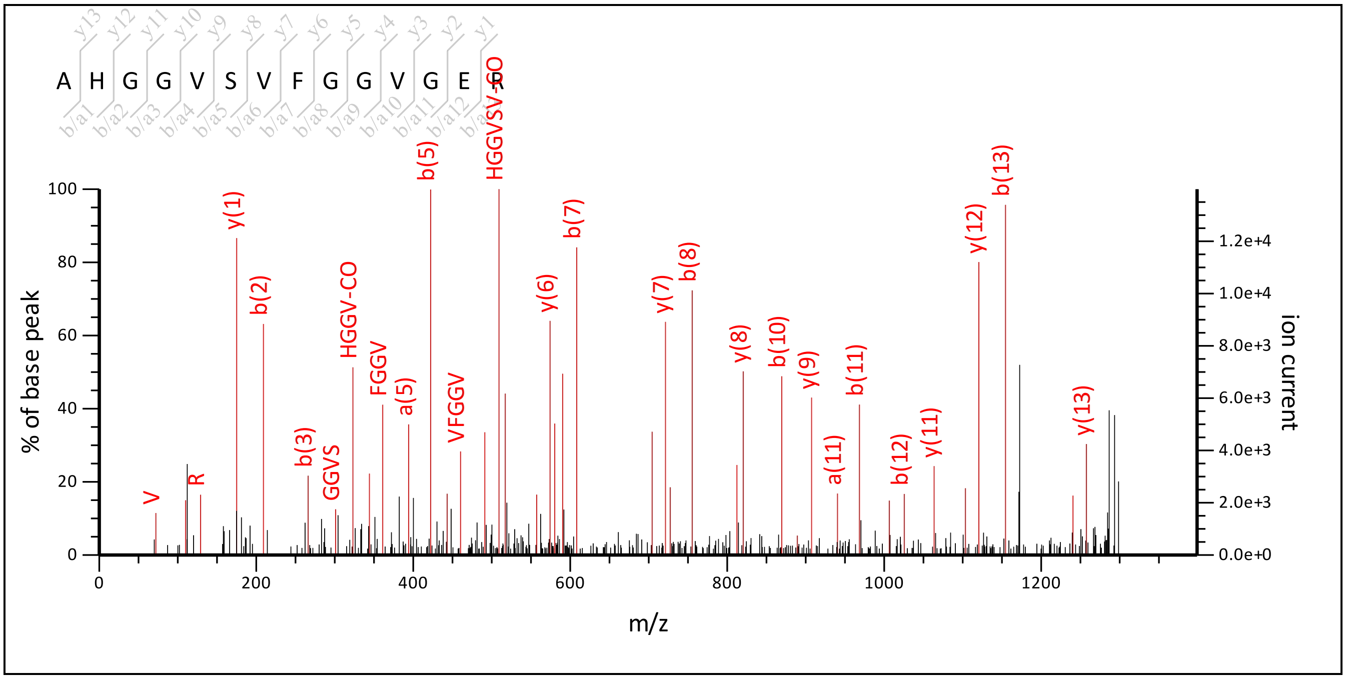


0 to 1398.59

Label all possible matches ○ Label matches used for scoring ◉

**Monoisotopic mass of neutral peptide Mr(calc):** 1327.6633
**Fixed modifications:** Carbamidomethyl (C) (apply to specified residues or termini only)
**Ions Score:** 128 **Expect:** 8.9e-10
**Matches :** 53/220 fragment ions using 52 most intense peaks (help)

| # | Immon. | a | $a^0$ | b | $b^0$ | d | Seq. | v | w | y | y* | $y^0$ | # |
|---|---|---|---|---|---|---|---|---|---|---|---|---|---|
| 1 | 44.0495 | 44.0495 | | ***72.0444*** | | 44.0495 | A | | | | | | 14 |
| 2 | 110.0713 | 181.1084 | | ***209.1033*** | | | H | 1175.5804 | | **1257.6335** | 1240.6069 | 1239.6229 | 13 |
| 3 | 30.0338 | 238.1299 | | ***266.1248*** | | | G | | | **1120.5745** | 1103.5480 | 1102.5640 | 12 |
| 4 | 30.0338 | 295.1513 | | ***323.1462*** | | | G | | | **1063.5531** | 1046.5265 | 1045.5425 | 11 |
| 5 | 72.0808 | **394.2197** | | ***422.2146*** | | 380.2041 | V | 962.4690 | 975.4894 | **1006.5316** | 989.5051 | 988.5211 | 10 |
| 6 | 60.0444 | 481.2518 | 463.2412 | ***509.2467*** | 491.2361 | 465.2568 | S | 875.4370 | 874.4417 | **907.4632** | 890.4367 | 889.4526 | 9 |
| 7 | 72.0808 | **580.3202** | 562.3096 | ***608.3151*** | 590.3045 | 566.3045 | V | 776.3686 | 789.3890 | **820.4312** | 803.4046 | 802.4206 | 8 |
| 8 | 120.0808 | **727.3886** | 709.3780 | ***755.3835*** | 737.3729 | | F | 629.3002 | | **721.3628** | 704.3362 | 703.3522 | 7 |
| 9 | 30.0338 | 784.4100 | 766.3995 | ***812.4050*** | 794.3944 | | G | | | **574.2944** | 557.2678 | 556.2838 | 6 |
| 10 | 30.0338 | 841.4315 | 823.4209 | ***869.4264*** | 851.4159 | | G | | | **517.2729** | 500.2463 | 499.2623 | 5 |
| 11 | 72.0808 | **940.4999** | 922.4894 | ***968.4948*** | 950.4843 | 926.4843 | V | 416.1888 | 429.2092 | **460.2514** | 443.2249 | 442.2409 | 4 |

| | | | | | | | | | | | | | |
|---|---|---|---|---|---|---|---|---|---|---|---|---|---|
| 12 | 30.0338 | 997.5214 | 979.5108 | *1025.5163* | 1007.5057 | | G | | | **361.1830** | 344.1565 | 343.1724 | 3 |
| 13 | 102.0550 | 1126.5640 | 1108.5534 | *1154.5589* | 1136.5483 | 1068.5585 | E | 230.1248 | 229.1295 | 304.1615 | 287.1350 | 286.1510 | 2 |
| 14 | 129.1135 | | | | | | R | 74.0237 | 73.0284 | **175.1190** | 158.0924 | | 1 |

| Seq | ya | yb | Seq | ya | yb | Seq | ya | yb |
|---|---|---|---|---|---|---|---|---|
| **HG** | 167.0927 | 195.0877 | **HGG** | 224.1142 | 252.1091 | **HGGV** | 323.1826 | 351.1775 |
| **HGGVS** | 410.2146 | 438.2096 | **HGGVSV** | 509.2831 | 537.2780 | **HGGVSVF** | 656.3515 | 684.3464 |
| **GG** | 87.0553 | 115.0502 | **GGV** | 186.1237 | 214.1186 | **GGVS** | 273.1557 | 301.1506 |
| **GGVSV** | 372.2241 | 400.2191 | **GGVSVF** | 519.2926 | 547.2875 | **GGVSVFG** | 576.3140 | 604.3089 |
| **GGVSVFGG** | 633.3355 | 661.3304 | **GV** | 129.1022 | 157.0972 | **GVS** | 216.1343 | 244.1292 |
| **GVSV** | 315.2027 | 343.1976 | **GVSVF** | 462.2711 | 490.2660 | **GVSVFG** | 519.2926 | 547.2875 |
| **GVSVFGG** | 576.3140 | 604.3089 | **GVSVFGGV** | 675.3824 | 703.3774 | **VS** | 159.1128 | 187.1077 |
| **VSV** | 258.1812 | 286.1761 | **VSVF** | 405.2496 | 433.2445 | **VSVFG** | 462.2711 | 490.2660 |
| **VSVFGG** | 519.2926 | 547.2875 | **VSVFGGV** | 618.3610 | 646.3559 | **VSVFGGVG** | 675.3824 | 703.3774 |
| **SV** | 159.1128 | 187.1077 | **SVF** | 306.1812 | 334.1761 | **SVFG** | 363.2027 | 391.1976 |
| **SVFGG** | 420.2241 | 448.2191 | **SVFGGV** | 519.2926 | 547.2875 | **SVFGGVG** | 576.3140 | 604.3089 |
| **VF** | 219.1492 | 247.1441 | **VFG** | 276.1707 | 304.1656 | **VFGG** | 333.1921 | 361.1870 |
| **VFGGV** | 432.2605 | 460.2554 | **VFGGVG** | 489.2820 | 517.2769 | **VFGGVGE** | 618.3246 | 646.3195 |
| **FG** | 177.1022 | 205.0972 | **FGG** | 234.1237 | 262.1186 | **FGGV** | 333.1921 | 361.1870 |
| **FGGVG** | 390.2136 | 418.2085 | **FGGVGE** | 519.2562 | 547.2511 | **GG** | 87.0553 | 115.0502 |
| **GGV** | 186.1237 | 214.1186 | **GGVG** | 243.1452 | 271.1401 | **GGVGE** | 372.1878 | 400.1827 |
| **GV** | 129.1022 | 157.0972 | **GVG** | 186.1237 | 214.1186 | **GVGE** | 315.1663 | 343.1612 |
| **VG** | 129.1022 | 157.0972 | **VGE** | 258.1448 | 286.1397 | **GE** | 159.0764 | 187.0713 |

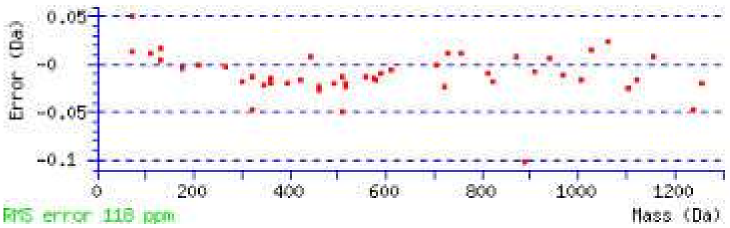


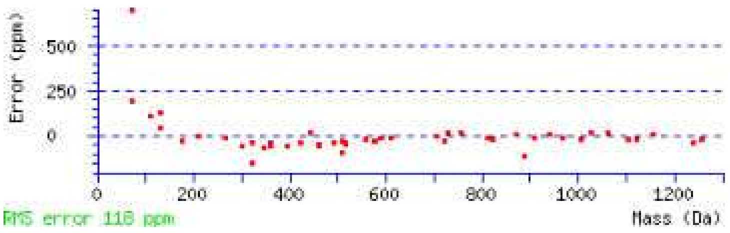


NCBI **BLAST** search of AHGGVSVFGGVGER
(Parameters: blastp, nr protein database, expect=20000, no filter, PAM30)
Other BLAST web gateways

**All matches to this query**

| Score | Mr(calc) | Delta | Sequence |
|---|---|---|---|
| 127.9 | 1327.6633 | 0.0826 | AHGGVSVFGGVGER |
| 127.9 | 1327.6633 | 0.0826 | AHGGVSVFGGVGER |
| 127.9 | 1327.6633 | 0.0826 | AHGGVSVFGGVGER |
| 127.9 | 1327.6633 | 0.0826 | AHGGVSVFGGVGER |

| | | | |
|---|---|---|---|
| 127.9 | 1327.6633 | 0.0826 | AHGGVSVFGGVGER |
| 127.9 | 1327.6633 | 0.0826 | AHGGVSVFGGVGER |
| 127.9 | 1327.6633 | 0.0826 | AHGGVSVFGGVGER |
| 127.9 | 1327.6633 | 0.0826 | AHGGVSVFGGVGER |
| 127.9 | 1327.6633 | 0.0826 | AHGGVSVFGGVGER |
| 127.9 | 1327.6633 | 0.0826 | AHGGVSVFGGVGER |

**Mascot:** http://www.matrixscience.com/

# MATRIX SCIENCE Mascot Search Results

## Peptide View **Spot no 87**

MS/MS Fragmentation of **IVGNEHYETAQR**
Found in **gi|11466794** in **NCBInr**, ATP synthase CF1 beta subunit [Oryza sativa Japonica Group]

Match to Query 12: 1415.766024 from(1416.773300,1+) intensity(0.0000) index(6)
Title: Label: D11, Spot_Id: 216640, Peak_List_Id: 221230, MSMS Job_Run_Id: 21346, Comment:
Data file C:\Documents and Settings\Administrator\Desktop\farah BHU\RAJESH\12-1-13 raj\ppw_D11_135997699500.txt

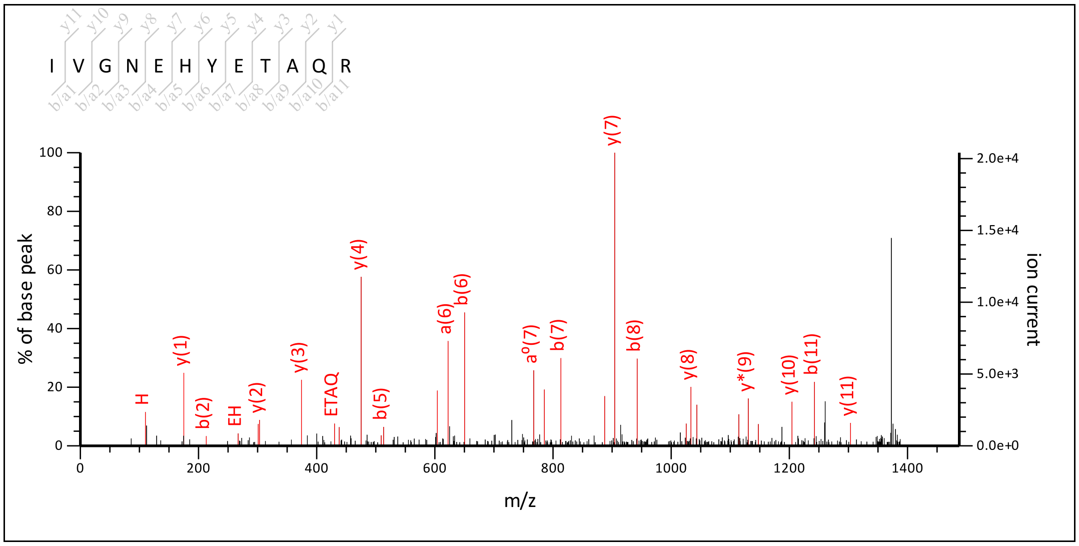


0 to 1487.61

Label all possible matches ○ Label matches used for scoring ◉

**Monoisotopic mass of neutral peptide Mr(calc):** 1415.6793
**Fixed modifications:** Carbamidomethyl (C) (apply to specified residues or termini only)
**Ions Score:** 95 **Expect:** 1.7e-06
**Matches :** 40/181 fragment ions using 41 most intense peaks (help)

| # | Immon. | a | a* | a⁰ | b | b* | b⁰ | d | d' | Seq. | v | w | w' | y | y* | |
|---|---|---|---|---|---|---|---|---|---|---|---|---|---|---|---|---|
| 1 | 86.0964 | 86.0964 | | | 114.0913 | | | 44.0495 | | I | | | | | | |
| 2 | 72.0808 | 185.1648 | | | **213.1598** | | | 171.1492 | | V | 1259.5400 | 1272.5604 | | ***1303.6026*** | 1286.5760 | 128 |
| 3 | 30.0338 | 242.1863 | | | 270.1812 | | | | | G | | | | ***1204.5341*** | 1187.5076 | 118 |
| 4 | 87.0553 | 356.2292 | 339.2027 | | 384.2241 | 367.1976 | | 313.2234 | | N | 1088.4756 | 1087.4803 | | ***1147.5127*** | 1130.4861 | 11 |
| 5 | 102.0550 | 485.2718 | 468.2453 | 467.2613 | **513.2667** | 496.2402 | 495.2562 | 427.2663 | | E | 959.4330 | 958.4377 | | ***1033.4697*** | 1016.4432 | 10 |
| 6 | 110.0713 | 622.3307 | 605.3042 | 604.3202 | **650.3256** | 633.2991 | 632.3151 | | | H | 822.3741 | | | ***904.4272*** | 887.4006 | 88 |
| 7 | 136.0757 | 785.3941 | 768.3675 | 767.3835 | **813.3890** | 796.3624 | 795.3784 | | | Y | 659.3107 | | | ***767.3682*** | 750.3417 | 74 |
| 8 | 102.0550 | 914.4367 | 897.4101 | 896.4261 | **942.4316** | 925.4050 | 924.4210 | 856.4312 | | E | 530.2681 | 529.2729 | | ***604.3049*** | 587.2784 | 58 |
| 9 | 74.0600 | 1015.4843 | 998.4578 | 997.4738 | **1043.4793** | 1026.4527 | 1025.4687 | 999.4894 | 1001.4687 | T | 429.2205 | 442.2409 | 444.2201 | ***475.2623*** | 458.2358 | 45 |
| 10 | 44.0495 | 1086.5214 | 1069.4949 | 1068.5109 | **1114.5164** | 1097.4898 | 1096.5058 | | | A | 358.1833 | | | ***374.2146*** | 357.1881 | |
| 11 | 101.0709 | 1214.5800 | 1197.5535 | 1196.5695 | **1242.5749** | 1225.5484 | 1224.5644 | 1157.5586 | | Q | 230.1248 | 229.1295 | | ***303.1775*** | 286.1510 | |
| 12 | 129.1135 | | | | | | | | | R | 74.0237 | 73.0284 | | ***175.1190*** | 158.0924 | |

| Seq | ya | yb | Seq | ya | yb | Seq | ya | yb |
|---|---|---|---|---|---|---|---|---|
| VG | 129.1022 | 157.0972 | VGN | 243.1452 | 271.1401 | VGNE | 372.1878 | 400.1827 |
| VGNEH | 509.2467 | 537.2416 | VGNEHY | 672.3100 | 700.3049 | GN | 144.0768 | 172.0717 |
| GNE | 273.1193 | 301.1143 | GNEH | 410.1783 | 438.1732 | GNEHY | 573.2416 | 601.2365 |
| NE | 216.0979 | 244.0928 | NEH | 353.1568 | 381.1517 | NEHY | 516.2201 | 544.2150 |
| NEHYE | 645.2627 | 673.2576 | EH | 239.1139 | 267.1088 | EHY | 402.1772 | 430.1721 |
| EHYE | 531.2198 | 559.2147 | EHYET | 632.2675 | 660.2624 | HY | 273.1346 | 301.1295 |
| HYE | 402.1772 | 430.1721 | HYET | 503.2249 | 531.2198 | HYETA | 574.2620 | 602.2569 |
| YE | 265.1183 | 293.1132 | YET | 366.1660 | 394.1609 | YETA | 437.2031 | 465.1980 |
| YETAQ | 565.2617 | 593.2566 | ET | 203.1026 | 231.0975 | ETA | 274.1397 | 302.1347 |

| | | | | | | | | |
|---|---|---|---|---|---|---|---|---|
| **ETAQ** | 402.1983 | 430.1932 | **TA** | 145.0972 | 173.0921 | **TAQ** | 273.1557 | 301.1506 |
| **AQ** | 172.1081 | 200.1030 | | | | | | |

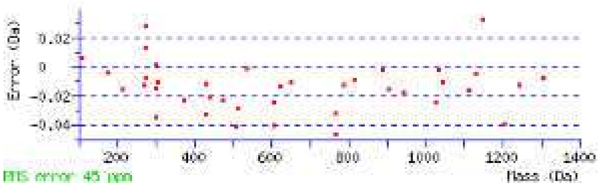


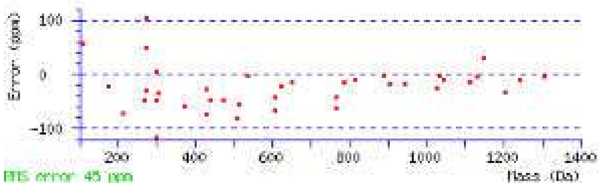


NCBI **BLAST** search of IVGNEHYETAQR
(Parameters: blastp, nr protein database, expect=20000, no filter, PAM30)
Other BLAST web gateways

**All matches to this query**

| Score | Mr(calc) | Delta | Sequence |
|---|---|---|---|
| 95.3 | 1415.6793 | 0.0867 | IVGNEHYETAQR |
| 95.3 | 1415.6793 | 0.0867 | IVGNEHYETAQR |
| 78.0 | 1415.7157 | 0.0503 | IVGDKHYETAQR |
| 78.0 | 1415.7157 | 0.0503 | IVGDKHYETAQR |
| 78.0 | 1415.6793 | 0.0867 | IVGDQHYETAQR |
| 78.0 | 1415.6793 | 0.0867 | IVGENHYETAQR |
| 78.0 | 1415.6793 | 0.0867 | IVGENHYETAQR |
| 78.0 | 1415.7157 | 0.0503 | IVGKDHYETAQR |
| 78.0 | 1415.7157 | 0.0503 | IVGKDHYETAQR |
| 39.4 | 1416.6633 | -0.8973 | IVGDEHYETAQR |

**Mascot:** http://www.matrixscience.com/

# Mascot Search Results

## Peptide View Spot no 87

MS/MS Fragmentation of **FVQAGSEVSALLGR**
Found in **gi|11466794** in **NCBInr**, ATP synthase CF1 beta subunit [Oryza sativa Japonica Group]

Match to Query 14: 1432.848424 from(1433.855700,1+) intensity(0.0000) index(7)
Title: Label: D11, Spot_Id: 216640, Peak_List_Id: 221233, MSMS Job_Run_Id: 21346, Comment:
Data file C:\Documents and Settings\Administrator\Desktop\farah BHU\RAJESH\12-1-13 raj\ppw_D11_135997699500.txt

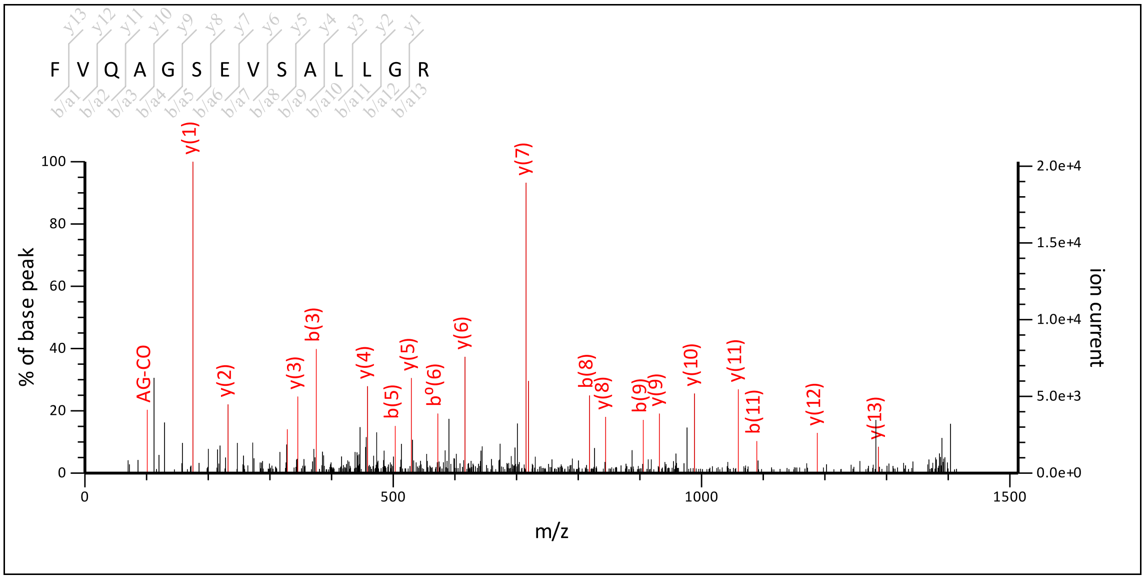


0 to 1513.41

Label all possible matches ○ Label matches used for scoring ◉

**Monoisotopic mass of neutral peptide Mr(calc):** 1432.7674
**Fixed modifications:** Carbamidomethyl (C) (apply to specified residues or termini only)
**Ions Score:** 131 **Expect:** 4.9e-10
**Matches :** 34/243 fragment ions using 28 most intense peaks (help)

| # | Immon. | a | a* | $a^0$ | b | b* | $b^0$ | d | Seq. | v | w | y | y* | $y^0$ | # |
|---|---|---|---|---|---|---|---|---|---|---|---|---|---|---|---|
| 1 | 120.0808 | 120.0808 | | | 148.0757 | | | 44.0495 | F | | | | | | 14 |
| 2 | 72.0808 | 219.1492 | | | 247.1441 | | | 205.1335 | V | 1242.6437 | 1255.6641 | ***1286.7063*** | 1269.6797 | 1268.6957 | 13 |
| 3 | 101.0709 | 347.2078 | 330.1812 | | **375.2027** | 358.1761 | | 290.1863 | Q | 1114.5851 | 1113.5899 | ***1187.6379*** | 1170.6113 | 1169.6273 | 12 |
| 4 | 44.0495 | 418.2449 | 401.2183 | | 446.2398 | 429.2132 | | | A | 1043.5480 | | ***1059.5793*** | 1042.5527 | 1041.5687 | 11 |
| 5 | 30.0338 | 475.2663 | 458.2398 | | **503.2613** | 486.2347 | | | G | | | ***988.5422*** | 971.5156 | 970.5316 | 10 |
| 6 | 60.0444 | 562.2984 | 545.2718 | 544.2878 | 590.2933 | 573.2667 | 572.2827 | 546.3035 | S | 899.4945 | 898.4993 | ***931.5207*** | 914.4942 | 913.5102 | 9 |
| 7 | 102.0550 | 691.3410 | 674.3144 | 673.3304 | **719.3359** | 702.3093 | 701.3253 | 633.3355 | E | 770.4519 | 769.4567 | ***844.4887*** | 827.4621 | 826.4781 | 8 |
| 8 | 72.0808 | 790.4094 | 773.3828 | 772.3988 | **818.4043** | 801.3777 | 800.3937 | 776.3937 | V | 671.3835 | 684.4039 | ***715.4461*** | 698.4196 | 697.4355 | 7 |
| 9 | 60.0444 | 877.4414 | 860.4149 | 859.4308 | **905.4363** | 888.4098 | 887.4258 | 861.4465 | S | 584.3515 | 583.3562 | ***616.3777*** | 599.3511 | 598.3671 | 6 |
| 10 | 44.0495 | 948.4785 | 931.4520 | 930.4680 | 976.4734 | 959.4469 | 958.4629 | | A | 513.3144 | | ***529.3457*** | 512.3191 | | 5 |
| 11 | 86.0964 | 1061.5626 | 1044.5360 | 1043.5520 | **1089.5575** | 1072.5310 | 1071.5469 | 1019.5156 | L | 400.2303 | 399.2350 | ***458.3085*** | 441.2820 | | 4 |
| 12 | 86.0964 | 1174.6466 | 1157.6201 | 1156.6361 | 1202.6416 | 1185.6150 | 1184.6310 | 1132.5997 | L | 287.1462 | 286.1510 | ***345.2245*** | 328.1979 | | 3 |
| 13 | 30.0338 | 1231.6681 | 1214.6416 | 1213.6575 | 1259.6630 | 1242.6365 | 1241.6525 | | G | | | ***232.1404*** | 215.1139 | | 2 |
| 14 | 129.1135 | | | | | | | | R | 74.0237 | 73.0284 | ***175.1190*** | 158.0924 | | 1 |

| Seq | ya | yb | Seq | ya | yb | Seq | ya | yb |
|---|---|---|---|---|---|---|---|---|
| VQ | 200.1394 | 228.1343 | VQA | 271.1765 | 299.1714 | VQAG | 328.1979 | 356.1928 |
| VQAGS | 415.2300 | 443.2249 | VQAGSE | 544.2726 | 572.2675 | VQAGSEV | 643.3410 | 671.3359 |
| QA | 172.1081 | 200.1030 | QAG | 229.1295 | 257.1244 | QAGS | 316.1615 | 344.1565 |
| QAGSE | 445.2041 | 473.1991 | QAGSEV | 544.2726 | 572.2675 | QAGSEVS | 631.3046 | 659.2995 |

| | | | | | | | | |
|---|---|---|---|---|---|---|---|---|
| **AG** | 101.0709 | 129.0659 | **AGS** | 188.1030 | 216.0979 | **AGSE** | 317.1456 | 345.1405 |
| **AGSEV** | 416.2140 | 444.2089 | **AGSEVS** | 503.2460 | 531.2409 | **AGSEVSA** | 574.2831 | 602.2780 |
| **AGSEVSAL** | 687.3672 | 715.3621 | **GS** | 117.0659 | 145.0608 | **GSE** | 246.1084 | 274.1034 |
| **GSEV** | 345.1769 | 373.1718 | **GSEVS** | 432.2089 | 460.2038 | **GSEVSA** | 503.2460 | 531.2409 |
| **GSEVSAL** | 616.3301 | 644.3250 | **SE** | 189.0870 | 217.0819 | **SEV** | 288.1554 | 316.1503 |
| **SEVS** | 375.1874 | 403.1823 | **SEVSA** | 446.2245 | 474.2195 | **SEVSAL** | 559.3086 | 587.3035 |
| **SEVSALL** | 672.3927 | 700.3876 | **EV** | 201.1234 | 229.1183 | **EVS** | 288.1554 | 316.1503 |
| **EVSA** | 359.1925 | 387.1874 | **EVSAL** | 472.2766 | 500.2715 | **EVSALL** | 585.3606 | 613.3556 |
| **EVSALLG** | 642.3821 | 670.3770 | **VS** | 159.1128 | 187.1077 | **VSA** | 230.1499 | 258.1448 |
| **VSAL** | 343.2340 | 371.2289 | **VSALL** | 456.3180 | 484.3130 | **VSALLG** | 513.3395 | 541.3344 |
| **SA** | 131.0815 | 159.0764 | **SAL** | 244.1656 | 272.1605 | **SALL** | 357.2496 | 385.2445 |
| **SALLG** | 414.2711 | 442.2660 | **AL** | 157.1335 | 185.1285 | **ALL** | 270.2176 | 298.2125 |
| **ALLG** | 327.2391 | 355.2340 | **LL** | 199.1805 | 227.1754 | **LLG** | 256.2020 | 284.1969 |
| **LG** | 143.1179 | 171.1128 | | | | | | |

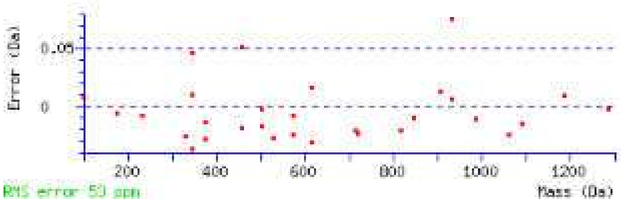


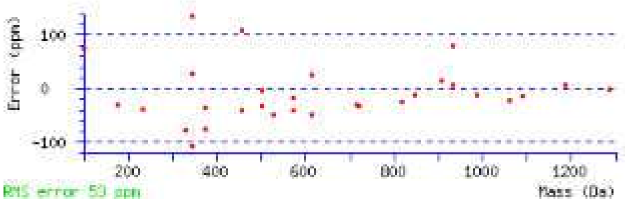


NCBI **BLAST** search of FVQAGSEVSALLGR
(Parameters: blastp, nr protein database, expect=20000, no filter, PAM30)
Other BLAST web gateways

**All matches to this query**

| Score | Mr(calc) | Delta | Sequence |
|---|---|---|---|
| 130.8 | 1432.7674 | 0.0810 | FVQAGSEVSAILGR |
| 130.8 | 1432.7674 | 0.0810 | FVQAGSEVSALIGR |
| 130.8 | 1432.7674 | 0.0810 | FVQAGSEVSALLGR |
| 130.8 | 1432.7674 | 0.0810 | FVQAGSEVSALLGR |
| 130.8 | 1432.7674 | 0.0810 | FVQAGSEVSALLGR |
| 130.8 | 1432.7674 | 0.0810 | FVQAGSEVSALLGR |
| 130.8 | 1432.7674 | 0.0810 | FVQAGSEVSALLGR |
| 130.8 | 1432.7674 | 0.0810 | FVQAGSEVSALLGR |
| 130.8 | 1432.7674 | 0.0810 | FVQAGSEVSALLGR |
| 130.8 | 1432.7674 | 0.0810 | FVQAGSEVSALLGR |

**Mascot:** http://www.matrixscience.com/

# MATRIX SCIENCE Mascot Search Results

## Peptide View **Spot no 87**

MS/MS Fragmentation of **VGLTALTMAEYFR**
Found in **gi|11466794** in **NCBInr**, ATP synthase CF1 beta subunit [Oryza sativa Japonica Group]

Match to Query 16: 1470.840124 from(1471.847400,1+) intensity(0.0000) index(8)
Title: Label: D11, Spot_Id: 216640, Peak_List_Id: 221227, MSMS Job_Run_Id: 21346, Comment:
Data file C:\Documents and Settings\Administrator\Desktop\farah BHU\RAJESH\12-1-13 raj\ppw_D11_135997699500.txt

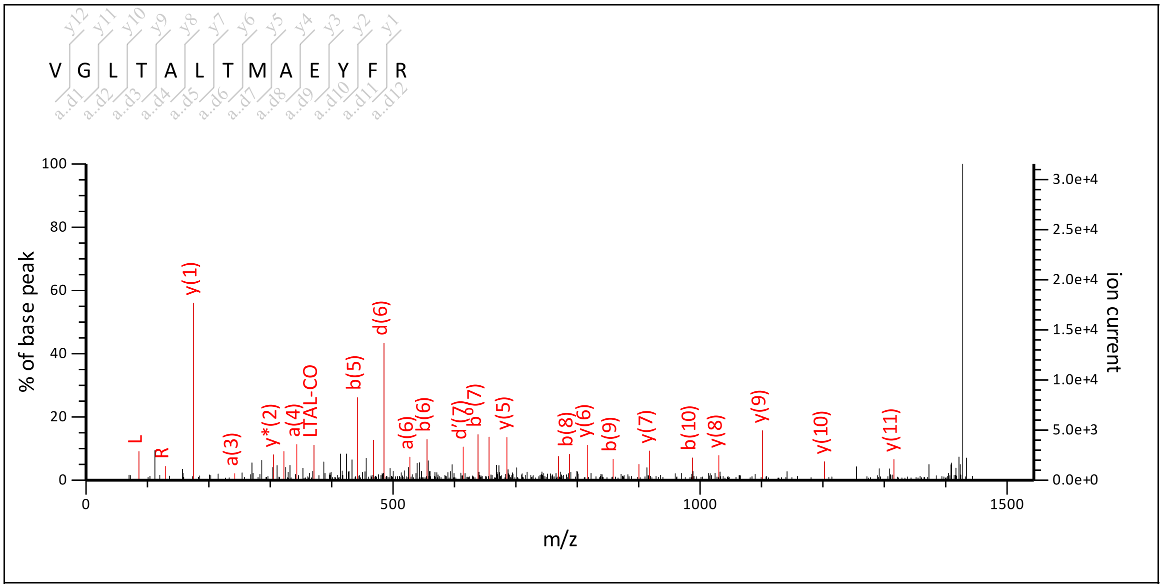


0 to 1543.55

Label all possible matches ○ Label matches used for scoring ◉

**Monoisotopic mass of neutral peptide Mr(calc):** 1470.7541
**Fixed modifications:** Carbamidomethyl (C) (apply to specified residues or termini only)
**Ions Score:** 88 **Expect:** 9.3e-06
**Matches :** 36/198 fragment ions using 42 most intense peaks (help)

| # | Immon. | a | $a^0$ | b | $b^0$ | d | d' | Seq. | v | w | w' | y | y* | $y^0$ | # |
|---|---|---|---|---|---|---|---|---|---|---|---|---|---|---|---|
| 1 | 72.0808 | 72.0808 | | 100.0757 | | 44.0495 | | V | | | | | | | 13 |
| 2 | 30.0338 | 129.1022 | | 157.0972 | | | | G | | | | 1372.6930 | 1355.6664 | 1354.6824 | 12 |
| 3 | 86.0964 | 242.1863 | | 270.1812 | | 200.1394 | | L | 1257.5932 | 1256.5980 | | 1315.6715 | 1298.6449 | 1297.6609 | 11 |
| 4 | 74.0600 | 343.2340 | 325.2234 | 371.2289 | 353.2183 | 327.2391 | 329.2183 | T | 1156.5456 | 1169.5660 | 1171.5452 | 1202.5874 | 1185.5609 | 1184.5769 | 10 |
| 5 | 44.0495 | 414.2711 | 396.2605 | 442.2660 | 424.2554 | | | A | 1085.5084 | | | 1101.5397 | 1084.5132 | 1083.5292 | 9 |
| 6 | 86.0964 | 527.3552 | 509.3446 | 555.3501 | 537.3395 | 485.3082 | | L | 972.4244 | 971.4291 | | 1030.5026 | 1013.4761 | 1012.4921 | 8 |
| 7 | 74.0600 | 628.4028 | 610.3923 | 656.3978 | 638.3872 | 612.4079 | 614.3872 | T | 871.3767 | 884.3971 | 886.3764 | 917.4186 | 900.3920 | 899.4080 | 7 |
| 8 | 104.0528 | 759.4433 | 741.4328 | 787.4382 | 769.4277 | 699.4400 | | M | 740.3362 | 739.3410 | | 816.3709 | 799.3443 | 798.3603 | 6 |
| 9 | 44.0495 | 830.4804 | 812.4699 | 858.4754 | 840.4648 | | | A | 669.2991 | | | 685.3304 | 668.3039 | 667.3198 | 5 |
| 10 | 102.0550 | 959.5230 | 941.5125 | 987.5179 | 969.5074 | 901.5176 | | E | 540.2565 | 539.2613 | | 614.2933 | 597.2667 | 596.2827 | 4 |
| 11 | 136.0757 | 1122.5864 | 1104.5758 | 1150.5813 | 1132.5707 | | | Y | 377.1932 | | | 485.2507 | 468.2241 | | 3 |
| 12 | 120.0808 | 1269.6548 | 1251.6442 | 1297.6497 | 1279.6391 | | | F | 230.1248 | | | 322.1874 | 305.1608 | | 2 |
| 13 | 129.1135 | | | | | | | R | 74.0237 | 73.0284 | | 175.1190 | 158.0924 | | 1 |

| Seq | ya | yb | Seq | ya | yb | Seq | ya | yb |
|---|---|---|---|---|---|---|---|---|
| GL | 143.1179 | 171.1128 | GLT | 244.1656 | 272.1605 | GLTA | 315.2027 | 343.1976 |
| GLTAL | 428.2867 | 456.2817 | GLTALT | 529.3344 | 557.3293 | GLTALTM | 660.3749 | 688.3698 |
| LT | 187.1441 | 215.1390 | LTA | 258.1812 | 286.1761 | LTAL | 371.2653 | 399.2602 |
| LTALT | 472.3130 | 500.3079 | LTALTM | 603.3534 | 631.3484 | LTALTMA | 674.3906 | 702.3855 |

| | | | | | | | | |
|---|---|---|---|---|---|---|---|---|
| **TA** | 145.0972 | 173.0921 | **TAL** | 258.1812 | 286.1761 | **TALT** | 359.2289 | 387.2238 |
| **TALTM** | 490.2694 | 518.2643 | **TALTMA** | 561.3065 | 589.3014 | **TALTMAE** | 690.3491 | 718.3440 |
| **AL** | 157.1335 | 185.1285 | **ALT** | 258.1812 | 286.1761 | **ALTM** | 389.2217 | 417.2166 |
| **ALTMA** | 460.2588 | 488.2537 | **ALTMAE** | 589.3014 | 617.2963 | **LT** | 187.1441 | 215.1390 |
| **LTM** | 318.1846 | 346.1795 | **LTMA** | 389.2217 | 417.2166 | **LTMAE** | 518.2643 | 546.2592 |
| **LTMAEY** | 681.3276 | 709.3225 | **TM** | 205.1005 | 233.0954 | **TMA** | 276.1376 | 304.1326 |
| **TMAE** | 405.1802 | 433.1751 | **TMAEY** | 568.2436 | 596.2385 | **MA** | 175.0900 | 203.0849 |
| **MAE** | 304.1326 | 332.1275 | **MAEY** | 467.1959 | 495.1908 | **MAEYF** | 614.2643 | 642.2592 |
| **AE** | 173.0921 | 201.0870 | **AEY** | 336.1554 | 364.1503 | **AEYF** | 483.2238 | 511.2187 |
| **EY** | 265.1183 | 293.1132 | **EYF** | 412.1867 | 440.1816 | **YF** | 283.1441 | 311.1390 |

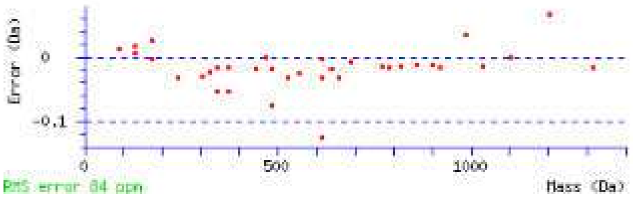


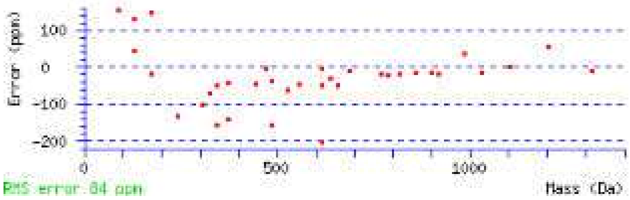


NCBI **BLAST** search of VGLTALTMAEYFR
(Parameters: blastp, nr protein database, expect=20000, no filter, PAM30)
Other BLAST web gateways

**All matches to this query**

| Score | Mr(calc) | Delta | Sequence |
|---|---|---|---|
| 88.0 | 1470.7541 | 0.0861 | VGITALTMAEYFR |
| 88.0 | 1470.7541 | 0.0861 | VGLTALTMAEYFR |
| 88.0 | 1470.7541 | 0.0861 | VGLTALTMAEYFR |
| 88.0 | 1470.7541 | 0.0861 | VGLTALTMAEYFR |
| 88.0 | 1470.7541 | 0.0861 | VGLTALTMAEYFR |
| 88.0 | 1470.7541 | 0.0861 | VGLTALTMAEYFR |
| 88.0 | 1470.7541 | 0.0861 | VGLTALTMAEYFR |
| 88.0 | 1470.7541 | 0.0861 | VGLTALTMAEYFR |
| 88.0 | 1470.7541 | 0.0861 | VGLTALTMAEYFR |
| 88.0 | 1470.7541 | 0.0861 | VGLTALTMAEYFR |

**Mascot:** http://www.matrixscience.com/

# MATRIX SCIENCE Mascot Search Results

## Peptide View Spot no 87

MS/MS Fragmentation of **TREGNDLYMEMK**
Found in **gi|11466794** in **NCBInr**, ATP synthase CF1 beta subunit [Oryza sativa Japonica Group]

Match to Query 18: 1485.767524 from(1486.774800,1+) intensity(0.0000) index(9)
Title: Label: D11, Spot_Id: 216640, Peak_List_Id: 221236, MSMS Job_Run_Id: 21346, Comment:
Data file C:\Documents and Settings\Administrator\Desktop\farah BHU\RAJESH\12-1-13 raj\ppw_D11_135997699500.txt

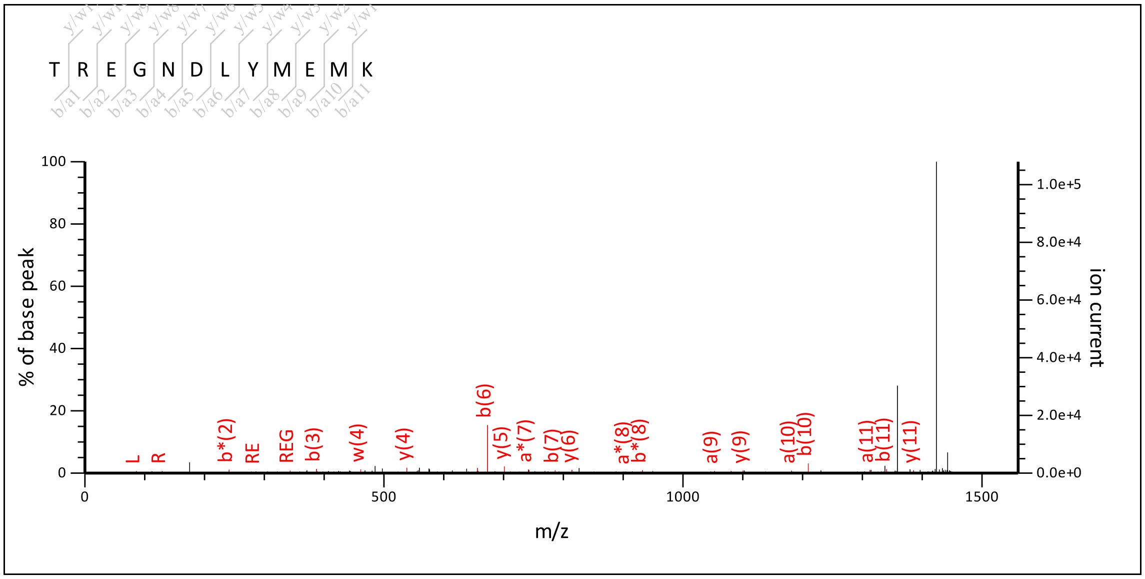


0 to 1560.45

Label all possible matches ○ Label matches used for scoring ◉

**Monoisotopic mass of neutral peptide Mr(calc):** 1485.6592
**Fixed modifications:** Carbamidomethyl (C) (apply to specified residues or termini only)
**Ions Score:** 42 **Expect:** 0.41
**Matches :** 47/201 fragment ions using 95 most intense peaks (help)

| # | Immon. | a | a* | a$^0$ | b | b* | b$^0$ | d | Seq. | v | w | y | y* | y$^0$ | # |
|---|---|---|---|---|---|---|---|---|---|---|---|---|---|---|---|
| 1 | 74.0600 | 74.0600 | | 56.0495 | 102.0550 | | 84.0444 | 44.0495 | T | | | | | | 12 |
| 2 | 129.1135 | 230.1612 | 213.1346 | 212.1506 | ***258.1561*** | 241.1295 | 240.1455 | 145.0972 | R | 1284.5235 | 1283.5283 | ***1385.6188*** | 1368.5922 | 1367.6082 | 11 |
| 3 | 102.0550 | **359.2037** | 342.1772 | 341.1932 | ***387.1987*** | 370.1721 | 369.1881 | 301.1983 | E | 1155.4809 | 1154.4857 | ***1229.5177*** | 1212.4911 | 1211.5071 | 10 |
| 4 | 30.0338 | 416.2252 | 399.1987 | 398.2146 | ***444.2201*** | 427.1936 | 426.2096 | | G | | | ***1100.4751*** | 1083.4485 | 1082.4645 | 9 |
| 5 | 87.0553 | 530.2681 | 513.2416 | 512.2576 | 558.2631 | 541.2365 | 540.2525 | 487.2623 | N | 984.4165 | 983.4213 | ***1043.4536*** | 1026.4271 | 1025.4431 | 8 |
| 6 | 88.0393 | 645.2951 | 628.2685 | 627.2845 | ***673.2900*** | 656.2634 | 655.2794 | 601.3052 | D | 869.3896 | 868.3943 | ***929.4107*** | 912.3842 | 911.4001 | 7 |
| 7 | 86.0964 | 758.3791 | 741.3526 | 740.3686 | ***786.3741*** | 769.3475 | 768.3635 | 716.3322 | L | 756.3055 | 755.3103 | ***814.3838*** | 797.3572 | 796.3732 | 6 |
| 8 | 136.0757 | 921.4425 | 904.4159 | 903.4319 | ***949.4374*** | 932.4108 | 931.4268 | | Y | 593.2422 | | ***701.2997*** | 684.2731 | 683.2891 | 5 |
| 9 | 104.0528 | **1052.4830** | 1035.4564 | 1034.4724 | ***1080.4779*** | 1063.4513 | 1062.4673 | 992.4796 | M | 462.2017 | 461.2064 | ***538.2364*** | 521.2098 | 520.2258 | 4 |
| 10 | 102.0550 | **1181.5255** | 1164.4990 | 1163.5150 | ***1209.5205*** | 1192.4939 | 1191.5099 | 1123.5201 | E | 333.1591 | 332.1639 | 407.1959 | 390.1693 | 389.1853 | 3 |
| 11 | 104.0528 | **1312.5660** | 1295.5395 | 1294.5555 | ***1340.5609*** | 1323.5344 | 1322.5504 | 1252.5627 | M | 202.1186 | 201.1234 | ***278.1533*** | 261.1267 | | 2 |
| 12 | 101.1073 | | | | | | | | K | 74.0237 | 73.0284 | 147.1128 | 130.0863 | | 1 |

| Seq | ya | yb | Seq | ya | yb | Seq | ya | yb |
|---|---|---|---|---|---|---|---|---|
| RE | 258.1561 | 286.1510 | REG | 315.1775 | 343.1724 | REGN | 429.2205 | 457.2154 |
| REGND | 544.2474 | 572.2423 | REGNDL | 657.3315 | 685.3264 | EG | 159.0764 | 187.0713 |
| EGN | 273.1193 | 301.1143 | EGND | 388.1463 | 416.1412 | EGNDL | 501.2304 | 529.2253 |
| EGNDLY | 664.2937 | 692.2886 | GN | 144.0768 | 172.0717 | GND | 259.1037 | 287.0986 |
| GNDL | 372.1878 | 400.1827 | GNDLY | 535.2511 | 563.2460 | GNDLYM | 666.2916 | 694.2865 |
| ND | 202.0822 | 230.0771 | NDL | 315.1663 | 343.1612 | NDLY | 478.2296 | 506.2245 |

| | | | | | | | | |
|---|---|---|---|---|---|---|---|---|
| **NDLYM** | 609.2701 | 637.2650 | **DL** | 201.1234 | 229.1183 | **DLY** | 364.1867 | 392.1816 |
| **DLYM** | 495.2272 | 523.2221 | **DLYME** | 624.2698 | 652.2647 | **LY** | 249.1598 | 277.1547 |
| **LYM** | 380.2002 | 408.1952 | **LYME** | 509.2428 | 537.2377 | **LYMEM** | 640.2833 | 668.2782 |
| **YM** | 267.1162 | 295.1111 | **YME** | 396.1588 | 424.1537 | **YMEM** | 527.1993 | 555.1942 |
| **ME** | 233.0954 | 261.0904 | **MEM** | 364.1359 | 392.1308 | **EM** | 233.0954 | 261.0904 |

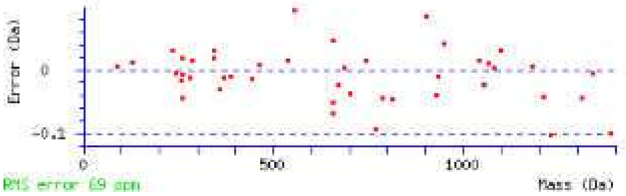


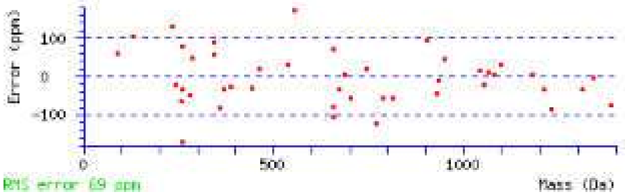


NCBI **BLAST** search of TREGNDLYMEMK
(Parameters: blastp, nr protein database, expect=20000, no filter, PAM30)
Other BLAST web gateways

**All matches to this query**

| Score | Mr(calc) | Delta | Sequence |
|---|---|---|---|
| 41.7 | 1485.6592 | 0.1084 | TREGNDLYMEMK |
| 41.7 | 1485.6592 | 0.1084 | TREGNDLYMEMK |
| 41.7 | 1485.6592 | 0.1084 | TREGNDLYMEMK |
| 41.7 | 1485.6592 | 0.1084 | TREGNDLYMEMK |
| 41.7 | 1485.6592 | 0.1084 | TREGNDLYMEMK |
| 41.7 | 1485.6592 | 0.1084 | TREGNDLYMEMK |
| 41.7 | 1485.6592 | 0.1084 | TREGNDLYMEMK |
| 41.7 | 1485.6592 | 0.1084 | TREGNDLYMEMK |
| 41.7 | 1485.6592 | 0.1084 | TREGNDLYMEMK |
| 35.4 | 1485.6262 | 0.1414 | TREGNDLMMEMK |

**Mascot:** http://www.matrixscience.com/

# Mascot Search Results

## Peptide View Spot no 87

MS/MS Fragmentation of **VALVYGQMNEPPGAR**
Found in **gi|11466794** in **NCBInr**, ATP synthase CF1 beta subunit [Oryza sativa Japonica Group]

Match to Query 20: 1600.889724 from(1601.897000,1+) intensity(0.0000) index(10)
Title: Label: D11, Spot_Id: 216640, Peak_List_Id: 221229, MSMS Job_Run_Id: 21346, Comment:
Data file C:\Documents and Settings\Administrator\Desktop\farah BHU\RAJESH\12-1-13 raj\ppw_D11_135997699500.txt

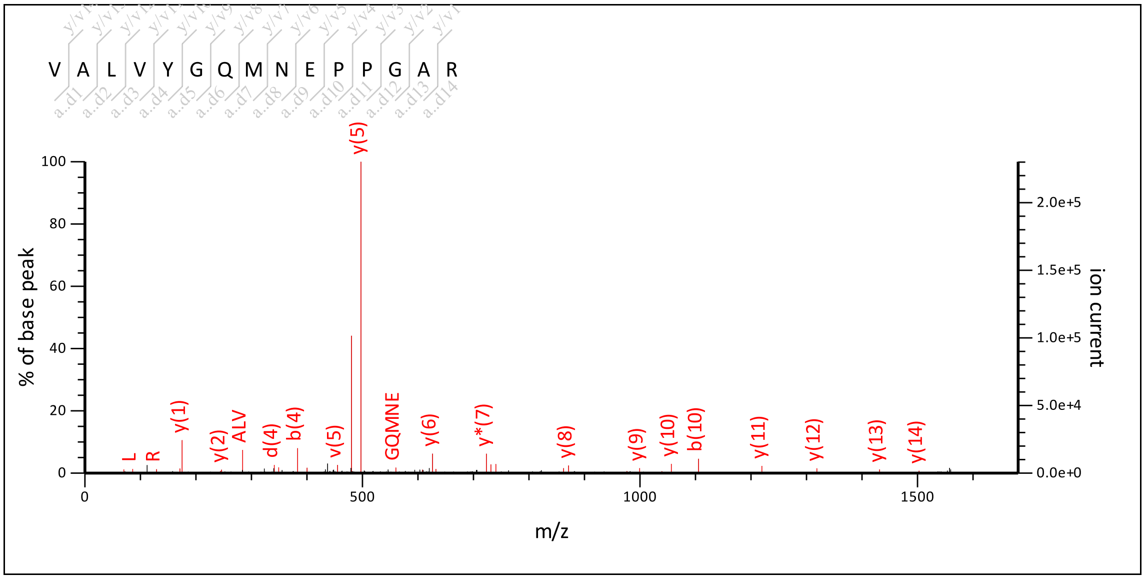


0 to 1681.32

Label all possible matches ○ Label matches used for scoring ◉

**Monoisotopic mass of neutral peptide Mr(calc):** 1600.8031
**Fixed modifications:** Carbamidomethyl (C) (apply to specified residues or termini only)
**Ions Score:** 100 **Expect:** 5.6e-07
**Matches :** 48/237 fragment ions using 61 most intense peaks (help)

| # | Immon. | a | a* | $a^0$ | b | b* | $b^0$ | d | Seq. | v | w | y | y* | $y^0$ | # |
|---|---|---|---|---|---|---|---|---|---|---|---|---|---|---|---|
| 1 | 72.0808 | 72.0808 | | | 100.0757 | | | 44.0495 | V | | | | | | 15 |
| 2 | 44.0495 | 143.1179 | | | **171.1128** | | | | A | 1486.7107 | | ***1502.7420*** | 1485.7155 | 1484.7315 | 14 |
| 3 | 86.0964 | 256.2020 | | | **284.1969** | | | 214.1550 | L | 1373.6267 | 1372.6314 | ***1431.7049*** | 1414.6784 | 1413.6943 | 13 |
| 4 | 72.0808 | 355.2704 | | | **383.2653** | | | 341.2547 | V | 1274.5582 | 1287.5786 | ***1318.6208*** | 1301.5943 | 1300.6103 | 12 |
| 5 | 136.0757 | 518.3337 | | | 546.3286 | | | | Y | 1111.4949 | | ***1219.5524*** | 1202.5259 | 1201.5419 | 11 |
| 6 | 30.0338 | 575.3552 | | | 603.3501 | | | | G | | | ***1056.4891*** | 1039.4626 | 1038.4785 | 10 |
| 7 | 101.0709 | 703.4137 | 686.3872 | | **731.4087** | 714.3821 | | 646.3923 | Q | 926.4149 | 925.4196 | ***999.4676*** | 982.4411 | 981.4571 | 9 |
| 8 | 104.0528 | 834.4542 | 817.4277 | | **862.4491** | 845.4226 | | 774.4509 | M | 795.3744 | 794.3791 | ***871.4091*** | 854.3825 | 853.3985 | 8 |
| 9 | 87.0553 | 948.4972 | 931.4706 | | **976.4921** | 959.4655 | | 905.4913 | N | 681.3315 | 680.3362 | ***740.3686*** | 723.3420 | 722.3580 | 7 |
| 10 | 102.0550 | 1077.5397 | 1060.5132 | 1059.5292 | **1105.5347** | 1088.5081 | 1087.5241 | 1019.5343 | E | 552.2889 | 551.2936 | ***626.3257*** | 609.2991 | 608.3151 | 6 |
| 11 | 70.0651 | 1174.5925 | 1157.5660 | 1156.5819 | **1202.5874** | 1185.5609 | 1184.5769 | 1148.5769 | P | 455.2361 | 454.2409 | ***497.2831*** | 480.2565 | | 5 |
| 12 | 70.0651 | 1271.6453 | 1254.6187 | 1253.6347 | 1299.6402 | 1282.6136 | 1281.6296 | 1245.6296 | P | 358.1833 | 357.1881 | ***400.2303*** | 383.2037 | | 4 |
| 13 | 30.0338 | 1328.6667 | 1311.6402 | 1310.6562 | 1356.6617 | 1339.6351 | 1338.6511 | | G | | | 303.1775 | 286.1510 | | 3 |
| 14 | 44.0495 | 1399.7038 | 1382.6773 | 1381.6933 | 1427.6988 | 1410.6722 | 1409.6882 | | A | 230.1248 | | ***246.1561*** | 229.1295 | | 2 |
| 15 | 129.1135 | | | | | | | | R | 74.0237 | 73.0284 | ***175.1190*** | 158.0924 | | 1 |

| Seq | ya | yb | Seq | ya | yb | Seq | ya | yb |
|---|---|---|---|---|---|---|---|---|
| AL | 157.1335 | 185.1285 | ALV | 256.2020 | 284.1969 | ALVY | 419.2653 | 447.2602 |
| ALVYG | 476.2867 | 504.2817 | ALVYGQ | 604.3453 | 632.3402 | LV | 185.1648 | 213.1598 |
| LVY | 348.2282 | 376.2231 | LVYG | 405.2496 | 433.2445 | LVYGQ | 533.3082 | 561.3031 |

| | | | | | | | | |
|---|---|---|---|---|---|---|---|---|
| **LVYGQM** | 664.3487 | 692.3436 | **VY** | 235.1441 | 263.1390 | **VYG** | 292.1656 | 320.1605 |
| **VYGQ** | 420.2241 | 448.2191 | **VYGQM** | 551.2646 | 579.2595 | **VYGQMN** | 665.3076 | 693.3025 |
| **YG** | 193.0972 | 221.0921 | **YGQ** | 321.1557 | 349.1506 | **YGQM** | 452.1962 | 480.1911 |
| **YGQMN** | 566.2391 | 594.2341 | **YGQMNE** | 695.2817 | 723.2767 | **GQ** | 158.0924 | 186.0873 |
| **GQM** | 289.1329 | 317.1278 | **GQMN** | 403.1758 | 431.1707 | **GQMNE** | 532.2184 | 560.2133 |
| **GQMNEP** | 629.2712 | 657.2661 | **QM** | 232.1114 | 260.1063 | **QMN** | 346.1544 | 374.1493 |
| **QMNE** | 475.1969 | 503.1919 | **QMNEP** | 572.2497 | 600.2446 | **QMNEPP** | 669.3025 | 697.2974 |
| **MN** | 218.0958 | 246.0907 | **MNE** | 347.1384 | 375.1333 | **MNEP** | 444.1911 | 472.1860 |
| **MNEPP** | 541.2439 | 569.2388 | **MNEPPG** | 598.2654 | 626.2603 | **MNEPPGA** | 669.3025 | 697.2974 |
| **NE** | 216.0979 | 244.0928 | **NEP** | 313.1506 | 341.1456 | **NEPP** | 410.2034 | 438.1983 |
| **NEPPG** | 467.2249 | 495.2198 | **NEPPGA** | 538.2620 | 566.2569 | **EP** | 199.1077 | 227.1026 |
| **EPP** | 296.1605 | 324.1554 | **EPPG** | 353.1819 | 381.1769 | **EPPGA** | 424.2191 | 452.2140 |
| **PP** | 167.1179 | 195.1128 | **PPG** | 224.1394 | 252.1343 | **PPGA** | 295.1765 | 323.1714 |
| **PG** | 127.0866 | 155.0815 | **PGA** | 198.1237 | 226.1186 | **GA** | 101.0709 | 129.0659 |

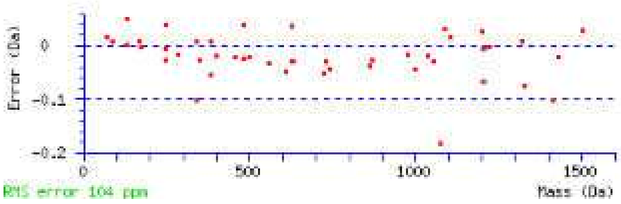


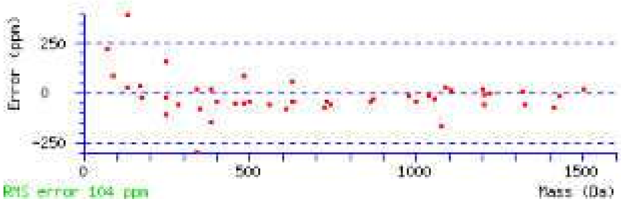


NCBI **BLAST** search of VALVYGQMNEPPGAR
(Parameters: blastp, nr protein database, expect=20000, no filter, PAM30)
Other BLAST web gateways

**All matches to this query**

| Score | Mr(calc) | Delta | Sequence |
|---|---|---|---|
| 99.6 | 1600.8031 | 0.0866 | VAIVYGQMNEPPGAR |
| 99.6 | 1600.8031 | 0.0866 | VAIVYGQMNEPPGAR |
| 99.6 | 1600.8395 | 0.0502 | VALVYGKMNEPPGAR |
| 99.6 | 1600.8031 | 0.0866 | VALVYGQMNEPPGAR |
| 99.6 | 1600.8031 | 0.0866 | VALVYGQMNEPPGAR |
| 99.6 | 1600.8031 | 0.0866 | VALVYGQMNEPPGAR |
| 99.6 | 1600.8031 | 0.0866 | VALVYGQMNEPPGAR |
| 99.6 | 1600.8031 | 0.0866 | VALVYGQMNEPPGAR |
| 99.6 | 1600.8031 | 0.0866 | VALVYGQMNEPPGAR |
| 99.6 | 1600.8031 | 0.0866 | VALVYGQMNEPPGAR |

**Mascot:** http://www.matrixscience.com/

# MATRIX SCIENCE Mascot Search Results

## Peptide View Spot no 87

MS/MS Fragmentation of QINVTCEVQQLLGNNR
Found in gi|11466794 in NCBInr, ATP synthase CF1 beta subunit [Oryza sativa Japonica Group]

Match to Query 23: 1885.043724 from(1886.051000,1+) intensity(0.0000) index(12)
Title: Label: D11, Spot_Id: 216640, Peak_List_Id: 221231, MSMS Job_Run_Id: 21346, Comment:
Data file C:\Documents and Settings\Administrator\Desktop\farah BHU\RAJESH\12-1-13 raj\ppw_D11_135997699500.txt

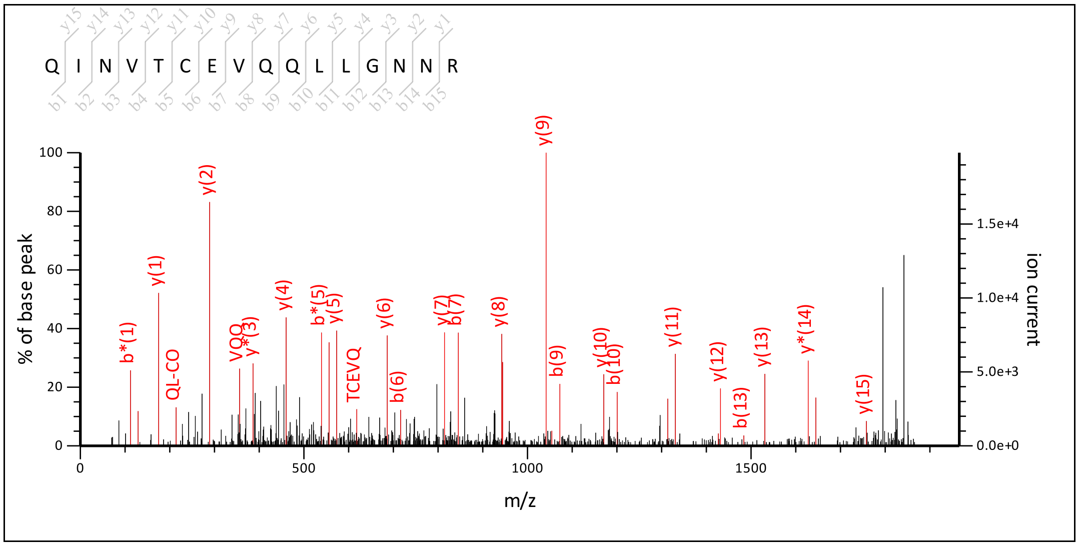


0 to 1965.69

Label all possible matches ○ Label matches used for scoring ◉

**Monoisotopic mass of neutral peptide Mr(calc):** 1884.9476
**Fixed modifications:** Carbamidomethyl (C) (apply to specified residues or termini only)
**Ions Score:** 143 **Expect:** 1.9e-11
**Matches :** 38/282 fragment ions using 36 most intense peaks (help)

| # | Immon. | a | a* | $a^0$ | b | b* | $b^0$ | d | d' | Seq. | v | w | w' | y | y* | |
|---|---|---|---|---|---|---|---|---|---|---|---|---|---|---|---|---|
| 1 | 101.0709 | 101.0709 | 84.0444 | | 129.0659 | 112.0393 | | 44.0495 | | Q | | | | | | |
| 2 | 86.0964 | 214.1550 | 197.1285 | | 242.1499 | 225.1234 | | 186.1237 | 200.1394 | I | 1699.8180 | 1712.8384 | 1726.8541 | 1757.8963 | 1740.8697 | [illegible] |
| 3 | 87.0553 | 328.1979 | 311.1714 | | 356.1928 | 339.1663 | | 285.1921 | | N | 1585.7751 | 1584.7799 | | 1644.8122 | 1627.7857 | [illegible] |
| 4 | 72.0808 | 427.2663 | 410.2398 | | 455.2613 | 438.2347 | | 413.2507 | | V | 1486.7067 | 1499.7271 | | 1530.7693 | 1513.7428 | [illegible] |
| 5 | 74.0600 | 528.3140 | 511.2875 | 510.3035 | 556.3089 | 539.2824 | 538.2984 | 512.3191 | 514.2984 | T | 1385.6590 | 1398.6794 | 1400.6587 | 1431.7009 | 1414.6743 | [illegible] |
| 6 | 133.0430 | 688.3447 | 671.3181 | 670.3341 | 716.3396 | 699.3130 | 698.3290 | 599.3511 | | C | 1225.6284 | 1224.6331 | | 1330.6532 | 1313.6267 | [illegible] |
| 7 | 102.0550 | 817.3873 | 800.3607 | 799.3767 | 845.3822 | 828.3556 | 827.3716 | 759.3818 | | E | 1096.5858 | 1095.5905 | | 1170.6226 | 1153.5960 | [illegible] |
| 8 | 72.0808 | 916.4557 | 899.4291 | 898.4451 | 944.4506 | 927.4240 | 926.4400 | 902.4400 | | V | 997.5174 | 1010.5378 | | 1041.5800 | 1024.5534 | |
| 9 | 101.0709 | 1044.5143 | 1027.4877 | 1026.5037 | 1072.5092 | 1055.4826 | 1054.4986 | 987.4928 | | Q | 869.4588 | 868.4635 | | 942.5116 | 925.4850 | |
| 10 | 101.0709 | 1172.5728 | 1155.5463 | 1154.5623 | 1200.5678 | 1183.5412 | 1182.5572 | 1115.5514 | | Q | 741.4002 | 740.4050 | | 814.4530 | 797.4264 | |
| 11 | 86.0964 | 1285.6569 | 1268.6304 | 1267.6463 | 1313.6518 | 1296.6253 | 1295.6412 | 1243.6099 | | L | 628.3161 | 627.3209 | | 686.3944 | 669.3678 | |
| 12 | 86.0964 | 1398.7410 | 1381.7144 | 1380.7304 | 1426.7359 | 1409.7093 | 1408.7253 | 1356.6940 | | L | 515.2321 | 514.2368 | | 573.3103 | 556.2838 | |
| 13 | 30.0338 | 1455.7624 | 1438.7359 | 1437.7519 | 1483.7573 | 1466.7308 | 1465.7468 | | | G | | | | 460.2263 | 443.1997 | |
| 14 | 87.0553 | 1569.8054 | 1552.7788 | 1551.7948 | 1597.8003 | 1580.7737 | 1579.7897 | 1526.7995 | | N | 344.1677 | 343.1724 | | 403.2048 | 386.1783 | |
| 15 | 87.0553 | 1683.8483 | 1666.8217 | 1665.8377 | 1711.8432 | 1694.8166 | 1693.8326 | 1640.8425 | | N | 230.1248 | 229.1295 | | 289.1619 | 272.1353 | |
| 16 | 129.1135 | | | | | | | | | R | 74.0237 | 73.0284 | | 175.1190 | 158.0924 | |

| Seq | ya | yb | Seq | ya | yb | Seq | ya | yb |
|---|---|---|---|---|---|---|---|---|
| IN | 200.1394 | 228.1343 | INV | 299.2078 | 327.2027 | INVT | 400.2554 | 428.2504 |
| INVTC | 560.2861 | 588.2810 | INVTCE | 689.3287 | 717.3236 | NV | 186.1237 | 214.1186 |
| NVT | 287.1714 | 315.1663 | NVTC | 447.2020 | 475.1969 | NVTCE | 576.2446 | 604.2395 |
| NVTCEV | 675.3130 | 703.3080 | VT | 173.1285 | 201.1234 | VTC | 333.1591 | 361.1540 |
| VTCE | 462.2017 | 490.1966 | VTCEV | 561.2701 | 589.2650 | VTCEVQ | 689.3287 | 717.3236 |

| | | | | | | | | |
|---|---|---|---|---|---|---|---|---|
| **TC** | 234.0907 | 262.0856 | **TCE** | 363.1333 | 391.1282 | **TCEV** | 462.2017 | 490.1966 |
| **TCEVQ** | 590.2603 | 618.2552 | **CE** | 262.0856 | 290.0805 | **CEV** | 361.1540 | 389.1489 |
| **CEVQ** | 489.2126 | 517.2075 | **CEVQQ** | 617.2712 | 645.2661 | **EV** | 201.1234 | 229.1183 |
| **EVQ** | 329.1819 | 357.1769 | **EVQQ** | 457.2405 | 485.2354 | **EVQQL** | 570.3246 | 598.3195 |
| **EVQQLL** | 683.4087 | 711.4036 | **VQ** | 200.1394 | 228.1343 | **VQQ** | 328.1979 | 356.1928 |
| **VQQL** | 441.2820 | 469.2769 | **VQQLL** | 554.3661 | 582.3610 | **VQQLLG** | 611.3875 | 639.3824 |
| **QQ** | 229.1295 | 257.1244 | **QQL** | 342.2136 | 370.2085 | **QQLL** | 455.2976 | 483.2926 |
| **QQLLG** | 512.3191 | 540.3140 | **QQLLGN** | 626.3620 | 654.3570 | **QL** | 214.1550 | 242.1499 |
| **QLL** | 327.2391 | 355.2340 | **QLLG** | 384.2605 | 412.2554 | **QLLGN** | 498.3035 | 526.2984 |
| **QLLGNN** | 612.3464 | 640.3413 | **LL** | 199.1805 | 227.1754 | **LLG** | 256.2020 | 284.1969 |
| **LLGN** | 370.2449 | 398.2398 | **LLGNN** | 484.2878 | 512.2827 | **LG** | 143.1179 | 171.1128 |
| **LGN** | 257.1608 | 285.1557 | **LGNN** | 371.2037 | 399.1987 | **GN** | 144.0768 | 172.0717 |
| **GNN** | 258.1197 | 286.1146 | **NN** | 201.0982 | 229.0931 | | | |

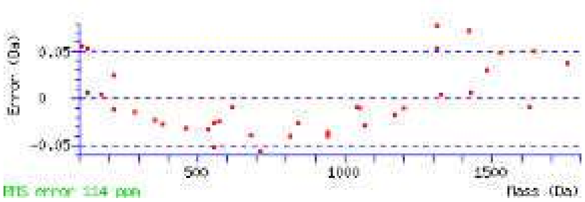


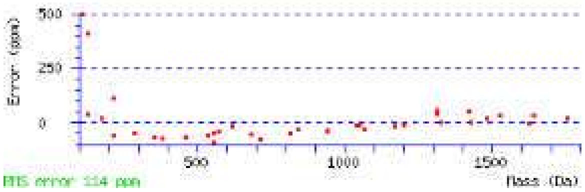


NCBI **BLAST** search of QINVTCEVQQLLGNNR
(Parameters: blastp, nr protein database, expect=20000, no filter, PAM30)
Other BLAST web gateways

**All matches to this query**

| Score | Mr(calc) | Delta | Sequence |
|---|---|---|---|
| 143.3 | 1884.9840 | 0.0598 | KINVTCEVQQLLGNNR |
| 143.3 | 1884.9476 | 0.0961 | QINVTCEVQQLLGNNR |
| 122.8 | 1885.9316 | -0.8879 | EINVTCEVQQLLGNNR |
| 30.0 | 1884.8927 | 0.1510 | AGLNSILQFDCEVYEK |
| 24.2 | 1885.9105 | -0.8667 | EGPTFSNIVQEMHKNR |
| 24.1 | 1886.0448 | -1.0010 | RISGMTLAIVEAPYIPR |
| 19.0 | 1885.9996 | -0.9559 | AISEEVESVKQLDAQLK |
| 17.8 | 1885.0673 | -0.0236 | IVGPISFRVDSGIAVDLK |
| 17.3 | 1883.9701 | 1.0736 | KQLEQGLEGGTAVGDLNR |
| 16.1 | 1884.9905 | 0.0533 | IQANGLTSGGAEELLKER |

**Mascot:** http://www.matrixscience.com/

# MATRIX SCIENCE Mascot Search Results

## Peptide View Spot no 87

MS/MS Fragmentation of **GIYPAVDPLDSTSTMLQPR**
Found in **gi|11466794** in **NCBInr**, ATP synthase CF1 beta subunit [Oryza sativa Japonica Group]

Match to Query 27: 2060.121624 from(2061.128900,1+) intensity(0.0000) index(14)
Title: Label: D11, Spot_Id: 216640, Peak_List_Id: 221232, MSMS Job_Run_Id: 21346, Comment:
Data file C:\Documents and Settings\Administrator\Desktop\farah BHU\RAJESH\12-1-13 raj\ppw_D11_135997699500.txt

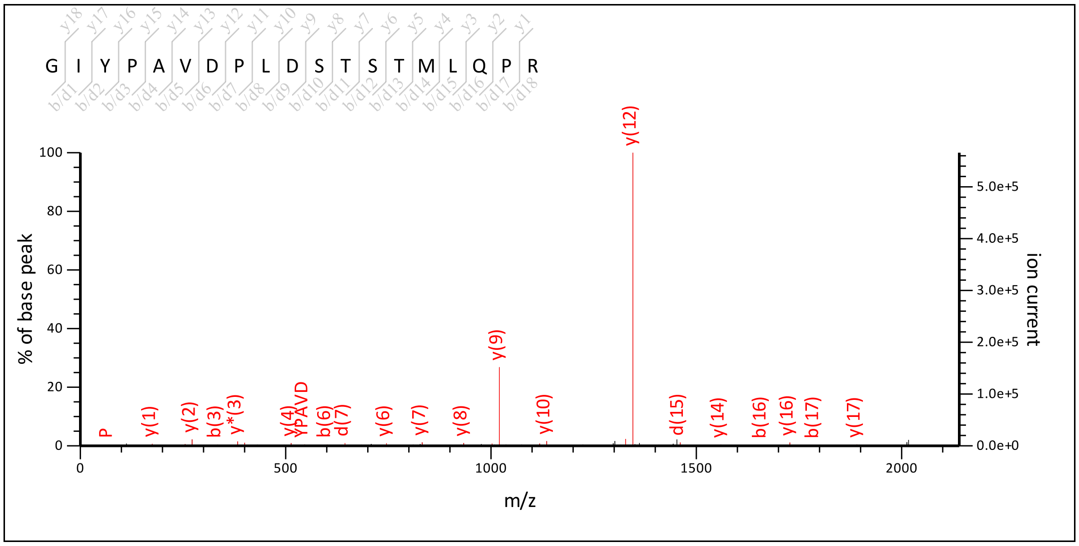


0 to 2140.33

Label all possible matches ○ Label matches used for scoring ◉

**Monoisotopic mass of neutral peptide Mr(calc):** 2060.0248
**Fixed modifications:** Carbamidomethyl (C) (apply to specified residues or termini only)
**Ions Score:** 152 **Expect:** 2.5e-12
**Matches :** 40/333 fragment ions using 40 most intense peaks (help)

| # | Immon. | a | a* | a⁰ | b | b* | b⁰ | d | d' | Seq. | v | w | w' | y | y* | |
|---|---|---|---|---|---|---|---|---|---|---|---|---|---|---|---|---|
| 1 | 30.0338 | 30.0338 | | | 58.0287 | | | 44.0495 | | G | | | | | | |
| 2 | 86.0964 | 143.1179 | | | 171.1128 | | | 115.0866 | 129.1022 | I | 1945.9324 | 1958.9528 | 1972.9685 | 2004.0107 | 1986.9841 | [illegible] |
| 3 | 136.0757 | 306.1812 | | | **334.1761** | | | | | Y | 1782.8691 | | | ***1890.9266*** | 1873.9000 | [illegible] |
| 4 | 70.0651 | 403.2340 | | | 431.2289 | | | 377.2183 | | P | 1685.8163 | 1684.8211 | | ***1727.8633*** | 1710.8367 | [illegible] |
| 5 | 44.0495 | 474.2711 | | | 502.2660 | | | | | A | 1614.7792 | | | ***1630.8105*** | 1613.7840 | [illegible] |
| 6 | 72.0808 | 573.3395 | | | **601.3344** | | | 559.3239 | | V | 1515.7108 | 1528.7312 | | ***1559.7734*** | 1542.7468 | [illegible] |
| 7 | 88.0393 | 688.3665 | | 670.3559 | 716.3614 | | 698.3508 | 644.3766 | | D | 1400.6838 | 1399.6886 | | ***1460.7050*** | 1443.6784 | [illegible] |
| 8 | 70.0651 | 785.4192 | | 767.4087 | 813.4141 | | 795.4036 | 759.4036 | | P | 1303.6311 | 1302.6358 | | ***1345.6780*** | 1328.6515 | [illegible] |
| 9 | 86.0964 | 898.5033 | | 880.4927 | **926.4982** | | 908.4876 | 856.4563 | | L | 1190.5470 | 1189.5518 | | 1248.6253 | 1231.5987 | [illegible] |
| 10 | 88.0393 | 1013.5302 | | 995.5197 | 1041.5251 | | 1023.5146 | 969.5404 | | D | 1075.5201 | 1074.5248 | | ***1135.5412*** | 1118.5147 | [illegible] |
| 11 | 60.0444 | 1100.5623 | | 1082.5517 | 1128.5572 | | 1110.5466 | 1084.5673 | | S | 988.4880 | 987.4928 | | ***1020.5143*** | 1003.4877 | [illegible] |
| 12 | 74.0600 | 1201.6099 | | 1183.5994 | 1229.6048 | | 1211.5943 | 1185.6150 | 1187.5943 | T | 887.4404 | 900.4608 | 902.4400 | ***933.4822*** | 916.4557 | [illegible] |
| 13 | 60.0444 | 1288.6420 | | 1270.6314 | 1316.6369 | | 1298.6263 | 1272.6470 | | S | 800.4083 | 799.4131 | | ***832.4345*** | 815.4080 | [illegible] |
| 14 | 74.0600 | 1389.6896 | | 1371.6791 | 1417.6846 | | 1399.6740 | 1373.6947 | 1375.6740 | T | 699.3607 | 712.3811 | 714.3603 | ***745.4025*** | 728.3760 | [illegible] |
| 15 | 104.0528 | 1520.7301 | | 1502.7196 | **1548.7250** | | 1530.7145 | 1460.7268 | | M | 568.3202 | 567.3249 | | ***644.3548*** | 627.3283 | |
| 16 | 86.0964 | 1633.8142 | | 1615.8036 | **1661.8091** | | 1643.7985 | 1591.7672 | | L | 455.2361 | 454.2409 | | ***513.3144*** | 496.2878 | |
| 17 | 101.0709 | 1761.8728 | 1744.8462 | 1743.8622 | **1789.8677** | 1772.8411 | 1771.8571 | 1704.8513 | | Q | 327.1775 | 326.1823 | | ***400.2303*** | 383.2037 | |
| 18 | 70.0651 | 1858.9255 | 1841.8990 | 1840.9150 | 1886.9204 | 1869.8939 | 1868.9099 | 1832.9099 | | P | 230.1248 | 229.1295 | | ***272.1717*** | 255.1452 | |
| 19 | 129.1135 | | | | | | | | | R | 74.0237 | 73.0284 | | ***175.1190*** | 158.0924 | |

| Seq | ya | yb | Seq | ya | yb | Seq | ya | yb |
|---|---|---|---|---|---|---|---|---|
| IY | 249.1598 | 277.1547 | IYP | 346.2125 | 374.2074 | IYPA | 417.2496 | 445.2445 |
| IYPAV | 516.3180 | 544.3130 | IYPAVD | 631.3450 | 659.3399 | YP | 233.1285 | 261.1234 |

| | | | | | | | | |
|---|---|---|---|---|---|---|---|---|
| YPA | 304.1656 | 332.1605 | YPAV | 403.2340 | 431.2289 | YPAVD | 518.2609 | 546.2558 |
| YPAVDP | 615.3137 | 643.3086 | PA | 141.1022 | 169.0972 | PAV | 240.1707 | 268.1656 |
| PAVD | 355.1976 | 383.1925 | PAVDP | 452.2504 | 480.2453 | PAVDPL | 565.3344 | 593.3293 |
| PAVDPLD | 680.3614 | 708.3563 | AV | 143.1179 | 171.1128 | AVD | 258.1448 | 286.1397 |
| AVDP | 355.1976 | 383.1925 | AVDPL | 468.2817 | 496.2766 | AVDPLD | 583.3086 | 611.3035 |
| AVDPLDS | 670.3406 | 698.3355 | VD | 187.1077 | 215.1026 | VDP | 284.1605 | 312.1554 |
| VDPL | 397.2445 | 425.2395 | VDPLD | 512.2715 | 540.2664 | VDPLDS | 599.3035 | 627.2984 |
| DP | 185.0921 | 213.0870 | DPL | 298.1761 | 326.1710 | DPLD | 413.2031 | 441.1980 |
| DPLDS | 500.2351 | 528.2300 | DPLDST | 601.2828 | 629.2777 | DPLDSTS | 688.3148 | 716.3097 |
| PL | 183.1492 | 211.1441 | PLD | 298.1761 | 326.1710 | PLDS | 385.2082 | 413.2031 |
| PLDST | 486.2558 | 514.2508 | PLDSTS | 573.2879 | 601.2828 | PLDSTST | 674.3355 | 702.3305 |
| LD | 201.1234 | 229.1183 | LDS | 288.1554 | 316.1503 | LDST | 389.2031 | 417.1980 |
| LDSTS | 476.2351 | 504.2300 | LDSTST | 577.2828 | 605.2777 | DS | 175.0713 | 203.0662 |
| DST | 276.1190 | 304.1139 | DSTS | 363.1510 | 391.1460 | DSTST | 464.1987 | 492.1936 |
| DSTSTM | 595.2392 | 623.2341 | ST | 161.0921 | 189.0870 | STS | 248.1241 | 276.1190 |
| STST | 349.1718 | 377.1667 | STSTM | 480.2123 | 508.2072 | STSTML | 593.2963 | 621.2912 |
| TS | 161.0921 | 189.0870 | TST | 262.1397 | 290.1347 | TSTM | 393.1802 | 421.1751 |
| TSTML | 506.2643 | 534.2592 | TSTMLQ | 634.3229 | 662.3178 | ST | 161.0921 | 189.0870 |
| STM | 292.1326 | 320.1275 | STML | 405.2166 | 433.2115 | STMLQ | 533.2752 | 561.2701 |
| STMLQP | 630.3280 | 658.3229 | TM | 205.1005 | 233.0954 | TML | 318.1846 | 346.1795 |
| TMLQ | 446.2432 | 474.2381 | TMLQP | 543.2959 | 571.2908 | ML | 217.1369 | 245.1318 |
| MLQ | 345.1955 | 373.1904 | MLQP | 442.2483 | 470.2432 | LQ | 214.1550 | 242.1499 |
| LQP | 311.2078 | 339.2027 | QP | 198.1237 | 226.1186 | | | |

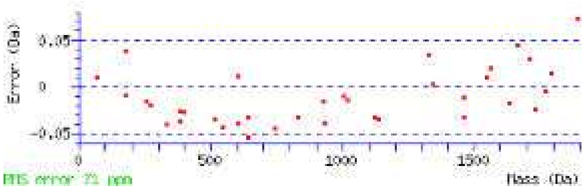


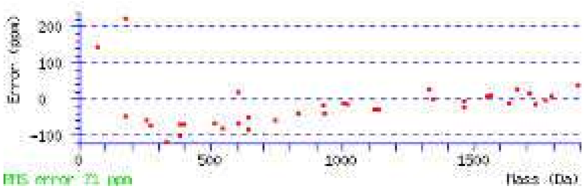


NCBI **BLAST** search of GIYPAVDPLDSTSTMLQPR
(Parameters: blastp, nr protein database, expect=20000, no filter, PAM30)
Other BLAST web gateways

**All matches to this query**

| Score | Mr(calc) | Delta | Sequence |
|---|---|---|---|
| 151.7 | 2060.0248 | 0.0968 | GIYPAVDPLDSTSTMIQPR |
| 151.7 | 2060.0612 | 0.0604 | GIYPAVDPLDSTSTMLKPR |
| 151.7 | 2060.0248 | 0.0968 | GIYPAVDPLDSTSTMLQPR |
| 151.7 | 2060.0248 | 0.0968 | GIYPAVDPLDSTSTMLQPR |
| 151.7 | 2060.0248 | 0.0968 | GIYPAVDPLDSTSTMLQPR |
| 151.7 | 2060.0248 | 0.0968 | GIYPAVDPLDSTSTMLQPR |
| 151.7 | 2060.0248 | 0.0968 | GIYPAVDPLDSTSTMLQPR |
| 151.7 | 2060.0248 | 0.0968 | GIYPAVDPLDSTSTMLQPR |
| 151.7 | 2060.0248 | 0.0968 | GIYPAVDPLDSTSTMLQPR |
| 151.7 | 2060.0248 | 0.0968 | GIYPAVDPLDSTSTMLQPR |

**Mascot:** http://www.matrixscience.com/

# MATRIX SCIENCE Mascot Search Results

## Peptide View Spot no 87

MS/MS Fragmentation of **GIYPAVDPLDSTSTMLQPR**
Found in **gi|11466794** in **NCBInr**, ATP synthase CF1 beta subunit [Oryza sativa Japonica Group]

Match to Query 29: 2076.112824 from(2077.120100,1+) intensity(0.0000) index(15)
Title: Label: D11, Spot_Id: 216640, Peak_List_Id: 221241, MSMS Job_Run_Id: 21346, Comment:
Data file C:\Documents and Settings\Administrator\Desktop\farah BHU\RAJESH\12-1-13 raj\ppw_D11_135997699500.txt

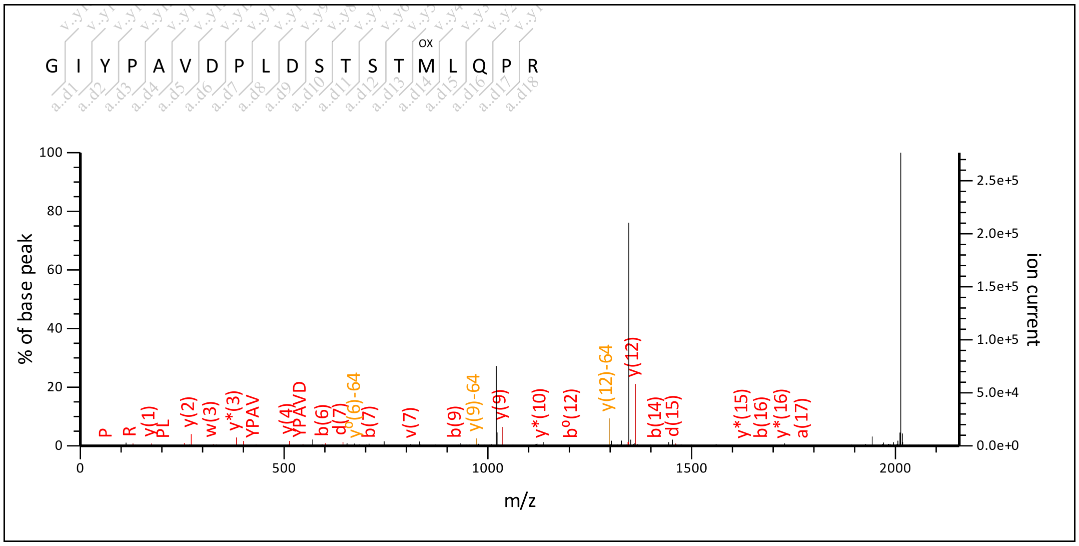


0 to 2156.66

Label all possible matches ○ Label matches used for scoring ◉

**Monoisotopic mass of neutral peptide Mr(calc):** 2076.0198
**Fixed modifications:** Carbamidomethyl (C) (apply to specified residues or termini only)
**Variable modifications:**
**M15** : Oxidation (M), with neutral losses 0.0000(shown in table), 63.9983
**Ions Score:** 36 **Expect:** 0.88
**Matches :** 90/459 fragment ions using 199 most intense peaks (help)

| # | Immon. | a | a* | $a^0$ | b | b* | $b^0$ | d | d' | Seq. | v | w | w' | y | y* | |
|---|---|---|---|---|---|---|---|---|---|---|---|---|---|---|---|---|
| 1 | 30.0338 | 30.0338 | | | 58.0287 | | | 44.0495 | | G | | | | | | |
| 2 | 86.0964 | 143.1179 | | | ***171.1128*** | | | 115.0866 | 129.1022 | I | 1961.9273 | 1974.9477 | 1988.9634 | 2020.0056 | 2002.9790 | [illegible] |
| 3 | 136.0757 | 306.1812 | | | ***334.1761*** | | | | | Y | 1798.8640 | | | 1906.9215 | 1889.8950 | [illegible] |
| 4 | 70.0651 | 403.2340 | | | ***431.2289*** | | | 377.2183 | | P | 1701.8112 | 1700.8160 | | 1743.8582 | 1726.8316 | [illegible] |
| 5 | 44.0495 | 474.2711 | | | ***502.2660*** | | | | | A | 1630.7741 | | | 1646.8054 | 1629.7789 | [illegible] |
| 6 | 72.0808 | 573.3395 | | | ***601.3344*** | | | 559.3239 | | V | 1531.7057 | 1544.7261 | | 1575.7683 | 1558.7418 | [illegible] |
| 7 | 88.0393 | 688.3665 | | 670.3559 | ***716.3614*** | | 698.3508 | 644.3766 | | D | 1416.6788 | 1415.6835 | | **1476.6999** | 1459.6733 | [illegible] |
| 8 | 70.0651 | 785.4192 | | 767.4087 | ***813.4141*** | | 795.4036 | 759.4036 | | P | 1319.6260 | 1318.6307 | | **1361.6729** | 1344.6464 | [illegible] |
| 9 | 86.0964 | 898.5033 | | 880.4927 | ***926.4982*** | | 908.4876 | 856.4563 | | L | 1206.5419 | 1205.5467 | | 1264.6202 | 1247.5936 | [illegible] |
| 10 | 88.0393 | 1013.5302 | | 995.5197 | 1041.5251 | | 1023.5146 | 969.5404 | | D | 1091.5150 | 1090.5197 | | 1151.5361 | 1134.5096 | [illegible] |
| 11 | 60.0444 | 1100.5623 | | 1082.5517 | ***1128.5572*** | | 1110.5466 | 1084.5673 | | S | 1004.4830 | 1003.4877 | | **1036.5092** | 1019.4826 | [illegible] |
| 12 | 74.0600 | 1201.6099 | | 1183.5994 | ***1229.6048*** | | 1211.5943 | 1185.6150 | 1187.5943 | T | 903.4353 | 916.4557 | 918.4349 | 949.4771 | 932.4506 | [illegible] |
| 13 | 60.0444 | 1288.6420 | | 1270.6314 | 1316.6369 | | 1298.6263 | 1272.6470 | | S | 816.4033 | 815.4080 | | 848.4295 | 831.4029 | [illegible] |
| 14 | 74.0600 | 1389.6896 | | 1371.6791 | ***1417.6846*** | | 1399.6740 | 1373.6947 | 1375.6740 | T | 715.3556 | 728.3760 | 730.3552 | 761.3974 | 744.3709 | [illegible] |
| 15 | 120.0478 | 1536.7250 | | 1518.7145 | 1564.7200 | | 1546.7094 | 1460.7268 | | M | 568.3202 | 567.3249 | | 660.3498 | 643.3232 | |
| 16 | 86.0964 | 1649.8091 | | 1631.7985 | ***1677.8040*** | | 1659.7935 | 1607.7622 | | L | 455.2361 | 454.2409 | | **513.3144** | 496.2878 | |
| 17 | 101.0709 | 1777.8677 | 1760.8411 | 1759.8571 | ***1805.8626*** | 1788.8360 | 1787.8520 | 1720.8462 | | Q | 327.1775 | 326.1823 | | **400.2303** | 383.2037 | |
| 18 | 70.0651 | 1874.9204 | 1857.8939 | 1856.9099 | 1902.9154 | 1885.8888 | 1884.9048 | 1848.9048 | | P | 230.1248 | 229.1295 | | **272.1717** | 255.1452 | |
| 19 | 129.1135 | | | | | | | | | R | 74.0237 | 73.0284 | | **175.1190** | 158.0924 | |

| Seq | ya | yb | Seq | ya | yb | Seq | ya | yb |
|---|---|---|---|---|---|---|---|---|
| IY | 249.1598 | 277.1547 | IYP | 346.2125 | 374.2074 | IYPA | 417.2496 | 445.2445 |
| IYPAV | 516.3180 | 544.3130 | IYPAVD | 631.3450 | 659.3399 | YP | 233.1285 | 261.1234 |
| YPA | 304.1656 | 332.1605 | YPAV | 403.2340 | 431.2289 | YPAVD | 518.2609 | 546.2558 |
| YPAVDP | 615.3137 | 643.3086 | PA | 141.1022 | 169.0972 | PAV | 240.1707 | 268.1656 |
| PAVD | 355.1976 | 383.1925 | PAVDP | 452.2504 | 480.2453 | PAVDPL | 565.3344 | 593.3293 |
| PAVDPLD | 680.3614 | 708.3563 | AV | 143.1179 | 171.1128 | AVD | 258.1448 | 286.1397 |
| AVDP | 355.1976 | 383.1925 | AVDPL | 468.2817 | 496.2766 | AVDPLD | 583.3086 | 611.3035 |
| AVDPLDS | 670.3406 | 698.3355 | VD | 187.1077 | 215.1026 | VDP | 284.1605 | 312.1554 |
| VDPL | 397.2445 | 425.2395 | VDPLD | 512.2715 | 540.2664 | VDPLDS | 599.3035 | 627.2984 |
| DP | 185.0921 | 213.0870 | DPL | 298.1761 | 326.1710 | DPLD | 413.2031 | 441.1980 |
| DPLDS | 500.2351 | 528.2300 | DPLDST | 601.2828 | 629.2777 | DPLDSTS | 688.3148 | 716.3097 |
| PL | 183.1492 | 211.1441 | PLD | 298.1761 | 326.1710 | PLDS | 385.2082 | 413.2031 |
| PLDST | 486.2558 | 514.2508 | PLDSTS | 573.2879 | 601.2828 | PLDSTST | 674.3355 | 702.3305 |
| LD | 201.1234 | 229.1183 | LDS | 288.1554 | 316.1503 | LDST | 389.2031 | 417.1980 |
| LDSTS | 476.2351 | 504.2300 | LDSTST | 577.2828 | 605.2777 | DS | 175.0713 | 203.0662 |
| DST | 276.1190 | 304.1139 | DSTS | 363.1510 | 391.1460 | DSTST | 464.1987 | 492.1936 |
| DSTSTM | 611.2341 | 639.2290 | ST | 161.0921 | 189.0870 | STS | 248.1241 | 276.1190 |
| STST | 349.1718 | 377.1667 | STSTM | 496.2072 | 524.2021 | STSTML | 609.2912 | 637.2862 |
| TS | 161.0921 | 189.0870 | TST | 262.1397 | 290.1347 | TSTM | 409.1751 | 437.1701 |
| TSTML | 522.2592 | 550.2541 | TSTMLQ | 650.3178 | 678.3127 | ST | 161.0921 | 189.0870 |
| STM | 308.1275 | 336.1224 | STML | 421.2115 | 449.2064 | STMLQ | 549.2701 | 577.2650 |
| STMLQP | 646.3229 | 674.3178 | TM | 221.0954 | 249.0904 | TML | 334.1795 | 362.1744 |
| TMLQ | 462.2381 | 490.2330 | TMLQP | 559.2908 | 587.2858 | ML | 233.1318 | 261.1267 |
| MLQ | 361.1904 | 389.1853 | MLQP | 458.2432 | 486.2381 | LQ | 214.1550 | 242.1499 |
| LQP | 311.2078 | 339.2027 | QP | 198.1237 | 226.1186 | | | |

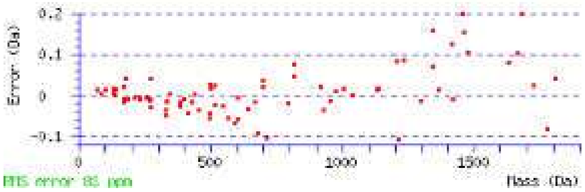


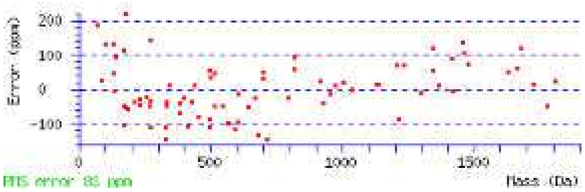


NCBI **BLAST** search of GIYPAVDPLDSTSTMLQPR
(Parameters: blastp, nr protein database, expect=20000, no filter, PAM30)
Other BLAST web gateways

**All matches to this query**

| Score | Mr(calc) | Delta | Sequence |
|---|---|---|---|
| 79.3 | 2075.9834 | 0.1295 | GEYPAVDPLDSTSTMLQPR |
| 79.3 | 2075.0357 | 1.0771 | GKYPAVDPLDSTSTMLQPR |
| 79.3 | 2074.9994 | 1.1135 | GQYPAVDPLDSTSTMLQPR |
| 64.6 | 2076.0198 | 0.0931 | GIYPSVDPLDSTSTMLQPR |
| 53.3 | 2074.9993 | 1.1135 | GIYPANDPLDSTSTMLQPR |
| 53.3 | 2075.9834 | 0.1295 | GIYPADDPLDSTSTMLQPR |
| 51.2 | 2076.0384 | 0.0744 | GIYPAVMPLDSTSTMLQPR |
| 40.2 | 2076.0384 | 0.0744 | GIYPAVDPLMSTSTMLQPR |
| 36.3 | 2076.0561 | 0.0567 | GIYPAVDPLDSTSTMLKPR |
| 36.3 | 2076.0198 | 0.0931 | GIYPAVDPLDSTSTMLQPR |

**Mascot:** http://www.matrixscience.com/

# MATRIX SCIENCE Mascot Search Results

## Peptide View Spot no 87

MS/MS Fragmentation of **GMEVIDTGAPLSVPVGGATLGR**
Found in **gi|11466794** in **NCBInr**, ATP synthase CF1 beta subunit [Oryza sativa Japonica Group]

Match to Query 31: 2096.196324 from(2097.203600,1+) intensity(0.0000) index(16)
Title: Label: D11, Spot_Id: 216640, Peak_List_Id: 221226, MSMS Job_Run_Id: 21346, Comment:
Data file C:\Documents and Settings\Administrator\Desktop\farah BHU\RAJESH\12-1-13 raj\ppw_D11_135997699500.txt

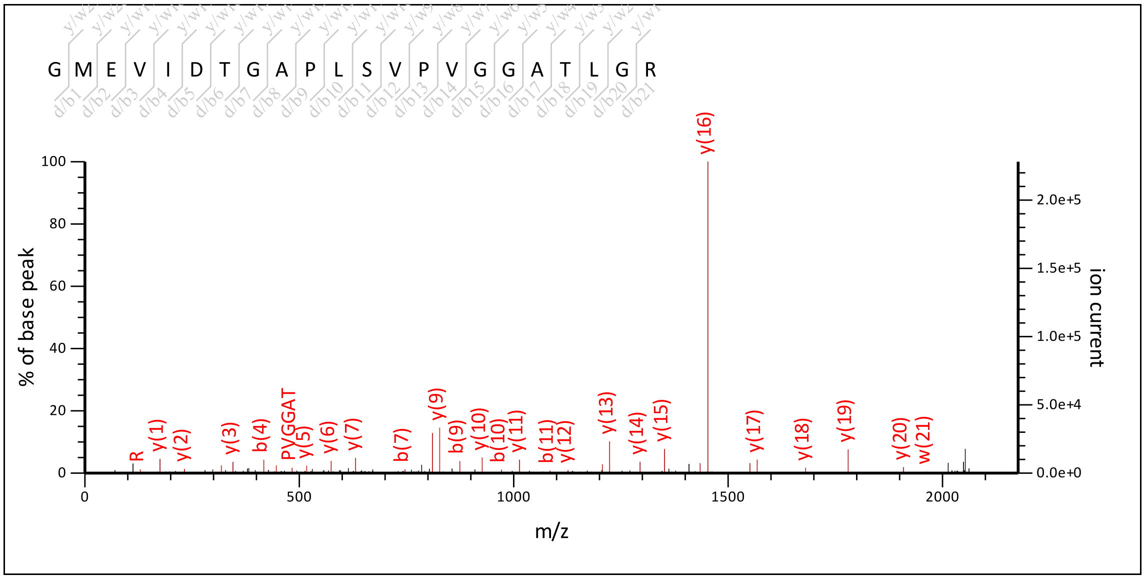


0 to 2176.41

Label all possible matches ○ Label matches used for scoring ◉

**Monoisotopic mass of neutral peptide Mr(calc):** 2096.0936
**Fixed modifications:** Carbamidomethyl (C) (apply to specified residues or termini only)
**Ions Score:** 185 **Expect:** 1.5e-15
**Matches :** 38/423 fragment ions using 42 most intense peaks (help)

| # | Immon. | a | $a^0$ | b | $b^0$ | d | d' | Seq. | v | w | w' | y | y* | $y^0$ | # |
|---|---|---|---|---|---|---|---|---|---|---|---|---|---|---|---|
| 1 | 30.0338 | 30.0338 | | 58.0287 | | 44.0495 | | G | | | | | | | 22 |
| 2 | 104.0528 | 161.0743 | | 189.0692 | | 101.0709 | | M | 1964.0447 | 1963.0495 | | 2040.0794 | 2023.0529 | 2022.0688 | 21 |
| 3 | 102.0550 | 290.1169 | 272.1063 | **318.1118** | 300.1013 | 232.1114 | | E | 1835.0021 | 1834.0069 | | **1909.0389** | 1892.0124 | 1891.0284 | 20 |
| 4 | 72.0808 | 389.1853 | 371.1748 | **417.1802** | 399.1697 | 375.1697 | | V | 1735.9337 | 1748.9541 | | **1779.9963** | 1762.9698 | 1761.9858 | 19 |
| 5 | 86.0964 | 502.2694 | 484.2588 | 530.2643 | 512.2537 | 474.2381 | 488.2537 | I | 1622.8497 | 1635.8701 | 1649.8857 | **1680.9279** | 1663.9014 | 1662.9173 | 18 |
| 6 | 88.0393 | 617.2963 | 599.2858 | 645.2912 | 627.2807 | 573.3065 | | D | 1507.8227 | 1506.8275 | | **1567.8438** | 1550.8173 | 1549.8333 | 17 |
| 7 | 74.0600 | 718.3440 | 700.3334 | **746.3389** | 728.3284 | 702.3491 | 704.3284 | T | 1406.7750 | 1419.7954 | 1421.7747 | **1452.8169** | 1435.7904 | 1434.8063 | 16 |
| 8 | 30.0338 | 775.3655 | 757.3549 | 803.3604 | 785.3498 | | | G | | | | **1351.7692** | 1334.7427 | 1333.7587 | 15 |
| 9 | 44.0495 | 846.4026 | 828.3920 | **874.3975** | 856.3869 | | | A | 1278.7165 | | | **1294.7478** | 1277.7212 | 1276.7372 | 14 |
| 10 | 70.0651 | 943.4553 | 925.4448 | **971.4503** | 953.4397 | 917.4397 | | P | 1181.6637 | 1180.6685 | | **1223.7106** | 1206.6841 | 1205.7001 | 13 |
| 11 | 86.0964 | 1056.5394 | 1038.5288 | **1084.5343** | 1066.5238 | 1014.4925 | | L | 1068.5796 | 1067.5844 | | **1126.6579** | 1109.6313 | 1108.6473 | 12 |
| 12 | 60.0444 | 1143.5714 | 1125.5609 | 1171.5664 | 1153.5558 | 1127.5765 | | S | 981.5476 | 980.5524 | | **1013.5738** | 996.5473 | 995.5633 | 11 |
| 13 | 72.0808 | 1242.6399 | 1224.6293 | 1270.6348 | 1252.6242 | 1228.6242 | | V | 882.4792 | 895.4996 | | **926.5418** | 909.5152 | 908.5312 | 10 |
| 14 | 70.0651 | 1339.6926 | 1321.6821 | 1367.6875 | 1349.6770 | 1313.6770 | | P | 785.4264 | 784.4312 | | **827.4734** | 810.4468 | 809.4628 | 9 |
| 15 | 72.0808 | 1438.7610 | 1420.7505 | 1466.7559 | 1448.7454 | 1424.7454 | | V | 686.3580 | 699.3784 | | 730.4206 | 713.3941 | 712.4100 | 8 |
| 16 | 30.0338 | 1495.7825 | 1477.7719 | 1523.7774 | 1505.7668 | | | G | | | | **631.3522** | 614.3257 | 613.3416 | 7 |
| 17 | 30.0338 | 1552.8040 | 1534.7934 | 1580.7989 | 1562.7883 | | | G | | | | **574.3307** | 557.3042 | 556.3202 | 6 |
| 18 | 44.0495 | 1623.8411 | 1605.8305 | 1651.8360 | 1633.8254 | | | A | 501.2780 | | | **517.3093** | 500.2827 | 499.2987 | 5 |
| 19 | 74.0600 | 1724.8888 | 1706.8782 | 1752.8837 | 1734.8731 | 1708.8938 | 1710.8731 | T | 400.2303 | 413.2507 | 415.2300 | **446.2722** | 429.2456 | 428.2616 | 4 |

| | | | | | | | | | | | | | | | |
|---|---|---|---|---|---|---|---|---|---|---|---|---|---|---|---|
| 20 | 86.0964 | 1837.9728 | 1819.9622 | 1865.9677 | 1847.9572 | 1795.9259 | | L | 287.1462 | 286.1510 | | 345.2245 | 328.1979 | | 3 |
| 21 | 30.0338 | 1894.9943 | 1876.9837 | 1922.9892 | 1904.9786 | | | G | | | | 232.1404 | 215.1139 | | 2 |
| 22 | 129.1135 | | | | | | | R | 74.0237 | 73.0284 | | 175.1190 | 158.0924 | | 1 |

| Seq | ya | yb | Seq | ya | yb | Seq | ya | yb |
|---|---|---|---|---|---|---|---|---|
| ME | 233.0954 | 261.0904 | MEV | 332.1639 | 360.1588 | MEVI | 445.2479 | 473.2428 |
| MEVID | 560.2749 | 588.2698 | MEVIDT | 661.3225 | 689.3175 | EV | 201.1234 | 229.1183 |
| EVI | 314.2074 | 342.2023 | EVID | 429.2344 | 457.2293 | EVIDT | 530.2821 | 558.2770 |
| EVIDTG | 587.3035 | 615.2984 | EVIDTGA | 658.3406 | 686.3355 | VI | 185.1648 | 213.1598 |
| VID | 300.1918 | 328.1867 | VIDT | 401.2395 | 429.2344 | VIDTG | 458.2609 | 486.2558 |
| VIDTGA | 529.2980 | 557.2930 | VIDTGAP | 626.3508 | 654.3457 | ID | 201.1234 | 229.1183 |
| IDT | 302.1710 | 330.1660 | IDTG | 359.1925 | 387.1874 | IDTGA | 430.2296 | 458.2245 |
| IDTGAP | 527.2824 | 555.2773 | IDTGAPL | 640.3665 | 668.3614 | DT | 189.0870 | 217.0819 |
| DTG | 246.1084 | 274.1034 | DTGA | 317.1456 | 345.1405 | DTGAP | 414.1983 | 442.1932 |
| DTGAPL | 527.2824 | 555.2773 | DTGAPLS | 614.3144 | 642.3093 | TG | 131.0815 | 159.0764 |
| TGA | 202.1186 | 230.1135 | TGAP | 299.1714 | 327.1663 | TGAPL | 412.2554 | 440.2504 |
| TGAPLS | 499.2875 | 527.2824 | TGAPLSV | 598.3559 | 626.3508 | TGAPLSVP | 695.4087 | 723.4036 |
| GA | 101.0709 | 129.0659 | GAP | 198.1237 | 226.1186 | GAPL | 311.2078 | 339.2027 |
| GAPLS | 398.2398 | 426.2347 | GAPLSV | 497.3082 | 525.3031 | GAPLSVP | 594.3610 | 622.3559 |
| GAPLSVPV | 693.4294 | 721.4243 | AP | 141.1022 | 169.0972 | APL | 254.1863 | 282.1812 |
| APLS | 341.2183 | 369.2132 | APLSV | 440.2867 | 468.2817 | APLSVP | 537.3395 | 565.3344 |
| APLSVPV | 636.4079 | 664.4028 | APLSVPVG | 693.4294 | 721.4243 | PL | 183.1492 | 211.1441 |
| PLS | 270.1812 | 298.1761 | PLSV | 369.2496 | 397.2445 | PLSVP | 466.3024 | 494.2973 |
| PLSVPV | 565.3708 | 593.3657 | PLSVPVG | 622.3923 | 650.3872 | PLSVPVGG | 679.4137 | 707.4087 |
| LS | 173.1285 | 201.1234 | LSV | 272.1969 | 300.1918 | LSVP | 369.2496 | 397.2445 |
| LSVPV | 468.3180 | 496.3130 | LSVPVG | 525.3395 | 553.3344 | LSVPVGG | 582.3610 | 610.3559 |
| LSVPVGGA | 653.3981 | 681.3930 | SV | 159.1128 | 187.1077 | SVP | 256.1656 | 284.1605 |
| SVPV | 355.2340 | 383.2289 | SVPVG | 412.2554 | 440.2504 | SVPVGG | 469.2769 | 497.2718 |
| SVPVGGA | 540.3140 | 568.3089 | SVPVGGAT | 641.3617 | 669.3566 | VP | 169.1335 | 197.1285 |
| VPV | 268.2020 | 296.1969 | VPVG | 325.2234 | 353.2183 | VPVGG | 382.2449 | 410.2398 |
| VPVGGA | 453.2820 | 481.2769 | VPVGGAT | 554.3297 | 582.3246 | VPVGGATL | 667.4137 | 695.4087 |
| PV | 169.1335 | 197.1285 | PVG | 226.1550 | 254.1499 | PVGG | 283.1765 | 311.1714 |
| PVGGA | 354.2136 | 382.2085 | PVGGAT | 455.2613 | 483.2562 | PVGGATL | 568.3453 | 596.3402 |
| PVGGATLG | 625.3668 | 653.3617 | VG | 129.1022 | 157.0972 | VGG | 186.1237 | 214.1186 |
| VGGA | 257.1608 | 285.1557 | VGGAT | 358.2085 | 386.2034 | VGGATL | 471.2926 | 499.2875 |
| VGGATLG | 528.3140 | 556.3089 | GG | 87.0553 | 115.0502 | GGA | 158.0924 | 186.0873 |
| GGAT | 259.1401 | 287.1350 | GGATL | 372.2241 | 400.2191 | GGATLG | 429.2456 | 457.2405 |
| GA | 101.0709 | 129.0659 | GAT | 202.1186 | 230.1135 | GATL | 315.2027 | 343.1976 |
| GATLG | 372.2241 | 400.2191 | AT | 145.0972 | 173.0921 | ATL | 258.1812 | 286.1761 |
| ATLG | 315.2027 | 343.1976 | TL | 187.1441 | 215.1390 | TLG | 244.1656 | 272.1605 |
| LG | 143.1179 | 171.1128 | | | | | | |

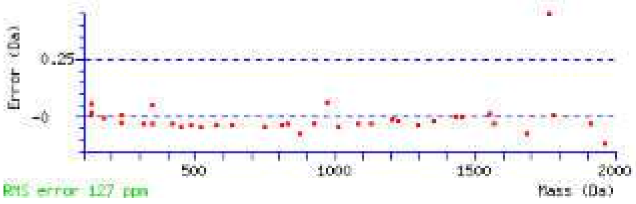


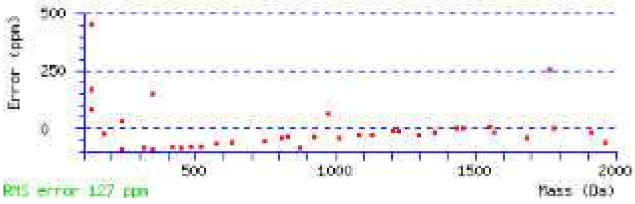


NCBI **BLAST** search of GMEVIDTGAPLSVPVGGATLGR
(Parameters: blastp, nr protein database, expect=20000, no filter, PAM30)
Other BLAST web gateways

**All matches to this query**

| Score | Mr(calc) | Delta | Sequence |
|---|---|---|---|
| 185.0 | 2096.0936 | 0.1027 | GMEVIDTGAPISVPVGGATLGR |
| 185.0 | 2096.0936 | 0.1027 | GMEVIDTGAPISVPVGGATLGR |
| 185.0 | 2096.0936 | 0.1027 | GMEVIDTGAPISVPVGGATLGR |
| 185.0 | 2096.0936 | 0.1027 | GMEVIDTGAPISVPVGGATLGR |
| 185.0 | 2096.0936 | 0.1027 | GMEVIDTGAPLSVPVGGATIGR |
| 185.0 | 2096.0936 | 0.1027 | GMEVIDTGAPLSVPVGGATLGR |
| 185.0 | 2096.0936 | 0.1027 | GMEVIDTGAPLSVPVGGATLGR |
| 185.0 | 2096.0936 | 0.1027 | GMEVIDTGAPLSVPVGGATLGR |
| 185.0 | 2096.0936 | 0.1027 | GMEVIDTGAPLSVPVGGATLGR |
| 185.0 | 2096.0936 | 0.1027 | GMEVIDTGAPLSVPVGGATLGR |

**Mascot:** http://www.matrixscience.com/

# MATRIX SCIENCE Mascot Search Results

## Peptide View Spot no 87

MS/MS Fragmentation of **GMEVIDTGAPLSVPVGGATLGR**
Found in **gi|11466794** in **NCBInr**, ATP synthase CF1 beta subunit [Oryza sativa Japonica Group]

Match to Query 33: 2112.183924 from(2113.191200,1+) intensity(0.0000) index(17)
Title: Label: D11, Spot_Id: 216640, Peak_List_Id: 221239, MSMS Job_Run_Id: 21346, Comment:
Data file C:\Documents and Settings\Administrator\Desktop\farah BHU\RAJESH\12-1-13 raj\ppw_D11_135997699500.txt

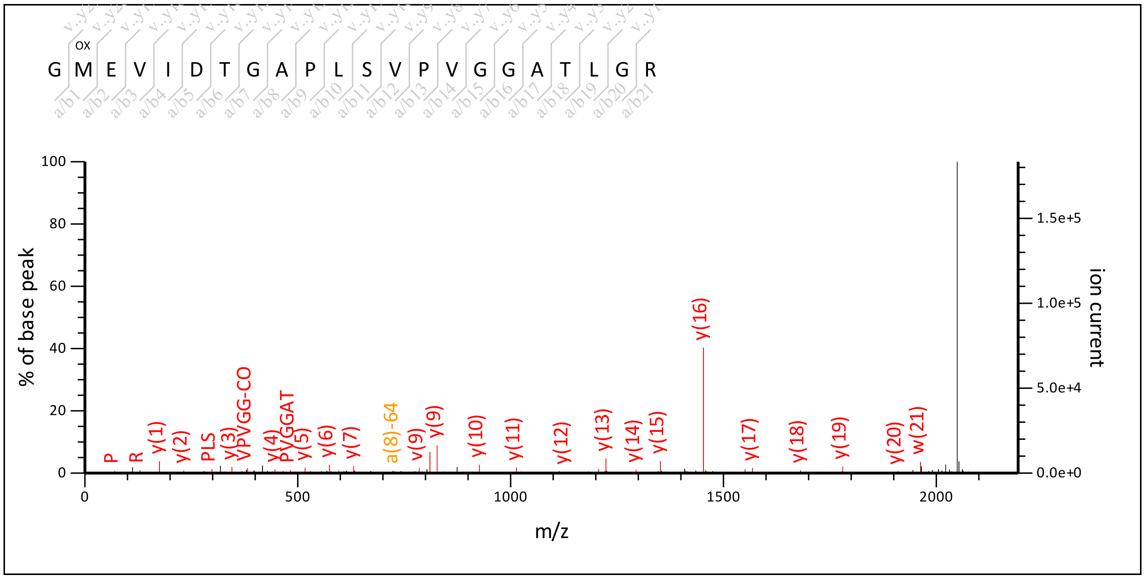


0 to 2192.25

Label all possible matches ○ Label matches used for scoring ◉

**Monoisotopic mass of neutral peptide Mr(calc):** 2112.0885
**Fixed modifications:** Carbamidomethyl (C) (apply to specified residues or termini only)
**Variable modifications:**
**M2** : Oxidation (M), with neutral losses 0.0000(shown in table), 63.9983
**Ions Score:** 141 **Expect:** 2.7e-11
**Matches :** 43/529 fragment ions using 63 most intense peaks (help)

| # | Immon. | a | $a^0$ | b | $b^0$ | d | d' | Seq. | v | w | w' | y | y* | $y^0$ | # |
|---|---|---|---|---|---|---|---|---|---|---|---|---|---|---|---|
| 1 | 30.0338 | 30.0338 | | 58.0287 | | 44.0495 | | G | | | | | | | 22 |
| 2 | 120.0478 | 177.0692 | | 205.0641 | | 101.0709 | | M | 1964.0447 | 1963.0495 | | 2056.0743 | 2039.0478 | 2038.0638 | 21 |
| 3 | 102.0550 | 306.1118 | 288.1013 | 334.1067 | 316.0962 | 248.1063 | | E | 1835.0021 | 1834.0069 | | ***1909.0389*** | 1892.0124 | 1891.0284 | 20 |
| 4 | 72.0808 | 405.1802 | 387.1697 | 433.1751 | 415.1646 | 391.1646 | | V | 1735.9337 | 1748.9541 | | ***1779.9963*** | 1762.9698 | 1761.9858 | 19 |
| 5 | 86.0964 | 518.2643 | 500.2537 | 546.2592 | 528.2486 | 490.2330 | 504.2486 | I | 1622.8497 | 1635.8701 | 1649.8857 | ***1680.9279*** | 1663.9014 | 1662.9173 | 18 |
| 6 | 88.0393 | 633.2912 | 615.2807 | 661.2862 | 643.2756 | 589.3014 | | D | 1507.8227 | 1506.8275 | | ***1567.8438*** | 1550.8173 | 1549.8333 | 17 |
| 7 | 74.0600 | 734.3389 | 716.3284 | 762.3338 | 744.3233 | 718.3440 | 720.3233 | T | 1406.7750 | 1419.7954 | 1421.7747 | ***1452.8169*** | 1435.7904 | 1434.8063 | 16 |
| 8 | 30.0338 | 791.3604 | 773.3498 | 819.3553 | 801.3447 | | | G | | | | ***1351.7692*** | 1334.7427 | 1333.7587 | 15 |
| 9 | 44.0495 | 862.3975 | 844.3869 | 890.3924 | 872.3818 | | | A | 1278.7165 | | | ***1294.7478*** | 1277.7212 | 1276.7372 | 14 |
| 10 | 70.0651 | 959.4503 | 941.4397 | 987.4452 | 969.4346 | 933.4346 | | P | 1181.6637 | 1180.6684 | | ***1223.7106*** | 1206.6841 | 1205.7001 | 13 |
| 11 | 86.0964 | 1072.5343 | 1054.5238 | 1100.5292 | 1082.5187 | 1030.4874 | | L | 1068.5796 | 1067.5844 | | ***1126.6579*** | 1109.6313 | 1108.6473 | 12 |
| 12 | 60.0444 | 1159.5664 | 1141.5558 | 1187.5613 | 1169.5507 | 1143.5714 | | S | 981.5476 | 980.5524 | | ***1013.5738*** | 996.5473 | 995.5633 | 11 |
| 13 | 72.0808 | 1258.6348 | 1240.6242 | 1286.6297 | 1268.6191 | 1244.6191 | | V | 882.4792 | 895.4996 | | ***926.5418*** | 909.5152 | 908.5312 | 10 |
| 14 | 70.0651 | 1355.6875 | 1337.6770 | 1383.6824 | 1365.6719 | 1329.6719 | | P | 785.4264 | 784.4312 | | ***827.4734*** | 810.4468 | 809.4628 | 9 |
| 15 | 72.0808 | 1454.7559 | 1436.7454 | 1482.7509 | 1464.7403 | 1440.7403 | | V | 686.3580 | 699.3784 | | 730.4206 | 713.3941 | 712.4100 | 8 |
| 16 | 30.0338 | 1511.7774 | 1493.7668 | 1539.7723 | 1521.7618 | | | G | | | | ***631.3522*** | 614.3257 | 613.3416 | 7 |
| 17 | 30.0338 | 1568.7989 | 1550.7883 | 1596.7938 | 1578.7832 | | | G | | | | ***574.3307*** | 557.3042 | 556.3202 | 6 |

| | | | | | | | | | | | | | | | |
|---|---|---|---|---|---|---|---|---|---|---|---|---|---|---|---|
| 18 | 44.0495 | 1639.8360 | 1621.8254 | 1667.8309 | 1649.8203 | | | A | 501.2780 | | | ***517.3093*** | 500.2827 | 499.2987 | 5 |
| 19 | 74.0600 | 1740.8837 | 1722.8731 | 1768.8786 | 1750.8680 | 1724.8888 | 1726.8680 | T | 400.2303 | 413.2507 | 415.2300 | ***446.2722*** | 429.2456 | 428.2616 | 4 |
| 20 | 86.0964 | 1853.9677 | 1835.9572 | 1881.9626 | 1863.9521 | 1811.9208 | | L | 287.1462 | 286.1510 | | ***345.2245*** | 328.1979 | | 3 |
| 21 | 30.0338 | 1910.9892 | 1892.9786 | 1938.9841 | 1920.9735 | | | G | | | | ***232.1404*** | 215.1139 | | 2 |
| 22 | 129.1135 | | | | | | | R | 74.0237 | 73.0284 | | ***175.1190*** | 158.0924 | | 1 |

| Seq | ya | yb | Seq | ya | yb | Seq | ya | yb |
|---|---|---|---|---|---|---|---|---|
| ME | 249.0904 | 277.0853 | MEV | 348.1588 | 376.1537 | MEVI | 461.2428 | 489.2377 |
| MEVID | 576.2698 | 604.2647 | MEVIDT | 677.3175 | 705.3124 | EV | 201.1234 | 229.1183 |
| EVI | 314.2074 | 342.2023 | EVID | 429.2344 | 457.2293 | EVIDT | 530.2821 | 558.2770 |
| EVIDTG | 587.3035 | 615.2984 | EVIDTGA | 658.3406 | 686.3355 | VI | 185.1648 | 213.1598 |
| VID | 300.1918 | 328.1867 | VIDT | 401.2395 | 429.2344 | VIDTG | 458.2609 | 486.2558 |
| VIDTGA | 529.2980 | 557.2930 | VIDTGAP | 626.3508 | 654.3457 | ID | 201.1234 | 229.1183 |
| IDT | 302.1710 | 330.1660 | IDTG | 359.1925 | 387.1874 | IDTGA | 430.2296 | 458.2245 |
| IDTGAP | 527.2824 | 555.2773 | IDTGAPL | 640.3665 | 668.3614 | DT | 189.0870 | 217.0819 |
| DTG | 246.1084 | 274.1034 | DTGA | 317.1456 | 345.1405 | DTGAP | 414.1983 | 442.1932 |
| DTGAPL | 527.2824 | 555.2773 | DTGAPLS | 614.3144 | 642.3093 | TG | 131.0815 | 159.0764 |
| TGA | 202.1186 | 230.1135 | TGAP | 299.1714 | 327.1663 | TGAPL | 412.2554 | 440.2504 |
| TGAPLS | 499.2875 | 527.2824 | TGAPLSV | 598.3559 | 626.3508 | TGAPLSVP | 695.4087 | 723.4036 |
| GA | 101.0709 | 129.0659 | GAP | 198.1237 | 226.1186 | GAPL | 311.2078 | 339.2027 |
| GAPLS | 398.2398 | 426.2347 | GAPLSV | 497.3082 | 525.3031 | GAPLSVP | 594.3610 | 622.3559 |
| GAPLSVPV | 693.4294 | 721.4243 | AP | 141.1022 | 169.0972 | APL | 254.1863 | 282.1812 |
| APLS | 341.2183 | 369.2132 | APLSV | 440.2867 | 468.2817 | APLSVP | 537.3395 | 565.3344 |
| APLSVPV | 636.4079 | 664.4028 | APLSVPVG | 693.4294 | 721.4243 | PL | 183.1492 | 211.1441 |
| PLS | 270.1812 | 298.1761 | PLSV | 369.2496 | 397.2445 | PLSVP | 466.3024 | 494.2973 |
| PLSVPV | 565.3708 | 593.3657 | PLSVPVG | 622.3923 | 650.3872 | PLSVPVGG | 679.4137 | 707.4087 |
| LS | 173.1285 | 201.1234 | LSV | 272.1969 | 300.1918 | LSVP | 369.2496 | 397.2445 |
| LSVPV | 468.3180 | 496.3130 | LSVPVG | 525.3395 | 553.3344 | LSVPVGG | 582.3610 | 610.3559 |
| LSVPVGGA | 653.3981 | 681.3930 | SV | 159.1128 | 187.1077 | SVP | 256.1656 | 284.1605 |
| SVPV | 355.2340 | 383.2289 | SVPVG | 412.2554 | 440.2504 | SVPVGG | 469.2769 | 497.2718 |
| SVPVGGA | 540.3140 | 568.3089 | SVPVGGAT | 641.3617 | 669.3566 | VP | 169.1335 | 197.1285 |
| VPV | 268.2020 | 296.1969 | VPVG | 325.2234 | 353.2183 | VPVGG | 382.2449 | 410.2398 |
| VPVGGA | 453.2820 | 481.2769 | VPVGGAT | 554.3297 | 582.3246 | VPVGGATL | 667.4137 | 695.4087 |
| PV | 169.1335 | 197.1285 | PVG | 226.1550 | 254.1499 | PVGG | 283.1765 | 311.1714 |
| PVGGA | 354.2136 | 382.2085 | PVGGAT | 455.2613 | 483.2562 | PVGGATL | 568.3453 | 596.3402 |
| PVGGATLG | 625.3668 | 653.3617 | VG | 129.1022 | 157.0972 | VGG | 186.1237 | 214.1186 |
| VGGA | 257.1608 | 285.1557 | VGGAT | 358.2085 | 386.2034 | VGGATL | 471.2926 | 499.2875 |
| VGGATLG | 528.3140 | 556.3089 | GG | 87.0553 | 115.0502 | GGA | 158.0924 | 186.0873 |
| GGAT | 259.1401 | 287.1350 | GGATL | 372.2241 | 400.2191 | GGATLG | 429.2456 | 457.2405 |
| GA | 101.0709 | 129.0659 | GAT | 202.1186 | 230.1135 | GATL | 315.2027 | 343.1976 |
| GATLG | 372.2241 | 400.2191 | AT | 145.0972 | 173.0921 | ATL | 258.1812 | 286.1761 |
| ATLG | 315.2027 | 343.1976 | TL | 187.1441 | 215.1390 | TLG | 244.1656 | 272.1605 |
| LG | 143.1179 | 171.1128 | | | | | | |

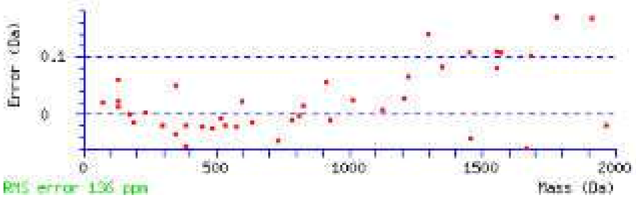


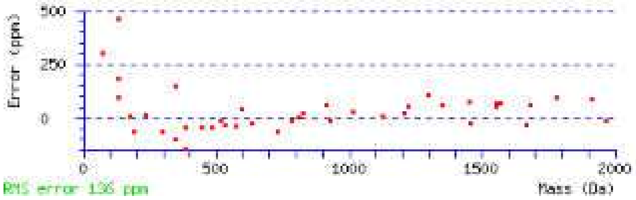


NCBI **BLAST** search of GMEVIDTGAPLSVPVGGATLGR
(Parameters: blastp, nr protein database, expect=20000, no filter, PAM30)
Other BLAST web gateways

**All matches to this query**

| Score | Mr(calc) | Delta | Sequence |
|---|---|---|---|
| 141.1 | 2112.0885 | 0.0954 | GMEVIDTGAPISVPVGGATLGR |
| 141.1 | 2112.0885 | 0.0954 | GMEVIDTGAPISVPVGGATLGR |
| 141.1 | 2112.0885 | 0.0954 | GMEVIDTGAPISVPVGGATLGR |
| 141.1 | 2112.0885 | 0.0954 | GMEVIDTGAPISVPVGGATLGR |
| 141.1 | 2112.0885 | 0.0954 | GMEVIDTGAPLSVPVGGATIGR |
| 141.1 | 2112.0885 | 0.0954 | GMEVIDTGAPLSVPVGGATLGR |
| 141.1 | 2112.0885 | 0.0954 | GMEVIDTGAPLSVPVGGATLGR |
| 141.1 | 2112.0885 | 0.0954 | GMEVIDTGAPLSVPVGGATLGR |
| 141.1 | 2112.0885 | 0.0954 | GMEVIDTGAPLSVPVGGATLGR |
| 141.1 | 2112.0885 | 0.0954 | GMEVIDTGAPLSVPVGGATLGR |

**Mascot:** http://www.matrixscience.com/

# MATRIX SCIENCE Mascot Search Results

## Peptide View **Spot no 87**

MS/MS Fragmentation of **DTDGKQINVTCEVQQLLGNNR**
Found in **gi|11466794** in **NCBInr**, ATP synthase CF1 beta subunit [Oryza sativa Japonica Group]

Match to Query 39: 2401.280124 from(2402.287400,1+) intensity(0.0000) index(19)
Title: Label: D11, Spot_Id: 216640, Peak_List_Id: 221235, MSMS Job_Run_Id: 21346, Comment:
Data file C:\Documents and Settings\Administrator\Desktop\farah BHU\RAJESH\12-1-13 raj\ppw_D11_135997699500.txt

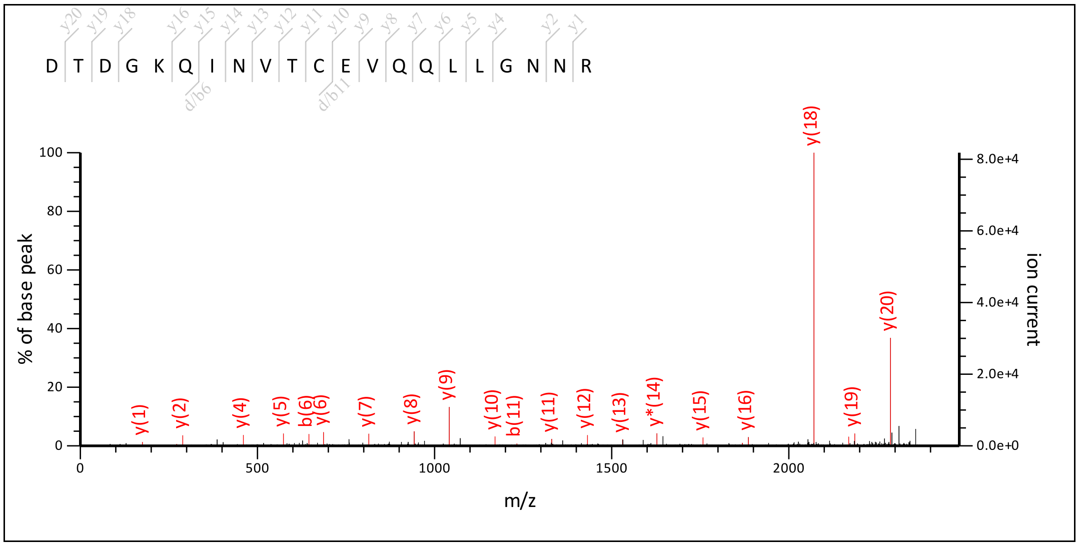


0 to 2481.19

Label all possible matches ○ Label matches used for scoring ◉

**Monoisotopic mass of neutral peptide Mr(calc):** 2401.1656
**Fixed modifications:** Carbamidomethyl (C) (apply to specified residues or termini only)
**Ions Score:** 196 **Expect:** 8.1e-17
**Matches :** 25/395 fragment ions using 23 most intense peaks (help)

| # | Immon. | a | a* | a0 | b | b* | b0 | d | d' | Seq. | v | w | w' | y | y* | |
|---|---|---|---|---|---|---|---|---|---|---|---|---|---|---|---|---|
| 1 | 88.0393 | 88.0393 | | 70.0287 | 116.0342 | | 98.0237 | 44.0495 | | D | | | | | | |
| 2 | 74.0600 | 189.0870 | | 171.0764 | 217.0819 | | 199.0713 | 173.0921 | 175.0713 | T | 2241.1041 | 2254.1245 | 2256.1037 | 2287.1459 | 2270.1194 | [illegible] |
| 3 | 88.0393 | 304.1139 | | 286.1034 | 332.1088 | | 314.0983 | 260.1241 | | D | 2126.0771 | 2125.0819 | | 2186.0982 | 2169.0717 | [illegible] |
| 4 | 30.0338 | 361.1354 | | 343.1248 | 389.1303 | | 371.1197 | | | G | | | | 2071.0713 | 2054.0447 | [illegible] |
| 5 | 101.1073 | 489.2304 | 472.2038 | 471.2198 | 517.2253 | 500.1987 | 499.2147 | 432.1725 | | K | 1940.9607 | 1939.9654 | | 2014.0498 | 1997.0233 | [illegible] |
| 6 | 101.0709 | 617.2889 | 600.2624 | 599.2784 | **645.2838** | 628.2573 | 627.2733 | 560.2675 | | Q | 1812.9021 | 1811.9069 | | 1885.9549 | 1868.9283 | [illegible] |
| 7 | 86.0964 | 730.3730 | 713.3464 | 712.3624 | 758.3679 | 741.3414 | 740.3573 | 702.3417 | 716.3573 | I | 1699.8180 | 1712.8384 | 1726.8541 | 1757.8963 | 1740.8697 | [illegible] |
| 8 | 87.0553 | 844.4159 | 827.3894 | 826.4054 | 872.4108 | 855.3843 | 854.4003 | 801.4101 | | N | 1585.7751 | 1584.7799 | | 1644.8122 | 1627.7857 | [illegible] |
| 9 | 72.0808 | 943.4843 | 926.4578 | 925.4738 | 971.4793 | 954.4527 | 953.4687 | 929.4687 | | V | 1486.7067 | 1499.7271 | | 1530.7693 | 1513.7428 | [illegible] |
| 10 | 74.0600 | 1044.5320 | 1027.5055 | 1026.5214 | 1072.5269 | 1055.5004 | 1054.5164 | 1028.5371 | 1030.5164 | T | 1385.6590 | 1398.6794 | 1400.6587 | 1431.7009 | 1414.6743 | [illegible] |
| 11 | 133.0430 | 1204.5627 | 1187.5361 | 1186.5521 | **1232.5576** | 1215.5310 | 1214.5470 | 1115.5691 | | C | 1225.6284 | 1224.6331 | | 1330.6532 | 1313.6267 | [illegible] |
| 12 | 102.0550 | 1333.6053 | 1316.5787 | 1315.5947 | 1361.6002 | 1344.5736 | 1343.5896 | 1275.5998 | | E | 1096.5858 | 1095.5905 | | 1170.6226 | 1153.5960 | [illegible] |
| 13 | 72.0808 | 1432.6737 | 1415.6471 | 1414.6631 | 1460.6686 | 1443.6420 | 1442.6580 | 1418.6580 | | V | 997.5174 | 1010.5378 | | 1041.5800 | 1024.5534 | |
| 14 | 101.0709 | 1560.7322 | 1543.7057 | 1542.7217 | 1588.7272 | 1571.7006 | 1570.7166 | 1503.7108 | | Q | 869.4588 | 868.4635 | | 942.5116 | 925.4850 | |
| 15 | 101.0709 | 1688.7908 | 1671.7643 | 1670.7803 | 1716.7857 | 1699.7592 | 1698.7752 | 1631.7694 | | Q | 741.4002 | 740.4050 | | 814.4530 | 797.4264 | |
| 16 | 86.0964 | 1801.8749 | 1784.8483 | 1783.8643 | 1829.8698 | 1812.8433 | 1811.8592 | 1759.8279 | | L | 628.3161 | 627.3209 | | 686.3944 | 669.3678 | |
| 17 | 86.0964 | 1914.9590 | 1897.9324 | 1896.9484 | 1942.9539 | 1925.9273 | 1924.9433 | 1872.9120 | | L | 515.2321 | 514.2368 | | 573.3103 | 556.2838 | |
| 18 | 30.0338 | 1971.9804 | 1954.9539 | 1953.9699 | 1999.9753 | 1982.9488 | 1981.9648 | | | G | | | | 460.2263 | 443.1997 | |
| 19 | 87.0553 | 2086.0233 | 2068.9968 | 2068.0128 | 2114.0183 | 2096.9917 | 2096.0077 | 2043.0175 | | N | 344.1677 | 343.1724 | | 403.2048 | 386.1783 | |
| 20 | 87.0553 | 2200.0663 | 2183.0397 | 2182.0557 | 2228.0612 | 2211.0346 | 2210.0506 | 2157.0605 | | N | 230.1248 | 229.1295 | | 289.1619 | 272.1353 | |
| 21 | 129.1135 | | | | | | | | | R | 74.0237 | 73.0284 | | 175.1190 | 158.0924 | |

| Seq | ya | yb | Seq | ya | yb | Seq | ya | yb |
|---|---|---|---|---|---|---|---|---|

| | | | | | | | | |
|---|---|---|---|---|---|---|---|---|
| **TD** | 189.0870 | 217.0819 | **TDG** | 246.1084 | 274.1034 | **TDGK** | 374.2034 | 402.1983 |
| **TDGKQ** | 502.2620 | 530.2569 | **TDGKQI** | 615.3461 | 643.3410 | **DG** | 145.0608 | 173.0557 |
| **DGK** | 273.1557 | 301.1506 | **DGKQ** | 401.2143 | 429.2092 | **DGKQI** | 514.2984 | 542.2933 |
| **DGKQIN** | 628.3413 | 656.3362 | **GK** | 158.1288 | 186.1237 | **GKQ** | 286.1874 | 314.1823 |
| **GKQI** | 399.2714 | 427.2663 | **GKQIN** | 513.3144 | 541.3093 | **GKQINV** | 612.3828 | 640.3777 |
| **KQ** | 229.1659 | 257.1608 | **KQI** | 342.2500 | 370.2449 | **KQIN** | 456.2929 | 484.2878 |
| **KQINV** | 555.3613 | 583.3562 | **KQINVT** | 656.4090 | 684.4039 | **QI** | 214.1550 | 242.1499 |
| **QIN** | 328.1979 | 356.1928 | **QINV** | 427.2663 | 455.2613 | **QINVT** | 528.3140 | 556.3089 |
| **QINVTC** | 688.3447 | 716.3396 | **IN** | 200.1394 | 228.1343 | **INV** | 299.2078 | 327.2027 |
| **INVT** | 400.2554 | 428.2504 | **INVTC** | 560.2861 | 588.2810 | **INVTCE** | 689.3287 | 717.3236 |
| **NV** | 186.1237 | 214.1186 | **NVT** | 287.1714 | 315.1663 | **NVTC** | 447.2020 | 475.1969 |
| **NVTCE** | 576.2446 | 604.2395 | **NVTCEV** | 675.3130 | 703.3080 | **VT** | 173.1285 | 201.1234 |
| **VTC** | 333.1591 | 361.1540 | **VTCE** | 462.2017 | 490.1966 | **VTCEV** | 561.2701 | 589.2650 |
| **VTCEVQ** | 689.3287 | 717.3236 | **TC** | 234.0907 | 262.0856 | **TCE** | 363.1333 | 391.1282 |
| **TCEV** | 462.2017 | 490.1966 | **TCEVQ** | 590.2603 | 618.2552 | **CE** | 262.0856 | 290.0805 |
| **CEV** | 361.1540 | 389.1489 | **CEVQ** | 489.2126 | 517.2075 | **CEVQQ** | 617.2712 | 645.2661 |
| **EV** | 201.1234 | 229.1183 | **EVQ** | 329.1819 | 357.1769 | **EVQQ** | 457.2405 | 485.2354 |
| **EVQQL** | 570.3246 | 598.3195 | **EVQQLL** | 683.4087 | 711.4036 | **VQ** | 200.1394 | 228.1343 |
| **VQQ** | 328.1979 | 356.1928 | **VQQL** | 441.2820 | 469.2769 | **VQQLL** | 554.3661 | 582.3610 |
| **VQQLLG** | 611.3875 | 639.3824 | **QQ** | 229.1295 | 257.1244 | **QQL** | 342.2136 | 370.2085 |
| **QQLL** | 455.2976 | 483.2926 | **QQLLG** | 512.3191 | 540.3140 | **QQLLGN** | 626.3620 | 654.3570 |
| **QL** | 214.1550 | 242.1499 | **QLL** | 327.2391 | 355.2340 | **QLLG** | 384.2605 | 412.2554 |
| **QLLGN** | 498.3035 | 526.2984 | **QLLGNN** | 612.3464 | 640.3413 | **LL** | 199.1805 | 227.1754 |
| **LLG** | 256.2020 | 284.1969 | **LLGN** | 370.2449 | 398.2398 | **LLGNN** | 484.2878 | 512.2827 |
| **LG** | 143.1179 | 171.1128 | **LGN** | 257.1608 | 285.1557 | **LGNN** | 371.2037 | 399.1987 |
| **GN** | 144.0768 | 172.0717 | **GNN** | 258.1197 | 286.1146 | **NN** | 201.0982 | 229.0931 |

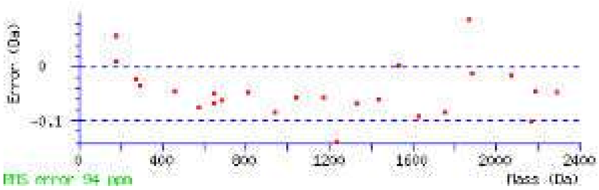

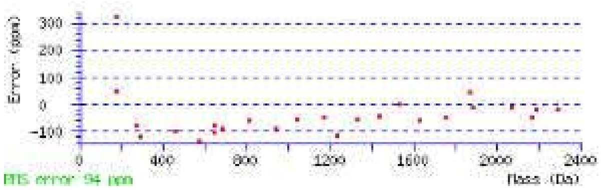


NCBI **BLAST** search of DTDGKQINVTCEVQQLLGNNR
(Parameters: blastp, nr protein database, expect=20000, no filter, PAM30)
Other BLAST web gateways

**All matches to this query**

| Score | Mr(calc) | Delta | Sequence |
|---|---|---|---|
| 195.6 | 2401.1656 | 0.1145 | DTDGKQINVTCEVQQLLGNNR |
| 195.6 | 2401.1292 | 0.1509 | DTDGQQINVTCEVQQLLGNNR |
| 195.6 | 2401.1292 | 0.1509 | DTDGQQINVTCEVQQLLGNNR |
| 149.7 | 2401.1543 | 0.1258 | DTLGEEINVTCEVQQLLGNNR |
| 149.7 | 2401.1292 | 0.1509 | DTNGQEINVTCEVQQLLGNNR |
| 146.3 | 2401.1842 | 0.0959 | MTVGQQINVTCEVQQLLGNNR |
| 141.4 | 2400.1452 | 1.1349 | DTNGQQINVTCEVQQLLGNNR |
| 126.0 | 2401.1292 | 0.1509 | DNTGQEINVTCEVQQLLGNNR |
| 124.4 | 2402.1132 | -0.8331 | DTDGEQINVTCEVQQLLGNNR |
| 121.6 | 2401.1478 | 0.1323 | DIAGQCINVTCEVQQLLGNNR |

**Mascot:** http://www.matrixscience.com/

# MATRIX SCIENCE Mascot Search Results

## Peptide View Spot no 87

MS/MS Fragmentation of **IFNVLGEPVDNLGPVDTSATFPIHR**
Found in **gi|11466794** in **NCBInr**, ATP synthase CF1 beta subunit [Oryza sativa Japonica Group]

Match to Query 41: 2707.533024 from(2708.540300,1+) intensity(0.0000) index(20)
Title: Label: D11, Spot_Id: 216640, Peak_List_Id: 221225, MSMS Job_Run_Id: 21346, Comment:
Data file C:\Documents and Settings\Administrator\Desktop\farah BHU\RAJESH\12-1-13 raj\ppw_D11_135997699500.txt

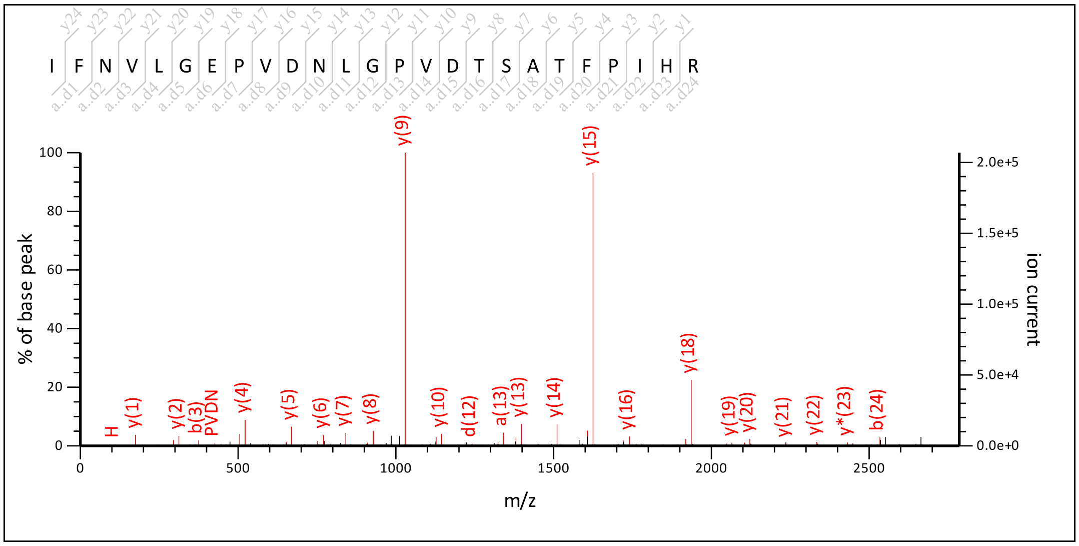


0 to 2785.71

Label all possible matches ○ Label matches used for scoring ◉

**Monoisotopic mass of neutral peptide Mr(calc):** 2707.3970
**Fixed modifications:** Carbamidomethyl (C) (apply to specified residues or termini only)
**Ions Score:** 177 **Expect:** 4.4e-15
**Matches :** 52/505 fragment ions using 54 most intense peaks (help)

| # | Immon. | a | a* | $a^0$ | b | b* | $b^0$ | d | d' | Seq. | v | w | w' | y | y* | |
|---|---|---|---|---|---|---|---|---|---|---|---|---|---|---|---|---|
| 1 | 86.0964 | 86.0964 | | | 114.0913 | | | 44.0495 | | I | | | | | | |
| 2 | 120.0808 | 233.1648 | | | 261.1598 | | | | | F | 2503.2576 | | | 2595.3202 | 2578.2936 | [illegible] |
| 3 | 87.0553 | 347.2078 | 330.1812 | | 375.2027 | 358.1761 | | 304.2020 | | N | 2389.2147 | 2388.2194 | | 2448.2518 | 2431.2252 | [illegible] |
| 4 | 72.0808 | 446.2762 | 429.2496 | | 474.2711 | 457.2445 | | 432.2605 | | V | 2290.1462 | 2303.1666 | | 2334.2088 | 2317.1823 | [illegible] |
| 5 | 86.0964 | 559.3602 | 542.3337 | | 587.3552 | 570.3286 | | 517.3133 | | L | 2177.0622 | 2176.0669 | | 2235.1404 | 2218.1139 | [illegible] |
| 6 | 30.0338 | 616.3817 | 599.3552 | | 644.3766 | 627.3501 | | | | G | | | | 2122.0564 | 2105.0298 | [illegible] |
| 7 | 102.0550 | 745.4243 | 728.3978 | 727.4137 | 773.4192 | 756.3927 | 755.4087 | 687.4188 | | E | 1990.9981 | 1990.0029 | | 2065.0349 | 2048.0083 | [illegible] |
| 8 | 70.0651 | 842.4771 | 825.4505 | 824.4665 | 870.4720 | 853.4454 | 852.4614 | 816.4614 | | P | 1893.9454 | 1892.9501 | | 1935.9923 | 1918.9658 | [illegible] |
| 9 | 72.0808 | 941.5455 | 924.5189 | 923.5349 | 969.5404 | 952.5138 | 951.5298 | 927.5298 | | V | 1794.8769 | 1807.8973 | | 1838.9395 | 1821.9130 | [illegible] |
| 10 | 88.0393 | 1056.5724 | 1039.5459 | 1038.5619 | 1084.5673 | 1067.5408 | 1066.5568 | 1012.5826 | | D | 1679.8500 | 1678.8547 | | 1739.8711 | 1722.8446 | [illegible] |
| 11 | 87.0553 | 1170.6153 | 1153.5888 | 1152.6048 | 1198.6103 | 1181.5837 | 1180.5997 | 1127.6095 | | N | 1565.8071 | 1564.8118 | | 1624.8442 | 1607.8176 | [illegible] |
| 12 | 86.0964 | 1283.6994 | 1266.6729 | 1265.6888 | 1311.6943 | 1294.6678 | 1293.6838 | 1241.6525 | | L | 1452.7230 | 1451.7278 | | 1510.8013 | 1493.7747 | [illegible] |
| 13 | 30.0338 | 1340.7209 | 1323.6943 | 1322.7103 | 1368.7158 | 1351.6892 | 1350.7052 | | | G | | | | 1397.7172 | 1380.6906 | [illegible] |
| 14 | 70.0651 | 1437.7736 | 1420.7471 | 1419.7631 | 1465.7686 | 1448.7420 | 1447.7580 | 1411.7580 | | P | 1298.6488 | 1297.6535 | | 1340.6957 | 1323.6692 | [illegible] |
| 15 | 72.0808 | 1536.8421 | 1519.8155 | 1518.8315 | 1564.8370 | 1547.8104 | 1546.8264 | 1522.8264 | | V | 1199.5804 | 1212.6008 | | 1243.6430 | 1226.6164 | [illegible] |
| 16 | 88.0393 | 1651.8690 | 1634.8424 | 1633.8584 | 1679.8639 | 1662.8374 | 1661.8533 | 1607.8792 | | D | 1084.5534 | 1083.5582 | | 1144.5745 | 1127.5480 | [illegible] |
| 17 | 74.0600 | 1752.9167 | 1735.8901 | 1734.9061 | 1780.9116 | 1763.8850 | 1762.9010 | 1736.9218 | 1738.9010 | T | 983.5057 | 996.5261 | 998.5054 | 1029.5476 | 1012.5211 | [illegible] |
| 18 | 60.0444 | 1839.9487 | 1822.9222 | 1821.9381 | 1867.9436 | 1850.9171 | 1849.9331 | 1823.9538 | | S | 896.4737 | 895.4785 | | 928.4999 | 911.4734 | [illegible] |
| 19 | 44.0495 | 1910.9858 | 1893.9593 | 1892.9753 | 1938.9807 | 1921.9542 | 1920.9702 | | | A | 825.4366 | | | 841.4679 | 824.4413 | [illegible] |
| 20 | 74.0600 | 2012.0335 | 1995.0069 | 1994.0229 | 2040.0284 | 2023.0019 | 2022.0178 | 1996.0386 | 1998.0178 | T | 724.3889 | 737.4093 | 739.3886 | 770.4308 | 753.4042 | [illegible] |
| 21 | 120.0808 | 2159.1019 | 2142.0754 | 2141.0913 | 2187.0968 | 2170.0703 | 2169.0863 | | | F | 577.3205 | | | 669.3831 | 652.3566 | |
| 22 | 70.0651 | 2256.1547 | 2239.1281 | 2238.1441 | 2284.1496 | 2267.1230 | 2266.1390 | 2230.1390 | | P | 480.2677 | 479.2725 | | 522.3147 | 505.2881 | |
| 23 | 86.0964 | 2369.2387 | 2352.2122 | 2351.2282 | 2397.2337 | 2380.2071 | 2379.2231 | 2341.2074 | 2355.2231 | I | 367.1837 | 380.2041 | 394.2197 | 425.2619 | 408.2354 | |

| | | | | | | | | | | | | | | | | |
|---|---|---|---|---|---|---|---|---|---|---|---|---|---|---|---|---|
| 24 | 110.0713 | 2506.2977 | 2489.2711 | 2488.2871 | 2534.2926 | 2517.2660 | 2516.2820 | | | H | 230.1248 | | | ***312.1779*** | 295.1513 | |
| 25 | 129.1135 | | | | | | | | | R | 74.0237 | 73.0284 | | ***175.1190*** | 158.0924 | |

| Seq | ya | yb | Seq | ya | yb | Seq | ya | yb |
|---|---|---|---|---|---|---|---|---|
| FN | 234.1237 | 262.1186 | FNV | 333.1921 | 361.1870 | FNVL | 446.2762 | 474.2711 |
| FNVLG | 503.2976 | 531.2926 | FNVLGE | 632.3402 | 660.3352 | NV | 186.1237 | 214.1186 |
| NVL | 299.2078 | 327.2027 | NVLG | 356.2292 | 384.2241 | NVLGE | 485.2718 | 513.2667 |
| NVLGEP | 582.3246 | 610.3195 | NVLGEPV | 681.3930 | 709.3879 | VL | 185.1648 | 213.1598 |
| VLG | 242.1863 | 270.1812 | VLGE | 371.2289 | 399.2238 | VLGEP | 468.2817 | 496.2766 |
| VLGEPV | 567.3501 | 595.3450 | VLGEPVD | 682.3770 | 710.3719 | LG | 143.1179 | 171.1128 |
| LGE | 272.1605 | 300.1554 | LGEP | 369.2132 | 397.2082 | LGEPV | 468.2817 | 496.2766 |
| LGEPVD | 583.3086 | 611.3035 | LGEPVDN | 697.3515 | 725.3464 | GE | 159.0764 | 187.0713 |
| GEP | 256.1292 | 284.1241 | GEPV | 355.1976 | 383.1925 | GEPVD | 470.2245 | 498.2195 |
| GEPVDN | 584.2675 | 612.2624 | GEPVDNL | 697.3515 | 725.3464 | EP | 199.1077 | 227.1026 |
| EPV | 298.1761 | 326.1710 | EPVD | 413.2031 | 441.1980 | EPVDN | 527.2460 | 555.2409 |
| EPVDNL | 640.3301 | 668.3250 | EPVDNLG | 697.3515 | 725.3464 | PV | 169.1335 | 197.1285 |
| PVD | 284.1605 | 312.1554 | PVDN | 398.2034 | 426.1983 | PVDNL | 511.2875 | 539.2824 |
| PVDNLG | 568.3089 | 596.3039 | PVDNLGP | 665.3617 | 693.3566 | VD | 187.1077 | 215.1026 |
| VDN | 301.1506 | 329.1456 | VDNL | 414.2347 | 442.2296 | VDNLG | 471.2562 | 499.2511 |
| VDNLGP | 568.3089 | 596.3039 | VDNLGPV | 667.3774 | 695.3723 | DN | 202.0822 | 230.0771 |
| DNL | 315.1663 | 343.1612 | DNLG | 372.1878 | 400.1827 | DNLGP | 469.2405 | 497.2354 |
| DNLGPV | 568.3089 | 596.3039 | DNLGPVD | 683.3359 | 711.3308 | NL | 200.1394 | 228.1343 |
| NLG | 257.1608 | 285.1557 | NLGP | 354.2136 | 382.2085 | NLGPV | 453.2820 | 481.2769 |
| NLGPVD | 568.3089 | 596.3039 | NLGPVDT | 669.3566 | 697.3515 | LG | 143.1179 | 171.1128 |
| LGP | 240.1707 | 268.1656 | LGPV | 339.2391 | 367.2340 | LGPVD | 454.2660 | 482.2609 |
| LGPVDT | 555.3137 | 583.3086 | LGPVDTS | 642.3457 | 670.3406 | GP | 127.0866 | 155.0815 |
| GPV | 226.1550 | 254.1499 | GPVD | 341.1819 | 369.1769 | GPVDT | 442.2296 | 470.2245 |
| GPVDTS | 529.2617 | 557.2566 | GPVDTSA | 600.2988 | 628.2937 | PV | 169.1335 | 197.1285 |
| PVD | 284.1605 | 312.1554 | PVDT | 385.2082 | 413.2031 | PVDTS | 472.2402 | 500.2351 |
| PVDTSA | 543.2773 | 571.2722 | PVDTSAT | 644.3250 | 672.3199 | VD | 187.1077 | 215.1026 |
| VDT | 288.1554 | 316.1503 | VDTS | 375.1874 | 403.1823 | VDTSA | 446.2245 | 474.2195 |
| VDTSAT | 547.2722 | 575.2671 | VDTSATF | 694.3406 | 722.3355 | DT | 189.0870 | 217.0819 |
| DTS | 276.1190 | 304.1139 | DTSA | 347.1561 | 375.1510 | DTSAT | 448.2038 | 476.1987 |
| DTSATF | 595.2722 | 623.2671 | DTSATFP | 692.3250 | 720.3199 | TS | 161.0921 | 189.0870 |
| TSA | 232.1292 | 260.1241 | TSAT | 333.1769 | 361.1718 | TSATF | 480.2453 | 508.2402 |
| TSATFP | 577.2980 | 605.2930 | TSATFPI | 690.3821 | 718.3770 | SA | 131.0815 | 159.0764 |
| SAT | 232.1292 | 260.1241 | SATF | 379.1976 | 407.1925 | SATFP | 476.2504 | 504.2453 |
| SATFPI | 589.3344 | 617.3293 | AT | 145.0972 | 173.0921 | ATF | 292.1656 | 320.1605 |
| ATFP | 389.2183 | 417.2132 | ATFPI | 502.3024 | 530.2973 | ATFPIH | 639.3613 | 667.3562 |
| TF | 221.1285 | 249.1234 | TFP | 318.1812 | 346.1761 | TFPI | 431.2653 | 459.2602 |
| TFPIH | 568.3242 | 596.3191 | FP | 217.1335 | 245.1285 | FPI | 330.2176 | 358.2125 |
| FPIH | 467.2765 | 495.2714 | PI | 183.1492 | 211.1441 | PIH | 320.2081 | 348.2030 |
| IH | 223.1553 | 251.1503 | | | | | | |

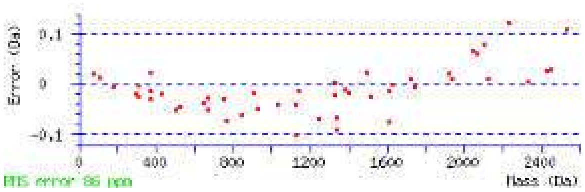


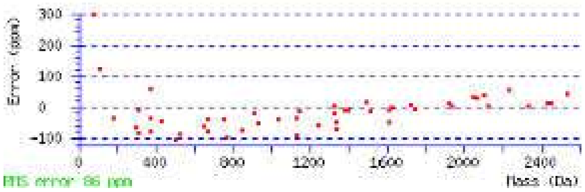


NCBI **BLAST** search of IFNVLGEPVDNLGPVDTSATFPIHR
(Parameters: blastp, nr protein database, expect=20000, no filter, PAM30)
Other BLAST web gateways

**All matches to this query**

| Score | Mr(calc) | Delta | Sequence |
|---|---|---|---|
| | | | |

| | | | |
|---|---|---|---|
| 177.0 | 2707.3970 | 0.1360 | IFNVLGEPVDNLGPVDTSATFPIHR |
| 160.7 | 2707.3970 | 0.1360 | IFNVLGEPVDNLGPVDTGTTFPIHR |
| 159.1 | 2707.3970 | 0.1361 | IFNVLGEPVDNLGPVESSATFPIHR |
| 158.0 | 2708.3559 | -0.8228 | NFNVLGEPVDNLGPVDTGTTFPIHR |
| 144.1 | 2707.3640 | 0.1691 | IFNVLGEPVDNLGPVDTSTMSPIHR |
| 143.6 | 2707.3970 | 0.1360 | IFNVLGEPVDNLGPVESGTTFPIHR |
| 142.7 | 2708.3558 | -0.8228 | NFNVLGEPVDNLGPVDASTTFPIHR |
| 142.7 | 2707.3970 | 0.1360 | IFNVIGEPVDNLGPVDASTTFPIHR |
| 142.7 | 2707.3970 | 0.1360 | IFNVLGEPVDNLGPVDASTTFPIHR |
| 142.7 | 2707.3970 | 0.1360 | IFNVLGEPVDNLGPVDASTTFPIHR |

**Mascot:** http://www.matrixscience.com/

# MATRIX SCIENCE Mascot Search Results

## Peptide View Spot no 90

MS/MS Fragmentation of **LANAVGVTLGPR**
Found in **gi|115488160** in **NCBInr**, Os12g0277500 [Oryza sativa Japonica Group]

Match to Query 41: 1166.729224 from(1167.736500,1+) intensity(0.0000) index(14)
Title: Label: D12, Spot_Id: 219853, Peak_List_Id: 227214, MSMS Job_Run_Id: 21955, Comment:
Data file C:\Documents and Settings\Administrator\Desktop\Farah-Garima 11May 2013\ppw_D12_136868220000.txt

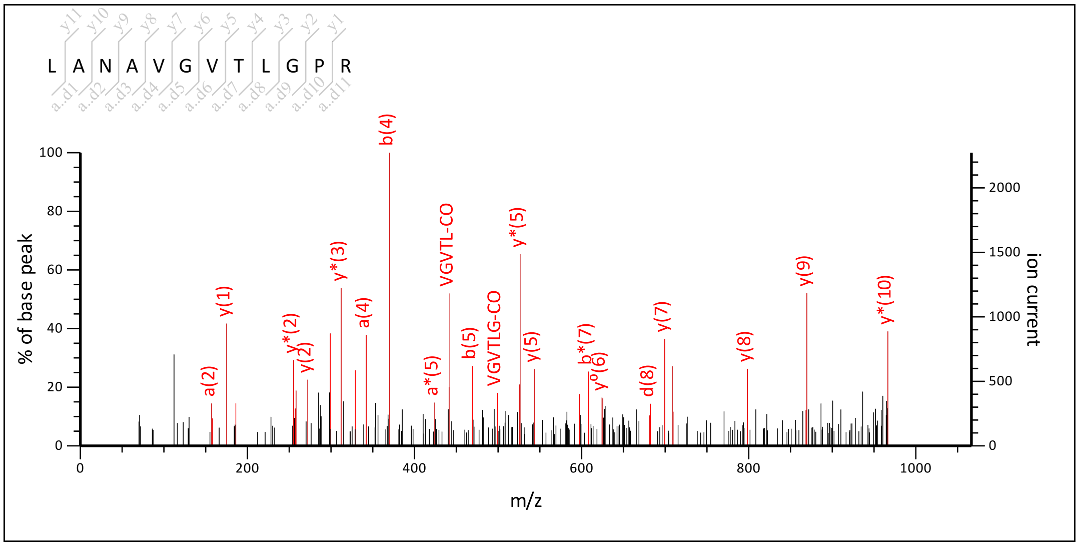


0 to 1066.56

Label all possible matches ○ Label matches used for scoring ◉

**Monoisotopic mass of neutral peptide Mr(calc):** 1166.6772
**Fixed modifications:** Carbamidomethyl (C) (apply to specified residues or termini only)
**Ions Score:** 61 **Expect:** 0.011
**Matches :** 52/196 fragment ions using 45 most intense peaks (help)

| # | Immon. | a | a* | $a^0$ | b | b* | $b^0$ | d | d' | Seq. | v | w | w' | y | y* | $y^0$ | # |
|---|---|---|---|---|---|---|---|---|---|---|---|---|---|---|---|---|---|
| 1 | 86.0964 | 86.0964 | | | 114.0913 | | | 44.0495 | | L | | | | | | | 12 |
| 2 | 44.0495 | **157.1335** | | | 185.1285 | | | | | A | 1038.5691 | | | 1054.6004 | 1037.5738 | 1036.5898 | 11 |
| 3 | 87.0553 | 271.1765 | 254.1499 | | **299.1714** | 282.1448 | | 228.1707 | | N | 924.5261 | 923.5309 | | 983.5633 | 966.5367 | 965.5527 | 10 |
| 4 | 44.0495 | **342.2136** | 325.1870 | | **370.2085** | 353.1819 | | | | A | 853.4890 | | | ***869.5203*** | 852.4938 | 851.5098 | 9 |
| 5 | 72.0808 | **441.2820** | 424.2554 | | **469.2769** | 452.2504 | | 427.2663 | | V | 754.4206 | 767.4410 | | ***798.4832*** | 781.4567 | 780.4727 | 8 |
| 6 | 30.0338 | 498.3035 | 481.2769 | | **526.2984** | 509.2718 | | | | G | | | | ***699.4148*** | 682.3883 | 681.4042 | 7 |
| 7 | 72.0808 | **597.3719** | 580.3453 | | **625.3668** | 608.3402 | | 583.3562 | | V | 598.3307 | 611.3511 | | 642.3933 | 625.3668 | 624.3828 | 6 |
| 8 | 74.0600 | 698.4196 | 681.3930 | 680.4090 | 726.4145 | 709.3879 | 708.4039 | 682.4246 | 684.4039 | T | 497.2831 | 510.3035 | 512.2827 | ***543.3249*** | 526.2984 | 525.3144 | 5 |
| 9 | 86.0964 | 811.5036 | 794.4771 | 793.4931 | 839.4985 | 822.4720 | 821.4880 | 769.4567 | | L | 384.1990 | 383.2037 | | ***442.2772*** | 425.2507 | | 4 |
| 10 | 30.0338 | **868.5251** | 851.4985 | 850.5145 | 896.5200 | 879.4934 | 878.5094 | | | G | | | | ***329.1932*** | 312.1666 | | 3 |
| 11 | 70.0651 | 965.5778 | 948.5513 | 947.5673 | 993.5728 | 976.5462 | 975.5622 | 939.5622 | | P | 230.1248 | 229.1295 | | ***272.1717*** | 255.1452 | | 2 |
| 12 | 129.1135 | | | | | | | | | R | 74.0237 | 73.0284 | | ***175.1190*** | 158.0924 | | 1 |

| Seq | ya | yb | Seq | ya | yb | Seq | ya | yb |
|---|---|---|---|---|---|---|---|---|
| AN | 158.0924 | 186.0873 | ANA | 229.1295 | 257.1244 | ANAV | 328.1979 | 356.1928 |
| ANAVG | 385.2194 | 413.2143 | ANAVGV | 484.2878 | 512.2827 | ANAVGVT | 585.3355 | 613.3304 |
| ANAVGVTL | 698.4196 | 726.4145 | NA | 158.0924 | 186.0873 | NAV | 257.1608 | 285.1557 |
| NAVG | 314.1823 | 342.1772 | NAVGV | 413.2507 | 441.2456 | NAVGVT | 514.2984 | 542.2933 |
| NAVGVTL | 627.3824 | 655.3774 | NAVGVTLG | 684.4039 | 712.3988 | AV | 143.1179 | 171.1128 |
| AVG | 200.1394 | 228.1343 | AVGV | 299.2078 | 327.2027 | AVGVT | 400.2554 | 428.2504 |
| AVGVTL | 513.3395 | 541.3344 | AVGVTLG | 570.3610 | 598.3559 | AVGVTLGP | 667.4137 | 695.4087 |
| VG | 129.1022 | 157.0972 | VGV | 228.1707 | 256.1656 | VGVT | 329.2183 | 357.2132 |
| VGVTL | 442.3024 | 470.2973 | VGVTLG | 499.3239 | 527.3188 | VGVTLGP | 596.3766 | 624.3715 |

| | | | | | | | | |
|---|---|---|---|---|---|---|---|---|
| **GV** | 129.1022 | 157.0972 | **GVT** | 230.1499 | 258.1448 | **GVTL** | 343.2340 | 371.2289 |
| **GVTLG** | 400.2554 | 428.2504 | **GVTLGP** | 497.3082 | 525.3031 | **VT** | 173.1285 | 201.1234 |
| **VTL** | 286.2125 | 314.2074 | **VTLG** | 343.2340 | 371.2289 | **VTLGP** | 440.2867 | 468.2817 |
| **TL** | 187.1441 | 215.1390 | **TLG** | 244.1656 | 272.1605 | **TLGP** | 341.2183 | 369.2132 |
| **LG** | 143.1179 | 171.1128 | **LGP** | 240.1707 | 268.1656 | **GP** | 127.0866 | 155.0815 |

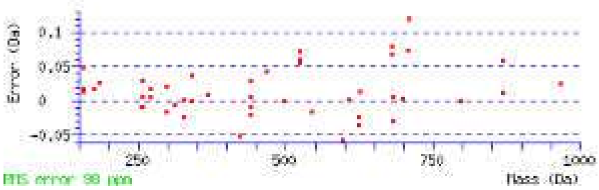


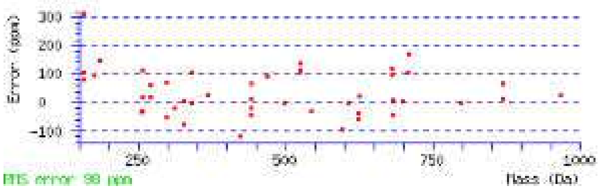


NCBI **BLAST** search of LANAVGVTLGPR
(Parameters: blastp, nr protein database, expect=20000, no filter, PAM30)
Other BLAST web gateways

**All matches to this query**

| Score | Mr(calc) | Delta | Sequence |
|---|---|---|---|
| 60.9 | 1166.6772 | 0.0521 | LANAVGVTLGPR |
| 42.9 | 1166.6771 | 0.0521 | AKVAVEQLGPR |
| 42.8 | 1166.6519 | 0.0773 | AAIAAAREAAPR |
| 39.5 | 1166.6408 | 0.0884 | VGGAGVIAVPDGR |
| 38.2 | 1166.6884 | 0.0408 | VRAAAGTVVAPR |
| 37.1 | 1166.6884 | 0.0408 | TRIAVQGIGPR |
| 36.2 | 1166.6884 | 0.0409 | VNAIAPVARTR |
| 35.4 | 1166.6771 | 0.0521 | GAQIVKELGPR |
| 34.6 | 1166.6520 | 0.0772 | AARVAAEAGVPR |
| 33.5 | 1166.6520 | 0.0772 | VGAAAEARGLPR |

**Mascot:** http://www.matrixscience.com/

# Mascot Search Results

## Peptide View Spot no 90

MS/MS Fragmentation of **GYISPQFVTNLEK**
Found in **gi|115488160** in **NCBInr**, Os12g0277500 [Oryza sativa Japonica Group]

Match to Query 56: 1494.815124 from(1495.822400,1+) intensity(0.0000) index(18)
Title: Label: D12, Spot_Id: 219853, Peak_List_Id: 227229, MSMS Job_Run_Id: 21955, Comment:
Data file C:\Documents and Settings\Administrator\Desktop\Farah-Garima 11May 2013\ppw_D12_136868220000.txt

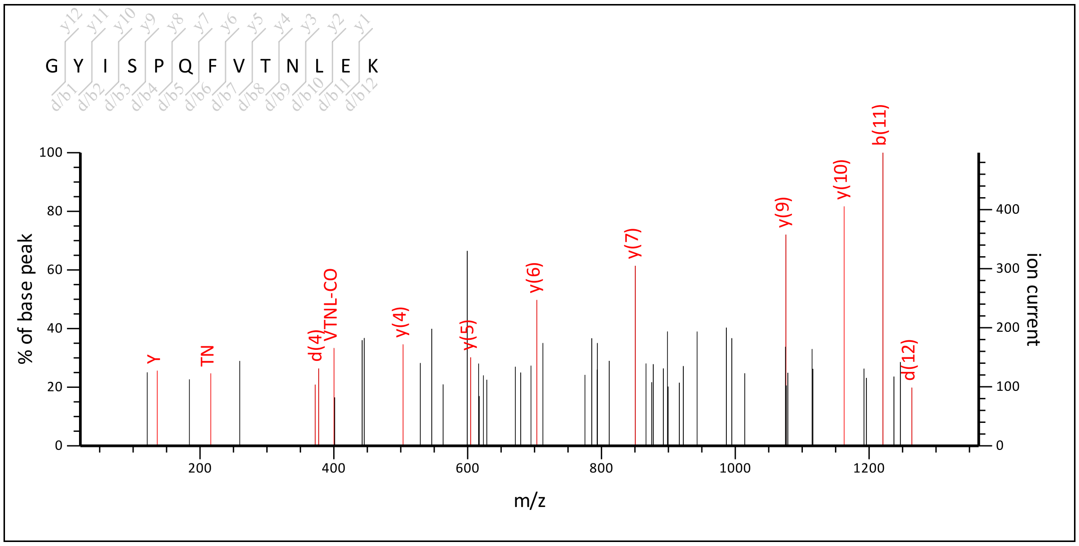


21.14 to 1363.82

Label all possible matches ○ Label matches used for scoring ◉

**Monoisotopic mass of neutral peptide Mr(calc):** 1494.7718
**Fixed modifications:** Carbamidomethyl (C) (apply to specified residues or termini only)
**Ions Score:** 19 **Expect:** 3.5e+02
**Matches :** 14/216 fragment ions using 34 most intense peaks (help)

| # | Immon. | a | a* | $a^0$ | b | b* | $b^0$ | d | d' | Seq. | v | w | w' | y | y* | |
|---|---|---|---|---|---|---|---|---|---|---|---|---|---|---|---|---|
| 1 | 30.0338 | 30.0338 | | | 58.0287 | | | 44.0495 | | G | | | | | | |
| 2 | 136.0757 | 193.0972 | | | 221.0921 | | | | | Y | 1330.7001 | | | 1438.7577 | 1421.7311 | 142 |
| 3 | 86.0964 | 306.1812 | | | 334.1761 | | | 278.1499 | 292.1656 | I | 1217.6161 | 1230.6365 | 1244.6521 | 1275.6943 | 1258.6678 | 125 |
| 4 | 60.0444 | 393.2132 | | 375.2027 | 421.2082 | | 403.1976 | 377.2183 | | S | 1130.5841 | 1129.5888 | | ***1162.6103*** | 1145.5837 | 114 |
| 5 | 70.0651 | 490.2660 | | 472.2554 | 518.2609 | | 500.2504 | 464.2504 | | P | 1033.5313 | 1032.5360 | | ***1075.5782*** | 1058.5517 | 105 |
| 6 | 101.0709 | 618.3246 | 601.2980 | 600.3140 | 646.3195 | 629.2930 | 628.3089 | 561.3031 | | Q | 905.4727 | 904.4775 | | 978.5255 | 961.4989 | 96 |
| 7 | 120.0808 | 765.3930 | 748.3665 | 747.3824 | 793.3879 | 776.3614 | 775.3774 | | | F | 758.4043 | | | ***850.4669*** | 833.4403 | 83 |
| 8 | 72.0808 | 864.4614 | 847.4349 | 846.4509 | 892.4563 | 875.4298 | 874.4458 | 850.4458 | | V | 659.3359 | 672.3563 | | ***703.3985*** | 686.3719 | 68 |
| 9 | 74.0600 | 965.5091 | 948.4825 | 947.4985 | 993.5040 | 976.4775 | 975.4934 | 949.5142 | 951.4934 | T | 558.2882 | 571.3086 | 573.2879 | ***604.3301*** | 587.3035 | 58 |
| 10 | 87.0553 | 1079.5520 | 1062.5255 | 1061.5415 | 1107.5469 | 1090.5204 | 1089.5364 | 1036.5462 | | N | 444.2453 | 443.2500 | | ***503.2824*** | 486.2558 | 48 |
| 11 | 86.0964 | 1192.6361 | 1175.6095 | 1174.6255 | 1220.6310 | 1203.6045 | 1202.6204 | 1150.5891 | | L | 331.1612 | 330.1660 | | 389.2395 | 372.2129 | 37 |
| 12 | 102.0550 | 1321.6787 | 1304.6521 | 1303.6681 | 1349.6736 | 1332.6470 | 1331.6630 | 1263.6732 | | E | 202.1186 | 201.1234 | | 276.1554 | 259.1288 | 25 |
| 13 | 101.1073 | | | | | | | | | K | 74.0237 | 73.0284 | | 147.1128 | 130.0863 | |

| Seq | ya | yb | Seq | ya | yb | Seq | ya | yb |
|---|---|---|---|---|---|---|---|---|
| YI | 249.1598 | 277.1547 | YIS | 336.1918 | 364.1867 | YISP | 433.2445 | 461.2395 |
| YISPQ | 561.3031 | 589.2980 | IS | 173.1285 | 201.1234 | ISP | 270.1812 | 298.1761 |
| ISPQ | 398.2398 | 426.2347 | ISPQF | 545.3082 | 573.3031 | ISPQFV | 644.3766 | 672.3715 |
| SP | 157.0972 | 185.0921 | SPQ | 285.1557 | 313.1506 | SPQF | 432.2241 | 460.2191 |
| SPQFV | 531.2926 | 559.2875 | SPQFVT | 632.3402 | 660.3352 | PQ | 198.1237 | 226.1186 |
| PQF | 345.1921 | 373.1870 | PQFV | 444.2605 | 472.2554 | PQFVT | 545.3082 | 573.3031 |
| PQFVTN | 659.3511 | 687.3461 | QF | 248.1394 | 276.1343 | QFV | 347.2078 | 375.2027 |
| QFVT | 448.2554 | 476.2504 | QFVTN | 562.2984 | 590.2933 | QFVTNL | 675.3824 | 703.3774 |

| | | | | | | | | |
|---|---|---|---|---|---|---|---|---|
| **FV** | 219.1492 | 247.1441 | **FVT** | 320.1969 | 348.1918 | **FVTN** | 434.2398 | 462.2347 |
| **FVTNL** | 547.3239 | 575.3188 | **FVTNLE** | 676.3665 | 704.3614 | **VT** | 173.1285 | 201.1234 |
| **VTN** | 287.1714 | 315.1663 | **VTNL** | 400.2554 | 428.2504 | **VTNLE** | 529.2980 | 557.2930 |
| **TN** | 188.1030 | 216.0979 | **TNL** | 301.1870 | 329.1819 | **TNLE** | 430.2296 | 458.2245 |
| **NL** | 200.1394 | 228.1343 | **NLE** | 329.1819 | 357.1769 | **LE** | 215.1390 | 243.1339 |

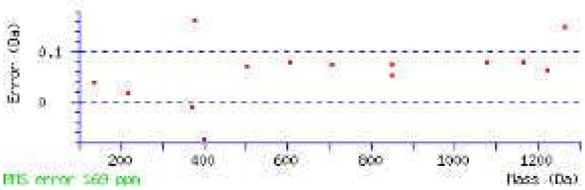


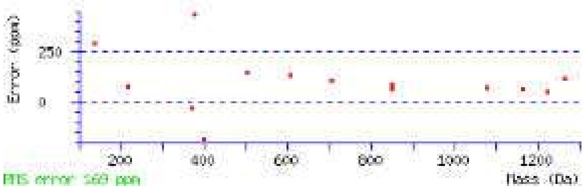


NCBI **BLAST** search of GYISPQFVTNLEK
(Parameters: blastp, nr protein database, expect=20000, no filter, PAM30)
Other BLAST web gateways

**All matches to this query**

| Score | Mr(calc) | Delta | Sequence |
|---|---|---|---|
| 19.2 | 1494.7718 | 0.0433 | GYISPQFVTNLEK |
| 18.4 | 1494.7718 | 0.0433 | FQPEKYVLNTEK |
| 10.2 | 1494.8770 | -0.0618 | GGDVISRVIPLLEK |
| 9.1 | 1494.7718 | 0.0433 | YVAPENGGLLAYTK |
| 8.4 | 1494.7235 | 0.0916 | SKALESDEVMAATK |
| 8.4 | 1494.7790 | 0.0361 | TLASEPPPGQNKTR |
| 8.3 | 1494.7712 | 0.0439 | MTTSTLRTTQDLK |
| 7.9 | 1494.7653 | 0.0498 | SLGYPGAMKWLTR |
| 7.6 | 1494.7565 | 0.0586 | QSTEKEVLAYAEK |
| 7.1 | 1494.7534 | 0.0617 | TMRNAMAITLTEK |

**Mascot:** http://www.matrixscience.com/

# MATRIX SCIENCE Mascot Search Results

## Peptide View Spot no 90

MS/MS Fragmentation of **AIELYDPMENAGAALIR**
Found in **gi|115488160** in **NCBInr**, Os12g0277500 [Oryza sativa Japonica Group]

Match to Query 63: 1846.017124 from(1847.024400,1+) intensity(0.0000) index(21)
Title: Label: D12, Spot_Id: 219853, Peak_List_Id: 227218, MSMS Job_Run_Id: 21955, Comment:
Data file C:\Documents and Settings\Administrator\Desktop\Farah-Garima 11May 2013\ppw_D12_136868220000.txt

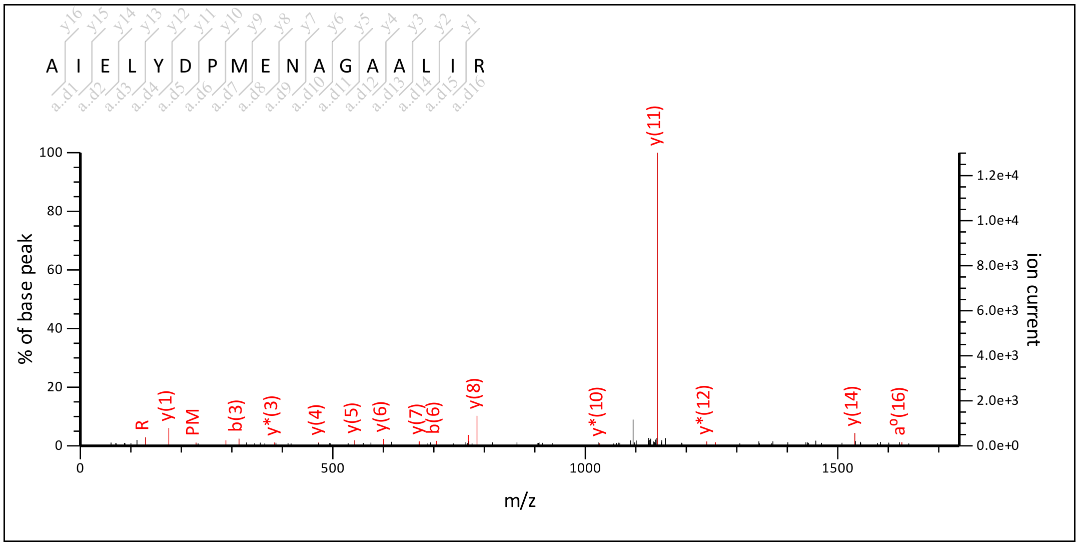


0 to 1740.62

Label all possible matches ○ Label matches used for scoring ◉

**Monoisotopic mass of neutral peptide Mr(calc):** 1845.9294
**Fixed modifications:** Carbamidomethyl (C) (apply to specified residues or termini only)
**Ions Score:** 65 **Expect:** 0.0073
**Matches :** 28/293 fragment ions using 32 most intense peaks (help)

| # | Immon. | a | a* | $a^0$ | b | b* | $b^0$ | d | d' | Seq. | v | w | w' | y | y* | |
|---|---|---|---|---|---|---|---|---|---|---|---|---|---|---|---|---|
| 1 | 44.0495 | 44.0495 | | | 72.0444 | | | 44.0495 | | A | | | | | | |
| 2 | 86.0964 | 157.1335 | | | 185.1285 | | | 129.1022 | 143.1179 | I | 1717.8214 | 1730.8418 | 1744.8574 | 1775.8996 | 1758.8731 | [illegible] |
| 3 | 102.0550 | 286.1761 | | 268.1656 | **314.1710** | | 296.1605 | 228.1707 | | E | 1588.7788 | 1587.7836 | | 1662.8156 | 1645.7890 | [illegible] |
| 4 | 86.0964 | 399.2602 | | 381.2496 | 427.2551 | | 409.2445 | 357.2132 | | L | 1475.6947 | 1474.6995 | | ***1533.7730*** | 1516.7464 | [illegible] |
| 5 | 136.0757 | 562.3235 | | 544.3130 | 590.3184 | | 572.3079 | | | Y | 1312.6314 | | | 1420.6889 | 1403.6624 | [illegible] |
| 6 | 88.0393 | 677.3505 | | 659.3399 | **705.3454** | | 687.3348 | 633.3606 | | D | 1197.6045 | 1196.6092 | | ***1257.6256*** | 1240.5990 | [illegible] |
| 7 | 70.0651 | 774.4032 | | 756.3927 | 802.3981 | | 784.3876 | 748.3876 | | P | 1100.5517 | 1099.5565 | | ***1142.5987*** | 1125.5721 | [illegible] |
| 8 | 104.0528 | 905.4437 | | 887.4332 | 933.4386 | | 915.4281 | 845.4403 | | M | 969.5112 | 968.5160 | | 1045.5459 | 1028.5193 | [illegible] |
| 9 | 102.0550 | 1034.4863 | | 1016.4757 | 1062.4812 | | 1044.4707 | 976.4808 | | E | 840.4686 | 839.4734 | | 914.5054 | 897.4789 | [illegible] |
| 10 | 87.0553 | 1148.5292 | 1131.5027 | 1130.5187 | 1176.5242 | 1159.4976 | 1158.5136 | 1105.5234 | | N | 726.4257 | 725.4305 | | ***785.4628*** | 768.4363 | |
| 11 | 44.0495 | 1219.5664 | 1202.5398 | 1201.5558 | 1247.5613 | 1230.5347 | 1229.5507 | | | A | 655.3886 | | | ***671.4199*** | 654.3933 | |
| 12 | 30.0338 | 1276.5878 | 1259.5613 | 1258.5772 | 1304.5827 | 1287.5562 | 1286.5722 | | | G | | | | ***600.3828*** | 583.3562 | |
| 13 | 44.0495 | 1347.6249 | 1330.5984 | 1329.6144 | 1375.6198 | 1358.5933 | 1357.6093 | | | A | 527.3300 | | | ***543.3613*** | 526.3348 | |
| 14 | 44.0495 | 1418.6620 | 1401.6355 | 1400.6515 | 1446.6570 | 1429.6304 | 1428.6464 | | | A | 456.2929 | | | ***472.3242*** | 455.2976 | |
| 15 | 86.0964 | 1531.7461 | 1514.7196 | 1513.7355 | 1559.7410 | 1542.7145 | 1541.7305 | 1489.6992 | | L | 343.2088 | 342.2136 | | 401.2871 | 384.2605 | |
| 16 | 86.0964 | 1644.8302 | 1627.8036 | 1626.8196 | 1672.8251 | 1655.7985 | 1654.8145 | 1616.7989 | 1630.8145 | I | 230.1248 | 243.1452 | 257.1608 | ***288.2030*** | 271.1765 | |
| 17 | 129.1135 | | | | | | | | | R | 74.0237 | 73.0284 | | ***175.1190*** | 158.0924 | |

| Seq | ya | yb | Seq | ya | yb | Seq | ya | yb |
|---|---|---|---|---|---|---|---|---|
| **IE** | 215.1390 | 243.1339 | **IEL** | 328.2231 | 356.2180 | **IELY** | 491.2864 | 519.2813 |
| **IELYD** | 606.3134 | 634.3083 | **EL** | 215.1390 | 243.1339 | **ELY** | 378.2023 | 406.1973 |
| **ELYD** | 493.2293 | 521.2242 | **ELYDP** | 590.2821 | 618.2770 | **LY** | 249.1598 | 277.1547 |
| **LYD** | 364.1867 | 392.1816 | **LYDP** | 461.2395 | 489.2344 | **LYDPM** | 592.2799 | 620.2749 |

| | | | | | | | | |
|---|---|---|---|---|---|---|---|---|
| **YD** | 251.1026 | 279.0975 | **YDP** | 348.1554 | 376.1503 | **YDPM** | 479.1959 | 507.1908 |
| **YDPME** | 608.2385 | 636.2334 | **DP** | 185.0921 | 213.0870 | **DPM** | 316.1326 | 344.1275 |
| **DPME** | 445.1751 | 473.1701 | **DPMEN** | 559.2181 | 587.2130 | **DPMENA** | 630.2552 | 658.2501 |
| **DPMENAG** | 687.2767 | 715.2716 | **PM** | 201.1056 | 229.1005 | **PME** | 330.1482 | 358.1431 |
| **PMEN** | 444.1911 | 472.1860 | **PMENA** | 515.2282 | 543.2232 | **PMENAG** | 572.2497 | 600.2446 |
| **PMENAGA** | 643.2868 | 671.2817 | **ME** | 233.0954 | 261.0904 | **MEN** | 347.1384 | 375.1333 |
| **MENA** | 418.1755 | 446.1704 | **MENAG** | 475.1969 | 503.1919 | **MENAGA** | 546.2341 | 574.2290 |
| **MENAGAA** | 617.2712 | 645.2661 | **EN** | 216.0979 | 244.0928 | **ENA** | 287.1350 | 315.1299 |
| **ENAG** | 344.1565 | 372.1514 | **ENAGA** | 415.1936 | 443.1885 | **ENAGAA** | 486.2307 | 514.2256 |
| **ENAGAAL** | 599.3148 | 627.3097 | **NA** | 158.0924 | 186.0873 | **NAG** | 215.1139 | 243.1088 |
| **NAGA** | 286.1510 | 314.1459 | **NAGAA** | 357.1881 | 385.1830 | **NAGAAL** | 470.2722 | 498.2671 |
| **NAGAALI** | 583.3562 | 611.3511 | **AG** | 101.0709 | 129.0659 | **AGA** | 172.1081 | 200.1030 |
| **AGAA** | 243.1452 | 271.1401 | **AGAAL** | 356.2292 | 384.2241 | **AGAALI** | 469.3133 | 497.3082 |
| **GA** | 101.0709 | 129.0659 | **GAA** | 172.1081 | 200.1030 | **GAAL** | 285.1921 | 313.1870 |
| **GAALI** | 398.2762 | 426.2711 | **AA** | 115.0866 | 143.0815 | **AAL** | 228.1707 | 256.1656 |
| **AALI** | 341.2547 | 369.2496 | **AL** | 157.1335 | 185.1285 | **ALI** | 270.2176 | 298.2125 |
| **LI** | 199.1805 | 227.1754 | | | | | | |

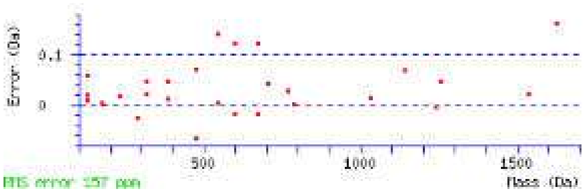


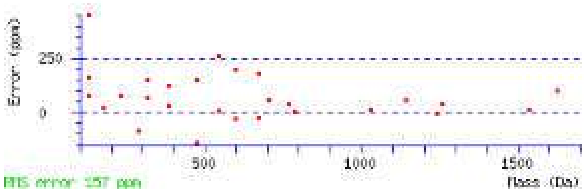


NCBI **BLAST** search of AIELYDPMENAGAALIR
(Parameters: blastp, nr protein database, expect=20000, no filter, PAM30)
Other BLAST web gateways

**All matches to this query**

| Score | Mr(calc) | Delta | Sequence |
|---|---|---|---|
| 65.4 | 1845.9294 | 0.0877 | AIELYDPMENAGAALIR |
| 28.4 | 1845.9155 | 0.1016 | CIVINAPAFRDNTEAR |
| 28.3 | 1845.8890 | 0.1281 | RMEASLDNENAKPDLK |
| 24.8 | 1845.9230 | 0.0942 | LDAPEMCPVFAGRVIR |
| 24.2 | 1846.0101 | 0.0071 | QAIYTAAFAAAPAGAALLR |
| 19.6 | 1845.9149 | 0.1023 | AELLYDVIDKSDFYR |
| 19.2 | 1845.8969 | 0.1202 | RWISEIENNGTATEAR |
| 17.7 | 1845.8527 | 0.1644 | DVNMGGDTPVSAAALQER |
| 17.4 | 1845.8817 | 0.1354 | VTLGGDGAEDARATTAADR |
| 17.2 | 1845.8832 | 0.1339 | GFDCGPANALLDAWALR |

**Mascot:** http://www.matrixscience.com/

# MATRIX SCIENCE Mascot Search Results

## Peptide View Spot no 90

MS/MS Fragmentation of **AIELYDPMENAGAALIR**
Found in **gi|115488160** in **NCBInr**, Os12g0277500 [Oryza sativa Japonica Group]

Match to Query 65: 1862.005224 from(1863.012500,1+) intensity(0.0000) index(22)
Title: Label: D12, Spot_Id: 219853, Peak_List_Id: 227217, MSMS Job_Run_Id: 21955, Comment:
Data file C:\Documents and Settings\Administrator\Desktop\Farah-Garima 11May 2013\ppw_D12_136868220000.txt

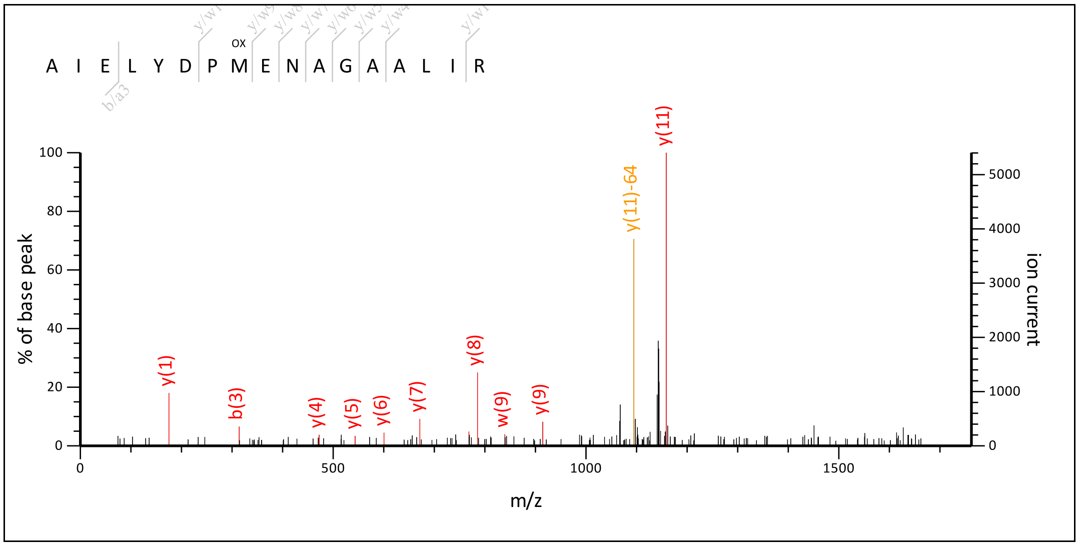


0 to 1762.25

Label all possible matches ○ Label matches used for scoring ◉

**Monoisotopic mass of neutral peptide Mr(calc):** 1861.9243
**Fixed modifications:** Carbamidomethyl (C) (apply to specified residues or termini only)
**Variable modifications:**
**M8** : Oxidation (M), with neutral losses 0.0000(shown in table), 63.9983
**Ions Score:** 33 **Expect:** 13
**Matches** : 14/419 fragment ions using 32 most intense peaks (help)

| # | Immon. | a | a* | a0 | b | b* | b0 | d | d' | Seq. | v | w | w' | y | y* | |
|---|---|---|---|---|---|---|---|---|---|---|---|---|---|---|---|---|
| 1 | 44.0495 | 44.0495 | | | 72.0444 | | | 44.0495 | | A | | | | | | |
| 2 | 86.0964 | 157.1335 | | | 185.1285 | | | 129.1022 | 143.1179 | I | 1733.8163 | 1746.8367 | 1760.8524 | 1791.8946 | 1774.8680 | [illegible] |
| 3 | 102.0550 | 286.1761 | | 268.1656 | 314.1710 | | 296.1605 | 228.1707 | | E | 1604.7737 | 1603.7785 | | 1678.8105 | 1661.7839 | [illegible] |
| 4 | 86.0964 | 399.2602 | | 381.2496 | 427.2551 | | 409.2445 | 357.2132 | | L | 1491.6897 | 1490.6944 | | 1549.7679 | 1532.7414 | [illegible] |
| 5 | 136.0757 | 562.3235 | | 544.3130 | 590.3184 | | 572.3079 | | | Y | 1328.6263 | | | 1436.6838 | 1419.6573 | [illegible] |
| 6 | 88.0393 | 677.3505 | | 659.3399 | 705.3454 | | 687.3348 | 633.3606 | | D | 1213.5994 | 1212.6041 | | 1273.6205 | 1256.5940 | [illegible] |
| 7 | 70.0651 | 774.4032 | | 756.3927 | 802.3981 | | 784.3876 | 748.3876 | | P | 1116.5466 | 1115.5514 | | ***1158.5936*** | 1141.5670 | [illegible] |
| 8 | 120.0478 | 921.4386 | | 903.4281 | 949.4335 | | 931.4230 | 845.4403 | | M | 969.5112 | 968.5160 | | 1061.5408 | 1044.5143 | [illegible] |
| 9 | 102.0550 | 1050.4812 | | 1032.4707 | 1078.4761 | | 1060.4656 | 992.4757 | | E | 840.4686 | 839.4734 | | ***914.5054*** | 897.4789 | [illegible] |
| 10 | 87.0553 | 1164.5242 | 1147.4976 | 1146.5136 | 1192.5191 | 1175.4925 | 1174.5085 | 1121.5183 | | N | 726.4257 | 725.4305 | | ***785.4628*** | 768.4363 | |
| 11 | 44.0495 | 1235.5613 | 1218.5347 | 1217.5507 | 1263.5562 | 1246.5296 | 1245.5456 | | | A | 655.3886 | | | ***671.4199*** | 654.3933 | |
| 12 | 30.0338 | 1292.5827 | 1275.5562 | 1274.5722 | 1320.5776 | 1303.5511 | 1302.5671 | | | G | | | | ***600.3828*** | 583.3562 | |
| 13 | 44.0495 | 1363.6198 | 1346.5933 | 1345.6093 | 1391.6148 | 1374.5882 | 1373.6042 | | | A | 527.3300 | | | ***543.3613*** | 526.3348 | |
| 14 | 44.0495 | 1434.6570 | 1417.6304 | 1416.6464 | 1462.6519 | 1445.6253 | 1444.6413 | | | A | 456.2929 | | | ***472.3242*** | 455.2976 | |
| 15 | 86.0964 | 1547.7410 | 1530.7145 | 1529.7305 | 1575.7359 | 1558.7094 | 1557.7254 | 1505.6941 | | L | 343.2088 | 342.2136 | | 401.2871 | 384.2605 | |
| 16 | 86.0964 | 1660.8251 | 1643.7985 | 1642.8145 | 1688.8200 | 1671.7935 | 1670.8094 | 1632.7938 | 1646.8094 | I | 230.1248 | 243.1452 | 257.1608 | 288.2030 | 271.1765 | |
| 17 | 129.1135 | | | | | | | | | R | 74.0237 | 73.0284 | | ***175.1190*** | 158.0924 | |

| Seq | ya | yb | Seq | ya | yb | Seq | ya | yb |
|---|---|---|---|---|---|---|---|---|
| IE | 215.1390 | 243.1339 | IEL | 328.2231 | 356.2180 | IELY | 491.2864 | 519.2813 |
| | | | | | | | | |

| | | | | | | | | |
|---|---|---|---|---|---|---|---|---|
| **IELYD** | 606.3134 | 634.3083 | **EL** | 215.1390 | 243.1339 | **ELY** | 378.2023 | 406.1973 |
| **ELYD** | 493.2293 | 521.2242 | **ELYDP** | 590.2821 | 618.2770 | **LY** | 249.1598 | 277.1547 |
| **LYD** | 364.1867 | 392.1816 | **LYDP** | 461.2395 | 489.2344 | **LYDPM** | 608.2749 | 636.2698 |
| **YD** | 251.1026 | 279.0975 | **YDP** | 348.1554 | 376.1503 | **YDPM** | 495.1908 | 523.1857 |
| **YDPME** | 624.2334 | 652.2283 | **DP** | 185.0921 | 213.0870 | **DPM** | 332.1275 | 360.1224 |
| **DPME** | 461.1701 | 489.1650 | **DPMEN** | 575.2130 | 603.2079 | **DPMENA** | 646.2501 | 674.2450 |
| **PM** | 217.1005 | 245.0954 | **PME** | 346.1431 | 374.1380 | **PMEN** | 460.1860 | 488.1810 |
| **PMENA** | 531.2232 | 559.2181 | **PMENAG** | 588.2446 | 616.2395 | **PMENAGA** | 659.2817 | 687.2767 |
| **ME** | 249.0904 | 277.0853 | **MEN** | 363.1333 | 391.1282 | **MENA** | 434.1704 | 462.1653 |
| **MENAG** | 491.1919 | 519.1868 | **MENAGA** | 562.2290 | 590.2239 | **MENAGAA** | 633.2661 | 661.2610 |
| **EN** | 216.0979 | 244.0928 | **ENA** | 287.1350 | 315.1299 | **ENAG** | 344.1565 | 372.1514 |
| **ENAGA** | 415.1936 | 443.1885 | **ENAGAA** | 486.2307 | 514.2256 | **ENAGAAL** | 599.3148 | 627.3097 |
| **NA** | 158.0924 | 186.0873 | **NAG** | 215.1139 | 243.1088 | **NAGA** | 286.1510 | 314.1459 |
| **NAGAA** | 357.1881 | 385.1830 | **NAGAAL** | 470.2722 | 498.2671 | **NAGAALI** | 583.3562 | 611.3511 |
| **AG** | 101.0709 | 129.0659 | **AGA** | 172.1081 | 200.1030 | **AGAA** | 243.1452 | 271.1401 |
| **AGAAL** | 356.2292 | 384.2241 | **AGAALI** | 469.3133 | 497.3082 | **GA** | 101.0709 | 129.0659 |
| **GAA** | 172.1081 | 200.1030 | **GAAL** | 285.1921 | 313.1870 | **GAALI** | 398.2762 | 426.2711 |
| **AA** | 115.0866 | 143.0815 | **AAL** | 228.1707 | 256.1656 | **AALI** | 341.2547 | 369.2496 |
| **AL** | 157.1335 | 185.1285 | **ALI** | 270.2176 | 298.2125 | **LI** | 199.1805 | 227.1754 |

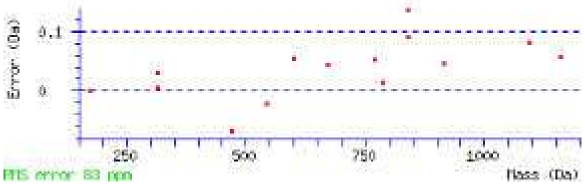


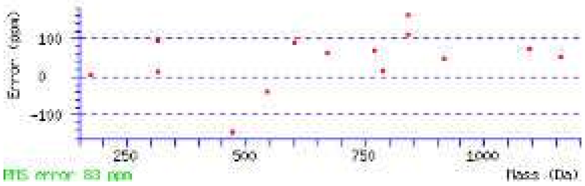


NCBI **BLAST** search of AIELYDPMENAGAALIR
(Parameters: blastp, nr protein database, expect=20000, no filter, PAM30)
Other BLAST web gateways

**All matches to this query**

| Score | Mr(calc) | Delta | Sequence |
|---|---|---|---|
| 33.0 | 1861.9243 | 0.0809 | AIELYDPMENAGAALIR |
| 15.5 | 1862.0737 | -0.0685 | GAGIIRVDIEPAGAALIAR |
| 14.2 | 1862.0513 | -0.0460 | VLAEAPAPVTEALEALIR |
| 13.7 | 1862.0196 | -0.0144 | SQMAAALLTHPAAGAVVVR |
| 13.4 | 1861.9871 | 0.0181 | SGGPAGGERPGTAPAGRVLR |
| 11.8 | 1861.9356 | 0.0696 | GAKTALENEMPNVGFLR |
| 11.4 | 1862.0951 | -0.0898 | SSLLASIIFILAIVGMAK |
| 10.2 | 1862.0486 | -0.0434 | LSGQNAGAAALPAVTLRPR |
| 10.0 | 1861.8961 | 0.1091 | MMKVSQNATSVTIHCR |
| 9.9 | 1861.9641 | 0.0411 | DSAILLTVIMLSACSPR |

**Mascot:** http://www.matrixscience.com/

# MATRIX SCIENCE Mascot Search Results

## Peptide View **Spot no 90**

MS/MS Fragmentation of **DLGLLVENATEEQLGTAR**
Found in **gi|115488160** in **NCBInr**, Os12g0277500 [Oryza sativa Japonica Group]

Match to Query 67: 1928.089624 from(1929.096900,1+) intensity(0.0000) index(23)
Title: Label: D12, Spot_Id: 219853, Peak_List_Id: 227213, MSMS Job_Run_Id: 21955, Comment:
Data file C:\Documents and Settings\Administrator\Desktop\Farah-Garima 11May 2013\ppw_D12_136868220000.txt

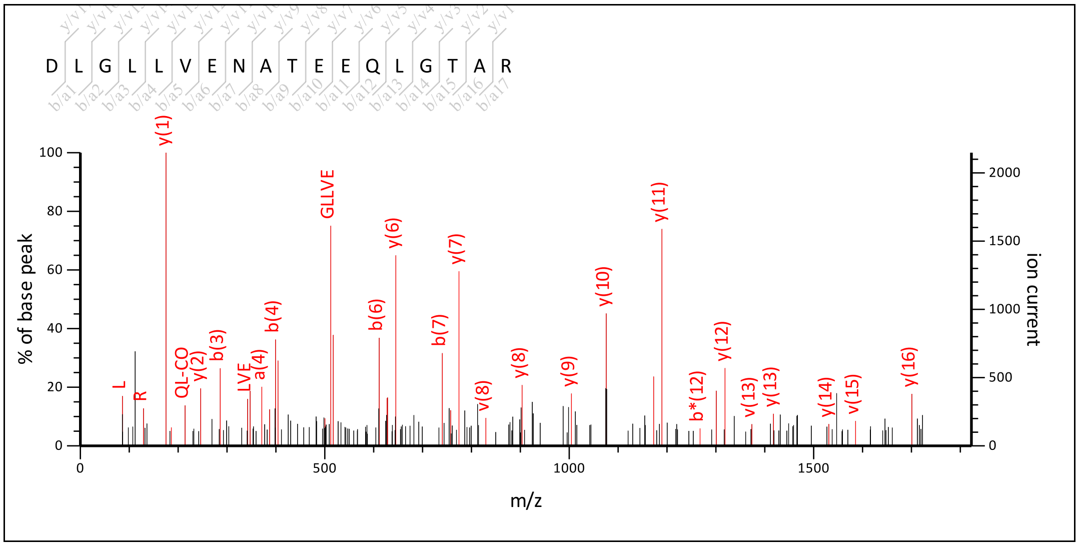


0 to 1822.72

Label all possible matches ○ Label matches used for scoring ◉

**Monoisotopic mass of neutral peptide Mr(calc):** 1927.9851
**Fixed modifications:** Carbamidomethyl (C) (apply to specified residues or termini only)
**Ions Score:** 103 **Expect:** 1e-06
**Matches :** 48/332 fragment ions using 68 most intense peaks (help)

| # | Immon. | a | a* | a⁰ | b | b* | b⁰ | d | d' | Seq. | v | w | w' | y | y* | |
|---|---|---|---|---|---|---|---|---|---|---|---|---|---|---|---|---|
| 1 | 88.0393 | 88.0393 | | 70.0287 | 116.0342 | | 98.0237 | 44.0495 | | D | | | | | | |
| 2 | 86.0964 | 201.1234 | | 183.1128 | 229.1183 | | 211.1077 | 159.0764 | | L | 1755.8872 | 1754.8919 | | 1813.9654 | 1796.9389 | [illegible] |
| 3 | 30.0338 | 258.1448 | | 240.1343 | **286.1397** | | 268.1292 | | | G | | | | **1700.8814** | 1683.8548 | [illegible] |
| 4 | 86.0964 | 371.2289 | | 353.2183 | **399.2238** | | 381.2132 | 329.1819 | | L | 1585.7816 | 1584.7864 | | 1643.8599 | 1626.8333 | [illegible] |
| 5 | 86.0964 | 484.3130 | | 466.3024 | **512.3079** | | 494.2973 | 442.2660 | | L | 1472.6976 | 1471.7023 | | **1530.7758** | 1513.7493 | [illegible] |
| 6 | 72.0808 | 583.3814 | | 565.3708 | **611.3763** | | 593.3657 | 569.3657 | | V | 1373.6292 | 1386.6496 | | **1417.6918** | 1400.6652 | [illegible] |
| 7 | 102.0550 | 712.4240 | | 694.4134 | **740.4189** | | 722.4083 | 654.4185 | | E | 1244.5866 | 1243.5913 | | **1318.6233** | 1301.5968 | [illegible] |
| 8 | 87.0553 | 826.4669 | 809.4403 | 808.4563 | 854.4618 | 837.4353 | 836.4512 | 783.4611 | | N | 1130.5436 | 1129.5484 | | **1189.5808** | 1172.5542 | [illegible] |
| 9 | 44.0495 | 897.5040 | 880.4775 | 879.4934 | 925.4989 | 908.4724 | 907.4884 | | | A | 1059.5065 | | | **1075.5378** | 1058.5113 | [illegible] |
| 10 | 74.0600 | 998.5517 | 981.5251 | 980.5411 | 1026.5466 | 1009.5201 | 1008.5360 | 982.5568 | 984.5360 | T | 958.4589 | 971.4793 | 973.4585 | **1004.5007** | 987.4742 | [illegible] |
| 11 | 102.0550 | 1127.5943 | 1110.5677 | 1109.5837 | 1155.5892 | 1138.5626 | 1137.5786 | 1069.5888 | | E | 829.4163 | 828.4210 | | **903.4530** | 886.4265 | [illegible] |
| 12 | 102.0550 | 1256.6369 | 1239.6103 | 1238.6263 | 1284.6318 | 1267.6052 | 1266.6212 | 1198.6314 | | E | 700.3737 | 699.3784 | | **774.4104** | 757.3839 | [illegible] |
| 13 | 101.0709 | 1384.6955 | 1367.6689 | 1366.6849 | 1412.6904 | 1395.6638 | 1394.6798 | 1327.6740 | | Q | 572.3151 | 571.3198 | | **645.3679** | 628.3413 | [illegible] |
| 14 | 86.0964 | 1497.7795 | 1480.7530 | 1479.7689 | 1525.7744 | 1508.7479 | 1507.7639 | 1455.7326 | | L | 459.2310 | 458.2358 | | **517.3093** | 500.2827 | [illegible] |
| 15 | 30.0338 | 1554.8010 | 1537.7744 | 1536.7904 | 1582.7959 | 1565.7693 | 1564.7853 | | | G | | | | **404.2252** | 387.1987 | [illegible] |
| 16 | 74.0600 | 1655.8487 | 1638.8221 | 1637.8381 | 1683.8436 | 1666.8170 | 1665.8330 | 1639.8537 | 1641.8330 | T | 301.1619 | 314.1823 | 316.1615 | **347.2037** | 330.1772 | [illegible] |
| 17 | 44.0495 | 1726.8858 | 1709.8592 | 1708.8752 | 1754.8807 | 1737.8541 | 1736.8701 | | | A | 230.1248 | | | **246.1561** | 229.1295 | |
| 18 | 129.1135 | | | | | | | | | R | 74.0237 | 73.0284 | | **175.1190** | 158.0924 | |

| Seq | ya | yb | Seq | ya | yb | Seq | ya | yb |
|---|---|---|---|---|---|---|---|---|
| LG | 143.1179 | 171.1128 | LGL | 256.2020 | 284.1969 | LGLL | 369.2860 | 397.2809 |
| LGLLV | 468.3544 | 496.3493 | LGLLVE | 597.3970 | 625.3919 | GL | 143.1179 | 171.1128 |
| GLL | 256.2020 | 284.1969 | GLLV | 355.2704 | 383.2653 | GLLVE | 484.3130 | 512.3079 |

| | | | | | | | | |
|---|---|---|---|---|---|---|---|---|
| **GLLVEN** | 598.3559 | 626.3508 | **GLLVENA** | 669.3930 | 697.3879 | **LL** | 199.1805 | 227.1754 |
| **LLV** | 298.2489 | 326.2438 | **LLVE** | 427.2915 | 455.2864 | **LLVEN** | 541.3344 | 569.3293 |
| **LLVENA** | 612.3715 | 640.3665 | **LV** | 185.1648 | 213.1598 | **LVE** | 314.2074 | 342.2023 |
| **LVEN** | 428.2504 | 456.2453 | **LVENA** | 499.2875 | 527.2824 | **LVENAT** | 600.3352 | 628.3301 |
| **VE** | 201.1234 | 229.1183 | **VEN** | 315.1663 | 343.1612 | **VENA** | 386.2034 | 414.1983 |
| **VENAT** | 487.2511 | 515.2460 | **VENATE** | 616.2937 | 644.2886 | **EN** | 216.0979 | 244.0928 |
| **ENA** | 287.1350 | 315.1299 | **ENAT** | 388.1827 | 416.1776 | **ENATE** | 517.2253 | 545.2202 |
| **ENATEE** | 646.2679 | 674.2628 | **NA** | 158.0924 | 186.0873 | **NAT** | 259.1401 | 287.1350 |
| **NATE** | 388.1827 | 416.1776 | **NATEE** | 517.2253 | 545.2202 | **NATEEQ** | 645.2838 | 673.2788 |
| **AT** | 145.0972 | 173.0921 | **ATE** | 274.1397 | 302.1347 | **ATEE** | 403.1823 | 431.1773 |
| **ATEEQ** | 531.2409 | 559.2358 | **ATEEQL** | 644.3250 | 672.3199 | **TE** | 203.1026 | 231.0975 |
| **TEE** | 332.1452 | 360.1401 | **TEEQ** | 460.2038 | 488.1987 | **TEEQL** | 573.2879 | 601.2828 |
| **TEEQLG** | 630.3093 | 658.3042 | **EE** | 231.0975 | 259.0925 | **EEQ** | 359.1561 | 387.1510 |
| **EEQL** | 472.2402 | 500.2351 | **EEQLG** | 529.2617 | 557.2566 | **EEQLGT** | 630.3093 | 658.3042 |
| **EQ** | 230.1135 | 258.1084 | **EQL** | 343.1976 | 371.1925 | **EQLG** | 400.2191 | 428.2140 |
| **EQLGT** | 501.2667 | 529.2617 | **EQLGTA** | 572.3039 | 600.2988 | **QL** | 214.1550 | 242.1499 |
| **QLG** | 271.1765 | 299.1714 | **QLGT** | 372.2241 | 400.2191 | **QLGTA** | 443.2613 | 471.2562 |
| **LG** | 143.1179 | 171.1128 | **LGT** | 244.1656 | 272.1605 | **LGTA** | 315.2027 | 343.1976 |
| **GT** | 131.0815 | 159.0764 | **GTA** | 202.1186 | 230.1135 | **TA** | 145.0972 | 173.0921 |

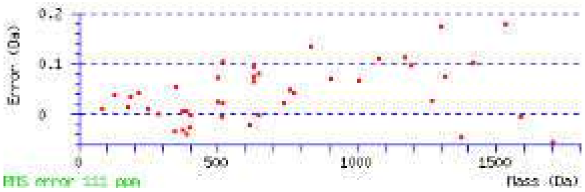


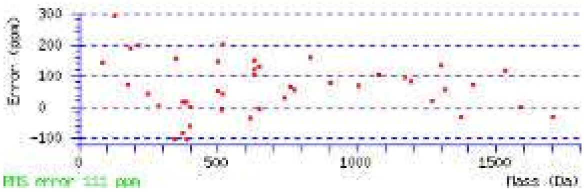


NCBI **BLAST** search of DLGLLVENATEEQLGTAR
(Parameters: blastp, nr protein database, expect=20000, no filter, PAM30)
Other BLAST web gateways

**All matches to this query**

| Score | Mr(calc) | Delta | Sequence |
|---|---|---|---|
| 103.2 | 1927.9851 | 0.1046 | DLGLLVENATEEQLGTAR |
| 42.6 | 1927.9673 | 0.1223 | DILAVVECVVGEEGANVR |
| 29.5 | 1928.0367 | 0.0529 | DIGIIIDYLRELQPDR |
| 26.1 | 1928.0513 | 0.0383 | QGIVLMKNQGSLPLSTAR |
| 25.1 | 1927.9599 | 0.1297 | ILGNNVNTLSEDEQAGVR |
| 24.7 | 1927.9673 | 0.1223 | AIVDLDTNTVLMDLSHR |
| 24.2 | 1928.0149 | 0.0747 | ATILIVEDNEANMRLAR |
| 23.7 | 1927.9309 | 0.1587 | LAVDLPADLSCDLGGDAAR |
| 22.9 | 1927.9262 | 0.1634 | DTEIETITATNEVTYTK |
| 22.9 | 1927.9309 | 0.1587 | AAMGYTGSASIEELQTKR |

**Mascot:** http://www.matrixscience.com/

# MATRIX SCIENCE Mascot Search Results

## Peptide View Spot no 90

MS/MS Fragmentation of **ELSETDSIYDTEKLAER**
Found in **gi|115488160** in **NCBInr**, Os12g0277500 [Oryza sativa Japonica Group]

Match to Query 69: 1998.051924 from(1999.059200,1+) intensity(0.0000) index(24)
Title: Label: D12, Spot_Id: 219853, Peak_List_Id: 227219, MSMS Job_Run_Id: 21955, Comment:
Data file C:\Documents and Settings\Administrator\Desktop\Farah-Garima 11May 2013\ppw_D12_136868220000.txt

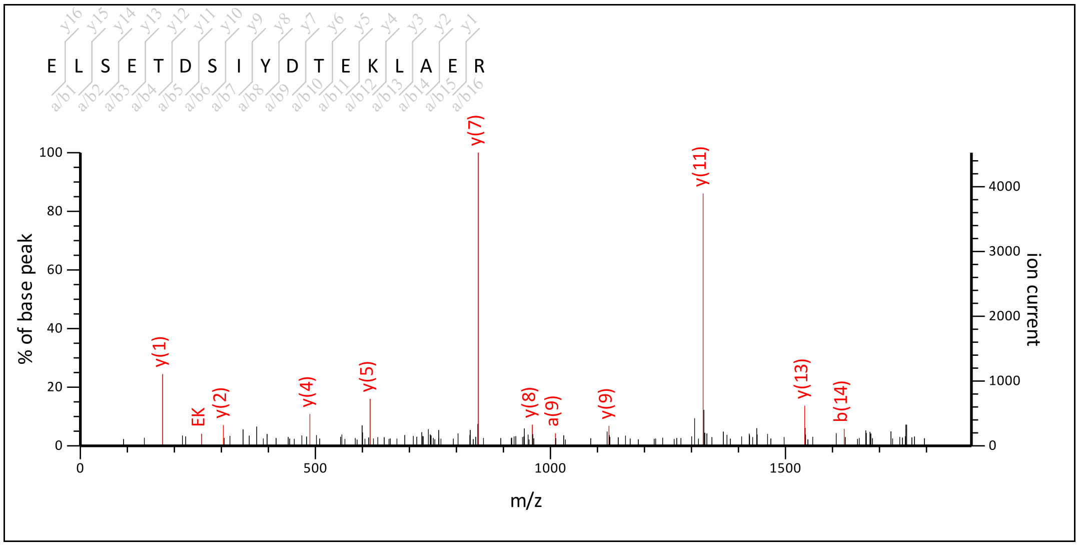


0 to 1895.61

Label all possible matches ○ Label matches used for scoring ◉

**Monoisotopic mass of neutral peptide Mr(calc):** 1997.9429
**Fixed modifications:** Carbamidomethyl (C) (apply to specified residues or termini only)
**Ions Score:** 58 **Expect:** 0.042
**Matches :** 14/300 fragment ions using 18 most intense peaks (help)

| # | Immon. | a | a* | a⁰ | b | b* | b⁰ | d | d' | Seq. | v | w | w' | y | y* |
|---|---|---|---|---|---|---|---|---|---|---|---|---|---|---|---|
| 1 | 102.0550 | 102.0550 | | 84.0444 | 130.0499 | | 112.0393 | 44.0495 | | E | | | | | |
| 2 | 86.0964 | 215.1390 | | 197.1285 | 243.1339 | | 225.1234 | 173.0921 | | L | 1811.8294 | 1810.8341 | | 1869.9076 | 1852.8811 |
| 3 | 60.0444 | 302.1710 | | 284.1605 | 330.1660 | | 312.1554 | 286.1761 | | S | 1724.7974 | 1723.8021 | | 1756.8236 | 1739.7970 |
| 4 | 102.0550 | 431.2136 | | 413.2031 | 459.2086 | | 441.1980 | 373.2082 | | E | 1595.7548 | 1594.7595 | | 1669.7915 | 1652.7650 |
| 5 | 74.0600 | 532.2613 | | 514.2508 | 560.2562 | | 542.2457 | 516.2664 | 518.2457 | T | 1494.7071 | 1507.7275 | 1509.7067 | ***1540.7489*** | 1523.7224 |
| 6 | 88.0393 | 647.2883 | | 629.2777 | 675.2832 | | 657.2726 | 603.2984 | | D | 1379.6801 | 1378.6849 | | 1439.7013 | 1422.6747 |
| 7 | 60.0444 | 734.3203 | | 716.3097 | 762.3152 | | 744.3046 | 718.3254 | | S | 1292.6481 | 1291.6529 | | ***1324.6743*** | 1307.6478 |
| 8 | 86.0964 | 847.4044 | | 829.3938 | 875.3993 | | 857.3887 | 819.3731 | 833.3887 | I | 1179.5640 | 1192.5844 | 1206.6001 | 1237.6423 | 1220.6157 |
| 9 | 136.0757 | 1010.4677 | | 992.4571 | 1038.4626 | | 1020.4520 | | | Y | 1016.5007 | | | ***1124.5582*** | 1107.5317 |
| 10 | 88.0393 | 1125.4946 | | 1107.4841 | 1153.4895 | | 1135.4790 | 1081.5048 | | D | 901.4738 | 900.4785 | | ***961.4949*** | 944.4684 |
| 11 | 74.0600 | 1226.5423 | | 1208.5317 | 1254.5372 | | 1236.5267 | 1210.5474 | 1212.5267 | T | 800.4261 | 813.4465 | 815.4258 | ***846.4680*** | 829.4414 |
| 12 | 102.0550 | 1355.5849 | | 1337.5743 | 1383.5798 | | 1365.5692 | 1297.5794 | | E | 671.3835 | 670.3883 | | 745.4203 | 728.3937 |
| 13 | 101.1073 | 1483.6799 | 1466.6533 | 1465.6693 | 1511.6748 | 1494.6482 | 1493.6642 | 1426.6220 | | K | 543.2885 | 542.2933 | | ***616.3777*** | 599.3511 |
| 14 | 86.0964 | 1596.7639 | 1579.7374 | 1578.7534 | 1624.7588 | 1607.7323 | 1606.7483 | 1554.7170 | | L | 430.2045 | 429.2092 | | ***488.2827*** | 471.2562 |
| 15 | 44.0495 | 1667.8010 | 1650.7745 | 1649.7905 | 1695.7960 | 1678.7694 | 1677.7854 | | | A | 359.1674 | | | 375.1987 | 358.1721 |
| 16 | 102.0550 | 1796.8436 | 1779.8171 | 1778.8331 | 1824.8385 | 1807.8120 | 1806.8280 | 1738.8382 | | E | 230.1248 | 229.1295 | | ***304.1615*** | 287.1350 |
| 17 | 129.1135 | | | | | | | | | R | 74.0237 | 73.0284 | | ***175.1190*** | 158.0924 |

| Seq | ya | yb | Seq | ya | yb | Seq | ya | yb |
|---|---|---|---|---|---|---|---|---|
| **LS** | 173.1285 | 201.1234 | **LSE** | 302.1710 | 330.1660 | **LSET** | 403.2187 | 431.2136 |
| **LSETD** | 518.2457 | 546.2406 | **LSETDS** | 605.2777 | 633.2726 | **SE** | 189.0870 | 217.0819 |
| **SET** | 290.1347 | 318.1296 | **SETD** | 405.1616 | 433.1565 | **SETDS** | 492.1936 | 520.1885 |
| **SETDSI** | 605.2777 | 633.2726 | **ET** | 203.1026 | 231.0975 | **ETD** | 318.1296 | 346.1245 |

| | | | | | | | | |
|---|---|---|---|---|---|---|---|---|
| **ETDS** | 405.1616 | 433.1565 | **ETDSI** | 518.2457 | 546.2406 | **ETDSIY** | 681.3090 | 709.3039 |
| **TD** | 189.0870 | 217.0819 | **TDS** | 276.1190 | 304.1139 | **TDSI** | 389.2031 | 417.1980 |
| **TDSIY** | 552.2664 | 580.2613 | **TDSIYD** | 667.2933 | 695.2883 | **DS** | 175.0713 | 203.0662 |
| **DSI** | 288.1554 | 316.1503 | **DSIY** | 451.2187 | 479.2136 | **DSIYD** | 566.2457 | 594.2406 |
| **DSIYDT** | 667.2933 | 695.2883 | **SI** | 173.1285 | 201.1234 | **SIY** | 336.1918 | 364.1867 |
| **SIYD** | 451.2187 | 479.2136 | **SIYDT** | 552.2664 | 580.2613 | **SIYDTE** | 681.3090 | 709.3039 |
| **IY** | 249.1598 | 277.1547 | **IYD** | 364.1867 | 392.1816 | **IYDT** | 465.2344 | 493.2293 |
| **IYDTE** | 594.2770 | 622.2719 | **YD** | 251.1026 | 279.0975 | **YDT** | 352.1503 | 380.1452 |
| **YDTE** | 481.1929 | 509.1878 | **YDTEK** | 609.2879 | 637.2828 | **DT** | 189.0870 | 217.0819 |
| **DTE** | 318.1296 | 346.1245 | **DTEK** | 446.2245 | 474.2195 | **DTEKL** | 559.3086 | 587.3035 |
| **DTEKLA** | 630.3457 | 658.3406 | **TE** | 203.1026 | 231.0975 | **TEK** | 331.1976 | 359.1925 |
| **TEKL** | 444.2817 | 472.2766 | **TEKLA** | 515.3188 | 543.3137 | **TEKLAE** | 644.3614 | 672.3563 |
| **EK** | 230.1499 | 258.1448 | **EKL** | 343.2340 | 371.2289 | **EKLA** | 414.2711 | 442.2660 |
| **EKLAE** | 543.3137 | 571.3086 | **KL** | 214.1914 | 242.1863 | **KLA** | 285.2285 | 313.2234 |
| **KLAE** | 414.2711 | 442.2660 | **LA** | 157.1335 | 185.1285 | **LAE** | 286.1761 | 314.1710 |
| **AE** | 173.0921 | 201.0870 | | | | | | |

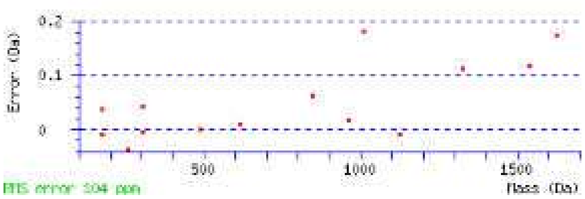


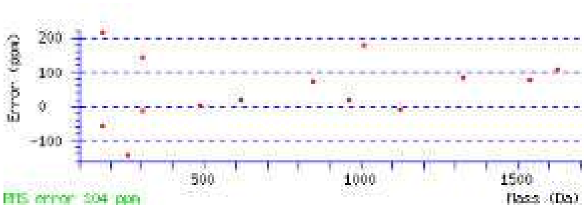


NCBI **BLAST** search of ELSETDSIYDTEKLAER
(Parameters: blastp, nr protein database, expect=20000, no filter, PAM30)
Other BLAST web gateways

**All matches to this query**

| Score | Mr(calc) | Delta | Sequence |
|---|---|---|---|
| 57.7 | 1997.9429 | 0.1090 | ELSETDSIYDTEKLAER |
| 28.5 | 1998.0786 | -0.0266 | KLDIQLFPPTTEQIAER |
| 22.1 | 1997.8744 | 0.1775 | IEMILDCPTDDKMTER |
| 21.1 | 1998.1837 | -0.1318 | IQSLTIRLLDLVTGTLSR |
| 20.0 | 1998.0105 | 0.0414 | SAHIWISSRDMVQLAER |
| 18.7 | 1997.8836 | 0.1683 | GLCQGQWHADLNTMPDR |
| 17.1 | 1997.8791 | 0.1728 | CVACVAKNADNMIETMR |
| 17.0 | 1998.0323 | 0.0197 | LQEQPWQGAIFAAAALER |
| 16.9 | 1998.0647 | -0.0127 | AQATALRVAGVDWATIAER |
| 16.9 | 1998.0316 | 0.0203 | LAARAPACNLDLLSDAATR |

**Mascot:** http://www.matrixscience.com/

# MATRIX SCIENCE Mascot Search Results

## Peptide View Spot no 90

MS/MS Fragmentation of **ALVAPASLIAHNAGVEGEVVVEK**
Found in **gi|115488160** in **NCBInr**, Os12g0277500 [Oryza sativa Japonica Group]

Match to Query 72: 2272.367024 from(2273.374300,1+) intensity(0.0000) index(25)
Title: Label: D12, Spot_Id: 219853, Peak_List_Id: 227230, MSMS Job_Run_Id: 21955, Comment:
Data file C:\Documents and Settings\Administrator\Desktop\Farah-Garima 11May 2013\ppw_D12_136868220000.txt

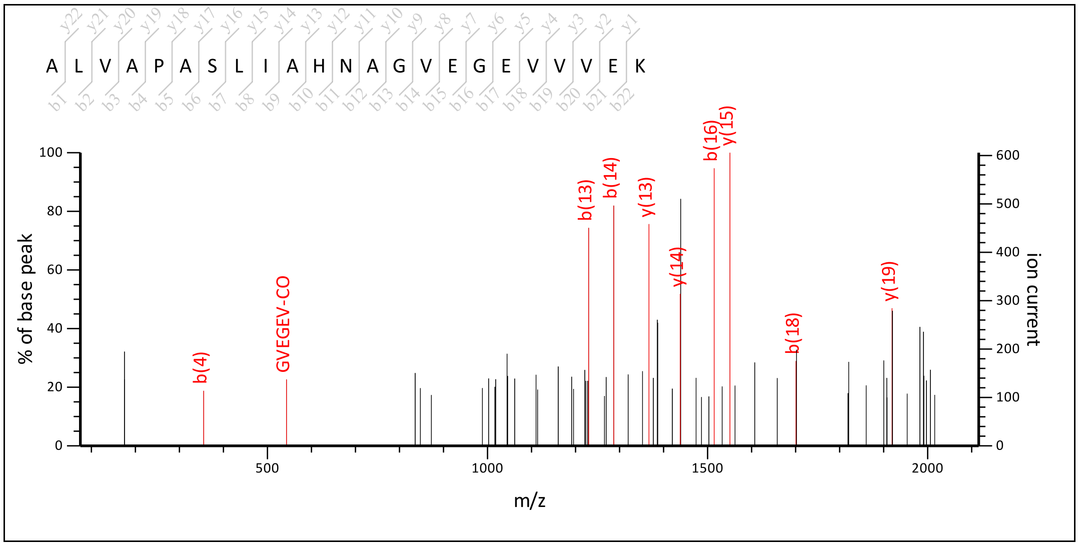


74.93 to 2115.99

Label all possible matches ○ Label matches used for scoring ◉

**Monoisotopic mass of neutral peptide Mr(calc):** 2272.2427
**Fixed modifications:** Carbamidomethyl (C) (apply to specified residues or termini only)
**Ions Score:** 18 **Expect:** 1.8e+02
**Matches :** 12/444 fragment ions using 28 most intense peaks (help)

| # | Immon. | a | a* | a$^0$ | b | b* | b$^0$ | d | d' | Seq. | v | w | w' | y | y* | |
|---|---|---|---|---|---|---|---|---|---|---|---|---|---|---|---|---|
| 1 | 44.0495 | 44.0495 | | | 72.0444 | | | 44.0495 | | A | | | | | | |
| 2 | 86.0964 | 157.1335 | | | 185.1285 | | | 115.0866 | | L | 2144.1346 | 2143.1394 | | 2202.2129 | 2185.1863 | 218 |
| 3 | 72.0808 | 256.2020 | | | 284.1969 | | | 242.1863 | | V | 2045.0662 | 2058.0866 | | 2089.1288 | 2072.1022 | 207 |
| 4 | 44.0495 | 327.2391 | | | ***355.2340*** | | | | | A | 1974.0291 | | | 1990.0604 | 1973.0338 | 197 |
| 5 | 70.0651 | 424.2918 | | | 452.2867 | | | 398.2762 | | P | 1876.9763 | 1875.9811 | | ***1919.0233*** | 1901.9967 | 190 |
| 6 | 44.0495 | 495.3289 | | | 523.3239 | | | | | A | 1805.9392 | | | 1821.9705 | 1804.9440 | 180 |
| 7 | 60.0444 | 582.3610 | | 564.3504 | 610.3559 | | 592.3453 | 566.3661 | | S | 1718.9072 | 1717.9119 | | 1750.9334 | 1733.9068 | 173 |
| 8 | 86.0964 | 695.4450 | | 677.4345 | 723.4400 | | 705.4294 | 653.3981 | | L | 1605.8231 | 1604.8279 | | 1663.9014 | 1646.8748 | 164 |
| 9 | 86.0964 | 808.5291 | | 790.5185 | 836.5240 | | 818.5135 | 780.4978 | 794.5135 | I | 1492.7390 | 1505.7594 | 1519.7751 | ***1550.8173*** | 1533.7907 | 153 |
| 10 | 44.0495 | 879.5662 | | 861.5557 | 907.5611 | | 889.5506 | | | A | 1421.7019 | | | ***1437.7332*** | 1420.7067 | 141 |
| 11 | 110.0713 | 1016.6251 | | 998.6146 | 1044.6200 | | 1026.6095 | | | H | 1284.6430 | | | ***1366.6961*** | 1349.6696 | 134 |
| 12 | 87.0553 | 1130.6681 | 1113.6415 | 1112.6575 | 1158.6630 | 1141.6364 | 1140.6524 | 1087.6622 | | N | 1170.6001 | 1169.6048 | | ***1229.6372*** | 1212.6107 | 121 |
| 13 | 44.0495 | 1201.7052 | 1184.6786 | 1183.6946 | ***1229.7001*** | 1212.6735 | 1211.6895 | | | A | 1099.5630 | | | 1115.5943 | 1098.5677 | 109 |
| 14 | 30.0338 | 1258.7266 | 1241.7001 | 1240.7161 | ***1286.7215*** | 1269.6950 | 1268.7110 | | | G | | | | 1044.5572 | 1027.5306 | 102 |
| 15 | 72.0808 | 1357.7950 | 1340.7685 | 1339.7845 | 1385.7900 | 1368.7634 | 1367.7794 | 1343.7794 | | V | 943.4731 | 956.4935 | | 987.5357 | 970.5092 | 96 |
| 16 | 102.0550 | 1486.8376 | 1469.8111 | 1468.8271 | ***1514.8326*** | 1497.8060 | 1496.8220 | 1428.8322 | | E | 814.4305 | 813.4353 | | 888.4673 | 871.4407 | 87 |
| 17 | 30.0338 | 1543.8591 | 1526.8326 | 1525.8485 | 1571.8540 | 1554.8275 | 1553.8435 | | | G | | | | 759.4247 | 742.3981 | 74 |
| 18 | 102.0550 | 1672.9017 | 1655.8751 | 1654.8911 | ***1700.8966*** | 1683.8701 | 1682.8860 | 1614.8962 | | E | 628.3665 | 627.3712 | | 702.4032 | 685.3767 | 68 |
| 19 | 72.0808 | 1771.9701 | 1754.9436 | 1753.9595 | 1799.9650 | 1782.9385 | 1781.9545 | 1757.9545 | | V | 529.2980 | 542.3184 | | 573.3606 | 556.3341 | 55 |
| 20 | 72.0808 | 1871.0385 | 1854.0120 | 1853.0280 | 1899.0334 | 1882.0069 | 1881.0229 | 1857.0229 | | V | 430.2296 | 443.2500 | | 474.2922 | 457.2657 | 45 |
| 21 | 72.0808 | 1970.1069 | 1953.0804 | 1952.0964 | 1998.1019 | 1981.0753 | 1980.0913 | 1956.0913 | | V | 331.1612 | 344.1816 | | 375.2238 | 358.1973 | 35 |
| 22 | 102.0550 | 2099.1495 | 2082.1230 | 2081.1390 | 2127.1444 | 2110.1179 | 2109.1339 | 2041.1441 | | E | 202.1186 | 201.1234 | | 276.1554 | 259.1288 | 25 |
| 23 | 101.1073 | | | | | | | | | K | 74.0237 | 73.0284 | | 147.1128 | 130.0863 | |

| Seq | ya | yb | Seq | ya | yb | Seq | ya | yb |
|---|---|---|---|---|---|---|---|---|
| LV | 185.1648 | 213.1598 | LVA | 256.2020 | 284.1969 | LVAP | 353.2547 | 381.2496 |
| LVAPA | 424.2918 | 452.2867 | LVAPAS | 511.3239 | 539.3188 | LVAPASL | 624.4079 | 652.4028 |
| VA | 143.1179 | 171.1128 | VAP | 240.1707 | 268.1656 | VAPA | 311.2078 | 339.2027 |
| VAPAS | 398.2398 | 426.2347 | VAPASL | 511.3239 | 539.3188 | VAPASLI | 624.4079 | 652.4028 |
| VAPASLIA | 695.4450 | 723.4400 | AP | 141.1022 | 169.0972 | APA | 212.1394 | 240.1343 |
| APAS | 299.1714 | 327.1663 | APASL | 412.2554 | 440.2504 | APASLI | 525.3395 | 553.3344 |
| APASLIA | 596.3766 | 624.3715 | PA | 141.1022 | 169.0972 | PAS | 228.1343 | 256.1292 |
| PASL | 341.2183 | 369.2132 | PASLI | 454.3024 | 482.2973 | PASLIA | 525.3395 | 553.3344 |
| PASLIAH | 662.3984 | 690.3933 | AS | 131.0815 | 159.0764 | ASL | 244.1656 | 272.1605 |
| ASLI | 357.2496 | 385.2445 | ASLIA | 428.2867 | 456.2817 | ASLIAH | 565.3457 | 593.3406 |
| ASLIAHN | 679.3886 | 707.3835 | SL | 173.1285 | 201.1234 | SLI | 286.2125 | 314.2074 |
| SLIA | 357.2496 | 385.2445 | SLIAH | 494.3085 | 522.3035 | SLIAHN | 608.3515 | 636.3464 |
| SLIAHNA | 679.3886 | 707.3835 | LI | 199.1805 | 227.1754 | LIA | 270.2176 | 298.2125 |
| LIAH | 407.2765 | 435.2714 | LIAHN | 521.3194 | 549.3144 | LIAHNA | 592.3566 | 620.3515 |
| LIAHNAG | 649.3780 | 677.3729 | IA | 157.1335 | 185.1285 | IAH | 294.1925 | 322.1874 |
| IAHN | 408.2354 | 436.2303 | IAHNA | 479.2725 | 507.2674 | IAHNAG | 536.2940 | 564.2889 |
| IAHNAGV | 635.3624 | 663.3573 | AH | 181.1084 | 209.1033 | AHN | 295.1513 | 323.1462 |
| AHNA | 366.1884 | 394.1833 | AHNAG | 423.2099 | 451.2048 | AHNAGV | 522.2783 | 550.2732 |
| AHNAGVE | 651.3209 | 679.3158 | HN | 224.1142 | 252.1091 | HNA | 295.1513 | 323.1462 |
| HNAG | 352.1728 | 380.1677 | HNAGV | 451.2412 | 479.2361 | HNAGVE | 580.2838 | 608.2787 |
| HNAGVEG | 637.3052 | 665.3002 | NA | 158.0924 | 186.0873 | NAG | 215.1139 | 243.1088 |
| NAGV | 314.1823 | 342.1772 | NAGVE | 443.2249 | 471.2198 | NAGVEG | 500.2463 | 528.2413 |
| NAGVEGE | 629.2889 | 657.2838 | AG | 101.0709 | 129.0659 | AGV | 200.1394 | 228.1343 |
| AGVE | 329.1819 | 357.1769 | AGVEG | 386.2034 | 414.1983 | AGVEGE | 515.2460 | 543.2409 |
| AGVEGEV | 614.3144 | 642.3093 | GV | 129.1022 | 157.0972 | GVE | 258.1448 | 286.1397 |
| GVEG | 315.1663 | 343.1612 | GVEGE | 444.2089 | 472.2038 | GVEGEV | 543.2773 | 571.2722 |
| GVEGEVV | 642.3457 | 670.3406 | VE | 201.1234 | 229.1183 | VEG | 258.1448 | 286.1397 |
| VEGE | 387.1874 | 415.1823 | VEGEV | 486.2558 | 514.2508 | VEGEVV | 585.3243 | 613.3192 |
| VEGEVVV | 684.3927 | 712.3876 | EG | 159.0764 | 187.0713 | EGE | 288.1190 | 316.1139 |
| EGEV | 387.1874 | 415.1823 | EGEVV | 486.2558 | 514.2508 | EGEVVV | 585.3243 | 613.3192 |
| GE | 159.0764 | 187.0713 | GEV | 258.1448 | 286.1397 | GEVV | 357.2132 | 385.2082 |
| GEVVV | 456.2817 | 484.2766 | GEVVVE | 585.3243 | 613.3192 | EV | 201.1234 | 229.1183 |
| EVV | 300.1918 | 328.1867 | EVVV | 399.2602 | 427.2551 | EVVVE | 528.3028 | 556.2977 |
| VV | 171.1492 | 199.1441 | VVV | 270.2176 | 298.2125 | VVVE | 399.2602 | 427.2551 |
| VV | 171.1492 | 199.1441 | VVE | 300.1918 | 328.1867 | VE | 201.1234 | 229.1183 |

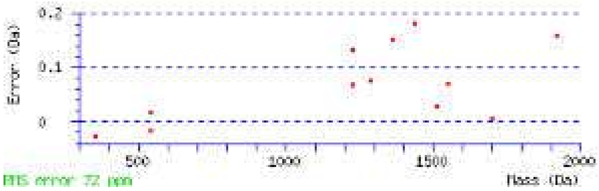


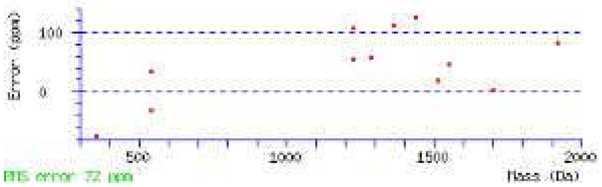


NCBI **BLAST** search of ALVAPASLIAHNAGVEGEVVVEK
(Parameters: blastp, nr protein database, expect=20000, no filter, PAM30)
Other BLAST web gateways

**All matches to this query**

| Score | Mr(calc) | Delta | Sequence |
|---|---|---|---|
| 17.8 | 2272.2427 | 0.1243 | ALVAPASLIAHNAGVEGEVVVEK |
| 10.0 | 2272.2501 | 0.1169 | LDLVMVILFKGIPLGSTDGER |
| 9.6 | 2272.3810 | -0.0140 | LLQVGVLLAVLYVGLVLFGSSI |
| 9.6 | 2272.1786 | 0.1884 | GRWLLPTYLEAERPADMVR |
| 9.3 | 2272.2215 | 0.1455 | SYIANIPDAVLALPQKQEFR |
| 7.8 | 2272.2290 | 0.1381 | QAQFLALAVTYFLSVLMVSR |
| | | | |

| | | | |
|---|---|---|---|
| 7.7 | 2272.3195 | 0.0476 | ILQALDVAAAFLLVVVALDYR |
| 7.7 | 2272.2144 | 0.1527 | IVAASVFFYDFLVTVPSEIR |
| 6.8 | 2272.1569 | 0.2102 | GMPELHGLIPALGSLMDRGHR |
| 6.4 | 2272.1825 | 0.1845 | TASGGGGGFVPKPVDEHHLVRR |

**Mascot:** http://www.matrixscience.com/

# MATRIX SCIENCE Mascot Search Results

## Peptide View **Spot no 92**

MS/MS Fragmentation of **RALSYPLK**
Found in **gi|15231255** in **NCBInr**, TCP-1/cpn60 chaperonin family protein [Arabidopsis thaliana]

Match to Query 45: 946.585864 from(947.593140,1+) intensity(0.0000) index(5)
Title: Label: E15, Spot_Id: 219902, Peak_List_Id: 228294, MSMS Job_Run_Id: 21986, Comment:
Data file C:\Documents and Settings\Administrator\Desktop\Farah-Garima 11May 2013\ppw_E15_136868276000.txt

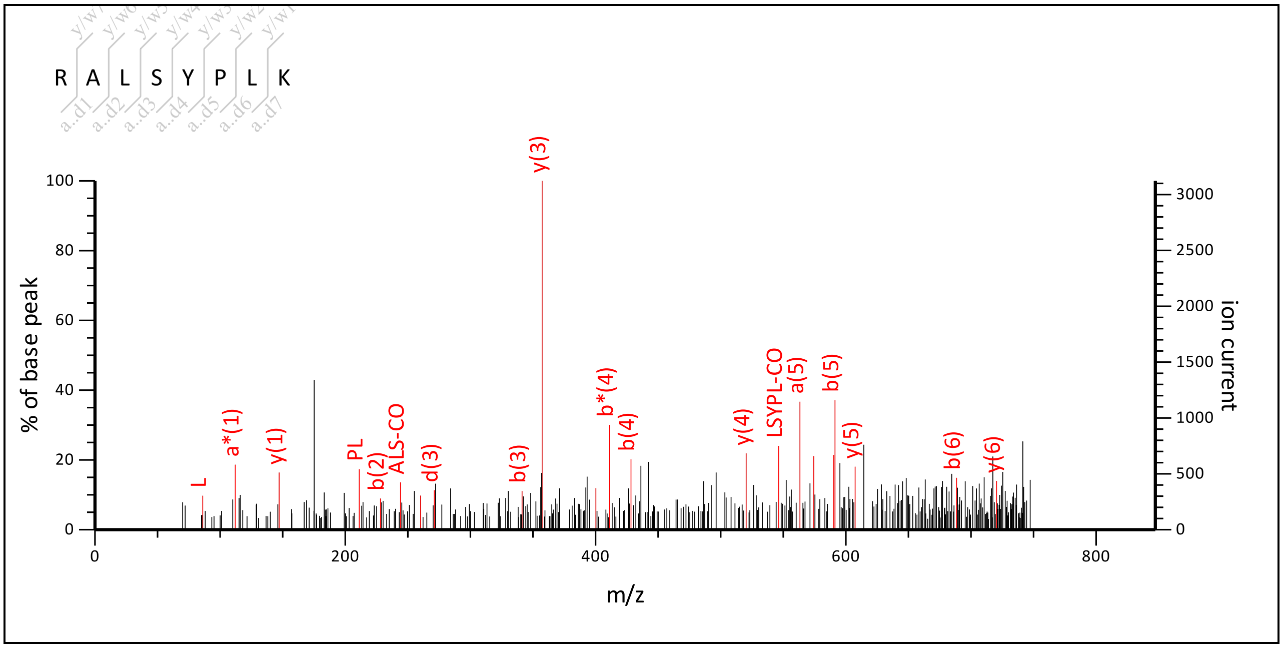


0 to 847.31

Label all possible matches ○ Label matches used for scoring ◉

**Monoisotopic mass of neutral peptide Mr(calc):** 946.5600
**Fixed modifications:** Carbamidomethyl (C) (apply to specified residues or termini only)
**Ions Score:** 26 **Expect:** 61
**Matches :** 30/108 fragment ions using 56 most intense peaks (help)

| # | Immon. | a | a* | $a^0$ | b | b* | $b^0$ | d | Seq. | v | w | y | y* | $y^0$ | # |
|---|---|---|---|---|---|---|---|---|---|---|---|---|---|---|---|
| 1 | 129.1135 | 129.1135 | 112.0869 | | 157.1084 | 140.0818 | | 44.0495 | R | | | | | | 8 |
| 2 | 44.0495 | 200.1506 | 183.1240 | | 228.1455 | 211.1190 | | | A | 775.4349 | | 791.4662 | 774.4396 | 773.4556 | 7 |
| 3 | 86.0964 | 313.2346 | 296.2081 | | 341.2296 | 324.2030 | | 271.1877 | L | 662.3508 | 661.3556 | 720.4291 | 703.4025 | 702.4185 | 6 |
| 4 | 60.0444 | 400.2667 | 383.2401 | 382.2561 | 428.2616 | 411.2350 | 410.2510 | 384.2718 | S | 575.3188 | 574.3235 | 607.3450 | 590.3184 | 589.3344 | 5 |
| 5 | 136.0757 | 563.3300 | 546.3035 | 545.3194 | 591.3249 | 574.2984 | 573.3144 | | Y | 412.2554 | | 520.3130 | 503.2864 | | 4 |
| 6 | 70.0651 | 660.3828 | 643.3562 | 642.3722 | 688.3777 | 671.3511 | 670.3671 | 634.3671 | P | 315.2027 | 314.2074 | 357.2496 | 340.2231 | | 3 |
| 7 | 86.0964 | 773.4668 | 756.4403 | 755.4563 | 801.4618 | 784.4352 | 783.4512 | 731.4199 | L | 202.1186 | 201.1234 | 260.1969 | 243.1703 | | 2 |
| 8 | 101.1073 | | | | | | | | K | 74.0237 | 73.0284 | 147.1128 | 130.0863 | | 1 |

| Seq | ya | yb | Seq | ya | yb | Seq | ya | yb |
|---|---|---|---|---|---|---|---|---|
| AL | 157.1335 | 185.1285 | ALS | 244.1656 | 272.1605 | ALSY | 407.2289 | 435.2238 |
| ALSYP | 504.2817 | 532.2766 | ALSYPL | 617.3657 | 645.3606 | LS | 173.1285 | 201.1234 |
| LSY | 336.1918 | 364.1867 | LSYP | 433.2445 | 461.2395 | LSYPL | 546.3286 | 574.3235 |
| SY | 223.1077 | 251.1026 | SYP | 320.1605 | 348.1554 | SYPL | 433.2445 | 461.2395 |

| | | | | | | | | |
|---|---|---|---|---|---|---|---|---|
| **YP** | 233.1285 | 261.1234 | **YPL** | 346.2125 | 374.2074 | **PL** | 183.1492 | 211.1441 |

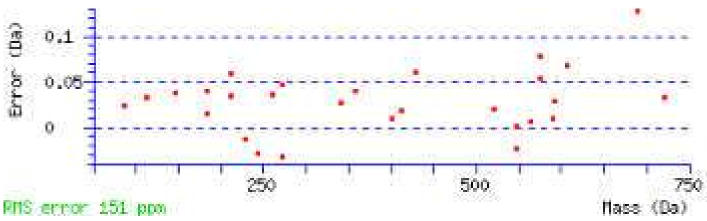


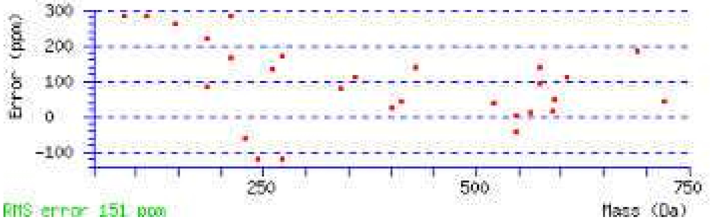


NCBI **BLAST** search of RALSYPLK
(Parameters: blastp, nr protein database, expect=20000, no filter, PAM30)
Other BLAST web gateways

**All matches to this query**

| Score | Mr(calc) | Delta | Sequence |
|---|---|---|---|
| 27.0 | 946.5487 | 0.0371 | KLDAYLPK |
| 27.0 | 946.5124 | 0.0735 | QLDAYIPK |
| 26.7 | 946.4946 | 0.0913 | QMPAYIPK |
| 26.4 | 946.5487 | 0.0371 | DLKYALPK |
| 26.0 | 946.5600 | 0.0259 | RALSYPIK |
| 26.0 | 946.5600 | 0.0259 | RALSYPLK |
| 25.5 | 946.5236 | 0.0623 | REAAYLPK |
| 25.3 | 946.5236 | 0.0623 | NGGKAYLPK |
| 25.1 | 946.5851 | 0.0007 | KVLSYLPK |
| 25.1 | 946.5487 | 0.0371 | INISYIPK |

**Mascot:** http://www.matrixscience.com/

# Mascot Search Results

## Peptide View Spot no 92

MS/MS Fragmentation of **IVNDGVTVAR**
Found in **gi|15231255** in **NCBInr**, TCP-1/cpn60 chaperonin family protein [Arabidopsis thaliana]

Match to Query 97: 1042.616524 from(1043.623800,1+) intensity(0.0000) index(9)
Title: Label: E15, Spot_Id: 219902, Peak_List_Id: 228297, MSMS Job_Run_Id: 21986, Comment:
Data file C:\Documents and Settings\Administrator\Desktop\Farah-Garima 11May 2013\ppw_E15_136868276000.txt

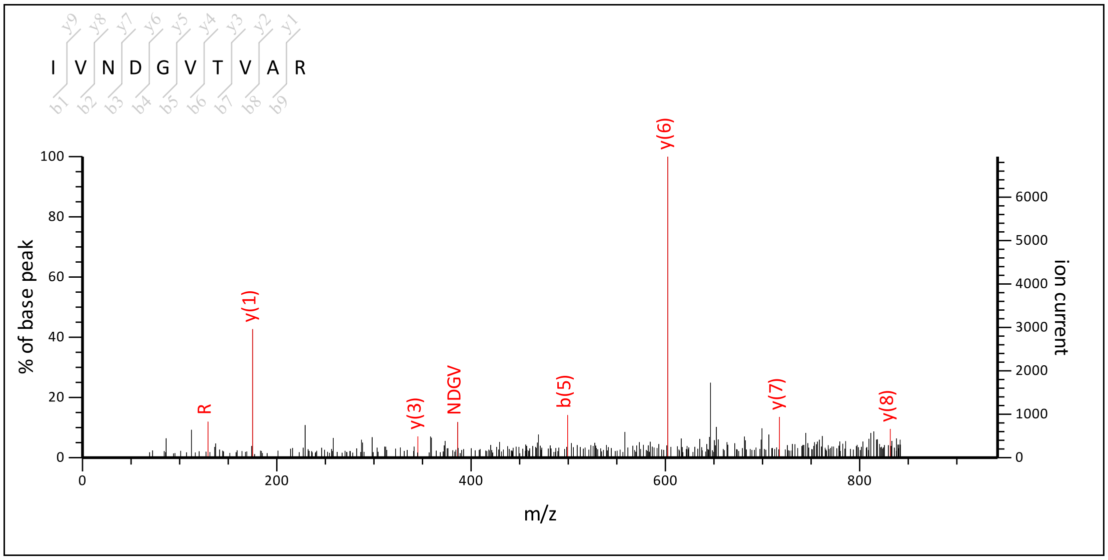


0 to 941.95

Label all possible matches ○ Label matches used for scoring ◉

**Monoisotopic mass of neutral peptide Mr(calc):** 1042.5771
**Fixed modifications:** Carbamidomethyl (C) (apply to specified residues or termini only)
**Ions Score:** 49 **Expect:** 0.44
**Matches :** 11/156 fragment ions using 8 most intense peaks (help)

| # | Immon. | a | a* | $a^0$ | b | b* | $b^0$ | d | d' | Seq. | v | w | w' | y | y* | $y^0$ | # |
|---|---|---|---|---|---|---|---|---|---|---|---|---|---|---|---|---|---|
| 1 | 86.0964 | 86.0964 | | | 114.0913 | | | 44.0495 | | I | | | | | | | 10 |
| 2 | 72.0808 | 185.1648 | | | 213.1598 | | | 171.1492 | | V | 886.4377 | 899.4581 | | 930.5003 | 913.4738 | 912.4898 | 9 |
| 3 | 87.0553 | 299.2078 | 282.1812 | | 327.2027 | 310.1761 | | 256.2020 | | N | 772.3948 | 771.3995 | | ***831.4319*** | 814.4054 | 813.4213 | 8 |
| 4 | 88.0393 | 414.2347 | 397.2082 | 396.2241 | 442.2296 | 425.2031 | 424.2191 | 370.2449 | | D | 657.3679 | 656.3726 | | ***717.3890*** | 700.3624 | 699.3784 | 7 |
| 5 | 30.0338 | 471.2562 | 454.2296 | 453.2456 | **499.2511** | 482.2245 | 481.2405 | | | G | | | | ***602.3620*** | 585.3355 | 584.3515 | 6 |
| 6 | 72.0808 | 570.3246 | 553.2980 | 552.3140 | 598.3195 | 581.2930 | 580.3089 | 556.3089 | | V | 501.2780 | 514.2984 | | 545.3406 | 528.3140 | 527.3300 | 5 |
| 7 | 74.0600 | 671.3723 | 654.3457 | 653.3617 | 699.3672 | 682.3406 | 681.3566 | 655.3774 | 657.3566 | T | 400.2303 | 413.2507 | 415.2300 | 446.2722 | 429.2456 | 428.2616 | 4 |
| 8 | 72.0808 | 770.4407 | 753.4141 | 752.4301 | 798.4356 | 781.4090 | 780.4250 | 756.4250 | | V | 301.1619 | 314.1823 | | ***345.2245*** | 328.1979 | | 3 |
| 9 | 44.0495 | 841.4778 | 824.4512 | 823.4672 | 869.4727 | 852.4462 | 851.4621 | | | A | 230.1248 | | | 246.1561 | 229.1295 | | 2 |
| 10 | 129.1135 | | | | | | | | | R | 74.0237 | 73.0284 | | ***175.1190*** | 158.0924 | | 1 |

| Seq | ya | yb | Seq | ya | yb | Seq | ya | yb |
|---|---|---|---|---|---|---|---|---|
| VN | 186.1237 | 214.1186 | VND | 301.1506 | 329.1456 | VNDG | 358.1721 | 386.1670 |
| VNDGV | 457.2405 | 485.2354 | VNDGVT | 558.2882 | 586.2831 | VNDGVTV | 657.3566 | 685.3515 |
| ND | 202.0822 | 230.0771 | NDG | 259.1037 | 287.0986 | NDGV | 358.1721 | 386.1670 |
| NDGVT | 459.2198 | 487.2147 | NDGVTV | 558.2882 | 586.2831 | NDGVTVA | 629.3253 | 657.3202 |
| DG | 145.0608 | 173.0557 | DGV | 244.1292 | 272.1241 | DGVT | 345.1769 | 373.1718 |
| DGVTV | 444.2453 | 472.2402 | DGVTVA | 515.2824 | 543.2773 | GV | 129.1022 | 157.0972 |
| GVT | 230.1499 | 258.1448 | GVTV | 329.2183 | 357.2132 | GVTVA | 400.2554 | 428.2504 |
| VT | 173.1285 | 201.1234 | VTV | 272.1969 | 300.1918 | VTVA | 343.2340 | 371.2289 |
| TV | 173.1285 | 201.1234 | TVA | 244.1656 | 272.1605 | VA | 143.1179 | 171.1128 |

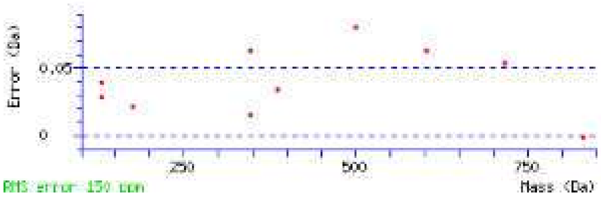


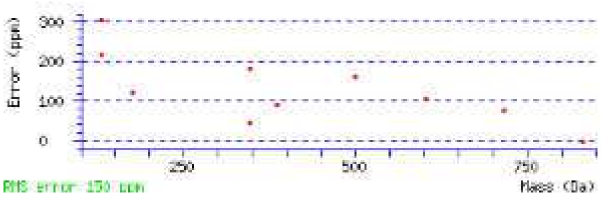


NCBI **BLAST** search of IVNDGVTVAR
(Parameters: blastp, nr protein database, expect=20000, no filter, PAM30)
Other BLAST web gateways

**All matches to this query**

| Score | Mr(calc) | Delta | Sequence |
|---|---|---|---|
| 48.9 | 1042.5771 | 0.0394 | IVNDGVTVAR |
| 32.2 | 1042.5407 | 0.0758 | VLNADGELGR |
| 31.8 | 1042.5883 | 0.0282 | IVNDGTRLR |
| 31.8 | 1042.5407 | 0.0758 | LVNDGLEQR |
| 31.8 | 1042.5407 | 0.0758 | LVNDINAER |
| 31.8 | 1042.5883 | 0.0282 | VLNDAISRR |
| 31.8 | 1042.5883 | 0.0282 | VLNDALSRR |
| 31.7 | 1042.5771 | 0.0394 | LVASAQEGLR |
| 31.7 | 1042.5771 | 0.0394 | LVSAAEQGIR |
| 31.7 | 1042.6135 | 0.0030 | LVTKQEIGR |

**Mascot:** http://www.matrixscience.com/

# MATRIX SCIENCE Mascot Search Results

## Peptide View Spot no 92

MS/MS Fragmentation of **LADLVGVTLGPK**
Found in **gi|15231255** in **NCBInr**, TCP-1/cpn60 chaperonin family protein [Arabidopsis thaliana]

Match to Query 157: 1181.733724 from(1182.741000,1+) intensity(0.0000) index(13)
Title: Label: E15, Spot_Id: 219902, Peak_List_Id: 228292, MSMS Job_Run_Id: 21986, Comment:
Data file C:\Documents and Settings\Administrator\Desktop\Farah-Garima 11May 2013\ppw_E15_136868276000.txt

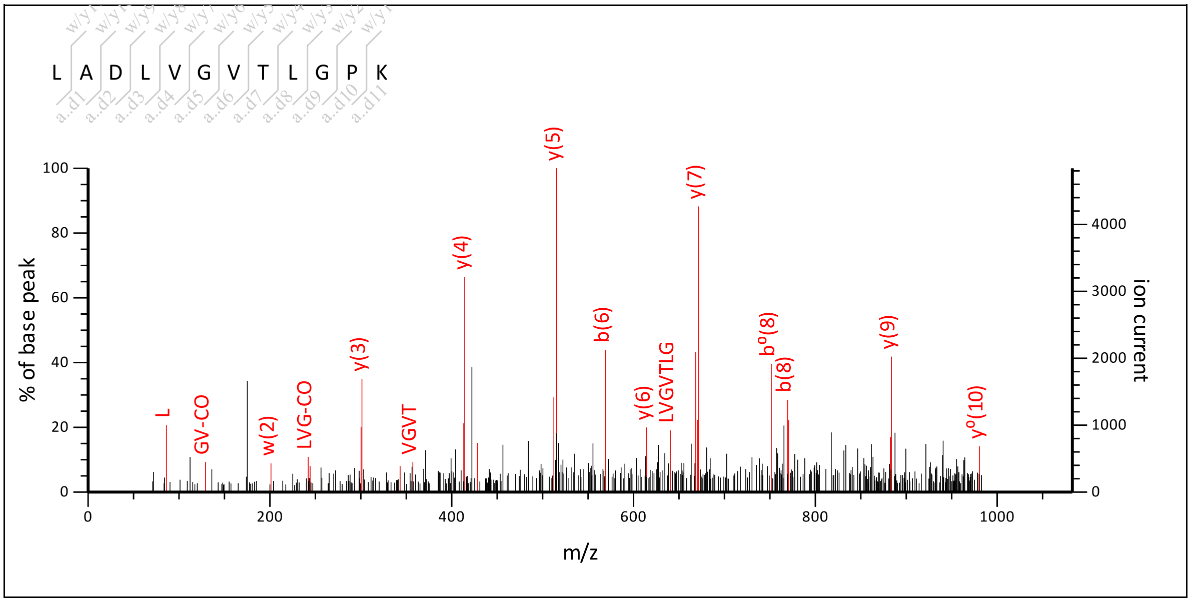


0 to 1082.71

Label all possible matches ○ Label matches used for scoring ◉

**Monoisotopic mass of neutral peptide Mr(calc):** 1181.7020
**Fixed modifications:** Carbamidomethyl (C) (apply to specified residues or termini only)
**Ions Score:** 55 **Expect:** 0.049
**Matches :** 40/186 fragment ions using 40 most intense peaks (help)

| # | Immon. | a | $a^0$ | b | $b^0$ | d | d' | Seq. | v | w | w' | y | y* | $y^0$ | # |
|---|---|---|---|---|---|---|---|---|---|---|---|---|---|---|---|
| 1 | 86.0964 | **86.0964** | | 114.0913 | | 44.0495 | | **L** | | | | | | | 12 |
| 2 | 44.0495 | 157.1335 | | 185.1285 | | | | **A** | 1053.5939 | | | 1069.6252 | 1052.5986 | 1051.6146 | 11 |
| 3 | 88.0393 | 272.1605 | 254.1499 | **300.1554** | 282.1448 | 228.1707 | | **D** | 938.5669 | 937.5717 | | 998.5881 | 981.5615 | 980.5775 | 10 |
| 4 | 86.0964 | 385.2445 | 367.2340 | **413.2395** | 395.2289 | 343.1976 | | **L** | 825.4829 | 824.4876 | | ***883.5611*** | 866.5346 | 865.5506 | 9 |
| 5 | 72.0808 | 484.3130 | 466.3024 | **512.3079** | 494.2973 | 470.2973 | | **V** | 726.4145 | 739.4349 | | ***770.4771*** | 753.4505 | 752.4665 | 8 |
| 6 | 30.0338 | 541.3344 | 523.3239 | **569.3293** | 551.3188 | | | **G** | | | | ***671.4087*** | 654.3821 | 653.3981 | 7 |
| 7 | 72.0808 | **640.4028** | 622.3923 | **668.3978** | 650.3872 | 626.3872 | | **V** | 570.3246 | 583.3450 | | ***614.3872*** | 597.3606 | 596.3766 | 6 |
| 8 | 74.0600 | 741.4505 | 723.4400 | **769.4454** | 751.4349 | 725.4556 | 727.4349 | **T** | 469.2769 | 482.2973 | 484.2766 | ***515.3188*** | 498.2922 | 497.3082 | 5 |
| 9 | 86.0964 | 854.5346 | 836.5240 | **882.5295** | 864.5189 | 812.4876 | | **L** | 356.1928 | 355.1976 | | ***414.2711*** | 397.2445 | | 4 |
| 10 | 30.0338 | 911.5560 | 893.5455 | 939.5510 | 921.5404 | | | **G** | | | | ***301.1870*** | 284.1605 | | 3 |
| 11 | 70.0651 | 1008.6088 | 990.5982 | 1036.6037 | 1018.5932 | 982.5932 | | **P** | 202.1186 | 201.1234 | | ***244.1656*** | 227.1390 | | 2 |
| 12 | 101.1073 | | | | | | | **K** | 74.0237 | 73.0284 | | 147.1128 | 130.0863 | | 1 |

| Seq | ya | yb | Seq | ya | yb | Seq | ya | yb |
|---|---|---|---|---|---|---|---|---|
| **AD** | 159.0764 | 187.0713 | **ADL** | 272.1605 | 300.1554 | **ADLV** | 371.2289 | 399.2238 |
| **ADLVG** | 428.2504 | 456.2453 | **ADLVGV** | 527.3188 | 555.3137 | **ADLVGVT** | 628.3665 | 656.3614 |
| **DL** | 201.1234 | 229.1183 | **DLV** | 300.1918 | 328.1867 | **DLVG** | 357.2132 | 385.2082 |
| **DLVGV** | 456.2817 | 484.2766 | **DLVGVT** | 557.3293 | 585.3243 | **DLVGVTL** | 670.4134 | 698.4083 |

| | | | | | | | | |
|---|---|---|---|---|---|---|---|---|
| **LV** | 185.1648 | 213.1598 | **LVG** | 242.1863 | 270.1812 | **LVGV** | 341.2547 | 369.2496 |
| **LVGVT** | 442.3024 | 470.2973 | **LVGVTL** | 555.3865 | 583.3814 | **LVGVTLG** | 612.4079 | 640.4028 |
| **VG** | 129.1022 | 157.0972 | **VGV** | 228.1707 | 256.1656 | **VGVT** | 329.2183 | 357.2132 |
| **VGVTL** | 442.3024 | 470.2973 | **VGVTLG** | 499.3239 | 527.3188 | **VGVTLGP** | 596.3766 | 624.3715 |
| **GV** | 129.1022 | 157.0972 | **GVT** | 230.1499 | 258.1448 | **GVTL** | 343.2340 | 371.2289 |
| **GVTLG** | 400.2554 | 428.2504 | **GVTLGP** | 497.3082 | 525.3031 | **VT** | 173.1285 | 201.1234 |
| **VTL** | 286.2125 | 314.2074 | **VTLG** | 343.2340 | 371.2289 | **VTLGP** | 440.2867 | 468.2817 |
| **TL** | 187.1441 | 215.1390 | **TLG** | 244.1656 | 272.1605 | **TLGP** | 341.2183 | 369.2132 |
| **LG** | 143.1179 | 171.1128 | **LGP** | 240.1707 | 268.1656 | **GP** | 127.0866 | 155.0815 |

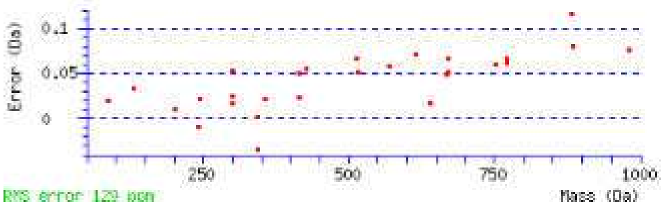


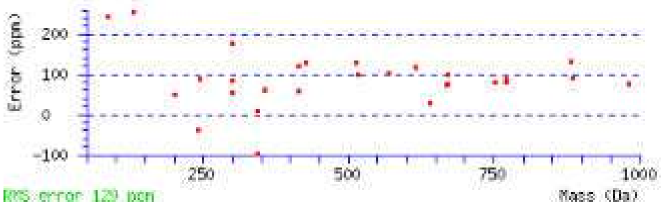


NCBI **BLAST** search of LADLVGVTLGPK
(Parameters: blastp, nr protein database, expect=20000, no filter, PAM30)
Other BLAST web gateways

**All matches to this query**

| Score | Mr(calc) | Delta | Sequence |
|---|---|---|---|
| 54.7 | 1181.7020 | 0.0317 | LADLVGVTLGPK |
| 46.5 | 1181.7244 | 0.0093 | LVATASRVLPR |
| 45.2 | 1181.7244 | 0.0093 | LSQLTRPVIR |
| 45.1 | 1181.6768 | 0.0569 | QITAEVGVLPR |
| 44.1 | 1181.6768 | 0.0569 | VGLDVLSINPR |
| 43.9 | 1181.7132 | 0.0205 | IASGLTGVVPIR |
| 43.4 | 1181.6404 | 0.0933 | LDVAGLVENPR |
| 38.1 | 1181.6880 | 0.0457 | DLVQSVRIPR |
| 37.9 | 1181.6517 | 0.0821 | AVISATRDPPR |
| 37.9 | 1181.6516 | 0.0821 | IANDERLVPR |

**Mascot:** http://www.matrixscience.com/

# MATRIX SCIENCE Mascot Search Results

## Peptide View Spot no 92

MS/MS Fragmentation of **IAALKAPGFGER**
Found in **gi|15231255** in **NCBInr**, TCP-1/cpn60 chaperonin family protein [Arabidopsis thaliana]

Match to Query 179: 1228.734624 from(1229.741900,1+) intensity(0.0000) index(15)
Title: Label: E15, Spot_Id: 219902, Peak_List_Id: 228286, MSMS Job_Run_Id: 21986, Comment:
Data file C:\Documents and Settings\Administrator\Desktop\Farah-Garima 11May 2013\ppw_E15_136868276000.txt

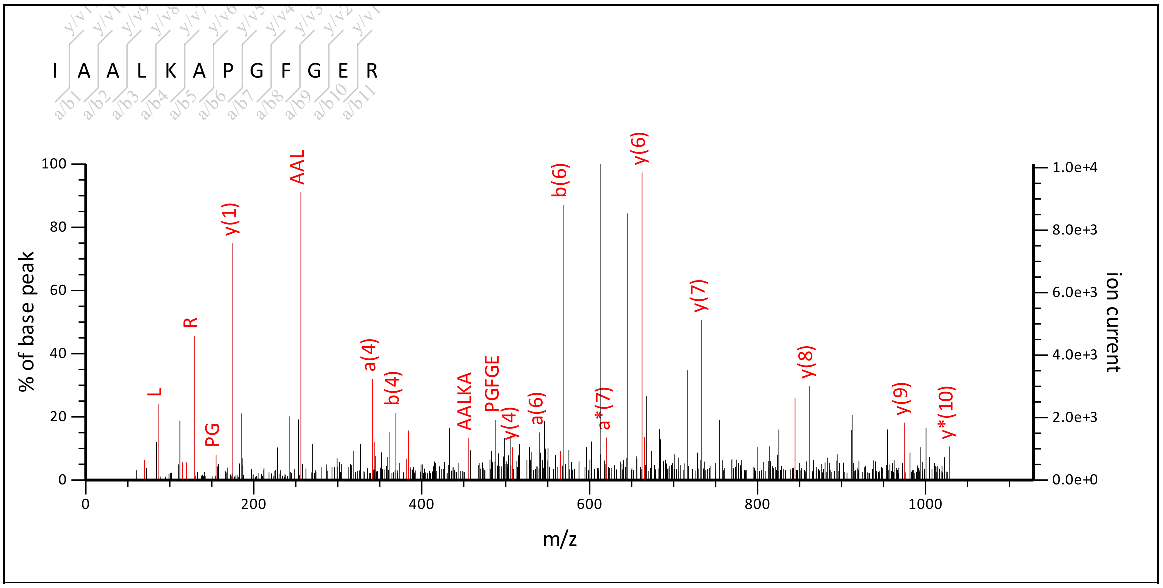


0 to 1128.68

Label all possible matches ○ Label matches used for scoring ◉

**Monoisotopic mass of neutral peptide Mr(calc):** 1228.6928
**Fixed modifications:** Carbamidomethyl (C) (apply to specified residues or termini only)
**Ions Score:** 35 **Expect:** 8.8
**Matches :** 42/179 fragment ions using 80 most intense peaks (help)

| # | Immon. | a | a* | $a^0$ | b | b* | $b^0$ | d | Seq. | v | w | y | y* | $y^0$ | # |
|---|---|---|---|---|---|---|---|---|---|---|---|---|---|---|---|
| 1 | 86.0964 | **86.0964** | | | 114.0913 | | | 44.0495 | I | | | | | | 12 |
| 2 | 44.0495 | 157.1335 | | | **185.1285** | | | | A | 1100.5847 | | 1116.6160 | 1099.5895 | 1098.6055 | 11 |
| 3 | 44.0495 | **228.1707** | | | **256.1656** | | | | A | 1029.5476 | | 1045.5789 | 1028.5524 | 1027.5683 | 10 |
| 4 | 86.0964 | **341.2547** | | | **369.2496** | | | 299.2078 | L | 916.4635 | 915.4683 | ***974.5418*** | 957.5152 | 956.5312 | 9 |
| 5 | 101.1073 | 469.3497 | 452.3231 | | 497.3446 | 480.3180 | | 412.2918 | K | 788.3686 | 787.3733 | ***861.4577*** | 844.4312 | 843.4472 | 8 |
| 6 | 44.0495 | **540.3868** | 523.3602 | | **568.3817** | 551.3552 | | | A | 717.3315 | | ***733.3628*** | 716.3362 | 715.3522 | 7 |
| 7 | 70.0651 | 637.4396 | 620.4130 | | **665.4345** | 648.4079 | | 611.4239 | P | 620.2787 | 619.2835 | ***662.3257*** | 645.2991 | 644.3151 | 6 |
| 8 | 30.0338 | 694.4610 | 677.4345 | | 722.4559 | 705.4294 | | | G | | | ***565.2729*** | 548.2463 | 547.2623 | 5 |
| 9 | 120.0808 | 841.5294 | 824.5029 | | 869.5244 | 852.4978 | | | F | 416.1888 | | ***508.2514*** | 491.2249 | 490.2409 | 4 |
| 10 | 30.0338 | 898.5509 | 881.5244 | | 926.5458 | 909.5193 | | | G | | | ***361.1830*** | 344.1565 | 343.1724 | 3 |
| 11 | 102.0550 | 1027.5935 | 1010.5669 | 1009.5829 | 1055.5884 | 1038.5619 | 1037.5778 | 969.5880 | E | 230.1248 | 229.1295 | 304.1615 | 287.1350 | 286.1510 | 2 |
| 12 | 129.1135 | | | | | | | | R | 74.0237 | 73.0284 | ***175.1190*** | 158.0924 | | 1 |

| Seq | ya | yb | Seq | ya | yb | Seq | ya | yb |
|---|---|---|---|---|---|---|---|---|
| AA | 115.0866 | 143.0815 | AAL | 228.1707 | 256.1656 | AALK | 356.2656 | 384.2605 |
| AALKA | 427.3027 | 455.2976 | AALKAP | 524.3555 | 552.3504 | AALKAPG | 581.3770 | 609.3719 |
| AL | 157.1335 | 185.1285 | ALK | 285.2285 | 313.2234 | ALKA | 356.2656 | 384.2605 |
| ALKAP | 453.3184 | 481.3133 | ALKAPG | 510.3398 | 538.3348 | ALKAPGF | 657.4083 | 685.4032 |
| LK | 214.1914 | 242.1863 | LKA | 285.2285 | 313.2234 | LKAP | 382.2813 | 410.2762 |

| | | | | | | | | |
|---|---|---|---|---|---|---|---|---|
| **LKAPG** | 439.3027 | 467.2976 | **LKAPGF** | 586.3711 | 614.3661 | **LKAPGFG** | 643.3926 | 671.3875 |
| **KA** | 172.1444 | 200.1394 | **KAP** | 269.1972 | 297.1921 | **KAPG** | 326.2187 | 354.2136 |
| **KAPGF** | 473.2871 | 501.2820 | **KAPGFG** | 530.3085 | 558.3035 | **KAPGFGE** | 659.3511 | 687.3461 |
| **AP** | 141.1022 | 169.0972 | **APG** | 198.1237 | 226.1186 | **APGF** | 345.1921 | 373.1870 |
| **APGFG** | 402.2136 | 430.2085 | **APGFGE** | 531.2562 | 559.2511 | **PG** | 127.0866 | 155.0815 |
| **PGF** | 274.1550 | 302.1499 | **PGFG** | 331.1765 | 359.1714 | **PGFGE** | 460.2191 | 488.2140 |
| **GF** | 177.1022 | 205.0972 | **GFG** | 234.1237 | 262.1186 | **GFGE** | 363.1663 | 391.1612 |
| **FG** | 177.1022 | 205.0972 | **FGE** | 306.1448 | 334.1397 | **GE** | 159.0764 | 187.0713 |

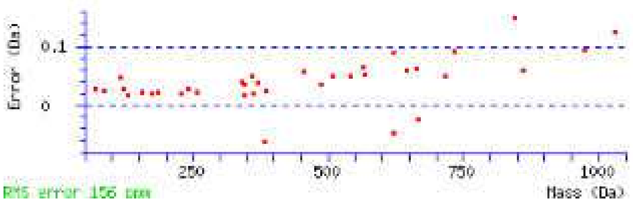


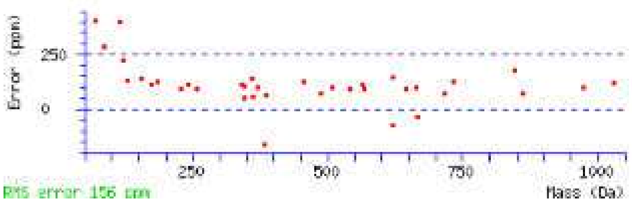


NCBI **BLAST** search of IAALKAPGFGER
(Parameters: blastp, nr protein database, expect=20000, no filter, PAM30)
Other BLAST web gateways

**All matches to this query**

| Score | Mr(calc) | Delta | Sequence |
|---|---|---|---|
| 36.6 | 1228.6928 | 0.0418 | GPTAKIPFAATR |
| 35.8 | 1228.6928 | 0.0418 | IAAIKAPGFGER |
| 35.4 | 1228.6775 | 0.0571 | AGIVNIVSATER |
| 35.0 | 1228.6928 | 0.0418 | IAALKAPGFGER |
| 34.6 | 1228.6663 | 0.0683 | VGLVNIGEEATK |
| 32.1 | 1228.6775 | 0.0571 | ALALAGASTLDAR |
| 31.3 | 1228.7325 | 0.0021 | IAAIKIGMLGSR |
| 29.8 | 1228.6775 | 0.0571 | LAAQLAESGITR |
| 29.7 | 1228.6710 | 0.0636 | TGPAGLIGRMTR |
| 29.6 | 1228.6775 | 0.0571 | ALAKALDADSVR |

**Mascot:** http://www.matrixscience.com/

# MATRIX SCIENCE Mascot Search Results

## Peptide View Spot no 92

MS/MS Fragmentation of **GYISPYFVTDSEK**
Found in **gi|15231255** in **NCBInr**, TCP-1/cpn60 chaperonin family protein [Arabidopsis thaliana]

Match to Query 256: 1504.756124 from(1505.763400,1+) intensity(0.0000) index(24)
Title: Label: E15, Spot_Id: 219902, Peak_List_Id: 228296, MSMS Job_Run_Id: 21986, Comment:
Data file C:\Documents and Settings\Administrator\Desktop\Farah-Garima 11May 2013\ppw_E15_136868276000.txt

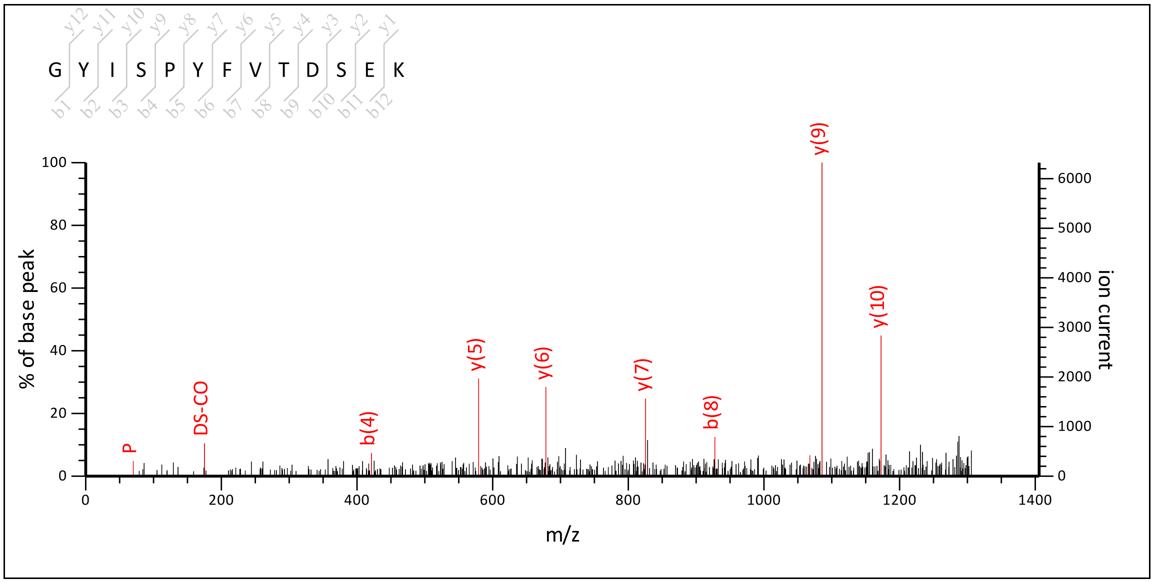


0 to 1405.75

Label all possible matches ○ Label matches used for scoring ◉

**Monoisotopic mass of neutral peptide Mr(calc):** 1504.7086
**Fixed modifications:** Carbamidomethyl (C) (apply to specified residues or termini only)
**Ions Score:** 33 **Expect:** 15
**Matches :** 10/199 fragment ions using 13 most intense peaks (help)

| # | Immon. | a | $a^0$ | b | $b^0$ | d | d' | Seq. | v | w | w' | y | y* | $y^0$ | # |
|---|---|---|---|---|---|---|---|---|---|---|---|---|---|---|---|
| 1 | 30.0338 | 30.0338 | | 58.0287 | | 44.0495 | | G | | | | | | | 13 |
| 2 | 136.0757 | 193.0972 | | 221.0921 | | | | Y | 1340.6369 | | | 1448.6944 | 1431.6678 | 1430.6838 | 12 |
| 3 | 86.0964 | 306.1812 | | 334.1761 | | 278.1499 | 292.1656 | I | 1227.5528 | 1240.5732 | 1254.5889 | 1285.6311 | 1268.6045 | 1267.6205 | 11 |
| 4 | 60.0444 | 393.2132 | 375.2027 | **421.2082** | 403.1976 | 377.2183 | | S | 1140.5208 | 1139.5255 | | ***1172.5470*** | 1155.5204 | 1154.5364 | 10 |
| 5 | 70.0651 | 490.2660 | 472.2554 | 518.2609 | 500.2504 | 464.2504 | | P | 1043.4680 | 1042.4728 | | ***1085.5150*** | 1068.4884 | 1067.5044 | 9 |
| 6 | 136.0757 | 653.3293 | 635.3188 | 681.3243 | 663.3137 | | | Y | 880.4047 | | | 988.4622 | 971.4357 | 970.4516 | 8 |
| 7 | 120.0808 | 800.3978 | 782.3872 | 828.3927 | 810.3821 | | | F | 733.3363 | | | ***825.3989*** | 808.3723 | 807.3883 | 7 |
| 8 | 72.0808 | 899.4662 | 881.4556 | **927.4611** | 909.4505 | 885.4505 | | V | 634.2679 | 647.2883 | | ***678.3305*** | 661.3039 | 660.3199 | 6 |
| 9 | 74.0600 | 1000.5138 | 982.5033 | 1028.5088 | 1010.4982 | 984.5189 | 986.4982 | T | 533.2202 | 546.2406 | 548.2198 | ***579.2620*** | 562.2355 | 561.2515 | 5 |
| 10 | 88.0393 | 1115.5408 | 1097.5302 | 1143.5357 | 1125.5251 | 1071.5510 | | D | 418.1932 | 417.1980 | | 478.2144 | 461.1878 | 460.2038 | 4 |
| 11 | 60.0444 | 1202.5728 | 1184.5623 | 1230.5677 | 1212.5572 | 1186.5779 | | S | 331.1612 | 330.1660 | | 363.1874 | 346.1609 | 345.1769 | 3 |
| 12 | 102.0550 | 1331.6154 | 1313.6048 | 1359.6103 | 1341.5998 | 1273.6099 | | E | 202.1186 | 201.1234 | | 276.1554 | 259.1288 | 258.1448 | 2 |
| 13 | 101.1073 | | | | | | | K | 74.0237 | 73.0284 | | 147.1128 | 130.0863 | | 1 |

| Seq | ya | yb | Seq | ya | yb | Seq | ya | yb |
|---|---|---|---|---|---|---|---|---|
| YI | 249.1598 | 277.1547 | YIS | 336.1918 | 364.1867 | YISP | 433.2445 | 461.2395 |
| YISPY | 596.3079 | 624.3028 | IS | 173.1285 | 201.1234 | ISP | 270.1812 | 298.1761 |
| ISPY | 433.2445 | 461.2395 | ISPYF | 580.3130 | 608.3079 | ISPYFV | 679.3814 | 707.3763 |
| SP | 157.0972 | 185.0921 | SPY | 320.1605 | 348.1554 | SPYF | 467.2289 | 495.2238 |

| | | | | | | | | |
|---|---|---|---|---|---|---|---|---|
| **SPYFV** | 566.2973 | 594.2922 | **SPYFVT** | 667.3450 | 695.3399 | **PY** | 233.1285 | 261.1234 |
| **PYF** | 380.1969 | 408.1918 | **PYFV** | 479.2653 | 507.2602 | **PYFVT** | 580.3130 | 608.3079 |
| **PYFVTD** | 695.3399 | 723.3348 | **YF** | 283.1441 | 311.1390 | **YFV** | 382.2125 | 410.2074 |
| **YFVT** | 483.2602 | 511.2551 | **YFVTD** | 598.2871 | 626.2821 | **YFVTDS** | 685.3192 | 713.3141 |
| **FV** | 219.1492 | 247.1441 | **FVT** | 320.1969 | 348.1918 | **FVTD** | 435.2238 | 463.2187 |
| **FVTDS** | 522.2558 | 550.2508 | **FVTDSE** | 651.2984 | 679.2933 | **VT** | 173.1285 | 201.1234 |
| **VTD** | 288.1554 | 316.1503 | **VTDS** | 375.1874 | 403.1823 | **VTDSE** | 504.2300 | 532.2249 |
| **TD** | 189.0870 | 217.0819 | **TDS** | 276.1190 | 304.1139 | **TDSE** | 405.1616 | 433.1565 |
| **DS** | 175.0713 | 203.0662 | **DSE** | 304.1139 | 332.1088 | **SE** | 189.0870 | 217.0819 |

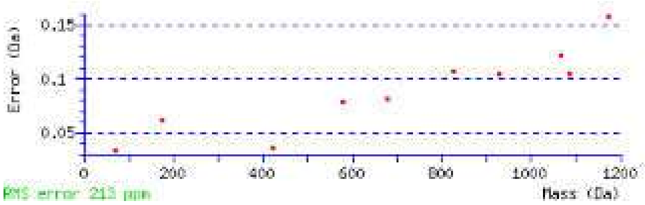


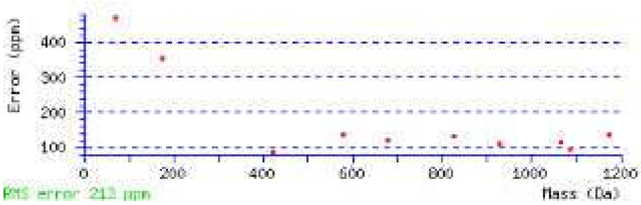


NCBI **BLAST** search of GYISPYFVTDSEK
(Parameters: blastp, nr protein database, expect=20000, no filter, PAM30)
Other BLAST web gateways

**All matches to this query**

| Score | Mr(calc) | Delta | Sequence |
|---|---|---|---|
| 33.5 | 1504.7086 | 0.0475 | GYISPYFVTDTDK |
| 33.5 | 1504.6756 | 0.0806 | GYISPYMVTDTDK |
| 33.5 | 1504.7086 | 0.0475 | GYLSPYFVTDTDK |
| 33.5 | 1504.6756 | 0.0806 | GYLSPYMVTDTDK |
| 33.5 | 1504.7086 | 0.0476 | GYISPYFVTDSEK |
| 33.5 | 1504.6756 | 0.0806 | GYISPYMVTDSEK |
| 33.5 | 1504.7086 | 0.0476 | GYLSPYFVTDSEK |
| 33.5 | 1504.6756 | 0.0806 | GYLSPYMVSDTEK |
| 33.5 | 1504.6756 | 0.0806 | GYLSPYMVTDSEK |
| 32.9 | 1504.7191 | 0.0370 | SMDSSADPLNVTLR |

**Mascot:** http://www.matrixscience.com/

# MATRIX SCIENCE Mascot Search Results

## Peptide View Spot no 92

MS/MS Fragmentation of **EVELEDPVENIGAK**
Found in **gi|15231255** in **NCBInr**, TCP-1/cpn60 chaperonin family protein [Arabidopsis thaliana]

Match to Query 262: 1540.817724 from(1541.825000,1+) intensity(0.0000) index(25)
Title: Label: E15, Spot_Id: 219902, Peak_List_Id: 228309, MSMS Job_Run_Id: 21986, Comment:
Data file C:\Documents and Settings\Administrator\Desktop\Farah-Garima 11May 2013\ppw_E15_136868276000.txt

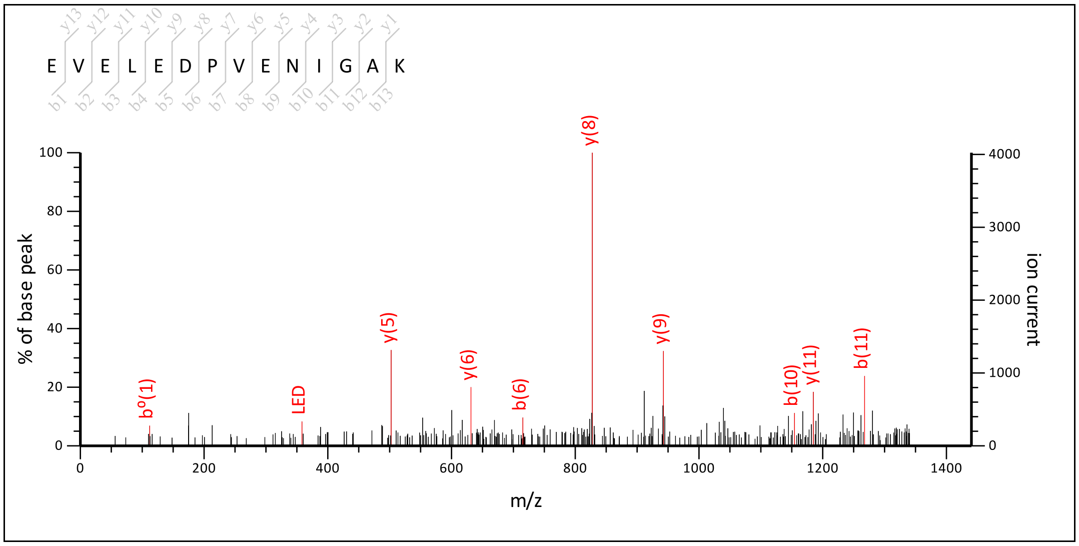


0 to 1440.41

Label all possible matches   Label matches used for scoring

**Monoisotopic mass of neutral peptide Mr(calc):** 1540.7620
**Fixed modifications:** Carbamidomethyl (C) (apply to specified residues or termini only)
**Ions Score:** 39 **Expect:** 3.6
**Matches :** 11/237 fragment ions using 13 most intense peaks (help)

| # | Immon. | a | a* | $a^0$ | b | b* | $b^0$ | d | d' | Seq. | v | w | w' | y | y* | |
|---|---|---|---|---|---|---|---|---|---|---|---|---|---|---|---|---|
| 1 | 102.0550 | 102.0550 | | 84.0444 | 130.0499 | | 112.0393 | 44.0495 | | E | | | | | | |
| 2 | 72.0808 | 201.1234 | | 183.1128 | 229.1183 | | 211.1077 | 187.1077 | | V | 1368.6642 | 1381.6846 | | 1412.7268 | 1395.7002 | 139[illegible] |
| 3 | 102.0550 | 330.1660 | | 312.1554 | ***358.1609*** | | 340.1503 | 272.1605 | | E | 1239.6216 | 1238.6263 | | 1313.6583 | 1296.6318 | 129[illegible] |
| 4 | 86.0964 | 443.2500 | | 425.2395 | 471.2449 | | 453.2344 | 401.2031 | | L | 1126.5375 | 1125.5422 | | ***1184.6157*** | 1167.5892 | 116[illegible] |
| 5 | 102.0550 | 572.2926 | | 554.2821 | 600.2875 | | 582.2770 | 514.2871 | | E | 997.4949 | 996.4997 | | 1071.5317 | 1054.5051 | 105[illegible] |
| 6 | 88.0393 | 687.3196 | | 669.3090 | ***715.3145*** | | 697.3039 | 643.3297 | | D | 882.4680 | 881.4727 | | ***942.4891*** | 925.4625 | 92[illegible] |
| 7 | 70.0651 | 784.3723 | | 766.3618 | 812.3672 | | 794.3567 | 758.3567 | | P | 785.4152 | 784.4199 | | ***827.4621*** | 810.4356 | 80[illegible] |
| 8 | 72.0808 | 883.4407 | | 865.4302 | 911.4357 | | 893.4251 | 869.4251 | | V | 686.3468 | 699.3672 | | 730.4094 | 713.3828 | 71[illegible] |
| 9 | 102.0550 | 1012.4833 | | 994.4728 | 1040.4782 | | 1022.4677 | 954.4779 | | E | 557.3042 | 556.3089 | | ***631.3410*** | 614.3144 | 61[illegible] |
| 10 | 87.0553 | 1126.5263 | 1109.4997 | 1108.5157 | ***1154.5212*** | 1137.4946 | 1136.5106 | 1083.5204 | | N | 443.2613 | 442.2660 | | ***502.2984*** | 485.2718 | |
| 11 | 86.0964 | 1239.6103 | 1222.5838 | 1221.5998 | ***1267.6052*** | 1250.5787 | 1249.5947 | 1211.5790 | 1225.5947 | I | 330.1772 | 343.1976 | 357.2132 | 388.2554 | 371.2289 | |
| 12 | 30.0338 | 1296.6318 | 1279.6052 | 1278.6212 | 1324.6267 | 1307.6002 | 1306.6161 | | | G | | | | 275.1714 | 258.1448 | |
| 13 | 44.0495 | 1367.6689 | 1350.6424 | 1349.6583 | 1395.6638 | 1378.6373 | 1377.6533 | | | A | 202.1186 | | | 218.1499 | 201.1234 | |
| 14 | 101.1073 | | | | | | | | | K | 74.0237 | 73.0284 | | 147.1128 | 130.0863 | |

| Seq | ya | yb | Seq | ya | yb | Seq | ya | yb |
|---|---|---|---|---|---|---|---|---|
| VE | 201.1234 | 229.1183 | VEL | 314.2074 | 342.2023 | VELE | 443.2500 | 471.2449 |
| VELED | 558.2770 | 586.2719 | VELEDP | 655.3297 | 683.3246 | EL | 215.1390 | 243.1339 |
| ELE | 344.1816 | 372.1765 | ELED | 459.2086 | 487.2035 | ELEDP | 556.2613 | 584.2562 |
| ELEDPV | 655.3297 | 683.3246 | LE | 215.1390 | 243.1339 | LED | 330.1660 | 358.1609 |
| LEDP | 427.2187 | 455.2136 | LEDPV | 526.2871 | 554.2821 | LEDPVE | 655.3297 | 683.3246 |
| ED | 217.0819 | 245.0768 | EDP | 314.1347 | 342.1296 | EDPV | 413.2031 | 441.1980 |
| EDPVE | 542.2457 | 570.2406 | EDPVEN | 656.2886 | 684.2835 | DP | 185.0921 | 213.0870 |

| | | | | | | | | |
|---|---|---|---|---|---|---|---|---|
| **DPV** | 284.1605 | 312.1554 | **DPVE** | 413.2031 | 441.1980 | **DPVEN** | 527.2460 | 555.2409 |
| **DPVENI** | 640.3301 | 668.3250 | **DPVENIG** | 697.3515 | 725.3464 | **PV** | 169.1335 | 197.1285 |
| **PVE** | 298.1761 | 326.1710 | **PVEN** | 412.2191 | 440.2140 | **PVENI** | 525.3031 | 553.2980 |
| **PVENIG** | 582.3246 | 610.3195 | **PVENIGA** | 653.3617 | 681.3566 | **VE** | 201.1234 | 229.1183 |
| **VEN** | 315.1663 | 343.1612 | **VENI** | 428.2504 | 456.2453 | **VENIG** | 485.2718 | 513.2667 |
| **VENIGA** | 556.3089 | 584.3039 | **EN** | 216.0979 | 244.0928 | **ENI** | 329.1819 | 357.1769 |
| **ENIG** | 386.2034 | 414.1983 | **ENIGA** | 457.2405 | 485.2354 | **NI** | 200.1394 | 228.1343 |
| **NIG** | 257.1608 | 285.1557 | **NIGA** | 328.1979 | 356.1928 | **IG** | 143.1179 | 171.1128 |
| **IGA** | 214.1550 | 242.1499 | **GA** | 101.0709 | 129.0659 | | | |

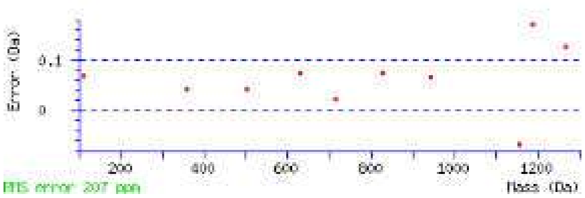


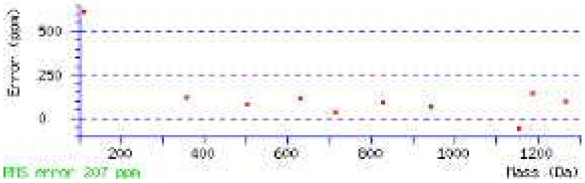


NCBI **BLAST** search of EVELEDPVENIGAK
(Parameters: blastp, nr protein database, expect=20000, no filter, PAM30)
Other BLAST web gateways

**All matches to this query**

| Score | Mr(calc) | Delta | Sequence |
|---|---|---|---|
| 39.3 | 1540.7620 | 0.0557 | EVELEDPVENIGAK |
| 28.2 | 1540.8322 | -0.0144 | ETSGQAGTPIVLRGR |
| 26.8 | 1540.8865 | -0.0687 | EPLYLVVPETLLR |
| 22.4 | 1540.7621 | 0.0557 | EDLLEDAGVDPAGLK |
| 21.9 | 1540.8474 | -0.0297 | VSTSKPFPVPNRGR |
| 21.8 | 1540.7409 | 0.0768 | FSYSDDPINDLKK |
| 20.1 | 1540.8322 | -0.0144 | SGIDAVGQVDLGVRR |
| 20.0 | 1540.7845 | 0.0332 | GTAGTDPDGILQAGLR |
| 19.0 | 1540.7998 | 0.0179 | WGEQVDPVKSQIR |
| 17.9 | 1540.8685 | -0.0508 | GLGSDATLVLINGRR |

**Mascot:** http://www.matrixscience.com/

# MATRIX SCIENCE Mascot Search Results

## Peptide View Spot no 92

MS/MS Fragmentation of **AAVEEGIVVGGGCTLLR**
Found in **gi|15231255** in **NCBInr**, TCP-1/cpn60 chaperonin family protein [Arabidopsis thaliana]

Match to Query 275: 1699.963424 from(1700.970700,1+) intensity(0.0000) index(27)
Title: Label: E15, Spot_Id: 219902, Peak_List_Id: 228285, MSMS Job_Run_Id: 21986, Comment:
Data file C:\Documents and Settings\Administrator\Desktop\Farah-Garima 11May 2013\ppw_E15_136868276000.txt

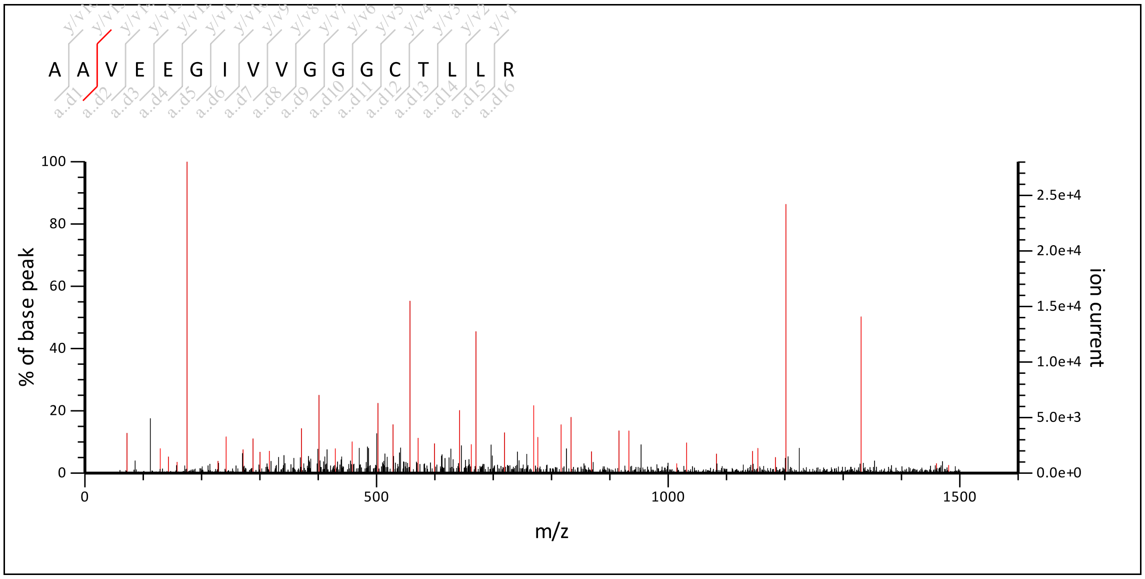


0 to 1600.02

Label all possible matches ○ Label matches used for scoring ◉

**Monoisotopic mass of neutral peptide Mr(calc):** 1699.8927
**Fixed modifications:** Carbamidomethyl (C) (apply to specified residues or termini only)
**Ions Score:** 111 **Expect:** 2.1e-07
**Matches :** 66/298 fragment ions using 60 most intense peaks (help)

| # | Immon. | a | a0 | b | b0 | d | d' | Seq. | v | w | w' | y | y* | y0 | # |
|---|---|---|---|---|---|---|---|---|---|---|---|---|---|---|---|
| 1 | 44.0495 | 44.0495 | | **72.0444** | | 44.0495 | | A | | | | | | | 17 |
| 2 | 44.0495 | 115.0866 | | **143.0815** | | | | A | 1613.8316 | | | 1629.8629 | 1612.8363 | 1611.8523 | 16 |
| 3 | 72.0808 | 214.1550 | | **242.1499** | | 200.1394 | | V | 1514.7632 | 1527.7836 | | 1558.8258 | 1541.7992 | 1540.8152 | 15 |
| 4 | 102.0550 | 343.1976 | 325.1870 | **371.1925** | 353.1819 | 285.1921 | | E | 1385.7206 | 1384.7253 | | ***1459.7573*** | 1442.7308 | 1441.7468 | 14 |
| 5 | 102.0550 | 472.2402 | 454.2296 | **500.2351** | 482.2245 | 414.2347 | | E | 1256.6780 | 1255.6827 | | ***1330.7147*** | 1313.6882 | 1312.7042 | 13 |
| 6 | 30.0338 | 529.2617 | 511.2511 | **557.2566** | 539.2460 | | | G | | | | ***1201.6722*** | 1184.6456 | 1183.6616 | 12 |
| 7 | 86.0964 | 642.3457 | 624.3352 | **670.3406** | 652.3301 | 614.3144 | 628.3301 | I | 1086.5724 | 1099.5928 | 1113.6085 | ***1144.6507*** | 1127.6241 | 1126.6401 | 11 |
| 8 | 72.0808 | 741.4141 | 723.4036 | **769.4090** | 751.3985 | 727.3985 | | V | 987.5040 | 1000.5244 | | ***1031.5666*** | 1014.5401 | 1013.5561 | 10 |
| 9 | 72.0808 | 840.4825 | 822.4720 | **868.4775** | 850.4669 | 826.4669 | | V | 888.4356 | 901.4560 | | ***932.4982*** | 915.4717 | 914.4876 | 9 |
| 10 | 30.0338 | 897.5040 | 879.4934 | 925.4989 | 907.4884 | | | G | | | | ***833.4298*** | 816.4033 | 815.4192 | 8 |
| 11 | 30.0338 | 954.5255 | 936.5149 | 982.5204 | 964.5098 | | | G | | | | ***776.4083*** | 759.3818 | 758.3978 | 7 |
| 12 | 30.0338 | 1011.5469 | 993.5364 | 1039.5419 | 1021.5313 | | | G | | | | ***719.3869*** | 702.3603 | 701.3763 | 6 |
| 13 | 133.0430 | 1171.5776 | 1153.5670 | 1199.5725 | 1181.5619 | 1082.5841 | | C | 557.3406 | 556.3453 | | ***662.3654*** | 645.3389 | 644.3548 | 5 |
| 14 | 74.0600 | 1272.6253 | 1254.6147 | 1300.6202 | 1282.6096 | 1256.6304 | 1258.6096 | T | 456.2929 | 469.3133 | 471.2926 | ***502.3348*** | 485.3082 | 484.3242 | 4 |
| 15 | 86.0964 | 1385.7093 | 1367.6988 | 1413.7042 | 1395.6937 | 1343.6624 | | L | 343.2088 | 342.2136 | | ***401.2871*** | 384.2605 | | 3 |
| 16 | 86.0964 | 1498.7934 | 1480.7828 | 1526.7883 | 1508.7777 | 1456.7464 | | L | 230.1248 | 229.1295 | | ***288.2030*** | 271.1765 | | 2 |
| 17 | 129.1135 | | | | | | | R | 74.0237 | 73.0284 | | ***175.1190*** | 158.0924 | | 1 |

| Seq | ya | yb | Seq | ya | yb | Seq | ya | yb |
|---|---|---|---|---|---|---|---|---|
| AV | 143.1179 | 171.1128 | AVE | 272.1605 | 300.1554 | AVEE | 401.2031 | 429.1980 |

| | | | | | | | | |
|---|---|---|---|---|---|---|---|---|
| AVEEG | 458.2245 | 486.2195 | AVEEGI | 571.3086 | 599.3035 | AVEEGIV | 670.3770 | 698.3719 |
| VE | 201.1234 | 229.1183 | VEE | 330.1660 | 358.1609 | VEEG | 387.1874 | 415.1823 |
| VEEGI | 500.2715 | 528.2664 | VEEGIV | 599.3399 | 627.3348 | VEEGIVV | 698.4083 | 726.4032 |
| EE | 231.0975 | 259.0925 | EEG | 288.1190 | 316.1139 | EEGI | 401.2031 | 429.1980 |
| EEGIV | 500.2715 | 528.2664 | EEGIVV | 599.3399 | 627.3348 | EEGIVVG | 656.3614 | 684.3563 |
| EG | 159.0764 | 187.0713 | EGI | 272.1605 | 300.1554 | EGIV | 371.2289 | 399.2238 |
| EGIVV | 470.2973 | 498.2922 | EGIVVG | 527.3188 | 555.3137 | EGIVVGG | 584.3402 | 612.3352 |
| EGIVVGGG | 641.3617 | 669.3566 | GI | 143.1179 | 171.1128 | GIV | 242.1863 | 270.1812 |
| GIVV | 341.2547 | 369.2496 | GIVVG | 398.2762 | 426.2711 | GIVVGG | 455.2976 | 483.2926 |
| GIVVGGG | 512.3191 | 540.3140 | GIVVGGGC | 672.3498 | 700.3447 | IV | 185.1648 | 213.1598 |
| IVV | 284.2333 | 312.2282 | IVVG | 341.2547 | 369.2496 | IVVGG | 398.2762 | 426.2711 |
| IVVGGG | 455.2976 | 483.2926 | IVVGGGC | 615.3283 | 643.3232 | VV | 171.1492 | 199.1441 |
| VVG | 228.1707 | 256.1656 | VVGG | 285.1921 | 313.1870 | VVGGG | 342.2136 | 370.2085 |
| VVGGGC | 502.2442 | 530.2391 | VVGGGCT | 603.2919 | 631.2868 | VG | 129.1022 | 157.0972 |
| VGG | 186.1237 | 214.1186 | VGGG | 243.1452 | 271.1401 | VGGGC | 403.1758 | 431.1707 |
| VGGGCT | 504.2235 | 532.2184 | VGGGCTL | 617.3076 | 645.3025 | GG | 87.0553 | 115.0502 |
| GGG | 144.0768 | 172.0717 | GGGC | 304.1074 | 332.1023 | GGGCT | 405.1551 | 433.1500 |
| GGGCTL | 518.2391 | 546.2341 | GGGCTLL | 631.3232 | 659.3181 | GG | 87.0553 | 115.0502 |
| GGC | 247.0859 | 275.0809 | GGCT | 348.1336 | 376.1285 | GGCTL | 461.2177 | 489.2126 |
| GGCTLL | 574.3017 | 602.2967 | GC | 190.0645 | 218.0594 | GCT | 291.1122 | 319.1071 |
| GCTL | 404.1962 | 432.1911 | GCTLL | 517.2803 | 545.2752 | CT | 234.0907 | 262.0856 |
| CTL | 347.1748 | 375.1697 | CTLL | 460.2588 | 488.2537 | TL | 187.1441 | 215.1390 |
| TLL | 300.2282 | 328.2231 | LL | 199.1805 | 227.1754 | | | |

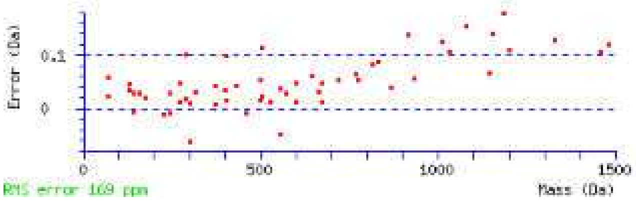


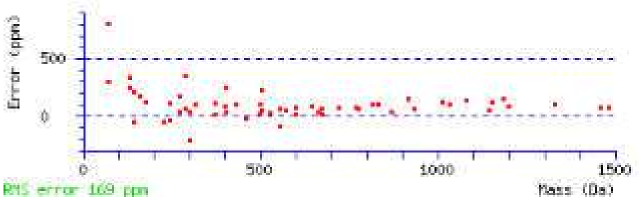


NCBI **BLAST** search of AAVEEGIVVGGGCTLLR
(Parameters: blastp, nr protein database, expect=20000, no filter, PAM30)
Other BLAST web gateways

**All matches to this query**

| Score | Mr(calc) | Delta | Sequence |
|---|---|---|---|
| 110.7 | 1699.8927 | 0.0707 | AAVEEGIVVGGGCTLIR |
| 110.7 | 1699.8927 | 0.0707 | AAVEEGIVVGGGCTLLR |
| 74.7 | 1699.8927 | 0.0707 | AAVEEGVVIGGGCTLLR |
| 57.2 | 1699.8927 | 0.0707 | AAVEEGIVLGGGCSLLR |
| 38.9 | 1699.9356 | 0.0278 | GLLDTGIVDISTDLLR |
| 36.3 | 1699.9046 | 0.0589 | EAFEGPWDLVRLIR |
| 33.8 | 1699.9178 | 0.0456 | AAVEEVIGVLLGMQAGK |
| 33.5 | 1699.9145 | 0.0490 | SSLIRPDDFIPIAEK |
| 33.2 | 1699.9551 | 0.0083 | LLAMVVAAGMVMLPLR |
| 32.4 | 1699.9356 | 0.0278 | AGLSIDLTDSIDVLLR |

**Mascot:** http://www.matrixscience.com/

# MATRIX SCIENCE Mascot Search Results

## Peptide View Spot no 96

MS/MS Fragmentation of **ACYECLR**
Found in **gi|11466795** in **NCBInr**, ribulose-1,5-bisphosphate carboxylase/oxygenase large subunit [Oryza sativa Japonica Group]

Match to Query 2: 970.467514 from(971.474790,1+) intensity(0.0000) index(0)
Title: Label: E11, Spot_Id: 216641, Peak_List_Id: 221252, MSMS Job_Run_Id: 21347, Comment:
Data file C:\Documents and Settings\Administrator\Desktop\farah BHU\RAJESH\12-1-13 raj\ppw_E11_135997701000.txt

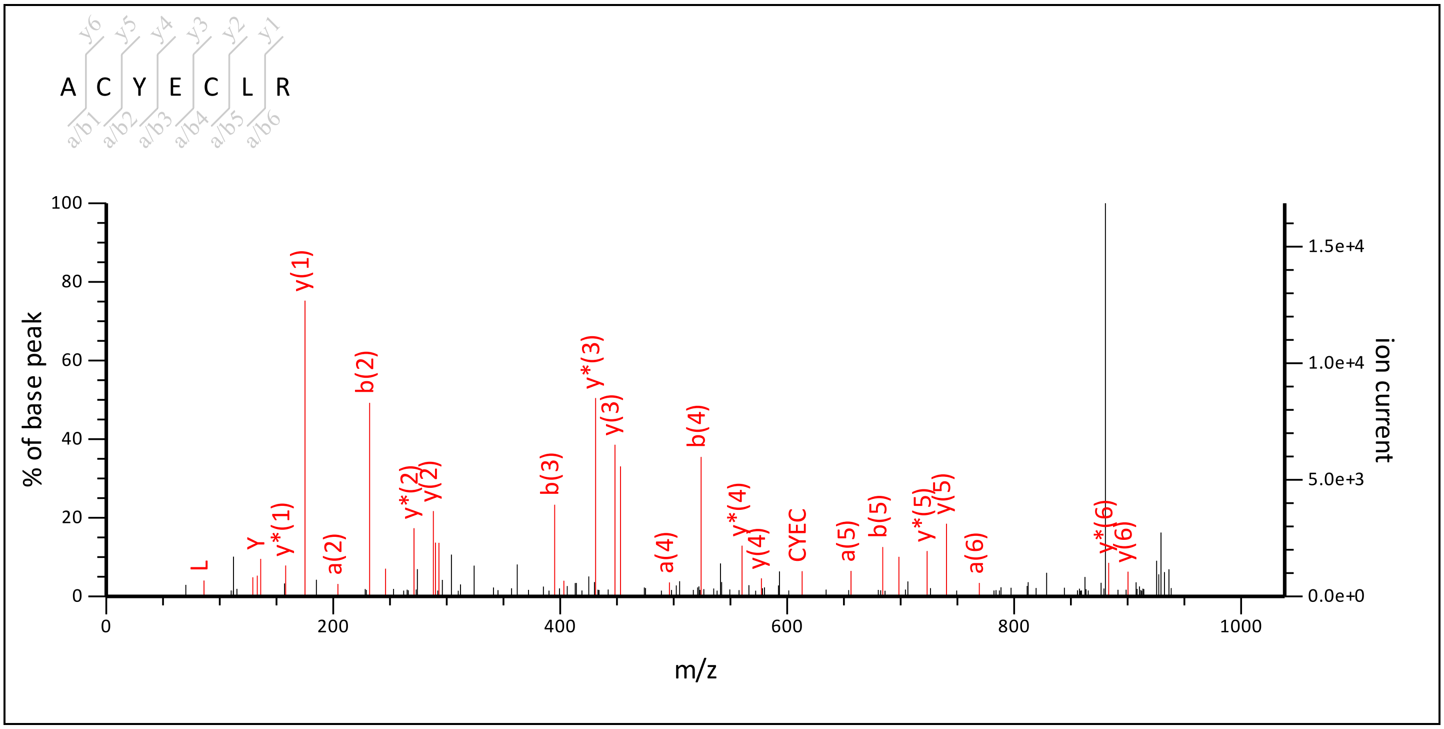


0 to 1038.4

Label all possible matches ○ Label matches used for scoring ●

**Monoisotopic mass of neutral peptide Mr(calc):** 970.4001
**Fixed modifications:** Carbamidomethyl (C) (apply to specified residues or termini only)
**Ions Score:** 36 **Expect:** 1.7
**Matches :** 35/75 fragment ions using 54 most intense peaks (help)

| # | Immon. | a | a0 | b | b0 | d | Seq. | v | w | y | y* | y0 | # |
|---|---|---|---|---|---|---|---|---|---|---|---|---|---|
| 1 | 44.0495 | 44.0495 | | 72.0444 | | 44.0495 | A | | | | | | 7 |
| 2 | 133.0430 | **204.0801** | | **232.0750** | | 115.0866 | C | 795.3454 | 794.3502 | ***900.3702*** | 883.3437 | 882.3597 | 6 |
| 3 | 136.0757 | 367.1435 | | **395.1384** | | | Y | 632.2821 | | ***740.3396*** | 723.3130 | 722.3290 | 5 |
| 4 | 102.0550 | **496.1860** | 478.1755 | **524.1810** | 506.1704 | 438.1806 | E | 503.2395 | 502.2442 | ***577.2763*** | 560.2497 | 559.2657 | 4 |
| 5 | 133.0430 | **656.2167** | 638.2061 | **684.2116** | 666.2010 | 567.2232 | C | 343.2088 | 342.2136 | ***448.2337*** | 431.2071 | | 3 |
| 6 | 86.0964 | **769.3008** | 751.2902 | 797.2957 | 779.2851 | 727.2538 | L | 230.1248 | 229.1295 | ***288.2030*** | 271.1765 | | 2 |
| 7 | 129.1135 | | | | | | R | 74.0237 | 73.0284 | ***175.1190*** | 158.0924 | | 1 |

| Seq | ya | yb | Seq | ya | yb | Seq | ya | yb |
|---|---|---|---|---|---|---|---|---|
| CY | 296.1063 | 324.1013 | CYE | 425.1489 | 453.1438 | CYEC | 585.1796 | 613.1745 |
| CYECL | 698.2636 | 726.2586 | YE | 265.1183 | 293.1132 | YEC | 425.1489 | 453.1438 |
| YECL | 538.2330 | 566.2279 | EC | 262.0856 | 290.0805 | ECL | 375.1697 | 403.1646 |
| CL | 246.1271 | 274.1220 | | | | | | |

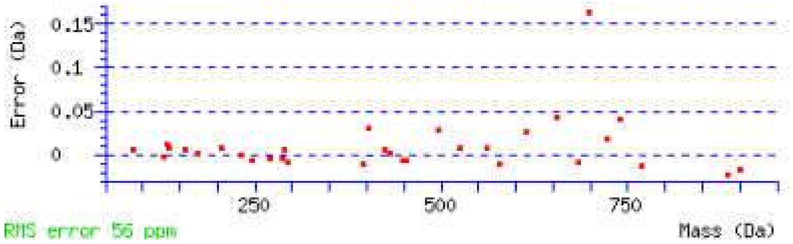


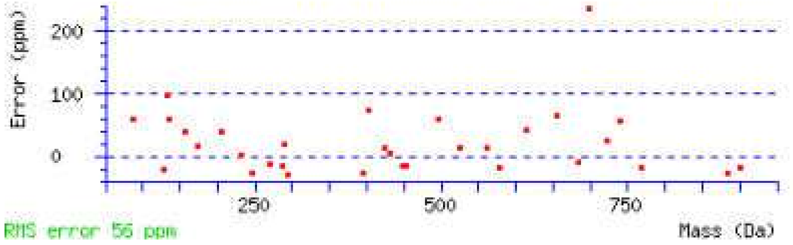


NCBI **BLAST** search of ACYECLR
(Parameters: blastp, nr protein database, expect=20000, no filter, PAM30)
Other BLAST web gateways

**All matches to this query**

| Score | Mr(calc) | Delta | Sequence |
|---|---|---|---|
| 35.9 | 970.4001 | 0.0675 | ACYECIR |
| 35.9 | 970.4001 | 0.0675 | ACYECLR |
| 35.9 | 970.4001 | 0.0675 | ACYECLR |
| 35.9 | 970.4001 | 0.0675 | ACYECLR |
| 35.9 | 970.4001 | 0.0675 | ACYECLR |
| 29.5 | 970.4807 | -0.0132 | ACFAPHLR |
| 22.4 | 970.4364 | 0.0311 | CVYMNIR |
| 22.4 | 969.4412 | 1.0263 | MVYECLR |
| 20.6 | 970.5382 | -0.0707 | LANPKCLR |
| 19.2 | 969.5243 | 0.9432 | AAATPAAELR |

**Mascot:** http://www.matrixscience.com/

# MATRIX SCIENCE Mascot Search Results

## Peptide View Spot no 96

MS/MS Fragmentation of **ALRLEDLR**
Found in **gi|11466795** in **NCBInr**, ribulose-1,5-bisphosphate carboxylase/oxygenase large subunit [Oryza sativa Japonica Group]

Match to Query 4: 984.639634 from(985.646910,1+) intensity(0.0000) index(1)
Title: Label: E11, Spot_Id: 216641, Peak_List_Id: 221259, MSMS Job_Run_Id: 21347, Comment:
Data file C:\Documents and Settings\Administrator\Desktop\farah BHU\RAJESH\12-1-13 raj\ppw_E11_135997701000.txt

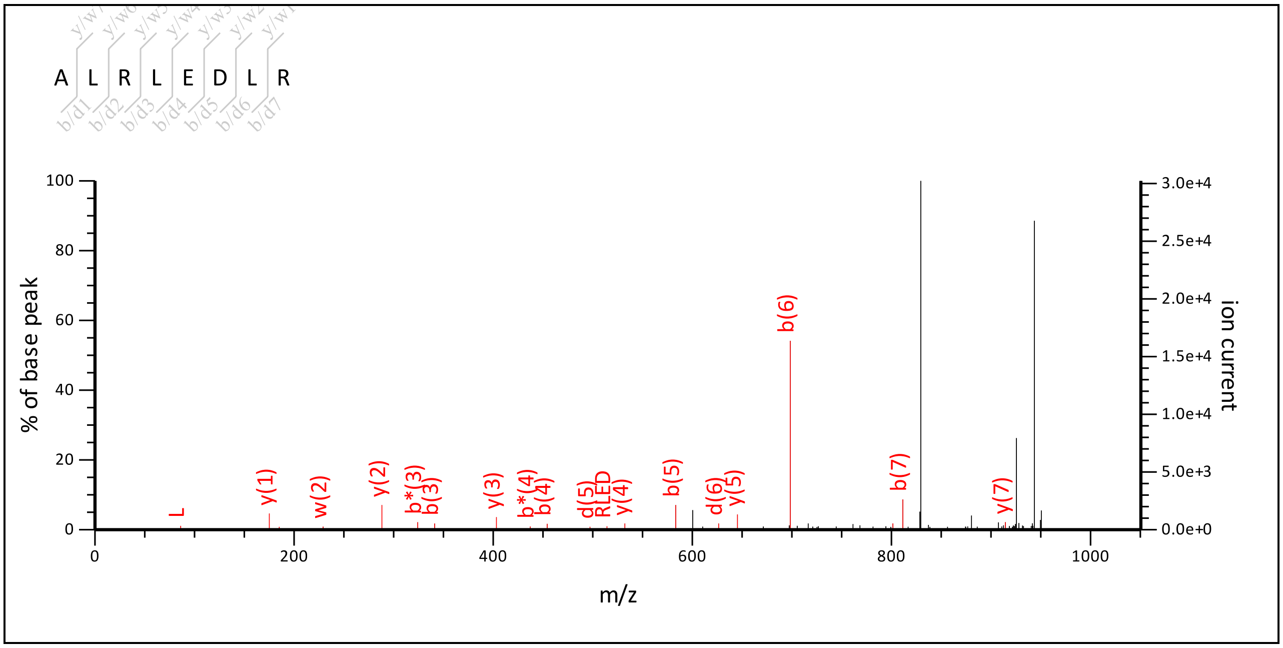


0 to 1050.54

Label all possible matches ○ Label matches used for scoring ◉

**Monoisotopic mass of neutral peptide Mr(calc):** 984.5716
**Fixed modifications:** Carbamidomethyl (C) (apply to specified residues or termini only)
**Ions Score:** 49 **Expect:** 0.092
**Matches :** 24/106 fragment ions using 34 most intense peaks (help)

| # | Immon. | a | a* | $a^0$ | b | b* | $b^0$ | d | Seq. | v | w | y | y* | $y^0$ | # |
|---|---|---|---|---|---|---|---|---|---|---|---|---|---|---|---|
| 1 | 44.0495 | 44.0495 | | | 72.0444 | | | 44.0495 | A | | | | | | 8 |
| 2 | 86.0964 | 157.1335 | | | **185.1285** | | | 115.0866 | L | 856.4635 | 855.4683 | ***914.5418*** | 897.5152 | 896.5312 | 7 |
| 3 | 129.1135 | 313.2346 | 296.2081 | | **341.2296** | 324.2030 | | 228.1707 | R | 700.3624 | 699.3672 | ***801.4577*** | 784.4312 | 783.4472 | 6 |
| 4 | 86.0964 | 426.3187 | 409.2922 | | **454.3136** | 437.2871 | | 384.2718 | L | 587.2784 | 586.2831 | ***645.3566*** | 628.3301 | 627.3461 | 5 |
| 5 | 102.0550 | 555.3613 | 538.3348 | 537.3507 | **583.3562** | 566.3297 | 565.3457 | 497.3558 | E | 458.2358 | 457.2405 | ***532.2726*** | 515.2460 | 514.2620 | 4 |
| 6 | 88.0393 | 670.3883 | 653.3617 | 652.3777 | **698.3832** | 681.3566 | 680.3726 | 626.3984 | D | 343.2088 | 342.2136 | ***403.2300*** | 386.2034 | 385.2194 | 3 |
| 7 | 86.0964 | 783.4723 | 766.4458 | 765.4617 | **811.4672** | 794.4407 | 793.4567 | 741.4254 | L | 230.1248 | 229.1295 | ***288.2030*** | 271.1765 | | 2 |
| 8 | 129.1135 | | | | | | | | R | 74.0237 | 73.0284 | ***175.1190*** | 158.0924 | | 1 |

| Seq | ya | yb | Seq | ya | yb | Seq | ya | yb |
|---|---|---|---|---|---|---|---|---|
| LR | 242.1975 | 270.1925 | LRL | 355.2816 | 383.2765 | LRLE | 484.3242 | 512.3191 |
| LRLED | 599.3511 | 627.3461 | RL | 242.1975 | 270.1925 | RLE | 371.2401 | 399.2350 |
| RLED | 486.2671 | 514.2620 | RLEDL | 599.3511 | 627.3461 | LE | 215.1390 | 243.1339 |
| LED | 330.1660 | 358.1609 | LEDL | 443.2500 | 471.2449 | ED | 217.0819 | 245.0768 |

| | | | | | | | | |
|---|---|---|---|---|---|---|---|---|
| **EDL** | 330.1660 | 358.1609 | **DL** | 201.1234 | 229.1183 | | | |

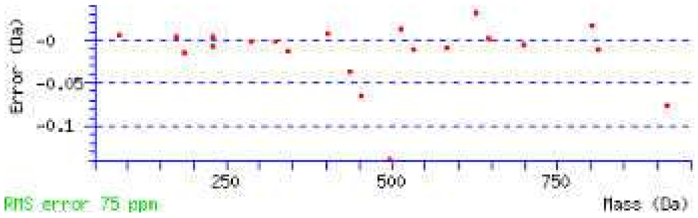


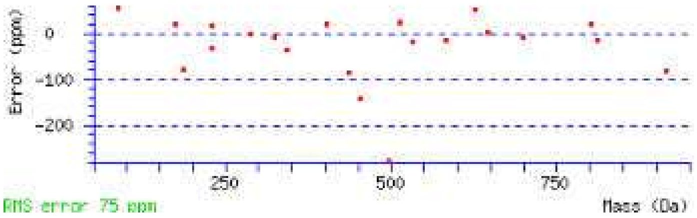


NCBI **BLAST** search of ALRLEDLR
(Parameters: blastp, nr protein database, expect=20000, no filter, PAM30)
Other BLAST web gateways

**All matches to this query**

| Score | Mr(calc) | Delta | Sequence |
|---|---|---|---|
| 50.9 | 984.5716 | 0.0680 | ALRIEDLR |
| 50.9 | 984.5716 | 0.0680 | ALRIEDLR |
| 50.7 | 984.0796 | 0.5600 | ALRLEBLR |
| 50.7 | 984.0796 | 0.5600 | ALRLEBLR |
| 49.1 | 984.5716 | 0.0680 | AIRLEDLR |
| 49.1 | 984.5716 | 0.0680 | AIRLEDLR |
| 49.1 | 984.5716 | 0.0680 | ALRLEDIR |
| 49.1 | 984.5716 | 0.0680 | ALRLEDIR |
| 49.1 | 984.5716 | 0.0680 | ALRLEDLR |
| 49.1 | 984.5716 | 0.0680 | ALRLEDLR |

**Mascot:** http://www.matrixscience.com/

# MATRIX SCIENCE Mascot Search Results

## Peptide View Spot no 96

MS/MS Fragmentation of **FVFCAEAIYK**
Found in **gi|11466795** in **NCBInr**, ribulose-1,5-bisphosphate carboxylase/oxygenase large subunit [Oryza sativa Japonica Group]

Match to Query 9: 1246.668124 from(1247.675400,1+) intensity(0.0000) index(4)
Title: Label: E11, Spot_Id: 216641, Peak_List_Id: 221255, MSMS Job_Run_Id: 21347, Comment:
Data file C:\Documents and Settings\Administrator\Desktop\farah BHU\RAJESH\12-1-13 raj\ppw_E11_135997701000.txt

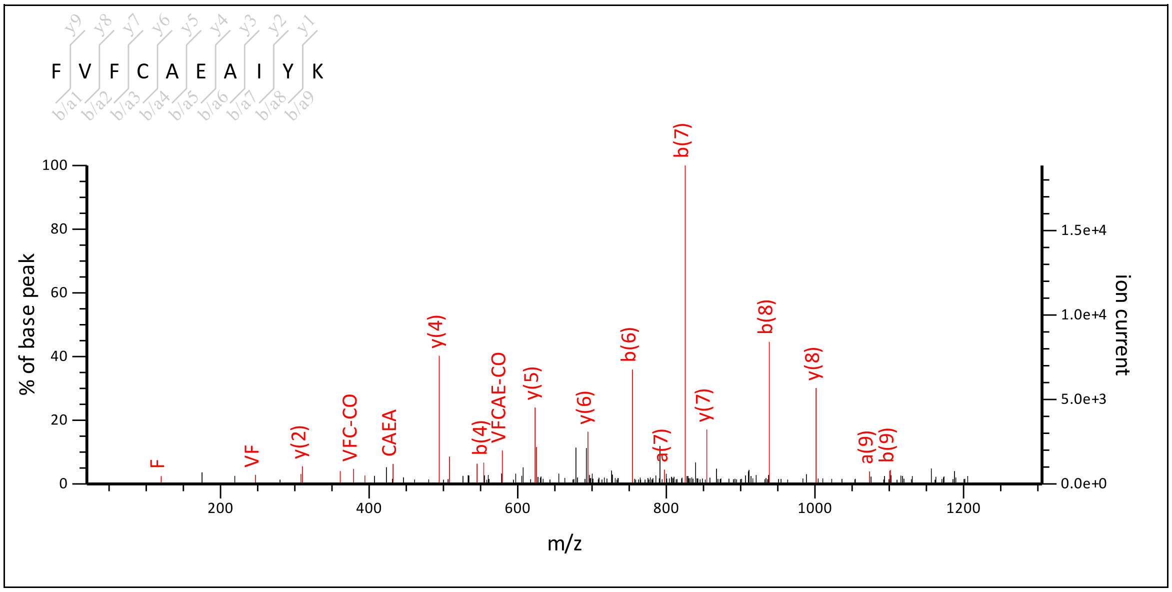


20.1 to 1305.58

Label all possible matches ○ Label matches used for scoring ◉

**Monoisotopic mass of neutral peptide Mr(calc):** 1246.6056
**Fixed modifications:** Carbamidomethyl (C) (apply to specified residues or termini only)
**Ions Score:** 68 **Expect:** 0.0011
**Matches :** 32/129 fragment ions using 33 most intense peaks (help)

| # | Immon. | a | a0 | b | b0 | d | d' | Seq. | v | w | w' | y | y* | y0 | # |
|---|---|---|---|---|---|---|---|---|---|---|---|---|---|---|---|
| 1 | 120.0808 | **120.0808** | | 148.0757 | | 44.0495 | | F | | | | | | | 10 |
| 2 | 72.0808 | **219.1492** | | ***247.1441*** | | 205.1335 | | V | 1056.4819 | 1069.5023 | | ***1100.5445*** | 1083.5179 | 1082.5339 | 9 |
| 3 | 120.0808 | 366.2176 | | ***394.2125*** | | | | F | 909.4135 | | | ***1001.4761*** | 984.4495 | 983.4655 | 8 |
| 4 | 133.0430 | 526.2483 | | ***554.2432*** | | 437.2547 | | C | 749.3828 | 748.3876 | | ***854.4077*** | 837.3811 | 836.3971 | 7 |
| 5 | 44.0495 | 597.2854 | | ***625.2803*** | | | | A | 678.3457 | | | ***694.3770*** | 677.3505 | 676.3665 | 6 |
| 6 | 102.0550 | 726.3280 | 708.3174 | ***754.3229*** | 736.3123 | 668.3225 | | E | 549.3031 | 548.3079 | | ***623.3399*** | 606.3134 | 605.3293 | 5 |
| 7 | 44.0495 | **797.3651** | 779.3545 | ***825.3600*** | 807.3494 | | | A | 478.2660 | | | ***494.2973*** | 477.2708 | | 4 |
| 8 | 86.0964 | 910.4491 | 892.4386 | ***938.4441*** | 920.4335 | 882.4178 | 896.4335 | I | 365.1819 | 378.2023 | 392.2180 | 423.2602 | 406.2336 | | 3 |
| 9 | 136.0757 | **1073.5125** | 1055.5019 | ***1101.5074*** | 1083.4968 | | | Y | 202.1186 | | | ***310.1761*** | 293.1496 | | 2 |
| 10 | 101.1073 | | | | | | | K | 74.0237 | 73.0284 | | 147.1128 | 130.0863 | | 1 |

| Seq | ya | yb | Seq | ya | yb | Seq | ya | yb |
|---|---|---|---|---|---|---|---|---|
| VF | 219.1492 | 247.1441 | VFC | 379.1798 | 407.1748 | VFCA | 450.2170 | 478.2119 |
| VFCAE | 579.2595 | 607.2545 | VFCAEA | 650.2967 | 678.2916 | FC | 280.1114 | 308.1063 |
| FCA | 351.1485 | 379.1435 | FCAE | 480.1911 | 508.1860 | FCAEA | 551.2282 | 579.2232 |
| FCAEAI | 664.3123 | 692.3072 | CA | 204.0801 | 232.0750 | CAE | 333.1227 | 361.1176 |
| CAEA | 404.1598 | 432.1547 | CAEAI | 517.2439 | 545.2388 | CAEAIY | 680.3072 | 708.3021 |
| AE | 173.0921 | 201.0870 | AEA | 244.1292 | 272.1241 | AEAI | 357.2132 | 385.2082 |

| | | | | | | | | |
|---|---|---|---|---|---|---|---|---|
| AEAIY | 520.2766 | 548.2715 | EA | 173.0921 | 201.0870 | EAI | 286.1761 | 314.1710 |
| EAIY | 449.2395 | 477.2344 | AI | 157.1335 | 185.1285 | AIY | 320.1969 | 348.1918 |
| IY | 249.1598 | 277.1547 | | | | | | |

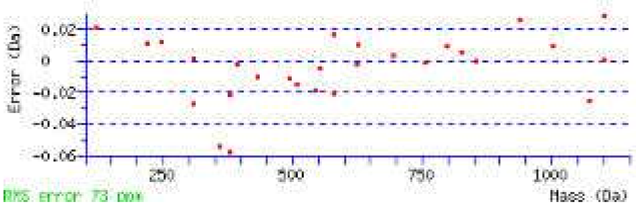


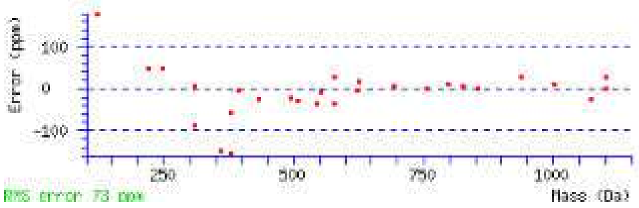


NCBI **BLAST** search of FVFCAEAIYK
(Parameters: blastp, nr protein database, expect=20000, no filter, PAM30)
Other BLAST web gateways

**All matches to this query**

| Score | Mr(calc) | Delta | Sequence |
|---|---|---|---|
| 67.5 | 1246.6056 | 0.0625 | FVFCAEAIYK |
| 67.5 | 1246.6056 | 0.0625 | FVFCAEAIYK |
| 67.5 | 1246.6056 | 0.0625 | FVFCAEAIYK |
| 67.5 | 1246.6056 | 0.0625 | FVFCAEAIYK |
| 67.5 | 1246.6056 | 0.0625 | FVFCAEAIYK |
| 67.5 | 1246.6056 | 0.0625 | FVFCAEAIYK |
| 67.5 | 1246.6056 | 0.0625 | FVFCAEAIYK |
| 67.5 | 1246.6056 | 0.0625 | FVFCAEALYK |
| 67.5 | 1246.6056 | 0.0625 | FVFCAEALYK |
| 67.5 | 1246.6056 | 0.0625 | FVFCAEALYK |

**Mascot:** http://www.matrixscience.com/

# MATRIX SCIENCE Mascot Search Results

## Peptide View **Spot no 96**

MS/MS Fragmentation of **TFQGPPHGIQVER**
Found in **gi|11466795** in **NCBInr**, ribulose-1,5-bisphosphate carboxylase/oxygenase large subunit [Oryza sativa Japonica Group]

Match to Query 11: 1464.832324 from(1465.839600,1+) intensity(0.0000) index(5)
Title: Label: E11, Spot_Id: 216641, Peak_List_Id: 221246, MSMS Job_Run_Id: 21347, Comment:
Data file C:\Documents and Settings\Administrator\Desktop\farah BHU\RAJESH\12-1-13 raj\ppw_E11_135997701000.txt

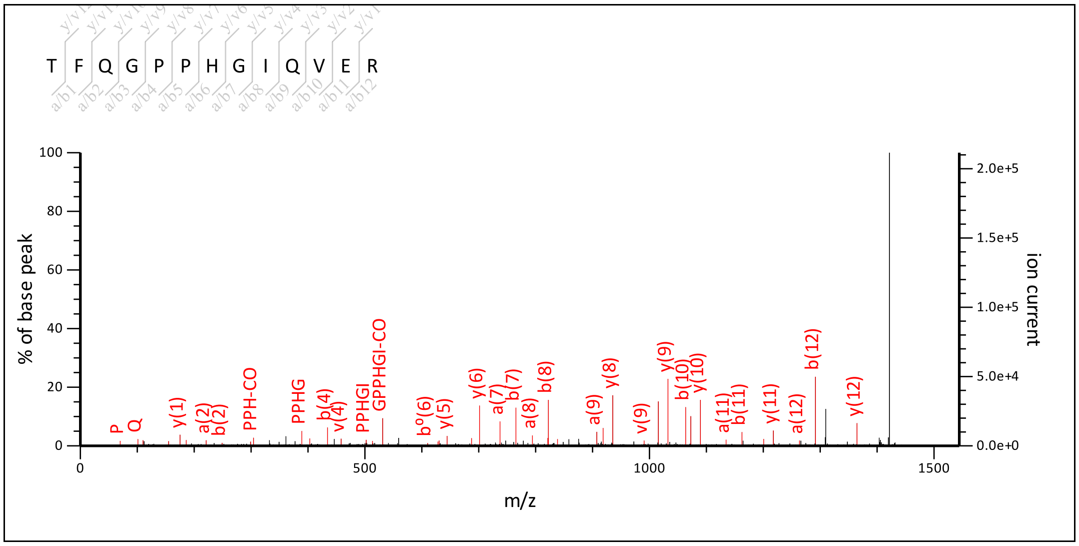


0 to 1544.37

Label all possible matches ○ Label matches used for scoring ◉

**Monoisotopic mass of neutral peptide Mr(calc):** 1464.7474
**Fixed modifications:** Carbamidomethyl (C) (apply to specified residues or termini only)
**Ions Score:** 117 **Expect:** 1.5e-08
**Matches :** 61/229 fragment ions using 56 most intense peaks (help)

| # | Immon. | a | a* | $a^0$ | b | b* | $b^0$ | d | d' | Seq. | v | w | w' | y | y* | |
|---|---|---|---|---|---|---|---|---|---|---|---|---|---|---|---|---|
| 1 | 74.0600 | 74.0600 | | 56.0495 | 102.0550 | | 84.0444 | 44.0495 | | T | | | | | | |
| 2 | 120.0808 | **221.1285** | | 203.1179 | **249.1234** | | 231.1128 | | | F | 1272.6444 | | | ***1364.7070*** | 1347.6804 | 134[illegible] |
| 3 | 101.0709 | 349.1870 | 332.1605 | 331.1765 | 377.1819 | 360.1554 | 359.1714 | 292.1656 | | Q | 1144.5858 | 1143.5905 | | ***1217.6385*** | 1200.6120 | 119[illegible] |
| 4 | 30.0338 | 406.2085 | 389.1819 | 388.1979 | **434.2034** | 417.1769 | 416.1928 | | | G | | | | ***1089.5800*** | 1072.5534 | 107[illegible] |
| 5 | 70.0651 | 503.2613 | 486.2347 | 485.2507 | **531.2562** | 514.2296 | 513.2456 | 477.2456 | | P | 990.5116 | 989.5163 | | ***1032.5585*** | 1015.5320 | 101[illegible] |
| 6 | 70.0651 | 600.3140 | 583.2875 | 582.3035 | **628.3089** | 611.2824 | 610.2984 | 574.2984 | | P | 893.4588 | 892.4635 | | ***935.5057*** | 918.4792 | 91[illegible] |
| 7 | 110.0713 | **737.3729** | 720.3464 | 719.3624 | **765.3679** | 748.3413 | 747.3573 | | | H | 756.3999 | | | ***838.4530*** | 821.4264 | 82[illegible] |
| 8 | 30.0338 | **794.3944** | 777.3679 | 776.3838 | **822.3893** | 805.3628 | 804.3787 | | | G | | | | ***701.3941*** | 684.3675 | 68[illegible] |
| 9 | 86.0964 | **907.4785** | 890.4519 | 889.4679 | **935.4734** | 918.4468 | 917.4628 | 879.4472 | 893.4628 | I | 586.2944 | 599.3148 | 613.3304 | ***644.3726*** | 627.3461 | 62[illegible] |
| 10 | 101.0709 | 1035.5370 | 1018.5105 | 1017.5265 | **1063.5320** | 1046.5054 | 1045.5214 | 978.5156 | | Q | 458.2358 | 457.2405 | | ***531.2885*** | 514.2620 | 51[illegible] |
| 11 | 72.0808 | **1134.6055** | 1117.5789 | 1116.5949 | **1162.6004** | 1145.5738 | 1144.5898 | 1120.5898 | | V | 359.1674 | 372.1878 | | ***403.2300*** | 386.2034 | 38[illegible] |
| 12 | 102.0550 | **1263.6480** | 1246.6215 | 1245.6375 | **1291.6430** | 1274.6164 | 1273.6324 | 1205.6426 | | E | 230.1248 | 229.1295 | | ***304.1615*** | 287.1350 | 28[illegible] |
| 13 | 129.1135 | | | | | | | | | R | 74.0237 | 73.0284 | | ***175.1190*** | 158.0924 | |

| Seq | ya | yb | Seq | ya | yb | Seq | ya | yb |
|---|---|---|---|---|---|---|---|---|
| FQ | 248.1394 | 276.1343 | FQG | 305.1608 | 333.1557 | FQGP | 402.2136 | 430.2085 |
| FQGPP | 499.2663 | 527.2613 | FQGPPH | 636.3253 | 664.3202 | FQGPPHG | 693.3467 | 721.3416 |
| QG | 158.0924 | 186.0873 | QGP | 255.1452 | 283.1401 | QGPP | 352.1979 | 380.1928 |
| QGPPH | 489.2568 | 517.2518 | QGPPHG | 546.2783 | 574.2732 | QGPPHGI | 659.3624 | 687.3573 |
| GP | 127.0866 | 155.0815 | GPP | 224.1394 | 252.1343 | GPPH | 361.1983 | 389.1932 |
| GPPHG | 418.2197 | 446.2146 | GPPHGI | 531.3038 | 559.2987 | GPPHGIQ | 659.3624 | 687.3573 |
| PP | 167.1179 | 195.1128 | PPH | 304.1768 | 332.1717 | PPHG | 361.1983 | 389.1932 |
| PPHGI | 474.2823 | 502.2772 | PPHGIQ | 602.3409 | 630.3358 | PH | 207.1240 | 235.1190 |

| | | | | | | | | |
|---|---|---|---|---|---|---|---|---|
| **PHG** | 264.1455 | 292.1404 | **PHGI** | 377.2296 | 405.2245 | **PHGIQ** | 505.2881 | 533.2831 |
| **PHGIQV** | 604.3566 | 632.3515 | **HG** | 167.0927 | 195.0877 | **HGI** | 280.1768 | 308.1717 |
| **HGIQ** | 408.2354 | 436.2303 | **HGIQV** | 507.3038 | 535.2987 | **HGIQVE** | 636.3464 | 664.3413 |
| **GI** | 143.1179 | 171.1128 | **GIQ** | 271.1765 | 299.1714 | **GIQV** | 370.2449 | 398.2398 |
| **GIQVE** | 499.2875 | 527.2824 | **IQ** | 214.1550 | 242.1499 | **IQV** | 313.2234 | 341.2183 |
| **IQVE** | 442.2660 | 470.2609 | **QV** | 200.1394 | 228.1343 | **QVE** | 329.1819 | 357.1769 |
| **VE** | 201.1234 | 229.1183 | | | | | | |

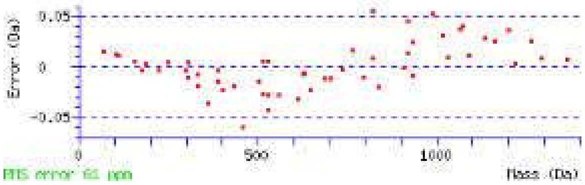


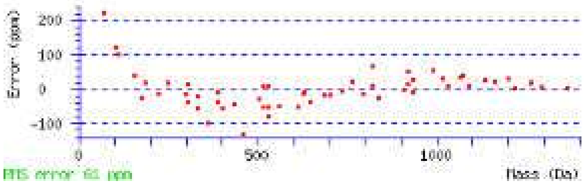


NCBI **BLAST** search of TFQGPPHGIQVER
(Parameters: blastp, nr protein database, expect=20000, no filter, PAM30)
Other BLAST web gateways

**All matches to this query**

| Score | Mr(calc) | Delta | Sequence |
|---|---|---|---|
| 116.9 | 1464.7838 | 0.0486 | TFKGPPHGIQVER |
| 116.9 | 1464.7838 | 0.0486 | TFKGPPHGIQVER |
| 116.9 | 1464.7838 | 0.0486 | TFQGPPHGIKVER |
| 116.9 | 1464.7838 | 0.0486 | TFQGPPHGIKVER |
| 116.9 | 1464.7474 | 0.0849 | TFQGPPHGIQVER |
| 116.9 | 1464.7474 | 0.0849 | TFQGPPHGIQVER |
| 116.9 | 1464.7474 | 0.0849 | TFQGPPHGIQVER |
| 116.9 | 1464.7474 | 0.0849 | TFQGPPHGIQVER |
| 116.9 | 1464.7474 | 0.0849 | TFQGPPHGIQVER |
| 116.9 | 1464.7474 | 0.0849 | TFQGPPHGIQVER |

**Mascot:** http://www.matrixscience.com/

# MATRIX SCIENCE Mascot Search Results

## Peptide View Spot no 96

MS/MS Fragmentation of **YGRPLLGCTIKPK**
Found in **gi|11466795** in **NCBInr**, ribulose-1,5-bisphosphate carboxylase/oxygenase large subunit [Oryza sativa Japonica Group]

Match to Query 13: 1501.927124 from(1502.934400,1+) intensity(0.0000) index(6)
Title: Label: E11, Spot_Id: 216641, Peak_List_Id: 221250, MSMS Job_Run_Id: 21347, Comment:
Data file C:\Documents and Settings\Administrator\Desktop\farah BHU\RAJESH\12-1-13 raj\ppw_E11_135997701000.txt

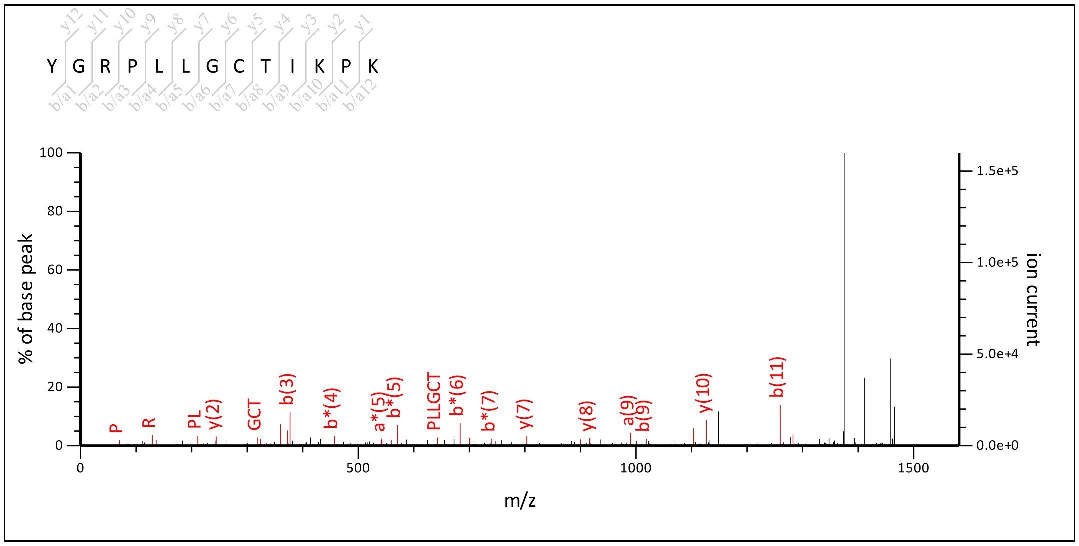


0 to 1581.78

Label all possible matches ○ Label matches used for scoring ◉

**Monoisotopic mass of neutral peptide Mr(calc):** 1501.8439
**Fixed modifications:** Carbamidomethyl (C) (apply to specified residues or termini only)
**Ions Score:** 40 **Expect:** 0.61
**Matches :** 33/211 fragment ions using 44 most intense peaks (help)

| # | Immon. | a | a* | $a^0$ | b | b* | $b^0$ | d | d' | Seq. | v | w | w' | y | y* | [illegible] |
|---|---|---|---|---|---|---|---|---|---|---|---|---|---|---|---|---|
| 1 | 136.0757 | **136.0757** | | | 164.0706 | | | 44.0495 | | **Y** | | | | | | |
| 2 | 30.0338 | 193.0972 | | | 221.0921 | | | | | **G** | | | | 1339.7879 | 1322.7613 | [illegible] |
| 3 | 129.1135 | 349.1983 | 332.1717 | | **377.1932** | 360.1666 | | 264.1343 | | **R** | 1181.6711 | 1180.6758 | | ***1282.7664*** | 1265.7398 | [illegible] |
| 4 | 70.0651 | 446.2510 | 429.2245 | | 474.2459 | 457.2194 | | 420.2354 | | **P** | 1084.6183 | 1083.6231 | | ***1126.6653*** | 1109.6387 | [illegible] |
| 5 | 86.0964 | **559.3351** | 542.3085 | | 587.3300 | 570.3035 | | 517.2881 | | **L** | 971.5343 | 970.5390 | | 1029.6125 | 1012.5860 | [illegible] |
| 6 | 86.0964 | 672.4192 | 655.3926 | | **700.4141** | 683.3875 | | 630.3722 | | **L** | 858.4502 | 857.4550 | | ***916.5285*** | 899.5019 | [illegible] |
| 7 | 30.0338 | 729.4406 | 712.4141 | | 757.4355 | 740.4090 | | | | **G** | | | | ***803.4444*** | 786.4178 | [illegible] |
| 8 | 133.0430 | 889.4713 | 872.4447 | | 917.4662 | 900.4396 | | 800.4777 | | **C** | 641.3981 | 640.4028 | | 746.4229 | 729.3964 | [illegible] |
| 9 | 74.0600 | **990.5189** | 973.4924 | 972.5084 | **1018.5139** | 1001.4873 | 1000.5033 | 974.5240 | 976.5033 | **T** | 540.3504 | 553.3708 | 555.3501 | ***586.3923*** | 569.3657 | [illegible] |
| 10 | 86.0964 | **1103.6030** | 1086.5765 | 1085.5924 | 1131.5979 | 1114.5714 | 1113.5874 | 1075.5717 | 1089.5874 | **I** | 427.2663 | 440.2867 | 454.3024 | 485.3446 | 468.3180 | |
| 11 | 101.1073 | 1231.6980 | 1214.6714 | 1213.6874 | **1259.6929** | 1242.6663 | 1241.6823 | 1174.6401 | | **K** | 299.1714 | 298.1761 | | ***372.2605*** | 355.2340 | |
| 12 | 70.0651 | 1328.7507 | 1311.7242 | 1310.7402 | 1356.7457 | 1339.7191 | 1338.7351 | 1302.7351 | | **P** | 202.1186 | 201.1234 | | ***244.1656*** | 227.1390 | |
| 13 | 101.1073 | | | | | | | | | **K** | 74.0237 | 73.0284 | | 147.1128 | 130.0863 | |

| Seq | ya | yb | Seq | ya | yb | Seq | ya | yb |
|---|---|---|---|---|---|---|---|---|
| **GR** | 186.1349 | 214.1299 | **GRP** | 283.1877 | 311.1826 | **GRPL** | 396.2718 | 424.2667 |
| **GRPLL** | 509.3558 | 537.3507 | **GRPLLG** | 566.3773 | 594.3722 | **RP** | 226.1662 | 254.1612 |
| **RPL** | 339.2503 | 367.2452 | **RPLL** | 452.3344 | 480.3293 | **RPLLG** | 509.3558 | 537.3507 |
| **RPLLGC** | 669.3865 | 697.3814 | **PL** | 183.1492 | 211.1441 | **PLL** | 296.2333 | 324.2282 |
| **PLLG** | 353.2547 | 381.2496 | **PLLGC** | 513.2854 | 541.2803 | **PLLGCT** | 614.3330 | 642.3280 |
| **LL** | 199.1805 | 227.1754 | **LLG** | 256.2020 | 284.1969 | **LLGC** | 416.2326 | 444.2275 |
| **LLGCT** | 517.2803 | 545.2752 | **LLGCTI** | 630.3643 | 658.3593 | **LG** | 143.1179 | 171.1128 |
| **LGC** | 303.1485 | 331.1435 | **LGCT** | 404.1962 | 432.1911 | **LGCTI** | 517.2803 | 545.2752 |

| | | | | | | | | |
|---|---|---|---|---|---|---|---|---|
| **LGCTIK** | 645.3752 | 673.3702 | **GC** | 190.0645 | 218.0594 | **GCT** | 291.1122 | 319.1071 |
| **GCTI** | 404.1962 | 432.1911 | **GCTIK** | 532.2912 | 560.2861 | **GCTIKP** | 629.3439 | 657.3389 |
| **CT** | 234.0907 | 262.0856 | **CTI** | 347.1748 | 375.1697 | **CTIK** | 475.2697 | 503.2646 |
| **CTIKP** | 572.3225 | 600.3174 | **TI** | 187.1441 | 215.1390 | **TIK** | 315.2391 | 343.2340 |
| **TIKP** | 412.2918 | 440.2867 | **IK** | 214.1914 | 242.1863 | **IKP** | 311.2442 | 339.2391 |
| **KP** | 198.1601 | 226.1550 | | | | | | |

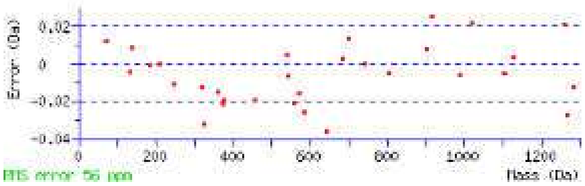


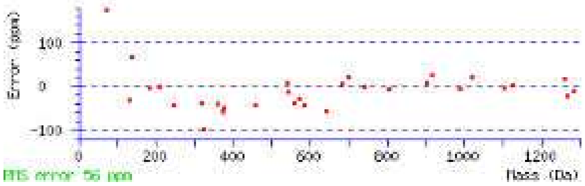


NCBI **BLAST** search of YGRPLLGCTIKPK
(Parameters: blastp, nr protein database, expect=20000, no filter, PAM30)
Other BLAST web gateways

**All matches to this query**

| Score | Mr(calc) | Delta | Sequence |
|---|---|---|---|
| 40.4 | 1501.8439 | 0.0832 | YGRPILGCTIKPK |
| 40.4 | 1501.8439 | 0.0832 | YGRPILGCTIKPK |
| 40.4 | 1501.8439 | 0.0832 | YGRPLIGCTIKPK |
| 40.4 | 1501.8439 | 0.0832 | YGRPLLGCTIKPK |
| 40.4 | 1501.8439 | 0.0832 | YGRPLLGCTIKPK |
| 40.4 | 1501.8439 | 0.0832 | YGRPLLGCTIKPK |
| 40.4 | 1501.8439 | 0.0832 | YGRPLLGCTIKPK |
| 40.4 | 1501.8439 | 0.0832 | YGRPLLGCTIKPK |
| 40.4 | 1501.8439 | 0.0832 | YGRPLLGCTIKPK |
| 40.4 | 1501.8439 | 0.0832 | YGRPLLGCTIKPK |

**Mascot:** http://www.matrixscience.com/

# MATRIX SCIENCE Mascot Search Results

## Peptide View Spot no 96

MS/MS Fragmentation of **DRFVFCAEAIYK**
Found in **gi|11466795** in **NCBInr**, ribulose-1,5-bisphosphate carboxylase/oxygenase large subunit [Oryza sativa Japonica Group]

Match to Query 15: 1517.823824 from(1518.831100,1+) intensity(0.0000) index(7)
Title: Label: E11, Spot_Id: 216641, Peak_List_Id: 221258, MSMS Job_Run_Id: 21347, Comment:
Data file C:\Documents and Settings\Administrator\Desktop\farah BHU\RAJESH\12-1-13 raj\ppw_E11_135997701000.txt

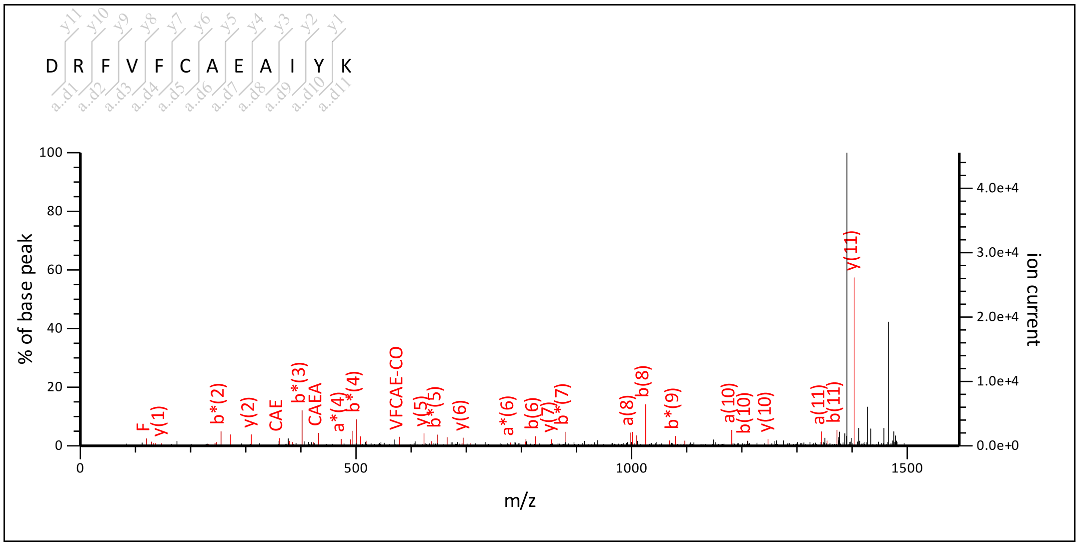


0 to 1594.38

Label all possible matches ○ Label matches used for scoring ◉

**Monoisotopic mass of neutral peptide Mr(calc):** 1517.7337
**Fixed modifications:** Carbamidomethyl (C) (apply to specified residues or termini only)
**Ions Score:** 51 **Expect:** 0.042
**Matches :** 59/194 fragment ions using 99 most intense peaks (help)

| # | Immon. | a | a* | $a^0$ | b | b* | $b^0$ | d | d' | Seq. | v | w | w' | y | y* | |
|---|---|---|---|---|---|---|---|---|---|---|---|---|---|---|---|---|
| 1 | 88.0393 | 88.0393 | | 70.0287 | 116.0342 | | 98.0237 | 44.0495 | | D | | | | | | |
| 2 | 129.1135 | **244.1404** | 227.1139 | 226.1299 | **272.1353** | 255.1088 | 254.1248 | 159.0764 | | R | 1302.6187 | 1301.6235 | | ***1403.7140*** | 1386.6875 | 138[illegible] |
| 3 | 120.0808 | 391.2088 | 374.1823 | 373.1983 | 419.2037 | 402.1772 | 401.1932 | | | F | 1155.5503 | | | ***1247.6129*** | 1230.5864 | 122[illegible] |
| 4 | 72.0808 | **490.2772** | 473.2507 | 472.2667 | **518.2722** | 501.2456 | 500.2616 | 476.2616 | | V | 1056.4819 | 1069.5023 | | 1100.5445 | 1083.5179 | 108[illegible] |
| 5 | 120.0808 | **637.3457** | 620.3191 | 619.3351 | **665.3406** | 648.3140 | 647.3300 | | | F | 909.4135 | | | ***1001.4761*** | 984.4495 | 98[illegible] |
| 6 | 133.0430 | 797.3763 | 780.3498 | 779.3657 | **825.3712** | 808.3447 | 807.3607 | 708.3828 | | C | 749.3828 | 748.3876 | | ***854.4077*** | 837.3811 | 83[illegible] |
| 7 | 44.0495 | 868.4134 | 851.3869 | 850.4029 | 896.4083 | 879.3818 | 878.3978 | | | A | 678.3457 | | | ***694.3770*** | 677.3505 | 6[illegible] |
| 8 | 102.0550 | **997.4560** | 980.4295 | 979.4454 | **1025.4509** | 1008.4244 | 1007.4404 | 939.4505 | | E | 549.3031 | 548.3079 | | ***623.3399*** | 606.3134 | 60[illegible] |
| 9 | 44.0495 | **1068.4931** | 1051.4666 | 1050.4826 | **1096.4880** | 1079.4615 | 1078.4775 | | | A | 478.2660 | | | ***494.2973*** | 477.2708 | |
| 10 | 86.0964 | **1181.5772** | 1164.5506 | 1163.5666 | **1209.5721** | 1192.5456 | 1191.5615 | 1153.5459 | 1167.5615 | I | 365.1819 | 378.2023 | 392.2180 | ***423.2602*** | 406.2336 | |
| 11 | 136.0757 | **1344.6405** | 1327.6140 | 1326.6300 | **1372.6354** | 1355.6089 | 1354.6249 | | | Y | 202.1186 | | | ***310.1761*** | 293.1496 | |
| 12 | 101.1073 | | | | | | | | | K | 74.0237 | 73.0284 | | ***147.1128*** | 130.0863 | |

| Seq | ya | yb | Seq | ya | yb | Seq | ya | yb |
|---|---|---|---|---|---|---|---|---|
| RF | 276.1819 | 304.1768 | RFV | 375.2503 | 403.2452 | RFVF | 522.3187 | 550.3136 |
| RFVFC | 682.3494 | 710.3443 | FV | 219.1492 | 247.1441 | FVF | 366.2176 | 394.2125 |
| FVFC | 526.2483 | 554.2432 | FVFCA | 597.2854 | 625.2803 | VF | 219.1492 | 247.1441 |
| VFC | 379.1798 | 407.1748 | VFCA | 450.2170 | 478.2119 | VFCAE | 579.2595 | 607.2545 |
| VFCAEA | 650.2967 | 678.2916 | FC | 280.1114 | 308.1063 | FCA | 351.1485 | 379.1435 |
| FCAE | 480.1911 | 508.1860 | FCAEA | 551.2282 | 579.2232 | FCAEAI | 664.3123 | 692.3072 |
| CA | 204.0801 | 232.0750 | CAE | 333.1227 | 361.1176 | CAEA | 404.1598 | 432.1547 |
| CAEAI | 517.2439 | 545.2388 | CAEAIY | 680.3072 | 708.3021 | AE | 173.0921 | 201.0870 |
| AEA | 244.1292 | 272.1241 | AEAI | 357.2132 | 385.2082 | AEAIY | 520.2766 | 548.2715 |

| | | | | | | | | |
|---|---|---|---|---|---|---|---|---|
| **EA** | 173.0921 | 201.0870 | **EAI** | 286.1761 | 314.1710 | **EAIY** | 449.2395 | 477.2344 |
| **AI** | 157.1335 | 185.1285 | **AIY** | 320.1969 | 348.1918 | **IY** | 249.1598 | 277.1547 |

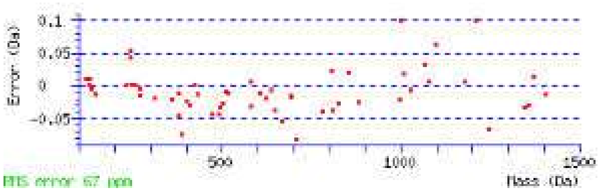


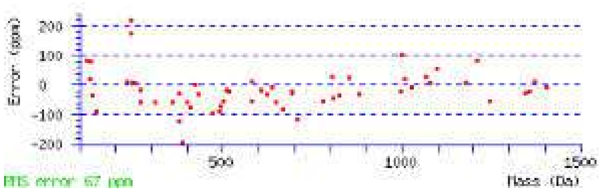


NCBI **BLAST** search of [DRFVFCAEAIYK]
(Parameters: blastp, nr protein database, expect=20000, no filter, PAM30)
Other BLAST [web gateways]

**All matches to this query**

| Score | Mr(calc) | Delta | Sequence |
|---|---|---|---|
| 52.6 | 1517.7007 | 0.1232 | DRFVMCAEAIYK |
| 52.6 | 1517.7007 | 0.1232 | DRMVFCAEALYK |
| 51.1 | 1517.7337 | 0.0902 | DRFVFCAEAIYK |
| 51.1 | 1517.7337 | 0.0902 | DRFVFCAEAIYK |
| 51.1 | 1517.7337 | 0.0902 | DRFVFCAEAIYK |
| 51.1 | 1517.7337 | 0.0902 | DRFVFCAEAIYK |
| 51.1 | 1517.7337 | 0.0902 | DRFVFCAEAIYK |
| 51.1 | 1517.7337 | 0.0902 | DRFVFCAEAIYK |
| 51.1 | 1517.7337 | 0.0902 | DRFVFCAEAIYK |
| 51.1 | 1517.7337 | 0.0902 | DRFVFCAEAIYK |

**Mascot:** http://www.matrixscience.com/

# MATRIX SCIENCE Mascot Search Results

## Peptide View Spot no 96

MS/MS Fragmentation of **WSPELAAACEIWK**
Found in **gi|11466795** in **NCBInr**, ribulose-1,5-bisphosphate carboxylase/oxygenase large subunit [Oryza sativa Japonica Group]

Match to Query 17: 1559.808024 from(1560.815300,1+) intensity(0.0000) index(8)
Title: Label: E11, Spot_Id: 216641, Peak_List_Id: 221257, MSMS Job_Run_Id: 21347, Comment:
Data file C:\Documents and Settings\Administrator\Desktop\farah BHU\RAJESH\12-1-13 raj\ppw_E11_135997701000.txt

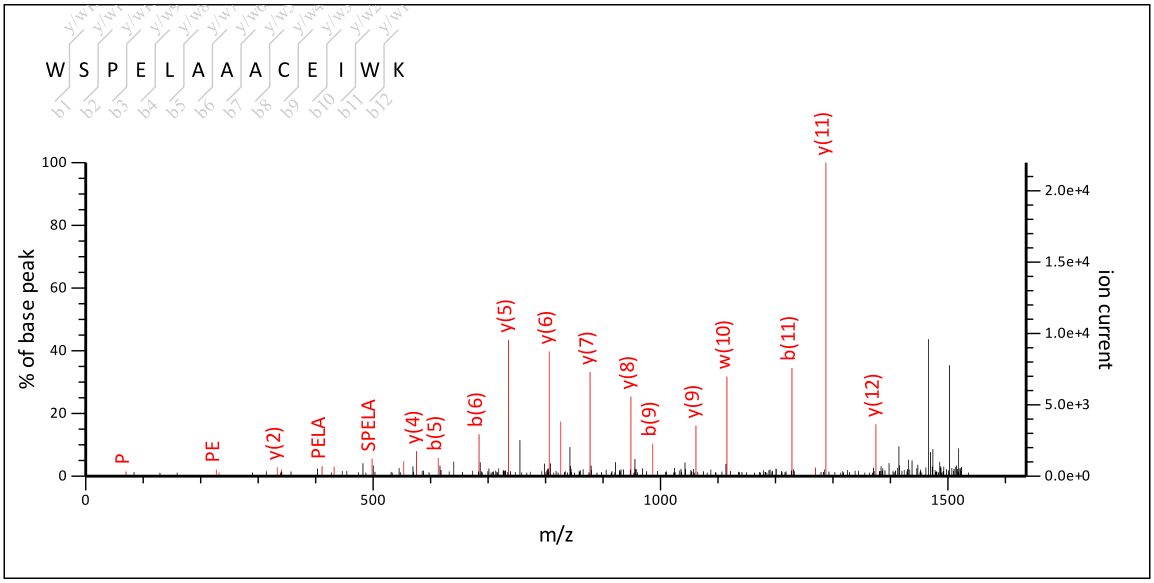


0 to 1636.08

Label all possible matches ○ Label matches used for scoring ◉

**Monoisotopic mass of neutral peptide Mr(calc):** 1559.7442
**Fixed modifications:** Carbamidomethyl (C) (apply to specified residues or termini only)
**Ions Score:** 74 **Expect:** 0.00022
**Matches :** 26/203 fragment ions using 30 most intense peaks (help)

| # | Immon. | a | $a^0$ | b | $b^0$ | d | d' | Seq. | v | w | w' | y | y* | $y^0$ | # |
|---|---|---|---|---|---|---|---|---|---|---|---|---|---|---|---|
| 1 | 159.0917 | 159.0917 | | 187.0866 | | 44.0495 | | W | | | | | | | 13 |
| 2 | 60.0444 | 246.1237 | 228.1131 | 274.1186 | 256.1081 | 230.1288 | | S | 1342.6460 | 1341.6508 | | ***1374.6722*** | 1357.6457 | 1356.6616 | 12 |
| 3 | 70.0651 | 343.1765 | 325.1659 | 371.1714 | 353.1608 | 317.1608 | | P | 1245.5932 | 1244.5980 | | ***1287.6402*** | 1270.6136 | 1269.6296 | 11 |
| 4 | 102.0550 | 472.2191 | 454.2085 | 500.2140 | 482.2034 | 414.2136 | | E | 1116.5506 | 1115.5554 | | 1190.5874 | 1173.5609 | 1172.5769 | 10 |
| 5 | 86.0964 | 585.3031 | 567.2926 | **613.2980** | 595.2875 | 543.2562 | | L | 1003.4666 | 1002.4713 | | ***1061.5448*** | 1044.5183 | 1043.5343 | 9 |
| 6 | 44.0495 | 656.3402 | 638.3297 | **684.3352** | 666.3246 | | | A | 932.4295 | | | ***948.4608*** | 931.4342 | 930.4502 | 8 |
| 7 | 44.0495 | 727.3774 | 709.3668 | 755.3723 | 737.3617 | | | A | 861.3924 | | | ***877.4237*** | 860.3971 | 859.4131 | 7 |
| 8 | 44.0495 | 798.4145 | 780.4039 | **826.4094** | 808.3988 | | | A | 790.3552 | | | ***806.3865*** | 789.3600 | 788.3760 | 6 |
| 9 | 133.0430 | 958.4451 | 940.4345 | **986.4400** | 968.4295 | 869.4516 | | C | 630.3246 | 629.3293 | | ***735.3494*** | 718.3229 | 717.3389 | 5 |
| 10 | 102.0550 | 1087.4877 | 1069.4771 | **1115.4826** | 1097.4721 | 1029.4822 | | E | 501.2820 | 500.2867 | | ***575.3188*** | 558.2922 | 557.3082 | 4 |
| 11 | 86.0964 | 1200.5718 | 1182.5612 | **1228.5667** | 1210.5561 | 1172.5405 | 1186.5561 | I | 388.1979 | 401.2183 | 415.2340 | 446.2762 | 429.2496 | | 3 |
| 12 | 159.0917 | 1386.6511 | 1368.6405 | 1414.6460 | 1396.6354 | | | W | 202.1186 | | | ***333.1921*** | 316.1656 | | 2 |
| 13 | 101.1073 | | | | | | | K | 74.0237 | 73.0284 | | 147.1128 | 130.0863 | | 1 |

| Seq | ya | yb | Seq | ya | yb | Seq | ya | yb |
|---|---|---|---|---|---|---|---|---|
| SP | 157.0972 | 185.0921 | SPE | 286.1397 | 314.1347 | SPEL | 399.2238 | 427.2187 |
| SPELA | 470.2609 | 498.2558 | SPELAA | 541.2980 | 569.2930 | SPELAAA | 612.3352 | 640.3301 |
| PE | 199.1077 | 227.1026 | PEL | 312.1918 | 340.1867 | PELA | 383.2289 | 411.2238 |
| PELAA | 454.2660 | 482.2609 | PELAAA | 525.3031 | 553.2980 | PELAAAC | 685.3338 | 713.3287 |

| | | | | | | | | |
|---|---|---|---|---|---|---|---|---|
| **EL** | 215.1390 | 243.1339 | **ELA** | 286.1761 | 314.1710 | **ELAA** | 357.2132 | 385.2082 |
| **ELAAA** | 428.2504 | 456.2453 | **ELAAAC** | 588.2810 | 616.2759 | **LA** | 157.1335 | 185.1285 |
| **LAA** | 228.1707 | 256.1656 | **LAAA** | 299.2078 | 327.2027 | **LAAAC** | 459.2384 | 487.2333 |
| **LAAACE** | 588.2810 | 616.2759 | **AA** | 115.0866 | 143.0815 | **AAA** | 186.1237 | 214.1186 |
| **AAAC** | 346.1544 | 374.1493 | **AAACE** | 475.1969 | 503.1919 | **AAACEI** | 588.2810 | 616.2759 |
| **AA** | 115.0866 | 143.0815 | **AAC** | 275.1172 | 303.1122 | **AACE** | 404.1598 | 432.1547 |
| **AACEI** | 517.2439 | 545.2388 | **AC** | 204.0801 | 232.0750 | **ACE** | 333.1227 | 361.1176 |
| **ACEI** | 446.2068 | 474.2017 | **ACEIW** | 632.2861 | 660.2810 | **CE** | 262.0856 | 290.0805 |
| **CEI** | 375.1697 | 403.1646 | **CEIW** | 561.2490 | 589.2439 | **EI** | 215.1390 | 243.1339 |
| **EIW** | 401.2183 | 429.2132 | **IW** | 272.1757 | 300.1707 | | | |

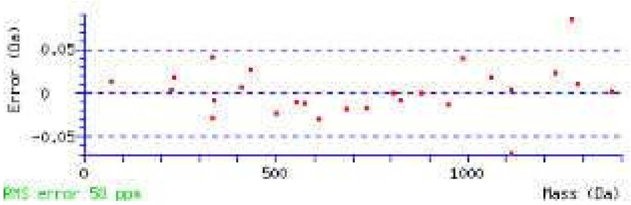


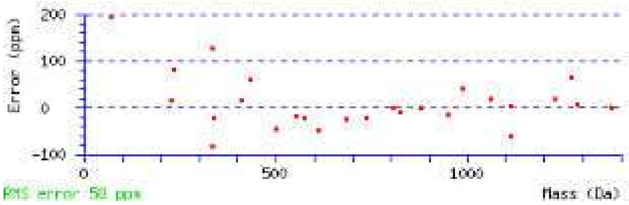


NCBI **BLAST** search of WSPELAAACEIWK
(Parameters: blastp, nr protein database, expect=20000, no filter, PAM30)
Other BLAST web gateways

**All matches to this query**

| Score | Mr(calc) | Delta | Sequence |
|---|---|---|---|
| 73.7 | 1559.7442 | 0.0638 | WSPEIAAACEIWK |
| 73.7 | 1559.7442 | 0.0638 | WSPELAAACEIWK |
| 73.7 | 1559.7442 | 0.0638 | WSPELAAACEIWK |
| 73.7 | 1559.7442 | 0.0638 | WSPELAAACEIWK |
| 73.7 | 1559.7442 | 0.0638 | WSPELAAACEIWK |
| 73.7 | 1559.7442 | 0.0638 | WSPELAAACEIWK |
| 73.7 | 1559.7442 | 0.0638 | WSPELAAACEIWK |
| 73.7 | 1559.7442 | 0.0638 | WSPELAAACEIWK |
| 73.7 | 1559.7442 | 0.0638 | WSPELAAACEIWK |
| 73.7 | 1559.7442 | 0.0638 | WSPELAAACEIWK |

**Mascot:** http://www.matrixscience.com/

# MATRIX SCIENCE Mascot Search Results

## Peptide View Spot no 96

MS/MS Fragmentation of **SACKWSPELAAACEIWK**
Found in **gi|11466795** in **NCBInr**, ribulose-1,5-bisphosphate carboxylase/oxygenase large subunit [Oryza sativa Japonica Group]

Match to Query 20: 2006.005524 from(2007.012800,1+) intensity(0.0000) index(10)
Title: Label: E11, Spot_Id: 216641, Peak_List_Id: 221262, MSMS Job_Run_Id: 21347, Comment:
Data file C:\Documents and Settings\Administrator\Desktop\farah BHU\RAJESH\12-1-13 raj\ppw_E11_135997701000.txt

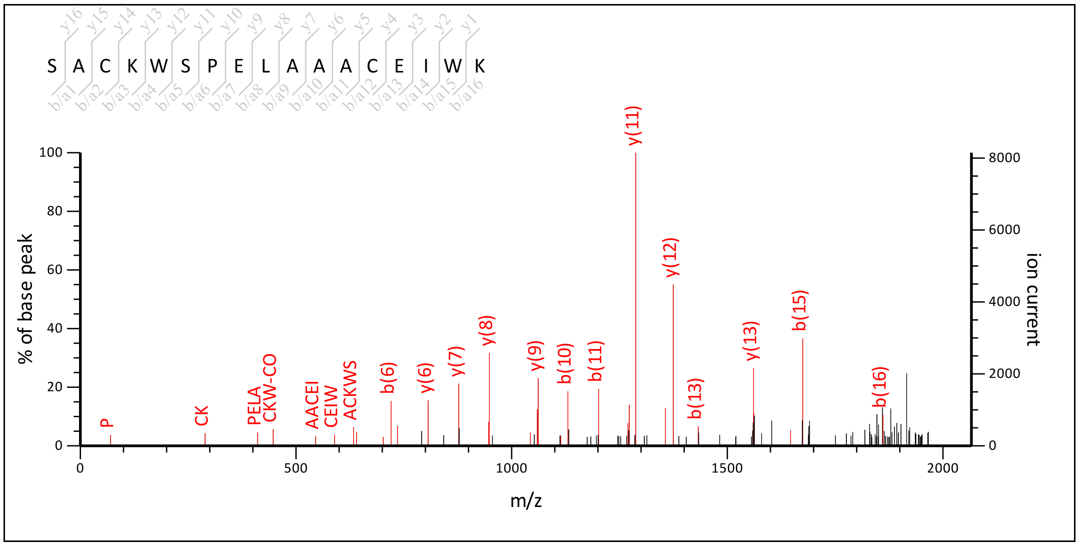


0 to 2066.02

Label all possible matches ○ Label matches used for scoring ◉

```
Monoisotopic mass of neutral peptide Mr(calc): 2005.9390
Fixed modifications: Carbamidomethyl (C) (apply to specified residues or termini only)
Ions Score: 84  Expect: 1.5e-05
Matches : 35/305 fragment ions using 47 most intense peaks   (help)
```

| # | Immon. | a | a* | a⁰ | b | b* | b⁰ | d | d' | Seq. | v | w | w' | y | y* | |
|---|---|---|---|---|---|---|---|---|---|---|---|---|---|---|---|---|
| 1 | 60.0444 | 60.0444 | | 42.0338 | 88.0393 | | 70.0287 | 44.0495 | | S | | | | | | |
| 2 | 44.0495 | 131.0815 | | 113.0709 | 159.0764 | | 141.0659 | | | A | 1903.8830 | | | 1919.9143 | 1902.8877 | 190 |
| 3 | 133.0430 | 291.1122 | | 273.1016 | 319.1071 | | 301.0965 | 202.1186 | | C | 1743.8523 | 1742.8571 | | 1848.8771 | 1831.8506 | 183 |
| 4 | 101.1073 | 419.2071 | 402.1806 | 401.1966 | ***447.2020*** | 430.1755 | 429.1915 | 362.1493 | | K | 1615.7573 | 1614.7621 | | 1688.8465 | 1671.8199 | 167 |
| 5 | 159.0917 | 605.2864 | 588.2599 | 587.2759 | ***633.2813*** | 616.2548 | 615.2708 | | | W | 1429.6780 | | | **1560.7515** | 1543.7250 | 154 |
| 6 | 60.0444 | 692.3185 | 675.2919 | 674.3079 | ***720.3134*** | 703.2868 | 702.3028 | 676.3235 | | S | 1342.6460 | 1341.6508 | | **1374.6722** | 1357.6457 | 135 |
| 7 | 70.0651 | 789.3712 | 772.3447 | 771.3607 | 817.3661 | 800.3396 | 799.3556 | 763.3556 | | P | 1245.5932 | 1244.5980 | | **1287.6402** | 1270.6136 | 126 |
| 8 | 102.0550 | 918.4138 | 901.3873 | 900.4032 | ***946.4087*** | 929.3822 | 928.3982 | 860.4083 | | E | 1116.5506 | 1115.5554 | | 1190.5874 | 1173.5609 | 117 |
| 9 | 86.0964 | 1031.4979 | 1014.4713 | 1013.4873 | ***1059.4928*** | 1042.4662 | 1041.4822 | 989.4509 | | L | 1003.4666 | 1002.4713 | | **1061.5448** | 1044.5183 | 104 |
| 10 | 44.0495 | 1102.5350 | 1085.5084 | 1084.5244 | ***1130.5299*** | 1113.5034 | 1112.5193 | | | A | 932.4295 | | | **948.4608** | 931.4342 | 93 |
| 11 | 44.0495 | 1173.5721 | 1156.5456 | 1155.5615 | ***1201.5670*** | 1184.5405 | 1183.5565 | | | A | 861.3924 | | | **877.4237** | 860.3971 | 85 |
| 12 | 44.0495 | 1244.6092 | 1227.5827 | 1226.5987 | ***1272.6041*** | 1255.5776 | 1254.5936 | | | A | 790.3552 | | | **806.3865** | 789.3600 | 78 |
| 13 | 133.0430 | 1404.6399 | 1387.6133 | 1386.6293 | ***1432.6348*** | 1415.6082 | 1414.6242 | 1315.6463 | | C | 630.3246 | 629.3293 | | **735.3494** | 718.3229 | 71 |
| 14 | 102.0550 | 1533.6825 | 1516.6559 | 1515.6719 | ***1561.6774*** | 1544.6508 | 1543.6668 | 1475.6770 | | E | 501.2820 | 500.2867 | | 575.3188 | 558.2922 | 55 |
| 15 | 86.0964 | 1646.7665 | 1629.7400 | 1628.7560 | ***1674.7614*** | 1657.7349 | 1656.7509 | 1618.7352 | 1632.7509 | I | 388.1979 | 401.2183 | 415.2340 | 446.2762 | 429.2496 | |
| 16 | 159.0917 | 1832.8458 | 1815.8193 | 1814.8353 | ***1860.8408*** | 1843.8142 | 1842.8302 | | | W | 202.1186 | | | 333.1921 | 316.1656 | |
| 17 | 101.1073 | | | | | | | | | K | 74.0237 | 73.0284 | | 147.1128 | 130.0863 | |

| Seq | ya | yb | Seq | ya | yb | Seq | ya | yb |
|---|---|---|---|---|---|---|---|---|
| AC | 204.0801 | 232.0750 | ACK | 332.1751 | 360.1700 | ACKW | 518.2544 | 546.2493 |
| ACKWS | 605.2864 | 633.2813 | CK | 261.1380 | 289.1329 | CKW | 447.2173 | 475.2122 |
| CKWS | 534.2493 | 562.2442 | CKWSP | 631.3021 | 659.2970 | KW | 287.1866 | 315.1816 |
| KWS | 374.2187 | 402.2136 | KWSP | 471.2714 | 499.2663 | KWSPE | 600.3140 | 628.3089 |

| | | | | | | | | |
|---|---|---|---|---|---|---|---|---|
| **WS** | 246.1237 | 274.1186 | **WSP** | 343.1765 | 371.1714 | **WSPE** | 472.2191 | 500.2140 |
| **WSPEL** | 585.3031 | 613.2980 | **WSPELA** | 656.3402 | 684.3352 | **SP** | 157.0972 | 185.0921 |
| **SPE** | 286.1397 | 314.1347 | **SPEL** | 399.2238 | 427.2187 | **SPELA** | 470.2609 | 498.2558 |
| **SPELAA** | 541.2980 | 569.2930 | **SPELAAA** | 612.3352 | 640.3301 | **PE** | 199.1077 | 227.1026 |
| **PEL** | 312.1918 | 340.1867 | **PELA** | 383.2289 | 411.2238 | **PELAA** | 454.2660 | 482.2609 |
| **PELAAA** | 525.3031 | 553.2980 | **PELAAAC** | 685.3338 | 713.3287 | **EL** | 215.1390 | 243.1339 |
| **ELA** | 286.1761 | 314.1710 | **ELAA** | 357.2132 | 385.2082 | **ELAAA** | 428.2504 | 456.2453 |
| **ELAAAC** | 588.2810 | 616.2759 | **LA** | 157.1335 | 185.1285 | **LAA** | 228.1707 | 256.1656 |
| **LAAA** | 299.2078 | 327.2027 | **LAAAC** | 459.2384 | 487.2333 | **LAAACE** | 588.2810 | 616.2759 |
| **AA** | 115.0866 | 143.0815 | **AAA** | 186.1237 | 214.1186 | **AAAC** | 346.1544 | 374.1493 |
| **AAACE** | 475.1969 | 503.1919 | **AAACEI** | 588.2810 | 616.2759 | **AA** | 115.0866 | 143.0815 |
| **AAC** | 275.1172 | 303.1122 | **AACE** | 404.1598 | 432.1547 | **AACEI** | 517.2439 | 545.2388 |
| **AC** | 204.0801 | 232.0750 | **ACE** | 333.1227 | 361.1176 | **ACEI** | 446.2068 | 474.2017 |
| **ACEIW** | 632.2861 | 660.2810 | **CE** | 262.0856 | 290.0805 | **CEI** | 375.1697 | 403.1646 |
| **CEIW** | 561.2490 | 589.2439 | **EI** | 215.1390 | 243.1339 | **EIW** | 401.2183 | 429.2132 |
| **IW** | 272.1757 | 300.1707 | | | | | | |

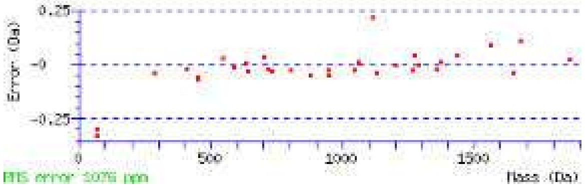


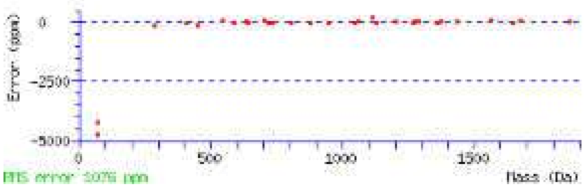


NCBI **BLAST** search of SACKWSPELAAACEIWK
(Parameters: blastp, nr protein database, expect=20000, no filter, PAM30)
Other BLAST web gateways

**All matches to this query**

| Score | Mr(calc) | Delta | Sequence |
|---|---|---|---|
| 84.1 | 2005.9390 | 0.0666 | SACKWSPELAAACEIWK |
| 62.1 | 2006.8866 | -0.8811 | SACKWSPELAAACEIWE |
| 41.2 | 2006.8866 | -0.8811 | EACSWSPELAAACEIWK |
| 36.8 | 2004.9615 | 1.0441 | ETSKWSPELAAACEIWK |
| 15.7 | 2006.0334 | -0.0278 | QKTGASFILPASAQHGHEK |
| 14.8 | 2006.1452 | -0.1397 | TKIVFSSNALLSIPEFLK |
| 14.4 | 2005.9487 | 0.0569 | MAAAANSSASAAGAGGGSAAAAGLK |
| 11.6 | 2005.9449 | 0.0607 | SGVGCKMEIVPETEQGSAK |
| 11.4 | 2004.9977 | 1.0079 | NSSRSYHIIAASESQSLR |
| 11.2 | 2005.9390 | 0.0665 | MDLVGTSNVKAWMGYFR |

**Mascot:** http://www.matrixscience.com/

# MATRIX SCIENCE Mascot Search Results

## Peptide View Spot no 96

MS/MS Fragmentation of **GGLDFTKDDENVNSQPFMR**
Found in **gi|11466795** in **NCBInr**, ribulose-1,5-bisphosphate carboxylase/oxygenase large subunit [Oryza sativa Japonica Group]

Match to Query 24: 2169.069124 from(2170.076400,1+) intensity(0.0000) index(12)
Title: Label: E11, Spot_Id: 216641, Peak_List_Id: 221248, MSMS Job_Run_Id: 21347, Comment:
Data file C:\Documents and Settings\Administrator\Desktop\farah BHU\RAJESH\12-1-13 raj\ppw_E11_135997701000.txt

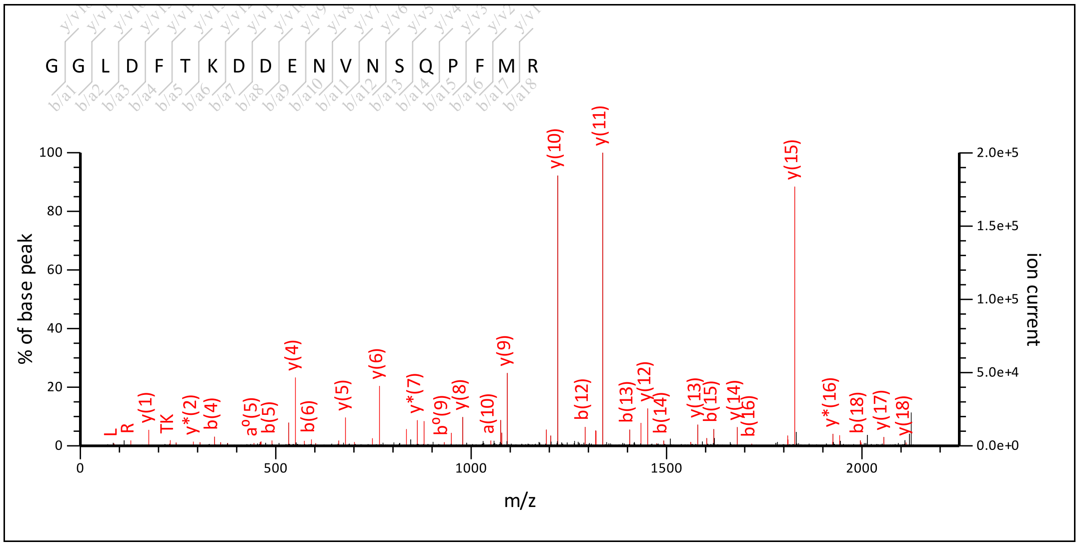


0 to 2249

Label all possible matches ○ Label matches used for scoring ◉

**Monoisotopic mass of neutral peptide Mr(calc):** 2168.9797
**Fixed modifications:** Carbamidomethyl (C) (apply to specified residues or termini only)
**Ions Score:** 138 **Expect:** 8e-11
**Matches :** 72/340 fragment ions using 84 most intense peaks (help)

| # | Immon. | a | a* | $a^0$ | b | b* | $b^0$ | d | d' | Seq. | v | w | w' | y | y* | |
|---|---|---|---|---|---|---|---|---|---|---|---|---|---|---|---|---|
| 1 | 30.0338 | 30.0338 | | | 58.0287 | | | 44.0495 | | G | | | | | | |
| 2 | 30.0338 | 87.0553 | | | 115.0502 | | | | | G | | | | 2112.9655 | 2095.9389 | 209 |
| 3 | 86.0964 | 200.1394 | | | 228.1343 | | | 158.0924 | | L | 1997.8658 | 1996.8705 | | 2055.9440 | 2038.9175 | 20[illegible] |
| 4 | 88.0393 | 315.1663 | | 297.1557 | 343.1612 | | 325.1506 | 271.1765 | | D | 1882.8388 | 1881.8436 | | 1942.8600 | 1925.8334 | 192 |
| 5 | 120.0808 | 462.2347 | | 444.2241 | 490.2296 | | 472.2191 | | | F | 1735.7704 | | | 1827.8330 | 1810.8065 | 180 |
| 6 | 74.0600 | 563.2824 | | 545.2718 | 591.2773 | | 573.2667 | 547.2875 | 549.2667 | T | 1634.7227 | 1647.7431 | 1649.7224 | 1680.7646 | 1663.7381 | 166 |
| 7 | 101.1073 | 691.3774 | 674.3508 | 673.3668 | 719.3723 | 702.3457 | 701.3617 | 634.3195 | | K | 1506.6278 | 1505.6325 | | 1579.7169 | 1562.6904 | 156 |
| 8 | 88.0393 | 806.4043 | 789.3777 | 788.3937 | 834.3992 | 817.3727 | 816.3886 | 762.4145 | | D | 1391.6008 | 1390.6056 | | 1451.6220 | 1434.5954 | 143 |
| 9 | 88.0393 | 921.4312 | 904.4047 | 903.4207 | 949.4262 | 932.3996 | 931.4156 | 877.4414 | | D | 1276.5739 | 1275.5786 | | 1336.5950 | 1319.5685 | 131 |
| 10 | 102.0550 | 1050.4738 | 1033.4473 | 1032.4633 | 1078.4687 | 1061.4422 | 1060.4582 | 992.4684 | | E | 1147.5313 | 1146.5361 | | 1221.5681 | 1204.5415 | 120 |
| 11 | 87.0553 | 1164.5168 | 1147.4902 | 1146.5062 | 1192.5117 | 1175.4851 | 1174.5011 | 1121.5109 | | N | 1033.4884 | 1032.4931 | | 1092.5255 | 1075.4989 | 107 |
| 12 | 72.0808 | 1263.5852 | 1246.5586 | 1245.5746 | 1291.5801 | 1274.5535 | 1273.5695 | 1249.5695 | | V | 934.4200 | 947.4404 | | 978.4826 | 961.4560 | 96 |
| 13 | 87.0553 | 1377.6281 | 1360.6016 | 1359.6175 | 1405.6230 | 1388.5965 | 1387.6124 | 1334.6223 | | N | 820.3770 | 819.3818 | | 879.4141 | 862.3876 | 86 |
| 14 | 60.0444 | 1464.6601 | 1447.6336 | 1446.6496 | 1492.6550 | 1475.6285 | 1474.6445 | 1448.6652 | | S | 733.3450 | 732.3498 | | 765.3712 | 748.3447 | 74 |
| 15 | 101.0709 | 1592.7187 | 1575.6922 | 1574.7081 | 1620.7136 | 1603.6871 | 1602.7031 | 1535.6972 | | Q | 605.2864 | 604.2912 | | 678.3392 | 661.3126 | |
| 16 | 70.0651 | 1689.7715 | 1672.7449 | 1671.7609 | 1717.7664 | 1700.7398 | 1699.7558 | 1663.7558 | | P | 508.2337 | 507.2384 | | 550.2806 | 533.2541 | |
| 17 | 120.0808 | 1836.8399 | 1819.8133 | 1818.8293 | 1864.8348 | 1847.8082 | 1846.8242 | | | F | 361.1653 | | | 453.2279 | 436.2013 | |
| 18 | 104.0528 | 1967.8804 | 1950.8538 | 1949.8698 | 1995.8753 | 1978.8487 | 1977.8647 | 1907.8770 | | M | 230.1248 | 229.1295 | | 306.1594 | 289.1329 | |
| 19 | 129.1135 | | | | | | | | | R | 74.0237 | 73.0284 | | 175.1190 | 158.0924 | |

| Seq | ya | yb | Seq | ya | yb | Seq | ya | yb |
|---|---|---|---|---|---|---|---|---|
| GL | 143.1179 | 171.1128 | GLD | 258.1448 | 286.1397 | GLDF | 405.2132 | 433.2082 |
| GLDFT | 506.2609 | 534.2558 | GLDFTK | 634.3559 | 662.3508 | LD | 201.1234 | 229.1183 |

| | | | | | | | | |
|---|---|---|---|---|---|---|---|---|
| LDF | 348.1918 | 376.1867 | LDFT | 449.2395 | 477.2344 | LDFTK | 577.3344 | 605.3293 |
| LDFTKD | 692.3614 | 720.3563 | DF | 235.1077 | 263.1026 | DFT | 336.1554 | 364.1503 |
| DFTK | 464.2504 | 492.2453 | DFTKD | 579.2773 | 607.2722 | DFTKDD | 694.3042 | 722.2992 |
| FT | 221.1285 | 249.1234 | FTK | 349.2234 | 377.2183 | FTKD | 464.2504 | 492.2453 |
| FTKDD | 579.2773 | 607.2722 | TK | 202.1550 | 230.1499 | TKD | 317.1819 | 345.1769 |
| TKDD | 432.2089 | 460.2038 | TKDDE | 561.2515 | 589.2464 | TKDDEN | 675.2944 | 703.2893 |
| KD | 216.1343 | 244.1292 | KDD | 331.1612 | 359.1561 | KDDE | 460.2038 | 488.1987 |
| KDDEN | 574.2467 | 602.2416 | KDDENV | 673.3151 | 701.3101 | DD | 203.0662 | 231.0612 |
| DDE | 332.1088 | 360.1038 | DDEN | 446.1518 | 474.1467 | DDENV | 545.2202 | 573.2151 |
| DDENVN | 659.2631 | 687.2580 | DE | 217.0819 | 245.0768 | DEN | 331.1248 | 359.1197 |
| DENV | 430.1932 | 458.1882 | DENVN | 544.2362 | 572.2311 | DENVNS | 631.2682 | 659.2631 |
| EN | 216.0979 | 244.0928 | ENV | 315.1663 | 343.1612 | ENVN | 429.2092 | 457.2041 |
| ENVNS | 516.2413 | 544.2362 | ENVNSQ | 644.2998 | 672.2947 | NV | 186.1237 | 214.1186 |
| NVN | 300.1666 | 328.1615 | NVNS | 387.1987 | 415.1936 | NVNSQ | 515.2572 | 543.2522 |
| NVNSQP | 612.3100 | 640.3049 | VN | 186.1237 | 214.1186 | VNS | 273.1557 | 301.1506 |
| VNSQ | 401.2143 | 429.2092 | VNSQP | 498.2671 | 526.2620 | VNSQPF | 645.3355 | 673.3304 |
| NS | 174.0873 | 202.0822 | NSQ | 302.1459 | 330.1408 | NSQP | 399.1987 | 427.1936 |
| NSQPF | 546.2671 | 574.2620 | NSQPFM | 677.3076 | 705.3025 | SQ | 188.1030 | 216.0979 |
| SQP | 285.1557 | 313.1506 | SQPF | 432.2241 | 460.2191 | SQPFM | 563.2646 | 591.2595 |
| QP | 198.1237 | 226.1186 | QPF | 345.1921 | 373.1870 | QPFM | 476.2326 | 504.2275 |
| PF | 217.1335 | 245.1285 | PFM | 348.1740 | 376.1689 | FM | 251.1213 | 279.1162 |

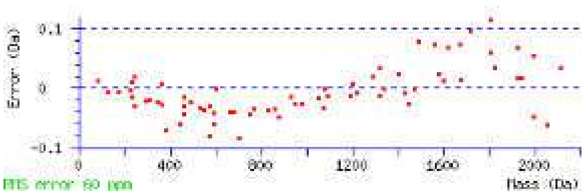


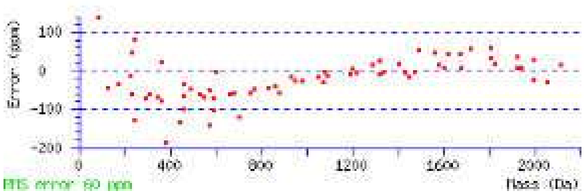


NCBI **BLAST** search of GGLDFTKDDENVNSQPFMR
(Parameters: blastp, nr protein database, expect=20000, no filter, PAM30)
Other BLAST web gateways

**All matches to this query**

| Score | Mr(calc) | Delta | Sequence |
|---|---|---|---|
| 138.1 | 2168.9797 | 0.0894 | GGIDFTKDDENVNSQPFMR |
| 138.1 | 2168.9797 | 0.0894 | GGIDFTKDDENVNSQPFMR |
| 138.1 | 2169.0161 | 0.0531 | GGLDFTKDDENVNSKPFMR |
| 138.1 | 2168.9797 | 0.0894 | GGLDFTKDDENVNSQPFMR |
| 138.1 | 2168.9797 | 0.0894 | GGLDFTKDDENVNSQPFMR |
| 138.1 | 2168.9797 | 0.0894 | GGLDFTKDDENVNSQPFMR |
| 138.1 | 2168.9797 | 0.0894 | GGLDFTKDDENVNSQPFMR |
| 138.1 | 2168.9797 | 0.0894 | GGLDFTKDDENVNSQPFMR |
| 138.1 | 2168.9797 | 0.0894 | GGLDFTKDDENVNSQPFMR |
| 138.1 | 2168.9797 | 0.0894 | GGLDFTKDDENVNSQPFMR |

**Mascot:** http://www.matrixscience.com/

# MATRIX SCIENCE Mascot Search Results

## Peptide View

MS/MS Fragmentation of **LTYYTPEYETKDTDILAAFR**
Found in **gi|11466795** in **NCBInr**, ribulose-1,5-bisphosphate carboxylase/oxygenase large subunit [Oryza sativa Japonica Group]

Match to Query 28: 2409.267424 from(2410.274700,1+) intensity(0.0000) index(14)
Title: Label: E11, Spot_Id: 216641, Peak_List_Id: 221247, MSMS Job_Run_Id: 21347, Comment:
Data file C:\Documents and Settings\Administrator\Desktop\farah BHU\RAJESH\12-1-13 raj\ppw_E11_135997701000.txt

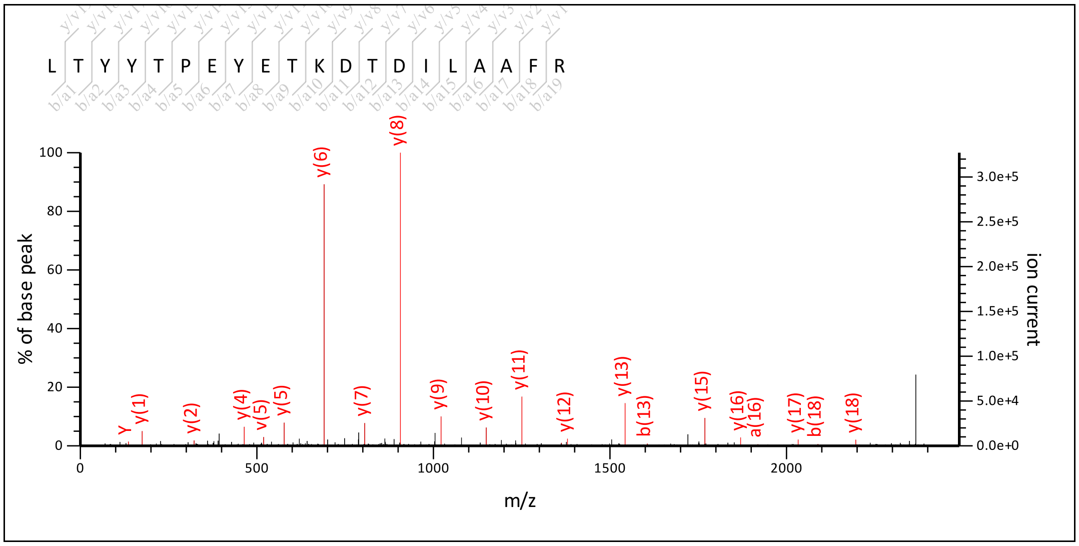


0 to 2489.35

Label all possible matches ○ Label matches used for scoring ◉

**Monoisotopic mass of neutral peptide Mr(calc):** 2409.1740
**Fixed modifications:** Carbamidomethyl (C) (apply to specified residues or termini only)
**Ions Score:** 162 **Expect:** 2.5e-13
**Matches :** 26/356 fragment ions using 24 most intense peaks (help)

| # | Immon. | a | a* | $a^0$ | b | b* | $b^0$ | d | d' | Seq. | v | w | w' | y | y* |
|---|---|---|---|---|---|---|---|---|---|---|---|---|---|---|---|
| 1 | 86.0964 | 86.0964 | | | 114.0913 | | | 44.0495 | | L | | | | | |
| 2 | 74.0600 | 187.1441 | | 169.1335 | 215.1390 | | 197.1285 | 171.1492 | 173.1285 | T | 2251.0554 | 2264.0758 | 2266.0550 | 2297.0972 | 2280.0707 |
| 3 | 136.0757 | 350.2074 | | 332.1969 | 378.2023 | | 360.1918 | | | Y | 2087.9920 | | | ***2196.0495*** | 2179.0230 |
| 4 | 136.0757 | 513.2708 | | 495.2602 | 541.2657 | | 523.2551 | | | Y | 1924.9287 | | | ***2032.9862*** | 2015.9597 |
| 5 | 74.0600 | 614.3184 | | 596.3079 | 642.3134 | | 624.3028 | 598.3235 | 600.3028 | T | 1823.8810 | 1836.9014 | 1838.8807 | ***1869.9229*** | 1852.8963 |
| 6 | 70.0651 | 711.3712 | | 693.3606 | 739.3661 | | 721.3556 | 685.3556 | | P | 1726.8283 | 1725.8330 | | ***1768.8752*** | 1751.8487 |
| 7 | 102.0550 | 840.4138 | | 822.4032 | 868.4087 | | 850.3981 | 782.4083 | | E | 1597.7857 | 1596.7904 | | 1671.8224 | 1654.7959 |
| 8 | 136.0757 | 1003.4771 | | 985.4666 | 1031.4720 | | 1013.4615 | | | Y | 1434.7223 | | | ***1542.7799*** | 1525.7533 |
| 9 | 102.0550 | 1132.5197 | | 1114.5092 | 1160.5146 | | 1142.5041 | 1074.5142 | | E | 1305.6797 | 1304.6845 | | ***1379.7165*** | 1362.6900 |
| 10 | 74.0600 | 1233.5674 | | 1215.5568 | 1261.5623 | | 1243.5517 | 1217.5725 | 1219.5517 | T | 1204.6321 | 1217.6525 | 1219.6317 | ***1250.6739*** | 1233.6474 |
| 11 | 101.1073 | 1361.6624 | 1344.6358 | 1343.6518 | 1389.6573 | 1372.6307 | 1371.6467 | 1304.6045 | | K | 1076.5371 | 1075.5419 | | ***1149.6262*** | 1132.5997 |
| 12 | 88.0393 | 1476.6893 | 1459.6628 | 1458.6787 | 1504.6842 | 1487.6577 | 1486.6737 | 1432.6995 | | D | 961.5102 | 960.5149 | | ***1021.5313*** | 1004.5047 |
| 13 | 74.0600 | 1577.7370 | 1560.7104 | 1559.7264 | **1605.7319** | 1588.7054 | 1587.7213 | 1561.7421 | 1563.7213 | T | 860.4625 | 873.4829 | 875.4621 | ***906.5043*** | 889.4778 |
| 14 | 88.0393 | 1692.7639 | 1675.7374 | 1674.7534 | 1720.7588 | 1703.7323 | 1702.7483 | 1648.7741 | | D | 745.4355 | 744.4403 | | ***805.4567*** | 788.4301 |
| 15 | 86.0964 | 1805.8480 | 1788.8214 | 1787.8374 | 1833.8429 | 1816.8164 | 1815.8323 | 1777.8167 | 1791.8323 | I | 632.3515 | 645.3719 | 659.3875 | ***690.4297*** | 673.4032 |
| 16 | 86.0964 | 1918.9321 | 1901.9055 | 1900.9215 | 1946.9270 | 1929.9004 | 1928.9164 | 1876.8851 | | L | 519.2674 | 518.2722 | | ***577.3457*** | 560.3191 |
| 17 | 44.0495 | 1989.9692 | 1972.9426 | 1971.9586 | 2017.9641 | 2000.9375 | 1999.9535 | | | A | 448.2303 | | | ***464.2616*** | 447.2350 |
| 18 | 44.0495 | 2061.0063 | 2043.9797 | 2042.9957 | **2089.0012** | 2071.9746 | 2070.9906 | | | A | 377.1932 | | | 393.2245 | 376.1979 |
| 19 | 120.0808 | 2208.0747 | 2191.0481 | 2190.0641 | 2236.0696 | 2219.0431 | 2218.0590 | | | F | 230.1248 | | | ***322.1874*** | 305.1608 |
| 20 | 129.1135 | | | | | | | | | R | 74.0237 | 73.0284 | | ***175.1190*** | 158.0924 |

| Seq | ya | yb | Seq | ya | yb | Seq | ya | yb |
|---|---|---|---|---|---|---|---|---|
| TY | 237.1234 | 265.1183 | TYY | 400.1867 | 428.1816 | TYYT | 501.2344 | 529.2293 |

| | | | | | | | | |
|---|---|---|---|---|---|---|---|---|
| **TYYTP** | 598.2871 | 626.2821 | **YY** | 299.1390 | 327.1339 | **YYT** | 400.1867 | 428.1816 |
| **YYTP** | 497.2395 | 525.2344 | **YYTPE** | 626.2821 | 654.2770 | **YT** | 237.1234 | 265.1183 |
| **YTP** | 334.1761 | 362.1710 | **YTPE** | 463.2187 | 491.2136 | **YTPEY** | 626.2821 | 654.2770 |
| **TP** | 171.1128 | 199.1077 | **TPE** | 300.1554 | 328.1503 | **TPEY** | 463.2187 | 491.2136 |
| **TPEYE** | 592.2613 | 620.2562 | **TPEYET** | 693.3090 | 721.3039 | **PE** | 199.1077 | 227.1026 |
| **PEY** | 362.1710 | 390.1660 | **PEYE** | 491.2136 | 519.2086 | **PEYET** | 592.2613 | 620.2562 |
| **EY** | 265.1183 | 293.1132 | **EYE** | 394.1609 | 422.1558 | **EYET** | 495.2086 | 523.2035 |
| **EYETK** | 623.3035 | 651.2984 | **YE** | 265.1183 | 293.1132 | **YET** | 366.1660 | 394.1609 |
| **YETK** | 494.2609 | 522.2558 | **YETKD** | 609.2879 | 637.2828 | **ET** | 203.1026 | 231.0975 |
| **ETK** | 331.1976 | 359.1925 | **ETKD** | 446.2245 | 474.2195 | **ETKDT** | 547.2722 | 575.2671 |
| **ETKDTD** | 662.2992 | 690.2941 | **TK** | 202.1550 | 230.1499 | **TKD** | 317.1819 | 345.1769 |
| **TKDT** | 418.2296 | 446.2245 | **TKDTD** | 533.2566 | 561.2515 | **TKDTDI** | 646.3406 | 674.3355 |
| **KD** | 216.1343 | 244.1292 | **KDT** | 317.1819 | 345.1769 | **KDTD** | 432.2089 | 460.2038 |
| **KDTDI** | 545.2930 | 573.2879 | **KDTDIL** | 658.3770 | 686.3719 | **DT** | 189.0870 | 217.0819 |
| **DTD** | 304.1139 | 332.1088 | **DTDI** | 417.1980 | 445.1929 | **DTDIL** | 530.2821 | 558.2770 |
| **DTDILA** | 601.3192 | 629.3141 | **DTDILAA** | 672.3563 | 700.3512 | **TD** | 189.0870 | 217.0819 |
| **TDI** | 302.1710 | 330.1660 | **TDIL** | 415.2551 | 443.2500 | **TDILA** | 486.2922 | 514.2871 |
| **TDILAA** | 557.3293 | 585.3243 | **DI** | 201.1234 | 229.1183 | **DIL** | 314.2074 | 342.2023 |
| **DILA** | 385.2445 | 413.2395 | **DILAA** | 456.2817 | 484.2766 | **DILAAF** | 603.3501 | 631.3450 |
| **IL** | 199.1805 | 227.1754 | **ILA** | 270.2176 | 298.2125 | **ILAA** | 341.2547 | 369.2496 |
| **ILAAF** | 488.3231 | 516.3180 | **LA** | 157.1335 | 185.1285 | **LAA** | 228.1707 | 256.1656 |
| **LAAF** | 375.2391 | 403.2340 | **AA** | 115.0866 | 143.0815 | **AAF** | 262.1550 | 290.1499 |
| **AF** | 191.1179 | 219.1128 | | | | | | |

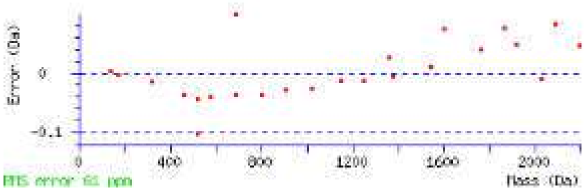


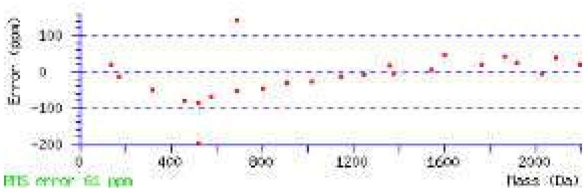


NCBI **BLAST** search of LTYYTPEYETKDTDILAAFR
(Parameters: blastp, nr protein database, expect=20000, no filter, PAM30)
Other BLAST web gateways

**All matches to this query**

| Score | Mr(calc) | Delta | Sequence |
|---|---|---|---|
| 161.7 | 2408.6820 | 0.5854 | ITYYTPEYETKDTBILAAFR |
| 161.7 | 2409.1740 | 0.0934 | ITYYTPEYETKDTDILAAFR |
| 161.7 | 2409.1740 | 0.0934 | ITYYTPEYETKDTDILAAFR |
| 161.7 | 2408.6820 | 0.5854 | LTYYTPEYETKDTBILAAFR |
| 161.7 | 2409.1740 | 0.0934 | LTYYTPEYETKDTDIIAAFR |
| 161.7 | 2409.1740 | 0.0934 | LTYYTPEYETKDTDILAAFR |
| 161.7 | 2409.1740 | 0.0934 | LTYYTPEYETKDTDILAAFR |
| 161.7 | 2409.1740 | 0.0934 | LTYYTPEYETKDTDILAAFR |
| 161.7 | 2409.1740 | 0.0934 | LTYYTPEYETKDTDILAAFR |
| 161.7 | 2409.1740 | 0.0934 | LTYYTPEYETKDTDILAAFR |

**Mascot:** http://www.matrixscience.com/

# MATRIX SCIENCE Mascot Search Results

## Peptide View Spot no 96

MS/MS Fragmentation of **VTPQPGVPPEEAGAAVAAESSTGTWTTVWTDGLTSLDR**
Found in **gi|11466795** in **NCBInr**, ribulose-1,5-bisphosphate carboxylase/oxygenase large subunit [Oryza sativa Japonica Group]

Match to Query 36: 3854.980224 from(3855.987500,1+) intensity(0.0000) index(17)
Title: Label: E11, Spot_Id: 216641, Peak_List_Id: 221253, MSMS Job_Run_Id: 21347, Comment:
Data file C:\Documents and Settings\Administrator\Desktop\farah BHU\RAJESH\12-1-13 raj\ppw_E11_135997701000.txt

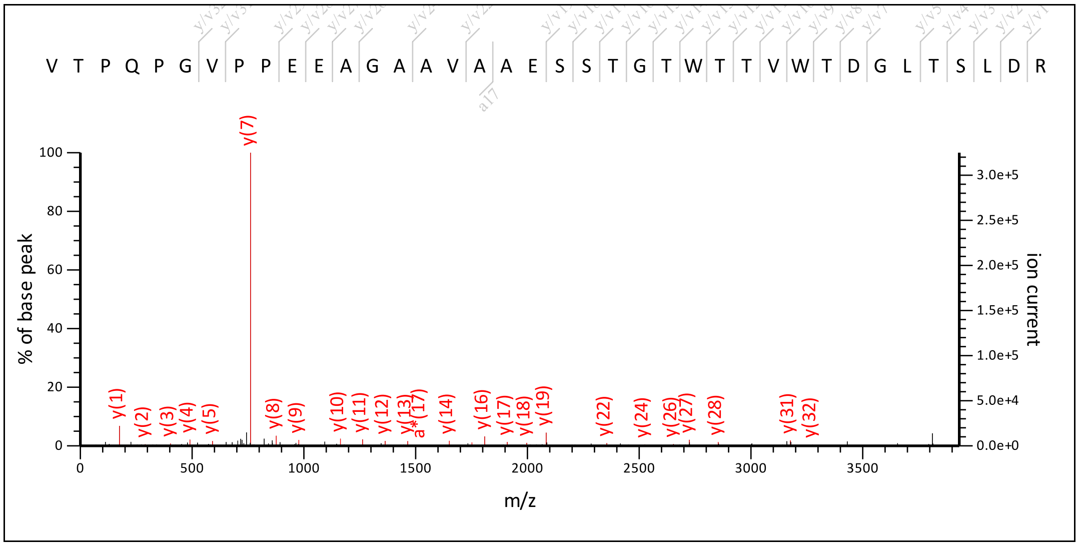


0 to 3931.96

Label all possible matches ○ Label matches used for scoring ◉

**Monoisotopic mass of neutral peptide Mr(calc):** 3853.8647
**Fixed modifications:** Carbamidomethyl (C) (apply to specified residues or termini only)
**Ions Score:** 208 **Expect:** 3.6e-18
**Matches :** 31/837 fragment ions using 38 most intense peaks (help)

| # | Immon. | a | a* | $a^0$ | b | b* | $b^0$ | d | d' | Seq. | v | w | w' | y | y* | |
|---|---|---|---|---|---|---|---|---|---|---|---|---|---|---|---|---|
| 1 | 72.0808 | 72.0808 | | | 100.0757 | | | 44.0495 | | V | | | | | | |
| 2 | 74.0600 | 173.1285 | | 155.1179 | 201.1234 | | 183.1128 | 157.1335 | 159.1128 | T | 3709.7616 | 3722.7820 | 3724.7613 | 3755.8035 | 3738.7770 | 37 |
| 3 | 70.0651 | 270.1812 | | 252.1707 | 298.1761 | | 280.1656 | 244.1656 | | P | 3612.7089 | 3611.7136 | | 3654.7558 | 3637.7293 | 36 |
| 4 | 101.0709 | 398.2398 | 381.2132 | 380.2292 | 426.2347 | 409.2082 | 408.2241 | 341.2183 | | Q | 3484.6503 | 3483.6551 | | 3557.7031 | 3540.6765 | 35 |
| 5 | 70.0651 | 495.2926 | 478.2660 | 477.2820 | 523.2875 | 506.2609 | 505.2769 | 469.2769 | | P | 3387.5975 | 3386.6023 | | 3429.6445 | 3412.6179 | 34 |
| 6 | 30.0338 | 552.3140 | 535.2875 | 534.3035 | 580.3089 | 563.2824 | 562.2984 | | | G | | | | 3332.5917 | 3315.5652 | 33 |
| 7 | 72.0808 | 651.3824 | 634.3559 | 633.3719 | 679.3774 | 662.3508 | 661.3668 | 637.3668 | | V | 3231.5077 | 3244.5281 | | ***3275.5703*** | 3258.5437 | 32 |
| 8 | 70.0651 | 748.4352 | 731.4087 | 730.4246 | 776.4301 | 759.4036 | 758.4196 | 722.4196 | | P | 3134.4549 | 3133.4596 | | ***3176.5018*** | 3159.4753 | 31 |
| 9 | 70.0651 | 845.4880 | 828.4614 | 827.4774 | 873.4829 | 856.4563 | 855.4723 | 819.4723 | | P | 3037.4021 | 3036.4069 | | 3079.4491 | 3062.4225 | 30 |
| 10 | 102.0550 | 974.5306 | 957.5040 | 956.5200 | 1002.5255 | 985.4989 | 984.5149 | 916.5251 | | E | 2908.3595 | 2907.3643 | | 2982.3963 | 2965.3698 | 29 |
| 11 | 102.0550 | 1103.5732 | 1086.5466 | 1085.5626 | 1131.5681 | 1114.5415 | 1113.5575 | 1045.5677 | | E | 2779.3169 | 2778.3217 | | ***2853.3537*** | 2836.3272 | 28 |
| 12 | 44.0495 | 1174.6103 | 1157.5837 | 1156.5997 | 1202.6052 | 1185.5786 | 1184.5946 | | | A | 2708.2798 | | | ***2724.3111*** | 2707.2846 | 27 |
| 13 | 30.0338 | 1231.6317 | 1214.6052 | 1213.6212 | 1259.6266 | 1242.6001 | 1241.6161 | | | G | | | | ***2653.2740*** | 2636.2475 | 26 |
| 14 | 44.0495 | 1302.6688 | 1285.6423 | 1284.6583 | 1330.6638 | 1313.6372 | 1312.6532 | | | A | 2580.2213 | | | 2596.2526 | 2579.2260 | 25 |
| 15 | 44.0495 | 1373.7060 | 1356.6794 | 1355.6954 | 1401.7009 | 1384.6743 | 1383.6903 | | | A | 2509.1841 | | | ***2525.2154*** | 2508.1889 | 25 |
| 16 | 72.0808 | 1472.7744 | 1455.7478 | 1454.7638 | 1500.7693 | 1483.7427 | 1482.7587 | 1458.7587 | | V | 2410.1157 | 2423.1361 | | 2454.1783 | 2437.1518 | 24 |
| 17 | 44.0495 | 1543.8115 | 1526.7849 | 1525.8009 | 1571.8064 | 1554.7799 | 1553.7958 | | | A | 2339.0786 | | | ***2355.1099*** | 2338.0834 | 23 |
| 18 | 44.0495 | 1614.8486 | 1597.8221 | 1596.8380 | 1642.8435 | 1625.8170 | 1624.8329 | | | A | 2268.0415 | | | 2284.0728 | 2267.0462 | 22 |
| 19 | 102.0550 | 1743.8912 | 1726.8646 | 1725.8806 | 1771.8861 | 1754.8596 | 1753.8755 | 1685.8857 | | E | 2138.9989 | 2138.0037 | | 2213.0357 | 2196.0091 | 21 |
| 20 | 60.0444 | 1830.9232 | 1813.8967 | 1812.9127 | 1858.9181 | 1841.8916 | 1840.9076 | 1814.9283 | | S | 2051.9669 | 2050.9716 | | ***2083.9931*** | 2066.9665 | 20 |
| 21 | 60.0444 | 1917.9552 | 1900.9287 | 1899.9447 | 1945.9502 | 1928.9236 | 1927.9396 | 1901.9603 | | S | 1964.9348 | 1963.9396 | | ***1996.9611*** | 1979.9345 | 19 |
| 22 | 74.0600 | 2019.0029 | 2001.9764 | 2000.9924 | 2046.9978 | 2029.9713 | 2028.9873 | 2003.0080 | 2004.9873 | T | 1863.8872 | 1876.9076 | 1878.8868 | ***1909.9290*** | 1892.9025 | 18 |

| | | | | | | | | | | | | | | | | |
|---|---|---|---|---|---|---|---|---|---|---|---|---|---|---|---|---|
| 23 | 30.0338 | 2076.0244 | 2058.9978 | 2058.0138 | 2104.0193 | 2086.9928 | 2086.0087 | | | G | | | | ***1808.8814*** | 1791.8548 | [illegible] |
| 24 | 74.0600 | 2177.0721 | 2160.0455 | 2159.0615 | 2205.0670 | 2188.0404 | 2187.0564 | 2161.0772 | 2163.0564 | T | 1705.8180 | 1718.8384 | 1720.8177 | ***1751.8599*** | 1734.8333 | [illegible] |
| 25 | 159.0917 | 2363.1514 | 2346.1248 | 2345.1408 | 2391.1463 | 2374.1197 | 2373.1357 | | | W | 1519.7387 | | | ***1650.8122*** | 1633.7857 | [illegible] |
| 26 | 74.0600 | 2464.1991 | 2447.1725 | 2446.1885 | 2492.1940 | 2475.1674 | 2474.1834 | 2448.2041 | 2450.1834 | T | 1418.6910 | 1431.7114 | 1433.6907 | ***1464.7329*** | 1447.7064 | [illegible] |
| 27 | 74.0600 | 2565.2467 | 2548.2202 | 2547.2362 | 2593.2417 | 2576.2151 | 2575.2311 | 2549.2518 | 2551.2311 | T | 1317.6434 | 1330.6638 | 1332.6430 | ***1363.6852*** | 1346.6587 | [illegible] |
| 28 | 72.0808 | 2664.3152 | 2647.2886 | 2646.3046 | 2692.3101 | 2675.2835 | 2674.2995 | 2650.2995 | | V | 1218.5749 | 1231.5953 | | ***1262.6375*** | 1245.6110 | [illegible] |
| 29 | 159.0917 | 2850.3945 | 2833.3679 | 2832.3839 | 2878.3894 | 2861.3628 | 2860.3788 | | | W | 1032.4956 | | | ***1163.5691*** | 1146.5426 | [illegible] |
| 30 | 74.0600 | 2951.4421 | 2934.4156 | 2933.4316 | 2979.4371 | 2962.4105 | 2961.4265 | 2935.4472 | 2937.4265 | T | 931.4480 | 944.4684 | 946.4476 | ***977.4898*** | 960.4633 | [illegible] |
| 31 | 88.0393 | 3066.4691 | 3049.4425 | 3048.4585 | 3094.4640 | 3077.4375 | 3076.4534 | 3022.4793 | | D | 816.4210 | 815.4258 | | ***876.4421*** | 859.4156 | [illegible] |
| 32 | 30.0338 | 3123.4906 | 3106.4640 | 3105.4800 | 3151.4855 | 3134.4589 | 3133.4749 | | | G | | | | ***761.4152*** | 744.3886 | [illegible] |
| 33 | 86.0964 | 3236.5746 | 3219.5481 | 3218.5641 | 3264.5695 | 3247.5430 | 3246.5590 | 3194.5277 | | L | 646.3155 | 645.3202 | | 704.3937 | 687.3672 | [illegible] |
| 34 | 74.0600 | 3337.6223 | 3320.5957 | 3319.6117 | 3365.6172 | 3348.5907 | 3347.6066 | 3321.6274 | 3323.6066 | T | 545.2678 | 558.2882 | 560.2675 | ***591.3097*** | 574.2831 | [illegible] |
| 35 | 60.0444 | 3424.6543 | 3407.6278 | 3406.6438 | 3452.6492 | 3435.6227 | 3434.6387 | 3408.6594 | | S | 458.2358 | 457.2405 | | ***490.2620*** | 473.2354 | [illegible] |
| 36 | 86.0964 | 3537.7384 | 3520.7118 | 3519.7278 | 3565.7333 | 3548.7068 | 3547.7227 | 3495.6914 | | L | 345.1517 | 344.1565 | | ***403.2300*** | 386.2034 | [illegible] |
| 37 | 88.0393 | 3652.7653 | 3635.7388 | 3634.7548 | 3680.7602 | 3663.7337 | 3662.7497 | 3608.7755 | | D | 230.1248 | 229.1295 | | ***290.1459*** | 273.1193 | [illegible] |
| 38 | 129.1135 | | | | | | | | | R | 74.0237 | 73.0284 | | ***175.1190*** | 158.0924 | |

| Seq | ya | yb | Seq | ya | yb | Seq | ya | yb |
|---|---|---|---|---|---|---|---|---|
| TP | 171.1128 | 199.1077 | TPQ | 299.1714 | 327.1663 | TPQP | 396.2241 | 424.2191 |
| TPQPG | 453.2456 | 481.2405 | TPQPGV | 552.3140 | 580.3089 | TPQPGVP | 649.3668 | 677.3617 |
| PQ | 198.1237 | 226.1186 | PQP | 295.1765 | 323.1714 | PQPG | 352.1979 | 380.1928 |
| PQPGV | 451.2663 | 479.2613 | PQPGVP | 548.3191 | 576.3140 | PQPGVPP | 645.3719 | 673.3668 |
| QP | 198.1237 | 226.1186 | QPG | 255.1452 | 283.1401 | QPGV | 354.2136 | 382.2085 |
| QPGVP | 451.2663 | 479.2613 | QPGVPP | 548.3191 | 576.3140 | QPGVPPE | 677.3617 | 705.3566 |
| PG | 127.0866 | 155.0815 | PGV | 226.1550 | 254.1499 | PGVP | 323.2078 | 351.2027 |
| PGVPP | 420.2605 | 448.2554 | PGVPPE | 549.3031 | 577.2980 | PGVPPEE | 678.3457 | 706.3406 |
| GV | 129.1022 | 157.0972 | GVP | 226.1550 | 254.1499 | GVPP | 323.2078 | 351.2027 |
| GVPPE | 452.2504 | 480.2453 | GVPPEE | 581.2930 | 609.2879 | GVPPEEA | 652.3301 | 680.3250 |
| VP | 169.1335 | 197.1285 | VPP | 266.1863 | 294.1812 | VPPE | 395.2289 | 423.2238 |
| VPPEE | 524.2715 | 552.2664 | VPPEEA | 595.3086 | 623.3035 | VPPEEAG | 652.3301 | 680.3250 |
| PP | 167.1179 | 195.1128 | PPE | 296.1605 | 324.1554 | PPEE | 425.2031 | 453.1980 |
| PPEEA | 496.2402 | 524.2351 | PPEEAG | 553.2617 | 581.2566 | PPEEAGA | 624.2988 | 652.2937 |
| PPEEAGAA | 695.3359 | 723.3308 | PE | 199.1077 | 227.1026 | PEE | 328.1503 | 356.1452 |
| PEEA | 399.1874 | 427.1823 | PEEAG | 456.2089 | 484.2038 | PEEAGA | 527.2460 | 555.2409 |
| PEEAGAA | 598.2831 | 626.2780 | PEEAGAAV | 697.3515 | 725.3464 | EE | 231.0975 | 259.0925 |
| EEA | 302.1347 | 330.1296 | EEAG | 359.1561 | 387.1510 | EEAGA | 430.1932 | 458.1882 |
| EEAGAA | 501.2304 | 529.2253 | EEAGAAV | 600.2988 | 628.2937 | EEAGAAVA | 671.3359 | 699.3308 |
| EA | 173.0921 | 201.0870 | EAG | 230.1135 | 258.1084 | EAGA | 301.1506 | 329.1456 |
| EAGAA | 372.1878 | 400.1827 | EAGAAV | 471.2562 | 499.2511 | EAGAAVA | 542.2933 | 570.2882 |
| EAGAAVAA | 613.3304 | 641.3253 | AG | 101.0709 | 129.0659 | AGA | 172.1081 | 200.1030 |
| AGAA | 243.1452 | 271.1401 | AGAAV | 342.2136 | 370.2085 | AGAAVA | 413.2507 | 441.2456 |
| AGAAVAA | 484.2878 | 512.2827 | AGAAVAAE | 613.3304 | 641.3253 | GA | 101.0709 | 129.0659 |
| GAA | 172.1081 | 200.1030 | GAAV | 271.1765 | 299.1714 | GAAVA | 342.2136 | 370.2085 |
| GAAVAA | 413.2507 | 441.2456 | GAAVAAE | 542.2933 | 570.2882 | GAAVAAES | 629.3253 | 657.3202 |
| AA | 115.0866 | 143.0815 | AAV | 214.1550 | 242.1499 | AAVA | 285.1921 | 313.1870 |
| AAVAA | 356.2292 | 384.2241 | AAVAAE | 485.2718 | 513.2667 | AAVAAES | 572.3039 | 600.2988 |
| AAVAAESS | 659.3359 | 687.3308 | AV | 143.1179 | 171.1128 | AVA | 214.1550 | 242.1499 |
| AVAA | 285.1921 | 313.1870 | AVAAE | 414.2347 | 442.2296 | AVAAES | 501.2667 | 529.2617 |
| AVAAESS | 588.2988 | 616.2937 | AVAAESST | 689.3464 | 717.3414 | VA | 143.1179 | 171.1128 |
| VAA | 214.1550 | 242.1499 | VAAE | 343.1976 | 371.1925 | VAAES | 430.2296 | 458.2245 |
| VAAESS | 517.2617 | 545.2566 | VAAESST | 618.3093 | 646.3042 | VAAESSTG | 675.3308 | 703.3257 |
| AA | 115.0866 | 143.0815 | AAE | 244.1292 | 272.1241 | AAES | 331.1612 | 359.1561 |
| AAESS | 418.1932 | 446.1882 | AAESST | 519.2409 | 547.2358 | AAESSTG | 576.2624 | 604.2573 |
| AAESSTGT | 677.3101 | 705.3050 | AE | 173.0921 | 201.0870 | AES | 260.1241 | 288.1190 |
| AESS | 347.1561 | 375.1510 | AESST | 448.2038 | 476.1987 | AESSTG | 505.2253 | 533.2202 |
| AESSTGT | 606.2729 | 634.2679 | ES | 189.0870 | 217.0819 | ESS | 276.1190 | 304.1139 |

| | | | | | | | | |
|---|---|---|---|---|---|---|---|---|
| **ESST** | 377.1667 | 405.1616 | **ESSTG** | 434.1882 | 462.1831 | **ESSTGT** | 535.2358 | 563.2307 |
| **SS** | 147.0764 | 175.0713 | **SST** | 248.1241 | 276.1190 | **SSTG** | 305.1456 | 333.1405 |
| **SSTGT** | 406.1932 | 434.1882 | **SSTGTW** | 592.2726 | 620.2675 | **SSTGTWT** | 693.3202 | 721.3151 |
| **ST** | 161.0921 | 189.0870 | **STG** | 218.1135 | 246.1084 | **STGT** | 319.1612 | 347.1561 |
| **STGTW** | 505.2405 | 533.2354 | **STGTWT** | 606.2882 | 634.2831 | **TG** | 131.0815 | 159.0764 |
| **TGT** | 232.1292 | 260.1241 | **TGTW** | 418.2085 | 446.2034 | **TGTWT** | 519.2562 | 547.2511 |
| **TGTWTT** | 620.3039 | 648.2988 | **GT** | 131.0815 | 159.0764 | **GTW** | 317.1608 | 345.1557 |
| **GTWT** | 418.2085 | 446.2034 | **GTWTT** | 519.2562 | 547.2511 | **GTWTTV** | 618.3246 | 646.3195 |
| **TW** | 260.1394 | 288.1343 | **TWT** | 361.1870 | 389.1819 | **TWTT** | 462.2347 | 490.2296 |
| **TWTTV** | 561.3031 | 589.2980 | **WT** | 260.1394 | 288.1343 | **WTT** | 361.1870 | 389.1819 |
| **WTTV** | 460.2554 | 488.2504 | **WTTVW** | 646.3348 | 674.3297 | **TT** | 175.1077 | 203.1026 |
| **TTV** | 274.1761 | 302.1710 | **TTVW** | 460.2554 | 488.2504 | **TTVWT** | 561.3031 | 589.2980 |
| **TTVWTD** | 676.3301 | 704.3250 | **TV** | 173.1285 | 201.1234 | **TVW** | 359.2078 | 387.2027 |
| **TVWT** | 460.2554 | 488.2504 | **TVWTD** | 575.2824 | 603.2773 | **TVWTDG** | 632.3039 | 660.2988 |
| **VW** | 258.1601 | 286.1550 | **VWT** | 359.2078 | 387.2027 | **VWTD** | 474.2347 | 502.2296 |
| **VWTDG** | 531.2562 | 559.2511 | **VWTDGL** | 644.3402 | 672.3352 | **WT** | 260.1394 | 288.1343 |
| **WTD** | 375.1663 | 403.1612 | **WTDG** | 432.1878 | 460.1827 | **WTDGL** | 545.2718 | 573.2667 |
| **WTDGLT** | 646.3195 | 674.3144 | **TD** | 189.0870 | 217.0819 | **TDG** | 246.1084 | 274.1034 |
| **TDGL** | 359.1925 | 387.1874 | **TDGLT** | 460.2402 | 488.2351 | **TDGLTS** | 547.2722 | 575.2671 |
| **TDGLTSL** | 660.3563 | 688.3512 | **DG** | 145.0608 | 173.0557 | **DGL** | 258.1448 | 286.1397 |
| **DGLT** | 359.1925 | 387.1874 | **DGLTS** | 446.2245 | 474.2195 | **DGLTSL** | 559.3086 | 587.3035 |
| **DGLTSLD** | 674.3355 | 702.3305 | **GL** | 143.1179 | 171.1128 | **GLT** | 244.1656 | 272.1605 |
| **GLTS** | 331.1976 | 359.1925 | **GLTSL** | 444.2817 | 472.2766 | **GLTSLD** | 559.3086 | 587.3035 |
| **LT** | 187.1441 | 215.1390 | **LTS** | 274.1761 | 302.1710 | **LTSL** | 387.2602 | 415.2551 |
| **LTSLD** | 502.2871 | 530.2821 | **TS** | 161.0921 | 189.0870 | **TSL** | 274.1761 | 302.1710 |
| **TSLD** | 389.2031 | 417.1980 | **SL** | 173.1285 | 201.1234 | **SLD** | 288.1554 | 316.1503 |
| **LD** | 201.1234 | 229.1183 | | | | | | |

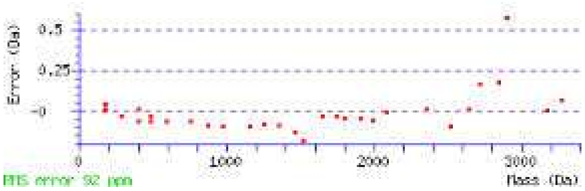


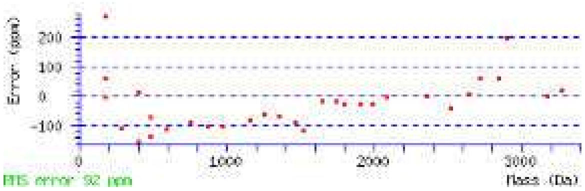


NCBI **BLAST** search of VTPQPGVPPEEAGAAVAAESSTGTWTTVWTDGLTSLDR
(Parameters: blastp, nr protein database, expect=20000, no filter, PAM30)
Other BLAST web gateways

**All matches to this query**

| Score | Mr(calc) | Delta | Sequence |
|---|---|---|---|
| 229.8 | 3854.8487 | 0.1315 | VTPEPGVPPEEAGAAVAAESSTGTWTTVWTDGLTSLDR |
| 229.8 | 3854.8487 | 0.1315 | VTPEPGVPPEEAGAAVAAESSTGTWTTVWTDGLTSLDR |
| 216.9 | 3854.9075 | 0.0727 | VTPQPGVPAREAGAAVAAESSTGTWTTVWTDGLTSLDR |
| 208.1 | 3854.8309 | 0.1493 | MTPPPGVPPEEAGAAVAAESSTGTWTTVWTDGLTSLDR |
| 208.1 | 3855.8439 | -0.8637 | TTPQPGVPPEEAGAAVAAESSTGTWTTVWTDGLTSLDR |
| 208.1 | 3853.9011 | 1.0792 | VTPKPGVPPEEAGAAVAAESSTGTWTTVWTDGLTSLDR |
| 208.1 | 3853.8647 | 1.1155 | VTPQPGVPPEEAGAAVAAESSTGTWTTVWTDGITSLDR |
| 208.1 | 3853.8647 | 1.1155 | VTPQPGVPPEEAGAAVAAESSTGTWTTVWTDGITSLDR |
| 208.1 | 3853.8647 | 1.1155 | VTPQPGVPPEEAGAAVAAESSTGTWTTVWTDGLTSIDR |
| 208.1 | 3853.8647 | 1.1155 | VTPQPGVPPEEAGAAVAAESSTGTWTTVWTDGLTSLDR |

**Mascot:** http://www.matrixscience.com/

# Mascot Search Results

## Peptide View Spot no 97

MS/MS Fragmentation of **QMSLLLR**
Found in **gi|11466784** in **NCBInr**, ATP synthase CF1 alpha subunit [Oryza sativa Japonica Group]

Match to Query 5: 859.545454 from(860.552730,1+) intensity(0.0000) index(0)
Title: Label: I11, Spot_Id: 216645, Peak_List_Id: 221405, MSMS Job_Run_Id: 21364, Comment:
Data file C:\Documents and Settings\Administrator\Desktop\farah BHU\Akansha BHU\RAJESH\12-1-13 raj\ppw_I11_136074508400.txt

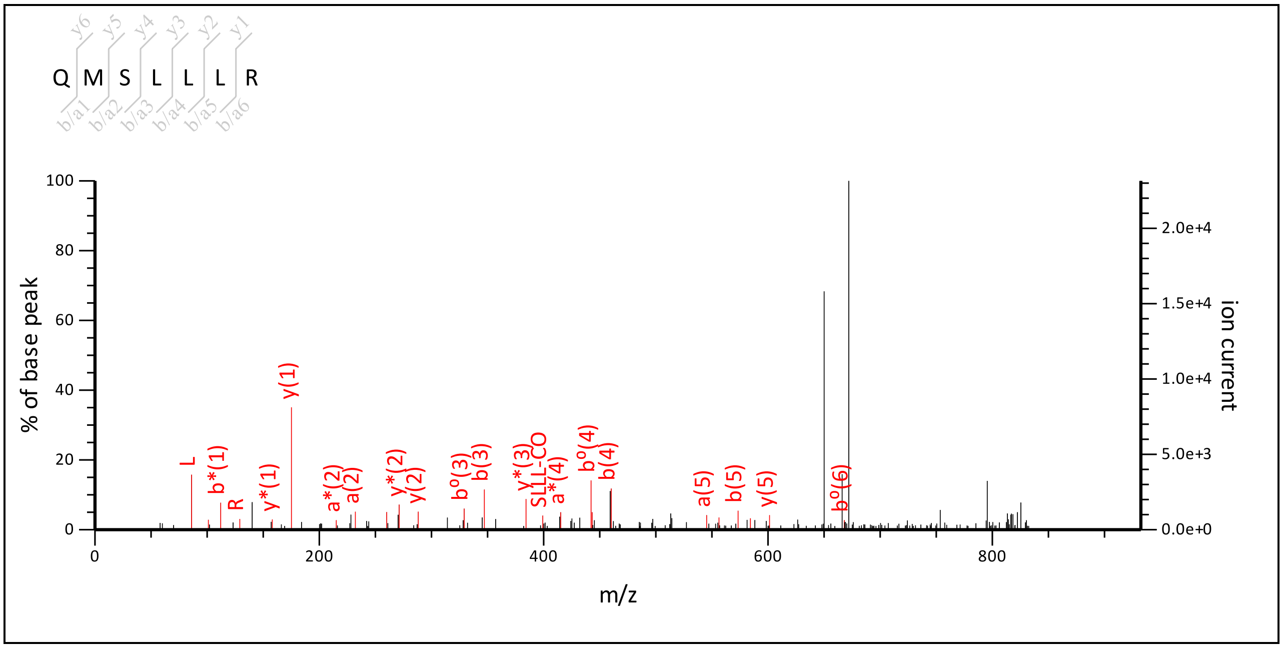


0 to 932.41

Label all possible matches Label matches used for scoring

**Monoisotopic mass of neutral peptide Mr(calc):** 859.4949
**Fixed modifications:** Carbamidomethyl (C) (apply to specified residues or termini only)
**Ions Score:** 28 **Expect:** 12
**Matches :** 29/91 fragment ions using 40 most intense peaks (help)

| # | Immon. | a | a* | a$^0$ | b | b* | b$^0$ | d | Seq. | v | w | y | y* | y$^0$ | # |
|---|---|---|---|---|---|---|---|---|---|---|---|---|---|---|---|
| 1 | 101.0709 | **101.0709** | 84.0444 | | ***129.0659*** | 112.0393 | | 44.0495 | **Q** | | | | | | 7 |
| 2 | 104.0528 | **232.1114** | 215.0849 | | ***260.1063*** | 243.0798 | | 172.1081 | **M** | 656.4090 | 655.4137 | 732.4437 | 715.4171 | 714.4331 | 6 |
| 3 | 60.0444 | 319.1435 | 302.1169 | 301.1329 | ***347.1384*** | 330.1118 | 329.1278 | 303.1485 | **S** | 569.3770 | 568.3817 | **601.4032** | 584.3766 | 583.3926 | 5 |
| 4 | 86.0964 | 432.2275 | 415.2010 | 414.2170 | ***460.2224*** | 443.1959 | 442.2119 | 390.1806 | **L** | 456.2929 | 455.2976 | 514.3711 | 497.3446 | | 4 |
| 5 | 86.0964 | **545.3116** | 528.2850 | 527.3010 | ***573.3065*** | 556.2799 | 555.2959 | 503.2646 | **L** | 343.2088 | 342.2136 | 401.2871 | 384.2605 | | 3 |
| 6 | 86.0964 | 658.3956 | 641.3691 | 640.3851 | 686.3906 | 669.3640 | 668.3800 | 616.3487 | **L** | 230.1248 | 229.1295 | **288.2030** | 271.1765 | | 2 |
| 7 | 129.1135 | | | | | | | | **R** | 74.0237 | 73.0284 | **175.1190** | 158.0924 | | 1 |

| Seq | ya | yb | Seq | ya | yb | Seq | ya | yb |
|---|---|---|---|---|---|---|---|---|
| **MS** | 191.0849 | 219.0798 | **MSL** | 304.1689 | 332.1639 | **MSLL** | 417.2530 | 445.2479 |
| **MSLLL** | 530.3371 | 558.3320 | **SL** | 173.1285 | 201.1234 | **SLL** | 286.2125 | 314.2074 |
| **SLLL** | 399.2966 | 427.2915 | **LL** | 199.1805 | 227.1754 | **LLL** | 312.2646 | 340.2595 |
| **LL** | 199.1805 | 227.1754 | | | | | | |

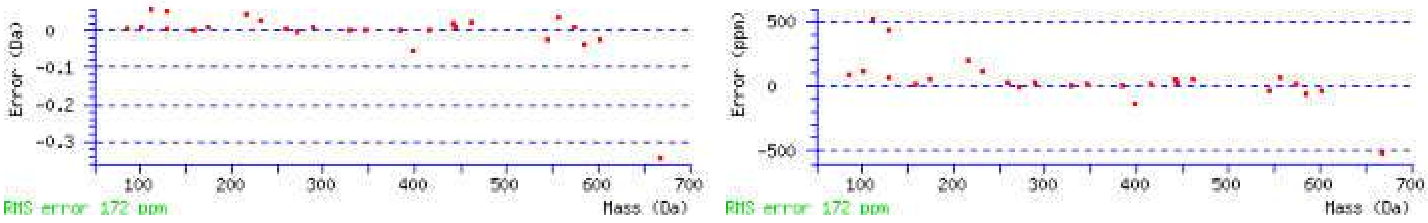


NCBI **BLAST** search of QMSLLLR
(Parameters: blastp, nr protein database, expect=20000, no filter, PAM30)
Other BLAST web gateways

**All matches to this query**

| Score | Mr(calc) | Delta | Sequence |
|---|---|---|---|
| 40.8 | 859.4586 | 0.0869 | MGVLIDGR |
| 30.2 | 859.4222 | 0.1233 | GMPDLATR |
| 28.4 | 859.4949 | 0.0505 | QMSLILR |
| 28.4 | 859.4949 | 0.0505 | QMSLLLR |
| 24.7 | 858.4923 | 1.0532 | ASKLLDGR |
| 22.6 | 858.4811 | 1.0644 | LEGGLAATK |
| 22.5 | 859.4586 | 0.0869 | GIVMIDGR |
| 22.3 | 859.4586 | 0.0869 | AVVMLDGR |
| 22.3 | 860.4352 | -0.8897 | GDKTIDGR |
| 22.3 | 859.4399 | 0.1055 | IGETLDGR |

**Mascot:** http://www.matrixscience.com/

# MATRIX SCIENCE Mascot Search Results

## Peptide View **Spot no 97**

MS/MS Fragmentation of **VVQVGDGIAR**
Found in **gi|11466784** in **NCBInr**, ATP synthase CF1 alpha subunit [Oryza sativa Japonica Group]

Match to Query 14: 1012.631024 from(1013.638300,1+) intensity(0.0000) index(3)
Title: Label: I11, Spot_Id: 216645, Peak_List_Id: 221421, MSMS Job_Run_Id: 21364, Comment:
Data file C:\Documents and Settings\Administrator\Desktop\farah BHU\Akansha BHU\RAJESH\12-1-13 raj\ppw_I11_136074508400.txt

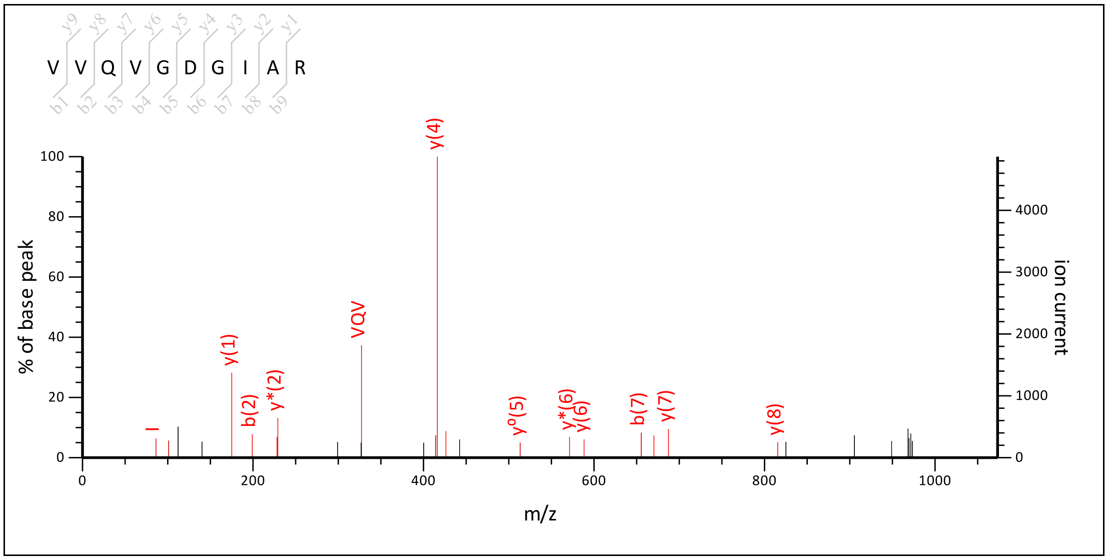


0 to 1073.36

Label all possible matches ○ Label matches used for scoring ◉

**Monoisotopic mass of neutral peptide Mr(calc):** 1012.5666
**Fixed modifications:** Carbamidomethyl (C) (apply to specified residues or termini only)
**Ions Score:** 40 **Expect:** 0.54
**Matches :** 24/148 fragment ions using 26 most intense peaks (help)

| # | Immon. | a | a* | $a^0$ | b | b* | $b^0$ | d | d' | Seq. | v | w | w' | y | y* | $y^0$ | # |
|---|---|---|---|---|---|---|---|---|---|---|---|---|---|---|---|---|---|
| 1 | 72.0808 | 72.0808 | | | 100.0757 | | | 44.0495 | | V | | | | | | | 10 |
| 2 | 72.0808 | 171.1492 | | | ***199.1441*** | | | 157.1335 | | V | 870.4428 | 883.4632 | | 914.5054 | 897.4789 | 896.4948 | 9 |
| 3 | 101.0709 | 299.2078 | 282.1812 | | ***327.2027*** | 310.1761 | | 242.1863 | | Q | 742.3842 | 741.3890 | | ***815.4370*** | 798.4104 | 797.4264 | 8 |
| 4 | 72.0808 | 398.2762 | 381.2496 | | ***426.2711*** | 409.2445 | | 384.2605 | | V | 643.3158 | 656.3362 | | ***687.3784*** | 670.3519 | 669.3679 | 7 |
| 5 | 30.0338 | 455.2976 | 438.2711 | | 483.2926 | 466.2660 | | | | G | | | | ***588.3100*** | 571.2835 | 570.2994 | 6 |
| 6 | 88.0393 | 570.3246 | 553.2980 | 552.3140 | 598.3195 | 581.2930 | 580.3089 | 526.3348 | | D | 471.2674 | 470.2722 | | 531.2885 | 514.2620 | 513.2780 | 5 |
| 7 | 30.0338 | 627.3461 | 610.3195 | 609.3355 | ***655.3410*** | 638.3144 | 637.3304 | | | G | | | | ***416.2616*** | 399.2350 | | 4 |
| 8 | 86.0964 | 740.4301 | 723.4036 | 722.4196 | 768.4250 | 751.3985 | 750.4145 | 712.3988 | 726.4145 | I | 301.1619 | 314.1823 | 328.1979 | 359.2401 | 342.2136 | | 3 |
| 9 | 44.0495 | 811.4672 | 794.4407 | 793.4567 | 839.4621 | 822.4356 | 821.4516 | | | A | 230.1248 | | | 246.1561 | 229.1295 | | 2 |
| 10 | 129.1135 | | | | | | | | | R | 74.0237 | 73.0284 | | ***175.1190*** | 158.0924 | | 1 |

| Seq | ya | yb | Seq | ya | yb | Seq | ya | yb |
|---|---|---|---|---|---|---|---|---|
| VQ | 200.1394 | 228.1343 | VQV | 299.2078 | 327.2027 | VQVG | 356.2292 | 384.2241 |
| VQVGD | 471.2562 | 499.2511 | VQVGDG | 528.2776 | 556.2726 | VQVGDGI | 641.3617 | 669.3566 |
| QV | 200.1394 | 228.1343 | QVG | 257.1608 | 285.1557 | QVGD | 372.1878 | 400.1827 |
| QVGDG | 429.2092 | 457.2041 | QVGDGI | 542.2933 | 570.2882 | QVGDGIA | 613.3304 | 641.3253 |
| VG | 129.1022 | 157.0972 | VGD | 244.1292 | 272.1241 | VGDG | 301.1506 | 329.1456 |
| VGDGI | 414.2347 | 442.2296 | VGDGIA | 485.2718 | 513.2667 | GD | 145.0608 | 173.0557 |
| GDG | 202.0822 | 230.0771 | GDGI | 315.1663 | 343.1612 | GDGIA | 386.2034 | 414.1983 |
| DG | 145.0608 | 173.0557 | DGI | 258.1448 | 286.1397 | DGIA | 329.1819 | 357.1769 |
| GI | 143.1179 | 171.1128 | GIA | 214.1550 | 242.1499 | IA | 157.1335 | 185.1285 |

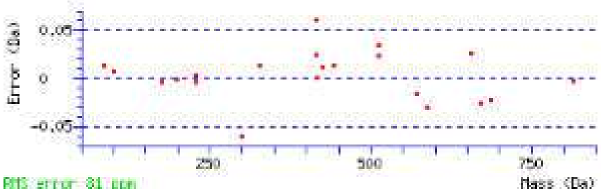


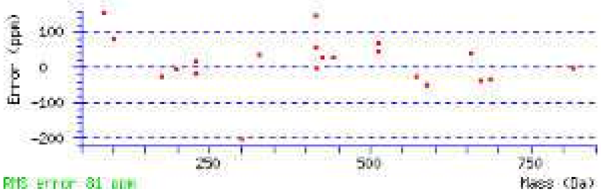


NCBI **BLAST** search of VVQVGDGIAR
(Parameters: blastp, nr protein database, expect=20000, no filter, PAM30)
Other BLAST web gateways

**All matches to this query**

| Score | Mr(calc) | Delta | Sequence |
|---|---|---|---|
| 40.3 | 1012.5666 | 0.0645 | VVQVGDGIAR |
| 32.8 | 1012.5666 | 0.0645 | VQVVTAGSPR |
| 25.6 | 1012.6393 | -0.0083 | LGGVVTKIAR |
| 24.6 | 1012.5777 | 0.0533 | RAVDAAAAIR |
| 22.6 | 1012.5665 | 0.0645 | NIVVSAPSAR |
| 22.4 | 1012.5665 | 0.0645 | LAAASVAAPSR |
| 21.6 | 1013.5730 | -0.9420 | AAGQVRGLSR |
| 21.4 | 1013.5869 | -0.9559 | LLVSGEVAAR |
| 21.4 | 1013.6022 | -0.9712 | ILGAFPLQR |
| 21.2 | 1012.5778 | 0.0532 | VVQRDAVAR |

**Mascot:** http://www.matrixscience.com/

# MATRIX SCIENCE Mascot Search Results

## Peptide View Spot no 97

MS/MS Fragmentation of **IAQIPVSEAYLGR**
Found in **gi|11466784** in **NCBInr**, ATP synthase CF1 alpha subunit [Oryza sativa Japonica Group]

Match to Query 40: 1415.861624 from(1416.868900,1+) intensity(0.0000) index(9)
Title: Label: I11, Spot_Id: 216645, Peak_List_Id: 221389, MSMS Job_Run_Id: 21364, Comment:
Data file C:\Documents and Settings\Administrator\Desktop\farah BHU\Akansha BHU\RAJESH\12-1-13 raj\ppw_I11_136074508400.txt

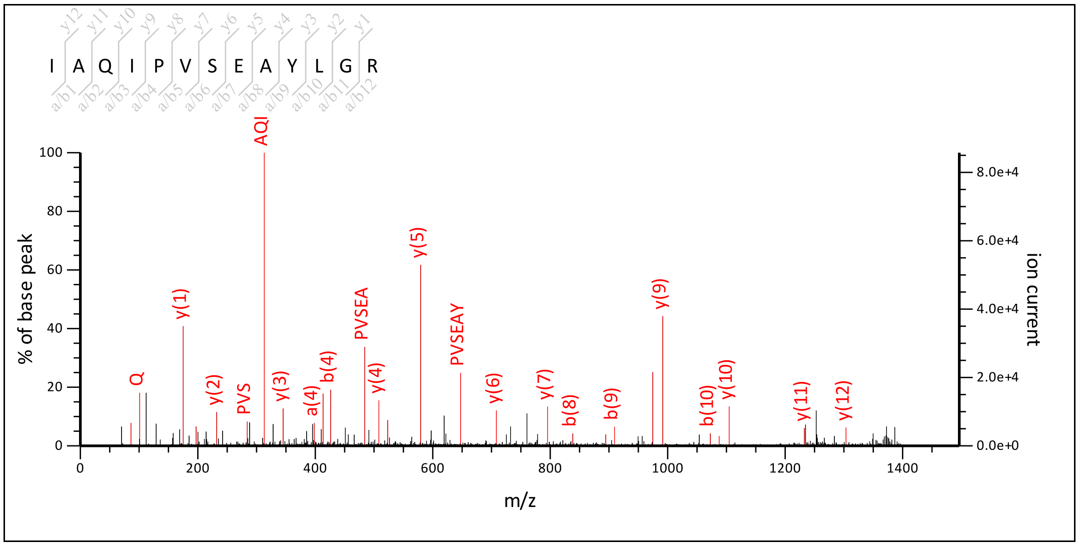


0 to 1496.46

Label all possible matches Label matches used for scoring

**Monoisotopic mass of neutral peptide Mr(calc):** 1415.7772
**Fixed modifications:** Carbamidomethyl (C) (apply to specified residues or termini only)
**Ions Score:** 106 **Expect:** 1.4e-07
**Matches :** 33/212 fragment ions using 42 most intense peaks (help)

| # | Immon. | a | a* | a0 | b | b* | b0 | d | d' | Seq. | v | w | w' | y | y* | |
|---|---|---|---|---|---|---|---|---|---|---|---|---|---|---|---|---|
| 1 | 86.0964 | 86.0964 | | | 114.0913 | | | 44.0495 | | I | | | | | | |
| 2 | 44.0495 | 157.1335 | | | 185.1285 | | | | | A | 1287.6692 | | | ***1303.7005*** | 1286.6739 | 128 |
| 3 | 101.0709 | 285.1921 | 268.1656 | | **313.1870** | 296.1605 | | 228.1707 | | Q | 1159.6106 | 1158.6154 | | ***1232.6634*** | 1215.6368 | 12 |
| 4 | 86.0964 | 398.2762 | 381.2496 | | **426.2711** | 409.2445 | | 370.2449 | 384.2605 | I | 1046.5265 | 1059.5469 | 1073.5626 | ***1104.6048*** | 1087.5782 | 108 |
| 5 | 70.0651 | 495.3289 | 478.3024 | | **523.3239** | 506.2973 | | 469.3133 | | P | 949.4738 | 948.4785 | | ***991.5207*** | 974.4942 | 97 |
| 6 | 72.0808 | 594.3974 | 577.3708 | | 622.3923 | 605.3657 | | 580.3817 | | V | 850.4054 | 863.4258 | | ***894.4680*** | 877.4414 | 87 |
| 7 | 60.0444 | 681.4294 | 664.4028 | 663.4188 | 709.4243 | 692.3978 | 691.4137 | 665.4345 | | S | 763.3733 | 762.3781 | | ***795.3995*** | 778.3730 | 77 |
| 8 | 102.0550 | 810.4720 | 793.4454 | 792.4614 | **838.4669** | 821.4403 | 820.4563 | 752.4665 | | E | 634.3307 | 633.3355 | | ***708.3675*** | 691.3410 | 69 |
| 9 | 44.0495 | 881.5091 | 864.4825 | 863.4985 | **909.5040** | 892.4775 | 891.4934 | | | A | 563.2936 | | | ***579.3249*** | 562.2984 | |
| 10 | 136.0757 | 1044.5724 | 1027.5459 | 1026.5619 | **1072.5673** | 1055.5408 | 1054.5568 | | | Y | 400.2303 | | | ***508.2878*** | 491.2613 | |
| 11 | 86.0964 | 1157.6565 | 1140.6299 | 1139.6459 | 1185.6514 | 1168.6249 | 1167.6408 | 1115.6095 | | L | 287.1462 | 286.1510 | | ***345.2245*** | 328.1979 | |
| 12 | 30.0338 | 1214.6780 | 1197.6514 | 1196.6674 | 1242.6729 | 1225.6463 | 1224.6623 | | | G | | | | ***232.1404*** | 215.1139 | |
| 13 | 129.1135 | | | | | | | | | R | 74.0237 | 73.0284 | | ***175.1190*** | 158.0924 | |

| Seq | ya | yb | Seq | ya | yb | Seq | ya | yb |
|---|---|---|---|---|---|---|---|---|
| AQ | 172.1081 | 200.1030 | AQI | 285.1921 | 313.1870 | AQIP | 382.2449 | 410.2398 |
| AQIPV | 481.3133 | 509.3082 | AQIPVS | 568.3453 | 596.3402 | AQIPVSE | 697.3879 | 725.3828 |
| QI | 214.1550 | 242.1499 | QIP | 311.2078 | 339.2027 | QIPV | 410.2762 | 438.2711 |
| QIPVS | 497.3082 | 525.3031 | QIPVSE | 626.3508 | 654.3457 | QIPVSEA | 697.3879 | 725.3828 |
| IP | 183.1492 | 211.1441 | IPV | 282.2176 | 310.2125 | IPVS | 369.2496 | 397.2445 |
| IPVSE | 498.2922 | 526.2871 | IPVSEA | 569.3293 | 597.3243 | PV | 169.1335 | 197.1285 |
| PVS | 256.1656 | 284.1605 | PVSE | 385.2082 | 413.2031 | PVSEA | 456.2453 | 484.2402 |
| PVSEAY | 619.3086 | 647.3035 | VS | 159.1128 | 187.1077 | VSE | 288.1554 | 316.1503 |

| | | | | | | | | |
|---|---|---|---|---|---|---|---|---|
| **VSEA** | 359.1925 | 387.1874 | **VSEAY** | 522.2558 | 550.2508 | **VSEAYL** | 635.3399 | 663.3348 |
| **VSEAYLG** | 692.3614 | 720.3563 | **SE** | 189.0870 | 217.0819 | **SEA** | 260.1241 | 288.1190 |
| **SEAY** | 423.1874 | 451.1823 | **SEAYL** | 536.2715 | 564.2664 | **SEAYLG** | 593.2930 | 621.2879 |
| **EA** | 173.0921 | 201.0870 | **EAY** | 336.1554 | 364.1503 | **EAYL** | 449.2395 | 477.2344 |
| **EAYLG** | 506.2609 | 534.2558 | **AY** | 207.1128 | 235.1077 | **AYL** | 320.1969 | 348.1918 |
| **AYLG** | 377.2183 | 405.2132 | **YL** | 249.1598 | 277.1547 | **YLG** | 306.1812 | 334.1761 |
| **LG** | 143.1179 | 171.1128 | | | | | | |

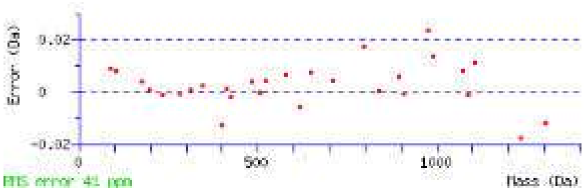


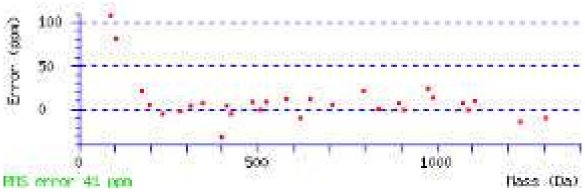


NCBI **BLAST** search of IAQIPVSEAYLGR
(Parameters: blastp, nr protein database, expect=20000, no filter, PAM30)
Other BLAST web gateways

**All matches to this query**

| Score | Mr(calc) | Delta | Sequence |
|---|---|---|---|
| 105.8 | 1415.7772 | 0.0844 | IAQIPVSEAYLGR |
| 103.6 | 1415.7772 | 0.0844 | LAQIPVSEAYLGR |
| 85.2 | 1415.7773 | 0.0844 | IAQIPVSESFLGR |
| 56.4 | 1415.7772 | 0.0844 | IAQIPIGESYIGR |
| 22.9 | 1415.7368 | 0.1248 | IAGALQADSEASRK |
| 20.7 | 1415.8136 | 0.0480 | LLAQELLQYGLR |
| 20.2 | 1416.7473 | -0.8857 | NPTNHQIELVPR |
| 20.1 | 1415.8500 | 0.0116 | KIAVALAEILYGR |
| 17.9 | 1415.7885 | 0.0731 | RGADPVLSYIAVR |
| 17.8 | 1415.7343 | 0.1273 | IKALHLYDCQR |

**Mascot:** http://www.matrixscience.com/

# MATRIX SCIENCE Mascot Search Results

## Peptide View **Spot no 97**

MS/MS Fragmentation of **EAYPGDVFYLHSR**
Found in **gi|11466784** in **NCBInr**, ATP synthase CF1 alpha subunit [Oryza sativa Japonica Group]

Match to Query 52: 1552.821724 from(1553.829000,1+) intensity(0.0000) index(14)
Title: Label: I11, Spot_Id: 216645, Peak_List_Id: 221390, MSMS Job_Run_Id: 21364, Comment:
Data file C:\Documents and Settings\Administrator\Desktop\farah BHU\Akansha BHU\RAJESH\12-1-13 raj\ppw_I11_136074508400.txt

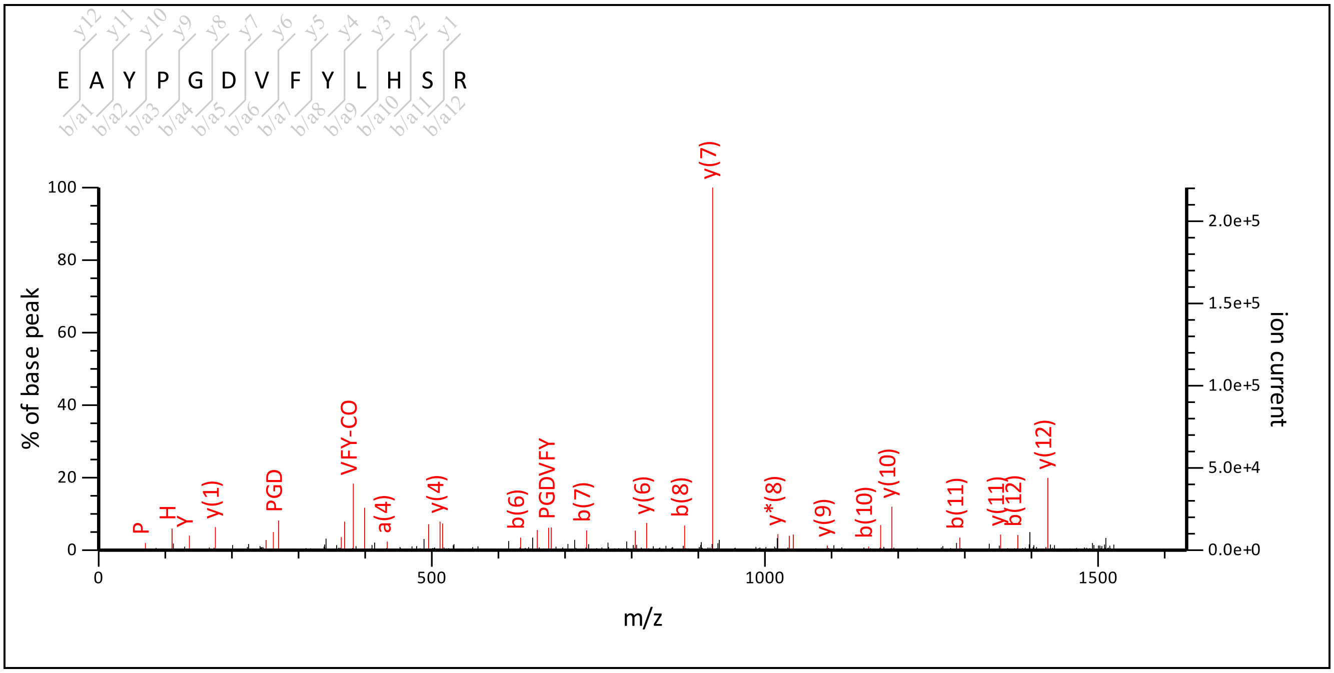


0 to 1632.96

Label all possible matches ○ Label matches used for scoring ◉

**Monoisotopic mass of neutral peptide Mr(calc):** 1552.7310
**Fixed modifications:** Carbamidomethyl (C) (apply to specified residues or termini only)
**Ions Score:** 112 **Expect:** 3.4e-08
**Matches :** 42/195 fragment ions using 45 most intense peaks (help)

| # | Immon. | a | $a^0$ | b | $b^0$ | d | Seq. | v | w | y | y* | $y^0$ | # |
|---|---|---|---|---|---|---|---|---|---|---|---|---|---|
| 1 | 102.0550 | 102.0550 | 84.0444 | 130.0499 | 112.0393 | 44.0495 | E | | | | | | 13 |
| 2 | 44.0495 | 173.0921 | 155.0815 | 201.0870 | 183.0764 | | A | 1408.6644 | | **1424.6957** | 1407.6692 | 1406.6852 | 12 |
| 3 | 136.0757 | 336.1554 | 318.1448 | **364.1503** | 346.1397 | | Y | 1245.6011 | | **1353.6586** | 1336.6321 | 1335.6480 | 11 |
| 4 | 70.0651 | 433.2082 | 415.1976 | 461.2031 | 443.1925 | 407.1925 | P | 1148.5483 | 1147.5531 | **1190.5953** | 1173.5687 | 1172.5847 | 10 |
| 5 | 30.0338 | 490.2296 | 472.2191 | 518.2245 | 500.2140 | | G | | | **1093.5425** | 1076.5160 | 1075.5320 | 9 |
| 6 | 88.0393 | 605.2566 | 587.2460 | **633.2515** | 615.2409 | 561.2667 | D | 976.4999 | 975.5047 | **1036.5211** | 1019.4945 | 1018.5105 | 8 |
| 7 | 72.0808 | 704.3250 | 686.3144 | **732.3199** | 714.3093 | 690.3093 | V | 877.4315 | 890.4519 | **921.4941** | 904.4676 | 903.4835 | 7 |
| 8 | 120.0808 | 851.3934 | 833.3828 | **879.3883** | 861.3777 | | F | 730.3631 | | **822.4257** | 805.3991 | 804.4151 | 6 |
| 9 | 136.0757 | 1014.4567 | 996.4462 | **1042.4516** | 1024.4411 | | Y | 567.2998 | | **675.3573** | 658.3307 | 657.3467 | 5 |
| 10 | 86.0964 | 1127.5408 | 1109.5302 | **1155.5357** | 1137.5251 | 1085.4938 | L | 454.2157 | 453.2205 | **512.2940** | 495.2674 | 494.2834 | 4 |
| 11 | 110.0713 | 1264.5997 | 1246.5891 | **1292.5946** | 1274.5841 | | H | 317.1568 | | **399.2099** | 382.1833 | 381.1993 | 3 |
| 12 | 60.0444 | 1351.6317 | 1333.6212 | **1379.6266** | 1361.6161 | 1335.6368 | S | 230.1248 | 229.1295 | **262.1510** | 245.1244 | 244.1404 | 2 |

| | | | | | | | | | | | | |
|---|---|---|---|---|---|---|---|---|---|---|---|---|
| 13 | 129.1135 | | | | | R | 74.0237 | 73.0284 | *175.1190* | 158.0924 | | 1 |

| Seq | ya | yb | Seq | ya | yb | Seq | ya | yb |
|---|---|---|---|---|---|---|---|---|
| AY | 207.1128 | 235.1077 | AYP | 304.1656 | 332.1605 | AYPG | 361.1870 | 389.1819 |
| AYPGD | 476.2140 | 504.2089 | AYPGDV | 575.2824 | 603.2773 | YP | 233.1285 | 261.1234 |
| YPG | 290.1499 | 318.1448 | YPGD | 405.1769 | 433.1718 | YPGDV | 504.2453 | 532.2402 |
| YPGDVF | 651.3137 | 679.3086 | PG | 127.0866 | 155.0815 | PGD | 242.1135 | 270.1084 |
| PGDV | 341.1819 | 369.1769 | PGDVF | 488.2504 | 516.2453 | PGDVFY | 651.3137 | 679.3086 |
| GD | 145.0608 | 173.0557 | GDV | 244.1292 | 272.1241 | GDVF | 391.1976 | 419.1925 |
| GDVFY | 554.2609 | 582.2558 | GDVFYL | 667.3450 | 695.3399 | DV | 187.1077 | 215.1026 |
| DVF | 334.1761 | 362.1710 | DVFY | 497.2395 | 525.2344 | DVFYL | 610.3235 | 638.3184 |
| VF | 219.1492 | 247.1441 | VFY | 382.2125 | 410.2074 | VFYL | 495.2966 | 523.2915 |
| VFYLH | 632.3555 | 660.3504 | FY | 283.1441 | 311.1390 | FYL | 396.2282 | 424.2231 |
| FYLH | 533.2871 | 561.2820 | FYLHS | 620.3191 | 648.3140 | YL | 249.1598 | 277.1547 |
| YLH | 386.2187 | 414.2136 | YLHS | 473.2507 | 501.2456 | LH | 223.1553 | 251.1503 |
| LHS | 310.1874 | 338.1823 | HS | 197.1033 | 225.0982 | | | |

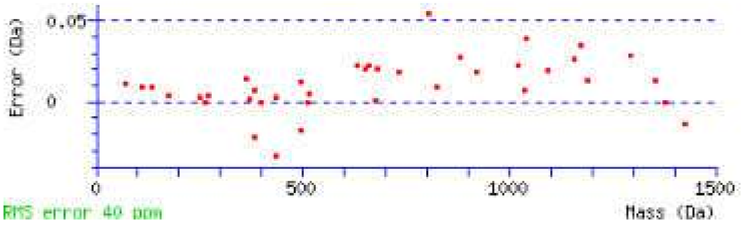


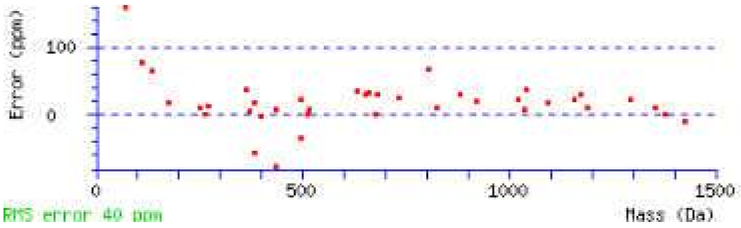


NCBI **BLAST** search of EAYPGDVFYLHSR
(Parameters: blastp, nr protein database, expect=20000, no filter, PAM30)
Other BLAST web gateways

**All matches to this query**

| Score | Mr(calc) | Delta | Sequence |
|---|---|---|---|
| 111.8 | 1552.7310 | 0.0907 | EAYPGDVFYIHSR |
| 111.8 | 1552.7310 | 0.0907 | EAYPGDVFYLHSR |
| 19.7 | 1552.7998 | 0.0219 | LRVYTVQDAAFDR |
| 17.3 | 1552.7191 | 0.1026 | LSYPEDLGMQASSR |
| 17.1 | 1552.8725 | -0.0508 | LRPRYFSISSSLK |
| 15.7 | 1552.7369 | 0.0848 | EAYSDSQLIEKDR |
| 15.3 | 1552.8249 | -0.0032 | EFSLVGRNIYIDK |
| 15.2 | 1552.8144 | 0.0073 | EGMDISVRHLLQR |
| 15.1 | 1552.7555 | 0.0662 | ISPLYSAEGTCLSR |
| 14.5 | 1552.7609 | 0.0608 | FLREWMLYHSR |

**Mascot:** http://www.matrixscience.com/

# MATRIX SCIENCE Mascot Search Results

## Peptide View **Spot no 97**

MS/MS Fragmentation of **GQDVICVYVAIGQR**
Found in **gi|11466784** in **NCBInr**, ATP synthase CF1 alpha subunit [Oryza sativa Japonica Group]

Match to Query 55: 1576.897524 from(1577.904800,1+) intensity(0.0000) index(15)
Title: Label: I11, Spot_Id: 216645, Peak_List_Id: 221403, MSMS Job_Run_Id: 21364, Comment:
Data file C:\Documents and Settings\Administrator\Desktop\farah BHU\Akansha BHU\RAJESH\12-1-13 raj\ppw_I11_136074508400.txt

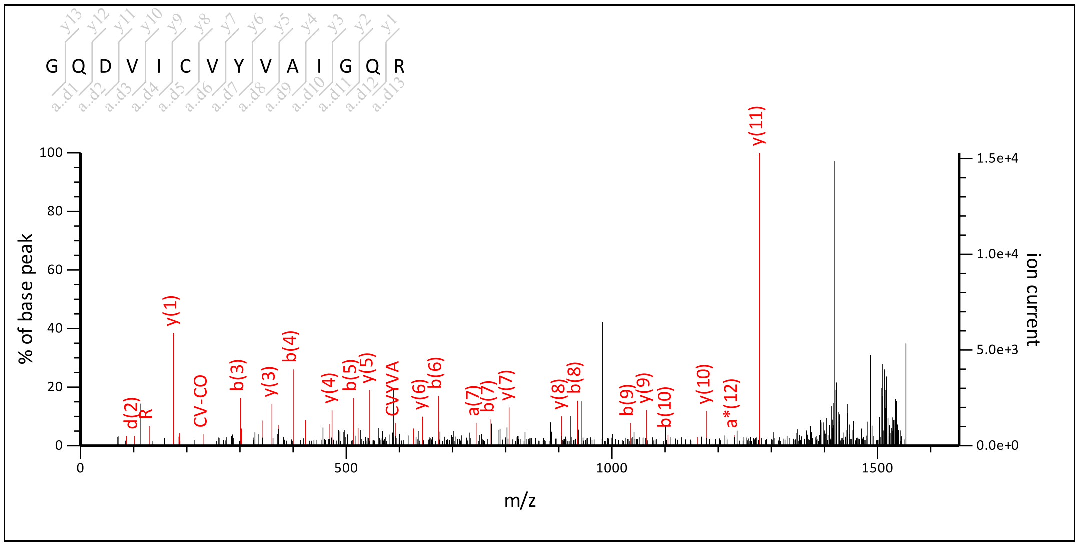


Label all possible matches ○ Label matches used for scoring ◉

**Monoisotopic mass of neutral peptide Mr(calc):** 1576.8032
**Fixed modifications:** Carbamidomethyl (C) (apply to specified residues or termini only)
**Ions Score:** 66 **Expect:** 0.0012
**Matches :** 43/233 fragment ions using 60 most intense peaks (help)

| # | Immon. | a | a* | a⁰ | b | b* | b⁰ | d | d' | Seq. | v | w | w' | y | y* | |
|---|---|---|---|---|---|---|---|---|---|---|---|---|---|---|---|---|
| 1 | 30.0338 | 30.0338 | | | 58.0287 | | | 44.0495 | | G | | | | | | |
| 2 | 101.0709 | 158.0924 | 141.0659 | | **186.0873** | 169.0608 | | 101.0709 | | Q | 1447.7362 | 1446.7410 | | 1520.7890 | 1503.7624 | [illegible] |
| 3 | 88.0393 | 273.1193 | 256.0928 | 255.1088 | **301.1143** | 284.0877 | 283.1037 | 229.1295 | | D | 1332.7093 | 1331.7140 | | 1392.7304 | 1375.7038 | [illegible] |
| 4 | 72.0808 | 372.1878 | 355.1612 | 354.1772 | **400.1827** | 383.1561 | 382.1721 | 358.1721 | | V | 1233.6409 | 1246.6613 | | ***1277.7035*** | 1260.6769 | |
| 5 | 86.0964 | 485.2718 | 468.2453 | 467.2613 | **513.2667** | 496.2402 | 495.2562 | 457.2405 | 471.2562 | I | 1120.5568 | 1133.5772 | 1147.5928 | ***1178.6350*** | 1161.6085 | |
| 6 | 133.0430 | 645.3025 | 628.2759 | 627.2919 | **673.2974** | 656.2708 | 655.2868 | 556.3089 | | C | 960.5261 | 959.5309 | | ***1065.5510*** | 1048.5244 | |
| 7 | 72.0808 | 744.3709 | 727.3443 | 726.3603 | **772.3658** | 755.3393 | 754.3552 | 730.3552 | | V | 861.4577 | 874.4781 | | ***905.5203*** | 888.4938 | |
| 8 | 136.0757 | 907.4342 | 890.4077 | 889.4237 | **935.4291** | 918.4026 | 917.4186 | | | Y | 698.3944 | | | ***806.4519*** | 789.4254 | |
| 9 | 72.0808 | 1006.5026 | 989.4761 | 988.4921 | **1034.4975** | 1017.4710 | 1016.4870 | 992.4870 | | V | 599.3260 | 612.3464 | | ***643.3886*** | 626.3620 | |
| 10 | 44.0495 | 1077.5397 | 1060.5132 | 1059.5292 | **1105.5347** | 1088.5081 | 1087.5241 | | | A | 528.2889 | | | ***544.3202*** | 527.2936 | |
| 11 | 86.0964 | 1190.6238 | 1173.5973 | 1172.6132 | 1218.6187 | 1201.5922 | 1200.6082 | 1162.5925 | 1176.6082 | I | 415.2048 | 428.2252 | 442.2409 | ***473.2831*** | 456.2565 | |
| 12 | 30.0338 | 1247.6453 | 1230.6187 | 1229.6347 | 1275.6402 | 1258.6136 | 1257.6296 | | | G | | | | ***360.1990*** | 343.1724 | |
| 13 | 101.0709 | 1375.7038 | 1358.6773 | 1357.6933 | 1403.6988 | 1386.6722 | 1385.6882 | 1318.6824 | | Q | 230.1248 | 229.1295 | | ***303.1775*** | 286.1510 | |
| 14 | 129.1135 | | | | | | | | | R | 74.0237 | 73.0284 | | ***175.1190*** | 158.0924 | |

| Seq | ya | yb | Seq | ya | yb | Seq | ya | yb |
|---|---|---|---|---|---|---|---|---|
| QD | 216.0979 | 244.0928 | QDV | 315.1663 | 343.1612 | QDVI | 428.2504 | 456.2453 |
| QDVIC | 588.2810 | 616.2759 | QDVICV | 687.3494 | 715.3443 | DV | 187.1077 | 215.1026 |
| DVI | 300.1918 | 328.1867 | DVIC | 460.2224 | 488.2173 | DVICV | 559.2908 | 587.2858 |
| VI | 185.1648 | 213.1598 | VIC | 345.1955 | 373.1904 | VICV | 444.2639 | 472.2588 |
| VICVY | 607.3272 | 635.3221 | IC | 246.1271 | 274.1220 | ICV | 345.1955 | 373.1904 |
| ICVY | 508.2588 | 536.2537 | ICVYV | 607.3272 | 635.3221 | ICVYVA | 678.3643 | 706.3593 |
| CV | 232.1114 | 260.1063 | CVY | 395.1748 | 423.1697 | CVYV | 494.2432 | 522.2381 |

| | | | | | | | | |
|---|---|---|---|---|---|---|---|---|
| **CVYVA** | 565.2803 | 593.2752 | **CVYVAI** | 678.3643 | 706.3593 | **VY** | 235.1441 | 263.1390 |
| **VYV** | 334.2125 | 362.2074 | **VYVA** | 405.2496 | 433.2445 | **VYVAI** | 518.3337 | 546.3286 |
| **VYVAIG** | 575.3552 | 603.3501 | **YV** | 235.1441 | 263.1390 | **YVA** | 306.1812 | 334.1761 |
| **YVAI** | 419.2653 | 447.2602 | **YVAIG** | 476.2867 | 504.2817 | **YVAIGQ** | 604.3453 | 632.3402 |
| **VA** | 143.1179 | 171.1128 | **VAI** | 256.2020 | 284.1969 | **VAIG** | 313.2234 | 341.2183 |
| **VAIGQ** | 441.2820 | 469.2769 | **AI** | 157.1335 | 185.1285 | **AIG** | 214.1550 | 242.1499 |
| **AIGQ** | 342.2136 | 370.2085 | **IG** | 143.1179 | 171.1128 | **IGQ** | 271.1765 | 299.1714 |
| **GQ** | 158.0924 | 186.0873 | | | | | | |

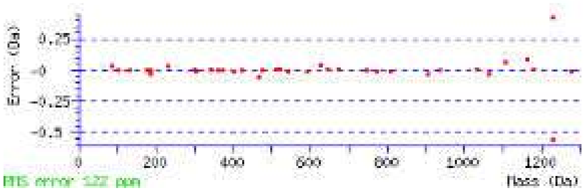


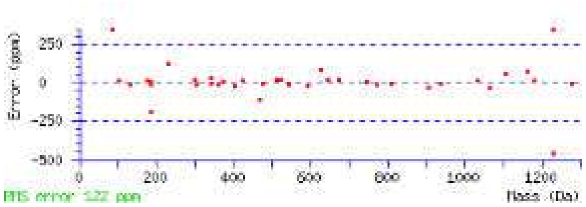


NCBI **BLAST** search of GQDVICVYVAIGQR
(Parameters: blastp, nr protein database, expect=20000, no filter, PAM30)
Other BLAST web gateways

**All matches to this query**

| Score | Mr(calc) | Delta | Sequence |
|---|---|---|---|
| 65.8 | 1576.8032 | 0.0943 | GQDVICVYVAIGQR |
| 65.8 | 1576.8032 | 0.0944 | GENVICVYVAIGQR |
| 53.8 | 1575.8192 | 1.0784 | GQNVICVYVAIGQR |
| 20.0 | 1577.7169 | -0.8193 | GAGDSLAEAAQSSESAK |
| 17.6 | 1577.6926 | -0.7951 | VNDVICTMSGSHSR |
| 17.0 | 1576.9413 | -0.0438 | QHLVLITATKLQGR |
| 16.7 | 1575.8290 | 1.0685 | DLMTTALAATTAKPR |
| 16.0 | 1575.7059 | 1.1916 | QMSDASGRAASDQPR |
| 15.0 | 1576.7733 | 0.1243 | ASAAVYPASEDELVR |
| 14.2 | 1576.8243 | 0.0732 | QTGLSGSSAIVIAAMR |

**Mascot:** http://www.matrixscience.com/

# MATRIX SCIENCE Mascot Search Results

## Peptide View **Spot no 97**

MS/MS Fragmentation of **HTLIIYDDLSKQAQAYR**
Found in **gi|11466784** in **NCBInr**, ATP synthase CF1 alpha subunit [Oryza sativa Japonica Group]

Match to Query 69: 2034.170424 from(2035.177700,1+) intensity(0.0000) index(19)
Title: Label: I11, Spot_Id: 216645, Peak_List_Id: 221401, MSMS Job_Run_Id: 21364, Comment:
Data file C:\Documents and Settings\Administrator\Desktop\farah BHU\Akansha BHU\RAJESH\12-1-13 raj\ppw_I11_136074508400.txt

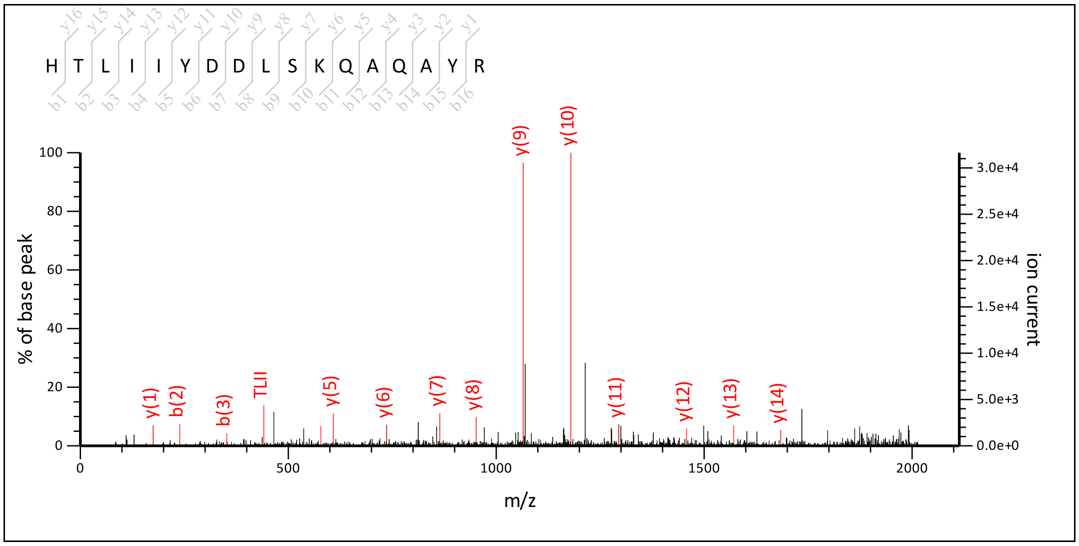


0 to 2113.44

Label all possible matches ○ Label matches used for scoring ◉

**Monoisotopic mass of neutral peptide Mr(calc):** 2034.0534
**Fixed modifications:** Carbamidomethyl (C) (apply to specified residues or termini only)
**Ions Score:** 90 **Expect:** 3.8e-06
**Matches :** 15/292 fragment ions using 20 most intense peaks (help)

| # | Immon. | a | a* | a⁰ | b | b* | b⁰ | d | d' | Seq. | v | w | w' | y | y* | |
|---|---|---|---|---|---|---|---|---|---|---|---|---|---|---|---|---|
| 1 | 110.0713 | 110.0713 | | | 138.0662 | | | 44.0495 | | H | | | | | | |
| 2 | 74.0600 | 211.1190 | | 193.1084 | **239.1139** | | 221.1033 | 195.1240 | 197.1033 | T | 1851.9599 | 1864.9803 | 1866.9596 | 1898.0018 | 1880.9753 | 18 |
| 3 | 86.0964 | 324.2030 | | 306.1925 | **352.1979** | | 334.1874 | 282.1561 | | L | 1738.8759 | 1737.8806 | | 1796.9541 | 1779.9276 | 17 |
| 4 | 86.0964 | 437.2871 | | 419.2765 | 465.2820 | | 447.2714 | 409.2558 | 423.2714 | I | 1625.7918 | 1638.8122 | 1652.8279 | ***1683.8701*** | 1666.8435 | 16 |
| 5 | 86.0964 | 550.3711 | | 532.3606 | **578.3661** | | 560.3555 | 522.3398 | 536.3555 | I | 1512.7077 | 1525.7281 | 1539.7438 | ***1570.7860*** | 1553.7594 | 15 |
| 6 | 136.0757 | 713.4345 | | 695.4239 | 741.4294 | | 723.4188 | | | Y | 1349.6444 | | | ***1457.7019*** | 1440.6754 | 14 |
| 7 | 88.0393 | 828.4614 | | 810.4509 | 856.4563 | | 838.4458 | 784.4716 | | D | 1234.6175 | 1233.6222 | | ***1294.6386*** | 1277.6121 | 12 |
| 8 | 88.0393 | 943.4884 | | 925.4778 | 971.4833 | | 953.4727 | 899.4985 | | D | 1119.5905 | 1118.5953 | | ***1179.6117*** | 1162.5851 | 11 |
| 9 | 86.0964 | 1056.5724 | | 1038.5619 | 1084.5673 | | 1066.5568 | 1014.5255 | | L | 1006.5065 | 1005.5112 | | ***1064.5847*** | 1047.5582 | 10 |
| 10 | 60.0444 | 1143.6045 | | 1125.5939 | 1171.5994 | | 1153.5888 | 1127.6095 | | S | 919.4744 | 918.4792 | | ***951.5007*** | 934.4741 | 9 |
| 11 | 101.1073 | 1271.6994 | 1254.6729 | 1253.6888 | 1299.6943 | 1282.6678 | 1281.6838 | 1214.6416 | | K | 791.3795 | 790.3842 | | ***864.4686*** | 847.4421 | |
| 12 | 101.0709 | 1399.7580 | 1382.7314 | 1381.7474 | 1427.7529 | 1410.7264 | 1409.7423 | 1342.7365 | | Q | 663.3209 | 662.3257 | | ***736.3737*** | 719.3471 | |
| 13 | 44.0495 | 1470.7951 | 1453.7686 | 1452.7845 | 1498.7900 | 1481.7635 | 1480.7795 | | | A | 592.2838 | | | ***608.3151*** | 591.2885 | |
| 14 | 101.0709 | 1598.8537 | 1581.8271 | 1580.8431 | 1626.8486 | 1609.8220 | 1608.8380 | 1541.8322 | | Q | 464.2252 | 463.2300 | | 537.2780 | 520.2514 | |
| 15 | 44.0495 | 1669.8908 | 1652.8642 | 1651.8802 | 1697.8857 | 1680.8592 | 1679.8751 | | | A | 393.1881 | | | 409.2194 | 392.1928 | |
| 16 | 136.0757 | 1832.9541 | 1815.9276 | 1814.9436 | 1860.9490 | 1843.9225 | 1842.9385 | | | Y | 230.1248 | | | 338.1823 | 321.1557 | |
| 17 | 129.1135 | | | | | | | | | R | 74.0237 | 73.0284 | | ***175.1190*** | 158.0924 | |

| Seq | ya | yb | Seq | ya | yb | Seq | ya | yb |
|---|---|---|---|---|---|---|---|---|
| TL | 187.1441 | 215.1390 | TLI | 300.2282 | 328.2231 | TLII | 413.3122 | 441.3071 |
| TLIIY | 576.3756 | 604.3705 | TLIIYD | 691.4025 | 719.3974 | LI | 199.1805 | 227.1754 |
| LII | 312.2646 | 340.2595 | LIIY | 475.3279 | 503.3228 | LIIYD | 590.3548 | 618.3497 |
| II | 199.1805 | 227.1754 | IIY | 362.2438 | 390.2387 | IIYD | 477.2708 | 505.2657 |

| | | | | | | | | |
|---|---|---|---|---|---|---|---|---|
| **IIYDD** | 592.2977 | 620.2926 | **IY** | 249.1598 | 277.1547 | **IYD** | 364.1867 | 392.1816 |
| **IYDD** | 479.2136 | 507.2086 | **IYDDL** | 592.2977 | 620.2926 | **IYDDLS** | 679.3297 | 707.3246 |
| **YD** | 251.1026 | 279.0975 | **YDD** | 366.1296 | 394.1245 | **YDDL** | 479.2136 | 507.2086 |
| **YDDLS** | 566.2457 | 594.2406 | **YDDLSK** | 694.3406 | 722.3355 | **DD** | 203.0662 | 231.0612 |
| **DDL** | 316.1503 | 344.1452 | **DDLS** | 403.1823 | 431.1773 | **DDLSK** | 531.2773 | 559.2722 |
| **DDLSKQ** | 659.3359 | 687.3308 | **DL** | 201.1234 | 229.1183 | **DLS** | 288.1554 | 316.1503 |
| **DLSK** | 416.2504 | 444.2453 | **DLSKQ** | 544.3089 | 572.3039 | **DLSKQA** | 615.3461 | 643.3410 |
| **LS** | 173.1285 | 201.1234 | **LSK** | 301.2234 | 329.2183 | **LSKQ** | 429.2820 | 457.2769 |
| **LSKQA** | 500.3191 | 528.3140 | **LSKQAQ** | 628.3777 | 656.3726 | **LSKQAQA** | 699.4148 | 727.4097 |
| **SK** | 188.1394 | 216.1343 | **SKQ** | 316.1979 | 344.1928 | **SKQA** | 387.2350 | 415.2300 |
| **SKQAQ** | 515.2936 | 543.2885 | **SKQAQA** | 586.3307 | 614.3257 | **KQ** | 229.1659 | 257.1608 |
| **KQA** | 300.2030 | 328.1979 | **KQAQ** | 428.2616 | 456.2565 | **KQAQA** | 499.2987 | 527.2936 |
| **KQAQAY** | 662.3620 | 690.3570 | **QA** | 172.1081 | 200.1030 | **QAQ** | 300.1666 | 328.1615 |
| **QAQA** | 371.2037 | 399.1987 | **QAQAY** | 534.2671 | 562.2620 | **AQ** | 172.1081 | 200.1030 |
| **AQA** | 243.1452 | 271.1401 | **AQAY** | 406.2085 | 434.2034 | **QA** | 172.1081 | 200.1030 |
| **QAY** | 335.1714 | 363.1663 | **AY** | 207.1128 | 235.1077 | | | |

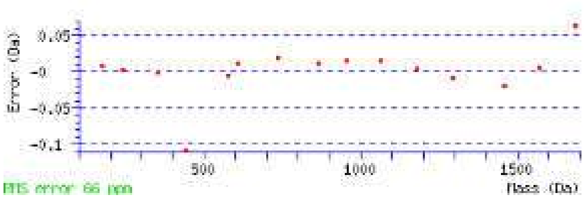


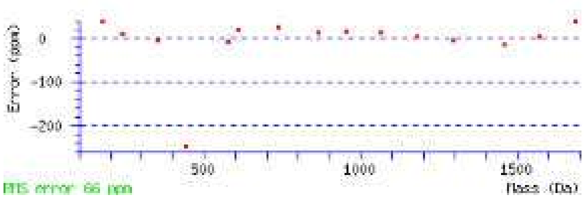


NCBI **BLAST** search of HTLIIYDDLSKQAQAYR
(Parameters: blastp, nr protein database, expect=20000, no filter, PAM30)
Other BLAST web gateways

**All matches to this query**

| Score | Mr(calc) | Delta | Sequence |
|---|---|---|---|
| 89.9 | 2034.0534 | 0.1170 | HTLIIYDDLSKQAQAYR |
| 13.3 | 2034.0534 | 0.1170 | HTLVIYDDLTKQAQAYR |
| 9.9 | 2033.0694 | 1.1010 | GSFYATDLDLVLHTRGIR |
| 9.7 | 2033.9768 | 0.1936 | LVFMGGGSYSFDLEDLLR |
| 8.9 | 2033.9906 | 0.1799 | TYSLPSSVNDQTPDLELR |
| 7.8 | 2033.0518 | 1.1186 | ASMKLAPFFFGPYLIMGK |
| 7.2 | 2035.0051 | -0.8346 | EHFDLSQPAFLQIAEYK |
| 6.6 | 2033.0438 | 1.1267 | YMPTLATAGLPAGMLAAAAAR |
| 6.6 | 2034.0357 | 0.1347 | VGEFKPPTPSGRMDFTLR |
| 6.4 | 2033.0188 | 1.1516 | ASMKLAPMFFGPYLIMGK |

**Mascot:** http://www.matrixscience.com/

# MATRIX SCIENCE Mascot Search Results

## Peptide View Spot no 97

MS/MS Fragmentation of **IIGLGEIMSGELVEFAEGTR**
Found in **gi|11466784** in **NCBInr**, ATP synthase CF1 alpha subunit [Oryza sativa Japonica Group]

Match to Query 78: 2120.200724 from(2121.208000,1+) intensity(0.0000) index(21)
Title: Label: I11, Spot_Id: 216645, Peak_List_Id: 221395, MSMS Job_Run_Id: 21364, Comment:
Data file C:\Documents and Settings\Administrator\Desktop\farah BHU\Akansha BHU\RAJESH\12-1-13 raj\ppw_I11_136074508400.txt

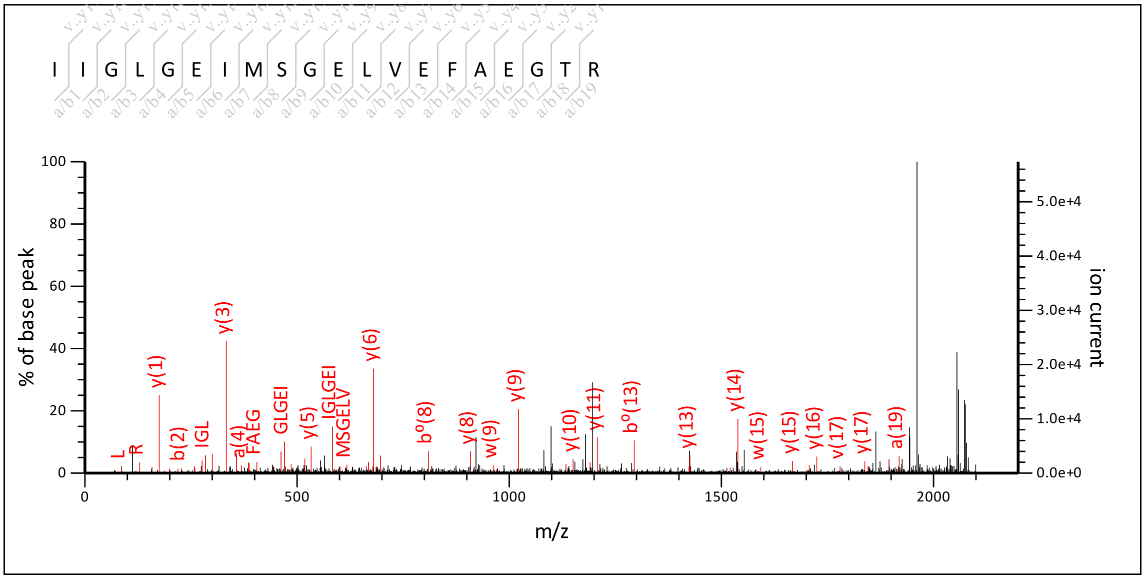


0 to 2199.42

Label all possible matches ○ Label matches used for scoring ◉

**Monoisotopic mass of neutral peptide Mr(calc):** 2120.0823
**Fixed modifications:** Carbamidomethyl (C) (apply to specified residues or termini only)
**Ions Score:** 107 **Expect:** 6.2e-08
**Matches :** 81/345 fragment ions using 126 most intense peaks (help)

| # | Immon. | a | a$^0$ | b | b$^0$ | d | d' | Seq. | v | w | w' | y | y* | y$^0$ | # |
|---|---|---|---|---|---|---|---|---|---|---|---|---|---|---|---|
| 1 | 86.0964 | **86.0964** | | 114.0913 | | 44.0495 | | I | | | | | | | 20 |
| 2 | 86.0964 | **199.1805** | | **227.1754** | | 171.1492 | 185.1648 | I | 1949.9273 | 1962.9477 | 1976.9634 | 2008.0056 | 1990.9790 | 1989.9950 | 19 |
| 3 | 30.0338 | 256.2020 | | **284.1969** | | | | G | | | | ***1894.9215*** | 1877.8950 | 1876.9109 | 18 |
| 4 | 86.0964 | **369.2860** | | 397.2809 | | 327.2391 | | L | 1779.8218 | 1778.8265 | | ***1837.9000*** | 1820.8735 | 1819.8895 | 17 |
| 5 | 30.0338 | 426.3075 | | 454.3024 | | | | G | | | | ***1724.8160*** | 1707.7894 | 1706.8054 | 16 |
| 6 | 102.0550 | **555.3501** | 537.3395 | **583.3450** | 565.3344 | 497.3446 | | E | 1593.7577 | 1592.7625 | | ***1667.7945*** | 1650.7680 | 1649.7839 | 15 |
| 7 | 86.0964 | **668.4341** | 650.4236 | **696.4291** | 678.4185 | 640.4028 | 654.4185 | I | 1480.6737 | 1493.6941 | 1507.7097 | ***1538.7519*** | 1521.7254 | 1520.7414 | 14 |
| 8 | 104.0528 | 799.4746 | 781.4641 | 827.4695 | 809.4590 | 739.4713 | | M | 1349.6332 | 1348.6379 | | ***1425.6679*** | 1408.6413 | 1407.6573 | 13 |
| 9 | 60.0444 | 886.5067 | 868.4961 | 914.5016 | 896.4910 | 870.5117 | | S | 1262.6012 | 1261.6059 | | ***1294.6274*** | 1277.6008 | 1276.6168 | 12 |
| 10 | 30.0338 | 943.5281 | 925.5176 | **971.5230** | 953.5125 | | | G | | | | ***1207.5953*** | 1190.5688 | 1189.5848 | 11 |
| 11 | 102.0550 | 1072.5707 | 1054.5601 | 1100.5656 | 1082.5551 | 1014.5652 | | E | 1076.5371 | 1075.5419 | | ***1150.5739*** | 1133.5473 | 1132.5633 | 10 |
| 12 | 86.0964 | 1185.6548 | 1167.6442 | 1213.6497 | 1195.6391 | 1143.6078 | | L | 963.4530 | 962.4578 | | ***1021.5313*** | 1004.5047 | 1003.5207 | 9 |
| 13 | 72.0808 | 1284.7232 | 1266.7126 | 1312.7181 | 1294.7075 | 1270.7075 | | V | 864.3846 | 877.4050 | | ***908.4472*** | 891.4207 | 890.4367 | 8 |
| 14 | 102.0550 | 1413.7658 | 1395.7552 | 1441.7607 | 1423.7501 | 1355.7603 | | E | 735.3420 | 734.3468 | | ***809.3788*** | 792.3523 | 791.3682 | 7 |
| 15 | 120.0808 | 1560.8342 | 1542.8236 | 1588.8291 | 1570.8185 | | | F | 588.2736 | | | ***680.3362*** | 663.3097 | 662.3257 | 6 |
| 16 | 44.0495 | 1631.8713 | 1613.8607 | 1659.8662 | 1641.8557 | | | A | 517.2365 | | | ***533.2678*** | 516.2413 | 515.2572 | 5 |
| 17 | 102.0550 | 1760.9139 | 1742.9033 | 1788.9088 | 1770.8983 | 1702.9084 | | E | 388.1939 | 387.1987 | | ***462.2307*** | 445.2041 | 444.2201 | 4 |
| 18 | 30.0338 | 1817.9354 | 1799.9248 | **1845.9303** | 1827.9197 | | | G | | | | ***333.1881*** | 316.1615 | 315.1775 | 3 |
| 19 | 74.0600 | **1918.9830** | 1900.9725 | 1946.9780 | 1928.9674 | 1902.9881 | 1904.9674 | T | 230.1248 | 243.1452 | 245.1244 | ***276.1666*** | 259.1401 | 258.1561 | 2 |

| | | | | | | | | | | | | | | | |
|---|---|---|---|---|---|---|---|---|---|---|---|---|---|---|---|
| 20 | 129.1135 | | | | | | | R | 74.0237 | 73.0284 | | *175.1190* | 158.0924 | | 1 |

| Seq | ya | yb | Seq | ya | yb | Seq | ya | yb |
|---|---|---|---|---|---|---|---|---|
| IG | 143.1179 | 171.1128 | IGL | 256.2020 | 284.1969 | IGLG | 313.2234 | 341.2183 |
| IGLGE | 442.2660 | 470.2609 | IGLGEI | 555.3501 | 583.3450 | IGLGEIM | 686.3906 | 714.3855 |
| GL | 143.1179 | 171.1128 | GLG | 200.1394 | 228.1343 | GLGE | 329.1819 | 357.1769 |
| GLGEI | 442.2660 | 470.2609 | GLGEIM | 573.3065 | 601.3014 | GLGEIMS | 660.3385 | 688.3334 |
| LG | 143.1179 | 171.1128 | LGE | 272.1605 | 300.1554 | LGEI | 385.2445 | 413.2395 |
| LGEIM | 516.2850 | 544.2799 | LGEIMS | 603.3171 | 631.3120 | LGEIMSG | 660.3385 | 688.3334 |
| GE | 159.0764 | 187.0713 | GEI | 272.1605 | 300.1554 | GEIM | 403.2010 | 431.1959 |
| GEIMS | 490.2330 | 518.2279 | GEIMSG | 547.2545 | 575.2494 | GEIMSGE | 676.2971 | 704.2920 |
| EI | 215.1390 | 243.1339 | EIM | 346.1795 | 374.1744 | EIMS | 433.2115 | 461.2064 |
| EIMSG | 490.2330 | 518.2279 | EIMSGE | 619.2756 | 647.2705 | IM | 217.1369 | 245.1318 |
| IMS | 304.1689 | 332.1639 | IMSG | 361.1904 | 389.1853 | IMSGE | 490.2330 | 518.2279 |
| IMSGEL | 603.3171 | 631.3120 | MS | 191.0849 | 219.0798 | MSG | 248.1063 | 276.1013 |
| MSGE | 377.1489 | 405.1438 | MSGEL | 490.2330 | 518.2279 | MSGELV | 589.3014 | 617.2963 |
| SG | 117.0659 | 145.0608 | SGE | 246.1084 | 274.1034 | SGEL | 359.1925 | 387.1874 |
| SGELV | 458.2609 | 486.2558 | SGELVE | 587.3035 | 615.2984 | GE | 159.0764 | 187.0713 |
| GEL | 272.1605 | 300.1554 | GELV | 371.2289 | 399.2238 | GELVE | 500.2715 | 528.2664 |
| GELVEF | 647.3399 | 675.3348 | EL | 215.1390 | 243.1339 | ELV | 314.2074 | 342.2023 |
| ELVE | 443.2500 | 471.2449 | ELVEF | 590.3184 | 618.3134 | ELVEFA | 661.3556 | 689.3505 |
| LV | 185.1648 | 213.1598 | LVE | 314.2074 | 342.2023 | LVEF | 461.2758 | 489.2708 |
| LVEFA | 532.3130 | 560.3079 | LVEFAE | 661.3556 | 689.3505 | VE | 201.1234 | 229.1183 |
| VEF | 348.1918 | 376.1867 | VEFA | 419.2289 | 447.2238 | VEFAE | 548.2715 | 576.2664 |
| VEFAEG | 605.2930 | 633.2879 | EF | 249.1234 | 277.1183 | EFA | 320.1605 | 348.1554 |
| EFAE | 449.2031 | 477.1980 | EFAEG | 506.2245 | 534.2195 | EFAEGT | 607.2722 | 635.2671 |
| FA | 191.1179 | 219.1128 | FAE | 320.1605 | 348.1554 | FAEG | 377.1819 | 405.1769 |
| FAEGT | 478.2296 | 506.2245 | AE | 173.0921 | 201.0870 | AEG | 230.1135 | 258.1084 |
| AEGT | 331.1612 | 359.1561 | EG | 159.0764 | 187.0713 | EGT | 260.1241 | 288.1190 |
| GT | 131.0815 | 159.0764 | | | | | | |

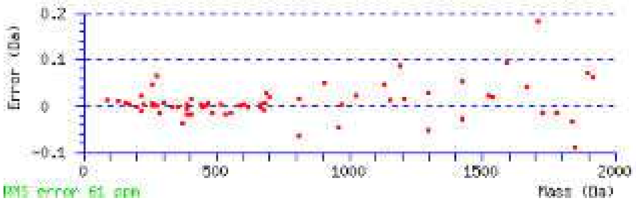


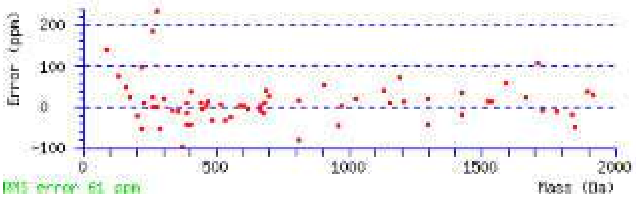


NCBI **BLAST** search of IIGLGEIMSGELVEFAEGTR
(Parameters: blastp, nr protein database, expect=20000, no filter, PAM30)
Other BLAST web gateways

**All matches to this query**

| Score | Mr(calc) | Delta | Sequence |
|---|---|---|---|
| 107.1 | 2120.0823 | 0.1184 | IIGLGEIMSGELVEFAEGTR |
| 57.8 | 2119.0983 | 1.1024 | IIGLGQIMSGELVEFAEGTR |
| 28.2 | 2119.1347 | 1.0660 | IIGLGEIMSGELVKFAEGTR |
| 28.2 | 2119.0983 | 1.1024 | IIGLGEIMSGELVQFAEGTR |
| 11.1 | 2119.1473 | 1.0534 | SWHRAGVLLLGAQSCLPVR |
| 10.9 | 2121.0717 | -0.8710 | WMHELNFKQVIFSLDSK |
| 10.8 | 2121.0875 | -0.8868 | LLDSGMEKLVDDTSTLLSGK |
| 10.7 | 2120.0969 | 0.1038 | CLLTLLMSTAAIPGGSESRK |
| 10.6 | 2121.2045 | -1.0038 | AGEVLIPTIDDSTLPIALRK |
| | | | |

| | | | |
|---|---|---|---|
| 10.3 | 2120.0175 | 0.1833 | TAPSGPWSATSGLDPISSFSR |

**Mascot:** http://www.matrixscience.com/

# MATRIX SCIENCE Mascot Search Results

## Peptide View Spot no 97

MS/MS Fragmentation of **IIGLGEIMSGELVEFAEGTR**
Found in **gi|11466784** in **NCBInr**, ATP synthase CF1 alpha subunit [Oryza sativa Japonica Group]

Match to Query 80: 2136.190724 from(2137.198000,1+) intensity(0.0000) index(22)
Title: Label: I11, Spot_Id: 216645, Peak_List_Id: 221413, MSMS Job_Run_Id: 21364, Comment:
Data file C:\Documents and Settings\Administrator\Desktop\farah BHU\Akansha BHU\RAJESH\12-1-13 raj\ppw_I11_136074508400.txt

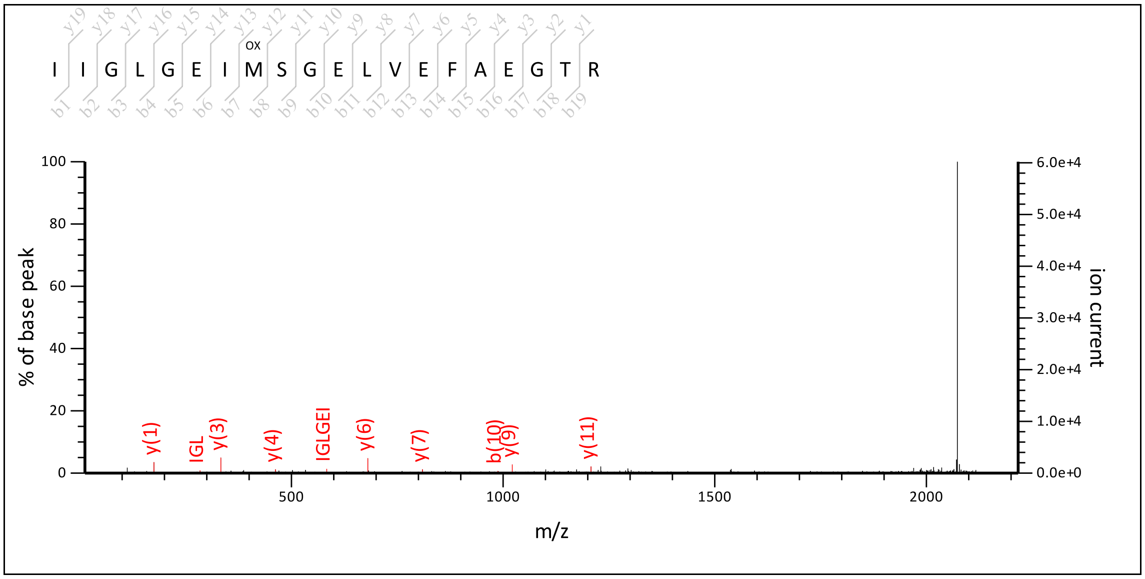


12.1 to 2216.75

Label all possible matches ○ Label matches used for scoring ◉

**Monoisotopic mass of neutral peptide Mr(calc):** 2136.0773
**Fixed modifications:** Carbamidomethyl (C) (apply to specified residues or termini only)
**Variable modifications:**
**M8** : Oxidation (M), with neutral losses 0.0000(shown in table), 63.9983
**Ions Score:** 37 **Expect:** 0.6
**Matches :** 15/473 fragment ions using 21 most intense peaks (help)

| # | Immon. | a | $a^0$ | b | $b^0$ | d | d' | Seq. | v | w | w' | y | y* | $y^0$ | # |
|---|---|---|---|---|---|---|---|---|---|---|---|---|---|---|---|
| 1 | 86.0964 | 86.0964 | | 114.0913 | | 44.0495 | | I | | | | | | | 20 |
| 2 | 86.0964 | 199.1805 | | 227.1754 | | 171.1492 | 185.1648 | I | 1965.9222 | 1978.9426 | 1992.9583 | 2024.0005 | 2006.9739 | 2005.9899 | 19 |
| 3 | 30.0338 | 256.2020 | | ***284.1969*** | | | | G | | | | 1910.9164 | 1893.8899 | 1892.9059 | 18 |
| 4 | 86.0964 | 369.2860 | | 397.2809 | | 327.2391 | | L | 1795.8167 | 1794.8215 | | 1853.8950 | 1836.8684 | 1835.8844 | 17 |
| 5 | 30.0338 | 426.3075 | | 454.3024 | | | | G | | | | 1740.8109 | 1723.7843 | 1722.8003 | 16 |
| 6 | 102.0550 | 555.3501 | 537.3395 | ***583.3450*** | 565.3344 | 497.3446 | | E | 1609.7527 | 1608.7574 | | 1683.7894 | 1666.7629 | 1665.7789 | 15 |
| 7 | 86.0964 | 668.4341 | 650.4236 | 696.4291 | 678.4185 | 640.4028 | 654.4185 | I | 1496.6686 | 1509.6890 | 1523.7046 | 1554.7468 | 1537.7203 | 1536.7363 | 14 |
| 8 | 120.0478 | 815.4695 | 797.4590 | 843.4645 | 825.4539 | 739.4713 | | M | 1349.6332 | 1348.6379 | | 1441.6628 | 1424.6362 | 1423.6522 | 13 |
| 9 | 60.0444 | 902.5016 | 884.4910 | 930.4965 | 912.4859 | 886.5067 | | S | 1262.6012 | 1261.6059 | | 1294.6274 | 1277.6008 | 1276.6168 | 12 |
| 10 | 30.0338 | 959.5230 | 941.5125 | ***987.5179*** | 969.5074 | | | G | | | | ***1207.5953*** | 1190.5688 | 1189.5848 | 11 |
| 11 | 102.0550 | 1088.5656 | 1070.5551 | 1116.5605 | 1098.5500 | 1030.5601 | | E | 1076.5371 | 1075.5419 | | 1150.5739 | 1133.5473 | 1132.5633 | 10 |
| 12 | 86.0964 | 1201.6497 | 1183.6391 | 1229.6446 | 1211.6340 | 1159.6027 | | L | 963.4530 | 962.4578 | | ***1021.5313*** | 1004.5047 | 1003.5207 | 9 |
| 13 | 72.0808 | 1300.7181 | 1282.7075 | 1328.7130 | 1310.7025 | 1286.7025 | | V | 864.3846 | 877.4050 | | 908.4472 | 891.4207 | 890.4367 | 8 |
| 14 | 102.0550 | 1429.7607 | 1411.7501 | 1457.7556 | 1439.7450 | 1371.7552 | | E | 735.3420 | 734.3468 | | ***809.3788*** | 792.3523 | 791.3682 | 7 |
| 15 | 120.0808 | 1576.8291 | 1558.8185 | 1604.8240 | 1586.8135 | | | F | 588.2736 | | | ***680.3362*** | 663.3097 | 662.3257 | 6 |
| 16 | 44.0495 | 1647.8662 | 1629.8557 | 1675.8611 | 1657.8506 | | | A | 517.2365 | | | 533.2678 | 516.2413 | 515.2572 | 5 |
| 17 | 102.0550 | 1776.9088 | 1758.8983 | 1804.9037 | 1786.8932 | 1718.9033 | | E | 388.1939 | 387.1987 | | ***462.2307*** | 445.2041 | 444.2201 | 4 |

| | | | | | | | | | | | | | | | | |
|---|---|---|---|---|---|---|---|---|---|---|---|---|---|---|---|---|
| 18 | 30.0338 | 1833.9303 | 1815.9197 | 1861.9252 | 1843.9146 | | | G | | | | ***333.1881*** | 316.1615 | 315.1775 | 3 |
| 19 | 74.0600 | 1934.9780 | 1916.9674 | 1962.9729 | 1944.9623 | 1918.9830 | 1920.9623 | T | 230.1248 | 243.1452 | 245.1244 | 276.1666 | 259.1401 | 258.1561 | 2 |
| 20 | 129.1135 | | | | | | | R | 74.0237 | 73.0284 | | ***175.1190*** | 158.0924 | | 1 |

| Seq | ya | yb | Seq | ya | yb | Seq | ya | yb |
|---|---|---|---|---|---|---|---|---|
| IG | 143.1179 | 171.1128 | IGL | 256.2020 | 284.1969 | IGLG | 313.2234 | 341.2183 |
| IGLGE | 442.2660 | 470.2609 | IGLGEI | 555.3501 | 583.3450 | GL | 143.1179 | 171.1128 |
| GLG | 200.1394 | 228.1343 | GLGE | 329.1819 | 357.1769 | GLGEI | 442.2660 | 470.2609 |
| GLGEIM | 589.3014 | 617.2963 | GLGEIMS | 676.3334 | 704.3284 | LG | 143.1179 | 171.1128 |
| LGE | 272.1605 | 300.1554 | LGEI | 385.2445 | 413.2395 | LGEIM | 532.2799 | 560.2749 |
| LGEIMS | 619.3120 | 647.3069 | LGEIMSG | 676.3334 | 704.3284 | GE | 159.0764 | 187.0713 |
| GEI | 272.1605 | 300.1554 | GEIM | 419.1959 | 447.1908 | GEIMS | 506.2279 | 534.2228 |
| GEIMSG | 563.2494 | 591.2443 | GEIMSGE | 692.2920 | 720.2869 | EI | 215.1390 | 243.1339 |
| EIM | 362.1744 | 390.1693 | EIMS | 449.2064 | 477.2014 | EIMSG | 506.2279 | 534.2228 |
| EIMSGE | 635.2705 | 663.2654 | IM | 233.1318 | 261.1267 | IMS | 320.1639 | 348.1588 |
| IMSG | 377.1853 | 405.1802 | IMSGE | 506.2279 | 534.2228 | IMSGEL | 619.3120 | 647.3069 |
| MS | 207.0798 | 235.0747 | MSG | 264.1013 | 292.0962 | MSGE | 393.1438 | 421.1388 |
| MSGEL | 506.2279 | 534.2228 | MSGELV | 605.2963 | 633.2912 | SG | 117.0659 | 145.0608 |
| SGE | 246.1084 | 274.1034 | SGEL | 359.1925 | 387.1874 | SGELV | 458.2609 | 486.2558 |
| SGELVE | 587.3035 | 615.2984 | GE | 159.0764 | 187.0713 | GEL | 272.1605 | 300.1554 |
| GELV | 371.2289 | 399.2238 | GELVE | 500.2715 | 528.2664 | GELVEF | 647.3399 | 675.3348 |
| EL | 215.1390 | 243.1339 | ELV | 314.2074 | 342.2023 | ELVE | 443.2500 | 471.2449 |
| ELVEF | 590.3184 | 618.3134 | ELVEFA | 661.3556 | 689.3505 | LV | 185.1648 | 213.1598 |
| LVE | 314.2074 | 342.2023 | LVEF | 461.2758 | 489.2708 | LVEFA | 532.3130 | 560.3079 |
| LVEFAE | 661.3556 | 689.3505 | VE | 201.1234 | 229.1183 | VEF | 348.1918 | 376.1867 |
| VEFA | 419.2289 | 447.2238 | VEFAE | 548.2715 | 576.2664 | VEFAEG | 605.2930 | 633.2879 |
| EF | 249.1234 | 277.1183 | EFA | 320.1605 | 348.1554 | EFAE | 449.2031 | 477.1980 |
| EFAEG | 506.2245 | 534.2195 | EFAEGT | 607.2722 | 635.2671 | FA | 191.1179 | 219.1128 |
| FAE | 320.1605 | 348.1554 | FAEG | 377.1819 | 405.1769 | FAEGT | 478.2296 | 506.2245 |
| AE | 173.0921 | 201.0870 | AEG | 230.1135 | 258.1084 | AEGT | 331.1612 | 359.1561 |
| EG | 159.0764 | 187.0713 | EGT | 260.1241 | 288.1190 | GT | 131.0815 | 159.0764 |

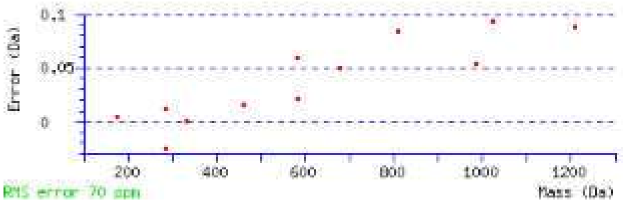


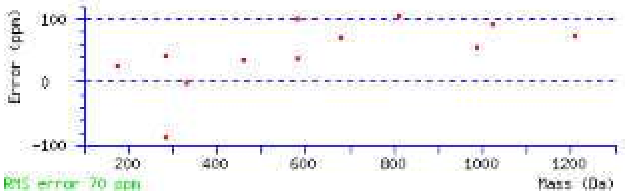


NCBI **BLAST** search of IIGLGEIMSGELVEFAEGTR
(Parameters: blastp, nr protein database, expect=20000, no filter, PAM30)
Other BLAST web gateways

**All matches to this query**

| Score | Mr(calc) | Delta | Sequence |
|---|---|---|---|
| 37.3 | 2136.0773 | 0.1135 | IIGLGEIMSGELVEFAEGTR |
| 27.2 | 2135.0932 | 1.0975 | IIGLGQIMSGELVEFAEGTR |
| 11.4 | 2136.2307 | -0.0399 | LATLALILVLVSFSHSPEAR |
| 9.5 | 2136.0898 | 0.1009 | SHGRVVVNASVESWLPMPR |
| 9.2 | 2136.0198 | 0.1709 | KDLFTGTYMPSTELTGGFR |
| 9.1 | 2135.9793 | 0.2114 | SNYAFSMLTGRGSEASISDK |
| 8.8 | 2137.1354 | -0.9447 | GPWKNPEVLEMIQVVNLR |
| 8.0 | 2135.0854 | 1.1054 | VIRGSFFANGWLWSWPGR |
| 7.7 | 2136.0496 | 0.1411 | QLIGPAMYFGLMGDGQPIGR |

| | | | |
|---|---|---|---|
| 7.7 | 2136.0125 | 0.1782 | SLMMPGSPSEAGLSRSLMNR |

**Mascot:** http://www.matrixscience.com/

# MATRIX SCIENCE Mascot Search Results

## Peptide View **Spot no 97**

MS/MS Fragmentation of **VINALAKPIDGRGEIVASESR**
Found in **gi|11466784** in **NCBInr**, ATP synthase CF1 alpha subunit [Oryza sativa Japonica Group]

Match to Query 88: 2194.327924 from(2195.335200,1+) intensity(0.0000) index(24)
Title: Label: I11, Spot_Id: 216645, Peak_List_Id: 221393, MSMS Job_Run_Id: 21364, Comment:
Data file C:\Documents and Settings\Administrator\Desktop\farah BHU\Akansha BHU\RAJESH\12-1-13 raj\ppw_I11_136074508400.txt

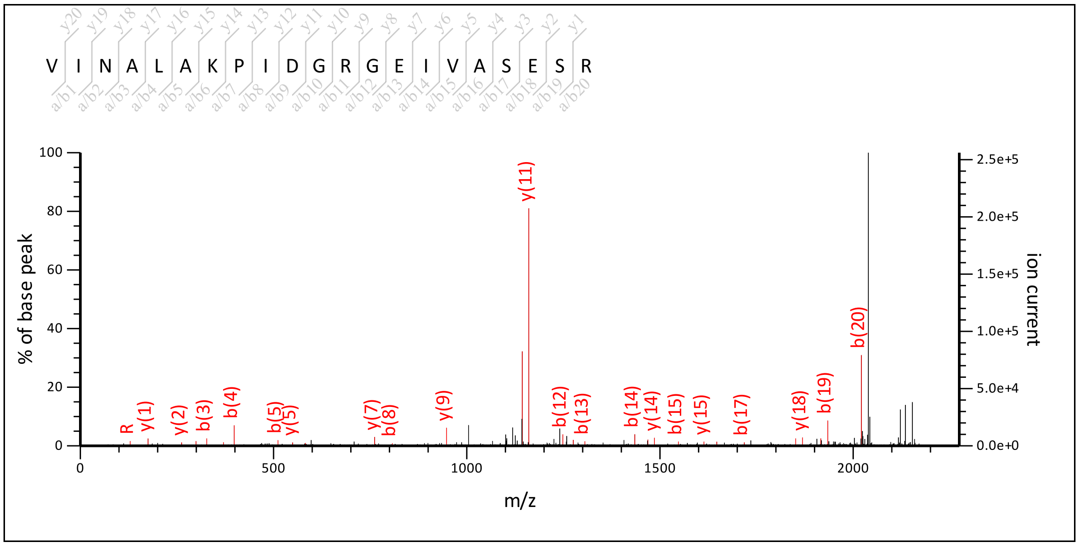


0 to 2274.56

Label all possible matches ○ Label matches used for scoring ◉

**Monoisotopic mass of neutral peptide Mr(calc):** 2194.2069
**Fixed modifications:** Carbamidomethyl (C) (apply to specified residues or termini only)
**Ions Score:** 98 **Expect:** 4.2e-07
**Matches :** 37/406 fragment ions using 44 most intense peaks (help)

| # | Immon. | a | a* | $a^0$ | b | b* | $b^0$ | d | d' | Seq. | v | w | w' | y | y* |
|---|---|---|---|---|---|---|---|---|---|---|---|---|---|---|---|
| 1 | 72.0808 | 72.0808 | | | 100.0757 | | | 44.0495 | | V | | | | | |
| 2 | 86.0964 | 185.1648 | | | 213.1598 | | | 157.1335 | 171.1492 | I | 2038.0676 | 2051.0880 | 2065.1036 | 2096.1458 | 2079.1193 |
| 3 | 87.0553 | 299.2078 | 282.1812 | | ***327.2027*** | 310.1761 | | 256.2020 | | N | 1924.0247 | 1923.0294 | | 1983.0618 | 1966.0352 |
| 4 | 44.0495 | 370.2449 | 353.2183 | | ***398.2398*** | 381.2132 | | | | A | 1852.9876 | | | ***1869.0189*** | 1851.9923 |
| 5 | 86.0964 | 483.3289 | 466.3024 | | ***511.3239*** | 494.2973 | | 441.2820 | | L | 1739.9035 | 1738.9082 | | 1797.9817 | 1780.9552 |
| 6 | 44.0495 | 554.3661 | 537.3395 | | ***582.3610*** | 565.3344 | | | | A | 1668.8664 | | | 1684.8977 | 1667.8711 |
| 7 | 101.1073 | 682.4610 | 665.4345 | | 710.4559 | 693.4294 | | 625.4032 | | K | 1540.7714 | 1539.7762 | | ***1613.8606*** | 1596.8340 |
| 8 | 70.0651 | 779.5138 | 762.4872 | | ***807.5087*** | 790.4822 | | 753.4981 | | P | 1443.7186 | 1442.7234 | | ***1485.7656*** | 1468.7390 |
| 9 | 86.0964 | 892.5978 | 875.5713 | | 920.5928 | 903.5662 | | 864.5665 | 878.5822 | I | 1330.6346 | 1343.6550 | 1357.6706 | 1388.7128 | 1371.6863 |
| 10 | 88.0393 | 1007.6248 | 990.5982 | 989.6142 | 1035.6197 | 1018.5932 | 1017.6091 | 963.6350 | | D | 1215.6076 | 1214.6124 | | ***1275.6288*** | 1258.6022 |
| 11 | 30.0338 | 1064.6463 | 1047.6197 | 1046.6357 | 1092.6412 | 1075.6146 | 1074.6306 | | | G | | | | ***1160.6018*** | 1143.5753 |
| 12 | 129.1135 | 1220.7474 | 1203.7208 | 1202.7368 | ***1248.7423*** | 1231.7157 | 1230.7317 | 1135.6834 | | R | 1002.4851 | 1001.4898 | | 1103.5804 | 1086.5538 |
| 13 | 30.0338 | 1277.7688 | 1260.7423 | 1259.7583 | ***1305.7637*** | 1288.7372 | 1287.7532 | | | G | | | | ***947.4793*** | 930.4527 |
| 14 | 102.0550 | 1406.8114 | 1389.7849 | 1388.8009 | ***1434.8063*** | 1417.7798 | 1416.7958 | 1348.8059 | | E | 816.4210 | 815.4258 | | 890.4578 | 873.4312 |
| 15 | 86.0964 | 1519.8955 | 1502.8689 | 1501.8849 | ***1547.8904*** | 1530.8639 | 1529.8798 | 1491.8642 | 1505.8798 | I | 703.3369 | 716.3573 | 730.3730 | ***761.4152*** | 744.3886 |
| 16 | 72.0808 | 1618.9639 | 1601.9374 | 1600.9533 | ***1646.9588*** | 1629.9323 | 1628.9483 | 1604.9483 | | V | 604.2685 | 617.2889 | | 648.3311 | 631.3046 |
| 17 | 44.0495 | 1690.0010 | 1672.9745 | 1671.9905 | ***1717.9959*** | 1700.9694 | 1699.9854 | | | A | 533.2314 | | | ***549.2627*** | 532.2362 |
| 18 | 60.0444 | 1777.0330 | 1760.0065 | 1759.0225 | 1805.0280 | 1788.0014 | 1787.0174 | 1761.0381 | | S | 446.1994 | 445.2041 | | 478.2256 | 461.1991 |
| 19 | 102.0550 | 1906.0756 | 1889.0491 | 1888.0651 | ***1934.0706*** | 1917.0440 | 1916.0600 | 1848.0702 | | E | 317.1568 | 316.1615 | | 391.1936 | 374.1670 |
| 20 | 60.0444 | 1993.1077 | 1976.0811 | 1975.0971 | ***2021.1026*** | 2004.0760 | 2003.0920 | 1977.1128 | | S | 230.1248 | 229.1295 | | ***262.1510*** | 245.1244 |
| 21 | 129.1135 | | | | | | | | | R | 74.0237 | 73.0284 | | ***175.1190*** | 158.0924 |

| Seq | ya | yb | Seq | ya | yb | Seq | ya | yb |
|---|---|---|---|---|---|---|---|---|

| | | | | | | | | |
|---|---|---|---|---|---|---|---|---|
| **IN** | 200.1394 | 228.1343 | **INA** | 271.1765 | 299.1714 | **INAL** | 384.2605 | 412.2554 |
| **INALA** | 455.2976 | 483.2926 | **INALAK** | 583.3926 | 611.3875 | **INALAKP** | 680.4454 | 708.4403 |
| **NA** | 158.0924 | 186.0873 | **NAL** | 271.1765 | 299.1714 | **NALA** | 342.2136 | 370.2085 |
| **NALAK** | 470.3085 | 498.3035 | **NALAKP** | 567.3613 | 595.3562 | **NALAKPI** | 680.4454 | 708.4403 |
| **AL** | 157.1335 | 185.1285 | **ALA** | 228.1707 | 256.1656 | **ALAK** | 356.2656 | 384.2605 |
| **ALAKP** | 453.3184 | 481.3133 | **ALAKPI** | 566.4024 | 594.3974 | **ALAKPID** | 681.4294 | 709.4243 |
| **LA** | 157.1335 | 185.1285 | **LAK** | 285.2285 | 313.2234 | **LAKP** | 382.2813 | 410.2762 |
| **LAKPI** | 495.3653 | 523.3602 | **LAKPID** | 610.3923 | 638.3872 | **LAKPIDG** | 667.4137 | 695.4087 |
| **AK** | 172.1444 | 200.1394 | **AKP** | 269.1972 | 297.1921 | **AKPI** | 382.2813 | 410.2762 |
| **AKPID** | 497.3082 | 525.3031 | **AKPIDG** | 554.3297 | 582.3246 | **KP** | 198.1601 | 226.1550 |
| **KPI** | 311.2442 | 339.2391 | **KPID** | 426.2711 | 454.2660 | **KPIDG** | 483.2926 | 511.2875 |
| **KPIDGR** | 639.3937 | 667.3886 | **KPIDGRG** | 696.4151 | 724.4100 | **PI** | 183.1492 | 211.1441 |
| **PID** | 298.1761 | 326.1710 | **PIDG** | 355.1976 | 383.1925 | **PIDGR** | 511.2987 | 539.2936 |
| **PIDGRG** | 568.3202 | 596.3151 | **PIDGRGE** | 697.3628 | 725.3577 | **ID** | 201.1234 | 229.1183 |
| **IDG** | 258.1448 | 286.1397 | **IDGR** | 414.2459 | 442.2409 | **IDGRG** | 471.2674 | 499.2623 |
| **IDGRGE** | 600.3100 | 628.3049 | **DG** | 145.0608 | 173.0557 | **DGR** | 301.1619 | 329.1568 |
| **DGRG** | 358.1833 | 386.1783 | **DGRGE** | 487.2259 | 515.2209 | **DGRGEI** | 600.3100 | 628.3049 |
| **DGRGEIV** | 699.3784 | 727.3733 | **GR** | 186.1349 | 214.1299 | **GRG** | 243.1564 | 271.1513 |
| **GRGE** | 372.1990 | 400.1939 | **GRGEI** | 485.2831 | 513.2780 | **GRGEIV** | 584.3515 | 612.3464 |
| **GRGEIVA** | 655.3886 | 683.3835 | **RG** | 186.1349 | 214.1299 | **RGE** | 315.1775 | 343.1724 |
| **RGEI** | 428.2616 | 456.2565 | **RGEIV** | 527.3300 | 555.3249 | **RGEIVA** | 598.3671 | 626.3620 |
| **RGEIVAS** | 685.3991 | 713.3941 | **GE** | 159.0764 | 187.0713 | **GEI** | 272.1605 | 300.1554 |
| **GEIV** | 371.2289 | 399.2238 | **GEIVA** | 442.2660 | 470.2609 | **GEIVAS** | 529.2980 | 557.2930 |
| **GEIVASE** | 658.3406 | 686.3355 | **EI** | 215.1390 | 243.1339 | **EIV** | 314.2074 | 342.2023 |
| **EIVA** | 385.2445 | 413.2395 | **EIVAS** | 472.2766 | 500.2715 | **EIVASE** | 601.3192 | 629.3141 |
| **EIVASES** | 688.3512 | 716.3461 | **IV** | 185.1648 | 213.1598 | **IVA** | 256.2020 | 284.1969 |
| **IVAS** | 343.2340 | 371.2289 | **IVASE** | 472.2766 | 500.2715 | **IVASES** | 559.3086 | 587.3035 |
| **VA** | 143.1179 | 171.1128 | **VAS** | 230.1499 | 258.1448 | **VASE** | 359.1925 | 387.1874 |
| **VASES** | 446.2245 | 474.2195 | **AS** | 131.0815 | 159.0764 | **ASE** | 260.1241 | 288.1190 |
| **ASES** | 347.1561 | 375.1510 | **SE** | 189.0870 | 217.0819 | **SES** | 276.1190 | 304.1139 |
| **ES** | 189.0870 | 217.0819 | | | | | | |

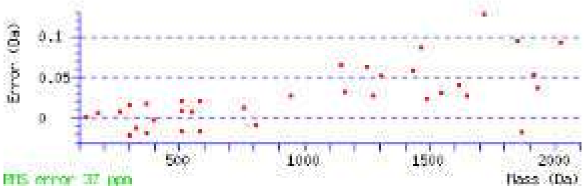


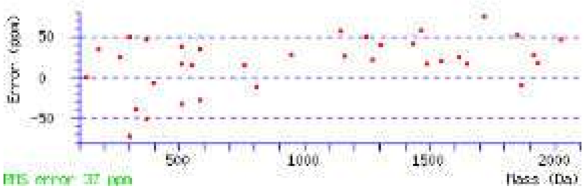


NCBI **BLAST** search of VINALAKPIDGRGEIVASESR
(Parameters: blastp, nr protein database, expect=20000, no filter, PAM30)
Other BLAST web gateways

**All matches to this query**

| Score | Mr(calc) | Delta | Sequence |
|---|---|---|---|
| 97.9 | 2194.2069 | 0.1210 | VINALAKPIDGRGEIVASESR |
| 16.6 | 2194.0807 | 0.2472 | SFADRAVLSLQSNPGWGFDK |
| 14.9 | 2194.0993 | 0.2286 | TWMNWPNITPPALQLPGSR |
| 12.7 | 2194.2223 | 0.1056 | LSRGTVFVVPSGHPIVVTSSR |
| 11.9 | 2194.1706 | 0.1574 | VINALGKPIDGRGEIPSSESR |
| 11.8 | 2194.0484 | 0.2796 | LFHAIFFVQGDEHASSDFK |
| 11.6 | 2194.2011 | 0.1269 | LHIINGIAQGLLYLHNYSR |
| 10.2 | 2194.0350 | 0.2930 | VGAGLTNLGSDGDASNGTSSLSSK |
| 10.2 | 2194.1899 | 0.1381 | RYVYLLPVLALDPSAHPDR |
| 9.0 | 2194.9491 | -0.6211 | AFDAMQAWDGVGGGHATYDAR |

**Mascot:** http://www.matrixscience.com/

# MATRIX SCIENCE Mascot Search Results

## Peptide View Spot no 97

MS/MS Fragmentation of **SVYEPLQTGLIAIDSMIPIGR**
Found in **gi|11466784** in **NCBInr**, ATP synthase CF1 alpha subunit [Oryza sativa Japonica Group]

Match to Query 90: 2272.342124 from(2273.349400,1+) intensity(0.0000) index(25)
Title: Label: I11, Spot_Id: 216645, Peak_List_Id: 221394, MSMS Job_Run_Id: 21364, Comment:
Data file C:\Documents and Settings\Administrator\Desktop\farah BHU\Akansha BHU\RAJESH\12-1-13 raj\ppw_I11_136074508400.txt

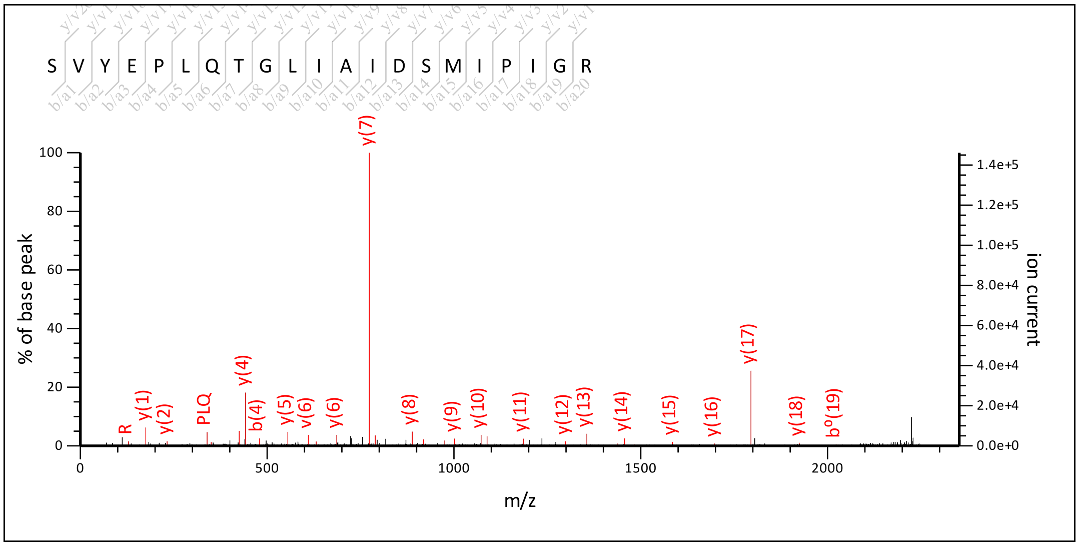


0 to 2352.6

Label all possible matches ○ Label matches used for scoring ◉

**Monoisotopic mass of neutral peptide Mr(calc):** 2272.2137
**Fixed modifications:** Carbamidomethyl (C) (apply to specified residues or termini only)
**Ions Score:** 144 **Expect:** 1e-11
**Matches :** 37/409 fragment ions using 44 most intense peaks (help)

| # | Immon. | a | a* | a0 | b | b* | b0 | d | d' | Seq. | v | w | w' | y | y* | |
|---|---|---|---|---|---|---|---|---|---|---|---|---|---|---|---|---|
| 1 | 60.0444 | 60.0444 | | 42.0338 | 88.0393 | | 70.0287 | 44.0495 | | S | | | | | | |
| 2 | 72.0808 | 159.1128 | | 141.1022 | 187.1077 | | 169.0972 | 145.0972 | | V | 2142.1264 | 2155.1468 | | 2186.1890 | 2169.1624 | [illegible] |
| 3 | 136.0757 | 322.1761 | | 304.1656 | **350.1710** | | 332.1605 | | | Y | 1979.0630 | | | 2087.1205 | 2070.0940 | [illegible] |
| 4 | 102.0550 | 451.2187 | | 433.2082 | **479.2136** | | 461.2031 | 393.2132 | | E | 1850.0204 | 1849.0252 | | ***1924.0572*** | 1907.0307 | [illegible] |
| 5 | 70.0651 | 548.2715 | | 530.2609 | 576.2664 | | 558.2558 | 522.2558 | | P | 1752.9677 | 1751.9724 | | ***1795.0146*** | 1777.9881 | [illegible] |
| 6 | 86.0964 | 661.3556 | | 643.3450 | 689.3505 | | 671.3399 | 619.3086 | | L | 1639.8836 | 1638.8884 | | ***1697.9619*** | 1680.9353 | [illegible] |
| 7 | 101.0709 | 789.4141 | 772.3876 | 771.4036 | 817.4090 | 800.3825 | 799.3985 | 732.3927 | | Q | 1511.8250 | 1510.8298 | | ***1584.8778*** | 1567.8512 | [illegible] |
| 8 | 74.0600 | 890.4618 | 873.4353 | 872.4512 | **918.4567** | 901.4302 | 900.4462 | 874.4669 | 876.4462 | T | 1410.7773 | 1423.7977 | 1425.7770 | ***1456.8192*** | 1439.7927 | [illegible] |
| 9 | 30.0338 | 947.4833 | 930.4567 | 929.4727 | **975.4782** | 958.4516 | 957.4676 | | | G | | | | ***1355.7715*** | 1338.7450 | [illegible] |
| 10 | 86.0964 | 1060.5673 | 1043.5408 | 1042.5568 | **1088.5623** | 1071.5357 | 1070.5517 | 1018.5204 | | L | 1240.6718 | 1239.6766 | | ***1298.7501*** | 1281.7235 | [illegible] |
| 11 | 86.0964 | 1173.6514 | 1156.6249 | 1155.6408 | 1201.6463 | 1184.6198 | 1183.6358 | 1145.6201 | 1159.6358 | I | 1127.5878 | 1140.6082 | 1154.6238 | ***1185.6660*** | 1168.6395 | [illegible] |
| 12 | 44.0495 | 1244.6885 | 1227.6620 | 1226.6780 | 1272.6834 | 1255.6569 | 1254.6729 | | | A | 1056.5506 | | | ***1072.5819*** | 1055.5554 | [illegible] |
| 13 | 86.0964 | 1357.7726 | 1340.7460 | 1339.7620 | 1385.7675 | 1368.7409 | 1367.7569 | 1329.7413 | 1343.7569 | I | 943.4666 | 956.4870 | 970.5026 | ***1001.5448*** | 984.5183 | [illegible] |
| 14 | 88.0393 | 1472.7995 | 1455.7730 | 1454.7890 | 1500.7944 | 1483.7679 | 1482.7839 | 1428.8097 | | D | 828.4396 | 827.4444 | | ***888.4608*** | 871.4342 | [illegible] |
| 15 | 60.0444 | 1559.8316 | 1542.8050 | 1541.8210 | 1587.8265 | 1570.7999 | 1569.8159 | 1543.8366 | | S | 741.4076 | 740.4124 | | ***773.4338*** | 756.4073 | [illegible] |
| 16 | 104.0528 | 1690.8720 | 1673.8455 | 1672.8615 | 1718.8670 | 1701.8404 | 1700.8564 | 1630.8687 | | M | 610.3671 | 609.3719 | | ***686.4018*** | 669.3752 | |
| 17 | 86.0964 | 1803.9561 | 1786.9296 | 1785.9455 | 1831.9510 | 1814.9245 | 1813.9404 | 1775.9248 | 1789.9405 | I | 497.2831 | 510.3035 | 524.3191 | ***555.3613*** | 538.3348 | |
| 18 | 70.0651 | 1901.0089 | 1883.9823 | 1882.9983 | 1929.0038 | 1911.9772 | 1910.9932 | 1874.9932 | | P | 400.2303 | 399.2350 | | ***442.2772*** | 425.2507 | |
| 19 | 86.0964 | 2014.0929 | 1997.0664 | 1996.0824 | 2042.0878 | 2025.0613 | 2024.0773 | 1986.0616 | 2000.0773 | I | 287.1462 | 300.1666 | 314.1823 | 345.2245 | 328.1979 | |
| 20 | 30.0338 | 2071.1144 | 2054.0878 | 2053.1038 | 2099.1093 | 2082.0828 | 2081.0987 | | | G | | | | ***232.1404*** | 215.1139 | |
| 21 | 129.1135 | | | | | | | | | R | 74.0237 | 73.0284 | | ***175.1190*** | 158.0924 | |

| Seq | ya | yb | Seq | ya | yb | Seq | ya | yb |
|---|---|---|---|---|---|---|---|---|

| | | | | | | | | |
|---|---|---|---|---|---|---|---|---|
| **VY** | 235.1441 | 263.1390 | **VYE** | 364.1867 | 392.1816 | **VYEP** | 461.2395 | 489.2344 |
| **VYEPL** | 574.3235 | 602.3184 | **YE** | 265.1183 | 293.1132 | **YEP** | 362.1710 | 390.1660 |
| **YEPL** | 475.2551 | 503.2500 | **YEPLQ** | 603.3137 | 631.3086 | **EP** | 199.1077 | 227.1026 |
| **EPL** | 312.1918 | 340.1867 | **EPLQ** | 440.2504 | 468.2453 | **EPLQT** | 541.2980 | 569.2930 |
| **EPLQTG** | 598.3195 | 626.3144 | **PL** | 183.1492 | 211.1441 | **PLQ** | 311.2078 | 339.2027 |
| **PLQT** | 412.2554 | 440.2504 | **PLQTG** | 469.2769 | 497.2718 | **PLQTGL** | 582.3610 | 610.3559 |
| **PLQTGLI** | 695.4450 | 723.4400 | **LQ** | 214.1550 | 242.1499 | **LQT** | 315.2027 | 343.1976 |
| **LQTG** | 372.2241 | 400.2191 | **LQTGL** | 485.3082 | 513.3031 | **LQTGLI** | 598.3923 | 626.3872 |
| **LQTGLIA** | 669.4294 | 697.4243 | **QT** | 202.1186 | 230.1135 | **QTG** | 259.1401 | 287.1350 |
| **QTGL** | 372.2241 | 400.2191 | **QTGLI** | 485.3082 | 513.3031 | **QTGLIA** | 556.3453 | 584.3402 |
| **QTGLIAI** | 669.4294 | 697.4243 | **TG** | 131.0815 | 159.0764 | **TGL** | 244.1656 | 272.1605 |
| **TGLI** | 357.2496 | 385.2445 | **TGLIA** | 428.2867 | 456.2817 | **TGLIAI** | 541.3708 | 569.3657 |
| **TGLIAID** | 656.3978 | 684.3927 | **GL** | 143.1179 | 171.1128 | **GLI** | 256.2020 | 284.1969 |
| **GLIA** | 327.2391 | 355.2340 | **GLIAI** | 440.3231 | 468.3180 | **GLIAID** | 555.3501 | 583.3450 |
| **GLIAIDS** | 642.3821 | 670.3770 | **LI** | 199.1805 | 227.1754 | **LIA** | 270.2176 | 298.2125 |
| **LIAI** | 383.3017 | 411.2966 | **LIAID** | 498.3286 | 526.3235 | **LIAIDS** | 585.3606 | 613.3556 |
| **IA** | 157.1335 | 185.1285 | **IAI** | 270.2176 | 298.2125 | **IAID** | 385.2445 | 413.2395 |
| **IAIDS** | 472.2766 | 500.2715 | **IAIDSM** | 603.3171 | 631.3120 | **AI** | 157.1335 | 185.1285 |
| **AID** | 272.1605 | 300.1554 | **AIDS** | 359.1925 | 387.1874 | **AIDSM** | 490.2330 | 518.2279 |
| **AIDSMI** | 603.3171 | 631.3120 | **ID** | 201.1234 | 229.1183 | **IDS** | 288.1554 | 316.1503 |
| **IDSM** | 419.1959 | 447.1908 | **IDSMI** | 532.2799 | 560.2749 | **IDSMIP** | 629.3327 | 657.3276 |
| **DS** | 175.0713 | 203.0662 | **DSM** | 306.1118 | 334.1067 | **DSMI** | 419.1959 | 447.1908 |
| **DSMIP** | 516.2486 | 544.2436 | **DSMIPI** | 629.3327 | 657.3276 | **DSMIPIG** | 686.3542 | 714.3491 |
| **SM** | 191.0849 | 219.0798 | **SMI** | 304.1689 | 332.1639 | **SMIP** | 401.2217 | 429.2166 |
| **SMIPI** | 514.3058 | 542.3007 | **SMIPIG** | 571.3272 | 599.3221 | **MI** | 217.1369 | 245.1318 |
| **MIP** | 314.1897 | 342.1846 | **MIPI** | 427.2737 | 455.2687 | **MIPIG** | 484.2952 | 512.2901 |
| **IP** | 183.1492 | 211.1441 | **IPI** | 296.2333 | 324.2282 | **IPIG** | 353.2547 | 381.2496 |
| **PI** | 183.1492 | 211.1441 | **PIG** | 240.1707 | 268.1656 | **IG** | 143.1179 | 171.1128 |

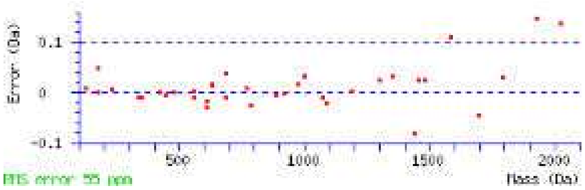


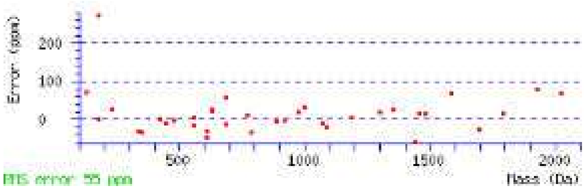


NCBI **BLAST** search of SVYEPLQTGLIAIDSMIPIGR
(Parameters: blastp, nr protein database, expect=20000, no filter, PAM30)
Other BLAST web gateways

**All matches to this query**

| Score | Mr(calc) | Delta | Sequence |
|---|---|---|---|
| 144.3 | 2272.2137 | 0.1284 | SVYEPIQTGLIAIDSMIPIGR |
| 144.3 | 2272.2137 | 0.1284 | SVYEPLQTGIIAIDSMIPIGR |
| 144.3 | 2272.2137 | 0.1284 | SVYEPLQTGLIAIDSMIPIGR |
| 144.3 | 2272.2137 | 0.1284 | SVYEPLQTGLIAIDSMIPIGR |
| 131.1 | 2272.2137 | 0.1284 | SVYEPLQTGIIAIDAMIPIGR |
| 131.1 | 2272.2137 | 0.1284 | SVYEPLQTGLIAIDAMIPIGR |
| 94.2 | 2272.2137 | 0.1284 | SVYEPLQTGILSIDAMIPIGR |
| 18.1 | 2272.1919 | 0.1502 | LMAKDLILSAIMSLAETHSGR |
| 17.7 | 2272.2137 | 0.1284 | SVFEPLQTGLIAIDSMIPIGR |
| 17.7 | 2272.1807 | 0.1614 | SVMEPLQTGLIAIDSMIPIGR |

**Mascot:** http://www.matrixscience.com/

# MATRIX SCIENCE Mascot Search Results

## Peptide View **Spot no 97**

MS/MS Fragmentation of **SVYEPLQTGLIAIDSMIPIGR**
Found in **gi|11466784** in **NCBInr**, ATP synthase CF1 alpha subunit [Oryza sativa Japonica Group]

Match to Query 92: 2288.339424 from(2289.346700,1+) intensity(0.0000) index(26)
Title: Label: I11, Spot_Id: 216645, Peak_List_Id: 221407, MSMS Job_Run_Id: 21364, Comment:
Data file C:\Documents and Settings\Administrator\Desktop\farah BHU\Akansha BHU\RAJESH\12-1-13 raj\ppw_I11_136074508400.txt

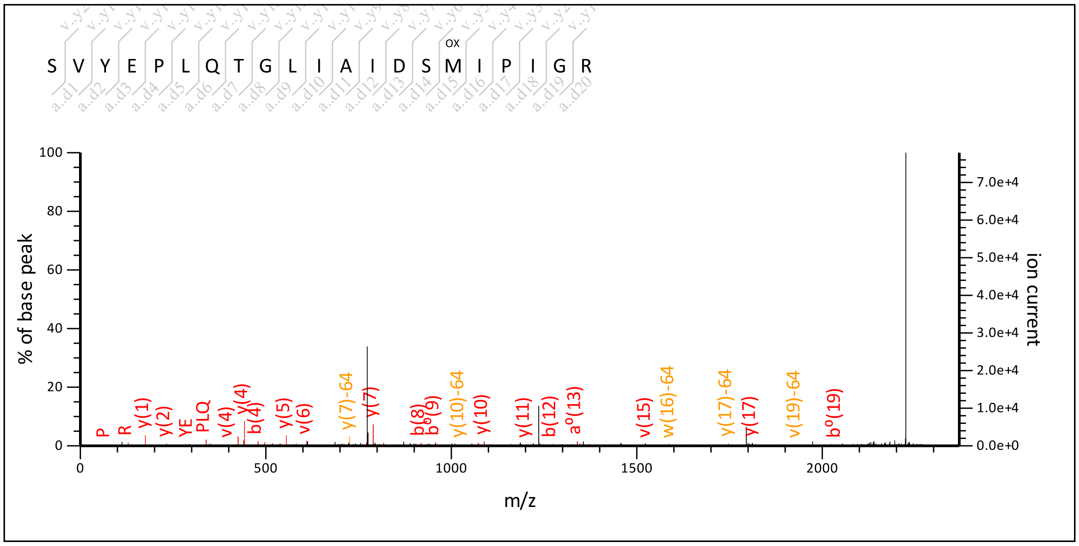


0 to 2369.31

Label all possible matches ○ Label matches used for scoring ◉

**Monoisotopic mass of neutral peptide Mr(calc):** 2288.2086
**Fixed modifications:** Carbamidomethyl (C) (apply to specified residues or termini only)
**Variable modifications:**
**M16** : Oxidation (M), with neutral losses 0.0000(shown in table), 63.9983
**Ions Score:** 41 **Expect:** 0.19
**Matches :** 86/553 fragment ions using 143 most intense peaks (help)

| # | Immon. | a | a* | $a^0$ | b | b* | $b^0$ | d | d' | Seq. | v | w | w' | y | y* | |
|---|---|---|---|---|---|---|---|---|---|---|---|---|---|---|---|---|
| 1 | 60.0444 | 60.0444 | | 42.0338 | 88.0393 | | 70.0287 | 44.0495 | | S | | | | | | |
| 2 | 72.0808 | 159.1128 | | 141.1022 | 187.1077 | | 169.0972 | 145.0972 | | V | 2158.1213 | 2171.1417 | | 2202.1839 | 2185.1573 | [illegible] |
| 3 | 136.0757 | 322.1761 | | 304.1656 | ***350.1710*** | | 332.1605 | | | Y | 1995.0579 | | | 2103.1155 | 2086.0889 | [illegible] |
| 4 | 102.0550 | 451.2187 | | 433.2082 | ***479.2136*** | | 461.2031 | 393.2132 | | E | 1866.0153 | 1865.0201 | | 1940.0521 | 1923.0256 | [illegible] |
| 5 | 70.0651 | 548.2715 | | 530.2609 | ***576.2664*** | | 558.2558 | 522.2558 | | P | 1768.9626 | 1767.9673 | | ***1811.0095*** | 1793.9830 | [illegible] |
| 6 | 86.0964 | 661.3556 | | 643.3450 | 689.3505 | | 671.3399 | 619.3086 | | L | 1655.8785 | 1654.8833 | | 1713.9568 | 1696.9302 | [illegible] |
| 7 | 101.0709 | 789.4141 | 772.3876 | 771.4036 | ***817.4090*** | 800.3825 | 799.3985 | 732.3927 | | Q | 1527.8199 | 1526.8247 | | 1600.8727 | 1583.8462 | [illegible] |
| 8 | 74.0600 | 890.4618 | 873.4353 | 872.4512 | ***918.4567*** | 901.4302 | 900.4462 | 874.4669 | 876.4462 | T | 1426.7723 | 1439.7927 | 1441.7719 | 1472.8141 | 1455.7876 | [illegible] |
| 9 | 30.0338 | 947.4833 | 930.4567 | 929.4727 | ***975.4782*** | 958.4516 | 957.4676 | | | G | | | | ***1371.7664*** | 1354.7399 | [illegible] |
| 10 | 86.0964 | 1060.5673 | 1043.5408 | 1042.5568 | ***1088.5623*** | 1071.5357 | 1070.5517 | 1018.5204 | | L | 1256.6667 | 1255.6715 | | 1314.7450 | 1297.7184 | [illegible] |
| 11 | 86.0964 | 1173.6514 | 1156.6249 | 1155.6408 | ***1201.6463*** | 1184.6198 | 1183.6358 | 1145.6201 | 1159.6358 | I | 1143.5827 | 1156.6031 | 1170.6187 | ***1201.6609*** | 1184.6344 | [illegible] |
| 12 | 44.0495 | 1244.6885 | 1227.6620 | 1226.6780 | ***1272.6834*** | 1255.6569 | 1254.6729 | | | A | 1072.5456 | | | ***1088.5769*** | 1071.5503 | [illegible] |
| 13 | 86.0964 | 1357.7726 | 1340.7460 | 1339.7620 | 1385.7675 | 1368.7409 | 1367.7569 | 1329.7413 | 1343.7569 | I | 959.4615 | 972.4819 | 986.4975 | 1017.5397 | 1000.5132 | [illegible] |
| 14 | 88.0393 | 1472.7995 | 1455.7730 | 1454.7890 | 1500.7944 | 1483.7679 | 1482.7839 | 1428.8097 | | D | 844.4345 | 843.4393 | | ***904.4557*** | 887.4291 | [illegible] |
| 15 | 60.0444 | 1559.8316 | 1542.8050 | 1541.8210 | 1587.8265 | 1570.7999 | 1569.8159 | 1543.8366 | | S | 757.4025 | 756.4073 | | ***789.4287*** | 772.4022 | [illegible] |
| 16 | 120.0478 | 1706.8670 | 1689.8404 | 1688.8564 | 1734.8619 | 1717.8353 | 1716.8513 | 1630.8687 | | M | 610.3671 | 609.3719 | | ***702.3967*** | 685.3702 | |
| 17 | 86.0964 | 1819.9510 | 1802.9245 | 1801.9404 | 1847.9459 | 1830.9194 | 1829.9354 | 1791.9197 | 1805.9354 | I | 497.2831 | 510.3035 | 524.3191 | ***555.3613*** | 538.3348 | |
| 18 | 70.0651 | 1917.0038 | 1899.9772 | 1898.9932 | 1944.9987 | 1927.9721 | 1926.9881 | 1890.9881 | | P | 400.2303 | 399.2350 | | ***442.2772*** | 425.2507 | |
| 19 | 86.0964 | 2030.0878 | 2013.0613 | 2012.0773 | 2058.0828 | 2041.0562 | 2040.0722 | 2002.0565 | 2016.0722 | I | 287.1462 | 300.1666 | 314.1823 | ***345.2245*** | 328.1979 | |
| 20 | 30.0338 | 2087.1093 | 2070.0828 | 2069.0987 | 2115.1042 | 2098.0777 | 2097.0937 | | | G | | | | ***232.1404*** | 215.1139 | |

| | | | | | | | | | | | | | | | |
|---|---|---|---|---|---|---|---|---|---|---|---|---|---|---|---|
| 21 | 129.1135 | | | | | | | | | R | 74.0237 | 73.0284 | | 175.1190 | 158.0924 |

| Seq | ya | yb | Seq | ya | yb | Seq | ya | yb |
|---|---|---|---|---|---|---|---|---|
| VY | 235.1441 | 263.1390 | VYE | 364.1867 | 392.1816 | VYEP | 461.2395 | 489.2344 |
| VYEPL | 574.3235 | 602.3184 | YE | 265.1183 | 293.1132 | YEP | 362.1710 | 390.1660 |
| YEPL | 475.2551 | 503.2500 | YEPLQ | 603.3137 | 631.3086 | EP | 199.1077 | 227.1026 |
| EPL | 312.1918 | 340.1867 | EPLQ | 440.2504 | 468.2453 | EPLQT | 541.2980 | 569.2930 |
| EPLQTG | 598.3195 | 626.3144 | PL | 183.1492 | 211.1441 | PLQ | 311.2078 | 339.2027 |
| PLQT | 412.2554 | 440.2504 | PLQTG | 469.2769 | 497.2718 | PLQTGL | 582.3610 | 610.3559 |
| PLQTGLI | 695.4450 | 723.4400 | LQ | 214.1550 | 242.1499 | LQT | 315.2027 | 343.1976 |
| LQTG | 372.2241 | 400.2191 | LQTGL | 485.3082 | 513.3031 | LQTGLI | 598.3923 | 626.3872 |
| LQTGLIA | 669.4294 | 697.4243 | QT | 202.1186 | 230.1135 | QTG | 259.1401 | 287.1350 |
| QTGL | 372.2241 | 400.2191 | QTGLI | 485.3082 | 513.3031 | QTGLIA | 556.3453 | 584.3402 |
| QTGLIAI | 669.4294 | 697.4243 | TG | 131.0815 | 159.0764 | TGL | 244.1656 | 272.1605 |
| TGLI | 357.2496 | 385.2445 | TGLIA | 428.2867 | 456.2817 | TGLIAI | 541.3708 | 569.3657 |
| TGLIAID | 656.3978 | 684.3927 | GL | 143.1179 | 171.1128 | GLI | 256.2020 | 284.1969 |
| GLIA | 327.2391 | 355.2340 | GLIAI | 440.3231 | 468.3180 | GLIAID | 555.3501 | 583.3450 |
| GLIAIDS | 642.3821 | 670.3770 | LI | 199.1805 | 227.1754 | LIA | 270.2176 | 298.2125 |
| LIAI | 383.3017 | 411.2966 | LIAID | 498.3286 | 526.3235 | LIAIDS | 585.3606 | 613.3556 |
| IA | 157.1335 | 185.1285 | IAI | 270.2176 | 298.2125 | IAID | 385.2445 | 413.2395 |
| IAIDS | 472.2766 | 500.2715 | IAIDSM | 619.3120 | 647.3069 | AI | 157.1335 | 185.1285 |
| AID | 272.1605 | 300.1554 | AIDS | 359.1925 | 387.1874 | AIDSM | 506.2279 | 534.2228 |
| AIDSMI | 619.3120 | 647.3069 | ID | 201.1234 | 229.1183 | IDS | 288.1554 | 316.1503 |
| IDSM | 435.1908 | 463.1857 | IDSMI | 548.2749 | 576.2698 | IDSMIP | 645.3276 | 673.3225 |
| DS | 175.0713 | 203.0662 | DSM | 322.1067 | 350.1016 | DSMI | 435.1908 | 463.1857 |
| DSMIP | 532.2436 | 560.2385 | DSMIPI | 645.3276 | 673.3225 | SM | 207.0798 | 235.0747 |
| SMI | 320.1639 | 348.1588 | SMIP | 417.2166 | 445.2115 | SMIPI | 530.3007 | 558.2956 |
| SMIPIG | 587.3221 | 615.3171 | MI | 233.1318 | 261.1267 | MIP | 330.1846 | 358.1795 |
| MIPI | 443.2687 | 471.2636 | MIPIG | 500.2901 | 528.2850 | IP | 183.1492 | 211.1441 |
| IPI | 296.2333 | 324.2282 | IPIG | 353.2547 | 381.2496 | PI | 183.1492 | 211.1441 |
| PIG | 240.1707 | 268.1656 | IG | 143.1179 | 171.1128 | | | |

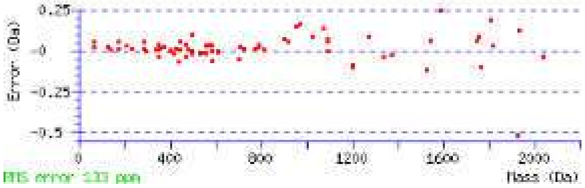


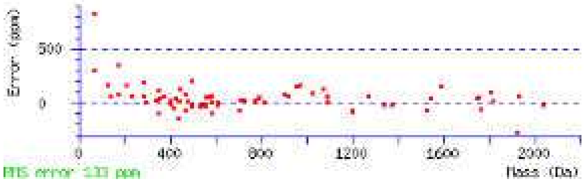


NCBI **BLAST** search of [SVYEPLQTGLIAIDSMIPIGR]()
(Parameters: blastp, nr protein database, expect=20000, no filter, PAM30)
Other BLAST [web gateways]()

**All matches to this query**

| Score | Mr(calc) | Delta | Sequence |
|---|---|---|---|
| 41.5 | 2288.2086 | 0.1308 | SVYEPIQTGLIAIDSMIPIGR |
| 41.5 | 2288.2086 | 0.1308 | SVYEPLQTGIIAIDSMIPIGR |
| 41.5 | 2288.2086 | 0.1308 | SVYEPLQTGLIAIDSMIPIGR |
| 41.5 | 2288.2086 | 0.1308 | SVYEPLQTGLIAIDSMIPIGR |
| 39.1 | 2288.2086 | 0.1308 | SVYEPLQTGILSIDAMIPIGR |
| 13.6 | 2288.1206 | 0.2189 | ENDIELSTTPLSPSEKQCIK |
| 10.8 | 2287.3007 | 1.0387 | TMILSVLSSPALVSGLMVVRAK |
| 8.7 | 2288.1042 | 0.2352 | RGVEPDVVTYTALMDGHCLR |
| 7.7 | 2288.1273 | 0.2121 | SADAVLVFMHQHPIATHWGR |
| 7.7 | 2288.1518 | 0.1876 | LGFGLDTHVCTSLVLANCRR |

**Mascot:** http://www.matrixscience.com/

# MATRIX SCIENCE Mascot Search Results

## Peptide View Spot no 97

MS/MS Fragmentation of **QSQANPLPVEEQIATIYIGTR**
Found in **gi|11466784** in **NCBInr**, ATP synthase CF1 alpha subunit [Oryza sativa Japonica Group]

Match to Query 97: 2327.341824 from(2328.349100,1+) intensity(0.0000) index(28)
Title: Label: I11, Spot_Id: 216645, Peak_List_Id: 221391, MSMS Job_Run_Id: 21364, Comment:
Data file C:\Documents and Settings\Administrator\Desktop\farah BHU\Akansha BHU\RAJESH\12-1-13 raj\ppw_I11_136074508400.txt

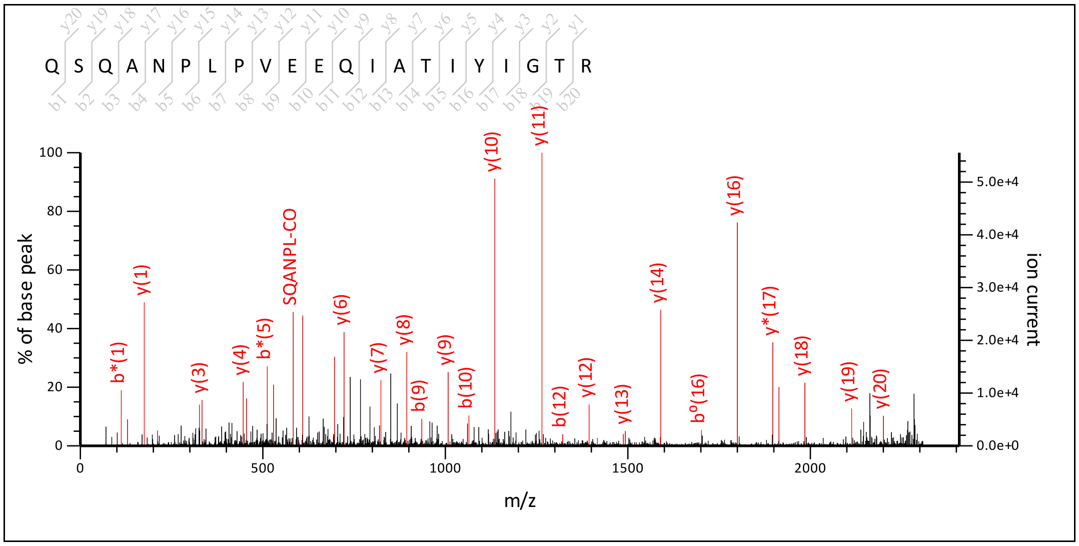


0 to 2408.04

Label all possible matches ○ Label matches used for scoring ◉

**Monoisotopic mass of neutral peptide Mr(calc):** 2327.2121
**Fixed modifications:** Carbamidomethyl (C) (apply to specified residues or termini only)
**Ions Score:** 167 **Expect:** 5.1e-14
**Matches :** 43/423 fragment ions using 46 most intense peaks (help)

| # | Immon. | a | a* | a⁰ | b | b* | b⁰ | d | d' | Seq. | v | w | w' | y | y* | |
|---|---|---|---|---|---|---|---|---|---|---|---|---|---|---|---|---|
| 1 | 101.0709 | 101.0709 | 84.0444 | | **129.0659** | 112.0393 | | 44.0495 | | Q | | | | | | |
| 2 | 60.0444 | 188.1030 | 171.0764 | 170.0924 | 216.0979 | 199.0713 | 198.0873 | 172.1081 | | S | 2168.1346 | 2167.1394 | | ***2200.1608*** | 2183.1343 | [illegible] |
| 3 | 101.0709 | 316.1615 | 299.1350 | 298.1510 | 344.1565 | 327.1299 | 326.1459 | 259.1401 | | Q | 2040.0760 | 2039.0808 | | ***2113.1288*** | 2096.1022 | [illegible] |
| 4 | 44.0495 | 387.1987 | 370.1721 | 369.1881 | 415.1936 | 398.1670 | 397.1830 | | | A | 1969.0389 | | | ***1985.0702*** | 1968.0437 | [illegible] |
| 5 | 87.0553 | 501.2416 | 484.2150 | 483.2310 | **529.2365** | 512.2100 | 511.2259 | 458.2358 | | N | 1854.9960 | 1854.0007 | | ***1914.0331*** | 1897.0066 | [illegible] |
| 6 | 70.0651 | 598.2944 | 581.2678 | 580.2838 | 626.2893 | 609.2627 | 608.2787 | 572.2787 | | P | 1757.9432 | 1756.9480 | | ***1799.9902*** | 1782.9636 | [illegible] |
| 7 | 86.0964 | 711.3784 | 694.3519 | 693.3678 | 739.3733 | 722.3468 | 721.3628 | 669.3315 | | L | 1644.8592 | 1643.8639 | | 1702.9374 | 1685.9109 | [illegible] |
| 8 | 70.0651 | 808.4312 | 791.4046 | 790.4206 | 836.4261 | 819.3995 | 818.4155 | 782.4155 | | P | 1547.8064 | 1546.8112 | | ***1589.8534*** | 1572.8268 | [illegible] |
| 9 | 72.0808 | 907.4996 | 890.4730 | 889.4890 | **935.4945** | 918.4680 | 917.4839 | 893.4839 | | V | 1448.7380 | 1461.7584 | | ***1492.8006*** | 1475.7740 | [illegible] |
| 10 | 102.0550 | 1036.5422 | 1019.5156 | 1018.5316 | **1064.5371** | 1047.5106 | 1046.5265 | 978.5367 | | E | 1319.6954 | 1318.7001 | | ***1393.7322*** | 1376.7056 | [illegible] |
| 11 | 102.0550 | 1165.5848 | 1148.5582 | 1147.5742 | 1193.5797 | 1176.5531 | 1175.5691 | 1107.5793 | | E | 1190.6528 | 1189.6576 | | ***1264.6896*** | 1247.6630 | [illegible] |
| 12 | 101.0709 | 1293.6434 | 1276.6168 | 1275.6328 | **1321.6383** | 1304.6117 | 1303.6277 | 1236.6219 | | Q | 1062.5942 | 1061.5990 | | ***1135.6470*** | 1118.6204 | [illegible] |
| 13 | 86.0964 | 1406.7274 | 1389.7009 | 1388.7169 | 1434.7223 | 1417.6958 | 1416.7118 | 1378.6961 | 1392.7118 | I | 949.5102 | 962.5306 | 976.5462 | ***1007.5884*** | 990.5619 | [illegible] |
| 14 | 44.0495 | 1477.7645 | 1460.7380 | 1459.7540 | 1505.7594 | 1488.7329 | 1487.7489 | | | A | 878.4730 | | | ***894.5043*** | 877.4778 | [illegible] |
| 15 | 74.0600 | 1578.8122 | 1561.7857 | 1560.8016 | 1606.8071 | 1589.7806 | 1588.7966 | 1562.8173 | 1564.7966 | T | 777.4254 | 790.4458 | 792.4250 | ***823.4672*** | 806.4407 | [illegible] |
| 16 | 86.0964 | 1691.8963 | 1674.8697 | 1673.8857 | 1719.8912 | 1702.8646 | 1701.8806 | 1663.8650 | 1677.8806 | I | 664.3413 | 677.3617 | 691.3774 | ***722.4196*** | 705.3930 | [illegible] |
| 17 | 136.0757 | 1854.9596 | 1837.9331 | 1836.9490 | 1882.9545 | 1865.9280 | 1864.9440 | | | Y | 501.2780 | | | ***609.3355*** | 592.3089 | [illegible] |
| 18 | 86.0964 | 1968.0437 | 1951.0171 | 1950.0331 | 1996.0386 | 1979.0120 | 1978.0280 | 1940.0124 | 1954.0280 | I | 388.1939 | 401.2143 | 415.2300 | ***446.2722*** | 429.2456 | [illegible] |
| 19 | 30.0338 | 2025.0651 | 2008.0386 | 2007.0546 | 2053.0600 | 2036.0335 | 2035.0495 | | | G | | | | ***333.1881*** | 316.1615 | [illegible] |
| 20 | 74.0600 | 2126.1128 | 2109.0863 | 2108.1022 | 2154.1077 | 2137.0812 | 2136.0972 | 2110.1179 | 2112.0972 | T | 230.1248 | 243.1452 | 245.1244 | 276.1666 | 259.1401 | [illegible] |
| 21 | 129.1135 | | | | | | | | | R | 74.0237 | 73.0284 | | ***175.1190*** | 158.0924 | |

| Seq | ya | yb | Seq | ya | yb | Seq | ya | yb |
|---|---|---|---|---|---|---|---|---|

| | | | | | | | | |
|---|---|---|---|---|---|---|---|---|
| SQ | 188.1030 | 216.0979 | SQA | 259.1401 | 287.1350 | SQAN | 373.1830 | 401.1779 |
| SQANP | 470.2358 | 498.2307 | SQANPL | 583.3198 | 611.3148 | SQANPLP | 680.3726 | 708.3675 |
| QA | 172.1081 | 200.1030 | QAN | 286.1510 | 314.1459 | QANP | 383.2037 | 411.1987 |
| QANPL | 496.2878 | 524.2827 | QANPLP | 593.3406 | 621.3355 | QANPLPV | 692.4090 | 720.4039 |
| AN | 158.0924 | 186.0873 | ANP | 255.1452 | 283.1401 | ANPL | 368.2292 | 396.2241 |
| ANPLP | 465.2820 | 493.2769 | ANPLPV | 564.3504 | 592.3453 | ANPLPVE | 693.3930 | 721.3879 |
| NP | 184.1081 | 212.1030 | NPL | 297.1921 | 325.1870 | NPLP | 394.2449 | 422.2398 |
| NPLPV | 493.3133 | 521.3082 | NPLPVE | 622.3559 | 650.3508 | PL | 183.1492 | 211.1441 |
| PLP | 280.2020 | 308.1969 | PLPV | 379.2704 | 407.2653 | PLPVE | 508.3130 | 536.3079 |
| PLPVEE | 637.3556 | 665.3505 | LP | 183.1492 | 211.1441 | LPV | 282.2176 | 310.2125 |
| LPVE | 411.2602 | 439.2551 | LPVEE | 540.3028 | 568.2977 | LPVEEQ | 668.3614 | 696.3563 |
| PV | 169.1335 | 197.1285 | PVE | 298.1761 | 326.1710 | PVEE | 427.2187 | 455.2136 |
| PVEEQ | 555.2773 | 583.2722 | PVEEQI | 668.3614 | 696.3563 | VE | 201.1234 | 229.1183 |
| VEE | 330.1660 | 358.1609 | VEEQ | 458.2245 | 486.2195 | VEEQI | 571.3086 | 599.3035 |
| VEEQIA | 642.3457 | 670.3406 | EE | 231.0975 | 259.0925 | EEQ | 359.1561 | 387.1510 |
| EEQI | 472.2402 | 500.2351 | EEQIA | 543.2773 | 571.2722 | EEQIAT | 644.3250 | 672.3199 |
| EQ | 230.1135 | 258.1084 | EQI | 343.1976 | 371.1925 | EQIA | 414.2347 | 442.2296 |
| EQIAT | 515.2824 | 543.2773 | EQIATI | 628.3665 | 656.3614 | QI | 214.1550 | 242.1499 |
| QIA | 285.1921 | 313.1870 | QIAT | 386.2398 | 414.2347 | QIATI | 499.3239 | 527.3188 |
| QIATIY | 662.3872 | 690.3821 | IA | 157.1335 | 185.1285 | IAT | 258.1812 | 286.1761 |
| IATI | 371.2653 | 399.2602 | IATIY | 534.3286 | 562.3235 | IATIYI | 647.4127 | 675.4076 |
| AT | 145.0972 | 173.0921 | ATI | 258.1812 | 286.1761 | ATIY | 421.2445 | 449.2395 |
| ATIYI | 534.3286 | 562.3235 | ATIYIG | 591.3501 | 619.3450 | ATIYIGT | 692.3978 | 720.3927 |
| TI | 187.1441 | 215.1390 | TIY | 350.2074 | 378.2023 | TIYI | 463.2915 | 491.2864 |
| TIYIG | 520.3130 | 548.3079 | TIYIGT | 621.3606 | 649.3556 | IY | 249.1598 | 277.1547 |
| IYI | 362.2438 | 390.2387 | IYIG | 419.2653 | 447.2602 | IYIGT | 520.3130 | 548.3079 |
| YI | 249.1598 | 277.1547 | YIG | 306.1812 | 334.1761 | YIGT | 407.2289 | 435.2238 |
| IG | 143.1179 | 171.1128 | IGT | 244.1656 | 272.1605 | GT | 131.0815 | 159.0764 |

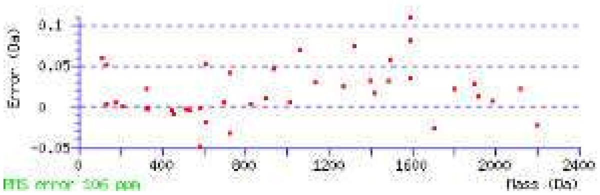


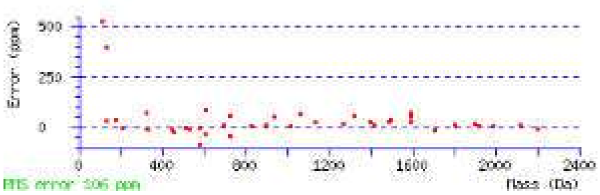


NCBI **BLAST** search of QSQANPLPVEEQIATIYIGTR
(Parameters: blastp, nr protein database, expect=20000, no filter, PAM30)
Other BLAST web gateways

**All matches to this query**

| Score | Mr(calc) | Delta | Sequence |
|---|---|---|---|
| 167.3 | 2327.2121 | 0.1297 | QSQANPLPVEEQIATIYIGTR |
| 17.5 | 2326.2218 | 1.1201 | TGTMSLFPLLMAGIVSIAYWR |
| 17.2 | 2327.2155 | 0.1263 | MTTVSTLTALAGETALRSSFVR |
| 16.4 | 2327.1427 | 0.1991 | TLENSHPDLKELDVSTNMLR |
| 13.7 | 2327.2824 | 0.0594 | QFMFGVKPTTLALHSITIPAR |
| 13.6 | 2326.1448 | 1.1970 | RQLNAALDVMNAATTGDGTHVR |
| 12.8 | 2328.2226 | -0.8808 | AGNRIPGLSIVIDEATWDFVR |
| 11.1 | 2327.0899 | 0.2519 | MYAQEGGMRLVSHPIAAHDGR |
| 10.8 | 2326.1917 | 1.1501 | QEDLIVYGQSVGSGPTLHLASR |
| 9.4 | 2326.1917 | 1.1501 | QEDVILYGQSVGSGPTLHLASR |

**Mascot:** http://www.matrixscience.com/

# MATRIX SCIENCE Mascot Search Results

## Peptide View Spot no 97

MS/MS Fragmentation of **TFTEEAEILLKEAIQEQLER**
Found in **gi|11466784** in **NCBInr**, ATP synthase CF1 alpha subunit [Oryza sativa Japonica Group]

Match to Query 99: 2389.375824 from(2390.383100,1+) intensity(0.0000) index(29)
Title: Label: I11, Spot_Id: 216645, Peak_List_Id: 221397, MSMS Job_Run_Id: 21364, Comment:
Data file C:\Documents and Settings\Administrator\Desktop\farah BHU\Akansha BHU\RAJESH\12-1-13 raj\ppw_I11_136074508400.txt

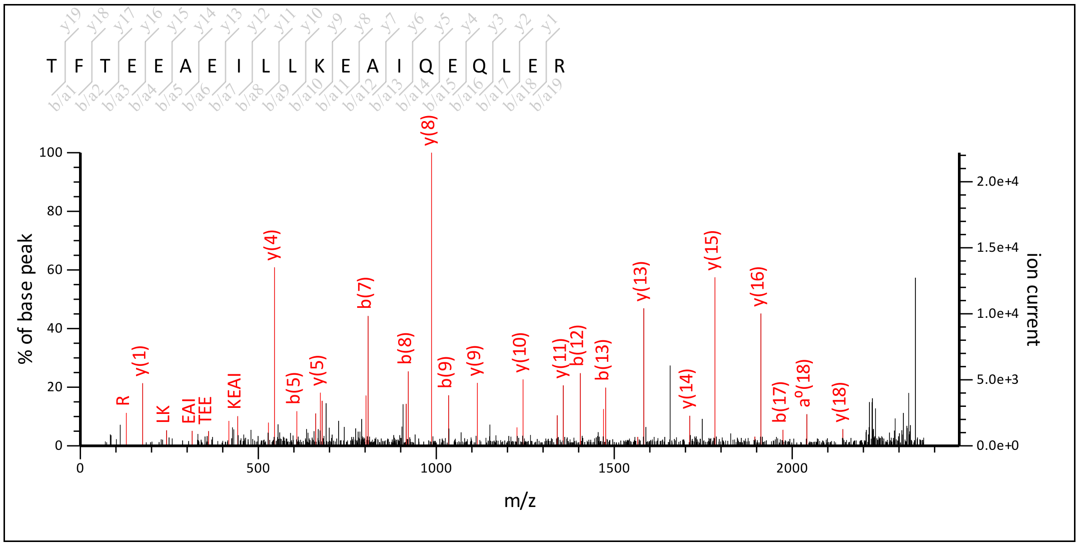


0 to 2469.35

Label all possible matches ○ Label matches used for scoring ◉

**Monoisotopic mass of neutral peptide Mr(calc):** 2389.2376
**Fixed modifications:** Carbamidomethyl (C) (apply to specified residues or termini only)
**Ions Score:** 152 **Expect:** 1.5e-12
**Matches :** 45/373 fragment ions using 46 most intense peaks (help)

| # | Immon. | a | a* | a0 | b | b* | b0 | d | d' | Seq. | v | w | w' | y | y* | |
|---|---|---|---|---|---|---|---|---|---|---|---|---|---|---|---|---|
| 1 | 74.0600 | 74.0600 | | 56.0495 | 102.0550 | | 84.0444 | 44.0495 | | T | | | | | | |
| 2 | 120.0808 | 221.1285 | | 203.1179 | 249.1234 | | 231.1128 | | | F | 2197.1347 | | | 2289.1973 | 2272.1707 | 22 |
| 3 | 74.0600 | 322.1761 | | 304.1656 | 350.1710 | | 332.1605 | 306.1812 | 308.1605 | T | 2096.0870 | 2109.1074 | 2111.0867 | **_2142.1289_** | 2125.1023 | 21 |
| 4 | 102.0550 | 451.2187 | | 433.2082 | 479.2136 | | 461.2031 | 393.2132 | | E | 1967.0444 | 1966.0491 | | **_2041.0812_** | 2024.0546 | 20 |
| 5 | 102.0550 | 580.2613 | | 562.2508 | **608.2562** | | 590.2457 | 522.2558 | | E | 1838.0018 | 1837.0066 | | **_1912.0386_** | 1895.0120 | 18 |
| 6 | 44.0495 | 651.2984 | | 633.2879 | **679.2933** | | 661.2828 | | | A | 1766.9647 | | | **_1782.9960_** | 1765.9694 | 17 |
| 7 | 102.0550 | 780.3410 | | 762.3305 | **808.3359** | | 790.3254 | 722.3355 | | E | 1637.9221 | 1636.9268 | | **_1711.9589_** | 1694.9323 | 16 |
| 8 | 86.0964 | 893.4251 | | 875.4145 | **921.4200** | | 903.4094 | 865.3938 | 879.4094 | I | 1524.8380 | 1537.8584 | 1551.8741 | **_1582.9163_** | 1565.8897 | 15 |
| 9 | 86.0964 | 1006.5092 | | 988.4986 | **1034.5041** | | 1016.4935 | 964.4622 | | L | 1411.7540 | 1410.7587 | | **_1469.8322_** | 1452.8057 | 14 |
| 10 | 86.0964 | 1119.5932 | | 1101.5827 | 1147.5881 | | 1129.5776 | 1077.5463 | | L | 1298.6699 | 1297.6747 | | **_1356.7482_** | 1339.7216 | 13 |
| 11 | 101.1073 | 1247.6882 | 1230.6616 | 1229.6776 | 1275.6831 | 1258.6565 | 1257.6725 | 1190.6303 | | K | 1170.5749 | 1169.5797 | | **_1243.6641_** | 1226.6375 | 12 |
| 12 | 102.0550 | 1376.7308 | 1359.7042 | 1358.7202 | **1404.7257** | 1387.6991 | 1386.7151 | 1318.7253 | | E | 1041.5324 | 1040.5371 | | **_1115.5691_** | 1098.5426 | 10 |
| 13 | 44.0495 | 1447.7679 | 1430.7413 | 1429.7573 | **1475.7628** | 1458.7363 | 1457.7522 | | | A | 970.4952 | | | **_986.5265_** | 969.5000 | 9 |
| 14 | 86.0964 | 1560.8520 | 1543.8254 | 1542.8414 | 1588.8469 | 1571.8203 | 1570.8363 | 1532.8207 | 1546.8363 | I | 857.4112 | 870.4316 | 884.4472 | **_915.4894_** | 898.4629 | 8 |
| 15 | 101.0709 | 1688.9105 | 1671.8840 | 1670.9000 | 1716.9054 | 1699.8789 | 1698.8949 | 1631.8891 | | Q | 729.3526 | 728.3573 | | **_802.4054_** | 785.3788 | 7 |
| 16 | 102.0550 | 1817.9531 | 1800.9266 | 1799.9426 | 1845.9480 | 1828.9215 | 1827.9375 | 1759.9476 | | E | 600.3100 | 599.3148 | | **_674.3468_** | 657.3202 | 6 |
| 17 | 101.0709 | 1946.0117 | 1928.9852 | 1928.0011 | **1974.0066** | 1956.9801 | 1955.9961 | 1888.9902 | | Q | 472.2514 | 471.2562 | | **_545.3042_** | 528.2776 | 5 |
| 18 | 86.0964 | 2059.0958 | 2042.0692 | 2041.0852 | 2087.0907 | 2070.0641 | 2069.0801 | 2017.0488 | | L | 359.1674 | 358.1721 | | **_417.2456_** | 400.2191 | 3 |
| 19 | 102.0550 | 2188.1384 | 2171.1118 | 2170.1278 | 2216.1333 | 2199.1067 | 2198.1227 | 2130.1329 | | E | 230.1248 | 229.1295 | | 304.1615 | 287.1350 | 2 |
| 20 | 129.1135 | | | | | | | | | R | 74.0237 | 73.0284 | | **_175.1190_** | 158.0924 | |

| Seq | ya | yb | Seq | ya | yb | Seq | ya | yb |
|---|---|---|---|---|---|---|---|---|
| FT | 221.1285 | 249.1234 | FTE | 350.1710 | 378.1660 | FTEE | 479.2136 | 507.2086 |

| | | | | | | | | |
|---|---|---|---|---|---|---|---|---|
| **FTEEA** | 550.2508 | 578.2457 | **FTEEAE** | 679.2933 | 707.2883 | **TE** | 203.1026 | 231.0975 |
| **TEE** | 332.1452 | 360.1401 | **TEEA** | 403.1823 | 431.1773 | **TEEAE** | 532.2249 | 560.2198 |
| **TEEAEI** | 645.3090 | 673.3039 | **EE** | 231.0975 | 259.0925 | **EEA** | 302.1347 | 330.1296 |
| **EEAE** | 431.1773 | 459.1722 | **EEAEI** | 544.2613 | 572.2562 | **EEAEIL** | 657.3454 | 685.3403 |
| **EA** | 173.0921 | 201.0870 | **EAE** | 302.1347 | 330.1296 | **EAEI** | 415.2187 | 443.2136 |
| **EAEIL** | 528.3028 | 556.2977 | **EAEILL** | 641.3869 | 669.3818 | **AE** | 173.0921 | 201.0870 |
| **AEI** | 286.1761 | 314.1710 | **AEIL** | 399.2602 | 427.2551 | **AEILL** | 512.3443 | 540.3392 |
| **AEILLK** | 640.4392 | 668.4341 | **EI** | 215.1390 | 243.1339 | **EIL** | 328.2231 | 356.2180 |
| **EILL** | 441.3071 | 469.3021 | **EILLK** | 569.4021 | 597.3970 | **EILLKE** | 698.4447 | 726.4396 |
| **IL** | 199.1805 | 227.1754 | **ILL** | 312.2646 | 340.2595 | **ILLK** | 440.3595 | 468.3544 |
| **ILLKE** | 569.4021 | 597.3970 | **ILLKEA** | 640.4392 | 668.4341 | **LL** | 199.1805 | 227.1754 |
| **LLK** | 327.2755 | 355.2704 | **LLKE** | 456.3180 | 484.3130 | **LLKEA** | 527.3552 | 555.3501 |
| **LLKEAI** | 640.4392 | 668.4341 | **LK** | 214.1914 | 242.1863 | **LKE** | 343.2340 | 371.2289 |
| **LKEA** | 414.2711 | 442.2660 | **LKEAI** | 527.3552 | 555.3501 | **LKEAIQ** | 655.4137 | 683.4087 |
| **KE** | 230.1499 | 258.1448 | **KEA** | 301.1870 | 329.1819 | **KEAI** | 414.2711 | 442.2660 |
| **KEAIQ** | 542.3297 | 570.3246 | **KEAIQE** | 671.3723 | 699.3672 | **EA** | 173.0921 | 201.0870 |
| **EAI** | 286.1761 | 314.1710 | **EAIQ** | 414.2347 | 442.2296 | **EAIQE** | 543.2773 | 571.2722 |
| **EAIQEQ** | 671.3359 | 699.3308 | **AI** | 157.1335 | 185.1285 | **AIQ** | 285.1921 | 313.1870 |
| **AIQE** | 414.2347 | 442.2296 | **AIQEQ** | 542.2933 | 570.2882 | **AIQEQL** | 655.3774 | 683.3723 |
| **IQ** | 214.1550 | 242.1499 | **IQE** | 343.1976 | 371.1925 | **IQEQ** | 471.2562 | 499.2511 |
| **IQEQL** | 584.3402 | 612.3352 | **QE** | 230.1135 | 258.1084 | **QEQ** | 358.1721 | 386.1670 |
| **QEQL** | 471.2562 | 499.2511 | **QEQLE** | 600.2988 | 628.2937 | **EQ** | 230.1135 | 258.1084 |
| **EQL** | 343.1976 | 371.1925 | **EQLE** | 472.2402 | 500.2351 | **QL** | 214.1550 | 242.1499 |
| **QLE** | 343.1976 | 371.1925 | **LE** | 215.1390 | 243.1339 | | | |

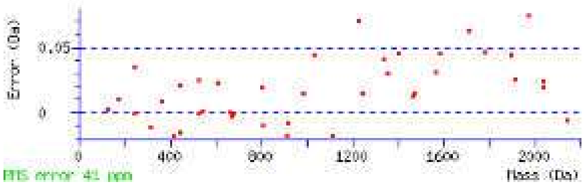


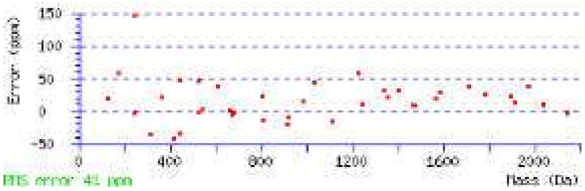


NCBI **BLAST** search of TFTEEAEILLKEAIQEQLER
(Parameters: blastp, nr protein database, expect=20000, no filter, PAM30)
Other BLAST web gateways

**All matches to this query**

| Score | Mr(calc) | Delta | Sequence |
|---|---|---|---|
| 152.3 | 2389.2376 | 0.1382 | TFTEEAEILLKEAIQEQIER |
| 152.3 | 2389.2376 | 0.1382 | TFTEEAEILLKEAIQEQLER |
| 93.3 | 2388.2536 | 1.1222 | TFTEQAEILLKEAIQEQLER |
| 59.0 | 2390.1965 | -0.8206 | TFTEEAENLLKEAIQEQIER |
| 22.2 | 2390.1675 | -0.7917 | ALCEKDSLPEAVALLDEFENK |
| 17.3 | 2389.1597 | 0.2161 | NLNHESIIRYCTSWIDVNR |
| 14.4 | 2390.0696 | -0.6938 | DNGTAEMVTDEGAIYFTKDAAR |
| 13.7 | 2388.9621 | 0.4138 | GDDMYMETSDDLIRHMQER |
| 13.6 | 2390.2515 | -0.8757 | GFNPESGLLVLVDKSLITMDSR |
| 11.8 | 2390.2151 | -0.8393 | IVVSQAELLLMEGNANPSTFNK |

**Mascot:** http://www.matrixscience.com/

# MATRIX SCIENCE Mascot Search Results

## Peptide View **Spot no 97**

MS/MS Fragmentation of **RSVYEPLQTGLIAIDSMIPIGR**
Found in **gi|11466784** in **NCBInr**, ATP synthase CF1 alpha subunit [Oryza sativa Japonica Group]

Match to Query 103: 2428.453424 from(2429.460700,1+) intensity(0.0000) index(31)
Title: Label: I11, Spot_Id: 216645, Peak_List_Id: 221402, MSMS Job_Run_Id: 21364, Comment:
Data file C:\Documents and Settings\Administrator\Desktop\farah BHU\Akansha BHU\RAJESH\12-1-13 raj\ppw_I11_136074508400.txt

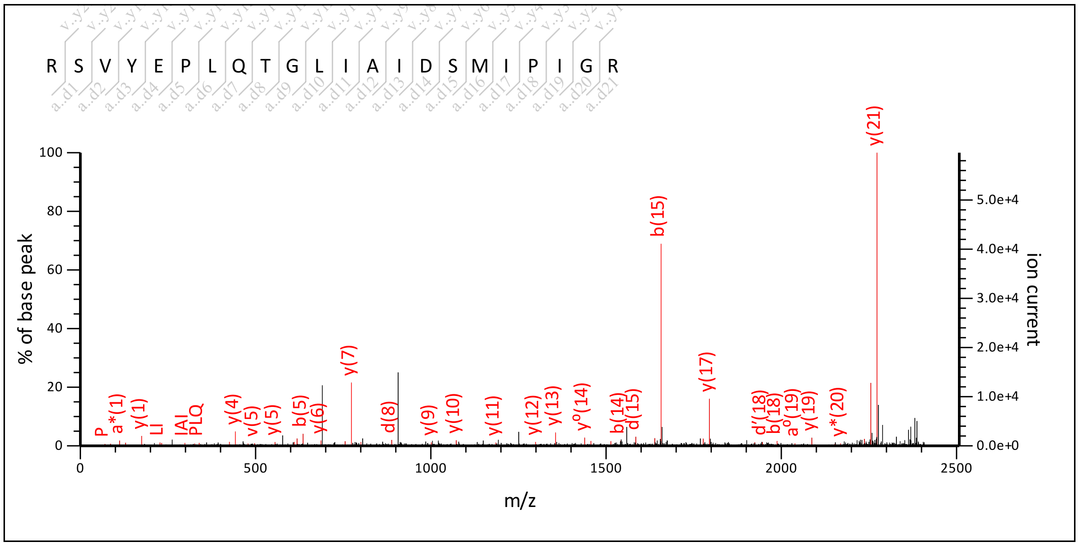


0 to 2507.8

Label all possible matches ○ Label matches used for scoring ◉

**Monoisotopic mass of neutral peptide Mr(calc):** 2428.3148
**Fixed modifications:** Carbamidomethyl (C) (apply to specified residues or termini only)
**Ions Score:** 100 **Expect:** 2.4e-07
**Matches :** 69/442 fragment ions using 96 most intense peaks (help)

| # | Immon. | a | a* | a0 | b | b* | b0 | d | d' | Seq. | v | w | w' | y | y* | |
|---|---|---|---|---|---|---|---|---|---|---|---|---|---|---|---|---|
| 1 | 129.1135 | 129.1135 | 112.0869 | | 157.1084 | 140.0818 | | 44.0495 | | R | | | | | | |
| 2 | 60.0444 | 216.1455 | 199.1190 | 198.1349 | 244.1404 | 227.1139 | 226.1298 | 200.1506 | | S | 2241.1948 | 2240.1995 | | 2273.2210 | 2256.1944 | [illegible] |
| 3 | 72.0808 | 315.2139 | 298.1874 | 297.2033 | 343.2088 | 326.1823 | 325.1983 | 301.1983 | | V | 2142.1264 | 2155.1468 | | 2186.1890 | 2169.1624 | [illegible] |
| 4 | 136.0757 | 478.2772 | 461.2507 | 460.2667 | 506.2722 | 489.2456 | 488.2616 | | | Y | 1979.0630 | | | 2087.1205 | 2070.0940 | [illegible] |
| 5 | 102.0550 | 607.3198 | 590.2933 | 589.3093 | 635.3148 | 618.2882 | 617.3042 | 549.3144 | | E | 1850.0204 | 1849.0252 | | 1924.0572 | 1907.0307 | [illegible] |
| 6 | 70.0651 | 704.3726 | 687.3461 | 686.3620 | 732.3675 | 715.3410 | 714.3570 | 678.3570 | | P | 1752.9677 | 1751.9724 | | 1795.0146 | 1777.9881 | [illegible] |
| 7 | 86.0964 | 817.4567 | 800.4301 | 799.4461 | 845.4516 | 828.4250 | 827.4410 | 775.4097 | | L | 1639.8836 | 1638.8884 | | 1697.9619 | 1680.9353 | [illegible] |
| 8 | 101.0709 | 945.5152 | 928.4887 | 927.5047 | 973.5102 | 956.4836 | 955.4996 | 888.4938 | | Q | 1511.8250 | 1510.8298 | | 1584.8778 | 1567.8512 | [illegible] |
| 9 | 74.0600 | 1046.5629 | 1029.5364 | 1028.5524 | 1074.5578 | 1057.5313 | 1056.5473 | 1030.5680 | 1032.5473 | T | 1410.7773 | 1423.7977 | 1425.7770 | 1456.8192 | 1439.7927 | [illegible] |
| 10 | 30.0338 | 1103.5844 | 1086.5578 | 1085.5738 | 1131.5793 | 1114.5528 | 1113.5687 | | | G | | | | 1355.7715 | 1338.7450 | [illegible] |
| 11 | 86.0964 | 1216.6684 | 1199.6419 | 1198.6579 | 1244.6634 | 1227.6368 | 1226.6528 | 1174.6215 | | L | 1240.6718 | 1239.6766 | | 1298.7501 | 1281.7235 | [illegible] |
| 12 | 86.0964 | 1329.7525 | 1312.7260 | 1311.7419 | 1357.7474 | 1340.7209 | 1339.7369 | 1301.7212 | 1315.7369 | I | 1127.5878 | 1140.6082 | 1154.6238 | 1185.6660 | 1168.6395 | [illegible] |
| 13 | 44.0495 | 1400.7896 | 1383.7631 | 1382.7791 | 1428.7845 | 1411.7580 | 1410.7740 | | | A | 1056.5506 | | | 1072.5819 | 1055.5554 | [illegible] |
| 14 | 86.0964 | 1513.8737 | 1496.8471 | 1495.8631 | 1541.8686 | 1524.8421 | 1523.8580 | 1485.8424 | 1499.8580 | I | 943.4666 | 956.4870 | 970.5026 | 1001.5448 | 984.5183 | [illegible] |
| 15 | 88.0393 | 1628.9006 | 1611.8741 | 1610.8901 | 1656.8955 | 1639.8690 | 1638.8850 | 1584.9108 | | D | 828.4396 | 827.4444 | | 888.4608 | 871.4342 | [illegible] |
| 16 | 60.0444 | 1715.9327 | 1698.9061 | 1697.9221 | 1743.9276 | 1726.9010 | 1725.9170 | 1699.9377 | | S | 741.4076 | 740.4124 | | 773.4338 | 756.4073 | [illegible] |
| 17 | 104.0528 | 1846.9731 | 1829.9466 | 1828.9626 | 1874.9681 | 1857.9415 | 1856.9575 | 1786.9698 | | M | 610.3671 | 609.3719 | | 686.4018 | 669.3752 | |
| 18 | 86.0964 | 1960.0572 | 1943.0307 | 1942.0466 | 1988.0521 | 1971.0256 | 1970.0416 | 1932.0259 | 1946.0416 | I | 497.2831 | 510.3035 | 524.3191 | 555.3613 | 538.3348 | |
| 19 | 70.0651 | 2057.1100 | 2040.0834 | 2039.0994 | 2085.1049 | 2068.0783 | 2067.0943 | 2031.0943 | | P | 400.2303 | 399.2350 | | 442.2772 | 425.2507 | |
| 20 | 86.0964 | 2170.1940 | 2153.1675 | 2152.1835 | 2198.1890 | 2181.1624 | 2180.1784 | 2142.1627 | 2156.1784 | I | 287.1462 | 300.1666 | 314.1823 | 345.2245 | 328.1979 | |
| 21 | 30.0338 | 2227.2155 | 2210.1890 | 2209.2049 | 2255.2104 | 2238.1839 | 2237.1999 | | | G | | | | 232.1404 | 215.1139 | |
| 22 | 129.1135 | | | | | | | | | R | 74.0237 | 73.0284 | | 175.1190 | 158.0924 | |

| Seq | ya | yb | Seq | ya | yb | Seq | ya | yb |
|---|---|---|---|---|---|---|---|---|
| SV | 159.1128 | 187.1077 | SVY | 322.1761 | 350.1710 | SVYE | 451.2187 | 479.2136 |
| SVYEP | 548.2715 | 576.2664 | SVYEPL | 661.3556 | 689.3505 | VY | 235.1441 | 263.1390 |
| VYE | 364.1867 | 392.1816 | VYEP | 461.2395 | 489.2344 | VYEPL | 574.3235 | 602.3184 |
| YE | 265.1183 | 293.1132 | YEP | 362.1710 | 390.1660 | YEPL | 475.2551 | 503.2500 |
| YEPLQ | 603.3137 | 631.3086 | EP | 199.1077 | 227.1026 | EPL | 312.1918 | 340.1867 |
| EPLQ | 440.2504 | 468.2453 | EPLQT | 541.2980 | 569.2930 | EPLQTG | 598.3195 | 626.3144 |
| PL | 183.1492 | 211.1441 | PLQ | 311.2078 | 339.2027 | PLQT | 412.2554 | 440.2504 |
| PLQTG | 469.2769 | 497.2718 | PLQTGL | 582.3610 | 610.3559 | PLQTGLI | 695.4450 | 723.4400 |
| LQ | 214.1550 | 242.1499 | LQT | 315.2027 | 343.1976 | LQTG | 372.2241 | 400.2191 |
| LQTGL | 485.3082 | 513.3031 | LQTGLI | 598.3923 | 626.3872 | LQTGLIA | 669.4294 | 697.4243 |
| QT | 202.1186 | 230.1135 | QTG | 259.1401 | 287.1350 | QTGL | 372.2241 | 400.2191 |
| QTGLI | 485.3082 | 513.3031 | QTGLIA | 556.3453 | 584.3402 | QTGLIAI | 669.4294 | 697.4243 |
| TG | 131.0815 | 159.0764 | TGL | 244.1656 | 272.1605 | TGLI | 357.2496 | 385.2445 |
| TGLIA | 428.2867 | 456.2817 | TGLIAI | 541.3708 | 569.3657 | TGLIAID | 656.3978 | 684.3927 |
| GL | 143.1179 | 171.1128 | GLI | 256.2020 | 284.1969 | GLIA | 327.2391 | 355.2340 |
| GLIAI | 440.3231 | 468.3180 | GLIAID | 555.3501 | 583.3450 | GLIAIDS | 642.3821 | 670.3770 |
| LI | 199.1805 | 227.1754 | LIA | 270.2176 | 298.2125 | LIAI | 383.3017 | 411.2966 |
| LIAID | 498.3286 | 526.3235 | LIAIDS | 585.3606 | 613.3556 | IA | 157.1335 | 185.1285 |
| IAI | 270.2176 | 298.2125 | IAID | 385.2445 | 413.2395 | IAIDS | 472.2766 | 500.2715 |
| IAIDSM | 603.3171 | 631.3120 | AI | 157.1335 | 185.1285 | AID | 272.1605 | 300.1554 |
| AIDS | 359.1925 | 387.1874 | AIDSM | 490.2330 | 518.2279 | AIDSMI | 603.3171 | 631.3120 |
| ID | 201.1234 | 229.1183 | IDS | 288.1554 | 316.1503 | IDSM | 419.1959 | 447.1908 |
| IDSMI | 532.2799 | 560.2749 | IDSMIP | 629.3327 | 657.3276 | DS | 175.0713 | 203.0662 |
| DSM | 306.1118 | 334.1067 | DSMI | 419.1959 | 447.1908 | DSMIP | 516.2486 | 544.2436 |
| DSMIPI | 629.3327 | 657.3276 | DSMIPIG | 686.3542 | 714.3491 | SM | 191.0849 | 219.0798 |
| SMI | 304.1689 | 332.1639 | SMIP | 401.2217 | 429.2166 | SMIPI | 514.3058 | 542.3007 |
| SMIPIG | 571.3272 | 599.3221 | MI | 217.1369 | 245.1318 | MIP | 314.1897 | 342.1846 |
| MIPI | 427.2737 | 455.2687 | MIPIG | 484.2952 | 512.2901 | IP | 183.1492 | 211.1441 |
| IPI | 296.2333 | 324.2282 | IPIG | 353.2547 | 381.2496 | PI | 183.1492 | 211.1441 |
| PIG | 240.1707 | 268.1656 | IG | 143.1179 | 171.1128 | | | |

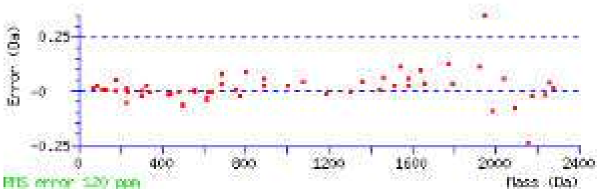


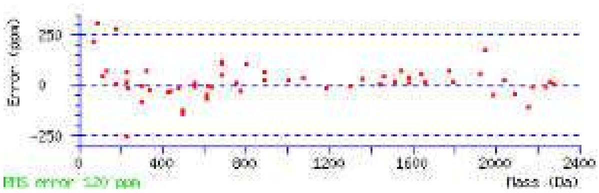


NCBI **BLAST** search of RSVYEPLQTGLIAIDSMIPIGR
(Parameters: blastp, nr protein database, expect=20000, no filter, PAM30)
Other BLAST web gateways

**All matches to this query**

| Score | Mr(calc) | Delta | Sequence |
|---|---|---|---|
| 100.4 | 2428.3148 | 0.1386 | RSVYEPIQTGLIAIDSMIPIGR |
| 100.4 | 2428.3148 | 0.1386 | RSVYEPLQTGIIAIDSMIPIGR |
| 100.4 | 2428.3148 | 0.1386 | RSVYEPLQTGLIAIDSMIPIGR |
| 100.4 | 2428.3148 | 0.1386 | RSVYEPLQTGLIAIDSMIPIGR |
| 88.6 | 2428.3148 | 0.1386 | RSVYEPLQTGIIAIDAMIPIGR |
| 88.6 | 2428.3148 | 0.1386 | RSVYEPLQTGLIAIDAMIPIGR |
| 65.3 | 2428.3148 | 0.1386 | RSVYEPLQTGILSIDAMIPIGR |
| 44.8 | 2428.1257 | 0.3278 | MTYYTPNYETKDTDILAAFR |
| 34.5 | 2429.1097 | -0.6563 | MTYYTPDYETKDTDILAAFR |
| 34.5 | 2429.1097 | -0.6563 | MTYYTPDYETKDTDILAAFR |

**Mascot:** http://www.matrixscience.com/

# Mascot Search Results

## Peptide View Spot no 101

MS/MS Fragmentation of **FLEYLDKDR**
Found in **gi|75114857** in **NCBInr**, RecName: Full=ATP-dependent zinc metalloprotease FTSH 2, chloroplastic; Short=OsFTSH2; Flags: Precursor

Match to Query 16: 1197.624024 from(1198.631300,1+) intensity(0.0000) index(6)
Title: Label: H1, Spot_Id: 219681, Peak_List_Id: 224997, MSMS Job_Run_Id: 21760, Comment:
Data file C:\Documents and Settings\Administrator\Desktop\farah BHU\spot set plate\Farah-Garima 11May 2013\ppw_H1_136842035700.txt

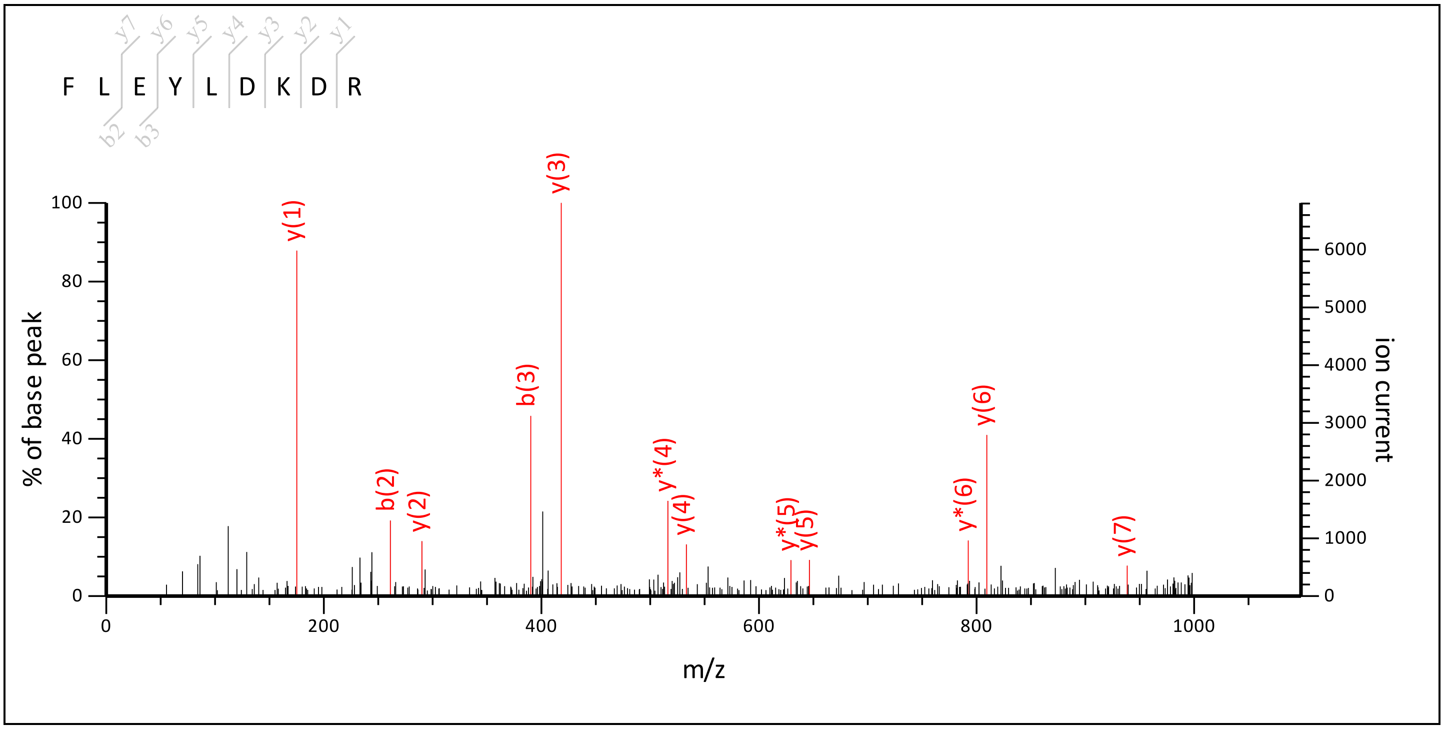


0 to 1098.23

Label all possible matches ○ Label matches used for scoring ◉

**Monoisotopic mass of neutral peptide Mr(calc):** 1197.6030
**Fixed modifications:** Carbamidomethyl (C) (apply to specified residues or termini only)
**Ions Score:** 50 **Expect:** 0.022
**Matches :** 12/36 fragment ions using 20 most intense peaks (help)

| # | a | a* | b | b* | Seq. | y | y* | # |
|---|---|---|---|---|---|---|---|---|
| 1 | 120.0808 | | 148.0757 | | F | | | 9 |
| 2 | 233.1648 | | **261.1598** | | L | 1051.5419 | 1034.5153 | 8 |
| 3 | 362.2074 | | **390.2023** | | E | ***938.4578*** | 921.4312 | 7 |
| 4 | 525.2708 | | 553.2657 | | Y | ***809.4152*** | 792.3886 | 6 |
| 5 | 638.3548 | | 666.3497 | | L | ***646.3519*** | 629.3253 | 5 |
| 6 | 753.3818 | | 781.3767 | | D | ***533.2678*** | 516.2413 | 4 |
| 7 | 881.4767 | 864.4502 | 909.4716 | 892.4451 | K | ***418.2409*** | 401.2143 | 3 |

| | | | | | | | | |
|---|---|---|---|---|---|---|---|---|
| 8 | 996.5037 | 979.4771 | 1024.4986 | 1007.4720 | D | *290.1459* | 273.1193 | 2 |
| 9 | | | | | R | *175.1190* | 158.0924 | 1 |

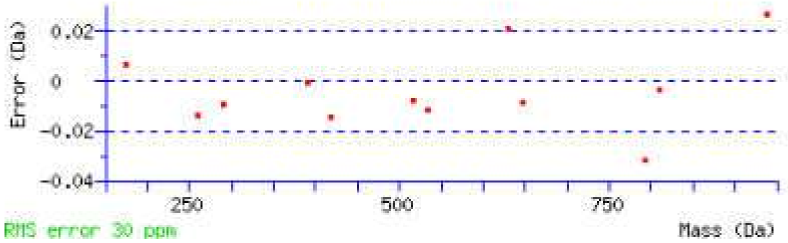


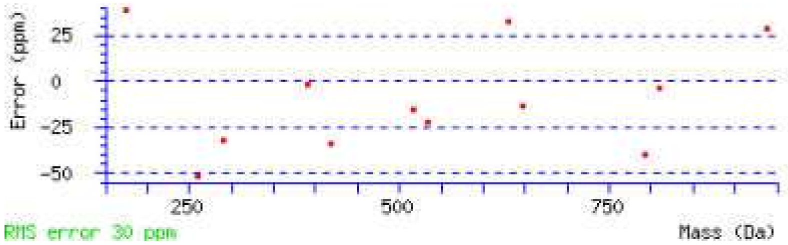


NCBI **BLAST** search of FLEYLDKDR
(Parameters: blastp, nr protein database, expect=20000, no filter, PAM30)
Other BLAST web gateways

**All matches to this query**

| Score | Mr(calc) | Delta | Sequence |
|---|---|---|---|
| 50.1 | 1197.6030 | 0.0211 | FLEYLDKDR |
| 22.8 | 1197.6030 | 0.0211 | LFELYKDDR |
| 22.0 | 1197.5302 | 0.0938 | IFEDFEAGDR |
| 22.0 | 1197.5302 | 0.0938 | LFEDFEAGDR |
| 20.2 | 1197.6030 | 0.0211 | LEFIYDAATR |
| 18.2 | 1197.5818 | 0.0422 | LFEYGGYPPR |
| 18.2 | 1197.5448 | 0.0792 | MEEYSRDLR |
| 17.6 | 1197.5812 | 0.0428 | MLASNLYTNR |
| 13.6 | 1197.5886 | 0.0354 | MLEIYVCVR |
| 13.5 | 1197.6104 | 0.0137 | MIEYLVTWK |

**Mascot:** http://www.matrixscience.com/

# MATRIX SCIENCE Mascot Search Results

## Peptide View Spot no 101

MS/MS Fragmentation of **LSDEAYEIALSQIR**
Found in **gi|75114857** in **NCBInr**, RecName: Full=ATP-dependent zinc metalloprotease FTSH 2, chloroplastic; Short=OsFTSH2; Flags: Precursor

Match to Query 20: 1606.863224 from(1607.870500,1+) intensity(0.0000) index(8)
Title: Label: H1, Spot_Id: 219681, Peak_List_Id: 225000, MSMS Job_Run_Id: 21760, Comment:
Data file C:\Documents and Settings\Administrator\Desktop\farah BHU\spot set plate\Farah-Garima 11May 2013\ppw_H1_136842035700.txt

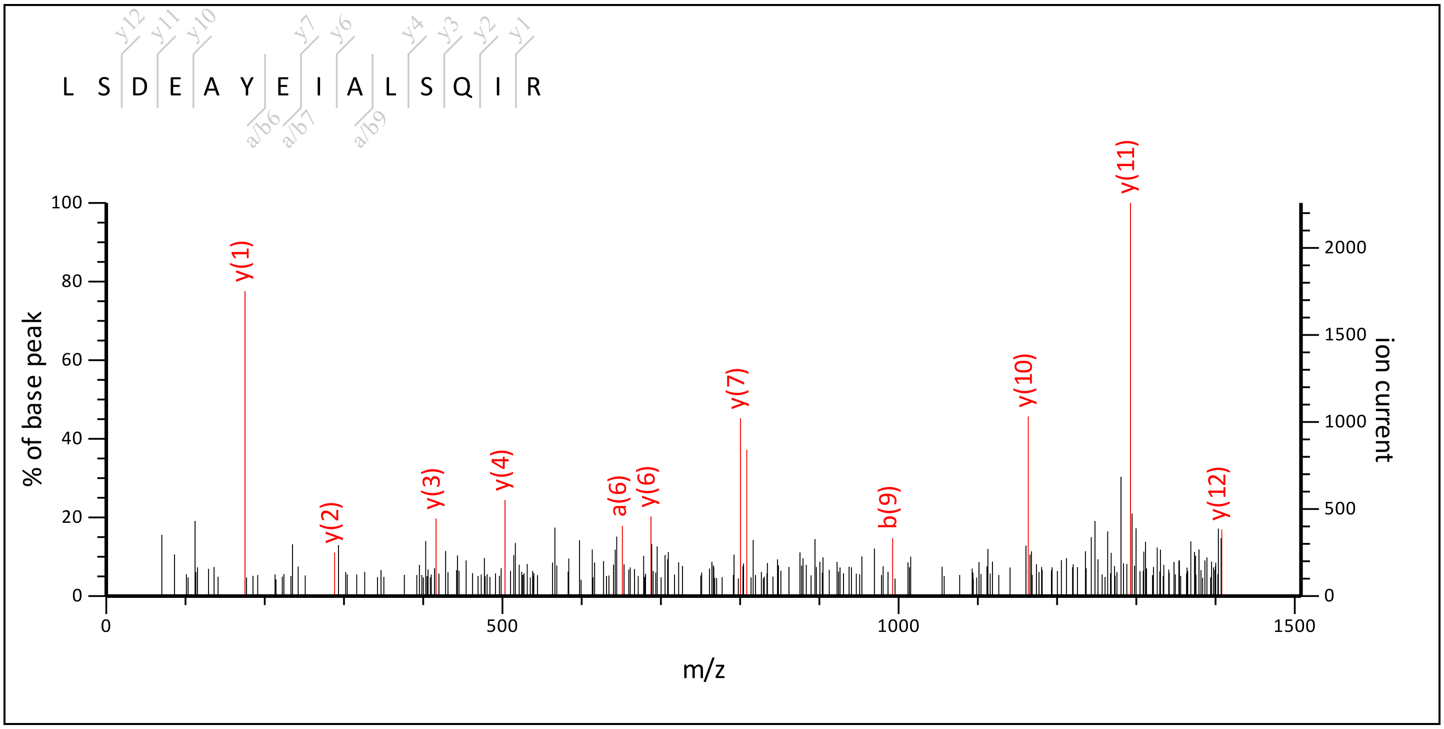


0 to 1507.84

Label all possible matches ○ Label matches used for scoring ◉

```
Monoisotopic mass of neutral peptide Mr(calc): 1606.8202
Fixed modifications: Carbamidomethyl (C) (apply to specified residues or termini only)
Ions Score: 48   Expect: 0.028
Matches : 14/56 fragment ions using 28 most intense peaks   (help)
```

| # | a | a* | b | b* | Seq. | y | y* | # |
|---|---|---|---|---|---|---|---|---|
| 1 | 86.0964 | | 114.0913 | | L | | | 14 |
| 2 | 173.1285 | | 201.1234 | | S | 1494.7435 | 1477.7169 | 13 |
| 3 | **288.1554** | | 316.1503 | | D | ***1407.7114*** | 1390.6849 | 12 |
| 4 | 417.1980 | | 445.1929 | | E | ***1292.6845*** | 1275.6579 | 11 |
| 5 | 488.2351 | | 516.2300 | | A | ***1163.6419*** | 1146.6154 | 10 |
| 6 | **651.2984** | | 679.2933 | | Y | 1092.6048 | 1075.5782 | 9 |
| 7 | 780.3410 | | **808.3359** | | E | 929.5415 | 912.5149 | 8 |

| | | | | | | | | |
|---|---|---|---|---|---|---|---|---|
| **8** | 893.4251 | | 921.4200 | | **I** | ***800.4989*** | 783.4723 | **7** |
| **9** | 964.4622 | | **992.4571** | | **A** | ***687.4148*** | 670.3883 | **6** |
| **10** | 1077.5463 | | 1105.5412 | | **L** | 616.3777 | 599.3511 | **5** |
| **11** | 1164.5783 | | 1192.5732 | | **S** | ***503.2936*** | 486.2671 | **4** |
| **12** | **1292.6369** | 1275.6103 | 1320.6318 | 1303.6052 | **Q** | ***416.2616*** | 399.2350 | **3** |
| **13** | 1405.7209 | 1388.6944 | 1433.7159 | 1416.6893 | **I** | ***288.2030*** | 271.1765 | **2** |
| **14** | | | | | **R** | ***175.1190*** | 158.0924 | **1** |

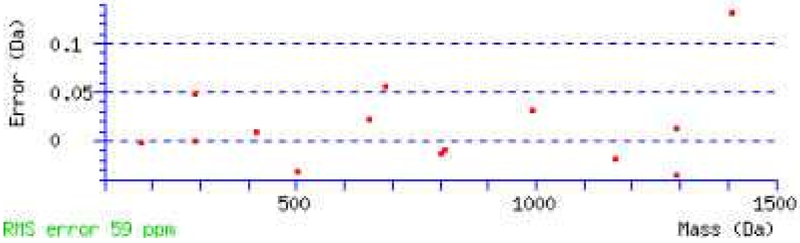


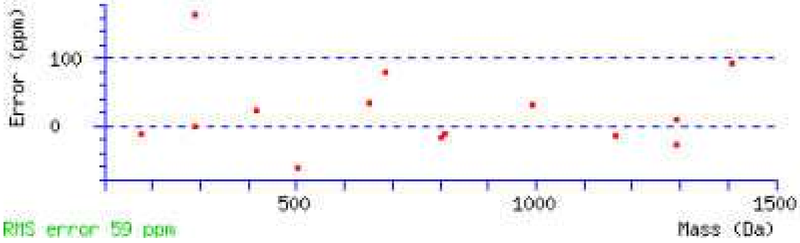


NCBI **BLAST** search of LSDEAYEIALSQIR
(Parameters: blastp, nr protein database, expect=20000, no filter, PAM30)
Other BLAST web gateways

**All matches to this query**

| Score | Mr(calc) | Delta | Sequence |
|---|---|---|---|
| 47.9 | 1606.8202 | 0.0430 | LSDEAYEIALSQIR |
| 20.5 | 1606.8177 | 0.0455 | SMAYYVINYSKLR |
| 11.2 | 1606.8865 | -0.0232 | MAFLLNNTALSKLR |
| 9.3 | 1606.8539 | 0.0093 | QAVADHANNLASKIR |
| 8.2 | 1606.9671 | -0.1039 | LVDIVVFLHRQIR |
| 8.0 | 1606.8250 | 0.0383 | LSDRGYVMVNGQIR |
| 7.9 | 1606.8791 | -0.0158 | EAGDASAILPPRAIAR |
| 7.8 | 1606.7813 | 0.0819 | ISDYMYRFLDLR |
| 7.2 | 1606.7621 | 0.1012 | LSDDGAVETRMELR |
| 6.7 | 1606.9195 | -0.0563 | LLEFPSLIAQHALR |

**Mascot:** http://www.matrixscience.com/

# Mascot Search Results

## Peptide View Spot no 101

MS/MS Fragmentation of **AQGGLGGPNGPGFPLGFGQSR**
Found in **gi|75114857** in **NCBInr**, RecName: Full=ATP-dependent zinc metalloprotease FTSH 2, chloroplastic; Short=OsFTSH2; Flags: Precursor

Match to Query 22: 1970.016724 from(1971.024000,1+) intensity(0.0000) index(9)
Title: Label: H1, Spot_Id: 219681, Peak_List_Id: 224998, MSMS Job_Run_Id: 21760, Comment:
Data file C:\Documents and Settings\Administrator\Desktop\farah BHU\spot set plate\Farah-Garima 11May 2013\ppw_H1_136842035700.txt

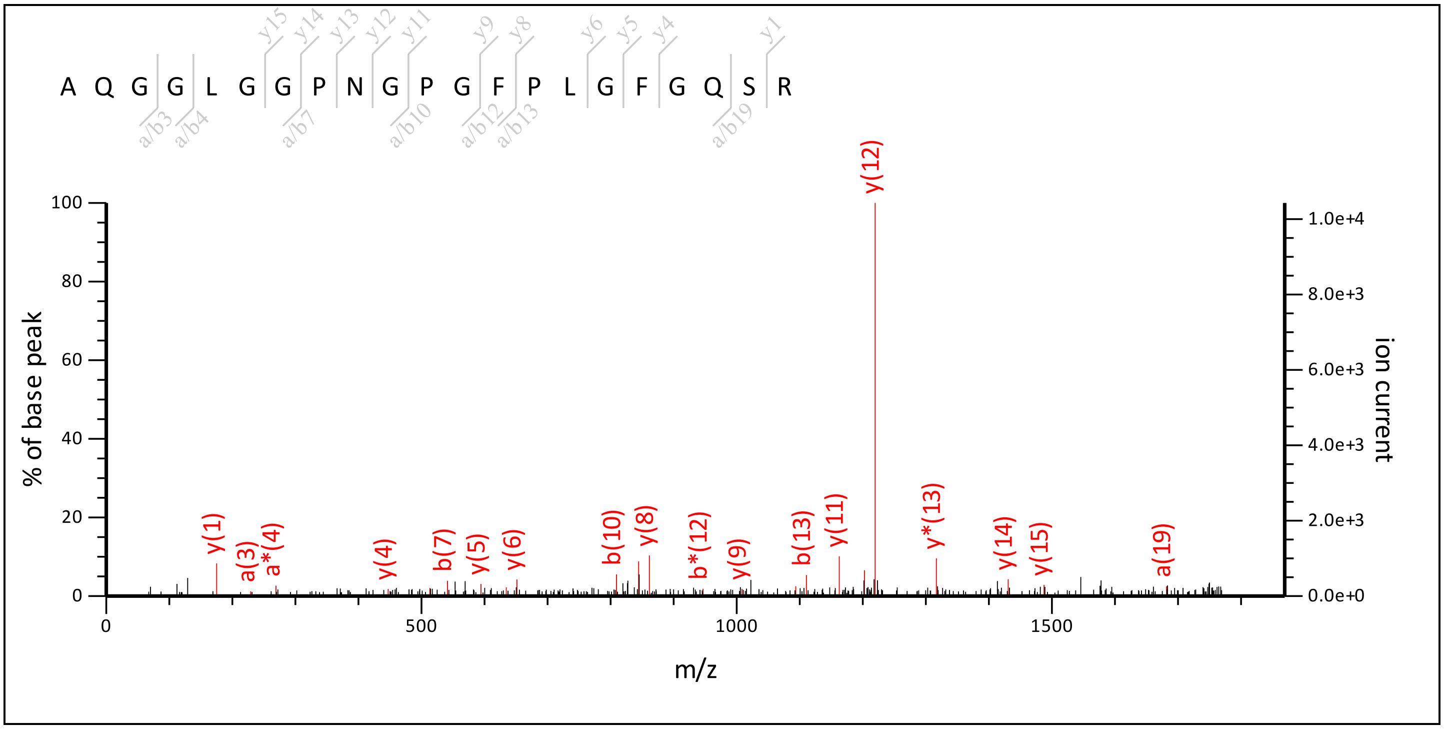


0 to 1869.23

Label all possible matches ○ Label matches used for scoring ◉

**Monoisotopic mass of neutral peptide Mr(calc):** 1969.9759
**Fixed modifications:** Carbamidomethyl (C) (apply to specified residues or termini only)
**Ions Score:** 22 **Expect:** 9.4
**Matches :** 25/118 fragment ions using 87 most intense peaks (help)

| # | a | a* | b | b* | Seq. | y | y* | # |
|---|---|---|---|---|---|---|---|---|
| 1 | 44.0495 | | 72.0444 | | A | | | 21 |
| 2 | 172.1081 | 155.0815 | 200.1030 | 183.0764 | Q | 1899.9460 | 1882.9195 | 20 |
| 3 | **229.1295** | 212.1030 | 257.1244 | 240.0979 | G | 1771.8874 | 1754.8609 | 19 |
| 4 | 286.1510 | 269.1244 | 314.1459 | 297.1193 | G | 1714.8660 | 1697.8394 | 18 |
| 5 | 399.2350 | 382.2085 | 427.2300 | 410.2034 | L | 1657.8445 | 1640.8180 | 17 |
| 6 | 456.2565 | 439.2300 | 484.2514 | 467.2249 | G | 1544.7605 | 1527.7339 | 16 |
| 7 | **513.2780** | 496.2514 | **541.2729** | 524.2463 | G | ***1487.7390*** | 1470.7124 | 15 |

| | | | | | | | | |
|---|---|---|---|---|---|---|---|---|
| 8 | 610.3307 | 593.3042 | 638.3257 | 621.2991 | P | ***1430.7175*** | 1413.6910 | 14 |
| 9 | 724.3737 | 707.3471 | 752.3686 | 735.3420 | N | 1333.6648 | 1316.6382 | 13 |
| 10 | 781.3951 | 764.3686 | **809.3900** | 792.3635 | G | ***1219.6218*** | 1202.5953 | 12 |
| 11 | 878.4479 | 861.4213 | 906.4428 | 889.4163 | P | ***1162.6004*** | 1145.5738 | 11 |
| 12 | 935.4694 | 918.4428 | 963.4643 | 946.4377 | G | 1065.5476 | 1048.5211 | 10 |
| 13 | 1082.5378 | 1065.5112 | **1110.5327** | 1093.5061 | F | ***1008.5261*** | 991.4996 | 9 |
| 14 | 1179.5905 | 1162.5640 | 1207.5854 | 1190.5589 | P | ***861.4577*** | 844.4312 | 8 |
| 15 | 1292.6746 | 1275.6480 | 1320.6695 | 1303.6430 | L | 764.4050 | 747.3784 | 7 |
| 16 | 1349.6961 | 1332.6695 | 1377.6910 | 1360.6644 | G | ***651.3209*** | 634.2944 | 6 |
| 17 | 1496.7645 | 1479.7379 | 1524.7594 | 1507.7328 | F | ***594.2994*** | 577.2729 | 5 |
| 18 | 1553.7859 | 1536.7594 | 1581.7809 | 1564.7543 | G | ***447.2310*** | 430.2045 | 4 |
| 19 | **1681.8445** | 1664.8180 | 1709.8394 | 1692.8129 | Q | 390.2096 | 373.1830 | 3 |
| 20 | 1768.8765 | 1751.8500 | 1796.8715 | 1779.8449 | S | 262.1510 | 245.1244 | 2 |
| 21 | | | | | R | ***175.1190*** | 158.0924 | 1 |

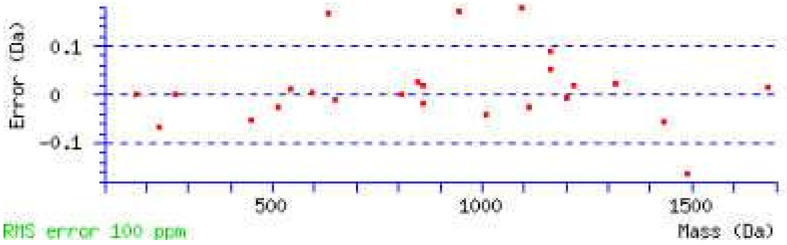


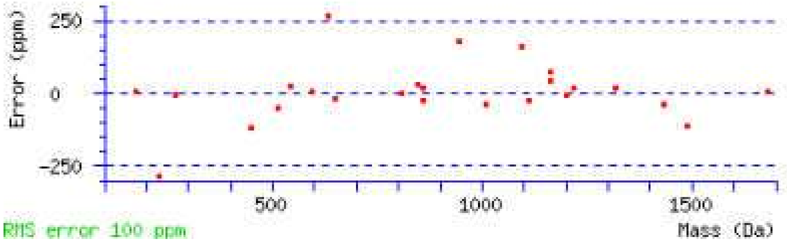


NCBI **BLAST** search of AQGGLGGPNGPGFPLGFGQSR
(Parameters: blastp, nr protein database, expect=20000, no filter, PAM30)
Other BLAST web gateways

**All matches to this query**

| Score | Mr(calc) | Delta | Sequence |
|---|---|---|---|
| 22.4 | 1969.9759 | 0.0408 | AQGGLGGPNGPGFPLGFGQSR |
| 4.7 | 1970.1676 | -0.1509 | KILALPTTRPQTLSLYR |
| 4.7 | 1970.0044 | 0.0123 | LSYLGPGGLTARTASFGMR |
| 4.5 | 1969.9350 | 0.0818 | RLGMGTNMYPSSSLLGDR |
| 4.5 | 1969.9350 | 0.0818 | RLGMGTNMYPSSSLLGDR |
| 4.5 | 1970.0334 | -0.0167 | GANPNVVIGAIVGGPDHLDR |
| 4.3 | 1969.9455 | 0.0712 | MIGTDAFQETPIVEFTR |
| 4.1 | 1969.9970 | 0.0197 | QIGGQTHQVAAKVDEYAR |
| 1.2 | 1969.8622 | 0.1545 | EVMVYGGGGGAMEREGAER |

| | | | |
|---|---|---|---|
| 0.9 | 1969.9567 | 0.0600 | IELPADLGNEWPSMDKR |

**Mascot:** http://www.matrixscience.com/

# MATRIX SCIENCE Mascot Search Results

## Peptide View **Spot no 102**

MS/MS Fragmentation of **SKFESLVNSLIER**
Found in **gi|115448989** in **NCBInr**, Os02g0774300 [Oryza sativa Japonica Group]

Match to Query 7: 1520.957424 from(1521.964700,1+) intensity(0.0000) index(2)
Title: Label: J5, Spot_Id: 228915, Peak_List_Id: 257482, MSMS Job_Run_Id: 24918, Comment:
Data file ppw_J5_138985127800.txt

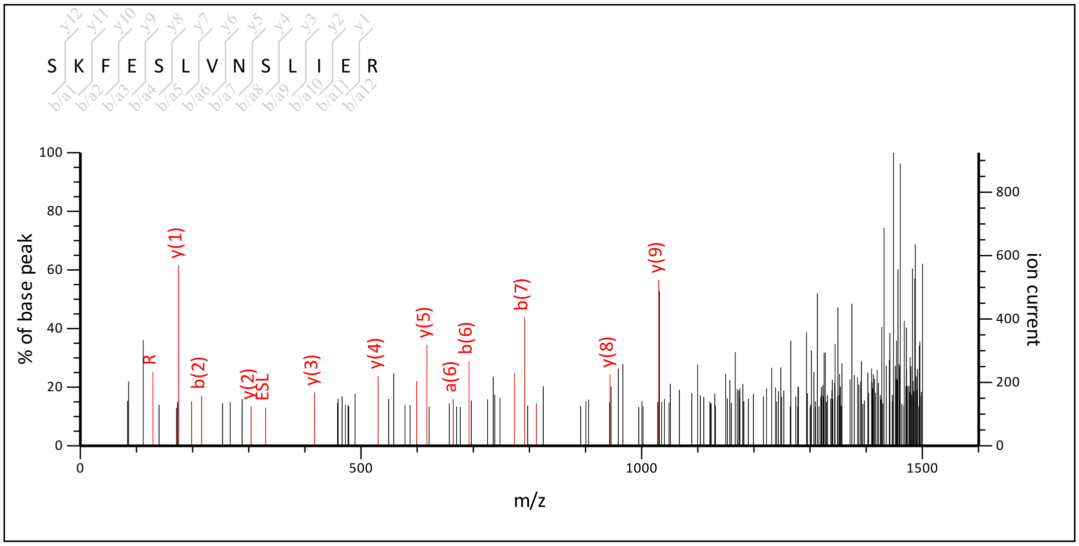


0 to 1600.43

Label all possible matches Label matches used for scoring

**Monoisotopic mass of neutral peptide Mr(calc):** 1520.8198
**Fixed modifications:** Carbamidomethyl (C) (apply to specified residues or termini only)
**Ions Score:** 23 **Expect:** 3
**Matches :** 17/234 fragment ions using 45 most intense peaks (help)

| # | Immon. | a | a* | $a^0$ | b | b* | $b^0$ | d | d' | Seq. | v | w | w' | y | y* | |
|---|---|---|---|---|---|---|---|---|---|---|---|---|---|---|---|---|
| 1 | 60.0444 | 60.0444 | | 42.0338 | 88.0393 | | 70.0287 | 44.0495 | | S | | | | | | |
| 2 | 101.1073 | 188.1394 | 171.1128 | 170.1288 | ***216.1343*** | 199.1077 | 198.1237 | 131.0815 | | K | 1361.7060 | 1360.7107 | | 1434.7951 | 1417.7686 | 14[illegible] |
| 3 | 120.0808 | 335.2078 | 318.1812 | 317.1972 | 363.2027 | 346.1761 | 345.1921 | | | F | 1214.6375 | | | 1306.7001 | 1289.6736 | 128[illegible] |
| 4 | 102.0550 | 464.2504 | 447.2238 | 446.2398 | 492.2453 | 475.2187 | 474.2347 | 406.2449 | | E | 1085.5949 | 1084.5997 | | 1159.6317 | 1142.6052 | 114[illegible] |
| 5 | 60.0444 | 551.2824 | 534.2558 | 533.2718 | 579.2773 | 562.2508 | 561.2667 | 535.2875 | | S | 998.5629 | 997.5677 | | ***1030.5891*** | 1013.5626 | 101[illegible] |
| 6 | 86.0964 | 664.3665 | 647.3399 | 646.3559 | ***692.3614*** | 675.3348 | 674.3508 | 622.3195 | | L | 885.4789 | 884.4836 | | ***943.5571*** | 926.5306 | 92[illegible] |
| 7 | 72.0808 | 763.4349 | 746.4083 | 745.4243 | ***791.4298*** | 774.4032 | 773.4192 | 749.4192 | | V | 786.4104 | 799.4308 | | 830.4730 | 813.4465 | 8[illegible] |
| 8 | 87.0553 | 877.4778 | 860.4512 | 859.4672 | 905.4727 | 888.4462 | 887.4621 | 834.4720 | | N | 672.3675 | 671.3723 | | 731.4046 | 714.3781 | 7[illegible] |
| 9 | 60.0444 | 964.5098 | 947.4833 | 946.4993 | 992.5047 | 975.4782 | 974.4942 | 948.5149 | | S | 585.3355 | 584.3402 | | ***617.3617*** | 600.3352 | 59[illegible] |
| 10 | 86.0964 | 1077.5939 | 1060.5673 | 1059.5833 | 1105.5888 | 1088.5622 | 1087.5782 | 1035.5469 | | L | 472.2514 | 471.2562 | | ***530.3297*** | 513.3031 | 51[illegible] |
| 11 | 86.0964 | 1190.6779 | 1173.6514 | 1172.6674 | 1218.6729 | 1201.6463 | 1200.6623 | 1162.6466 | 1176.6623 | I | 359.1674 | 372.1878 | 386.2034 | ***417.2456*** | 400.2191 | 39[illegible] |
| 12 | 102.0550 | 1319.7205 | 1302.6940 | 1301.7100 | 1347.7155 | 1330.6889 | 1329.7049 | 1261.7151 | | E | 230.1248 | 229.1295 | | ***304.1615*** | 287.1350 | 28[illegible] |
| 13 | 129.1135 | | | | | | | | | R | 74.0237 | 73.0284 | | ***175.1190*** | 158.0924 | |

| Seq | ya | yb | Seq | ya | yb | Seq | ya | yb |
|---|---|---|---|---|---|---|---|---|
| KF | 248.1757 | 276.1707 | KFE | 377.2183 | 405.2132 | KFES | 464.2504 | 492.2453 |
| KFESL | 577.3344 | 605.3293 | KFESLV | 676.4028 | 704.3978 | FE | 249.1234 | 277.1183 |
| FES | 336.1554 | 364.1503 | FESL | 449.2395 | 477.2344 | FESLV | 548.3079 | 576.3028 |
| FESLVN | 662.3508 | 690.3457 | ES | 189.0870 | 217.0819 | ESL | 302.1710 | 330.1660 |
| ESLV | 401.2395 | 429.2344 | ESLVN | 515.2824 | 543.2773 | ESLVNS | 602.3144 | 630.3093 |
| SL | 173.1285 | 201.1234 | SLV | 272.1969 | 300.1918 | SLVN | 386.2398 | 414.2347 |
| SLVNS | 473.2718 | 501.2667 | SLVNSL | 586.3559 | 614.3508 | SLVNSLI | 699.4399 | 727.4349 |

| | | | | | | | | |
|---|---|---|---|---|---|---|---|---|
| **LV** | 185.1648 | 213.1598 | **LVN** | 299.2078 | 327.2027 | **LVNS** | 386.2398 | 414.2347 |
| **LVNSL** | 499.3239 | 527.3188 | **LVNSLI** | 612.4079 | 640.4028 | **VN** | 186.1237 | 214.1186 |
| **VNS** | 273.1557 | 301.1506 | **VNSL** | 386.2398 | 414.2347 | **VNSLI** | 499.3239 | 527.3188 |
| **VNSLIE** | 628.3665 | 656.3614 | **NS** | 174.0873 | 202.0822 | **NSL** | 287.1714 | 315.1663 |
| **NSLI** | 400.2554 | 428.2504 | **NSLIE** | 529.2980 | 557.2930 | **SL** | 173.1285 | 201.1234 |
| **SLI** | 286.2125 | 314.2074 | **SLIE** | 415.2551 | 443.2500 | **LI** | 199.1805 | 227.1754 |
| **LIE** | 328.2231 | 356.2180 | **IE** | 215.1390 | 243.1339 | | | |

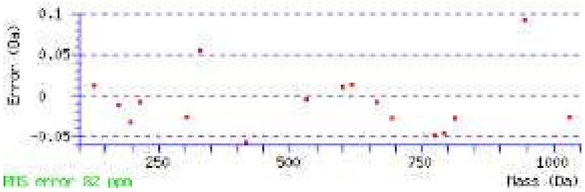


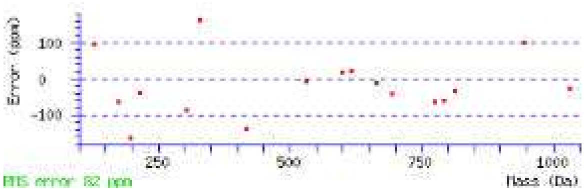


NCBI **BLAST** search of SKFESLVNSLIER
(Parameters: blastp, nr protein database, expect=20000, no filter, PAM30)
Other BLAST web gateways

**All matches to this query**

| Score | Mr(calc) | Delta | Sequence |
|---|---|---|---|
| 23.2 | 1520.8198 | 0.1376 | SKFESLVNSLIER |
| 17.6 | 1520.8674 | 0.0900 | KQVSLEDAIIHIR |
| 15.0 | 1520.8198 | 0.1376 | SKFESLVSNLIER |
| 13.4 | 1520.8463 | 0.1111 | AGQVAKAFGAYGILR |
| 9.2 | 1520.8311 | 0.1264 | QRTLATAYSLIER |
| 7.7 | 1520.8423 | 0.1151 | QLNLARSAGPPELR |
| 6.5 | 1520.8133 | 0.1441 | LAMELSRLSQAFR |
| 5.0 | 1520.8059 | 0.1515 | NIQISHDPSKVQR |
| 4.9 | 1520.8385 | 0.1190 | SPCSSLVVKIYLR |
| 3.9 | 1520.9039 | 0.0536 | GLQGVVPKVLNEIR |

**Mascot:** http://www.matrixscience.com/

# MATRIX SCIENCE Mascot Search Results

## Peptide View **Spot no 102**

MS/MS Fragmentation of **TTPSVVAFNQKGER**
Found in **gi|115448989** in **NCBInr**, Os02g0774300 [Oryza sativa Japonica Group]

Match to Query 9: 1532.939124 from(1533.946400,1+) intensity(0.0000) index(3)
Title: Label: J5, Spot_Id: 228915, Peak_List_Id: 257483, MSMS Job_Run_Id: 24918, Comment:
Data file ppw_J5_138985127800.txt

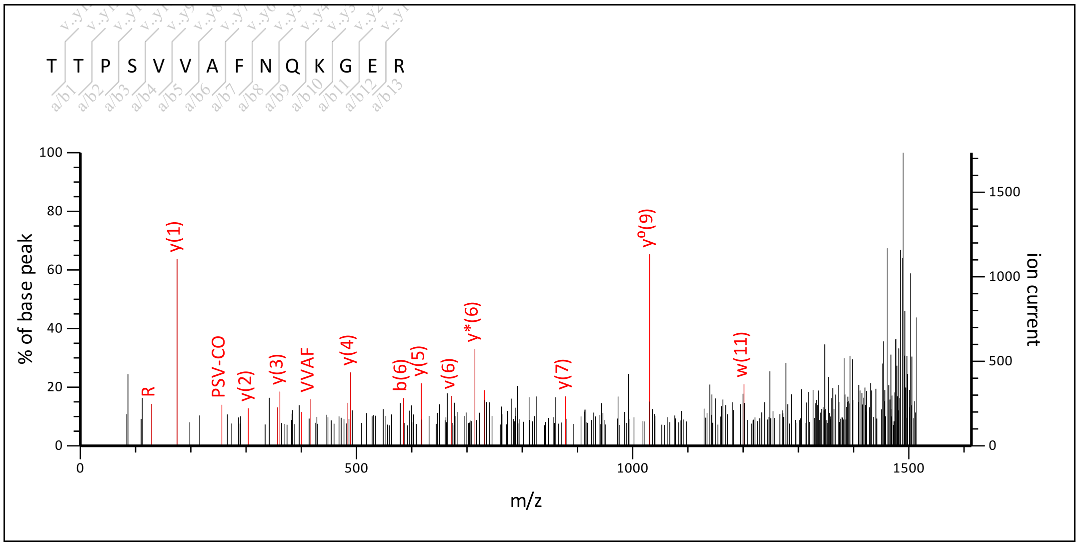


0 to 1613.33

Label all possible matches ○ Label matches used for scoring ◉

**Monoisotopic mass of neutral peptide Mr(calc):** 1532.7947
**Fixed modifications:** Carbamidomethyl (C) (apply to specified residues or termini only)
**Ions Score:** 15 **Expect:** 30
**Matches :** 20/239 fragment ions using 60 most intense peaks (help)

| # | Immon. | a | a* | a⁰ | b | b* | b⁰ | d | d' | Seq. | v | w | w' | y | y* | |
|---|---|---|---|---|---|---|---|---|---|---|---|---|---|---|---|---|
| 1 | 74.0600 | 74.0600 | | 56.0495 | 102.0550 | | 84.0444 | 44.0495 | | T | | | | | | |
| 2 | 74.0600 | 175.1077 | | 157.0972 | 203.1026 | | 185.0921 | 159.1128 | 161.0921 | T | 1386.7124 | 1399.7328 | 1401.7121 | 1432.7543 | 1415.7278 | 14[illegible] |
| 3 | 70.0651 | 272.1605 | | 254.1499 | 300.1554 | | 282.1448 | 246.1448 | | P | 1289.6597 | 1288.6644 | | 1331.7066 | 1314.6801 | 13[illegible] |
| 4 | 60.0444 | 359.1925 | | 341.1819 | 387.1874 | | 369.1769 | 343.1976 | | S | 1202.6276 | 1201.6324 | | 1234.6539 | 1217.6273 | 12[illegible] |
| 5 | 72.0808 | 458.2609 | | 440.2504 | 486.2558 | | 468.2453 | 444.2453 | | V | 1103.5592 | 1116.5796 | | 1147.6218 | 1130.5953 | 11[illegible] |
| 6 | 72.0808 | 557.3293 | | 539.3188 | 585.3243 | | 567.3137 | 543.3137 | | V | 1004.4908 | 1017.5112 | | 1048.5534 | 1031.5269 | 10[illegible] |
| 7 | 44.0495 | 628.3665 | | 610.3559 | 656.3614 | | 638.3508 | | | A | 933.4537 | | | 949.4850 | 932.4585 | 9[illegible] |
| 8 | 120.0808 | 775.4349 | | 757.4243 | 803.4298 | | 785.4192 | | | F | 786.3853 | | | ***878.4479*** | 861.4213 | 8[illegible] |
| 9 | 87.0553 | 889.4778 | 872.4512 | 871.4672 | 917.4727 | 900.4462 | 899.4621 | 846.4720 | | N | 672.3424 | 671.3471 | | ***731.3795*** | 714.3529 | 7[illegible] |
| 10 | 101.0709 | 1017.5364 | 1000.5098 | 999.5258 | 1045.5313 | 1028.5047 | 1027.5207 | 960.5149 | | Q | 544.2838 | 543.2885 | | ***617.3366*** | 600.3100 | 5[illegible] |
| 11 | 101.1073 | 1145.6313 | 1128.6048 | 1127.6208 | 1173.6263 | 1156.5997 | 1155.6157 | 1088.5735 | | K | 416.1888 | 415.1936 | | ***489.2780*** | 472.2514 | 4[illegible] |
| 12 | 30.0338 | 1202.6528 | 1185.6263 | 1184.6422 | 1230.6477 | 1213.6212 | 1212.6371 | | | G | | | | ***361.1830*** | 344.1565 | 3[illegible] |
| 13 | 102.0550 | 1331.6954 | 1314.6688 | 1313.6848 | 1359.6903 | 1342.6638 | 1341.6797 | 1273.6899 | | E | 230.1248 | 229.1295 | | ***304.1615*** | 287.1350 | 2[illegible] |
| 14 | 129.1135 | | | | | | | | | R | 74.0237 | 73.0284 | | ***175.1190*** | 158.0924 | |

| Seq | ya | yb | Seq | ya | yb | Seq | ya | yb |
|---|---|---|---|---|---|---|---|---|
| **TP** | 171.1128 | 199.1077 | **TPS** | 258.1448 | 286.1397 | **TPSV** | 357.2132 | 385.2082 |
| **TPSVV** | 456.2817 | 484.2766 | **TPSVVA** | 527.3188 | 555.3137 | **TPSVVAF** | 674.3872 | 702.3821 |
| **PS** | 157.0972 | 185.0921 | **PSV** | 256.1656 | 284.1605 | **PSVV** | 355.2340 | 383.2289 |
| **PSVVA** | 426.2711 | 454.2660 | **PSVVAF** | 573.3395 | 601.3344 | **PSVVAFN** | 687.3824 | 715.3774 |
| **SV** | 159.1128 | 187.1077 | **SVV** | 258.1812 | 286.1761 | **SVVA** | 329.2183 | 357.2132 |
| **SVVAF** | 476.2867 | 504.2817 | **SVVAFN** | 590.3297 | 618.3246 | **VV** | 171.1492 | 199.1441 |

| | | | | | | | | |
|---|---|---|---|---|---|---|---|---|
| **VVA** | 242.1863 | 270.1812 | **VVAF** | 389.2547 | 417.2496 | **VVAFN** | 503.2976 | 531.2926 |
| **VVAFNQ** | 631.3562 | 659.3511 | **VA** | 143.1179 | 171.1128 | **VAF** | 290.1863 | 318.1812 |
| **VAFN** | 404.2292 | 432.2241 | **VAFNQ** | 532.2878 | 560.2827 | **VAFNQK** | 660.3828 | 688.3777 |
| **AF** | 191.1179 | 219.1128 | **AFN** | 305.1608 | 333.1557 | **AFNQ** | 433.2194 | 461.2143 |
| **AFNQK** | 561.3144 | 589.3093 | **AFNQKG** | 618.3358 | 646.3307 | **FN** | 234.1237 | 262.1186 |
| **FNQ** | 362.1823 | 390.1772 | **FNQK** | 490.2772 | 518.2722 | **FNQKG** | 547.2987 | 575.2936 |
| **FNQKGE** | 676.3413 | 704.3362 | **NQ** | 215.1139 | 243.1088 | **NQK** | 343.2088 | 371.2037 |
| **NQKG** | 400.2303 | 428.2252 | **NQKGE** | 529.2729 | 557.2678 | **QK** | 229.1659 | 257.1608 |
| **QKG** | 286.1874 | 314.1823 | **QKGE** | 415.2300 | 443.2249 | **KG** | 158.1288 | 186.1237 |
| **KGE** | 287.1714 | 315.1663 | **GE** | 159.0764 | 187.0713 | | | |

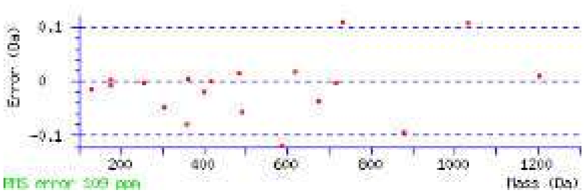


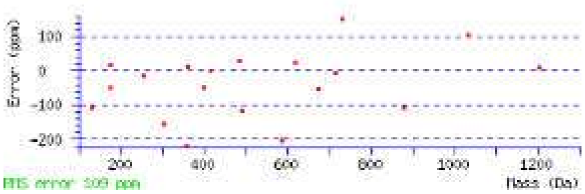


NCBI **BLAST** search of TTPSVVAFNQKGER
(Parameters: blastp, nr protein database, expect=20000, no filter, PAM30)
Other BLAST web gateways

**All matches to this query**

| Score | Mr(calc) | Delta | Sequence |
|---|---|---|---|
| 15.5 | 1532.8423 | 0.0968 | VSINFSRLQNLSR |
| 15.2 | 1532.7947 | 0.1444 | TTPSVVAFNQKGER |
| 8.4 | 1532.7947 | 0.1444 | VSFGLSVEQAAAAQR |
| 8.3 | 1532.7868 | 0.1523 | KAGVELLDMGSLER |
| 7.0 | 1532.8602 | 0.0789 | SVIFPIIFEALER |
| 6.3 | 1532.8351 | 0.1040 | VSLFQDILGLSWR |
| 6.2 | 1532.8306 | 0.1085 | KSMVVADVIPVMTK |
| 5.9 | 1532.8055 | 0.1337 | VSEAVLLMGLCVSR |
| 5.0 | 1532.7981 | 0.1410 | GMSSVLLVNSVGQAR |
| 4.8 | 1532.8133 | 0.1258 | AGGFLRLGVMDLER |

**Mascot:** http://www.matrixscience.com/

# MATRIX SCIENCE Mascot Search Results

## Peptide View **Spot no 102**

MS/MS Fragmentation of **AVITVPAYFNDAQR**
Found in **gi|115448989** in **NCBInr**, Os02g0774300 [Oryza sativa Japonica Group]

Match to Query 11: 1563.948424 from(1564.955700,1+) intensity(0.0000) index(4)
Title: Label: J5, Spot_Id: 228915, Peak_List_Id: 257481, MSMS Job_Run_Id: 24918, Comment:
Data file ppw_J5_138985127800.txt

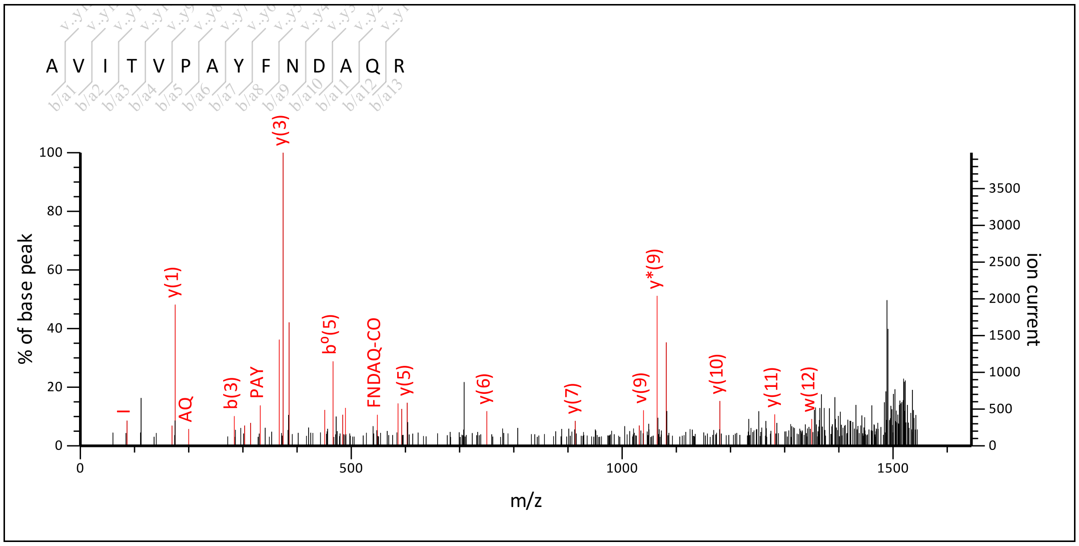


0 to 1644.68

Label all possible matches ○ Label matches used for scoring ◉

**Monoisotopic mass of neutral peptide Mr(calc):** 1563.8045
**Fixed modifications:** Carbamidomethyl (C) (apply to specified residues or termini only)
**Ions Score:** 50 **Expect:** 0.013
**Matches :** 31/226 fragment ions using 60 most intense peaks (help)

| # | Immon. | a | a* | $a^0$ | b | b* | $b^0$ | d | d' | Seq. | v | w | w' | y | y* | |
|---|---|---|---|---|---|---|---|---|---|---|---|---|---|---|---|---|
| 1 | 44.0495 | 44.0495 | | | 72.0444 | | | 44.0495 | | A | | | | | | |
| 2 | 72.0808 | 143.1179 | | | 171.1128 | | | 129.1022 | | V | 1449.7121 | 1462.7325 | | 1493.7747 | 1476.7482 | 14[illegible] |
| 3 | 86.0964 | 256.2020 | | | **284.1969** | | | 228.1707 | 242.1863 | I | 1336.6280 | 1349.6484 | 1363.6641 | 1394.7063 | 1377.6797 | 13[illegible] |
| 4 | 74.0600 | 357.2496 | | 339.2391 | **385.2445** | | 367.2340 | 341.2547 | 343.2340 | T | 1235.5804 | 1248.6008 | 1250.5800 | ***1281.6222*** | 1264.5957 | 12[illegible] |
| 5 | 72.0808 | 456.3180 | | 438.3075 | **484.3130** | | 466.3024 | 442.3024 | | V | 1136.5119 | 1149.5324 | | ***1180.5745*** | 1163.5480 | 11[illegible] |
| 6 | 70.0651 | 553.3708 | | 535.3602 | 581.3657 | | 563.3552 | 527.3552 | | P | 1039.4592 | 1038.4639 | | ***1081.5061*** | 1064.4796 | 10[illegible] |
| 7 | 44.0495 | 624.4079 | | 606.3974 | 652.4028 | | 634.3923 | | | A | 968.4221 | | | 984.4534 | 967.4268 | 9[illegible] |
| 8 | 136.0757 | 787.4713 | | 769.4607 | 815.4662 | | 797.4556 | | | Y | 805.3587 | | | ***913.4163*** | 896.3897 | 8[illegible] |
| 9 | 120.0808 | 934.5397 | | 916.5291 | 962.5346 | | 944.5240 | | | F | 658.2903 | | | ***750.3529*** | 733.3264 | 7[illegible] |
| 10 | 87.0553 | 1048.5826 | 1031.5560 | 1030.5720 | 1076.5775 | 1059.5510 | 1058.5669 | 1005.5768 | | N | 544.2474 | 543.2522 | | ***603.2845*** | 586.2580 | 5[illegible] |
| 11 | 88.0393 | 1163.6095 | 1146.5830 | 1145.5990 | 1191.6045 | 1174.5779 | 1173.5939 | 1119.6197 | | D | 429.2205 | 428.2252 | | ***489.2416*** | 472.2150 | 4[illegible] |
| 12 | 44.0495 | 1234.6467 | 1217.6201 | 1216.6361 | 1262.6416 | 1245.6150 | 1244.6310 | | | A | 358.1833 | | | ***374.2146*** | 357.1881 | |
| 13 | 101.0709 | 1362.7052 | 1345.6787 | 1344.6947 | 1390.7001 | 1373.6736 | 1372.6896 | 1305.6838 | | Q | 230.1248 | 229.1295 | | ***303.1775*** | 286.1510 | |
| 14 | 129.1135 | | | | | | | | | R | 74.0237 | 73.0284 | | ***175.1190*** | 158.0924 | |

| Seq | ya | yb | Seq | ya | yb | Seq | ya | yb |
|---|---|---|---|---|---|---|---|---|
| VI | 185.1648 | 213.1598 | VIT | 286.2125 | 314.2074 | VITV | 385.2809 | 413.2758 |
| VITVP | 482.3337 | 510.3286 | VITVPA | 553.3708 | 581.3657 | IT | 187.1441 | 215.1390 |
| ITV | 286.2125 | 314.2074 | ITVP | 383.2653 | 411.2602 | ITVPA | 454.3024 | 482.2973 |
| ITVPAY | 617.3657 | 645.3606 | TV | 173.1285 | 201.1234 | TVP | 270.1812 | 298.1761 |
| TVPA | 341.2183 | 369.2132 | TVPAY | 504.2817 | 532.2766 | TVPAYF | 651.3501 | 679.3450 |
| VP | 169.1335 | 197.1285 | VPA | 240.1707 | 268.1656 | VPAY | 403.2340 | 431.2289 |

| | | | | | | | | |
|---|---|---|---|---|---|---|---|---|
| **VPAYF** | 550.3024 | 578.2973 | **VPAYFN** | 664.3453 | 692.3402 | **PA** | 141.1022 | 169.0972 |
| **PAY** | 304.1656 | 332.1605 | **PAYF** | 451.2340 | 479.2289 | **PAYFN** | 565.2769 | 593.2718 |
| **PAYFND** | 680.3039 | 708.2988 | **AY** | 207.1128 | 235.1077 | **AYF** | 354.1812 | 382.1761 |
| **AYFN** | 468.2241 | 496.2191 | **AYFND** | 583.2511 | 611.2460 | **AYFNDA** | 654.2882 | 682.2831 |
| **YF** | 283.1441 | 311.1390 | **YFN** | 397.1870 | 425.1819 | **YFND** | 512.2140 | 540.2089 |
| **YFNDA** | 583.2511 | 611.2460 | **FN** | 234.1237 | 262.1186 | **FND** | 349.1506 | 377.1456 |
| **FNDA** | 420.1878 | 448.1827 | **FNDAQ** | 548.2463 | 576.2413 | **ND** | 202.0822 | 230.0771 |
| **NDA** | 273.1193 | 301.1143 | **NDAQ** | 401.1779 | 429.1728 | **DA** | 159.0764 | 187.0713 |
| **DAQ** | 287.1350 | 315.1299 | **AQ** | 172.1081 | 200.1030 | | | |

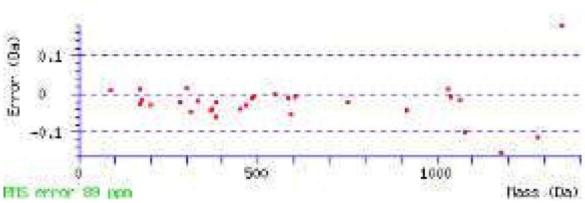


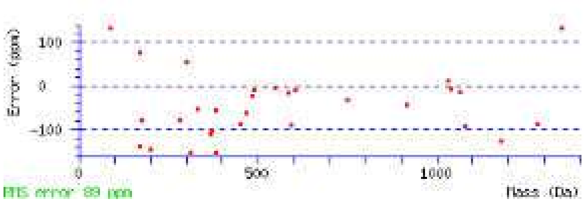


NCBI **BLAST** search of AVITVPAYFNDAQR
(Parameters: blastp, nr protein database, expect=20000, no filter, PAM30)
Other BLAST web gateways

**All matches to this query**

| Score | Mr(calc) | Delta | Sequence |
|---|---|---|---|
| 49.5 | 1563.8045 | 0.1439 | AVITVPAYFNDAQR |
| 8.0 | 1563.7926 | 0.1558 | MLSTTSSASGGISLPR |
| 6.2 | 1563.8117 | 0.1367 | KSVPASSSAPHNVQR |
| 4.9 | 1563.8005 | 0.1479 | IDIAASSDRFVTNR |
| 4.5 | 1563.8521 | 0.0963 | IEAAVHVWNLKER |
| 4.0 | 1563.8113 | 0.1372 | SIMAEALVTTMGKGR |
| 3.7 | 1563.8153 | 0.1332 | LAEAMLLFEKMPR |
| 3.2 | 1563.7927 | 0.1558 | ATAASGDIVVSSTMVR |
| 3.2 | 1563.8620 | 0.0864 | ATGSIKSLVYSPSVR |
| 3.1 | 1563.8555 | 0.0929 | VTALVNTSVAFMRR |

**Mascot:** http://www.matrixscience.com/

# MATRIX SCIENCE Mascot Search Results

## Peptide View **Spot no 102**

MS/MS Fragmentation of **QAVTNPQNTFFGTKR**
Found in **gi|115448989** in **NCBInr**, Os02g0774300 [Oryza sativa Japonica Group]

Match to Query 15: 1708.022024 from(1709.029300,1+) intensity(0.0000) index(6)
Title: Label: J5, Spot_Id: 228915, Peak_List_Id: 257485, MSMS Job_Run_Id: 24918, Comment:
Data file ppw_J5_138985127800.txt

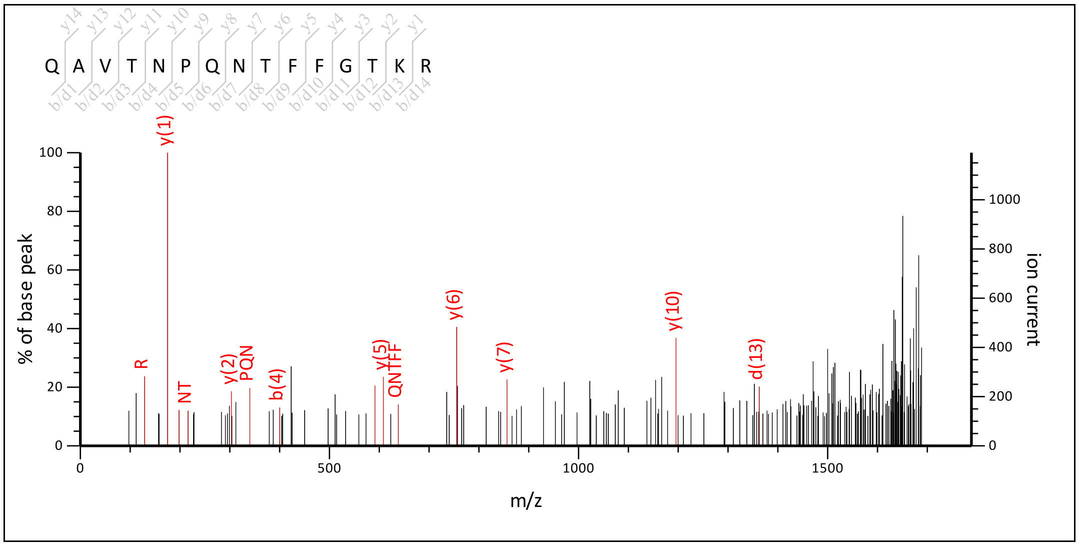


0 to 1788.57

Label all possible matches ○ Label matches used for scoring ◉

**Monoisotopic mass of neutral peptide Mr(calc):** 1707.8693
**Fixed modifications:** Carbamidomethyl (C) (apply to specified residues or termini only)
**Ions Score:** 19 **Expect:** 37
**Matches :** 17/270 fragment ions using 32 most intense peaks (help)

| # | Immon. | a | a* | $a^0$ | b | b* | $b^0$ | d | d' | Seq. | v | w | w' | y | y* |
|---|---|---|---|---|---|---|---|---|---|---|---|---|---|---|---|
| 1 | 101.0709 | 101.0709 | 84.0444 | | **129.0659** | 112.0393 | | 44.0495 | | Q | | | | | |
| 2 | 44.0495 | 172.1081 | 155.0815 | | 200.1030 | 183.0764 | | | | A | 1564.7867 | | | 1580.8180 | 1563.7914 |
| 3 | 72.0808 | 271.1765 | 254.1499 | | 299.1714 | 282.1448 | | 257.1608 | | V | 1465.7183 | 1478.7387 | | 1509.7809 | 1492.7543 |
| 4 | 74.0600 | 372.2241 | 355.1976 | 354.2136 | **400.2191** | 383.1925 | 382.2085 | 356.2292 | 358.2085 | T | 1364.6706 | 1377.6910 | 1379.6702 | 1410.7124 | 1393.6859 |
| 5 | 87.0553 | 486.2671 | 469.2405 | 468.2565 | 514.2620 | 497.2354 | 496.2514 | 443.2613 | | N | 1250.6276 | 1249.6324 | | 1309.6648 | 1292.6382 |
| 6 | 70.0651 | 583.3198 | 566.2933 | 565.3093 | 611.3148 | 594.2882 | 593.3042 | 557.3042 | | P | 1153.5749 | 1152.5796 | | ***1195.6218*** | 1178.5953 |
| 7 | 101.0709 | 711.3784 | 694.3519 | 693.3679 | 739.3733 | 722.3468 | 721.3628 | 654.3570 | | Q | 1025.5163 | 1024.5211 | | 1098.5691 | 1081.5425 |
| 8 | 87.0553 | 825.4213 | 808.3948 | 807.4108 | 853.4163 | 836.3897 | 835.4057 | 782.4155 | | N | 911.4734 | 910.4781 | | 970.5105 | 953.4839 |
| 9 | 74.0600 | 926.4690 | 909.4425 | 908.4585 | 954.4639 | 937.4374 | 936.4534 | 910.4741 | 912.4534 | T | 810.4257 | 823.4461 | 825.4254 | ***856.4676*** | 839.4410 |
| 10 | 120.0808 | 1073.5374 | 1056.5109 | 1055.5269 | 1101.5324 | 1084.5058 | 1083.5218 | | | F | 663.3573 | | | ***755.4199*** | 738.3933 |
| 11 | 120.0808 | 1220.6058 | 1203.5793 | 1202.5953 | 1248.6008 | 1231.5742 | 1230.5902 | | | F | 516.2889 | | | ***608.3515*** | 591.3249 |
| 12 | 30.0338 | 1277.6273 | 1260.6008 | 1259.6167 | 1305.6222 | 1288.5957 | 1287.6117 | | | G | | | | 461.2831 | 444.2565 |
| 13 | 74.0600 | 1378.6750 | 1361.6484 | 1360.6644 | 1406.6699 | 1389.6434 | 1388.6593 | 1362.6801 | 1364.6593 | T | 358.2197 | 371.2401 | 373.2194 | 404.2616 | 387.2350 |
| 14 | 101.1073 | 1506.7700 | 1489.7434 | 1488.7594 | 1534.7649 | 1517.7383 | 1516.7543 | 1449.7121 | | K | 230.1248 | 229.1295 | | ***303.2139*** | 286.1874 |
| 15 | 129.1135 | | | | | | | | | R | 74.0237 | 73.0284 | | ***175.1190*** | 158.0924 |

| Seq | ya | yb | Seq | ya | yb | Seq | ya | yb |
|---|---|---|---|---|---|---|---|---|
| AV | 143.1179 | 171.1128 | AVT | 244.1656 | 272.1605 | AVTN | 358.2085 | 386.2034 |
| AVTNP | 455.2613 | 483.2562 | AVTNPQ | 583.3198 | 611.3148 | AVTNPQN | 697.3628 | 725.3577 |
| VT | 173.1285 | 201.1234 | VTN | 287.1714 | 315.1663 | VTNP | 384.2241 | 412.2191 |
| VTNPQ | 512.2827 | 540.2776 | VTNPQN | 626.3257 | 654.3206 | TN | 188.1030 | 216.0979 |
| TNP | 285.1557 | 313.1506 | TNPQ | 413.2143 | 441.2092 | TNPQN | 527.2572 | 555.2522 |

| | | | | | | | | |
|---|---|---|---|---|---|---|---|---|
| **TNPQNT** | 628.3049 | 656.2998 | **NP** | 184.1081 | 212.1030 | **NPQ** | 312.1666 | 340.1615 |
| **NPQN** | 426.2096 | 454.2045 | **NPQNT** | 527.2572 | 555.2522 | **NPQNTF** | 674.3257 | 702.3206 |
| **PQ** | 198.1237 | 226.1186 | **PQN** | 312.1666 | 340.1615 | **PQNT** | 413.2143 | 441.2092 |
| **PQNTF** | 560.2827 | 588.2776 | **QN** | 215.1139 | 243.1088 | **QNT** | 316.1615 | 344.1565 |
| **QNTF** | 463.2300 | 491.2249 | **QNTFF** | 610.2984 | 638.2933 | **QNTFFG** | 667.3198 | 695.3148 |
| **NT** | 188.1030 | 216.0979 | **NTF** | 335.1714 | 363.1663 | **NTFF** | 482.2398 | 510.2347 |
| **NTFFG** | 539.2613 | 567.2562 | **NTFFGT** | 640.3089 | 668.3039 | **TF** | 221.1285 | 249.1234 |
| **TFF** | 368.1969 | 396.1918 | **TFFG** | 425.2183 | 453.2132 | **TFFGT** | 526.2660 | 554.2609 |
| **TFFGTK** | 654.3610 | 682.3559 | **FF** | 267.1492 | 295.1441 | **FFG** | 324.1707 | 352.1656 |
| **FFGT** | 425.2183 | 453.2132 | **FFGTK** | 553.3133 | 581.3082 | **FG** | 177.1022 | 205.0972 |
| **FGT** | 278.1499 | 306.1448 | **FGTK** | 406.2449 | 434.2398 | **GT** | 131.0815 | 159.0764 |
| **GTK** | 259.1765 | 287.1714 | **TK** | 202.1550 | 230.1499 | | | |

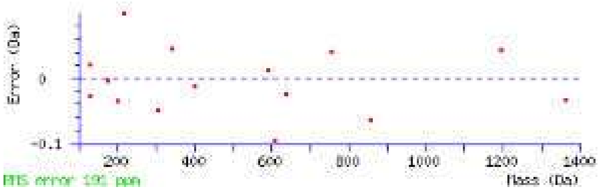


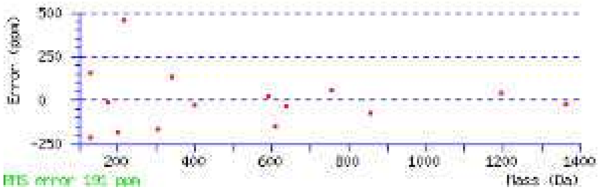


NCBI **BLAST** search of QAVTNPQNTFFGTKR
(Parameters: blastp, nr protein database, expect=20000, no filter, PAM30)
Other BLAST web gateways

**All matches to this query**

| Score | Mr(calc) | Delta | Sequence |
|---|---|---|---|
| 18.9 | 1707.8693 | 0.1527 | QAVTNPQNTFFGTKR |
| 11.5 | 1707.9189 | 0.1031 | QSSLAISMASSLTLKR |
| 5.7 | 1707.9090 | 0.1130 | TVKSLLNSMPGATPHR |
| 5.7 | 1707.8904 | 0.1316 | GSQVVPGAKAVEEGPAGR |
| 5.5 | 1707.9229 | 0.0991 | ILIMQGEEITKVYR |
| 5.1 | 1707.8726 | 0.1494 | LCGPTGTIQAAAGAPAPR |
| 4.5 | 1707.8540 | 0.1681 | EEQRDSKPIPEPQR |
| 4.4 | 1707.9056 | 0.1164 | SRIWLGSYSTAVAAAR |
| 4.1 | 1707.9169 | 0.1052 | ISRPGLRVYSNYQR |
| 3.6 | 1707.8766 | 0.1454 | GWLFSMLNTNLLQR |

**Mascot:** http://www.matrixscience.com/

# MATRIX SCIENCE Mascot Search Results

## Peptide View **Spot no 102**

MS/MS Fragmentation of **SQVFSTAADNQTQVGIR**
Found in **gi|115448989** in **NCBInr**, Os02g0774300 [Oryza sativa Japonica Group]

Match to Query 17: 1821.065524 from(1822.072800,1+) intensity(0.0000) index(7)
Title: Label: J5, Spot_Id: 228915, Peak_List_Id: 257484, MSMS Job_Run_Id: 24918, Comment:
Data file ppw_J5_138985127800.txt

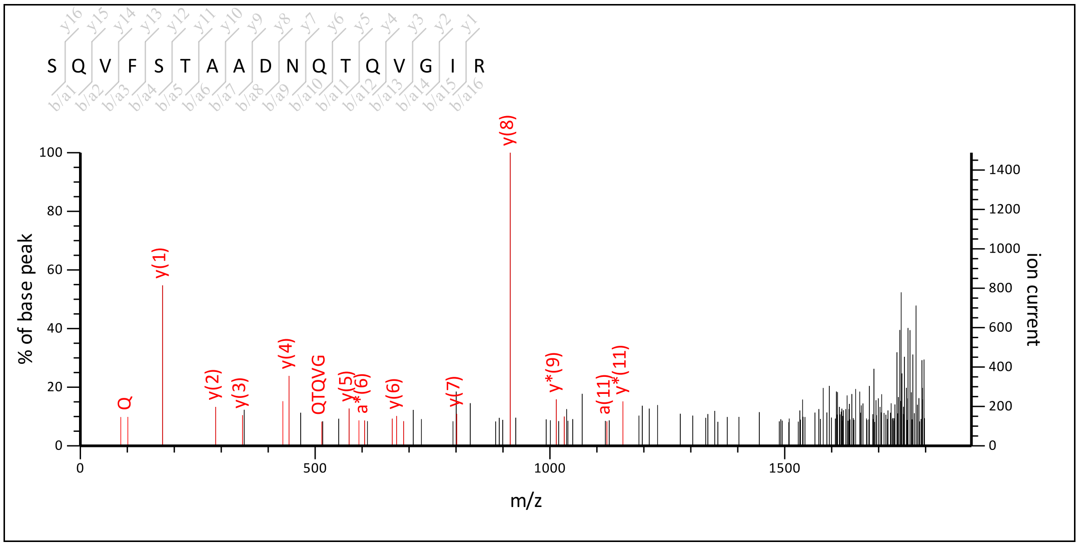


0 to 1897.59

Label all possible matches ○ Label matches used for scoring ◉

**Monoisotopic mass of neutral peptide Mr(calc):** 1820.9017
**Fixed modifications:** Carbamidomethyl (C) (apply to specified residues or termini only)
**Ions Score:** 25 **Expect:** 7.8
**Matches :** 24/326 fragment ions using 62 most intense peaks (help)

| # | Immon. | a | a* | a0 | b | b* | b0 | d | d' | Seq. | v | w | w' | y | y* | [illegible] |
|---|---|---|---|---|---|---|---|---|---|---|---|---|---|---|---|---|
| 1 | 60.0444 | 60.0444 | | 42.0338 | 88.0393 | | 70.0287 | 44.0495 | | S | | | | | | |
| 2 | 101.0709 | 188.1030 | 171.0764 | 170.0924 | 216.0979 | 199.0713 | 198.0873 | 131.0815 | | Q | 1661.8242 | 1660.8289 | | 1734.8769 | 1717.8504 | [illegible] |
| 3 | 72.0808 | 287.1714 | 270.1448 | 269.1608 | 315.1663 | 298.1397 | 297.1557 | 273.1557 | | V | 1562.7558 | 1575.7762 | | 1606.8184 | 1589.7918 | [illegible] |
| 4 | 120.0808 | 434.2398 | 417.2132 | 416.2292 | 462.2347 | 445.2082 | 444.2241 | | | F | 1415.6873 | | | 1507.7499 | 1490.7234 | [illegible] |
| 5 | 60.0444 | 521.2718 | 504.2453 | 503.2613 | 549.2667 | 532.2402 | 531.2562 | 505.2769 | | S | 1328.6553 | 1327.6601 | | 1360.6815 | 1343.6550 | [illegible] |
| 6 | 74.0600 | 622.3195 | 605.2930 | 604.3089 | 650.3144 | 633.2879 | 632.3039 | 606.3246 | 608.3039 | T | 1227.6076 | 1240.6280 | 1242.6073 | 1273.6495 | 1256.6230 | [illegible] |
| 7 | 44.0495 | 693.3566 | 676.3301 | 675.3461 | 721.3515 | 704.3250 | 703.3410 | | | A | 1156.5705 | | | 1172.6018 | 1155.5753 | [illegible] |
| 8 | 44.0495 | 764.3937 | 747.3672 | 746.3832 | 792.3886 | 775.3621 | 774.3781 | | | A | 1085.5334 | | | 1101.5647 | 1084.5382 | [illegible] |
| 9 | 88.0393 | 879.4207 | 862.3941 | 861.4101 | 907.4156 | 890.3890 | 889.4050 | 835.4308 | | D | 970.5065 | 969.5112 | | ***1030.5276*** | 1013.5010 | [illegible] |
| 10 | 87.0553 | 993.4636 | 976.4371 | 975.4530 | 1021.4585 | 1004.4320 | 1003.4479 | 950.4578 | | N | 856.4635 | 855.4683 | | ***915.5007*** | 898.4741 | [illegible] |
| 11 | 101.0709 | 1121.5222 | 1104.4956 | 1103.5116 | 1149.5171 | 1132.4905 | 1131.5065 | 1064.5007 | | Q | 728.4050 | 727.4097 | | ***801.4577*** | 784.4312 | [illegible] |
| 12 | 74.0600 | 1222.5699 | 1205.5433 | 1204.5593 | 1250.5648 | 1233.5382 | 1232.5542 | 1206.5749 | 1208.5542 | T | 627.3573 | 640.3777 | 642.3570 | ***673.3992*** | 656.3726 | [illegible] |
| 13 | 101.0709 | 1350.6284 | 1333.6019 | 1332.6179 | 1378.6234 | 1361.5968 | 1360.6128 | 1293.6070 | | Q | 499.2987 | 498.3035 | | ***572.3515*** | 555.3249 | |
| 14 | 72.0808 | 1449.6968 | 1432.6703 | 1431.6863 | 1477.6918 | 1460.6652 | 1459.6812 | 1435.6812 | | V | 400.2303 | 413.2507 | | ***444.2929*** | 427.2663 | |
| 15 | 30.0338 | 1506.7183 | 1489.6918 | 1488.7077 | 1534.7132 | 1517.6867 | 1516.7027 | | | G | | | | ***345.2245*** | 328.1979 | |
| 16 | 86.0964 | 1619.8024 | 1602.7758 | 1601.7918 | 1647.7973 | 1630.7707 | 1629.7867 | 1591.7711 | 1605.7867 | I | 230.1248 | 243.1452 | 257.1608 | ***288.2030*** | 271.1765 | |
| 17 | 129.1135 | | | | | | | | | R | 74.0237 | 73.0284 | | ***175.1190*** | 158.0924 | |

| Seq | ya | yb | Seq | ya | yb | Seq | ya | yb |
|---|---|---|---|---|---|---|---|---|
| QV | 200.1394 | 228.1343 | QVF | 347.2078 | 375.2027 | QVFS | 434.2398 | 462.2347 |
| QVFST | 535.2875 | 563.2824 | QVFSTA | 606.3246 | 634.3195 | QVFSTAA | 677.3617 | 705.3566 |
| VF | 219.1492 | 247.1441 | VFS | 306.1812 | 334.1761 | VFST | 407.2289 | 435.2238 |

| | | | | | | | | |
|---|---|---|---|---|---|---|---|---|
| **VFSTA** | 478.2660 | 506.2609 | **VFSTAA** | 549.3031 | 577.2980 | **VFSTAAD** | 664.3301 | 692.3250 |
| **FS** | 207.1128 | 235.1077 | **FST** | 308.1605 | 336.1554 | **FSTA** | 379.1976 | 407.1925 |
| **FSTAA** | 450.2347 | 478.2296 | **FSTAAD** | 565.2617 | 593.2566 | **FSTAADN** | 679.3046 | 707.2995 |
| **ST** | 161.0921 | 189.0870 | **STA** | 232.1292 | 260.1241 | **STAA** | 303.1663 | 331.1612 |
| **STAAD** | 418.1932 | 446.1882 | **STAADN** | 532.2362 | 560.2311 | **STAADNQ** | 660.2947 | 688.2897 |
| **TA** | 145.0972 | 173.0921 | **TAA** | 216.1343 | 244.1292 | **TAAD** | 331.1612 | 359.1561 |
| **TAADN** | 445.2041 | 473.1991 | **TAADNQ** | 573.2627 | 601.2576 | **TAADNQT** | 674.3104 | 702.3053 |
| **AA** | 115.0866 | 143.0815 | **AAD** | 230.1135 | 258.1084 | **AADN** | 344.1565 | 372.1514 |
| **AADNQ** | 472.2150 | 500.2100 | **AADNQT** | 573.2627 | 601.2576 | **AD** | 159.0764 | 187.0713 |
| **ADN** | 273.1193 | 301.1143 | **ADNQ** | 401.1779 | 429.1728 | **ADNQT** | 502.2256 | 530.2205 |
| **ADNQTQ** | 630.2842 | 658.2791 | **DN** | 202.0822 | 230.0771 | **DNQ** | 330.1408 | 358.1357 |
| **DNQT** | 431.1885 | 459.1834 | **DNQTQ** | 559.2471 | 587.2420 | **DNQTQV** | 658.3155 | 686.3104 |
| **NQ** | 215.1139 | 243.1088 | **NQT** | 316.1615 | 344.1565 | **NQTQ** | 444.2201 | 472.2150 |
| **NQTQV** | 543.2885 | 571.2835 | **NQTQVG** | 600.3100 | 628.3049 | **QT** | 202.1186 | 230.1135 |
| **QTQ** | 330.1772 | 358.1721 | **QTQV** | 429.2456 | 457.2405 | **QTQVG** | 486.2671 | 514.2620 |
| **QTQVGI** | 599.3511 | 627.3461 | **TQ** | 202.1186 | 230.1135 | **TQV** | 301.1870 | 329.1819 |
| **TQVG** | 358.2085 | 386.2034 | **TQVGI** | 471.2926 | 499.2875 | **QV** | 200.1394 | 228.1343 |
| **QVG** | 257.1608 | 285.1557 | **QVGI** | 370.2449 | 398.2398 | **VG** | 129.1022 | 157.0972 |
| **VGI** | 242.1863 | 270.1812 | **GI** | 143.1179 | 171.1128 | | | |

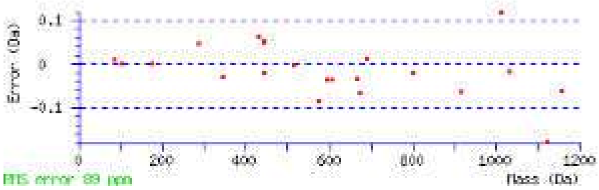


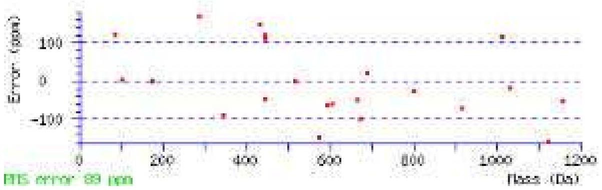


NCBI **BLAST** search of SQVFSTAADNQTQVGIR
(Parameters: blastp, nr protein database, expect=20000, no filter, PAM30)
Other BLAST web gateways

**All matches to this query**

| Score | Mr(calc) | Delta | Sequence |
|---|---|---|---|
| 24.5 | 1820.9017 | 0.1638 | SQVFSTAADNQTQVGIR |
| 11.6 | 1820.9322 | 0.1333 | AWWSQTFDIAATRLR |
| 9.6 | 1820.8945 | 0.1710 | GLGFPAEDLEVSGFDLR |
| 8.8 | 1820.9930 | 0.0725 | MIRAYTQSAATLNLLR |
| 8.0 | 1820.9454 | 0.1201 | EGLAPINLVIETEMHR |
| 7.1 | 1820.9421 | 0.1235 | WEDYSLINKGSIQLR |
| 6.6 | 1820.9376 | 0.1279 | KVLMDDSMVLLGDTIR |
| 6.1 | 1820.8913 | 0.1742 | ALRLMEEMPYKPDGR |
| 6.0 | 1820.9706 | 0.0949 | ITMYPALTELQEKIR |
| 5.9 | 1821.0182 | 0.0473 | VKGTMLNYSTVLVQLR |

**Mascot:** http://www.matrixscience.com/

# MATRIX SCIENCE Mascot Search Results

## Peptide View **Spot no 104**

MS/MS Fragmentation of **IAGLEVLR**
Found in **gi|6746592** in **NCBInr**, heat shock protein 70 [Arabidopsis thaliana]

Match to Query 2: 869.582934 from(870.590210,1+) intensity(0.0000) index(0)
Title: Label: F1, Spot_Id: 219679, Peak_List_Id: 224947, MSMS Job_Run_Id: 21758, Comment:
Data file C:\Documents and Settings\Administrator\Desktop\farah BHU\spot set plate\Farah-Garima 11May 2013\ppw_F1_136842030500.txt

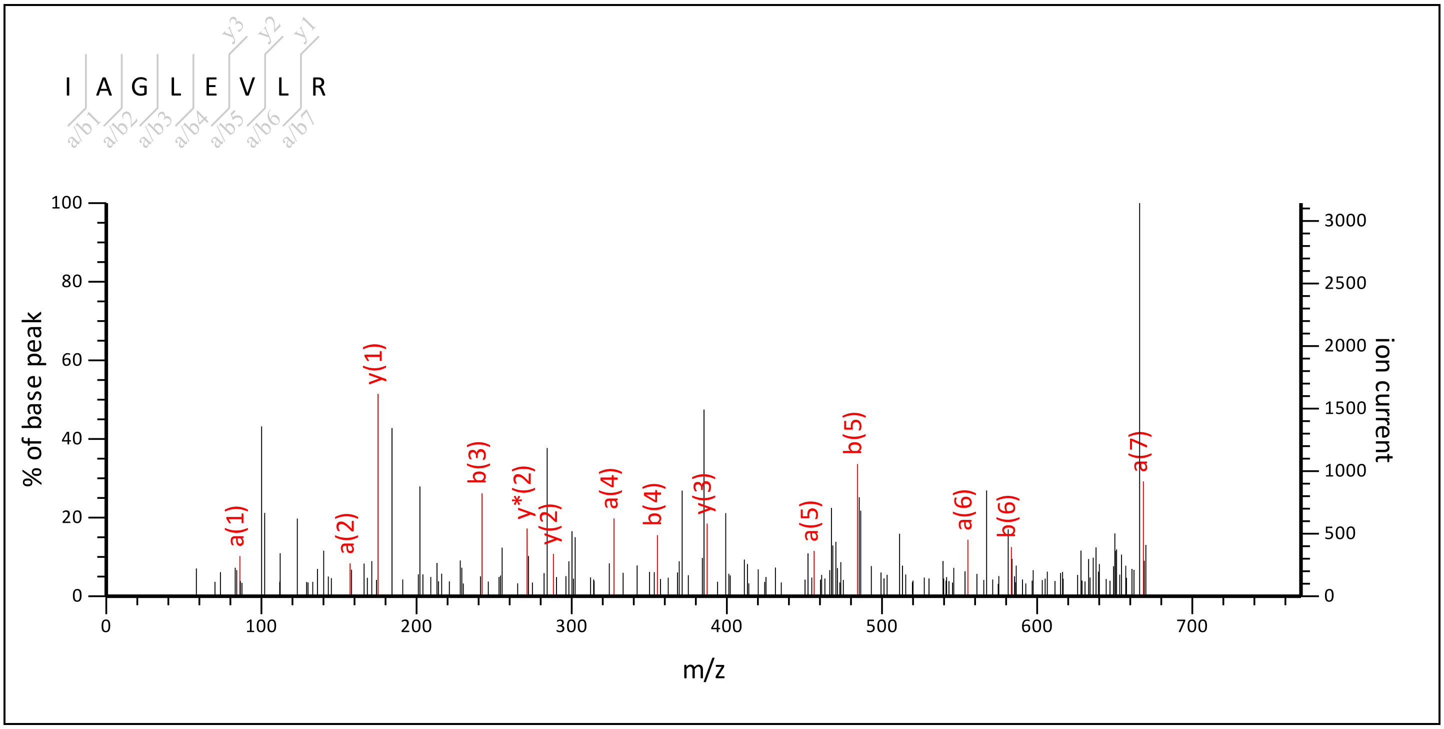


0 to 770.05

Label all possible matches ○ Label matches used for scoring ◉

**Monoisotopic mass of neutral peptide Mr(calc):** 869.5334
**Fixed modifications:** Carbamidomethyl (C) (apply to specified residues or termini only)
**Ions Score:** 20 **Expect:** 7.1
**Matches :** 14/28 fragment ions using 48 most intense peaks (help)

| # | a | b | Seq. | y | y* | # |
|---|---|---|---|---|---|---|
| 1 | *86.0964* | 114.0913 | I | | | 8 |
| 2 | *157.1335* | 185.1285 | A | 757.4567 | 740.4301 | 7 |
| 3 | 214.1550 | **242.1499** | G | 686.4196 | 669.3930 | 6 |
| 4 | *327.2391* | **355.2340** | L | 629.3981 | 612.3715 | 5 |
| 5 | *456.2817* | **484.2766** | E | 516.3140 | 499.2875 | 4 |
| 6 | *555.3501* | **583.3450** | V | **387.2714** | 370.2449 | 3 |
| 7 | *668.4341* | 696.4291 | L | **288.2030** | 271.1765 | 2 |
| 8 | | | R | **175.1190** | 158.0924 | 1 |

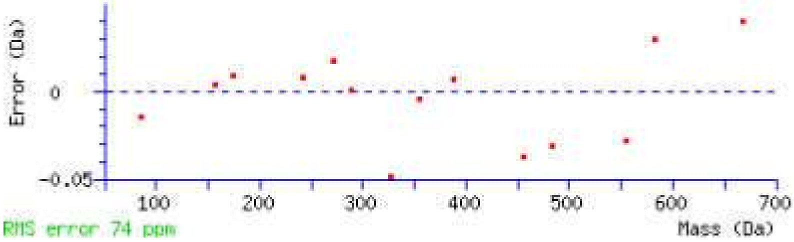


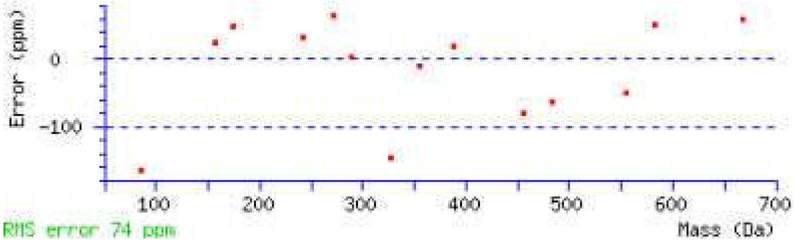


NCBI **BLAST** search of IAGLEVLR
(Parameters: blastp, nr protein database, expect=20000, no filter, PAM30)
Other BLAST web gateways

**All matches to this query**

| Score | Mr(calc) | Delta | Sequence |
|---|---|---|---|
| 25.1 | 869.5083 | 0.0747 | EIRLPSR |
| 25.1 | 869.5083 | 0.0747 | ELRLPSR |
| 25.1 | 869.5083 | 0.0747 | ERILPSR |
| 25.1 | 869.5083 | 0.0747 | ERLIPSR |
| 22.6 | 869.5083 | 0.0746 | IQGNAVLR |
| 20.2 | 869.5334 | 0.0495 | IAGLEVLR |
| 19.3 | 869.5334 | 0.0495 | VALDALLR |
| 19.1 | 869.5195 | 0.0634 | RGGGGVVLR |
| 19.0 | 869.5335 | 0.0495 | TVVVSPIR |
| 18.2 | 869.5157 | 0.0673 | LAMAVPLR |

**Mascot:** http://www.matrixscience.com/

# MATRIX SCIENCE Mascot Search Results

## Peptide View **Spot no 104**

MS/MS Fragmentation of **DIDEVILVGGSTR**
Found in **gi|6746592** in **NCBInr**, heat shock protein 70 [Arabidopsis thaliana]

Match to Query 82: 1372.761024 from(1373.768300,1+) intensity(0.0000) index(16)
Title: Label: F1, Spot_Id: 219679, Peak_List_Id: 224942, MSMS Job_Run_Id: 21758, Comment:
Data file C:\Documents and Settings\Administrator\Desktop\farah BHU\spot set plate\Farah-Garima 11May 2013\ppw_F1_136842030500.txt

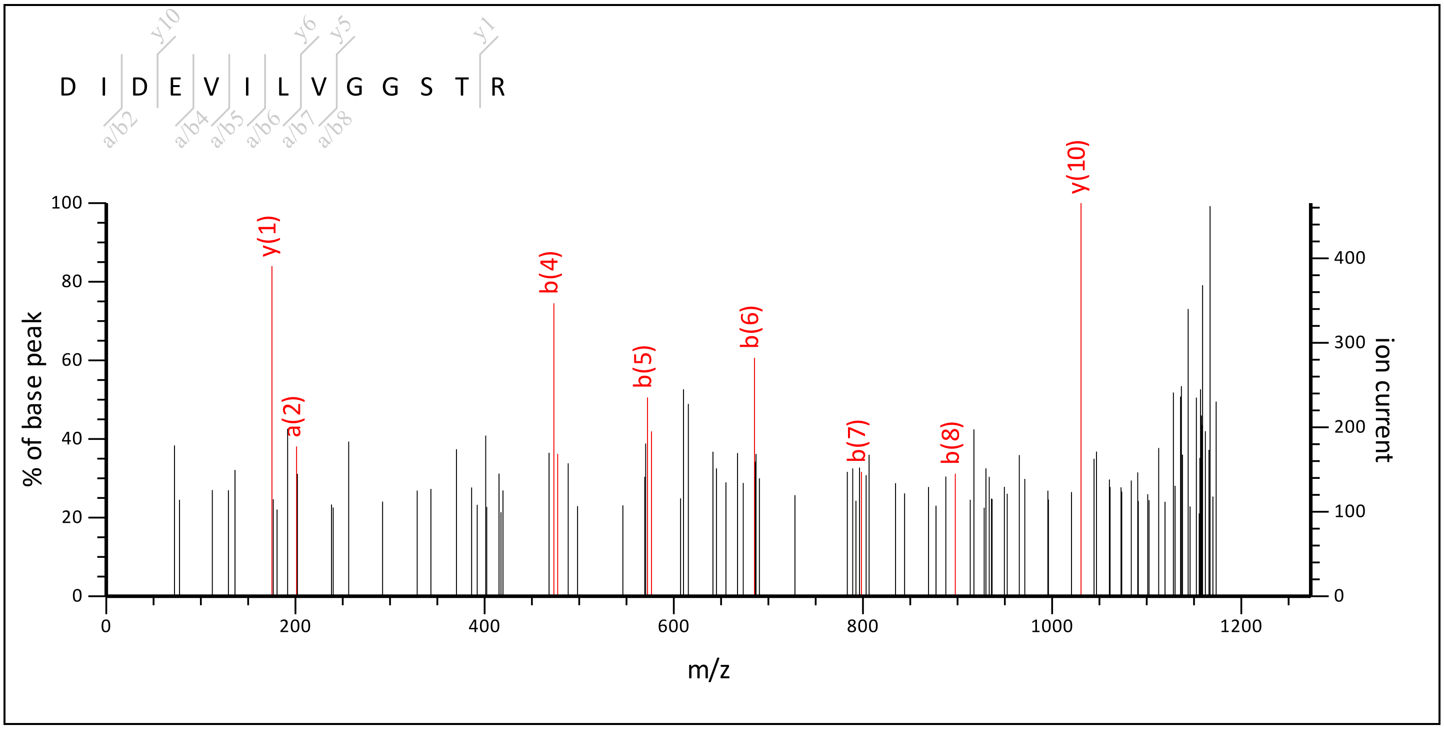


0 to 1273.42

Label all possible matches ○ Label matches used for scoring ◉

**Monoisotopic mass of neutral peptide Mr(calc):** 1372.7198
**Fixed modifications:** Carbamidomethyl (C) (apply to specified residues or termini only)
**Ions Score:** 6 **Expect:** 4.5e+02
**Matches :** 10/48 fragment ions using 45 most intense peaks (help)

| # | a | b | Seq. | y | y* | # |
|---|---|---|---|---|---|---|
| 1 | 88.0393 | 116.0342 | D | | | 13 |
| 2 | 201.1234 | 229.1183 | I | 1258.7001 | 1241.6736 | 12 |
| 3 | 316.1503 | 344.1452 | D | 1145.6161 | 1128.5895 | 11 |
| 4 | 445.1929 | ***473.1878*** | E | ***1030.5891*** | 1013.5626 | 10 |
| 5 | 544.2613 | ***572.2562*** | V | 901.5465 | 884.5200 | 9 |
| 6 | 657.3454 | ***685.3403*** | I | 802.4781 | 785.4516 | 8 |
| 7 | 770.4294 | ***798.4244*** | L | 689.3941 | 672.3675 | 7 |
| 8 | 869.4979 | ***897.4928*** | V | ***576.3100*** | 559.2835 | 6 |

| | | | | | | |
|---|---|---|---|---|---|---|
| **9** | 926.5193 | 954.5142 | **G** | *477.2416* | 460.2150 | **5** |
| **10** | 983.5408 | 1011.5357 | **G** | 420.2201 | 403.1936 | **4** |
| **11** | 1070.5728 | 1098.5677 | **S** | 363.1987 | 346.1721 | **3** |
| **12** | 1171.6205 | 1199.6154 | **T** | 276.1666 | 259.1401 | **2** |
| **13** | | | **R** | *175.1190* | 158.0924 | **1** |

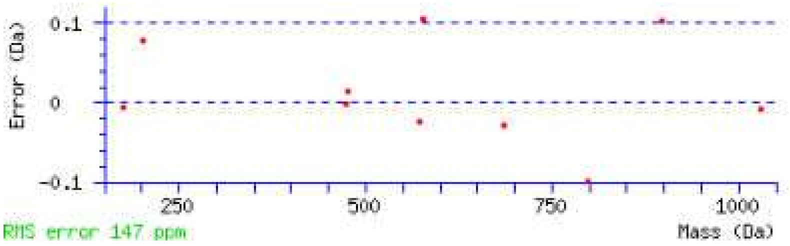


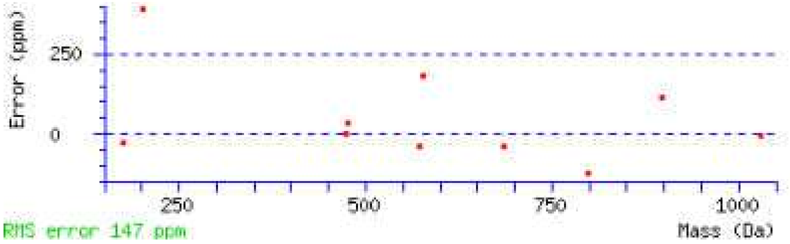


NCBI **BLAST** search of [DIDEVILVGGSTR]
(Parameters: blastp, nr protein database, expect=20000, no filter, PAM30)
Other BLAST [web gateways]

**All matches to this query**

| Score | Mr(calc) | Delta | Sequence |
|---|---|---|---|
| 9.7 | 1372.7133 | 0.0478 | VLNASEVGMALNR |
| 6.5 | 1372.6834 | 0.0776 | AESIEELEAQVR |
| 6.3 | 1372.7198 | 0.0412 | DIDEVILVGGSTR |
| 6.3 | 1372.7198 | 0.0412 | DIDEVILVGGSTR |
| 6.3 | 1372.7198 | 0.0412 | DLDEVILVGGSTR |
| 5.9 | 1372.7463 | 0.0147 | QYANVLDKPLGR |
| 3.6 | 1372.7350 | 0.0260 | ETLEIFAPLANR |
| 2.8 | 1372.6371 | 0.1239 | QSKESFGGYTNR |
| 2.5 | 1372.7939 | -0.0329 | AGYLRQLVGGIAR |
| 2.5 | 1372.7939 | -0.0329 | LGRQLVFSNLAR |

**Mascot:** http://www.matrixscience.com/

# MATRIX SCIENCE Mascot Search Results

## Peptide View Spot no 104

MS/MS Fragmentation of **QFAAEEISAQVLR**
Found in **gi|6746592** in **NCBInr**, heat shock protein 70 [Arabidopsis thaliana]

Match to Query 90: 1460.814724 from(1461.822000,1+) intensity(0.0000) index(19)
Title: Label: F1, Spot_Id: 219679, Peak_List_Id: 224932, MSMS Job_Run_Id: 21758, Comment:
Data file C:\Documents and Settings\Administrator\Desktop\farah BHU\spot set plate\Farah-Garima 11May 2013\ppw_F1_136842030500.txt

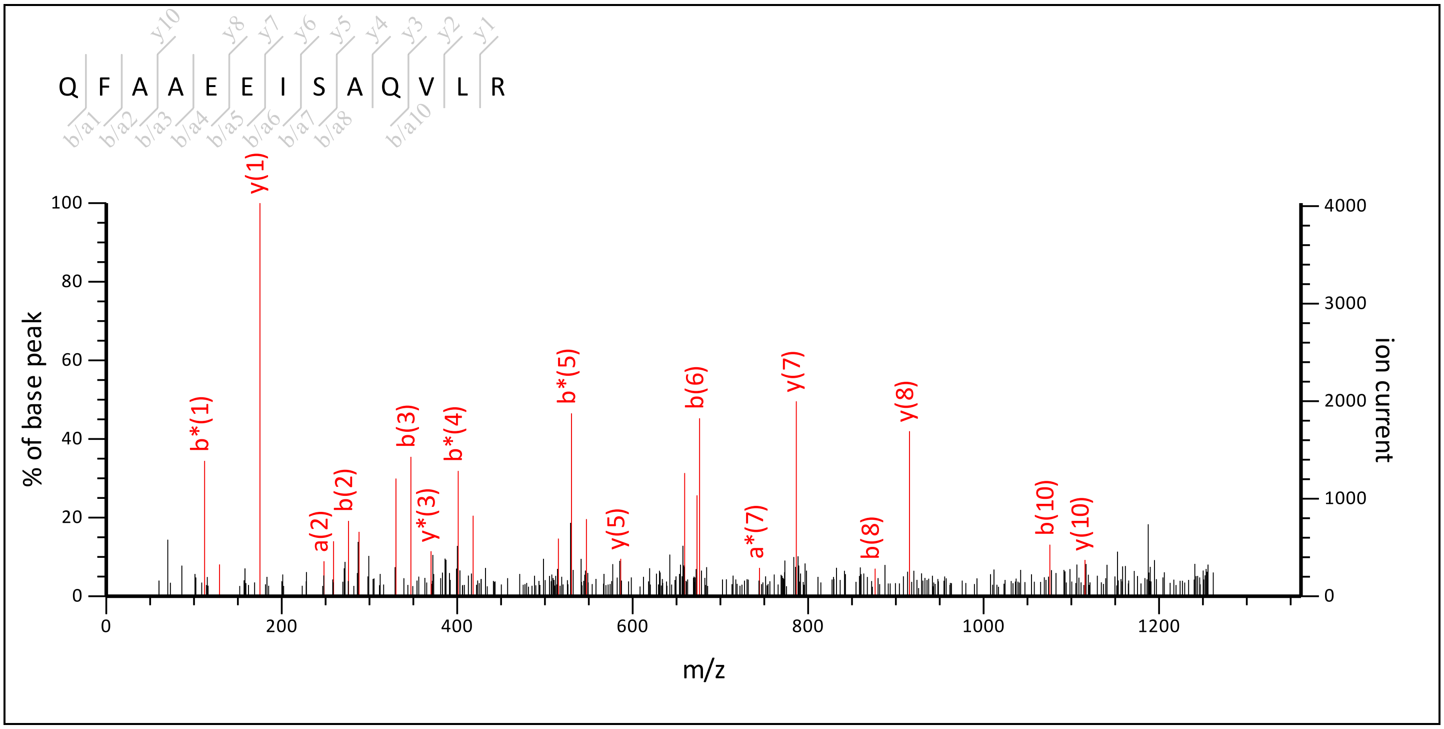


0 to 1361.77

Label all possible matches ○ Label matches used for scoring ◉

**Monoisotopic mass of neutral peptide Mr(calc):** 1460.7623
**Fixed modifications:** Carbamidomethyl (C) (apply to specified residues or termini only)
**Ions Score:** 54 **Expect:** 0.0072
**Matches :** 25/72 fragment ions using 49 most intense peaks (help)

| # | a | a* | b | b* | Seq. | y | y* | # |
|---|---|---|---|---|---|---|---|---|
| 1 | 101.0709 | 84.0444 | ***129.0659*** | 112.0393 | Q | | | 13 |
| 2 | 248.1394 | 231.1128 | ***276.1343*** | 259.1077 | F | 1333.7110 | 1316.6845 | 12 |
| 3 | 319.1765 | 302.1499 | ***347.1714*** | 330.1448 | A | 1186.6426 | 1169.6161 | 11 |
| 4 | 390.2136 | 373.1870 | ***418.2085*** | 401.1819 | A | ***1115.6055*** | 1098.5790 | 10 |
| 5 | 519.2562 | 502.2296 | ***547.2511*** | 530.2245 | E | 1044.5684 | 1027.5419 | 9 |
| 6 | 648.2988 | 631.2722 | ***676.2937*** | 659.2671 | E | ***915.5258*** | 898.4993 | 8 |
| 7 | 761.3828 | 744.3563 | 789.3777 | 772.3512 | I | ***786.4832*** | 769.4567 | 7 |
| 8 | 848.4149 | 831.3883 | ***876.4098*** | 859.3832 | S | ***673.3991*** | 656.3726 | 6 |

| | | | | | | | | |
|---|---|---|---|---|---|---|---|---|
| **9** | 919.4520 | 902.4254 | 947.4469 | 930.4203 | **A** | ***586.3671*** | 569.3406 | **5** |
| **10** | 1047.5106 | 1030.4840 | ***1075.5055*** | 1058.4789 | **Q** | ***515.3300*** | 498.3035 | **4** |
| **11** | 1146.5790 | 1129.5524 | 1174.5739 | 1157.5473 | **V** | 387.2714 | 370.2449 | **3** |
| **12** | 1259.6630 | 1242.6365 | 1287.6579 | 1270.6314 | **L** | ***288.2030*** | 271.1765 | **2** |
| **13** | | | | | **R** | ***175.1190*** | 158.0924 | **1** |

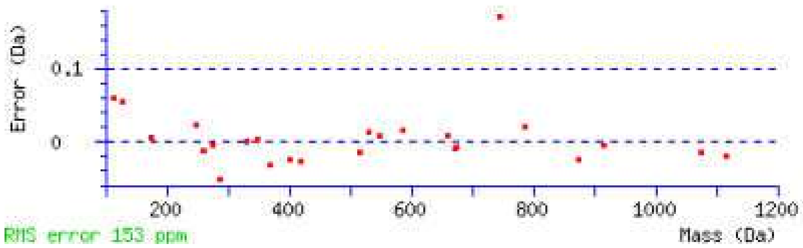


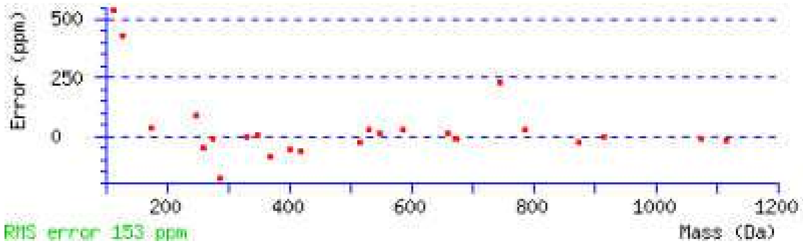


NCBI **BLAST** search of QFAAEEISAQVLR
(Parameters: blastp, nr protein database, expect=20000, no filter, PAM30)
Other BLAST web gateways

**All matches to this query**

| Score | Mr(calc) | Delta | Sequence |
|---|---|---|---|
| 54.3 | 1460.7623 | 0.0524 | QFAAEEISAQVLR |
| 36.8 | 1460.7194 | 0.0953 | EAWCEVLRATAR |
| 29.7 | 1460.8351 | -0.0204 | KFAAEQISSLVLR |
| 23.7 | 1460.7405 | 0.0742 | MQSEEALVRAATR |
| 15.2 | 1460.7848 | 0.0299 | QVIPHTGGSKANPR |
| 13.0 | 1460.7293 | 0.0854 | IDGDMKEAGQLLR |
| 11.9 | 1460.8095 | 0.0053 | LSLDLIMMAVLAR |
| 11.6 | 1460.7293 | 0.0854 | MSAINGVIEEKDR |
| 10.5 | 1460.7657 | 0.0490 | SLGEPISMTKQVR |
| 10.3 | 1460.6929 | 0.1218 | ESCIGQQEILER |

**Mascot:** http://www.matrixscience.com/

# MATRIX SCIENCE Mascot Search Results

## Peptide View **Spot no 104**

MS/MS Fragmentation of **AVITVPAYFNDSQR**
Found in **gi|6746592** in **NCBInr**, heat shock protein 70 [Arabidopsis thaliana]

Match to Query 99: 1579.854624 from(1580.861900,1+) intensity(0.0000) index(21)
Title: Label: F1, Spot_Id: 219679, Peak_List_Id: 224931, MSMS Job_Run_Id: 21758, Comment:
Data file C:\Documents and Settings\Administrator\Desktop\farah BHU\spot set plate\Farah-Garima 11May 2013\ppw_F1_136842030500.txt

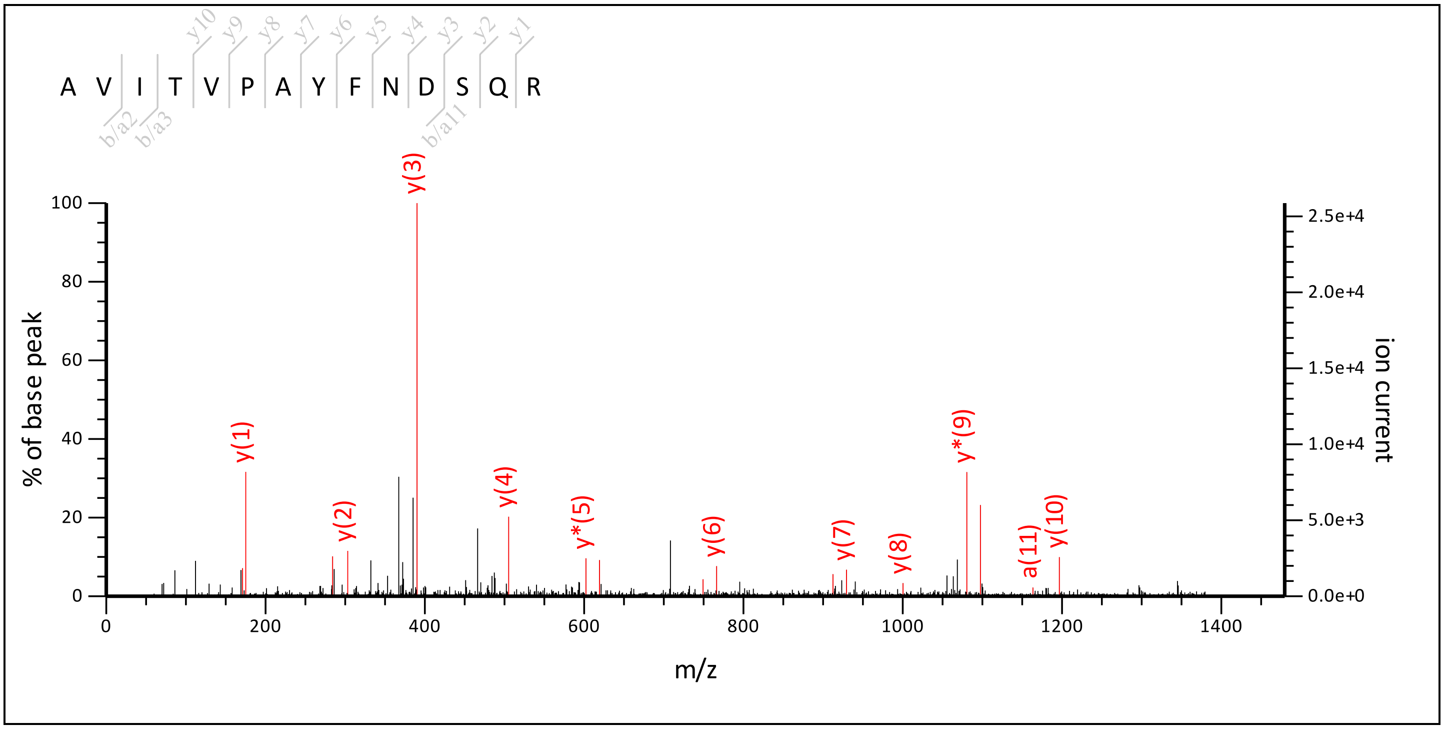


0 to 1479.56

Label all possible matches ○ Label matches used for scoring ●

**Monoisotopic mass of neutral peptide Mr(calc):** 1579.7995
**Fixed modifications:** Carbamidomethyl (C) (apply to specified residues or termini only)
**Ions Score:** 70 **Expect:** 0.00018
**Matches :** 17/60 fragment ions using 28 most intense peaks (help)

| # | a | a* | b | b* | Seq. | y | y* | # |
|---|---|---|---|---|---|---|---|---|
| 1 | 44.0495 | | 72.0444 | | A | | | 14 |
| 2 | 143.1179 | | **171.1128** | | V | 1509.7696 | 1492.7431 | 13 |
| 3 | 256.2020 | | **284.1969** | | I | 1410.7012 | 1393.6747 | 12 |
| 4 | 357.2496 | | 385.2445 | | T | 1297.6171 | 1280.5906 | 11 |
| 5 | 456.3180 | | 484.3130 | | V | ***1196.5695*** | 1179.5429 | 10 |
| 6 | 553.3708 | | 581.3657 | | P | ***1097.5010*** | 1080.4745 | 9 |
| 7 | 624.4079 | | 652.4028 | | A | ***1000.4483*** | 983.4217 | 8 |
| 8 | 787.4713 | | 815.4662 | | Y | ***929.4112*** | 912.3846 | 7 |

| | | | | | | | | |
|---|---|---|---|---|---|---|---|---|
| **9** | 934.5397 | | 962.5346 | | **F** | ***766.3478*** | 749.3213 | **6** |
| **10** | 1048.5826 | 1031.5560 | 1076.5775 | 1059.5510 | **N** | ***619.2794*** | 602.2529 | **5** |
| **11** | 1163.6095 | 1146.5830 | 1191.6045 | 1174.5779 | **D** | ***505.2365*** | 488.2100 | **4** |
| **12** | 1250.6416 | 1233.6150 | 1278.6365 | 1261.6099 | **S** | ***390.2096*** | 373.1830 | **3** |
| **13** | 1378.7001 | 1361.6736 | 1406.6951 | 1389.6685 | **Q** | ***303.1775*** | 286.1510 | **2** |
| **14** | | | | | **R** | ***175.1190*** | 158.0924 | **1** |

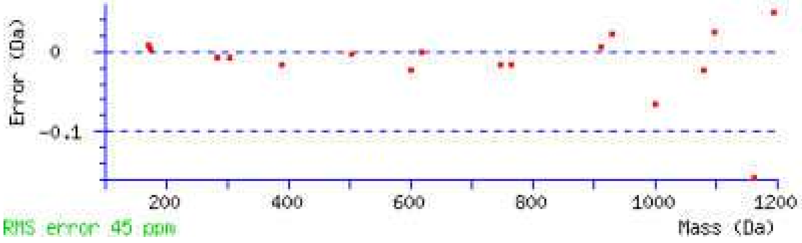


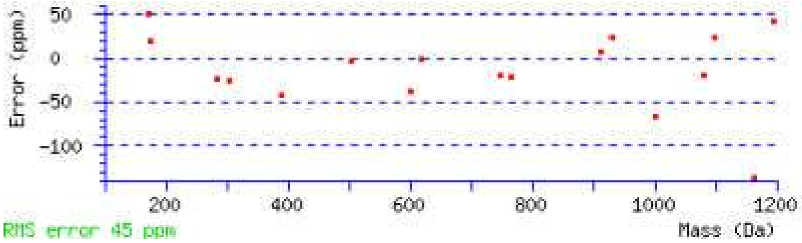


NCBI **BLAST** search of AVITVPAYFNDSQR
(Parameters: blastp, nr protein database, expect=20000, no filter, PAM30)
Other BLAST web gateways

**All matches to this query**

| Score | Mr(calc) | Delta | Sequence |
|---|---|---|---|
| 70.1 | 1579.7995 | 0.0552 | AVITVPAYFNDSQR |
| 11.7 | 1579.8392 | 0.0154 | GITMTVGVAVFDSKR |
| 11.7 | 1579.7413 | 0.1133 | MGVLDGAERFDSQR |
| 11.3 | 1579.7671 | 0.0876 | IIYFDYYNDAKR |
| 11.1 | 1579.9297 | -0.0751 | AVLTVPAGLGELTALR |
| 9.9 | 1579.7399 | 0.1147 | SSKSDEIENSLPMK |
| 9.9 | 1579.8028 | 0.0518 | KVLDMFDNLVSQR |
| 9.7 | 1579.8206 | 0.0340 | VTPSSSSLGVFESKR |
| 9.5 | 1579.8318 | 0.0228 | VNHSDVVEVVLQSR |
| 9.3 | 1579.7876 | 0.0671 | MAKSGGVSSSASSSLPK |

**Mascot:** http://www.matrixscience.com/

# MATRIX SCIENCE Mascot Search Results

## Peptide View **Spot no 108**

MS/MS Fragmentation of **APFDLFDTR**
Found in **gi|39104468** in **NCBInr**, heat shock protein 90 [Oryza sativa Japonica Group]

Match to Query 39: 1080.553724 from(1081.561000,1+) intensity(0.0000) index(13)
Title: Label: C2, Spot_Id: 219692, Peak_List_Id: 224833, MSMS Job_Run_Id: 21755, Comment:
Data file C:\Documents and Settings\Administrator\Desktop\farah BHU\spot set plate\Farah-Garima 11May 2013\ppw_C2_136842094000.txt

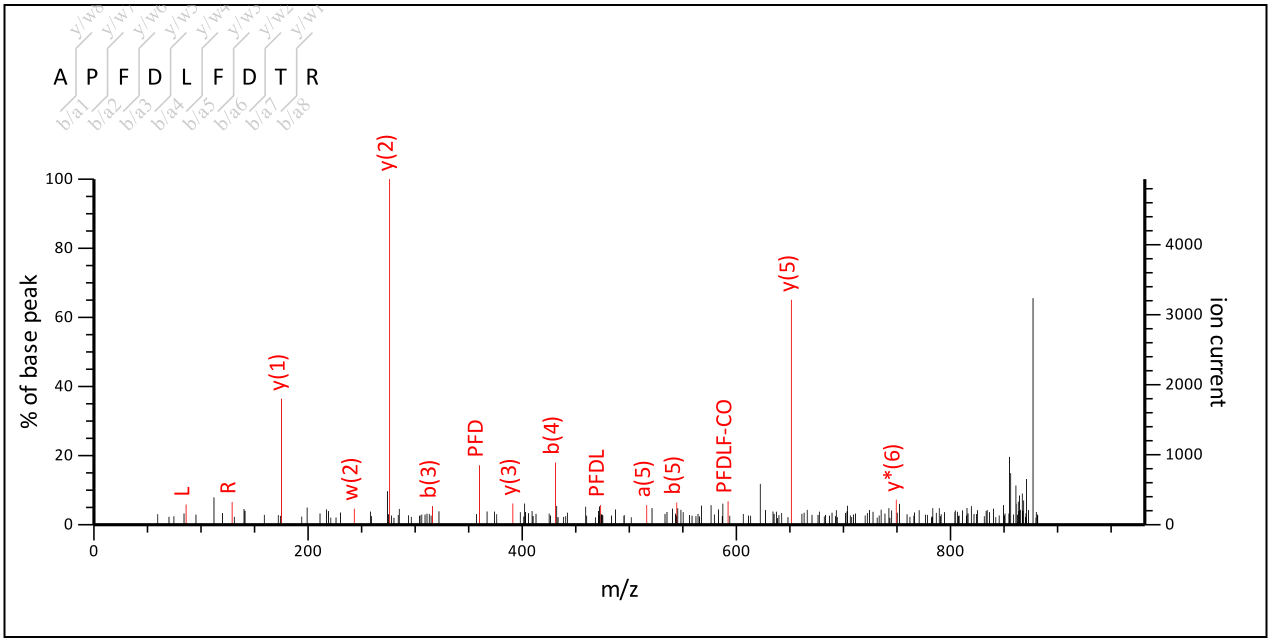


0 to 981.47

Label all possible matches ○ Label matches used for scoring ◉

**Monoisotopic mass of neutral peptide Mr(calc):** 1080.5240
**Fixed modifications:** Carbamidomethyl (C) (apply to specified residues or termini only)
**Ions Score:** 21 **Expect:** 16
**Matches :** 17/116 fragment ions using 27 most intense peaks (help)

| # | Immon. | a | $a^0$ | b | $b^0$ | d | d' | Seq. | v | w | w' | y | y* | $y^0$ | # |
|---|---|---|---|---|---|---|---|---|---|---|---|---|---|---|---|
| 1 | 44.0495 | 44.0495 | | 72.0444 | | 44.0495 | | A | | | | | | | 9 |
| 2 | 70.0651 | 141.1022 | | 169.0972 | | 115.0866 | | P | 968.4472 | 967.4520 | | 1010.4942 | 993.4676 | 992.4836 | 8 |
| 3 | 120.0808 | 288.1707 | | ***316.1656*** | | | | F | 821.3788 | | | 913.4414 | 896.4149 | 895.4308 | 7 |
| 4 | 88.0393 | 403.1976 | 385.1870 | ***431.1925*** | 413.1819 | 359.2078 | | D | 706.3519 | 705.3566 | | 766.3730 | 749.3464 | 748.3624 | 6 |
| 5 | 86.0964 | 516.2817 | 498.2711 | ***544.2766*** | 526.2660 | 474.2347 | | L | 593.2678 | 592.2726 | | ***651.3461*** | 634.3195 | 633.3355 | 5 |
| 6 | 120.0808 | 663.3501 | 645.3395 | 691.3450 | 673.3344 | | | F | 446.1994 | | | 538.2620 | 521.2354 | 520.2514 | 4 |
| 7 | 88.0393 | 778.3770 | 760.3665 | 806.3719 | 788.3614 | 734.3872 | | D | 331.1724 | 330.1772 | | ***391.1936*** | 374.1670 | 373.1830 | 3 |
| 8 | 74.0600 | 879.4247 | 861.4141 | 907.4196 | 889.4090 | 863.4298 | 865.4090 | T | 230.1248 | 243.1452 | 245.1244 | ***276.1666*** | 259.1401 | 258.1561 | 2 |
| 9 | 129.1135 | | | | | | | R | 74.0237 | 73.0284 | | ***175.1190*** | 158.0924 | | 1 |

| Seq | ya | yb | Seq | ya | yb | Seq | ya | yb |
|---|---|---|---|---|---|---|---|---|
| PF | 217.1335 | 245.1285 | PFD | 332.1605 | 360.1554 | PFDL | 445.2445 | 473.2395 |
| PFDLF | 592.3130 | 620.3079 | FD | 235.1077 | 263.1026 | FDL | 348.1918 | 376.1867 |
| FDLF | 495.2602 | 523.2551 | FDLFD | 610.2871 | 638.2821 | DL | 201.1234 | 229.1183 |
| | | | | | | | | |

| | | | | | | | | |
|---|---|---|---|---|---|---|---|---|
| **DLF** | 348.1918 | 376.1867 | **DLFD** | 463.2187 | 491.2136 | **DLFDT** | 564.2664 | 592.2613 |
| **LF** | 233.1648 | 261.1598 | **LFD** | 348.1918 | 376.1867 | **LFDT** | 449.2395 | 477.2344 |
| **FD** | 235.1077 | 263.1026 | **FDT** | 336.1554 | 364.1503 | **DT** | 189.0870 | 217.0819 |

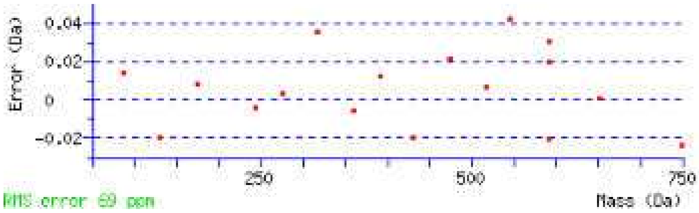


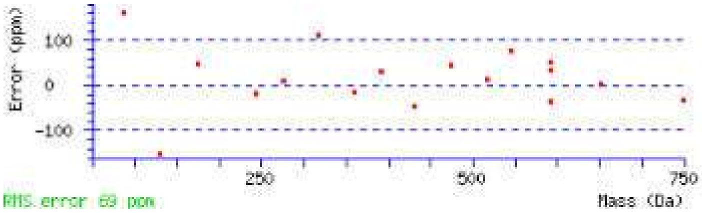


NCBI **BLAST** search of [APFDLFDTR]
(Parameters: blastp, nr protein database, expect=20000, no filter, PAM30)
Other BLAST [web gateways]

**All matches to this query**

| Score | Mr(calc) | Delta | Sequence |
|---|---|---|---|
| 21.0 | 1080.5240 | 0.0297 | APFDLFDTR |
| 15.0 | 1080.5386 | 0.0151 | LLGFCTGGTR |
| 15.0 | 1080.5096 | 0.0441 | LMWIDMTR |
| 15.0 | 1080.5386 | 0.0151 | MATYALQQR |
| 14.7 | 1080.5234 | 0.0304 | MAVAATGSTTR |
| 13.8 | 1080.4870 | 0.0668 | ETSINMNTR |
| 13.7 | 1080.5056 | 0.0481 | DMKMIQQR |
| 13.7 | 1080.5200 | 0.0337 | FDTSSLQQR |
| 12.6 | 1080.6403 | -0.0866 | QALKPAIQGR |
| 12.0 | 1080.5274 | 0.0263 | GLMLYPDTR |

**Mascot:** http://www.matrixscience.com/

# MATRIX SCIENCE Mascot Search Results

## Peptide View **Spot no 108**

MS/MS Fragmentation of **RAPFDLFDTR**
Found in **gi|39104468** in **NCBInr**, heat shock protein 90 [Oryza sativa Japonica Group]

Match to Query 55: 1236.646424 from(1237.653700,1+) intensity(0.0000) index(17)
Title: Label: C2, Spot_Id: 219692, Peak_List_Id: 224827, MSMS Job_Run_Id: 21755, Comment:
Data file C:\Documents and Settings\Administrator\Desktop\farah BHU\spot set plate\Farah-Garima 11May 2013\ppw_C2_136842094000.txt

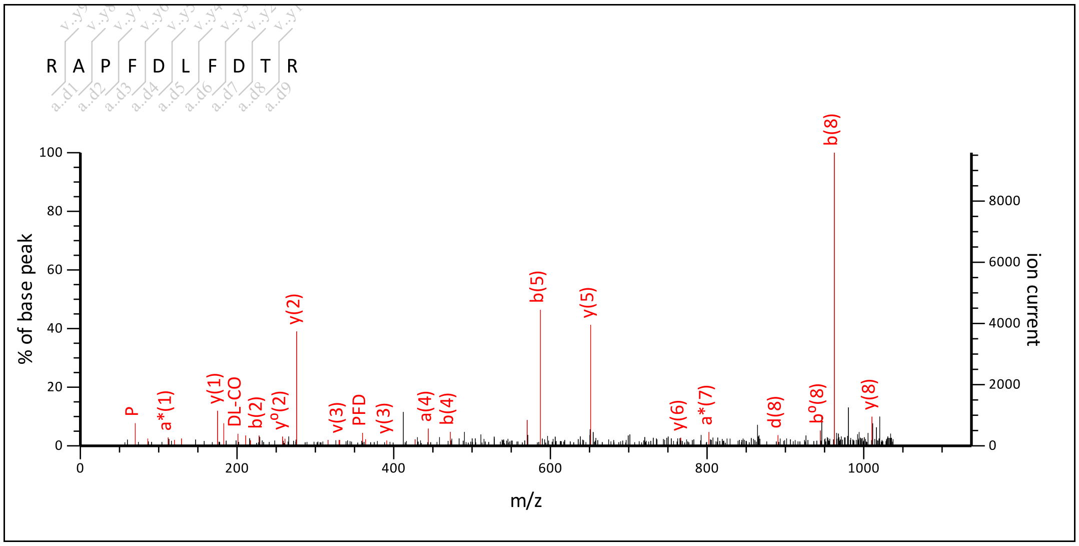


0 to 1137.47

Label all possible matches ○ Label matches used for scoring ◉

**Monoisotopic mass of neutral peptide Mr(calc):** 1236.6251
**Fixed modifications:** Carbamidomethyl (C) (apply to specified residues or termini only)
**Ions Score:** 19 **Expect:** 27
**Matches :** 37/151 fragment ions using 80 most intense peaks (help)

| # | Immon. | a | a* | $a^0$ | b | b* | $b^0$ | d | d' | Seq. | v | w | w' | y | y* | [illegible] |
|---|---|---|---|---|---|---|---|---|---|---|---|---|---|---|---|---|
| 1 | 129.1135 | **129.1135** | 112.0869 | | 157.1084 | 140.0818 | | 44.0495 | | **R** | | | | | | |
| 2 | 44.0495 | 200.1506 | 183.1240 | | **228.1455** | 211.1190 | | | | **A** | 1065.5000 | | | 1081.5313 | 1064.5047 | 106[illegible] |
| 3 | 70.0651 | 297.2034 | 280.1768 | | 325.1983 | 308.1717 | | 271.1877 | | **P** | 968.4472 | 967.4520 | | ***1010.4942*** | 993.4676 | 99[illegible] |
| 4 | 120.0808 | **444.2718** | 427.2452 | | **472.2667** | 455.2401 | | | | **F** | 821.3788 | | | 913.4414 | 896.4149 | 89[illegible] |
| 5 | 88.0393 | 559.2987 | 542.2722 | 541.2881 | **587.2936** | 570.2671 | 569.2831 | 515.3089 | | **D** | 706.3519 | 705.3566 | | ***766.3730*** | 749.3464 | 74[illegible] |
| 6 | 86.0964 | 672.3828 | 655.3562 | 654.3722 | 700.3777 | 683.3511 | 682.3671 | 630.3358 | | **L** | 593.2678 | 592.2726 | | ***651.3461*** | 634.3195 | 63[illegible] |
| 7 | 120.0808 | 819.4512 | 802.4246 | 801.4406 | 847.4461 | 830.4196 | 829.4355 | | | **F** | 446.1994 | | | 538.2620 | 521.2354 | 52[illegible] |
| 8 | 88.0393 | 934.4781 | 917.4516 | 916.4676 | **962.4730** | 945.4465 | 944.4625 | 890.4883 | | **D** | 331.1724 | 330.1772 | | ***391.1936*** | 374.1670 | 37[illegible] |
| 9 | 74.0600 | 1035.5258 | 1018.4993 | 1017.5152 | 1063.5207 | 1046.4942 | 1045.5102 | 1019.5309 | 1021.5102 | **T** | 230.1248 | 243.1452 | 245.1244 | ***276.1666*** | 259.1401 | 25[illegible] |
| 10 | 129.1135 | | | | | | | | | **R** | 74.0237 | 73.0284 | | ***175.1190*** | 158.0924 | |

| Seq | ya | yb | Seq | ya | yb | Seq | ya | yb |
|---|---|---|---|---|---|---|---|---|
| **AP** | 141.1022 | 169.0972 | **APF** | 288.1707 | 316.1656 | **APFD** | 403.1976 | 431.1925 |
| **APFDL** | 516.2817 | 544.2766 | **APFDLF** | 663.3501 | 691.3450 | **PF** | 217.1335 | 245.1285 |
| **PFD** | 332.1605 | 360.1554 | **PFDL** | 445.2445 | 473.2395 | **PFDLF** | 592.3130 | 620.3079 |
| **FD** | 235.1077 | 263.1026 | **FDL** | 348.1918 | 376.1867 | **FDLF** | 495.2602 | 523.2551 |
| **FDLFD** | 610.2871 | 638.2821 | **DL** | 201.1234 | 229.1183 | **DLF** | 348.1918 | 376.1867 |
| **DLFD** | 463.2187 | 491.2136 | **DLFDT** | 564.2664 | 592.2613 | **LF** | 233.1648 | 261.1598 |
| **LFD** | 348.1918 | 376.1867 | **LFDT** | 449.2395 | 477.2344 | **FD** | 235.1077 | 263.1026 |
| **FDT** | 336.1554 | 364.1503 | **DT** | 189.0870 | 217.0819 | | | |

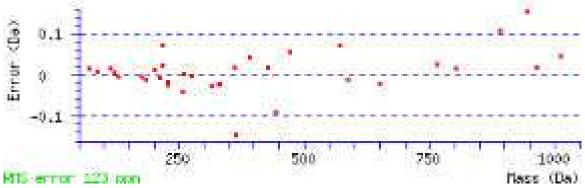


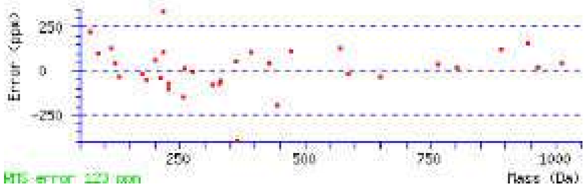


NCBI **BLAST** search of RAPFDLFDTR
(Parameters: blastp, nr protein database, expect=20000, no filter, PAM30)
Other BLAST web gateways

**All matches to this query**

| Score | Mr(calc) | Delta | Sequence |
|---|---|---|---|
| 27.9 | 1236.6132 | 0.0332 | SMGSGVGEKITR |
| 19.5 | 1236.6251 | 0.0213 | RAPFDLFDTR |
| 17.2 | 1236.6067 | 0.0397 | MSTRKPTCTR |
| 16.2 | 1236.5907 | 0.0557 | KVTEEDMLEK |
| 16.0 | 1236.7190 | -0.0726 | KVLVPPSGPSTR |
| 16.0 | 1236.6132 | 0.0332 | SMGSVGGEKITR |
| 16.0 | 1236.5470 | 0.0995 | SSTATADAEQEK |
| 15.7 | 1236.5366 | 0.1098 | NLPMDLMDEK |
| 15.4 | 1236.5622 | 0.0842 | NNTGLDEFAEK |
| 15.2 | 1236.7303 | -0.0838 | VGVRAPATPLTR |

**Mascot:** http://www.matrixscience.com/

# MATRIX SCIENCE Mascot Search Results

## Peptide View **Spot no 108**

MS/MS Fragmentation of **SDLVNNLGTIAR**
Found in **gi|39104468** in **NCBInr**, heat shock protein 90 [Oryza sativa Japonica Group]

Match to Query 60: 1271.722724 from(1272.730000,1+) intensity(0.0000) index(18)
Title: Label: C2, Spot_Id: 219692, Peak_List_Id: 224835, MSMS Job_Run_Id: 21755, Comment:
Data file C:\Documents and Settings\Administrator\Desktop\farah BHU\spot set plate\Farah-Garima 11May 2013\ppw_C2_136842094000.txt

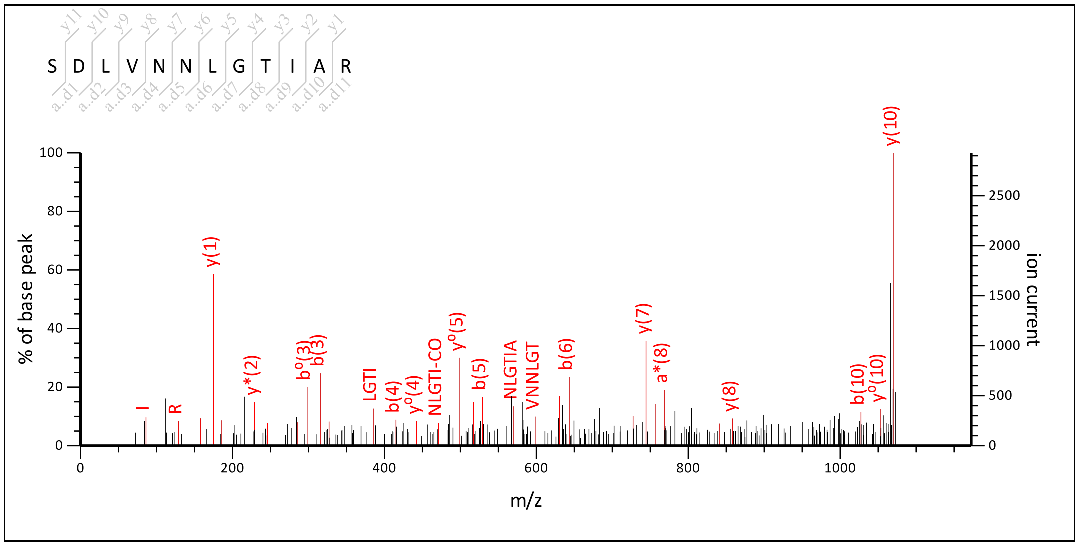


0 to 1172.54

Label all possible matches Label matches used for scoring

```
Monoisotopic mass of neutral peptide Mr(calc): 1271.6834
Fixed modifications: Carbamidomethyl (C) (apply to specified residues or termini only)
Ions Score: 39  Expect: 0.22
Matches : 43/207 fragment ions using 51 most intense peaks  (help)
```

| # | Immon. | a | a* | a$^0$ | b | b* | b$^0$ | d | d' | Seq. | v | w | w' | y | y* | y |
|---|---|---|---|---|---|---|---|---|---|---|---|---|---|---|---|---|
| 1 | 60.0444 | 60.0444 | | 42.0338 | 88.0393 | | 70.0287 | 44.0495 | | S | | | | | | |
| 2 | 88.0393 | 175.0713 | | 157.0608 | 203.0662 | | 185.0557 | 131.0815 | | D | 1125.6375 | 1124.6422 | | 1185.6586 | 1168.6321 | 1167. |
| 3 | 86.0964 | 288.1554 | | 270.1448 | 316.1503 | | 298.1397 | 246.1084 | | L | 1012.5534 | 1011.5582 | | 1070.6317 | 1053.6051 | 1052. |
| 4 | 72.0808 | 387.2238 | | 369.2132 | 415.2187 | | 397.2082 | 373.2082 | | V | 913.4850 | 926.5054 | | 957.5476 | 940.5211 | 939. |
| 5 | 87.0553 | 501.2667 | 484.2402 | 483.2562 | 529.2617 | 512.2351 | 511.2511 | 458.2609 | | N | 799.4421 | 798.4468 | | 858.4792 | 841.4526 | 840. |
| 6 | 87.0553 | 615.3097 | 598.2831 | 597.2991 | 643.3046 | 626.2780 | 625.2940 | 572.3039 | | N | 685.3992 | 684.4039 | | 744.4363 | 727.4097 | 726. |
| 7 | 86.0964 | 728.3937 | 711.3672 | 710.3832 | 756.3886 | 739.3621 | 738.3781 | 686.3468 | | L | 572.3151 | 571.3198 | | 630.3933 | 613.3668 | 612. |
| 8 | 30.0338 | 785.4152 | 768.3886 | 767.4046 | 813.4101 | 796.3836 | 795.3995 | | | G | | | | 517.3093 | 500.2827 | 499. |
| 9 | 74.0600 | 886.4629 | 869.4363 | 868.4523 | 914.4578 | 897.4312 | 896.4472 | 870.4680 | 872.4472 | T | 414.2459 | 427.2663 | 429.2456 | 460.2878 | 443.2613 | 442. |
| 10 | 86.0964 | 999.5469 | 982.5204 | 981.5364 | 1027.5419 | 1010.5153 | 1009.5313 | 971.5156 | 985.5313 | I | 301.1619 | 314.1823 | 328.1979 | 359.2401 | 342.2136 | |
| 11 | 44.0495 | 1070.5840 | 1053.5575 | 1052.5735 | 1098.5790 | 1081.5524 | 1080.5684 | | | A | 230.1248 | | | 246.1561 | 229.1295 | |
| 12 | 129.1135 | | | | | | | | | R | 74.0237 | 73.0284 | | 175.1190 | 158.0924 | |

| Seq | ya | yb | Seq | ya | yb | Seq | ya | yb |
|---|---|---|---|---|---|---|---|---|
| DL | 201.1234 | 229.1183 | DLV | 300.1918 | 328.1867 | DLVN | 414.2347 | 442.2296 |
| DLVNN | 528.2776 | 556.2726 | DLVNNL | 641.3617 | 669.3566 | DLVNNLG | 698.3832 | 726.3781 |
| LV | 185.1648 | 213.1598 | LVN | 299.2078 | 327.2027 | LVNN | 413.2507 | 441.2456 |
| LVNNL | 526.3348 | 554.3297 | LVNNLG | 583.3562 | 611.3511 | LVNNLGT | 684.4039 | 712.3988 |
| VN | 186.1237 | 214.1186 | VNN | 300.1666 | 328.1615 | VNNL | 413.2507 | 441.2456 |
| VNNLG | 470.2722 | 498.2671 | VNNLGT | 571.3198 | 599.3148 | VNNLGTI | 684.4039 | 712.3988 |
| NN | 201.0982 | 229.0931 | NNL | 314.1823 | 342.1772 | NNLG | 371.2037 | 399.1987 |
| NNLGT | 472.2514 | 500.2463 | NNLGTI | 585.3355 | 613.3304 | NNLGTIA | 656.3726 | 684.3675 |
| NL | 200.1394 | 228.1343 | NLG | 257.1608 | 285.1557 | NLGT | 358.2085 | 386.2034 |

| | | | | | | | | |
|---|---|---|---|---|---|---|---|---|
| **NLGTI** | 471.2926 | 499.2875 | **NLGTIA** | 542.3297 | 570.3246 | **LG** | 143.1179 | 171.1128 |
| **LGT** | 244.1656 | 272.1605 | **LGTI** | 357.2496 | 385.2445 | **LGTIA** | 428.2867 | 456.2817 |
| **GT** | 131.0815 | 159.0764 | **GTI** | 244.1656 | 272.1605 | **GTIA** | 315.2027 | 343.1976 |
| **TI** | 187.1441 | 215.1390 | **TIA** | 258.1812 | 286.1761 | **IA** | 157.1335 | 185.1285 |

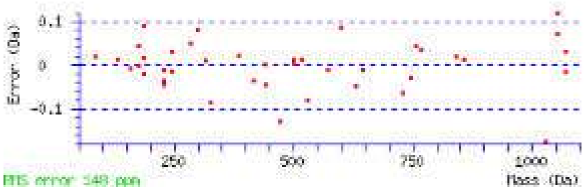


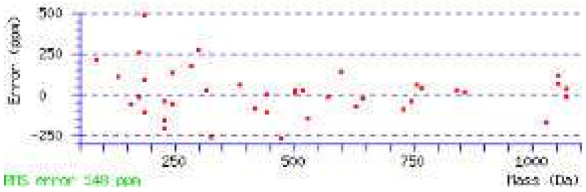


NCBI **BLAST** search of SDLVNNLGTIAR
(Parameters: blastp, nr protein database, expect=20000, no filter, PAM30)
Other BLAST web gateways

**All matches to this query**

| Score | Mr(calc) | Delta | Sequence |
|---|---|---|---|
| 39.1 | 1271.6834 | 0.0394 | SDLVNNLGTIAR |
| 30.3 | 1271.6834 | 0.0394 | SDLVNNLATIGR |
| 28.2 | 1271.6470 | 0.0758 | QLIDSNTPAASR |
| 26.2 | 1271.7020 | 0.0207 | VSIAVGMPKGSAR |
| 24.5 | 1271.7085 | 0.0142 | TTLPETLIGTAR |
| 21.7 | 1271.6833 | 0.0394 | TTPLAEANTKAR |
| 21.5 | 1271.6292 | 0.0935 | MADPGASVNIAAR |
| 20.4 | 1271.6833 | 0.0394 | IAAATAAEEAKAR |
| 19.9 | 1271.7197 | 0.0030 | LAAEVSSQIKAR |
| 18.4 | 1271.6833 | 0.0394 | IVAEELERSAR |

**Mascot:** http://www.matrixscience.com/

# MATRIX SCIENCE Mascot Search Results

## Peptide View **Spot no 108**

MS/MS Fragmentation of **GIVDSEDLPLNISR**
Found in **gi|39104468** in **NCBInr**, heat shock protein 90 [Oryza sativa Japonica Group]

Match to Query 80: 1526.843524 from(1527.850800,1+) intensity(0.0000) index(25)
Title: Label: C2, Spot_Id: 219692, Peak_List_Id: 224828, MSMS Job_Run_Id: 21755, Comment:
Data file C:\Documents and Settings\Administrator\Desktop\farah BHU\spot set plate\Farah-Garima 11May 2013\ppw_C2_136842094000.txt

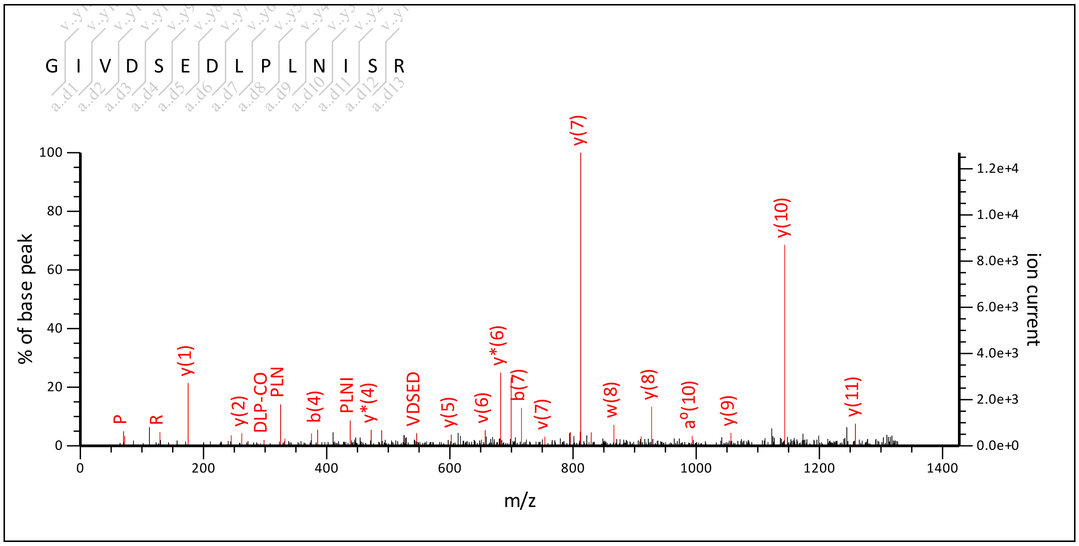


0 to 1427.16

Label all possible matches ○ Label matches used for scoring ◉

**Monoisotopic mass of neutral peptide Mr(calc):** 1526.7940
**Fixed modifications:** Carbamidomethyl (C) (apply to specified residues or termini only)
**Ions Score:** 81 **Expect:** 1.3e-05
**Matches :** 44/237 fragment ions using 52 most intense peaks (help)

| # | Immon. | a | a* | a0 | b | b* | b0 | d | d' | Seq. | v | w | w' | y | y* | |
|---|---|---|---|---|---|---|---|---|---|---|---|---|---|---|---|---|
| 1 | 30.0338 | 30.0338 | | | 58.0287 | | | 44.0495 | | G | | | | | | |
| 2 | 86.0964 | 143.1179 | | | 171.1128 | | | 115.0866 | 129.1022 | I | 1412.7016 | 1425.7220 | 1439.7376 | 1470.7798 | 1453.7533 | [illegible] |
| 3 | 72.0808 | 242.1863 | | | 270.1812 | | | 228.1707 | | V | 1313.6332 | 1326.6536 | | 1357.6958 | 1340.6692 | [illegible] |
| 4 | 88.0393 | 357.2132 | | 339.2027 | **385.2082** | | 367.1976 | 313.2234 | | D | 1198.6062 | 1197.6110 | | ***1258.6274*** | 1241.6008 | [illegible] |
| 5 | 60.0444 | 444.2453 | | 426.2347 | **472.2402** | | 454.2296 | 428.2504 | | S | 1111.5742 | 1110.5790 | | ***1143.6004*** | 1126.5739 | [illegible] |
| 6 | 102.0550 | 573.2879 | | 555.2773 | 601.2828 | | 583.2722 | 515.2824 | | E | 982.5316 | 981.5364 | | ***1056.5684*** | 1039.5419 | [illegible] |
| 7 | 88.0393 | 688.3148 | | 670.3042 | **716.3097** | | 698.2992 | 644.3250 | | D | 867.5047 | 866.5094 | | ***927.5258*** | 910.4993 | [illegible] |
| 8 | 86.0964 | 801.3989 | | 783.3883 | **829.3938** | | 811.3832 | 759.3519 | | L | 754.4206 | 753.4254 | | ***812.4989*** | 795.4723 | [illegible] |
| 9 | 70.0651 | 898.4516 | | 880.4411 | 926.4466 | | 908.4360 | 872.4360 | | P | 657.3678 | 656.3726 | | ***699.4148*** | 682.3882 | [illegible] |
| 10 | 86.0964 | 1011.5357 | | 993.5251 | 1039.5306 | | 1021.5201 | 969.4888 | | L | 544.2838 | 543.2885 | | ***602.3620*** | 585.3355 | [illegible] |
| 11 | 87.0553 | 1125.5786 | 1108.5521 | 1107.5681 | 1153.5735 | 1136.5470 | 1135.5630 | 1082.5728 | | N | 430.2409 | 429.2456 | | ***489.2780*** | 472.2514 | [illegible] |
| 12 | 86.0964 | 1238.6627 | 1221.6361 | 1220.6521 | 1266.6576 | 1249.6311 | 1248.6470 | 1210.6314 | 1224.6470 | I | 317.1568 | 330.1772 | 344.1928 | ***375.2350*** | 358.2085 | [illegible] |
| 13 | 60.0444 | 1325.6947 | 1308.6682 | 1307.6842 | 1353.6896 | 1336.6631 | 1335.6791 | 1309.6998 | | S | 230.1248 | 229.1295 | | ***262.1510*** | 245.1244 | [illegible] |
| 14 | 129.1135 | | | | | | | | | R | 74.0237 | 73.0284 | | ***175.1190*** | 158.0924 | |

| Seq | ya | yb | Seq | ya | yb | Seq | ya | yb |
|---|---|---|---|---|---|---|---|---|
| IV | 185.1648 | 213.1598 | IVD | 300.1918 | 328.1867 | IVDS | 387.2238 | 415.2187 |
| IVDSE | 516.2664 | 544.2613 | IVDSED | 631.2933 | 659.2883 | VD | 187.1077 | 215.1026 |
| VDS | 274.1397 | 302.1347 | VDSE | 403.1823 | 431.1773 | VDSED | 518.2093 | 546.2042 |
| VDSEDL | 631.2933 | 659.2883 | DS | 175.0713 | 203.0662 | DSE | 304.1139 | 332.1088 |
| DSED | 419.1409 | 447.1358 | DSEDL | 532.2249 | 560.2198 | DSEDLP | 629.2777 | 657.2726 |
| SE | 189.0870 | 217.0819 | SED | 304.1139 | 332.1088 | SEDL | 417.1980 | 445.1929 |
| SEDLP | 514.2508 | 542.2457 | SEDLPL | 627.3348 | 655.3297 | ED | 217.0819 | 245.0768 |

| | | | | | | | | |
|---|---|---|---|---|---|---|---|---|
| **EDL** | 330.1660 | 358.1609 | **EDLP** | 427.2187 | 455.2136 | **EDLPL** | 540.3028 | 568.2977 |
| **EDLPLN** | 654.3457 | 682.3406 | **DL** | 201.1234 | 229.1183 | **DLP** | 298.1761 | 326.1710 |
| **DLPL** | 411.2602 | 439.2551 | **DLPLN** | 525.3031 | 553.2980 | **DLPLNI** | 638.3872 | 666.3821 |
| **LP** | 183.1492 | 211.1441 | **LPL** | 296.2333 | 324.2282 | **LPLN** | 410.2762 | 438.2711 |
| **LPLNI** | 523.3602 | 551.3552 | **LPLNIS** | 610.3923 | 638.3872 | **PL** | 183.1492 | 211.1441 |
| **PLN** | 297.1921 | 325.1870 | **PLNI** | 410.2762 | 438.2711 | **PLNIS** | 497.3082 | 525.3031 |
| **LN** | 200.1394 | 228.1343 | **LNI** | 313.2234 | 341.2183 | **LNIS** | 400.2554 | 428.2504 |
| **NI** | 200.1394 | 228.1343 | **NIS** | 287.1714 | 315.1663 | **IS** | 173.1285 | 201.1234 |

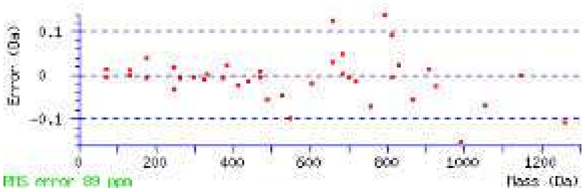


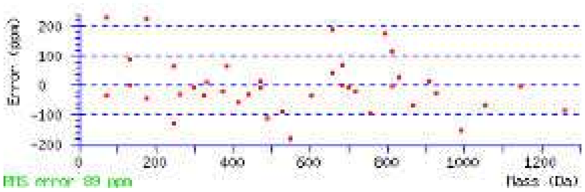


NCBI **BLAST** search of GIVDSEDLPLNISR
(Parameters: blastp, nr protein database, expect=20000, no filter, PAM30)
Other BLAST web gateways

**All matches to this query**

| Score | Mr(calc) | Delta | Sequence |
|---|---|---|---|
| 81.2 | 1526.7940 | 0.0495 | GIVDSEDLPLNISR |
| 25.5 | 1526.8052 | 0.0383 | DRISNLPDELISR |
| 22.8 | 1526.8973 | -0.0538 | FVPKVQLPFSPLR |
| 21.3 | 1526.8239 | 0.0196 | QMALQQLVAGSPLR |
| 19.8 | 1526.8205 | 0.0230 | NPFNENLPVKISR |
| 15.6 | 1526.8165 | 0.0270 | RNSAGETLSVQIPR |
| 15.3 | 1526.8165 | 0.0271 | IRAAESALNLEQGR |
| 15.0 | 1526.7664 | 0.0772 | AVVDSNMVHHYKK |
| 14.1 | 1526.7073 | 0.1362 | NRNDTSTYSNISR |
| 13.7 | 1526.7913 | 0.0522 | TVSSPRNSSSPRPR |

**Mascot:** http://www.matrixscience.com/

# MATRIX SCIENCE Mascot Search Results

## Peptide View **Spot no 108**

MS/MS Fragmentation of **SGDELTSLKDYVTR**
Found in **gi|39104468** in **NCBInr**, heat shock protein 90 [Oryza sativa Japonica Group]

Match to Query 88: 1582.835724 from(1583.843000,1+) intensity(0.0000) index(28)
Title: Label: C2, Spot_Id: 219692, Peak_List_Id: 224832, MSMS Job_Run_Id: 21755, Comment:
Data file C:\Documents and Settings\Administrator\Desktop\farah BHU\spot set plate\Farah-Garima 11May 2013\ppw_C2_136842094000.txt

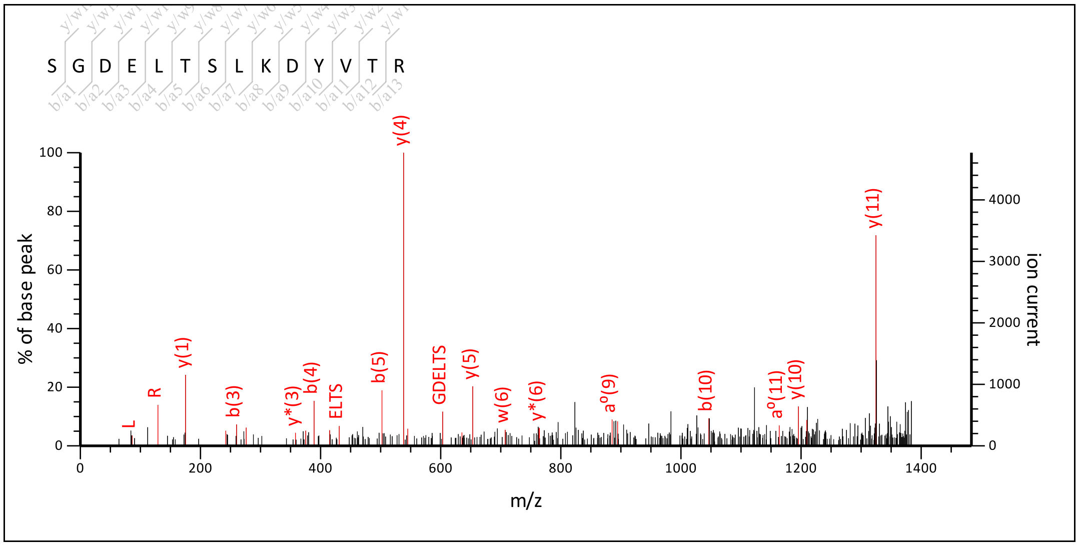


0 to 1483.7

Label all possible matches Label matches used for scoring

**Monoisotopic mass of neutral peptide Mr(calc):** 1582.7839
**Fixed modifications:** Carbamidomethyl (C) (apply to specified residues or termini only)
**Ions Score:** 35 **Expect:** 0.6
**Matches :** 33/240 fragment ions using 56 most intense peaks (help)

| # | Immon. | a | a* | $a^0$ | b | b* | $b^0$ | d | d' | Seq. | v | w | w' | y | y* | |
|---|---|---|---|---|---|---|---|---|---|---|---|---|---|---|---|---|
| 1 | 60.0444 | 60.0444 | | 42.0338 | 88.0393 | | 70.0287 | 44.0495 | | S | | | | | | |
| 2 | 30.0338 | 117.0659 | | 99.0553 | 145.0608 | | 127.0502 | | | G | | | | 1496.7591 | 1479.7326 | [illegible] |
| 3 | 88.0393 | 232.0928 | | 214.0822 | ***260.0877*** | | 242.0771 | 188.1030 | | D | 1379.7165 | 1378.7213 | | 1439.7377 | 1422.7111 | [illegible] |
| 4 | 102.0550 | 361.1354 | | 343.1248 | ***389.1303*** | | 371.1197 | 303.1299 | | E | 1250.6739 | 1249.6787 | | ***1324.7107*** | 1307.6842 | [illegible] |
| 5 | 86.0964 | 474.2195 | | 456.2089 | ***502.2144*** | | 484.2038 | 432.1725 | | L | 1137.5899 | 1136.5946 | | ***1195.6681*** | 1178.6416 | [illegible] |
| 6 | 74.0600 | 575.2671 | | 557.2566 | ***603.2620*** | | 585.2515 | 559.2722 | 561.2515 | T | 1036.5422 | 1049.5626 | 1051.5419 | 1082.5841 | 1065.5575 | [illegible] |
| 7 | 60.0444 | 662.2992 | | 644.2886 | 690.2941 | | 672.2835 | 646.3042 | | S | 949.5102 | 948.5149 | | 981.5364 | 964.5098 | [illegible] |
| 8 | 86.0964 | 775.3832 | | 757.3727 | 803.3781 | | 785.3676 | 733.3363 | | L | 836.4261 | 835.4308 | | ***894.5043*** | 877.4778 | [illegible] |
| 9 | 101.1073 | 903.4782 | 886.4516 | 885.4676 | 931.4731 | 914.4466 | 913.4625 | 846.4203 | | K | 708.3311 | 707.3359 | | 781.4203 | 764.3937 | [illegible] |
| 10 | 88.0393 | 1018.5051 | 1001.4786 | 1000.4946 | ***1046.5000*** | 1029.4735 | 1028.4895 | 974.5153 | | D | 593.3042 | 592.3089 | | ***653.3253*** | 636.2988 | [illegible] |
| 11 | 136.0757 | 1181.5685 | 1164.5419 | 1163.5579 | ***1209.5634*** | 1192.5368 | 1191.5528 | | | Y | 430.2409 | | | ***538.2984*** | 521.2718 | [illegible] |
| 12 | 72.0808 | 1280.6369 | 1263.6103 | 1262.6263 | 1308.6318 | 1291.6052 | 1290.6212 | 1266.6212 | | V | 331.1724 | 344.1928 | | ***375.2350*** | 358.2085 | [illegible] |
| 13 | 74.0600 | 1381.6846 | 1364.6580 | 1363.6740 | 1409.6795 | 1392.6529 | 1391.6689 | 1365.6896 | 1367.6689 | T | 230.1248 | 243.1452 | 245.1244 | ***276.1666*** | 259.1401 | [illegible] |
| 14 | 129.1135 | | | | | | | | | R | 74.0237 | 73.0284 | | ***175.1190*** | 158.0924 | |

| Seq | ya | yb | Seq | ya | yb | Seq | ya | yb |
|---|---|---|---|---|---|---|---|---|
| **GD** | 145.0608 | 173.0557 | **GDE** | 274.1034 | 302.0983 | **GDEL** | 387.1874 | 415.1823 |
| **GDELT** | 488.2351 | 516.2300 | **GDELTS** | 575.2671 | 603.2620 | **GDELTSL** | 688.3512 | 716.3461 |
| **DE** | 217.0819 | 245.0768 | **DEL** | 330.1660 | 358.1609 | **DELT** | 431.2136 | 459.2086 |
| **DELTS** | 518.2457 | 546.2406 | **DELTSL** | 631.3297 | 659.3246 | **EL** | 215.1390 | 243.1339 |
| **ELT** | 316.1867 | 344.1816 | **ELTS** | 403.2187 | 431.2136 | **ELTSL** | 516.3028 | 544.2977 |
| **ELTSLK** | 644.3978 | 672.3927 | **LT** | 187.1441 | 215.1390 | **LTS** | 274.1761 | 302.1710 |
| **LTSL** | 387.2602 | 415.2551 | **LTSLK** | 515.3552 | 543.3501 | **LTSLKD** | 630.3821 | 658.3770 |

| | | | | | | | | |
|---|---|---|---|---|---|---|---|---|
| **TS** | 161.0921 | 189.0870 | **TSL** | 274.1761 | 302.1710 | **TSLK** | 402.2711 | 430.2660 |
| **TSLKD** | 517.2980 | 545.2930 | **TSLKDY** | 680.3614 | 708.3563 | **SL** | 173.1285 | 201.1234 |
| **SLK** | 301.2234 | 329.2183 | **SLKD** | 416.2504 | 444.2453 | **SLKDY** | 579.3137 | 607.3086 |
| **SLKDYV** | 678.3821 | 706.3770 | **LK** | 214.1914 | 242.1863 | **LKD** | 329.2183 | 357.2132 |
| **LKDY** | 492.2817 | 520.2766 | **LKDYV** | 591.3501 | 619.3450 | **LKDYVT** | 692.3978 | 720.3927 |
| **KD** | 216.1343 | 244.1292 | **KDY** | 379.1976 | 407.1925 | **KDYV** | 478.2660 | 506.2609 |
| **KDYVT** | 579.3137 | 607.3086 | **DY** | 251.1026 | 279.0975 | **DYV** | 350.1710 | 378.1660 |
| **DYVT** | 451.2187 | 479.2136 | **YV** | 235.1441 | 263.1390 | **YVT** | 336.1918 | 364.1867 |
| **VT** | 173.1285 | 201.1234 | | | | | | |

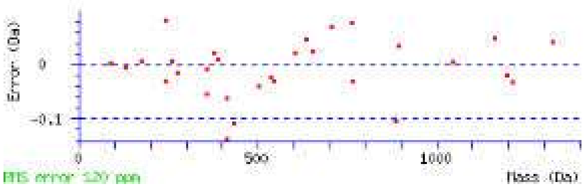


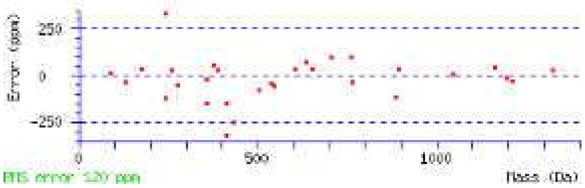


NCBI **BLAST** search of SGDELTSLKDYVTR
(Parameters: blastp, nr protein database, expect=20000, no filter, PAM30)
Other BLAST web gateways

**All matches to this query**

| Score | Mr(calc) | Delta | Sequence |
|---|---|---|---|
| 34.5 | 1582.7839 | 0.0519 | SGDELTSLKDYVTR |
| 24.6 | 1582.7839 | 0.0519 | SGEDLTSLKDYVTR |
| 23.6 | 1582.9658 | -0.1300 | SITSILKPLEILTR |
| 18.5 | 1582.7773 | 0.0584 | KLHMPATTSPDDVR |
| 15.4 | 1582.8468 | -0.0110 | SLTAHFSPPVTGITR |
| 14.7 | 1582.7369 | 0.0988 | TSSLSSSGGGCTAQRK |
| 14.2 | 1582.6900 | 0.1458 | DGSESWVEVQYER |
| 13.2 | 1582.8243 | 0.0114 | DTSLLQPGTFYLTK |
| 12.8 | 1582.8905 | -0.0548 | KLQFGMVYVLTLR |
| 12.7 | 1582.6901 | 0.1456 | MEVLMNERMNER |

**Mascot:** http://www.matrixscience.com/

# MATRIX SCIENCE Mascot Search Results

**Peptide View** **Spot no 108**

MS/MS Fragmentation of **KPEEITKEEYAAFYK**
Found in **gi|39104468** in **NCBInr**, heat shock protein 90 [Oryza sativa Japonica Group]

Match to Query 94: 1844.956424 from(1845.963700,1+) intensity(0.0000) index(31)
Title: Label: C2, Spot_Id: 219692, Peak_List_Id: 224829, MSMS Job_Run_Id: 21755, Comment:
Data file C:\Documents and Settings\Administrator\Desktop\farah BHU\spot set plate\Farah-Garima 11May 2013\ppw_C2_136842094000.txt

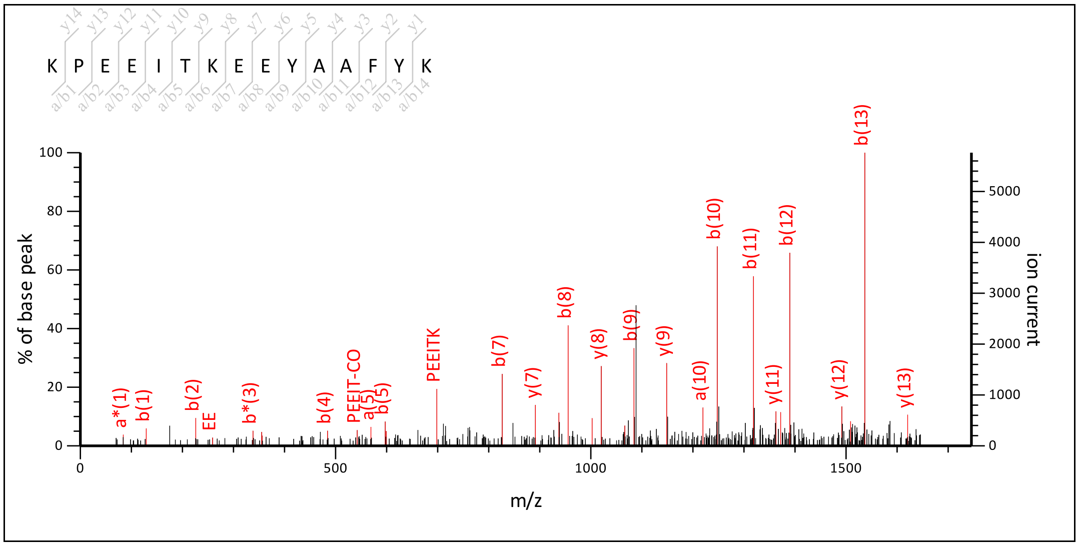


0 to 1745.61

Label all possible matches ○ Label matches used for scoring ◉

**Monoisotopic mass of neutral peptide Mr(calc):** 1844.9196
**Fixed modifications:** Carbamidomethyl (C) (apply to specified residues or termini only)
**Ions Score:** 103 **Expect:** 1.1e-07
**Matches :** 35/257 fragment ions using 48 most intense peaks (help)

| # | Immon. | a | a* | a0 | b | b* | b0 | d | d' | Seq. | v | w | w' | y | y* | |
|---|---|---|---|---|---|---|---|---|---|---|---|---|---|---|---|---|
| 1 | 101.1073 | 101.1073 | 84.0808 | | ***129.1022*** | 112.0757 | | 44.0495 | | K | | | | | | |
| 2 | 70.0651 | 198.1601 | 181.1335 | | ***226.1550*** | 209.1285 | | 172.1444 | | P | 1675.7850 | 1674.7897 | | 1717.8319 | 1700.8054 | 169 |
| 3 | 102.0550 | 327.2027 | 310.1761 | 309.1921 | ***355.1976*** | 338.1710 | 337.1870 | 269.1972 | | E | 1546.7424 | 1545.7472 | | **1620.7792** | 1603.7526 | 160 |
| 4 | 102.0550 | 456.2453 | 439.2187 | 438.2347 | ***484.2402*** | 467.2136 | 466.2296 | 398.2398 | | E | 1417.6998 | 1416.7046 | | **1491.7366** | 1474.7100 | 147 |
| 5 | 86.0964 | **569.3293** | 552.3028 | 551.3188 | ***597.3243*** | 580.2977 | 579.3137 | 541.2980 | 555.3137 | I | 1304.6157 | 1317.6361 | 1331.6518 | **1362.6940** | 1345.6674 | 134 |
| 6 | 74.0600 | 670.3770 | 653.3505 | 652.3665 | ***698.3719*** | 681.3454 | 680.3614 | 654.3821 | 656.3614 | T | 1203.5681 | 1216.5885 | 1218.5677 | 1249.6099 | 1232.5834 | 123 |
| 7 | 101.1073 | 798.4720 | 781.4454 | 780.4614 | ***826.4669*** | 809.4403 | 808.4563 | 741.4141 | | K | 1075.4731 | 1074.4779 | | **1148.5623** | 1131.5357 | 113 |
| 8 | 102.0550 | 927.5146 | 910.4880 | 909.5040 | ***955.5095*** | 938.4829 | 937.4989 | 869.5091 | | E | 946.4305 | 945.4353 | | **1020.4673** | 1003.4407 | 100 |
| 9 | 102.0550 | 1056.5572 | 1039.5306 | 1038.5466 | ***1084.5521*** | 1067.5255 | 1066.5415 | 998.5517 | | E | 817.3879 | 816.3927 | | **891.4247** | 874.3981 | 87 |
| 10 | 136.0757 | **1219.6205** | 1202.5939 | 1201.6099 | ***1247.6154*** | 1230.5889 | 1229.6048 | | | Y | 654.3246 | | | 762.3821 | 745.3556 | |
| 11 | 44.0495 | 1290.6576 | 1273.6311 | 1272.6470 | ***1318.6525*** | 1301.6260 | 1300.6420 | | | A | 583.2875 | | | **599.3188** | 582.2922 | |
| 12 | 44.0495 | 1361.6947 | 1344.6682 | 1343.6842 | ***1389.6896*** | 1372.6631 | 1371.6791 | | | A | 512.2504 | | | 528.2817 | 511.2551 | |
| 13 | 120.0808 | **1508.7631** | 1491.7366 | 1490.7526 | ***1536.7581*** | 1519.7315 | 1518.7475 | | | F | 365.1819 | | | 457.2445 | 440.2180 | |
| 14 | 136.0757 | 1671.8265 | 1654.7999 | 1653.8159 | 1699.8214 | 1682.7948 | 1681.8108 | | | Y | 202.1186 | | | 310.1761 | 293.1496 | |
| 15 | 101.1073 | | | | | | | | | K | 74.0237 | 73.0284 | | 147.1128 | 130.0863 | |

| Seq | ya | yb | Seq | ya | yb | Seq | ya | yb |
|---|---|---|---|---|---|---|---|---|
| PE | 199.1077 | 227.1026 | PEE | 328.1503 | 356.1452 | PEEI | 441.2344 | 469.2293 |
| PEEIT | 542.2821 | 570.2770 | PEEITK | 670.3770 | 698.3719 | EE | 231.0975 | 259.0925 |
| EEI | 344.1816 | 372.1765 | EEIT | 445.2293 | 473.2242 | EEITK | 573.3243 | 601.3192 |
| EI | 215.1390 | 243.1339 | EIT | 316.1867 | 344.1816 | EITK | 444.2817 | 472.2766 |
| EITKE | 573.3243 | 601.3192 | IT | 187.1441 | 215.1390 | ITK | 315.2391 | 343.2340 |
| ITKE | 444.2817 | 472.2766 | ITKEE | 573.3243 | 601.3192 | TK | 202.1550 | 230.1499 |

| | | | | | | | | |
|---|---|---|---|---|---|---|---|---|
| **TKE** | 331.1976 | 359.1925 | **TKEE** | 460.2402 | 488.2351 | **TKEEY** | 623.3035 | 651.2984 |
| **TKEEYA** | 694.3406 | 722.3355 | **KE** | 230.1499 | 258.1448 | **KEE** | 359.1925 | 387.1874 |
| **KEEY** | 522.2558 | 550.2508 | **KEEYA** | 593.2930 | 621.2879 | **KEEYAA** | 664.3301 | 692.3250 |
| **EE** | 231.0975 | 259.0925 | **EEY** | 394.1609 | 422.1558 | **EEYA** | 465.1980 | 493.1929 |
| **EEYAA** | 536.2351 | 564.2300 | **EEYAAF** | 683.3035 | 711.2984 | **EY** | 265.1183 | 293.1132 |
| **EYA** | 336.1554 | 364.1503 | **EYAA** | 407.1925 | 435.1874 | **EYAAF** | 554.2609 | 582.2558 |
| **YA** | 207.1128 | 235.1077 | **YAA** | 278.1499 | 306.1448 | **YAAF** | 425.2183 | 453.2132 |
| **YAAFY** | 588.2817 | 616.2766 | **AA** | 115.0866 | 143.0815 | **AAF** | 262.1550 | 290.1499 |
| **AAFY** | 425.2183 | 453.2132 | **AF** | 191.1179 | 219.1128 | **AFY** | 354.1812 | 382.1761 |
| **FY** | 283.1441 | 311.1390 | | | | | | |

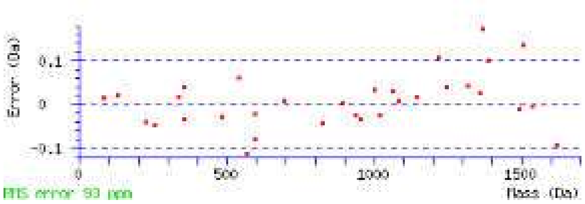


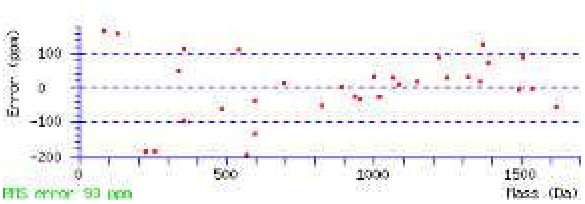


NCBI **BLAST** search of [KPEEITKEEYAAFYK]
(Parameters: blastp, nr protein database, expect=20000, no filter, PAM30)
Other BLAST web gateways

**All matches to this query**

| Score | Mr(calc) | Delta | Sequence |
|---|---|---|---|
| 103.1 | 1844.9196 | 0.0369 | KPEEITKEEYAAFYK |
| 19.3 | 1844.8580 | 0.0984 | NPEEITRDEYAAFYK |
| 11.9 | 1844.9818 | -0.0254 | VMLPAVAKAGQIEEAFR |
| 11.5 | 1844.8913 | 0.0651 | MFLEKPQPPSMPNSAR |
| 9.8 | 1844.9025 | 0.0539 | KPSSAASLFGRMAMGFR |
| 8.6 | 1845.0546 | -0.0982 | KPIMFVGGAGVGKTQLVK |
| 8.5 | 1844.8767 | 0.0797 | QYVEAGDWKAAVAMYK |
| 8.5 | 1844.9917 | -0.0353 | RLILTQEEMEEVVLK |
| 8.2 | 1844.9169 | 0.0395 | LVPFASTWAQQANEGAR |
| 8.1 | 1845.0294 | -0.0730 | KLLELMVLDNHLHVR |

**Mascot:** http://www.matrixscience.com/

# MATRIX SCIENCE Mascot Search Results

## Peptide View **Spot no 108**

MS/MS Fragmentation of **MKEGQSEIYYITGESK**
Found in **gi|39104468** in **NCBInr**, heat shock protein 90 [Oryza sativa Japonica Group]

Match to Query 96: 1877.903324 from(1878.910600,1+) intensity(0.0000) index(32)
Title: Label: C2, Spot_Id: 219692, Peak_List_Id: 224847, MSMS Job_Run_Id: 21755, Comment:
Data file C:\Documents and Settings\Administrator\Desktop\farah BHU\spot set plate\Farah-Garima 11May 2013\ppw_C2_136842094000.txt

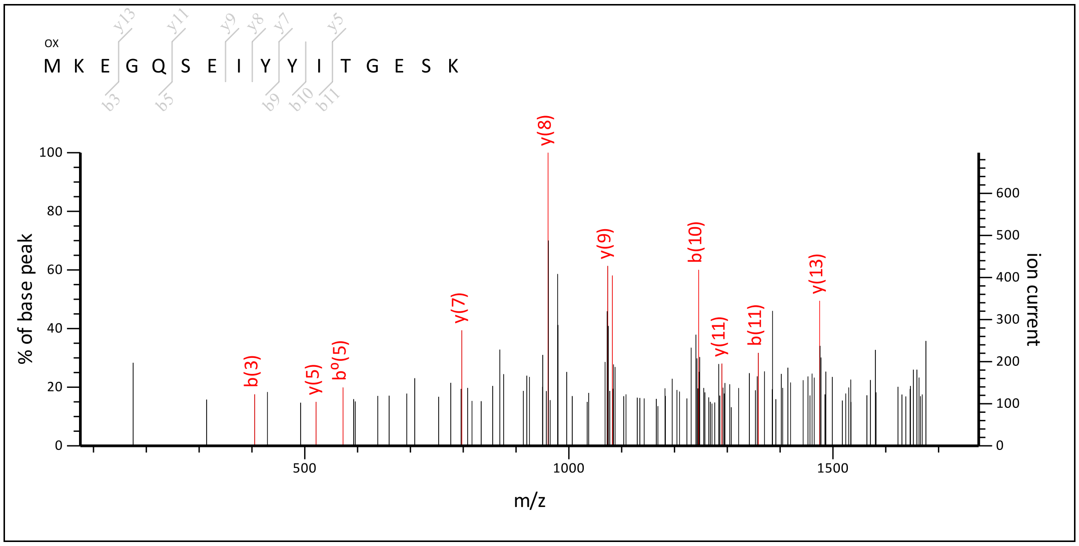


75.12 to 1775.99

Label all possible matches ○ Label matches used for scoring ◉

**Monoisotopic mass of neutral peptide Mr(calc):** 1877.8717
**Fixed modifications:** Carbamidomethyl (C) (apply to specified residues or termini only)
**Variable modifications:**
**M1** : Oxidation (M), with neutral losses 0.0000(shown in table), 63.9983
**Ions Score:** 17 **Expect:** 32
**Matches :** 11/385 fragment ions using 29 most intense peaks (help)

| # | Immon. | a | a* | $a^0$ | b | b* | $b^0$ | d | d' | Seq. | v | w | w' | y | y* |
|---|---|---|---|---|---|---|---|---|---|---|---|---|---|---|---|
| 1 | 120.0478 | 120.0478 | | | 148.0427 | | | 44.0495 | | M | | | | | |
| 2 | 101.1073 | 248.1427 | 231.1162 | | 276.1376 | 259.1111 | | 191.0849 | | K | 1658.7544 | 1657.7592 | | 1731.8436 | 1714.8170 |
| 3 | 102.0550 | 377.1853 | 360.1588 | 359.1748 | **405.1802** | 388.1537 | 387.1697 | 319.1798 | | E | 1529.7118 | 1528.7166 | | 1603.7486 | 1586.7221 |
| 4 | 30.0338 | 434.2068 | 417.1802 | 416.1962 | 462.2017 | 445.1751 | 444.1911 | | | G | | | | ***1474.7060*** | 1457.6795 |
| 5 | 101.0709 | 562.2654 | 545.2388 | 544.2548 | 590.2603 | 573.2337 | 572.2497 | 505.2439 | | Q | 1344.6318 | 1343.6365 | | 1417.6846 | 1400.6580 |
| 6 | 60.0444 | 649.2974 | 632.2708 | 631.2868 | 677.2923 | 660.2658 | 659.2817 | 633.3025 | | S | 1257.5998 | 1256.6045 | | ***1289.6260*** | 1272.5994 |
| 7 | 102.0550 | 778.3400 | 761.3134 | 760.3294 | 806.3349 | 789.3083 | 788.3243 | 720.3345 | | E | 1128.5572 | 1127.5619 | | 1202.5939 | 1185.5674 |
| 8 | 86.0964 | 891.4240 | 874.3975 | 873.4135 | 919.4190 | 902.3924 | 901.4084 | 863.3927 | 877.4084 | I | 1015.4731 | 1028.4935 | 1042.5092 | ***1073.5514*** | 1056.5248 |
| 9 | 136.0757 | 1054.4874 | 1037.4608 | 1036.4768 | **1082.4823** | 1065.4557 | 1064.4717 | | | Y | 852.4098 | | | ***960.4673*** | 943.4407 |
| 10 | 136.0757 | 1217.5507 | 1200.5242 | 1199.5401 | **1245.5456** | 1228.5191 | 1227.5351 | | | Y | 689.3464 | | | ***797.4040*** | 780.3774 |
| 11 | 86.0964 | 1330.6348 | 1313.6082 | 1312.6242 | **1358.6297** | 1341.6031 | 1340.6191 | 1302.6035 | 1316.6191 | I | 576.2624 | 589.2828 | 603.2984 | 634.3406 | 617.3141 |
| 12 | 74.0600 | 1431.6824 | 1414.6559 | 1413.6719 | 1459.6774 | 1442.6508 | 1441.6668 | 1415.6875 | 1417.6668 | T | 475.2147 | 488.2351 | 490.2144 | ***521.2566*** | 504.2300 |
| 13 | 30.0338 | 1488.7039 | 1471.6774 | 1470.6933 | 1516.6988 | 1499.6723 | 1498.6883 | | | G | | | | 420.2089 | 403.1823 |
| 14 | 102.0550 | 1617.7465 | 1600.7200 | 1599.7359 | 1645.7414 | 1628.7149 | 1627.7309 | 1559.7410 | | E | 289.1506 | 288.1554 | | 363.1874 | 346.1609 |
| 15 | 60.0444 | 1704.7785 | 1687.7520 | 1686.7680 | 1732.7734 | 1715.7469 | 1714.7629 | 1688.7836 | | S | 202.1186 | 201.1234 | | 234.1448 | 217.1183 |
| 16 | 101.1073 | | | | | | | | | K | 74.0237 | 73.0284 | | 147.1128 | 130.0863 |

| Seq | ya | yb | Seq | ya | yb | Seq | ya | yb |
|---|---|---|---|---|---|---|---|---|
| KE | 230.1499 | 258.1448 | KEG | 287.1714 | 315.1663 | KEGQ | 415.2300 | 443.2249 |
| KEGQS | 502.2620 | 530.2569 | KEGQSE | 631.3046 | 659.2995 | EG | 159.0764 | 187.0713 |
| | | | | | | | | |

| | | | | | | | | |
|---|---|---|---|---|---|---|---|---|
| **EGQ** | 287.1350 | 315.1299 | **EGQS** | 374.1670 | 402.1619 | **EGQSE** | 503.2096 | 531.2045 |
| **EGQSEI** | 616.2937 | 644.2886 | **GQ** | 158.0924 | 186.0873 | **GQS** | 245.1244 | 273.1193 |
| **GQSE** | 374.1670 | 402.1619 | **GQSEI** | 487.2511 | 515.2460 | **GQSEIY** | 650.3144 | 678.3093 |
| **QS** | 188.1030 | 216.0979 | **QSE** | 317.1456 | 345.1405 | **QSEI** | 430.2296 | 458.2245 |
| **QSEIY** | 593.2930 | 621.2879 | **SE** | 189.0870 | 217.0819 | **SEI** | 302.1710 | 330.1660 |
| **SEIY** | 465.2344 | 493.2293 | **SEIYY** | 628.2977 | 656.2926 | **EI** | 215.1390 | 243.1339 |
| **EIY** | 378.2023 | 406.1973 | **EIYY** | 541.2657 | 569.2606 | **EIYYI** | 654.3497 | 682.3447 |
| **IY** | 249.1598 | 277.1547 | **IYY** | 412.2231 | 440.2180 | **IYYI** | 525.3071 | 553.3021 |
| **IYYIT** | 626.3548 | 654.3497 | **IYYITG** | 683.3763 | 711.3712 | **YY** | 299.1390 | 327.1339 |
| **YYI** | 412.2231 | 440.2180 | **YYIT** | 513.2708 | 541.2657 | **YYITG** | 570.2922 | 598.2871 |
| **YYITGE** | 699.3348 | 727.3297 | **YI** | 249.1598 | 277.1547 | **YIT** | 350.2074 | 378.2023 |
| **YITG** | 407.2289 | 435.2238 | **YITGE** | 536.2715 | 564.2664 | **YITGES** | 623.3035 | 651.2984 |
| **IT** | 187.1441 | 215.1390 | **ITG** | 244.1656 | 272.1605 | **ITGE** | 373.2082 | 401.2031 |
| **ITGES** | 460.2402 | 488.2351 | **TG** | 131.0815 | 159.0764 | **TGE** | 260.1241 | 288.1190 |
| **TGES** | 347.1561 | 375.1510 | **GE** | 159.0764 | 187.0713 | **GES** | 246.1084 | 274.1034 |
| **ES** | 189.0870 | 217.0819 | | | | | | |

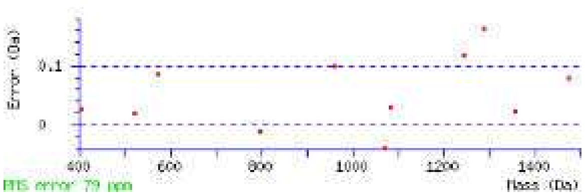


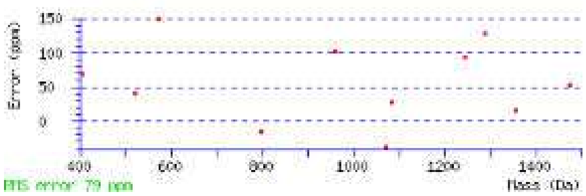


NCBI **BLAST** search of MKEGQSEIYYITGESK
(Parameters: blastp, nr protein database, expect=20000, no filter, PAM30)
Other BLAST web gateways

**All matches to this query**

| Score | Mr(calc) | Delta | Sequence |
|---|---|---|---|
| 17.2 | 1877.8717 | 0.0317 | MKEGQSEIYYITGESK |
| 17.2 | 1877.8717 | 0.0317 | MKEGQSEIYYITGESK |
| 17.2 | 1877.8353 | 0.0680 | MQEGQSEIYYITGESK |
| 15.4 | 1878.0251 | -0.1217 | ILFFIGDLEVASAEAKR |
| 7.3 | 1878.0615 | -0.1581 | SSVLALILRFYDPISGK |
| 6.4 | 1877.8764 | 0.0269 | HGFEEAEMVKVMVSNR |
| 6.4 | 1877.8248 | 0.0786 | DCAGDDDAVAAGIICSLR |
| 6.4 | 1877.8877 | 0.0157 | GKVHVATCPHGTTCEPK |
| 5.2 | 1877.9339 | -0.0306 | SSMAKVCDELAQVIGVR |
| 4.9 | 1877.9345 | -0.0312 | VYEELNALMPFSPNVR |

**Mascot:** http://www.matrixscience.com/

# MATRIX SCIENCE Mascot Search Results

## Peptide View **Spot no 110**

MS/MS Fragmentation of **LDEMIVFR**
Found in **gi|18423214** in **NCBInr**, ATP-dependent Clp protease ATP-binding subunit ClpC [Arabidopsis thaliana]

Match to Query 88: 1021.553424 from(1022.560700,1+) intensity(0.0000) index(3)
Title: Label: C9, Spot_Id: 219804, Peak_List_Id: 226663, MSMS Job_Run_Id: 21852, Comment:
Data file C:\Documents and Settings\Administrator\Desktop\Farah-Garima 11May 2013\ppw_C9_136859938300.txt

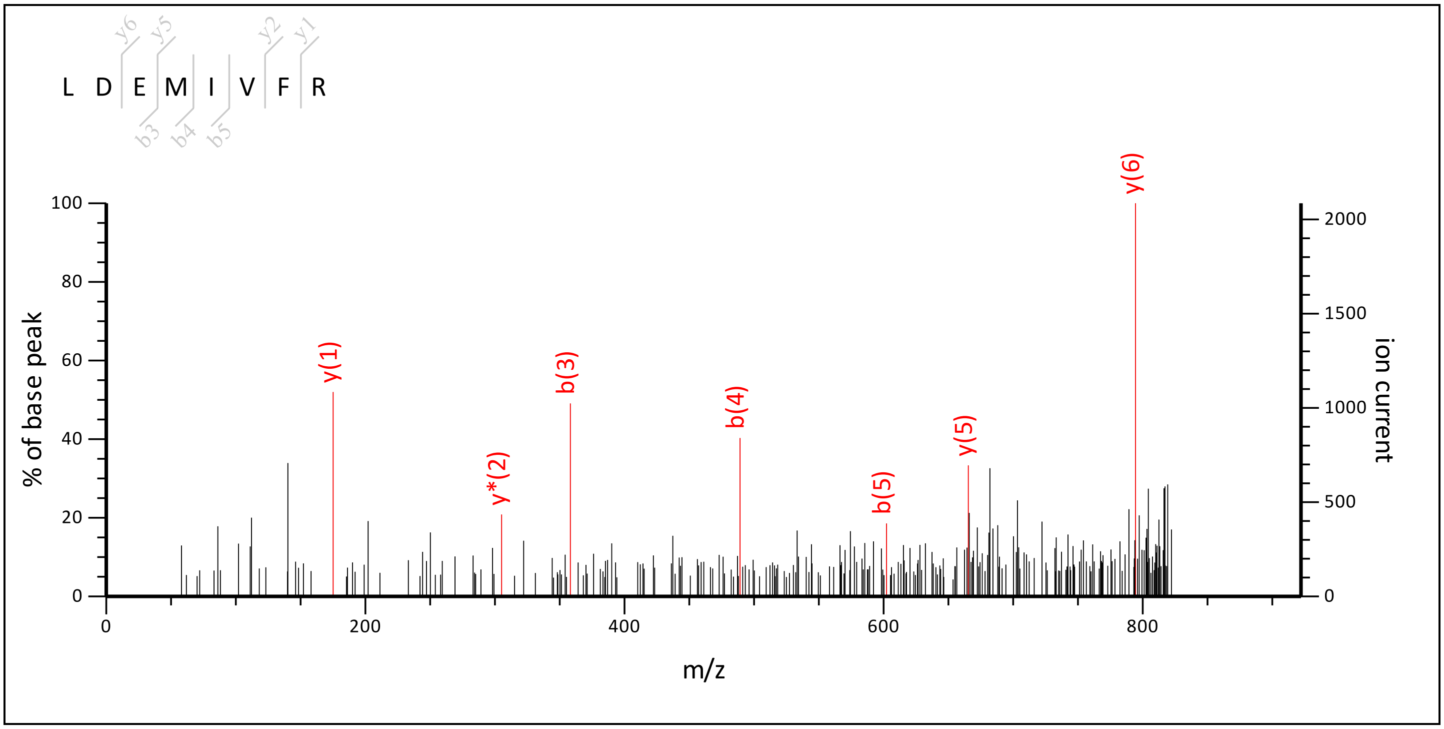


0 to 922.15

Label all possible matches ○ Label matches used for scoring ◉

**Monoisotopic mass of neutral peptide Mr(calc):** 1021.5266
**Fixed modifications:** Carbamidomethyl (C) (apply to specified residues or termini only)
**Ions Score:** 29 **Expect:** 48
**Matches :** 7/28 fragment ions using 8 most intense peaks (help)

| # | a | b | Seq. | y | y* | # |
|---|---|---|---|---|---|---|
| 1 | 86.0964 | 114.0913 | L | | | 8 |
| 2 | 201.1234 | 229.1183 | D | 909.4499 | 892.4233 | 7 |
| 3 | 330.1660 | ***358.1609*** | E | ***794.4229*** | 777.3964 | 6 |
| 4 | 461.2064 | ***489.2014*** | M | ***665.3803*** | 648.3538 | 5 |
| 5 | 574.2905 | ***602.2854*** | I | 534.3398 | 517.3133 | 4 |
| 6 | 673.3589 | 701.3538 | V | 421.2558 | 404.2292 | 3 |
| 7 | 820.4273 | 848.4223 | F | 322.1874 | 305.1608 | 2 |
| 8 | | | R | ***175.1190*** | 158.0924 | 1 |

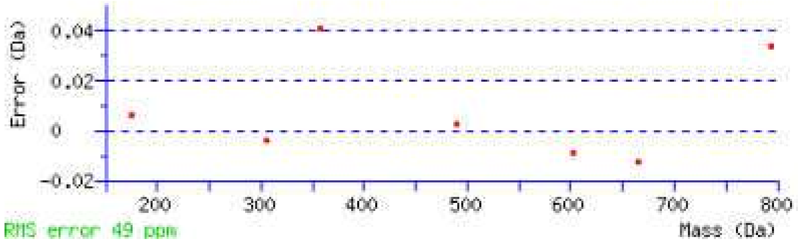


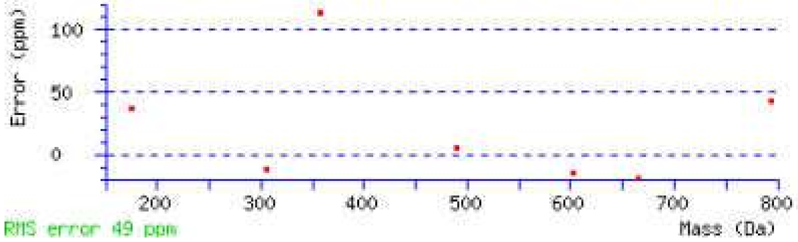


NCBI **BLAST** search of LDEMIVFR
(Parameters: blastp, nr protein database, expect=20000, no filter, PAM30)
Other BLAST web gateways

**All matches to this query**

| Score | Mr(calc) | Delta | Sequence |
|---|---|---|---|
| 28.9 | 1021.5266 | 0.0268 | IDEMLVFR |
| 28.9 | 1021.5266 | 0.0268 | LDEMIVFR |
| 25.8 | 1021.4539 | 0.0996 | DLEMPDFR |
| 23.3 | 1021.4716 | 0.0818 | EVEDIDFR |
| 17.6 | 1021.4936 | 0.0598 | DLELMVMR |
| 17.6 | 1021.4716 | 0.0818 | EVEDEVFR |
| 17.6 | 1021.4903 | 0.0632 | EVEMPVFR |
| 17.6 | 1021.5055 | 0.0479 | MPEPFVFR |
| 17.6 | 1021.4685 | 0.0850 | NNELMVMR |
| 17.6 | 1021.4903 | 0.0632 | VEEMPVFR |

**Mascot:** http://www.matrixscience.com/

# MATRIX SCIENCE Mascot Search Results

## Peptide View **Spot no 110**

MS/MS Fragmentation of **LAEEGKLDPVVGR**
Found in **gi|18423214** in **NCBInr**, ATP-dependent Clp protease ATP-binding subunit ClpC [Arabidopsis thaliana]

Match to Query 197: 1381.789724 from(1382.797000,1+) intensity(0.0000) index(15)
Title: Label: C9, Spot_Id: 219804, Peak_List_Id: 226664, MSMS Job_Run_Id: 21852, Comment:
Data file C:\Documents and Settings\Administrator\Desktop\Farah-Garima 11May 2013\ppw_C9_136859938300.txt

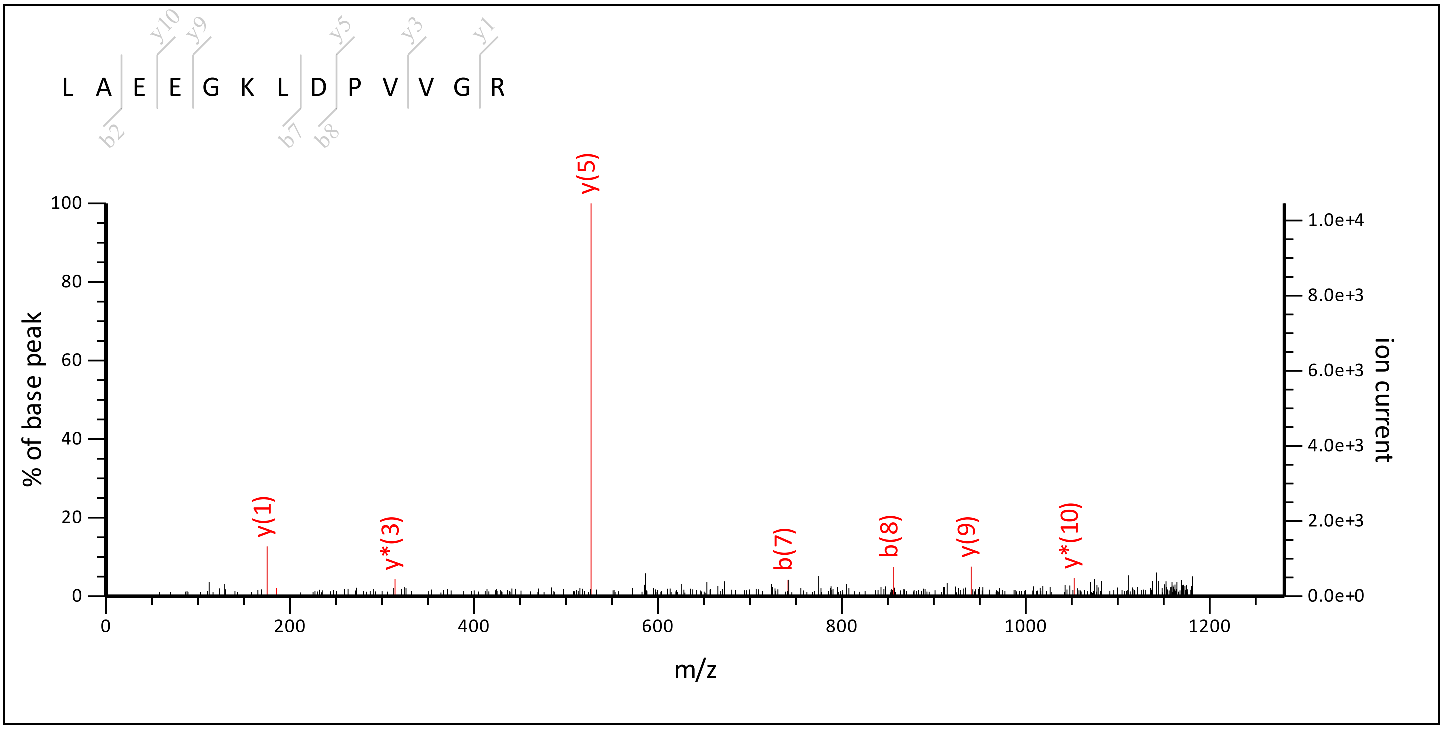


0 to 1281.23

Label all possible matches ○ Label matches used for scoring ◉

**Monoisotopic mass of neutral peptide Mr(calc):** 1381.7565
**Fixed modifications:** Carbamidomethyl (C) (apply to specified residues or termini only)
**Ions Score:** 18 **Expect:** 4.3e+02
**Matches :** 10/62 fragment ions using 24 most intense peaks (help)

| # | a | a* | b | b* | Seq. | y | y* | # |
|---|---|---|---|---|---|---|---|---|
| 1 | 86.0964 | | 114.0913 | | L | | | 13 |
| 2 | 157.1335 | | ***185.1285*** | | A | 1269.6797 | 1252.6532 | 12 |
| 3 | 286.1761 | | ***314.1710*** | | E | 1198.6426 | 1181.6161 | 11 |
| 4 | 415.2187 | | 443.2136 | | E | 1069.6000 | 1052.5735 | 10 |
| 5 | 472.2402 | | 500.2351 | | G | ***940.5574*** | 923.5309 | 9 |
| 6 | 600.3352 | 583.3086 | 628.3301 | 611.3035 | K | 883.5360 | 866.5094 | 8 |
| 7 | 713.4192 | 696.3927 | ***741.4141*** | 724.3876 | L | 755.4410 | 738.4145 | 7 |
| 8 | 828.4462 | 811.4196 | ***856.4411*** | 839.4145 | D | 642.3570 | 625.3304 | 6 |

| | | | | | | | | |
|---|---|---|---|---|---|---|---|---|
| **9** | 925.4989 | 908.4724 | 953.4938 | 936.4673 | **P** | ***527.3300*** | 510.3035 | **5** |
| **10** | 1024.5673 | 1007.5408 | ***1052.5623*** | 1035.5357 | **V** | 430.2772 | 413.2507 | **4** |
| **11** | 1123.6358 | 1106.6092 | 1151.6307 | 1134.6041 | **V** | 331.2088 | 314.1823 | **3** |
| **12** | 1180.6572 | 1163.6307 | 1208.6521 | 1191.6256 | **G** | 232.1404 | 215.1139 | **2** |
| **13** | | | | | **R** | ***175.1190*** | 158.0924 | **1** |

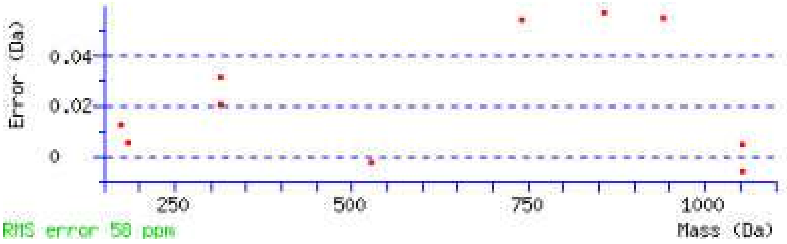


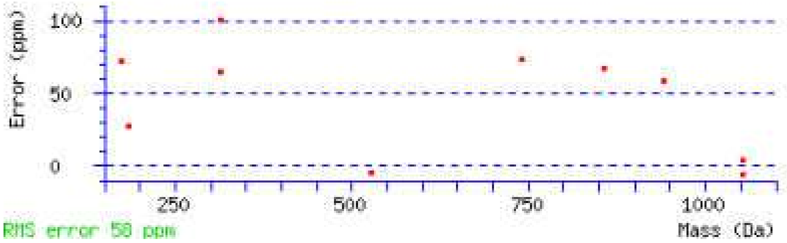


NCBI **BLAST** search of LAEEGKLDPVVGR
(Parameters: blastp, nr protein database, expect=20000, no filter, PAM30)
Other BLAST web gateways

**All matches to this query**

| Score | Mr(calc) | Delta | Sequence |
|---|---|---|---|
| 18.0 | 1381.7354 | 0.0543 | ISPKTNGVTYFR |
| 17.9 | 1381.7565 | 0.0332 | LAEEGKLDPVVGR |
| 17.9 | 1381.7215 | 0.0683 | LAPSASNRQPWR |
| 17.4 | 1381.7426 | 0.0471 | IGTALANRGPEAGR |
| 15.8 | 1381.7426 | 0.0471 | NEAVLNRPQVSR |
| 15.8 | 1381.7466 | 0.0431 | SIAAALNRTFYR |
| 15.3 | 1381.7062 | 0.0835 | AGSITPNRPAADGR |
| 14.9 | 1381.7790 | 0.0107 | AVIDAGGGRVLGAAR |
| 13.9 | 1381.8154 | -0.0256 | SVKIVSRPVNQR |
| 13.9 | 1381.7677 | 0.0220 | QKGEQTAIAIAPR |

**Mascot:** http://www.matrixscience.com/

# MATRIX SCIENCE Mascot Search Results

## Peptide View  Spot no 110

MS/MS Fragmentation of **GSGFVAVEIPFTPR**
Found in **gi|18423214** in **NCBInr**, ATP-dependent Clp protease ATP-binding subunit ClpC [Arabidopsis thaliana]

Match to Query 213: 1475.807424 from(1476.814700,1+) intensity(0.0000) index(18)
Title: Label: C9, Spot_Id: 219804, Peak_List_Id: 226647, MSMS Job_Run_Id: 21852, Comment:
Data file C:\Documents and Settings\Administrator\Desktop\Farah-Garima 11May 2013\ppw_C9_136859938300.txt

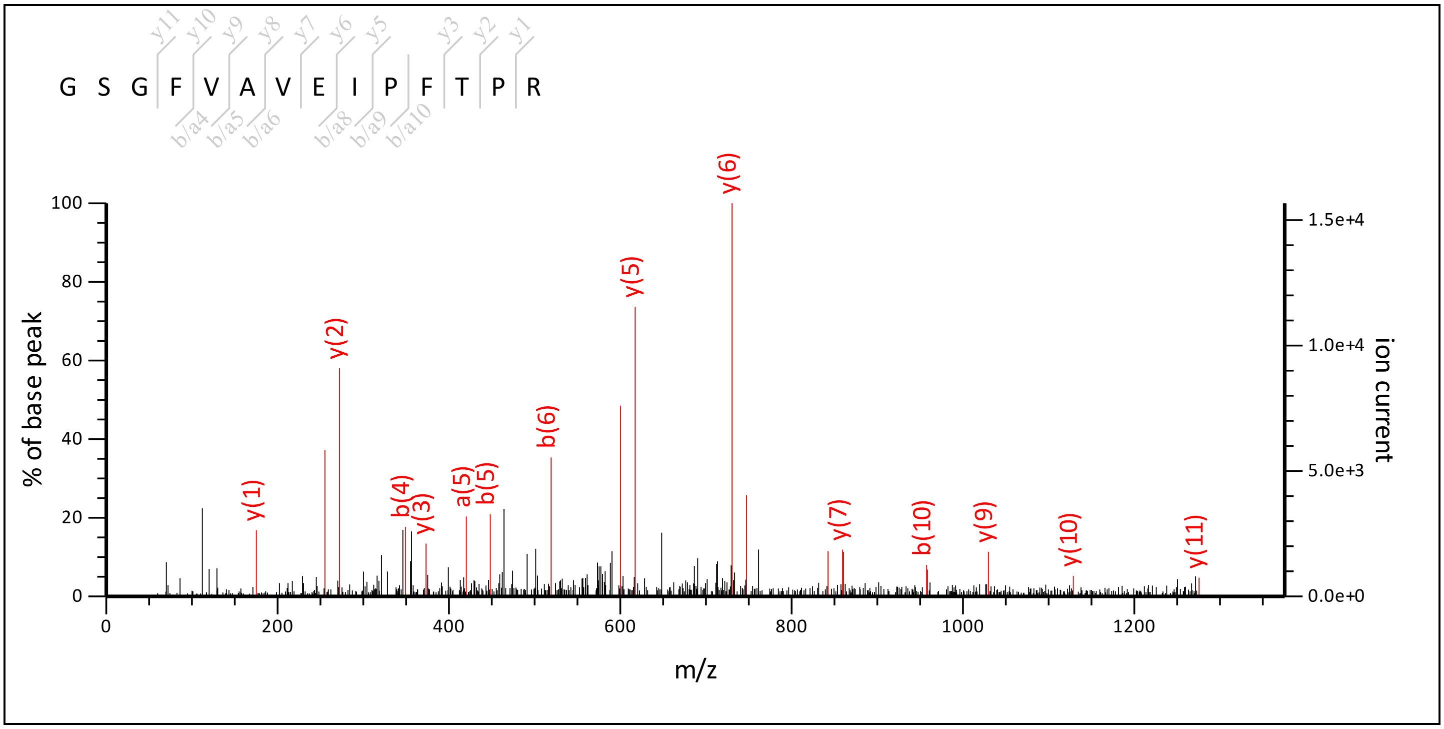


0 to 1375.54

Label all possible matches ○ Label matches used for scoring ◉

**Monoisotopic mass of neutral peptide Mr(calc):** 1475.7773
**Fixed modifications:** Carbamidomethyl (C) (apply to specified residues or termini only)
**Ions Score:** 62 **Expect:** 0.02
**Matches :** 20/52 fragment ions using 39 most intense peaks (help)

| # | a | b | Seq. | y | y* | # |
|---|---|---|---|---|---|---|
| 1 | 30.0338 | 58.0287 | G | | | 14 |
| 2 | 117.0659 | 145.0608 | S | 1419.7631 | 1402.7365 | 13 |
| 3 | 174.0873 | 202.0822 | G | 1332.7311 | 1315.7045 | 12 |
| 4 | 321.1557 | **349.1506** | F | ***1275.7096*** | 1258.6830 | 11 |
| 5 | 420.2241 | **448.2191** | V | ***1128.6412*** | 1111.6146 | 10 |
| 6 | 491.2613 | **519.2562** | A | ***1029.5728*** | 1012.5462 | 9 |
| 7 | 590.3297 | 618.3246 | V | ***958.5356*** | 941.5091 | 8 |
| 8 | 719.3723 | **747.3672** | E | ***859.4672*** | 842.4407 | 7 |

| | | | | | | |
|---|---|---|---|---|---|---|
| **9** | 832.4563 | **860.4512** | **I** | ***730.4246*** | 713.3981 | **6** |
| **10** | 929.5091 | **957.5040** | **P** | ***617.3406*** | 600.3140 | **5** |
| **11** | 1076.5775 | 1104.5724 | **F** | 520.2878 | 503.2613 | **4** |
| **12** | 1177.6252 | 1205.6201 | **T** | ***373.2194*** | 356.1928 | **3** |
| **13** | 1274.6780 | 1302.6729 | **P** | ***272.1717*** | 255.1452 | **2** |
| **14** | | | **R** | ***175.1190*** | 158.0924 | **1** |

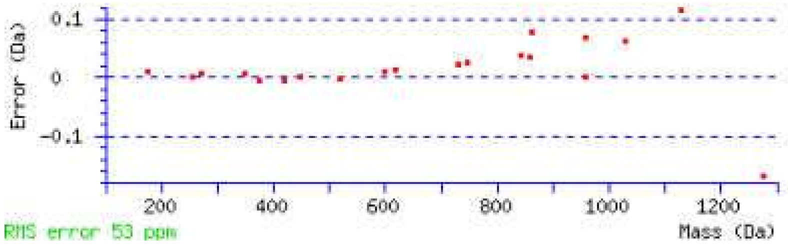


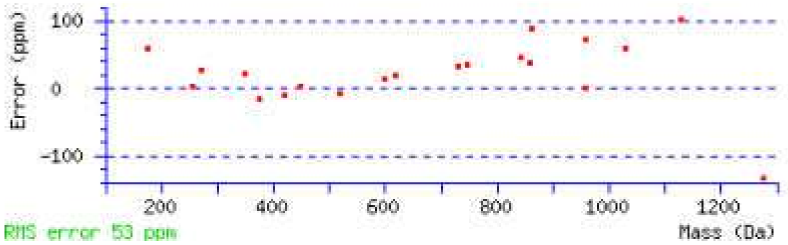


NCBI **BLAST** search of GSGFVAVEIPFTPR
(Parameters: blastp, nr protein database, expect=20000, no filter, PAM30)
Other BLAST web gateways

**All matches to this query**

| Score | Mr(calc) | Delta | Sequence |
|---|---|---|---|
| 62.1 | 1475.7773 | 0.0301 | GSGFVAVEIPFTPR |
| 41.0 | 1475.7409 | 0.0665 | GSGFTPAEIPFTPR |
| 17.2 | 1475.7766 | 0.0308 | RAGVSMELLSEIR |
| 16.8 | 1475.7224 | 0.0850 | RVMETLEQMAPR |
| 16.7 | 1475.7845 | 0.0230 | QNSSVTLRTFAPR |
| 16.2 | 1475.7256 | 0.0818 | FLEEVELEQGQR |
| 15.2 | 1475.7265 | 0.0810 | MKFPPLPETMNR |
| 15.2 | 1475.6926 | 0.1148 | EMLQDLIDTENR |
| 15.1 | 1475.7038 | 0.1036 | NSMVQPKTSSEPR |
| 14.9 | 1475.7620 | 0.0454 | VEYQPLVSLSEGR |

**Mascot:** http://www.matrixscience.com/

# Mascot Search Results

## Peptide View

**Spot no 110**

MS/MS Fragmentation of **RPYTVVLFDEIEK**
Found in **gi|18423214** in **NCBInr**, ATP-dependent Clp protease ATP-binding subunit ClpC [Arabidopsis thaliana]

Match to Query 236: 1607.891724 from(1608.899000,1+) intensity(0.0000) index(22)
Title: Label: C9, Spot_Id: 219804, Peak_List_Id: 226658, MSMS Job_Run_Id: 21852, Comment:
Data file C:\Documents and Settings\Administrator\Desktop\Farah-Garima 11May 2013\ppw_C9_136859938300.txt

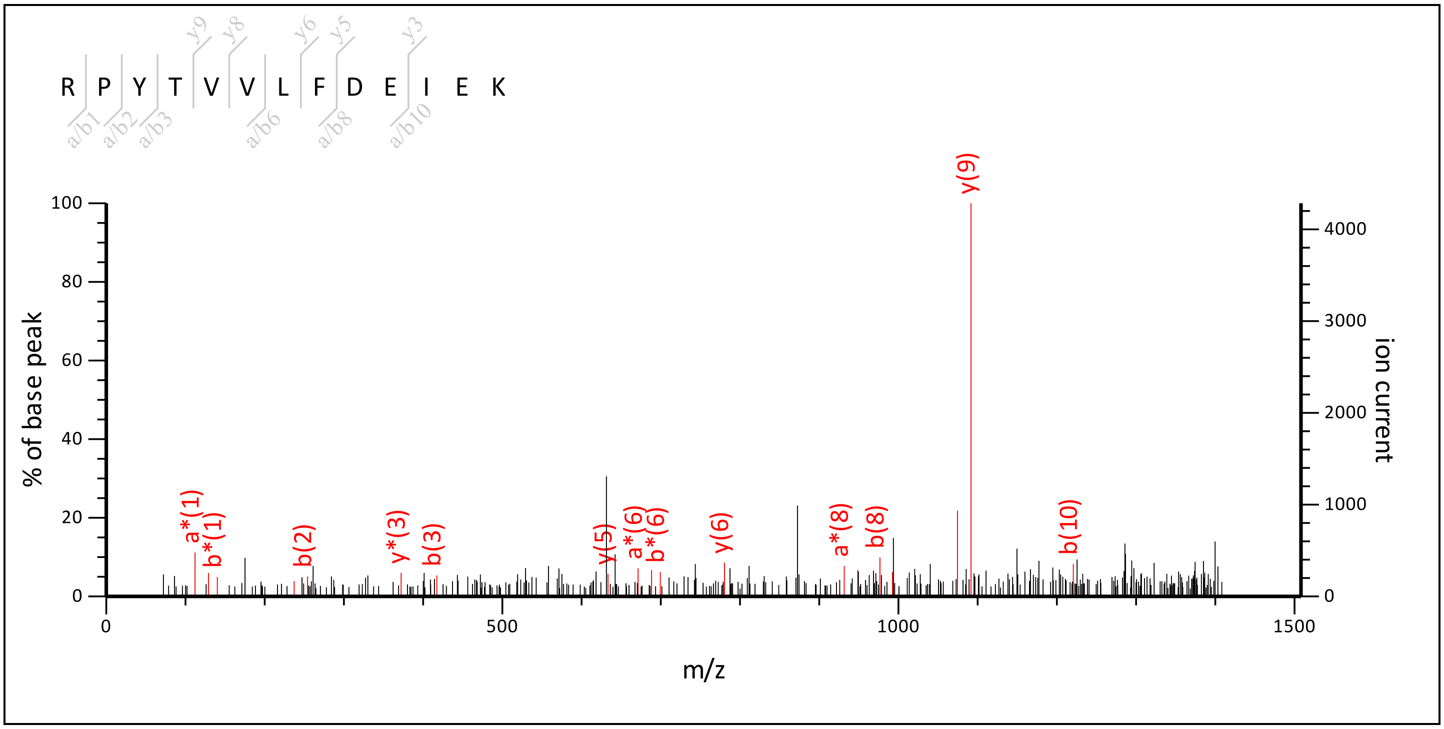


0 to 1508.17

Label all possible matches ○ Label matches used for scoring ◉

**Monoisotopic mass of neutral peptide Mr(calc):** 1607.8559
**Fixed modifications:** Carbamidomethyl (C) (apply to specified residues or termini only)
**Ions Score:** 7 **Expect:** 5.9e+03
**Matches :** 22/72 fragment ions using 70 most intense peaks (help)

| # | a | a* | b | b* | Seq. | y | y* | # |
|---|---|---|---|---|---|---|---|---|
| 1 | **129.1135** | 112.0869 | 157.1084 | 140.0818 | R | | | 13 |
| 2 | 226.1662 | 209.1397 | ***254.1612*** | 237.1346 | P | 1452.7621 | 1435.7355 | 12 |
| 3 | 389.2296 | 372.2030 | ***417.2245*** | 400.1979 | Y | 1355.7093 | 1338.6828 | 11 |
| 4 | 490.2772 | 473.2507 | 518.2722 | 501.2456 | T | 1192.6460 | 1175.6194 | 10 |
| 5 | 589.3457 | 572.3191 | 617.3406 | 600.3140 | V | ***1091.5983*** | 1074.5718 | 9 |
| 6 | **688.4141** | 671.3875 | 716.4090 | 699.3824 | V | ***992.5299*** | 975.5033 | 8 |
| 7 | 801.4981 | 784.4716 | 829.4931 | 812.4665 | L | 893.4615 | 876.4349 | 7 |
| 8 | **948.5666** | 931.5400 | ***976.5615*** | 959.5349 | F | ***780.3774*** | 763.3509 | 6 |

| | | | | | | | | |
|---|---|---|---|---|---|---|---|---|
| 9 | 1063.5935 | 1046.5669 | ***1091.5884*** | 1074.5619 | D | ***633.3090*** | 616.2824 | 5 |
| 10 | 1192.6361 | 1175.6095 | ***1220.6310*** | 1203.6045 | E | 518.2821 | 501.2555 | 4 |
| 11 | 1305.7201 | 1288.6936 | 1333.7151 | 1316.6885 | I | 389.2395 | 372.2129 | 3 |
| 12 | 1434.7627 | 1417.7362 | 1462.7577 | 1445.7311 | E | 276.1554 | 259.1288 | 2 |
| 13 | | | | | K | 147.1128 | 130.0863 | 1 |

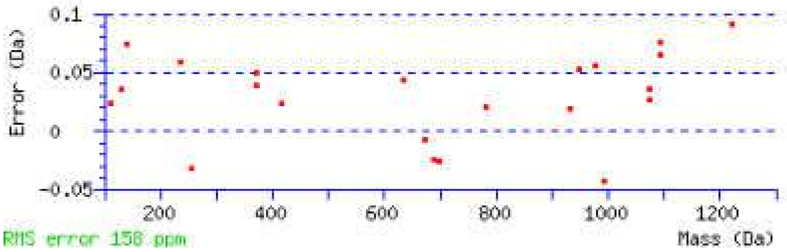


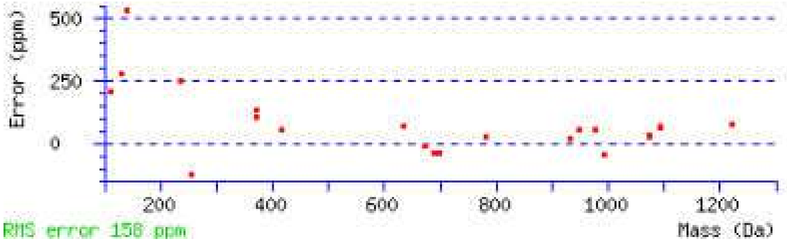


NCBI **BLAST** search of RPYTVVLFDEIEK
(Parameters: blastp, nr protein database, expect=20000, no filter, PAM30)
Other BLAST web gateways

**All matches to this query**

| Score | Mr(calc) | Delta | Sequence |
|---|---|---|---|
| 13.6 | 1607.8090 | 0.0828 | ARGSADLSAFMPLTR |
| 11.1 | 1607.7348 | 0.1569 | IDTLKEEMDEAGNK |
| 11.1 | 1607.7348 | 0.1569 | IESLKEEMDEAGNK |
| 9.8 | 1607.7804 | 0.1113 | AGRWAEAHPDETLR |
| 7.8 | 1607.8341 | 0.0576 | SELPMRTYIETLR |
| 7.3 | 1607.8090 | 0.0827 | ATIMTGQYGQINGVR |
| 6.9 | 1607.8559 | 0.0358 | RPYSVILFDEIEK |
| 6.9 | 1607.8229 | 0.0689 | RPYSVILMDEIEK |
| 6.9 | 1607.8559 | 0.0358 | RPYSVLLFDEIEK |
| 6.9 | 1607.8559 | 0.0358 | RPYTVVLFDEIEK |

**Mascot:** http://www.matrixscience.com/

# Mascot Search Results

## Peptide View

**Spot no 110**

MS/MS Fragmentation of **ALAAYYFGSEEAMIR**
Found in **gi|18423214** in **NCBInr**, ATP-dependent Clp protease ATP-binding subunit ClpC [Arabidopsis thaliana]

Match to Query 242: 1690.842624 from(1691.849900,1+) intensity(0.0000) index(23)
Title: Label: C9, Spot_Id: 219804, Peak_List_Id: 226649, MSMS Job_Run_Id: 21852, Comment:
Data file C:\Documents and Settings\Administrator\Desktop\Farah-Garima 11May 2013\ppw_C9_136859938300.txt

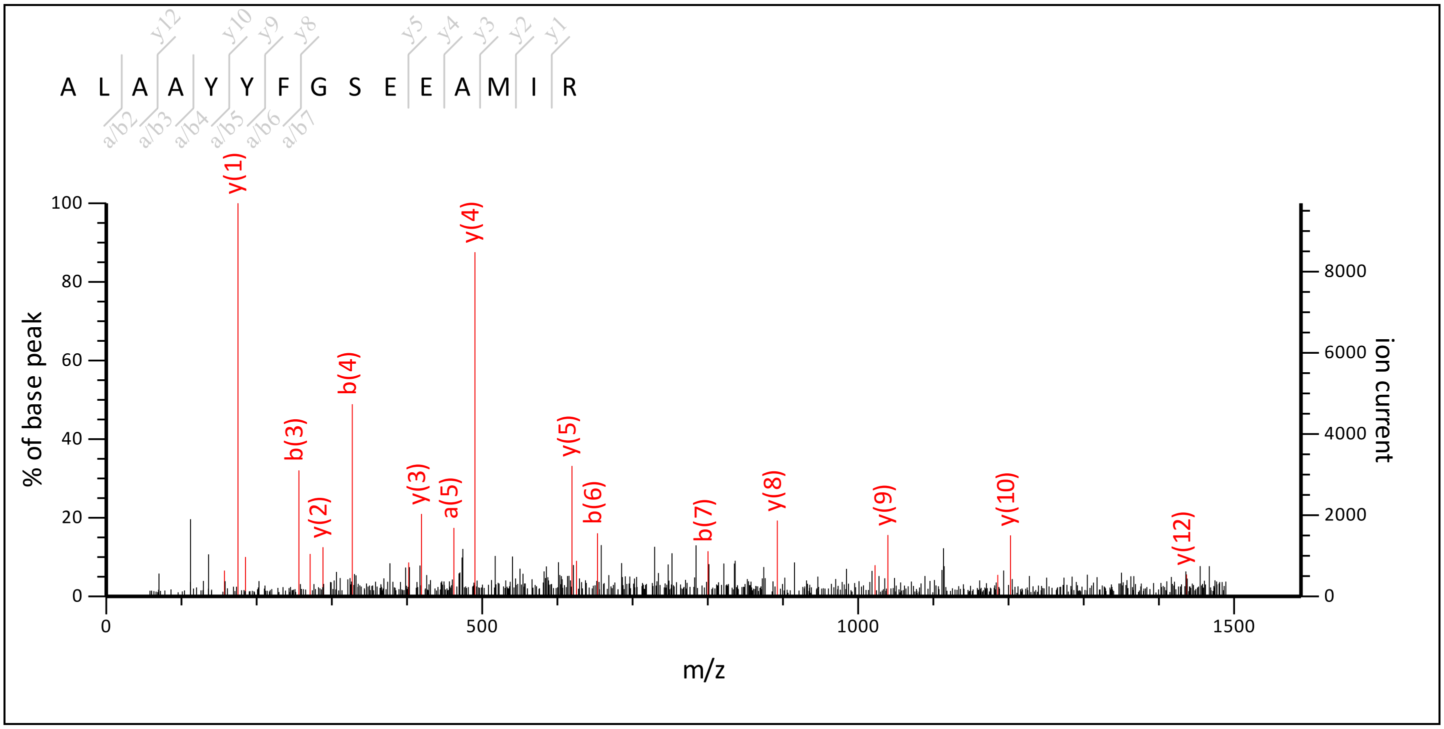


0 to 1588.97

Label all possible matches ○ Label matches used for scoring ◉

**Monoisotopic mass of neutral peptide Mr(calc):** 1690.8024
**Fixed modifications:** Carbamidomethyl (C) (apply to specified residues or termini only)
**Ions Score:** 47 **Expect:** 0.71
**Matches :** 23/56 fragment ions using 45 most intense peaks (help)

| # | a | b | Seq. | y | y* | # |
|---|---|---|---|---|---|---|
| 1 | 44.0495 | 72.0444 | A | | | 15 |
| 2 | **157.1335** | **185.1285** | L | 1620.7727 | 1603.7461 | 14 |
| 3 | 228.1707 | **256.1656** | A | 1507.6886 | 1490.6620 | 13 |
| 4 | 299.2078 | **327.2027** | A | **1436.6515** | 1419.6249 | 12 |
| 5 | **462.2711** | **490.2660** | Y | 1365.6144 | 1348.5878 | 11 |
| 6 | **625.3344** | **653.3293** | Y | **1202.5510** | 1185.5245 | 10 |
| 7 | 772.4028 | **800.3978** | F | **1039.4877** | 1022.4612 | 9 |
| 8 | 829.4243 | 857.4192 | G | **892.4193** | 875.3927 | 8 |

| | | | | | | |
|---|---|---|---|---|---|---|
| 9 | 916.4563 | 944.4512 | S | 835.3978 | 818.3713 | 7 |
| 10 | 1045.4989 | 1073.4938 | E | 748.3658 | 731.3393 | 6 |
| 11 | 1174.5415 | ***1202.5364*** | E | ***619.3232*** | 602.2967 | 5 |
| 12 | 1245.5786 | 1273.5735 | A | ***490.2806*** | 473.2541 | 4 |
| 13 | 1376.6191 | 1404.6140 | M | ***419.2435*** | 402.2170 | 3 |
| 14 | 1489.7032 | 1517.6981 | I | ***288.2030*** | 271.1765 | 2 |
| 15 | | | R | ***175.1190*** | 158.0924 | 1 |

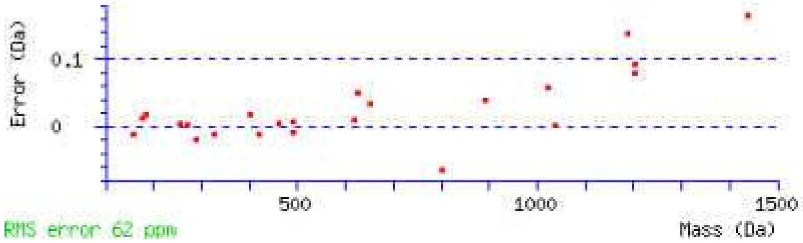


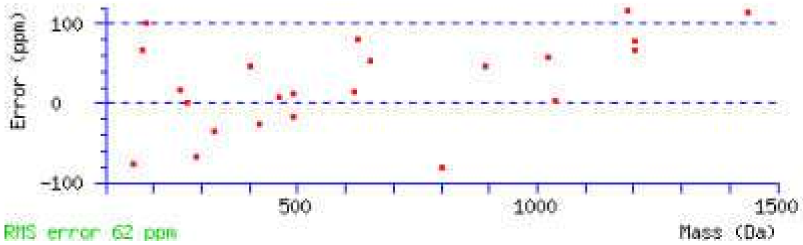


NCBI **BLAST** search of ALAAYYFGSEEAMIR
(Parameters: blastp, nr protein database, expect=20000, no filter, PAM30)
Other BLAST web gateways

**All matches to this query**

| Score | Mr(calc) | Delta | Sequence |
|---|---|---|---|
| 46.5 | 1690.8024 | 0.0402 | ALAAYYFGSEEAMIR |
| 31.4 | 1690.8025 | 0.0402 | ALASYFFGSEEAMIR |
| 29.2 | 1690.8025 | 0.0402 | SLAAYFFGSEEAMIR |
| 16.0 | 1690.7001 | 0.1426 | MNYLDMLCESIDR |
| 14.4 | 1690.9770 | -0.1344 | SPAVLGKFYLAGSLLR |
| 13.6 | 1690.7984 | 0.0442 | RVAYTSETMYEGIR |
| 12.9 | 1690.8092 | 0.0334 | KLLMSFMSTMDSLR |
| 12.9 | 1690.8092 | 0.0334 | KLLMSFMSTMDSLR |
| 12.8 | 1690.9406 | -0.0980 | VGYVAAPAALTVEFRK |
| 12.5 | 1690.8613 | -0.0187 | YNLVYNHPKGSMIR |

**Mascot:** http://www.matrixscience.com/

# MATRIX SCIENCE Mascot Search Results

## Peptide View **Spot no 110**

MS/MS Fragmentation of **FLPDKAIDLIDEAGSR**
Found in **gi|18423214** in **NCBInr**, ATP-dependent Clp protease ATP-binding subunit ClpC [Arabidopsis thaliana]

Match to Query 250: 1758.953624 from(1759.960900,1+) intensity(0.0000) index(24)
Title: Label: C9, Spot_Id: 219804, Peak_List_Id: 226653, MSMS Job_Run_Id: 21852, Comment:
Data file C:\Documents and Settings\Administrator\Desktop\Farah-Garima 11May 2013\ppw_C9_136859938300.txt

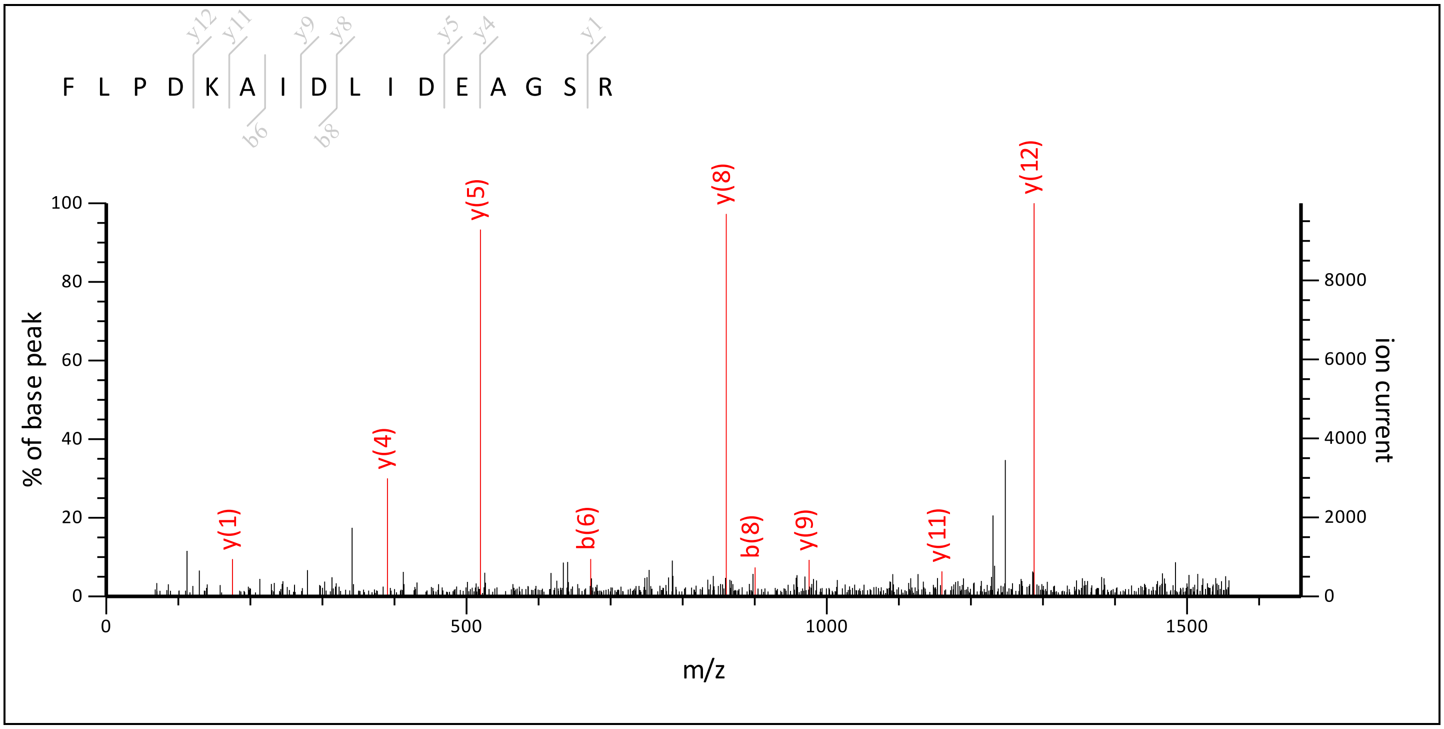


0 to 1658.11

Label all possible matches ○ Label matches used for scoring ◉

**Monoisotopic mass of neutral peptide Mr(calc):** 1758.9152
**Fixed modifications:** Carbamidomethyl (C) (apply to specified residues or termini only)
**Ions Score:** 45 **Expect:** 0.91
**Matches :** 9/82 fragment ions using 15 most intense peaks (help)

| # | a | a* | b | b* | Seq. | y | y* | # |
|---|---|---|---|---|---|---|---|---|
| 1 | 120.0808 | | 148.0757 | | F | | | 16 |
| 2 | 233.1648 | | 261.1598 | | L | 1612.8541 | 1595.8275 | 15 |
| 3 | 330.2176 | | 358.2125 | | P | 1499.7700 | 1482.7435 | 14 |
| 4 | 445.2445 | | 473.2395 | | D | 1402.7172 | 1385.6907 | 13 |
| 5 | 573.3395 | 556.3130 | 601.3344 | 584.3079 | K | *1287.6903* | 1270.6638 | 12 |
| 6 | 644.3766 | 627.3501 | **672.3715** | 655.3450 | A | *1159.5953* | 1142.5688 | 11 |
| 7 | 757.4607 | 740.4341 | 785.4556 | 768.4291 | I | 1088.5582 | 1071.5317 | 10 |
| 8 | 872.4876 | 855.4611 | **900.4825** | 883.4560 | D | *975.4742* | 958.4476 | 9 |

| | | | | | | | | |
|---|---|---|---|---|---|---|---|---|
| 9 | 985.5717 | 968.5451 | 1013.5666 | 996.5401 | L | 860.4472 | 843.4207 | 8 |
| 10 | 1098.6558 | 1081.6292 | 1126.6507 | 1109.6241 | I | 747.3632 | 730.3366 | 7 |
| 11 | 1213.6827 | 1196.6562 | 1241.6776 | 1224.6511 | D | 634.2791 | 617.2525 | 6 |
| 12 | 1342.7253 | 1325.6987 | 1370.7202 | 1353.6937 | E | 519.2522 | 502.2256 | 5 |
| 13 | 1413.7624 | 1396.7359 | 1441.7573 | 1424.7308 | A | 390.2096 | 373.1830 | 4 |
| 14 | 1470.7839 | 1453.7573 | 1498.7788 | 1481.7522 | G | 319.1724 | 302.1459 | 3 |
| 15 | 1557.8159 | 1540.7894 | 1585.8108 | 1568.7843 | S | 262.1510 | 245.1244 | 2 |
| 16 | | | | | R | 175.1190 | 158.0924 | 1 |

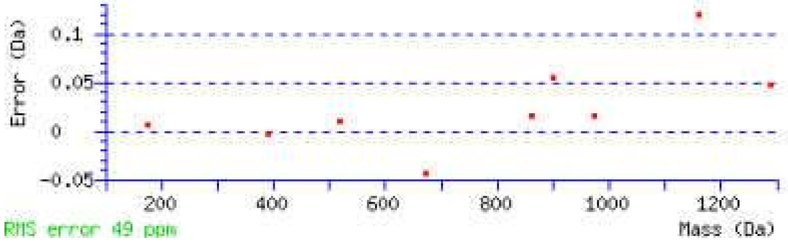


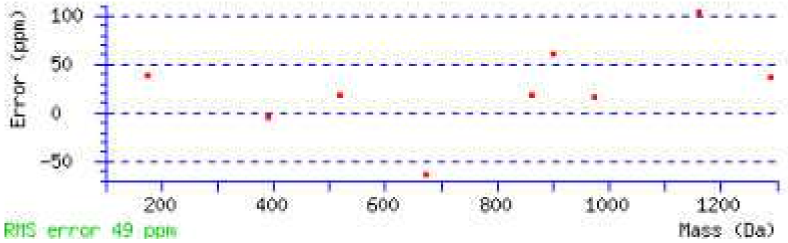


NCBI **BLAST** search of FLPDKAIDLIDEAGSR
(Parameters: blastp, nr protein database, expect=20000, no filter, PAM30)
Other BLAST web gateways

**All matches to this query**

| Score | Mr(calc) | Delta | Sequence |
|---|---|---|---|
| 44.8 | 1758.9152 | 0.0384 | FLPDKAIDIIDEAGSR |
| 44.8 | 1758.9152 | 0.0384 | FLPDKAIDLIDEAGSR |
| 44.8 | 1758.9152 | 0.0384 | FLPDKAIDLIDEGSAR |
| 44.8 | 1758.9152 | 0.0384 | FLPDKAIDLLDEAGSR |
| 20.4 | 1758.9152 | 0.0384 | FLPDKAIDLVDEAASR |
| 20.4 | 1758.9152 | 0.0384 | FLPDKAIDLVDEASAR |
| 20.4 | 1758.9152 | 0.0384 | FLPDKAIDLVDESAAR |
| 20.4 | 1758.9152 | 0.0384 | FLPDKAIDVIDEASAR |
| 20.4 | 1758.8822 | 0.0715 | MLPDKAIDLVDEAASR |
| 17.3 | 1758.8114 | 0.1422 | FELEHFGDYQRYR |

**Mascot:** http://www.matrixscience.com/

# MATRIX SCIENCE Mascot Search Results

## Peptide View **Spot no 110**

MS/MS Fragmentation of **GELQCIGATTLDEYRK**
Found in **gi|18423214** in **NCBInr**, ATP-dependent Clp protease ATP-binding subunit ClpC [Arabidopsis thaliana]

Match to Query 263: 1852.939724 from(1853.947000,1+) intensity(0.0000) index(25)
Title: Label: C9, Spot_Id: 219804, Peak_List_Id: 226651, MSMS Job_Run_Id: 21852, Comment:
Data file C:\Documents and Settings\Administrator\Desktop\Farah-Garima 11May 2013\ppw_C9_136859938300.txt

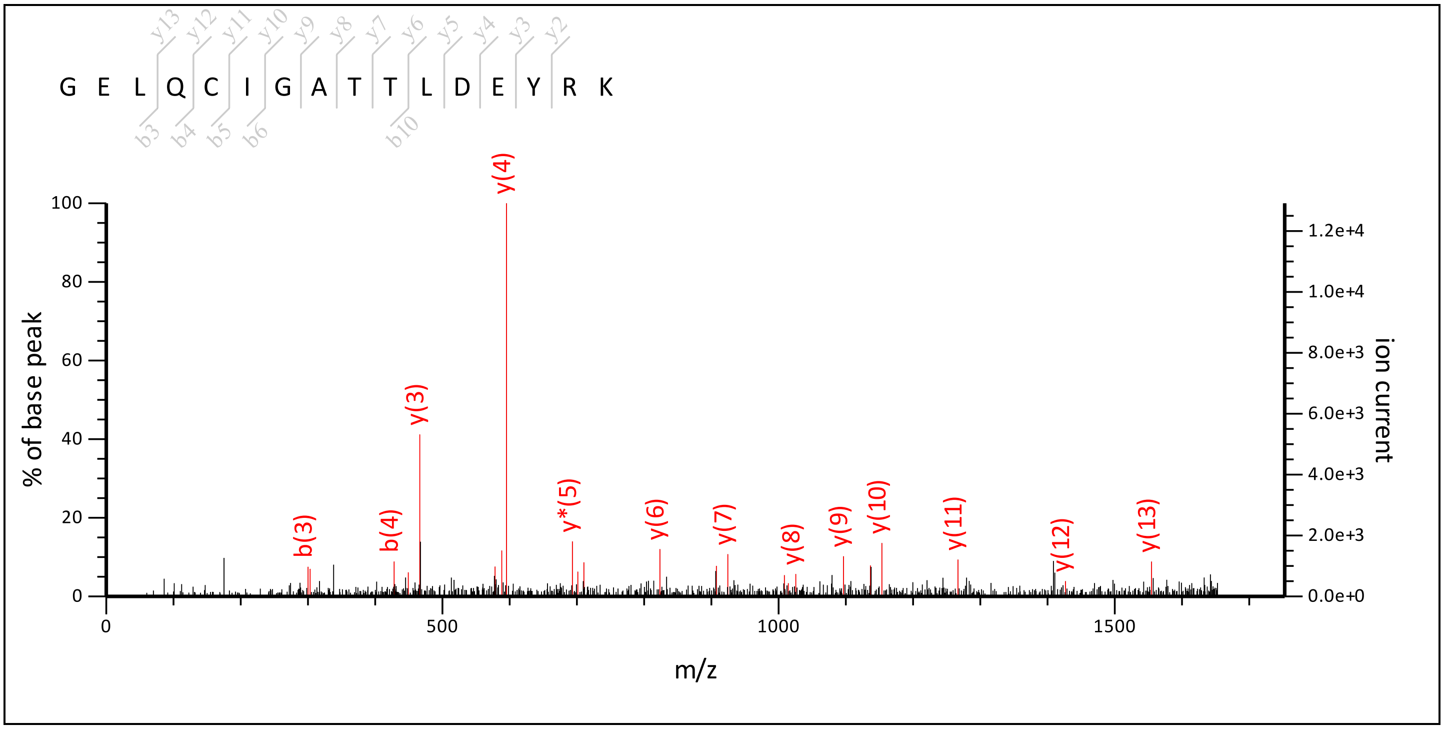


0 to 1752.74

Label all possible matches ○ Label matches used for scoring ◉

**Monoisotopic mass of neutral peptide Mr(calc):** 1852.8989
**Fixed modifications:** Carbamidomethyl (C) (apply to specified residues or termini only)
**Ions Score:** 72 **Expect:** 0.0019
**Matches :** 23/84 fragment ions using 48 most intense peaks (help)

| # | a | a* | b | b* | Seq. | y | y* | # |
|---|---|---|---|---|---|---|---|---|
| 1 | 30.0338 | | 58.0287 | | G | | | 16 |
| 2 | 159.0764 | | 187.0713 | | E | 1796.8847 | 1779.8582 | 15 |
| 3 | 272.1605 | | **300.1554** | | L | 1667.8421 | 1650.8156 | 14 |
| 4 | 400.2191 | 383.1925 | **428.2140** | 411.1874 | Q | ***1554.7581*** | 1537.7315 | 13 |
| 5 | 560.2497 | 543.2232 | **588.2446** | 571.2181 | C | ***1426.6995*** | 1409.6729 | 12 |
| 6 | 673.3338 | 656.3072 | **701.3287** | 684.3021 | I | ***1266.6688*** | 1249.6423 | 11 |
| 7 | 730.3552 | 713.3287 | 758.3502 | 741.3236 | G | ***1153.5848*** | 1136.5582 | 10 |
| 8 | 801.3924 | 784.3658 | 829.3873 | 812.3607 | A | ***1096.5633*** | 1079.5368 | 9 |

| | | | | | | | | |
|---|---|---|---|---|---|---|---|---|
| **9** | 902.4400 | 885.4135 | 930.4349 | 913.4084 | **T** | ***1025.5262*** | 1008.4997 | **8** |
| **10** | 1003.4877 | 986.4612 | 1031.4826 | 1014.4561 | **T** | ***924.4785*** | 907.4520 | **7** |
| **11** | 1116.5718 | 1099.5452 | 1144.5667 | 1127.5401 | **L** | ***823.4308*** | 806.4043 | **6** |
| **12** | 1231.5987 | 1214.5722 | 1259.5936 | 1242.5671 | **D** | ***710.3468*** | 693.3202 | **5** |
| **13** | 1360.6413 | 1343.6148 | 1388.6362 | 1371.6097 | **E** | ***595.3198*** | 578.2933 | **4** |
| **14** | 1523.7046 | 1506.6781 | 1551.6996 | 1534.6730 | **Y** | ***466.2772*** | 449.2507 | **3** |
| **15** | 1679.8057 | 1662.7792 | 1707.8007 | 1690.7741 | **R** | ***303.2139*** | 286.1874 | **2** |
| **16** | | | | | **K** | 147.1128 | 130.0863 | **1** |

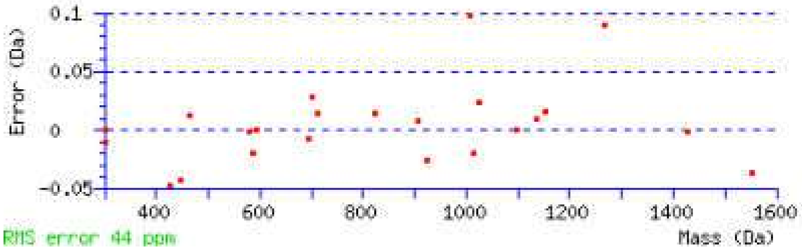


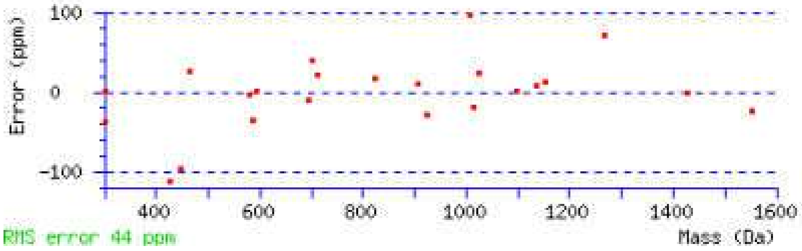


NCBI **BLAST** search of GELQCIGATTLDEYRK
(Parameters: blastp, nr protein database, expect=20000, no filter, PAM30)
Other BLAST web gateways

**All matches to this query**

| Score | Mr(calc) | Delta | Sequence |
|---|---|---|---|
| 84.7 | 1852.8989 | 0.0408 | GELQCLGATTLDEYKR |
| 71.7 | 1852.8989 | 0.0408 | GEIQCIGATTIDEYRK |
| 71.7 | 1852.8989 | 0.0408 | GEIQCIGATTLDEYRK |
| 71.7 | 1852.8989 | 0.0408 | GELQCIGATTIDEYRK |
| 71.7 | 1852.8989 | 0.0408 | GELQCIGATTLDEYRK |
| 71.7 | 1852.8989 | 0.0408 | GELQCLGATTIDEYRK |
| 71.7 | 1852.8989 | 0.0408 | GELQCLGATTLDEYRK |
| 66.5 | 1852.8737 | 0.0660 | GELQCIGATTNNEYRK |
| 57.8 | 1852.8989 | 0.0408 | GELQCLGATTVEEYRK |
| 23.4 | 1852.8228 | 0.1169 | GVGGIDSWGSDVEEQYR |

**Mascot:** http://www.matrixscience.com/

# Mascot Search Results

## Peptide View Spot no 110

MS/MS Fragmentation of **NPNRPIASFIFSGPTGVGK**
Found in **gi|18423214** in **NCBInr**, ATP-dependent Clp protease ATP-binding subunit ClpC [Arabidopsis thaliana]

Match to Query 274: 1958.077924 from(1959.085200,1+) intensity(0.0000) index(27)
Title: Label: C9, Spot_Id: 219804, Peak_List_Id: 226648, MSMS Job_Run_Id: 21852, Comment:
Data file C:\Documents and Settings\Administrator\Desktop\Farah-Garima 11May 2013\ppw_C9_136859938300.txt

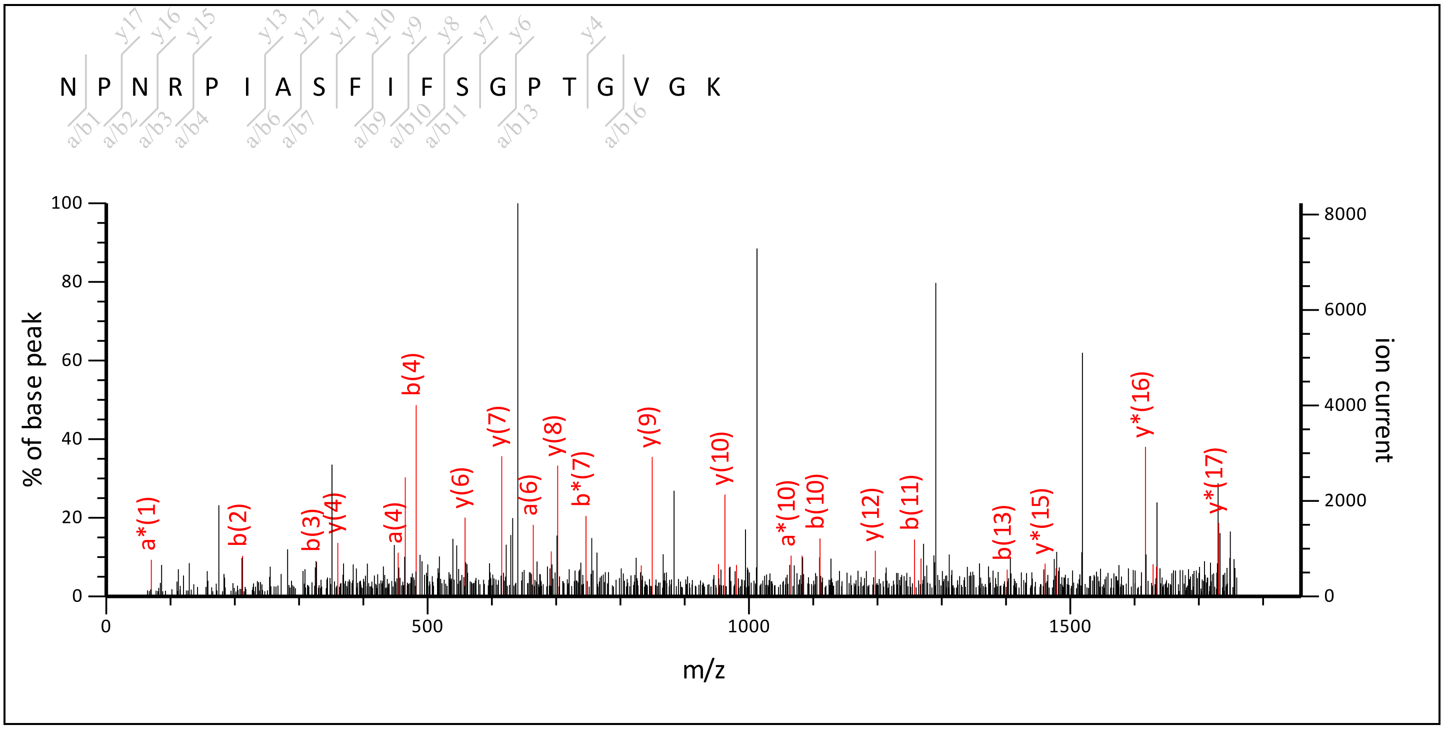


0 to 1858.94

Label all possible matches Label matches used for scoring

**Monoisotopic mass of neutral peptide Mr(calc):** 1958.0374
**Fixed modifications:** Carbamidomethyl (C) (apply to specified residues or termini only)
**Ions Score:** 31 **Expect:** 21
**Matches :** 32/108 fragment ions using 102 most intense peaks (help)

| # | a | a* | b | b* | Seq. | y | y* | # |
|---|---|---|---|---|---|---|---|---|
| 1 | 87.0553 | 70.0287 | 115.0502 | 98.0237 | N | | | 19 |
| 2 | 184.1081 | 167.0815 | **212.1030** | 195.0764 | P | 1845.0017 | 1827.9752 | 18 |
| 3 | 298.1510 | 281.1244 | **326.1459** | 309.1193 | N | 1747.9490 | 1730.9224 | 17 |
| 4 | **454.2521** | 437.2255 | **482.2470** | 465.2205 | R | ***1633.9061*** | 1616.8795 | 16 |
| 5 | 551.3049 | 534.2783 | 579.2998 | 562.2732 | P | ***1477.8049*** | 1460.7784 | 15 |
| 6 | **664.3889** | 647.3624 | **692.3838** | 675.3573 | I | 1380.7522 | 1363.7256 | 14 |
| 7 | 735.4260 | 718.3995 | 763.4209 | 746.3944 | A | ***1267.6681*** | 1250.6416 | 13 |
| 8 | 822.4581 | 805.4315 | 850.4530 | 833.4264 | S | ***1196.6310*** | 1179.6045 | 12 |

| | | | | | | | | |
|---|---|---|---|---|---|---|---|---|
| 9 | 969.5265 | 952.4999 | 997.5214 | 980.4948 | F | 1109.5990 | 1092.5724 | 11 |
| 10 | 1082.6105 | 1065.5840 | 1110.6055 | 1093.5789 | I | 962.5306 | 945.5040 | 10 |
| 11 | 1229.6790 | 1212.6524 | 1257.6739 | 1240.6473 | F | 849.4465 | 832.4199 | 9 |
| 12 | 1316.7110 | 1299.6844 | 1344.7059 | 1327.6793 | S | 702.3781 | 685.3515 | 8 |
| 13 | 1373.7324 | 1356.7059 | 1401.7274 | 1384.7008 | G | 615.3461 | 598.3195 | 7 |
| 14 | 1470.7852 | 1453.7587 | 1498.7801 | 1481.7536 | P | 558.3246 | 541.2980 | 6 |
| 15 | 1571.8329 | 1554.8063 | 1599.8278 | 1582.8013 | T | 461.2718 | 444.2453 | 5 |
| 16 | 1628.8544 | 1611.8278 | 1656.8493 | 1639.8227 | G | 360.2241 | 343.1976 | 4 |
| 17 | 1727.9228 | 1710.8962 | 1755.9177 | 1738.8911 | V | 303.2027 | 286.1761 | 3 |
| 18 | 1784.9442 | 1767.9177 | 1812.9391 | 1795.9126 | G | 204.1343 | 187.1077 | 2 |
| 19 | | | | | K | 147.1128 | 130.0863 | 1 |

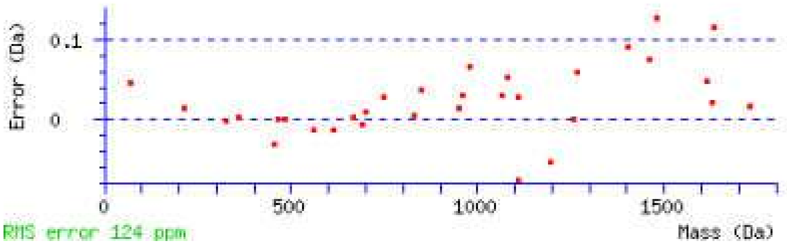


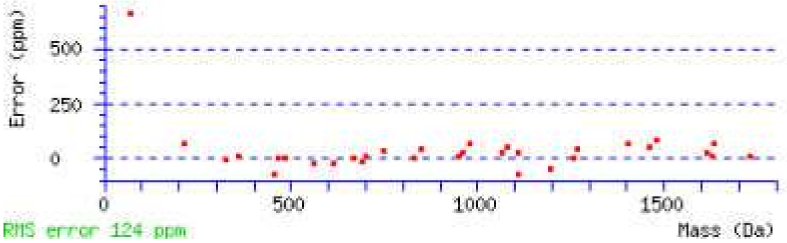


NCBI **BLAST** search of NPNRPIASFIFSGPTGVGK
(Parameters: blastp, nr protein database, expect=20000, no filter, PAM30)
Other BLAST web gateways

**All matches to this query**

| Score | Mr(calc) | Delta | Sequence |
|---|---|---|---|
| 30.5 | 1958.0374 | 0.0405 | NPNRPIASFIFSGPTGVGK |
| 30.5 | 1958.0374 | 0.0405 | NPNRPIASFLFSGPTGVGK |
| 14.2 | 1957.9001 | 0.1778 | FHYKICFNMPDIAMR |
| 12.8 | 1957.9646 | 0.1133 | TQIENYDFVFGQSRVR |
| 10.6 | 1958.0594 | 0.0186 | AGTLRVAAMPAMAIGFLPR |
| 10.6 | 1958.0594 | 0.0186 | AGTLRVAAMPAMAIGFLPR |
| 9.8 | 1957.9785 | 0.0994 | TQLDVVYQEFLVYADR |
| 9.5 | 1957.9381 | 0.1398 | GATIYAELAGEGNSLSSYR |
| 8.6 | 1957.8873 | 0.1906 | TISANMAPDEFTQKSMR |
| 8.4 | 1957.9857 | 0.0922 | DNLTEEEQRVLALWSR |

**Mascot:** http://www.matrixscience.com/

# MATRIX SCIENCE Mascot Search Results

## Peptide View **Spot no 110**

MS/MS Fragmentation of **QLGHNYIGSEHLLLGLLR**
Found in **gi|18423214** in **NCBInr**, ATP-dependent Clp protease ATP-binding subunit ClpC [Arabidopsis thaliana]

Match to Query 280: 2032.161224 from(2033.168500,1+) intensity(0.0000) index(29)
Title: Label: C9, Spot_Id: 219804, Peak_List_Id: 226666, MSMS Job_Run_Id: 21852, Comment:
Data file C:\Documents and Settings\Administrator\Desktop\Farah-Garima 11May 2013\ppw_C9_136859938300.txt

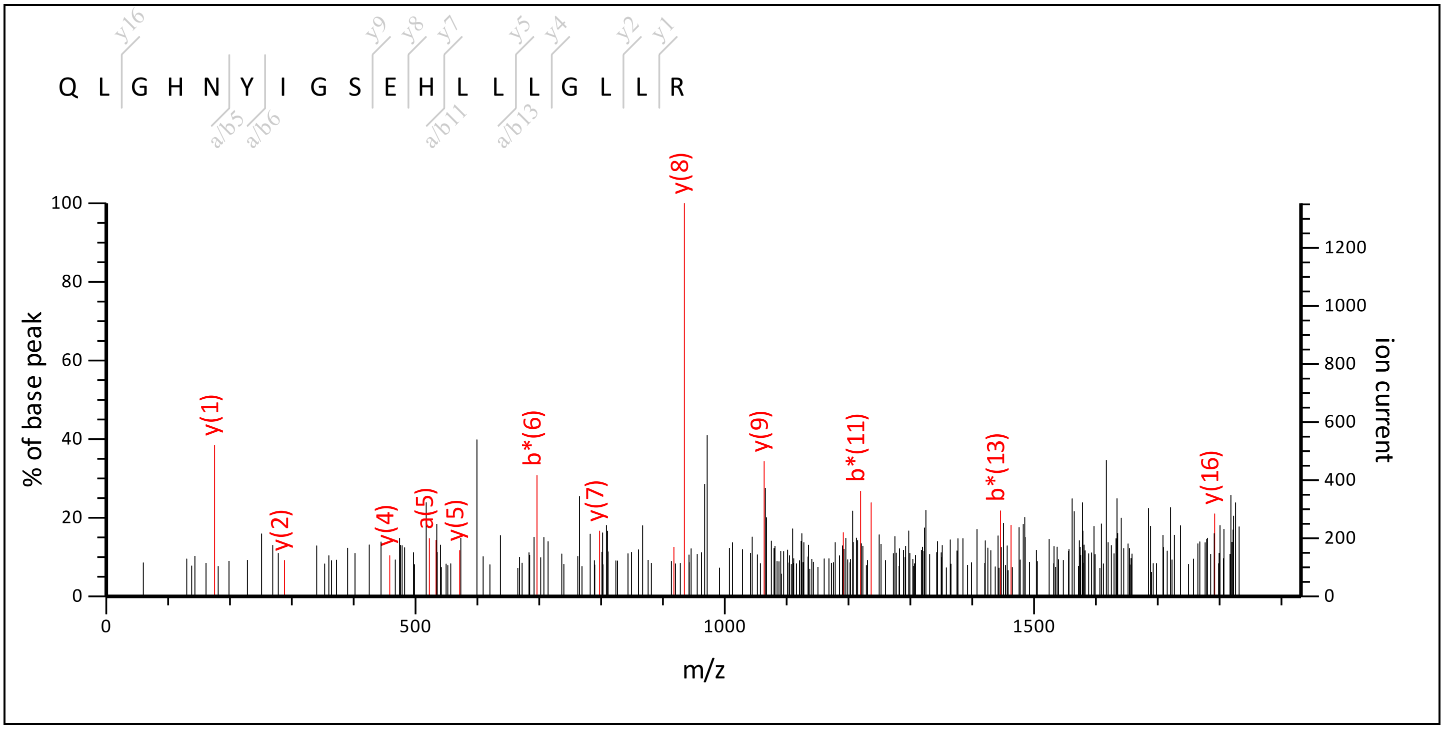


0 to 1931.57

Label all possible matches ○ Label matches used for scoring ◉

**Monoisotopic mass of neutral peptide Mr(calc):** 2032.1218
**Fixed modifications:** Carbamidomethyl (C) (apply to specified residues or termini only)
**Ions Score:** 5 **Expect:** 6.8e+03
**Matches :** 17/102 fragment ions using 89 most intense peaks (help)

| # | a | a* | b | b* | Seq. | y | y* | # |
|---|---|---|---|---|---|---|---|---|
| 1 | 101.0709 | 84.0444 | 129.0659 | 112.0393 | Q | | | 18 |
| 2 | 214.1550 | 197.1285 | 242.1499 | 225.1234 | L | 1905.0705 | 1888.0439 | 17 |
| 3 | 271.1765 | 254.1499 | 299.1714 | 282.1448 | G | ***1791.9864*** | 1774.9599 | 16 |
| 4 | 408.2354 | 391.2088 | 436.2303 | 419.2037 | H | 1734.9650 | 1717.9384 | 15 |
| 5 | 522.2783 | 505.2518 | 550.2732 | 533.2467 | N | 1597.9061 | 1580.8795 | 14 |
| 6 | 685.3416 | 668.3151 | 713.3365 | 696.3100 | Y | 1483.8631 | 1466.8366 | 13 |
| 7 | 798.4257 | 781.3991 | 826.4206 | 809.3941 | I | 1320.7998 | 1303.7732 | 12 |
| 8 | 855.4472 | 838.4206 | 883.4421 | 866.4155 | G | 1207.7157 | 1190.6892 | 11 |

| | | | | | | | | |
|---|---|---|---|---|---|---|---|---|
| 9 | 942.4792 | 925.4526 | 970.4741 | 953.4476 | S | 1150.6943 | 1133.6677 | 10 |
| 10 | 1071.5218 | 1054.4952 | 1099.5167 | 1082.4902 | E | ***1063.6622*** | 1046.6357 | 9 |
| 11 | 1208.5807 | 1191.5541 | **1236.5756** | 1219.5491 | H | ***934.6196*** | 917.5931 | 8 |
| 12 | 1321.6648 | 1304.6382 | 1349.6597 | 1332.6331 | L | ***797.5607*** | 780.5342 | 7 |
| 13 | 1434.7488 | 1417.7223 | **1462.7437** | 1445.7172 | L | 684.4767 | 667.4501 | 6 |
| 14 | 1547.8329 | 1530.8063 | 1575.8278 | 1558.8013 | L | ***571.3926*** | 554.3661 | 5 |
| 15 | 1604.8544 | 1587.8278 | 1632.8493 | 1615.8227 | G | ***458.3085*** | 441.2820 | 4 |
| 16 | 1717.9384 | 1700.9119 | 1745.9333 | 1728.9068 | L | 401.2871 | 384.2605 | 3 |
| 17 | 1831.0225 | 1813.9959 | 1859.0174 | 1841.9908 | L | ***288.2030*** | 271.1765 | 2 |
| 18 | | | | | R | ***175.1190*** | 158.0924 | 1 |

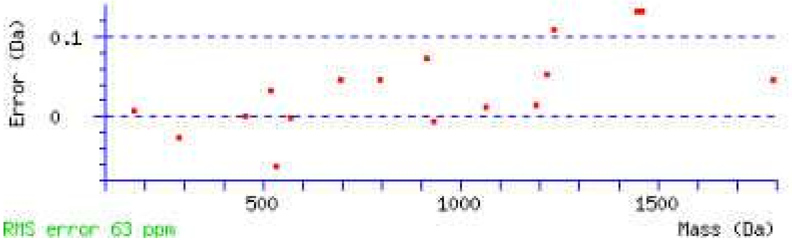


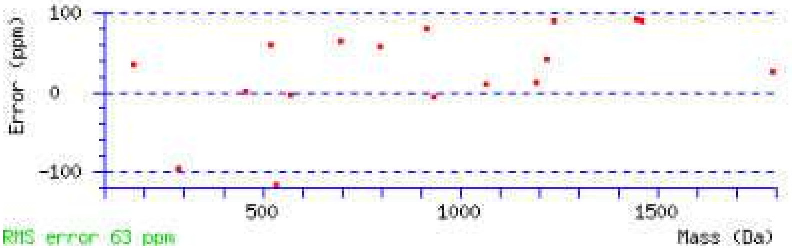


NCBI **BLAST** search of QLGHNYIGSEHLLLGLLR
(Parameters: blastp, nr protein database, expect=20000, no filter, PAM30)
Other BLAST web gateways

**All matches to this query**

| Score | Mr(calc) | Delta | Sequence |
|---|---|---|---|
| 4.6 | 2032.1582 | 0.0031 | KLGHNYVGTEHILLGLIR |
| 4.6 | 2032.1218 | 0.0395 | QLGHNYIGSEHLLLGLLR |
| 4.6 | 2032.1218 | 0.0394 | QLGHNYVGTEHLLLGLIR |
| 4.0 | 2032.0313 | 0.1300 | FYMSATPRNHVPTAAVVR |
| 2.0 | 2032.1470 | 0.0143 | GIFVLQRIGDLVGYIDVR |
| 0.7 | 2032.0888 | 0.0724 | VMGLPLPRISDLAHGADVR |
| 0.6 | 2032.0663 | 0.0949 | AVFVSMPDSLSPLLTAVNR |
| 0.4 | 2031.9644 | 0.1969 | AAQNTLSSPTQMLNSSPGGR |
| 0.3 | 2031.9585 | 0.2027 | NTPMHIYAGNGNFVGDGIR |
| 0.1 | 2032.0272 | 0.1340 | LAQCPGPHTSATAPERALR |

**Mascot:** http://www.matrixscience.com/

# MATRIX SCIENCE Mascot Search Results

## Peptide View **Spot no 110**

MS/MS Fragmentation of **LIGSPPGYVGYTEGGQLTEAVR**
Found in **gi|18423214** in **NCBInr**, ATP-dependent Clp protease ATP-binding subunit ClpC [Arabidopsis thaliana]

Match to Query 295: 2263.184624 from(2264.191900,1+) intensity(0.0000) index(32)
Title: Label: C9, Spot_Id: 219804, Peak_List_Id: 226650, MSMS Job_Run_Id: 21852, Comment:
Data file C:\Documents and Settings\Administrator\Desktop\Farah-Garima 11May 2013\ppw_C9_136859938300.txt

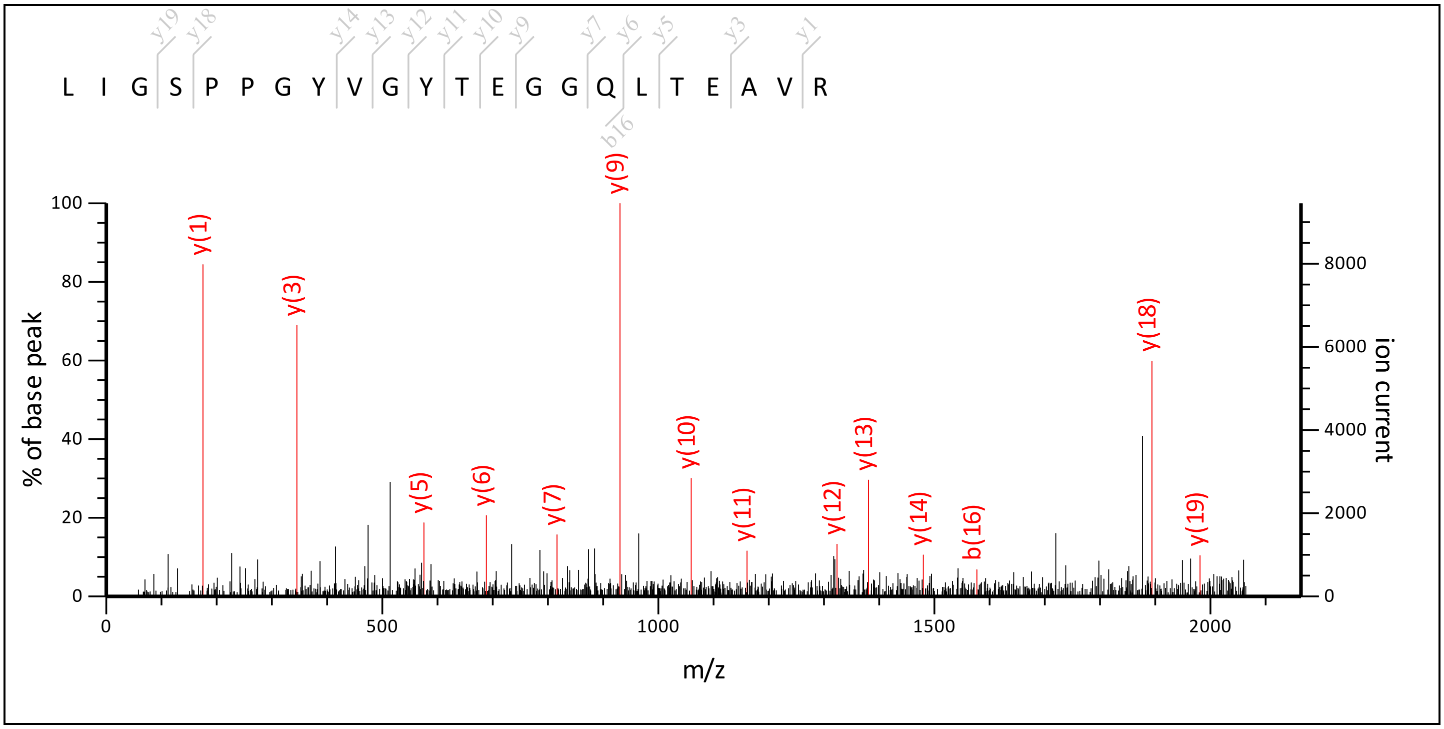


0 to 2164.02

Label all possible matches ○ Label matches used for scoring ◉

**Monoisotopic mass of neutral peptide Mr(calc):** 2263.1485
**Fixed modifications:** Carbamidomethyl (C) (apply to specified residues or termini only)
**Ions Score:** 98 **Expect:** 3.7e-06
**Matches :** 14/96 fragment ions using 21 most intense peaks (help)

| # | a | a* | b | b* | Seq. | y | y* | # |
|---|---|---|---|---|---|---|---|---|
| 1 | 86.0964 | | 114.0913 | | L | | | 22 |
| 2 | 199.1805 | | 227.1754 | | I | 2151.0717 | 2134.0451 | 21 |
| 3 | 256.2020 | | 284.1969 | | G | 2037.9876 | 2020.9611 | 20 |
| 4 | 343.2340 | | 371.2289 | | S | ***1980.9662*** | 1963.9396 | 19 |
| 5 | 440.2867 | | 468.2817 | | P | ***1893.9341*** | 1876.9076 | 18 |
| 6 | 537.3395 | | 565.3344 | | P | 1796.8814 | 1779.8548 | 17 |
| 7 | 594.3610 | | 622.3559 | | G | 1699.8286 | 1682.8020 | 16 |
| 8 | 757.4243 | | 785.4192 | | Y | 1642.8071 | 1625.7806 | 15 |

| | | | | | | | | |
|---|---|---|---|---|---|---|---|---|
| 9 | 856.4927 | | 884.4876 | | V | 1479.7438 | 1462.7173 | 14 |
| 10 | 913.5142 | | 941.5091 | | G | 1380.6754 | 1363.6488 | 13 |
| 11 | 1076.5775 | | 1104.5724 | | Y | 1323.6539 | 1306.6274 | 12 |
| 12 | 1177.6252 | | 1205.6201 | | T | 1160.5906 | 1143.5640 | 11 |
| 13 | 1306.6678 | | 1334.6627 | | E | 1059.5429 | 1042.5164 | 10 |
| 14 | 1363.6892 | | 1391.6842 | | G | 930.5003 | 913.4738 | 9 |
| 15 | 1420.7107 | | 1448.7056 | | G | 873.4789 | 856.4523 | 8 |
| 16 | 1548.7693 | 1531.7427 | 1576.7642 | 1559.7377 | Q | 816.4574 | 799.4308 | 7 |
| 17 | 1661.8534 | 1644.8268 | 1689.8483 | 1672.8217 | L | 688.3988 | 671.3723 | 6 |
| 18 | 1762.9010 | 1745.8745 | 1790.8959 | 1773.8694 | T | 575.3148 | 558.2882 | 5 |
| 19 | 1891.9436 | 1874.9171 | 1919.9385 | 1902.9120 | E | 474.2671 | 457.2405 | 4 |
| 20 | 1962.9807 | 1945.9542 | 1990.9757 | 1973.9491 | A | 345.2245 | 328.1979 | 3 |
| 21 | 2062.0492 | 2045.0226 | 2090.0441 | 2073.0175 | V | 274.1874 | 257.1608 | 2 |
| 22 | | | | | R | 175.1190 | 158.0924 | 1 |

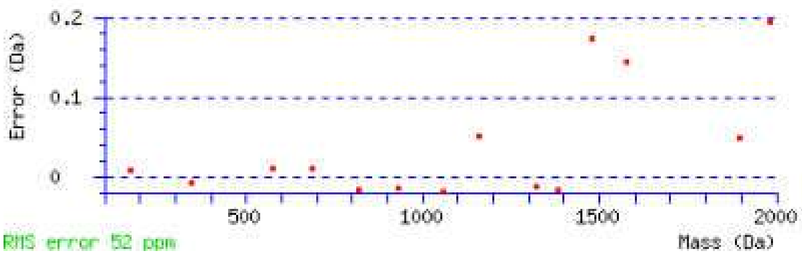


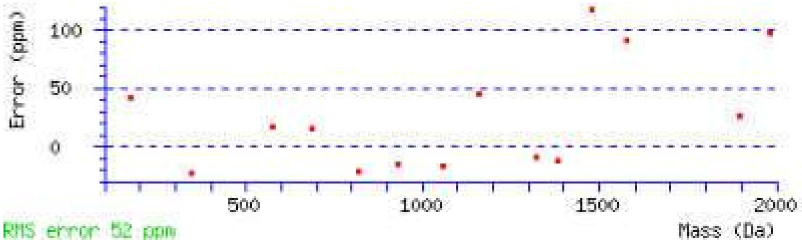


NCBI **BLAST** search of LIGSPPGYVGYTEGGQLTEAVR
(Parameters: blastp, nr protein database, expect=20000, no filter, PAM30)
Other BLAST web gateways

**All matches to this query**

| Score | Mr(calc) | Delta | Sequence |
|---|---|---|---|
| 97.9 | 2263.1485 | 0.0362 | LIGSPPGYVGYTEGGQLTEAVR |
| 58.0 | 2263.1121 | 0.0725 | LIGSPPGYVGYDDGGQLTEAVR |
| 58.0 | 2263.1485 | 0.0362 | LIGSPPGYIGYSEGGQLTEAVR |
| 33.5 | 2263.1484 | 0.0362 | LIGAAPGYIGYEEGGQLTEAVR |
| 33.5 | 2263.1597 | 0.0249 | LIGAPPGYIGHEEGGQLTEAVR |
| 33.5 | 2263.1485 | 0.0362 | LIGTAPGFVGYEEGGQLTEAVR |
| 23.1 | 2263.1121 | 0.0725 | LVGSPPGYVGYDEGGQLTEAVR |
| 16.6 | 2263.1484 | 0.0362 | LIGAPAGYIGYDEAGQLTEAVR |
| 16.6 | 2263.1484 | 0.0362 | LIGAPAGYVGYEEAGQLTEAVR |
| | | | |

| | | | |
|---|---|---|---|
| 16.6 | 2263.1597 | 0.0249 | LIGAPPGYVGHEEAGQLTEAVR |

**Mascot:** http://www.matrixscience.com/

# Mascot Search Results

## Peptide View **Spot no 110**

MS/MS Fragmentation of **YTDESLVAAAQLSYQYISDR**
Found in **gi|18423214** in **NCBInr**, ATP-dependent Clp protease ATP-binding subunit ClpC [Arabidopsis thaliana]

Match to Query 300: 2292.128924 from(2293.136200,1+) intensity(0.0000) index(33)
Title: Label: C9, Spot_Id: 219804, Peak_List_Id: 226656, MSMS Job_Run_Id: 21852, Comment:
Data file C:\Documents and Settings\Administrator\Desktop\Farah-Garima 11May 2013\ppw_C9_136859938300.txt

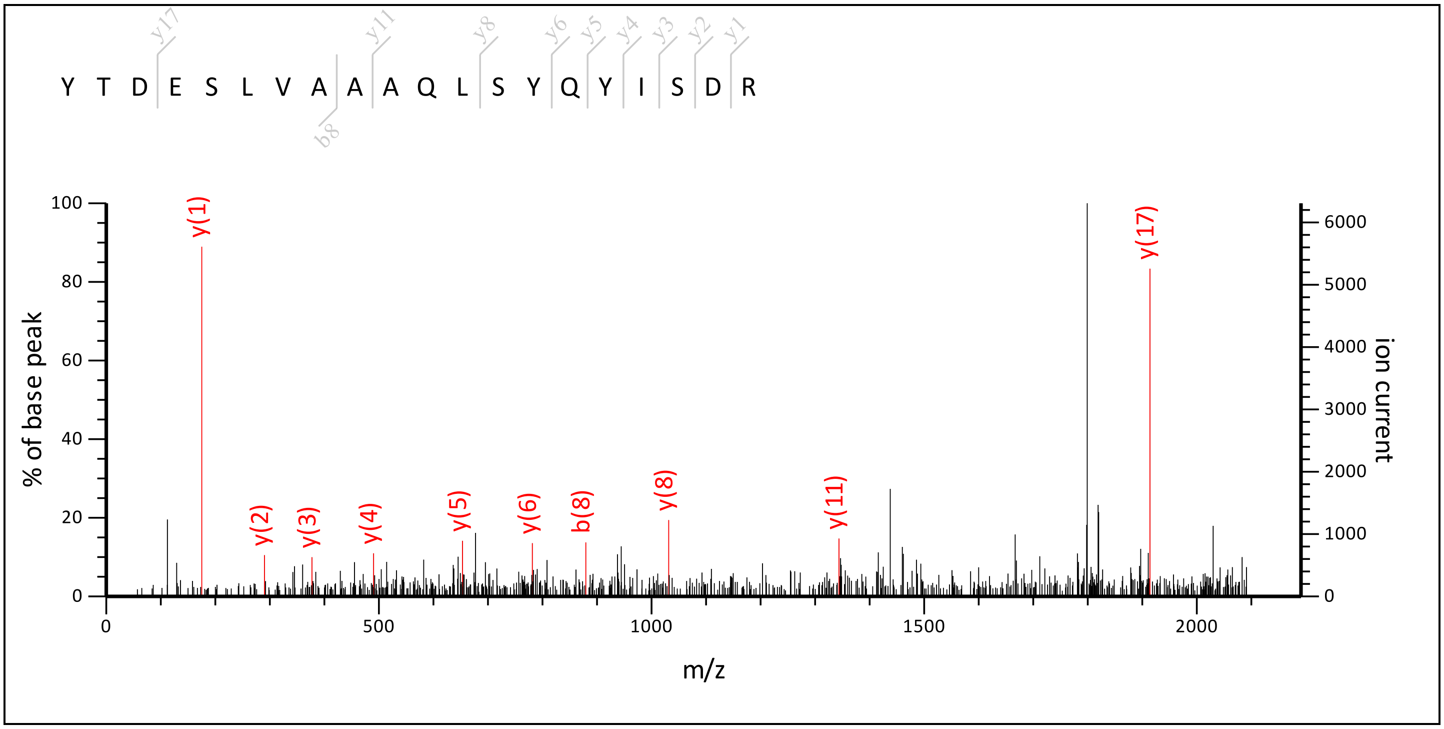


0 to 2191.06

Label all possible matches ○ Label matches used for scoring ◉

**Monoisotopic mass of neutral peptide Mr(calc):** 2292.0910
**Fixed modifications:** Carbamidomethyl (C) (apply to specified residues or termini only)
**Ions Score:** 43 **Expect:** 1.2
**Matches** : 10/94 fragment ions using 21 most intense peaks (help)

| # | a | a* | b | b* | Seq. | y | y* | # |
|---|---|---|---|---|---|---|---|---|
| 1 | 136.0757 | | 164.0706 | | Y | | | 20 |
| 2 | 237.1234 | | 265.1183 | | T | 2130.0350 | 2113.0084 | 19 |
| 3 | 352.1503 | | 380.1452 | | D | 2028.9873 | 2011.9607 | 18 |
| 4 | 481.1929 | | 509.1878 | | E | ***1913.9603*** | 1896.9338 | 17 |
| 5 | 568.2249 | | 596.2198 | | S | 1784.9177 | 1767.8912 | 16 |
| 6 | 681.3090 | | 709.3039 | | L | 1697.8857 | 1680.8592 | 15 |
| 7 | 780.3774 | | 808.3723 | | V | 1584.8016 | 1567.7751 | 14 |
| 8 | 851.4145 | | 879.4094 | | A | 1485.7332 | 1468.7067 | 13 |

| | | | | | | | | |
|---|---|---|---|---|---|---|---|---|
| 9 | 922.4516 | | 950.4466 | | A | 1414.6961 | 1397.6696 | 12 |
| 10 | 993.4888 | | 1021.4837 | | A | *1343.6590* | 1326.6325 | 11 |
| 11 | 1121.5473 | 1104.5208 | 1149.5422 | 1132.5157 | Q | 1272.6219 | 1255.5953 | 10 |
| 12 | 1234.6314 | 1217.6048 | 1262.6263 | 1245.5998 | L | 1144.5633 | 1127.5368 | 9 |
| 13 | 1321.6634 | 1304.6369 | 1349.6583 | 1332.6318 | S | *1031.4793* | 1014.4527 | 8 |
| 14 | 1484.7268 | 1467.7002 | 1512.7217 | 1495.6951 | Y | 944.4472 | 927.4207 | 7 |
| 15 | 1612.7853 | 1595.7588 | 1640.7802 | 1623.7537 | Q | *781.3839* | 764.3573 | 6 |
| 16 | 1775.8487 | 1758.8221 | 1803.8436 | 1786.8170 | Y | *653.3253* | 636.2988 | 5 |
| 17 | 1888.9327 | 1871.9062 | 1916.9276 | 1899.9011 | I | *490.2620* | 473.2354 | 4 |
| 18 | 1975.9648 | 1958.9382 | 2003.9597 | 1986.9331 | S | *377.1779* | 360.1514 | 3 |
| 19 | 2090.9917 | 2073.9651 | 2118.9866 | 2101.9601 | D | *290.1459* | 273.1193 | 2 |
| 20 | | | | | R | *175.1190* | 158.0924 | 1 |

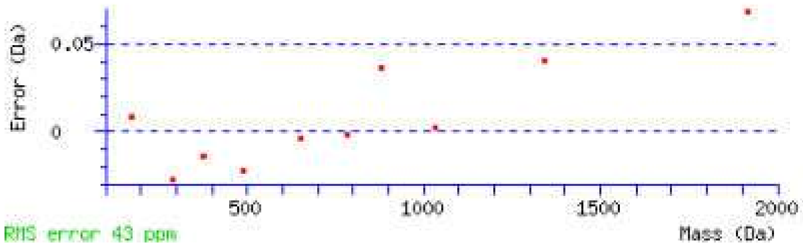


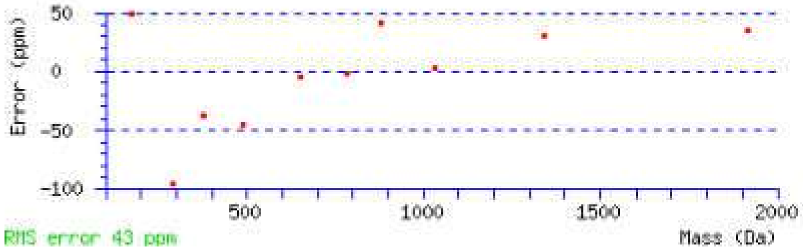


NCBI **BLAST** search of YTDESLVAAAQLSYQYISDR
(Parameters: blastp, nr protein database, expect=20000, no filter, PAM30)
Other BLAST web gateways

**All matches to this query**

| Score | Mr(calc) | Delta | Sequence |
|---|---|---|---|
| 58.5 | 2292.1274 | 0.0016 | YTDDSLIAAAKLSYQYISDR |
| 58.5 | 2292.0910 | 0.0380 | YTDDSLIAAAQLSYQYISDR |
| 56.0 | 2292.1274 | 0.0016 | YTDDALISAAKLSYQYISDR |
| 42.7 | 2292.0910 | 0.0380 | YTDEALVSAAQLSYQYISDR |
| 42.7 | 2292.0910 | 0.0380 | YTDESLVAAAQLSYQYISDR |
| 12.1 | 2292.0830 | 0.0460 | VDRTISAIENVDTDSGATASDR |
| 11.9 | 2292.1249 | 0.0041 | FYDPIQALNTMFAKYISDR |
| 10.3 | 2292.0991 | 0.0299 | QNVINIDHTYSVEAMMKAGR |
| 9.0 | 2292.2161 | -0.0872 | VHPPVPPPPTAAPRTTNMQIR |
| 8.6 | 2292.1168 | 0.0121 | EVSIVPRQAQGLSMQEYADR |

**Mascot:** http://www.matrixscience.com/

# Mascot Search Results

## Peptide View Spot no 112

MS/MS Fragmentation of **NPYWFNR**
Found in **gi|28190676** in **NCBInr**, putative transketolase [Oryza sativa Japonica Group]

Match to Query 41: 995.498884 from(996.506160,1+) intensity(0.0000) index(4)
Title: Label: E1, Spot_Id: 219678, Peak_List_Id: 224896, MSMS Job_Run_Id: 21757, Comment:
Data file C:\Documents and Settings\Administrator\Desktop\farah BHU\spot set plate\Farah-Garima 11May 2013\ppw_E1_136842029000.txt

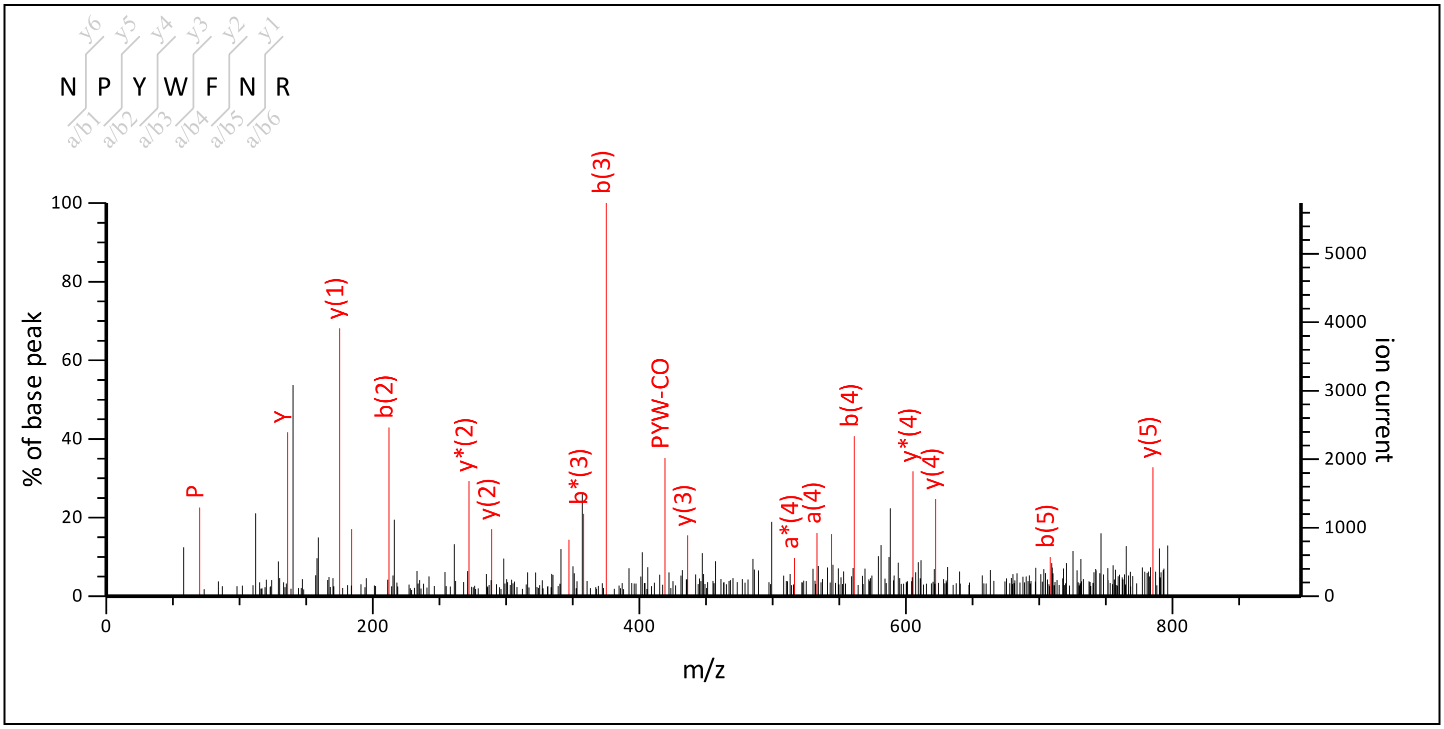


0 to 896.43

Label all possible matches Label matches used for scoring

**Monoisotopic mass of neutral peptide Mr(calc):** 995.4613
**Fixed modifications:** Carbamidomethyl (C) (apply to specified residues or termini only)
**Ions Score:** 29 **Expect:** 2.2
**Matches :** 22/74 fragment ions using 32 most intense peaks (help)

| # | Immon. | a | a* | b | b* | d | Seq. | v | w | y | y* | # |
|---|---|---|---|---|---|---|---|---|---|---|---|---|
| 1 | 87.0553 | 87.0553 | 70.0287 | 115.0502 | 98.0237 | 44.0495 | N | | | | | 7 |
| 2 | 70.0651 | **184.1081** | 167.0815 | **212.1030** | 195.0764 | 158.0924 | P | 840.3787 | 839.3835 | 882.4257 | 865.3992 | 6 |
| 3 | 136.0757 | **347.1714** | 330.1448 | **375.1663** | 358.1397 | | Y | 677.3154 | | ***785.3729*** | 768.3464 | 5 |
| 4 | 159.0917 | **533.2507** | 516.2241 | **561.2456** | 544.2191 | | W | 491.2361 | | ***622.3096*** | 605.2831 | 4 |
| 5 | 120.0808 | 680.3191 | 663.2926 | **708.3140** | 691.2875 | | F | 344.1677 | | ***436.2303*** | 419.2037 | 3 |
| 6 | 87.0553 | 794.3620 | 777.3355 | 822.3569 | 805.3304 | 751.3562 | N | 230.1248 | 229.1295 | ***289.1619*** | 272.1353 | 2 |
| 7 | 129.1135 | | | | | | R | 74.0237 | 73.0284 | ***175.1190*** | 158.0924 | 1 |

| Seq | ya | yb | Seq | ya | yb | Seq | ya | yb |
|---|---|---|---|---|---|---|---|---|
| PY | 233.1285 | 261.1234 | PYW | 419.2078 | 447.2027 | PYWF | 566.2762 | 594.2711 |
| PYWFN | 680.3191 | 708.3140 | YW | 322.1550 | 350.1499 | YWF | 469.2234 | 497.2183 |
| YWFN | 583.2663 | 611.2613 | WF | 306.1601 | 334.1550 | WFN | 420.2030 | 448.1979 |
| FN | 234.1237 | 262.1186 | | | | | | |

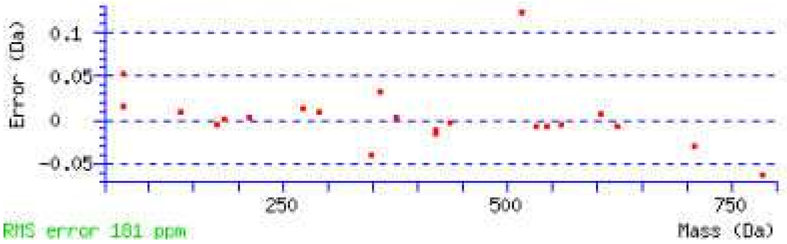


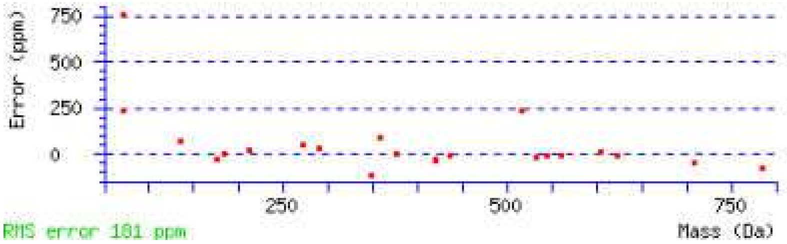


NCBI **BLAST** search of NPYWFNR
(Parameters: blastp, nr protein database, expect=20000, no filter, PAM30)
Other BLAST web gateways

**All matches to this query**

| Score | Mr(calc) | Delta | Sequence |
|---|---|---|---|
| 29.4 | 995.4613 | 0.0376 | NPYWFNR |
| 16.4 | 995.4825 | 0.0164 | VGSGYPFNR |
| 16.0 | 995.5036 | -0.0047 | GVPSGTEPPR |
| 15.3 | 995.5261 | -0.0272 | SLSSGRHPR |
| 14.9 | 995.4494 | 0.0494 | ISAECFNR |
| 14.9 | 995.4436 | 0.0553 | WGWMFNR |
| 14.7 | 995.5876 | -0.0887 | HGLKVSLSR |
| 14.5 | 995.4573 | 0.0416 | RGYSDWGR |
| 14.3 | 995.4494 | 0.0495 | FNAMAAAER |
| 14.0 | 995.4421 | 0.0568 | DRDSSFGGR |

**Mascot:** http://www.matrixscience.com/

# MATRIX SCIENCE Mascot Search Results

## Peptide View

MS/MS Fragmentation of **KRPSVLALSR**
Found in **gi|28190676** in **NCBInr**, putative transketolase [Oryza sativa Japonica Group]

Match to Query 75: 1125.736224 from(1126.743500,1+) intensity(0.0000) index(6)
Title: Label: E1, Spot_Id: 219678, Peak_List_Id: 224900, MSMS Job_Run_Id: 21757, Comment:
Data file C:\Documents and Settings\Administrator\Desktop\farah BHU\spot set plate\Farah-Garima 11May 2013\ppw_E1_136842029000.txt

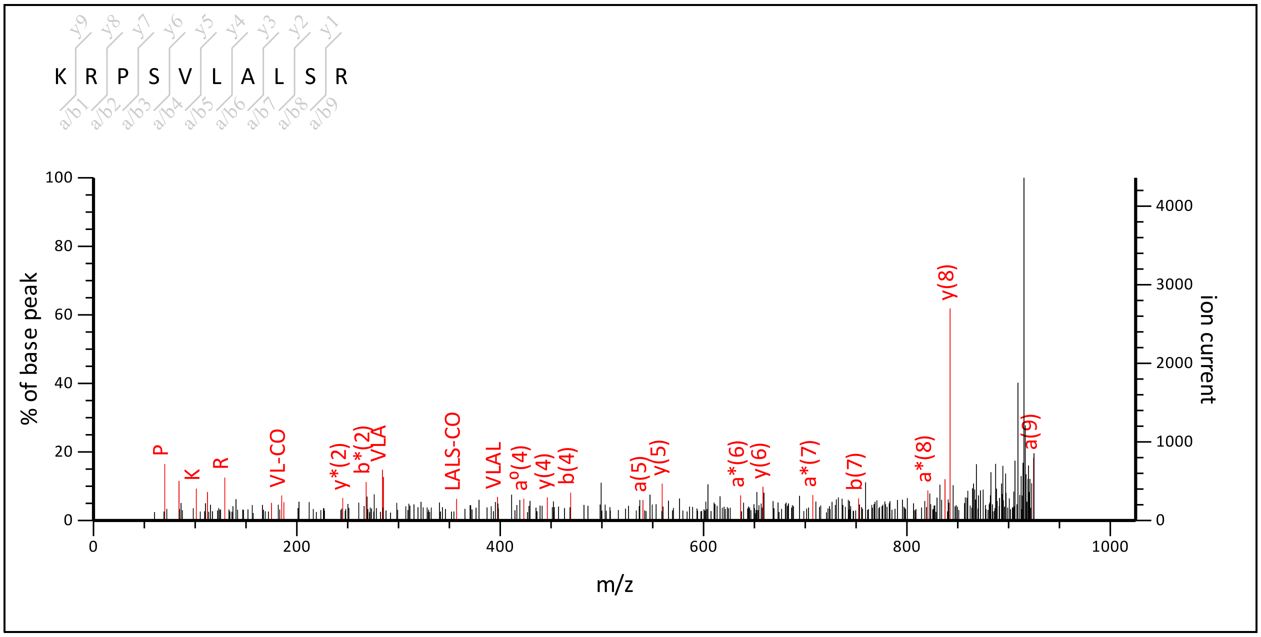


0 to 1024.94

Label all possible matches ○ Label matches used for scoring ◉

**Monoisotopic mass of neutral peptide Mr(calc):** 1125.6982
**Fixed modifications:** Carbamidomethyl (C) (apply to specified residues or termini only)
**Ions Score:** 21 **Expect:** 4.2
**Matches :** 36/161 fragment ions using 54 most intense peaks (help)

| # | Immon. | a | a* | $a^0$ | b | b* | $b^0$ | d | Seq. | v | w | y | y* | $y^0$ | # |
|---|---|---|---|---|---|---|---|---|---|---|---|---|---|---|---|
| 1 | 101.1073 | ***101.1073*** | 84.0808 | | **129.1022** | 112.0757 | | 44.0495 | K | | | | | | 10 |
| 2 | 129.1135 | 257.2084 | 240.1819 | | **285.2034** | 268.1768 | | 172.1444 | R | 897.5152 | 896.5200 | 998.6105 | 981.5840 | 980.6000 | 9 |
| 3 | 70.0651 | 354.2612 | 337.2346 | | 382.2561 | 365.2296 | | 328.2455 | P | 800.4625 | 799.4672 | ***842.5094*** | 825.4829 | 824.4989 | 8 |
| 4 | 60.0444 | 441.2932 | 424.2667 | 423.2827 | **469.2881** | 452.2616 | 451.2776 | 425.2983 | S | 713.4305 | 712.4352 | 745.4567 | 728.4301 | 727.4461 | 7 |
| 5 | 72.0808 | ***540.3616*** | 523.3351 | 522.3511 | 568.3566 | 551.3300 | 550.3460 | 526.3460 | V | 614.3620 | 627.3824 | ***658.4246*** | 641.3981 | 640.4141 | 6 |
| 6 | 86.0964 | 653.4457 | 636.4192 | 635.4351 | 681.4406 | 664.4141 | 663.4301 | 611.3988 | L | 501.2780 | 500.2827 | ***559.3562*** | 542.3297 | 541.3457 | 5 |
| 7 | 44.0495 | 724.4828 | 707.4563 | 706.4723 | **752.4777** | 735.4512 | 734.4672 | | A | 430.2409 | | ***446.2722*** | 429.2456 | 428.2616 | 4 |
| 8 | 86.0964 | ***837.5669*** | 820.5403 | 819.5563 | 865.5618 | 848.5352 | 847.5512 | 795.5199 | L | 317.1568 | 316.1615 | 375.2350 | 358.2085 | 357.2245 | 3 |
| 9 | 60.0444 | ***924.5989*** | 907.5724 | 906.5883 | 952.5938 | 935.5673 | 934.5833 | 908.6040 | S | 230.1248 | 229.1295 | 262.1510 | 245.1244 | 244.1404 | 2 |
| 10 | 129.1135 | | | | | | | | R | 74.0237 | 73.0284 | ***175.1190*** | 158.0924 | | 1 |

| Seq | ya | yb | Seq | ya | yb | Seq | ya | yb |
|---|---|---|---|---|---|---|---|---|
| RP | 226.1662 | 254.1612 | RPS | 313.1983 | 341.1932 | RPSV | 412.2667 | 440.2616 |
| RPSVL | 525.3507 | 553.3457 | RPSVLA | 596.3879 | 624.3828 | PS | 157.0972 | 185.0921 |
| PSV | 256.1656 | 284.1605 | PSVL | 369.2496 | 397.2445 | PSVLA | 440.2867 | 468.2817 |

| | | | | | | | | |
|---|---|---|---|---|---|---|---|---|
| **PSVLAL** | 553.3708 | 581.3657 | **PSVLALS** | 640.4028 | 668.3978 | **SV** | 159.1128 | 187.1077 |
| **SVL** | 272.1969 | 300.1918 | **SVLA** | 343.2340 | 371.2289 | **SVLAL** | 456.3180 | 484.3130 |
| **SVLALS** | 543.3501 | 571.3450 | **VL** | 185.1648 | 213.1598 | **VLA** | 256.2020 | 284.1969 |
| **VLAL** | 369.2860 | 397.2809 | **VLALS** | 456.3180 | 484.3130 | **LA** | 157.1335 | 185.1285 |
| **LAL** | 270.2176 | 298.2125 | **LALS** | 357.2496 | 385.2445 | **AL** | 157.1335 | 185.1285 |
| **ALS** | 244.1656 | 272.1605 | **LS** | 173.1285 | 201.1234 | | | |

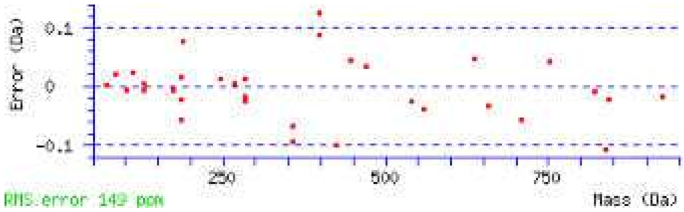


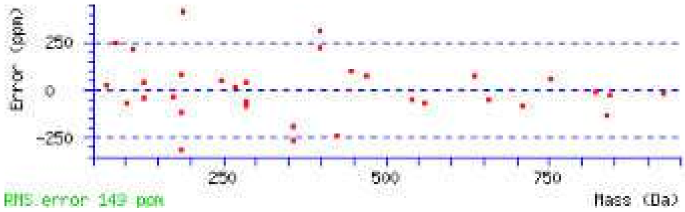


NCBI **BLAST** search of KRPSVLALSR
(Parameters: blastp, nr protein database, expect=20000, no filter, PAM30)
Other BLAST web gateways

**All matches to this query**

| Score | Mr(calc) | Delta | Sequence |
|---|---|---|---|
| 21.1 | 1125.6982 | 0.0380 | KRPSVIALSR |
| 21.1 | 1125.6982 | 0.0380 | KRPSVLALSR |
| 21.1 | 1125.6618 | 0.0744 | QRPSVLALSR |
| 13.7 | 1125.6618 | 0.0744 | RQVIASPISR |
| 11.2 | 1125.6982 | 0.0380 | QRAIVLSALR |
| 8.6 | 1125.6328 | 0.1034 | VGKAMHGVLAK |
| 7.8 | 1125.6254 | 0.1108 | RQLPDAALSR |
| 7.4 | 1125.7121 | 0.0241 | GGGLLAVLVSLK |
| 7.0 | 1125.6506 | 0.0856 | AGRILGPSLDK |
| 7.0 | 1125.6506 | 0.0856 | AGRLLGPSLDK |

**Mascot:** http://www.matrixscience.com/

# MATRIX SCIENCE Mascot Search Results

## Peptide View **Spot no 112**

MS/MS Fragmentation of **YTPESPADATR**
Found in **gi|28190676** in **NCBInr**, putative transketolase [Oryza sativa Japonica Group]

Match to Query 84: 1206.597824 from(1207.605100,1+) intensity(0.0000) index(8)
Title: Label: E1, Spot_Id: 219678, Peak_List_Id: 224904, MSMS Job_Run_Id: 21757, Comment:
Data file C:\Documents and Settings\Administrator\Desktop\farah BHU\spot set plate\Farah-Garima 11May 2013\ppw_E1_136842029000.txt

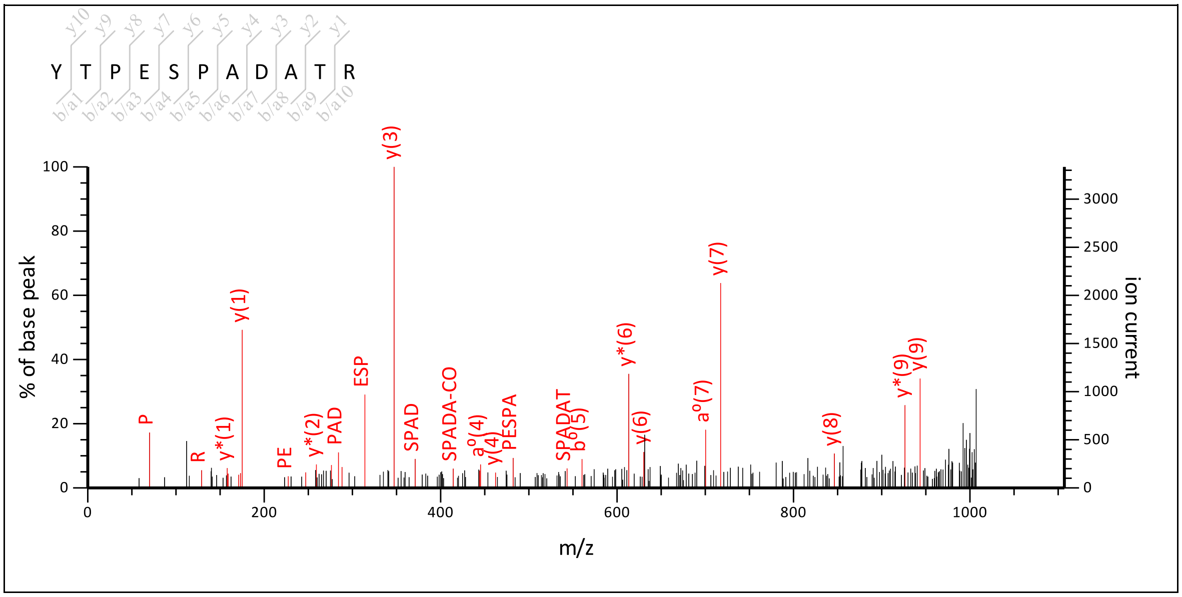


0 to 1107.04

Label all possible matches ○ Label matches used for scoring ◉

**Monoisotopic mass of neutral peptide Mr(calc):** 1206.5517
**Fixed modifications:** Carbamidomethyl (C) (apply to specified residues or termini only)
**Ions Score:** 41 **Expect:** 0.16
**Matches :** 36/174 fragment ions using 60 most intense peaks (help)

| # | Immon. | a | $a^0$ | b | $b^0$ | d | d' | Seq. | v | w | w' | y | y* | $y^0$ | # |
|---|---|---|---|---|---|---|---|---|---|---|---|---|---|---|---|
| 1 | 136.0757 | 136.0757 | | 164.0706 | | 44.0495 | | Y | | | | | | | 11 |
| 2 | 74.0600 | 237.1234 | 219.1128 | 265.1183 | 247.1077 | 221.1285 | 223.1077 | T | 998.4538 | 1011.4742 | 1013.4534 | 1044.4956 | 1027.4691 | 1026.4851 | 10 |
| 3 | 70.0651 | 334.1761 | 316.1656 | 362.1710 | 344.1605 | 308.1605 | | P | 901.4010 | 900.4058 | | ***943.4480*** | 926.4214 | 925.4374 | 9 |
| 4 | 102.0550 | 463.2187 | 445.2082 | 491.2136 | 473.2031 | 405.2132 | | E | 772.3584 | 771.3632 | | ***846.3952*** | 829.3686 | 828.3846 | 8 |
| 5 | 60.0444 | 550.2508 | 532.2402 | 578.2457 | 560.2351 | 534.2558 | | S | 685.3264 | 684.3311 | | ***717.3526*** | 700.3260 | 699.3420 | 7 |
| 6 | 70.0651 | 647.3035 | 629.2930 | 675.2984 | 657.2879 | 621.2879 | | P | 588.2736 | 587.2784 | | ***630.3206*** | 613.2940 | 612.3100 | 6 |
| 7 | 44.0495 | 718.3406 | 700.3301 | 746.3355 | 728.3250 | | | A | 517.2365 | | | 533.2678 | 516.2413 | 515.2572 | 5 |
| 8 | 88.0393 | 833.3676 | 815.3570 | 861.3625 | 843.3519 | 789.3777 | | D | 402.2096 | 401.2143 | | ***462.2307*** | 445.2041 | 444.2201 | 4 |
| 9 | 44.0495 | 904.4047 | 886.3941 | 932.3996 | 914.3890 | | | A | 331.1724 | | | ***347.2037*** | 330.1772 | 329.1932 | 3 |
| 10 | 74.0600 | 1005.4524 | 987.4418 | 1033.4473 | 1015.4367 | 989.4575 | 991.4367 | T | 230.1248 | 243.1452 | 245.1244 | ***276.1666*** | 259.1401 | 258.1561 | 2 |
| 11 | 129.1135 | | | | | | | R | 74.0237 | 73.0284 | | ***175.1190*** | 158.0924 | | 1 |

| Seq | ya | yb | Seq | ya | yb | Seq | ya | yb |
|---|---|---|---|---|---|---|---|---|
| TP | 171.1128 | 199.1077 | TPE | 300.1554 | 328.1503 | TPES | 387.1874 | 415.1823 |
| TPESP | 484.2402 | 512.2351 | TPESPA | 555.2773 | 583.2722 | TPESPAD | 670.3042 | 698.2992 |
| PE | 199.1077 | 227.1026 | PES | 286.1397 | 314.1347 | PESP | 383.1925 | 411.1874 |
| PESPA | 454.2296 | 482.2245 | PESPAD | 569.2566 | 597.2515 | PESPADA | 640.2937 | 668.2886 |
| ES | 189.0870 | 217.0819 | ESP | 286.1397 | 314.1347 | ESPA | 357.1769 | 385.1718 |

| | | | | | | | | |
|---|---|---|---|---|---|---|---|---|
| ESPAD | 472.2038 | 500.1987 | ESPADA | 543.2409 | 571.2358 | ESPADAT | 644.2886 | 672.2835 |
| SP | 157.0972 | 185.0921 | SPA | 228.1343 | 256.1292 | SPAD | 343.1612 | 371.1561 |
| SPADA | 414.1983 | 442.1932 | SPADAT | 515.2460 | 543.2409 | PA | 141.1022 | 169.0972 |
| PAD | 256.1292 | 284.1241 | PADA | 327.1663 | 355.1612 | PADAT | 428.2140 | 456.2089 |
| AD | 159.0764 | 187.0713 | ADA | 230.1135 | 258.1084 | ADAT | 331.1612 | 359.1561 |
| DA | 159.0764 | 187.0713 | DAT | 260.1241 | 288.1190 | AT | 145.0972 | 173.0921 |

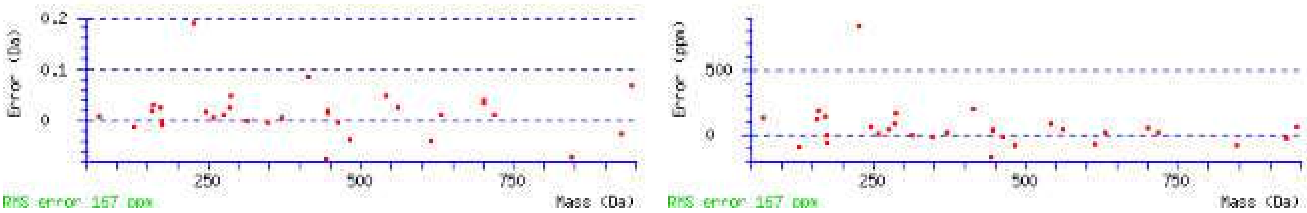


NCBI **BLAST** search of YTPESPADATR
(Parameters: blastp, nr protein database, expect=20000, no filter, PAM30)
Other BLAST web gateways

**All matches to this query**

| Score | Mr(calc) | Delta | Sequence |
|---|---|---|---|
| 41.3 | 1206.5517 | 0.0462 | YTPESPADATR |
| 16.6 | 1206.6721 | -0.0743 | TTTARGAVFVGK |
| 15.1 | 1206.5373 | 0.0606 | ISPEMQEAMR |
| 14.1 | 1206.6720 | -0.0742 | YTRSILEGIR |
| 13.8 | 1206.5373 | 0.0606 | ISPEMQEAMR |
| 13.6 | 1206.5914 | 0.0064 | SIPEALTSMSR |
| 13.6 | 1206.6026 | -0.0048 | AESLLSEMRR |
| 12.2 | 1206.5914 | 0.0064 | IISEGMVDATR |
| 11.2 | 1206.5881 | 0.0097 | FEDVSTPGVTR |
| 11.2 | 1206.5411 | 0.0567 | TMEEGQSGGRR |

**Mascot:** http://www.matrixscience.com/

# MATRIX SCIENCE Mascot Search Results

## Peptide View Spot no 112

MS/MS Fragmentation of **NPYWFNRDR**
Found in **gi|28190676** in **NCBInr**, putative transketolase [Oryza sativa Japonica Group]

Match to Query 93: 1266.632824 from(1267.640100,1+) intensity(0.0000) index(10)
Title: Label: E1, Spot_Id: 219678, Peak_List_Id: 224897, MSMS Job_Run_Id: 21757, Comment:
Data file C:\Documents and Settings\Administrator\Desktop\farah BHU\spot set plate\Farah-Garima 11May 2013\ppw_E1_136842029000.txt

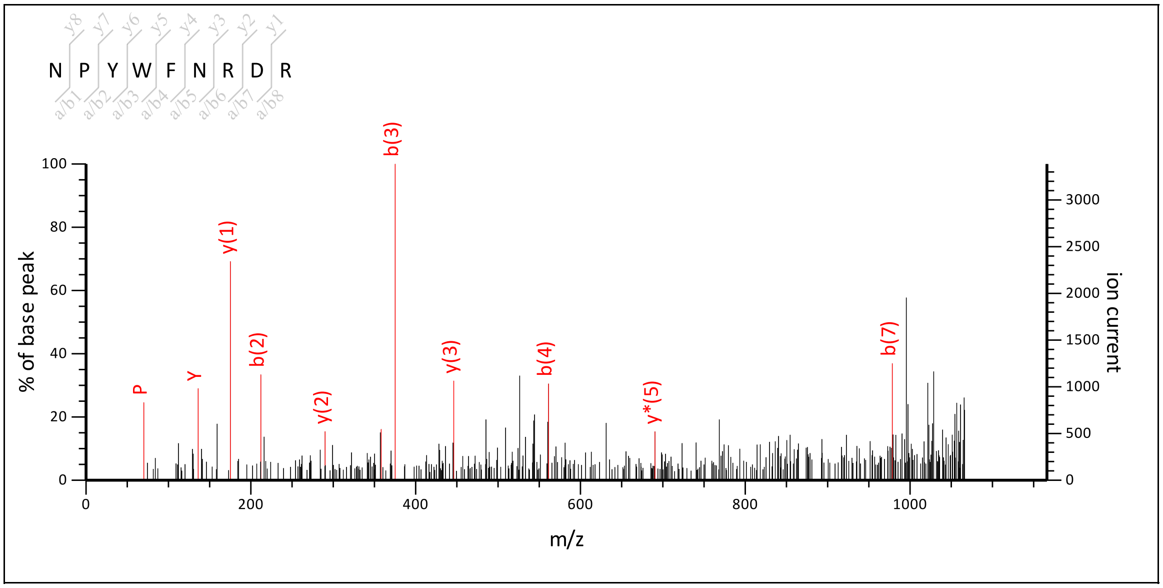


0 to 1166.12

Label all possible matches ○ Label matches used for scoring ◉

```
Monoisotopic mass of neutral peptide Mr(calc): 1266.5894
Fixed modifications: Carbamidomethyl (C) (apply to specified residues or termini only)
Ions Score: 20  Expect: 19
Matches : 13/116 fragment ions using 20 most intense peaks   (help)
```

| # | Immon. | a | a* | $a^0$ | b | b* | $b^0$ | d | Seq. | v | w | y | y* | $y^0$ | # |
|---|---|---|---|---|---|---|---|---|---|---|---|---|---|---|---|
| 1 | 87.0553 | 87.0553 | 70.0287 | | 115.0502 | 98.0237 | | 44.0495 | N | | | | | | 9 |
| 2 | 70.0651 | 184.1081 | 167.0815 | | ***212.1030*** | 195.0764 | | 158.0924 | P | 1111.5068 | 1110.5116 | 1153.5538 | 1136.5272 | 1135.5432 | 8 |
| 3 | 136.0757 | 347.1714 | 330.1448 | | ***375.1663*** | 358.1397 | | | Y | 948.4435 | | 1056.5010 | 1039.4744 | 1038.4904 | 7 |
| 4 | 159.0917 | 533.2507 | 516.2241 | | ***561.2456*** | 544.2191 | | | W | 762.3642 | | 893.4377 | 876.4111 | 875.4271 | 6 |
| 5 | 120.0808 | 680.3191 | 663.2926 | | 708.3140 | 691.2875 | | | F | 615.2957 | | 707.3583 | 690.3318 | 689.3478 | 5 |
| 6 | 87.0553 | 794.3620 | 777.3355 | | 822.3569 | 805.3304 | | 751.3562 | N | 501.2528 | 500.2576 | 560.2899 | 543.2634 | 542.2794 | 4 |
| 7 | 129.1135 | 950.4631 | 933.4366 | | ***978.4581*** | 961.4315 | | 865.3991 | R | 345.1517 | 344.1565 | ***446.2470*** | 429.2205 | 428.2364 | 3 |
| 8 | 88.0393 | 1065.4901 | 1048.4635 | 1047.4795 | 1093.4850 | 1076.4585 | 1075.4744 | 1021.5003 | D | 230.1248 | 229.1295 | ***290.1459*** | 273.1193 | 272.1353 | 2 |
| 9 | 129.1135 | | | | | | | | R | 74.0237 | 73.0284 | ***175.1190*** | 158.0924 | | 1 |

| Seq | ya | yb | Seq | ya | yb | Seq | ya | yb |
|---|---|---|---|---|---|---|---|---|
| PY | 233.1285 | 261.1234 | PYW | 419.2078 | 447.2027 | PYWF | 566.2762 | 594.2711 |
| PYWFN | 680.3191 | 708.3140 | YW | 322.1550 | 350.1499 | YWF | 469.2234 | 497.2183 |
| YWFN | 583.2663 | 611.2613 | WF | 306.1601 | 334.1550 | WFN | 420.2030 | 448.1979 |
| WFNR | 576.3041 | 604.2990 | WFNRD | 691.3311 | 719.3260 | FN | 234.1237 | 262.1186 |
| FNR | 390.2248 | 418.2197 | FNRD | 505.2518 | 533.2467 | NR | 243.1564 | 271.1513 |
| NRD | 358.1833 | 386.1783 | RD | 244.1404 | 272.1353 | | | |

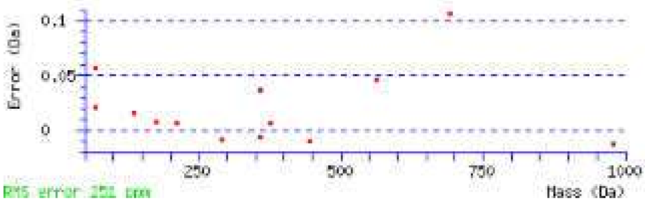


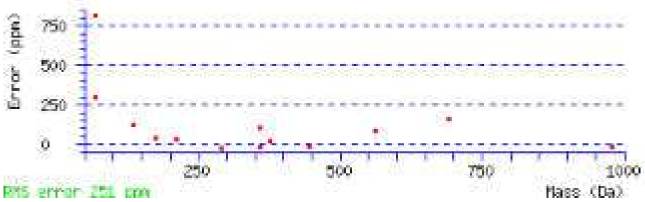


NCBI **BLAST** search of NPYWFNRDR
(Parameters: blastp, nr protein database, expect=20000, no filter, PAM30)
Other BLAST web gateways

**All matches to this query**

| Score | Mr(calc) | Delta | Sequence |
|---|---|---|---|
| 20.3 | 1266.5894 | 0.0434 | NPYWFNRDR |
| 16.6 | 1266.6292 | 0.0037 | RLGFPCFNTR |
| 15.8 | 1266.6932 | -0.0604 | NEALAVGIIPDR |
| 15.4 | 1266.7197 | -0.0869 | VRFAPPLPTNR |
| 13.8 | 1266.5762 | 0.0567 | TLMESEDASLR |
| 12.5 | 1266.6391 | -0.0062 | CVGEVPPPGLSR |
| 12.5 | 1266.6568 | -0.0239 | EKVAAAPEPAER |
| 12.0 | 1266.7157 | -0.0828 | TNIVAVGQGRPR |
| 12.0 | 1266.6164 | 0.0164 | ETRSSTSSVASR |
| 11.9 | 1266.6092 | 0.0236 | DSSLPYTELSR |

| **Mascot:** http://www.matrixscience.com/ |
|---|

# MATRIX SCIENCE Mascot Search Results

## Peptide View **Spot no 112**

MS/MS Fragmentation of **NLSQQCLNALAK**
Found in **gi|28190676** in **NCBInr**, putative transketolase [Oryza sativa Japonica Group]

Match to Query 100: 1358.729324 from(1359.736600,1+) intensity(0.0000) index(12)
Title: Label: E1, Spot_Id: 219678, Peak_List_Id: 224912, MSMS Job_Run_Id: 21757, Comment:
Data file C:\Documents and Settings\Administrator\Desktop\farah BHU\spot set plate\Farah-Garima 11May 2013\ppw_E1_136842029000.txt

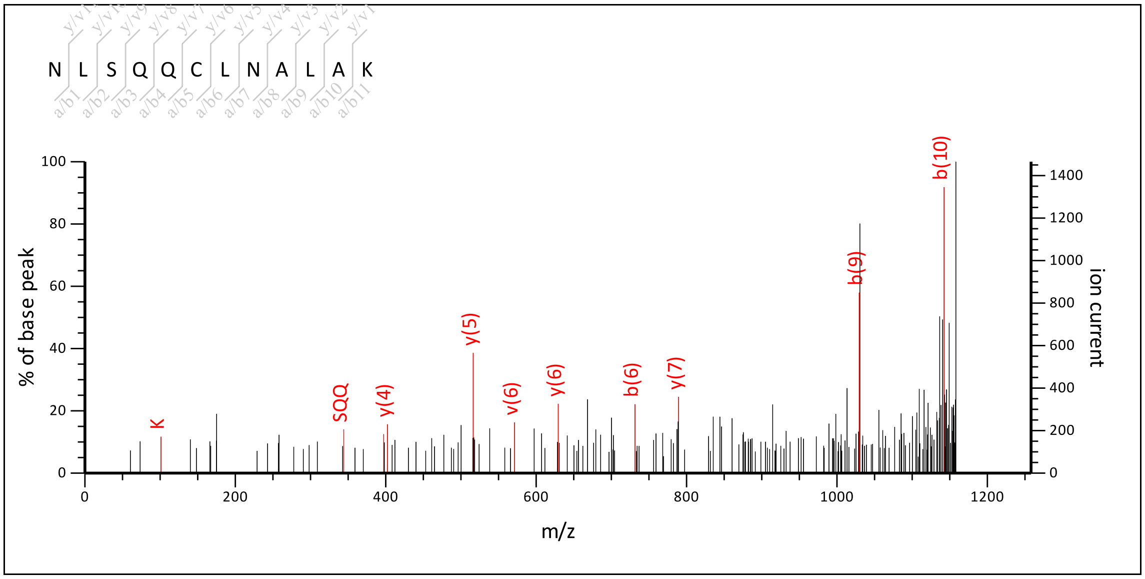


0 to 1258.09

Label all possible matches Label matches used for scoring

**Monoisotopic mass of neutral peptide Mr(calc):** 1358.6976
**Fixed modifications:** Carbamidomethyl (C) (apply to specified residues or termini only)
**Ions Score:** 17 **Expect:** 41
**Matches :** 16/191 fragment ions using 22 most intense peaks (help)

| # | Immon. | a | a* | a$^0$ | b | b* | b$^0$ | d | Seq. | v | w | y | y* | y$^0$ | # |
|---|---|---|---|---|---|---|---|---|---|---|---|---|---|---|---|
| 1 | 87.0553 | 87.0553 | 70.0287 | | 115.0502 | 98.0237 | | 44.0495 | N | | | | | | 12 |
| 2 | 86.0964 | 200.1394 | 183.1128 | | 228.1343 | 211.1077 | | 158.0924 | L | 1187.5837 | 1186.5885 | 1245.6620 | 1228.6354 | 1227.6514 | 11 |
| 3 | 60.0444 | 287.1714 | 270.1448 | 269.1608 | 315.1663 | 298.1397 | 297.1557 | 271.1765 | S | 1100.5517 | 1099.5565 | 1132.5779 | 1115.5514 | 1114.5674 | 10 |
| 4 | 101.0709 | 415.2300 | 398.2034 | 397.2194 | 443.2249 | 426.1983 | 425.2143 | 358.2085 | Q | 972.4931 | 971.4979 | 1045.5459 | 1028.5193 | | 9 |
| 5 | 101.0709 | 543.2885 | 526.2620 | 525.2780 | ***571.2835*** | 554.2569 | 553.2729 | 486.2671 | Q | 844.4346 | 843.4393 | 917.4873 | 900.4608 | | 8 |
| 6 | 133.0430 | 703.3192 | 686.2926 | 685.3086 | ***731.3141*** | 714.2876 | 713.3035 | 614.3256 | C | 684.4039 | 683.4087 | ***789.4287*** | 772.4022 | | 7 |
| 7 | 86.0964 | 816.4032 | 799.3767 | 798.3927 | 844.3982 | 827.3716 | 826.3876 | 774.3563 | L | 571.3198 | 570.3246 | ***629.3981*** | 612.3715 | | 6 |
| 8 | 87.0553 | 930.4462 | 913.4196 | 912.4356 | 958.4411 | 941.4145 | 940.4305 | 887.4404 | N | 457.2769 | 456.2817 | ***516.3140*** | 499.2875 | | 5 |
| 9 | 44.0495 | 1001.4833 | 984.4567 | 983.4727 | ***1029.4782*** | 1012.4517 | 1011.4676 | | A | 386.2398 | | ***402.2711*** | 385.2445 | | 4 |
| 10 | 86.0964 | 1114.5674 | 1097.5408 | 1096.5568 | ***1142.5623*** | 1125.5357 | 1124.5517 | 1072.5204 | L | 273.1557 | 272.1605 | 331.2340 | 314.2074 | | 3 |
| 11 | 44.0495 | 1185.6045 | 1168.5779 | 1167.5939 | 1213.5994 | 1196.5728 | 1195.5888 | | A | 202.1186 | | 218.1499 | 201.1234 | | 2 |
| 12 | 101.1073 | | | | | | | | K | 74.0237 | 73.0284 | 147.1128 | 130.0863 | | 1 |

| Seq | ya | yb | Seq | ya | yb | Seq | ya | yb |
|---|---|---|---|---|---|---|---|---|
| LS | 173.1285 | 201.1234 | LSQ | 301.1870 | 329.1819 | LSQQ | 429.2456 | 457.2405 |
| LSQQC | 589.2763 | 617.2712 | SQ | 188.1030 | 216.0979 | SQQ | 316.1615 | 344.1565 |
| SQQC | 476.1922 | 504.1871 | SQQCL | 589.2763 | 617.2712 | QQ | 229.1295 | 257.1244 |
| QQC | 389.1602 | 417.1551 | QQCL | 502.2442 | 530.2391 | QQCLN | 616.2872 | 644.2821 |
| QQCLNA | 687.3243 | 715.3192 | QC | 261.1016 | 289.0965 | QCL | 374.1857 | 402.1806 |
| QCLN | 488.2286 | 516.2235 | QCLNA | 559.2657 | 587.2606 | QCLNAL | 672.3498 | 700.3447 |

| | | | | | | | | |
|---|---|---|---|---|---|---|---|---|
| **CL** | 246.1271 | 274.1220 | **CLN** | 360.1700 | 388.1649 | **CLNA** | 431.2071 | 459.2020 |
| **CLNAL** | 544.2912 | 572.2861 | **CLNALA** | 615.3283 | 643.3232 | **LN** | 200.1394 | 228.1343 |
| **LNA** | 271.1765 | 299.1714 | **LNAL** | 384.2605 | 412.2554 | **LNALA** | 455.2976 | 483.2926 |
| **NA** | 158.0924 | 186.0873 | **NAL** | 271.1765 | 299.1714 | **NALA** | 342.2136 | 370.2085 |
| **AL** | 157.1335 | 185.1285 | **ALA** | 228.1707 | 256.1656 | **LA** | 157.1335 | 185.1285 |

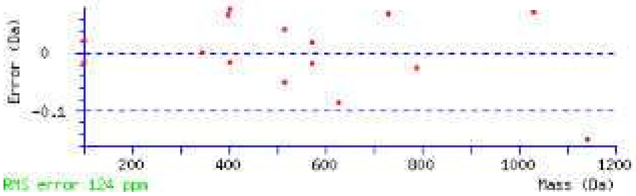


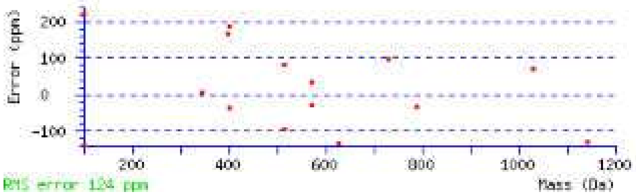


NCBI **BLAST** search of NLSQQCLNALAK
(Parameters: blastp, nr protein database, expect=20000, no filter, PAM30)
Other BLAST web gateways

**All matches to this query**

| Score | Mr(calc) | Delta | Sequence |
|---|---|---|---|
| 19.9 | 1358.6677 | 0.0616 | QELAETQNDLAK |
| 18.8 | 1358.7405 | -0.0112 | ELSVAESKVLER |
| 16.9 | 1358.6976 | 0.0317 | NISQQCLNALAK |
| 16.9 | 1358.6976 | 0.0317 | NLSQQCLNAIAK |
| 16.9 | 1358.6976 | 0.0317 | NLSQQCLNALAK |
| 15.8 | 1358.7154 | 0.0140 | SGKENLVADALSR |
| 15.2 | 1358.6691 | 0.0603 | DAAELASHYARR |
| 14.5 | 1358.6976 | 0.0317 | AGAMNALLESLNR |
| 13.0 | 1358.7129 | 0.0164 | ERVWCLDIIR |
| 12.0 | 1358.6691 | 0.0602 | KGGSVGGGGEVGGWR |

**Mascot:** http://www.matrixscience.com/

# MATRIX SCIENCE Mascot Search Results

## Peptide View **Spot no 112**

MS/MS Fragmentation of **VSLEAGSTLGWQK**
Found in **gi|28190676** in **NCBInr**, putative transketolase [Oryza sativa Japonica Group]

Match to Query 102: 1374.747524 from(1375.754800,1+) intensity(0.0000) index(13)
Title: Label: E1, Spot_Id: 219678, Peak_List_Id: 224914, MSMS Job_Run_Id: 21757, Comment:
Data file C:\Documents and Settings\Administrator\Desktop\farah BHU\spot set plate\Farah-Garima 11May 2013\ppw_E1_136842029000.txt

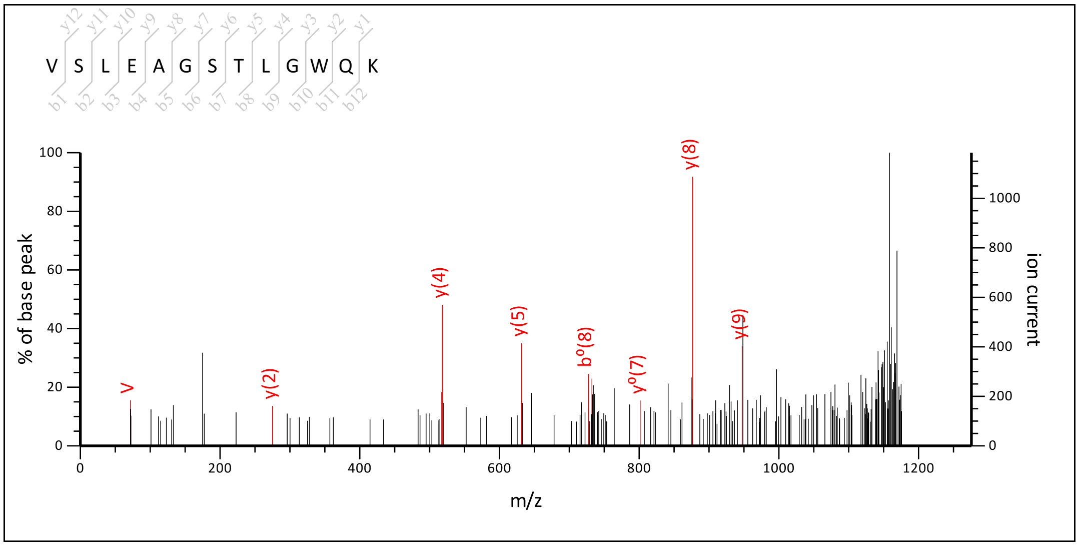


0 to 1275.67

Label all possible matches  Label matches used for scoring

**Monoisotopic mass of neutral peptide Mr(calc):** 1374.7143
**Fixed modifications:** Carbamidomethyl (C) (apply to specified residues or termini only)
**Ions Score:** 13 **Expect:** 98
**Matches :** 12/208 fragment ions using 35 most intense peaks (help)

| # | Immon. | a | a* | $a^0$ | b | b* | $b^0$ | d | d' | Seq. | v | w | w' | y | y* | |
|---|---|---|---|---|---|---|---|---|---|---|---|---|---|---|---|---|
| 1 | 72.0808 | 72.0808 | | | 100.0757 | | | 44.0495 | | V | | | | | | |
| 2 | 60.0444 | 159.1128 | | 141.1022 | 187.1077 | | 169.0972 | 143.1179 | | S | 1244.6270 | 1243.6317 | | 1276.6532 | 1259.6266 | 125[illegible] |
| 3 | 86.0964 | 272.1969 | | 254.1863 | 300.1918 | | 282.1812 | 230.1499 | | L | 1131.5429 | 1130.5477 | | 1189.6212 | 1172.5946 | 117[illegible] |
| 4 | 102.0550 | 401.2395 | | 383.2289 | 429.2344 | | 411.2238 | 343.2340 | | E | 1002.5003 | 1001.5051 | | 1076.5371 | 1059.5106 | 105[illegible] |
| 5 | 44.0495 | 472.2766 | | 454.2660 | 500.2715 | | 482.2609 | | | A | 931.4632 | | | ***947.4945*** | 930.4680 | 92[illegible] |
| 6 | 30.0338 | 529.2980 | | 511.2875 | 557.2930 | | 539.2824 | | | G | | | | ***876.4574*** | 859.4308 | 85[illegible] |
| 7 | 60.0444 | 616.3301 | | 598.3195 | 644.3250 | | 626.3144 | 600.3352 | | S | 787.4097 | 786.4145 | | 819.4359 | 802.4094 | 80[illegible] |
| 8 | 74.0600 | 717.3777 | | 699.3672 | 745.3727 | | 727.3621 | 701.3828 | 703.3621 | T | 686.3620 | 699.3824 | 701.3617 | ***732.4039*** | 715.3774 | 71[illegible] |
| 9 | 86.0964 | 830.4618 | | 812.4512 | 858.4567 | | 840.4462 | 788.4149 | | L | 573.2780 | 572.2827 | | ***631.3562*** | 614.3297 | |
| 10 | 30.0338 | 887.4833 | | 869.4727 | 915.4782 | | 897.4676 | | | G | | | | ***518.2722*** | 501.2456 | |
| 11 | 159.0917 | 1073.5626 | | 1055.5520 | 1101.5575 | | 1083.5469 | | | W | 330.1772 | | | 461.2507 | 444.2241 | |
| 12 | 101.0709 | 1201.6212 | 1184.5946 | 1183.6106 | 1229.6161 | 1212.5895 | 1211.6055 | 1144.5997 | | Q | 202.1186 | 201.1234 | | ***275.1714*** | 258.1448 | |
| 13 | 101.1073 | | | | | | | | | K | 74.0237 | 73.0284 | | 147.1128 | 130.0863 | |

| Seq | ya | yb | Seq | ya | yb | Seq | ya | yb |
|---|---|---|---|---|---|---|---|---|
| **SL** | 173.1285 | 201.1234 | **SLE** | 302.1710 | 330.1660 | **SLEA** | 373.2082 | 401.2031 |
| **SLEAG** | 430.2296 | 458.2245 | **SLEAGS** | 517.2617 | 545.2566 | **SLEAGST** | 618.3093 | 646.3042 |
| **LE** | 215.1390 | 243.1339 | **LEA** | 286.1761 | 314.1710 | **LEAG** | 343.1976 | 371.1925 |
| **LEAGS** | 430.2296 | 458.2245 | **LEAGST** | 531.2773 | 559.2722 | **LEAGSTL** | 644.3614 | 672.3563 |
| **EA** | 173.0921 | 201.0870 | **EAG** | 230.1135 | 258.1084 | **EAGS** | 317.1456 | 345.1405 |
| **EAGST** | 418.1932 | 446.1882 | **EAGSTL** | 531.2773 | 559.2722 | **EAGSTLG** | 588.2988 | 616.2937 |
| **AG** | 101.0709 | 129.0659 | **AGS** | 188.1030 | 216.0979 | **AGST** | 289.1506 | 317.1456 |
| **AGSTL** | 402.2347 | 430.2296 | **AGSTLG** | 459.2562 | 487.2511 | **AGSTLGW** | 645.3355 | 673.3304 |

| | | | | | | | | |
|---|---|---|---|---|---|---|---|---|
| **GS** | 117.0659 | 145.0608 | **GST** | 218.1135 | 246.1084 | **GSTL** | 331.1976 | 359.1925 |
| **GSTLG** | 388.2191 | 416.2140 | **GSTLGW** | 574.2984 | 602.2933 | **ST** | 161.0921 | 189.0870 |
| **STL** | 274.1761 | 302.1710 | **STLG** | 331.1976 | 359.1925 | **STLGW** | 517.2769 | 545.2718 |
| **STLGWQ** | 645.3355 | 673.3304 | **TL** | 187.1441 | 215.1390 | **TLG** | 244.1656 | 272.1605 |
| **TLGW** | 430.2449 | 458.2398 | **TLGWQ** | 558.3035 | 586.2984 | **LG** | 143.1179 | 171.1128 |
| **LGW** | 329.1972 | 357.1921 | **LGWQ** | 457.2558 | 485.2507 | **GW** | 216.1131 | 244.1081 |
| **GWQ** | 344.1717 | 372.1666 | **WQ** | 287.1503 | 315.1452 | | | |

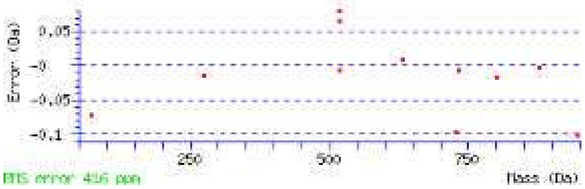


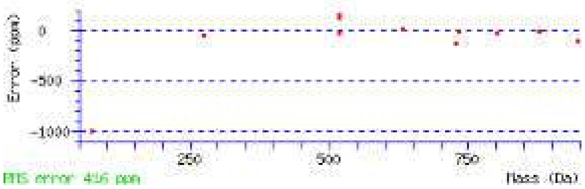


NCBI **BLAST** search of VSLEAGSTLGWQK
(Parameters: blastp, nr protein database, expect=20000, no filter, PAM30)
Other BLAST web gateways

**All matches to this query**

| Score | Mr(calc) | Delta | Sequence |
|---|---|---|---|
| 13.0 | 1374.7143 | 0.0332 | VSLEAGSTLGWQK |
| 11.3 | 1374.6991 | 0.0485 | VALSESTGLDQKQ |
| 8.7 | 1374.6925 | 0.0550 | GEELARAMETLR |
| 8.7 | 1374.7177 | 0.0298 | VAITDMKVDVER |
| 6.8 | 1374.7363 | 0.0112 | AKALVPMMETIR |
| 6.4 | 1374.6813 | 0.0662 | VQIMQELDDIR |
| 6.1 | 1374.7004 | 0.0471 | GAKAATGSQSVWGR |
| 5.4 | 1374.7103 | 0.0373 | ASNASSLEQLSLR |
| 5.1 | 1374.6561 | 0.0914 | AVEEAMDELGRR |
| 5.1 | 1374.6991 | 0.0485 | LSDTELASSSPLR |

**Mascot:** http://www.matrixscience.com/

# MATRIX SCIENCE Mascot Search Results

## Peptide View Spot no 112

MS/MS Fragmentation of **ANSYSVHGSALGTK**
Found in **gi|28190676** in **NCBInr**, putative transketolase [Oryza sativa Japonica Group]

Match to Query 105: 1390.736224 from(1391.743500,1+) intensity(0.0000) index(14)
Title: Label: E1, Spot_Id: 219678, Peak_List_Id: 224906, MSMS Job_Run_Id: 21757, Comment:
Data file C:\Documents and Settings\Administrator\Desktop\farah BHU\spot set plate\Farah-Garima 11May 2013\ppw_E1_136842029000.txt

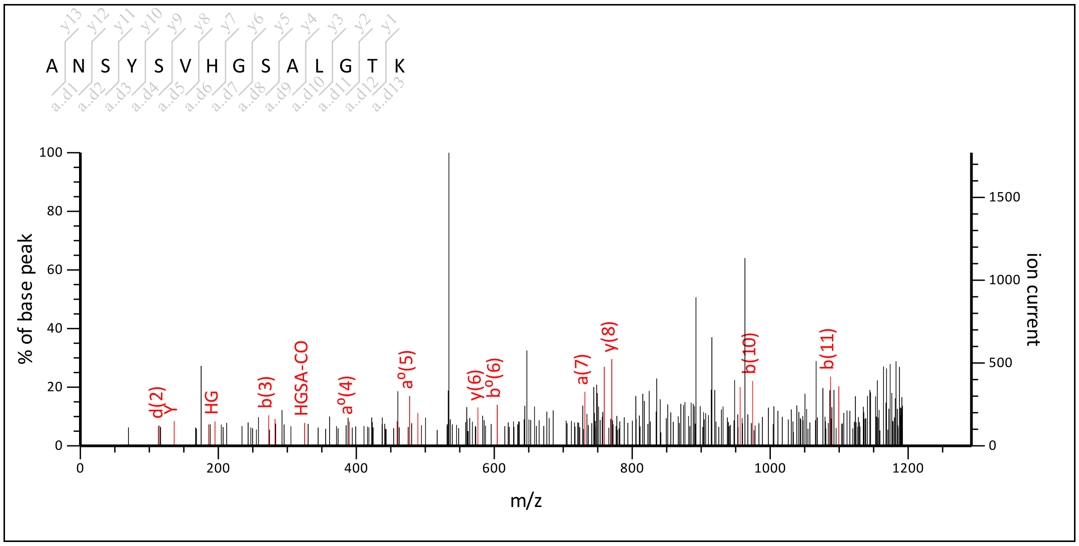


Label all possible matches Label matches used for scoring

**Monoisotopic mass of neutral peptide Mr(calc):** 1390.6841
**Fixed modifications:** Carbamidomethyl (C) (apply to specified residues or termini only)
**Ions Score:** 8 **Expect:** 3.2e+02
**Matches :** 25/253 fragment ions using 72 most intense peaks (help)

| # | Immon. | a | a* | $a^0$ | b | b* | $b^0$ | d | d' | Seq. | v | w | w' | y | y* | |
|---|---|---|---|---|---|---|---|---|---|---|---|---|---|---|---|---|
| 1 | 44.0495 | 44.0495 | | | 72.0444 | | | 44.0495 | | A | | | | | | |
| 2 | 87.0553 | 158.0924 | 141.0659 | | 186.0873 | 169.0608 | | 115.0866 | | N | 1261.6171 | 1260.6219 | | 1320.6543 | 1303.6277 | [illegible] |
| 3 | 60.0444 | 245.1244 | 228.0979 | 227.1139 | 273.1193 | 256.0928 | 255.1088 | 229.1295 | | S | 1174.5851 | 1173.5899 | | 1206.6113 | 1189.5848 | [illegible] |
| 4 | 136.0757 | 408.1878 | 391.1612 | 390.1772 | 436.1827 | 419.1561 | 418.1721 | | | Y | 1011.5218 | | | 1119.5793 | 1102.5528 | [illegible] |
| 5 | 60.0444 | 495.2198 | 478.1932 | 477.2092 | 523.2147 | 506.1882 | 505.2041 | 479.2249 | | S | 924.4898 | 923.4945 | | 956.5160 | 939.4894 | [illegible] |
| 6 | 72.0808 | 594.2882 | 577.2617 | 576.2776 | 622.2831 | 605.2566 | 604.2726 | 580.2726 | | V | 825.4213 | 838.4417 | | 869.4839 | 852.4574 | [illegible] |
| 7 | 110.0713 | 731.3471 | 714.3206 | 713.3365 | 759.3420 | 742.3155 | 741.3315 | | | H | 688.3624 | | | 770.4155 | 753.3890 | [illegible] |
| 8 | 30.0338 | 788.3686 | 771.3420 | 770.3580 | 816.3635 | 799.3369 | 798.3529 | | | G | | | | 633.3566 | 616.3301 | [illegible] |
| 9 | 60.0444 | 875.4006 | 858.3741 | 857.3900 | 903.3955 | 886.3690 | 885.3850 | 859.4057 | | S | 544.3089 | 543.3137 | | 576.3352 | 559.3086 | [illegible] |
| 10 | 44.0495 | 946.4377 | 929.4112 | 928.4272 | 974.4326 | 957.4061 | 956.4221 | | | A | 473.2718 | | | 489.3031 | 472.2766 | [illegible] |
| 11 | 86.0964 | 1059.5218 | 1042.4952 | 1041.5112 | 1087.5167 | 1070.4901 | 1069.5061 | 1017.4748 | | L | 360.1878 | 359.1925 | | 418.2660 | 401.2395 | [illegible] |
| 12 | 30.0338 | 1116.5432 | 1099.5167 | 1098.5327 | 1144.5382 | 1127.5116 | 1126.5276 | | | G | | | | 305.1819 | 288.1554 | [illegible] |
| 13 | 74.0600 | 1217.5909 | 1200.5644 | 1199.5804 | 1245.5858 | 1228.5593 | 1227.5753 | 1201.5960 | 1203.5753 | T | 202.1186 | 215.1390 | 217.1183 | 248.1605 | 231.1339 | [illegible] |
| 14 | 101.1073 | | | | | | | | | K | 74.0237 | 73.0284 | | 147.1128 | 130.0863 | |

| Seq | ya | yb | Seq | ya | yb | Seq | ya | yb |
|---|---|---|---|---|---|---|---|---|
| NS | 174.0873 | 202.0822 | NSY | 337.1506 | 365.1456 | NSYS | 424.1827 | 452.1776 |
| NSYSV | 523.2511 | 551.2460 | NSYSVH | 660.3100 | 688.3049 | SY | 223.1077 | 251.1026 |
| SYS | 310.1397 | 338.1347 | SYSV | 409.2082 | 437.2031 | SYSVH | 546.2671 | 574.2620 |
| SYSVHG | 603.2885 | 631.2835 | SYSVHGS | 690.3206 | 718.3155 | YS | 223.1077 | 251.1026 |
| YSV | 322.1761 | 350.1710 | YSVH | 459.2350 | 487.2300 | YSVHG | 516.2565 | 544.2514 |
| YSVHGS | 603.2885 | 631.2835 | YSVHGSA | 674.3256 | 702.3206 | SV | 159.1128 | 187.1077 |
| SVH | 296.1717 | 324.1666 | SVHG | 353.1932 | 381.1881 | SVHGS | 440.2252 | 468.2201 |

| | | | | | | | | |
|---|---|---|---|---|---|---|---|---|
| **SVHGSA** | 511.2623 | 539.2572 | **SVHGSAL** | 624.3464 | 652.3413 | **SVHGSALG** | 681.3678 | 709.3628 |
| **VH** | 209.1397 | 237.1346 | **VHG** | 266.1612 | 294.1561 | **VHGS** | 353.1932 | 381.1881 |
| **VHGSA** | 424.2303 | 452.2252 | **VHGSAL** | 537.3144 | 565.3093 | **VHGSALG** | 594.3358 | 622.3307 |
| **VHGSALGT** | 695.3835 | 723.3784 | **HG** | 167.0927 | 195.0877 | **HGS** | 254.1248 | 282.1197 |
| **HGSA** | 325.1619 | 353.1568 | **HGSAL** | 438.2459 | 466.2409 | **HGSALG** | 495.2674 | 523.2623 |
| **HGSALGT** | 596.3151 | 624.3100 | **GS** | 117.0659 | 145.0608 | **GSA** | 188.1030 | 216.0979 |
| **GSAL** | 301.1870 | 329.1819 | **GSALG** | 358.2085 | 386.2034 | **GSALGT** | 459.2562 | 487.2511 |
| **SA** | 131.0815 | 159.0764 | **SAL** | 244.1656 | 272.1605 | **SALG** | 301.1870 | 329.1819 |
| **SALGT** | 402.2347 | 430.2296 | **AL** | 157.1335 | 185.1285 | **ALG** | 214.1550 | 242.1499 |
| **ALGT** | 315.2027 | 343.1976 | **LG** | 143.1179 | 171.1128 | **LGT** | 244.1656 | 272.1605 |
| **GT** | 131.0815 | 159.0764 | | | | | | |

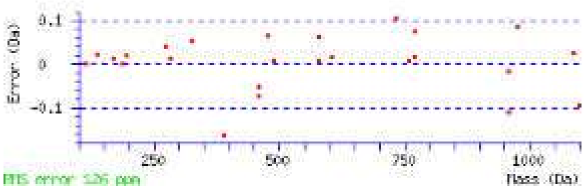


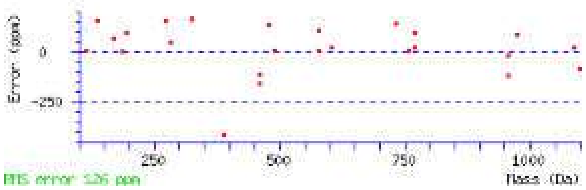


NCBI **BLAST** search of ANSYSVHGSALGTK
(Parameters: blastp, nr protein database, expect=20000, no filter, PAM30)
Other BLAST web gateways

**All matches to this query**

| Score | Mr(calc) | Delta | Sequence |
|---|---|---|---|
| 11.5 | 1390.7126 | 0.0236 | VKATESELMDLR |
| 11.3 | 1390.6298 | 0.1064 | GRGGTQSSGGGSGGGGR |
| 10.6 | 1390.6407 | 0.0955 | RYMIMTTNMSK |
| 8.8 | 1390.6407 | 0.0955 | RYMIMTTNMSK |
| 8.6 | 1390.6510 | 0.0852 | NEGQAEMGSAILR |
| 8.2 | 1390.6841 | 0.0521 | ANSYSVHGSALGTK |
| 7.6 | 1390.6623 | 0.0739 | RASASSTSPSGMPR |
| 7.3 | 1390.6576 | 0.0786 | IAKEDSTDDIER |
| 7.2 | 1390.6623 | 0.0739 | GTLLDGSDMNGRR |
| 6.8 | 1390.7431 | -0.0069 | HPISVFLYMLR |

**Mascot:** http://www.matrixscience.com/

# Mascot Search Results

## Peptide View    Spot no 112

MS/MS Fragmentation of **YNPKNPYWFNR**
Found in **gi|28190676** in **NCBInr**, putative transketolase [Oryza sativa Japonica Group]

Match to Query 115: 1497.774024 from(1498.781300,1+) intensity(0.0000) index(17)
Title: Label: E1, Spot_Id: 219678, Peak_List_Id: 224902, MSMS Job_Run_Id: 21757, Comment:
Data file C:\Documents and Settings\Administrator\Desktop\farah BHU\spot set plate\Farah-Garima 11May 2013\ppw_E1_136842029000.txt

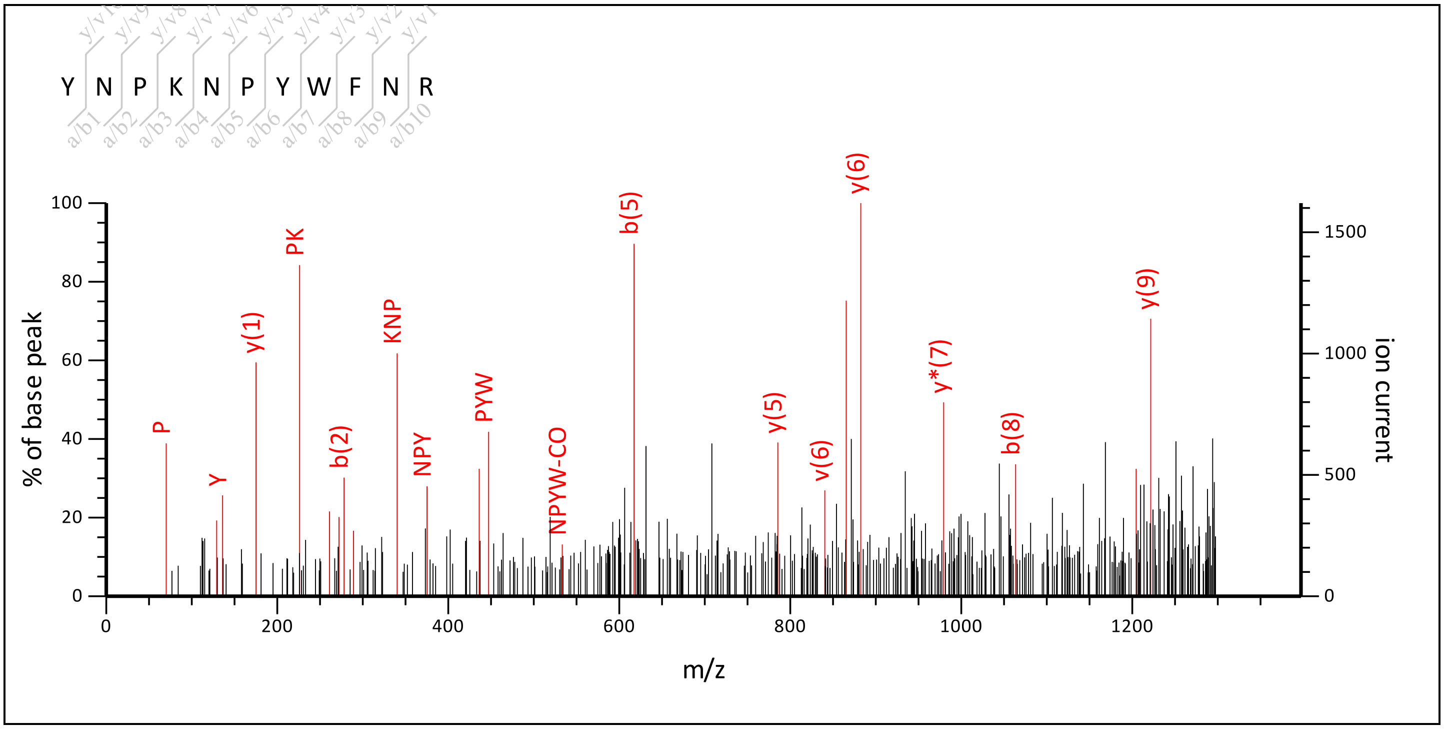


0 to 1397.32

Label all possible matches ○ Label matches used for scoring ◉

**Monoisotopic mass of neutral peptide Mr(calc):** 1497.7153
**Fixed modifications:** Carbamidomethyl (C) (apply to specified residues or termini only)
**Ions Score:** 20 **Expect:** 18
**Matches :** 32/144 fragment ions using 52 most intense peaks (help)

| # | Immon. | a | a* | b | b* | d | Seq. | v | w | y | y* | # |
|---|---|---|---|---|---|---|---|---|---|---|---|---|
| 1 | 136.0757 | 136.0757 | | 164.0706 | | 44.0495 | Y | | | | | 11 |
| 2 | 87.0553 | 250.1186 | 233.0921 | **278.1135** | 261.0870 | 207.1128 | N | 1276.6222 | 1275.6269 | 1335.6593 | 1318.6327 | 10 |
| 3 | 70.0651 | 347.1714 | 330.1448 | **375.1663** | 358.1397 | 321.1557 | P | 1179.5694 | 1178.5742 | ***1221.6164*** | 1204.5898 | 9 |
| 4 | 101.1073 | 475.2663 | 458.2398 | 503.2613 | 486.2347 | 418.2085 | K | 1051.4744 | 1050.4792 | 1124.5636 | 1107.5370 | 8 |
| 5 | 87.0553 | 589.3093 | 572.2827 | **617.3042** | 600.2776 | 546.3035 | N | 937.4315 | 936.4363 | 996.4686 | 979.4421 | 7 |
| 6 | 70.0651 | 686.3620 | 669.3355 | 714.3570 | 697.3304 | 660.3464 | P | 840.3787 | 839.3835 | ***882.4257*** | 865.3992 | 6 |
| 7 | 136.0757 | 849.4254 | 832.3988 | 877.4203 | 860.3937 | | Y | 677.3154 | | ***785.3729*** | 768.3464 | 5 |
| 8 | 159.0917 | 1035.5047 | 1018.4781 | **1063.4996** | 1046.4730 | | W | 491.2361 | | 622.3096 | 605.2831 | 4 |

| | | | | | | | | | | | | |
|---|---|---|---|---|---|---|---|---|---|---|---|---|
| **9** | 120.0808 | 1182.5731 | 1165.5465 | 1210.5680 | 1193.5415 | | **F** | 344.1677 | | ***436.2303*** | 419.2037 | **3** |
| **10** | 87.0553 | 1296.6160 | 1279.5895 | 1324.6109 | 1307.5844 | 1253.6102 | **N** | 230.1248 | 229.1295 | ***289.1619*** | 272.1353 | **2** |
| **11** | 129.1135 | | | | | | **R** | 74.0237 | 73.0284 | ***175.1190*** | 158.0924 | **1** |

| Seq | ya | yb | Seq | ya | yb | Seq | ya | yb |
|---|---|---|---|---|---|---|---|---|
| **NP** | 184.1081 | 212.1030 | **NPK** | 312.2030 | 340.1979 | **NPKN** | 426.2459 | 454.2409 |
| **NPKNP** | 523.2987 | 551.2936 | **NPKNPY** | 686.3620 | 714.3570 | **PK** | 198.1601 | 226.1550 |
| **PKN** | 312.2030 | 340.1979 | **PKNP** | 409.2558 | 437.2507 | **PKNPY** | 572.3191 | 600.3140 |
| **KN** | 215.1503 | 243.1452 | **KNP** | 312.2030 | 340.1979 | **KNPY** | 475.2663 | 503.2613 |
| **KNPYW** | 661.3457 | 689.3406 | **NP** | 184.1081 | 212.1030 | **NPY** | 347.1714 | 375.1663 |
| **NPYW** | 533.2507 | 561.2456 | **NPYWF** | 680.3191 | 708.3140 | **PY** | 233.1285 | 261.1234 |
| **PYW** | 419.2078 | 447.2027 | **PYWF** | 566.2762 | 594.2711 | **PYWFN** | 680.3191 | 708.3140 |
| **YW** | 322.1550 | 350.1499 | **YWF** | 469.2234 | 497.2183 | **YWFN** | 583.2663 | 611.2613 |
| **WF** | 306.1601 | 334.1550 | **WFN** | 420.2030 | 448.1979 | **FN** | 234.1237 | 262.1186 |

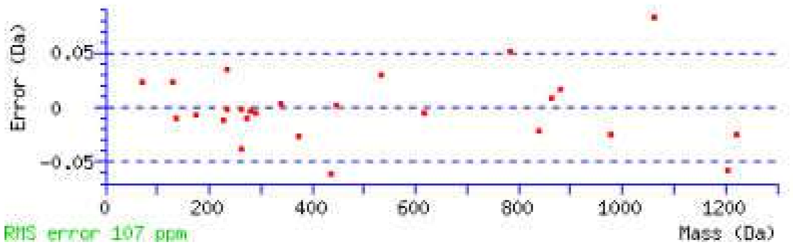


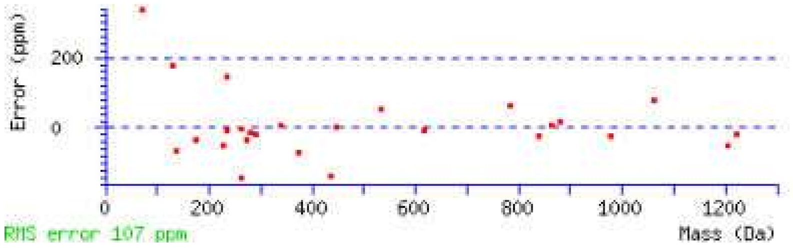


NCBI **BLAST** search of YNPKNPYWFNR
(Parameters: blastp, nr protein database, expect=20000, no filter, PAM30)
Other BLAST web gateways

**All matches to this query**

| Score | Mr(calc) | Delta | Sequence |
|---|---|---|---|
| 20.3 | 1497.7153 | 0.0587 | YNPKNPYWFNR |
| 13.2 | 1497.8416 | -0.0676 | RPLPDYVQKVQR |
| 10.7 | 1497.7834 | -0.0094 | MAVGGRGPAVGGGLQR |
| 9.0 | 1497.7762 | -0.0022 | KLIAQCWEPTPR |
| 9.0 | 1497.6751 | 0.0989 | MLRCHAPPQCGR |
| 8.2 | 1497.6332 | 0.1409 | NYEDAVGSGSGSAER |
| 7.9 | 1497.7333 | 0.0407 | MGGLPPPVHGHMPR |
| 7.5 | 1497.7113 | 0.0627 | NTWRQVYDFNR |
| 7.5 | 1497.8629 | -0.0889 | QPVILFSPMIPLK |
| | | | |

| | | | |
|---|---|---|---|
| 7.4 | 1497.7609 | 0.0131 | MEEGLGPLQQQIR |

**Mascot:** http://www.matrixscience.com/

# MATRIX SCIENCE Mascot Search Results

## Peptide View Spot no 112

MS/MS Fragmentation of **AIGIDKFGASAPAGK**
Found in **gi|28190676** in **NCBInr**, putative transketolase [Oryza sativa Japonica Group]

Match to Query 108: 1401.796924 from(1402.804200,1+) intensity(0.0000) index(15)
Title: Label: E1, Spot_Id: 219678, Peak_List_Id: 224911, MSMS Job_Run_Id: 21757, Comment:
Data file C:\Documents and Settings\Administrator\Desktop\farah BHU\spot set plate\Farah-Garima 11May 2013\ppw_E1_136842029000.txt

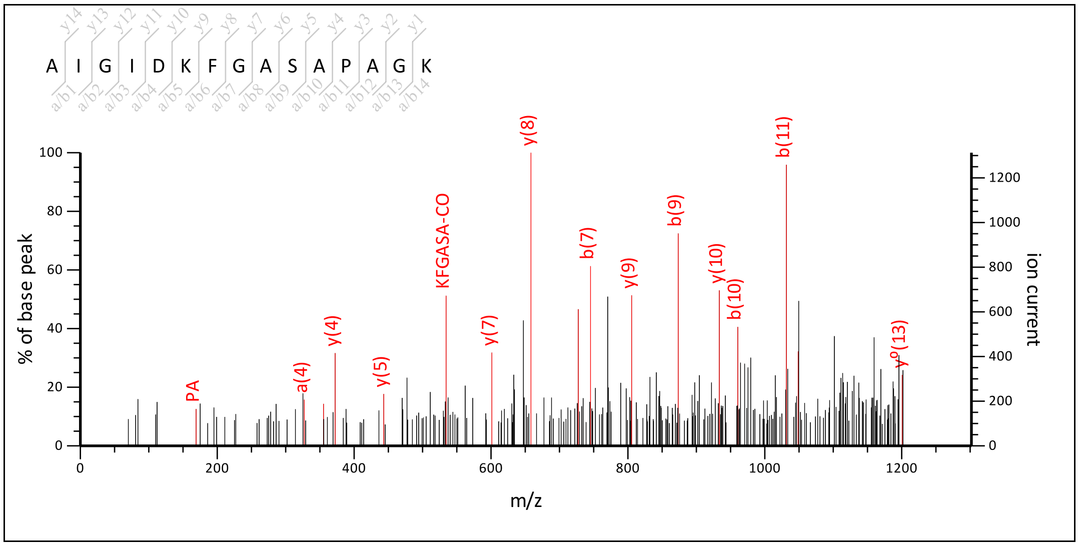


0 to 1301.89

Label all possible matches ○ Label matches used for scoring ◉

**Monoisotopic mass of neutral peptide Mr(calc):** 1401.7616
**Fixed modifications:** Carbamidomethyl (C) (apply to specified residues or termini only)
**Ions Score:** 32 **Expect:** 1.2
**Matches :** 23/260 fragment ions using 36 most intense peaks (help)

| # | Immon. | a | a* | $a^0$ | b | b* | $b^0$ | d | d' | Seq. | v | w | w' | y | y* | |
|---|---|---|---|---|---|---|---|---|---|---|---|---|---|---|---|---|
| 1 | 44.0495 | 44.0495 | | | 72.0444 | | | 44.0495 | | A | | | | | | |
| 2 | 86.0964 | 157.1335 | | | 185.1285 | | | 129.1022 | 143.1179 | I | 1273.6535 | 1286.6739 | 1300.6896 | 1331.7318 | 1314.7052 | 13[illegible] |
| 3 | 30.0338 | 214.1550 | | | 242.1499 | | | | | G | | | | 1218.6477 | 1201.6212 | 12[illegible] |
| 4 | 86.0964 | 327.2391 | | | **355.2340** | | | 299.2078 | 313.2234 | I | 1103.5480 | 1116.5684 | 1130.5841 | 1161.6263 | 1144.5997 | 11[illegible] |
| 5 | 88.0393 | 442.2660 | | 424.2554 | 470.2609 | | 452.2504 | 398.2762 | | D | 988.5211 | 987.5258 | | ***1048.5422*** | 1031.5156 | 10[illegible] |
| 6 | 101.1073 | 570.3610 | 553.3344 | 552.3504 | 598.3559 | 581.3293 | 580.3453 | 513.3031 | | K | 860.4261 | 859.4308 | | ***933.5152*** | 916.4887 | 9[illegible] |
| 7 | 120.0808 | 717.4294 | 700.4028 | 699.4188 | **745.4243** | 728.3978 | 727.4137 | | | F | 713.3577 | | | ***805.4203*** | 788.3937 | 7[illegible] |
| 8 | 30.0338 | 774.4509 | 757.4243 | 756.4403 | 802.4458 | 785.4192 | 784.4352 | | | G | | | | ***658.3519*** | 641.3253 | 6[illegible] |
| 9 | 44.0495 | 845.4880 | 828.4614 | 827.4774 | **873.4829** | 856.4563 | 855.4723 | | | A | 585.2991 | | | ***601.3304*** | 584.3039 | 5[illegible] |
| 10 | 60.0444 | 932.5200 | 915.4934 | 914.5094 | **960.5149** | 943.4884 | 942.5043 | 916.5251 | | S | 498.2671 | 497.2718 | | 530.2933 | 513.2667 | 5[illegible] |
| 11 | 44.0495 | 1003.5571 | 986.5306 | 985.5465 | **1031.5520** | 1014.5255 | 1013.5415 | | | A | 427.2300 | | | ***443.2613*** | 426.2347 | |
| 12 | 70.0651 | 1100.6099 | 1083.5833 | 1082.5993 | 1128.6048 | 1111.5782 | 1110.5942 | 1074.5942 | | P | 330.1772 | 329.1819 | | ***372.2241*** | 355.1976 | |
| 13 | 44.0495 | 1171.6470 | 1154.6204 | 1153.6364 | 1199.6419 | 1182.6154 | 1181.6313 | | | A | 259.1401 | | | 275.1714 | 258.1448 | |
| 14 | 30.0338 | 1228.6684 | 1211.6419 | 1210.6579 | 1256.6634 | 1239.6368 | 1238.6528 | | | G | | | | 204.1343 | 187.1077 | |
| 15 | 101.1073 | | | | | | | | | K | 74.0237 | 73.0284 | | 147.1128 | 130.0863 | |

| Seq | ya | yb | Seq | ya | yb | Seq | ya | yb |
|---|---|---|---|---|---|---|---|---|
| IG | 143.1179 | 171.1128 | IGI | 256.2020 | 284.1969 | IGID | 371.2289 | 399.2238 |
| IGIDK | 499.3239 | 527.3188 | IGIDKF | 646.3923 | 674.3872 | GI | 143.1179 | 171.1128 |
| GID | 258.1448 | 286.1397 | GIDK | 386.2398 | 414.2347 | GIDKF | 533.3082 | 561.3031 |
| GIDKFG | 590.3297 | 618.3246 | GIDKFGA | 661.3668 | 689.3617 | ID | 201.1234 | 229.1183 |
| IDK | 329.2183 | 357.2132 | IDKF | 476.2867 | 504.2817 | IDKFG | 533.3082 | 561.3031 |
| IDKFGA | 604.3453 | 632.3402 | IDKFGAS | 691.3774 | 719.3723 | DK | 216.1343 | 244.1292 |

| | | | | | | | | |
|---|---|---|---|---|---|---|---|---|
| **DKF** | 363.2027 | 391.1976 | **DKFG** | 420.2241 | 448.2191 | **DKFGA** | 491.2613 | 519.2562 |
| **DKFGAS** | 578.2933 | 606.2882 | **DKFGASA** | 649.3304 | 677.3253 | **KF** | 248.1757 | 276.1707 |
| **KFG** | 305.1972 | 333.1921 | **KFGA** | 376.2343 | 404.2292 | **KFGAS** | 463.2663 | 491.2613 |
| **KFGASA** | 534.3035 | 562.2984 | **KFGASAP** | 631.3562 | 659.3511 | **FG** | 177.1022 | 205.0972 |
| **FGA** | 248.1394 | 276.1343 | **FGAS** | 335.1714 | 363.1663 | **FGASA** | 406.2085 | 434.2034 |
| **FGASAP** | 503.2613 | 531.2562 | **FGASAPA** | 574.2984 | 602.2933 | **FGASAPAG** | 631.3198 | 659.3148 |
| **GA** | 101.0709 | 129.0659 | **GAS** | 188.1030 | 216.0979 | **GASA** | 259.1401 | 287.1350 |
| **GASAP** | 356.1928 | 384.1878 | **GASAPA** | 427.2300 | 455.2249 | **GASAPAG** | 484.2514 | 512.2463 |
| **AS** | 131.0815 | 159.0764 | **ASA** | 202.1186 | 230.1135 | **ASAP** | 299.1714 | 327.1663 |
| **ASAPA** | 370.2085 | 398.2034 | **ASAPAG** | 427.2300 | 455.2249 | **SA** | 131.0815 | 159.0764 |
| **SAP** | 228.1343 | 256.1292 | **SAPA** | 299.1714 | 327.1663 | **SAPAG** | 356.1928 | 384.1878 |
| **AP** | 141.1022 | 169.0972 | **APA** | 212.1394 | 240.1343 | **APAG** | 269.1608 | 297.1557 |
| **PA** | 141.1022 | 169.0972 | **PAG** | 198.1237 | 226.1186 | **AG** | 101.0709 | 129.0659 |

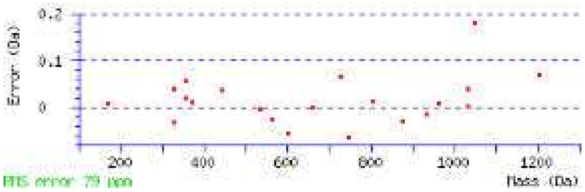


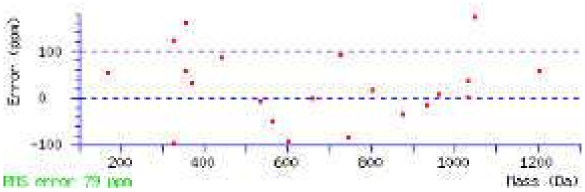


NCBI **BLAST** search of AIGIDKFGASAPAGK
(Parameters: blastp, nr protein database, expect=20000, no filter, PAM30)
Other BLAST web gateways

**All matches to this query**

| Score | Mr(calc) | Delta | Sequence |
|---|---|---|---|
| 31.7 | 1401.7616 | 0.0353 | AIGIDKFGASAPAGK |
| 22.3 | 1401.7073 | 0.0897 | NTRAQANAVSQSR |
| 17.0 | 1401.7517 | 0.0452 | HAYGTKWSLIAR |
| 13.8 | 1401.6888 | 0.1081 | WDDIAGLEEAKR |
| 12.6 | 1401.6597 | 0.1373 | KDQAGQGQDSNVR |
| 12.5 | 1401.7324 | 0.0645 | QSREGQSQLITR |
| 11.8 | 1401.6960 | 0.1009 | DSIGVEGSNRVNR |
| 11.3 | 1401.7728 | 0.0241 | VGPLYQSRLVDR |
| 9.3 | 1401.8344 | -0.0375 | RQSLLFTVTIPK |
| 8.6 | 1401.6848 | 0.1121 | DADAKQGSNELVR |

**Mascot:** http://www.matrixscience.com/

# MATRIX SCIENCE Mascot Search Results

## Peptide View Spot no 112

MS/MS Fragmentation of **MFGDFQKDTPEER**
Found in **gi|28190676** in **NCBInr**, putative transketolase [Oryza sativa Japonica Group]

Match to Query 132: 1598.764824 from(1599.772100,1+) intensity(0.0000) index(21)
Title: Label: E1, Spot_Id: 219678, Peak_List_Id: 224901, MSMS Job_Run_Id: 21757, Comment:
Data file C:\Documents and Settings\Administrator\Desktop\farah BHU\spot set plate\Farah-Garima 11May 2013\ppw_E1_136842029000.txt

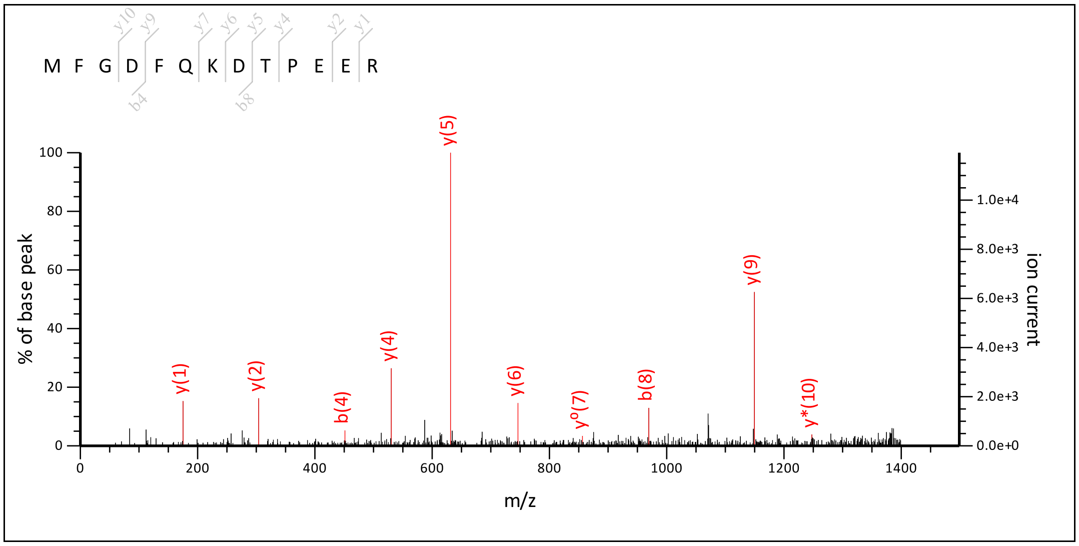


0 to 1498.97

Label all possible matches ○ Label matches used for scoring ◉

**Monoisotopic mass of neutral peptide Mr(calc):** 1598.7035
**Fixed modifications:** Carbamidomethyl (C) (apply to specified residues or termini only)
**Ions Score:** 41 **Expect:** 0.14
**Matches :** 11/210 fragment ions using 14 most intense peaks (help)

| # | Immon. | a | a* | $a^0$ | b | b* | $b^0$ | d | d' | Seq. | v | w | w' | y | y* | |
|---|---|---|---|---|---|---|---|---|---|---|---|---|---|---|---|---|
| 1 | 104.0528 | 104.0528 | | | 132.0478 | | | 44.0495 | | M | | | | | | |
| 2 | 120.0808 | 251.1213 | | | 279.1162 | | | | | F | 1376.6077 | | | 1468.6703 | 1451.6438 | 14 |
| 3 | 30.0338 | 308.1427 | | | 336.1376 | | | | | G | | | | 1321.6019 | 1304.5753 | 13 |
| 4 | 88.0393 | 423.1697 | | 405.1591 | **451.1646** | | 433.1540 | 379.1798 | | D | 1204.5593 | 1203.5640 | | 1264.5804 | 1247.5539 | 12 |
| 5 | 120.0808 | 570.2381 | | 552.2275 | 598.2330 | | 580.2224 | | | F | 1057.4909 | | | ***1149.5535*** | 1132.5269 | 11 |
| 6 | 101.0709 | 698.2967 | 681.2701 | 680.2861 | 726.2916 | 709.2650 | 708.2810 | 641.2752 | | Q | 929.4323 | 928.4371 | | 1002.4851 | 985.4585 | 98 |
| 7 | 101.1073 | 826.3916 | 809.3651 | 808.3811 | 854.3865 | 837.3600 | 836.3760 | 769.3338 | | K | 801.3373 | 800.3421 | | 874.4265 | 857.3999 | 85 |
| 8 | 88.0393 | 941.4186 | 924.3920 | 923.4080 | **969.4135** | 952.3869 | 951.4029 | 897.4287 | | D | 686.3104 | 685.3151 | | ***746.3315*** | 729.3050 | 72 |
| 9 | 74.0600 | 1042.4662 | 1025.4397 | 1024.4557 | 1070.4612 | 1053.4346 | 1052.4506 | 1026.4713 | 1028.4506 | T | 585.2627 | 598.2831 | 600.2624 | ***631.3046*** | 614.2780 | 61 |
| 10 | 70.0651 | 1139.5190 | 1122.4925 | 1121.5084 | 1167.5139 | 1150.4874 | 1149.5034 | 1113.5034 | | P | 488.2100 | 487.2147 | | ***530.2569*** | 513.2304 | 51 |
| 11 | 102.0550 | 1268.5616 | 1251.5351 | 1250.5510 | 1296.5565 | 1279.5300 | 1278.5460 | 1210.5561 | | E | 359.1674 | 358.1721 | | 433.2041 | 416.1776 | 41 |
| 12 | 102.0550 | 1397.6042 | 1380.5776 | 1379.5936 | 1425.5991 | 1408.5726 | 1407.5885 | 1339.5987 | | E | 230.1248 | 229.1295 | | ***304.1615*** | 287.1350 | 28 |
| 13 | 129.1135 | | | | | | | | | R | 74.0237 | 73.0284 | | ***175.1190*** | 158.0924 | |

| Seq | ya | yb | Seq | ya | yb | Seq | ya | yb |
|---|---|---|---|---|---|---|---|---|
| FG | 177.1022 | 205.0972 | FGD | 292.1292 | 320.1241 | FGDF | 439.1976 | 467.1925 |
| FGDFQ | 567.2562 | 595.2511 | FGDFQK | 695.3511 | 723.3461 | GD | 145.0608 | 173.0557 |
| GDF | 292.1292 | 320.1241 | GDFQ | 420.1878 | 448.1827 | GDFQK | 548.2827 | 576.2776 |
| GDFQKD | 663.3097 | 691.3046 | DF | 235.1077 | 263.1026 | DFQ | 363.1663 | 391.1612 |
| DFQK | 491.2613 | 519.2562 | DFQKD | 606.2882 | 634.2831 | FQ | 248.1394 | 276.1343 |
| FQK | 376.2343 | 404.2292 | FQKD | 491.2613 | 519.2562 | FQKDT | 592.3089 | 620.3039 |
| FQKDTP | 689.3617 | 717.3566 | QK | 229.1659 | 257.1608 | QKD | 344.1928 | 372.1878 |
| QKDT | 445.2405 | 473.2354 | QKDTP | 542.2933 | 570.2882 | QKDTPE | 671.3359 | 699.3308 |

| | | | | | | | | |
|---|---|---|---|---|---|---|---|---|
| **KD** | 216.1343 | 244.1292 | **KDT** | 317.1819 | 345.1769 | **KDTP** | 414.2347 | 442.2296 |
| **KDTPE** | 543.2773 | 571.2722 | **KDTPEE** | 672.3199 | 700.3148 | **DT** | 189.0870 | 217.0819 |
| **DTP** | 286.1397 | 314.1347 | **DTPE** | 415.1823 | 443.1773 | **DTPEE** | 544.2249 | 572.2198 |
| **TP** | 171.1128 | 199.1077 | **TPE** | 300.1554 | 328.1503 | **TPEE** | 429.1980 | 457.1929 |
| **PE** | 199.1077 | 227.1026 | **PEE** | 328.1503 | 356.1452 | **EE** | 231.0975 | 259.0925 |

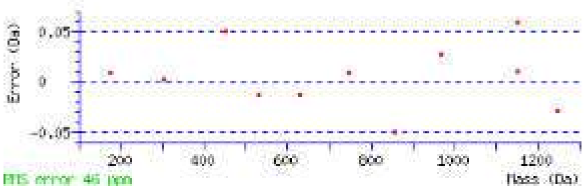


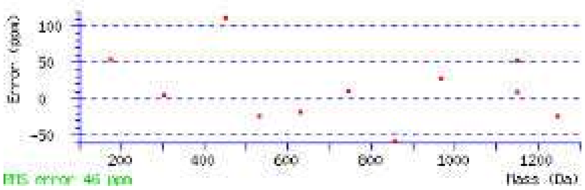


NCBI **BLAST** search of MFGDFQKDTPEER
(Parameters: blastp, nr protein database, expect=20000, no filter, PAM30)
Other BLAST web gateways

**All matches to this query**

| Score | Mr(calc) | Delta | Sequence |
|---|---|---|---|
| 41.3 | 1598.7035 | 0.0613 | MFGDFQKDTPEER |
| 15.8 | 1598.7134 | 0.0514 | LYSMEDVDEVVER |
| 15.3 | 1598.8053 | -0.0404 | LSGSLLFHNTEPER |
| 14.5 | 1598.8991 | -0.1343 | KAATSATDLKPIVER |
| 14.4 | 1598.9032 | -0.1384 | LLALDGSALPVVFER |
| 14.1 | 1598.8569 | -0.0920 | LQNFKLNYQYLR |
| 13.8 | 1598.7974 | -0.0326 | MSIDSPQDPVLLER |
| 13.5 | 1598.8879 | -0.1231 | EGKTLVILDDVLER |
| 13.1 | 1598.8127 | -0.0478 | TTLAFFAGGMLGEIR |
| 12.8 | 1598.8165 | -0.0517 | SQDKAQHAFGLLER |

**Mascot:** http://www.matrixscience.com/

# MATRIX SCIENCE Mascot Search Results

## Peptide View Spot no 112

MS/MS Fragmentation of **MFGDFQKDTPEER**
Found in **gi|28190676** in **NCBInr**, putative transketolase [Oryza sativa Japonica Group]

Match to Query 134: 1614.742524 from(1615.749800,1+) intensity(0.0000) index(22)
Title: Label: E1, Spot_Id: 219678, Peak_List_Id: 224898, MSMS Job_Run_Id: 21757, Comment:
Data file C:\Documents and Settings\Administrator\Desktop\farah BHU\spot set plate\Farah-Garima 11May 2013\ppw_E1_136842029000.txt

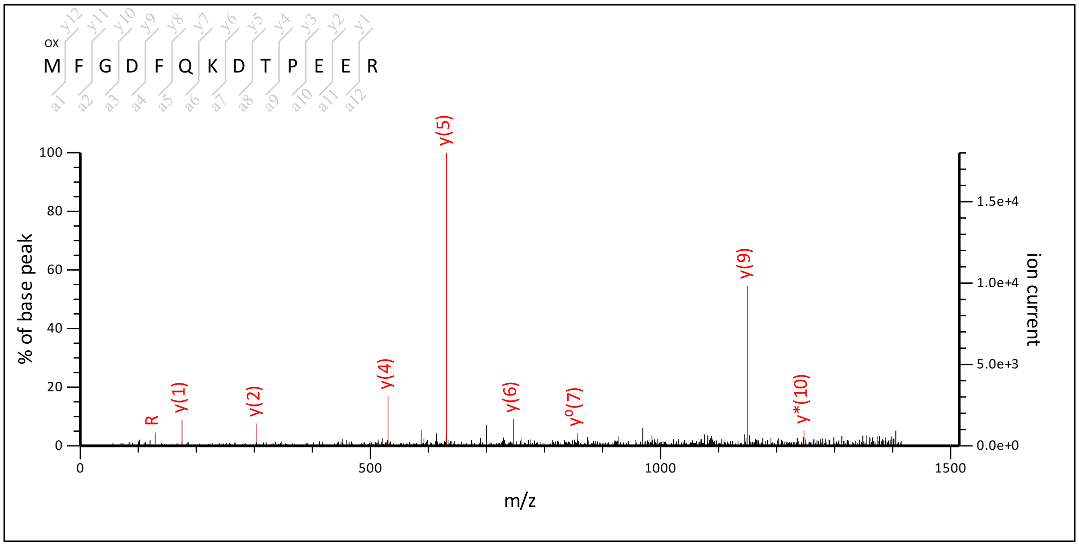


0 to 1515.04

Label all possible matches Label matches used for scoring

**Monoisotopic mass of neutral peptide Mr(calc):** 1614.6984
**Fixed modifications:** Carbamidomethyl (C) (apply to specified residues or termini only)
**Variable modifications:**
**M1** : Oxidation (M), with neutral losses 0.0000(shown in table), 63.9983
**Ions Score:** 39 **Expect:** 0.22
**Matches :** 10/276 fragment ions using 14 most intense peaks (help)

| # | Immon. | a | a* | a$^0$ | b | b* | b$^0$ | d | d' | Seq. | v | w | w' | y | y* | |
|---|---|---|---|---|---|---|---|---|---|---|---|---|---|---|---|---|
| 1 | 120.0478 | 120.0478 | | | 148.0427 | | | 44.0495 | | M | | | | | | |
| 2 | 120.0808 | 267.1162 | | | 295.1111 | | | | | F | 1376.6077 | | | 1468.6703 | 1451.6438 | 14[illegible] |
| 3 | 30.0338 | 324.1376 | | | 352.1326 | | | | | G | | | | 1321.6019 | 1304.5753 | 13[illegible] |
| 4 | 88.0393 | 439.1646 | | 421.1540 | 467.1595 | | 449.1489 | 395.1748 | | D | 1204.5593 | 1203.5640 | | 1264.5804 | 1247.5539 | 12[illegible] |
| 5 | 120.0808 | 586.2330 | | 568.2224 | 614.2279 | | 596.2173 | | | F | 1057.4909 | | | ***1149.5535*** | 1132.5269 | 11[illegible] |
| 6 | 101.0709 | 714.2916 | 697.2650 | 696.2810 | 742.2865 | 725.2599 | 724.2759 | 657.2701 | | Q | 929.4323 | 928.4371 | | 1002.4851 | 985.4585 | 9[illegible] |
| 7 | 101.1073 | 842.3865 | 825.3600 | 824.3760 | 870.3815 | 853.3549 | 852.3709 | 785.3287 | | K | 801.3373 | 800.3421 | | 874.4265 | 857.3999 | 8[illegible] |
| 8 | 88.0393 | 957.4135 | 940.3869 | 939.4029 | 985.4084 | 968.3818 | 967.3978 | 913.4237 | | D | 686.3104 | 685.3151 | | ***746.3315*** | 729.3050 | 7[illegible] |
| 9 | 74.0600 | 1058.4612 | 1041.4346 | 1040.4506 | 1086.4561 | 1069.4295 | 1068.4455 | 1042.4662 | 1044.4455 | T | 585.2627 | 598.2831 | 600.2624 | ***631.3046*** | 614.2780 | 6[illegible] |
| 10 | 70.0651 | 1155.5139 | 1138.4874 | 1137.5034 | 1183.5088 | 1166.4823 | 1165.4983 | 1129.4983 | | P | 488.2100 | 487.2147 | | ***530.2569*** | 513.2304 | 5[illegible] |
| 11 | 102.0550 | 1284.5565 | 1267.5300 | 1266.5460 | 1312.5514 | 1295.5249 | 1294.5409 | 1226.5510 | | E | 359.1674 | 358.1721 | | 433.2041 | 416.1776 | 4[illegible] |
| 12 | 102.0550 | 1413.5991 | 1396.5726 | 1395.5885 | 1441.5940 | 1424.5675 | 1423.5835 | 1355.5936 | | E | 230.1248 | 229.1295 | | ***304.1615*** | 287.1350 | 2[illegible] |
| 13 | 129.1135 | | | | | | | | | R | 74.0237 | 73.0284 | | ***175.1190*** | 158.0924 | |

| Seq | ya | yb | Seq | ya | yb | Seq | ya | yb |
|---|---|---|---|---|---|---|---|---|
| FG | 177.1022 | 205.0972 | FGD | 292.1292 | 320.1241 | FGDF | 439.1976 | 467.1925 |
| FGDFQ | 567.2562 | 595.2511 | FGDFQK | 695.3511 | 723.3461 | GD | 145.0608 | 173.0557 |
| GDF | 292.1292 | 320.1241 | GDFQ | 420.1878 | 448.1827 | GDFQK | 548.2827 | 576.2776 |
| GDFQKD | 663.3097 | 691.3046 | DF | 235.1077 | 263.1026 | DFQ | 363.1663 | 391.1612 |
| DFQK | 491.2613 | 519.2562 | DFQKD | 606.2882 | 634.2831 | FQ | 248.1394 | 276.1343 |
| | | | | | | | | |

| | | | | | | | | |
|---|---|---|---|---|---|---|---|---|
| **FQK** | 376.2343 | 404.2292 | **FQKD** | 491.2613 | 519.2562 | **FQKDT** | 592.3089 | 620.3039 |
| **FQKDTP** | 689.3617 | 717.3566 | **QK** | 229.1659 | 257.1608 | **QKD** | 344.1928 | 372.1878 |
| **QKDT** | 445.2405 | 473.2354 | **QKDTP** | 542.2933 | 570.2882 | **QKDTPE** | 671.3359 | 699.3308 |
| **KD** | 216.1343 | 244.1292 | **KDT** | 317.1819 | 345.1769 | **KDTP** | 414.2347 | 442.2296 |
| **KDTPE** | 543.2773 | 571.2722 | **KDTPEE** | 672.3199 | 700.3148 | **DT** | 189.0870 | 217.0819 |
| **DTP** | 286.1397 | 314.1347 | **DTPE** | 415.1823 | 443.1773 | **DTPEE** | 544.2249 | 572.2198 |
| **TP** | 171.1128 | 199.1077 | **TPE** | 300.1554 | 328.1503 | **TPEE** | 429.1980 | 457.1929 |
| **PE** | 199.1077 | 227.1026 | **PEE** | 328.1503 | 356.1452 | **EE** | 231.0975 | 259.0925 |

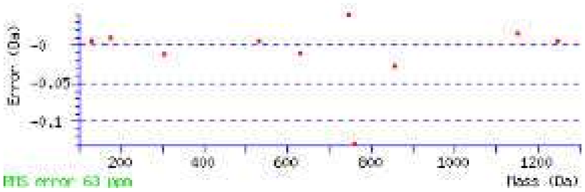


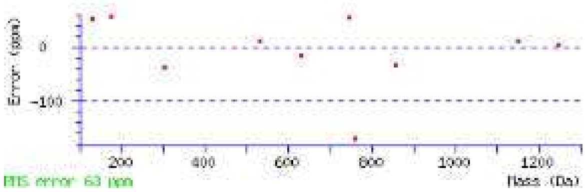


NCBI **BLAST** search of MFGDFQKDTPEER
(Parameters: blastp, nr protein database, expect=20000, no filter, PAM30)
Other BLAST web gateways

**All matches to this query**

| Score | Mr(calc) | Delta | Sequence |
|---|---|---|---|
| 38.9 | 1614.6984 | 0.0441 | MFGDFQKDTPEER |
| 38.9 | 1614.6984 | 0.0441 | MYGDFQKDTPEER |
| 23.4 | 1614.7599 | -0.0174 | SFFVEMDETIIER |
| 21.1 | 1614.8049 | -0.0624 | RVIQAGCATHAFER |
| 15.9 | 1614.7083 | 0.0342 | ESSEPESMTFTNLK |
| 15.9 | 1614.6885 | 0.0540 | QVLAYALDXTXDER |
| 15.8 | 1614.6791 | 0.0634 | TTSGNMDSSNKESNK |
| 15.6 | 1614.8213 | -0.0788 | ALAISNDNGEIEITR |
| 15.6 | 1614.7485 | -0.0060 | LEDNGVLDEDANGVR |
| 14.3 | 1614.7494 | -0.0069 | SIDGDVTAPMMLHGR |

**Mascot:** http://www.matrixscience.com/

# MATRIX SCIENCE Mascot Search Results

## Peptide View Spot no 112

MS/MS Fragmentation of **HVPQGAAFEADWNAK**
Found in **gi|28190676** in **NCBInr**, putative transketolase [Oryza sativa Japonica Group]

Match to Query 138: 1639.816324 from(1640.823600,1+) intensity(0.0000) index(23)
Title: Label: E1, Spot_Id: 219678, Peak_List_Id: 224908, MSMS Job_Run_Id: 21757, Comment:
Data file C:\Documents and Settings\Administrator\Desktop\farah BHU\spot set plate\Farah-Garima 11May 2013\ppw_E1_136842029000.txt

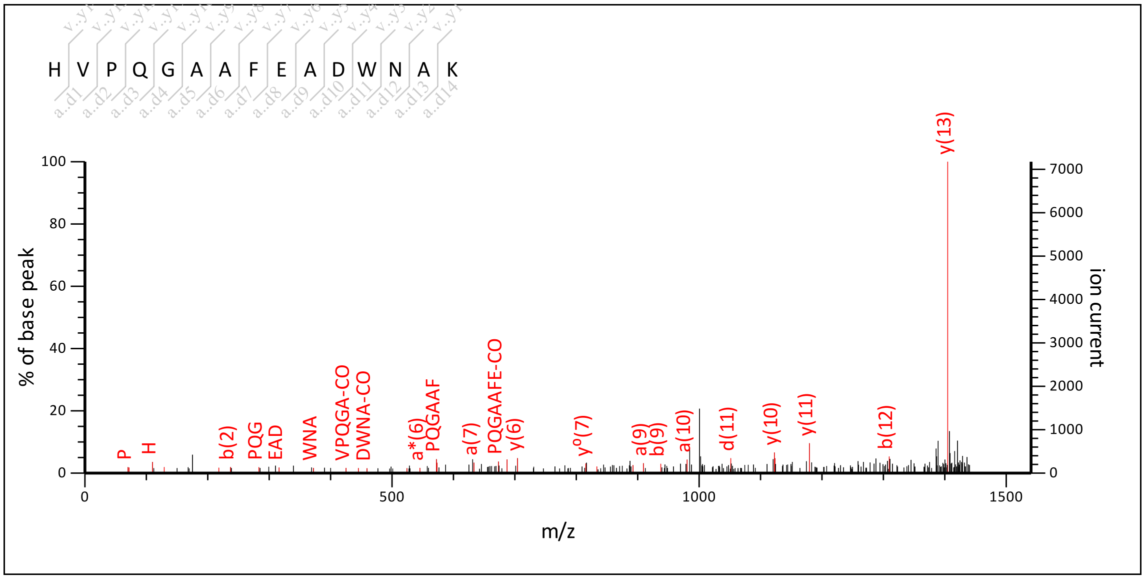


0 to 1540.43

Label all possible matches ○ Label matches used for scoring ◉

**Monoisotopic mass of neutral peptide Mr(calc):** 1639.7743
**Fixed modifications:** Carbamidomethyl (C) (apply to specified residues or termini only)
**Ions Score:** 28 **Expect:** 2.8
**Matches :** 41/246 fragment ions using 94 most intense peaks (help)

| # | Immon. | a | a* | $a^0$ | b | b* | $b^0$ | d | Seq. | v | w | y | y* | $y^0$ | # |
|---|---|---|---|---|---|---|---|---|---|---|---|---|---|---|---|
| 1 | 110.0713 | **110.0713** | | | 138.0662 | | | 44.0495 | H | | | | | | 15 |
| 2 | 72.0808 | 209.1397 | | | **237.1346** | | | 195.1240 | V | 1459.6601 | 1472.6805 | 1503.7227 | 1486.6961 | 1485.7121 | 14 |
| 3 | 70.0651 | 306.1925 | | | 334.1874 | | | 280.1768 | P | 1362.6073 | 1361.6121 | ***1404.6543*** | 1387.6277 | 1386.6437 | 13 |
| 4 | 101.0709 | 434.2510 | 417.2245 | | 462.2459 | 445.2194 | | 377.2296 | Q | 1234.5487 | 1233.5535 | 1307.6015 | 1290.5749 | 1289.5909 | 12 |
| 5 | 30.0338 | 491.2725 | 474.2459 | | 519.2674 | 502.2409 | | | G | | | ***1179.5429*** | 1162.5164 | 1161.5323 | 11 |
| 6 | 44.0495 | 562.3096 | 545.2831 | | 590.3045 | 573.2780 | | | A | 1106.4901 | | ***1122.5214*** | 1105.4949 | 1104.5109 | 10 |
| 7 | 44.0495 | **633.3467** | 616.3202 | | 661.3416 | 644.3151 | | | A | 1035.4530 | | ***1051.4843*** | 1034.4578 | 1033.4738 | 9 |
| 8 | 120.0808 | 780.4151 | 763.3886 | | 808.4100 | 791.3835 | | | F | 888.3846 | | ***980.4472*** | 963.4207 | 962.4367 | 8 |
| 9 | 102.0550 | **909.4577** | 892.4312 | 891.4472 | **937.4526** | 920.4261 | 919.4421 | 851.4522 | E | 759.3420 | 758.3468 | ***833.3788*** | 816.3523 | 815.3682 | 7 |
| 10 | 44.0495 | **980.4948** | 963.4683 | 962.4843 | 1008.4898 | 991.4632 | 990.4792 | | A | 688.3049 | | ***704.3362*** | 687.3097 | 686.3256 | 6 |
| 11 | 88.0393 | 1095.5218 | 1078.4952 | 1077.5112 | **1123.5167** | 1106.4902 | 1105.5061 | 1051.5320 | D | 573.2780 | 572.2827 | ***633.2991*** | 616.2726 | 615.2885 | 5 |
| 12 | 159.0917 | 1281.6011 | 1264.5745 | 1263.5905 | **1309.5960** | 1292.5695 | 1291.5854 | | W | 387.1987 | | 518.2722 | 501.2456 | | 4 |
| 13 | 87.0553 | 1395.6440 | 1378.6175 | 1377.6335 | 1423.6389 | 1406.6124 | 1405.6284 | 1352.6382 | N | 273.1557 | 272.1605 | 332.1928 | 315.1663 | | 3 |
| 14 | 44.0495 | 1466.6811 | 1449.6546 | 1448.6706 | 1494.6761 | 1477.6495 | 1476.6655 | | A | 202.1186 | | ***218.1499*** | 201.1234 | | 2 |
| 15 | 101.1073 | | | | | | | | K | 74.0237 | 73.0284 | 147.1128 | 130.0863 | | 1 |

| Seq | ya | yb | Seq | ya | yb | Seq | ya | yb |
|---|---|---|---|---|---|---|---|---|
| VP | 169.1335 | 197.1285 | VPQ | 297.1921 | 325.1870 | VPQG | 354.2136 | 382.2085 |
| VPQGA | 425.2507 | 453.2456 | VPQGAA | 496.2878 | 524.2827 | VPQGAAF | 643.3562 | 671.3511 |
| PQ | 198.1237 | 226.1186 | PQG | 255.1452 | 283.1401 | PQGA | 326.1823 | 354.1772 |

| | | | | | | | | |
|---|---|---|---|---|---|---|---|---|
| **PQGAA** | 397.2194 | 425.2143 | **PQGAAF** | 544.2878 | 572.2827 | **PQGAAFE** | 673.3304 | 701.3253 |
| **QG** | 158.0924 | 186.0873 | **QGA** | 229.1295 | 257.1244 | **QGAA** | 300.1666 | 328.1615 |
| **QGAAF** | 447.2350 | 475.2300 | **QGAAFE** | 576.2776 | 604.2726 | **QGAAFEA** | 647.3148 | 675.3097 |
| **GA** | 101.0709 | 129.0659 | **GAA** | 172.1081 | 200.1030 | **GAAF** | 319.1765 | 347.1714 |
| **GAAFE** | 448.2191 | 476.2140 | **GAAFEA** | 519.2562 | 547.2511 | **GAAFEAD** | 634.2831 | 662.2780 |
| **AA** | 115.0866 | 143.0815 | **AAF** | 262.1550 | 290.1499 | **AAFE** | 391.1976 | 419.1925 |
| **AAFEA** | 462.2347 | 490.2296 | **AAFEAD** | 577.2617 | 605.2566 | **AF** | 191.1179 | 219.1128 |
| **AFE** | 320.1605 | 348.1554 | **AFEA** | 391.1976 | 419.1925 | **AFEAD** | 506.2245 | 534.2195 |
| **AFEADW** | 692.3039 | 720.2988 | **FE** | 249.1234 | 277.1183 | **FEA** | 320.1605 | 348.1554 |
| **FEAD** | 435.1874 | 463.1823 | **FEADW** | 621.2667 | 649.2617 | **EA** | 173.0921 | 201.0870 |
| **EAD** | 288.1190 | 316.1139 | **EADW** | 474.1983 | 502.1932 | **EADWN** | 588.2413 | 616.2362 |
| **EADWNA** | 659.2784 | 687.2733 | **AD** | 159.0764 | 187.0713 | **ADW** | 345.1557 | 373.1506 |
| **ADWN** | 459.1987 | 487.1936 | **ADWNA** | 530.2358 | 558.2307 | **DW** | 274.1186 | 302.1135 |
| **DWN** | 388.1615 | 416.1565 | **DWNA** | 459.1987 | 487.1936 | **WN** | 273.1346 | 301.1295 |
| **WNA** | 344.1717 | 372.1666 | **NA** | 158.0924 | 186.0873 | | | |

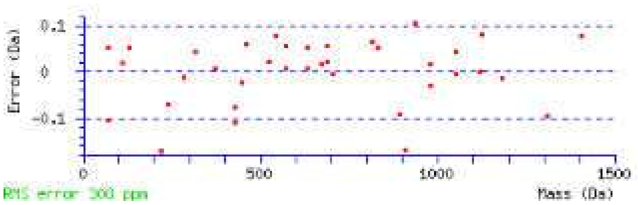


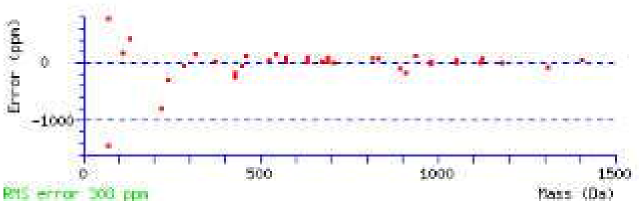


NCBI **BLAST** search of HVPQGAAFEADWNAK
(Parameters: blastp, nr protein database, expect=20000, no filter, PAM30)
Other BLAST web gateways

**All matches to this query**

| Score | Mr(calc) | Delta | Sequence |
|---|---|---|---|
| 27.9 | 1639.7743 | 0.0420 | HVPQGAAFEADWNAK |
| 19.0 | 1639.8174 | -0.0011 | MSIHTALLCASPSPR |
| 10.9 | 1639.7348 | 0.0815 | VHVHVTGNCMEWR |
| 10.3 | 1639.8716 | -0.0552 | HVALMSELSAVISQR |
| 9.4 | 1639.7069 | 0.1094 | AQVEMEMEAQIDSK |
| 8.6 | 1639.8781 | -0.0617 | AAAASSLSAPAAPISLDK |
| 8.4 | 1639.8907 | -0.0743 | HARPGSPVPTLAPGGAR |
| 8.1 | 1639.7698 | 0.0465 | MAIPTSDFLQMNTR |
| 7.5 | 1639.8682 | -0.0519 | HVFSLGEGSPLTVAAR |
| 7.4 | 1639.7916 | 0.0247 | AFLHGDLTETVYMK |

**Mascot:** http://www.matrixscience.com/

# MATRIX SCIENCE Mascot Search Results

## Peptide View **Spot no 112**

MS/MS Fragmentation of **SIVSGELPAGWADALPK**
Found in **gi|28190676** in **NCBInr**, putative transketolase [Oryza sativa Japonica Group]

Match to Query 145: 1709.934824 from(1710.942100,1+) intensity(0.0000) index(25)
Title: Label: E1, Spot_Id: 219678, Peak_List_Id: 224909, MSMS Job_Run_Id: 21757, Comment:
Data file C:\Documents and Settings\Administrator\Desktop\farah BHU\spot set plate\Farah-Garima 11May 2013\ppw_E1_136842029000.txt

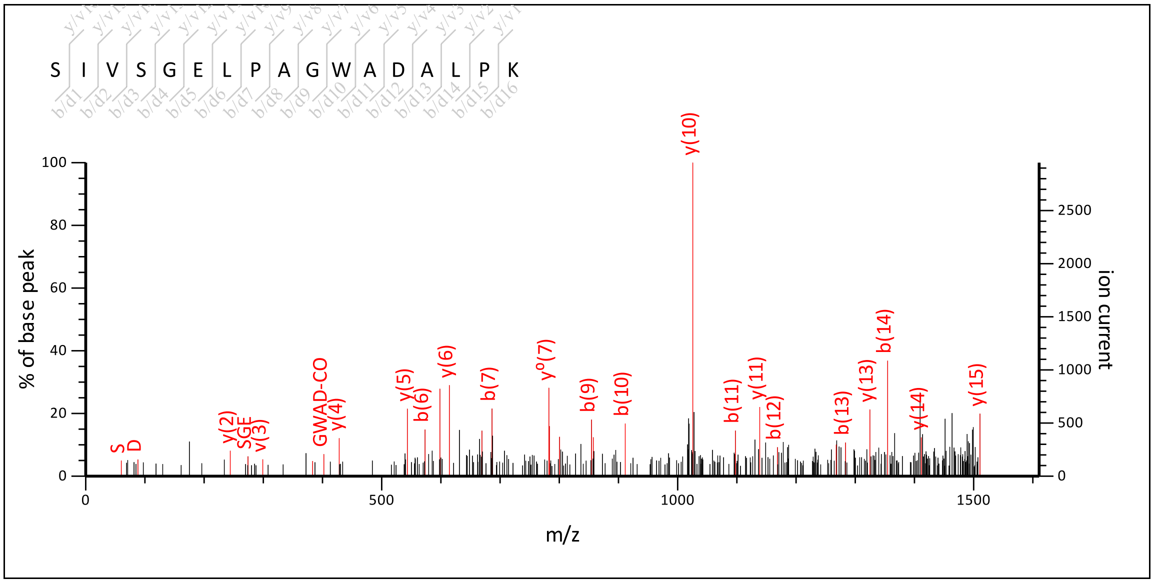


0 to 1610.65

Label all possible matches ○ Label matches used for scoring ◉

**Monoisotopic mass of neutral peptide Mr(calc):** 1709.8988
**Fixed modifications:** Carbamidomethyl (C) (apply to specified residues or termini only)
**Ions Score:** 62 **Expect:** 0.0011
**Matches :** 41/296 fragment ions using 75 most intense peaks (help)

| # | Immon. | a | $a^0$ | b | $b^0$ | d | d' | Seq. | v | w | w' | y | y* | $y^0$ | # |
|---|---|---|---|---|---|---|---|---|---|---|---|---|---|---|---|
| 1 | 60.0444 | 60.0444 | 42.0338 | ***88.0393*** | 70.0287 | 44.0495 | | S | | | | | | | 17 |
| 2 | 86.0964 | 173.1285 | 155.1179 | 201.1234 | 183.1128 | 145.0972 | 159.1128 | I | 1565.7958 | 1578.8162 | 1592.8319 | 1623.8741 | 1606.8475 | 1605.8635 | 16 |
| 3 | 72.0808 | 272.1969 | 254.1863 | 300.1918 | 282.1812 | 258.1812 | | V | 1466.7274 | 1479.7478 | | ***1510.7900*** | 1493.7635 | 1492.7795 | 15 |
| 4 | 60.0444 | 359.2289 | 341.2183 | 387.2238 | 369.2132 | 343.2340 | | S | 1379.6954 | 1378.7001 | | ***1411.7216*** | 1394.6951 | 1393.7110 | 14 |
| 5 | 30.0338 | 416.2504 | 398.2398 | 444.2453 | 426.2347 | | | G | | | | ***1324.6896*** | 1307.6630 | 1306.6790 | 13 |
| 6 | 102.0550 | 545.2930 | 527.2824 | ***573.2879*** | 555.2773 | 487.2875 | | E | 1193.6313 | 1192.6361 | | ***1267.6681*** | 1250.6416 | 1249.6575 | 12 |
| 7 | 86.0964 | 658.3770 | 640.3665 | ***686.3719*** | 668.3614 | 616.3301 | | L | 1080.5473 | 1079.5520 | | ***1138.6255*** | 1121.5990 | 1120.6150 | 11 |
| 8 | 70.0651 | 755.4298 | 737.4192 | ***783.4247*** | 765.4141 | 729.4141 | | P | 983.4945 | 982.4993 | | ***1025.5415*** | 1008.5149 | 1007.5309 | 10 |
| 9 | 44.0495 | 826.4669 | 808.4563 | ***854.4618*** | 836.4512 | | | A | 912.4574 | | | 928.4887 | 911.4621 | 910.4781 | 9 |
| 10 | 30.0338 | 883.4884 | 865.4778 | ***911.4833*** | 893.4727 | | | G | | | | ***857.4516*** | 840.4250 | 839.4410 | 8 |
| 11 | 159.0917 | 1069.5677 | 1051.5571 | ***1097.5626*** | 1079.5520 | | | W | 669.3566 | | | ***800.4301*** | 783.4036 | 782.4196 | 7 |
| 12 | 44.0495 | 1140.6048 | 1122.5942 | ***1168.5997*** | 1150.5891 | | | A | 598.3195 | | | ***614.3508*** | 597.3243 | 596.3402 | 6 |
| 13 | 88.0393 | 1255.6317 | 1237.6212 | ***1283.6266*** | 1265.6161 | 1211.6419 | | D | 483.2926 | 482.2973 | | ***543.3137*** | 526.2871 | 525.3031 | 5 |
| 14 | 44.0495 | 1326.6688 | 1308.6583 | ***1354.6638*** | 1336.6532 | | | A | 412.2554 | | | ***428.2867*** | 411.2602 | | 4 |
| 15 | 86.0964 | 1439.7529 | 1421.7423 | 1467.7478 | 1449.7373 | 1397.7060 | | L | 299.1714 | 298.1761 | | 357.2496 | 340.2231 | | 3 |
| 16 | 70.0651 | 1536.8057 | 1518.7951 | 1564.8006 | 1546.7900 | 1510.7900 | | P | 202.1186 | 201.1234 | | ***244.1656*** | 227.1390 | | 2 |
| 17 | 101.1073 | | | | | | | K | 74.0237 | 73.0284 | | 147.1128 | 130.0863 | | 1 |

| Seq | ya | yb | Seq | ya | yb | Seq | ya | yb |
|---|---|---|---|---|---|---|---|---|
| | | | | | | | | |

| | | | | | | | | |
|---|---|---|---|---|---|---|---|---|
| **IV** | 185.1648 | 213.1598 | **IVS** | 272.1969 | 300.1918 | **IVSG** | 329.2183 | 357.2132 |
| **IVSGE** | 458.2609 | 486.2558 | **IVSGEL** | 571.3450 | 599.3399 | **IVSGELP** | 668.3978 | 696.3927 |
| **VS** | 159.1128 | 187.1077 | **VSG** | 216.1343 | 244.1292 | **VSGE** | 345.1769 | 373.1718 |
| **VSGEL** | 458.2609 | 486.2558 | **VSGELP** | 555.3137 | 583.3086 | **VSGELPA** | 626.3508 | 654.3457 |
| **VSGELPAG** | 683.3723 | 711.3672 | **SG** | 117.0659 | 145.0608 | **SGE** | 246.1084 | 274.1034 |
| **SGEL** | 359.1925 | 387.1874 | **SGELP** | 456.2453 | 484.2402 | **SGELPA** | 527.2824 | 555.2773 |
| **SGELPAG** | 584.3039 | 612.2988 | **GE** | 159.0764 | 187.0713 | **GEL** | 272.1605 | 300.1554 |
| **GELP** | 369.2132 | 397.2082 | **GELPA** | 440.2504 | 468.2453 | **GELPAG** | 497.2718 | 525.2667 |
| **GELPAGW** | 683.3511 | 711.3461 | **EL** | 215.1390 | 243.1339 | **ELP** | 312.1918 | 340.1867 |
| **ELPA** | 383.2289 | 411.2238 | **ELPAG** | 440.2504 | 468.2453 | **ELPAGW** | 626.3297 | 654.3246 |
| **ELPAGWA** | 697.3668 | 725.3617 | **LP** | 183.1492 | 211.1441 | **LPA** | 254.1863 | 282.1812 |
| **LPAG** | 311.2078 | 339.2027 | **LPAGW** | 497.2871 | 525.2820 | **LPAGWA** | 568.3242 | 596.3191 |
| **LPAGWAD** | 683.3511 | 711.3461 | **PA** | 141.1022 | 169.0972 | **PAG** | 198.1237 | 226.1186 |
| **PAGW** | 384.2030 | 412.1979 | **PAGWA** | 455.2401 | 483.2350 | **PAGWAD** | 570.2671 | 598.2620 |
| **PAGWADA** | 641.3042 | 669.2991 | **AG** | 101.0709 | 129.0659 | **AGW** | 287.1503 | 315.1452 |
| **AGWA** | 358.1874 | 386.1823 | **AGWAD** | 473.2143 | 501.2092 | **AGWADA** | 544.2514 | 572.2463 |
| **AGWADAL** | 657.3355 | 685.3304 | **GW** | 216.1131 | 244.1081 | **GWA** | 287.1503 | 315.1452 |
| **GWAD** | 402.1772 | 430.1721 | **GWADA** | 473.2143 | 501.2092 | **GWADAL** | 586.2984 | 614.2933 |
| **GWADALP** | 683.3511 | 711.3461 | **WA** | 230.1288 | 258.1237 | **WAD** | 345.1557 | 373.1506 |
| **WADA** | 416.1928 | 444.1878 | **WADAL** | 529.2769 | 557.2718 | **WADALP** | 626.3297 | 654.3246 |
| **AD** | 159.0764 | 187.0713 | **ADA** | 230.1135 | 258.1084 | **ADAL** | 343.1976 | 371.1925 |
| **ADALP** | 440.2504 | 468.2453 | **DA** | 159.0764 | 187.0713 | **DAL** | 272.1605 | 300.1554 |
| **DALP** | 369.2132 | 397.2082 | **AL** | 157.1335 | 185.1285 | **ALP** | 254.1863 | 282.1812 |
| **LP** | 183.1492 | 211.1441 | | | | | | |

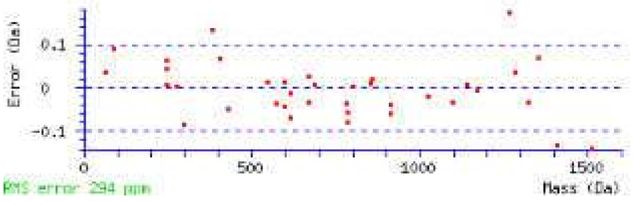


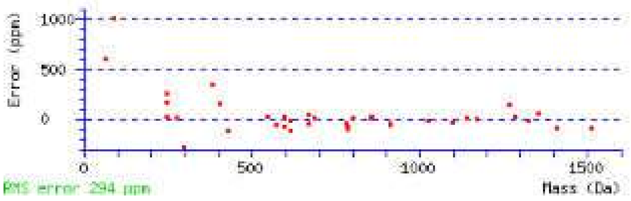


NCBI **BLAST** search of SIVSGELPAGWADALPK
(Parameters: blastp, nr protein database, expect=20000, no filter, PAM30)
Other BLAST web gateways

**All matches to this query**

| Score | Mr(calc) | Delta | Sequence |
|---|---|---|---|
| 61.8 | 1709.8988 | 0.0360 | SIVSGELPAGWADALPK |
| 38.2 | 1709.8697 | 0.0652 | VSDLSSPDQPASPRVR |
| 27.2 | 1709.9213 | 0.0135 | GLDADAATPVARWLVR |
| 21.2 | 1709.8696 | 0.0652 | SLEAEAGITGPAADPRR |
| 19.0 | 1709.9617 | -0.0269 | GWLSLALVIGSPWGVR |
| 19.0 | 1709.7721 | 0.1627 | MPMSMSTMLPGSPRR |
| 19.0 | 1709.7721 | 0.1627 | MPMSMSTMLPGSPRR |
| 17.2 | 1709.7721 | 0.1627 | MPMSMSTMLPGSPRR |
| 16.1 | 1709.9213 | 0.0135 | DAGALLGAIPPHPAAPSR |
| 16.1 | 1709.9788 | -0.0440 | ANVTGIILDQGKAVGVR |

**Mascot:** http://www.matrixscience.com/

# MATRIX SCIENCE Mascot Search Results

## Peptide View **Spot no 112**

MS/MS Fragmentation of **IYQEYGITAENVIATAK**
Found in **gi|28190676** in **NCBInr**, putative transketolase [Oryza sativa Japonica Group]

Match to Query 155: 1883.010524 from(1884.017800,1+) intensity(0.0000) index(28)
Title: Label: E1, Spot_Id: 219678, Peak_List_Id: 224905, MSMS Job_Run_Id: 21757, Comment:
Data file C:\Documents and Settings\Administrator\Desktop\farah BHU\spot set plate\Farah-Garima 11May 2013\ppw_E1_136842029000.txt

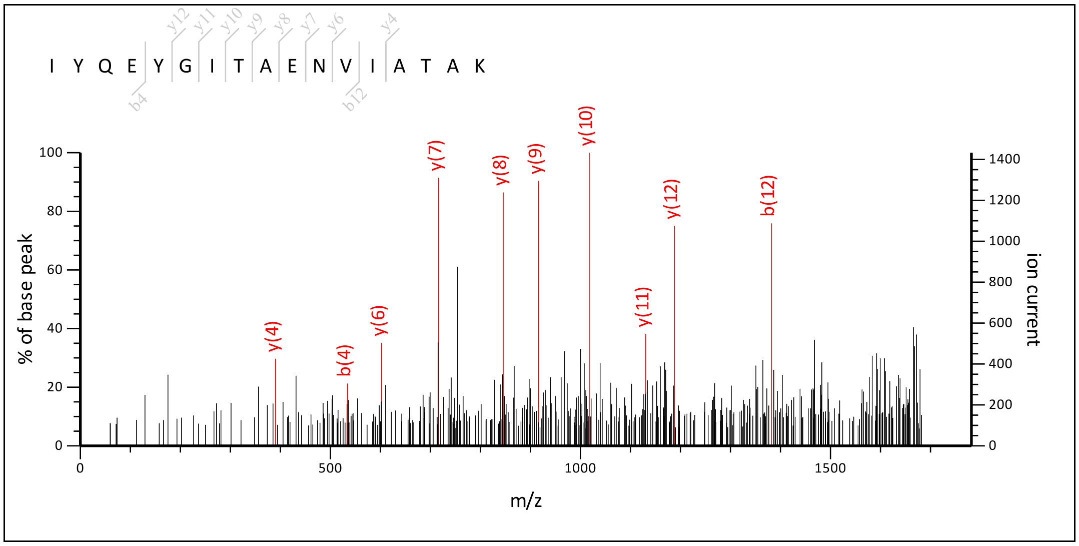


0 to 1781.86

Label all possible matches ○ Label matches used for scoring ◉

**Monoisotopic mass of neutral peptide Mr(calc):** 1882.9676
**Fixed modifications:** Carbamidomethyl (C) (apply to specified residues or termini only)
**Ions Score:** 52 **Expect:** 0.0095
**Matches :** 10/318 fragment ions using 17 most intense peaks (help)

| # | Immon. | a | a* | a⁰ | b | b* | b⁰ | d | d' | Seq. | v | w | w' | y | y* |
|---|---|---|---|---|---|---|---|---|---|---|---|---|---|---|---|
| 1 | 86.0964 | 86.0964 | | | 114.0913 | | | 44.0495 | | I | | | | | |
| 2 | 136.0757 | 249.1598 | | | 277.1547 | | | | | Y | 1662.8333 | | | 1770.8909 | 1753.8643 |
| 3 | 101.0709 | 377.2183 | 360.1918 | | 405.2132 | 388.1867 | | 320.1969 | | Q | 1534.7748 | 1533.7795 | | 1607.8275 | 1590.8010 |
| 4 | 102.0550 | 506.2609 | 489.2344 | 488.2504 | **534.2558** | 517.2293 | 516.2453 | 448.2554 | | E | 1405.7322 | 1404.7369 | | 1479.7690 | 1462.7424 |
| 5 | 136.0757 | 669.3243 | 652.2977 | 651.3137 | 697.3192 | 680.2926 | 679.3086 | | | Y | 1242.6688 | | | 1350.7264 | 1333.6998 |
| 6 | 30.0338 | 726.3457 | 709.3192 | 708.3352 | 754.3406 | 737.3141 | 736.3301 | | | G | | | | ***1187.6630*** | 1170.6365 |
| 7 | 86.0964 | 839.4298 | 822.4032 | 821.4192 | 867.4247 | 850.3981 | 849.4141 | 811.3985 | 825.4141 | I | 1072.5633 | 1085.5837 | 1099.5994 | ***1130.6416*** | 1113.6150 |
| 8 | 74.0600 | 940.4775 | 923.4509 | 922.4669 | 968.4724 | 951.4458 | 950.4618 | 924.4825 | 926.4618 | T | 971.5156 | 984.5360 | 986.5153 | ***1017.5575*** | 1000.5310 |
| 9 | 44.0495 | 1011.5146 | 994.4880 | 993.5040 | 1039.5095 | 1022.4829 | 1021.4989 | | | A | 900.4785 | | | ***916.5098*** | 899.4833 |
| 10 | 102.0550 | 1140.5572 | 1123.5306 | 1122.5466 | 1168.5521 | 1151.5255 | 1150.5415 | 1082.5517 | | E | 771.4359 | 770.4407 | | ***845.4727*** | 828.4462 |
| 11 | 87.0553 | 1254.6001 | 1237.5735 | 1236.5895 | 1282.5950 | 1265.5685 | 1264.5844 | 1211.5943 | | N | 657.3930 | 656.3978 | | ***716.4301*** | 699.4036 |
| 12 | 72.0808 | 1353.6685 | 1336.6420 | 1335.6579 | **1381.6634** | 1364.6369 | 1363.6529 | 1339.6529 | | V | 558.3246 | 571.3450 | | ***602.3872*** | 585.3606 |
| 13 | 86.0964 | 1466.7526 | 1449.7260 | 1448.7420 | 1494.7475 | 1477.7209 | 1476.7369 | 1438.7213 | 1452.7369 | I | 445.2405 | 458.2609 | 472.2766 | 503.3188 | 486.2922 |
| 14 | 44.0495 | 1537.7897 | 1520.7631 | 1519.7791 | 1565.7846 | 1548.7581 | 1547.7740 | | | A | 374.2034 | | | ***390.2347*** | 373.2082 |
| 15 | 74.0600 | 1638.8374 | 1621.8108 | 1620.8268 | 1666.8323 | 1649.8057 | 1648.8217 | 1622.8425 | 1624.8217 | T | 273.1557 | 286.1761 | 288.1554 | 319.1976 | 302.1710 |
| 16 | 44.0495 | 1709.8745 | 1692.8479 | 1691.8639 | 1737.8694 | 1720.8428 | 1719.8588 | | | A | 202.1186 | | | 218.1499 | 201.1234 |
| 17 | 101.1073 | | | | | | | | | K | 74.0237 | 73.0284 | | 147.1128 | 130.0863 |

| Seq | ya | yb | Seq | ya | yb | Seq | ya | yb |
|---|---|---|---|---|---|---|---|---|
| YQ | 264.1343 | 292.1292 | YQE | 393.1769 | 421.1718 | YQEY | 556.2402 | 584.2351 |
| YQEYG | 613.2617 | 641.2566 | QE | 230.1135 | 258.1084 | QEY | 393.1769 | 421.1718 |
| QEYG | 450.1983 | 478.1932 | QEYGI | 563.2824 | 591.2773 | QEYGIT | 664.3301 | 692.3250 |
| EY | 265.1183 | 293.1132 | EYG | 322.1397 | 350.1347 | EYGI | 435.2238 | 463.2187 |

| | | | | | | | | |
|---|---|---|---|---|---|---|---|---|
| **EYGIT** | 536.2715 | 564.2664 | **EYGITA** | 607.3086 | 635.3035 | **YG** | 193.0972 | 221.0921 |
| **YGI** | 306.1812 | 334.1761 | **YGIT** | 407.2289 | 435.2238 | **YGITA** | 478.2660 | 506.2609 |
| **YGITAE** | 607.3086 | 635.3035 | **GI** | 143.1179 | 171.1128 | **GIT** | 244.1656 | 272.1605 |
| **GITA** | 315.2027 | 343.1976 | **GITAE** | 444.2453 | 472.2402 | **GITAEN** | 558.2882 | 586.2831 |
| **GITAENV** | 657.3566 | 685.3515 | **IT** | 187.1441 | 215.1390 | **ITA** | 258.1812 | 286.1761 |
| **ITAE** | 387.2238 | 415.2187 | **ITAEN** | 501.2667 | 529.2617 | **ITAENV** | 600.3352 | 628.3301 |
| **TA** | 145.0972 | 173.0921 | **TAE** | 274.1397 | 302.1347 | **TAEN** | 388.1827 | 416.1776 |
| **TAENV** | 487.2511 | 515.2460 | **TAENVI** | 600.3352 | 628.3301 | **TAENVIA** | 671.3723 | 699.3672 |
| **AE** | 173.0921 | 201.0870 | **AEN** | 287.1350 | 315.1299 | **AENV** | 386.2034 | 414.1983 |
| **AENVI** | 499.2875 | 527.2824 | **AENVIA** | 570.3246 | 598.3195 | **AENVIAT** | 671.3723 | 699.3672 |
| **EN** | 216.0979 | 244.0928 | **ENV** | 315.1663 | 343.1612 | **ENVI** | 428.2504 | 456.2453 |
| **ENVIA** | 499.2875 | 527.2824 | **ENVIAT** | 600.3352 | 628.3301 | **ENVIATA** | 671.3723 | 699.3672 |
| **NV** | 186.1237 | 214.1186 | **NVI** | 299.2078 | 327.2027 | **NVIA** | 370.2449 | 398.2398 |
| **NVIAT** | 471.2926 | 499.2875 | **NVIATA** | 542.3297 | 570.3246 | **VI** | 185.1648 | 213.1598 |
| **VIA** | 256.2020 | 284.1969 | **VIAT** | 357.2496 | 385.2445 | **VIATA** | 428.2867 | 456.2817 |
| **IA** | 157.1335 | 185.1285 | **IAT** | 258.1812 | 286.1761 | **IATA** | 329.2183 | 357.2132 |
| **AT** | 145.0972 | 173.0921 | **ATA** | 216.1343 | 244.1292 | **TA** | 145.0972 | 173.0921 |

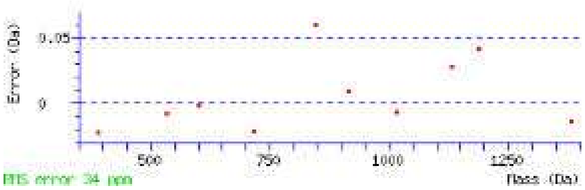


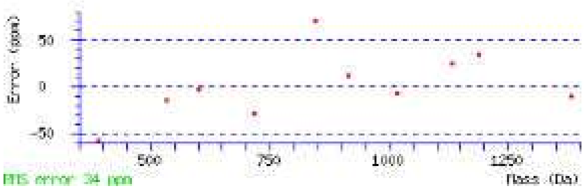


NCBI **BLAST** search of IYQEYGITAENVIATAK
(Parameters: blastp, nr protein database, expect=20000, no filter, PAM30)
Other BLAST web gateways

**All matches to this query**

| Score | Mr(calc) | Delta | Sequence |
|---|---|---|---|
| 52.2 | 1882.9676 | 0.0429 | IYQEYGITAENVIATAK |
| 15.5 | 1883.0815 | -0.0710 | LPLPLYGLAQIMVRQR |
| 12.6 | 1882.8657 | 0.1448 | TTAENGDPSATSTNKFSR |
| 9.3 | 1882.8843 | 0.1262 | RMDSSTNEPLFTNVTR |
| 8.1 | 1882.9206 | 0.0899 | KEAPAAPAMTAAEAAAAEGR |
| 7.6 | 1882.8666 | 0.1440 | NGECGDGLRYLVEMVR |
| 7.6 | 1882.9312 | 0.0793 | NVYGPVTAAKTIYEDDK |
| 6.5 | 1883.0000 | 0.0105 | QGTLDPALATAEAGQTVIK |
| 6.3 | 1882.9975 | 0.0130 | ALTFSFSLAAVVVMGTNR |
| 6.1 | 1882.9458 | 0.0647 | MFVKEAQSATIASSIER |

**Mascot:** http://www.matrixscience.com/

# MATRIX SCIENCE Mascot Search Results

## Peptide View **Spot no 112**

MS/MS Fragmentation of **EVEATRENLGWPYEPFFVPEDVK**
Found in **gi|28190676** in **NCBInr**, putative transketolase [Oryza sativa Japonica Group]

Match to Query 174: 2750.426324 from(2751.433600,1+) intensity(0.0000) index(34)
Title: Label: E1, Spot_Id: 219678, Peak_List_Id: 224930, MSMS Job_Run_Id: 21757, Comment:
Data file C:\Documents and Settings\Administrator\Desktop\farah BHU\spot set plate\Farah-Garima 11May 2013\ppw_E1_136842029000.txt

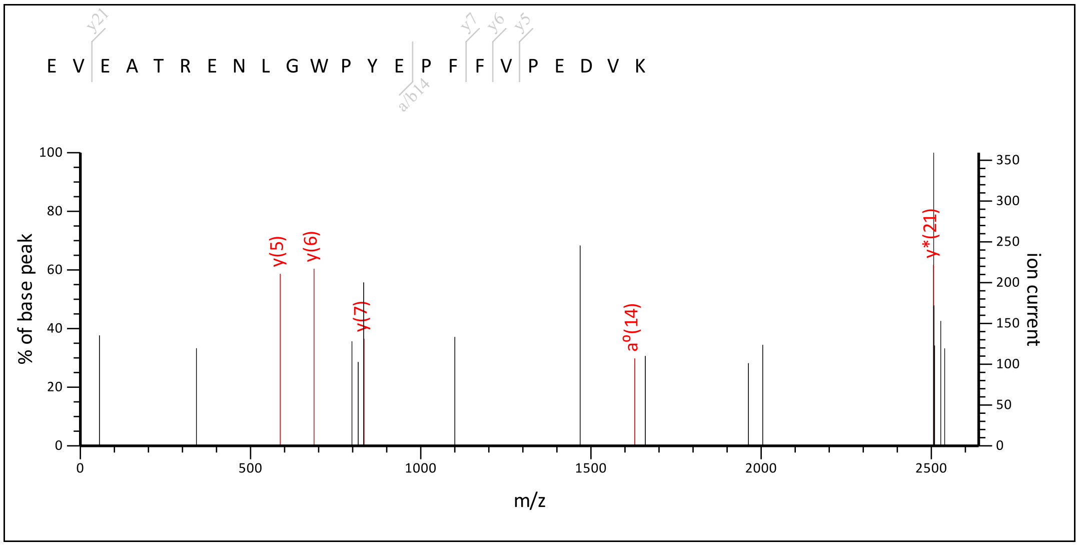


0 to 2639.17

Label all possible matches ○ Label matches used for scoring ◉

**Monoisotopic mass of neutral peptide Mr(calc):** 2750.3228
**Fixed modifications:** Carbamidomethyl (C) (apply to specified residues or termini only)
**Ions Score:** 3 **Expect:** 5.4e+02
**Matches :** 9/425 fragment ions using 14 most intense peaks (help)

| # | Immon. | a | a* | $a^0$ | b | b* | $b^0$ | d | d' | Seq. | v | w | w' | y | y* | |
|---|---|---|---|---|---|---|---|---|---|---|---|---|---|---|---|---|
| 1 | 102.0550 | 102.0550 | | 84.0444 | 130.0499 | | 112.0393 | 44.0495 | | E | | | | | | |
| 2 | 72.0808 | 201.1234 | | 183.1128 | 229.1183 | | 211.1077 | 187.1077 | | V | 2578.2249 | 2591.2453 | | 2622.2875 | 2605.2609 | 26 |
| 3 | 102.0550 | 330.1660 | | 312.1554 | 358.1609 | | 340.1503 | 272.1605 | | E | 2449.1823 | 2448.1870 | | 2523.2191 | 2506.1925 | 25 |
| 4 | 44.0495 | 401.2031 | | 383.1925 | 429.1980 | | 411.1874 | | | A | 2378.1452 | | | 2394.1765 | 2377.1499 | 23 |
| 5 | 74.0600 | 502.2508 | | 484.2402 | 530.2457 | | 512.2351 | 486.2558 | 488.2351 | T | 2277.0975 | 2290.1179 | 2292.0972 | 2323.1394 | 2306.1128 | 23 |
| 6 | 129.1135 | 658.3519 | 641.3253 | 640.3413 | ***686.3468*** | 669.3202 | 668.3362 | 573.2879 | | R | 2120.9964 | 2120.0011 | | 2222.0917 | 2205.0651 | 22 |
| 7 | 102.0550 | 787.3945 | 770.3679 | 769.3839 | 815.3894 | 798.3628 | 797.3788 | 729.3890 | | E | 1991.9538 | 1990.9585 | | 2065.9906 | 2048.9640 | 20 |
| 8 | 87.0553 | 901.4374 | 884.4108 | 883.4268 | 929.4323 | 912.4058 | 911.4217 | 858.4316 | | N | 1877.9109 | 1876.9156 | | 1936.9480 | 1919.9214 | 19 |
| 9 | 86.0964 | 1014.5214 | 997.4949 | 996.5109 | 1042.5164 | 1025.4898 | 1024.5058 | 972.4745 | | L | 1764.8268 | 1763.8316 | | 1822.9051 | 1805.8785 | 18 |
| 10 | 30.0338 | 1071.5429 | 1054.5164 | 1053.5323 | 1099.5378 | 1082.5113 | 1081.5273 | | | G | | | | 1709.8210 | 1692.7944 | 16 |
| 11 | 159.0917 | 1257.6222 | 1240.5957 | 1239.6117 | 1285.6171 | 1268.5906 | 1267.6066 | | | W | 1521.7260 | | | 1652.7995 | 1635.7730 | 16 |
| 12 | 70.0651 | 1354.6750 | 1337.6484 | 1336.6644 | 1382.6699 | 1365.6434 | 1364.6593 | 1328.6593 | | P | 1424.6733 | 1423.6780 | | 1466.7202 | 1449.6937 | 14 |
| 13 | 136.0757 | 1517.7383 | 1500.7118 | 1499.7278 | 1545.7332 | 1528.7067 | 1527.7227 | | | Y | 1261.6099 | | | 1369.6674 | 1352.6409 | 13 |
| 14 | 102.0550 | 1646.7809 | 1629.7544 | 1628.7703 | 1674.7758 | 1657.7493 | 1656.7653 | 1588.7754 | | E | 1132.5673 | 1131.5721 | | 1206.6041 | 1189.5776 | 11 |
| 15 | 70.0651 | 1743.8337 | 1726.8071 | 1725.8231 | 1771.8286 | 1754.8020 | 1753.8180 | 1717.8180 | | P | 1035.5146 | 1034.5193 | | 1077.5615 | 1060.5350 | 10 |
| 16 | 120.0808 | 1890.9021 | 1873.8755 | 1872.8915 | 1918.8970 | 1901.8705 | 1900.8864 | | | F | 888.4462 | | | 980.5088 | 963.4822 | 9 |
| 17 | 120.0808 | 2037.9705 | 2020.9440 | 2019.9599 | 2065.9654 | 2048.9389 | 2047.9549 | | | F | 741.3777 | | | ***833.4403*** | 816.4138 | 8 |
| 18 | 72.0808 | 2137.0389 | 2120.0124 | 2119.0284 | 2165.0338 | 2148.0073 | 2147.0233 | 2123.0233 | | V | 642.3093 | 655.3297 | | ***686.3719*** | 669.3454 | 6 |
| 19 | 70.0651 | 2234.0917 | 2217.0651 | 2216.0811 | 2262.0866 | 2245.0600 | 2244.0760 | 2208.0760 | | P | 545.2566 | 544.2613 | | ***587.3035*** | 570.2770 | 5 |
| 20 | 102.0550 | 2363.1343 | 2346.1077 | 2345.1237 | 2391.1292 | 2374.1026 | 2373.1186 | 2305.1288 | | E | 416.2140 | 415.2187 | | 490.2508 | 473.2242 | 4 |
| 21 | 88.0393 | 2478.1612 | 2461.1347 | 2460.1507 | ***2506.1561*** | 2489.1296 | 2488.1456 | 2434.1714 | | D | 301.1870 | 300.1918 | | 361.2082 | 344.1816 | 3 |
| 22 | 72.0808 | 2577.2296 | 2560.2031 | 2559.2191 | 2605.2245 | 2588.1980 | 2587.2140 | 2563.2140 | | V | 202.1186 | 215.1390 | | 246.1812 | 229.1547 | |
| 23 | 101.1073 | | | | | | | | | K | 74.0237 | 73.0284 | | 147.1128 | 130.0863 | |

| Seq | ya | yb | Seq | ya | yb | Seq | ya | yb |
|---|---|---|---|---|---|---|---|---|
| VE | 201.1234 | 229.1183 | VEA | 272.1605 | 300.1554 | VEAT | 373.2082 | 401.2031 |
| VEATR | 529.3093 | 557.3042 | VEATRE | 658.3519 | 686.3468 | EA | 173.0921 | 201.0870 |
| EAT | 274.1397 | 302.1347 | EATR | 430.2409 | 458.2358 | EATRE | 559.2835 | 587.2784 |
| EATREN | 673.3264 | 701.3213 | AT | 145.0972 | 173.0921 | ATR | 301.1983 | 329.1932 |
| ATRE | 430.2409 | 458.2358 | ATREN | 544.2838 | 572.2787 | ATRENL | 657.3678 | 685.3628 |
| TR | 230.1612 | 258.1561 | TRE | 359.2037 | 387.1987 | TREN | 473.2467 | 501.2416 |
| TRENL | 586.3307 | 614.3256 | TRENLG | 643.3522 | 671.3471 | RE | 258.1561 | 286.1510 |
| REN | 372.1990 | 400.1939 | RENL | 485.2831 | 513.2780 | RENLG | 542.3045 | 570.2994 |
| EN | 216.0979 | 244.0928 | ENL | 329.1819 | 357.1769 | ENLG | 386.2034 | 414.1983 |
| ENLGW | 572.2827 | 600.2776 | ENLGWP | 669.3355 | 697.3304 | NL | 200.1394 | 228.1343 |
| NLG | 257.1608 | 285.1557 | NLGW | 443.2401 | 471.2350 | NLGWP | 540.2929 | 568.2878 |
| LG | 143.1179 | 171.1128 | LGW | 329.1972 | 357.1921 | LGWP | 426.2500 | 454.2449 |
| LGWPY | 589.3133 | 617.3082 | GW | 216.1131 | 244.1081 | GWP | 313.1659 | 341.1608 |
| GWPY | 476.2292 | 504.2241 | GWPYE | 605.2718 | 633.2667 | WP | 256.1444 | 284.1394 |
| WPY | 419.2078 | 447.2027 | WPYE | 548.2504 | 576.2453 | WPYEP | 645.3031 | 673.2980 |
| PY | 233.1285 | 261.1234 | PYE | 362.1710 | 390.1660 | PYEP | 459.2238 | 487.2187 |
| PYEPF | 606.2922 | 634.2871 | YE | 265.1183 | 293.1132 | YEP | 362.1710 | 390.1660 |
| YEPF | 509.2395 | 537.2344 | YEPFF | 656.3079 | 684.3028 | EP | 199.1077 | 227.1026 |
| EPF | 346.1761 | 374.1710 | EPFF | 493.2445 | 521.2395 | EPFFV | 592.3130 | 620.3079 |
| EPFFVP | 689.3657 | 717.3606 | PF | 217.1335 | 245.1285 | PFF | 364.2020 | 392.1969 |
| PFFV | 463.2704 | 491.2653 | PFFVP | 560.3231 | 588.3180 | PFFVPE | 689.3657 | 717.3606 |
| FF | 267.1492 | 295.1441 | FFV | 366.2176 | 394.2125 | FFVP | 463.2704 | 491.2653 |
| FFVPE | 592.3130 | 620.3079 | FV | 219.1492 | 247.1441 | FVP | 316.2020 | 344.1969 |
| FVPE | 445.2445 | 473.2395 | FVPED | 560.2715 | 588.2664 | FVPEDV | 659.3399 | 687.3348 |
| VP | 169.1335 | 197.1285 | VPE | 298.1761 | 326.1710 | VPED | 413.2031 | 441.1980 |
| VPEDV | 512.2715 | 540.2664 | PE | 199.1077 | 227.1026 | PED | 314.1347 | 342.1296 |
| PEDV | 413.2031 | 441.1980 | ED | 217.0819 | 245.0768 | EDV | 316.1503 | 344.1452 |
| DV | 187.1077 | 215.1026 | | | | | | |

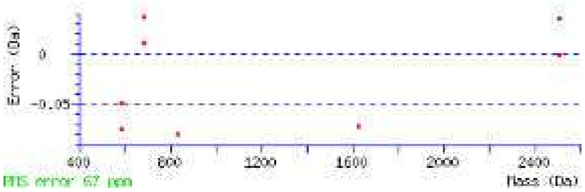


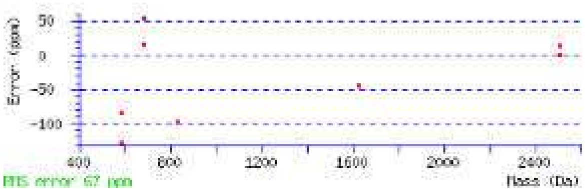


NCBI **BLAST** search of EVEATRENLGWPYEPFFVPEDVK
(Parameters: blastp, nr protein database, expect=20000, no filter, PAM30)
Other BLAST web gateways

**All matches to this query**

| Score | Mr(calc) | Delta | Sequence |
|---|---|---|---|
| 2.8 | 2750.3228 | 0.1035 | EVEATRENLGWPYEPFFVPEDVK |
| 2.2 | 2750.3421 | 0.0842 | FSGTFAGRRPCYFNTGVMVLDLAR |
| 0.8 | 2750.3746 | 0.0517 | MAAWWCFALLLVLCAPAGADVSKGK |
| 0.8 | 2750.4136 | 0.0128 | TDIVASSTGFGPPVMPIFAKMLVNSR |
| 0.8 | 2750.2543 | 0.1721 | VECILMGGTFMSLPADYHDYFIR |
| 0.3 | 2750.3320 | 0.0943 | DGDGIEVFLALDESSMLTLQQNLDK |
| 0.3 | 2750.4643 | -0.0380 | ELEKVLGGKPYLGNDTFGFVDIALR |
| 0.3 | 2750.4778 | -0.0515 | GVIGLFVFFFFVGVAFDKLWTWR |
| 0.3 | 2750.5862 | -0.1599 | LIFAAVVFMGLAFLAFWLIVRPTR |
| 0.3 | 2750.6114 | -0.1851 | LIFAAVVFMGLAFLVFWLIVRPTK |

**Mascot:** http://www.matrixscience.com/

# MATRIX SCIENCE Mascot Search Results

## Peptide View **Spot no 113**

MS/MS Fragmentation of **NPYWFNR**
Found in **gi|28190676** in **NCBInr**, putative transketolase [Oryza sativa Japonica Group]

Match to Query 13: 995.495594 from(996.502870,1+) intensity(0.0000) index(4)
Title: Label: D1, Spot_Id: 219677, Peak_List_Id: 224867, MSMS Job_Run_Id: 21756, Comment:
Data file C:\Documents and Settings\Administrator\Desktop\farah BHU\spot set plate\Farah-Garima 11May 2013\ppw_D1_136842020900.txt

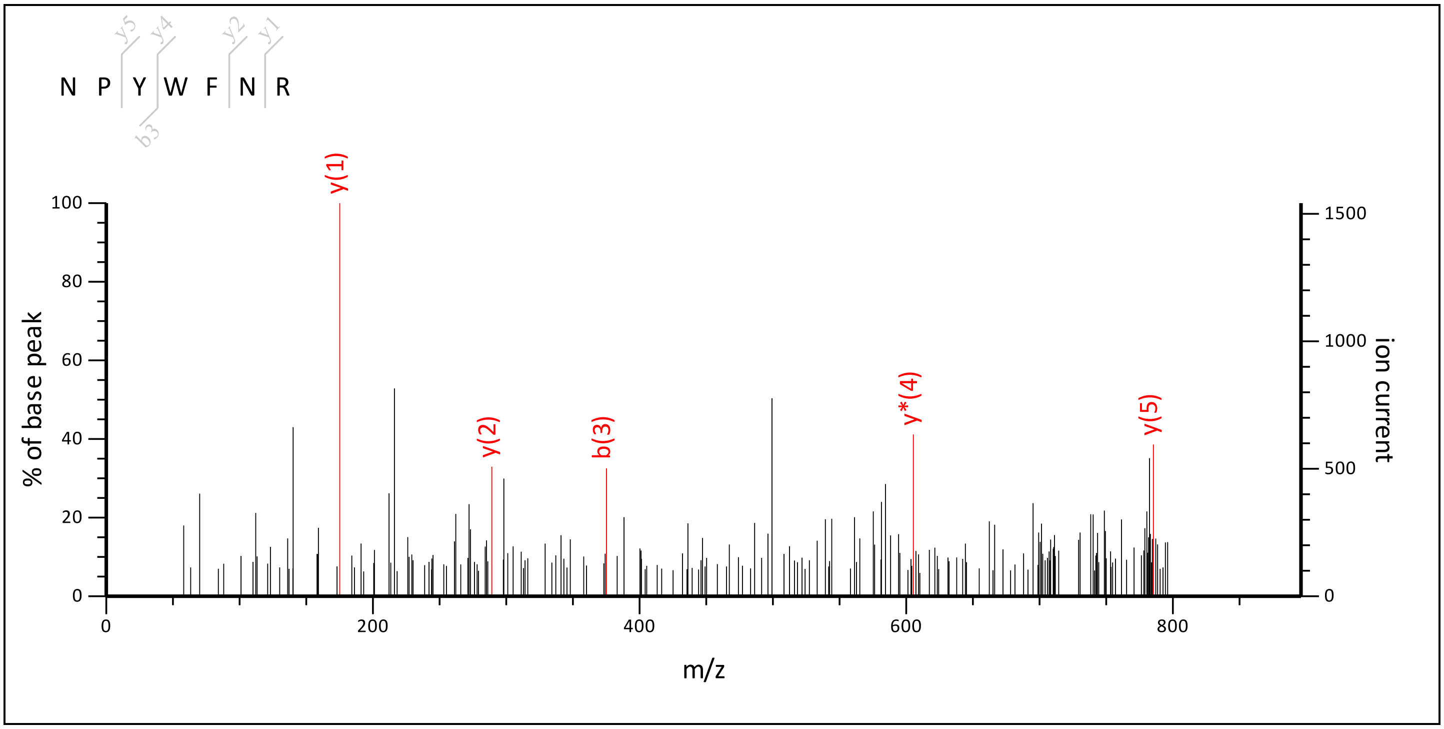


0 to 896.09

Label all possible matches ○ Label matches used for scoring ◉

**Monoisotopic mass of neutral peptide Mr(calc):** 995.4613
**Fixed modifications:** Carbamidomethyl (C) (apply to specified residues or termini only)
**Ions Score:** 13 **Expect:** 94
**Matches :** 5/74 fragment ions using 8 most intense peaks (help)

| # | Immon. | a | a* | b | b* | d | Seq. | v | w | y | y* | # |
|---|---|---|---|---|---|---|---|---|---|---|---|---|
| 1 | 87.0553 | 87.0553 | 70.0287 | 115.0502 | 98.0237 | 44.0495 | N | | | | | 7 |
| 2 | 70.0651 | 184.1081 | 167.0815 | 212.1030 | 195.0764 | 158.0924 | P | 840.3787 | 839.3835 | 882.4257 | 865.3992 | 6 |
| 3 | 136.0757 | 347.1714 | 330.1448 | **375.1663** | 358.1397 | | Y | 677.3154 | | ***785.3729*** | 768.3464 | 5 |
| 4 | 159.0917 | 533.2507 | 516.2241 | 561.2456 | 544.2191 | | W | 491.2361 | | 622.3096 | 605.2831 | 4 |
| 5 | 120.0808 | 680.3191 | 663.2926 | 708.3140 | 691.2875 | | F | 344.1677 | | 436.2303 | 419.2037 | 3 |
| 6 | 87.0553 | 794.3620 | 777.3355 | 822.3569 | 805.3304 | 751.3562 | N | 230.1248 | 229.1295 | ***289.1619*** | 272.1353 | 2 |
| 7 | 129.1135 | | | | | | R | 74.0237 | 73.0284 | ***175.1190*** | 158.0924 | 1 |

| Seq | ya | yb | Seq | ya | yb | Seq | ya | yb |
|---|---|---|---|---|---|---|---|---|
| PY | 233.1285 | 261.1234 | PYW | 419.2078 | 447.2027 | PYWF | 566.2762 | 594.2711 |
| PYWFN | 680.3191 | 708.3140 | YW | 322.1550 | 350.1499 | YWF | 469.2234 | 497.2183 |
| YWFN | 583.2663 | 611.2613 | WF | 306.1601 | 334.1550 | WFN | 420.2030 | 448.1979 |
| FN | 234.1237 | 262.1186 | | | | | | |

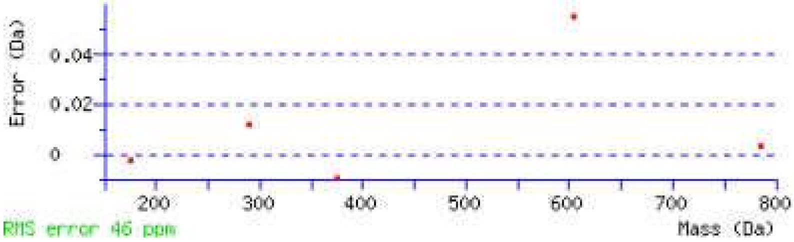


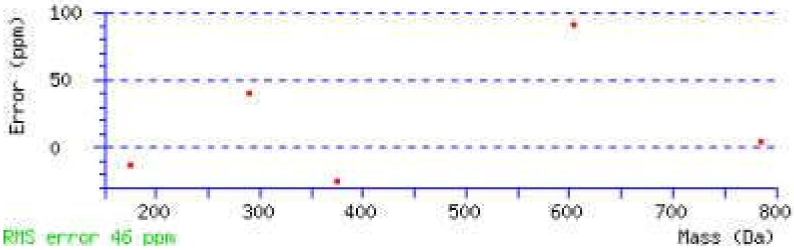


NCBI **BLAST** search of NPYWFNR
(Parameters: blastp, nr protein database, expect=20000, no filter, PAM30)
Other BLAST web gateways

**All matches to this query**

| Score | Mr(calc) | Delta | Sequence |
|---|---|---|---|
| 17.6 | 995.5625 | -0.0669 | TITHRVNR |
| 15.2 | 995.5148 | -0.0192 | TLSRSYNR |
| 13.1 | 995.4613 | 0.0343 | NPYWFNR |
| 12.0 | 995.5036 | -0.0080 | QSTFSATVR |
| 11.9 | 995.4131 | 0.0825 | CSSEPFNR |
| 11.3 | 995.4825 | 0.0131 | VGSGYPFNR |
| 11.1 | 995.4971 | -0.0015 | MNLGGVHPR |
| 11.0 | 995.4091 | 0.0865 | GTGGSGTGSCR |
| 10.9 | 995.4495 | 0.0461 | GEIFNTCR |
| 10.7 | 995.5036 | -0.0080 | TISSAFTNR |

**Mascot:** http://www.matrixscience.com/

# MATRIX SCIENCE Mascot Search Results

## Peptide View Spot no 113

MS/MS Fragmentation of **YTPESPADATR**
Found in **gi|28190676** in **NCBInr**, putative transketolase [Oryza sativa Japonica Group]

Match to Query 45: 1206.583424 from(1207.590700,1+) intensity(0.0000) index(13)
Title: Label: D1, Spot_Id: 219677, Peak_List_Id: 224868, MSMS Job_Run_Id: 21756, Comment:
Data file C:\Documents and Settings\Administrator\Desktop\farah BHU\spot set plate\Farah-Garima 11May 2013\ppw_D1_136842020900.txt

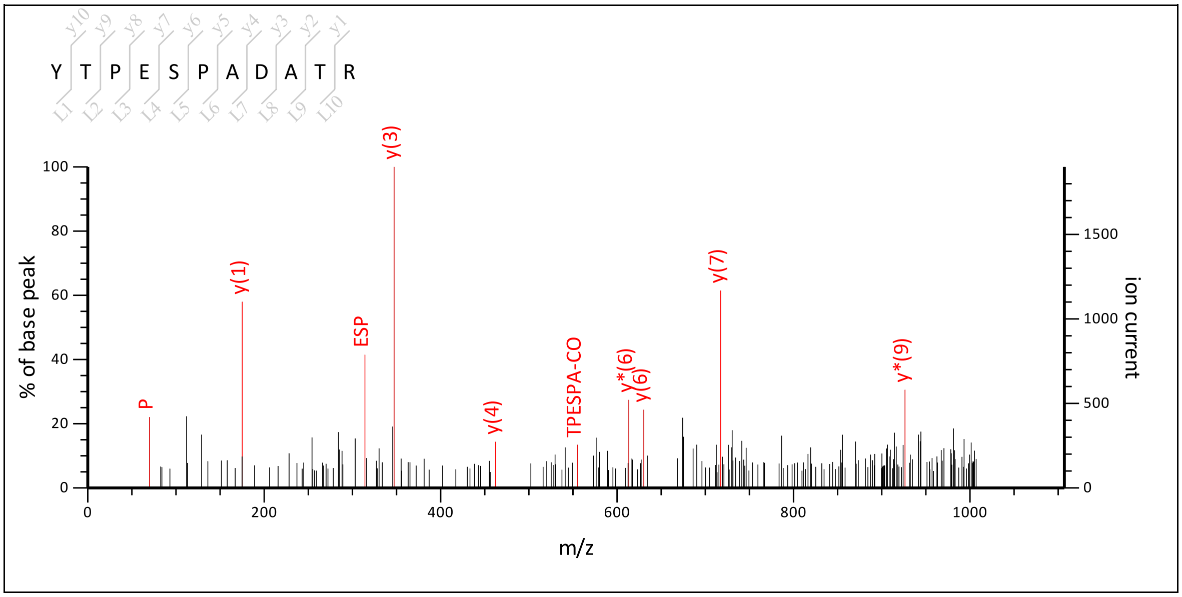


0 to 1107.02

Label all possible matches ○ Label matches used for scoring ◉

**Monoisotopic mass of neutral peptide Mr(calc):** 1206.5517
**Fixed modifications:** Carbamidomethyl (C) (apply to specified residues or termini only)
**Ions Score:** 24 **Expect:** 9
**Matches :** 12/174 fragment ions using 20 most intense peaks (help)

| # | Immon. | a | $a^0$ | b | $b^0$ | d | d' | Seq. | v | w | w' | y | y* | $y^0$ | # |
|---|---|---|---|---|---|---|---|---|---|---|---|---|---|---|---|
| 1 | 136.0757 | 136.0757 | | 164.0706 | | 44.0495 | | Y | | | | | | | 11 |
| 2 | 74.0600 | 237.1234 | 219.1128 | 265.1183 | 247.1077 | 221.1285 | 223.1077 | T | 998.4538 | 1011.4742 | 1013.4534 | 1044.4956 | 1027.4691 | 1026.4851 | 10 |
| 3 | 70.0651 | 334.1761 | 316.1656 | 362.1710 | 344.1605 | 308.1605 | | P | 901.4010 | 900.4058 | | 943.4480 | 926.4214 | 925.4374 | 9 |
| 4 | 102.0550 | 463.2187 | 445.2082 | 491.2136 | 473.2031 | 405.2132 | | E | 772.3584 | 771.3632 | | 846.3952 | 829.3686 | 828.3846 | 8 |
| 5 | 60.0444 | 550.2508 | 532.2402 | 578.2457 | 560.2351 | 534.2558 | | S | 685.3264 | 684.3311 | | ***717.3526*** | 700.3260 | 699.3420 | 7 |
| 6 | 70.0651 | 647.3035 | 629.2930 | 675.2984 | 657.2879 | 621.2879 | | P | 588.2736 | 587.2784 | | ***630.3206*** | 613.2940 | 612.3100 | 6 |
| 7 | 44.0495 | 718.3406 | 700.3301 | 746.3355 | 728.3250 | | | A | 517.2365 | | | 533.2678 | 516.2413 | 515.2572 | 5 |
| 8 | 88.0393 | 833.3676 | 815.3570 | 861.3625 | 843.3519 | 789.3777 | | D | 402.2096 | 401.2143 | | ***462.2307*** | 445.2041 | 444.2201 | 4 |
| 9 | 44.0495 | 904.4047 | 886.3941 | 932.3996 | 914.3890 | | | A | 331.1724 | | | ***347.2037*** | 330.1772 | 329.1932 | 3 |
| 10 | 74.0600 | 1005.4524 | 987.4418 | 1033.4473 | 1015.4367 | 989.4575 | 991.4367 | T | 230.1248 | 243.1452 | 245.1244 | 276.1666 | 259.1401 | 258.1561 | 2 |
| 11 | 129.1135 | | | | | | | R | 74.0237 | 73.0284 | | ***175.1190*** | 158.0924 | | 1 |

| Seq | ya | yb | Seq | ya | yb | Seq | ya | yb |
|---|---|---|---|---|---|---|---|---|
| **TP** | 171.1128 | 199.1077 | **TPE** | 300.1554 | 328.1503 | **TPES** | 387.1874 | 415.1823 |
| **TPESP** | 484.2402 | 512.2351 | **TPESPA** | 555.2773 | 583.2722 | **TPESPAD** | 670.3042 | 698.2992 |
| **PE** | 199.1077 | 227.1026 | **PES** | 286.1397 | 314.1347 | **PESP** | 383.1925 | 411.1874 |
| **PESPA** | 454.2296 | 482.2245 | **PESPAD** | 569.2566 | 597.2515 | **PESPADA** | 640.2937 | 668.2886 |
| **ES** | 189.0870 | 217.0819 | **ESP** | 286.1397 | 314.1347 | **ESPA** | 357.1769 | 385.1718 |

| | | | | | | | | |
|---|---|---|---|---|---|---|---|---|
| **ESPAD** | 472.2038 | 500.1987 | **ESPADA** | 543.2409 | 571.2358 | **ESPADAT** | 644.2886 | 672.2835 |
| **SP** | 157.0972 | 185.0921 | **SPA** | 228.1343 | 256.1292 | **SPAD** | 343.1612 | 371.1561 |
| **SPADA** | 414.1983 | 442.1932 | **SPADAT** | 515.2460 | 543.2409 | **PA** | 141.1022 | 169.0972 |
| **PAD** | 256.1292 | 284.1241 | **PADA** | 327.1663 | 355.1612 | **PADAT** | 428.2140 | 456.2089 |
| **AD** | 159.0764 | 187.0713 | **ADA** | 230.1135 | 258.1084 | **ADAT** | 331.1612 | 359.1561 |
| **DA** | 159.0764 | 187.0713 | **DAT** | 260.1241 | 288.1190 | **AT** | 145.0972 | 173.0921 |

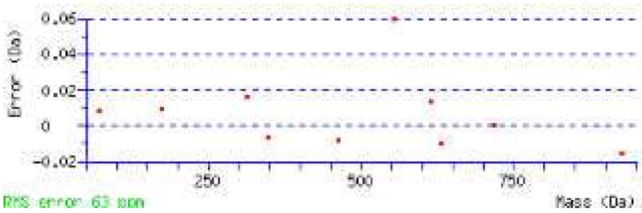

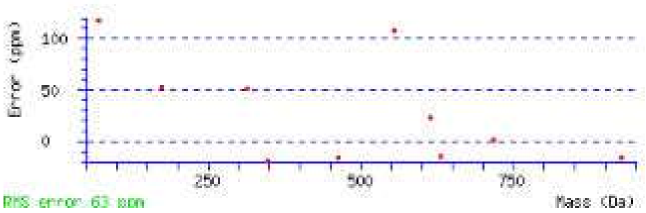


NCBI **BLAST** search of [YTPESPADATR]
(Parameters: blastp, nr protein database, expect=20000, no filter, PAM30)
Other BLAST [web gateways]

**All matches to this query**

| Score | Mr(calc) | Delta | Sequence |
|---|---|---|---|
| 23.7 | 1206.5517 | 0.0318 | YTPESPADATR |
| 13.9 | 1206.5881 | -0.0046 | LLDAVFSDDGR |
| 8.9 | 1206.6218 | -0.0383 | TNHHSVGKTAR |
| 8.0 | 1206.6721 | -0.0886 | QVRASTSLAFK |
| 7.9 | 1206.5153 | 0.0681 | EWDDSIDATR |
| 7.8 | 1206.6357 | -0.0523 | TFEIRTIGDR |
| 7.8 | 1206.6357 | -0.0523 | TFELRTIGDR |
| 7.5 | 1206.6244 | -0.0410 | EFLTQLEATR |
| 7.5 | 1206.5014 | 0.0820 | GHVHDEADGDR |
| 7.5 | 1206.5485 | 0.0349 | GMIQMIGGGGDR |

**Mascot:** http://www.matrixscience.com/

# MATRIX SCIENCE Mascot Search Results

**Peptide View** **Spot no 113**

MS/MS Fragmentation of **AIGIDKFGASAPAGK**
Found in **gi|28190676** in **NCBInr**, putative transketolase [Oryza sativa Japonica Group]

Match to Query 68: 1401.770324 from(1402.777600,1+) intensity(0.0000) index(23)
Title: Label: D1, Spot_Id: 219677, Peak_List_Id: 224877, MSMS Job_Run_Id: 21756, Comment:
Data file C:\Documents and Settings\Administrator\Desktop\farah BHU\spot set plate\Farah-Garima 11May 2013\ppw_D1_136842020900.txt

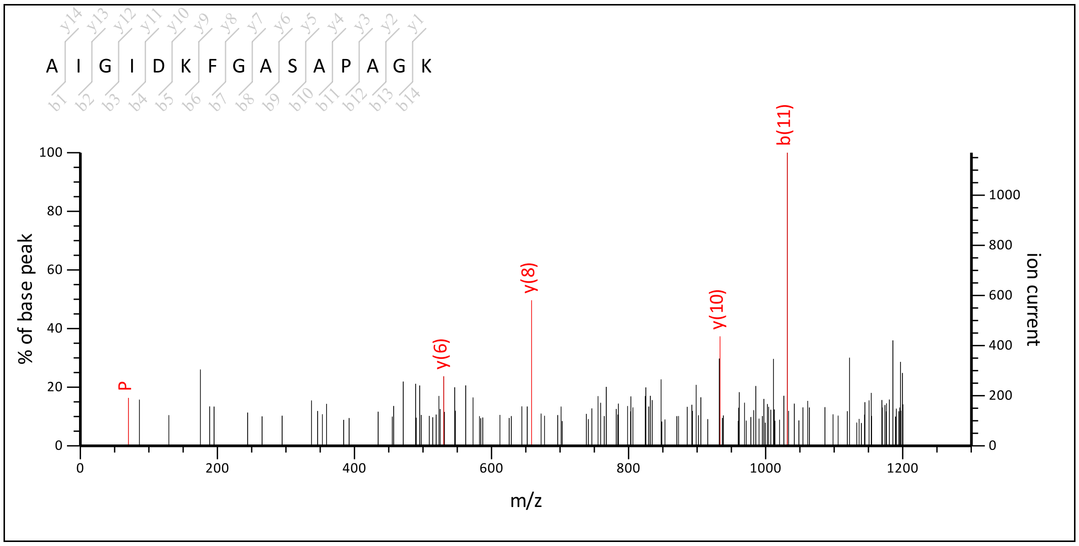


0 to 1300.29

Label all possible matches ○ Label matches used for scoring ◉

**Monoisotopic mass of neutral peptide Mr(calc):** 1401.7616
**Fixed modifications:** Carbamidomethyl (C) (apply to specified residues or termini only)
**Ions Score:** 5 **Expect:** 6e+02
**Matches :** 6/260 fragment ions using 12 most intense peaks (help)

| # | Immon. | a | a* | a0 | b | b* | b0 | d | d' | Seq. | v | w | w' | y | y* | [illegible] |
|---|---|---|---|---|---|---|---|---|---|---|---|---|---|---|---|---|
| 1 | 44.0495 | 44.0495 | | | 72.0444 | | | 44.0495 | | A | | | | | | |
| 2 | 86.0964 | 157.1335 | | | 185.1285 | | | 129.1022 | 143.1179 | I | 1273.6535 | 1286.6739 | 1300.6896 | 1331.7318 | 1314.7052 | [illegible] |
| 3 | 30.0338 | 214.1550 | | | 242.1499 | | | | | G | | | | 1218.6477 | 1201.6212 | [illegible] |
| 4 | 86.0964 | 327.2391 | | | 355.2340 | | | 299.2078 | 313.2234 | I | 1103.5480 | 1116.5684 | 1130.5841 | 1161.6263 | 1144.5997 | [illegible] |
| 5 | 88.0393 | 442.2660 | | 424.2554 | 470.2609 | | 452.2504 | 398.2762 | | D | 988.5211 | 987.5258 | | 1048.5422 | 1031.5156 | [illegible] |
| 6 | 101.1073 | 570.3610 | 553.3344 | 552.3504 | 598.3559 | 581.3293 | 580.3453 | 513.3031 | | K | 860.4261 | 859.4308 | | ***933.5152*** | 916.4887 | [illegible] |
| 7 | 120.0808 | 717.4294 | 700.4028 | 699.4188 | 745.4243 | 728.3978 | 727.4137 | | | F | 713.3577 | | | 805.4203 | 788.3937 | [illegible] |
| 8 | 30.0338 | 774.4509 | 757.4243 | 756.4403 | 802.4458 | 785.4192 | 784.4352 | | | G | | | | ***658.3519*** | 641.3253 | [illegible] |
| 9 | 44.0495 | 845.4880 | 828.4614 | 827.4774 | 873.4829 | 856.4563 | 855.4723 | | | A | 585.2991 | | | 601.3304 | 584.3039 | [illegible] |
| 10 | 60.0444 | 932.5200 | 915.4934 | 914.5094 | 960.5149 | 943.4884 | 942.5043 | 916.5251 | | S | 498.2671 | 497.2718 | | ***530.2933*** | 513.2667 | [illegible] |
| 11 | 44.0495 | 1003.5571 | 986.5306 | 985.5465 | **1031.5520** | 1014.5255 | 1013.5415 | | | A | 427.2300 | | | 443.2613 | 426.2347 | |
| 12 | 70.0651 | 1100.6099 | 1083.5833 | 1082.5993 | 1128.6048 | 1111.5782 | 1110.5942 | 1074.5942 | | P | 330.1772 | 329.1819 | | 372.2241 | 355.1976 | |
| 13 | 44.0495 | 1171.6470 | 1154.6204 | 1153.6364 | 1199.6419 | 1182.6154 | 1181.6313 | | | A | 259.1401 | | | 275.1714 | 258.1448 | |
| 14 | 30.0338 | 1228.6684 | 1211.6419 | 1210.6579 | 1256.6634 | 1239.6368 | 1238.6528 | | | G | | | | 204.1343 | 187.1077 | |
| 15 | 101.1073 | | | | | | | | | K | 74.0237 | 73.0284 | | 147.1128 | 130.0863 | |

| Seq | ya | yb | Seq | ya | yb | Seq | ya | yb |
|---|---|---|---|---|---|---|---|---|
| IG | 143.1179 | 171.1128 | IGI | 256.2020 | 284.1969 | IGID | 371.2289 | 399.2238 |
| IGIDK | 499.3239 | 527.3188 | IGIDKF | 646.3923 | 674.3872 | GI | 143.1179 | 171.1128 |
| GID | 258.1448 | 286.1397 | GIDK | 386.2398 | 414.2347 | GIDKF | 533.3082 | 561.3031 |
| GIDKFG | 590.3297 | 618.3246 | GIDKFGA | 661.3668 | 689.3617 | ID | 201.1234 | 229.1183 |
| IDK | 329.2183 | 357.2132 | IDKF | 476.2867 | 504.2817 | IDKFG | 533.3082 | 561.3031 |
| IDKFGA | 604.3453 | 632.3402 | IDKFGAS | 691.3774 | 719.3723 | DK | 216.1343 | 244.1292 |

| | | | | | | | | |
|---|---|---|---|---|---|---|---|---|
| **DKF** | 363.2027 | 391.1976 | **DKFG** | 420.2241 | 448.2191 | **DKFGA** | 491.2613 | 519.2562 |
| **DKFGAS** | 578.2933 | 606.2882 | **DKFGASA** | 649.3304 | 677.3253 | **KF** | 248.1757 | 276.1707 |
| **KFG** | 305.1972 | 333.1921 | **KFGA** | 376.2343 | 404.2292 | **KFGAS** | 463.2663 | 491.2613 |
| **KFGASA** | 534.3035 | 562.2984 | **KFGASAP** | 631.3562 | 659.3511 | **FG** | 177.1022 | 205.0972 |
| **FGA** | 248.1394 | 276.1343 | **FGAS** | 335.1714 | 363.1663 | **FGASA** | 406.2085 | 434.2034 |
| **FGASAP** | 503.2613 | 531.2562 | **FGASAPA** | 574.2984 | 602.2933 | **FGASAPAG** | 631.3198 | 659.3148 |
| **GA** | 101.0709 | 129.0659 | **GAS** | 188.1030 | 216.0979 | **GASA** | 259.1401 | 287.1350 |
| **GASAP** | 356.1928 | 384.1878 | **GASAPA** | 427.2300 | 455.2249 | **GASAPAG** | 484.2514 | 512.2463 |
| **AS** | 131.0815 | 159.0764 | **ASA** | 202.1186 | 230.1135 | **ASAP** | 299.1714 | 327.1663 |
| **ASAPA** | 370.2085 | 398.2034 | **ASAPAG** | 427.2300 | 455.2249 | **SA** | 131.0815 | 159.0764 |
| **SAP** | 228.1343 | 256.1292 | **SAPA** | 299.1714 | 327.1663 | **SAPAG** | 356.1928 | 384.1878 |
| **AP** | 141.1022 | 169.0972 | **APA** | 212.1394 | 240.1343 | **APAG** | 269.1608 | 297.1557 |
| **PA** | 141.1022 | 169.0972 | **PAG** | 198.1237 | 226.1186 | **AG** | 101.0709 | 129.0659 |

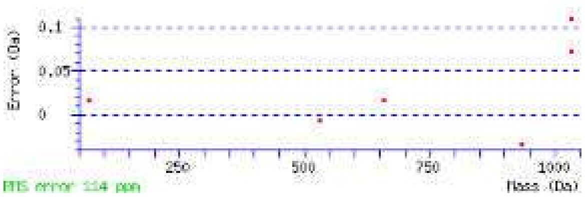


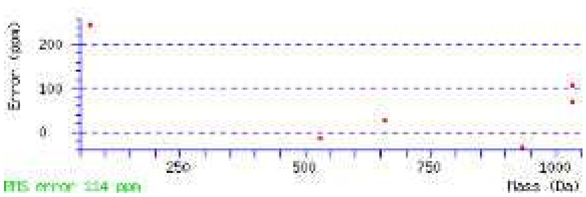


NCBI **BLAST** search of AIGIDKFGASAPAGK
(Parameters: blastp, nr protein database, expect=20000, no filter, PAM30)
Other BLAST web gateways

**All matches to this query**

| Score | Mr(calc) | Delta | Sequence |
|---|---|---|---|
| 11.3 | 1401.7252 | 0.0451 | KGWSASIDSVGPAK |
| 7.2 | 1401.7326 | 0.0377 | MEIPGVTFGVPAGK |
| 6.9 | 1401.7034 | 0.0669 | ILDRAMEVENGR |
| 6.9 | 1401.6671 | 0.1033 | GCGPGANTISIAER |
| 6.2 | 1401.6848 | 0.0855 | AQGDPSSTAAATVAR |
| 5.9 | 1401.6671 | 0.1033 | GCGPASASSVGLSPR |
| 5.8 | 1401.6484 | 0.1219 | GEANVGSGDSSPAVR |
| 5.2 | 1401.7616 | 0.0087 | TPQASAFAVELIR |
| 5.2 | 1401.6381 | 0.1322 | LHGALCDDMGLGK |
| 5.1 | 1401.7616 | 0.0087 | AIGIDKFGASAPAGK |

**Mascot:** http://www.matrixscience.com/

# MATRIX SCIENCE Mascot Search Results

## Peptide View **Spot no 113**

MS/MS Fragmentation of **MFGDFQKDTPEER**
Found in **gi|28190676** in **NCBInr**, putative transketolase [Oryza sativa Japonica Group]

Match to Query 77: 1598.731824 from(1599.739100,1+) intensity(0.0000) index(26)
Title: Label: D1, Spot_Id: 219677, Peak_List_Id: 224863, MSMS Job_Run_Id: 21756, Comment:
Data file C:\Documents and Settings\Administrator\Desktop\farah BHU\spot set plate\Farah-Garima 11May 2013\ppw_D1_136842020900.txt

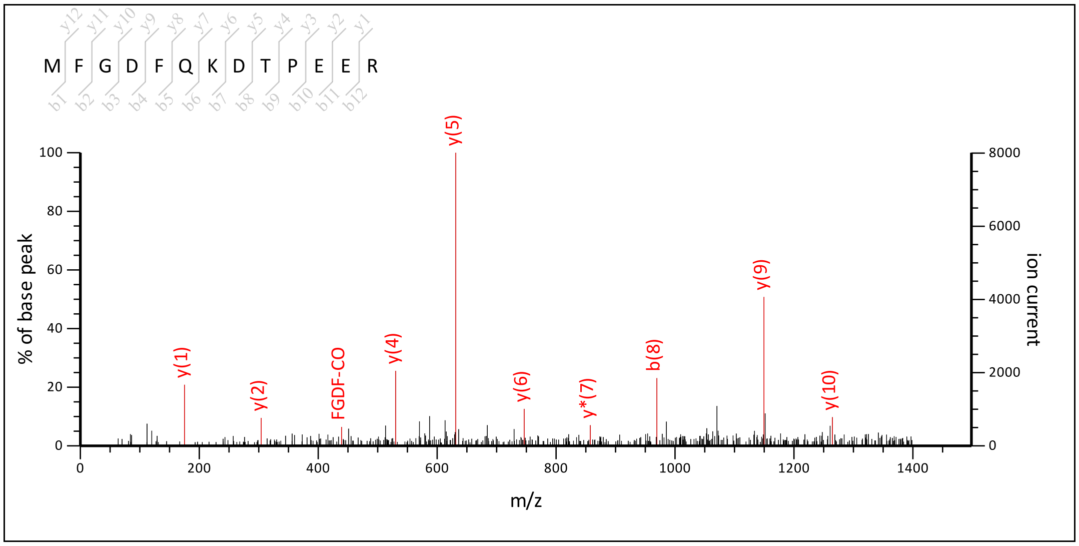


0 to 1498.45

Label all possible matches ○ Label matches used for scoring ◉

**Monoisotopic mass of neutral peptide Mr(calc):** 1598.7035
**Fixed modifications:** Carbamidomethyl (C) (apply to specified residues or termini only)
**Ions Score:** 51 **Expect:** 0.012
**Matches :** 11/210 fragment ions using 14 most intense peaks (help)

| # | Immon. | a | a* | $a^0$ | b | b* | $b^0$ | d | d' | Seq. | v | w | w' | y | y* | |
|---|---|---|---|---|---|---|---|---|---|---|---|---|---|---|---|---|
| 1 | 104.0528 | 104.0528 | | | 132.0478 | | | 44.0495 | | M | | | | | | |
| 2 | 120.0808 | 251.1213 | | | 279.1162 | | | | | F | 1376.6077 | | | 1468.6703 | 1451.6438 | 14[illegible] |
| 3 | 30.0338 | 308.1427 | | | 336.1376 | | | | | G | | | | 1321.6019 | 1304.5753 | 13[illegible] |
| 4 | 88.0393 | 423.1697 | | 405.1591 | 451.1646 | | 433.1540 | 379.1798 | | D | 1204.5593 | 1203.5640 | | ***1264.5804*** | 1247.5539 | 12[illegible] |
| 5 | 120.0808 | 570.2381 | | 552.2275 | 598.2330 | | 580.2224 | | | F | 1057.4909 | | | ***1149.5535*** | 1132.5269 | 11[illegible] |
| 6 | 101.0709 | 698.2967 | 681.2701 | 680.2861 | 726.2916 | 709.2650 | 708.2810 | 641.2752 | | Q | 929.4323 | 928.4371 | | 1002.4851 | 985.4585 | 9[illegible] |
| 7 | 101.1073 | 826.3916 | 809.3651 | 808.3811 | 854.3865 | 837.3600 | 836.3760 | 769.3338 | | K | 801.3373 | 800.3421 | | 874.4265 | 857.3999 | 8[illegible] |
| 8 | 88.0393 | 941.4186 | 924.3920 | 923.4080 | **969.4135** | 952.3869 | 951.4029 | 897.4287 | | D | 686.3104 | 685.3151 | | ***746.3315*** | 729.3050 | 7[illegible] |
| 9 | 74.0600 | 1042.4662 | 1025.4397 | 1024.4557 | 1070.4612 | 1053.4346 | 1052.4506 | 1026.4713 | 1028.4506 | T | 585.2627 | 598.2831 | 600.2624 | ***631.3046*** | 614.2780 | 6[illegible] |
| 10 | 70.0651 | 1139.5190 | 1122.4925 | 1121.5084 | 1167.5139 | 1150.4874 | 1149.5034 | 1113.5034 | | P | 488.2100 | 487.2147 | | ***530.2569*** | 513.2304 | 5[illegible] |
| 11 | 102.0550 | 1268.5616 | 1251.5351 | 1250.5510 | 1296.5565 | 1279.5300 | 1278.5460 | 1210.5561 | | E | 359.1674 | 358.1721 | | 433.2041 | 416.1776 | 4[illegible] |
| 12 | 102.0550 | 1397.6042 | 1380.5776 | 1379.5936 | 1425.5991 | 1408.5726 | 1407.5885 | 1339.5987 | | E | 230.1248 | 229.1295 | | ***304.1615*** | 287.1350 | 2[illegible] |
| 13 | 129.1135 | | | | | | | | | R | 74.0237 | 73.0284 | | ***175.1190*** | 158.0924 | |

| Seq | ya | yb | Seq | ya | yb | Seq | ya | yb |
|---|---|---|---|---|---|---|---|---|
| FG | 177.1022 | 205.0972 | FGD | 292.1292 | 320.1241 | FGDF | 439.1976 | 467.1925 |
| FGDFQ | 567.2562 | 595.2511 | FGDFQK | 695.3511 | 723.3461 | GD | 145.0608 | 173.0557 |
| GDF | 292.1292 | 320.1241 | GDFQ | 420.1878 | 448.1827 | GDFQK | 548.2827 | 576.2776 |
| GDFQKD | 663.3097 | 691.3046 | DF | 235.1077 | 263.1026 | DFQ | 363.1663 | 391.1612 |
| DFQK | 491.2613 | 519.2562 | DFQKD | 606.2882 | 634.2831 | FQ | 248.1394 | 276.1343 |
| FQK | 376.2343 | 404.2292 | FQKD | 491.2613 | 519.2562 | FQKDT | 592.3089 | 620.3039 |
| FQKDTP | 689.3617 | 717.3566 | QK | 229.1659 | 257.1608 | QKD | 344.1928 | 372.1878 |
| QKDT | 445.2405 | 473.2354 | QKDTP | 542.2933 | 570.2882 | QKDTPE | 671.3359 | 699.3308 |

| | | | | | | | | |
|---|---|---|---|---|---|---|---|---|
| **KD** | 216.1343 | 244.1292 | **KDT** | 317.1819 | 345.1769 | **KDTP** | 414.2347 | 442.2296 |
| **KDTPE** | 543.2773 | 571.2722 | **KDTPEE** | 672.3199 | 700.3148 | **DT** | 189.0870 | 217.0819 |
| **DTP** | 286.1397 | 314.1347 | **DTPE** | 415.1823 | 443.1773 | **DTPEE** | 544.2249 | 572.2198 |
| **TP** | 171.1128 | 199.1077 | **TPE** | 300.1554 | 328.1503 | **TPEE** | 429.1980 | 457.1929 |
| **PE** | 199.1077 | 227.1026 | **PEE** | 328.1503 | 356.1452 | **EE** | 231.0975 | 259.0925 |

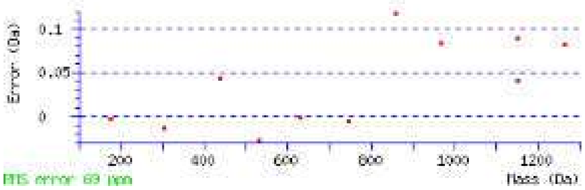

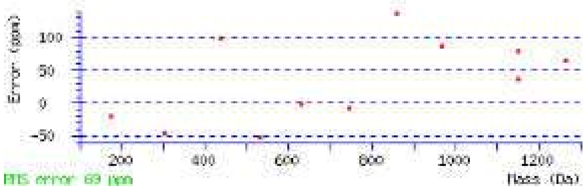


NCBI **BLAST** search of MFGDFQKDTPEER
(Parameters: blastp, nr protein database, expect=20000, no filter, PAM30)
Other BLAST web gateways

**All matches to this query**

| Score | Mr(calc) | Delta | Sequence |
|---|---|---|---|
| 51.5 | 1598.7035 | 0.0283 | MFGDFQKDTPEER |
| 14.8 | 1598.8879 | -0.1561 | EGKTLVILDDVLER |
| 13.4 | 1598.7134 | 0.0184 | LYSMEDVDEVVER |
| 13.3 | 1598.7974 | -0.0656 | MSIDSPQDPVLLER |
| 12.8 | 1598.7722 | -0.0404 | GAMVAHVSEDGEILR |
| 12.3 | 1598.8165 | -0.0847 | SQDKAQHAFGLLER |
| 11.6 | 1598.7909 | -0.0591 | MKPQGGSVAPICNPK |
| 11.5 | 1598.8053 | -0.0734 | LSGSLLFHNTEPER |
| 10.5 | 1598.7954 | -0.0636 | VFPWNNAPSGGGAIGR |
| 9.5 | 1598.6990 | 0.0328 | AMELIDEMEVTMR |

**Mascot:** http://www.matrixscience.com/

# MATRIX SCIENCE Mascot Search Results

## Peptide View Spot no 113

MS/MS Fragmentation of **MFGDFQKDTPEER**
Found in **gi|28190676** in **NCBInr**, putative transketolase [Oryza sativa Japonica Group]

Match to Query 79: 1614.710724 from(1615.718000,1+) intensity(0.0000) index(27)
Title: Label: D1, Spot_Id: 219677, Peak_List_Id: 224862, MSMS Job_Run_Id: 21756, Comment:
Data file C:\Documents and Settings\Administrator\Desktop\farah BHU\spot set plate\Farah-Garima 11May 2013\ppw_D1_136842020900.txt

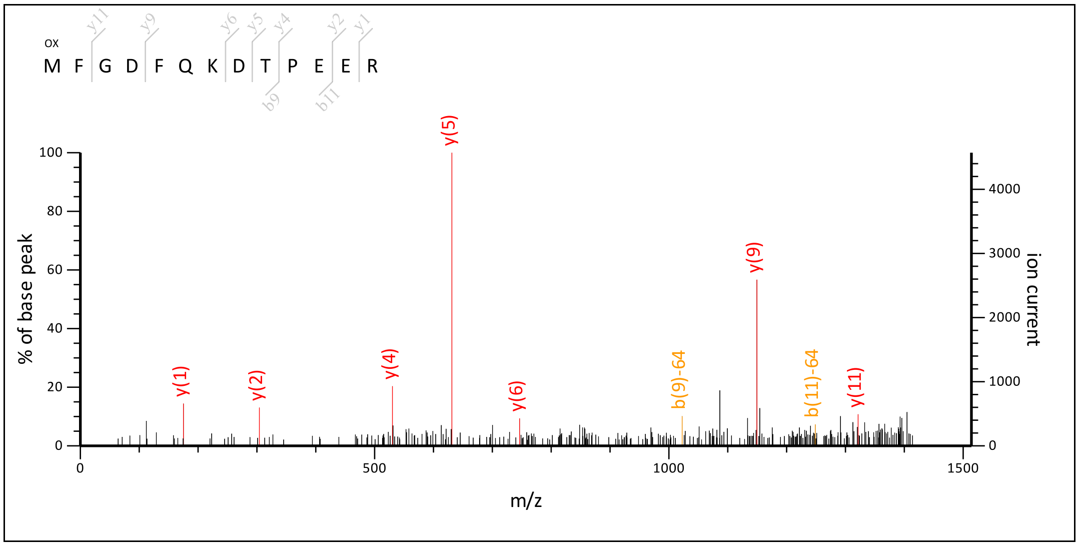


0 to 1514.04

Label all possible matches ○ Label matches used for scoring ◉

**Monoisotopic mass of neutral peptide Mr(calc):** 1614.6984
**Fixed modifications:** Carbamidomethyl (C) (apply to specified residues or termini only)
**Variable modifications:**
**M1** : Oxidation (M), with neutral losses 63.9983(shown in table), 0.0000
**Ions Score:** 47 **Expect:** 0.028
**Matches** : 9/276 fragment ions using 14 most intense peaks (help)

| # | Immon. | a | a* | $a^0$ | b | b* | $b^0$ | d | d' | Seq. | v | w | w' | y | y* | [illegible] |
|---|---|---|---|---|---|---|---|---|---|---|---|---|---|---|---|---|
| 1 | 56.0495 | 56.0495 | | | 84.0444 | | | 44.0495 | | M | | | | | | |
| 2 | 120.0808 | 203.1179 | | | 231.1128 | | | | | F | 1376.6077 | | | 1468.6703 | 1451.6438 | 145[illegible] |
| 3 | 30.0338 | 260.1394 | | | 288.1343 | | | | | G | | | | ***1321.6019*** | 1304.5753 | 130[illegible] |
| 4 | 88.0393 | 375.1663 | | 357.1557 | 403.1612 | | 385.1506 | 331.1765 | | D | 1204.5593 | 1203.5640 | | 1264.5804 | 1247.5539 | 124[illegible] |
| 5 | 120.0808 | 522.2347 | | 504.2241 | 550.2296 | | 532.2191 | | | F | 1057.4909 | | | ***1149.5535*** | 1132.5269 | 113[illegible] |
| 6 | 101.0709 | 650.2933 | 633.2667 | 632.2827 | 678.2882 | 661.2617 | 660.2776 | 593.2718 | | Q | 929.4323 | 928.4371 | | 1002.4851 | 985.4585 | 98[illegible] |
| 7 | 101.1073 | 778.3883 | 761.3617 | 760.3777 | 806.3832 | 789.3566 | 788.3726 | 721.3304 | | K | 801.3373 | 800.3421 | | 874.4265 | 857.3999 | 85[illegible] |
| 8 | 88.0393 | 893.4152 | 876.3886 | 875.4046 | 921.4101 | 904.3836 | 903.3995 | 849.4254 | | D | 686.3104 | 685.3151 | | ***746.3315*** | 729.3050 | 72[illegible] |
| 9 | 74.0600 | 994.4629 | 977.4363 | 976.4523 | **1022.4578** | 1005.4312 | 1004.4472 | 978.4680 | 980.4472 | T | 585.2627 | 598.2831 | 600.2624 | ***631.3046*** | 614.2780 | 61[illegible] |
| 10 | 70.0651 | 1091.5156 | 1074.4891 | 1073.5051 | 1119.5106 | 1102.4840 | 1101.5000 | 1065.5000 | | P | 488.2100 | 487.2147 | | ***530.2569*** | 513.2304 | 51[illegible] |
| 11 | 102.0550 | 1220.5582 | 1203.5317 | 1202.5477 | **1248.5531** | 1231.5266 | 1230.5426 | 1162.5528 | | E | 359.1674 | 358.1721 | | 433.2041 | 416.1776 | 41[illegible] |
| 12 | 102.0550 | 1349.6008 | 1332.5743 | 1331.5903 | 1377.5957 | 1360.5692 | 1359.5852 | 1291.5953 | | E | 230.1248 | 229.1295 | | ***304.1615*** | 287.1350 | 28[illegible] |
| 13 | 129.1135 | | | | | | | | | R | 74.0237 | 73.0284 | | ***175.1190*** | 158.0924 | |

| Seq | ya | yb | Seq | ya | yb | Seq | ya | yb |
|---|---|---|---|---|---|---|---|---|
| FG | 177.1022 | 205.0972 | FGD | 292.1292 | 320.1241 | FGDF | 439.1976 | 467.1925 |
| FGDFQ | 567.2562 | 595.2511 | FGDFQK | 695.3511 | 723.3461 | GD | 145.0608 | 173.0557 |
| GDF | 292.1292 | 320.1241 | GDFQ | 420.1878 | 448.1827 | GDFQK | 548.2827 | 576.2776 |
| GDFQKD | 663.3097 | 691.3046 | DF | 235.1077 | 263.1026 | DFQ | 363.1663 | 391.1612 |
| DFQK | 491.2613 | 519.2562 | DFQKD | 606.2882 | 634.2831 | FQ | 248.1394 | 276.1343 |
| | | | | | | | | |

| | | | | | | | | |
|---|---|---|---|---|---|---|---|---|
| **FQK** | 376.2343 | 404.2292 | **FQKD** | 491.2613 | 519.2562 | **FQKDT** | 592.3089 | 620.3039 |
| **FQKDTP** | 689.3617 | 717.3566 | **QK** | 229.1659 | 257.1608 | **QKD** | 344.1928 | 372.1878 |
| **QKDT** | 445.2405 | 473.2354 | **QKDTP** | 542.2933 | 570.2882 | **QKDTPE** | 671.3359 | 699.3308 |
| **KD** | 216.1343 | 244.1292 | **KDT** | 317.1819 | 345.1769 | **KDTP** | 414.2347 | 442.2296 |
| **KDTPE** | 543.2773 | 571.2722 | **KDTPEE** | 672.3199 | 700.3148 | **DT** | 189.0870 | 217.0819 |
| **DTP** | 286.1397 | 314.1347 | **DTPE** | 415.1823 | 443.1773 | **DTPEE** | 544.2249 | 572.2198 |
| **TP** | 171.1128 | 199.1077 | **TPE** | 300.1554 | 328.1503 | **TPEE** | 429.1980 | 457.1929 |
| **PE** | 199.1077 | 227.1026 | **PEE** | 328.1503 | 356.1452 | **EE** | 231.0975 | 259.0925 |

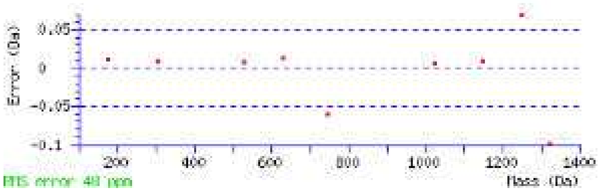


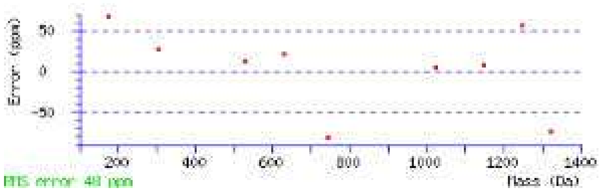


NCBI **BLAST** search of MFGDFQKDTPEER
(Parameters: blastp, nr protein database, expect=20000, no filter, PAM30)
Other BLAST web gateways

**All matches to this query**

| Score | Mr(calc) | Delta | Sequence |
|---|---|---|---|
| 47.2 | 1614.6984 | 0.0123 | MFGDFQKDTPEER |
| 40.6 | 1614.6984 | 0.0123 | MYGDFQKDTPEER |
| 25.2 | 1614.7242 | -0.0135 | MMRANTFTAQGAER |
| 12.0 | 1614.8076 | -0.0968 | FGSEGSKIFMVLER |
| 10.7 | 1614.7204 | -0.0097 | MFQKMEELCVER |
| 10.4 | 1614.6885 | 0.0222 | QVLAYALDXTXDER |
| 10.3 | 1614.7083 | 0.0024 | ESSEPESMTFTNLK |
| 10.3 | 1614.7712 | -0.0605 | CFQEGLSYTIIER |
| 10.2 | 1614.7599 | -0.0492 | SFFVEMDETIIER |
| 10.2 | 1614.6879 | 0.0229 | MMAQEHAHSSAVER |

**Mascot:** http://www.matrixscience.com/

# MATRIX SCIENCE Mascot Search Results

## Peptide View **Spot no 113**

MS/MS Fragmentation of **IYQEYGITAENVIATAK**
Found in **gi|28190676** in **NCBInr**, putative transketolase [Oryza sativa Japonica Group]

Match to Query 88: 1882.988524 from(1883.995800,1+) intensity(0.0000) index(30)
Title: Label: D1, Spot_Id: 219677, Peak_List_Id: 224891, MSMS Job_Run_Id: 21756, Comment:
Data file C:\Documents and Settings\Administrator\Desktop\farah BHU\spot set plate\Farah-Garima 11May 2013\ppw_D1_136842020900.txt

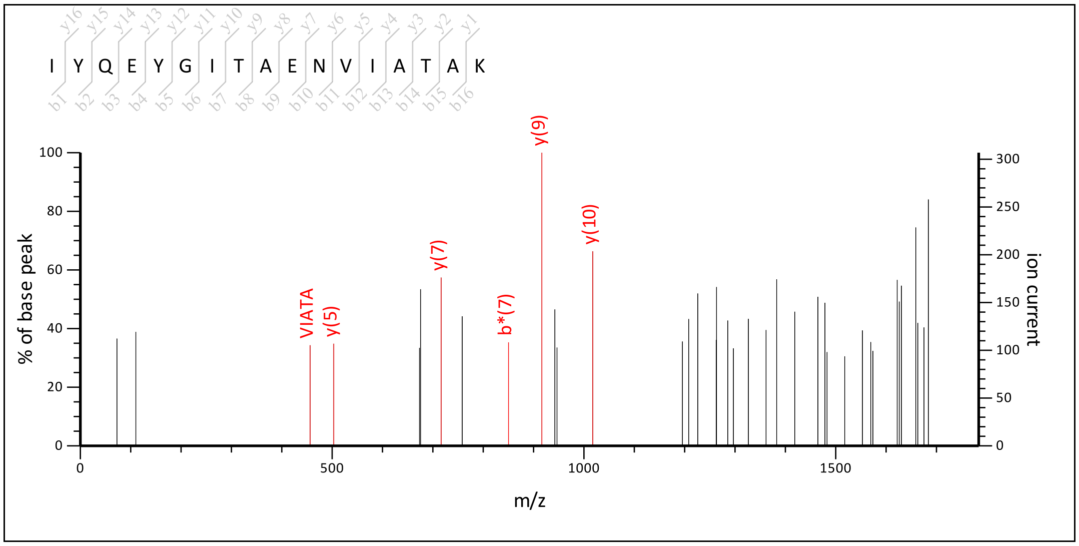


0 to 1783.92

Label all possible matches ○ Label matches used for scoring ◉

**Monoisotopic mass of neutral peptide Mr(calc):** 1882.9676
**Fixed modifications:** Carbamidomethyl (C) (apply to specified residues or termini only)
**Ions Score:** 10 **Expect:** 1.5e+02
**Matches :** 7/318 fragment ions using 13 most intense peaks (help)

| # | Immon. | a | a* | $a^0$ | b | b* | $b^0$ | d | d' | Seq. | v | w | w' | y | y* | |
|---|---|---|---|---|---|---|---|---|---|---|---|---|---|---|---|---|
| 1 | 86.0964 | 86.0964 | | | 114.0913 | | | 44.0495 | | I | | | | | | |
| 2 | 136.0757 | 249.1598 | | | 277.1547 | | | | | Y | 1662.8333 | | | 1770.8909 | 1753.8643 | [illegible] |
| 3 | 101.0709 | 377.2183 | 360.1918 | | 405.2132 | 388.1867 | | 320.1969 | | Q | 1534.7748 | 1533.7795 | | 1607.8275 | 1590.8010 | [illegible] |
| 4 | 102.0550 | 506.2609 | 489.2344 | 488.2504 | 534.2558 | 517.2293 | 516.2453 | 448.2554 | | E | 1405.7322 | 1404.7369 | | 1479.7690 | 1462.7424 | [illegible] |
| 5 | 136.0757 | 669.3243 | 652.2977 | 651.3137 | 697.3192 | 680.2926 | 679.3086 | | | Y | 1242.6688 | | | 1350.7264 | 1333.6998 | [illegible] |
| 6 | 30.0338 | 726.3457 | 709.3192 | 708.3352 | 754.3406 | 737.3141 | 736.3301 | | | G | | | | 1187.6630 | 1170.6365 | [illegible] |
| 7 | 86.0964 | 839.4298 | 822.4032 | 821.4192 | 867.4247 | 850.3981 | 849.4141 | 811.3985 | 825.4141 | I | 1072.5633 | 1085.5837 | 1099.5994 | 1130.6416 | 1113.6150 | [illegible] |
| 8 | 74.0600 | 940.4775 | 923.4509 | 922.4669 | 968.4724 | 951.4458 | 950.4618 | 924.4825 | 926.4618 | T | 971.5156 | 984.5360 | 986.5153 | ***1017.5575*** | 1000.5310 | [illegible] |
| 9 | 44.0495 | 1011.5146 | 994.4880 | 993.5040 | 1039.5095 | 1022.4829 | 1021.4989 | | | A | 900.4785 | | | ***916.5098*** | 899.4833 | [illegible] |
| 10 | 102.0550 | 1140.5572 | 1123.5306 | 1122.5466 | 1168.5521 | 1151.5255 | 1150.5415 | 1082.5517 | | E | 771.4359 | 770.4407 | | 845.4727 | 828.4462 | [illegible] |
| 11 | 87.0553 | 1254.6001 | 1237.5735 | 1236.5895 | 1282.5950 | 1265.5685 | 1264.5844 | 1211.5943 | | N | 657.3930 | 656.3978 | | ***716.4301*** | 699.4036 | [illegible] |
| 12 | 72.0808 | 1353.6685 | 1336.6420 | 1335.6579 | 1381.6634 | 1364.6369 | 1363.6529 | 1339.6529 | | V | 558.3246 | 571.3450 | | 602.3872 | 585.3606 | [illegible] |
| 13 | 86.0964 | 1466.7526 | 1449.7260 | 1448.7420 | 1494.7475 | 1477.7209 | 1476.7369 | 1438.7213 | 1452.7369 | I | 445.2405 | 458.2609 | 472.2766 | ***503.3188*** | 486.2922 | [illegible] |
| 14 | 44.0495 | 1537.7897 | 1520.7631 | 1519.7791 | 1565.7846 | 1548.7581 | 1547.7740 | | | A | 374.2034 | | | 390.2347 | 373.2082 | [illegible] |
| 15 | 74.0600 | 1638.8374 | 1621.8108 | 1620.8268 | 1666.8323 | 1649.8057 | 1648.8217 | 1622.8425 | 1624.8217 | T | 273.1557 | 286.1761 | 288.1554 | 319.1976 | 302.1710 | [illegible] |
| 16 | 44.0495 | 1709.8745 | 1692.8479 | 1691.8639 | 1737.8694 | 1720.8428 | 1719.8588 | | | A | 202.1186 | | | 218.1499 | 201.1234 | |
| 17 | 101.1073 | | | | | | | | | K | 74.0237 | 73.0284 | | 147.1128 | 130.0863 | |

| Seq | ya | yb | Seq | ya | yb | Seq | ya | yb |
|---|---|---|---|---|---|---|---|---|
| YQ | 264.1343 | 292.1292 | YQE | 393.1769 | 421.1718 | YQEY | 556.2402 | 584.2351 |
| YQEYG | 613.2617 | 641.2566 | QE | 230.1135 | 258.1084 | QEY | 393.1769 | 421.1718 |
| QEYG | 450.1983 | 478.1932 | QEYGI | 563.2824 | 591.2773 | QEYGIT | 664.3301 | 692.3250 |
| EY | 265.1183 | 293.1132 | EYG | 322.1397 | 350.1347 | EYGI | 435.2238 | 463.2187 |

| | | | | | | | | |
|---|---|---|---|---|---|---|---|---|
| **EYGIT** | 536.2715 | 564.2664 | **EYGITA** | 607.3086 | 635.3035 | **YG** | 193.0972 | 221.0921 |
| **YGI** | 306.1812 | 334.1761 | **YGIT** | 407.2289 | 435.2238 | **YGITA** | 478.2660 | 506.2609 |
| **YGITAE** | 607.3086 | 635.3035 | **GI** | 143.1179 | 171.1128 | **GIT** | 244.1656 | 272.1605 |
| **GITA** | 315.2027 | 343.1976 | **GITAE** | 444.2453 | 472.2402 | **GITAEN** | 558.2882 | 586.2831 |
| **GITAENV** | 657.3566 | 685.3515 | **IT** | 187.1441 | 215.1390 | **ITA** | 258.1812 | 286.1761 |
| **ITAE** | 387.2238 | 415.2187 | **ITAEN** | 501.2667 | 529.2617 | **ITAENV** | 600.3352 | 628.3301 |
| **TA** | 145.0972 | 173.0921 | **TAE** | 274.1397 | 302.1347 | **TAEN** | 388.1827 | 416.1776 |
| **TAENV** | 487.2511 | 515.2460 | **TAENVI** | 600.3352 | 628.3301 | **TAENVIA** | 671.3723 | 699.3672 |
| **AE** | 173.0921 | 201.0870 | **AEN** | 287.1350 | 315.1299 | **AENV** | 386.2034 | 414.1983 |
| **AENVI** | 499.2875 | 527.2824 | **AENVIA** | 570.3246 | 598.3195 | **AENVIAT** | 671.3723 | 699.3672 |
| **EN** | 216.0979 | 244.0928 | **ENV** | 315.1663 | 343.1612 | **ENVI** | 428.2504 | 456.2453 |
| **ENVIA** | 499.2875 | 527.2824 | **ENVIAT** | 600.3352 | 628.3301 | **ENVIATA** | 671.3723 | 699.3672 |
| **NV** | 186.1237 | 214.1186 | **NVI** | 299.2078 | 327.2027 | **NVIA** | 370.2449 | 398.2398 |
| **NVIAT** | 471.2926 | 499.2875 | **NVIATA** | 542.3297 | 570.3246 | **VI** | 185.1648 | 213.1598 |
| **VIA** | 256.2020 | 284.1969 | **VIAT** | 357.2496 | 385.2445 | **VIATA** | 428.2867 | 456.2817 |
| **IA** | 157.1335 | 185.1285 | **IAT** | 258.1812 | 286.1761 | **IATA** | 329.2183 | 357.2132 |
| **AT** | 145.0972 | 173.0921 | **ATA** | 216.1343 | 244.1292 | **TA** | 145.0972 | 173.0921 |

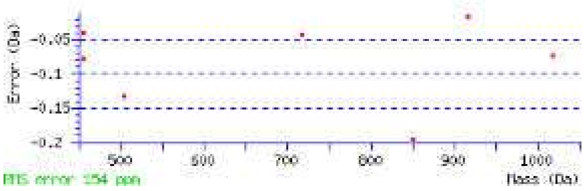


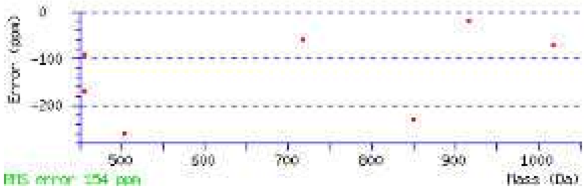


NCBI **BLAST** search of IYQEYGITAENVIATAK
(Parameters: blastp, nr protein database, expect=20000, no filter, PAM30)
Other BLAST web gateways

**All matches to this query**

| Score | Mr(calc) | Delta | Sequence |
|---|---|---|---|
| 10.4 | 1882.9676 | 0.0209 | IYQEYGITAENVIATAK |
| 10.4 | 1882.8730 | 0.1155 | QIKNMIAETEQDYER |
| 6.8 | 1883.0265 | -0.0379 | ISPTASGAALVSLKFEHR |
| 2.2 | 1882.9135 | 0.0750 | TVSVPMSLETYSWDLR |
| 1.7 | 1882.8731 | 0.1154 | NMAHGTSNSTTYTELLK |
| 1.6 | 1883.0000 | -0.0115 | QGTLDPALATAEAGQTVIK |
| 1.1 | 1883.0364 | -0.0479 | SLDSVVPVNGAALSEKLGK |
| 0.7 | 1882.9346 | 0.0539 | SILYQTMDISLVENNK |
| 0.1 | 1882.9724 | 0.0162 | LSAVCGGASFFERLLTR |

**Mascot:** http://www.matrixscience.com/

# MATRIX SCIENCE Mascot Search Results

## Peptide View **Spot no 114**

MS/MS Fragmentation of **VVDLLAPYRR**
Found in **gi|11583** in **NCBInr**, ATPase, beta subunit [Hordeum vulgare]

Match to Query 127: 1200.726724 from(1201.734000,1+) intensity(0.0000) index(14)
Title: Label: I1, Spot_Id: 219682, Peak_List_Id: 225014, MSMS Job_Run_Id: 21761, Comment:
Data file C:\Documents and Settings\Administrator\Desktop\farah BHU\spot set plate\Farah-Garima 11May 2013\ppw_I1_136842037600.txt

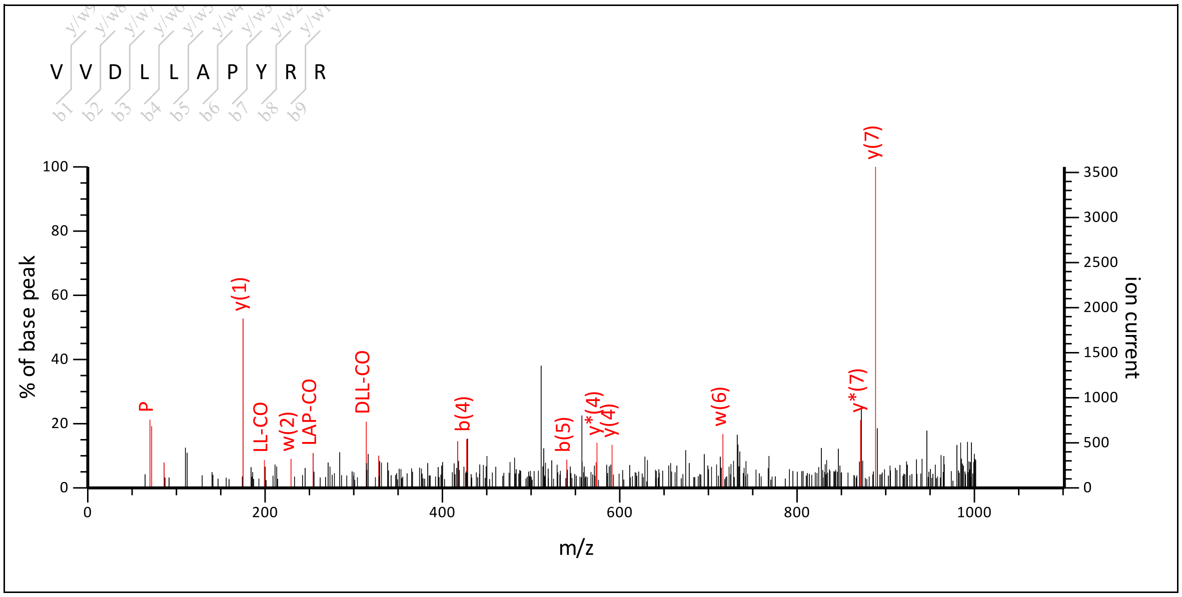


0 to 1101.48

Label all possible matches ○ Label matches used for scoring ◉

**Monoisotopic mass of neutral peptide Mr(calc):** 1200.6979
**Fixed modifications:** Carbamidomethyl (C) (apply to specified residues or termini only)
**Ions Score:** 15 **Expect:** 50
**Matches :** 27/136 fragment ions using 50 most intense peaks (help)

| # | Immon. | a | a* | a0 | b | b* | b0 | d | Seq. | v | w | y | y* | y0 | # |
|---|---|---|---|---|---|---|---|---|---|---|---|---|---|---|---|
| 1 | 72.0808 | 72.0808 | | | 100.0757 | | | 44.0495 | V | | | | | | 10 |
| 2 | 72.0808 | 171.1492 | | | ***199.1441*** | | | 157.1335 | V | 1058.5742 | 1071.5946 | 1102.6368 | 1085.6102 | 1084.6262 | 9 |
| 3 | 88.0393 | 286.1761 | | 268.1656 | ***314.1710*** | | 296.1605 | 242.1863 | D | 943.5472 | 942.5520 | 1003.5683 | 986.5418 | 985.5578 | 8 |
| 4 | 86.0964 | 399.2602 | | 381.2496 | ***427.2551*** | | 409.2445 | 357.2132 | L | 830.4631 | 829.4679 | ***888.5414*** | 871.5148 | | 7 |
| 5 | 86.0964 | 512.3443 | | 494.3337 | ***540.3392*** | | 522.3286 | 470.2973 | L | 717.3791 | 716.3838 | 775.4573 | 758.4308 | | 6 |
| 6 | 44.0495 | 583.3814 | | 565.3708 | 611.3763 | | 593.3657 | | A | 646.3420 | | 662.3733 | 645.3467 | | 5 |
| 7 | 70.0651 | 680.4341 | | 662.4236 | 708.4291 | | 690.4185 | 654.4185 | P | 549.2892 | 548.2940 | ***591.3362*** | 574.3096 | | 4 |
| 8 | 136.0757 | 843.4975 | | 825.4869 | ***871.4924*** | | 853.4818 | | Y | 386.2259 | | 494.2834 | 477.2568 | | 3 |
| 9 | 129.1135 | 999.5986 | 982.5720 | 981.5880 | 1027.5935 | 1010.5669 | 1009.5829 | 914.5346 | R | 230.1248 | 229.1295 | 331.2201 | 314.1935 | | 2 |
| 10 | 129.1135 | | | | | | | | R | 74.0237 | 73.0284 | ***175.1190*** | 158.0924 | | 1 |

| Seq | ya | yb | Seq | ya | yb | Seq | ya | yb |
|---|---|---|---|---|---|---|---|---|
| VD | 187.1077 | 215.1026 | VDL | 300.1918 | 328.1867 | VDLL | 413.2758 | 441.2708 |
| VDLLA | 484.3130 | 512.3079 | VDLLAP | 581.3657 | 609.3606 | DL | 201.1234 | 229.1183 |
| DLL | 314.2074 | 342.2023 | DLLA | 385.2445 | 413.2395 | DLLAP | 482.2973 | 510.2922 |
| DLLAPY | 645.3606 | 673.3556 | LL | 199.1805 | 227.1754 | LLA | 270.2176 | 298.2125 |
| LLAP | 367.2704 | 395.2653 | LLAPY | 530.3337 | 558.3286 | LLAPYR | 686.4348 | 714.4297 |
| LA | 157.1335 | 185.1285 | LAP | 254.1863 | 282.1812 | LAPY | 417.2496 | 445.2445 |

| | | | | | | | | |
|---|---|---|---|---|---|---|---|---|
| **LAPYR** | 573.3507 | 601.3457 | **AP** | 141.1022 | 169.0972 | **APY** | 304.1656 | 332.1605 |
| **APYR** | 460.2667 | 488.2616 | **PY** | 233.1285 | 261.1234 | **PYR** | 389.2296 | 417.2245 |
| **YR** | 292.1768 | 320.1717 | | | | | | |

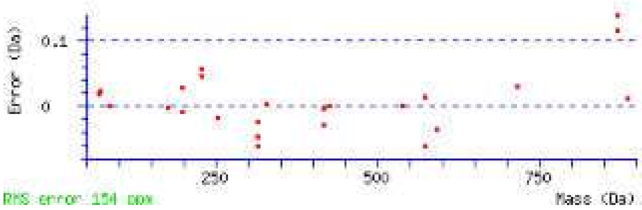


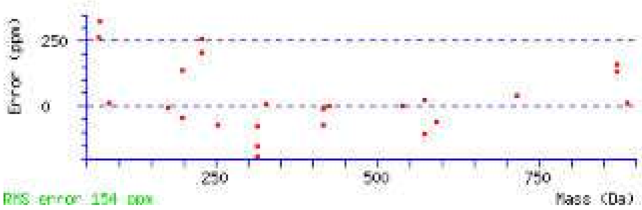


NCBI **BLAST** search of VVDLLAPYRR
(Parameters: blastp, nr protein database, expect=20000, no filter, PAM30)
Other BLAST web gateways

**All matches to this query**

| Score | Mr(calc) | Delta | Sequence |
|---|---|---|---|
| 15.4 | 1200.6979 | 0.0288 | VVDILAPYRR |
| 15.4 | 1200.6979 | 0.0288 | VVDLLAPYRR |
| 15.4 | 1200.6979 | 0.0288 | VVDLLAPYRR |
| 15.4 | 1200.6979 | 0.0288 | VVDLLAPYRR |
| 15.4 | 1200.6979 | 0.0288 | VVDLLAPYRR |
| 15.4 | 1200.6979 | 0.0288 | VVDLLAPYRR |
| 15.4 | 1200.6979 | 0.0288 | VVDLLAPYRR |
| 15.4 | 1200.6979 | 0.0288 | VVDLLAPYRR |
| 15.4 | 1200.6979 | 0.0288 | VVDLLAPYRR |
| 15.3 | 1200.6211 | 0.1056 | QAVTNAQNTVR |

**Mascot:** http://www.matrixscience.com/

# MATRIX SCIENCE Mascot Search Results

## Peptide View **Spot no 114**

MS/MS Fragmentation of **AHGGVSVFGGVGER**
Found in **gi|11583** in **NCBInr**, ATPase, beta subunit [Hordeum vulgare]

Match to Query 156: 1327.698024 from(1328.705300,1+) intensity(0.0000) index(16)
Title: Label: I1, Spot_Id: 219682, Peak_List_Id: 225009, MSMS Job_Run_Id: 21761, Comment:
Data file C:\Documents and Settings\Administrator\Desktop\farah BHU\spot set plate\Farah-Garima 11May 2013\ppw_I1_136842037600.txt

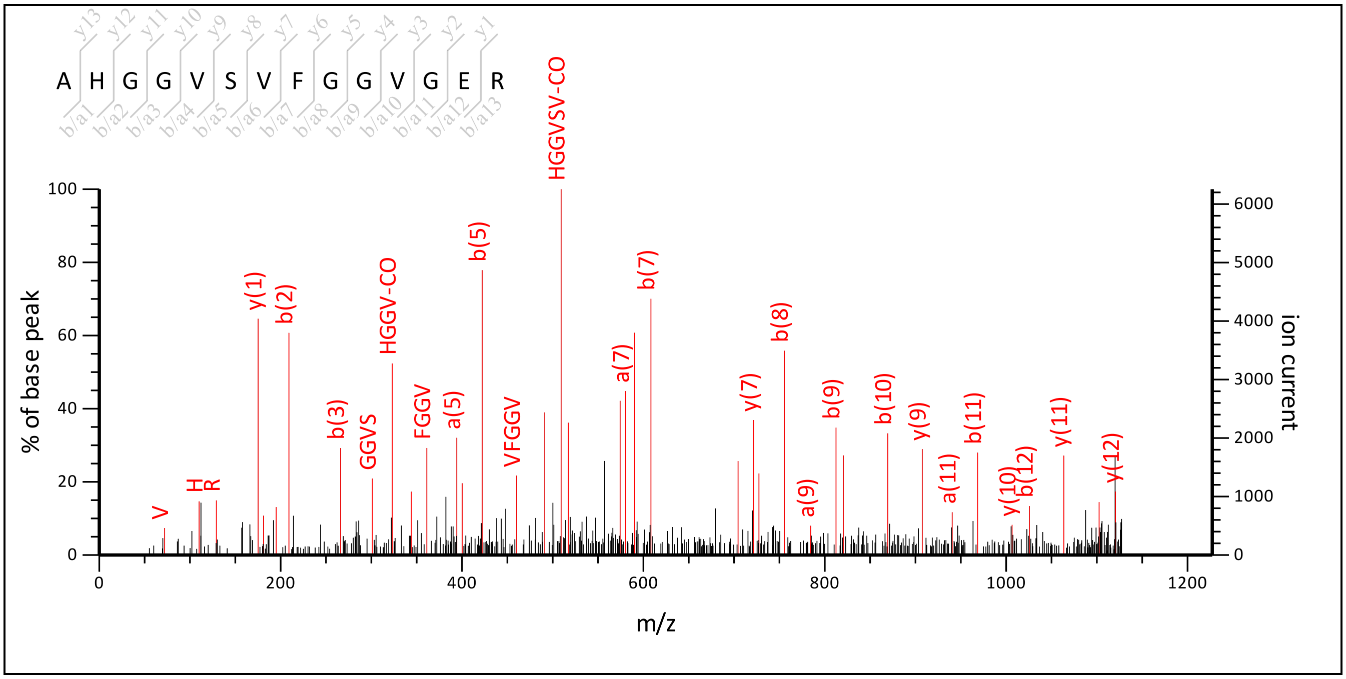


0 to 1227.25

Label all possible matches ○ Label matches used for scoring ◉

**Monoisotopic mass of neutral peptide Mr(calc):** 1327.6633
**Fixed modifications:** Carbamidomethyl (C) (apply to specified residues or termini only)
**Ions Score:** 124 **Expect:** 8.7e-10
**Matches :** 52/220 fragment ions using 44 most intense peaks (help)

| # | Immon. | a | $a^0$ | b | $b^0$ | d | Seq. | v | w | y | y* | $y^0$ | # |
|---|---|---|---|---|---|---|---|---|---|---|---|---|---|
| 1 | 44.0495 | 44.0495 | | 72.0444 | | 44.0495 | A | | | | | | 14 |
| 2 | 110.0713 | 181.1084 | | 209.1033 | | | H | 1175.5804 | | 1257.6335 | 1240.6069 | 1239.6229 | 13 |
| 3 | 30.0338 | 238.1299 | | 266.1248 | | | G | | | 1120.5745 | 1103.5480 | 1102.5640 | 12 |
| 4 | 30.0338 | 295.1513 | | 323.1462 | | | G | | | 1063.5531 | 1046.5265 | 1045.5425 | 11 |
| 5 | 72.0808 | 394.2197 | | 422.2146 | | 380.2041 | V | 962.4690 | 975.4894 | 1006.5316 | 989.5051 | 988.5211 | 10 |
| 6 | 60.0444 | 481.2518 | 463.2412 | 509.2467 | 491.2361 | 465.2568 | S | 875.4370 | 874.4417 | 907.4632 | 890.4367 | 889.4526 | 9 |
| 7 | 72.0808 | 580.3202 | 562.3096 | 608.3151 | 590.3045 | 566.3045 | V | 776.3686 | 789.3890 | 820.4312 | 803.4046 | 802.4206 | 8 |
| 8 | 120.0808 | 727.3886 | 709.3780 | 755.3835 | 737.3729 | | F | 629.3002 | | 721.3628 | 704.3362 | 703.3522 | 7 |
| 9 | 30.0338 | 784.4100 | 766.3995 | 812.4050 | 794.3944 | | G | | | 574.2944 | 557.2678 | 556.2838 | 6 |
| 10 | 30.0338 | 841.4315 | 823.4209 | 869.4264 | 851.4159 | | G | | | 517.2729 | 500.2463 | 499.2623 | 5 |
| 11 | 72.0808 | 940.4999 | 922.4894 | 968.4948 | 950.4843 | 926.4843 | V | 416.1888 | 429.2092 | 460.2514 | 443.2249 | 442.2409 | 4 |

| | | | | | | | | | | | | | |
|---|---|---|---|---|---|---|---|---|---|---|---|---|---|
| 12 | 30.0338 | 997.5214 | 979.5108 | ***1025.5163*** | 1007.5057 | | G | | | ***361.1830*** | 344.1565 | 343.1724 | 3 |
| 13 | 102.0550 | 1126.5640 | 1108.5534 | 1154.5589 | 1136.5483 | 1068.5585 | E | 230.1248 | 229.1295 | 304.1615 | 287.1350 | 286.1510 | 2 |
| 14 | 129.1135 | | | | | | R | 74.0237 | 73.0284 | ***175.1190*** | 158.0924 | | 1 |

| Seq | ya | yb | Seq | ya | yb | Seq | ya | yb |
|---|---|---|---|---|---|---|---|---|
| HG | 167.0927 | 195.0877 | HGG | 224.1142 | 252.1091 | HGGV | 323.1826 | 351.1775 |
| HGGVS | 410.2146 | 438.2096 | HGGVSV | 509.2831 | 537.2780 | HGGVSVF | 656.3515 | 684.3464 |
| GG | 87.0553 | 115.0502 | GGV | 186.1237 | 214.1186 | GGVS | 273.1557 | 301.1506 |
| GGVSV | 372.2241 | 400.2191 | GGVSVF | 519.2926 | 547.2875 | GGVSVFG | 576.3140 | 604.3089 |
| GGVSVFGG | 633.3355 | 661.3304 | GV | 129.1022 | 157.0972 | GVS | 216.1343 | 244.1292 |
| GVSV | 315.2027 | 343.1976 | GVSVF | 462.2711 | 490.2660 | GVSVFG | 519.2926 | 547.2875 |
| GVSVFGG | 576.3140 | 604.3089 | GVSVFGGV | 675.3824 | 703.3774 | VS | 159.1128 | 187.1077 |
| VSV | 258.1812 | 286.1761 | VSVF | 405.2496 | 433.2445 | VSVFG | 462.2711 | 490.2660 |
| VSVFGG | 519.2926 | 547.2875 | VSVFGGV | 618.3610 | 646.3559 | VSVFGGVG | 675.3824 | 703.3774 |
| SV | 159.1128 | 187.1077 | SVF | 306.1812 | 334.1761 | SVFG | 363.2027 | 391.1976 |
| SVFGG | 420.2241 | 448.2191 | SVFGGV | 519.2926 | 547.2875 | SVFGGVG | 576.3140 | 604.3089 |
| VF | 219.1492 | 247.1441 | VFG | 276.1707 | 304.1656 | VFGG | 333.1921 | 361.1870 |
| VFGGV | 432.2605 | 460.2554 | VFGGVG | 489.2820 | 517.2769 | VFGGVGE | 618.3246 | 646.3195 |
| FG | 177.1022 | 205.0972 | FGG | 234.1237 | 262.1186 | FGGV | 333.1921 | 361.1870 |
| FGGVG | 390.2136 | 418.2085 | FGGVGE | 519.2562 | 547.2511 | GG | 87.0553 | 115.0502 |
| GGV | 186.1237 | 214.1186 | GGVG | 243.1452 | 271.1401 | GGVGE | 372.1878 | 400.1827 |
| GV | 129.1022 | 157.0972 | GVG | 186.1237 | 214.1186 | GVGE | 315.1663 | 343.1612 |
| VG | 129.1022 | 157.0972 | VGE | 258.1448 | 286.1397 | GE | 159.0764 | 187.0713 |

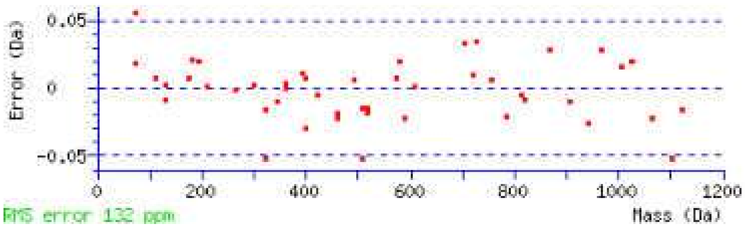


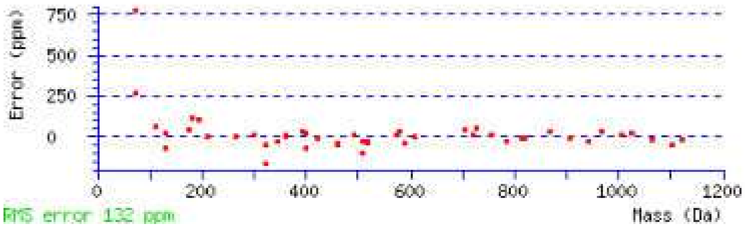


NCBI **BLAST** search of AHGGVSVFGGVGER
(Parameters: blastp, nr protein database, expect=20000, no filter, PAM30)
Other BLAST web gateways

**All matches to this query**

| Score | Mr(calc) | Delta | Sequence |
|---|---|---|---|
| 124.2 | 1327.6633 | 0.0347 | AHGGVSVFGGVGER |
| 124.2 | 1327.6633 | 0.0347 | AHGGVSVFGGVGER |
| 124.2 | 1327.6633 | 0.0347 | AHGGVSVFGGVGER |
| 124.2 | 1327.6633 | 0.0347 | AHGGVSVFGGVGER |

| | | | |
|---|---|---|---|
| 124.2 | 1327.6633 | 0.0347 | AHGGVSVFGGVGER |
| 124.2 | 1327.6633 | 0.0347 | AHGGVSVFGGVGER |
| 124.2 | 1327.6633 | 0.0347 | AHGGVSVFGGVGER |
| 124.2 | 1327.6633 | 0.0347 | AHGGVSVFGGVGER |
| 124.2 | 1327.6633 | 0.0347 | AHGGVSVFGGVGER |
| 124.2 | 1327.6633 | 0.0347 | AHGGVSVFGGVGER |

**Mascot:** http://www.matrixscience.com/

# MATRIX SCIENCE Mascot Search Results

## Peptide View **Spot no 114**

MS/MS Fragmentation of **IVGNEHYETAQR**
Found in **gi|11583** in **NCBInr**, ATPase, beta subunit [Hordeum vulgare]

Match to Query 175: 1415.721924 from(1416.729200,1+) intensity(0.0000) index(18)
Title: Label: I1, Spot_Id: 219682, Peak_List_Id: 225010, MSMS Job_Run_Id: 21761, Comment:
Data file C:\Documents and Settings\Administrator\Desktop\farah BHU\spot set plate\Farah-Garima 11May 2013\ppw_I1_136842037600.txt

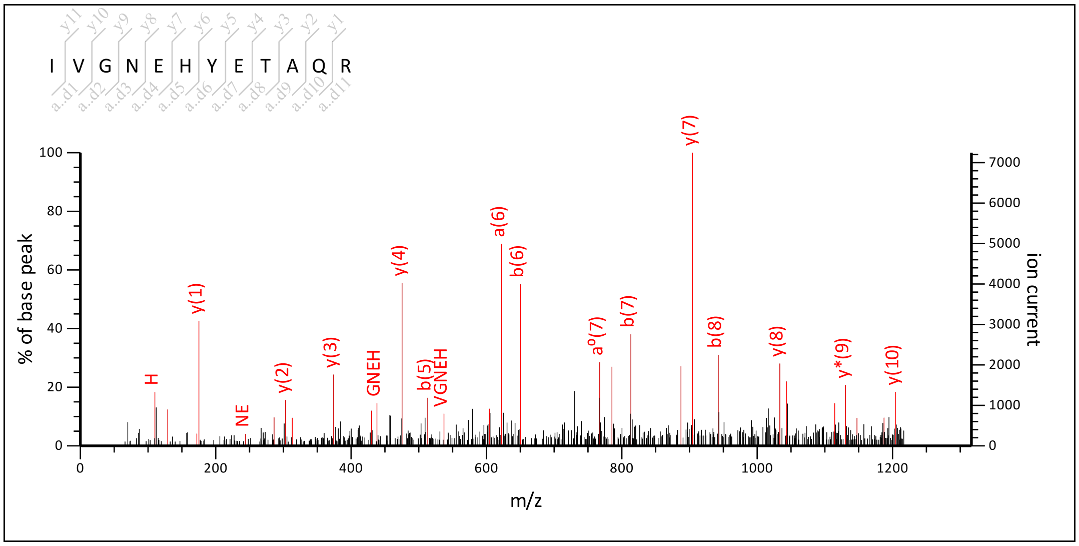


0 to 1316.55

Label all possible matches Label matches used for scoring

**Monoisotopic mass of neutral peptide Mr(calc):** 1415.6793
**Fixed modifications:** Carbamidomethyl (C) (apply to specified residues or termini only)
**Ions Score:** 93 **Expect:** 1.1e-06
**Matches :** 36/181 fragment ions using 36 most intense peaks (help)

| # | Immon. | a | a* | $a^0$ | b | b* | $b^0$ | d | d' | Seq. | v | w | w' | y | y* | |
|---|---|---|---|---|---|---|---|---|---|---|---|---|---|---|---|---|
| 1 | 86.0964 | 86.0964 | | | 114.0913 | | | 44.0495 | | I | | | | | | |
| 2 | 72.0808 | 185.1648 | | | 213.1598 | | | 171.1492 | | V | 1259.5400 | 1272.5604 | | 1303.6026 | 1286.5760 | [illegible] |
| 3 | 30.0338 | 242.1863 | | | 270.1812 | | | | | G | | | | 1204.5341 | 1187.5076 | [illegible] |
| 4 | 87.0553 | 356.2292 | 339.2027 | | 384.2241 | 367.1976 | | 313.2234 | | N | 1088.4756 | 1087.4803 | | 1147.5127 | 1130.4861 | [illegible] |
| 5 | 102.0550 | 485.2718 | 468.2453 | 467.2613 | 513.2667 | 496.2402 | 495.2562 | 427.2663 | | E | 959.4330 | 958.4377 | | 1033.4697 | 1016.4432 | [illegible] |
| 6 | 110.0713 | 622.3307 | 605.3042 | 604.3202 | 650.3256 | 633.2991 | 632.3151 | | | H | 822.3741 | | | 904.4272 | 887.4006 | [illegible] |
| 7 | 136.0757 | 785.3941 | 768.3675 | 767.3835 | 813.3890 | 796.3624 | 795.3784 | | | Y | 659.3107 | | | 767.3682 | 750.3417 | [illegible] |
| 8 | 102.0550 | 914.4367 | 897.4101 | 896.4261 | 942.4316 | 925.4050 | 924.4210 | 856.4312 | | E | 530.2681 | 529.2729 | | 604.3049 | 587.2784 | [illegible] |
| 9 | 74.0600 | 1015.4843 | 998.4578 | 997.4738 | 1043.4793 | 1026.4527 | 1025.4687 | 999.4894 | 1001.4687 | T | 429.2205 | 442.2409 | 444.2201 | 475.2623 | 458.2358 | [illegible] |
| 10 | 44.0495 | 1086.5214 | 1069.4949 | 1068.5109 | 1114.5164 | 1097.4898 | 1096.5058 | | | A | 358.1833 | | | 374.2146 | 357.1881 | |
| 11 | 101.0709 | 1214.5800 | 1197.5535 | 1196.5695 | 1242.5749 | 1225.5484 | 1224.5644 | 1157.5586 | | Q | 230.1248 | 229.1295 | | 303.1775 | 286.1510 | |
| 12 | 129.1135 | | | | | | | | | R | 74.0237 | 73.0284 | | 175.1190 | 158.0924 | |

| Seq | ya | yb | Seq | ya | yb | Seq | ya | yb |
|---|---|---|---|---|---|---|---|---|
| VG | 129.1022 | 157.0972 | VGN | 243.1452 | 271.1401 | VGNE | 372.1878 | 400.1827 |
| VGNEH | 509.2467 | 537.2416 | VGNEHY | 672.3100 | 700.3049 | GN | 144.0768 | 172.0717 |
| GNE | 273.1193 | 301.1143 | GNEH | 410.1783 | 438.1732 | GNEHY | 573.2416 | 601.2365 |
| NE | 216.0979 | 244.0928 | NEH | 353.1568 | 381.1517 | NEHY | 516.2201 | 544.2150 |
| NEHYE | 645.2627 | 673.2576 | EH | 239.1139 | 267.1088 | EHY | 402.1772 | 430.1721 |
| EHYE | 531.2198 | 559.2147 | EHYET | 632.2675 | 660.2624 | HY | 273.1346 | 301.1295 |
| HYE | 402.1772 | 430.1721 | HYET | 503.2249 | 531.2198 | HYETA | 574.2620 | 602.2569 |
| YE | 265.1183 | 293.1132 | YET | 366.1660 | 394.1609 | YETA | 437.2031 | 465.1980 |
| YETAQ | 565.2617 | 593.2566 | ET | 203.1026 | 231.0975 | ETA | 274.1397 | 302.1347 |

| | | | | | | | | |
|---|---|---|---|---|---|---|---|---|
| **ETAQ** | 402.1983 | 430.1932 | **TA** | 145.0972 | 173.0921 | **TAQ** | 273.1557 | 301.1506 |
| **AQ** | 172.1081 | 200.1030 | | | | | | |

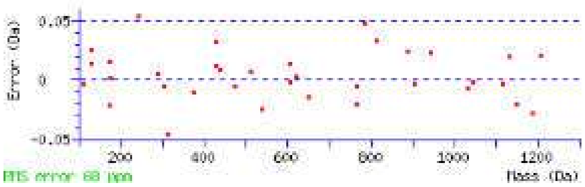


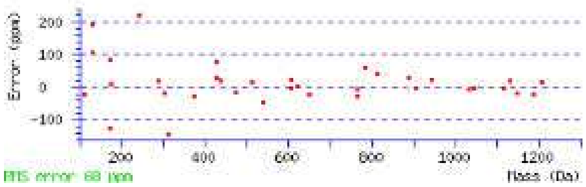


NCBI **BLAST** search of IVGNEHYETAQR
(Parameters: blastp, nr protein database, expect=20000, no filter, PAM30)
Other BLAST web gateways

**All matches to this query**

| Score | Mr(calc) | Delta | Sequence |
|---|---|---|---|
| 92.6 | 1415.6793 | 0.0426 | IVGNEHYETAQR |
| 92.6 | 1415.6793 | 0.0426 | IVGNEHYETAQR |
| 76.7 | 1415.7157 | 0.0062 | IVGDKHYETAQR |
| 76.7 | 1415.7157 | 0.0062 | IVGDKHYETAQR |
| 76.7 | 1415.6793 | 0.0426 | IVGDQHYETAQR |
| 76.7 | 1415.6793 | 0.0426 | IVGENHYETAQR |
| 76.7 | 1415.6793 | 0.0426 | IVGENHYETAQR |
| 76.7 | 1415.7157 | 0.0062 | IVGKDHYETAQR |
| 76.7 | 1415.7157 | 0.0062 | IVGKDHYETAQR |
| 27.4 | 1415.7157 | 0.0062 | IVGDEHYKTAQR |

**Mascot:** http://www.matrixscience.com/

# MATRIX SCIENCE Mascot Search Results

## Peptide View **Spot no 114**

MS/MS Fragmentation of **FVQAGSEVSALLGR**
Found in **gi|11583** in **NCBInr**, ATPase, beta subunit [Hordeum vulgare]

Match to Query 181: 1432.815824 from(1433.823100,1+) intensity(0.0000) index(19)
Title: Label: I1, Spot_Id: 219682, Peak_List_Id: 225016, MSMS Job_Run_Id: 21761, Comment:
Data file C:\Documents and Settings\Administrator\Desktop\farah BHU\spot set plate\Farah-Garima 11May 2013\ppw_I1_136842037600.txt

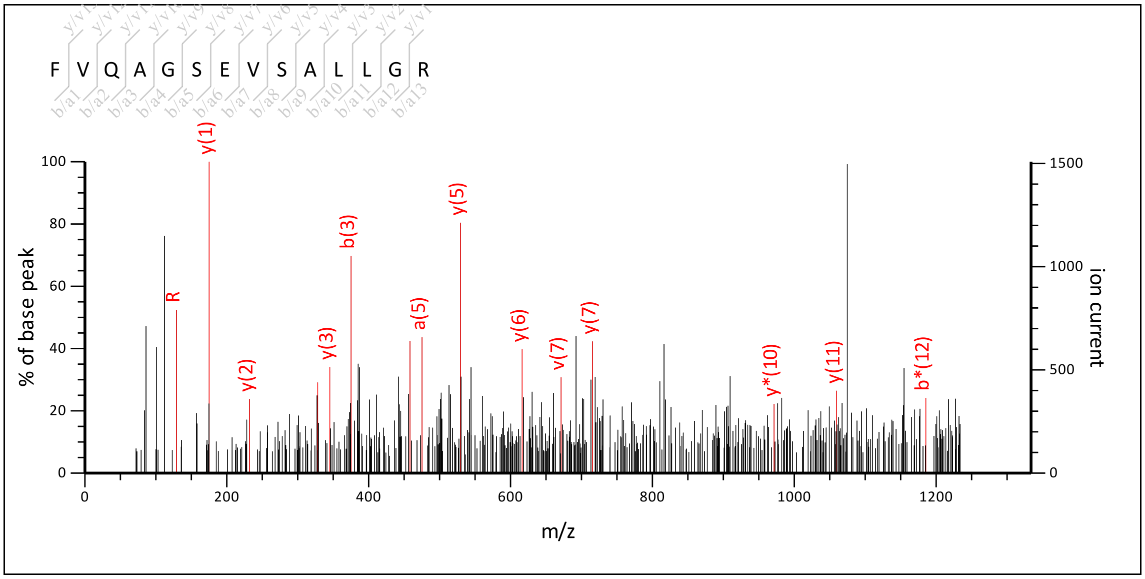


0 to 1333.81

Label all possible matches Label matches used for scoring

**Monoisotopic mass of neutral peptide Mr(calc):** 1432.7674
**Fixed modifications:** Carbamidomethyl (C) (apply to specified residues or termini only)
**Ions Score:** 47 **Expect:** 0.037
**Matches :** 23/243 fragment ions using 24 most intense peaks (help)

| # | Immon. | a | a* | a⁰ | b | b* | b⁰ | d | Seq. | v | w | y | y* | y⁰ | # |
|---|---|---|---|---|---|---|---|---|---|---|---|---|---|---|---|
| 1 | 120.0808 | 120.0808 | | | 148.0757 | | | 44.0495 | F | | | | | | 14 |
| 2 | 72.0808 | 219.1492 | | | 247.1441 | | | 205.1335 | V | 1242.6437 | 1255.6641 | 1286.7063 | 1269.6797 | 1268.6957 | 13 |
| 3 | 101.0709 | 347.2078 | 330.1812 | | 375.2027 | 358.1761 | | 290.1863 | Q | 1114.5851 | 1113.5899 | 1187.6379 | 1170.6113 | 1169.6273 | 12 |
| 4 | 44.0495 | 418.2449 | 401.2183 | | 446.2398 | 429.2132 | | | A | 1043.5480 | | ***1059.5793*** | 1042.5527 | 1041.5687 | 11 |
| 5 | 30.0338 | 475.2663 | 458.2398 | | 503.2613 | 486.2347 | | | G | | | 988.5422 | 971.5156 | 970.5316 | 10 |
| 6 | 60.0444 | 562.2984 | 545.2718 | 544.2878 | 590.2933 | 573.2667 | 572.2827 | 546.3035 | S | 899.4945 | 898.4993 | 931.5207 | 914.4942 | 913.5102 | 9 |
| 7 | 102.0550 | 691.3410 | 674.3144 | 673.3304 | 719.3359 | 702.3093 | 701.3253 | 633.3355 | E | 770.4519 | 769.4567 | 844.4887 | 827.4621 | 826.4781 | 8 |
| 8 | 72.0808 | 790.4094 | 773.3828 | 772.3988 | 818.4043 | 801.3777 | 800.3937 | 776.3937 | V | 671.3835 | 684.4039 | ***715.4461*** | 698.4196 | 697.4355 | 7 |
| 9 | 60.0444 | 877.4414 | 860.4149 | 859.4308 | 905.4363 | 888.4098 | 887.4258 | 861.4465 | S | 584.3515 | 583.3562 | ***616.3777*** | 599.3511 | 598.3671 | 6 |
| 10 | 44.0495 | 948.4785 | 931.4520 | 930.4680 | 976.4734 | 959.4469 | 958.4629 | | A | 513.3144 | | ***529.3457*** | 512.3191 | | 5 |
| 11 | 86.0964 | 1061.5626 | 1044.5360 | 1043.5520 | 1089.5575 | 1072.5310 | 1071.5469 | 1019.5156 | L | 400.2303 | 399.2350 | ***458.3085*** | 441.2820 | | 4 |
| 12 | 86.0964 | 1174.6466 | 1157.6201 | 1156.6361 | 1202.6416 | 1185.6150 | 1184.6310 | 1132.5997 | L | 287.1462 | 286.1510 | ***345.2245*** | 328.1979 | | 3 |
| 13 | 30.0338 | 1231.6681 | 1214.6416 | 1213.6575 | 1259.6630 | 1242.6365 | 1241.6525 | | G | | | ***232.1404*** | 215.1139 | | 2 |
| 14 | 129.1135 | | | | | | | | R | 74.0237 | 73.0284 | ***175.1190*** | 158.0924 | | 1 |

| Seq | ya | yb | Seq | ya | yb | Seq | ya | yb |
|---|---|---|---|---|---|---|---|---|
| VQ | 200.1394 | 228.1343 | VQA | 271.1765 | 299.1714 | VQAG | 328.1979 | 356.1928 |
| VQAGS | 415.2300 | 443.2249 | VQAGSE | 544.2726 | 572.2675 | VQAGSEV | 643.3410 | 671.3359 |
| QA | 172.1081 | 200.1030 | QAG | 229.1295 | 257.1244 | QAGS | 316.1615 | 344.1565 |
| QAGSE | 445.2041 | 473.1991 | QAGSEV | 544.2726 | 572.2675 | QAGSEVS | 631.3046 | 659.2995 |

| | | | | | | | | |
|---|---|---|---|---|---|---|---|---|
| **AG** | 101.0709 | 129.0659 | **AGS** | 188.1030 | 216.0979 | **AGSE** | 317.1456 | 345.1405 |
| **AGSEV** | 416.2140 | 444.2089 | **AGSEVS** | 503.2460 | 531.2409 | **AGSEVSA** | 574.2831 | 602.2780 |
| **AGSEVSAL** | 687.3672 | 715.3621 | **GS** | 117.0659 | 145.0608 | **GSE** | 246.1084 | 274.1034 |
| **GSEV** | 345.1769 | 373.1718 | **GSEVS** | 432.2089 | 460.2038 | **GSEVSA** | 503.2460 | 531.2409 |
| **GSEVSAL** | 616.3301 | 644.3250 | **SE** | 189.0870 | 217.0819 | **SEV** | 288.1554 | 316.1503 |
| **SEVS** | 375.1874 | 403.1823 | **SEVSA** | 446.2245 | 474.2195 | **SEVSAL** | 559.3086 | 587.3035 |
| **SEVSALL** | 672.3927 | 700.3876 | **EV** | 201.1234 | 229.1183 | **EVS** | 288.1554 | 316.1503 |
| **EVSA** | 359.1925 | 387.1874 | **EVSAL** | 472.2766 | 500.2715 | **EVSALL** | 585.3606 | 613.3556 |
| **EVSALLG** | 642.3821 | 670.3770 | **VS** | 159.1128 | 187.1077 | **VSA** | 230.1499 | 258.1448 |
| **VSAL** | 343.2340 | 371.2289 | **VSALL** | 456.3180 | 484.3130 | **VSALLG** | 513.3395 | 541.3344 |
| **SA** | 131.0815 | 159.0764 | **SAL** | 244.1656 | 272.1605 | **SALL** | 357.2496 | 385.2445 |
| **SALLG** | 414.2711 | 442.2660 | **AL** | 157.1335 | 185.1285 | **ALL** | 270.2176 | 298.2125 |
| **ALLG** | 327.2391 | 355.2340 | **LL** | 199.1805 | 227.1754 | **LLG** | 256.2020 | 284.1969 |
| **LG** | 143.1179 | 171.1128 | | | | | | |

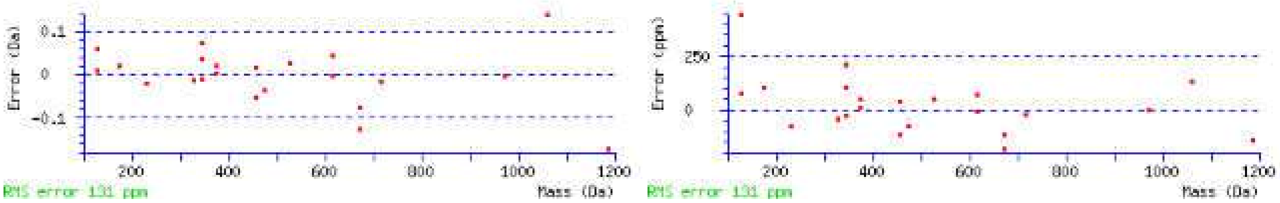


NCBI **BLAST** search of FVQAGSEVSALLGR
(Parameters: blastp, nr protein database, expect=20000, no filter, PAM30)
Other BLAST web gateways

**All matches to this query**

| Score | Mr(calc) | Delta | Sequence |
|---|---|---|---|
| 47.4 | 1432.7674 | 0.0484 | FVQAGSEVSAILGR |
| 47.4 | 1432.7674 | 0.0484 | FVQAGSEVSALIGR |
| 47.4 | 1432.7674 | 0.0484 | FVQAGSEVSALLGR |
| 47.4 | 1432.7674 | 0.0484 | FVQAGSEVSALLGR |
| 47.4 | 1432.7674 | 0.0484 | FVQAGSEVSALLGR |
| 47.4 | 1432.7674 | 0.0484 | FVQAGSEVSALLGR |
| 47.4 | 1432.7674 | 0.0484 | FVQAGSEVSALLGR |
| 47.4 | 1432.7674 | 0.0484 | FVQAGSEVSALLGR |
| 47.4 | 1432.7674 | 0.0484 | FVQAGSEVSALLGR |
| 47.4 | 1432.7674 | 0.0484 | FVQAGSEVSALLGR |

**Mascot:** http://www.matrixscience.com/

# MATRIX SCIENCE Mascot Search Results

## Peptide View **Spot no 114**

MS/MS Fragmentation of **GIYPAVDPLDSTSTMLQPR**
Found in **gi|11583** in **NCBInr**, ATPase, beta subunit [Hordeum vulgare]

Match to Query 232: 2076.058624 from(2077.065900,1+) intensity(0.0000) index(31)
Title: Label: I1, Spot_Id: 219682, Peak_List_Id: 225028, MSMS Job_Run_Id: 21761, Comment:
Data file C:\Documents and Settings\Administrator\Desktop\farah BHU\spot set plate\Farah-Garima 11May 2013\ppw_I1_136842037600.txt

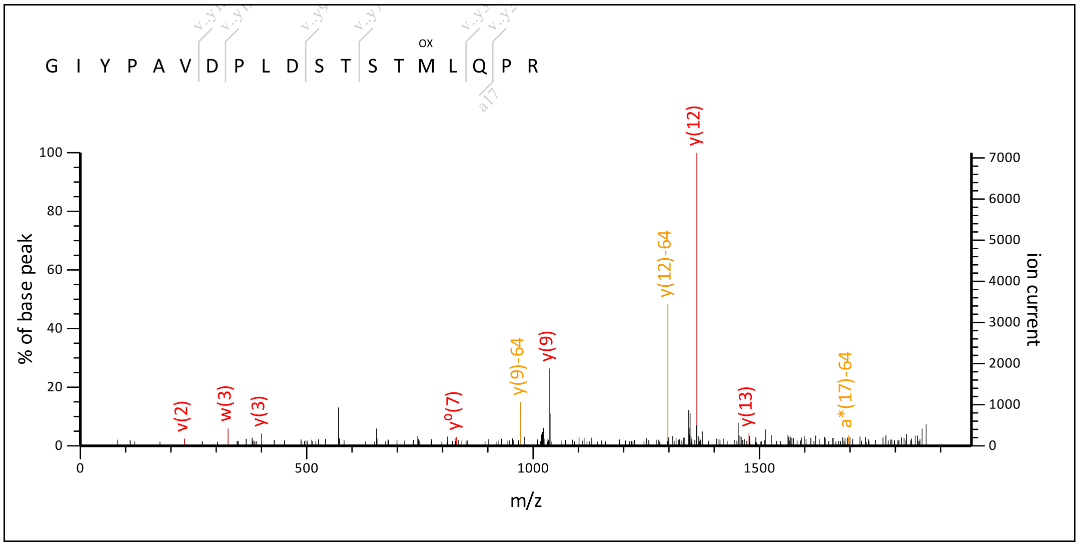


0 to 1968.07

Label all possible matches ○ Label matches used for scoring ◉

**Monoisotopic mass of neutral peptide Mr(calc):** 2076.0198
**Fixed modifications:** Carbamidomethyl (C) (apply to specified residues or termini only)
**Variable modifications:**
**M15** : Oxidation (M), with neutral losses 0.0000(shown in table), 63.9983
**Ions Score:** 2 **Expect:** 9.9e+02
**Matches** : 15/459 fragment ions using 36 most intense peaks (help)

| # | Immon. | a | a* | $a^0$ | b | b* | $b^0$ | d | d' | Seq. | v | w | w' | y | y* | |
|---|---|---|---|---|---|---|---|---|---|---|---|---|---|---|---|---|
| 1 | 30.0338 | 30.0338 | | | 58.0287 | | | 44.0495 | | G | | | | | | |
| 2 | 86.0964 | 143.1179 | | | 171.1128 | | | 115.0866 | 129.1022 | I | 1961.9273 | 1974.9477 | 1988.9634 | 2020.0056 | 2002.9790 | [illegible] |
| 3 | 136.0757 | 306.1812 | | | 334.1761 | | | | | Y | 1798.8640 | | | 1906.9215 | 1889.8950 | [illegible] |
| 4 | 70.0651 | 403.2340 | | | 431.2289 | | | 377.2183 | | P | 1701.8112 | 1700.8160 | | 1743.8582 | 1726.8316 | [illegible] |
| 5 | 44.0495 | 474.2711 | | | 502.2660 | | | | | A | 1630.7741 | | | 1646.8054 | 1629.7789 | [illegible] |
| 6 | 72.0808 | 573.3395 | | | 601.3344 | | | 559.3239 | | V | 1531.7057 | 1544.7261 | | 1575.7683 | 1558.7418 | [illegible] |
| 7 | 88.0393 | 688.3665 | | 670.3559 | 716.3614 | | 698.3508 | 644.3766 | | D | 1416.6788 | 1415.6835 | | ***1476.6999*** | 1459.6733 | [illegible] |
| 8 | 70.0651 | 785.4192 | | 767.4087 | 813.4141 | | 795.4036 | 759.4036 | | P | 1319.6260 | 1318.6307 | | ***1361.6729*** | 1344.6464 | [illegible] |
| 9 | 86.0964 | 898.5033 | | 880.4927 | 926.4982 | | 908.4876 | 856.4563 | | L | 1206.5419 | 1205.5467 | | 1264.6202 | 1247.5936 | [illegible] |
| 10 | 88.0393 | 1013.5302 | | 995.5197 | 1041.5251 | | 1023.5146 | 969.5404 | | D | 1091.5150 | 1090.5197 | | 1151.5361 | 1134.5096 | [illegible] |
| 11 | 60.0444 | 1100.5623 | | 1082.5517 | 1128.5572 | | 1110.5466 | 1084.5673 | | S | 1004.4830 | 1003.4877 | | ***1036.5092*** | 1019.4826 | [illegible] |
| 12 | 74.0600 | 1201.6099 | | 1183.5994 | 1229.6048 | | 1211.5943 | 1185.6150 | 1187.5943 | T | 903.4353 | 916.4557 | 918.4349 | 949.4771 | 932.4506 | [illegible] |
| 13 | 60.0444 | 1288.6420 | | 1270.6314 | 1316.6369 | | 1298.6263 | 1272.6470 | | S | 816.4033 | 815.4080 | | 848.4295 | 831.4029 | [illegible] |
| 14 | 74.0600 | 1389.6896 | | 1371.6791 | 1417.6846 | | 1399.6740 | 1373.6947 | 1375.6740 | T | 715.3556 | 728.3760 | 730.3552 | 761.3974 | 744.3709 | [illegible] |
| 15 | 120.0478 | 1536.7250 | | 1518.7145 | 1564.7200 | | 1546.7094 | 1460.7268 | | M | 568.3202 | 567.3249 | | 660.3498 | 643.3232 | |
| 16 | 86.0964 | 1649.8091 | | 1631.7985 | 1677.8040 | | 1659.7935 | 1607.7622 | | L | 455.2361 | 454.2409 | | 513.3144 | 496.2878 | |
| 17 | 101.0709 | 1777.8677 | 1760.8411 | 1759.8571 | 1805.8626 | 1788.8360 | 1787.8520 | 1720.8462 | | Q | 327.1775 | 326.1823 | | ***400.2303*** | 383.2037 | |
| 18 | 70.0651 | 1874.9204 | 1857.8939 | 1856.9099 | 1902.9154 | 1885.8888 | 1884.9048 | 1848.9048 | | P | 230.1248 | 229.1295 | | 272.1717 | 255.1452 | |
| 19 | 129.1135 | | | | | | | | | R | 74.0237 | 73.0284 | | 175.1190 | 158.0924 | |

| Seq | ya | yb | Seq | ya | yb | Seq | ya | yb |
|---|---|---|---|---|---|---|---|---|
| IY | 249.1598 | 277.1547 | IYP | 346.2125 | 374.2074 | IYPA | 417.2496 | 445.2445 |
| IYPAV | 516.3180 | 544.3130 | IYPAVD | 631.3450 | 659.3399 | YP | 233.1285 | 261.1234 |
| YPA | 304.1656 | 332.1605 | YPAV | 403.2340 | 431.2289 | YPAVD | 518.2609 | 546.2558 |
| YPAVDP | 615.3137 | 643.3086 | PA | 141.1022 | 169.0972 | PAV | 240.1707 | 268.1656 |
| PAVD | 355.1976 | 383.1925 | PAVDP | 452.2504 | 480.2453 | PAVDPL | 565.3344 | 593.3293 |
| PAVDPLD | 680.3614 | 708.3563 | AV | 143.1179 | 171.1128 | AVD | 258.1448 | 286.1397 |
| AVDP | 355.1976 | 383.1925 | AVDPL | 468.2817 | 496.2766 | AVDPLD | 583.3086 | 611.3035 |
| AVDPLDS | 670.3406 | 698.3355 | VD | 187.1077 | 215.1026 | VDP | 284.1605 | 312.1554 |
| VDPL | 397.2445 | 425.2395 | VDPLD | 512.2715 | 540.2664 | VDPLDS | 599.3035 | 627.2984 |
| DP | 185.0921 | 213.0870 | DPL | 298.1761 | 326.1710 | DPLD | 413.2031 | 441.1980 |
| DPLDS | 500.2351 | 528.2300 | DPLDST | 601.2828 | 629.2777 | DPLDSTS | 688.3148 | 716.3097 |
| PL | 183.1492 | 211.1441 | PLD | 298.1761 | 326.1710 | PLDS | 385.2082 | 413.2031 |
| PLDST | 486.2558 | 514.2508 | PLDSTS | 573.2879 | 601.2828 | PLDSTST | 674.3355 | 702.3305 |
| LD | 201.1234 | 229.1183 | LDS | 288.1554 | 316.1503 | LDST | 389.2031 | 417.1980 |
| LDSTS | 476.2351 | 504.2300 | LDSTST | 577.2828 | 605.2777 | DS | 175.0713 | 203.0662 |
| DST | 276.1190 | 304.1139 | DSTS | 363.1510 | 391.1460 | DSTST | 464.1987 | 492.1936 |
| DSTSTM | 611.2341 | 639.2290 | ST | 161.0921 | 189.0870 | STS | 248.1241 | 276.1190 |
| STST | 349.1718 | 377.1667 | STSTM | 496.2072 | 524.2021 | STSTML | 609.2912 | 637.2862 |
| TS | 161.0921 | 189.0870 | TST | 262.1397 | 290.1347 | TSTM | 409.1751 | 437.1701 |
| TSTML | 522.2592 | 550.2541 | TSTMLQ | 650.3178 | 678.3127 | ST | 161.0921 | 189.0870 |
| STM | 308.1275 | 336.1224 | STML | 421.2115 | 449.2064 | STMLQ | 549.2701 | 577.2650 |
| STMLQP | 646.3229 | 674.3178 | TM | 221.0954 | 249.0904 | TML | 334.1795 | 362.1744 |
| TMLQ | 462.2381 | 490.2330 | TMLQP | 559.2908 | 587.2858 | ML | 233.1318 | 261.1267 |
| MLQ | 361.1904 | 389.1853 | MLQP | 458.2432 | 486.2381 | LQ | 214.1550 | 242.1499 |
| LQP | 311.2078 | 339.2027 | QP | 198.1237 | 226.1186 | | | |

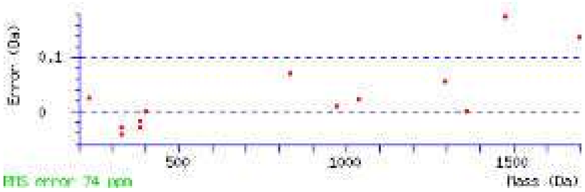


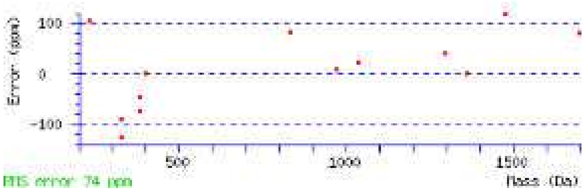


NCBI **BLAST** search of GIYPAVDPLDSTSTMLQPR
(Parameters: blastp, nr protein database, expect=20000, no filter, PAM30)
Other BLAST web gateways

**All matches to this query**

| Score | Mr(calc) | Delta | Sequence |
|---|---|---|---|
| 3.9 | 2076.0595 | -0.0009 | TQRMILTQEEMEEVVLK |
| 2.0 | 2076.0198 | 0.0389 | GIYPAVDPLDSTSTMIQPR |
| 2.0 | 2076.0561 | 0.0025 | GIYPAVDPLDSTSTMLKPR |
| 2.0 | 2076.0198 | 0.0389 | GIYPAVDPLDSTSTMLQPR |
| 2.0 | 2076.0198 | 0.0389 | GIYPAVDPLDSTSTMLQPR |
| 2.0 | 2076.0198 | 0.0389 | GIYPAVDPLDSTSTMLQPR |
| 2.0 | 2076.0198 | 0.0389 | GIYPAVDPLDSTSTMLQPR |
| 2.0 | 2076.0198 | 0.0389 | GIYPAVDPLDSTSTMLQPR |
| 2.0 | 2076.0198 | 0.0389 | GIYPAVDPLDSTSTMLQPR |
| 2.0 | 2076.0198 | 0.0389 | GIYPAVDPLDSTSTMLQPR |

**Mascot:** http://www.matrixscience.com/

# MATRIX SCIENCE Mascot Search Results

## Peptide View **Spot no 114**

MS/MS Fragmentation of **GMEVIDTGAPLSVPVGGATLGR**
Found in **gi|11583** in **NCBInr**, ATPase, beta subunit [Hordeum vulgare]

Match to Query 234: 2112.123324 from(2113.130600,1+) intensity(0.0000) index(32)
Title: Label: I1, Spot_Id: 219682, Peak_List_Id: 225041, MSMS Job_Run_Id: 21761, Comment:
Data file C:\Documents and Settings\Administrator\Desktop\farah BHU\spot set plate\Farah-Garima 11May 2013\ppw_I1_136842037600.txt

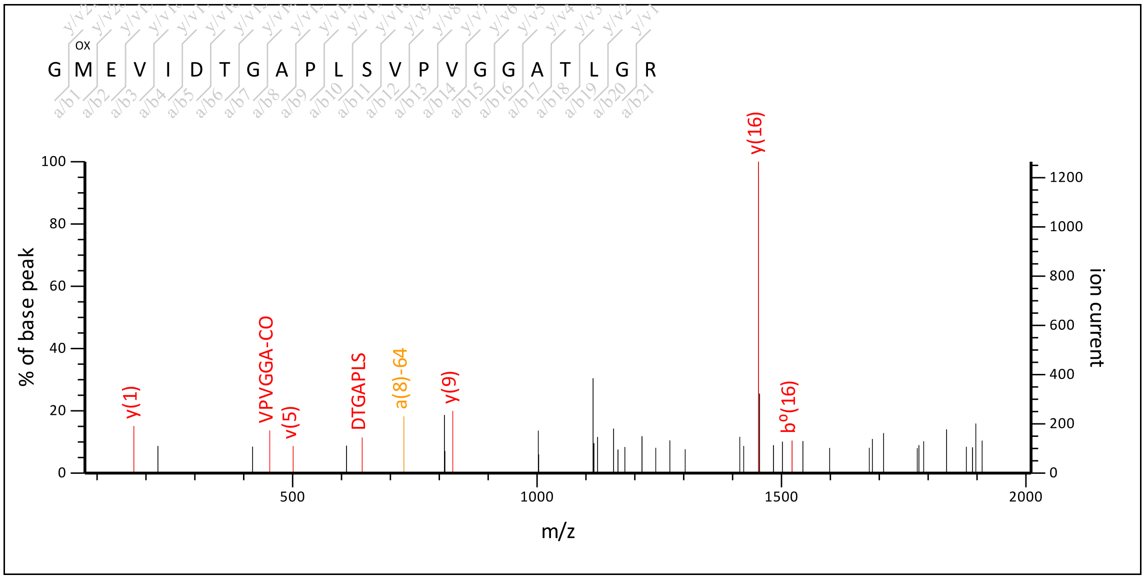


75.13 to 2010.5

Label all possible matches ○ Label matches used for scoring ◉

**Monoisotopic mass of neutral peptide Mr(calc):** 2112.0885
**Fixed modifications:** Carbamidomethyl (C) (apply to specified residues or termini only)
**Variable modifications:**
**M2** : Oxidation (M), with neutral losses 63.9983(shown in table), 0.0000
**Ions Score:** 3 **Expect:** 6.6e+02
**Matches :** 8/535 fragment ions using 16 most intense peaks (help)

| # | Immon. | a | $a^0$ | b | $b^0$ | d | d' | Seq. | v | w | w' | y | y* | $y^0$ | # |
|---|---|---|---|---|---|---|---|---|---|---|---|---|---|---|---|
| 1 | 30.0338 | 30.0338 | | 58.0287 | | 44.0495 | | G | | | | | | | 22 |
| 2 | 56.0495 | 113.0709 | | 141.0659 | | 101.0709 | | M | 1964.0447 | 1963.0495 | | 1992.0760 | 1975.0495 | 1974.0655 | 21 |
| 3 | 102.0550 | 242.1135 | 224.1030 | 270.1084 | 252.0979 | 184.1081 | | E | 1835.0021 | 1834.0069 | | 1909.0389 | 1892.0124 | 1891.0284 | 20 |
| 4 | 72.0808 | 341.1819 | 323.1714 | 369.1769 | 351.1663 | 327.1663 | | V | 1735.9337 | 1748.9541 | | 1779.9963 | 1762.9698 | 1761.9858 | 19 |
| 5 | 86.0964 | 454.2660 | 436.2554 | 482.2609 | 464.2504 | 426.2347 | 440.2504 | I | 1622.8497 | 1635.8701 | 1649.8857 | 1680.9279 | 1663.9014 | 1662.9173 | 18 |
| 6 | 88.0393 | 569.2930 | 551.2824 | 597.2879 | 579.2773 | 525.3031 | | D | 1507.8227 | 1506.8275 | | 1567.8438 | 1550.8173 | 1549.8333 | 17 |
| 7 | 74.0600 | 670.3406 | 652.3301 | 698.3355 | 680.3250 | 654.3457 | 656.3250 | T | 1406.7750 | 1419.7954 | 1421.7747 | ***1452.8169*** | 1435.7904 | 1434.8063 | 16 |
| 8 | 30.0338 | 727.3621 | 709.3515 | 755.3570 | 737.3464 | | | G | | | | 1351.7692 | 1334.7427 | 1333.7587 | 15 |
| 9 | 44.0495 | 798.3992 | 780.3886 | 826.3941 | 808.3836 | | | A | 1278.7165 | | | 1294.7478 | 1277.7212 | 1276.7372 | 14 |
| 10 | 70.0651 | 895.4520 | 877.4414 | 923.4469 | 905.4363 | 869.4363 | | P | 1181.6637 | 1180.6684 | | 1223.7106 | 1206.6841 | 1205.7001 | 13 |
| 11 | 86.0964 | 1008.5360 | 990.5255 | 1036.5310 | 1018.5204 | 966.4891 | | L | 1068.5796 | 1067.5844 | | 1126.6579 | 1109.6313 | 1108.6473 | 12 |
| 12 | 60.0444 | 1095.5681 | 1077.5575 | 1123.5630 | 1105.5524 | 1079.5732 | | S | 981.5476 | 980.5524 | | 1013.5738 | 996.5473 | 995.5633 | 11 |
| 13 | 72.0808 | 1194.6365 | 1176.6259 | 1222.6314 | 1204.6208 | 1180.6208 | | V | 882.4792 | 895.4996 | | 926.5418 | 909.5152 | 908.5312 | 10 |
| 14 | 70.0651 | 1291.6892 | 1273.6787 | 1319.6842 | 1301.6736 | 1265.6736 | | P | 785.4264 | 784.4312 | | ***827.4734*** | 810.4468 | 809.4628 | 9 |
| 15 | 72.0808 | 1390.7577 | 1372.7471 | 1418.7526 | 1400.7420 | 1376.7420 | | V | 686.3580 | 699.3784 | | 730.4206 | 713.3941 | 712.4100 | 8 |
| 16 | 30.0338 | 1447.7791 | 1429.7686 | 1475.7740 | 1457.7635 | | | G | | | | 631.3522 | 614.3257 | 613.3416 | 7 |
| 17 | 30.0338 | 1504.8006 | 1486.7900 | 1532.7955 | 1514.7849 | | | G | | | | 574.3307 | 557.3042 | 556.3202 | 6 |

| | | | | | | | | | | | | | | | |
|---|---|---|---|---|---|---|---|---|---|---|---|---|---|---|---|
| 18 | 44.0495 | 1575.8377 | 1557.8271 | 1603.8326 | 1585.8221 | | | A | 501.2780 | | | 517.3093 | 500.2827 | 499.2987 | 5 |
| 19 | 74.0600 | 1676.8854 | 1658.8748 | 1704.8803 | 1686.8697 | 1660.8905 | 1662.8697 | T | 400.2303 | 413.2507 | 415.2300 | 446.2722 | 429.2456 | 428.2616 | 4 |
| 20 | 86.0964 | 1789.9694 | 1771.9589 | 1817.9644 | 1799.9538 | 1747.9225 | | L | 287.1462 | 286.1510 | | 345.2245 | 328.1979 | | 3 |
| 21 | 30.0338 | 1846.9909 | 1828.9803 | 1874.9858 | 1856.9753 | | | G | | | | 232.1404 | 215.1139 | | 2 |
| 22 | 129.1135 | | | | | | | R | 74.0237 | 73.0284 | | *175.1190* | 158.0924 | | 1 |

| Seq | ya | yb | Seq | ya | yb | Seq | ya | yb |
|---|---|---|---|---|---|---|---|---|
| **ME** | 185.0921 | 213.0870 | **MEV** | 284.1605 | 312.1554 | **MEVI** | 397.2445 | 425.2395 |
| **MEVID** | 512.2715 | 540.2664 | **MEVIDT** | 613.3192 | 641.3141 | **MEVIDTG** | 670.3406 | 698.3355 |
| **EV** | 201.1234 | 229.1183 | **EVI** | 314.2074 | 342.2023 | **EVID** | 429.2344 | 457.2293 |
| **EVIDT** | 530.2821 | 558.2770 | **EVIDTG** | 587.3035 | 615.2984 | **EVIDTGA** | 658.3406 | 686.3355 |
| **VI** | 185.1648 | 213.1598 | **VID** | 300.1918 | 328.1867 | **VIDT** | 401.2395 | 429.2344 |
| **VIDTG** | 458.2609 | 486.2558 | **VIDTGA** | 529.2980 | 557.2930 | **VIDTGAP** | 626.3508 | 654.3457 |
| **ID** | 201.1234 | 229.1183 | **IDT** | 302.1710 | 330.1660 | **IDTG** | 359.1925 | 387.1874 |
| **IDTGA** | 430.2296 | 458.2245 | **IDTGAP** | 527.2824 | 555.2773 | **IDTGAPL** | 640.3665 | 668.3614 |
| **DT** | 189.0870 | 217.0819 | **DTG** | 246.1084 | 274.1034 | **DTGA** | 317.1456 | 345.1405 |
| **DTGAP** | 414.1983 | 442.1932 | **DTGAPL** | 527.2824 | 555.2773 | **DTGAPLS** | 614.3144 | 642.3093 |
| **TG** | 131.0815 | 159.0764 | **TGA** | 202.1186 | 230.1135 | **TGAP** | 299.1714 | 327.1663 |
| **TGAPL** | 412.2554 | 440.2504 | **TGAPLS** | 499.2875 | 527.2824 | **TGAPLSV** | 598.3559 | 626.3508 |
| **TGAPLSVP** | 695.4087 | 723.4036 | **GA** | 101.0709 | 129.0659 | **GAP** | 198.1237 | 226.1186 |
| **GAPL** | 311.2078 | 339.2027 | **GAPLS** | 398.2398 | 426.2347 | **GAPLSV** | 497.3082 | 525.3031 |
| **GAPLSVP** | 594.3610 | 622.3559 | **GAPLSVPV** | 693.4294 | 721.4243 | **AP** | 141.1022 | 169.0972 |
| **APL** | 254.1863 | 282.1812 | **APLS** | 341.2183 | 369.2132 | **APLSV** | 440.2867 | 468.2817 |
| **APLSVP** | 537.3395 | 565.3344 | **APLSVPV** | 636.4079 | 664.4028 | **APLSVPVG** | 693.4294 | 721.4243 |
| **PL** | 183.1492 | 211.1441 | **PLS** | 270.1812 | 298.1761 | **PLSV** | 369.2496 | 397.2445 |
| **PLSVP** | 466.3024 | 494.2973 | **PLSVPV** | 565.3708 | 593.3657 | **PLSVPVG** | 622.3923 | 650.3872 |
| **PLSVPVGG** | 679.4137 | 707.4087 | **LS** | 173.1285 | 201.1234 | **LSV** | 272.1969 | 300.1918 |
| **LSVP** | 369.2496 | 397.2445 | **LSVPV** | 468.3180 | 496.3130 | **LSVPVG** | 525.3395 | 553.3344 |
| **LSVPVGG** | 582.3610 | 610.3559 | **LSVPVGGA** | 653.3981 | 681.3930 | **SV** | 159.1128 | 187.1077 |
| **SVP** | 256.1656 | 284.1605 | **SVPV** | 355.2340 | 383.2289 | **SVPVG** | 412.2554 | 440.2504 |
| **SVPVGG** | 469.2769 | 497.2718 | **SVPVGGA** | 540.3140 | 568.3089 | **SVPVGGAT** | 641.3617 | 669.3566 |
| **VP** | 169.1335 | 197.1285 | **VPV** | 268.2020 | 296.1969 | **VPVG** | 325.2234 | 353.2183 |
| **VPVGG** | 382.2449 | 410.2398 | **VPVGGA** | 453.2820 | 481.2769 | **VPVGGAT** | 554.3297 | 582.3246 |
| **VPVGGATL** | 667.4137 | 695.4087 | **PV** | 169.1335 | 197.1285 | **PVG** | 226.1550 | 254.1499 |
| **PVGG** | 283.1765 | 311.1714 | **PVGGA** | 354.2136 | 382.2085 | **PVGGAT** | 455.2613 | 483.2562 |
| **PVGGATL** | 568.3453 | 596.3402 | **PVGGATLG** | 625.3668 | 653.3617 | **VG** | 129.1022 | 157.0972 |
| **VGG** | 186.1237 | 214.1186 | **VGGA** | 257.1608 | 285.1557 | **VGGAT** | 358.2085 | 386.2034 |
| **VGGATL** | 471.2926 | 499.2875 | **VGGATLG** | 528.3140 | 556.3089 | **GG** | 87.0553 | 115.0502 |
| **GGA** | 158.0924 | 186.0873 | **GGAT** | 259.1401 | 287.1350 | **GGATL** | 372.2241 | 400.2191 |
| **GGATLG** | 429.2456 | 457.2405 | **GA** | 101.0709 | 129.0659 | **GAT** | 202.1186 | 230.1135 |
| **GATL** | 315.2027 | 343.1976 | **GATLG** | 372.2241 | 400.2191 | **AT** | 145.0972 | 173.0921 |
| **ATL** | 258.1812 | 286.1761 | **ATLG** | 315.2027 | 343.1976 | **TL** | 187.1441 | 215.1390 |
| **TLG** | 244.1656 | 272.1605 | **LG** | 143.1179 | 171.1128 | | | |

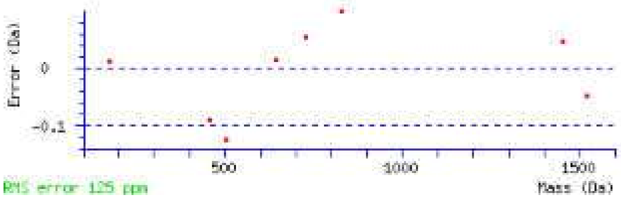


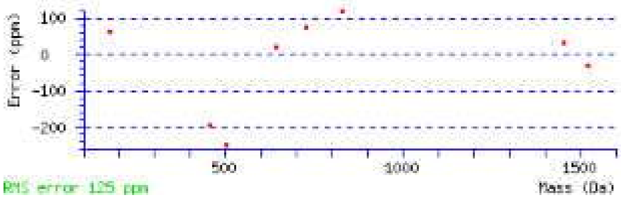


NCBI **BLAST** search of GMEVIDTGAPLSVPVGGATLGR
(Parameters: blastp, nr protein database, expect=20000, no filter, PAM30)
Other BLAST web gateways

**All matches to this query**

| Score | Mr(calc) | Delta | Sequence |
|---|---|---|---|
| 6.2 | 2112.0819 | 0.0414 | EMGAQKPQIQNPTAIMIAR |
| 5.2 | 2111.9793 | 0.1440 | SSTSNDMFQLIQQLTEDR |
| 3.4 | 2112.0885 | 0.0348 | GMEVIDTGAPISVPVGGATLGR |
| 3.4 | 2112.0885 | 0.0348 | GMEVIDTGAPISVPVGGATLGR |
| 3.4 | 2112.0885 | 0.0348 | GMEVIDTGAPISVPVGGATLGR |
| 3.4 | 2112.0885 | 0.0348 | GMEVIDTGAPISVPVGGATLGR |
| 3.4 | 2112.0885 | 0.0348 | GMEVIDTGAPLSVPVGGATIGR |
| 3.4 | 2112.0885 | 0.0348 | GMEVIDTGAPLSVPVGGATLGR |
| 3.4 | 2112.0885 | 0.0348 | GMEVIDTGAPLSVPVGGATLGR |
| 3.4 | 2112.0885 | 0.0348 | GMEVIDTGAPLSVPVGGATLGR |

**Mascot:** http://www.matrixscience.com/

# MATRIX SCIENCE Mascot Search Results

## Peptide View Spot no 116

MS/MS Fragmentation of **LGCIEDTGNHKR**
Found in **gi|115477014** in **NCBInr**, Os08g0487800 [Oryza sativa Japonica Group]

Match to Query 38: 1398.791424 from(1399.798700,1+) intensity(0.0000) index(13)
Title: Label: M6, Spot_Id: 219766, Peak_List_Id: 226016, MSMS Job_Run_Id: 21831, Comment:
Data file C:\Documents and Settings\Administrator\Desktop\Farah-Garima 11May 2013\ppw_M6_136859901000.txt

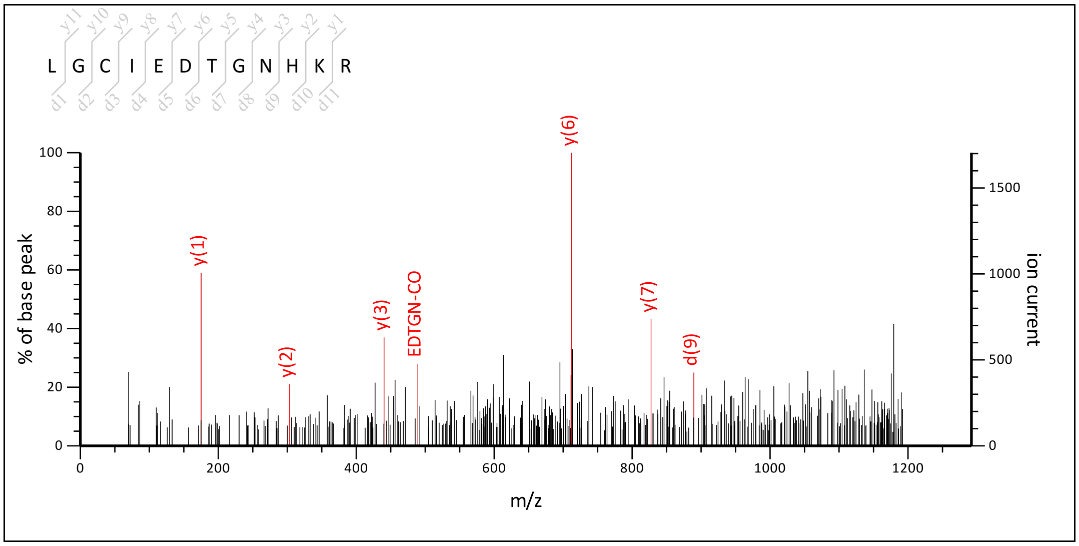


0 to 1291.96

Label all possible matches Label matches used for scoring

```
Monoisotopic mass of neutral peptide Mr(calc): 1398.6674
Fixed modifications: Carbamidomethyl (C) (apply to specified residues or termini only)
Ions Score: 25  Expect: 1e+02
Matches : 9/181 fragment ions using 12 most intense peaks   (help)
```

| # | Immon. | a | a* | $a^0$ | b | b* | $b^0$ | d | d' | Seq. | v | w | w' | y | y* | [illegible] |
|---|---|---|---|---|---|---|---|---|---|---|---|---|---|---|---|---|
| 1 | 86.0964 | 86.0964 | | | 114.0913 | | | 44.0495 | | L | | | | | | |
| 2 | 30.0338 | 143.1179 | | | 171.1128 | | | | | G | | | | 1286.5906 | 1269.5641 | [illegible] |
| 3 | 133.0430 | **303.1485** | | | 331.1435 | | | 214.1550 | | C | 1124.5443 | 1123.5491 | | 1229.5691 | 1212.5426 | [illegible] |
| 4 | 86.0964 | 416.2326 | | | 444.2275 | | | 388.2013 | 402.2170 | I | 1011.4602 | 1024.4806 | 1038.4963 | 1069.5385 | 1052.5119 | [illegible] |
| 5 | 102.0550 | 545.2752 | | 527.2646 | 573.2701 | | 555.2595 | 487.2697 | | E | 882.4177 | 881.4224 | | 956.4544 | 939.4279 | [illegible] |
| 6 | 88.0393 | 660.3021 | | 642.2916 | 688.2971 | | 670.2865 | 616.3123 | | D | 767.3907 | 766.3955 | | ***827.4118*** | 810.3853 | [illegible] |
| 7 | 74.0600 | 761.3498 | | 743.3393 | 789.3447 | | 771.3342 | 745.3549 | 747.3342 | T | 666.3430 | 679.3634 | 681.3427 | ***712.3849*** | 695.3583 | [illegible] |
| 8 | 30.0338 | 818.3713 | | 800.3607 | 846.3662 | | 828.3556 | | | G | | | | 611.3372 | 594.3107 | |
| 9 | 87.0553 | 932.4142 | 915.3877 | 914.4036 | 960.4091 | 943.3826 | 942.3986 | 889.4084 | | N | 495.2786 | 494.2834 | | 554.3158 | 537.2892 | |
| 10 | 110.0713 | 1069.4731 | 1052.4466 | 1051.4626 | 1097.4680 | 1080.4415 | 1079.4575 | | | H | 358.2197 | | | ***440.2728*** | 423.2463 | |
| 11 | 101.1073 | 1197.5681 | 1180.5415 | 1179.5575 | 1225.5630 | 1208.5364 | 1207.5524 | 1140.5102 | | K | 230.1248 | 229.1295 | | ***303.2139*** | 286.1874 | |
| 12 | 129.1135 | | | | | | | | | R | 74.0237 | 73.0284 | | ***175.1190*** | 158.0924 | |

| Seq | ya | yb | Seq | ya | yb | Seq | ya | yb |
|---|---|---|---|---|---|---|---|---|
| GC | 190.0645 | 218.0594 | GCI | 303.1485 | 331.1435 | GCIE | 432.1911 | 460.1860 |
| GCIED | 547.2181 | 575.2130 | GCIEDT | 648.2658 | 676.2607 | CI | 246.1271 | 274.1220 |
| CIE | 375.1697 | 403.1646 | CIED | 490.1966 | 518.1915 | CIEDT | 591.2443 | 619.2392 |
| CIEDTG | 648.2658 | 676.2607 | IE | 215.1390 | 243.1339 | IED | 330.1660 | 358.1609 |
| IEDT | 431.2136 | 459.2086 | IEDTG | 488.2351 | 516.2300 | IEDTGN | 602.2780 | 630.2729 |
| ED | 217.0819 | 245.0768 | EDT | 318.1296 | 346.1245 | EDTG | 375.1510 | 403.1460 |
| EDTGN | 489.1940 | 517.1889 | EDTGNH | 626.2529 | 654.2478 | DT | 189.0870 | 217.0819 |
| DTG | 246.1084 | 274.1034 | DTGN | 360.1514 | 388.1463 | DTGNH | 497.2103 | 525.2052 |
| DTGNHK | 625.3052 | 653.3002 | TG | 131.0815 | 159.0764 | TGN | 245.1244 | 273.1193 |

| | | | | | | | | |
|---|---|---|---|---|---|---|---|---|
| **TGNH** | 382.1833 | 410.1783 | **TGNHK** | 510.2783 | 538.2732 | **GN** | 144.0768 | 172.0717 |
| **GNH** | 281.1357 | 309.1306 | **GNHK** | 409.2306 | 437.2255 | **NH** | 224.1142 | 252.1091 |
| **NHK** | 352.2092 | 380.2041 | **HK** | 238.1662 | 266.1612 | | | |

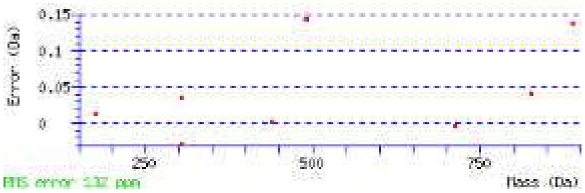


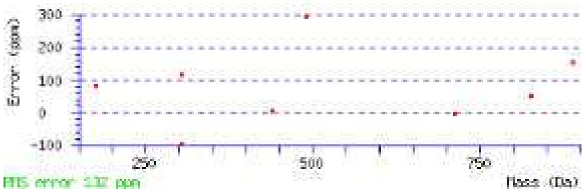


NCBI **BLAST** search of LGCIEDTGNHKR
(Parameters: blastp, nr protein database, expect=20000, no filter, PAM30)
Other BLAST web gateways

**All matches to this query**

| Score | Mr(calc) | Delta | Sequence |
|---|---|---|---|
| 28.1 | 1398.7078 | 0.0836 | MDVQVPSGPWKR |
| 25.3 | 1398.6674 | 0.1240 | LGCIEDTGNHKR |
| 22.3 | 1398.6640 | 0.1274 | LWSDADSGAGHKR |
| 22.2 | 1398.6537 | 0.1378 | GRPMMDVYFQR |
| 16.3 | 1398.8194 | -0.0280 | KASALAAVDTLIAR |
| 15.8 | 1398.7467 | 0.0447 | GITEATDPVAAAKR |
| 14.8 | 1398.6852 | 0.1063 | DETQVGVDPRAGR |
| 14.8 | 1398.8380 | -0.0466 | ITMINNAILLKR |
| 14.4 | 1398.6852 | 0.1063 | AGTTSAGQLGEHVR |
| 14.2 | 1398.7467 | 0.0447 | SLERSDVDLPLR |

**Mascot:** http://www.matrixscience.com/

# MATRIX SCIENCE Mascot Search Results

## Peptide View Spot no 116

MS/MS Fragmentation of **GVVDSNDLPLNVSR**
Found in **gi|115477014** in **NCBInr**, Os08g0487800 [Oryza sativa Japonica Group]

Match to Query 46: 1483.886124 from(1484.893400,1+) intensity(0.0000) index(16)
Title: Label: M6, Spot_Id: 219766, Peak_List_Id: 226005, MSMS Job_Run_Id: 21831, Comment:
Data file C:\Documents and Settings\Administrator\Desktop\Farah-Garima 11May 2013\ppw_M6_136859901000.txt

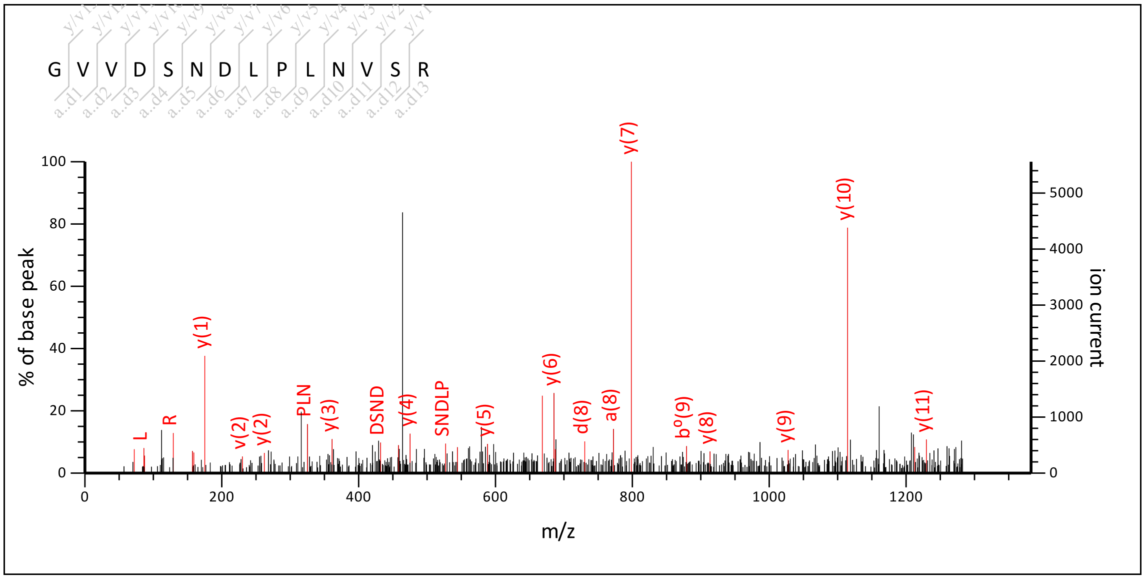


Label all possible matches ○ Label matches used for scoring ◉

**Monoisotopic mass of neutral peptide Mr(calc):** 1483.7631
**Fixed modifications:** Carbamidomethyl (C) (apply to specified residues or termini only)
**Ions Score:** 60 **Expect:** 0.022
**Matches :** 39/243 fragment ions using 65 most intense peaks (help)

| # | Immon. | a | a* | $a^0$ | b | b* | $b^0$ | d | Seq. | v | w | y | y* | $y^0$ | # |
|---|---|---|---|---|---|---|---|---|---|---|---|---|---|---|---|
| 1 | 30.0338 | 30.0338 | | | 58.0287 | | | 44.0495 | G | | | | | | 14 |
| 2 | 72.0808 | **129.1022** | | | **157.0972** | | | 115.0866 | V | 1383.6863 | 1396.7067 | 1427.7489 | 1410.7223 | 1409.7383 | 13 |
| 3 | 72.0808 | 228.1707 | | | 256.1656 | | | 214.1550 | V | 1284.6179 | 1297.6383 | 1328.6805 | 1311.6539 | 1310.6699 | 12 |
| 4 | 88.0393 | 343.1976 | | 325.1870 | 371.1925 | | 353.1819 | 299.2078 | D | 1169.5909 | 1168.5957 | ***1229.6121*** | 1212.5855 | 1211.6015 | 11 |
| 5 | 60.0444 | 430.2296 | | 412.2191 | **458.2245** | | 440.2140 | 414.2347 | S | 1082.5589 | 1081.5636 | ***1114.5851*** | 1097.5586 | 1096.5745 | 10 |
| 6 | 87.0553 | **544.2726** | 527.2460 | 526.2620 | 572.2675 | 555.2409 | 554.2569 | 501.2667 | N | 968.5160 | 967.5207 | ***1027.5531*** | 1010.5265 | 1009.5425 | 9 |
| 7 | 88.0393 | 659.2995 | 642.2729 | 641.2889 | **687.2944** | 670.2679 | 669.2838 | 615.3097 | D | 853.4890 | 852.4938 | ***913.5102*** | 896.4836 | 895.4996 | 8 |
| 8 | 86.0964 | **772.3836** | 755.3570 | 754.3730 | 800.3785 | 783.3519 | 782.3679 | 730.3366 | L | 740.4050 | 739.4097 | ***798.4832*** | 781.4567 | 780.4726 | 7 |
| 9 | 70.0651 | 869.4363 | 852.4098 | 851.4258 | 897.4312 | 880.4047 | 879.4207 | 843.4207 | P | 643.3522 | 642.3570 | ***685.3991*** | 668.3726 | 667.3886 | 6 |
| 10 | 86.0964 | 982.5204 | 965.4938 | 964.5098 | 1010.5153 | 993.4888 | 992.5047 | 940.4734 | L | 530.2681 | 529.2729 | ***588.3464*** | 571.3198 | 570.3358 | 5 |
| 11 | 87.0553 | 1096.5633 | 1079.5368 | 1078.5527 | 1124.5582 | 1107.5317 | 1106.5477 | 1053.5575 | N | 416.2252 | 415.2300 | ***475.2623*** | 458.2358 | 457.2518 | 4 |
| 12 | 72.0808 | 1195.6317 | 1178.6052 | 1177.6212 | 1223.6266 | 1206.6001 | 1205.6161 | 1181.6161 | V | 317.1568 | 330.1772 | ***361.2194*** | 344.1928 | 343.2088 | 3 |
| 13 | 60.0444 | 1282.6638 | 1265.6372 | 1264.6532 | 1310.6587 | 1293.6321 | 1292.6481 | 1266.6688 | S | 230.1248 | 229.1295 | ***262.1510*** | 245.1244 | 244.1404 | 2 |
| 14 | 129.1135 | | | | | | | | R | 74.0237 | 73.0284 | ***175.1190*** | 158.0924 | | 1 |

| Seq | ya | yb | Seq | ya | yb | Seq | ya | yb |
|---|---|---|---|---|---|---|---|---|
| VV | 171.1492 | 199.1441 | VVD | 286.1761 | 314.1710 | VVDS | 373.2082 | 401.2031 |
| VVDSN | 487.2511 | 515.2460 | VVDSND | 602.2780 | 630.2729 | VD | 187.1077 | 215.1026 |
| VDS | 274.1397 | 302.1347 | VDSN | 388.1827 | 416.1776 | VDSND | 503.2096 | 531.2045 |
| VDSNDL | 616.2937 | 644.2886 | DS | 175.0713 | 203.0662 | DSN | 289.1143 | 317.1092 |

| | | | | | | | | |
|---|---|---|---|---|---|---|---|---|
| **DSND** | 404.1412 | 432.1361 | **DSNDL** | 517.2253 | 545.2202 | **DSNDLP** | 614.2780 | 642.2729 |
| **SN** | 174.0873 | 202.0822 | **SND** | 289.1143 | 317.1092 | **SNDL** | 402.1983 | 430.1932 |
| **SNDLP** | 499.2511 | 527.2460 | **SNDLPL** | 612.3352 | 640.3301 | **ND** | 202.0822 | 230.0771 |
| **NDL** | 315.1663 | 343.1612 | **NDLP** | 412.2191 | 440.2140 | **NDLPL** | 525.3031 | 553.2980 |
| **NDLPLN** | 639.3461 | 667.3410 | **DL** | 201.1234 | 229.1183 | **DLP** | 298.1761 | 326.1710 |
| **DLPL** | 411.2602 | 439.2551 | **DLPLN** | 525.3031 | 553.2980 | **DLPLNV** | 624.3715 | 652.3665 |
| **LP** | 183.1492 | 211.1441 | **LPL** | 296.2333 | 324.2282 | **LPLN** | 410.2762 | 438.2711 |
| **LPLNV** | 509.3446 | 537.3395 | **LPLNVS** | 596.3766 | 624.3715 | **PL** | 183.1492 | 211.1441 |
| **PLN** | 297.1921 | 325.1870 | **PLNV** | 396.2605 | 424.2554 | **PLNVS** | 483.2926 | 511.2875 |
| **LN** | 200.1394 | 228.1343 | **LNV** | 299.2078 | 327.2027 | **LNVS** | 386.2398 | 414.2347 |
| **NV** | 186.1237 | 214.1186 | **NVS** | 273.1557 | 301.1506 | **VS** | 159.1128 | 187.1077 |

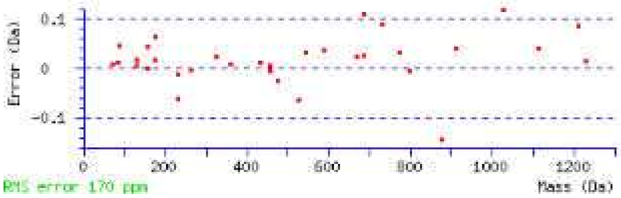


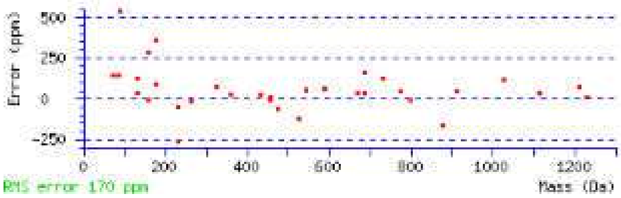


NCBI **BLAST** search of GVVDSNDLPLNVSR
(Parameters: blastp, nr protein database, expect=20000, no filter, PAM30)
Other BLAST web gateways

**All matches to this query**

| Score | Mr(calc) | Delta | Sequence |
|---|---|---|---|
| 60.3 | 1483.7631 | 0.1230 | GVVDSNDLPLNVSR |
| 30.3 | 1483.7783 | 0.1078 | VLSAVDQLPHSFR |
| 27.6 | 1483.7743 | 0.1118 | IADADAAAVRAVADR |
| 26.4 | 1483.7995 | 0.0867 | GLVDTNSLPLNVSR |
| 23.1 | 1483.7631 | 0.1231 | IADDLGLQPSNVSR |
| 21.3 | 1483.8107 | 0.0754 | GVISIDQSPIRGSR |
| 20.0 | 1483.7994 | 0.0867 | DISKENIPINSVR |
| 19.8 | 1483.8107 | 0.0754 | VDRNEIAVGLGVSR |
| 19.4 | 1483.8511 | 0.0351 | IAENALTLAKVWR |
| 19.1 | 1483.8470 | 0.0391 | LAAELNVSLVGRSR |

**Mascot:** http://www.matrixscience.com/

# MATRIX SCIENCE Mascot Search Results

## Peptide View **Spot no 114**

MS/MS Fragmentation of **ELVSNASDALDKLR**
Found in **gi|115477014** in **NCBInr**, Os08g0487800 [Oryza sativa Japonica Group]

Match to Query 50: 1529.926724 from(1530.934000,1+) intensity(0.0000) index(18)
Title: Label: M6, Spot_Id: 219766, Peak_List_Id: 226013, MSMS Job_Run_Id: 21831, Comment:
Data file C:\Documents and Settings\Administrator\Desktop\Farah-Garima 11May 2013\ppw_M6_136859901000.txt

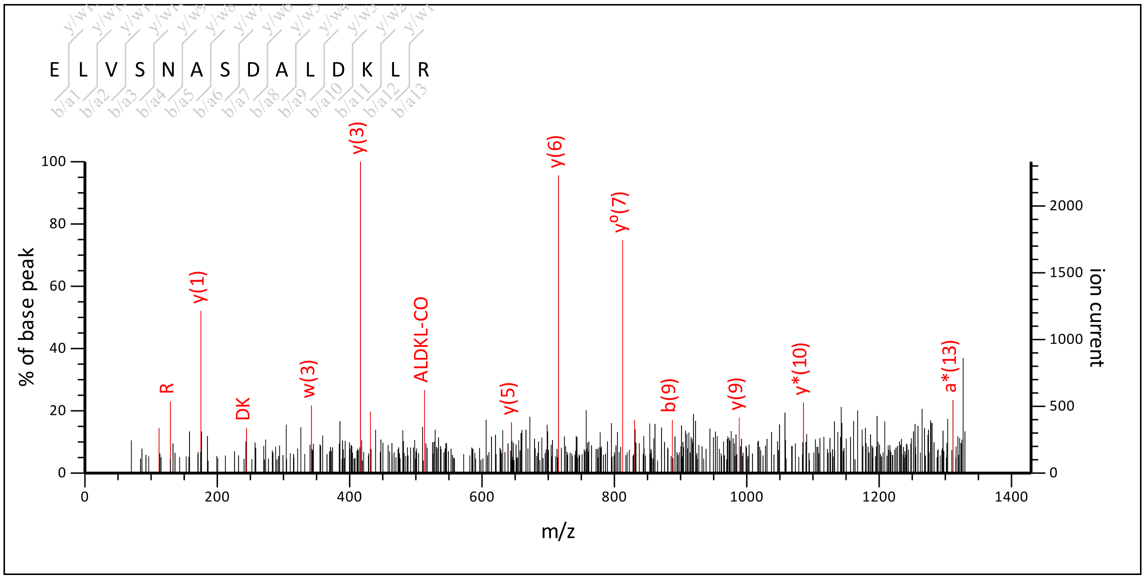


0 to 1429.53

Label all possible matches ○ Label matches used for scoring ◉

**Monoisotopic mass of neutral peptide Mr(calc):** 1529.8049
**Fixed modifications:** Carbamidomethyl (C) (apply to specified residues or termini only)
**Ions Score:** 30 **Expect:** 21
**Matches :** 20/254 fragment ions using 26 most intense peaks (help)

| # | Immon. | a | a* | $a^0$ | b | b* | $b^0$ | d | Seq. | v | w | y | y* | $y^0$ | # |
|---|---|---|---|---|---|---|---|---|---|---|---|---|---|---|---|
| 1 | 102.0550 | 102.0550 | | 84.0444 | 130.0499 | | 112.0393 | 44.0495 | E | | | | | | 14 |
| 2 | 86.0964 | 215.1390 | | 197.1285 | 243.1339 | | 225.1234 | 173.0921 | L | 1343.6914 | 1342.6961 | 1401.7696 | 1384.7431 | 1383.7591 | 13 |
| 3 | 72.0808 | 314.2074 | | 296.1969 | **342.2023** | | 324.1918 | 300.1918 | V | 1244.6230 | 1257.6434 | 1288.6856 | 1271.6590 | 1270.6750 | 12 |
| 4 | 60.0444 | 401.2395 | | 383.2289 | 429.2344 | | 411.2238 | 385.2445 | S | 1157.5909 | 1156.5957 | 1189.6171 | 1172.5906 | 1171.6066 | 11 |
| 5 | 87.0553 | 515.2824 | 498.2558 | 497.2718 | 543.2773 | 526.2508 | 525.2667 | 472.2766 | N | 1043.5480 | 1042.5527 | 1102.5851 | 1085.5586 | 1084.5745 | 10 |
| 6 | 44.0495 | 586.3195 | 569.2930 | 568.3089 | 614.3144 | 597.2879 | 596.3039 | | A | 972.5109 | | ***988.5422*** | 971.5156 | 970.5316 | 9 |
| 7 | 60.0444 | 673.3515 | 656.3250 | 655.3410 | 701.3464 | 684.3199 | 683.3359 | 657.3566 | S | 885.4789 | 884.4836 | 917.5051 | 900.4785 | 899.4945 | 8 |
| 8 | 88.0393 | 788.3785 | 771.3519 | 770.3679 | 816.3734 | 799.3468 | 798.3628 | 744.3886 | D | 770.4519 | 769.4567 | ***830.4730*** | 813.4465 | 812.4625 | 7 |
| 9 | 44.0495 | 859.4156 | 842.3890 | 841.4050 | **887.4105** | 870.3840 | 869.3999 | | A | 699.4148 | | ***715.4461*** | 698.4196 | 697.4355 | 6 |
| 10 | 86.0964 | 972.4996 | 955.4731 | 954.4891 | 1000.4946 | 983.4680 | 982.4840 | 930.4527 | L | 586.3307 | 585.3355 | ***644.4090*** | 627.3824 | 626.3984 | 5 |
| 11 | 88.0393 | 1087.5266 | 1070.5000 | 1069.5160 | 1115.5215 | 1098.4950 | 1097.5109 | 1043.5368 | D | 471.3038 | 470.3085 | 531.3249 | 514.2984 | 513.3144 | 4 |
| 12 | 101.1073 | 1215.6216 | 1198.5950 | 1197.6110 | 1243.6165 | 1226.5899 | 1225.6059 | 1158.5637 | K | 343.2088 | 342.2136 | ***416.2980*** | 399.2714 | | 3 |
| 13 | 86.0964 | 1328.7056 | 1311.6791 | 1310.6951 | 1356.7005 | 1339.6740 | 1338.6900 | 1286.6587 | L | 230.1248 | 229.1295 | 288.2030 | 271.1765 | | 2 |
| 14 | 129.1135 | | | | | | | | R | 74.0237 | 73.0284 | ***175.1190*** | 158.0924 | | 1 |

| Seq | ya | yb | Seq | ya | yb | Seq | ya | yb |
|---|---|---|---|---|---|---|---|---|
| LV | 185.1648 | 213.1598 | LVS | 272.1969 | 300.1918 | LVSN | 386.2398 | 414.2347 |
| LVSNA | 457.2769 | 485.2718 | LVSNAS | 544.3089 | 572.3039 | LVSNASD | 659.3359 | 687.3308 |
| VS | 159.1128 | 187.1077 | VSN | 273.1557 | 301.1506 | VSNA | 344.1928 | 372.1878 |
| VSNAS | 431.2249 | 459.2198 | VSNASD | 546.2518 | 574.2467 | VSNASDA | 617.2889 | 645.2838 |

| | | | | | | | | |
|---|---|---|---|---|---|---|---|---|
| **SN** | 174.0873 | 202.0822 | **SNA** | 245.1244 | 273.1193 | **SNAS** | 332.1565 | 360.1514 |
| **SNASD** | 447.1834 | 475.1783 | **SNASDA** | 518.2205 | 546.2154 | **SNASDAL** | 631.3046 | 659.2995 |
| **NA** | 158.0924 | 186.0873 | **NAS** | 245.1244 | 273.1193 | **NASD** | 360.1514 | 388.1463 |
| **NASDA** | 431.1885 | 459.1834 | **NASDAL** | 544.2726 | 572.2675 | **NASDALD** | 659.2995 | 687.2944 |
| **AS** | 131.0815 | 159.0764 | **ASD** | 246.1084 | 274.1034 | **ASDA** | 317.1456 | 345.1405 |
| **ASDAL** | 430.2296 | 458.2245 | **ASDALD** | 545.2566 | 573.2515 | **ASDALDK** | 673.3515 | 701.3464 |
| **SD** | 175.0713 | 203.0662 | **SDA** | 246.1084 | 274.1034 | **SDAL** | 359.1925 | 387.1874 |
| **SDALD** | 474.2195 | 502.2144 | **SDALDK** | 602.3144 | 630.3093 | **DA** | 159.0764 | 187.0713 |
| **DAL** | 272.1605 | 300.1554 | **DALD** | 387.1874 | 415.1823 | **DALDK** | 515.2824 | 543.2773 |
| **DALDKL** | 628.3665 | 656.3614 | **AL** | 157.1335 | 185.1285 | **ALD** | 272.1605 | 300.1554 |
| **ALDK** | 400.2554 | 428.2504 | **ALDKL** | 513.3395 | 541.3344 | **LD** | 201.1234 | 229.1183 |
| **LDK** | 329.2183 | 357.2132 | **LDKL** | 442.3024 | 470.2973 | **DK** | 216.1343 | 244.1292 |
| **DKL** | 329.2183 | 357.2132 | **KL** | 214.1914 | 242.1863 | | | |

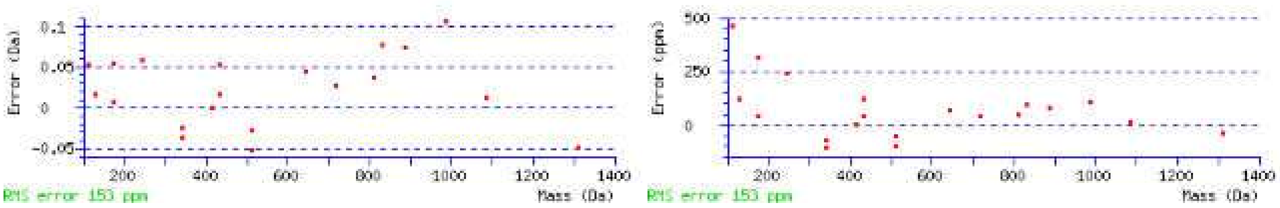


NCBI **BLAST** search of ELVSNASDALDKLR
(Parameters: blastp, nr protein database, expect=20000, no filter, PAM30)
Other BLAST web gateways

**All matches to this query**

| Score | Mr(calc) | Delta | Sequence |
|---|---|---|---|
| 31.2 | 1529.8413 | 0.0854 | TTLQDIADRVGITK |
| 29.9 | 1529.8049 | 0.1218 | EIVSNASDALDKIR |
| 29.9 | 1529.8049 | 0.1218 | ELVSNASDAIDKLR |
| 29.9 | 1529.8049 | 0.1218 | ELVSNASDALDKIR |
| 29.9 | 1529.8049 | 0.1218 | ELVSNASDALDKLR |
| 29.9 | 1529.8049 | 0.1218 | EVISNASDALDKIR |
| 29.9 | 1529.8049 | 0.1218 | EVISNSADALDKIR |
| 26.4 | 1529.7872 | 0.1396 | QDRLMAPGESLSVK |
| 25.8 | 1529.8202 | 0.1065 | FLQGPGTDKDALLR |
| 25.7 | 1529.8348 | 0.0919 | MLSAARLDGTVQIR |

**Mascot:** http://www.matrixscience.com/

# MATRIX SCIENCE Mascot Search Results

## Peptide View **Spot no 114**

MS/MS Fragmentation of **RIFEINPDHPIVK**
Found in **gi|115477014** in **NCBInr**, Os08g0487800 [Oryza sativa Japonica Group]

Match to Query 58: 1577.003924 from(1578.011200,1+) intensity(0.0000) index(21)
Title: Label: M6, Spot_Id: 219766, Peak_List_Id: 226006, MSMS Job_Run_Id: 21831, Comment:
Data file C:\Documents and Settings\Administrator\Desktop\Farah-Garima 11May 2013\ppw_M6_136859901000.txt

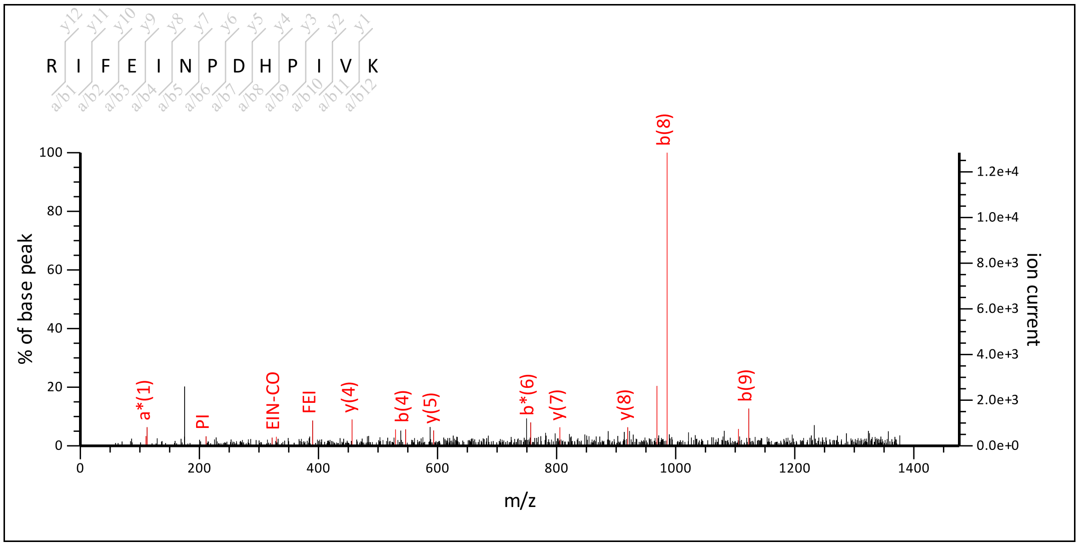


0 to 1476.33

Label all possible matches ○ Label matches used for scoring ◉

**Monoisotopic mass of neutral peptide Mr(calc):** 1576.8725
**Fixed modifications:** Carbamidomethyl (C) (apply to specified residues or termini only)
**Ions Score:** 15 **Expect:** 2.2e+02
**Matches :** 19/225 fragment ions using 28 most intense peaks (help)

| # | Immon. | a | a* | a⁰ | b | b* | b⁰ | d | d' | Seq. | v | w | w' | y | y* | |
|---|---|---|---|---|---|---|---|---|---|---|---|---|---|---|---|---|
| 1 | 129.1135 | 129.1135 | 112.0869 | | 157.1084 | 140.0818 | | 44.0495 | | R | | | | | | |
| 2 | 86.0964 | 242.1975 | 225.1710 | | 270.1925 | 253.1659 | | 214.1662 | 228.1819 | I | 1363.7005 | 1376.7209 | 1390.7365 | 1421.7787 | 1404.7522 | [illegible] |
| 3 | 120.0808 | 389.2660 | 372.2394 | | 417.2609 | 400.2343 | | | | F | 1216.6321 | | | 1308.6947 | 1291.6681 | [illegible] |
| 4 | 102.0550 | 518.3085 | 501.2820 | 500.2980 | ***546.3035*** | 529.2769 | 528.2929 | 460.3031 | | E | 1087.5895 | 1086.5942 | | 1161.6262 | 1144.5997 | [illegible] |
| 5 | 86.0964 | 631.3926 | 614.3661 | 613.3820 | 659.3875 | 642.3610 | 641.3770 | 603.3613 | 617.3770 | I | 974.5054 | 987.5258 | 1001.5415 | 1032.5837 | 1015.5571 | [illegible] |
| 6 | 87.0553 | 745.4355 | 728.4090 | 727.4250 | 773.4304 | 756.4039 | 755.4199 | 702.4297 | | N | 860.4625 | 859.4672 | | ***919.4996*** | 902.4730 | [illegible] |
| 7 | 70.0651 | 842.4883 | 825.4617 | 824.4777 | 870.4832 | 853.4567 | 852.4726 | 816.4726 | | P | 763.4097 | 762.4145 | | ***805.4567*** | 788.4301 | [illegible] |
| 8 | 88.0393 | 957.5152 | 940.4887 | 939.5047 | ***985.5102*** | 968.4836 | 967.4996 | 913.5254 | | D | 648.3828 | 647.3875 | | 708.4039 | 691.3774 | [illegible] |
| 9 | 110.0713 | 1094.5742 | 1077.5476 | 1076.5636 | ***1122.5691*** | 1105.5425 | 1104.5585 | | | H | 511.3239 | | | ***593.3770*** | 576.3504 | |
| 10 | 70.0651 | 1191.6269 | 1174.6004 | 1173.6164 | 1219.6218 | 1202.5953 | 1201.6113 | 1165.6113 | | P | 414.2711 | 413.2758 | | ***456.3180*** | 439.2915 | |
| 11 | 86.0964 | 1304.7110 | 1287.6844 | 1286.7004 | 1332.7059 | 1315.6793 | 1314.6953 | 1276.6797 | 1290.6953 | I | 301.1870 | 314.2074 | 328.2231 | 359.2653 | 342.2387 | |
| 12 | 72.0808 | 1403.7794 | 1386.7528 | 1385.7688 | 1431.7743 | 1414.7478 | 1413.7637 | 1389.7637 | | V | 202.1186 | 215.1390 | | 246.1812 | 229.1547 | |
| 13 | 101.1073 | | | | | | | | | K | 74.0237 | 73.0284 | | 147.1128 | 130.0863 | |

| Seq | ya | yb | Seq | ya | yb | Seq | ya | yb |
|---|---|---|---|---|---|---|---|---|
| IF | 233.1648 | 261.1598 | IFE | 362.2074 | 390.2023 | IFEI | 475.2915 | 503.2864 |
| IFEIN | 589.3344 | 617.3293 | IFEINP | 686.3872 | 714.3821 | FE | 249.1234 | 277.1183 |
| FEI | 362.2074 | 390.2023 | FEIN | 476.2504 | 504.2453 | FEINP | 573.3031 | 601.2980 |
| FEINPD | 688.3301 | 716.3250 | EI | 215.1390 | 243.1339 | EIN | 329.1819 | 357.1769 |
| EINP | 426.2347 | 454.2296 | EINPD | 541.2617 | 569.2566 | EINPDH | 678.3206 | 706.3155 |
| IN | 200.1394 | 228.1343 | INP | 297.1921 | 325.1870 | INPD | 412.2191 | 440.2140 |
| INPDH | 549.2780 | 577.2729 | INPDHP | 646.3307 | 674.3256 | NP | 184.1081 | 212.1030 |
| NPD | 299.1350 | 327.1299 | NPDH | 436.1939 | 464.1888 | NPDHP | 533.2467 | 561.2416 |

| | | | | | | | | |
|---|---|---|---|---|---|---|---|---|
| **NPDHPI** | 646.3307 | 674.3256 | **PD** | 185.0921 | 213.0870 | **PDH** | 322.1510 | 350.1459 |
| **PDHP** | 419.2037 | 447.1987 | **PDHPI** | 532.2878 | 560.2827 | **PDHPIV** | 631.3562 | 659.3511 |
| **DH** | 225.0982 | 253.0931 | **DHP** | 322.1510 | 350.1459 | **DHPI** | 435.2350 | 463.2300 |
| **DHPIV** | 534.3035 | 562.2984 | **HP** | 207.1240 | 235.1190 | **HPI** | 320.2081 | 348.2030 |
| **HPIV** | 419.2765 | 447.2714 | **PI** | 183.1492 | 211.1441 | **PIV** | 282.2176 | 310.2125 |
| **IV** | 185.1648 | 213.1598 | | | | | | |

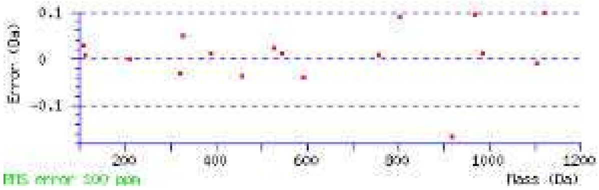


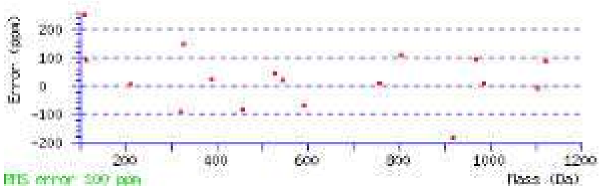


NCBI **BLAST** search of RIFEINPDHPIVK
(Parameters: blastp, nr protein database, expect=20000, no filter, PAM30)
Other BLAST web gateways

**All matches to this query**

| Score | Mr(calc) | Delta | Sequence |
|---|---|---|---|
| 17.1 | 1576.8838 | 0.1202 | KNVNHIVWADLIR |
| 15.3 | 1576.9049 | 0.0990 | VERAHALLTLDLAR |
| 15.3 | 1576.8725 | 0.1314 | RIFEINPDHPIVK |
| 14.8 | 1576.9566 | 0.0474 | LPIIRVLFGIQHR |
| 13.5 | 1576.8508 | 0.1532 | MRPAHAVIDLDALR |
| 12.0 | 1576.8725 | 0.1314 | LPTALAALAYRDFR |
| 11.7 | 1576.8759 | 0.1280 | MAKILVVDDHPAIR |
| 11.5 | 1576.8685 | 0.1354 | REPPIVEVERPTR |
| 11.5 | 1576.8573 | 0.1466 | QPLHTVIADVAELR |
| 11.4 | 1576.9300 | 0.0739 | IKSSIEITIHIAPR |

**Mascot:** http://www.matrixscience.com/

# MATRIX SCIENCE Mascot Search Results

## Peptide View Spot no 114

MS/MS Fragmentation of **VFISDDFDGELFPR**
Found in **gi|115477014** in **NCBInr**, Os08g0487800 [Oryza sativa Japonica Group]

Match to Query 62: 1655.925424 from(1656.932700,1+) intensity(0.0000) index(22)
Title: Label: M6, Spot_Id: 219766, Peak_List_Id: 226001, MSMS Job_Run_Id: 21831, Comment:
Data file C:\Documents and Settings\Administrator\Desktop\Farah-Garima 11May 2013\ppw_M6_136859901000.txt

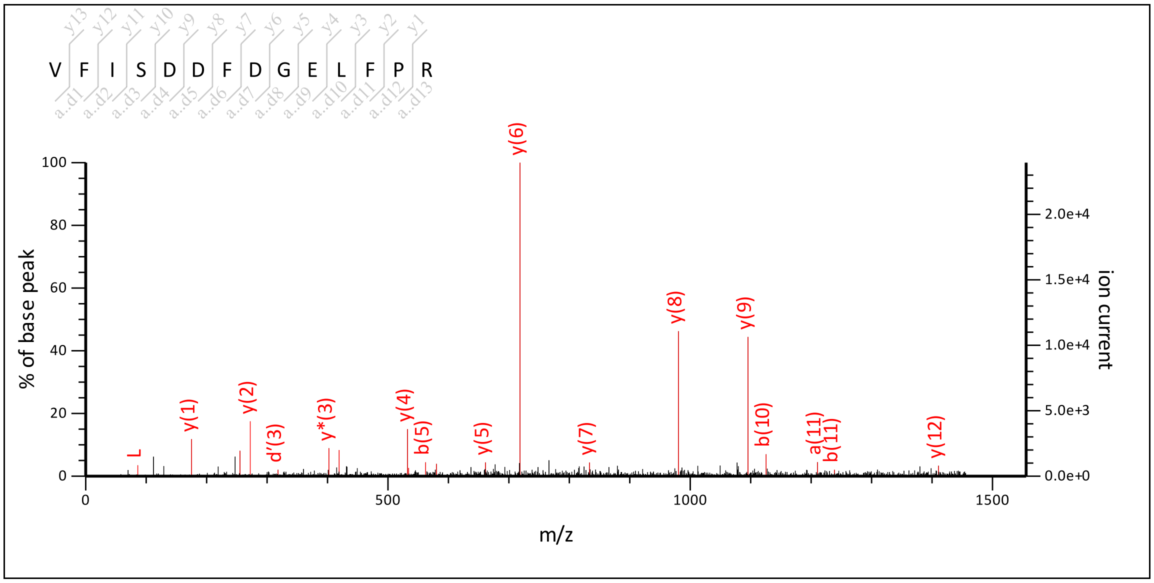


0 to 1555.67

Label all possible matches ○ Label matches used for scoring ◉

**Monoisotopic mass of neutral peptide Mr(calc):** 1655.7832
**Fixed modifications:** Carbamidomethyl (C) (apply to specified residues or termini only)
**Ions Score:** 94 **Expect:** 1.3e-05
**Matches :** 31/215 fragment ions using 28 most intense peaks (help)

| # | Immon. | a | $a^0$ | b | $b^0$ | d | d' | Seq. | v | w | w' | y | y* | $y^0$ | # |
|---|---|---|---|---|---|---|---|---|---|---|---|---|---|---|---|
| 1 | 72.0808 | 72.0808 | | 100.0757 | | 44.0495 | | V | | | | | | | 14 |
| 2 | 120.0808 | 219.1492 | | 247.1441 | | | | F | 1465.6594 | | | 1557.7220 | 1540.6955 | 1539.7114 | 13 |
| 3 | 86.0964 | 332.2333 | | 360.2282 | | 304.2020 | 318.2176 | I | 1352.5753 | 1365.5957 | 1379.6114 | ***1410.6536*** | 1393.6270 | 1392.6430 | 12 |
| 4 | 60.0444 | **419.2653** | 401.2547 | 447.2602 | 429.2496 | 403.2704 | | S | 1265.5433 | 1264.5481 | | 1297.5695 | 1280.5430 | 1279.5590 | 11 |
| 5 | 88.0393 | **534.2922** | 516.2817 | **562.2871** | 544.2766 | 490.3024 | | D | 1150.5164 | 1149.5211 | | ***1210.5375*** | 1193.5109 | 1192.5269 | 10 |
| 6 | 88.0393 | 649.3192 | 631.3086 | 677.3141 | 659.3035 | 605.3293 | | D | 1035.4894 | 1034.4942 | | ***1095.5106*** | 1078.4840 | 1077.5000 | 9 |
| 7 | 120.0808 | 796.3876 | 778.3770 | 824.3825 | 806.3719 | | | F | 888.4210 | | | ***980.4836*** | 963.4571 | 962.4730 | 8 |
| 8 | 88.0393 | 911.4145 | 893.4040 | 939.4094 | 921.3989 | 867.4247 | | D | 773.3941 | 772.3988 | | ***833.4152*** | 816.3886 | 815.4046 | 7 |
| 9 | 30.0338 | 968.4360 | 950.4254 | 996.4309 | 978.4203 | | | G | | | | ***718.3883*** | 701.3617 | 700.3777 | 6 |
| 10 | 102.0550 | 1097.4786 | 1079.4680 | **1125.4735** | 1107.4629 | 1039.4731 | | E | 587.3300 | 586.3348 | | ***661.3668*** | 644.3402 | 643.3562 | 5 |
| 11 | 86.0964 | **1210.5626** | 1192.5521 | **1238.5576** | 1220.5470 | 1168.5157 | | L | 474.2459 | 473.2507 | | ***532.3242*** | 515.2976 | | 4 |
| 12 | 120.0808 | 1357.6311 | 1339.6205 | 1385.6260 | 1367.6154 | | | F | 327.1775 | | | ***419.2401*** | 402.2136 | | 3 |
| 13 | 70.0651 | 1454.6838 | 1436.6733 | 1482.6787 | 1464.6682 | 1428.6682 | | P | 230.1248 | 229.1295 | | ***272.1717*** | 255.1452 | | 2 |
| 14 | 129.1135 | | | | | | | R | 74.0237 | 73.0284 | | ***175.1190*** | 158.0924 | | 1 |

| Seq | ya | yb | Seq | ya | yb | Seq | ya | yb |
|---|---|---|---|---|---|---|---|---|
| FI | 233.1648 | 261.1598 | FIS | 320.1969 | 348.1918 | FISD | 435.2238 | 463.2187 |
| FISDD | 550.2508 | 578.2457 | FISDDF | 697.3192 | 725.3141 | IS | 173.1285 | 201.1234 |
| ISD | 288.1554 | 316.1503 | ISDD | 403.1823 | 431.1773 | ISDDF | 550.2508 | 578.2457 |

| | | | | | | | | |
|---|---|---|---|---|---|---|---|---|
| **ISDDFD** | 665.2777 | 693.2726 | **SD** | 175.0713 | 203.0662 | **SDD** | 290.0983 | 318.0932 |
| **SDDF** | 437.1667 | 465.1616 | **SDDFD** | 552.1936 | 580.1885 | **SDDFDG** | 609.2151 | 637.2100 |
| **DD** | 203.0662 | 231.0612 | **DDF** | 350.1347 | 378.1296 | **DDFD** | 465.1616 | 493.1565 |
| **DDFDG** | 522.1831 | 550.1780 | **DDFDGE** | 651.2257 | 679.2206 | **DF** | 235.1077 | 263.1026 |
| **DFD** | 350.1347 | 378.1296 | **DFDG** | 407.1561 | 435.1510 | **DFDGE** | 536.1987 | 564.1936 |
| **DFDGEL** | 649.2828 | 677.2777 | **FD** | 235.1077 | 263.1026 | **FDG** | 292.1292 | 320.1241 |
| **FDGE** | 421.1718 | 449.1667 | **FDGEL** | 534.2558 | 562.2508 | **FDGELF** | 681.3243 | 709.3192 |
| **DG** | 145.0608 | 173.0557 | **DGE** | 274.1034 | 302.0983 | **DGEL** | 387.1874 | 415.1823 |
| **DGELF** | 534.2558 | 562.2508 | **DGELFP** | 631.3086 | 659.3035 | **GE** | 159.0764 | 187.0713 |
| **GEL** | 272.1605 | 300.1554 | **GELF** | 419.2289 | 447.2238 | **GELFP** | 516.2817 | 544.2766 |
| **EL** | 215.1390 | 243.1339 | **ELF** | 362.2074 | 390.2023 | **ELFP** | 459.2602 | 487.2551 |
| **LF** | 233.1648 | 261.1598 | **LFP** | 330.2176 | 358.2125 | **FP** | 217.1335 | 245.1285 |

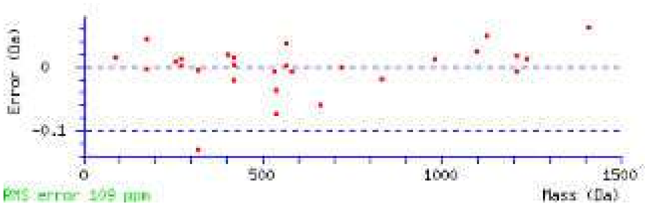


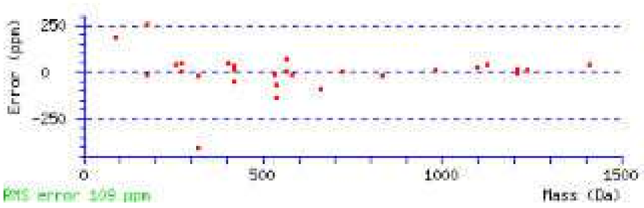


NCBI **BLAST** search of VFISDDFDGELFPR
(Parameters: blastp, nr protein database, expect=20000, no filter, PAM30)
Other BLAST web gateways

**All matches to this query**

| Score | Mr(calc) | Delta | Sequence |
|---|---|---|---|
| 93.9 | 1655.7832 | 0.1423 | VFISDDFDGELFPR |
| 33.9 | 1655.8757 | 0.0497 | QALHARTLGFHHPR |
| 33.9 | 1655.8757 | 0.0497 | QALHARTLGFHHPR |
| 27.6 | 1655.7791 | 0.1463 | FDIREYDSVLDER |
| 27.6 | 1655.8076 | 0.1178 | EVPPEIEITDDMIR |
| 26.4 | 1655.8606 | 0.0648 | NCALFVFRDLFVR |
| 23.9 | 1655.8155 | 0.1099 | VFTVDNSSSTLFPSR |
| 22.5 | 1655.8883 | 0.0371 | IPIHTFGITDSNTLK |
| 22.5 | 1655.8406 | 0.0848 | LDAILGYFTEDKSGK |
| 21.4 | 1655.8065 | 0.1190 | DRFLFWMQNIMR |

**Mascot:** http://www.matrixscience.com/

# MATRIX SCIENCE Mascot Search Results

## Peptide View **Spot no 114**

MS/MS Fragmentation of **RVFISDDFDGELFPR**
Found in **gi|115477014** in **NCBInr**, Os08g0487800 [Oryza sativa Japonica Group]

Match to Query 71: 1812.035424 from(1813.042700,1+) intensity(0.0000) index(26)
Title: Label: M6, Spot_Id: 219766, Peak_List_Id: 226011, MSMS Job_Run_Id: 21831, Comment:
Data file C:\Documents and Settings\Administrator\Desktop\Farah-Garima 11May 2013\ppw_M6_136859901000.txt

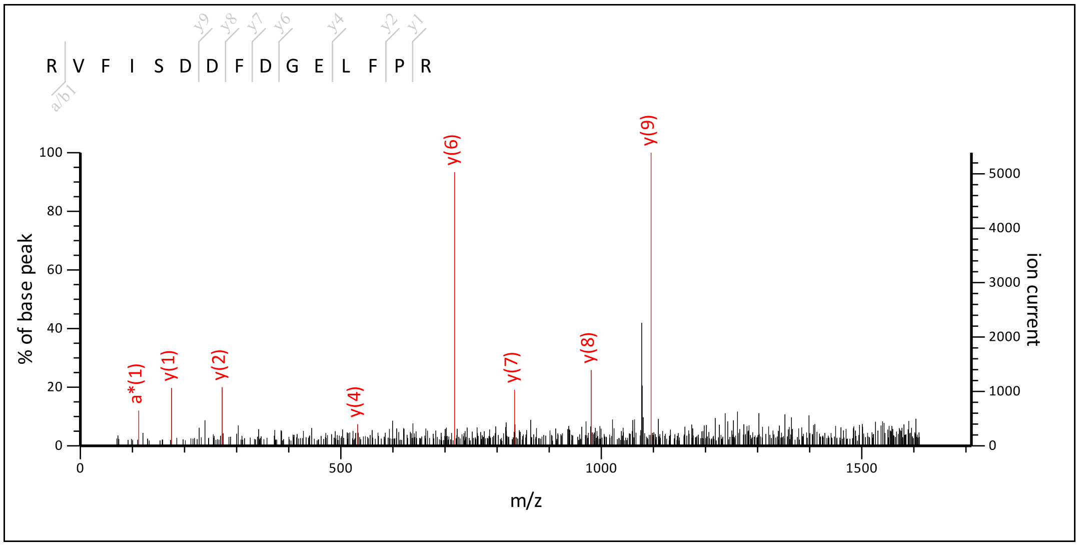


0 to 1710.5

Label all possible matches ○ Label matches used for scoring ◉

**Monoisotopic mass of neutral peptide Mr(calc):** 1811.8843
**Fixed modifications:** Carbamidomethyl (C) (apply to specified residues or termini only)
**Ions Score:** 34 **Expect:** 10
**Matches :** 14/262 fragment ions using 16 most intense peaks (help)

| # | Immon. | a | a* | $a^0$ | b | b* | $b^0$ | d | d' | Seq. | v | w | w' | y | y* | [illegible] |
|---|---|---|---|---|---|---|---|---|---|---|---|---|---|---|---|---|
| 1 | 129.1135 | 129.1135 | 112.0869 | | 157.1084 | 140.0818 | | 44.0495 | | R | | | | | | |
| 2 | 72.0808 | 228.1819 | 211.1553 | | 256.1768 | 239.1503 | | 214.1662 | | V | 1612.7278 | 1625.7482 | | 1656.7904 | 1639.7639 | 16[illegible] |
| 3 | 120.0808 | 375.2503 | 358.2238 | | 403.2452 | 386.2187 | | | | F | 1465.6594 | | | 1557.7220 | 1540.6955 | 15[illegible] |
| 4 | 86.0964 | 488.3344 | 471.3078 | | 516.3293 | 499.3027 | | 460.3031 | 474.3187 | I | 1352.5753 | 1365.5957 | 1379.6114 | 1410.6536 | 1393.6270 | 13[illegible] |
| 5 | 60.0444 | 575.3664 | 558.3398 | 557.3558 | 603.3613 | 586.3348 | 585.3507 | 559.3715 | | S | 1265.5433 | 1264.5481 | | 1297.5695 | 1280.5430 | 12[illegible] |
| 6 | 88.0393 | 690.3933 | 673.3668 | 672.3828 | **718.3882** | 701.3617 | 700.3777 | 646.4035 | | D | 1150.5164 | 1149.5211 | | 1210.5375 | 1193.5109 | 11[illegible] |
| 7 | 88.0393 | 805.4203 | 788.3937 | 787.4097 | **833.4152** | 816.3886 | 815.4046 | 761.4304 | | D | 1035.4894 | 1034.4942 | | ***1095.5106*** | 1078.4840 | 10[illegible] |
| 8 | 120.0808 | 952.4887 | 935.4621 | 934.4781 | **980.4836** | 963.4571 | 962.4730 | | | F | 888.4210 | | | ***980.4836*** | 963.4571 | 9[illegible] |
| 9 | 88.0393 | 1067.5156 | 1050.4891 | 1049.5051 | **1095.5105** | 1078.4840 | 1077.5000 | 1023.5258 | | D | 773.3941 | 772.3988 | | ***833.4152*** | 816.3886 | 8[illegible] |
| 10 | 30.0338 | 1124.5371 | 1107.5105 | 1106.5265 | 1152.5320 | 1135.5055 | 1134.5214 | | | G | | | | ***718.3883*** | 701.3617 | 7[illegible] |
| 11 | 102.0550 | 1253.5797 | 1236.5531 | 1235.5691 | 1281.5746 | 1264.5481 | 1263.5640 | 1195.5742 | | E | 587.3300 | 586.3348 | | 661.3668 | 644.3402 | 6[illegible] |
| 12 | 86.0964 | 1366.6638 | 1349.6372 | 1348.6532 | 1394.6587 | 1377.6321 | 1376.6481 | 1324.6168 | | L | 474.2459 | 473.2507 | | ***532.3242*** | 515.2976 | |
| 13 | 120.0808 | 1513.7322 | 1496.7056 | 1495.7216 | 1541.7271 | 1524.7005 | 1523.7165 | | | F | 327.1775 | | | 419.2401 | 402.2136 | |
| 14 | 70.0651 | 1610.7849 | 1593.7584 | 1592.7744 | 1638.7798 | 1621.7533 | 1620.7693 | 1584.7693 | | P | 230.1248 | 229.1295 | | ***272.1717*** | 255.1452 | |
| 15 | 129.1135 | | | | | | | | | R | 74.0237 | 73.0284 | | ***175.1190*** | 158.0924 | |

| Seq | ya | yb | Seq | ya | yb | Seq | ya | yb |
|---|---|---|---|---|---|---|---|---|
| VF | 219.1492 | 247.1441 | VFI | 332.2333 | 360.2282 | VFIS | 419.2653 | 447.2602 |
| VFISD | 534.2922 | 562.2871 | VFISDD | 649.3192 | 677.3141 | FI | 233.1648 | 261.1598 |
| FIS | 320.1969 | 348.1918 | FISD | 435.2238 | 463.2187 | FISDD | 550.2508 | 578.2457 |
| FISDDF | 697.3192 | 725.3141 | IS | 173.1285 | 201.1234 | ISD | 288.1554 | 316.1503 |
| ISDD | 403.1823 | 431.1773 | ISDDF | 550.2508 | 578.2457 | ISDDFD | 665.2777 | 693.2726 |
| SD | 175.0713 | 203.0662 | SDD | 290.0983 | 318.0932 | SDDF | 437.1667 | 465.1616 |

| | | | | | | | | |
|---|---|---|---|---|---|---|---|---|
| **SDDFD** | 552.1936 | 580.1885 | **SDDFDG** | 609.2151 | 637.2100 | **DD** | 203.0662 | 231.0612 |
| **DDF** | 350.1347 | 378.1296 | **DDFD** | 465.1616 | 493.1565 | **DDFDG** | 522.1831 | 550.1780 |
| **DDFDGE** | 651.2257 | 679.2206 | **DF** | 235.1077 | 263.1026 | **DFD** | 350.1347 | 378.1296 |
| **DFDG** | 407.1561 | 435.1510 | **DFDGE** | 536.1987 | 564.1936 | **DFDGEL** | 649.2828 | 677.2777 |
| **FD** | 235.1077 | 263.1026 | **FDG** | 292.1292 | 320.1241 | **FDGE** | 421.1718 | 449.1667 |
| **FDGEL** | 534.2558 | 562.2508 | **FDGELF** | 681.3243 | 709.3192 | **DG** | 145.0608 | 173.0557 |
| **DGE** | 274.1034 | 302.0983 | **DGEL** | 387.1874 | 415.1823 | **DGELF** | 534.2558 | 562.2508 |
| **DGELFP** | 631.3086 | 659.3035 | **GE** | 159.0764 | 187.0713 | **GEL** | 272.1605 | 300.1554 |
| **GELF** | 419.2289 | 447.2238 | **GELFP** | 516.2817 | 544.2766 | **EL** | 215.1390 | 243.1339 |
| **ELF** | 362.2074 | 390.2023 | **ELFP** | 459.2602 | 487.2551 | **LF** | 233.1648 | 261.1598 |
| **LFP** | 330.2176 | 358.2125 | **FP** | 217.1335 | 245.1285 | | | |

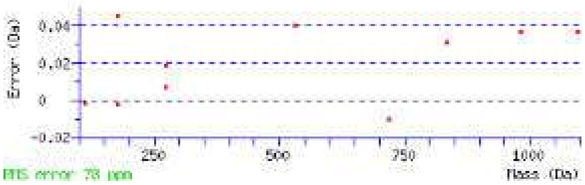


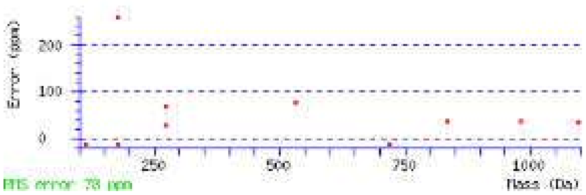


NCBI **BLAST** search of RVFISDDFDGELFPR
(Parameters: blastp, nr protein database, expect=20000, no filter, PAM30)
Other BLAST web gateways

**All matches to this query**

| Score | Mr(calc) | Delta | Sequence |
|---|---|---|---|
| 34.1 | 1811.8843 | 0.1512 | RVFISDDFDGELFPR |
| 20.3 | 1811.9755 | 0.0599 | ILHGTPVDPGQVRNGPR |
| 19.9 | 1811.8836 | 0.1518 | VGPVKADELADASGCAPR |
| 18.2 | 1811.9741 | 0.0613 | KNILGILLDDVSENNR |
| 16.7 | 1811.8802 | 0.1552 | AGDLGPGDLLAPHPDDPR |
| 16.2 | 1811.9027 | 0.1327 | HHAAVDADVHQLAEKR |
| 16.2 | 1811.9027 | 0.1327 | HHAAVDADVHQLAEKR |
| 15.7 | 1811.8955 | 0.1399 | IGFTPWDGHPLAQSVR |
| 15.3 | 1811.9027 | 0.1327 | ALANIGGRAPSSWDGGAGR |
| 14.8 | 1811.9265 | 0.1089 | IVTTHADIEDKDQTVK |

**Mascot:** http://www.matrixscience.com/

# MATRIX SCIENCE Mascot Search Results

## Peptide View Spot no 116

MS/MS Fragmentation of **FLGVTDSSLLADGGELEIR**
Found in **gi|115477014** in **NCBInr**, Os08g0487800 [Oryza sativa Japonica Group]

Match to Query 74: 1991.202924 from(1992.210200,1+) intensity(0.0000) index(27)
Title: Label: M6, Spot_Id: 219766, Peak_List_Id: 226014, MSMS Job_Run_Id: 21831, Comment:
Data file C:\Documents and Settings\Administrator\Desktop\Farah-Garima 11May 2013\ppw_M6_136859901000.txt

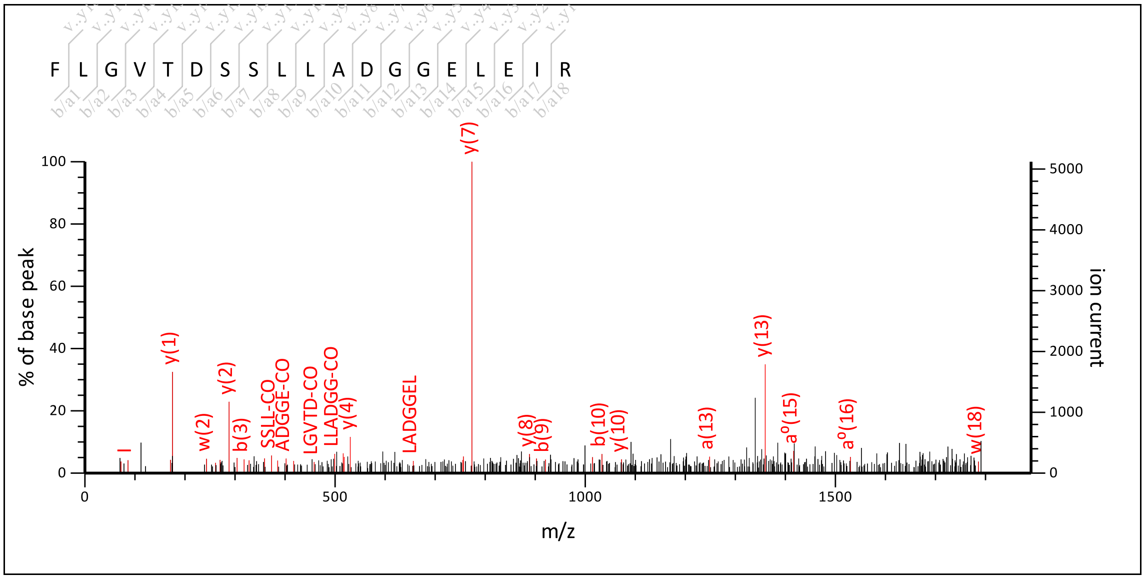


0 to 1891.03

Label all possible matches ○ Label matches used for scoring ◉

**Monoisotopic mass of neutral peptide Mr(calc):** 1991.0211
**Fixed modifications:** Carbamidomethyl (C) (apply to specified residues or termini only)
**Ions Score:** 18 **Expect:** 2.4e+02
**Matches :** 54/342 fragment ions using 125 most intense peaks (help)

| # | Immon. | a | $a^0$ | b | $b^0$ | d | d' | Seq. | v | w | w' | y | y* | $y^0$ | # |
|---|---|---|---|---|---|---|---|---|---|---|---|---|---|---|---|
| 1 | 120.0808 | 120.0808 | | 148.0757 | | 44.0495 | | F | | | | | | | 19 |
| 2 | 86.0964 | 233.1648 | | ***261.1598*** | | 191.1179 | | L | 1786.8817 | 1785.8865 | | 1844.9600 | 1827.9334 | 1826.9494 | 18 |
| 3 | 30.0338 | 290.1863 | | ***318.1812*** | | | | G | | | | 1731.8759 | 1714.8494 | 1713.8654 | 17 |
| 4 | 72.0808 | 389.2547 | | ***417.2496*** | | 375.2391 | | V | 1630.7919 | 1643.8123 | | 1674.8545 | 1657.8279 | 1656.8439 | 16 |
| 5 | 74.0600 | 490.3024 | 472.2918 | ***518.2973*** | 500.2867 | 474.3075 | 476.2867 | T | 1529.7442 | 1542.7646 | 1544.7439 | 1575.7861 | 1558.7595 | 1557.7755 | 15 |
| 6 | 88.0393 | 605.3293 | 587.3188 | 633.3243 | 615.3137 | 561.3395 | | D | 1414.7172 | 1413.7220 | | 1474.7384 | 1457.7118 | 1456.7278 | 14 |
| 7 | 60.0444 | 692.3614 | 674.3508 | 720.3563 | 702.3457 | 676.3665 | | S | 1327.6852 | 1326.6900 | | ***1359.7114*** | 1342.6849 | 1341.7009 | 13 |
| 8 | 60.0444 | 779.3934 | 761.3828 | 807.3883 | 789.3777 | 763.3985 | | S | 1240.6532 | 1239.6579 | | 1272.6794 | 1255.6529 | 1254.6688 | 12 |
| 9 | 86.0964 | 892.4775 | 874.4669 | ***920.4724*** | 902.4618 | 850.4305 | | L | 1127.5691 | 1126.5739 | | 1185.6474 | 1168.6208 | 1167.6368 | 11 |
| 10 | 86.0964 | 1005.5615 | 987.5510 | ***1033.5564*** | 1015.5459 | 963.5146 | | L | 1014.4851 | 1013.4898 | | ***1072.5633*** | 1055.5368 | 1054.5527 | 10 |
| 11 | 44.0495 | 1076.5986 | 1058.5881 | 1104.5936 | 1086.5830 | | | A | 943.4480 | | | 959.4793 | 942.4527 | 941.4687 | 9 |
| 12 | 88.0393 | 1191.6256 | 1173.6150 | 1219.6205 | 1201.6099 | 1147.6358 | | D | 828.4210 | 827.4258 | | ***888.4421*** | 871.4156 | 870.4316 | 8 |
| 13 | 30.0338 | 1248.6470 | 1230.6365 | 1276.6420 | 1258.6314 | | | G | | | | ***773.4152*** | 756.3886 | 755.4046 | 7 |
| 14 | 30.0338 | 1305.6685 | 1287.6579 | 1333.6634 | 1315.6529 | | | G | | | | 716.3937 | 699.3672 | 698.3832 | 6 |
| 15 | 102.0550 | 1434.7111 | 1416.7005 | 1462.7060 | 1444.6955 | 1376.7056 | | E | 585.3355 | 584.3402 | | 659.3723 | 642.3457 | 641.3617 | 5 |
| 16 | 86.0964 | 1547.7952 | 1529.7846 | 1575.7901 | 1557.7795 | 1505.7482 | | L | 472.2514 | 471.2562 | | ***530.3297*** | 513.3031 | 512.3191 | 4 |
| 17 | 102.0550 | 1676.8378 | 1658.8272 | 1704.8327 | 1686.8221 | 1618.8323 | | E | 343.2088 | 342.2136 | | ***417.2456*** | 400.2191 | 399.2350 | 3 |
| 18 | 86.0964 | 1789.9218 | 1771.9113 | 1817.9167 | 1799.9062 | 1761.8905 | 1775.9062 | I | 230.1248 | 243.1452 | 257.1608 | ***288.2030*** | 271.1765 | | 2 |
| 19 | 129.1135 | | | | | | | R | 74.0237 | 73.0284 | | ***175.1190*** | 158.0924 | | 1 |

| Seq | ya | yb | Seq | ya | yb | Seq | ya | yb |
|---|---|---|---|---|---|---|---|---|
| LG | 143.1179 | 171.1128 | LGV | 242.1863 | 270.1812 | LGVT | 343.2340 | 371.2289 |
| LGVTD | 458.2609 | 486.2558 | LGVTDS | 545.2930 | 573.2879 | LGVTDSS | 632.3250 | 660.3199 |
| GV | 129.1022 | 157.0972 | GVT | 230.1499 | 258.1448 | GVTD | 345.1769 | 373.1718 |
| GVTDS | 432.2089 | 460.2038 | GVTDSS | 519.2409 | 547.2358 | GVTDSSL | 632.3250 | 660.3199 |
| VT | 173.1285 | 201.1234 | VTD | 288.1554 | 316.1503 | VTDS | 375.1874 | 403.1823 |
| VTDSS | 462.2195 | 490.2144 | VTDSSL | 575.3035 | 603.2984 | VTDSSLL | 688.3876 | 716.3825 |
| TD | 189.0870 | 217.0819 | TDS | 276.1190 | 304.1139 | TDSS | 363.1510 | 391.1460 |
| TDSSL | 476.2351 | 504.2300 | TDSSLL | 589.3192 | 617.3141 | TDSSLLA | 660.3563 | 688.3512 |
| DS | 175.0713 | 203.0662 | DSS | 262.1034 | 290.0983 | DSSL | 375.1874 | 403.1823 |
| DSSLL | 488.2715 | 516.2664 | DSSLLA | 559.3086 | 587.3035 | DSSLLAD | 674.3355 | 702.3305 |
| SS | 147.0764 | 175.0713 | SSL | 260.1605 | 288.1554 | SSLL | 373.2445 | 401.2395 |
| SSLLA | 444.2817 | 472.2766 | SSLLAD | 559.3086 | 587.3035 | SSLLADG | 616.3301 | 644.3250 |
| SSLLADGG | 673.3515 | 701.3464 | SL | 173.1285 | 201.1234 | SLL | 286.2125 | 314.2074 |
| SLLA | 357.2496 | 385.2445 | SLLAD | 472.2766 | 500.2715 | SLLADG | 529.2980 | 557.2930 |
| SLLADGG | 586.3195 | 614.3144 | LL | 199.1805 | 227.1754 | LLA | 270.2176 | 298.2125 |
| LLAD | 385.2445 | 413.2395 | LLADG | 442.2660 | 470.2609 | LLADGG | 499.2875 | 527.2824 |
| LLADGGE | 628.3301 | 656.3250 | LA | 157.1335 | 185.1285 | LAD | 272.1605 | 300.1554 |
| LADG | 329.1819 | 357.1769 | LADGG | 386.2034 | 414.1983 | LADGGE | 515.2460 | 543.2409 |
| LADGGEL | 628.3301 | 656.3250 | AD | 159.0764 | 187.0713 | ADG | 216.0979 | 244.0928 |
| ADGG | 273.1193 | 301.1143 | ADGGE | 402.1619 | 430.1569 | ADGGEL | 515.2460 | 543.2409 |
| ADGGELE | 644.2886 | 672.2835 | DG | 145.0608 | 173.0557 | DGG | 202.0822 | 230.0771 |
| DGGE | 331.1248 | 359.1197 | DGGEL | 444.2089 | 472.2038 | DGGELE | 573.2515 | 601.2464 |
| DGGELEI | 686.3355 | 714.3305 | GG | 87.0553 | 115.0502 | GGE | 216.0979 | 244.0928 |
| GGEL | 329.1819 | 357.1769 | GGELE | 458.2245 | 486.2195 | GGELEI | 571.3086 | 599.3035 |
| GE | 159.0764 | 187.0713 | GEL | 272.1605 | 300.1554 | GELE | 401.2031 | 429.1980 |
| GELEI | 514.2871 | 542.2821 | EL | 215.1390 | 243.1339 | ELE | 344.1816 | 372.1765 |
| ELEI | 457.2657 | 485.2606 | LE | 215.1390 | 243.1339 | LEI | 328.2231 | 356.2180 |
| EI | 215.1390 | 243.1339 | | | | | | |

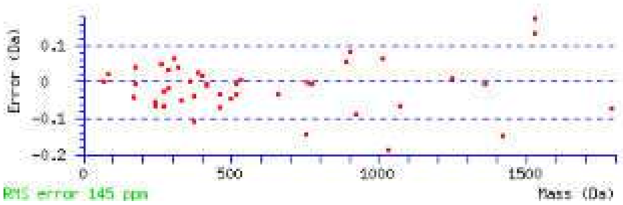


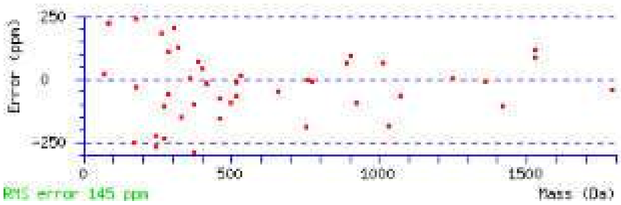


NCBI **BLAST** search of FLGVTDSSLLADGGELEIR
(Parameters: blastp, nr protein database, expect=20000, no filter, PAM30)
Other BLAST web gateways

**All matches to this query**

| Score | Mr(calc) | Delta | Sequence |
|---|---|---|---|
| 17.6 | 1991.0211 | 0.1818 | FLGVTDSSLLADGGELEIR |
| 14.4 | 1991.0701 | 0.1328 | HRAEQIVAASGVPWTTLR |
| 12.9 | 1991.0265 | 0.1764 | SNFNVAAPILFWGDTAIR |
| 12.0 | 1991.0800 | 0.1230 | VAPPGRSLADLAADAALDLR |
| 11.0 | 1991.0490 | 0.1540 | ALHKFQPNALVHFGEQR |
| 10.7 | 1991.0258 | 0.1771 | IMYSLHNGTSSISAKNLR |
| 9.9 | 1991.0225 | 0.1804 | GASQKGVYIDTVYGHQIR |
| 9.5 | 1991.0476 | 0.1553 | YSIPPENIIGHSDIAPLR |
| 9.3 | 1991.0258 | 0.1771 | IDLVLSTHNDQSMHLLR |
| 8.9 | 1991.0106 | 0.1923 | TGSNLCDVTVTAGKQTALR |

**Mascot:** http://www.matrixscience.com/

# MATRIX SCIENCE Mascot Search Results

## Peptide View Spot no 116

MS/MS Fragmentation of **AFNEFLDPLAYTHFTTEGEVEFR**
Found in **gi|115477014** in **NCBInr**, Os08g0487800 [Oryza sativa Japonica Group]

Match to Query 87: 2732.527924 from(2733.535200,1+) intensity(0.0000) index(33)
Title: Label: M6, Spot_Id: 219766, Peak_List_Id: 226009, MSMS Job_Run_Id: 21831, Comment:
Data file C:\Documents and Settings\Administrator\Desktop\Farah-Garima 11May 2013\ppw_M6_136859901000.txt

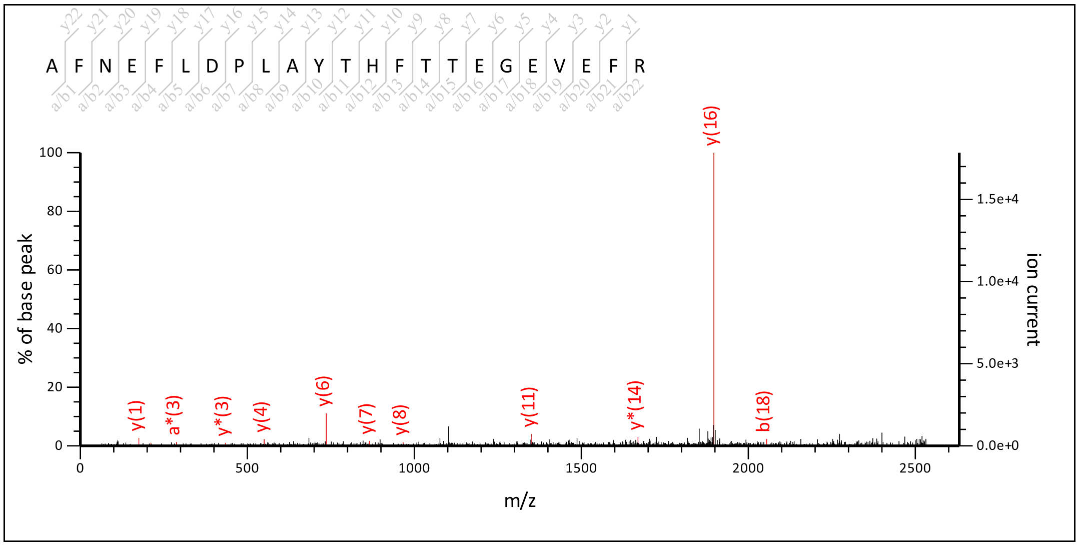


0 to 2631.64

Label all possible matches ○ Label matches used for scoring ◉

**Monoisotopic mass of neutral peptide Mr(calc):** 2732.2758
**Fixed modifications:** Carbamidomethyl (C) (apply to specified residues or termini only)
**Ions Score:** 10 **Expect:** 1.9e+03
**Matches :** 17/434 fragment ions using 50 most intense peaks (help)

| # | Immon. | a | a* | a⁰ | b | b* | b⁰ | d | d' | Seq. | v | w | w' | y | y* |
|---|---|---|---|---|---|---|---|---|---|---|---|---|---|---|---|
| 1 | 44.0495 | 44.0495 | | | 72.0444 | | | 44.0495 | | A | | | | | |
| 2 | 120.0808 | 191.1179 | | | 219.1128 | | | | | F | 2570.1834 | | | 2662.2460 | 2645.2195 |
| 3 | 87.0553 | 305.1608 | 288.1343 | | 333.1557 | 316.1292 | | 262.1550 | | N | 2456.1405 | 2455.1452 | | 2515.1776 | 2498.1510 |
| 4 | 102.0550 | 434.2034 | 417.1769 | 416.1928 | 462.1983 | 445.1718 | 444.1878 | 376.1979 | | E | 2327.0979 | 2326.1026 | | 2401.1347 | 2384.1081 |
| 5 | 120.0808 | 581.2718 | 564.2453 | 563.2613 | 609.2667 | 592.2402 | 591.2562 | | | F | 2180.0295 | | | 2272.0921 | 2255.0655 |
| 6 | 86.0964 | 694.3559 | 677.3293 | 676.3453 | 722.3508 | 705.3243 | 704.3402 | 652.3089 | | L | 2066.9454 | 2065.9502 | | 2125.0237 | 2107.9971 |
| 7 | 88.0393 | 809.3828 | 792.3563 | 791.3723 | 837.3777 | 820.3512 | 819.3672 | 765.3930 | | D | 1951.9185 | 1950.9232 | | 2011.9396 | 1994.9131 |
| 8 | 70.0651 | 906.4356 | 889.4090 | 888.4250 | 934.4305 | 917.4040 | 916.4199 | 880.4199 | | P | 1854.8657 | 1853.8705 | | ***1896.9127*** | 1879.8861 |
| 9 | 86.0964 | 1019.5197 | 1002.4931 | 1001.5091 | 1047.5146 | 1030.4880 | 1029.5040 | 977.4727 | | L | 1741.7816 | 1740.7864 | | 1799.8599 | 1782.8333 |
| 10 | 44.0495 | 1090.5568 | 1073.5302 | 1072.5462 | 1118.5517 | 1101.5251 | 1100.5411 | | | A | 1670.7445 | | | 1686.7758 | 1669.7493 |
| 11 | 136.0757 | 1253.6201 | 1236.5936 | 1235.6095 | 1281.6150 | 1264.5885 | 1263.6045 | | | Y | 1507.6812 | | | 1615.7387 | 1598.7122 |
| 12 | 74.0600 | 1354.6678 | 1337.6412 | 1336.6572 | 1382.6627 | 1365.6361 | 1364.6521 | 1338.6729 | 1340.6521 | T | 1406.6335 | 1419.6539 | 1421.6332 | 1452.6754 | 1435.6488 |
| 13 | 110.0713 | 1491.7267 | 1474.7001 | 1473.7161 | 1519.7216 | 1502.6951 | 1501.7110 | | | H | 1269.5746 | | | ***1351.6277*** | 1334.6012 |
| 14 | 120.0808 | 1638.7951 | 1621.7686 | 1620.7845 | 1666.7900 | 1649.7635 | 1648.7795 | | | F | 1122.5062 | | | 1214.5688 | 1197.5422 |
| 15 | 74.0600 | 1739.8428 | 1722.8162 | 1721.8322 | 1767.8377 | 1750.8112 | 1749.8271 | 1723.8479 | 1725.8271 | T | 1021.4585 | 1034.4789 | 1036.4582 | 1067.5004 | 1050.4738 |
| 16 | 74.0600 | 1840.8905 | 1823.8639 | 1822.8799 | 1868.8854 | 1851.8588 | 1850.8748 | 1824.8955 | 1826.8748 | T | 920.4108 | 933.4312 | 935.4105 | ***966.4527*** | 949.4262 |
| 17 | 102.0550 | 1969.9331 | 1952.9065 | 1951.9225 | 1997.9280 | 1980.9014 | 1979.9174 | 1911.9276 | | E | 791.3682 | 790.3730 | | ***865.4050*** | 848.3785 |
| 18 | 30.0338 | 2026.9545 | 2009.9280 | 2008.9440 | 2054.9494 | 2037.9229 | 2036.9389 | | | G | | | | ***736.3624*** | 719.3359 |
| 19 | 102.0550 | 2155.9971 | 2138.9706 | 2137.9865 | 2183.9920 | 2166.9655 | 2165.9815 | 2097.9916 | | E | 605.3042 | 604.3089 | | 679.3410 | 662.3144 |
| 20 | 72.0808 | 2255.0655 | 2238.0390 | 2237.0550 | 2283.0604 | 2266.0339 | 2265.0499 | 2241.0499 | | V | 506.2358 | 519.2562 | | ***550.2984*** | 533.2718 |
| 21 | 102.0550 | 2384.1081 | 2367.0816 | 2366.0976 | 2412.1030 | 2395.0765 | 2394.0925 | 2326.1026 | | E | 377.1932 | 376.1979 | | 451.2300 | 434.2034 |
| 22 | 120.0808 | 2531.1765 | 2514.1500 | 2513.1660 | 2559.1714 | 2542.1449 | 2541.1609 | | | F | 230.1248 | | | 322.1874 | 305.1608 |
| 23 | 129.1135 | | | | | | | | | R | 74.0237 | 73.0284 | | ***175.1190*** | 158.0924 |

| Seq | ya | yb | Seq | ya | yb | Seq | ya | yb |
|---|---|---|---|---|---|---|---|---|
| FN | 234.1237 | 262.1186 | FNE | 363.1663 | 391.1612 | FNEF | 510.2347 | 538.2296 |
| FNEFL | 623.3188 | 651.3137 | NE | 216.0979 | 244.0928 | NEF | 363.1663 | 391.1612 |
| NEFL | 476.2504 | 504.2453 | NEFLD | 591.2773 | 619.2722 | NEFLDP | 688.3301 | 716.3250 |
| EF | 249.1234 | 277.1183 | EFL | 362.2074 | 390.2023 | EFLD | 477.2344 | 505.2293 |
| EFLDP | 574.2871 | 602.2821 | EFLDPL | 687.3712 | 715.3661 | FL | 233.1648 | 261.1598 |
| FLD | 348.1918 | 376.1867 | FLDP | 445.2445 | 473.2395 | FLDPL | 558.3286 | 586.3235 |
| FLDPLA | 629.3657 | 657.3606 | LD | 201.1234 | 229.1183 | LDP | 298.1761 | 326.1710 |
| LDPL | 411.2602 | 439.2551 | LDPLA | 482.2973 | 510.2922 | LDPLAY | 645.3606 | 673.3556 |
| DP | 185.0921 | 213.0870 | DPL | 298.1761 | 326.1710 | DPLA | 369.2132 | 397.2082 |
| DPLAY | 532.2766 | 560.2715 | DPLAYT | 633.3243 | 661.3192 | PL | 183.1492 | 211.1441 |
| PLA | 254.1863 | 282.1812 | PLAY | 417.2496 | 445.2445 | PLAYT | 518.2973 | 546.2922 |
| PLAYTH | 655.3562 | 683.3511 | LA | 157.1335 | 185.1285 | LAY | 320.1969 | 348.1918 |
| LAYT | 421.2445 | 449.2395 | LAYTH | 558.3035 | 586.2984 | AY | 207.1128 | 235.1077 |
| AYT | 308.1605 | 336.1554 | AYTH | 445.2194 | 473.2143 | AYTHF | 592.2878 | 620.2827 |
| AYTHFT | 693.3355 | 721.3304 | YT | 237.1234 | 265.1183 | YTH | 374.1823 | 402.1772 |
| YTHF | 521.2507 | 549.2456 | YTHFT | 622.2984 | 650.2933 | TH | 211.1190 | 239.1139 |
| THF | 358.1874 | 386.1823 | THFT | 459.2350 | 487.2300 | THFTT | 560.2827 | 588.2776 |
| THFTTE | 689.3253 | 717.3202 | HF | 257.1397 | 285.1346 | HFT | 358.1874 | 386.1823 |
| HFTT | 459.2350 | 487.2300 | HFTTE | 588.2776 | 616.2726 | HFTTEG | 645.2991 | 673.2940 |
| FT | 221.1285 | 249.1234 | FTT | 322.1761 | 350.1710 | FTTE | 451.2187 | 479.2136 |
| FTTEG | 508.2402 | 536.2351 | FTTEGE | 637.2828 | 665.2777 | TT | 175.1077 | 203.1026 |
| TTE | 304.1503 | 332.1452 | TTEG | 361.1718 | 389.1667 | TTEGE | 490.2144 | 518.2093 |
| TTEGEV | 589.2828 | 617.2777 | TE | 203.1026 | 231.0975 | TEG | 260.1241 | 288.1190 |
| TEGE | 389.1667 | 417.1616 | TEGEV | 488.2351 | 516.2300 | TEGEVE | 617.2777 | 645.2726 |
| EG | 159.0764 | 187.0713 | EGE | 288.1190 | 316.1139 | EGEV | 387.1874 | 415.1823 |
| EGEVE | 516.2300 | 544.2249 | EGEVEF | 663.2984 | 691.2933 | GE | 159.0764 | 187.0713 |
| GEV | 258.1448 | 286.1397 | GEVE | 387.1874 | 415.1823 | GEVEF | 534.2558 | 562.2508 |
| EV | 201.1234 | 229.1183 | EVE | 330.1660 | 358.1609 | EVEF | 477.2344 | 505.2293 |
| VE | 201.1234 | 229.1183 | VEF | 348.1918 | 376.1867 | EF | 249.1234 | 277.1183 |

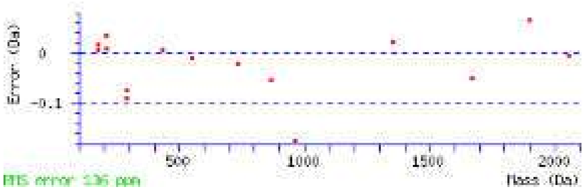


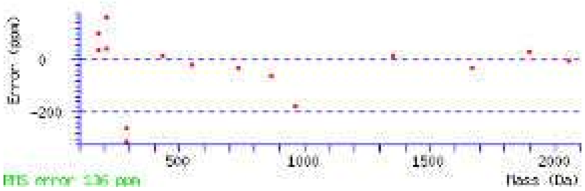


NCBI **BLAST** search of AFNEFLDPLAYTHFTTEGEVEFR
(Parameters: blastp, nr protein database, expect=20000, no filter, PAM30)
Other BLAST web gateways

**All matches to this query**

| Score | Mr(calc) | Delta | Sequence |
|---|---|---|---|
| 9.8 | 2732.2758 | 0.2521 | AFNEFLDPLAYTHFTTEGEVEFR |
| 7.4 | 2732.3446 | 0.1833 | LQYGDLEHVLDTGLHDYLTDFLR |
| 6.5 | 2732.4650 | 0.0629 | HTITNLDPPLVAPFDGYSLHKILR |
| 6.1 | 2732.3592 | 0.1687 | MGYGTAGQALAVLLGTDGHQLEVFER |
| 6.0 | 2732.3895 | 0.1384 | QEGFDAVVNAANAWLRPGGGVAGAIHR |
| 5.7 | 2732.3446 | 0.1833 | LEAFFAGTDDGTKLPHVDEWLALR |
| 5.4 | 2732.3379 | 0.1900 | GHEQSQIGAHGATPFSDAEVARQALR |
| 5.0 | 2732.3340 | 0.1939 | DKLIEGVQFHPESIASEHGHAMLR |
| 4.9 | 2732.4650 | 0.0629 | KTHPPVLTDLNEGFLYVTGHLPIR |
| 4.0 | 2732.3055 | 0.2224 | SLDFDSARLPNPHIRPEHEEWR |

**Mascot:** http://www.matrixscience.com/

# MATRIX SCIENCE Mascot Search Results

## Peptide View **Spot no 118**

MS/MS Fragmentation of **TYPQQAGTIR**
Found in **gi|108711192** in **NCBInr**, Eukaryotic translation initiation factor 5A-2, putative, expressed [Oryza sativa Japonica Group]

Match to Query 30: 1133.637124 from(1134.644400,1+) intensity(0.0000) index(8)
Title: Label: N8, Spot_Id: 219799, Peak_List_Id: 226504, MSMS Job_Run_Id: 21847, Comment:
Data file C:\Documents and Settings\Administrator\Desktop\Farah-Garima 11May 2013\ppw_N8_136859933900.txt

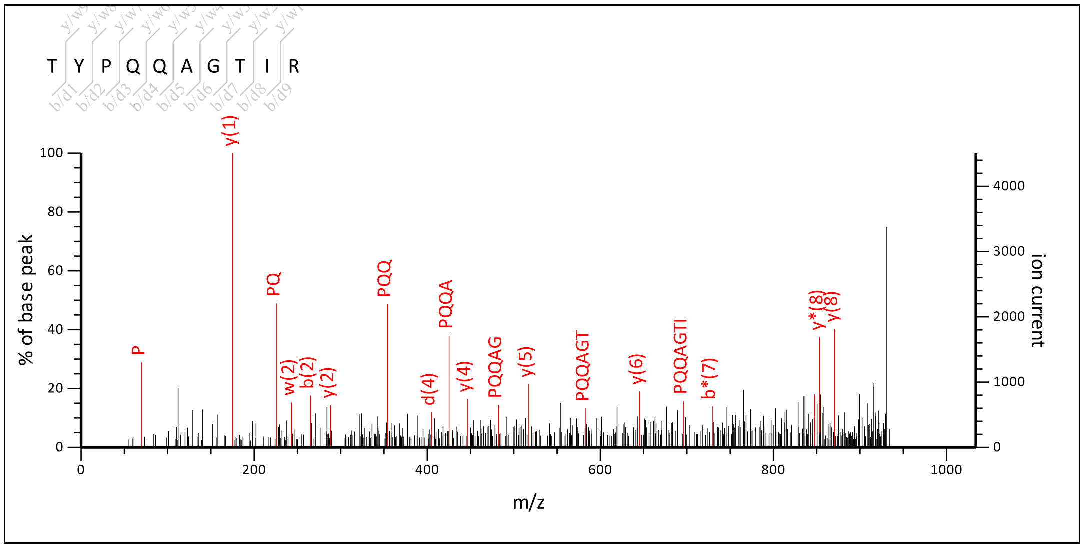


0 to 1033.86

Label all possible matches ○ Label matches used for scoring ◉

**Monoisotopic mass of neutral peptide Mr(calc):** 1133.5829
**Fixed modifications:** Carbamidomethyl (C) (apply to specified residues or termini only)
**Ions Score:** 40 **Expect:** 3.5
**Matches :** 21/159 fragment ions using 27 most intense peaks (help)

| # | Immon. | a | a* | a0 | b | b* | b0 | d | d' | Seq. | v | w | w' | y | y* | y0 | # |
|---|---|---|---|---|---|---|---|---|---|---|---|---|---|---|---|---|---|
| 1 | 74.0600 | 74.0600 | | 56.0495 | 102.0550 | | 84.0444 | 44.0495 | | T | | | | | | | 10 |
| 2 | 136.0757 | 237.1234 | | 219.1128 | **265.1183** | | 247.1077 | | | Y | 925.4850 | | | 1033.5425 | 1016.5160 | 1015.5320 | 9 |
| 3 | 70.0651 | 334.1761 | | 316.1656 | 362.1710 | | 344.1605 | 308.1605 | | P | 828.4322 | 827.4370 | | ***870.4792*** | 853.4526 | 852.4686 | 8 |
| 4 | 101.0709 | 462.2347 | 445.2082 | 444.2241 | 490.2296 | 473.2031 | 472.2191 | 405.2132 | | Q | 700.3737 | 699.3784 | | 773.4264 | 756.3999 | 755.4159 | 7 |
| 5 | 101.0709 | 590.2933 | 573.2667 | 572.2827 | 618.2882 | 601.2617 | 600.2776 | 533.2718 | | Q | 572.3151 | 571.3198 | | ***645.3679*** | 628.3413 | 627.3573 | 6 |
| 6 | 44.0495 | 661.3304 | 644.3039 | 643.3198 | 689.3253 | 672.2988 | 671.3148 | | | A | 501.2780 | | | ***517.3093*** | 500.2827 | 499.2987 | 5 |
| 7 | 30.0338 | 718.3519 | 701.3253 | 700.3413 | 746.3468 | 729.3202 | 728.3362 | | | G | | | | ***446.2722*** | 429.2456 | 428.2616 | 4 |
| 8 | 74.0600 | 819.3995 | 802.3730 | 801.3890 | **847.3945** | 830.3679 | 829.3839 | 803.4046 | 805.3839 | T | 343.2088 | 356.2292 | 358.2085 | 389.2507 | 372.2241 | 371.2401 | 3 |
| 9 | 86.0964 | 932.4836 | 915.4571 | 914.4730 | 960.4785 | 943.4520 | 942.4680 | 904.4523 | 918.4680 | I | 230.1248 | 243.1452 | 257.1608 | ***288.2030*** | 271.1765 | | 2 |
| 10 | 129.1135 | | | | | | | | | R | 74.0237 | 73.0284 | | ***175.1190*** | 158.0924 | | 1 |

| Seq | ya | yb | Seq | ya | yb | Seq | ya | yb |
|---|---|---|---|---|---|---|---|---|
| YP | 233.1285 | 261.1234 | YPQ | 361.1870 | 389.1819 | YPQQ | 489.2456 | 517.2405 |
| YPQQA | 560.2827 | 588.2776 | YPQQAG | 617.3042 | 645.2991 | PQ | 198.1237 | 226.1186 |
| PQQ | 326.1823 | 354.1772 | PQQA | 397.2194 | 425.2143 | PQQAG | 454.2409 | 482.2358 |
| PQQAGT | 555.2885 | 583.2835 | PQQAGTI | 668.3726 | 696.3675 | QQ | 229.1295 | 257.1244 |
| QQA | 300.1666 | 328.1615 | QQAG | 357.1881 | 385.1830 | QQAGT | 458.2358 | 486.2307 |
| QQAGTI | 571.3198 | 599.3148 | QA | 172.1081 | 200.1030 | QAG | 229.1295 | 257.1244 |
| QAGT | 330.1772 | 358.1721 | QAGTI | 443.2613 | 471.2562 | AG | 101.0709 | 129.0659 |
| AGT | 202.1186 | 230.1135 | AGTI | 315.2027 | 343.1976 | GT | 131.0815 | 159.0764 |
| GTI | 244.1656 | 272.1605 | TI | 187.1441 | 215.1390 | | | |

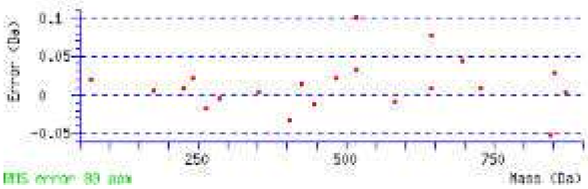


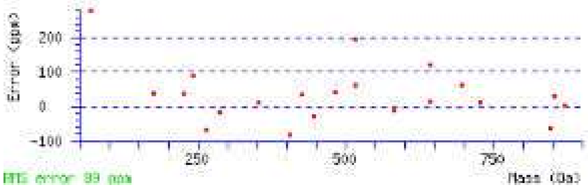


NCBI **BLAST** search of TYPQQAGTIR
(Parameters: blastp, nr protein database, expect=20000, no filter, PAM30)
Other BLAST web gateways

**All matches to this query**

| Score | Mr(calc) | Delta | Sequence |
|---|---|---|---|
| 40.2 | 1133.5829 | 0.0542 | TYPQQAGTIR |
| 24.8 | 1133.5499 | 0.0873 | GAAAMAEAASIR |
| 22.5 | 1133.6669 | -0.0298 | QAVAGLLGHLR |
| 20.3 | 1133.5802 | 0.0569 | APGAGAGHGAGRR |
| 20.2 | 1133.6379 | -0.0008 | KPKAMAGFLR |
| 19.8 | 1133.6557 | -0.0186 | ILDPIHGTIR |
| 19.3 | 1133.5651 | 0.0720 | MKETYGHIR |
| 19.1 | 1133.6193 | 0.0178 | AAGKPGYTTLR |
| 18.8 | 1133.6040 | 0.0331 | GETTTQTKIR |
| 18.2 | 1133.6227 | 0.0145 | NALAMTKTLR |

**Mascot:** http://www.matrixscience.com/

# MATRIX SCIENCE Mascot Search Results

## Peptide View Spot no 118

MS/MS Fragmentation of **CHFVAIDIFNGK**
Found in **gi|108711192** in **NCBInr**, Eukaryotic translation initiation factor 5A-2, putative, expressed [Oryza sativa Japonica Group]

Match to Query 61: 1419.753224 from(1420.760500,1+) intensity(0.0000) index(17)
Title: Label: N8, Spot_Id: 219799, Peak_List_Id: 226513, MSMS Job_Run_Id: 21847, Comment:
Data file C:\Documents and Settings\Administrator\Desktop\Farah-Garima 11May 2013\ppw_N8_136859933900.txt

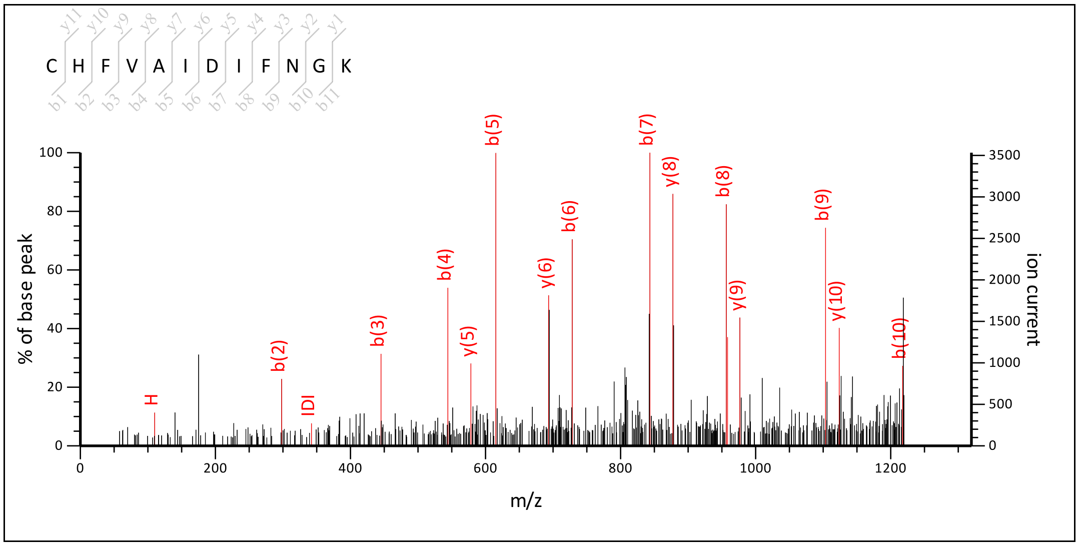


0 to 1319.53

Label all possible matches ○ Label matches used for scoring ◉

**Monoisotopic mass of neutral peptide Mr(calc):** 1419.6969
**Fixed modifications:** Carbamidomethyl (C) (apply to specified residues or termini only)
**Ions Score:** 73 **Expect:** 0.0017
**Matches :** 17/172 fragment ions using 24 most intense peaks (help)

| # | Immon. | a | a* | a0 | b | b* | b0 | d | d' | Seq. | v | w | w' | y | y* | y |
|---|---|---|---|---|---|---|---|---|---|---|---|---|---|---|---|---|
| 1 | 133.0430 | 133.0430 | | | 161.0379 | | | 44.0495 | | C | | | | | | |
| 2 | 110.0713 | 270.1019 | | | **298.0968** | | | | | H | 1178.6204 | | | 1260.6735 | 1243.6470 | 1242. |
| 3 | 120.0808 | 417.1703 | | | **445.1653** | | | | | F | 1031.5520 | | | **1123.6146** | 1106.5881 | 1105. |
| 4 | 72.0808 | 516.2387 | | | **544.2337** | | | 502.2231 | | V | 932.4836 | 945.5040 | | **976.5462** | 959.5197 | 958. |
| 5 | 44.0495 | 587.2759 | | | **615.2708** | | | | | A | 861.4465 | | | **877.4778** | 860.4512 | 859. |
| 6 | 86.0964 | 700.3599 | | | **728.3548** | | | 672.3286 | 686.3443 | I | 748.3624 | 761.3828 | 775.3985 | 806.4407 | 789.4141 | 788. |
| 7 | 88.0393 | 815.3869 | | 797.3763 | **843.3818** | | 825.3712 | 771.3970 | | D | 633.3355 | 632.3402 | | **693.3566** | 676.3301 | 675. |
| 8 | 86.0964 | 928.4709 | | 910.4604 | **956.4658** | | 938.4553 | 900.4396 | 914.4553 | I | 520.2514 | 533.2718 | 547.2875 | **578.3297** | 561.3031 | |
| 9 | 120.0808 | 1075.5393 | | 1057.5288 | **1103.5343** | | 1085.5237 | | | F | 373.1830 | | | 465.2456 | 448.2191 | |
| 10 | 87.0553 | 1189.5823 | 1172.5557 | 1171.5717 | **1217.5772** | 1200.5506 | 1199.5666 | 1146.5765 | | N | 259.1401 | 258.1448 | | 318.1772 | 301.1506 | |
| 11 | 30.0338 | 1246.6037 | 1229.5772 | 1228.5932 | 1274.5987 | 1257.5721 | 1256.5881 | | | G | | | | 204.1343 | 187.1077 | |
| 12 | 101.1073 | | | | | | | | | K | 74.0237 | 73.0284 | | 147.1128 | 130.0863 | |

| Seq | ya | yb | Seq | ya | yb | Seq | ya | yb |
|---|---|---|---|---|---|---|---|---|
| HF | 257.1397 | 285.1346 | HFV | 356.2081 | 384.2030 | HFVA | 427.2452 | 455.2401 |
| HFVAI | 540.3293 | 568.3242 | HFVAID | 655.3562 | 683.3511 | FV | 219.1492 | 247.1441 |
| FVA | 290.1863 | 318.1812 | FVAI | 403.2704 | 431.2653 | FVAID | 518.2973 | 546.2922 |
| FVAIDI | 631.3814 | 659.3763 | VA | 143.1179 | 171.1128 | VAI | 256.2020 | 284.1969 |
| VAID | 371.2289 | 399.2238 | VAIDI | 484.3130 | 512.3079 | VAIDIF | 631.3814 | 659.3763 |
| AI | 157.1335 | 185.1285 | AID | 272.1605 | 300.1554 | AIDI | 385.2445 | 413.2395 |
| AIDIF | 532.3130 | 560.3079 | AIDIFN | 646.3559 | 674.3508 | ID | 201.1234 | 229.1183 |
| IDI | 314.2074 | 342.2023 | IDIF | 461.2758 | 489.2708 | IDIFN | 575.3188 | 603.3137 |
| IDIFNG | 632.3402 | 660.3352 | DI | 201.1234 | 229.1183 | DIF | 348.1918 | 376.1867 |

| | | | | | | | | |
|---|---|---|---|---|---|---|---|---|
| **DIFN** | 462.2347 | 490.2296 | **DIFNG** | 519.2562 | 547.2511 | **IF** | 233.1648 | 261.1598 |
| **IFN** | 347.2078 | 375.2027 | **IFNG** | 404.2292 | 432.2241 | **FN** | 234.1237 | 262.1186 |
| **FNG** | 291.1452 | 319.1401 | **NG** | 144.0768 | 172.0717 | | | |

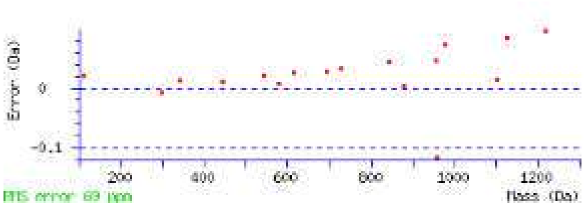


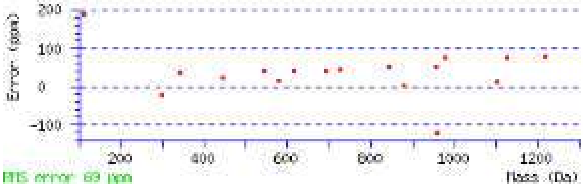


NCBI **BLAST** search of CHFVAIDIFNGK
(Parameters: blastp, nr protein database, expect=20000, no filter, PAM30)
Other BLAST web gateways

**All matches to this query**

| Score | Mr(calc) | Delta | Sequence |
|---|---|---|---|
| 73.0 | 1419.6969 | 0.0563 | CHFVAIDIFNGK |
| 73.0 | 1419.6969 | 0.0563 | CHFVALDIFNGK |
| 44.2 | 1419.6664 | 0.0868 | VGSDNAVLDMIDR |
| 43.3 | 1419.7511 | 0.0022 | DPLFAVITFNQR |
| 40.2 | 1419.6969 | 0.0563 | CHFVAIDVFNAK |
| 39.9 | 1419.6552 | 0.0981 | DDLEGLIDLMDR |
| 36.6 | 1419.6882 | 0.0651 | LEDDLGIDIFDR |
| 33.6 | 1419.7106 | 0.0426 | HADAALAALAPEDR |
| 33.1 | 1419.7246 | 0.0287 | DSDLFDILDLVR |
| 32.6 | 1419.7544 | -0.0012 | LSPFVALGCITSR |

**Mascot:** http://www.matrixscience.com/

# MATRIX SCIENCE Mascot Search Results

## Peptide View **Spot no 118**

MS/MS Fragmentation of **KLEDIVPSSHNCDVPHVNR**
Found in **gi|108711192** in **NCBInr**, Eukaryotic translation initiation factor 5A-2, putative, expressed [Oryza sativa Japonica Group]

Match to Query 106: 2215.193424 from(2216.200700,1+) intensity(0.0000) index(32)
Title: Label: N8, Spot_Id: 219799, Peak_List_Id: 226503, MSMS Job_Run_Id: 21847, Comment:
Data file C:\Documents and Settings\Administrator\Desktop\Farah-Garima 11May 2013\ppw_N8_136859933900.txt

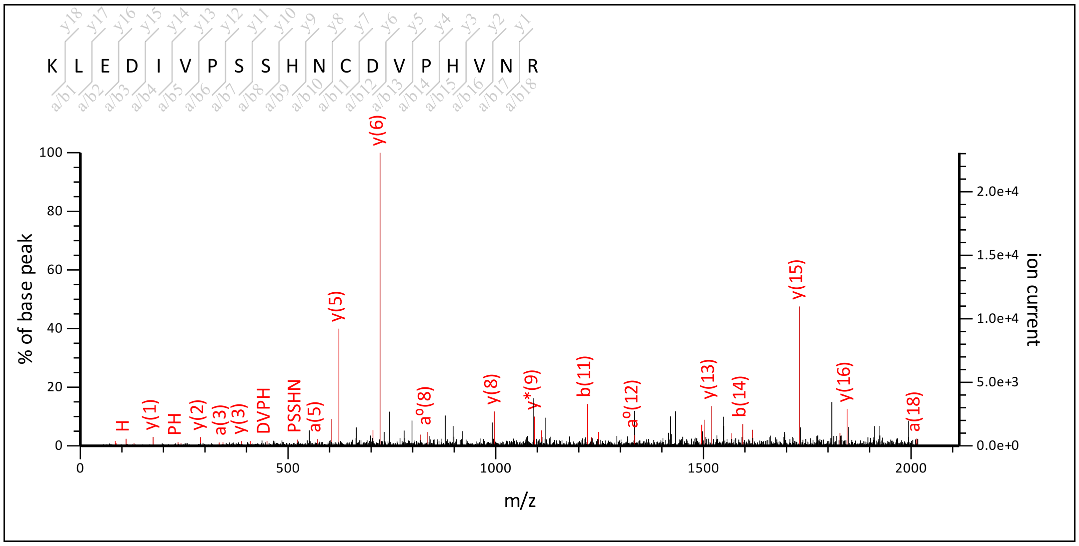


0 to 2115.94

Label all possible matches ○ Label matches used for scoring ◉

**Monoisotopic mass of neutral peptide Mr(calc):** 2215.0804
**Fixed modifications:** Carbamidomethyl (C) (apply to specified residues or termini only)
**Ions Score:** 75 **Expect:** 0.00073
**Matches :** 49/358 fragment ions using 80 most intense peaks (help)

| # | Immon. | a | a* | $a^0$ | b | b* | $b^0$ | d | d' | Seq. | v | w | w' | y | y* | [illegible] |
|---|---|---|---|---|---|---|---|---|---|---|---|---|---|---|---|---|
| 1 | 101.1073 | 101.1073 | 84.0808 | | **129.1022** | 112.0757 | | 44.0495 | | K | | | | | | |
| 2 | 86.0964 | 214.1914 | 197.1648 | | **242.1863** | 225.1598 | | 172.1444 | | L | 2029.9145 | 2028.9192 | | 2087.9927 | 2070.9662 | 206[illegible] |
| 3 | 102.0550 | **343.2340** | 326.2074 | 325.2234 | 371.2289 | 354.2023 | 353.2183 | 285.2285 | | E | 1900.8719 | 1899.8766 | | 1974.9086 | 1957.8821 | 19[illegible] |
| 4 | 88.0393 | 458.2609 | 441.2344 | 440.2504 | 486.2558 | 469.2293 | 468.2453 | 414.2711 | | D | 1785.8449 | 1784.8497 | | ***1845.8661*** | 1828.8395 | 18[illegible] |
| 5 | 86.0964 | **571.3450** | 554.3184 | 553.3344 | 599.3399 | 582.3134 | 581.3293 | 543.3137 | 557.3293 | I | 1672.7609 | 1685.7813 | 1699.7969 | ***1730.8391*** | 1713.8126 | 17[illegible] |
| 6 | 72.0808 | 670.4134 | 653.3869 | 652.4028 | 698.4083 | 681.3818 | 680.3978 | 656.3978 | | V | 1573.6924 | 1586.7128 | | ***1617.7550*** | 1600.7285 | 15[illegible] |
| 7 | 70.0651 | 767.4662 | 750.4396 | 749.4556 | 795.4611 | 778.4345 | 777.4505 | 741.4505 | | P | 1476.6397 | 1475.6444 | | ***1518.6866*** | 1501.6601 | 15[illegible] |
| 8 | 60.0444 | 854.4982 | 837.4716 | 836.4876 | 882.4931 | 865.4666 | 864.4825 | 838.5033 | | S | 1389.6077 | 1388.6124 | | 1421.6339 | 1404.6073 | 14[illegible] |
| 9 | 60.0444 | 941.5302 | 924.5037 | 923.5197 | 969.5251 | 952.4986 | 951.5146 | 925.5353 | | S | 1302.5756 | 1301.5804 | | ***1334.6018*** | 1317.5753 | 13[illegible] |
| 10 | 110.0713 | 1078.5891 | 1061.5626 | 1060.5786 | 1106.5840 | 1089.5575 | 1088.5735 | | | H | 1165.5167 | | | ***1247.5698*** | 1230.5433 | 12[illegible] |
| 11 | 87.0553 | 1192.6321 | 1175.6055 | 1174.6215 | **1220.6270** | 1203.6004 | 1202.6164 | 1149.6262 | | N | 1051.4738 | 1050.4785 | | ***1110.5109*** | 1093.4844 | 10[illegible] |
| 12 | 133.0430 | 1352.6627 | 1335.6362 | 1334.6521 | 1380.6576 | 1363.6311 | 1362.6471 | 1263.6692 | | C | 891.4431 | 890.4479 | | ***996.4680*** | 979.4414 | 9[illegible] |
| 13 | 88.0393 | 1467.6897 | 1450.6631 | 1449.6791 | **1495.6846** | 1478.6580 | 1477.6740 | 1423.6998 | | D | 776.4162 | 775.4209 | | ***836.4373*** | 819.4108 | 8[illegible] |
| 14 | 72.0808 | **1566.7581** | 1549.7315 | 1548.7475 | **1594.7530** | 1577.7264 | 1576.7424 | 1552.7424 | | V | 677.3478 | 690.3682 | | ***721.4104*** | 704.3838 | |
| 15 | 70.0651 | 1663.8108 | 1646.7843 | 1645.8003 | 1691.8057 | 1674.7792 | 1673.7952 | 1637.7952 | | P | 580.2950 | 579.2998 | | ***622.3420*** | 605.3154 | |
| 16 | 110.0713 | 1800.8697 | 1783.8432 | 1782.8592 | **1828.8647** | 1811.8381 | 1810.8541 | | | H | 443.2361 | | | 525.2892 | 508.2627 | |
| 17 | 72.0808 | 1899.9382 | 1882.9116 | 1881.9276 | 1927.9331 | 1910.9065 | 1909.9225 | 1885.9225 | | V | 344.1677 | 357.1881 | | ***388.2303*** | 371.2037 | |
| 18 | 87.0553 | **2013.9811** | 1996.9545 | 1995.9705 | 2041.9760 | 2024.9495 | 2023.9654 | 1970.9753 | | N | 230.1248 | 229.1295 | | ***289.1619*** | 272.1353 | |
| 19 | 129.1135 | | | | | | | | | R | 74.0237 | 73.0284 | | ***175.1190*** | 158.0924 | |

| Seq | ya | yb | Seq | ya | yb | Seq | ya | yb |
|---|---|---|---|---|---|---|---|---|
| LE | 215.1390 | 243.1339 | LED | 330.1660 | 358.1609 | LEDI | 443.2500 | 471.2449 |
| LEDIV | 542.3184 | 570.3134 | LEDIVP | 639.3712 | 667.3661 | ED | 217.0819 | 245.0768 |

| | | | | | | | | |
|---|---|---|---|---|---|---|---|---|
| **EDI** | 330.1660 | 358.1609 | **EDIV** | 429.2344 | 457.2293 | **EDIVP** | 526.2871 | 554.2821 |
| **EDIVPS** | 613.3192 | 641.3141 | **DI** | 201.1234 | 229.1183 | **DIV** | 300.1918 | 328.1867 |
| **DIVP** | 397.2445 | 425.2395 | **DIVPS** | 484.2766 | 512.2715 | **DIVPSS** | 571.3086 | 599.3035 |
| **IV** | 185.1648 | 213.1598 | **IVP** | 282.2176 | 310.2125 | **IVPS** | 369.2496 | 397.2445 |
| **IVPSS** | 456.2817 | 484.2766 | **IVPSSH** | 593.3406 | 621.3355 | **VP** | 169.1335 | 197.1285 |
| **VPS** | 256.1656 | 284.1605 | **VPSS** | 343.1976 | 371.1925 | **VPSSH** | 480.2565 | 508.2514 |
| **VPSSHN** | 594.2994 | 622.2943 | **PS** | 157.0972 | 185.0921 | **PSS** | 244.1292 | 272.1241 |
| **PSSH** | 381.1881 | 409.1830 | **PSSHN** | 495.2310 | 523.2259 | **PSSHNC** | 655.2617 | 683.2566 |
| **SS** | 147.0764 | 175.0713 | **SSH** | 284.1353 | 312.1302 | **SSHN** | 398.1783 | 426.1732 |
| **SSHNC** | 558.2089 | 586.2038 | **SSHNCD** | 673.2358 | 701.2308 | **SH** | 197.1033 | 225.0982 |
| **SHN** | 311.1462 | 339.1411 | **SHNC** | 471.1769 | 499.1718 | **SHNCD** | 586.2038 | 614.1987 |
| **SHNCDV** | 685.2722 | 713.2671 | **HN** | 224.1142 | 252.1091 | **HNC** | 384.1448 | 412.1398 |
| **HNCD** | 499.1718 | 527.1667 | **HNCDV** | 598.2402 | 626.2351 | **HNCDVP** | 695.2930 | 723.2879 |
| **NC** | 247.0859 | 275.0809 | **NCD** | 362.1129 | 390.1078 | **NCDV** | 461.1813 | 489.1762 |
| **NCDVP** | 558.2341 | 586.2290 | **NCDVPH** | 695.2930 | 723.2879 | **CD** | 248.0700 | 276.0649 |
| **CDV** | 347.1384 | 375.1333 | **CDVP** | 444.1911 | 472.1860 | **CDVPH** | 581.2500 | 609.2450 |
| **CDVPHV** | 680.3185 | 708.3134 | **DV** | 187.1077 | 215.1026 | **DVP** | 284.1605 | 312.1554 |
| **DVPH** | 421.2194 | 449.2143 | **DVPHV** | 520.2878 | 548.2827 | **DVPHVN** | 634.3307 | 662.3257 |
| **VP** | 169.1335 | 197.1285 | **VPH** | 306.1925 | 334.1874 | **VPHV** | 405.2609 | 433.2558 |
| **VPHVN** | 519.3038 | 547.2987 | **PH** | 207.1240 | 235.1190 | **PHV** | 306.1925 | 334.1874 |
| **PHVN** | 420.2354 | 448.2303 | **HV** | 209.1397 | 237.1346 | **HVN** | 323.1826 | 351.1775 |
| **VN** | 186.1237 | 214.1186 | | | | | | |

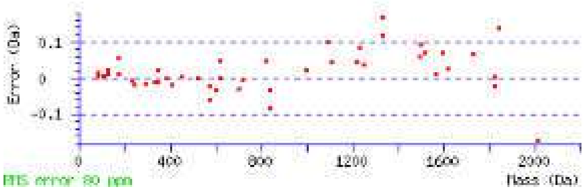


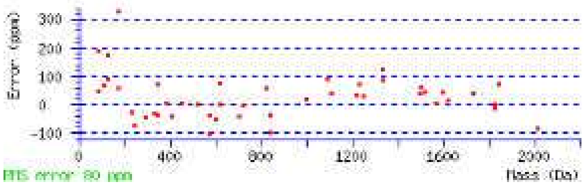


NCBI **BLAST** search of KLEDIVPSSHNCDVPHVNR
(Parameters: blastp, nr protein database, expect=20000, no filter, PAM30)
Other BLAST web gateways

**All matches to this query**

| Score | Mr(calc) | Delta | Sequence |
|---|---|---|---|
| 74.9 | 2215.0804 | 0.1130 | KLEDIVPSSHNCDVPHVNR |
| 23.0 | 2215.1848 | 0.0086 | NDQVVDIVLEDLAAVHQIPK |
| 17.4 | 2215.1233 | 0.0701 | HVGNDVVVLIFKEQSSSSDR |
| 17.2 | 2215.0918 | 0.1016 | MEMTLFLNESYIFHRLR |
| 15.2 | 2215.1518 | 0.0416 | DLAAKGEVDTVLVALVDMQGR |
| 14.8 | 2215.0914 | 0.1021 | MIMVGTMAITLWVIGWAMK |
| 14.3 | 2215.0651 | 0.1283 | QTIAHMSTLNQRLESAEDR |
| 13.8 | 2215.1056 | 0.0879 | VHRMEDTTWIPLVASTTSR |
| 13.8 | 2215.0918 | 0.1016 | MEMTLFLNESYIFHRLR |
| 13.2 | 2215.1129 | 0.0805 | DIPHEKVMILECIFSVNR |

**Mascot:** http://www.matrixscience.com/

# MATRIX SCIENCE Mascot Search Results

## Peptide View **Spot no 120**

MS/MS Fragmentation of **VTFLDDAQVK**
Found in **gi|4239821** in **NCBInr**, germin-like protein 1 [Oryza sativa]

Match to Query 115: 1134.630224 from(1135.637500,1+) intensity(0.0000) index(13)
Title: Label: G18, Spot_Id: 219952, Peak_List_Id: 228894, MSMS Job_Run_Id: 22004, Comment:
Data file C:\Documents and Settings\Administrator\Desktop\Farah-Garima 11May 2013\ppw_G18_136868326500.txt

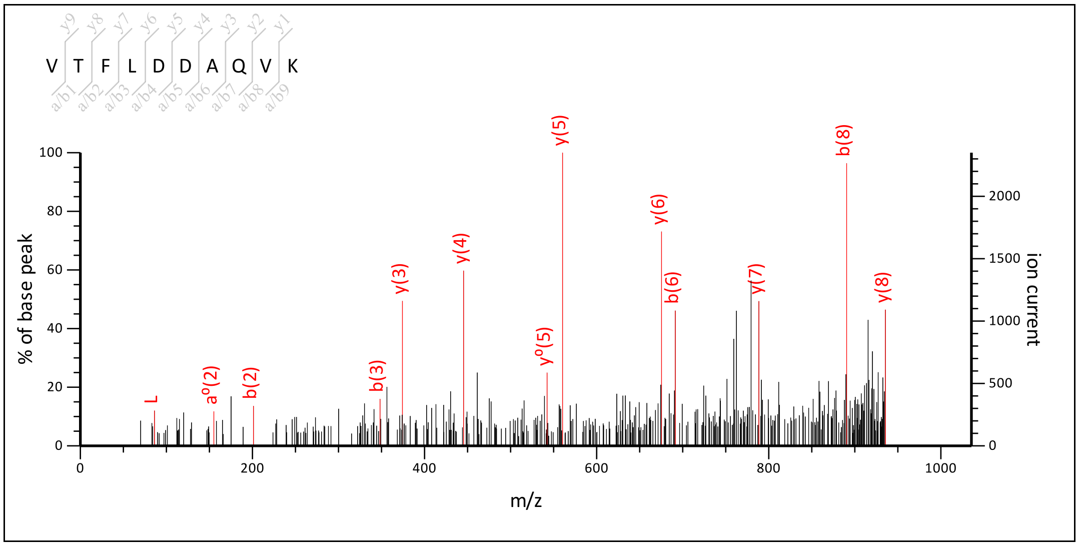


0 to 1035.52

Label all possible matches ○ Label matches used for scoring ◉

**Monoisotopic mass of neutral peptide Mr(calc):** 1134.5921
**Fixed modifications:** Carbamidomethyl (C) (apply to specified residues or termini only)
**Ions Score:** 41 **Expect:** 3.1
**Matches :** 15/146 fragment ions using 18 most intense peaks (help)

| # | Immon. | a | a* | a⁰ | b | b* | b⁰ | d | d' | Seq. | v | w | w' | y | y* | y⁰ | # |
|---|---|---|---|---|---|---|---|---|---|---|---|---|---|---|---|---|---|
| 1 | 72.0808 | 72.0808 | | | 100.0757 | | | 44.0495 | | V | | | | | | | [illegible] |
| 2 | 74.0600 | 173.1285 | | 155.1179 | **201.1234** | | 183.1128 | 157.1335 | 159.1128 | T | 990.4891 | 1003.5095 | 1005.4888 | 1036.5310 | 1019.5044 | 1018.5204 | [illegible] |
| 3 | 120.0808 | 320.1969 | | 302.1863 | **348.1918** | | 330.1812 | | | F | 843.4207 | | | ***935.4833*** | 918.4567 | 917.4727 | [illegible] |
| 4 | 86.0964 | 433.2809 | | 415.2704 | 461.2758 | | 443.2653 | 391.2340 | | L | 730.3366 | 729.3414 | | ***788.4149*** | 771.3883 | 770.4043 | [illegible] |
| 5 | 88.0393 | 548.3079 | | 530.2973 | 576.3028 | | 558.2922 | 504.3180 | | D | 615.3097 | 614.3144 | | ***675.3308*** | 658.3042 | 657.3202 | [illegible] |
| 6 | 88.0393 | 663.3348 | | 645.3243 | **691.3297** | | 673.3192 | 619.3450 | | D | 500.2827 | 499.2875 | | ***560.3039*** | 543.2773 | 542.2933 | [illegible] |
| 7 | 44.0495 | 734.3719 | | 716.3614 | 762.3668 | | 744.3563 | | | A | 429.2456 | | | ***445.2769*** | 428.2504 | | [illegible] |
| 8 | 101.0709 | 862.4305 | 845.4040 | 844.4199 | **890.4254** | 873.3989 | 872.4149 | 805.4090 | | Q | 301.1870 | 300.1918 | | ***374.2398*** | 357.2132 | | [illegible] |
| 9 | 72.0808 | 961.4989 | 944.4724 | 943.4884 | 989.4938 | 972.4673 | 971.4833 | 947.4833 | | V | 202.1186 | 215.1390 | | 246.1812 | 229.1547 | | [illegible] |
| 10 | 101.1073 | | | | | | | | | K | 74.0237 | 73.0284 | | 147.1128 | 130.0863 | | |

| Seq | ya | yb | Seq | ya | yb | Seq | ya | yb |
|---|---|---|---|---|---|---|---|---|
| TF | 221.1285 | 249.1234 | TFL | 334.2125 | 362.2074 | TFLD | 449.2395 | 477.2344 |
| TFLDD | 564.2664 | 592.2613 | TFLDDA | 635.3035 | 663.2984 | FL | 233.1648 | 261.1598 |
| FLD | 348.1918 | 376.1867 | FLDD | 463.2187 | 491.2136 | FLDDA | 534.2558 | 562.2508 |
| FLDDAQ | 662.3144 | 690.3093 | LD | 201.1234 | 229.1183 | LDD | 316.1503 | 344.1452 |
| LDDA | 387.1874 | 415.1823 | LDDAQ | 515.2460 | 543.2409 | LDDAQV | 614.3144 | 642.3093 |
| DD | 203.0662 | 231.0612 | DDA | 274.1034 | 302.0983 | DDAQ | 402.1619 | 430.1569 |
| DDAQV | 501.2304 | 529.2253 | DA | 159.0764 | 187.0713 | DAQ | 287.1350 | 315.1299 |
| DAQV | 386.2034 | 414.1983 | AQ | 172.1081 | 200.1030 | AQV | 271.1765 | 299.1714 |
| QV | 200.1394 | 228.1343 | | | | | | |

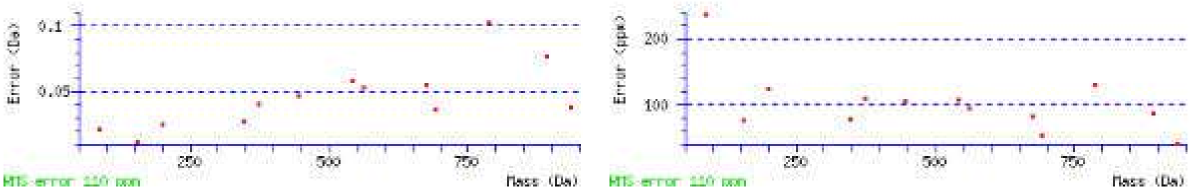


NCBI **BLAST** search of VTFLDDAQVK
(Parameters: blastp, nr protein database, expect=20000, no filter, PAM30)
Other BLAST web gateways

**All matches to this query**

| Score | Mr(calc) | Delta | Sequence |
|---|---|---|---|
| 41.0 | 1134.5921 | 0.0381 | VTFLDDAQVK |
| 37.7 | 1134.5418 | 0.0884 | AEFIDDGGRR |
| 34.8 | 1134.5591 | 0.0711 | TVMPDSDKVK |
| 33.9 | 1134.5855 | 0.0447 | YLPSVMAGAAR |
| 32.1 | 1134.5306 | 0.0996 | TVFPDTGDGAR |
| 32.0 | 1134.6219 | 0.0083 | IDFIAMAKAR |
| 30.4 | 1134.6761 | -0.0458 | LSLALYTKAR |
| 29.4 | 1134.5669 | 0.0633 | EAFTELAAQR |
| 29.1 | 1134.6067 | 0.0236 | SLLMDTTKAR |
| 29.1 | 1134.5404 | 0.0898 | LETEDDKSAK |

**Mascot:** http://www.matrixscience.com/

# MATRIX SCIENCE Mascot Search Results

## Peptide View **Spot no 120**

MS/MS Fragmentation of **VTFLDDAQVKK**
Found in **gi|4239821** in **NCBInr**, germin-like protein 1 [Oryza sativa]

Match to Query 127: 1262.729324 from(1263.736600,1+) intensity(0.0000) index(16)
Title: Label: G18, Spot_Id: 219952, Peak_List_Id: 228895, MSMS Job_Run_Id: 22004, Comment:
Data file C:\Documents and Settings\Administrator\Desktop\Farah-Garima 11May 2013\ppw_G18_136868326500.txt

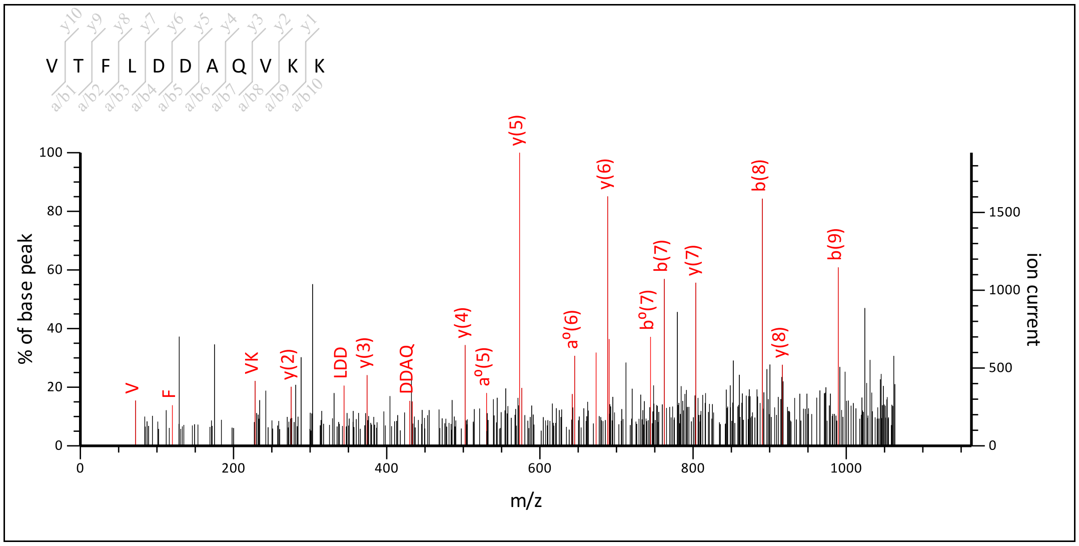


0 to 1163.44

Label all possible matches ○ Label matches used for scoring ◉

**Monoisotopic mass of neutral peptide Mr(calc):** 1262.6871
**Fixed modifications:** Carbamidomethyl (C) (apply to specified residues or termini only)
**Ions Score:** 33 **Expect:** 16
**Matches :** 27/168 fragment ions using 50 most intense peaks (help)

| # | Immon. | a | a* | $a^0$ | b | b* | $b^0$ | d | d' | Seq. | v | w | w' | y | y* | [illegible] |
|---|---|---|---|---|---|---|---|---|---|---|---|---|---|---|---|---|
| 1 | 72.0808 | **72.0808** | | | 100.0757 | | | 44.0495 | | V | | | | | | |
| 2 | 74.0600 | 173.1285 | | 155.1179 | 201.1234 | | 183.1128 | 157.1335 | 159.1128 | T | 1118.5841 | 1131.6045 | 1133.5837 | 1164.6259 | 1147.5994 | [illegible] |
| 3 | 120.0808 | 320.1969 | | 302.1863 | 348.1918 | | 330.1812 | | | F | 971.5156 | | | 1063.5782 | 1046.5517 | [illegible] |
| 4 | 86.0964 | **433.2809** | | 415.2704 | 461.2758 | | 443.2653 | 391.2340 | | L | 858.4316 | 857.4363 | | ***916.5098*** | 899.4833 | [illegible] |
| 5 | 88.0393 | 548.3079 | | 530.2973 | **576.3028** | | 558.2922 | 504.3180 | | D | 743.4046 | 742.4094 | | ***803.4258*** | 786.3992 | [illegible] |
| 6 | 88.0393 | 663.3348 | | 645.3243 | 691.3297 | | 673.3192 | 619.3450 | | D | 628.3777 | 627.3824 | | ***688.3988*** | 671.3723 | [illegible] |
| 7 | 44.0495 | 734.3719 | | 716.3614 | **762.3668** | | 744.3563 | | | A | 557.3406 | | | ***573.3719*** | 556.3453 | |
| 8 | 101.0709 | 862.4305 | 845.4040 | 844.4199 | **890.4254** | 873.3989 | 872.4149 | 805.4090 | | Q | 429.2820 | 428.2867 | | ***502.3348*** | 485.3082 | |
| 9 | 72.0808 | 961.4989 | 944.4724 | 943.4884 | **989.4938** | 972.4673 | 971.4833 | 947.4833 | | V | 330.2136 | 343.2340 | | ***374.2762*** | 357.2496 | |
| 10 | 101.1073 | 1089.5939 | 1072.5673 | 1071.5833 | 1117.5888 | 1100.5623 | 1099.5782 | 1032.5360 | | K | 202.1186 | 201.1234 | | ***275.2078*** | 258.1812 | |
| 11 | 101.1073 | | | | | | | | | K | 74.0237 | 73.0284 | | 147.1128 | 130.0863 | |

| Seq | ya | yb | Seq | ya | yb | Seq | ya | yb |
|---|---|---|---|---|---|---|---|---|
| TF | 221.1285 | 249.1234 | TFL | 334.2125 | 362.2074 | TFLD | 449.2395 | 477.2344 |
| TFLDD | 564.2664 | 592.2613 | TFLDDA | 635.3035 | 663.2984 | FL | 233.1648 | 261.1598 |
| FLD | 348.1918 | 376.1867 | FLDD | 463.2187 | 491.2136 | FLDDA | 534.2558 | 562.2508 |
| FLDDAQ | 662.3144 | 690.3093 | LD | 201.1234 | 229.1183 | LDD | 316.1503 | 344.1452 |
| LDDA | 387.1874 | 415.1823 | LDDAQ | 515.2460 | 543.2409 | LDDAQV | 614.3144 | 642.3093 |
| DD | 203.0662 | 231.0612 | DDA | 274.1034 | 302.0983 | DDAQ | 402.1619 | 430.1569 |
| DDAQV | 501.2304 | 529.2253 | DDAQVK | 629.3253 | 657.3202 | DA | 159.0764 | 187.0713 |
| DAQ | 287.1350 | 315.1299 | DAQV | 386.2034 | 414.1983 | DAQVK | 514.2984 | 542.2933 |
| AQ | 172.1081 | 200.1030 | AQV | 271.1765 | 299.1714 | AQVK | 399.2714 | 427.2663 |
| QV | 200.1394 | 228.1343 | QVK | 328.2343 | 356.2292 | VK | 200.1757 | 228.1707 |

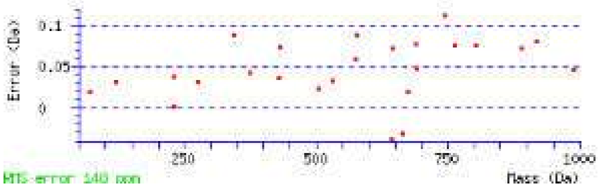

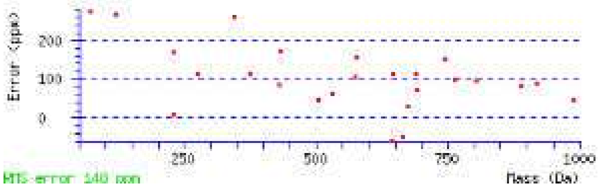

NCBI **BLAST** search of VTFLDDAQVKK
(Parameters: blastp, nr protein database, expect=20000, no filter, PAM30)
Other BLAST web gateways

**All matches to this query**

| Score | Mr(calc) | Delta | Sequence |
|---|---|---|---|
| 45.6 | 1262.6289 | 0.1004 | MTDVIDAKAQR |
| 35.7 | 1262.7234 | 0.0059 | IIFTTDAAKGVK |
| 33.2 | 1262.6871 | 0.0423 | VTFLDDAQVKK |
| 33.0 | 1262.6255 | 0.1038 | VFEEADAKAQR |
| 32.1 | 1262.6077 | 0.1216 | ELMDSWKAAGR |
| 31.9 | 1262.6329 | 0.0964 | LPEGFMAEVVR |
| 31.8 | 1262.6448 | 0.0845 | QFILWDWQK |
| 31.6 | 1262.6077 | 0.1216 | ELMADWKAAGR |
| 28.8 | 1262.6363 | 0.0931 | MEKLMGPSVQK |
| 28.7 | 1262.6983 | 0.0311 | LFLSVSGEQKR |

**Mascot:** http://www.matrixscience.com/

# MATRIX SCIENCE Mascot Search Results

## Peptide View **Spot no 120**

MS/MS Fragmentation of **AAVTPAFVGQFPGVNGLGISAAR**
Found in **gi|4239821** in **NCBInr**, germin-like protein 1 [Oryza sativa]

Match to Query 150: 2199.290324 from(2200.297600,1+) intensity(0.0000) index(23)
Title: Label: G18, Spot_Id: 219952, Peak_List_Id: 228893, MSMS Job_Run_Id: 22004, Comment:
Data file C:\Documents and Settings\Administrator\Desktop\Farah-Garima 11May 2013\ppw_G18_136868326500.txt

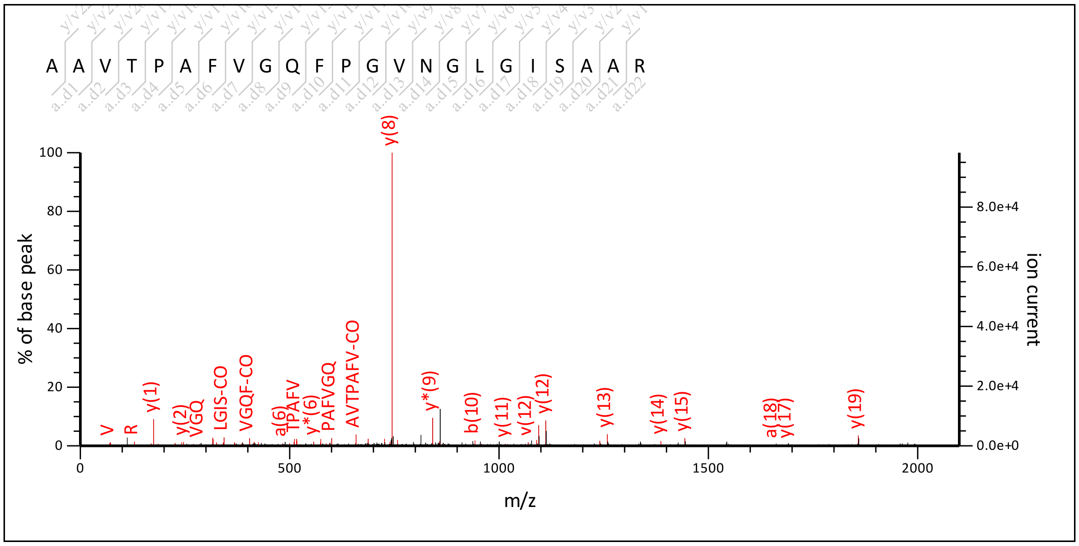


0 to 2099.29

Label all possible matches ○ Label matches used for scoring ◉

**Monoisotopic mass of neutral peptide Mr(calc):** 2199.1801
**Fixed modifications:** Carbamidomethyl (C) (apply to specified residues or termini only)
**Ions Score:** 77 **Expect:** 0.00032
**Matches :** 105/447 fragment ions using 160 most intense peaks (help)

| # | Immon. | a | a* | a0 | b | b* | b0 | d | d' | Seq. | v | w | w' | y | y* | |
|---|---|---|---|---|---|---|---|---|---|---|---|---|---|---|---|---|
| 1 | 44.0495 | 44.0495 | | | **72.0444** | | | 44.0495 | | A | | | | | | |
| 2 | 44.0495 | **115.0866** | | | **143.0815** | | | | | A | 2113.1189 | | | 2129.1502 | 2112.1237 | 2 |
| 3 | 72.0808 | 214.1550 | | | **242.1499** | | | 200.1394 | | V | 2014.0505 | 2027.0709 | | 2058.1131 | 2041.0865 | 20 |
| 4 | 74.0600 | 315.2027 | | 297.1921 | **343.1976** | | 325.1870 | 299.2078 | 301.1870 | T | 1913.0028 | 1926.0232 | 1928.0025 | 1959.0447 | 1942.0181 | 19 |
| 5 | 70.0651 | 412.2554 | | 394.2449 | 440.2504 | | 422.2398 | 386.2398 | | P | 1815.9500 | 1814.9548 | | ***1857.9970*** | 1840.9704 | 18 |
| 6 | 44.0495 | **483.2926** | | 465.2820 | **511.2875** | | 493.2769 | | | A | 1744.9129 | | | 1760.9442 | 1743.9177 | 17 |
| 7 | 120.0808 | 630.3610 | | 612.3504 | **658.3559** | | 640.3453 | | | F | 1597.8445 | | | ***1689.9071*** | 1672.8806 | 16 |
| 8 | 72.0808 | 729.4294 | | 711.4188 | **757.4243** | | 739.4137 | 715.4137 | | V | 1498.7761 | 1511.7965 | | 1542.8387 | 1525.8122 | 15 |
| 9 | 30.0338 | 786.4509 | | 768.4403 | 814.4458 | | 796.4352 | | | G | | | | ***1443.7703*** | 1426.7437 | 14 |
| 10 | 101.0709 | 914.5094 | 897.4829 | 896.4989 | **942.5043** | 925.4778 | 924.4938 | 857.4880 | | Q | 1313.6961 | 1312.7008 | | ***1386.7488*** | 1369.7223 | 13 |
| 11 | 120.0808 | 1061.5778 | 1044.5513 | 1043.5673 | **1089.5728** | 1072.5462 | 1071.5622 | | | F | 1166.6276 | | | ***1258.6902*** | 1241.6637 | 12 |
| 12 | 70.0651 | 1158.6306 | 1141.6041 | 1140.6200 | 1186.6255 | 1169.5990 | 1168.6150 | 1132.6150 | | P | 1069.5749 | 1068.5796 | | ***1111.6218*** | 1094.5953 | 10 |
| 13 | 30.0338 | 1215.6521 | 1198.6255 | 1197.6415 | 1243.6470 | 1226.6204 | 1225.6364 | | | G | | | | ***1014.5691*** | 997.5425 | 9 |
| 14 | 72.0808 | 1314.7205 | 1297.6939 | 1296.7099 | 1342.7154 | 1325.6889 | 1324.7048 | 1300.7048 | | V | 913.4850 | 926.5054 | | ***957.5476*** | 940.5211 | 9 |
| 15 | 87.0553 | 1428.7634 | 1411.7369 | 1410.7528 | 1456.7583 | 1439.7318 | 1438.7478 | 1385.7576 | | N | 799.4421 | 798.4468 | | ***858.4792*** | 841.4526 | 8 |
| 16 | 30.0338 | 1485.7849 | 1468.7583 | 1467.7743 | 1513.7798 | 1496.7532 | 1495.7692 | | | G | | | | ***744.4363*** | 727.4097 | 7 |
| 17 | 86.0964 | 1598.8689 | 1581.8424 | 1580.8584 | 1626.8639 | 1609.8373 | 1608.8533 | 1556.8220 | | L | 629.3365 | 628.3413 | | ***687.4148*** | 670.3883 | 6 |
| 18 | 30.0338 | **1655.8904** | 1638.8639 | 1637.8798 | 1683.8853 | 1666.8588 | 1665.8748 | | | G | | | | ***574.3307*** | 557.3042 | 5 |
| 19 | 86.0964 | 1768.9745 | 1751.9479 | 1750.9639 | 1796.9694 | 1779.9428 | 1778.9588 | 1740.9432 | 1754.9588 | I | 459.2310 | 472.2514 | 486.2671 | ***517.3093*** | 500.2827 | 4 |
| 20 | 60.0444 | 1856.0065 | 1838.9799 | 1837.9959 | 1884.0014 | 1866.9749 | 1865.9908 | 1840.0116 | | S | 372.1990 | 371.2037 | | ***404.2252*** | 387.1987 | 3 |
| 21 | 44.0495 | 1927.0436 | 1910.0171 | 1909.0330 | 1955.0385 | 1938.0120 | 1937.0280 | | | A | 301.1619 | | | ***317.1932*** | 300.1666 | |
| 22 | 44.0495 | 1998.0807 | 1981.0542 | 1980.0702 | 2026.0756 | 2009.0491 | 2008.0651 | | | A | 230.1248 | | | ***246.1561*** | 229.1295 | |
| 23 | 129.1135 | | | | | | | | | R | 74.0237 | 73.0284 | | ***175.1190*** | 158.0924 | |

| Seq | ya | yb | Seq | ya | yb | Seq | ya | yb |
|---|---|---|---|---|---|---|---|---|
| AV | 143.1179 | 171.1128 | AVT | 244.1656 | 272.1605 | AVTP | 341.2183 | 369.2132 |
| AVTPA | 412.2554 | 440.2504 | AVTPAF | 559.3239 | 587.3188 | AVTPAFV | 658.3923 | 686.3872 |
| VT | 173.1285 | 201.1234 | VTP | 270.1812 | 298.1761 | VTPA | 341.2183 | 369.2132 |
| VTPAF | 488.2867 | 516.2817 | VTPAFV | 587.3552 | 615.3501 | VTPAFVG | 644.3766 | 672.3715 |
| TP | 171.1128 | 199.1077 | TPA | 242.1499 | 270.1448 | TPAF | 389.2183 | 417.2132 |
| TPAFV | 488.2867 | 516.2817 | TPAFVG | 545.3082 | 573.3031 | TPAFVGQ | 673.3668 | 701.3617 |
| PA | 141.1022 | 169.0972 | PAF | 288.1707 | 316.1656 | PAFV | 387.2391 | 415.2340 |
| PAFVG | 444.2605 | 472.2554 | PAFVGQ | 572.3191 | 600.3140 | AF | 191.1179 | 219.1128 |
| AFV | 290.1863 | 318.1812 | AFVG | 347.2078 | 375.2027 | AFVGQ | 475.2663 | 503.2613 |
| AFVGQF | 622.3348 | 650.3297 | FV | 219.1492 | 247.1441 | FVG | 276.1707 | 304.1656 |
| FVGQ | 404.2292 | 432.2241 | FVGQF | 551.2976 | 579.2926 | FVGQFP | 648.3504 | 676.3453 |
| VG | 129.1022 | 157.0972 | VGQ | 257.1608 | 285.1557 | VGQF | 404.2292 | 432.2241 |
| VGQFP | 501.2820 | 529.2769 | VGQFPG | 558.3035 | 586.2984 | VGQFPGV | 657.3719 | 685.3668 |
| GQ | 158.0924 | 186.0873 | GQF | 305.1608 | 333.1557 | GQFP | 402.2136 | 430.2085 |
| GQFPG | 459.2350 | 487.2300 | GQFPGV | 558.3035 | 586.2984 | GQFPGVN | 672.3464 | 700.3413 |
| QF | 248.1394 | 276.1343 | QFP | 345.1921 | 373.1870 | QFPG | 402.2136 | 430.2085 |
| QFPGV | 501.2820 | 529.2769 | QFPGVN | 615.3249 | 643.3198 | QFPGVNG | 672.3464 | 700.3413 |
| FP | 217.1335 | 245.1285 | FPG | 274.1550 | 302.1499 | FPGV | 373.2234 | 401.2183 |
| FPGVN | 487.2663 | 515.2613 | FPGVNG | 544.2878 | 572.2827 | FPGVNGL | 657.3719 | 685.3668 |
| PG | 127.0866 | 155.0815 | PGV | 226.1550 | 254.1499 | PGVN | 340.1979 | 368.1928 |
| PGVNG | 397.2194 | 425.2143 | PGVNGL | 510.3035 | 538.2984 | PGVNGLG | 567.3249 | 595.3198 |
| PGVNGLGI | 680.4090 | 708.4039 | GV | 129.1022 | 157.0972 | GVN | 243.1452 | 271.1401 |
| GVNG | 300.1666 | 328.1615 | GVNGL | 413.2507 | 441.2456 | GVNGLG | 470.2722 | 498.2671 |
| GVNGLGI | 583.3562 | 611.3511 | GVNGLGIS | 670.3882 | 698.3832 | VN | 186.1237 | 214.1186 |
| VNG | 243.1452 | 271.1401 | VNGL | 356.2292 | 384.2241 | VNGLG | 413.2507 | 441.2456 |
| VNGLGI | 526.3348 | 554.3297 | VNGLGIS | 613.3668 | 641.3617 | VNGLGISA | 684.4039 | 712.3988 |
| NG | 144.0768 | 172.0717 | NGL | 257.1608 | 285.1557 | NGLG | 314.1823 | 342.1772 |
| NGLGI | 427.2663 | 455.2613 | NGLGIS | 514.2984 | 542.2933 | NGLGISA | 585.3355 | 613.3304 |
| NGLGISAA | 656.3726 | 684.3675 | GL | 143.1179 | 171.1128 | GLG | 200.1394 | 228.1343 |
| GLGI | 313.2234 | 341.2183 | GLGIS | 400.2554 | 428.2504 | GLGISA | 471.2926 | 499.2875 |
| GLGISAA | 542.3297 | 570.3246 | LG | 143.1179 | 171.1128 | LGI | 256.2020 | 284.1969 |
| LGIS | 343.2340 | 371.2289 | LGISA | 414.2711 | 442.2660 | LGISAA | 485.3082 | 513.3031 |
| GI | 143.1179 | 171.1128 | GIS | 230.1499 | 258.1448 | GISA | 301.1870 | 329.1819 |
| GISAA | 372.2241 | 400.2191 | IS | 173.1285 | 201.1234 | ISA | 244.1656 | 272.1605 |
| ISAA | 315.2027 | 343.1976 | SA | 131.0815 | 159.0764 | SAA | 202.1186 | 230.1135 |
| AA | 115.0866 | 143.0815 | | | | | | |

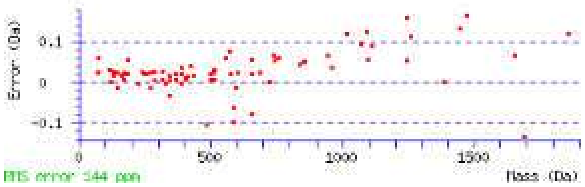


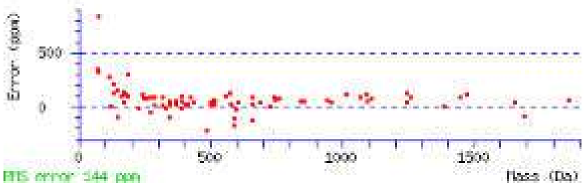


NCBI **BLAST** search of AAVTPAFVGQFPGVNGLGISAAR
(Parameters: blastp, nr protein database, expect=20000, no filter, PAM30)
Other BLAST web gateways

**All matches to this query**

| Score | Mr(calc) | Delta | Sequence |
|---|---|---|---|
| 76.9 | 2199.1801 | 0.1103 | AAVTPAFVGQFPGVNGLGISAAR |
| 27.2 | 2199.1800 | 0.1103 | AAVTPAFAAQFPGLNGLGLSAAR |
| 18.2 | 2199.1746 | 0.1157 | SNLSAAIDDVDKIIDLLSAAR |
| 18.2 | 2199.2164 | 0.0739 | IQTWQATIWLASGLKTAVGR |
| 15.8 | 2199.0920 | 0.1984 | LISDIDERNATLSNEWAPR |
| | | | |

| | | | |
|---|---|---|---|
| 15.0 | 2199.1106 | 0.1797 | INDAMGHAVGDDFLKIVTQR |
| 14.3 | 2199.1430 | 0.1473 | GMVEAGLITRDDVSVGTRPAR |
| 14.2 | 2199.1800 | 0.1103 | ELAFVPDAVVHYQLRSTVR |
| 14.1 | 2199.1131 | 0.1772 | NQAETISEQKAQETPVTGIR |
| 13.2 | 2199.1933 | 0.0970 | IEPLGMTGAGGLTEILAKVTGR |

**Mascot:** http://www.matrixscience.com/

# Mascot Search Results

## Peptide View **Spot no 125**

MS/MS Fragmentation of **IGINGFGR**
Found in **gi|968996** in **NCBInr**, glyceraldehyde-3-phosphate dehydrogenase [Oryza sativa]

Match to Query 5: 833.447684 from(834.454960,1+) intensity(0.0000) index(1)
Title: Label: P10, Spot_Id: 216636, Peak_List_Id: 221672, MSMS Job_Run_Id: 21387, Comment:
Data file C:\Documents and Settings\Administrator\Desktop\farah BHU\Cotton leaves\RAJESH\12-1-13 raj\ppw_P10_136117687500.txt

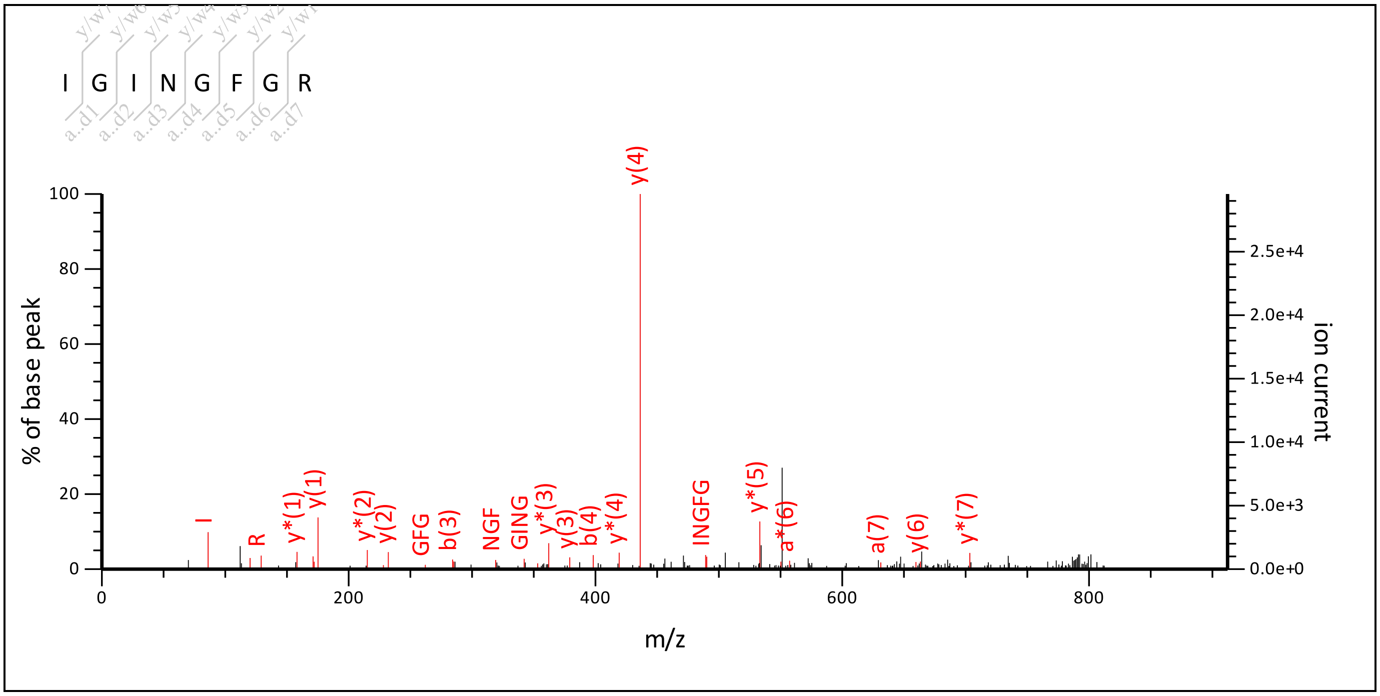


0 to 912.28

Label all possible matches Label matches used for scoring

**Monoisotopic mass of neutral peptide Mr(calc):** 832.4555
**Fixed modifications:** Carbamidomethyl (C) (apply to specified residues or termini only)
**Ions Score:** 28 **Expect:** 11
**Matches :** 40/86 fragment ions using 72 most intense peaks (help)

| # | Immon. | a | a* | b | b* | d | d' | Seq. | v | w | w' | y | y* | # |
|---|---|---|---|---|---|---|---|---|---|---|---|---|---|---|
| 1 | 86.0964 | **86.0964** | | 114.0913 | | 44.0495 | | I | | | | | | 8 |
| 2 | 30.0338 | **143.1179** | | **171.1128** | | | | G | | | | 720.3787 | 703.3522 | 7 |
| 3 | 86.0964 | 256.2020 | | **284.1969** | | 228.1707 | 242.1863 | I | 605.2790 | 618.2994 | 632.3151 | ***663.3573*** | 646.3307 | 6 |
| 4 | 87.0553 | 370.2449 | 353.2183 | **398.2398** | 381.2132 | 327.2391 | | N | 491.2361 | 490.2409 | | ***550.2732*** | 533.2467 | 5 |
| 5 | 30.0338 | 427.2663 | 410.2398 | 455.2613 | 438.2347 | | | G | | | | ***436.2303*** | 419.2037 | 4 |
| 6 | 120.0808 | 574.3348 | 557.3082 | 602.3297 | 585.3031 | | | F | 287.1462 | | | ***379.2088*** | 362.1823 | 3 |
| 7 | 30.0338 | **631.3562** | 614.3297 | **659.3511** | 642.3246 | | | G | | | | ***232.1404*** | 215.1139 | 2 |
| 8 | 129.1135 | | | | | | | R | 74.0237 | 73.0284 | | ***175.1190*** | 158.0924 | 1 |

| Seq | ya | yb | Seq | ya | yb | Seq | ya | yb |
|---|---|---|---|---|---|---|---|---|
| GI | 143.1179 | 171.1128 | GIN | 257.1608 | 285.1557 | GING | 314.1823 | 342.1772 |

| | | | | | | | | |
|---|---|---|---|---|---|---|---|---|
| **GINGF** | 461.2507 | 489.2456 | **GINGFG** | 518.2722 | 546.2671 | **IN** | 200.1394 | 228.1343 |
| **ING** | 257.1608 | 285.1557 | **INGF** | 404.2292 | 432.2241 | **INGFG** | 461.2507 | 489.2456 |
| **NG** | 144.0768 | 172.0717 | **NGF** | 291.1452 | 319.1401 | **NGFG** | 348.1666 | 376.1615 |
| **GF** | 177.1022 | 205.0972 | **GFG** | 234.1237 | 262.1186 | **FG** | 177.1022 | 205.0972 |

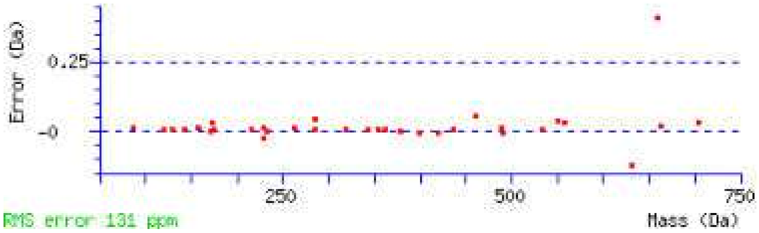


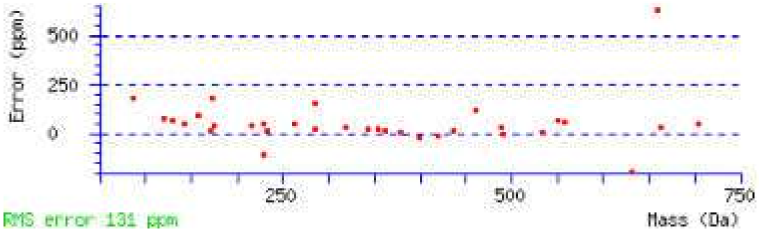


NCBI **BLAST** search of IGINGFGR
(Parameters: blastp, nr protein database, expect=20000, no filter, PAM30)
Other BLAST web gateways

**All matches to this query**

| Score | Mr(calc) | Delta | Sequence |
|---|---|---|---|
| 35.8 | 832.4555 | 0.9921 | VAINGFGR |
| 31.4 | 833.4065 | 0.0411 | GILDTCR |
| 31.4 | 833.4395 | 0.0081 | IGLDFNR |
| 29.6 | 833.4396 | 0.0081 | VADLGFGR |
| 28.4 | 832.4555 | 0.9921 | IGINGFGR |
| 28.1 | 834.3984 | -0.9507 | GNIDFNR |
| 27.7 | 832.4555 | 0.9921 | LGINGFGR |
| 27.2 | 833.4256 | 0.0220 | QRGGGFGR |
| 26.6 | 834.4195 | -0.9719 | TSNTLSGR |
| 25.9 | 833.4144 | 0.0333 | IGGGGFGGGR |

**Mascot:** http://www.matrixscience.com/

# MATRIX SCIENCE Mascot Search Results

## Peptide View **Spot no 125**

MS/MS Fragmentation of **TVDGPSSKDWR**
Found in **gi|968996** in **NCBInr**, glyceraldehyde-3-phosphate dehydrogenase [Oryza sativa]

Match to Query 25: 1246.611824 from(1247.619100,1+) intensity(0.0000) index(7)
Title: Label: P10, Spot_Id: 216636, Peak_List_Id: 221687, MSMS Job_Run_Id: 21387, Comment:
Data file C:\Documents and Settings\Administrator\Desktop\farah BHU\Cotton leaves\RAJESH\12-1-13 raj\ppw_P10_136117687500.txt

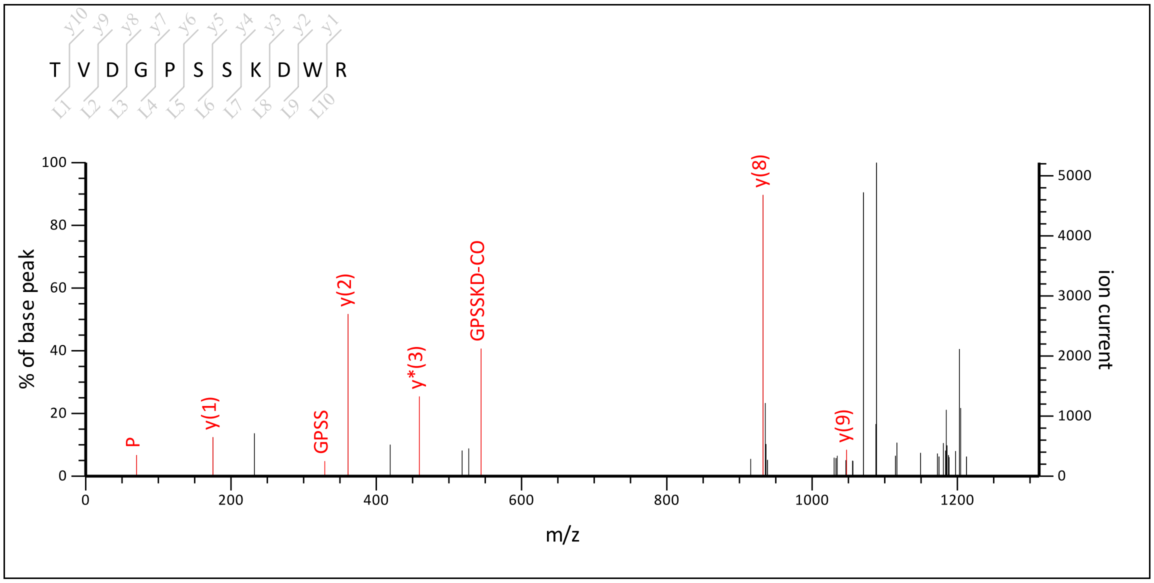


0 to 1312.55

Label all possible matches ○ Label matches used for scoring ◉

**Monoisotopic mass of neutral peptide Mr(calc):** 1246.5942
**Fixed modifications:** Carbamidomethyl (C) (apply to specified residues or termini only)
**Ions Score:** 16 **Expect:** 1.7e+02
**Matches :** 11/173 fragment ions using 17 most intense peaks (help)

| # | Immon. | a | a* | $a^0$ | b | b* | $b^0$ | d | Seq. | v | w | y | y* | $y^0$ | # |
|---|---|---|---|---|---|---|---|---|---|---|---|---|---|---|---|
| 1 | 74.0600 | 74.0600 | | 56.0495 | 102.0550 | | 84.0444 | 44.0495 | T | | | | | | 11 |
| 2 | 72.0808 | 173.1285 | | 155.1179 | 201.1234 | | 183.1128 | 159.1128 | V | 1102.4912 | 1115.5116 | 1146.5538 | 1129.5273 | 1128.5432 | 10 |
| 3 | 88.0393 | 288.1554 | | 270.1448 | 316.1503 | | 298.1397 | 244.1656 | D | 987.4643 | 986.4690 | ***1047.4854*** | 1030.4588 | 1029.4748 | 9 |
| 4 | 30.0338 | 345.1769 | | 327.1663 | 373.1718 | | 355.1612 | | G | | | ***932.4585*** | 915.4319 | 914.4479 | 8 |
| 5 | 70.0651 | 442.2296 | | 424.2191 | 470.2245 | | 452.2140 | 416.2140 | P | 833.3900 | 832.3948 | 875.4370 | 858.4104 | 857.4264 | 7 |
| 6 | 60.0444 | 529.2617 | | 511.2511 | 557.2566 | | 539.2460 | 513.2667 | S | 746.3580 | 745.3628 | 778.3842 | 761.3577 | 760.3737 | 6 |
| 7 | 60.0444 | 616.2937 | | 598.2831 | 644.2886 | | 626.2780 | 600.2988 | S | 659.3260 | 658.3307 | 691.3522 | 674.3256 | 673.3416 | 5 |
| 8 | 101.1073 | 744.3886 | 727.3621 | 726.3781 | 772.3836 | 755.3570 | 754.3730 | 687.3308 | K | 531.2310 | 530.2358 | 604.3202 | 587.2936 | 586.3096 | 4 |
| 9 | 88.0393 | 859.4156 | 842.3890 | 841.4050 | 887.4105 | 870.3840 | 869.3999 | 815.4258 | D | 416.2041 | 415.2088 | 476.2252 | 459.1987 | 458.2146 | 3 |
| 10 | 159.0917 | 1045.4949 | 1028.4684 | 1027.4843 | 1073.4898 | 1056.4633 | 1055.4792 | | W | 230.1248 | | ***361.1983*** | 344.1717 | | 2 |
| 11 | 129.1135 | | | | | | | | R | 74.0237 | 73.0284 | ***175.1190*** | 158.0924 | | 1 |

| Seq | ya | yb | Seq | ya | yb | Seq | ya | yb |
|---|---|---|---|---|---|---|---|---|
| VD | 187.1077 | 215.1026 | VDG | 244.1292 | 272.1241 | VDGP | 341.1819 | 369.1769 |
| VDGPS | 428.2140 | 456.2089 | VDGPSS | 515.2460 | 543.2409 | VDGPSSK | 643.3410 | 671.3359 |
| DG | 145.0608 | 173.0557 | DGP | 242.1135 | 270.1084 | DGPS | 329.1456 | 357.1405 |
| DGPSS | 416.1776 | 444.1725 | DGPSSK | 544.2726 | 572.2675 | DGPSSKD | 659.2995 | 687.2944 |
| GP | 127.0866 | 155.0815 | GPS | 214.1186 | 242.1135 | GPSS | 301.1506 | 329.1456 |
| GPSSK | 429.2456 | 457.2405 | GPSSKD | 544.2726 | 572.2675 | PS | 157.0972 | 185.0921 |
| | | | | | | | | |

| | | | | | | | | |
|---|---|---|---|---|---|---|---|---|
| **PSS** | 244.1292 | 272.1241 | **PSSK** | 372.2241 | 400.2191 | **PSSKD** | 487.2511 | 515.2460 |
| **PSSKDW** | 673.3304 | 701.3253 | **SS** | 147.0764 | 175.0713 | **SSK** | 275.1714 | 303.1663 |
| **SSKD** | 390.1983 | 418.1932 | **SSKDW** | 576.2776 | 604.2726 | **SK** | 188.1394 | 216.1343 |
| **SKD** | 303.1663 | 331.1612 | **SKDW** | 489.2456 | 517.2405 | **KD** | 216.1343 | 244.1292 |
[truncated: 373,553 more chars]
