# Supplementary material for: Sulfur alleviates arsenic toxicity by reducing its accumulation and modulating proteome, amino acids and thiol metabolism in rice leaves
Source: Sci Rep. 2015 Nov 10;5:16205. doi: 10.1038/srep16205 (PMC4639781; doi:10.1038/srep16205)

**Sulfur alleviates arsenic toxicity by reducing its accumulation and modulating proteome, amino acids and thiol metabolism in rice leaves**

Garima Dixit^1^, Amit Pal Singh^1^, Amit Kumar^1^, Sanjay Dwivedi^1^, Farah Deeba^1^, Smita Kumar^1^, Shankar Suman^2^, Bijan Adhikari^3^, Yogeshwar Shukla^2^, Prabodh Kumar Trivedi^1^, Vivek Pandey^1^, Rudra Deo Tripathi^1^*

*^1^CSIR-National Botanical Research Institute, Rana Pratap Marg, Lucknow – 226001, Uttar Pradesh, India*

*^2^CSIR-Indian Institute of Toxicology Research, Rana Pratap Marg, Lucknow – 226001, Uttar Pradesh, India*

*^3^Rice Research Station, Government of West Bengal, Chinsurah, Hooghly – 712102, West Bengal, India*

*Corresponding author

**Email addresses:**

Garima Dixit: [gariimaa21@gmail.com](mailto:gariimaa21@gmail.com)

Amit Pal Singh: [amitcomrade@gmail.com](mailto:amitcomrade@gmail.com)

Amit Kumar: [amit_gene@yahoo.com](mailto:amit_gene@yahoo.com)

Sanjay Dwivedi: [drs_dwivedi@yahoo.co.in](mailto:drs_dwivedi@yahoo.co.in)

Farah Deeba: [farahnbri@gmail.com](mailto:farahnbri@gmail.com)

Smita Kumar: [smitabiochem@gmail.com](mailto:smitabiochem@gmail.com)

Shankar Suman: [shankarsuman.suman@gmail.com](mailto:shankarsuman.suman@gmail.com)

Bijan Adhikari: [adhikaribijan@hotmail.com](mailto:adhikaribijan@hotmail.com)

Yogeshwer Shukla: [yshukla@itrc.res.in](mailto:yshukla@itrc.res.in)

Prabodh Kumar Trivedi: [prabodht@nbri.res.in](mailto:prabodht@nbri.res.in)

Vivek Pandey: [vivekpandey64@yahoo.com](mailto:vivekpandey64@yahoo.com)

Rudra Deo Tripathi* (corresponding author): [tripathird@gmail.com](mailto:tripathird@gmail.com), [tripathi_rd@rediffmail.com](mailto:tripathi_rd@rediffmail.com) (Telephone: +91 (0)522 2297825; Fax: +91 (0)522 2205836 / 2205839

**Supplemental Information 3**

**Text-S1: Material and methods**

# Text-S1: A- Arsenic quantification and quality control

For the estimation of As, 0.5 g oven dried tissue was taken and digested in 3 ml of HNO_3_ at 80 °C for 2 h and 100 °C for 4 h. Then filtered in 10 ml of Milli Q water and stored at 4 ^o^C till the estimation. Arsenic was quantified with the help of Inductively Coupled Plasma Mass Spectrometer (ICP-MS, Agilent 7500 cx). The standard solution material of As (Agilent, Part # 8500–6940) was used for the calibration and quality assurance for each analytical batch. Recovery of As rice flour NIST 1568a was used as a reference material with known spiked samples and recovery of total As were 95.3% (±2.8; *n*=5) and 92.5% (±3.1; *n*=5) respectively. The detection limit of As was 1 µg L^-1^.

Total sulfur was estimated by Chesnin, L. and Yien, C.H. (1951). A suitable aliquot was taken in 50 ml test tube and volume was made 2.5 ml with glass distilled water. To this aliquot, 2.5 ml sodium acetate buffer pH 4.8, 2.0 ml of 50% glycerol and 5.0 ml of 20% barium chloride were added one after the addition of barium chloride. A reagent blank was also run with each set of estimation. The turbidity of the solution was measured within half an hour of reaction using violet filter on spectrophotometer. Concentration of sulfur was estimated by referring the reading to a standard calibration curve prepared from AR grade sodium sulfate within the range of 50-250 µg sulfur.

**Text-S1: B-** **Amino Acid Profiling**

The pico tag kit from Waters (Milford, MA, USA) was used for estimation of AAs on Waters–HPLC system (Bidlingmeyer *et al.* 1984). 0.2 g of homogenized rice plant samples were hydrolysed in 10 ml of 6 N HCl in an oven for 1 hour at 150 ºC. 10 µl of filtered hydrolysed samples (Flow chart) and of standard (2.5 µmoles/ml in 0.1 N HCl) were derivatised with phenylisothiocynate (PITC) in vacuum oven at 55 ºC for 30 mins at 75 milli torr after three rounds of drying and redrying. The derivatised samples were then diluted with pico tag sample diluent and filtered with syringe filters. 20 µl of this was then injected into the system. Chromatographic analysis of the extracts was performed with a Waters Binary gradient HPLC system with accessories module 2475, (Waters, Milford, MA, USA) equipped with a degasser (DG2), a binary pump module (515), Temperature control module (TC2), Pump control module (PC2) and a photodiode array detector (Waters 2998). The separation was carried out at 40°C using a Pico Tag amino acid C18 column (3.9 x 15 cm; 5 µm). For each sample, 20 µl of extract was injected and the column was eluted at 1 ml min^-1^, with an optimized gradient established using solvents A (0.14 M sodium acetate, containing 0.05% triethylamine and 6% acetonitrile, pH 6.40) and B (60% acetonitrile in water). A step-by-step gradient was used with an increase of proportion of solvent B until it reached 46% during 10 min, followed by an increase upto 100% in 5 min, with a flux of 1 ml min^-1^. The column was then cleared and optimised to 100% A for 8 min at 1 ml min^-1^.

The AAs analysed were aspartic acid (Asp), glutamic acid (Glu), serine (Ser), glycine (Gly), histidine (His), aginine (Arg), threonine (Thr), alanine (Ala), proline (Pro), tyrosine (Tyr), valine (Val), methionine (Met), cysteine (Cys), isoleucine (Ile), leucine (Leu), phenalanine (Phe) and lysine (Lys). Tryptophan (Trp), asparagine (Asn) and glutamine (Gln) could not be analysed by this procedure as these are heat labile. Chromatograms were integrated using Empower 2 HPLC software v6.0.

**Text-S1: C- Estimation of thiol compounds and enzymes**

For the estimation of cysteine, plant material (500 mg) was homogenized in 5% chilled perchloric acid and centrifuged at 10000g for 10 min at 4^o^C. Cysteine content was measured in supernatant using acid–ninhydrin reagent at 560 nm according to the method of Gaitonde (1967). NP-SH content was measured by following the method of Ellman (1959). For which, plant material (700 mg) was homogenized in 6.67% 50-sulfosalicylic acid. After centrifugation at 10 000g for 10 min at 4 °C, NP-SH content was measured in the supernatant by reaction with Ellman reagent. The level of GSH was measured by following the protocol of Hissin and Hilf (1976). Plant material (500 mg) was frozen in liquid nitrogen homogenized in 0.1 M sodium phosphate buffer (pH 8.0) containing 25% meta-phosphoric acid. The homogenate as centrifuged at 20000g for 20 min at 4^o^C and GSH content was determined fluorometrically in the supernatant after 15 min incubation with o-phthaldialdehyde (OPT). Fluorescence intensity was recorded at 420 nm after excitation at 350 nm on Hitachi Fluorescence Spectrophotometer (F-7000).

Assay of CS (EC 2.5.1.47) activity, plant material (1 g) was extracted in 50 mM phosphate buffer (pH 7.5) containing 1 mM EDTA, 5 mM MgCl_2_, 2 mM dithiotheritol (DTT), 0.1% Triton X-100 and 0.5 mM PMSF (Harada *et al.* 2001). The homogenate was subjected to centrifugation at 16,000 × g for 15 min at 4ºC and the supernatant was used for enzymatic assay. The CS activity was determined in the reaction mixture (0.5 ml) containing 50 mM phosphate buffer (pH 8.0), 4 mM sodium sulfide (Na_2_S), 12.5 mM*O*-acetyl L-serine (OAS) and a suitable aliquot of enzyme extract (Saito *et al.* 1994). The incubation was performed at 30ºC for 20 min and reaction was terminated by the addition of 0.1 ml of 7.5% TCA. The amount of cysteine synthesized in the resulting mixture was determined spectrophotometrically by the method of Gaitonde (1967) as described in section 5.1.1. The activity of CS is expressed as nmol cysteine min^-1^ mg^-1^ protein.

Activity of GR (EC 1.6.4.2) was assayed by following the method of Smith *et al.* (1988). The reaction mixture contained 1.0 mL of 0.2 M potassium phosphate buffer (pH 7.5) containing 1 mM EDTA, 0.5 mL 3 mM 5,5 0-dithiobis (2-nitrobenzoic acid) in 0.01 M phosphate buffer (pH 7.5), 0.25 mL H2O, 0.1 mL 2 mM NADPH, 0.05 mL enzyme extract and 0.1 mL 20 mM GSSG. The components were added in the order as above directly to a cuvette and the reaction was started by the addition of GSSG. The increase in absorbance was monitored for 5 min at 412 nm. The rate of enzyme activity was calculated using standard curve prepared by known amounts of GR (Sigma, USA). The rate of enzyme activity was expressed as l moles GSSG reduced min^-1^ mg^-1^ protein.

Glutathione S-transferase (GST) activity was assayed in fresh roots and leaves. Sample extracts (10%) were prepared in 0.1 M phosphate buffer (pH ¼ 6.5). A reaction mixture containing 2.25 mM GSH and 2.25 mM 1-chloro-2,4-dinitrobenzene (CDNB) was freshly prepared and used within 20 min of preparation. The mixture (200 mL) was transferred into each microplate well followed by 25 mL aliquot of the sample. Absorbance was measured at 340 nm for 6 min at 37 C using a Spectramax 190
ultraviolet (UV) spectrophotometer. Measurement intervals of 15 s with 5 s shaking before each reading were programmed. Reagent blanks, containing phosphate buffer instead of sample, were also prepared and measured similar to samples. All samples and blanks were assayed in triplicate.

For the assay of γECS (EC 6.3.2.2), plants were homogenized in 100 mM Tris–HCl (pH 8.0) containing 5 mM EDTA, and 1% polyvinylpyrrolidone (PVP). The reaction mixture contained 0.1 M Tris–HCl (pH 8.0), 150 mM KCl, 2 mM EDTA, 20 mM MgCl2, 5 mM Na2ATP, 2 mM phosphoenol pyruvate, 10 mM L-glutamate, 10 mM L-α-aminobutyrate, 0.2 mM NADH, 7 Uml^−1^ pyruvate kinase (ICN, USA), and 10 Uml^−1^ L-lactic dehydrogenase (Sigma, USA; Seelig and Meister 1984). The definition of 1 U activity of the enzymes used for this and other assays is the amount of enzyme catalyzing the conversion of 1 µmol of substrate to product per minute. The reaction was initiated by the addition of enzyme extract. The activity of γECS was determined from the rate of formation of ADP (assumed to be equal to the rate of NADH oxidation; ε=6.2 mM^−1^ cm^−1^, monitored at 340 nm).

**Reference**

Bidlingmeyer B. A., Cohen S. A., & Tarvin T. L. (1984) Rapid analysis of amino acids using pre-column derivatization. *Journal of Chromatography B: Biomedical Sciences and Applications* *336*, 93-104.

Ellman G. L. (1959) Tissue sulfhydryl groups. *Archives of biochemistry and biophysics* *82*, 70-77.

Gaitonde M. K. (1967) A spectrophotometric method for the direct determination of cysteine in the presence of other naturally occurring amino acids. *Biochem. J* *104*, 627-633.

Harada E., Choi Y. E., Tsuchisaka A., Obata H., & Sano H. (2001) Transgenic tobacco plants expressing a rice cysteine synthase gene are tolerant to toxic levels of cadmium. *Journal of Plant Physiology* *158*, 655-661.

Hissin P. J., & Hilf R. (1976) A fluorometric method for determination of oxidized and reduced glutathione in tissues. *Analytical biochemistry* *74*, 214-226.

Saito K., Kurosawa M., Tatsuguchi K., Takagi Y., & Murakoshi I. (1994) Modulation of cysteine biosynthesis in chloroplasts of transgenic tobacco over expressing cysteine synthase [O-acetylserine (thiol)-Iyase]. *Plant Physiology* *106*, 887-895.

Seelig G. F. & Meister A. (1984) Gamma-glutamylcysteine synthetase. Interactions of an essential sulfhydryl group. *Journal of Biological Chemistry* *259*, 3534-3538.

Smith I. K., Vierheller T. L., & Thorne C. A. (1989) Properties and functions of glutathione reductase in plants. *Physiologia Plantarum* *77*, 449-456.

Caldana C., Scheible W. R., Mueller-Roeber B., Ruzicic S. (2007) A quantitative RT-PCR platform for high-throughput expression profiling of 2500 rice transcription factors. *Plant Methods* **3**, 7.

Table S1: Primer sequences for qRT-PCR analysis

| Protein number | Description | Gene ID | Primers |
| --- | --- | --- | --- |
| 2 | photosystem II 10 kDa polypeptide | Os08g10020 F | CCTTCCGTCCATCTCCCGC |
|  |  | Os08g10020 R | GCGAGTAGATTGGGCTGTA |
| 16 | 2Fe-2S iron-sulfur cluster binding domain containing protein | Os08g01380 F | GGTTGCCGATGTCCCTGCG |
|  |  | Os08g01380 R | ATCCCTTCCTCCTCCGCCTG |
| 28 | triosephosphate isomerase | Os09g36450 F | GGAAGTGCAATGGAACGAAGGAC |
|  |  | Os09g36450 R | GCAGCCAATGTCCACCAAT |
| 29 | ribulose-phosphate 3-epimerase | Os03g07300 F | TGGAGGTGGCAGGATGTGACT |
|  |  | Os03g07300 R | GTAGATGGATGGTTGACGATTGC |
| 39 | glyceraldehyde-3-phosphate dehydrogenase | Os04g38600 F | GAATGCCACCTCCGACGACT |
|  |  | Os04g38600 R | TACTTGAGGAGGTGCGACG |
| 45 | oxygen-evolving enhancer protein 1 | Os01g31690 F | GGCTTACCTTCGACGAGATTC |
|  |  | Os01g31690 R | GGGTCATGAGCTTGGTCTT |
| 50 | ferredoxin--NADP reductase | Os02g01340 F | GGAGGTGACTACTAAGGTGGAGAAG |
|  |  | Os02g01340 R | ATGGAGTAGAGGCGGAGCTTGTG |
| 56 | phosphoribulokinase/Uridine kinase family protein | Os02g47020 F | AGCAACAGGAGGGGTAGCAC |
|  |  | Os02g47020 R | GAGTGTGTTTGAGTCTGGGTTGCC |
| 58 | fructose-bisphospate aldolase isozyme | Os11g07020 F | ATGGCGTCTGCTACTCTCCTCAAG |
|  |  | Os11g07020 R | CCCCTTCCTGGTGATGCAA |
| 64 | aminotransferase, classes I and II, domain containing protein | Os07g01760 F | AGGTGCTTACAGTGATTCCCG |
|  |  | Os07g01760 R | GGAAATGGCAGCAGAATAAAGCGGG |
| 66 | glutamine synthetase, catalytic domain containing protein | Os04g56400 F | CTCAGAAGCAAATCAAGGAC |
|  |  | Os04g56400 R | CGTTTGTTAGTAGGGATGGG |
| 69 | glutamine synthetase, catalytic domain containing protein | Os02g50240 F | TCCCCACCAACAAGAGGCA |
|  |  | Os02g50240 R | CAGGGAAGCCACCAACAGG |
| 85 | ATP synthase | Os06g45120 F | GGTGGAGATCTATATGCTACTGTG |
|  |  | Os06g45120 R | AGAAGAGGCGTGTCAGCAGC |
| 125 | glyceraldehyde-3-phosphate dehydrogenase | Os03g03720 F | TGCTGGCACGAACGCGAGAAC |
|  |  | Os03g03720 R | GAGCAGTGATGATGACCTTCTTCGC |
| 136 | aminotransferase | Os08g41990 F | GCCCATTGTGTTTGATTCTGTG |
|  |  | Os08g41990 R | CTCAGCCAACACATTCTCCAACAC |
| Control | Actin | Rice actin F | GGAAGTACAGTGTCTGGATTGGAG |
|  |  | Rice actin R | TCTTGGCTTAGCATTCTTGGGT |

**Fig S1**: Observable effects of different S doses under arsenic stress on leaves of rice (*Oryza sativa* L.) plant. The photographs of the same samples were taken at each treatment concentration point, and photographed enlarged.

**Fig. S2**: Effect of different S doses on arsenite stress in leaves of rice (*Oryza sativa* L.) plant at (A) length; (B) weight; (C), values are the average of 15–20 plants. Chlorophyll a; (D) chlorophyll b; (E) total chlorophyll; (F) carotenoid. All the values are means of triplicate ± S.D. ANOVA significant at *p ≤* 0.01. Different letters indicate significantly different values at a particular treatment (DMRT, *p ≤* 0.05).

**Fig. S3:** Expressional changes of genes (proteins of glycolytic pathway, photosynthesis and energy metabolism) at the mRNA level in rice (*Oryza sativa* L.) leaves. The x-axis stands for locus ids of genes and their corresponding protein spots are given in Table S1. The values are means of three replicates ± SD. ANOVA significant at *p ≤* 0.01. Different letters indicate significantly different values at a particular treatment (DMRT, *p ≤* 0.05).

**Fig. S4:** Hierarchical clustering of S responsive proteins, analysis was conducted using MeV software.


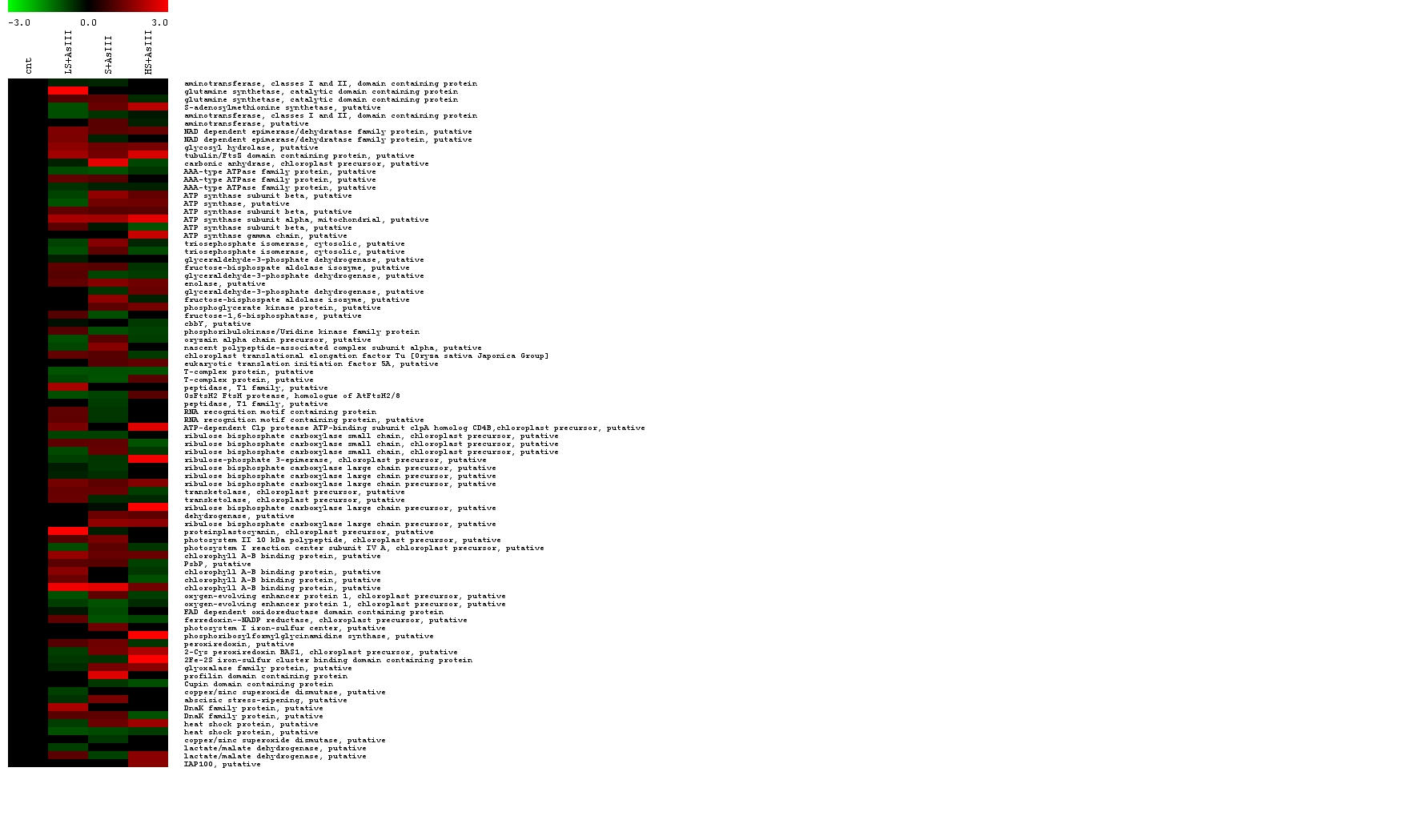

Supplement: Supplemental Information 3 [file srep16205-s3.doc]
